# Molecular fossils illuminate the evolution of retroviruses following a macroevolutionary transition from land to water

**DOI:** 10.1371/journal.ppat.1009730

**Published:** 2021-07-12

**Authors:** Jialu Zheng, Jianhua Wang, Zhen Gong, Guan-Zhu Han

**Affiliations:** Jiangsu Key Laboratory for Microbes and Functional Genomics, College of Life Sciences, Nanjing Normal University, Nanjing, China; University of North Carolina at Chapel Hill, UNITED STATES

## Abstract

The ancestor of cetaceans underwent a macroevolutionary transition from land to water early in the Eocene Period >50 million years ago. However, little is known about how diverse retroviruses evolved during this shift from terrestrial to aquatic environments. Did retroviruses transition into water accompanying their hosts? Did retroviruses infect cetaceans through cross-species transmission after cetaceans invaded the aquatic environments? Endogenous retroviruses (ERVs) provide important molecular fossils for tracing the evolution of retroviruses during this macroevolutionary transition. Here, we use a phylogenomic approach to study the origin and evolution of ERVs in cetaceans. We identify a total of 8,724 ERVs within the genomes of 25 cetaceans, and phylogenetic analyses suggest these ERVs cluster into 315 independent lineages, each of which represents one or more independent endogenization events. We find that cetacean ERVs originated through two possible routes. 298 ERV lineages may derive from retrovirus endogenization that occurred before or during the transition from land to water of cetaceans, and most of these cetacean ERVs were reaching evolutionary dead-ends. 17 ERV lineages are likely to arise from independent retrovirus endogenization events that occurred after the split of mysticetes and odontocetes, indicating that diverse retroviruses infected cetaceans through cross-species transmission from non-cetacean mammals after the transition to aquatic life of cetaceans. Both integration time and synteny analyses support the recent or ongoing activity of multiple retroviral lineages in cetaceans, some of which proliferated into hundreds of copies within the host genomes. Although ERVs only recorded a proportion of past retroviral infections, our findings illuminate the complex evolution of retroviruses during one of the most marked macroevolutionary transitions in vertebrate history.

## Author summary

The ancestor of cetaceans invaded the aquatic environments >50 million years ago. Recent comparative genomic studies have begun to reveal the molecular mechanisms underlying the morphological and ecological transformation of cetaceans. However, it has been largely obscure how retroviruses evolved during this land-to-water transition. Endogenous retroviruses (ERVs) recorded past retroviral infections. Here, we systematically mined and analyzed ERVs in the genomes of 25 cetaceans, and found that cetacean ERVs clustered into 315 distinct lineages. Among these cetacean ERV lineages, 298 lineages may originate through retrovirus endogenization before or during the colonization of aquatic environments by cetaceans, and 17 lineages may arise from endogenization of retroviruses that infected cetaceans through cross-species transmission from non-cetacean mammals after the transition to aquatic life of cetaceans. Taken together, our study provides a unique snapshot of the evolution of retroviruses during a major macroevolutionary transition.

## Introduction

The ancestors of modern cetaceans (whales, dolphins, and porpoises) underwent a macroevolutionary transition from terrestrial to aquatic environments early in the Eocene >50 million years ago [1–4]. Pakicetids, the earliest known cetaceans that existed in the early Eocene, are like to be aquatic waders [3, 4]. During the transition from land to water, cetaceans evolved a range of morphological and behavioral innovations, including streamlined bodies, filter-feeding, echolocation, as well as loss of hindlimbs, body hair, and dermal glands [1, 2, 5]. Phylogenetic analyses reveal that cetaceans are closely related to and fall within the diversity of even-toed ungulates (Artiodactyla) [6]. Therefore, Cetacea and Artiodactyla have been sometimes united into Cetartiodactyla [6]. Within Cetartiodactyla, Hippopotamidae has been placed to be the sister group of Cetacea [6]. Modern cetacean species can be further divided into two clades, namely mysticetes (baleen whales) and odontocetes (toothed whales) [3, 7]. Mysticetes and odontocetes have been estimated to diverge from each other in the Late Eocene (~36 million years ago) [6].

Diverse viruses have been reported to infect cetaceans, including adenoviruses [8], astroviruses [9], circoviruses [10], coronaviruses [11], enteroviruses [12], herpesviruses [13], influenza viruses [14], morbilliviruses [15, 16], papillomaviruses [17], pegiviruses [18], pestiviruses [19], poxviruses [20], and rhabdoviruses [21]. However, it remains largely obscure how these viruses originated in cetaceans, and how these viruses evolved during the shift from land to water.

Retroviruses have been known to infect vertebrates, including cetaceans [22–27]. The replication of retroviruses requires reverse transcription and integration of viral genomes into host genomes. On occasion, retroviruses infect germ line cells, and the integrated retroviruses may become vertically inherited, forming the so-called endogenous retroviruses (ERVs) [25–27]. ERVs recorded past retroviral infections, providing molecular fossils for studying the macroevolution of retroviruses. Therefore, ERVs represent a unique resource to explore the evolution of retroviruses during the macroevolutionary transition from land to water of cetaceans.

The International Committee on Viral Classification (ICTV) classifies exogenous retroviruses into seven genera, including *Alpharetrovirus*, *Betaretrovirus*, *Gammaretrovirus*, *Deltaretrovirus*, *Epsilonretrovirus*, *Lentivirus*, and *Spumaretrovirus* (foamy viruses). Based on their relationship with exogenous retroviruses, ERVs have been grouped into three classes, namely Class I, Class II, and Class III ERVs [28–30]. Class I ERVs are closely related to gammaretroviruses and epsilonretroviruses, Class II ERVs are closely related to betaretroviruses, and Class III ERVs are closely related to foamy viruses [26, 27].

ERVs proliferate within the host genomes through three modes: ERVs in germ line cells or somatic cells produce virus particles to infect germ line cells, namely reinfection [31, 32]; ERVs can also increase in copy number within the cell either by retrotransposition in *cis* (viruses use their own proteins for mobilization) or by complementation in *trans* (viruses use proteins produced by other transposable elements within the same cell) [33–35]. Reinfection can also occur by complementation in *trans*, that is, retroviruses without functional *env* genes can produce virus particles to infect germline cells by “hitchhiking” *env* gene of other retroviruses [36]. Reinfection requires the three core genes (*gag*, *pol*, and *env*) to be functional, and thus all the three core genes are subject to purifying selection, as indicated by a nonsynonymous to synonymous substitution rate ratio (dN/dS) of < 1 [31, 32]. ERV transposition in *cis* does not require a functional *env* gene, and ERV proliferation by complementation in *trans* does not require any functional gene of its own [31, 32]. Different ERVs increase in copy number through different ways; for example, while human ERV family HERV-K (HML2) members proliferate mainly by reinfection [31], intracisternal A-type particles (IAPs) proliferate mainly by retrotransposition in *cis* [36].

In this study, we used a phylogenomic approach to trace the origin and evolution of ERVs along the course of cetacean evolution, and identified a total of 8,724 ERVs in 25 cetacean genomes, which cluster into 315 distinct ERV lineages. We hypothesize that cetacean ERVs originated through two possible routes, through either land-to-water transition or secondary host switching. Our study provides novel insights into the evolution of retroviruses during one of the most remarkable macroevolutionary transitions in vertebrate history.

## Results

### Identification and classification of ERVs in cetaceans

To explore the evolution of retroviruses in cetaceans, we used a similarity search and phylogenetic analysis combined approach to systematically identify ERVs within the genomes of 25 cetaceans, including 6 mysticetes and 19 odontocetes (S1 Table). We found the presence of ERVs in all the cetaceans, and identified a total of 8,724 ERVs, which is consistent with the ubiquitous distribution of ERVs in vertebrates [26, 27]. The copy numbers of ERVs in cetaceans are relatively low, varying from 222 in *Physeter catodon* to 627 in *Lagenorhynchus obliquidens*. However, the estimates of ERV copy numbers should be taken with caution, because the quality and completeness of genome assemblies might affect the number of ERVs detected [37]. Moreover, our mining approach is based on reverse transcriptase (RT) proteins, and fragmented ERVs without RT proteins might not be identified.

Next, we performed a large-scale phylogenetic analysis of cetacean ERVs, representative vertebrate ERVs, and representative exogenous retroviruses to identify distinct ERV lineages in cetaceans. Based on the phylogenetic analyses and host species distribution of ERVs, the cetacean ERVs identified were classified into 315 distinct lineages (S1 and S2 Figs), including an ERV closely related to deltaretroviruses within the genome of *Platanista minor* as previously described [22]. To confirm the classification of cetacean ERV lineages and investigate the origin of cetacean retroviruses, we further screened for ERVs that are closely related to each ERV lineage within the vertebrate genomes. We found that each cetacean retroviral lineage identified in this study forms a monophyletic group and nests within the diversity of retroviruses from non-cetacean mammals, suggesting that each cetacean ERV lineage represents one or more independent invasion events (S2 Fig). Phylogenetic analysis shows that lineages 1 to 282 belong to Class I ERVs, among which lineages 1 to 123 and 124 to 282 are closely related to gammaretroviruses and epsilonretroviruses, respectively (S1 Fig). Lineages 283 to 304 belong to Class III ERVs, and lineages 305 to 315 belong to Class II ERVs (S1 Fig). Taken together, these results suggest a wide variety of retroviruses infected cetaceans and/or their ancestors.

### Scenarios of retrovirus evolution in cetaceans

**Fig 1:**
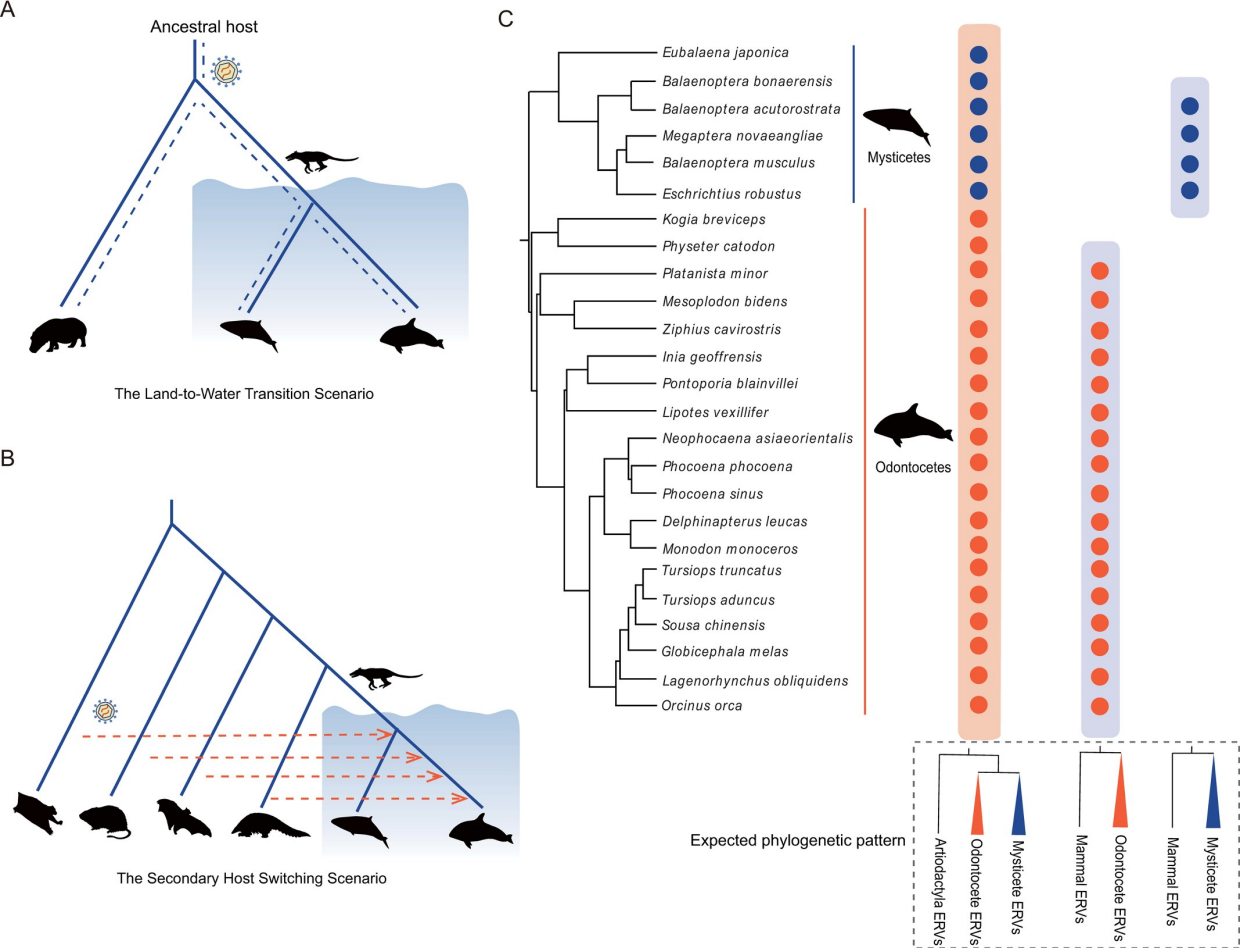
Scenarios of retrovirus evolution in cetaceans. (**A**) The land-to-water transition (LTW) scenario. A retrovirus infected and became integrated in the ancestor of cetaceans before or during the conquest of aquatic environment and transitioned into water with their ancient cetacean hosts. (**B**) The secondary host switching (SHS) scenario. Retroviruses infected and became endogenized in cetaceans through cross-species transmission from diverse sources after cetaceans became fully aquatic. (**C**) The distribution and expected phylogenetic pattern of cetacean ERVs under two scenarios. Under the LTW scenario, the ERV should be identified in the genomes of nearly all the cetaceans. Most of the cetacean ERVs are expected to be closely related to *H*. *amphibious* ERVs, while others are most closely related to artiodactyla (except the *H*. *amphibious*). Under the SHS scenario, the ERV should only be identified in a sub-lineage of cetaceans. The ERV is not expected to be closely related to ERVs from any certain vertebrate species. The phylogenetic relationships of cetaceans are based on TimeTree [64] and literatures [65, 66]. Illustrations of mysticetes, odontocetes, and *Pakicetus* courtesy by Chris Huh, Chris Huh, and Conty, respectively.

We hypothesize that cetacean retroviruses originated through two possible evolutionary scenarios, the land-to-water transition (LTW) scenario and the secondary host switching (SHS) scenario (Fig 1). In the LTW scenario (Fig 1A), a retrovirus infected the ancestor of cetaceans, integrated into its genome before or during (discussed below) the conquest of aquatic environment, and transited into water with their ancient cetacean hosts. Then, the ERV remnants (including solo-long terminal repeat [solo-LTR], if the ERV internal region was deleted due to recombination between two LTRs) should be identified in the genomes of nearly all the modern cetaceans. Most of these cetacean ERVs are expected to be closely related to ERVs from *Hippopotamus amphibious* (Fig 1C). It should be noted that some ERVs might be lost during the evolutionary course of hippopotamuses or cetaceans, and some retroviruses might infect the ancestor of modern cetaceans during cetaceans invaded the aquatic environments but after cetaceans and hippopotamuses diverged. In the SHS scenario (Fig 1B), retroviruses infected cetaceans through cross-species transmission after the conquest of aquatic environments by cetaceans, and became integrated into the host genome. Then, the ERV can be only identified in a sub-lineage of cetaceans and might proliferate to a high copy number (Fig 1C). The ERV is not expected to be closely related to ERVs from a certain vertebrate species. Moreover, the species whose ERVs are identified to be closely related to the cetacean ERVs might not represent the “actual” source of the cetacean retroviruses.

### Origins of retroviruses through land-to-water transition

**Fig 2:**
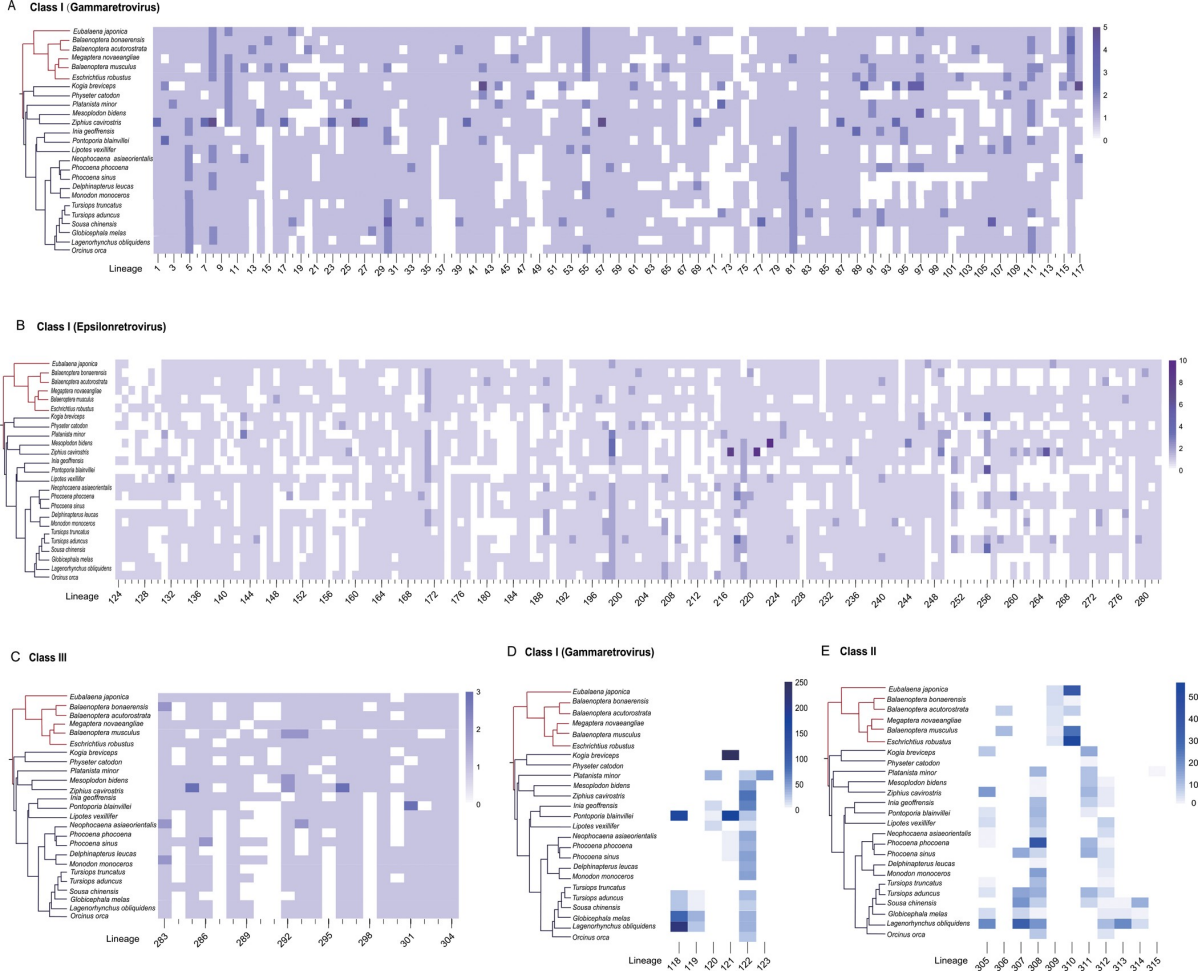
The copy numbers of distinct cetacean ERV lineages. (**A**), (**B**), and (**C**) show the copy numbers of ERVs in the LTW lineages that belong to Class I ERVs (gammaretrovirus), Class I ERVs (epsilonretrovirus), and Class III ERVs, respectively. (**D**) and (**E**) show the copy numbers of ERVs in the SHS lineages that belong to Class I ERVs (gammaretrovirus) and Class II ERVs, respectively. Phylogenetic relationship of cetaceans is shown on the left. Odontocetes and mysticetes are highlighted in blue and red, respectively.

**Fig 3:**
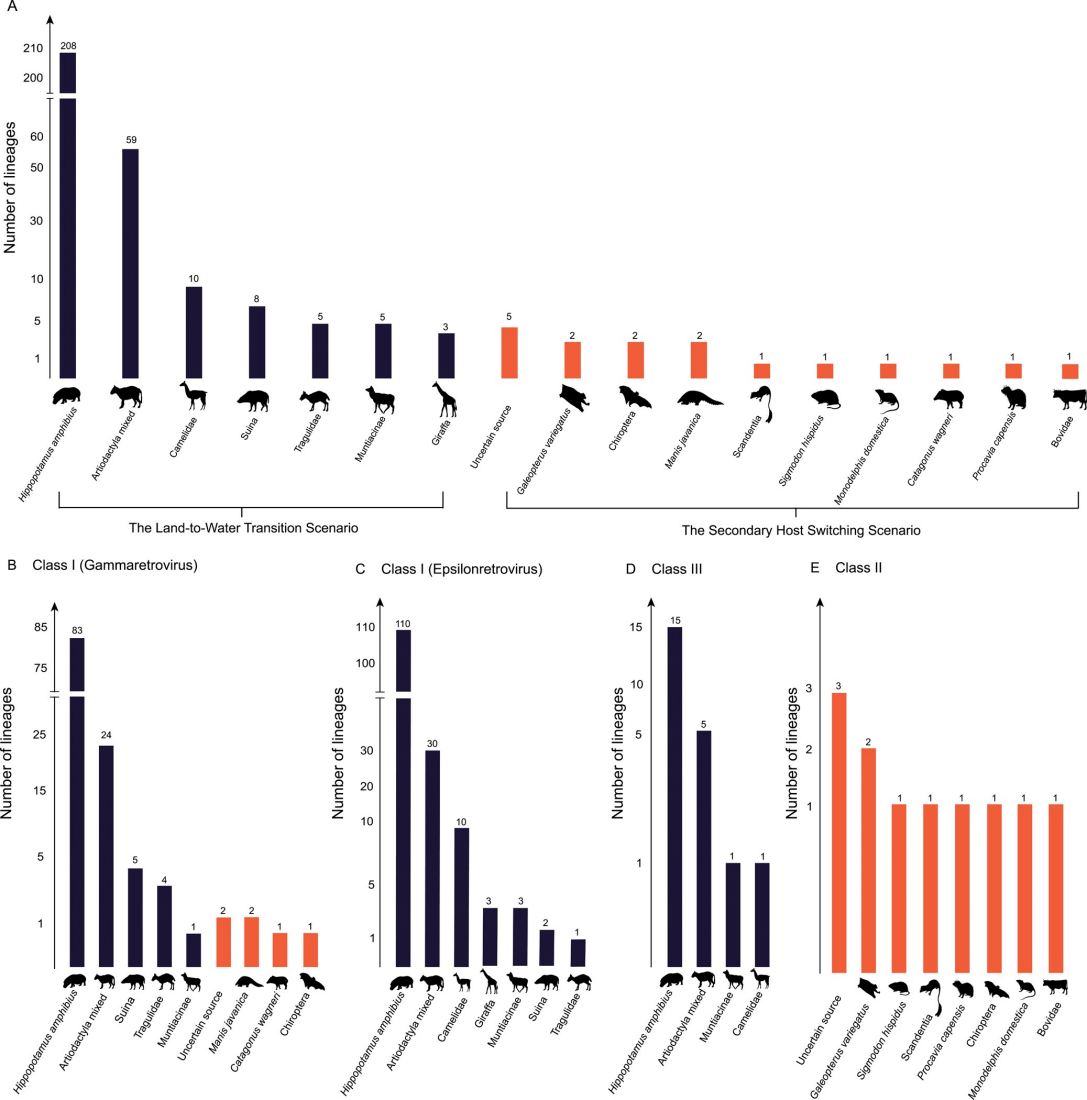
Potential sources of cetacean ERV lineages. (**A**) An overview of potential sources of all the 315 cetacean ERV lineages. Boxes in blue and orange indicate the numbers of ERVs under the land-to-water transition scenario and under the secondary host switching scenario, respectively. (**B to E**) Potential sources of cetacean ERV lineages that belong to Class I (gammaretrovirus), Class I (epsilonretrovirus), Class III, and Class II ERVs. Boxes in blue and orange indicate the numbers of ERVs under the LTW scenario and under the SHS scenario, respectively. Illustrations of Artiodactyla, Tragulidae, and *Monodelphis domestica* courtesy by Zimices, Zimices, and Sarah Werning, respectively.

Consistent with the LTW scenario, 298 (94.60%) out of 315 ERV lineages were found to be distributed in both mysticetes and odontocetes (Fig 2), implying that these ERV lineages were present in the last common ancestor of modern cetaceans. Class I ERVs account for a major proportion (276/298, 92.62%) of the LTW ERV lineages, among which lineages 1 to 117 and lineages 124 to 282 are closely related to gammaretroviruses and epsilonretroviruses, respectively (Fig 2). The remaining LTW ERV lineages (lineages 283 to 304) belong to Class III ERVs. The copy numbers of these LTW ERV lineages within a cetacean genome are generally very low (usually one copy in one genome) (Fig 2), suggesting that most of the LTW ERVs were not active after transiting to water along with their hosts. Some of these ERV lineages might be absent in certain species, due to internal region removal through recombination between the two LTRs of an ERV, degradation due to the absence of functional constraints, or occasionally sequencing error. To further elucidate the origin and evolutionary history of distinct ERV lineages in cetaceans, we performed phylogenetic analyses of cetacean ERVs and ERVs closely related within the vertebrate genomes for each ERV lineage. Interestingly, for 208 (69.80%) of these LTW ERV lineages, cetacean ERVs are closely related to ERVs from *H*. *amphibius*, indicating that these retrovirus endogenization events occurred before the last common ancestor of cetaceans and hippopotamuses. For the remaining 90 (30.20%) of these LTW ERV lineages, cetacean ERVs cluster with ERVs from diverse even-toed ungulates other than *H*. *amphibious* (Fig 3). This pattern can be explained by ERV removal in the lineage leading to *H*. *amphibious*, or cross-species transmission from even-toed ungulates other than *H*. *amphibious* to cetaceans after cetaceans and hippopotamuses diverged.

**Fig 4:**
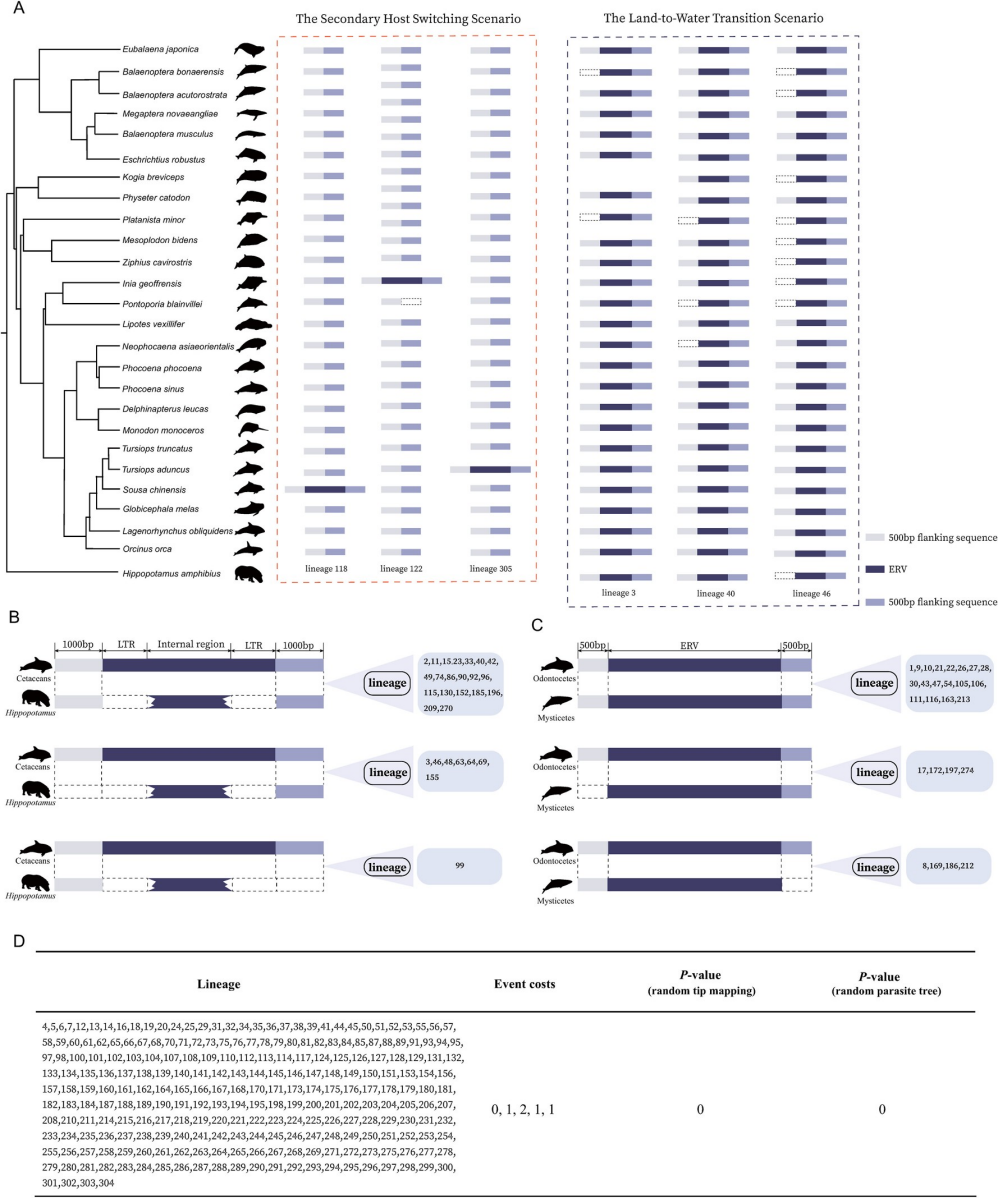
ERV orthologous insertions in cetaceans. (**A**) Examples of orthologous insertions for the SHS and the LTW ERV lineages. The phylogenetic relationship of cetaceans is shown on the left. The dotted boxes in red and blue show orthologous ERV insertions for the SHS and the LTW scenarios, respectively. The white and mauve rectangles represent 500 bp sequences flanking complete ERVs. The deep purple rectangles represent complete ERVs. Illustrations of cetacean species courtesy by Chris Huh. (**B**) ERV orthologous insertions between cetaceans and *H*. *amphibious*. Rectangles from left to right represent 1,000 bp flanking sequence, 5’-LTR, internal genes of an ERV, 3’-LTR, and 1,000 bp flanking sequence, respectively. Dashed boxes indicate missing of the corresponding regions. (**C**) ERV orthologous insertions between mysticetes and odontocetes. Rectangles from left to right represent 500 bp flanking sequence, ERV, and 500 bp flanking sequence, respectively. Dashed boxes indicate missing of the corresponding regions. (**D**) ERV and cetacean phylogeny congruence test. The event cost scheme (0, 1, 2, 1, 1) is for cospeciation, duplication, duplication with host switch, loss, and failure to diverge, respectively.

Moreover, we also performed synteny analyses for these LTW ERV lineages. For 28 LTW ERV lineages, we found orthologous ERV insertions between cetaceans and *H*. *amphibious* (Fig 4A and 4B and S6 Table). For 26 LTW lineages, we found orthologous ERV insertions between odontocetes and mysticetes (Fig 4C and S7 Table). For the remaining 244 LTW ERV lineages, no complete ERV was identified, which makes it difficult to distinguish the host-ERV boundary to establish orthologous relationships. For these 244 LTW ERV lineages without full length ERVs, we used an event-based method to quantitatively compare phylogenetic congruence between ERVs and their cetacean hosts. For all the 244 lineages, we found ERV phylogenies are statistically congruent with the cetacean phylogeny (*P* < 0.01) (Fig 4D and S8 Table). These results further confirmed that these 298 ERV linages arose through retrovirus endogenization events that occurred before the last common ancestor of modern cetaceans (before or during the evolutionary transition from land to water of cetaceans), and these ERVs transitioned to aquatic environments within their host genomes.

### Origins of retroviruses through secondary host switching

Consistent with the SHS scenario, we found 17 (5.40%) out of 315 cetacean ERV lineages are distributed in the genomes of species within a sub-lineage of cetaceans. Lineages 306, 309, 310 were only identified within the genomes of mysticetes, and lineages 118 to 123, 305, 307–308, 311 to 315 were only identified within the genomes of odontocetes (Fig 2). Lineages 118 to 123 belong to Class I ERVs and are closely related to gammaretroviruses, and lineages 305 to 315 belong to Class II ERVs (Figs 2 and S1) [22]. The copy numbers of these SHS ERV lineages are generally higher than those of the LTW ERV lineages; for example, lineage 121 ERVs reach 251 copies in the genome of *Kogia breviceps*. The SHS ERV lineages are closely related to ERVs from various mammals, including Chiroptera, *Galeopterus variegatus*, *Manis javanica*, *Catagonus wagneri*, *Sigmodon hispidus*, Scandentia, *Monodelphis domestica*, *Procavia capensis*, and Bovidae (Fig 3) [22]. However, five SHS ERV lineages are closely related to ERVs of mammals but their closest relatives could not be accurately identified. Once again, it should be noted that the species in which the ERVs closely related to a SHS ERV lineage were identified might not represent the “actual” source. Moreover, the possibility that one SHS ERV lineage arose through multiple endogenization of closely related retroviruses cannot formally excluded. Nevertheless, our results indicate that these 17 ERV lineages may derive from the endogenization of retroviruses which infected cetaceans through cross-species transmission from non-cetacean mammals after the land-to-water transition of cetaceans.

### Temporal dynamics of cetacean ERV amplification

**Fig 5:**
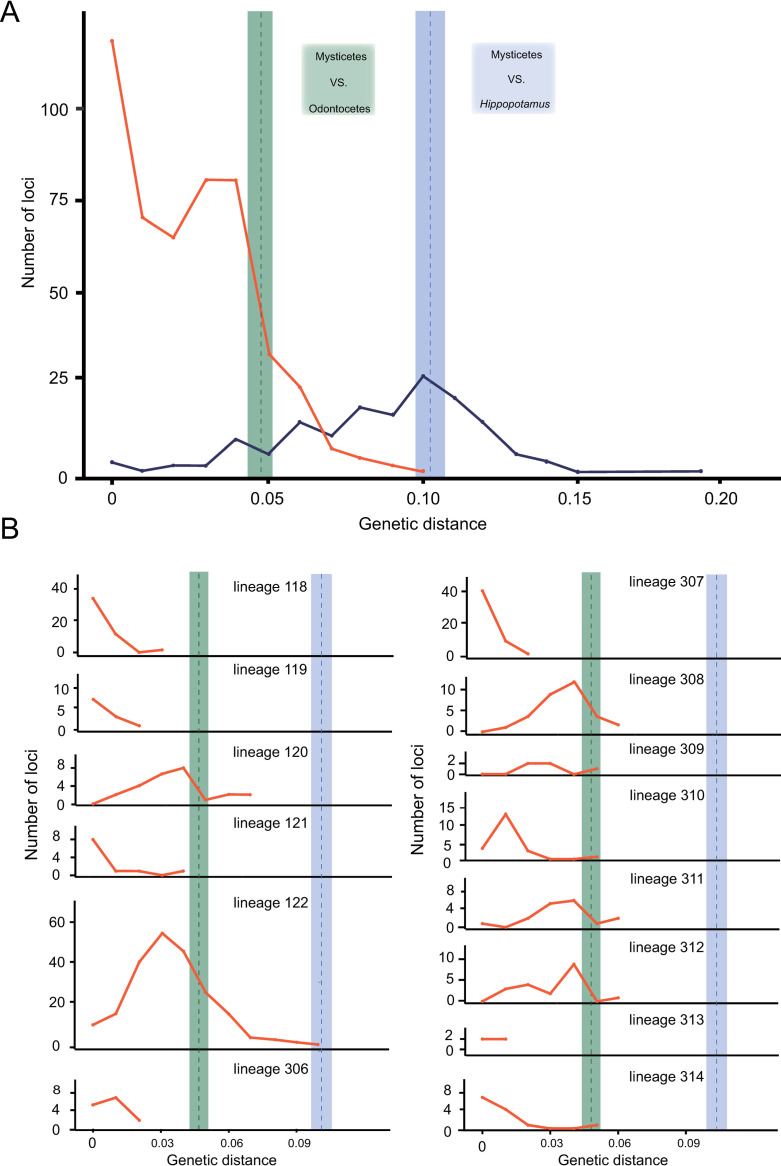
Evolutionary dynamics of cetacean ERVs. (**A**) Distribution of the genetic distance between 5’- and 3’ LTRs of complete ERVs in 315 cetacean ERV lineages. Blue and orange lines represent the distribution of the genetic distance between 5’- and 3’ LTRs of complete ERVs in the LTW and the SHS ERV lineages, respectively. The purple and green boxes represent 95% highest posterior distributions (HPD) of the genetic distance of introns between *B*. *acutorostrata* and *H*. *amphibious* (reflecting the divergence between cetaceans and hippopotamuses) and between *B*. *acutorostrata* and *O*. *orca* (reflecting the divergence between mysticetes and odontocetes) with dashed lines as the means. (**B**) Distribution of the genetic distance between 5’- and 3’- LTRs of complete ERVs in different cetacean ERV lineages under the SHS scenario. The purple and green boxes represent 95% HPD of the genetic distance of introns between *B*. *acutorostrata* and *H*. *amphibious* and between *B*. *acutorostrata* and *O*. *orca*, respectively, with dashed lines as the means.

The long terminal repeats (LTRs) on both sides of a provirus are identical at the beginning of virus integration, followed by divergence due to neutral evolution in the host genome. Therefore, the timing of a single ERV integration event can be estimated by measuring the genetic distance between LTR sequences. The genetic distance between 5’- and 3’-LTRs of an ERV increases with its integration time [37, 38]. To explore the temporal dynamics of the LTW ERV lineages, we retrieved all the complete LTW ERVs and calculated the genetic distance between their 5’- and 3’-LTRs (Fig 5A). The traditional estimation of ERV ages requires host neutral evolutionary rates to translate genetic distance into absolute time (in years). However, host neutral evolutionary rates based on known mammal rates might not be accurate for cetaceans. Instead, in this study, we directly compared the genetic distance of LTRs with that of cetacean neutrally evolving regions (introns used in this study) [39, 40]. We first estimated the genetic distance of orthologous introns between *Balaenoptera acutorostrata* and *H*. *amphibious* (reflecting the divergence between cetaceans and hippopotamuses) as well as the genetic distance of orthologous introns between *B*. *acutorostrata* and *Orcinus orca* (reflecting the divergence between mysticetes and odontocetes). The peak of genetic distance between 5’- and 3’-LTRs of the LTW ERVs overlaps the mean genetic distance of introns between cetacean and hippopotamus and is much greater than the mean genetic distance of introns between mysticetes and odontocetes (Fig 5A). These analyses have two caveats: (I) Gene conversion might occur between 5’- and 3’-LTRs, which decreases their genetic distance [41, 42]. Therefore, all the LTR sequences involving recombination or gene conversion were excluded in this study. (II) ERVs might not evolve at a similar rate as introns. Nevertheless, these results further support that most of the LTW ERVs invaded host genomes before the last common ancestor of the modern cetaceans.

We also investigated the temporal dynamics of the SHS ERV lineages by retrieving a total of 485 complete ERVs and calculating the genetic distance between 5’- and 3’-LTRs for each ERV (Fig 5A). Unlike the LTW ERVs, the genetic distance between 5’- and 3’-LTRs of these SHS ERVs peaks at 0, suggesting that a majority of the SHS ERVs proliferated in relative recent time. Then, we mapped 5’- and 3’-LTR distance distribution for 14 SHS ERV lineages (lineages 123, 305 and 315 were excluded due to their limited number of complete ERVs) (Fig 5B). We found that the genetic distance between 5’- and 3’-LTRs for all these 14 SHS ERV lineages peaked after the divergence of mysticetes and odontocetes, and seven SHS ERV lineages (lineages 118, 119, 121, 307 and 314) peak at 0. Ten SHS ERV lineages (lineages 118, 119, 121, 122, 306, 307, 310, 311, 313, and 314) contain ERVs with identical LTRs, suggesting that these ERV lineages might still actively proliferate. The genetic distances between 5’-and 3’-LTRs for all the SHS ERV lineages are less than the genetic distance of introns between cetaceans and hippopotamuses, and are less or around the genetic distance of introns between mysticetes and odontocetes, with lineage 122 as an exception (Fig 5B). For lineage 122, we found closely related ERV sequences in *Elephantulus edwardii* but not in the mysticetes (S2 Fig). Thus it is possible that the large 5’- and 3’-LTR distance might be due to local elevated evolutionary rates, but other possibilities cannot be formally excluded. Moreover, we identified orthologous ERV insertions in some but not all the cetaceans for several SHS ERV lineages, further supporting these ERVs were still active after the divergence between mysticetes and odontocetes (see three examples in Fig 4A). Taken together, all these lines of evidence suggest that the SHS ERV lineages might originate independently from recent cross-species transmissions and have been actively transposing in cetaceans in relatively recent time after the divergence between mysticetes and odontocetes.

### Modes of ERV proliferation in cetaceans

**Fig 6:**
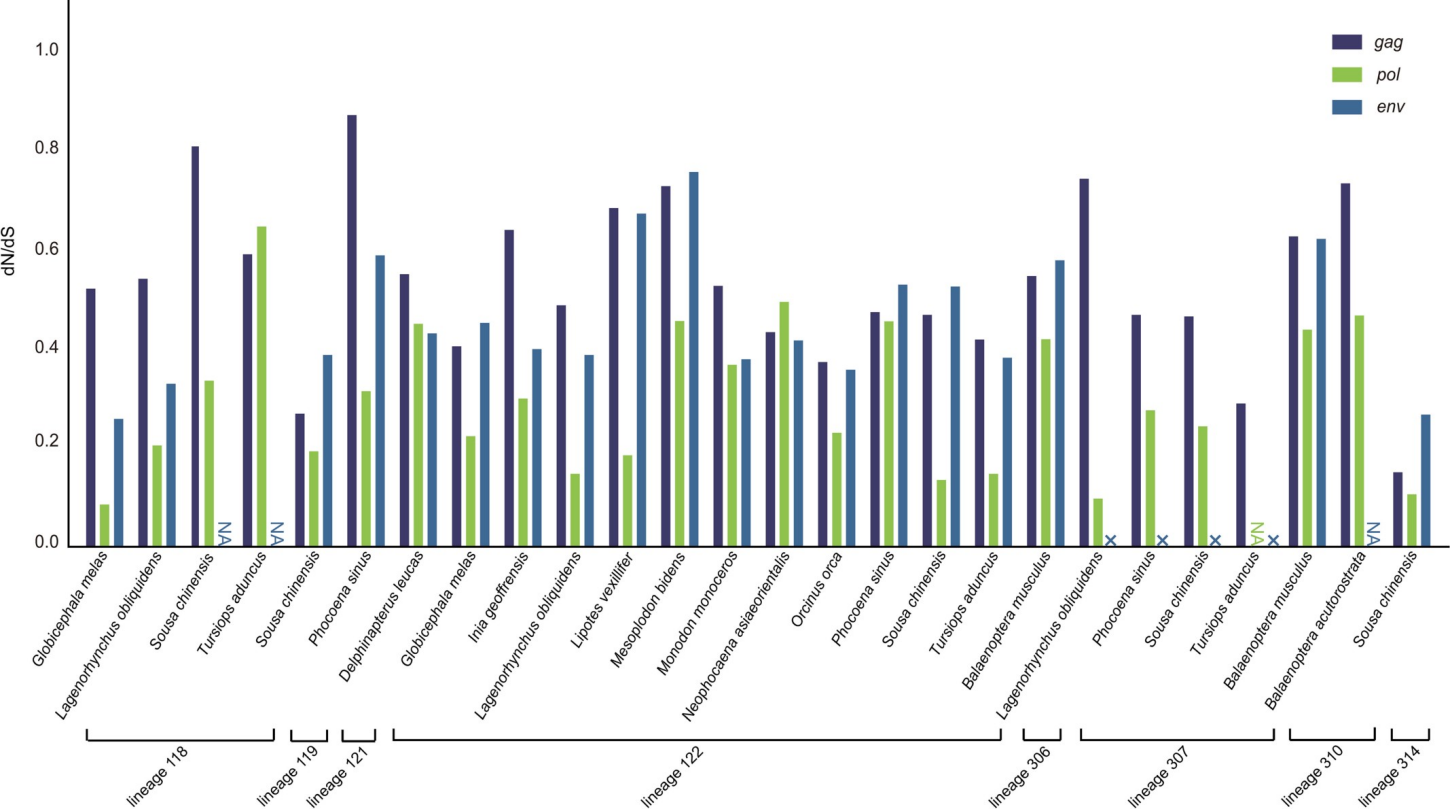
Selection pressure on the genes of cetacean ERVs. The dN/dS ratio values of three retroviral genes (*gag*, *pol*, and *env*) of the SHS ERV lineages in a certain species are shown. NA indicates not applicable, because information is not enough for the dN/dS calculation. × represents the loss of the corresponding gene.

For the LTW ERV lineages, the ERV copy numbers within a single cetacean genome are generally low, further supporting that most of these lineages have not been active. However, for the SHS ERV lineages, the ERV copy numbers within a single genome are generally high, sometimes reaching hundreds of copies. ERVs have been thought to proliferate in the host genomes through either reinfection or retrotransposition. Under different proliferation modes, the three core genes (*gag*, *pol*, and *env*) are subject to different selection pressure. To explore the proliferation modes for the SHS ERV lineages, we performed selection pressure analyses of retroviral genes for eight lineages with greater than four ERVs in a certain species by estimating dN/dS ratios for internal branches [43]. For seven lineages (lineages 118, 119, 121, 122, 306, 310, and 314), we found that all the three retroviral genes are subject to purifying selection (dN/dS <1), indicating that these SHS ERVs might proliferate mainly through reinfection. Interestingly, the ERVs of lineage 307 lose *env* gene, and the *gag* and *pol* genes of lineage 307 ERVs underwent purifying selection (Fig 6). The proliferation of this lineage may be mainly through retrotransposition in *cis* or complementation by hitchhiking of the functional *env* gene of a co-infecting retrovirus [31, 36].

## Discussion

In this study, we investigated the evolutionary histories of ERVs within the cetaceans, a group of mammals that underwent a macroevolutionary transition from terrestrial to aquatic environments >50 million years ago. We identified a total of 315 distinct ERV lineages that belong to Class I, II, and III, suggesting that diverse retroviruses infected cetaceans and their ancestors. We found two major routes through which retroviruses evolved during the macroevolutionary transition from land to water by cetaceans, namely the land-to-water transition scenario and the secondary host switching scenario. A majority (about 95%) of ERV lineages as genomic loci (not exogenous retroviruses) appear to have undergone a shift from land to water with their cetacean hosts. The LTW scenario actually includes retroviruses that infected and became endogenized in the terrestrial ancestor of cetaceans (before the conquest of aquatic environment) and retroviruses that infected the semiaquatic or aquatic ancestor of cetaceans before the last common ancestor of mysticetes and odontocetes (during the conquest of aquatic environment), which cannot be clearly distinguished based on the current data. The ERV copy numbers for these ERV lineages are generally low within a single host genome, suggesting the activity of ERVs was not high upon transiting into water. These retrovirus lineages seem to await degradation after evolutionary journey to aquatic environment, namely evolutionary dead-ends.

Interestingly, we identified 17 ERVs lineages that are only present in a sub-lineage of cetaceans, either within mysticetes or within odontocetes. Synteny analyses and integration time analyses show that these ERVs derived from recent retroviral integrations. Phylogenetic analyses indicate these retroviral lineages might originate from cross-species transmissions after the colonization of aquatic environments of cetaceans. These ERVs might represent a proportion of retroviruses currently circulating in cetaceans, given not all the retroviruses in cetacean have been endogenized. Many of these cetacean ERVs are closely related to mammal species other than even-toed ungulates. Actually, cetaceans have more interaction with terrestrial and semi-aquatic mammals than intuitively thought; for example, killer wales have been seen feeding on terrestrial mammals and seals [44–46]. Indeed, our previous studies found retroviruses of aquatic and terrestrial origins are frequently interconnected with each other [27]. Therefore, the land-water interfaces might not present a strict barrier for retrovirus transmission [27]. However, no evidence that these ERVs originated from cross-species transmissions from fishes was found, although cetaceans have been feeding on fishes for long. The identical LTRs of many SHS ERVs suggest they integrated into the host genomes very recently, and the non-zero peaks of LTR distance distribution for some SHS ERV lineages indicate they might be still proliferating. The pathogenicity of these potentially active retroviruses remains to be explored. Understanding the diversity of retroviruses might have implications in the conservation biology of cetaceans.

The LTW ERV lineages belong to Class I and III ERVs, but the SHS ERV lineages belong to Class I and II ERVs, reflecting a changed retrovirus spectrum after diving into the aquatic environments. Given that the 17 SHS ERV lineages originated independently, our results suggest at least 17 cross-species transmission events from non-cetacean mammals to cetaceans occurred after cetaceans invaded aquatic environments but during the evolutionary course of the modern cetaceans. However, these ERVs only represent a proportion of retroviruses currently circulating in cetaceans, because not all the retroviruses in cetaceans have been endogenized. It is possible that host-switching from non-cetacean mammals to cetaceans might be more frequently than appreciated.

Dozens of viruses, both DNA viruses and RNA viruses, have been described in cetaceans. Due to the under-sampling of viruses in cetaceans and more generally wild mammals, it is difficult to explore how these DNA and RNA viruses originated in cetaceans, as well as how viruses evolved during the evolutionary transition from terrestrial to aquatic environments. In contrast, ERVs provide one of the best models to study these questions. Our study provides novel insights into the complex evolution of retroviruses, and possibly viruses in general, during the macroevolutionary transition of cetaceans.

## Materials and methods

### ERV mining

All the cetacean genomes were retrieved from NCBI genome resources (https://www.ncbi.nlm.nih.gov/genome/), including 6 Mysticeti species and 19 Odontoceti species (S1 Table). We used a similarity search and phylogenetic analysis combined approach to identify ERVs in the cetacean genomes [27]. Briefly, we first used the tBLASTn algorithm to search the cetacean genomes with representative RT proteins as queries and an *e* cut-off value of 10^−5^ and a length cut-off value of 150 amino acids. Due to homology shared between retroviruses and retrotransposons, we performed phylogenetic analysis of the significant hits and RT proteins of representative retroviruses and retrotransposons [27]. Sequences forming a monophyletic group with representative retroviruses are authentic ERVs. We also used the forementioned method to mine ERVs in representative vertebrates (S2 Table). We performed large-scale phylogenetic analyses of RT proteins from cetacean ERVs, representative vertebrate ERVs, and representative exogenous retroviruses (S4 Table). Protein sequences were aligned using MAFFT 7.450 [47]. Initial large-scale phylogenetic analyses were performed using an approximate maximum likelihood method implemented in FastTree 2.1.10 [48]. A monophyletic group of cetacean ERVs was treated as a distinct lineage for the subsequent analyses.

### Classification of cetacean ERVs

To explore the relationship between cetacean ERVs and retroviruses, one representative sequence was selected for each cetacean ERV lineage. The RT sequences of cetacean ERVs and representative endogenous and exogenous retroviruses were aligned using MAFFT 7.450 [47] (S3 and S4 Tables). Phylogenetic analysis was performed using a maximum likelihood method implemented in IQ-tree 2 [49] (S1 Fig). ModelFinder implemented in IQ-tree 2 was used to determine the best-fitting model [50]. The node supports were evaluated using the ultrafast bootstrap method with 1,000 replicates [51, 52].

### Identifying putative sources of cetacean ERVs

To confirm the distribution of each cetacean ERV lineage in cetaceans and to identify the putative source of each cetacean ERV lineage, we used the BLASTn algorithm to search against the cetacean genomes and all the currently available vertebrate genomes with representative cetacean ERV RT sequences as queries and an *e* cut-off value of 10^−5^. All the sequences were aligned using the L-INS-i strategy implemented in MAFFT 7.450 [47] and then manually refined. Phylogenetic analyses for each cetacean ERV lineage were performed using a maximum likelihood method implemented in IQ-tree 2 [49] (S2 Fig).

### Dating the invasion time of cetacean ERVs

To identify complete ERVs, we bidirectionally extended all the cetacean ERV RT sequences and used LTRfinder [53] and LTRharvest [54] to identify the LTRs. The complete ERVs were annotated using Conserved Domain search [55] and the BLASTp algorithm [56]. For each cetacean ERV lineage, the 5’- and 3’-LTRs was aligned using MUSCLE [57], and phylogenetic analyses were performed using IQ-tree 2 [49]. The ERVs whose 5’- and 3’-LTRs cluster together were retrieved for further analyses[58]. The 3SEQ algorithm implemented in RDP4 [59, 60] was used to detect recombination among LTR sequences. GeneConv [61] was used to detect gene conversion occurring in LTR sequences [58]. LTR sequences with signals of recombination or gene conversion were excluded from the dating analyses. The genetic distance between 5’-LTR and 3’-LTR was estimated with the Kimura two-parameter substitution model [62]. To get a cetacean evolutionary time scale, 116 random orthologous introns of *B*. *acutorostrata* and *H*. *amphibian* and 100 random orthologous introns of *Balaenoptera acutorostrata* and *Orcinus orca* were retrieved and aligned using MAFFT 7.450 [47].

### Identification of orthologous ERV insertions

To identify the orthologous insertions of a complete ERV between cetaceans and *H*. *amphibious* or between mysticetes and odontocetes, we bidirectionally extended 500–1,000 bp sequences flanking the ERV (S5–S7 Tables). We used the BLASTn algorithm to search against the genomes of cetaceans and *H*. *amphibious* with the flanking sequences and the ERV as the queries. If the two flanking sequences are connected to each other, there is no ERV insertion.

### Test of the congruence between ERV and cetacean phylogenies

To assess whether ERV phylogenies are congruent with the cetacean phylogeny, we used an event-based approach implemented in Jane 4 [63]. The cost scheme of cospeciation-duplication-duplication with host switching-loss-failure to diverge was set as 0-1-2-1-1 [27]. Sample size for random parasitic tree and random tip mapping analyses was set to 50 (S8 Table). The cetacean phylogeny used in this study was based on TimeTree [64] and literatures [65, 66].

### Selection pressure analyses of cetacean ERV genes

Within a single cetacean species, all the complete ERV sequences for each SHS ERV lineage were retrieved and aligned with the L-INS-i strategy using MAFFT 7.450 [47]. Datasets with less than four sequences or with sequences with a common disruptive mutation were excluded. The ORF Finder (https://www.ncbi.nlm.nih.gov/orffinder/) was used to predict ORFs. The Conserved domain (CD) Search [55] and the BLASTp algorithm [56] were used to determine the retroviral genes, namely *gag*, *pol*, and *env*. Premature stop codons were removed. The dN/dS ratio was estimated using the CodeML program in PAML 4.9 [67]. The "one-ratio" model is used to calculate the overall dN/dS ratio, and the "two-ratio" model is used to estimate the dN/dS ratios for internal and terminal branches. The likelihood ratio test was used to evaluate the significance of the difference between the "one-ratio" model and the "two-ratio" model.

## Supporting information

S1 Table The information of the cetacean genomes used in this study. (PDF)

S2 Table The abbreviation of the mammal names used in this study. (PDF)

S3 Table The information of the representative cetacean ERV RT sequences used in S1 Fig. (PDF)

S4 Table The information of the representative retrovirus RT protein sequences used for phylogenetic analyses. (PDF)

S5 Table The information of the orthologous insertion examples in Fig 4A. (PDF)

S6 Table The information of the LTW ERV orthologous insertions in cetaceans and *Hippopotamus*. (PDF)

S7 Table The information of the LTW ERV orthologous insertions in mysticetes and odontocetes. (PDF)

S8 Table The results of ERV-cetacean phylogeny congruence test for the LTW ERV lineages. (PDF)

S1 Fig Phylogenetic analyses of 315 cetacean ERV lineages. Reference retrovirus sequences are highlighted in red. The 315 cetacean ERV lineages are classified into Class I (in blue and green boxes), Class II (in red box), and Class III (in purple box) ERVs. The support values (ultrafast bootstrap approximation) are shown for selected nodes. The information of the representative cetacean ERVs is available in S3 Table. The information of the representative retroviruses is available in S4 Table.
(PDF)

S2 Fig Phylogenetic analyses of cetacean ERV lineages 1 to 314. The cetacean ERV sequences are highlighted in purple. The support values are shown for selected nodes. Abbreviations are listed in S1 and S2 Tables.
(PDF)

## References

[1] Huelsmann M, Hecker N, Springer MS, Gatesy J, Sharma V, Hiller M. Genes lost during the transition from land to water in cetaceans highlight genomic changes associated with aquatic adaptations. Sci Adv. 2019; 5(9). 10.1126/sciadv.aaw6671 . 31579821 PMC6760925

[2] McGowen MR, Gatesy J, Wildman DE. Molecular evolution tracks macroevolutionary transitions in Cetacea. Trends Ecol Evol. 2014; 29(6): 336–46. 10.1016/j.tree.2014.04.001 . 24794916

[3] Pyenson ND. The Ecological Rise of Whales Chronicled by the Fossil Record. Current Biology. 2017;27(11): R558–R64. 10.1016/j.cub.2017.05.001 . 28586693

[4] Thewissen JG, Cooper LN, Clementz MT, Bajpai S, Tiwari BN. Whales originated from aquatic artiodactyls in the Eocene epoch of India. Nature. 2007; 450(7173): 1190–4. 10.1038/nature06343 . 18097400

[5] Gatesy J, O’Leary MA. Deciphering whale origins with molecules and fossils. Trends Ecol Evol. 2001; 16(11): 562–570. 10.1016/S0169-5347(01)02236-4.

[6] Zurano JP, Magalhaes FM, Asato AE, Silva G, Bidau CJ, Mesquita DO, et al. Cetartiodactyla: Updating a time-calibrated molecular phylogeny. Molecular Phylogenetics and Evolution. 2019; 133: 256–62. 10.1016/j.ympev.2018.12.015 . 30562611

[7] Zhou X, Sun D, Guang X, et al. Molecular Footprints of Aquatic Adaptation Including Bone Mass Changes in Cetaceans. Genome Biol Evol. 2018; 10(3):967–975. 10.1093/gbe/evy062 . 29608729 PMC5952927

[8] Standorf K, Cortes-Hinojosa G, Venn-Watson S, Rivera R, Archer LL, Wellehan JFX. Phylogenetic Analysis of the Genome of an Enteritis-Associated Bottlenose Dolphin Mastadenovirus Supports a Clade Infecting the Cetartiodactyla. J Wildlife Dis. 2018; 54(1): 112–21. 10.7589/2017-03-052 . 29077545

[9] Rivera R, Nollens HH, Venn-Watson S, Gulland FMD, Wellehan JF Jr. Characterization of phylogenetically diverse astroviruses of marine mammals. J Gen Virol. 2010; 91: 166–73. 10.1099/vir.0.015222-0 . 19759240

[10] Landrau-Giovannetti N, Subramaniam K, Brown MA, Ng TFF, Rotstein DS, West K, et al. Genomic characterization of a novel circovirus from a stranded Longman’s beaked whale (Indopacetus pacificus). Virus Res. 2020; 277. 10.1016/j.virusres.2019.197826 . 31790774

[11] Wang L, Maddox C, Terio K, Lanka S, Fredrickson R, Novick B, et al. Detection and Characterization of New Coronavirus in Bottlenose Dolphin, United States, 2019. Emerg Infect Dis. 2020; 26(7): 1610–2. 10.3201/eid2607.200093 . 32568058 PMC7323548

[12] Nollens HH, Rivera R, Palacios G, Wellehan JFX, Saliki JT, Caseltine SL, et al. New recognition of Enterovirus infections in bottlenose dolphins (Tursiops truncatus). Vet Microbiol. 2009; 139(1–2): 170–5. 10.1016/j.vetmic.2009.05.010 . 19581059 PMC4310689

[13] Bento MC, Canha R, Eira C, Vingada J, Nicolau L, Ferreira M, et al. Herpesvirus infection in marine mammals: A retrospective molecular survey of stranded cetaceans in the Portuguese coastline. Infect Genet Evol. 2019; 67: 222–33. 10.1016/j.meegid.2018.11.013 . 30445114

[14] Runstadler JA, Puryear W. A Brief Introduction to Influenza A Virus in Marine Mammals. Methods Mol Biol. 2020; 2123: 429–50. 10.1007/978-1-0716-0346-8_33 . 32170708

[15] Di Guardo G, Mazzariol S. Cetacean morbillivirus: A Land-to-Sea Journey and Back? Virologica Sinica. 2019; 34(3): 240–2. 10.1007/s12250-019-00128-x . 31093883 PMC6599500

[16] Ohishi K, Maruyama T, Seki F, Takeda M. Marine Morbilliviruses: Diversity and Interaction with Signaling Lymphocyte Activation Molecules. Viruses. 2019; 11(7): 606. 10.3390/v11070606 . 31277275 PMC6669707

[17] Borveto F, Bravos IG, Willemsen A. Papillomaviruses infecting cetaceans exhibit signs of genome adaptation following a recombination event. Virus Evol. 2020; 6(1): veaa038. 10.1093/ve/veaa038 . 32665861 PMC7326301

[18] Rodrigues TCS, Subramaniam K, McCulloch SD, Goldstein JD, Schaefer AM, Fair PA, et al. Genomic characterization of a novel pegivirus species from free-ranging bottlenose dolphins (Tursiops truncatus) in the Indian River Lagoon, Florida. Virus Res. 2019; 263: 98–101. 10.1016/j.virusres.2019.01.002 . 30633958

[19] Jo WK, van Elk C, van de Bildt M, van Run P, Petry M, Jesse ST, et al. An evolutionary divergent pestivirus lacking the Npro gene systemically infects a whale species. Emerg Microbes Infect. 2019; 8(1): 1383–92. 10.1080/22221751.2019.1664940 . 31526243 PMC6758615

[20] Rodrigues TCS, Subramaniam K, Varsani A, McFadden G, Schaefer AM, Bossart GD, et al. Genome characterization of cetaceanpox virus from a managed Indo-Pacific bottlenose dolphin (Tursiops aduncus). Virus Res. 2020; 278. 10.1016/j.virusres.2020.197861 . 31923559

[21] Emelianchik A, Rodrigues TCS, Subramaniam K, Nielsen O, Burek-Huntington KA, Rotstein D, et al. Characterization of a novel rhabdovirus isolated from a stranded harbour porpoise (Phocoena phocoena). Virus Res. 2019; 273. 10.1016/j.virusres.2019.197742 . 31499088

[22] Hron T, Elleder D, Gifford RJ. Deltaretroviruses have circulated since at least the Paleogene and infected a broad range of mammalian species. Retrovirology. 2019; 16(1). 10.1186/s12977-019-0495-9 . 31775783 PMC6882180

[23] LaMere SA, St Leger JA, Schrenzel MD, Anthony SJ, Rideout BA, Salomon DR. Molecular Characterization of a Novel Gammaretrovirus in Killer Whales (Orcinus orca). Journal of Virology. 2009; 83(24): 12956–67. 10.1128/JVI.01354-09 . 19812152 PMC2786842

[24] Wang L, Yin Q, He G, Rossiter SJ, Holmes EC, Cui J. Ancient invasion of an extinct gammaretrovirus in cetaceans. Virology. 2013; 441(1): 66–9. 10.1016/j.virol.2013.03.006 . 23545142

[25] Wang J, Han GZ. A sister lineage of sampled retroviruses corroborates the complex evolution of retroviruses. Molecular Biology and Evolution. 2021. 10.1093/molbev/msaa272 . 33249491 PMC7947760

[26] Hayward A, Cornwallis CK, Jern P. Pan-vertebrate comparative genomics unmasks retrovirus macroevolution. Proc Natl Acad Sci USA. 2015; 112(2): 464–9. 10.1073/pnas.1414980112 . 25535393 PMC4299219

[27] Xu X, Zhao H, Gong Z, Han GZ. Endogenous retroviruses of non-avian/mammalian vertebrates illuminate diversity and deep history of retroviruses. Plos Pathogens. 2018; 14(6). 10.1371/journal.ppat.1007072 . 29902269 PMC6001957

[28] International Committee on Taxonomy of Viruses. Virus Taxonomy: 2013 Release.

[29] Weiss RA. The discovery of endogenous retroviruses. Retrovirology. 2006. 10.1186/1742-4690-3-67 . 17018135 PMC1617120

[30] Jern P, Sperber GO, Blomberg J. Use of Endogenous Retroviral Sequences (ERVs) and structural markers for retroviral phylogenetic inference and taxonomy. Retrovirology. 2005. 10.1186/1742-4690-2-50 . 16092962 PMC1224870

[31] Belshaw R, Pereira V, Katzourakis A, Talbot G, Paces J, Burt A, et al. Long-term reinfection of the human genome by endogenous retroviruses. Proc Natl Acad Sci USA. 2004; 101(14): 4894–9. 10.1073/pnas.0307800101 . 15044706 PMC387345

[32] Belshaw R, Katzourakis A, Paces J, Burt A, Tristem M. High copy number in human endogenous retrovirus families is associated with copying mechanisms in addition to reinfection. Mol Biol Evol. 2005; 22(4): 814–7. 10.1093/molbev/msi088 . 15659556

[33] Bannert N, Kurth R. The evolutionary dynamics of human endogenous retroviral families. Annu Rev Genom Hum G. 2006; 7: 149–73. 10.1146/annurev.genom.7.080505.115700 . 16722807

[34] Katzourakis A, Rambaut A, Pybus OG. The evolutionary dynamics of endogenous retroviruses. Trends Microbiol. 2005; 13(10): 463–8. 10.1016/j.tim.2005.08.004 . 16109487

[35] Yang N, Kazazian HH Jr. L1 retrotransposition is suppressed by endogenously encoded small interfering RNAs in human cultured cells. Nat Struct Mol Biol. 2006; 13(9): 763–71. 10.1038/nsmb1141 . 16936727

[36] Magiorkinis G, Gifford RJ, Katzourakis A, De Ranter J, Belshaw R. Env-less endogenous retroviruses are genomic superspreaders. Proc Natl Acad Sci USA. 2012; 109(19): 7385–90. 10.1073/pnas.1200913109 . 22529376 PMC3358877

[37] Liu Y, ul Qamar MT, Feng JW, Ding YD, Wang S, Wu GZ, et al. Comparative analysis of miniature inverted-repeat transposable elements (MITEs) and long terminal repeat (LTR) retrotransposons in six Citrus species. BMC Plant Biol. 2019; 19 (1):140. 10.1186/s12870-019-1757-3 . 30987586 PMC6466647

[38] Hou F, Ma B, Xin Y, Kuang L, He N, Bureau TE. Horizontal transfers of LTR retrotransposons in seven species of Rosales. Genome. 2018; 61(8):587–94. 10.1139/gen-2017-0208 . 29958091

[39] Roy SW. How Common Is Parallel Intron Gain? Rapid Evolution Versus Independent Creation in Recently Created Introns in Daphnia. Mol Biol Evol. 2016; 33(8):1902–6. 10.1093/molbev/msw091 . 27189562

[40] Poverennaya IV, Roytberg MA. Spliceosomal Introns: Features, Functions, and Evolution. Biochemistry (Mosc). 2020; 85(7):725–734. 10.1134/S0006297920070019 . 33040717

[41] Fawcett JA, Innan H. The Role of Gene Conversion between Transposable Elements in Rewiring Regulatory Networks. Genome Biol Evol. 2019; 11(7):1723–1729. 10.1093/gbe/evz124 . 31209488 PMC6598467

[42] Hughes JF, Coffin JM. Evidence for genomic rearrangements mediated by human endogenous retroviruses during primate evolution. Nat Genet. 2001; 29(4):487–9. 10.1038/ng775 . 11704760

[43] Angelis K, dos Reis M, Yang Z. Bayesian estimation of nonsynonymous/synonymous rate ratios for pairwise sequence comparisons. Molecular Biology and Evolution. 2014; 31(7): 1902–13. 10.1093/molbev/msu142 . 24748652 PMC4069626

[44] Jefferson T.A., Stacey P.J. and Baird R.W., A review of Killer Whale interactions with other marine mammals: predation to co-existence. Mammal Review, 1991; 21: 151–180. 10.1111/j.1365-2907.1991.tb00291.x.

[45] Jourdain E, Vongraven D, Bisther A, Karoliussen R. First longitudinal study of seal-feeding killer whales (Orcinus orca) in Norwegian coastal waters. PLoS One. 2017; 12(6): e0180099. 10.1371/journal.pone.0180099 . 28666015 PMC5493372

[46] Samarra FIP, Bassoi M, Béesau J, Elíasdóttir MÓ, Gunnarsson K, Mrusczok MT, Rasmussen M, Rempel JN, Thorvaldsson B, Víkingsson GA. Prey of killer whales (Orcinus orca) in Iceland. PLoS One. 2018; 13(12): e0207287. 10.1371/journal.pone.0207287 . 30540762 PMC6291266

[47] Katoh K, Standley DM. MAFFT Multiple Sequence Alignment Software Version 7: Improvements in Performance and Usability. Molecular Biology and Evolution. 2013; 30(4): 772–80. 10.1093/molbev/mst010 23329690 PMC3603318

[48] Price MN, Dehal PS, Arkin AP. FastTree 2-Approximately Maximum-Likelihood Trees for Large Alignments. Plos One. 2010; 5(3): e9490. 10.1371/journal.pone.0009490 . 20224823 PMC2835736

[49] Minh BQ, Schmidt HA, Chernomor O, Schrempf D, Woodhams MD, von Haeseler A, et al. IQ-TREE 2: New Models and Efficient Methods for Phylogenetic Inference in the Genomic Era. Molecular Biology and Evolution. 2020; 37(5): 1530–4. 10.1093/molbev/msaa015 . 32011700 PMC7182206

[50] Kalyaanamoorthy S, Minh BQ, Wong TKF, von Haeseler A, Jermiin LS. ModelFinder: fast model selection for accurate phylogenetic estimates. Nat Methods. 2017; 14(6): 587–589. 10.1038/nmeth.4285 . 28481363 PMC5453245

[51] Hoang DT, Chernomor O, von Haeseler A, Minh BQ, Vinh LS. UFBoot2: Improving the Ultrafast Bootstrap Approximation. Molecular Biology and Evolution. 2018; 35(2): 518–22. 10.1093/molbev/msx281 . 29077904 PMC5850222

[52] Minh BQ, Nguyen MAT, von Haeseler A. Ultrafast Approximation for Phylogenetic Bootstrap. Molecular Biology and Evolution. 2013; 30(5): 1188–95. 10.1093/molbev/mst024 . 23418397 PMC3670741

[53] Xu Z, Wang H. LTR_FINDER: an efficient tool for the prediction of full-length LTR retrotransposons. Nucleic Acids Res. 2007; 35: W265–8. 10.1093/nar/gkm286 . 17485477 PMC1933203

[54] Ellinghaus D, Kurtz S, Willhoeft U. LTRharvest, an efficient and flexible software for de novo detection of LTR retrotransposons. BMC Bioinformatics. 2008; 9: 18. 10.1186/1471-2105-9-18 . 18194517 PMC2253517

[55] Lu SN, Wang JY, Chitsaz F, Derbyshire MK, Geer RC, Gonzales NR, et al. CDD/SPARCLE: the conserved domain database in 2020. Nucleic Acids Res. 2020; 48(D1): D265–D268. 10.1093/nar/gkz991 . 31777944 PMC6943070

[56] Altschul SF, Wootton JC, Gertz EM, Agarwala R, Morgulis A, Schaffer AA, et al. Protein database searches using compositionally adjusted substitution matrices. Febs J. 2005; 272(20): 5101–9. 10.1111/j.1742-4658.2005.04945.x . 16218944 PMC1343503

[57] Edgar RC. MUSCLE: multiple sequence alignment with high accuracy and high throughput. Nucleic Acids Res. 2004; 32(5): 1792–7. 10.1093/nar/gkh340 15034147 PMC390337

[58] Hughes JF, Coffin JM. Evidence for genomic rearrangements mediated by humanendogenous retroviruses during primate evolution. Nat Genet. 2001; 29(4): 487–9. 10.1038/ng775 . 11704760

[59] Martin D, Rybicki E. RDP: detection of recombination amongst aligned sequences. Bioinformatics. 2000; 16(6): 562–3. 10.1093/bioinformatics/16.6.562 . 10980155

[60] Lam HM, Ratmann O, Boni MF. Improved Algorithmic Complexity for the 3SEQ Recombination Detection Algorithm. Mol Biol Evol. 2018; 35(1): 247–251. 10.1093/molbev/msx263 29029186 PMC5850291

[61] Padidam M, Sawyer S, Fauquet CM. Possible emergence of new geminiviruses by frequent recombination. Virology. 1999; 265(2): 218–25. 10.1006/viro.1999.0056 . 10600594

[62] Kimura M. A simple method for estimating evolutionary rates of base substitutions through comparative studies of nucleotide sequences. J Mol Evol. 1980; 16: 111–20. 10.1007/BF01731581 . 7463489

[63] Conow C, Fielder D, Ovadia Y, Libeskind-Hadas R. Jane: a new tool for the cophylogeny reconstruction problem. Algorithms Mol Biol. 2010; 5: 16. 10.1186/1748-7188-5-16 20181081 PMC2830923

[64] Kumar S, Stecher G, Suleski M, Hedges SB. TimeTree: A Resource for Timelines, Timetrees, and Divergence Times. Molecular Biology and Evolution. 2017; 34(7): 1812–9. 10.1093/molbev/msx116 . 28387841

[65] McGowen MR, Tsagkogeorga G, Alvarez-Carretero S, dos Reis M, Struebig M, Deaville R, et al. Phylogenomic Resolution of the Cetacean Tree of Life Using Target Sequence Capture. Systematic Biology. 2020; 69(3): 479–501. 10.1093/sysbio/syz068 . 31633766 PMC7164366

[66] Arnason U, Lammers F, Kumar V, Nilsson MA, Janke A. Whole-genome sequencing of the blue whale and other rorquals finds signatures for introgressive gene flow. Sci Adv. 2018; 4(4): eaap9873. 10.1126/sciadv.aap9873 . 29632892 PMC5884691

[67] Yang ZH. PAML 4: Phylogenetic analysis by maximum likelihood. Molecular Biology and Evolution. 2007; 24(8): 1586–91. 10.1093/molbev/msm088 . 17483113

