# Supplementary material for: Molecular fossils illuminate the evolution of retroviruses following a macroevolutionary transition from land to water
Source: PLoS Pathog. 2021 Jul 12;17(7):e1009730. doi: 10.1371/journal.ppat.1009730 (PMC8297934; doi:10.1371/journal.ppat.1009730)
Supplement: S1 Table — (PDF) [file ppat.1009730.s001.pdf]

S1 Table. The information of the cetacean genomes used in this study

Organism Name	Order	Assembly	Level	Size (Mb)	Scaffolds	Abbreviation
Balaenoptera acutorostrata	Cetartiodactyla	GCF_000493695.1	Scaffold	2431.69	10776	Balacu
Balaenoptera bonaerensis	Cetartiodactyla	GCA_000978805.1	Scaffold	2234.64	421444	Balbon
Balaenoptera musculus	Cetartiodactyla	GCA_008658375.2	Scaffold	1,835.39	10,996	Balmus
Delphinapterus leucas	Cetartiodactyla	GCF_002288925.1	Scaffold	2358.52	6972	Delleu
Eschrichtius robustus	Cetartiodactyla	GCA_002189225.1	Scaffold	2849.45	57203	Escrob
Eubalaena japonica	Cetartiodactyla	GCA_004363455.1	Scaffold	2693.58	1353963	Eubjap
Inia geoffrensis	Cetartiodactyla	GCA_004363515.1	Scaffold	2596.28	1213610	Inigeo
Kogia breviceps	Cetartiodactyla	GCA_004363705.1	Scaffold	2759.7	1252072	Kogbre
Lagenorhynchus obliquidens	Cetartiodactyla	GCF_003676395.1	Scaffold	2334.47	5422	Lagobl
Lipotes vexillifer	Cetartiodactyla	GCF_000442215.1	Scaffold	2429.21	30713	Lipvex
Megaptera novaeangliae	Cetartiodactyla	GCA_004329385.1	Scaffold	2265.79	2558	Megnov
Mesoplodon bidens	Cetartiodactyla	GCA_004027085.1	Scaffold	2797.69	1801720	Mesbid
Monodon monoceros	Cetartiodactyla	GCF_005190385.1	Scaffold	2355.57	6972	Monmon
Neophocaena siaeorientalis asiaeorientalis	Cetartiodactyla	GCF_003031525.1	Scaffold	2284.63	13699	Neoasi
Orcinus orca	Cetartiodactyla	GCF_000331955.2	Scaffold	2372.92	1668	Orcorc
Phocoena phocoena	Cetartiodactyla	GCA_003071005.1	Scaffold	2441.14	142029	Phopho
Physeter catodon	Cetartiodactyla	GCF_002837175.2	Chromosome	2512.15	14677	Phycat
Platanista minor	Cetartiodactyla	GCA_004363435.1	Scaffold	2671.07	1098790	Plamin
Pontoporia blainvillei	Cetartiodactyla	GCA_004363935.1	Scaffold	1685.1	1885058	Ponbla
Sousa chinensis	Cetartiodactyla	GCA_003521335.2	Scaffold	2339.09	20903	Souchi
Tursiops aduncus	Cetartiodactyla	GCA_003227395.1	Scaffold	2503.93	16249	Turadu
Tursiops truncatus	Cetartiodactyla	GCF_001922835.1	Scaffold	2132.52	2648	Turtru
Ziphius cavirostris	Cetartiodactyla	GCA_004364475.1	Scaffold	3150.42	3758276	Zipcav
Globicephala melas	Cetartiodactyla	GCA_006547405.1	Scaffold	2333.87	18102937	Glomel
Phocoena sinus	Cetartiodactyla	GCA_008692025.1	Chromosome	2371.52	64	Phosin
