# Supplementary material for: Molecular fossils illuminate the evolution of retroviruses following a macroevolutionary transition from land to water
Source: PLoS Pathog. 2021 Jul 12;17(7):e1009730. doi: 10.1371/journal.ppat.1009730 (PMC8297934; doi:10.1371/journal.ppat.1009730)
Supplement: S2 Table — (PDF) [file ppat.1009730.s002.pdf]

S2 Table. The abbreviation of the mammal names used in this study

Organism Name	Order	abbreviation
Canis lupus dingo	Carnivora	Canlup
Leptonychotes weddellii	Carnivora	Lepwed
Mustela putorius furo	Carnivora	Musput
Odobenus rosmarus divergens	Carnivora	Odoros
Ursus maritimus	Carnivora	Ursmar
Aepyceros melampus	Cetartiodactyla	Aepmel
Alcelaphus buselaphus	Cetartiodactyla	Alcbus
Ammotragus lervia	Cetartiodactyla	Ammler
Axis porcinus	Cetartiodactyla	Axipor
Beatragus hunteri	Cetartiodactyla	Beahun
Bison bison bison	Cetartiodactyla	Bisbis
Bos grunniens	Cetartiodactyla	Bosgru
Bos indicus	Cetartiodactyla	Bosind
Bos mutus	Cetartiodactyla	Bosmut
Bos taurus	Cetartiodactyla	Bostau
Bubalus bubalis	Cetartiodactyla	Bubbub
Camelus bactrianus	Cetartiodactyla	Cambac
Camelus dromedarius	Cetartiodactyla	Camdro
Camelus ferus	Cetartiodactyla	Camfer
Capra aegagrus	Cetartiodactyla	Capaeg
Capra hircus	Cetartiodactyla	Caphir
Capra ibex	Cetartiodactyla	Capibe
Capra sibirica	Cetartiodactyla	Capsib
Capreolus capreolus	Cetartiodactyla	Capcap
Catagonus wagneri	Cetartiodactyla	Catwag
Cephalophus harveyi	Cetartiodactyla	Cephar
Cervus elaphus hippelaphus	Cetartiodactyla	Cerela
Connochaetes taurinus	Cetartiodactyla	Contau
Damaliscus lunatus	Cetartiodactyla	Damlun
Elaphurus davidianus	Cetartiodactyla	Eladav
Eudorcas thomsonii	Cetartiodactyla	Eudtho
Giraffa camelopardalis	Cetartiodactyla	Gircam
Giraffa tippelskirchi	Cetartiodactyla	Girtip
Hemitragus hylocrius	Cetartiodactyla	Hemhyl
Hippopotamus amphibius	Cetartiodactyla	Hipamp
Hydropotes inermis	Cetartiodactyla	Hydine
Kobus ellipsiprymnus	Cetartiodactyla	Kobell
Litocranius walleri	Cetartiodactyla	Litwal
Madoqua kirkii	Cetartiodactyla	Madkir
Moschus berezovskii	Cetartiodactyla	Mosber
Moschus chrysogaster	Cetartiodactyla	Moschr
Moschus moschiferus	Cetartiodactyla	Mosmos
Muntiacus crinifrons	Cetartiodactyla	Muncri
Muntiacus muntjak	Cetartiodactyla	Munmun

Muntiacus reevesi	Cetartiodactyla	Munree
Nanger granti	Cetartiodactyla	Nangra
Neotragus moschatus	Cetartiodactyla	Neomos
Neotragus pygmaeus	Cetartiodactyla	Neopyg
Odocoileus hemionus hemionus	Cetartiodactyla	Odohem
Odocoileus virginianus texanus	Cetartiodactyla	Odovir
Okapia johnstoni	Cetartiodactyla	Okajoh
Oreotragus oreotragus	Cetartiodactyla	Oreore
Oryx gazella	Cetartiodactyla	Orygaz
Ourebia ourebi	Cetartiodactyla	Ourour
Ovis ammon	Cetartiodactyla	Oviamm
Ovis aries	Cetartiodactyla	Oviari
Ovis canadensis	Cetartiodactyla	Ovican
Pantholops hodgsonii	Cetartiodactyla	Panhod
Philantomba maxwellii	Cetartiodactyla	Phimax
Procapra przewalskii	Cetartiodactyla	Proprz
Przewalskium albirostris	Cetartiodactyla	Przalb
Pseudois nayaur	Cetartiodactyla	Psenay
Rangifer tarandus	Cetartiodactyla	Rantar
Raphicerus campestris	Cetartiodactyla	Rapcam
Redunca redunca	Cetartiodactyla	Redred
Saiga tatarica	Cetartiodactyla	Saitat
Sus scrofa	Cetartiodactyla	Susscr
Sylvicapra grimmia	Cetartiodactyla	Sylgri
Syncerus caffer	Cetartiodactyla	Syncaf
Tragelaphus buxtoni	Cetartiodactyla	Trabux
Tragelaphus eurycerus	Cetartiodactyla	Traeur
Tragelaphus imberbis	Cetartiodactyla	Traimb
Tragelaphus scriptus	Cetartiodactyla	Trascr
Tragelaphus spekii	Cetartiodactyla	Traspe
Tragelaphus strepsiceros	Cetartiodactyla	Trastr
Tragulus javanicus	Cetartiodactyla	Trajav
Tragulus kanchil	Cetartiodactyla	Trakan
Vicugna pacos	Cetartiodactyla	Vicpac
Antidorcas marsupialis	Cetartiodactyla	Antmar
Antilocapra americana	Cetartiodactyla	Antame
Miniopterus natalensis	Chiroptera	Minnat
Galeopterus variegatus	Dermoptera	Galvar
Phascolarctos cinereus	Diprotodontia	Phacin
Erinaceus europaeus	Eulipotyphla	Erieur
Heterohyrax brucei	Hyracoidea	Hetbru
Procavia capensis	Hyracoidea	Procap
Lepus americanus	Lagomorpha	Lepame
Ochotona princeps	Lagomorpha	Ochpri
Oryctolagus cuniculus	Lagomorpha	Orycun
Elephantulus edwardii	Macroscelidea	Eleedw
Equus caballus	Perissodactyla	Equcab

Manis javanica	Pholidota	Manjav
Manis pentadactyla	Pholidota	Manpen
Myrmecophaga tridactyla	Pilosa	Myrtri
Homo sapiens	Primates	Homsap
Loxodonta africana	Proboscidea	Loxafr
Rattus norvegicus	Rodentia	Ratnor
Tupaia belangeri	Scandentia	Tupbel
Tupaia chinensis	Scandentia	Tupchi
Tupaia tana	Scandentia	Tuptan
Trichechus manatus latirostris	Sirenia	Triman
Orycteropus afer afer	Tubulidentata	Oryafe
