# Supplementary material for: Molecular fossils illuminate the evolution of retroviruses following a macroevolutionary transition from land to water
Source: PLoS Pathog. 2021 Jul 12;17(7):e1009730. doi: 10.1371/journal.ppat.1009730 (PMC8297934; doi:10.1371/journal.ppat.1009730)
Supplement: S3 Table — (PDF) [file ppat.1009730.s003.pdf]

S3 Table. The information of the representative cetacean ERV RT sequences used in S1 Fig

Species Name	Abbreviation	Accession No.	Location	Lineage
Inia geoffrensis	Inigeo	RJWO010024734.1	10221-10811	259
		RJWO010007610.1	40435-40953	258
		RJWO010003727.1	5962-6441	204
		RJWO010072929.1	3587-4111	146
		RJWO010026905.1	8915-9613	120
		RJWO010011130.1	39782-40231	110
		RJWO010036056.1	6446-6997	106
		RJWO010001162.1	49248-49901	90
		RJWO010014446.1	8744-9301	91
		RJWO010044333.1	4924-5496	44
		RJWO010001397.1	62382-63065	29
		RJWO010007272.1	49769-50245	3
Platanista minor	Plamin	RJWK010033051.1	159-851	123
		RJWK010064335.1	3087-3641	292
		RJWK010077719.1	6186-6650	263
		RJWK010000758.1	31649-32152	250
		RJWK010052619.1	13319-13999	236
		RJWK010088250.1	6215-6817	217
		RJWK010064231.1	1911-1297	218
		RJWK010047772.1	7660-8331	315
		RJWK010034037.1	7005-7694	18
		RJWK010041956.1	7655-8128	75
		RJWK010085578.1	4377-5078	122
		RJWK010041956.1	7655-8128	73
		RJWK010006755.1	35300-35830	174
Eubalaena japonica	Eubjap	RJWP010032195.1	19304-19774	4
		RJWP010147549.1	6-671	310
		RJWP010005863.1	25938-26348	278
		RJWP010082889.1	2176-2862	309
		RJWP010002746.1	53102-53713	1
		RJWP010001663.1	105866-106333	298
		RJWP010025932.1	4165-4617	267
		RJWP010009417.1	39298-39924	269
		RJWP010005135.1	54658-55314	233
		RJWP010002763.1	22962-23648	223
		RJWP010052173.1	7349-7996	212
		RJWP010000849.1	124515-125186	200
		RJWP010016884.1	31262-31774	195
		RJWP010001564.1	56538-57173	177
		RJWP010007489.1	40469-41140	142
		RJWP010003749.1	81503-81970	112
		RJWP010006526.1	65977-66501	97
		RJWP010003444.1	31634-32116	45
		RJWP010018736.1	14006-14473	41
		RJWP010027413.1	1444-2127	16

		RJWP010029314.1	9788-10255	14
Balaenoptera bonaerensis	Balbon	DF481028.1	6039-6716	2
		DF422475.1	17814-18506	282
		DF455354.1	19756-20355	238
		DF693794.1	549-1226	198
		DF490461.1	14241-14774	160
		DF419293.1	70577-71182	57
Kogia breviceps	Kogbre	RJWL010001341.1	19489-20145	6
		RJWL010000762.1	112779-113267	272
		RJWL010013686.1	14827-15333	203
		RJWL010003151.1	48421-49041	266
		RJWL010004193.1	67462-68088	186
		RJWL010016279.1	13760-13146	78
		RJWL010049126.1	10034-10708	72
		RJWL010011225.1	9491-9964	194
		RJWL010015998.1	34502-35059	66
		RJWL010041332.1	14257-14856	48
Lipotes vexillifer	Lipvex	NW_006782744.1	206091-206564	178
		NW_006770215.1	269982-270578	241
		NW_006792921.1	1162371-1161886	224
		NW_006784707.1	1091191-1091865	209
		NW_006787679.1	1334282-1334764	254
		NW_006791528.1	441515-441982	113
		NW_006793703.1	7736702-7737238	68
		NW_006785059.1	797145-797675	39
		NW_006799533.1	2888857-2889504	38
		NW_006796981.1	677271-677906	28
Orcinus orca	Orcorc	NW_004438440.1	8604852-8604301	8
		NW_004438536.1	3172885-3173463	175
		NW_004438493.1	262997-263701	64
		NW_004438546.1	4809258-4808638	183
		NW_004438493.1	4277662-4278321	162
		NW_004438470.1	2207701-2207027	157
		NW_004438435.1	13315569-13316027	149
		NW_004438531.1	5967028_5967714	88
		NW_004438498.1	10056694-10057302	74
		NW_004438472.1	1425008-1425466	168
		NW_004438435.1	12325653-12326339	69
		NW_004438570.1	87533-88195	54
		NW_004438505.1	673547-674239	33
		NW_004438450.1	11991291-11991770	25
		NW_004438437.1	20376859-20377320	22
Eschrichtius robustus	Escrob	NIPP01010710.1	37858-38451	9
		NIPP01002879.1	46433-47014	260
		NIPP01009433.1	14337-14795	228
		NIPP01005022.1	158876-159505	255
		NIPP01010143.1	42168-42815	190
		NIPP01012288.1	47832-48461	239

		NIPP01000242.1	149206-149751	232
		NIPP01011418.1	66280-66963	229
		NIPP01010401.1	77759-78256	154
		NIPP01000003.1	127540-128073	173
		NIPP01014594.1	5174-5629	115
		NIPP01000194.1	655477-655953	111
		NIPP01003157.1	112106-112810	109
		NIPP01002041.1	73276-73956	105
		NIPP01001728.1	109511-110026	101
		NIPP01000471.1	132741-133415	79
		NIPP01022642.1	14273-14749	62
		NIPP01003182.1	198636-199262	49
		NIPP01003265.1	201795-202403	34
		NIPP01002795.1	212307-213002	30
		NIPP01000350.1	141409-141951	21
Phocoena phocoena	Phopho	PKGA01141510.1	1793744-1794199	10
		PKGA01139369.1	20966104-20966745	303
		PKGA01132689.1	8520786-8521319	249
		PKGA01136281.1	827973-828539	214
		PKGA01132982.1	19457250-19457945	207
		PKGA01133207.1	15959218-15959889	189
		PKGA01133086.1	6978275-6978736	179
		PKGA01133770.1	5845825-5846331	144
		PKGA01137354.1	11894894-11895547	147
		PKGA01137354.1	6231863-6232534	129
		PKGA01138879.1	47291966-47292346	131
		PKGA01137958.1	510874-511530	117
		PKGA01139552.1	11931307-11931795	61
		PKGA01000564.1	13431185-13431877	56
		PKGA01135373.1	3928138-3928641	50
		PKGA01142022.1	12371779-12372447	181
		PKGA01137354.1	8349483-8349662	256
		PKGA01000085.1	3027880-3028398	76
		PKGA01132541.1	1436054-1436506	95
		PKGA01003081.1	33917-34507	13
Mesoplodon bidens	Mesbid	PVJJ010003276.1	64033-64500	11
		PVJJ010012643.1	10938-11624	308
		PVJJ010001485.1	77273-77761	304
		PVJJ010013433.1	39932-40459	302
		PVJJ010044995.1	7661-8197	294
		PVJJ010006096.1	57517-58068	293
		PVJJ010007911.1	42707-43243	286
		PVJJ010001918.1	7091-7780	277
		PVJJ010030338.1	17071-17712	273
		PVJJ010016476.1	29941-30597	201
		PVJJ010018355.1	23328-23798	127
		PVJJ010061819.1	3588-4211	107
		PVJJ010037101.1	8487-8999	103

		PVJJ010005374.1	35942-36730	98
		PVJJ010004348.1	35357-35908	71
		PVJJ010006375.1	5825-6382	275
		PVJJ010008227.1	57788-58489	40
Ziphius cavirostris	Zipcav	RJWS010127954.1	2456-3028	15
		RJWS010029576.1	1855-2379	246
		RJWS010003832.1	13335-14012	240
		RJWS010281672.1	411-866	231
		RJWS010014054.1	10058-10702	220
		RJWS010040220.1	1512-1979	187
		RJWS010031782.1	3622-4209	251
		RJWS010108033.1	1069-1698	145
		RJWS010027261.1	13602-14255	99
Delphinapterus leucas	Delleu	NW_019160856.1	72493499-72494017	19
		NW_019160955.1	5412793-5412125	247
		NW_019160904.1	13973078-13973590	193
		NW_019160872.1	21076531-21076992	166
		NW_019160867.1	4916565-4917212	152
		NW_019160964.1	4706073-4706612	65
		NW_019160947.1	102737-103393	126
		NW_019160964.1	4706073-4706612	84
		NW_019160931.1	177961-178428	165
		NW_019160868.1	13259443-13259991	52
		NW_019160971.1	2125619-2126296	37
		NW_019160864.1	21601003-21601698	17
Globicephala melas	Glomel	ML658796.1	9395413-9396114	26
		ML658806.1	25761794-25762420	313
		ML658816.1	1830205-1830822	257
		ML658775.1	26652976-26653647	163
		SWEB01016123.1	1221-1940	118
Sousa chinensis	Souchi	RWJT01018868.1	10913402-10914052	31
		RWJT01016124.1	3553500-3554120	280
		RWJT01002282.1	17910999-17911664	314
		RWJT01015552.1	2585841-2586395	288
		RWJT01019919.1	13513980-13514498	284
		RWJT01020743.1	4496081-4496566	237
		RWJT01022727.1	2268993-2269556	210
		RWJT01002386.1	4625010-4625597	206
		RWJT01008232.1	127862-128440	199
		RWJT01015473.1	8701361-8701876	191
		RWJT01021215.1	10600354-10600944	170
		RWJT01015975.1	14176246-14176932	70
		RWJT01003978.1	634196-634867	125
		RWJT01015824.1	6815169-6815852	119
		RWJT01006909.1	12734201-12734800	114
		RWJT01023066.1	6193643-6194122	100
		RWJT01001056.1	2709053-2708604	93
		RWJT01019965.1	389618-390241	311

		RWJT01022217.1	175644-176279	169
Neophocaena asiaeorientalis asiaeorientalis	Neoasi	NW_020174258.1	134892-135578	32
		NW_020174135.1	6633368-6633961	274
		NW_020172733.1	916376-917071	116
		NW_020173687.1	9268727-9269260	87
		NW_020172725.1	314052-313426	67
		NW_020175827.1	765173-765808	20
		NW_020172176.1	966214-966885	208
		NW_020173377.1	748039-748719	43
Physeter catodon	Phycat	NC_041229.1	76657711-76658259	35
		NC_041232.1	44660102-44660578	130
		NC_041220.1	27275252-27275899	139
		NC_041232.1	64360246-64360812	242
		NC_041225.1	68636023-68636697	226
		NC_041234.1	43580153-43580893	230
		NC_041231.1	5975465-5976076	222
		NC_041215.1	23334861-23335424	213
		NC_041219.1	20717188-20717706	150
		NC_041225.1	2682483-2683031	148
		NC_041217.1	76623116-76623808	94
		NC_041232.1	34542729-34543313	86
		NC_041224.1	144567431-144568102	81
Monodon monoceros	Monmon	NW_021703772.1	89294436-89294897	46
		NW_021701183.1	205250-205792	262
		NW_021697799.1	149269-149907	221
		NW_021703779.1	68010258-68010914	192
		NW_021703774.1	26485210-26485896	23
		NW_021703777.1	58016-58654	155
		NW_021703779.1	46048232-46048729	290
		NW_021703785.1	70208952-70209419	85
		NW_021703777.1	63709874-63710380	77
		NW_021703780.1	64886112-64886807	63
Tursiops aduncus	Turadu	NCQN01000416.1	714012-714560	47
		NCQN01000077.1	3191528-3191986	184
		NCQN01002131.1	126608-127261	312
		NCQN01000121.1	1407743-1408372	305
		NCQN01000021.1	1637763-1638299	297
		NCQN01016174.1	1350921-1351457	261
		NCQN01000393.1	2064016-2064534	243
		NCQN01016155.1	733569-734228	244
		NCQN01000266.1	1269723-1270250	245
		NCQN01000330.1	1473764-1474384	307
		NCQN01000340.1	721698-722357	128
		NCQN01000303.1	1531567-1532019	188
		NCQN01000338.1	589904-590563	161
		NCQN01001079.1	629504-630004	172
		NCQN01001576.1	135236-135814	171
		NCQN01001957.1	147716-148201	164

		NCQN01001063.1	419868-420491	140
		NCQN01001056.1	488610-489152	141
		NCQN01000705.1	651588-652040	136
		NCQN01001175.1	262933-263493	92
		NCQN01002814.1	85184-85855	89
		NCQN01000028.1	3539204-3539842	59
		NCQN01002938.1	317248-317742	176
		NCQN01016191.1	301951-302580	133
		NCQN01000581.1	417246-417941	55
Tursiops truncatus	Turtru	NW_017844396.1	1596377-1596925	132
		NW_017843875.1	18044622-18045296	215
		NW_017842782.1	2426924-2427526	234
		NW_017842989.1	7632642-7633169	235
		NW_017842814.1	18162937-18163551	270
		NW_017844429.1	14243533-14242877	281
		NW_017842131.1	13577361-13578044	83
		NW_017844399.1	2536-3102	102
		NW_017843227.1	5875732-5876271	104
		NW_017843160.1	13274831-13275403	134
Balaenoptera acutorostrata	Balacu	NW_006733789.1	2939185-2939745	96
		NW_006733900.1	3794145-3794816	153
		NW_006727476.1	132711-133391	180
		NW_006727242.1	688357-689025	202
		NW_006725743.1	4537957-4538634	211
		NW_006727242.1	3935906-3936529	27
		NW_006725632.1	4482404-4483069	306
		NW_006726220.1	5084575-5085168	289
		NW_006726898.1	1107419-1108000	296
		NW_006725699.1	1184311-1184964	58
Lagenorhynchus obliquidens	Lagobl	NW_020837946.1	88299661-88300320	196
		NW_020837993.1	13810104-13810562	227
		NW_020837960.1	9382563-9383186	248
		NW_020837953.1	33177632-33178159	265
		NW_020837948.1	36292432-36292986	158
		NW_020837954.1	4386136-4386585	300
		NW_020837953.1	21067551-21068090	108
		NW_020837981.1	18335928-18336494	135
Pontoporia blainvillei	Ponbla	RJWI010176290.1	1264-1929	279
		RJWI010013874.1	4827-5345	53
		RJWI010091791.1	569-1192	82
		RJWI010108960.1	2160-2819	185
		RJWI010050908.1	339-1013	225
		RJWI010061552.1	1108-1512	295
		RJWI010017925.1	3993-4514	7
Megaptera novaeangliae	Megnov	RYZJ01001732.1	4551352-4552023	216
		RYZJ01000241.1	11246372-11246992	219
		RYZJ01000501.1	1166111-1165521	252
		RYZJ01000437.1	1608585-1609169	253

		RYZJ01000770.1	5792061-5792573	264
		RYZJ01001616.1	178413-179051	276
		RYZJ01001441.1	3812431-3811868	268
		RYZJ01000348.1	328910-329551	271
		RYZJ01000139.1	2043956-2044597	283
		RYZJ01002407.1	442664-443317	285
		RYZJ01001001.1	1960588-1961136	205
		RYZJ01000131.1	926248-926844	287
		RYZJ01000922.1	2618744-2619229	299
		RYZJ01001772.1	3723137-3723775	301
		RYZJ01000684.1	1489740-1490429	5
		RYZJ01001573.1	15173037-15173603	12
		RYZJ01000786.1	167572-166883	24
		RYZJ01001153.1	8105586-8106269	36
		RYZJ01001010.1	1286791-1287351	42
		RYZJ01000773.1	3374415-3374990	51
		RYZJ01000410.1	1508966-1509418	60
		RYZJ01000575.1	9892435_9893079	80
		RYZJ01001793.1	21955121-21955738	124
		RYZJ01000921.1	325588-326256	137
		RYZJ01000128.1	18336870-18337409	138
		RYZJ01000503.1	4977438-4978073	143
		RYZJ01000280.1	8404986-8405639	151
		RYZJ01001342.1	23149456-23149944	156
		RYZJ01001453.1	14089888-14090547	159
		RYZJ01000503.1	4827488-4827961	167
		RYZJ01000148.1	6912996-6913667	182
		RYZJ01000011.1	217333-217995	197
Phocoena sinus	Phosin	VOSU01000006.1	21433409-21434128	121
Balaenoptera musculus	Balmus	VNFC01000008.1	95815471-95815641	291
