# Supplementary material for: Molecular fossils illuminate the evolution of retroviruses following a macroevolutionary transition from land to water
Source: PLoS Pathog. 2021 Jul 12;17(7):e1009730. doi: 10.1371/journal.ppat.1009730 (PMC8297934; doi:10.1371/journal.ppat.1009730)
Supplement: S4 Table — (PDF) [file ppat.1009730.s004.pdf]

S4 Table. The information of the representative retrovirus RT protein sequences used for phylogenetic analyses

Name	Abbreviation	Accession No./Source
Mus musculus intracisternal A-particle	M17551.1_ IAP	M17551.1
Mus musculus intracisternal A-particle	AB099818.1_IAP	AB099818.1
Snakehead retrovirus	SnRV	NC_001724.1
Chinese hamster ovary retrovirus	RCHO-K1	Ref.1
Koala retrovirus	KoRV	AF151794.2
Sus scrofa porcine endogenous retrovirus	PERV-MSL	AF038600
Python molurus endogenous retrovirus	PyERV	AF500296.1
Danio rerio endogenous retrovirus	ZFERV	AF503912.1
Mus dunni endogenous virus	MdEV	AF053745.1
Xenopus laevis endogenous retrovirus 1	XEN1	AJ506107.1
Human endogenous retrovirus E	HERV-E	Ref.2
Baboon endogenous virus	BAEVM	D10032.1
Retrovirus-like element-isoleucine	RTVL-Ia	Ref.3
Human immunodeficiency virus type 1	HIV-1	EF029066.1
Simian immunodeficiency retrovirus	SIVAGM	Ref.4
Simian immunodeficiency retrovirus of macaques	SIVMAC	Ref.4
Simian immunodeficiency virus of mandrill	SIVMND	Ref.4
Human immunodeficiency virus type 2	HIV-2	HM245790.1
Simian retrovirus 1	SRV-1	M11841.1
Squirrel monkey retrovirus H	SMRV-H	M23385.1
Rous sarcoma virus	RSV	NC_001407.1
Bovine immunodeficiency virus	BIV	NC_001413.1
Bovine leukemia virus	BLV	NC_001414.1
Human T-cell leukemia virus type I	HTLV-1	NC_001436.1
Equine infectious anemia virus	EIAV	NC_001450.1
Caprine arthritis encephalitis virus	CAEV	NC_001463.1
Feline immunodeficiency virus	FIV	NC_001482.1
Human T-lymphotropic virus 2	HTLV-2	NC_001488.1
Jaagsiekte sheep retrovirus	JSRV	NC_001494.1
Human foamy virus	HFV	Y07725
Mouse mammary tumor virus	MMTV	NC_001503.1
Simian foamy virus type 1	SFV-1	Ref.4
Danio rerio foamy virus type 1	DrFV-1	Ref.4
Zebrafish endogenous retrovirus	ZFERV-2	Ref.4
Bovine foamy virus	BFV	NC_001831.1
Walleye dermal sarcoma virus	WDSV	NC_001867.1
Gibbon ape leukemia virus	GaLV	NC_001885.3
Feline leukemia virus	FeLV	NC_001940.1
Equine foamy virus	EFV	NC_002201.1
Reticuloendotheliosis virus	REV	NC_006934
Ovine maedi visna virus	SA-OMVV	Ref.4
Maedi visna virus	VMV	NC_001452
Simian T-lymphotropic virus	STcLV2PP1664	Ref.4
Lymphoproliferative disease virus	LPDV	Ref.4
Simian endogenous retrovirus of mandrill	SERV	AF164894.1
Feline foamy virus	FFV	NC_001871.1
Mouse mammary tumor viruses	MMTV	Ref.4
Human endogenous retrovirus K10	HERV-K10	Ref.4

Murine endogenous retrovirus L	MuERV-L	Y12713.1
Human endogenous retrovirus K50D	K-HERV	DQ112152.1
Mason-Pfizer monkey virus	MPMV	NC_001550.1
Atlantic salmon swim bladder sarcoma virus	ASSBSV	Ref.4

References

1. Lie YS, Penuel EM, Low MA, Nguyen TP, Mangahas JO, Anderson KP, Petropoulos CJ. (1994). Chinese hamster ovary cells contain transcriptionally active full-length type C proviruses. *J Virol*. 68(12):7840-9.

2. Vargiu L, Rodriguez-Tomé P, Sperber GO, Cadeddu M, Grandi N, Blikstad V, Tramontano E, Blomberg J. (2016). Classification and characterization of human endogenous retroviruses; mosaic forms are common. *Retrovirology* 13:7.

3. Maeda N. (1985). Nucleotide equence of the haptoglobin and haptoglobin-related gene pair. The haptoglobin-related gene contains a retrovirus-like element. *J Biol Chem.* 260:6698-709.

4. Llorens C, Futami R, Covelli L, Dominguez-Escriba L, Viu JM, Tamarit D, Aguilar-Rodriguez J, Vicente-Ripolles M, Fuster G, Bernet GP, Maumus F, Munoz-Pomer A, Sempere JM, LaTorre A, Moya A. (2011). The Gypsy Database (GyDB) of mobile genetic elements: release 2.0. *Nucleic Acids Res.* 39: D70-D74.
