# Supplementary material for: Molecular fossils illuminate the evolution of retroviruses following a macroevolutionary transition from land to water
Source: PLoS Pathog. 2021 Jul 12;17(7):e1009730. doi: 10.1371/journal.ppat.1009730 (PMC8297934; doi:10.1371/journal.ppat.1009730)
Supplement: S5 Table — (PDF) [file ppat.1009730.s005.pdf]

S5 Table. The information of the orthologous insertion examples in Fig. 4A

Species Name	Accession No.	Location	Lineage
Eubalaena japonica	RWJP010001190.1	101373-106252	3
Monodon monoceros	NW_021703782.1	87454460-87456347	40
Neophocaena asiaeorientalis asiaeorientalis	NW_020173124.1	30137-37761	46
Sousa chinensis	RWJT01005720.1	8164481-8171103	118
Inia geoffrensis	RJWO010004527.1	69868-75135	122
Tursiops aduncus	NCQN01000121.1	1402865-1414137	305
