# Supplementary material for: Molecular fossils illuminate the evolution of retroviruses following a macroevolutionary transition from land to water
Source: PLoS Pathog. 2021 Jul 12;17(7):e1009730. doi: 10.1371/journal.ppat.1009730 (PMC8297934; doi:10.1371/journal.ppat.1009730)
Supplement: S6 Table — (PDF) [file ppat.1009730.s006.pdf]

S6 Table. The information of the LTW ERV orthologous insertions in cetaceans and *Hippopotamus*

Lineage	Cetaceans				*Hippopotamus*			% identity
	Species	Accession No.	5' flanking sequence	3' flanking sequence	Accession No.	5' flanking sequence	3' flanking sequence	
2	*Platanista minor*	RJWK010069829.1	6742-7242	8844-9344	PVJP02910652.1	12309849-12310249	12311453-12311934	84.44%
3	*Eubalaena japonica*	RJWP010001190.1	100873-101873	105752-106252	PVJP02910455.1		9971561-9972062	84.49%
11	*Orcinus orca*	NW_004438513.1	7271745-7272745	7280258-7280758	PVJP02021330.1	5566722-5567087	5569816-5570325	81.85%
15	*Eubalaena japonica*	RJWP010036371.1	6715-7215	12715-13715	PVJP02910616.1	291693-292216	297447-298209	86.71%
23	*Platanista minor*	RJWK010010417.1	16661-17661	26514-27014	PVJP02910680.1	1694426-1694849	1677634-1678129	91.02%
33	*Tursiops aduncus*	NCQN01000042.1	1299751-1300251	1308357-1308857	PVJP02910647.1	2370434-2370934	2378875-2379154	88.29%
40	*Monodon monoceros*	NW_021703782.1	87453960-87454460	87456347-87456847	PVJP02910177.1	1703047-1703557	1701095-1701576	89.43%
42	*Balaenoptera musculus*	VNFC01000022.1	51605959-51606459	51610214-51610714	PVJP02910622.1	29087318-29087820	29084531-29085029	86.68%
46	*Neophocaena asiaeorientalis*	NW_020173124.1	29637-30637	37261-37761	PVJP02910812.1		2151301-2151725	89.65%
48	*Sousa chinensis*	RWJT01022005.1	2049816-2050316	2052373-2052873	PVJP02018429.1		1491456-1491946	88.82%
49	*Balaenoptera bonaerensis*	DF450602.1	9519-10019	20493-20993	PVJP02910746.1	1437874-1438358	1452632-1453102	91.37%
63	*Phocoena phocoena*	PKGA01132106.1	1-501	2673-3173	PVJP02021229.1		1544012-1544298	87.29%
64	*Neophocaena asiaeorientalis*	NW_020173124.1	29637-30637	37261-37761	PVJP02910812.1		2151301-2151725	89.65%
69	*Pontoporia blainvillei*	RJWI010018877.1	4075-4575	6739-7739	PVJP02910652.1		5356719-5357178	85.77%
74	*Mesoplodon bidens*	PVJJ010007725.1	20323-20823	26934-27434	PVJP02910477.1	8382761-8383188	8389155-8389657	87.90%

86	*Lagenorhynchus obliquidens*	NW_020837976.1	251917-252917	259698-260198	PVJP02910233.1	8169563-8170131	8161051-8161547	86.76%
90	*Tursiops aduncus*	NCQN01000390.1	1311869-1312369	1314002-1314502	PVJP02910263.1	3198124-3198534	3196381-3196723	86.89%
92	*Platanista minor*	RJWK010031508.1	1-501	9597-10097	PVJP02910136.1	118820-119061	108607-109079	85.57%
96	*Lagenorhynchus obliquidens*	NW_020838007.1	5199458-5199958	5202076-5202576	PVJP02910233.1	14719214-14719589	14717326-14717797	86.46%
99	*Inia geoffrensis*	RJWO010015840.1	17427-18427	25125-26125	PVJP02910622.1	17280238-17281209		79.72%
115	*Physeter catodon*	NC_041231.1	62360484-62360984	62368332-62368832	PVJP02000497.1	228786-229288	218819-219322	91.25%
130	*Kogia breviceps*	RJWL010001846.1	4385-5385	6909-7909	PVJP02910237.1	9113944-9114540	9109308-9109740	83.00%
152	*Orcinus orca*	NW_004438494.1	599555-600055	602142-602642	PVJP02910290.1	750798-751292	747682-748168	85.17%
155	*Kogia breviceps*	RJWL010001269.1	22177-23177	27604-28604	PVJP02910473.1		632982-633934	84.79%
185	*Eubalaena japonica*	RJWP010013032.1	25776-26276	31022-31522	PVJP02910697.1	4081376-4081891	4072688-4073135	89.36%
196	*Eubalaena japonica*	RJWP010006344.1	7055-7555	13518-14018	PVJP02910812.1	2805344-2805769	2799490-2799956	91.55%
209	*Physeter catodon*	NC_041222.1	89808511-89809011	89816969-89817469	PVJP02011199.1	1845573-1846076	1833254-1833648	84.41%
270	*Kogia breviceps*	RJWL010001368.1	77957-78457	83048-83548	PVJP02910233.1	5971575-5972079	5964898-5965363	89.57%
