# Supplementary material for: Molecular fossils illuminate the evolution of retroviruses following a macroevolutionary transition from land to water
Source: PLoS Pathog. 2021 Jul 12;17(7):e1009730. doi: 10.1371/journal.ppat.1009730 (PMC8297934; doi:10.1371/journal.ppat.1009730)
Supplement: S7 Table — (PDF) [file ppat.1009730.s007.pdf]

S7 Table. The information of the LTW ERV orthologous insertions in mysticetes and odontocetes

Lineage	odontocetes				mysticetes				% identity
	Species	Accession No.	5' flanking sequence	3' flanking sequence	Species	Accession No.	5' flanking sequence	3' flanking sequence	
1	*Platanista minor*	RJWK010003858.1	23902-24402	27216-27716	Eubalaena japonica	RJWP010002746.1	46873-47346	49584-50059	94.53%
8	*Megaptera novaeangliae*	RYZJ01000543.1	3767618-3768118	3778763-3779263	Eschrichtius robustus	NIPP01001315.1	245382-245881		98.40%
9	*Sousa chinensis*	RWJT01012213.1	720213-720714	710470-710776	Eubalaena japonica	RJWP010000329.1	72747-73247	82940-83440	95.83%
10	*Platanista minor*	RJWK010010280.1	11638-12138	20924-21424	Megaptera novaeangliae	RYZJ01001395.1	5870035-5870566	5832044-5832320	89.49%
17	*Orcinus orca*	NW_004438505.1	3358251-3358751	3368995-3369495	Eschrichtius robustus	NIPP01024888.1		3847-4344	93.98%
21	*Inia geoffrensis*	RJWO010002978.1	26094-26594	34289-34789	Eschrichtius robustus	NIPP01000350.1	143061-143532	134803-135313	95.70%
22	*Ziphius cavirostris*	RJWS010013713.1	4460-4973	13649-14157	Eubalaena japonica	RJWP010018522.1	8694-9194	17819-18319	97.45%
26	*Tursiops aduncus*	NCQN01000191.1	944686-945186	946522-947022	Eschrichtius robustus	NIPP01013532.1	11446-11894	12873-13367	94.95%
27	*Platanista minor*	RJWK010010417.1	17161-17661	26514-27014	Megaptera novaeangliae	RYZJ01001385.1	2417170-2417589	2407560-2408056	96.60%
28	*Orcinus orca*	NW_004438495.1	1899797-1900297	1909838-1910338	Eschrichtius robustus	NIPP01004754.1	39722-40222	29425-29924	95.63%
30	*Platanista minor*	RJWK010050847.1	4202-4702	14896-15396	Megaptera novaeangliae	RYZJ01001286.1	5653257-5653766	5642090-5642590	94.82%
43	*Physeter catodon*	NW_021145386.1	4658-5658	9830-10330	Eschrichtius robustus	NIPP01000275.1	501549-502065	506148-506658	94.91%
47	*Orcinus orca*	NW_004438529.1	1063659-1064159	1074055-1074555	Megaptera novaeangliae	RYZJ01001558.1	1684436-1684922	1674034-1674532	96.12%
54	*Ziphius cavirostris*	RJWS010002865.1	1676-2176	10552-11052	Eubalaena japonica	RJWP010009589.1	35776-36265	26637-27160	94.44%
105	*Phocoena phocoena*	RJWQ010002051.1	105126-105626	112501-113001	Eschrichtius robustus	NIPP01002041.1	83499-83994	70396-70896	96.81%

106	*Sousa chinensis*	RWJT01022673.1	194401-194901	207566-208066	Eubalaena japonica	RJWP010040171.1	5615-6114	7344-7848	96.04%
111	*Neophocaena asiaeorientalis*	NW_020174553.1	1592624-1593124	1597374-1597874	Eschrichtius robustus	NIPP01000194.1	655244-655491	651038-651529	97.16%
116	*Neophocaena asiaeorientalis*	NW_020172733.1	912918-913418	921344-921844	Eschrichtius robustus	NIPP01016527.1	1173-1673	9727-10237	96.01%
163	*Inia geoffrensis*	RJWO010028147.1	956-1456	7539-8039	Megaptera novaeangliae	RYZJ01000218.1	11800924-11801192	11794835-11795255	81.80%
169	*Mesoplodon bidens*	PVJJ010027994.1	301-801	7141-7641	Eubalaena japonica	RJWP010055779.1	5078-5578		97.41%
172	*Kogia breviceps*	RJWL010001269.1	22453-22953	27604-28104	Eschrichtius robustus	NIPP01011487.1		43002-43449	93.99%
186	*Physeter catodon*	NC_041224.1	3166915-3167415	3169856-3170356	Eschrichtius robustus	NIPP01007054.1	28005-28494		96.53%
197	*Lipotes vexillifer*	NW_006769631.1	1798544-1799044	1802148-1802648	Megaptera novaeangliae	RYZJ01000011.1		219169-219526	96.09%
212	*Platanista minor*	RJWK010015805.1	1-501	2105-2605	Eschrichtius robustus	NIPP01003645.1	32568-33054		94.87%
213	*Lagenorhynchus obliquidens*	NW_020837991.1	16397719-16398208	16400955-16401455	Eubalaena japonica	RJWP010039255.1	4910-5410	8797-9797	96.21%
274	*Phocoena sinus*	CM018169.1	6832284-6832784	6834904-6835404	Eubalaena japonica	RJWP010014354.1		35907-36424	86.95%
