# Supplementary material for: Molecular fossils illuminate the evolution of retroviruses following a macroevolutionary transition from land to water
Source: PLoS Pathog. 2021 Jul 12;17(7):e1009730. doi: 10.1371/journal.ppat.1009730 (PMC8297934; doi:10.1371/journal.ppat.1009730)
Supplement: S8 Table — (PDF) [file ppat.1009730.s008.pdf]

S8 Table. The results of ERV-cetacean phylogeny congruence test for the LTW ERV lineages.

Lineage	Cospeciation	Duplication	Duplication & host switching	Loss	Failure to diverge	Total cost	*P*-value (Random Tip mapping)	*P*-value (Random parasite tree)
4	13-15	0	8-10	2-6	0	22	0	0
5	16-19	1-2	15-19	4-11	0	43	0	0
6	12	0	9	0	0	18	0	0
7	19	2	4	4	0	14	0	0
12	17-18	0	8-9	3-5	0	21	0	0
13	12	0	2	2	0	6	0	0
14	13	1	6	1	0	14	0	0
16	16	0	8	5	0	21	0	0
18	14	2	6	4	0	18	0	0
19	10-11	0	5-6	3-5	0	15	0	0
20	12	0	1	1	0	3	0	0
24	15	0	2	1	0	5	0	0
25	14	1	10	4	0	25	0	0
29	19	0	5	5	0	15	0	0
31	17	0	2	4	0	8	0	0
32	13	0	4	2	0	10	0	0
34	17	1	7	9	0	24	0	0
35	16	0	8	9	0	25	0	0
36	7	0	5	1	0	11	0	0
37	9-10	0	4-5	1-3	0	11	0	0
38	15	0	3	3	0	9	0	0
39	15-17	2	7-9	1-5	0	21	0	0
41	17	0	5	4	0	14	0	0
44	9	1	4	2	0	11	0	0

45	8	0	5	3	0	13	0	0
50	10	0	2	1	0	5	0	0
51	12-14	0	7-9	4-8	0	22	0	0
52	14	1	10	1	0	22	0	0
53	12	1	11	2	0	25	0	0
55	13	3	13	5	0	34	0	0
56	14-15	0	7-8	2-4	0	18	0	0
57	17-18	4	5-6	2-4	0	18	0	0
58	17	1	8	7	0	24	0	0
59	19	0	5	7	0	17	0	0
60	12-13	0	9-10	2-4	0	22	0	0
61	14	2	6	6	0	20	0	0
62	14	0	7	4	0	18	0	0
65	17	0	3	4	0	10	0	0
66	13-14	0	6-7	3-5	0	17	0	0
67	10	0	1	0	0	2	0	0
68	14-16	0-1	5-8	1-6	0	17	0	0
70	15	0	4	4	0	12	0	0
71	8	0	1	1	0	3	0	0
72	8	2	4	3	0	13	0	0
73	7	0	2	2	0	6	0	0
75	6	0	5	2	0	12	0	0
76	10	0	2	0	0	4	0	0
77	13	2	10	3	0	25	0	0
78	16	0	8	1	0	17	0	0
79	16	0	5	5	0	15	0	0
80	18	1	6	3	0	16	0	0
81	19-20	2	15-16	3-5	0	37	0	0
82	16	0	6	6	0	18	0	0

83	16-18	1-2	5-8	1-6	0	18	0	0
84	16	0	8	4	0	20	0	0
85	9	0	6	2	0	14	0	0
87	15-16	2	8-9	3-5	0	23	0	0
88	16	0	8	2	0	18	0	0
89	14-16	3	9-11	2-6	0	27	0	0
91	11-14	0-1	10-14	2-9	0	30	0	0
93	10	0	3	2	0	8	0	0
94	18	2	7	2	0	18	0	0
95	7	1	5	1	0	12	0	0
97	12-13	5-6	10-11	2-4	0	30	0	0
98	12	0	9	4	0	22	0	0
100	14	0	9	3	0	21	0	0
101	6-7	0	2-3	0-2	0	6	0	0
102	14-15	1-2	2-3	2-4	0	9	0	0
103	15	0	4	3	0	11	0	0
104	14	1	6	3	0	16	0	0
107	19	0	3	3	0	9	0	0
108	16	1	6	2	0	15	0	0
109	13	0	11	4	0	26	0	0
110	18	1	6	2	0	15	0	0
112	15	0	6	3	0	15	0	0
113	16	0	5	3	0	13	0	0
114	6	0	1	1	0	3	0	0
117	13	4	3	2	0	12	0	0
124	14	0	3	4	0	10	0	0
125	12	0	2	2	0	6	0	0
126	9	0	2	4	0	8	0	0
127	11	0	3	2	0	8	0	0

128	10-11	0	5-6	2-4	0	14	0	0
129	9	0	2	2	0	6	0	0
131	13	0	5	2	0	12	0	0
132	15	0	5	4	0	14	0	0
133	15-16	0	5-6	3-5	0	15	0	0
134	10	0	5	3	0	13	0	0
135	10	0	4	5	0	13	0	0
136	14-15	0	6-7	1-3	0	15	0	0
137	14	0	8	2	0	18	0	0
138	14	0	8	4	0	20	0	0
139	10-11	0	3-4	3-5	0	11	0	0
140	15	0	3	6	0	12	0	0
141	11	0	9	5	0	23	0	0
142	6-7	0	3-4	0-2	0	8	0	0
143	16	1	9	2	0	21	0	0
144	14	0	7	5	0	19	0	0
145	12	1	6	4	0	17	0	0
146	8-10	0	6-8	1-5	0	17	0	0
147	16-18	0	6-8	4-8	0	20	0	0
148	6	1	2	2	0	7	0	0
149	15	0	4	4	0	12	0	0
150	16-17	0	6-7	3-5	0	17	0	0
151	6	0	3	3	0	9	0	0
153	7	0	4	3	0	11	0	0
154	17	0	4	6	0	14	0	0
156	4	0	2	1	0	5	0	0
157	7	0	2	2	0	6	0	0
158	10	1	7	2	0	17	0	0
159	5	1	6	0	0	13	0	0

160	13	0	3	1	0	7	0	0
161	10	0	4	3	0	11	0	0
162	17	0	4	4	0	12	0	0
164	15-16	0	6-7	4-6	0	18	0	0
165	13	0	4	1	0	9	0	0
166	12-13	0-1	2-4	4-7	0	12	0	0
167	16	0	7	4	0	18	0	0
168	10	0	7	1	0	15	0	0
170	16	0	8	5	0	21	0	0
171	18-19	1-2	17-18	2-4	0	39	0	0
173	18	0	6	4	0	16	0	0
174	6	0	3	1	0	7	0	0
175	10	0	9	4	0	22	0	0
176	13	0	8	2	0	18	0	0
177	15-17	0	5-7	3-7	0	17	0	0
178	3-5	0	2-4	0-4	0	8	0	0
179	13	0	2	4	0	8	0	0
180	10	0	7	4	0	18	0	0
181	12	0	4	5	0	13	0	0
182	8	0	0	0	0	0	0	0
183	7	0	1	1	0	3	0	0
184	10	0	5	3	0	13	0	0
187	8	0	0	0	0	0	0	0
188	13	0	3	2	0	8	0	0
189	14-15	4	10-11	6-8	0	32	0	0
190	11	0	2	3	0	7	0	0
191	14	0	7	6	0	20	0	0
192	10	0	1	1	0	3	0	0
193	10	0	8	2	0	18	0	0

194	13	0	8	4	0	20	0	0
195	15	0	7	7	0	21	0	0
198	16	1	8	3	0	20	0	0
199	26	5	9	8	0	31	0	0
200	13-14	0	8-9	4-6	0	22	0	0
201	18	1	5	6	0	17	0	0
202	12-13	2	9-10	1-3	0	23	0	0
203	17	0	6	7	0	19	0	0
204	12	1	11	2	0	25	0	0
205	6	0	6	2	0	14	0	0
206	13	0	4	2	0	10	0	0
207	15	2	9	6	0	26	0	0
208	11	0	1	1	0	3	0	0
210	5	0	3	1	0	7	0	0
211	6	0	1	1	0	3	0	0
214	6-7	0	2-3	1-3	0	7	0	0
215	8	0-1	1-2	4-5	0	8	0	0
216	19	0	5	3	0	13	0	0
217	14-16	7	8-10	2-6	0	29	0	0
218	17-20	1	10-13	3-9	0	30	0	0
219	15-17	0	13-15	2-6	0	32	0	0
220	14-15	0	10-11	3-5	0	25	0	0
221	18	8	2	4	0	16	0	0
222	18	0	5	7	0	17	0	0
223	14	9	8	3	0	28	0	0
224	15	0	6	6	0	18	0	0
225	17	0	9	7	0	25	0	0
226	17	1	5	4	0	15	0	0
227	10	0	6	2	0	14	0	0

228	7	0	2	1	0	5	0	0
229	17	0	3	3	0	9	0	0
230	18	0	3	3	0	9	0	0
231	12	0	3	3	0	9	0	0
232	13	0	3	1	0	7	0	0
233	16-17	0-1	6-8	4-7	0	20	0	0
234	14-17	0	7-10	2-8	0	22	0	0
235	16	0	7	3	0	17	0	0
236	16	0	2	2	0	6	0	0
237	14	0	7	2	0	16	0	0
238	16	0	7	5	0	19	0	0
239	14	0	9	7	0	25	0	0
240	19	1	6	3	0	16	0	0
241	15-16	0	7-8	3-5	0	19	0	0
242	18	1	6	4	0	17	0	0
243	9-11	0	3-5	1-5	0	11	0	0
244	15-17	2	6-8	3-7	0	21	0	0
245	16-17	0	5-6	2-4	0	14	0	0
246	16	1	8	4	0	21	0	0
247	7	0	2	1	0	5	0	0
248	16	0	5	2	0	12	0	0
249	15-17	0	10-12	4-8	0	28	0	0
250	4	0	2	1	0	5	0	0
251	11-12	0	5-6	3-5	0	15	0	0
252	9	0	3	1	0	7	0	0
253	4	1	5	0	0	11	0	0
254	6	0	2	2	0	6	0	0
255	8	0	2	0	0	4	0	0
256	17	8	5	5	0	23	0	0

257	16	0	6	5	0	17	0	0
258	11	0	3	3	0	9	0	0
259	11	0	2	1	0	5	0	0
260	15	2	8	3	0	21	0	0
261	9	0	2	2	0	6	0	0
262	11	1	4	4	0	13	0	0
263	9	0	2	2	0	6	0	0
264	10	0	2	1	0	5	0	0
265	12	2	10	4	0	26	0	0
266	10	2	3	3	0	11	0	0
267	3	1	1	1	0	4	0	0
268	10	0	3	2	0	8	0	0
269	12-13	0	9-10	4-6	0	24	0	0
271	14	0	5	3	0	13	0	0
272	8-9	0	3-4	1-3	0	9	0	0
273	17	0	6	4	0	16	0	0
275	7	0	4	2	0	10	0	0
276	10	0	2	1	0	5	0	0
277	18	2	6	4	0	18	0	0
278	6	0	1	1	0	3	0	0
279	15-17	0	8-10	2-6	0	22	0	0
280	17-18	0	6-7	3-5	0	17	0	0
281	14	0	8	5	0	21	0	0
282	16	0	7	4	0	18	0	0
283	21	0	6	4	0	16	0	0
284	5	0	5	0	0	10	0	0
285	17	2	6	3	0	17	0	0
286	18	1	4	2	0	11	0	0
287	4-6	0	1-3	0-4	0	6	0	0

288	12-14	0	9-11	2-6	0	24	0	0
289	10	0	5	2	0	12	0	0
290	7	0	1	0	0	2	0	0
291	4	0	2	1	0	5	0	0
292	15	1-2	8-9	3-4	0	22	0	0
293	11-12	1-2	9-11	2-5	0	25	0	0
294	18	1	5	5	0	16	0	0
295	5-6	0	5-6	0-2	0	12	0	0
296	19	0	7	7	0	21	0	0
297	19	0	5	6	0	16	0	0
298	6	0	2	0	0	4	0	0
299	14	0	10	1	0	21	0	0
300	9	0	3	3	0	9	0	0
301	19	2	4	3	0	13	0	0
302	15	0	7	5	0	19	0	0
303	14-15	0	8-9	6-8	0	24	0	0
304	17	0	7	8	0	22	0	0
