# Supplementary material for: Molecular fossils illuminate the evolution of retroviruses following a macroevolutionary transition from land to water
Source: PLoS Pathog. 2021 Jul 12;17(7):e1009730. doi: 10.1371/journal.ppat.1009730 (PMC8297934; doi:10.1371/journal.ppat.1009730)
Supplement: S1 Fig — Reference retrovirus sequences are highlighted in red. The 315 cetacean ERV lineages are classified into Class I (in blue and green boxes), Class II (in red box), and Class III (in purple box) ERVs. The support values (ultrafast bootstrap approximation) are shown for selected nodes. The information of the representative cetacean ERVs is available in S3 Table. The information of the representative retroviruses is available in S4 Table. (PDF) [file ppat.1009730.s009.pdf]

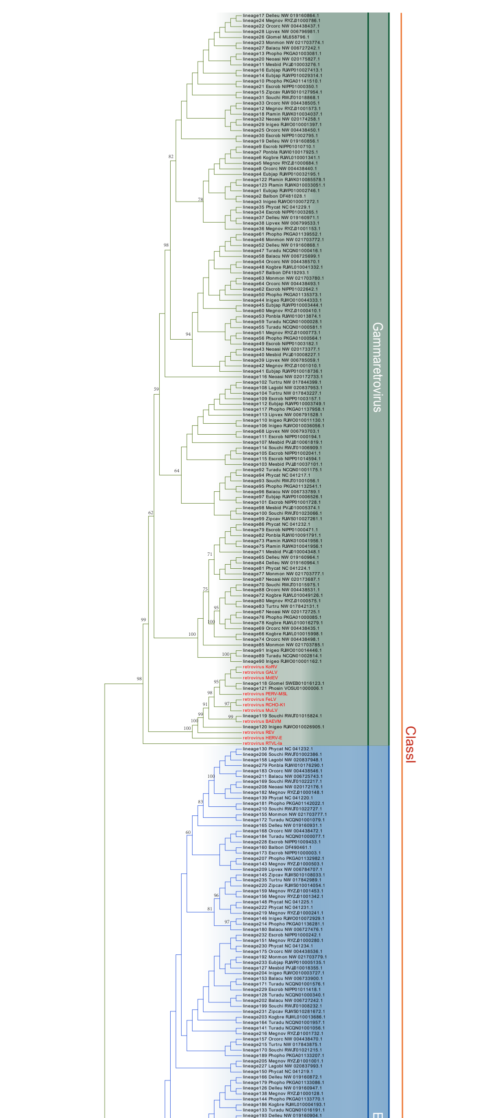

S1 Fig. Phylogenetic analyses of 315 cetacean ERV lineages. Reference retrovirus sequences are highlighted in red. The 315 cetacean ERV lineages are classified into Class I (in blue and green boxes), Class II (in red box), and Class III (in purple box) ERVs. The support values (ultrafast bootstrap approximation) are shown for selected nodes. The information of the representative cetacean ERVs is available in S3 Table. The information of the representative retroviruses is available in S4 Table.
