# Supplementary material for: Molecular fossils illuminate the evolution of retroviruses following a macroevolutionary transition from land to water
Source: PLoS Pathog. 2021 Jul 12;17(7):e1009730. doi: 10.1371/journal.ppat.1009730 (PMC8297934; doi:10.1371/journal.ppat.1009730)
Supplement: S2 Fig — The cetacean ERV sequences are highlighted in purple. The support values are shown for selected nodes. Abbreviations are listed in S1 and S2 Tables. (PDF) [file ppat.1009730.s010.pdf]

S2 Fig. Phylogenetic analyses of cetacean ERV lineages 1 to 314. The cetacean ERV sequences are highlighted in purple. The support values are shown for selected nodes. Abbreviations are listed in S1 and S2 Tables.

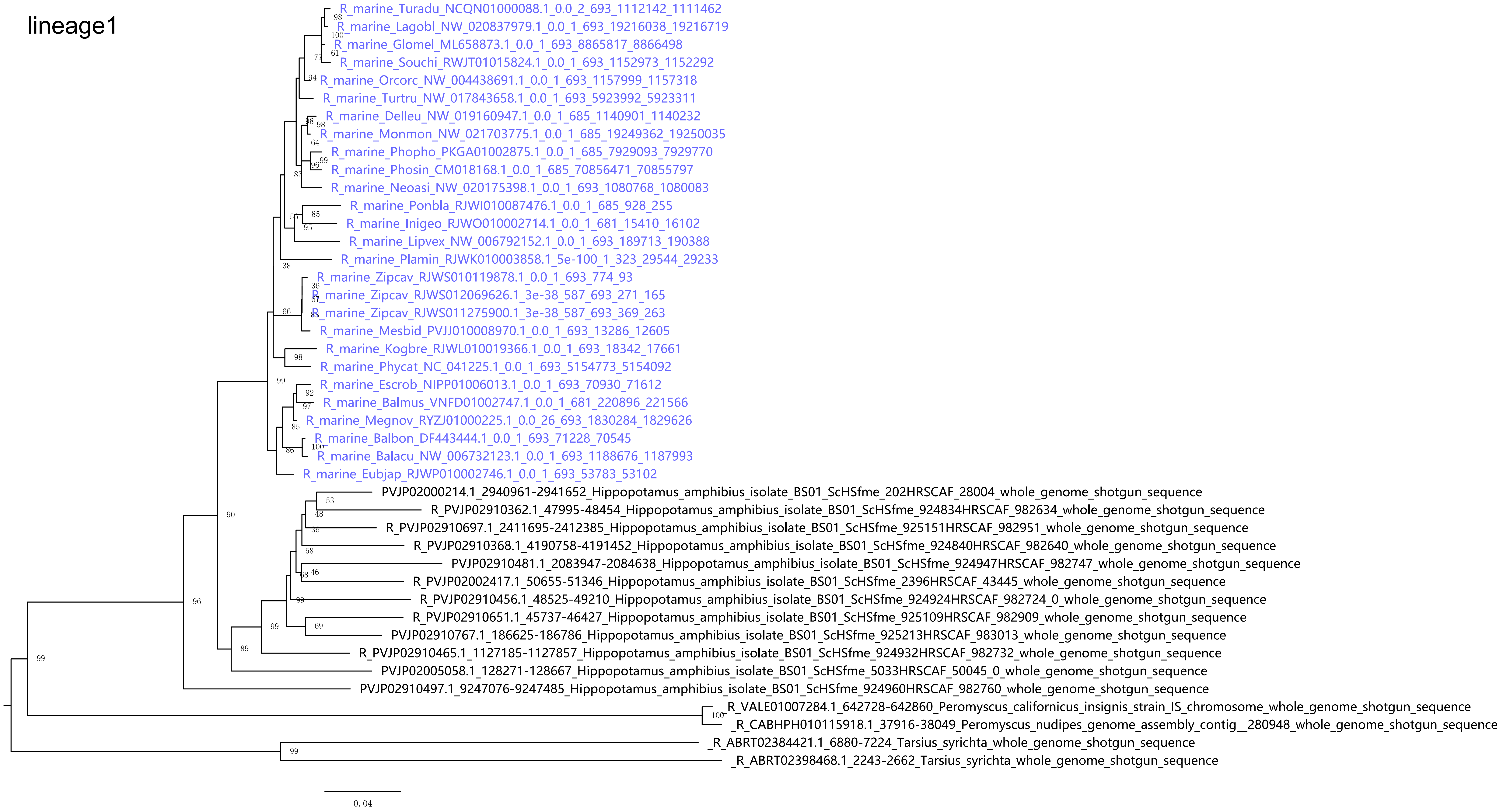

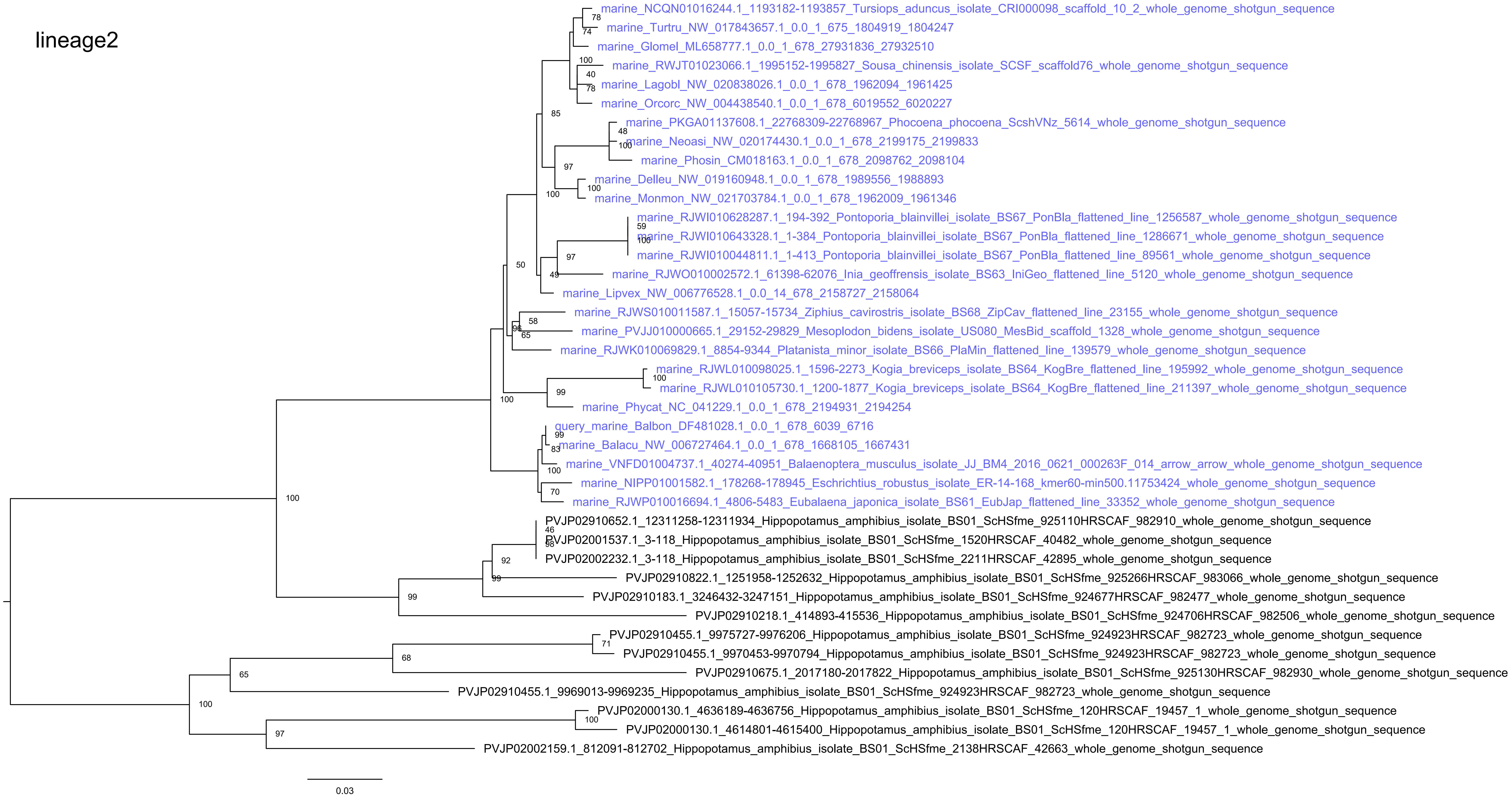
lineage2
marine_NCQN01016244.1_1193182-1193857_Tursiops_aduncus_isolate_CRI000098_scaffold_10_2_whole_genome_shotgun_sequence
marine_Turtru_NW_017843657.1_0.0_1_675_1804919_1804247
marine_Glomel_ML658777.1_0.0_1_678_27931836_27932510
marine_RWJT01023066.1_1995152-1995827_Sousa_chinensis_isolate_SCSF_scaffold76_whole_genome_shotgun_sequence
marine_Lagobl_NW_020838026.1_0.0_1_678_1962094_1961425
marine_Orcorc_NW_004438540.1_0.0_1_678_6019552_6020227
marine_PKGA01137608.1_22768309-22768967_Phocoena_phocoena_ScshVNz_5614_whole_genome_shotgun_sequence
marine_Neoasi_NW_020174430.1_0.0_1_678_2199175_2199833
marine_Phosin_CM018163.1_0.0_1_678_2098762_2098104
marine_Delleu_NW_019160948.1_0.0_1_678_1989556_1988893
marine_Monmon_NW_021703784.1_0.0_1_678_1962009_1961346
marine_RJWI010628287.1_194-392_Pontoporia_blainvillei_isolate_BS67_PonBla_flattened_line_1256587_whole_genome_shotgun_sequence
marine_RJWI010643328.1_1-384_Pontoporia_blainvillei_isolate_BS67_PonBla_flattened_line_1286671_whole_genome_shotgun_sequence
marine_RJWI010044811.1_1-413_Pontoporia_blainvillei_isolate_BS67_PonBla_flattened_line_89561_whole_genome_shotgun_sequence
marine_RJWO010002572.1_61398-62076_Inia_geoffrensis_isolate_BS63_IniGeo_flattened_line_5120_whole_genome_shotgun_sequence
marine_Lipvex_NW_006776528.1_0.0_14_678_2158727_2158064
marine_RJWS010011587.1_15057-15734_Ziphius_cavirostris_isolate_BS68_ZipCav_flattened_line_23155_whole_genome_shotgun_sequence
marine_PVJJ010000665.1_29152-29829_Mesoplodon_bidens_isolate_US080_MesBid_scaffold_1328_whole_genome_shotgun_sequence
marine_RJWK010069829.1_8854-9344_Platanista_minor_isolate_BS66_PlaMin_flattened_line_139579_whole_genome_shotgun_sequence
marine_RJWL010098025.1_1596-2273_Kogia_breviceps_isolate_BS64_KogBre_flattened_line_195992_whole_genome_shotgun_sequence
marine_RJWL010105730.1_1200-1877_Kogia_breviceps_isolate_BS64_KogBre_flattened_line_211397_whole_genome_shotgun_sequence
marine_Phycat_NC_041229.1_0.0_1_678_2194931_2194254
query_marine_Balbon_DF481028.1_0.0_1_678_6039_6716
marine_Balacu_NW_006727464.1_0.0_1_678_1668105_1667431
marine_VNFD01004737.1_40274-40951_Balaenoptera_musculus_isolate_JJ_BM4_2016_0621_000263F_014_arrow_arrow_whole_genome_shotgun_sequence
marine_NIPP01001582.1_178268-178945_Eschrichtius_robustus_isolate_ER-14-168_kmer60-min500.11753424_whole_genome_shotgun_sequence
marine_RJWP010016694.1_4806-5483_Eubalaena_japonica_isolate_BS61_EubJap_flattened_line_33352_whole_genome_shotgun_sequence
PVJP02910652.1_12311258-12311934_Hippopotamus_amphibius_isolate_BS01_ScHSfme_925110HRSCAF_982910_whole_genome_shotgun_sequence
PVJP02001537.1_3-118_Hippopotamus_amphibius_isolate_BS01_ScHSfme_1520HRSCAF_40482_whole_genome_shotgun_sequence
PVJP02002232.1_3-118_Hippopotamus_amphibius_isolate_BS01_ScHSfme_2211HRSCAF_42895_whole_genome_shotgun_sequence
PVJP02910822.1_1251958-1252632_Hippopotamus_amphibius_isolate_BS01_ScHSfme_925266HRSCAF_983066_whole_genome_shotgun_sequence
PVJP02910183.1_3246432-3247151_Hippopotamus_amphibius_isolate_BS01_ScHSfme_924677HRSCAF_982477_whole_genome_shotgun_sequence
PVJP02910218.1_414893-415536_Hippopotamus_amphibius_isolate_BS01_ScHSfme_924706HRSCAF_982506_whole_genome_shotgun_sequence
PVJP02910455.1_9975727-9976206_Hippopotamus_amphibius_isolate_BS01_ScHSfme_924923HRSCAF_982723_whole_genome_shotgun_sequence
PVJP02910455.1_9970453-9970794_Hippopotamus_amphibius_isolate_BS01_ScHSfme_924923HRSCAF_982723_whole_genome_shotgun_sequence
PVJP02910675.1_2017180-2017822_Hippopotamus_amphibius_isolate_BS01_ScHSfme_925130HRSCAF_982930_whole_genome_shotgun_sequence
PVJP02910455.1_9969013-9969235_Hippopotamus_amphibius_isolate_BS01_ScHSfme_924923HRSCAF_982723_whole_genome_shotgun_sequence
PVJP02000130.1_4636189-4636756_Hippopotamus_amphibius_isolate_BS01_ScHSfme_120HRSCAF_19457_1_whole_genome_shotgun_sequence
PVJP02000130.1_4614801-4615400_Hippopotamus_amphibius_isolate_BS01_ScHSfme_120HRSCAF_19457_1_whole_genome_shotgun_sequence
PVJP02002159.1_812091-812702_Hippopotamus_amphibius_isolate_BS01_ScHSfme_2138HRSCAF_42663_whole_genome_shotgun_sequence
0.03

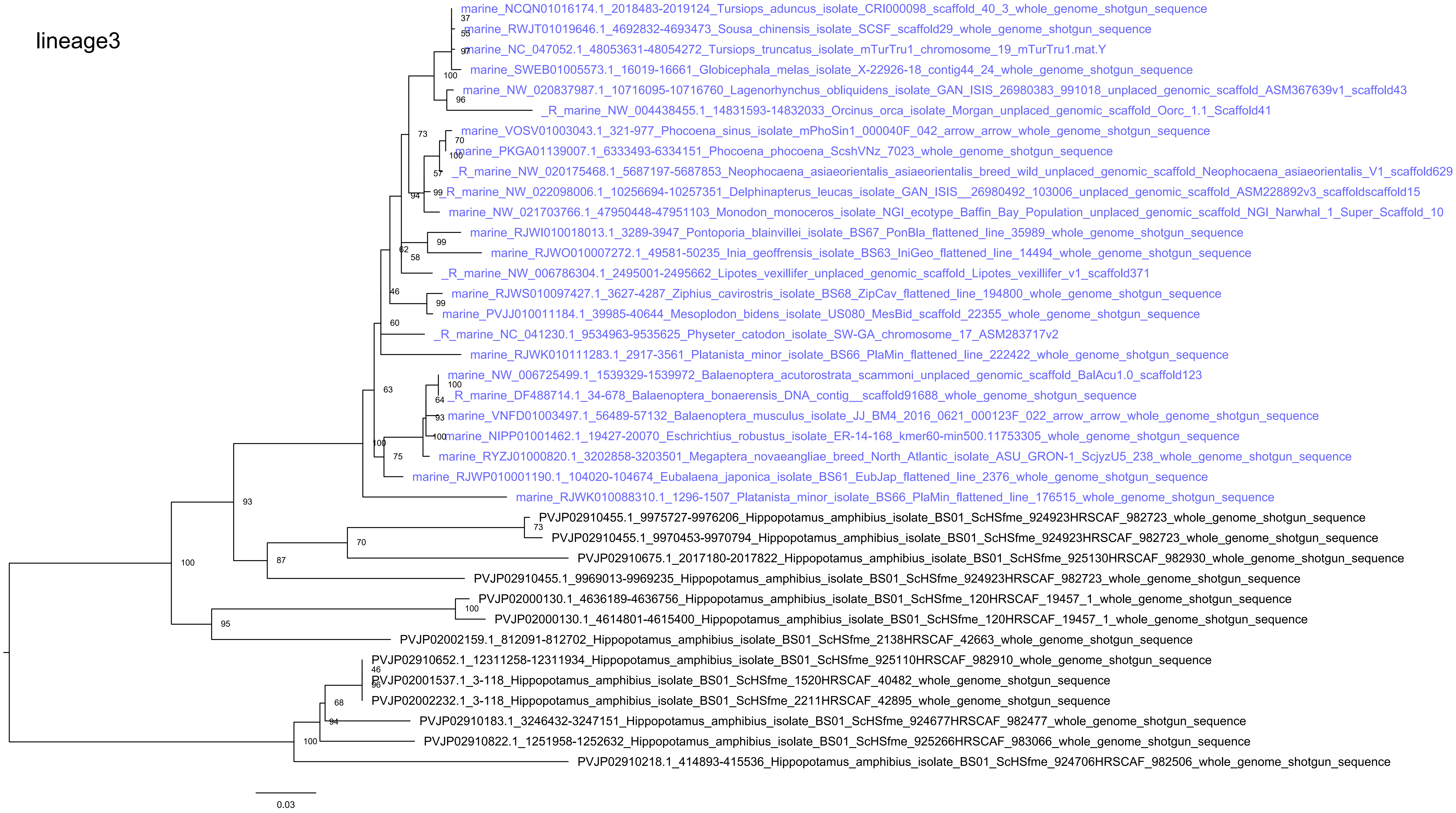
lineage3
marine_NCQN01016174.1_2018483-2019124_Tursiops_aduncus_isolate_CRI000098_scaffold_40_3_whole_genome_shotgun_sequence
marine_RWJT01019646.1_4692832-4693473_Sousa_chinensis_isolate_SCSF_scaffold29_whole_genome_shotgun_sequence
marine_NC_047052.1_48053631-48054272_Tursiops_truncatus_isolate_mTurTru1_chromosome_19_mTurTru1.mat.Y
marine_SWEB01005573.1_16019-16661_Globicephala_melas_isolate_X-22926-18_contig44_24_whole_genome_shotgun_sequence
marine_NW_020837987.1_10716095-10716760_Lagenorhynchus_obliquidens_isolate_GAN_ISIS_26980383_991018_unplaced_genomic_scaffold_ASM367639v1_scaffold43
_R_marine_NW_004438455.1_14831593-14832033_Orcinus_orca_isolate_Morgan_unplaced_genomic_scaffold_Oorc_1.1_Scaffold41
marine_VOSV01003043.1_321-977_Phocoena_sinus_isolate_mPhoSin1_000040F_042_arrow_arrow_whole_genome_shotgun_sequence
marine_PKGA01139007.1_6333493-6334151_Phocoena_phocoena_ScshVNz_7023_whole_genome_shotgun_sequence
_R_marine_NW_020175468.1_5687197-5687853_Neophocaena_asiaeorientalis_asiaeorientalis_breed_wild_unplaced_genomic_scaffold_Neophocaena_asiaeorientalis_V1_scaffold629
_R_marine_NW_022098006.1_10256694-10257351_Delphinapterus_leucas_isolate_GAN_ISIS__26980492_103006_unplaced_genomic_scaffold_ASM228892v3_scaffoldscaffold15
marine_NW_021703766.1_47950448-47951103_Monodon_monoceros_isolate_NGI_ecotype_Baffin_Bay_Population_unplaced_genomic_scaffold_NGI_Narwhal_1_Super_Scaffold_10
marine_RJWI010018013.1_3289-3947_Pontoporia_blainvillei_isolate_BS67_PonBla_flattened_line_35989_whole_genome_shotgun_sequence
marine_RJWO010007272.1_49581-50235_Inia_geoffrensis_isolate_BS63_IniGeo_flattened_line_14494_whole_genome_shotgun_sequence
_R_marine_NW_006786304.1_2495001-2495662_Lipotes_vexillifer_unplaced_genomic_scaffold_Lipotes_vexillifer_v1_scaffold371
marine_RJWS010097427.1_3627-4287_Ziphius_cavirostris_isolate_BS68_ZipCav_flattened_line_194800_whole_genome_shotgun_sequence
marine_PVJJ010011184.1_39985-40644_Mesoplodon_bidens_isolate_US080_MesBid_scaffold_22355_whole_genome_shotgun_sequence
_R_marine_NC_041230.1_9534963-9535625_Physeter_catodon_isolate_SW-GA_chromosome_17_ASM283717v2
marine_RJWK010111283.1_2917-3561_Platanista_minor_isolate_BS66_PlaMin_flattened_line_222422_whole_genome_shotgun_sequence
marine_NW_006725499.1_1539329-1539972_Balaenoptera_acutorostrata_scammoni_unplaced_genomic_scaffold_BalAcu1.0_scaffold123
_R_marine_DF488714.1_34-678_Balaenoptera_bonaerensis_DNA_contig__scaffold91688_whole_genome_shotgun_sequence
marine_VNFD01003497.1_56489-57132_Balaenoptera_musculus_isolate_JJ_BM4_2016_0621_000123F_022_arrow_arrow_whole_genome_shotgun_sequence
marine_NIPP01001462.1_19427-20070_Eschrichtius_robustus_isolate_ER-14-168_kmer60-min500.11753305_whole_genome_shotgun_sequence
marine_RYZJ01000820.1_3202858-3203501_Megaptera_novaeangliae_breed_North_Atlantic_isolate_ASU_GRON-1_ScjyzU5_238_whole_genome_shotgun_sequence
marine_RJWP010001190.1_104020-104674_Eubalaena_japonica_isolate_BS61_EubJap_flattened_line_2376_whole_genome_shotgun_sequence
marine_RJWK010088310.1_1296-1507_Platanista_minor_isolate_BS66_PlaMin_flattened_line_176515_whole_genome_shotgun_sequence
PVJP02910455.1_9975727-9976206_Hippopotamus_amphibius_isolate_BS01_ScHSfme_924923HRSCAF_982723_whole_genome_shotgun_sequence
PVJP02910455.1_9970453-9970794_Hippopotamus_amphibius_isolate_BS01_ScHSfme_924923HRSCAF_982723_whole_genome_shotgun_sequence
PVJP02910675.1_2017180-2017822_Hippopotamus_amphibius_isolate_BS01_ScHSfme_925130HRSCAF_982930_whole_genome_shotgun_sequence
PVJP02910455.1_9969013-9969235_Hippopotamus_amphibius_isolate_BS01_ScHSfme_924923HRSCAF_982723_whole_genome_shotgun_sequence
PVJP02000130.1_4636189-4636756_Hippopotamus_amphibius_isolate_BS01_ScHSfme_120HRSCAF_19457_1_whole_genome_shotgun_sequence
PVJP02000130.1_4614801-4615400_Hippopotamus_amphibius_isolate_BS01_ScHSfme_120HRSCAF_19457_1_whole_genome_shotgun_sequence
PVJP02002159.1_812091-812702_Hippopotamus_amphibius_isolate_BS01_ScHSfme_2138HRSCAF_42663_whole_genome_shotgun_sequence
PVJP02910652.1_12311258-12311934_Hippopotamus_amphibius_isolate_BS01_ScHSfme_925110HRSCAF_982910_whole_genome_shotgun_sequence
PVJP02001537.1_3-118_Hippopotamus_amphibius_isolate_BS01_ScHSfme_1520HRSCAF_40482_whole_genome_shotgun_sequence
PVJP02002232.1_3-118_Hippopotamus_amphibius_isolate_BS01_ScHSfme_2211HRSCAF_42895_whole_genome_shotgun_sequence
PVJP02910183.1_3246432-3247151_Hippopotamus_amphibius_isolate_BS01_ScHSfme_924677HRSCAF_982477_whole_genome_shotgun_sequence
PVJP02910822.1_1251958-1252632_Hippopotamus_amphibius_isolate_BS01_ScHSfme_925266HRSCAF_983066_whole_genome_shotgun_sequence
PVJP02910218.1_414893-415536_Hippopotamus_amphibius_isolate_BS01_ScHSfme_924706HRSCAF_982506_whole_genome_shotgun_sequence
0.03

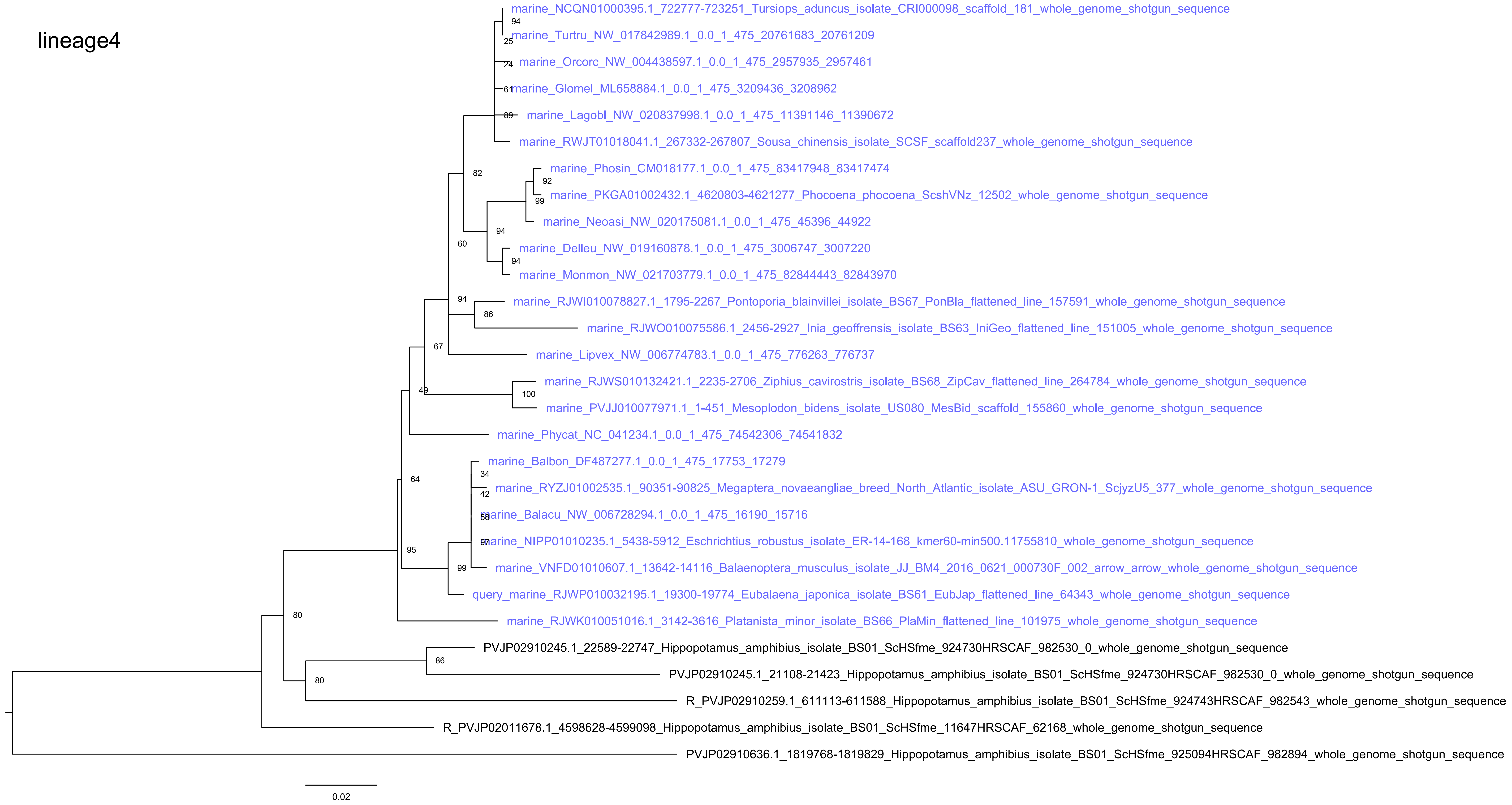
lineage4
marine_NCQN01000395.1_722777-723251_Tursiops_aduncus_isolate_CRI000098_scaffold_181_whole_genome_shotgun_sequence
marine_Turtru_NW_017842989.1_0.0_1_475_20761683_20761209
marine_Orcorc_NW_004438597.1_0.0_1_475_2957935_2957461
marine_Glomel_ML658884.1_0.0_1_475_3209436_3208962
marine_Lagobl_NW_020837998.1_0.0_1_475_11391146_11390672
marine_RWJT01018041.1_267332-267807_Sousa_chinensis_isolate_SCSF_scaffold237_whole_genome_shotgun_sequence
marine_Phosin_CM018177.1_0.0_1_475_83417948_83417474
marine_PKGA01002432.1_4620803-4621277_Phocoena_phocoena_ScshVNz_12502_whole_genome_shotgun_sequence
marine_Neoasi_NW_020175081.1_0.0_1_475_45396_44922
marine_Delleu_NW_019160878.1_0.0_1_475_3006747_3007220
marine_Monmon_NW_021703779.1_0.0_1_475_82844443_82843970
marine_RJWI010078827.1_1795-2267_Pontoporia_blainvillei_isolate_BS67_PonBla_flattened_line_157591_whole_genome_shotgun_sequence
marine_RJWO010075586.1_2456-2927_Inia_geoffrensis_isolate_BS63_IniGeo_flattened_line_151005_whole_genome_shotgun_sequence
marine_Lipvex_NW_006774783.1_0.0_1_475_776263_776737
marine_RJWS010132421.1_2235-2706_Ziphius_cavirostris_isolate_BS68_ZipCav_flattened_line_264784_whole_genome_shotgun_sequence
marine_PVJJ010077971.1_1-451_Mesoplodon_bidens_isolate_US080_MesBid_scaffold_155860_whole_genome_shotgun_sequence
marine_Phycat_NC_041234.1_0.0_1_475_74542306_74541832
marine_Balbon_DF487277.1_0.0_1_475_17753_17279
marine_RYZJ01002535.1_90351-90825_Megaptera_novaeangliae_breed_North_Atlantic_isolate_ASU_GRON-1_ScjyzU5_377_whole_genome_shotgun_sequence
marine_Balacu_NW_006728294.1_0.0_1_475_16190_15716
marine_NIPP01010235.1_5438-5912_Eschrichtius_robustus_isolate_ER-14-168_kmer60-min500.11755810_whole_genome_shotgun_sequence
marine_VNFD01010607.1_13642-14116_Balaenoptera_musculus_isolate_JJ_BM4_2016_0621_000730F_002_arrow_arrow_whole_genome_shotgun_sequence
query_marine_RJWP010032195.1_19300-19774_Eubalaena_japonica_isolate_BS61_EubJap_flattened_line_64343_whole_genome_shotgun_sequence
marine_RJWK010051016.1_3142-3616_Platanista_minor_isolate_BS66_PlaMin_flattened_line_101975_whole_genome_shotgun_sequence
PVJP02910245.1_22589-22747_Hippopotamus_amphibius_isolate_BS01_ScHSfme_924730HRSCAF_982530_0_whole_genome_shotgun_sequence
PVJP02910245.1_21108-21423_Hippopotamus_amphibius_isolate_BS01_ScHSfme_924730HRSCAF_982530_0_whole_genome_shotgun_sequence
R_PVJP02910259.1_611113-611588_Hippopotamus_amphibius_isolate_BS01_ScHSfme_924743HRSCAF_982543_whole_genome_shotgun_sequence
R_PVJP02011678.1_4598628-4599098_Hippopotamus_amphibius_isolate_BS01_ScHSfme_11647HRSCAF_62168_whole_genome_shotgun_sequence
PVJP02910636.1_1819768-1819829_Hippopotamus_amphibius_isolate_BS01_ScHSfme_925094HRSCAF_982894_whole_genome_shotgun_sequence
0.02

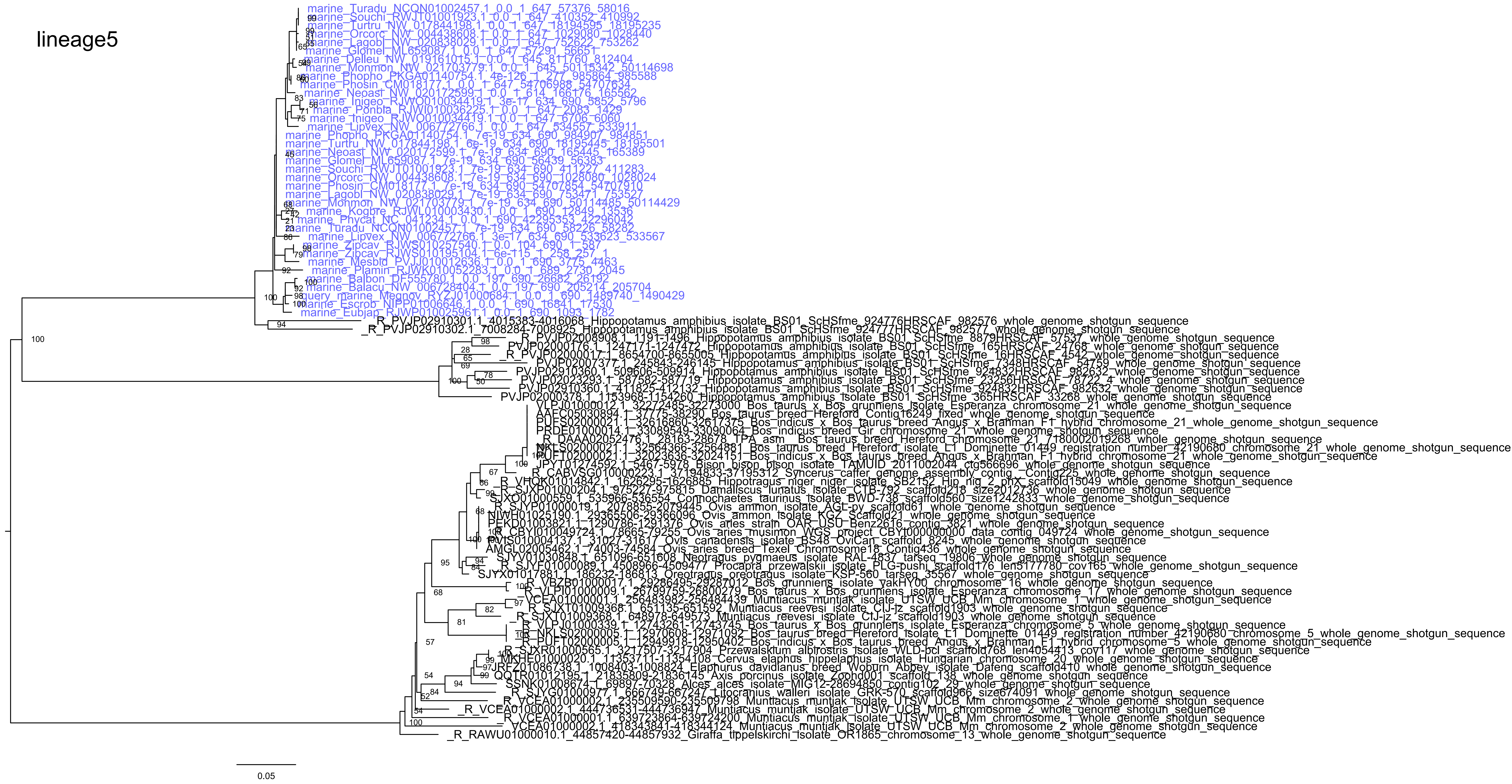

lineage5
marine_Turadu_NCQN01002457.1_0.0_1_647_57376_58016
marine_Souchi_RWJT01001923.1_0.0_1_647_410352_410992
marine_Turtru_NW_017844198.1_0.0_1_647_18194595_18195235
marine_Orcorc_NW_004438608.1_0.0_1_647_1029080_1028440
marine_Lagobl_NW_020838029.1_0.0_1_647_752622_753262
marine_Glomel_ML659087.1_0.0_1_647_57291_56651
marine_Delleu_NW_019161015.1_0.0_1_645_811760_812404
marine_Monmon_NW_021703779.1_0.0_1_645_50115342_50114698
marine_Phopho_PKGA01140754.1_4e-126_1_277_985864_985588
marine_Phosin_CM018177.1_0.0_1_647_54706988_54707634
marine_Neoasi_NW_020172599.1_0.0_1_614_166176_165562
marine_Inigeo_RJWO010034419.1_3e-17_634_690_5852_5796
marine_Ponbla_RJWI010036225.1_0.0_1_647_2083_1429
marine_Inigeo_RJWO010034419.1_0.0_1_647_6706_6060
marine_Lipvex_NW_006772766.1_0.0_1_647_534557_533911
marine_Phopho_PKGA01140754.1_7e-19_634_690_984907_984851
marine_Turtru_NW_017844198.1_6e-19_634_690_18195445_18195501
marine_Neoasi_NW_020172599.1_7e-19_634_690_165445_165389
marine_Glomel_ML659087.1_7e-19_634_690_56439_56383
marine_Souchi_RWJT01001923.1_7e-19_634_690_411227_411283
marine_Orcorc_NW_004438608.1_7e-19_634_690_1028080_1028024
marine_Phosin_CM018177.1_7e-19_634_690_54707854_54707910
marine_Lagobl_NW_020838029.1_7e-19_634_690_753471_753527
marine_Monmon_NW_021703779.1_7e-19_634_690_50114485_50114429
marine_Kogbre_RJWL010003430.1_0.0_1_690_12849_13536
marine_Phycat_NC_041234.1_0.0_1_690_42295353_42296042
marine_Turadu_NCQN01002457.1_7e-19_634_690_58226_58282
marine_Lipvex_NW_006772766.1_3e-17_634_690_533623_533567
marine_Zipcav_RJWS010257540.1_0.0_104_690_1_587
marine_Zipcav_RJWS010195104.1_6e-115_1_258_257_1
marine_Mesbid_PVJJ010012636.1_0.0_1_690_3775_4463
marine_Plamin_RJWK010052283.1_0.0_1_689_2730_2045
marine_Balbon_DF555780.1_0.0_197_690_26682_26192
marine_Balacu_NW_006728404.1_0.0_197_690_205214_205704
query_marine_Megnov_RYZJ01000684.1_0.0_1_690_1489740_1490429
marine_Escrob_NIPP01006646.1_0.0_1_690_16841_17530
marine_Eubjap_RJWP010025961.1_0.0_1_690_1093_1782
_R_PVJP02910301.1_4015383-4016068_Hippopotamus_amphibius_isolate_BS01_ScHSfme_924776HRSCAF_982576_whole_genome_shotgun_sequence
_R_PVJP02910302.1_7008284-7008925_Hippopotamus_amphibius_isolate_BS01_ScHSfme_924777HRSCAF_982577_whole_genome_shotgun_sequence
_R_PVJP02008908.1_1191-1496_Hippopotamus_amphibius_isolate_BS01_ScHSfme_8879HRSCAF_57537_whole_genome_shotgun_sequence
PVJP02000176.1_1247171-1247472_Hippopotamus_amphibius_isolate_BS01_ScHSfme_165HRSCAF_24768_whole_genome_shotgun_sequence
_R_PVJP02000017.1_8654700-8655005_Hippopotamus_amphibius_isolate_BS01_ScHSfme_16HRSCAF_4542_whole_genome_shotgun_sequence
PVJP02007377.1_245843-246145_Hippopotamus_amphibius_isolate_BS01_ScHSfme_7348HRSCAF_54759_whole_genome_shotgun_sequence
PVJP02910360.1_509606-509914_Hippopotamus_amphibius_isolate_BS01_ScHSfme_924832HRSCAF_982632_whole_genome_shotgun_sequence
PVJP02023293.1_587582-587719_Hippopotamus_amphibius_isolate_BS01_ScHSfme_23256HRSCAF_78722_4_whole_genome_shotgun_sequence
PVJP02910360.1_411825-412132_Hippopotamus_amphibius_isolate_BS01_ScHSfme_924832HRSCAF_982632_whole_genome_shotgun_sequence
PVJP02000378.1_1153968-1154260_Hippopotamus_amphibius_isolate_BS01_ScHSfme_365HRSCAF_33268_whole_genome_shotgun_sequence
VLPJ01000012.1_32272485-32273000_Bos_grunniens_x_Bos_taurus_isolate_Esperanza_chromosome_21_whole_genome_shotgun_sequence
AAFC05030894.1_37775-38290_Bos_taurus_breed_Hereford_Contig16249_fixed_whole_genome_shotgun_sequence
PUFS02000021.1_32616860-32617375_Bos_indicus_x_Bos_taurus_breed_Angus_x_Brahman_F1_hybrid_chromosome_21_whole_genome_shotgun_sequence
PRDE01000014.1_33089549-33090064_Bos_indicus_breed_Gir_chromosome_21_whole_genome_shotgun_sequence
_R_DAAA02052476.1_28163-28678_TPA_asm:_Bos_taurus_breed_Hereford_chromosome_21_7180002019268_whole_genome_shotgun_sequence
NKLS02000021.1_32564366-32564881_Bos_taurus_breed_Hereford_isolate_L1_Dominette_01449_registration_number_42190680_chromosome_21_whole_genome_shotgun_sequence
PUFT02000021.1_32023636-32024151_Bos_indicus_x_Bos_taurus_breed_Angus_x_Brahman_F1_hybrid_chromosome_21_whole_genome_shotgun_sequence
JPYT01274592.1_5467-5978_Bison_bison_bison_isolate_TAMUID_2011002044_ctg566696_whole_genome_shotgun_sequence
_R_CABVSG010000223.1_37194833-37195312_Syncerus_caffer_genome_assembly_contig_Contig225_whole_genome_shotgun_sequence
_R_VHQK01014842.1_1626295-1626885_Hippotragus_niger_niger_isolate_SB2152_Hip_nig_2_pnX_scaffold15049_whole_genome_shotgun_sequence
_R_SJXP01000204.1_975227-975815_Damaliscus_lunatus_isolate_CTB-792_scaffold218_size2012736_whole_genome_shotgun_sequence
SJXO01000559.1_535966-536554_Connochaetes_taurinus_isolate_BWD-738_scaffold560_size1242833_whole_genome_shotgun_sequence
_R_SJYP01000019.1_2078855-2079445_Ovis_ammon_isolate_AGL-py_scaffold61_whole_genome_shotgun_sequence
NIWH01025190.1_29365506-29366096_Ovis_ammon_isolate_KGZ_Scaffold21_whole_genome_shotgun_sequence
PEKD01003821.1_1290786-1291376_Ovis_aries_strain_OAR_USU_Benz2616_contig_3821_whole_genome_shotgun_sequence
_R_CBYI010049724.1_78665-79255_Ovis_aries_musimon_WGS_project_CBYI000000000_data_contig_049724_whole_genome_shotgun_sequence
PVIS010004137.1_31027-31617_Ovis_canadensis_isolate_BS48_OviCan_scaffold_8245_whole_genome_shotgun_sequence
AMGL02005462.1_74003-74584_Ovis_aries_breed_Texel_Chromosome18_Contig436_whole_genome_shotgun_sequence
SJYV01030848.1_651096-651608_Neotragus_pygmaeus_isolate_RAL-4837_tarseq_19806_whole_genome_shotgun_sequence
_R_SJYF01000089.1_4508966-4509477_Procapra_przewalskii_isolate_PLG-pushi_scaffold176_len5177780_cov165_whole_genome_shotgun_sequence
SJYX01017881.1_186232-186813_Oreotragus_oreotragus_isolate_KSP-560_tarseq_35567_whole_genome_shotgun_sequence
_R_VBZB01000017.1_29286495-29287012_Bos_grunniens_isolate_yakHY00_chromosome_16_whole_genome_shotgun_sequence
_R_VLPI01000009.1_26799759-26800279_Bos_taurus_x_Bos_grunniens_isolate_Esperanza_chromosome_17_whole_genome_shotgun_sequence
VCEA01000001.1_256483982-256484439_Muntiacus_muntjak_isolate_UTSW_UCB_Mm_chromosome_1_whole_genome_shotgun_sequence
_R_SJXT01009368.1_651135-651592_Muntiacus_reevesi_isolate_CIJ-jz_scaffold1903_whole_genome_shotgun_sequence
_R_SJXT01009368.1_648978-649573_Muntiacus_reevesi_isolate_CIJ-jz_scaffold1903_whole_genome_shotgun_sequence
_R_VLPJ01000339.1_12743261-12743745_Bos_taurus_x_Bos_grunniens_isolate_Esperanza_chromosome_5_whole_genome_shotgun_sequence
_R_NKLS02000005.1_12970608-12971092_Bos_taurus_breed_Hereford_isolate_L1_Dominette_01449_registration_number_42190680_chromosome_5_whole_genome_shotgun_sequence
_R_PUFT02000005.1_12949918-12950402_Bos_indicus_x_Bos_taurus_breed_Angus_x_Brahman_F1_hybrid_chromosome_5_whole_genome_shotgun_sequence
_R_SJXR01000565.1_3217507-3217904_Przewalskium_albirostris_isolate_WLD-bcl_scaffold768_len4054413_cov117_whole_genome_shotgun_sequence
MKHE01000020.1_11353711-11354108_Cervus_elaphus_hippelaphus_isolate_Hungarian_chromosome_20_whole_genome_shotgun_sequence
JRFZ01086738.1_1008403-1008824_Elaphurus_davidianus_breed_Woburn_Abbey_isolate_Dafeng_scaffold410_whole_genome_shotgun_sequence
QQTR01012195.1_21835809-21836145_Axis_porcinus_isolate_Zoohd001_scaffold_138_whole_genome_shotgun_sequence
SSNK01008674.1_69897-70328_Alces_alces_isolate_MIG12-28694850_contig102_29_whole_genome_shotgun_sequence
_R_SJYG01000977.1_666749-667247_Litocranius_walleri_isolate_GRK-570_scaffold966_size674091_whole_genome_shotgun_sequence
_R_VCEA01000002.1_235509590-235509798_Muntiacus_muntjak_isolate_UTSW_UCB_Mm_chromosome_2_whole_genome_shotgun_sequence
_R_VCEA01000002.1_444736531-444736947_Muntiacus_muntjak_isolate_UTSW_UCB_Mm_chromosome_2_whole_genome_shotgun_sequence
_R_VCEA01000001.1_639723864-639724200_Muntiacus_muntjak_isolate_UTSW_UCB_Mm_chromosome_1_whole_genome_shotgun_sequence
VCEA01000002.1_418343841-418344124_Muntiacus_muntjak_isolate_UTSW_UCB_Mm_chromosome_2_whole_genome_shotgun_sequence
_R_RAWU01000010.1_44857420-44857932_Giraffa_tippelskirchi_isolate_OR1865_chromosome_13_whole_genome_shotgun_sequence
0.05

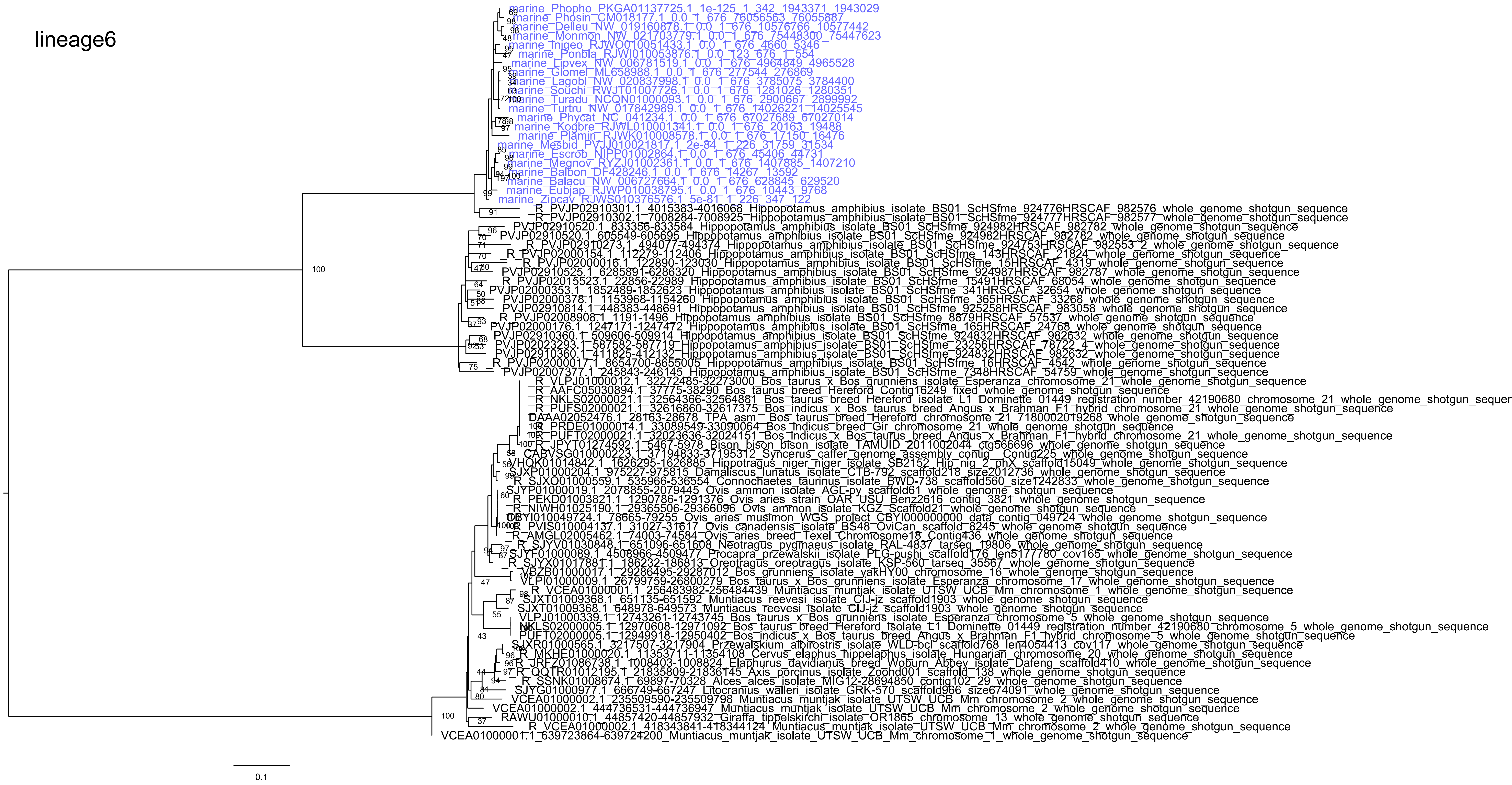
lineage6
marine_Phopho_PKGA01137725.1_1e-125_1_342_1943371_1943029
marine_Phosin_CM018177.1_0.0_1_676_76056563_76055887
marine_Delleu_NW_019160878.1_0.0_1_676_10576766_10577442
marine_Monmon_NW_021703779.1_0.0_1_676_75448300_75447623
marine_Inigeo_RJWO010051433.1_0.0_1_676_4660_5346
marine_Ponbla_RJWI010053876.1_0.0_123_676_1_554
marine_Lipvex_NW_006781519.1_0.0_1_676_4964849_4965528
marine_Glomel_ML658988.1_0.0_1_676_277544_276869
marine_Lagobl_NW_020837998.1_0.0_1_676_3785075_3784400
marine_Souchi_RWJT01007726.1_0.0_1_676_1281026_1280351
marine_Turadu_NCQN01000093.1_0.0_1_676_2900667_2899992
marine_Turtru_NW_017842989.1_0.0_1_676_14026221_14025545
marine_Phycat_NC_041234.1_0.0_1_676_67027689_67027014
marine_Kogbre_RJWL010001341.1_0.0_1_676_20163_19488
marine_Plamin_RJWK010008578.1_0.0_1_676_17150_16476
marine_Mesbid_PVJJ010021817.1_2e-84_1_226_31759_31534
marine_Escrob_NIPP01002864.1_0.0_1_676_45406_44731
marine_Megnov_RYZJ01002361.1_0.0_1_676_1407885_1407210
marine_Balbon_DF428246.1_0.0_1_676_14267_13592
marine_Balacu_NW_006727664.1_0.0_1_676_628845_629520
marine_Eubjap_RJWP010038795.1_0.0_1_676_10443_9768
marine_Zipcav_RJWS010376576.1_5e-81_1_226_347_122
_R_PVJP02910301.1_4015383-4016068_Hippopotamus_amphibius_isolate_BS01_ScHSfme_924776HRSCAF_982576_whole_genome_shotgun_sequence
_R_PVJP02910302.1_7008284-7008925_Hippopotamus_amphibius_isolate_BS01_ScHSfme_924777HRSCAF_982577_whole_genome_shotgun_sequence
PVJP02910520.1_833356-833584_Hippopotamus_amphibius_isolate_BS01_ScHSfme_924982HRSCAF_982782_whole_genome_shotgun_sequence
PVJP02910520.1_605549-605695_Hippopotamus_amphibius_isolate_BS01_ScHSfme_924982HRSCAF_982782_whole_genome_shotgun_sequence
_R_PVJP02910273.1_494077-494374_Hippopotamus_amphibius_isolate_BS01_ScHSfme_924753HRSCAF_982553_2_whole_genome_shotgun_sequence
_R_PVJP02000154.1_112279-112406_Hippopotamus_amphibius_isolate_BS01_ScHSfme_143HRSCAF_21824_whole_genome_shotgun_sequence
_R_PVJP02000016.1_122890-123030_Hippopotamus_amphibius_isolate_BS01_ScHSfme_15HRSCAF_4319_whole_genome_shotgun_sequence
PVJP02910525.1_6285891-6286320_Hippopotamus_amphibius_isolate_BS01_ScHSfme_924987HRSCAF_982787_whole_genome_shotgun_sequence
_R_PVJP02015523.1_22856-22989_Hippopotamus_amphibius_isolate_BS01_ScHSfme_15491HRSCAF_68054_whole_genome_shotgun_sequence
PVJP02000353.1_1852489-1852623_Hippopotamus_amphibius_isolate_BS01_ScHSfme_341HRSCAF_32654_whole_genome_shotgun_sequence
PVJP02000378.1_1153968-1154260_Hippopotamus_amphibius_isolate_BS01_ScHSfme_365HRSCAF_33268_whole_genome_shotgun_sequence
PVJP02910814.1_448383-448691_Hippopotamus_amphibius_isolate_BS01_ScHSfme_925258HRSCAF_983058_whole_genome_shotgun_sequence
_R_PVJP02008908.1_1191-1496_Hippopotamus_amphibius_isolate_BS01_ScHSfme_8879HRSCAF_57537_whole_genome_shotgun_sequence
PVJP02000176.1_1247171-1247472_Hippopotamus_amphibius_isolate_BS01_ScHSfme_165HRSCAF_24768_whole_genome_shotgun_sequence
PVJP02910360.1_509606-509914_Hippopotamus_amphibius_isolate_BS01_ScHSfme_924832HRSCAF_982632_whole_genome_shotgun_sequence
PVJP02023293.1_587582-587719_Hippopotamus_amphibius_isolate_BS01_ScHSfme_23256HRSCAF_78722_4_whole_genome_shotgun_sequence
PVJP02910360.1_411825-412132_Hippopotamus_amphibius_isolate_BS01_ScHSfme_924832HRSCAF_982632_whole_genome_shotgun_sequence
_R_PVJP02000017.1_8654700-8655005_Hippopotamus_amphibius_isolate_BS01_ScHSfme_16HRSCAF_4542_whole_genome_shotgun_sequence
PVJP02007377.1_245843-246145_Hippopotamus_amphibius_isolate_BS01_ScHSfme_7348HRSCAF_54759_whole_genome_shotgun_sequence
_R_VLPJ01000012.1_32272485-32273000_Bos_taurus_x_Bos_grunniens_isolate_Esperanza_chromosome_21_whole_genome_shotgun_sequence
_R_AAFC05030894.1_37775-38290_Bos_taurus_breed_Hereford_Contig16249_fixed_whole_genome_shotgun_sequence
_R_NKLS02000021.1_32564366-32564881_Bos_taurus_breed_Hereford_isolate_L1_Dominette_01449_registration_number_42190680_chromosome_21_whole_genome_shotgun_sequen
_R_PUFS02000021.1_32616860-32617375_Bos_indicus_x_Bos_taurus_breed_Angus_x_Brahman_F1_hybrid_chromosome_21_whole_genome_shotgun_sequence
DAAA02052476.1_28163-28678_TPA_asm:_Bos_taurus_breed_Hereford_chromosome_21_7180002019268_whole_genome_shotgun_sequence
_R_PRDE01000014.1_33089549-33090064_Bos_indicus_breed_Gir_chromosome_21_whole_genome_shotgun_sequence
_R_PUFT02000021.1_32023636-32024151_Bos_indicus_x_Bos_taurus_breed_Angus_x_Brahman_F1_hybrid_chromosome_21_whole_genome_shotgun_sequence
_R_JPYT01274592.1_5467-5978_Bison_bison_bison_isolate_TAMUID_2011002044_ctg566696_whole_genome_shotgun_sequence
CABVSG010000223.1_37194833-37195312_Syncerus_caffer_genome_assembly,_contig:_Contig225,_whole_genome_shotgun_sequence
VHQK01014842.1_1626295-1626885_Hippotragus_niger_niger_isolate_SB2152_Hip_nig_2_phX_scaffold15049_whole_genome_shotgun_sequence
SJXP01000204.1_975227-975815_Damaliscus_lunatus_isolate_CTB-792_scaffold218_size2012736_whole_genome_shotgun_sequence
_R_SJXO01000559.1_535966-536554_Connochaetes_taurinus_isolate_BWD-738_scaffold560_size1242833_whole_genome_shotgun_sequence
SJYP01000019.1_2078855-2079445_Ovis_ammon_isolate_AGL-py_scaffold61_whole_genome_shotgun_sequence
_R_PEKD01003821.1_1290786-1291376_Ovis_aries_strain_OAR_USU_Benz2616_contig_3821_whole_genome_shotgun_sequence
_R_NIWH01025190.1_29365506-29366096_Ovis_ammon_isolate_KGZ_Scaffold21_whole_genome_shotgun_sequence
CBYI010049724.1_78665-79255_Ovis_aries_musimon_WGS_project_CBYI000000000_data,_contig_049724,_whole_genome_shotgun_sequence
_R_PVIS01000413 7.1_31027-31617_Ovis_canadensis_isolate_BS48_OviCan_scaffold_8245_whole_genome_shotgun_sequence
_R_AMGL02005462.1_74003-74584_Ovis_aries_breed_Texel_Chromosome18_Contig436_whole_genome_shotgun_sequence
_R_SJYV01030848.1_651096-651608_Neotragus_pygmaeus_isolate_RAL-4837_tarseq_19806_whole_genome_shotgun_sequence
SJYF01000089.1_4508966-4509477_Procapra_przewalskii_isolate_PLG-pushi_scaffold176_len5177780_cov165_whole_genome_shotgun_sequence
_R_SJYX01017881.1_186232-186813_Oreotragus_oreotragus_isolate_KSP-560_tarseq_35567_whole_genome_shotgun_sequence
VBZB01000017.1_29286495-29287012_Bos_grunniens_isolate_yakHY00_chromosome_16_whole_genome_shotgun_sequence
VLPI01000009.1_26799759-26800279_Bos_taurus_x_Bos_grunniens_isolate_Esperanza_chromosome_17_whole_genome_shotgun_sequence
_R_VCEA01000001.1_256483982-256484439_Muntiacus_muntjak_isolate_UTSW_UCB_Mm_chromosome_1_whole_genome_shotgun_sequence
SJXT01009368.1_651135-651592_Muntiacus_reevesi_isolate_CIJ-jz_scaffold1903_whole_genome_shotgun_sequence
SJXT01009368.1_648978-649573_Muntiacus_reevesi_isolate_CIJ-jz_scaffold1903_whole_genome_shotgun_sequence
VLPJ01000339.1_12743261-12743745_Bos_taurus_x_Bos_grunniens_isolate_Esperanza_chromosome_5_whole_genome_shotgun_sequence
NKLS02000005.1_12970608-12971092_Bos_taurus_breed_Hereford_isolate_L1_Dominette_01449_registration_number_42190680_chromosome_5_whole_genome_shotgun_sequence
PUFT02000005.1_12949918-12950402_Bos_indicus_x_Bos_taurus_breed_Angus_x_Brahman_F1_hybrid_chromosome_5_whole_genome_shotgun_sequence
SJXR01000565.1_3217507-3217904_Przewalskium_albirostris_isolate_WLD-bcl_scaffold768_len4054413_cov117_whole_genome_shotgun_sequence
_R_MKHE01000020.1_11353711-11354108_Cervus_elaphus_hippelaphus_isolate_Hungarian_chromosome_20_whole_genome_shotgun_sequence
_R_JRFZ01086738.1_1008403-1008824_Elaphurus_davidianus_breed_Woburn_Abbey_isolate_Dafeng_scaffold410_whole_genome_shotgun_sequence
_R_QQTR01012195.1_21835809-21836145_Axis_porcinus_isolate_Zoohd001_scaffold_138_whole_genome_shotgun_sequence
_R_SSNK01008674.1_69897-70328_Alces_alces_isolate_MIG12-28694850_contig102_29_whole_genome_shotgun_sequence
SJYG01000977.1_666749-667247_Litocranius_walleri_isolate_GRK-570_scaffold966_size674091_whole_genome_shotgun_sequence
VCEA01000002.1_235509590-235509798_Muntiacus_muntjak_isolate_UTSW_UCB_Mm_chromosome_2_whole_genome_shotgun_sequence
VCEA01000002.1_444736531-444736947_Muntiacus_muntjak_isolate_UTSW_UCB_Mm_chromosome_2_whole_genome_shotgun_sequence
RAWU01000010.1_44857420-44857932_Giraffa_tippelskirchi_isolate_OR1865_chromosome_13_whole_genome_shotgun_sequence
_R_VCEA01000002.1_418343841-418344124_Muntiacus_muntjak_isolate_UTSW_UCB_Mm_chromosome_2_whole_genome_shotgun_sequence
VCEA01000001.1_639723864-639724200_Muntiacus_muntjak_isolate_UTSW_UCB_Mm_chromosome_1_whole_genome_shotgun_sequence
0.1

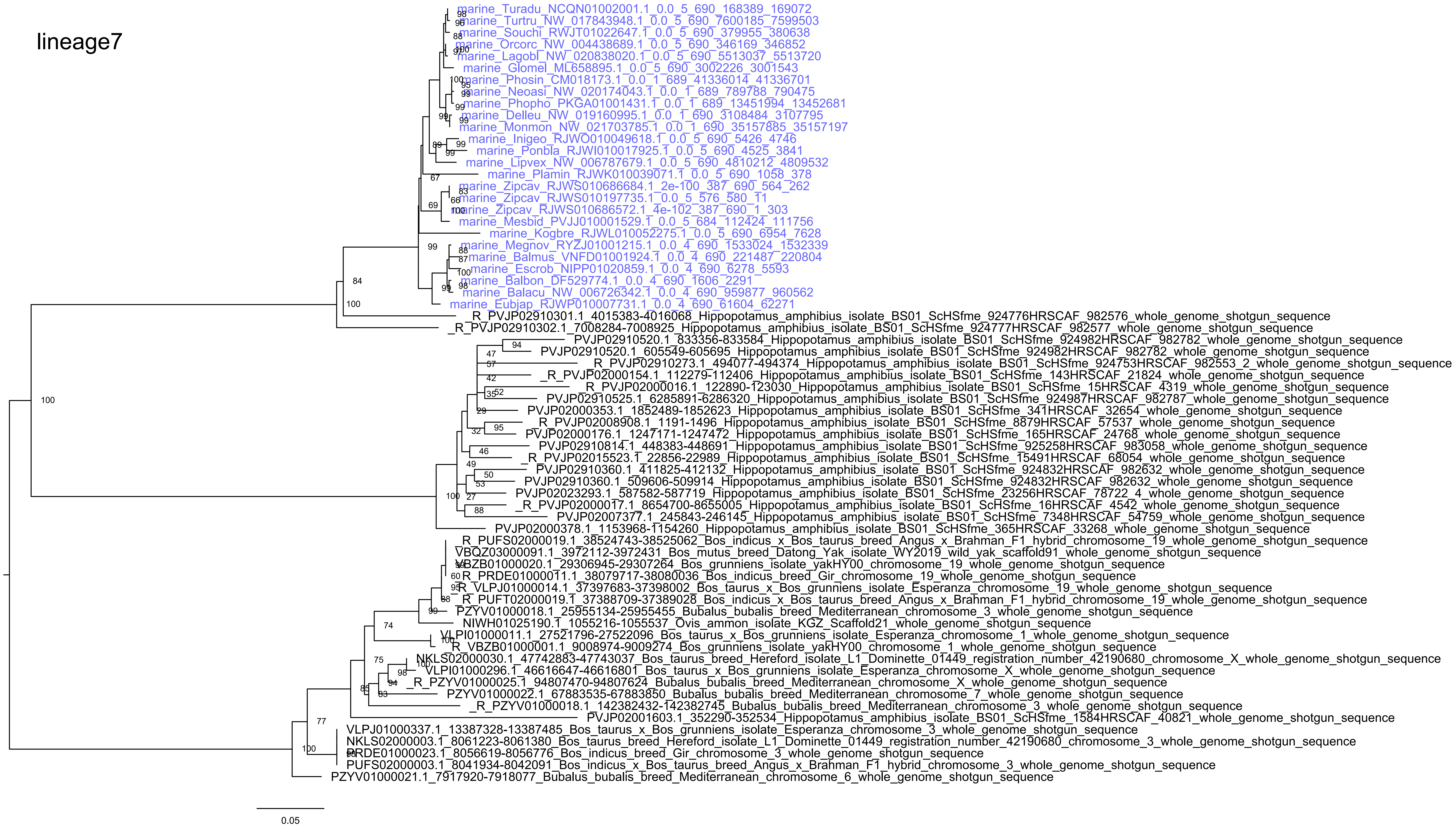
lineage7
marine_Turadu_NCQN01002001.1_0.0_5_690_168389_169072
marine_Turtru_NW_017843948.1_0.0_5_690_7600185_7599503
marine_Souchi_RWJT01022647.1_0.0_5_690_379955_380638
marine_Orcorc_NW_004438689.1_0.0_5_690_346169_346852
marine_Lagobl_NW_020838020.1_0.0_5_690_5513037_5513720
marine_Glomel_ML658895.1_0.0_5_690_3002226_3001543
marine_Phosin_CM018173.1_0.0_1_689_41336014_41336701
marine_Neoasi_NW_020174043.1_0.0_1_689_789788_790475
marine_Phopho_PKGA01001431.1_0.0_1_689_13451994_13452681
marine_Delleu_NW_019160995.1_0.0_1_690_3108484_3107795
marine_Monmon_NW_021703785.1_0.0_1_690_35157885_35157197
marine_Inigeo_RJWO010049618.1_0.0_5_690_5426_4746
marine_Ponbla_RJWI010017925.1_0.0_5_690_4525_3841
marine_Lipvex_NW_006787679.1_0.0_5_690_4810212_4809532
marine_Plamin_RJWK010039071.1_0.0_5_690_1058_378
marine_Zipcav_RJWS010686684.1_2e-100_387_690_564_262
marine_Zipcav_RJWS010197735.1_0.0_5_576_580_11
marine_Zipcav_RJWS010686572.1_4e-102_387_690_1_303
marine_Mesbid_PVJJ010001529.1_0.0_5_684_112424_111756
marine_Kogbre_RJWL010052275.1_0.0_5_690_6954_7628
marine_Megnov_RYZJ01001215.1_0.0_4_690_1533024_1532339
marine_Balmus_VNFD01001924.1_0.0_4_690_221487_220804
marine_Escrob_NIPP01020859.1_0.0_4_690_6278_5593
marine_Balbon_DF529774.1_0.0_4_690_1606_2291
marine_Balacu_NW_006726342.1_0.0_4_690_959877_960562
marine_Eubjap_RJWP010007731.1_0.0_4_690_61604_62271
_R_PVJP02910301.1_4015383-4016068_Hippopotamus_amphibius_isolate_BS01_ScHSfme_924776HRSCAF_982576_whole_genome_shotgun_sequence
_R_PVJP02910302.1_7008284-7008925_Hippopotamus_amphibius_isolate_BS01_ScHSfme_924777HRSCAF_982577_whole_genome_shotgun_sequence
PVJP02910520.1_833356-833584_Hippopotamus_amphibius_isolate_BS01_ScHSfme_924982HRSCAF_982782_whole_genome_shotgun_sequence
PVJP02910520.1_605549-605695_Hippopotamus_amphibius_isolate_BS01_ScHSfme_924982HRSCAF_982782_whole_genome_shotgun_sequence
_R_PVJP02910273.1_494077-494374_Hippopotamus_amphibius_isolate_BS01_ScHSfme_924753HRSCAF_982553_2_whole_genome_shotgun_sequence
_R_PVJP02000154.1_112279-112406_Hippopotamus_amphibius_isolate_BS01_ScHSfme_143HRSCAF_21824_whole_genome_shotgun_sequence
_R_PVJP02000016.1_122890-123030_Hippopotamus_amphibius_isolate_BS01_ScHSfme_15HRSCAF_4319_whole_genome_shotgun_sequence
PVJP02910525.1_6285891-6286320_Hippopotamus_amphibius_isolate_BS01_ScHSfme_924987HRSCAF_982787_whole_genome_shotgun_sequence
PVJP02000353.1_1852489-1852623_Hippopotamus_amphibius_isolate_BS01_ScHSfme_341HRSCAF_32654_whole_genome_shotgun_sequence
_R_PVJP02008908.1_1191-1496_Hippopotamus_amphibius_isolate_BS01_ScHSfme_8879HRSCAF_57537_whole_genome_shotgun_sequence
PVJP02000176.1_1247171-1247472_Hippopotamus_amphibius_isolate_BS01_ScHSfme_165HRSCAF_24768_whole_genome_shotgun_sequence
PVJP02910814.1_448383-448691_Hippopotamus_amphibius_isolate_BS01_ScHSfme_925258HRSCAF_983058_whole_genome_shotgun_sequence
_R_PVJP02015523.1_22856-22989_Hippopotamus_amphibius_isolate_BS01_ScHSfme_15491HRSCAF_68054_whole_genome_shotgun_sequence
PVJP02910360.1_411825-412132_Hippopotamus_amphibius_isolate_BS01_ScHSfme_924832HRSCAF_982632_whole_genome_shotgun_sequence
PVJP02910360.1_509606-509914_Hippopotamus_amphibius_isolate_BS01_ScHSfme_924832HRSCAF_982632_whole_genome_shotgun_sequence
PVJP02023293.1_587582-587719_Hippopotamus_amphibius_isolate_BS01_ScHSfme_23256HRSCAF_78722_4_whole_genome_shotgun_sequence
_R_PVJP02000017.1_8654700-8655005_Hippopotamus_amphibius_isolate_BS01_ScHSfme_16HRSCAF_4542_whole_genome_shotgun_sequence
PVJP02007377.1_245843-246145_Hippopotamus_amphibius_isolate_BS01_ScHSfme_7348HRSCAF_54759_whole_genome_shotgun_sequence
PVJP02000378.1_1153968-1154260_Hippopotamus_amphibius_isolate_BS01_ScHSfme_365HRSCAF_33268_whole_genome_shotgun_sequence
_R_PUFS02000019.1_38524743-38525062_Bos_indicus_x_Bos_taurus_breed_Angus_x_Brahman_F1_hybrid_chromosome_19_whole_genome_shotgun_sequence
VBQZ03000091.1_3972112-3972431_Bos_mutus_breed_Datong_Yak_isolate_WY2019_wild_yak_scaffold91_whole_genome_shotgun_sequence
VBZB01000020.1_29306945-29307264_Bos_grunniens_isolate_yakHY00_chromosome_19_whole_genome_shotgun_sequence
_R_PRDE01000011.1_38079717-38080036_Bos_indicus_breed_Gir_chromosome_19_whole_genome_shotgun_sequence
_R_VLPJ01000014.1_37397683-37398002_Bos_taurus_x_Bos_grunniens_isolate_Esperanza_chromosome_19_whole_genome_shotgun_sequence
_R_PUFT02000019.1_37388709-37389028_Bos_indicus_x_Bos_taurus_breed_Angus_x_Brahman_F1_hybrid_chromosome_19_whole_genome_shotgun_sequence
PZYV01000018.1_25955134-25955455_Bubalus_bubalis_breed_Mediterranean_chromosome_3_whole_genome_shotgun_sequence
NIWH01025190.1_1055216-1055537_Ovis_ammon_isolate_KGZ_Scaffold21_whole_genome_shotgun_sequence
VLPI01000011.1_27521796-27522096_Bos_taurus_x_Bos_grunniens_isolate_Esperanza_chromosome_1_whole_genome_shotgun_sequence
_R_VBZB01000001.1_9008974-9009274_Bos_grunniens_isolate_yakHY00_chromosome_1_whole_genome_shotgun_sequence
NKLS02000030.1_47742883-47743037_Bos_taurus_breed_Hereford_isolate_L1_Dominette_01449_registration_number_42190680_chromosome_X_whole_genome_shotgun_sequence
VLPI01000296.1_46616647-46616801_Bos_taurus_x_Bos_grunniens_isolate_Esperanza_chromosome_X_whole_genome_shotgun_sequence
_R_PZYV01000025.1_94807470-94807624_Bubalus_bubalis_breed_Mediterranean_chromosome_X_whole_genome_shotgun_sequence
PZYV01000022.1_67883535-67883850_Bubalus_bubalis_breed_Mediterranean_chromosome_7_whole_genome_shotgun_sequence
_R_PZYV01000018.1_142382432-142382745_Bubalus_bubalis_breed_Mediterranean_chromosome_3_whole_genome_shotgun_sequence
PVJP02001603.1_352290-352534_Hippopotamus_amphibius_isolate_BS01_ScHSfme_1584HRSCAF_40821_whole_genome_shotgun_sequence
VLPJ01000337.1_13387328-13387485_Bos_taurus_x_Bos_grunniens_isolate_Esperanza_chromosome_3_whole_genome_shotgun_sequence
NKLS02000003.1_8061223-8061380_Bos_taurus_breed_Hereford_isolate_L1_Dominette_01449_registration_number_42190680_chromosome_3_whole_genome_shotgun_sequence
PRDE01000023.1_8056619-8056776_Bos_indicus_breed_Gir_chromosome_3_whole_genome_shotgun_sequence
PUFS02000003.1_8041934-8042091_Bos_indicus_x_Bos_taurus_breed_Angus_x_Brahman_F1_hybrid_chromosome_3_whole_genome_shotgun_sequence
PZYV01000021.1_7917920-7918077_Bubalus_bubalis_breed_Mediterranean_chromosome_6_whole_genome_shotgun_sequence
0.05

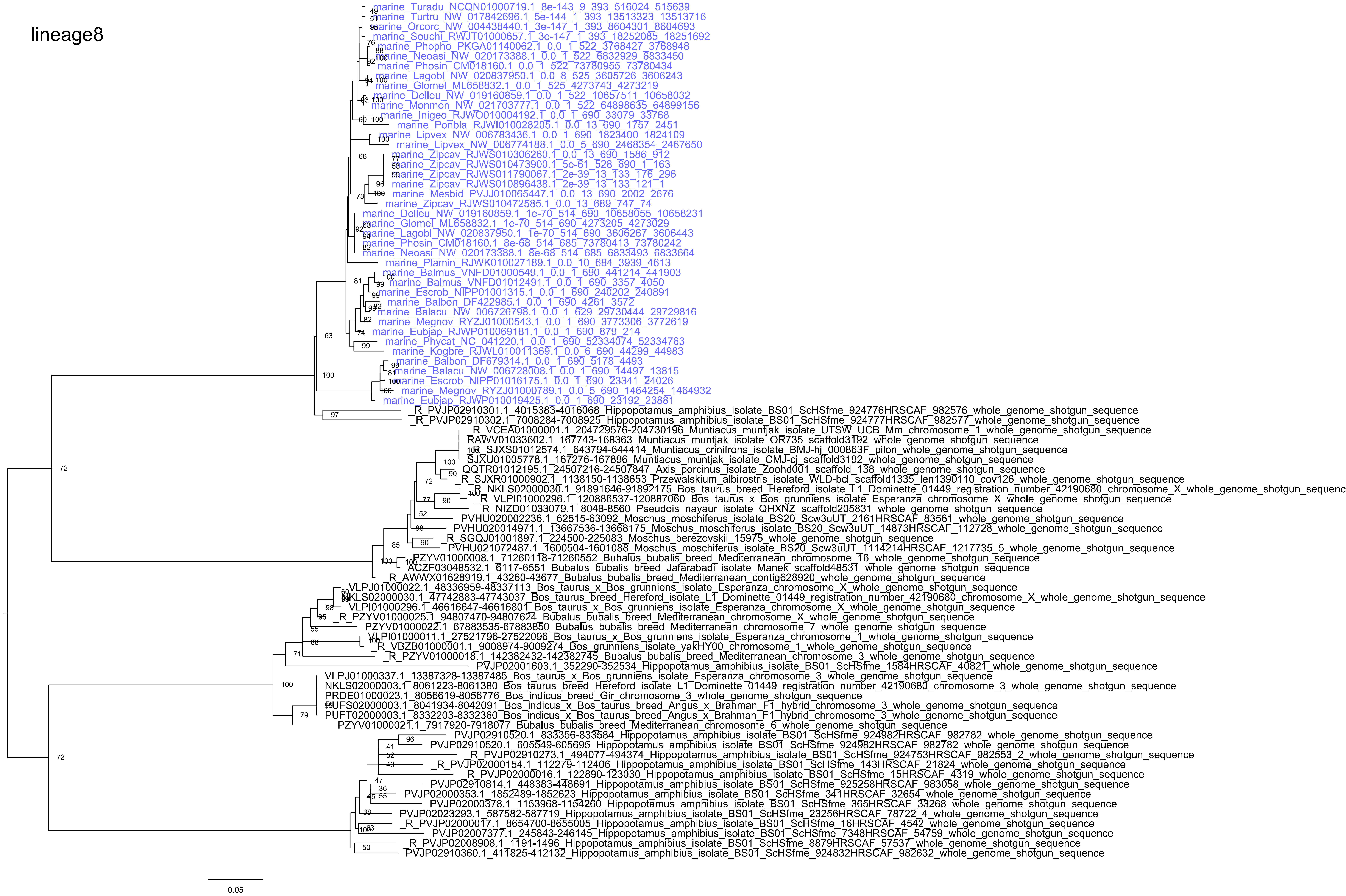
lineage8
marine_Turadu_NCQN01000719.1_8e-143_9_393_516024_515639
marine_Turtru_NW_017842696.1_5e-144_1_393_13513323_13513716
marine_Orcorc_NW_004438440.1_3e-147_1_393_8604301_8604693
marine_Souchi_RWJT01000657.1_3e-147_1_393_18252085_18251692
marine_Phopho_PKGA01140062.1_0.0_1_522_3768427_3768948
marine_Neoasi_NW_020173388.1_0.0_1_522_6832929_6833450
marine_Phosin_CM018160.1_0.0_1_522_73780955_73780434
marine_Lagobl_NW_020837950.1_0.0_8_525_3605726_3606243
marine_Glomel_ML658832.1_0.0_1_525_4273743_4273219
marine_Delleu_NW_019160859.1_0.0_1_522_10657511_10658032
marine_Monmon_NW_021703777.1_0.0_1_522_64898635_64899156
marine_Inigeo_RJWO010004192.1_0.0_1_690_33079_33768
marine_Ponbla_RJWI010028205.1_0.0_13_690_1757_2451
marine_Lipvex_NW_006783436.1_0.0_1_690_1823400_1824109
marine_Lipvex_NW_006774188.1_0.0_5_690_2468354_2467650
marine_Zipcav_RJWS010306260.1_0.0_13_690_1586_912
marine_Zipcav_RJWS010473900.1_5e-61_528_690_1_163
marine_Zipcav_RJWS011790067.1_2e-39_13_133_176_296
marine_Zipcav_RJWS010896438.1_2e-39_13_133_121_1
marine_Mesbid_PVJJ010065447.1_0.0_13_690_2002_2676
marine_Zipcav_RJWS010472585.1_0.0_13_689_747_74
marine_Delleu_NW_019160859.1_1e-70_514_690_10658055_10658231
marine_Glomel_ML658832.1_1e-70_514_690_4273205_4273029
marine_Lagobl_NW_020837950.1_1e-70_514_690_3606267_3606443
marine_Phosin_CM018160.1_8e-68_514_685_73780413_73780242
marine_Neoasi_NW_020173388.1_8e-68_514_685_6833493_6833664
marine_Plamin_RJWK010027189.1_0.0_10_684_3939_4613
marine_Balmus_VNFD01000549.1_0.0_1_690_441214_441903
marine_Balmus_VNFD01012491.1_0.0_1_690_3357_4050
marine_Escrob_NIPP01001315.1_0.0_1_690_240202_240891
marine_Balbon_DF422985.1_0.0_1_690_4261_3572
marine_Balacu_NW_006726798.1_0.0_1_629_29730444_29729816
marine_Megnov_RYZJ01000543.1_0.0_1_690_3773306_3772619
marine_Eubjap_RJWP010069181.1_0.0_1_690_879_214
marine_Phycat_NC_041220.1_0.0_1_690_52334074_52334763
marine_Kogbre_RJWL010011369.1_0.0_6_690_44299_44983
marine_Balbon_DF679314.1_0.0_1_690_5178_4493
marine_Balacu_NW_006728008.1_0.0_1_690_14497_13815
marine_Escrob_NIPP01016175.1_0.0_1_690_23341_24026
marine_Megnov_RYZJ01000789.1_0.0_5_690_1464254_1464932
marine_Eubjap_RJWP010019425.1_0.0_1_690_23192_23881
_R_PVJP02910301.1_4015383-4016068_Hippopotamus_amphibius_isolate_BS01_ScHSfme_924776HRSCAF_982576_whole_genome_shotgun_sequence
_R_PVJP02910302.1_7008284-7008925_Hippopotamus_amphibius_isolate_BS01_ScHSfme_924777HRSCAF_982577_whole_genome_shotgun_sequence
_R_VCEA01000001.1_204729576-204730196_Muntiacus_muntjak_isolate_UTSW_UCB_Mm_chromosome_1_whole_genome_shotgun_sequence
RAWV01033602.1_167743-168363_Muntiacus_muntjak_isolate_OR735_scaffold3192_whole_genome_shotgun_sequence
_R_SJXS01012574.1_643794-644414_Muntiacus_crinifrons_isolate_BMJ-hj_000863F_pilon_whole_genome_shotgun_sequence
SJXU01005778.1_167276-167896_Muntiacus_muntjak_isolate_CMJ-cj_scaffold3192_whole_genome_shotgun_sequence
QQTR01012195.1_24507216-24507847_Axis_porcinus_isolate_Zoohd001_scaffold_138_whole_genome_shotgun_sequence
_R_SJXR01000902.1_1138150-1138653_Przewalskium_albirostris_isolate_WLD-bcl_scaffold1335_len1390110_cov126_whole_genome_shotgun_sequence
_R_NKLS02000030.1_91891646-91892175_Bos_taurus_breed_Hereford_isolate_L1_Dominette_01449_registration_number_42190680_chromosome_X_whole_genome_shotgun_sequenc
_R_VLPI01000296.1_120886537-120887060_Bos_taurus_x_Bos_grunniens_isolate_Esperanza_chromosome_X_whole_genome_shotgun_sequence
_R_NIZD01033079.1_8048-8560_Pseudois_nayaur_isolate_QHXNZ_scaffold205831_whole_genome_shotgun_sequence
PVHU020002236.1_62515-63092_Moschus_moschiferus_isolate_BS20_Scw3uUT_2161HRSCAF_83561_whole_genome_shotgun_sequence
PVHU020014971.1_13667536-13668175_Moschus_moschiferus_isolate_BS20_Scw3uUT_14873HRSCAF_112728_whole_genome_shotgun_sequence
_R_SGQJ01001897.1_224500-225083_Moschus_berezovskii_15975_whole_genome_shotgun_sequence
PVHU021072487.1_1600504-1601088_Moschus_moschiferus_isolate_BS20_Scw3uUT_1114214HRSCAF_1217735_5_whole_genome_shotgun_sequence
PZYV01000008.1_71260118-71260552_Bubalus_bubalis_breed_Mediterranean_chromosome_16_whole_genome_shotgun_sequence
ACZF03048532.1_6117-6551_Bubalus_bubalis_breed_Jafarabadi_isolate_Manek_scaffold48531_whole_genome_shotgun_sequence
_R_AWWX01628919.1_43260-43677_Bubalus_bubalis_breed_Mediterranean_contig628920_whole_genome_shotgun_sequence
VLPJ01000022.1_48336959-48337113_Bos_taurus_x_Bos_grunniens_isolate_Esperanza_chromosome_X_whole_genome_shotgun_sequence
NKLS02000030.1_47742883-47743037_Bos_taurus_breed_Hereford_isolate_L1_Dominette_01449_registration_number_42190680_chromosome_X_whole_genome_shotgun_sequence
VLPI01000296.1_46616647-46616801_Bos_taurus_x_Bos_grunniens_isolate_Esperanza_chromosome_X_whole_genome_shotgun_sequence
_R_PZYV01000025.1_94807470-94807624_Bubalus_bubalis_breed_Mediterranean_chromosome_X_whole_genome_shotgun_sequence
PZYV01000022.1_67883535-67883850_Bubalus_bubalis_breed_Mediterranean_chromosome_7_whole_genome_shotgun_sequence
VLPI01000011.1_27521796-27522096_Bos_taurus_x_Bos_grunniens_isolate_Esperanza_chromosome_1_whole_genome_shotgun_sequence
_R_VBZB01000001.1_9008974-9009274_Bos_grunniens_isolate_yakHY00_chromosome_1_whole_genome_shotgun_sequence
_R_PZYV01000018.1_142382432-142382745_Bubalus_bubalis_breed_Mediterranean_chromosome_3_whole_genome_shotgun_sequence
PVJP02001603.1_352290-352534_Hippopotamus_amphibius_isolate_BS01_ScHSfme_1584HRSCAF_40821_whole_genome_shotgun_sequence
VLPJ01000337.1_13387328-13387485_Bos_taurus_x_Bos_grunniens_isolate_Esperanza_chromosome_3_whole_genome_shotgun_sequence
NKLS02000003.1_8061223-8061380_Bos_taurus_breed_Hereford_isolate_L1_Dominette_01449_registration_number_42190680_chromosome_3_whole_genome_shotgun_sequence
PRDE01000023.1_8056619-8056776_Bos_indicus_breed_Gir_chromosome_3_whole_genome_shotgun_sequence
PUFS02000003.1_8041934-8042091_Bos_indicus_x_Bos_taurus_breed_Angus_x_Brahman_F1_hybrid_chromosome_3_whole_genome_shotgun_sequence
PUFT02000003.1_8332203-8332360_Bos_indicus_x_Bos_taurus_breed_Angus_x_Brahman_F1_hybrid_chromosome_3_whole_genome_shotgun_sequence
PZYV01000021.1_7917920-7918077_Bubalus_bubalis_breed_Mediterranean_chromosome_6_whole_genome_shotgun_sequence
PVJP02910520.1_833356-833584_Hippopotamus_amphibius_isolate_BS01_ScHSfme_924982HRSCAF_982782_whole_genome_shotgun_sequence
PVJP02910520.1_605549-605695_Hippopotamus_amphibius_isolate_BS01_ScHSfme_924982HRSCAF_982782_whole_genome_shotgun_sequence
_R_PVJP02910273.1_494077-494374_Hippopotamus_amphibius_isolate_BS01_ScHSfme_924753HRSCAF_982553_2_whole_genome_shotgun_sequence
_R_PVJP02000154.1_112279-112406_Hippopotamus_amphibius_isolate_BS01_ScHSfme_143HRSCAF_21824_whole_genome_shotgun_sequence
_R_PVJP02000016.1_122890-123030_Hippopotamus_amphibius_isolate_BS01_ScHSfme_15HRSCAF_4319_whole_genome_shotgun_sequence
PVJP02910814.1_448383-448691_Hippopotamus_amphibius_isolate_BS01_ScHSfme_925258HRSCAF_983058_whole_genome_shotgun_sequence
PVJP02000353.1_1852489-1852623_Hippopotamus_amphibius_isolate_BS01_ScHSfme_341HRSCAF_32654_whole_genome_shotgun_sequence
PVJP02000378.1_1153968-1154260_Hippopotamus_amphibius_isolate_BS01_ScHSfme_365HRSCAF_33268_whole_genome_shotgun_sequence
PVJP02023293.1_587582-587719_Hippopotamus_amphibius_isolate_BS01_ScHSfme_23256HRSCAF_78722_4_whole_genome_shotgun_sequence
_R_PVJP02000017.1_8654700-8655005_Hippopotamus_amphibius_isolate_BS01_ScHSfme_16HRSCAF_4542_whole_genome_shotgun_sequence
PVJP02007377.1_245843-246145_Hippopotamus_amphibius_isolate_BS01_ScHSfme_7348HRSCAF_54759_whole_genome_shotgun_sequence
_R_PVJP02008908.1_1191-1496_Hippopotamus_amphibius_isolate_BS01_ScHSfme_8879HRSCAF_57537_whole_genome_shotgun_sequence
PVJP02910360.1_411825-412132_Hippopotamus_amphibius_isolate_BS01_ScHSfme_924832HRSCAF_982632_whole_genome_shotgun_sequence
0.05

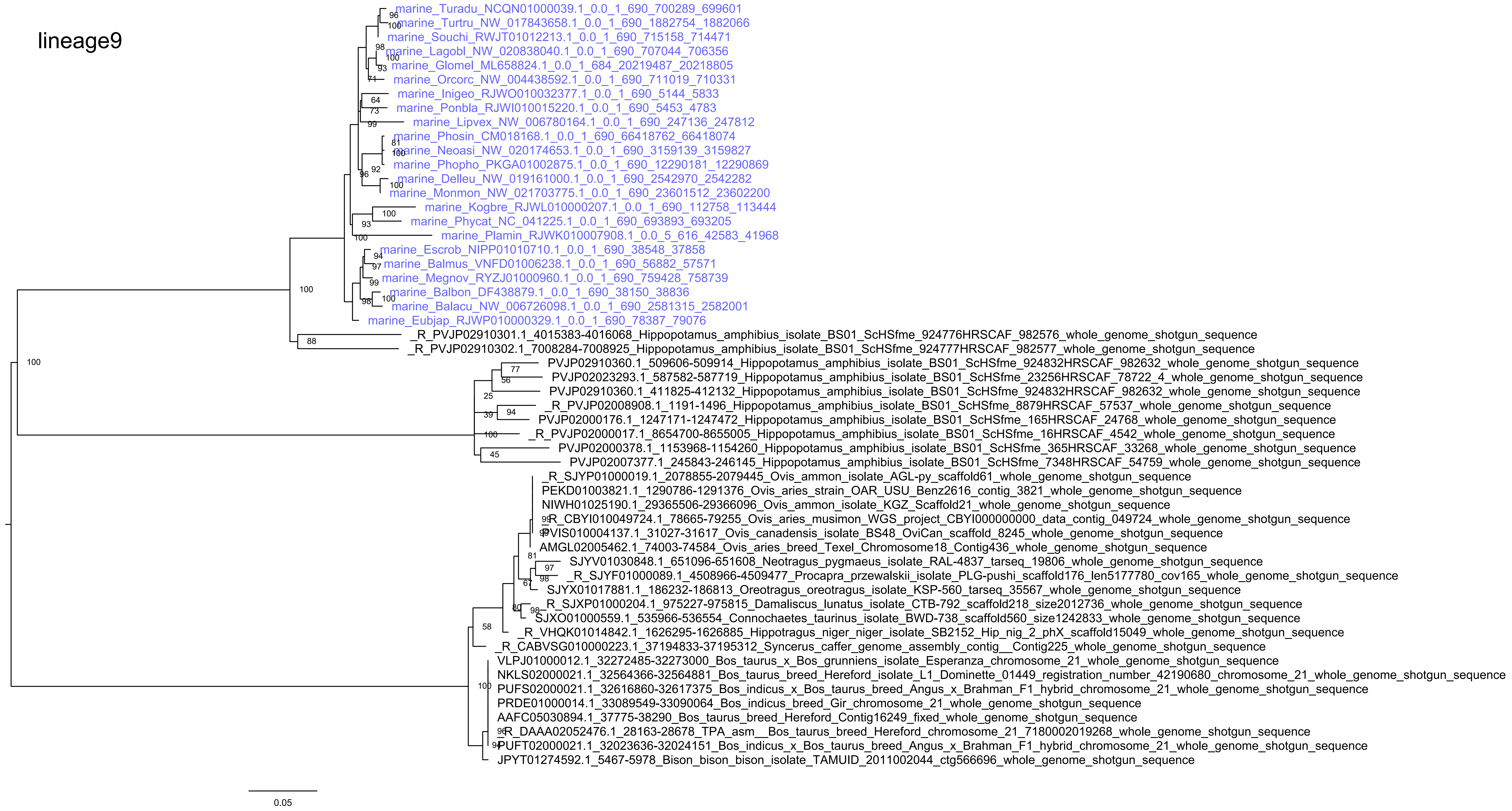

lineage9
marine_Turadu_NCQN01000039.1_0.0_1_690_700289_699601
marine_Turtru_NW_017843658.1_0.0_1_690_1882754_1882066
marine_Souchi_RWJT01012213.1_0.0_1_690_715158_714471
marine_Lagobl_NW_020838040.1_0.0_1_690_707044_706356
marine_Glomel_ML658824.1_0.0_1_684_20219487_20218805
marine_Orcorc_NW_004438592.1_0.0_1_690_711019_710331
marine_Inigeo_RJWO010032377.1_0.0_1_690_5144_5833
marine_Ponbla_RJWI010015220.1_0.0_1_690_5453_4783
marine_Lipvex_NW_006780164.1_0.0_1_690_247136_247812
marine_Phosin_CM018168.1_0.0_1_690_66418762_66418074
marine_Neoasi_NW_020174653.1_0.0_1_690_3159139_3159827
marine_Phopho_PKGA01002875.1_0.0_1_690_12290181_12290869
marine_Delleu_NW_019161000.1_0.0_1_690_2542970_2542282
marine_Monmon_NW_021703775.1_0.0_1_690_23601512_23602200
marine_Kogbre_RJWL010000207.1_0.0_1_690_112758_113444
marine_Phycat_NC_041225.1_0.0_1_690_693893_693205
marine_Plamin_RJWK010007908.1_0.0_5_616_42583_41968
marine_Escrob_NIPP01010710.1_0.0_1_690_38548_37858
marine_Balmus_VNFD01006238.1_0.0_1_690_56882_57571
marine_Megnov_RYZJ01000960.1_0.0_1_690_759428_758739
marine_Balbon_DF438879.1_0.0_1_690_38150_38836
marine_Balacu_NW_006726098.1_0.0_1_690_2581315_2582001
marine_Eubjap_RJWP010000329.1_0.0_1_690_78387_79076
_R_PVJP02910301.1_4015383-4016068_Hippopotamus_amphibius_isolate_BS01_ScHSfme_924776HRSCAF_982576_whole_genome_shotgun_sequence
_R_PVJP02910302.1_7008284-7008925_Hippopotamus_amphibius_isolate_BS01_ScHSfme_924777HRSCAF_982577_whole_genome_shotgun_sequence
PVJP02910360.1_509606-509914_Hippopotamus_amphibius_isolate_BS01_ScHSfme_924832HRSCAF_982632_whole_genome_shotgun_sequence
PVJP02023293.1_587582-587719_Hippopotamus_amphibius_isolate_BS01_ScHSfme_23256HRSCAF_78722_4_whole_genome_shotgun_sequence
PVJP02910360.1_411825-412132_Hippopotamus_amphibius_isolate_BS01_ScHSfme_924832HRSCAF_982632_whole_genome_shotgun_sequence
_R_PVJP02008908.1_1191-1496_Hippopotamus_amphibius_isolate_BS01_ScHSfme_8879HRSCAF_57537_whole_genome_shotgun_sequence
PVJP02000176.1_1247171-1247472_Hippopotamus_amphibius_isolate_BS01_ScHSfme_165HRSCAF_24768_whole_genome_shotgun_sequence
_R_PVJP02000017.1_8654700-8655005_Hippopotamus_amphibius_isolate_BS01_ScHSfme_16HRSCAF_4542_whole_genome_shotgun_sequence
PVJP02000378.1_1153968-1154260_Hippopotamus_amphibius_isolate_BS01_ScHSfme_365HRSCAF_33268_whole_genome_shotgun_sequence
PVJP02007377.1_245843-246145_Hippopotamus_amphibius_isolate_BS01_ScHSfme_7348HRSCAF_54759_whole_genome_shotgun_sequence
_R_SJYP01000019.1_2078855-2079445_Ovis_ammon_isolate_AGL-py_scaffold61_whole_genome_shotgun_sequence
PEKD01003821.1_1290786-1291376_Ovis_aries_strain_OAR_USU_Benz2616_contig_3821_whole_genome_shotgun_sequence
NIWH01025190.1_29365506-29366096_Ovis_ammon_isolate_KGZ_Scaffold21_whole_genome_shotgun_sequence
_R_CBYI010049724.1_78665-79255_Ovis_aries_musimon_WGS_project_CBYI000000000_data_contig_049724_whole_genome_shotgun_sequence
PVIS010004137.1_31027-31617_Ovis_canadensis_isolate_BS48_OviCan_scaffold_8245_whole_genome_shotgun_sequence
AMGL02005462.1_74003-74584_Ovis_aries_breed_Texel_Chromosome18_Contig436_whole_genome_shotgun_sequence
SJYV01030848.1_651096-651608_Neotragus_pygmaeus_isolate_RAL-4837_tarseq_19806_whole_genome_shotgun_sequence
_R_SJYF01000089.1_4508966-4509477_Procapra_przewalskii_isolate_PLG-pushi_scaffold176_len5177780_cov165_whole_genome_shotgun_sequence
SJYX01017881.1_186232-186813_Oreotragus_oreotragus_isolate_KSP-560_tarseq_35567_whole_genome_shotgun_sequence
_R_SJXP01000204.1_975227-975815_Damaliscus_lunatus_isolate_CTB-792_scaffold218_size2012736_whole_genome_shotgun_sequence
SJXO01000559.1_535966-536554_Connochaetes_taurinus_isolate_BWD-738_scaffold560_size1242833_whole_genome_shotgun_sequence
_R_VHQK01014842.1_1626295-1626885_Hippotragus_niger_niger_isolate_SB2152_Hip_nig_2_phX_scaffold15049_whole_genome_shotgun_sequence
_R_CABVSG010000223.1_37194833-37195312_Syncerus_caffer_genome_assembly_contig__Contig225_whole_genome_shotgun_sequence
VLPJ01000012.1_32272485-32273000_Bos_taurus_x_Bos_grunniens_isolate_Esperanza_chromosome_21_whole_genome_shotgun_sequence
NKLS02000021.1_32564366-32564881_Bos_taurus_breed_Hereford_isolate_L1_Dominette_01449_registration_number_42190680_chromosome_21_whole_genome_shotgun_sequence
PUFS02000021.1_32616860-32617375_Bos_indicus_x_Bos_taurus_breed_Angus_x_Brahman_F1_hybrid_chromosome_21_whole_genome_shotgun_sequence
PRDE01000014.1_33089549-33090064_Bos_indicus_breed_Gir_chromosome_21_whole_genome_shotgun_sequence
AAFC05030894.1_37775-38290_Bos_taurus_breed_Hereford_Contig16249_fixed_whole_genome_shotgun_sequence
_R_DAAA02052476.1_28163-28678_TPA_asm__Bos_taurus_breed_Hereford_chromosome_21_7180002019268_whole_genome_shotgun_sequence
PUFT02000021.1_32023636-32024151_Bos_indicus_x_Bos_taurus_breed_Angus_x_Brahman_F1_hybrid_chromosome_21_whole_genome_shotgun_sequence
JPYT01274592.1_5467-5978_Bison_bison_bison_isolate_TAMUID_2011002044_ctg566696_whole_genome_shotgun_sequence
0.05

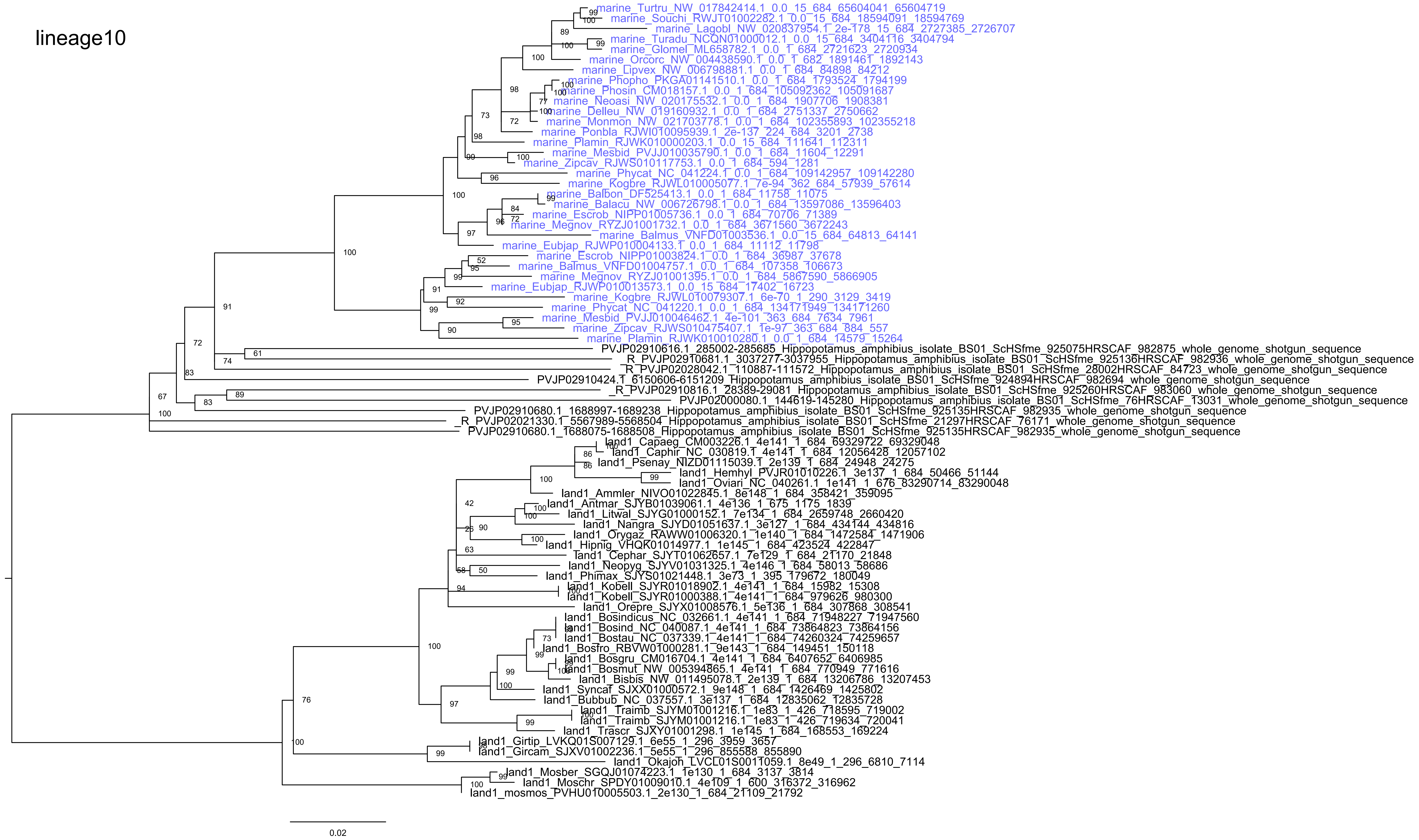
lineage10
marine_Turtru_NW_017842414.1_0.0_15_684_65604041_65604719
marine_Souchi_RWJT01002282.1_0.0_15_684_18594091_18594769
marine_Lagobl_NW_020837954.1_2e-178_15_684_2727385_2726707
marine_Turadu_NCQN01000012.1_0.0_15_684_3404116_3404794
marine_Glomel_ML658782.1_0.0_1_684_2721623_2720934
marine_Orcorc_NW_004438590.1_0.0_1_682_1891461_1892143
marine_Lipvex_NW_006798881.1_0.0_1_684_84898_84212
marine_Phopho_PKGA01141510.1_0.0_1_684_1793524_1794199
marine_Phosin_CM018157.1_0.0_1_684_105092362_105091687
marine_Neoasi_NW_020175532.1_0.0_1_684_1907706_1908381
marine_Delleu_NW_019160932.1_0.0_1_684_2751337_2750662
marine_Monmon_NW_021703778.1_0.0_1_684_102355893_102355218
marine_Ponbla_RJWI010095939.1_2e-137_224_684_3201_2738
marine_Plamin_RJWK010000203.1_0.0_15_684_111641_112311
marine_Mesbid_PVJJ010035790.1_0.0_1_684_11604_12291
marine_Zipcav_RJWS010117753.1_0.0_1_684_594_1281
marine_Phycat_NC_041224.1_0.0_1_684_109142957_109142280
marine_Kogbre_RJWL010005077.1_7e-94_362_684_57939_57614
marine_Balbon_DF525413.1_0.0_1_684_11758_11075
marine_Balacu_NW_006726798.1_0.0_1_684_13597086_13596403
marine_Escrob_NIPP01005736.1_0.0_1_684_70706_71389
marine_Megnov_RYZJ01001732.1_0.0_1_684_3671560_3672243
marine_Balmus_VNFD01003536.1_0.0_15_684_64813_64141
marine_Eubjap_RJWP010004133.1_0.0_1_684_11112_11798
marine_Escrob_NIPP01003824.1_0.0_1_684_36987_37678
marine_Balmus_VNFD01004757.1_0.0_1_684_107358_106673
marine_Megnov_RYZJ01001395.1_0.0_1_684_5867590_5866905
marine_Eubjap_RJWP010013573.1_0.0_15_684_17402_16723
marine_Kogbre_RJWL010079307.1_6e-70_1_290_3129_3419
marine_Phycat_NC_041220.1_0.0_1_684_134171949_134171260
marine_Mesbid_PVJJ010046462.1_4e-101_363_684_7634_7961
marine_Zipcav_RJWS010475407.1_1e-97_363_684_884_557
marine_Plamin_RJWK010010280.1_0.0_1_684_14579_15264
PVJP02910616.1_285002-285685_Hippopotamus_amphibius_isolate_BS01_ScHSfme_925075HRSCAF_982875_whole_genome_shotgun_sequence
_R_PVJP02910681.1_3037277-3037955_Hippopotamus_amphibius_isolate_BS01_ScHSfme_925136HRSCAF_982936_whole_genome_shotgun_sequence
_R_PVJP02028042.1_110887-111572_Hippopotamus_amphibius_isolate_BS01_ScHSfme_28002HRSCAF_84723_whole_genome_shotgun_sequence
PVJP02910424.1_6150606-6151209_Hippopotamus_amphibius_isolate_BS01_ScHSfme_924894HRSCAF_982694_whole_genome_shotgun_sequence
_R_PVJP02910816.1_28389-29081_Hippopotamus_amphibius_isolate_BS01_ScHSfme_925260HRSCAF_983060_whole_genome_shotgun_sequence
PVJP02000080.1_144619-145280_Hippopotamus_amphibius_isolate_BS01_ScHSfme_76HRSCAF_13031_whole_genome_shotgun_sequence
PVJP02910680.1_1688997-1689238_Hippopotamus_amphibius_isolate_BS01_ScHSfme_925135HRSCAF_982935_whole_genome_shotgun_sequence
_R_PVJP02021330.1_5567989-5568504_Hippopotamus_amphibius_isolate_BS01_ScHSfme_21297HRSCAF_76171_whole_genome_shotgun_sequence
PVJP02910680.1_1688075-1688508_Hippopotamus_amphibius_isolate_BS01_ScHSfme_925135HRSCAF_982935_whole_genome_shotgun_sequence
land1_Capaeg_CM003226.1_4e141_1_684_69329722_69329048
land1_Caphir_NC_030819.1_4e141_1_684_12056428_12057102
land1_Psenay_NIZD01115039.1_2e139_1_684_24948_24275
land1_Hemhyl_PVJR01010226.1_3e137_1_684_50466_51144
land1_Oviari_NC_040261.1_1e141_1_676_83290714_83290048
land1_Ammler_NIVO01022845.1_8e148_1_684_358421_359095
land1_Antmar_SJYB01039061.1_4e136_1_675_1175_1839
land1_Litwal_SJYG01000152.1_7e134_1_684_2659748_2660420
land1_Nangra_SJYD01051637.1_3e127_1_684_434144_434816
land1_Orygaz_RAWW01006320.1_1e140_1_684_1472584_1471906
land1_Hipnig_VHQK01014977.1_1e145_1_684_423524_422847
land1_Cephar_SJYT01062657.1_7e129_1_684_21170_21848
land1_Neopyg_SJYV01031325.1_4e146_1_684_58013_58686
land1_Phimax_SJYS01021448.1_3e73_1_395_179672_180049
land1_Kobell_SJYR01018902.1_4e141_1_684_15982_15308
land1_Kobell_SJYR01000388.1_4e141_1_684_979626_980300
land1_Orepre_SJYX01008576.1_5e136_1_684_307868_308541
land1_Bosindicus_NC_032661.1_4e141_1_684_71948227_71947560
land1_Bosind_NC_040087.1_4e141_1_684_73864823_73864156
land1_Bostau_NC_037339.1_4e141_1_684_74260324_74259657
land1_Bosfro_RBVW01000281.1_9e143_1_684_149451_150118
land1_Bosgru_CM016704.1_4e141_1_684_6407652_6406985
land1_Bosmut_NW_005394865.1_4e141_1_684_770949_771616
land1_Bisbis_NW_011495078.1_2e139_1_684_13206786_13207453
land1_Syncaf_SJXX01000572.1_9e148_1_684_1426469_1425802
land1_Bubbub_NC_037557.1_3e137_1_684_12835062_12835728
land1_Traimb_SJYM01001216.1_1e83_1_426_718595_719002
land1_Traimb_SJYM01001216.1_1e83_1_426_719634_720041
land1_Trascr_SJXY01001298.1_1e145_1_684_168553_169224
land1_Girtip_LVKQ01S007129.1_6e55_1_296_3959_3657
land1_Gircam_SJXV01002236.1_5e55_1_296_855588_855890
land1_Okajoh_LVCL01S0011059.1_8e49_1_296_6810_7114
land1_Mosber_SGQJ01074223.1_1e130_1_684_3137_3814
land1_Moschr_SPDY01009010.1_4e109_1_600_316372_316962
land1_mosmos_PVHU010005503.1_2e130_1_684_21109_21792
0.02

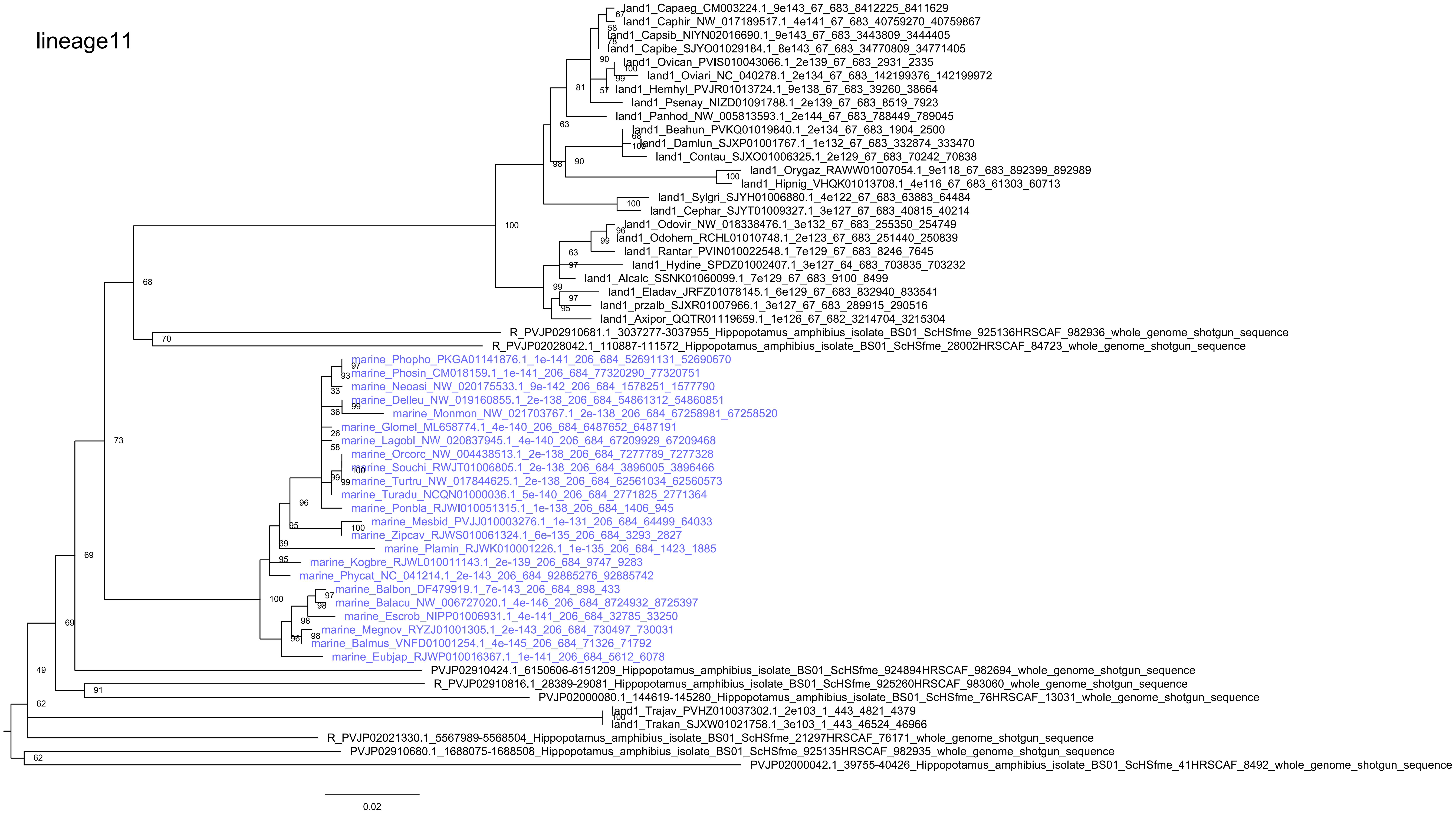
lineage11
land1_Capaeg_CM003224.1_9e143_67_683_8412225_8411629
land1_Caphir_NW_017189517.1_4e141_67_683_40759270_40759867
land1_Capsib_NIYN02016690.1_9e143_67_683_3443809_3444405
land1_Capibe_SJYO01029184.1_8e143_67_683_34770809_34771405
land1_Ovican_PVIS010043066.1_2e139_67_683_2931_2335
land1_Oviari_NC_040278.1_2e134_67_683_142199376_142199972
land1_Hemhyl_PVJR01013724.1_9e138_67_683_39260_38664
land1_Psenay_NIZD01091788.1_2e139_67_683_8519_7923
land1_Panhod_NW_005813593.1_2e144_67_683_788449_789045
land1_Beahun_PVKQ01019840.1_2e134_67_683_1904_2500
land1_Damlun_SJXP01001767.1_1e132_67_683_332874_333470
land1_Contau_SJXO01006325.1_2e129_67_683_70242_70838
land1_Orygaz_RAWW01007054.1_9e118_67_683_892399_892989
land1_Hipnig_VHQK01013708.1_4e116_67_683_61303_60713
land1_Sylgri_SJYH01006880.1_4e122_67_683_63883_64484
land1_Cephar_SJYT01009327.1_3e127_67_683_40815_40214
land1_Odovir_NW_018338476.1_3e132_67_683_255350_254749
land1_Odohem_RCHL01010748.1_2e123_67_683_251440_250839
land1_Rantar_PVIN010022548.1_7e129_67_683_8246_7645
land1_Hydine_SPDZ01002407.1_3e127_64_683_703835_703232
land1_Alcalc_SSNK01060099.1_7e129_67_683_9100_8499
land1_Eladav_JRFZ01078145.1_6e129_67_683_832940_833541
land1_przalb_SJXR01007966.1_3e127_67_683_289915_290516
land1_Axipor_QQTR01119659.1_1e126_67_682_3214704_3215304
R_PVJP02910681.1_3037277-3037955_Hippopotamus_amphibius_isolate_BS01_ScHSfme_925136HRSCAF_982936_whole_genome_shotgun_sequence
R_PVJP02028042.1_110887-111572_Hippopotamus_amphibius_isolate_BS01_ScHSfme_28002HRSCAF_84723_whole_genome_shotgun_sequence
marine_Phopho_PKGA01141876.1_1e-141_206_684_52691131_52690670
marine_Phosin_CM018159.1_1e-141_206_684_77320290_77320751
marine_Neoasi_NW_020175533.1_9e-142_206_684_1578251_1577790
marine_Delleu_NW_019160855.1_2e-138_206_684_54861312_54860851
marine_Monmon_NW_021703767.1_2e-138_206_684_67258981_67258520
marine_Glomel_ML658774.1_4e-140_206_684_6487652_6487191
marine_Lagobl_NW_020837945.1_4e-140_206_684_67209929_67209468
marine_Orcorc_NW_004438513.1_2e-138_206_684_7277789_7277328
marine_Souchi_RWJT01006805.1_2e-138_206_684_3896005_3896466
marine_Turtru_NW_017844625.1_2e-138_206_684_62561034_62560573
marine_Turadu_NCQN01000036.1_5e-140_206_684_2771825_2771364
marine_Ponbla_RJWI010051315.1_1e-138_206_684_1406_945
marine_Mesbid_PVJJ010003276.1_1e-131_206_684_64499_64033
marine_Zipcav_RJWS010061324.1_6e-135_206_684_3293_2827
marine_Plamin_RJWK010001226.1_1e-135_206_684_1423_1885
marine_Kogbre_RJWL010011143.1_2e-139_206_684_9747_9283
marine_Phycat_NC_041214.1_2e-143_206_684_92885276_92885742
marine_Balbon_DF479919.1_7e-143_206_684_898_433
marine_Balacu_NW_006727020.1_4e-146_206_684_8724932_8725397
marine_Escrob_NIPP01006931.1_4e-141_206_684_32785_33250
marine_Megnov_RYZJ01001305.1_2e-143_206_684_730497_730031
marine_Balmus_VNFD01001254.1_4e-145_206_684_71326_71792
marine_Eubjap_RJWP010016367.1_1e-141_206_684_5612_6078
PVJP02910424.1_6150606-6151209_Hippopotamus_amphibius_isolate_BS01_ScHSfme_924894HRSCAF_982694_whole_genome_shotgun_sequence
R_PVJP02910816.1_28389-29081_Hippopotamus_amphibius_isolate_BS01_ScHSfme_925260HRSCAF_983060_whole_genome_shotgun_sequence
PVJP02000080.1_144619-145280_Hippopotamus_amphibius_isolate_BS01_ScHSfme_76HRSCAF_13031_whole_genome_shotgun_sequence
land1_Trajav_PVHZ010037302.1_2e103_1_443_4821_4379
land1_Trakan_SJXW01021758.1_3e103_1_443_46524_46966
R_PVJP02021330.1_5567989-5568504_Hippopotamus_amphibius_isolate_BS01_ScHSfme_21297HRSCAF_76171_whole_genome_shotgun_sequence
PVJP02910680.1_1688075-1688508_Hippopotamus_amphibius_isolate_BS01_ScHSfme_925135HRSCAF_982935_whole_genome_shotgun_sequence
PVJP02000042.1_39755-40426_Hippopotamus_amphibius_isolate_BS01_ScHSfme_41HRSCAF_8492_whole_genome_shotgun_sequence
0.02

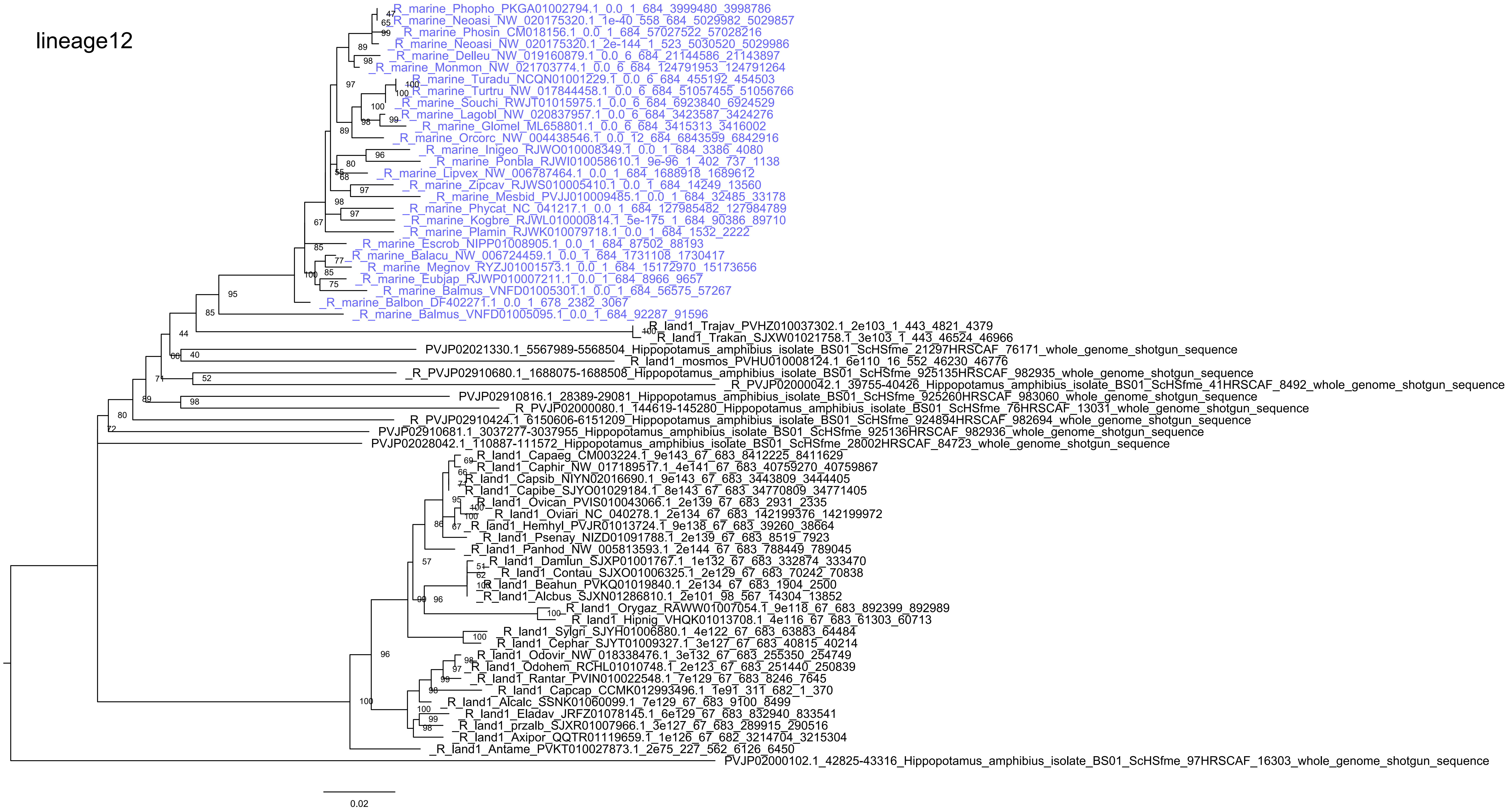

lineage12
_R_marine_Phopho_PKGA01002794.1_0.0_1_684_3999480_3998786
_R_marine_Neoasi_NW_020175320.1_1e-40_558_684_5029982_5029857
_R_marine_Phosin_CM018156.1_0.0_1_684_57027522_57028216
_R_marine_Neoasi_NW_020175320.1_2e-144_1_523_5030520_5029986
_R_marine_Delleu_NW_019160879.1_0.0_6_684_21144586_21143897
_R_marine_Monmon_NW_021703774.1_0.0_6_684_124791953_124791264
_R_marine_Turadu_NCQN01001229.1_0.0_6_684_455192_454503
_R_marine_Turtru_NW_017844458.1_0.0_6_684_51057455_51056766
_R_marine_Souchi_RWJT01015975.1_0.0_6_684_6923840_6924529
_R_marine_Lagobl_NW_020837957.1_0.0_6_684_3423587_3424276
_R_marine_Glomel_ML658801.1_0.0_6_684_3415313_3416002
_R_marine_Orcorc_NW_004438546.1_0.0_12_684_6843599_6842916
_R_marine_Inigeo_RJWO010008349.1_0.0_1_684_3386_4080
_R_marine_Ponbla_RJWI010058610.1_9e-96_1_402_737_1138
_R_marine_Lipvex_NW_006787464.1_0.0_1_684_1688918_1689612
_R_marine_Zipcav_RJWS010005410.1_0.0_1_684_14249_13560
_R_marine_Mesbid_PVJJ010009485.1_0.0_1_684_32485_33178
_R_marine_Phycat_NC_041217.1_0.0_1_684_127985482_127984789
_R_marine_Kogbre_RJWL010000814.1_5e-175_1_684_90386_89710
_R_marine_Plamin_RJWK010079718.1_0.0_1_684_1532_2222
_R_marine_Escrob_NIPP01008905.1_0.0_1_684_87502_88193
_R_marine_Balacu_NW_006724459.1_0.0_1_684_1731108_1730417
_R_marine_Megnov_RYZJ01001573.1_0.0_1_684_15172970_15173656
_R_marine_Eubjap_RJWP010007211.1_0.0_1_684_8966_9657
_R_marine_Balmus_VNFD01005301.1_0.0_1_684_56575_57267
_R_marine_Balbon_DF402271.1_0.0_1_678_2382_3067
_R_marine_Balmus_VNFD01005095.1_0.0_1_684_92287_91596
_R_land1_Trajav_PVHZ010037302.1_2e103_1_443_4821_4379
_R_land1_Trakan_SJXW01021758.1_3e103_1_443_46524_46966
PVJP02021330.1_5567989-5568504_Hippopotamus_amphibius_isolate_BS01_ScHSfme_21297HRSCAF_76171_whole_genome_shotgun_sequence
_R_land1_mosmos_PVHU010008124.1_6e110_16_552_46230_46776
_R_PVJP02910680.1_1688075-1688508_Hippopotamus_amphibius_isolate_BS01_ScHSfme_925135HRSCAF_982935_whole_genome_shotgun_sequence
_R_PVJP02000042.1_39755-40426_Hippopotamus_amphibius_isolate_BS01_ScHSfme_41HRSCAF_8492_whole_genome_shotgun_sequence
PVJP02910816.1_28389-29081_Hippopotamus_amphibius_isolate_BS01_ScHSfme_925260HRSCAF_983060_whole_genome_shotgun_sequence
_R_PVJP02000080.1_144619-145280_Hippopotamus_amphibius_isolate_BS01_ScHSfme_76HRSCAF_13031_whole_genome_shotgun_sequence
_R_PVJP02910424.1_6150606-6151209_Hippopotamus_amphibius_isolate_BS01_ScHSfme_924894HRSCAF_982694_whole_genome_shotgun_sequence
PVJP02910681.1_3037277-3037955_Hippopotamus_amphibius_isolate_BS01_ScHSfme_925136HRSCAF_982936_whole_genome_shotgun_sequence
PVJP02028042.1_110887-111572_Hippopotamus_amphibius_isolate_BS01_ScHSfme_28002HRSCAF_84723_whole_genome_shotgun_sequence
_R_land1_Capaeg_CM003224.1_9e143_67_683_8412225_8411629
_R_land1_Caphir_NW_017189517.1_4e141_67_683_40759270_40759867
_R_land1_Capsib_NIYN02016690.1_9e143_67_683_3443809_3444405
_R_land1_Capibe_SJYO01029184.1_8e143_67_683_34770809_34771405
_R_land1_Ovican_PVIS010043066.1_2e139_67_683_2931_2335
_R_land1_Oviari_NC_040278.1_2e134_67_683_142199376_142199972
_R_land1_Hemhyl_PVJR01013724.1_9e138_67_683_39260_38664
_R_land1_Psenay_NIZD01091788.1_2e139_67_683_8519_7923
_R_land1_Panhod_NW_005813593.1_2e144_67_683_788449_789045
_R_land1_Damlun_SJXP01001767.1_1e132_67_683_332874_333470
_R_land1_Contau_SJXO01006325.1_2e129_67_683_70242_70838
_R_land1_Beahun_PVKQ01019840.1_2e134_67_683_1904_2500
_R_land1_Alcbus_SJXN01286810.1_2e101_98_567_14304_13852
_R_land1_Orygaz_RAWW01007054.1_9e118_67_683_892399_892989
_R_land1_Hipnig_VHQK01013708.1_4e116_67_683_61303_60713
_R_land1_Sylgri_SJYH01006880.1_4e122_67_683_63883_64484
_R_land1_Cephar_SJYT01009327.1_3e127_67_683_40815_40214
_R_land1_Odovir_NW_018338476.1_3e132_67_683_255350_254749
_R_land1_Odohem_RCHL01010748.1_2e123_67_683_251440_250839
_R_land1_Rantar_PVIN010022548.1_7e129_67_683_8246_7645
_R_land1_Capcap_CCMK012993496.1_1e91_311_682_1_370
_R_land1_Alcalc_SSNK01060099.1_7e129_67_683_9100_8499
_R_land1_Eladav_JRFZ01078145.1_6e129_67_683_832940_833541
_R_land1_przalb_SJXR01007966.1_3e127_67_683_289915_290516
_R_land1_Axipor_QQTR01119659.1_1e126_67_682_3214704_3215304
_R_land1_Antame_PVKT010027873.1_2e75_227_562_6126_6450
PVJP02000102.1_42825-43316_Hippopotamus_amphibius_isolate_BS01_ScHSfme_97HRSCAF_16303_whole_genome_shotgun_sequence
0.02

lineage13

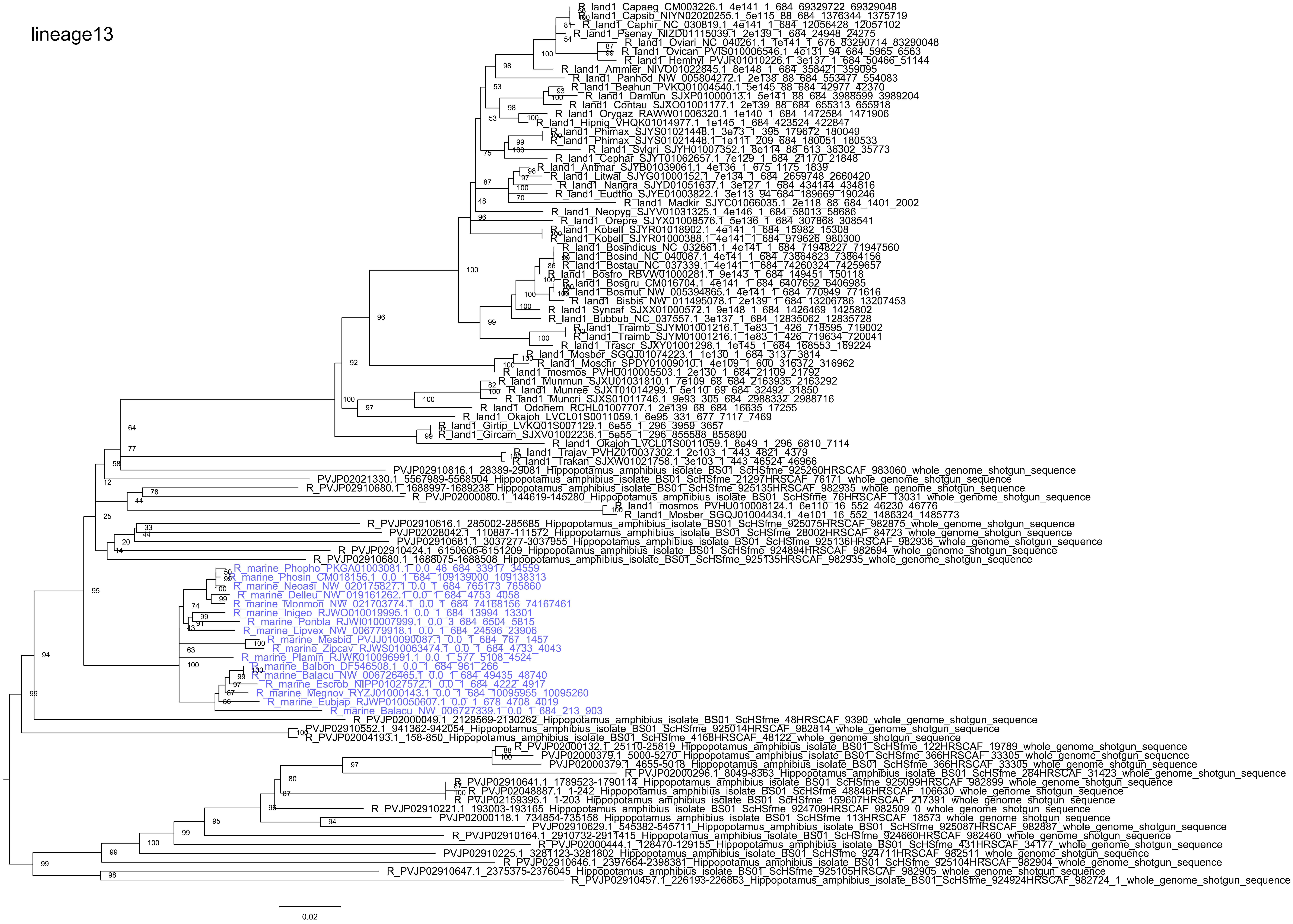
R_land1_Capaeg_CM003226.1_4e141_1_684_69329722_69329048
R_land1_Capsib_NIYN02020255.1_5e115_88_684_1376344_1375719
R_land1_Caphir_NC_030819.1_4e141_1_684_12056428_12057102
R_land1_Psenay_NIZD01115039.1_2e139_1_684_24948_24275
R_land1_Oviari_NC_040261.1_1e141_1_676_83290714_83290048
R_land1_Ovican_PVIS010006546.1_4e131_94_684_5965_6563
R_land1_Hemhyl_PVJR01010226.1_3e137_1_684_50466_51144
R_land1_Ammler_NIVO01022845.1_8e148_1_684_358421_359095
R_land1_Panhod_NW_005804272.1_2e138_88_684_553477_554083
R_land1_Beahun_PVKQ01004540.1_5e145_88_684_42977_42370
R_land1_Damlun_SJXP01000013.1_5e141_88_684_3988599_3989204
R_land1_Contau_SJXO01001177.1_2e139_88_684_655313_655918
R_land1_Orygaz_RAWW01006320.1_1e140_1_684_1472584_1471906
R_land1_Hipnig_VHQK01014977.1_1e145_1_684_423524_422847
R_land1_Phimax_SJYS01021448.1_3e73_1_395_179672_180049
R_land1_Phimax_SJYS01021448.1_1e111_209_684_180051_180533
R_land1_Sylgri_SJYH01007352.1_8e114_88_613_36302_35773
R_land1_Cephar_SJYT01062657.1_7e129_1_684_21170_21848
R_land1_Antmar_SJYB01039061.1_4e136_1_675_1175_1839
R_land1_Litwal_SJYG01000152.1_7e134_1_684_2659748_2660420
R_land1_Nangra_SJYD01051637.1_3e127_1_684_434144_434816
R_land1_Eudtho_SJYE01003822.1_3e113_94_684_189669_190246
R_land1_Madkir_SJYC01066035.1_2e118_88_684_1401_2002
R_land1_Neopyg_SJYV01031325.1_4e146_1_684_58013_58686
R_land1_Orepre_SJYX01008576.1_5e136_1_684_307868_308541
R_land1_Kobell_SJYR01018902.1_4e141_1_684_15982_15308
R_land1_Kobell_SJYR01000388.1_4e141_1_684_979626_980300
R_land1_Bosindicus_NC_032661.1_4e141_1_684_71948227_71947560
R_land1_Bosind_NC_040087.1_4e141_1_684_73864823_73864156
R_land1_Bostau_NC_037339.1_4e141_1_684_74260324_74259657
R_land1_Bosfro_RBVW01000281.1_9e143_1_684_149451_150118
R_land1_Bosgru_CM016704.1_4e141_1_684_6407652_6406985
R_land1_Bosmut_NW_005394865.1_4e141_1_684_770949_771616
R_land1_Bisbis_NW_011495078.1_2e139_1_684_13206786_13207453
R_land1_Syncaf_SJXX01000572.1_9e148_1_684_1426469_1425802
R_land1_Bubbub_NC_037557.1_3e137_1_684_12835062_12835728
R_land1_Traimb_SJYM01001216.1_1e83_1_426_718595_719002
R_land1_Traimb_SJYM01001216.1_1e83_1_426_719634_720041
R_land1_Trascr_SJXY01001298.1_1e145_1_684_168553_169224
R_land1_Mosber_SGQJ01074223.1_1e130_1_684_3137_3814
R_land1_Moschr_SPDY01009010.1_4e109_1_600_316372_316962
R_land1_mosmos_PVHU010005503.1_2e130_1_684_21109_21792
R_land1_Munmun_SJXU01031810.1_7e109_68_684_2163935_2163292
R_land1_Munree_SJXT01014299.1_5e110_69_684_32492_31850
R_land1_Muncri_SJXS01011746.1_9e93_305_684_2988332_2988716
R_land1_Odohem_RCHL01007707.1_2e139_68_684_16635_17255
R_land1_Okajoh_LVCL01S0011059.1_6e95_331_677_7117_7469
R_land1_Girtip_LVKQ01S007129.1_6e55_1_296_3959_3657
R_land1_Gircam_SJXV01002236.1_5e55_1_296_855588_855890
R_land1_Okajoh_LVCL01S0011059.1_8e49_1_296_6810_7114
R_land1_Trajav_PVHZ010037302.1_2e103_1_443_4821_4379
R_land1_Trakan_SJXW01021758.1_3e103_1_443_46524_46966
PVJP02910816.1_28389-29081_Hippopotamus_amphibius_isolate_BS01_ScHSfme_925260HRSCAF_983060_whole_genome_shotgun_sequence
PVJP02021330.1_5567989-5568504_Hippopotamus_amphibius_isolate_BS01_ScHSfme_21297HRSCAF_76171_whole_genome_shotgun_sequence
R_PVJP02910680.1_1688997-1689238_Hippopotamus_amphibius_isolate_BS01_ScHSfme_925135HRSCAF_982935_whole_genome_shotgun_sequence
R_PVJP02000080.1_144619-145280_Hippopotamus_amphibius_isolate_BS01_ScHSfme_76HRSCAF_13031_whole_genome_shotgun_sequence
R_land1_mosmos_PVHU010008124.1_6e110_16_552_46230_46776
R_land1_Mosber_SGQJ01004434.1_4e101_16_552_1486324_1485773
R_PVJP02910616.1_285002-285685_Hippopotamus_amphibius_isolate_BS01_ScHSfme_925075HRSCAF_982875_whole_genome_shotgun_sequence
PVJP02028042.1_110887-111572_Hippopotamus_amphibius_isolate_BS01_ScHSfme_28002HRSCAF_84723_whole_genome_shotgun_sequence
PVJP02910681.1_3037277-3037955_Hippopotamus_amphibius_isolate_BS01_ScHSfme_925136HRSCAF_982936_whole_genome_shotgun_sequence
R_PVJP02910424.1_6150606-6151209_Hippopotamus_amphibius_isolate_BS01_ScHSfme_924894HRSCAF_982694_whole_genome_shotgun_sequence
R_PVJP02910680.1_1688075-1688508_Hippopotamus_amphibius_isolate_BS01_ScHSfme_925135HRSCAF_982935_whole_genome_shotgun_sequence
R_marine_Phopho_PKGA01003081.1_0.0_46_684_33917_34559
R_marine_Phosin_CM018156.1_0.0_1_684_109139000_109138313
R_marine_Neoasi_NW_020175827.1_0.0_1_684_765173_765860
R_marine_Delleu_NW_019161262.1_0.0_1_684_4753_4058
R_marine_Monmon_NW_021703774.1_0.0_1_684_74168156_74167461
R_marine_Inigeo_RJWO010019995.1_0.0_1_684_13994_13301
R_marine_Ponbla_RJWI010007999.1_0.0_3_684_6504_5815
R_marine_Lipvex_NW_006779918.1_0.0_1_684_24596_23906
R_marine_Mesbid_PVJJ010090087.1_0.0_1_684_767_1457
R_marine_Zipcav_RJWS010063474.1_0.0_1_684_4733_4043
R_marine_Plamin_RJWK010096991.1_0.0_1_577_5108_4524
R_marine_Balbon_DF546508.1_0.0_1_684_961_266
R_marine_Balacu_NW_006726465.1_0.0_1_684_49435_48740
R_marine_Escrob_NIPP01027572.1_0.0_1_684_4222_4917
R_marine_Megnov_RYZJ01000143.1_0.0_1_684_10095955_10095260
R_marine_Eubjap_RJWP010050607.1_0.0_1_678_4708_4019
R_marine_Balacu_NW_006727339.1_0.0_1_684_213_903
R_PVJP02000049.1_2129569-2130262_Hippopotamus_amphibius_isolate_BS01_ScHSfme_48HRSCAF_9390_whole_genome_shotgun_sequence
PVJP02910552.1_941362-942054_Hippopotamus_amphibius_isolate_BS01_ScHSfme_925014HRSCAF_982814_whole_genome_shotgun_sequence
R_PVJP02004193.1_158-850_Hippopotamus_amphibius_isolate_BS01_ScHSfme_4168HRSCAF_48122_whole_genome_shotgun_sequence
R_PVJP02000132.1_25110-25819_Hippopotamus_amphibius_isolate_BS01_ScHSfme_122HRSCAF_19789_whole_genome_shotgun_sequence
PVJP02000379.1_5000-5270_Hippopotamus_amphibius_isolate_BS01_ScHSfme_366HRSCAF_33305_whole_genome_shotgun_sequence
PVJP02000379.1_4655-5018_Hippopotamus_amphibius_isolate_BS01_ScHSfme_366HRSCAF_33305_whole_genome_shotgun_sequence
R_PVJP02000296.1_8049-8363_Hippopotamus_amphibius_isolate_BS01_ScHSfme_284HRSCAF_31423_whole_genome_shotgun_sequence
R_PVJP02910641.1_1789523-1790114_Hippopotamus_amphibius_isolate_BS01_ScHSfme_925099HRSCAF_982899_whole_genome_shotgun_sequence
R_PVJP02048887.1_1-242_Hippopotamus_amphibius_isolate_BS01_ScHSfme_48846HRSCAF_106630_whole_genome_shotgun_sequence
R_PVJP02159395.1_1-203_Hippopotamus_amphibius_isolate_BS01_ScHSfme_159607HRSCAF_217391_whole_genome_shotgun_sequence
R_PVJP02910221.1_193003-193165_Hippopotamus_amphibius_isolate_BS01_ScHSfme_924709HRSCAF_982509_0_whole_genome_shotgun_sequence
PVJP02000118.1_734854-735158_Hippopotamus_amphibius_isolate_BS01_ScHSfme_113HRSCAF_18573_whole_genome_shotgun_sequence
PVJP02910629.1_545382-545711_Hippopotamus_amphibius_isolate_BS01_ScHSfme_925087HRSCAF_982887_whole_genome_shotgun_sequence
R_PVJP02910164.1_2910732-2911415_Hippopotamus_amphibius_isolate_BS01_ScHSfme_924660HRSCAF_982460_whole_genome_shotgun_sequence
R_PVJP02000444.1_128470-129155_Hippopotamus_amphibius_isolate_BS01_ScHSfme_431HRSCAF_34177_whole_genome_shotgun_sequence
PVJP02910225.1_3281123-3281802_Hippopotamus_amphibius_isolate_BS01_ScHSfme_924711HRSCAF_982511_whole_genome_shotgun_sequence
R_PVJP02910646.1_2397664-2398381_Hippopotamus_amphibius_isolate_BS01_ScHSfme_925104HRSCAF_982904_whole_genome_shotgun_sequence
R_PVJP02910647.1_2375375-2376045_Hippopotamus_amphibius_isolate_BS01_ScHSfme_925105HRSCAF_982905_whole_genome_shotgun_sequence
R_PVJP02910457.1_226193-226863_Hippopotamus_amphibius_isolate_BS01_ScHSfme_924924HRSCAF_982724_1_whole_genome_shotgun_sequence
0.02

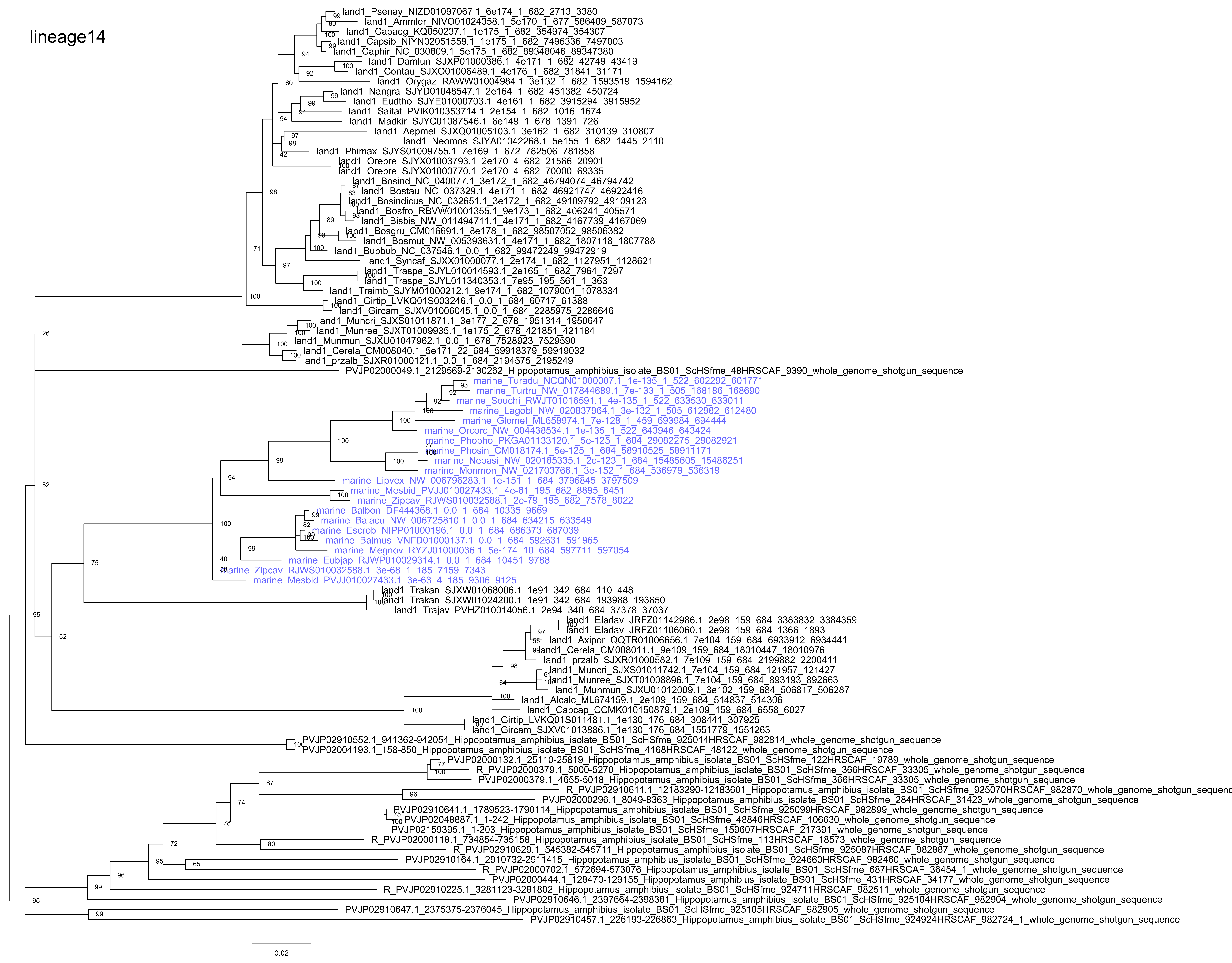
lineage14
land1_Psenay_NIZD01097067.1_6e174_1_682_2713_3380
land1_Ammler_NIVO01024358.1_5e170_1_677_586409_587073
land1_Capaeg_KQ050237.1_1e175_1_682_354974_354307
land1_Capsib_NIYN02051559.1_1e175_1_682_7496336_7497003
land1_Caphir_NC_030809.1_5e175_1_682_89348046_89347380
land1_Damlun_SJXP01000386.1_4e171_1_682_42749_43419
land1_Contau_SJXO01006489.1_4e176_1_682_31841_31171
land1_Orygaz_RAWW01004984.1_3e132_1_682_1593519_1594162
land1_Nangra_SJYD01048547.1_2e164_1_682_451382_450724
land1_Eudtho_SJYE01000703.1_4e161_1_682_3915294_3915952
land1_Saitat_PVIK010353714.1_2e154_1_682_1016_1674
land1_Madkir_SJYC01087546.1_6e149_1_678_1391_726
land1_Aepmel_SJXQ01005103.1_3e162_1_682_310139_310807
land1_Neomos_SJYA01042268.1_5e155_1_682_1445_2110
land1_Phimax_SJYS01009755.1_7e169_1_672_782506_781858
land1_Orepre_SJYX01003793.1_2e170_4_682_21566_20901
land1_Orepre_SJYX01000770.1_2e170_4_682_70000_69335
land1_Bosind_NC_040077.1_3e172_1_682_46794074_46794742
land1_Bostau_NC_037329.1_4e171_1_682_46921747_46922416
land1_Bosindicus_NC_032651.1_3e172_1_682_49109792_49109123
land1_Bosfro_RBVW01001355.1_9e173_1_682_406241_405571
land1_Bisbis_NW_011494711.1_4e171_1_682_4167739_4167069
land1_Bosgru_CM016691.1_8e178_1_682_98507052_98506382
land1_Bosmut_NW_005393631.1_4e171_1_682_1807118_1807788
land1_Bubbub_NC_037546.1_0.0_1_682_99472249_99472919
land1_Syncaf_SJXX01000077.1_2e174_1_682_1127951_1128621
land1_Traspe_SJYL010014593.1_2e165_1_682_7964_7297
land1_Traspe_SJYL011340353.1_7e95_195_561_1_363
land1_Traimb_SJYM01000212.1_9e174_1_682_1079001_1078334
land1_Girtip_LVKQ01S003246.1_0.0_1_684_60717_61388
land1_Gircam_SJXV01006045.1_0.0_1_684_2285975_2286646
land1_Muncri_SJXS01011871.1_3e177_2_678_1951314_1950647
land1_Munree_SJXT01009935.1_1e175_2_678_421851_421184
land1_Munmun_SJXU01047962.1_0.0_1_678_7528923_7529590
land1_Cerela_CM008040.1_5e171_22_684_59918379_59919032
land1_przalb_SJXR01000121.1_0.0_1_684_2194575_2195249
PVJP02000049.1_2129569-2130262_Hippopotamus_amphibius_isolate_BS01_ScHSfme_48HRSCAF_9390_whole_genome_shotgun_sequence
marine_Turadu_NCQN01000007.1_1e-135_1_522_602292_601771
marine_Turtru_NW_017844689.1_7e-133_1_505_168186_168690
marine_Souchi_RWJT01016591.1_4e-135_1_522_633530_633011
marine_Lagobl_NW_020837964.1_3e-132_1_505_612982_612480
marine_Glomel_ML658974.1_7e-128_1_459_693984_694444
marine_Orcorc_NW_004438534.1_1e-135_1_522_643946_643424
marine_Phopho_PKGA01133120.1_5e-125_1_684_29082275_29082921
marine_Phosin_CM018174.1_5e-125_1_684_58910525_58911171
marine_Neoasi_NW_020185335.1_2e-123_1_684_15485605_15486251
marine_Monmon_NW_021703766.1_3e-152_1_684_536979_536319
marine_Lipvex_NW_006796283.1_1e-151_1_684_3796845_3797509
marine_Mesbid_PVJJ010027433.1_4e-81_195_682_8895_8451
marine_Zipcav_RJWS010032588.1_2e-79_195_682_7578_8022
marine_Balbon_DF444368.1_0.0_1_684_10335_9669
marine_Balacu_NW_006725810.1_0.0_1_684_634215_633549
marine_Escrob_NIPP01000196.1_0.0_1_684_686373_687039
marine_Balmus_VNFD01000137.1_0.0_1_684_592631_591965
marine_Megnov_RYZJ01000036.1_5e-174_10_684_597711_597054
marine_Eubjap_RJWP010029314.1_0.0_1_684_10451_9788
marine_Zipcav_RJWS010032588.1_3e-68_1_185_7159_7343
marine_Mesbid_PVJJ010027433.1_3e-63_4_185_9306_9125
land1_Trakan_SJXW01068006.1_1e91_342_684_110_448
land1_Trakan_SJXW01024200.1_1e91_342_684_193988_193650
land1_Trajav_PVHZ010014056.1_2e94_340_684_37378_37037
land1_Eladav_JRFZ01142986.1_2e98_159_684_3383832_3384359
land1_Eladav_JRFZ01106060.1_2e98_159_684_1366_1893
land1_Axipor_QQTR01006656.1_7e104_159_684_6933912_6934441
land1_Cerela_CM008011.1_9e109_159_684_18010447_18010976
land1_przalb_SJXR01000582.1_7e109_159_684_2199882_2200411
land1_Muncri_SJXS01011742.1_7e104_159_684_121957_121427
land1_Munree_SJXT01008896.1_7e104_159_684_893193_892663
land1_Munmun_SJXU01012009.1_3e102_159_684_506817_506287
land1_Alcalc_ML674159.1_2e109_159_684_514837_514306
land1_Capcap_CCMK010150879.1_2e109_159_684_6558_6027
land1_Girtip_LVKQ01S011481.1_1e130_176_684_308441_307925
land1_Gircam_SJXV01013886.1_1e130_176_684_1551779_1551263
PVJP02910552.1_941362-942054_Hippopotamus_amphibius_isolate_BS01_ScHSfme_925014HRSCAF_982814_whole_genome_shotgun_sequence
PVJP02004193.1_158-850_Hippopotamus_amphibius_isolate_BS01_ScHSfme_4168HRSCAF_48122_whole_genome_shotgun_sequence
PVJP02000132.1_25110-25819_Hippopotamus_amphibius_isolate_BS01_ScHSfme_122HRSCAF_19789_whole_genome_shotgun_sequence
R_PVJP02000379.1_5000-5270_Hippopotamus_amphibius_isolate_BS01_ScHSfme_366HRSCAF_33305_whole_genome_shotgun_sequence
PVJP02000379.1_4655-5018_Hippopotamus_amphibius_isolate_BS01_ScHSfme_366HRSCAF_33305_whole_genome_shotgun_sequence
R_PVJP02910611.1_12183290-12183601_Hippopotamus_amphibius_isolate_BS01_ScHSfme_925070HRSCAF_982870_whole_genome_shotgun_sequenc
PVJP02000296.1_8049-8363_Hippopotamus_amphibius_isolate_BS01_ScHSfme_284HRSCAF_31423_whole_genome_shotgun_sequence
PVJP02910641.1_1789523-1790114_Hippopotamus_amphibius_isolate_BS01_ScHSfme_925099HRSCAF_982899_whole_genome_shotgun_sequence
PVJP02048887.1_1-242_Hippopotamus_amphibius_isolate_BS01_ScHSfme_48846HRSCAF_106630_whole_genome_shotgun_sequence
PVJP02159395.1_1-203_Hippopotamus_amphibius_isolate_BS01_ScHSfme_159607HRSCAF_217391_whole_genome_shotgun_sequence
R_PVJP02000118.1_734854-735158_Hippopotamus_amphibius_isolate_BS01_ScHSfme_113HRSCAF_18573_whole_genome_shotgun_sequence
R_PVJP02910629.1_545382-545711_Hippopotamus_amphibius_isolate_BS01_ScHSfme_925087HRSCAF_982887_whole_genome_shotgun_sequence
PVJP02910164.1_2910732-2911415_Hippopotamus_amphibius_isolate_BS01_ScHSfme_924660HRSCAF_982460_whole_genome_shotgun_sequence
R_PVJP02000702.1_572694-573076_Hippopotamus_amphibius_isolate_BS01_ScHSfme_687HRSCAF_36454_1_whole_genome_shotgun_sequence
PVJP02000444.1_128470-129155_Hippopotamus_amphibius_isolate_BS01_ScHSfme_431HRSCAF_34177_whole_genome_shotgun_sequence
R_PVJP02910225.1_3281123-3281802_Hippopotamus_amphibius_isolate_BS01_ScHSfme_924711HRSCAF_982511_whole_genome_shotgun_sequence
PVJP02910646.1_2397664-2398381_Hippopotamus_amphibius_isolate_BS01_ScHSfme_925104HRSCAF_982904_whole_genome_shotgun_sequence
PVJP02910647.1_2375375-2376045_Hippopotamus_amphibius_isolate_BS01_ScHSfme_925105HRSCAF_982905_whole_genome_shotgun_sequence
PVJP02910457.1_226193-226863_Hippopotamus_amphibius_isolate_BS01_ScHSfme_924924HRSCAF_982724_1_whole_genome_shotgun_sequence
0.02

lineage15

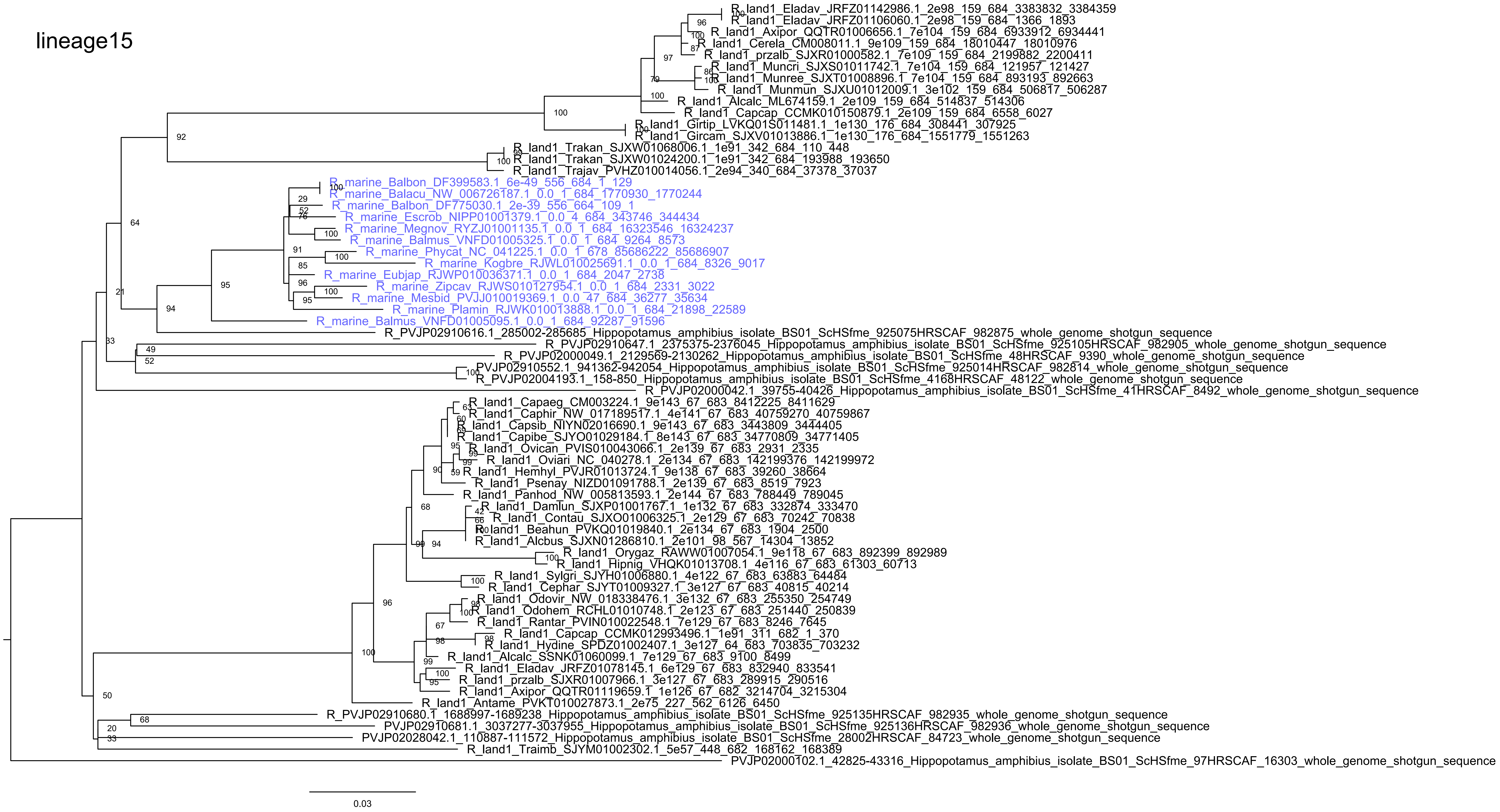
R_land1_Eladav_JRFZ01142986.1_2e98_159_684_3383832_3384359
R_land1_Eladav_JRFZ01106060.1_2e98_159_684_1366_1893
R_land1_Axipor_QQTR01006656.1_7e104_159_684_6933912_6934441
R_land1_Cerela_CM008011.1_9e109_159_684_18010447_18010976
R_land1_przalb_SJXR01000582.1_7e109_159_684_2199882_2200411
R_land1_Muncri_SJXS01011742.1_7e104_159_684_121957_121427
R_land1_Munree_SJXT01008896.1_7e104_159_684_893193_892663
R_land1_Munmun_SJXU01012009.1_3e102_159_684_506817_506287
R_land1_Alcalc_ML674159.1_2e109_159_684_514837_514306
R_land1_Capcap_CCMK010150879.1_2e109_159_684_6558_6027
R_land1_Girtip_LVKQ01S011481.1_1e130_176_684_308441_307925
R_land1_Gircam_SJXV01013886.1_1e130_176_684_1551779_1551263
R_land1_Trakan_SJXW01068006.1_1e91_342_684_110_448
R_land1_Trakan_SJXW01024200.1_1e91_342_684_193988_193650
R_land1_Trajav_PVHZ010014056.1_2e94_340_684_37378_37037
R_marine_Balbon_DF399583.1_6e-49_556_684_1_129
R_marine_Balacu_NW_006726187.1_0.0_1_684_1770930_1770244
R_marine_Balbon_DF775030.1_2e-39_556_664_109_1
R_marine_Escrob_NIPP01001379.1_0.0_4_684_343746_344434
R_marine_Megnov_RYZJ01001135.1_0.0_1_684_16323546_16324237
R_marine_Balmus_VNFD01005325.1_0.0_1_684_9264_8573
R_marine_Phycat_NC_041225.1_0.0_1_678_85686222_85686907
R_marine_Kogbre_RJWL010025691.1_0.0_1_684_8326_9017
R_marine_Eubjap_RJWP010036371.1_0.0_1_684_2047_2738
R_marine_Zipcav_RJWS010127954.1_0.0_1_684_2331_3022
R_marine_Mesbid_PVJJ010019369.1_0.0_47_684_36277_35634
R_marine_Plamin_RJWK010013888.1_0.0_1_684_21898_22589
R_marine_Balmus_VNFD01005095.1_0.0_1_684_92287_91596
R_PVJP02910616.1_285002-285685_Hippopotamus_amphibius_isolate_BS01_ScHSfme_925075HRSCAF_982875_whole_genome_shotgun_sequence
R_PVJP02910647.1_2375375-2376045_Hippopotamus_amphibius_isolate_BS01_ScHSfme_925105HRSCAF_982905_whole_genome_shotgun_sequence
R_PVJP02000049.1_2129569-2130262_Hippopotamus_amphibius_isolate_BS01_ScHSfme_48HRSCAF_9390_whole_genome_shotgun_sequence
PVJP02910552.1_941362-942054_Hippopotamus_amphibius_isolate_BS01_ScHSfme_925014HRSCAF_982814_whole_genome_shotgun_sequence
R_PVJP02004193.1_158-850_Hippopotamus_amphibius_isolate_BS01_ScHSfme_4168HRSCAF_48122_whole_genome_shotgun_sequence
R_PVJP02000042.1_39755-40426_Hippopotamus_amphibius_isolate_BS01_ScHSfme_41HRSCAF_8492_whole_genome_shotgun_sequence
R_land1_Capaeg_CM003224.1_9e143_67_683_8412225_8411629
R_land1_Caphir_NW_017189517.1_4e141_67_683_40759270_40759867
R_land1_Capsib_NIYN02016690.1_9e143_67_683_3443809_3444405
R_land1_Capibe_SJYO01029184.1_8e143_67_683_34770809_34771405
R_land1_Ovican_PVIS010043066.1_2e139_67_683_2931_2335
R_land1_Oviari_NC_040278.1_2e134_67_683_142199376_142199972
R_land1_Hemhyl_PVJR01013724.1_9e138_67_683_39260_38664
R_land1_Psenay_NIZD01091788.1_2e139_67_683_8519_7923
R_land1_Panhod_NW_005813593.1_2e144_67_683_788449_789045
R_land1_Damlun_SJXP01001767.1_1e132_67_683_332874_333470
R_land1_Contau_SJXO01006325.1_2e129_67_683_70242_70838
R_land1_Beahun_PVKQ01019840.1_2e134_67_683_1904_2500
R_land1_Alcbus_SJXN01286810.1_2e101_98_567_14304_13852
R_land1_Orygaz_RAWW01007054.1_9e118_67_683_892399_892989
R_land1_Hipnig_VHQK01013708.1_4e116_67_683_61303_60713
R_land1_Sylgri_SJYH01006880.1_4e122_67_683_63883_64484
R_land1_Cephar_SJYT01009327.1_3e127_67_683_40815_40214
R_land1_Odovir_NW_018338476.1_3e132_67_683_255350_254749
R_land1_Odohem_RCHL01010748.1_2e123_67_683_251440_250839
R_land1_Rantar_PVIN010022548.1_7e129_67_683_8246_7645
R_land1_Capcap_CCMK012993496.1_1e91_311_682_1_370
R_land1_Hydine_SPDZ01002407.1_3e127_64_683_703835_703232
R_land1_Alcalc_SSNK01060099.1_7e129_67_683_9100_8499
R_land1_Eladav_JRFZ01078145.1_6e129_67_683_832940_833541
R_land1_przalb_SJXR01007966.1_3e127_67_683_289915_290516
R_land1_Axipor_QQTR01119659.1_1e126_67_682_3214704_3215304
R_land1_Antame_PVKT010027873.1_2e75_227_562_6126_6450
R_PVJP02910680.1_1688997-1689238_Hippopotamus_amphibius_isolate_BS01_ScHSfme_925135HRSCAF_982935_whole_genome_shotgun_sequence
PVJP02910681.1_3037277-3037955_Hippopotamus_amphibius_isolate_BS01_ScHSfme_925136HRSCAF_982936_whole_genome_shotgun_sequence
PVJP02028042.1_110887-111572_Hippopotamus_amphibius_isolate_BS01_ScHSfme_28002HRSCAF_84723_whole_genome_shotgun_sequence
R_land1_Traimb_SJYM01002302.1_5e57_448_682_168162_168389
PVJP02000102.1_42825-43316_Hippopotamus_amphibius_isolate_BS01_ScHSfme_97HRSCAF_16303_whole_genome_shotgun_sequence
0.03

lineage16

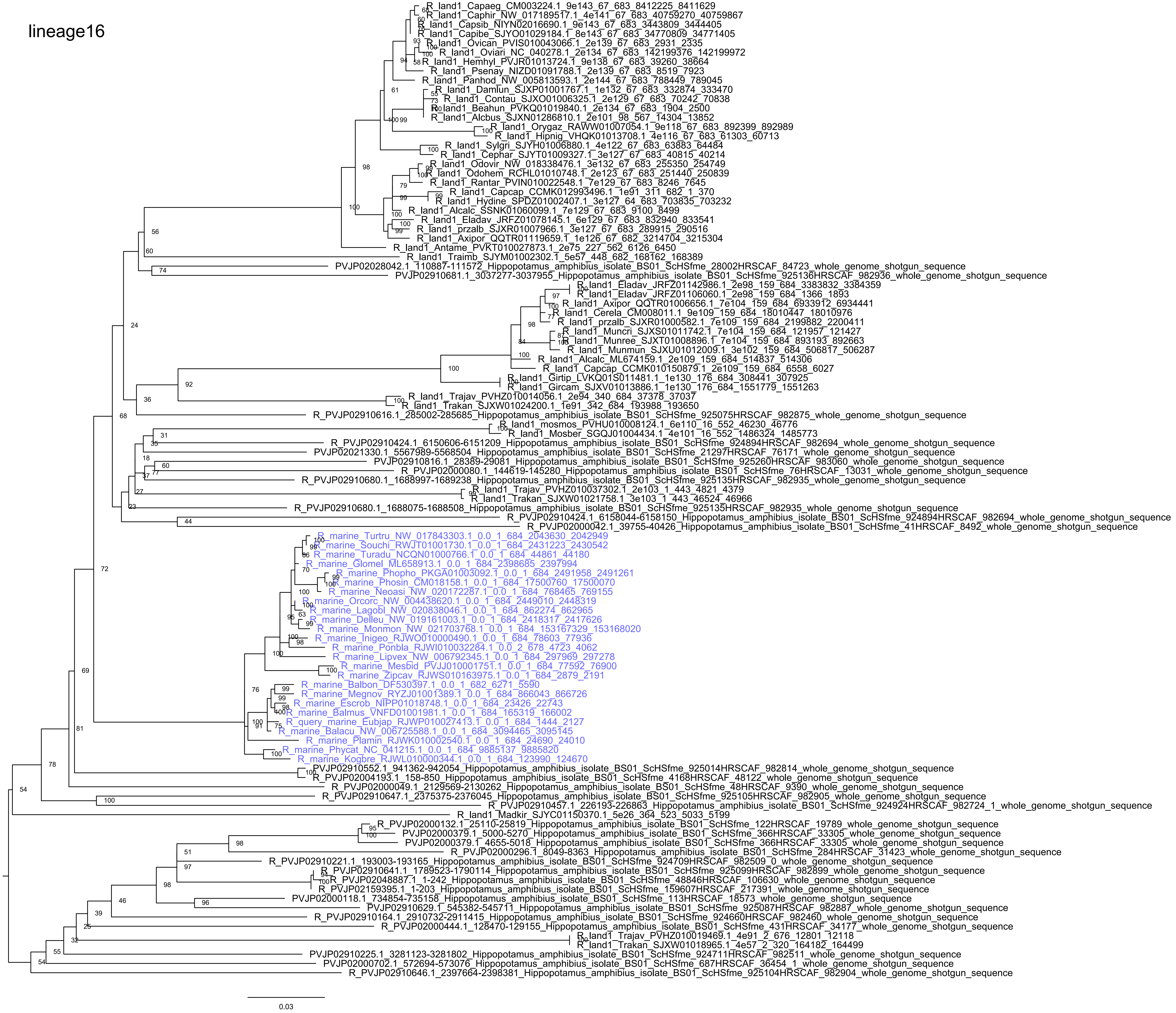
R_land1_Capaeg_CM003224.1_9e143_67_683_8412225_8411629
R_land1_Caphir_NW_017189517.1_4e141_67_683_40759270_40759867
R_land1_Capsib_NIYN02016690.1_9e143_67_683_3443809_3444405
R_land1_Capibe_SJYO01029184.1_8e143_67_683_34770809_34771405
R_land1_Ovican_PVIS010043066.1_2e139_67_683_2931_2335
R_land1_Oviari_NC_040278.1_2e134_67_683_142199376_142199972
R_land1_Hemhyl_PVJR01013724.1_9e138_67_683_39260_38664
R_land1_Psenay_NIZD01091788.1_2e139_67_683_8519_7923
R_land1_Panhod_NW_005813593.1_2e144_67_683_788449_789045
R_land1_Damlun_SJXP01001767.1_1e132_67_683_332874_333470
R_land1_Contau_SJXO01006325.1_2e129_67_683_70242_70838
R_land1_Beahun_PVKQ01019840.1_2e134_67_683_1904_2500
R_land1_Alcbus_SJXN01286810.1_2e101_98_567_14304_13852
R_land1_Orygaz_RAWW01007054.1_9e118_67_683_892399_892989
R_land1_Hipnig_VHQK01013708.1_4e116_67_683_61303_60713
R_land1_Sylgri_SJYH01006880.1_4e122_67_683_63883_64484
R_land1_Cephar_SJYT01009327.1_3e127_67_683_40815_40214
R_land1_Odovir_NW_018338476.1_3e132_67_683_255350_254749
R_land1_Odohem_RCHL01010748.1_2e123_67_683_251440_250839
R_land1_Rantar_PVIN010022548.1_7e129_67_683_8246_7645
R_land1_Capcap_CCMK012993496.1_1e91_311_682_1_370
R_land1_Hydine_SPDZ01002407.1_3e127_64_683_703835_703232
R_land1_Alcalc_SSNK01060099.1_7e129_67_683_9100_8499
R_land1_Eladav_JRFZ01078145.1_6e129_67_683_832940_833541
R_land1_przalb_SJXR01007966.1_3e127_67_683_289915_290516
R_land1_Axipor_QQTR01119659.1_1e126_67_682_3214704_3215304
R_land1_Antame_PVKT010027873.1_2e75_227_562_6126_6450
R_land1_Traimb_SJYM01002302.1_5e57_448_682_168162_168389
PVJP02028042.1_110887-111572_Hippopotamus_amphibius_isolate_BS01_ScHSfme_28002HRSCAF_84723_whole_genome_shotgun_sequence
PVJP02910681.1_3037277-3037955_Hippopotamus_amphibius_isolate_BS01_ScHSfme_925136HRSCAF_982936_whole_genome_shotgun_sequence
R_land1_Eladav_JRFZ01142986.1_2e98_159_684_3383832_3384359
R_land1_Eladav_JRFZ01106060.1_2e98_159_684_1366_1893
R_land1_Axipor_QQTR01006656.1_7e104_159_684_6933912_6934441
R_land1_Cerela_CM008011.1_9e109_159_684_18010447_18010976
R_land1_przalb_SJXR01000582.1_7e109_159_684_2199882_2200411
R_land1_Muncri_SJXS01011742.1_7e104_159_684_121957_121427
R_land1_Munree_SJXT01008896.1_7e104_159_684_893193_892663
R_land1_Munmun_SJXU01012009.1_3e102_159_684_506817_506287
R_land1_Alcalc_ML674159.1_2e109_159_684_514837_514306
R_land1_Capcap_CCMK010150879.1_2e109_159_684_6558_6027
R_land1_Girtip_LVKQ01S011481.1_1e130_176_684_308441_307925
R_land1_Gircam_SJXV01013886.1_1e130_176_684_1551779_1551263
R_land1_Trajav_PVHZ010014056.1_2e94_340_684_37378_37037
R_land1_Trakan_SJXW01024200.1_1e91_342_684_193988_193650
R_PVJP02910616.1_285002-285685_Hippopotamus_amphibius_isolate_BS01_ScHSfme_925075HRSCAF_982875_whole_genome_shotgun_sequence
R_land1_mosmos_PVHU010008124.1_6e110_16_552_46230_46776
R_land1_Mosber_SGQJ01004434.1_4e101_16_552_1486324_1485773
R_PVJP02910424.1_6150606-6151209_Hippopotamus_amphibius_isolate_BS01_ScHSfme_924894HRSCAF_982694_whole_genome_shotgun_sequence
PVJP02021330.1_5567989-5568504_Hippopotamus_amphibius_isolate_BS01_ScHSfme_21297HRSCAF_76171_whole_genome_shotgun_sequence
PVJP02910816.1_28389-29081_Hippopotamus_amphibius_isolate_BS01_ScHSfme_925260HRSCAF_983060_whole_genome_shotgun_sequence
R_PVJP02000080.1_144619-145280_Hippopotamus_amphibius_isolate_BS01_ScHSfme_76HRSCAF_13031_whole_genome_shotgun_sequence
R_PVJP02910680.1_1688997-1689238_Hippopotamus_amphibius_isolate_BS01_ScHSfme_925135HRSCAF_982935_whole_genome_shotgun_sequence
R_land1_Trajav_PVHZ010037302.1_2e103_1_443_4821_4379
R_land1_Trakan_SJXW01021758.1_3e103_1_443_46524_46966
R_PVJP02910680.1_1688075-1688508_Hippopotamus_amphibius_isolate_BS01_ScHSfme_925135HRSCAF_982935_whole_genome_shotgun_sequence
R_PVJP02910424.1_6158044-6158150_Hippopotamus_amphibius_isolate_BS01_ScHSfme_924894HRSCAF_982694_whole_genome_shotgun_sequence
R_PVJP02000042.1_39755-40426_Hippopotamus_amphibius_isolate_BS01_ScHSfme_41HRSCAF_8492_whole_genome_shotgun_sequence
R_marine_Turtru_NW_017843303.1_0.0_1_684_2043630_2042949
R_marine_Souchi_RWJT01001730.1_0.0_1_684_2431223_2430542
R_marine_Turadu_NCQN01000766.1_0.0_1_684_44861_44180
R_marine_Glomel_ML658913.1_0.0_1_684_2398685_2397994
R_marine_Phopho_PKGA01003092.1_0.0_1_684_2491958_2491261
R_marine_Phosin_CM018158.1_0.0_1_684_17500760_17500070
R_marine_Neoasi_NW_020172287.1_0.0_1_684_768465_769155
R_marine_Orcorc_NW_004438620.1_0.0_1_684_2449010_2448319
R_marine_Lagobl_NW_020838046.1_0.0_1_684_862274_862965
R_marine_Delleu_NW_019161003.1_0.0_1_684_2418317_2417626
R_marine_Monmon_NW_021703768.1_0.0_1_684_153167329_153168020
R_marine_Inigeo_RJWO010000490.1_0.0_1_684_78603_77936
R_marine_Ponbla_RJWI010032284.1_0.0_2_678_4723_4062
R_marine_Lipvex_NW_006792345.1_0.0_1_684_297969_297278
R_marine_Mesbid_PVJJ01000175.1_0.0_1_684_77592_76900
R_marine_Zipcav_RJWS010163975.1_0.0_1_684_2879_2191
R_marine_Balbon_DF530397.1_0.0_1_682_6271_5590
R_marine_Megnov_RYZJ01001389.1_0.0_1_684_866043_866726
R_marine_Escrob_NIPP01018748.1_0.0_1_684_23426_22743
R_marine_Balmus_VNFD01001981.1_0.0_1_684_165319_166002
R_query_marine_Eubjap_RJWP010027413.1_0.0_1_684_1444_2127
R_marine_Balacu_NW_006725588.1_0.0_1_684_3094465_3095145
R_marine_Plamin_RJWK010002540.1_0.0_1_684_24690_24010
R_marine_Phycat_NC_041215.1_0.0_1_684_9885137_9885820
R_marine_Kogbre_RJWL010000344.1_0.0_1_684_123990_124670
PVJP02910552.1_941362-942054_Hippopotamus_amphibius_isolate_BS01_ScHSfme_925014HRSCAF_982814_whole_genome_shotgun_sequence
R_PVJP02004193.1_158-850_Hippopotamus_amphibius_isolate_BS01_ScHSfme_4168HRSCAF_48122_whole_genome_shotgun_sequence
R_PVJP02000049.1_2129569-2130262_Hippopotamus_amphibius_isolate_BS01_ScHSfme_48HRSCAF_9390_whole_genome_shotgun_sequence
R_PVJP02910647.1_2375375-2376045_Hippopotamus_amphibius_isolate_BS01_ScHSfme_925105HRSCAF_982905_whole_genome_shotgun_sequence
R_PVJP02910457.1_226193-226863_Hippopotamus_amphibius_isolate_BS01_ScHSfme_924924HRSCAF_982724_1_whole_genome_shotgun_sequence
R_land1_Madkir_SJYC01150370.1_5e26_364_523_5033_5199
R_PVJP02000132.1_25110-25819_Hippopotamus_amphibius_isolate_BS01_ScHSfme_122HRSCAF_19789_whole_genome_shotgun_sequence
PVJP02000379.1_5000-5270_Hippopotamus_amphibius_isolate_BS01_ScHSfme_366HRSCAF_33305_whole_genome_shotgun_sequence
PVJP02000379.1_4655-5018_Hippopotamus_amphibius_isolate_BS01_ScHSfme_366HRSCAF_33305_whole_genome_shotgun_sequence
R_PVJP02000296.1_8049-8363_Hippopotamus_amphibius_isolate_BS01_ScHSfme_284HRSCAF_31423_whole_genome_shotgun_sequence
R_PVJP02910221.1_193003-193165_Hippopotamus_amphibius_isolate_BS01_ScHSfme_924709HRSCAF_982509_0_whole_genome_shotgun_sequence
R_PVJP02910641.1_1789523-1790114_Hippopotamus_amphibius_isolate_BS01_ScHSfme_925099HRSCAF_982899_whole_genome_shotgun_sequence
R_PVJP02048887.1_1-242_Hippopotamus_amphibius_isolate_BS01_ScHSfme_48846HRSCAF_106630_whole_genome_shotgun_sequence
R_PVJP02159395.1_1-203_Hippopotamus_amphibius_isolate_BS01_ScHSfme_159607HRSCAF_217391_whole_genome_shotgun_sequence
PVJP02000118.1_734854-735158_Hippopotamus_amphibius_isolate_BS01_ScHSfme_113HRSCAF_18573_whole_genome_shotgun_sequence
PVJP02910629.1_545382-545711_Hippopotamus_amphibius_isolate_BS01_ScHSfme_925087HRSCAF_982887_whole_genome_shotgun_sequence
R_PVJP02910164.1_2910732-2911415_Hippopotamus_amphibius_isolate_BS01_ScHSfme_924660HRSCAF_982460_whole_genome_shotgun_sequence
R_PVJP02000444.1_128470-129155_Hippopotamus_amphibius_isolate_BS01_ScHSfme_431HRSCAF_34177_whole_genome_shotgun_sequence
R_land1_Trajav_PVHZ010019469.1_4e91_2_676_12801_12118
R_land1_Trakan_SJXW01018965.1_4e57_2_320_164182_164499
PVJP02910225.1_3281123-3281802_Hippopotamus_amphibius_isolate_BS01_ScHSfme_924711HRSCAF_982511_whole_genome_shotgun_sequence
PVJP02000702.1_572694-573076_Hippopotamus_amphibius_isolate_BS01_ScHSfme_687HRSCAF_36454_1_whole_genome_shotgun_sequence
R_PVJP02910646.1_2397664-2398381_Hippopotamus_amphibius_isolate_BS01_ScHSfme_925104HRSCAF_982904_whole_genome_shotgun_sequence
0.03

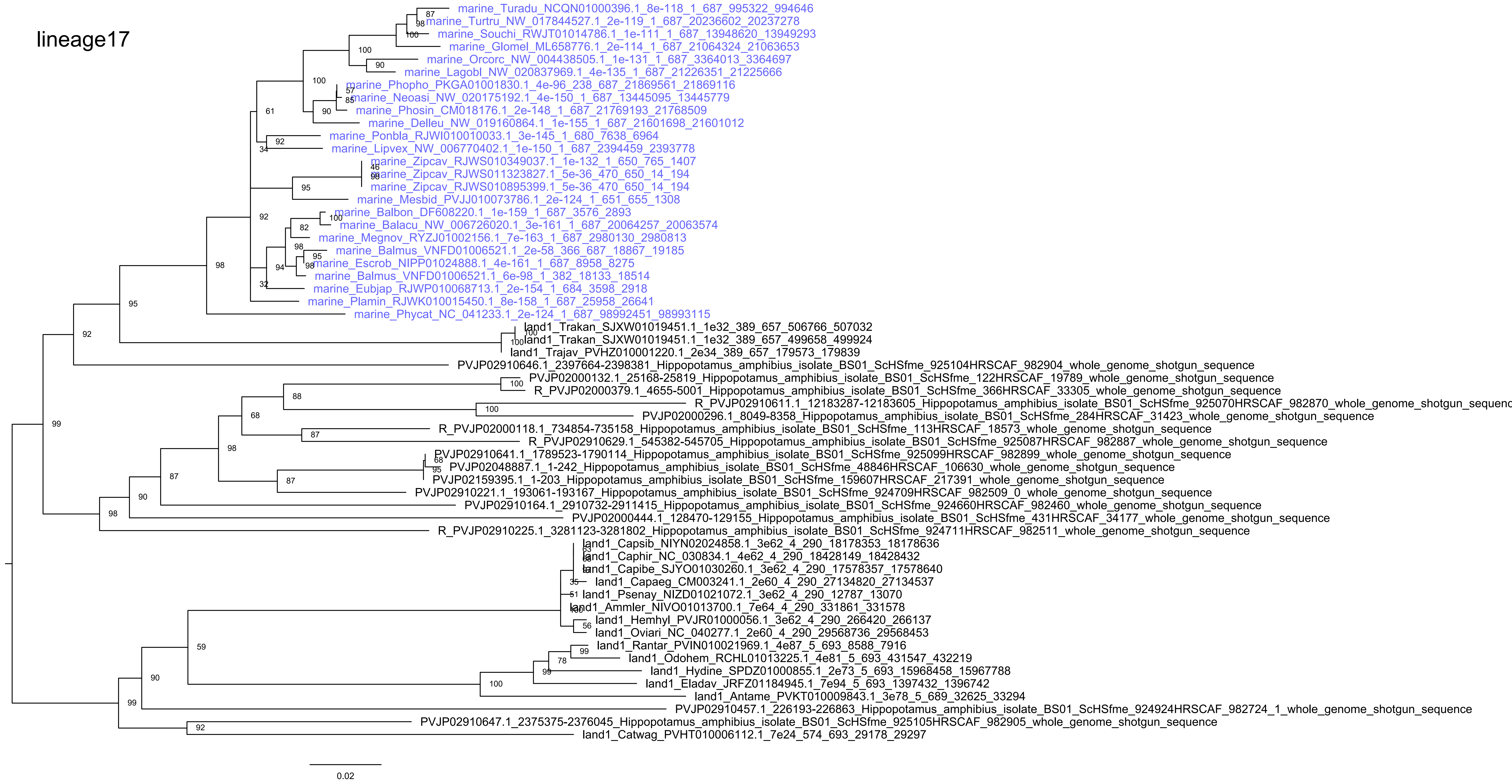
lineage17
marine_Turadu_NCQN01000396.1_8e-118_1_687_995322_994646
marine_Turtru_NW_017844527.1_2e-119_1_687_20236602_20237278
marine_Souchi_RWJT01014786.1_1e-111_1_687_13948620_13949293
marine_Glomel_ML658776.1_2e-114_1_687_21064324_21063653
marine_Orcorc_NW_004438505.1_1e-131_1_687_3364013_3364697
marine_Lagobl_NW_020837969.1_4e-135_1_687_21226351_21225666
marine_Phopho_PKGA01001830.1_4e-96_238_687_21869561_21869116
marine_Neoasi_NW_020175192.1_4e-150_1_687_13445095_13445779
marine_Phosin_CM018176.1_2e-148_1_687_21769193_21768509
marine_Delleu_NW_019160864.1_1e-155_1_687_21601698_21601012
marine_Ponbla_RJWI010010033.1_3e-145_1_680_7638_6964
marine_Lipvex_NW_006770402.1_1e-150_1_687_2394459_2393778
marine_Zipcav_RJWS010349037.1_1e-132_1_650_765_1407
marine_Zipcav_RJWS011323827.1_5e-36_470_650_14_194
marine_Zipcav_RJWS010895399.1_5e-36_470_650_14_194
marine_Mesbid_PVJJ010073786.1_2e-124_1_651_655_1308
marine_Balbon_DF608220.1_1e-159_1_687_3576_2893
marine_Balacu_NW_006726020.1_3e-161_1_687_20064257_20063574
marine_Megnov_RYZJ01002156.1_7e-163_1_687_2980130_2980813
marine_Balmus_VNFD01006521.1_2e-58_366_687_18867_19185
marine_Escrob_NIPP01024888.1_4e-161_1_687_8958_8275
marine_Balmus_VNFD01006521.1_6e-98_1_382_18133_18514
marine_Eubjap_RJWP010068713.1_2e-154_1_684_3598_2918
marine_Plamin_RJWK010015450.1_8e-158_1_687_25958_26641
marine_Phycat_NC_041233.1_2e-124_1_687_98992451_98993115
land1_Trakan_SJXW01019451.1_1e32_389_657_506766_507032
land1_Trakan_SJXW01019451.1_1e32_389_657_499658_499924
land1_Trajav_PVHZ010001220.1_2e34_389_657_179573_179839
PVJP02910646.1_2397664-2398381_Hippopotamus_amphibius_isolate_BS01_ScHSfme_925104HRSCAF_982904_whole_genome_shotgun_sequence
PVJP02000132.1_25168-25819_Hippopotamus_amphibius_isolate_BS01_ScHSfme_122HRSCAF_19789_whole_genome_shotgun_sequence
R_PVJP02000379.1_4655-5001_Hippopotamus_amphibius_isolate_BS01_ScHSfme_366HRSCAF_33305_whole_genome_shotgun_sequence
R_PVJP02910611.1_12183287-12183605_Hippopotamus_amphibius_isolate_BS01_ScHSfme_925070HRSCAF_982870_whole_genome_shotgun_sequenc
PVJP02000296.1_8049-8358_Hippopotamus_amphibius_isolate_BS01_ScHSfme_284HRSCAF_31423_whole_genome_shotgun_sequence
R_PVJP02000118.1_734854-735158_Hippopotamus_amphibius_isolate_BS01_ScHSfme_113HRSCAF_18573_whole_genome_shotgun_sequence
R_PVJP02910629.1_545382-545705_Hippopotamus_amphibius_isolate_BS01_ScHSfme_925087HRSCAF_982887_whole_genome_shotgun_sequence
PVJP02910641.1_1789523-1790114_Hippopotamus_amphibius_isolate_BS01_ScHSfme_925099HRSCAF_982899_whole_genome_shotgun_sequence
PVJP02048887.1_1-242_Hippopotamus_amphibius_isolate_BS01_ScHSfme_48846HRSCAF_106630_whole_genome_shotgun_sequence
PVJP02159395.1_1-203_Hippopotamus_amphibius_isolate_BS01_ScHSfme_159607HRSCAF_217391_whole_genome_shotgun_sequence
PVJP02910221.1_193061-193167_Hippopotamus_amphibius_isolate_BS01_ScHSfme_924709HRSCAF_982509_0_whole_genome_shotgun_sequence
PVJP02910164.1_2910732-2911415_Hippopotamus_amphibius_isolate_BS01_ScHSfme_924660HRSCAF_982460_whole_genome_shotgun_sequence
PVJP02000444.1_128470-129155_Hippopotamus_amphibius_isolate_BS01_ScHSfme_431HRSCAF_34177_whole_genome_shotgun_sequence
R_PVJP02910225.1_3281123-3281802_Hippopotamus_amphibius_isolate_BS01_ScHSfme_924711HRSCAF_982511_whole_genome_shotgun_sequence
land1_Capsib_NIYN02024858.1_3e62_4_290_18178353_18178636
land1_Caphir_NC_030834.1_4e62_4_290_18428149_18428432
land1_Capibe_SJYO01030260.1_3e62_4_290_17578357_17578640
land1_Capaeg_CM003241.1_2e60_4_290_27134820_27134537
land1_Psenay_NIZD01021072.1_3e62_4_290_12787_13070
land1_Ammler_NIVO01013700.1_7e64_4_290_331861_331578
land1_Hemhyl_PVJR01000056.1_3e62_4_290_266420_266137
land1_Oviari_NC_040277.1_2e60_4_290_29568736_29568453
land1_Rantar_PVIN010021969.1_4e87_5_693_8588_7916
land1_Odohem_RCHL01013225.1_4e81_5_693_431547_432219
land1_Hydine_SPDZ01000855.1_2e73_5_693_15968458_15967788
land1_Eladav_JRFZ01184945.1_7e94_5_693_1397432_1396742
land1_Antame_PVKT010009843.1_3e78_5_689_32625_33294
PVJP02910457.1_226193-226863_Hippopotamus_amphibius_isolate_BS01_ScHSfme_924924HRSCAF_982724_1_whole_genome_shotgun_sequence
PVJP02910647.1_2375375-2376045_Hippopotamus_amphibius_isolate_BS01_ScHSfme_925105HRSCAF_982905_whole_genome_shotgun_sequence
land1_Catwag_PVHT010006112.1_7e24_574_693_29178_29297
0.02

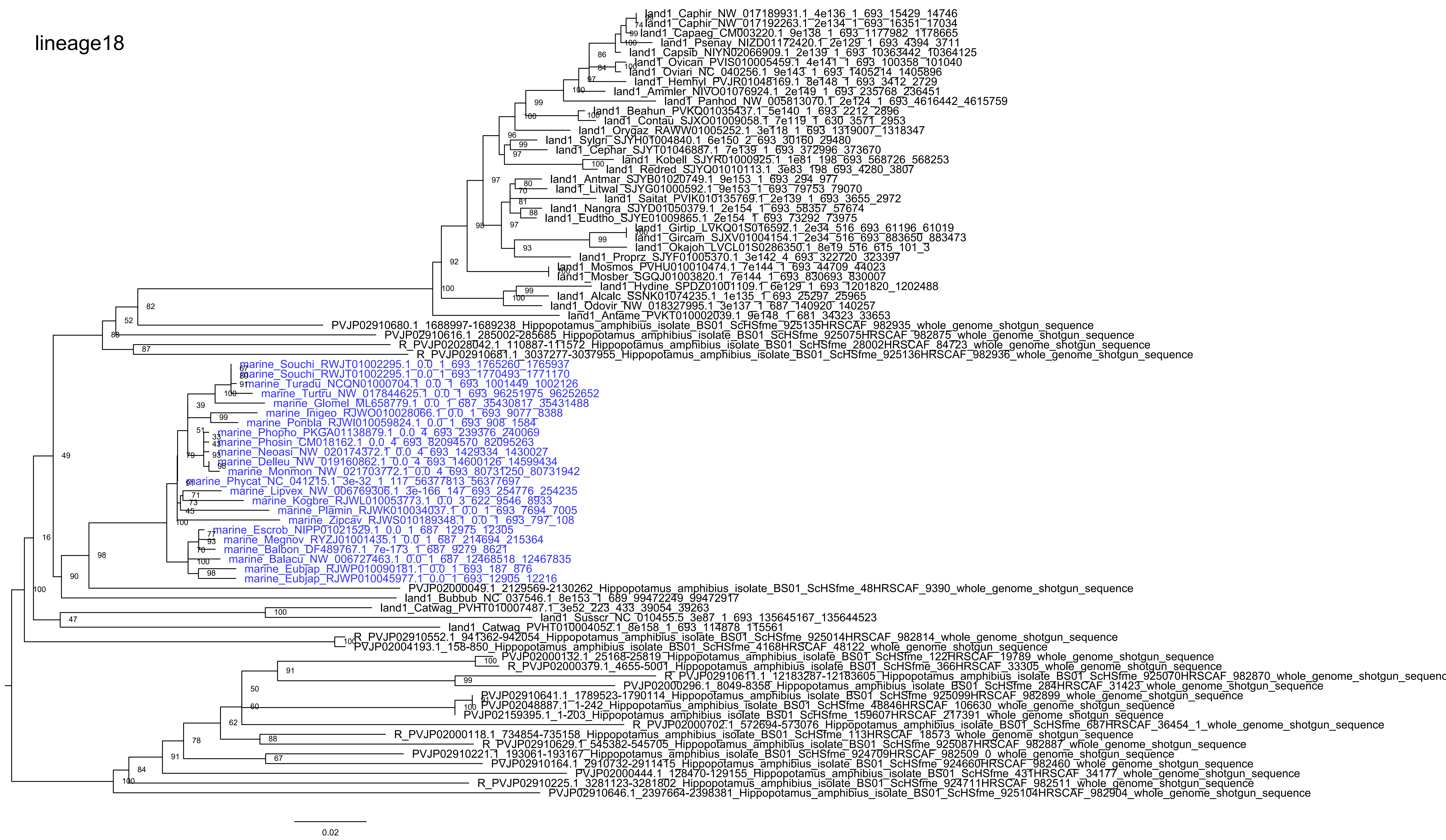
lineage18
land1_Caphir_NW_017189931.1_4e136_1_693_15429_14746
land1_Caphir_NW_017192263.1_2e134_1_693_16351_17034
land1_Capaeg_CM003220.1_9e138_1_693_1177982_1178665
land1_Psenay_NIZD01172420.1_2e129_1_693_4394_3711
land1_Capsib_NIYN02066909.1_2e139_1_693_10363442_10364125
land1_Ovican_PVIS010005459.1_4e141_1_693_100358_101040
land1_Oviari_NC_040256.1_9e143_1_693_1405214_1405896
land1_Hemhyl_PVJR01048169.1_8e148_1_693_3412_2729
land1_Ammler_NIVO01076924.1_2e149_1_693_235768_236451
land1_Panhod_NW_005813070.1_2e124_1_693_4616442_4615759
land1_Beahun_PVKQ01035437.1_5e140_1_693_2212_2896
land1_Contau_SJXO01009058.1_7e119_1_630_3571_2953
land1_Orygaz_RAWW01005252.1_3e118_1_693_1319007_1318347
land1_Sylgri_SJYH01004840.1_6e150_2_693_30160_29480
land1_Cephar_SJYT01046887.1_7e139_1_693_372996_373670
land1_Kobell_SJYR01000925.1_1e81_198_693_568726_568253
land1_Redred_SJYQ01010113.1_3e83_198_693_4280_3807
land1_Antmar_SJYB01020749.1_9e153_1_693_294_977
land1_Litwal_SJYG01000592.1_9e153_1_693_79753_79070
land1_Saitat_PVIK010135769.1_2e139_1_693_3655_2972
land1_Nangra_SJYD01050379.1_2e154_1_693_58357_57674
land1_Eudtho_SJYE01009865.1_2e154_1_693_73292_73975
land1_Girtip_LVKQ01S016592.1_2e34_516_693_61196_61019
land1_Gircam_SJXV01004154.1_2e34_516_693_883650_883473
land1_Okajoh_LVCL01S0286350.1_8e19_516_615_101_3
land1_Proprz_SJYF01005370.1_3e142_4_693_322720_323397
land1_Mosmos_PVHU010010474.1_7e144_1_693_44709_44023
land1_Mosber_SGQJ01003820.1_7e144_1_693_830693_830007
land1_Hydine_SPDZ01001109.1_6e129_1_693_1201820_1202488
land1_Alcalc_SSNK01074235.1_1e135_1_693_25297_25965
land1_Odovir_NW_018327995.1_3e137_1_687_140920_140257
land1_Antame_PVKT010002039.1_9e148_1_681_34323_33653
PVJP02910680.1_1688997-1689238_Hippopotamus_amphibius_isolate_BS01_ScHSfme_925135HRSCAF_982935_whole_genome_shotgun_sequence
PVJP02910616.1_285002-285685_Hippopotamus_amphibius_isolate_BS01_ScHSfme_925075HRSCAF_982875_whole_genome_shotgun_sequence
R_PVJP02028042.1_110887-111572_Hippopotamus_amphibius_isolate_BS01_ScHSfme_28002HRSCAF_84723_whole_genome_shotgun_sequence
R_PVJP02910681.1_3037277-3037955_Hippopotamus_amphibius_isolate_BS01_ScHSfme_925136HRSCAF_982936_whole_genome_shotgun_sequence
marine_Souchi_RWJT01002295.1_0.0_1_693_1765260_1765937
marine_Souchi_RWJT01002295.1_0.0_1_693_1770493_1771170
marine_Turadu_NCQN01000704.1_0.0_1_693_1001449_1002126
marine_Turtru_NW_017844625.1_0.0_1_693_96251975_96252652
marine_Glomel_ML658779.1_0.0_1_687_35430817_35431488
marine_Inigeo_RJWO010028066.1_0.0_1_693_9077_8388
marine_Ponbla_RJWI010059824.1_0.0_1_693_908_1584
marine_Phopho_PKGA01138879.1_0.0_4_693_239376_240069
marine_Phosin_CM018162.1_0.0_4_693_82094570_82095263
marine_Neoasi_NW_020174372.1_0.0_4_693_1429334_1430027
marine_Delleu_NW_019160862.1_0.0_4_693_14600126_14599434
marine_Monmon_NW_021703772.1_0.0_4_693_80731250_80731942
marine_Phycat_NC_041215.1_3e-32_1_117_56377813_56377697
marine_Lipvex_NW_006769306.1_3e-166_147_693_254776_254235
marine_Kogbre_RJWL010053773.1_0.0_3_622_9546_8933
marine_Plamin_RJWK010034037.1_0.0_1_693_7694_7005
marine_Zipcav_RJWS010189348.1_0.0_1_693_797_108
marine_Escrob_NIPP01021529.1_0.0_1_687_12975_12305
marine_Megnov_RYZJ01001435.1_0.0_1_687_214694_215364
marine_Balbon_DF489767.1_7e-173_1_687_9279_8621
marine_Balacu_NW_006727463.1_0.0_1_687_12468518_12467835
marine_Eubjap_RJWP010090181.1_0.0_1_693_187_876
marine_Eubjap_RJWP010045977.1_0.0_1_693_12905_12216
PVJP02000049.1_2129569-2130262_Hippopotamus_amphibius_isolate_BS01_ScHSfme_48HRSCAF_9390_whole_genome_shotgun_sequence
land1_Bubbub_NC_037546.1_8e153_1_689_99472249_99472917
land1_Catwag_PVHT010007487.1_3e52_223_433_39054_39263
land1_Susscr_NC_010455.5_3e87_1_693_135645167_135644523
land1_Catwag_PVHT010004052.1_8e158_1_693_114878_115561
R_PVJP02910552.1_941362-942054_Hippopotamus_amphibius_isolate_BS01_ScHSfme_925014HRSCAF_982814_whole_genome_shotgun_sequence
PVJP02004193.1_158-850_Hippopotamus_amphibius_isolate_BS01_ScHSfme_4168HRSCAF_48122_whole_genome_shotgun_sequence
PVJP02000132.1_25168-25819_Hippopotamus_amphibius_isolate_BS01_ScHSfme_122HRSCAF_19789_whole_genome_shotgun_sequence
R_PVJP02000379.1_4655-5001_Hippopotamus_amphibius_isolate_BS01_ScHSfme_366HRSCAF_33305_whole_genome_shotgun_sequence
R_PVJP02910611.1_12183287-12183605_Hippopotamus_amphibius_isolate_BS01_ScHSfme_925070HRSCAF_982870_whole_genome_shotgun_sequence
PVJP02000296.1_8049-8358_Hippopotamus_amphibius_isolate_BS01_ScHSfme_284HRSCAF_31423_whole_genome_shotgun_sequence
PVJP02910641.1_1789523-1790114_Hippopotamus_amphibius_isolate_BS01_ScHSfme_925099HRSCAF_982899_whole_genome_shotgun_sequence
PVJP02048887.1_1-242_Hippopotamus_amphibius_isolate_BS01_ScHSfme_48846HRSCAF_106630_whole_genome_shotgun_sequence
PVJP02159395.1_1-203_Hippopotamus_amphibius_isolate_BS01_ScHSfme_159607HRSCAF_217391_whole_genome_shotgun_sequence
R_PVJP02000702.1_572694-573076_Hippopotamus_amphibius_isolate_BS01_ScHSfme_687HRSCAF_36454_1_whole_genome_shotgun_sequence
R_PVJP02000118.1_734854-735158_Hippopotamus_amphibius_isolate_BS01_ScHSfme_113HRSCAF_18573_whole_genome_shotgun_sequence
R_PVJP02910629.1_545382-545705_Hippopotamus_amphibius_isolate_BS01_ScHSfme_925087HRSCAF_982887_whole_genome_shotgun_sequence
PVJP02910221.1_193061-193167_Hippopotamus_amphibius_isolate_BS01_ScHSfme_924709HRSCAF_982509_0_whole_genome_shotgun_sequence
PVJP02910164.1_2910732-2911415_Hippopotamus_amphibius_isolate_BS01_ScHSfme_924660HRSCAF_982460_whole_genome_shotgun_sequence
PVJP02000444.1_128470-129155_Hippopotamus_amphibius_isolate_BS01_ScHSfme_431HRSCAF_34177_whole_genome_shotgun_sequence
R_PVJP02910225.1_3281123-3281802_Hippopotamus_amphibius_isolate_BS01_ScHSfme_924711HRSCAF_982511_whole_genome_shotgun_sequence
PVJP02910646.1_2397664-2398381_Hippopotamus_amphibius_isolate_BS01_ScHSfme_925104HRSCAF_982904_whole_genome_shotgun_sequence
0.02

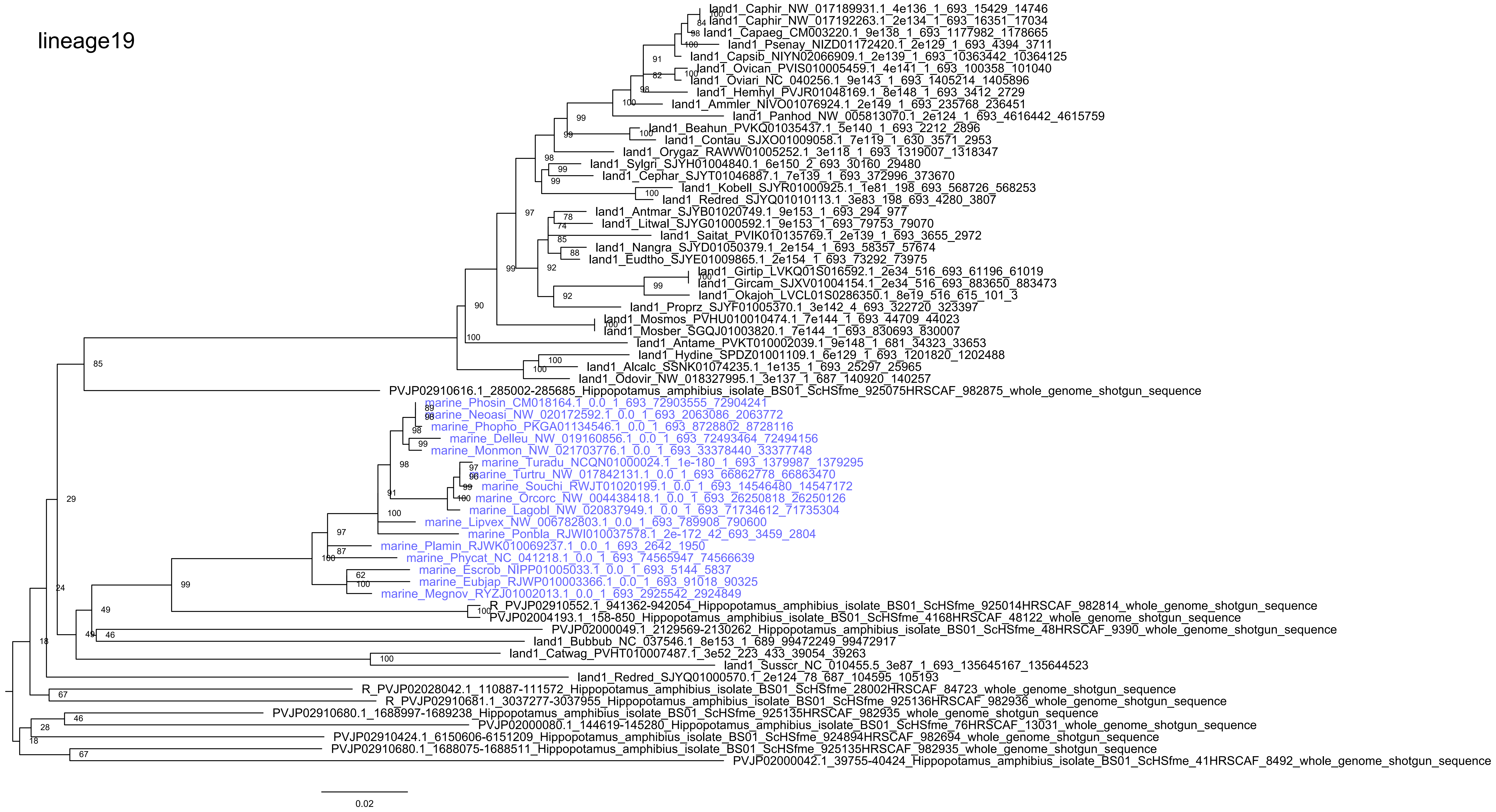

lineage19
land1_Caphir_NW_017189931.1_4e136_1_693_15429_14746
land1_Caphir_NW_017192263.1_2e134_1_693_16351_17034
land1_Capaeg_CM003220.1_9e138_1_693_1177982_1178665
land1_Psenay_NIZD01172420.1_2e129_1_693_4394_3711
land1_Capsib_NIYN02066909.1_2e139_1_693_10363442_10364125
land1_Ovican_PVIS010005459.1_4e141_1_693_100358_101040
land1_Oviari_NC_040256.1_9e143_1_693_1405214_1405896
land1_Hemhyl_PVJR01048169.1_8e148_1_693_3412_2729
land1_Ammler_NIVO01076924.1_2e149_1_693_235768_236451
land1_Panhod_NW_005813070.1_2e124_1_693_4616442_4615759
land1_Beahun_PVKQ01035437.1_5e140_1_693_2212_2896
land1_Contau_SJXO01009058.1_7e119_1_630_3571_2953
land1_Orygaz_RAWW01005252.1_3e118_1_693_1319007_1318347
land1_Sylgri_SJYH01004840.1_6e150_2_693_30160_29480
land1_Cephar_SJYT01046887.1_7e139_1_693_372996_373670
land1_Kobell_SJYR01000925.1_1e81_198_693_568726_568253
land1_Redred_SJYQ01010113.1_3e83_198_693_4280_3807
land1_Antmar_SJYB01020749.1_9e153_1_693_294_977
land1_Litwal_SJYG01000592.1_9e153_1_693_79753_79070
land1_Saitat_PVIK010135769.1_2e139_1_693_3655_2972
land1_Nangra_SJYD01050379.1_2e154_1_693_58357_57674
land1_Eudtho_SJYE01009865.1_2e154_1_693_73292_73975
land1_Girtip_LVKQ01S016592.1_2e34_516_693_61196_61019
land1_Gircam_SJXV01004154.1_2e34_516_693_883650_883473
land1_Okajoh_LVCL01S0286350.1_8e19_516_615_101_3
land1_Proprz_SJYF01005370.1_3e142_4_693_322720_323397
land1_Mosmos_PVHU010010474.1_7e144_1_693_44709_44023
land1_Mosber_SGQJ01003820.1_7e144_1_693_830693_830007
land1_Antame_PVKT010002039.1_9e148_1_681_34323_33653
land1_Hydine_SPDZ01001109.1_6e129_1_693_1201820_1202488
land1_Alcalc_SSNK01074235.1_1e135_1_693_25297_25965
land1_Odovir_NW_018327995.1_3e137_1_687_140920_140257
PVJP02910616.1_285002-285685_Hippopotamus_amphibius_isolate_BS01_ScHSfme_925075HRSCAF_982875_whole_genome_shotgun_sequence
marine_Phosin_CM018164.1_0.0_1_693_72903555_72904241
marine_Neoasi_NW_020172592.1_0.0_1_693_2063086_2063772
marine_Phopho_PKGA01134546.1_0.0_1_693_8728802_8728116
marine_Delleu_NW_019160856.1_0.0_1_693_72493464_72494156
marine_Monmon_NW_021703776.1_0.0_1_693_33378440_33377748
marine_Turadu_NCQN01000024.1_1e-180_1_693_1379987_1379295
marine_Turtru_NW_017842131.1_0.0_1_693_66862778_66863470
marine_Souchi_RWJT01020199.1_0.0_1_693_14546480_14547172
marine_Orcorc_NW_004438418.1_0.0_1_693_26250818_26250126
marine_Lagobl_NW_020837949.1_0.0_1_693_71734612_71735304
marine_Lipvex_NW_006782803.1_0.0_1_693_789908_790600
marine_Ponbla_RJWI010037578.1_2e-172_42_693_3459_2804
marine_Plamin_RJWK010069237.1_0.0_1_693_2642_1950
marine_Phycat_NC_041218.1_0.0_1_693_74565947_74566639
marine_Escrob_NIPP01005033.1_0.0_1_693_5144_5837
marine_Eubjap_RJWP010003366.1_0.0_1_693_91018_90325
marine_Megnov_RYZJ01002013.1_0.0_1_693_2925542_2924849
R_PVJP02910552.1_941362-942054_Hippopotamus_amphibius_isolate_BS01_ScHSfme_925014HRSCAF_982814_whole_genome_shotgun_sequence
PVJP02004193.1_158-850_Hippopotamus_amphibius_isolate_BS01_ScHSfme_4168HRSCAF_48122_whole_genome_shotgun_sequence
PVJP02000049.1_2129569-2130262_Hippopotamus_amphibius_isolate_BS01_ScHSfme_48HRSCAF_9390_whole_genome_shotgun_sequence
land1_Bubbub_NC_037546.1_8e153_1_689_99472249_99472917
land1_Catwag_PVHT010007487.1_3e52_223_433_39054_39263
land1_Susscr_NC_010455.5_3e87_1_693_135645167_135644523
land1_Redred_SJYQ01000570.1_2e124_78_687_104595_105193
R_PVJP02028042.1_110887-111572_Hippopotamus_amphibius_isolate_BS01_ScHSfme_28002HRSCAF_84723_whole_genome_shotgun_sequence
R_PVJP02910681.1_3037277-3037955_Hippopotamus_amphibius_isolate_BS01_ScHSfme_925136HRSCAF_982936_whole_genome_shotgun_sequence
PVJP02910680.1_1688997-1689238_Hippopotamus_amphibius_isolate_BS01_ScHSfme_925135HRSCAF_982935_whole_genome_shotgun_sequence
PVJP02000080.1_144619-145280_Hippopotamus_amphibius_isolate_BS01_ScHSfme_76HRSCAF_13031_whole_genome_shotgun_sequence
PVJP02910424.1_6150606-6151209_Hippopotamus_amphibius_isolate_BS01_ScHSfme_924894HRSCAF_982694_whole_genome_shotgun_sequence
PVJP02910680.1_1688075-1688511_Hippopotamus_amphibius_isolate_BS01_ScHSfme_925135HRSCAF_982935_whole_genome_shotgun_sequence
PVJP02000042.1_39755-40424_Hippopotamus_amphibius_isolate_BS01_ScHSfme_41HRSCAF_8492_whole_genome_shotgun_sequence
0.02

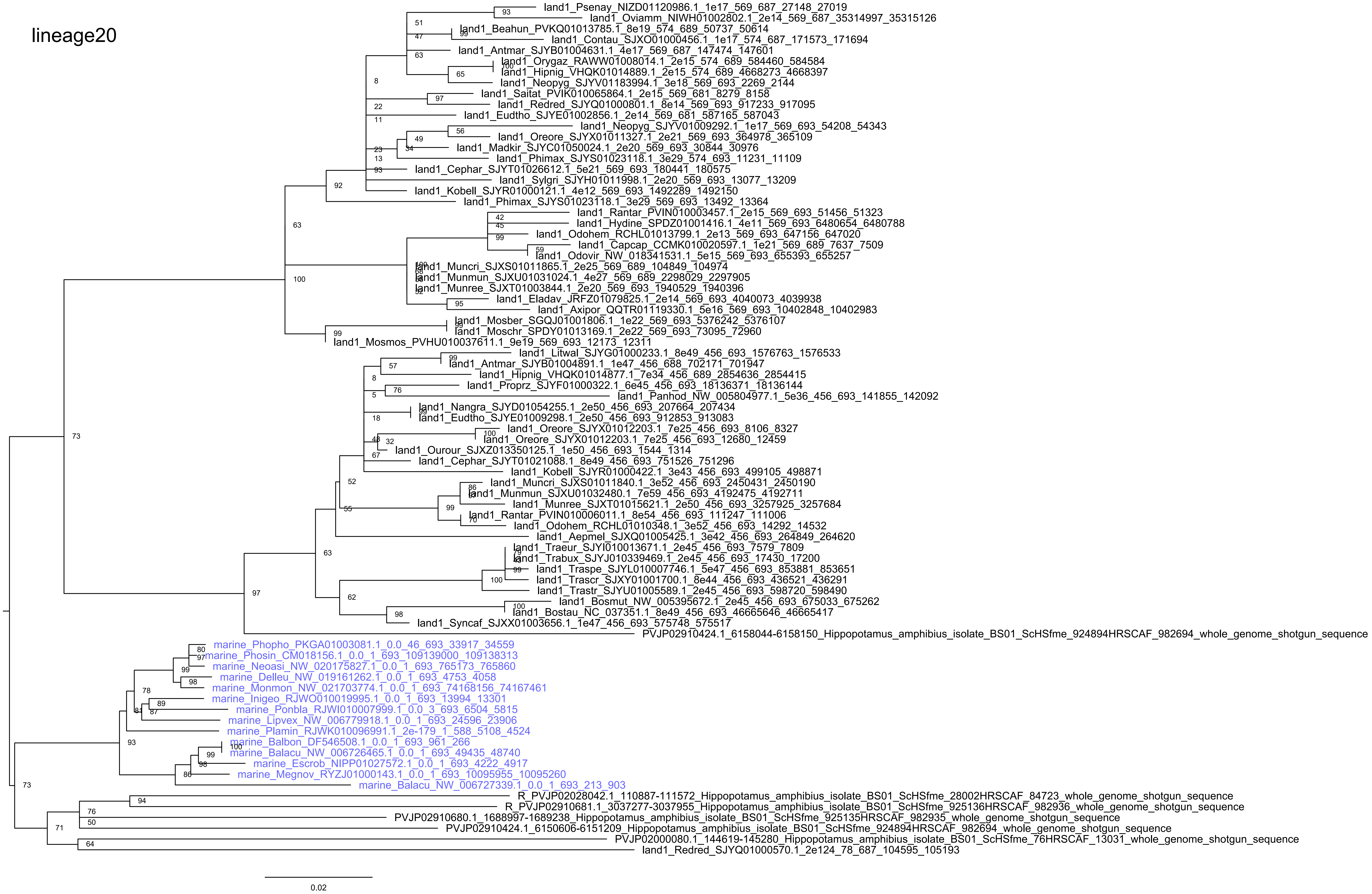
lineage20
land1_Psenay_NIZD01120986.1_1e17_569_687_27148_27019
land1_Oviamm_NIWH01002802.1_2e14_569_687_35314997_35315126
land1_Beahun_PVKQ01013785.1_8e19_574_689_50737_50614
land1_Contau_SJXO01000456.1_1e17_574_687_171573_171694
land1_Antmar_SJYB01004631.1_4e17_569_687_147474_147601
land1_Orygaz_RAWW01008014.1_2e15_574_689_584460_584584
land1_Hipnig_VHQK01014889.1_2e15_574_689_4668273_4668397
land1_Neopyg_SJYV01183994.1_3e18_569_693_2269_2144
land1_Saitat_PVIK010065864.1_2e15_569_681_8279_8158
land1_Redred_SJYQ01000801.1_8e14_569_693_917233_917095
land1_Eudtho_SJYE01002856.1_2e14_569_681_587165_587043
land1_Neopyg_SJYV01009292.1_1e17_569_693_54208_54343
land1_Oreore_SJYX01011327.1_2e21_569_693_364978_365109
land1_Madkir_SJYC01050024.1_2e20_569_693_30844_30976
land1_Phimax_SJYS01023118.1_3e29_574_693_11231_11109
land1_Cephar_SJYT01026612.1_5e21_569_693_180441_180575
land1_Sylgri_SJYH01011998.1_2e20_569_693_13077_13209
land1_Kobell_SJYR01000121.1_4e12_569_693_1492289_1492150
land1_Phimax_SJYS01023118.1_3e29_569_693_13492_13364
land1_Rantar_PVIN010003457.1_2e15_569_693_51456_51323
land1_Hydine_SPDZ01001416.1_4e11_569_693_6480654_6480788
land1_Odohem_RCHL01013799.1_2e13_569_693_647156_647020
land1_Capcap_CCMK010020597.1_1e21_569_689_7637_7509
land1_Odovir_NW_018341531.1_5e15_569_693_655393_655257
land1_Muncri_SJXS01011865.1_2e25_569_689_104849_104974
land1_Munmun_SJXU01031024.1_4e27_569_689_2298029_2297905
land1_Munree_SJXT01003844.1_2e20_569_693_1940529_1940396
land1_Eladav_JRFZ01079825.1_2e14_569_693_4040073_4039938
land1_Axipor_QQTR01119330.1_5e16_569_693_10402848_10402983
land1_Mosber_SGQJ01001806.1_1e22_569_693_5376242_5376107
land1_Moschr_SPDY01013169.1_2e22_569_693_73095_72960
land1_Mosmos_PVHU010037611.1_9e19_569_693_12173_12311
land1_Litwal_SJYG01000233.1_8e49_456_693_1576763_1576533
land1_Antmar_SJYB01004891.1_1e47_456_688_702171_701947
land1_Hipnig_VHQK01014877.1_7e34_456_689_2854636_2854415
land1_Proprz_SJYF01000322.1_6e45_456_693_18136371_18136144
land1_Panhod_NW_005804977.1_5e36_456_693_141855_142092
land1_Nangra_SJYD01054255.1_2e50_456_693_207664_207434
land1_Eudtho_SJYE01009298.1_2e50_456_693_912853_913083
land1_Oreore_SJYX01012203.1_7e25_456_693_8106_8327
land1_Oreore_SJYX01012203.1_7e25_456_693_12680_12459
land1_Ourour_SJXZ013350125.1_1e50_456_693_1544_1314
land1_Cephar_SJYT01021088.1_8e49_456_693_751526_751296
land1_Kobell_SJYR01000422.1_3e43_456_693_499105_498871
land1_Muncri_SJXS01011840.1_3e52_456_693_2450431_2450190
land1_Munmun_SJXU01032480.1_7e59_456_693_4192475_4192711
land1_Munree_SJXT01015621.1_2e50_456_693_3257925_3257684
land1_Rantar_PVIN010006011.1_8e54_456_693_111247_111006
land1_Odohem_RCHL01010348.1_3e52_456_693_14292_14532
land1_Aepmel_SJXQ01005425.1_3e42_456_693_264849_264620
land1_Traeur_SJYI010013671.1_2e45_456_693_7579_7809
land1_Trabux_SJYJ010339469.1_2e45_456_693_17430_17200
land1_Traspe_SJYL010007746.1_5e47_456_693_853881_853651
land1_Trascr_SJXY01001700.1_8e44_456_693_436521_436291
land1_Trastr_SJYU01005589.1_2e45_456_693_598720_598490
land1_Bosmut_NW_005395672.1_2e45_456_693_675033_675262
land1_Bostau_NC_037351.1_8e49_456_693_46665646_46665417
land1_Syncaf_SJXX01003656.1_1e47_456_693_575748_575517
PVJP02910424.1_6158044-6158150_Hippopotamus_amphibius_isolate_BS01_ScHSfme_924894HRSCAF_982694_whole_genome_shotgun_sequence
marine_Phopho_PKGA01003081.1_0.0_46_693_33917_34559
marine_Phosin_CM018156.1_0.0_1_693_109139000_109138313
marine_Neoasi_NW_020175827.1_0.0_1_693_765173_765860
marine_Delleu_NW_019161262.1_0.0_1_693_4753_4058
marine_Monmon_NW_021703774.1_0.0_1_693_74168156_74167461
marine_Inigeo_RJWO010019995.1_0.0_1_693_13994_13301
marine_Ponbla_RJWI010007999.1_0.0_3_693_6504_5815
marine_Lipvex_NW_006779918.1_0.0_1_693_24596_23906
marine_Plamin_RJWK010096991.1_2e-179_1_588_5108_4524
marine_Balbon_DF546508.1_0.0_1_693_961_266
marine_Balacu_NW_006726465.1_0.0_1_693_49435_48740
marine_Escrob_NIPP01027572.1_0.0_1_693_4222_4917
marine_Megnov_RYZJ01000143.1_0.0_1_693_10095955_10095260
marine_Balacu_NW_006727339.1_0.0_1_693_213_903
R_PVJP02028042.1_110887-111572_Hippopotamus_amphibius_isolate_BS01_ScHSfme_28002HRSCAF_84723_whole_genome_shotgun_sequence
R_PVJP02910681.1_3037277-3037955_Hippopotamus_amphibius_isolate_BS01_ScHSfme_925136HRSCAF_982936_whole_genome_shotgun_sequence
PVJP02910680.1_1688997-1689238_Hippopotamus_amphibius_isolate_BS01_ScHSfme_925135HRSCAF_982935_whole_genome_shotgun_sequence
PVJP02910424.1_6150606-6151209_Hippopotamus_amphibius_isolate_BS01_ScHSfme_924894HRSCAF_982694_whole_genome_shotgun_sequence
PVJP02000080.1_144619-145280_Hippopotamus_amphibius_isolate_BS01_ScHSfme_76HRSCAF_13031_whole_genome_shotgun_sequence
land1_Redred_SJYQ01000570.1_2e124_78_687_104595_105193
0.02

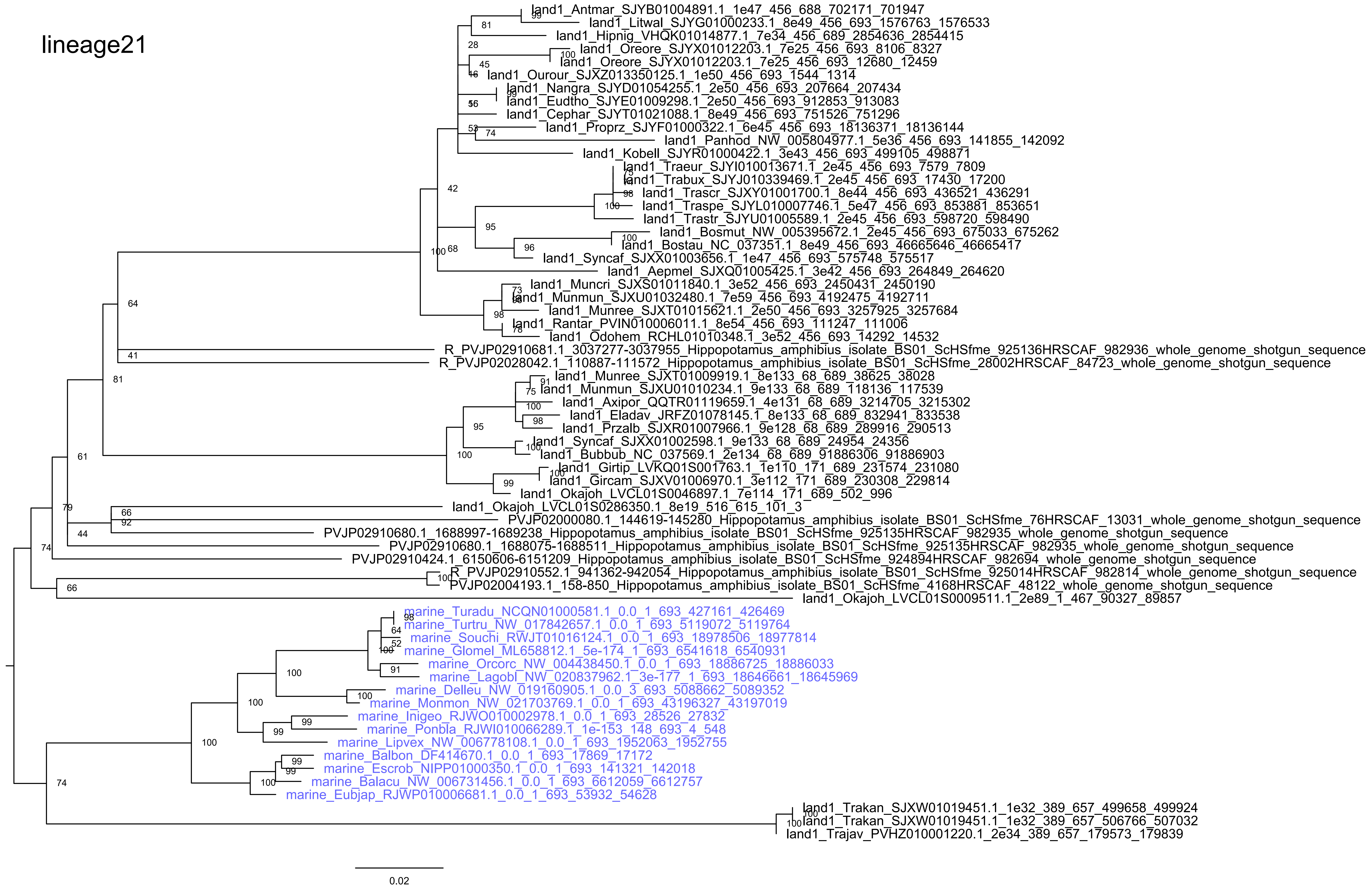
lineage21
land1_Antmar_SJYB01004891.1_1e47_456_688_702171_701947
land1_Litwal_SJYG01000233.1_8e49_456_693_1576763_1576533
land1_Hipnig_VHQK01014877.1_7e34_456_689_2854636_2854415
land1_Oreore_SJYX01012203.1_7e25_456_693_8106_8327
land1_Oreore_SJYX01012203.1_7e25_456_693_12680_12459
land1_Ourour_SJXZ013350125.1_1e50_456_693_1544_1314
land1_Nangra_SJYD01054255.1_2e50_456_693_207664_207434
land1_Eudtho_SJYE01009298.1_2e50_456_693_912853_913083
land1_Cephar_SJYT01021088.1_8e49_456_693_751526_751296
land1_Proprz_SJYF01000322.1_6e45_456_693_18136371_18136144
land1_Panhod_NW_005804977.1_5e36_456_693_141855_142092
land1_Kobell_SJYR01000422.1_3e43_456_693_499105_498871
land1_Traeur_SJYI010013671.1_2e45_456_693_7579_7809
land1_Trabux_SJYJ010339469.1_2e45_456_693_17430_17200
land1_Trascr_SJXY01001700.1_8e44_456_693_436521_436291
land1_Traspe_SJYL010007746.1_5e47_456_693_853881_853651
land1_Trastr_SJYU01005589.1_2e45_456_693_598720_598490
land1_Bosmut_NW_005395672.1_2e45_456_693_675033_675262
land1_Bostau_NC_037351.1_8e49_456_693_46665646_46665417
land1_Syncaf_SJXX01003656.1_1e47_456_693_575748_575517
land1_Aepmel_SJXQ01005425.1_3e42_456_693_264849_264620
land1_Muncri_SJXS01011840.1_3e52_456_693_2450431_2450190
land1_Munmun_SJXU01032480.1_7e59_456_693_4192475_4192711
land1_Munree_SJXT01015621.1_2e50_456_693_3257925_3257684
land1_Rantar_PVIN010006011.1_8e54_456_693_111247_111006
land1_Odohem_RCHL01010348.1_3e52_456_693_14292_14532
R_PVJP02910681.1_3037277-3037955_Hippopotamus_amphibius_isolate_BS01_ScHSfme_925136HRSCAF_982936_whole_genome_shotgun_sequence
R_PVJP02028042.1_110887-111572_Hippopotamus_amphibius_isolate_BS01_ScHSfme_28002HRSCAF_84723_whole_genome_shotgun_sequence
land1_Munree_SJXT01009919.1_8e133_68_689_38625_38028
land1_Munmun_SJXU01010234.1_9e133_68_689_118136_117539
land1_Axipor_QQTR01119659.1_4e131_68_689_3214705_3215302
land1_Eladav_JRFZ01078145.1_8e133_68_689_832941_833538
land1_Przalb_SJXR01007966.1_9e128_68_689_289916_290513
land1_Syncaf_SJXX01002598.1_9e133_68_689_24954_24356
land1_Bubbub_NC_037569.1_2e134_68_689_91886306_91886903
land1_Girtip_LVKQ01S001763.1_1e110_171_689_231574_231080
land1_Gircam_SJXV01006970.1_3e112_171_689_230308_229814
land1_Okajoh_LVCL01S0046897.1_7e114_171_689_502_996
land1_Okajoh_LVCL01S0286350.1_8e19_516_615_101_3
PVJP02000080.1_144619-145280_Hippopotamus_amphibius_isolate_BS01_ScHSfme_76HRSCAF_13031_whole_genome_shotgun_sequence
PVJP02910680.1_1688997-1689238_Hippopotamus_amphibius_isolate_BS01_ScHSfme_925135HRSCAF_982935_whole_genome_shotgun_sequence
PVJP02910680.1_1688075-1688511_Hippopotamus_amphibius_isolate_BS01_ScHSfme_925135HRSCAF_982935_whole_genome_shotgun_sequence
PVJP02910424.1_6150606-6151209_Hippopotamus_amphibius_isolate_BS01_ScHSfme_924894HRSCAF_982694_whole_genome_shotgun_sequence
R_PVJP02910552.1_941362-942054_Hippopotamus_amphibius_isolate_BS01_ScHSfme_925014HRSCAF_982814_whole_genome_shotgun_sequence
PVJP02004193.1_158-850_Hippopotamus_amphibius_isolate_BS01_ScHSfme_4168HRSCAF_48122_whole_genome_shotgun_sequence
land1_Okajoh_LVCL01S0009511.1_2e89_1_467_90327_89857
marine_Turadu_NCQN01000581.1_0.0_1_693_427161_426469
marine_Turtru_NW_017842657.1_0.0_1_693_5119072_5119764
marine_Souchi_RWJT01016124.1_0.0_1_693_18978506_18977814
marine_Glomel_ML658812.1_5e-174_1_693_6541618_6540931
marine_Orcorc_NW_004438450.1_0.0_1_693_18886725_18886033
marine_Lagobl_NW_020837962.1_3e-177_1_693_18646661_18645969
marine_Delleu_NW_019160905.1_0.0_3_693_5088662_5089352
marine_Monmon_NW_021703769.1_0.0_1_693_43196327_43197019
marine_Inigeo_RJWO010002978.1_0.0_1_693_28526_27832
marine_Ponbla_RJWI010066289.1_1e-153_148_693_4_548
marine_Lipvex_NW_006778108.1_0.0_1_693_1952063_1952755
marine_Balbon_DF414670.1_0.0_1_693_17869_17172
marine_Escrob_NIPP01000350.1_0.0_1_693_141321_142018
marine_Balacu_NW_006731456.1_0.0_1_693_6612059_6612757
marine_Eubjap_RJWP010006681.1_0.0_1_693_53932_54628
land1_Trakan_SJXW01019451.1_1e32_389_657_499658_499924
land1_Trakan_SJXW01019451.1_1e32_389_657_506766_507032
land1_Trajav_PVHZ010001220.1_2e34_389_657_179573_179839
0.02

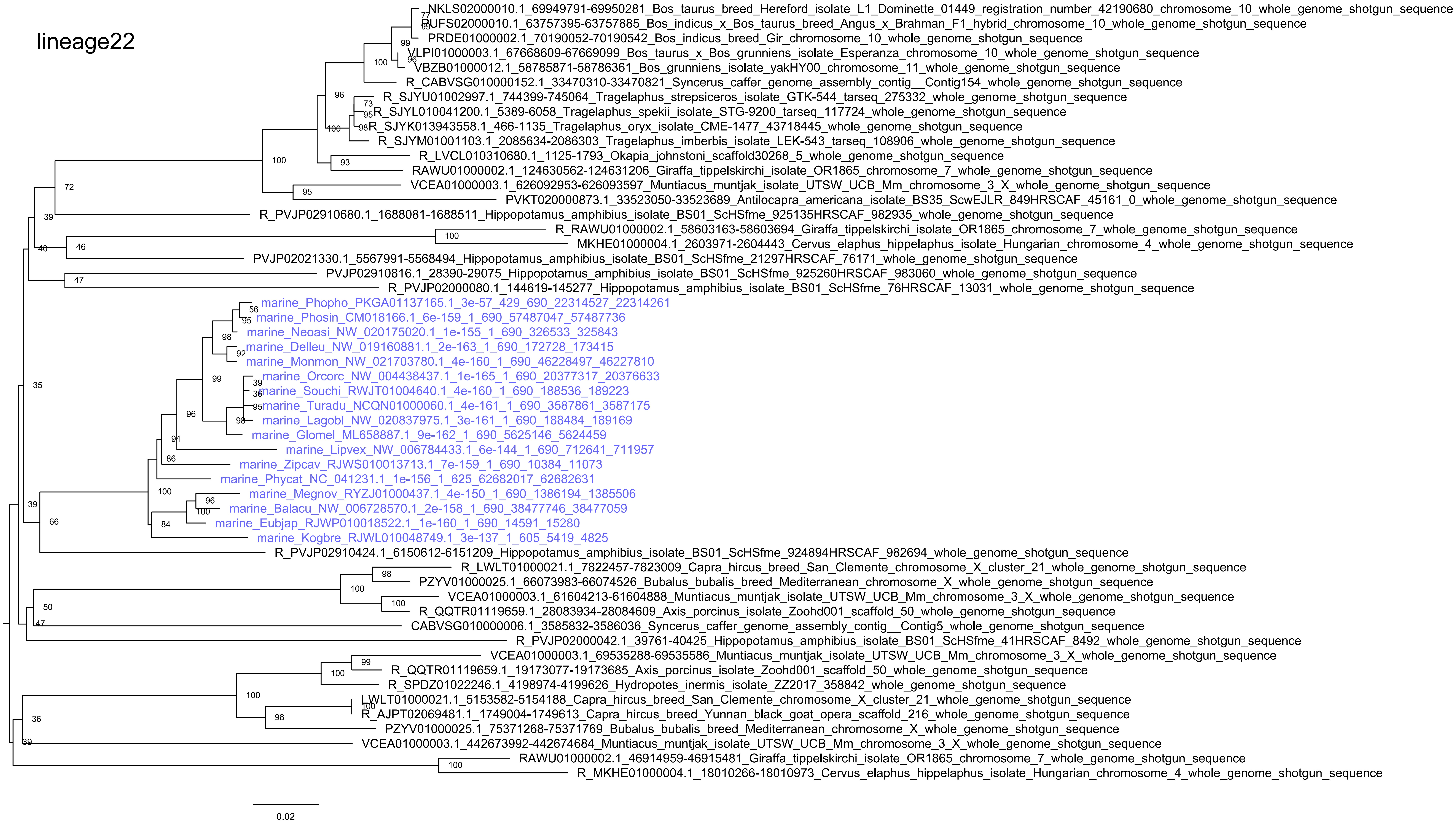
lineage22
NKLS02000010.1_69949791-69950281_Bos_taurus_breed_Hereford_isolate_L1_Dominette_01449_registration_number_42190680_chromosome_10_whole_genome_shotgun_sequence
RUFS02000010.1_63757395-63757885_Bos_indicus_x_Bos_taurus_breed_Angus_x_Brahman_F1_hybrid_chromosome_10_whole_genome_shotgun_sequence
PRDE01000002.1_70190052-70190542_Bos_indicus_breed_Gir_chromosome_10_whole_genome_shotgun_sequence
VLPI01000003.1_67668609-67669099_Bos_taurus_x_Bos_grunniens_isolate_Esperanza_chromosome_10_whole_genome_shotgun_sequence
VBZB01000012.1_58785871-58786361_Bos_grunniens_isolate_yakHY00_chromosome_11_whole_genome_shotgun_sequence
R_CABVSG010000152.1_33470310-33470821_Syncerus_caffer_genome_assembly_contig__Contig154_whole_genome_shotgun_sequence
R_SJYU01002997.1_744399-745064_Tragelaphus_strepsiceros_isolate_GTK-544_tarseq_275332_whole_genome_shotgun_sequence
R_SJYL010041200.1_5389-6058_Tragelaphus_spekii_isolate_STG-9200_tarseq_117724_whole_genome_shotgun_sequence
R_SJYK013943558.1_466-1135_Tragelaphus_oryx_isolate_CME-1477_43718445_whole_genome_shotgun_sequence
R_SJYM01001103.1_2085634-2086303_Tragelaphus_imberbis_isolate_LEK-543_tarseq_108906_whole_genome_shotgun_sequence
R_LVCL010310680.1_1125-1793_Okapia_johnstoni_scaffold30268_5_whole_genome_shotgun_sequence
RAWU01000002.1_124630562-124631206_Giraffa_tippelskirchi_isolate_OR1865_chromosome_7_whole_genome_shotgun_sequence
VCEA01000003.1_626092953-626093597_Muntiacus_muntjak_isolate_UTSW_UCB_Mm_chromosome_3_X_whole_genome_shotgun_sequence
PVKT020000873.1_33523050-33523689_Antilocapra_americana_isolate_BS35_ScwEJLR_849HRSCAF_45161_0_whole_genome_shotgun_sequence
R_PVJP02910680.1_1688081-1688511_Hippopotamus_amphibius_isolate_BS01_ScHSfme_925135HRSCAF_982935_whole_genome_shotgun_sequence
R_RAWU01000002.1_58603163-58603694_Giraffa_tippelskirchi_isolate_OR1865_chromosome_7_whole_genome_shotgun_sequence
MKHE01000004.1_2603971-2604443_Cervus_elaphus_hippelaphus_isolate_Hungarian_chromosome_4_whole_genome_shotgun_sequence
PVJP02021330.1_5567991-5568494_Hippopotamus_amphibius_isolate_BS01_ScHSfme_21297HRSCAF_76171_whole_genome_shotgun_sequence
PVJP02910816.1_28390-29075_Hippopotamus_amphibius_isolate_BS01_ScHSfme_925260HRSCAF_983060_whole_genome_shotgun_sequence
R_PVJP02000080.1_144619-145277_Hippopotamus_amphibius_isolate_BS01_ScHSfme_76HRSCAF_13031_whole_genome_shotgun_sequence
marine_Phopho_PKGA01137165.1_3e-57_429_690_22314527_22314261
marine_Phosin_CM018166.1_6e-159_1_690_57487047_57487736
marine_Neoasi_NW_020175020.1_1e-155_1_690_326533_325843
marine_Delleu_NW_019160881.1_2e-163_1_690_172728_173415
marine_Monmon_NW_021703780.1_4e-160_1_690_46228497_46227810
marine_Orcorc_NW_004438437.1_1e-165_1_690_20377317_20376633
marine_Souchi_RWJT01004640.1_4e-160_1_690_188536_189223
marine_Turadu_NCQN01000060.1_4e-161_1_690_3587861_3587175
marine_Lagobl_NW_020837975.1_3e-161_1_690_188484_189169
marine_Glomel_ML658887.1_9e-162_1_690_5625146_5624459
marine_Lipvex_NW_006784433.1_6e-144_1_690_712641_711957
marine_Zipcav_RJWS010013713.1_7e-159_1_690_10384_11073
marine_Phycat_NC_041231.1_1e-156_1_625_62682017_62682631
marine_Megnov_RYZJ01000437.1_4e-150_1_690_1386194_1385506
marine_Balacu_NW_006728570.1_2e-158_1_690_38477746_38477059
marine_Eubjap_RJWP010018522.1_1e-160_1_690_14591_15280
marine_Kogbre_RJWL010048749.1_3e-137_1_605_5419_4825
R_PVJP02910424.1_6150612-6151209_Hippopotamus_amphibius_isolate_BS01_ScHSfme_924894HRSCAF_982694_whole_genome_shotgun_sequence
R_LWLT01000021.1_7822457-7823009_Capra_hircus_breed_San_Clemente_chromosome_X_cluster_21_whole_genome_shotgun_sequence
PZYV01000025.1_66073983-66074526_Bubalus_bubalis_breed_Mediterranean_chromosome_X_whole_genome_shotgun_sequence
VCEA01000003.1_61604213-61604888_Muntiacus_muntjak_isolate_UTSW_UCB_Mm_chromosome_3_X_whole_genome_shotgun_sequence
R_QQTR01119659.1_28083934-28084609_Axis_porcinus_isolate_Zoohd001_scaffold_50_whole_genome_shotgun_sequence
CABVSG010000006.1_3585832-3586036_Syncerus_caffer_genome_assembly_contig__Contig5_whole_genome_shotgun_sequence
R_PVJP02000042.1_39761-40425_Hippopotamus_amphibius_isolate_BS01_ScHSfme_41HRSCAF_8492_whole_genome_shotgun_sequence
VCEA01000003.1_69535288-69535586_Muntiacus_muntjak_isolate_UTSW_UCB_Mm_chromosome_3_X_whole_genome_shotgun_sequence
R_QQTR01119659.1_19173077-19173685_Axis_porcinus_isolate_Zoohd001_scaffold_50_whole_genome_shotgun_sequence
R_SPDZ01022246.1_4198974-4199626_Hydropotes_inermis_isolate_ZZ2017_358842_whole_genome_shotgun_sequence
LWLT01000021.1_5153582-5154188_Capra_hircus_breed_San_Clemente_chromosome_X_cluster_21_whole_genome_shotgun_sequence
R_AJPT02069481.1_1749004-1749613_Capra_hircus_breed_Yunnan_black_goat_opera_scaffold_216_whole_genome_shotgun_sequence
PZYV01000025.1_75371268-75371769_Bubalus_bubalis_breed_Mediterranean_chromosome_X_whole_genome_shotgun_sequence
VCEA01000003.1_442673992-442674684_Muntiacus_muntjak_isolate_UTSW_UCB_Mm_chromosome_3_X_whole_genome_shotgun_sequence
RAWU01000002.1_46914959-46915481_Giraffa_tippelskirchi_isolate_OR1865_chromosome_7_whole_genome_shotgun_sequence
R_MKHE01000004.1_18010266-18010973_Cervus_elaphus_hippelaphus_isolate_Hungarian_chromosome_4_whole_genome_shotgun_sequence
0.02

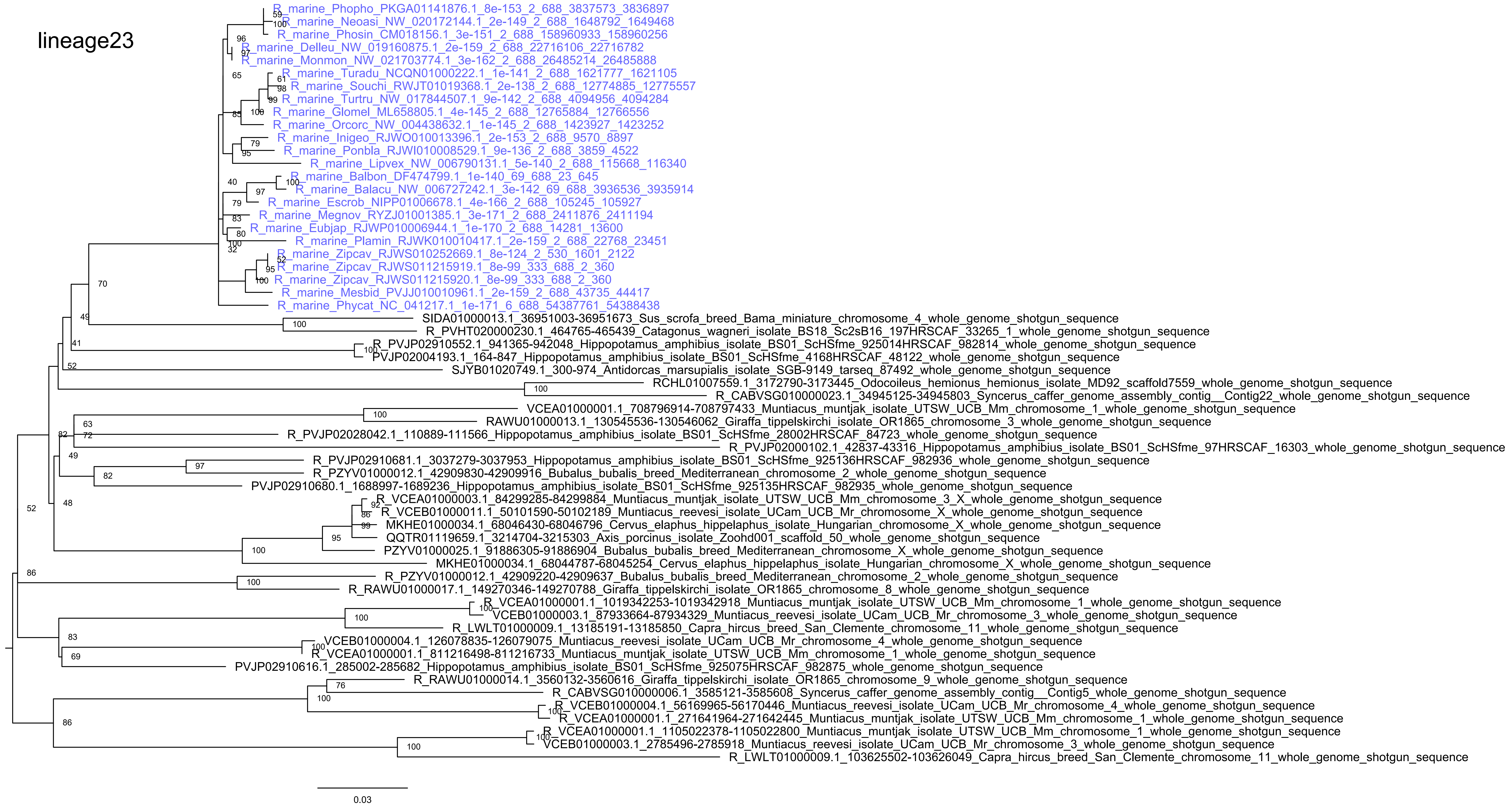
lineage23
R_marine_Phopho_PKGA01141876.1_8e-153_2_688_3837573_3836897
R_marine_Neoasi_NW_020172144.1_2e-149_2_688_1648792_1649468
R_marine_Phosin_CM018156.1_3e-151_2_688_158960933_158960256
R_marine_Delleu_NW_019160875.1_2e-159_2_688_22716106_22716782
R_marine_Monmon_NW_021703774.1_3e-162_2_688_26485214_26485888
R_marine_Turadu_NCQN01000222.1_1e-141_2_688_1621777_1621105
R_marine_Souchi_RWJT01019368.1_2e-138_2_688_12774885_12775557
R_marine_Turtru_NW_017844507.1_9e-142_2_688_4094956_4094284
R_marine_Glomel_ML658805.1_4e-145_2_688_12765884_12766556
R_marine_Orcorc_NW_004438632.1_1e-145_2_688_1423927_1423252
R_marine_Inigeo_RJWO010013396.1_2e-153_2_688_9570_8897
R_marine_Ponbla_RJWI010008529.1_9e-136_2_688_3859_4522
R_marine_Lipvex_NW_006790131.1_5e-140_2_688_115668_116340
R_marine_Balbon_DF474799.1_1e-140_69_688_23_645
R_marine_Balacu_NW_006727242.1_3e-142_69_688_3936536_3935914
R_marine_Escrob_NIPP01006678.1_4e-166_2_688_105245_105927
R_marine_Megnov_RYZJ01001385.1_3e-171_2_688_2411876_2411194
R_marine_Eubjap_RJWP010006944.1_1e-170_2_688_14281_13600
R_marine_Plamin_RJWK010010417.1_2e-159_2_688_22768_23451
R_marine_Zipcav_RJWS010252669.1_8e-124_2_530_1601_2122
R_marine_Zipcav_RJWS011215919.1_8e-99_333_688_2_360
R_marine_Zipcav_RJWS011215920.1_8e-99_333_688_2_360
R_marine_Mesbid_PVJJ010010961.1_2e-159_2_688_43735_44417
R_marine_Phycat_NC_041217.1_1e-171_6_688_54387761_54388438
SIDA01000013.1_36951003-36951673_Sus_scrofa_breed_Bama_miniature_chromosome_4_whole_genome_shotgun_sequence
R_PVHT020000230.1_464765-465439_Catagonus_wagneri_isolate_BS18_Sc2sB16_197HRSCAF_33265_1_whole_genome_shotgun_sequence
R_PVJP02910552.1_941365-942048_Hippopotamus_amphibius_isolate_BS01_ScHSfme_925014HRSCAF_982814_whole_genome_shotgun_sequence
PVJP02004193.1_164-847_Hippopotamus_amphibius_isolate_BS01_ScHSfme_4168HRSCAF_48122_whole_genome_shotgun_sequence
SJYB01020749.1_300-974_Antidorcas_marsupialis_isolate_SGB-9149_tarseq_87492_whole_genome_shotgun_sequence
RCHL01007559.1_3172790-3173445_Odocoileus_hemionus_hemionus_isolate_MD92_scaffold7559_whole_genome_shotgun_sequence
R_CABVSG010000023.1_34945125-34945803_Syncerus_caffer_genome_assembly_contig__Contig22_whole_genome_shotgun_sequence
VCEA01000001.1_708796914-708797433_Muntiacus_muntjak_isolate_UTSW_UCB_Mm_chromosome_1_whole_genome_shotgun_sequence
RAWU01000013.1_130545536-130546062_Giraffa_tippelskirchi_isolate_OR1865_chromosome_3_whole_genome_shotgun_sequence
R_PVJP02028042.1_110889-111566_Hippopotamus_amphibius_isolate_BS01_ScHSfme_28002HRSCAF_84723_whole_genome_shotgun_sequence
R_PVJP02000102.1_42837-43316_Hippopotamus_amphibius_isolate_BS01_ScHSfme_97HRSCAF_16303_whole_genome_shotgun_sequence
R_PVJP02910681.1_3037279-3037953_Hippopotamus_amphibius_isolate_BS01_ScHSfme_925136HRSCAF_982936_whole_genome_shotgun_sequence
R_PZYV01000012.1_42909830-42909916_Bubalus_bubalis_breed_Mediterranean_chromosome_2_whole_genome_shotgun_sequence
PVJP02910680.1_1688997-1689236_Hippopotamus_amphibius_isolate_BS01_ScHSfme_925135HRSCAF_982935_whole_genome_shotgun_sequence
R_VCEA01000003.1_84299285-84299884_Muntiacus_muntjak_isolate_UTSW_UCB_Mm_chromosome_3_X_whole_genome_shotgun_sequence
R_VCEB01000011.1_50101590-50102189_Muntiacus_reevesi_isolate_UCam_UCB_Mr_chromosome_X_whole_genome_shotgun_sequence
MKHE01000034.1_68046430-68046796_Cervus_elaphus_hippelaphus_isolate_Hungarian_chromosome_X_whole_genome_shotgun_sequence
QQTR01119659.1_3214704-3215303_Axis_porcinus_isolate_Zoohd001_scaffold_50_whole_genome_shotgun_sequence
PZYV01000025.1_91886305-91886904_Bubalus_bubalis_breed_Mediterranean_chromosome_X_whole_genome_shotgun_sequence
MKHE01000034.1_68044787-68045254_Cervus_elaphus_hippelaphus_isolate_Hungarian_chromosome_X_whole_genome_shotgun_sequence
R_PZYV01000012.1_42909220-42909637_Bubalus_bubalis_breed_Mediterranean_chromosome_2_whole_genome_shotgun_sequence
R_RAWU01000017.1_149270346-149270788_Giraffa_tippelskirchi_isolate_OR1865_chromosome_8_whole_genome_shotgun_sequence
R_VCEA01000001.1_1019342253-1019342918_Muntiacus_muntjak_isolate_UTSW_UCB_Mm_chromosome_1_whole_genome_shotgun_sequence
VCEB01000003.1_87933664-87934329_Muntiacus_reevesi_isolate_UCam_UCB_Mr_chromosome_3_whole_genome_shotgun_sequence
R_LWLT01000009.1_13185191-13185850_Capra_hircus_breed_San_Clemente_chromosome_11_whole_genome_shotgun_sequence
VCEB01000004.1_126078835-126079075_Muntiacus_reevesi_isolate_UCam_UCB_Mr_chromosome_4_whole_genome_shotgun_sequence
R_VCEA01000001.1_811216498-811216733_Muntiacus_muntjak_isolate_UTSW_UCB_Mm_chromosome_1_whole_genome_shotgun_sequence
PVJP02910616.1_285002-285682_Hippopotamus_amphibius_isolate_BS01_ScHSfme_925075HRSCAF_982875_whole_genome_shotgun_sequence
R_RAWU01000014.1_3560132-3560616_Giraffa_tippelskirchi_isolate_OR1865_chromosome_9_whole_genome_shotgun_sequence
R_CABVSG010000006.1_3585121-3585608_Syncerus_caffer_genome_assembly_contig__Contig5_whole_genome_shotgun_sequence
R_VCEB01000004.1_56169965-56170446_Muntiacus_reevesi_isolate_UCam_UCB_Mr_chromosome_4_whole_genome_shotgun_sequence
R_VCEA01000001.1_271641964-271642445_Muntiacus_muntjak_isolate_UTSW_UCB_Mm_chromosome_1_whole_genome_shotgun_sequence
R_VCEA01000001.1_1105022378-1105022800_Muntiacus_muntjak_isolate_UTSW_UCB_Mm_chromosome_1_whole_genome_shotgun_sequence
VCEB01000003.1_2785496-2785918_Muntiacus_reevesi_isolate_UCam_UCB_Mr_chromosome_3_whole_genome_shotgun_sequence
R_LWLT01000009.1_103625502-103626049_Capra_hircus_breed_San_Clemente_chromosome_11_whole_genome_shotgun_sequence
0.03

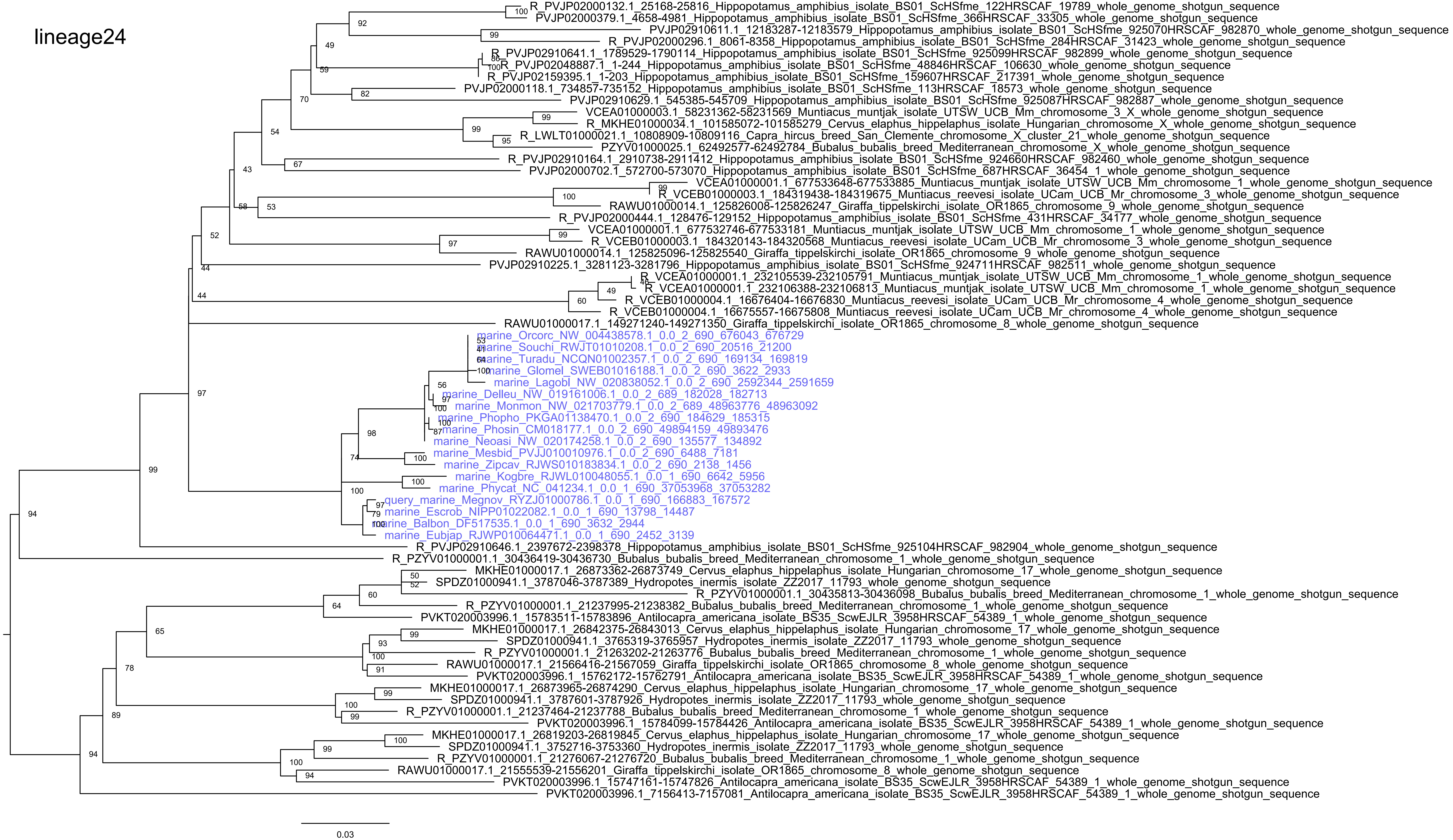
lineage24
R_PVJP02000132.1_25168-25816_Hippopotamus_amphibius_isolate_BS01_ScHSfme_122HRSCAF_19789_whole_genome_shotgun_sequence
PVJP02000379.1_4658-4981_Hippopotamus_amphibius_isolate_BS01_ScHSfme_366HRSCAF_33305_whole_genome_shotgun_sequence
PVJP02910611.1_12183287-12183579_Hippopotamus_amphibius_isolate_BS01_ScHSfme_925070HRSCAF_982870_whole_genome_shotgun_sequence
R_PVJP02000296.1_8061-8358_Hippopotamus_amphibius_isolate_BS01_ScHSfme_284HRSCAF_31423_whole_genome_shotgun_sequence
R_PVJP02910641.1_1789529-1790114_Hippopotamus_amphibius_isolate_BS01_ScHSfme_925099HRSCAF_982899_whole_genome_shotgun_sequence
R_PVJP02048887.1_1-244_Hippopotamus_amphibius_isolate_BS01_ScHSfme_48846HRSCAF_106630_whole_genome_shotgun_sequence
R_PVJP02159395.1_1-203_Hippopotamus_amphibius_isolate_BS01_ScHSfme_159607HRSCAF_217391_whole_genome_shotgun_sequence
PVJP02000118.1_734857-735152_Hippopotamus_amphibius_isolate_BS01_ScHSfme_113HRSCAF_18573_whole_genome_shotgun_sequence
PVJP02910629.1_545385-545709_Hippopotamus_amphibius_isolate_BS01_ScHSfme_925087HRSCAF_982887_whole_genome_shotgun_sequence
VCEA01000003.1_58231362-58231569_Muntiacus_muntjak_isolate_UTSW_UCB_Mm_chromosome_3_X_whole_genome_shotgun_sequence
R_MKHE01000034.1_101585072-101585279_Cervus_elaphus_hippelaphus_isolate_Hungarian_chromosome_X_whole_genome_shotgun_sequence
R_LWLT01000021.1_10808909-10809116_Capra_hircus_breed_San_Clemente_chromosome_X_cluster_21_whole_genome_shotgun_sequence
PZYV01000025.1_62492577-62492784_Bubalus_bubalis_breed_Mediterranean_chromosome_X_whole_genome_shotgun_sequence
R_PVJP02910164.1_2910738-2911412_Hippopotamus_amphibius_isolate_BS01_ScHSfme_924660HRSCAF_982460_whole_genome_shotgun_sequence
PVJP02000702.1_572700-573070_Hippopotamus_amphibius_isolate_BS01_ScHSfme_687HRSCAF_36454_1_whole_genome_shotgun_sequence
VCEA01000001.1_677533648-677533885_Muntiacus_muntjak_isolate_UTSW_UCB_Mm_chromosome_1_whole_genome_shotgun_sequence
R_VCEB01000003.1_184319438-184319675_Muntiacus_reevesi_isolate_UCam_UCB_Mr_chromosome_3_whole_genome_shotgun_sequence
RAWU01000014.1_125826008-125826247_Giraffa_tippelskirchi_isolate_OR1865_chromosome_9_whole_genome_shotgun_sequence
R_PVJP02000444.1_128476-129152_Hippopotamus_amphibius_isolate_BS01_ScHSfme_431HRSCAF_34177_whole_genome_shotgun_sequence
VCEA01000001.1_677532746-677533181_Muntiacus_muntjak_isolate_UTSW_UCB_Mm_chromosome_1_whole_genome_shotgun_sequence
R_VCEB01000003.1_184320143-184320568_Muntiacus_reevesi_isolate_UCam_UCB_Mr_chromosome_3_whole_genome_shotgun_sequence
RAWU01000014.1_125825096-125825540_Giraffa_tippelskirchi_isolate_OR1865_chromosome_9_whole_genome_shotgun_sequence
PVJP02910225.1_3281123-3281796_Hippopotamus_amphibius_isolate_BS01_ScHSfme_924711HRSCAF_982511_whole_genome_shotgun_sequence
R_VCEA01000001.1_232105539-232105791_Muntiacus_muntjak_isolate_UTSW_UCB_Mm_chromosome_1_whole_genome_shotgun_sequence
R_VCEA01000001.1_232106388-232106813_Muntiacus_muntjak_isolate_UTSW_UCB_Mm_chromosome_1_whole_genome_shotgun_sequence
R_VCEB01000004.1_16676404-16676830_Muntiacus_reevesi_isolate_UCam_UCB_Mr_chromosome_4_whole_genome_shotgun_sequence
R_VCEB01000004.1_16675557-16675808_Muntiacus_reevesi_isolate_UCam_UCB_Mr_chromosome_4_whole_genome_shotgun_sequence
RAWU01000017.1_149271240-149271350_Giraffa_tippelskirchi_isolate_OR1865_chromosome_8_whole_genome_shotgun_sequence
marine_Orcorc_NW_004438578.1_0.0_2_690_676043_676729
marine_Souchi_RWJT01010208.1_0.0_2_690_20516_21200
marine_Turadu_NCQN01002357.1_0.0_2_690_169134_169819
marine_Glomel_SWEB01016188.1_0.0_2_690_3622_2933
marine_Lagobl_NW_020838052.1_0.0_2_690_2592344_2591659
marine_Delleu_NW_019161006.1_0.0_2_689_182028_182713
marine_Monmon_NW_021703779.1_0.0_2_689_48963776_48963092
marine_Phopho_PKGA01138470.1_0.0_2_690_184629_185315
marine_Phosin_CM018177.1_0.0_2_690_49894159_49893476
marine_Neoasi_NW_020174258.1_0.0_2_690_135577_134892
marine_Mesbid_PVJJ010010976.1_0.0_2_690_6488_7181
marine_Zipcav_RJWS010183834.1_0.0_2_690_2138_1456
marine_Kogbre_RJWL010048055.1_0.0_1_690_6642_5956
marine_Phycat_NC_041234.1_0.0_1_690_37053968_37053282
query_marine_Megnov_RYZJ01000786.1_0.0_1_690_166883_167572
marine_Escrob_NIPP01022082.1_0.0_1_690_13798_14487
marine_Balbon_DF517535.1_0.0_1_690_3632_2944
marine_Eubjap_RJWP010064471.1_0.0_1_690_2452_3139
R_PVJP02910646.1_2397672-2398378_Hippopotamus_amphibius_isolate_BS01_ScHSfme_925104HRSCAF_982904_whole_genome_shotgun_sequence
R_PZYV01000001.1_30436419-30436730_Bubalus_bubalis_breed_Mediterranean_chromosome_1_whole_genome_shotgun_sequence
MKHE01000017.1_26873362-26873749_Cervus_elaphus_hippelaphus_isolate_Hungarian_chromosome_17_whole_genome_shotgun_sequence
SPDZ01000941.1_3787046-3787389_Hydropotes_inermis_isolate_ZZ2017_11793_whole_genome_shotgun_sequence
R_PZYV01000001.1_30435813-30436098_Bubalus_bubalis_breed_Mediterranean_chromosome_1_whole_genome_shotgun_sequence
R_PZYV01000001.1_21237995-21238382_Bubalus_bubalis_breed_Mediterranean_chromosome_1_whole_genome_shotgun_sequence
PVKT020003996.1_15783511-15783896_Antilocapra_americana_isolate_BS35_ScwEJLR_3958HRSCAF_54389_1_whole_genome_shotgun_sequence
MKHE01000017.1_26842375-26843013_Cervus_elaphus_hippelaphus_isolate_Hungarian_chromosome_17_whole_genome_shotgun_sequence
SPDZ01000941.1_3765319-3765957_Hydropotes_inermis_isolate_ZZ2017_11793_whole_genome_shotgun_sequence
R_PZYV01000001.1_21263202-21263776_Bubalus_bubalis_breed_Mediterranean_chromosome_1_whole_genome_shotgun_sequence
RAWU01000017.1_21566416-21567059_Giraffa_tippelskirchi_isolate_OR1865_chromosome_8_whole_genome_shotgun_sequence
PVKT020003996.1_15762172-15762791_Antilocapra_americana_isolate_BS35_ScwEJLR_3958HRSCAF_54389_1_whole_genome_shotgun_sequence
MKHE01000017.1_26873965-26874290_Cervus_elaphus_hippelaphus_isolate_Hungarian_chromosome_17_whole_genome_shotgun_sequence
SPDZ01000941.1_3787601-3787926_Hydropotes_inermis_isolate_ZZ2017_11793_whole_genome_shotgun_sequence
R_PZYV01000001.1_21237464-21237788_Bubalus_bubalis_breed_Mediterranean_chromosome_1_whole_genome_shotgun_sequence
PVKT020003996.1_15784099-15784426_Antilocapra_americana_isolate_BS35_ScwEJLR_3958HRSCAF_54389_1_whole_genome_shotgun_sequence
MKHE01000017.1_26819203-26819845_Cervus_elaphus_hippelaphus_isolate_Hungarian_chromosome_17_whole_genome_shotgun_sequence
SPDZ01000941.1_3752716-3753360_Hydropotes_inermis_isolate_ZZ2017_11793_whole_genome_shotgun_sequence
R_PZYV01000001.1_21276067-21276720_Bubalus_bubalis_breed_Mediterranean_chromosome_1_whole_genome_shotgun_sequence
RAWU01000017.1_21555539-21556201_Giraffa_tippelskirchi_isolate_OR1865_chromosome_8_whole_genome_shotgun_sequence
PVKT020003996.1_15747161-15747826_Antilocapra_americana_isolate_BS35_ScwEJLR_3958HRSCAF_54389_1_whole_genome_shotgun_sequence
PVKT020003996.1_7156413-7157081_Antilocapra_americana_isolate_BS35_ScwEJLR_3958HRSCAF_54389_1_whole_genome_shotgun_sequence
0.03

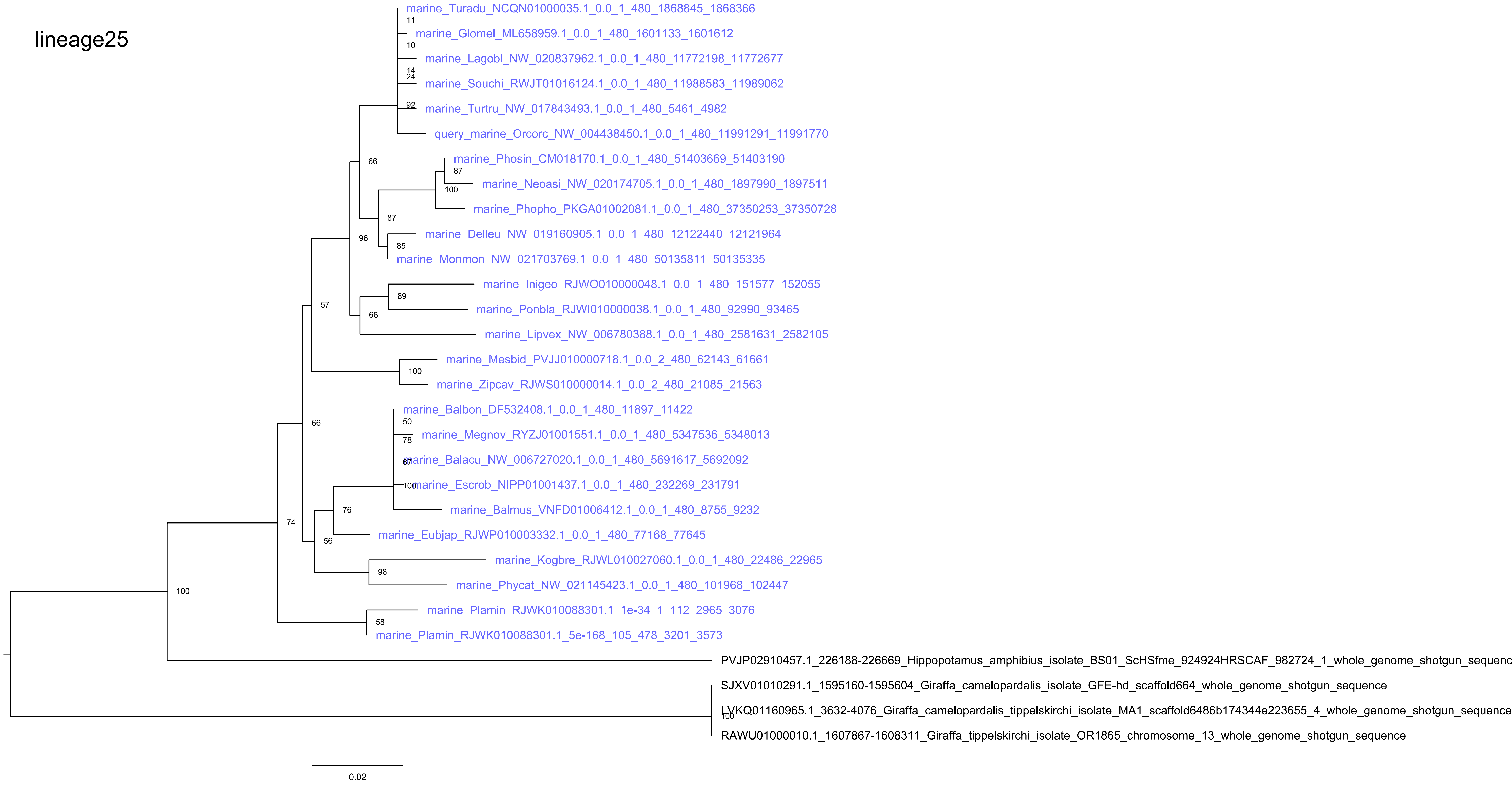
lineage25
marine_Turadu_NCQN01000035.1_0.0_1_480_1868845_1868366
marine_Glomel_ML658959.1_0.0_1_480_1601133_1601612
marine_Lagobl_NW_020837962.1_0.0_1_480_11772198_11772677
marine_Souchi_RWJT01016124.1_0.0_1_480_11988583_11989062
marine_Turtru_NW_017843493.1_0.0_1_480_5461_4982
query_marine_Orcorc_NW_004438450.1_0.0_1_480_11991291_11991770
marine_Phosin_CM018170.1_0.0_1_480_51403669_51403190
marine_Neoasi_NW_020174705.1_0.0_1_480_1897990_1897511
marine_Phopho_PKGA01002081.1_0.0_1_480_37350253_37350728
marine_Delleu_NW_019160905.1_0.0_1_480_12122440_12121964
marine_Monmon_NW_021703769.1_0.0_1_480_50135811_50135335
marine_Inigeo_RJWO010000048.1_0.0_1_480_151577_152055
marine_Ponbla_RJWI010000038.1_0.0_1_480_92990_93465
marine_Lipvex_NW_006780388.1_0.0_1_480_2581631_2582105
marine_Mesbid_PVJJ010000718.1_0.0_2_480_62143_61661
marine_Zipcav_RJWS010000014.1_0.0_2_480_21085_21563
marine_Balbon_DF532408.1_0.0_1_480_11897_11422
marine_Megnov_RYZJ01001551.1_0.0_1_480_5347536_5348013
marine_Balacu_NW_006727020.1_0.0_1_480_5691617_5692092
marine_Escrob_NIPP01001437.1_0.0_1_480_232269_231791
marine_Balmus_VNFD01006412.1_0.0_1_480_8755_9232
marine_Eubjap_RJWP010003332.1_0.0_1_480_77168_77645
marine_Kogbre_RJWL010027060.1_0.0_1_480_22486_22965
marine_Phycat_NW_021145423.1_0.0_1_480_101968_102447
marine_Plamin_RJWK010088301.1_1e-34_1_112_2965_3076
marine_Plamin_RJWK010088301.1_5e-168_105_478_3201_3573
PVJP02910457.1_226188-226669_Hippopotamus_amphibius_isolate_BS01_ScHSfme_924924HRSCAF_982724_1_whole_genome_shotgun_sequenc
SJXV01010291.1_1595160-1595604_Giraffa_camelopardalis_isolate_GFE-hd_scaffold664_whole_genome_shotgun_sequence
LVKQ01160965.1_3632-4076_Giraffa_camelopardalis_tippelskirchi_isolate_MA1_scaffold6486b174344e223655_4_whole_genome_shotgun_sequence
RAWU01000010.1_1607867-1608311_Giraffa_tippelskirchi_isolate_OR1865_chromosome_13_whole_genome_shotgun_sequence
0.02
11
10
14
24
92
66
87
100
87
96
85
89
57
66
100
66
50
78
67
100
76
74
56
98
100
58
100

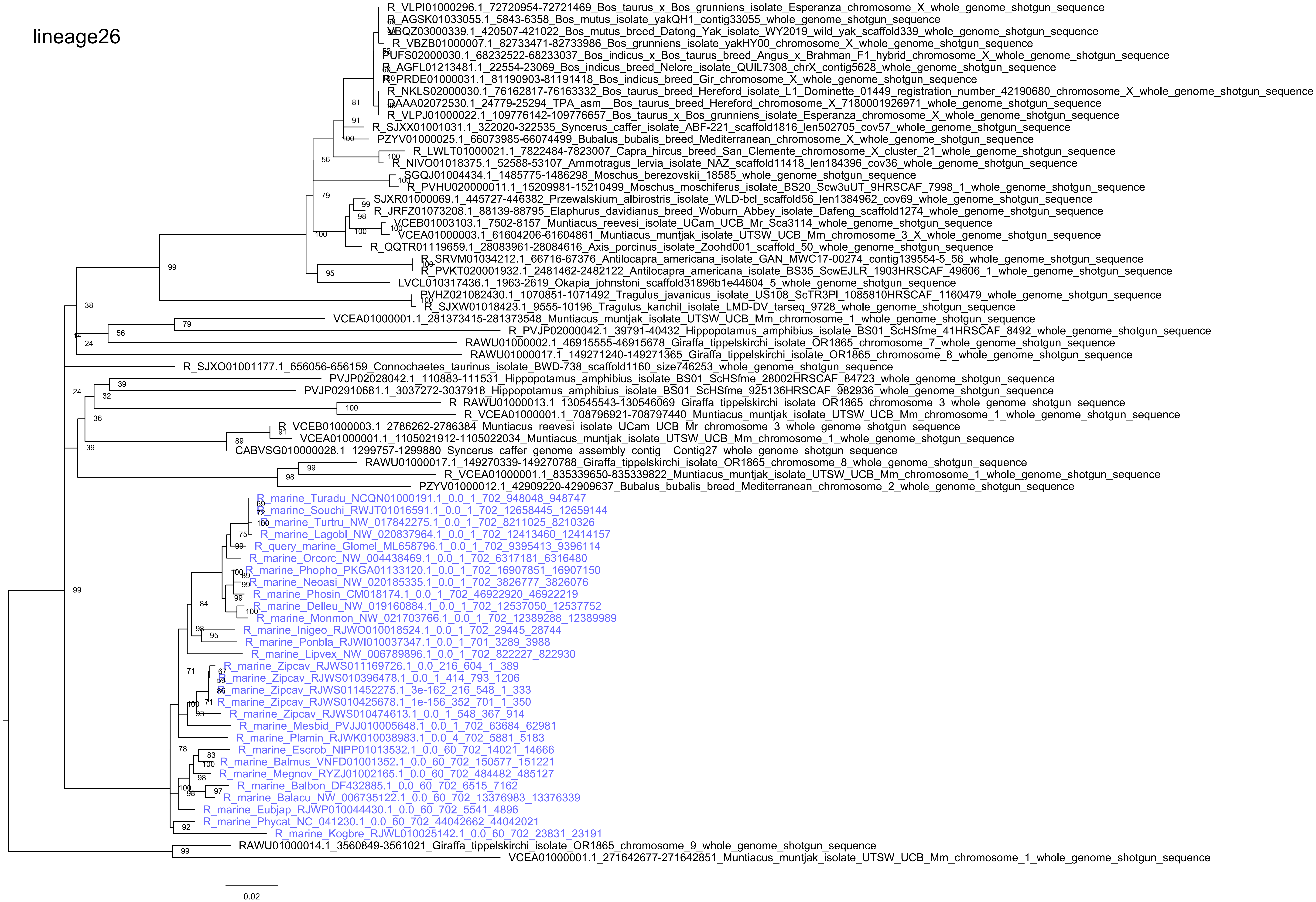

lineage26
R_VLPI01000296.1_72720954-72721469_Bos_taurus_x_Bos_grunniens_isolate_Esperanza_chromosome_X_whole_genome_shotgun_sequence
R_AGSK01033055.1_5843-6358_Bos_mutus_isolate_yakQH1_contig33055_whole_genome_shotgun_sequence
VBQZ03000339.1_420507-421022_Bos_mutus_breed_Datong_Yak_isolate_WY2019_wild_yak_scaffold339_whole_genome_shotgun_sequence
R_VBZB01000007.1_82733471-82733986_Bos_grunniens_isolate_yakHY00_chromosome_X_whole_genome_shotgun_sequence
PUFS02000030.1_68232522-68233037_Bos_indicus_x_Bos_taurus_breed_Angus_x_Brahman_F1_hybrid_chromosome_X_whole_genome_shotgun_sequence
R_AGFL01213481.1_22554-23069_Bos_indicus_breed_Nelore_isolate_QUIL7308_chrX_contig5628_whole_genome_shotgun_sequence
R_PRDE01000031.1_81190903-81191418_Bos_indicus_breed_Gir_chromosome_X_whole_genome_shotgun_sequence
R_NKLS02000030.1_76162817-76163332_Bos_taurus_breed_Hereford_isolate_L1_Dominette_01449_registration_number_42190680_chromosome_X_whole_genome_shotgun_sequence
DAAA02072530.1_24779-25294_TPA_asm__Bos_taurus_breed_Hereford_chromosome_X_7180001926971_whole_genome_shotgun_sequence
R_VLPJ01000022.1_109776142-109776657_Bos_taurus_x_Bos_grunniens_isolate_Esperanza_chromosome_X_whole_genome_shotgun_sequence
R_SJXX01001031.1_322020-322535_Syncerus_caffer_isolate_ABF-221_scaffold1816_len502705_cov57_whole_genome_shotgun_sequence
PZYV01000025.1_66073985-66074499_Bubalus_bubalis_breed_Mediterranean_chromosome_X_whole_genome_shotgun_sequence
R_LWLT01000021.1_7822484-7823007_Capra_hircus_breed_San_Clemente_chromosome_X_cluster_21_whole_genome_shotgun_sequence
R_NIVO01018375.1_52588-53107_Ammotragus_lervia_isolate_NAZ_scaffold11418_len184396_cov36_whole_genome_shotgun_sequence
SGQJ01004434.1_1485775-1486298_Moschus_berezovskii_18585_whole_genome_shotgun_sequence
R_PVHU020000011.1_15209981-15210499_Moschus_moschiferus_isolate_BS20_Scw3uUT_9HRSCAF_7998_1_whole_genome_shotgun_sequence
SJXR01000069.1_445727-446382_Przewalskium_albirostris_isolate_WLD-bcl_scaffold56_len1384962_cov69_whole_genome_shotgun_sequence
R_JRFZ01073208.1_88139-88795_Elaphurus_davidianus_breed_Woburn_Abbey_isolate_Dafeng_scaffold1274_whole_genome_shotgun_sequence
VCEB01003103.1_7502-8157_Muntiacus_reevesi_isolate_UCam_UCB_Mr_Sca3114_whole_genome_shotgun_sequence
VCEA01000003.1_61604206-61604861_Muntiacus_muntjak_isolate_UTSW_UCB_Mm_chromosome_3_X_whole_genome_shotgun_sequence
R_QQTR01119659.1_28083961-28084616_Axis_porcinus_isolate_Zoohd001_scaffold_50_whole_genome_shotgun_sequence
R_SRVM01034212.1_66716-67376_Antilocapra_americana_isolate_GAN_MWC17-00274_contig139554-5_56_whole_genome_shotgun_sequence
R_PVKT020001932.1_2481462-2482122_Antilocapra_americana_isolate_BS35_ScwEJLR_1903HRSCAF_49606_1_whole_genome_shotgun_sequence
LVCL010317436.1_1963-2619_Okapia_johnstoni_scaffold31896b1e44604_5_whole_genome_shotgun_sequence
PVHZ021082430.1_1070851-1071492_Tragulus_javanicus_isolate_US108_ScTR3PI_1085810HRSCAF_1160479_whole_genome_shotgun_sequence
R_SJXW01018423.1_9555-10196_Tragulus_kanchil_isolate_LMD-DV_tarseq_9728_whole_genome_shotgun_sequence
VCEA01000001.1_281373415-281373548_Muntiacus_muntjak_isolate_UTSW_UCB_Mm_chromosome_1_whole_genome_shotgun_sequence
R_PVJP02000042.1_39791-40432_Hippopotamus_amphibius_isolate_BS01_ScHSfme_41HRSCAF_8492_whole_genome_shotgun_sequence
RAWU01000002.1_46915555-46915678_Giraffa_tippelskirchi_isolate_OR1865_chromosome_7_whole_genome_shotgun_sequence
RAWU01000017.1_149271240-149271365_Giraffa_tippelskirchi_isolate_OR1865_chromosome_8_whole_genome_shotgun_sequence
R_SJXO01001177.1_656056-656159_Connochaetes_taurinus_isolate_BWD-738_scaffold1160_size746253_whole_genome_shotgun_sequence
PVJP02028042.1_110883-111531_Hippopotamus_amphibius_isolate_BS01_ScHSfme_28002HRSCAF_84723_whole_genome_shotgun_sequence
PVJP02910681.1_3037272-3037918_Hippopotamus_amphibius_isolate_BS01_ScHSfme_925136HRSCAF_982936_whole_genome_shotgun_sequence
R_RAWU01000013.1_130545543-130546069_Giraffa_tippelskirchi_isolate_OR1865_chromosome_3_whole_genome_shotgun_sequence
R_VCEA01000001.1_708796921-708797440_Muntiacus_muntjak_isolate_UTSW_UCB_Mm_chromosome_1_whole_genome_shotgun_sequence
R_VCEB01000003.1_2786262-2786384_Muntiacus_reevesi_isolate_UCam_UCB_Mr_chromosome_3_whole_genome_shotgun_sequence
VCEA01000001.1_1105021912-1105022034_Muntiacus_muntjak_isolate_UTSW_UCB_Mm_chromosome_1_whole_genome_shotgun_sequence
CABVSG010000028.1_1299757-1299880_Syncerus_caffer_genome_assembly_contig__Contig27_whole_genome_shotgun_sequence
RAWU01000017.1_149270339-149270788_Giraffa_tippelskirchi_isolate_OR1865_chromosome_8_whole_genome_shotgun_sequence
R_VCEA01000001.1_835339650-835339822_Muntiacus_muntjak_isolate_UTSW_UCB_Mm_chromosome_1_whole_genome_shotgun_sequence
PZYV01000012.1_42909220-42909637_Bubalus_bubalis_breed_Mediterranean_chromosome_2_whole_genome_shotgun_sequence
R_marine_Turadu_NCQN01000191.1_0.0_1_702_948048_948747
R_marine_Souchi_RWJT01016591.1_0.0_1_702_12658445_12659144
R_marine_Turtru_NW_017842275.1_0.0_1_702_8211025_8210326
R_marine_Lagobl_NW_020837964.1_0.0_1_702_12413460_12414157
R_query_marine_Glomel_ML658796.1_0.0_1_702_9395413_9396114
R_marine_Orcorc_NW_004438469.1_0.0_1_702_6317181_6316480
R_marine_Phopho_PKGA01133120.1_0.0_1_702_16907851_16907150
R_marine_Neoasi_NW_020185335.1_0.0_1_702_3826777_3826076
R_marine_Phosin_CM018174.1_0.0_1_702_46922920_46922219
R_marine_Delleu_NW_019160884.1_0.0_1_702_12537050_12537752
R_marine_Monmon_NW_021703766.1_0.0_1_702_12389288_12389989
R_marine_Inigeo_RJWO010018524.1_0.0_1_702_29445_28744
R_marine_Ponbla_RJWI010037347.1_0.0_1_701_3289_3988
R_marine_Lipvex_NW_006789896.1_0.0_1_702_822227_822930
R_marine_Zipcav_RJWS011169726.1_0.0_216_604_1_389
R_marine_Zipcav_RJWS010396478.1_0.0_1_414_793_1206
R_marine_Zipcav_RJWS011452275.1_3e-162_216_548_1_333
R_marine_Zipcav_RJWS010425678.1_1e-156_352_701_1_350
R_marine_Zipcav_RJWS010474613.1_0.0_1_548_367_914
R_marine_Mesbid_PVJJ010005648.1_0.0_1_702_63684_62981
R_marine_Plamin_RJWK010038983.1_0.0_4_702_5881_5183
R_marine_Escrob_NIPP01013532.1_0.0_60_702_14021_14666
R_marine_Balmus_VNFD01001352.1_0.0_60_702_150577_151221
R_marine_Megnov_RYZJ01002165.1_0.0_60_702_484482_485127
R_marine_Balbon_DF432885.1_0.0_60_702_6515_7162
R_marine_Balacu_NW_006735122.1_0.0_60_702_13376983_13376339
R_marine_Eubjap_RJWP010044430.1_0.0_60_702_5541_4896
R_marine_Phycat_NC_041230.1_0.0_60_702_44042662_44042021
R_marine_Kogbre_RJWL010025142.1_0.0_60_702_23831_23191
RAWU01000014.1_3560849-3561021_Giraffa_tippelskirchi_isolate_OR1865_chromosome_9_whole_genome_shotgun_sequence
VCEA01000001.1_271642677-271642851_Muntiacus_muntjak_isolate_UTSW_UCB_Mm_chromosome_1_whole_genome_shotgun_sequence
0.02

lineage27

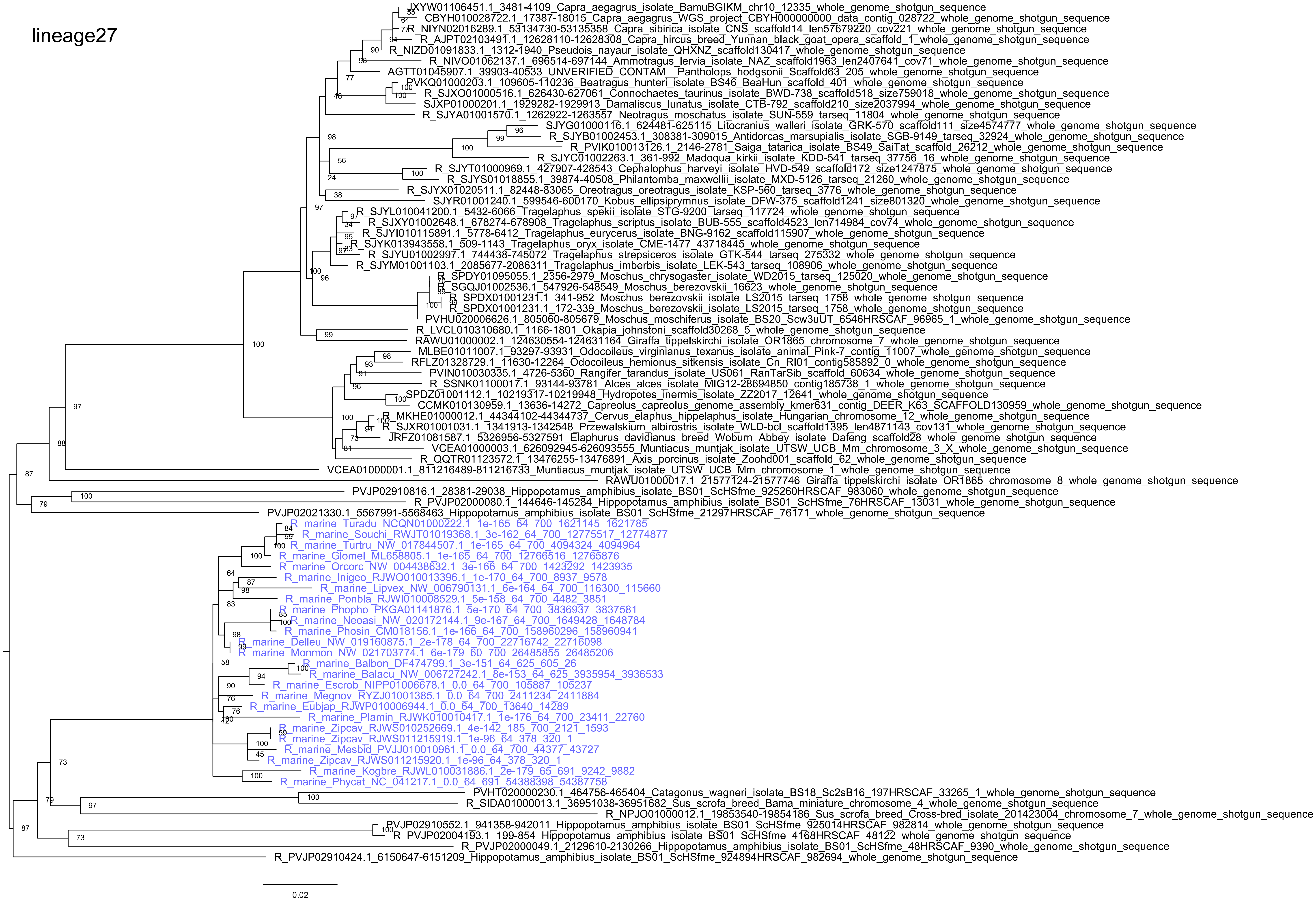
JXYW01106451.1_3481-4109_Capra_aegagrus_isolate_BamuBGIKM_chr10_12335_whole_genome_shotgun_sequence
CBYH010028722.1_17387-18015_Capra_aegagrus_WGS_project_CBYH000000000_data_contig_028722_whole_genome_shotgun_sequence
R_NIYN02016289.1_53134730-53135358_Capra_sibirica_isolate_CNS_scaffold14_len57679220_cov221_whole_genome_shotgun_sequence
R_AJPT02103491.1_12628110-12628308_Capra_hircus_breed_Yunnan_black_goat_opera_scaffold_1_whole_genome_shotgun_sequence
R_NIZD01091833.1_1312-1940_Pseudois_nayaur_isolate_QHXNZ_scaffold130417_whole_genome_shotgun_sequence
R_NIVO01062137.1_696514-697144_Ammotragus_lervia_isolate_NAZ_scaffold1963_len2407641_cov71_whole_genome_shotgun_sequence
AGTT01045907.1_39903-40533_UNVERIFIED_CONTAM__Pantholops_hodgsonii_Scaffold63_205_whole_genome_shotgun_sequence
PVKQ01000203.1_109605-110236_Beatragus_hunteri_isolate_BS46_BeaHun_scaffold_401_whole_genome_shotgun_sequence
R_SJXO01000516.1_626430-627061_Connochaetes_taurinus_isolate_BWD-738_scaffold518_size759018_whole_genome_shotgun_sequence
SJXP01000201.1_1929282-1929913_Damaliscus_lunatus_isolate_CTB-792_scaffold210_size2037994_whole_genome_shotgun_sequence
R_SJYA01001570.1_1262922-1263557_Neotragus_moschatus_isolate_SUN-559_tarseq_11804_whole_genome_shotgun_sequence
SJYG01000116.1_624481-625115_Litocranius_walleri_isolate_GRK-570_scaffold111_size4574777_whole_genome_shotgun_sequence
R_SJYB01002453.1_308381-309015_Antidorcas_marsupialis_isolate_SGB-9149_tarseq_32924_whole_genome_shotgun_sequence
R_PVIK010013126.1_2146-2781_Saiga_tatarica_isolate_BS49_SaiTat_scaffold_26212_whole_genome_shotgun_sequence
R_SJYC01002263.1_361-992_Madoqua_kirkii_isolate_KDD-541_tarseq_37756_16_whole_genome_shotgun_sequence
R_SJYT01000969.1_427907-428543_Cephalophus_harveyi_isolate_HVD-549_scaffold172_size1247875_whole_genome_shotgun_sequence
R_SJYS01018855.1_39874-40508_Philantomba_maxwellii_isolate_MXD-5126_tarseq_21260_whole_genome_shotgun_sequence
R_SJYX01020511.1_82448-83065_Oreotragus_oreotragus_isolate_KSP-560_tarseq_3776_whole_genome_shotgun_sequence
SJYR01001240.1_599546-600170_Kobus_ellipsiprymnus_isolate_DFW-375_scaffold1241_size801320_whole_genome_shotgun_sequence
R_SJYL010041200.1_5432-6066_Tragelaphus_spekii_isolate_STG-9200_tarseq_117724_whole_genome_shotgun_sequence
R_SJXY01002648.1_678274-678908_Tragelaphus_scriptus_isolate_BUB-555_scaffold4523_len714984_cov74_whole_genome_shotgun_sequence
R_SJYI010115891.1_5778-6412_Tragelaphus_eurycerus_isolate_BNG-9162_scaffold115907_whole_genome_shotgun_sequence
R_SJYK013943558.1_509-1143_Tragelaphus_oryx_isolate_CME-1477_43718445_whole_genome_shotgun_sequence
R_SJYU01002997.1_744438-745072_Tragelaphus_strepsiceros_isolate_GTK-544_tarseq_275332_whole_genome_shotgun_sequence
R_SJYM01001103.1_2085677-2086311_Tragelaphus_imberbis_isolate_LEK-543_tarseq_108906_whole_genome_shotgun_sequence
R_SPDY01095055.1_2356-2979_Moschus_chrysogaster_isolate_WD2015_tarseq_125020_whole_genome_shotgun_sequence
R_SGGQ01002536.1_547926-548549_Moschus_berezovskii_16623_whole_genome_shotgun_sequence
R_SPDX01001231.1_341-952_Moschus_berezovskii_isolate_LS2015_tarseq_1758_whole_genome_shotgun_sequence
R_SPDX01001231.1_172-339_Moschus_berezovskii_isolate_LS2015_tarseq_1758_whole_genome_shotgun_sequence
PVHU020006626.1_805060-805679_Moschus_moschiferus_isolate_BS20_Scw3uUT_6546HRSCAF_96965_1_whole_genome_shotgun_sequence
R_LVCL010310680.1_1166-1801_Okapia_johnstoni_scaffold30268_5_whole_genome_shotgun_sequence
RAWU01000002.1_124630554-124631164_Giraffa_tippelskirchi_isolate_OR1865_chromosome_7_whole_genome_shotgun_sequence
MLBE01011007.1_93297-93931_Odocoileus_virginianus_texanus_isolate_animal_Pink-7_contig_11007_whole_genome_shotgun_sequence
RFLZ01328729.1_11630-12264_Odocoileus_hemionus_sitkensis_isolate_Cn_RI01_contig585892_0_whole_genome_shotgun_sequence
PVIN010030335.1_4726-5360_Rangifer_tarandus_isolate_US061_RanTarSib_scaffold_60634_whole_genome_shotgun_sequence
R_SSNK01100017.1_93144-93781_Alces_alces_isolate_MIG12-28694850_contig185738_1_whole_genome_shotgun_sequence
SPDZ01001112.1_10219317-10219948_Hydropotes_inermis_isolate_ZZ2017_12641_whole_genome_shotgun_sequence
CCMK010130959.1_13636-14272_Capreolus_capreolus_genome_assembly_kmer631_contig_DEER_K63_SCAFFOLD130959_whole_genome_shotgun_sequence
R_MKHE01000012.1_44344102-44344737_Cervus_elaphus_hippelaphus_isolate_Hungarian_chromosome_12_whole_genome_shotgun_sequence
R_SJXR01001031.1_1341913-1342548_Przewalskium_albirostris_isolate_WLD-bcl_scaffold1395_len4871143_cov131_whole_genome_shotgun_sequence
JRFZ01081587.1_5326956-5327591_Elaphurus_davidianus_breed_Woburn_Abbey_isolate_Dafeng_scaffold28_whole_genome_shotgun_sequence
VCEA01000003.1_626092945-626093555_Muntiacus_muntjak_isolate_UTSW_UCB_Mm_chromosome_3_X_whole_genome_shotgun_sequence
R_QQTR01123572.1_13476255-13476891_Axis_porcinus_isolate_Zoohd001_scaffold_62_whole_genome_shotgun_sequence
VCEA01000001.1_811216489-811216733_Muntiacus_muntjak_isolate_UTSW_UCB_Mm_chromosome_1_whole_genome_shotgun_sequence
RAWU01000017.1_21577124-21577746_Giraffa_tippelskirchi_isolate_OR1865_chromosome_8_whole_genome_shotgun_sequence
PVJP02910816.1_28381-29038_Hippopotamus_amphibius_isolate_BS01_ScHSfme_925260HRSCAF_983060_whole_genome_shotgun_sequence
R_PVJP02000080.1_144646-145284_Hippopotamus_amphibius_isolate_BS01_ScHSfme_76HRSCAF_13031_whole_genome_shotgun_sequence
PVJP02021330.1_5567991-5568463_Hippopotamus_amphibius_isolate_BS01_ScHSfme_21297HRSCAF_76171_whole_genome_shotgun_sequence
R_marine_Turadu_NCQN01000222.1_1e-165_64_700_1621145_1621785
R_marine_Souchi_RWJT01019368.1_3e-162_64_700_12775517_12774877
R_marine_Turtru_NW_017844507.1_1e-165_64_700_4094324_4094964
R_marine_Glomel_ML658805.1_1e-165_64_700_12766516_12765876
R_marine_Orcorc_NW_004438632.1_3e-166_64_700_1423292_1423935
R_marine_Inigeo_RJWO010013396.1_1e-170_64_700_8937_9578
R_marine_Lipvex_NW_006790131.1_6e-164_64_700_116300_115660
R_marine_Ponbla_RJWI010008529.1_5e-158_64_700_4482_3851
R_marine_Phopho_PKGA01141876.1_5e-170_64_700_3836937_3837581
R_marine_Neoasi_NW_020172144.1_9e-167_64_700_1649428_1648784
R_marine_Phosin_CM018156.1_1e-166_64_700_158960296_158960941
R_marine_Delleu_NW_019160875.1_2e-178_64_700_22716742_22716098
R_marine_Monmon_NW_021703774.1_6e-179_60_700_26485855_26485206
R_marine_Balbon_DF474799.1_3e-151_64_625_605_26
R_marine_Balacu_NW_006727242.1_8e-153_64_625_3935954_3936533
R_marine_Escrob_NIPP01006678.1_0.0_64_700_105887_105237
R_marine_Megnov_RYZJ01001385.1_0.0_64_700_2411234_2411884
R_marine_Eubjap_RJWP010006944.1_0.0_64_700_13640_14289
R_marine_Plamin_RJWK010010417.1_1e-176_64_700_23411_22760
R_marine_Zipcav_RJWS010252669.1_4e-142_185_700_2121_1593
R_marine_Zipcav_RJWS011215919.1_1e-96_64_378_320_1
R_marine_Mesbid_PVJJ010010961.1_0.0_64_700_44377_43727
R_marine_Zipcav_RJWS011215920.1_1e-96_64_378_320_1
R_marine_Kogbre_RJWL010031886.1_2e-179_65_691_9242_9882
R_marine_Phycat_NC_041217.1_0.0_64_691_54388398_54387758
PVHT020000230.1_464756-465404_Catagonus_wagneri_isolate_BS18_Sc2sB16_197HRSCAF_33265_1_whole_genome_shotgun_sequence
R_SIDA01000013.1_36951038-36951682_Sus_scrofa_breed_Bama_miniature_chromosome_4_whole_genome_shotgun_sequence
R_NPJO01000012.1_19853540-19854186_Sus_scrofa_breed_Cross-bred_isolate_201423004_chromosome_7_whole_genome_shotgun_sequence
PVJP02910552.1_941358-942011_Hippopotamus_amphibius_isolate_BS01_ScHSfme_925014HRSCAF_982814_whole_genome_shotgun_sequence
R_PVJP02004193.1_199-854_Hippopotamus_amphibius_isolate_BS01_ScHSfme_4168HRSCAF_48122_whole_genome_shotgun_sequence
R_PVJP02000049.1_2129610-2130266_Hippopotamus_amphibius_isolate_BS01_ScHSfme_48HRSCAF_9390_whole_genome_shotgun_sequence
R_PVJP02910424.1_6150647-6151209_Hippopotamus_amphibius_isolate_BS01_ScHSfme_924894HRSCAF_982694_whole_genome_shotgun_sequence
0.02

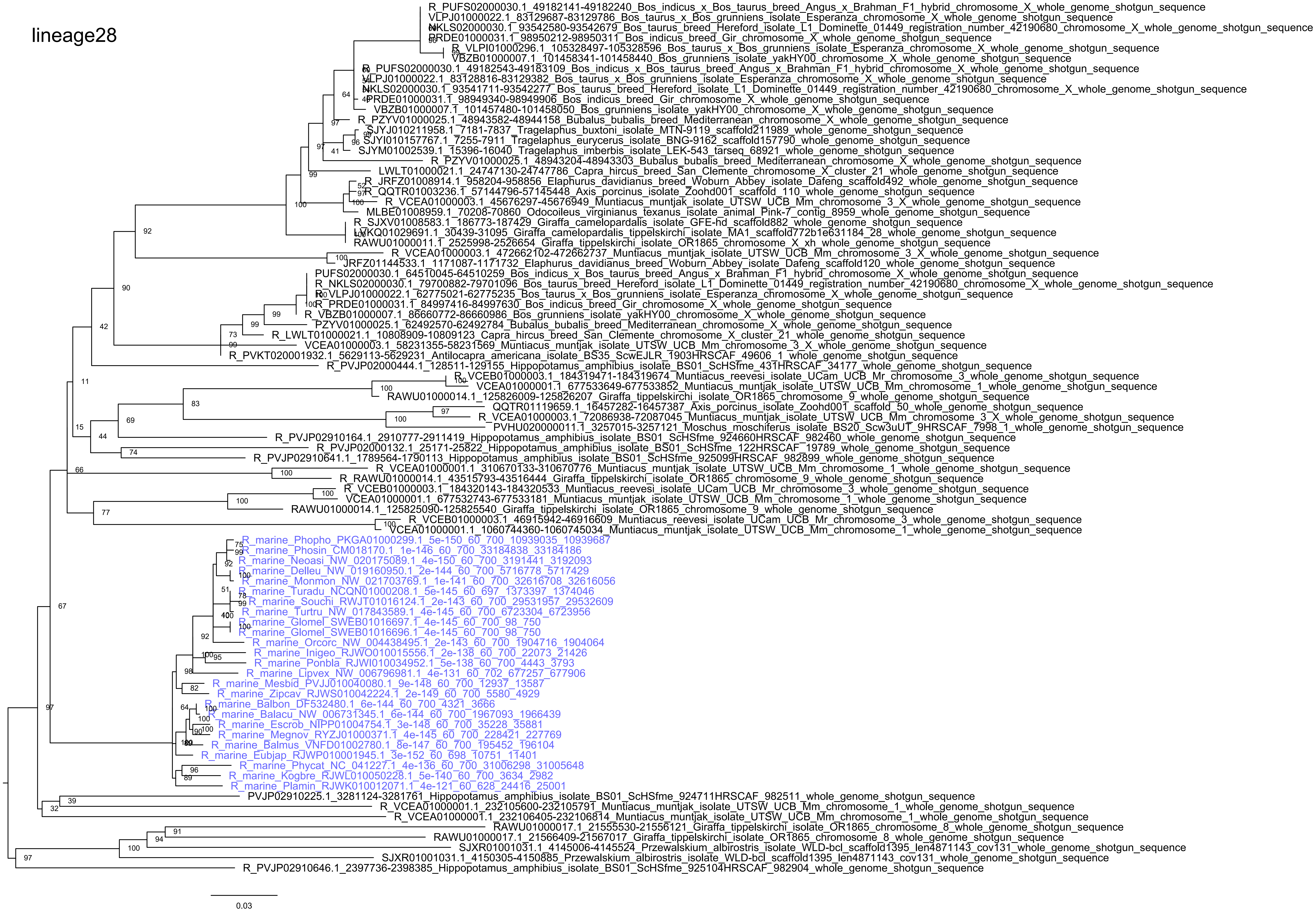
lineage28
R_PUFS02000030.1_49182141-49182240_Bos_indicus_x_Bos_taurus_breed_Angus_x_Brahman_F1_hybrid_chromosome_X_whole_genome_shotgun_sequence
VLPJ01000022.1_83129687-83129786_Bos_taurus_x_Bos_grunniens_isolate_Esperanza_chromosome_X_whole_genome_shotgun_sequence
NKLS02000030.1_93542580-93542679_Bos_taurus_breed_Hereford_isolate_L1_Dominette_01449_registration_number_42190680_chromosome_X_whole_genome_shotgun_sequence
PRDE01000031.1_98950212-98950311_Bos_indicus_breed_Gir_chromosome_X_whole_genome_shotgun_sequence
R_VLPI01000296.1_105328497-105328596_Bos_taurus_x_Bos_grunniens_isolate_Esperanza_chromosome_X_whole_genome_shotgun_sequence
VBZB01000007.1_101458341-101458440_Bos_grunniens_isolate_yakHY00_chromosome_X_whole_genome_shotgun_sequence
R_PUFS02000030.1_49182543-49183109_Bos_indicus_x_Bos_taurus_breed_Angus_x_Brahman_F1_hybrid_chromosome_X_whole_genome_shotgun_sequence
VLPJ01000022.1_83128816-83129382_Bos_taurus_x_Bos_grunniens_isolate_Esperanza_chromosome_X_whole_genome_shotgun_sequence
NKLS02000030.1_93541711-93542277_Bos_taurus_breed_Hereford_isolate_L1_Dominette_01449_registration_number_42190680_chromosome_X_whole_genome_shotgun_sequence
PRDE01000031.1_98949340-98949906_Bos_indicus_breed_Gir_chromosome_X_whole_genome_shotgun_sequence
VBZB01000007.1_101457480-101458050_Bos_grunniens_isolate_yakHY00_chromosome_X_whole_genome_shotgun_sequence
R_PZYV01000025.1_48943582-48944158_Bubalus_bubalis_breed_Mediterranean_chromosome_X_whole_genome_shotgun_sequence
SJYJ010211958.1_7181-7837_Tragelaphus_buxtoni_isolate_MTN-9119_scaffold211989_whole_genome_shotgun_sequence
SJYI010157767.1_7255-7911_Tragelaphus_eurycerus_isolate_BNG-9162_scaffold157790_whole_genome_shotgun_sequence
SJYM01002539.1_15396-16040_Tragelaphus_imberbis_isolate_LEK-543_tarseq_68921_whole_genome_shotgun_sequence
R_PZYV01000025.1_48943204-48943303_Bubalus_bubalis_breed_Mediterranean_chromosome_X_whole_genome_shotgun_sequence
LWLT01000021.1_24747130-24747786_Capra_hircus_breed_San_Clemente_chromosome_X_cluster_21_whole_genome_shotgun_sequence
R_JRFZ01008914.1_958204-958856_Elaphurus_davidianus_breed_Woburn_Abbey_isolate_Dafeng_scaffold492_whole_genome_shotgun_sequence
R_QQTR01003236.1_57144796-57145448_Axis_porcinus_isolate_Zoohd001_scaffold_110_whole_genome_shotgun_sequence
R_VCEA01000003.1_45676297-45676949_Muntiacus_muntjak_isolate_UTSW_UCB_Mm_chromosome_3_X_whole_genome_shotgun_sequence
MLBE01008959.1_70208-70860_Odocoileus_virginianus_texanus_isolate_animal_Pink-7_contig_8959_whole_genome_shotgun_sequence
R_SJXV01008583.1_186773-187429_Giraffa_camelopardalis_isolate_GFE-hd_scaffold882_whole_genome_shotgun_sequence
LVKQ01029691.1_30439-31095_Giraffa_camelopardalis_tippelskirchi_isolate_MA1_scaffold772b1e631184_28_whole_genome_shotgun_sequence
RAWU01000011.1_2525998-2526654_Giraffa_tippelskirchi_isolate_OR1865_chromosome_X_xh_whole_genome_shotgun_sequence
R_VCEA01000003.1_472662102-472662737_Muntiacus_muntjak_isolate_UTSW_UCB_Mm_chromosome_3_X_whole_genome_shotgun_sequence
JRFZ01144533.1_1171087-1171732_Elaphurus_davidianus_breed_Woburn_Abbey_isolate_Dafeng_scaffold120_whole_genome_shotgun_sequence
PUFS02000030.1_64510045-64510259_Bos_indicus_x_Bos_taurus_breed_Angus_x_Brahman_F1_hybrid_chromosome_X_whole_genome_shotgun_sequence
R_NKLS02000030.1_79700882-79701096_Bos_taurus_breed_Hereford_isolate_L1_Dominette_01449_registration_number_42190680_chromosome_X_whole_genome_shotgun_sequence
R_VLPJ01000022.1_62775021-62775235_Bos_taurus_x_Bos_grunniens_isolate_Esperanza_chromosome_X_whole_genome_shotgun_sequence
R_PRDE01000031.1_84997416-84997630_Bos_indicus_breed_Gir_chromosome_X_whole_genome_shotgun_sequence
R_VBZB01000007.1_86660772-86660986_Bos_grunniens_isolate_yakHY00_chromosome_X_whole_genome_shotgun_sequence
PZYV01000025.1_62492570-62492784_Bubalus_bubalis_breed_Mediterranean_chromosome_X_whole_genome_shotgun_sequence
R_LWLT01000021.1_10808909-10809123_Capra_hircus_breed_San_Clemente_chromosome_X_cluster_21_whole_genome_shotgun_sequence
VCEA01000003.1_58231355-58231569_Muntiacus_muntjak_isolate_UTSW_UCB_Mm_chromosome_3_X_whole_genome_shotgun_sequence
R_PVKT020001932.1_5629113-5629231_Antilocapra_americana_isolate_BS35_ScwEJLR_1903HRSCAF_49606_1_whole_genome_shotgun_sequence
R_PVJP02000444.1_128511-129155_Hippopotamus_amphibius_isolate_BS01_ScHSfme_431HRSCAF_34177_whole_genome_shotgun_sequence
R_VCEB01000003.1_184319471-184319674_Muntiacus_reevesi_isolate_UCam_UCB_Mr_chromosome_3_whole_genome_shotgun_sequence
VCEA01000001.1_677533649-677533852_Muntiacus_muntjak_isolate_UTSW_UCB_Mm_chromosome_1_whole_genome_shotgun_sequence
RAWU01000014.1_125826009-125826207_Giraffa_tippelskirchi_isolate_OR1865_chromosome_9_whole_genome_shotgun_sequence
QQTR01119659.1_16457282-16457387_Axis_porcinus_isolate_Zoohd001_scaffold_50_whole_genome_shotgun_sequence
R_VCEA01000003.1_72086938-72087045_Muntiacus_muntjak_isolate_UTSW_UCB_Mm_chromosome_3_X_whole_genome_shotgun_sequence
PVHU020000011.1_3257015-3257121_Moschus_moschiferus_isolate_BS20_Scw3uUT_9HRSCAF_7998_1_whole_genome_shotgun_sequence
R_PVJP02910164.1_2910777-2911419_Hippopotamus_amphibius_isolate_BS01_ScHSfme_924660HRSCAF_982460_whole_genome_shotgun_sequence
R_PVJP02000132.1_25171-25822_Hippopotamus_amphibius_isolate_BS01_ScHSfme_122HRSCAF_19789_whole_genome_shotgun_sequence
R_PVJP02910641.1_1789564-1790113_Hippopotamus_amphibius_isolate_BS01_ScHSfme_925099HRSCAF_982899_whole_genome_shotgun_sequence
R_VCEA01000001.1_310670133-310670776_Muntiacus_muntjak_isolate_UTSW_UCB_Mm_chromosome_1_whole_genome_shotgun_sequence
R_RAWU01000014.1_43515793-43516444_Giraffa_tippelskirchi_isolate_OR1865_chromosome_9_whole_genome_shotgun_sequence
R_VCEB01000003.1_184320143-184320533_Muntiacus_reevesi_isolate_UCam_UCB_Mr_chromosome_3_whole_genome_shotgun_sequence
VCEA01000001.1_677532743-677533181_Muntiacus_muntjak_isolate_UTSW_UCB_Mm_chromosome_1_whole_genome_shotgun_sequence
RAWU01000014.1_125825090-125825540_Giraffa_tippelskirchi_isolate_OR1865_chromosome_9_whole_genome_shotgun_sequence
R_VCEB01000003.1_46915942-46916609_Muntiacus_reevesi_isolate_UCam_UCB_Mr_chromosome_3_whole_genome_shotgun_sequence
VCEA01000001.1_1060744360-1060745034_Muntiacus_muntjak_isolate_UTSW_UCB_Mm_chromosome_1_whole_genome_shotgun_sequence
R_marine_Phopho_PKGA01000299.1_5e-150_60_700_10939035_10939687
R_marine_Phosin_CM018170.1_1e-146_60_700_33184838_33184186
R_marine_Neoasi_NW_020175089.1_4e-150_60_700_3191441_3192093
R_marine_Delleu_NW_019160950.1_2e-144_60_700_5716778_5717429
R_marine_Monmon_NW_021703769.1_1e-141_60_700_32616708_32616056
R_marine_Turadu_NCQN01000208.1_5e-145_60_697_1373397_1374046
R_marine_Souchi_RWJT01016124.1_2e-143_60_700_29531957_29532609
R_marine_Turtru_NW_017843589.1_4e-145_60_700_6723304_6723956
R_marine_Glomel_SWEB01016697.1_4e-145_60_700_98_750
R_marine_Glomel_SWEB01016696.1_4e-145_60_700_98_750
R_marine_Orcorc_NW_004438495.1_2e-143_60_700_1904716_1904064
R_marine_Inigeo_RJWO010015556.1_2e-138_60_700_22073_21426
R_marine_Ponbla_RJWI010034952.1_5e-138_60_700_4443_3793
R_marine_Lipvex_NW_006796981.1_4e-131_60_702_677257_677906
R_marine_Mesbid_PVJJ010040080.1_9e-148_60_700_12937_13587
R_marine_Zipcav_RJWS010042224.1_2e-149_60_700_5580_4929
R_marine_Balbon_DF532480.1_6e-144_60_700_4321_3666
R_marine_Balacu_NW_006731345.1_6e-144_60_700_1967093_1966439
R_marine_Escrob_NIPP01004754.1_3e-148_60_700_35228_35881
R_marine_Megnov_RYZJ01000371.1_4e-145_60_700_228421_227769
R_marine_Balmus_VNFD01002780.1_8e-147_60_700_195452_196104
R_marine_Eubjap_RJWP010001945.1_3e-152_60_698_10751_11401
R_marine_Phycat_NC_041227.1_4e-136_60_700_31006298_31005648
R_marine_Kogbre_RJWL010050228.1_5e-140_60_700_3634_2982
R_marine_Plamin_RJWK010012071.1_4e-121_60_628_24416_25001
PVJP02910225.1_3281124-3281761_Hippopotamus_amphibius_isolate_BS01_ScHSfme_924711HRSCAF_982511_whole_genome_shotgun_sequence
R_VCEA01000001.1_232105600-232105791_Muntiacus_muntjak_isolate_UTSW_UCB_Mm_chromosome_1_whole_genome_shotgun_sequence
R_VCEA01000001.1_232106405-232106814_Muntiacus_muntjak_isolate_UTSW_UCB_Mm_chromosome_1_whole_genome_shotgun_sequence
RAWU01000017.1_21555530-21556121_Giraffa_tippelskirchi_isolate_OR1865_chromosome_8_whole_genome_shotgun_sequence
RAWU01000017.1_21566409-21567017_Giraffa_tippelskirchi_isolate_OR1865_chromosome_8_whole_genome_shotgun_sequence
SJXR01001031.1_4145006-4145524_Przewalskium_albirostris_isolate_WLD-bcl_scaffold1395_len4871143_cov131_whole_genome_shotgun_sequence
SJXR01001031.1_4150305-4150885_Przewalskium_albirostris_isolate_WLD-bcl_scaffold1395_len4871143_cov131_whole_genome_shotgun_sequence
R_PVJP02910646.1_2397736-2398385_Hippopotamus_amphibius_isolate_BS01_ScHSfme_925104HRSCAF_982904_whole_genome_shotgun_sequence
0.03

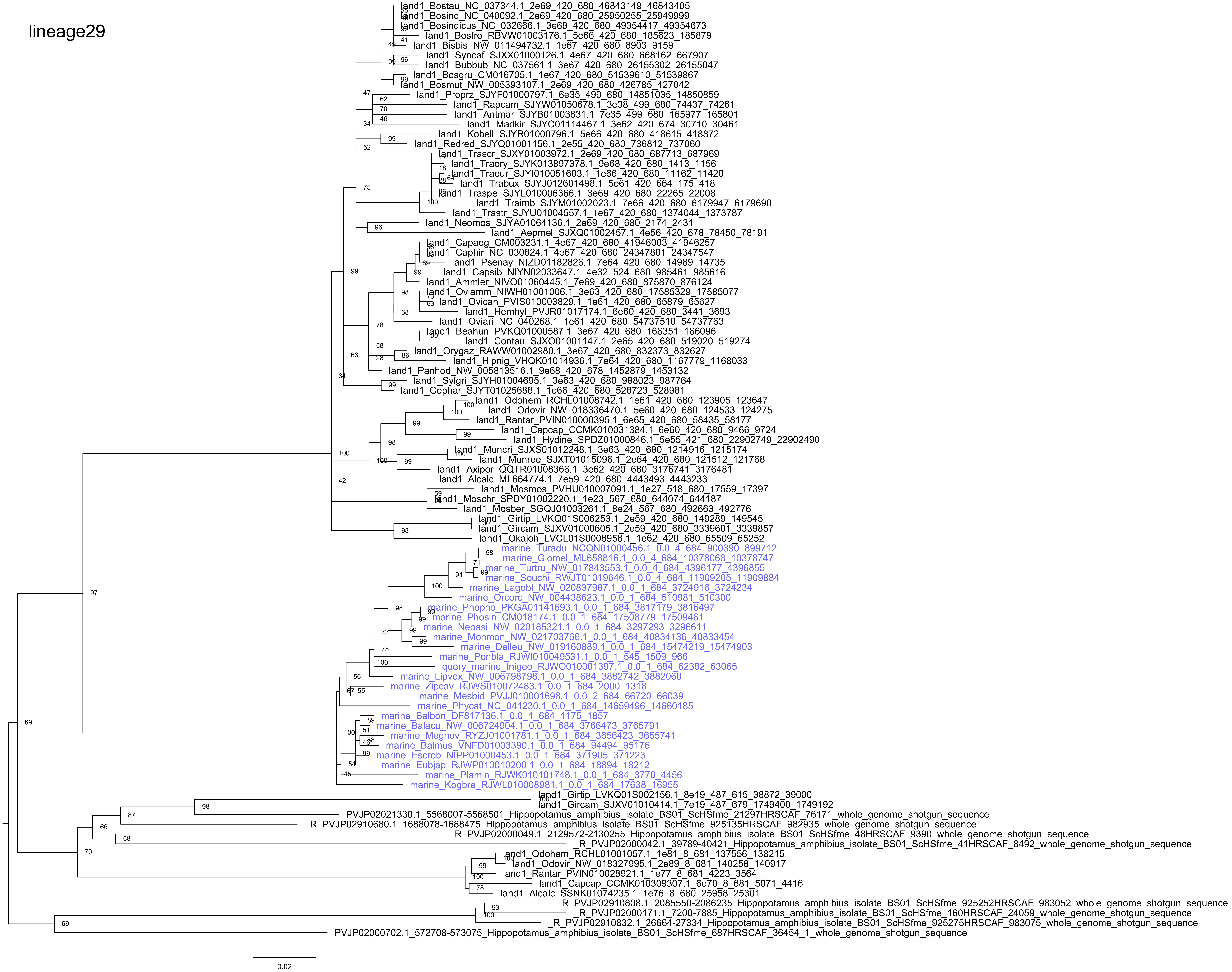
lineage29
land1_Bostau_NC_037344.1_2e69_420_680_46843149_46843405
land1_Bosind_NC_040092.1_2e69_420_680_25950255_25949999
land1_Bosindicus_NC_032666.1_3e68_420_680_49354417_49354673
land1_Bosfro_RBVW01003176.1_5e66_420_680_185623_185879
land1_Bisbis_NW_011494732.1_1e67_420_680_8903_9159
land1_Syncaf_SJXX01000126.1_4e67_420_680_668162_667907
land1_Bubbub_NC_037561.1_3e67_420_680_26155302_26155047
land1_Bosgru_CM016705.1_1e67_420_680_51539610_51539867
land1_Bosmut_NW_005393107.1_2e69_420_680_426785_427042
land1_Proprz_SJYF01000797.1_6e35_499_680_14851035_14850859
land1_Rapcam_SJYW01050678.1_3e38_499_680_74437_74261
land1_Antmar_SJYB01003831.1_7e35_499_680_165977_165801
land1_Madkir_SJYC01114467.1_3e62_420_674_30710_30461
land1_Kobell_SJYR01000796.1_5e66_420_680_418615_418872
land1_Redred_SJYQ01001156.1_2e55_420_680_736812_737060
land1_Trascr_SJXY01003972.1_2e69_420_680_687713_687969
land1_Traory_SJYK013897378.1_9e68_420_680_1413_1156
land1_Traeur_SJYI010051603.1_1e66_420_680_11162_11420
land1_Trabux_SJYJ012601498.1_5e61_420_664_175_418
land1_Traspe_SJYL010006366.1_3e69_420_680_22265_22008
land1_Traimb_SJYM01002023.1_7e66_420_680_6179947_6179690
land1_Trastr_SJYU01004557.1_1e67_420_680_1374044_1373787
land1_Neomos_SJYA01064136.1_2e69_420_680_2174_2431
land1_Aepmel_SJXQ01002457.1_4e56_420_678_78450_78191
land1_Capaeg_CM003231.1_4e67_420_680_41946003_41946257
land1_Caphir_NC_030824.1_4e67_420_680_24347801_24347547
land1_Psenay_NIZD01182826.1_7e64_420_680_14989_14735
land1_Capsib_NIYN02033647.1_4e32_524_680_985461_985616
land1_Ammler_NIVO01060445.1_7e69_420_680_875870_876124
land1_Oviamm_NIWH01001006.1_3e63_420_680_17585329_17585077
land1_Ovican_PVIS010003829.1_1e61_420_680_65879_65627
land1_Hemhyl_PVJR01017174.1_6e60_420_680_3441_3693
land1_Oviari_NC_040268.1_1e61_420_680_54737510_54737763
land1_Beahun_PVKQ01000587.1_3e67_420_680_166351_166096
land1_Contau_SJXO01001147.1_2e65_420_680_519020_519274
land1_Orygaz_RAWW01002980.1_3e67_420_680_832373_832627
land1_Hipnig_VHQK01014936.1_7e64_420_680_1167779_1168033
land1_Panhod_NW_005813516.1_9e68_420_678_1452879_1453132
land1_Sylgri_SJYH01004695.1_3e63_420_680_988023_987764
land1_Cephar_SJYT01025688.1_1e66_420_680_528723_528981
land1_Odohem_RCHL01008742.1_1e61_420_680_123905_123647
land1_Odovir_NW_018336470.1_5e60_420_680_124533_124275
land1_Rantar_PVIN010000395.1_6e65_420_680_58435_58177
land1_Capcap_CCMK010031384.1_6e60_420_680_9466_9724
land1_Hydine_SPDZ01000846.1_5e55_421_680_22902749_22902490
land1_Muncri_SJXS01012248.1_3e63_420_680_1214916_1215174
land1_Munree_SJXT01015096.1_2e64_420_680_121512_121768
land1_Axipor_QQTR01008366.1_3e62_420_680_3176741_3176481
land1_Alcalc_ML664774.1_7e59_420_680_4443493_4443233
land1_Mosmos_PVHU010007091.1_1e27_518_680_17559_17397
land1_Moschr_SPDY01002220.1_1e23_567_680_644074_644187
land1_Mosber_SGQJ01003261.1_8e24_567_680_492663_492776
land1_Girtip_LVKQ01S006253.1_2e59_420_680_149289_149545
land1_Gircam_SJXV01000605.1_2e59_420_680_3339601_3339857
land1_Okajoh_LVCL01S0008958.1_1e62_420_680_65509_65252
marine_Turadu_NCQN01000456.1_0.0_4_684_900390_899712
marine_Glomel_ML658816.1_0.0_4_684_10378068_10378747
marine_Turtru_NW_017843553.1_0.0_4_684_4396177_4396855
marine_Souchi_RWJT01019646.1_0.0_4_684_11909205_11909884
marine_Lagobl_NW_020837987.1_0.0_1_684_3724916_3724234
marine_Orcorc_NW_004438623.1_0.0_1_684_510981_510300
marine_Phopho_PKGA01141693.1_0.0_1_684_3817179_3816497
marine_Phosin_CM018174.1_0.0_1_684_17508779_17509461
marine_Neoasi_NW_020185321.1_0.0_1_684_3297293_3296611
marine_Monmon_NW_021703766.1_0.0_1_684_40834136_40833454
marine_Delleu_NW_019160889.1_0.0_1_684_15474219_15474903
marine_Ponbla_RJWI010049531.1_0.0_1_545_1509_966
query_marine_Inigeo_RJWO010001397.1_0.0_1_684_62382_63065
marine_Lipvex_NW_006798798.1_0.0_1_684_3882742_3882060
marine_Zipcav_RJWS010072483.1_0.0_1_684_2000_1318
marine_Mesbid_PVJJ010001698.1_0.0_2_684_66720_66039
marine_Phycat_NC_041230.1_0.0_1_684_14659496_14660185
marine_Balbon_DF817136.1_0.0_1_684_1175_1857
marine_Balacu_NW_006724904.1_0.0_1_684_3766473_3765791
marine_Megnov_RYZJ01001781.1_0.0_1_684_3656423_3655741
marine_Balmus_VNFD01003390.1_0.0_1_684_94494_95176
marine_Escrob_NIPP01000453.1_0.0_1_684_371905_371223
marine_Eubjap_RJWP010010200.1_0.0_1_684_18894_18212
marine_Plamin_RJWK010101748.1_0.0_1_684_3770_4456
marine_Kogbre_RJWL010008981.1_0.0_1_684_17638_16955
land1_Girtip_LVKQ01S002156.1_8e19_487_615_38872_39000
land1_Gircam_SJXV01010414.1_7e19_487_679_1749400_1749192
PVJP02021330.1_5568007-5568501_Hippopotamus_amphibius_isolate_BS01_ScHSfme_21297HRSCAF_76171_whole_genome_shotgun_sequence
_R_PVJP02910680.1_1688078-1688475_Hippopotamus_amphibius_isolate_BS01_ScHSfme_925135HRSCAF_982935_whole_genome_shotgun_sequence
_R_PVJP02000049.1_2129572-2130255_Hippopotamus_amphibius_isolate_BS01_ScHSfme_48HRSCAF_9390_whole_genome_shotgun_sequence
_R_PVJP02000042.1_39789-40421_Hippopotamus_amphibius_isolate_BS01_ScHSfme_41HRSCAF_8492_whole_genome_shotgun_sequence
land1_Odohem_RCHL01001057.1_1e81_8_681_137556_138215
land1_Odovir_NW_018327995.1_2e89_8_681_140258_140917
land1_Rantar_PVIN010028921.1_1e77_8_681_4223_3564
land1_Capcap_CCMK010309307.1_6e70_8_681_5071_4416
land1_Alcalc_SSNK01074235.1_1e76_8_680_25958_25301
_R_PVJP02910808.1_2085550-2086235_Hippopotamus_amphibius_isolate_BS01_ScHSfme_925252HRSCAF_983052_whole_genome_shotgun_sequence
_R_PVJP02000171.1_7200-7885_Hippopotamus_amphibius_isolate_BS01_ScHSfme_160HRSCAF_24059_whole_genome_shotgun_sequence
_R_PVJP02910832.1_26664-27334_Hippopotamus_amphibius_isolate_BS01_ScHSfme_925275HRSCAF_983075_whole_genome_shotgun_sequence
PVJP02000702.1_572708-573075_Hippopotamus_amphibius_isolate_BS01_ScHSfme_687HRSCAF_36454_1_whole_genome_shotgun_sequence
0.02

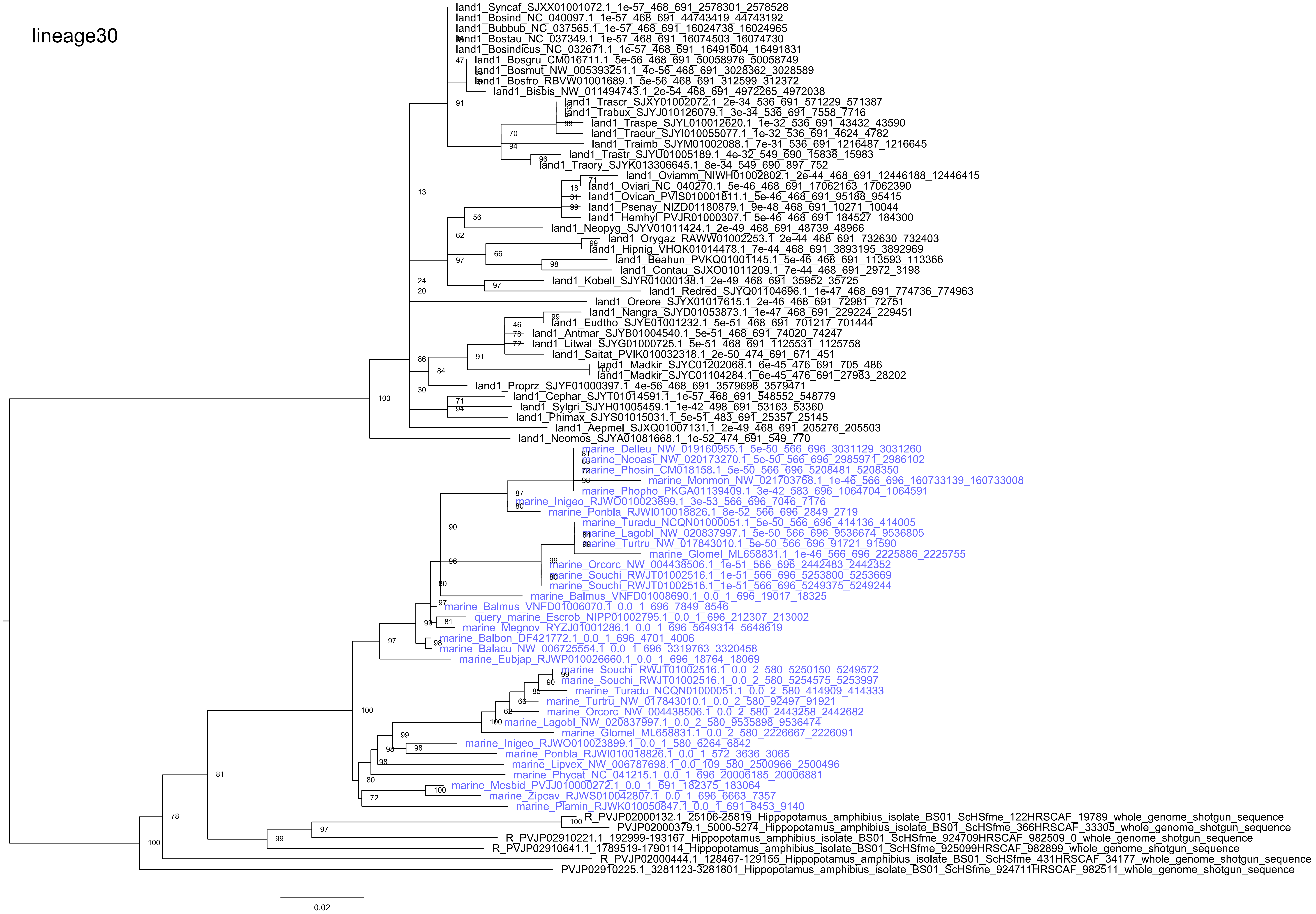
lineage30
land1_Syncaf_SJXX01001072.1_1e-57_468_691_2578301_2578528
land1_Bosind_NC_040097.1_1e-57_468_691_44743419_44743192
land1_Bubbub_NC_037565.1_1e-57_468_691_16024738_16024965
land1_Bostau_NC_037349.1_1e-57_468_691_16074503_16074730
land1_Bosindicus_NC_032671.1_1e-57_468_691_16491604_16491831
land1_Bosgru_CM016711.1_5e-56_468_691_50058976_50058749
land1_Bosmut_NW_005393251.1_4e-56_468_691_3028362_3028589
land1_Bosfro_RBVW01001689.1_5e-56_468_691_312599_312372
land1_Bisbis_NW_011494743.1_2e-54_468_691_4972265_4972038
land1_Trascr_SJXY01002072.1_2e-34_536_691_571229_571387
land1_Trabux_SJYJ010126079.1_3e-34_536_691_7558_7716
land1_Traspe_SJYL010012620.1_1e-32_536_691_43432_43590
land1_Traeur_SJYI010055077.1_1e-32_536_691_4624_4782
land1_Traimb_SJYM01002088.1_7e-31_536_691_1216487_1216645
land1_Trastr_SJYU01005189.1_4e-32_549_690_15838_15983
land1_Traory_SJYK013306645.1_8e-34_549_690_897_752
land1_Oviamm_NIWH01002802.1_2e-44_468_691_12446188_12446415
land1_Oviari_NC_040270.1_5e-46_468_691_17062163_17062390
land1_Ovican_PVIS010001811.1_5e-46_468_691_95188_95415
land1_Psenay_NIZD01180879.1_9e-48_468_691_10271_10044
land1_Hemhyl_PVJR01000307.1_5e-46_468_691_184527_184300
land1_Neopyg_SJYV01011424.1_2e-49_468_691_48739_48966
land1_Orygaz_RAWW01002253.1_2e-44_468_691_732630_732403
land1_Hipnig_VHQK01014478.1_7e-44_468_691_3893195_3892969
land1_Beahun_PVKQ01001145.1_5e-46_468_691_113593_113366
land1_Contau_SJXO01011209.1_7e-44_468_691_2972_3198
land1_Kobell_SJYR01000138.1_2e-49_468_691_35952_35725
land1_Redred_SJYQ01104696.1_1e-47_468_691_774736_774963
land1_Oreore_SJYX01017615.1_2e-46_468_691_72981_72751
land1_Nangra_SJYD01053873.1_1e-47_468_691_229224_229451
land1_Eudtho_SJYE01001232.1_5e-51_468_691_701217_701444
land1_Antmar_SJYB01004540.1_5e-51_468_691_74020_74247
land1_Litwal_SJYG01000725.1_5e-51_468_691_1125531_1125758
land1_Saitat_PVIK010032318.1_2e-50_474_691_671_451
land1_Madkir_SJYC01202068.1_6e-45_476_691_705_486
land1_Madkir_SJYC01104284.1_6e-45_476_691_27983_28202
land1_Proprz_SJYF01000397.1_4e-56_468_691_3579698_3579471
land1_Cephar_SJYT01014591.1_1e-57_468_691_548552_548779
land1_Sylgri_SJYH01005459.1_1e-42_498_691_53163_53360
land1_Phimax_SJYS01015031.1_5e-51_483_691_25357_25145
land1_Aepmel_SJXQ01007131.1_2e-49_468_691_205276_205503
land1_Neomos_SJYA01081668.1_1e-52_474_691_549_770
marine_Delleu_NW_019160955.1_5e-50_566_696_3031129_3031260
marine_Neoasi_NW_020173270.1_5e-50_566_696_2985971_2986102
marine_Phosin_CM018158.1_5e-50_566_696_5208481_5208350
marine_Monmon_NW_021703768.1_1e-46_566_696_160733139_160733008
marine_Phopho_PKGA01139409.1_3e-42_583_696_1064704_1064591
marine_Inigeo_RJWO010023899.1_3e-53_566_696_7046_7176
marine_Ponbla_RJWI010018826.1_8e-52_566_696_2849_2719
marine_Turadu_NCQN01000051.1_5e-50_566_696_414136_414005
marine_Lagobl_NW_020837997.1_5e-50_566_696_9536674_9536805
marine_Turtru_NW_017843010.1_5e-50_566_696_91721_91590
marine_Glomel_ML658831.1_1e-46_566_696_2225886_2225755
marine_Orcorc_NW_004438506.1_1e-51_566_696_2442483_2442352
marine_Souchi_RWJT01002516.1_1e-51_566_696_5253800_5253669
marine_Souchi_RWJT01002516.1_1e-51_566_696_5249375_5249244
marine_Balmus_VNFD01008690.1_0.0_1_696_19017_18325
marine_Balmus_VNFD01006070.1_0.0_1_696_7849_8546
query_marine_Escrob_NIPP01002795.1_0.0_1_696_212307_213002
marine_Megnov_RYZJ01001286.1_0.0_1_696_5649314_5648619
marine_Balbon_DF421772.1_0.0_1_696_4701_4006
marine_Balacu_NW_006725554.1_0.0_1_696_3319763_3320458
marine_Eubjap_RJWP010026660.1_0.0_1_696_18764_18069
marine_Souchi_RWJT01002516.1_0.0_2_580_5250150_5249572
marine_Souchi_RWJT01002516.1_0.0_2_580_5254575_5253997
marine_Turadu_NCQN01000051.1_0.0_2_580_414909_414333
marine_Turtru_NW_017843010.1_0.0_2_580_92497_91921
marine_Orcorc_NW_004438506.1_0.0_2_580_2443258_2442682
marine_Lagobl_NW_020837997.1_0.0_2_580_9535898_9536474
marine_Glomel_ML658831.1_0.0_2_580_2226667_2226091
marine_Inigeo_RJWO010023899.1_0.0_1_580_6264_6842
marine_Ponbla_RJWI010018826.1_0.0_1_572_3636_3065
marine_Lipvex_NW_006787698.1_0.0_109_580_2500966_2500496
marine_Phycat_NC_041215.1_0.0_1_696_20006185_20006881
marine_Mesbid_PVJJ010000272.1_0.0_1_691_182375_183064
marine_Zipcav_RJWS010042807.1_0.0_1_696_6663_7357
marine_Plamin_RJWK010050847.1_0.0_1_691_8453_9140
R_PVJP02000132.1_25106-25819_Hippopotamus_amphibius_isolate_BS01_ScHSfme_122HRSCAF_19789_whole_genome_shotgun_sequence
PVJP02000379.1_5000-5274_Hippopotamus_amphibius_isolate_BS01_ScHSfme_366HRSCAF_33305_whole_genome_shotgun_sequence
R_PVJP02910221.1_192999-193167_Hippopotamus_amphibius_isolate_BS01_ScHSfme_924709HRSCAF_982509_0_whole_genome_shotgun_sequence
R_PVJP02910641.1_1789519-1790114_Hippopotamus_amphibius_isolate_BS01_ScHSfme_925099HRSCAF_982899_whole_genome_shotgun_sequence
R_PVJP02000444.1_128467-129155_Hippopotamus_amphibius_isolate_BS01_ScHSfme_431HRSCAF_34177_whole_genome_shotgun_sequence
PVJP02910225.1_3281123-3281801_Hippopotamus_amphibius_isolate_BS01_ScHSfme_924711HRSCAF_982511_whole_genome_shotgun_sequence
0.02

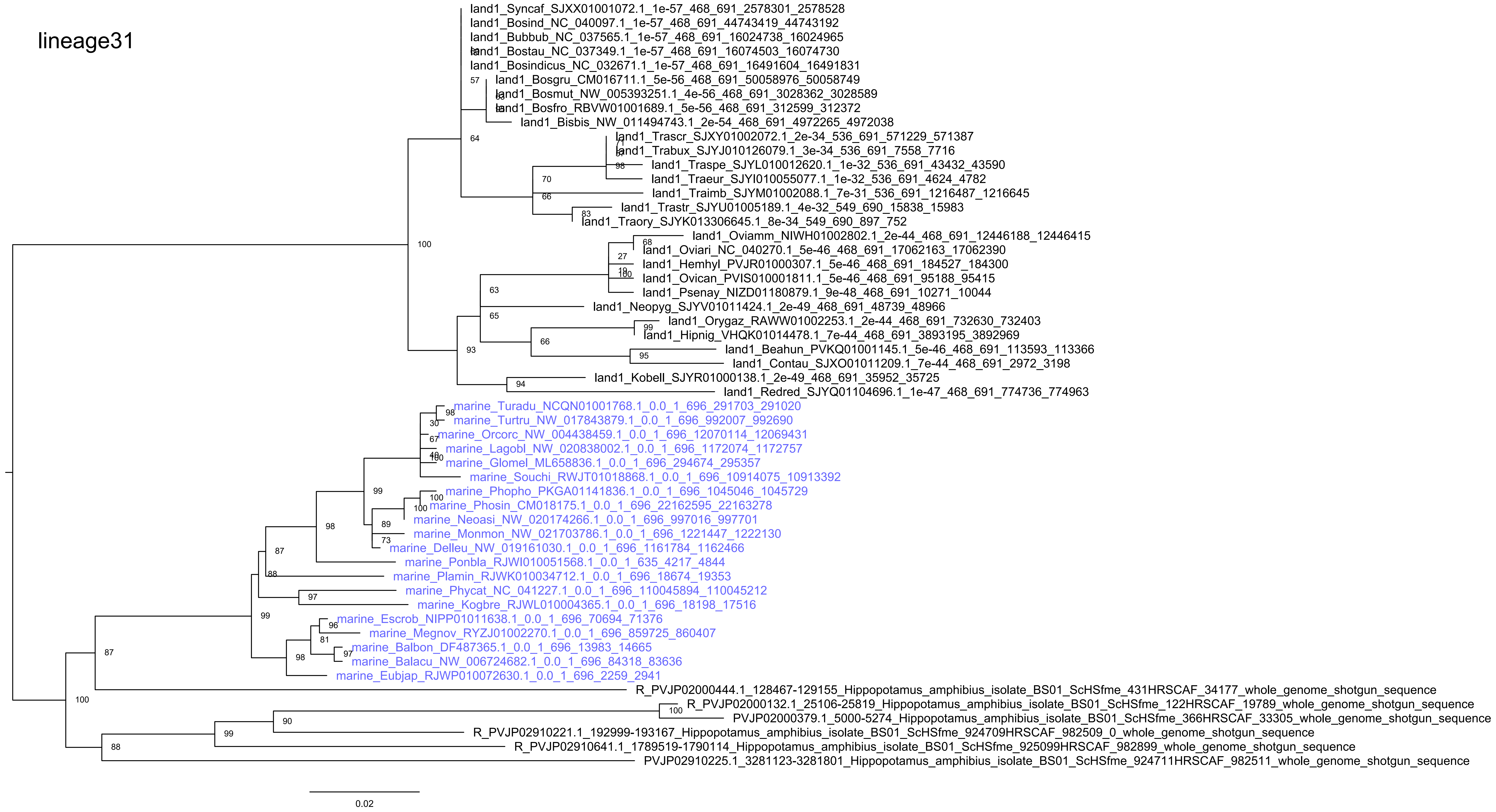
lineage31
land1_Syncaf_SJXX01001072.1_1e-57_468_691_2578301_2578528
land1_Bosind_NC_040097.1_1e-57_468_691_44743419_44743192
land1_Bubbub_NC_037565.1_1e-57_468_691_16024738_16024965
land1_Bostau_NC_037349.1_1e-57_468_691_16074503_16074730
land1_Bosindicus_NC_032671.1_1e-57_468_691_16491604_16491831
land1_Bosgru_CM016711.1_5e-56_468_691_50058976_50058749
land1_Bosmut_NW_005393251.1_4e-56_468_691_3028362_3028589
land1_Bosfro_RBVW01001689.1_5e-56_468_691_312599_312372
land1_Bisbis_NW_011494743.1_2e-54_468_691_4972265_4972038
land1_Trascr_SJXY01002072.1_2e-34_536_691_571229_571387
land1_Trabux_SJYJ010126079.1_3e-34_536_691_7558_7716
land1_Traspe_SJYL010012620.1_1e-32_536_691_43432_43590
land1_Traeur_SJYI010055077.1_1e-32_536_691_4624_4782
land1_Traimb_SJYM01002088.1_7e-31_536_691_1216487_1216645
land1_Trastr_SJYU01005189.1_4e-32_549_690_15838_15983
land1_Traory_SJYK013306645.1_8e-34_549_690_897_752
land1_Oviamm_NIWH01002802.1_2e-44_468_691_12446188_12446415
land1_Oviari_NC_040270.1_5e-46_468_691_17062163_17062390
land1_Hemhyl_PVJR01000307.1_5e-46_468_691_184527_184300
land1_Ovican_PVIS010001811.1_5e-46_468_691_95188_95415
land1_Psenay_NIZD01180879.1_9e-48_468_691_10271_10044
land1_Neopyg_SJYV01011424.1_2e-49_468_691_48739_48966
land1_Orygaz_RAWW01002253.1_2e-44_468_691_732630_732403
land1_Hipnig_VHQK01014478.1_7e-44_468_691_3893195_3892969
land1_Beahun_PVKQ01001145.1_5e-46_468_691_113593_113366
land1_Contau_SJXO01011209.1_7e-44_468_691_2972_3198
land1_Kobell_SJYR01000138.1_2e-49_468_691_35952_35725
land1_Redred_SJYQ01104696.1_1e-47_468_691_774736_774963
marine_Turadu_NCQN01001768.1_0.0_1_696_291703_291020
marine_Turtru_NW_017843879.1_0.0_1_696_992007_992690
marine_Orcorc_NW_004438459.1_0.0_1_696_12070114_12069431
marine_Lagobl_NW_020838002.1_0.0_1_696_1172074_1172757
marine_Glomel_ML658836.1_0.0_1_696_294674_295357
marine_Souchi_RWJT01018868.1_0.0_1_696_10914075_10913392
marine_Phopho_PKGA01141836.1_0.0_1_696_1045046_1045729
marine_Phosin_CM018175.1_0.0_1_696_22162595_22163278
marine_Neoasi_NW_020174266.1_0.0_1_696_997016_997701
marine_Monmon_NW_021703786.1_0.0_1_696_1221447_1222130
marine_Delleu_NW_019161030.1_0.0_1_696_1161784_1162466
marine_Ponbla_RJWI010051568.1_0.0_1_635_4217_4844
marine_Plamin_RJWK010034712.1_0.0_1_696_18674_19353
marine_Phycat_NC_041227.1_0.0_1_696_110045894_110045212
marine_Kogbre_RJWL010004365.1_0.0_1_696_18198_17516
marine_Escrob_NIPP01011638.1_0.0_1_696_70694_71376
marine_Megnov_RYZJ01002270.1_0.0_1_696_859725_860407
marine_Balbon_DF487365.1_0.0_1_696_13983_14665
marine_Balacu_NW_006724682.1_0.0_1_696_84318_83636
marine_Eubjap_RJWP010072630.1_0.0_1_696_2259_2941
R_PVJP02000444.1_128467-129155_Hippopotamus_amphibius_isolate_BS01_ScHSfme_431HRSCAF_34177_whole_genome_shotgun_sequence
R_PVJP02000132.1_25106-25819_Hippopotamus_amphibius_isolate_BS01_ScHSfme_122HRSCAF_19789_whole_genome_shotgun_sequence
PVJP02000379.1_5000-5274_Hippopotamus_amphibius_isolate_BS01_ScHSfme_366HRSCAF_33305_whole_genome_shotgun_sequence
R_PVJP02910221.1_192999-193167_Hippopotamus_amphibius_isolate_BS01_ScHSfme_924709HRSCAF_982509_0_whole_genome_shotgun_sequence
R_PVJP02910641.1_1789519-1790114_Hippopotamus_amphibius_isolate_BS01_ScHSfme_925099HRSCAF_982899_whole_genome_shotgun_sequence
PVJP02910225.1_3281123-3281801_Hippopotamus_amphibius_isolate_BS01_ScHSfme_924711HRSCAF_982511_whole_genome_shotgun_sequence
0.02

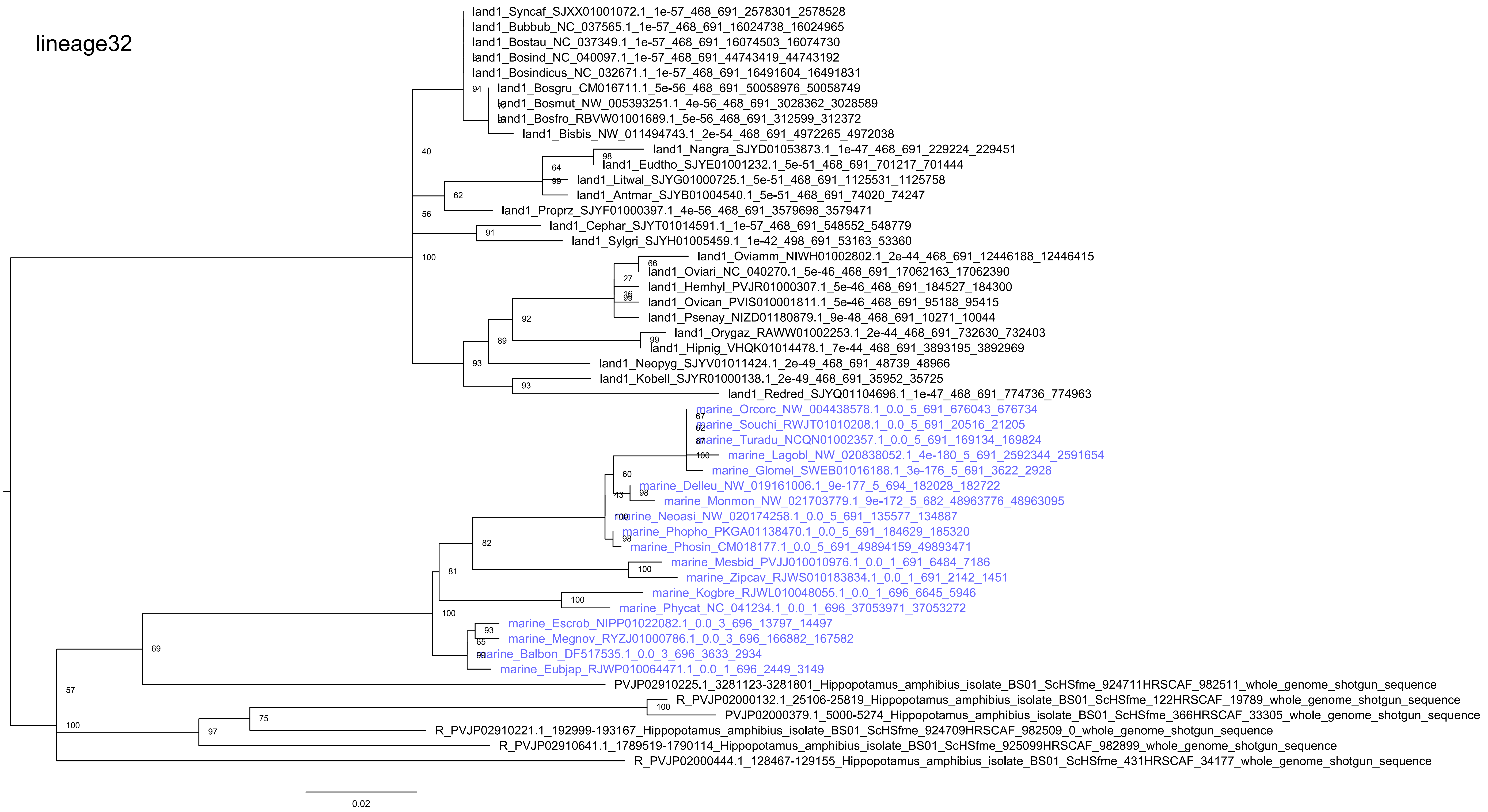
lineage32
land1_Syncaf_SJXX01001072.1_1e-57_468_691_2578301_2578528
land1_Bubbub_NC_037565.1_1e-57_468_691_16024738_16024965
land1_Bostau_NC_037349.1_1e-57_468_691_16074503_16074730
land1_Bosind_NC_040097.1_1e-57_468_691_44743419_44743192
land1_Bosindicus_NC_032671.1_1e-57_468_691_16491604_16491831
land1_Bosgru_CM016711.1_5e-56_468_691_50058976_50058749
land1_Bosmut_NW_005393251.1_4e-56_468_691_3028362_3028589
land1_Bosfro_RBVW01001689.1_5e-56_468_691_312599_312372
land1_Bisbis_NW_011494743.1_2e-54_468_691_4972265_4972038
land1_Nangra_SJYD01053873.1_1e-47_468_691_229224_229451
land1_Eudtho_SJYE01001232.1_5e-51_468_691_701217_701444
land1_Litwal_SJYG01000725.1_5e-51_468_691_1125531_1125758
land1_Antmar_SJYB01004540.1_5e-51_468_691_74020_74247
land1_Proprz_SJYF01000397.1_4e-56_468_691_3579698_3579471
land1_Cephar_SJYT01014591.1_1e-57_468_691_548552_548779
land1_Sylgri_SJYH01005459.1_1e-42_498_691_53163_53360
land1_Oviamm_NIWH01002802.1_2e-44_468_691_12446188_12446415
land1_Oviari_NC_040270.1_5e-46_468_691_17062163_17062390
land1_Hemhyl_PVJR01000307.1_5e-46_468_691_184527_184300
land1_Ovican_PVIS010001811.1_5e-46_468_691_95188_95415
land1_Psenay_NIZD01180879.1_9e-48_468_691_10271_10044
land1_Orygaz_RAWW01002253.1_2e-44_468_691_732630_732403
land1_Hipnig_VHQK01014478.1_7e-44_468_691_3893195_3892969
land1_Neopyg_SJYV01011424.1_2e-49_468_691_48739_48966
land1_Kobell_SJYR01000138.1_2e-49_468_691_35952_35725
land1_Redred_SJYQ01104696.1_1e-47_468_691_774736_774963
marine_Orcorc_NW_004438578.1_0.0_5_691_676043_676734
marine_Souchi_RWJT01010208.1_0.0_5_691_20516_21205
marine_Turadu_NCQN01002357.1_0.0_5_691_169134_169824
marine_Lagobl_NW_020838052.1_4e-180_5_691_2592344_2591654
marine_Glomel_SWEB01016188.1_3e-176_5_691_3622_2928
marine_Delleu_NW_019161006.1_9e-177_5_694_182028_182722
marine_Monmon_NW_021703779.1_9e-172_5_682_48963776_48963095
marine_Neoasi_NW_020174258.1_0.0_5_691_135577_134887
marine_Phopho_PKGA01138470.1_0.0_5_691_184629_185320
marine_Phosin_CM018177.1_0.0_5_691_49894159_49893471
marine_Mesbid_PVJJ010010976.1_0.0_1_691_6484_7186
marine_Zipcav_RJWS010183834.1_0.0_1_691_2142_1451
marine_Kogbre_RJWL010048055.1_0.0_1_696_6645_5946
marine_Phycat_NC_041234.1_0.0_1_696_37053971_37053272
marine_Escrob_NIPP01022082.1_0.0_3_696_13797_14497
marine_Megnov_RYZJ01000786.1_0.0_3_696_166882_167582
marine_Balbon_DF517535.1_0.0_3_696_3633_2934
marine_Eubjap_RJWP010064471.1_0.0_1_696_2449_3149
PVJP02910225.1_3281123-3281801_Hippopotamus_amphibius_isolate_BS01_ScHSfme_924711HRSCAF_982511_whole_genome_shotgun_sequence
R_PVJP02000132.1_25106-25819_Hippopotamus_amphibius_isolate_BS01_ScHSfme_122HRSCAF_19789_whole_genome_shotgun_sequence
PVJP02000379.1_5000-5274_Hippopotamus_amphibius_isolate_BS01_ScHSfme_366HRSCAF_33305_whole_genome_shotgun_sequence
R_PVJP02910221.1_192999-193167_Hippopotamus_amphibius_isolate_BS01_ScHSfme_924709HRSCAF_982509_0_whole_genome_shotgun_sequence
R_PVJP02910641.1_1789519-1790114_Hippopotamus_amphibius_isolate_BS01_ScHSfme_925099HRSCAF_982899_whole_genome_shotgun_sequence
R_PVJP02000444.1_128467-129155_Hippopotamus_amphibius_isolate_BS01_ScHSfme_431HRSCAF_34177_whole_genome_shotgun_sequence
0.02

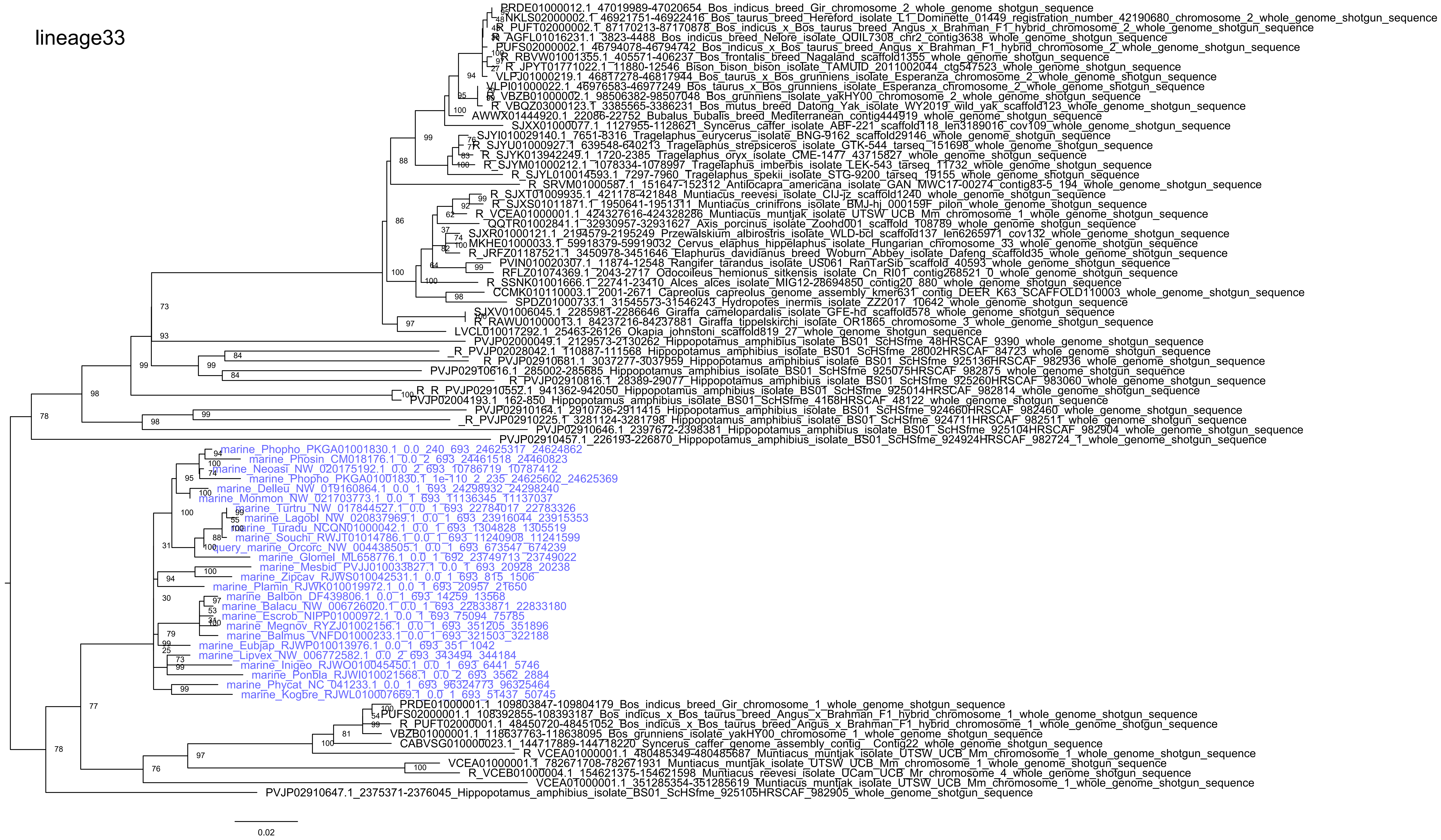
lineage33
PRDE01000012.1_47019989-47020654_Bos_indicus_breed_Gir_chromosome_2_whole_genome_shotgun_sequence
NKLS02000002.1_46921751-46922416_Bos_taurus_breed_Hereford_isolate_L1_Dominette_01449_registration_number_42190680_chromosome_2_whole_genome_shotgun_sequence
R_PUFT02000002.1_87170213-87170878_Bos_indicus_x_Bos_taurus_breed_Angus_x_Brahman_F1_hybrid_chromosome_2_whole_genome_shotgun_sequence
R_AGFL01016231.1_3823-4488_Bos_indicus_breed_Nelore_isolate_QUIL7308_chr2_contig3638_whole_genome_shotgun_sequence
PUFS02000002.1_46794078-46794742_Bos_indicus_x_Bos_taurus_breed_Angus_x_Brahman_F1_hybrid_chromosome_2_whole_genome_shotgun_sequence
R_RBVW01001355.1_405571-406237_Bos_frontalis_breed_Nagaland_scaffold1355_whole_genome_shotgun_sequence
R_JPYT01771022.1_11880-12546_Bison_bison_bison_isolate_TAMUID_2011002044_ctg547523_whole_genome_shotgun_sequence
VLPJ01000219.1_46817278-46817944_Bos_taurus_x_Bos_grunniens_isolate_Esperanza_chromosome_2_whole_genome_shotgun_sequence
VLPI01000022.1_46976583-46977249_Bos_taurus_x_Bos_grunniens_isolate_Esperanza_chromosome_2_whole_genome_shotgun_sequence
R_VBZB01000002.1_98506382-98507048_Bos_grunniens_isolate_yakHY00_chromosome_2_whole_genome_shotgun_sequence
R_VBQZ03000123.1_3385565-3386231_Bos_mutus_breed_Datong_Yak_isolate_WY2019_wild_yak_scaffold123_whole_genome_shotgun_sequence
AWWX01444920.1_22086-22752_Bubalus_bubalis_breed_Mediterranean_contig444919_whole_genome_shotgun_sequence
SJXX01000077.1_1127955-1128621_Syncerus_caffer_isolate_ABF-221_scaffold118_len3189016_cov109_whole_genome_shotgun_sequence
SJYI010029140.1_7651-8316_Tragelaphus_eurycerus_isolate_BNG-9162_scaffold29146_whole_genome_shotgun_sequence
R_SJYU01000927.1_639548-640213_Tragelaphus_strepsiceros_isolate_GTK-544_tarseq_151698_whole_genome_shotgun_sequence
R_SJYK013942249.1_1720-2385_Tragelaphus_oryx_isolate_CME-1477_43715827_whole_genome_shotgun_sequence
R_SJYM01000212.1_1078334-1078997_Tragelaphus_imberbis_isolate_LEK-543_tarseq_11732_whole_genome_shotgun_sequence
R_SJYL010014593.1_7297-7960_Tragelaphus_spekii_isolate_STG-9200_tarseq_19155_whole_genome_shotgun_sequence
R_SRVM01000587.1_151647-152312_Antilocapra_americana_isolate_GAN_MWC17-00274_contig83-5_194_whole_genome_shotgun_sequence
R_SJXT01009935.1_421178-421848_Muntiacus_reevesi_isolate_CIJ-jz_scaffold1240_whole_genome_shotgun_sequence
R_SJXS01011871.1_1950641-1951311_Muntiacus_crinifrons_isolate_BMJ-hj_000159F_pilon_whole_genome_shotgun_sequence
R_VCEA01000001.1_424327616-424328286_Muntiacus_muntjak_isolate_UTSW_UCB_Mm_chromosome_1_whole_genome_shotgun_sequence
QQTR01002841.1_32930957-32931627_Axis_porcinus_isolate_Zoohd001_scaffold_108789_whole_genome_shotgun_sequence
SJXR01000121.1_2194579-2195249_Przewalskium_albirostris_isolate_WLD-bcl_scaffold137_len6265971_cov132_whole_genome_shotgun_sequence
MKHE01000033.1_59918379-59919032_Cervus_elaphus_hippelaphus_isolate_Hungarian_chromosome_33_whole_genome_shotgun_sequence
R_JRFZ01187521.1_3450978-3451646_Elaphurus_davidianus_breed_Woburn_Abbey_isolate_Dafeng_scaffold35_whole_genome_shotgun_sequence
PVIN010020307.1_11874-12548_Rangifer_tarandus_isolate_US061_RanTarSib_scaffold_40593_whole_genome_shotgun_sequence
RFLZ01074369.1_2043-2717_Odocoileus_hemionus_sitkensis_isolate_Cn_RI01_contig268521_0_whole_genome_shotgun_sequence
R_SSNK01001666.1_22741-23410_Alces_alces_isolate_MIG12-28694850_contig20_880_whole_genome_shotgun_sequence
CCMK010110003.1_2001-2671_Capreolus_capreolus_genome_assembly_kmer631_contig_DEER_K63_SCAFFOLD110003_whole_genome_shotgun_sequence
SPDZ01000733.1_31545573-31546243_Hydropotes_inermis_isolate_ZZ2017_10642_whole_genome_shotgun_sequence
SJXV01006045.1_2285981-2286646_Giraffa_camelopardalis_isolate_GFE-hd_scaffold578_whole_genome_shotgun_sequence
R_RAWU01000013.1_84237216-84237881_Giraffa_tippelskirchi_isolate_OR1865_chromosome_3_whole_genome_shotgun_sequence
LVCL010017292.1_25463-26126_Okapia_johnstoni_scaffold819_27_whole_genome_shotgun_sequence
PVJP02000049.1_2129573-2130262_Hippopotamus_amphibius_isolate_BS01_ScHSfme_48HRSCAF_9390_whole_genome_shotgun_sequence
_R_PVJP02028042.1_110887-111568_Hippopotamus_amphibius_isolate_BS01_ScHSfme_28002HRSCAF_84723_whole_genome_shotgun_sequence
R_PVJP02910681.1_3037277-3037959_Hippopotamus_amphibius_isolate_BS01_ScHSfme_925136HRSCAF_982936_whole_genome_shotgun_sequence
PVJP02910616.1_285002-285685_Hippopotamus_amphibius_isolate_BS01_ScHSfme_925075HRSCAF_982875_whole_genome_shotgun_sequence
R_PVJP02910816.1_28389-29077_Hippopotamus_amphibius_isolate_BS01_ScHSfme_925260HRSCAF_983060_whole_genome_shotgun_sequence
R_R_PVJP02910552.1_941362-942050_Hippopotamus_amphibius_isolate_BS01_ScHSfme_925014HRSCAF_982814_whole_genome_shotgun_sequence
PVJP02004193.1_162-850_Hippopotamus_amphibius_isolate_BS01_ScHSfme_4168HRSCAF_48122_whole_genome_shotgun_sequence
PVJP02910164.1_2910736-2911415_Hippopotamus_amphibius_isolate_BS01_ScHSfme_924660HRSCAF_982460_whole_genome_shotgun_sequence
_R_PVJP02910225.1_3281124-3281798_Hippopotamus_amphibius_isolate_BS01_ScHSfme_924711HRSCAF_982511_whole_genome_shotgun_sequence
PVJP02910646.1_2397672-2398381_Hippopotamus_amphibius_isolate_BS01_ScHSfme_925104HRSCAF_982904_whole_genome_shotgun_sequence
PVJP02910457.1_226193-226870_Hippopotamus_amphibius_isolate_BS01_ScHSfme_924924HRSCAF_982724_1_whole_genome_shotgun_sequence
marine_Phopho_PKGA01001830.1_0.0_240_693_24625317_24624862
marine_Phosin_CM018176.1_0.0_2_693_24461518_24460823
marine_Neoasi_NW_020175192.1_0.0_2_693_10786719_10787412
marine_Phopho_PKGA01001830.1_1e-110_2_235_24625602_24625369
marine_Delleu_NW_019160864.1_0.0_1_693_24298932_24298240
marine_Monmon_NW_021703773.1_0.0_1_693_11136345_11137037
marine_Turtru_NW_017844527.1_0.0_1_693_22784017_22783326
marine_Lagobl_NW_020837969.1_0.0_1_693_23916044_23915353
marine_Turadu_NCQN01000042.1_0.0_1_693_1304828_1305519
marine_Souchi_RWJT01014786.1_0.0_1_693_11240908_11241599
query_marine_Orcorc_NW_004438505.1_0.0_1_693_673547_674239
marine_Glomel_ML658776.1_0.0_1_692_23749713_23749022
marine_Mesbid_PVJJ010033827.1_0.0_1_693_20928_20238
marine_Zipcav_RJWS010042531.1_0.0_1_693_815_1506
marine_Plamin_RJWK010019972.1_0.0_1_693_20957_21650
marine_Balbon_DF439806.1_0.0_1_693_14259_13568
marine_Balacu_NW_006726020.1_0.0_1_693_22833871_22833180
marine_Escrob_NIPP01000972.1_0.0_1_693_75094_75785
marine_Megnov_RYZJ01002156.1_0.0_1_693_351205_351896
marine_Balmus_VNFD01000233.1_0.0_1_693_321503_322188
marine_Eubjap_RJWP010013976.1_0.0_1_693_351_1042
marine_Lipvex_NW_006772582.1_0.0_2_693_343494_344184
marine_Inigeo_RJWO010045450.1_0.0_1_693_6441_5746
marine_Ponbla_RJWI010021568.1_0.0_2_693_3562_2884
marine_Phycat_NC_041233.1_0.0_1_693_96324773_96325464
marine_Kogbre_RJWL010007669.1_0.0_1_693_51437_50745
PRDE01000001.1_109803847-109804179_Bos_indicus_breed_Gir_chromosome_1_whole_genome_shotgun_sequence
PUFS02000001.1_108392855-108393187_Bos_indicus_x_Bos_taurus_breed_Angus_x_Brahman_F1_hybrid_chromosome_1_whole_genome_shotgun_sequence
R_PUFT02000001.1_48450720-48451052_Bos_indicus_x_Bos_taurus_breed_Angus_x_Brahman_F1_hybrid_chromosome_1_whole_genome_shotgun_sequence
VBZB01000001.1_118637763-118638095_Bos_grunniens_isolate_yakHY00_chromosome_1_whole_genome_shotgun_sequence
CABVSG010000023.1_144717889-144718220_Syncerus_caffer_genome_assembly_contig__Contig22_whole_genome_shotgun_sequence
R_VCEA01000001.1_480485349-480485687_Muntiacus_muntjak_isolate_UTSW_UCB_Mm_chromosome_1_whole_genome_shotgun_sequence
VCEA01000001.1_782671708-782671931_Muntiacus_muntjak_isolate_UTSW_UCB_Mm_chromosome_1_whole_genome_shotgun_sequence
R_VCEB01000004.1_154621375-154621598_Muntiacus_reevesi_isolate_UCam_UCB_Mr_chromosome_4_whole_genome_shotgun_sequence
VCEA01000001.1_351285354-351285619_Muntiacus_muntjak_isolate_UTSW_UCB_Mm_chromosome_1_whole_genome_shotgun_sequence
PVJP02910647.1_2375371-2376045_Hippopotamus_amphibius_isolate_BS01_ScHSfme_925105HRSCAF_982905_whole_genome_shotgun_sequence
0.02

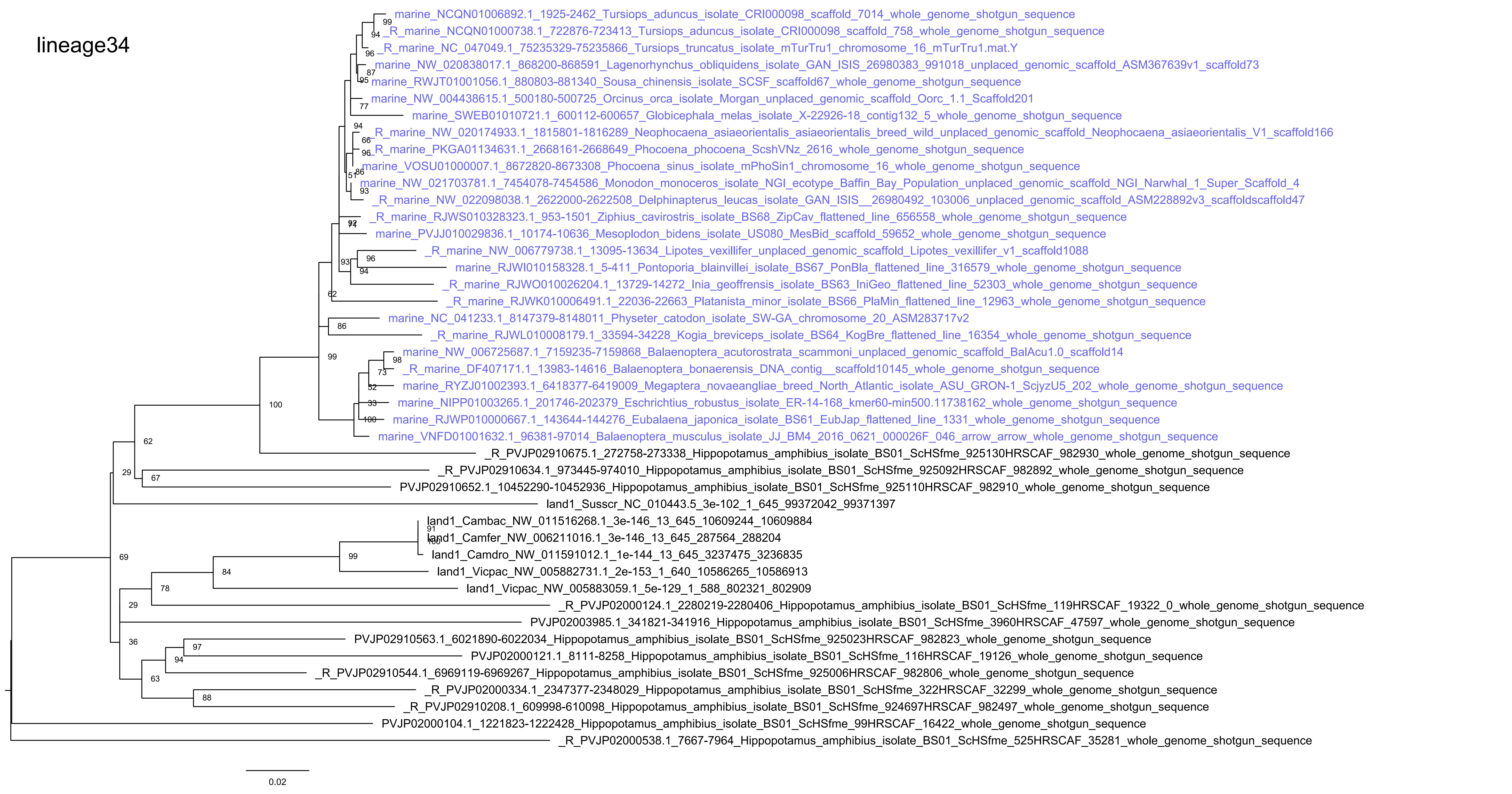
lineage34
marine_NCQN01006892.1_1925-2462_Tursiops_aduncus_isolate_CRI000098_scaffold_7014_whole_genome_shotgun_sequence
_R_marine_NCQN01000738.1_722876-723413_Tursiops_aduncus_isolate_CRI000098_scaffold_758_whole_genome_shotgun_sequence
_R_marine_NC_047049.1_75235329-75235866_Tursiops_truncatus_isolate_mTurTru1_chromosome_16_mTurTru1.mat.Y
marine_NW_020838017.1_868200-868591_Lagenorhynchus_obliquidens_isolate_GAN_ISIS_26980383_991018_unplaced_genomic_scaffold_ASM367639v1_scaffold73
marine_RWJT01001056.1_880803-881340_Sousa_chinensis_isolate_SCSF_scaffold67_whole_genome_shotgun_sequence
marine_NW_004438615.1_500180-500725_Orcinus_orca_isolate_Morgan_unplaced_genomic_scaffold_Oorc_1.1_Scaffold201
marine_SWEB01010721.1_600112-600657_Globicephala_melas_isolate_X-22926-18_contig132_5_whole_genome_shotgun_sequence
_R_marine_NW_020174933.1_1815801-1816289_Neophocaena_asiaeorientalis_asiaeorientalis_breed_wild_unplaced_genomic_scaffold_Neophocaena_asiaeorientalis_V1_scaffold166
_R_marine_PKGA01134631.1_2668161-2668649_Phocoena_phocoena_ScshVNz_2616_whole_genome_shotgun_sequence
marine_VOSU01000007.1_8672820-8673308_Phocoena_sinus_isolate_mPhoSin1_chromosome_16_whole_genome_shotgun_sequence
marine_NW_021703781.1_7454078-7454586_Monodon_monoceros_isolate_NGI_ecotype_Baffin_Bay_Population_unplaced_genomic_scaffold_NGI_Narwhal_1_Super_Scaffold_4
_R_marine_NW_022098038.1_2622000-2622508_Delphinapterus_leucas_isolate_GAN_ISIS__26980492_103006_unplaced_genomic_scaffold_ASM228892v3_scaffoldscaffold47
_R_marine_RJWS010328323.1_953-1501_Ziphius_cavirostris_isolate_BS68_ZipCav_flattened_line_656558_whole_genome_shotgun_sequence
marine_PVJJ010029836.1_10174-10636_Mesoplodon_bidens_isolate_US080_MesBid_scaffold_59652_whole_genome_shotgun_sequence
_R_marine_NW_006779738.1_13095-13634_Lipotes_vexillifer_unplaced_genomic_scaffold_Lipotes_vexillifer_v1_scaffold1088
marine_RJWI010158328.1_5-411_Pontoporia_blainvillei_isolate_BS67_PonBla_flattened_line_316579_whole_genome_shotgun_sequence
_R_marine_RJWO010026204.1_13729-14272_Inia_geoffrensis_isolate_BS63_IniGeo_flattened_line_52303_whole_genome_shotgun_sequence
_R_marine_RJWK010006491.1_22036-22663_Platanista_minor_isolate_BS66_PlaMin_flattened_line_12963_whole_genome_shotgun_sequence
marine_NC_041233.1_8147379-8148011_Physeter_catodon_isolate_SW-GA_chromosome_20_ASM283717v2
_R_marine_RJWL010008179.1_33594-34228_Kogia_breviceps_isolate_BS64_KogBre_flattened_line_16354_whole_genome_shotgun_sequence
marine_NW_006725687.1_7159235-7159868_Balaenoptera_acutorostrata_scammoni_unplaced_genomic_scaffold_BalAcu1.0_scaffold14
_R_marine_DF407171.1_13983-14616_Balaenoptera_bonaerensis_DNA_contig__scaffold10145_whole_genome_shotgun_sequence
marine_RYZJ01002393.1_6418377-6419009_Megaptera_novaeangliae_breed_North_Atlantic_isolate_ASU_GRON-1_ScjyzU5_202_whole_genome_shotgun_sequence
marine_NIPP01003265.1_201746-202379_Eschrichtius_robustus_isolate_ER-14-168_kmer60-min500.11738162_whole_genome_shotgun_sequence
marine_RJWP010000667.1_143644-144276_Eubalaena_japonica_isolate_BS61_EubJap_flattened_line_1331_whole_genome_shotgun_sequence
marine_VNFD01001632.1_96381-97014_Balaenoptera_musculus_isolate_JJ_BM4_2016_0621_000026F_046_arrow_arrow_whole_genome_shotgun_sequence
_R_PVJP02910675.1_272758-273338_Hippopotamus_amphibius_isolate_BS01_ScHSfme_925130HRSCAF_982930_whole_genome_shotgun_sequence
_R_PVJP02910634.1_973445-974010_Hippopotamus_amphibius_isolate_BS01_ScHSfme_925092HRSCAF_982892_whole_genome_shotgun_sequence
PVJP02910652.1_10452290-10452936_Hippopotamus_amphibius_isolate_BS01_ScHSfme_925110HRSCAF_982910_whole_genome_shotgun_sequence
land1_Susscr_NC_010443.5_3e-102_1_645_99372042_99371397
land1_Cambac_NW_011516268.1_3e-146_13_645_10609244_10609884
land1_Camfer_NW_006211016.1_3e-146_13_645_287564_288204
land1_Camdro_NW_011591012.1_1e-144_13_645_3237475_3236835
land1_Vicpac_NW_005882731.1_2e-153_1_640_10586265_10586913
land1_Vicpac_NW_005883059.1_5e-129_1_588_802321_802909
_R_PVJP02000124.1_2280219-2280406_Hippopotamus_amphibius_isolate_BS01_ScHSfme_119HRSCAF_19322_0_whole_genome_shotgun_sequence
PVJP02003985.1_341821-341916_Hippopotamus_amphibius_isolate_BS01_ScHSfme_3960HRSCAF_47597_whole_genome_shotgun_sequence
PVJP02910563.1_6021890-6022034_Hippopotamus_amphibius_isolate_BS01_ScHSfme_925023HRSCAF_982823_whole_genome_shotgun_sequence
PVJP02000121.1_8111-8258_Hippopotamus_amphibius_isolate_BS01_ScHSfme_116HRSCAF_19126_whole_genome_shotgun_sequence
_R_PVJP02910544.1_6969119-6969267_Hippopotamus_amphibius_isolate_BS01_ScHSfme_925006HRSCAF_982806_whole_genome_shotgun_sequence
_R_PVJP02000334.1_2347377-2348029_Hippopotamus_amphibius_isolate_BS01_ScHSfme_322HRSCAF_32299_whole_genome_shotgun_sequence
_R_PVJP02910208.1_609998-610098_Hippopotamus_amphibius_isolate_BS01_ScHSfme_924697HRSCAF_982497_whole_genome_shotgun_sequence
PVJP02000104.1_1221823-1222428_Hippopotamus_amphibius_isolate_BS01_ScHSfme_99HRSCAF_16422_whole_genome_shotgun_sequence
_R_PVJP02000538.1_7667-7964_Hippopotamus_amphibius_isolate_BS01_ScHSfme_525HRSCAF_35281_whole_genome_shotgun_sequence
0.02

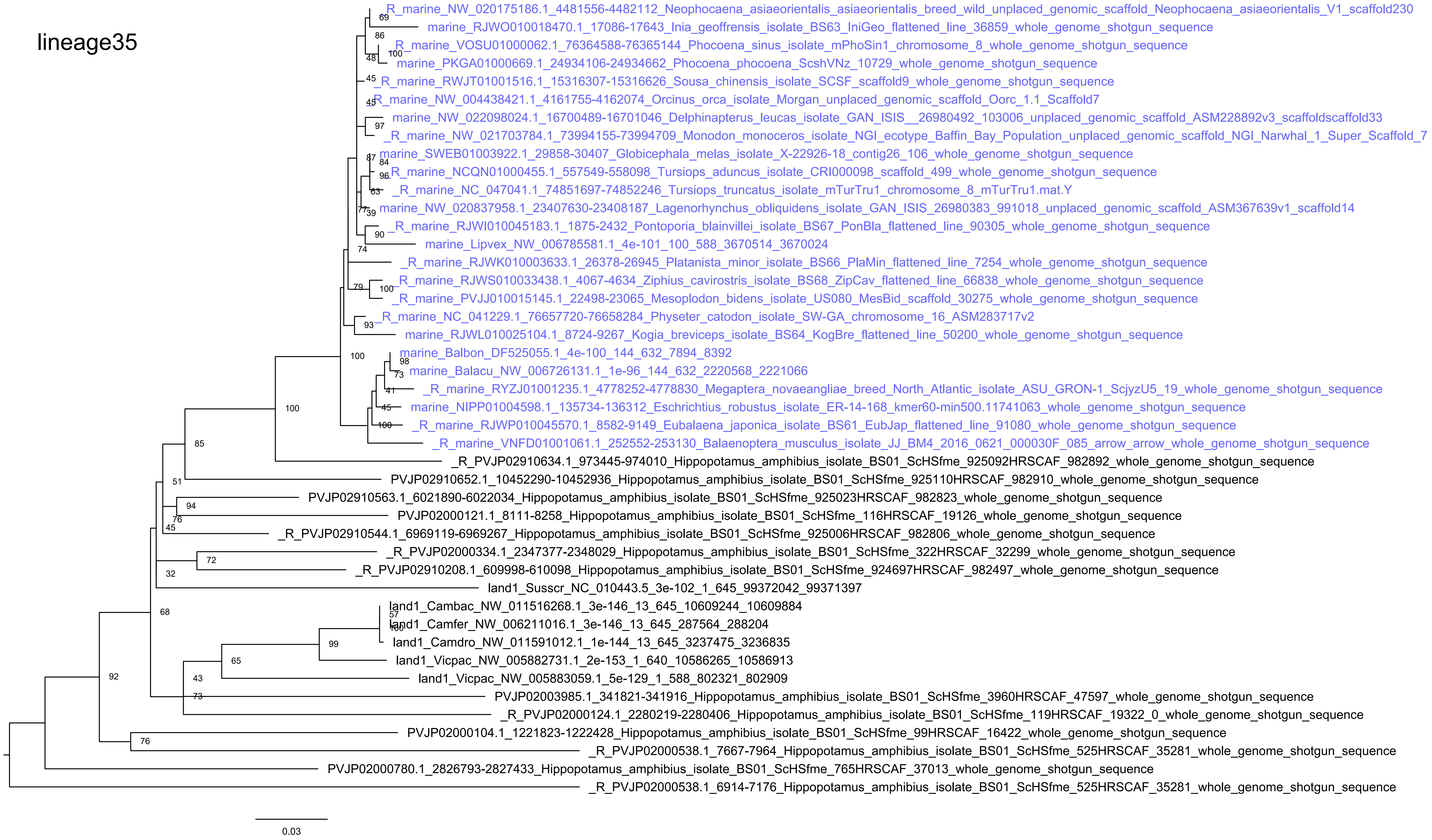
lineage35
_R_marine_NW_020175186.1_4481556-4482112_Neophocaena_asiaeorientalis_asiaeorientalis_breed_wild_unplaced_genomic_scaffold_Neophocaena_asiaeorientalis_V1_scaffold230
marine_RJWO010018470.1_17086-17643_Inia_geoffrensis_isolate_BS63_IniGeo_flattened_line_36859_whole_genome_shotgun_sequence
_R_marine_VOSU01000062.1_76364588-76365144_Phocoena_sinus_isolate_mPhoSin1_chromosome_8_whole_genome_shotgun_sequence
marine_PKGA01000669.1_24934106-24934662_Phocoena_phocoena_ScshVNz_10729_whole_genome_shotgun_sequence
_R_marine_RWJT01001516.1_15316307-15316626_Sousa_chinensis_isolate_SCSF_scaffold9_whole_genome_shotgun_sequence
_R_marine_NW_004438421.1_4161755-4162074_Orcinus_orca_isolate_Morgan_unplaced_genomic_scaffold_Oorc_1.1_Scaffold7
marine_NW_022098024.1_16700489-16701046_Delphinapterus_leucas_isolate_GAN_ISIS__26980492_103006_unplaced_genomic_scaffold_ASM228892v3_scaffoldscaffold33
_R_marine_NW_021703784.1_73994155-73994709_Monodon_monoceros_isolate_NGI_ecotype_Baffin_Bay_Population_unplaced_genomic_scaffold_NGI_Narwhal_1_Super_Scaffold_7
marine_SWEB01003922.1_29858-30407_Globicephala_melas_isolate_X-22926-18_contig26_106_whole_genome_shotgun_sequence
_R_marine_NCQN01000455.1_557549-558098_Tursiops_aduncus_isolate_CRI000098_scaffold_499_whole_genome_shotgun_sequence
_R_marine_NC_047041.1_74851697-74852246_Tursiops_truncatus_isolate_mTurTru1_chromosome_8_mTurTru1.mat.Y
marine_NW_020837958.1_23407630-23408187_Lagenorhynchus_obliquidens_isolate_GAN_ISIS_26980383_991018_unplaced_genomic_scaffold_ASM367639v1_scaffold14
_R_marine_RJWI010045183.1_1875-2432_Pontoporia_blainvillei_isolate_BS67_PonBla_flattened_line_90305_whole_genome_shotgun_sequence
marine_Lipvex_NW_006785581.1_4e-101_100_588_3670514_3670024
_R_marine_RJWK010003633.1_26378-26945_Platanista_minor_isolate_BS66_PlaMin_flattened_line_7254_whole_genome_shotgun_sequence
_R_marine_RJWS010033438.1_4067-4634_Ziphius_cavirostris_isolate_BS68_ZipCav_flattened_line_66838_whole_genome_shotgun_sequence
_R_marine_PVJJ010015145.1_22498-23065_Mesoplodon_bidens_isolate_US080_MesBid_scaffold_30275_whole_genome_shotgun_sequence
_R_marine_NC_041229.1_76657720-76658284_Physeter_catodon_isolate_SW-GA_chromosome_16_ASM283717v2
marine_RJWL010025104.1_8724-9267_Kogia_breviceps_isolate_BS64_KogBre_flattened_line_50200_whole_genome_shotgun_sequence
marine_Balbon_DF525055.1_4e-100_144_632_7894_8392
marine_Balacu_NW_006726131.1_1e-96_144_632_2220568_2221066
_R_marine_RYZJ01001235.1_4778252-4778830_Megaptera_novaeangliae_breed_North_Atlantic_isolate_ASU_GRON-1_ScjyzU5_19_whole_genome_shotgun_sequence
marine_NIPP01004598.1_135734-136312_Eschrichtius_robustus_isolate_ER-14-168_kmer60-min500.11741063_whole_genome_shotgun_sequence
_R_marine_RJWP010045570.1_8582-9149_Eubalaena_japonica_isolate_BS61_EubJap_flattened_line_91080_whole_genome_shotgun_sequence
_R_marine_VNFD01001061.1_252552-253130_Balaenoptera_musculus_isolate_JJ_BM4_2016_0621_000030F_085_arrow_arrow_whole_genome_shotgun_sequence
_R_PVJP02910634.1_973445-974010_Hippopotamus_amphibius_isolate_BS01_ScHSfme_925092HRSCAF_982892_whole_genome_shotgun_sequence
PVJP02910652.1_10452290-10452936_Hippopotamus_amphibius_isolate_BS01_ScHSfme_925110HRSCAF_982910_whole_genome_shotgun_sequence
PVJP02910563.1_6021890-6022034_Hippopotamus_amphibius_isolate_BS01_ScHSfme_925023HRSCAF_982823_whole_genome_shotgun_sequence
PVJP02000121.1_8111-8258_Hippopotamus_amphibius_isolate_BS01_ScHSfme_116HRSCAF_19126_whole_genome_shotgun_sequence
_R_PVJP02910544.1_6969119-6969267_Hippopotamus_amphibius_isolate_BS01_ScHSfme_925006HRSCAF_982806_whole_genome_shotgun_sequence
_R_PVJP02000334.1_2347377-2348029_Hippopotamus_amphibius_isolate_BS01_ScHSfme_322HRSCAF_32299_whole_genome_shotgun_sequence
_R_PVJP02910208.1_609998-610098_Hippopotamus_amphibius_isolate_BS01_ScHSfme_924697HRSCAF_982497_whole_genome_shotgun_sequence
land1_Susscr_NC_010443.5_3e-102_1_645_99372042_99371397
land1_Cambac_NW_011516268.1_3e-146_13_645_10609244_10609884
land1_Camfer_NW_006211016.1_3e-146_13_645_287564_288204
land1_Camdro_NW_011591012.1_1e-144_13_645_3237475_3236835
land1_Vicpac_NW_005882731.1_2e-153_1_640_10586265_10586913
land1_Vicpac_NW_005883059.1_5e-129_1_588_802321_802909
PVJP02003985.1_341821-341916_Hippopotamus_amphibius_isolate_BS01_ScHSfme_3960HRSCAF_47597_whole_genome_shotgun_sequence
_R_PVJP02000124.1_2280219-2280406_Hippopotamus_amphibius_isolate_BS01_ScHSfme_119HRSCAF_19322_0_whole_genome_shotgun_sequence
PVJP02000104.1_1221823-1222428_Hippopotamus_amphibius_isolate_BS01_ScHSfme_99HRSCAF_16422_whole_genome_shotgun_sequence
_R_PVJP02000538.1_7667-7964_Hippopotamus_amphibius_isolate_BS01_ScHSfme_525HRSCAF_35281_whole_genome_shotgun_sequence
PVJP02000780.1_2826793-2827433_Hippopotamus_amphibius_isolate_BS01_ScHSfme_765HRSCAF_37013_whole_genome_shotgun_sequence
_R_PVJP02000538.1_6914-7176_Hippopotamus_amphibius_isolate_BS01_ScHSfme_525HRSCAF_35281_whole_genome_shotgun_sequence
0.03

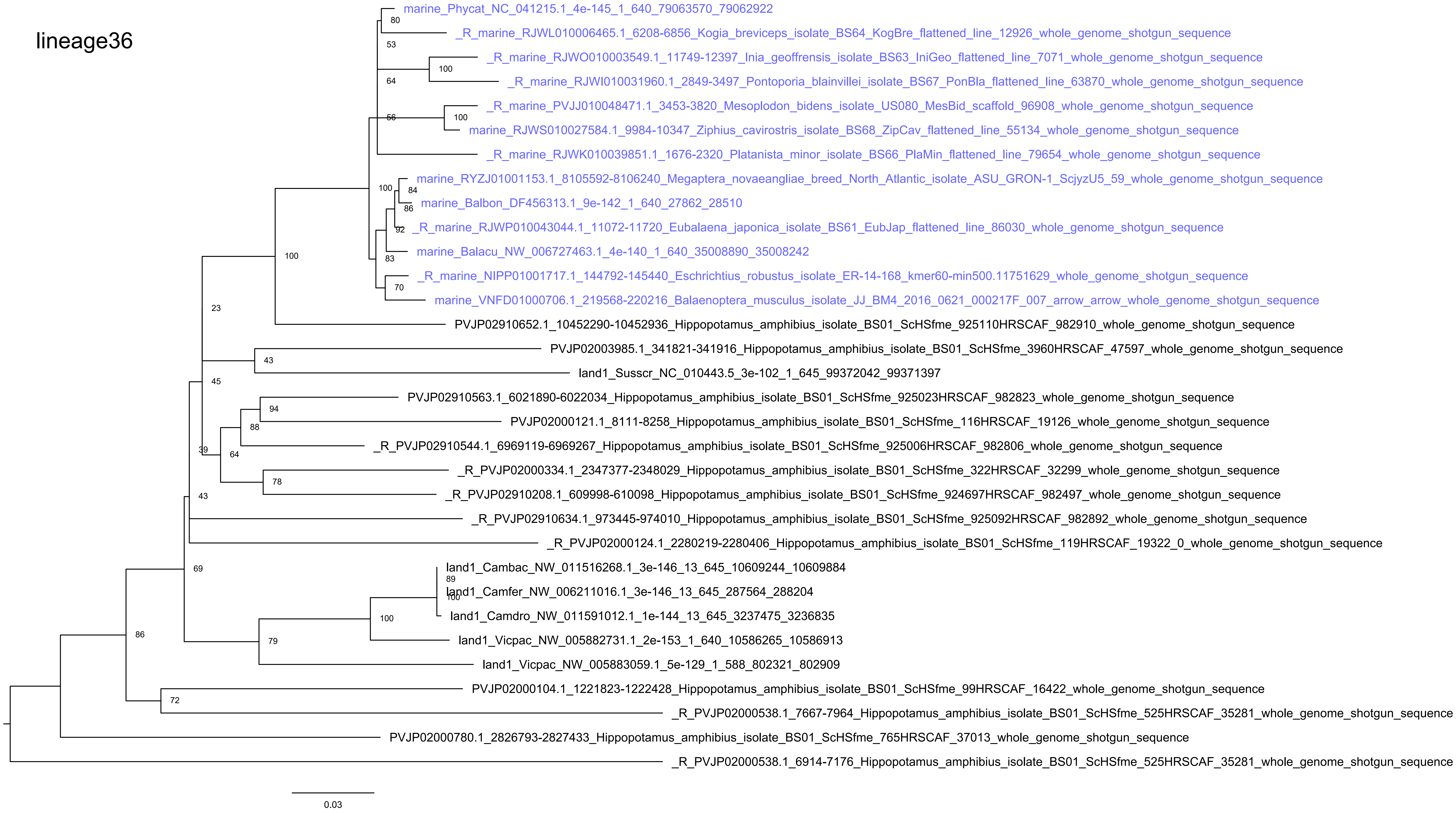

lineage36
marine_Phycat_NC_041215.1_4e-145_1_640_79063570_79062922
_R_marine_RJWL010006465.1_6208-6856_Kogia_breviceps_isolate_BS64_KogBre_flattened_line_12926_whole_genome_shotgun_sequence
_R_marine_RJWO010003549.1_11749-12397_Inia_geoffrensis_isolate_BS63_IniGeo_flattened_line_7071_whole_genome_shotgun_sequence
_R_marine_RJWI010031960.1_2849-3497_Pontoporia_blainvillei_isolate_BS67_PonBla_flattened_line_63870_whole_genome_shotgun_sequence
_R_marine_PVJJ010048471.1_3453-3820_Mesoplodon_bidens_isolate_US080_MesBid_scaffold_96908_whole_genome_shotgun_sequence
marine_RJWS010027584.1_9984-10347_Ziphius_cavirostris_isolate_BS68_ZipCav_flattened_line_55134_whole_genome_shotgun_sequence
_R_marine_RJWK010039851.1_1676-2320_Platanista_minor_isolate_BS66_PlaMin_flattened_line_79654_whole_genome_shotgun_sequence
marine_RYZJ01001153.1_8105592-8106240_Megaptera_novaeangliae_breed_North_Atlantic_isolate_ASU_GRON-1_ScjyzU5_59_whole_genome_shotgun_sequence
marine_Balbon_DF456313.1_9e-142_1_640_27862_28510
_R_marine_RJWP010043044.1_11072-11720_Eubalaena_japonica_isolate_BS61_EubJap_flattened_line_86030_whole_genome_shotgun_sequence
marine_Balacu_NW_006727463.1_4e-140_1_640_35008890_35008242
_R_marine_NIPP01001717.1_144792-145440_Eschrichtius_robustus_isolate_ER-14-168_kmer60-min500.11751629_whole_genome_shotgun_sequence
marine_VNFD01000706.1_219568-220216_Balaenoptera_musculus_isolate_JJ_BM4_2016_0621_000217F_007_arrow_arrow_whole_genome_shotgun_sequence
PVJP02910652.1_10452290-10452936_Hippopotamus_amphibius_isolate_BS01_ScHSfme_925110HRSCAF_982910_whole_genome_shotgun_sequence
PVJP02003985.1_341821-341916_Hippopotamus_amphibius_isolate_BS01_ScHSfme_3960HRSCAF_47597_whole_genome_shotgun_sequence
land1_Susscr_NC_010443.5_3e-102_1_645_99372042_99371397
PVJP02910563.1_6021890-6022034_Hippopotamus_amphibius_isolate_BS01_ScHSfme_925023HRSCAF_982823_whole_genome_shotgun_sequence
PVJP02000121.1_8111-8258_Hippopotamus_amphibius_isolate_BS01_ScHSfme_116HRSCAF_19126_whole_genome_shotgun_sequence
_R_PVJP02910544.1_6969119-6969267_Hippopotamus_amphibius_isolate_BS01_ScHSfme_925006HRSCAF_982806_whole_genome_shotgun_sequence
_R_PVJP02000334.1_2347377-2348029_Hippopotamus_amphibius_isolate_BS01_ScHSfme_322HRSCAF_32299_whole_genome_shotgun_sequence
_R_PVJP02910208.1_609998-610098_Hippopotamus_amphibius_isolate_BS01_ScHSfme_924697HRSCAF_982497_whole_genome_shotgun_sequence
_R_PVJP02910634.1_973445-974010_Hippopotamus_amphibius_isolate_BS01_ScHSfme_925092HRSCAF_982892_whole_genome_shotgun_sequence
_R_PVJP02000124.1_2280219-2280406_Hippopotamus_amphibius_isolate_BS01_ScHSfme_119HRSCAF_19322_0_whole_genome_shotgun_sequence
land1_Cambac_NW_011516268.1_3e-146_13_645_10609244_10609884
land1_Camfer_NW_006211016.1_3e-146_13_645_287564_288204
land1_Camdro_NW_011591012.1_1e-144_13_645_3237475_3236835
land1_Vicpac_NW_005882731.1_2e-153_1_640_10586265_10586913
land1_Vicpac_NW_005883059.1_5e-129_1_588_802321_802909
PVJP02000104.1_1221823-1222428_Hippopotamus_amphibius_isolate_BS01_ScHSfme_99HRSCAF_16422_whole_genome_shotgun_sequence
_R_PVJP02000538.1_7667-7964_Hippopotamus_amphibius_isolate_BS01_ScHSfme_525HRSCAF_35281_whole_genome_shotgun_sequence
PVJP02000780.1_2826793-2827433_Hippopotamus_amphibius_isolate_BS01_ScHSfme_765HRSCAF_37013_whole_genome_shotgun_sequence
_R_PVJP02000538.1_6914-7176_Hippopotamus_amphibius_isolate_BS01_ScHSfme_525HRSCAF_35281_whole_genome_shotgun_sequence
0.03

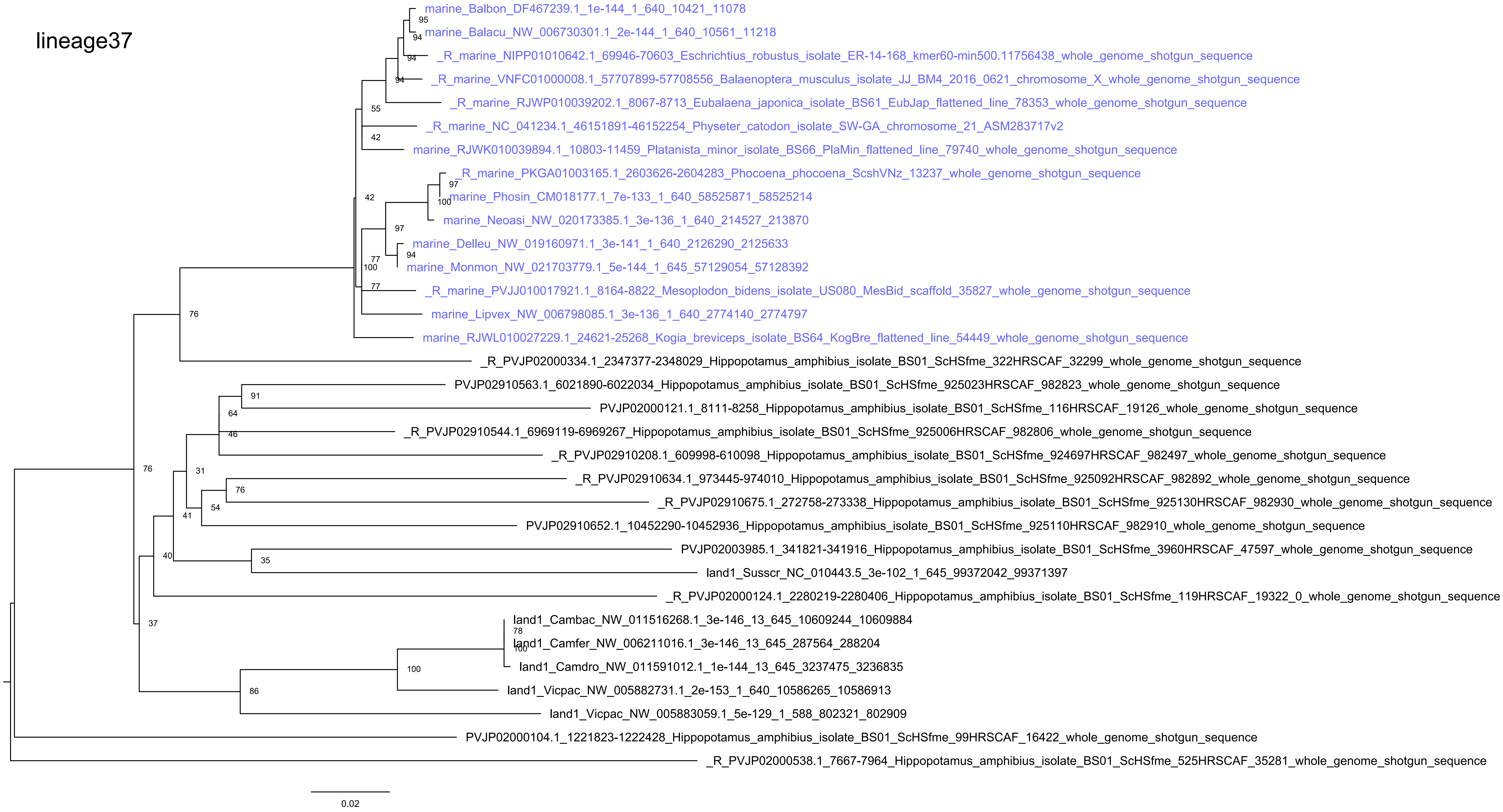
lineage37
marine_Balbon_DF467239.1_1e-144_1_640_10421_11078
marine_Balacu_NW_006730301.1_2e-144_1_640_10561_11218
_R_marine_NIPP01010642.1_69946-70603_Eschrichtius_robustus_isolate_ER-14-168_kmer60-min500.11756438_whole_genome_shotgun_sequence
_R_marine_VNFC01000008.1_57707899-57708556_Balaenoptera_musculus_isolate_JJ_BM4_2016_0621_chromosome_X_whole_genome_shotgun_sequence
_R_marine_RJWP010039202.1_8067-8713_Eubalaena_japonica_isolate_BS61_EubJap_flattened_line_78353_whole_genome_shotgun_sequence
_R_marine_NC_041234.1_46151891-46152254_Physeter_catodon_isolate_SW-GA_chromosome_21_ASM283717v2
marine_RJWK010039894.1_10803-11459_Platanista_minor_isolate_BS66_PlaMin_flattened_line_79740_whole_genome_shotgun_sequence
_R_marine_PKGA01003165.1_2603626-2604283_Phocoena_phocoena_ScshVNz_13237_whole_genome_shotgun_sequence
marine_Phosin_CM018177.1_7e-133_1_640_58525871_58525214
marine_Neoasi_NW_020173385.1_3e-136_1_640_214527_213870
marine_Delleu_NW_019160971.1_3e-141_1_640_2126290_2125633
marine_Monmon_NW_021703779.1_5e-144_1_645_57129054_57128392
_R_marine_PVJJ010017921.1_8164-8822_Mesoplodon_bidens_isolate_US080_MesBid_scaffold_35827_whole_genome_shotgun_sequence
marine_Lipvex_NW_006798085.1_3e-136_1_640_2774140_2774797
marine_RJWL010027229.1_24621-25268_Kogia_breviceps_isolate_BS64_KogBre_flattened_line_54449_whole_genome_shotgun_sequence
_R_PVJP02000334.1_2347377-2348029_Hippopotamus_amphibius_isolate_BS01_ScHSfme_322HRSCAF_32299_whole_genome_shotgun_sequence
PVJP02910563.1_6021890-6022034_Hippopotamus_amphibius_isolate_BS01_ScHSfme_925023HRSCAF_982823_whole_genome_shotgun_sequence
PVJP02000121.1_8111-8258_Hippopotamus_amphibius_isolate_BS01_ScHSfme_116HRSCAF_19126_whole_genome_shotgun_sequence
_R_PVJP02910544.1_6969119-6969267_Hippopotamus_amphibius_isolate_BS01_ScHSfme_925006HRSCAF_982806_whole_genome_shotgun_sequence
_R_PVJP02910208.1_609998-610098_Hippopotamus_amphibius_isolate_BS01_ScHSfme_924697HRSCAF_982497_whole_genome_shotgun_sequence
_R_PVJP02910634.1_973445-974010_Hippopotamus_amphibius_isolate_BS01_ScHSfme_925092HRSCAF_982892_whole_genome_shotgun_sequence
_R_PVJP02910675.1_272758-273338_Hippopotamus_amphibius_isolate_BS01_ScHSfme_925130HRSCAF_982930_whole_genome_shotgun_sequence
PVJP02910652.1_10452290-10452936_Hippopotamus_amphibius_isolate_BS01_ScHSfme_925110HRSCAF_982910_whole_genome_shotgun_sequence
PVJP02003985.1_341821-341916_Hippopotamus_amphibius_isolate_BS01_ScHSfme_3960HRSCAF_47597_whole_genome_shotgun_sequence
land1_Susscr_NC_010443.5_3e-102_1_645_99372042_99371397
_R_PVJP02000124.1_2280219-2280406_Hippopotamus_amphibius_isolate_BS01_ScHSfme_119HRSCAF_19322_0_whole_genome_shotgun_sequence
land1_Cambac_NW_011516268.1_3e-146_13_645_10609244_10609884
land1_Camfer_NW_006211016.1_3e-146_13_645_287564_288204
land1_Camdro_NW_011591012.1_1e-144_13_645_3237475_3236835
land1_Vicpac_NW_005882731.1_2e-153_1_640_10586265_10586913
land1_Vicpac_NW_005883059.1_5e-129_1_588_802321_802909
PVJP02000104.1_1221823-1222428_Hippopotamus_amphibius_isolate_BS01_ScHSfme_99HRSCAF_16422_whole_genome_shotgun_sequence
_R_PVJP02000538.1_7667-7964_Hippopotamus_amphibius_isolate_BS01_ScHSfme_525HRSCAF_35281_whole_genome_shotgun_sequence
0.02

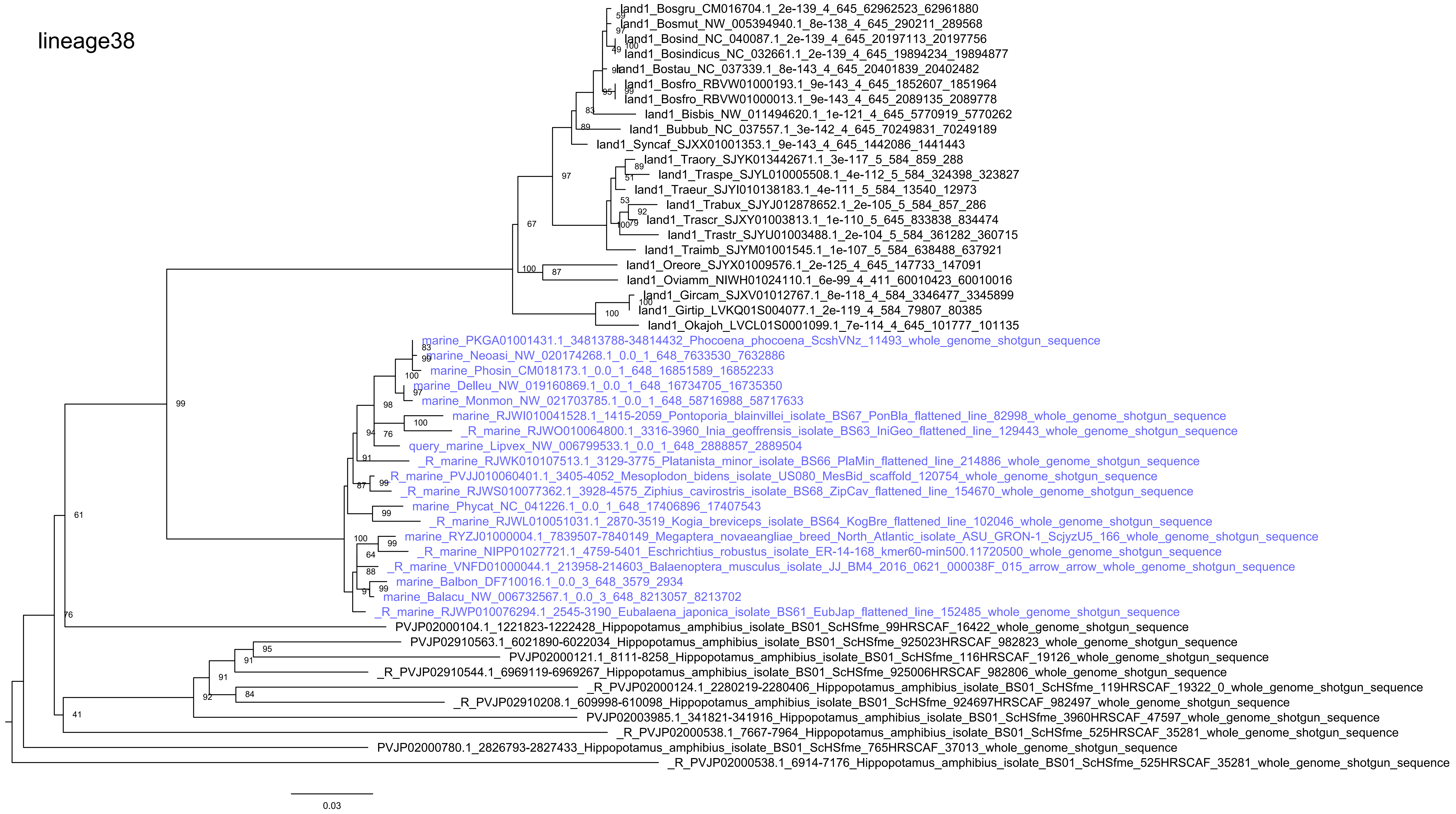
lineage38
land1_Bosgru_CM016704.1_2e-139_4_645_62962523_62961880
land1_Bosmut_NW_005394940.1_8e-138_4_645_290211_289568
land1_Bosind_NC_040087.1_2e-139_4_645_20197113_20197756
land1_Bosindicus_NC_032661.1_2e-139_4_645_19894234_19894877
land1_Bostau_NC_037339.1_8e-143_4_645_20401839_20402482
land1_Bosfro_RBVW01000193.1_9e-143_4_645_1852607_1851964
land1_Bosfro_RBVW01000013.1_9e-143_4_645_2089135_2089778
land1_Bisbis_NW_011494620.1_1e-121_4_645_5770919_5770262
land1_Bubbub_NC_037557.1_3e-142_4_645_70249831_70249189
land1_Syncaf_SJXX01001353.1_9e-143_4_645_1442086_1441443
land1_Traory_SJYK013442671.1_3e-117_5_584_859_288
land1_Traspe_SJYL010005508.1_4e-112_5_584_324398_323827
land1_Traeur_SJYI010138183.1_4e-111_5_584_13540_12973
land1_Trabux_SJYJ012878652.1_2e-105_5_584_857_286
land1_Trascr_SJXY01003813.1_1e-110_5_645_833838_834474
land1_Trastr_SJYU01003488.1_2e-104_5_584_361282_360715
land1_Traimb_SJYM01001545.1_1e-107_5_584_638488_637921
land1_Oreore_SJYX01009576.1_2e-125_4_645_147733_147091
land1_Oviamm_NIWH01024110.1_6e-99_4_411_60010423_60010016
land1_Gircam_SJXV01012767.1_8e-118_4_584_3346477_3345899
land1_Girtip_LVKQ01S004077.1_2e-119_4_584_79807_80385
land1_Okajoh_LVCL01S0001099.1_7e-114_4_645_101777_101135
marine_PKGA01001431.1_34813788-34814432_Phocoena_phocoena_ScshVNz_11493_whole_genome_shotgun_sequence
marine_Neoasi_NW_020174268.1_0.0_1_648_7633530_7632886
marine_Phosin_CM018173.1_0.0_1_648_16851589_16852233
marine_Delleu_NW_019160869.1_0.0_1_648_16734705_16735350
marine_Monmon_NW_021703785.1_0.0_1_648_58716988_58717633
marine_RJWI010041528.1_1415-2059_Pontoporia_blainvillei_isolate_BS67_PonBla_flattened_line_82998_whole_genome_shotgun_sequence
_R_marine_RJWO010064800.1_3316-3960_Inia_geoffrensis_isolate_BS63_IniGeo_flattened_line_129443_whole_genome_shotgun_sequence
query_marine_Lipvex_NW_006799533.1_0.0_1_648_2888857_2889504
_R_marine_RJWK010107513.1_3129-3775_Platanista_minor_isolate_BS66_PlaMin_flattened_line_214886_whole_genome_shotgun_sequence
_R_marine_PVJJ010060401.1_3405-4052_Mesoplodon_bidens_isolate_US080_MesBid_scaffold_120754_whole_genome_shotgun_sequence
_R_marine_RJWS010077362.1_3928-4575_Ziphius_cavirostris_isolate_BS68_ZipCav_flattened_line_154670_whole_genome_shotgun_sequence
marine_Phycat_NC_041226.1_0.0_1_648_17406896_17407543
_R_marine_RJWL010051031.1_2870-3519_Kogia_breviceps_isolate_BS64_KogBre_flattened_line_102046_whole_genome_shotgun_sequence
marine_RYZJ01000004.1_7839507-7840149_Megaptera_novaeangliae_breed_North_Atlantic_isolate_ASU_GRON-1_ScjyzU5_166_whole_genome_shotgun_sequence
_R_marine_NIPP01027721.1_4759-5401_Eschrichtius_robustus_isolate_ER-14-168_kmer60-min500.11720500_whole_genome_shotgun_sequence
_R_marine_VNFD01000044.1_213958-214603_Balaenoptera_musculus_isolate_JJ_BM4_2016_0621_000038F_015_arrow_arrow_whole_genome_shotgun_sequence
marine_Balbon_DF710016.1_0.0_3_648_3579_2934
marine_Balacu_NW_006732567.1_0.0_3_648_8213057_8213702
_R_marine_RJWP010076294.1_2545-3190_Eubalaena_japonica_isolate_BS61_EubJap_flattened_line_152485_whole_genome_shotgun_sequence
PVJP02000104.1_1221823-1222428_Hippopotamus_amphibius_isolate_BS01_ScHSfme_99HRSCAF_16422_whole_genome_shotgun_sequence
PVJP02910563.1_6021890-6022034_Hippopotamus_amphibius_isolate_BS01_ScHSfme_925023HRSCAF_982823_whole_genome_shotgun_sequence
PVJP02000121.1_8111-8258_Hippopotamus_amphibius_isolate_BS01_ScHSfme_116HRSCAF_19126_whole_genome_shotgun_sequence
_R_PVJP02910544.1_6969119-6969267_Hippopotamus_amphibius_isolate_BS01_ScHSfme_925006HRSCAF_982806_whole_genome_shotgun_sequence
_R_PVJP02000124.1_2280219-2280406_Hippopotamus_amphibius_isolate_BS01_ScHSfme_119HRSCAF_19322_0_whole_genome_shotgun_sequence
_R_PVJP02910208.1_609998-610098_Hippopotamus_amphibius_isolate_BS01_ScHSfme_924697HRSCAF_982497_whole_genome_shotgun_sequence
PVJP02003985.1_341821-341916_Hippopotamus_amphibius_isolate_BS01_ScHSfme_3960HRSCAF_47597_whole_genome_shotgun_sequence
_R_PVJP02000538.1_7667-7964_Hippopotamus_amphibius_isolate_BS01_ScHSfme_525HRSCAF_35281_whole_genome_shotgun_sequence
PVJP02000780.1_2826793-2827433_Hippopotamus_amphibius_isolate_BS01_ScHSfme_765HRSCAF_37013_whole_genome_shotgun_sequence
_R_PVJP02000538.1_6914-7176_Hippopotamus_amphibius_isolate_BS01_ScHSfme_525HRSCAF_35281_whole_genome_shotgun_sequence
0.03

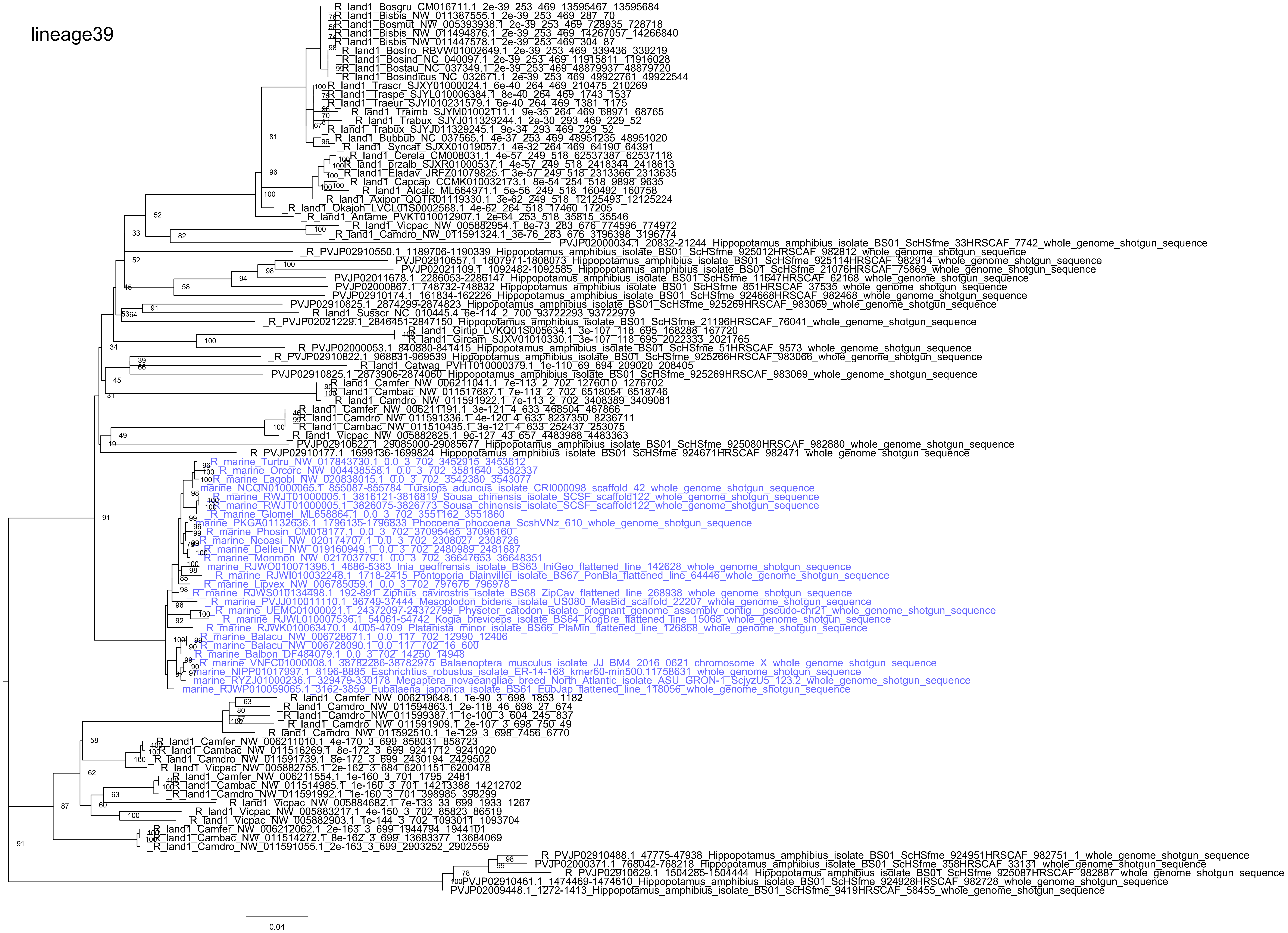
lineage39
_R_land1_Bosgru_CM016711.1_2e-39_253_469_13595467_13595684
_R_land1_Bisbis_NW_011387555.1_2e-39_253_469_287_70
_R_land1_Bosmut_NW_005393938.1_2e-39_253_469_728935_728718
_R_land1_Bisbis_NW_011494876.1_2e-39_253_469_14267057_14266840
_R_land1_Bisbis_NW_011447578.1_2e-39_253_469_304_87
_R_land1_Bosfro_RBVW01002649.1_2e-39_253_469_339436_339219
_R_land1_Bosind_NC_040097.1_2e-39_253_469_11915811_11916028
_R_land1_Bostau_NC_037349.1_2e-39_253_469_48879937_48879720
_R_land1_Bosindicus_NC_032671.1_2e-39_253_469_49922761_49922544
R_land1_Trascr_SJXY01000024.1_6e-40_264_469_210475_210269
_R_land1_Traspe_SJYL010006384.1_8e-40_264_469_1743_1537
_R_land1_Traeur_SJYI010231579.1_6e-40_264_469_1381_1175
_R_land1_Traimb_SJYM01002111.1_9e-35_264_469_68971_68765
_R_land1_Trabux_SJYJ011329244.1_2e-30_293_469_229_52
_R_land1_Trabux_SJYJ011329245.1_9e-34_293_469_229_52
_R_land1_Bubbub_NC_037565.1_4e-37_253_469_48951235_48951020
_R_land1_Syncaf_SJXX01019057.1_4e-32_264_469_64190_64391
_R_land1_Cerela_CM008031.1_4e-57_249_518_62537387_62537118
R_land1_przalb_SJXR01000537.1_4e-57_249_518_2418344_2418613
_R_land1_Eladav_JRFZ01079825.1_3e-57_249_518_2313366_2313635
_R_land1_Capcap_CCMK010032173.1_8e-54_254_518_9898_9635
_R_land1_Alcalc_ML664971.1_5e-56_249_518_160492_160758
R_land1_Axipor_QQTR01119330.1_3e-62_249_518_12125493_12125224
_R_land1_Okajoh_LVCL01S0002568.1_4e-62_264_518_17460_17205
_R_land1_Antame_PVKT010012907.1_2e-64_253_518_35815_35546
_R_land1_Vicpac_NW_005882954.1_8e-73_283_676_774596_774972
_R_land1_Camdro_NW_011591324.1_3e-76_283_676_3196398_3196774
PVJP02000034.1_20832-21244_Hippopotamus_amphibius_isolate_BS01_ScHSfme_33HRSCAF_7742_whole_genome_shotgun_sequence
_R_PVJP02910550.1_1189706-1190339_Hippopotamus_amphibius_isolate_BS01_ScHSfme_925012HRSCAF_982812_whole_genome_shotgun_sequence
PVJP02910657.1_1807971-1808073_Hippopotamus_amphibius_isolate_BS01_ScHSfme_925114HRSCAF_982914_whole_genome_shotgun_sequence
PVJP02021109.1_1092482-1092585_Hippopotamus_amphibius_isolate_BS01_ScHSfme_21076HRSCAF_75869_whole_genome_shotgun_sequence
PVJP02011678.1_2286053-2286147_Hippopotamus_amphibius_isolate_BS01_ScHSfme_11647HRSCAF_62168_whole_genome_shotgun_sequence
PVJP02000867.1_748732-748832_Hippopotamus_amphibius_isolate_BS01_ScHSfme_851HRSCAF_37535_whole_genome_shotgun_sequence
PVJP02910174.1_161834-162226_Hippopotamus_amphibius_isolate_BS01_ScHSfme_924668HRSCAF_982468_whole_genome_shotgun_sequence
PVJP02910825.1_2874299-2874823_Hippopotamus_amphibius_isolate_BS01_ScHSfme_925269HRSCAF_983069_whole_genome_shotgun_sequence
R_land1_Susscr_NC_010445.4_6e-114_2_700_93722293_93722979
_R_PVJP02021229.1_2846451-2847150_Hippopotamus_amphibius_isolate_BS01_ScHSfme_21196HRSCAF_76041_whole_genome_shotgun_sequence
R_land1_Girtip_LVKQ01S005634.1_3e-107_118_695_168288_167720
R_land1_Gircam_SJXV01010330.1_3e-107_118_695_2022333_2021765
R_PVJP02000053.1_840880-841415_Hippopotamus_amphibius_isolate_BS01_ScHSfme_51HRSCAF_9573_whole_genome_shotgun_sequence
_R_PVJP02910822.1_968831-969539_Hippopotamus_amphibius_isolate_BS01_ScHSfme_925266HRSCAF_983066_whole_genome_shotgun_sequence
R_land1_Catwag_PVHT010000379.1_1e-110_69_694_209020_208405
PVJP02910825.1_2873906-2874060_Hippopotamus_amphibius_isolate_BS01_ScHSfme_925269HRSCAF_983069_whole_genome_shotgun_sequence
R_land1_Camfer_NW_006211041.1_7e-113_2_702_1276010_1276702
R_land1_Cambac_NW_011517687.1_7e-113_2_702_6518054_6518746
R_land1_Camdro_NW_011591922.1_7e-113_2_702_3408389_3409081
R_land1_Camfer_NW_006211191.1_3e-121_4_633_468504_467866
R_land1_Camdro_NW_011591336.1_4e-120_4_633_8237350_8236711
R_land1_Cambac_NW_011510435.1_3e-121_4_633_252437_253075
R_land1_Vicpac_NW_005882825.1_9e-127_43_657_4483988_4483363
PVJP02910622.1_29085000-29085677_Hippopotamus_amphibius_isolate_BS01_ScHSfme_925080HRSCAF_982880_whole_genome_shotgun_sequence
_R_PVJP02910177.1_1699136-1699824_Hippopotamus_amphibius_isolate_BS01_ScHSfme_924671HRSCAF_982471_whole_genome_shotgun_sequence
R_marine_Turtru_NW_017843730.1_0.0_3_702_3452915_3453612
R_marine_Orcorc_NW_004438558.1_0.0_3_702_3581640_3582337
R_marine_Lagobl_NW_020838015.1_0.0_3_702_3542380_3543077
marine_NCQN01000065.1_855087-855784_Tursiops_aduncus_isolate_CRI000098_scaffold_42_whole_genome_shotgun_sequence
R_marine_RWJT01000005.1_3816121-3816819_Sousa_chinensis_isolate_SCSF_scaffold122_whole_genome_shotgun_sequence
R_marine_RWJT01000005.1_3826075-3826773_Sousa_chinensis_isolate_SCSF_scaffold122_whole_genome_shotgun_sequence
_R_marine_Glomel_ML658864.1_0.0_3_702_3551162_3551860
marine_PKGA01132636.1_1796135-1796833_Phocoena_phocoena_ScshVNz_610_whole_genome_shotgun_sequence
R_marine_Phosin_CM018177.1_0.0_3_702_37095465_37096160
R_marine_Neoasi_NW_020174707.1_0.0_3_702_2308027_2308726
R_marine_Delleu_NW_019160949.1_0.0_3_702_2480989_2481687
R_marine_Monmon_NW_021703779.1_0.0_3_702_36647653_36648351
marine_RJWO010071396.1_4686-5383_Inia_geoffrensis_isolate_BS63_IniGeo_flattened_line_142628_whole_genome_shotgun_sequence
_R_marine_RJWI010032248.1_1718-2415_Pontoporia_blainvillei_isolate_BS67_PonBla_flattened_line_64446_whole_genome_shotgun_sequence
R_marine_Lipvex_NW_006785059.1_0.0_3_702_797676_796978
_R_marine_RJWS010134498.1_192-891_Ziphius_cavirostris_isolate_BS68_ZipCav_flattened_line_268938_whole_genome_shotgun_sequence
_R_marine_PVJJ010011110.1_36749-37444_Mesoplodon_bidens_isolate_US080_MesBid_scaffold_22207_whole_genome_shotgun_sequence
_R_marine_UEMC01000021.1_24372097-24372799_Physeter_catodon_isolate_pregnant_genome_assembly_contig__pseudo-chr21_whole_genome_shotgun_sequence
_R_marine_RJWL010007536.1_54061-54742_Kogia_breviceps_isolate_BS64_KogBre_flattened_line_15068_whole_genome_shotgun_sequence
_R_marine_RJWK010063470.1_4005-4709_Platanista_minor_isolate_BS66_PlaMin_flattened_line_126868_whole_genome_shotgun_sequence
R_marine_Balacu_NW_006728671.1_0.0_117_702_12990_12406
R_marine_Balacu_NW_006728090.1_0.0_117_702_16_600
R_marine_Balbon_DF484079.1_0.0_3_702_14250_14948
R_marine_VNFC01000008.1_38782286-38782975_Balaenoptera_musculus_isolate_JJ_BM4_2016_0621_chromosome_X_whole_genome_shotgun_sequence
marine_NIPP01017997.1_8196-8885_Eschrichtius_robustus_isolate_ER-14-168_kmer60-min500.11758631_whole_genome_shotgun_sequence
marine_RYZJ01000236.1_329479-330178_Megaptera_novaeangliae_breed_North_Atlantic_isolate_ASU_GRON-1_ScjyzU5_123.2_whole_genome_shotgun_sequence
marine_RJWP010059065.1_3162-3859_Eubalaena_japonica_isolate_BS61_EubJap_flattened_line_118056_whole_genome_shotgun_sequence
R_land1_Camfer_NW_006219648.1_1e-90_3_698_1853_1182
_R_land1_Camdro_NW_011594863.1_2e-118_46_698_27_674
_R_land1_Camdro_NW_011599387.1_1e-100_3_604_245_837
_R_land1_Camdro_NW_011591909.1_2e-107_3_698_750_49
R_land1_Camdro_NW_011592510.1_1e-129_3_698_7456_6770
R_land1_Camfer_NW_006211010.1_4e-170_3_699_858031_858723
R_land1_Cambac_NW_011516269.1_8e-172_3_699_9241712_9241020
_R_land1_Camdro_NW_011591739.1_8e-172_3_699_2430194_2429502
_R_land1_Vicpac_NW_005882755.1_2e-162_3_684_6201151_6200478
_R_land1_Camfer_NW_006211554.1_1e-160_3_701_1795_2481
R_land1_Cambac_NW_011514985.1_1e-160_3_701_14213388_14212702
_R_land1_Camdro_NW_011591992.1_1e-160_3_701_398985_398299
R_land1_Vicpac_NW_005884682.1_7e-133_33_699_1933_1267
R_land1_Vicpac_NW_005883217.1_4e-150_3_702_85823_86519
R_land1_Vicpac_NW_005882903.1_1e-144_3_702_1093011_1093704
R_land1_Camfer_NW_006212062.1_2e-163_3_699_1944794_1944101
R_land1_Cambac_NW_011514272.1_8e-162_3_699_13683377_13684069
_R_land1_Camdro_NW_011591055.1_2e-163_3_699_2903252_2902559
R_PVJP02910488.1_47775-47938_Hippopotamus_amphibius_isolate_BS01_ScHSfme_924951HRSCAF_982751_1_whole_genome_shotgun_sequence
PVJP02000371.1_768042-768218_Hippopotamus_amphibius_isolate_BS01_ScHSfme_358HRSCAF_33131_whole_genome_shotgun_sequence
R_PVJP02910629.1_1504285-1504444_Hippopotamus_amphibius_isolate_BS01_ScHSfme_925087HRSCAF_982887_whole_genome_shotgun_sequence
PVJP02910461.1_1474469-1474610_Hippopotamus_amphibius_isolate_BS01_ScHSfme_924928HRSCAF_982728_whole_genome_shotgun_sequence
PVJP02009448.1_1272-1413_Hippopotamus_amphibius_isolate_BS01_ScHSfme_9419HRSCAF_58455_whole_genome_shotgun_sequence
0.04

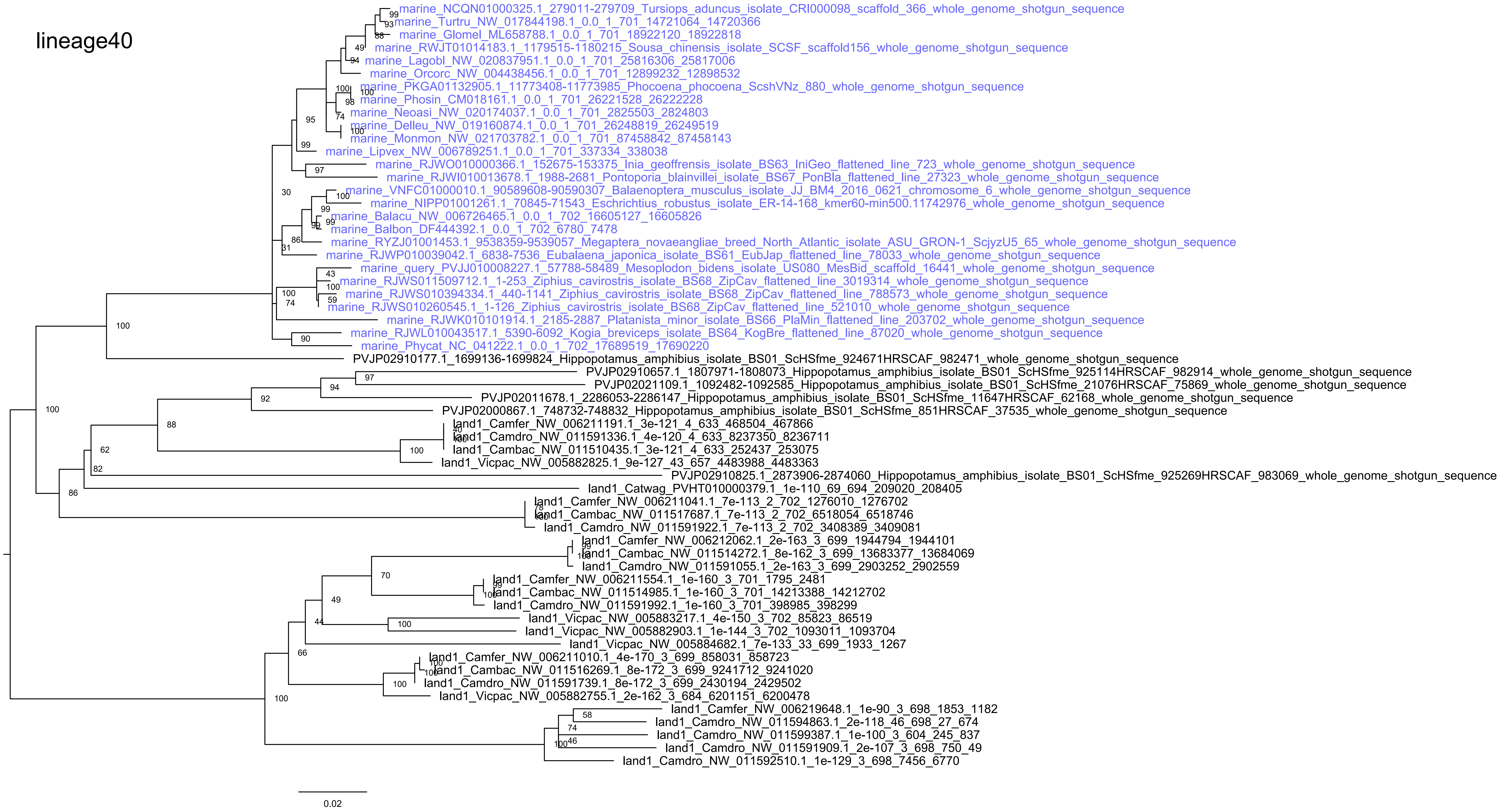

lineage40
marine_NCQN01000325.1_279011-279709_Tursiops_aduncus_isolate_CRI000098_scaffold_366_whole_genome_shotgun_sequence
marine_Turtru_NW_017844198.1_0.0_1_701_14721064_14720366
marine_Glomel_ML658788.1_0.0_1_701_18922120_18922818
marine_RWJT01014183.1_1179515-1180215_Sousa_chinensis_isolate_SCSF_scaffold156_whole_genome_shotgun_sequence
marine_Lagobl_NW_020837951.1_0.0_1_701_25816306_25817006
marine_Orcorc_NW_004438456.1_0.0_1_701_12899232_12898532
marine_PKGA01132905.1_11773408-11773985_Phocoena_phocoena_ScshVNz_880_whole_genome_shotgun_sequence
marine_Phosin_CM018161.1_0.0_1_701_26221528_26222228
marine_Neoasi_NW_020174037.1_0.0_1_701_2825503_2824803
marine_Delleu_NW_019160874.1_0.0_1_701_26248819_26249519
marine_Monmon_NW_021703782.1_0.0_1_701_87458842_87458143
marine_Lipvex_NW_006789251.1_0.0_1_701_337334_338038
marine_RJWO010000366.1_152675-153375_Inia_geoffrensis_isolate_BS63_IniGeo_flattened_line_723_whole_genome_shotgun_sequence
marine_RJWI010013678.1_1988-2681_Pontoporia_blainvillei_isolate_BS67_PonBla_flattened_line_27323_whole_genome_shotgun_sequence
marine_VNFC01000010.1_90589608-90590307_Balaenoptera_musculus_isolate_JJ_BM4_2016_0621_chromosome_6_whole_genome_shotgun_sequence
marine_NIPP01001261.1_70845-71543_Eschrichtius_robustus_isolate_ER-14-168_kmer60-min500.11742976_whole_genome_shotgun_sequence
marine_Balacu_NW_006726465.1_0.0_1_702_16605127_16605826
marine_Balbon_DF444392.1_0.0_1_702_6780_7478
marine_RYZJ01001453.1_9538359-9539057_Megaptera_novaeangliae_breed_North_Atlantic_isolate_ASU_GRON-1_ScjyzU5_65_whole_genome_shotgun_sequence
marine_RJWP010039042.1_6838-7536_Eubalaena_japonica_isolate_BS61_EubJap_flattened_line_78033_whole_genome_shotgun_sequence
marine_query_PVJJ010008227.1_57788-58489_Mesoplodon_bidens_isolate_US080_MesBid_scaffold_16441_whole_genome_shotgun_sequence
marine_RJWS011509712.1_1-253_Ziphius_cavirostris_isolate_BS68_ZipCav_flattened_line_3019314_whole_genome_shotgun_sequence
marine_RJWS010394334.1_440-1141_Ziphius_cavirostris_isolate_BS68_ZipCav_flattened_line_788573_whole_genome_shotgun_sequence
marine_RJWS010260545.1_1-126_Ziphius_cavirostris_isolate_BS68_ZipCav_flattened_line_521010_whole_genome_shotgun_sequence
marine_RJWK010101914.1_2185-2887_Platanista_minor_isolate_BS66_PlaMin_flattened_line_203702_whole_genome_shotgun_sequence
marine_RJWL010043517.1_5390-6092_Kogia_breviceps_isolate_BS64_KogBre_flattened_line_87020_whole_genome_shotgun_sequence
marine_Phycat_NC_041222.1_0.0_1_702_17689519_17690220
PVJP02910177.1_1699136-1699824_Hippopotamus_amphibius_isolate_BS01_ScHSfme_924671HRSCAF_982471_whole_genome_shotgun_sequence
PVJP02910657.1_1807971-1808073_Hippopotamus_amphibius_isolate_BS01_ScHSfme_925114HRSCAF_982914_whole_genome_shotgun_sequence
PVJP02021109.1_1092482-1092585_Hippopotamus_amphibius_isolate_BS01_ScHSfme_21076HRSCAF_75869_whole_genome_shotgun_sequence
PVJP02011678.1_2286053-2286147_Hippopotamus_amphibius_isolate_BS01_ScHSfme_11647HRSCAF_62168_whole_genome_shotgun_sequence
PVJP02000867.1_748732-748832_Hippopotamus_amphibius_isolate_BS01_ScHSfme_851HRSCAF_37535_whole_genome_shotgun_sequence
land1_Camfer_NW_006211191.1_3e-121_4_633_468504_467866
land1_Camdro_NW_011591336.1_4e-120_4_633_8237350_8236711
land1_Cambac_NW_011510435.1_3e-121_4_633_252437_253075
land1_Vicpac_NW_005882825.1_9e-127_43_657_4483988_4483363
PVJP02910825.1_2873906-2874060_Hippopotamus_amphibius_isolate_BS01_ScHSfme_925269HRSCAF_983069_whole_genome_shotgun_sequence
land1_Catwag_PVHT010000379.1_1e-110_69_694_209020_208405
land1_Camfer_NW_006211041.1_7e-113_2_702_1276010_1276702
land1_Cambac_NW_011517687.1_7e-113_2_702_6518054_6518746
land1_Camdro_NW_011591922.1_7e-113_2_702_3408389_3409081
land1_Camfer_NW_006212062.1_2e-163_3_699_1944794_1944101
land1_Cambac_NW_011514272.1_8e-162_3_699_13683377_13684069
land1_Camdro_NW_011591055.1_2e-163_3_699_2903252_2902559
land1_Camfer_NW_006211554.1_1e-160_3_701_1795_2481
land1_Cambac_NW_011514985.1_1e-160_3_701_14213388_14212702
land1_Camdro_NW_011591992.1_1e-160_3_701_398985_398299
land1_Vicpac_NW_005883217.1_4e-150_3_702_85823_86519
land1_Vicpac_NW_005882903.1_1e-144_3_702_1093011_1093704
land1_Vicpac_NW_005884682.1_7e-133_33_699_1933_1267
land1_Camfer_NW_006211010.1_4e-170_3_699_858031_858723
land1_Cambac_NW_011516269.1_8e-172_3_699_9241712_9241020
land1_Camdro_NW_011591739.1_8e-172_3_699_2430194_2429502
land1_Vicpac_NW_005882755.1_2e-162_3_684_6201151_6200478
land1_Camfer_NW_006219648.1_1e-90_3_698_1853_1182
land1_Camdro_NW_011594863.1_2e-118_46_698_27_674
land1_Camdro_NW_011599387.1_1e-100_3_604_245_837
land1_Camdro_NW_011591909.1_2e-107_3_698_750_49
land1_Camdro_NW_011592510.1_1e-129_3_698_7456_6770
0.02

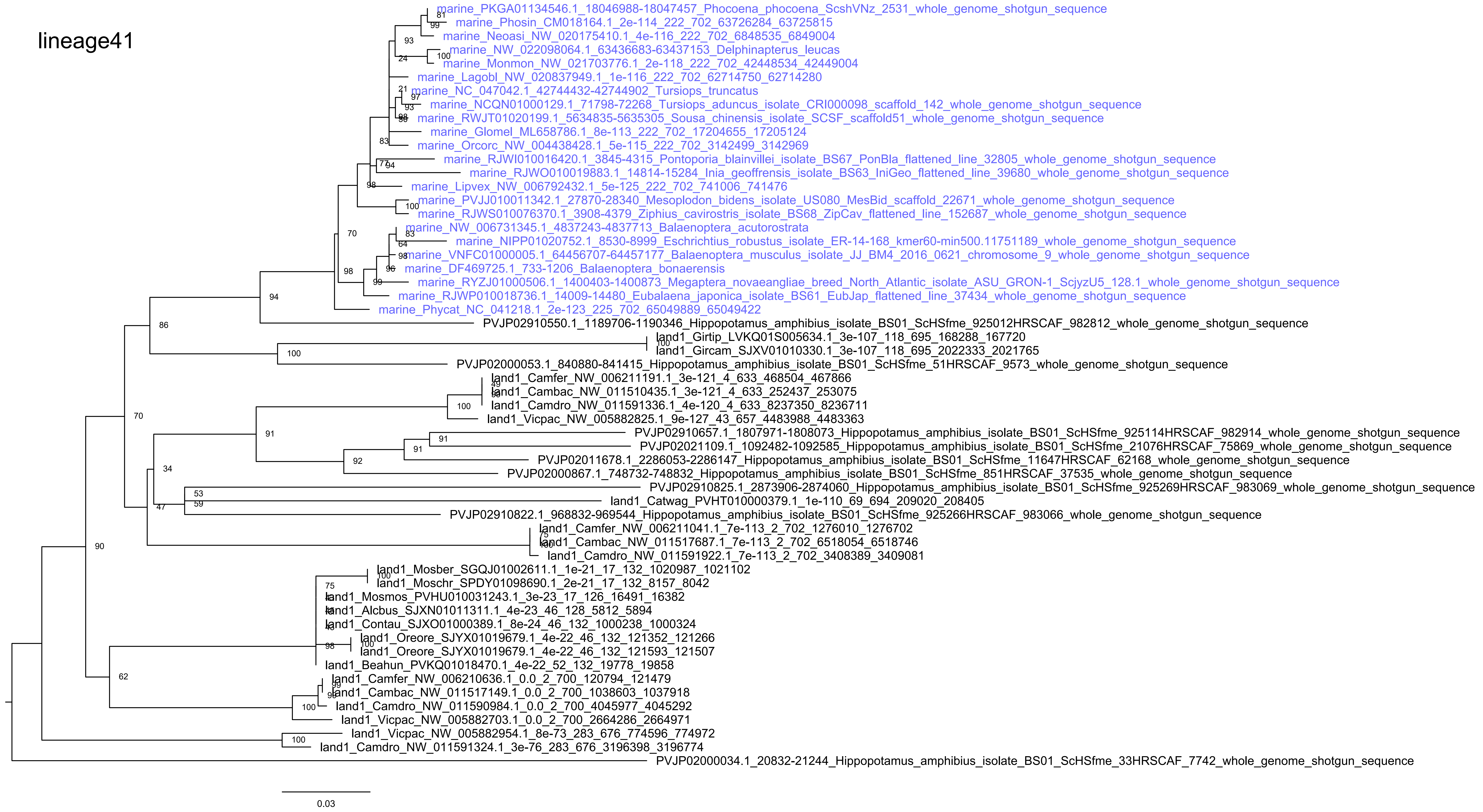
lineage41
marine_PKGA01134546.1_18046988-18047457_Phocoena_phocoena_ScshVNz_2531_whole_genome_shotgun_sequence
marine_Phosin_CM018164.1_2e-114_222_702_63726284_63725815
marine_Neoasi_NW_020175410.1_4e-116_222_702_6848535_6849004
marine_NW_022098064.1_63436683-63437153_Delphinapterus_leucas
marine_Monmon_NW_021703776.1_2e-118_222_702_42448534_42449004
marine_Lagobl_NW_020837949.1_1e-116_222_702_62714750_62714280
marine_NC_047042.1_42744432-42744902_Tursiops_truncatus
marine_NCQN01000129.1_71798-72268_Tursiops_aduncus_isolate_CRI000098_scaffold_142_whole_genome_shotgun_sequence
marine_RWJT01020199.1_5634835-5635305_Sousa_chinensis_isolate_SCSF_scaffold51_whole_genome_shotgun_sequence
marine_Glomel_ML658786.1_8e-113_222_702_17204655_17205124
marine_Orcorc_NW_004438428.1_5e-115_222_702_3142499_3142969
marine_RJWI010016420.1_3845-4315_Pontoporia_blainvillei_isolate_BS67_PonBla_flattened_line_32805_whole_genome_shotgun_sequence
marine_RJWO010019883.1_14814-15284_Inia_geoffrensis_isolate_BS63_IniGeo_flattened_line_39680_whole_genome_shotgun_sequence
marine_Lipvex_NW_006792432.1_5e-125_222_702_741006_741476
marine_PVJJ010011342.1_27870-28340_Mesoplodon_bidens_isolate_US080_MesBid_scaffold_22671_whole_genome_shotgun_sequence
marine_RJWS010076370.1_3908-4379_Ziphius_cavirostris_isolate_BS68_ZipCav_flattened_line_152687_whole_genome_shotgun_sequence
marine_NW_006731345.1_4837243-4837713_Balaenoptera_acutorostrata
marine_NIPP01020752.1_8530-8999_Eschrichtius_robustus_isolate_ER-14-168_kmer60-min500.11751189_whole_genome_shotgun_sequence
marine_VNFC01000005.1_64456707-64457177_Balaenoptera_musculus_isolate_JJ_BM4_2016_0621_chromosome_9_whole_genome_shotgun_sequence
marine_DF469725.1_733-1206_Balaenoptera_bonaerensis
marine_RYZJ01000506.1_1400403-1400873_Megaptera_novaeangliae_breed_North_Atlantic_isolate_ASU_GRON-1_ScjyzU5_128.1_whole_genome_shotgun_sequence
marine_RJWP010018736.1_14009-14480_Eubalaena_japonica_isolate_BS61_EubJap_flattened_line_37434_whole_genome_shotgun_sequence
marine_Phycat_NC_041218.1_2e-123_225_702_65049889_65049422
PVJP02910550.1_1189706-1190346_Hippopotamus_amphibius_isolate_BS01_ScHSfme_925012HRSCAF_982812_whole_genome_shotgun_sequence
land1_Girtip_LVKQ01S005634.1_3e-107_118_695_168288_167720
land1_Gircam_SJXV01010330.1_3e-107_118_695_2022333_2021765
PVJP02000053.1_840880-841415_Hippopotamus_amphibius_isolate_BS01_ScHSfme_51HRSCAF_9573_whole_genome_shotgun_sequence
land1_Camfer_NW_006211191.1_3e-121_4_633_468504_467866
land1_Cambac_NW_011510435.1_3e-121_4_633_252437_253075
land1_Camdro_NW_011591336.1_4e-120_4_633_8237350_8236711
land1_Vicpac_NW_005882825.1_9e-127_43_657_4483988_4483363
PVJP02910657.1_1807971-1808073_Hippopotamus_amphibius_isolate_BS01_ScHSfme_925114HRSCAF_982914_whole_genome_shotgun_sequence
PVJP02021109.1_1092482-1092585_Hippopotamus_amphibius_isolate_BS01_ScHSfme_21076HRSCAF_75869_whole_genome_shotgun_sequence
PVJP02011678.1_2286053-2286147_Hippopotamus_amphibius_isolate_BS01_ScHSfme_11647HRSCAF_62168_whole_genome_shotgun_sequence
PVJP02000867.1_748732-748832_Hippopotamus_amphibius_isolate_BS01_ScHSfme_851HRSCAF_37535_whole_genome_shotgun_sequence
PVJP02910825.1_2873906-2874060_Hippopotamus_amphibius_isolate_BS01_ScHSfme_925269HRSCAF_983069_whole_genome_shotgun_sequence
land1_Catwag_PVHT010000379.1_1e-110_69_694_209020_208405
PVJP02910822.1_968832-969544_Hippopotamus_amphibius_isolate_BS01_ScHSfme_925266HRSCAF_983066_whole_genome_shotgun_sequence
land1_Camfer_NW_006211041.1_7e-113_2_702_1276010_1276702
land1_Cambac_NW_011517687.1_7e-113_2_702_6518054_6518746
land1_Camdro_NW_011591922.1_7e-113_2_702_3408389_3409081
land1_Mosber_SGQJ01002611.1_1e-21_17_132_1020987_1021102
land1_Moschr_SPDY01098690.1_2e-21_17_132_8157_8042
land1_Mosmos_PVHU010031243.1_3e-23_17_126_16491_16382
land1_Alcbus_SJXN01011311.1_4e-23_46_128_5812_5894
land1_Contau_SJXO01000389.1_8e-24_46_132_1000238_1000324
land1_Oreore_SJYX01019679.1_4e-22_46_132_121352_121266
land1_Oreore_SJYX01019679.1_4e-22_46_132_121593_121507
land1_Beahun_PVKQ01018470.1_4e-22_52_132_19778_19858
land1_Camfer_NW_006210636.1_0.0_2_700_120794_121479
land1_Cambac_NW_011517149.1_0.0_2_700_1038603_1037918
land1_Camdro_NW_011590984.1_0.0_2_700_4045977_4045292
land1_Vicpac_NW_005882703.1_0.0_2_700_2664286_2664971
land1_Vicpac_NW_005882954.1_8e-73_283_676_774596_774972
land1_Camdro_NW_011591324.1_3e-76_283_676_3196398_3196774
PVJP02000034.1_20832-21244_Hippopotamus_amphibius_isolate_BS01_ScHSfme_33HRSCAF_7742_whole_genome_shotgun_sequence
0.03

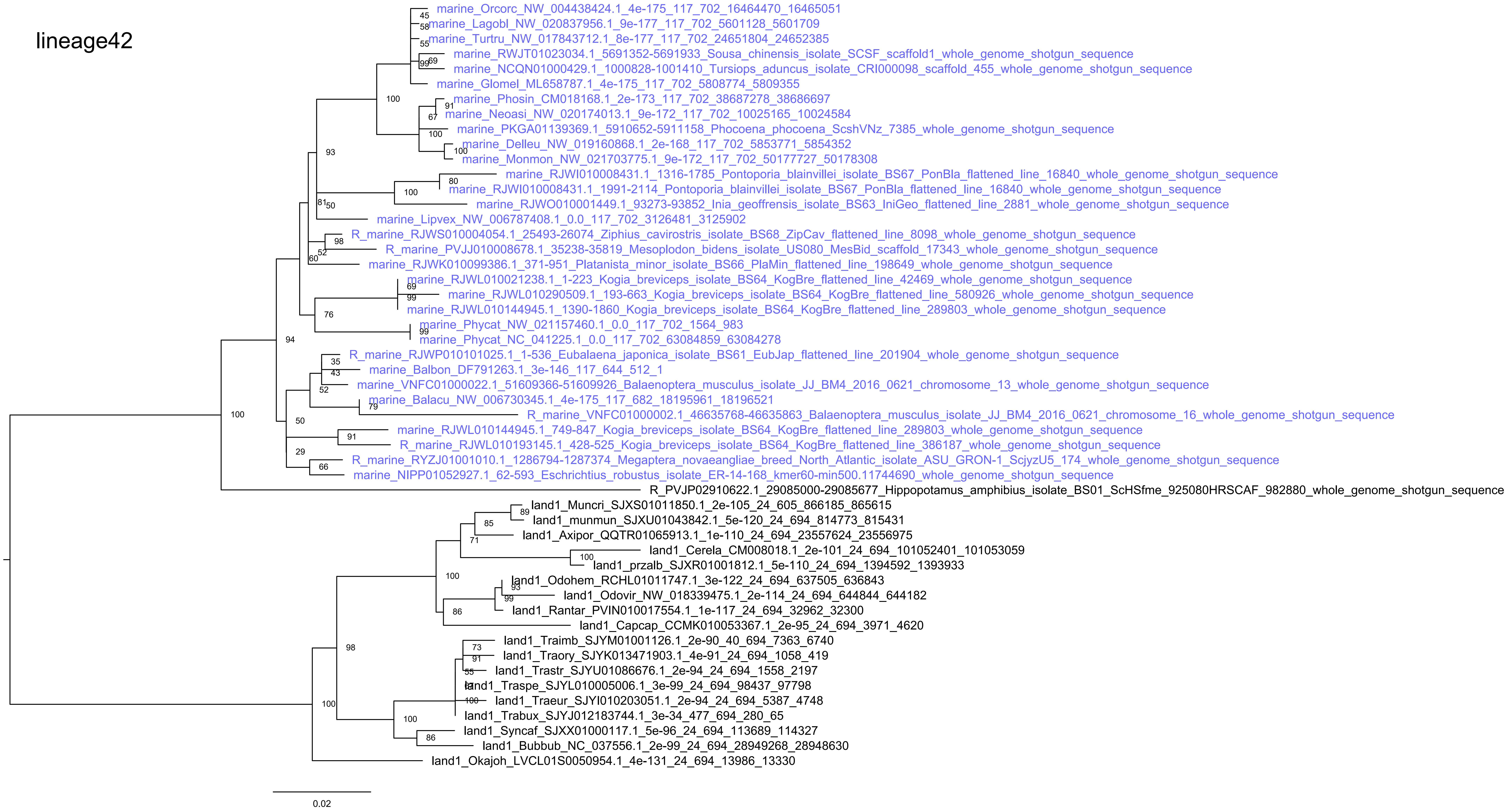
lineage42
marine_Orcorc_NW_004438424.1_4e-175_117_702_16464470_16465051
marine_Lagobl_NW_020837956.1_9e-177_117_702_5601128_5601709
marine_Turtru_NW_017843712.1_8e-177_117_702_24651804_24652385
marine_RWJT01023034.1_5691352-5691933_Sousa_chinensis_isolate_SCSF_scaffold1_whole_genome_shotgun_sequence
marine_NCQN01000429.1_1000828-1001410_Tursiops_aduncus_isolate_CRI000098_scaffold_455_whole_genome_shotgun_sequence
marine_Glomel_ML658787.1_4e-175_117_702_5808774_5809355
marine_Phosin_CM018168.1_2e-173_117_702_38687278_38686697
marine_Neoasi_NW_020174013.1_9e-172_117_702_10025165_10024584
marine_PKGA01139369.1_5910652-5911158_Phocoena_phocoena_ScshVNz_7385_whole_genome_shotgun_sequence
marine_Delleu_NW_019160868.1_2e-168_117_702_5853771_5854352
marine_Monmon_NW_021703775.1_9e-172_117_702_50177727_50178308
marine_RJWI010008431.1_1316-1785_Pontoporia_blainvillei_isolate_BS67_PonBla_flattened_line_16840_whole_genome_shotgun_sequence
marine_RJWI010008431.1_1991-2114_Pontoporia_blainvillei_isolate_BS67_PonBla_flattened_line_16840_whole_genome_shotgun_sequence
marine_RJWO010001449.1_93273-93852_Inia_geoffrensis_isolate_BS63_IniGeo_flattened_line_2881_whole_genome_shotgun_sequence
marine_Lipvex_NW_006787408.1_0.0_117_702_3126481_3125902
R_marine_RJWS010004054.1_25493-26074_Ziphius_cavirostris_isolate_BS68_ZipCav_flattened_line_8098_whole_genome_shotgun_sequence
R_marine_PVJJ010008678.1_35238-35819_Mesoplodon_bidens_isolate_US080_MesBid_scaffold_17343_whole_genome_shotgun_sequence
marine_RJWK010099386.1_371-951_Platanista_minor_isolate_BS66_PlaMin_flattened_line_198649_whole_genome_shotgun_sequence
marine_RJWL010021238.1_1-223_Kogia_breviceps_isolate_BS64_KogBre_flattened_line_42469_whole_genome_shotgun_sequence
marine_RJWL010290509.1_193-663_Kogia_breviceps_isolate_BS64_KogBre_flattened_line_580926_whole_genome_shotgun_sequence
marine_RJWL010144945.1_1390-1860_Kogia_breviceps_isolate_BS64_KogBre_flattened_line_289803_whole_genome_shotgun_sequence
marine_Phycat_NW_021157460.1_0.0_117_702_1564_983
marine_Phycat_NC_041225.1_0.0_117_702_63084859_63084278
R_marine_RJWP010101025.1_1-536_Eubalaena_japonica_isolate_BS61_EubJap_flattened_line_201904_whole_genome_shotgun_sequence
marine_Balbon_DF791263.1_3e-146_117_644_512_1
marine_VNFC01000022.1_51609366-51609926_Balaenoptera_musculus_isolate_JJ_BM4_2016_0621_chromosome_13_whole_genome_shotgun_sequence
marine_Balacu_NW_006730345.1_4e-175_117_682_18195961_18196521
R_marine_VNFC01000002.1_46635768-46635863_Balaenoptera_musculus_isolate_JJ_BM4_2016_0621_chromosome_16_whole_genome_shotgun_sequence
marine_RJWL010144945.1_749-847_Kogia_breviceps_isolate_BS64_KogBre_flattened_line_289803_whole_genome_shotgun_sequence
R_marine_RJWL010193145.1_428-525_Kogia_breviceps_isolate_BS64_KogBre_flattened_line_386187_whole_genome_shotgun_sequence
R_marine_RYZJ01001010.1_1286794-1287374_Megaptera_novaeangliae_breed_North_Atlantic_isolate_ASU_GRON-1_ScjyzU5_174_whole_genome_shotgun_sequence
marine_NIPP01052927.1_62-593_Eschrichtius_robustus_isolate_ER-14-168_kmer60-min500.11744690_whole_genome_shotgun_sequence
R_PVJP02910622.1_29085000-29085677_Hippopotamus_amphibius_isolate_BS01_ScHSfme_925080HRSCAF_982880_whole_genome_shotgun_sequence
land1_Muncri_SJXS01011850.1_2e-105_24_605_866185_865615
land1_munmun_SJXU01043842.1_5e-120_24_694_814773_815431
land1_Axipor_QQTR01065913.1_1e-110_24_694_23557624_23556975
land1_Cerela_CM008018.1_2e-101_24_694_101052401_101053059
land1_przalb_SJXR01001812.1_5e-110_24_694_1394592_1393933
land1_Odohem_RCHL01011747.1_3e-122_24_694_637505_636843
land1_Odovir_NW_018339475.1_2e-114_24_694_644844_644182
land1_Rantar_PVIN010017554.1_1e-117_24_694_32962_32300
land1_Capcap_CCMK010053367.1_2e-95_24_694_3971_4620
land1_Traimb_SJYM01001126.1_2e-90_40_694_7363_6740
land1_Traory_SJYK013471903.1_4e-91_24_694_1058_419
land1_Trastr_SJYU01086676.1_2e-94_24_694_1558_2197
land1_Traspe_SJYL010005006.1_3e-99_24_694_98437_97798
land1_Traeur_SJYI010203051.1_2e-94_24_694_5387_4748
land1_Trabux_SJYJ012183744.1_3e-34_477_694_280_65
land1_Syncaf_SJXX01000117.1_5e-96_24_694_113689_114327
land1_Bubbub_NC_037556.1_2e-99_24_694_28948268_28948630
land1_Okajoh_LVCL01S0050954.1_4e-131_24_694_13986_13330
0.02

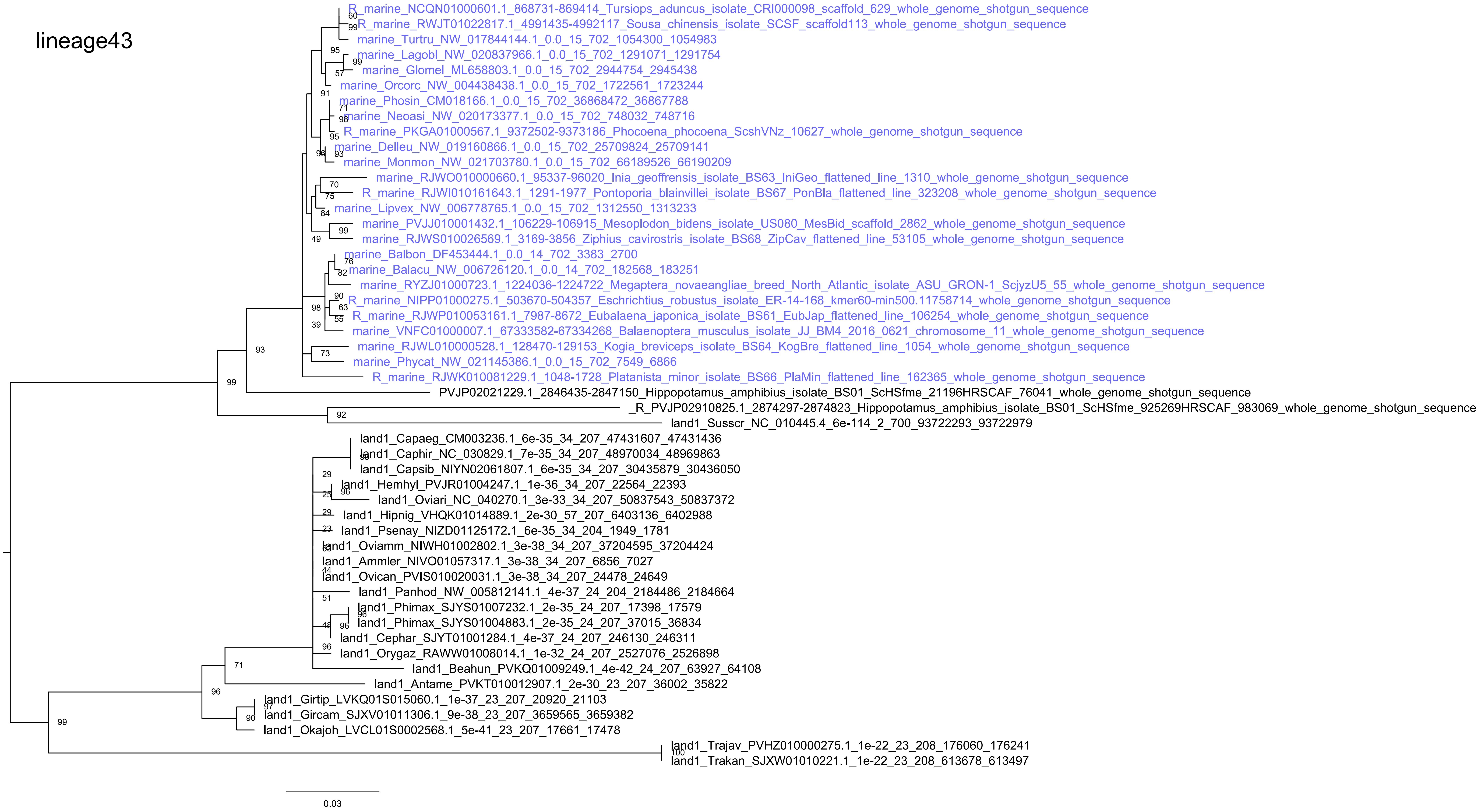
lineage43
R_marine_NCQN01000601.1_868731-869414_Tursiops_aduncus_isolate_CRI000098_scaffold_629_whole_genome_shotgun_sequence
R_marine_RWJT01022817.1_4991435-4992117_Sousa_chinensis_isolate_SCSF_scaffold113_whole_genome_shotgun_sequence
marine_Turtru_NW_017844144.1_0.0_15_702_1054300_1054983
marine_Lagobl_NW_020837966.1_0.0_15_702_1291071_1291754
marine_Glomel_ML658803.1_0.0_15_702_2944754_2945438
marine_Orcorc_NW_004438438.1_0.0_15_702_1722561_1723244
marine_Phosin_CM018166.1_0.0_15_702_36868472_36867788
marine_Neoasi_NW_020173377.1_0.0_15_702_748032_748716
R_marine_PKGA01000567.1_9372502-9373186_Phocoena_phocoena_ScshVNz_10627_whole_genome_shotgun_sequence
marine_Delleu_NW_019160866.1_0.0_15_702_25709824_25709141
marine_Monmon_NW_021703780.1_0.0_15_702_66189526_66190209
marine_RJWO010000660.1_95337-96020_Inia_geoffrensis_isolate_BS63_IniGeo_flattened_line_1310_whole_genome_shotgun_sequence
R_marine_RJWI010161643.1_1291-1977_Pontoporia_blainvillei_isolate_BS67_PonBla_flattened_line_323208_whole_genome_shotgun_sequence
marine_Lipvex_NW_006778765.1_0.0_15_702_1312550_1313233
marine_PVJJ010001432.1_106229-106915_Mesoplodon_bidens_isolate_US080_MesBid_scaffold_2862_whole_genome_shotgun_sequence
marine_RJWS010026569.1_3169-3856_Ziphius_cavirostris_isolate_BS68_ZipCav_flattened_line_53105_whole_genome_shotgun_sequence
marine_Balbon_DF453444.1_0.0_14_702_3383_2700
marine_Balacu_NW_006726120.1_0.0_14_702_182568_183251
marine_RYZJ01000723.1_1224036-1224722_Megaptera_novaeangliae_breed_North_Atlantic_isolate_ASU_GRON-1_ScjyzU5_55_whole_genome_shotgun_sequence
R_marine_NIPP01000275.1_503670-504357_Eschrichtius_robustus_isolate_ER-14-168_kmer60-min500.11758714_whole_genome_shotgun_sequence
R_marine_RJWP010053161.1_7987-8672_Eubalaena_japonica_isolate_BS61_EubJap_flattened_line_106254_whole_genome_shotgun_sequence
marine_VNFC01000007.1_67333582-67334268_Balaenoptera_musculus_isolate_JJ_BM4_2016_0621_chromosome_11_whole_genome_shotgun_sequence
marine_RJWL010000528.1_128470-129153_Kogia_breviceps_isolate_BS64_KogBre_flattened_line_1054_whole_genome_shotgun_sequence
marine_Phycat_NW_021145386.1_0.0_15_702_7549_6866
R_marine_RJWK010081229.1_1048-1728_Platanista_minor_isolate_BS66_PlaMin_flattened_line_162365_whole_genome_shotgun_sequence
PVJP02021229.1_2846435-2847150_Hippopotamus_amphibius_isolate_BS01_ScHSfme_21196HRSCAF_76041_whole_genome_shotgun_sequence
_R_PVJP02910825.1_2874297-2874823_Hippopotamus_amphibius_isolate_BS01_ScHSfme_925269HRSCAF_983069_whole_genome_shotgun_sequence
land1_Susscr_NC_010445.4_6e-114_2_700_93722293_93722979
land1_Capaeg_CM003236.1_6e-35_34_207_47431607_47431436
land1_Caphir_NC_030829.1_7e-35_34_207_48970034_48969863
land1_Capsib_NIYN02061807.1_6e-35_34_207_30435879_30436050
land1_Hemhyl_PVJR01004247.1_1e-36_34_207_22564_22393
land1_Oviari_NC_040270.1_3e-33_34_207_50837543_50837372
land1_Hipnig_VHQK01014889.1_2e-30_57_207_6403136_6402988
land1_Psenay_NIZD01125172.1_6e-35_34_204_1949_1781
land1_Oviamm_NIWH01002802.1_3e-38_34_207_37204595_37204424
land1_Ammler_NIVO01057317.1_3e-38_34_207_6856_7027
land1_Ovican_PVIS010020031.1_3e-38_34_207_24478_24649
land1_Panhod_NW_005812141.1_4e-37_24_204_2184486_2184664
land1_Phimax_SJYS01007232.1_2e-35_24_207_17398_17579
land1_Phimax_SJYS01004883.1_2e-35_24_207_37015_36834
land1_Cephar_SJYT01001284.1_4e-37_24_207_246130_246311
land1_Orygaz_RAWW01008014.1_1e-32_24_207_2527076_2526898
land1_Beahun_PVKQ01009249.1_4e-42_24_207_63927_64108
land1_Antame_PVKT010012907.1_2e-30_23_207_36002_35822
land1_Girtip_LVKQ01S015060.1_1e-37_23_207_20920_21103
land1_Gircam_SJXV01011306.1_9e-38_23_207_3659565_3659382
land1_Okajoh_LVCL01S0002568.1_5e-41_23_207_17661_17478
land1_Trajav_PVHZ010000275.1_1e-22_23_208_176060_176241
land1_Trakan_SJXW01010221.1_1e-22_23_208_613678_613497
0.03

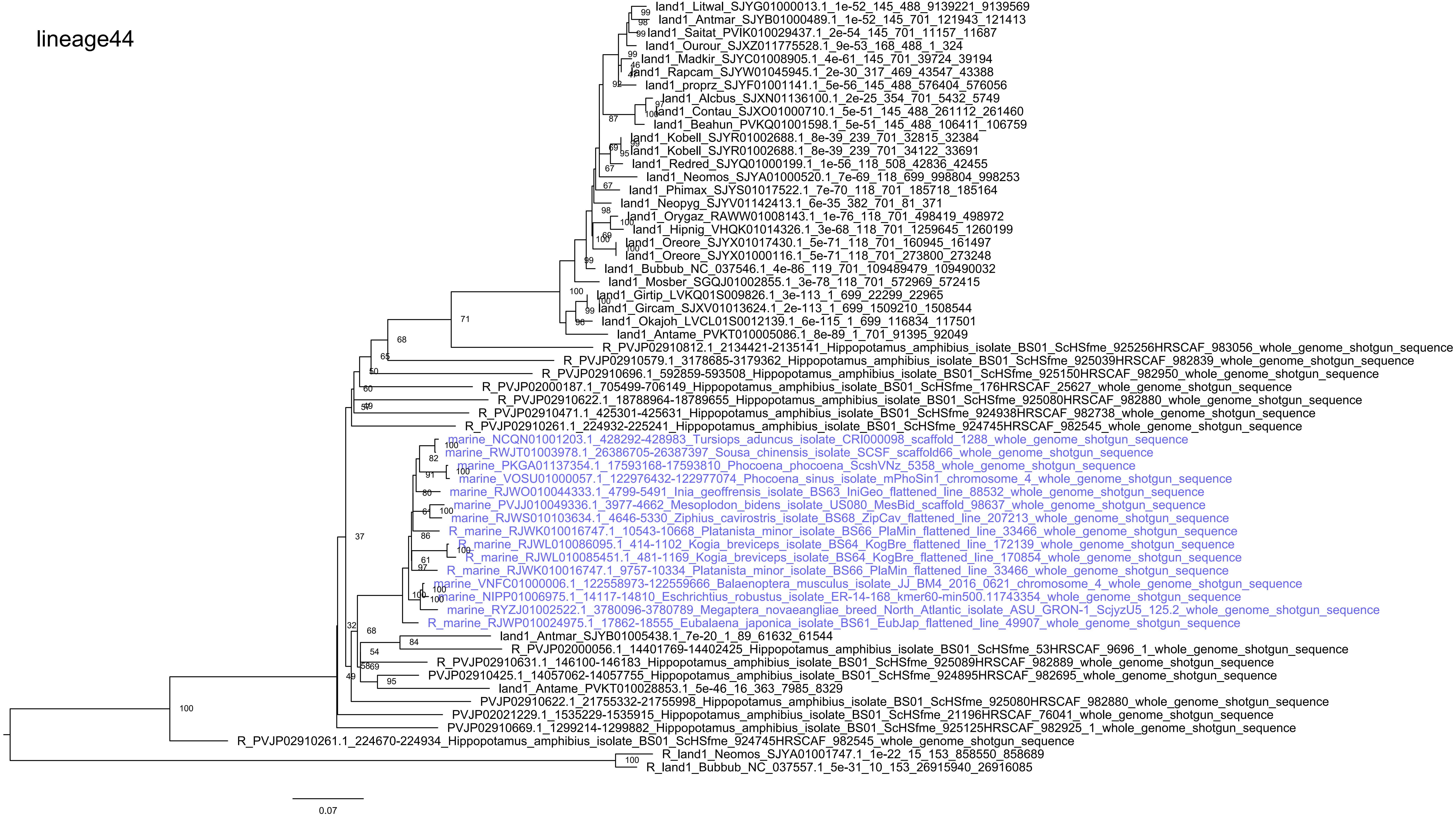
lineage44
land1_Litwal_SJYG01000013.1_1e-52_145_488_9139221_9139569
land1_Antmar_SJYB01000489.1_1e-52_145_701_121943_121413
land1_Saitat_PVIK010029437.1_2e-54_145_701_11157_11687
land1_Ourour_SJXZ011775528.1_9e-53_168_488_1_324
land1_Madkir_SJYC01008905.1_4e-61_145_701_39724_39194
land1_Rapcam_SJYW01045945.1_2e-30_317_469_43547_43388
land1_proprz_SJYF01001141.1_5e-56_145_488_576404_576056
land1_Alcbus_SJXN01136100.1_2e-25_354_701_5432_5749
land1_Contau_SJXO01000710.1_5e-51_145_488_261112_261460
land1_Beahun_PVKQ01001598.1_5e-51_145_488_106411_106759
land1_Kobell_SJYR01002688.1_8e-39_239_701_32815_32384
land1_Kobell_SJYR01002688.1_8e-39_239_701_34122_33691
land1_Redred_SJYQ01000199.1_1e-56_118_508_42836_42455
land1_Neomos_SJYA01000520.1_7e-69_118_699_998804_998253
land1_Phimax_SJYS01017522.1_7e-70_118_701_185718_185164
land1_Neopyg_SJYV01142413.1_6e-35_382_701_81_371
land1_Orygaz_RAWW01008143.1_1e-76_118_701_498419_498972
land1_Hipnig_VHQK01014326.1_3e-68_118_701_1259645_1260199
land1_Oreore_SJYX01017430.1_5e-71_118_701_160945_161497
land1_Oreore_SJYX01000116.1_5e-71_118_701_273800_273248
land1_Bubbub_NC_037546.1_4e-86_119_701_109489479_109490032
land1_Mosber_SGQJ01002855.1_3e-78_118_701_572969_572415
land1_Girtip_LVKQ01S009826.1_3e-113_1_699_22299_22965
land1_Gircam_SJXV01013624.1_2e-113_1_699_1509210_1508544
land1_Okajoh_LVCL01S0012139.1_6e-115_1_699_116834_117501
land1_Antame_PVKT010005086.1_8e-89_1_701_91395_92049
R_PVJP02910812.1_2134421-2135141_Hippopotamus_amphibius_isolate_BS01_ScHSfme_925256HRSCAF_983056_whole_genome_shotgun_sequence
R_PVJP02910579.1_3178685-3179362_Hippopotamus_amphibius_isolate_BS01_ScHSfme_925039HRSCAF_982839_whole_genome_shotgun_sequence
R_PVJP02910696.1_592859-593508_Hippopotamus_amphibius_isolate_BS01_ScHSfme_925150HRSCAF_982950_whole_genome_shotgun_sequence
R_PVJP02000187.1_705499-706149_Hippopotamus_amphibius_isolate_BS01_ScHSfme_176HRSCAF_25627_whole_genome_shotgun_sequence
R_PVJP02910622.1_18788964-18789655_Hippopotamus_amphibius_isolate_BS01_ScHSfme_925080HRSCAF_982880_whole_genome_shotgun_sequence
R_PVJP02910471.1_425301-425631_Hippopotamus_amphibius_isolate_BS01_ScHSfme_924938HRSCAF_982738_whole_genome_shotgun_sequence
R_PVJP02910261.1_224932-225241_Hippopotamus_amphibius_isolate_BS01_ScHSfme_924745HRSCAF_982545_whole_genome_shotgun_sequence
marine_NCQN01001203.1_428292-428983_Tursiops_aduncus_isolate_CRI000098_scaffold_1288_whole_genome_shotgun_sequence
marine_RWJT01003978.1_26386705-26387397_Sousa_chinensis_isolate_SCSF_scaffold66_whole_genome_shotgun_sequence
marine_PKGA01137354.1_17593168-17593810_Phocoena_phocoena_ScshVNz_5358_whole_genome_shotgun_sequence
marine_VOSU01000057.1_122976432-122977074_Phocoena_sinus_isolate_mPhoSin1_chromosome_4_whole_genome_shotgun_sequence
marine_RJWO010044333.1_4799-5491_Inia_geoffrensis_isolate_BS63_IniGeo_flattened_line_88532_whole_genome_shotgun_sequence
marine_PVJJ010049336.1_3977-4662_Mesoplodon_bidens_isolate_US080_MesBid_scaffold_98637_whole_genome_shotgun_sequence
marine_RJWS010103634.1_4646-5330_Ziphius_cavirostris_isolate_BS68_ZipCav_flattened_line_207213_whole_genome_shotgun_sequence
R_marine_RJWK010016747.1_10543-10668_Platanista_minor_isolate_BS66_PlaMin_flattened_line_33466_whole_genome_shotgun_sequence
R_marine_RJWL010086095.1_414-1102_Kogia_breviceps_isolate_BS64_KogBre_flattened_line_172139_whole_genome_shotgun_sequence
R_marine_RJWL010085451.1_481-1169_Kogia_breviceps_isolate_BS64_KogBre_flattened_line_170854_whole_genome_shotgun_sequence
R_marine_RJWK010016747.1_9757-10334_Platanista_minor_isolate_BS66_PlaMin_flattened_line_33466_whole_genome_shotgun_sequence
marine_VNFC01000006.1_122558973-122559666_Balaenoptera_musculus_isolate_JJ_BM4_2016_0621_chromosome_4_whole_genome_shotgun_sequence
marine_NIPP01006975.1_14117-14810_Eschrichtius_robustus_isolate_ER-14-168_kmer60-min500.11743354_whole_genome_shotgun_sequence
marine_RYZJ01002522.1_3780096-3780789_Megaptera_novaeangliae_breed_North_Atlantic_isolate_ASU_GRON-1_ScjyzU5_125.2_whole_genome_shotgun_sequence
R_marine_RJWP010024975.1_17862-18555_Eubalaena_japonica_isolate_BS61_EubJap_flattened_line_49907_whole_genome_shotgun_sequence
land1_Antmar_SJYB01005438.1_7e-20_1_89_61632_61544
R_PVJP02000056.1_14401769-14402425_Hippopotamus_amphibius_isolate_BS01_ScHSfme_53HRSCAF_9696_1_whole_genome_shotgun_sequence
R_PVJP02910631.1_146100-146183_Hippopotamus_amphibius_isolate_BS01_ScHSfme_925089HRSCAF_982889_whole_genome_shotgun_sequence
PVJP02910425.1_14057062-14057755_Hippopotamus_amphibius_isolate_BS01_ScHSfme_924895HRSCAF_982695_whole_genome_shotgun_sequence
land1_Antame_PVKT010028853.1_5e-46_16_363_7985_8329
PVJP02910622.1_21755332-21755998_Hippopotamus_amphibius_isolate_BS01_ScHSfme_925080HRSCAF_982880_whole_genome_shotgun_sequence
PVJP02021229.1_1535229-1535915_Hippopotamus_amphibius_isolate_BS01_ScHSfme_21196HRSCAF_76041_whole_genome_shotgun_sequence
PVJP02910669.1_1299214-1299882_Hippopotamus_amphibius_isolate_BS01_ScHSfme_925125HRSCAF_982925_1_whole_genome_shotgun_sequence
R_PVJP02910261.1_224670-224934_Hippopotamus_amphibius_isolate_BS01_ScHSfme_924745HRSCAF_982545_whole_genome_shotgun_sequence
R_land1_Neomos_SJYA01001747.1_1e-22_15_153_858550_858689
R_land1_Bubbub_NC_037557.1_5e-31_10_153_26915940_26916085
0.07

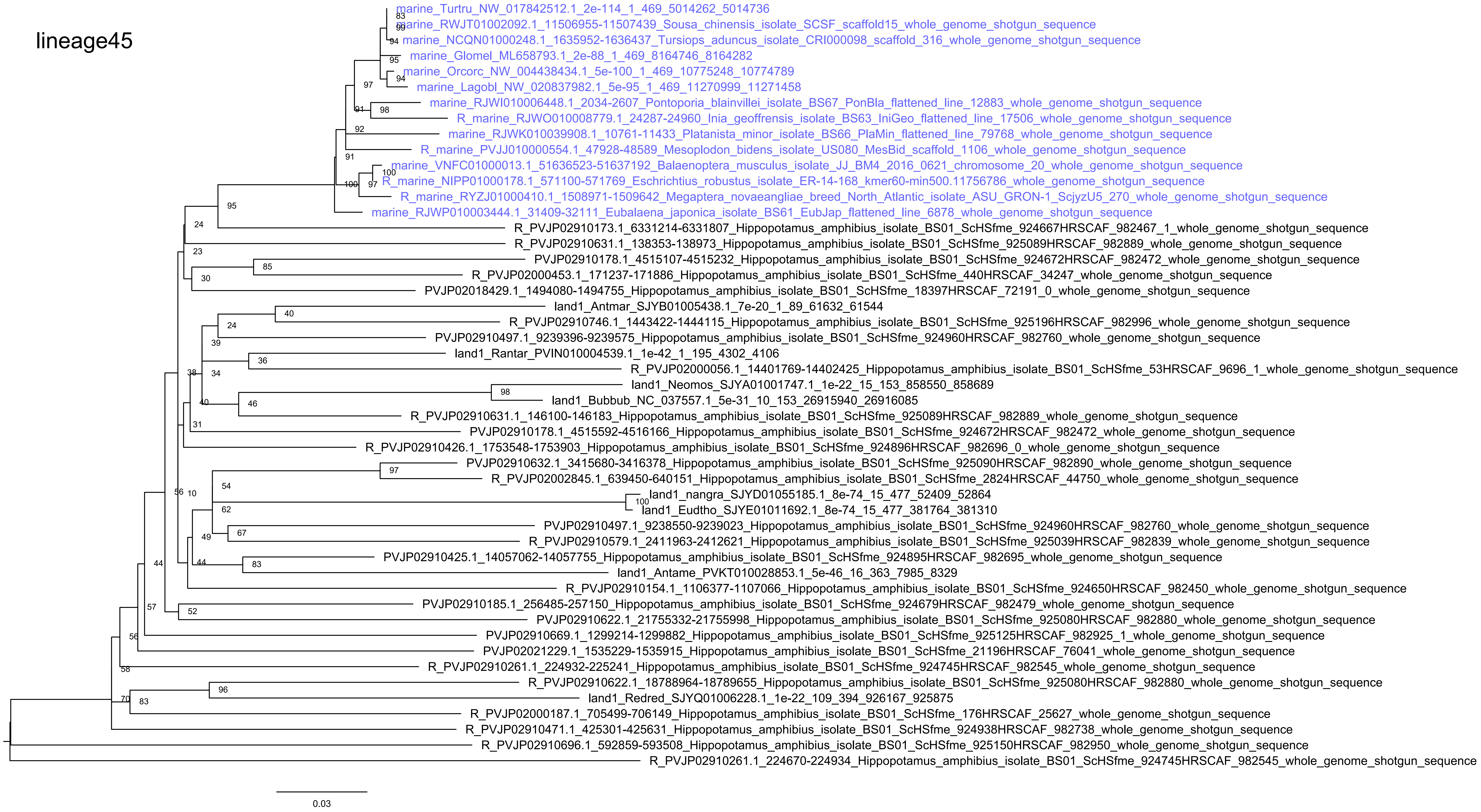
lineage45
marine_Turtru_NW_017842512.1_2e-114_1_469_5014262_5014736
marine_RWJT01002092.1_11506955-11507439_Sousa_chinensis_isolate_SCSF_scaffold15_whole_genome_shotgun_sequence
marine_NCQN01000248.1_1635952-1636437_Tursiops_aduncus_isolate_CRI000098_scaffold_316_whole_genome_shotgun_sequence
marine_Glomel_ML658793.1_2e-88_1_469_8164746_8164282
marine_Orcorc_NW_004438434.1_5e-100_1_469_10775248_10774789
marine_Lagobl_NW_020837982.1_5e-95_1_469_11270999_11271458
marine_RJWI010006448.1_2034-2607_Pontoporia_blainvillei_isolate_BS67_PonBla_flattened_line_12883_whole_genome_shotgun_sequence
R_marine_RJWO010008779.1_24287-24960_Inia_geoffrensis_isolate_BS63_IniGeo_flattened_line_17506_whole_genome_shotgun_sequence
marine_RJWK010039908.1_10761-11433_Platanista_minor_isolate_BS66_PlaMin_flattened_line_79768_whole_genome_shotgun_sequence
R_marine_PVJJ010000554.1_47928-48589_Mesoplodon_bidens_isolate_US080_MesBid_scaffold_1106_whole_genome_shotgun_sequence
marine_VNFC01000013.1_51636523-51637192_Balaenoptera_musculus_isolate_JJ_BM4_2016_0621_chromosome_20_whole_genome_shotgun_sequence
R_marine_NIPP01000178.1_571100-571769_Eschrichtius_robustus_isolate_ER-14-168_kmer60-min500.11756786_whole_genome_shotgun_sequence
R_marine_RYZJ01000410.1_1508971-1509642_Megaptera_novaeangliae_breed_North_Atlantic_isolate_ASU_GRON-1_ScjyzU5_270_whole_genome_shotgun_sequence
marine_RJWP010003444.1_31409-32111_Eubalaena_japonica_isolate_BS61_EubJap_flattened_line_6878_whole_genome_shotgun_sequence
R_PVJP02910173.1_6331214-6331807_Hippopotamus_amphibius_isolate_BS01_ScHSfme_924667HRSCAF_982467_1_whole_genome_shotgun_sequence
R_PVJP02910631.1_138353-138973_Hippopotamus_amphibius_isolate_BS01_ScHSfme_925089HRSCAF_982889_whole_genome_shotgun_sequence
PVJP02910178.1_4515107-4515232_Hippopotamus_amphibius_isolate_BS01_ScHSfme_924672HRSCAF_982472_whole_genome_shotgun_sequence
R_PVJP02000453.1_171237-171886_Hippopotamus_amphibius_isolate_BS01_ScHSfme_440HRSCAF_34247_whole_genome_shotgun_sequence
PVJP02018429.1_1494080-1494755_Hippopotamus_amphibius_isolate_BS01_ScHSfme_18397HRSCAF_72191_0_whole_genome_shotgun_sequence
land1_Antmar_SJYB01005438.1_7e-20_1_89_61632_61544
R_PVJP02910746.1_1443422-1444115_Hippopotamus_amphibius_isolate_BS01_ScHSfme_925196HRSCAF_982996_whole_genome_shotgun_sequence
PVJP02910497.1_9239396-9239575_Hippopotamus_amphibius_isolate_BS01_ScHSfme_924960HRSCAF_982760_whole_genome_shotgun_sequence
land1_Rantar_PVIN010004539.1_1e-42_1_195_4302_4106
R_PVJP02000056.1_14401769-14402425_Hippopotamus_amphibius_isolate_BS01_ScHSfme_53HRSCAF_9696_1_whole_genome_shotgun_sequence
land1_Neomos_SJYA01001747.1_1e-22_15_153_858550_858689
land1_Bubbub_NC_037557.1_5e-31_10_153_26915940_26916085
R_PVJP02910631.1_146100-146183_Hippopotamus_amphibius_isolate_BS01_ScHSfme_925089HRSCAF_982889_whole_genome_shotgun_sequence
PVJP02910178.1_4515592-4516166_Hippopotamus_amphibius_isolate_BS01_ScHSfme_924672HRSCAF_982472_whole_genome_shotgun_sequence
R_PVJP02910426.1_1753548-1753903_Hippopotamus_amphibius_isolate_BS01_ScHSfme_924896HRSCAF_982696_0_whole_genome_shotgun_sequence
PVJP02910632.1_3415680-3416378_Hippopotamus_amphibius_isolate_BS01_ScHSfme_925090HRSCAF_982890_whole_genome_shotgun_sequence
R_PVJP02002845.1_639450-640151_Hippopotamus_amphibius_isolate_BS01_ScHSfme_2824HRSCAF_44750_whole_genome_shotgun_sequence
land1_nangra_SJYD01055185.1_8e-74_15_477_52409_52864
land1_Eudtho_SJYE01011692.1_8e-74_15_477_381764_381310
PVJP02910497.1_9238550-9239023_Hippopotamus_amphibius_isolate_BS01_ScHSfme_924960HRSCAF_982760_whole_genome_shotgun_sequence
R_PVJP02910579.1_2411963-2412621_Hippopotamus_amphibius_isolate_BS01_ScHSfme_925039HRSCAF_982839_whole_genome_shotgun_sequence
PVJP02910425.1_14057062-14057755_Hippopotamus_amphibius_isolate_BS01_ScHSfme_924895HRSCAF_982695_whole_genome_shotgun_sequence
land1_Antame_PVKT010028853.1_5e-46_16_363_7985_8329
R_PVJP02910154.1_1106377-1107066_Hippopotamus_amphibius_isolate_BS01_ScHSfme_924650HRSCAF_982450_whole_genome_shotgun_sequence
PVJP02910185.1_256485-257150_Hippopotamus_amphibius_isolate_BS01_ScHSfme_924679HRSCAF_982479_whole_genome_shotgun_sequence
PVJP02910622.1_21755332-21755998_Hippopotamus_amphibius_isolate_BS01_ScHSfme_925080HRSCAF_982880_whole_genome_shotgun_sequence
PVJP02910669.1_1299214-1299882_Hippopotamus_amphibius_isolate_BS01_ScHSfme_925125HRSCAF_982925_1_whole_genome_shotgun_sequence
PVJP02021229.1_1535229-1535915_Hippopotamus_amphibius_isolate_BS01_ScHSfme_21196HRSCAF_76041_whole_genome_shotgun_sequence
R_PVJP02910261.1_224932-225241_Hippopotamus_amphibius_isolate_BS01_ScHSfme_924745HRSCAF_982545_whole_genome_shotgun_sequence
R_PVJP02910622.1_18788964-18789655_Hippopotamus_amphibius_isolate_BS01_ScHSfme_925080HRSCAF_982880_whole_genome_shotgun_sequence
land1_Redred_SJYQ01006228.1_1e-22_109_394_926167_925875
R_PVJP02000187.1_705499-706149_Hippopotamus_amphibius_isolate_BS01_ScHSfme_176HRSCAF_25627_whole_genome_shotgun_sequence
R_PVJP02910471.1_425301-425631_Hippopotamus_amphibius_isolate_BS01_ScHSfme_924938HRSCAF_982738_whole_genome_shotgun_sequence
R_PVJP02910696.1_592859-593508_Hippopotamus_amphibius_isolate_BS01_ScHSfme_925150HRSCAF_982950_whole_genome_shotgun_sequence
R_PVJP02910261.1_224670-224934_Hippopotamus_amphibius_isolate_BS01_ScHSfme_924745HRSCAF_982545_whole_genome_shotgun_sequence
0.03

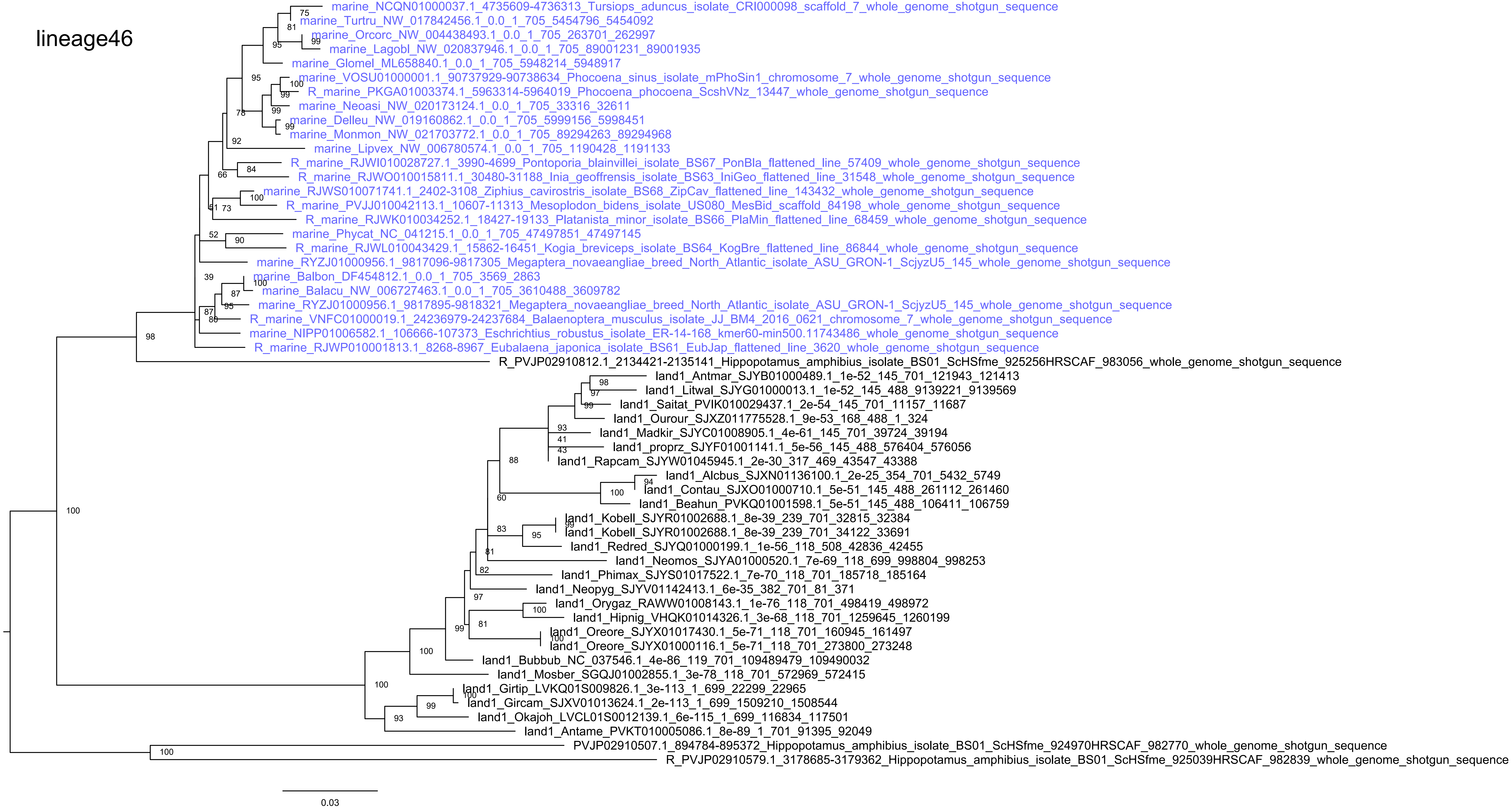

lineage46
marine_NCQN01000037.1_4735609-4736313_Tursiops_aduncus_isolate_CRI000098_scaffold_7_whole_genome_shotgun_sequence
marine_Turtru_NW_017842456.1_0.0_1_705_5454796_5454092
marine_Orcorc_NW_004438493.1_0.0_1_705_263701_262997
marine_Lagobl_NW_020837946.1_0.0_1_705_89001231_89001935
marine_Glomel_ML658840.1_0.0_1_705_5948214_5948917
marine_VOSU01000001.1_90737929-90738634_Phocoena_sinus_isolate_mPhoSin1_chromosome_7_whole_genome_shotgun_sequence
R_marine_PKGA01003374.1_5963314-5964019_Phocoena_phocoena_ScshVNz_13447_whole_genome_shotgun_sequence
marine_Neoasi_NW_020173124.1_0.0_1_705_33316_32611
marine_Delleu_NW_019160862.1_0.0_1_705_5999156_5998451
marine_Monmon_NW_021703772.1_0.0_1_705_89294263_89294968
marine_Lipvex_NW_006780574.1_0.0_1_705_1190428_1191133
R_marine_RJWI010028727.1_3990-4699_Pontoporia_blainvillei_isolate_BS67_PonBla_flattened_line_57409_whole_genome_shotgun_sequence
R_marine_RJWO010015811.1_30480-31188_Inia_geoffrensis_isolate_BS63_IniGeo_flattened_line_31548_whole_genome_shotgun_sequence
marine_RJWS010071741.1_2402-3108_Ziphius_cavirostris_isolate_BS68_ZipCav_flattened_line_143432_whole_genome_shotgun_sequence
R_marine_PVJJ010042113.1_10607-11313_Mesoplodon_bidens_isolate_US080_MesBid_scaffold_84198_whole_genome_shotgun_sequence
R_marine_RJWK010034252.1_18427-19133_Platanista_minor_isolate_BS66_PlaMin_flattened_line_68459_whole_genome_shotgun_sequence
marine_Phycat_NC_041215.1_0.0_1_705_47497851_47497145
R_marine_RJWL010043429.1_15862-16451_Kogia_breviceps_isolate_BS64_KogBre_flattened_line_86844_whole_genome_shotgun_sequence
marine_RYZJ01000956.1_9817096-9817305_Megaptera_novaeangliae_breed_North_Atlantic_isolate_ASU_GRON-1_ScjyzU5_145_whole_genome_shotgun_sequence
marine_Balbon_DF454812.1_0.0_1_705_3569_2863
marine_Balacu_NW_006727463.1_0.0_1_705_3610488_3609782
marine_RYZJ01000956.1_9817895-9818321_Megaptera_novaeangliae_breed_North_Atlantic_isolate_ASU_GRON-1_ScjyzU5_145_whole_genome_shotgun_sequence
R_marine_VNFC01000019.1_24236979-24237684_Balaenoptera_musculus_isolate_JJ_BM4_2016_0621_chromosome_7_whole_genome_shotgun_sequence
marine_NIPP01006582.1_106666-107373_Eschrichtius_robustus_isolate_ER-14-168_kmer60-min500.11743486_whole_genome_shotgun_sequence
R_marine_RJWP010001813.1_8268-8967_Eubalaena_japonica_isolate_BS61_EubJap_flattened_line_3620_whole_genome_shotgun_sequence
R_PVJP02910812.1_2134421-2135141_Hippopotamus_amphibius_isolate_BS01_ScHSfme_925256HRSCAF_983056_whole_genome_shotgun_sequence
land1_Antmar_SJYB01000489.1_1e-52_145_701_121943_121413
land1_Litwal_SJYG01000013.1_1e-52_145_488_9139221_9139569
land1_Saitat_PVIK010029437.1_2e-54_145_701_11157_11687
land1_Ourour_SJXZ011775528.1_9e-53_168_488_1_324
land1_Madkir_SJYC01008905.1_4e-61_145_701_39724_39194
land1_proprz_SJYF01001141.1_5e-56_145_488_576404_576056
land1_Rapcam_SJYW01045945.1_2e-30_317_469_43547_43388
land1_Alcbus_SJXN01136100.1_2e-25_354_701_5432_5749
land1_Contau_SJXO01000710.1_5e-51_145_488_261112_261460
land1_Beahun_PVKQ01001598.1_5e-51_145_488_106411_106759
land1_Kobell_SJYR01002688.1_8e-39_239_701_32815_32384
land1_Kobell_SJYR01002688.1_8e-39_239_701_34122_33691
land1_Redred_SJYQ01000199.1_1e-56_118_508_42836_42455
land1_Neomos_SJYA01000520.1_7e-69_118_699_998804_998253
land1_Phimax_SJYS01017522.1_7e-70_118_701_185718_185164
land1_Neopyg_SJYV01142413.1_6e-35_382_701_81_371
land1_Orygaz_RAWW01008143.1_1e-76_118_701_498419_498972
land1_Hipnig_VHQK01014326.1_3e-68_118_701_1259645_1260199
land1_Oreore_SJYX01017430.1_5e-71_118_701_160945_161497
land1_Oreore_SJYX01000116.1_5e-71_118_701_273800_273248
land1_Bubbub_NC_037546.1_4e-86_119_701_109489479_109490032
land1_Mosber_SGQJ01002855.1_3e-78_118_701_572969_572415
land1_Girtip_LVKQ01S009826.1_3e-113_1_699_22299_22965
land1_Gircam_SJXV01013624.1_2e-113_1_699_1509210_1508544
land1_Okajoh_LVCL01S0012139.1_6e-115_1_699_116834_117501
land1_Antame_PVKT010005086.1_8e-89_1_701_91395_92049
PVJP02910507.1_894784-895372_Hippopotamus_amphibius_isolate_BS01_ScHSfme_924970HRSCAF_982770_whole_genome_shotgun_sequence
R_PVJP02910579.1_3178685-3179362_Hippopotamus_amphibius_isolate_BS01_ScHSfme_925039HRSCAF_982839_whole_genome_shotgun_sequence
0.03

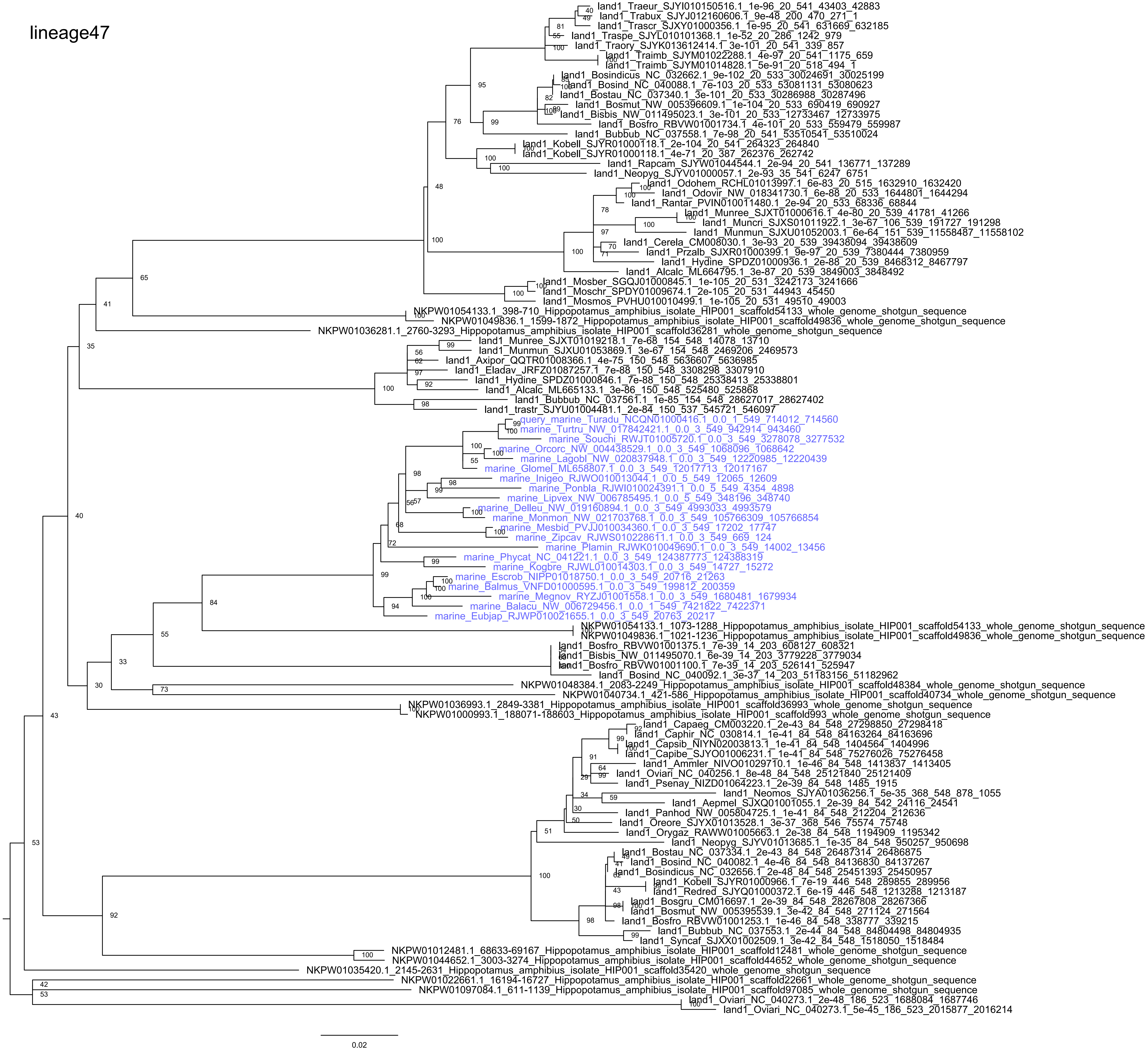
lineage47
land1_Traeur_SJYI010150516.1_1e-96_20_541_43403_42883
land1_Trabux_SJYJ012160606.1_9e-48_200_470_271_1
land1_Trascr_SJXY01000356.1_1e-95_20_541_631669_632185
land1_Traspe_SJYL010101368.1_1e-52_20_286_1242_979
land1_Traory_SJYK013612414.1_3e-101_20_541_339_857
land1_Traimb_SJYM01022288.1_4e-97_20_541_1175_659
land1_Traimb_SJYM01014828.1_5e-91_20_518_494_1
land1_Bosindicus_NC_032662.1_9e-102_20_533_30024691_30025199
land1_Bosind_NC_040088.1_7e-103_20_533_53081131_53080623
land1_Bostau_NC_037340.1_3e-101_20_533_30286988_30287496
land1_Bosmut_NW_005396609.1_1e-104_20_533_690419_690927
land1_Bisbis_NW_011495023.1_3e-101_20_533_12733467_12733975
land1_Bosfro_RBVW01001734.1_4e-101_20_533_559479_559987
land1_Bubbub_NC_037558.1_7e-98_20_541_53510541_53510024
land1_Kobell_SJYR01000118.1_2e-104_20_541_264323_264840
land1_Kobell_SJYR01000118.1_4e-71_20_387_262376_262742
land1_Rapcam_SJYW01044544.1_2e-94_20_541_136771_137289
land1_Neopyg_SJYV01000057.1_2e-93_35_541_6247_6751
land1_Odohem_RCHL01013997.1_6e-83_20_515_1632910_1632420
land1_Odovir_NW_018341730.1_6e-88_20_533_1644801_1644294
land1_Rantar_PVIN010011480.1_2e-94_20_533_68336_68844
land1_Munree_SJXT01000616.1_4e-80_20_539_41781_41266
land1_Muncri_SJXS01011922.1_3e-67_106_539_191727_191298
land1_Munmun_SJXU01052003.1_6e-64_151_539_11558487_11558102
land1_Cerela_CM008030.1_3e-93_20_539_39438094_39438609
land1_Przalb_SJXR01000399.1_9e-97_20_539_7380444_7380959
land1_Hydine_SPDZ01000936.1_2e-88_20_539_8468312_8467797
land1_Alcalc_ML664795.1_3e-87_20_539_3849003_3848492
land1_Mosber_SGQJ01000845.1_1e-105_20_531_3242173_3241666
land1_Moschr_SPDY01009674.1_2e-105_20_531_44943_45450
land1_Mosmos_PVHU010010499.1_1e-105_20_531_49510_49003
NKPW01054133.1_398-710_Hippopotamus_amphibius_isolate_HIP001_scaffold54133_whole_genome_shotgun_sequence
NKPW01049836.1_1599-1872_Hippopotamus_amphibius_isolate_HIP001_scaffold49836_whole_genome_shotgun_sequence
NKPW01036281.1_2760-3293_Hippopotamus_amphibius_isolate_HIP001_scaffold36281_whole_genome_shotgun_sequence
land1_Munree_SJXT01019218.1_7e-68_154_548_14078_13710
land1_Munmun_SJXU01053869.1_3e-67_154_548_2469206_2469573
land1_Axipor_QQTR01008366.1_4e-75_150_548_5636607_5636985
land1_Eladav_JRFZ01087257.1_7e-88_150_548_3308298_3307910
land1_Hydine_SPDZ01000846.1_7e-88_150_548_25338413_25338801
land1_Alcalc_ML665133.1_3e-86_150_548_525480_525868
land1_Bubbub_NC_037561.1_1e-85_154_548_28627017_28627402
land1_trastr_SJYU01004481.1_2e-84_150_537_545721_546097
query_marine_Turadu_NCQN01000416.1_0.0_1_549_714012_714560
marine_Turtru_NW_017842421.1_0.0_3_549_942914_943460
marine_Souchi_RWJT01005720.1_0.0_3_549_3278078_3277532
marine_Orcorc_NW_004438529.1_0.0_3_549_1068096_1068642
marine_Lagobl_NW_020837948.1_0.0_3_549_12220985_12220439
marine_Glomel_ML658807.1_0.0_3_549_12017713_12017167
marine_Inigeo_RJWO010013044.1_0.0_5_549_12065_12609
marine_Ponbla_RJWI010024391.1_0.0_5_549_4354_4898
marine_Lipvex_NW_006785495.1_0.0_5_549_348196_348740
marine_Delleu_NW_019160894.1_0.0_3_549_4993033_4993579
marine_Monmon_NW_021703768.1_0.0_3_549_105766309_105766854
marine_Mesbid_PVJJ010034360.1_0.0_3_549_17202_17747
marine_Zipcav_RJWS0102286.1.1_0.0_3_549_669_124
marine_Plamin_RJWK010049690.1_0.0_3_549_14002_13456
marine_Phycat_NC_041221.1_0.0_3_549_124387773_124388319
marine_Kogbre_RJWL010014303.1_0.0_3_549_14727_15272
marine_Escrob_NIPP01018750.1_0.0_3_549_20716_21263
marine_Balmus_VNFD01000595.1_0.0_3_549_199812_200359
marine_Megnov_RYZJ01001558.1_0.0_3_549_1680481_1679934
marine_Balacu_NW_006729456.1_0.0_1_549_7421822_7422371
marine_Eubjap_RJWP010021655.1_0.0_3_549_20763_20217
NKPW01054133.1_1073-1288_Hippopotamus_amphibius_isolate_HIP001_scaffold54133_whole_genome_shotgun_sequence
NKPW01049836.1_1021-1236_Hippopotamus_amphibius_isolate_HIP001_scaffold49836_whole_genome_shotgun_sequence
land1_Bosfro_RBVW01001375.1_7e-39_14_203_608127_608321
land1_Bisbis_NW_011495070.1_6e-39_14_203_3779228_3779034
land1_Bosfro_RBVW01001100.1_7e-39_14_203_526141_525947
land1_Bosind_NC_040092.1_3e-37_14_203_51183156_51182962
NKPW01048384.1_2083-2249_Hippopotamus_amphibius_isolate_HIP001_scaffold48384_whole_genome_shotgun_sequence
NKPW01040734.1_421-586_Hippopotamus_amphibius_isolate_HIP001_scaffold40734_whole_genome_shotgun_sequence
NKPW01036993.1_2849-3381_Hippopotamus_amphibius_isolate_HIP001_scaffold36993_whole_genome_shotgun_sequence
NKPW01000993.1_188071-188603_Hippopotamus_amphibius_isolate_HIP001_scaffold993_whole_genome_shotgun_sequence
land1_Capaeg_CM003220.1_2e-43_84_548_27298850_27298418
land1_Caphir_NC_030814.1_1e-41_84_548_84163264_84163696
land1_Capsib_NIYN02003813.1_1e-41_84_548_1404564_1404996
land1_Capibe_SJYO01006231.1_1e-41_84_548_75276026_75276458
land1_Ammler_NIVO01029710.1_1e-46_84_548_1413837_1413405
land1_Oviari_NC_040256.1_8e-48_84_548_25121840_25121409
land1_Psenay_NIZD01064223.1_2e-39_84_548_1485_1915
land1_Neomos_SJYA01036256.1_5e-35_368_548_878_1055
land1_Aepmel_SJXQ01001055.1_2e-39_84_542_24116_24541
land1_Panhod_NW_005804725.1_1e-41_84_548_212204_212636
land1_Oreore_SJYX01013528.1_3e-37_368_546_75574_75748
land1_Orygaz_RAWW01005663.1_2e-38_84_548_1194909_1195342
land1_Neopyg_SJYV01013685.1_1e-35_84_548_950257_950698
land1_Bostau_NC_037334.1_2e-43_84_548_26487314_26486875
land1_Bosind_NC_040082.1_4e-46_84_548_84136830_84137267
land1_Bosindicus_NC_032656.1_2e-48_84_548_25451393_25450957
land1_Kobell_SJYR01000966.1_7e-19_446_548_289855_289956
land1_Redred_SJYQ01000372.1_6e-19_446_548_1213288_1213187
land1_Bosgru_CM016697.1_2e-39_84_548_28267808_28267366
land1_Bosmut_NW_005395539.1_3e-42_84_548_271124_271564
land1_Bosfro_RBVW01001253.1_1e-46_84_548_338777_339215
land1_Bubbub_NC_037553.1_2e-44_84_548_84804498_84804935
land1_Syncaf_SJXX01002509.1_3e-42_84_548_1518050_1518484
NKPW01012481.1_68633-69167_Hippopotamus_amphibius_isolate_HIP001_scaffold12481_whole_genome_shotgun_sequence
NKPW01044652.1_3003-3274_Hippopotamus_amphibius_isolate_HIP001_scaffold44652_whole_genome_shotgun_sequence
NKPW01035420.1_2145-2631_Hippopotamus_amphibius_isolate_HIP001_scaffold35420_whole_genome_shotgun_sequence
NKPW01022661.1_16194-16727_Hippopotamus_amphibius_isolate_HIP001_scaffold22661_whole_genome_shotgun_sequence
NKPW01097084.1_611-1139_Hippopotamus_amphibius_isolate_HIP001_scaffold97085_whole_genome_shotgun_sequence
land1_Oviari_NC_040273.1_2e-48_186_523_1688084_1687746
land1_Oviari_NC_040273.1_5e-45_186_523_2015877_2016214
0.02

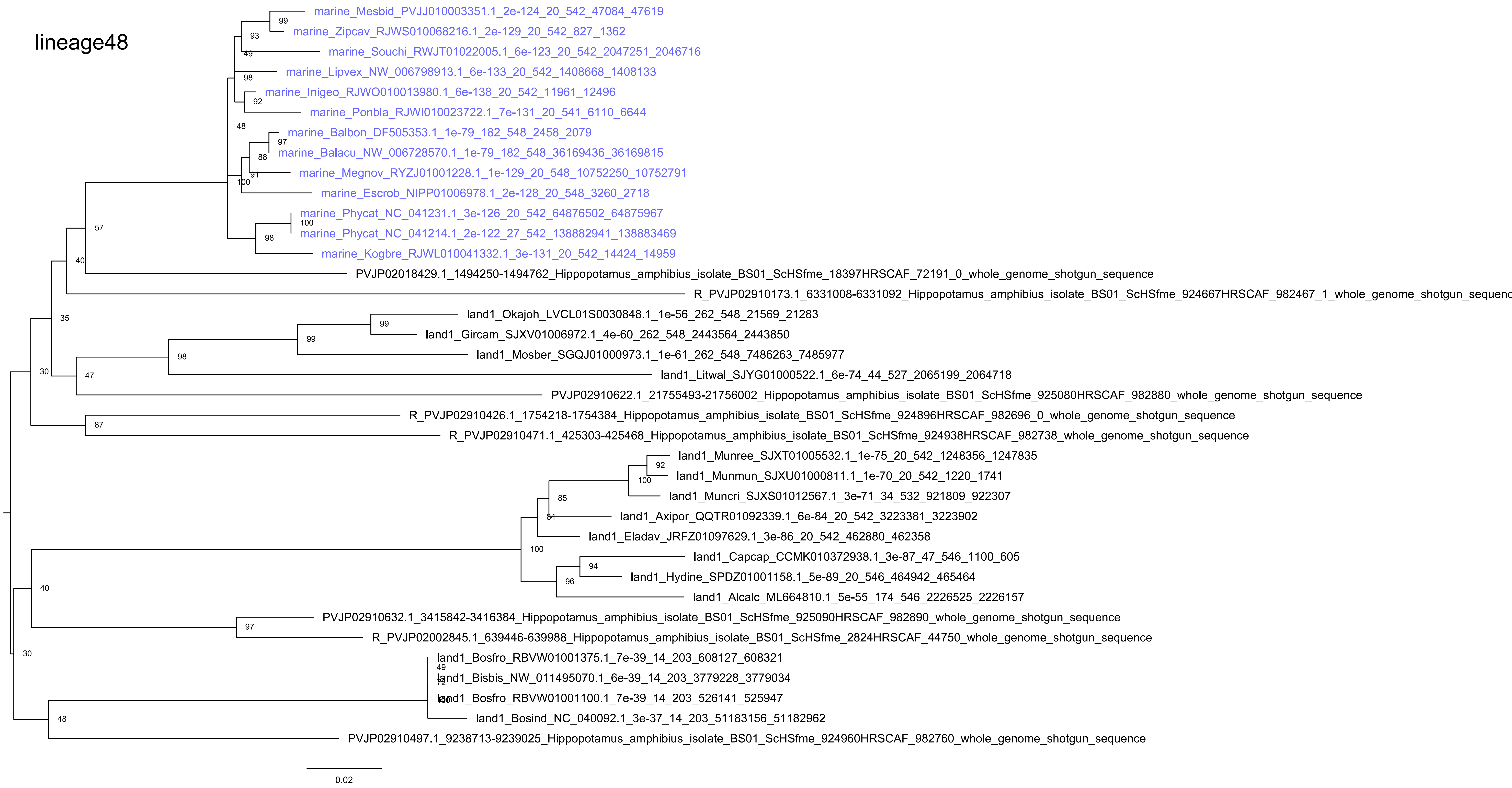
lineage48
marine_Mesbid_PVJJ010003351.1_2e-124_20_542_47084_47619
marine_Zipcav_RJWS010068216.1_2e-129_20_542_827_1362
marine_Souchi_RWJT01022005.1_6e-123_20_542_2047251_2046716
marine_Lipvex_NW_006798913.1_6e-133_20_542_1408668_1408133
marine_Inigeo_RJWO010013980.1_6e-138_20_542_11961_12496
marine_Ponbla_RJWI010023722.1_7e-131_20_541_6110_6644
marine_Balbon_DF505353.1_1e-79_182_548_2458_2079
marine_Balacu_NW_006728570.1_1e-79_182_548_36169436_36169815
marine_Megnov_RYZJ01001228.1_1e-129_20_548_10752250_10752791
marine_Escrob_NIPP01006978.1_2e-128_20_548_3260_2718
marine_Phycat_NC_041231.1_3e-126_20_542_64876502_64875967
marine_Phycat_NC_041214.1_2e-122_27_542_138882941_138883469
marine_Kogbre_RJWL010041332.1_3e-131_20_542_14424_14959
PVJP02018429.1_1494250-1494762_Hippopotamus_amphibius_isolate_BS01_ScHSfme_18397HRSCAF_72191_0_whole_genome_shotgun_sequence
R_PVJP02910173.1_6331008-6331092_Hippopotamus_amphibius_isolate_BS01_ScHSfme_924667HRSCAF_982467_1_whole_genome_shotgun_sequence
land1_Okajoh_LVCL01S0030848.1_1e-56_262_548_21569_21283
land1_Gircam_SJXV01006972.1_4e-60_262_548_2443564_2443850
land1_Mosber_SGQJ01000973.1_1e-61_262_548_7486263_7485977
land1_Litwal_SJYG01000522.1_6e-74_44_527_2065199_2064718
PVJP02910622.1_21755493-21756002_Hippopotamus_amphibius_isolate_BS01_ScHSfme_925080HRSCAF_982880_whole_genome_shotgun_sequence
R_PVJP02910426.1_1754218-1754384_Hippopotamus_amphibius_isolate_BS01_ScHSfme_924896HRSCAF_982696_0_whole_genome_shotgun_sequence
R_PVJP02910471.1_425303-425468_Hippopotamus_amphibius_isolate_BS01_ScHSfme_924938HRSCAF_982738_whole_genome_shotgun_sequence
land1_Munree_SJXT01005532.1_1e-75_20_542_1248356_1247835
land1_Munmun_SJXU01000811.1_1e-70_20_542_1220_1741
land1_Muncri_SJXS01012567.1_3e-71_34_532_921809_922307
land1_Axipor_QQTR01092339.1_6e-84_20_542_3223381_3223902
land1_Eladav_JRFZ01097629.1_3e-86_20_542_462880_462358
land1_Capcap_CCMK010372938.1_3e-87_47_546_1100_605
land1_Hydine_SPDZ01001158.1_5e-89_20_546_464942_465464
land1_Alcalc_ML664810.1_5e-55_174_546_2226525_2226157
PVJP02910632.1_3415842-3416384_Hippopotamus_amphibius_isolate_BS01_ScHSfme_925090HRSCAF_982890_whole_genome_shotgun_sequence
R_PVJP02002845.1_639446-639988_Hippopotamus_amphibius_isolate_BS01_ScHSfme_2824HRSCAF_44750_whole_genome_shotgun_sequence
land1_Bosfro_RBVW01001375.1_7e-39_14_203_608127_608321
land1_Bisbis_NW_011495070.1_6e-39_14_203_3779228_3779034
land1_Bosfro_RBVW01001100.1_7e-39_14_203_526141_525947
land1_Bosind_NC_040092.1_3e-37_14_203_51183156_51182962
PVJP02910497.1_9238713-9239025_Hippopotamus_amphibius_isolate_BS01_ScHSfme_924960HRSCAF_982760_whole_genome_shotgun_sequence
99
93
49
98
92
48
97
88
91
100
100
98
57
40
35
99
99
98
47
30
87
92
100
85
84
100
94
96
40
97
30
49
72
100
48
0.02

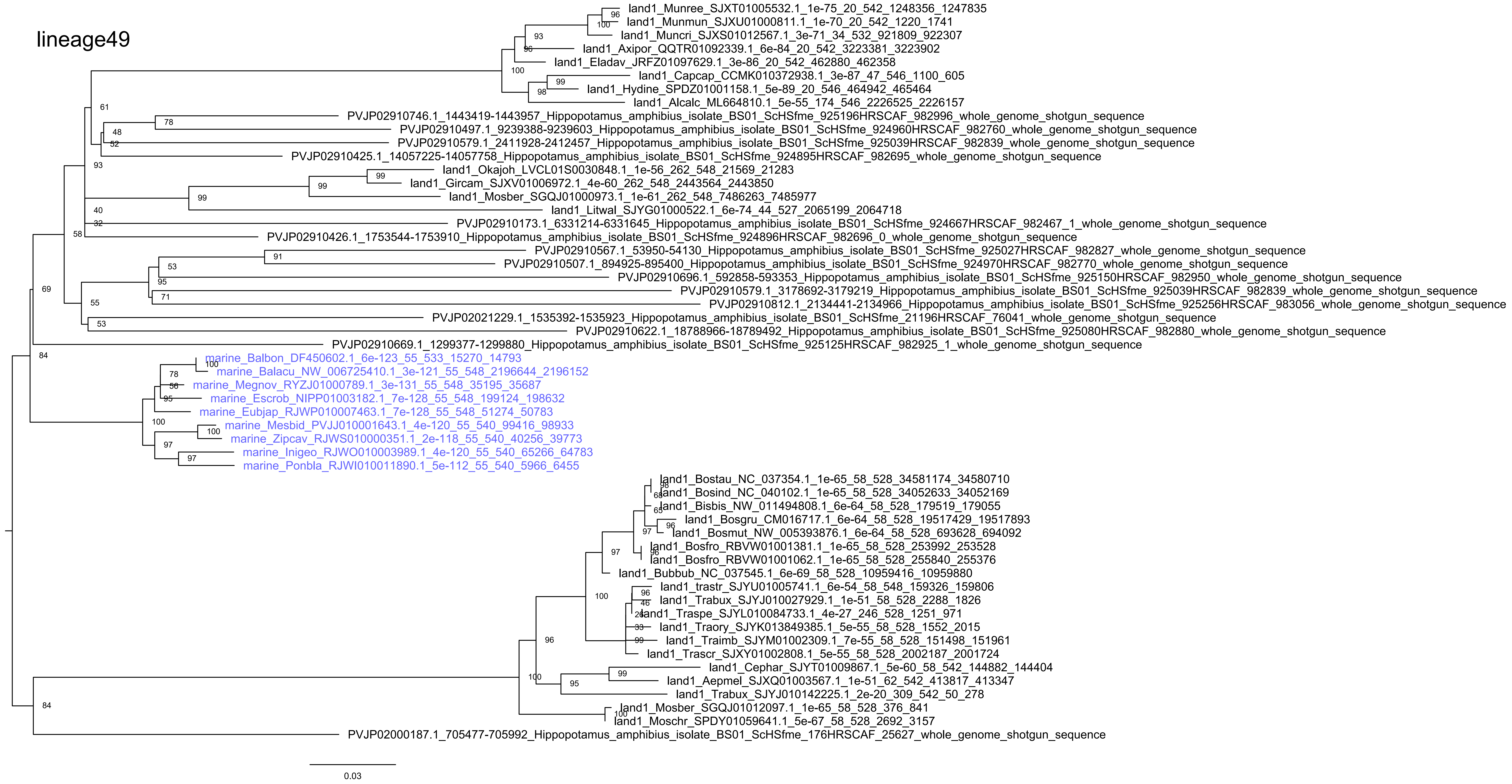
lineage49
land1_Munree_SJXT01005532.1_1e-75_20_542_1248356_1247835
land1_Munmun_SJXU01000811.1_1e-70_20_542_1220_1741
land1_Muncri_SJXS01012567.1_3e-71_34_532_921809_922307
land1_Axipor_QQTR01092339.1_6e-84_20_542_3223381_3223902
land1_Eladav_JRFZ01097629.1_3e-86_20_542_462880_462358
land1_Capcap_CCMK010372938.1_3e-87_47_546_1100_605
land1_Hydine_SPDZ01001158.1_5e-89_20_546_464942_465464
land1_Alcalc_ML664810.1_5e-55_174_546_2226525_2226157
PVJP02910746.1_1443419-1443957_Hippopotamus_amphibius_isolate_BS01_ScHSfme_925196HRSCAF_982996_whole_genome_shotgun_sequence
PVJP02910497.1_9239388-9239603_Hippopotamus_amphibius_isolate_BS01_ScHSfme_924960HRSCAF_982760_whole_genome_shotgun_sequence
PVJP02910579.1_2411928-2412457_Hippopotamus_amphibius_isolate_BS01_ScHSfme_925039HRSCAF_982839_whole_genome_shotgun_sequence
PVJP02910425.1_14057225-14057758_Hippopotamus_amphibius_isolate_BS01_ScHSfme_924895HRSCAF_982695_whole_genome_shotgun_sequence
land1_Okajoh_LVCL01S0030848.1_1e-56_262_548_21569_21283
land1_Gircam_SJXV01006972.1_4e-60_262_548_2443564_2443850
land1_Mosber_SGQJ01000973.1_1e-61_262_548_7486263_7485977
land1_Litwal_SJYG01000522.1_6e-74_44_527_2065199_2064718
PVJP02910173.1_6331214-6331645_Hippopotamus_amphibius_isolate_BS01_ScHSfme_924667HRSCAF_982467_1_whole_genome_shotgun_sequence
PVJP02910426.1_1753544-1753910_Hippopotamus_amphibius_isolate_BS01_ScHSfme_924896HRSCAF_982696_0_whole_genome_shotgun_sequence
PVJP02910567.1_53950-54130_Hippopotamus_amphibius_isolate_BS01_ScHSfme_925027HRSCAF_982827_whole_genome_shotgun_sequence
PVJP02910507.1_894925-895400_Hippopotamus_amphibius_isolate_BS01_ScHSfme_924970HRSCAF_982770_whole_genome_shotgun_sequence
PVJP02910696.1_592858-593353_Hippopotamus_amphibius_isolate_BS01_ScHSfme_925150HRSCAF_982950_whole_genome_shotgun_sequence
PVJP02910579.1_3178692-3179219_Hippopotamus_amphibius_isolate_BS01_ScHSfme_925039HRSCAF_982839_whole_genome_shotgun_sequence
PVJP02910812.1_2134441-2134966_Hippopotamus_amphibius_isolate_BS01_ScHSfme_925256HRSCAF_983056_whole_genome_shotgun_sequence
PVJP02021229.1_1535392-1535923_Hippopotamus_amphibius_isolate_BS01_ScHSfme_21196HRSCAF_76041_whole_genome_shotgun_sequence
PVJP02910622.1_18788966-18789492_Hippopotamus_amphibius_isolate_BS01_ScHSfme_925080HRSCAF_982880_whole_genome_shotgun_sequence
PVJP02910669.1_1299377-1299880_Hippopotamus_amphibius_isolate_BS01_ScHSfme_925125HRSCAF_982925_1_whole_genome_shotgun_sequence
marine_Balbon_DF450602.1_6e-123_55_533_15270_14793
marine_Balacu_NW_006725410.1_3e-121_55_548_2196644_2196152
marine_Megnov_RYZJ01000789.1_3e-131_55_548_35195_35687
marine_Escrob_NIPP01003182.1_7e-128_55_548_199124_198632
marine_Eubjap_RJWP010007463.1_7e-128_55_548_51274_50783
marine_Mesbid_PVJJ010001643.1_4e-120_55_540_99416_98933
marine_Zipcav_RJWS010000351.1_2e-118_55_540_40256_39773
marine_Inigeo_RJWO010003989.1_4e-120_55_540_65266_64783
marine_Ponbla_RJWI010011890.1_5e-112_55_540_5966_6455
land1_Bostau_NC_037354.1_1e-65_58_528_34581174_34580710
land1_Bosind_NC_040102.1_1e-65_58_528_34052633_34052169
land1_Bisbis_NW_011494808.1_6e-64_58_528_179519_179055
land1_Bosgru_CM016717.1_6e-64_58_528_19517429_19517893
land1_Bosmut_NW_005393876.1_6e-64_58_528_693628_694092
land1_Bosfro_RBVW01001381.1_1e-65_58_528_253992_253528
land1_Bosfro_RBVW01001062.1_1e-65_58_528_255840_255376
land1_Bubbub_NC_037545.1_6e-69_58_528_10959416_10959880
land1_trastr_SJYU01005741.1_6e-54_58_548_159326_159806
land1_Trabux_SJYJ010027929.1_1e-51_58_528_2288_1826
land1_Traspe_SJYL010084733.1_4e-27_246_528_1251_971
land1_Traory_SJYK013849385.1_5e-55_58_528_1552_2015
land1_Traimb_SJYM01002309.1_7e-55_58_528_151498_151961
land1_Trascr_SJXY01002808.1_5e-55_58_528_2002187_2001724
land1_Cephar_SJYT01009867.1_5e-60_58_542_144882_144404
land1_Aepmel_SJXQ01003567.1_1e-51_62_542_413817_413347
land1_Trabux_SJYJ010142225.1_2e-20_309_542_50_278
land1_Mosber_SGQJ01012097.1_1e-65_58_528_376_841
land1_Moschr_SPDY01059641.1_5e-67_58_528_2692_3157
PVJP02000187.1_705477-705992_Hippopotamus_amphibius_isolate_BS01_ScHSfme_176HRSCAF_25627_whole_genome_shotgun_sequence
0.03

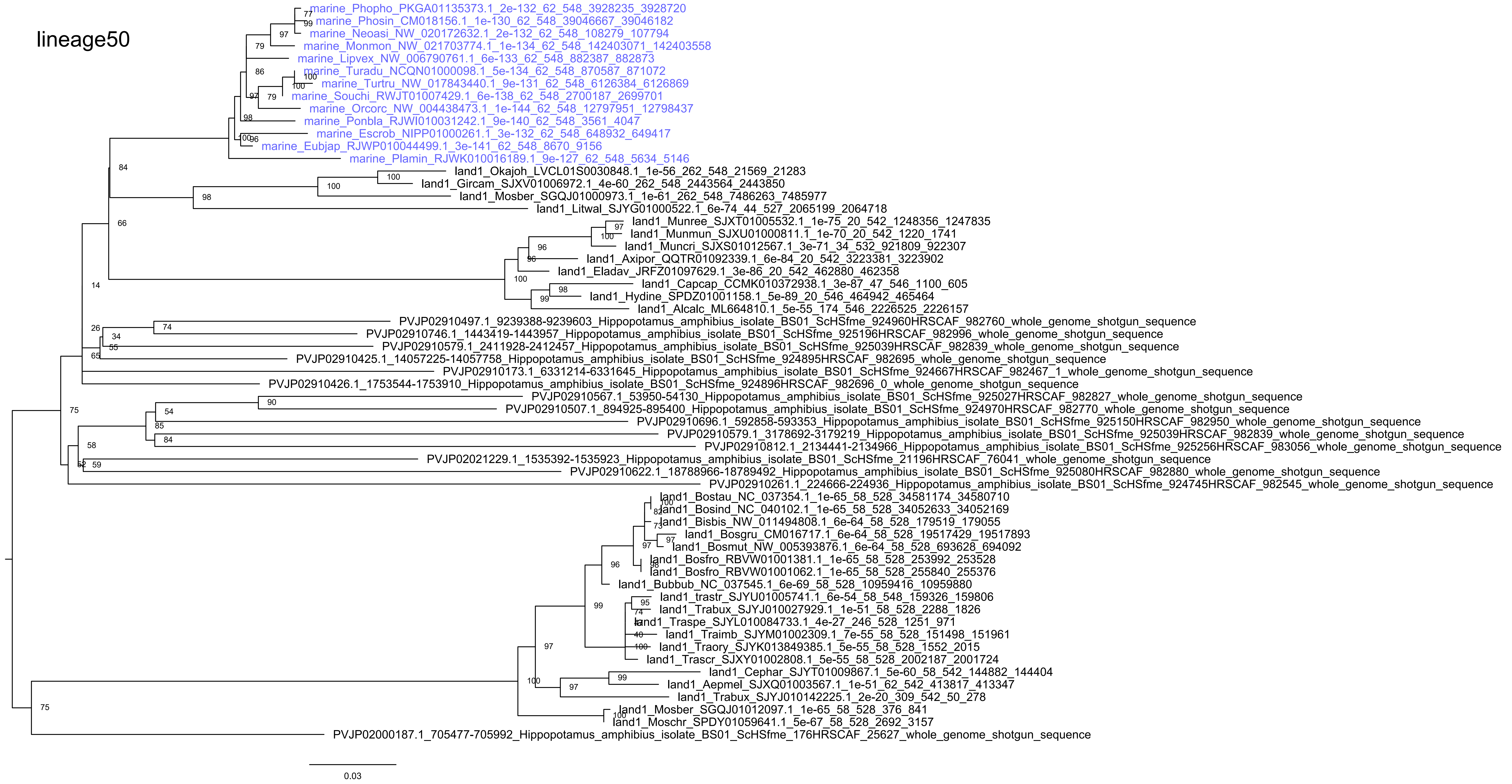

lineage50
marine_Phopho_PKGA01135373.1_2e-132_62_548_3928235_3928720
marine_Phosin_CM018156.1_1e-130_62_548_39046667_39046182
marine_Neoasi_NW_020172632.1_2e-132_62_548_108279_107794
marine_Monmon_NW_021703774.1_1e-134_62_548_142403071_142403558
marine_Lipvex_NW_006790761.1_6e-133_62_548_882387_882873
marine_Turadu_NCQN01000098.1_5e-134_62_548_870587_871072
marine_Turtru_NW_017843440.1_9e-131_62_548_6126384_6126869
marine_Souchi_RWJT01007429.1_6e-138_62_548_2700187_2699701
marine_Orcorc_NW_004438473.1_1e-144_62_548_12797951_12798437
marine_Ponbla_RJWI010031242.1_9e-140_62_548_3561_4047
marine_Escrob_NIPP01000261.1_3e-132_62_548_648932_649417
marine_Eubjap_RJWP010044499.1_3e-141_62_548_8670_9156
marine_Plamin_RJWK010016189.1_9e-127_62_548_5634_5146
land1_Okajoh_LVCL01S0030848.1_1e-56_262_548_21569_21283
land1_Gircam_SJXV01006972.1_4e-60_262_548_2443564_2443850
land1_Mosber_SGQJ01000973.1_1e-61_262_548_7486263_7485977
land1_Litwal_SJYG01000522.1_6e-74_44_527_2065199_2064718
land1_Munree_SJXT01005532.1_1e-75_20_542_1248356_1247835
land1_Munmun_SJXU01000811.1_1e-70_20_542_1220_1741
land1_Muncri_SJXS01012567.1_3e-71_34_532_921809_922307
land1_Axipor_QQTR01092339.1_6e-84_20_542_3223381_3223902
land1_Eladav_JRFZ01097629.1_3e-86_20_542_462880_462358
land1_Capcap_CCMK010372938.1_3e-87_47_546_1100_605
land1_Hydine_SPDZ01001158.1_5e-89_20_546_464942_465464
land1_Alcalc_ML664810.1_5e-55_174_546_2226525_2226157
PVJP02910497.1_9239388-9239603_Hippopotamus_amphibius_isolate_BS01_ScHSfme_924960HRSCAF_982760_whole_genome_shotgun_sequence
PVJP02910746.1_1443419-1443957_Hippopotamus_amphibius_isolate_BS01_ScHSfme_925196HRSCAF_982996_whole_genome_shotgun_sequence
PVJP02910579.1_2411928-2412457_Hippopotamus_amphibius_isolate_BS01_ScHSfme_925039HRSCAF_982839_whole_genome_shotgun_sequence
PVJP02910425.1_14057225-14057758_Hippopotamus_amphibius_isolate_BS01_ScHSfme_924895HRSCAF_982695_whole_genome_shotgun_sequence
PVJP02910173.1_6331214-6331645_Hippopotamus_amphibius_isolate_BS01_ScHSfme_924667HRSCAF_982467_1_whole_genome_shotgun_sequence
PVJP02910426.1_1753544-1753910_Hippopotamus_amphibius_isolate_BS01_ScHSfme_924896HRSCAF_982696_0_whole_genome_shotgun_sequence
PVJP02910567.1_53950-54130_Hippopotamus_amphibius_isolate_BS01_ScHSfme_925027HRSCAF_982827_whole_genome_shotgun_sequence
PVJP02910507.1_894925-895400_Hippopotamus_amphibius_isolate_BS01_ScHSfme_924970HRSCAF_982770_whole_genome_shotgun_sequence
PVJP02910696.1_592858-593353_Hippopotamus_amphibius_isolate_BS01_ScHSfme_925150HRSCAF_982950_whole_genome_shotgun_sequence
PVJP02910579.1_3178692-3179219_Hippopotamus_amphibius_isolate_BS01_ScHSfme_925039HRSCAF_982839_whole_genome_shotgun_sequence
PVJP02910812.1_2134441-2134966_Hippopotamus_amphibius_isolate_BS01_ScHSfme_925256HRSCAF_983056_whole_genome_shotgun_sequence
PVJP02021229.1_1535392-1535923_Hippopotamus_amphibius_isolate_BS01_ScHSfme_21196HRSCAF_76041_whole_genome_shotgun_sequence
PVJP02910622.1_18788966-18789492_Hippopotamus_amphibius_isolate_BS01_ScHSfme_925080HRSCAF_982880_whole_genome_shotgun_sequence
PVJP02910261.1_224666-224936_Hippopotamus_amphibius_isolate_BS01_ScHSfme_924745HRSCAF_982545_whole_genome_shotgun_sequence
land1_Bostau_NC_037354.1_1e-65_58_528_34581174_34580710
land1_Bosind_NC_040102.1_1e-65_58_528_34052633_34052169
land1_Bisbis_NW_011494808.1_6e-64_58_528_179519_179055
land1_Bosgru_CM016717.1_6e-64_58_528_19517429_19517893
land1_Bosmut_NW_005393876.1_6e-64_58_528_693628_694092
land1_Bosfro_RBVW01001381.1_1e-65_58_528_253992_253528
land1_Bosfro_RBVW01001062.1_1e-65_58_528_255840_255376
land1_Bubbub_NC_037545.1_6e-69_58_528_10959416_10959880
land1_trastr_SJYU01005741.1_6e-54_58_548_159326_159806
land1_Trabux_SJYJ010027929.1_1e-51_58_528_2288_1826
land1_Traspe_SJYL010084733.1_4e-27_246_528_1251_971
land1_Traimb_SJYM01002309.1_7e-55_58_528_151498_151961
land1_Traory_SJYK013849385.1_5e-55_58_528_1552_2015
land1_Trascr_SJXY01002808.1_5e-55_58_528_2002187_2001724
land1_Cephar_SJYT01009867.1_5e-60_58_542_144882_144404
land1_Aepmel_SJXQ01003567.1_1e-51_62_542_413817_413347
land1_Trabux_SJYJ010142225.1_2e-20_309_542_50_278
land1_Mosber_SGQJ01012097.1_1e-65_58_528_376_841
land1_Moschr_SPDY01059641.1_5e-67_58_528_2692_3157
PVJP02000187.1_705477-705992_Hippopotamus_amphibius_isolate_BS01_ScHSfme_176HRSCAF_25627_whole_genome_shotgun_sequence
0.03

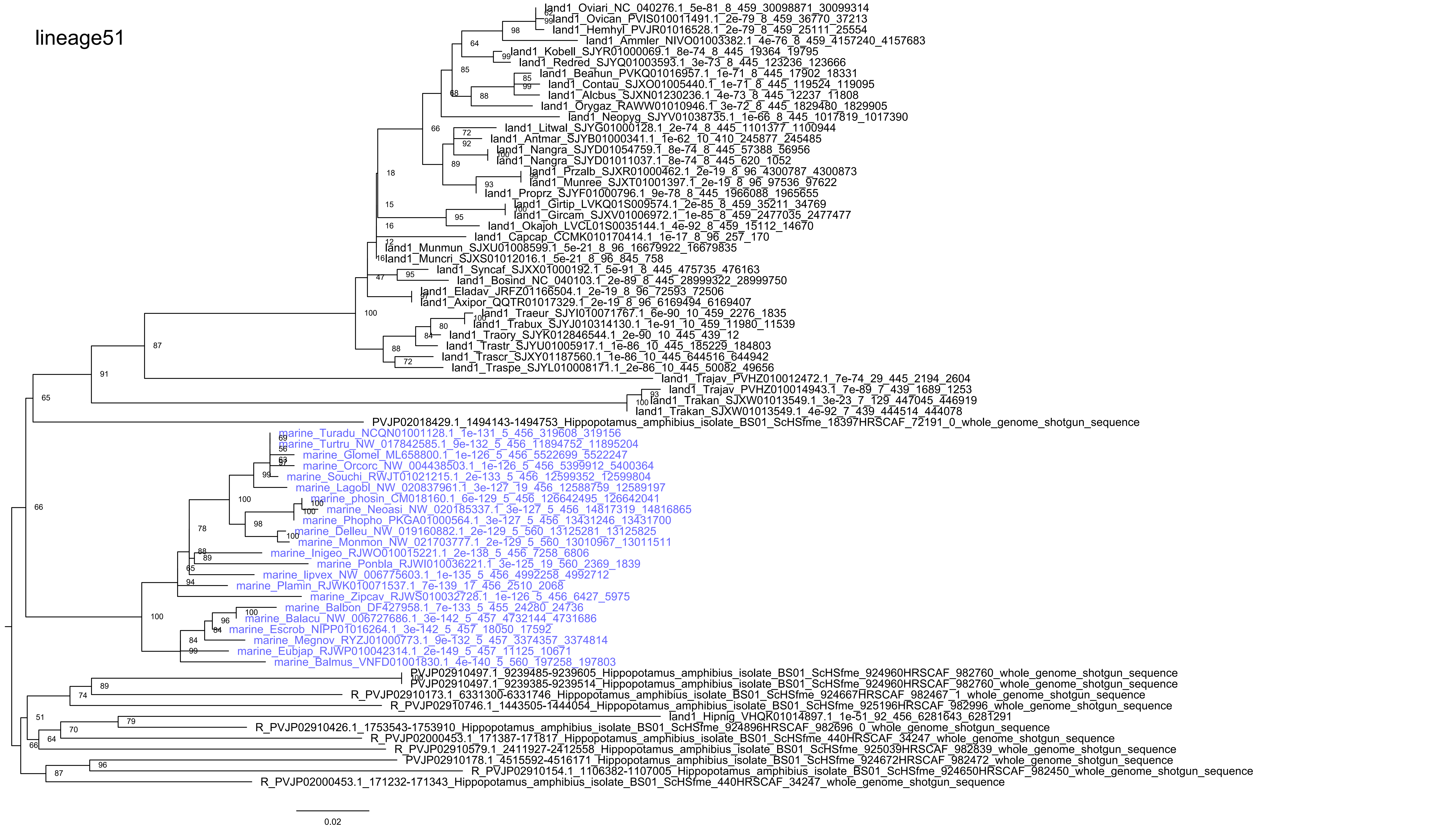
lineage51
land1_Oviari_NC_040276.1_5e-81_8_459_30098871_30099314
land1_Ovican_PVIS010011491.1_2e-79_8_459_36770_37213
land1_Hemhyl_PVJR01016528.1_2e-79_8_459_25111_25554
land1_Ammler_NIVO01003382.1_4e-76_8_459_4157240_4157683
land1_Kobell_SJYR01000069.1_8e-74_8_445_19364_19795
land1_Redred_SJYQ01003593.1_3e-73_8_445_123236_123666
land1_Beahun_PVKQ01016957.1_1e-71_8_445_17902_18331
land1_Contau_SJXO01005440.1_1e-71_8_445_119524_119095
land1_Alcbus_SJXN01230236.1_4e-73_8_445_12237_11808
land1_Orygaz_RAWW01010946.1_3e-72_8_445_1829480_1829905
land1_Neopyg_SJYV01038735.1_1e-66_8_445_1017819_1017390
land1_Litwal_SJYG01000128.1_2e-74_8_445_1101377_1100944
land1_Antmar_SJYB01000341.1_1e-62_10_410_245877_245485
land1_Nangra_SJYD01054759.1_8e-74_8_445_57388_56956
land1_Nangra_SJYD01011037.1_8e-74_8_445_620_1052
land1_Przalb_SJXR01000462.1_2e-19_8_96_4300787_4300873
land1_Munree_SJXT01001397.1_2e-19_8_96_97536_97622
land1_Proprz_SJYF01000796.1_9e-78_8_445_1966088_1965655
land1_Girtip_LVKQ01S009574.1_2e-85_8_459_35211_34769
land1_Gircam_SJXV01006972.1_1e-85_8_459_2477035_2477477
land1_Okajoh_LVCL01S0035144.1_4e-92_8_459_15112_14670
land1_Capcap_CCMK010170414.1_1e-17_8_96_257_170
land1_Munmun_SJXU01008599.1_5e-21_8_96_16679922_16679835
land1_Muncri_SJXS01012016.1_5e-21_8_96_845_758
land1_Syncaf_SJXX01000192.1_5e-91_8_445_475735_476163
land1_Bosind_NC_040103.1_2e-89_8_445_28999322_28999750
land1_Eladav_JRFZ01166504.1_2e-19_8_96_72593_72506
land1_Axipor_QQTR01017329.1_2e-19_8_96_6169494_6169407
land1_Traeur_SJYI010071767.1_6e-90_10_459_2276_1835
land1_Trabux_SJYJ010314130.1_1e-91_10_459_11980_11539
land1_Traory_SJYK012846544.1_2e-90_10_445_439_12
land1_Trastr_SJYU01005917.1_1e-86_10_445_185229_184803
land1_Trascr_SJXY01187560.1_1e-86_10_445_644516_644942
land1_Traspe_SJYL010008171.1_2e-86_10_445_50082_49656
land1_Trajav_PVHZ010012472.1_7e-74_29_445_2194_2604
land1_Trajav_PVHZ010014943.1_7e-89_7_439_1689_1253
land1_Trakan_SJXW01013549.1_3e-23_7_129_447045_446919
land1_Trakan_SJXW01013549.1_4e-92_7_439_444514_444078
PVJP02018429.1_1494143-1494753_Hippopotamus_amphibius_isolate_BS01_ScHSfme_18397HRSCAF_72191_0_whole_genome_shotgun_sequence
marine_Turadu_NCQN01001128.1_1e-131_5_456_319608_319156
marine_Turtru_NW_017842585.1_9e-132_5_456_11894752_11895204
marine_Glomel_ML658800.1_1e-126_5_456_5522699_5522247
marine_Orcorc_NW_004438503.1_1e-126_5_456_5399912_5400364
marine_Souchi_RWJT01021215.1_2e-133_5_456_12599352_12599804
marine_Lagobl_NW_020837961.1_3e-127_19_456_12588759_12589197
marine_phosin_CM018160.1_6e-129_5_456_126642495_126642041
marine_Neoasi_NW_020185337.1_3e-127_5_456_14817319_14816865
marine_Phopho_PKGA01000564.1_3e-127_5_456_13431246_13431700
marine_Delleu_NW_019160882.1_2e-129_5_560_13125281_13125825
marine_Monmon_NW_021703777.1_2e-129_5_560_13010967_13011511
marine_Inigeo_RJWO010015221.1_2e-138_5_456_7258_6806
marine_Ponbla_RJWI010036221.1_3e-125_19_560_2369_1839
marine_lipvex_NW_006775603.1_1e-135_5_456_4992258_4992712
marine_Plamin_RJWK010071537.1_7e-139_17_456_2510_2068
marine_Zipcav_RJWS010032728.1_1e-126_5_456_6427_5975
marine_Balbon_DF427958.1_7e-133_5_455_24280_24736
marine_Balacu_NW_006727686.1_3e-142_5_457_4732144_4731686
marine_Escrob_NIPP01016264.1_3e-142_5_457_18050_17592
marine_Megnov_RYZJ01000773.1_9e-132_5_457_3374357_3374814
marine_Eubjap_RJWP010042314.1_2e-149_5_457_11125_10671
marine_Balmus_VNFD01001830.1_4e-140_5_560_197258_197803
PVJP02910497.1_9239485-9239605_Hippopotamus_amphibius_isolate_BS01_ScHSfme_924960HRSCAF_982760_whole_genome_shotgun_sequence
PVJP02910497.1_9239385-9239514_Hippopotamus_amphibius_isolate_BS01_ScHSfme_924960HRSCAF_982760_whole_genome_shotgun_sequence
R_PVJP02910173.1_6331300-6331746_Hippopotamus_amphibius_isolate_BS01_ScHSfme_924667HRSCAF_982467_1_whole_genome_shotgun_sequence
R_PVJP02910746.1_1443505-1444054_Hippopotamus_amphibius_isolate_BS01_ScHSfme_925196HRSCAF_982996_whole_genome_shotgun_sequence
land1_Hipnig_VHQK01014897.1_1e-51_92_456_6281643_6281291
R_PVJP02910426.1_1753543-1753910_Hippopotamus_amphibius_isolate_BS01_ScHSfme_924896HRSCAF_982696_0_whole_genome_shotgun_sequence
R_PVJP02000453.1_171387-171817_Hippopotamus_amphibius_isolate_BS01_ScHSfme_440HRSCAF_34247_whole_genome_shotgun_sequence
R_PVJP02910579.1_2411927-2412558_Hippopotamus_amphibius_isolate_BS01_ScHSfme_925039HRSCAF_982839_whole_genome_shotgun_sequence
PVJP02910178.1_4515592-4516171_Hippopotamus_amphibius_isolate_BS01_ScHSfme_924672HRSCAF_982472_whole_genome_shotgun_sequence
R_PVJP02910154.1_1106382-1107005_Hippopotamus_amphibius_isolate_BS01_ScHSfme_924650HRSCAF_982450_whole_genome_shotgun_sequence
R_PVJP02000453.1_171232-171343_Hippopotamus_amphibius_isolate_BS01_ScHSfme_440HRSCAF_34247_whole_genome_shotgun_sequence
0.02

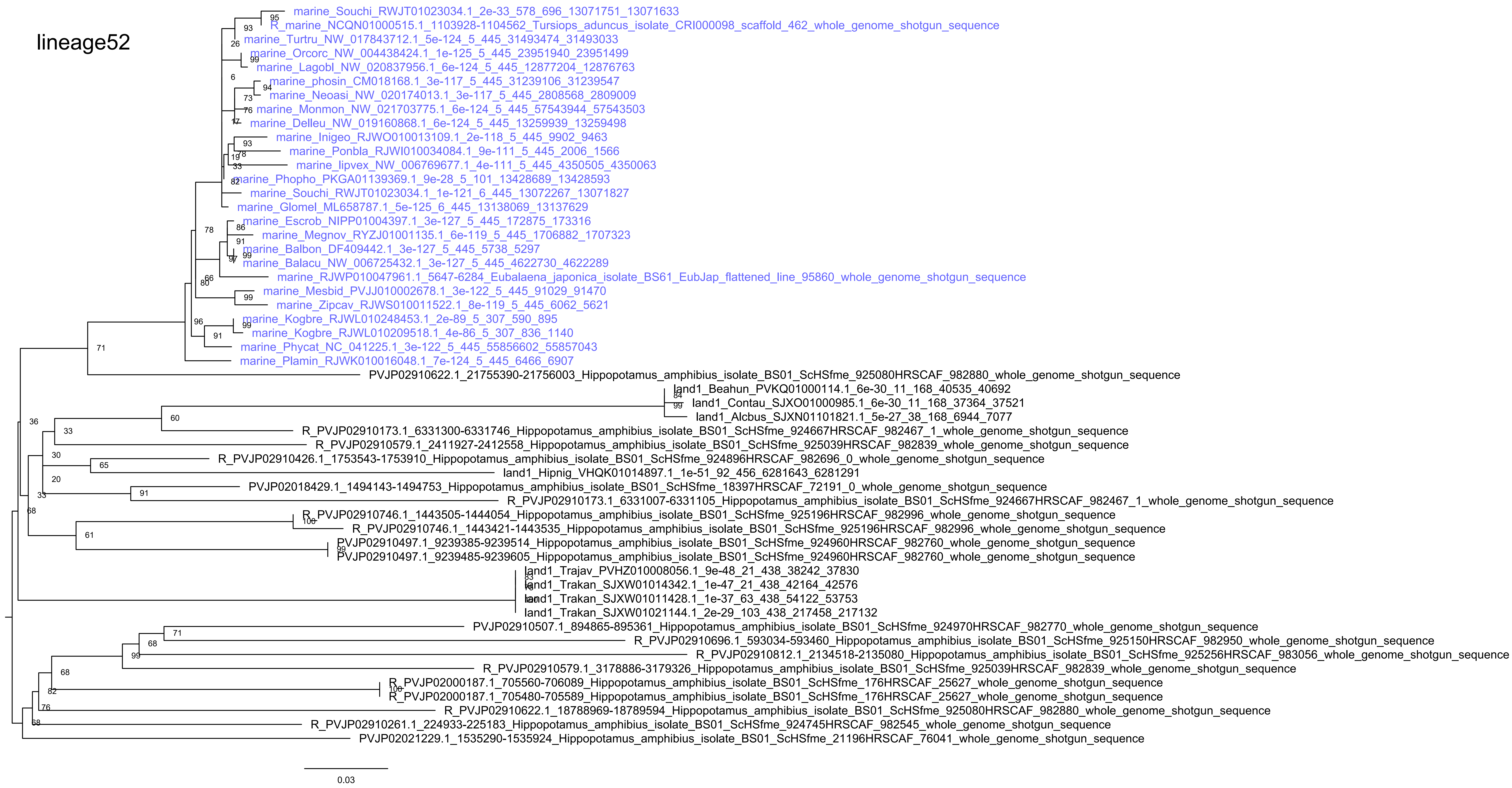
lineage52
marine_Souchi_RWJT01023034.1_2e-33_578_696_13071751_13071633
R_marine_NCQN01000515.1_1103928-1104562_Tursiops_aduncus_isolate_CRI000098_scaffold_462_whole_genome_shotgun_sequence
marine_Turtru_NW_017843712.1_5e-124_5_445_31493474_31493033
marine_Orcorc_NW_004438424.1_1e-125_5_445_23951940_23951499
marine_Lagobl_NW_020837956.1_6e-124_5_445_12877204_12876763
marine_phosin_CM018168.1_3e-117_5_445_31239106_31239547
marine_Neoasi_NW_020174013.1_3e-117_5_445_2808568_2809009
marine_Monmon_NW_021703775.1_6e-124_5_445_57543944_57543503
marine_Delleu_NW_019160868.1_6e-124_5_445_13259939_13259498
marine_Inigeo_RJWO010013109.1_2e-118_5_445_9902_9463
marine_Ponbla_RJWI010034084.1_9e-111_5_445_2006_1566
marine_lipvex_NW_006769677.1_4e-111_5_445_4350505_4350063
marine_Phopho_PKGA01139369.1_9e-28_5_101_13428689_13428593
marine_Souchi_RWJT01023034.1_1e-121_6_445_13072267_13071827
marine_Glomel_ML658787.1_5e-125_6_445_13138069_13137629
marine_Escrob_NIPP01004397.1_3e-127_5_445_172875_173316
marine_Megnov_RYZJ01001135.1_6e-119_5_445_1706882_1707323
marine_Balbon_DF409442.1_3e-127_5_445_5738_5297
marine_Balacu_NW_006725432.1_3e-127_5_445_4622730_4622289
marine_RJWP010047961.1_5647-6284_Eubalaena_japonica_isolate_BS61_EubJap_flattened_line_95860_whole_genome_shotgun_sequence
marine_Mesbid_PVJJ010002678.1_3e-122_5_445_91029_91470
marine_Zipcav_RJWS010011522.1_8e-119_5_445_6062_5621
marine_Kogbre_RJWL010248453.1_2e-89_5_307_590_895
marine_Kogbre_RJWL010209518.1_4e-86_5_307_836_1140
marine_Phycat_NC_041225.1_3e-122_5_445_55856602_55857043
marine_Plamin_RJWK010016048.1_7e-124_5_445_6466_6907
PVJP02910622.1_21755390-21756003_Hippopotamus_amphibius_isolate_BS01_ScHSfme_925080HRSCAF_982880_whole_genome_shotgun_sequence
land1_Beahun_PVKQ01000114.1_6e-30_11_168_40535_40692
land1_Contau_SJXO01000985.1_6e-30_11_168_37364_37521
land1_Alcbus_SJXN01101821.1_5e-27_38_168_6944_7077
R_PVJP02910173.1_6331300-6331746_Hippopotamus_amphibius_isolate_BS01_ScHSfme_924667HRSCAF_982467_1_whole_genome_shotgun_sequence
R_PVJP02910579.1_2411927-2412558_Hippopotamus_amphibius_isolate_BS01_ScHSfme_925039HRSCAF_982839_whole_genome_shotgun_sequence
R_PVJP02910426.1_1753543-1753910_Hippopotamus_amphibius_isolate_BS01_ScHSfme_924896HRSCAF_982696_0_whole_genome_shotgun_sequence
land1_Hipnig_VHQK01014897.1_1e-51_92_456_6281643_6281291
PVJP02018429.1_1494143-1494753_Hippopotamus_amphibius_isolate_BS01_ScHSfme_18397HRSCAF_72191_0_whole_genome_shotgun_sequence
R_PVJP02910173.1_6331007-6331105_Hippopotamus_amphibius_isolate_BS01_ScHSfme_924667HRSCAF_982467_1_whole_genome_shotgun_sequence
R_PVJP02910746.1_1443505-1444054_Hippopotamus_amphibius_isolate_BS01_ScHSfme_925196HRSCAF_982996_whole_genome_shotgun_sequence
R_PVJP02910746.1_1443421-1443535_Hippopotamus_amphibius_isolate_BS01_ScHSfme_925196HRSCAF_982996_whole_genome_shotgun_sequence
PVJP02910497.1_9239385-9239514_Hippopotamus_amphibius_isolate_BS01_ScHSfme_924960HRSCAF_982760_whole_genome_shotgun_sequence
PVJP02910497.1_9239485-9239605_Hippopotamus_amphibius_isolate_BS01_ScHSfme_924960HRSCAF_982760_whole_genome_shotgun_sequence
land1_Trajav_PVHZ010008056.1_9e-48_21_438_38242_37830
land1_Trakan_SJXW01014342.1_1e-47_21_438_42164_42576
land1_Trakan_SJXW01011428.1_1e-37_63_438_54122_53753
land1_Trakan_SJXW01021144.1_2e-29_103_438_217458_217132
PVJP02910507.1_894865-895361_Hippopotamus_amphibius_isolate_BS01_ScHSfme_924970HRSCAF_982770_whole_genome_shotgun_sequence
R_PVJP02910696.1_593034-593460_Hippopotamus_amphibius_isolate_BS01_ScHSfme_925150HRSCAF_982950_whole_genome_shotgun_sequence
R_PVJP02910812.1_2134518-2135080_Hippopotamus_amphibius_isolate_BS01_ScHSfme_925256HRSCAF_983056_whole_genome_shotgun_sequence
R_PVJP02910579.1_3178886-3179326_Hippopotamus_amphibius_isolate_BS01_ScHSfme_925039HRSCAF_982839_whole_genome_shotgun_sequence
R_PVJP02000187.1_705560-706089_Hippopotamus_amphibius_isolate_BS01_ScHSfme_176HRSCAF_25627_whole_genome_shotgun_sequence
R_PVJP02000187.1_705480-705589_Hippopotamus_amphibius_isolate_BS01_ScHSfme_176HRSCAF_25627_whole_genome_shotgun_sequence
R_PVJP02910622.1_18788969-18789594_Hippopotamus_amphibius_isolate_BS01_ScHSfme_925080HRSCAF_982880_whole_genome_shotgun_sequence
R_PVJP02910261.1_224933-225183_Hippopotamus_amphibius_isolate_BS01_ScHSfme_924745HRSCAF_982545_whole_genome_shotgun_sequence
PVJP02021229.1_1535290-1535924_Hippopotamus_amphibius_isolate_BS01_ScHSfme_21196HRSCAF_76041_whole_genome_shotgun_sequence
0.03

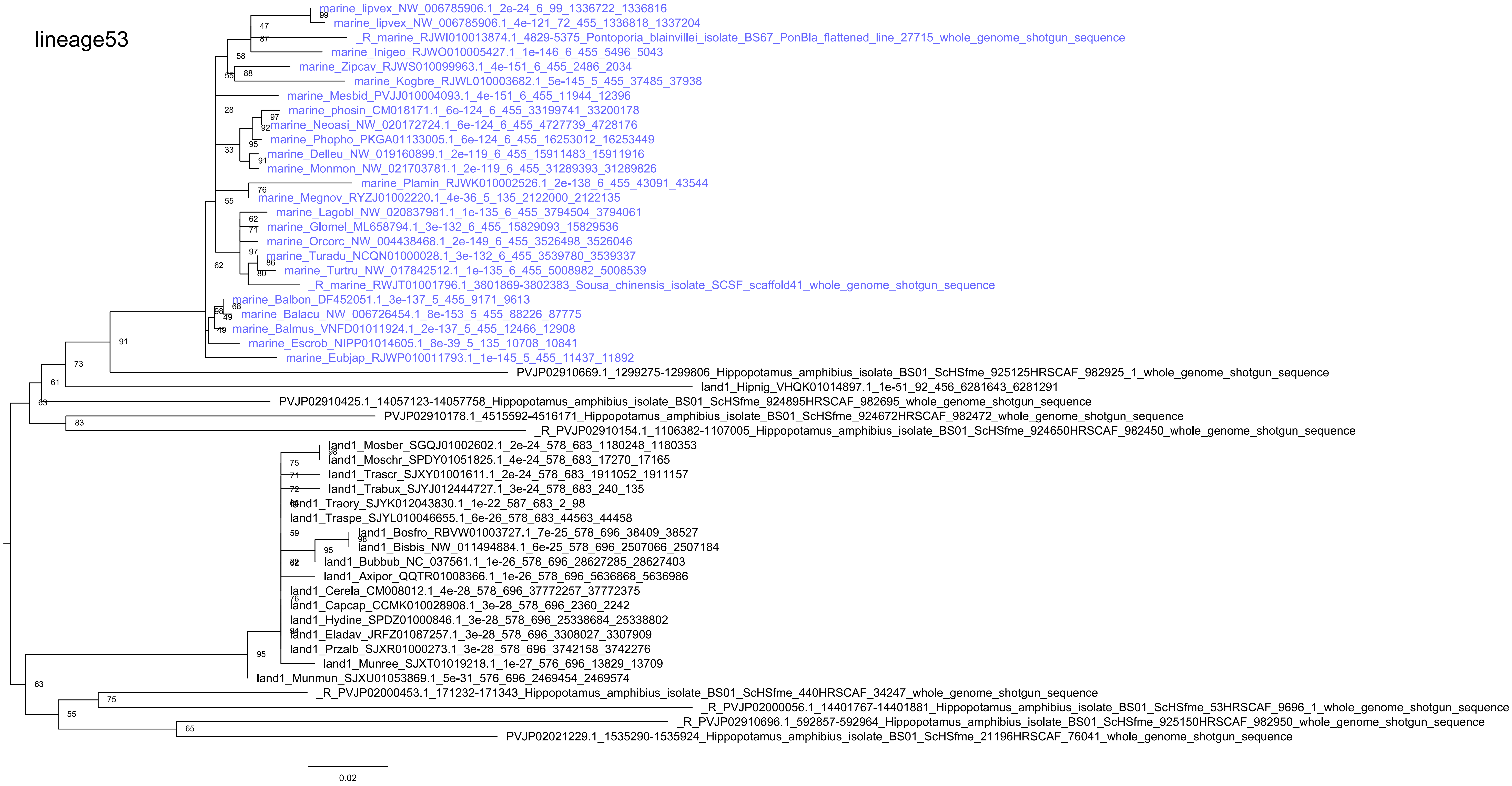
lineage53
marine_lipvex_NW_006785906.1_2e-24_6_99_1336722_1336816
marine_lipvex_NW_006785906.1_4e-121_72_455_1336818_1337204
_R_marine_RJWI010013874.1_4829-5375_Pontoporia_blainvillei_isolate_BS67_PonBla_flattened_line_27715_whole_genome_shotgun_sequence
marine_Inigeo_RJWO010005427.1_1e-146_6_455_5496_5043
marine_Zipcav_RJWS010099963.1_4e-151_6_455_2486_2034
marine_Kogbre_RJWL010003682.1_5e-145_5_455_37485_37938
marine_Mesbid_PVJJ010004093.1_4e-151_6_455_11944_12396
marine_phosin_CM018171.1_6e-124_6_455_33199741_33200178
marine_Neoasi_NW_020172724.1_6e-124_6_455_4727739_4728176
marine_Phopho_PKGA01133005.1_6e-124_6_455_16253012_16253449
marine_Delleu_NW_019160899.1_2e-119_6_455_15911483_15911916
marine_Monmon_NW_021703781.1_2e-119_6_455_31289393_31289826
marine_Plamin_RJWK010002526.1_2e-138_6_455_43091_43544
marine_Megnov_RYZJ01002220.1_4e-36_5_135_2122000_2122135
marine_Lagobl_NW_020837981.1_1e-135_6_455_3794504_3794061
marine_Glomel_ML658794.1_3e-132_6_455_15829093_15829536
marine_Orcorc_NW_004438468.1_2e-149_6_455_3526498_3526046
marine_Turadu_NCQN01000028.1_3e-132_6_455_3539780_3539337
marine_Turtru_NW_017842512.1_1e-135_6_455_5008982_5008539
_R_marine_RWJT01001796.1_3801869-3802383_Sousa_chinensis_isolate_SCSF_scaffold41_whole_genome_shotgun_sequence
marine_Balbon_DF452051.1_3e-137_5_455_9171_9613
marine_Balacu_NW_006726454.1_8e-153_5_455_88226_87775
marine_Balmus_VNFD01011924.1_2e-137_5_455_12466_12908
marine_Escrob_NIPP01014605.1_8e-39_5_135_10708_10841
marine_Eubjap_RJWP010011793.1_1e-145_5_455_11437_11892
PVJP02910669.1_1299275-1299806_Hippopotamus_amphibius_isolate_BS01_ScHSfme_925125HRSCAF_982925_1_whole_genome_shotgun_sequence
land1_Hipnig_VHQK01014897.1_1e-51_92_456_6281643_6281291
PVJP02910425.1_14057123-14057758_Hippopotamus_amphibius_isolate_BS01_ScHSfme_924895HRSCAF_982695_whole_genome_shotgun_sequence
PVJP02910178.1_4515592-4516171_Hippopotamus_amphibius_isolate_BS01_ScHSfme_924672HRSCAF_982472_whole_genome_shotgun_sequence
_R_PVJP02910154.1_1106382-1107005_Hippopotamus_amphibius_isolate_BS01_ScHSfme_924650HRSCAF_982450_whole_genome_shotgun_sequence
land1_Mosber_SGQJ01002602.1_2e-24_578_683_1180248_1180353
land1_Moschr_SPDY01051825.1_4e-24_578_683_17270_17165
land1_Trascr_SJXY01001611.1_2e-24_578_683_1911052_1911157
land1_Trabux_SJYJ012444727.1_3e-24_578_683_240_135
land1_Traory_SJYK012043830.1_1e-22_587_683_2_98
land1_Traspe_SJYL010046655.1_6e-26_578_683_44563_44458
land1_Bosfro_RBVW01003727.1_7e-25_578_696_38409_38527
land1_Bisbis_NW_011494884.1_6e-25_578_696_2507066_2507184
land1_Bubbub_NC_037561.1_1e-26_578_696_28627285_28627403
land1_Axipor_QQTR01008366.1_1e-26_578_696_5636868_5636986
land1_Cerela_CM008012.1_4e-28_578_696_37772257_37772375
land1_Capcap_CCMK010028908.1_3e-28_578_696_2360_2242
land1_Hydine_SPDZ01000846.1_3e-28_578_696_25338684_25338802
land1_Eladav_JRFZ01087257.1_3e-28_578_696_3308027_3307909
land1_Przalb_SJXR01000273.1_3e-28_578_696_3742158_3742276
land1_Munree_SJXT01019218.1_1e-27_576_696_13829_13709
land1_Munmun_SJXU01053869.1_5e-31_576_696_2469454_2469574
_R_PVJP02000453.1_171232-171343_Hippopotamus_amphibius_isolate_BS01_ScHSfme_440HRSCAF_34247_whole_genome_shotgun_sequence
_R_PVJP02000056.1_14401767-14401881_Hippopotamus_amphibius_isolate_BS01_ScHSfme_53HRSCAF_9696_1_whole_genome_shotgun_sequence
_R_PVJP02910696.1_592857-592964_Hippopotamus_amphibius_isolate_BS01_ScHSfme_925150HRSCAF_982950_whole_genome_shotgun_sequence
PVJP02021229.1_1535290-1535924_Hippopotamus_amphibius_isolate_BS01_ScHSfme_21196HRSCAF_76041_whole_genome_shotgun_sequence
0.02

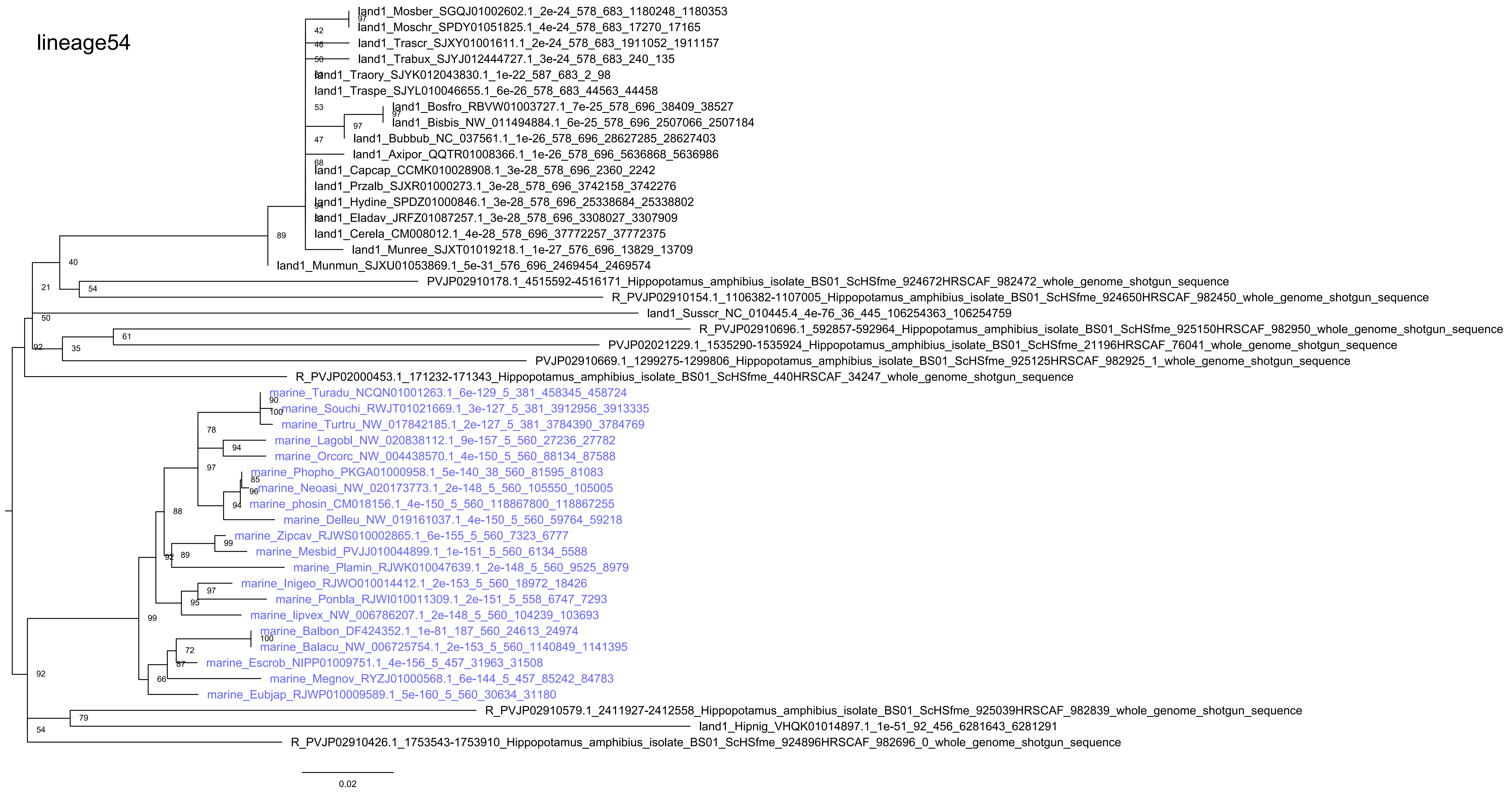
lineage54
land1_Mosber_SGQJ01002602.1_2e-24_578_683_1180248_1180353
land1_Moschr_SPDY01051825.1_4e-24_578_683_17270_17165
land1_Trascr_SJXY01001611.1_2e-24_578_683_1911052_1911157
land1_Trabux_SJYJ012444727.1_3e-24_578_683_240_135
land1_Traory_SJYK012043830.1_1e-22_587_683_2_98
land1_Traspe_SJYL010046655.1_6e-26_578_683_44563_44458
land1_Bosfro_RBVW01003727.1_7e-25_578_696_38409_38527
land1_Bisbis_NW_011494884.1_6e-25_578_696_2507066_2507184
land1_Bubbub_NC_037561.1_1e-26_578_696_28627285_28627403
land1_Axipor_QQTR01008366.1_1e-26_578_696_5636868_5636986
land1_Capcap_CCMK010028908.1_3e-28_578_696_2360_2242
land1_Przalb_SJXR01000273.1_3e-28_578_696_3742158_3742276
land1_Hydine_SPDZ01000846.1_3e-28_578_696_25338684_25338802
land1_Eladav_JRFZ01087257.1_3e-28_578_696_3308027_3307909
land1_Cerela_CM008012.1_4e-28_578_696_37772257_37772375
land1_Munree_SJXT01019218.1_1e-27_576_696_13829_13709
land1_Munmun_SJXU01053869.1_5e-31_576_696_2469454_2469574
PVJP02910178.1_4515592-4516171_Hippopotamus_amphibius_isolate_BS01_ScHSfme_924672HRSCAF_982472_whole_genome_shotgun_sequence
R_PVJP02910154.1_1106382-1107005_Hippopotamus_amphibius_isolate_BS01_ScHSfme_924650HRSCAF_982450_whole_genome_shotgun_sequence
land1_Susscr_NC_010445.4_4e-76_36_445_106254363_106254759
R_PVJP02910696.1_592857-592964_Hippopotamus_amphibius_isolate_BS01_ScHSfme_925150HRSCAF_982950_whole_genome_shotgun_sequence
PVJP02021229.1_1535290-1535924_Hippopotamus_amphibius_isolate_BS01_ScHSfme_21196HRSCAF_76041_whole_genome_shotgun_sequence
PVJP02910669.1_1299275-1299806_Hippopotamus_amphibius_isolate_BS01_ScHSfme_925125HRSCAF_982925_1_whole_genome_shotgun_sequence
R_PVJP02000453.1_171232-171343_Hippopotamus_amphibius_isolate_BS01_ScHSfme_440HRSCAF_34247_whole_genome_shotgun_sequence
marine_Turadu_NCQN01001263.1_6e-129_5_381_458345_458724
marine_Souchi_RWJT01021669.1_3e-127_5_381_3912956_3913335
marine_Turtru_NW_017842185.1_2e-127_5_381_3784390_3784769
marine_Lagobl_NW_020838112.1_9e-157_5_560_27236_27782
marine_Orcorc_NW_004438570.1_4e-150_5_560_88134_87588
marine_Phopho_PKGA01000958.1_5e-140_38_560_81595_81083
marine_Neoasi_NW_020173773.1_2e-148_5_560_105550_105005
marine_phosin_CM018156.1_4e-150_5_560_118867800_118867255
marine_Delleu_NW_019161037.1_4e-150_5_560_59764_59218
marine_Zipcav_RJWS010002865.1_6e-155_5_560_7323_6777
marine_Mesbid_PVJJ010044899.1_1e-151_5_560_6134_5588
marine_Plamin_RJWK010047639.1_2e-148_5_560_9525_8979
marine_Inigeo_RJWO010014412.1_2e-153_5_560_18972_18426
marine_Ponbla_RJWI010011309.1_2e-151_5_558_6747_7293
marine_lipvex_NW_006786207.1_2e-148_5_560_104239_103693
marine_Balbon_DF424352.1_1e-81_187_560_24613_24974
marine_Balacu_NW_006725754.1_2e-153_5_560_1140849_1141395
marine_Escrob_NIPP01009751.1_4e-156_5_457_31963_31508
marine_Megnov_RYZJ01000568.1_6e-144_5_457_85242_84783
marine_Eubjap_RJWP010009589.1_5e-160_5_560_30634_31180
R_PVJP02910579.1_2411927-2412558_Hippopotamus_amphibius_isolate_BS01_ScHSfme_925039HRSCAF_982839_whole_genome_shotgun_sequence
land1_Hipnig_VHQK01014897.1_1e-51_92_456_6281643_6281291
R_PVJP02910426.1_1753543-1753910_Hippopotamus_amphibius_isolate_BS01_ScHSfme_924896HRSCAF_982696_0_whole_genome_shotgun_sequence
0.02

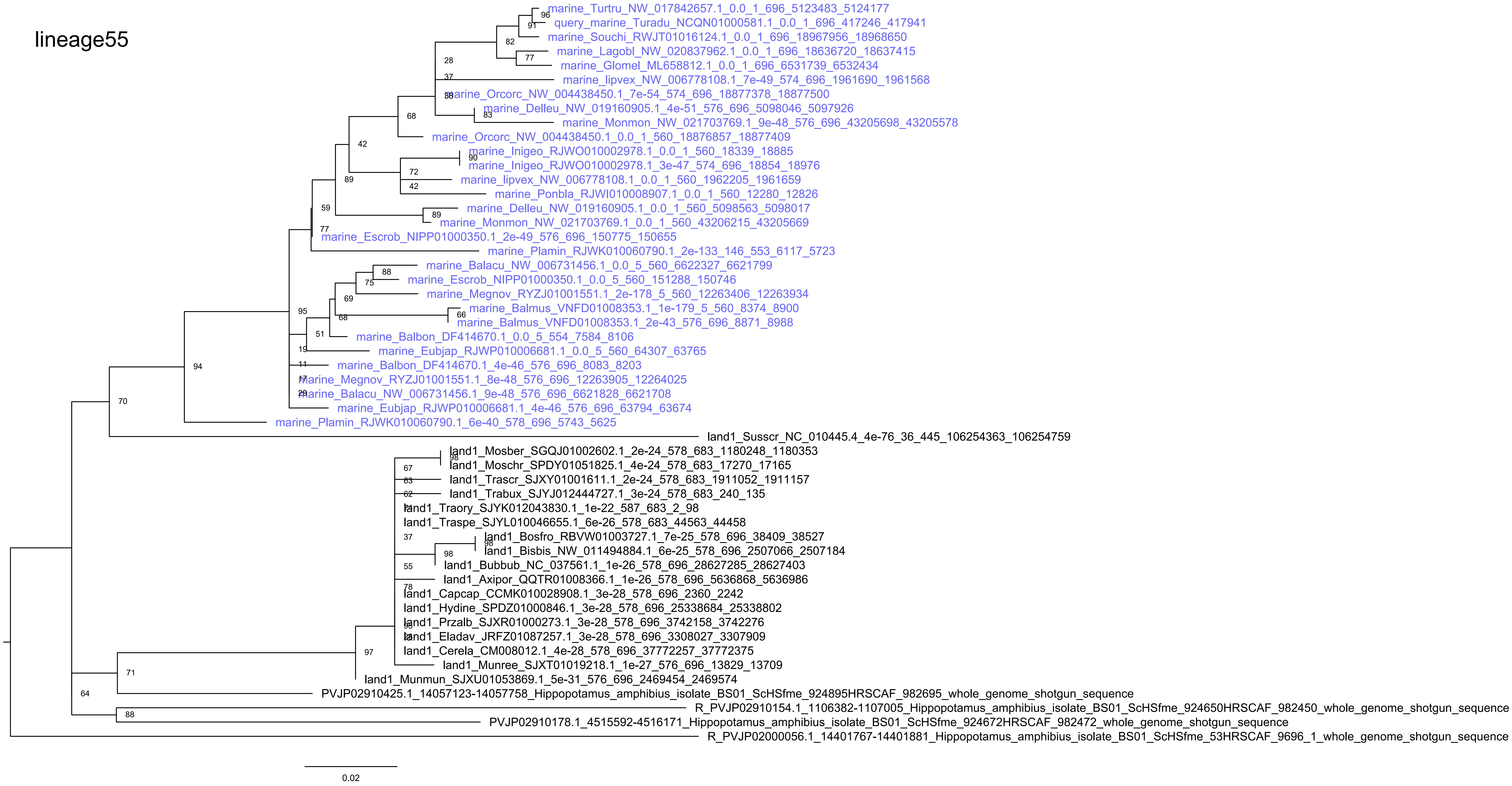
lineage55
marine_Turtru_NW_017842657.1_0.0_1_696_5123483_5124177
query_marine_Turadu_NCQN01000581.1_0.0_1_696_417246_417941
marine_Souchi_RWJT01016124.1_0.0_1_696_18967956_18968650
marine_Lagobl_NW_020837962.1_0.0_1_696_18636720_18637415
marine_Glomel_ML658812.1_0.0_1_696_6531739_6532434
marine_lipvex_NW_006778108.1_7e-49_574_696_1961690_1961568
marine_Orcorc_NW_004438450.1_7e-54_574_696_18877378_18877500
marine_Delleu_NW_019160905.1_4e-51_576_696_5098046_5097926
marine_Monmon_NW_021703769.1_9e-48_576_696_43205698_43205578
marine_Orcorc_NW_004438450.1_0.0_1_560_18876857_18877409
marine_Inigeo_RJWO010002978.1_0.0_1_560_18339_18885
marine_Inigeo_RJWO010002978.1_3e-47_574_696_18854_18976
marine_lipvex_NW_006778108.1_0.0_1_560_1962205_1961659
marine_Ponbla_RJWI010008907.1_0.0_1_560_12280_12826
marine_Delleu_NW_019160905.1_0.0_1_560_5098563_5098017
marine_Monmon_NW_021703769.1_0.0_1_560_43206215_43205669
marine_Escrob_NIPP01000350.1_2e-49_576_696_150775_150655
marine_Plamin_RJWK010060790.1_2e-133_146_553_6117_5723
marine_Balacu_NW_006731456.1_0.0_5_560_6622327_6621799
marine_Escrob_NIPP01000350.1_0.0_5_560_151288_150746
marine_Megnov_RYZJ01001551.1_2e-178_5_560_12263406_12263934
marine_Balmus_VNFD01008353.1_1e-179_5_560_8374_8900
marine_Balmus_VNFD01008353.1_2e-43_576_696_8871_8988
marine_Balbon_DF414670.1_0.0_5_554_7584_8106
marine_Eubjap_RJWP010006681.1_0.0_5_560_64307_63765
marine_Balbon_DF414670.1_4e-46_576_696_8083_8203
marine_Megnov_RYZJ01001551.1_8e-48_576_696_12263905_12264025
marine_Balacu_NW_006731456.1_9e-48_576_696_6621828_6621708
marine_Eubjap_RJWP010006681.1_4e-46_576_696_63794_63674
marine_Plamin_RJWK010060790.1_6e-40_578_696_5743_5625
land1_Susscr_NC_010445.4_4e-76_36_445_106254363_106254759
land1_Mosber_SGQJ01002602.1_2e-24_578_683_1180248_1180353
land1_Moschr_SPDY01051825.1_4e-24_578_683_17270_17165
land1_Trascr_SJXY01001611.1_2e-24_578_683_1911052_1911157
land1_Trabux_SJYJ012444727.1_3e-24_578_683_240_135
land1_Traory_SJYK012043830.1_1e-22_587_683_2_98
land1_Traspe_SJYL010046655.1_6e-26_578_683_44563_44458
land1_Bosfro_RBVW01003727.1_7e-25_578_696_38409_38527
land1_Bisbis_NW_011494884.1_6e-25_578_696_2507066_2507184
land1_Bubbub_NC_037561.1_1e-26_578_696_28627285_28627403
land1_Axipor_QQTR01008366.1_1e-26_578_696_5636868_5636986
land1_Capcap_CCMK010028908.1_3e-28_578_696_2360_2242
land1_Hydine_SPDZ01000846.1_3e-28_578_696_25338684_25338802
land1_Przalb_SJXR01000273.1_3e-28_578_696_3742158_3742276
land1_Eladav_JRFZ01087257.1_3e-28_578_696_3308027_3307909
land1_Cerela_CM008012.1_4e-28_578_696_37772257_37772375
land1_Munree_SJXT01019218.1_1e-27_576_696_13829_13709
land1_Munmun_SJXU01053869.1_5e-31_576_696_2469454_2469574
PVJP02910425.1_14057123-14057758_Hippopotamus_amphibius_isolate_BS01_ScHSfme_924895HRSCAF_982695_whole_genome_shotgun_sequence
R_PVJP02910154.1_1106382-1107005_Hippopotamus_amphibius_isolate_BS01_ScHSfme_924650HRSCAF_982450_whole_genome_shotgun_sequence
PVJP02910178.1_4515592-4516171_Hippopotamus_amphibius_isolate_BS01_ScHSfme_924672HRSCAF_982472_whole_genome_shotgun_sequence
R_PVJP02000056.1_14401767-14401881_Hippopotamus_amphibius_isolate_BS01_ScHSfme_53HRSCAF_9696_1_whole_genome_shotgun_sequence
0.02

lineage56

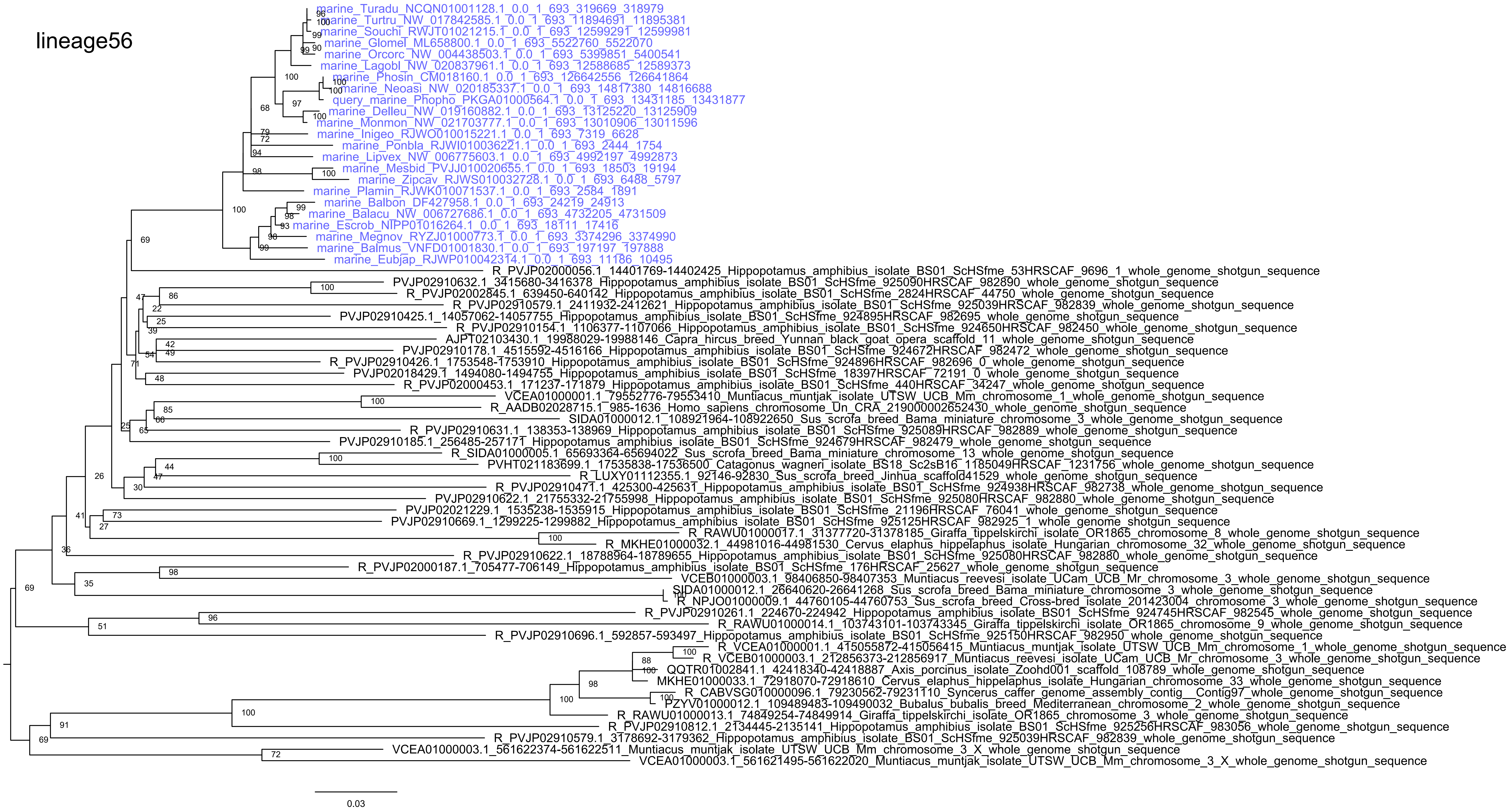

marine_Turadu_NCQN01001128.1_0.0_1_693_319669_318979
marine_Turtru_NW_017842585.1_0.0_1_693_11894691_11895381
marine_Souchi_RWJT01021215.1_0.0_1_693_12599291_12599981
marine_Glomel_ML658800.1_0.0_1_693_5522760_5522070
marine_Orcorc_NW_004438503.1_0.0_1_693_5399851_5400541
marine_Lagobl_NW_020837961.1_0.0_1_693_12588685_12589373
marine_Phosin_CM018160.1_0.0_1_693_126642556_126641864
marine_Neoasi_NW_020185337.1_0.0_1_693_14817380_14816688
query_marine_Phopho_PKGA01000564.1_0.0_1_693_13431185_13431877
marine_Delleu_NW_019160882.1_0.0_1_693_13125220_13125909
marine_Monmon_NW_021703777.1_0.0_1_693_13010906_13011596
marine_Inigeo_RJWO010015221.1_0.0_1_693_7319_6628
marine_Ponbla_RJWI010036221.1_0.0_1_693_2444_1754
marine_Lipvex_NW_006775603.1_0.0_1_693_4992197_4992873
marine_Mesbid_PVJJ010020655.1_0.0_1_693_18503_19194
marine_Zipcav_RJWS010032728.1_0.0_1_693_6488_5797
marine_Plamin_RJWK010071537.1_0.0_1_693_2584_1891
marine_Balbon_DF427958.1_0.0_1_693_24219_24913
marine_Balacu_NW_006727686.1_0.0_1_693_4732205_4731509
marine_Escrob_NIPP01016264.1_0.0_1_693_18111_17416
marine_Megnov_RYZJ01000773.1_0.0_1_693_3374296_3374990
marine_Balmus_VNFD01001830.1_0.0_1_693_197197_197888
marine_Eubjap_RJWP010042314.1_0.0_1_693_11186_10495
R_PVJP02000056.1_14401769-14402425_Hippopotamus_amphibius_isolate_BS01_ScHSfme_53HRSCAF_9696_1_whole_genome_shotgun_sequence
PVJP02910632.1_3415680-3416378_Hippopotamus_amphibius_isolate_BS01_ScHSfme_925090HRSCAF_982890_whole_genome_shotgun_sequence
R_PVJP02002845.1_639450-640142_Hippopotamus_amphibius_isolate_BS01_ScHSfme_2824HRSCAF_44750_whole_genome_shotgun_sequence
R_PVJP02910579.1_2411932-2412621_Hippopotamus_amphibius_isolate_BS01_ScHSfme_925039HRSCAF_982839_whole_genome_shotgun_sequence
PVJP02910425.1_14057062-14057755_Hippopotamus_amphibius_isolate_BS01_ScHSfme_924895HRSCAF_982695_whole_genome_shotgun_sequence
R_PVJP02910154.1_1106377-1107066_Hippopotamus_amphibius_isolate_BS01_ScHSfme_924650HRSCAF_982450_whole_genome_shotgun_sequence
AJPT02103430.1_19988029-19988146_Capra_hircus_breed_Yunnan_black_goat_opera_scaffold_11_whole_genome_shotgun_sequence
PVJP02910178.1_4515592-4516166_Hippopotamus_amphibius_isolate_BS01_ScHSfme_924672HRSCAF_982472_whole_genome_shotgun_sequence
R_PVJP02910426.1_1753548-1753910_Hippopotamus_amphibius_isolate_BS01_ScHSfme_924896HRSCAF_982696_0_whole_genome_shotgun_sequence
PVJP02018429.1_1494080-1494755_Hippopotamus_amphibius_isolate_BS01_ScHSfme_18397HRSCAF_72191_0_whole_genome_shotgun_sequence
R_PVJP02000453.1_171237-171879_Hippopotamus_amphibius_isolate_BS01_ScHSfme_440HRSCAF_34247_whole_genome_shotgun_sequence
VCEA01000001.1_79552776-79553410_Muntiacus_muntjak_isolate_UTSW_UCB_Mm_chromosome_1_whole_genome_shotgun_sequence
R_AADB02028715.1_985-1636_Homo_sapiens_chromosome_Un_CRA_219000002652430_whole_genome_shotgun_sequence
SIDA01000012.1_108921964-108922650_Sus_scrofa_breed_Bama_miniature_chromosome_3_whole_genome_shotgun_sequence
R_PVJP02910631.1_138353-138969_Hippopotamus_amphibius_isolate_BS01_ScHSfme_925089HRSCAF_982889_whole_genome_shotgun_sequence
PVJP02910185.1_256485-257171_Hippopotamus_amphibius_isolate_BS01_ScHSfme_924679HRSCAF_982479_whole_genome_shotgun_sequence
R_SIDA01000005.1_65693364-65694022_Sus_scrofa_breed_Bama_miniature_chromosome_13_whole_genome_shotgun_sequence
PVHT021183699.1_17535838-17536500_Catagonus_wagneri_isolate_BS18_Sc2sB16_1185049HRSCAF_1231756_whole_genome_shotgun_sequence
R_LUXY01112355.1_92146-92830_Sus_scrofa_breed_Jinhua_scaffold41529_whole_genome_shotgun_sequence
R_PVJP02910471.1_425300-425631_Hippopotamus_amphibius_isolate_BS01_ScHSfme_924938HRSCAF_982738_whole_genome_shotgun_sequence
PVJP02910622.1_21755332-21755998_Hippopotamus_amphibius_isolate_BS01_ScHSfme_925080HRSCAF_982880_whole_genome_shotgun_sequence
PVJP02021229.1_1535238-1535915_Hippopotamus_amphibius_isolate_BS01_ScHSfme_21196HRSCAF_76041_whole_genome_shotgun_sequence
PVJP02910669.1_1299225-1299882_Hippopotamus_amphibius_isolate_BS01_ScHSfme_925125HRSCAF_982925_1_whole_genome_shotgun_sequence
R_RAWU01000017.1_31377720-31378185_Giraffa_tippelskirchi_isolate_OR1865_chromosome_8_whole_genome_shotgun_sequence
R_MKHE01000032.1_44981016-44981530_Cervus_elaphus_hippelaphus_isolate_Hungarian_chromosome_32_whole_genome_shotgun_sequence
R_PVJP02910622.1_18788964-18789655_Hippopotamus_amphibius_isolate_BS01_ScHSfme_925080HRSCAF_982880_whole_genome_shotgun_sequence
R_PVJP02000187.1_705477-706149_Hippopotamus_amphibius_isolate_BS01_ScHSfme_176HRSCAF_25627_whole_genome_shotgun_sequence
VCEB01000003.1_98406850-98407353_Muntiacus_reevesi_isolate_UCam_UCB_Mr_chromosome_3_whole_genome_shotgun_sequence
SIDA01000012.1_26640620-26641268_Sus_scrofa_breed_Bama_miniature_chromosome_3_whole_genome_shotgun_sequence
R_NPJO01000009.1_44760105-44760753_Sus_scrofa_breed_Cross-bred_isolate_201423004_chromosome_3_whole_genome_shotgun_sequence
R_PVJP02910261.1_224670-224942_Hippopotamus_amphibius_isolate_BS01_ScHSfme_924745HRSCAF_982545_whole_genome_shotgun_sequence
R_RAWU01000014.1_103743101-103743345_Giraffa_tippelskirchi_isolate_OR1865_chromosome_9_whole_genome_shotgun_sequence
R_PVJP02910696.1_592857-593497_Hippopotamus_amphibius_isolate_BS01_ScHSfme_925150HRSCAF_982950_whole_genome_shotgun_sequence
R_VCEA01000001.1_415055872-415056415_Muntiacus_muntjak_isolate_UTSW_UCB_Mm_chromosome_1_whole_genome_shotgun_sequence
R_VCEB01000003.1_212856373-212856917_Muntiacus_reevesi_isolate_UCam_UCB_Mr_chromosome_3_whole_genome_shotgun_sequence
QQTR01002841.1_42418340-42418887_Axis_porcinus_isolate_Zoohd001_scaffold_108789_whole_genome_shotgun_sequence
MKHE01000033.1_72918070-72918610_Cervus_elaphus_hippelaphus_isolate_Hungarian_chromosome_33_whole_genome_shotgun_sequence
R_CABVSG010000096.1_79230562-79231110_Syncerus_caffer_genome_assembly_contig__Contig97_whole_genome_shotgun_sequence
PZYV01000012.1_109489483-109490032_Bubalus_bubalis_breed_Mediterranean_chromosome_2_whole_genome_shotgun_sequence
R_RAWU01000013.1_74849254-74849914_Giraffa_tippelskirchi_isolate_OR1865_chromosome_3_whole_genome_shotgun_sequence
R_PVJP02910812.1_2134445-2135141_Hippopotamus_amphibius_isolate_BS01_ScHSfme_925256HRSCAF_983056_whole_genome_shotgun_sequence
R_PVJP02910579.1_3178692-3179362_Hippopotamus_amphibius_isolate_BS01_ScHSfme_925039HRSCAF_982839_whole_genome_shotgun_sequence
VCEA01000003.1_561622374-561622511_Muntiacus_muntjak_isolate_UTSW_UCB_Mm_chromosome_3_X_whole_genome_shotgun_sequence
VCEA01000003.1_561621495-561622020_Muntiacus_muntjak_isolate_UTSW_UCB_Mm_chromosome_3_X_whole_genome_shotgun_sequence
0.03

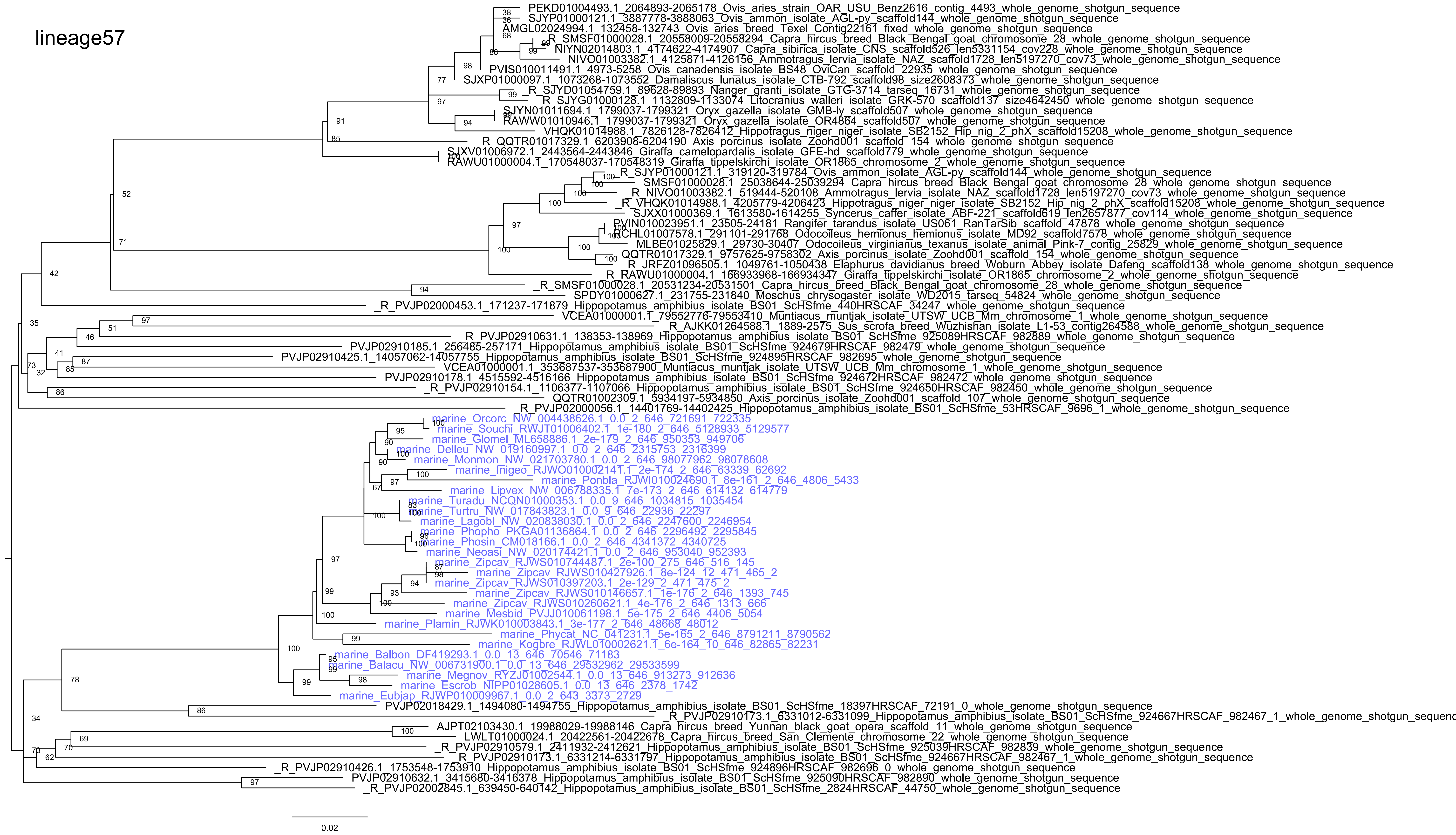
lineage57
PEKD01004493.1_2064893-2065178_Ovis_aries_strain_OAR_USU_Benz2616_contig_4493_whole_genome_shotgun_sequence
SJYP01000121.1_3887778-3888063_Ovis_ammon_isolate_AGL-py_scaffold144_whole_genome_shotgun_sequence
AMGL02024994.1_132458-132743_Ovis_aries_breed_Texel_Contig22161_fixed_whole_genome_shotgun_sequence
_R_SMSF01000028.1_20558009-20558294_Capra_hircus_breed_Black_Bengal_goat_chromosome_28_whole_genome_shotgun_sequence
NIYN02014803.1_4174622-4174907_Capra_sibirica_isolate_CNS_scaffold526_len5331154_cov228_whole_genome_shotgun_sequence
NIVO01003382.1_4125871-4126156_Ammotragus_lervia_isolate_NAZ_scaffold1728_len5197270_cov73_whole_genome_shotgun_sequence
PVIS010011491.1_4973-5258_Ovis_canadensis_isolate_BS48_OviCan_scaffold_22935_whole_genome_shotgun_sequence
SJXP01000097.1_1073268-1073552_Damaliscus_lunatus_isolate_CTB-792_scaffold98_size2608373_whole_genome_shotgun_sequence
_R_SJYD01054759.1_89628-89893_Nanger_granti_isolate_GTG-3714_tarseq_16731_whole_genome_shotgun_sequence
_R_SJYG01000128.1_1132809-1133074_Litocranius_walleri_isolate_GRK-570_scaffold137_size4642450_whole_genome_shotgun_sequence
SJYN01011694.1_1799037-1799321_Oryx_gazella_isolate_GMB-ly_scaffold507_whole_genome_shotgun_sequence
RAWW01010946.1_1799037-1799321_Oryx_gazella_isolate_OR4864_scaffold507_whole_genome_shotgun_sequence
VHQK01014988.1_7826128-7826412_Hippotragus_niger_niger_isolate_SB2152_Hip_nig_2_phX_scaffold15208_whole_genome_shotgun_sequence
_R_QQTR01017329.1_6203908-6204190_Axis_porcinus_isolate_Zoohd001_scaffold_154_whole_genome_shotgun_sequence
SJXV01006972.1_2443564-2443846_Giraffa_camelopardalis_isolate_GFE-hd_scaffold779_whole_genome_shotgun_sequence
RAWU01000004.1_170548037-170548319_Giraffa_tippelskirchi_isolate_OR1865_chromosome_2_whole_genome_shotgun_sequence
_R_SJYP01000121.1_319120-319784_Ovis_ammon_isolate_AGL-py_scaffold144_whole_genome_shotgun_sequence
SMSF01000028.1_25038644-25039294_Capra_hircus_breed_Black_Bengal_goat_chromosome_28_whole_genome_shotgun_sequence
_R_NIVO01003382.1_519444-520108_Ammotragus_lervia_isolate_NAZ_scaffold1728_len5197270_cov73_whole_genome_shotgun_sequence
_R_VHQK01014988.1_4205779-4206423_Hippotragus_niger_niger_isolate_SB2152_Hip_nig_2_phX_scaffold15208_whole_genome_shotgun_sequence
SJXX01000369.1_1613580-1614255_Syncerus_caffer_isolate_ABF-221_scaffold619_len2657877_cov114_whole_genome_shotgun_sequence
PVIN010023951.1_23505-24181_Rangifer_tarandus_isolate_US061_RanTarSib_scaffold_47878_whole_genome_shotgun_sequence
RCHL01007578.1_291101-291768_Odocoileus_hemionus_hemionus_isolate_MD92_scaffold7578_whole_genome_shotgun_sequence
MLBE01025829.1_29730-30407_Odocoileus_virginianus_texanus_isolate_animal_Pink-7_contig_25829_whole_genome_shotgun_sequence
QQTR01017329.1_9757625-9758302_Axis_porcinus_isolate_Zoohd001_scaffold_154_whole_genome_shotgun_sequence
_R_JRFZ01096505.1_1049761-1050438_Elaphurus_davidianus_breed_Woburn_Abbey_isolate_Dafeng_scaffold138_whole_genome_shotgun_sequence
_R_RAWU01000004.1_166933968-166934347_Giraffa_tippelskirchi_isolate_OR1865_chromosome_2_whole_genome_shotgun_sequence
_R_SMSF01000028.1_20531234-20531501_Capra_hircus_breed_Black_Bengal_goat_chromosome_28_whole_genome_shotgun_sequence
SPDY01000627.1_231755-231840_Moschus_chrysogaster_isolate_WD2015_tarseq_54824_whole_genome_shotgun_sequence
_R_PVJP02000453.1_171237-171879_Hippopotamus_amphibius_isolate_BS01_ScHSfme_440HRSCAF_34247_whole_genome_shotgun_sequence
VCEA01000001.1_79552776-79553410_Muntiacus_muntjak_isolate_UTSW_UCB_Mm_chromosome_1_whole_genome_shotgun_sequence
_R_AJKK01264588.1_1889-2575_Sus_scrofa_breed_Wuzhishan_isolate_L1-53_contig264588_whole_genome_shotgun_sequence
_R_PVJP02910631.1_138353-138969_Hippopotamus_amphibius_isolate_BS01_ScHSfme_925089HRSCAF_982889_whole_genome_shotgun_sequence
PVJP02910185.1_256485-257171_Hippopotamus_amphibius_isolate_BS01_ScHSfme_924679HRSCAF_982479_whole_genome_shotgun_sequence
PVJP02910425.1_14057062-14057755_Hippopotamus_amphibius_isolate_BS01_ScHSfme_924895HRSCAF_982695_whole_genome_shotgun_sequence
VCEA01000001.1_353687537-353687900_Muntiacus_muntjak_isolate_UTSW_UCB_Mm_chromosome_1_whole_genome_shotgun_sequence
PVJP02910178.1_4515592-4516166_Hippopotamus_amphibius_isolate_BS01_ScHSfme_924672HRSCAF_982472_whole_genome_shotgun_sequence
_R_PVJP02910154.1_1106377-1107066_Hippopotamus_amphibius_isolate_BS01_ScHSfme_924650HRSCAF_982450_whole_genome_shotgun_sequence
QQTR01002309.1_5934197-5934850_Axis_porcinus_isolate_Zoohd001_scaffold_107_whole_genome_shotgun_sequence
_R_PVJP02000056.1_14401769-14402425_Hippopotamus_amphibius_isolate_BS01_ScHSfme_53HRSCAF_9696_1_whole_genome_shotgun_sequence
marine_Orcorc_NW_004438626.1_0.0_2_646_721691_722335
marine_Souchi_RWJT01006402.1_1e-180_2_646_5128933_5129577
marine_Glomel_ML658886.1_2e-179_2_646_950353_949706
marine_Delleu_NW_019160997.1_0.0_2_646_2315753_2316399
marine_Monmon_NW_021703780.1_0.0_2_646_98077962_98078608
marine_Inigeo_RJWO010002141.1_2e-174_2_646_63339_62692
marine_Ponbla_RJWI010024690.1_8e-161_2_646_4806_5433
marine_Lipvex_NW_006788335.1_7e-173_2_646_614132_614779
marine_Turadu_NCQN01000353.1_0.0_9_646_1034815_1035454
marine_Turtru_NW_017843823.1_0.0_9_646_22936_22297
marine_Lagobl_NW_020838030.1_0.0_2_646_2247600_2246954
marine_Phopho_PKGA01136864.1_0.0_2_646_2296492_2295845
marine_Phosin_CM018166.1_0.0_2_646_4341372_4340725
marine_Neoasi_NW_020174421.1_0.0_2_646_953040_952393
marine_Zipcav_RJWS010744487.1_2e-100_275_646_516_145
marine_Zipcav_RJWS010427926.1_8e-124_12_471_465_2
marine_Zipcav_RJWS010397203.1_2e-129_2_471_475_2
marine_Zipcav_RJWS010146657.1_1e-176_2_646_1393_745
marine_Zipcav_RJWS010260621.1_4e-176_2_646_1313_666
marine_Mesbid_PVJJ010061198.1_5e-175_2_646_4406_5054
marine_Plamin_RJWK010003843.1_3e-177_2_646_48668_48012
marine_Phycat_NC_041231.1_5e-165_2_646_8791211_8790562
marine_Kogbre_RJWL010002621.1_6e-164_10_646_82865_82231
marine_Balbon_DF419293.1_0.0_13_646_70546_71183
marine_Balacu_NW_006731900.1_0.0_13_646_29532962_29533599
marine_Megnov_RYZJ01002544.1_0.0_13_646_913273_912636
marine_Escrob_NIPP01028605.1_0.0_13_646_2378_1742
marine_Eubjap_RJWP010009967.1_0.0_2_643_3373_2729
PVJP02018429.1_1494080-1494755_Hippopotamus_amphibius_isolate_BS01_ScHSfme_18397HRSCAF_72191_0_whole_genome_shotgun_sequence
_R_PVJP02910173.1_6331012-6331099_Hippopotamus_amphibius_isolate_BS01_ScHSfme_924667HRSCAF_982467_1_whole_genome_shotgun_sequenc
AJPT02103430.1_19988029-19988146_Capra_hircus_breed_Yunnan_black_goat_opera_scaffold_11_whole_genome_shotgun_sequence
LWLT01000024.1_20422561-20422678_Capra_hircus_breed_San_Clemente_chromosome_22_whole_genome_shotgun_sequence
_R_PVJP02910579.1_2411932-2412621_Hippopotamus_amphibius_isolate_BS01_ScHSfme_925039HRSCAF_982839_whole_genome_shotgun_sequence
_R_PVJP02910173.1_6331214-6331797_Hippopotamus_amphibius_isolate_BS01_ScHSfme_924667HRSCAF_982467_1_whole_genome_shotgun_sequence
_R_PVJP02910426.1_1753548-1753910_Hippopotamus_amphibius_isolate_BS01_ScHSfme_924896HRSCAF_982696_0_whole_genome_shotgun_sequence
PVJP02910632.1_3415680-3416378_Hippopotamus_amphibius_isolate_BS01_ScHSfme_925090HRSCAF_982890_whole_genome_shotgun_sequence
_R_PVJP02002845.1_639450-640142_Hippopotamus_amphibius_isolate_BS01_ScHSfme_2824HRSCAF_44750_whole_genome_shotgun_sequence
0.02

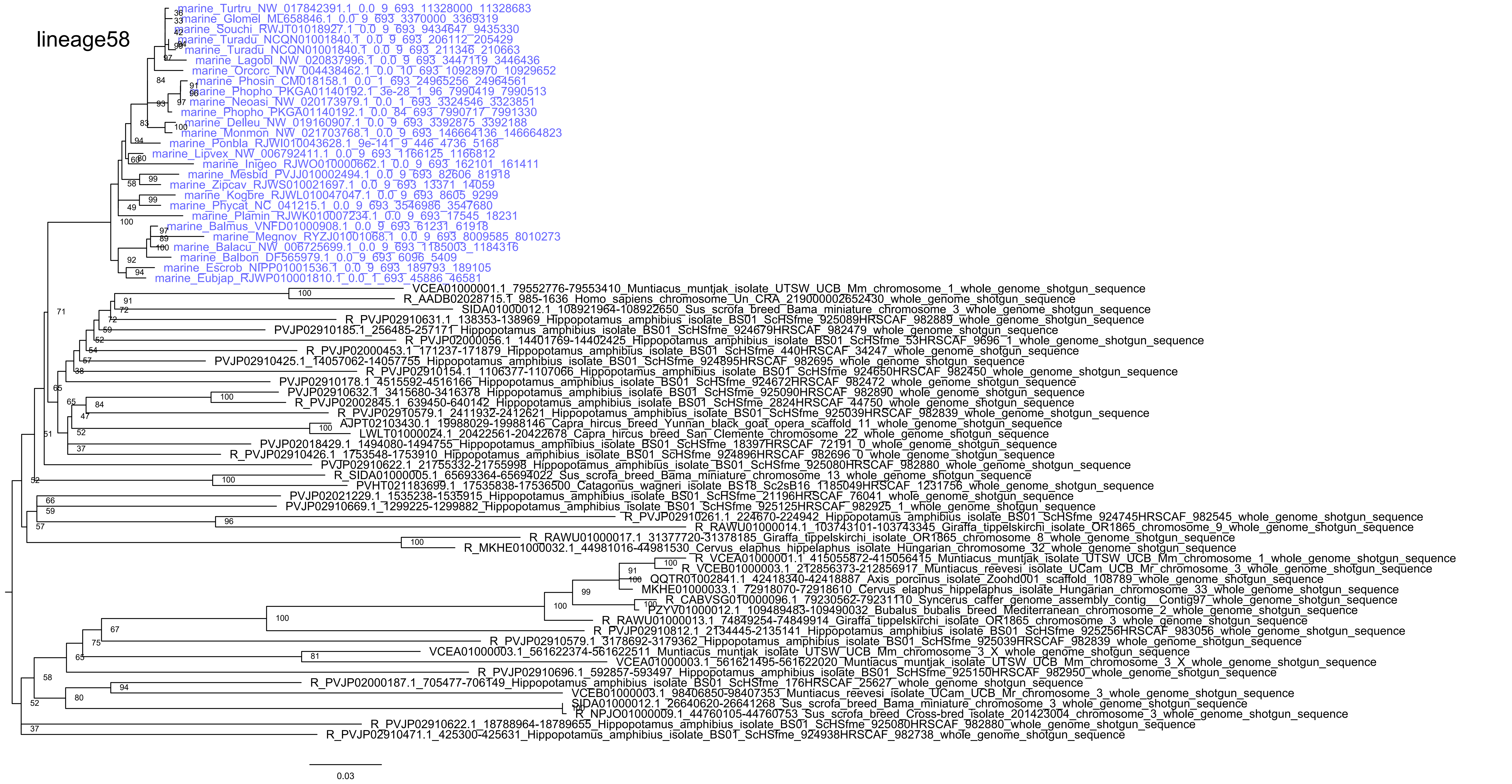
lineage58
marine_Turtru_NW_017842391.1_0.0_9_693_11328000_11328683
marine_Glomel_ML658846.1_0.0_9_693_3370000_3369319
marine_Souchi_RWJT01018927.1_0.0_9_693_9434647_9435330
marine_Turadu_NCQN01001840.1_0.0_9_693_206112_205429
marine_Turadu_NCQN01001840.1_0.0_9_693_211346_210663
marine_Lagobl_NW_020837996.1_0.0_9_693_3447119_3446436
marine_Orcorc_NW_004438462.1_0.0_10_693_10928970_10929652
marine_Phosin_CM018158.1_0.0_1_693_24965256_24964561
marine_Phopho_PKGA01140192.1_3e-28_1_96_7990419_7990513
marine_Neoasi_NW_020173979.1_0.0_1_693_3324546_3323851
marine_Phopho_PKGA01140192.1_0.0_84_693_7990717_7991330
marine_Delleu_NW_019160907.1_0.0_9_693_3392875_3392188
marine_Monmon_NW_021703768.1_0.0_9_693_146664136_146664823
marine_Ponbla_RJWI010043628.1_9e-141_9_446_4736_5168
marine_Lipvex_NW_006792411.1_0.0_9_693_1166125_1166812
marine_Inigeo_RJWO010000662.1_0.0_9_693_162101_161411
marine_Mesbid_PVJJ010002494.1_0.0_9_693_82606_81918
marine_Zipcav_RJWS010021697.1_0.0_9_693_13371_14059
marine_Kogbre_RJWL010047047.1_0.0_9_693_8605_9299
marine_Phycat_NC_041215.1_0.0_9_693_3546986_3547680
marine_Plamin_RJWK010007234.1_0.0_9_693_17545_18231
marine_Balmus_VNFD01000908.1_0.0_9_693_61231_61918
marine_Megnov_RYZJ01001068.1_0.0_9_693_8009585_8010273
marine_Balacu_NW_006725699.1_0.0_9_693_1185003_1184316
marine_Balbon_DF565979.1_0.0_9_693_6096_5409
marine_Escrob_NIPP01001536.1_0.0_9_693_189793_189105
marine_Eubjap_RJWP010001810.1_0.0_1_693_45886_46581
VCEA01000001.1_79552776-79553410_Muntiacus_muntjak_isolate_UTSW_UCB_Mm_chromosome_1_whole_genome_shotgun_sequence
R_AADB02028715.1_985-1636_Homo_sapiens_chromosome_Un_CRA_219000002652430_whole_genome_shotgun_sequence
SIDA01000012.1_108921964-108922650_Sus_scrofa_breed_Bama_miniature_chromosome_3_whole_genome_shotgun_sequence
R_PVJP02910631.1_138353-138969_Hippopotamus_amphibius_isolate_BS01_ScHSfme_925089HRSCAF_982889_whole_genome_shotgun_sequence
PVJP02910185.1_256485-257171_Hippopotamus_amphibius_isolate_BS01_ScHSfme_924679HRSCAF_982479_whole_genome_shotgun_sequence
R_PVJP02000056.1_14401769-14402425_Hippopotamus_amphibius_isolate_BS01_ScHSfme_53HRSCAF_9696_1_whole_genome_shotgun_sequence
R_PVJP02000453.1_171237-171879_Hippopotamus_amphibius_isolate_BS01_ScHSfme_440HRSCAF_34247_whole_genome_shotgun_sequence
PVJP02910425.1_14057062-14057755_Hippopotamus_amphibius_isolate_BS01_ScHSfme_924895HRSCAF_982695_whole_genome_shotgun_sequence
R_PVJP02910154.1_1106377-1107066_Hippopotamus_amphibius_isolate_BS01_ScHSfme_924650HRSCAF_982450_whole_genome_shotgun_sequence
PVJP02910178.1_4515592-4516166_Hippopotamus_amphibius_isolate_BS01_ScHSfme_924672HRSCAF_982472_whole_genome_shotgun_sequence
PVJP02910632.1_3415680-3416378_Hippopotamus_amphibius_isolate_BS01_ScHSfme_925090HRSCAF_982890_whole_genome_shotgun_sequence
R_PVJP02002845.1_639450-640142_Hippopotamus_amphibius_isolate_BS01_ScHSfme_2824HRSCAF_44750_whole_genome_shotgun_sequence
R_PVJP02910579.1_2411932-2412621_Hippopotamus_amphibius_isolate_BS01_ScHSfme_925039HRSCAF_982839_whole_genome_shotgun_sequence
AJPT02103430.1_19988029-19988146_Capra_hircus_breed_Yunnan_black_goat_opera_scaffold_11_whole_genome_shotgun_sequence
LWLT01000024.1_20422561-20422678_Capra_hircus_breed_San_Clemente_chromosome_22_whole_genome_shotgun_sequence
PVJP02018429.1_1494080-1494755_Hippopotamus_amphibius_isolate_BS01_ScHSfme_18397HRSCAF_72191_0_whole_genome_shotgun_sequence
R_PVJP02910426.1_1753548-1753910_Hippopotamus_amphibius_isolate_BS01_ScHSfme_924896HRSCAF_982696_0_whole_genome_shotgun_sequence
PVJP02910622.1_21755332-21755998_Hippopotamus_amphibius_isolate_BS01_ScHSfme_925080HRSCAF_982880_whole_genome_shotgun_sequence
R_SIDA01000005.1_65693364-65694022_Sus_scrofa_breed_Bama_miniature_chromosome_13_whole_genome_shotgun_sequence
PVHT021183699.1_17535838-17536500_Catagonus_wagneri_isolate_BS18_Sc2sB16_1185049HRSCAF_1231756_whole_genome_shotgun_sequence
PVJP02021229.1_1535238-1535915_Hippopotamus_amphibius_isolate_BS01_ScHSfme_21196HRSCAF_76041_whole_genome_shotgun_sequence
PVJP02910669.1_1299225-1299882_Hippopotamus_amphibius_isolate_BS01_ScHSfme_925125HRSCAF_982925_1_whole_genome_shotgun_sequence
R_PVJP02910261.1_224670-224942_Hippopotamus_amphibius_isolate_BS01_ScHSfme_924745HRSCAF_982545_whole_genome_shotgun_sequence
R_RAWU01000014.1_103743101-103743345_Giraffa_tippelskirchi_isolate_OR1865_chromosome_9_whole_genome_shotgun_sequence
R_RAWU01000017.1_31377720-31378185_Giraffa_tippelskirchi_isolate_OR1865_chromosome_8_whole_genome_shotgun_sequence
R_MKHE01000032.1_44981016-44981530_Cervus_elaphus_hippelaphus_isolate_Hungarian_chromosome_32_whole_genome_shotgun_sequence
R_VCEA01000001.1_415055872-415056415_Muntiacus_muntjak_isolate_UTSW_UCB_Mm_chromosome_1_whole_genome_shotgun_sequence
R_VCEB01000003.1_212856373-212856917_Muntiacus_reevesi_isolate_UCam_UCB_Mr_chromosome_3_whole_genome_shotgun_sequence
QQTR01002841.1_42418340-42418887_Axis_porcinus_isolate_Zoohd001_scaffold_108789_whole_genome_shotgun_sequence
MKHE01000033.1_72918070-72918610_Cervus_elaphus_hippelaphus_isolate_Hungarian_chromosome_33_whole_genome_shotgun_sequence
R_CABVSG010000096.1_79230562-79231110_Syncerus_caffer_genome_assembly_contig__Contig97_whole_genome_shotgun_sequence
PZYV01000012.1_109489483-109490032_Bubalus_bubalis_breed_Mediterranean_chromosome_2_whole_genome_shotgun_sequence
R_RAWU01000013.1_74849254-74849914_Giraffa_tippelskirchi_isolate_OR1865_chromosome_3_whole_genome_shotgun_sequence
R_PVJP02910812.1_2134445-2135141_Hippopotamus_amphibius_isolate_BS01_ScHSfme_925256HRSCAF_983056_whole_genome_shotgun_sequence
R_PVJP02910579.1_3178692-3179362_Hippopotamus_amphibius_isolate_BS01_ScHSfme_925039HRSCAF_982839_whole_genome_shotgun_sequence
VCEA01000003.1_561622374-561622511_Muntiacus_muntjak_isolate_UTSW_UCB_Mm_chromosome_3_X_whole_genome_shotgun_sequence
VCEA01000003.1_561621495-561622020_Muntiacus_muntjak_isolate_UTSW_UCB_Mm_chromosome_3_X_whole_genome_shotgun_sequence
R_PVJP02910696.1_592857-593497_Hippopotamus_amphibius_isolate_BS01_ScHSfme_925150HRSCAF_982950_whole_genome_shotgun_sequence
R_PVJP02000187.1_705477-706149_Hippopotamus_amphibius_isolate_BS01_ScHSfme_176HRSCAF_25627_whole_genome_shotgun_sequence
VCEB01000003.1_98406850-98407353_Muntiacus_reevesi_isolate_UCam_UCB_Mr_chromosome_3_whole_genome_shotgun_sequence
SIDA01000012.1_26640620-26641268_Sus_scrofa_breed_Bama_miniature_chromosome_3_whole_genome_shotgun_sequence
R_NPJO01000009.1_44760105-44760753_Sus_scrofa_breed_Cross-bred_isolate_201423004_chromosome_3_whole_genome_shotgun_sequence
R_PVJP02910622.1_18788964-18789655_Hippopotamus_amphibius_isolate_BS01_ScHSfme_925080HRSCAF_982880_whole_genome_shotgun_sequence
R_PVJP02910471.1_425300-425631_Hippopotamus_amphibius_isolate_BS01_ScHSfme_924938HRSCAF_982738_whole_genome_shotgun_sequence
0.03

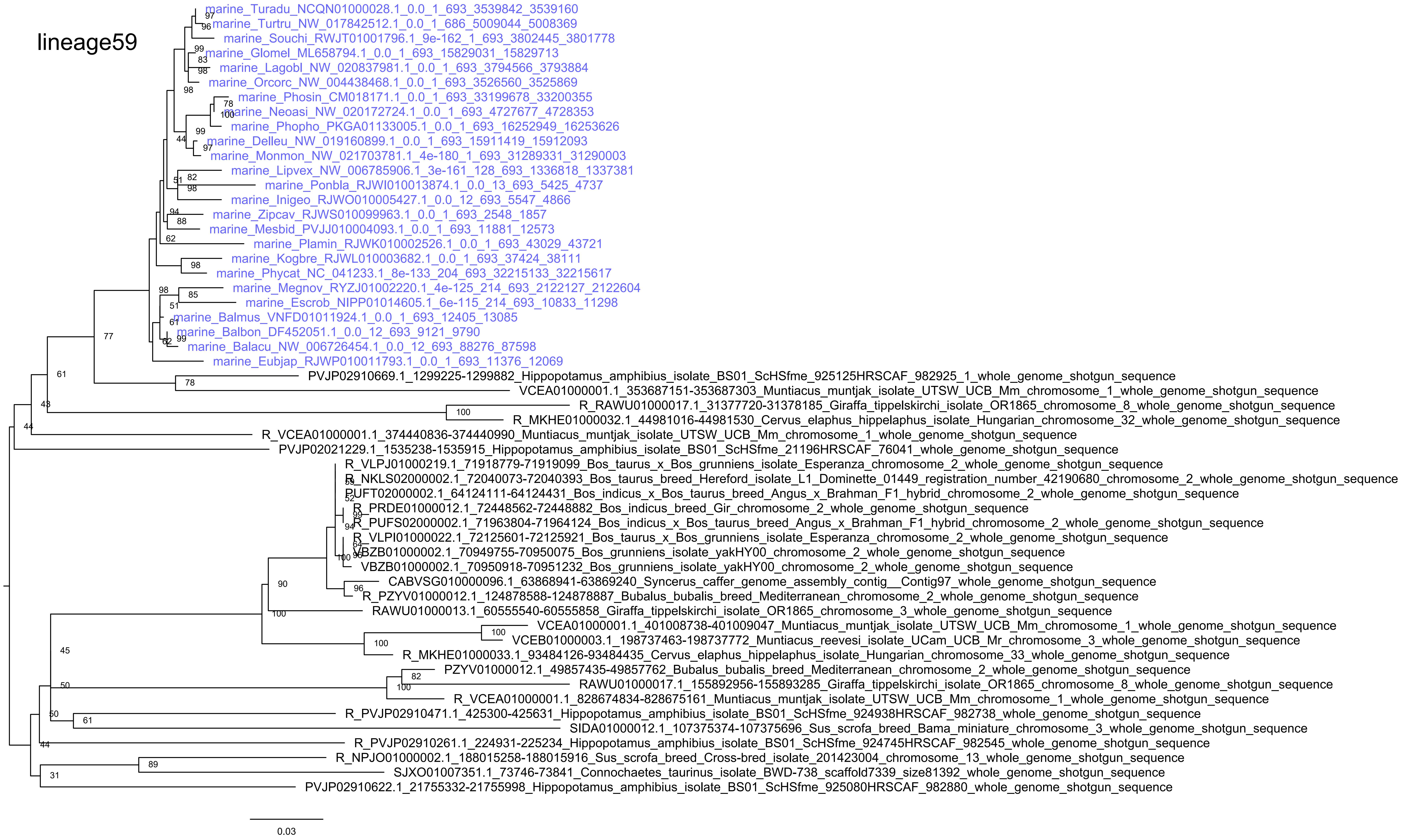

lineage59
marine_Turadu_NCQN01000028.1_0.0_1_693_3539842_3539160
marine_Turtru_NW_017842512.1_0.0_1_686_5009044_5008369
marine_Souchi_RWJT01001796.1_9e-162_1_693_3802445_3801778
marine_Glomel_ML658794.1_0.0_1_693_15829031_15829713
marine_Lagobl_NW_020837981.1_0.0_1_693_3794566_3793884
marine_Orcorc_NW_004438468.1_0.0_1_693_3526560_3525869
marine_Phosin_CM018171.1_0.0_1_693_33199678_33200355
marine_Neoasi_NW_020172724.1_0.0_1_693_4727677_4728353
marine_Phopho_PKGA01133005.1_0.0_1_693_16252949_16253626
marine_Delleu_NW_019160899.1_0.0_1_693_15911419_15912093
marine_Monmon_NW_021703781.1_4e-180_1_693_31289331_31290003
marine_Lipvex_NW_006785906.1_3e-161_128_693_1336818_1337381
marine_Ponbla_RJWI010013874.1_0.0_13_693_5425_4737
marine_Inigeo_RJWO010005427.1_0.0_12_693_5547_4866
marine_Zipcav_RJWS010099963.1_0.0_1_693_2548_1857
marine_Mesbid_PVJJ010004093.1_0.0_1_693_11881_12573
marine_Plamin_RJWK010002526.1_0.0_1_693_43029_43721
marine_Kogbre_RJWL010003682.1_0.0_1_693_37424_38111
marine_Phycat_NC_041233.1_8e-133_204_693_32215133_32215617
marine_Megnov_RYZJ01002220.1_4e-125_214_693_2122127_2122604
marine_Escrob_NIPP01014605.1_6e-115_214_693_10833_11298
marine_Balmus_VNFD01011924.1_0.0_1_693_12405_13085
marine_Balbon_DF452051.1_0.0_12_693_9121_9790
marine_Balacu_NW_006726454.1_0.0_12_693_88276_87598
marine_Eubjap_RJWP010011793.1_0.0_1_693_11376_12069
PVJP02910669.1_1299225-1299882_Hippopotamus_amphibius_isolate_BS01_ScHSfme_925125HRSCAF_982925_1_whole_genome_shotgun_sequence
VCEA01000001.1_353687151-353687303_Muntiacus_muntjak_isolate_UTSW_UCB_Mm_chromosome_1_whole_genome_shotgun_sequence
R_RAWU01000017.1_31377720-31378185_Giraffa_tippelskirchi_isolate_OR1865_chromosome_8_whole_genome_shotgun_sequence
R_MKHE01000032.1_44981016-44981530_Cervus_elaphus_hippelaphus_isolate_Hungarian_chromosome_32_whole_genome_shotgun_sequence
R_VCEA01000001.1_374440836-374440990_Muntiacus_muntjak_isolate_UTSW_UCB_Mm_chromosome_1_whole_genome_shotgun_sequence
PVJP02021229.1_1535238-1535915_Hippopotamus_amphibius_isolate_BS01_ScHSfme_21196HRSCAF_76041_whole_genome_shotgun_sequence
R_VLPJ01000219.1_71918779-71919099_Bos_taurus_x_Bos_grunniens_isolate_Esperanza_chromosome_2_whole_genome_shotgun_sequence
R_NKLS02000002.1_72040073-72040393_Bos_taurus_breed_Hereford_isolate_L1_Dominette_01449_registration_number_42190680_chromosome_2_whole_genome_shotgun_sequence
PUFT02000002.1_64124111-64124431_Bos_indicus_x_Bos_taurus_breed_Angus_x_Brahman_F1_hybrid_chromosome_2_whole_genome_shotgun_sequence
R_PRDE01000012.1_72448562-72448882_Bos_indicus_breed_Gir_chromosome_2_whole_genome_shotgun_sequence
R_PUFS02000002.1_71963804-71964124_Bos_indicus_x_Bos_taurus_breed_Angus_x_Brahman_F1_hybrid_chromosome_2_whole_genome_shotgun_sequence
R_VLPI01000022.1_72125601-72125921_Bos_taurus_x_Bos_grunniens_isolate_Esperanza_chromosome_2_whole_genome_shotgun_sequence
VBZB01000002.1_70949755-70950075_Bos_grunniens_isolate_yakHY00_chromosome_2_whole_genome_shotgun_sequence
VBZB01000002.1_70950918-70951232_Bos_grunniens_isolate_yakHY00_chromosome_2_whole_genome_shotgun_sequence
CABVSG010000096.1_63868941-63869240_Syncerus_caffer_genome_assembly_contig__Contig97_whole_genome_shotgun_sequence
R_PZYV01000012.1_124878588-124878887_Bubalus_bubalis_breed_Mediterranean_chromosome_2_whole_genome_shotgun_sequence
RAWU01000013.1_60555540-60555858_Giraffa_tippelskirchi_isolate_OR1865_chromosome_3_whole_genome_shotgun_sequence
VCEA01000001.1_401008738-401009047_Muntiacus_muntjak_isolate_UTSW_UCB_Mm_chromosome_1_whole_genome_shotgun_sequence
VCEB01000003.1_198737463-198737772_Muntiacus_reevesi_isolate_UCam_UCB_Mr_chromosome_3_whole_genome_shotgun_sequence
R_MKHE01000033.1_93484126-93484435_Cervus_elaphus_hippelaphus_isolate_Hungarian_chromosome_33_whole_genome_shotgun_sequence
PZYV01000012.1_49857435-49857762_Bubalus_bubalis_breed_Mediterranean_chromosome_2_whole_genome_shotgun_sequence
RAWU01000017.1_155892956-155893285_Giraffa_tippelskirchi_isolate_OR1865_chromosome_8_whole_genome_shotgun_sequence
R_VCEA01000001.1_828674834-828675161_Muntiacus_muntjak_isolate_UTSW_UCB_Mm_chromosome_1_whole_genome_shotgun_sequence
R_PVJP02910471.1_425300-425631_Hippopotamus_amphibius_isolate_BS01_ScHSfme_924938HRSCAF_982738_whole_genome_shotgun_sequence
SIDA01000012.1_107375374-107375696_Sus_scrofa_breed_Bama_miniature_chromosome_3_whole_genome_shotgun_sequence
R_PVJP02910261.1_224931-225234_Hippopotamus_amphibius_isolate_BS01_ScHSfme_924745HRSCAF_982545_whole_genome_shotgun_sequence
R_NPJO01000002.1_188015258-188015916_Sus_scrofa_breed_Cross-bred_isolate_201423004_chromosome_13_whole_genome_shotgun_sequence
SJXO01007351.1_73746-73841_Connochaetes_taurinus_isolate_BWD-738_scaffold7339_size81392_whole_genome_shotgun_sequence
PVJP02910622.1_21755332-21755998_Hippopotamus_amphibius_isolate_BS01_ScHSfme_925080HRSCAF_982880_whole_genome_shotgun_sequence
0.03

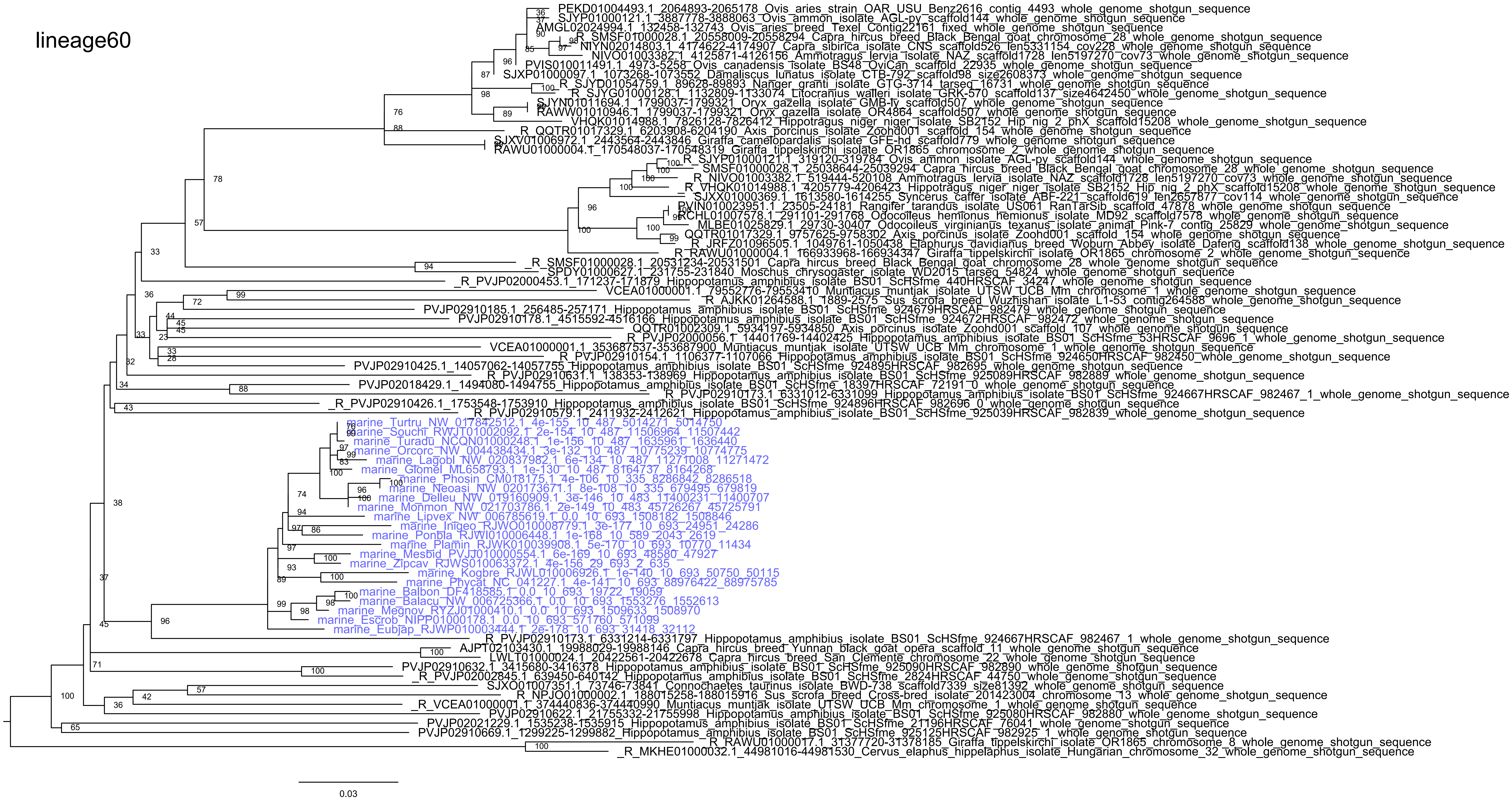
lineage60
PEKD01004493.1_2064893-2065178_Ovis_aries_strain_OAR_USU_Benz2616_contig_4493_whole_genome_shotgun_sequence
SJYP01000121.1_3887778-3888063_Ovis_ammon_isolate_AGL-py_scaffold144_whole_genome_shotgun_sequence
AMGL02024994.1_132458-132743_Ovis_aries_breed_Texel_Contig22161_fixed_whole_genome_shotgun_sequence
_R_SMSF01000028.1_20558009-20558294_Capra_hircus_breed_Black_Bengal_goat_chromosome_28_whole_genome_shotgun_sequence
NIYN02014803.1_4174622-4174907_Capra_sibirica_isolate_CNS_scaffold526_len5331154_cov228_whole_genome_shotgun_sequence
NIVO01003382.1_4125871-4126156_Ammotragus_lervia_isolate_NAZ_scaffold1728_len5197270_cov73_whole_genome_shotgun_sequence
PVIS010011491.1_4973-5258_Ovis_canadensis_isolate_BS48_OviCan_scaffold_22935_whole_genome_shotgun_sequence
SJXP01000097.1_1073268-1073552_Damaliscus_lunatus_isolate_CTB-792_scaffold98_size2608373_whole_genome_shotgun_sequence
_R_SJYD01054759.1_89628-89893_Nanger_granti_isolate_GTG-3714_tarseq_16731_whole_genome_shotgun_sequence
_R_SJYG01000128.1_1132809-1133074_Litocranius_walleri_isolate_GRK-570_scaffold137_size4642450_whole_genome_shotgun_sequence
SJYN01011694.1_1799037-1799321_Oryx_gazella_isolate_GMB-ty_scaffold507_whole_genome_shotgun_sequence
RAWW01010946.1_1799037-1799321_Oryx_gazella_isolate_OR4864_scaffold507_whole_genome_shotgun_sequence
VHQK01014988.1_7826128-7826412_Hippotragus_niger_niger_isolate_SB2152_Hip_nig_2_phX_scaffold15208_whole_genome_shotgun_sequence
_R_QQTR01017329.1_6203908-6204190_Axis_porcinus_isolate_Zoohd001_scaffold_154_whole_genome_shotgun_sequence
SJXV01006972.1_2443564-2443846_Giraffa_camelopardalis_isolate_GFE-hd_scaffold779_whole_genome_shotgun_sequence
RAWU01000004.1_170548037-170548319_Giraffa_tippelskirchi_isolate_OR1865_chromosome_2_whole_genome_shotgun_sequence
_R_SJYP01000121.1_319120-319784_Ovis_ammon_isolate_AGL-py_scaffold144_whole_genome_shotgun_sequence
SMSF01000028.1_25038644-25039294_Capra_hircus_breed_Black_Bengal_goat_chromosome_28_whole_genome_shotgun_sequence
_R_NIVO01003382.1_519444-520108_Ammotragus_lervia_isolate_NAZ_scaffold1728_len5197270_cov73_whole_genome_shotgun_sequence
_R_VHQK01014988.1_4205779-4206423_Hippotragus_niger_niger_isolate_SB2152_Hip_nig_2_phX_scaffold15208_whole_genome_shotgun_sequence
_SJXX01000369.1_1613580-1614255_Syncerus_caffer_isolate_ABF-221_scaffold619_len2657877_cov114_whole_genome_shotgun_sequence
PVIN010023951.1_23505-24181_Rangifer_tarandus_isolate_US061_RanTarSib_scaffold_47878_whole_genome_shotgun_sequence
RCHL01007578.1_291101-291768_Odocoileus_hemionus_hemionus_isolate_MD92_scaffold7578_whole_genome_shotgun_sequence
MLBE01025829.1_29730-30407_Odocoileus_virginianus_texanus_isolate_animal_Pink-7_contig_25829_whole_genome_shotgun_sequence
QQTR01017329.1_9757625-9758302_Axis_porcinus_isolate_Zoohd001_scaffold_154_whole_genome_shotgun_sequence
_R_JRFZ01096505.1_1049761-1050438_Elaphurus_davidianus_breed_Woburn_Abbey_isolate_Dafeng_scaffold138_whole_genome_shotgun_sequence
_R_RAWU01000004.1_166933968-166934347_Giraffa_tippelskirchi_isolate_OR1865_chromosome_2_whole_genome_shotgun_sequence
_R_SMSF01000028.1_20531234-20531501_Capra_hircus_breed_Black_Bengal_goat_chromosome_28_whole_genome_shotgun_sequence
SPDY01000627.1_231755-231840_Moschus_chrysogaster_isolate_WD2015_tarseq_54824_whole_genome_shotgun_sequence
_R_PVJP02000453.1_171237-171879_Hippopotamus_amphibius_isolate_BS01_ScHSfme_440HRSCAF_34247_whole_genome_shotgun_sequence
VCEA01000001.1_79552776-79553410_Muntiacus_muntjak_isolate_UTSW_UCB_Mm_chromosome_1_whole_genome_shotgun_sequence
_R_AJKK01264588.1_1889-2575_Sus_scrofa_breed_Wuzhishan_isolate_L1-53_contig264588_whole_genome_shotgun_sequence
PVJP02910185.1_256485-257171_Hippopotamus_amphibius_isolate_BS01_ScHSfme_924679HRSCAF_982479_whole_genome_shotgun_sequence
PVJP02910178.1_4515592-4516166_Hippopotamus_amphibius_isolate_BS01_ScHSfme_924672HRSCAF_982472_whole_genome_shotgun_sequence
QQTR01002309.1_5934197-5934850_Axis_porcinus_isolate_Zoohd001_scaffold_107_whole_genome_shotgun_sequence
_R_PVJP02000056.1_14401769-14402425_Hippopotamus_amphibius_isolate_BS01_ScHSfme_53HRSCAF_9696_1_whole_genome_shotgun_sequence
VCEA01000001.1_353687537-353687900_Muntiacus_muntjak_isolate_UTSW_UCB_Mm_chromosome_1_whole_genome_shotgun_sequence
_R_PVJP02910154.1_1106377-1107066_Hippopotamus_amphibius_isolate_BS01_ScHSfme_924650HRSCAF_982450_whole_genome_shotgun_sequence
PVJP02910425.1_14057062-14057755_Hippopotamus_amphibius_isolate_BS01_ScHSfme_924895HRSCAF_982695_whole_genome_shotgun_sequence
_R_PVJP02910631.1_138353-138969_Hippopotamus_amphibius_isolate_BS01_ScHSfme_925089HRSCAF_982889_whole_genome_shotgun_sequence
PVJP02018429.1_1494080-1494755_Hippopotamus_amphibius_isolate_BS01_ScHSfme_18397HRSCAF_72191_0_whole_genome_shotgun_sequence
_R_PVJP02910173.1_6331012-6331099_Hippopotamus_amphibius_isolate_BS01_ScHSfme_924667HRSCAF_982467_1_whole_genome_shotgun_sequence
_R_PVJP02910426.1_1753548-1753910_Hippopotamus_amphibius_isolate_BS01_ScHSfme_924896HRSCAF_982696_0_whole_genome_shotgun_sequence
_R_PVJP02910579.1_2411932-2412621_Hippopotamus_amphibius_isolate_BS01_ScHSfme_925039HRSCAF_982839_whole_genome_shotgun_sequence
marine_Turtru_NW_017842512.1_4e-155_10_487_5014271_5014750
marine_Souchi_RWJT01002092.1_2e-154_10_487_11506964_11507442
marine_Turadu_NCQN01000248.1_1e-156_10_487_1635961_1636440
marine_Orcorc_NW_004438434.1_3e-132_10_487_10775239_10774775
marine_Lagobl_NW_020837982.1_6e-134_10_487_11271008_11271472
marine_Glomel_ML658793.1_1e-130_10_487_8164737_8164268
marine_Phosin_CM018175.1_4e-106_10_335_8286842_8286518
marine_Neoasi_NW_020173671.1_8e-108_10_335_679495_679819
marine_Delleu_NW_019160909.1_3e-146_10_483_11400231_11400707
marine_Monmon_NW_021703786.1_2e-149_10_483_45726267_45725791
marine_Lipvex_NW_006785619.1_0.0_10_693_1508182_1508846
marine_Inigeo_RJWO010008779.1_3e-177_10_693_24951_24286
marine_Ponbla_RJWI010006448.1_1e-168_10_589_2043_2619
marine_Plamin_RJWK010039908.1_5e-170_10_693_10770_11434
marine_Mesbid_PVJJ010000554.1_6e-169_10_693_48580_47927
marine_Zipcav_RJWS010063372.1_4e-156_29_693_2_635
marine_Kogbre_RJWL010006926.1_1e-140_10_693_50750_50115
marine_Phycat_NC_041227.1_4e-141_10_693_88976422_88975785
marine_Balbon_DF418585.1_0.0_10_693_19722_19059
marine_Balacu_NW_006725366.1_0.0_10_693_1553276_1552613
marine_Megnov_RYZJ01000410.1_0.0_10_693_1509633_1508970
marine_Escrob_NIPP01000178.1_0.0_10_693_571760_571099
marine_Eubjap_RJWP010003444.1_2e-178_10_693_31418_32112
_R_PVJP02910173.1_6331214-6331797_Hippopotamus_amphibius_isolate_BS01_ScHSfme_924667HRSCAF_982467_1_whole_genome_shotgun_sequence
AJPT02103430.1_19988029-19988146_Capra_hircus_breed_Yunnan_black_goat_opera_scaffold_11_whole_genome_shotgun_sequence
LWLT01000024.1_20422561-20422678_Capra_hircus_breed_San_Clemente_chromosome_22_whole_genome_shotgun_sequence
PVJP02910632.1_3415680-3416378_Hippopotamus_amphibius_isolate_BS01_ScHSfme_925090HRSCAF_982890_whole_genome_shotgun_sequence
_R_PVJP02002845.1_639450-640142_Hippopotamus_amphibius_isolate_BS01_ScHSfme_2824HRSCAF_44750_whole_genome_shotgun_sequence
SJXO01007351.1_73746-73841_Connochaetes_taurinus_isolate_BWD-738_scaffold7339_size81392_whole_genome_shotgun_sequence
_R_NPJO01000002.1_188015258-188015916_Sus_scrofa_breed_Cross-bred_isolate_201423004_chromosome_13_whole_genome_shotgun_sequence
_R_VCEA01000001.1_374440836-374440990_Muntiacus_muntjak_isolate_UTSW_UCB_Mm_chromosome_1_whole_genome_shotgun_sequence
PVJP02910622.1_21755332-21755998_Hippopotamus_amphibius_isolate_BS01_ScHSfme_925080HRSCAF_982880_whole_genome_shotgun_sequence
PVJP02021229.1_1535238-1535915_Hippopotamus_amphibius_isolate_BS01_ScHSfme_21196HRSCAF_76041_whole_genome_shotgun_sequence
PVJP02910669.1_1299225-1299882_Hippopotamus_amphibius_isolate_BS01_ScHSfme_925125HRSCAF_982925_1_whole_genome_shotgun_sequence
_R_RAWU01000017.1_31377720-31378185_Giraffa_tippelskirchi_isolate_OR1865_chromosome_8_whole_genome_shotgun_sequence
_R_MKHE01000032.1_44981016-44981530_Cervus_elaphus_hippelaphus_isolate_Hungarian_chromosome_32_whole_genome_shotgun_sequence
0.03

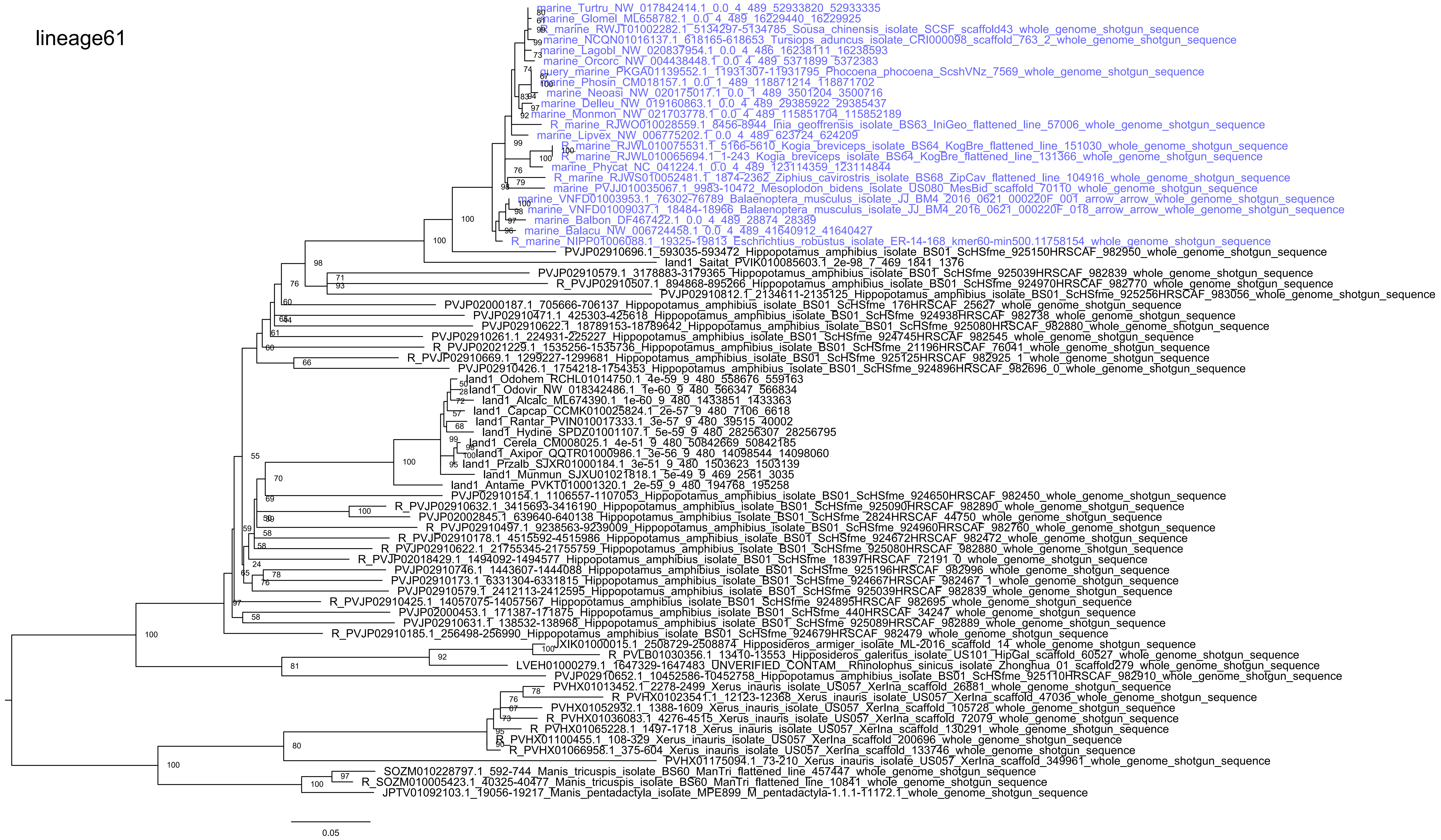
lineage61
marine_Turtru_NW_017842414.1_0.0_4_489_52933820_52933335
marine_Glomel_ML658782.1_0.0_4_489_16229440_16229925
R_marine_RWJT01002282.1_5134297-5134785_Sousa_chinensis_isolate_SCSF_scaffold43_whole_genome_shotgun_sequence
marine_NCQN01016137.1_618165-618653_Tursiops_aduncus_isolate_CRI000098_scaffold_763_2_whole_genome_shotgun_sequence
marine_Lagobl_NW_020837954.1_0.0_4_486_16238111_16238593
marine_Orcorc_NW_004438448.1_0.0_4_489_5371899_5372383
query_marine_PKGA01139552.1_11931307-11931795_Phocoena_phocoena_ScshVNz_7569_whole_genome_shotgun_sequence
marine_Phosin_CM018157.1_0.0_1_489_118871214_118871702
marine_Neoasi_NW_020175017.1_0.0_1_489_3501204_3500716
marine_Delleu_NW_019160863.1_0.0_4_489_29385922_29385437
marine_Monmon_NW_021703778.1_0.0_4_489_115851704_115852189
R_marine_RJWO010028559.1_8456-8944_Inia_geoffrensis_isolate_BS63_IniGeo_flattened_line_57006_whole_genome_shotgun_sequence
marine_Lipvex_NW_006775202.1_0.0_4_489_623724_624209
R_marine_RJWL010075531.1_5166-5610_Kogia_breviceps_isolate_BS64_KogBre_flattened_line_151030_whole_genome_shotgun_sequence
R_marine_RJWL010065694.1_1-243_Kogia_breviceps_isolate_BS64_KogBre_flattened_line_131366_whole_genome_shotgun_sequence
marine_Phycat_NC_041224.1_0.0_4_489_123114359_123114844
R_marine_RJWS010052481.1_1874-2362_Ziphius_cavirostris_isolate_BS68_ZipCav_flattened_line_104916_whole_genome_shotgun_sequence
marine_PVJJ010035067.1_9983-10472_Mesoplodon_bidens_isolate_US080_MesBid_scaffold_70110_whole_genome_shotgun_sequence
marine_VNFD01003953.1_76302-76789_Balaenoptera_musculus_isolate_JJ_BM4_2016_0621_000220F_001_arrow_arrow_whole_genome_shotgun_sequence
marine_VNFD01009037.1_18484-18966_Balaenoptera_musculus_isolate_JJ_BM4_2016_0621_000220F_018_arrow_arrow_whole_genome_shotgun_sequence
marine_Balbon_DF467422.1_0.0_4_489_28874_28389
marine_Balacu_NW_006724458.1_0.0_4_489_41640912_41640427
R_marine_NIPP01006088.1_19325-19813_Eschrichtius_robustus_isolate_ER-14-168_kmer60-min500.11758154_whole_genome_shotgun_sequence
PVJP02910696.1_593035-593472_Hippopotamus_amphibius_isolate_BS01_ScHSfme_925150HRSCAF_982950_whole_genome_shotgun_sequence
land1_Saitat_PVIK010085603.1_2e-98_7_469_1841_1376
PVJP02910579.1_3178883-3179365_Hippopotamus_amphibius_isolate_BS01_ScHSfme_925039HRSCAF_982839_whole_genome_shotgun_sequence
R_PVJP02910507.1_894868-895266_Hippopotamus_amphibius_isolate_BS01_ScHSfme_924970HRSCAF_982770_whole_genome_shotgun_sequence
PVJP02910812.1_2134611-2135125_Hippopotamus_amphibius_isolate_BS01_ScHSfme_925256HRSCAF_983056_whole_genome_shotgun_sequence
PVJP02000187.1_705666-706137_Hippopotamus_amphibius_isolate_BS01_ScHSfme_176HRSCAF_25627_whole_genome_shotgun_sequence
PVJP02910471.1_425303-425618_Hippopotamus_amphibius_isolate_BS01_ScHSfme_924938HRSCAF_982738_whole_genome_shotgun_sequence
PVJP02910622.1_18789153-18789642_Hippopotamus_amphibius_isolate_BS01_ScHSfme_925080HRSCAF_982880_whole_genome_shotgun_sequence
PVJP02910261.1_224931-225227_Hippopotamus_amphibius_isolate_BS01_ScHSfme_924745HRSCAF_982545_whole_genome_shotgun_sequence
R_PVJP02021229.1_1535256-1535736_Hippopotamus_amphibius_isolate_BS01_ScHSfme_21196HRSCAF_76041_whole_genome_shotgun_sequence
R_PVJP02910669.1_1299227-1299681_Hippopotamus_amphibius_isolate_BS01_ScHSfme_925125HRSCAF_982925_1_whole_genome_shotgun_sequence
PVJP02910426.1_1754218-1754353_Hippopotamus_amphibius_isolate_BS01_ScHSfme_924896HRSCAF_982696_0_whole_genome_shotgun_sequence
land1_Odohem_RCHL01014750.1_4e-59_9_480_558676_559163
land1_Odovir_NW_018342486.1_1e-60_9_480_566347_566834
land1_Alcalc_ML674390.1_1e-60_9_480_1433851_1433363
land1_Capcap_CCMK010025824.1_2e-57_9_480_7106_6618
land1_Rantar_PVIN010017333.1_3e-57_9_480_39515_40002
land1_Hydine_SPDZ01001107.1_5e-59_9_480_28256307_28256795
land1_Cerela_CM008025.1_4e-51_9_480_50842669_50842185
land1_Axipor_QQTR01000986.1_3e-56_9_480_14098544_14098060
land1_Przalb_SJXR01000184.1_3e-51_9_480_1503623_1503139
land1_Munmun_SJXU01021818.1_5e-49_9_469_2561_3035
land1_Antame_PVKT010001320.1_2e-59_9_480_194768_195258
PVJP02910154.1_1106557-1107053_Hippopotamus_amphibius_isolate_BS01_ScHSfme_924650HRSCAF_982450_whole_genome_shotgun_sequence
R_PVJP02910632.1_3415693-3416190_Hippopotamus_amphibius_isolate_BS01_ScHSfme_925090HRSCAF_982890_whole_genome_shotgun_sequence
PVJP02002845.1_639640-640138_Hippopotamus_amphibius_isolate_BS01_ScHSfme_2824HRSCAF_44750_whole_genome_shotgun_sequence
R_PVJP02910497.1_9238563-9239009_Hippopotamus_amphibius_isolate_BS01_ScHSfme_924960HRSCAF_982760_whole_genome_shotgun_sequence
R_PVJP02910178.1_4515592-4515986_Hippopotamus_amphibius_isolate_BS01_ScHSfme_924672HRSCAF_982472_whole_genome_shotgun_sequence
R_PVJP02910622.1_21755345-21755759_Hippopotamus_amphibius_isolate_BS01_ScHSfme_925080HRSCAF_982880_whole_genome_shotgun_sequence
R_PVJP02018429.1_1494092-1494577_Hippopotamus_amphibius_isolate_BS01_ScHSfme_18397HRSCAF_72191_0_whole_genome_shotgun_sequence
PVJP02910746.1_1443607-1444088_Hippopotamus_amphibius_isolate_BS01_ScHSfme_925196HRSCAF_982996_whole_genome_shotgun_sequence
PVJP02910173.1_6331304-6331815_Hippopotamus_amphibius_isolate_BS01_ScHSfme_924667HRSCAF_982467_1_whole_genome_shotgun_sequence
PVJP02910579.1_2412113-2412595_Hippopotamus_amphibius_isolate_BS01_ScHSfme_925039HRSCAF_982839_whole_genome_shotgun_sequence
R_PVJP02910425.1_14057075-14057567_Hippopotamus_amphibius_isolate_BS01_ScHSfme_924895HRSCAF_982695_whole_genome_shotgun_sequence
PVJP02000453.1_171387-171875_Hippopotamus_amphibius_isolate_BS01_ScHSfme_440HRSCAF_34247_whole_genome_shotgun_sequence
PVJP02910631.1_138532-138968_Hippopotamus_amphibius_isolate_BS01_ScHSfme_925089HRSCAF_982889_whole_genome_shotgun_sequence
R_PVJP02910185.1_256498-256990_Hippopotamus_amphibius_isolate_BS01_ScHSfme_924679HRSCAF_982479_whole_genome_shotgun_sequence
JXIK01000015.1_2508729-2508874_Hipposideros_armiger_isolate_ML-2016_scaffold_14_whole_genome_shotgun_sequence
R_PVLB01030356.1_13410-13553_Hipposideros_galeritus_isolate_US101_HipGal_scaffold_60527_whole_genome_shotgun_sequence
LVEH01000279.1_1647329-1647483_UNVERIFIED_CONTAM__Rhinolophus_sinicus_isolate_Zhonghua_01_scaffold279_whole_genome_shotgun_sequence
PVJP02910652.1_10452586-10452758_Hippopotamus_amphibius_isolate_BS01_ScHSfme_925110HRSCAF_982910_whole_genome_shotgun_sequence
PVHX01013452.1_2278-2499_Xerus_inauris_isolate_US057_XerIna_scaffold_26881_whole_genome_shotgun_sequence
R_PVHX01023541.1_12123-12368_Xerus_inauris_isolate_US057_XerIna_scaffold_47036_whole_genome_shotgun_sequence
PVHX01052932.1_1388-1609_Xerus_inauris_isolate_US057_XerIna_scaffold_105728_whole_genome_shotgun_sequence
R_PVHX01036083.1_4276-4515_Xerus_inauris_isolate_US057_XerIna_scaffold_72079_whole_genome_shotgun_sequence
R_PVHX01065228.1_1497-1718_Xerus_inauris_isolate_US057_XerIna_scaffold_130291_whole_genome_shotgun_sequence
R_PVHX01100455.1_108-329_Xerus_inauris_isolate_US057_XerIna_scaffold_200696_whole_genome_shotgun_sequence
R_PVHX01066958.1_375-604_Xerus_inauris_isolate_US057_XerIna_scaffold_133746_whole_genome_shotgun_sequence
PVHX01175094.1_73-210_Xerus_inauris_isolate_US057_XerIna_scaffold_349961_whole_genome_shotgun_sequence
SOZM010228797.1_592-744_Manis_tricuspis_isolate_BS60_ManTri_flattened_line_457447_whole_genome_shotgun_sequence
R_SOZM010005423.1_40325-40477_Manis_tricuspis_isolate_BS60_ManTri_flattened_line_10841_whole_genome_shotgun_sequence
JPTV01092103.1_19056-19217_Manis_pentadactyla_isolate_MPE899_M_pentadactyla-1.1.1-T1172.1_whole_genome_shotgun_sequence
0.05

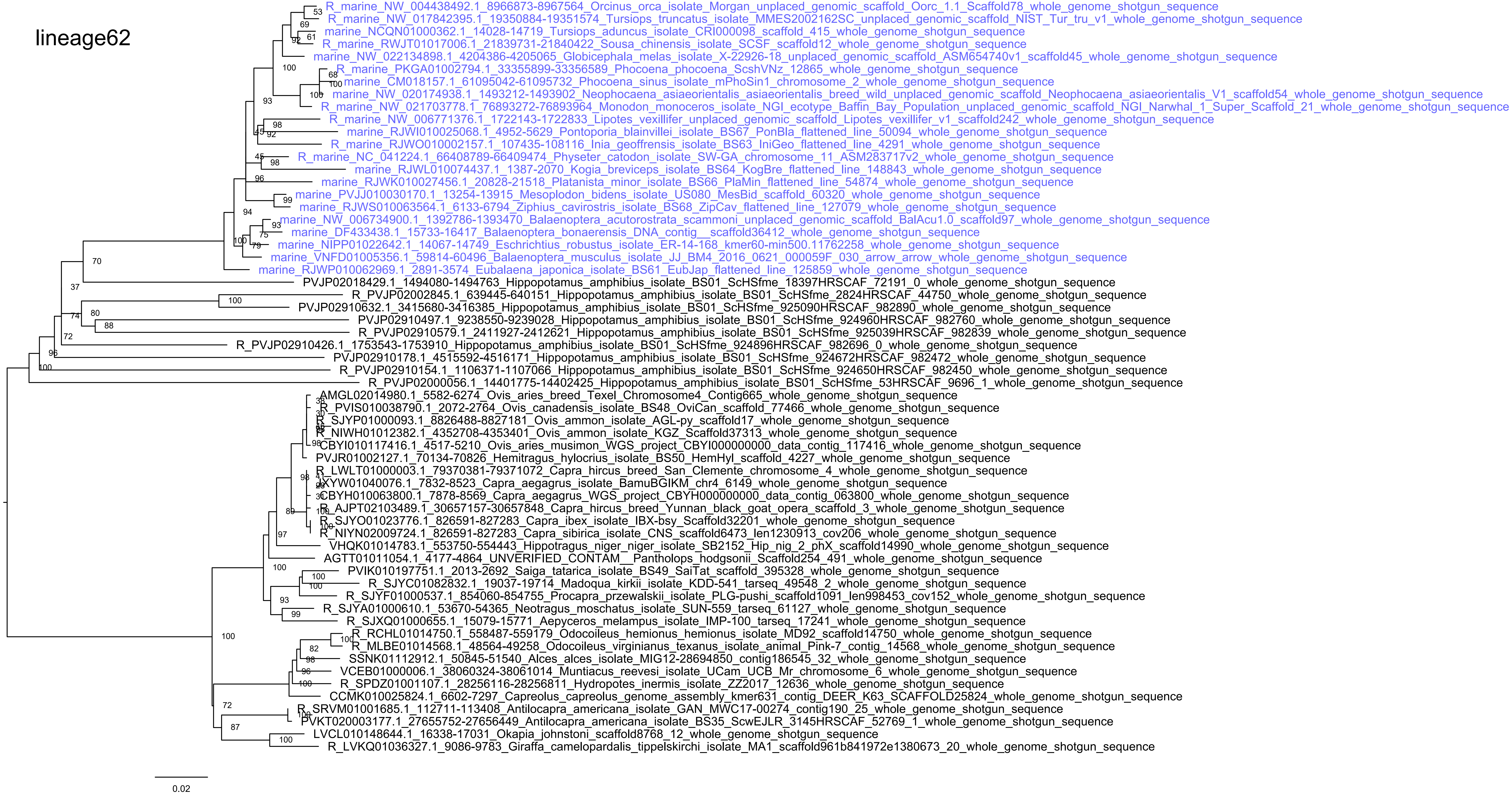

lineage62
R_marine_NW_004438492.1_8966873-8967564_Orcinus_orca_isolate_Morgan_unplaced_genomic_scaffold_Oorc_1.1_Scaffold78_whole_genome_shotgun_sequence
R_marine_NW_017842395.1_19350884-19351574_Tursiops_truncatus_isolate_MMES2002162SC_unplaced_genomic_scaffold_NIST_Tur_tru_v1_whole_genome_shotgun_sequence
marine_NCQN01000362.1_14028-14719_Tursiops_aduncus_isolate_CRI000098_scaffold_415_whole_genome_shotgun_sequence
R_marine_RWJT01017006.1_21839731-21840422_Sousa_chinensis_isolate_SCSF_scaffold12_whole_genome_shotgun_sequence
marine_NW_022134898.1_4204386-4205065_Globicephala_melas_isolate_X-22926-18_unplaced_genomic_scaffold_ASM654740v1_scaffold45_whole_genome_shotgun_sequence
R_marine_PKGA01002794.1_33355899-33356589_Phocoena_phocoena_ScshVNz_12865_whole_genome_shotgun_sequence
marine_CM018157.1_61095042-61095732_Phocoena_sinus_isolate_mPhoSin1_chromosome_2_whole_genome_shotgun_sequence
marine_NW_020174938.1_1493212-1493902_Neophocaena_asiaeorientalis_asiaeorientalis_breed_wild_unplaced_genomic_scaffold_Neophocaena_asiaeorientalis_V1_scaffold54_whole_genome_shotgun_sequence
R_marine_NW_021703778.1_76893272-76893964_Monodon_monoceros_isolate_NGI_ecotype_Baffin_Bay_Population_unplaced_genomic_scaffold_NGI_Narwhal_1_Super_Scaffold_21_whole_genome_shotgun_sequence
R_marine_NW_006771376.1_1722143-1722833_Lipotes_vexillifer_unplaced_genomic_scaffold_Lipotes_vexillifer_v1_scaffold242_whole_genome_shotgun_sequence
marine_RJWI010025068.1_4952-5629_Pontoporia_blainvillei_isolate_BS67_PonBla_flattened_line_50094_whole_genome_shotgun_sequence
R_marine_RJWO010002157.1_107435-108116_Inia_geoffrensis_isolate_BS63_IniGeo_flattened_line_4291_whole_genome_shotgun_sequence
R_marine_NC_041224.1_66408789-66409474_Physeter_catodon_isolate_SW-GA_chromosome_11_ASM283717v2_whole_genome_shotgun_sequence
marine_RJWL010074437.1_1387-2070_Kogia_breviceps_isolate_BS64_KogBre_flattened_line_148843_whole_genome_shotgun_sequence
marine_RJWK010027456.1_20828-21518_Platanista_minor_isolate_BS66_PlaMin_flattened_line_54874_whole_genome_shotgun_sequence
marine_PVJJ010030170.1_13254-13915_Mesoplodon_bidens_isolate_US080_MesBid_scaffold_60320_whole_genome_shotgun_sequence
marine_RJWS010063564.1_6133-6794_Ziphius_cavirostris_isolate_BS68_ZipCav_flattened_line_127079_whole_genome_shotgun_sequence
marine_NW_006734900.1_1392786-1393470_Balaenoptera_acutorostrata_scammoni_unplaced_genomic_scaffold_BalAcu1.0_scaffold97_whole_genome_shotgun_sequence
marine_DF433438.1_15733-16417_Balaenoptera_bonaerensis_DNA_contig__scaffold36412_whole_genome_shotgun_sequence
marine_NIPP01022642.1_14067-14749_Eschrichtius_robustus_isolate_ER-14-168_kmer60-min500.11762258_whole_genome_shotgun_sequence
marine_VNFD01005356.1_59814-60496_Balaenoptera_musculus_isolate_JJ_BM4_2016_0621_000059F_030_arrow_arrow_whole_genome_shotgun_sequence
marine_RJWP010062969.1_2891-3574_Eubalaena_japonica_isolate_BS61_EubJap_flattened_line_125859_whole_genome_shotgun_sequence
PVJP02018429.1_1494080-1494763_Hippopotamus_amphibius_isolate_BS01_ScHSfme_18397HRSCAF_72191_0_whole_genome_shotgun_sequence
R_PVJP02002845.1_639445-640151_Hippopotamus_amphibius_isolate_BS01_ScHSfme_2824HRSCAF_44750_whole_genome_shotgun_sequence
PVJP02910632.1_3415680-3416385_Hippopotamus_amphibius_isolate_BS01_ScHSfme_925090HRSCAF_982890_whole_genome_shotgun_sequence
PVJP02910497.1_9238550-9239028_Hippopotamus_amphibius_isolate_BS01_ScHSfme_924960HRSCAF_982760_whole_genome_shotgun_sequence
R_PVJP02910579.1_2411927-2412621_Hippopotamus_amphibius_isolate_BS01_ScHSfme_925039HRSCAF_982839_whole_genome_shotgun_sequence
R_PVJP02910426.1_1753543-1753910_Hippopotamus_amphibius_isolate_BS01_ScHSfme_924896HRSCAF_982696_0_whole_genome_shotgun_sequence
PVJP02910178.1_4515592-4516171_Hippopotamus_amphibius_isolate_BS01_ScHSfme_924672HRSCAF_982472_whole_genome_shotgun_sequence
R_PVJP02910154.1_1106371-1107066_Hippopotamus_amphibius_isolate_BS01_ScHSfme_924650HRSCAF_982450_whole_genome_shotgun_sequence
R_PVJP02000056.1_14401775-14402425_Hippopotamus_amphibius_isolate_BS01_ScHSfme_53HRSCAF_9696_1_whole_genome_shotgun_sequence
AMGL02014980.1_5582-6274_Ovis_aries_breed_Texel_Chromosome4_Contig665_whole_genome_shotgun_sequence
R_PVIS010038790.1_2072-2764_Ovis_canadensis_isolate_BS48_OviCan_scaffold_77466_whole_genome_shotgun_sequence
R_SJYP01000093.1_8826488-8827181_Ovis_ammon_isolate_AGL-py_scaffold17_whole_genome_shotgun_sequence
R_NIWH01012382.1_4352708-4353401_Ovis_ammon_isolate_KGZ_Scaffold37313_whole_genome_shotgun_sequence
CBYI010117416.1_4517-5210_Ovis_aries_musimon_WGS_project_CBYI000000000_data_contig_117416_whole_genome_shotgun_sequence
PVJR01002127.1_70134-70826_Hemitragus_hylocrius_isolate_BS50_HemHyl_scaffold_4227_whole_genome_shotgun_sequence
R_LWLT01000003.1_79370381-79371072_Capra_hircus_breed_San_Clemente_chromosome_4_whole_genome_shotgun_sequence
JXYW01040076.1_7832-8523_Capra_aegagrus_isolate_BamuBGIKM_chr4_6149_whole_genome_shotgun_sequence
CBYH010063800.1_7878-8569_Capra_aegagrus_WGS_project_CBYH000000000_data_contig_063800_whole_genome_shotgun_sequence
R_AJPT02103489.1_30657157-30657848_Capra_hircus_breed_Yunnan_black_goat_opera_scaffold_3_whole_genome_shotgun_sequence
R_SJYO01023776.1_826591-827283_Capra_ibex_isolate_IBX-bsy_Scaffold32201_whole_genome_shotgun_sequence
R_NIYN02009724.1_826591-827283_Capra_sibirica_isolate_CNS_scaffold6473_len1230913_cov206_whole_genome_shotgun_sequence
VHQK01014783.1_553750-554443_Hippotragus_niger_niger_isolate_SB2152_Hip_nig_2_phX_scaffold14990_whole_genome_shotgun_sequence
AGTT01011054.1_4177-4864_UNVERIFIED_CONTAM__Pantholops_hodgsonii_Scaffold254_491_whole_genome_shotgun_sequence
PVIK010197751.1_2013-2692_Saiga_tatarica_isolate_BS49_SaiTat_scaffold_395328_whole_genome_shotgun_sequence
R_SJYC01082832.1_19037-19714_Madoqua_kirkii_isolate_KDD-541_tarseq_49548_2_whole_genome_shotgun_sequence
R_SJYF01000537.1_854060-854755_Procapra_przewalskii_isolate_PLG-pushi_scaffold1091_len998453_cov152_whole_genome_shotgun_sequence
R_SJYA01000610.1_53670-54365_Neotragus_moschatus_isolate_SUN-559_tarseq_61127_whole_genome_shotgun_sequence
R_SJXQ01000655.1_15079-15771_Aepyceros_melampus_isolate_IMP-100_tarseq_17241_whole_genome_shotgun_sequence
R_RCHL01014750.1_558487-559179_Odocoileus_hemionus_hemionus_isolate_MD92_scaffold14750_whole_genome_shotgun_sequence
R_MLBE01014568.1_48564-49258_Odocoileus_virginianus_texanus_isolate_animal_Pink-7_contig_14568_whole_genome_shotgun_sequence
SSNK01112912.1_50845-51540_Alces_alces_isolate_MIG12-28694850_contig186545_32_whole_genome_shotgun_sequence
VCEB01000006.1_38060324-38061014_Muntiacus_reevesi_isolate_UCam_UCB_Mr_chromosome_6_whole_genome_shotgun_sequence
R_SPDZ01001107.1_28256116-28256811_Hydropotes_inermis_isolate_ZZ2017_12636_whole_genome_shotgun_sequence
CCMK010025824.1_6602-7297_Capreolus_capreolus_genome_assembly_kmer631_contig_DEER_K63_SCAFFOLD25824_whole_genome_shotgun_sequence
R_SRVM01001685.1_112711-113408_Antilocapra_americana_isolate_GAN_MWC17-00274_contig190_25_whole_genome_shotgun_sequence
PVKT020003177.1_27655752-27656449_Antilocapra_americana_isolate_BS35_ScwEJLR_3145HRSCAF_52769_1_whole_genome_shotgun_sequence
LVCL010148644.1_16338-17031_Okapia_johnstoni_scaffold8768_12_whole_genome_shotgun_sequence
R_LVKQ01036327.1_9086-9783_Giraffa_camelopardalis_tippelskirchi_isolate_MA1_scaffold961b841972e1380673_20_whole_genome_shotgun_sequence
0.02

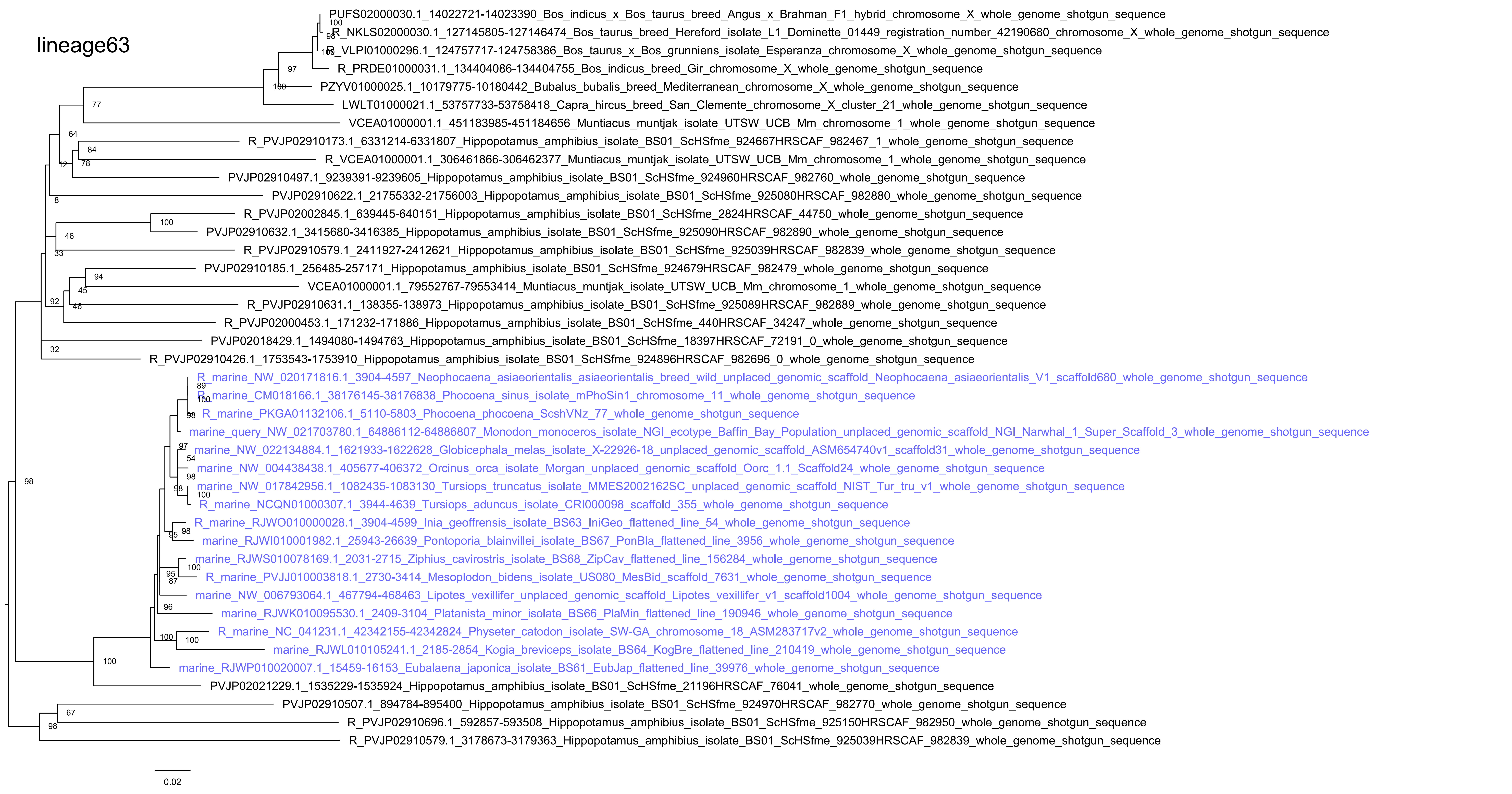
lineage63
PUFS02000030.1_14022721-14023390_Bos_indicus_x_Bos_taurus_breed_Angus_x_Brahman_F1_hybrid_chromosome_X_whole_genome_shotgun_sequence
R_NKLS02000030.1_127145805-127146474_Bos_taurus_breed_Hereford_isolate_L1_Dominette_01449_registration_number_42190680_chromosome_X_whole_genome_shotgun_sequence
R_VLPI01000296.1_124757717-124758386_Bos_taurus_x_Bos_grunniens_isolate_Esperanza_chromosome_X_whole_genome_shotgun_sequence
R_PRDE01000031.1_134404086-134404755_Bos_indicus_breed_Gir_chromosome_X_whole_genome_shotgun_sequence
PZYV01000025.1_10179775-10180442_Bubalus_bubalis_breed_Mediterranean_chromosome_X_whole_genome_shotgun_sequence
LWLT01000021.1_53757733-53758418_Capra_hircus_breed_San_Clemente_chromosome_X_cluster_21_whole_genome_shotgun_sequence
VCEA01000001.1_451183985-451184656_Muntiacus_muntjak_isolate_UTSW_UCB_Mm_chromosome_1_whole_genome_shotgun_sequence
R_PVJP02910173.1_6331214-6331807_Hippopotamus_amphibius_isolate_BS01_ScHSfme_924667HRSCAF_982467_1_whole_genome_shotgun_sequence
R_VCEA01000001.1_306461866-306462377_Muntiacus_muntjak_isolate_UTSW_UCB_Mm_chromosome_1_whole_genome_shotgun_sequence
PVJP02910497.1_9239391-9239605_Hippopotamus_amphibius_isolate_BS01_ScHSfme_924960HRSCAF_982760_whole_genome_shotgun_sequence
PVJP02910622.1_21755332-21756003_Hippopotamus_amphibius_isolate_BS01_ScHSfme_925080HRSCAF_982880_whole_genome_shotgun_sequence
R_PVJP02002845.1_639445-640151_Hippopotamus_amphibius_isolate_BS01_ScHSfme_2824HRSCAF_44750_whole_genome_shotgun_sequence
PVJP02910632.1_3415680-3416385_Hippopotamus_amphibius_isolate_BS01_ScHSfme_925090HRSCAF_982890_whole_genome_shotgun_sequence
R_PVJP02910579.1_2411927-2412621_Hippopotamus_amphibius_isolate_BS01_ScHSfme_925039HRSCAF_982839_whole_genome_shotgun_sequence
PVJP02910185.1_256485-257171_Hippopotamus_amphibius_isolate_BS01_ScHSfme_924679HRSCAF_982479_whole_genome_shotgun_sequence
VCEA01000001.1_79552767-79553414_Muntiacus_muntjak_isolate_UTSW_UCB_Mm_chromosome_1_whole_genome_shotgun_sequence
R_PVJP02910631.1_138355-138973_Hippopotamus_amphibius_isolate_BS01_ScHSfme_925089HRSCAF_982889_whole_genome_shotgun_sequence
R_PVJP02000453.1_171232-171886_Hippopotamus_amphibius_isolate_BS01_ScHSfme_440HRSCAF_34247_whole_genome_shotgun_sequence
PVJP02018429.1_1494080-1494763_Hippopotamus_amphibius_isolate_BS01_ScHSfme_18397HRSCAF_72191_0_whole_genome_shotgun_sequence
R_PVJP02910426.1_1753543-1753910_Hippopotamus_amphibius_isolate_BS01_ScHSfme_924896HRSCAF_982696_0_whole_genome_shotgun_sequence
R_marine_NW_020171816.1_3904-4597_Neophocaena_asiaeorientalis_asiaeorientalis_breed_wild_unplaced_genomic_scaffold_Neophocaena_asiaeorientalis_V1_scaffold680_whole_genome_shotgun_sequence
R_marine_CM018166.1_38176145-38176838_Phocoena_sinus_isolate_mPhoSin1_chromosome_11_whole_genome_shotgun_sequence
R_marine_PKGA01132106.1_5110-5803_Phocoena_phocoena_ScshVNz_77_whole_genome_shotgun_sequence
marine_query_NW_021703780.1_64886112-64886807_Monodon_monoceros_isolate_NGI_ecotype_Baffin_Bay_Population_unplaced_genomic_scaffold_NGI_Narwhal_1_Super_Scaffold_3_whole_genome_shotgun_sequence
marine_NW_022134884.1_1621933-1622628_Globicephala_melas_isolate_X-22926-18_unplaced_genomic_scaffold_ASM654740v1_scaffold31_whole_genome_shotgun_sequence
marine_NW_004438438.1_405677-406372_Orcinus_orca_isolate_Morgan_unplaced_genomic_scaffold_Oorc_1.1_Scaffold24_whole_genome_shotgun_sequence
marine_NW_017842956.1_1082435-1083130_Tursiops_truncatus_isolate_MMES2002162SC_unplaced_genomic_scaffold_NIST_Tur_tru_v1_whole_genome_shotgun_sequence
R_marine_NCQN01000307.1_3944-4639_Tursiops_aduncus_isolate_CRI000098_scaffold_355_whole_genome_shotgun_sequence
R_marine_RJWO010000028.1_3904-4599_Inia_geoffrensis_isolate_BS63_IniGeo_flattened_line_54_whole_genome_shotgun_sequence
marine_RJWI010001982.1_25943-26639_Pontoporia_blainvillei_isolate_BS67_PonBla_flattened_line_3956_whole_genome_shotgun_sequence
marine_RJWS010078169.1_2031-2715_Ziphius_cavirostris_isolate_BS68_ZipCav_flattened_line_156284_whole_genome_shotgun_sequence
R_marine_PVJJ010003818.1_2730-3414_Mesoplodon_bidens_isolate_US080_MesBid_scaffold_7631_whole_genome_shotgun_sequence
marine_NW_006793064.1_467794-468463_Lipotes_vexillifer_unplaced_genomic_scaffold_Lipotes_vexillifer_v1_scaffold1004_whole_genome_shotgun_sequence
marine_RJWK010095530.1_2409-3104_Platanista_minor_isolate_BS66_PlaMin_flattened_line_190946_whole_genome_shotgun_sequence
R_marine_NC_041231.1_42342155-42342824_Physeter_catodon_isolate_SW-GA_chromosome_18_ASM283717v2_whole_genome_shotgun_sequence
marine_RJWL010105241.1_2185-2854_Kogia_breviceps_isolate_BS64_KogBre_flattened_line_210419_whole_genome_shotgun_sequence
marine_RJWP010020007.1_15459-16153_Eubalaena_japonica_isolate_BS61_EubJap_flattened_line_39976_whole_genome_shotgun_sequence
PVJP02021229.1_1535229-1535924_Hippopotamus_amphibius_isolate_BS01_ScHSfme_21196HRSCAF_76041_whole_genome_shotgun_sequence
PVJP02910507.1_894784-895400_Hippopotamus_amphibius_isolate_BS01_ScHSfme_924970HRSCAF_982770_whole_genome_shotgun_sequence
R_PVJP02910696.1_592857-593508_Hippopotamus_amphibius_isolate_BS01_ScHSfme_925150HRSCAF_982950_whole_genome_shotgun_sequence
R_PVJP02910579.1_3178673-3179363_Hippopotamus_amphibius_isolate_BS01_ScHSfme_925039HRSCAF_982839_whole_genome_shotgun_sequence
0.02

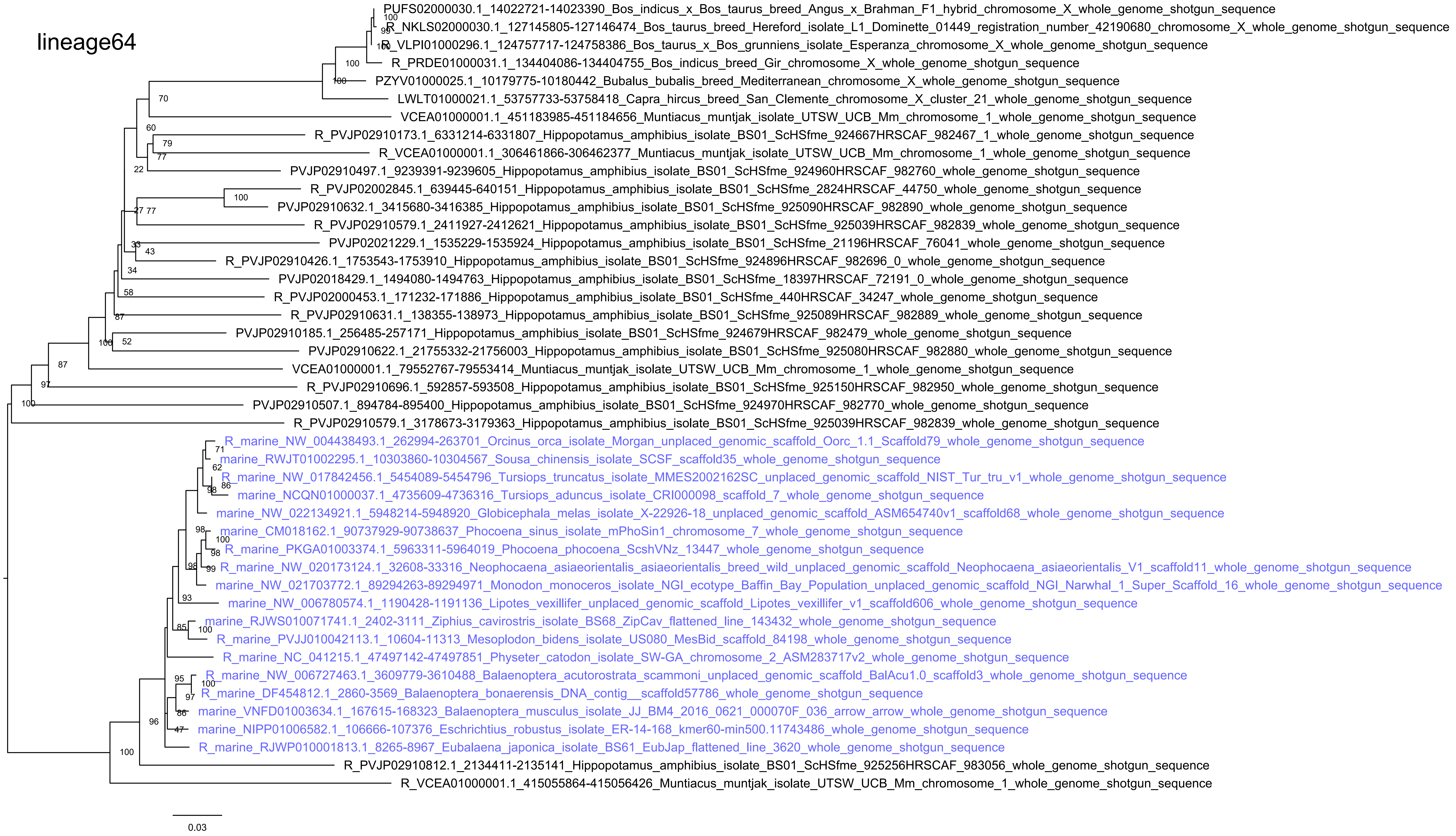
lineage64
PUFS02000030.1_14022721-14023390_Bos_indicus_x_Bos_taurus_breed_Angus_x_Brahman_F1_hybrid_chromosome_X_whole_genome_shotgun_sequence
R_NKLS02000030.1_127145805-127146474_Bos_taurus_breed_Hereford_isolate_L1_Dominette_01449_registration_number_42190680_chromosome_X_whole_genome_shotgun_sequence
R_VLPI01000296.1_124757717-124758386_Bos_taurus_x_Bos_grunniens_isolate_Esperanza_chromosome_X_whole_genome_shotgun_sequence
R_PRDE01000031.1_134404086-134404755_Bos_indicus_breed_Gir_chromosome_X_whole_genome_shotgun_sequence
PZYV01000025.1_10179775-10180442_Bubalus_bubalis_breed_Mediterranean_chromosome_X_whole_genome_shotgun_sequence
LWLT01000021.1_53757733-53758418_Capra_hircus_breed_San_Clemente_chromosome_X_cluster_21_whole_genome_shotgun_sequence
VCEA01000001.1_451183985-451184656_Muntiacus_muntjak_isolate_UTSW_UCB_Mm_chromosome_1_whole_genome_shotgun_sequence
R_PVJP02910173.1_6331214-6331807_Hippopotamus_amphibius_isolate_BS01_ScHSfme_924667HRSCAF_982467_1_whole_genome_shotgun_sequence
R_VCEA01000001.1_306461866-306462377_Muntiacus_muntjak_isolate_UTSW_UCB_Mm_chromosome_1_whole_genome_shotgun_sequence
PVJP02910497.1_9239391-9239605_Hippopotamus_amphibius_isolate_BS01_ScHSfme_924960HRSCAF_982760_whole_genome_shotgun_sequence
R_PVJP02002845.1_639445-640151_Hippopotamus_amphibius_isolate_BS01_ScHSfme_2824HRSCAF_44750_whole_genome_shotgun_sequence
PVJP02910632.1_3415680-3416385_Hippopotamus_amphibius_isolate_BS01_ScHSfme_925090HRSCAF_982890_whole_genome_shotgun_sequence
R_PVJP02910579.1_2411927-2412621_Hippopotamus_amphibius_isolate_BS01_ScHSfme_925039HRSCAF_982839_whole_genome_shotgun_sequence
PVJP02021229.1_1535229-1535924_Hippopotamus_amphibius_isolate_BS01_ScHSfme_21196HRSCAF_76041_whole_genome_shotgun_sequence
R_PVJP02910426.1_1753543-1753910_Hippopotamus_amphibius_isolate_BS01_ScHSfme_924896HRSCAF_982696_0_whole_genome_shotgun_sequence
PVJP02018429.1_1494080-1494763_Hippopotamus_amphibius_isolate_BS01_ScHSfme_18397HRSCAF_72191_0_whole_genome_shotgun_sequence
R_PVJP02000453.1_171232-171886_Hippopotamus_amphibius_isolate_BS01_ScHSfme_440HRSCAF_34247_whole_genome_shotgun_sequence
R_PVJP02910631.1_138355-138973_Hippopotamus_amphibius_isolate_BS01_ScHSfme_925089HRSCAF_982889_whole_genome_shotgun_sequence
PVJP02910185.1_256485-257171_Hippopotamus_amphibius_isolate_BS01_ScHSfme_924679HRSCAF_982479_whole_genome_shotgun_sequence
PVJP02910622.1_21755332-21756003_Hippopotamus_amphibius_isolate_BS01_ScHSfme_925080HRSCAF_982880_whole_genome_shotgun_sequence
VCEA01000001.1_79552767-79553414_Muntiacus_muntjak_isolate_UTSW_UCB_Mm_chromosome_1_whole_genome_shotgun_sequence
R_PVJP02910696.1_592857-593508_Hippopotamus_amphibius_isolate_BS01_ScHSfme_925150HRSCAF_982950_whole_genome_shotgun_sequence
PVJP02910507.1_894784-895400_Hippopotamus_amphibius_isolate_BS01_ScHSfme_924970HRSCAF_982770_whole_genome_shotgun_sequence
R_PVJP02910579.1_3178673-3179363_Hippopotamus_amphibius_isolate_BS01_ScHSfme_925039HRSCAF_982839_whole_genome_shotgun_sequence
R_marine_NW_004438493.1_262994-263701_Orcinus_orca_isolate_Morgan_unplaced_genomic_scaffold_Oorc_1.1_Scaffold79_whole_genome_shotgun_sequence
marine_RWJT01002295.1_10303860-10304567_Sousa_chinensis_isolate_SCSF_scaffold35_whole_genome_shotgun_sequence
R_marine_NW_017842456.1_5454089-5454796_Tursiops_truncatus_isolate_MMES2002162SC_unplaced_genomic_scaffold_NIST_Tur_tru_v1_whole_genome_shotgun_sequence
marine_NCQN01000037.1_4735609-4736316_Tursiops_aduncus_isolate_CRI000098_scaffold_7_whole_genome_shotgun_sequence
marine_NW_022134921.1_5948214-5948920_Globicephala_melas_isolate_X-22926-18_unplaced_genomic_scaffold_ASM654740v1_scaffold68_whole_genome_shotgun_sequence
marine_CM018162.1_90737929-90738637_Phocoena_sinus_isolate_mPhoSin1_chromosome_7_whole_genome_shotgun_sequence
R_marine_PKGA01003374.1_5963311-5964019_Phocoena_phocoena_ScshVNz_13447_whole_genome_shotgun_sequence
R_marine_NW_020173124.1_32608-33316_Neophocaena_asiaeorientalis_asiaeorientalis_breed_wild_unplaced_genomic_scaffold_Neophocaena_asiaeorientalis_V1_scaffold11_whole_genome_shotgun_sequence
marine_NW_021703772.1_89294263-89294971_Monodon_monoceros_isolate_NGI_ecotype_Baffin_Bay_Population_unplaced_genomic_scaffold_NGI_Narwhal_1_Super_Scaffold_16_whole_genome_shotgun_sequence
marine_NW_006780574.1_1190428-1191136_Lipotes_vexillifer_unplaced_genomic_scaffold_Lipotes_vexillifer_v1_scaffold606_whole_genome_shotgun_sequence
marine_RJWS010071741.1_2402-3111_Ziphius_cavirostris_isolate_BS68_ZipCav_flattened_line_143432_whole_genome_shotgun_sequence
R_marine_PVJJ010042113.1_10604-11313_Mesoplodon_bidens_isolate_US080_MesBid_scaffold_84198_whole_genome_shotgun_sequence
R_marine_NC_041215.1_47497142-47497851_Physeter_catodon_isolate_SW-GA_chromosome_2_ASM283717v2_whole_genome_shotgun_sequence
R_marine_NW_006727463.1_3609779-3610488_Balaenoptera_acutorostrata_scammoni_unplaced_genomic_scaffold_BalAcu1.0_scaffold3_whole_genome_shotgun_sequence
R_marine_DF454812.1_2860-3569_Balaenoptera_bonaerensis_DNA_contig__scaffold57786_whole_genome_shotgun_sequence
marine_VNFD01003634.1_167615-168323_Balaenoptera_musculus_isolate_JJ_BM4_2016_0621_000070F_036_arrow_arrow_whole_genome_shotgun_sequence
marine_NIPP01006582.1_106666-107376_Eschrichtius_robustus_isolate_ER-14-168_kmer60-min500.11743486_whole_genome_shotgun_sequence
R_marine_RJWP010001813.1_8265-8967_Eubalaena_japonica_isolate_BS61_EubJap_flattened_line_3620_whole_genome_shotgun_sequence
R_PVJP02910812.1_2134411-2135141_Hippopotamus_amphibius_isolate_BS01_ScHSfme_925256HRSCAF_983056_whole_genome_shotgun_sequence
R_VCEA01000001.1_415055864-415056426_Muntiacus_muntjak_isolate_UTSW_UCB_Mm_chromosome_1_whole_genome_shotgun_sequence
0.03

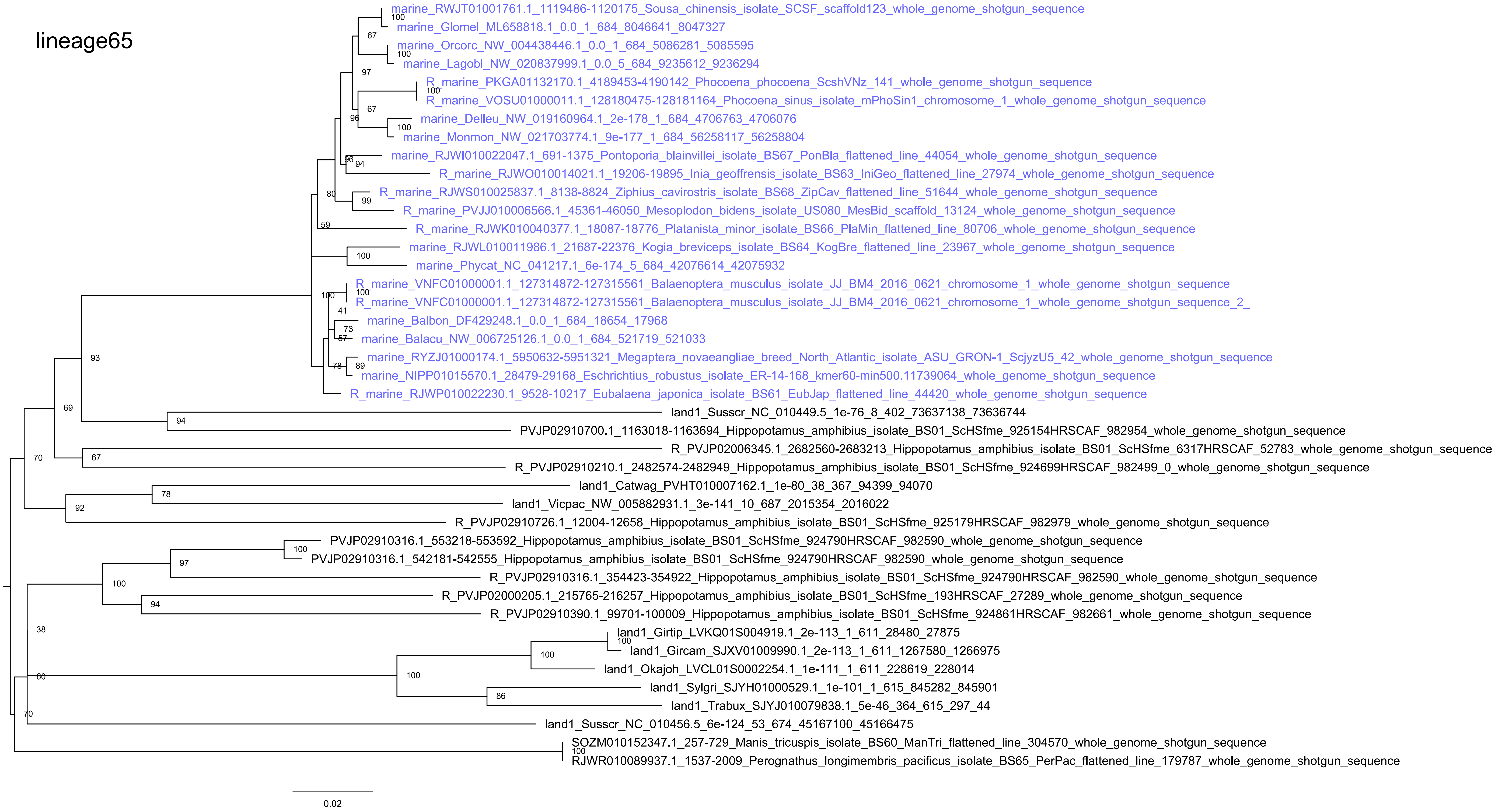
lineage65
marine_RWJT01001761.1_1119486-1120175_Sousa_chinensis_isolate_SCSF_scaffold123_whole_genome_shotgun_sequence
marine_Glomel_ML658818.1_0.0_1_684_8046641_8047327
marine_Orcorc_NW_004438446.1_0.0_1_684_5086281_5085595
marine_Lagobl_NW_020837999.1_0.0_5_684_9235612_9236294
R_marine_PKGA01132170.1_4189453-4190142_Phocoena_phocoena_ScshVNz_141_whole_genome_shotgun_sequence
R_marine_VOSU01000011.1_128180475-128181164_Phocoena_sinus_isolate_mPhoSin1_chromosome_1_whole_genome_shotgun_sequence
marine_Delleu_NW_019160964.1_2e-178_1_684_4706763_4706076
marine_Monmon_NW_021703774.1_9e-177_1_684_56258117_56258804
marine_RJWI010022047.1_691-1375_Pontoporia_blainvillei_isolate_BS67_PonBla_flattened_line_44054_whole_genome_shotgun_sequence
R_marine_RJWO010014021.1_19206-19895_Inia_geoffrensis_isolate_BS63_IniGeo_flattened_line_27974_whole_genome_shotgun_sequence
R_marine_RJWS010025837.1_8138-8824_Ziphius_cavirostris_isolate_BS68_ZipCav_flattened_line_51644_whole_genome_shotgun_sequence
R_marine_PVJJ010006566.1_45361-46050_Mesoplodon_bidens_isolate_US080_MesBid_scaffold_13124_whole_genome_shotgun_sequence
R_marine_RJWK010040377.1_18087-18776_Platanista_minor_isolate_BS66_PlaMin_flattened_line_80706_whole_genome_shotgun_sequence
marine_RJWL010011986.1_21687-22376_Kogia_breviceps_isolate_BS64_KogBre_flattened_line_23967_whole_genome_shotgun_sequence
marine_Phycat_NC_041217.1_6e-174_5_684_42076614_42075932
R_marine_VNFC01000001.1_127314872-127315561_Balaenoptera_musculus_isolate_JJ_BM4_2016_0621_chromosome_1_whole_genome_shotgun_sequence
R_marine_VNFC01000001.1_127314872-127315561_Balaenoptera_musculus_isolate_JJ_BM4_2016_0621_chromosome_1_whole_genome_shotgun_sequence_2_
marine_Balbon_DF429248.1_0.0_1_684_18654_17968
marine_Balacu_NW_006725126.1_0.0_1_684_521719_521033
marine_RYZJ01000174.1_5950632-5951321_Megaptera_novaeangliae_breed_North_Atlantic_isolate_ASU_GRON-1_ScjyzU5_42_whole_genome_shotgun_sequence
marine_NIPP01015570.1_28479-29168_Eschrichtius_robustus_isolate_ER-14-168_kmer60-min500.11739064_whole_genome_shotgun_sequence
R_marine_RJWP010022230.1_9528-10217_Eubalaena_japonica_isolate_BS61_EubJap_flattened_line_44420_whole_genome_shotgun_sequence
land1_Susscr_NC_010449.5_1e-76_8_402_73637138_73636744
PVJP02910700.1_1163018-1163694_Hippopotamus_amphibius_isolate_BS01_ScHSfme_925154HRSCAF_982954_whole_genome_shotgun_sequence
R_PVJP02006345.1_2682560-2683213_Hippopotamus_amphibius_isolate_BS01_ScHSfme_6317HRSCAF_52783_whole_genome_shotgun_sequence
R_PVJP02910210.1_2482574-2482949_Hippopotamus_amphibius_isolate_BS01_ScHSfme_924699HRSCAF_982499_0_whole_genome_shotgun_sequence
land1_Catwag_PVHT010007162.1_1e-80_38_367_94399_94070
land1_Vicpac_NW_005882931.1_3e-141_10_687_2015354_2016022
R_PVJP02910726.1_12004-12658_Hippopotamus_amphibius_isolate_BS01_ScHSfme_925179HRSCAF_982979_whole_genome_shotgun_sequence
PVJP02910316.1_553218-553592_Hippopotamus_amphibius_isolate_BS01_ScHSfme_924790HRSCAF_982590_whole_genome_shotgun_sequence
PVJP02910316.1_542181-542555_Hippopotamus_amphibius_isolate_BS01_ScHSfme_924790HRSCAF_982590_whole_genome_shotgun_sequence
R_PVJP02910316.1_354423-354922_Hippopotamus_amphibius_isolate_BS01_ScHSfme_924790HRSCAF_982590_whole_genome_shotgun_sequence
R_PVJP02000205.1_215765-216257_Hippopotamus_amphibius_isolate_BS01_ScHSfme_193HRSCAF_27289_whole_genome_shotgun_sequence
R_PVJP02910390.1_99701-100009_Hippopotamus_amphibius_isolate_BS01_ScHSfme_924861HRSCAF_982661_whole_genome_shotgun_sequence
land1_Girtip_LVKQ01S004919.1_2e-113_1_611_28480_27875
land1_Gircam_SJXV01009990.1_2e-113_1_611_1267580_1266975
land1_Okajoh_LVCL01S0002254.1_1e-111_1_611_228619_228014
land1_Sylgri_SJYH01000529.1_1e-101_1_615_845282_845901
land1_Trabux_SJYJ010079838.1_5e-46_364_615_297_44
land1_Susscr_NC_010456.5_6e-124_53_674_45167100_45166475
SOZM010152347.1_257-729_Manis_tricuspis_isolate_BS60_ManTri_flattened_line_304570_whole_genome_shotgun_sequence
RJWR010089937.1_1537-2009_Perognathus_longimembris_pacificus_isolate_BS65_PerPac_flattened_line_179787_whole_genome_shotgun_sequence
0.02

lineage66

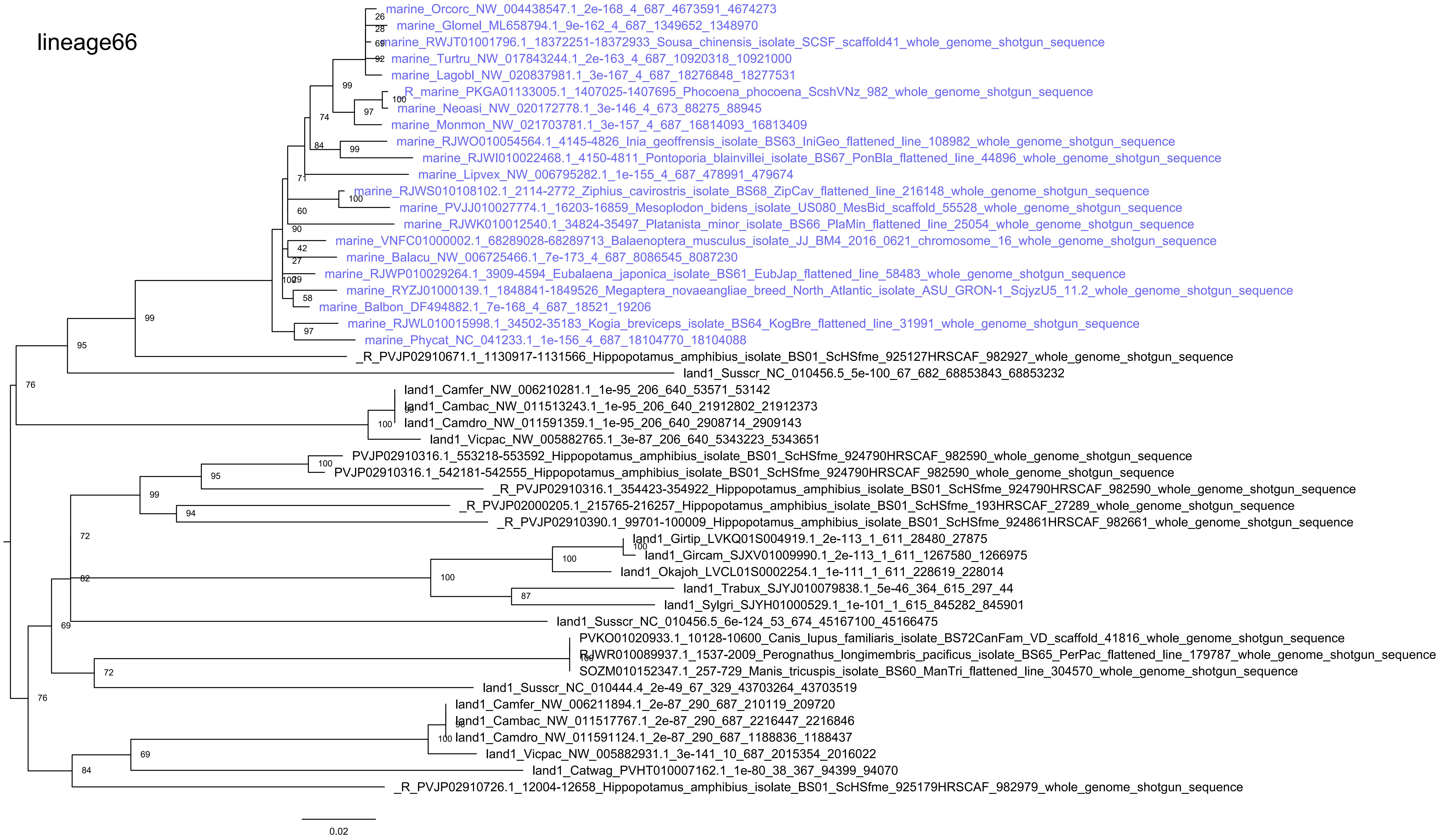

marine_Orcorc_NW_004438547.1_2e-168_4_687_4673591_4674273
marine_Glomel_ML658794.1_9e-162_4_687_1349652_1348970
marine_RWJT01001796.1_18372251-18372933_Sousa_chinensis_isolate_SCSF_scaffold41_whole_genome_shotgun_sequence
marine_Turtru_NW_017843244.1_2e-163_4_687_10920318_10921000
marine_Lagobl_NW_020837981.1_3e-167_4_687_18276848_18277531
R_marine_PKGA01133005.1_1407025-1407695_Phocoena_phocoena_ScshVNz_982_whole_genome_shotgun_sequence
marine_Neoasi_NW_020172778.1_3e-146_4_673_88275_88945
marine_Monmon_NW_021703781.1_3e-157_4_687_16814093_16813409
marine_RJWO010054564.1_4145-4826_Inia_geoffrensis_isolate_BS63_IniGeo_flattened_line_108982_whole_genome_shotgun_sequence
marine_RJWI010022468.1_4150-4811_Pontoporia_blainvillei_isolate_BS67_PonBla_flattened_line_44896_whole_genome_shotgun_sequence
marine_Lipvex_NW_006795282.1_1e-155_4_687_478991_479674
marine_RJWS010108102.1_2114-2772_Ziphius_cavirostris_isolate_BS68_ZipCav_flattened_line_216148_whole_genome_shotgun_sequence
marine_PVJJ010027774.1_16203-16859_Mesoplodon_bidens_isolate_US080_MesBid_scaffold_55528_whole_genome_shotgun_sequence
marine_RJWK010012540.1_34824-35497_Platanista_minor_isolate_BS66_PlaMin_flattened_line_25054_whole_genome_shotgun_sequence
marine_VNFC01000002.1_68289028-68289713_Balaenoptera_musculus_isolate_JJ_BM4_2016_0621_chromosome_16_whole_genome_shotgun_sequence
marine_Balacu_NW_006725466.1_7e-173_4_687_8086545_8087230
marine_RJWP010029264.1_3909-4594_Eubalaena_japonica_isolate_BS61_EubJap_flattened_line_58483_whole_genome_shotgun_sequence
marine_RYZJ01000139.1_1848841-1849526_Megaptera_novaeangliae_breed_North_Atlantic_isolate_ASU_GRON-1_ScjyzU5_11.2_whole_genome_shotgun_sequence
marine_Balbon_DF494882.1_7e-168_4_687_18521_19206
marine_RJWL010015998.1_34502-35183_Kogia_breviceps_isolate_BS64_KogBre_flattened_line_31991_whole_genome_shotgun_sequence
marine_Phycat_NC_041233.1_1e-156_4_687_18104770_18104088
_R_PVJP02910671.1_1130917-1131566_Hippopotamus_amphibius_isolate_BS01_ScHSfme_925127HRSCAF_982927_whole_genome_shotgun_sequence
land1_Susscr_NC_010456.5_5e-100_67_682_68853843_68853232
land1_Camfer_NW_006210281.1_1e-95_206_640_53571_53142
land1_Cambac_NW_011513243.1_1e-95_206_640_21912802_21912373
land1_Camdro_NW_011591359.1_1e-95_206_640_2908714_2909143
land1_Vicpac_NW_005882765.1_3e-87_206_640_5343223_5343651
PVJP02910316.1_553218-553592_Hippopotamus_amphibius_isolate_BS01_ScHSfme_924790HRSCAF_982590_whole_genome_shotgun_sequence
PVJP02910316.1_542181-542555_Hippopotamus_amphibius_isolate_BS01_ScHSfme_924790HRSCAF_982590_whole_genome_shotgun_sequence
_R_PVJP02910316.1_354423-354922_Hippopotamus_amphibius_isolate_BS01_ScHSfme_924790HRSCAF_982590_whole_genome_shotgun_sequence
_R_PVJP02000205.1_215765-216257_Hippopotamus_amphibius_isolate_BS01_ScHSfme_193HRSCAF_27289_whole_genome_shotgun_sequence
_R_PVJP02910390.1_99701-100009_Hippopotamus_amphibius_isolate_BS01_ScHSfme_924861HRSCAF_982661_whole_genome_shotgun_sequence
land1_Girtip_LVKQ01S004919.1_2e-113_1_611_28480_27875
land1_Gircam_SJXV01009990.1_2e-113_1_611_1267580_1266975
land1_Okajoh_LVCL01S0002254.1_1e-111_1_611_228619_228014
land1_Trabux_SJYJ010079838.1_5e-46_364_615_297_44
land1_Sylgri_SJYH01000529.1_1e-101_1_615_845282_845901
land1_Susscr_NC_010456.5_6e-124_53_674_45167100_45166475
PVKO01020933.1_10128-10600_Canis_lupus_familiaris_isolate_BS72CanFam_VD_scaffold_41816_whole_genome_shotgun_sequence
RJWR010089937.1_1537-2009_Perognathus_longimembris_pacificus_isolate_BS65_PerPac_flattened_line_179787_whole_genome_shotgun_sequence
SOZM010152347.1_257-729_Manis_tricuspis_isolate_BS60_ManTri_flattened_line_304570_whole_genome_shotgun_sequence
land1_Susscr_NC_010444.4_2e-49_67_329_43703264_43703519
land1_Camfer_NW_006211894.1_2e-87_290_687_210119_209720
land1_Cambac_NW_011517767.1_2e-87_290_687_2216447_2216846
land1_Camdro_NW_011591124.1_2e-87_290_687_1188836_1188437
land1_Vicpac_NW_005882931.1_3e-141_10_687_2015354_2016022
land1_Catwag_PVHT010007162.1_1e-80_38_367_94399_94070
_R_PVJP02910726.1_12004-12658_Hippopotamus_amphibius_isolate_BS01_ScHSfme_925179HRSCAF_982979_whole_genome_shotgun_sequence
0.02

lineage67

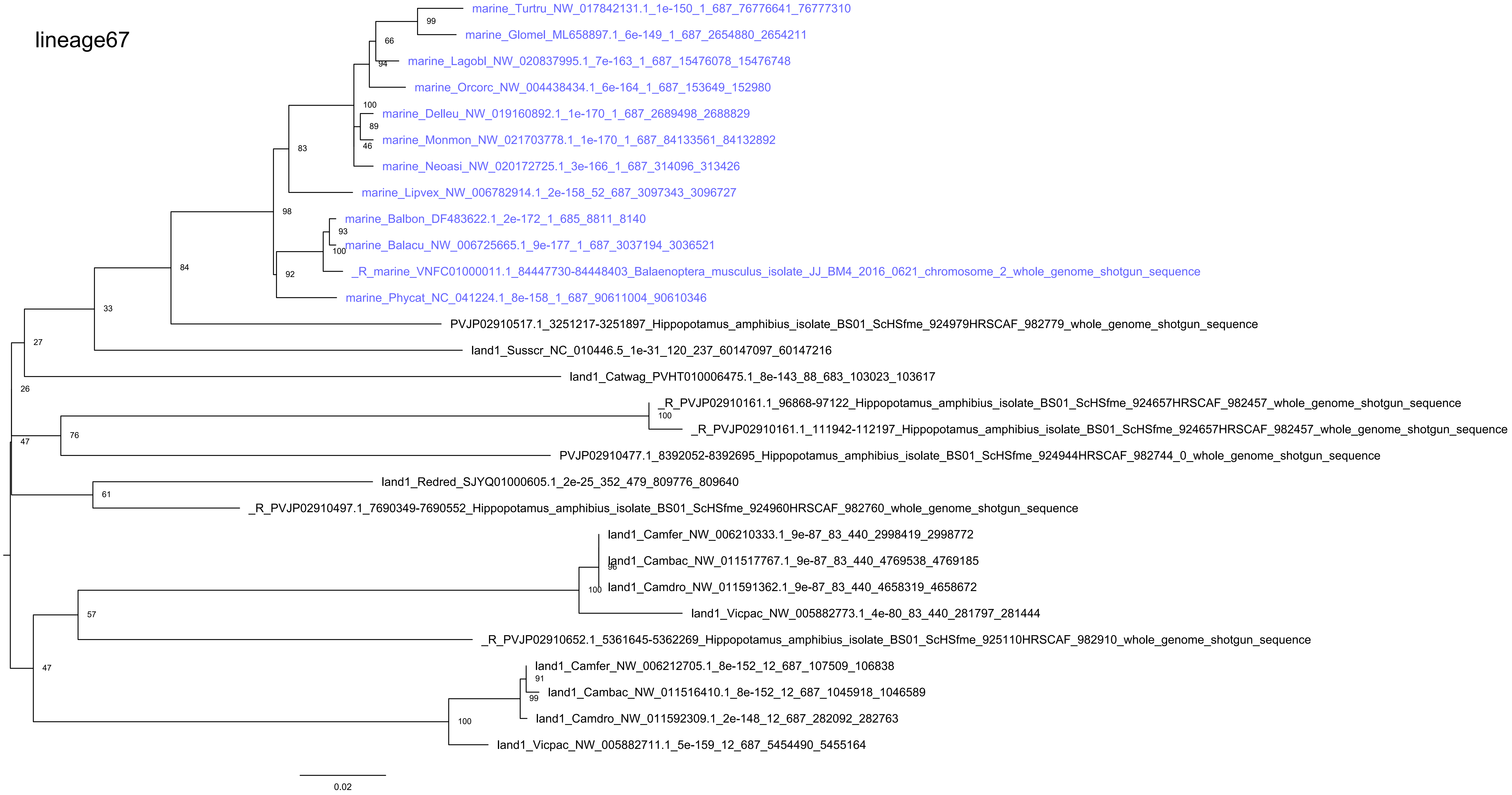
marine_Turtru_NW_017842131.1_1e-150_1_687_76776641_76777310
marine_Glomel_ML658897.1_6e-149_1_687_2654880_2654211
marine_Lagobl_NW_020837995.1_7e-163_1_687_15476078_15476748
marine_Orcorc_NW_004438434.1_6e-164_1_687_153649_152980
marine_Delleu_NW_019160892.1_1e-170_1_687_2689498_2688829
marine_Monmon_NW_021703778.1_1e-170_1_687_84133561_84132892
marine_Neoasi_NW_020172725.1_3e-166_1_687_314096_313426
marine_Lipvex_NW_006782914.1_2e-158_52_687_3097343_3096727
marine_Balbon_DF483622.1_2e-172_1_685_8811_8140
marine_Balacu_NW_006725665.1_9e-177_1_687_3037194_3036521
_R_marine_VNFC01000011.1_84447730-84448403_Balaenoptera_musculus_isolate_JJ_BM4_2016_0621_chromosome_2_whole_genome_shotgun_sequence
marine_Phycat_NC_041224.1_8e-158_1_687_90611004_90610346
PVJP02910517.1_3251217-3251897_Hippopotamus_amphibius_isolate_BS01_ScHSfme_924979HRSCAF_982779_whole_genome_shotgun_sequence
land1_Susscr_NC_010446.5_1e-31_120_237_60147097_60147216
land1_Catwag_PVHT010006475.1_8e-143_88_683_103023_103617
_R_PVJP02910161.1_96868-97122_Hippopotamus_amphibius_isolate_BS01_ScHSfme_924657HRSCAF_982457_whole_genome_shotgun_sequence
_R_PVJP02910161.1_111942-112197_Hippopotamus_amphibius_isolate_BS01_ScHSfme_924657HRSCAF_982457_whole_genome_shotgun_sequence
PVJP02910477.1_8392052-8392695_Hippopotamus_amphibius_isolate_BS01_ScHSfme_924944HRSCAF_982744_0_whole_genome_shotgun_sequence
land1_Redred_SJYQ01000605.1_2e-25_352_479_809776_809640
_R_PVJP02910497.1_7690349-7690552_Hippopotamus_amphibius_isolate_BS01_ScHSfme_924960HRSCAF_982760_whole_genome_shotgun_sequence
land1_Camfer_NW_006210333.1_9e-87_83_440_2998419_2998772
land1_Cambac_NW_011517767.1_9e-87_83_440_4769538_4769185
land1_Camdro_NW_011591362.1_9e-87_83_440_4658319_4658672
land1_Vicpac_NW_005882773.1_4e-80_83_440_281797_281444
_R_PVJP02910652.1_5361645-5362269_Hippopotamus_amphibius_isolate_BS01_ScHSfme_925110HRSCAF_982910_whole_genome_shotgun_sequence
land1_Camfer_NW_006212705.1_8e-152_12_687_107509_106838
land1_Cambac_NW_011516410.1_8e-152_12_687_1045918_1046589
land1_Camdro_NW_011592309.1_2e-148_12_687_282092_282763
land1_Vicpac_NW_005882711.1_5e-159_12_687_5454490_5455164
0.02

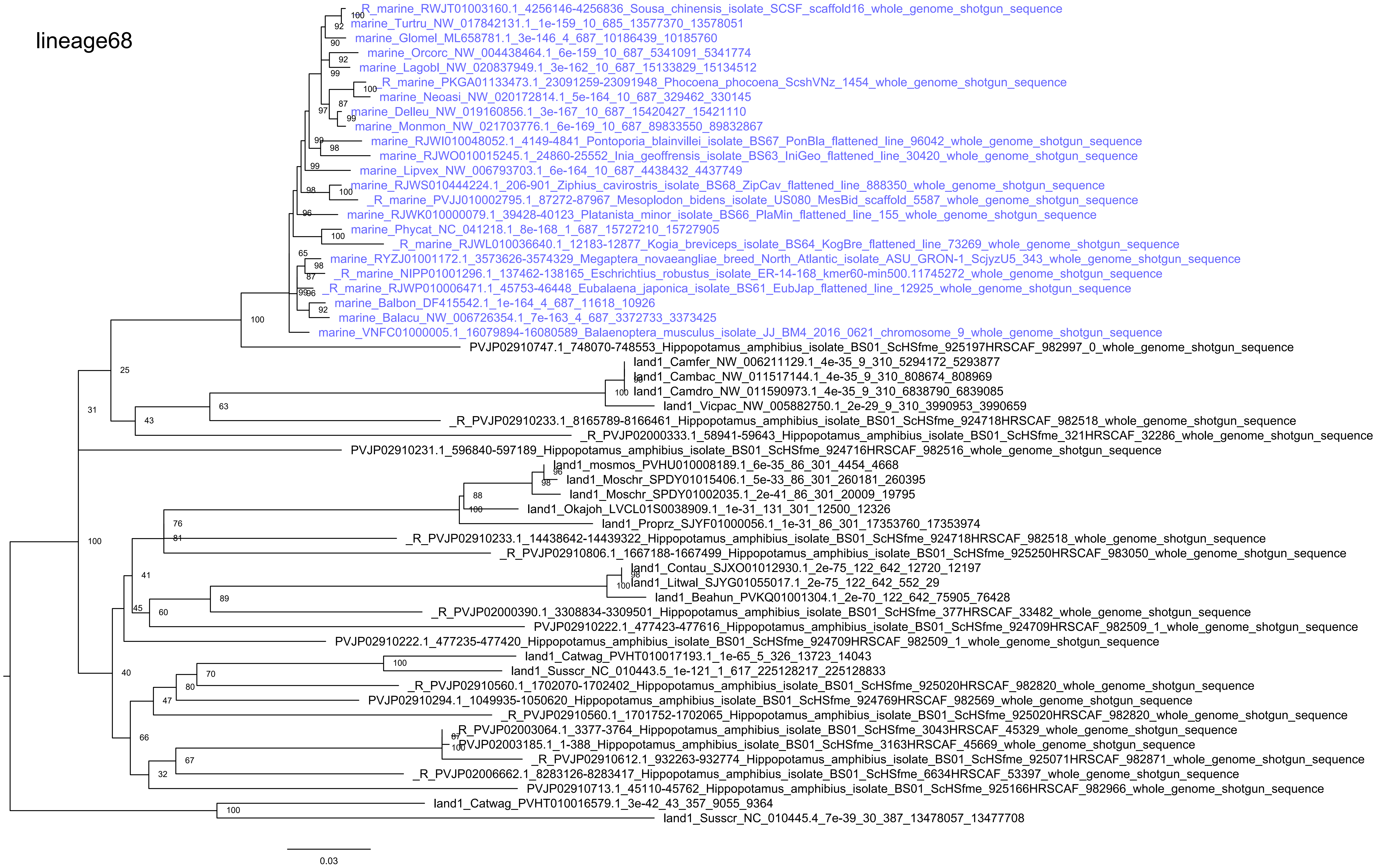
lineage68
_R_marine_RWJT01003160.1_4256146-4256836_Sousa_chinensis_isolate_SCSF_scaffold16_whole_genome_shotgun_sequence
marine_Turtru_NW_017842131.1_1e-159_10_685_13577370_13578051
marine_Glomel_ML658781.1_3e-146_4_687_10186439_10185760
marine_Orcorc_NW_004438464.1_6e-159_10_687_5341091_5341774
marine_Lagobl_NW_020837949.1_3e-162_10_687_15133829_15134512
_R_marine_PKGA01133473.1_23091259-23091948_Phocoena_phocoena_ScshVNz_1454_whole_genome_shotgun_sequence
marine_Neoasi_NW_020172814.1_5e-164_10_687_329462_330145
marine_Delleu_NW_019160856.1_3e-167_10_687_15420427_15421110
marine_Monmon_NW_021703776.1_6e-169_10_687_89833550_89832867
marine_RJWI010048052.1_4149-4841_Pontoporia_blainvillei_isolate_BS67_PonBla_flattened_line_96042_whole_genome_shotgun_sequence
marine_RJWO010015245.1_24860-25552_Inia_geoffrensis_isolate_BS63_IniGeo_flattened_line_30420_whole_genome_shotgun_sequence
marine_Lipvex_NW_006793703.1_6e-164_10_687_4438432_4437749
marine_RJWS010444224.1_206-901_Ziphius_cavirostris_isolate_BS68_ZipCav_flattened_line_888350_whole_genome_shotgun_sequence
_R_marine_PVJJ010002795.1_87272-87967_Mesoplodon_bidens_isolate_US080_MesBid_scaffold_5587_whole_genome_shotgun_sequence
marine_RJWK010000079.1_39428-40123_Platanista_minor_isolate_BS66_PlaMin_flattened_line_155_whole_genome_shotgun_sequence
marine_Phycat_NC_041218.1_8e-168_1_687_15727210_15727905
_R_marine_RJWL010036640.1_12183-12877_Kogia_breviceps_isolate_BS64_KogBre_flattened_line_73269_whole_genome_shotgun_sequence
marine_RYZJ01001172.1_3573626-3574329_Megaptera_novaeangliae_breed_North_Atlantic_isolate_ASU_GRON-1_ScjyzU5_343_whole_genome_shotgun_sequence
_R_marine_NIPP01001296.1_137462-138165_Eschrichtius_robustus_isolate_ER-14-168_kmer60-min500.11745272_whole_genome_shotgun_sequence
_R_marine_RJWP010006471.1_45753-46448_Eubalaena_japonica_isolate_BS61_EubJap_flattened_line_12925_whole_genome_shotgun_sequence
marine_Balbon_DF415542.1_1e-164_4_687_11618_10926
marine_Balacu_NW_006726354.1_7e-163_4_687_3372733_3373425
marine_VNFC01000005.1_16079894-16080589_Balaenoptera_musculus_isolate_JJ_BM4_2016_0621_chromosome_9_whole_genome_shotgun_sequence
PVJP02910747.1_748070-748553_Hippopotamus_amphibius_isolate_BS01_ScHSfme_925197HRSCAF_982997_0_whole_genome_shotgun_sequence
land1_Camfer_NW_006211129.1_4e-35_9_310_5294172_5293877
land1_Cambac_NW_011517144.1_4e-35_9_310_808674_808969
land1_Camdro_NW_011590973.1_4e-35_9_310_6838790_6839085
land1_Vicpac_NW_005882750.1_2e-29_9_310_3990953_3990659
_R_PVJP02910233.1_8165789-8166461_Hippopotamus_amphibius_isolate_BS01_ScHSfme_924718HRSCAF_982518_whole_genome_shotgun_sequence
_R_PVJP02000333.1_58941-59643_Hippopotamus_amphibius_isolate_BS01_ScHSfme_321HRSCAF_32286_whole_genome_shotgun_sequence
PVJP02910231.1_596840-597189_Hippopotamus_amphibius_isolate_BS01_ScHSfme_924716HRSCAF_982516_whole_genome_shotgun_sequence
land1_mosmos_PVHU010008189.1_6e-35_86_301_4454_4668
land1_Moschr_SPDY01015406.1_5e-33_86_301_260181_260395
land1_Moschr_SPDY01002035.1_2e-41_86_301_20009_19795
land1_Okajoh_LVCL01S0038909.1_1e-31_131_301_12500_12326
land1_Proprz_SJYF01000056.1_1e-31_86_301_17353760_17353974
_R_PVJP02910233.1_14438642-14439322_Hippopotamus_amphibius_isolate_BS01_ScHSfme_924718HRSCAF_982518_whole_genome_shotgun_sequence
_R_PVJP02910806.1_1667188-1667499_Hippopotamus_amphibius_isolate_BS01_ScHSfme_925250HRSCAF_983050_whole_genome_shotgun_sequence
land1_Contau_SJXO01012930.1_2e-75_122_642_12720_12197
land1_Litwal_SJYG01055017.1_2e-75_122_642_552_29
land1_Beahun_PVKQ01001304.1_2e-70_122_642_75905_76428
_R_PVJP02000390.1_3308834-3309501_Hippopotamus_amphibius_isolate_BS01_ScHSfme_377HRSCAF_33482_whole_genome_shotgun_sequence
PVJP02910222.1_477423-477616_Hippopotamus_amphibius_isolate_BS01_ScHSfme_924709HRSCAF_982509_1_whole_genome_shotgun_sequence
PVJP02910222.1_477235-477420_Hippopotamus_amphibius_isolate_BS01_ScHSfme_924709HRSCAF_982509_1_whole_genome_shotgun_sequence
land1_Catwag_PVHT010017193.1_1e-65_5_326_13723_14043
land1_Susscr_NC_010443.5_1e-121_1_617_225128217_225128833
_R_PVJP02910560.1_1702070-1702402_Hippopotamus_amphibius_isolate_BS01_ScHSfme_925020HRSCAF_982820_whole_genome_shotgun_sequence
PVJP02910294.1_1049935-1050620_Hippopotamus_amphibius_isolate_BS01_ScHSfme_924769HRSCAF_982569_whole_genome_shotgun_sequence
_R_PVJP02910560.1_1701752-1702065_Hippopotamus_amphibius_isolate_BS01_ScHSfme_925020HRSCAF_982820_whole_genome_shotgun_sequence
_R_PVJP02003064.1_3377-3764_Hippopotamus_amphibius_isolate_BS01_ScHSfme_3043HRSCAF_45329_whole_genome_shotgun_sequence
PVJP02003185.1_1-388_Hippopotamus_amphibius_isolate_BS01_ScHSfme_3163HRSCAF_45669_whole_genome_shotgun_sequence
_R_PVJP02910612.1_932263-932774_Hippopotamus_amphibius_isolate_BS01_ScHSfme_925071HRSCAF_982871_whole_genome_shotgun_sequence
_R_PVJP02006662.1_8283126-8283417_Hippopotamus_amphibius_isolate_BS01_ScHSfme_6634HRSCAF_53397_whole_genome_shotgun_sequence
PVJP02910713.1_45110-45762_Hippopotamus_amphibius_isolate_BS01_ScHSfme_925166HRSCAF_982966_whole_genome_shotgun_sequence
land1_Catwag_PVHT010016579.1_3e-42_43_357_9055_9364
land1_Susscr_NC_010445.4_7e-39_30_387_13478057_13477708
0.03

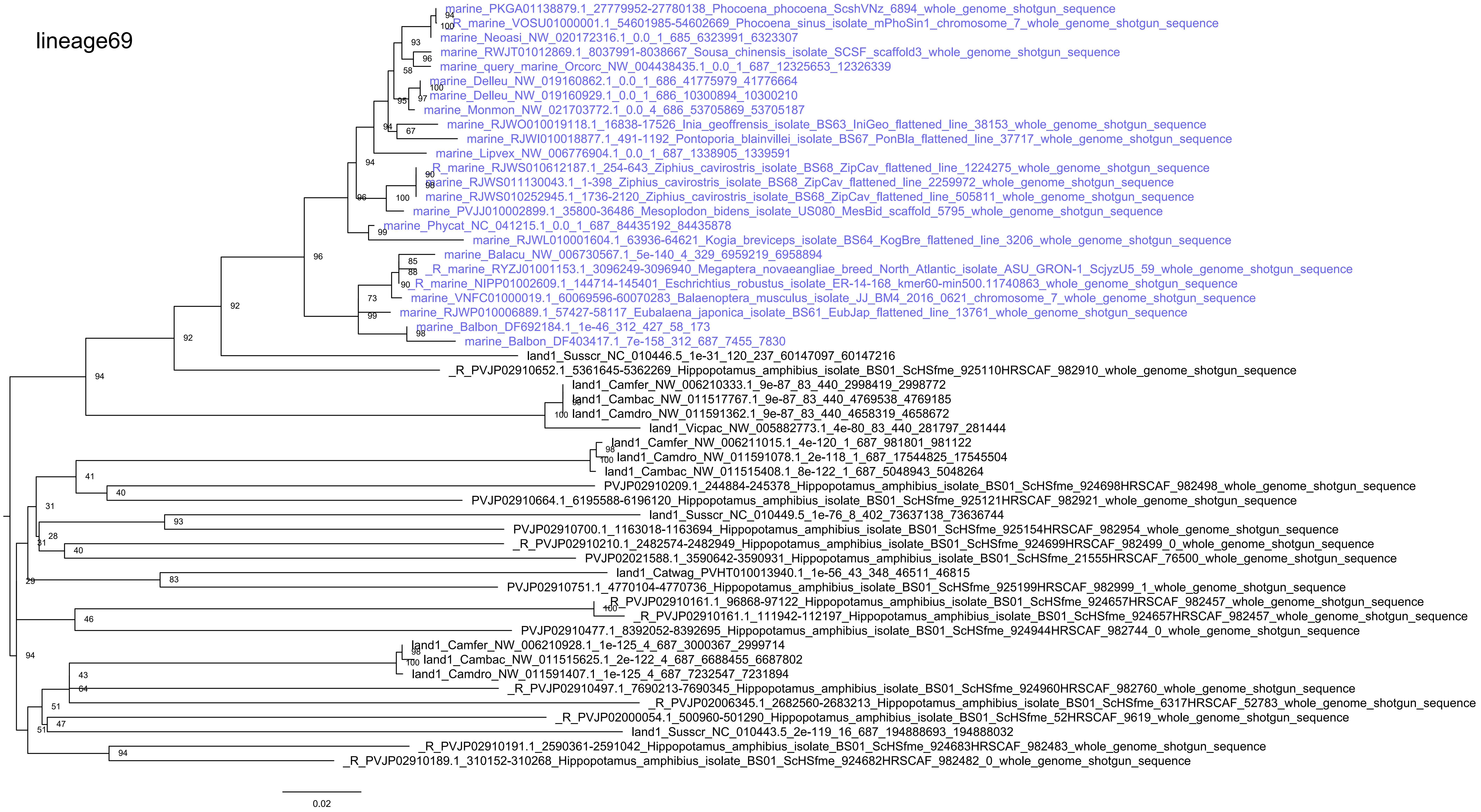
lineage69
marine_PKGA01138879.1_27779952-27780138_Phocoena_phocoena_ScshVNz_6894_whole_genome_shotgun_sequence
_R_marine_VOSU01000001.1_54601985-54602669_Phocoena_sinus_isolate_mPhoSin1_chromosome_7_whole_genome_shotgun_sequence
marine_Neoasi_NW_020172316.1_0.0_1_685_6323991_6323307
marine_RWJT01012869.1_8037991-8038667_Sousa_chinensis_isolate_SCSF_scaffold3_whole_genome_shotgun_sequence
marine_query_marine_Orcorc_NW_004438435.1_0.0_1_687_12325653_12326339
marine_Delleu_NW_019160862.1_0.0_1_686_41775979_41776664
marine_Delleu_NW_019160929.1_0.0_1_686_10300894_10300210
marine_Monmon_NW_021703772.1_0.0_4_686_53705869_53705187
marine_RJWO010019118.1_16838-17526_Inia_geoffrensis_isolate_BS63_IniGeo_flattened_line_38153_whole_genome_shotgun_sequence
marine_RJWI010018877.1_491-1192_Pontoporia_blainvillei_isolate_BS67_PonBla_flattened_line_37717_whole_genome_shotgun_sequence
marine_Lipvex_NW_006776904.1_0.0_1_687_1338905_1339591
_R_marine_RJWS010612187.1_254-643_Ziphius_cavirostris_isolate_BS68_ZipCav_flattened_line_1224275_whole_genome_shotgun_sequence
marine_RJWS011130043.1_1-398_Ziphius_cavirostris_isolate_BS68_ZipCav_flattened_line_2259972_whole_genome_shotgun_sequence
marine_RJWS010252945.1_1736-2120_Ziphius_cavirostris_isolate_BS68_ZipCav_flattened_line_505811_whole_genome_shotgun_sequence
marine_PVJJ010002899.1_35800-36486_Mesoplodon_bidens_isolate_US080_MesBid_scaffold_5795_whole_genome_shotgun_sequence
marine_Phycat_NC_041215.1_0.0_1_687_84435192_84435878
marine_RJWL010001604.1_63936-64621_Kogia_breviceps_isolate_BS64_KogBre_flattened_line_3206_whole_genome_shotgun_sequence
marine_Balacu_NW_006730567.1_5e-140_4_329_6959219_6958894
_R_marine_RYZJ01001153.1_3096249-3096940_Megaptera_novaeangliae_breed_North_Atlantic_isolate_ASU_GRON-1_ScjyzU5_59_whole_genome_shotgun_sequence
_R_marine_NIPP01002609.1_144714-145401_Eschrichtius_robustus_isolate_ER-14-168_kmer60-min500.11740863_whole_genome_shotgun_sequence
marine_VNFC01000019.1_60069596-60070283_Balaenoptera_musculus_isolate_JJ_BM4_2016_0621_chromosome_7_whole_genome_shotgun_sequence
marine_RJWP010006889.1_57427-58117_Eubalaena_japonica_isolate_BS61_EubJap_flattened_line_13761_whole_genome_shotgun_sequence
marine_Balbon_DF692184.1_1e-46_312_427_58_173
marine_Balbon_DF403417.1_7e-158_312_687_7455_7830
land1_Susscr_NC_010446.5_1e-31_120_237_60147097_60147216
_R_PVJP02910652.1_5361645-5362269_Hippopotamus_amphibius_isolate_BS01_ScHSfme_925110HRSCAF_982910_whole_genome_shotgun_sequence
land1_Camfer_NW_006210333.1_9e-87_83_440_2998419_2998772
land1_Cambac_NW_011517767.1_9e-87_83_440_4769538_4769185
land1_Camdro_NW_011591362.1_9e-87_83_440_4658319_4658672
land1_Vicpac_NW_005882773.1_4e-80_83_440_281797_281444
land1_Camfer_NW_006211015.1_4e-120_1_687_981801_981122
land1_Camdro_NW_011591078.1_2e-118_1_687_17544825_17545504
land1_Cambac_NW_011515408.1_8e-122_1_687_5048943_5048264
PVJP02910209.1_244884-245378_Hippopotamus_amphibius_isolate_BS01_ScHSfme_924698HRSCAF_982498_whole_genome_shotgun_sequence
PVJP02910664.1_6195588-6196120_Hippopotamus_amphibius_isolate_BS01_ScHSfme_925121HRSCAF_982921_whole_genome_shotgun_sequence
land1_Susscr_NC_010449.5_1e-76_8_402_73637138_73636744
PVJP02910700.1_1163018-1163694_Hippopotamus_amphibius_isolate_BS01_ScHSfme_925154HRSCAF_982954_whole_genome_shotgun_sequence
_R_PVJP02910210.1_2482574-2482949_Hippopotamus_amphibius_isolate_BS01_ScHSfme_924699HRSCAF_982499_0_whole_genome_shotgun_sequence
PVJP02021588.1_3590642-3590931_Hippopotamus_amphibius_isolate_BS01_ScHSfme_21555HRSCAF_76500_whole_genome_shotgun_sequence
land1_Catwag_PVHT010013940.1_1e-56_43_348_46511_46815
PVJP02910751.1_4770104-4770736_Hippopotamus_amphibius_isolate_BS01_ScHSfme_925199HRSCAF_982999_1_whole_genome_shotgun_sequence
_R_PVJP02910161.1_96868-97122_Hippopotamus_amphibius_isolate_BS01_ScHSfme_924657HRSCAF_982457_whole_genome_shotgun_sequence
_R_PVJP02910161.1_111942-112197_Hippopotamus_amphibius_isolate_BS01_ScHSfme_924657HRSCAF_982457_whole_genome_shotgun_sequence
PVJP02910477.1_8392052-8392695_Hippopotamus_amphibius_isolate_BS01_ScHSfme_924944HRSCAF_982744_0_whole_genome_shotgun_sequence
land1_Camfer_NW_006210928.1_1e-125_4_687_3000367_2999714
land1_Cambac_NW_011515625.1_2e-122_4_687_6688455_6687802
land1_Camdro_NW_011591407.1_1e-125_4_687_7232547_7231894
_R_PVJP02910497.1_7690213-7690345_Hippopotamus_amphibius_isolate_BS01_ScHSfme_924960HRSCAF_982760_whole_genome_shotgun_sequence
_R_PVJP02006345.1_2682560-2683213_Hippopotamus_amphibius_isolate_BS01_ScHSfme_6317HRSCAF_52783_whole_genome_shotgun_sequence
_R_PVJP02000054.1_500960-501290_Hippopotamus_amphibius_isolate_BS01_ScHSfme_52HRSCAF_9619_whole_genome_shotgun_sequence
land1_Susscr_NC_010443.5_2e-119_16_687_194888693_194888032
_R_PVJP02910191.1_2590361-2591042_Hippopotamus_amphibius_isolate_BS01_ScHSfme_924683HRSCAF_982483_whole_genome_shotgun_sequence
_R_PVJP02910189.1_310152-310268_Hippopotamus_amphibius_isolate_BS01_ScHSfme_924682HRSCAF_982482_0_whole_genome_shotgun_sequence
0.02

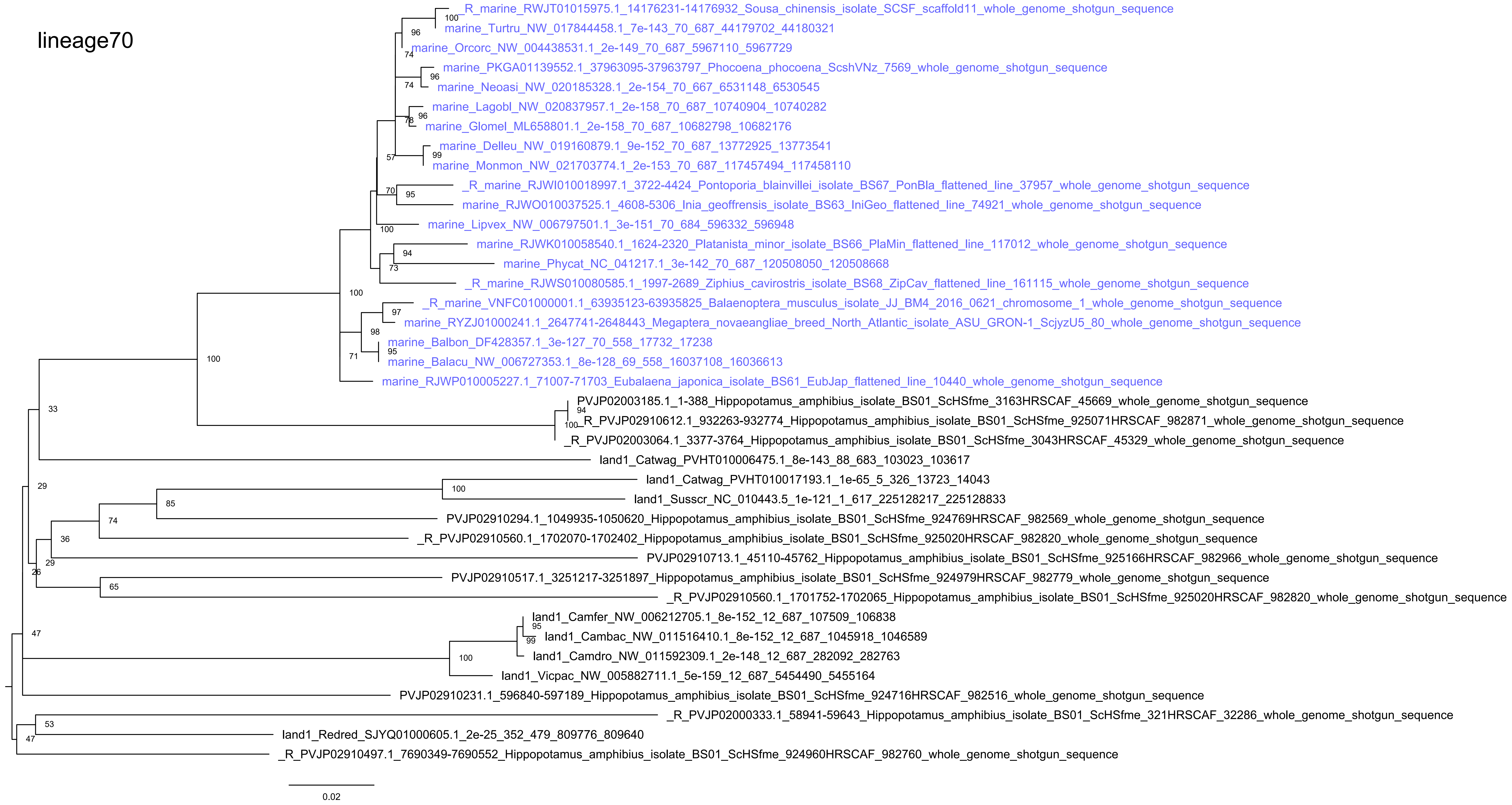
lineage70
_R_marine_RWJT01015975.1_14176231-14176932_Sousa_chinensis_isolate_SCSF_scaffold11_whole_genome_shotgun_sequence
marine_Turtru_NW_017844458.1_7e-143_70_687_44179702_44180321
marine_Orcorc_NW_004438531.1_2e-149_70_687_5967110_5967729
marine_PKGA01139552.1_37963095-37963797_Phocoena_phocoena_ScshVNz_7569_whole_genome_shotgun_sequence
marine_Neoasi_NW_020185328.1_2e-154_70_667_6531148_6530545
marine_Lagobl_NW_020837957.1_2e-158_70_687_10740904_10740282
marine_Glomel_ML658801.1_2e-158_70_687_10682798_10682176
marine_Delleu_NW_019160879.1_9e-152_70_687_13772925_13773541
marine_Monmon_NW_021703774.1_2e-153_70_687_117457494_117458110
_R_marine_RJWI010018997.1_3722-4424_Pontoporia_blainvillei_isolate_BS67_PonBla_flattened_line_37957_whole_genome_shotgun_sequence
marine_RJWO010037525.1_4608-5306_Inia_geoffrensis_isolate_BS63_IniGeo_flattened_line_74921_whole_genome_shotgun_sequence
marine_Lipvex_NW_006797501.1_3e-151_70_684_596332_596948
marine_RJWK010058540.1_1624-2320_Platanista_minor_isolate_BS66_PlaMin_flattened_line_117012_whole_genome_shotgun_sequence
marine_Phycat_NC_041217.1_3e-142_70_687_120508050_120508668
_R_marine_RJWS010080585.1_1997-2689_Ziphius_cavirostris_isolate_BS68_ZipCav_flattened_line_161115_whole_genome_shotgun_sequence
_R_marine_VNFC01000001.1_63935123-63935825_Balaenoptera_musculus_isolate_JJ_BM4_2016_0621_chromosome_1_whole_genome_shotgun_sequence
marine_RYZJ01000241.1_2647741-2648443_Megaptera_novaeangliae_breed_North_Atlantic_isolate_ASU_GRON-1_ScjyzU5_80_whole_genome_shotgun_sequence
marine_Balbon_DF428357.1_3e-127_70_558_17732_17238
marine_Balacu_NW_006727353.1_8e-128_69_558_16037108_16036613
marine_RJWP010005227.1_71007-71703_Eubalaena_japonica_isolate_BS61_EubJap_flattened_line_10440_whole_genome_shotgun_sequence
PVJP02003185.1_1-388_Hippopotamus_amphibius_isolate_BS01_ScHSfme_3163HRSCAF_45669_whole_genome_shotgun_sequence
_R_PVJP02910612.1_932263-932774_Hippopotamus_amphibius_isolate_BS01_ScHSfme_925071HRSCAF_982871_whole_genome_shotgun_sequence
_R_PVJP02003064.1_3377-3764_Hippopotamus_amphibius_isolate_BS01_ScHSfme_3043HRSCAF_45329_whole_genome_shotgun_sequence
land1_Catwag_PVHT010006475.1_8e-143_88_683_103023_103617
land1_Catwag_PVHT010017193.1_1e-65_5_326_13723_14043
land1_Susscr_NC_010443.5_1e-121_1_617_225128217_225128833
PVJP02910294.1_1049935-1050620_Hippopotamus_amphibius_isolate_BS01_ScHSfme_924769HRSCAF_982569_whole_genome_shotgun_sequence
_R_PVJP02910560.1_1702070-1702402_Hippopotamus_amphibius_isolate_BS01_ScHSfme_925020HRSCAF_982820_whole_genome_shotgun_sequence
PVJP02910713.1_45110-45762_Hippopotamus_amphibius_isolate_BS01_ScHSfme_925166HRSCAF_982966_whole_genome_shotgun_sequence
PVJP02910517.1_3251217-3251897_Hippopotamus_amphibius_isolate_BS01_ScHSfme_924979HRSCAF_982779_whole_genome_shotgun_sequence
_R_PVJP02910560.1_1701752-1702065_Hippopotamus_amphibius_isolate_BS01_ScHSfme_925020HRSCAF_982820_whole_genome_shotgun_sequence
land1_Camfer_NW_006212705.1_8e-152_12_687_107509_106838
land1_Cambac_NW_011516410.1_8e-152_12_687_1045918_1046589
land1_Camdro_NW_011592309.1_2e-148_12_687_282092_282763
land1_Vicpac_NW_005882711.1_5e-159_12_687_5454490_5455164
PVJP02910231.1_596840-597189_Hippopotamus_amphibius_isolate_BS01_ScHSfme_924716HRSCAF_982516_whole_genome_shotgun_sequence
_R_PVJP02000333.1_58941-59643_Hippopotamus_amphibius_isolate_BS01_ScHSfme_321HRSCAF_32286_whole_genome_shotgun_sequence
land1_Redred_SJYQ01000605.1_2e-25_352_479_809776_809640
_R_PVJP02910497.1_7690349-7690552_Hippopotamus_amphibius_isolate_BS01_ScHSfme_924960HRSCAF_982760_whole_genome_shotgun_sequence
0.02

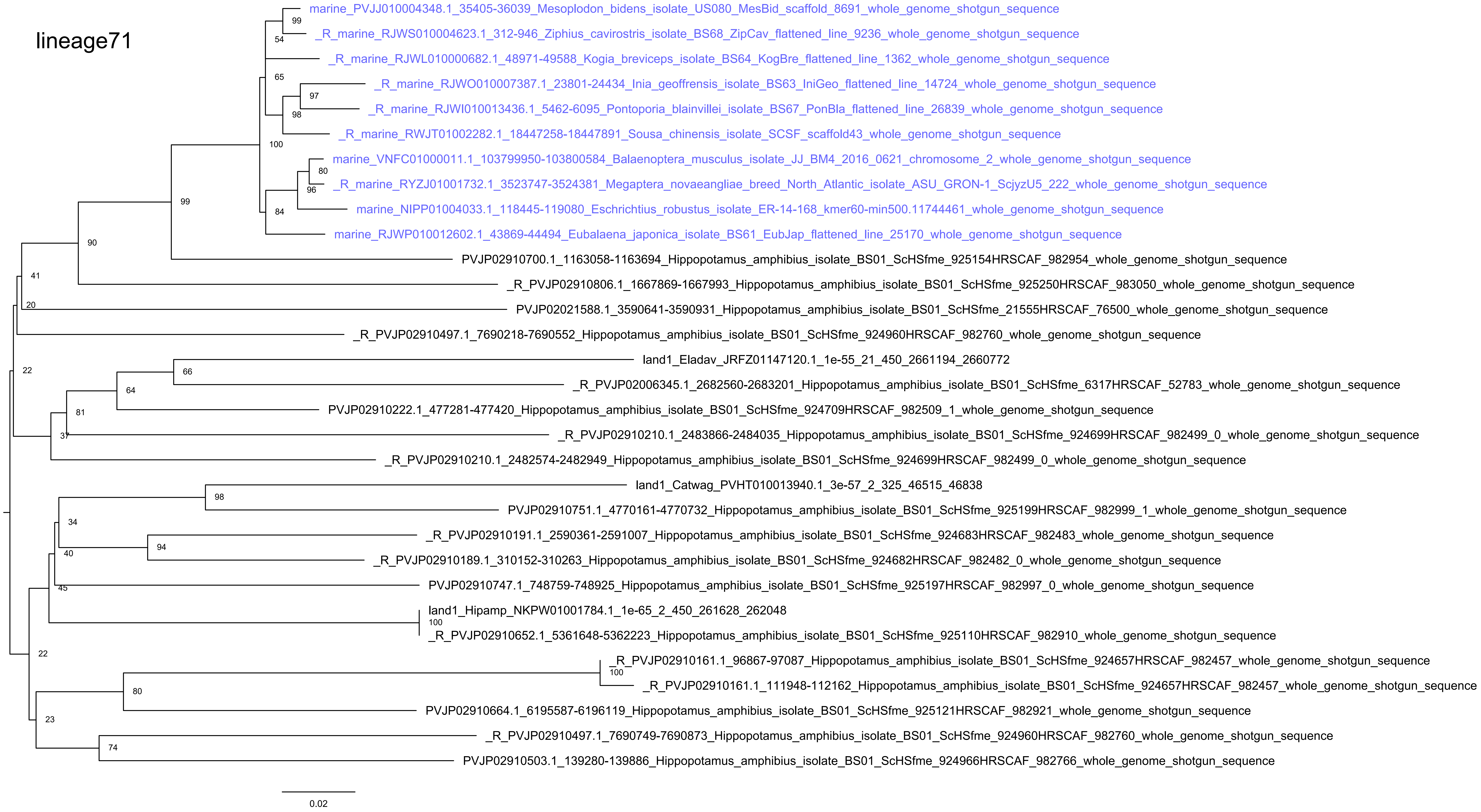

lineage71
marine_PVJJ010004348.1_35405-36039_Mesoplodon_bidens_isolate_US080_MesBid_scaffold_8691_whole_genome_shotgun_sequence
_R_marine_RJWS010004623.1_312-946_Ziphius_cavirostris_isolate_BS68_ZipCav_flattened_line_9236_whole_genome_shotgun_sequence
_R_marine_RJWL010000682.1_48971-49588_Kogia_breviceps_isolate_BS64_KogBre_flattened_line_1362_whole_genome_shotgun_sequence
_R_marine_RJWO010007387.1_23801-24434_Inia_geoffrensis_isolate_BS63_IniGeo_flattened_line_14724_whole_genome_shotgun_sequence
_R_marine_RJWI010013436.1_5462-6095_Pontoporia_blainvillei_isolate_BS67_PonBla_flattened_line_26839_whole_genome_shotgun_sequence
_R_marine_RWJT01002282.1_18447258-18447891_Sousa_chinensis_isolate_SCSF_scaffold43_whole_genome_shotgun_sequence
marine_VNFC01000011.1_103799950-103800584_Balaenoptera_musculus_isolate_JJ_BM4_2016_0621_chromosome_2_whole_genome_shotgun_sequence
_R_marine_RYZJ01001732.1_3523747-3524381_Megaptera_novaeangliae_breed_North_Atlantic_isolate_ASU_GRON-1_ScjyzU5_222_whole_genome_shotgun_sequence
marine_NIPP01004033.1_118445-119080_Eschrichtius_robustus_isolate_ER-14-168_kmer60-min500.11744461_whole_genome_shotgun_sequence
marine_RJWP010012602.1_43869-44494_Eubalaena_japonica_isolate_BS61_EubJap_flattened_line_25170_whole_genome_shotgun_sequence
PVJP02910700.1_1163058-1163694_Hippopotamus_amphibius_isolate_BS01_ScHSfme_925154HRSCAF_982954_whole_genome_shotgun_sequence
_R_PVJP02910806.1_1667869-1667993_Hippopotamus_amphibius_isolate_BS01_ScHSfme_925250HRSCAF_983050_whole_genome_shotgun_sequence
PVJP02021588.1_3590641-3590931_Hippopotamus_amphibius_isolate_BS01_ScHSfme_21555HRSCAF_76500_whole_genome_shotgun_sequence
_R_PVJP02910497.1_7690218-7690552_Hippopotamus_amphibius_isolate_BS01_ScHSfme_924960HRSCAF_982760_whole_genome_shotgun_sequence
land1_Eladav_JRFZ01147120.1_1e-55_21_450_2661194_2660772
_R_PVJP02006345.1_2682560-2683201_Hippopotamus_amphibius_isolate_BS01_ScHSfme_6317HRSCAF_52783_whole_genome_shotgun_sequence
PVJP02910222.1_477281-477420_Hippopotamus_amphibius_isolate_BS01_ScHSfme_924709HRSCAF_982509_1_whole_genome_shotgun_sequence
_R_PVJP02910210.1_2483866-2484035_Hippopotamus_amphibius_isolate_BS01_ScHSfme_924699HRSCAF_982499_0_whole_genome_shotgun_sequence
_R_PVJP02910210.1_2482574-2482949_Hippopotamus_amphibius_isolate_BS01_ScHSfme_924699HRSCAF_982499_0_whole_genome_shotgun_sequence
land1_Catwag_PVHT010013940.1_3e-57_2_325_46515_46838
PVJP02910751.1_4770161-4770732_Hippopotamus_amphibius_isolate_BS01_ScHSfme_925199HRSCAF_982999_1_whole_genome_shotgun_sequence
_R_PVJP02910191.1_2590361-2591007_Hippopotamus_amphibius_isolate_BS01_ScHSfme_924683HRSCAF_982483_whole_genome_shotgun_sequence
_R_PVJP02910189.1_310152-310263_Hippopotamus_amphibius_isolate_BS01_ScHSfme_924682HRSCAF_982482_0_whole_genome_shotgun_sequence
PVJP02910747.1_748759-748925_Hippopotamus_amphibius_isolate_BS01_ScHSfme_925197HRSCAF_982997_0_whole_genome_shotgun_sequence
land1_Hipamp_NKPW01001784.1_1e-65_2_450_261628_262048
_R_PVJP02910652.1_5361648-5362223_Hippopotamus_amphibius_isolate_BS01_ScHSfme_925110HRSCAF_982910_whole_genome_shotgun_sequence
_R_PVJP02910161.1_96867-97087_Hippopotamus_amphibius_isolate_BS01_ScHSfme_924657HRSCAF_982457_whole_genome_shotgun_sequence
_R_PVJP02910161.1_111948-112162_Hippopotamus_amphibius_isolate_BS01_ScHSfme_924657HRSCAF_982457_whole_genome_shotgun_sequence
PVJP02910664.1_6195587-6196119_Hippopotamus_amphibius_isolate_BS01_ScHSfme_925121HRSCAF_982921_whole_genome_shotgun_sequence
_R_PVJP02910497.1_7690749-7690873_Hippopotamus_amphibius_isolate_BS01_ScHSfme_924960HRSCAF_982760_whole_genome_shotgun_sequence
PVJP02910503.1_139280-139886_Hippopotamus_amphibius_isolate_BS01_ScHSfme_924966HRSCAF_982766_whole_genome_shotgun_sequence
0.02

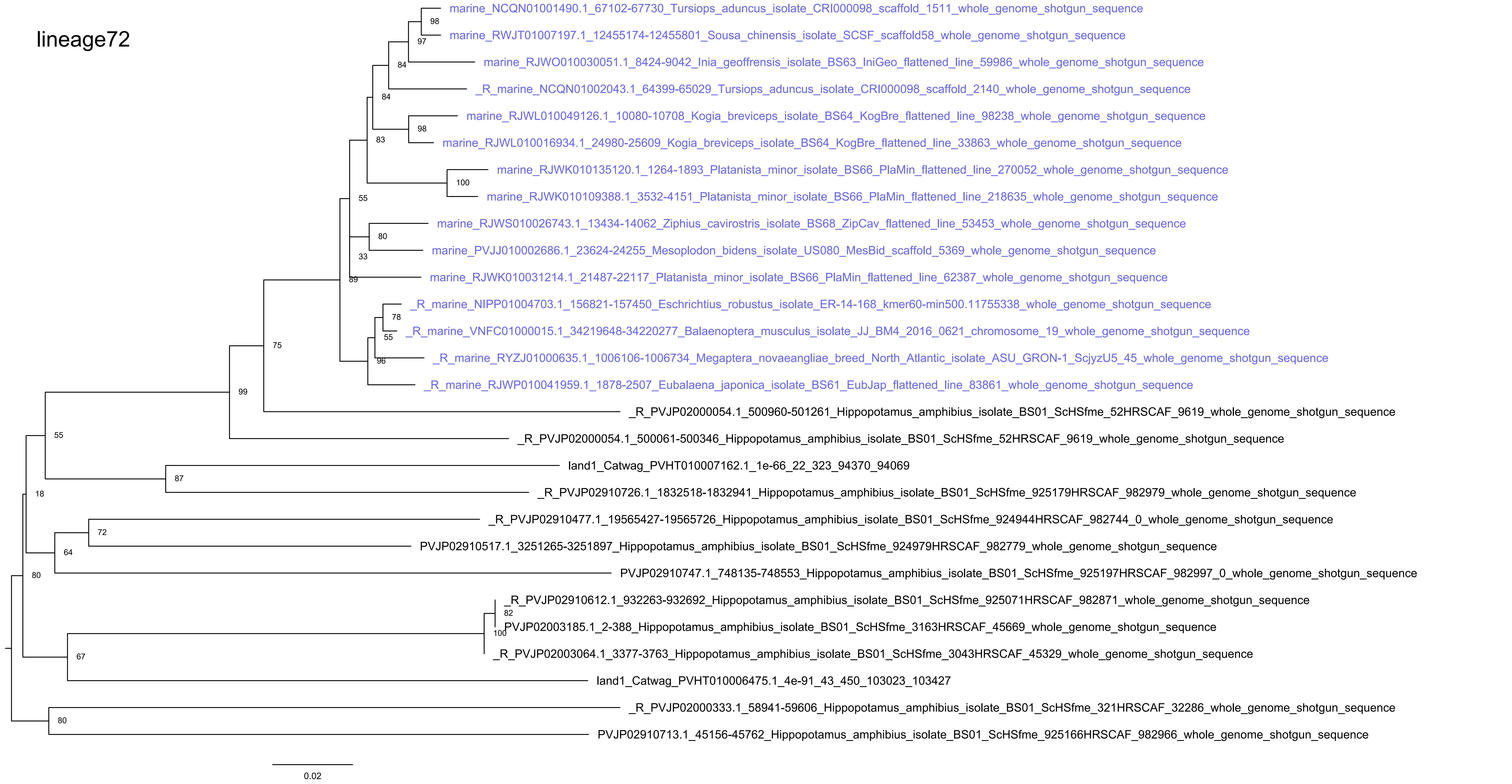

lineage72
marine_NCQN01001490.1_67102-67730_Tursiops_aduncus_isolate_CRI000098_scaffold_1511_whole_genome_shotgun_sequence
marine_RWJT01007197.1_12455174-12455801_Sousa_chinensis_isolate_SCSF_scaffold58_whole_genome_shotgun_sequence
marine_RJWO010030051.1_8424-9042_Inia_geoffrensis_isolate_BS63_IniGeo_flattened_line_59986_whole_genome_shotgun_sequence
_R_marine_NCQN01002043.1_64399-65029_Tursiops_aduncus_isolate_CRI000098_scaffold_2140_whole_genome_shotgun_sequence
marine_RJWL010049126.1_10080-10708_Kogia_breviceps_isolate_BS64_KogBre_flattened_line_98238_whole_genome_shotgun_sequence
marine_RJWL010016934.1_24980-25609_Kogia_breviceps_isolate_BS64_KogBre_flattened_line_33863_whole_genome_shotgun_sequence
marine_RJWK010135120.1_1264-1893_Platanista_minor_isolate_BS66_PlaMin_flattened_line_270052_whole_genome_shotgun_sequence
marine_RJWK010109388.1_3532-4151_Platanista_minor_isolate_BS66_PlaMin_flattened_line_218635_whole_genome_shotgun_sequence
marine_RJWS010026743.1_13434-14062_Ziphius_cavirostris_isolate_BS68_ZipCav_flattened_line_53453_whole_genome_shotgun_sequence
marine_PVJJ010002686.1_23624-24255_Mesoplodon_bidens_isolate_US080_MesBid_scaffold_5369_whole_genome_shotgun_sequence
marine_RJWK010031214.1_21487-22117_Platanista_minor_isolate_BS66_PlaMin_flattened_line_62387_whole_genome_shotgun_sequence
_R_marine_NIPP01004703.1_156821-157450_Eschrichtius_robustus_isolate_ER-14-168_kmer60-min500.11755338_whole_genome_shotgun_sequence
_R_marine_VNFC01000015.1_34219648-34220277_Balaenoptera_musculus_isolate_JJ_BM4_2016_0621_chromosome_19_whole_genome_shotgun_sequence
_R_marine_RYZJ01000635.1_1006106-1006734_Megaptera_novaeangliae_breed_North_Atlantic_isolate_ASU_GRON-1_ScjyzU5_45_whole_genome_shotgun_sequence
_R_marine_RJWP010041959.1_1878-2507_Eubalaena_japonica_isolate_BS61_EubJap_flattened_line_83861_whole_genome_shotgun_sequence
_R_PVJP02000054.1_500960-501261_Hippopotamus_amphibius_isolate_BS01_ScHSfme_52HRSCAF_9619_whole_genome_shotgun_sequence
_R_PVJP02000054.1_500061-500346_Hippopotamus_amphibius_isolate_BS01_ScHSfme_52HRSCAF_9619_whole_genome_shotgun_sequence
land1_Catwag_PVHT010007162.1_1e-66_22_323_94370_94069
_R_PVJP02910726.1_1832518-1832941_Hippopotamus_amphibius_isolate_BS01_ScHSfme_925179HRSCAF_982979_whole_genome_shotgun_sequence
_R_PVJP02910477.1_19565427-19565726_Hippopotamus_amphibius_isolate_BS01_ScHSfme_924944HRSCAF_982744_0_whole_genome_shotgun_sequence
PVJP02910517.1_3251265-3251897_Hippopotamus_amphibius_isolate_BS01_ScHSfme_924979HRSCAF_982779_whole_genome_shotgun_sequence
PVJP02910747.1_748135-748553_Hippopotamus_amphibius_isolate_BS01_ScHSfme_925197HRSCAF_982997_0_whole_genome_shotgun_sequence
_R_PVJP02910612.1_932263-932692_Hippopotamus_amphibius_isolate_BS01_ScHSfme_925071HRSCAF_982871_whole_genome_shotgun_sequence
PVJP02003185.1_2-388_Hippopotamus_amphibius_isolate_BS01_ScHSfme_3163HRSCAF_45669_whole_genome_shotgun_sequence
_R_PVJP02003064.1_3377-3763_Hippopotamus_amphibius_isolate_BS01_ScHSfme_3043HRSCAF_45329_whole_genome_shotgun_sequence
land1_Catwag_PVHT010006475.1_4e-91_43_450_103023_103427
_R_PVJP02000333.1_58941-59606_Hippopotamus_amphibius_isolate_BS01_ScHSfme_321HRSCAF_32286_whole_genome_shotgun_sequence
PVJP02910713.1_45156-45762_Hippopotamus_amphibius_isolate_BS01_ScHSfme_925166HRSCAF_982966_whole_genome_shotgun_sequence
0.02

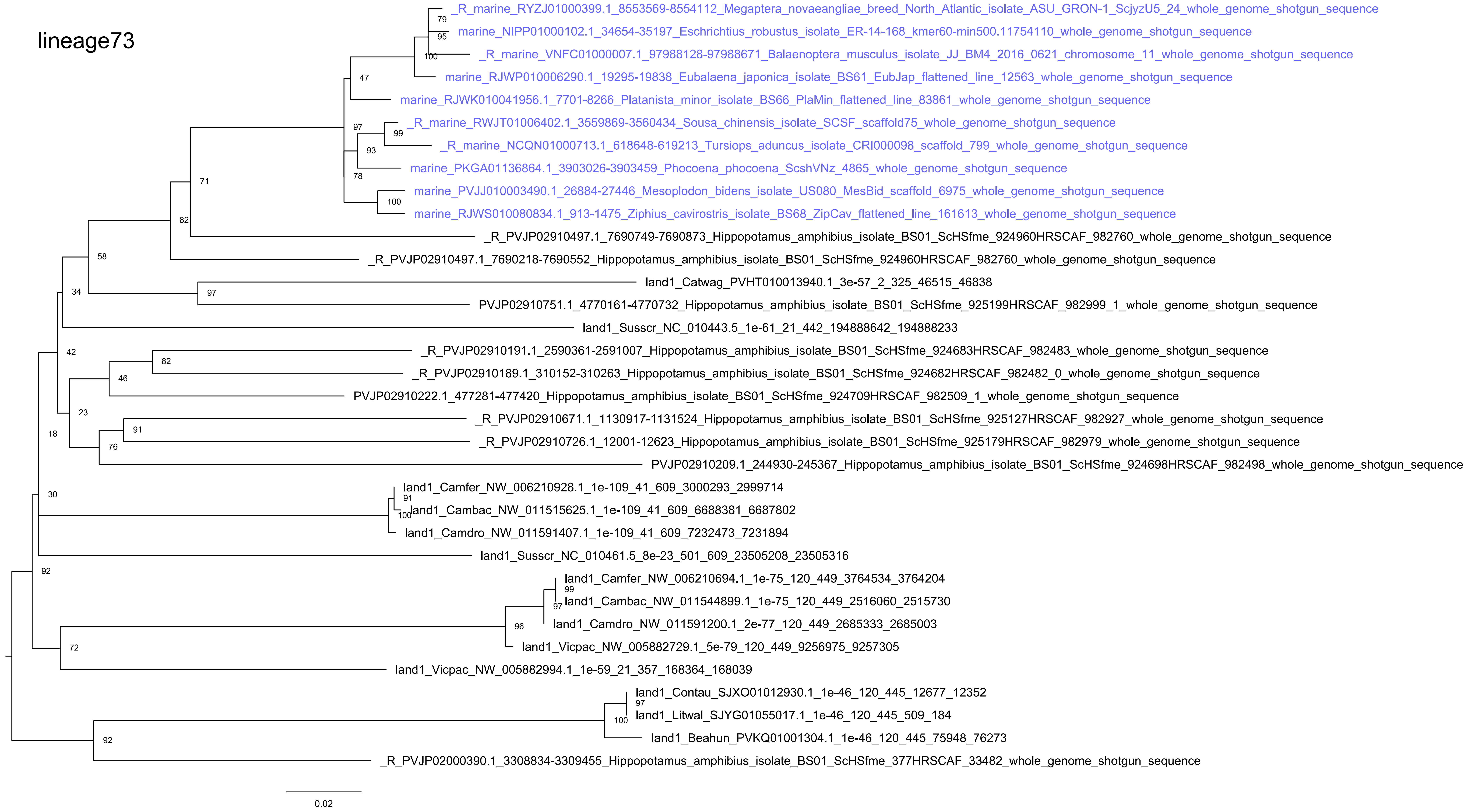
lineage73
_R_marine_RYZJ01000399.1_8553569-8554112_Megaptera_novaeangliae_breed_North_Atlantic_isolate_ASU_GRON-1_ScjyzU5_24_whole_genome_shotgun_sequence
marine_NIPP01000102.1_34654-35197_Eschrichtius_robustus_isolate_ER-14-168_kmer60-min500.11754110_whole_genome_shotgun_sequence
_R_marine_VNFC01000007.1_97988128-97988671_Balaenoptera_musculus_isolate_JJ_BM4_2016_0621_chromosome_11_whole_genome_shotgun_sequence
marine_RJWP010006290.1_19295-19838_Eubalaena_japonica_isolate_BS61_EubJap_flattened_line_12563_whole_genome_shotgun_sequence
marine_RJWK010041956.1_7701-8266_Platanista_minor_isolate_BS66_PlaMin_flattened_line_83861_whole_genome_shotgun_sequence
_R_marine_RWJT01006402.1_3559869-3560434_Sousa_chinensis_isolate_SCSF_scaffold75_whole_genome_shotgun_sequence
_R_marine_NCQN01000713.1_618648-619213_Tursiops_aduncus_isolate_CRI000098_scaffold_799_whole_genome_shotgun_sequence
marine_PKGA01136864.1_3903026-3903459_Phocoena_phocoena_ScshVNz_4865_whole_genome_shotgun_sequence
marine_PVJJ010003490.1_26884-27446_Mesoplodon_bidens_isolate_US080_MesBid_scaffold_6975_whole_genome_shotgun_sequence
marine_RJWS010080834.1_913-1475_Ziphius_cavirostris_isolate_BS68_ZipCav_flattened_line_161613_whole_genome_shotgun_sequence
_R_PVJP02910497.1_7690749-7690873_Hippopotamus_amphibius_isolate_BS01_ScHSfme_924960HRSCAF_982760_whole_genome_shotgun_sequence
_R_PVJP02910497.1_7690218-7690552_Hippopotamus_amphibius_isolate_BS01_ScHSfme_924960HRSCAF_982760_whole_genome_shotgun_sequence
land1_Catwag_PVHT010013940.1_3e-57_2_325_46515_46838
PVJP02910751.1_4770161-4770732_Hippopotamus_amphibius_isolate_BS01_ScHSfme_925199HRSCAF_982999_1_whole_genome_shotgun_sequence
land1_Susscr_NC_010443.5_1e-61_21_442_194888642_194888233
_R_PVJP02910191.1_2590361-2591007_Hippopotamus_amphibius_isolate_BS01_ScHSfme_924683HRSCAF_982483_whole_genome_shotgun_sequence
_R_PVJP02910189.1_310152-310263_Hippopotamus_amphibius_isolate_BS01_ScHSfme_924682HRSCAF_982482_0_whole_genome_shotgun_sequence
PVJP02910222.1_477281-477420_Hippopotamus_amphibius_isolate_BS01_ScHSfme_924709HRSCAF_982509_1_whole_genome_shotgun_sequence
_R_PVJP02910671.1_1130917-1131524_Hippopotamus_amphibius_isolate_BS01_ScHSfme_925127HRSCAF_982927_whole_genome_shotgun_sequence
_R_PVJP02910726.1_12001-12623_Hippopotamus_amphibius_isolate_BS01_ScHSfme_925179HRSCAF_982979_whole_genome_shotgun_sequence
PVJP02910209.1_244930-245367_Hippopotamus_amphibius_isolate_BS01_ScHSfme_924698HRSCAF_982498_whole_genome_shotgun_sequence
land1_Camfer_NW_006210928.1_1e-109_41_609_3000293_2999714
land1_Cambac_NW_011515625.1_1e-109_41_609_6688381_6687802
land1_Camdro_NW_011591407.1_1e-109_41_609_7232473_7231894
land1_Susscr_NC_010461.5_8e-23_501_609_23505208_23505316
land1_Camfer_NW_006210694.1_1e-75_120_449_3764534_3764204
land1_Cambac_NW_011544899.1_1e-75_120_449_2516060_2515730
land1_Camdro_NW_011591200.1_2e-77_120_449_2685333_2685003
land1_Vicpac_NW_005882729.1_5e-79_120_449_9256975_9257305
land1_Vicpac_NW_005882994.1_1e-59_21_357_168364_168039
land1_Contau_SJXO01012930.1_1e-46_120_445_12677_12352
land1_Litwal_SJYG01055017.1_1e-46_120_445_509_184
land1_Beahun_PVKQ01001304.1_1e-46_120_445_75948_76273
_R_PVJP02000390.1_3308834-3309455_Hippopotamus_amphibius_isolate_BS01_ScHSfme_377HRSCAF_33482_whole_genome_shotgun_sequence
0.02

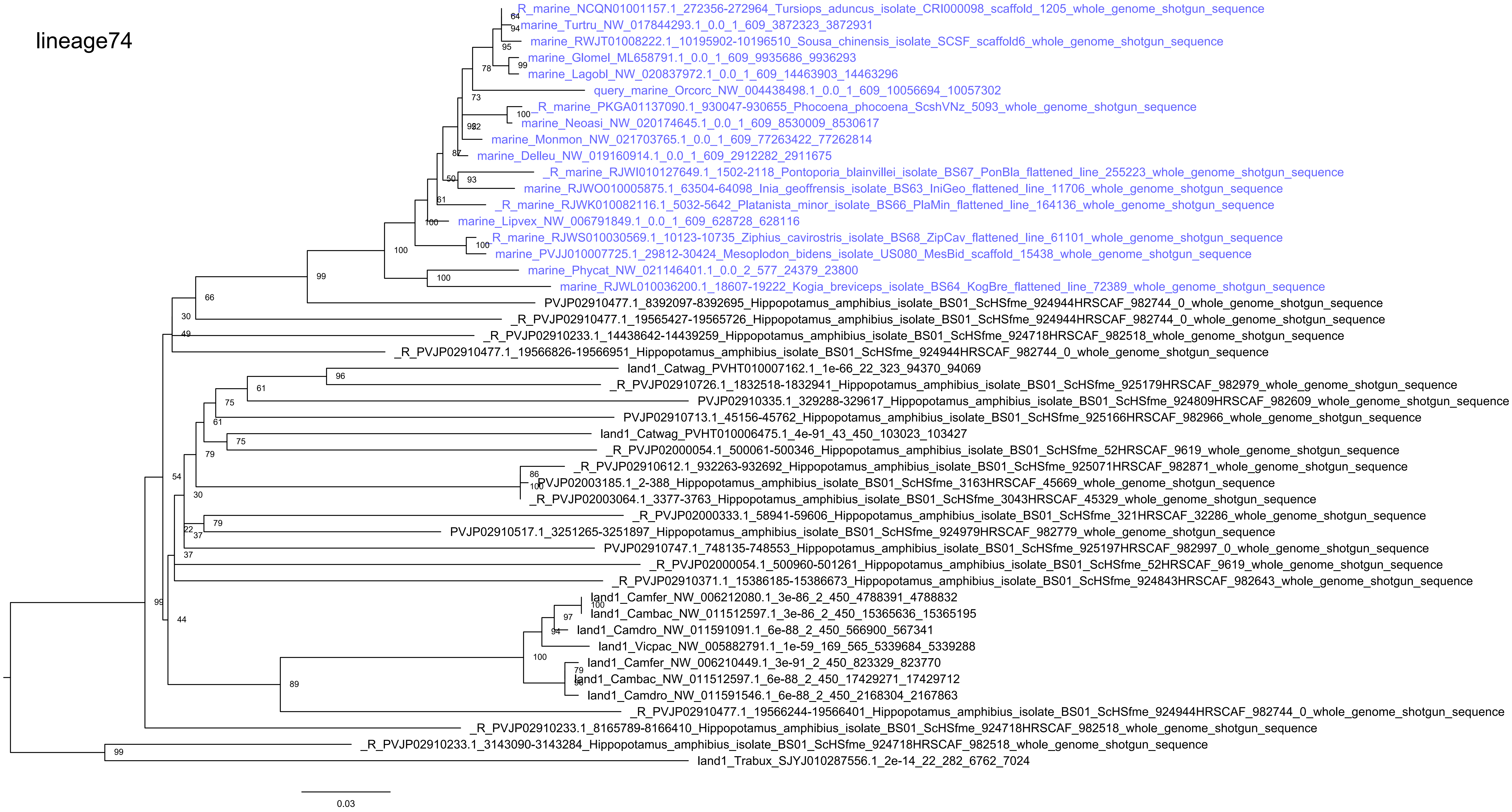

lineage74
_R_marine_NCQN01001157.1_272356-272964_Tursiops_aduncus_isolate_CRI000098_scaffold_1205_whole_genome_shotgun_sequence
marine_Turtru_NW_017844293.1_0.0_1_609_3872323_3872931
marine_RWJT01008222.1_10195902-10196510_Sousa_chinensis_isolate_SCSF_scaffold6_whole_genome_shotgun_sequence
marine_Glomel_ML658791.1_0.0_1_609_9935686_9936293
marine_Lagobl_NW_020837972.1_0.0_1_609_14463903_14463296
query_marine_Orcorc_NW_004438498.1_0.0_1_609_10056694_10057302
_R_marine_PKGA01137090.1_930047-930655_Phocoena_phocoena_ScshVNz_5093_whole_genome_shotgun_sequence
marine_Neoasi_NW_020174645.1_0.0_1_609_8530009_8530617
marine_Monmon_NW_021703765.1_0.0_1_609_77263422_77262814
marine_Delleu_NW_019160914.1_0.0_1_609_2912282_2911675
_R_marine_RJWI010127649.1_1502-2118_Pontoporia_blainvillei_isolate_BS67_PonBla_flattened_line_255223_whole_genome_shotgun_sequence
marine_RJWO010005875.1_63504-64098_Inia_geoffrensis_isolate_BS63_IniGeo_flattened_line_11706_whole_genome_shotgun_sequence
_R_marine_RJWK010082116.1_5032-5642_Platanista_minor_isolate_BS66_PlaMin_flattened_line_164136_whole_genome_shotgun_sequence
marine_Lipvex_NW_006791849.1_0.0_1_609_628728_628116
_R_marine_RJWS010030569.1_10123-10735_Ziphius_cavirostris_isolate_BS68_ZipCav_flattened_line_61101_whole_genome_shotgun_sequence
marine_PVJJ010007725.1_29812-30424_Mesoplodon_bidens_isolate_US080_MesBid_scaffold_15438_whole_genome_shotgun_sequence
marine_Phycat_NW_021146401.1_0.0_2_577_24379_23800
marine_RJWL010036200.1_18607-19222_Kogia_breviceps_isolate_BS64_KogBre_flattened_line_72389_whole_genome_shotgun_sequence
PVJP02910477.1_8392097-8392695_Hippopotamus_amphibius_isolate_BS01_ScHSfme_924944HRSCAF_982744_0_whole_genome_shotgun_sequence
_R_PVJP02910477.1_19565427-19565726_Hippopotamus_amphibius_isolate_BS01_ScHSfme_924944HRSCAF_982744_0_whole_genome_shotgun_sequence
_R_PVJP02910233.1_14438642-14439259_Hippopotamus_amphibius_isolate_BS01_ScHSfme_924718HRSCAF_982518_whole_genome_shotgun_sequence
_R_PVJP02910477.1_19566826-19566951_Hippopotamus_amphibius_isolate_BS01_ScHSfme_924944HRSCAF_982744_0_whole_genome_shotgun_sequence
land1_Catwag_PVHT010007162.1_1e-66_22_323_94370_94069
_R_PVJP02910726.1_1832518-1832941_Hippopotamus_amphibius_isolate_BS01_ScHSfme_925179HRSCAF_982979_whole_genome_shotgun_sequence
PVJP02910335.1_329288-329617_Hippopotamus_amphibius_isolate_BS01_ScHSfme_924809HRSCAF_982609_whole_genome_shotgun_sequence
PVJP02910713.1_45156-45762_Hippopotamus_amphibius_isolate_BS01_ScHSfme_925166HRSCAF_982966_whole_genome_shotgun_sequence
land1_Catwag_PVHT010006475.1_4e-91_43_450_103023_103427
_R_PVJP02000054.1_500061-500346_Hippopotamus_amphibius_isolate_BS01_ScHSfme_52HRSCAF_9619_whole_genome_shotgun_sequence
_R_PVJP02910612.1_932263-932692_Hippopotamus_amphibius_isolate_BS01_ScHSfme_925071HRSCAF_982871_whole_genome_shotgun_sequence
PVJP02003185.1_2-388_Hippopotamus_amphibius_isolate_BS01_ScHSfme_3163HRSCAF_45669_whole_genome_shotgun_sequence
_R_PVJP02003064.1_3377-3763_Hippopotamus_amphibius_isolate_BS01_ScHSfme_3043HRSCAF_45329_whole_genome_shotgun_sequence
_R_PVJP02000333.1_58941-59606_Hippopotamus_amphibius_isolate_BS01_ScHSfme_321HRSCAF_32286_whole_genome_shotgun_sequence
PVJP02910517.1_3251265-3251897_Hippopotamus_amphibius_isolate_BS01_ScHSfme_924979HRSCAF_982779_whole_genome_shotgun_sequence
PVJP02910747.1_748135-748553_Hippopotamus_amphibius_isolate_BS01_ScHSfme_925197HRSCAF_982997_0_whole_genome_shotgun_sequence
_R_PVJP02000054.1_500960-501261_Hippopotamus_amphibius_isolate_BS01_ScHSfme_52HRSCAF_9619_whole_genome_shotgun_sequence
_R_PVJP02910371.1_15386185-15386673_Hippopotamus_amphibius_isolate_BS01_ScHSfme_924843HRSCAF_982643_whole_genome_shotgun_sequence
land1_Camfer_NW_006212080.1_3e-86_2_450_4788391_4788832
land1_Cambac_NW_011512597.1_3e-86_2_450_15365636_15365195
land1_Camdro_NW_011591091.1_6e-88_2_450_566900_567341
land1_Vicpac_NW_005882791.1_1e-59_169_565_5339684_5339288
land1_Camfer_NW_006210449.1_3e-91_2_450_823329_823770
land1_Cambac_NW_011512597.1_6e-88_2_450_17429271_17429712
land1_Camdro_NW_011591546.1_6e-88_2_450_2168304_2167863
_R_PVJP02910477.1_19566244-19566401_Hippopotamus_amphibius_isolate_BS01_ScHSfme_924944HRSCAF_982744_0_whole_genome_shotgun_sequence
_R_PVJP02910233.1_8165789-8166410_Hippopotamus_amphibius_isolate_BS01_ScHSfme_924718HRSCAF_982518_whole_genome_shotgun_sequence
_R_PVJP02910233.1_3143090-3143284_Hippopotamus_amphibius_isolate_BS01_ScHSfme_924718HRSCAF_982518_whole_genome_shotgun_sequence
land1_Trabux_SJYJ010287556.1_2e-14_22_282_6762_7024
0.03

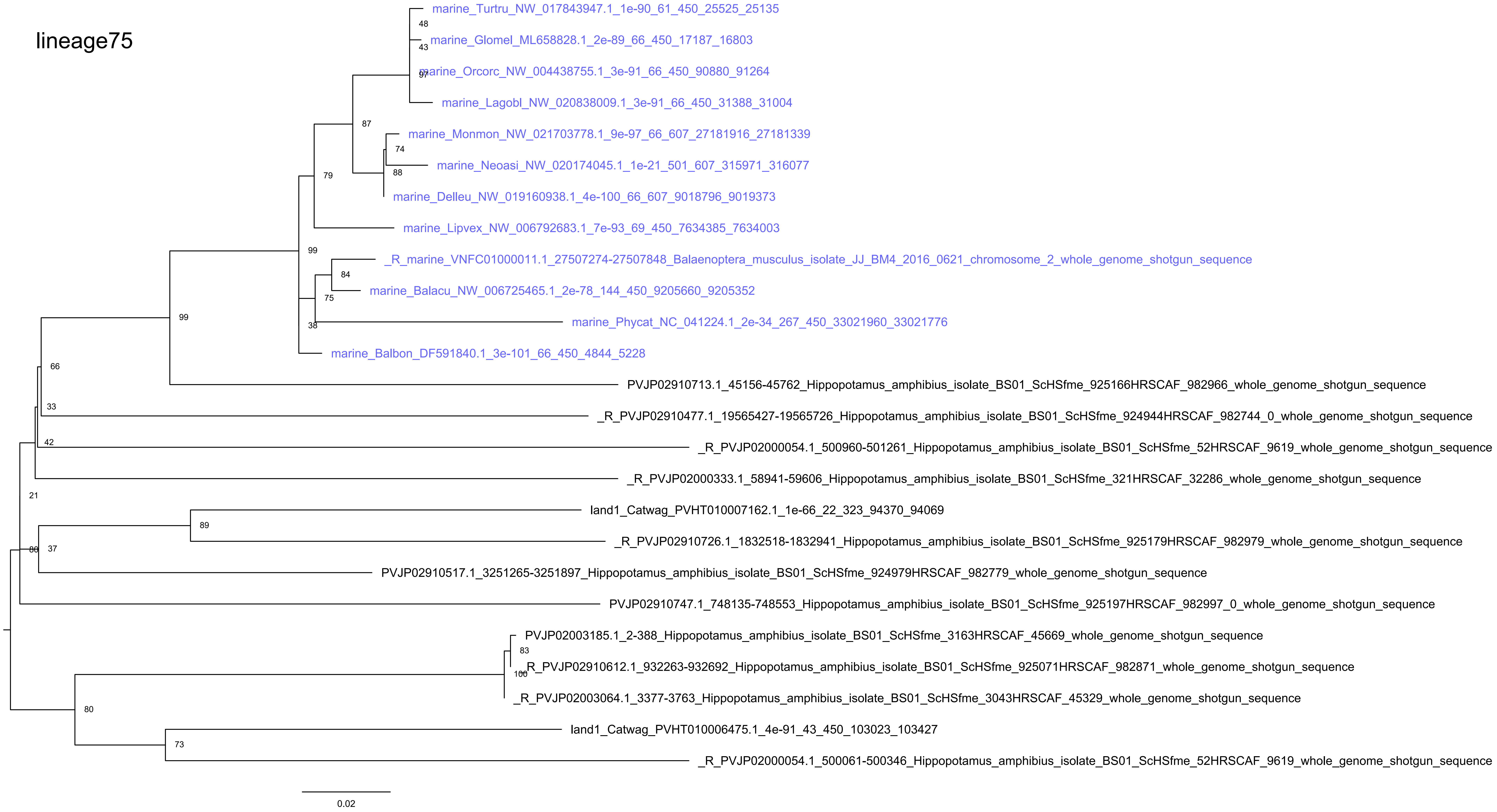

lineage75
marine_Turtru_NW_017843947.1_1e-90_61_450_25525_25135
marine_Glomel_ML658828.1_2e-89_66_450_17187_16803
marine_Orcorc_NW_004438755.1_3e-91_66_450_90880_91264
marine_Lagobl_NW_020838009.1_3e-91_66_450_31388_31004
marine_Monmon_NW_021703778.1_9e-97_66_607_27181916_27181339
marine_Neoasi_NW_020174045.1_1e-21_501_607_315971_316077
marine_Delleu_NW_019160938.1_4e-100_66_607_9018796_9019373
marine_Lipvex_NW_006792683.1_7e-93_69_450_7634385_7634003
_R_marine_VNFC01000011.1_27507274-27507848_Balaenoptera_musculus_isolate_JJ_BM4_2016_0621_chromosome_2_whole_genome_shotgun_sequence
marine_Balacu_NW_006725465.1_2e-78_144_450_9205660_9205352
marine_Phycat_NC_041224.1_2e-34_267_450_33021960_33021776
marine_Balbon_DF591840.1_3e-101_66_450_4844_5228
PVJP02910713.1_45156-45762_Hippopotamus_amphibius_isolate_BS01_ScHSfme_925166HRSCAF_982966_whole_genome_shotgun_sequence
_R_PVJP02910477.1_19565427-19565726_Hippopotamus_amphibius_isolate_BS01_ScHSfme_924944HRSCAF_982744_0_whole_genome_shotgun_sequence
_R_PVJP02000054.1_500960-501261_Hippopotamus_amphibius_isolate_BS01_ScHSfme_52HRSCAF_9619_whole_genome_shotgun_sequence
_R_PVJP02000333.1_58941-59606_Hippopotamus_amphibius_isolate_BS01_ScHSfme_321HRSCAF_32286_whole_genome_shotgun_sequence
land1_Catwag_PVHT010007162.1_1e-66_22_323_94370_94069
_R_PVJP02910726.1_1832518-1832941_Hippopotamus_amphibius_isolate_BS01_ScHSfme_925179HRSCAF_982979_whole_genome_shotgun_sequence
PVJP02910517.1_3251265-3251897_Hippopotamus_amphibius_isolate_BS01_ScHSfme_924979HRSCAF_982779_whole_genome_shotgun_sequence
PVJP02910747.1_748135-748553_Hippopotamus_amphibius_isolate_BS01_ScHSfme_925197HRSCAF_982997_0_whole_genome_shotgun_sequence
PVJP02003185.1_2-388_Hippopotamus_amphibius_isolate_BS01_ScHSfme_3163HRSCAF_45669_whole_genome_shotgun_sequence
_R_PVJP02910612.1_932263-932692_Hippopotamus_amphibius_isolate_BS01_ScHSfme_925071HRSCAF_982871_whole_genome_shotgun_sequence
_R_PVJP02003064.1_3377-3763_Hippopotamus_amphibius_isolate_BS01_ScHSfme_3043HRSCAF_45329_whole_genome_shotgun_sequence
land1_Catwag_PVHT010006475.1_4e-91_43_450_103023_103427
_R_PVJP02000054.1_500061-500346_Hippopotamus_amphibius_isolate_BS01_ScHSfme_52HRSCAF_9619_whole_genome_shotgun_sequence
48
43
97
87
74
88
79
99
84
75
38
99
66
33
42
21
89
37
80
83
100
80
73
0.02

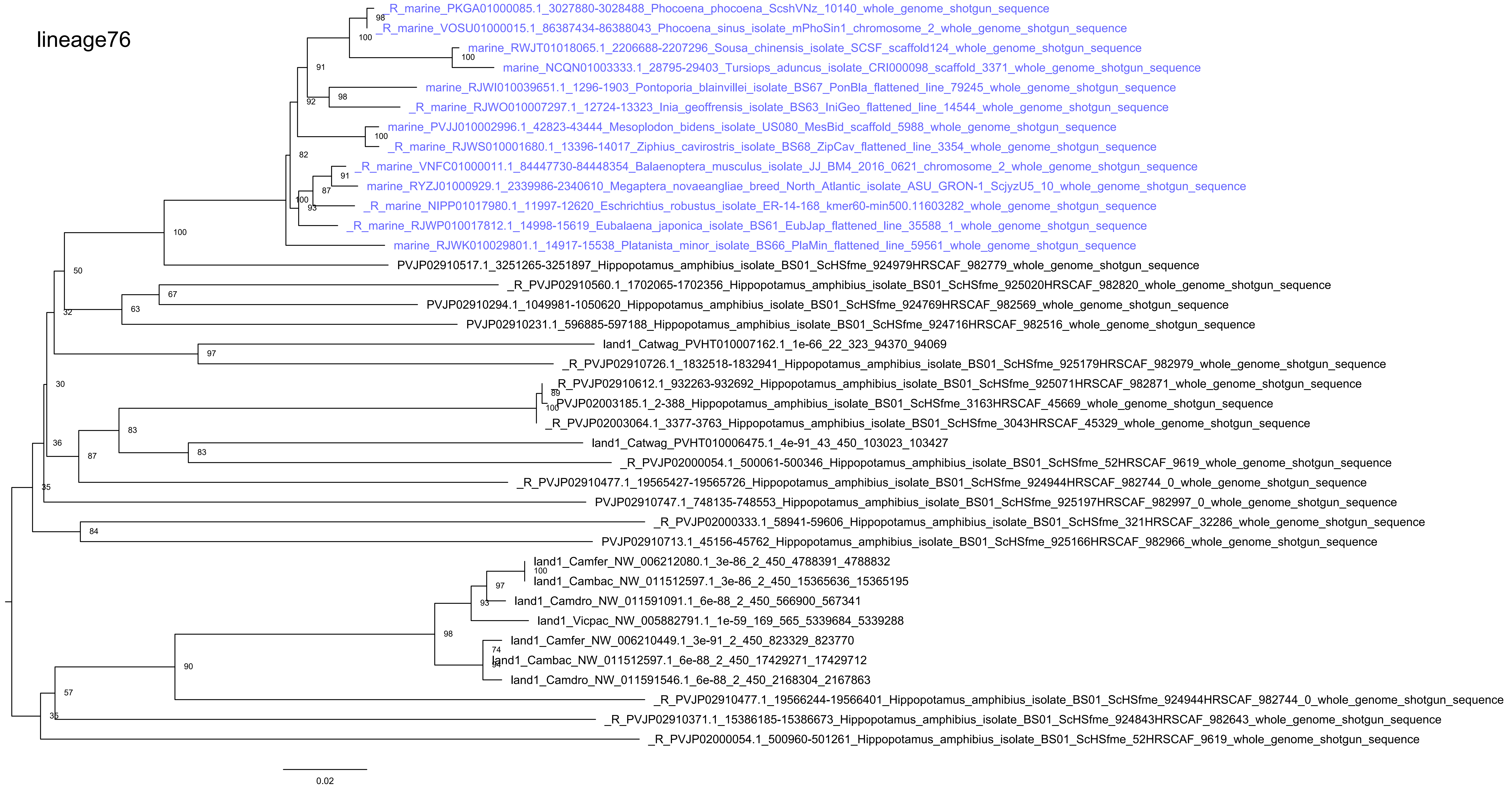
lineage76
_R_marine_PKGA01000085.1_3027880-3028488_Phocoena_phocoena_ScshVNz_10140_whole_genome_shotgun_sequence
_R_marine_VOSU01000015.1_86387434-86388043_Phocoena_sinus_isolate_mPhoSin1_chromosome_2_whole_genome_shotgun_sequence
marine_RWJT01018065.1_2206688-2207296_Sousa_chinensis_isolate_SCSF_scaffold124_whole_genome_shotgun_sequence
marine_NCQN01003333.1_28795-29403_Tursiops_aduncus_isolate_CRI000098_scaffold_3371_whole_genome_shotgun_sequence
marine_RJWI010039651.1_1296-1903_Pontoporia_blainvillei_isolate_BS67_PonBla_flattened_line_79245_whole_genome_shotgun_sequence
_R_marine_RJWO010007297.1_12724-13323_Inia_geoffrensis_isolate_BS63_IniGeo_flattened_line_14544_whole_genome_shotgun_sequence
marine_PVJJ010002996.1_42823-43444_Mesoplodon_bidens_isolate_US080_MesBid_scaffold_5988_whole_genome_shotgun_sequence
_R_marine_RJWS010001680.1_13396-14017_Ziphius_cavirostris_isolate_BS68_ZipCav_flattened_line_3354_whole_genome_shotgun_sequence
_R_marine_VNFC01000011.1_84447730-84448354_Balaenoptera_musculus_isolate_JJ_BM4_2016_0621_chromosome_2_whole_genome_shotgun_sequence
marine_RYZJ01000929.1_2339986-2340610_Megaptera_novaeangliae_breed_North_Atlantic_isolate_ASU_GRON-1_ScjyzU5_10_whole_genome_shotgun_sequence
_R_marine_NIPP01017980.1_11997-12620_Eschrichtius_robustus_isolate_ER-14-168_kmer60-min500.11603282_whole_genome_shotgun_sequence
_R_marine_RJWP010017812.1_14998-15619_Eubalaena_japonica_isolate_BS61_EubJap_flattened_line_35588_1_whole_genome_shotgun_sequence
marine_RJWK010029801.1_14917-15538_Platanista_minor_isolate_BS66_PlaMin_flattened_line_59561_whole_genome_shotgun_sequence
PVJP02910517.1_3251265-3251897_Hippopotamus_amphibius_isolate_BS01_ScHSfme_924979HRSCAF_982779_whole_genome_shotgun_sequence
_R_PVJP02910560.1_1702065-1702356_Hippopotamus_amphibius_isolate_BS01_ScHSfme_925020HRSCAF_982820_whole_genome_shotgun_sequence
PVJP02910294.1_1049981-1050620_Hippopotamus_amphibius_isolate_BS01_ScHSfme_924769HRSCAF_982569_whole_genome_shotgun_sequence
PVJP02910231.1_596885-597188_Hippopotamus_amphibius_isolate_BS01_ScHSfme_924716HRSCAF_982516_whole_genome_shotgun_sequence
land1_Catwag_PVHT010007162.1_1e-66_22_323_94370_94069
_R_PVJP02910726.1_1832518-1832941_Hippopotamus_amphibius_isolate_BS01_ScHSfme_925179HRSCAF_982979_whole_genome_shotgun_sequence
_R_PVJP02910612.1_932263-932692_Hippopotamus_amphibius_isolate_BS01_ScHSfme_925071HRSCAF_982871_whole_genome_shotgun_sequence
PVJP02003185.1_2-388_Hippopotamus_amphibius_isolate_BS01_ScHSfme_3163HRSCAF_45669_whole_genome_shotgun_sequence
_R_PVJP02003064.1_3377-3763_Hippopotamus_amphibius_isolate_BS01_ScHSfme_3043HRSCAF_45329_whole_genome_shotgun_sequence
land1_Catwag_PVHT010006475.1_4e-91_43_450_103023_103427
_R_PVJP02000054.1_500061-500346_Hippopotamus_amphibius_isolate_BS01_ScHSfme_52HRSCAF_9619_whole_genome_shotgun_sequence
_R_PVJP02910477.1_19565427-19565726_Hippopotamus_amphibius_isolate_BS01_ScHSfme_924944HRSCAF_982744_0_whole_genome_shotgun_sequence
PVJP02910747.1_748135-748553_Hippopotamus_amphibius_isolate_BS01_ScHSfme_925197HRSCAF_982997_0_whole_genome_shotgun_sequence
_R_PVJP02000333.1_58941-59606_Hippopotamus_amphibius_isolate_BS01_ScHSfme_321HRSCAF_32286_whole_genome_shotgun_sequence
PVJP02910713.1_45156-45762_Hippopotamus_amphibius_isolate_BS01_ScHSfme_925166HRSCAF_982966_whole_genome_shotgun_sequence
land1_Camfer_NW_006212080.1_3e-86_2_450_4788391_4788832
land1_Cambac_NW_011512597.1_3e-86_2_450_15365636_15365195
land1_Camdro_NW_011591091.1_6e-88_2_450_566900_567341
land1_Vicpac_NW_005882791.1_1e-59_169_565_5339684_5339288
land1_Camfer_NW_006210449.1_3e-91_2_450_823329_823770
land1_Cambac_NW_011512597.1_6e-88_2_450_17429271_17429712
land1_Camdro_NW_011591546.1_6e-88_2_450_2168304_2167863
_R_PVJP02910477.1_19566244-19566401_Hippopotamus_amphibius_isolate_BS01_ScHSfme_924944HRSCAF_982744_0_whole_genome_shotgun_sequence
_R_PVJP02910371.1_15386185-15386673_Hippopotamus_amphibius_isolate_BS01_ScHSfme_924843HRSCAF_982643_whole_genome_shotgun_sequence
_R_PVJP02000054.1_500960-501261_Hippopotamus_amphibius_isolate_BS01_ScHSfme_52HRSCAF_9619_whole_genome_shotgun_sequence
0.02

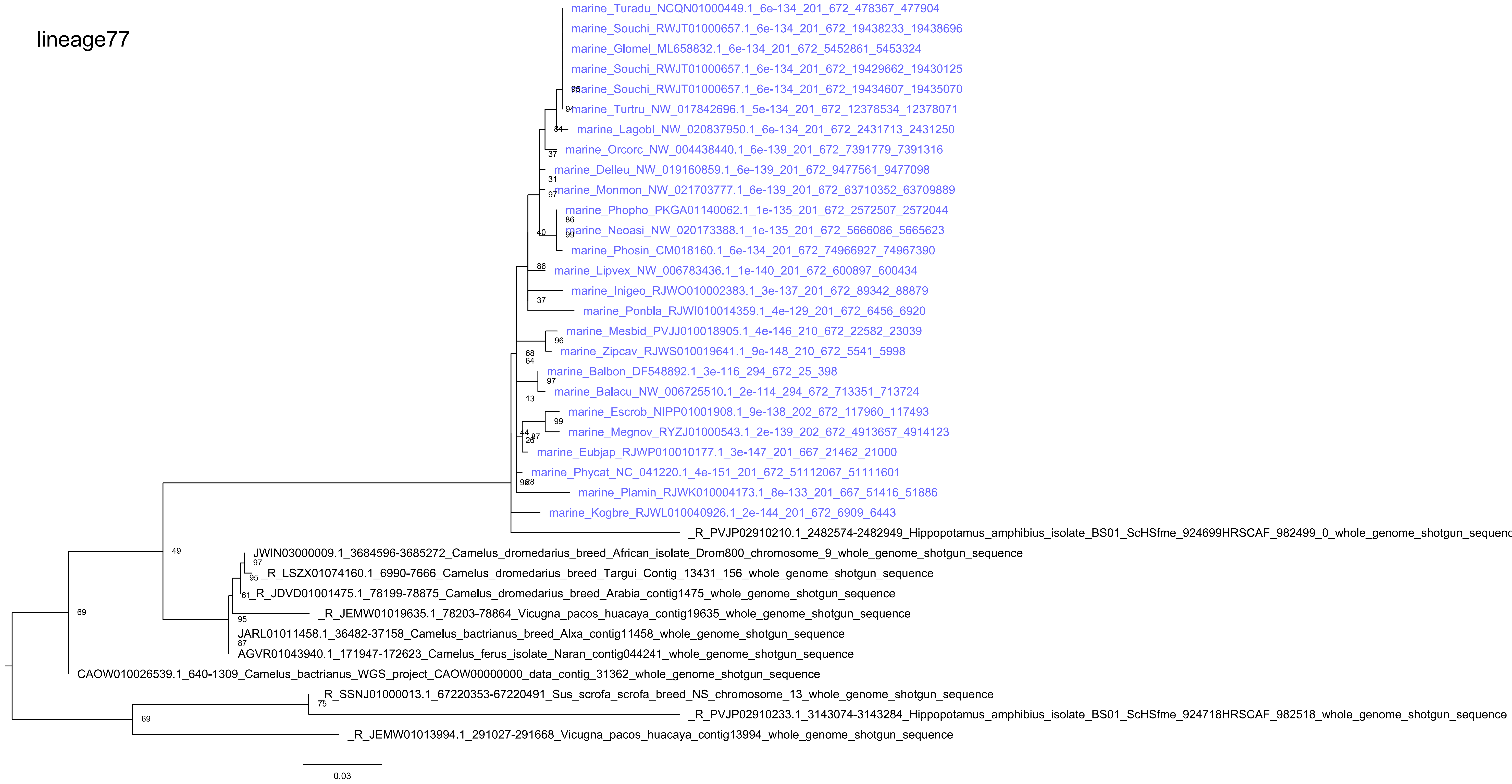
lineage77
marine_Turadu_NCQN01000449.1_6e-134_201_672_478367_477904
marine_Souchi_RWJT01000657.1_6e-134_201_672_19438233_19438696
marine_Glomel_ML658832.1_6e-134_201_672_5452861_5453324
marine_Souchi_RWJT01000657.1_6e-134_201_672_19429662_19430125
marine_Souchi_RWJT01000657.1_6e-134_201_672_19434607_19435070
marine_Turtru_NW_017842696.1_5e-134_201_672_12378534_12378071
marine_Lagobl_NW_020837950.1_6e-134_201_672_2431713_2431250
marine_Orcorc_NW_004438440.1_6e-139_201_672_7391779_7391316
marine_Delleu_NW_019160859.1_6e-139_201_672_9477561_9477098
marine_Monmon_NW_021703777.1_6e-139_201_672_63710352_63709889
marine_Phopho_PKGA01140062.1_1e-135_201_672_2572507_2572044
marine_Neoasi_NW_020173388.1_1e-135_201_672_5666086_5665623
marine_Phosin_CM018160.1_6e-134_201_672_74966927_74967390
marine_Lipvex_NW_006783436.1_1e-140_201_672_600897_600434
marine_Inigeo_RJWO010002383.1_3e-137_201_672_89342_88879
marine_Ponbla_RJWI010014359.1_4e-129_201_672_6456_6920
marine_Mesbid_PVJJ010018905.1_4e-146_210_672_22582_23039
marine_Zipcav_RJWS010019641.1_9e-148_210_672_5541_5998
marine_Balbon_DF548892.1_3e-116_294_672_25_398
marine_Balacu_NW_006725510.1_2e-114_294_672_713351_713724
marine_Escrob_NIPP01001908.1_9e-138_202_672_117960_117493
marine_Megnov_RYZJ01000543.1_2e-139_202_672_4913657_4914123
marine_Eubjap_RJWP010010177.1_3e-147_201_667_21462_21000
marine_Phycat_NC_041220.1_4e-151_201_672_51112067_51111601
marine_Plamin_RJWK010004173.1_8e-133_201_667_51416_51886
marine_Kogbre_RJWL010040926.1_2e-144_201_672_6909_6443
_R_PVJP02910210.1_2482574-2482949_Hippopotamus_amphibius_isolate_BS01_ScHSfme_924699HRSCAF_982499_0_whole_genome_shotgun_sequenc
JWIN03000009.1_3684596-3685272_Camelus_dromedarius_breed_African_isolate_Drom800_chromosome_9_whole_genome_shotgun_sequence
_R_LSZX01074160.1_6990-7666_Camelus_dromedarius_breed_Targui_Contig_13431_156_whole_genome_shotgun_sequence
_R_JDVD01001475.1_78199-78875_Camelus_dromedarius_breed_Arabia_contig1475_whole_genome_shotgun_sequence
_R_JEMW01019635.1_78203-78864_Vicugna_pacos_huacaya_contig19635_whole_genome_shotgun_sequence
JARL01011458.1_36482-37158_Camelus_bactrianus_breed_Alxa_contig11458_whole_genome_shotgun_sequence
AGVR01043940.1_171947-172623_Camelus_ferus_isolate_Naran_contig044241_whole_genome_shotgun_sequence
CAOW010026539.1_640-1309_Camelus_bactrianus_WGS_project_CAOW00000000_data_contig_31362_whole_genome_shotgun_sequence
_R_SSNJ01000013.1_67220353-67220491_Sus_scrofa_scrofa_breed_NS_chromosome_13_whole_genome_shotgun_sequence
_R_PVJP02910233.1_3143074-3143284_Hippopotamus_amphibius_isolate_BS01_ScHSfme_924718HRSCAF_982518_whole_genome_shotgun_sequence
_R_JEMW01013994.1_291027-291668_Vicugna_pacos_huacaya_contig13994_whole_genome_shotgun_sequence
0.03

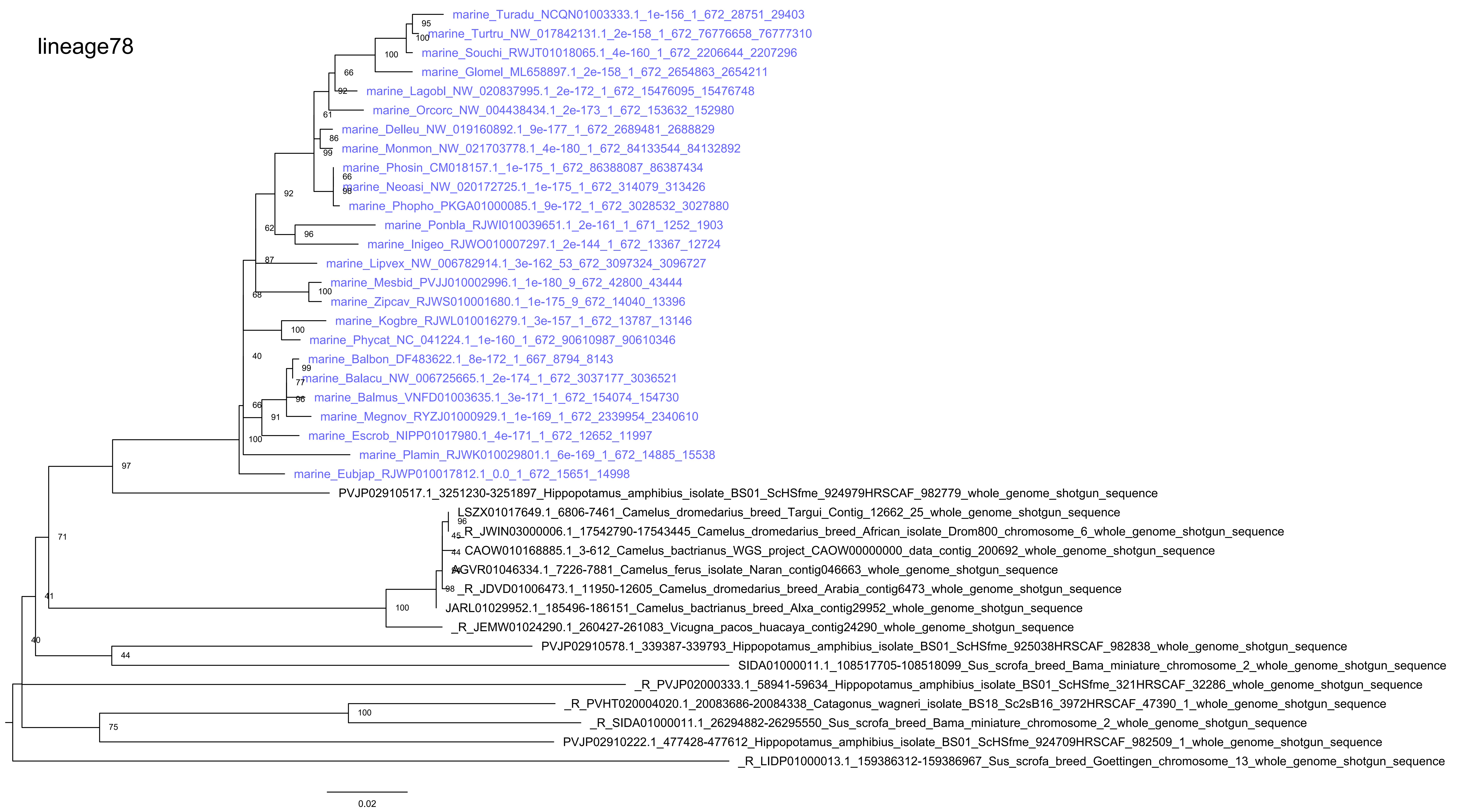
lineage78
marine_Turadu_NCQN01003333.1_1e-156_1_672_28751_29403
marine_Turtru_NW_017842131.1_2e-158_1_672_76776658_76777310
marine_Souchi_RWJT01018065.1_4e-160_1_672_2206644_2207296
marine_Glomel_ML658897.1_2e-158_1_672_2654863_2654211
marine_Lagobl_NW_020837995.1_2e-172_1_672_15476095_15476748
marine_Orcorc_NW_004438434.1_2e-173_1_672_153632_152980
marine_Delleu_NW_019160892.1_9e-177_1_672_2689481_2688829
marine_Monmon_NW_021703778.1_4e-180_1_672_84133544_84132892
marine_Phosin_CM018157.1_1e-175_1_672_86388087_86387434
marine_Neoasi_NW_020172725.1_1e-175_1_672_314079_313426
marine_Phopho_PKGA01000085.1_9e-172_1_672_3028532_3027880
marine_Ponbla_RJWI010039651.1_2e-161_1_671_1252_1903
marine_Inigeo_RJWO010007297.1_2e-144_1_672_13367_12724
marine_Lipvex_NW_006782914.1_3e-162_53_672_3097324_3096727
marine_Mesbid_PVJJ010002996.1_1e-180_9_672_42800_43444
marine_Zipcav_RJWS010001680.1_1e-175_9_672_14040_13396
marine_Kogbre_RJWL010016279.1_3e-157_1_672_13787_13146
marine_Phycat_NC_041224.1_1e-160_1_672_90610987_90610346
marine_Balbon_DF483622.1_8e-172_1_667_8794_8143
marine_Balacu_NW_006725665.1_2e-174_1_672_3037177_3036521
marine_Balmus_VNFD01003635.1_3e-171_1_672_154074_154730
marine_Megnov_RYZJ01000929.1_1e-169_1_672_2339954_2340610
marine_Escrob_NIPP01017980.1_4e-171_1_672_12652_11997
marine_Plamin_RJWK010029801.1_6e-169_1_672_14885_15538
marine_Eubjap_RJWP010017812.1_0.0_1_672_15651_14998
PVJP02910517.1_3251230-3251897_Hippopotamus_amphibius_isolate_BS01_ScHSfme_924979HRSCAF_982779_whole_genome_shotgun_sequence
LSZX01017649.1_6806-7461_Camelus_dromedarius_breed_Targui_Contig_12662_25_whole_genome_shotgun_sequence
_R_JWIN03000006.1_17542790-17543445_Camelus_dromedarius_breed_African_isolate_Drom800_chromosome_6_whole_genome_shotgun_sequence
CAOW010168885.1_3-612_Camelus_bactrianus_WGS_project_CAOW00000000_data_contig_200692_whole_genome_shotgun_sequence
AGVR01046334.1_7226-7881_Camelus_ferus_isolate_Naran_contig046663_whole_genome_shotgun_sequence
_R_JDVD01006473.1_11950-12605_Camelus_dromedarius_breed_Arabia_contig6473_whole_genome_shotgun_sequence
JARL01029952.1_185496-186151_Camelus_bactrianus_breed_Alxa_contig29952_whole_genome_shotgun_sequence
_R_JEMW01024290.1_260427-261083_Vicugna_pacos_huacaya_contig24290_whole_genome_shotgun_sequence
PVJP02910578.1_339387-339793_Hippopotamus_amphibius_isolate_BS01_ScHSfme_925038HRSCAF_982838_whole_genome_shotgun_sequence
SIDA01000011.1_108517705-108518099_Sus_scrofa_breed_Bama_miniature_chromosome_2_whole_genome_shotgun_sequence
_R_PVJP02000333.1_58941-59634_Hippopotamus_amphibius_isolate_BS01_ScHSfme_321HRSCAF_32286_whole_genome_shotgun_sequence
_R_PVHT020004020.1_20083686-20084338_Catagonus_wagneri_isolate_BS18_Sc2sB16_3972HRSCAF_47390_1_whole_genome_shotgun_sequence
_R_SIDA01000011.1_26294882-26295550_Sus_scrofa_breed_Bama_miniature_chromosome_2_whole_genome_shotgun_sequence
PVJP02910222.1_477428-477612_Hippopotamus_amphibius_isolate_BS01_ScHSfme_924709HRSCAF_982509_1_whole_genome_shotgun_sequence
_R_LIDP01000013.1_159386312-159386967_Sus_scrofa_breed_Goettingen_chromosome_13_whole_genome_shotgun_sequence
0.02

lineage79

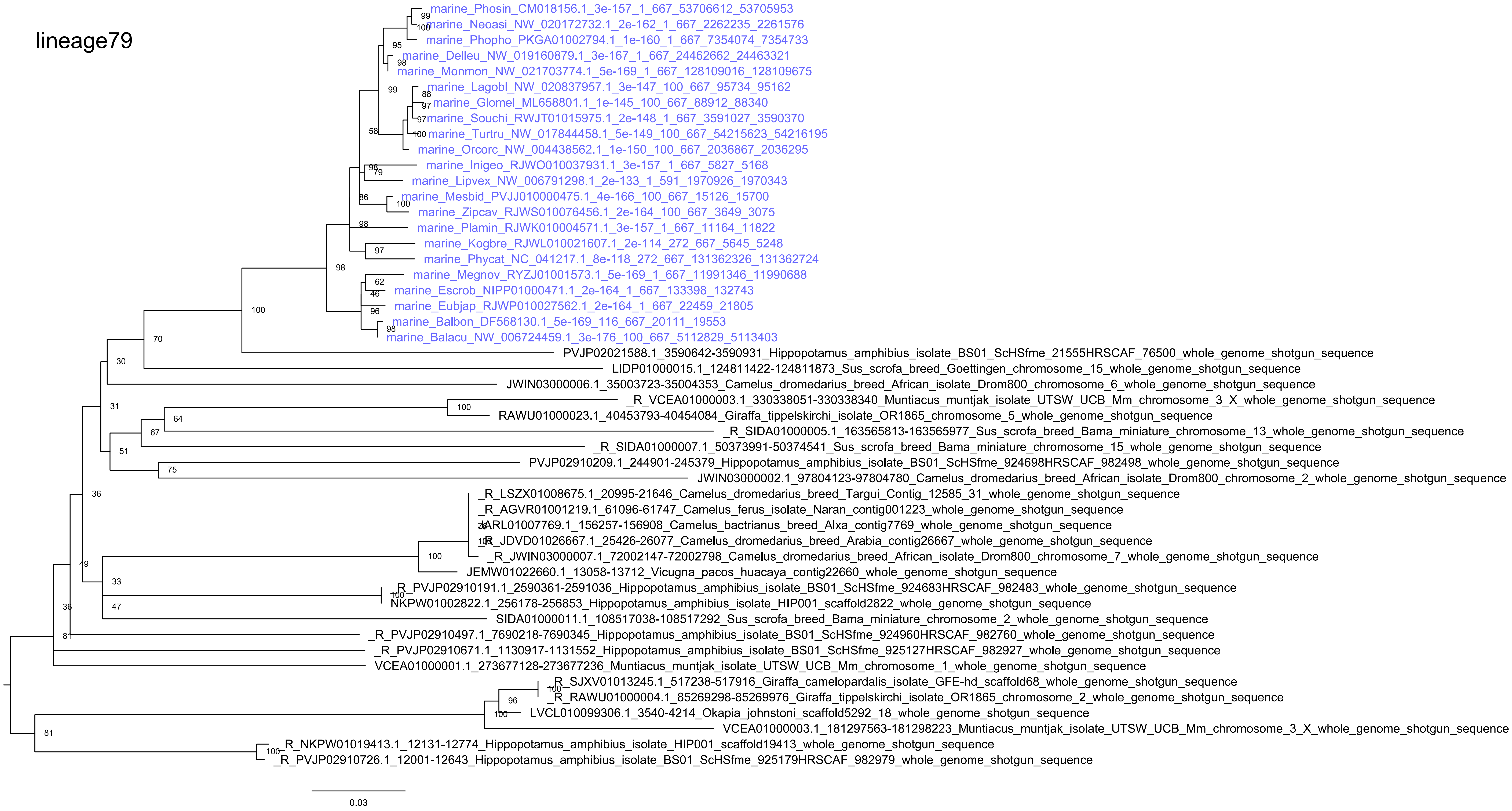

marine_Phosin_CM018156.1_3e-157_1_667_53706612_53705953
marine_Neoasi_NW_020172732.1_2e-162_1_667_2262235_2261576
marine_Phopho_PKGA01002794.1_1e-160_1_667_7354074_7354733
marine_Delleu_NW_019160879.1_3e-167_1_667_24462662_24463321
marine_Monmon_NW_021703774.1_5e-169_1_667_128109016_128109675
marine_Lagobl_NW_020837957.1_3e-147_100_667_95734_95162
marine_Glomel_ML658801.1_1e-145_100_667_88912_88340
marine_Souchi_RWJT01015975.1_2e-148_1_667_3591027_3590370
marine_Turtru_NW_017844458.1_5e-149_100_667_54215623_54216195
marine_Orcorc_NW_004438562.1_1e-150_100_667_2036867_2036295
marine_Inigeo_RJWO010037931.1_3e-157_1_667_5827_5168
marine_Lipvex_NW_006791298.1_2e-133_1_591_1970926_1970343
marine_Mesbid_PVJJ010000475.1_4e-166_100_667_15126_15700
marine_Zipcav_RJWS010076456.1_2e-164_100_667_3649_3075
marine_Plamin_RJWK010004571.1_3e-157_1_667_11164_11822
marine_Kogbre_RJWL010021607.1_2e-114_272_667_5645_5248
marine_Phycat_NC_041217.1_8e-118_272_667_131362326_131362724
marine_Megnov_RYZJ01001573.1_5e-169_1_667_11991346_11990688
marine_Escrob_NIPP01000471.1_2e-164_1_667_133398_132743
marine_Eubjap_RJWP010027562.1_2e-164_1_667_22459_21805
marine_Balbon_DF568130.1_5e-169_116_667_20111_19553
marine_Balacu_NW_006724459.1_3e-176_100_667_5112829_5113403
PVJP02021588.1_3590642-3590931_Hippopotamus_amphibius_isolate_BS01_ScHSfme_21555HRSCAF_76500_whole_genome_shotgun_sequence
LIDP01000015.1_124811422-124811873_Sus_scrofa_breed_Goettingen_chromosome_15_whole_genome_shotgun_sequence
JWIN03000006.1_35003723-35004353_Camelus_dromedarius_breed_African_isolate_Drom800_chromosome_6_whole_genome_shotgun_sequence
_R_VCEA01000003.1_330338051-330338340_Muntiacus_muntjak_isolate_UTSW_UCB_Mm_chromosome_3_X_whole_genome_shotgun_sequence
RAWU01000023.1_40453793-40454084_Giraffa_tippelskirchi_isolate_OR1865_chromosome_5_whole_genome_shotgun_sequence
_R_SIDA01000005.1_163565813-163565977_Sus_scrofa_breed_Bama_miniature_chromosome_13_whole_genome_shotgun_sequence
_R_SIDA01000007.1_50373991-50374541_Sus_scrofa_breed_Bama_miniature_chromosome_15_whole_genome_shotgun_sequence
PVJP02910209.1_244901-245379_Hippopotamus_amphibius_isolate_BS01_ScHSfme_924698HRSCAF_982498_whole_genome_shotgun_sequence
JWIN03000002.1_97804123-97804780_Camelus_dromedarius_breed_African_isolate_Drom800_chromosome_2_whole_genome_shotgun_sequence
_R_LSZX01008675.1_20995-21646_Camelus_dromedarius_breed_Targui_Contig_12585_31_whole_genome_shotgun_sequence
_R_AGVR01001219.1_61096-61747_Camelus_ferus_isolate_Naran_contig001223_whole_genome_shotgun_sequence
JARL01007769.1_156257-156908_Camelus_bactrianus_breed_Alxa_contig7769_whole_genome_shotgun_sequence
_R_JDVD01026667.1_25426-26077_Camelus_dromedarius_breed_Arabia_contig26667_whole_genome_shotgun_sequence
_R_JWIN03000007.1_72002147-72002798_Camelus_dromedarius_breed_African_isolate_Drom800_chromosome_7_whole_genome_shotgun_sequence
JEMW01022660.1_13058-13712_Vicugna_pacos_huacaya_contig22660_whole_genome_shotgun_sequence
_R_PVJP02910191.1_2590361-2591036_Hippopotamus_amphibius_isolate_BS01_ScHSfme_924683HRSCAF_982483_whole_genome_shotgun_sequence
NKPW01002822.1_256178-256853_Hippopotamus_amphibius_isolate_HIP001_scaffold2822_whole_genome_shotgun_sequence
SIDA01000011.1_108517038-108517292_Sus_scrofa_breed_Bama_miniature_chromosome_2_whole_genome_shotgun_sequence
_R_PVJP02910497.1_7690218-7690345_Hippopotamus_amphibius_isolate_BS01_ScHSfme_924960HRSCAF_982760_whole_genome_shotgun_sequence
_R_PVJP02910671.1_1130917-1131552_Hippopotamus_amphibius_isolate_BS01_ScHSfme_925127HRSCAF_982927_whole_genome_shotgun_sequence
VCEA01000001.1_273677128-273677236_Muntiacus_muntjak_isolate_UTSW_UCB_Mm_chromosome_1_whole_genome_shotgun_sequence
_R_SJXV01013245.1_517238-517916_Giraffa_camelopardalis_isolate_GFE-hd_scaffold68_whole_genome_shotgun_sequence
_R_RAWU01000004.1_85269298-85269976_Giraffa_tippelskirchi_isolate_OR1865_chromosome_2_whole_genome_shotgun_sequence
LVCL010099306.1_3540-4214_Okapia_johnstoni_scaffold5292_18_whole_genome_shotgun_sequence
VCEA01000003.1_181297563-181298223_Muntiacus_muntjak_isolate_UTSW_UCB_Mm_chromosome_3_X_whole_genome_shotgun_sequence
_R_NKPW01019413.1_12131-12774_Hippopotamus_amphibius_isolate_HIP001_scaffold19413_whole_genome_shotgun_sequence
_R_PVJP02910726.1_12001-12643_Hippopotamus_amphibius_isolate_BS01_ScHSfme_925179HRSCAF_982979_whole_genome_shotgun_sequence
0.03

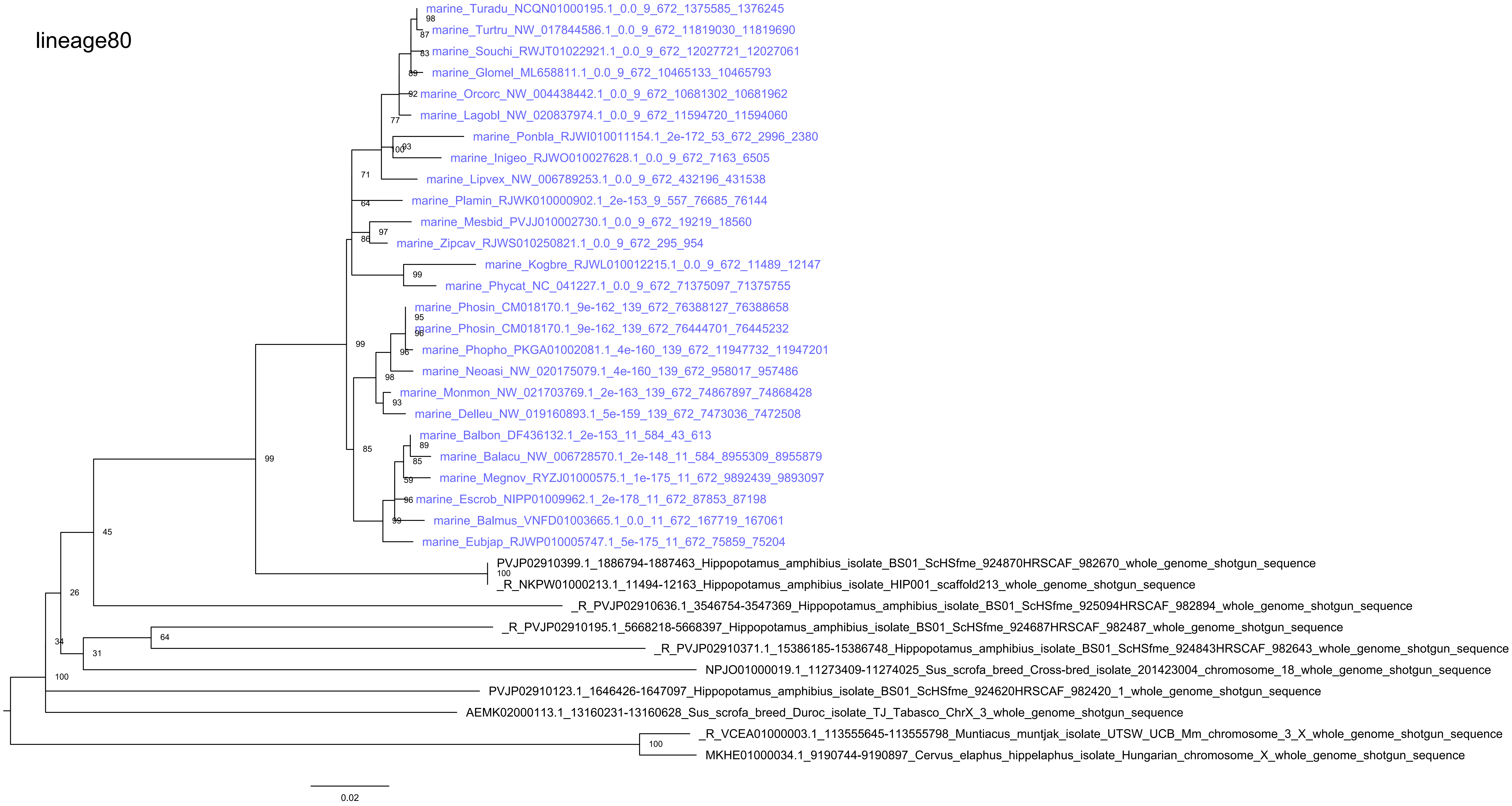
lineage80
marine_Turadu_NCQN01000195.1_0.0_9_672_1375585_1376245
marine_Turtru_NW_017844586.1_0.0_9_672_11819030_11819690
marine_Souchi_RWJT01022921.1_0.0_9_672_12027721_12027061
marine_Glomel_ML658811.1_0.0_9_672_10465133_10465793
marine_Orcorc_NW_004438442.1_0.0_9_672_10681302_10681962
marine_Lagobl_NW_020837974.1_0.0_9_672_11594720_11594060
marine_Ponbla_RJWI010011154.1_2e-172_53_672_2996_2380
marine_Inigeo_RJWO010027628.1_0.0_9_672_7163_6505
marine_Lipvex_NW_006789253.1_0.0_9_672_432196_431538
marine_Plamin_RJWK010000902.1_2e-153_9_557_76685_76144
marine_Mesbid_PVJJ010002730.1_0.0_9_672_19219_18560
marine_Zipcav_RJWS010250821.1_0.0_9_672_295_954
marine_Kogbre_RJWL010012215.1_0.0_9_672_11489_12147
marine_Phycat_NC_041227.1_0.0_9_672_71375097_71375755
marine_Phosin_CM018170.1_9e-162_139_672_76388127_76388658
marine_Phosin_CM018170.1_9e-162_139_672_76444701_76445232
marine_Phopho_PKGA01002081.1_4e-160_139_672_11947732_11947201
marine_Neoasi_NW_020175079.1_4e-160_139_672_958017_957486
marine_Monmon_NW_021703769.1_2e-163_139_672_74867897_74868428
marine_Delleu_NW_019160893.1_5e-159_139_672_7473036_7472508
marine_Balbon_DF436132.1_2e-153_11_584_43_613
marine_Balacu_NW_006728570.1_2e-148_11_584_8955309_8955879
marine_Megnov_RYZJ01000575.1_1e-175_11_672_9892439_9893097
marine_Escrob_NIPP01009962.1_2e-178_11_672_87853_87198
marine_Balmus_VNFD01003665.1_0.0_11_672_167719_167061
marine_Eubjap_RJWP010005747.1_5e-175_11_672_75859_75204
PVJP02910399.1_1886794-1887463_Hippopotamus_amphibius_isolate_BS01_ScHSfme_924870HRSCAF_982670_whole_genome_shotgun_sequence
_R_NKPW01000213.1_11494-12163_Hippopotamus_amphibius_isolate_HIP001_scaffold213_whole_genome_shotgun_sequence
_R_PVJP02910636.1_3546754-3547369_Hippopotamus_amphibius_isolate_BS01_ScHSfme_925094HRSCAF_982894_whole_genome_shotgun_sequence
_R_PVJP02910195.1_5668218-5668397_Hippopotamus_amphibius_isolate_BS01_ScHSfme_924687HRSCAF_982487_whole_genome_shotgun_sequence
_R_PVJP02910371.1_15386185-15386748_Hippopotamus_amphibius_isolate_BS01_ScHSfme_924843HRSCAF_982643_whole_genome_shotgun_sequence
NPJO01000019.1_11273409-11274025_Sus_scrofa_breed_Cross-bred_isolate_201423004_chromosome_18_whole_genome_shotgun_sequence
PVJP02910123.1_1646426-1647097_Hippopotamus_amphibius_isolate_BS01_ScHSfme_924620HRSCAF_982420_1_whole_genome_shotgun_sequence
AEMK02000113.1_13160231-13160628_Sus_scrofa_breed_Duroc_isolate_TJ_Tabasco_ChrX_3_whole_genome_shotgun_sequence
_R_VCEA01000003.1_113555645-113555798_Muntiacus_muntjak_isolate_UTSW_UCB_Mm_chromosome_3_X_whole_genome_shotgun_sequence
MKHE01000034.1_9190744-9190897_Cervus_elaphus_hippelaphus_isolate_Hungarian_chromosome_X_whole_genome_shotgun_sequence
0.02

lineage81

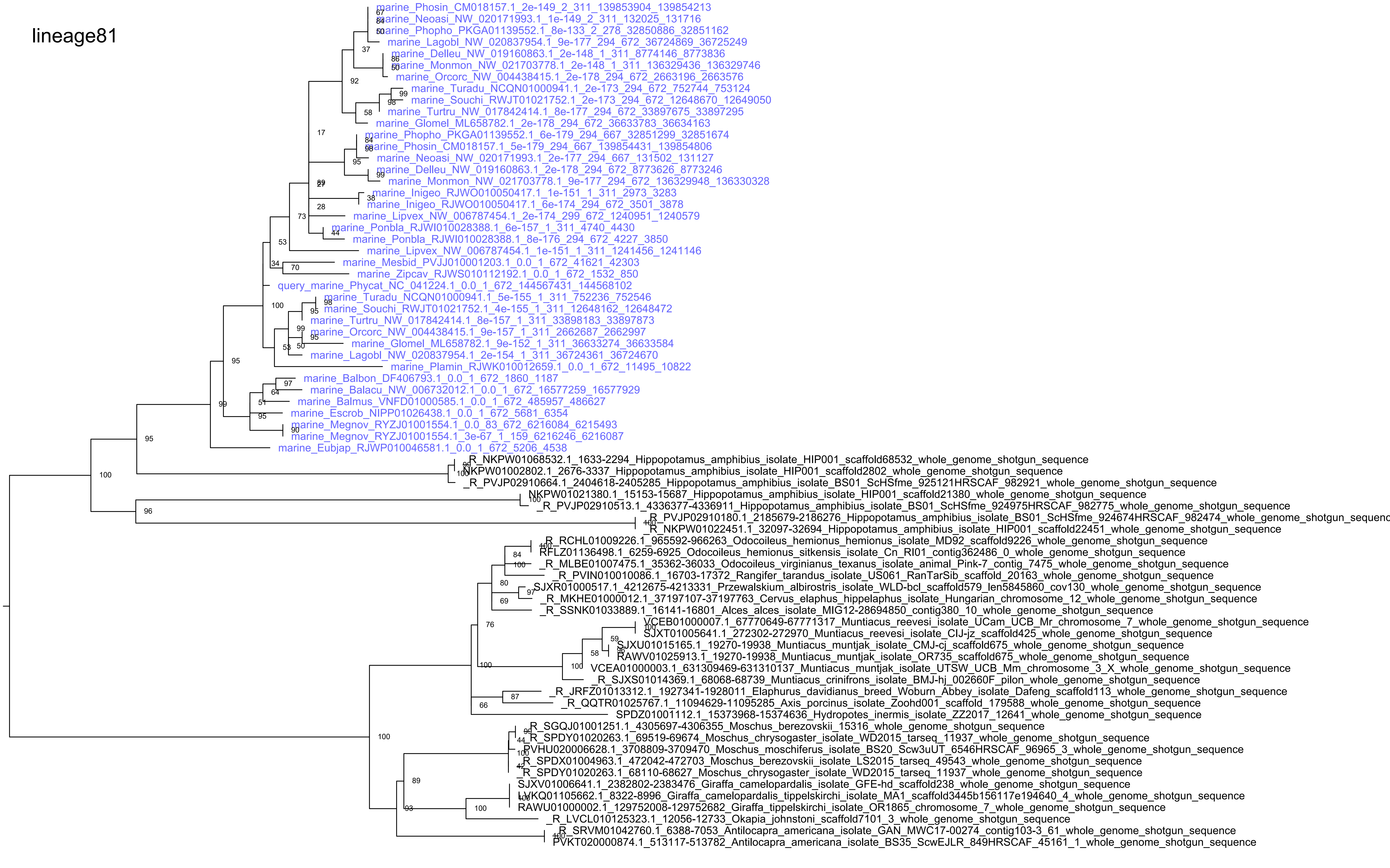

0.02

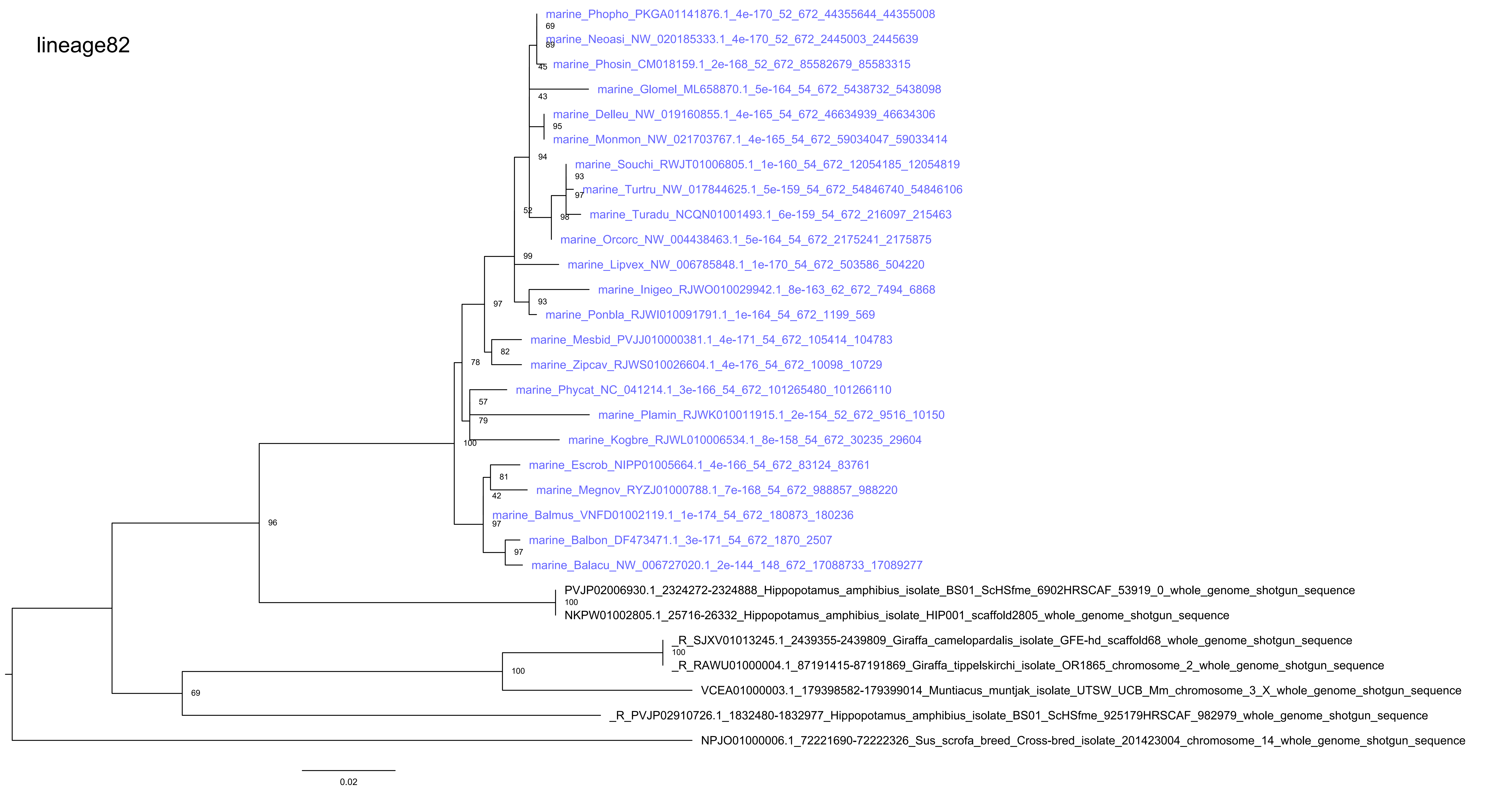

lineage82
marine_Phopho_PKGA01141876.1_4e-170_52_672_44355644_44355008
marine_Neoasi_NW_020185333.1_4e-170_52_672_2445003_2445639
marine_Phosin_CM018159.1_2e-168_52_672_85582679_85583315
marine_Glomel_ML658870.1_5e-164_54_672_5438732_5438098
marine_Delleu_NW_019160855.1_4e-165_54_672_46634939_46634306
marine_Monmon_NW_021703767.1_4e-165_54_672_59034047_59033414
marine_Souchi_RWJT01006805.1_1e-160_54_672_12054185_12054819
marine_Turtru_NW_017844625.1_5e-159_54_672_54846740_54846106
marine_Turadu_NCQN01001493.1_6e-159_54_672_216097_215463
marine_Orcorc_NW_004438463.1_5e-164_54_672_2175241_2175875
marine_Lipvex_NW_006785848.1_1e-170_54_672_503586_504220
marine_Inigeo_RJWO010029942.1_8e-163_62_672_7494_6868
marine_Ponbla_RJWI010091791.1_1e-164_54_672_1199_569
marine_Mesbid_PVJJ010000381.1_4e-171_54_672_105414_104783
marine_Zipcav_RJWS010026604.1_4e-176_54_672_10098_10729
marine_Phycat_NC_041214.1_3e-166_54_672_101265480_101266110
marine_Plamin_RJWK010011915.1_2e-154_52_672_9516_10150
marine_Kogbre_RJWL010006534.1_8e-158_54_672_30235_29604
marine_Escrob_NIPP01005664.1_4e-166_54_672_83124_83761
marine_Megnov_RYZJ01000788.1_7e-168_54_672_988857_988220
marine_Balmus_VNFD01002119.1_1e-174_54_672_180873_180236
marine_Balbon_DF473471.1_3e-171_54_672_1870_2507
marine_Balacu_NW_006727020.1_2e-144_148_672_17088733_17089277
PVJP02006930.1_2324272-2324888_Hippopotamus_amphibius_isolate_BS01_ScHSfme_6902HRSCAF_53919_0_whole_genome_shotgun_sequence
NKPW01002805.1_25716-26332_Hippopotamus_amphibius_isolate_HIP001_scaffold2805_whole_genome_shotgun_sequence
_R_SJXV01013245.1_2439355-2439809_Giraffa_camelopardalis_isolate_GFE-hd_scaffold68_whole_genome_shotgun_sequence
_R_RAWU01000004.1_87191415-87191869_Giraffa_tippelskirchi_isolate_OR1865_chromosome_2_whole_genome_shotgun_sequence
VCEA01000003.1_179398582-179399014_Muntiacus_muntjak_isolate_UTSW_UCB_Mm_chromosome_3_X_whole_genome_shotgun_sequence
_R_PVJP02910726.1_1832480-1832977_Hippopotamus_amphibius_isolate_BS01_ScHSfme_925179HRSCAF_982979_whole_genome_shotgun_sequence
NPJO01000006.1_72221690-72222326_Sus_scrofa_breed_Cross-bred_isolate_201423004_chromosome_14_whole_genome_shotgun_sequence
0.02

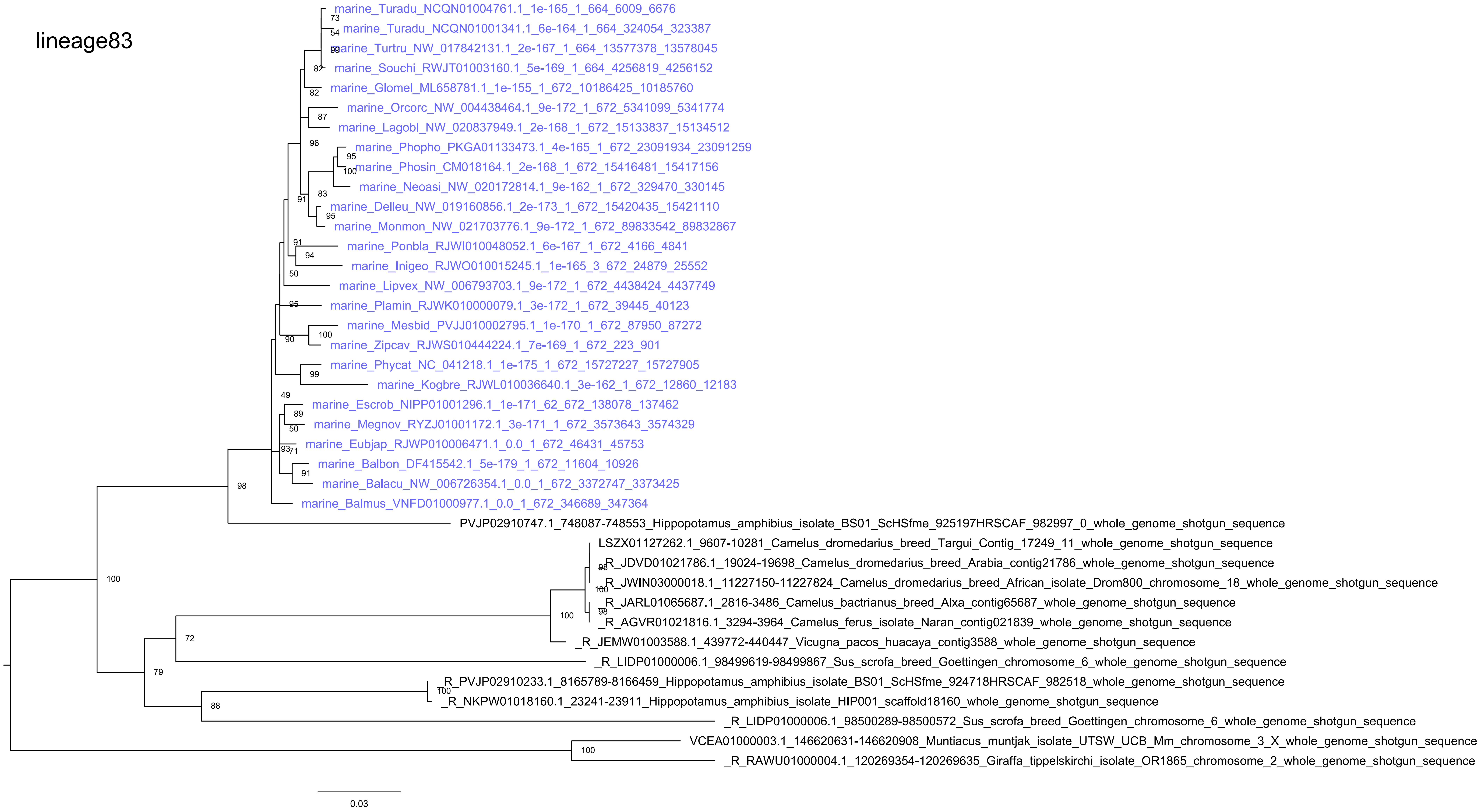
lineage83
marine_Turadu_NCQN01004761.1_1e-165_1_664_6009_6676
marine_Turadu_NCQN01001341.1_6e-164_1_664_324054_323387
marine_Turtru_NW_017842131.1_2e-167_1_664_13577378_13578045
marine_Souchi_RWJT01003160.1_5e-169_1_664_4256819_4256152
marine_Glomel_ML658781.1_1e-155_1_672_10186425_10185760
marine_Orcorc_NW_004438464.1_9e-172_1_672_5341099_5341774
marine_Lagobl_NW_020837949.1_2e-168_1_672_15133837_15134512
marine_Phopho_PKGA01133473.1_4e-165_1_672_23091934_23091259
marine_Phosin_CM018164.1_2e-168_1_672_15416481_15417156
marine_Neoasi_NW_020172814.1_9e-162_1_672_329470_330145
marine_Delleu_NW_019160856.1_2e-173_1_672_15420435_15421110
marine_Monmon_NW_021703776.1_9e-172_1_672_89833542_89832867
marine_Ponbla_RJWI010048052.1_6e-167_1_672_4166_4841
marine_Inigeo_RJWO010015245.1_1e-165_3_672_24879_25552
marine_Lipvex_NW_006793703.1_9e-172_1_672_4438424_4437749
marine_Plamin_RJWK010000079.1_3e-172_1_672_39445_40123
marine_Mesbid_PVJJ010002795.1_1e-170_1_672_87950_87272
marine_Zipcav_RJWS010444224.1_7e-169_1_672_223_901
marine_Phycat_NC_041218.1_1e-175_1_672_15727227_15727905
marine_Kogbre_RJWL010036640.1_3e-162_1_672_12860_12183
marine_Escrob_NIPP01001296.1_1e-171_62_672_138078_137462
marine_Megnov_RYZJ01001172.1_3e-171_1_672_3573643_3574329
marine_Eubjap_RJWP010006471.1_0.0_1_672_46431_45753
marine_Balbon_DF415542.1_5e-179_1_672_11604_10926
marine_Balacu_NW_006726354.1_0.0_1_672_3372747_3373425
marine_Balmus_VNFD01000977.1_0.0_1_672_346689_347364
PVJP02910747.1_748087-748553_Hippopotamus_amphibius_isolate_BS01_ScHSfme_925197HRSCAF_982997_0_whole_genome_shotgun_sequence
LSZX01127262.1_9607-10281_Camelus_dromedarius_breed_Targui_Contig_17249_11_whole_genome_shotgun_sequence
_R_JDVD01021786.1_19024-19698_Camelus_dromedarius_breed_Arabia_contig21786_whole_genome_shotgun_sequence
_R_JWIN03000018.1_11227150-11227824_Camelus_dromedarius_breed_African_isolate_Drom800_chromosome_18_whole_genome_shotgun_sequence
_R_JARL01065687.1_2816-3486_Camelus_bactrianus_breed_Alxa_contig65687_whole_genome_shotgun_sequence
_R_AGVR01021816.1_3294-3964_Camelus_ferus_isolate_Naran_contig021839_whole_genome_shotgun_sequence
_R_JEMW01003588.1_439772-440447_Vicugna_pacos_huacaya_contig3588_whole_genome_shotgun_sequence
_R_LIDP01000006.1_98499619-98499867_Sus_scrofa_breed_Goettingen_chromosome_6_whole_genome_shotgun_sequence
_R_PVJP02910233.1_8165789-8166459_Hippopotamus_amphibius_isolate_BS01_ScHSfme_924718HRSCAF_982518_whole_genome_shotgun_sequence
_R_NKPW01018160.1_23241-23911_Hippopotamus_amphibius_isolate_HIP001_scaffold18160_whole_genome_shotgun_sequence
_R_LIDP01000006.1_98500289-98500572_Sus_scrofa_breed_Goettingen_chromosome_6_whole_genome_shotgun_sequence
VCEA01000003.1_146620631-146620908_Muntiacus_muntjak_isolate_UTSW_UCB_Mm_chromosome_3_X_whole_genome_shotgun_sequence
_R_RAWU01000004.1_120269354-120269635_Giraffa_tippelskirchi_isolate_OR1865_chromosome_2_whole_genome_shotgun_sequence
0.03

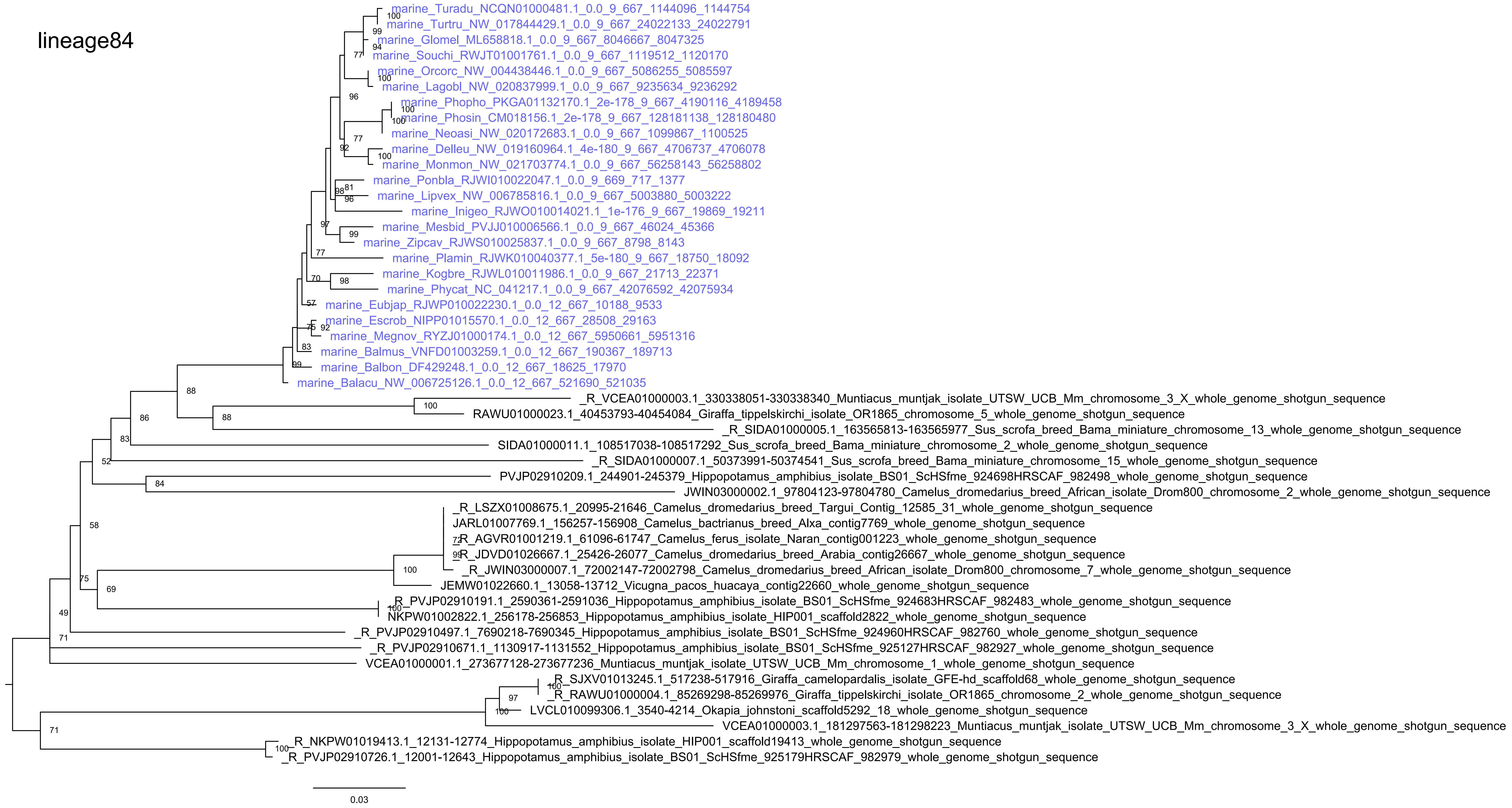
lineage84
marine_Turadu_NCQN01000481.1_0.0_9_667_1144096_1144754
marine_Turtru_NW_017844429.1_0.0_9_667_24022133_24022791
marine_Glomel_ML658818.1_0.0_9_667_8046667_8047325
marine_Souchi_RWJT01001761.1_0.0_9_667_1119512_1120170
marine_Orcorc_NW_004438446.1_0.0_9_667_5086255_5085597
marine_Lagobl_NW_020837999.1_0.0_9_667_9235634_9236292
marine_Phopho_PKGA01132170.1_2e-178_9_667_4190116_4189458
marine_Phosin_CM018156.1_2e-178_9_667_128181138_128180480
marine_Neoasi_NW_020172683.1_0.0_9_667_1099867_1100525
marine_Delleu_NW_019160964.1_4e-180_9_667_4706737_4706078
marine_Monmon_NW_021703774.1_0.0_9_667_56258143_56258802
marine_Ponbla_RJWI010022047.1_0.0_9_669_717_1377
marine_Lipvex_NW_006785816.1_0.0_9_667_5003880_5003222
marine_Inigeo_RJWO010014021.1_1e-176_9_667_19869_19211
marine_Mesbid_PVJJ010006566.1_0.0_9_667_46024_45366
marine_Zipcav_RJWS010025837.1_0.0_9_667_8798_8143
marine_Plamin_RJWK010040377.1_5e-180_9_667_18750_18092
marine_Kogbre_RJWL010011986.1_0.0_9_667_21713_22371
marine_Phycat_NC_041217.1_0.0_9_667_42076592_42075934
marine_Eubjap_RJWP010022230.1_0.0_12_667_10188_9533
marine_Escrob_NIPP01015570.1_0.0_12_667_28508_29163
marine_Megnov_RYZJ01000174.1_0.0_12_667_5950661_5951316
marine_Balmus_VNFD01003259.1_0.0_12_667_190367_189713
marine_Balbon_DF429248.1_0.0_12_667_18625_17970
marine_Balacu_NW_006725126.1_0.0_12_667_521690_521035
_R_VCEA01000003.1_330338051-330338340_Muntiacus_muntjak_isolate_UTSW_UCB_Mm_chromosome_3_X_whole_genome_shotgun_sequence
RAWU01000023.1_40453793-40454084_Giraffa_tippelskirchi_isolate_OR1865_chromosome_5_whole_genome_shotgun_sequence
_R_SIDA01000005.1_163565813-163565977_Sus_scrofa_breed_Bama_miniature_chromosome_13_whole_genome_shotgun_sequence
SIDA01000011.1_108517038-108517292_Sus_scrofa_breed_Bama_miniature_chromosome_2_whole_genome_shotgun_sequence
_R_SIDA01000007.1_50373991-50374541_Sus_scrofa_breed_Bama_miniature_chromosome_15_whole_genome_shotgun_sequence
PVJP02910209.1_244901-245379_Hippopotamus_amphibius_isolate_BS01_ScHSfme_924698HRSCAF_982498_whole_genome_shotgun_sequence
JWIN03000002.1_97804123-97804780_Camelus_dromedarius_breed_African_isolate_Drom800_chromosome_2_whole_genome_shotgun_sequence
_R_LSZX01008675.1_20995-21646_Camelus_dromedarius_breed_Targui_Contig_12585_31_whole_genome_shotgun_sequence
JARL01007769.1_156257-156908_Camelus_bactrianus_breed_Alxa_contig7769_whole_genome_shotgun_sequence
_R_AGVR01001219.1_61096-61747_Camelus_ferus_isolate_Naran_contig001223_whole_genome_shotgun_sequence
_R_JDVD01026667.1_25426-26077_Camelus_dromedarius_breed_Arabia_contig26667_whole_genome_shotgun_sequence
_R_JWIN03000007.1_72002147-72002798_Camelus_dromedarius_breed_African_isolate_Drom800_chromosome_7_whole_genome_shotgun_sequence
JEMW01022660.1_13058-13712_Vicugna_pacos_huacaya_contig22660_whole_genome_shotgun_sequence
_R_PVJP02910191.1_2590361-2591036_Hippopotamus_amphibius_isolate_BS01_ScHSfme_924683HRSCAF_982483_whole_genome_shotgun_sequence
NKPW01002822.1_256178-256853_Hippopotamus_amphibius_isolate_HIP001_scaffold2822_whole_genome_shotgun_sequence
_R_PVJP02910497.1_7690218-7690345_Hippopotamus_amphibius_isolate_BS01_ScHSfme_924960HRSCAF_982760_whole_genome_shotgun_sequence
_R_PVJP02910671.1_1130917-1131552_Hippopotamus_amphibius_isolate_BS01_ScHSfme_925127HRSCAF_982927_whole_genome_shotgun_sequence
VCEA01000001.1_273677128-273677236_Muntiacus_muntjak_isolate_UTSW_UCB_Mm_chromosome_1_whole_genome_shotgun_sequence
_R_SJXV01013245.1_517238-517916_Giraffa_camelopardalis_isolate_GFE-hd_scaffold68_whole_genome_shotgun_sequence
_R_RAWU01000004.1_85269298-85269976_Giraffa_tippelskirchi_isolate_OR1865_chromosome_2_whole_genome_shotgun_sequence
LVCL010099306.1_3540-4214_Okapia_johnstoni_scaffold5292_18_whole_genome_shotgun_sequence
VCEA01000003.1_181297563-181298223_Muntiacus_muntjak_isolate_UTSW_UCB_Mm_chromosome_3_X_whole_genome_shotgun_sequence
_R_NKPW01019413.1_12131-12774_Hippopotamus_amphibius_isolate_HIP001_scaffold19413_whole_genome_shotgun_sequence
_R_PVJP02910726.1_12001-12643_Hippopotamus_amphibius_isolate_BS01_ScHSfme_925179HRSCAF_982979_whole_genome_shotgun_sequence
0.03

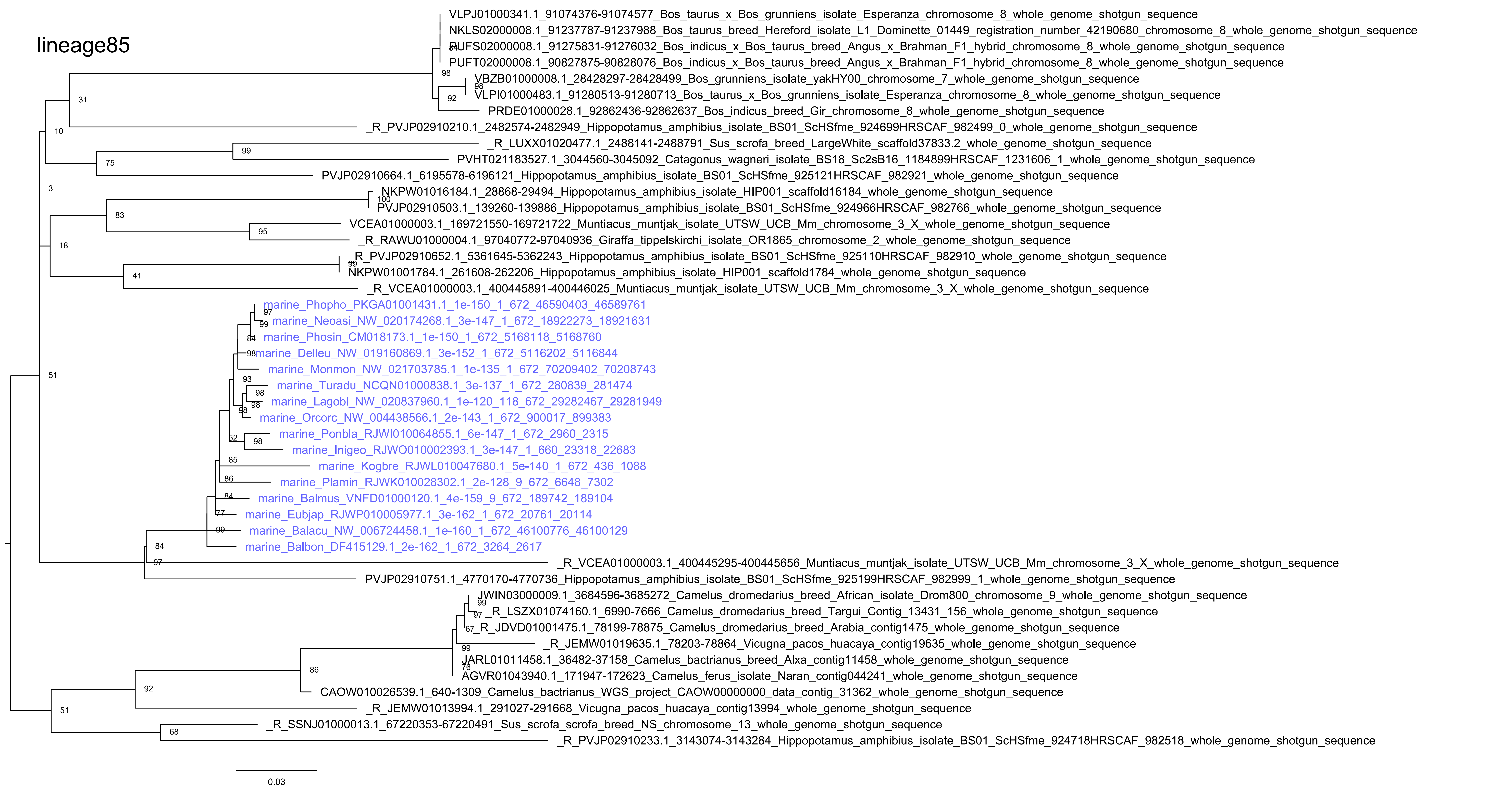
lineage85
VLPJ01000341.1_91074376-91074577_Bos_taurus_x_Bos_grunniens_isolate_Esperanza_chromosome_8_whole_genome_shotgun_sequence
NKLS02000008.1_91237787-91237988_Bos_taurus_breed_Hereford_isolate_L1_Dominette_01449_registration_number_42190680_chromosome_8_whole_genome_shotgun_sequence
PUFS02000008.1_91275831-91276032_Bos_indicus_x_Bos_taurus_breed_Angus_x_Brahman_F1_hybrid_chromosome_8_whole_genome_shotgun_sequence
PUFT02000008.1_90827875-90828076_Bos_indicus_x_Bos_taurus_breed_Angus_x_Brahman_F1_hybrid_chromosome_8_whole_genome_shotgun_sequence
VBZB01000008.1_28428297-28428499_Bos_grunniens_isolate_yakHY00_chromosome_7_whole_genome_shotgun_sequence
VLPI01000483.1_91280513-91280713_Bos_taurus_x_Bos_grunniens_isolate_Esperanza_chromosome_8_whole_genome_shotgun_sequence
PRDE01000028.1_92862436-92862637_Bos_indicus_breed_Gir_chromosome_8_whole_genome_shotgun_sequence
_R_PVJP02910210.1_2482574-2482949_Hippopotamus_amphibius_isolate_BS01_ScHSfme_924699HRSCAF_982499_0_whole_genome_shotgun_sequence
_R_LUXX01020477.1_2488141-2488791_Sus_scrofa_breed_LargeWhite_scaffold37833.2_whole_genome_shotgun_sequence
PVHT021183527.1_3044560-3045092_Catagonus_wagneri_isolate_BS18_Sc2sB16_1184899HRSCAF_1231606_1_whole_genome_shotgun_sequence
PVJP02910664.1_6195578-6196121_Hippopotamus_amphibius_isolate_BS01_ScHSfme_925121HRSCAF_982921_whole_genome_shotgun_sequence
NKPW01016184.1_28868-29494_Hippopotamus_amphibius_isolate_HIP001_scaffold16184_whole_genome_shotgun_sequence
PVJP02910503.1_139260-139886_Hippopotamus_amphibius_isolate_BS01_ScHSfme_924966HRSCAF_982766_whole_genome_shotgun_sequence
VCEA01000003.1_169721550-169721722_Muntiacus_muntjak_isolate_UTSW_UCB_Mm_chromosome_3_X_whole_genome_shotgun_sequence
_R_RAWU01000004.1_97040772-97040936_Giraffa_tippelskirchi_isolate_OR1865_chromosome_2_whole_genome_shotgun_sequence
_R_PVJP02910652.1_5361645-5362243_Hippopotamus_amphibius_isolate_BS01_ScHSfme_925110HRSCAF_982910_whole_genome_shotgun_sequence
NKPW01001784.1_261608-262206_Hippopotamus_amphibius_isolate_HIP001_scaffold1784_whole_genome_shotgun_sequence
_R_VCEA01000003.1_400445891-400446025_Muntiacus_muntjak_isolate_UTSW_UCB_Mm_chromosome_3_X_whole_genome_shotgun_sequence
marine_Phopho_PKGA01001431.1_1e-150_1_672_46590403_46589761
marine_Neoasi_NW_020174268.1_3e-147_1_672_18922273_18921631
marine_Phosin_CM018173.1_1e-150_1_672_5168118_5168760
marine_Delleu_NW_019160869.1_3e-152_1_672_5116202_5116844
marine_Monmon_NW_021703785.1_1e-135_1_672_70209402_70208743
marine_Turadu_NCQN01000838.1_3e-137_1_672_280839_281474
marine_Lagobl_NW_020837960.1_1e-120_118_672_29282467_29281949
marine_Orcorc_NW_004438566.1_2e-143_1_672_900017_899383
marine_Ponbla_RJWI010064855.1_6e-147_1_672_2960_2315
marine_Inigeo_RJWO010002393.1_3e-147_1_660_23318_22683
marine_Kogbre_RJWL010047680.1_5e-140_1_672_436_1088
marine_Plamin_RJWK010028302.1_2e-128_9_672_6648_7302
marine_Balmus_VNFD01000120.1_4e-159_9_672_189742_189104
marine_Eubjap_RJWP010005977.1_3e-162_1_672_20761_20114
marine_Balacu_NW_006724458.1_1e-160_1_672_46100776_46100129
marine_Balbon_DF415129.1_2e-162_1_672_3264_2617
_R_VCEA01000003.1_400445295-400445656_Muntiacus_muntjak_isolate_UTSW_UCB_Mm_chromosome_3_X_whole_genome_shotgun_sequence
PVJP02910751.1_4770170-4770736_Hippopotamus_amphibius_isolate_BS01_ScHSfme_925199HRSCAF_982999_1_whole_genome_shotgun_sequence
JWIN03000009.1_3684596-3685272_Camelus_dromedarius_breed_African_isolate_Drom800_chromosome_9_whole_genome_shotgun_sequence
_R_LSZX01074160.1_6990-7666_Camelus_dromedarius_breed_Targui_Contig_13431_156_whole_genome_shotgun_sequence
_R_JDVD01001475.1_78199-78875_Camelus_dromedarius_breed_Arabia_contig1475_whole_genome_shotgun_sequence
_R_JEMW01019635.1_78203-78864_Vicugna_pacos_huacaya_contig19635_whole_genome_shotgun_sequence
JARL01011458.1_36482-37158_Camelus_bactrianus_breed_Alxa_contig11458_whole_genome_shotgun_sequence
AGVR01043940.1_171947-172623_Camelus_ferus_isolate_Naran_contig044241_whole_genome_shotgun_sequence
CAOW010026539.1_640-1309_Camelus_bactrianus_WGS_project_CAOW00000000_data_contig_31362_whole_genome_shotgun_sequence
_R_JEMW01013994.1_291027-291668_Vicugna_pacos_huacaya_contig13994_whole_genome_shotgun_sequence
_R_SSNJ01000013.1_67220353-67220491_Sus_scrofa_scrofa_breed_NS_chromosome_13_whole_genome_shotgun_sequence
_R_PVJP02910233.1_3143074-3143284_Hippopotamus_amphibius_isolate_BS01_ScHSfme_924718HRSCAF_982518_whole_genome_shotgun_sequence
0.03

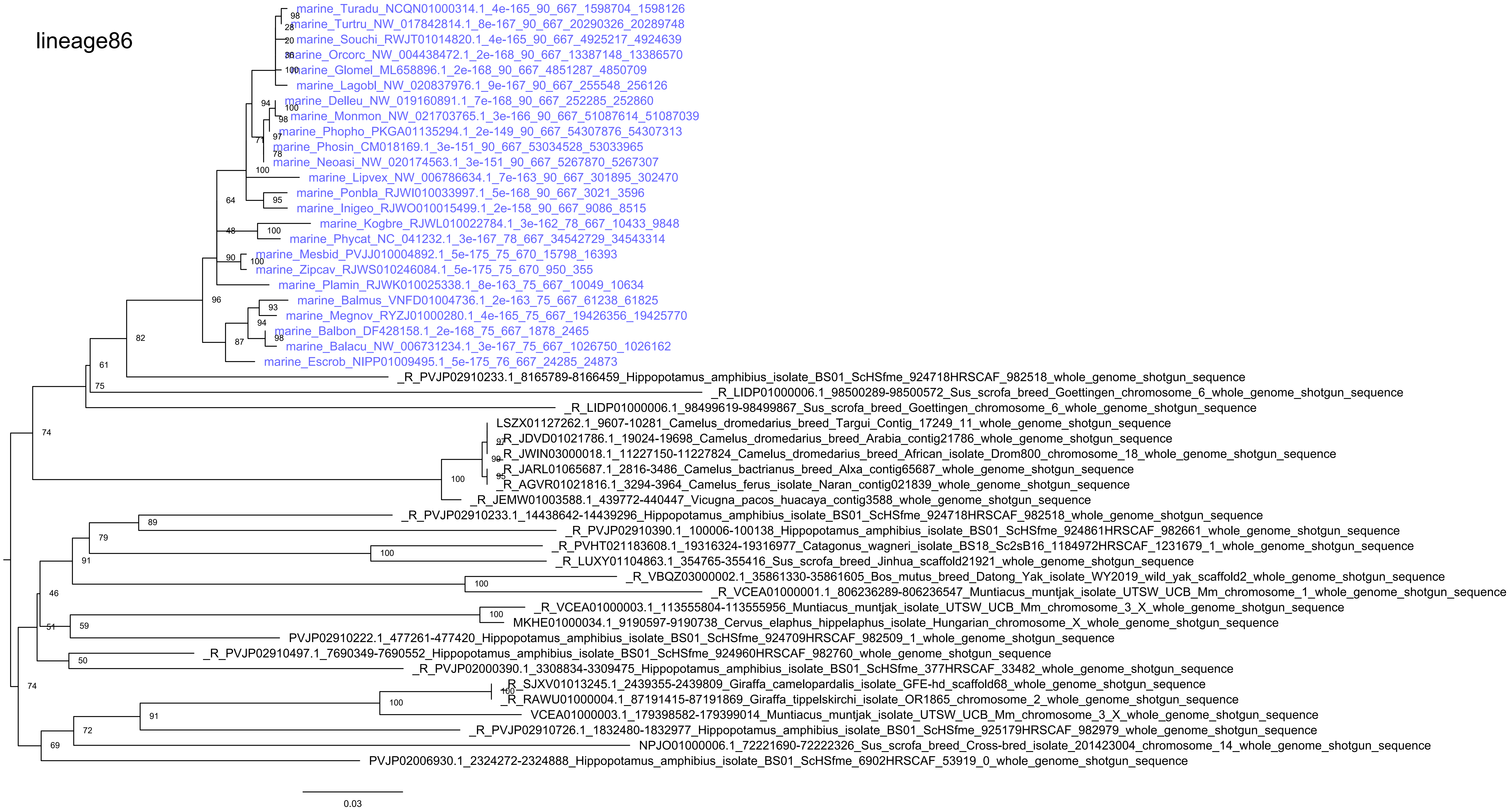
lineage86
marine_Turadu_NCQN01000314.1_4e-165_90_667_1598704_1598126
marine_Turtru_NW_017842814.1_8e-167_90_667_20290326_20289748
marine_Souchi_RWJT01014820.1_4e-165_90_667_4925217_4924639
marine_Orcorc_NW_004438472.1_2e-168_90_667_13387148_13386570
marine_Glomel_ML658896.1_2e-168_90_667_4851287_4850709
marine_Lagobl_NW_020837976.1_9e-167_90_667_255548_256126
marine_Delleu_NW_019160891.1_7e-168_90_667_252285_252860
marine_Monmon_NW_021703765.1_3e-166_90_667_51087614_51087039
marine_Phopho_PKGA01135294.1_2e-149_90_667_54307876_54307313
marine_Phosin_CM018169.1_3e-151_90_667_53034528_53033965
marine_Neoasi_NW_020174563.1_3e-151_90_667_5267870_5267307
marine_Lipvex_NW_006786634.1_7e-163_90_667_301895_302470
marine_Ponbla_RJWI010033997.1_5e-168_90_667_3021_3596
marine_Inigeo_RJWO010015499.1_2e-158_90_667_9086_8515
marine_Kogbre_RJWL010022784.1_3e-162_78_667_10433_9848
marine_Phycat_NC_041232.1_3e-167_78_667_34542729_34543314
marine_Mesbid_PVJJ010004892.1_5e-175_75_670_15798_16393
marine_Zipcav_RJWS010246084.1_5e-175_75_670_950_355
marine_Plamin_RJWK010025338.1_8e-163_75_667_10049_10634
marine_Balmus_VNFD01004736.1_2e-163_75_667_61238_61825
marine_Megnov_RYZJ01000280.1_4e-165_75_667_19426356_19425770
marine_Balbon_DF428158.1_2e-168_75_667_1878_2465
marine_Balacu_NW_006731234.1_3e-167_75_667_1026750_1026162
marine_Escrob_NIPP01009495.1_5e-175_76_667_24285_24873
_R_PVJP02910233.1_8165789-8166459_Hippopotamus_amphibius_isolate_BS01_ScHSfme_924718HRSCAF_982518_whole_genome_shotgun_sequence
_R_LIDP01000006.1_98500289-98500572_Sus_scrofa_breed_Goettingen_chromosome_6_whole_genome_shotgun_sequence
_R_LIDP01000006.1_98499619-98499867_Sus_scrofa_breed_Goettingen_chromosome_6_whole_genome_shotgun_sequence
LSZX01127262.1_9607-10281_Camelus_dromedarius_breed_Targui_Contig_17249_11_whole_genome_shotgun_sequence
_R_JDVD01021786.1_19024-19698_Camelus_dromedarius_breed_Arabia_contig21786_whole_genome_shotgun_sequence
_R_JWIN03000018.1_11227150-11227824_Camelus_dromedarius_breed_African_isolate_Drom800_chromosome_18_whole_genome_shotgun_sequence
_R_JARL01065687.1_2816-3486_Camelus_bactrianus_breed_Alxa_contig65687_whole_genome_shotgun_sequence
_R_AGVR01021816.1_3294-3964_Camelus_ferus_isolate_Naran_contig021839_whole_genome_shotgun_sequence
_R_JEMW01003588.1_439772-440447_Vicugna_pacos_huacaya_contig3588_whole_genome_shotgun_sequence
_R_PVJP02910233.1_14438642-14439296_Hippopotamus_amphibius_isolate_BS01_ScHSfme_924718HRSCAF_982518_whole_genome_shotgun_sequence
_R_PVJP02910390.1_100006-100138_Hippopotamus_amphibius_isolate_BS01_ScHSfme_924861HRSCAF_982661_whole_genome_shotgun_sequence
_R_PVHT021183608.1_19316324-19316977_Catagonus_wagneri_isolate_BS18_Sc2sB16_1184972HRSCAF_1231679_1_whole_genome_shotgun_sequence
_R_LUXY01104863.1_354765-355416_Sus_scrofa_breed_Jinhua_scaffold21921_whole_genome_shotgun_sequence
_R_VBQZ03000002.1_35861330-35861605_Bos_mutus_breed_Datong_Yak_isolate_WY2019_wild_yak_scaffold2_whole_genome_shotgun_sequence
_R_VCEA01000001.1_806236289-806236547_Muntiacus_muntjak_isolate_UTSW_UCB_Mm_chromosome_1_whole_genome_shotgun_sequence
_R_VCEA01000003.1_113555804-113555956_Muntiacus_muntjak_isolate_UTSW_UCB_Mm_chromosome_3_X_whole_genome_shotgun_sequence
MKHE01000034.1_9190597-9190738_Cervus_elaphus_hippelaphus_isolate_Hungarian_chromosome_X_whole_genome_shotgun_sequence
PVJP02910222.1_477261-477420_Hippopotamus_amphibius_isolate_BS01_ScHSfme_924709HRSCAF_982509_1_whole_genome_shotgun_sequence
_R_PVJP02910497.1_7690349-7690552_Hippopotamus_amphibius_isolate_BS01_ScHSfme_924960HRSCAF_982760_whole_genome_shotgun_sequence
_R_PVJP02000390.1_3308834-3309475_Hippopotamus_amphibius_isolate_BS01_ScHSfme_377HRSCAF_33482_whole_genome_shotgun_sequence
_R_SJXV01013245.1_2439355-2439809_Giraffa_camelopardalis_isolate_GFE-hd_scaffold68_whole_genome_shotgun_sequence
_R_RAWU01000004.1_87191415-87191869_Giraffa_tippelskirchi_isolate_OR1865_chromosome_2_whole_genome_shotgun_sequence
VCEA01000003.1_179398582-179399014_Muntiacus_muntjak_isolate_UTSW_UCB_Mm_chromosome_3_X_whole_genome_shotgun_sequence
_R_PVJP02910726.1_1832480-1832977_Hippopotamus_amphibius_isolate_BS01_ScHSfme_925179HRSCAF_982979_whole_genome_shotgun_sequence
NPJO01000006.1_72221690-72222326_Sus_scrofa_breed_Cross-bred_isolate_201423004_chromosome_14_whole_genome_shotgun_sequence
PVJP02006930.1_2324272-2324888_Hippopotamus_amphibius_isolate_BS01_ScHSfme_6902HRSCAF_53919_0_whole_genome_shotgun_sequence
0.03

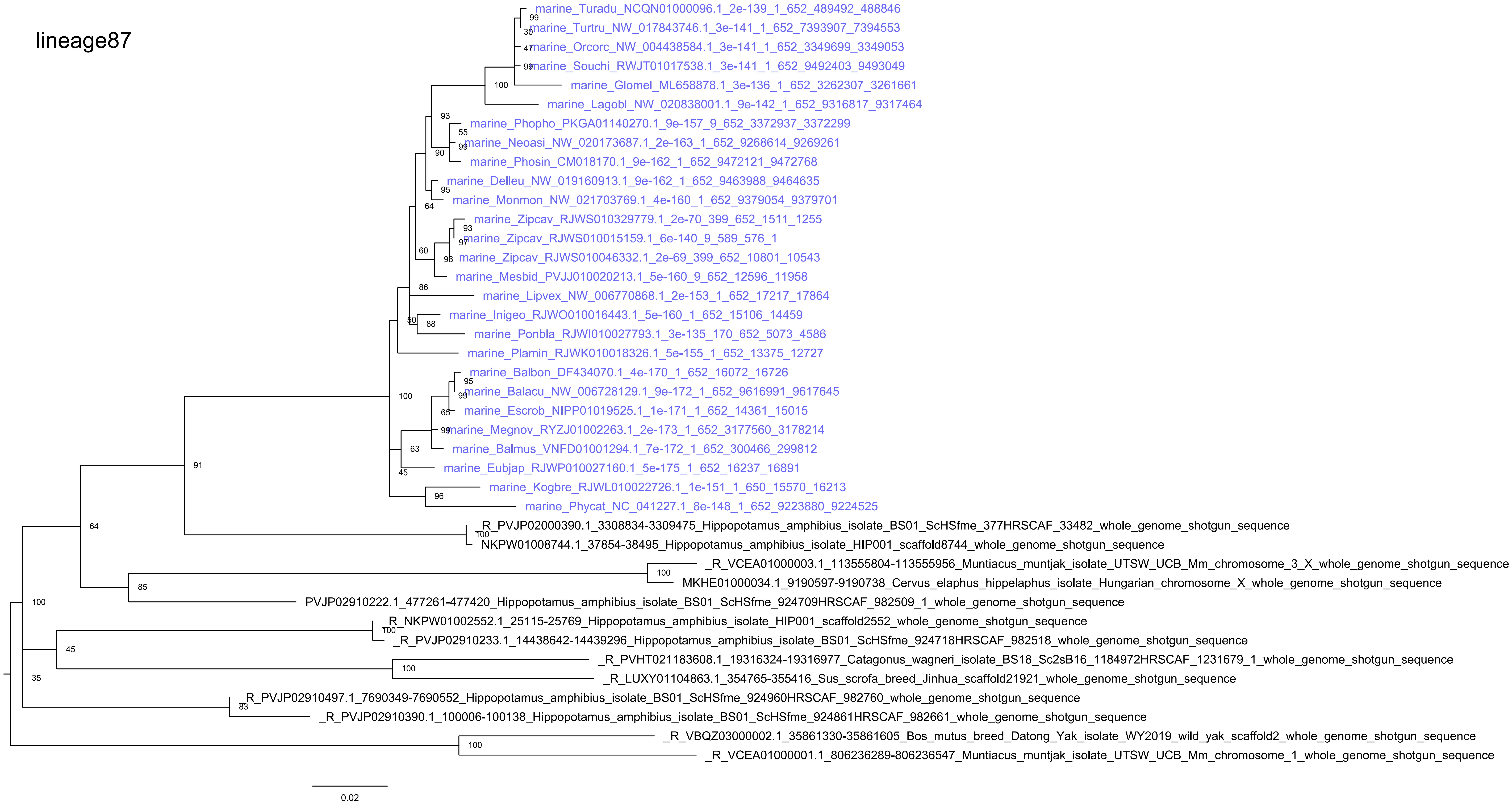
lineage87
marine_Turadu_NCQN01000096.1_2e-139_1_652_489492_488846
marine_Turtru_NW_017843746.1_3e-141_1_652_7393907_7394553
marine_Orcorc_NW_004438584.1_3e-141_1_652_3349699_3349053
marine_Souchi_RWJT01017538.1_3e-141_1_652_9492403_9493049
marine_Glomel_ML658878.1_3e-136_1_652_3262307_3261661
marine_Lagobl_NW_020838001.1_9e-142_1_652_9316817_9317464
marine_Phopho_PKGA01140270.1_9e-157_9_652_3372937_3372299
marine_Neoasi_NW_020173687.1_2e-163_1_652_9268614_9269261
marine_Phosin_CM018170.1_9e-162_1_652_9472121_9472768
marine_Delleu_NW_019160913.1_9e-162_1_652_9463988_9464635
marine_Monmon_NW_021703769.1_4e-160_1_652_9379054_9379701
marine_Zipcav_RJWS010329779.1_2e-70_399_652_1511_1255
marine_Zipcav_RJWS010015159.1_6e-140_9_589_576_1
marine_Zipcav_RJWS010046332.1_2e-69_399_652_10801_10543
marine_Mesbid_PVJJ010020213.1_5e-160_9_652_12596_11958
marine_Lipvex_NW_006770868.1_2e-153_1_652_17217_17864
marine_Inigeo_RJWO010016443.1_5e-160_1_652_15106_14459
marine_Ponbla_RJWI010027793.1_3e-135_170_652_5073_4586
marine_Plamin_RJWK010018326.1_5e-155_1_652_13375_12727
marine_Balbon_DF434070.1_4e-170_1_652_16072_16726
marine_Balacu_NW_006728129.1_9e-172_1_652_9616991_9617645
marine_Escrob_NIPP01019525.1_1e-171_1_652_14361_15015
marine_Megnov_RYZJ01002263.1_2e-173_1_652_3177560_3178214
marine_Balmus_VNFD01001294.1_7e-172_1_652_300466_299812
marine_Eubjap_RJWP010027160.1_5e-175_1_652_16237_16891
marine_Kogbre_RJWL010022726.1_1e-151_1_650_15570_16213
marine_Phycat_NC_041227.1_8e-148_1_652_9223880_9224525
_R_PVJP02000390.1_3308834-3309475_Hippopotamus_amphibius_isolate_BS01_ScHSfme_377HRSCAF_33482_whole_genome_shotgun_sequence
NKPW01008744.1_37854-38495_Hippopotamus_amphibius_isolate_HIP001_scaffold8744_whole_genome_shotgun_sequence
_R_VCEA01000003.1_113555804-113555956_Muntiacus_muntjak_isolate_UTSW_UCB_Mm_chromosome_3_X_whole_genome_shotgun_sequence
MKHE01000034.1_9190597-9190738_Cervus_elaphus_hippelaphus_isolate_Hungarian_chromosome_X_whole_genome_shotgun_sequence
PVJP02910222.1_477261-477420_Hippopotamus_amphibius_isolate_BS01_ScHSfme_924709HRSCAF_982509_1_whole_genome_shotgun_sequence
_R_NKPW01002552.1_25115-25769_Hippopotamus_amphibius_isolate_HIP001_scaffold2552_whole_genome_shotgun_sequence
_R_PVJP02910233.1_14438642-14439296_Hippopotamus_amphibius_isolate_BS01_ScHSfme_924718HRSCAF_982518_whole_genome_shotgun_sequence
_R_PVHT021183608.1_19316324-19316977_Catagonus_wagneri_isolate_BS18_Sc2sB16_1184972HRSCAF_1231679_1_whole_genome_shotgun_sequence
_R_LUXY01104863.1_354765-355416_Sus_scrofa_breed_Jinhua_scaffold21921_whole_genome_shotgun_sequence
_R_PVJP02910497.1_7690349-7690552_Hippopotamus_amphibius_isolate_BS01_ScHSfme_924960HRSCAF_982760_whole_genome_shotgun_sequence
_R_PVJP02910390.1_100006-100138_Hippopotamus_amphibius_isolate_BS01_ScHSfme_924861HRSCAF_982661_whole_genome_shotgun_sequence
_R_VBQZ03000002.1_35861330-35861605_Bos_mutus_breed_Datong_Yak_isolate_WY2019_wild_yak_scaffold2_whole_genome_shotgun_sequence
_R_VCEA01000001.1_806236289-806236547_Muntiacus_muntjak_isolate_UTSW_UCB_Mm_chromosome_1_whole_genome_shotgun_sequence
0.02

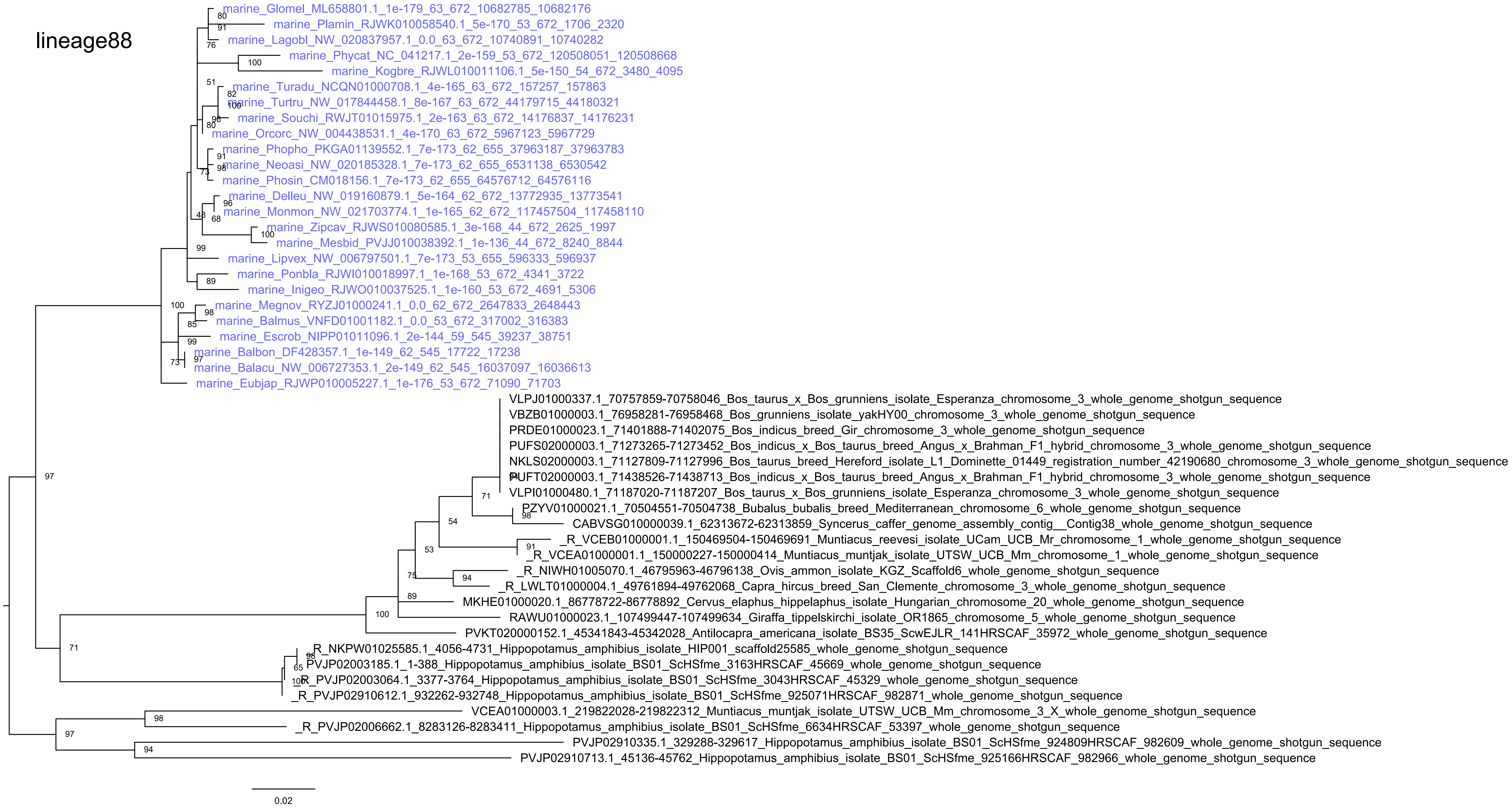

lineage88
marine_Glomel_ML658801.1_1e-179_63_672_10682785_10682176
marine_Plamin_RJWK010058540.1_5e-170_53_672_1706_2320
marine_Lagobl_NW_020837957.1_0.0_63_672_10740891_10740282
marine_Phycat_NC_041217.1_2e-159_53_672_120508051_120508668
marine_Kogbre_RJWL010011106.1_5e-150_54_672_3480_4095
marine_Turadu_NCQN01000708.1_4e-165_63_672_157257_157863
marine_Turtru_NW_017844458.1_8e-167_63_672_44179715_44180321
marine_Souchi_RWJT01015975.1_2e-163_63_672_14176837_14176231
marine_Orcorc_NW_004438531.1_4e-170_63_672_5967123_5967729
marine_Phopho_PKGA01139552.1_7e-173_62_655_37963187_37963783
marine_Neoasi_NW_020185328.1_7e-173_62_655_6531138_6530542
marine_Phosin_CM018156.1_7e-173_62_655_64576712_64576116
marine_Delleu_NW_019160879.1_5e-164_62_672_13772935_13773541
marine_Monmon_NW_021703774.1_1e-165_62_672_117457504_117458110
marine_Zipcav_RJWS010080585.1_3e-168_44_672_2625_1997
marine_Mesbid_PVJJ010038392.1_1e-136_44_672_8240_8844
marine_Lipvex_NW_006797501.1_7e-173_53_655_596333_596937
marine_Ponbla_RJWI010018997.1_1e-168_53_672_4341_3722
marine_Inigeo_RJWO010037525.1_1e-160_53_672_4691_5306
marine_Megnov_RYZJ01000241.1_0.0_62_672_2647833_2648443
marine_Balmus_VNFD01001182.1_0.0_53_672_317002_316383
marine_Escrob_NIPP01011096.1_2e-144_59_545_39237_38751
marine_Balbon_DF428357.1_1e-149_62_545_17722_17238
marine_Balacu_NW_006727353.1_2e-149_62_545_16037097_16036613
marine_Eubjap_RJWP010005227.1_1e-176_53_672_71090_71703
VLPJ01000337.1_70757859-70758046_Bos_taurus_x_Bos_grunniens_isolate_Esperanza_chromosome_3_whole_genome_shotgun_sequence
VBZB01000003.1_76958281-76958468_Bos_grunniens_isolate_yakHY00_chromosome_3_whole_genome_shotgun_sequence
PRDE01000023.1_71401888-71402075_Bos_indicus_breed_Gir_chromosome_3_whole_genome_shotgun_sequence
PUFS02000003.1_71273265-71273452_Bos_indicus_x_Bos_taurus_breed_Angus_x_Brahman_F1_hybrid_chromosome_3_whole_genome_shotgun_sequence
NKLS02000003.1_71127809-71127996_Bos_taurus_breed_Hereford_isolate_L1_Dominette_01449_registration_number_42190680_chromosome_3_whole_genome_shotgun_sequence
PUFT02000003.1_71438526-71438713_Bos_indicus_x_Bos_taurus_breed_Angus_x_Brahman_F1_hybrid_chromosome_3_whole_genome_shotgun_sequence
VLPI01000480.1_71187020-71187207_Bos_taurus_x_Bos_grunniens_isolate_Esperanza_chromosome_3_whole_genome_shotgun_sequence
PZYV01000021.1_70504551-70504738_Bubalus_bubalis_breed_Mediterranean_chromosome_6_whole_genome_shotgun_sequence
CABVSG010000039.1_62313672-62313859_Syncerus_caffer_genome_assembly_contig__Contig38_whole_genome_shotgun_sequence
_R_VCEB01000001.1_150469504-150469691_Muntiacus_reevesi_isolate_UCam_UCB_Mr_chromosome_1_whole_genome_shotgun_sequence
_R_VCEA01000001.1_150000227-150000414_Muntiacus_muntjak_isolate_UTSW_UCB_Mm_chromosome_1_whole_genome_shotgun_sequence
_R_NIWH01005070.1_46795963-46796138_Ovis_ammon_isolate_KGZ_Scaffold6_whole_genome_shotgun_sequence
_R_LWLT01000004.1_49761894-49762068_Capra_hircus_breed_San_Clemente_chromosome_3_whole_genome_shotgun_sequence
MKHE01000020.1_86778722-86778892_Cervus_elaphus_hippelaphus_isolate_Hungarian_chromosome_20_whole_genome_shotgun_sequence
RAWU01000023.1_107499447-107499634_Giraffa_tippelskirchi_isolate_OR1865_chromosome_5_whole_genome_shotgun_sequence
PVKT020000152.1_45341843-45342028_Antilocapra_americana_isolate_BS35_ScwEJLR_141HRSCAF_35972_whole_genome_shotgun_sequence
_R_NKPW01025585.1_4056-4731_Hippopotamus_amphibius_isolate_HIP001_scaffold25585_whole_genome_shotgun_sequence
PVJP02003185.1_1-388_Hippopotamus_amphibius_isolate_BS01_ScHSfme_3163HRSCAF_45669_whole_genome_shotgun_sequence
_R_PVJP02003064.1_3377-3764_Hippopotamus_amphibius_isolate_BS01_ScHSfme_3043HRSCAF_45329_whole_genome_shotgun_sequence
_R_PVJP02910612.1_932262-932748_Hippopotamus_amphibius_isolate_BS01_ScHSfme_925071HRSCAF_982871_whole_genome_shotgun_sequence
VCEA01000003.1_219822028-219822312_Muntiacus_muntjak_isolate_UTSW_UCB_Mm_chromosome_3_X_whole_genome_shotgun_sequence
_R_PVJP02006662.1_8283126-8283411_Hippopotamus_amphibius_isolate_BS01_ScHSfme_6634HRSCAF_53397_whole_genome_shotgun_sequence
PVJP02910335.1_329288-329617_Hippopotamus_amphibius_isolate_BS01_ScHSfme_924809HRSCAF_982609_whole_genome_shotgun_sequence
PVJP02910713.1_45136-45762_Hippopotamus_amphibius_isolate_BS01_ScHSfme_925166HRSCAF_982966_whole_genome_shotgun_sequence
0.02

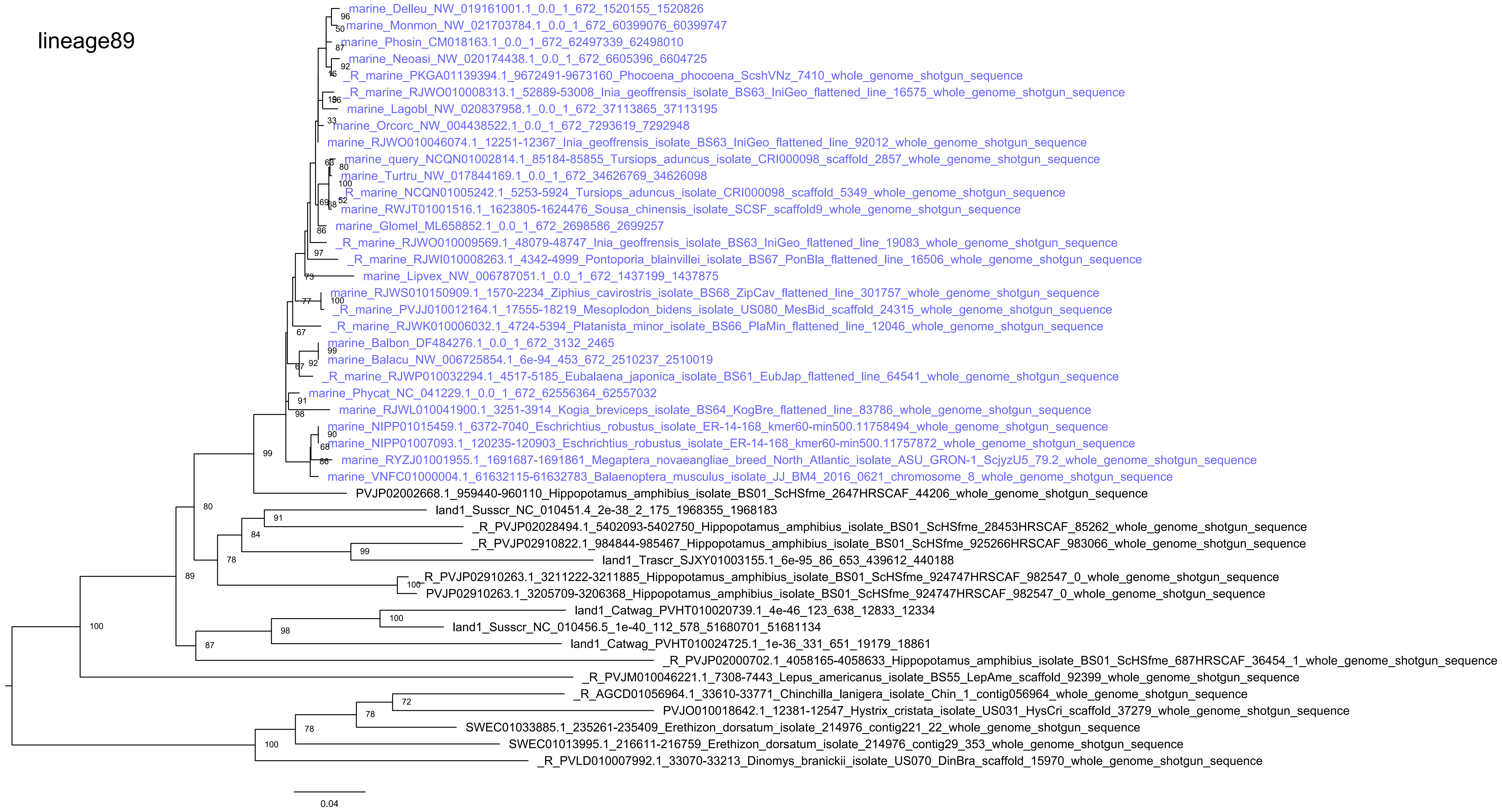

lineage89
marine_Delleu_NW_019161001.1_0.0_1_672_1520155_1520826
marine_Monmon_NW_021703784.1_0.0_1_672_60399076_60399747
marine_Phosin_CM018163.1_0.0_1_672_62497339_62498010
marine_Neoasi_NW_020174438.1_0.0_1_672_6605396_6604725
_R_marine_PKGA01139394.1_9672491-9673160_Phocoena_phocoena_ScshVNz_7410_whole_genome_shotgun_sequence
_R_marine_RJWO010008313.1_52889-53008_Inia_geoffrensis_isolate_BS63_IniGeo_flattened_line_16575_whole_genome_shotgun_sequence
marine_Lagobl_NW_020837958.1_0.0_1_672_37113865_37113195
marine_Orcorc_NW_004438522.1_0.0_1_672_7293619_7292948
marine_RJWO010046074.1_12251-12367_Inia_geoffrensis_isolate_BS63_IniGeo_flattened_line_92012_whole_genome_shotgun_sequence
marine_query_NCQN01002814.1_85184-85855_Tursiops_aduncus_isolate_CRI000098_scaffold_2857_whole_genome_shotgun_sequence
marine_Turtru_NW_017844169.1_0.0_1_672_34626769_34626098
_R_marine_NCQN01005242.1_5253-5924_Tursiops_aduncus_isolate_CRI000098_scaffold_5349_whole_genome_shotgun_sequence
marine_RWJT01001516.1_1623805-1624476_Sousa_chinensis_isolate_SCSF_scaffold9_whole_genome_shotgun_sequence
marine_Glomel_ML658852.1_0.0_1_672_2698586_2699257
_R_marine_RJWO010009569.1_48079-48747_Inia_geoffrensis_isolate_BS63_IniGeo_flattened_line_19083_whole_genome_shotgun_sequence
_R_marine_RJWI010008263.1_4342-4999_Pontoporia_blainvillei_isolate_BS67_PonBla_flattened_line_16506_whole_genome_shotgun_sequence
marine_Lipvex_NW_006787051.1_0.0_1_672_1437199_1437875
marine_RJWS010150909.1_1570-2234_Ziphius_cavirostris_isolate_BS68_ZipCav_flattened_line_301757_whole_genome_shotgun_sequence
_R_marine_PVJJ010012164.1_17555-18219_Mesoplodon_bidens_isolate_US080_MesBid_scaffold_24315_whole_genome_shotgun_sequence
_R_marine_RJWK010006032.1_4724-5394_Platanista_minor_isolate_BS66_PlaMin_flattened_line_12046_whole_genome_shotgun_sequence
marine_Balbon_DF484276.1_0.0_1_672_3132_2465
marine_Balacu_NW_006725854.1_6e-94_453_672_2510237_2510019
_R_marine_RJWP010032294.1_4517-5185_Eubalaena_japonica_isolate_BS61_EubJap_flattened_line_64541_whole_genome_shotgun_sequence
marine_Phycat_NC_041229.1_0.0_1_672_62556364_62557032
marine_RJWL010041900.1_3251-3914_Kogia_breviceps_isolate_BS64_KogBre_flattened_line_83786_whole_genome_shotgun_sequence
marine_NIPP01015459.1_6372-7040_Eschrichtius_robustus_isolate_ER-14-168_kmer60-min500.11758494_whole_genome_shotgun_sequence
marine_NIPP01007093.1_120235-120903_Eschrichtius_robustus_isolate_ER-14-168_kmer60-min500.11757872_whole_genome_shotgun_sequence
marine_RYZJ01001955.1_1691687-1691861_Megaptera_novaeangliae_breed_North_Atlantic_isolate_ASU_GRON-1_ScjyzU5_79.2_whole_genome_shotgun_sequence
marine_VNFC01000004.1_61632115-61632783_Balaenoptera_musculus_isolate_JJ_BM4_2016_0621_chromosome_8_whole_genome_shotgun_sequence
PVJP02002668.1_959440-960110_Hippopotamus_amphibius_isolate_BS01_ScHSfme_2647HRSCAF_44206_whole_genome_shotgun_sequence
land1_Susscr_NC_010451.4_2e-38_2_175_1968355_1968183
_R_PVJP02028494.1_5402093-5402750_Hippopotamus_amphibius_isolate_BS01_ScHSfme_28453HRSCAF_85262_whole_genome_shotgun_sequence
_R_PVJP02910822.1_984844-985467_Hippopotamus_amphibius_isolate_BS01_ScHSfme_925266HRSCAF_983066_whole_genome_shotgun_sequence
land1_Trascr_SJXY01003155.1_6e-95_86_653_439612_440188
R_PVJP02910263.1_3211222-3211885_Hippopotamus_amphibius_isolate_BS01_ScHSfme_924747HRSCAF_982547_0_whole_genome_shotgun_sequence
PVJP02910263.1_3205709-3206368_Hippopotamus_amphibius_isolate_BS01_ScHSfme_924747HRSCAF_982547_0_whole_genome_shotgun_sequence
land1_Catwag_PVHT010020739.1_4e-46_123_638_12833_12334
land1_Susscr_NC_010456.5_1e-40_112_578_51680701_51681134
land1_Catwag_PVHT010024725.1_1e-36_331_651_19179_18861
_R_PVJP02000702.1_4058165-4058633_Hippopotamus_amphibius_isolate_BS01_ScHSfme_687HRSCAF_36454_1_whole_genome_shotgun_sequence
_R_PVJM010046221.1_7308-7443_Lepus_americanus_isolate_BS55_LepAme_scaffold_92399_whole_genome_shotgun_sequence
_R_AGCD01056964.1_33610-33771_Chinchilla_lanigera_isolate_Chin_1_contig056964_whole_genome_shotgun_sequence
PVJO010018642.1_12381-12547_Hystrix_cristata_isolate_US031_HysCri_scaffold_37279_whole_genome_shotgun_sequence
SWEC01033885.1_235261-235409_Erethizon_dorsatum_isolate_214976_contig221_22_whole_genome_shotgun_sequence
SWEC01013995.1_216611-216759_Erethizon_dorsatum_isolate_214976_contig29_353_whole_genome_shotgun_sequence
_R_PVLD010007992.1_33070-33213_Dinomys_branickii_isolate_US070_DinBra_scaffold_15970_whole_genome_shotgun_sequence
0.04

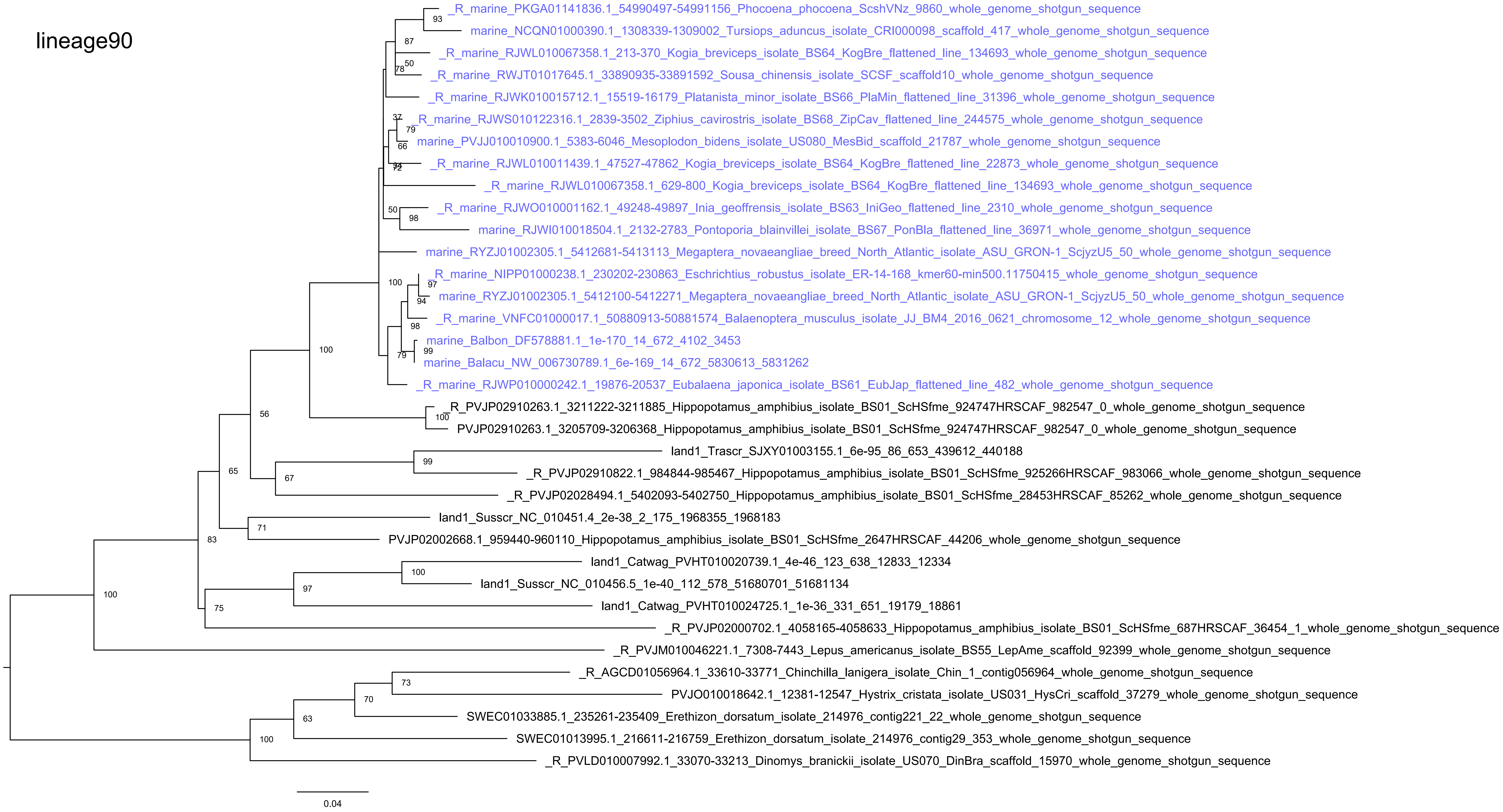
lineage90
_R_marine_PKGA01141836.1_54990497-54991156_Phocoena_phocoena_ScshVNz_9860_whole_genome_shotgun_sequence
marine_NCQN01000390.1_1308339-1309002_Tursiops_aduncus_isolate_CRI000098_scaffold_417_whole_genome_shotgun_sequence
_R_marine_RJWL010067358.1_213-370_Kogia_breviceps_isolate_BS64_KogBre_flattened_line_134693_whole_genome_shotgun_sequence
_R_marine_RWJT01017645.1_33890935-33891592_Sousa_chinensis_isolate_SCSF_scaffold10_whole_genome_shotgun_sequence
_R_marine_RJWK010015712.1_15519-16179_Platanista_minor_isolate_BS66_PlaMin_flattened_line_31396_whole_genome_shotgun_sequence
_R_marine_RJWS010122316.1_2839-3502_Ziphius_cavirostris_isolate_BS68_ZipCav_flattened_line_244575_whole_genome_shotgun_sequence
marine_PVJJ010010900.1_5383-6046_Mesoplodon_bidens_isolate_US080_MesBid_scaffold_21787_whole_genome_shotgun_sequence
_R_marine_RJWL010011439.1_47527-47862_Kogia_breviceps_isolate_BS64_KogBre_flattened_line_22873_whole_genome_shotgun_sequence
_R_marine_RJWL010067358.1_629-800_Kogia_breviceps_isolate_BS64_KogBre_flattened_line_134693_whole_genome_shotgun_sequence
_R_marine_RJWO010001162.1_49248-49897_Inia_geoffrensis_isolate_BS63_IniGeo_flattened_line_2310_whole_genome_shotgun_sequence
marine_RJWI010018504.1_2132-2783_Pontoporia_blainvillei_isolate_BS67_PonBla_flattened_line_36971_whole_genome_shotgun_sequence
marine_RYZJ01002305.1_5412681-5413113_Megaptera_novaeangliae_breed_North_Atlantic_isolate_ASU_GRON-1_ScjyzU5_50_whole_genome_shotgun_sequence
_R_marine_NIPP01000238.1_230202-230863_Eschrichtius_robustus_isolate_ER-14-168_kmer60-min500.11750415_whole_genome_shotgun_sequence
marine_RYZJ01002305.1_5412100-5412271_Megaptera_novaeangliae_breed_North_Atlantic_isolate_ASU_GRON-1_ScjyzU5_50_whole_genome_shotgun_sequence
_R_marine_VNFC01000017.1_50880913-50881574_Balaenoptera_musculus_isolate_JJ_BM4_2016_0621_chromosome_12_whole_genome_shotgun_sequence
marine_Balbon_DF578881.1_1e-170_14_672_4102_3453
marine_Balacu_NW_006730789.1_6e-169_14_672_5830613_5831262
_R_marine_RJWP010000242.1_19876-20537_Eubalaena_japonica_isolate_BS61_EubJap_flattened_line_482_whole_genome_shotgun_sequence
_R_PVJP02910263.1_3211222-3211885_Hippopotamus_amphibius_isolate_BS01_ScHSfme_924747HRSCAF_982547_0_whole_genome_shotgun_sequence
PVJP02910263.1_3205709-3206368_Hippopotamus_amphibius_isolate_BS01_ScHSfme_924747HRSCAF_982547_0_whole_genome_shotgun_sequence
land1_Trascr_SJXY01003155.1_6e-95_86_653_439612_440188
_R_PVJP02910822.1_984844-985467_Hippopotamus_amphibius_isolate_BS01_ScHSfme_925266HRSCAF_983066_whole_genome_shotgun_sequence
_R_PVJP02028494.1_5402093-5402750_Hippopotamus_amphibius_isolate_BS01_ScHSfme_28453HRSCAF_85262_whole_genome_shotgun_sequence
land1_Susscr_NC_010451.4_2e-38_2_175_1968355_1968183
PVJP02002668.1_959440-960110_Hippopotamus_amphibius_isolate_BS01_ScHSfme_2647HRSCAF_44206_whole_genome_shotgun_sequence
land1_Catwag_PVHT010020739.1_4e-46_123_638_12833_12334
land1_Susscr_NC_010456.5_1e-40_112_578_51680701_51681134
land1_Catwag_PVHT010024725.1_1e-36_331_651_19179_18861
_R_PVJP02000702.1_4058165-4058633_Hippopotamus_amphibius_isolate_BS01_ScHSfme_687HRSCAF_36454_1_whole_genome_shotgun_sequence
_R_PVJM010046221.1_7308-7443_Lepus_americanus_isolate_BS55_LepAme_scaffold_92399_whole_genome_shotgun_sequence
_R_AGCD01056964.1_33610-33771_Chinchilla_lanigera_isolate_Chin_1_contig056964_whole_genome_shotgun_sequence
PVJO010018642.1_12381-12547_Hystrix_cristata_isolate_US031_HysCri_scaffold_37279_whole_genome_shotgun_sequence
SWEC01033885.1_235261-235409_Erethizon_dorsatum_isolate_214976_contig221_22_whole_genome_shotgun_sequence
SWEC01013995.1_216611-216759_Erethizon_dorsatum_isolate_214976_contig29_353_whole_genome_shotgun_sequence
_R_PVLD010007992.1_33070-33213_Dinomys_branickii_isolate_US070_DinBra_scaffold_15970_whole_genome_shotgun_sequence
0.04

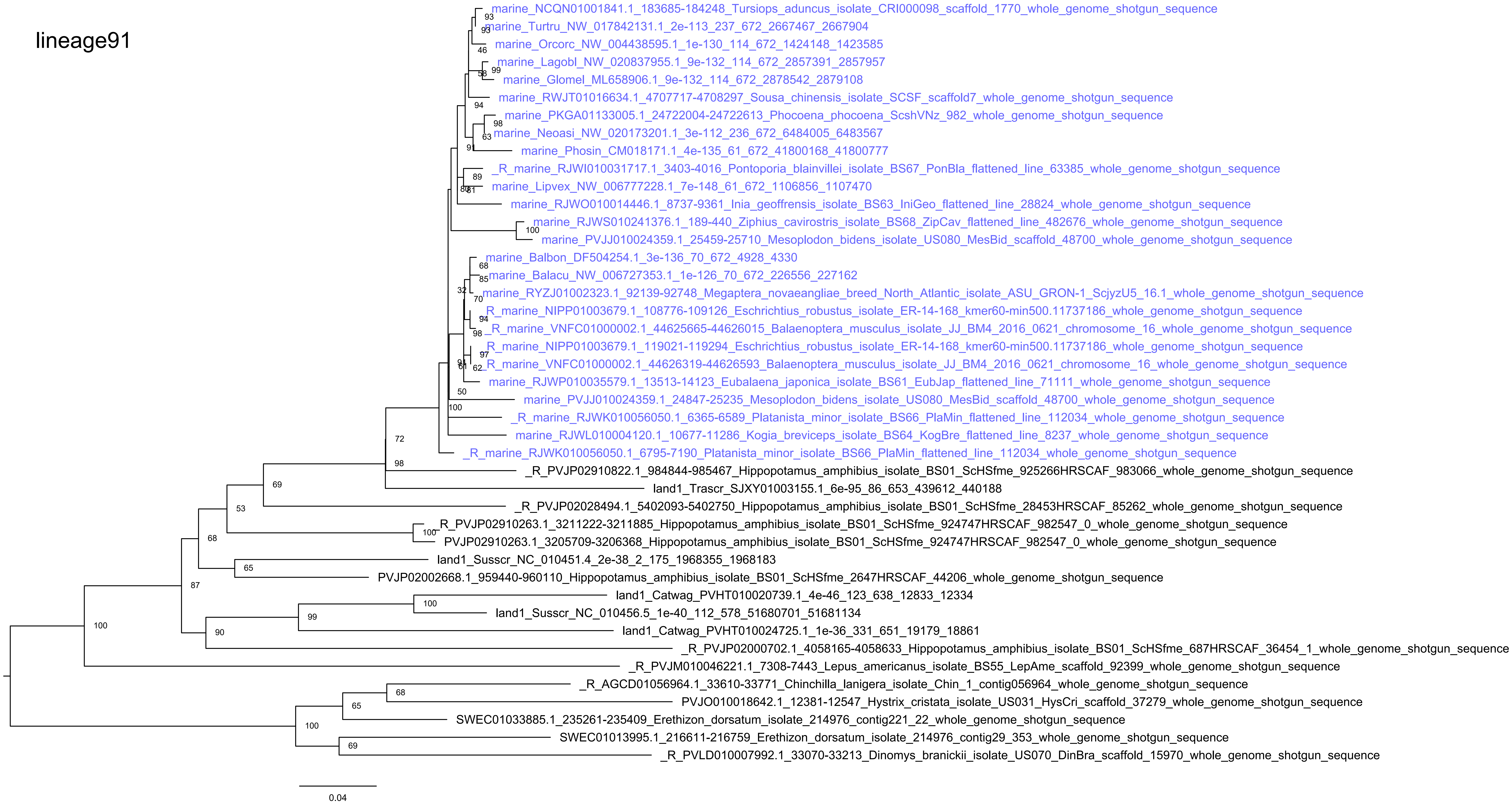
lineage91
marine_NCQN01001841.1_183685-184248_Tursiops_aduncus_isolate_CRI000098_scaffold_1770_whole_genome_shotgun_sequence
marine_Turtru_NW_017842131.1_2e-113_237_672_2667467_2667904
marine_Orcorc_NW_004438595.1_1e-130_114_672_1424148_1423585
marine_Lagobl_NW_020837955.1_9e-132_114_672_2857391_2857957
marine_Glomel_ML658906.1_9e-132_114_672_2878542_2879108
marine_RWJT01016634.1_4707717-4708297_Sousa_chinensis_isolate_SCSF_scaffold7_whole_genome_shotgun_sequence
marine_PKGA01133005.1_24722004-24722613_Phocoena_phocoena_ScshVNz_982_whole_genome_shotgun_sequence
marine_Neoasi_NW_020173201.1_3e-112_236_672_6484005_6483567
marine_Phosin_CM018171.1_4e-135_61_672_41800168_41800777
_R_marine_RJWI010031717.1_3403-4016_Pontoporia_blainvillei_isolate_BS67_PonBla_flattened_line_63385_whole_genome_shotgun_sequence
marine_Lipvex_NW_006777228.1_7e-148_61_672_1106856_1107470
marine_RJWO010014446.1_8737-9361_Inia_geoffrensis_isolate_BS63_IniGeo_flattened_line_28824_whole_genome_shotgun_sequence
marine_RJWS010241376.1_189-440_Ziphius_cavirostris_isolate_BS68_ZipCav_flattened_line_482676_whole_genome_shotgun_sequence
marine_PVJJ010024359.1_25459-25710_Mesoplodon_bidens_isolate_US080_MesBid_scaffold_48700_whole_genome_shotgun_sequence
marine_Balbon_DF504254.1_3e-136_70_672_4928_4330
marine_Balacu_NW_006727353.1_1e-126_70_672_226556_227162
marine_RYZJ01002323.1_92139-92748_Megaptera_novaeangliae_breed_North_Atlantic_isolate_ASU_GRON-1_ScjyzU5_16.1_whole_genome_shotgun_sequence
_R_marine_NIPP01003679.1_108776-109126_Eschrichtius_robustus_isolate_ER-14-168_kmer60-min500.11737186_whole_genome_shotgun_sequence
_R_marine_VNFC01000002.1_44625665-44626015_Balaenoptera_musculus_isolate_JJ_BM4_2016_0621_chromosome_16_whole_genome_shotgun_sequence
_R_marine_NIPP01003679.1_119021-119294_Eschrichtius_robustus_isolate_ER-14-168_kmer60-min500.11737186_whole_genome_shotgun_sequence
_R_marine_VNFC01000002.1_44626319-44626593_Balaenoptera_musculus_isolate_JJ_BM4_2016_0621_chromosome_16_whole_genome_shotgun_sequence
marine_RJWP010035579.1_13513-14123_Eubalaena_japonica_isolate_BS61_EubJap_flattened_line_71111_whole_genome_shotgun_sequence
marine_PVJJ010024359.1_24847-25235_Mesoplodon_bidens_isolate_US080_MesBid_scaffold_48700_whole_genome_shotgun_sequence
_R_marine_RJWK010056050.1_6365-6589_Platanista_minor_isolate_BS66_PlaMin_flattened_line_112034_whole_genome_shotgun_sequence
marine_RJWL010004120.1_10677-11286_Kogia_breviceps_isolate_BS64_KogBre_flattened_line_8237_whole_genome_shotgun_sequence
_R_marine_RJWK010056050.1_6795-7190_Platanista_minor_isolate_BS66_PlaMin_flattened_line_112034_whole_genome_shotgun_sequence
_R_PVJP02910822.1_984844-985467_Hippopotamus_amphibius_isolate_BS01_ScHSfme_925266HRSCAF_983066_whole_genome_shotgun_sequence
land1_Trascr_SJXY01003155.1_6e-95_86_653_439612_440188
_R_PVJP02028494.1_5402093-5402750_Hippopotamus_amphibius_isolate_BS01_ScHSfme_28453HRSCAF_85262_whole_genome_shotgun_sequence
_R_PVJP02910263.1_3211222-3211885_Hippopotamus_amphibius_isolate_BS01_ScHSfme_924747HRSCAF_982547_0_whole_genome_shotgun_sequence
PVJP02910263.1_3205709-3206368_Hippopotamus_amphibius_isolate_BS01_ScHSfme_924747HRSCAF_982547_0_whole_genome_shotgun_sequence
land1_Susscr_NC_010451.4_2e-38_2_175_1968355_1968183
PVJP02002668.1_959440-960110_Hippopotamus_amphibius_isolate_BS01_ScHSfme_2647HRSCAF_44206_whole_genome_shotgun_sequence
land1_Catwag_PVHT010020739.1_4e-46_123_638_12833_12334
land1_Susscr_NC_010456.5_1e-40_112_578_51680701_51681134
land1_Catwag_PVHT010024725.1_1e-36_331_651_19179_18861
_R_PVJP02000702.1_4058165-4058633_Hippopotamus_amphibius_isolate_BS01_ScHSfme_687HRSCAF_36454_1_whole_genome_shotgun_sequence
_R_PVJM010046221.1_7308-7443_Lepus_americanus_isolate_BS55_LepAme_scaffold_92399_whole_genome_shotgun_sequence
_R_AGCD01056964.1_33610-33771_Chinchilla_lanigera_isolate_Chin_1_contig056964_whole_genome_shotgun_sequence
PVJO010018642.1_12381-12547_Hystrix_cristata_isolate_US031_HysCri_scaffold_37279_whole_genome_shotgun_sequence
SWEC01033885.1_235261-235409_Erethizon_dorsatum_isolate_214976_contig221_22_whole_genome_shotgun_sequence
SWEC01013995.1_216611-216759_Erethizon_dorsatum_isolate_214976_contig29_353_whole_genome_shotgun_sequence
_R_PVLD010007992.1_33070-33213_Dinomys_branickii_isolate_US070_DinBra_scaffold_15970_whole_genome_shotgun_sequence
0.04

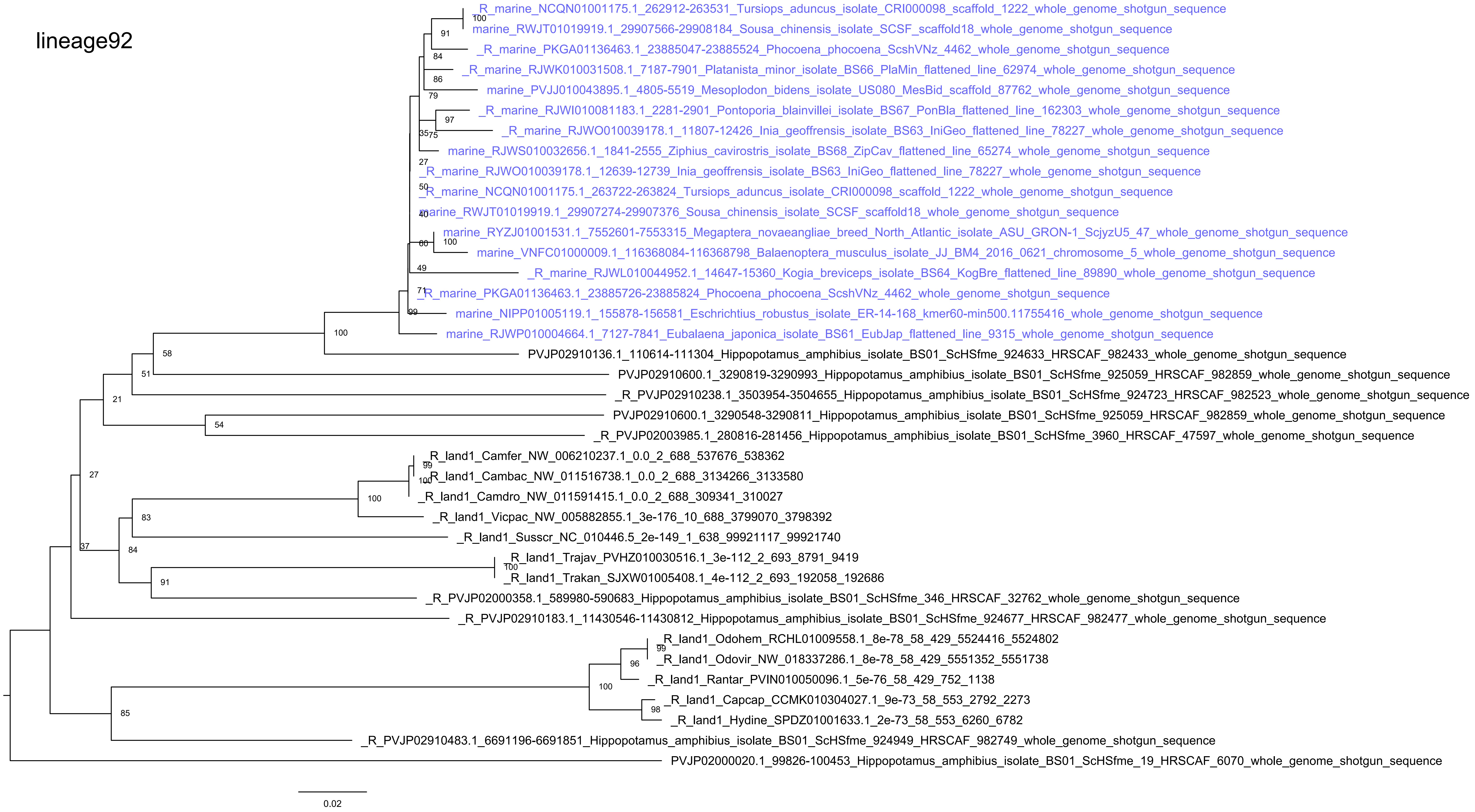

lineage92
_R_marine_NCQN01001175.1_262912-263531_Tursiops_aduncus_isolate_CRI000098_scaffold_1222_whole_genome_shotgun_sequence
marine_RWJT01019919.1_29907566-29908184_Sousa_chinensis_isolate_SCSF_scaffold18_whole_genome_shotgun_sequence
_R_marine_PKGA01136463.1_23885047-23885524_Phocoena_phocoena_ScshVNz_4462_whole_genome_shotgun_sequence
_R_marine_RJWK010031508.1_7187-7901_Platanista_minor_isolate_BS66_PlaMin_flattened_line_62974_whole_genome_shotgun_sequence
marine_PVJJ010043895.1_4805-5519_Mesoplodon_bidens_isolate_US080_MesBid_scaffold_87762_whole_genome_shotgun_sequence
_R_marine_RJWI010081183.1_2281-2901_Pontoporia_blainvillei_isolate_BS67_PonBla_flattened_line_162303_whole_genome_shotgun_sequence
_R_marine_RJWO010039178.1_11807-12426_Inia_geoffrensis_isolate_BS63_IniGeo_flattened_line_78227_whole_genome_shotgun_sequence
marine_RJWS010032656.1_1841-2555_Ziphius_cavirostris_isolate_BS68_ZipCav_flattened_line_65274_whole_genome_shotgun_sequence
_R_marine_RJWO010039178.1_12639-12739_Inia_geoffrensis_isolate_BS63_IniGeo_flattened_line_78227_whole_genome_shotgun_sequence
_R_marine_NCQN01001175.1_263722-263824_Tursiops_aduncus_isolate_CRI000098_scaffold_1222_whole_genome_shotgun_sequence
marine_RWJT01019919.1_29907274-29907376_Sousa_chinensis_isolate_SCSF_scaffold18_whole_genome_shotgun_sequence
marine_RYZJ01001531.1_7552601-7553315_Megaptera_novaeangliae_breed_North_Atlantic_isolate_ASU_GRON-1_ScjyzU5_47_whole_genome_shotgun_sequence
marine_VNFC01000009.1_116368084-116368798_Balaenoptera_musculus_isolate_JJ_BM4_2016_0621_chromosome_5_whole_genome_shotgun_sequence
_R_marine_RJWL010044952.1_14647-15360_Kogia_breviceps_isolate_BS64_KogBre_flattened_line_89890_whole_genome_shotgun_sequence
_R_marine_PKGA01136463.1_23885726-23885824_Phocoena_phocoena_ScshVNz_4462_whole_genome_shotgun_sequence
marine_NIPP01005119.1_155878-156581_Eschrichtius_robustus_isolate_ER-14-168_kmer60-min500.11755416_whole_genome_shotgun_sequence
marine_RJWP010004664.1_7127-7841_Eubalaena_japonica_isolate_BS61_EubJap_flattened_line_9315_whole_genome_shotgun_sequence
PVJP02910136.1_110614-111304_Hippopotamus_amphibius_isolate_BS01_ScHSfme_924633_HRSCAF_982433_whole_genome_shotgun_sequence
PVJP02910600.1_3290819-3290993_Hippopotamus_amphibius_isolate_BS01_ScHSfme_925059_HRSCAF_982859_whole_genome_shotgun_sequence
_R_PVJP02910238.1_3503954-3504655_Hippopotamus_amphibius_isolate_BS01_ScHSfme_924723_HRSCAF_982523_whole_genome_shotgun_sequence
PVJP02910600.1_3290548-3290811_Hippopotamus_amphibius_isolate_BS01_ScHSfme_925059_HRSCAF_982859_whole_genome_shotgun_sequence
_R_PVJP02003985.1_280816-281456_Hippopotamus_amphibius_isolate_BS01_ScHSfme_3960_HRSCAF_47597_whole_genome_shotgun_sequence
_R_land1_Camfer_NW_006210237.1_0.0_2_688_537676_538362
_R_land1_Cambac_NW_011516738.1_0.0_2_688_3134266_3133580
_R_land1_Camdro_NW_011591415.1_0.0_2_688_309341_310027
_R_land1_Vicpac_NW_005882855.1_3e-176_10_688_3799070_3798392
_R_land1_Susscr_NC_010446.5_2e-149_1_638_99921117_99921740
_R_land1_Trajav_PVHZ010030516.1_3e-112_2_693_8791_9419
_R_land1_Trakan_SJXW01005408.1_4e-112_2_693_192058_192686
_R_PVJP02000358.1_589980-590683_Hippopotamus_amphibius_isolate_BS01_ScHSfme_346_HRSCAF_32762_whole_genome_shotgun_sequence
_R_PVJP02910183.1_11430546-11430812_Hippopotamus_amphibius_isolate_BS01_ScHSfme_924677_HRSCAF_982477_whole_genome_shotgun_sequence
_R_land1_Odohem_RCHL01009558.1_8e-78_58_429_5524416_5524802
_R_land1_Odovir_NW_018337286.1_8e-78_58_429_5551352_5551738
_R_land1_Rantar_PVIN010050096.1_5e-76_58_429_752_1138
_R_land1_Capcap_CCMK010304027.1_9e-73_58_553_2792_2273
_R_land1_Hydine_SPDZ01001633.1_2e-73_58_553_6260_6782
_R_PVJP02910483.1_6691196-6691851_Hippopotamus_amphibius_isolate_BS01_ScHSfme_924949_HRSCAF_982749_whole_genome_shotgun_sequence
PVJP02000020.1_99826-100453_Hippopotamus_amphibius_isolate_BS01_ScHSfme_19_HRSCAF_6070_whole_genome_shotgun_sequence
0.02

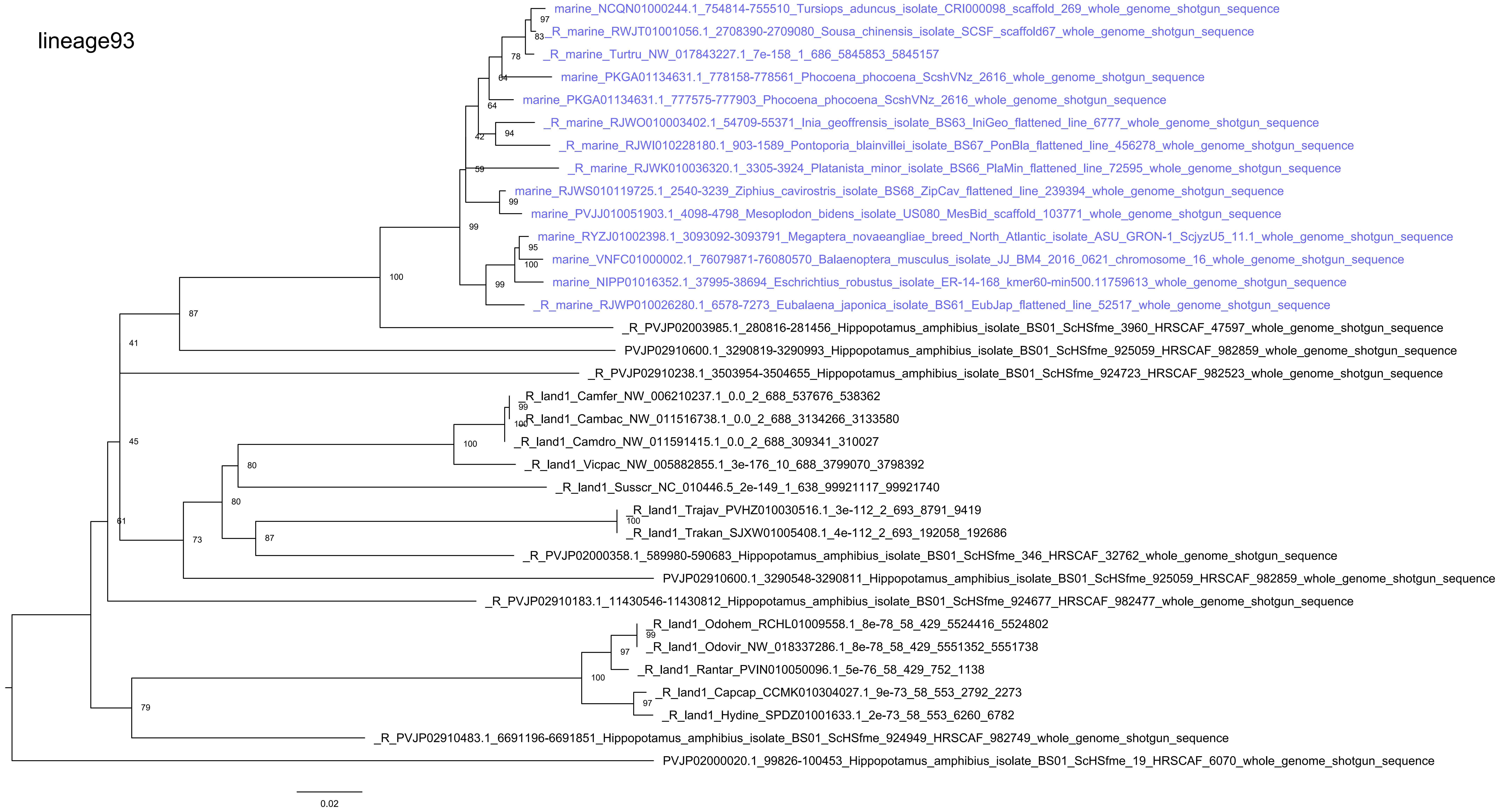
lineage93
marine_NCQN01000244.1_754814-755510_Tursiops_aduncus_isolate_CRI000098_scaffold_269_whole_genome_shotgun_sequence
_R_marine_RWJT01001056.1_2708390-2709080_Sousa_chinensis_isolate_SCSF_scaffold67_whole_genome_shotgun_sequence
_R_marine_Turtru_NW_017843227.1_7e-158_1_686_5845853_5845157
marine_PKGA01134631.1_778158-778561_Phocoena_phocoena_ScshVNz_2616_whole_genome_shotgun_sequence
marine_PKGA01134631.1_777575-777903_Phocoena_phocoena_ScshVNz_2616_whole_genome_shotgun_sequence
_R_marine_RJWO010003402.1_54709-55371_Inia_geoffrensis_isolate_BS63_IniGeo_flattened_line_6777_whole_genome_shotgun_sequence
_R_marine_RJWI010228180.1_903-1589_Pontoporia_blainvillei_isolate_BS67_PonBla_flattened_line_456278_whole_genome_shotgun_sequence
_R_marine_RJWK010036320.1_3305-3924_Platanista_minor_isolate_BS66_PlaMin_flattened_line_72595_whole_genome_shotgun_sequence
marine_RJWS010119725.1_2540-3239_Ziphius_cavirostris_isolate_BS68_ZipCav_flattened_line_239394_whole_genome_shotgun_sequence
marine_PVJJ010051903.1_4098-4798_Mesoplodon_bidens_isolate_US080_MesBid_scaffold_103771_whole_genome_shotgun_sequence
marine_RYZJ01002398.1_3093092-3093791_Megaptera_novaeangliae_breed_North_Atlantic_isolate_ASU_GRON-1_ScjyzU5_11.1_whole_genome_shotgun_sequence
marine_VNFC01000002.1_76079871-76080570_Balaenoptera_musculus_isolate_JJ_BM4_2016_0621_chromosome_16_whole_genome_shotgun_sequence
marine_NIPP01016352.1_37995-38694_Eschrichtius_robustus_isolate_ER-14-168_kmer60-min500.11759613_whole_genome_shotgun_sequence
_R_marine_RJWP010026280.1_6578-7273_Eubalaena_japonica_isolate_BS61_EubJap_flattened_line_52517_whole_genome_shotgun_sequence
_R_PVJP02003985.1_280816-281456_Hippopotamus_amphibius_isolate_BS01_ScHSfme_3960_HRSCAF_47597_whole_genome_shotgun_sequence
PVJP02910600.1_3290819-3290993_Hippopotamus_amphibius_isolate_BS01_ScHSfme_925059_HRSCAF_982859_whole_genome_shotgun_sequence
_R_PVJP02910238.1_3503954-3504655_Hippopotamus_amphibius_isolate_BS01_ScHSfme_924723_HRSCAF_982523_whole_genome_shotgun_sequence
_R_land1_Camfer_NW_006210237.1_0.0_2_688_537676_538362
_R_land1_Cambac_NW_011516738.1_0.0_2_688_3134266_3133580
_R_land1_Camdro_NW_011591415.1_0.0_2_688_309341_310027
_R_land1_Vicpac_NW_005882855.1_3e-176_10_688_3799070_3798392
_R_land1_Susscr_NC_010446.5_2e-149_1_638_99921117_99921740
_R_land1_Trajav_PVHZ010030516.1_3e-112_2_693_8791_9419
_R_land1_Trakan_SJXW01005408.1_4e-112_2_693_192058_192686
_R_PVJP02000358.1_589980-590683_Hippopotamus_amphibius_isolate_BS01_ScHSfme_346_HRSCAF_32762_whole_genome_shotgun_sequence
PVJP02910600.1_3290548-3290811_Hippopotamus_amphibius_isolate_BS01_ScHSfme_925059_HRSCAF_982859_whole_genome_shotgun_sequence
_R_PVJP02910183.1_11430546-11430812_Hippopotamus_amphibius_isolate_BS01_ScHSfme_924677_HRSCAF_982477_whole_genome_shotgun_sequence
_R_land1_Odohem_RCHL01009558.1_8e-78_58_429_5524416_5524802
_R_land1_Odovir_NW_018337286.1_8e-78_58_429_5551352_5551738
_R_land1_Rantar_PVIN010050096.1_5e-76_58_429_752_1138
_R_land1_Capcap_CCMK010304027.1_9e-73_58_553_2792_2273
_R_land1_Hydine_SPDZ01001633.1_2e-73_58_553_6260_6782
_R_PVJP02910483.1_6691196-6691851_Hippopotamus_amphibius_isolate_BS01_ScHSfme_924949_HRSCAF_982749_whole_genome_shotgun_sequence
PVJP02000020.1_99826-100453_Hippopotamus_amphibius_isolate_BS01_ScHSfme_19_HRSCAF_6070_whole_genome_shotgun_sequence
0.02

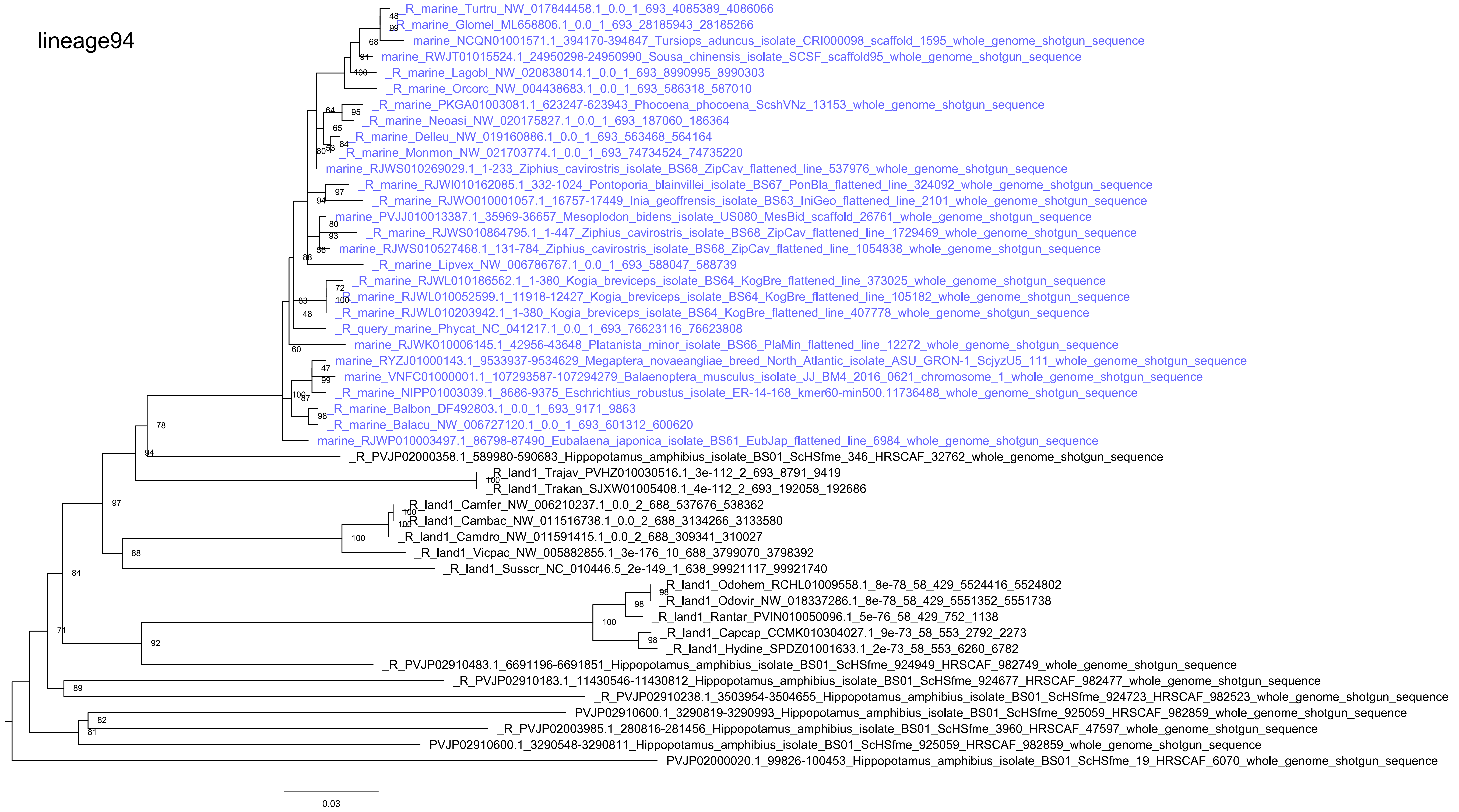
lineage94
_R_marine_Turtru_NW_017844458.1_0.0_1_693_4085389_4086066
_R_marine_Glomel_ML658806.1_0.0_1_693_28185943_28185266
marine_NCQN01001571.1_394170-394847_Tursiops_aduncus_isolate_CRI000098_scaffold_1595_whole_genome_shotgun_sequence
marine_RWJT01015524.1_24950298-24950990_Sousa_chinensis_isolate_SCSF_scaffold95_whole_genome_shotgun_sequence
_R_marine_Lagobl_NW_020838014.1_0.0_1_693_8990995_8990303
_R_marine_Orcorc_NW_004438683.1_0.0_1_693_586318_587010
_R_marine_PKGA01003081.1_623247-623943_Phocoena_phocoena_ScshVNz_13153_whole_genome_shotgun_sequence
_R_marine_Neoasi_NW_020175827.1_0.0_1_693_187060_186364
_R_marine_Delleu_NW_019160886.1_0.0_1_693_563468_564164
_R_marine_Monmon_NW_021703774.1_0.0_1_693_74734524_74735220
marine_RJWS010269029.1_1-233_Ziphius_cavirostris_isolate_BS68_ZipCav_flattened_line_537976_whole_genome_shotgun_sequence
_R_marine_RJWI010162085.1_332-1024_Pontoporia_blainvillei_isolate_BS67_PonBla_flattened_line_324092_whole_genome_shotgun_sequence
_R_marine_RJWO010001057.1_16757-17449_Inia_geoffrensis_isolate_BS63_IniGeo_flattened_line_2101_whole_genome_shotgun_sequence
marine_PVJJ010013387.1_35969-36657_Mesoplodon_bidens_isolate_US080_MesBid_scaffold_26761_whole_genome_shotgun_sequence
_R_marine_RJWS010864795.1_1-447_Ziphius_cavirostris_isolate_BS68_ZipCav_flattened_line_1729469_whole_genome_shotgun_sequence
marine_RJWS010527468.1_131-784_Ziphius_cavirostris_isolate_BS68_ZipCav_flattened_line_1054838_whole_genome_shotgun_sequence
_R_marine_Lipvex_NW_006786767.1_0.0_1_693_588047_588739
_R_marine_RJWL010186562.1_1-380_Kogia_breviceps_isolate_BS64_KogBre_flattened_line_373025_whole_genome_shotgun_sequence
_R_marine_RJWL010052599.1_11918-12427_Kogia_breviceps_isolate_BS64_KogBre_flattened_line_105182_whole_genome_shotgun_sequence
_R_marine_RJWL010203942.1_1-380_Kogia_breviceps_isolate_BS64_KogBre_flattened_line_407778_whole_genome_shotgun_sequence
_R_query_marine_Phycat_NC_041217.1_0.0_1_693_76623116_76623808
marine_RJWK010006145.1_42956-43648_Platanista_minor_isolate_BS66_PlaMin_flattened_line_12272_whole_genome_shotgun_sequence
marine_RYZJ01000143.1_9533937-9534629_Megaptera_novaeangliae_breed_North_Atlantic_isolate_ASU_GRON-1_ScjyzU5_111_whole_genome_shotgun_sequence
marine_VNFC01000001.1_107293587-107294279_Balaenoptera_musculus_isolate_JJ_BM4_2016_0621_chromosome_1_whole_genome_shotgun_sequence
_R_marine_NIPP01003039.1_8686-9375_Eschrichtius_robustus_isolate_ER-14-168_kmer60-min500.11736488_whole_genome_shotgun_sequence
_R_marine_Balbon_DF492803.1_0.0_1_693_9171_9863
_R_marine_Balacu_NW_006727120.1_0.0_1_693_601312_600620
marine_RJWP010003497.1_86798-87490_Eubalaena_japonica_isolate_BS61_EubJap_flattened_line_6984_whole_genome_shotgun_sequence
_R_PVJP02000358.1_589980-590683_Hippopotamus_amphibius_isolate_BS01_ScHSfme_346_HRSCAF_32762_whole_genome_shotgun_sequence
_R_land1_Trajav_PVHZ010030516.1_3e-112_2_693_8791_9419
_R_land1_Trakan_SJXW01005408.1_4e-112_2_693_192058_192686
_R_land1_Camfer_NW_006210237.1_0.0_2_688_537676_538362
_R_land1_Cambac_NW_011516738.1_0.0_2_688_3134266_3133580
_R_land1_Camdro_NW_011591415.1_0.0_2_688_309341_310027
_R_land1_Vicpac_NW_005882855.1_3e-176_10_688_3799070_3798392
_R_land1_Susscr_NC_010446.5_2e-149_1_638_99921117_99921740
_R_land1_Odohem_RCHL01009558.1_8e-78_58_429_5524416_5524802
_R_land1_Odovir_NW_018337286.1_8e-78_58_429_5551352_5551738
_R_land1_Rantar_PVIN010050096.1_5e-76_58_429_752_1138
_R_land1_Capcap_CCMK010304027.1_9e-73_58_553_2792_2273
_R_land1_Hydine_SPDZ01001633.1_2e-73_58_553_6260_6782
_R_PVJP02910483.1_6691196-6691851_Hippopotamus_amphibius_isolate_BS01_ScHSfme_924949_HRSCAF_982749_whole_genome_shotgun_sequence
_R_PVJP02910183.1_11430546-11430812_Hippopotamus_amphibius_isolate_BS01_ScHSfme_924677_HRSCAF_982477_whole_genome_shotgun_sequence
_R_PVJP02910238.1_3503954-3504655_Hippopotamus_amphibius_isolate_BS01_ScHSfme_924723_HRSCAF_982523_whole_genome_shotgun_sequence
PVJP02910600.1_3290819-3290993_Hippopotamus_amphibius_isolate_BS01_ScHSfme_925059_HRSCAF_982859_whole_genome_shotgun_sequence
_R_PVJP02003985.1_280816-281456_Hippopotamus_amphibius_isolate_BS01_ScHSfme_3960_HRSCAF_47597_whole_genome_shotgun_sequence
PVJP02910600.1_3290548-3290811_Hippopotamus_amphibius_isolate_BS01_ScHSfme_925059_HRSCAF_982859_whole_genome_shotgun_sequence
PVJP02000020.1_99826-100453_Hippopotamus_amphibius_isolate_BS01_ScHSfme_19_HRSCAF_6070_whole_genome_shotgun_sequence
0.03

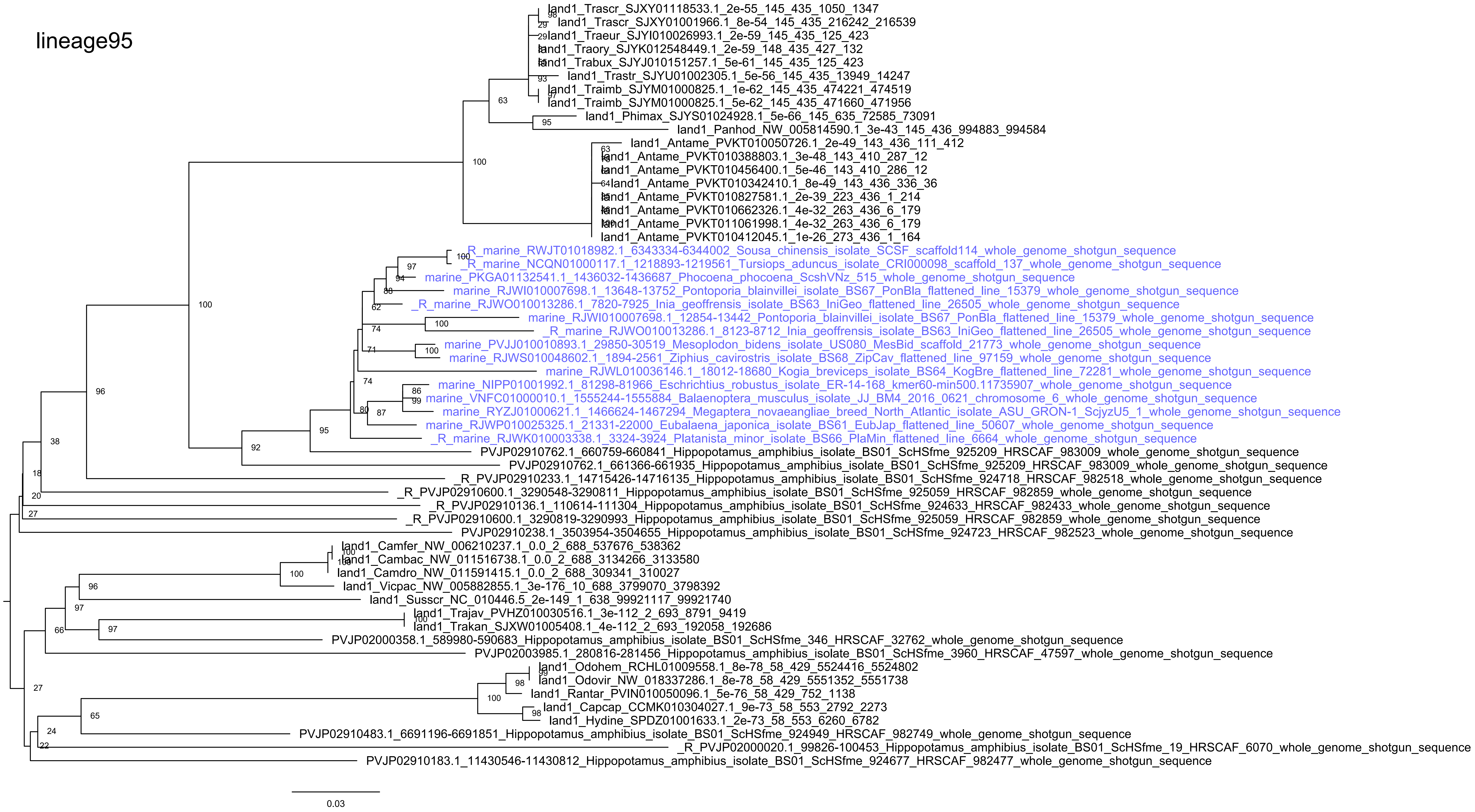

lineage95
land1_Trascr_SJXY01118533.1_2e-55_145_435_1050_1347
land1_Trascr_SJXY01001966.1_8e-54_145_435_216242_216539
land1_Traeur_SJYI010026993.1_2e-59_145_435_125_423
land1_Traory_SJYK012548449.1_2e-59_148_435_427_132
land1_Trabux_SJYJ010151257.1_5e-61_145_435_125_423
land1_Trastr_SJYU01002305.1_5e-56_145_435_13949_14247
land1_Traimb_SJYM01000825.1_1e-62_145_435_474221_474519
land1_Traimb_SJYM01000825.1_5e-62_145_435_471660_471956
land1_Phimax_SJYS01024928.1_5e-66_145_635_72585_73091
land1_Panhod_NW_005814590.1_3e-43_145_436_994883_994584
land1_Antame_PVKT010050726.1_2e-49_143_436_111_412
land1_Antame_PVKT010388803.1_3e-48_143_410_287_12
land1_Antame_PVKT010456400.1_5e-46_143_410_286_12
land1_Antame_PVKT010342410.1_8e-49_143_436_336_36
land1_Antame_PVKT010827581.1_2e-39_223_436_1_214
land1_Antame_PVKT010662326.1_4e-32_263_436_6_179
land1_Antame_PVKT011061998.1_4e-32_263_436_6_179
land1_Antame_PVKT010412045.1_1e-26_273_436_1_164
_R_marine_RWJT01018982.1_6343334-6344002_Sousa_chinensis_isolate_SCSF_scaffold114_whole_genome_shotgun_sequence
_R_marine_NCQN01000117.1_1218893-1219561_Tursiops_aduncus_isolate_CRI000098_scaffold_137_whole_genome_shotgun_sequence
marine_PKGA01132541.1_1436032-1436687_Phocoena_phocoena_ScshVNz_515_whole_genome_shotgun_sequence
marine_RJWI010007698.1_13648-13752_Pontoporia_blainvillei_isolate_BS67_PonBla_flattened_line_15379_whole_genome_shotgun_sequence
_R_marine_RJWO010013286.1_7820-7925_Inia_geoffrensis_isolate_BS63_IniGeo_flattened_line_26505_whole_genome_shotgun_sequence
marine_RJWI010007698.1_12854-13442_Pontoporia_blainvillei_isolate_BS67_PonBla_flattened_line_15379_whole_genome_shotgun_sequence
_R_marine_RJWO010013286.1_8123-8712_Inia_geoffrensis_isolate_BS63_IniGeo_flattened_line_26505_whole_genome_shotgun_sequence
marine_PVJJ010010893.1_29850-30519_Mesoplodon_bidens_isolate_US080_MesBid_scaffold_21773_whole_genome_shotgun_sequence
marine_RJWS010048602.1_1894-2561_Ziphius_cavirostris_isolate_BS68_ZipCav_flattened_line_97159_whole_genome_shotgun_sequence
marine_RJWL010036146.1_18012-18680_Kogia_breviceps_isolate_BS64_KogBre_flattened_line_72281_whole_genome_shotgun_sequence
marine_NIPP01001992.1_81298-81966_Eschrichtius_robustus_isolate_ER-14-168_kmer60-min500.11735907_whole_genome_shotgun_sequence
marine_VNFC01000010.1_1555244-1555884_Balaenoptera_musculus_isolate_JJ_BM4_2016_0621_chromosome_6_whole_genome_shotgun_sequence
marine_RYZJ01000621.1_1466624-1467294_Megaptera_novaeangliae_breed_North_Atlantic_isolate_ASU_GRON-1_ScjyzU5_1_whole_genome_shotgun_sequence
marine_RJWP010025325.1_21331-22000_Eubalaena_japonica_isolate_BS61_EubJap_flattened_line_50607_whole_genome_shotgun_sequence
_R_marine_RJWK010003338.1_3324-3924_Platanista_minor_isolate_BS66_PlaMin_flattened_line_6664_whole_genome_shotgun_sequence
PVJP02910762.1_660759-660841_Hippopotamus_amphibius_isolate_BS01_ScHSfme_925209_HRSCAF_983009_whole_genome_shotgun_sequence
PVJP02910762.1_661366-661935_Hippopotamus_amphibius_isolate_BS01_ScHSfme_925209_HRSCAF_983009_whole_genome_shotgun_sequence
_R_PVJP02910233.1_14715426-14716135_Hippopotamus_amphibius_isolate_BS01_ScHSfme_924718_HRSCAF_982518_whole_genome_shotgun_sequence
_R_PVJP02910600.1_3290548-3290811_Hippopotamus_amphibius_isolate_BS01_ScHSfme_925059_HRSCAF_982859_whole_genome_shotgun_sequence
_R_PVJP02910136.1_110614-111304_Hippopotamus_amphibius_isolate_BS01_ScHSfme_924633_HRSCAF_982433_whole_genome_shotgun_sequence
_R_PVJP02910600.1_3290819-3290993_Hippopotamus_amphibius_isolate_BS01_ScHSfme_925059_HRSCAF_982859_whole_genome_shotgun_sequence
PVJP02910238.1_3503954-3504655_Hippopotamus_amphibius_isolate_BS01_ScHSfme_924723_HRSCAF_982523_whole_genome_shotgun_sequence
land1_Camfer_NW_006210237.1_0.0_2_688_537676_538362
land1_Cambac_NW_011516738.1_0.0_2_688_3134266_3133580
land1_Camdro_NW_011591415.1_0.0_2_688_309341_310027
land1_Vicpac_NW_005882855.1_3e-176_10_688_3799070_3798392
land1_Susscr_NC_010446.5_2e-149_1_638_99921117_99921740
land1_Trajav_PVHZ010030516.1_3e-112_2_693_8791_9419
land1_Trakan_SJXW01005408.1_4e-112_2_693_192058_192686
PVJP02000358.1_589980-590683_Hippopotamus_amphibius_isolate_BS01_ScHSfme_346_HRSCAF_32762_whole_genome_shotgun_sequence
PVJP02003985.1_280816-281456_Hippopotamus_amphibius_isolate_BS01_ScHSfme_3960_HRSCAF_47597_whole_genome_shotgun_sequence
land1_Odohem_RCHL01009558.1_8e-78_58_429_5524416_5524802
land1_Odovir_NW_018337286.1_8e-78_58_429_5551352_5551738
land1_Rantar_PVIN010050096.1_5e-76_58_429_752_1138
land1_Capcap_CCMK010304027.1_9e-73_58_553_2792_2273
land1_Hydine_SPDZ01001633.1_2e-73_58_553_6260_6782
PVJP02910483.1_6691196-6691851_Hippopotamus_amphibius_isolate_BS01_ScHSfme_924949_HRSCAF_982749_whole_genome_shotgun_sequence
_R_PVJP02000020.1_99826-100453_Hippopotamus_amphibius_isolate_BS01_ScHSfme_19_HRSCAF_6070_whole_genome_shotgun_sequence
PVJP02910183.1_11430546-11430812_Hippopotamus_amphibius_isolate_BS01_ScHSfme_924677_HRSCAF_982477_whole_genome_shotgun_sequence
0.03

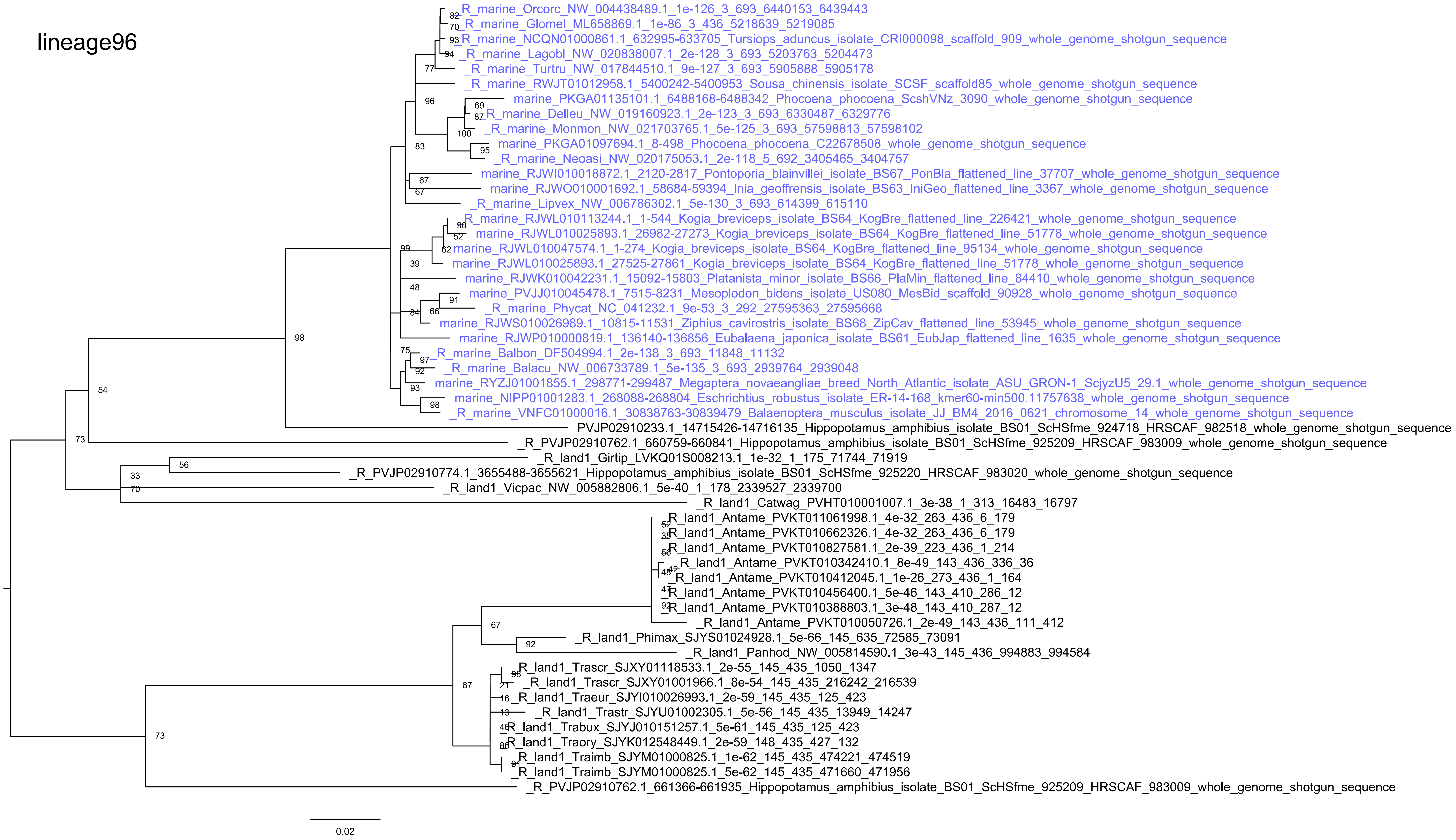
lineage96
R_marine_Orcorc_NW_004438489.1_1e-126_3_693_6440153_6439443
_R_marine_Glomel_ML658869.1_1e-86_3_436_5218639_5219085
R_marine_NCQN01000861.1_632995-633705_Tursiops_aduncus_isolate_CRI000098_scaffold_909_whole_genome_shotgun_sequence
_R_marine_Lagobl_NW_020838007.1_2e-128_3_693_5203763_5204473
_R_marine_Turtru_NW_017844510.1_9e-127_3_693_5905888_5905178
_R_marine_RWJT01012958.1_5400242-5400953_Sousa_chinensis_isolate_SCSF_scaffold85_whole_genome_shotgun_sequence
marine_PKGA01135101.1_6488168-6488342_Phocoena_phocoena_ScshVNz_3090_whole_genome_shotgun_sequence
R_marine_Delleu_NW_019160923.1_2e-123_3_693_6330487_6329776
_R_marine_Monmon_NW_021703765.1_5e-125_3_693_57598813_57598102
marine_PKGA01097694.1_8-498_Phocoena_phocoena_C22678508_whole_genome_shotgun_sequence
_R_marine_Neoasi_NW_020175053.1_2e-118_5_692_3405465_3404757
marine_RJWI010018872.1_2120-2817_Pontoporia_blainvillei_isolate_BS67_PonBla_flattened_line_37707_whole_genome_shotgun_sequence
marine_RJWO010001692.1_58684-59394_Inia_geoffrensis_isolate_BS63_IniGeo_flattened_line_3367_whole_genome_shotgun_sequence
_R_marine_Lipvex_NW_006786302.1_5e-130_3_693_614399_615110
R_marine_RJWL010113244.1_1-544_Kogia_breviceps_isolate_BS64_KogBre_flattened_line_226421_whole_genome_shotgun_sequence
marine_RJWL010025893.1_26982-27273_Kogia_breviceps_isolate_BS64_KogBre_flattened_line_51778_whole_genome_shotgun_sequence
marine_RJWL010047574.1_1-274_Kogia_breviceps_isolate_BS64_KogBre_flattened_line_95134_whole_genome_shotgun_sequence
marine_RJWL010025893.1_27525-27861_Kogia_breviceps_isolate_BS64_KogBre_flattened_line_51778_whole_genome_shotgun_sequence
marine_RJWK010042231.1_15092-15803_Platanista_minor_isolate_BS66_PlaMin_flattened_line_84410_whole_genome_shotgun_sequence
marine_PVJJ010045478.1_7515-8231_Mesoplodon_bidens_isolate_US080_MesBid_scaffold_90928_whole_genome_shotgun_sequence
_R_marine_Phycat_NC_041232.1_9e-53_3_292_27595363_27595668
marine_RJWS010026989.1_10815-11531_Ziphius_cavirostris_isolate_BS68_ZipCav_flattened_line_53945_whole_genome_shotgun_sequence
marine_RJWP010000819.1_136140-136856_Eubalaena_japonica_isolate_BS61_EubJap_flattened_line_1635_whole_genome_shotgun_sequence
R_marine_Balbon_DF504994.1_2e-138_3_693_11848_11132
_R_marine_Balacu_NW_006733789.1_5e-135_3_693_2939764_2939048
marine_RYZJ01001855.1_298771-299487_Megaptera_novaeangliae_breed_North_Atlantic_isolate_ASU_GRON-1_ScjyzU5_29.1_whole_genome_shotgun_sequence
marine_NIPP01001283.1_268088-268804_Eschrichtius_robustus_isolate_ER-14-168_kmer60-min500.11757638_whole_genome_shotgun_sequence
_R_marine_VNFC01000016.1_30838763-30839479_Balaenoptera_musculus_isolate_JJ_BM4_2016_0621_chromosome_14_whole_genome_shotgun_sequence
PVJP02910233.1_14715426-14716135_Hippopotamus_amphibius_isolate_BS01_ScHSfme_924718_HRSCAF_982518_whole_genome_shotgun_sequence
_R_PVJP02910762.1_660759-660841_Hippopotamus_amphibius_isolate_BS01_ScHSfme_925209_HRSCAF_983009_whole_genome_shotgun_sequence
_R_land1_Girtip_LVKQ01S008213.1_1e-32_1_175_71744_71919
_R_PVJP02910774.1_3655488-3655621_Hippopotamus_amphibius_isolate_BS01_ScHSfme_925220_HRSCAF_983020_whole_genome_shotgun_sequence
_R_land1_Vicpac_NW_005882806.1_5e-40_1_178_2339527_2339700
_R_land1_Catwag_PVHT010001007.1_3e-38_1_313_16483_16797
R_land1_Antame_PVKT011061998.1_4e-32_263_436_6_179
R_land1_Antame_PVKT010662326.1_4e-32_263_436_6_179
R_land1_Antame_PVKT010827581.1_2e-39_223_436_1_214
R_land1_Antame_PVKT010342410.1_8e-49_143_436_336_36
R_land1_Antame_PVKT010412045.1_1e-26_273_436_1_164
R_land1_Antame_PVKT010456400.1_5e-46_143_410_286_12
R_land1_Antame_PVKT010388803.1_3e-48_143_410_287_12
_R_land1_Antame_PVKT010050726.1_2e-49_143_436_111_412
_R_land1_Phimax_SJYS01024928.1_5e-66_145_635_72585_73091
_R_land1_Panhod_NW_005814590.1_3e-43_145_436_994883_994584
R_land1_Trascr_SJXY01118533.1_2e-55_145_435_1050_1347
_R_land1_Trascr_SJXY01001966.1_8e-54_145_435_216242_216539
_R_land1_Traeur_SJYI010026993.1_2e-59_145_435_125_423
_R_land1_Trastr_SJYU01002305.1_5e-56_145_435_13949_14247
R_land1_Trabux_SJYJ010151257.1_5e-61_145_435_125_423
R_land1_Traory_SJYK012548449.1_2e-59_148_435_427_132
R_land1_Traimb_SJYM01000825.1_1e-62_145_435_474221_474519
_R_land1_Traimb_SJYM01000825.1_5e-62_145_435_471660_471956
_R_PVJP02910762.1_661366-661935_Hippopotamus_amphibius_isolate_BS01_ScHSfme_925209_HRSCAF_983009_whole_genome_shotgun_sequence
0.02

lineage97

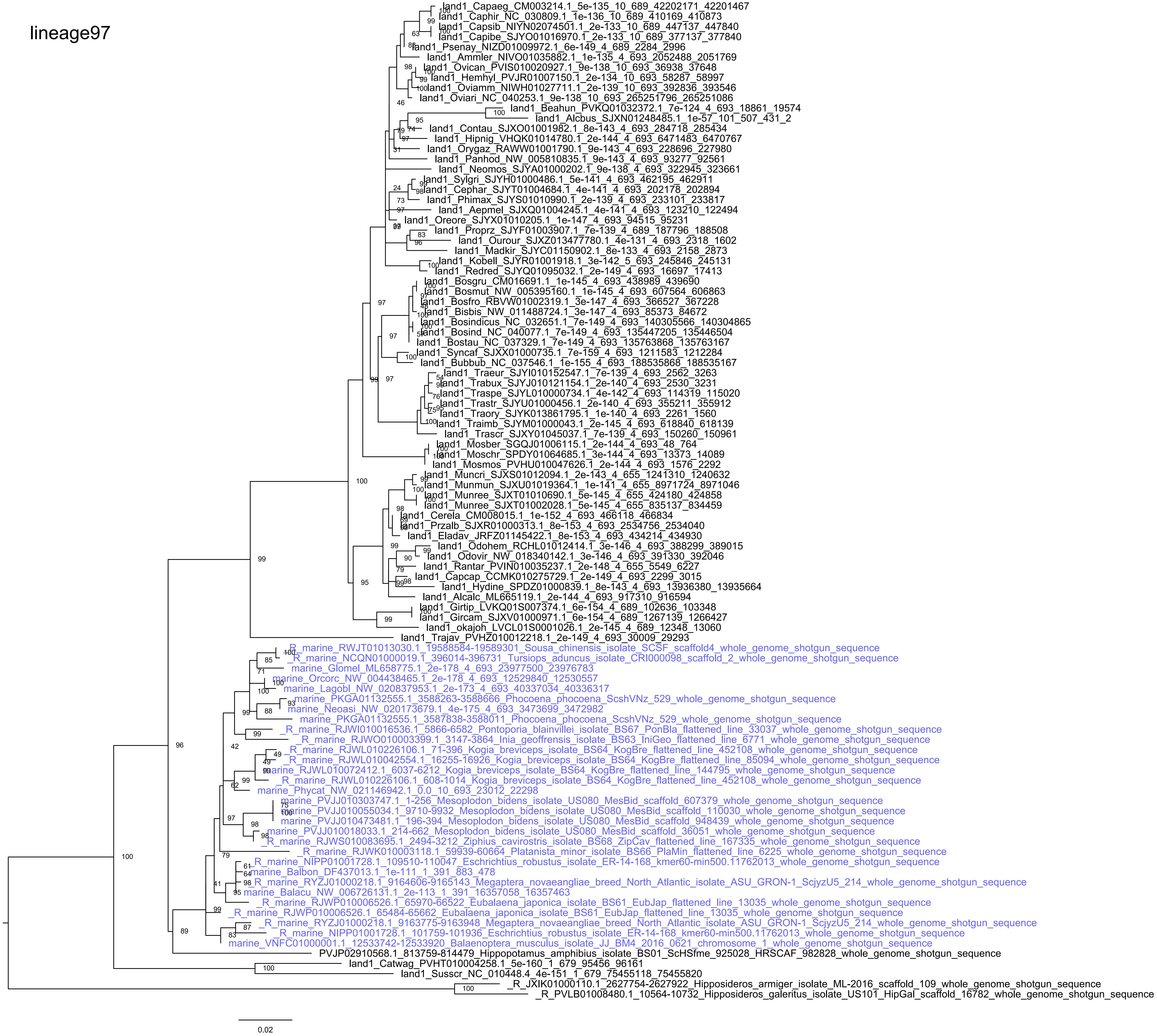
land1_Capaeg_CM003214.1_5e-135_10_689_42202171_42201467
land1_Caphir_NC_030809.1_1e-136_10_689_410169_410873
land1_Capsib_NIYN02074501.1_2e-133_10_689_447137_447840
land1_Capibe_SJYO01016970.1_2e-133_10_689_377137_377840
land1_Psenay_NIZD01009972.1_6e-149_4_689_2284_2996
land1_Ammler_NIVO01035882.1_1e-135_4_693_2052488_2051769
land1_Ovican_PVIS010020927.1_9e-138_10_693_36938_37648
land1_Hemhyl_PVJR01007150.1_2e-134_10_693_58287_58997
land1_Oviamm_NIWH01027711.1_2e-139_10_693_392836_393546
land1_Oviari_NC_040253.1_9e-138_10_693_265251796_265251086
land1_Beahun_PVKQ01032372.1_7e-124_4_693_18861_19574
land1_Alcbus_SJXN01248485.1_1e-57_101_507_431_2
land1_Contau_SJXO01001982.1_8e-143_4_693_284718_285434
land1_Hipnig_VHQK01014780.1_2e-144_4_693_6471483_6470767
land1_Orygaz_RAWW01001790.1_9e-143_4_693_228696_227980
land1_Panhod_NW_005810835.1_9e-143_4_693_93277_92561
land1_Neomos_SJYA01000202.1_9e-138_4_693_322945_323661
land1_Sylgri_SJYH01000486.1_5e-141_4_693_462195_462911
land1_Cephar_SJYT01004684.1_4e-141_4_693_202178_202894
land1_Phimax_SJYS01010990.1_2e-139_4_693_233101_233817
land1_Aepmel_SJXQ01004245.1_4e-141_4_693_123210_122494
land1_Oreore_SJYX01010205.1_1e-147_4_693_94515_95231
land1_Proprz_SJYF01003907.1_7e-139_4_689_187796_188508
land1_Ourour_SJXZ013477780.1_4e-131_4_693_2318_1602
land1_Madkir_SJYC01150902.1_8e-133_4_693_2158_2873
land1_Kobell_SJYR01001918.1_3e-142_5_693_245846_245131
land1_Redred_SJYQ01095032.1_2e-149_4_693_16697_17413
land1_Bosgru_CM016691.1_1e-145_4_693_438989_439690
land1_Bosmut_NW_005395160.1_1e-145_4_693_607564_606863
land1_Bosfro_RBVW01002319.1_3e-147_4_693_366527_367228
land1_Bisbis_NW_011488724.1_3e-147_4_693_85373_84672
land1_Bosindicus_NC_032651.1_7e-149_4_693_140305566_140304865
land1_Bosind_NC_040077.1_7e-149_4_693_135447205_135446504
land1_Bostau_NC_037329.1_7e-149_4_693_135763868_135763167
land1_Syncaf_SJXX01000735.1_7e-159_4_693_1211583_1212284
land1_Bubbub_NC_037546.1_1e-155_4_693_188535868_188535167
land1_Traeur_SJYI010152547.1_7e-139_4_693_2562_3263
land1_Trabux_SJYJ010121154.1_2e-140_4_693_2530_3231
land1_Traspe_SJYL010000734.1_4e-142_4_693_114319_115020
land1_Trastr_SJYU01000456.1_2e-140_4_693_355211_355912
land1_Traory_SJYK013861795.1_1e-140_4_693_2261_1560
land1_Traimb_SJYM01000043.1_2e-145_4_693_618840_618139
land1_Trascr_SJXY01045037.1_7e-139_4_693_150260_150961
land1_Mosber_SGQJ01006115.1_2e-144_4_693_48_764
land1_Moschr_SPDY01064685.1_3e-144_4_693_13373_14089
land1_Mosmos_PVHU010047626.1_2e-144_4_693_1576_2292
land1_Muncri_SJXS01012094.1_2e-143_4_655_1241310_1240632
land1_Munmun_SJXU01019364.1_1e-141_4_655_8971724_8971046
land1_Munree_SJXT01010690.1_5e-145_4_655_424180_424858
land1_Munree_SJXT01002028.1_5e-145_4_655_835137_834459
land1_Cerela_CM008015.1_1e-152_4_693_466118_466834
land1_Przalb_SJXR01000313.1_8e-153_4_693_2534756_2534040
land1_Eladav_JRFZ01145422.1_8e-153_4_693_434214_434930
land1_Odohem_RCHL01012414.1_3e-146_4_693_388299_389015
land1_Odovir_NW_018340142.1_3e-146_4_693_391330_392046
land1_Rantar_PVIN010035237.1_2e-148_4_655_5549_6227
land1_Capcap_CCMK010275729.1_2e-149_4_693_2299_3015
land1_Hydine_SPDZ01000839.1_8e-143_4_693_13936380_13935664
land1_Alcalc_ML665119.1_2e-144_4_693_917310_916594
land1_Girtip_LVKQ01S007374.1_6e-154_4_689_102636_103348
land1_Gircam_SJXV01000971.1_6e-154_4_689_1267139_1266427
land1_okajoh_LVCL01S0001026.1_2e-145_4_689_12348_13060
land1_Trajav_PVHZ010012218.1_2e-149_4_693_30009_29293
_R_marine_RWJT01013030.1_19588584-19589301_Sousa_chinensis_isolate_SCSF_scaffold4_whole_genome_shotgun_sequence
_R_marine_NCQN01000019.1_396014-396731_Tursiops_aduncus_isolate_CRI000098_scaffold_2_whole_genome_shotgun_sequence
marine_Glomel_ML658775.1_2e-178_4_693_23977500_23976783
marine_Orcorc_NW_004438465.1_2e-178_4_693_12529840_12530557
marine_Lagobl_NW_020837953.1_2e-173_4_693_40337034_40336317
marine_PKGA01132555.1_3588263-3588666_Phocoena_phocoena_ScshVNz_529_whole_genome_shotgun_sequence
marine_Neoasi_NW_020173679.1_4e-175_4_693_3473699_3472982
marine_PKGA01132555.1_3587838-3588011_Phocoena_phocoena_ScshVNz_529_whole_genome_shotgun_sequence
_R_marine_RJWI010016536.1_5866-6582_Pontoporia_blainvillei_isolate_BS67_PonBla_flattened_line_33037_whole_genome_shotgun_sequence
_R_marine_RJWO010003399.1_3147-3864_Inia_geoffrensis_isolate_BS63_IniGeo_flattened_line_6771_whole_genome_shotgun_sequence
_R_marine_RJWL010226106.1_71-396_Kogia_breviceps_isolate_BS64_KogBre_flattened_line_452108_whole_genome_shotgun_sequence
_R_marine_RJWL010042554.1_16255-16926_Kogia_breviceps_isolate_BS64_KogBre_flattened_line_85094_whole_genome_shotgun_sequence
marine_RJWL010072412.1_6037-6212_Kogia_breviceps_isolate_BS64_KogBre_flattened_line_144795_whole_genome_shotgun_sequence
_R_marine_RJWL010226106.1_608-1014_Kogia_breviceps_isolate_BS64_KogBre_flattened_line_452108_whole_genome_shotgun_sequence
marine_Phycat_NW_021146942.1_0.0_10_693_23012_22298
marine_PVJJ010303747.1_1-256_Mesoplodon_bidens_isolate_US080_MesBid_scaffold_607379_whole_genome_shotgun_sequence
marine_PVJJ010055034.1_9710-9932_Mesoplodon_bidens_isolate_US080_MesBid_scaffold_110030_whole_genome_shotgun_sequence
marine_PVJJ010473481.1_196-394_Mesoplodon_bidens_isolate_US080_MesBid_scaffold_948439_whole_genome_shotgun_sequence
marine_PVJJ010018033.1_214-662_Mesoplodon_bidens_isolate_US080_MesBid_scaffold_36051_whole_genome_shotgun_sequence
_R_marine_RJWS010083695.1_2494-3212_Ziphius_cavirostris_isolate_BS68_ZipCav_flattened_line_167335_whole_genome_shotgun_sequence
_R_marine_RJWK010003118.1_59939-60664_Platanista_minor_isolate_BS66_PlaMin_flattened_line_6225_whole_genome_shotgun_sequence
_R_marine_NIPP01001728.1_109510-110047_Eschrichtius_robustus_isolate_ER-14-168_kmer60-min500.11762013_whole_genome_shotgun_sequence
marine_Balbon_DF437013.1_1e-111_1_391_883_478
_R_marine_RYZJ01000218.1_9164606-9165143_Megaptera_novaeangliae_breed_North_Atlantic_isolate_ASU_GRON-1_ScjyzU5_214_whole_genome_shotgun_sequence
marine_Balacu_NW_006726131.1_2e-113_1_391_16357058_16357463
_R_marine_RJWP010006526.1_65970-66522_Eubalaena_japonica_isolate_BS61_EubJap_flattened_line_13035_whole_genome_shotgun_sequence
_R_marine_RJWP010006526.1_65484-65662_Eubalaena_japonica_isolate_BS61_EubJap_flattened_line_13035_whole_genome_shotgun_sequence
_R_marine_RYZJ01000218.1_9163775-9163948_Megaptera_novaeangliae_breed_North_Atlantic_isolate_ASU_GRON-1_ScjyzU5_214_whole_genome_shotgun_sequence
_R_marine_NIPP01001728.1_101759-101936_Eschrichtius_robustus_isolate_ER-14-168_kmer60-min500.11762013_whole_genome_shotgun_sequence
marine_VNFC01000001.1_12533742-12533920_Balaenoptera_musculus_isolate_JJ_BM4_2016_0621_chromosome_1_whole_genome_shotgun_sequence
PVJP02910568.1_813759-814479_Hippopotamus_amphibius_isolate_BS01_ScHSfme_925028_HRSCAF_982828_whole_genome_shotgun_sequence
land1_Catwag_PVHT010004258.1_5e-160_1_679_95456_96161
land1_Susscr_NC_010448.4_4e-151_1_679_75455118_75455820
_R_JXIK01000110.1_2627754-2627922_Hipposideros_armiger_isolate_ML-2016_scaffold_109_whole_genome_shotgun_sequence
_R_PVLB01008480.1_10564-10732_Hipposideros_galeritus_isolate_US101_HipGal_scaffold_16782_whole_genome_shotgun_sequence
0.02

lineage98

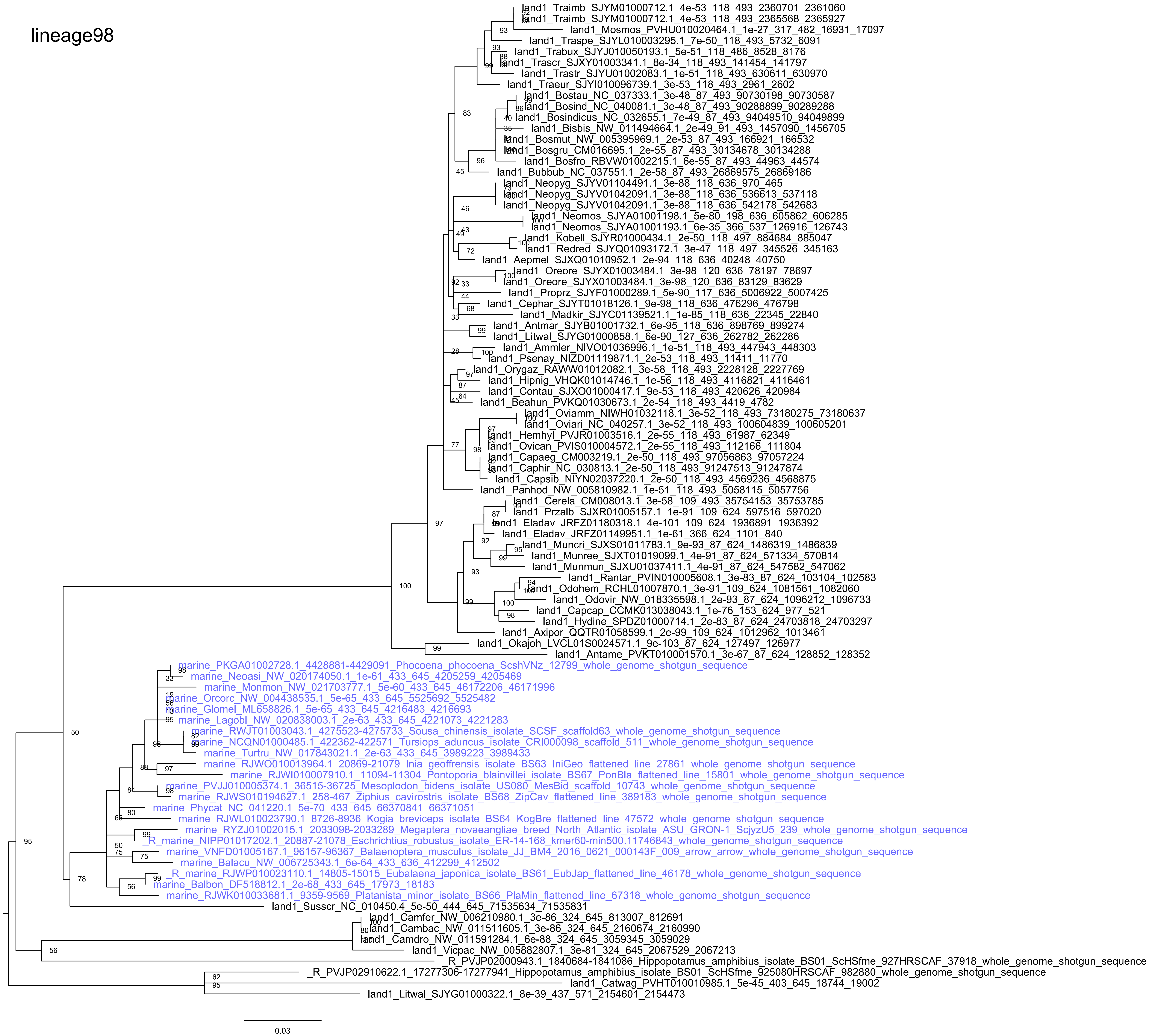

land1_Traimb_SJYM01000712.1_4e-53_118_493_2360701_2361060
land1_Traimb_SJYM01000712.1_4e-53_118_493_2365568_2365927
land1_Mosmos_PVHU010020464.1_1e-27_317_482_16931_17097
land1_Traspe_SJYL010003295.1_7e-50_118_493_5732_6091
land1_Trabux_SJYJ010050193.1_5e-51_118_486_8528_8176
land1_Trascr_SJXY01003341.1_8e-34_118_493_141454_141797
land1_Trastr_SJYU01002083.1_1e-51_118_493_630611_630970
land1_Traeur_SJYI010096739.1_3e-53_118_493_2961_2602
land1_Bostau_NC_037333.1_3e-48_87_493_90730198_90730587
land1_Bosind_NC_040081.1_3e-48_87_493_90288899_90289288
land1_Bosindicus_NC_032655.1_7e-49_87_493_94049510_94049899
land1_Bisbis_NW_011494664.1_2e-49_91_493_1457090_1456705
land1_Bosmut_NW_005395969.1_2e-53_87_493_166921_166532
land1_Bosgru_CM016695.1_2e-55_87_493_30134678_30134288
land1_Bosfro_RBVW01002215.1_6e-55_87_493_44963_44574
land1_Bubbub_NC_037551.1_2e-58_87_493_26869575_26869186
land1_Neopyg_SJYV01104491.1_3e-88_118_636_970_465
land1_Neopyg_SJYV01042091.1_3e-88_118_636_536613_537118
land1_Neopyg_SJYV01042091.1_3e-88_118_636_542178_542683
land1_Neomos_SJYA01001198.1_5e-80_198_636_605862_606285
land1_Neomos_SJYA01001193.1_6e-35_366_537_126916_126743
land1_Kobell_SJYR01000434.1_2e-50_118_497_884684_885047
land1_Redred_SJYQ01093172.1_3e-47_118_497_345526_345163
land1_Aepmel_SJXQ01010952.1_2e-94_118_636_40248_40750
land1_Oreore_SJYX01003484.1_3e-98_120_636_78197_78697
land1_Oreore_SJYX01003484.1_3e-98_120_636_83129_83629
land1_Proprz_SJYF01000289.1_5e-90_117_636_5006922_5007425
land1_Cephar_SJYT01018126.1_9e-98_118_636_476296_476798
land1_Madkir_SJYC01139521.1_1e-85_118_636_22345_22840
land1_Antmar_SJYB01001732.1_6e-95_118_636_898769_899274
land1_Litwal_SJYG01000858.1_6e-90_127_636_262782_262286
land1_Ammler_NIVO01036996.1_1e-51_118_493_447943_448303
land1_Psenay_NIZD01119871.1_2e-53_118_493_11411_11770
land1_Orygaz_RAWW01012082.1_3e-58_118_493_2228128_2227769
land1_Hipnig_VHQK01014746.1_1e-56_118_493_4116821_4116461
land1_Contau_SJXO01000417.1_9e-53_118_493_420626_420984
land1_Beahun_PVKQ01030673.1_2e-54_118_493_4419_4782
land1_Oviamm_NIWH01032118.1_3e-52_118_493_73180275_73180637
land1_Oviari_NC_040257.1_3e-52_118_493_100604839_100605201
land1_Hemhyl_PVJR01003516.1_2e-55_118_493_61987_62349
land1_Ovican_PVIS010004572.1_2e-55_118_493_112166_111804
land1_Capaeg_CM003219.1_2e-50_118_493_97056863_97057224
land1_Caphir_NC_030813.1_2e-50_118_493_91247513_91247874
land1_Capsib_NIYN02037220.1_2e-50_118_493_4569236_4568875
land1_Panhod_NW_005810982.1_1e-51_118_493_5058115_5057756
land1_Cerela_CM008013.1_3e-58_109_493_35754153_35753785
land1_Przalb_SJXR01005157.1_1e-91_109_624_597516_597020
land1_Eladav_JRFZ01180318.1_4e-101_109_624_1936891_1936392
land1_Eladav_JRFZ01149951.1_1e-61_366_624_1101_840
land1_Muncri_SJXS01011783.1_9e-93_87_624_1486319_1486839
land1_Munree_SJXT01019099.1_4e-91_87_624_571334_570814
land1_Munmun_SJXU01037411.1_4e-91_87_624_547582_547062
land1_Rantar_PVIN010005608.1_3e-83_87_624_103104_102583
land1_Odohem_RCHL01007870.1_3e-91_109_624_1081561_1082060
land1_Odovir_NW_018335598.1_2e-93_87_624_1096212_1096733
land1_Capcap_CCMK013038043.1_1e-76_153_624_977_521
land1_Hydine_SPDZ01000714.1_2e-83_87_624_24703818_24703297
land1_Axipor_QQTR01058599.1_2e-99_109_624_1012962_1013461
land1_Okajoh_LVCL01S0024571.1_9e-103_87_624_127497_126977
land1_Antame_PVKT010001570.1_3e-67_87_624_128852_128352
marine_PKGA01002728.1_4428881-4429091_Phocoena_phocoena_ScshVNz_12799_whole_genome_shotgun_sequence
marine_Neoasi_NW_020174050.1_1e-61_433_645_4205259_4205469
marine_Monmon_NW_021703777.1_5e-60_433_645_46172206_46171996
marine_Orcorc_NW_004438535.1_5e-65_433_645_5525692_5525482
marine_Glomel_ML658826.1_5e-65_433_645_4216483_4216693
marine_Lagobl_NW_020838003.1_2e-63_433_645_4221073_4221283
marine_RWJT01003043.1_4275523-4275733_Sousa_chinensis_isolate_SCSF_scaffold63_whole_genome_shotgun_sequence
marine_NCQN01000485.1_422362-422571_Tursiops_aduncus_isolate_CRI000098_scaffold_511_whole_genome_shotgun_sequence
marine_Turtru_NW_017843021.1_2e-63_433_645_3989223_3989433
marine_RJWO010013964.1_20869-21079_Inia_geoffrensis_isolate_BS63_IniGeo_flattened_line_27861_whole_genome_shotgun_sequence
marine_RJWI010007910.1_11094-11304_Pontoporia_blainvillei_isolate_BS67_PonBla_flattened_line_15801_whole_genome_shotgun_sequence
marine_PVJJ010005374.1_36515-36725_Mesoplodon_bidens_isolate_US080_MesBid_scaffold_10743_whole_genome_shotgun_sequence
marine_RJWS010194627.1_258-467_Ziphius_cavirostris_isolate_BS68_ZipCav_flattened_line_389183_whole_genome_shotgun_sequence
marine_Phycat_NC_041220.1_5e-70_433_645_66370841_66371051
marine_RJWL010023790.1_8726-8936_Kogia_breviceps_isolate_BS64_KogBre_flattened_line_47572_whole_genome_shotgun_sequence
marine_RYZJ01002015.1_2033098-2033289_Megaptera_novaeangliae_breed_North_Atlantic_isolate_ASU_GRON-1_ScjyzU5_239_whole_genome_shotgun_sequence
_R_marine_NIPP01017202.1_20887-21078_Eschrichtius_robustus_isolate_ER-14-168_kmer60-min500.11746843_whole_genome_shotgun_sequence
marine_VNFD01005167.1_96157-96367_Balaenoptera_musculus_isolate_JJ_BM4_2016_0621_000143F_009_arrow_arrow_whole_genome_shotgun_sequence
marine_Balacu_NW_006725343.1_6e-64_433_636_412299_412502
_R_marine_RJWP010023110.1_14805-15015_Eubalaena_japonica_isolate_BS61_EubJap_flattened_line_46178_whole_genome_shotgun_sequence
marine_Balbon_DF518812.1_2e-68_433_645_17973_18183
marine_RJWK010033681.1_9359-9569_Platanista_minor_isolate_BS66_PlaMin_flattened_line_67318_whole_genome_shotgun_sequence
land1_Susscr_NC_010450.4_5e-50_444_645_71535634_71535831
land1_Camfer_NW_006210980.1_3e-86_324_645_813007_812691
land1_Cambac_NW_011511605.1_3e-86_324_645_2160674_2160990
land1_Camdro_NW_011591284.1_6e-88_324_645_3059345_3059029
land1_Vicpac_NW_005882807.1_3e-81_324_645_2067529_2067213
_R_PVJP02000943.1_1840684-1841086_Hippopotamus_amphibius_isolate_BS01_ScHSfme_927HRSCAF_37918_whole_genome_shotgun_sequence
_R_PVJP02910622.1_17277306-17277941_Hippopotamus_amphibius_isolate_BS01_ScHSfme_925080HRSCAF_982880_whole_genome_shotgun_sequence
land1_Catwag_PVHT010010985.1_5e-45_403_645_18744_19002
land1_Litwal_SJYG01000322.1_8e-39_437_571_2154601_2154473
0.03

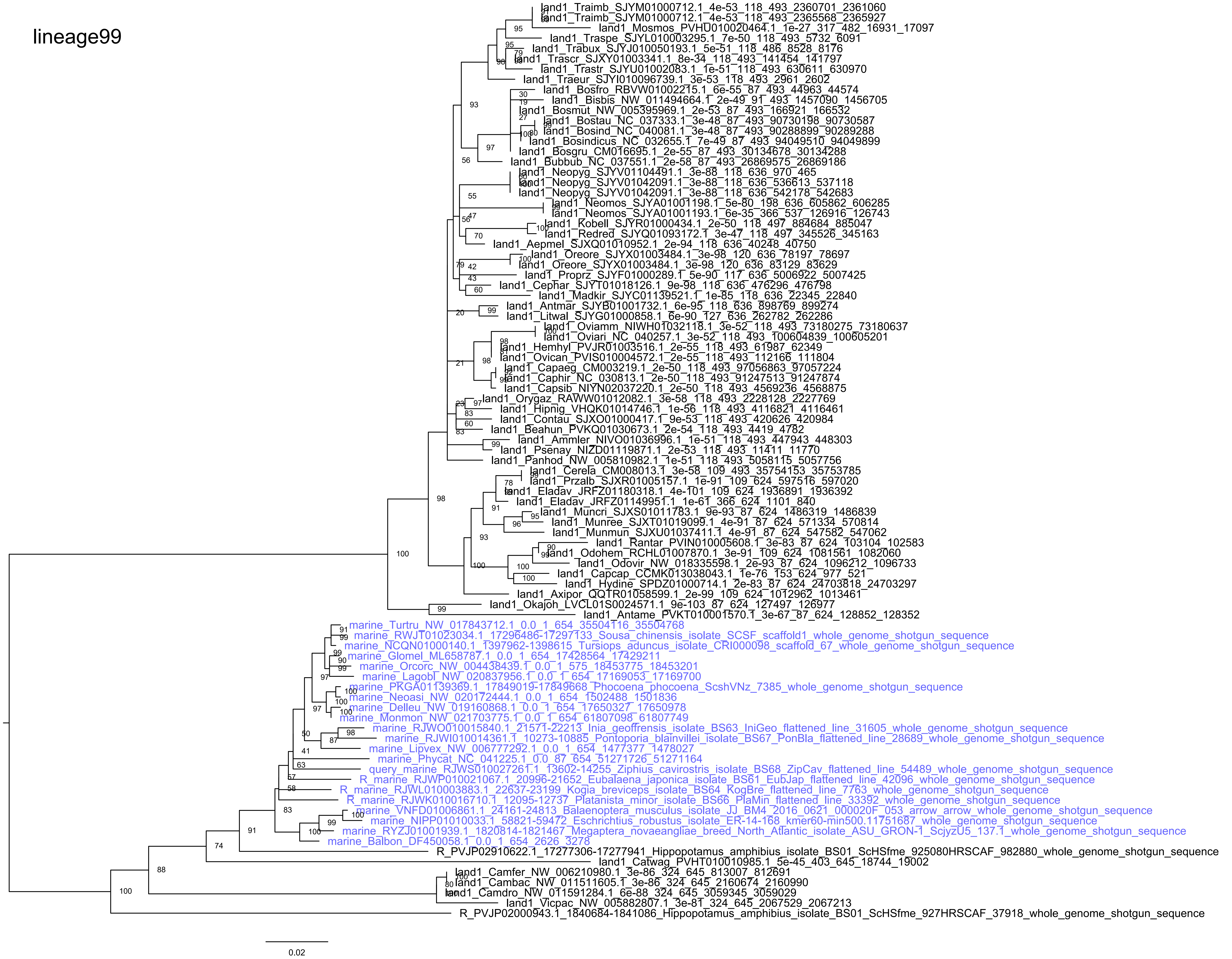
lineage99
land1_Traimb_SJYM01000712.1_4e-53_118_493_2360701_2361060
land1_Traimb_SJYM01000712.1_4e-53_118_493_2365568_2365927
land1_Mosmos_PVHU010020464.1_1e-27_317_482_16931_17097
land1_Traspe_SJYL010003295.1_7e-50_118_493_5732_6091
land1_Trabux_SJYJ010050193.1_5e-51_118_486_8528_8176
land1_Trascr_SJXY01003341.1_8e-34_118_493_141454_141797
land1_Trastr_SJYU01002083.1_1e-51_118_493_630611_630970
land1_Traeur_SJYI010096739.1_3e-53_118_493_2961_2602
land1_Bosfro_RBVW01002215.1_6e-55_87_493_44963_44574
land1_Bisbis_NW_011494664.1_2e-49_91_493_1457090_1456705
land1_Bosmut_NW_005395969.1_2e-53_87_493_166921_166532
land1_Bostau_NC_037333.1_3e-48_87_493_90730198_90730587
land1_Bosind_NC_040081.1_3e-48_87_493_90288899_90289288
land1_Bosindicus_NC_032655.1_7e-49_87_493_94049510_94049899
land1_Bosgru_CM016695.1_2e-55_87_493_30134678_30134288
land1_Bubbub_NC_037551.1_2e-58_87_493_26869575_26869186
land1_Neopyg_SJYV01104491.1_3e-88_118_636_970_465
land1_Neopyg_SJYV01042091.1_3e-88_118_636_536613_537118
land1_Neopyg_SJYV01042091.1_3e-88_118_636_542178_542683
land1_Neomos_SJYA01001198.1_5e-80_198_636_605862_606285
land1_Neomos_SJYA01001193.1_6e-35_366_537_126916_126743
land1_Kobell_SJYR01000434.1_2e-50_118_497_884684_885047
land1_Redred_SJYQ01093172.1_3e-47_118_497_345526_345163
land1_Aepmel_SJXQ01010952.1_2e-94_118_636_40248_40750
land1_Oreore_SJYX01003484.1_3e-98_120_636_78197_78697
land1_Oreore_SJYX01003484.1_3e-98_120_636_83129_83629
land1_Proprz_SJYF01000289.1_5e-90_117_636_5006922_5007425
land1_Cephar_SJYT01018126.1_9e-98_118_636_476296_476798
land1_Madkir_SJYC01139521.1_1e-85_118_636_22345_22840
land1_Antmar_SJYB01001732.1_6e-95_118_636_898769_899274
land1_Litwal_SJYG01000858.1_6e-90_127_636_262782_262286
land1_Oviamm_NIWH01032118.1_3e-52_118_493_73180275_73180637
land1_Oviari_NC_040257.1_3e-52_118_493_100604839_100605201
land1_Hemhyl_PVJR01003516.1_2e-55_118_493_61987_62349
land1_Ovican_PVIS010004572.1_2e-55_118_493_112166_111804
land1_Capaeg_CM003219.1_2e-50_118_493_97056863_97057224
land1_Caphir_NC_030813.1_2e-50_118_493_91247513_91247874
land1_Capsib_NIYN02037220.1_2e-50_118_493_4569236_4568875
land1_Orygaz_RAWW01012082.1_3e-58_118_493_2228128_2227769
land1_Hipnig_VHQK01014746.1_1e-56_118_493_4116821_4116461
land1_Contau_SJXO01000417.1_9e-53_118_493_420626_420984
land1_Beahun_PVKQ01030673.1_2e-54_118_493_4419_4782
land1_Ammler_NIVO01036996.1_1e-51_118_493_447943_448303
land1_Psenay_NIZD01119871.1_2e-53_118_493_11411_11770
land1_Panhod_NW_005810982.1_1e-51_118_493_5058115_5057756
land1_Cerela_CM008013.1_3e-58_109_493_35754153_35753785
land1_Przalb_SJXR01005157.1_1e-91_109_624_597516_597020
land1_Eladav_JRFZ01180318.1_4e-101_109_624_1936891_1936392
land1_Eladav_JRFZ01149951.1_1e-61_366_624_1101_840
land1_Muncri_SJXS01011783.1_9e-93_87_624_1486319_1486839
land1_Munree_SJXT01019099.1_4e-91_87_624_571334_570814
land1_Munmun_SJXU01037411.1_4e-91_87_624_547582_547062
land1_Rantar_PVIN010005608.1_3e-83_87_624_103104_102583
land1_Odohem_RCHL01007870.1_3e-91_109_624_1081561_1082060
land1_Odovir_NW_018335598.1_2e-93_87_624_1096212_1096733
land1_Capcap_CCMK013038043.1_1e-76_153_624_977_521
land1_Hydine_SPDZ01000714.1_2e-83_87_624_24703818_24703297
land1_Axipor_QQTR01058599.1_2e-99_109_624_1012962_1013461
land1_Okajoh_LVCL01S0024571.1_9e-103_87_624_127497_126977
land1_Antame_PVKT010001570.1_3e-67_87_624_128852_128352
marine_Turtru_NW_017843712.1_0.0_1_654_35504116_35504768
marine_RWJT01023034.1_17296486-17297133_Sousa_chinensis_isolate_SCSF_scaffold1_whole_genome_shotgun_sequence
marine_NCQN01000140.1_1397962-1398615_Tursiops_aduncus_isolate_CRI000098_scaffold_67_whole_genome_shotgun_sequence
marine_Glomel_ML658787.1_0.0_1_654_17428564_17429211
marine_Orcorc_NW_004438439.1_0.0_1_575_18453775_18453201
marine_Lagobl_NW_020837956.1_0.0_1_654_17169053_17169700
marine_PKGA01139369.1_17849019-17849668_Phocoena_phocoena_ScshVNz_7385_whole_genome_shotgun_sequence
marine_Neoasi_NW_020172444.1_0.0_1_654_1502488_1501836
marine_Delleu_NW_019160868.1_0.0_1_654_17650327_17650978
marine_Monmon_NW_021703775.1_0.0_1_654_61807098_61807749
marine_RJWO010015840.1_21571-22213_Inia_geoffrensis_isolate_BS63_IniGeo_flattened_line_31605_whole_genome_shotgun_sequence
marine_RJWI010014361.1_10273-10885_Pontoporia_blainvillei_isolate_BS67_PonBla_flattened_line_28689_whole_genome_shotgun_sequence
marine_Lipvex_NW_006777292.1_0.0_1_654_1477377_1478027
marine_Phycat_NC_041225.1_0.0_87_654_51271726_51271164
query_marine_RJWS010027261.1_13602-14255_Ziphius_cavirostris_isolate_BS68_ZipCav_flattened_line_54489_whole_genome_shotgun_sequence
R_marine_RJWP010021067.1_20996-21652_Eubalaena_japonica_isolate_BS61_EubJap_flattened_line_42096_whole_genome_shotgun_sequence
R_marine_RJWL010003883.1_22637-23199_Kogia_breviceps_isolate_BS64_KogBre_flattened_line_7763_whole_genome_shotgun_sequence
R_marine_RJWK010016710.1_12095-12737_Platanista_minor_isolate_BS66_PlaMin_flattened_line_33392_whole_genome_shotgun_sequence
marine_VNFD01006861.1_24161-24813_Balaenoptera_musculus_isolate_JJ_BM4_2016_0621_000020F_053_arrow_arrow_whole_genome_shotgun_sequence
marine_NIPP01010033.1_58821-59472_Eschrichtius_robustus_isolate_ER-14-168_kmer60-min500.11751687_whole_genome_shotgun_sequence
marine_RYZJ01001939.1_1820814-1821467_Megaptera_novaeangliae_breed_North_Atlantic_isolate_ASU_GRON-1_ScjyzU5_137.1_whole_genome_shotgun_sequence
marine_Balbon_DF450058.1_0.0_1_654_2626_3278
R_PVJP02910622.1_17277306-17277941_Hippopotamus_amphibius_isolate_BS01_ScHSfme_925080HRSCAF_982880_whole_genome_shotgun_sequence
land1_Catwag_PVHT010010985.1_5e-45_403_645_18744_19002
land1_Camfer_NW_006210980.1_3e-86_324_645_813007_812691
land1_Cambac_NW_011511605.1_3e-86_324_645_2160674_2160990
land1_Camdro_NW_011591284.1_6e-88_324_645_3059345_3059029
land1_Vicpac_NW_005882807.1_3e-81_324_645_2067529_2067213
R_PVJP02000943.1_1840684-1841086_Hippopotamus_amphibius_isolate_BS01_ScHSfme_927HRSCAF_37918_whole_genome_shotgun_sequence
0.02

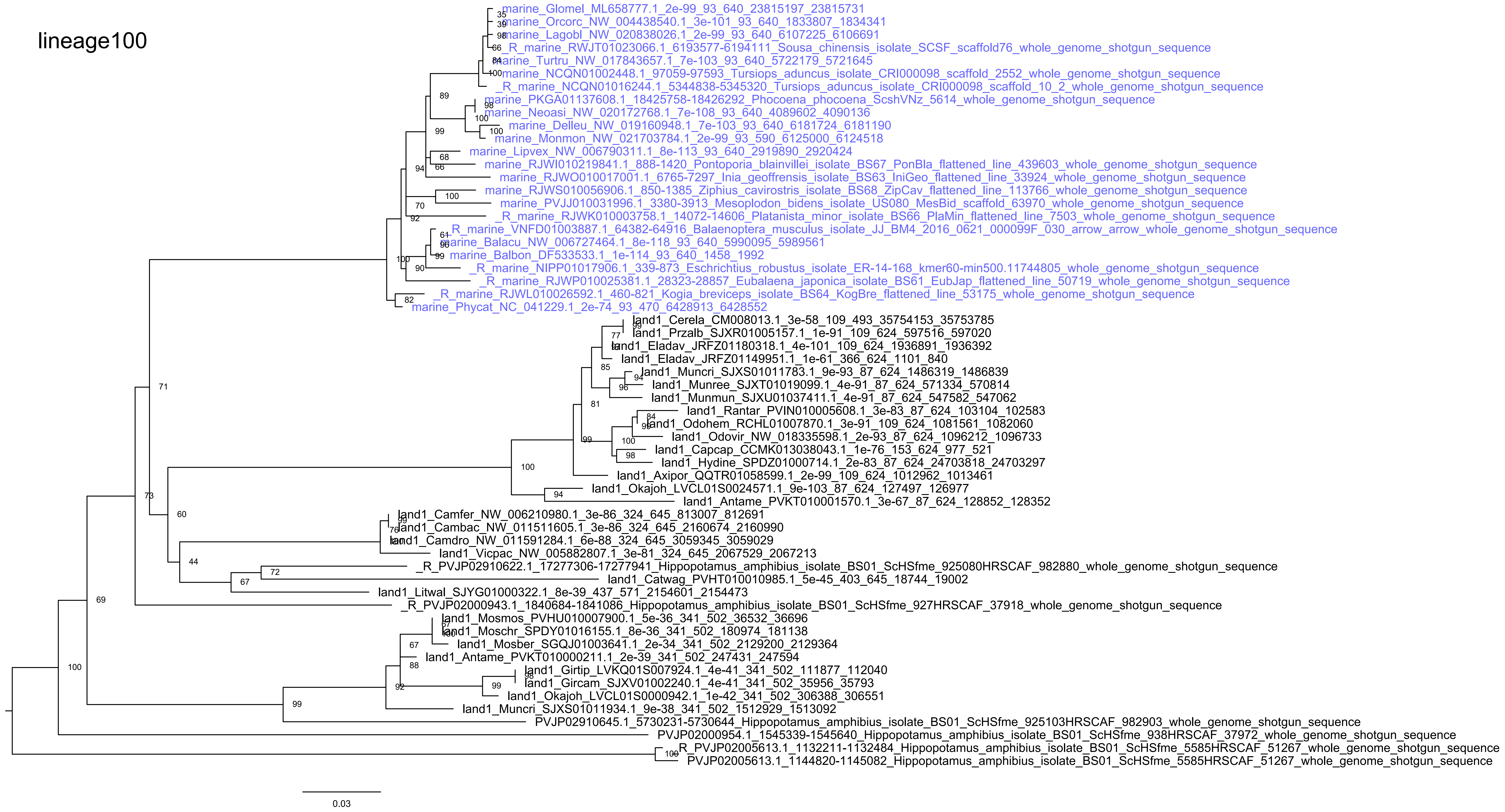
lineage100
marine_Glomel_ML658777.1_2e-99_93_640_23815197_23815731
marine_Orcorc_NW_004438540.1_3e-101_93_640_1833807_1834341
marine_Lagobl_NW_020838026.1_2e-99_93_640_6107225_6106691
_R_marine_RWJT01023066.1_6193577-6194111_Sousa_chinensis_isolate_SCSF_scaffold76_whole_genome_shotgun_sequence
marine_Turtru_NW_017843657.1_7e-103_93_640_5722179_5721645
marine_NCQN01002448.1_97059-97593_Tursiops_aduncus_isolate_CRI000098_scaffold_2552_whole_genome_shotgun_sequence
_R_marine_NCQN01016244.1_5344838-5345320_Tursiops_aduncus_isolate_CRI000098_scaffold_10_2_whole_genome_shotgun_sequence
marine_PKGA01137608.1_18425758-18426292_Phocoena_phocoena_ScshVNz_5614_whole_genome_shotgun_sequence
marine_Neoasi_NW_020172768.1_7e-108_93_640_4089602_4090136
marine_Delleu_NW_019160948.1_7e-103_93_640_6181724_6181190
marine_Monmon_NW_021703784.1_2e-99_93_590_6125000_6124518
marine_Lipvex_NW_006790311.1_8e-113_93_640_2919890_2920424
marine_RJWI010219841.1_888-1420_Pontoporia_blainvillei_isolate_BS67_PonBla_flattened_line_439603_whole_genome_shotgun_sequence
marine_RJWO010017001.1_6765-7297_Inia_geoffrensis_isolate_BS63_IniGeo_flattened_line_33924_whole_genome_shotgun_sequence
marine_RJWS010056906.1_850-1385_Ziphius_cavirostris_isolate_BS68_ZipCav_flattened_line_113766_whole_genome_shotgun_sequence
marine_PVJJ010031996.1_3380-3913_Mesoplodon_bidens_isolate_US080_MesBid_scaffold_63970_whole_genome_shotgun_sequence
_R_marine_RJWK010003758.1_14072-14606_Platanista_minor_isolate_BS66_PlaMin_flattened_line_7503_whole_genome_shotgun_sequence
_R_marine_VNFD01003887.1_64382-64916_Balaenoptera_musculus_isolate_JJ_BM4_2016_0621_000099F_030_arrow_arrow_whole_genome_shotgun_sequence
marine_Balacu_NW_006727464.1_8e-118_93_640_5990095_5989561
marine_Balbon_DF533533.1_1e-114_93_640_1458_1992
_R_marine_NIPP01017906.1_339-873_Eschrichtius_robustus_isolate_ER-14-168_kmer60-min500.11744805_whole_genome_shotgun_sequence
_R_marine_RJWP010025381.1_28323-28857_Eubalaena_japonica_isolate_BS61_EubJap_flattened_line_50719_whole_genome_shotgun_sequence
_R_marine_RJWL010026592.1_460-821_Kogia_breviceps_isolate_BS64_KogBre_flattened_line_53175_whole_genome_shotgun_sequence
marine_Phycat_NC_041229.1_2e-74_93_470_6428913_6428552
land1_Cerela_CM008013.1_3e-58_109_493_35754153_35753785
land1_Przalb_SJXR01005157.1_1e-91_109_624_597516_597020
land1_Eladav_JRFZ01180318.1_4e-101_109_624_1936891_1936392
land1_Eladav_JRFZ01149951.1_1e-61_366_624_1101_840
land1_Muncri_SJXS01011783.1_9e-93_87_624_1486319_1486839
land1_Munree_SJXT01019099.1_4e-91_87_624_571334_570814
land1_Munmun_SJXU01037411.1_4e-91_87_624_547582_547062
land1_Rantar_PVIN010005608.1_3e-83_87_624_103104_102583
land1_Odohem_RCHL01007870.1_3e-91_109_624_1081561_1082060
land1_Odovir_NW_018335598.1_2e-93_87_624_1096212_1096733
land1_Capcap_CCMK013038043.1_1e-76_153_624_977_521
land1_Hydine_SPDZ01000714.1_2e-83_87_624_24703818_24703297
land1_Axipor_QQTR01058599.1_2e-99_109_624_1012962_1013461
land1_Okajoh_LVCL01S0024571.1_9e-103_87_624_127497_126977
land1_Antame_PVKT010001570.1_3e-67_87_624_128852_128352
land1_Camfer_NW_006210980.1_3e-86_324_645_813007_812691
land1_Cambac_NW_011511605.1_3e-86_324_645_2160674_2160990
land1_Camdro_NW_011591284.1_6e-88_324_645_3059345_3059029
land1_Vicpac_NW_005882807.1_3e-81_324_645_2067529_2067213
_R_PVJP02910622.1_17277306-17277941_Hippopotamus_amphibius_isolate_BS01_ScHSfme_925080HRSCAF_982880_whole_genome_shotgun_sequence
land1_Catwag_PVHT010010985.1_5e-45_403_645_18744_19002
land1_Litwal_SJYG01000322.1_8e-39_437_571_2154601_2154473
_R_PVJP02000943.1_1840684-1841086_Hippopotamus_amphibius_isolate_BS01_ScHSfme_927HRSCAF_37918_whole_genome_shotgun_sequence
land1_Mosmos_PVHU010007900.1_5e-36_341_502_36532_36696
land1_Moschr_SPDY01016155.1_8e-36_341_502_180974_181138
land1_Mosber_SGQJ01003641.1_2e-34_341_502_2129200_2129364
land1_Antame_PVKT010000211.1_2e-39_341_502_247431_247594
land1_Girtip_LVKQ01S007924.1_4e-41_341_502_111877_112040
land1_Gircam_SJXV01002240.1_4e-41_341_502_35956_35793
land1_Okajoh_LVCL01S0000942.1_1e-42_341_502_306388_306551
land1_Muncri_SJXS01011934.1_9e-38_341_502_1512929_1513092
PVJP02910645.1_5730231-5730644_Hippopotamus_amphibius_isolate_BS01_ScHSfme_925103HRSCAF_982903_whole_genome_shotgun_sequence
PVJP02000954.1_1545339-1545640_Hippopotamus_amphibius_isolate_BS01_ScHSfme_938HRSCAF_37972_whole_genome_shotgun_sequence
_R_PVJP02005613.1_1132211-1132484_Hippopotamus_amphibius_isolate_BS01_ScHSfme_5585HRSCAF_51267_whole_genome_shotgun_sequence
PVJP02005613.1_1144820-1145082_Hippopotamus_amphibius_isolate_BS01_ScHSfme_5585HRSCAF_51267_whole_genome_shotgun_sequence
0.03

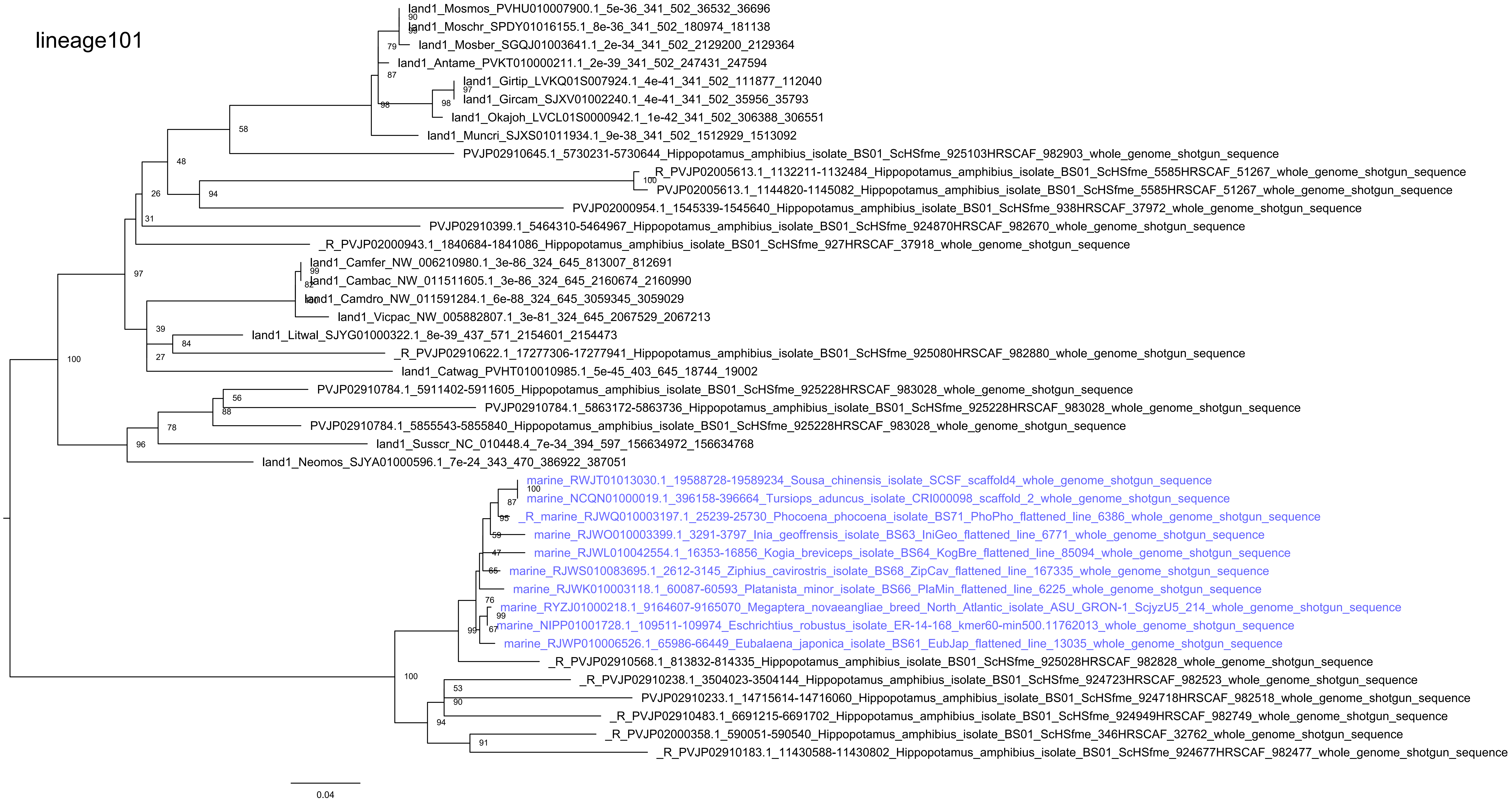

lineage101
land1_Mosmos_PVHU010007900.1_5e-36_341_502_36532_36696
land1_Moschr_SPDY01016155.1_8e-36_341_502_180974_181138
land1_Mosber_SGQJ01003641.1_2e-34_341_502_2129200_2129364
land1_Antame_PVKT010000211.1_2e-39_341_502_247431_247594
land1_Girtip_LVKQ01S007924.1_4e-41_341_502_111877_112040
land1_Gircam_SJXV01002240.1_4e-41_341_502_35956_35793
land1_Okajoh_LVCL01S0000942.1_1e-42_341_502_306388_306551
land1_Muncri_SJXS01011934.1_9e-38_341_502_1512929_1513092
PVJP02910645.1_5730231-5730644_Hippopotamus_amphibius_isolate_BS01_ScHSfme_925103HRSCAF_982903_whole_genome_shotgun_sequence
R_PVJP02005613.1_1132211-1132484_Hippopotamus_amphibius_isolate_BS01_ScHSfme_5585HRSCAF_51267_whole_genome_shotgun_sequence
PVJP02005613.1_1144820-1145082_Hippopotamus_amphibius_isolate_BS01_ScHSfme_5585HRSCAF_51267_whole_genome_shotgun_sequence
PVJP02000954.1_1545339-1545640_Hippopotamus_amphibius_isolate_BS01_ScHSfme_938HRSCAF_37972_whole_genome_shotgun_sequence
PVJP02910399.1_5464310-5464967_Hippopotamus_amphibius_isolate_BS01_ScHSfme_924870HRSCAF_982670_whole_genome_shotgun_sequence
_R_PVJP02000943.1_1840684-1841086_Hippopotamus_amphibius_isolate_BS01_ScHSfme_927HRSCAF_37918_whole_genome_shotgun_sequence
land1_Camfer_NW_006210980.1_3e-86_324_645_813007_812691
land1_Cambac_NW_011511605.1_3e-86_324_645_2160674_2160990
land1_Camdro_NW_011591284.1_6e-88_324_645_3059345_3059029
land1_Vicpac_NW_005882807.1_3e-81_324_645_2067529_2067213
land1_Litwal_SJYG01000322.1_8e-39_437_571_2154601_2154473
_R_PVJP02910622.1_17277306-17277941_Hippopotamus_amphibius_isolate_BS01_ScHSfme_925080HRSCAF_982880_whole_genome_shotgun_sequence
land1_Catwag_PVHT010010985.1_5e-45_403_645_18744_19002
PVJP02910784.1_5911402-5911605_Hippopotamus_amphibius_isolate_BS01_ScHSfme_925228HRSCAF_983028_whole_genome_shotgun_sequence
PVJP02910784.1_5863172-5863736_Hippopotamus_amphibius_isolate_BS01_ScHSfme_925228HRSCAF_983028_whole_genome_shotgun_sequence
PVJP02910784.1_5855543-5855840_Hippopotamus_amphibius_isolate_BS01_ScHSfme_925228HRSCAF_983028_whole_genome_shotgun_sequence
land1_Susscr_NC_010448.4_7e-34_394_597_156634972_156634768
land1_Neomos_SJYA01000596.1_7e-24_343_470_386922_387051
marine_RWJT01013030.1_19588728-19589234_Sousa_chinensis_isolate_SCSF_scaffold4_whole_genome_shotgun_sequence
marine_NCQN01000019.1_396158-396664_Tursiops_aduncus_isolate_CRI000098_scaffold_2_whole_genome_shotgun_sequence
_R_marine_RJWQ010003197.1_25239-25730_Phocoena_phocoena_isolate_BS71_PhoPho_flattened_line_6386_whole_genome_shotgun_sequence
marine_RJWO010003399.1_3291-3797_Inia_geoffrensis_isolate_BS63_IniGeo_flattened_line_6771_whole_genome_shotgun_sequence
marine_RJWL010042554.1_16353-16856_Kogia_breviceps_isolate_BS64_KogBre_flattened_line_85094_whole_genome_shotgun_sequence
marine_RJWS010083695.1_2612-3145_Ziphius_cavirostris_isolate_BS68_ZipCav_flattened_line_167335_whole_genome_shotgun_sequence
marine_RJWK010003118.1_60087-60593_Platanista_minor_isolate_BS66_PlaMin_flattened_line_6225_whole_genome_shotgun_sequence
marine_RYZJ01000218.1_9164607-9165070_Megaptera_novaeangliae_breed_North_Atlantic_isolate_ASU_GRON-1_ScjyzU5_214_whole_genome_shotgun_sequence
marine_NIPP01001728.1_109511-109974_Eschrichtius_robustus_isolate_ER-14-168_kmer60-min500.11762013_whole_genome_shotgun_sequence
marine_RJWP010006526.1_65986-66449_Eubalaena_japonica_isolate_BS61_EubJap_flattened_line_13035_whole_genome_shotgun_sequence
_R_PVJP02910568.1_813832-814335_Hippopotamus_amphibius_isolate_BS01_ScHSfme_925028HRSCAF_982828_whole_genome_shotgun_sequence
_R_PVJP02910238.1_3504023-3504144_Hippopotamus_amphibius_isolate_BS01_ScHSfme_924723HRSCAF_982523_whole_genome_shotgun_sequence
PVJP02910233.1_14715614-14716060_Hippopotamus_amphibius_isolate_BS01_ScHSfme_924718HRSCAF_982518_whole_genome_shotgun_sequence
_R_PVJP02910483.1_6691215-6691702_Hippopotamus_amphibius_isolate_BS01_ScHSfme_924949HRSCAF_982749_whole_genome_shotgun_sequence
_R_PVJP02000358.1_590051-590540_Hippopotamus_amphibius_isolate_BS01_ScHSfme_346HRSCAF_32762_whole_genome_shotgun_sequence
_R_PVJP02910183.1_11430588-11430802_Hippopotamus_amphibius_isolate_BS01_ScHSfme_924677HRSCAF_982477_whole_genome_shotgun_sequence
90
99
79
87
97
98
98
58
48
100
94
26
31
97
99
82
100
39
84
27
100
56
88
78
96
100
87
95
59
47
65
76
99
67
99
100
53
90
94
91
0.04

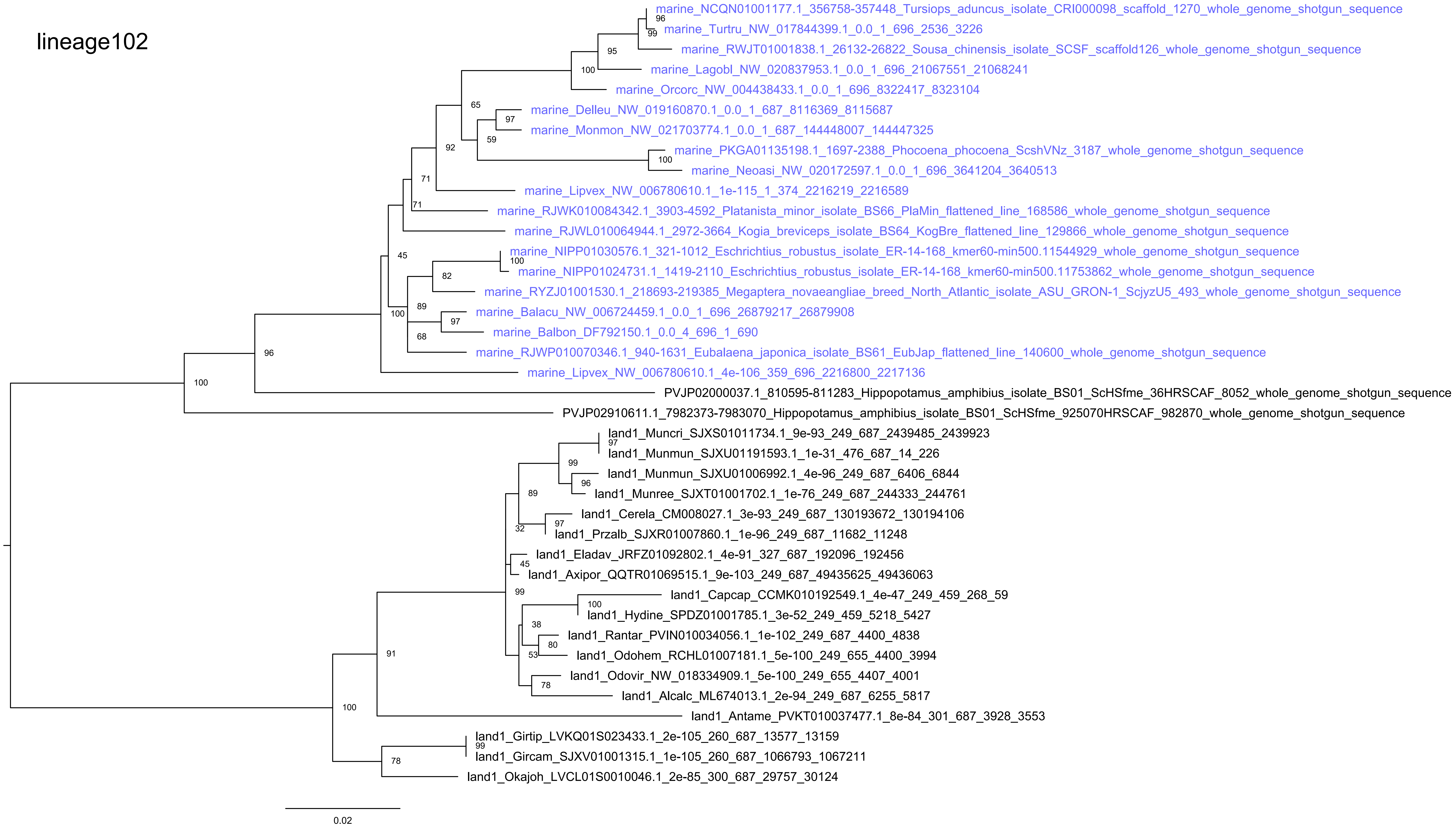
lineage102
marine_NCQN01001177.1_356758-357448_Tursiops_aduncus_isolate_CRI000098_scaffold_1270_whole_genome_shotgun_sequence
marine_Turtru_NW_017844399.1_0.0_1_696_2536_3226
marine_RWJT01001838.1_26132-26822_Sousa_chinensis_isolate_SCSF_scaffold126_whole_genome_shotgun_sequence
marine_Lagobl_NW_020837953.1_0.0_1_696_21067551_21068241
marine_Orcorc_NW_004438433.1_0.0_1_696_8322417_8323104
marine_Delleu_NW_019160870.1_0.0_1_687_8116369_8115687
marine_Monmon_NW_021703774.1_0.0_1_687_144448007_144447325
marine_PKGA01135198.1_1697-2388_Phocoena_phocoena_ScshVNz_3187_whole_genome_shotgun_sequence
marine_Neoasi_NW_020172597.1_0.0_1_696_3641204_3640513
marine_Lipvex_NW_006780610.1_1e-115_1_374_2216219_2216589
marine_RJWK010084342.1_3903-4592_Platanista_minor_isolate_BS66_PlaMin_flattened_line_168586_whole_genome_shotgun_sequence
marine_RJWL010064944.1_2972-3664_Kogia_breviceps_isolate_BS64_KogBre_flattened_line_129866_whole_genome_shotgun_sequence
marine_NIPP01030576.1_321-1012_Eschrichtius_robustus_isolate_ER-14-168_kmer60-min500.11544929_whole_genome_shotgun_sequence
marine_NIPP01024731.1_1419-2110_Eschrichtius_robustus_isolate_ER-14-168_kmer60-min500.11753862_whole_genome_shotgun_sequence
marine_RYZJ01001530.1_218693-219385_Megaptera_novaeangliae_breed_North_Atlantic_isolate_ASU_GRON-1_ScjyzU5_493_whole_genome_shotgun_sequence
marine_Balacu_NW_006724459.1_0.0_1_696_26879217_26879908
marine_Balbon_DF792150.1_0.0_4_696_1_690
marine_RJWP010070346.1_940-1631_Eubalaena_japonica_isolate_BS61_EubJap_flattened_line_140600_whole_genome_shotgun_sequence
marine_Lipvex_NW_006780610.1_4e-106_359_696_2216800_2217136
PVJP02000037.1_810595-811283_Hippopotamus_amphibius_isolate_BS01_ScHSfme_36HRSCAF_8052_whole_genome_shotgun_sequence
PVJP02910611.1_7982373-7983070_Hippopotamus_amphibius_isolate_BS01_ScHSfme_925070HRSCAF_982870_whole_genome_shotgun_sequence
land1_Muncri_SJXS01011734.1_9e-93_249_687_2439485_2439923
land1_Munmun_SJXU01191593.1_1e-31_476_687_14_226
land1_Munmun_SJXU01006992.1_4e-96_249_687_6406_6844
land1_Munree_SJXT01001702.1_1e-76_249_687_244333_244761
land1_Cerela_CM008027.1_3e-93_249_687_130193672_130194106
land1_Przalb_SJXR01007860.1_1e-96_249_687_11682_11248
land1_Eladav_JRFZ01092802.1_4e-91_327_687_192096_192456
land1_Axipor_QQTR01069515.1_9e-103_249_687_49435625_49436063
land1_Capcap_CCMK010192549.1_4e-47_249_459_268_59
land1_Hydine_SPDZ01001785.1_3e-52_249_459_5218_5427
land1_Rantar_PVIN010034056.1_1e-102_249_687_4400_4838
land1_Odohem_RCHL01007181.1_5e-100_249_655_4400_3994
land1_Odovir_NW_018334909.1_5e-100_249_655_4407_4001
land1_Alcalc_ML674013.1_2e-94_249_687_6255_5817
land1_Antame_PVKT010037477.1_8e-84_301_687_3928_3553
land1_Girtip_LVKQ01S023433.1_2e-105_260_687_13577_13159
land1_Gircam_SJXV01001315.1_1e-105_260_687_1066793_1067211
land1_Okajoh_LVCL01S0010046.1_2e-85_300_687_29757_30124
96
99
95
100
65
97
59
100
92
71
71
45
100
82
89
97
68
100
96
100
97
99
96
89
97
32
45
99
100
38
80
53
78
91
100
99
78
0.02

lineage103

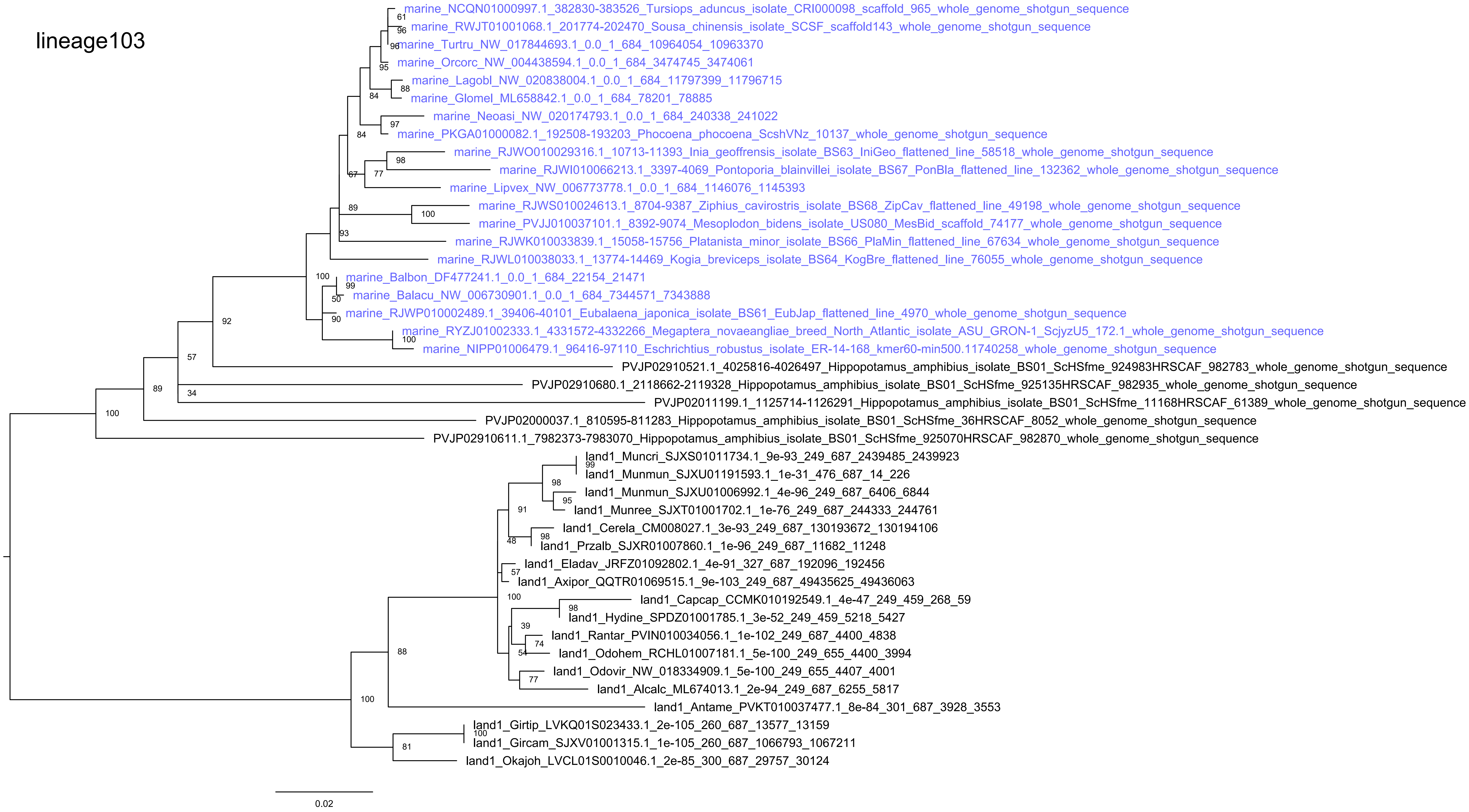
marine_NCQN01000997.1_382830-383526_Tursiops_aduncus_isolate_CRI000098_scaffold_965_whole_genome_shotgun_sequence
marine_RWJT01001068.1_201774-202470_Sousa_chinensis_isolate_SCSF_scaffold143_whole_genome_shotgun_sequence
marine_Turtru_NW_017844693.1_0.0_1_684_10964054_10963370
marine_Orcorc_NW_004438594.1_0.0_1_684_3474745_3474061
marine_Lagobl_NW_020838004.1_0.0_1_684_11797399_11796715
marine_Glomel_ML658842.1_0.0_1_684_78201_78885
marine_Neoasi_NW_020174793.1_0.0_1_684_240338_241022
marine_PKGA01000082.1_192508-193203_Phocoena_phocoena_ScshVNz_10137_whole_genome_shotgun_sequence
marine_RJWO010029316.1_10713-11393_Inia_geoffrensis_isolate_BS63_IniGeo_flattened_line_58518_whole_genome_shotgun_sequence
marine_RJWI010066213.1_3397-4069_Pontoporia_blainvillei_isolate_BS67_PonBla_flattened_line_132362_whole_genome_shotgun_sequence
marine_Lipvex_NW_006773778.1_0.0_1_684_1146076_1145393
marine_RJWS010024613.1_8704-9387_Ziphius_cavirostris_isolate_BS68_ZipCav_flattened_line_49198_whole_genome_shotgun_sequence
marine_PVJJ010037101.1_8392-9074_Mesoplodon_bidens_isolate_US080_MesBid_scaffold_74177_whole_genome_shotgun_sequence
marine_RJWK010033839.1_15058-15756_Platanista_minor_isolate_BS66_PlaMin_flattened_line_67634_whole_genome_shotgun_sequence
marine_RJWL010038033.1_13774-14469_Kogia_breviceps_isolate_BS64_KogBre_flattened_line_76055_whole_genome_shotgun_sequence
marine_Balbon_DF477241.1_0.0_1_684_22154_21471
marine_Balacu_NW_006730901.1_0.0_1_684_7344571_7343888
marine_RJWP010002489.1_39406-40101_Eubalaena_japonica_isolate_BS61_EubJap_flattened_line_4970_whole_genome_shotgun_sequence
marine_RYZJ01002333.1_4331572-4332266_Megaptera_novaeangliae_breed_North_Atlantic_isolate_ASU_GRON-1_ScjyzU5_172.1_whole_genome_shotgun_sequence
marine_NIPP01006479.1_96416-97110_Eschrichtius_robustus_isolate_ER-14-168_kmer60-min500.11740258_whole_genome_shotgun_sequence
PVJP02910521.1_4025816-4026497_Hippopotamus_amphibius_isolate_BS01_ScHSfme_924983HRSCAF_982783_whole_genome_shotgun_sequence
PVJP02910680.1_2118662-2119328_Hippopotamus_amphibius_isolate_BS01_ScHSfme_925135HRSCAF_982935_whole_genome_shotgun_sequence
PVJP02011199.1_1125714-1126291_Hippopotamus_amphibius_isolate_BS01_ScHSfme_11168HRSCAF_61389_whole_genome_shotgun_sequence
PVJP02000037.1_810595-811283_Hippopotamus_amphibius_isolate_BS01_ScHSfme_36HRSCAF_8052_whole_genome_shotgun_sequence
PVJP02910611.1_7982373-7983070_Hippopotamus_amphibius_isolate_BS01_ScHSfme_925070HRSCAF_982870_whole_genome_shotgun_sequence
land1_Muncri_SJXS01011734.1_9e-93_249_687_2439485_2439923
land1_Munmun_SJXU01191593.1_1e-31_476_687_14_226
land1_Munmun_SJXU01006992.1_4e-96_249_687_6406_6844
land1_Munree_SJXT01001702.1_1e-76_249_687_244333_244761
land1_Cerela_CM008027.1_3e-93_249_687_130193672_130194106
land1_Przalb_SJXR01007860.1_1e-96_249_687_11682_11248
land1_Eladav_JRFZ01092802.1_4e-91_327_687_192096_192456
land1_Axipor_QQTR01069515.1_9e-103_249_687_49435625_49436063
land1_Capcap_CCMK010192549.1_4e-47_249_459_268_59
land1_Hydine_SPDZ01001785.1_3e-52_249_459_5218_5427
land1_Rantar_PVIN010034056.1_1e-102_249_687_4400_4838
land1_Odohem_RCHL01007181.1_5e-100_249_655_4400_3994
land1_Odovir_NW_018334909.1_5e-100_249_655_4407_4001
land1_Alcalc_ML674013.1_2e-94_249_687_6255_5817
land1_Antame_PVKT010037477.1_8e-84_301_687_3928_3553
land1_Girtip_LVKQ01S023433.1_2e-105_260_687_13577_13159
land1_Gircam_SJXV01001315.1_1e-105_260_687_1066793_1067211
land1_Okajoh_LVCL01S0010046.1_2e-85_300_687_29757_30124
0.02

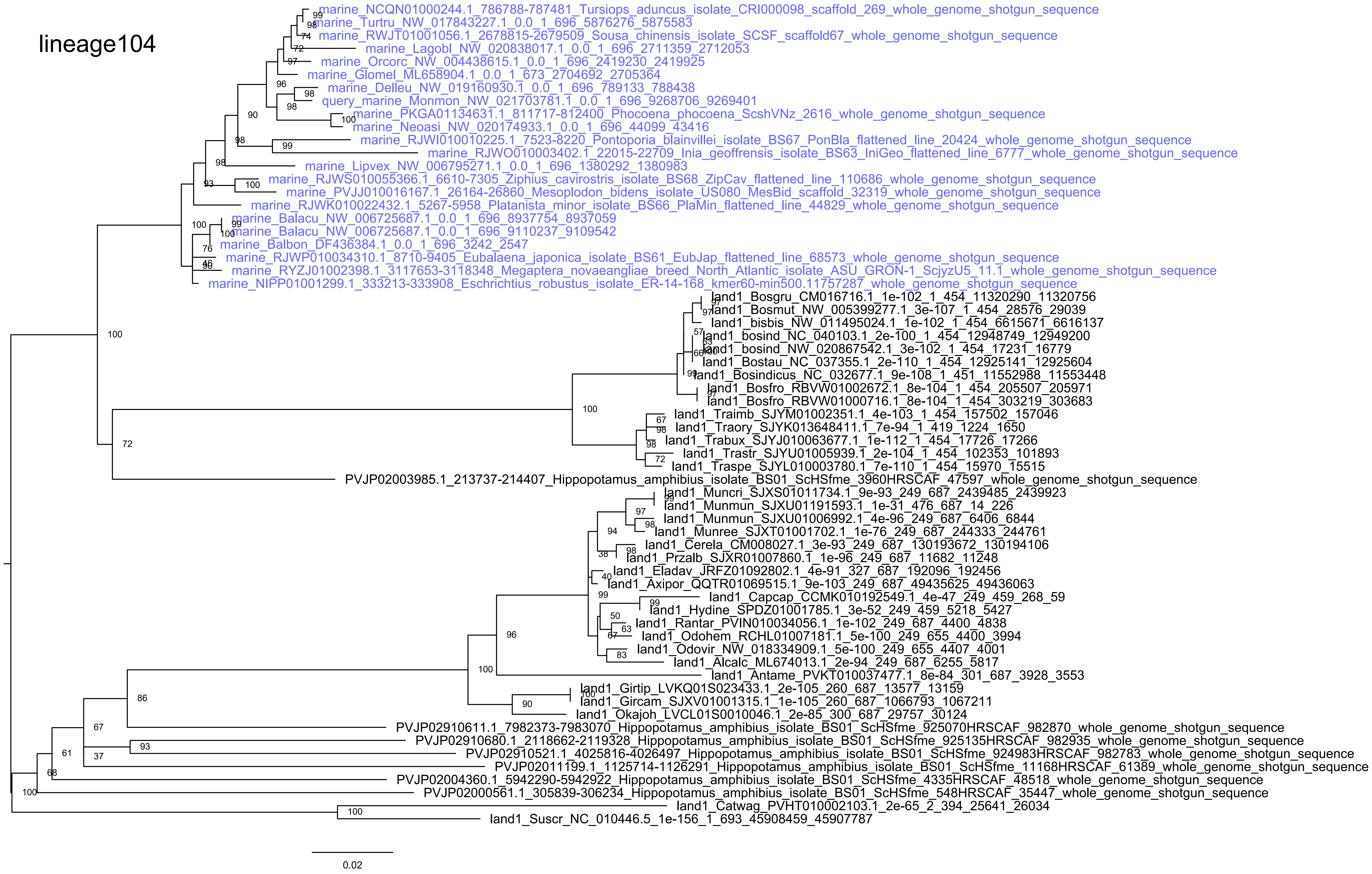
lineage104
marine_NCQN01000244.1_786788-787481_Tursiops_aduncus_isolate_CRI000098_scaffold_269_whole_genome_shotgun_sequence
marine_Turtru_NW_017843227.1_0.0_1_696_5876276_5875583
marine_RWJT01001056.1_2678815-2679509_Sousa_chinensis_isolate_SCSF_scaffold67_whole_genome_shotgun_sequence
marine_Lagobl_NW_020838017.1_0.0_1_696_2711359_2712053
marine_Orcorc_NW_004438615.1_0.0_1_696_2419230_2419925
marine_Glomel_ML658904.1_0.0_1_673_2704692_2705364
marine_Delleu_NW_019160930.1_0.0_1_696_789133_788438
query_marine_Monmon_NW_021703781.1_0.0_1_696_9268706_9269401
marine_PKGA01134631.1_811717-812400_Phocoena_phocoena_ScshVNz_2616_whole_genome_shotgun_sequence
marine_Neoasi_NW_020174933.1_0.0_1_696_44099_43416
marine_RJWI010010225.1_7523-8220_Pontoporia_blainvillei_isolate_BS67_PonBla_flattened_line_20424_whole_genome_shotgun_sequence
marine_RJWO010003402.1_22015-22709_Inia_geoffrensis_isolate_BS63_IniGeo_flattened_line_6777_whole_genome_shotgun_sequence
marine_Lipvex_NW_006795271.1_0.0_1_696_1380292_1380983
marine_RJWS010055366.1_6610-7305_Ziphius_cavirostris_isolate_BS68_ZipCav_flattened_line_110686_whole_genome_shotgun_sequence
marine_PVJJ010016167.1_26164-26860_Mesoplodon_bidens_isolate_US080_MesBid_scaffold_32319_whole_genome_shotgun_sequence
marine_RJWK010022432.1_5267-5958_Platanista_minor_isolate_BS66_PlaMin_flattened_line_44829_whole_genome_shotgun_sequence
marine_Balacu_NW_006725687.1_0.0_1_696_8937754_8937059
marine_Balacu_NW_006725687.1_0.0_1_696_9110237_9109542
marine_Balbon_DF436384.1_0.0_1_696_3242_2547
marine_RJWP010034310.1_8710-9405_Eubalaena_japonica_isolate_BS61_EubJap_flattened_line_68573_whole_genome_shotgun_sequence
marine_RYZJ01002398.1_3117653-3118348_Megaptera_novaeangliae_breed_North_Atlantic_isolate_ASU_GRON-1_ScjyzU5_11.1_whole_genome_shotgun_sequence
marine_NIPP01001299.1_333213-333908_Eschrichtius_robustus_isolate_ER-14-168_kmer60-min500.11757287_whole_genome_shotgun_sequence
land1_Bosgru_CM016716.1_1e-102_1_454_11320290_11320756
land1_Bosmut_NW_005399277.1_3e-107_1_454_28576_29039
land1_bisbis_NW_011495024.1_1e-102_1_454_6615671_6616137
land1_bosind_NC_040103.1_2e-100_1_454_12948749_12949200
land1_bosind_NW_020867542.1_3e-102_1_454_17231_16779
land1_Bostau_NC_037355.1_2e-110_1_454_12925141_12925604
land1_Bosindicus_NC_032677.1_9e-108_1_451_11552988_11553448
land1_Bosfro_RBVW01002672.1_8e-104_1_454_205507_205971
land1_Bosfro_RBVW01000716.1_8e-104_1_454_303219_303683
land1_Traimb_SJYM01002351.1_4e-103_1_454_157502_157046
land1_Traory_SJYK013648411.1_7e-94_1_419_1224_1650
land1_Trabux_SJYJ010063677.1_1e-112_1_454_17726_17266
land1_Trastr_SJYU01005939.1_2e-104_1_454_102353_101893
land1_Traspe_SJYL010003780.1_7e-110_1_454_15970_15515
PVJP02003985.1_213737-214407_Hippopotamus_amphibius_isolate_BS01_ScHSfme_3960HRSCAF_47597_whole_genome_shotgun_sequence
land1_Muncri_SJXS01011734.1_9e-93_249_687_2439485_2439923
land1_Munmun_SJXU01191593.1_1e-31_476_687_14_226
land1_Munmun_SJXU01006992.1_4e-96_249_687_6406_6844
land1_Munree_SJXT01001702.1_1e-76_249_687_244333_244761
land1_Cerela_CM008027.1_3e-93_249_687_130193672_130194106
land1_Przalb_SJXR01007860.1_1e-96_249_687_11682_11248
land1_Eladav_JRFZ01092802.1_4e-91_327_687_192096_192456
land1_Axipor_QQTR01069515.1_9e-103_249_687_49435625_49436063
land1_Capcap_CCMK010192549.1_4e-47_249_459_268_59
land1_Hydine_SPDZ01001785.1_3e-52_249_459_5218_5427
land1_Rantar_PVIN010034056.1_1e-102_249_687_4400_4838
land1_Odohem_RCHL01007181.1_5e-100_249_655_4400_3994
land1_Odovir_NW_018334909.1_5e-100_249_655_4407_4001
land1_Alcalc_ML674013.1_2e-94_249_687_6255_5817
land1_Antame_PVKT010037477.1_8e-84_301_687_3928_3553
land1_Girtip_LVKQ01S023433.1_2e-105_260_687_13577_13159
land1_Gircam_SJXV01001315.1_1e-105_260_687_1066793_1067211
land1_Okajoh_LVCL01S0010046.1_2e-85_300_687_29757_30124
PVJP02910611.1_7982373-7983070_Hippopotamus_amphibius_isolate_BS01_ScHSfme_925070HRSCAF_982870_whole_genome_shotgun_sequence
PVJP02910680.1_2118662-2119328_Hippopotamus_amphibius_isolate_BS01_ScHSfme_925135HRSCAF_982935_whole_genome_shotgun_sequence
PVJP02910521.1_4025816-4026497_Hippopotamus_amphibius_isolate_BS01_ScHSfme_924983HRSCAF_982783_whole_genome_shotgun_sequence
PVJP02011199.1_1125714-1126291_Hippopotamus_amphibius_isolate_BS01_ScHSfme_11168HRSCAF_61389_whole_genome_shotgun_sequence
PVJP02004360.1_5942290-5942922_Hippopotamus_amphibius_isolate_BS01_ScHSfme_4335HRSCAF_48518_whole_genome_shotgun_sequence
PVJP02000561.1_305839-306234_Hippopotamus_amphibius_isolate_BS01_ScHSfme_548HRSCAF_35447_whole_genome_shotgun_sequence
land1_Catwag_PVHT010002103.1_2e-65_2_394_25641_26034
land1_Suscr_NC_010446.5_1e-156_1_693_45908459_45907787
0.02

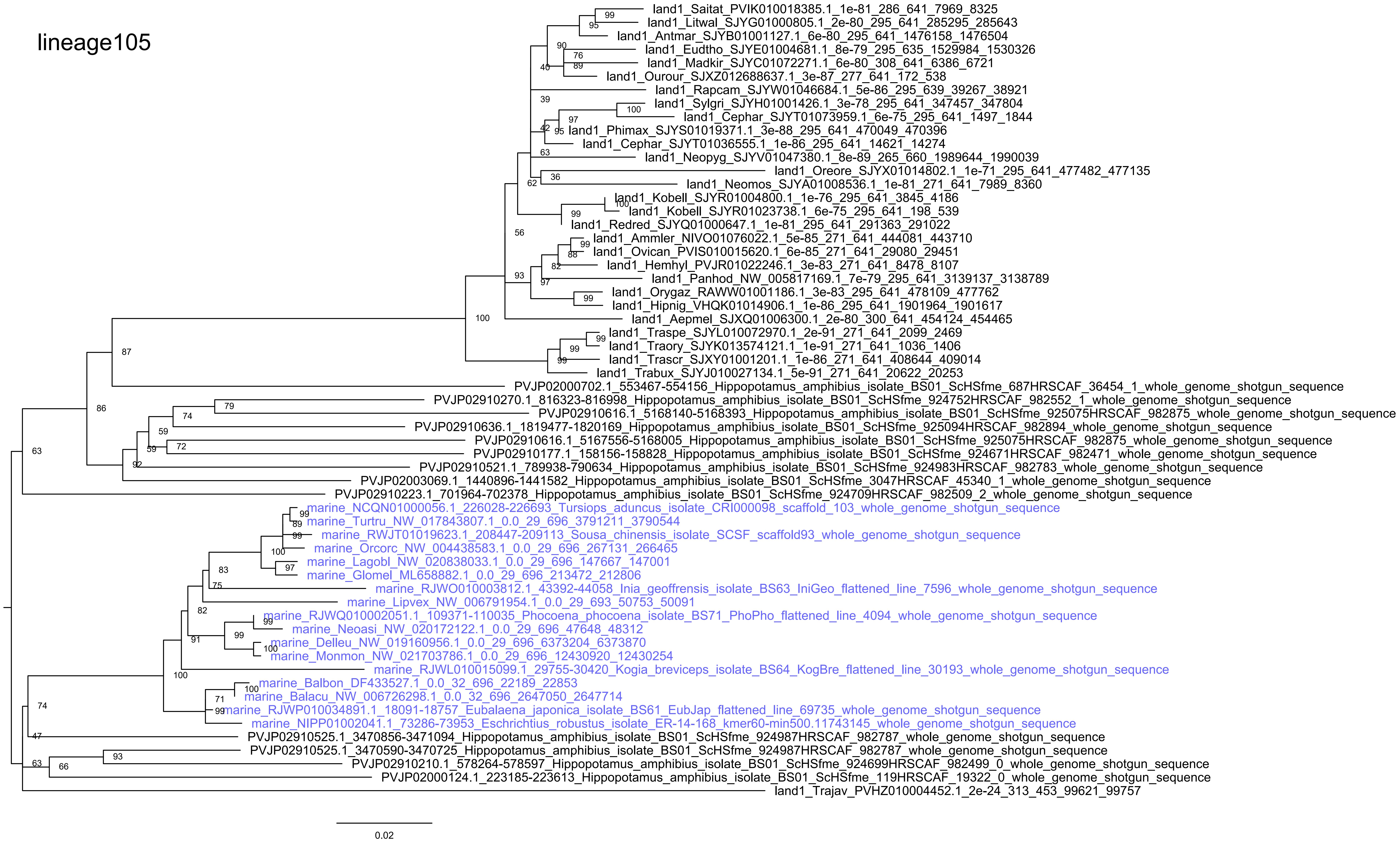
lineage105
land1_Saitat_PVIK010018385.1_1e-81_286_641_7969_8325
land1_Litwal_SJYG01000805.1_2e-80_295_641_285295_285643
land1_Antmar_SJYB01001127.1_6e-80_295_641_1476158_1476504
land1_Eudtho_SJYE01004681.1_8e-79_295_635_1529984_1530326
land1_Madkir_SJYC01072271.1_6e-80_308_641_6386_6721
land1_Ourour_SJXZ012688637.1_3e-87_277_641_172_538
land1_Rapcam_SJYW01046684.1_5e-86_295_639_39267_38921
land1_Sylgri_SJYH01001426.1_3e-78_295_641_347457_347804
land1_Cephar_SJYT01073959.1_6e-75_295_641_1497_1844
land1_Phimax_SJYS01019371.1_3e-88_295_641_470049_470396
land1_Cephar_SJYT01036555.1_1e-86_295_641_14621_14274
land1_Neopyg_SJYV01047380.1_8e-89_265_660_1989644_1990039
land1_Oreore_SJYX01014802.1_1e-71_295_641_477482_477135
land1_Neomos_SJYA01008536.1_1e-81_271_641_7989_8360
land1_Kobell_SJYR01004800.1_1e-76_295_641_3845_4186
land1_Kobell_SJYR01023738.1_6e-75_295_641_198_539
land1_Redred_SJYQ01000647.1_1e-81_295_641_291363_291022
land1_Ammler_NIVO01076022.1_5e-85_271_641_444081_443710
land1_Ovican_PVIS010015620.1_6e-85_271_641_29080_29451
land1_Hemhyl_PVJR01022246.1_3e-83_271_641_8478_8107
land1_Panhod_NW_005817169.1_7e-79_295_641_3139137_3138789
land1_Orygaz_RAWW01001186.1_3e-83_295_641_478109_477762
land1_Hipnig_VHQK01014906.1_1e-86_295_641_1901964_1901617
land1_Aepmel_SJXQ01006300.1_2e-80_300_641_454124_454465
land1_Traspe_SJYL010072970.1_2e-91_271_641_2099_2469
land1_Traory_SJYK013574121.1_1e-91_271_641_1036_1406
land1_Trascr_SJXY01001201.1_1e-86_271_641_408644_409014
land1_Trabux_SJYJ010027134.1_5e-91_271_641_20622_20253
PVJP02000702.1_553467-554156_Hippopotamus_amphibius_isolate_BS01_ScHSfme_687HRSCAF_36454_1_whole_genome_shotgun_sequence
PVJP02910270.1_816323-816998_Hippopotamus_amphibius_isolate_BS01_ScHSfme_924752HRSCAF_982552_1_whole_genome_shotgun_sequence
PVJP02910616.1_5168140-5168393_Hippopotamus_amphibius_isolate_BS01_ScHSfme_925075HRSCAF_982875_whole_genome_shotgun_sequence
PVJP02910636.1_1819477-1820169_Hippopotamus_amphibius_isolate_BS01_ScHSfme_925094HRSCAF_982894_whole_genome_shotgun_sequence
PVJP02910616.1_5167556-5168005_Hippopotamus_amphibius_isolate_BS01_ScHSfme_925075HRSCAF_982875_whole_genome_shotgun_sequence
PVJP02910177.1_158156-158828_Hippopotamus_amphibius_isolate_BS01_ScHSfme_924671HRSCAF_982471_whole_genome_shotgun_sequence
PVJP02910521.1_789938-790634_Hippopotamus_amphibius_isolate_BS01_ScHSfme_924983HRSCAF_982783_whole_genome_shotgun_sequence
PVJP02003069.1_1440896-1441582_Hippopotamus_amphibius_isolate_BS01_ScHSfme_3047HRSCAF_45340_1_whole_genome_shotgun_sequence
PVJP02910223.1_701964-702378_Hippopotamus_amphibius_isolate_BS01_ScHSfme_924709HRSCAF_982509_2_whole_genome_shotgun_sequence
marine_NCQN01000056.1_226028-226693_Tursiops_aduncus_isolate_CRI000098_scaffold_103_whole_genome_shotgun_sequence
marine_Turtru_NW_017843807.1_0.0_29_696_3791211_3790544
marine_RWJT01019623.1_208447-209113_Sousa_chinensis_isolate_SCSF_scaffold93_whole_genome_shotgun_sequence
marine_Orcorc_NW_004438583.1_0.0_29_696_267131_266465
marine_Lagobl_NW_020838033.1_0.0_29_696_147667_147001
marine_Glomel_ML658882.1_0.0_29_696_213472_212806
marine_RJWO010003812.1_43392-44058_Inia_geoffrensis_isolate_BS63_IniGeo_flattened_line_7596_whole_genome_shotgun_sequence
marine_Lipvex_NW_006791954.1_0.0_29_693_50753_50091
marine_RJWQ010002051.1_109371-110035_Phocoena_phocoena_isolate_BS71_PhoPho_flattened_line_4094_whole_genome_shotgun_sequence
marine_Neoasi_NW_020172122.1_0.0_29_696_47648_48312
marine_Delleu_NW_019160956.1_0.0_29_696_6373204_6373870
marine_Monmon_NW_021703786.1_0.0_29_696_12430920_12430254
marine_RJWL010015099.1_29755-30420_Kogia_breviceps_isolate_BS64_KogBre_flattened_line_30193_whole_genome_shotgun_sequence
marine_Balbon_DF433527.1_0.0_32_696_22189_22853
marine_Balacu_NW_006726298.1_0.0_32_696_2647050_2647714
marine_RJWP010034891.1_18091-18757_Eubalaena_japonica_isolate_BS61_EubJap_flattened_line_69735_whole_genome_shotgun_sequence
marine_NIPP01002041.1_73286-73953_Eschrichtius_robustus_isolate_ER-14-168_kmer60-min500.11743145_whole_genome_shotgun_sequence
PVJP02910525.1_3470856-3471094_Hippopotamus_amphibius_isolate_BS01_ScHSfme_924987HRSCAF_982787_whole_genome_shotgun_sequence
PVJP02910525.1_3470590-3470725_Hippopotamus_amphibius_isolate_BS01_ScHSfme_924987HRSCAF_982787_whole_genome_shotgun_sequence
PVJP02910210.1_578264-578597_Hippopotamus_amphibius_isolate_BS01_ScHSfme_924699HRSCAF_982499_0_whole_genome_shotgun_sequence
PVJP02000124.1_223185-223613_Hippopotamus_amphibius_isolate_BS01_ScHSfme_119HRSCAF_19322_0_whole_genome_shotgun_sequence
land1_Trajav_PVHZ010004452.1_2e-24_313_453_99621_99757
0.02

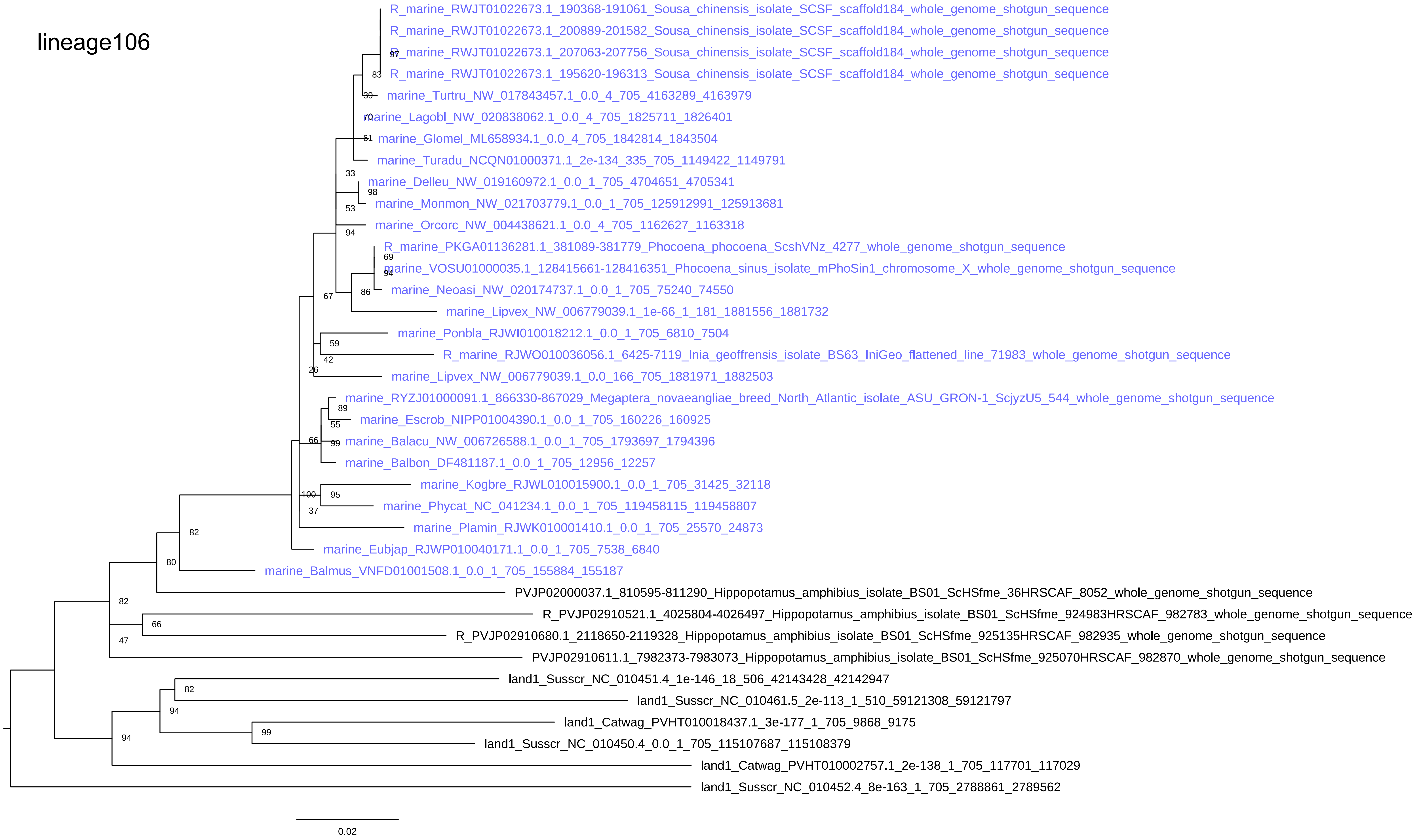
lineage106
R_marine_RWJT01022673.1_190368-191061_Sousa_chinensis_isolate_SCSF_scaffold184_whole_genome_shotgun_sequence
R_marine_RWJT01022673.1_200889-201582_Sousa_chinensis_isolate_SCSF_scaffold184_whole_genome_shotgun_sequence
R_marine_RWJT01022673.1_207063-207756_Sousa_chinensis_isolate_SCSF_scaffold184_whole_genome_shotgun_sequence
R_marine_RWJT01022673.1_195620-196313_Sousa_chinensis_isolate_SCSF_scaffold184_whole_genome_shotgun_sequence
marine_Turtru_NW_017843457.1_0.0_4_705_4163289_4163979
marine_Lagobl_NW_020838062.1_0.0_4_705_1825711_1826401
marine_Glomel_ML658934.1_0.0_4_705_1842814_1843504
marine_Turadu_NCQN01000371.1_2e-134_335_705_1149422_1149791
marine_Delleu_NW_019160972.1_0.0_1_705_4704651_4705341
marine_Monmon_NW_021703779.1_0.0_1_705_125912991_125913681
marine_Orcorc_NW_004438621.1_0.0_4_705_1162627_1163318
R_marine_PKGA01136281.1_381089-381779_Phocoena_phocoena_ScshVNz_4277_whole_genome_shotgun_sequence
marine_VOSU01000035.1_128415661-128416351_Phocoena_sinus_isolate_mPhoSin1_chromosome_X_whole_genome_shotgun_sequence
marine_Neoasi_NW_020174737.1_0.0_1_705_75240_74550
marine_Lipvex_NW_006779039.1_1e-66_1_181_1881556_1881732
marine_Ponbla_RJWI010018212.1_0.0_1_705_6810_7504
R_marine_RJWO010036056.1_6425-7119_Inia_geoffrensis_isolate_BS63_IniGeo_flattened_line_71983_whole_genome_shotgun_sequence
marine_Lipvex_NW_006779039.1_0.0_166_705_1881971_1882503
marine_RYZJ01000091.1_866330-867029_Megaptera_novaeangliae_breed_North_Atlantic_isolate_ASU_GRON-1_ScjyzU5_544_whole_genome_shotgun_sequence
marine_Escrob_NIPP01004390.1_0.0_1_705_160226_160925
marine_Balacu_NW_006726588.1_0.0_1_705_1793697_1794396
marine_Balbon_DF481187.1_0.0_1_705_12956_12257
marine_Kogbre_RJWL010015900.1_0.0_1_705_31425_32118
marine_Phycat_NC_041234.1_0.0_1_705_119458115_119458807
marine_Plamin_RJWK010001410.1_0.0_1_705_25570_24873
marine_Eubjap_RJWP010040171.1_0.0_1_705_7538_6840
marine_Balmus_VNFD01001508.1_0.0_1_705_155884_155187
PVJP02000037.1_810595-811290_Hippopotamus_amphibius_isolate_BS01_ScHSfme_36HRSCAF_8052_whole_genome_shotgun_sequence
R_PVJP02910521.1_4025804-4026497_Hippopotamus_amphibius_isolate_BS01_ScHSfme_924983HRSCAF_982783_whole_genome_shotgun_sequence
R_PVJP02910680.1_2118650-2119328_Hippopotamus_amphibius_isolate_BS01_ScHSfme_925135HRSCAF_982935_whole_genome_shotgun_sequence
PVJP02910611.1_7982373-7983073_Hippopotamus_amphibius_isolate_BS01_ScHSfme_925070HRSCAF_982870_whole_genome_shotgun_sequence
land1_Susscr_NC_010451.4_1e-146_18_506_42143428_42142947
land1_Susscr_NC_010461.5_2e-113_1_510_59121308_59121797
land1_Catwag_PVHT010018437.1_3e-177_1_705_9868_9175
land1_Susscr_NC_010450.4_0.0_1_705_115107687_115108379
land1_Catwag_PVHT010002757.1_2e-138_1_705_117701_117029
land1_Susscr_NC_010452.4_8e-163_1_705_2788861_2789562
0.02

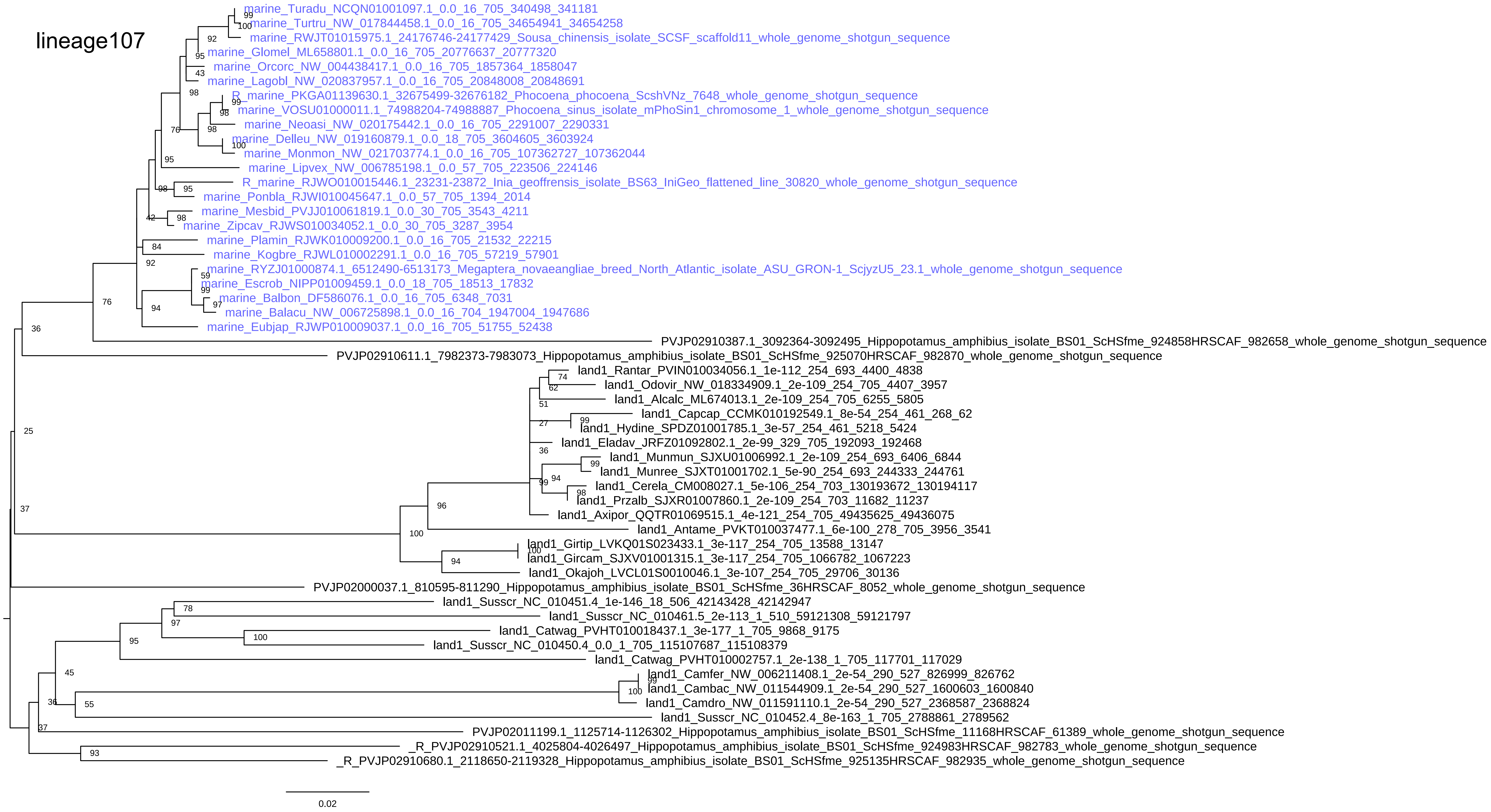
lineage107
marine_Turadu_NCQN01001097.1_0.0_16_705_340498_341181
marine_Turtru_NW_017844458.1_0.0_16_705_34654941_34654258
marine_RWJT01015975.1_24176746-24177429_Sousa_chinensis_isolate_SCSF_scaffold11_whole_genome_shotgun_sequence
marine_Glomel_ML658801.1_0.0_16_705_20776637_20777320
marine_Orcorc_NW_004438417.1_0.0_16_705_1857364_1858047
marine_Lagobl_NW_020837957.1_0.0_16_705_20848008_20848691
R_marine_PKGA01139630.1_32675499-32676182_Phocoena_phocoena_ScshVNz_7648_whole_genome_shotgun_sequence
marine_VOSU01000011.1_74988204-74988887_Phocoena_sinus_isolate_mPhoSin1_chromosome_1_whole_genome_shotgun_sequence
marine_Neoasi_NW_020175442.1_0.0_16_705_2291007_2290331
marine_Delleu_NW_019160879.1_0.0_18_705_3604605_3603924
marine_Monmon_NW_021703774.1_0.0_16_705_107362727_107362044
marine_Lipvex_NW_006785198.1_0.0_57_705_223506_224146
R_marine_RJWO010015446.1_23231-23872_Inia_geoffrensis_isolate_BS63_IniGeo_flattened_line_30820_whole_genome_shotgun_sequence
marine_Ponbla_RJWI010045647.1_0.0_57_705_1394_2014
marine_Mesbid_PVJJ010061819.1_0.0_30_705_3543_4211
marine_Zipcav_RJWS010034052.1_0.0_30_705_3287_3954
marine_Plamin_RJWK010009200.1_0.0_16_705_21532_22215
marine_Kogbre_RJWL010002291.1_0.0_16_705_57219_57901
marine_RYZJ01000874.1_6512490-6513173_Megaptera_novaeangliae_breed_North_Atlantic_isolate_ASU_GRON-1_ScjyzU5_23.1_whole_genome_shotgun_sequence
marine_Escrob_NIPP01009459.1_0.0_18_705_18513_17832
marine_Balbon_DF586076.1_0.0_16_705_6348_7031
marine_Balacu_NW_006725898.1_0.0_16_704_1947004_1947686
marine_Eubjap_RJWP010009037.1_0.0_16_705_51755_52438
PVJP02910387.1_3092364-3092495_Hippopotamus_amphibius_isolate_BS01_ScHSfme_924858HRSCAF_982658_whole_genome_shotgun_sequence
PVJP02910611.1_7982373-7983073_Hippopotamus_amphibius_isolate_BS01_ScHSfme_925070HRSCAF_982870_whole_genome_shotgun_sequence
land1_Rantar_PVIN010034056.1_1e-112_254_693_4400_4838
land1_Odovir_NW_018334909.1_2e-109_254_705_4407_3957
land1_Alcalc_ML674013.1_2e-109_254_705_6255_5805
land1_Capcap_CCMK010192549.1_8e-54_254_461_268_62
land1_Hydine_SPDZ01001785.1_3e-57_254_461_5218_5424
land1_Eladav_JRFZ01092802.1_2e-99_329_705_192093_192468
land1_Munmun_SJXU01006992.1_2e-109_254_693_6406_6844
land1_Munree_SJXT01001702.1_5e-90_254_693_244333_244761
land1_Cerela_CM008027.1_5e-106_254_703_130193672_130194117
land1_Przalb_SJXR01007860.1_2e-109_254_703_11682_11237
land1_Axipor_QQTR01069515.1_4e-121_254_705_49435625_49436075
land1_Antame_PVKT010037477.1_6e-100_278_705_3956_3541
land1_Girtip_LVKQ01S023433.1_3e-117_254_705_13588_13147
land1_Gircam_SJXV01001315.1_3e-117_254_705_1066782_1067223
land1_Okajoh_LVCL01S0010046.1_3e-107_254_705_29706_30136
PVJP02000037.1_810595-811290_Hippopotamus_amphibius_isolate_BS01_ScHSfme_36HRSCAF_8052_whole_genome_shotgun_sequence
land1_Susscr_NC_010451.4_1e-146_18_506_42143428_42142947
land1_Susscr_NC_010461.5_2e-113_1_510_59121308_59121797
land1_Catwag_PVHT010018437.1_3e-177_1_705_9868_9175
land1_Susscr_NC_010450.4_0.0_1_705_115107687_115108379
land1_Catwag_PVHT010002757.1_2e-138_1_705_117701_117029
land1_Camfer_NW_006211408.1_2e-54_290_527_826999_826762
land1_Cambac_NW_011544909.1_2e-54_290_527_1600603_1600840
land1_Camdro_NW_011591110.1_2e-54_290_527_2368587_2368824
land1_Susscr_NC_010452.4_8e-163_1_705_2788861_2789562
PVJP02011199.1_1125714-1126302_Hippopotamus_amphibius_isolate_BS01_ScHSfme_11168HRSCAF_61389_whole_genome_shotgun_sequence
_R_PVJP02910521.1_4025804-4026497_Hippopotamus_amphibius_isolate_BS01_ScHSfme_924983HRSCAF_982783_whole_genome_shotgun_sequence
_R_PVJP02910680.1_2118650-2119328_Hippopotamus_amphibius_isolate_BS01_ScHSfme_925135HRSCAF_982935_whole_genome_shotgun_sequence
0.02

lineage108

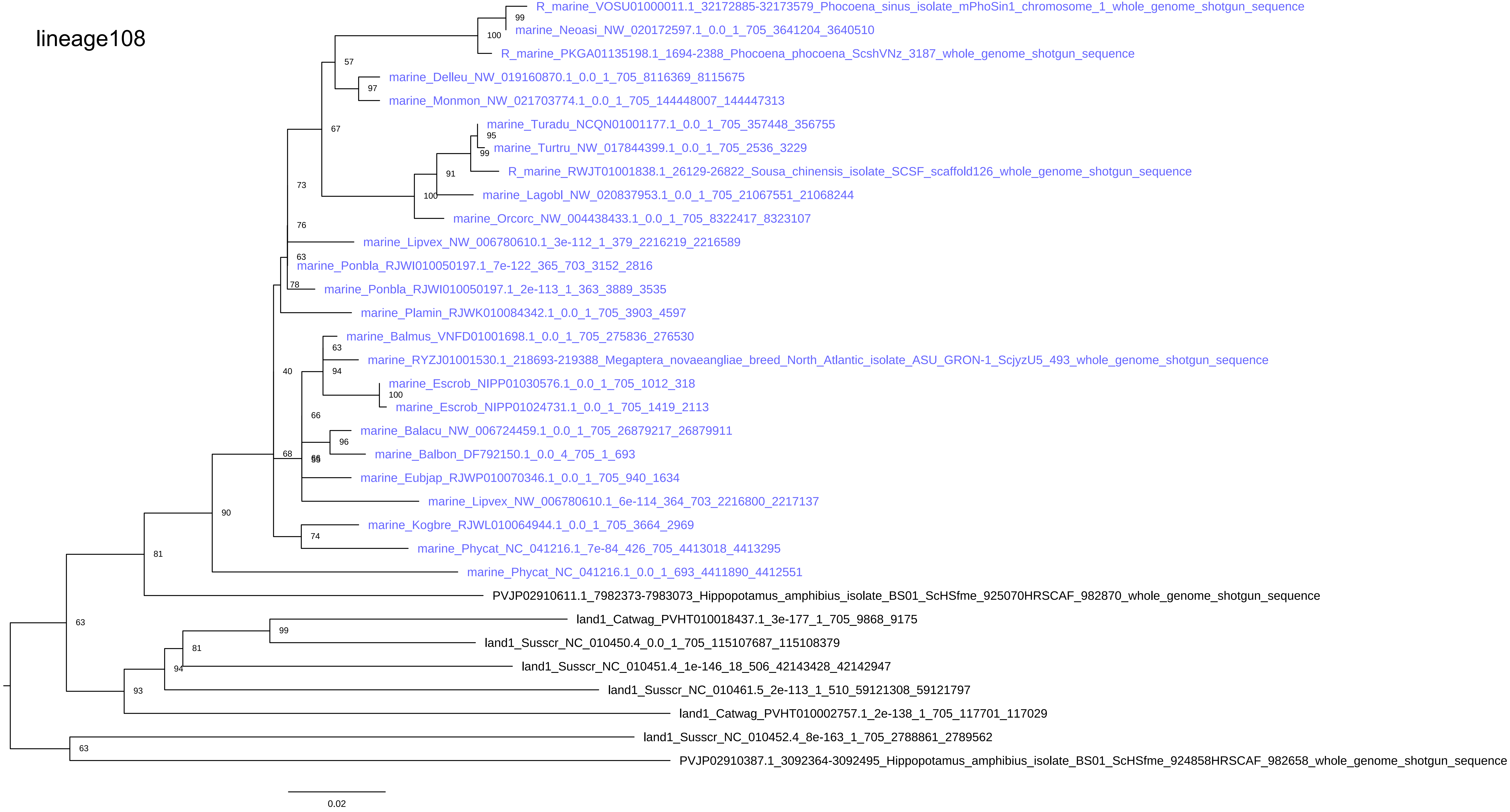
R_marine_VOSU01000011.1_32172885-32173579_Phocoena_sinus_isolate_mPhoSin1_chromosome_1_whole_genome_shotgun_sequence
marine_Neoasi_NW_020172597.1_0.0_1_705_3641204_3640510
R_marine_PKGA01135198.1_1694-2388_Phocoena_phocoena_ScshVNz_3187_whole_genome_shotgun_sequence
marine_Delleu_NW_019160870.1_0.0_1_705_8116369_8115675
marine_Monmon_NW_021703774.1_0.0_1_705_144448007_144447313
marine_Turadu_NCQN01001177.1_0.0_1_705_357448_356755
marine_Turtru_NW_017844399.1_0.0_1_705_2536_3229
R_marine_RWJT01001838.1_26129-26822_Sousa_chinensis_isolate_SCSF_scaffold126_whole_genome_shotgun_sequence
marine_Lagobl_NW_020837953.1_0.0_1_705_21067551_21068244
marine_Orcorc_NW_004438433.1_0.0_1_705_8322417_8323107
marine_Lipvex_NW_006780610.1_3e-112_1_379_2216219_2216589
marine_Ponbla_RJWI010050197.1_7e-122_365_703_3152_2816
marine_Ponbla_RJWI010050197.1_2e-113_1_363_3889_3535
marine_Plamin_RJWK010084342.1_0.0_1_705_3903_4597
marine_Balmus_VNFD01001698.1_0.0_1_705_275836_276530
marine_RYZJ01001530.1_218693-219388_Megaptera_novaeangliae_breed_North_Atlantic_isolate_ASU_GRON-1_ScjyzU5_493_whole_genome_shotgun_sequence
marine_Escrob_NIPP01030576.1_0.0_1_705_1012_318
marine_Escrob_NIPP01024731.1_0.0_1_705_1419_2113
marine_Balacu_NW_006724459.1_0.0_1_705_26879217_26879911
marine_Balbon_DF792150.1_0.0_4_705_1_693
marine_Eubjap_RJWP010070346.1_0.0_1_705_940_1634
marine_Lipvex_NW_006780610.1_6e-114_364_703_2216800_2217137
marine_Kogbre_RJWL010064944.1_0.0_1_705_3664_2969
marine_Phycat_NC_041216.1_7e-84_426_705_4413018_4413295
marine_Phycat_NC_041216.1_0.0_1_693_4411890_4412551
PVJP02910611.1_7982373-7983073_Hippopotamus_amphibius_isolate_BS01_ScHSfme_925070HRSCAF_982870_whole_genome_shotgun_sequence
land1_Catwag_PVHT010018437.1_3e-177_1_705_9868_9175
land1_Susscr_NC_010450.4_0.0_1_705_115107687_115108379
land1_Susscr_NC_010451.4_1e-146_18_506_42143428_42142947
land1_Susscr_NC_010461.5_2e-113_1_510_59121308_59121797
land1_Catwag_PVHT010002757.1_2e-138_1_705_117701_117029
land1_Susscr_NC_010452.4_8e-163_1_705_2788861_2789562
PVJP02910387.1_3092364-3092495_Hippopotamus_amphibius_isolate_BS01_ScHSfme_924858HRSCAF_982658_whole_genome_shotgun_sequence
99
100
57
97
67
95
99
91
100
73
76
63
78
63
40
94
100
66
96
68
90
74
81
63
99
81
94
93
63
0.02

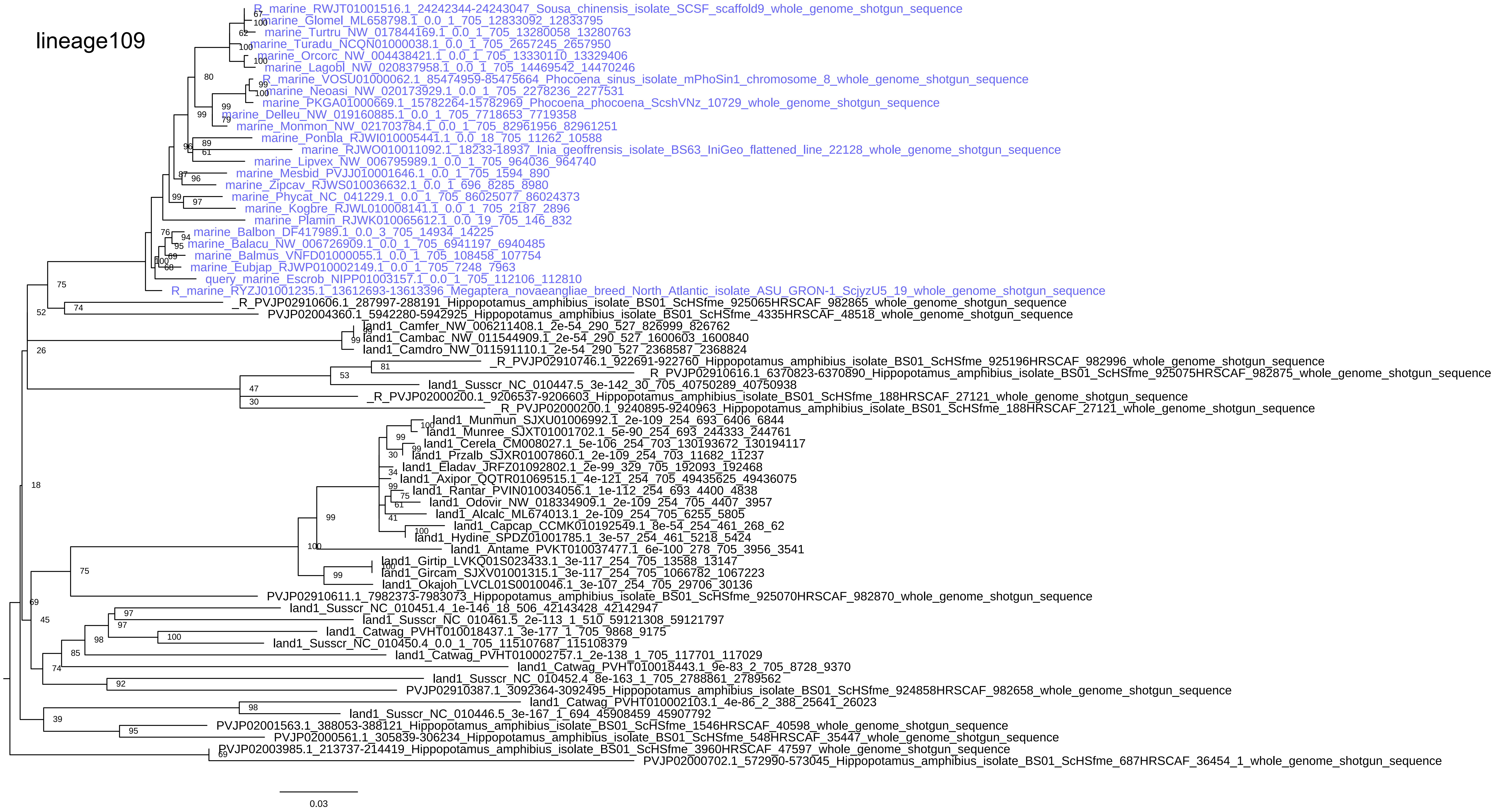
lineage109
R_marine_RWJT01001516.1_24242344-24243047_Sousa_chinensis_isolate_SCSF_scaffold9_whole_genome_shotgun_sequence
marine_Glomel_ML658798.1_0.0_1_705_12833092_12833795
marine_Turtru_NW_017844169.1_0.0_1_705_13280058_13280763
marine_Turadu_NCQN01000038.1_0.0_1_705_2657245_2657950
marine_Orcorc_NW_004438421.1_0.0_1_705_13330110_13329406
marine_Lagobl_NW_020837958.1_0.0_1_705_14469542_14470246
R_marine_VOSU01000062.1_85474959-85475664_Phocoena_sinus_isolate_mPhoSin1_chromosome_8_whole_genome_shotgun_sequence
marine_Neoasi_NW_020173929.1_0.0_1_705_2278236_2277531
marine_PKGA01000669.1_15782264-15782969_Phocoena_phocoena_ScshVNz_10729_whole_genome_shotgun_sequence
marine_Delleu_NW_019160885.1_0.0_1_705_7718653_7719358
marine_Monmon_NW_021703784.1_0.0_1_705_82961956_82961251
marine_Ponbla_RJWI010005441.1_0.0_18_705_11262_10588
marine_RJWO010011092.1_18233-18937_Inia_geoffrensis_isolate_BS63_IniGeo_flattened_line_22128_whole_genome_shotgun_sequence
marine_Lipvex_NW_006795989.1_0.0_1_705_964036_964740
marine_Mesbid_PVJJ010001646.1_0.0_1_705_1594_890
marine_Zipcav_RJWS010036632.1_0.0_1_696_8285_8980
marine_Phycat_NC_041229.1_0.0_1_705_86025077_86024373
marine_Kogbre_RJWL010008141.1_0.0_1_705_2187_2896
marine_Plamin_RJWK010065612.1_0.0_19_705_146_832
marine_Balbon_DF417989.1_0.0_3_705_14934_14225
marine_Balacu_NW_006726909.1_0.0_1_705_6941197_6940485
marine_Balmus_VNFD01000055.1_0.0_1_705_108458_107754
marine_Eubjap_RJWP010002149.1_0.0_1_705_7248_7963
query_marine_Escrob_NIPP01003157.1_0.0_1_705_112106_112810
R_marine_RYZJ01001235.1_13612693-13613396_Megaptera_novaeangliae_breed_North_Atlantic_isolate_ASU_GRON-1_ScjyzU5_19_whole_genome_shotgun_sequence
_R_PVJP02910606.1_287997-288191_Hippopotamus_amphibius_isolate_BS01_ScHSfme_925065HRSCAF_982865_whole_genome_shotgun_sequence
PVJP02004360.1_5942280-5942925_Hippopotamus_amphibius_isolate_BS01_ScHSfme_4335HRSCAF_48518_whole_genome_shotgun_sequence
land1_Camfer_NW_006211408.1_2e-54_290_527_826999_826762
land1_Cambac_NW_011544909.1_2e-54_290_527_1600603_1600840
land1_Camdro_NW_011591110.1_2e-54_290_527_2368587_2368824
_R_PVJP02910746.1_922691-922760_Hippopotamus_amphibius_isolate_BS01_ScHSfme_925196HRSCAF_982996_whole_genome_shotgun_sequence
_R_PVJP02910616.1_6370823-6370890_Hippopotamus_amphibius_isolate_BS01_ScHSfme_925075HRSCAF_982875_whole_genome_shotgun_sequence
land1_Susscr_NC_010447.5_3e-142_30_705_40750289_40750938
_R_PVJP02000200.1_9206537-9206603_Hippopotamus_amphibius_isolate_BS01_ScHSfme_188HRSCAF_27121_whole_genome_shotgun_sequence
_R_PVJP02000200.1_9240895-9240963_Hippopotamus_amphibius_isolate_BS01_ScHSfme_188HRSCAF_27121_whole_genome_shotgun_sequence
land1_Munmun_SJXU01006992.1_2e-109_254_693_6406_6844
land1_Munree_SJXT01001702.1_5e-90_254_693_244333_244761
land1_Cerela_CM008027.1_5e-106_254_703_130193672_130194117
land1_Przalb_SJXR01007860.1_2e-109_254_703_11682_11237
land1_Eladav_JRFZ01092802.1_2e-99_329_705_192093_192468
land1_Axipor_QQTR01069515.1_4e-121_254_705_49435625_49436075
land1_Rantar_PVIN010034056.1_1e-112_254_693_4400_4838
land1_Odovir_NW_018334909.1_2e-109_254_705_4407_3957
land1_Alcalc_ML674013.1_2e-109_254_705_6255_5805
land1_Capcap_CCMK010192549.1_8e-54_254_461_268_62
land1_Hydine_SPDZ01001785.1_3e-57_254_461_5218_5424
land1_Antame_PVKT010037477.1_6e-100_278_705_3956_3541
land1_Girtip_LVKQ01S023433.1_3e-117_254_705_13588_13147
land1_Gircam_SJXV01001315.1_3e-117_254_705_1066782_1067223
land1_Okajoh_LVCL01S0010046.1_3e-107_254_705_29706_30136
PVJP02910611.1_7982373-7983073_Hippopotamus_amphibius_isolate_BS01_ScHSfme_925070HRSCAF_982870_whole_genome_shotgun_sequence
land1_Susscr_NC_010451.4_1e-146_18_506_42143428_42142947
land1_Susscr_NC_010461.5_2e-113_1_510_59121308_59121797
land1_Catwag_PVHT010018437.1_3e-177_1_705_9868_9175
land1_Susscr_NC_010450.4_0.0_1_705_115107687_115108379
land1_Catwag_PVHT010002757.1_2e-138_1_705_117701_117029
land1_Catwag_PVHT010018443.1_9e-83_2_705_8728_9370
land1_Susscr_NC_010452.4_8e-163_1_705_2788861_2789562
PVJP02910387.1_3092364-3092495_Hippopotamus_amphibius_isolate_BS01_ScHSfme_924858HRSCAF_982658_whole_genome_shotgun_sequence
land1_Catwag_PVHT010002103.1_4e-86_2_388_25641_26023
land1_Susscr_NC_010446.5_3e-167_1_694_45908459_45907792
PVJP02001563.1_388053-388121_Hippopotamus_amphibius_isolate_BS01_ScHSfme_1546HRSCAF_40598_whole_genome_shotgun_sequence
PVJP02000561.1_305839-306234_Hippopotamus_amphibius_isolate_BS01_ScHSfme_548HRSCAF_35447_whole_genome_shotgun_sequence
PVJP02003985.1_213737-214419_Hippopotamus_amphibius_isolate_BS01_ScHSfme_3960HRSCAF_47597_whole_genome_shotgun_sequence
PVJP02000702.1_572990-573045_Hippopotamus_amphibius_isolate_BS01_ScHSfme_687HRSCAF_36454_1_whole_genome_shotgun_sequence
0.03

lineage110

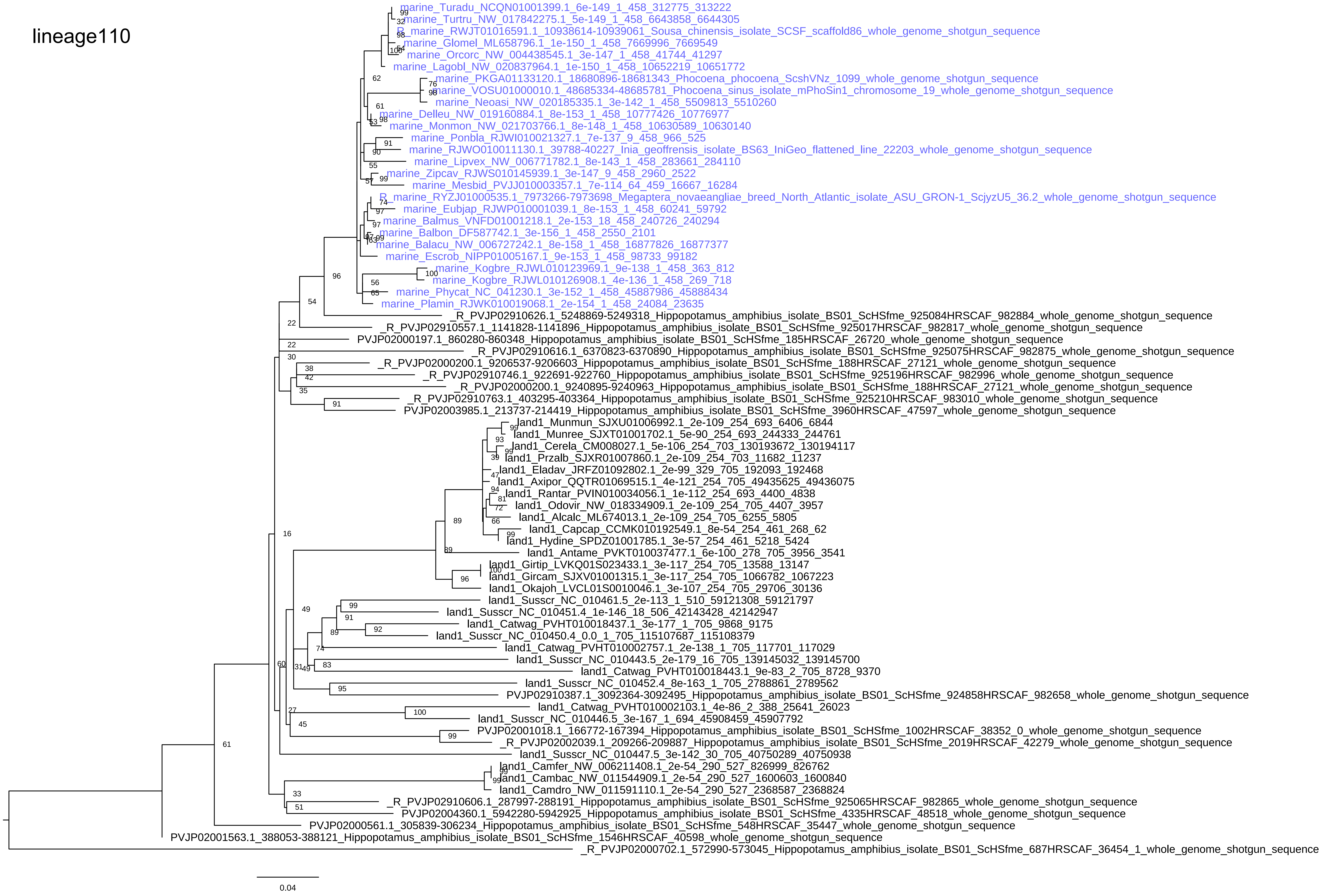
marine_Turadu_NCQN01001399.1_6e-149_1_458_312775_313222
marine_Turtru_NW_017842275.1_5e-149_1_458_6643858_6644305
R_marine_RWJT01016591.1_10938614-10939061_Sousa_chinensis_isolate_SCSF_scaffold86_whole_genome_shotgun_sequence
marine_Glomel_ML658796.1_1e-150_1_458_7669996_7669549
marine_Orcorc_NW_004438545.1_3e-147_1_458_41744_41297
marine_Lagobl_NW_020837964.1_1e-150_1_458_10652219_10651772
marine_PKGA01133120.1_18680896-18681343_Phocoena_phocoena_ScshVNz_1099_whole_genome_shotgun_sequence
marine_VOSU01000010.1_48685334-48685781_Phocoena_sinus_isolate_mPhoSin1_chromosome_19_whole_genome_shotgun_sequence
marine_Neoasi_NW_020185335.1_3e-142_1_458_5509813_5510260
marine_Delleu_NW_019160884.1_8e-153_1_458_10777426_10776977
marine_Monmon_NW_021703766.1_8e-148_1_458_10630589_10630140
marine_Ponbla_RJWI010021327.1_7e-137_9_458_966_525
marine_RJWO010011130.1_39788-40227_Inia_geoffrensis_isolate_BS63_IniGeo_flattened_line_22203_whole_genome_shotgun_sequence
marine_Lipvex_NW_006771782.1_8e-143_1_458_283661_284110
marine_Zipcav_RJWS010145939.1_3e-147_9_458_2960_2522
marine_Mesbid_PVJJ010003357.1_7e-114_64_459_16667_16284
R_marine_RYZJ01000535.1_7973266-7973698_Megaptera_novaeangliae_breed_North_Atlantic_isolate_ASU_GRON-1_ScjyzU5_36.2_whole_genome_shotgun_sequence
marine_Eubjap_RJWP010001039.1_8e-153_1_458_60241_59792
marine_Balmus_VNFD01001218.1_2e-153_18_458_240726_240294
marine_Balbon_DF587742.1_3e-156_1_458_2550_2101
marine_Balacu_NW_006727242.1_8e-158_1_458_16877826_16877377
marine_Escrob_NIPP01005167.1_9e-153_1_458_98733_99182
marine_Kogbre_RJWL010123969.1_9e-138_1_458_363_812
marine_Kogbre_RJWL010126908.1_4e-136_1_458_269_718
marine_Phycat_NC_041230.1_3e-152_1_458_45887986_45888434
marine_Plamin_RJWK010019068.1_2e-154_1_458_24084_23635
_R_PVJP02910626.1_5248869-5249318_Hippopotamus_amphibius_isolate_BS01_ScHSfme_925084HRSCAF_982884_whole_genome_shotgun_sequence
_R_PVJP02910557.1_1141828-1141896_Hippopotamus_amphibius_isolate_BS01_ScHSfme_925017HRSCAF_982817_whole_genome_shotgun_sequence
PVJP02000197.1_860280-860348_Hippopotamus_amphibius_isolate_BS01_ScHSfme_185HRSCAF_26720_whole_genome_shotgun_sequence
_R_PVJP02910616.1_6370823-6370890_Hippopotamus_amphibius_isolate_BS01_ScHSfme_925075HRSCAF_982875_whole_genome_shotgun_sequence
_R_PVJP02000200.1_9206537-9206603_Hippopotamus_amphibius_isolate_BS01_ScHSfme_188HRSCAF_27121_whole_genome_shotgun_sequence
_R_PVJP02910746.1_922691-922760_Hippopotamus_amphibius_isolate_BS01_ScHSfme_925196HRSCAF_982996_whole_genome_shotgun_sequence
_R_PVJP02000200.1_9240895-9240963_Hippopotamus_amphibius_isolate_BS01_ScHSfme_188HRSCAF_27121_whole_genome_shotgun_sequence
_R_PVJP02910763.1_403295-403364_Hippopotamus_amphibius_isolate_BS01_ScHSfme_925210HRSCAF_983010_whole_genome_shotgun_sequence
PVJP02003985.1_213737-214419_Hippopotamus_amphibius_isolate_BS01_ScHSfme_3960HRSCAF_47597_whole_genome_shotgun_sequence
land1_Munmun_SJXU01006992.1_2e-109_254_693_6406_6844
land1_Munree_SJXT01001702.1_5e-90_254_693_244333_244761
land1_Cerela_CM008027.1_5e-106_254_703_130193672_130194117
land1_Przalb_SJXR01007860.1_2e-109_254_703_11682_11237
land1_Eladav_JRFZ01092802.1_2e-99_329_705_192093_192468
land1_Axipor_QQTR01069515.1_4e-121_254_705_49435625_49436075
land1_Rantar_PVIN010034056.1_1e-112_254_693_4400_4838
land1_Odovir_NW_018334909.1_2e-109_254_705_4407_3957
land1_Alcalc_ML674013.1_2e-109_254_705_6255_5805
land1_Capcap_CCMK010192549.1_8e-54_254_461_268_62
land1_Hydine_SPDZ01001785.1_3e-57_254_461_5218_5424
land1_Antame_PVKT010037477.1_6e-100_278_705_3956_3541
land1_Girtip_LVKQ01S023433.1_3e-117_254_705_13588_13147
land1_Gircam_SJXV01001315.1_3e-117_254_705_1066782_1067223
land1_Okajoh_LVCL01S0010046.1_3e-107_254_705_29706_30136
land1_Susscr_NC_010461.5_2e-113_1_510_59121308_59121797
land1_Susscr_NC_010451.4_1e-146_18_506_42143428_42142947
land1_Catwag_PVHT010018437.1_3e-177_1_705_9868_9175
land1_Susscr_NC_010450.4_0.0_1_705_115107687_115108379
land1_Catwag_PVHT010002757.1_2e-138_1_705_117701_117029
land1_Susscr_NC_010443.5_2e-179_16_705_139145032_139145700
land1_Catwag_PVHT010018443.1_9e-83_2_705_8728_9370
land1_Susscr_NC_010452.4_8e-163_1_705_2788861_2789562
PVJP02910387.1_3092364-3092495_Hippopotamus_amphibius_isolate_BS01_ScHSfme_924858HRSCAF_982658_whole_genome_shotgun_sequence
land1_Catwag_PVHT010002103.1_4e-86_2_388_25641_26023
land1_Susscr_NC_010446.5_3e-167_1_694_45908459_45907792
PVJP02001018.1_166772-167394_Hippopotamus_amphibius_isolate_BS01_ScHSfme_1002HRSCAF_38352_0_whole_genome_shotgun_sequence
_R_PVJP02002039.1_209266-209887_Hippopotamus_amphibius_isolate_BS01_ScHSfme_2019HRSCAF_42279_whole_genome_shotgun_sequence
land1_Susscr_NC_010447.5_3e-142_30_705_40750289_40750938
land1_Camfer_NW_006211408.1_2e-54_290_527_826999_826762
land1_Cambac_NW_011544909.1_2e-54_290_527_1600603_1600840
land1_Camdro_NW_011591110.1_2e-54_290_527_2368587_2368824
_R_PVJP02910606.1_287997-288191_Hippopotamus_amphibius_isolate_BS01_ScHSfme_925065HRSCAF_982865_whole_genome_shotgun_sequence
PVJP02004360.1_5942280-5942925_Hippopotamus_amphibius_isolate_BS01_ScHSfme_4335HRSCAF_48518_whole_genome_shotgun_sequence
PVJP02000561.1_305839-306234_Hippopotamus_amphibius_isolate_BS01_ScHSfme_548HRSCAF_35447_whole_genome_shotgun_sequence
PVJP02001563.1_388053-388121_Hippopotamus_amphibius_isolate_BS01_ScHSfme_1546HRSCAF_40598_whole_genome_shotgun_sequence
_R_PVJP02000702.1_572990-573045_Hippopotamus_amphibius_isolate_BS01_ScHSfme_687HRSCAF_36454_1_whole_genome_shotgun_sequence
0.04

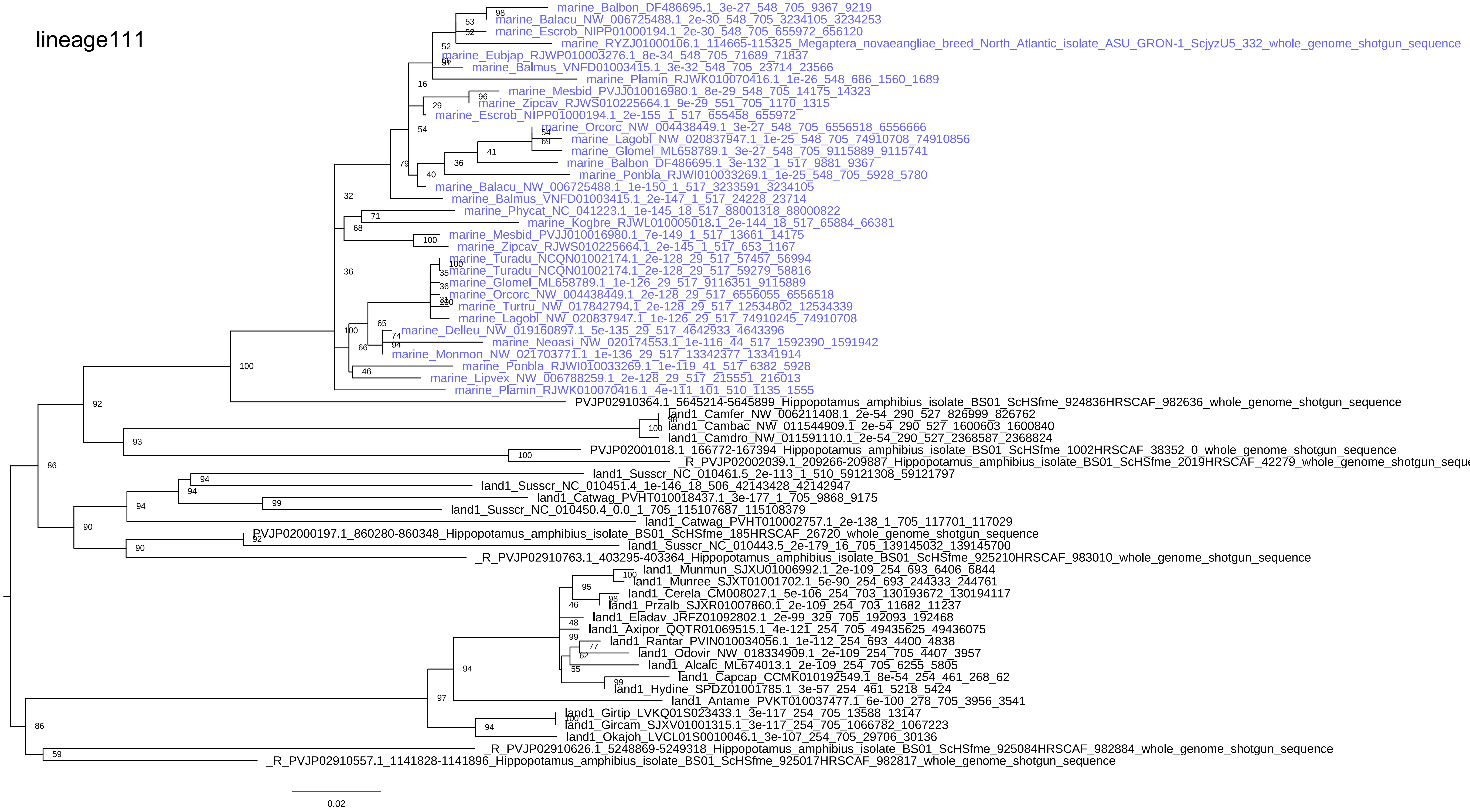
lineage111
marine_Balbon_DF486695.1_3e-27_548_705_9367_9219
marine_Balacu_NW_006725488.1_2e-30_548_705_3234105_3234253
marine_Escrob_NIPP01000194.1_2e-30_548_705_655972_656120
marine_RYZJ01000106.1_114665-115325_Megaptera_novaeangliae_breed_North_Atlantic_isolate_ASU_GRON-1_ScjyzU5_332_whole_genome_shotgun_sequence
marine_Eubjap_RJWP010003276.1_8e-34_548_705_71689_71837
marine_Balmus_VNFD01003415.1_3e-32_548_705_23714_23566
marine_Plamin_RJWK010070416.1_1e-26_548_686_1560_1689
marine_Mesbid_PVJJ010016980.1_8e-29_548_705_14175_14323
marine_Zipcav_RJWS010225664.1_9e-29_551_705_1170_1315
marine_Escrob_NIPP01000194.1_2e-155_1_517_655458_655972
marine_Orcorc_NW_004438449.1_3e-27_548_705_6556518_6556666
marine_Lagobl_NW_020837947.1_1e-25_548_705_74910708_74910856
marine_Glomel_ML658789.1_3e-27_548_705_9115889_9115741
marine_Balbon_DF486695.1_3e-132_1_517_9881_9367
marine_Ponbla_RJWI010033269.1_1e-25_548_705_5928_5780
marine_Balacu_NW_006725488.1_1e-150_1_517_3233591_3234105
marine_Balmus_VNFD01003415.1_2e-147_1_517_24228_23714
marine_Phycat_NC_041223.1_1e-145_18_517_88001318_88000822
marine_Kogbre_RJWL010005018.1_2e-144_18_517_65884_66381
marine_Mesbid_PVJJ010016980.1_7e-149_1_517_13661_14175
marine_Zipcav_RJWS010225664.1_2e-145_1_517_653_1167
marine_Turadu_NCQN01002174.1_2e-128_29_517_57457_56994
marine_Turadu_NCQN01002174.1_2e-128_29_517_59279_58816
marine_Glomel_ML658789.1_1e-126_29_517_9116351_9115889
marine_Orcorc_NW_004438449.1_2e-128_29_517_6556055_6556518
marine_Turtru_NW_017842794.1_2e-128_29_517_12534802_12534339
marine_Lagobl_NW_020837947.1_1e-126_29_517_74910245_74910708
marine_Delleu_NW_019160897.1_5e-135_29_517_4642933_4643396
marine_Neoasi_NW_020174553.1_1e-116_44_517_1592390_1591942
marine_Monmon_NW_021703771.1_1e-136_29_517_13342377_13341914
marine_Ponbla_RJWI010033269.1_1e-119_41_517_6382_5928
marine_Lipvex_NW_006788259.1_2e-128_29_517_215551_216013
marine_Plamin_RJWK010070416.1_4e-111_101_510_1135_1555
PVJP02910364.1_5645214-5645899_Hippopotamus_amphibius_isolate_BS01_ScHSfme_924836HRSCAF_982636_whole_genome_shotgun_sequence
land1_Camfer_NW_006211408.1_2e-54_290_527_826999_826762
land1_Cambac_NW_011544909.1_2e-54_290_527_1600603_1600840
land1_Camdro_NW_011591110.1_2e-54_290_527_2368587_2368824
PVJP02001018.1_166772-167394_Hippopotamus_amphibius_isolate_BS01_ScHSfme_1002HRSCAF_38352_0_whole_genome_shotgun_sequence
_R_PVJP02002039.1_209266-209887_Hippopotamus_amphibius_isolate_BS01_ScHSfme_2019HRSCAF_42279_whole_genome_shotgun_sequence
land1_Susscr_NC_010461.5_2e-113_1_510_59121308_59121797
land1_Susscr_NC_010451.4_1e-146_18_506_42143428_42142947
land1_Catwag_PVHT010018437.1_3e-177_1_705_9868_9175
land1_Susscr_NC_010450.4_0.0_1_705_115107687_115108379
land1_Catwag_PVHT010002757.1_2e-138_1_705_117701_117029
PVJP02000197.1_860280-860348_Hippopotamus_amphibius_isolate_BS01_ScHSfme_185HRSCAF_26720_whole_genome_shotgun_sequence
land1_Susscr_NC_010443.5_2e-179_16_705_139145032_139145700
_R_PVJP02910763.1_403295-403364_Hippopotamus_amphibius_isolate_BS01_ScHSfme_925210HRSCAF_983010_whole_genome_shotgun_sequence
land1_Munmun_SJXU01006992.1_2e-109_254_693_6406_6844
land1_Munree_SJXT01001702.1_5e-90_254_693_244333_244761
land1_Cerela_CM008027.1_5e-106_254_703_130193672_130194117
land1_Przalb_SJXR01007860.1_2e-109_254_703_11682_11237
land1_Eladav_JRFZ01092802.1_2e-99_329_705_192093_192468
land1_Axipor_QQTR01069515.1_4e-121_254_705_49435625_49436075
land1_Rantar_PVIN010034056.1_1e-112_254_693_4400_4838
land1_Odovir_NW_018334909.1_2e-109_254_705_4407_3957
land1_Alcalc_ML674013.1_2e-109_254_705_6255_5805
land1_Capcap_CCMK010192549.1_8e-54_254_461_268_62
land1_Hydine_SPDZ01001785.1_3e-57_254_461_5218_5424
land1_Antame_PVKT010037477.1_6e-100_278_705_3956_3541
land1_Girtip_LVKQ01S023433.1_3e-117_254_705_13588_13147
land1_Gircam_SJXV01001315.1_3e-117_254_705_1066782_1067223
land1_Okajoh_LVCL01S0010046.1_3e-107_254_705_29706_30136
_R_PVJP02910626.1_5248869-5249318_Hippopotamus_amphibius_isolate_BS01_ScHSfme_925084HRSCAF_982884_whole_genome_shotgun_sequence
_R_PVJP02910557.1_1141828-1141896_Hippopotamus_amphibius_isolate_BS01_ScHSfme_925017HRSCAF_982817_whole_genome_shotgun_sequence
0.02

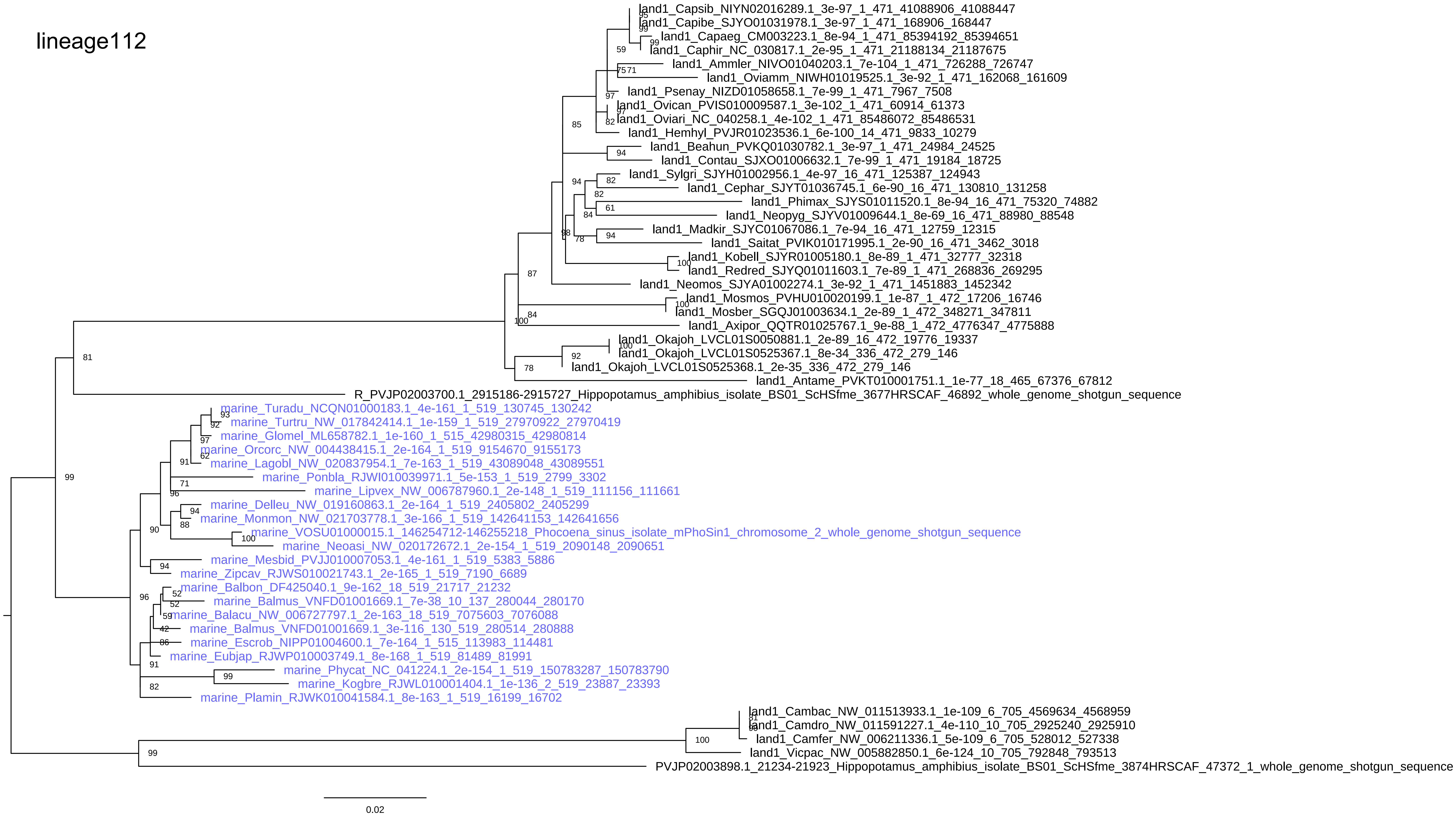
lineage112
land1_Capsib_NIYN02016289.1_3e-97_1_471_41088906_41088447
land1_Capibe_SJYO01031978.1_3e-97_1_471_168906_168447
land1_Capaeg_CM003223.1_8e-94_1_471_85394192_85394651
land1_Caphir_NC_030817.1_2e-95_1_471_21188134_21187675
land1_Ammler_NIVO01040203.1_7e-104_1_471_726288_726747
land1_Oviamm_NIWH01019525.1_3e-92_1_471_162068_161609
land1_Psenay_NIZD01058658.1_7e-99_1_471_7967_7508
land1_Ovican_PVIS010009587.1_3e-102_1_471_60914_61373
land1_Oviari_NC_040258.1_4e-102_1_471_85486072_85486531
land1_Hemhyl_PVJR01023536.1_6e-100_14_471_9833_10279
land1_Beahun_PVKQ01030782.1_3e-97_1_471_24984_24525
land1_Contau_SJXO01006632.1_7e-99_1_471_19184_18725
land1_Sylgri_SJYH01002956.1_4e-97_16_471_125387_124943
land1_Cephar_SJYT01036745.1_6e-90_16_471_130810_131258
land1_Phimax_SJYS01011520.1_8e-94_16_471_75320_74882
land1_Neopyg_SJYV01009644.1_8e-69_16_471_88980_88548
land1_Madkir_SJYC01067086.1_7e-94_16_471_12759_12315
land1_Saitat_PVIK010171995.1_2e-90_16_471_3462_3018
land1_Kobell_SJYR01005180.1_8e-89_1_471_32777_32318
land1_Redred_SJYQ01011603.1_7e-89_1_471_268836_269295
land1_Neomos_SJYA01002274.1_3e-92_1_471_1451883_1452342
land1_Mosmos_PVHU010020199.1_1e-87_1_472_17206_16746
land1_Mosber_SGQJ01003634.1_2e-89_1_472_348271_347811
land1_Axipor_QQTR01025767.1_9e-88_1_472_4776347_4775888
land1_Okajoh_LVCL01S0050881.1_2e-89_16_472_19776_19337
land1_Okajoh_LVCL01S0525367.1_8e-34_336_472_279_146
land1_Okajoh_LVCL01S0525368.1_2e-35_336_472_279_146
land1_Antame_PVKT010001751.1_1e-77_18_465_67376_67812
R_PVJP02003700.1_2915186-2915727_Hippopotamus_amphibius_isolate_BS01_ScHSfme_3677HRSCAF_46892_whole_genome_shotgun_sequence
marine_Turadu_NCQN01000183.1_4e-161_1_519_130745_130242
marine_Turtru_NW_017842414.1_1e-159_1_519_27970922_27970419
marine_Glomel_ML658782.1_1e-160_1_515_42980315_42980814
marine_Orcorc_NW_004438415.1_2e-164_1_519_9154670_9155173
marine_Lagobl_NW_020837954.1_7e-163_1_519_43089048_43089551
marine_Ponbla_RJWI010039971.1_5e-153_1_519_2799_3302
marine_Lipvex_NW_006787960.1_2e-148_1_519_111156_111661
marine_Delleu_NW_019160863.1_2e-164_1_519_2405802_2405299
marine_Monmon_NW_021703778.1_3e-166_1_519_142641153_142641656
marine_VOSU01000015.1_146254712-146255218_Phocoena_sinus_isolate_mPhoSin1_chromosome_2_whole_genome_shotgun_sequence
marine_Neoasi_NW_020172672.1_2e-154_1_519_2090148_2090651
marine_Mesbid_PVJJ010007053.1_4e-161_1_519_5383_5886
marine_Zipcav_RJWS010021743.1_2e-165_1_519_7190_6689
marine_Balbon_DF425040.1_9e-162_18_519_21717_21232
marine_Balmus_VNFD01001669.1_7e-38_10_137_280044_280170
marine_Balacu_NW_006727797.1_2e-163_18_519_7075603_7076088
marine_Balmus_VNFD01001669.1_3e-116_130_519_280514_280888
marine_Escrob_NIPP01004600.1_7e-164_1_515_113983_114481
marine_Eubjap_RJWP010003749.1_8e-168_1_519_81489_81991
marine_Phycat_NC_041224.1_2e-154_1_519_150783287_150783790
marine_Kogbre_RJWL010001404.1_1e-136_2_519_23887_23393
marine_Plamin_RJWK010041584.1_8e-163_1_519_16199_16702
land1_Cambac_NW_011513933.1_1e-109_6_705_4569634_4568959
land1_Camdro_NW_011591227.1_4e-110_10_705_2925240_2925910
land1_Camfer_NW_006211336.1_5e-109_6_705_528012_527338
land1_Vicpac_NW_005882850.1_6e-124_10_705_792848_793513
PVJP02003898.1_21234-21923_Hippopotamus_amphibius_isolate_BS01_ScHSfme_3874HRSCAF_47372_1_whole_genome_shotgun_sequence
0.02

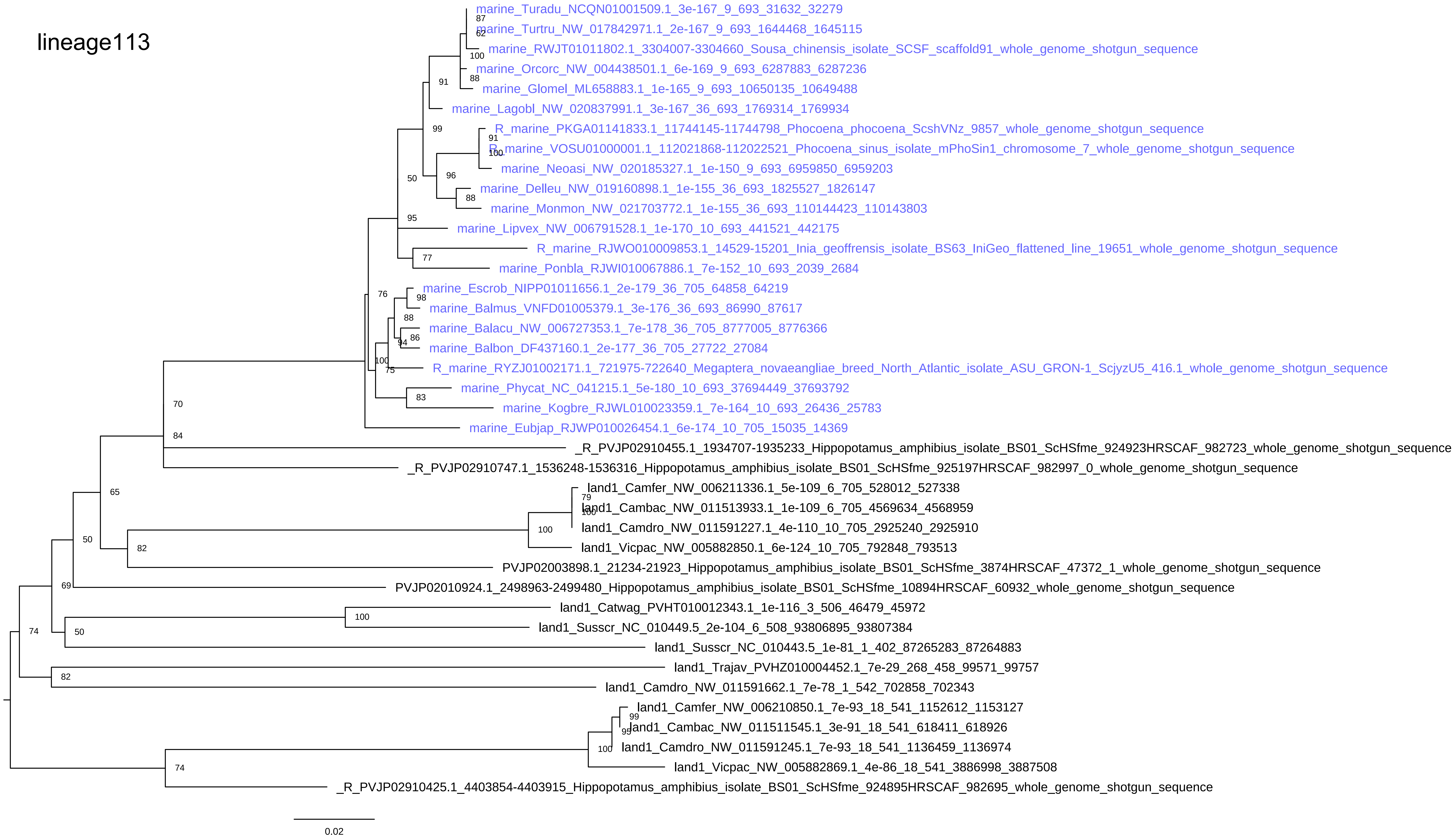
lineage113
marine_Turadu_NCQN01001509.1_3e-167_9_693_31632_32279
marine_Turtru_NW_017842971.1_2e-167_9_693_1644468_1645115
marine_RWJT01011802.1_3304007-3304660_Sousa_chinensis_isolate_SCSF_scaffold91_whole_genome_shotgun_sequence
marine_Orcorc_NW_004438501.1_6e-169_9_693_6287883_6287236
marine_Glomel_ML658883.1_1e-165_9_693_10650135_10649488
marine_Lagobl_NW_020837991.1_3e-167_36_693_1769314_1769934
R_marine_PKGA01141833.1_11744145-11744798_Phocoena_phocoena_ScshVNz_9857_whole_genome_shotgun_sequence
R_marine_VOSU01000001.1_112021868-112022521_Phocoena_sinus_isolate_mPhoSin1_chromosome_7_whole_genome_shotgun_sequence
marine_Neoasi_NW_020185327.1_1e-150_9_693_6959850_6959203
marine_Delleu_NW_019160898.1_1e-155_36_693_1825527_1826147
marine_Monmon_NW_021703772.1_1e-155_36_693_110144423_110143803
marine_Lipvex_NW_006791528.1_1e-170_10_693_441521_442175
R_marine_RJWO010009853.1_14529-15201_Inia_geoffrensis_isolate_BS63_IniGeo_flattened_line_19651_whole_genome_shotgun_sequence
marine_Ponbla_RJWI010067886.1_7e-152_10_693_2039_2684
marine_Escrob_NIPP01011656.1_2e-179_36_705_64858_64219
marine_Balmus_VNFD01005379.1_3e-176_36_693_86990_87617
marine_Balacu_NW_006727353.1_7e-178_36_705_8777005_8776366
marine_Balbon_DF437160.1_2e-177_36_705_27722_27084
R_marine_RYZJ01002171.1_721975-722640_Megaptera_novaeangliae_breed_North_Atlantic_isolate_ASU_GRON-1_ScjyzU5_416.1_whole_genome_shotgun_sequence
marine_Phycat_NC_041215.1_5e-180_10_693_37694449_37693792
marine_Kogbre_RJWL010023359.1_7e-164_10_693_26436_25783
marine_Eubjap_RJWP010026454.1_6e-174_10_705_15035_14369
_R_PVJP02910455.1_1934707-1935233_Hippopotamus_amphibius_isolate_BS01_ScHSfme_924923HRSCAF_982723_whole_genome_shotgun_sequence
_R_PVJP02910747.1_1536248-1536316_Hippopotamus_amphibius_isolate_BS01_ScHSfme_925197HRSCAF_982997_0_whole_genome_shotgun_sequence
land1_Camfer_NW_006211336.1_5e-109_6_705_528012_527338
land1_Cambac_NW_011513933.1_1e-109_6_705_4569634_4568959
land1_Camdro_NW_011591227.1_4e-110_10_705_2925240_2925910
land1_Vicpac_NW_005882850.1_6e-124_10_705_792848_793513
PVJP02003898.1_21234-21923_Hippopotamus_amphibius_isolate_BS01_ScHSfme_3874HRSCAF_47372_1_whole_genome_shotgun_sequence
PVJP02010924.1_2498963-2499480_Hippopotamus_amphibius_isolate_BS01_ScHSfme_10894HRSCAF_60932_whole_genome_shotgun_sequence
land1_Catwag_PVHT010012343.1_1e-116_3_506_46479_45972
land1_Susscr_NC_010449.5_2e-104_6_508_93806895_93807384
land1_Susscr_NC_010443.5_1e-81_1_402_87265283_87264883
land1_Trajav_PVHZ010004452.1_7e-29_268_458_99571_99757
land1_Camdro_NW_011591662.1_7e-78_1_542_702858_702343
land1_Camfer_NW_006210850.1_7e-93_18_541_1152612_1153127
land1_Cambac_NW_011511545.1_3e-91_18_541_618411_618926
land1_Camdro_NW_011591245.1_7e-93_18_541_1136459_1136974
land1_Vicpac_NW_005882869.1_4e-86_18_541_3886998_3887508
_R_PVJP02910425.1_4403854-4403915_Hippopotamus_amphibius_isolate_BS01_ScHSfme_924895HRSCAF_982695_whole_genome_shotgun_sequence
0.02

lineage114

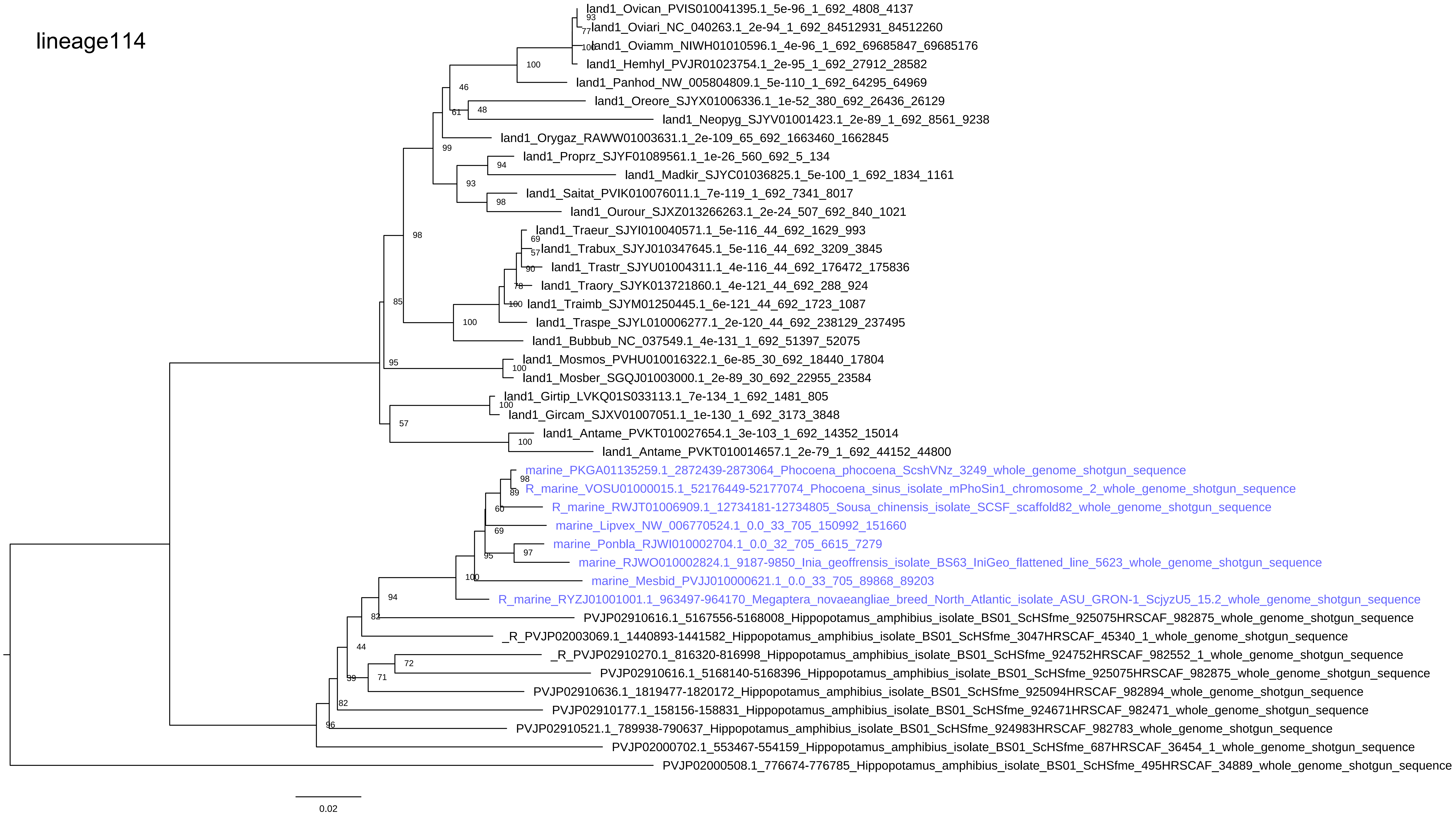

land1_Ovican_PVIS010041395.1_5e-96_1_692_4808_4137
land1_Oviari_NC_040263.1_2e-94_1_692_84512931_84512260
land1_Oviamm_NIWH01010596.1_4e-96_1_692_69685847_69685176
land1_Hemhyl_PVJR01023754.1_2e-95_1_692_27912_28582
land1_Panhod_NW_005804809.1_5e-110_1_692_64295_64969
land1_Oreore_SJYX01006336.1_1e-52_380_692_26436_26129
land1_Neopyg_SJYV01001423.1_2e-89_1_692_8561_9238
land1_Orygaz_RAWW01003631.1_2e-109_65_692_1663460_1662845
land1_Proprz_SJYF01089561.1_1e-26_560_692_5_134
land1_Madkir_SJYC01036825.1_5e-100_1_692_1834_1161
land1_Saitat_PVIK010076011.1_7e-119_1_692_7341_8017
land1_Ourour_SJXZ013266263.1_2e-24_507_692_840_1021
land1_Traeur_SJYI010040571.1_5e-116_44_692_1629_993
land1_Trabux_SJYJ010347645.1_5e-116_44_692_3209_3845
land1_Trastr_SJYU01004311.1_4e-116_44_692_176472_175836
land1_Traory_SJYK013721860.1_4e-121_44_692_288_924
land1_Traimb_SJYM01250445.1_6e-121_44_692_1723_1087
land1_Traspe_SJYL010006277.1_2e-120_44_692_238129_237495
land1_Bubbub_NC_037549.1_4e-131_1_692_51397_52075
land1_Mosmos_PVHU010016322.1_6e-85_30_692_18440_17804
land1_Mosber_SGQJ01003000.1_2e-89_30_692_22955_23584
land1_Girtip_LVKQ01S033113.1_7e-134_1_692_1481_805
land1_Gircam_SJXV01007051.1_1e-130_1_692_3173_3848
land1_Antame_PVKT010027654.1_3e-103_1_692_14352_15014
land1_Antame_PVKT010014657.1_2e-79_1_692_44152_44800
marine_PKGA01135259.1_2872439-2873064_Phocoena_phocoena_ScshVNz_3249_whole_genome_shotgun_sequence
R_marine_VOSU01000015.1_52176449-52177074_Phocoena_sinus_isolate_mPhoSin1_chromosome_2_whole_genome_shotgun_sequence
R_marine_RWJT01006909.1_12734181-12734805_Sousa_chinensis_isolate_SCSF_scaffold82_whole_genome_shotgun_sequence
marine_Lipvex_NW_006770524.1_0.0_33_705_150992_151660
marine_Ponbla_RJWI010002704.1_0.0_32_705_6615_7279
marine_RJWO010002824.1_9187-9850_Inia_geoffrensis_isolate_BS63_IniGeo_flattened_line_5623_whole_genome_shotgun_sequence
marine_Mesbid_PVJJ010000621.1_0.0_33_705_89868_89203
R_marine_RYZJ01001001.1_963497-964170_Megaptera_novaeangliae_breed_North_Atlantic_isolate_ASU_GRON-1_ScjyzU5_15.2_whole_genome_shotgun_sequence
PVJP02910616.1_5167556-5168008_Hippopotamus_amphibius_isolate_BS01_ScHSfme_925075HRSCAF_982875_whole_genome_shotgun_sequence
_R_PVJP02003069.1_1440893-1441582_Hippopotamus_amphibius_isolate_BS01_ScHSfme_3047HRSCAF_45340_1_whole_genome_shotgun_sequence
_R_PVJP02910270.1_816320-816998_Hippopotamus_amphibius_isolate_BS01_ScHSfme_924752HRSCAF_982552_1_whole_genome_shotgun_sequence
PVJP02910616.1_5168140-5168396_Hippopotamus_amphibius_isolate_BS01_ScHSfme_925075HRSCAF_982875_whole_genome_shotgun_sequence
PVJP02910636.1_1819477-1820172_Hippopotamus_amphibius_isolate_BS01_ScHSfme_925094HRSCAF_982894_whole_genome_shotgun_sequence
PVJP02910177.1_158156-158831_Hippopotamus_amphibius_isolate_BS01_ScHSfme_924671HRSCAF_982471_whole_genome_shotgun_sequence
PVJP02910521.1_789938-790637_Hippopotamus_amphibius_isolate_BS01_ScHSfme_924983HRSCAF_982783_whole_genome_shotgun_sequence
PVJP02000702.1_553467-554159_Hippopotamus_amphibius_isolate_BS01_ScHSfme_687HRSCAF_36454_1_whole_genome_shotgun_sequence
PVJP02000508.1_776674-776785_Hippopotamus_amphibius_isolate_BS01_ScHSfme_495HRSCAF_34889_whole_genome_shotgun_sequence
0.02

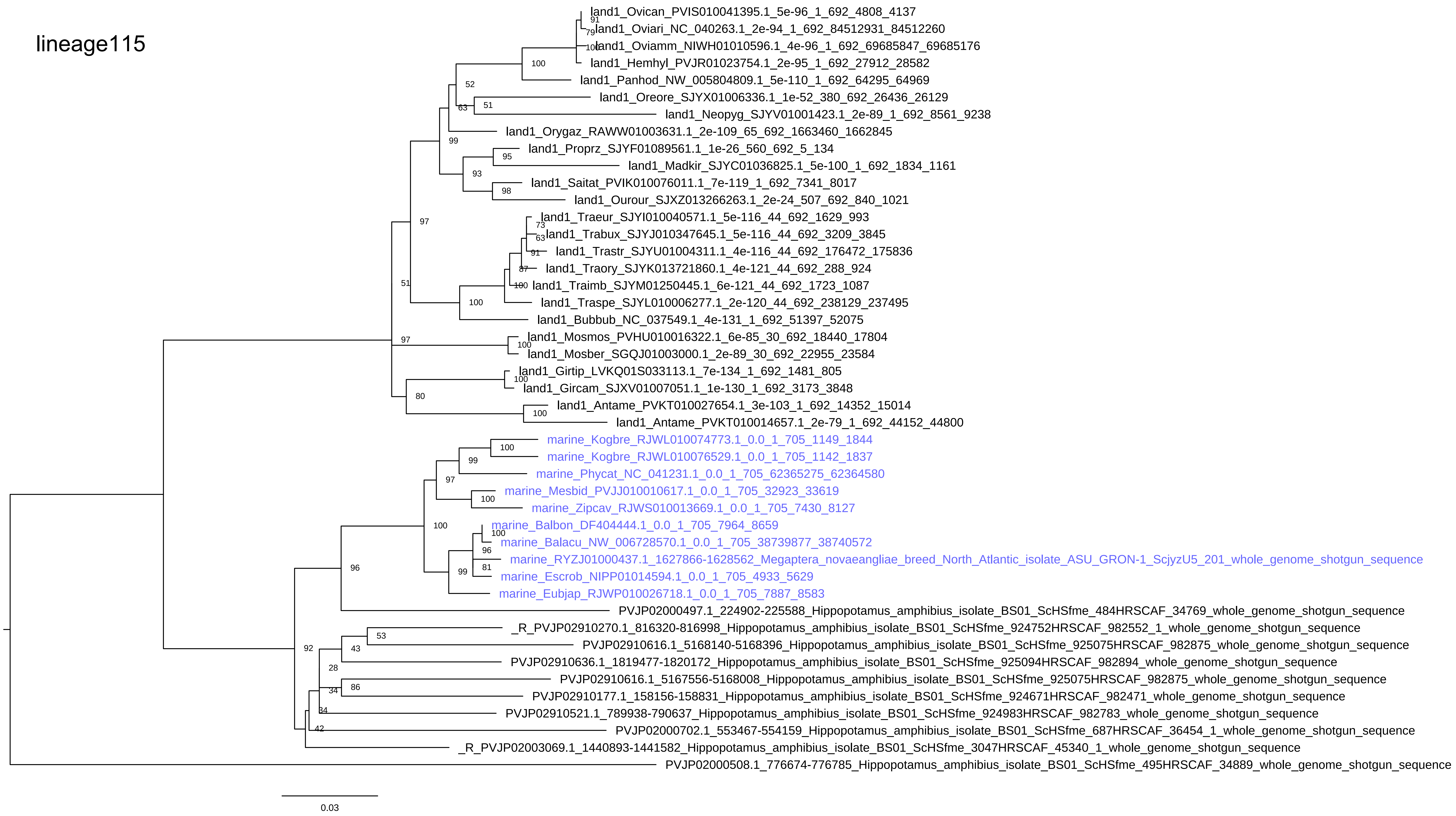
lineage115
land1_Ovican_PVIS010041395.1_5e-96_1_692_4808_4137
land1_Oviari_NC_040263.1_2e-94_1_692_84512931_84512260
land1_Oviamm_NIWH01010596.1_4e-96_1_692_69685847_69685176
land1_Hemhyl_PVJR01023754.1_2e-95_1_692_27912_28582
land1_Panhod_NW_005804809.1_5e-110_1_692_64295_64969
land1_Oreore_SJYX01006336.1_1e-52_380_692_26436_26129
land1_Neopyg_SJYV01001423.1_2e-89_1_692_8561_9238
land1_Orygaz_RAWW01003631.1_2e-109_65_692_1663460_1662845
land1_Proprz_SJYF01089561.1_1e-26_560_692_5_134
land1_Madkir_SJYC01036825.1_5e-100_1_692_1834_1161
land1_Saitat_PVIK010076011.1_7e-119_1_692_7341_8017
land1_Ourour_SJXZ013266263.1_2e-24_507_692_840_1021
land1_Traeur_SJYI010040571.1_5e-116_44_692_1629_993
land1_Trabux_SJYJ010347645.1_5e-116_44_692_3209_3845
land1_Trastr_SJYU01004311.1_4e-116_44_692_176472_175836
land1_Traory_SJYK013721860.1_4e-121_44_692_288_924
land1_Traimb_SJYM01250445.1_6e-121_44_692_1723_1087
land1_Traspe_SJYL010006277.1_2e-120_44_692_238129_237495
land1_Bubbub_NC_037549.1_4e-131_1_692_51397_52075
land1_Mosmos_PVHU010016322.1_6e-85_30_692_18440_17804
land1_Mosber_SGQJ01003000.1_2e-89_30_692_22955_23584
land1_Girtip_LVKQ01S033113.1_7e-134_1_692_1481_805
land1_Gircam_SJXV01007051.1_1e-130_1_692_3173_3848
land1_Antame_PVKT010027654.1_3e-103_1_692_14352_15014
land1_Antame_PVKT010014657.1_2e-79_1_692_44152_44800
marine_Kogbre_RJWL010074773.1_0.0_1_705_1149_1844
marine_Kogbre_RJWL010076529.1_0.0_1_705_1142_1837
marine_Phycat_NC_041231.1_0.0_1_705_62365275_62364580
marine_Mesbid_PVJJ010010617.1_0.0_1_705_32923_33619
marine_Zipcav_RJWS010013669.1_0.0_1_705_7430_8127
marine_Balbon_DF404444.1_0.0_1_705_7964_8659
marine_Balacu_NW_006728570.1_0.0_1_705_38739877_38740572
marine_RYZJ01000437.1_1627866-1628562_Megaptera_novaeangliae_breed_North_Atlantic_isolate_ASU_GRON-1_ScjyzU5_201_whole_genome_shotgun_sequence
marine_Escrob_NIPP01014594.1_0.0_1_705_4933_5629
marine_Eubjap_RJWP010026718.1_0.0_1_705_7887_8583
PVJP02000497.1_224902-225588_Hippopotamus_amphibius_isolate_BS01_ScHSfme_484HRSCAF_34769_whole_genome_shotgun_sequence
_R_PVJP02910270.1_816320-816998_Hippopotamus_amphibius_isolate_BS01_ScHSfme_924752HRSCAF_982552_1_whole_genome_shotgun_sequence
PVJP02910616.1_5168140-5168396_Hippopotamus_amphibius_isolate_BS01_ScHSfme_925075HRSCAF_982875_whole_genome_shotgun_sequence
PVJP02910636.1_1819477-1820172_Hippopotamus_amphibius_isolate_BS01_ScHSfme_925094HRSCAF_982894_whole_genome_shotgun_sequence
PVJP02910616.1_5167556-5168008_Hippopotamus_amphibius_isolate_BS01_ScHSfme_925075HRSCAF_982875_whole_genome_shotgun_sequence
PVJP02910177.1_158156-158831_Hippopotamus_amphibius_isolate_BS01_ScHSfme_924671HRSCAF_982471_whole_genome_shotgun_sequence
PVJP02910521.1_789938-790637_Hippopotamus_amphibius_isolate_BS01_ScHSfme_924983HRSCAF_982783_whole_genome_shotgun_sequence
PVJP02000702.1_553467-554159_Hippopotamus_amphibius_isolate_BS01_ScHSfme_687HRSCAF_36454_1_whole_genome_shotgun_sequence
_R_PVJP02003069.1_1440893-1441582_Hippopotamus_amphibius_isolate_BS01_ScHSfme_3047HRSCAF_45340_1_whole_genome_shotgun_sequence
PVJP02000508.1_776674-776785_Hippopotamus_amphibius_isolate_BS01_ScHSfme_495HRSCAF_34889_whole_genome_shotgun_sequence
0.03

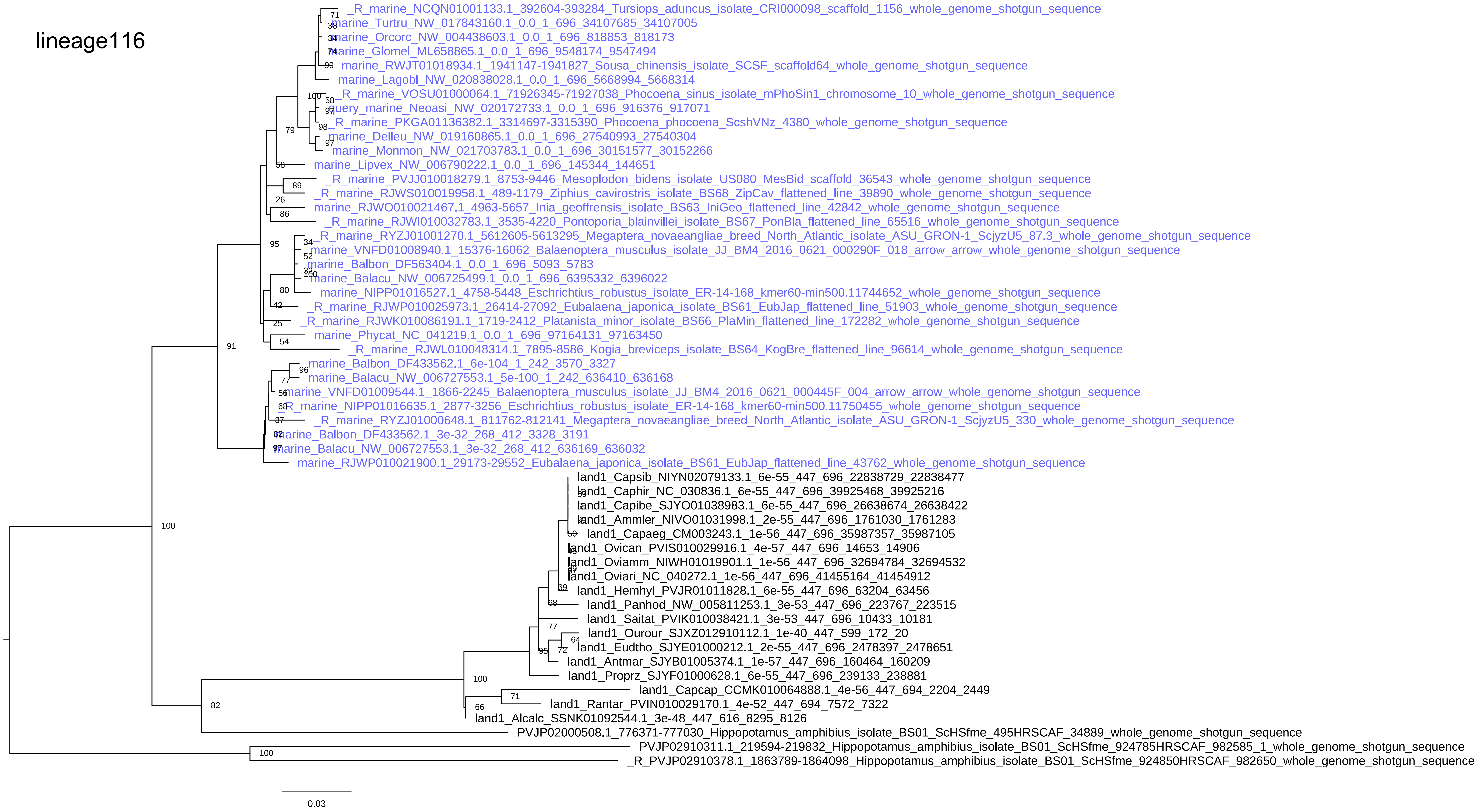
lineage116
_R_marine_NCQN01001133.1_392604-393284_Tursiops_aduncus_isolate_CRI000098_scaffold_1156_whole_genome_shotgun_sequence
marine_Turtru_NW_017843160.1_0.0_1_696_34107685_34107005
marine_Orcorc_NW_004438603.1_0.0_1_696_818853_818173
marine_Glomel_ML658865.1_0.0_1_696_9548174_9547494
marine_RWJT01018934.1_1941147-1941827_Sousa_chinensis_isolate_SCSF_scaffold64_whole_genome_shotgun_sequence
marine_Lagobl_NW_020838028.1_0.0_1_696_5668994_5668314
_R_marine_VOSU01000064.1_71926345-71927038_Phocoena_sinus_isolate_mPhoSin1_chromosome_10_whole_genome_shotgun_sequence
query_marine_Neoasi_NW_020172733.1_0.0_1_696_916376_917071
_R_marine_PKGA01136382.1_3314697-3315390_Phocoena_phocoena_ScshVNz_4380_whole_genome_shotgun_sequence
marine_Delleu_NW_019160865.1_0.0_1_696_27540993_27540304
marine_Monmon_NW_021703783.1_0.0_1_696_30151577_30152266
marine_Lipvex_NW_006790222.1_0.0_1_696_145344_144651
_R_marine_PVJJ010018279.1_8753-9446_Mesoplodon_bidens_isolate_US080_MesBid_scaffold_36543_whole_genome_shotgun_sequence
_R_marine_RJWS010019958.1_489-1179_Ziphius_cavirostris_isolate_BS68_ZipCav_flattened_line_39890_whole_genome_shotgun_sequence
marine_RJWO010021467.1_4963-5657_Inia_geoffrensis_isolate_BS63_IniGeo_flattened_line_42842_whole_genome_shotgun_sequence
_R_marine_RJWI010032783.1_3535-4220_Pontoporia_blainvillei_isolate_BS67_PonBla_flattened_line_65516_whole_genome_shotgun_sequence
_R_marine_RYZJ01001270.1_5612605-5613295_Megaptera_novaeangliae_breed_North_Atlantic_isolate_ASU_GRON-1_ScjyzU5_87.3_whole_genome_shotgun_sequence
marine_VNFD01008940.1_15376-16062_Balaenoptera_musculus_isolate_JJ_BM4_2016_0621_000290F_018_arrow_arrow_whole_genome_shotgun_sequence
marine_Balbon_DF563404.1_0.0_1_696_5093_5783
marine_Balacu_NW_006725499.1_0.0_1_696_6395332_6396022
marine_NIPP01016527.1_4758-5448_Eschrichtius_robustus_isolate_ER-14-168_kmer60-min500.11744652_whole_genome_shotgun_sequence
_R_marine_RJWP010025973.1_26414-27092_Eubalaena_japonica_isolate_BS61_EubJap_flattened_line_51903_whole_genome_shotgun_sequence
_R_marine_RJWK010086191.1_1719-2412_Platanista_minor_isolate_BS66_PlaMin_flattened_line_172282_whole_genome_shotgun_sequence
marine_Phycat_NC_041219.1_0.0_1_696_97164131_97163450
_R_marine_RJWL010048314.1_7895-8586_Kogia_breviceps_isolate_BS64_KogBre_flattened_line_96614_whole_genome_shotgun_sequence
marine_Balbon_DF433562.1_6e-104_1_242_3570_3327
marine_Balacu_NW_006727553.1_5e-100_1_242_636410_636168
marine_VNFD01009544.1_1866-2245_Balaenoptera_musculus_isolate_JJ_BM4_2016_0621_000445F_004_arrow_arrow_whole_genome_shotgun_sequence
_R_marine_NIPP01016635.1_2877-3256_Eschrichtius_robustus_isolate_ER-14-168_kmer60-min500.11750455_whole_genome_shotgun_sequence
_R_marine_RYZJ01000648.1_811762-812141_Megaptera_novaeangliae_breed_North_Atlantic_isolate_ASU_GRON-1_ScjyzU5_330_whole_genome_shotgun_sequence
marine_Balbon_DF433562.1_3e-32_268_412_3328_3191
marine_Balacu_NW_006727553.1_3e-32_268_412_636169_636032
marine_RJWP010021900.1_29173-29552_Eubalaena_japonica_isolate_BS61_EubJap_flattened_line_43762_whole_genome_shotgun_sequence
land1_Capsib_NIYN02079133.1_6e-55_447_696_22838729_22838477
land1_Caphir_NC_030836.1_6e-55_447_696_39925468_39925216
land1_Capibe_SJYO01038983.1_6e-55_447_696_26638674_26638422
land1_Ammler_NIVO01031998.1_2e-55_447_696_1761030_1761283
land1_Capaeg_CM003243.1_1e-56_447_696_35987357_35987105
land1_Ovican_PVIS010029916.1_4e-57_447_696_14653_14906
land1_Oviamm_NIWH01019901.1_1e-56_447_696_32694784_32694532
land1_Oviari_NC_040272.1_1e-56_447_696_41455164_41454912
land1_Hemhyl_PVJR01011828.1_6e-55_447_696_63204_63456
land1_Panhod_NW_005811253.1_3e-53_447_696_223767_223515
land1_Saitat_PVIK010038421.1_3e-53_447_696_10433_10181
land1_Ourour_SJXZ012910112.1_1e-40_447_599_172_20
land1_Eudtho_SJYE01000212.1_2e-55_447_696_2478397_2478651
land1_Antmar_SJYB01005374.1_1e-57_447_696_160464_160209
land1_Proprz_SJYF01000628.1_6e-55_447_696_239133_238881
land1_Capcap_CCMK010064888.1_4e-56_447_694_2204_2449
land1_Rantar_PVIN010029170.1_4e-52_447_694_7572_7322
land1_Alcalc_SSNK01092544.1_3e-48_447_616_8295_8126
PVJP02000508.1_776371-777030_Hippopotamus_amphibius_isolate_BS01_ScHSfme_495HRSCAF_34889_whole_genome_shotgun_sequence
PVJP02910311.1_219594-219832_Hippopotamus_amphibius_isolate_BS01_ScHSfme_924785HRSCAF_982585_1_whole_genome_shotgun_sequence
_R_PVJP02910378.1_1863789-1864098_Hippopotamus_amphibius_isolate_BS01_ScHSfme_924850HRSCAF_982650_whole_genome_shotgun_sequence
0.03

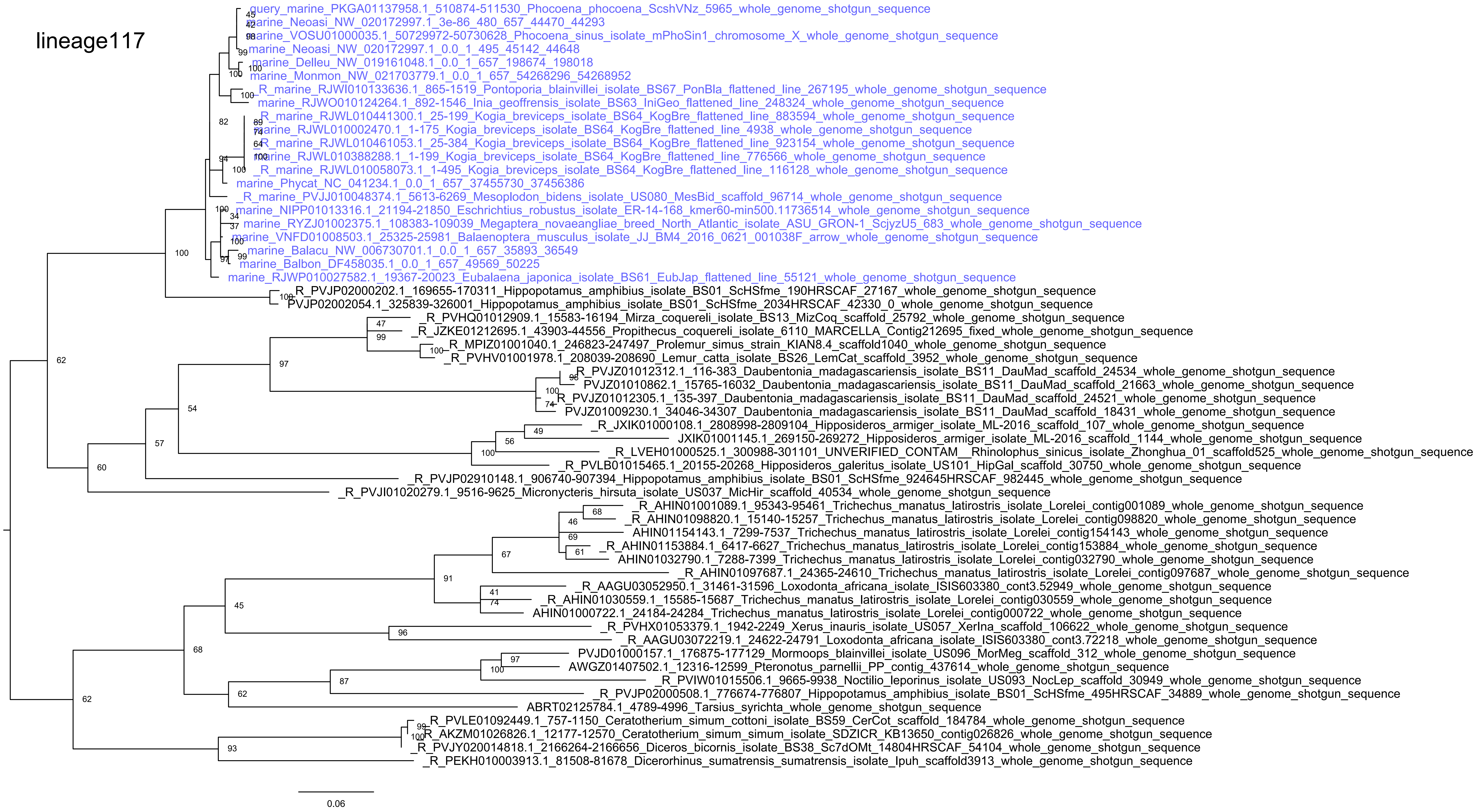
lineage117
query_marine_PKGA01137958.1_510874-511530_Phocoena_phocoena_ScshVNz_5965_whole_genome_shotgun_sequence
marine_Neoasi_NW_020172997.1_3e-86_480_657_44470_44293
marine_VOSU01000035.1_50729972-50730628_Phocoena_sinus_isolate_mPhoSin1_chromosome_X_whole_genome_shotgun_sequence
marine_Neoasi_NW_020172997.1_0.0_1_495_45142_44648
marine_Delleu_NW_019161048.1_0.0_1_657_198674_198018
marine_Monmon_NW_021703779.1_0.0_1_657_54268296_54268952
_R_marine_RJWI010133636.1_865-1519_Pontoporia_blainvillei_isolate_BS67_PonBla_flattened_line_267195_whole_genome_shotgun_sequence
marine_RJWO010124264.1_892-1546_Inia_geoffrensis_isolate_BS63_IniGeo_flattened_line_248324_whole_genome_shotgun_sequence
_R_marine_RJWL010441300.1_25-199_Kogia_breviceps_isolate_BS64_KogBre_flattened_line_883594_whole_genome_shotgun_sequence
marine_RJWL010002470.1_1-175_Kogia_breviceps_isolate_BS64_KogBre_flattened_line_4938_whole_genome_shotgun_sequence
_R_marine_RJWL010461053.1_25-384_Kogia_breviceps_isolate_BS64_KogBre_flattened_line_923154_whole_genome_shotgun_sequence
marine_RJWL010388288.1_1-199_Kogia_breviceps_isolate_BS64_KogBre_flattened_line_776566_whole_genome_shotgun_sequence
_R_marine_RJWL010058073.1_1-495_Kogia_breviceps_isolate_BS64_KogBre_flattened_line_116128_whole_genome_shotgun_sequence
marine_Phycat_NC_041234.1_0.0_1_657_37455730_37456386
_R_marine_PVJJ010048374.1_5613-6269_Mesoplodon_bidens_isolate_US080_MesBid_scaffold_96714_whole_genome_shotgun_sequence
marine_NIPP01013316.1_21194-21850_Eschrichtius_robustus_isolate_ER-14-168_kmer60-min500.11736514_whole_genome_shotgun_sequence
marine_RYZJ01002375.1_108383-109039_Megaptera_novaeangliae_breed_North_Atlantic_isolate_ASU_GRON-1_ScjyzU5_683_whole_genome_shotgun_sequence
marine_VNFD01008503.1_25325-25981_Balaenoptera_musculus_isolate_JJ_BM4_2016_0621_001038F_arrow_whole_genome_shotgun_sequence
marine_Balacu_NW_006730701.1_0.0_1_657_35893_36549
marine_Balbon_DF458035.1_0.0_1_657_49569_50225
marine_RJWP010027582.1_19367-20023_Eubalaena_japonica_isolate_BS61_EubJap_flattened_line_55121_whole_genome_shotgun_sequence
_R_PVJP02000202.1_169655-170311_Hippopotamus_amphibius_isolate_BS01_ScHSfme_190HRSCAF_27167_whole_genome_shotgun_sequence
PVJP02002054.1_325839-326001_Hippopotamus_amphibius_isolate_BS01_ScHSfme_2034HRSCAF_42330_0_whole_genome_shotgun_sequence
_R_PVHQ01012909.1_15583-16194_Mirza_coquereli_isolate_BS13_MizCoq_scaffold_25792_whole_genome_shotgun_sequence
_R_JZKE01212695.1_43903-44556_Propithecus_coquereli_isolate_6110_MARCELLA_Contig212695_fixed_whole_genome_shotgun_sequence
_R_MPIZ01001040.1_246823-247497_Prolemur_simus_strain_KIAN8.4_scaffold1040_whole_genome_shotgun_sequence
_R_PVHV01001978.1_208039-208690_Lemur_catta_isolate_BS26_LemCat_scaffold_3952_whole_genome_shotgun_sequence
_R_PVJZ01012312.1_116-383_Daubentonia_madagascariensis_isolate_BS11_DauMad_scaffold_24534_whole_genome_shotgun_sequence
PVJZ01010862.1_15765-16032_Daubentonia_madagascariensis_isolate_BS11_DauMad_scaffold_21663_whole_genome_shotgun_sequence
_R_PVJZ01012305.1_135-397_Daubentonia_madagascariensis_isolate_BS11_DauMad_scaffold_24521_whole_genome_shotgun_sequence
PVJZ01009230.1_34046-34307_Daubentonia_madagascariensis_isolate_BS11_DauMad_scaffold_18431_whole_genome_shotgun_sequence
_R_JXIK01000108.1_2808998-2809104_Hipposideros_armiger_isolate_ML-2016_scaffold_107_whole_genome_shotgun_sequence
JXIK01001145.1_269150-269272_Hipposideros_armiger_isolate_ML-2016_scaffold_1144_whole_genome_shotgun_sequence
_R_LVEH01000525.1_300988-301101_UNVERIFIED_CONTAM__Rhinolophus_sinicus_isolate_Zhonghua_01_scaffold525_whole_genome_shotgun_sequence
_R_PVLB01015465.1_20155-20268_Hipposideros_galeritus_isolate_US101_HipGal_scaffold_30750_whole_genome_shotgun_sequence
_R_PVJP02910148.1_906740-907394_Hippopotamus_amphibius_isolate_BS01_ScHSfme_924645HRSCAF_982445_whole_genome_shotgun_sequence
_R_PVJI01020279.1_9516-9625_Micronycteris_hirsuta_isolate_US037_MicHir_scaffold_40534_whole_genome_shotgun_sequence
_R_AHIN01001089.1_95343-95461_Trichechus_manatus_latirostris_isolate_Lorelei_contig001089_whole_genome_shotgun_sequence
_R_AHIN01098820.1_15140-15257_Trichechus_manatus_latirostris_isolate_Lorelei_contig098820_whole_genome_shotgun_sequence
AHIN01154143.1_7299-7537_Trichechus_manatus_latirostris_isolate_Lorelei_contig154143_whole_genome_shotgun_sequence
_R_AHIN01153884.1_6417-6627_Trichechus_manatus_latirostris_isolate_Lorelei_contig153884_whole_genome_shotgun_sequence
AHIN01032790.1_7288-7399_Trichechus_manatus_latirostris_isolate_Lorelei_contig032790_whole_genome_shotgun_sequence
_R_AHIN01097687.1_24365-24610_Trichechus_manatus_latirostris_isolate_Lorelei_contig097687_whole_genome_shotgun_sequence
_R_AAGU03052950.1_31461-31596_Loxodonta_africana_isolate_ISIS603380_cont3.52949_whole_genome_shotgun_sequence
_R_AHIN01030559.1_15585-15687_Trichechus_manatus_latirostris_isolate_Lorelei_contig030559_whole_genome_shotgun_sequence
AHIN01000722.1_24184-24284_Trichechus_manatus_latirostris_isolate_Lorelei_contig000722_whole_genome_shotgun_sequence
_R_PVHX01053379.1_1942-2249_Xerus_inauris_isolate_US057_XerIna_scaffold_106622_whole_genome_shotgun_sequence
_R_AAGU03072219.1_24622-24791_Loxodonta_africana_isolate_ISIS603380_cont3.72218_whole_genome_shotgun_sequence
PVJD01000157.1_176875-177129_Mormoops_blainvillei_isolate_US096_MorMeg_scaffold_312_whole_genome_shotgun_sequence
AWGZ01407502.1_12316-12599_Pteronotus_parnellii_PP_contig_437614_whole_genome_shotgun_sequence
_R_PVIW01015506.1_9665-9938_Noctilio_leporinus_isolate_US093_NocLep_scaffold_30949_whole_genome_shotgun_sequence
_R_PVJP02000508.1_776674-776807_Hippopotamus_amphibius_isolate_BS01_ScHSfme_495HRSCAF_34889_whole_genome_shotgun_sequence
ABRT02125784.1_4789-4996_Tarsius_syrichta_whole_genome_shotgun_sequence
_R_PVLE01092449.1_757-1150_Ceratotherium_simum_cottoni_isolate_BS59_CerCot_scaffold_184784_whole_genome_shotgun_sequence
_R_AKZM01026826.1_12177-12570_Ceratotherium_simum_simum_isolate_SDZICR_KB13650_contig026826_whole_genome_shotgun_sequence
_R_PVJY020014818.1_2166264-2166656_Diceros_bicornis_isolate_BS38_Sc7dOMt_14804HRSCAF_54104_whole_genome_shotgun_sequence
_R_PEKH010003913.1_81508-81678_Dicerorhinus_sumatrensis_sumatrensis_isolate_Ipuh_scaffold3913_whole_genome_shotgun_sequence
0.06

lineage118

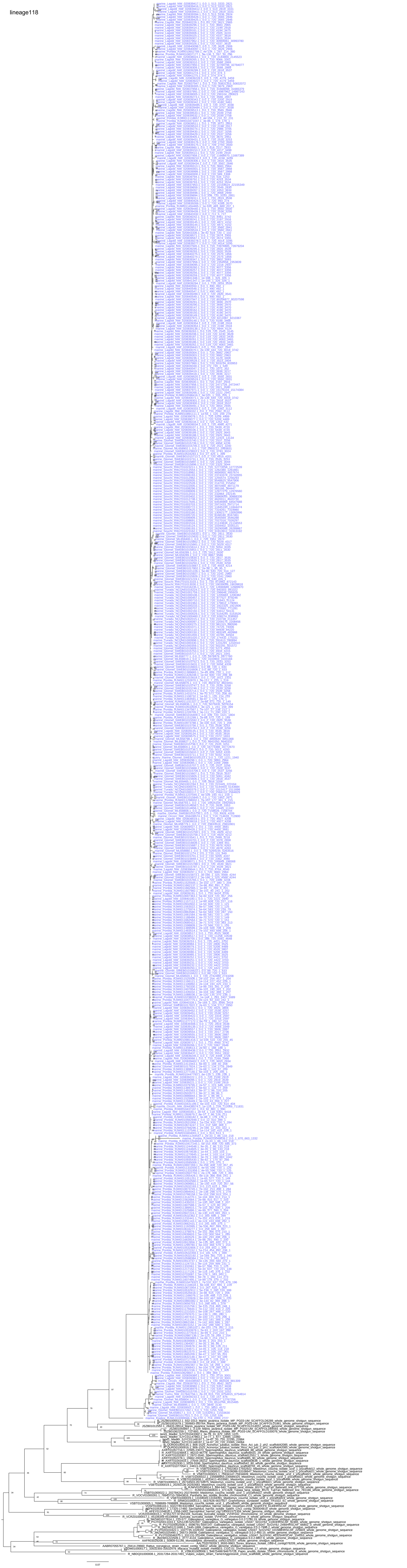

lineage119

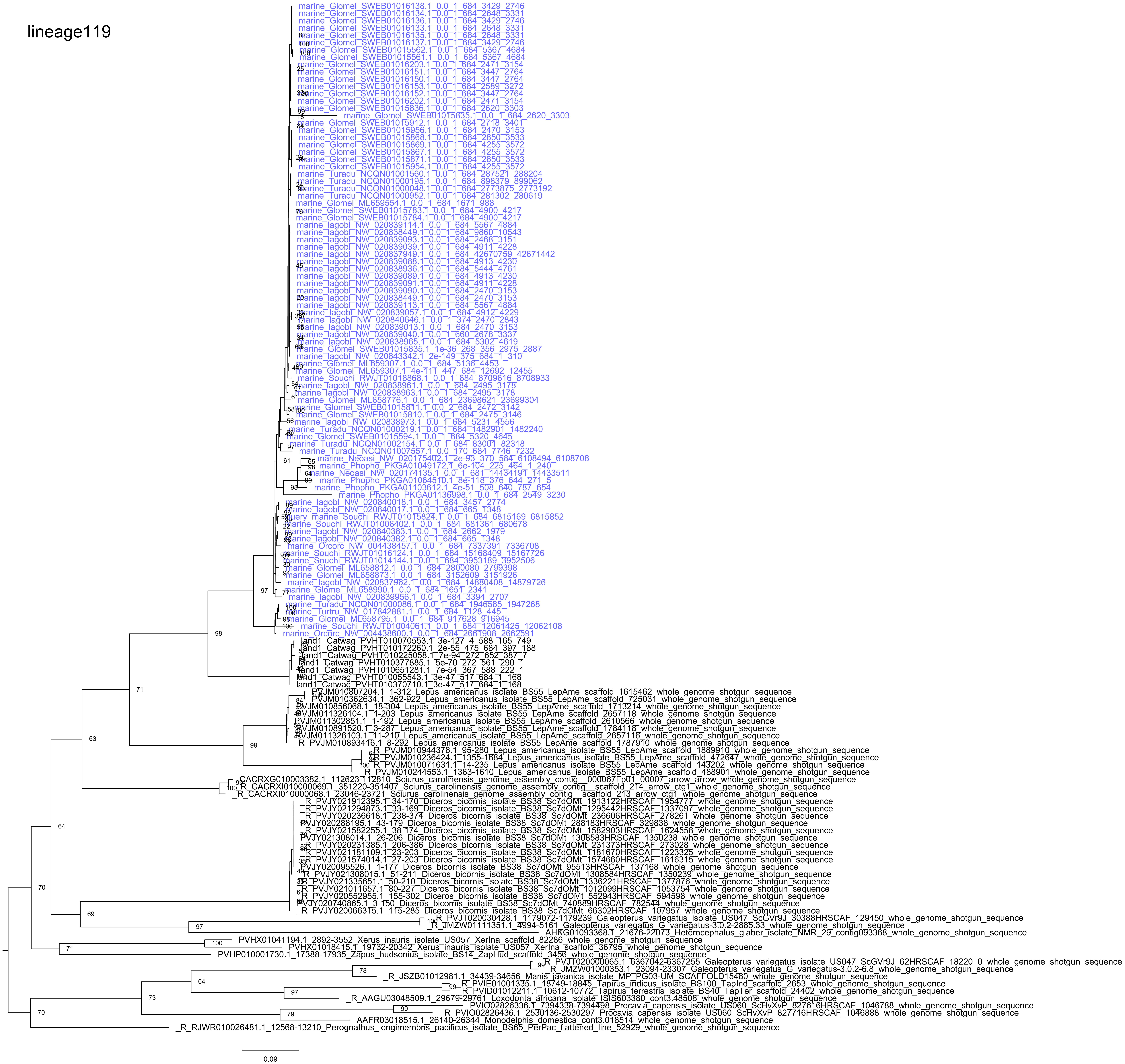
marine_Glomel_SWEB01016138.1_0.0_1_684_3429_2746
marine_Glomel_SWEB01016134.1_0.0_1_684_2648_3331
marine_Glomel_SWEB01016136.1_0.0_1_684_3429_2746
marine_Glomel_SWEB01016133.1_0.0_1_684_2648_3331
marine_Glomel_SWEB01016135.1_0.0_1_684_2648_3331
marine_Glomel_SWEB01016137.1_0.0_1_684_3429_2746
marine_Glomel_SWEB01015562.1_0.0_1_684_5367_4684
marine_Glomel_SWEB01015561.1_0.0_1_684_5367_4684
marine_Glomel_SWEB01016203.1_0.0_1_684_2471_3154
marine_Glomel_SWEB01016151.1_0.0_1_684_3447_2764
marine_Glomel_SWEB01016150.1_0.0_1_684_3447_2764
marine_Glomel_SWEB01016153.1_0.0_1_684_2589_3272
marine_Glomel_SWEB01016152.1_0.0_1_684_3447_2764
marine_Glomel_SWEB01016202.1_0.0_1_684_2471_3154
marine_Glomel_SWEB01015836.1_0.0_1_684_2620_3303
marine_Glomel_SWEB01015835.1_0.0_1_684_2620_3303
marine_Glomel_SWEB01015912.1_0.0_1_684_2718_3401
marine_Glomel_SWEB01015956.1_0.0_1_684_2470_3153
marine_Glomel_SWEB01015868.1_0.0_1_684_2850_3533
marine_Glomel_SWEB01015869.1_0.0_1_684_4255_3572
marine_Glomel_SWEB01015867.1_0.0_1_684_4255_3572
marine_Glomel_SWEB01015871.1_0.0_1_684_2850_3533
marine_Glomel_SWEB01015954.1_0.0_1_684_4255_3572
marine_Turadu_NCQN01001560.1_0.0_1_684_287521_288204
marine_Turadu_NCQN01000195.1_0.0_1_684_898379_899062
marine_Turadu_NCQN01000048.1_0.0_1_684_2773875_2773192
marine_Turadu_NCQN01000952.1_0.0_1_684_281302_280619
marine_Glomel_ML659554.1_0.0_1_684_1671_988
marine_Glomel_SWEB01015783.1_0.0_1_684_4900_4217
marine_Glomel_SWEB01015784.1_0.0_1_684_4900_4217
marine_lagobl_NW_020839114.1_0.0_1_684_5567_4884
marine_lagobl_NW_020838449.1_0.0_1_684_9860_10543
marine_lagobl_NW_020839093.1_0.0_1_684_2468_3151
marine_lagobl_NW_020839039.1_0.0_1_684_4911_4228
marine_lagobl_NW_020837949.1_0.0_1_684_42670759_42671442
marine_lagobl_NW_020839088.1_0.0_1_684_4913_4230
marine_lagobl_NW_020838936.1_0.0_1_684_5444_4761
marine_lagobl_NW_020839089.1_0.0_1_684_4913_4230
marine_lagobl_NW_020839091.1_0.0_1_684_4911_4228
marine_lagobl_NW_020839090.1_0.0_1_684_2470_3153
marine_lagobl_NW_020838449.1_0.0_1_684_2470_3153
marine_lagobl_NW_020839113.1_0.0_1_684_5567_4884
marine_lagobl_NW_020839057.1_0.0_1_684_4912_4229
marine_lagobl_NW_020840646.1_0.0_1_374_2470_2843
marine_lagobl_NW_020839013.1_0.0_1_684_2470_3153
marine_lagobl_NW_020839040.1_0.0_1_660_2678_3337
marine_lagobl_NW_020838965.1_0.0_1_684_5302_4619
marine_Glomel_SWEB01015835.1_1e-36_268_356_2975_2887
marine_lagobl_NW_020843342.1_2e-149_375_684_1_310
marine_Glomel_ML659307.1_0.0_1_684_5136_4453
marine_Glomel_ML659307.1_4e-111_447_684_12692_12455
marine_Souchi_RWJT01018868.1_0.0_1_684_8709616_8708933
marine_lagobl_NW_020838961.1_0.0_1_684_2495_3178
marine_lagobl_NW_020838963.1_0.0_1_684_2495_3178
marine_Glomel_ML658776.1_0.0_1_684_23698621_23699304
marine_Glomel_SWEB01015811.1_0.0_2_684_2472_3142
marine_Glomel_SWEB01015810.1_0.0_1_684_2475_3146
marine_lagobl_NW_020838973.1_0.0_1_684_5231_4556
marine_Turadu_NCQN01000219.1_0.0_1_684_1482901_1482240
marine_Glomel_SWEB01015594.1_0.0_1_684_5320_4645
marine_Turadu_NCQN01002154.1_0.0_1_684_83001_82318
marine_Turadu_NCQN01007557.1_0.0_170_684_7746_7232
marine_Neoasi_NW_020175402.1_2e-93_370_584_6108494_6108708
marine_Phopho_PKGA01049172.1_6e-104_225_464_1_240
marine_Neoasi_NW_020174135.1_0.0_1_681_14434191_14433511
marine_Phopho_PKGA01064510.1_8e-118_376_644_271_5
marine_Phopho_PKGA01103612.1_4e-51_508_640_787_654
marine_Phopho_PKGA01136998.1_0.0_1_684_2549_3230
marine_lagobl_NW_020840018.1_0.0_1_684_3457_2774
marine_lagobl_NW_020840017.1_0.0_1_684_665_1348
query_marine_Souchi_RWJT01015824.1_0.0_1_684_6815169_6815852
marine_Souchi_RWJT01006402.1_0.0_1_684_681361_680678
marine_lagobl_NW_020840383.1_0.0_1_684_2662_1979
marine_lagobl_NW_020840382.1_0.0_1_684_665_1348
marine_Orcorc_NW_004438457.1_0.0_1_684_7337391_7336708
marine_Souchi_RWJT01016124.1_0.0_1_684_15168409_15167726
marine_Souchi_RWJT01014144.1_0.0_1_684_3953189_3952506
marine_Glomel_ML658812.1_0.0_1_684_2800080_2799398
marine_Glomel_ML658873.1_0.0_1_684_3152609_3151926
marine_lagobl_NW_020837962.1_0.0_1_684_14880408_14879726
marine_Glomel_ML658990.1_0.0_1_684_1651_2341
marine_lagobl_NW_020839956.1_0.0_1_684_3394_2707
marine_Turadu_NCQN01000086.1_0.0_1_684_1946585_1947268
marine_Turtru_NW_017842881.1_0.0_1_684_1128_445
marine_Glomel_ML658795.1_0.0_1_684_917628_916945
marine_Souchi_RWJT01004061.1_0.0_1_684_12061425_12062108
marine_Orcorc_NW_004438600.1_0.0_1_684_2661908_2662591
land1_Catwag_PVHT010070553.1_3e-127_4_588_165_749
land1_Catwag_PVHT010172260.1_2e-55_475_684_397_188
land1_Catwag_PVHT010225058.1_7e-94_272_652_387_7
land1_Catwag_PVHT010377885.1_5e-70_272_561_290_1
land1_Catwag_PVHT010651281.1_7e-54_367_588_222_1
land1_Catwag_PVHT010055543.1_3e-47_517_684_1_168
land1_Catwag_PVHT010370710.1_3e-47_517_684_1_168
PVJM010807204.1_1-312_Lepus_americanus_isolate_BS55_LepAme_scaffold_1615462_whole_genome_shotgun_sequence
PVJM010362634.1_362-922_Lepus_americanus_isolate_BS55_LepAme_scaffold_725031_whole_genome_shotgun_sequence
PVJM010856068.1_18-304_Lepus_americanus_isolate_BS55_LepAme_scaffold_1713214_whole_genome_shotgun_sequence
PVJM011326104.1_1-203_Lepus_americanus_isolate_BS55_LepAme_scaffold_2657118_whole_genome_shotgun_sequence
PVJM011302851.1_1-192_Lepus_americanus_isolate_BS55_LepAme_scaffold_2610566_whole_genome_shotgun_sequence
PVJM010891520.1_3-287_Lepus_americanus_isolate_BS55_LepAme_scaffold_1784118_whole_genome_shotgun_sequence
PVJM011326103.1_11-210_Lepus_americanus_isolate_BS55_LepAme_scaffold_2657116_whole_genome_shotgun_sequence
_R_PVJM010893416.1_8-292_Lepus_americanus_isolate_BS55_LepAme_scaffold_1787910_whole_genome_shotgun_sequence
_R_PVJM010944378.1_95-280_Lepus_americanus_isolate_BS55_LepAme_scaffold_1889910_whole_genome_shotgun_sequence
_R_PVJM010236424.1_1355-1684_Lepus_americanus_isolate_BS55_LepAme_scaffold_472647_whole_genome_shotgun_sequence
_R_PVJM010071631.1_14-235_Lepus_americanus_isolate_BS55_LepAme_scaffold_143202_whole_genome_shotgun_sequence
_R_PVJM010244553.1_1363-1610_Lepus_americanus_isolate_BS55_LepAme_scaffold_488901_whole_genome_shotgun_sequence
CACRXG010003382.1_112623-112810_Sciurus_carolinensis_genome_assembly_contig__000067Fp01_00007_arrow_arrow_whole_genome_shotgun_sequence
_R_CACRXI010000069.1_351220-351407_Sciurus_carolinensis_genome_assembly_contig__scaffold_214_arrow_ctg1_whole_genome_shotgun_sequence
_R_CACRXI010000068.1_23046-23721_Sciurus_carolinensis_genome_assembly_contig__scaffold_213_arrow_ctg1_whole_genome_shotgun_sequence
_R_PVJY021912395.1_34-170_Diceros_bicornis_isolate_BS38_Sc7dOMt_1913122HRSCAF_1954777_whole_genome_shotgun_sequence
_R_PVJY021294873.1_33-169_Diceros_bicornis_isolate_BS38_Sc7dOMt_1295442HRSCAF_1337097_whole_genome_shotgun_sequence
_R_PVJY020236618.1_238-374_Diceros_bicornis_isolate_BS38_Sc7dOMt_236606HRSCAF_278261_whole_genome_shotgun_sequence
PVJY020288195.1_43-179_Diceros_bicornis_isolate_BS38_Sc7dOMt_288183HRSCAF_329838_whole_genome_shotgun_sequence
_R_PVJY021582255.1_38-174_Diceros_bicornis_isolate_BS38_Sc7dOMt_1582903HRSCAF_1624558_whole_genome_shotgun_sequence
PVJY021308014.1_26-206_Diceros_bicornis_isolate_BS38_Sc7dOMt_1308583HRSCAF_1350238_whole_genome_shotgun_sequence
_R_PVJY020231385.1_206-336_Diceros_bicornis_isolate_BS38_Sc7dOMt_231373HRSCAF_273028_whole_genome_shotgun_sequence
_R_PVJY021181109.1_23-203_Diceros_bicornis_isolate_BS38_Sc7dOMt_1181670HRSCAF_1223325_whole_genome_shotgun_sequence
_R_PVJY021574014.1_27-203_Diceros_bicornis_isolate_BS38_Sc7dOMt_1574660HRSCAF_1616315_whole_genome_shotgun_sequence
PVJY020095526.1_1-177_Diceros_bicornis_isolate_BS38_Sc7dOMt_95513HRSCAF_137168_whole_genome_shotgun_sequence
_R_PVJY021308015.1_51-211_Diceros_bicornis_isolate_BS38_Sc7dOMt_1308584HRSCAF_1350239_whole_genome_shotgun_sequence
_R_PVJY021335651.1_50-210_Diceros_bicornis_isolate_BS38_Sc7dOMt_1336221HRSCAF_1377876_whole_genome_shotgun_sequence
_R_PVJY021011657.1_80-227_Diceros_bicornis_isolate_BS38_Sc7dOMt_1012099HRSCAF_1053754_whole_genome_shotgun_sequence
_R_PVJY020552955.1_155-302_Diceros_bicornis_isolate_BS38_Sc7dOMt_552943HRSCAF_594598_whole_genome_shotgun_sequence
PVJY020740865.1_3-150_Diceros_bicornis_isolate_BS38_Sc7dOMt_740889HRSCAF_782544_whole_genome_shotgun_sequence
_R_PVJY020066315.1_115-285_Diceros_bicornis_isolate_BS38_Sc7dOMt_66302HRSCAF_107957_whole_genome_shotgun_sequence
_R_PVJT020030428.1_1179072-1179239_Galeopterus_variegatus_isolate_US047_ScGVr9J_30388HRSCAF_129450_whole_genome_shotgun_sequence
_R_JMZW01111351.1_4994-5161_Galeopterus_variegatus_G_variegatus-3.0.2-2885.33_whole_genome_shotgun_sequence
AHKG01093368.1_21676-22073_Heterocephalus_glaber_isolate_NMR_29_contig093368_whole_genome_shotgun_sequence
PVHX01041194.1_2892-3552_Xerus_inauris_isolate_US057_XerIna_scaffold_82286_whole_genome_shotgun_sequence
PVHX01018415.1_19732-20342_Xerus_inauris_isolate_US057_XerIna_scaffold_36795_whole_genome_shotgun_sequence
PVHP010001730.1_17388-17935_Zapus_hudsonius_isolate_BS14_ZapHud_scaffold_3456_whole_genome_shotgun_sequence
_R_PVJT020000065.1_6367042-6367255_Galeopterus_variegatus_isolate_US047_ScGVr9J_62HRSCAF_18220_0_whole_genome_shotgun_sequence
_R_JMZW01000353.1_23094-23307_Galeopterus_variegatus_G_variegatus-3.0.2-6.8_whole_genome_shotgun_sequence
_R_JSZB01012981.1_34439-34656_Manis_javanica_isolate_MP_PG03-UM_SCAFFOLD15480_whole_genome_shotgun_sequence
_R_PVIE01001335.1_18749-18845_Tapirus_indicus_isolate_BS100_TapInd_scaffold_2653_whole_genome_shotgun_sequence
_R_PVID01012211.1_10612-10772_Tapirus_terrestris_isolate_BS40_TapTer_scaffold_24402_whole_genome_shotgun_sequence
_R_AAGU03048509.1_29679-29761_Loxodonta_africana_isolate_ISIS603380_cont3.48508_whole_genome_shotgun_sequence
PVIO02826336.1_7394338-7394498_Procavia_capensis_isolate_US060_ScHvXvP_827616HRSCAF_1046788_whole_genome_shotgun_sequence
_R_PVIO02826436.1_2530136-2530297_Procavia_capensis_isolate_US060_ScHvXvP_827716HRSCAF_1046888_whole_genome_shotgun_sequence
AAFR03018515.1_26140-26344_Monodelphis_domestica_cont3.018514_whole_genome_shotgun_sequence
_R_RJWR010026481.1_12568-13210_Perognathus_longimembris_pacificus_isolate_BS65_PerPac_flattened_line_52929_whole_genome_shotgun_sequence

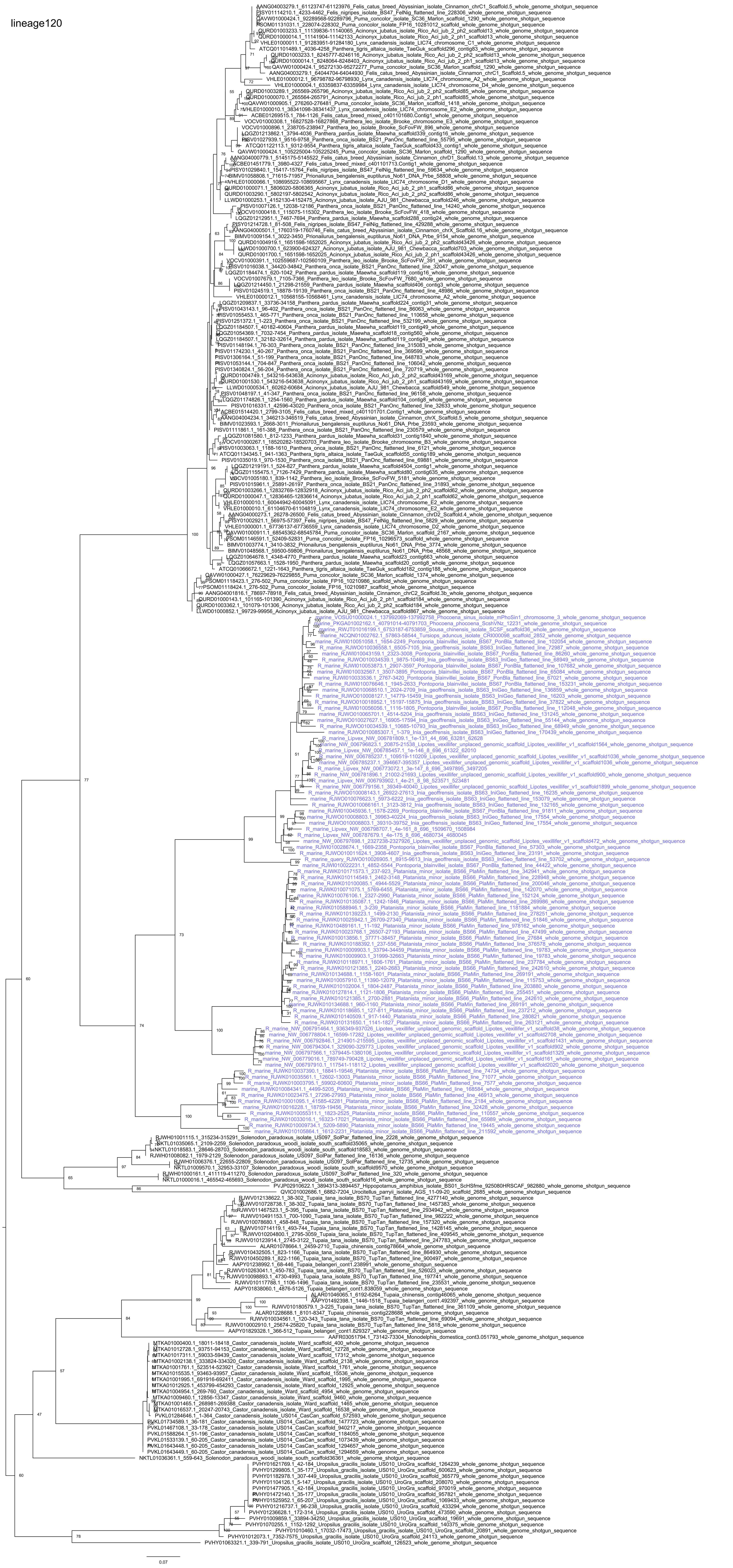
lineage120
0.07

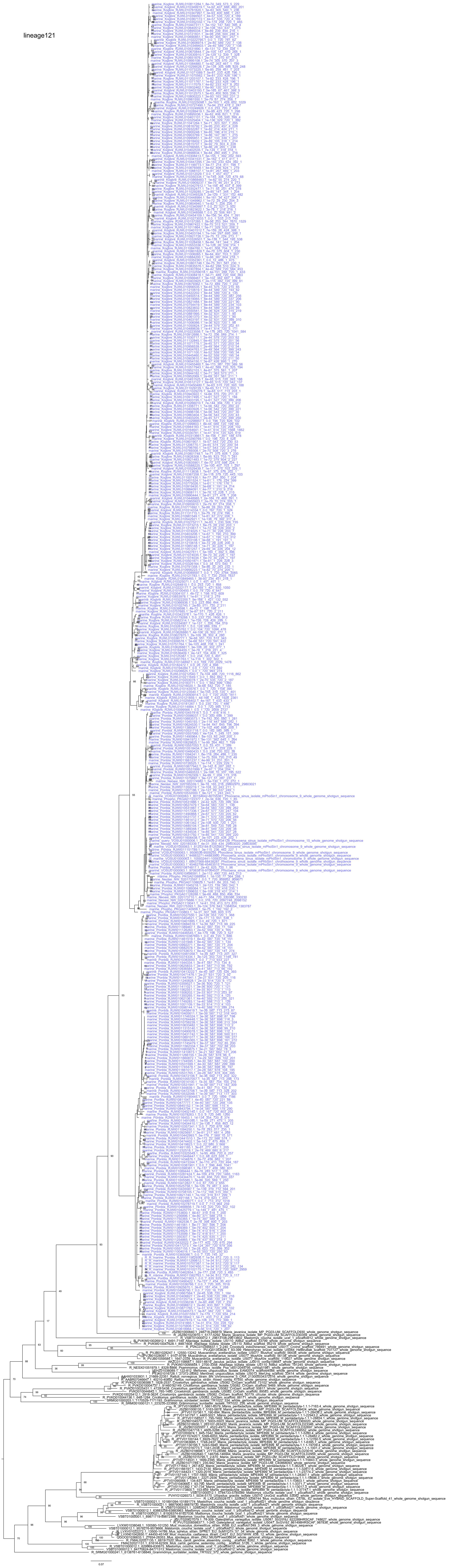
lineage121
0.07

lineage122

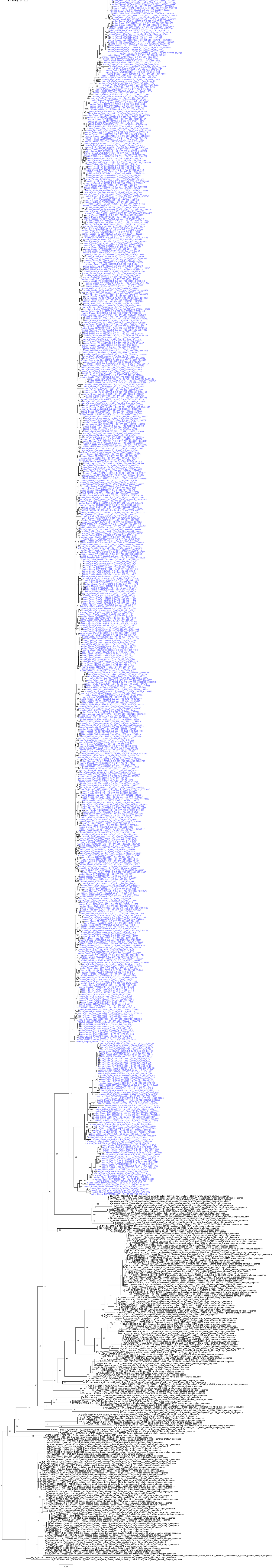

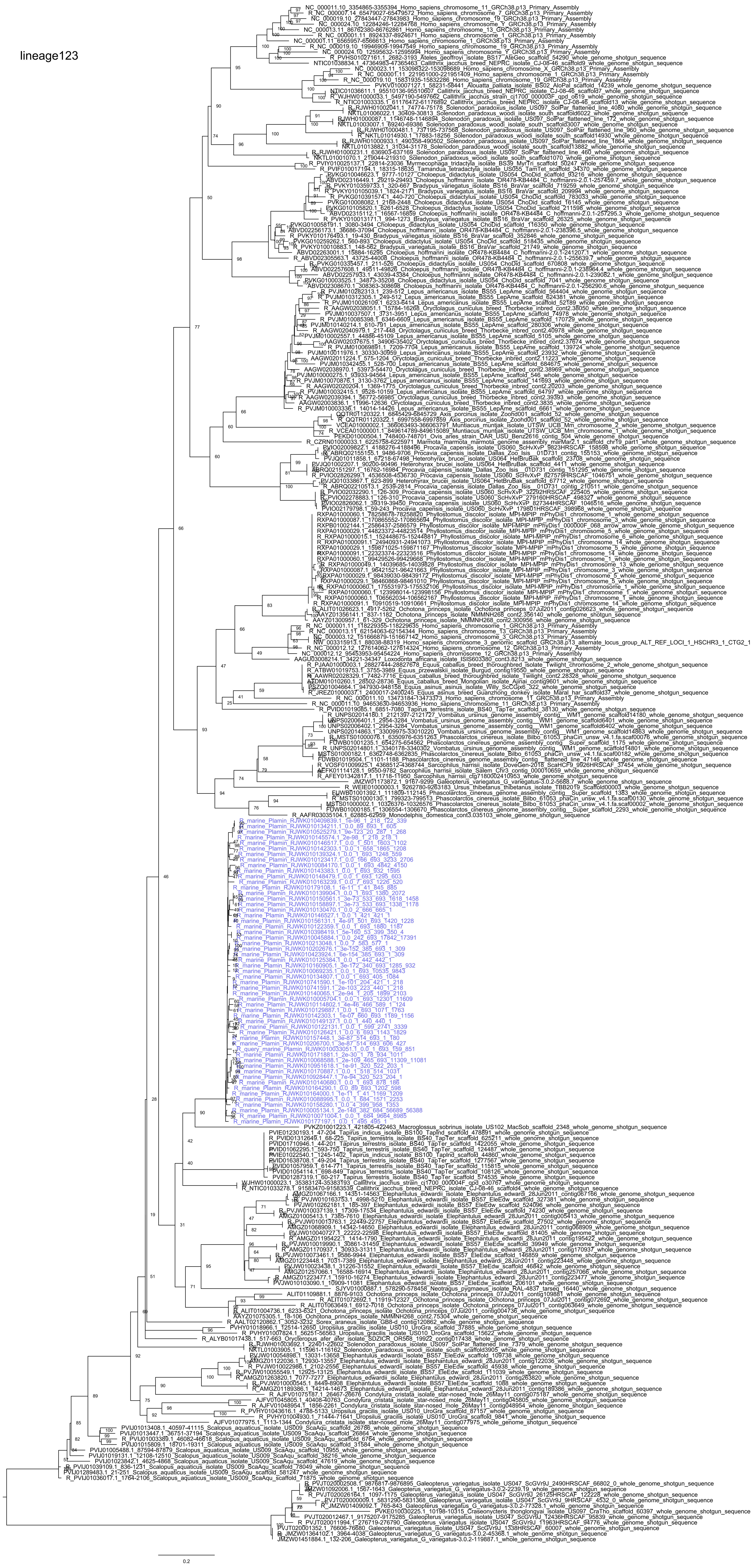
lineage123
0.2

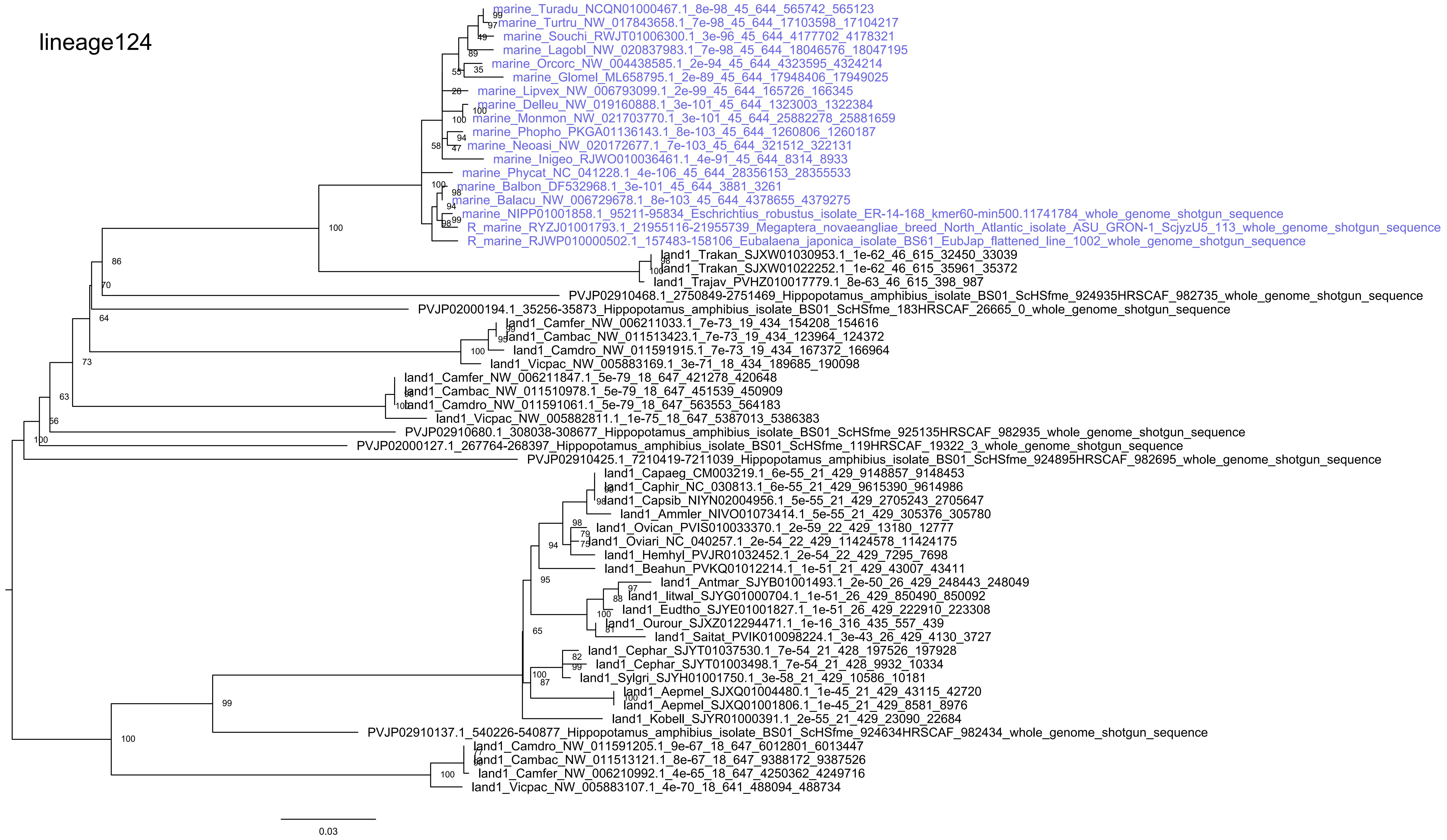

lineage124
marine_Turadu_NCQN01000467.1_8e-98_45_644_565742_565123
marine_Turtru_NW_017843658.1_7e-98_45_644_17103598_17104217
marine_Souchi_RWJT01006300.1_3e-96_45_644_4177702_4178321
marine_Lagobl_NW_020837983.1_7e-98_45_644_18046576_18047195
marine_Orcorc_NW_004438585.1_2e-94_45_644_4323595_4324214
marine_Glomel_ML658795.1_2e-89_45_644_17948406_17949025
marine_Lipvex_NW_006793099.1_2e-99_45_644_165726_166345
marine_Delleu_NW_019160888.1_3e-101_45_644_1323003_1322384
marine_Monmon_NW_021703770.1_3e-101_45_644_25882278_25881659
marine_Phopho_PKGA01136143.1_8e-103_45_644_1260806_1260187
marine_Neoasi_NW_020172677.1_7e-103_45_644_321512_322131
marine_Inigeo_RJWO010036461.1_4e-91_45_644_8314_8933
marine_Phycat_NC_041228.1_4e-106_45_644_28356153_28355533
marine_Balbon_DF532968.1_3e-101_45_644_3881_3261
marine_Balacu_NW_006729678.1_8e-103_45_644_4378655_4379275
marine_NIPP01001858.1_95211-95834_Eschrichtius_robustus_isolate_ER-14-168_kmer60-min500.11741784_whole_genome_shotgun_sequence
R_marine_RYZJ01001793.1_21955116-21955739_Megaptera_novaeangliae_breed_North_Atlantic_isolate_ASU_GRON-1_ScjyzU5_113_whole_genome_shotgun_sequence
R_marine_RJWP010000502.1_157483-158106_Eubalaena_japonica_isolate_BS61_EubJap_flattened_line_1002_whole_genome_shotgun_sequence
land1_Trakan_SJXW01030953.1_1e-62_46_615_32450_33039
land1_Trakan_SJXW01022252.1_1e-62_46_615_35961_35372
land1_Trajav_PVHZ010017779.1_8e-63_46_615_398_987
PVJP02910468.1_2750849-2751469_Hippopotamus_amphibius_isolate_BS01_ScHSfme_924935HRSCAF_982735_whole_genome_shotgun_sequence
PVJP02000194.1_35256-35873_Hippopotamus_amphibius_isolate_BS01_ScHSfme_183HRSCAF_26665_0_whole_genome_shotgun_sequence
land1_Camfer_NW_006211033.1_7e-73_19_434_154208_154616
land1_Cambac_NW_011513423.1_7e-73_19_434_123964_124372
land1_Camdro_NW_011591915.1_7e-73_19_434_167372_166964
land1_Vicpac_NW_005883169.1_3e-71_18_434_189685_190098
land1_Camfer_NW_006211847.1_5e-79_18_647_421278_420648
land1_Cambac_NW_011510978.1_5e-79_18_647_451539_450909
land1_Camdro_NW_011591061.1_5e-79_18_647_563553_564183
land1_Vicpac_NW_005882811.1_1e-75_18_647_5387013_5386383
PVJP02910680.1_308038-308677_Hippopotamus_amphibius_isolate_BS01_ScHSfme_925135HRSCAF_982935_whole_genome_shotgun_sequence
PVJP02000127.1_267764-268397_Hippopotamus_amphibius_isolate_BS01_ScHSfme_119HRSCAF_19322_3_whole_genome_shotgun_sequence
PVJP02910425.1_7210419-7211039_Hippopotamus_amphibius_isolate_BS01_ScHSfme_924895HRSCAF_982695_whole_genome_shotgun_sequence
land1_Capaeg_CM003219.1_6e-55_21_429_9148857_9148453
land1_Caphir_NC_030813.1_6e-55_21_429_9615390_9614986
land1_Capsib_NIYN02004956.1_5e-55_21_429_2705243_2705647
land1_Ammler_NIVO01073414.1_5e-55_21_429_305376_305780
land1_Ovican_PVIS010033370.1_2e-59_22_429_13180_12777
land1_Oviari_NC_040257.1_2e-54_22_429_11424578_11424175
land1_Hemhyl_PVJR01032452.1_2e-54_22_429_7295_7698
land1_Beahun_PVKQ01012214.1_1e-51_21_429_43007_43411
land1_Antmar_SJYB01001493.1_2e-50_26_429_248443_248049
land1_litwal_SJYG01000704.1_1e-51_26_429_850490_850092
land1_Eudtho_SJYE01001827.1_1e-51_26_429_222910_223308
land1_Ourour_SJXZ012294471.1_1e-16_316_435_557_439
land1_Saitat_PVIK010098224.1_3e-43_26_429_4130_3727
land1_Cephar_SJYT01037530.1_7e-54_21_428_197526_197928
land1_Cephar_SJYT01003498.1_7e-54_21_428_9932_10334
land1_Sylgri_SJYH01001750.1_3e-58_21_429_10586_10181
land1_Aepmel_SJXQ01004480.1_1e-45_21_429_43115_42720
land1_Aepmel_SJXQ01001806.1_1e-45_21_429_8581_8976
land1_Kobell_SJYR01000391.1_2e-55_21_429_23090_22684
PVJP02910137.1_540226-540877_Hippopotamus_amphibius_isolate_BS01_ScHSfme_924634HRSCAF_982434_whole_genome_shotgun_sequence
land1_Camdro_NW_011591205.1_9e-67_18_647_6012801_6013447
land1_Cambac_NW_011513121.1_8e-67_18_647_9388172_9387526
land1_Camfer_NW_006210992.1_4e-65_18_647_4250362_4249716
land1_Vicpac_NW_005883107.1_4e-70_18_641_488094_488734
0.03

lineage125

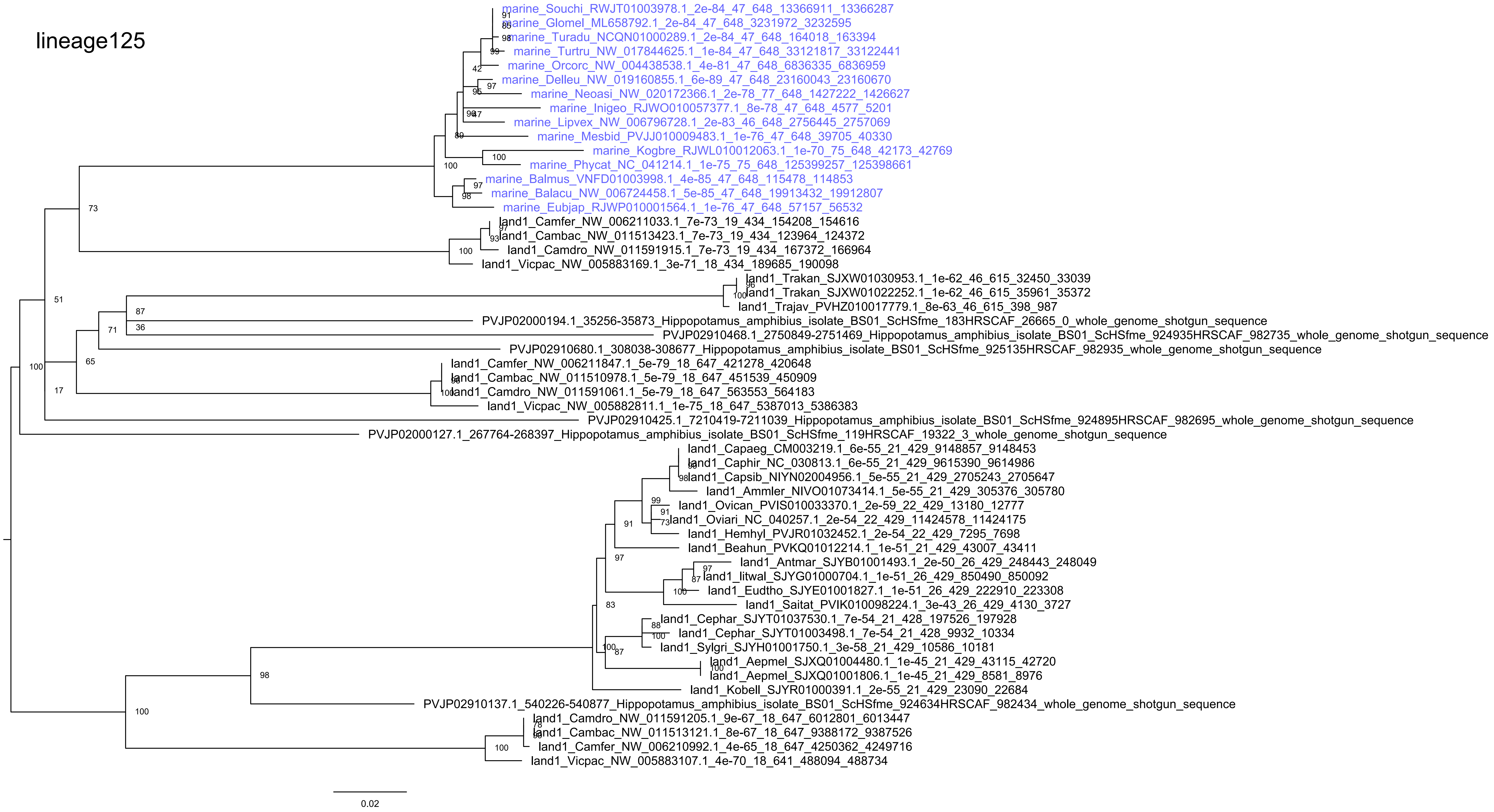
marine_Souchi_RWJT01003978.1_2e-84_47_648_13366911_13366287
marine_Glomel_ML658792.1_2e-84_47_648_3231972_3232595
marine_Turadu_NCQN01000289.1_2e-84_47_648_164018_163394
marine_Turtru_NW_017844625.1_1e-84_47_648_33121817_33122441
marine_Orcorc_NW_004438538.1_4e-81_47_648_6836335_6836959
marine_Delleu_NW_019160855.1_6e-89_47_648_23160043_23160670
marine_Neoasi_NW_020172366.1_2e-78_77_648_1427222_1426627
marine_Inigeo_RJWO010057377.1_8e-78_47_648_4577_5201
marine_Lipvex_NW_006796728.1_2e-83_46_648_2756445_2757069
marine_Mesbid_PVJJ010009483.1_1e-76_47_648_39705_40330
marine_Kogbre_RJWL010012063.1_1e-70_75_648_42173_42769
marine_Phycat_NC_041214.1_1e-75_75_648_125399257_125398661
marine_Balmus_VNFD01003998.1_4e-85_47_648_115478_114853
marine_Balacu_NW_006724458.1_5e-85_47_648_19913432_19912807
marine_Eubjap_RJWP010001564.1_1e-76_47_648_57157_56532
land1_Camfer_NW_006211033.1_7e-73_19_434_154208_154616
land1_Cambac_NW_011513423.1_7e-73_19_434_123964_124372
land1_Camdro_NW_011591915.1_7e-73_19_434_167372_166964
land1_Vicpac_NW_005883169.1_3e-71_18_434_189685_190098
land1_Trakan_SJXW01030953.1_1e-62_46_615_32450_33039
land1_Trakan_SJXW01022252.1_1e-62_46_615_35961_35372
land1_Trajav_PVHZ010017779.1_8e-63_46_615_398_987
PVJP02000194.1_35256-35873_Hippopotamus_amphibius_isolate_BS01_ScHSfme_183HRSCAF_26665_0_whole_genome_shotgun_sequence
PVJP02910468.1_2750849-2751469_Hippopotamus_amphibius_isolate_BS01_ScHSfme_924935HRSCAF_982735_whole_genome_shotgun_sequence
PVJP02910680.1_308038-308677_Hippopotamus_amphibius_isolate_BS01_ScHSfme_925135HRSCAF_982935_whole_genome_shotgun_sequence
land1_Camfer_NW_006211847.1_5e-79_18_647_421278_420648
land1_Cambac_NW_011510978.1_5e-79_18_647_451539_450909
land1_Camdro_NW_011591061.1_5e-79_18_647_563553_564183
land1_Vicpac_NW_005882811.1_1e-75_18_647_5387013_5386383
PVJP02910425.1_7210419-7211039_Hippopotamus_amphibius_isolate_BS01_ScHSfme_924895HRSCAF_982695_whole_genome_shotgun_sequence
PVJP02000127.1_267764-268397_Hippopotamus_amphibius_isolate_BS01_ScHSfme_119HRSCAF_19322_3_whole_genome_shotgun_sequence
land1_Capaeg_CM003219.1_6e-55_21_429_9148857_9148453
land1_Caphir_NC_030813.1_6e-55_21_429_9615390_9614986
land1_Capsib_NIYN02004956.1_5e-55_21_429_2705243_2705647
land1_Ammler_NIVO01073414.1_5e-55_21_429_305376_305780
land1_Ovican_PVIS010033370.1_2e-59_22_429_13180_12777
land1_Oviari_NC_040257.1_2e-54_22_429_11424578_11424175
land1_Hemhyl_PVJR01032452.1_2e-54_22_429_7295_7698
land1_Beahun_PVKQ01012214.1_1e-51_21_429_43007_43411
land1_Antmar_SJYB01001493.1_2e-50_26_429_248443_248049
land1_litwal_SJYG01000704.1_1e-51_26_429_850490_850092
land1_Eudtho_SJYE01001827.1_1e-51_26_429_222910_223308
land1_Saitat_PVIK010098224.1_3e-43_26_429_4130_3727
land1_Cephar_SJYT01037530.1_7e-54_21_428_197526_197928
land1_Cephar_SJYT01003498.1_7e-54_21_428_9932_10334
land1_Sylgri_SJYH01001750.1_3e-58_21_429_10586_10181
land1_Aepmel_SJXQ01004480.1_1e-45_21_429_43115_42720
land1_Aepmel_SJXQ01001806.1_1e-45_21_429_8581_8976
land1_Kobell_SJYR01000391.1_2e-55_21_429_23090_22684
PVJP02910137.1_540226-540877_Hippopotamus_amphibius_isolate_BS01_ScHSfme_924634HRSCAF_982434_whole_genome_shotgun_sequence
land1_Camdro_NW_011591205.1_9e-67_18_647_6012801_6013447
land1_Cambac_NW_011513121.1_8e-67_18_647_9388172_9387526
land1_Camfer_NW_006210992.1_4e-65_18_647_4250362_4249716
land1_Vicpac_NW_005883107.1_4e-70_18_641_488094_488734
0.02

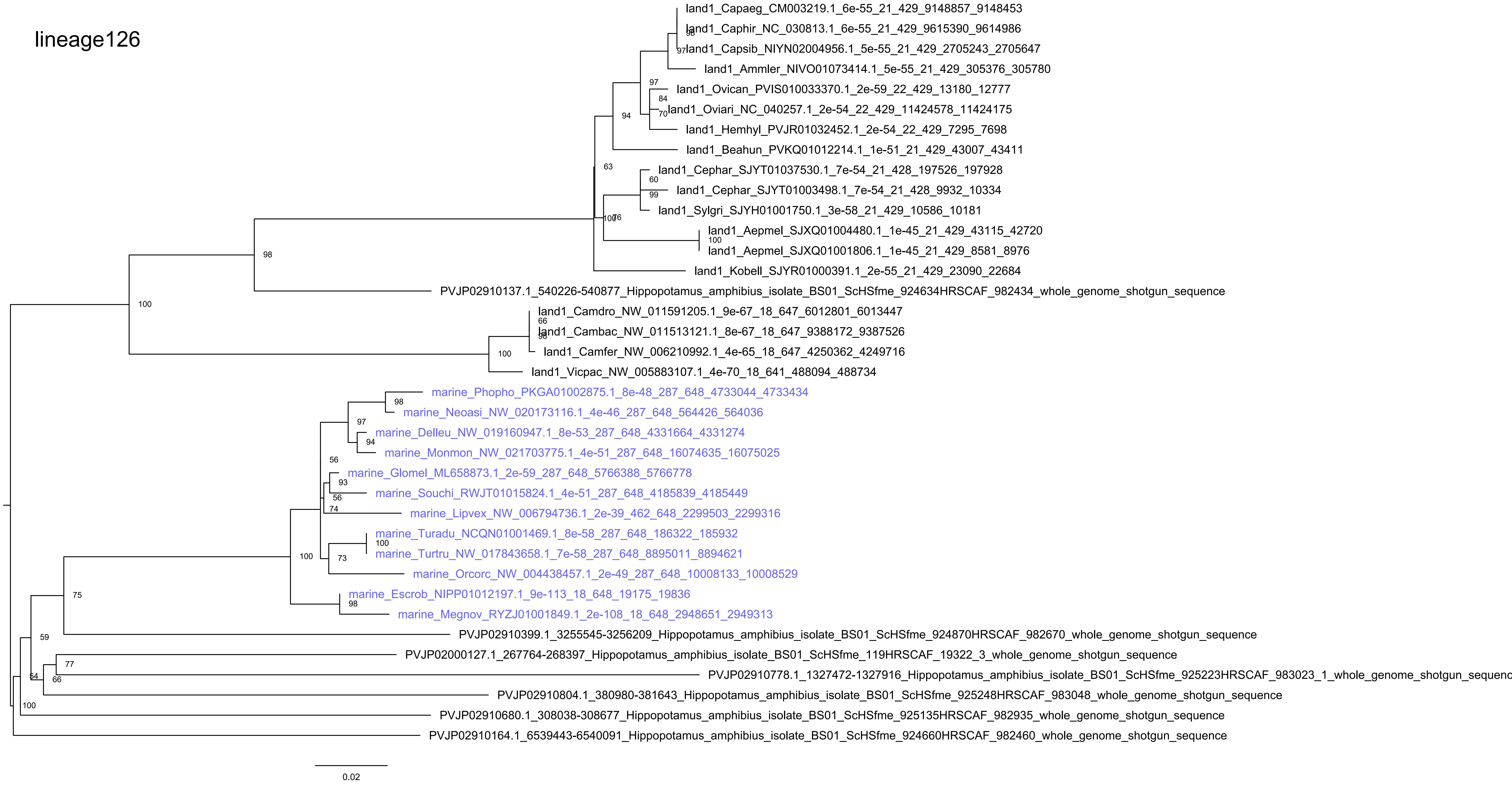
lineage126
land1_Capaeg_CM003219.1_6e-55_21_429_9148857_9148453
land1_Caphir_NC_030813.1_6e-55_21_429_9615390_9614986
land1_Capsib_NIYN02004956.1_5e-55_21_429_2705243_2705647
land1_Ammler_NIVO01073414.1_5e-55_21_429_305376_305780
land1_Ovican_PVIS010033370.1_2e-59_22_429_13180_12777
land1_Oviari_NC_040257.1_2e-54_22_429_11424578_11424175
land1_Hemhyl_PVJR01032452.1_2e-54_22_429_7295_7698
land1_Beahun_PVKQ01012214.1_1e-51_21_429_43007_43411
land1_Cephar_SJYT01037530.1_7e-54_21_428_197526_197928
land1_Cephar_SJYT01003498.1_7e-54_21_428_9932_10334
land1_Sylgri_SJYH01001750.1_3e-58_21_429_10586_10181
land1_Aepmel_SJXQ01004480.1_1e-45_21_429_43115_42720
land1_Aepmel_SJXQ01001806.1_1e-45_21_429_8581_8976
land1_Kobell_SJYR01000391.1_2e-55_21_429_23090_22684
PVJP02910137.1_540226-540877_Hippopotamus_amphibius_isolate_BS01_ScHSfme_924634HRSCAF_982434_whole_genome_shotgun_sequence
land1_Camdro_NW_011591205.1_9e-67_18_647_6012801_6013447
land1_Cambac_NW_011513121.1_8e-67_18_647_9388172_9387526
land1_Camfer_NW_006210992.1_4e-65_18_647_4250362_4249716
land1_Vicpac_NW_005883107.1_4e-70_18_641_488094_488734
marine_Phopho_PKGA01002875.1_8e-48_287_648_4733044_4733434
marine_Neoasi_NW_020173116.1_4e-46_287_648_564426_564036
marine_Delleu_NW_019160947.1_8e-53_287_648_4331664_4331274
marine_Monmon_NW_021703775.1_4e-51_287_648_16074635_16075025
marine_Glomel_ML658873.1_2e-59_287_648_5766388_5766778
marine_Souchi_RWJT01015824.1_4e-51_287_648_4185839_4185449
marine_Lipvex_NW_006794736.1_2e-39_462_648_2299503_2299316
marine_Turadu_NCQN01001469.1_8e-58_287_648_186322_185932
marine_Turtru_NW_017843658.1_7e-58_287_648_8895011_8894621
marine_Orcorc_NW_004438457.1_2e-49_287_648_10008133_10008529
marine_Escrob_NIPP01012197.1_9e-113_18_648_19175_19836
marine_Megnov_RYZJ01001849.1_2e-108_18_648_2948651_2949313
PVJP02910399.1_3255545-3256209_Hippopotamus_amphibius_isolate_BS01_ScHSfme_924870HRSCAF_982670_whole_genome_shotgun_sequence
PVJP02000127.1_267764-268397_Hippopotamus_amphibius_isolate_BS01_ScHSfme_119HRSCAF_19322_3_whole_genome_shotgun_sequence
PVJP02910778.1_1327472-1327916_Hippopotamus_amphibius_isolate_BS01_ScHSfme_925223HRSCAF_983023_1_whole_genome_shotgun_sequenc
PVJP02910804.1_380980-381643_Hippopotamus_amphibius_isolate_BS01_ScHSfme_925248HRSCAF_983048_whole_genome_shotgun_sequence
PVJP02910680.1_308038-308677_Hippopotamus_amphibius_isolate_BS01_ScHSfme_925135HRSCAF_982935_whole_genome_shotgun_sequence
PVJP02910164.1_6539443-6540091_Hippopotamus_amphibius_isolate_BS01_ScHSfme_924660HRSCAF_982460_whole_genome_shotgun_sequence
0.02

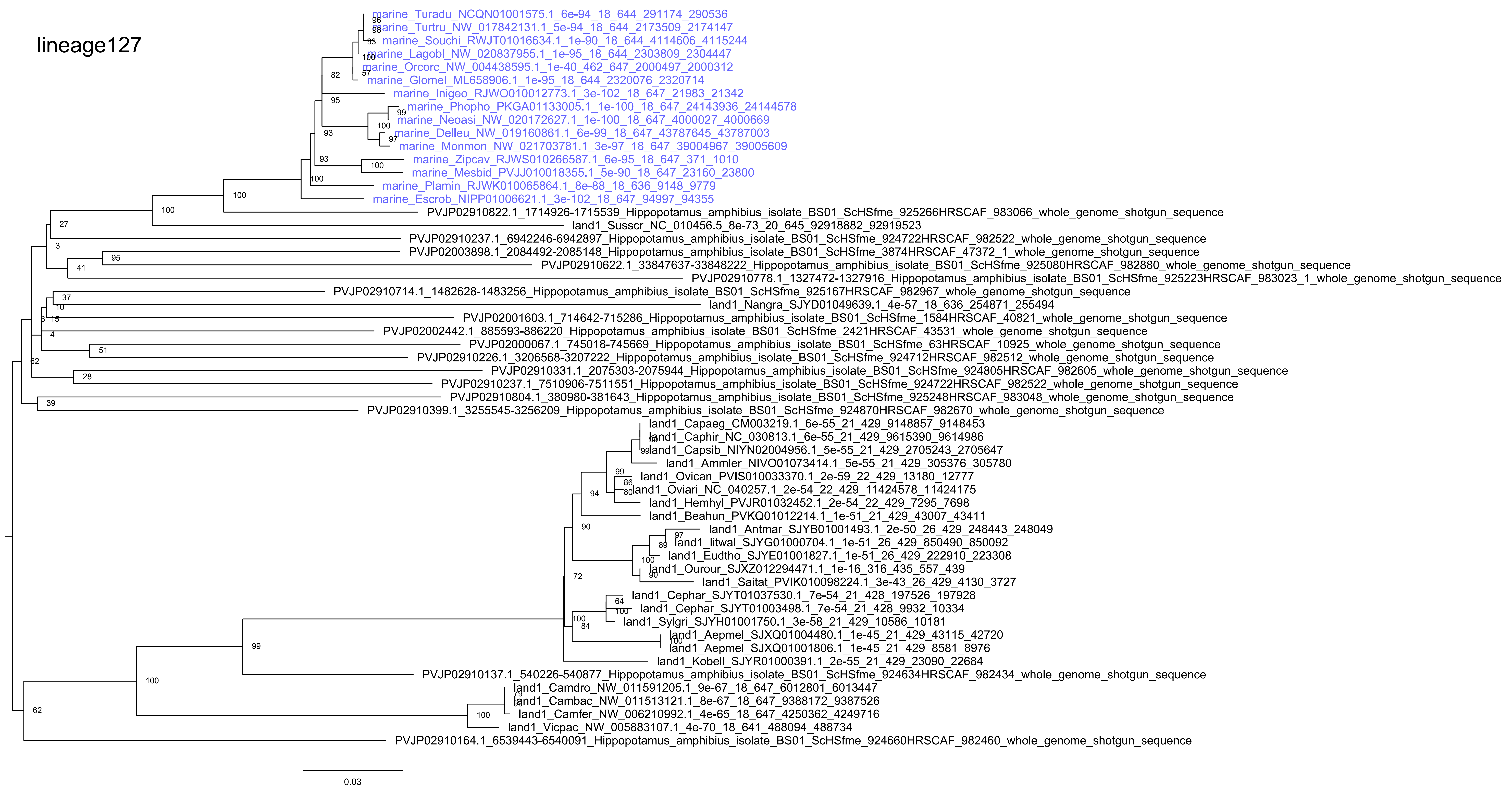
lineage127
marine_Turadu_NCQN01001575.1_6e-94_18_644_291174_290536
marine_Turtru_NW_017842131.1_5e-94_18_644_2173509_2174147
marine_Souchi_RWJT01016634.1_1e-90_18_644_4114606_4115244
marine_Lagobl_NW_020837955.1_1e-95_18_644_2303809_2304447
marine_Orcorc_NW_004438595.1_1e-40_462_647_2000497_2000312
marine_Glomel_ML658906.1_1e-95_18_644_2320076_2320714
marine_Inigeo_RJWO010012773.1_3e-102_18_647_21983_21342
marine_Phopho_PKGA01133005.1_1e-100_18_647_24143936_24144578
marine_Neoasi_NW_020172627.1_1e-100_18_647_4000027_4000669
marine_Delleu_NW_019160861.1_6e-99_18_647_43787645_43787003
marine_Monmon_NW_021703781.1_3e-97_18_647_39004967_39005609
marine_Zipcav_RJWS010266587.1_6e-95_18_647_371_1010
marine_Mesbid_PVJJ010018355.1_5e-90_18_647_23160_23800
marine_Plamin_RJWK010065864.1_8e-88_18_636_9148_9779
marine_Escrob_NIPP01006621.1_3e-102_18_647_94997_94355
PVJP02910822.1_1714926-1715539_Hippopotamus_amphibius_isolate_BS01_ScHSfme_925266HRSCAF_983066_whole_genome_shotgun_sequence
land1_Susscr_NC_010456.5_8e-73_20_645_92918882_92919523
PVJP02910237.1_6942246-6942897_Hippopotamus_amphibius_isolate_BS01_ScHSfme_924722HRSCAF_982522_whole_genome_shotgun_sequence
PVJP02003898.1_2084492-2085148_Hippopotamus_amphibius_isolate_BS01_ScHSfme_3874HRSCAF_47372_1_whole_genome_shotgun_sequence
PVJP02910622.1_33847637-33848222_Hippopotamus_amphibius_isolate_BS01_ScHSfme_925080HRSCAF_982880_whole_genome_shotgun_sequence
PVJP02910778.1_1327472-1327916_Hippopotamus_amphibius_isolate_BS01_ScHSfme_925223HRSCAF_983023_1_whole_genome_shotgun_sequence
PVJP02910714.1_1482628-1483256_Hippopotamus_amphibius_isolate_BS01_ScHSfme_925167HRSCAF_982967_whole_genome_shotgun_sequence
land1_Nangra_SJYD01049639.1_4e-57_18_636_254871_255494
PVJP02001603.1_714642-715286_Hippopotamus_amphibius_isolate_BS01_ScHSfme_1584HRSCAF_40821_whole_genome_shotgun_sequence
PVJP02002442.1_885593-886220_Hippopotamus_amphibius_isolate_BS01_ScHSfme_2421HRSCAF_43531_whole_genome_shotgun_sequence
PVJP02000067.1_745018-745669_Hippopotamus_amphibius_isolate_BS01_ScHSfme_63HRSCAF_10925_whole_genome_shotgun_sequence
PVJP02910226.1_3206568-3207222_Hippopotamus_amphibius_isolate_BS01_ScHSfme_924712HRSCAF_982512_whole_genome_shotgun_sequence
PVJP02910331.1_2075303-2075944_Hippopotamus_amphibius_isolate_BS01_ScHSfme_924805HRSCAF_982605_whole_genome_shotgun_sequence
PVJP02910237.1_7510906-7511551_Hippopotamus_amphibius_isolate_BS01_ScHSfme_924722HRSCAF_982522_whole_genome_shotgun_sequence
PVJP02910804.1_380980-381643_Hippopotamus_amphibius_isolate_BS01_ScHSfme_925248HRSCAF_983048_whole_genome_shotgun_sequence
PVJP02910399.1_3255545-3256209_Hippopotamus_amphibius_isolate_BS01_ScHSfme_924870HRSCAF_982670_whole_genome_shotgun_sequence
land1_Capaeg_CM003219.1_6e-55_21_429_9148857_9148453
land1_Caphir_NC_030813.1_6e-55_21_429_9615390_9614986
land1_Capsib_NIYN02004956.1_5e-55_21_429_2705243_2705647
land1_Ammler_NIVO01073414.1_5e-55_21_429_305376_305780
land1_Ovican_PVIS010033370.1_2e-59_22_429_13180_12777
land1_Oviari_NC_040257.1_2e-54_22_429_11424578_11424175
land1_Hemhyl_PVJR01032452.1_2e-54_22_429_7295_7698
land1_Beahun_PVKQ01012214.1_1e-51_21_429_43007_43411
land1_Antmar_SJYB01001493.1_2e-50_26_429_248443_248049
land1_litwal_SJYG01000704.1_1e-51_26_429_850490_850092
land1_Eudtho_SJYE01001827.1_1e-51_26_429_222910_223308
land1_Ourour_SJXZ012294471.1_1e-16_316_435_557_439
land1_Saitat_PVIK010098224.1_3e-43_26_429_4130_3727
land1_Cephar_SJYT01037530.1_7e-54_21_428_197526_197928
land1_Cephar_SJYT01003498.1_7e-54_21_428_9932_10334
land1_Sylgri_SJYH01001750.1_3e-58_21_429_10586_10181
land1_Aepmel_SJXQ01004480.1_1e-45_21_429_43115_42720
land1_Aepmel_SJXQ01001806.1_1e-45_21_429_8581_8976
land1_Kobell_SJYR01000391.1_2e-55_21_429_23090_22684
PVJP02910137.1_540226-540877_Hippopotamus_amphibius_isolate_BS01_ScHSfme_924634HRSCAF_982434_whole_genome_shotgun_sequence
land1_Camdro_NW_011591205.1_9e-67_18_647_6012801_6013447
land1_Cambac_NW_011513121.1_8e-67_18_647_9388172_9387526
land1_Camfer_NW_006210992.1_4e-65_18_647_4250362_4249716
land1_Vicpac_NW_005883107.1_4e-70_18_641_488094_488734
PVJP02910164.1_6539443-6540091_Hippopotamus_amphibius_isolate_BS01_ScHSfme_924660HRSCAF_982460_whole_genome_shotgun_sequence
0.03

lineage128

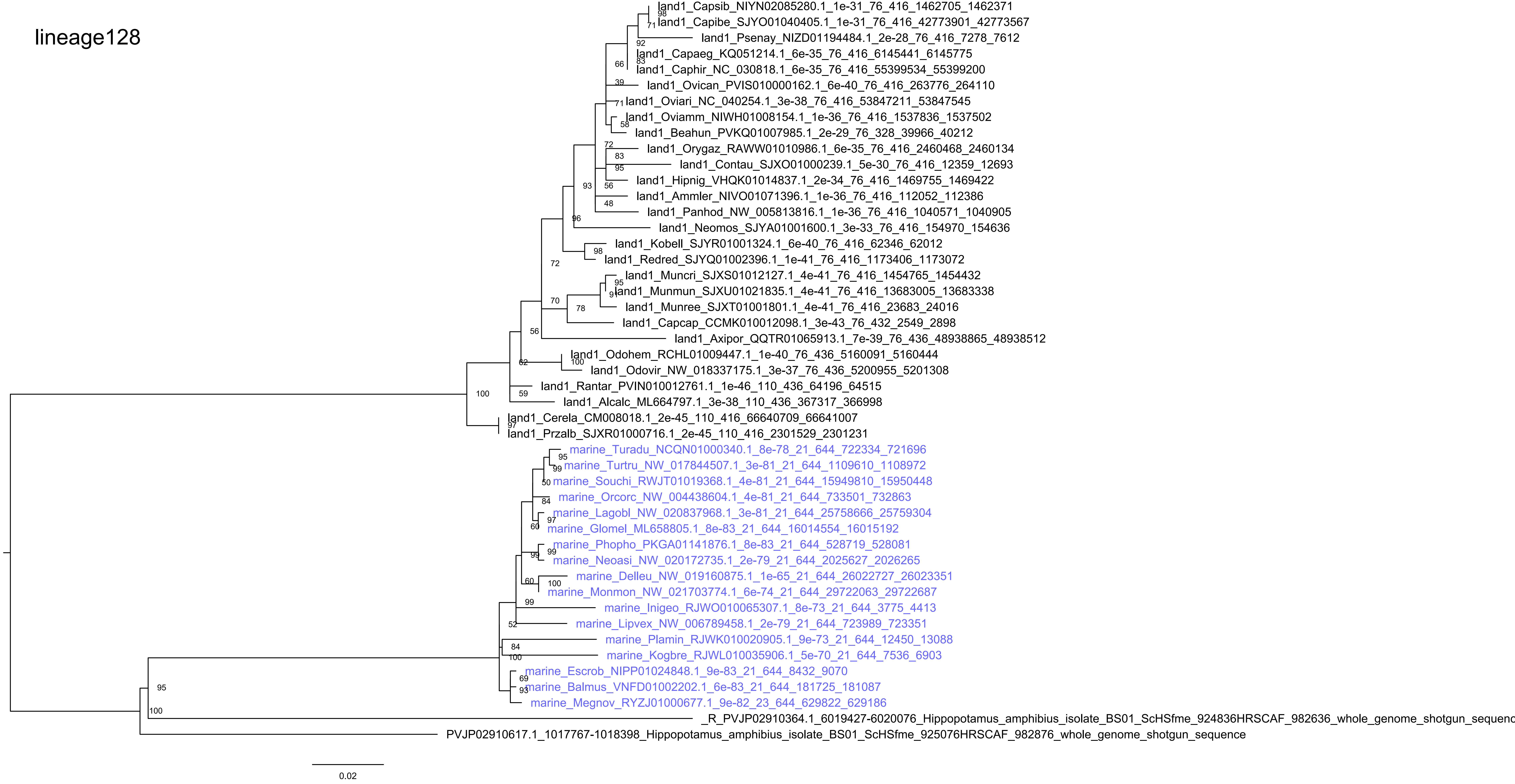
land1_Capsib_NIYN02085280.1_1e-31_76_416_1462705_1462371
land1_Capibe_SJYO01040405.1_1e-31_76_416_42773901_42773567
land1_Psenay_NIZD01194484.1_2e-28_76_416_7278_7612
land1_Capaeg_KQ051214.1_6e-35_76_416_6145441_6145775
land1_Caphir_NC_030818.1_6e-35_76_416_55399534_55399200
land1_Ovican_PVIS010000162.1_6e-40_76_416_263776_264110
land1_Oviari_NC_040254.1_3e-38_76_416_53847211_53847545
land1_Oviamm_NIWH01008154.1_1e-36_76_416_1537836_1537502
land1_Beahun_PVKQ01007985.1_2e-29_76_328_39966_40212
land1_Orygaz_RAWW01010986.1_6e-35_76_416_2460468_2460134
land1_Contau_SJXO01000239.1_5e-30_76_416_12359_12693
land1_Hipnig_VHQK01014837.1_2e-34_76_416_1469755_1469422
land1_Ammler_NIVO01071396.1_1e-36_76_416_112052_112386
land1_Panhod_NW_005813816.1_1e-36_76_416_1040571_1040905
land1_Neomos_SJYA01001600.1_3e-33_76_416_154970_154636
land1_Kobell_SJYR01001324.1_6e-40_76_416_62346_62012
land1_Redred_SJYQ01002396.1_1e-41_76_416_1173406_1173072
land1_Muncri_SJXS01012127.1_4e-41_76_416_1454765_1454432
land1_Munmun_SJXU01021835.1_4e-41_76_416_13683005_13683338
land1_Munree_SJXT01001801.1_4e-41_76_416_23683_24016
land1_Capcap_CCMK010012098.1_3e-43_76_432_2549_2898
land1_Axipor_QQTR01065913.1_7e-39_76_436_48938865_48938512
land1_Odohem_RCHL01009447.1_1e-40_76_436_5160091_5160444
land1_Odovir_NW_018337175.1_3e-37_76_436_5200955_5201308
land1_Rantar_PVIN010012761.1_1e-46_110_436_64196_64515
land1_Alcalc_ML664797.1_3e-38_110_436_367317_366998
land1_Cerela_CM008018.1_2e-45_110_416_66640709_66641007
land1_Przalb_SJXR01000716.1_2e-45_110_416_2301529_2301231
marine_Turadu_NCQN01000340.1_8e-78_21_644_722334_721696
marine_Turtru_NW_017844507.1_3e-81_21_644_1109610_1108972
marine_Souchi_RWJT01019368.1_4e-81_21_644_15949810_15950448
marine_Orcorc_NW_004438604.1_4e-81_21_644_733501_732863
marine_Lagobl_NW_020837968.1_3e-81_21_644_25758666_25759304
marine_Glomel_ML658805.1_8e-83_21_644_16014554_16015192
marine_Phopho_PKGA01141876.1_8e-83_21_644_528719_528081
marine_Neoasi_NW_020172735.1_2e-79_21_644_2025627_2026265
marine_Delleu_NW_019160875.1_1e-65_21_644_26022727_26023351
marine_Monmon_NW_021703774.1_6e-74_21_644_29722063_29722687
marine_Inigeo_RJWO010065307.1_8e-73_21_644_3775_4413
marine_Lipvex_NW_006789458.1_2e-79_21_644_723989_723351
marine_Plamin_RJWK010020905.1_9e-73_21_644_12450_13088
marine_Kogbre_RJWL010035906.1_5e-70_21_644_7536_6903
marine_Escrob_NIPP01024848.1_9e-83_21_644_8432_9070
marine_Balmus_VNFD01002202.1_6e-83_21_644_181725_181087
marine_Megnov_RYZJ01000677.1_9e-82_23_644_629822_629186
_R_PVJP02910364.1_6019427-6020076_Hippopotamus_amphibius_isolate_BS01_ScHSfme_924836HRSCAF_982636_whole_genome_shotgun_sequenc
PVJP02910617.1_1017767-1018398_Hippopotamus_amphibius_isolate_BS01_ScHSfme_925076HRSCAF_982876_whole_genome_shotgun_sequence
0.02

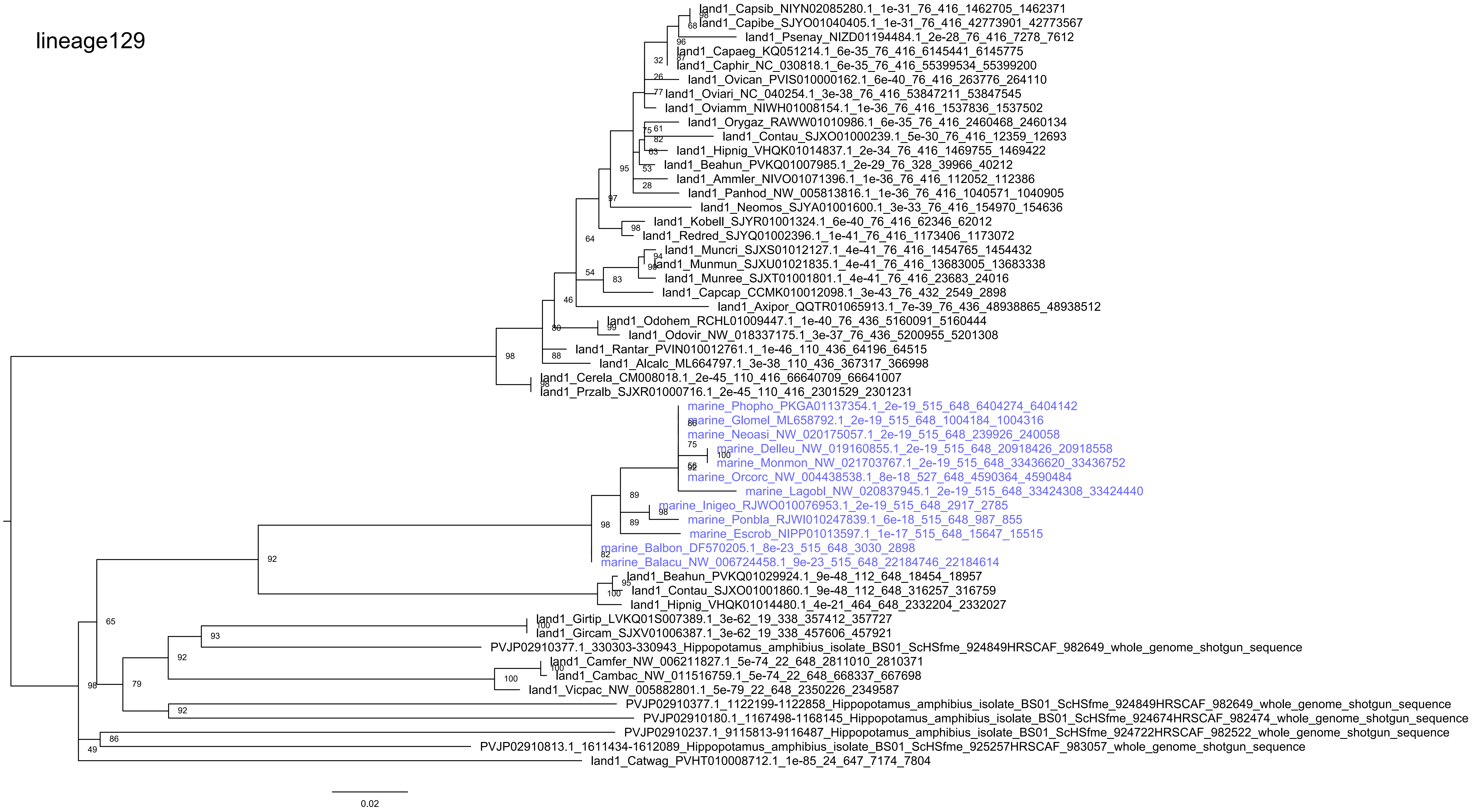
lineage129
land1_Capsib_NIYN02085280.1_1e-31_76_416_1462705_1462371
land1_Capibe_SJYO01040405.1_1e-31_76_416_42773901_42773567
land1_Psenay_NIZD01194484.1_2e-28_76_416_7278_7612
land1_Capaeg_KQ051214.1_6e-35_76_416_6145441_6145775
land1_Caphir_NC_030818.1_6e-35_76_416_55399534_55399200
land1_Ovican_PVIS010000162.1_6e-40_76_416_263776_264110
land1_Oviari_NC_040254.1_3e-38_76_416_53847211_53847545
land1_Oviamm_NIWH01008154.1_1e-36_76_416_1537836_1537502
land1_Orygaz_RAWW01010986.1_6e-35_76_416_2460468_2460134
land1_Contau_SJXO01000239.1_5e-30_76_416_12359_12693
land1_Hipnig_VHQK01014837.1_2e-34_76_416_1469755_1469422
land1_Beahun_PVKQ01007985.1_2e-29_76_328_39966_40212
land1_Ammler_NIVO01071396.1_1e-36_76_416_112052_112386
land1_Panhod_NW_005813816.1_1e-36_76_416_1040571_1040905
land1_Neomos_SJYA01001600.1_3e-33_76_416_154970_154636
land1_Kobell_SJYR01001324.1_6e-40_76_416_62346_62012
land1_Redred_SJYQ01002396.1_1e-41_76_416_1173406_1173072
land1_Muncri_SJXS01012127.1_4e-41_76_416_1454765_1454432
land1_Munmun_SJXU01021835.1_4e-41_76_416_13683005_13683338
land1_Munree_SJXT01001801.1_4e-41_76_416_23683_24016
land1_Capcap_CCMK010012098.1_3e-43_76_432_2549_2898
land1_Axipor_QQTR01065913.1_7e-39_76_436_48938865_48938512
land1_Odohem_RCHL01009447.1_1e-40_76_436_5160091_5160444
land1_Odovir_NW_018337175.1_3e-37_76_436_5200955_5201308
land1_Rantar_PVIN010012761.1_1e-46_110_436_64196_64515
land1_Alcalc_ML664797.1_3e-38_110_436_367317_366998
land1_Cerela_CM008018.1_2e-45_110_416_66640709_66641007
land1_Przalb_SJXR01000716.1_2e-45_110_416_2301529_2301231
marine_Phopho_PKGA01137354.1_2e-19_515_648_6404274_6404142
marine_Glomel_ML658792.1_2e-19_515_648_1004184_1004316
marine_Neoasi_NW_020175057.1_2e-19_515_648_239926_240058
marine_Delleu_NW_019160855.1_2e-19_515_648_20918426_20918558
marine_Monmon_NW_021703767.1_2e-19_515_648_33436620_33436752
marine_Orcorc_NW_004438538.1_8e-18_527_648_4590364_4590484
marine_Lagobl_NW_020837945.1_2e-19_515_648_33424308_33424440
marine_Inigeo_RJWO010076953.1_2e-19_515_648_2917_2785
marine_Ponbla_RJWI010247839.1_6e-18_515_648_987_855
marine_Escrob_NIPP01013597.1_1e-17_515_648_15647_15515
marine_Balbon_DF570205.1_8e-23_515_648_3030_2898
marine_Balacu_NW_006724458.1_9e-23_515_648_22184746_22184614
land1_Beahun_PVKQ01029924.1_9e-48_112_648_18454_18957
land1_Contau_SJXO01001860.1_9e-48_112_648_316257_316759
land1_Hipnig_VHQK01014480.1_4e-21_464_648_2332204_2332027
land1_Girtip_LVKQ01S007389.1_3e-62_19_338_357412_357727
land1_Gircam_SJXV01006387.1_3e-62_19_338_457606_457921
PVJP02910377.1_330303-330943_Hippopotamus_amphibius_isolate_BS01_ScHSfme_924849HRSCAF_982649_whole_genome_shotgun_sequence
land1_Camfer_NW_006211827.1_5e-74_22_648_2811010_2810371
land1_Cambac_NW_011516759.1_5e-74_22_648_668337_667698
land1_Vicpac_NW_005882801.1_5e-79_22_648_2350226_2349587
PVJP02910377.1_1122199-1122858_Hippopotamus_amphibius_isolate_BS01_ScHSfme_924849HRSCAF_982649_whole_genome_shotgun_sequence
PVJP02910180.1_1167498-1168145_Hippopotamus_amphibius_isolate_BS01_ScHSfme_924674HRSCAF_982474_whole_genome_shotgun_sequence
PVJP02910237.1_9115813-9116487_Hippopotamus_amphibius_isolate_BS01_ScHSfme_924722HRSCAF_982522_whole_genome_shotgun_sequence
PVJP02910813.1_1611434-1612089_Hippopotamus_amphibius_isolate_BS01_ScHSfme_925257HRSCAF_983057_whole_genome_shotgun_sequence
land1_Catwag_PVHT010008712.1_1e-85_24_647_7174_7804
0.02

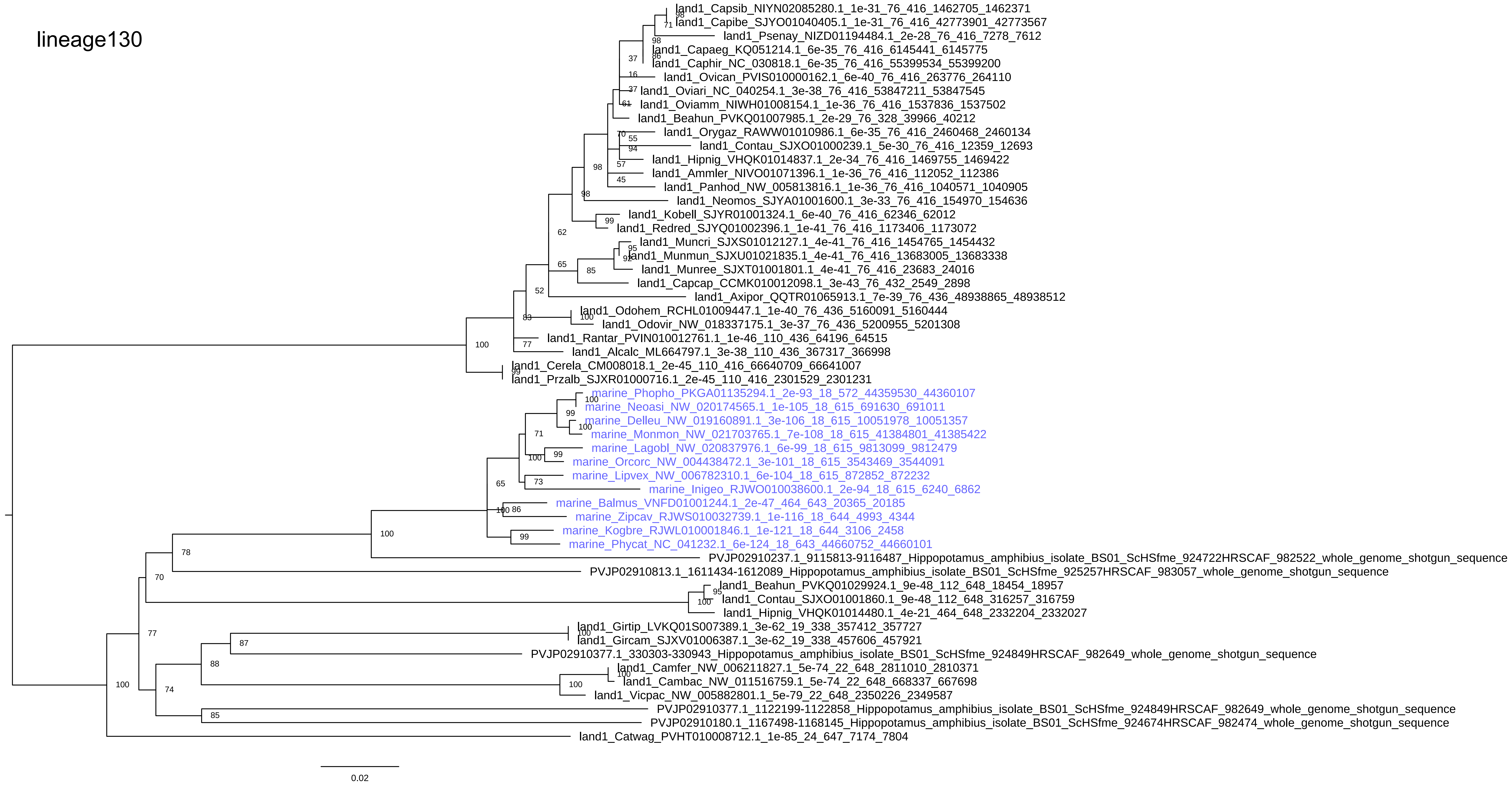
lineage130
land1_Capsib_NIYN02085280.1_1e-31_76_416_1462705_1462371
land1_Capibe_SJYO01040405.1_1e-31_76_416_42773901_42773567
land1_Psenay_NIZD01194484.1_2e-28_76_416_7278_7612
land1_Capaeg_KQ051214.1_6e-35_76_416_6145441_6145775
land1_Caphir_NC_030818.1_6e-35_76_416_55399534_55399200
land1_Ovican_PVIS010000162.1_6e-40_76_416_263776_264110
land1_Oviari_NC_040254.1_3e-38_76_416_53847211_53847545
land1_Oviamm_NIWH01008154.1_1e-36_76_416_1537836_1537502
land1_Beahun_PVKQ01007985.1_2e-29_76_328_39966_40212
land1_Orygaz_RAWW01010986.1_6e-35_76_416_2460468_2460134
land1_Contau_SJXO01000239.1_5e-30_76_416_12359_12693
land1_Hipnig_VHQK01014837.1_2e-34_76_416_1469755_1469422
land1_Ammler_NIVO01071396.1_1e-36_76_416_112052_112386
land1_Panhod_NW_005813816.1_1e-36_76_416_1040571_1040905
land1_Neomos_SJYA01001600.1_3e-33_76_416_154970_154636
land1_Kobell_SJYR01001324.1_6e-40_76_416_62346_62012
land1_Redred_SJYQ01002396.1_1e-41_76_416_1173406_1173072
land1_Muncri_SJXS01012127.1_4e-41_76_416_1454765_1454432
land1_Munmun_SJXU01021835.1_4e-41_76_416_13683005_13683338
land1_Munree_SJXT01001801.1_4e-41_76_416_23683_24016
land1_Capcap_CCMK010012098.1_3e-43_76_432_2549_2898
land1_Axipor_QQTR01065913.1_7e-39_76_436_48938865_48938512
land1_Odohem_RCHL01009447.1_1e-40_76_436_5160091_5160444
land1_Odovir_NW_018337175.1_3e-37_76_436_5200955_5201308
land1_Rantar_PVIN010012761.1_1e-46_110_436_64196_64515
land1_Alcalc_ML664797.1_3e-38_110_436_367317_366998
land1_Cerela_CM008018.1_2e-45_110_416_66640709_66641007
land1_Przalb_SJXR01000716.1_2e-45_110_416_2301529_2301231
marine_Phopho_PKGA01135294.1_2e-93_18_572_44359530_44360107
marine_Neoasi_NW_020174565.1_1e-105_18_615_691630_691011
marine_Delleu_NW_019160891.1_3e-106_18_615_10051978_10051357
marine_Monmon_NW_021703765.1_7e-108_18_615_41384801_41385422
marine_Lagobl_NW_020837976.1_6e-99_18_615_9813099_9812479
marine_Orcorc_NW_004438472.1_3e-101_18_615_3543469_3544091
marine_Lipvex_NW_006782310.1_6e-104_18_615_872852_872232
marine_Inigeo_RJWO010038600.1_2e-94_18_615_6240_6862
marine_Balmus_VNFD01001244.1_2e-47_464_643_20365_20185
marine_Zipcav_RJWS010032739.1_1e-116_18_644_4993_4344
marine_Kogbre_RJWL010001846.1_1e-121_18_644_3106_2458
marine_Phycat_NC_041232.1_6e-124_18_643_44660752_44660101
PVJP02910237.1_9115813-9116487_Hippopotamus_amphibius_isolate_BS01_ScHSfme_924722HRSCAF_982522_whole_genome_shotgun_sequence
PVJP02910813.1_1611434-1612089_Hippopotamus_amphibius_isolate_BS01_ScHSfme_925257HRSCAF_983057_whole_genome_shotgun_sequence
land1_Beahun_PVKQ01029924.1_9e-48_112_648_18454_18957
land1_Contau_SJXO01001860.1_9e-48_112_648_316257_316759
land1_Hipnig_VHQK01014480.1_4e-21_464_648_2332204_2332027
land1_Girtip_LVKQ01S007389.1_3e-62_19_338_357412_357727
land1_Gircam_SJXV01006387.1_3e-62_19_338_457606_457921
PVJP02910377.1_330303-330943_Hippopotamus_amphibius_isolate_BS01_ScHSfme_924849HRSCAF_982649_whole_genome_shotgun_sequence
land1_Camfer_NW_006211827.1_5e-74_22_648_2811010_2810371
land1_Cambac_NW_011516759.1_5e-74_22_648_668337_667698
land1_Vicpac_NW_005882801.1_5e-79_22_648_2350226_2349587
PVJP02910377.1_1122199-1122858_Hippopotamus_amphibius_isolate_BS01_ScHSfme_924849HRSCAF_982649_whole_genome_shotgun_sequence
PVJP02910180.1_1167498-1168145_Hippopotamus_amphibius_isolate_BS01_ScHSfme_924674HRSCAF_982474_whole_genome_shotgun_sequence
land1_Catwag_PVHT010008712.1_1e-85_24_647_7174_7804
0.02

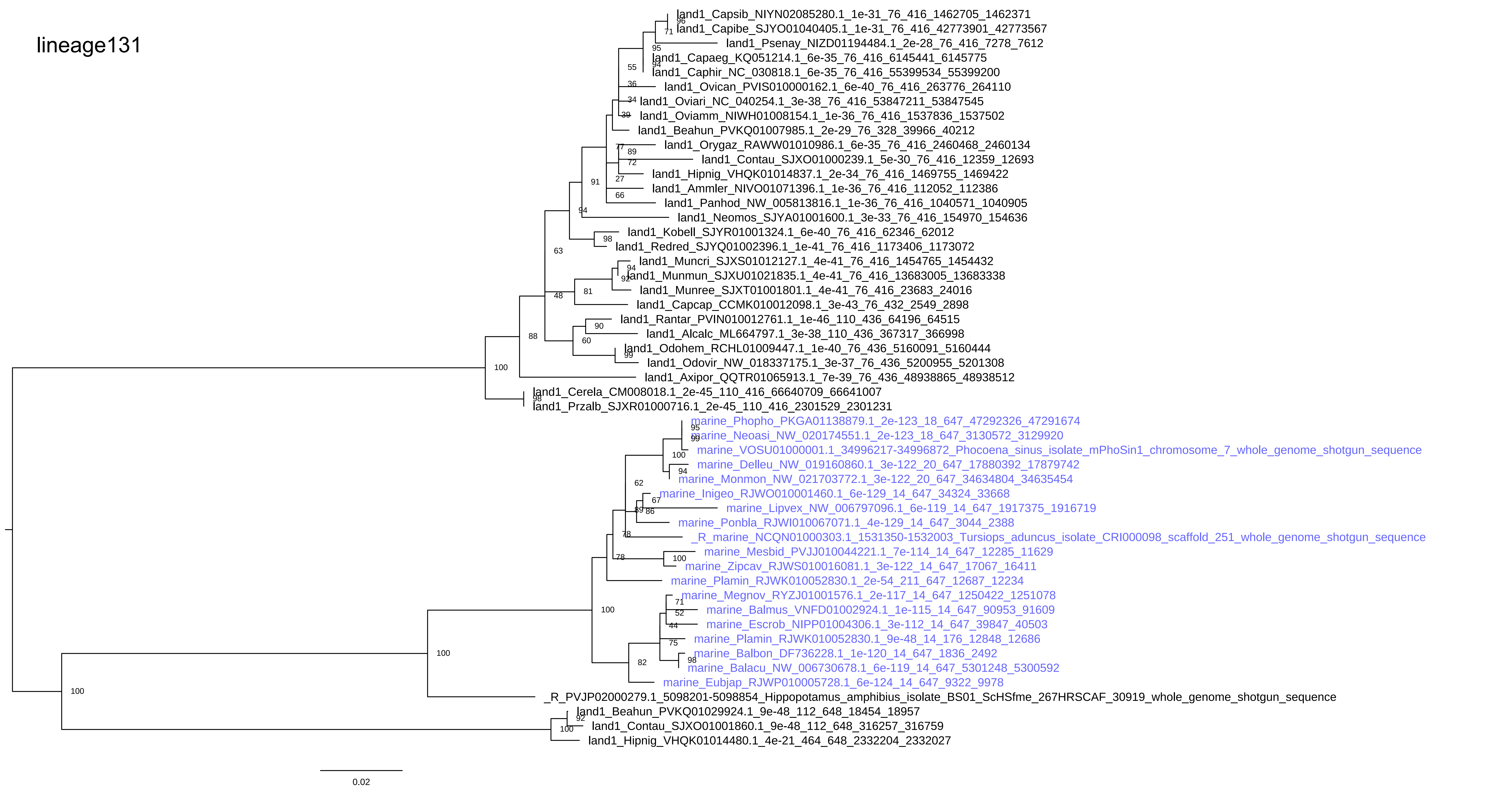

lineage131
land1_Capsib_NIYN02085280.1_1e-31_76_416_1462705_1462371
land1_Capibe_SJYO01040405.1_1e-31_76_416_42773901_42773567
land1_Psenay_NIZD01194484.1_2e-28_76_416_7278_7612
land1_Capaeg_KQ051214.1_6e-35_76_416_6145441_6145775
land1_Caphir_NC_030818.1_6e-35_76_416_55399534_55399200
land1_Ovican_PVIS010000162.1_6e-40_76_416_263776_264110
land1_Oviari_NC_040254.1_3e-38_76_416_53847211_53847545
land1_Oviamm_NIWH01008154.1_1e-36_76_416_1537836_1537502
land1_Beahun_PVKQ01007985.1_2e-29_76_328_39966_40212
land1_Orygaz_RAWW01010986.1_6e-35_76_416_2460468_2460134
land1_Contau_SJXO01000239.1_5e-30_76_416_12359_12693
land1_Hipnig_VHQK01014837.1_2e-34_76_416_1469755_1469422
land1_Ammler_NIVO01071396.1_1e-36_76_416_112052_112386
land1_Panhod_NW_005813816.1_1e-36_76_416_1040571_1040905
land1_Neomos_SJYA01001600.1_3e-33_76_416_154970_154636
land1_Kobell_SJYR01001324.1_6e-40_76_416_62346_62012
land1_Redred_SJYQ01002396.1_1e-41_76_416_1173406_1173072
land1_Muncri_SJXS01012127.1_4e-41_76_416_1454765_1454432
land1_Munmun_SJXU01021835.1_4e-41_76_416_13683005_13683338
land1_Munree_SJXT01001801.1_4e-41_76_416_23683_24016
land1_Capcap_CCMK010012098.1_3e-43_76_432_2549_2898
land1_Rantar_PVIN010012761.1_1e-46_110_436_64196_64515
land1_Alcalc_ML664797.1_3e-38_110_436_367317_366998
land1_Odohem_RCHL01009447.1_1e-40_76_436_5160091_5160444
land1_Odovir_NW_018337175.1_3e-37_76_436_5200955_5201308
land1_Axipor_QQTR01065913.1_7e-39_76_436_48938865_48938512
land1_Cerela_CM008018.1_2e-45_110_416_66640709_66641007
land1_Przalb_SJXR01000716.1_2e-45_110_416_2301529_2301231
marine_Phopho_PKGA01138879.1_2e-123_18_647_47292326_47291674
marine_Neoasi_NW_020174551.1_2e-123_18_647_3130572_3129920
marine_VOSU01000001.1_34996217-34996872_Phocoena_sinus_isolate_mPhoSin1_chromosome_7_whole_genome_shotgun_sequence
marine_Delleu_NW_019160860.1_3e-122_20_647_17880392_17879742
marine_Monmon_NW_021703772.1_3e-122_20_647_34634804_34635454
marine_Inigeo_RJWO010001460.1_6e-129_14_647_34324_33668
marine_Lipvex_NW_006797096.1_6e-119_14_647_1917375_1916719
marine_Ponbla_RJWI010067071.1_4e-129_14_647_3044_2388
_R_marine_NCQN01000303.1_1531350-1532003_Tursiops_aduncus_isolate_CRI000098_scaffold_251_whole_genome_shotgun_sequence
marine_Mesbid_PVJJ010044221.1_7e-114_14_647_12285_11629
marine_Zipcav_RJWS010016081.1_3e-122_14_647_17067_16411
marine_Plamin_RJWK010052830.1_2e-54_211_647_12687_12234
marine_Megnov_RYZJ01001576.1_2e-117_14_647_1250422_1251078
marine_Balmus_VNFD01002924.1_1e-115_14_647_90953_91609
marine_Escrob_NIPP01004306.1_3e-112_14_647_39847_40503
marine_Plamin_RJWK010052830.1_9e-48_14_176_12848_12686
marine_Balbon_DF736228.1_1e-120_14_647_1836_2492
marine_Balacu_NW_006730678.1_6e-119_14_647_5301248_5300592
marine_Eubjap_RJWP010005728.1_6e-124_14_647_9322_9978
_R_PVJP02000279.1_5098201-5098854_Hippopotamus_amphibius_isolate_BS01_ScHSfme_267HRSCAF_30919_whole_genome_shotgun_sequence
land1_Beahun_PVKQ01029924.1_9e-48_112_648_18454_18957
land1_Contau_SJXO01001860.1_9e-48_112_648_316257_316759
land1_Hipnig_VHQK01014480.1_4e-21_464_648_2332204_2332027
0.02

lineage132

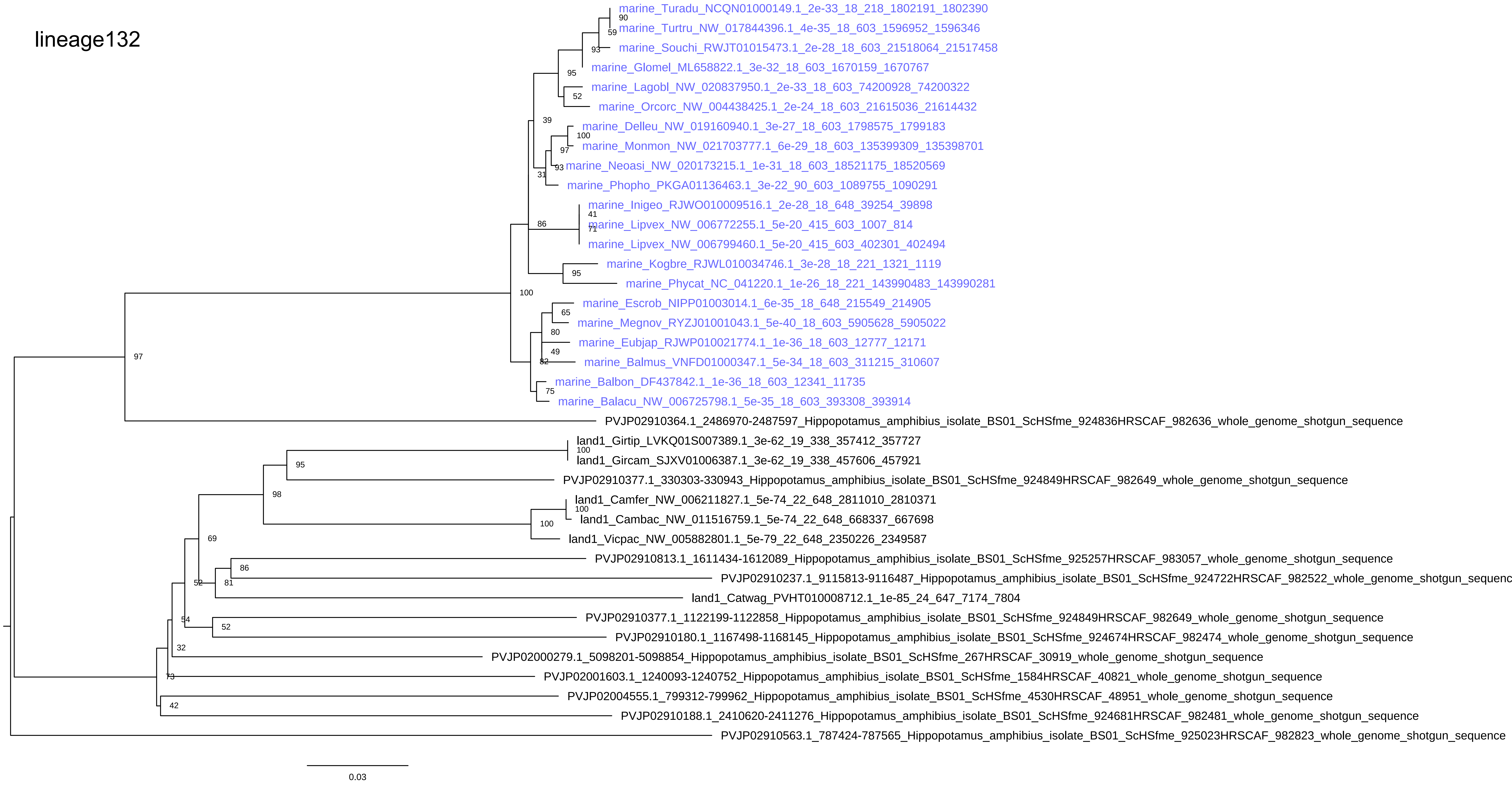

marine_Turadu_NCQN01000149.1_2e-33_18_218_1802191_1802390
marine_Turtru_NW_017844396.1_4e-35_18_603_1596952_1596346
marine_Souchi_RWJT01015473.1_2e-28_18_603_21518064_21517458
marine_Glomel_ML658822.1_3e-32_18_603_1670159_1670767
marine_Lagobl_NW_020837950.1_2e-33_18_603_74200928_74200322
marine_Orcorc_NW_004438425.1_2e-24_18_603_21615036_21614432
marine_Delleu_NW_019160940.1_3e-27_18_603_1798575_1799183
marine_Monmon_NW_021703777.1_6e-29_18_603_135399309_135398701
marine_Neoasi_NW_020173215.1_1e-31_18_603_18521175_18520569
marine_Phopho_PKGA01136463.1_3e-22_90_603_1089755_1090291
marine_Inigeo_RJWO010009516.1_2e-28_18_648_39254_39898
marine_Lipvex_NW_006772255.1_5e-20_415_603_1007_814
marine_Lipvex_NW_006799460.1_5e-20_415_603_402301_402494
marine_Kogbre_RJWL010034746.1_3e-28_18_221_1321_1119
marine_Phycat_NC_041220.1_1e-26_18_221_143990483_143990281
marine_Escrob_NIPP01003014.1_6e-35_18_648_215549_214905
marine_Megnov_RYZJ01001043.1_5e-40_18_603_5905628_5905022
marine_Eubjap_RJWP010021774.1_1e-36_18_603_12777_12171
marine_Balmus_VNFD01000347.1_5e-34_18_603_311215_310607
marine_Balbon_DF437842.1_1e-36_18_603_12341_11735
marine_Balacu_NW_006725798.1_5e-35_18_603_393308_393914
PVJP02910364.1_2486970-2487597_Hippopotamus_amphibius_isolate_BS01_ScHSfme_924836HRSCAF_982636_whole_genome_shotgun_sequence
land1_Girtip_LVKQ01S007389.1_3e-62_19_338_357412_357727
land1_Gircam_SJXV01006387.1_3e-62_19_338_457606_457921
PVJP02910377.1_330303-330943_Hippopotamus_amphibius_isolate_BS01_ScHSfme_924849HRSCAF_982649_whole_genome_shotgun_sequence
land1_Camfer_NW_006211827.1_5e-74_22_648_2811010_2810371
land1_Cambac_NW_011516759.1_5e-74_22_648_668337_667698
land1_Vicpac_NW_005882801.1_5e-79_22_648_2350226_2349587
PVJP02910813.1_1611434-1612089_Hippopotamus_amphibius_isolate_BS01_ScHSfme_925257HRSCAF_983057_whole_genome_shotgun_sequence
PVJP02910237.1_9115813-9116487_Hippopotamus_amphibius_isolate_BS01_ScHSfme_924722HRSCAF_982522_whole_genome_shotgun_sequenc
land1_Catwag_PVHT010008712.1_1e-85_24_647_7174_7804
PVJP02910377.1_1122199-1122858_Hippopotamus_amphibius_isolate_BS01_ScHSfme_924849HRSCAF_982649_whole_genome_shotgun_sequence
PVJP02910180.1_1167498-1168145_Hippopotamus_amphibius_isolate_BS01_ScHSfme_924674HRSCAF_982474_whole_genome_shotgun_sequence
PVJP02000279.1_5098201-5098854_Hippopotamus_amphibius_isolate_BS01_ScHSfme_267HRSCAF_30919_whole_genome_shotgun_sequence
PVJP02001603.1_1240093-1240752_Hippopotamus_amphibius_isolate_BS01_ScHSfme_1584HRSCAF_40821_whole_genome_shotgun_sequence
PVJP02004555.1_799312-799962_Hippopotamus_amphibius_isolate_BS01_ScHSfme_4530HRSCAF_48951_whole_genome_shotgun_sequence
PVJP02910188.1_2410620-2411276_Hippopotamus_amphibius_isolate_BS01_ScHSfme_924681HRSCAF_982481_whole_genome_shotgun_sequence
PVJP02910563.1_787424-787565_Hippopotamus_amphibius_isolate_BS01_ScHSfme_925023HRSCAF_982823_whole_genome_shotgun_sequence
0.03

lineage133

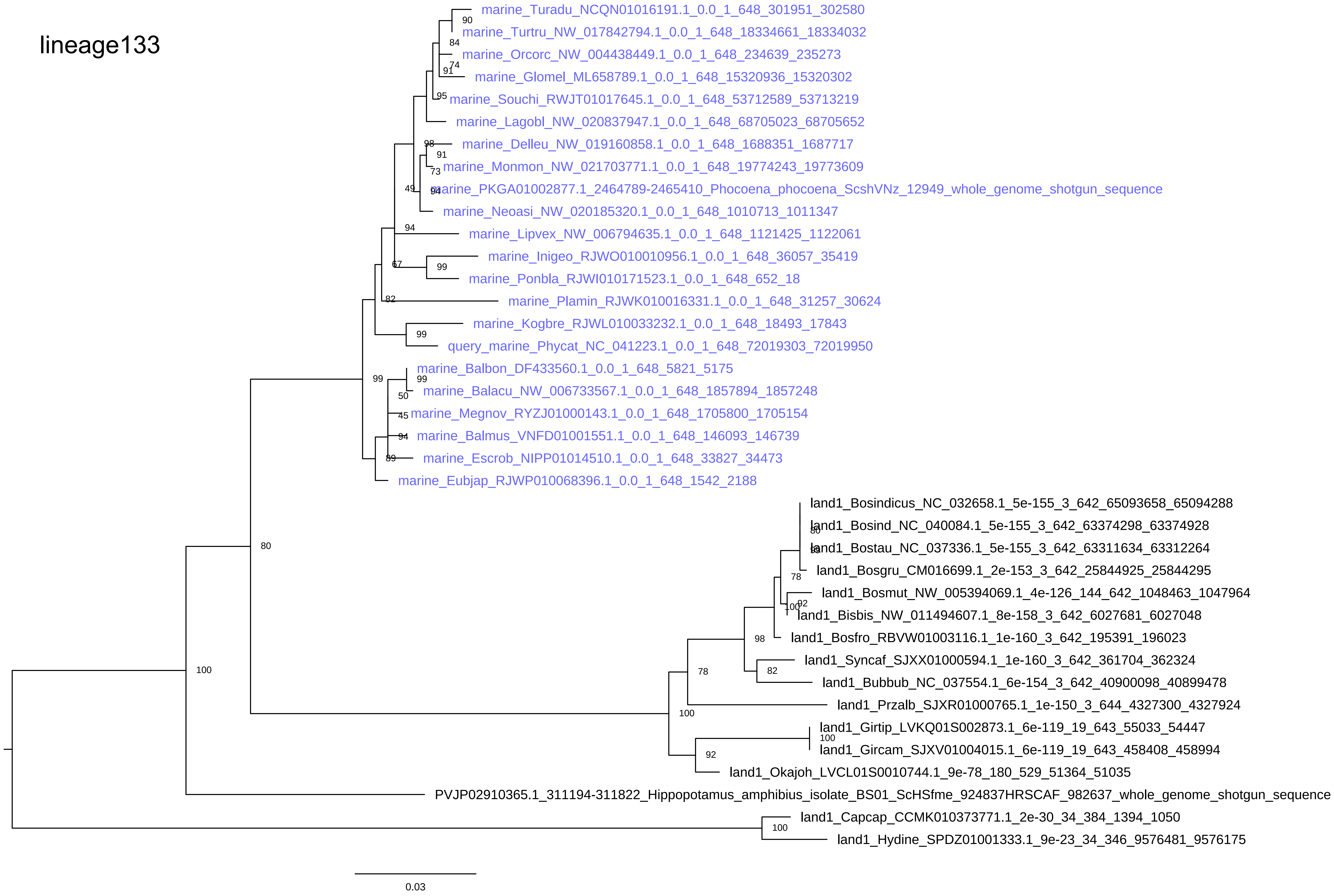
marine_Turadu_NCQN01016191.1_0.0_1_648_301951_302580
marine_Turtru_NW_017842794.1_0.0_1_648_18334661_18334032
marine_Orcorc_NW_004438449.1_0.0_1_648_234639_235273
marine_Glomel_ML658789.1_0.0_1_648_15320936_15320302
marine_Souchi_RWJT01017645.1_0.0_1_648_53712589_53713219
marine_Lagobl_NW_020837947.1_0.0_1_648_68705023_68705652
marine_Delleu_NW_019160858.1_0.0_1_648_1688351_1687717
marine_Monmon_NW_021703771.1_0.0_1_648_19774243_19773609
marine_PKGA01002877.1_2464789-2465410_Phocoena_phocoena_ScshVNz_12949_whole_genome_shotgun_sequence
marine_Neoasi_NW_020185320.1_0.0_1_648_1010713_1011347
marine_Lipvex_NW_006794635.1_0.0_1_648_1121425_1122061
marine_Inigeo_RJWO010010956.1_0.0_1_648_36057_35419
marine_Ponbla_RJWI010171523.1_0.0_1_648_652_18
marine_Plamin_RJWK010016331.1_0.0_1_648_31257_30624
marine_Kogbre_RJWL010033232.1_0.0_1_648_18493_17843
query_marine_Phycat_NC_041223.1_0.0_1_648_72019303_72019950
marine_Balbon_DF433560.1_0.0_1_648_5821_5175
marine_Balacu_NW_006733567.1_0.0_1_648_1857894_1857248
marine_Megnov_RYZJ01000143.1_0.0_1_648_1705800_1705154
marine_Balmus_VNFD01001551.1_0.0_1_648_146093_146739
marine_Escrob_NIPP01014510.1_0.0_1_648_33827_34473
marine_Eubjap_RJWP010068396.1_0.0_1_648_1542_2188
land1_Bosindicus_NC_032658.1_5e-155_3_642_65093658_65094288
land1_Bosind_NC_040084.1_5e-155_3_642_63374298_63374928
land1_Bostau_NC_037336.1_5e-155_3_642_63311634_63312264
land1_Bosgru_CM016699.1_2e-153_3_642_25844925_25844295
land1_Bosmut_NW_005394069.1_4e-126_144_642_1048463_1047964
land1_Bisbis_NW_011494607.1_8e-158_3_642_6027681_6027048
land1_Bosfro_RBVW01003116.1_1e-160_3_642_195391_196023
land1_Syncaf_SJXX01000594.1_1e-160_3_642_361704_362324
land1_Bubbub_NC_037554.1_6e-154_3_642_40900098_40899478
land1_Przalb_SJXR01000765.1_1e-150_3_644_4327300_4327924
land1_Girtip_LVKQ01S002873.1_6e-119_19_643_55033_54447
land1_Gircam_SJXV01004015.1_6e-119_19_643_458408_458994
land1_Okajoh_LVCL01S0010744.1_9e-78_180_529_51364_51035
PVJP02910365.1_311194-311822_Hippopotamus_amphibius_isolate_BS01_ScHSfme_924837HRSCAF_982637_whole_genome_shotgun_sequence
land1_Capcap_CCMK010373771.1_2e-30_34_384_1394_1050
land1_Hydine_SPDZ01001333.1_9e-23_34_346_9576481_9576175
0.03

lineage134

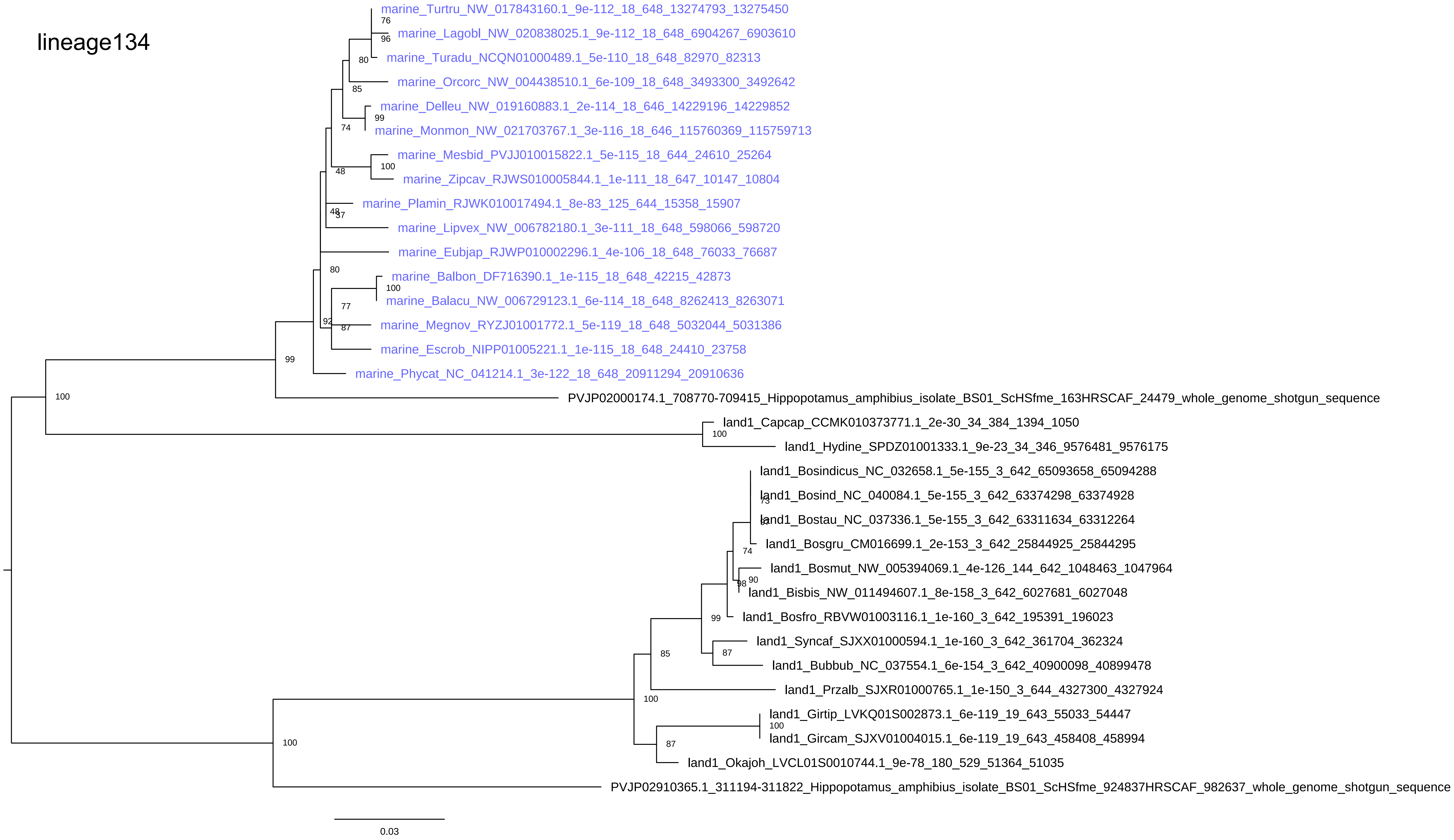

marine_Turtru_NW_017843160.1_9e-112_18_648_13274793_13275450
marine_Lagobl_NW_020838025.1_9e-112_18_648_6904267_6903610
marine_Turadu_NCQN01000489.1_5e-110_18_648_82970_82313
marine_Orcorc_NW_004438510.1_6e-109_18_648_3493300_3492642
marine_Delleu_NW_019160883.1_2e-114_18_646_14229196_14229852
marine_Monmon_NW_021703767.1_3e-116_18_646_115760369_115759713
marine_Mesbid_PVJJ010015822.1_5e-115_18_644_24610_25264
marine_Zipcav_RJWS010005844.1_1e-111_18_647_10147_10804
marine_Plamin_RJWK010017494.1_8e-83_125_644_15358_15907
marine_Lipvex_NW_006782180.1_3e-111_18_648_598066_598720
marine_Eubjap_RJWP010002296.1_4e-106_18_648_76033_76687
marine_Balbon_DF716390.1_1e-115_18_648_42215_42873
marine_Balacu_NW_006729123.1_6e-114_18_648_8262413_8263071
marine_Megnov_RYZJ01001772.1_5e-119_18_648_5032044_5031386
marine_Escrob_NIPP01005221.1_1e-115_18_648_24410_23758
marine_Phycat_NC_041214.1_3e-122_18_648_20911294_20910636
PVJP02000174.1_708770-709415_Hippopotamus_amphibius_isolate_BS01_ScHSfme_163HRSCAF_24479_whole_genome_shotgun_sequence
land1_Capcap_CCMK010373771.1_2e-30_34_384_1394_1050
land1_Hydine_SPDZ01001333.1_9e-23_34_346_9576481_9576175
land1_Bosindicus_NC_032658.1_5e-155_3_642_65093658_65094288
land1_Bosind_NC_040084.1_5e-155_3_642_63374298_63374928
land1_Bostau_NC_037336.1_5e-155_3_642_63311634_63312264
land1_Bosgru_CM016699.1_2e-153_3_642_25844925_25844295
land1_Bosmut_NW_005394069.1_4e-126_144_642_1048463_1047964
land1_Bisbis_NW_011494607.1_8e-158_3_642_6027681_6027048
land1_Bosfro_RBVW01003116.1_1e-160_3_642_195391_196023
land1_Syncaf_SJXX01000594.1_1e-160_3_642_361704_362324
land1_Bubbub_NC_037554.1_6e-154_3_642_40900098_40899478
land1_Przalb_SJXR01000765.1_1e-150_3_644_4327300_4327924
land1_Girtip_LVKQ01S002873.1_6e-119_19_643_55033_54447
land1_Gircam_SJXV01004015.1_6e-119_19_643_458408_458994
land1_Okajoh_LVCL01S0010744.1_9e-78_180_529_51364_51035
PVJP02910365.1_311194-311822_Hippopotamus_amphibius_isolate_BS01_ScHSfme_924837HRSCAF_982637_whole_genome_shotgun_sequence
0.03

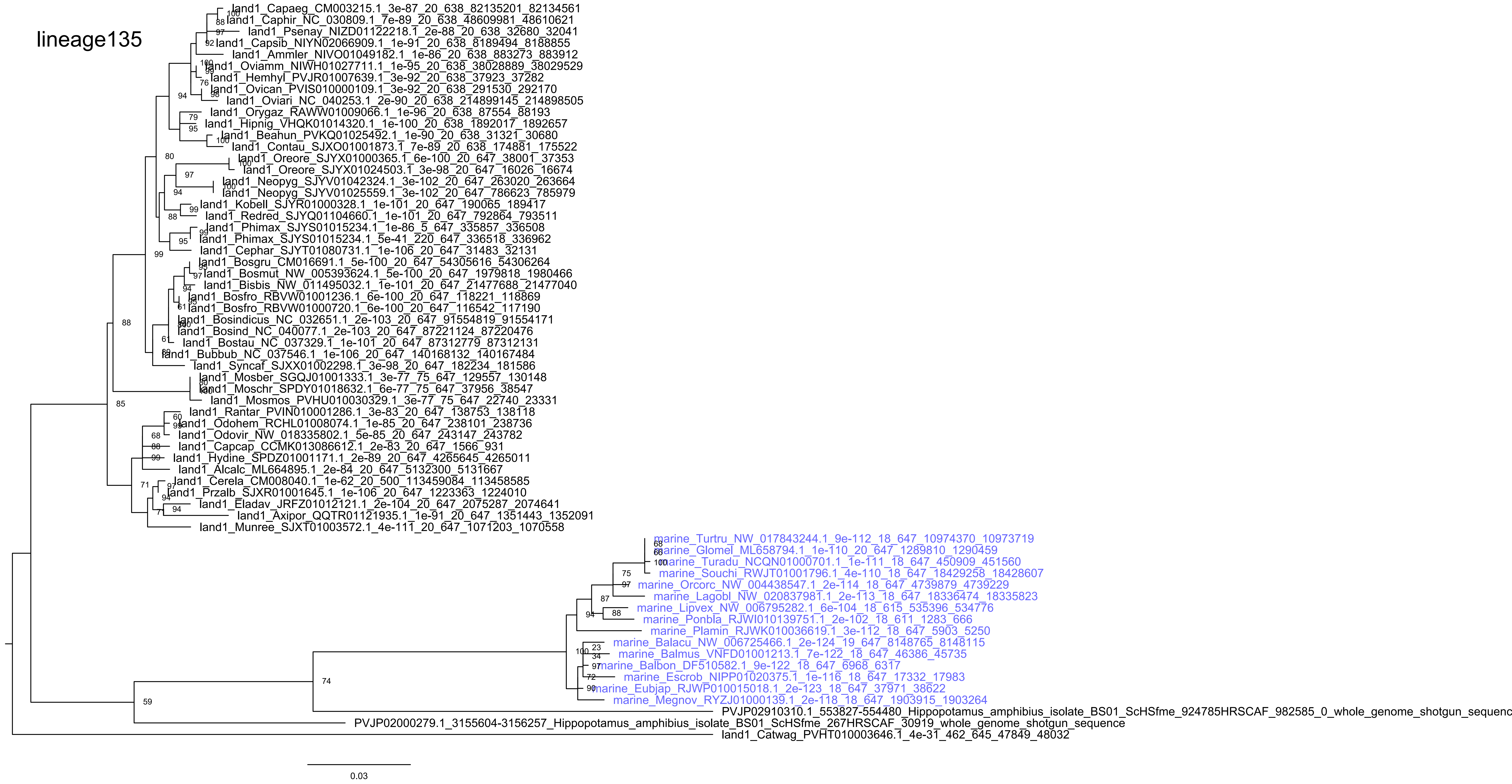

lineage135
land1_Capaeg_CM003215.1_3e-87_20_638_82135201_82134561
land1_Caphir_NC_030809.1_7e-89_20_638_48609981_48610621
land1_Psenay_NIZD01122218.1_2e-88_20_638_32680_32041
land1_Capsib_NIYN02066909.1_1e-91_20_638_8189494_8188855
land1_Ammler_NIVO01049182.1_1e-86_20_638_883273_883912
land1_Oviamm_NIWH01027711.1_1e-95_20_638_38028889_38029529
land1_Hemhyl_PVJR01007639.1_3e-92_20_638_37923_37282
land1_Ovican_PVIS010000109.1_3e-92_20_638_291530_292170
land1_Oviari_NC_040253.1_2e-90_20_638_214899145_214898505
land1_Orygaz_RAWW01009066.1_1e-96_20_638_87554_88193
land1_Hipnig_VHQK01014320.1_1e-100_20_638_1892017_1892657
land1_Beahun_PVKQ01025492.1_1e-90_20_638_31321_30680
land1_Contau_SJXO01001873.1_7e-89_20_638_174881_175522
land1_Oreore_SJYX01000365.1_6e-100_20_647_38001_37353
land1_Oreore_SJYX01024503.1_3e-98_20_647_16026_16674
land1_Neopyg_SJYV01042324.1_3e-102_20_647_263020_263664
land1_Neopyg_SJYV01025559.1_3e-102_20_647_786623_785979
land1_Kobell_SJYR01000328.1_1e-101_20_647_190065_189417
land1_Redred_SJYQ01104660.1_1e-101_20_647_792864_793511
land1_Phimax_SJYS01015234.1_1e-86_5_647_335857_336508
land1_Phimax_SJYS01015234.1_5e-41_220_647_336518_336962
land1_Cephar_SJYT01080731.1_1e-106_20_647_31483_32131
land1_Bosgru_CM016691.1_5e-100_20_647_54305616_54306264
land1_Bosmut_NW_005393624.1_5e-100_20_647_1979818_1980466
land1_Bisbis_NW_011495032.1_1e-101_20_647_21477688_21477040
land1_Bosfro_RBVW01001236.1_6e-100_20_647_118221_118869
land1_Bosfro_RBVW01000720.1_6e-100_20_647_116542_117190
land1_Bosindicus_NC_032651.1_2e-103_20_647_91554819_91554171
land1_Bosind_NC_040077.1_2e-103_20_647_87221124_87220476
land1_Bostau_NC_037329.1_1e-101_20_647_87312779_87312131
land1_Bubbub_NC_037546.1_1e-106_20_647_140168132_140167484
land1_Syncaf_SJXX01002298.1_3e-98_20_647_182234_181586
land1_Mosber_SGQJ01001333.1_3e-77_75_647_129557_130148
land1_Moschr_SPDY01018632.1_6e-77_75_647_37956_38547
land1_Mosmos_PVHU010030329.1_3e-77_75_647_22740_23331
land1_Rantar_PVIN010001286.1_3e-83_20_647_138753_138118
land1_Odohem_RCHL01008074.1_1e-85_20_647_238101_238736
land1_Odovir_NW_018335802.1_5e-85_20_647_243147_243782
land1_Capcap_CCMK013086612.1_2e-83_20_647_1566_931
land1_Hydine_SPDZ01001171.1_2e-89_20_647_4265645_4265011
land1_Alcalc_ML664895.1_2e-84_20_647_5132300_5131667
land1_Cerela_CM008040.1_1e-62_20_500_113459084_113458585
land1_Przalb_SJXR01001645.1_1e-106_20_647_1223363_1224010
land1_Eladav_JRFZ01012121.1_2e-104_20_647_2075287_2074641
land1_Axipor_QQTR01121935.1_1e-91_20_647_1351443_1352091
land1_Munree_SJXT01003572.1_4e-111_20_647_1071203_1070558
marine_Turtru_NW_017843244.1_9e-112_18_647_10974370_10973719
marine_Glomel_ML658794.1_1e-110_20_647_1289810_1290459
marine_Turadu_NCQN01000701.1_1e-111_18_647_450909_451560
marine_Souchi_RWJT01001796.1_4e-110_18_647_18429258_18428607
marine_Orcorc_NW_004438547.1_2e-114_18_647_4739879_4739229
marine_Lagobl_NW_020837981.1_2e-113_18_647_18336474_18335823
marine_Lipvex_NW_006795282.1_6e-104_18_615_535396_534776
marine_Ponbla_RJWI010139751.1_2e-102_18_611_1283_666
marine_Plamin_RJWK010036619.1_3e-112_18_647_5903_5250
marine_Balacu_NW_006725466.1_2e-124_19_647_8148765_8148115
marine_Balmus_VNFD01001213.1_7e-122_18_647_46386_45735
marine_Balbon_DF510582.1_9e-122_18_647_6968_6317
marine_Escrob_NIPP01020375.1_1e-116_18_647_17332_17983
marine_Eubjap_RJWP010015018.1_2e-123_18_647_37971_38622
marine_Megnov_RYZJ01000139.1_2e-118_18_647_1903915_1903264
PVJP02910310.1_553827-554480_Hippopotamus_amphibius_isolate_BS01_ScHSfme_924785HRSCAF_982585_0_whole_genome_shotgun_sequenc
PVJP02000279.1_3155604-3156257_Hippopotamus_amphibius_isolate_BS01_ScHSfme_267HRSCAF_30919_whole_genome_shotgun_sequence
land1_Catwag_PVHT010003646.1_4e-31_462_645_47849_48032
0.03

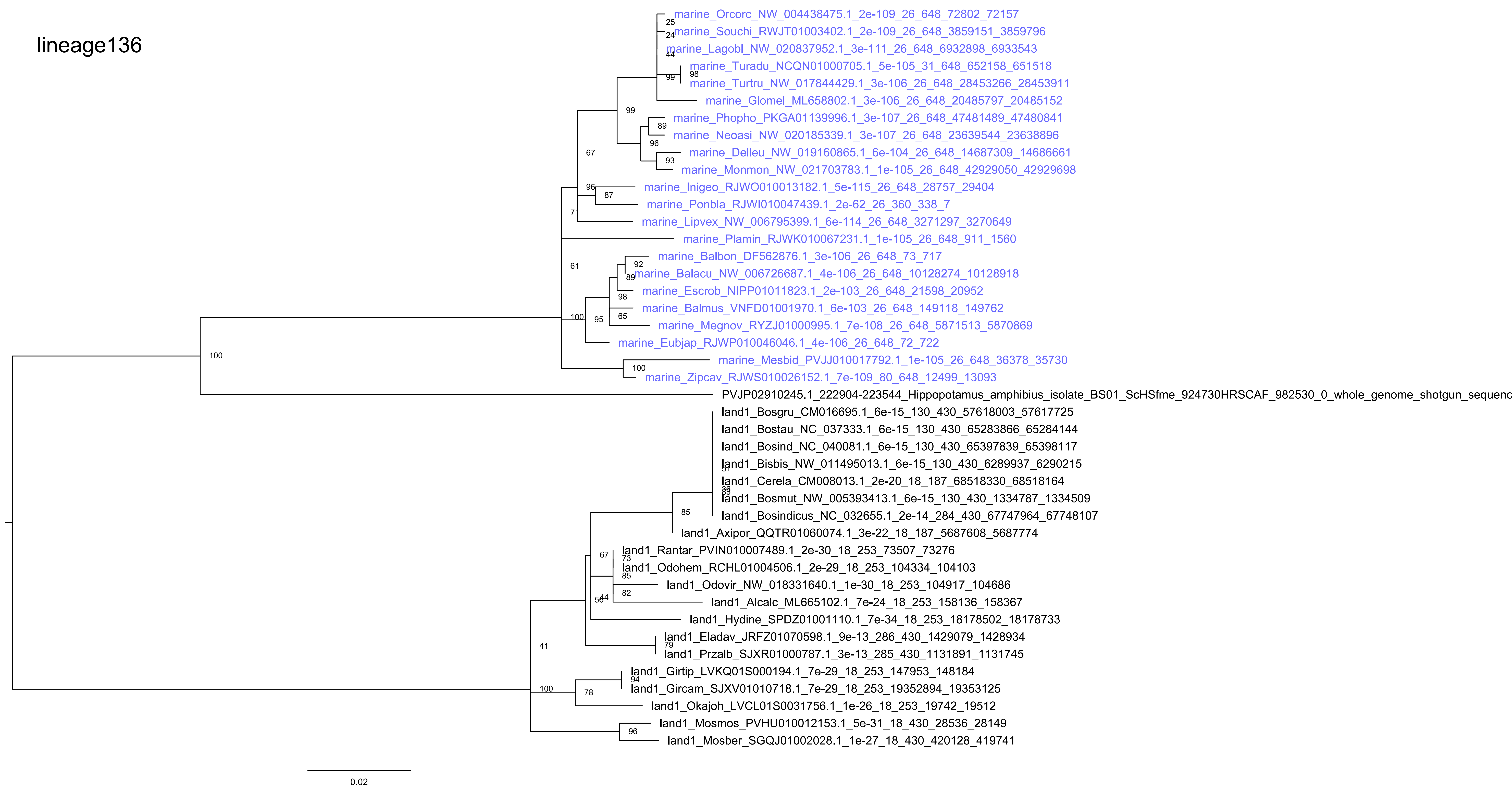
lineage136
marine_Orcorc_NW_004438475.1_2e-109_26_648_72802_72157
marine_Souchi_RWJT01003402.1_2e-109_26_648_3859151_3859796
marine_Lagobl_NW_020837952.1_3e-111_26_648_6932898_6933543
marine_Turadu_NCQN01000705.1_5e-105_31_648_652158_651518
marine_Turtru_NW_017844429.1_3e-106_26_648_28453266_28453911
marine_Glomel_ML658802.1_3e-106_26_648_20485797_20485152
marine_Phopho_PKGA01139996.1_3e-107_26_648_47481489_47480841
marine_Neoasi_NW_020185339.1_3e-107_26_648_23639544_23638896
marine_Delleu_NW_019160865.1_6e-104_26_648_14687309_14686661
marine_Monmon_NW_021703783.1_1e-105_26_648_42929050_42929698
marine_Inigeo_RJWO010013182.1_5e-115_26_648_28757_29404
marine_Ponbla_RJWI010047439.1_2e-62_26_360_338_7
marine_Lipvex_NW_006795399.1_6e-114_26_648_3271297_3270649
marine_Plamin_RJWK010067231.1_1e-105_26_648_911_1560
marine_Balbon_DF562876.1_3e-106_26_648_73_717
marine_Balacu_NW_006726687.1_4e-106_26_648_10128274_10128918
marine_Escrob_NIPP01011823.1_2e-103_26_648_21598_20952
marine_Balmus_VNFD01001970.1_6e-103_26_648_149118_149762
marine_Megnov_RYZJ01000995.1_7e-108_26_648_5871513_5870869
marine_Eubjap_RJWP010046046.1_4e-106_26_648_72_722
marine_Mesbid_PVJJ010017792.1_1e-105_26_648_36378_35730
marine_Zipcav_RJWS010026152.1_7e-109_80_648_12499_13093
PVJP02910245.1_222904-223544_Hippopotamus_amphibius_isolate_BS01_ScHSfme_924730HRSCAF_982530_0_whole_genome_shotgun_sequenc
land1_Bosgru_CM016695.1_6e-15_130_430_57618003_57617725
land1_Bostau_NC_037333.1_6e-15_130_430_65283866_65284144
land1_Bosind_NC_040081.1_6e-15_130_430_65397839_65398117
land1_Bisbis_NW_011495013.1_6e-15_130_430_6289937_6290215
land1_Cerela_CM008013.1_2e-20_18_187_68518330_68518164
land1_Bosmut_NW_005393413.1_6e-15_130_430_1334787_1334509
land1_Bosindicus_NC_032655.1_2e-14_284_430_67747964_67748107
land1_Axipor_QQTR01060074.1_3e-22_18_187_5687608_5687774
land1_Rantar_PVIN010007489.1_2e-30_18_253_73507_73276
land1_Odohem_RCHL01004506.1_2e-29_18_253_104334_104103
land1_Odovir_NW_018331640.1_1e-30_18_253_104917_104686
land1_Alcalc_ML665102.1_7e-24_18_253_158136_158367
land1_Hydine_SPDZ01001110.1_7e-34_18_253_18178502_18178733
land1_Eladav_JRFZ01070598.1_9e-13_286_430_1429079_1428934
land1_Przalb_SJXR01000787.1_3e-13_285_430_1131891_1131745
land1_Girtip_LVKQ01S000194.1_7e-29_18_253_147953_148184
land1_Gircam_SJXV01010718.1_7e-29_18_253_19352894_19353125
land1_Okajoh_LVCL01S0031756.1_1e-26_18_253_19742_19512
land1_Mosmos_PVHU010012153.1_5e-31_18_430_28536_28149
land1_Mosber_SGQJ01002028.1_1e-27_18_430_420128_419741
0.02

lineage137

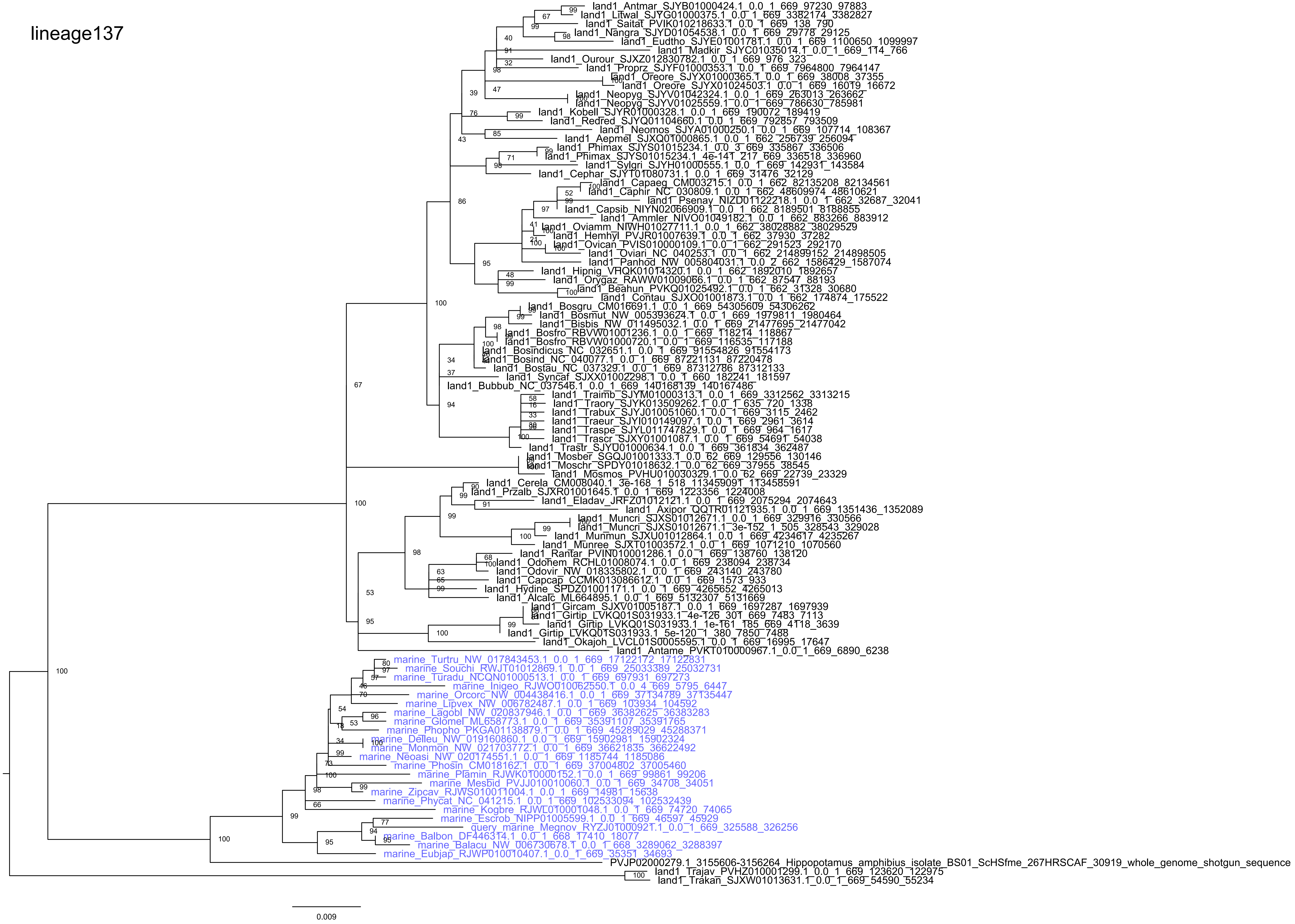
land1_Antmar_SJYB01000424.1_0.0_1_669_97230_97883
land1_Litwal_SJYG01000375.1_0.0_1_669_3382174_3382827
land1_Saitat_PVIK010218633.1_0.0_1_669_138_790
land1_Nangra_SJYD01054538.1_0.0_1_669_29778_29125
land1_Eudtho_SJYE01001781.1_0.0_1_669_1100650_1099997
land1_Madkir_SJYC01035014.1_0.0_1_669_114_766
land1_Ourour_SJXZ012830782.1_0.0_1_669_976_323
land1_Proprz_SJYF01000353.1_0.0_1_669_7964800_7964147
land1_Oreore_SJYX01000365.1_0.0_1_669_38008_37355
land1_Oreore_SJYX01024503.1_0.0_1_669_16019_16672
land1_Neopyg_SJYV01042324.1_0.0_1_669_263013_263662
land1_Neopyg_SJYV01025559.1_0.0_1_669_786630_785981
land1_Kobell_SJYR01000328.1_0.0_1_669_190072_189419
land1_Redred_SJYQ01104660.1_0.0_1_669_792857_793509
land1_Neomos_SJYA01000250.1_0.0_1_669_107714_108367
land1_Aepmel_SJXQ01000865.1_0.0_1_662_256739_256094
land1_Phimax_SJYS01015234.1_0.0_3_669_335867_336506
land1_Phimax_SJYS01015234.1_4e-141_217_669_336518_336960
land1_Sylgri_SJYH01000555.1_0.0_1_669_142931_143584
land1_Cephar_SJYT01080731.1_0.0_1_669_31476_32129
land1_Capaeg_CM003215.1_0.0_1_662_82135208_82134561
land1_Caphir_NC_030809.1_0.0_1_662_48609974_48610621
land1_Psenay_NIZD01122218.1_0.0_1_662_32687_32041
land1_Capsib_NIYN02066909.1_0.0_1_662_8189501_8188855
land1_Ammler_NIVO01049182.1_0.0_1_662_883266_883912
land1_Oviamm_NIWH01027711.1_0.0_1_662_38028882_38029529
land1_Hemhyl_PVJR01007639.1_0.0_1_662_37930_37282
land1_Ovican_PVIS010000109.1_0.0_1_662_291523_292170
land1_Oviari_NC_040253.1_0.0_1_662_214899152_214898505
land1_Panhod_NW_005804031.1_0.0_2_662_1586429_1587074
land1_Hipnig_VHQK01014320.1_0.0_1_662_1892010_1892657
land1_Orygaz_RAWW01009066.1_0.0_1_662_87547_88193
land1_Beahun_PVKQ01025492.1_0.0_1_662_31328_30680
land1_Contau_SJXO01001873.1_0.0_1_662_174874_175522
land1_Bosgru_CM016691.1_0.0_1_669_54305609_54306262
land1_Bosmut_NW_005393624.1_0.0_1_669_1979811_1980464
land1_Bisbis_NW_011495032.1_0.0_1_669_21477695_21477042
land1_Bosfro_RBVW01001236.1_0.0_1_669_118214_118867
land1_Bosfro_RBVW01000720.1_0.0_1_669_116535_117188
land1_Bosindicus_NC_032651.1_0.0_1_669_91554826_91554173
land1_Bosind_NC_040077.1_0.0_1_669_87221131_87220478
land1_Bostau_NC_037329.1_0.0_1_669_87312786_87312133
land1_Syncaf_SJXX01002298.1_0.0_1_660_182241_181597
land1_Bubbub_NC_037546.1_0.0_1_669_140168139_140167486
land1_Traimb_SJYM01000313.1_0.0_1_669_3312562_3313215
land1_Traory_SJYK013509262.1_0.0_1_635_720_1338
land1_Trabux_SJYJ010051060.1_0.0_1_669_3115_2462
land1_Traeur_SJYI010149097.1_0.0_1_669_2961_3614
land1_Traspe_SJYL011747829.1_0.0_1_669_964_1617
land1_Trascr_SJXY01001087.1_0.0_1_669_54691_54038
land1_Trastr_SJYU01000634.1_0.0_1_669_361834_362487
land1_Mosber_SGQJ01001333.1_0.0_62_669_129556_130146
land1_Moschr_SPDY01018632.1_0.0_62_669_37955_38545
land1_Mosmos_PVHU010030329.1_0.0_62_669_22739_23329
land1_Cerela_CM008040.1_3e-168_1_518_113459091_113458591
land1_Przalb_SJXR01001645.1_0.0_1_669_1223356_1224008
land1_Eladav_JRFZ01012121.1_0.0_1_669_2075294_2074643
land1_Axipor_QQTR01121935.1_0.0_1_669_1351436_1352089
land1_Muncri_SJXS01012671.1_0.0_1_669_329916_330566
land1_Muncri_SJXS01012671.1_3e-152_1_505_328543_329028
land1_Munmun_SJXU01012864.1_0.0_1_669_4234617_4235267
land1_Munree_SJXT01003572.1_0.0_1_669_1071210_1070560
land1_Rantar_PVIN010001286.1_0.0_1_669_138760_138120
land1_Odohem_RCHL01008074.1_0.0_1_669_238094_238734
land1_Odovir_NW_018335802.1_0.0_1_669_243140_243780
land1_Capcap_CCMK013086612.1_0.0_1_669_1573_933
land1_Hydine_SPDZ01001171.1_0.0_1_669_4265652_4265013
land1_Alcalc_ML664895.1_0.0_1_669_5132307_5131669
land1_Gircam_SJXV01005187.1_0.0_1_669_1697287_1697939
land1_Girtip_LVKQ01S031933.1_4e-126_301_669_7483_7113
land1_Girtip_LVKQ01S031933.1_1e-161_185_669_4118_3639
land1_Girtip_LVKQ01S031933.1_5e-120_1_380_7850_7488
land1_Okajoh_LVCL01S0005595.1_0.0_1_669_16995_17647
land1_Antame_PVKT010000967.1_0.0_1_669_6890_6238
marine_Turtru_NW_017843453.1_0.0_1_669_17122172_17122831
marine_Souchi_RWJT01012869.1_0.0_1_669_25033389_25032731
marine_Turadu_NCQN01000513.1_0.0_1_669_697931_697273
marine_Inigeo_RJWO010062550.1_0.0_4_669_5795_6447
marine_Orcorc_NW_004438416.1_0.0_1_669_37134789_37135447
marine_Lipvex_NW_006782487.1_0.0_1_669_103934_104592
marine_Lagobl_NW_020837946.1_0.0_1_669_36382625_36383283
marine_Glomel_ML658773.1_0.0_1_669_35391107_35391765
marine_Phopho_PKGA01138879.1_0.0_1_669_45289029_45288371
marine_Delleu_NW_019160860.1_0.0_1_669_15902981_15902324
marine_Monmon_NW_021703772.1_0.0_1_669_36621835_36622492
marine_Neoasi_NW_020174551.1_0.0_1_669_1185744_1185086
marine_Phosin_CM018162.1_0.0_1_669_37004802_37005460
marine_Plamin_RJWK01000152.1_0.0_1_669_99861_99206
marine_Mesbid_PVJJ010010060.1_0.0_1_669_34708_34051
marine_Zipcav_RJWS010011004.1_0.0_1_669_14981_15638
marine_Phycat_NC_041215.1_0.0_1_669_102533094_102532439
marine_Kogbre_RJWL010001048.1_0.0_1_669_74720_74065
marine_Escrob_NIPP01005599.1_0.0_1_669_46597_45929
query_marine_Megnov_RYZJ01000921.1_0.0_1_669_325588_326256
marine_Balbon_DF446314.1_0.0_1_668_17410_18077
marine_Balacu_NW_006730678.1_0.0_1_668_3289062_3288397
marine_Eubjap_RJWP010010407.1_0.0_1_669_35351_34693
PVJP02000279.1_3155606-3156264_Hippopotamus_amphibius_isolate_BS01_ScHSfme_267HRSCAF_30919_whole_genome_shotgun_sequence
land1_Trajav_PVHZ010001299.1_0.0_1_669_123620_122975
land1_Trakan_SJXW01013631.1_0.0_1_669_54590_55234
0.009

lineage138

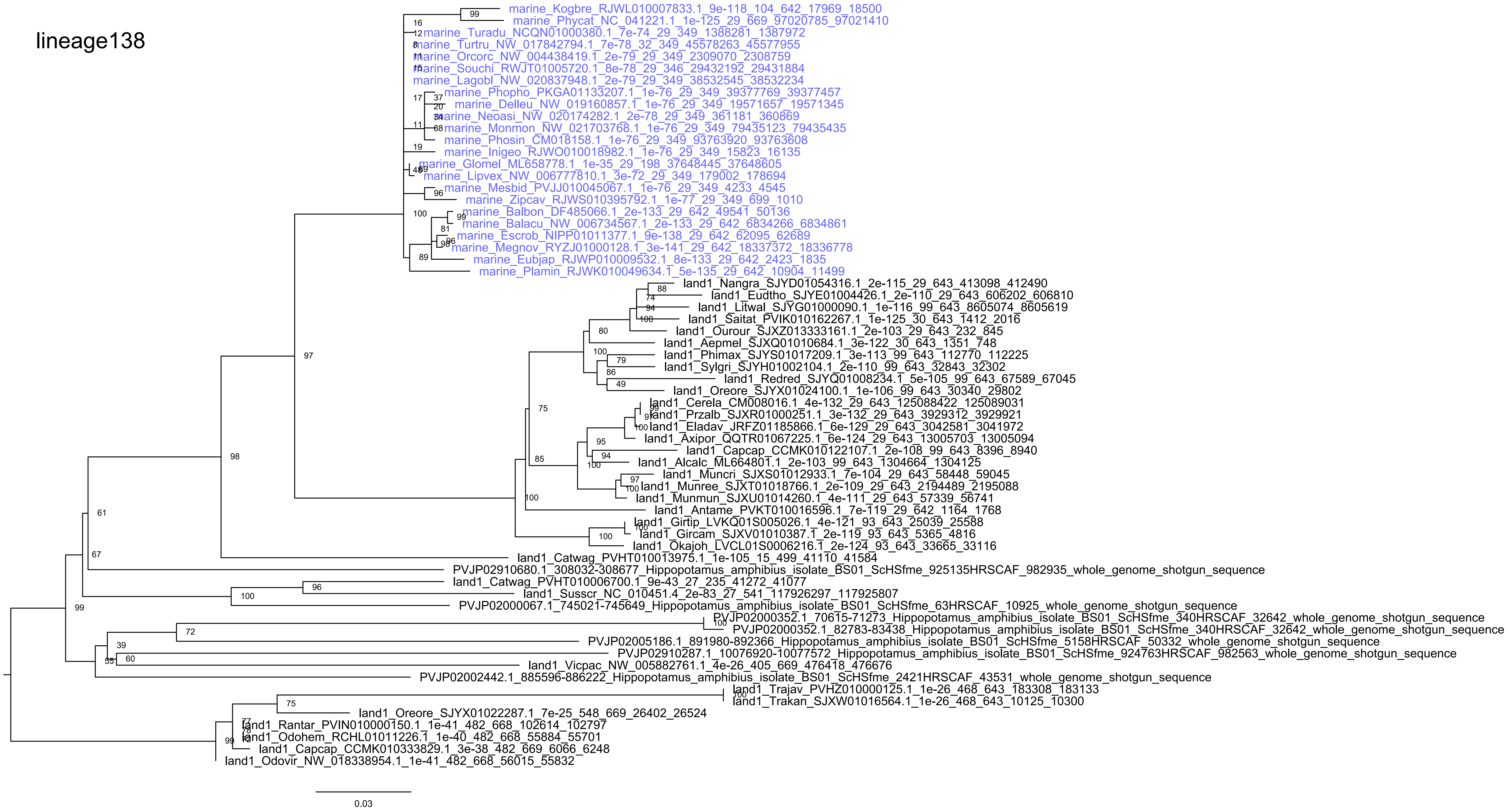

marine_Kogbre_RJWL010007833.1_9e-118_104_642_17969_18500
marine_Phycat_NC_041221.1_1e-125_29_669_97020785_97021410
marine_Turadu_NCQN01000380.1_7e-74_29_349_1388281_1387972
marine_Turtru_NW_017842794.1_7e-78_32_349_45578263_45577955
marine_Orcorc_NW_004438419.1_2e-79_29_349_2309070_2308759
marine_Souchi_RWJT01005720.1_8e-78_29_346_29432192_29431884
marine_Lagobl_NW_020837948.1_2e-79_29_349_38532545_38532234
marine_Phopho_PKGA01133207.1_1e-76_29_349_39377769_39377457
marine_Delleu_NW_019160857.1_1e-76_29_349_19571657_19571345
marine_Neoasi_NW_020174282.1_2e-78_29_349_361181_360869
marine_Monmon_NW_021703768.1_1e-76_29_349_79435123_79435435
marine_Phosin_CM018158.1_1e-76_29_349_93763920_93763608
marine_Inigeo_RJWO010018982.1_1e-76_29_349_15823_16135
marine_Glomel_ML658778.1_1e-35_29_198_37648445_37648605
marine_Lipvex_NW_006777810.1_3e-72_29_349_179002_178694
marine_Mesbid_PVJJ010045067.1_1e-76_29_349_4233_4545
marine_Zipcav_RJWS010395792.1_1e-77_29_349_699_1010
marine_Balbon_DF485066.1_2e-133_29_642_49541_50136
marine_Balacu_NW_006734567.1_2e-133_29_642_6834266_6834861
marine_Escrob_NIPP01011377.1_9e-138_29_642_62095_62689
marine_Megnov_RYZJ01000128.1_3e-141_29_642_18337372_18336778
marine_Eubjap_RJWP010009532.1_8e-133_29_642_2423_1835
marine_Plamin_RJWK010049634.1_5e-135_29_642_10904_11499
land1_Nangra_SJYD01054316.1_2e-115_29_643_413098_412490
land1_Eudtho_SJYE01004426.1_2e-110_29_643_606202_606810
land1_Litwal_SJYG01000090.1_1e-116_99_643_8605074_8605619
land1_Saitat_PVIK010162267.1_1e-125_30_643_1412_2016
land1_Ourour_SJXZ013333161.1_2e-103_29_643_232_845
land1_Aepmel_SJXQ01010684.1_3e-122_30_643_1351_748
land1_Phimax_SJYS01017209.1_3e-113_99_643_112770_112225
land1_Sylgri_SJYH01002104.1_2e-110_99_643_32843_32302
land1_Redred_SJYQ01008234.1_5e-105_99_643_67589_67045
land1_Oreore_SJYX01024100.1_1e-106_99_643_30340_29802
land1_Cerela_CM008016.1_4e-132_29_643_125088422_125089031
land1_Przalb_SJXR01000251.1_3e-132_29_643_3929312_3929921
land1_Eladav_JRFZ01185866.1_6e-129_29_643_3042581_3041972
land1_Axipor_QQTR01067225.1_6e-124_29_643_13005703_13005094
land1_Capcap_CCMK010122107.1_2e-108_99_643_8396_8940
land1_Alcalc_ML664801.1_2e-103_99_643_1304664_1304125
land1_Muncri_SJXS01012933.1_7e-104_29_643_58448_59045
land1_Munree_SJXT01018766.1_2e-109_29_643_2194489_2195088
land1_Munmun_SJXU01014260.1_4e-111_29_643_57339_56741
land1_Antame_PVKT010016596.1_7e-119_29_642_1164_1768
land1_Girtip_LVKQ01S005026.1_4e-121_93_643_25039_25588
land1_Gircam_SJXV01010387.1_2e-119_93_643_5365_4816
land1_Okajoh_LVCL01S0006216.1_2e-124_93_643_33665_33116
land1_Catwag_PVHT010013975.1_1e-105_15_499_41110_41584
PVJP02910680.1_308032-308677_Hippopotamus_amphibius_isolate_BS01_ScHSfme_925135HRSCAF_982935_whole_genome_shotgun_sequence
land1_Catwag_PVHT010006700.1_9e-43_27_235_41272_41077
land1_Susscr_NC_010451.4_2e-83_27_541_117926297_117925807
PVJP02000067.1_745021-745649_Hippopotamus_amphibius_isolate_BS01_ScHSfme_63HRSCAF_10925_whole_genome_shotgun_sequence
PVJP02000352.1_70615-71273_Hippopotamus_amphibius_isolate_BS01_ScHSfme_340HRSCAF_32642_whole_genome_shotgun_sequence
PVJP02000352.1_82783-83438_Hippopotamus_amphibius_isolate_BS01_ScHSfme_340HRSCAF_32642_whole_genome_shotgun_sequence
PVJP02005186.1_891980-892366_Hippopotamus_amphibius_isolate_BS01_ScHSfme_5158HRSCAF_50332_whole_genome_shotgun_sequence
PVJP02910287.1_10076920-10077572_Hippopotamus_amphibius_isolate_BS01_ScHSfme_924763HRSCAF_982563_whole_genome_shotgun_sequence
land1_Vicpac_NW_005882761.1_4e-26_405_669_476418_476676
PVJP02002442.1_885596-886222_Hippopotamus_amphibius_isolate_BS01_ScHSfme_2421HRSCAF_43531_whole_genome_shotgun_sequence
land1_Trajav_PVHZ010000125.1_1e-26_468_643_183308_183133
land1_Trakan_SJXW01016564.1_1e-26_468_643_10125_10300
land1_Oreore_SJYX01022287.1_7e-25_548_669_26402_26524
land1_Rantar_PVIN010000150.1_1e-41_482_668_102614_102797
land1_Odohem_RCHL01011226.1_1e-40_482_668_55884_55701
land1_Capcap_CCMK010333829.1_3e-38_482_669_6066_6248
land1_Odovir_NW_018338954.1_1e-41_482_668_56015_55832
0.03

lineage139

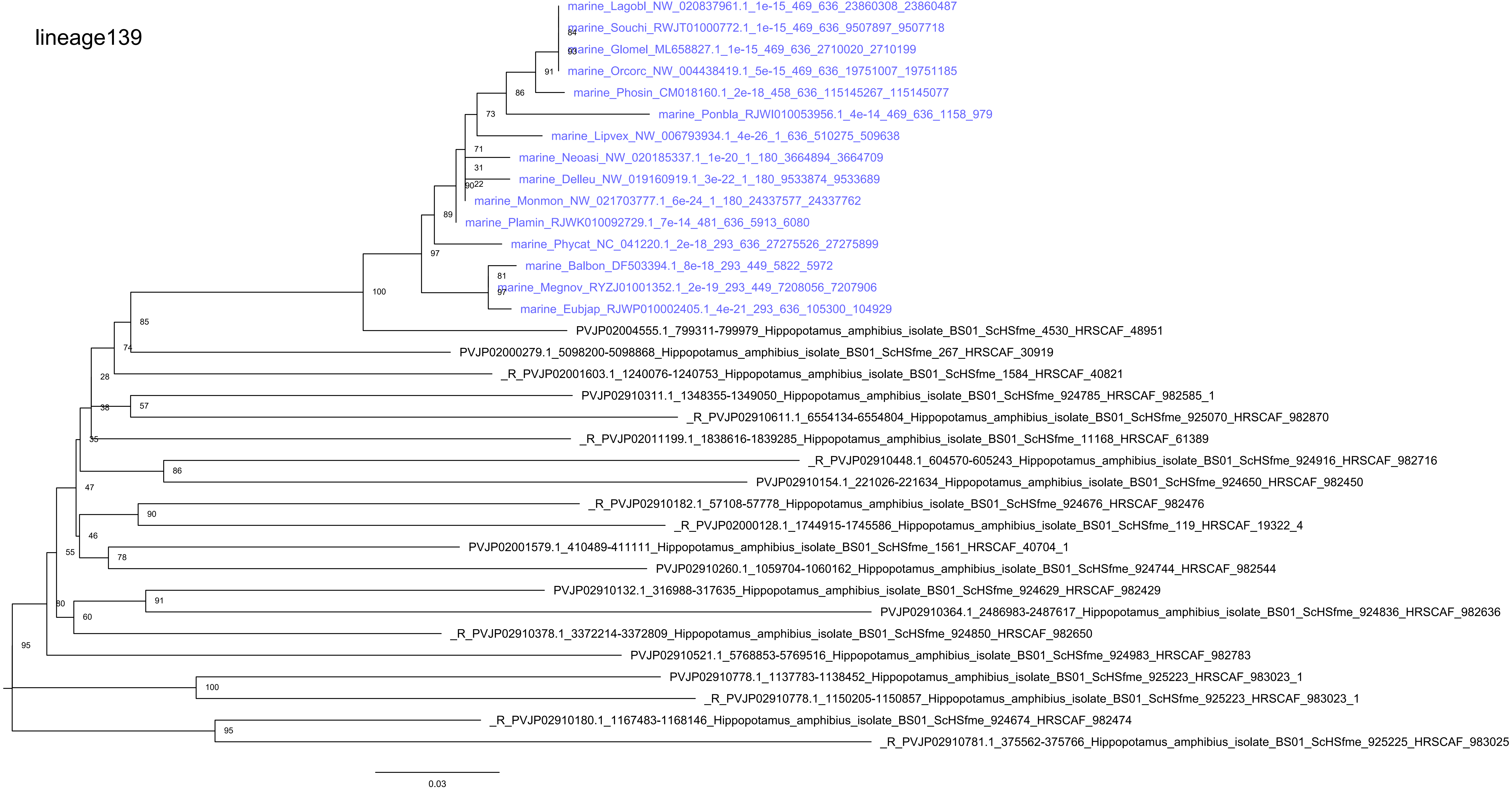
marine_Lagobl_NW_020837961.1_1e-15_469_636_23860308_23860487
marine_Souchi_RWJT01000772.1_1e-15_469_636_9507897_9507718
marine_Glomel_ML658827.1_1e-15_469_636_2710020_2710199
marine_Orcorc_NW_004438419.1_5e-15_469_636_19751007_19751185
marine_Phosin_CM018160.1_2e-18_458_636_115145267_115145077
marine_Ponbla_RJWI010053956.1_4e-14_469_636_1158_979
marine_Lipvex_NW_006793934.1_4e-26_1_636_510275_509638
marine_Neoasi_NW_020185337.1_1e-20_1_180_3664894_3664709
marine_Delleu_NW_019160919.1_3e-22_1_180_9533874_9533689
marine_Monmon_NW_021703777.1_6e-24_1_180_24337577_24337762
marine_Plamin_RJWK010092729.1_7e-14_481_636_5913_6080
marine_Phycat_NC_041220.1_2e-18_293_636_27275526_27275899
marine_Balbon_DF503394.1_8e-18_293_449_5822_5972
marine_Megnov_RYZJ01001352.1_2e-19_293_449_7208056_7207906
marine_Eubjap_RJWP010002405.1_4e-21_293_636_105300_104929
PVJP02004555.1_799311-799979_Hippopotamus_amphibius_isolate_BS01_ScHSfme_4530_HRSCAF_48951
PVJP02000279.1_5098200-5098868_Hippopotamus_amphibius_isolate_BS01_ScHSfme_267_HRSCAF_30919
_R_PVJP02001603.1_1240076-1240753_Hippopotamus_amphibius_isolate_BS01_ScHSfme_1584_HRSCAF_40821
PVJP02910311.1_1348355-1349050_Hippopotamus_amphibius_isolate_BS01_ScHSfme_924785_HRSCAF_982585_1
_R_PVJP02910611.1_6554134-6554804_Hippopotamus_amphibius_isolate_BS01_ScHSfme_925070_HRSCAF_982870
_R_PVJP02011199.1_1838616-1839285_Hippopotamus_amphibius_isolate_BS01_ScHSfme_11168_HRSCAF_61389
_R_PVJP02910448.1_604570-605243_Hippopotamus_amphibius_isolate_BS01_ScHSfme_924916_HRSCAF_982716
PVJP02910154.1_221026-221634_Hippopotamus_amphibius_isolate_BS01_ScHSfme_924650_HRSCAF_982450
_R_PVJP02910182.1_57108-57778_Hippopotamus_amphibius_isolate_BS01_ScHSfme_924676_HRSCAF_982476
_R_PVJP02000128.1_1744915-1745586_Hippopotamus_amphibius_isolate_BS01_ScHSfme_119_HRSCAF_19322_4
PVJP02001579.1_410489-411111_Hippopotamus_amphibius_isolate_BS01_ScHSfme_1561_HRSCAF_40704_1
PVJP02910260.1_1059704-1060162_Hippopotamus_amphibius_isolate_BS01_ScHSfme_924744_HRSCAF_982544
PVJP02910132.1_316988-317635_Hippopotamus_amphibius_isolate_BS01_ScHSfme_924629_HRSCAF_982429
PVJP02910364.1_2486983-2487617_Hippopotamus_amphibius_isolate_BS01_ScHSfme_924836_HRSCAF_982636
_R_PVJP02910378.1_3372214-3372809_Hippopotamus_amphibius_isolate_BS01_ScHSfme_924850_HRSCAF_982650
PVJP02910521.1_5768853-5769516_Hippopotamus_amphibius_isolate_BS01_ScHSfme_924983_HRSCAF_982783
PVJP02910778.1_1137783-1138452_Hippopotamus_amphibius_isolate_BS01_ScHSfme_925223_HRSCAF_983023_1
_R_PVJP02910778.1_1150205-1150857_Hippopotamus_amphibius_isolate_BS01_ScHSfme_925223_HRSCAF_983023_1
_R_PVJP02910180.1_1167483-1168146_Hippopotamus_amphibius_isolate_BS01_ScHSfme_924674_HRSCAF_982474
_R_PVJP02910781.1_375562-375766_Hippopotamus_amphibius_isolate_BS01_ScHSfme_925225_HRSCAF_983025
84
93
91
86
73
71
31
90
22
89
97
81
97
100
85
74
28
57
38
35
86
47
90
46
78
55
91
80
60
95
100
95
0.03

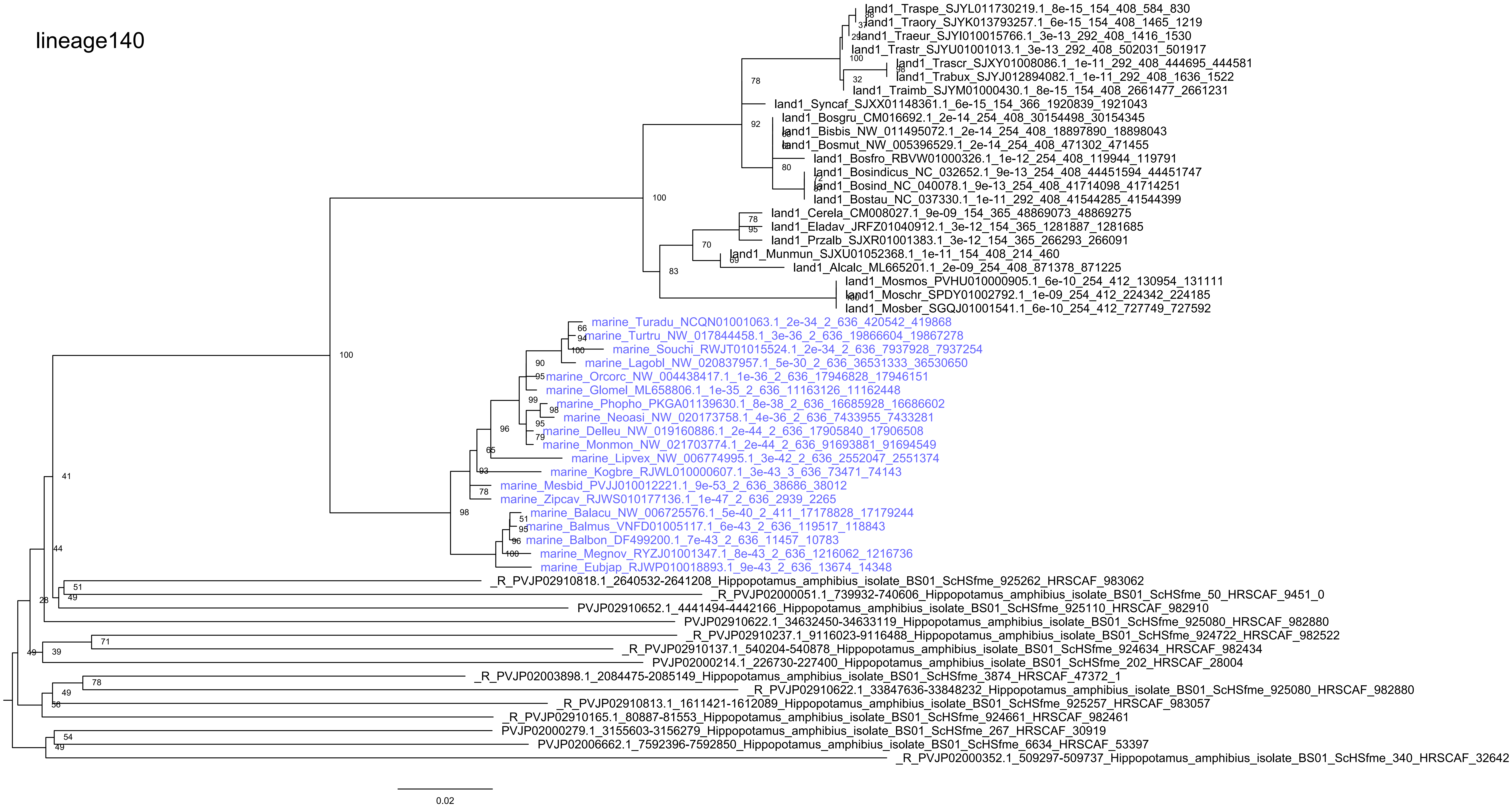
lineage140
land1_Traspe_SJYL011730219.1_8e-15_154_408_584_830
land1_Traory_SJYK013793257.1_6e-15_154_408_1465_1219
land1_Traeur_SJYI010015766.1_3e-13_292_408_1416_1530
land1_Trastr_SJYU01001013.1_3e-13_292_408_502031_501917
land1_Trascr_SJXY01008086.1_1e-11_292_408_444695_444581
land1_Trabux_SJYJ012894082.1_1e-11_292_408_1636_1522
land1_Traimb_SJYM01000430.1_8e-15_154_408_2661477_2661231
land1_Syncaf_SJXX01148361.1_6e-15_154_366_1920839_1921043
land1_Bosgru_CM016692.1_2e-14_254_408_30154498_30154345
land1_Bisbis_NW_011495072.1_2e-14_254_408_18897890_18898043
land1_Bosmut_NW_005396529.1_2e-14_254_408_471302_471455
land1_Bosfro_RBVW01000326.1_1e-12_254_408_119944_119791
land1_Bosindicus_NC_032652.1_9e-13_254_408_44451594_44451747
land1_Bosind_NC_040078.1_9e-13_254_408_41714098_41714251
land1_Bostau_NC_037330.1_1e-11_292_408_41544285_41544399
land1_Cerela_CM008027.1_9e-09_154_365_48869073_48869275
land1_Eladav_JRFZ01040912.1_3e-12_154_365_1281887_1281685
land1_Przalb_SJXR01001383.1_3e-12_154_365_266293_266091
land1_Munmun_SJXU01052368.1_1e-11_154_408_214_460
land1_Alcalc_ML665201.1_2e-09_254_408_871378_871225
land1_Mosmos_PVHU010000905.1_6e-10_254_412_130954_131111
land1_Moschr_SPDY01002792.1_1e-09_254_412_224342_224185
land1_Mosber_SGQJ01001541.1_6e-10_254_412_727749_727592
marine_Turadu_NCQN01001063.1_2e-34_2_636_420542_419868
marine_Turtru_NW_017844458.1_3e-36_2_636_19866604_19867278
marine_Souchi_RWJT01015524.1_2e-34_2_636_7937928_7937254
marine_Lagobl_NW_020837957.1_5e-30_2_636_36531333_36530650
marine_Orcorc_NW_004438417.1_1e-36_2_636_17946828_17946151
marine_Glomel_ML658806.1_1e-35_2_636_11163126_11162448
marine_Phopho_PKGA01139630.1_8e-38_2_636_16685928_16686602
marine_Neoasi_NW_020173758.1_4e-36_2_636_7433955_7433281
marine_Delleu_NW_019160886.1_2e-44_2_636_17905840_17906508
marine_Monmon_NW_021703774.1_2e-44_2_636_91693881_91694549
marine_Lipvex_NW_006774995.1_3e-42_2_636_2552047_2551374
marine_Kogbre_RJWL010000607.1_3e-43_3_636_73471_74143
marine_Mesbid_PVJJ010012221.1_9e-53_2_636_38686_38012
marine_Zipcav_RJWS010177136.1_1e-47_2_636_2939_2265
marine_Balacu_NW_006725576.1_5e-40_2_411_17178828_17179244
marine_Balmus_VNFD01005117.1_6e-43_2_636_119517_118843
marine_Balbon_DF499200.1_7e-43_2_636_11457_10783
marine_Megnov_RYZJ01001347.1_8e-43_2_636_1216062_1216736
marine_Eubjap_RJWP010018893.1_9e-43_2_636_13674_14348
_R_PVJP02910818.1_2640532-2641208_Hippopotamus_amphibius_isolate_BS01_ScHSfme_925262_HRSCAF_983062
_R_PVJP02000051.1_739932-740606_Hippopotamus_amphibius_isolate_BS01_ScHSfme_50_HRSCAF_9451_0
PVJP02910652.1_4441494-4442166_Hippopotamus_amphibius_isolate_BS01_ScHSfme_925110_HRSCAF_982910
PVJP02910622.1_34632450-34633119_Hippopotamus_amphibius_isolate_BS01_ScHSfme_925080_HRSCAF_982880
_R_PVJP02910237.1_9116023-9116488_Hippopotamus_amphibius_isolate_BS01_ScHSfme_924722_HRSCAF_982522
_R_PVJP02910137.1_540204-540878_Hippopotamus_amphibius_isolate_BS01_ScHSfme_924634_HRSCAF_982434
PVJP02000214.1_226730-227400_Hippopotamus_amphibius_isolate_BS01_ScHSfme_202_HRSCAF_28004
_R_PVJP02003898.1_2084475-2085149_Hippopotamus_amphibius_isolate_BS01_ScHSfme_3874_HRSCAF_47372_1
_R_PVJP02910622.1_33847636-33848232_Hippopotamus_amphibius_isolate_BS01_ScHSfme_925080_HRSCAF_982880
_R_PVJP02910813.1_1611421-1612089_Hippopotamus_amphibius_isolate_BS01_ScHSfme_925257_HRSCAF_983057
_R_PVJP02910165.1_80887-81553_Hippopotamus_amphibius_isolate_BS01_ScHSfme_924661_HRSCAF_982461
PVJP02000279.1_3155603-3156279_Hippopotamus_amphibius_isolate_BS01_ScHSfme_267_HRSCAF_30919
PVJP02006662.1_7592396-7592850_Hippopotamus_amphibius_isolate_BS01_ScHSfme_6634_HRSCAF_53397
_R_PVJP02000352.1_509297-509737_Hippopotamus_amphibius_isolate_BS01_ScHSfme_340_HRSCAF_32642
0.02

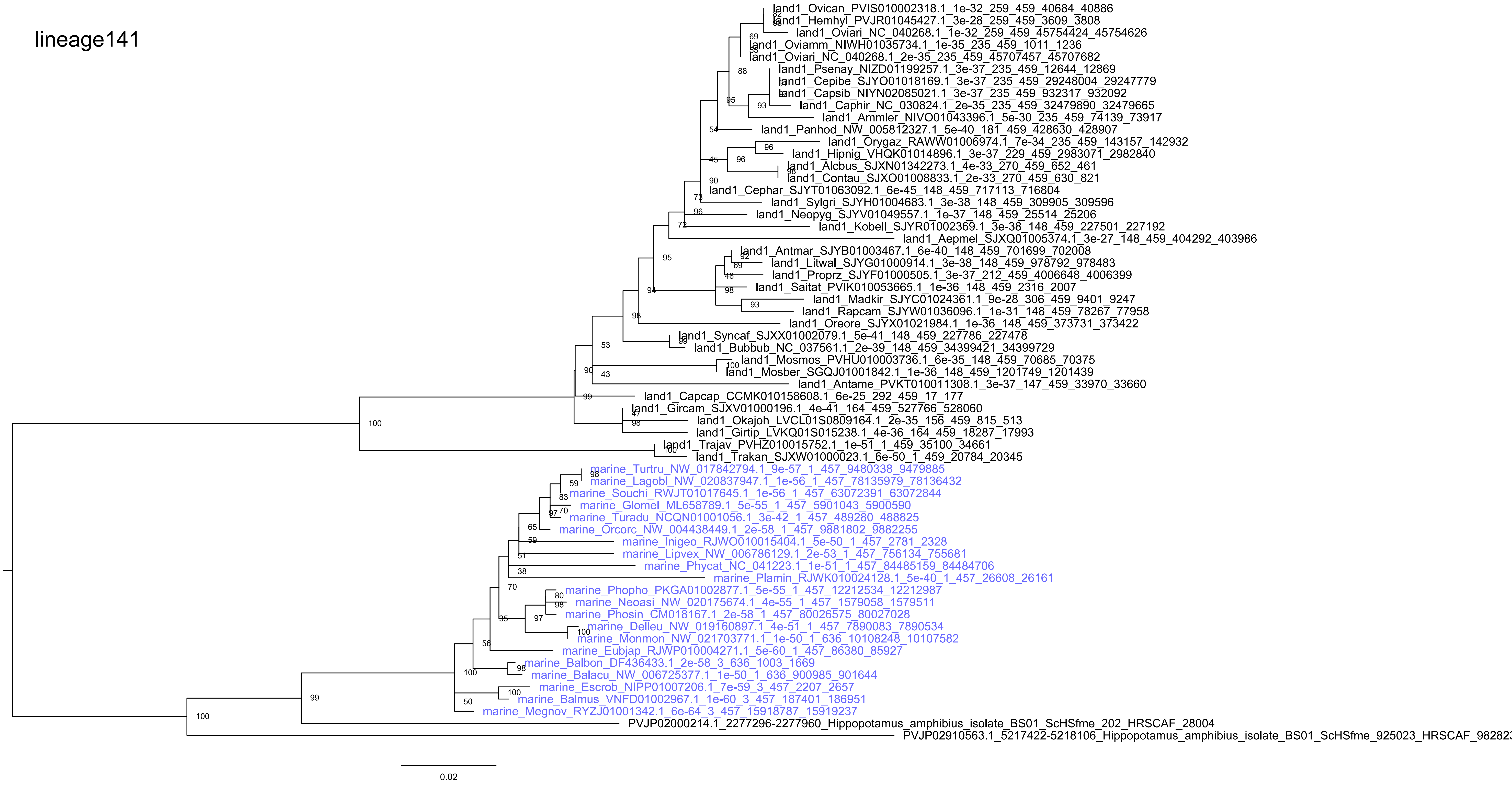
lineage141
land1_Ovican_PVIS010002318.1_1e-32_259_459_40684_40886
land1_Hemhyl_PVJR01045427.1_3e-28_259_459_3609_3808
land1_Oviari_NC_040268.1_1e-32_259_459_45754424_45754626
land1_Oviamm_NIWH01035734.1_1e-35_235_459_1011_1236
land1_Oviari_NC_040268.1_2e-35_235_459_45707457_45707682
land1_Psenay_NIZD01199257.1_3e-37_235_459_12644_12869
land1_Cepibe_SJYO01018169.1_3e-37_235_459_29248004_29247779
land1_Capsib_NIYN02085021.1_3e-37_235_459_932317_932092
land1_Caphir_NC_030824.1_2e-35_235_459_32479890_32479665
land1_Ammler_NIVO01043396.1_5e-30_235_459_74139_73917
land1_Panhod_NW_005812327.1_5e-40_181_459_428630_428907
land1_Orygaz_RAWW01006974.1_7e-34_235_459_143157_142932
land1_Hipnig_VHQK01014896.1_3e-37_229_459_2983071_2982840
land1_Alcbus_SJXN01342273.1_4e-33_270_459_652_461
land1_Contau_SJXO01008833.1_2e-33_270_459_630_821
land1_Cephar_SJYT01063092.1_6e-45_148_459_717113_716804
land1_Sylgri_SJYH01004683.1_3e-38_148_459_309905_309596
land1_Neopyg_SJYV01049557.1_1e-37_148_459_25514_25206
land1_Kobell_SJYR01002369.1_3e-38_148_459_227501_227192
land1_Aepmel_SJXQ01005374.1_3e-27_148_459_404292_403986
land1_Antmar_SJYB01003467.1_6e-40_148_459_701699_702008
land1_Litwal_SJYG01000914.1_3e-38_148_459_978792_978483
land1_Proprz_SJYF01000505.1_3e-37_212_459_4006648_4006399
land1_Saitat_PVIK010053665.1_1e-36_148_459_2316_2007
land1_Madkir_SJYC01024361.1_9e-28_306_459_9401_9247
land1_Rapcam_SJYW01036096.1_1e-31_148_459_78267_77958
land1_Oreore_SJYX01021984.1_1e-36_148_459_373731_373422
land1_Syncaf_SJXX01002079.1_5e-41_148_459_227786_227478
land1_Bubbub_NC_037561.1_2e-39_148_459_34399421_34399729
land1_Mosmos_PVHU010003736.1_6e-35_148_459_70685_70375
land1_Mosber_SGQJ01001842.1_1e-36_148_459_1201749_1201439
land1_Antame_PVKT010011308.1_3e-37_147_459_33970_33660
land1_Capcap_CCMK010158608.1_6e-25_292_459_17_177
land1_Gircam_SJXV01000196.1_4e-41_164_459_527766_528060
land1_Okajoh_LVCL01S0809164.1_2e-35_156_459_815_513
land1_Girtip_LVKQ01S015238.1_4e-36_164_459_18287_17993
land1_Trajav_PVHZ010015752.1_1e-51_1_459_35100_34661
land1_Trakan_SJXW01000023.1_6e-50_1_459_20784_20345
marine_Turtru_NW_017842794.1_9e-57_1_457_9480338_9479885
marine_Lagobl_NW_020837947.1_1e-56_1_457_78135979_78136432
marine_Souchi_RWJT01017645.1_1e-56_1_457_63072391_63072844
marine_Glomel_ML658789.1_5e-55_1_457_5901043_5900590
marine_Turadu_NCQN01001056.1_3e-42_1_457_489280_488825
marine_Orcorc_NW_004438449.1_2e-58_1_457_9881802_9882255
marine_Inigeo_RJWO010015404.1_5e-50_1_457_2781_2328
marine_Lipvex_NW_006786129.1_2e-53_1_457_756134_755681
marine_Phycat_NC_041223.1_1e-51_1_457_84485159_84484706
marine_Plamin_RJWK010024128.1_5e-40_1_457_26608_26161
marine_Phopho_PKGA01002877.1_5e-55_1_457_12212534_12212987
marine_Neoasi_NW_020175674.1_4e-55_1_457_1579058_1579511
marine_Phosin_CM018167.1_2e-58_1_457_80026575_80027028
marine_Delleu_NW_019160897.1_4e-51_1_457_7890083_7890534
marine_Monmon_NW_021703771.1_1e-50_1_636_10108248_10107582
marine_Eubjap_RJWP010004271.1_5e-60_1_457_86380_85927
marine_Balbon_DF436433.1_2e-58_3_636_1003_1669
marine_Balacu_NW_006725377.1_1e-50_1_636_900985_901644
marine_Escrob_NIPP01007206.1_7e-59_3_457_2207_2657
marine_Balmus_VNFD01002967.1_1e-60_3_457_187401_186951
marine_Megnov_RYZJ01001342.1_6e-64_3_457_15918787_15919237
PVJP02000214.1_2277296-2277960_Hippopotamus_amphibius_isolate_BS01_ScHSfme_202_HRSCAF_28004
PVJP02910563.1_5217422-5218106_Hippopotamus_amphibius_isolate_BS01_ScHSfme_925023_HRSCAF_98282
0.02

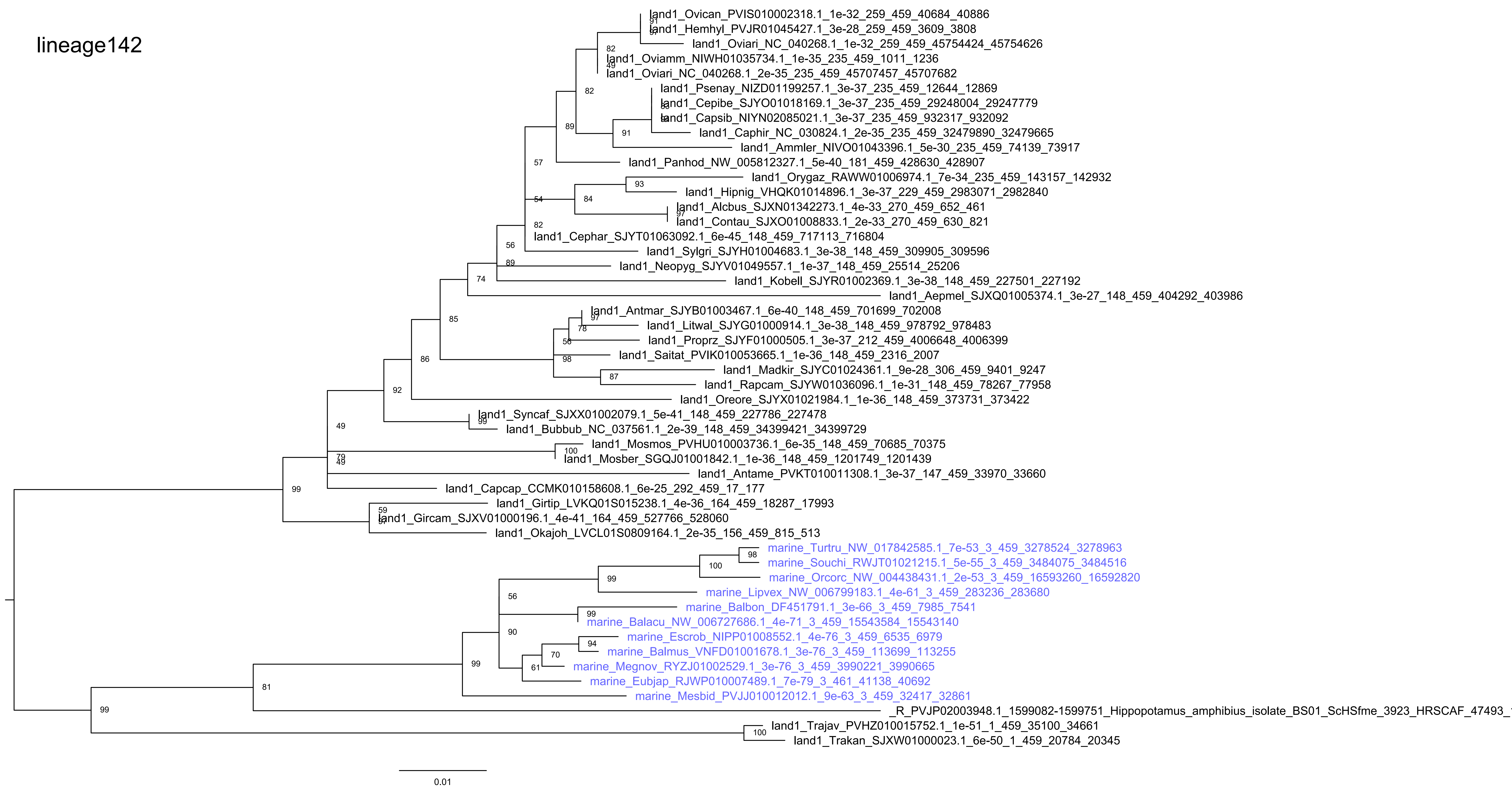
lineage142
land1_Ovican_PVIS010002318.1_1e-32_259_459_40684_40886
land1_Hemhyl_PVJR01045427.1_3e-28_259_459_3609_3808
land1_Oviari_NC_040268.1_1e-32_259_459_45754424_45754626
land1_Oviamm_NIWH01035734.1_1e-35_235_459_1011_1236
land1_Oviari_NC_040268.1_2e-35_235_459_45707457_45707682
land1_Psenay_NIZD01199257.1_3e-37_235_459_12644_12869
land1_Cepibe_SJYO01018169.1_3e-37_235_459_29248004_29247779
land1_Capsib_NIYN02085021.1_3e-37_235_459_932317_932092
land1_Caphir_NC_030824.1_2e-35_235_459_32479890_32479665
land1_Ammler_NIVO01043396.1_5e-30_235_459_74139_73917
land1_Panhod_NW_005812327.1_5e-40_181_459_428630_428907
land1_Orygaz_RAWW01006974.1_7e-34_235_459_143157_142932
land1_Hipnig_VHQK01014896.1_3e-37_229_459_2983071_2982840
land1_Alcbus_SJXN01342273.1_4e-33_270_459_652_461
land1_Contau_SJXO01008833.1_2e-33_270_459_630_821
land1_Cephar_SJYT01063092.1_6e-45_148_459_717113_716804
land1_Sylgri_SJYH01004683.1_3e-38_148_459_309905_309596
land1_Neopyg_SJYV01049557.1_1e-37_148_459_25514_25206
land1_Kobell_SJYR01002369.1_3e-38_148_459_227501_227192
land1_Aepmel_SJXQ01005374.1_3e-27_148_459_404292_403986
land1_Antmar_SJYB01003467.1_6e-40_148_459_701699_702008
land1_Litwal_SJYG01000914.1_3e-38_148_459_978792_978483
land1_Proprz_SJYF01000505.1_3e-37_212_459_4006648_4006399
land1_Saitat_PVIK010053665.1_1e-36_148_459_2316_2007
land1_Madkir_SJYC01024361.1_9e-28_306_459_9401_9247
land1_Rapcam_SJYW01036096.1_1e-31_148_459_78267_77958
land1_Oreore_SJYX01021984.1_1e-36_148_459_373731_373422
land1_Syncaf_SJXX01002079.1_5e-41_148_459_227786_227478
land1_Bubbub_NC_037561.1_2e-39_148_459_34399421_34399729
land1_Mosmos_PVHU010003736.1_6e-35_148_459_70685_70375
land1_Mosber_SGQJ01001842.1_1e-36_148_459_1201749_1201439
land1_Antame_PVKT010011308.1_3e-37_147_459_33970_33660
land1_Capcap_CCMK010158608.1_6e-25_292_459_17_177
land1_Girtip_LVKQ01S015238.1_4e-36_164_459_18287_17993
land1_Gircam_SJXV01000196.1_4e-41_164_459_527766_528060
land1_Okajoh_LVCL01S0809164.1_2e-35_156_459_815_513
marine_Turtru_NW_017842585.1_7e-53_3_459_3278524_3278963
marine_Souchi_RWJT01021215.1_5e-55_3_459_3484075_3484516
marine_Orcorc_NW_004438431.1_2e-53_3_459_16593260_16592820
marine_Lipvex_NW_006799183.1_4e-61_3_459_283236_283680
marine_Balbon_DF451791.1_3e-66_3_459_7985_7541
marine_Balacu_NW_006727686.1_4e-71_3_459_15543584_15543140
marine_Escrob_NIPP01008552.1_4e-76_3_459_6535_6979
marine_Balmus_VNFD01001678.1_3e-76_3_459_113699_113255
marine_Megnov_RYZJ01002529.1_3e-76_3_459_3990221_3990665
marine_Eubjap_RJWP010007489.1_7e-79_3_461_41138_40692
marine_Mesbid_PVJJ010012012.1_9e-63_3_459_32417_32861
_R_PVJP02003948.1_1599082-1599751_Hippopotamus_amphibius_isolate_BS01_ScHSfme_3923_HRSCAF_47493_
land1_Trajav_PVHZ010015752.1_1e-51_1_459_35100_34661
land1_Trakan_SJXW01000023.1_6e-50_1_459_20784_20345
0.01

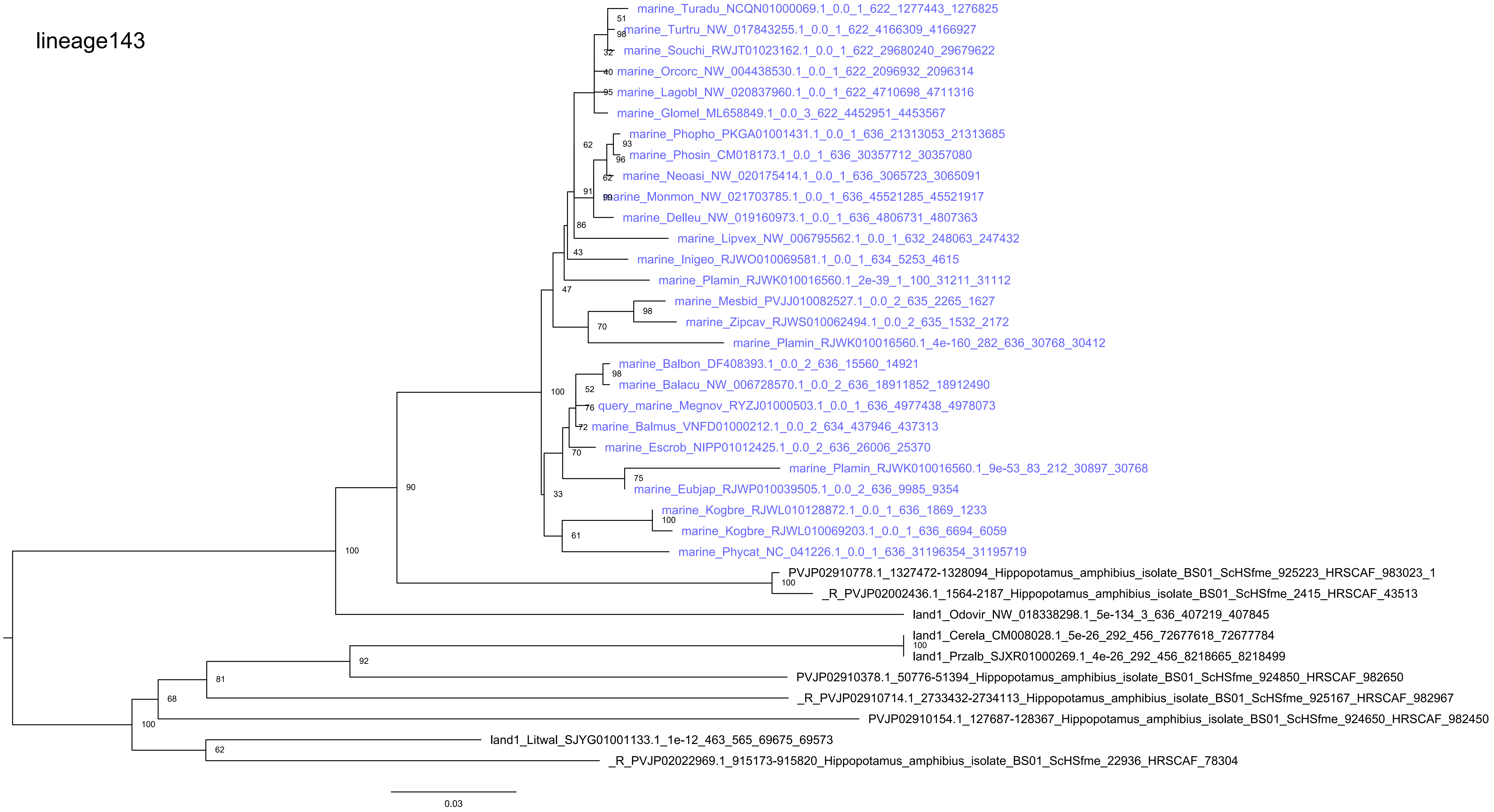
lineage143
marine_Turadu_NCQN01000069.1_0.0_1_622_1277443_1276825
marine_Turtru_NW_017843255.1_0.0_1_622_4166309_4166927
marine_Souchi_RWJT01023162.1_0.0_1_622_29680240_29679622
marine_Orcorc_NW_004438530.1_0.0_1_622_2096932_2096314
marine_Lagobl_NW_020837960.1_0.0_1_622_4710698_4711316
marine_Glomel_ML658849.1_0.0_3_622_4452951_4453567
marine_Phopho_PKGA01001431.1_0.0_1_636_21313053_21313685
marine_Phosin_CM018173.1_0.0_1_636_30357712_30357080
marine_Neoasi_NW_020175414.1_0.0_1_636_3065723_3065091
marine_Monmon_NW_021703785.1_0.0_1_636_45521285_45521917
marine_Delleu_NW_019160973.1_0.0_1_636_4806731_4807363
marine_Lipvex_NW_006795562.1_0.0_1_632_248063_247432
marine_Inigeo_RJWO010069581.1_0.0_1_634_5253_4615
marine_Plamin_RJWK010016560.1_2e-39_1_100_31211_31112
marine_Mesbid_PVJJ010082527.1_0.0_2_635_2265_1627
marine_Zipcav_RJWS010062494.1_0.0_2_635_1532_2172
marine_Plamin_RJWK010016560.1_4e-160_282_636_30768_30412
marine_Balbon_DF408393.1_0.0_2_636_15560_14921
marine_Balacu_NW_006728570.1_0.0_2_636_18911852_18912490
query_marine_Megnov_RYZJ01000503.1_0.0_1_636_4977438_4978073
marine_Balmus_VNFD01000212.1_0.0_2_634_437946_437313
marine_Escrob_NIPP01012425.1_0.0_2_636_26006_25370
marine_Plamin_RJWK010016560.1_9e-53_83_212_30897_30768
marine_Eubjap_RJWP010039505.1_0.0_2_636_9985_9354
marine_Kogbre_RJWL010128872.1_0.0_1_636_1869_1233
marine_Kogbre_RJWL010069203.1_0.0_1_636_6694_6059
marine_Phycat_NC_041226.1_0.0_1_636_31196354_31195719
PVJP02910778.1_1327472-1328094_Hippopotamus_amphibius_isolate_BS01_ScHSfme_925223_HRSCAF_983023_1
_R_PVJP02002436.1_1564-2187_Hippopotamus_amphibius_isolate_BS01_ScHSfme_2415_HRSCAF_43513
land1_Odovir_NW_018338298.1_5e-134_3_636_407219_407845
land1_Cerela_CM008028.1_5e-26_292_456_72677618_72677784
land1_Przalb_SJXR01000269.1_4e-26_292_456_8218665_8218499
PVJP02910378.1_50776-51394_Hippopotamus_amphibius_isolate_BS01_ScHSfme_924850_HRSCAF_982650
_R_PVJP02910714.1_2733432-2734113_Hippopotamus_amphibius_isolate_BS01_ScHSfme_925167_HRSCAF_982967
PVJP02910154.1_127687-128367_Hippopotamus_amphibius_isolate_BS01_ScHSfme_924650_HRSCAF_982450
land1_Litwal_SJYG01001133.1_1e-12_463_565_69675_69573
_R_PVJP02022969.1_915173-915820_Hippopotamus_amphibius_isolate_BS01_ScHSfme_22936_HRSCAF_78304
0.03

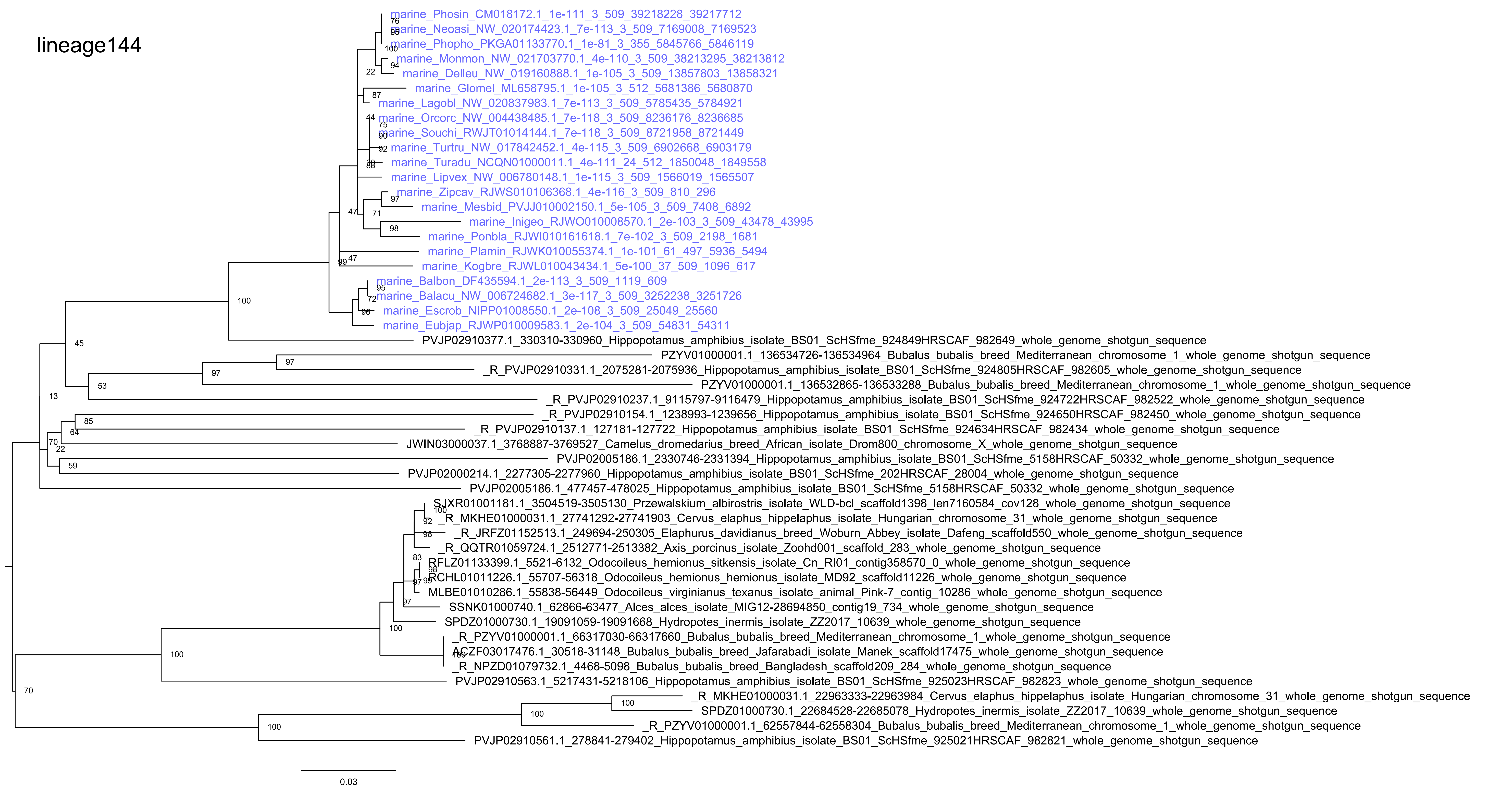
lineage144
marine_Phosin_CM018172.1_1e-111_3_509_39218228_39217712
marine_Neoasi_NW_020174423.1_7e-113_3_509_7169008_7169523
marine_Phopho_PKGA01133770.1_1e-81_3_355_5845766_5846119
marine_Monmon_NW_021703770.1_4e-110_3_509_38213295_38213812
marine_Delleu_NW_019160888.1_1e-105_3_509_13857803_13858321
marine_Glomel_ML658795.1_1e-105_3_512_5681386_5680870
marine_Lagobl_NW_020837983.1_7e-113_3_509_5785435_5784921
marine_Orcorc_NW_004438485.1_7e-118_3_509_8236176_8236685
marine_Souchi_RWJT01014144.1_7e-118_3_509_8721958_8721449
marine_Turtru_NW_017842452.1_4e-115_3_509_6902668_6903179
marine_Turadu_NCQN01000011.1_4e-111_24_512_1850048_1849558
marine_Lipvex_NW_006780148.1_1e-115_3_509_1566019_1565507
marine_Zipcav_RJWS010106368.1_4e-116_3_509_810_296
marine_Mesbid_PVJJ010002150.1_5e-105_3_509_7408_6892
marine_Inigeo_RJWO010008570.1_2e-103_3_509_43478_43995
marine_Ponbla_RJWI010161618.1_7e-102_3_509_2198_1681
marine_Plamin_RJWK010055374.1_1e-101_61_497_5936_5494
marine_Kogbre_RJWL010043434.1_5e-100_37_509_1096_617
marine_Balbon_DF435594.1_2e-113_3_509_1119_609
marine_Balacu_NW_006724682.1_3e-117_3_509_3252238_3251726
marine_Escrob_NIPP01008550.1_2e-108_3_509_25049_25560
marine_Eubjap_RJWP010009583.1_2e-104_3_509_54831_54311
PVJP02910377.1_330310-330960_Hippopotamus_amphibius_isolate_BS01_ScHSfme_924849HRSCAF_982649_whole_genome_shotgun_sequence
PZYV01000001.1_136534726-136534964_Bubalus_bubalis_breed_Mediterranean_chromosome_1_whole_genome_shotgun_sequence
_R_PVJP02910331.1_2075281-2075936_Hippopotamus_amphibius_isolate_BS01_ScHSfme_924805HRSCAF_982605_whole_genome_shotgun_sequence
PZYV01000001.1_136532865-136533288_Bubalus_bubalis_breed_Mediterranean_chromosome_1_whole_genome_shotgun_sequence
_R_PVJP02910237.1_9115797-9116479_Hippopotamus_amphibius_isolate_BS01_ScHSfme_924722HRSCAF_982522_whole_genome_shotgun_sequence
_R_PVJP02910154.1_1238993-1239656_Hippopotamus_amphibius_isolate_BS01_ScHSfme_924650HRSCAF_982450_whole_genome_shotgun_sequence
_R_PVJP02910137.1_127181-127722_Hippopotamus_amphibius_isolate_BS01_ScHSfme_924634HRSCAF_982434_whole_genome_shotgun_sequence
JWIN03000037.1_3768887-3769527_Camelus_dromedarius_breed_African_isolate_Drom800_chromosome_X_whole_genome_shotgun_sequence
PVJP02005186.1_2330746-2331394_Hippopotamus_amphibius_isolate_BS01_ScHSfme_5158HRSCAF_50332_whole_genome_shotgun_sequence
PVJP02000214.1_2277305-2277960_Hippopotamus_amphibius_isolate_BS01_ScHSfme_202HRSCAF_28004_whole_genome_shotgun_sequence
PVJP02005186.1_477457-478025_Hippopotamus_amphibius_isolate_BS01_ScHSfme_5158HRSCAF_50332_whole_genome_shotgun_sequence
SJXR01001181.1_3504519-3505130_Przewalskium_albirostris_isolate_WLD-bcl_scaffold1398_len7160584_cov128_whole_genome_shotgun_sequence
_R_MKHE01000031.1_27741292-27741903_Cervus_elaphus_hippelaphus_isolate_Hungarian_chromosome_31_whole_genome_shotgun_sequence
_R_JRFZ01152513.1_249694-250305_Elaphurus_davidianus_breed_Woburn_Abbey_isolate_Dafeng_scaffold550_whole_genome_shotgun_sequence
_R_QQTR01059724.1_2512771-2513382_Axis_porcinus_isolate_Zoohd001_scaffold_283_whole_genome_shotgun_sequence
RFLZ01133399.1_5521-6132_Odocoileus_hemionus_sitkensis_isolate_Cn_RI01_contig358570_0_whole_genome_shotgun_sequence
RCHL01011226.1_55707-56318_Odocoileus_hemionus_hemionus_isolate_MD92_scaffold11226_whole_genome_shotgun_sequence
MLBE01010286.1_55838-56449_Odocoileus_virginianus_texanus_isolate_animal_Pink-7_contig_10286_whole_genome_shotgun_sequence
SSNK01000740.1_62866-63477_Alces_alces_isolate_MIG12-28694850_contig19_734_whole_genome_shotgun_sequence
SPDZ01000730.1_19091059-19091668_Hydropotes_inermis_isolate_ZZ2017_10639_whole_genome_shotgun_sequence
_R_PZYV01000001.1_66317030-66317660_Bubalus_bubalis_breed_Mediterranean_chromosome_1_whole_genome_shotgun_sequence
ACZF03017476.1_30518-31148_Bubalus_bubalis_breed_Jafarabadi_isolate_Manek_scaffold17475_whole_genome_shotgun_sequence
_R_NPZD01079732.1_4468-5098_Bubalus_bubalis_breed_Bangladesh_scaffold209_284_whole_genome_shotgun_sequence
PVJP02910563.1_5217431-5218106_Hippopotamus_amphibius_isolate_BS01_ScHSfme_925023HRSCAF_982823_whole_genome_shotgun_sequence
_R_MKHE01000031.1_22963333-22963984_Cervus_elaphus_hippelaphus_isolate_Hungarian_chromosome_31_whole_genome_shotgun_sequence
SPDZ01000730.1_22684528-22685078_Hydropotes_inermis_isolate_ZZ2017_10639_whole_genome_shotgun_sequence
_R_PZYV01000001.1_62557844-62558304_Bubalus_bubalis_breed_Mediterranean_chromosome_1_whole_genome_shotgun_sequence
PVJP02910561.1_278841-279402_Hippopotamus_amphibius_isolate_BS01_ScHSfme_925021HRSCAF_982821_whole_genome_shotgun_sequence
0.03

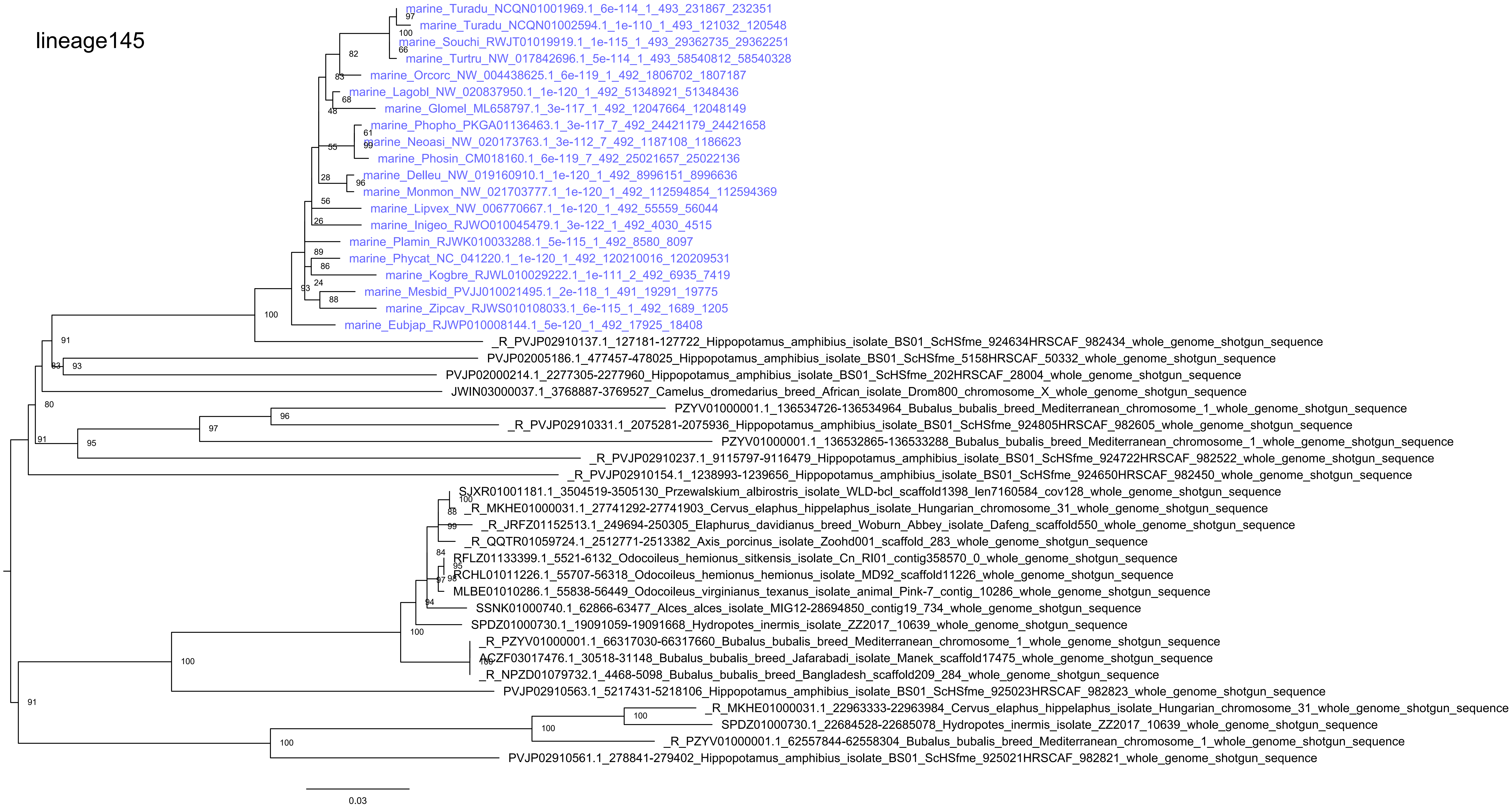
lineage145
marine_Turadu_NCQN01001969.1_6e-114_1_493_231867_232351
marine_Turadu_NCQN01002594.1_1e-110_1_493_121032_120548
marine_Souchi_RWJT01019919.1_1e-115_1_493_29362735_29362251
marine_Turtru_NW_017842696.1_5e-114_1_493_58540812_58540328
marine_Orcorc_NW_004438625.1_6e-119_1_492_1806702_1807187
marine_Lagobl_NW_020837950.1_1e-120_1_492_51348921_51348436
marine_Glomel_ML658797.1_3e-117_1_492_12047664_12048149
marine_Phopho_PKGA01136463.1_3e-117_7_492_24421179_24421658
marine_Neoasi_NW_020173763.1_3e-112_7_492_1187108_1186623
marine_Phosin_CM018160.1_6e-119_7_492_25021657_25022136
marine_Delleu_NW_019160910.1_1e-120_1_492_8996151_8996636
marine_Monmon_NW_021703777.1_1e-120_1_492_112594854_112594369
marine_Lipvex_NW_006770667.1_1e-120_1_492_55559_56044
marine_Inigeo_RJWO010045479.1_3e-122_1_492_4030_4515
marine_Plamin_RJWK010033288.1_5e-115_1_492_8580_8097
marine_Phycat_NC_041220.1_1e-120_1_492_120210016_120209531
marine_Kogbre_RJWL010029222.1_1e-111_2_492_6935_7419
marine_Mesbid_PVJJ010021495.1_2e-118_1_491_19291_19775
marine_Zipcav_RJWS010108033.1_6e-115_1_492_1689_1205
marine_Eubjap_RJWP010008144.1_5e-120_1_492_17925_18408
_R_PVJP02910137.1_127181-127722_Hippopotamus_amphibius_isolate_BS01_ScHSfme_924634HRSCAF_982434_whole_genome_shotgun_sequence
PVJP02005186.1_477457-478025_Hippopotamus_amphibius_isolate_BS01_ScHSfme_5158HRSCAF_50332_whole_genome_shotgun_sequence
PVJP02000214.1_2277305-2277960_Hippopotamus_amphibius_isolate_BS01_ScHSfme_202HRSCAF_28004_whole_genome_shotgun_sequence
JWIN03000037.1_3768887-3769527_Camelus_dromedarius_breed_African_isolate_Drom800_chromosome_X_whole_genome_shotgun_sequence
PZYV01000001.1_136534726-136534964_Bubalus_bubalis_breed_Mediterranean_chromosome_1_whole_genome_shotgun_sequence
_R_PVJP02910331.1_2075281-2075936_Hippopotamus_amphibius_isolate_BS01_ScHSfme_924805HRSCAF_982605_whole_genome_shotgun_sequence
PZYV01000001.1_136532865-136533288_Bubalus_bubalis_breed_Mediterranean_chromosome_1_whole_genome_shotgun_sequence
_R_PVJP02910237.1_9115797-9116479_Hippopotamus_amphibius_isolate_BS01_ScHSfme_924722HRSCAF_982522_whole_genome_shotgun_sequence
_R_PVJP02910154.1_1238993-1239656_Hippopotamus_amphibius_isolate_BS01_ScHSfme_924650HRSCAF_982450_whole_genome_shotgun_sequence
SJXR01001181.1_3504519-3505130_Przewalskium_albirostris_isolate_WLD-bcl_scaffold1398_len7160584_cov128_whole_genome_shotgun_sequence
_R_MKHE01000031.1_27741292-27741903_Cervus_elaphus_hippelaphus_isolate_Hungarian_chromosome_31_whole_genome_shotgun_sequence
_R_JRFZ01152513.1_249694-250305_Elaphurus_davidianus_breed_Woburn_Abbey_isolate_Dafeng_scaffold550_whole_genome_shotgun_sequence
_R_QQTR01059724.1_2512771-2513382_Axis_porcinus_isolate_Zoohd001_scaffold_283_whole_genome_shotgun_sequence
RFLZ01133399.1_5521-6132_Odocoileus_hemionus_sitkensis_isolate_Cn_RI01_contig358570_0_whole_genome_shotgun_sequence
RCHL01011226.1_55707-56318_Odocoileus_hemionus_hemionus_isolate_MD92_scaffold11226_whole_genome_shotgun_sequence
MLBE01010286.1_55838-56449_Odocoileus_virginianus_texanus_isolate_animal_Pink-7_contig_10286_whole_genome_shotgun_sequence
SSNK01000740.1_62866-63477_Alces_alces_isolate_MIG12-28694850_contig19_734_whole_genome_shotgun_sequence
SPDZ01000730.1_19091059-19091668_Hydropotes_inermis_isolate_ZZ2017_10639_whole_genome_shotgun_sequence
_R_PZYV01000001.1_66317030-66317660_Bubalus_bubalis_breed_Mediterranean_chromosome_1_whole_genome_shotgun_sequence
ACZF03017476.1_30518-31148_Bubalus_bubalis_breed_Jafarabadi_isolate_Manek_scaffold17475_whole_genome_shotgun_sequence
_R_NPZD01079732.1_4468-5098_Bubalus_bubalis_breed_Bangladesh_scaffold209_284_whole_genome_shotgun_sequence
PVJP02910563.1_5217431-5218106_Hippopotamus_amphibius_isolate_BS01_ScHSfme_925023HRSCAF_982823_whole_genome_shotgun_sequence
_R_MKHE01000031.1_22963333-22963984_Cervus_elaphus_hippelaphus_isolate_Hungarian_chromosome_31_whole_genome_shotgun_sequence
SPDZ01000730.1_22684528-22685078_Hydropotes_inermis_isolate_ZZ2017_10639_whole_genome_shotgun_sequence
_R_PZYV01000001.1_62557844-62558304_Bubalus_bubalis_breed_Mediterranean_chromosome_1_whole_genome_shotgun_sequence
PVJP02910561.1_278841-279402_Hippopotamus_amphibius_isolate_BS01_ScHSfme_925021HRSCAF_982821_whole_genome_shotgun_sequence
0.03

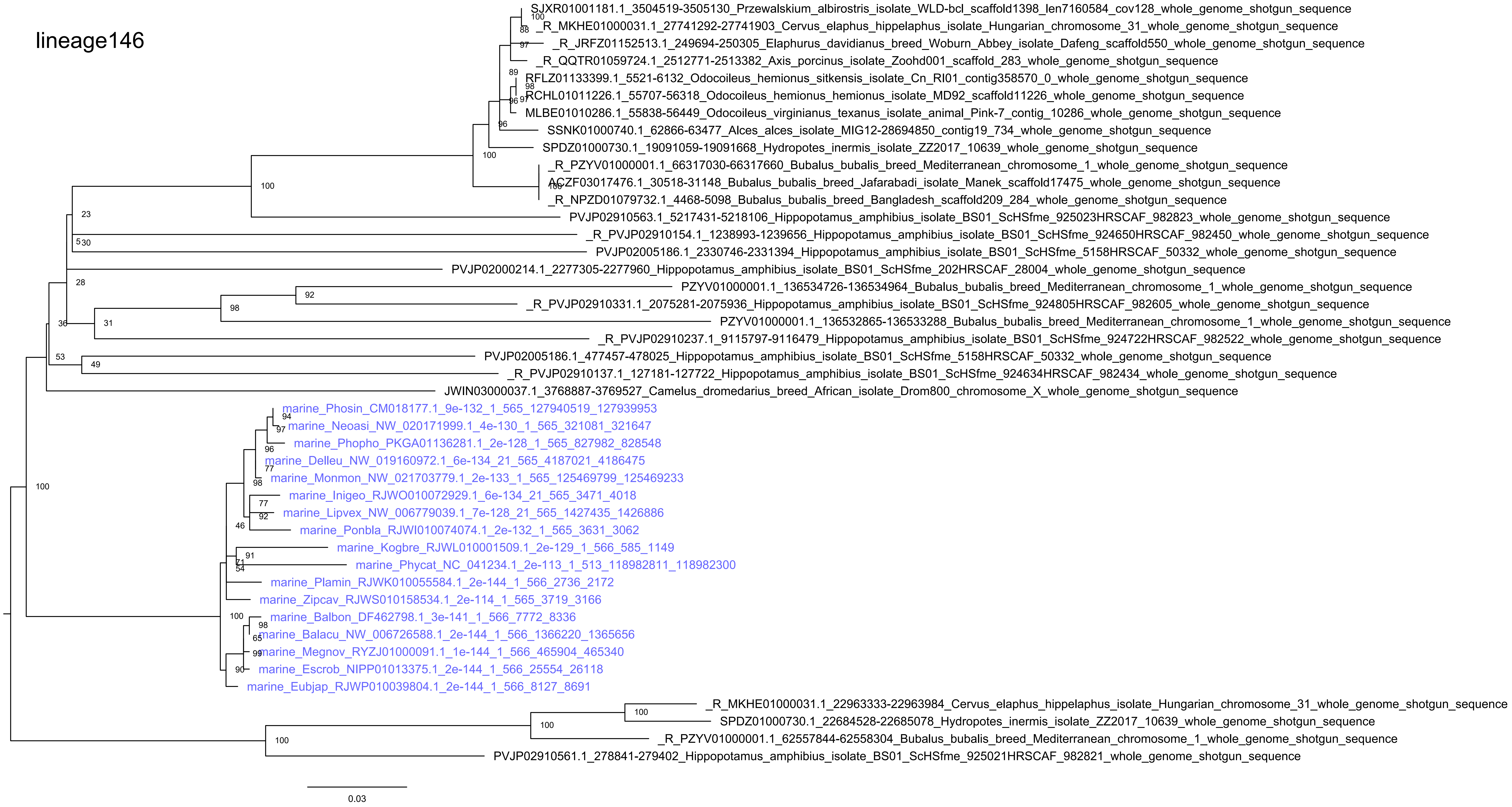
lineage146
SJXR01001181.1_3504519-3505130_Przewalskium_albirostris_isolate_WLD-bcl_scaffold1398_len7160584_cov128_whole_genome_shotgun_sequence
_R_MKHE01000031.1_27741292-27741903_Cervus_elaphus_hippelaphus_isolate_Hungarian_chromosome_31_whole_genome_shotgun_sequence
_R_JRFZ01152513.1_249694-250305_Elaphurus_davidianus_breed_Woburn_Abbey_isolate_Dafeng_scaffold550_whole_genome_shotgun_sequence
_R_QQTR01059724.1_2512771-2513382_Axis_porcinus_isolate_Zoohd001_scaffold_283_whole_genome_shotgun_sequence
RFLZ01133399.1_5521-6132_Odocoileus_hemionus_sitkensis_isolate_Cn_RI01_contig358570_0_whole_genome_shotgun_sequence
RCHL01011226.1_55707-56318_Odocoileus_hemionus_hemionus_isolate_MD92_scaffold11226_whole_genome_shotgun_sequence
MLBE01010286.1_55838-56449_Odocoileus_virginianus_texanus_isolate_animal_Pink-7_contig_10286_whole_genome_shotgun_sequence
SSNK01000740.1_62866-63477_Alces_alces_isolate_MIG12-28694850_contig19_734_whole_genome_shotgun_sequence
SPDZ01000730.1_19091059-19091668_Hydropotes_inermis_isolate_ZZ2017_10639_whole_genome_shotgun_sequence
_R_PZYV01000001.1_66317030-66317660_Bubalus_bubalis_breed_Mediterranean_chromosome_1_whole_genome_shotgun_sequence
ACZF03017476.1_30518-31148_Bubalus_bubalis_breed_Jafarabadi_isolate_Manek_scaffold17475_whole_genome_shotgun_sequence
_R_NPZD01079732.1_4468-5098_Bubalus_bubalis_breed_Bangladesh_scaffold209_284_whole_genome_shotgun_sequence
PVJP02910563.1_5217431-5218106_Hippopotamus_amphibius_isolate_BS01_ScHSfme_925023HRSCAF_982823_whole_genome_shotgun_sequence
_R_PVJP02910154.1_1238993-1239656_Hippopotamus_amphibius_isolate_BS01_ScHSfme_924650HRSCAF_982450_whole_genome_shotgun_sequence
PVJP02005186.1_2330746-2331394_Hippopotamus_amphibius_isolate_BS01_ScHSfme_5158HRSCAF_50332_whole_genome_shotgun_sequence
PVJP02000214.1_2277305-2277960_Hippopotamus_amphibius_isolate_BS01_ScHSfme_202HRSCAF_28004_whole_genome_shotgun_sequence
PZYV01000001.1_136534726-136534964_Bubalus_bubalis_breed_Mediterranean_chromosome_1_whole_genome_shotgun_sequence
_R_PVJP02910331.1_2075281-2075936_Hippopotamus_amphibius_isolate_BS01_ScHSfme_924805HRSCAF_982605_whole_genome_shotgun_sequence
PZYV01000001.1_136532865-136533288_Bubalus_bubalis_breed_Mediterranean_chromosome_1_whole_genome_shotgun_sequence
_R_PVJP02910237.1_9115797-9116479_Hippopotamus_amphibius_isolate_BS01_ScHSfme_924722HRSCAF_982522_whole_genome_shotgun_sequence
PVJP02005186.1_477457-478025_Hippopotamus_amphibius_isolate_BS01_ScHSfme_5158HRSCAF_50332_whole_genome_shotgun_sequence
_R_PVJP02910137.1_127181-127722_Hippopotamus_amphibius_isolate_BS01_ScHSfme_924634HRSCAF_982434_whole_genome_shotgun_sequence
JWIN03000037.1_3768887-3769527_Camelus_dromedarius_breed_African_isolate_Drom800_chromosome_X_whole_genome_shotgun_sequence
marine_Phosin_CM018177.1_9e-132_1_565_127940519_127939953
marine_Neoasi_NW_020171999.1_4e-130_1_565_321081_321647
marine_Phopho_PKGA01136281.1_2e-128_1_565_827982_828548
marine_Delleu_NW_019160972.1_6e-134_21_565_4187021_4186475
marine_Monmon_NW_021703779.1_2e-133_1_565_125469799_125469233
marine_Inigeo_RJWO010072929.1_6e-134_21_565_3471_4018
marine_Lipvex_NW_006779039.1_7e-128_21_565_1427435_1426886
marine_Ponbla_RJWI010074074.1_2e-132_1_565_3631_3062
marine_Kogbre_RJWL010001509.1_2e-129_1_566_585_1149
marine_Phycat_NC_041234.1_2e-113_1_513_118982811_118982300
marine_Plamin_RJWK010055584.1_2e-144_1_566_2736_2172
marine_Zipcav_RJWS010158534.1_2e-114_1_565_3719_3166
marine_Balbon_DF462798.1_3e-141_1_566_7772_8336
marine_Balacu_NW_006726588.1_2e-144_1_566_1366220_1365656
marine_Megnov_RYZJ01000091.1_1e-144_1_566_465904_465340
marine_Escrob_NIPP01013375.1_2e-144_1_566_25554_26118
marine_Eubjap_RJWP010039804.1_2e-144_1_566_8127_8691
_R_MKHE01000031.1_22963333-22963984_Cervus_elaphus_hippelaphus_isolate_Hungarian_chromosome_31_whole_genome_shotgun_sequence
SPDZ01000730.1_22684528-22685078_Hydropotes_inermis_isolate_ZZ2017_10639_whole_genome_shotgun_sequence
_R_PZYV01000001.1_62557844-62558304_Bubalus_bubalis_breed_Mediterranean_chromosome_1_whole_genome_shotgun_sequence
PVJP02910561.1_278841-279402_Hippopotamus_amphibius_isolate_BS01_ScHSfme_925021HRSCAF_982821_whole_genome_shotgun_sequence
0.03

lineage147

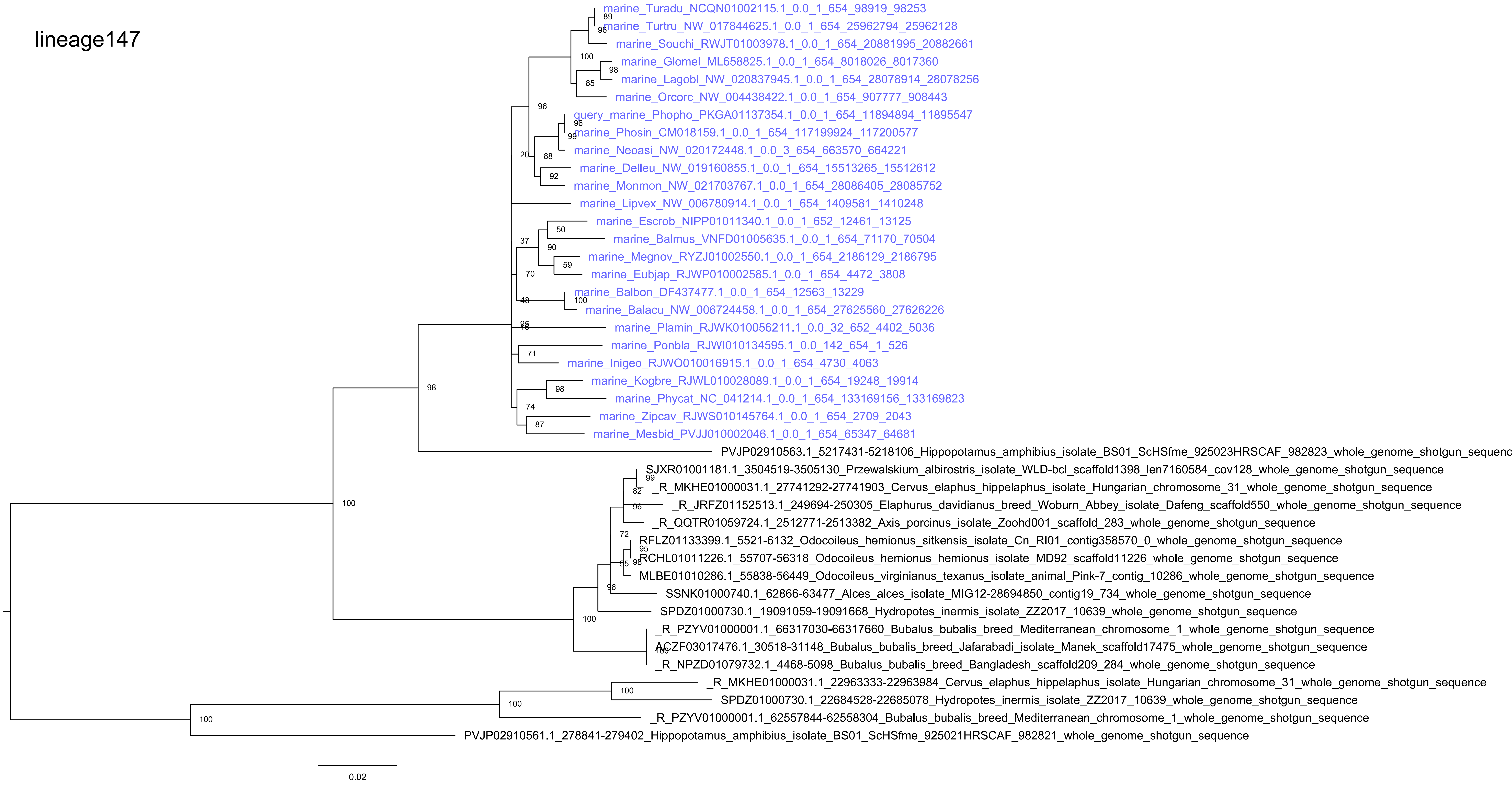

marine_Turadu_NCQN01002115.1_0.0_1_654_98919_98253
marine_Turtru_NW_017844625.1_0.0_1_654_25962794_25962128
marine_Souchi_RWJT01003978.1_0.0_1_654_20881995_20882661
marine_Glomel_ML658825.1_0.0_1_654_8018026_8017360
marine_Lagobl_NW_020837945.1_0.0_1_654_28078914_28078256
marine_Orcorc_NW_004438422.1_0.0_1_654_907777_908443
query_marine_Phopho_PKGA01137354.1_0.0_1_654_11894894_11895547
marine_Phosin_CM018159.1_0.0_1_654_117199924_117200577
marine_Neoasi_NW_020172448.1_0.0_3_654_663570_664221
marine_Delleu_NW_019160855.1_0.0_1_654_15513265_15512612
marine_Monmon_NW_021703767.1_0.0_1_654_28086405_28085752
marine_Lipvex_NW_006780914.1_0.0_1_654_1409581_1410248
marine_Escrob_NIPP01011340.1_0.0_1_652_12461_13125
marine_Balmus_VNFD01005635.1_0.0_1_654_71170_70504
marine_Megnov_RYZJ01002550.1_0.0_1_654_2186129_2186795
marine_Eubjap_RJWP010002585.1_0.0_1_654_4472_3808
marine_Balbon_DF437477.1_0.0_1_654_12563_13229
marine_Balacu_NW_006724458.1_0.0_1_654_27625560_27626226
marine_Plamin_RJWK010056211.1_0.0_32_652_4402_5036
marine_Ponbla_RJWI010134595.1_0.0_142_654_1_526
marine_Inigeo_RJWO010016915.1_0.0_1_654_4730_4063
marine_Kogbre_RJWL010028089.1_0.0_1_654_19248_19914
marine_Phycat_NC_041214.1_0.0_1_654_133169156_133169823
marine_Zipcav_RJWS010145764.1_0.0_1_654_2709_2043
marine_Mesbid_PVJJ010002046.1_0.0_1_654_65347_64681
PVJP02910563.1_5217431-5218106_Hippopotamus_amphibius_isolate_BS01_ScHSfme_925023HRSCAF_982823_whole_genome_shotgun_sequenc
SJXR01001181.1_3504519-3505130_Przewalskium_albirostris_isolate_WLD-bcl_scaffold1398_len7160584_cov128_whole_genome_shotgun_sequence
_R_MKHE01000031.1_27741292-27741903_Cervus_elaphus_hippelaphus_isolate_Hungarian_chromosome_31_whole_genome_shotgun_sequence
_R_JRFZ01152513.1_249694-250305_Elaphurus_davidianus_breed_Woburn_Abbey_isolate_Dafeng_scaffold550_whole_genome_shotgun_sequence
_R_QQTR01059724.1_2512771-2513382_Axis_porcinus_isolate_Zoohd001_scaffold_283_whole_genome_shotgun_sequence
RFLZ01133399.1_5521-6132_Odocoileus_hemionus_sitkensis_isolate_Cn_RI01_contig358570_0_whole_genome_shotgun_sequence
RCHL01011226.1_55707-56318_Odocoileus_hemionus_hemionus_isolate_MD92_scaffold11226_whole_genome_shotgun_sequence
MLBE01010286.1_55838-56449_Odocoileus_virginianus_texanus_isolate_animal_Pink-7_contig_10286_whole_genome_shotgun_sequence
SSNK01000740.1_62866-63477_Alces_alces_isolate_MIG12-28694850_contig19_734_whole_genome_shotgun_sequence
SPDZ01000730.1_19091059-19091668_Hydropotes_inermis_isolate_ZZ2017_10639_whole_genome_shotgun_sequence
_R_PZYV01000001.1_66317030-66317660_Bubalus_bubalis_breed_Mediterranean_chromosome_1_whole_genome_shotgun_sequence
ACZF03017476.1_30518-31148_Bubalus_bubalis_breed_Jafarabadi_isolate_Manek_scaffold17475_whole_genome_shotgun_sequence
_R_NPZD01079732.1_4468-5098_Bubalus_bubalis_breed_Bangladesh_scaffold209_284_whole_genome_shotgun_sequence
_R_MKHE01000031.1_22963333-22963984_Cervus_elaphus_hippelaphus_isolate_Hungarian_chromosome_31_whole_genome_shotgun_sequence
SPDZ01000730.1_22684528-22685078_Hydropotes_inermis_isolate_ZZ2017_10639_whole_genome_shotgun_sequence
_R_PZYV01000001.1_62557844-62558304_Bubalus_bubalis_breed_Mediterranean_chromosome_1_whole_genome_shotgun_sequence
PVJP02910561.1_278841-279402_Hippopotamus_amphibius_isolate_BS01_ScHSfme_925021HRSCAF_982821_whole_genome_shotgun_sequence
0.02

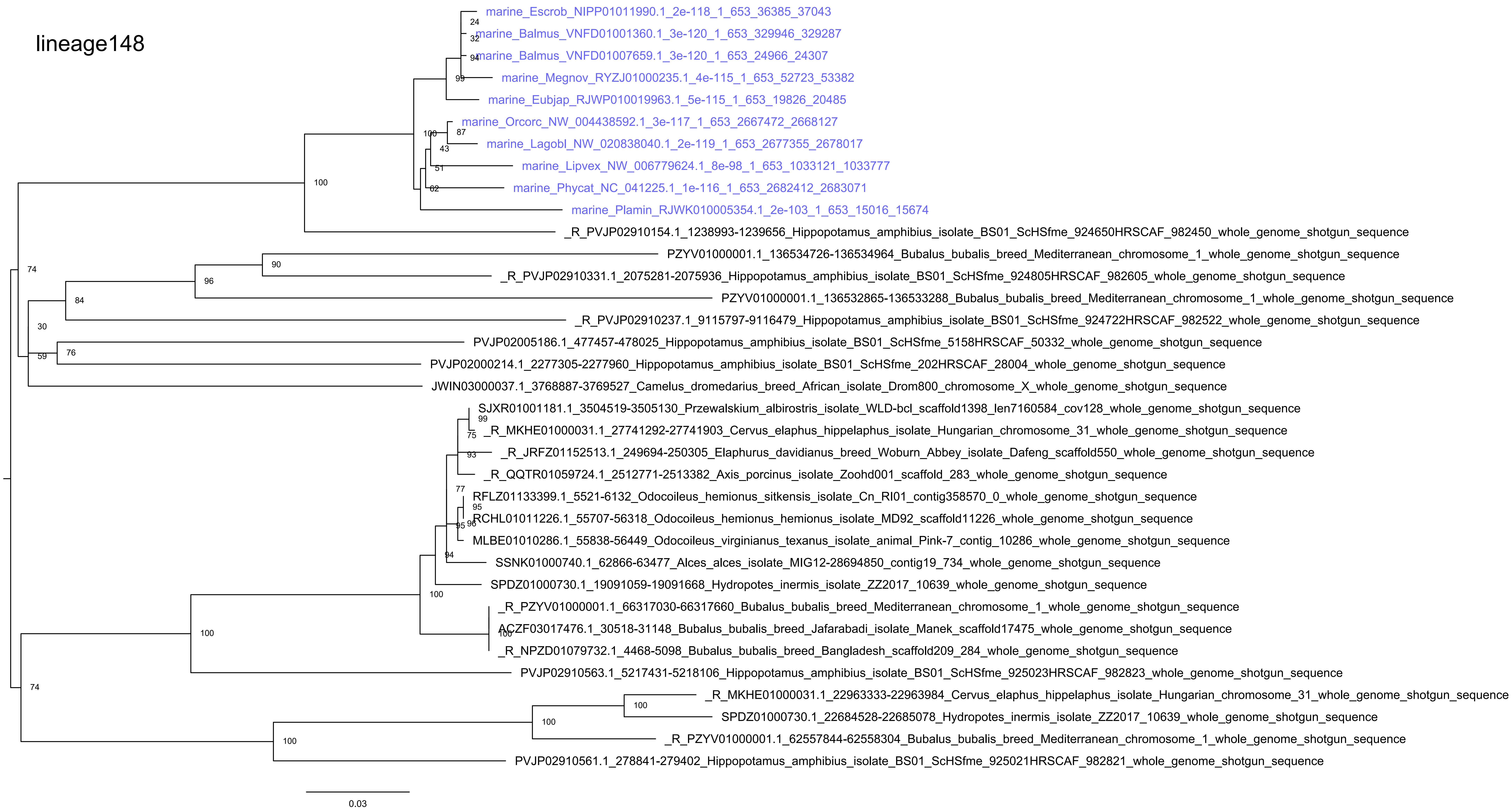

lineage148
marine_Escrob_NIPP01011990.1_2e-118_1_653_36385_37043
marine_Balmus_VNFD01001360.1_3e-120_1_653_329946_329287
marine_Balmus_VNFD01007659.1_3e-120_1_653_24966_24307
marine_Megnov_RYZJ01000235.1_4e-115_1_653_52723_53382
marine_Eubjap_RJWP010019963.1_5e-115_1_653_19826_20485
marine_Orcorc_NW_004438592.1_3e-117_1_653_2667472_2668127
marine_Lagobl_NW_020838040.1_2e-119_1_653_2677355_2678017
marine_Lipvex_NW_006779624.1_8e-98_1_653_1033121_1033777
marine_Phycat_NC_041225.1_1e-116_1_653_2682412_2683071
marine_Plamin_RJWK010005354.1_2e-103_1_653_15016_15674
_R_PVJP02910154.1_1238993-1239656_Hippopotamus_amphibius_isolate_BS01_ScHSfme_924650HRSCAF_982450_whole_genome_shotgun_sequence
PZYV01000001.1_136534726-136534964_Bubalus_bubalis_breed_Mediterranean_chromosome_1_whole_genome_shotgun_sequence
_R_PVJP02910331.1_2075281-2075936_Hippopotamus_amphibius_isolate_BS01_ScHSfme_924805HRSCAF_982605_whole_genome_shotgun_sequence
PZYV01000001.1_136532865-136533288_Bubalus_bubalis_breed_Mediterranean_chromosome_1_whole_genome_shotgun_sequence
_R_PVJP02910237.1_9115797-9116479_Hippopotamus_amphibius_isolate_BS01_ScHSfme_924722HRSCAF_982522_whole_genome_shotgun_sequence
PVJP02005186.1_477457-478025_Hippopotamus_amphibius_isolate_BS01_ScHSfme_5158HRSCAF_50332_whole_genome_shotgun_sequence
PVJP02000214.1_2277305-2277960_Hippopotamus_amphibius_isolate_BS01_ScHSfme_202HRSCAF_28004_whole_genome_shotgun_sequence
JWIN03000037.1_3768887-3769527_Camelus_dromedarius_breed_African_isolate_Drom800_chromosome_X_whole_genome_shotgun_sequence
SJXR01001181.1_3504519-3505130_Przewalskium_albirostris_isolate_WLD-bcl_scaffold1398_len7160584_cov128_whole_genome_shotgun_sequence
_R_MKHE01000031.1_27741292-27741903_Cervus_elaphus_hippelaphus_isolate_Hungarian_chromosome_31_whole_genome_shotgun_sequence
_R_JRFZ01152513.1_249694-250305_Elaphurus_davidianus_breed_Woburn_Abbey_isolate_Dafeng_scaffold550_whole_genome_shotgun_sequence
_R_QQTR01059724.1_2512771-2513382_Axis_porcinus_isolate_Zoohd001_scaffold_283_whole_genome_shotgun_sequence
RFLZ01133399.1_5521-6132_Odocoileus_hemionus_sitkensis_isolate_Cn_RI01_contig358570_0_whole_genome_shotgun_sequence
RCHL01011226.1_55707-56318_Odocoileus_hemionus_hemionus_isolate_MD92_scaffold11226_whole_genome_shotgun_sequence
MLBE01010286.1_55838-56449_Odocoileus_virginianus_texanus_isolate_animal_Pink-7_contig_10286_whole_genome_shotgun_sequence
SSNK01000740.1_62866-63477_Alces_alces_isolate_MIG12-28694850_contig19_734_whole_genome_shotgun_sequence
SPDZ01000730.1_19091059-19091668_Hydropotes_inermis_isolate_ZZ2017_10639_whole_genome_shotgun_sequence
_R_PZYV01000001.1_66317030-66317660_Bubalus_bubalis_breed_Mediterranean_chromosome_1_whole_genome_shotgun_sequence
ACZF03017476.1_30518-31148_Bubalus_bubalis_breed_Jafarabadi_isolate_Manek_scaffold17475_whole_genome_shotgun_sequence
_R_NPZD01079732.1_4468-5098_Bubalus_bubalis_breed_Bangladesh_scaffold209_284_whole_genome_shotgun_sequence
PVJP02910563.1_5217431-5218106_Hippopotamus_amphibius_isolate_BS01_ScHSfme_925023HRSCAF_982823_whole_genome_shotgun_sequence
_R_MKHE01000031.1_22963333-22963984_Cervus_elaphus_hippelaphus_isolate_Hungarian_chromosome_31_whole_genome_shotgun_sequence
SPDZ01000730.1_22684528-22685078_Hydropotes_inermis_isolate_ZZ2017_10639_whole_genome_shotgun_sequence
_R_PZYV01000001.1_62557844-62558304_Bubalus_bubalis_breed_Mediterranean_chromosome_1_whole_genome_shotgun_sequence
PVJP02910561.1_278841-279402_Hippopotamus_amphibius_isolate_BS01_ScHSfme_925021HRSCAF_982821_whole_genome_shotgun_sequence
0.03

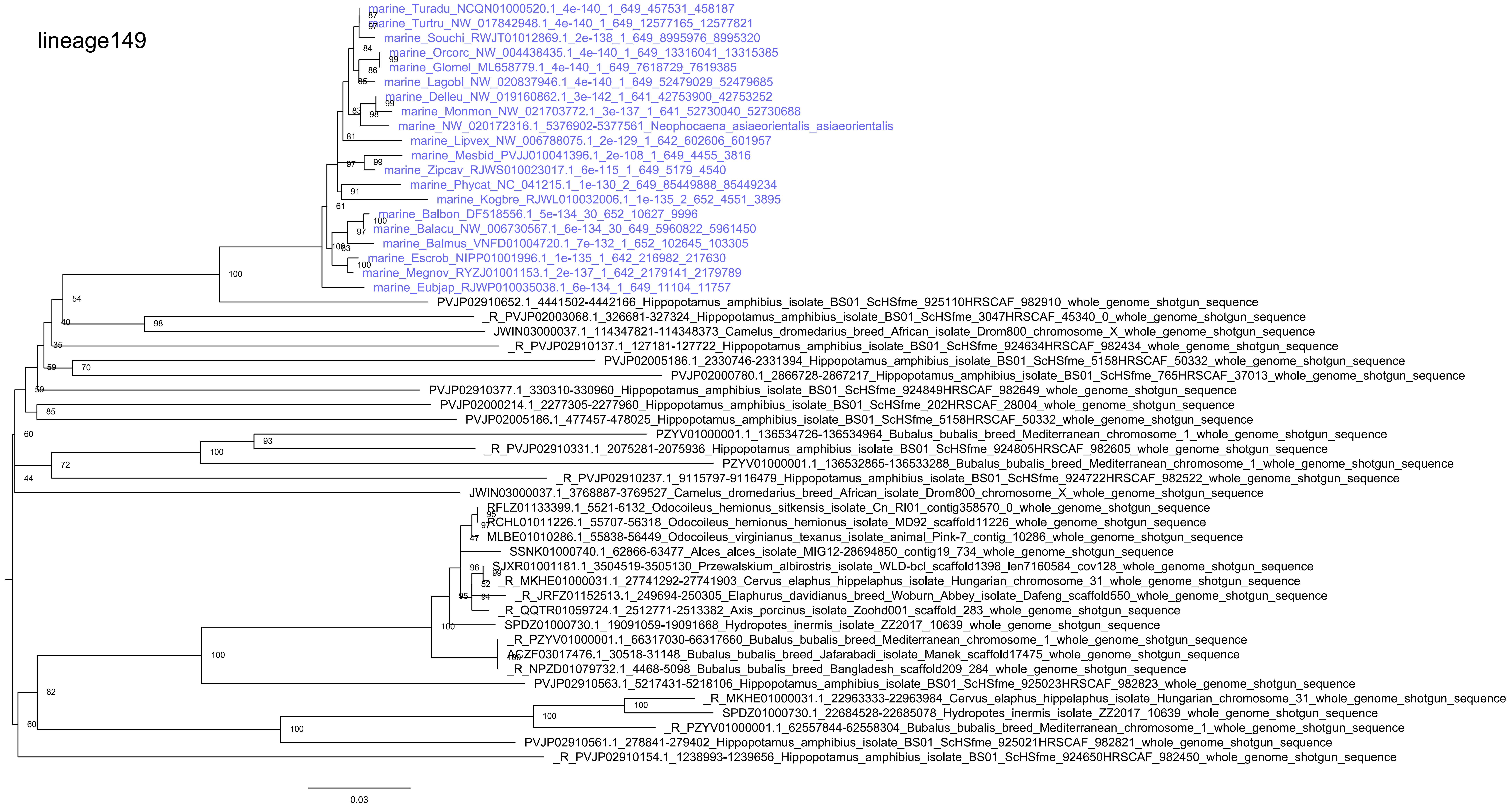
lineage149
marine_Turadu_NCQN01000520.1_4e-140_1_649_457531_458187
marine_Turtru_NW_017842948.1_4e-140_1_649_12577165_12577821
marine_Souchi_RWJT01012869.1_2e-138_1_649_8995976_8995320
marine_Orcorc_NW_004438435.1_4e-140_1_649_13316041_13315385
marine_Glomel_ML658779.1_4e-140_1_649_7618729_7619385
marine_Lagobl_NW_020837946.1_4e-140_1_649_52479029_52479685
marine_Delleu_NW_019160862.1_3e-142_1_641_42753900_42753252
marine_Monmon_NW_021703772.1_3e-137_1_641_52730040_52730688
marine_NW_020172316.1_5376902-5377561_Neophocaena_asiaeorientalis_asiaeorientalis
marine_Lipvex_NW_006788075.1_2e-129_1_642_602606_601957
marine_Mesbid_PVJJ010041396.1_2e-108_1_649_4455_3816
marine_Zipcav_RJWS010023017.1_6e-115_1_649_5179_4540
marine_Phycat_NC_041215.1_1e-130_2_649_85449888_85449234
marine_Kogbre_RJWL010032006.1_1e-135_2_652_4551_3895
marine_Balbon_DF518556.1_5e-134_30_652_10627_9996
marine_Balacu_NW_006730567.1_6e-134_30_649_5960822_5961450
marine_Balmus_VNFD01004720.1_7e-132_1_652_102645_103305
marine_Escrob_NIPP01001996.1_1e-135_1_642_216982_217630
marine_Megnov_RYZJ01001153.1_2e-137_1_642_2179141_2179789
marine_Eubjap_RJWP010035038.1_6e-134_1_649_11104_11757
PVJP02910652.1_4441502-4442166_Hippopotamus_amphibius_isolate_BS01_ScHSfme_925110HRSCAF_982910_whole_genome_shotgun_sequence
_R_PVJP02003068.1_326681-327324_Hippopotamus_amphibius_isolate_BS01_ScHSfme_3047HRSCAF_45340_0_whole_genome_shotgun_sequence
JWIN03000037.1_114347821-114348373_Camelus_dromedarius_breed_African_isolate_Drom800_chromosome_X_whole_genome_shotgun_sequence
_R_PVJP02910137.1_127181-127722_Hippopotamus_amphibius_isolate_BS01_ScHSfme_924634HRSCAF_982434_whole_genome_shotgun_sequence
PVJP02005186.1_2330746-2331394_Hippopotamus_amphibius_isolate_BS01_ScHSfme_5158HRSCAF_50332_whole_genome_shotgun_sequence
PVJP02000780.1_2866728-2867217_Hippopotamus_amphibius_isolate_BS01_ScHSfme_765HRSCAF_37013_whole_genome_shotgun_sequence
PVJP02910377.1_330310-330960_Hippopotamus_amphibius_isolate_BS01_ScHSfme_924849HRSCAF_982649_whole_genome_shotgun_sequence
PVJP02000214.1_2277305-2277960_Hippopotamus_amphibius_isolate_BS01_ScHSfme_202HRSCAF_28004_whole_genome_shotgun_sequence
PVJP02005186.1_477457-478025_Hippopotamus_amphibius_isolate_BS01_ScHSfme_5158HRSCAF_50332_whole_genome_shotgun_sequence
PZYV01000001.1_136534726-136534964_Bubalus_bubalis_breed_Mediterranean_chromosome_1_whole_genome_shotgun_sequence
_R_PVJP02910331.1_2075281-2075936_Hippopotamus_amphibius_isolate_BS01_ScHSfme_924805HRSCAF_982605_whole_genome_shotgun_sequence
PZYV01000001.1_136532865-136533288_Bubalus_bubalis_breed_Mediterranean_chromosome_1_whole_genome_shotgun_sequence
_R_PVJP02910237.1_9115797-9116479_Hippopotamus_amphibius_isolate_BS01_ScHSfme_924722HRSCAF_982522_whole_genome_shotgun_sequence
JWIN03000037.1_3768887-3769527_Camelus_dromedarius_breed_African_isolate_Drom800_chromosome_X_whole_genome_shotgun_sequence
RFLZ01133399.1_5521-6132_Odocoileus_hemionus_sitkensis_isolate_Cn_RI01_contig358570_0_whole_genome_shotgun_sequence
RCHL01011226.1_55707-56318_Odocoileus_hemionus_hemionus_isolate_MD92_scaffold11226_whole_genome_shotgun_sequence
MLBE01010286.1_55838-56449_Odocoileus_virginianus_texanus_isolate_animal_Pink-7_contig_10286_whole_genome_shotgun_sequence
SSNK01000740.1_62866-63477_Alces_alces_isolate_MIG12-28694850_contig19_734_whole_genome_shotgun_sequence
SJXR01001181.1_3504519-3505130_Przewalskium_albirostris_isolate_WLD-bcl_scaffold1398_len7160584_cov128_whole_genome_shotgun_sequence
_R_MKHE01000031.1_27741292-27741903_Cervus_elaphus_hippelaphus_isolate_Hungarian_chromosome_31_whole_genome_shotgun_sequence
_R_JRFZ01152513.1_249694-250305_Elaphurus_davidianus_breed_Woburn_Abbey_isolate_Dafeng_scaffold550_whole_genome_shotgun_sequence
_R_QQTR01059724.1_2512771-2513382_Axis_porcinus_isolate_Zoohd001_scaffold_283_whole_genome_shotgun_sequence
SPDZ01000730.1_19091059-19091668_Hydropotes_inermis_isolate_ZZ2017_10639_whole_genome_shotgun_sequence
_R_PZYV01000001.1_66317030-66317660_Bubalus_bubalis_breed_Mediterranean_chromosome_1_whole_genome_shotgun_sequence
ACZF03017476.1_30518-31148_Bubalus_bubalis_breed_Jafarabadi_isolate_Manek_scaffold17475_whole_genome_shotgun_sequence
_R_NPZD01079732.1_4468-5098_Bubalus_bubalis_breed_Bangladesh_scaffold209_284_whole_genome_shotgun_sequence
PVJP02910563.1_5217431-5218106_Hippopotamus_amphibius_isolate_BS01_ScHSfme_925023HRSCAF_982823_whole_genome_shotgun_sequence
_R_MKHE01000031.1_22963333-22963984_Cervus_elaphus_hippelaphus_isolate_Hungarian_chromosome_31_whole_genome_shotgun_sequence
SPDZ01000730.1_22684528-22685078_Hydropotes_inermis_isolate_ZZ2017_10639_whole_genome_shotgun_sequence
_R_PZYV01000001.1_62557844-62558304_Bubalus_bubalis_breed_Mediterranean_chromosome_1_whole_genome_shotgun_sequence
PVJP02910561.1_278841-279402_Hippopotamus_amphibius_isolate_BS01_ScHSfme_925021HRSCAF_982821_whole_genome_shotgun_sequence
_R_PVJP02910154.1_1238993-1239656_Hippopotamus_amphibius_isolate_BS01_ScHSfme_924650HRSCAF_982450_whole_genome_shotgun_sequence
0.03

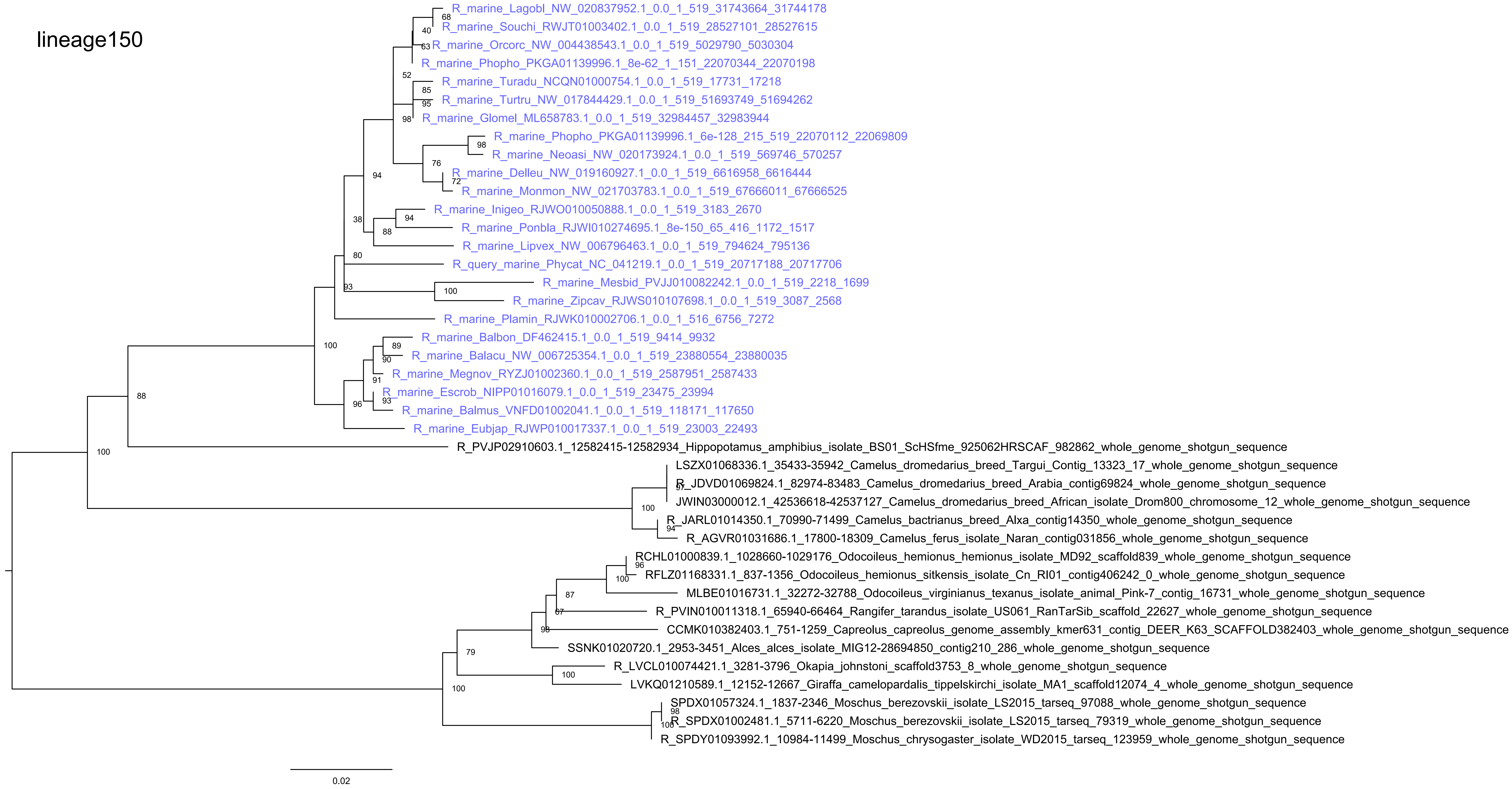
lineage150
R_marine_Lagobl_NW_020837952.1_0.0_1_519_31743664_31744178
R_marine_Souchi_RWJT01003402.1_0.0_1_519_28527101_28527615
R_marine_Orcorc_NW_004438543.1_0.0_1_519_5029790_5030304
R_marine_Phopho_PKGA01139996.1_8e-62_1_151_22070344_22070198
R_marine_Turadu_NCQN01000754.1_0.0_1_519_17731_17218
R_marine_Turtru_NW_017844429.1_0.0_1_519_51693749_51694262
R_marine_Glomel_ML658783.1_0.0_1_519_32984457_32983944
R_marine_Phopho_PKGA01139996.1_6e-128_215_519_22070112_22069809
R_marine_Neoasi_NW_020173924.1_0.0_1_519_569746_570257
R_marine_Delleu_NW_019160927.1_0.0_1_519_6616958_6616444
R_marine_Monmon_NW_021703783.1_0.0_1_519_67666011_67666525
R_marine_Inigeo_RJWO010050888.1_0.0_1_519_3183_2670
R_marine_Ponbla_RJWI010274695.1_8e-150_65_416_1172_1517
R_marine_Lipvex_NW_006796463.1_0.0_1_519_794624_795136
R_query_marine_Phycat_NC_041219.1_0.0_1_519_20717188_20717706
R_marine_Mesbid_PVJJ010082242.1_0.0_1_519_2218_1699
R_marine_Zipcav_RJWS010107698.1_0.0_1_519_3087_2568
R_marine_Plamin_RJWK010002706.1_0.0_1_516_6756_7272
R_marine_Balbon_DF462415.1_0.0_1_519_9414_9932
R_marine_Balacu_NW_006725354.1_0.0_1_519_23880554_23880035
R_marine_Megnov_RYZJ01002360.1_0.0_1_519_2587951_2587433
R_marine_Escrob_NIPP01016079.1_0.0_1_519_23475_23994
R_marine_Balmus_VNFD01002041.1_0.0_1_519_118171_117650
R_marine_Eubjap_RJWP010017337.1_0.0_1_519_23003_22493
R_PVJP02910603.1_12582415-12582934_Hippopotamus_amphibius_isolate_BS01_ScHSfme_925062HRSCAF_982862_whole_genome_shotgun_sequence
LSZX01068336.1_35433-35942_Camelus_dromedarius_breed_Targui_Contig_13323_17_whole_genome_shotgun_sequence
R_JDVD01069824.1_82974-83483_Camelus_dromedarius_breed_Arabia_contig69824_whole_genome_shotgun_sequence
JWIN03000012.1_42536618-42537127_Camelus_dromedarius_breed_African_isolate_Drom800_chromosome_12_whole_genome_shotgun_sequence
R_JARL01014350.1_70990-71499_Camelus_bactrianus_breed_Alxa_contig14350_whole_genome_shotgun_sequence
R_AGVR01031686.1_17800-18309_Camelus_ferus_isolate_Naran_contig031856_whole_genome_shotgun_sequence
RCHL01000839.1_1028660-1029176_Odocoileus_hemionus_hemionus_isolate_MD92_scaffold839_whole_genome_shotgun_sequence
RFLZ01168331.1_837-1356_Odocoileus_hemionus_sitkensis_isolate_Cn_RI01_contig406242_0_whole_genome_shotgun_sequence
MLBE01016731.1_32272-32788_Odocoileus_virginianus_texanus_isolate_animal_Pink-7_contig_16731_whole_genome_shotgun_sequence
R_PVIN010011318.1_65940-66464_Rangifer_tarandus_isolate_US061_RanTarSib_scaffold_22627_whole_genome_shotgun_sequence
CCMK010382403.1_751-1259_Capreolus_capreolus_genome_assembly_kmer631_contig_DEER_K63_SCAFFOLD382403_whole_genome_shotgun_sequence
SSNK01020720.1_2953-3451_Alces_alces_isolate_MIG12-28694850_contig210_286_whole_genome_shotgun_sequence
R_LVCL010074421.1_3281-3796_Okapia_johnstoni_scaffold3753_8_whole_genome_shotgun_sequence
LVKQ01210589.1_12152-12667_Giraffa_camelopardalis_tippelskirchi_isolate_MA1_scaffold12074_4_whole_genome_shotgun_sequence
SPDX01057324.1_1837-2346_Moschus_berezovskii_isolate_LS2015_tarseq_97088_whole_genome_shotgun_sequence
R_SPDX01002481.1_5711-6220_Moschus_berezovskii_isolate_LS2015_tarseq_79319_whole_genome_shotgun_sequence
R_SPDY01093992.1_10984-11499_Moschus_chrysogaster_isolate_WD2015_tarseq_123959_whole_genome_shotgun_sequence
0.02

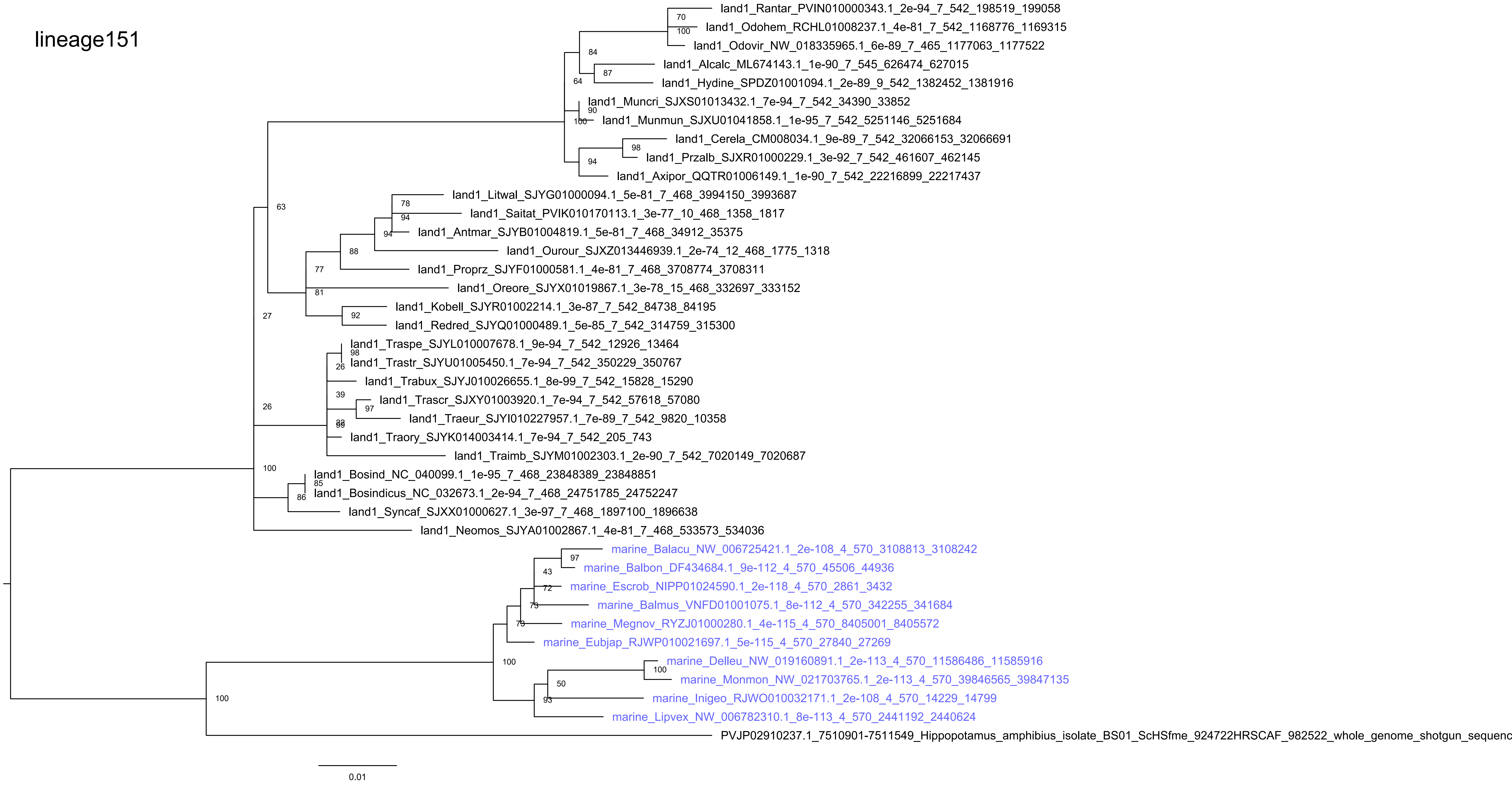
lineage151
land1_Rantar_PVIN010000343.1_2e-94_7_542_198519_199058
land1_Odohem_RCHL01008237.1_4e-81_7_542_1168776_1169315
land1_Odovir_NW_018335965.1_6e-89_7_465_1177063_1177522
land1_Alcalc_ML674143.1_1e-90_7_545_626474_627015
land1_Hydine_SPDZ01001094.1_2e-89_9_542_1382452_1381916
land1_Muncri_SJXS01013432.1_7e-94_7_542_34390_33852
land1_Munmun_SJXU01041858.1_1e-95_7_542_5251146_5251684
land1_Cerela_CM008034.1_9e-89_7_542_32066153_32066691
land1_Przalb_SJXR01000229.1_3e-92_7_542_461607_462145
land1_Axipor_QQTR01006149.1_1e-90_7_542_22216899_22217437
land1_Litwal_SJYG01000094.1_5e-81_7_468_3994150_3993687
land1_Saitat_PVIK010170113.1_3e-77_10_468_1358_1817
land1_Antmar_SJYB01004819.1_5e-81_7_468_34912_35375
land1_Ourour_SJXZ013446939.1_2e-74_12_468_1775_1318
land1_Proprz_SJYF01000581.1_4e-81_7_468_3708774_3708311
land1_Oreore_SJYX01019867.1_3e-78_15_468_332697_333152
land1_Kobell_SJYR01002214.1_3e-87_7_542_84738_84195
land1_Redred_SJYQ01000489.1_5e-85_7_542_314759_315300
land1_Traspe_SJYL010007678.1_9e-94_7_542_12926_13464
land1_Trastr_SJYU01005450.1_7e-94_7_542_350229_350767
land1_Trabux_SJYJ010026655.1_8e-99_7_542_15828_15290
land1_Trascr_SJXY01003920.1_7e-94_7_542_57618_57080
land1_Traeur_SJYI010227957.1_7e-89_7_542_9820_10358
land1_Traory_SJYK014003414.1_7e-94_7_542_205_743
land1_Traimb_SJYM01002303.1_2e-90_7_542_7020149_7020687
land1_Bosind_NC_040099.1_1e-95_7_468_23848389_23848851
land1_Bosindicus_NC_032673.1_2e-94_7_468_24751785_24752247
land1_Syncaf_SJXX01000627.1_3e-97_7_468_1897100_1896638
land1_Neomos_SJYA01002867.1_4e-81_7_468_533573_534036
marine_Balacu_NW_006725421.1_2e-108_4_570_3108813_3108242
marine_Balbon_DF434684.1_9e-112_4_570_45506_44936
marine_Escrob_NIPP01024590.1_2e-118_4_570_2861_3432
marine_Balmus_VNFD01001075.1_8e-112_4_570_342255_341684
marine_Megnov_RYZJ01000280.1_4e-115_4_570_8405001_8405572
marine_Eubjap_RJWP010021697.1_5e-115_4_570_27840_27269
marine_Delleu_NW_019160891.1_2e-113_4_570_11586486_11585916
marine_Monmon_NW_021703765.1_2e-113_4_570_39846565_39847135
marine_Inigeo_RJWO010032171.1_2e-108_4_570_14229_14799
marine_Lipvex_NW_006782310.1_8e-113_4_570_2441192_2440624
PVJP02910237.1_7510901-7511549_Hippopotamus_amphibius_isolate_BS01_ScHSfme_924722HRSCAF_982522_whole_genome_shotgun_sequenc
0.01

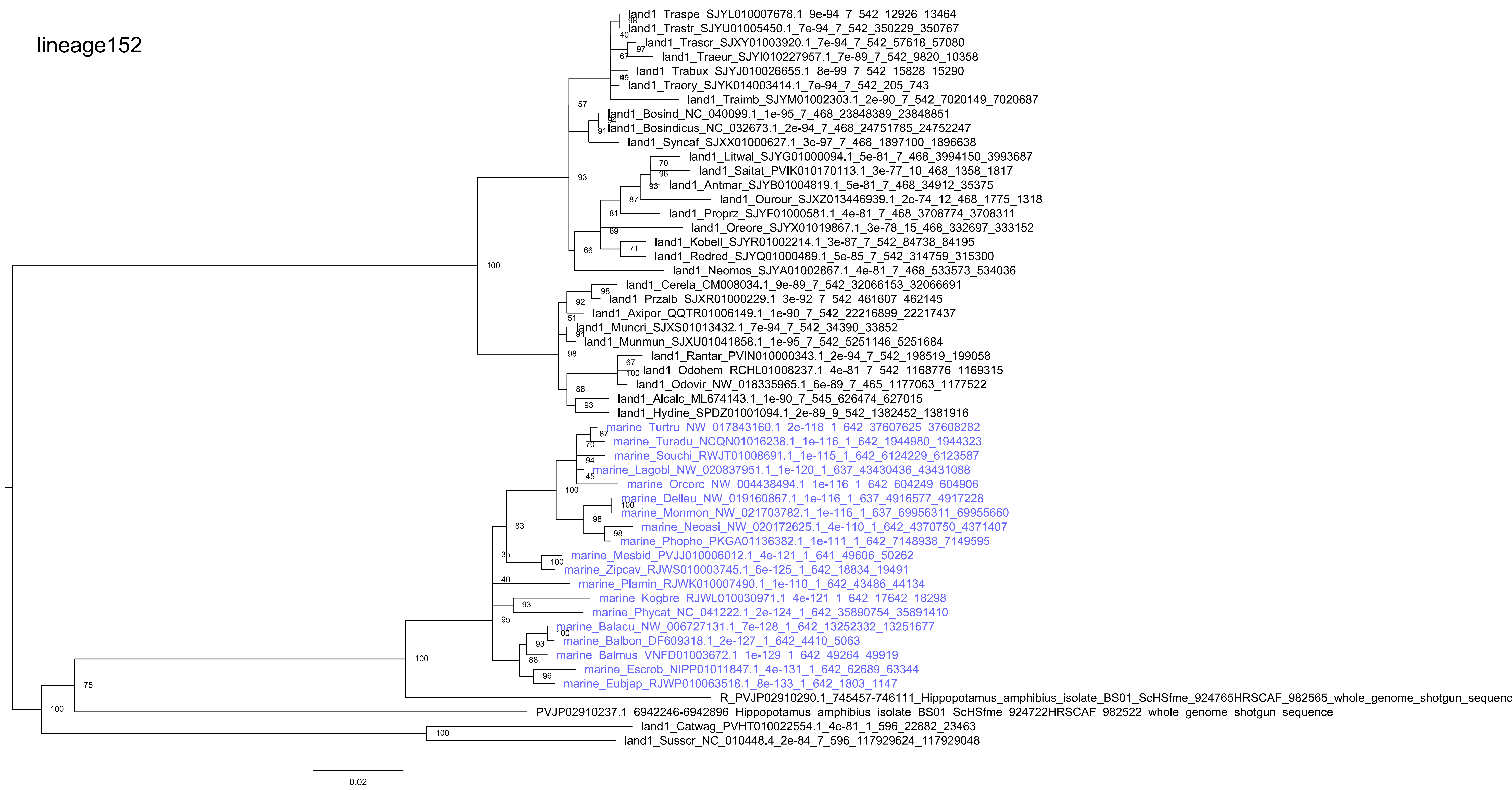

lineage152
land1_Traspe_SJYL010007678.1_9e-94_7_542_12926_13464
land1_Trastr_SJYU01005450.1_7e-94_7_542_350229_350767
land1_Trascr_SJXY01003920.1_7e-94_7_542_57618_57080
land1_Traeur_SJYI010227957.1_7e-89_7_542_9820_10358
land1_Trabux_SJYJ010026655.1_8e-99_7_542_15828_15290
land1_Traory_SJYK014003414.1_7e-94_7_542_205_743
land1_Traimb_SJYM01002303.1_2e-90_7_542_7020149_7020687
land1_Bosind_NC_040099.1_1e-95_7_468_23848389_23848851
land1_Bosindicus_NC_032673.1_2e-94_7_468_24751785_24752247
land1_Syncaf_SJXX01000627.1_3e-97_7_468_1897100_1896638
land1_Litwal_SJYG01000094.1_5e-81_7_468_3994150_3993687
land1_Saitat_PVIK010170113.1_3e-77_10_468_1358_1817
land1_Antmar_SJYB01004819.1_5e-81_7_468_34912_35375
land1_Ourour_SJXZ013446939.1_2e-74_12_468_1775_1318
land1_Proprz_SJYF01000581.1_4e-81_7_468_3708774_3708311
land1_Oreore_SJYX01019867.1_3e-78_15_468_332697_333152
land1_Kobell_SJYR01002214.1_3e-87_7_542_84738_84195
land1_Redred_SJYQ01000489.1_5e-85_7_542_314759_315300
land1_Neomos_SJYA01002867.1_4e-81_7_468_533573_534036
land1_Cerela_CM008034.1_9e-89_7_542_32066153_32066691
land1_Przalb_SJXR01000229.1_3e-92_7_542_461607_462145
land1_Axipor_QQTR01006149.1_1e-90_7_542_22216899_22217437
land1_Muncri_SJXS01013432.1_7e-94_7_542_34390_33852
land1_Munmun_SJXU01041858.1_1e-95_7_542_5251146_5251684
land1_Rantar_PVIN010000343.1_2e-94_7_542_198519_199058
land1_Odohem_RCHL01008237.1_4e-81_7_542_1168776_1169315
land1_Odovir_NW_018335965.1_6e-89_7_465_1177063_1177522
land1_Alcalc_ML674143.1_1e-90_7_545_626474_627015
land1_Hydine_SPDZ01001094.1_2e-89_9_542_1382452_1381916
marine_Turtru_NW_017843160.1_2e-118_1_642_37607625_37608282
marine_Turadu_NCQN01016238.1_1e-116_1_642_1944980_1944323
marine_Souchi_RWJT01008691.1_1e-115_1_642_6124229_6123587
marine_Lagobl_NW_020837951.1_1e-120_1_637_43430436_43431088
marine_Orcorc_NW_004438494.1_1e-116_1_642_604249_604906
marine_Delleu_NW_019160867.1_1e-116_1_637_4916577_4917228
marine_Monmon_NW_021703782.1_1e-116_1_637_69956311_69955660
marine_Neoasi_NW_020172625.1_4e-110_1_642_4370750_4371407
marine_Phopho_PKGA01136382.1_1e-111_1_642_7148938_7149595
marine_Mesbid_PVJJ010006012.1_4e-121_1_641_49606_50262
marine_Zipcav_RJWS010003745.1_6e-125_1_642_18834_19491
marine_Plamin_RJWK010007490.1_1e-110_1_642_43486_44134
marine_Kogbre_RJWL010030971.1_4e-121_1_642_17642_18298
marine_Phycat_NC_041222.1_2e-124_1_642_35890754_35891410
marine_Balacu_NW_006727131.1_7e-128_1_642_13252332_13251677
marine_Balbon_DF609318.1_2e-127_1_642_4410_5063
marine_Balmus_VNFD01003672.1_1e-129_1_642_49264_49919
marine_Escrob_NIPP01011847.1_4e-131_1_642_62689_63344
marine_Eubjap_RJWP010063518.1_8e-133_1_642_1803_1147
R_PVJP02910290.1_745457-746111_Hippopotamus_amphibius_isolate_BS01_ScHSfme_924765HRSCAF_982565_whole_genome_shotgun_sequenc
PVJP02910237.1_6942246-6942896_Hippopotamus_amphibius_isolate_BS01_ScHSfme_924722HRSCAF_982522_whole_genome_shotgun_sequence
land1_Catwag_PVHT010022554.1_4e-81_1_596_22882_23463
land1_Susscr_NC_010448.4_2e-84_7_596_117929624_117929048
98
40
97
67
57
94
91
70
96
93
93
87
81
69
71
66
100
98
92
51
94
98
67
100
88
93
87
70
94
45
100
100
98
98
83
35
100
40
93
95
100
93
88
96
100
75
100
100
0.02

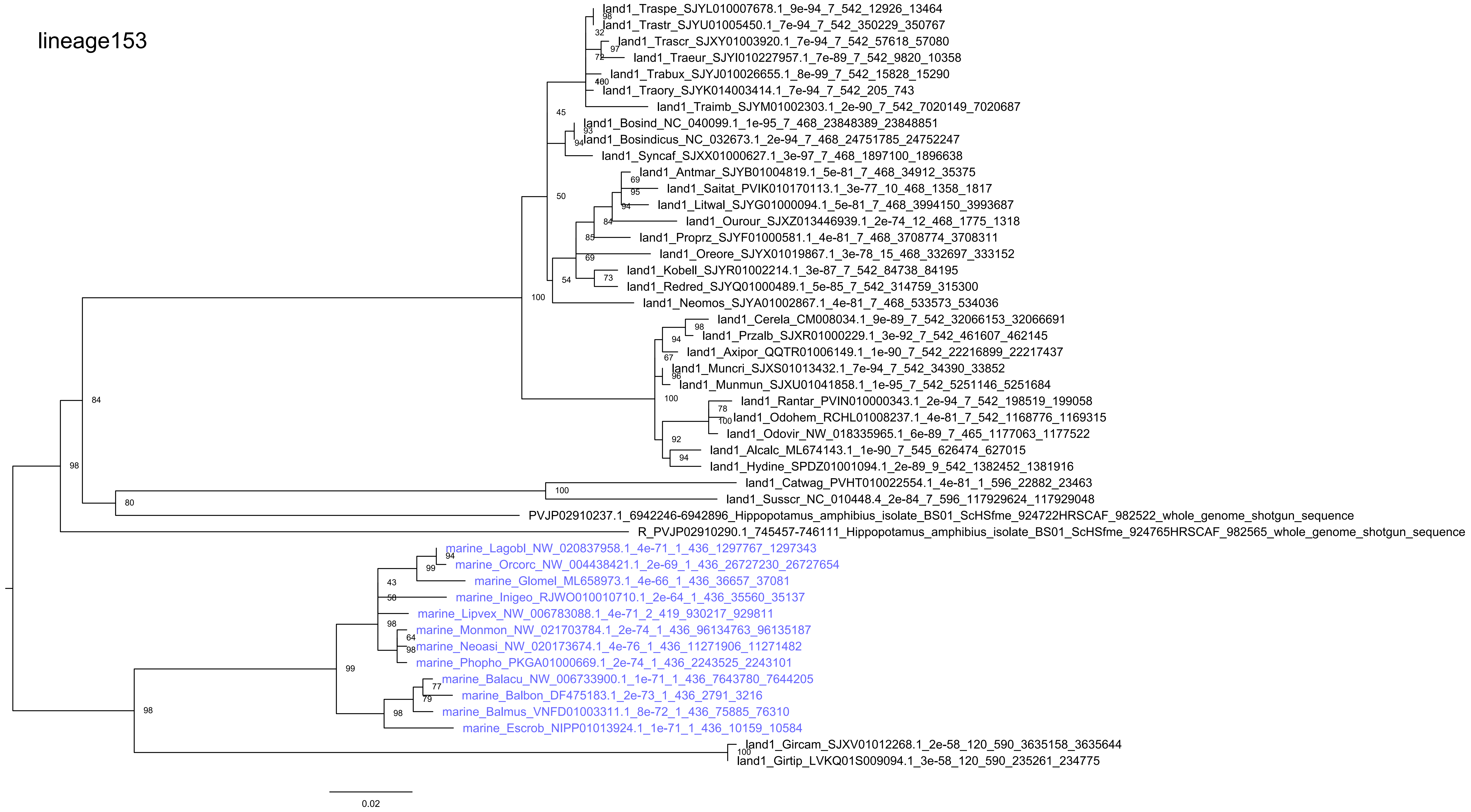
lineage153
land1_Traspe_SJYL010007678.1_9e-94_7_542_12926_13464
land1_Trastr_SJYU01005450.1_7e-94_7_542_350229_350767
land1_Trascr_SJXY01003920.1_7e-94_7_542_57618_57080
land1_Traeur_SJYI010227957.1_7e-89_7_542_9820_10358
land1_Trabux_SJYJ010026655.1_8e-99_7_542_15828_15290
land1_Traory_SJYK014003414.1_7e-94_7_542_205_743
land1_Traimb_SJYM01002303.1_2e-90_7_542_7020149_7020687
land1_Bosind_NC_040099.1_1e-95_7_468_23848389_23848851
land1_Bosindicus_NC_032673.1_2e-94_7_468_24751785_24752247
land1_Syncaf_SJXX01000627.1_3e-97_7_468_1897100_1896638
land1_Antmar_SJYB01004819.1_5e-81_7_468_34912_35375
land1_Saitat_PVIK010170113.1_3e-77_10_468_1358_1817
land1_Litwal_SJYG01000094.1_5e-81_7_468_3994150_3993687
land1_Ourour_SJXZ013446939.1_2e-74_12_468_1775_1318
land1_Proprz_SJYF01000581.1_4e-81_7_468_3708774_3708311
land1_Oreore_SJYX01019867.1_3e-78_15_468_332697_333152
land1_Kobell_SJYR01002214.1_3e-87_7_542_84738_84195
land1_Redred_SJYQ01000489.1_5e-85_7_542_314759_315300
land1_Neomos_SJYA01002867.1_4e-81_7_468_533573_534036
land1_Cerela_CM008034.1_9e-89_7_542_32066153_32066691
land1_Przalb_SJXR01000229.1_3e-92_7_542_461607_462145
land1_Axipor_QQTR01006149.1_1e-90_7_542_22216899_22217437
land1_Muncri_SJXS01013432.1_7e-94_7_542_34390_33852
land1_Munmun_SJXU01041858.1_1e-95_7_542_5251146_5251684
land1_Rantar_PVIN010000343.1_2e-94_7_542_198519_199058
land1_Odohem_RCHL01008237.1_4e-81_7_542_1168776_1169315
land1_Odovir_NW_018335965.1_6e-89_7_465_1177063_1177522
land1_Alcalc_ML674143.1_1e-90_7_545_626474_627015
land1_Hydine_SPDZ01001094.1_2e-89_9_542_1382452_1381916
land1_Catwag_PVHT010022554.1_4e-81_1_596_22882_23463
land1_Susscr_NC_010448.4_2e-84_7_596_117929624_117929048
PVJP02910237.1_6942246-6942896_Hippopotamus_amphibius_isolate_BS01_ScHSfme_924722HRSCAF_982522_whole_genome_shotgun_sequence
R_PVJP02910290.1_745457-746111_Hippopotamus_amphibius_isolate_BS01_ScHSfme_924765HRSCAF_982565_whole_genome_shotgun_sequence
marine_Lagobl_NW_020837958.1_4e-71_1_436_1297767_1297343
marine_Orcorc_NW_004438421.1_2e-69_1_436_26727230_26727654
marine_Glomel_ML658973.1_4e-66_1_436_36657_37081
marine_Inigeo_RJWO010010710.1_2e-64_1_436_35560_35137
marine_Lipvex_NW_006783088.1_4e-71_2_419_930217_929811
marine_Monmon_NW_021703784.1_2e-74_1_436_96134763_96135187
marine_Neoasi_NW_020173674.1_4e-76_1_436_11271906_11271482
marine_Phopho_PKGA01000669.1_2e-74_1_436_2243525_2243101
marine_Balacu_NW_006733900.1_1e-71_1_436_7643780_7644205
marine_Balbon_DF475183.1_2e-73_1_436_2791_3216
marine_Balmus_VNFD01003311.1_8e-72_1_436_75885_76310
marine_Escrob_NIPP01013924.1_1e-71_1_436_10159_10584
land1_Gircam_SJXV01012268.1_2e-58_120_590_3635158_3635644
land1_Girtip_LVKQ01S009094.1_3e-58_120_590_235261_234775
0.02

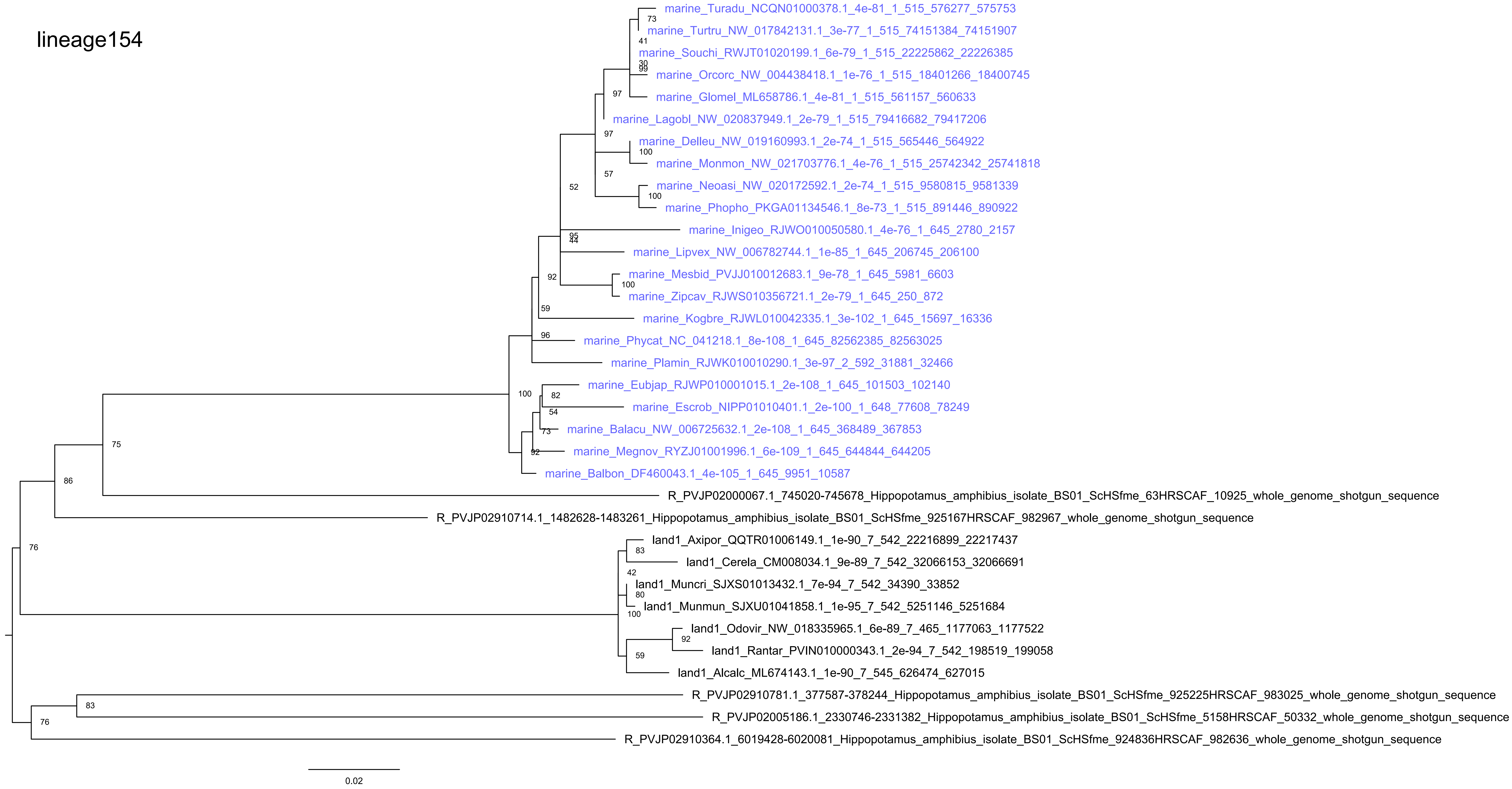
lineage154
marine_Turadu_NCQN01000378.1_4e-81_1_515_576277_575753
marine_Turtru_NW_017842131.1_3e-77_1_515_74151384_74151907
marine_Souchi_RWJT01020199.1_6e-79_1_515_22225862_22226385
marine_Orcorc_NW_004438418.1_1e-76_1_515_18401266_18400745
marine_Glomel_ML658786.1_4e-81_1_515_561157_560633
marine_Lagobl_NW_020837949.1_2e-79_1_515_79416682_79417206
marine_Delleu_NW_019160993.1_2e-74_1_515_565446_564922
marine_Monmon_NW_021703776.1_4e-76_1_515_25742342_25741818
marine_Neoasi_NW_020172592.1_2e-74_1_515_9580815_9581339
marine_Phopho_PKGA01134546.1_8e-73_1_515_891446_890922
marine_Inigeo_RJWO010050580.1_4e-76_1_645_2780_2157
marine_Lipvex_NW_006782744.1_1e-85_1_645_206745_206100
marine_Mesbid_PVJJ010012683.1_9e-78_1_645_5981_6603
marine_Zipcav_RJWS010356721.1_2e-79_1_645_250_872
marine_Kogbre_RJWL010042335.1_3e-102_1_645_15697_16336
marine_Phycat_NC_041218.1_8e-108_1_645_82562385_82563025
marine_Plamin_RJWK010010290.1_3e-97_2_592_31881_32466
marine_Eubjap_RJWP010001015.1_2e-108_1_645_101503_102140
marine_Escrob_NIPP01010401.1_2e-100_1_648_77608_78249
marine_Balacu_NW_006725632.1_2e-108_1_645_368489_367853
marine_Megnov_RYZJ01001996.1_6e-109_1_645_644844_644205
marine_Balbon_DF460043.1_4e-105_1_645_9951_10587
R_PVJP02000067.1_745020-745678_Hippopotamus_amphibius_isolate_BS01_ScHSfme_63HRSCAF_10925_whole_genome_shotgun_sequence
R_PVJP02910714.1_1482628-1483261_Hippopotamus_amphibius_isolate_BS01_ScHSfme_925167HRSCAF_982967_whole_genome_shotgun_sequence
land1_Axipor_QQTR01006149.1_1e-90_7_542_22216899_22217437
land1_Cerela_CM008034.1_9e-89_7_542_32066153_32066691
land1_Muncri_SJXS01013432.1_7e-94_7_542_34390_33852
land1_Munmun_SJXU01041858.1_1e-95_7_542_5251146_5251684
land1_Odovir_NW_018335965.1_6e-89_7_465_1177063_1177522
land1_Rantar_PVIN010000343.1_2e-94_7_542_198519_199058
land1_Alcalc_ML674143.1_1e-90_7_545_626474_627015
R_PVJP02910781.1_377587-378244_Hippopotamus_amphibius_isolate_BS01_ScHSfme_925225HRSCAF_983025_whole_genome_shotgun_sequence
R_PVJP02005186.1_2330746-2331382_Hippopotamus_amphibius_isolate_BS01_ScHSfme_5158HRSCAF_50332_whole_genome_shotgun_sequence
R_PVJP02910364.1_6019428-6020081_Hippopotamus_amphibius_isolate_BS01_ScHSfme_924836HRSCAF_982636_whole_genome_shotgun_sequence
0.02

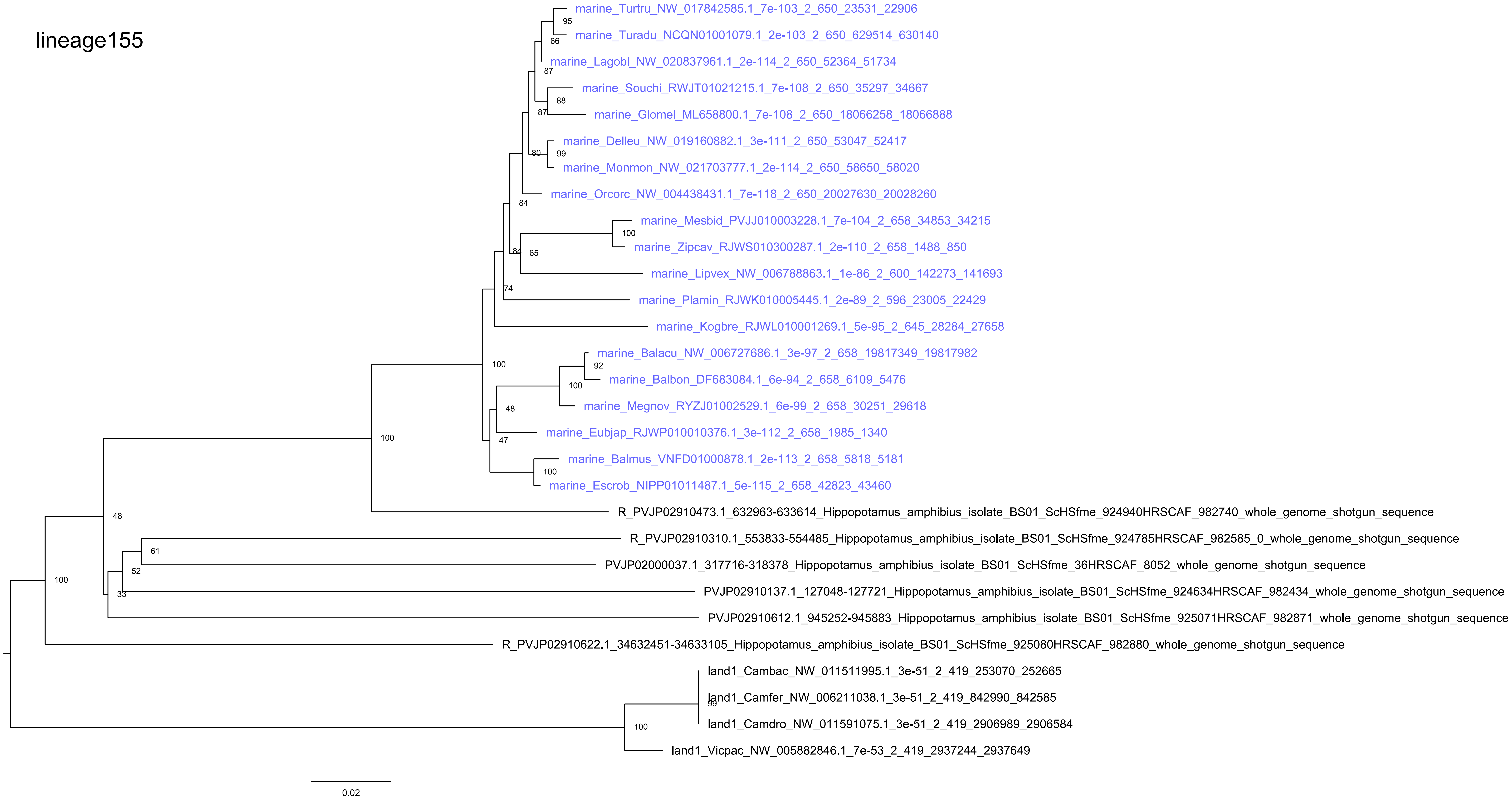
lineage155
marine_Turtru_NW_017842585.1_7e-103_2_650_23531_22906
marine_Turadu_NCQN01001079.1_2e-103_2_650_629514_630140
marine_Lagobl_NW_020837961.1_2e-114_2_650_52364_51734
marine_Souchi_RWJT01021215.1_7e-108_2_650_35297_34667
marine_Glomel_ML658800.1_7e-108_2_650_18066258_18066888
marine_Delleu_NW_019160882.1_3e-111_2_650_53047_52417
marine_Monmon_NW_021703777.1_2e-114_2_650_58650_58020
marine_Orcorc_NW_004438431.1_7e-118_2_650_20027630_20028260
marine_Mesbid_PVJJ010003228.1_7e-104_2_658_34853_34215
marine_Zipcav_RJWS010300287.1_2e-110_2_658_1488_850
marine_Lipvex_NW_006788863.1_1e-86_2_600_142273_141693
marine_Plamin_RJWK010005445.1_2e-89_2_596_23005_22429
marine_Kogbre_RJWL010001269.1_5e-95_2_645_28284_27658
marine_Balacu_NW_006727686.1_3e-97_2_658_19817349_19817982
marine_Balbon_DF683084.1_6e-94_2_658_6109_5476
marine_Megnov_RYZJ01002529.1_6e-99_2_658_30251_29618
marine_Eubjap_RJWP010010376.1_3e-112_2_658_1985_1340
marine_Balmus_VNFD01000878.1_2e-113_2_658_5818_5181
marine_Escrob_NIPP01011487.1_5e-115_2_658_42823_43460
R_PVJP02910473.1_632963-633614_Hippopotamus_amphibius_isolate_BS01_ScHSfme_924940HRSCAF_982740_whole_genome_shotgun_sequence
R_PVJP02910310.1_553833-554485_Hippopotamus_amphibius_isolate_BS01_ScHSfme_924785HRSCAF_982585_0_whole_genome_shotgun_sequence
PVJP02000037.1_317716-318378_Hippopotamus_amphibius_isolate_BS01_ScHSfme_36HRSCAF_8052_whole_genome_shotgun_sequence
PVJP02910137.1_127048-127721_Hippopotamus_amphibius_isolate_BS01_ScHSfme_924634HRSCAF_982434_whole_genome_shotgun_sequence
PVJP02910612.1_945252-945883_Hippopotamus_amphibius_isolate_BS01_ScHSfme_925071HRSCAF_982871_whole_genome_shotgun_sequence
R_PVJP02910622.1_34632451-34633105_Hippopotamus_amphibius_isolate_BS01_ScHSfme_925080HRSCAF_982880_whole_genome_shotgun_sequence
land1_Cambac_NW_011511995.1_3e-51_2_419_253070_252665
land1_Camfer_NW_006211038.1_3e-51_2_419_842990_842585
land1_Camdro_NW_011591075.1_3e-51_2_419_2906989_2906584
land1_Vicpac_NW_005882846.1_7e-53_2_419_2937244_2937649
95
66
87
88
87
80
99
84
100
84
65
74
100
92
100
48
47
100
100
48
61
52
33
100
100
0.02

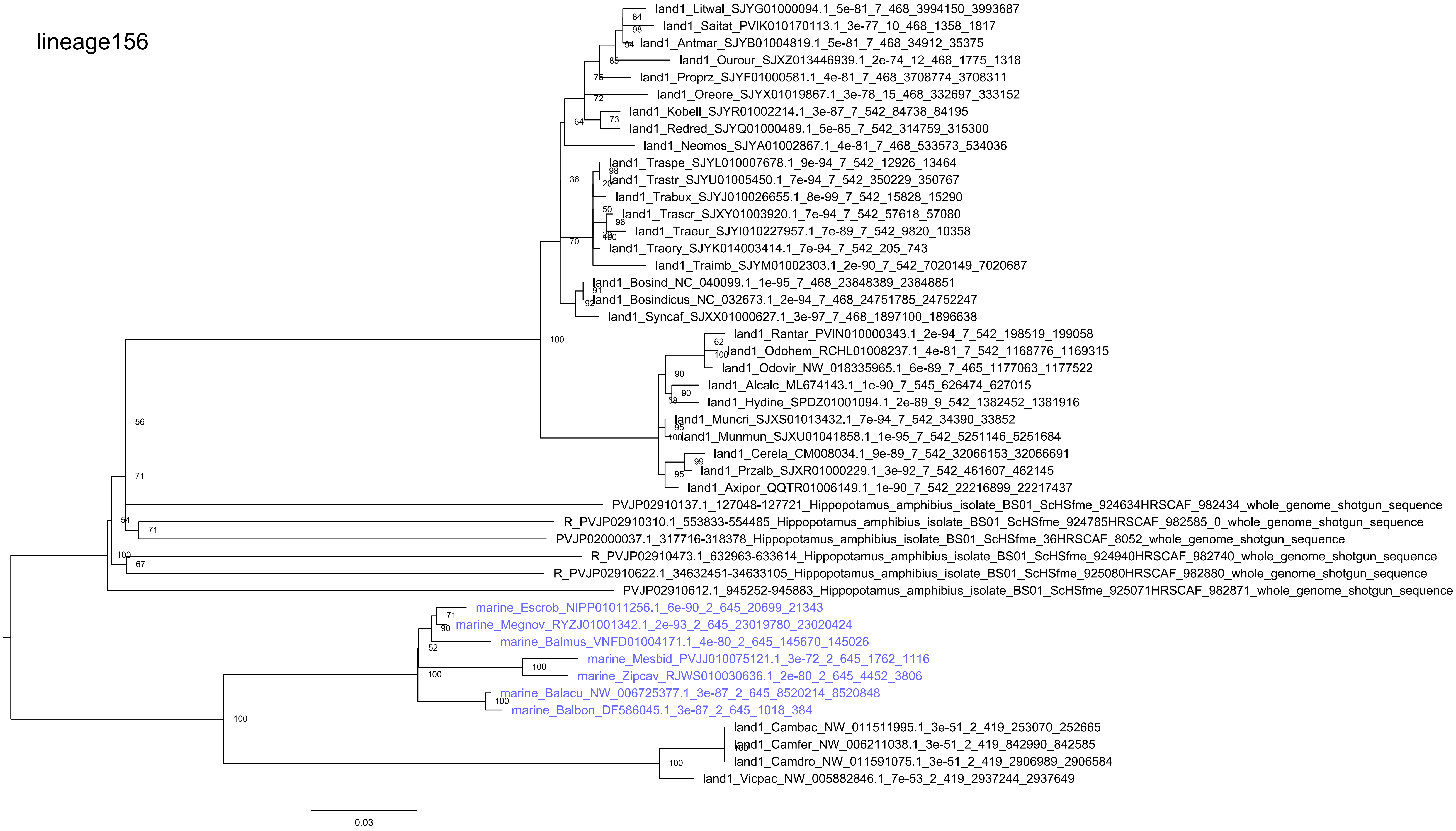
lineage156
land1_Litwal_SJYG01000094.1_5e-81_7_468_3994150_3993687
land1_Saitat_PVIK010170113.1_3e-77_10_468_1358_1817
land1_Antmar_SJYB01004819.1_5e-81_7_468_34912_35375
land1_Ourour_SJXZ013446939.1_2e-74_12_468_1775_1318
land1_Proprz_SJYF01000581.1_4e-81_7_468_3708774_3708311
land1_Oreore_SJYX01019867.1_3e-78_15_468_332697_333152
land1_Kobell_SJYR01002214.1_3e-87_7_542_84738_84195
land1_Redred_SJYQ01000489.1_5e-85_7_542_314759_315300
land1_Neomos_SJYA01002867.1_4e-81_7_468_533573_534036
land1_Traspe_SJYL010007678.1_9e-94_7_542_12926_13464
land1_Trastr_SJYU01005450.1_7e-94_7_542_350229_350767
land1_Trabux_SJYJ010026655.1_8e-99_7_542_15828_15290
land1_Trascr_SJXY01003920.1_7e-94_7_542_57618_57080
land1_Traeur_SJYI010227957.1_7e-89_7_542_9820_10358
land1_Traory_SJYK014003414.1_7e-94_7_542_205_743
land1_Traimb_SJYM01002303.1_2e-90_7_542_7020149_7020687
land1_Bosind_NC_040099.1_1e-95_7_468_23848389_23848851
land1_Bosindicus_NC_032673.1_2e-94_7_468_24751785_24752247
land1_Syncaf_SJXX01000627.1_3e-97_7_468_1897100_1896638
land1_Rantar_PVIN010000343.1_2e-94_7_542_198519_199058
land1_Odohem_RCHL01008237.1_4e-81_7_542_1168776_1169315
land1_Odovir_NW_018335965.1_6e-89_7_465_1177063_1177522
land1_Alcalc_ML674143.1_1e-90_7_545_626474_627015
land1_Hydine_SPDZ01001094.1_2e-89_9_542_1382452_1381916
land1_Muncri_SJXS01013432.1_7e-94_7_542_34390_33852
land1_Munmun_SJXU01041858.1_1e-95_7_542_5251146_5251684
land1_Cerela_CM008034.1_9e-89_7_542_32066153_32066691
land1_Przalb_SJXR01000229.1_3e-92_7_542_461607_462145
land1_Axipor_QQTR01006149.1_1e-90_7_542_22216899_22217437
PVJP02910137.1_127048-127721_Hippopotamus_amphibius_isolate_BS01_ScHSfme_924634HRSCAF_982434_whole_genome_shotgun_sequence
R_PVJP02910310.1_553833-554485_Hippopotamus_amphibius_isolate_BS01_ScHSfme_924785HRSCAF_982585_0_whole_genome_shotgun_sequence
PVJP02000037.1_317716-318378_Hippopotamus_amphibius_isolate_BS01_ScHSfme_36HRSCAF_8052_whole_genome_shotgun_sequence
R_PVJP02910473.1_632963-633614_Hippopotamus_amphibius_isolate_BS01_ScHSfme_924940HRSCAF_982740_whole_genome_shotgun_sequence
R_PVJP02910622.1_34632451-34633105_Hippopotamus_amphibius_isolate_BS01_ScHSfme_925080HRSCAF_982880_whole_genome_shotgun_sequence
PVJP02910612.1_945252-945883_Hippopotamus_amphibius_isolate_BS01_ScHSfme_925071HRSCAF_982871_whole_genome_shotgun_sequence
marine_Escrob_NIPP01011256.1_6e-90_2_645_20699_21343
marine_Megnov_RYZJ01001342.1_2e-93_2_645_23019780_23020424
marine_Balmus_VNFD01004171.1_4e-80_2_645_145670_145026
marine_Mesbid_PVJJ010075121.1_3e-72_2_645_1762_1116
marine_Zipcav_RJWS010030636.1_2e-80_2_645_4452_3806
marine_Balacu_NW_006725377.1_3e-87_2_645_8520214_8520848
marine_Balbon_DF586045.1_3e-87_2_645_1018_384
land1_Cambac_NW_011511995.1_3e-51_2_419_253070_252665
land1_Camfer_NW_006211038.1_3e-51_2_419_842990_842585
land1_Camdro_NW_011591075.1_3e-51_2_419_2906989_2906584
land1_Vicpac_NW_005882846.1_7e-53_2_419_2937244_2937649
0.03

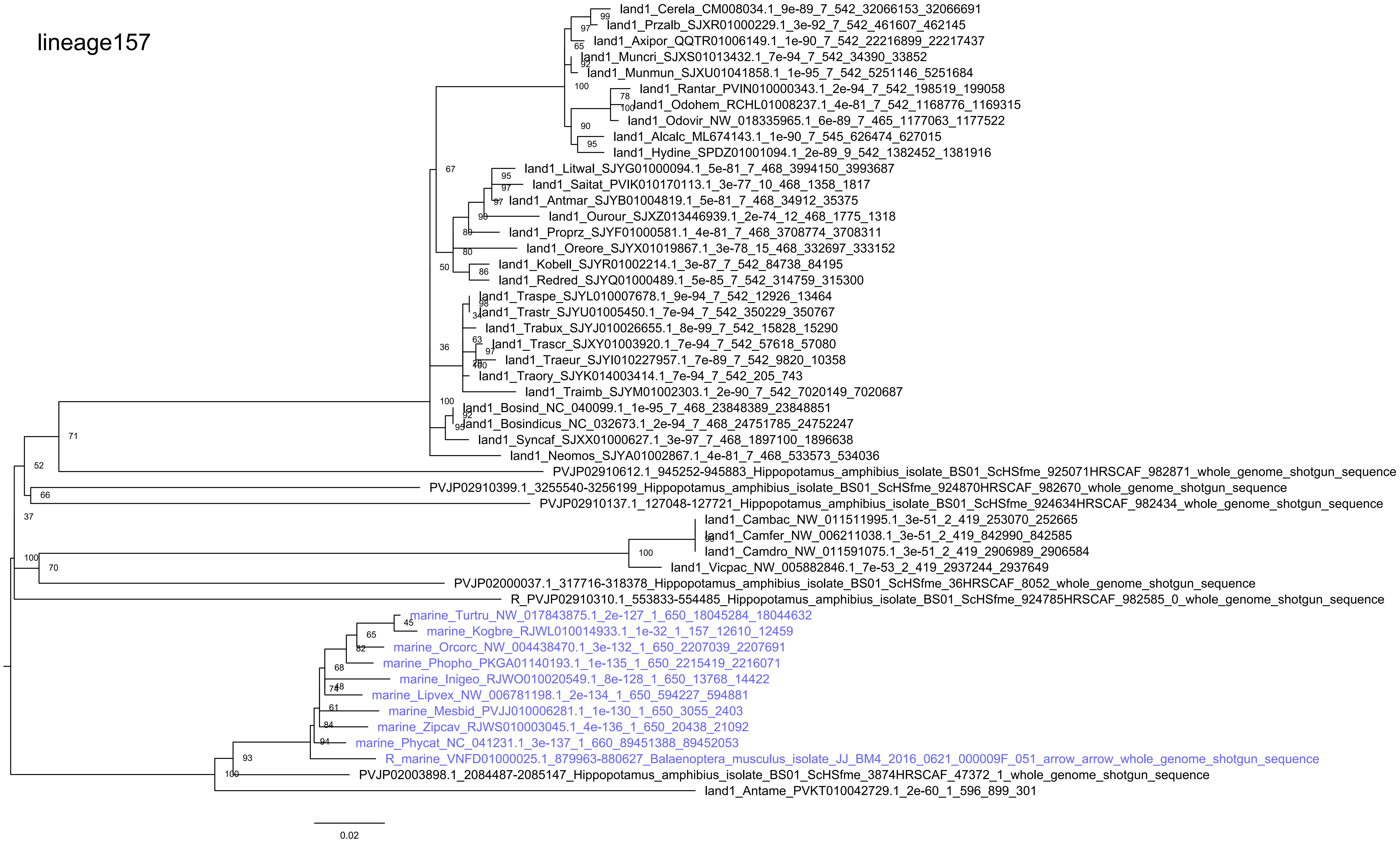

lineage157
land1_Cerela_CM008034.1_9e-89_7_542_32066153_32066691
land1_Przalb_SJXR01000229.1_3e-92_7_542_461607_462145
land1_Axipor_QQTR01006149.1_1e-90_7_542_22216899_22217437
land1_Muncri_SJXS01013432.1_7e-94_7_542_34390_33852
land1_Munmun_SJXU01041858.1_1e-95_7_542_5251146_5251684
land1_Rantar_PVIN010000343.1_2e-94_7_542_198519_199058
land1_Odohem_RCHL01008237.1_4e-81_7_542_1168776_1169315
land1_Odovir_NW_018335965.1_6e-89_7_465_1177063_1177522
land1_Alcalc_ML674143.1_1e-90_7_545_626474_627015
land1_Hydine_SPDZ01001094.1_2e-89_9_542_1382452_1381916
land1_Litwal_SJYG01000094.1_5e-81_7_468_3994150_3993687
land1_Saitat_PVIK010170113.1_3e-77_10_468_1358_1817
land1_Antmar_SJYB01004819.1_5e-81_7_468_34912_35375
land1_Ourour_SJXZ013446939.1_2e-74_12_468_1775_1318
land1_Proprz_SJYF01000581.1_4e-81_7_468_3708774_3708311
land1_Oreore_SJYX01019867.1_3e-78_15_468_332697_333152
land1_Kobell_SJYR01002214.1_3e-87_7_542_84738_84195
land1_Redred_SJYQ01000489.1_5e-85_7_542_314759_315300
land1_Traspe_SJYL010007678.1_9e-94_7_542_12926_13464
land1_Trastr_SJYU01005450.1_7e-94_7_542_350229_350767
land1_Trabux_SJYJ010026655.1_8e-99_7_542_15828_15290
land1_Trascr_SJXY01003920.1_7e-94_7_542_57618_57080
land1_Traeur_SJYI010227957.1_7e-89_7_542_9820_10358
land1_Traory_SJYK014003414.1_7e-94_7_542_205_743
land1_Traimb_SJYM01002303.1_2e-90_7_542_7020149_7020687
land1_Bosind_NC_040099.1_1e-95_7_468_23848389_23848851
land1_Bosindicus_NC_032673.1_2e-94_7_468_24751785_24752247
land1_Syncaf_SJXX01000627.1_3e-97_7_468_1897100_1896638
land1_Neomos_SJYA01002867.1_4e-81_7_468_533573_534036
PVJP02910612.1_945252-945883_Hippopotamus_amphibius_isolate_BS01_ScHSfme_925071HRSCAF_982871_whole_genome_shotgun_sequence
PVJP02910399.1_3255540-3256199_Hippopotamus_amphibius_isolate_BS01_ScHSfme_924870HRSCAF_982670_whole_genome_shotgun_sequence
PVJP02910137.1_127048-127721_Hippopotamus_amphibius_isolate_BS01_ScHSfme_924634HRSCAF_982434_whole_genome_shotgun_sequence
land1_Cambac_NW_011511995.1_3e-51_2_419_253070_252665
land1_Camfer_NW_006211038.1_3e-51_2_419_842990_842585
land1_Camdro_NW_011591075.1_3e-51_2_419_2906989_2906584
land1_Vicpac_NW_005882846.1_7e-53_2_419_2937244_2937649
PVJP02000037.1_317716-318378_Hippopotamus_amphibius_isolate_BS01_ScHSfme_36HRSCAF_8052_whole_genome_shotgun_sequence
R_PVJP02910310.1_553833-554485_Hippopotamus_amphibius_isolate_BS01_ScHSfme_924785HRSCAF_982585_0_whole_genome_shotgun_sequence
marine_Turtru_NW_017843875.1_2e-127_1_650_18045284_18044632
marine_Kogbre_RJWL010014933.1_1e-32_1_157_12610_12459
marine_Orcorc_NW_004438470.1_3e-132_1_650_2207039_2207691
marine_Phopho_PKGA01140193.1_1e-135_1_650_2215419_2216071
marine_Inigeo_RJWO010020549.1_8e-128_1_650_13768_14422
marine_Lipvex_NW_006781198.1_2e-134_1_650_594227_594881
marine_Mesbid_PVJJ010006281.1_1e-130_1_650_3055_2403
marine_Zipcav_RJWS010003045.1_4e-136_1_650_20438_21092
marine_Phycat_NC_041231.1_3e-137_1_660_89451388_89452053
R_marine_VNFD01000025.1_879963-880627_Balaenoptera_musculus_isolate_JJ_BM4_2016_0621_000009F_051_arrow_arrow_whole_genome_shotgun_sequence
PVJP02003898.1_2084487-2085147_Hippopotamus_amphibius_isolate_BS01_ScHSfme_3874HRSCAF_47372_1_whole_genome_shotgun_sequence
land1_Antame_PVKT010042729.1_2e-60_1_596_899_301
0.02

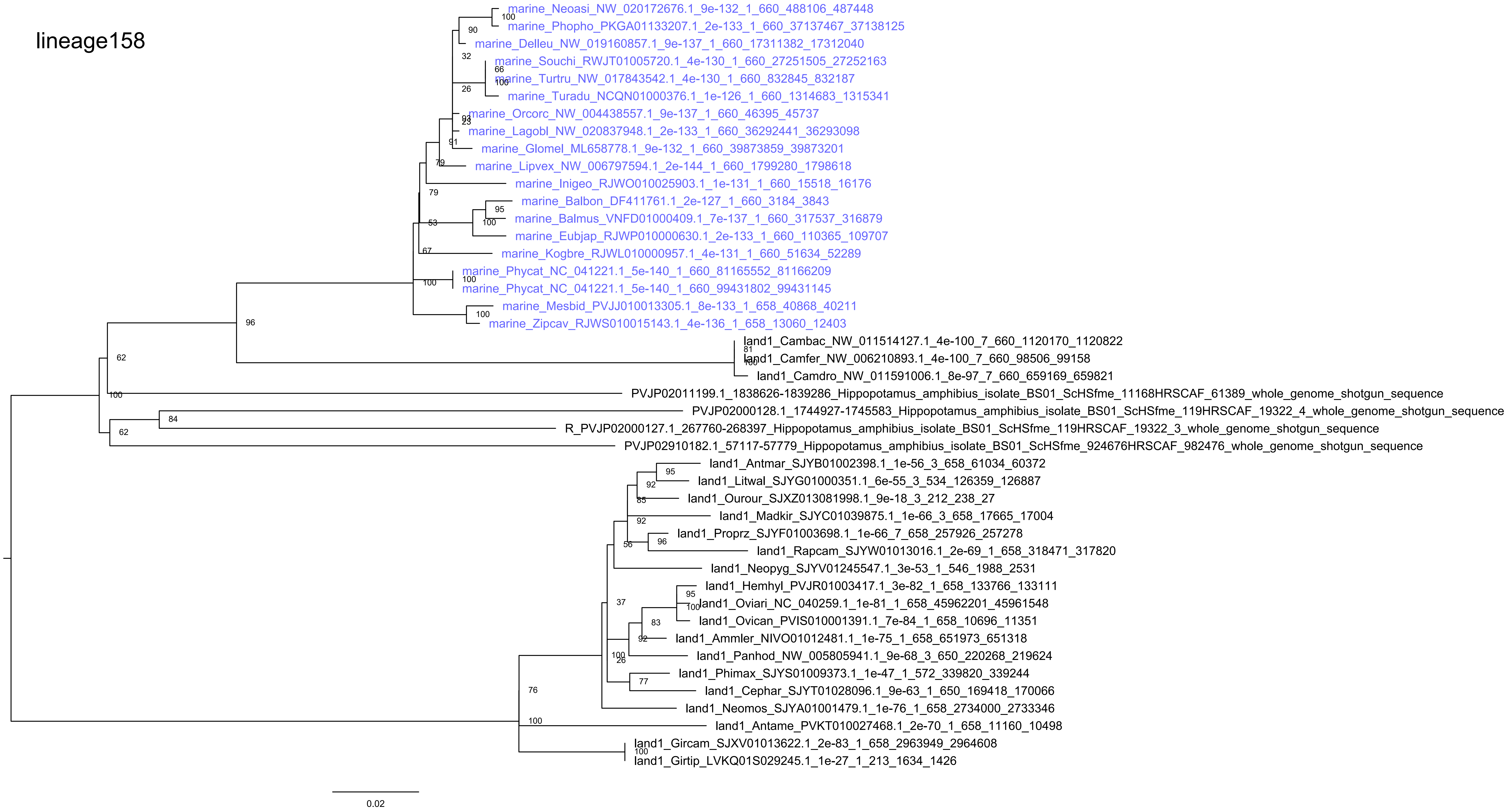

lineage158
marine_Neoasi_NW_020172676.1_9e-132_1_660_488106_487448
marine_Phopho_PKGA01133207.1_2e-133_1_660_37137467_37138125
marine_Delleu_NW_019160857.1_9e-137_1_660_17311382_17312040
marine_Souchi_RWJT01005720.1_4e-130_1_660_27251505_27252163
marine_Turtru_NW_017843542.1_4e-130_1_660_832845_832187
marine_Turadu_NCQN01000376.1_1e-126_1_660_1314683_1315341
marine_Orcorc_NW_004438557.1_9e-137_1_660_46395_45737
marine_Lagobl_NW_020837948.1_2e-133_1_660_36292441_36293098
marine_Glomel_ML658778.1_9e-132_1_660_39873859_39873201
marine_Lipvex_NW_006797594.1_2e-144_1_660_1799280_1798618
marine_Inigeo_RJWO010025903.1_1e-131_1_660_15518_16176
marine_Balbon_DF411761.1_2e-127_1_660_3184_3843
marine_Balmus_VNFD01000409.1_7e-137_1_660_317537_316879
marine_Eubjap_RJWP010000630.1_2e-133_1_660_110365_109707
marine_Kogbre_RJWL010000957.1_4e-131_1_660_51634_52289
marine_Phycat_NC_041221.1_5e-140_1_660_81165552_81166209
marine_Phycat_NC_041221.1_5e-140_1_660_99431802_99431145
marine_Mesbid_PVJJ010013305.1_8e-133_1_658_40868_40211
marine_Zipcav_RJWS010015143.1_4e-136_1_658_13060_12403
land1_Cambac_NW_011514127.1_4e-100_7_660_1120170_1120822
land1_Camfer_NW_006210893.1_4e-100_7_660_98506_99158
land1_Camdro_NW_011591006.1_8e-97_7_660_659169_659821
PVJP02011199.1_1838626-1839286_Hippopotamus_amphibius_isolate_BS01_ScHSfme_11168HRSCAF_61389_whole_genome_shotgun_sequence
PVJP02000128.1_1744927-1745583_Hippopotamus_amphibius_isolate_BS01_ScHSfme_119HRSCAF_19322_4_whole_genome_shotgun_sequence
R_PVJP02000127.1_267760-268397_Hippopotamus_amphibius_isolate_BS01_ScHSfme_119HRSCAF_19322_3_whole_genome_shotgun_sequence
PVJP02910182.1_57117-57779_Hippopotamus_amphibius_isolate_BS01_ScHSfme_924676HRSCAF_982476_whole_genome_shotgun_sequence
land1_Antmar_SJYB01002398.1_1e-56_3_658_61034_60372
land1_Litwal_SJYG01000351.1_6e-55_3_534_126359_126887
land1_Ourour_SJXZ013081998.1_9e-18_3_212_238_27
land1_Madkir_SJYC01039875.1_1e-66_3_658_17665_17004
land1_Proprz_SJYF01003698.1_1e-66_7_658_257926_257278
land1_Rapcam_SJYW01013016.1_2e-69_1_658_318471_317820
land1_Neopyg_SJYV01245547.1_3e-53_1_546_1988_2531
land1_Hemhyl_PVJR01003417.1_3e-82_1_658_133766_133111
land1_Oviari_NC_040259.1_1e-81_1_658_45962201_45961548
land1_Ovican_PVIS010001391.1_7e-84_1_658_10696_11351
land1_Ammler_NIVO01012481.1_1e-75_1_658_651973_651318
land1_Panhod_NW_005805941.1_9e-68_3_650_220268_219624
land1_Phimax_SJYS01009373.1_1e-47_1_572_339820_339244
land1_Cephar_SJYT01028096.1_9e-63_1_650_169418_170066
land1_Neomos_SJYA01001479.1_1e-76_1_658_2734000_2733346
land1_Antame_PVKT010027468.1_2e-70_1_658_11160_10498
land1_Gircam_SJXV01013622.1_2e-83_1_658_2963949_2964608
land1_Girtip_LVKQ01S029245.1_1e-27_1_213_1634_1426
0.02

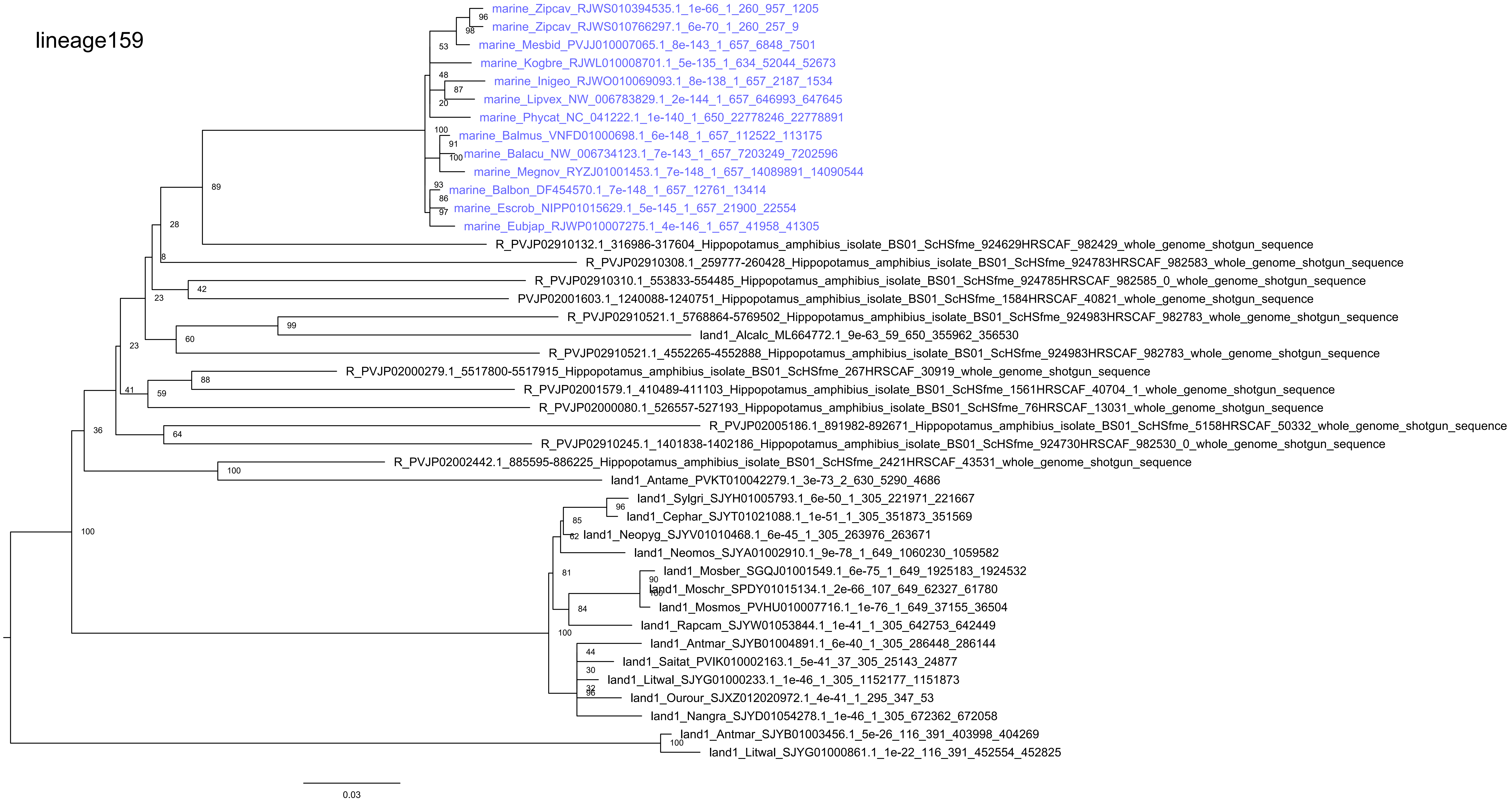
lineage159
marine_Zipcav_RJWS010394535.1_1e-66_1_260_957_1205
marine_Zipcav_RJWS010766297.1_6e-70_1_260_257_9
marine_Mesbid_PVJJ010007065.1_8e-143_1_657_6848_7501
marine_Kogbre_RJWL010008701.1_5e-135_1_634_52044_52673
marine_Inigeo_RJWO010069093.1_8e-138_1_657_2187_1534
marine_Lipvex_NW_006783829.1_2e-144_1_657_646993_647645
marine_Phycat_NC_041222.1_1e-140_1_650_22778246_22778891
marine_Balmus_VNFD01000698.1_6e-148_1_657_112522_113175
marine_Balacu_NW_006734123.1_7e-143_1_657_7203249_7202596
marine_Megnov_RYZJ01001453.1_7e-148_1_657_14089891_14090544
marine_Balbon_DF454570.1_7e-148_1_657_12761_13414
marine_Escrob_NIPP01015629.1_5e-145_1_657_21900_22554
marine_Eubjap_RJWP010007275.1_4e-146_1_657_41958_41305
R_PVJP02910132.1_316986-317604_Hippopotamus_amphibius_isolate_BS01_ScHSfme_924629HRSCAF_982429_whole_genome_shotgun_sequence
R_PVJP02910308.1_259777-260428_Hippopotamus_amphibius_isolate_BS01_ScHSfme_924783HRSCAF_982583_whole_genome_shotgun_sequence
R_PVJP02910310.1_553833-554485_Hippopotamus_amphibius_isolate_BS01_ScHSfme_924785HRSCAF_982585_0_whole_genome_shotgun_sequence
PVJP02001603.1_1240088-1240751_Hippopotamus_amphibius_isolate_BS01_ScHSfme_1584HRSCAF_40821_whole_genome_shotgun_sequence
R_PVJP02910521.1_5768864-5769502_Hippopotamus_amphibius_isolate_BS01_ScHSfme_924983HRSCAF_982783_whole_genome_shotgun_sequence
land1_Alcalc_ML664772.1_9e-63_59_650_355962_356530
R_PVJP02910521.1_4552265-4552888_Hippopotamus_amphibius_isolate_BS01_ScHSfme_924983HRSCAF_982783_whole_genome_shotgun_sequence
R_PVJP02000279.1_5517800-5517915_Hippopotamus_amphibius_isolate_BS01_ScHSfme_267HRSCAF_30919_whole_genome_shotgun_sequence
R_PVJP02001579.1_410489-411103_Hippopotamus_amphibius_isolate_BS01_ScHSfme_1561HRSCAF_40704_1_whole_genome_shotgun_sequence
R_PVJP02000080.1_526557-527193_Hippopotamus_amphibius_isolate_BS01_ScHSfme_76HRSCAF_13031_whole_genome_shotgun_sequence
R_PVJP02005186.1_891982-892671_Hippopotamus_amphibius_isolate_BS01_ScHSfme_5158HRSCAF_50332_whole_genome_shotgun_sequence
R_PVJP02910245.1_1401838-1402186_Hippopotamus_amphibius_isolate_BS01_ScHSfme_924730HRSCAF_982530_0_whole_genome_shotgun_sequence
R_PVJP02002442.1_885595-886225_Hippopotamus_amphibius_isolate_BS01_ScHSfme_2421HRSCAF_43531_whole_genome_shotgun_sequence
land1_Antame_PVKT010042279.1_3e-73_2_630_5290_4686
land1_Sylgri_SJYH01005793.1_6e-50_1_305_221971_221667
land1_Cephar_SJYT01021088.1_1e-51_1_305_351873_351569
land1_Neopyg_SJYV01010468.1_6e-45_1_305_263976_263671
land1_Neomos_SJYA01002910.1_9e-78_1_649_1060230_1059582
land1_Mosber_SGQJ01001549.1_6e-75_1_649_1925183_1924532
land1_Moschr_SPDY01015134.1_2e-66_107_649_62327_61780
land1_Mosmos_PVHU010007716.1_1e-76_1_649_37155_36504
land1_Rapcam_SJYW01053844.1_1e-41_1_305_642753_642449
land1_Antmar_SJYB01004891.1_6e-40_1_305_286448_286144
land1_Saitat_PVIK010002163.1_5e-41_37_305_25143_24877
land1_Litwal_SJYG01000233.1_1e-46_1_305_1152177_1151873
land1_Ourour_SJXZ012020972.1_4e-41_1_295_347_53
land1_Nangra_SJYD01054278.1_1e-46_1_305_672362_672058
land1_Antmar_SJYB01003456.1_5e-26_116_391_403998_404269
land1_Litwal_SJYG01000861.1_1e-22_116_391_452554_452825
0.03

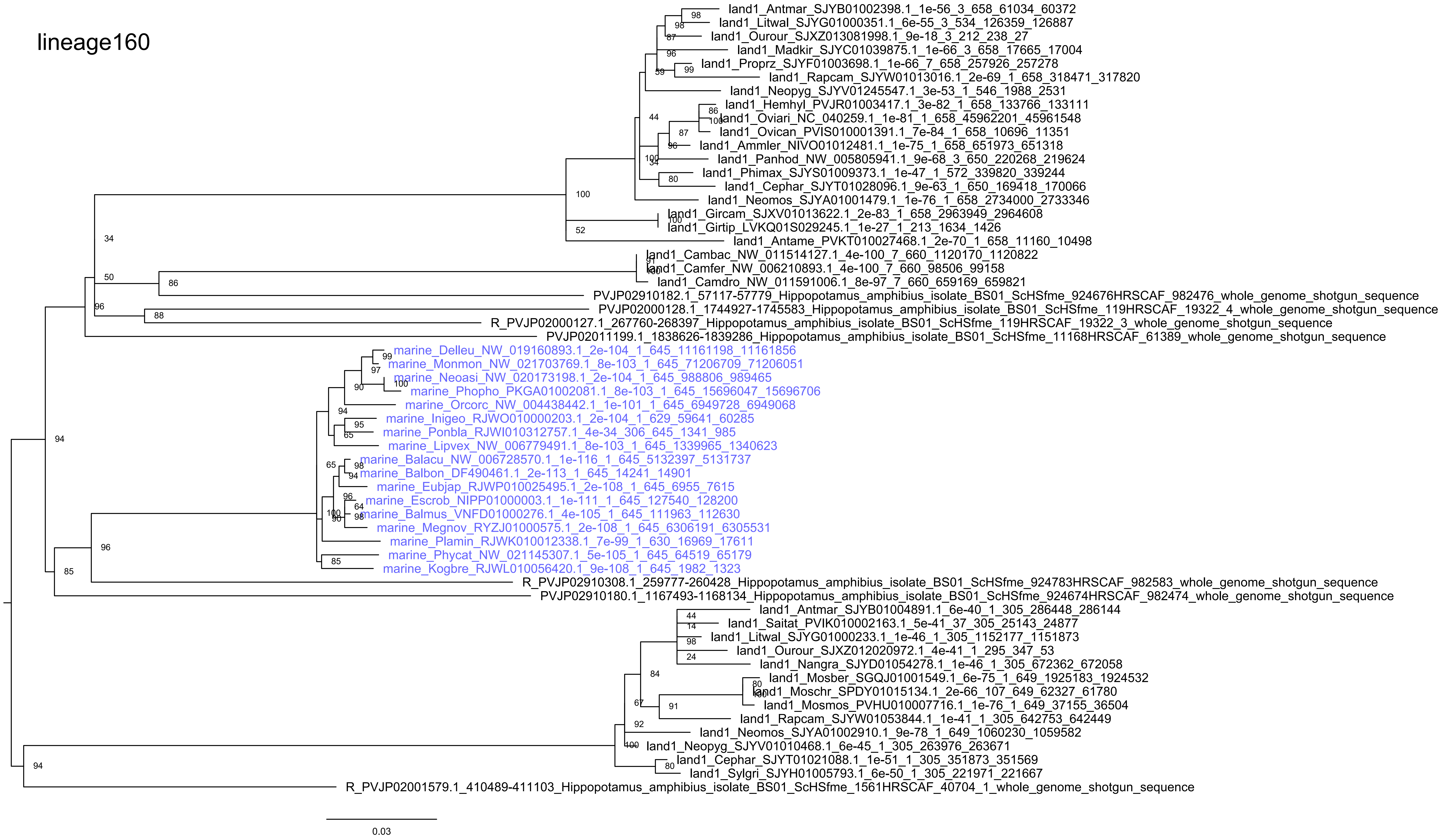
lineage160
land1_Antmar_SJYB01002398.1_1e-56_3_658_61034_60372
land1_Litwal_SJYG01000351.1_6e-55_3_534_126359_126887
land1_Ourour_SJXZ013081998.1_9e-18_3_212_238_27
land1_Madkir_SJYC01039875.1_1e-66_3_658_17665_17004
land1_Proprz_SJYF01003698.1_1e-66_7_658_257926_257278
land1_Rapcam_SJYW01013016.1_2e-69_1_658_318471_317820
land1_Neopyg_SJYV01245547.1_3e-53_1_546_1988_2531
land1_Hemhyl_PVJR01003417.1_3e-82_1_658_133766_133111
land1_Oviari_NC_040259.1_1e-81_1_658_45962201_45961548
land1_Ovican_PVIS010001391.1_7e-84_1_658_10696_11351
land1_Ammler_NIVO01012481.1_1e-75_1_658_651973_651318
land1_Panhod_NW_005805941.1_9e-68_3_650_220268_219624
land1_Phimax_SJYS01009373.1_1e-47_1_572_339820_339244
land1_Cephar_SJYT01028096.1_9e-63_1_650_169418_170066
land1_Neomos_SJYA01001479.1_1e-76_1_658_2734000_2733346
land1_Gircam_SJXV01013622.1_2e-83_1_658_2963949_2964608
land1_Girtip_LVKQ01S029245.1_1e-27_1_213_1634_1426
land1_Antame_PVKT010027468.1_2e-70_1_658_11160_10498
land1_Cambac_NW_011514127.1_4e-100_7_660_1120170_1120822
land1_Camfer_NW_006210893.1_4e-100_7_660_98506_99158
land1_Camdro_NW_011591006.1_8e-97_7_660_659169_659821
PVJP02910182.1_57117-57779_Hippopotamus_amphibius_isolate_BS01_ScHSfme_924676HRSCAF_982476_whole_genome_shotgun_sequence
PVJP02000128.1_1744927-1745583_Hippopotamus_amphibius_isolate_BS01_ScHSfme_119HRSCAF_19322_4_whole_genome_shotgun_sequence
R_PVJP02000127.1_267760-268397_Hippopotamus_amphibius_isolate_BS01_ScHSfme_119HRSCAF_19322_3_whole_genome_shotgun_sequence
PVJP02011199.1_1838626-1839286_Hippopotamus_amphibius_isolate_BS01_ScHSfme_11168HRSCAF_61389_whole_genome_shotgun_sequence
marine_Delleu_NW_019160893.1_2e-104_1_645_11161198_11161856
marine_Monmon_NW_021703769.1_8e-103_1_645_71206709_71206051
marine_Neoasi_NW_020173198.1_2e-104_1_645_988806_989465
marine_Phopho_PKGA01002081.1_8e-103_1_645_15696047_15696706
marine_Orcorc_NW_004438442.1_1e-101_1_645_6949728_6949068
marine_Inigeo_RJWO010000203.1_2e-104_1_629_59641_60285
marine_Ponbla_RJWI010312757.1_4e-34_306_645_1341_985
marine_Lipvex_NW_006779491.1_8e-103_1_645_1339965_1340623
marine_Balacu_NW_006728570.1_1e-116_1_645_5132397_5131737
marine_Balbon_DF490461.1_2e-113_1_645_14241_14901
marine_Eubjap_RJWP010025495.1_2e-108_1_645_6955_7615
marine_Escrob_NIPP01000003.1_1e-111_1_645_127540_128200
marine_Balmus_VNFD01000276.1_4e-105_1_645_111963_112630
marine_Megnov_RYZJ01000575.1_2e-108_1_645_6306191_6305531
marine_Plamin_RJWK010012338.1_7e-99_1_630_16969_17611
marine_Phycat_NW_021145307.1_5e-105_1_645_64519_65179
marine_Kogbre_RJWL010056420.1_9e-108_1_645_1982_1323
R_PVJP02910308.1_259777-260428_Hippopotamus_amphibius_isolate_BS01_ScHSfme_924783HRSCAF_982583_whole_genome_shotgun_sequence
PVJP02910180.1_1167493-1168134_Hippopotamus_amphibius_isolate_BS01_ScHSfme_924674HRSCAF_982474_whole_genome_shotgun_sequence
land1_Antmar_SJYB01004891.1_6e-40_1_305_286448_286144
land1_Saitat_PVIK010002163.1_5e-41_37_305_25143_24877
land1_Litwal_SJYG01000233.1_1e-46_1_305_1152177_1151873
land1_Ourour_SJXZ012020972.1_4e-41_1_295_347_53
land1_Nangra_SJYD01054278.1_1e-46_1_305_672362_672058
land1_Mosber_SGQJ01001549.1_6e-75_1_649_1925183_1924532
land1_Moschr_SPDY01015134.1_2e-66_107_649_62327_61780
land1_Mosmos_PVHU010007716.1_1e-76_1_649_37155_36504
land1_Rapcam_SJYW01053844.1_1e-41_1_305_642753_642449
land1_Neomos_SJYA01002910.1_9e-78_1_649_1060230_1059582
land1_Neopyg_SJYV01010468.1_6e-45_1_305_263976_263671
land1_Cephar_SJYT01021088.1_1e-51_1_305_351873_351569
land1_Sylgri_SJYH01005793.1_6e-50_1_305_221971_221667
R_PVJP02001579.1_410489-411103_Hippopotamus_amphibius_isolate_BS01_ScHSfme_1561HRSCAF_40704_1_whole_genome_shotgun_sequence
0.03

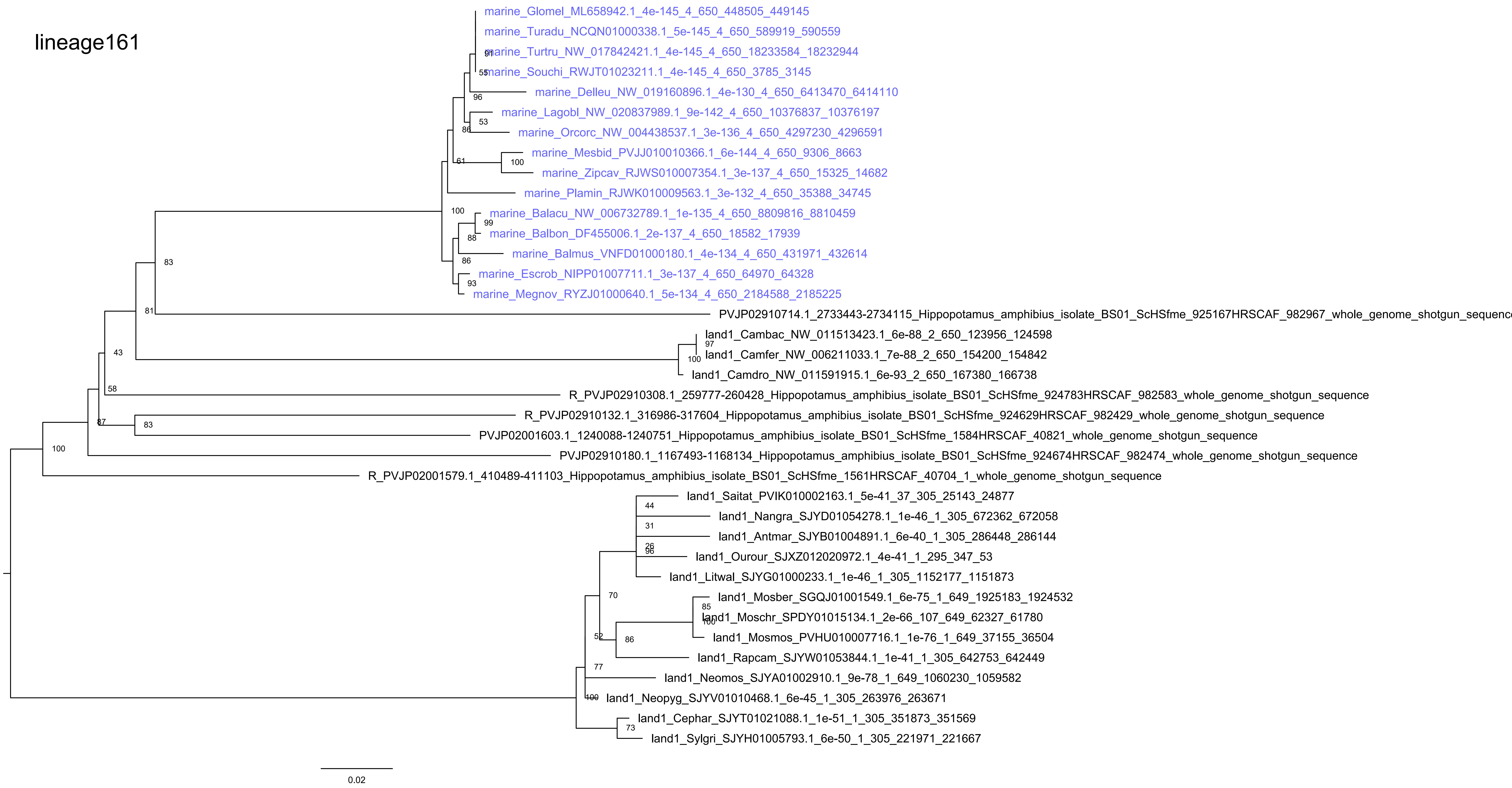
lineage161
marine_Glomel_ML658942.1_4e-145_4_650_448505_449145
marine_Turadu_NCQN01000338.1_5e-145_4_650_589919_590559
marine_Turtru_NW_017842421.1_4e-145_4_650_18233584_18232944
marine_Souchi_RWJT01023211.1_4e-145_4_650_3785_3145
marine_Delleu_NW_019160896.1_4e-130_4_650_6413470_6414110
marine_Lagobl_NW_020837989.1_9e-142_4_650_10376837_10376197
marine_Orcorc_NW_004438537.1_3e-136_4_650_4297230_4296591
marine_Mesbid_PVJJ010010366.1_6e-144_4_650_9306_8663
marine_Zipcav_RJWS010007354.1_3e-137_4_650_15325_14682
marine_Plamin_RJWK010009563.1_3e-132_4_650_35388_34745
marine_Balacu_NW_006732789.1_1e-135_4_650_8809816_8810459
marine_Balbon_DF455006.1_2e-137_4_650_18582_17939
marine_Balmus_VNFD01000180.1_4e-134_4_650_431971_432614
marine_Escrob_NIPP01007711.1_3e-137_4_650_64970_64328
marine_Megnov_RYZJ01000640.1_5e-134_4_650_2184588_2185225
PVJP02910714.1_2733443-2734115_Hippopotamus_amphibius_isolate_BS01_ScHSfme_925167HRSCAF_982967_whole_genome_shotgun_sequenc
land1_Cambac_NW_011513423.1_6e-88_2_650_123956_124598
land1_Camfer_NW_006211033.1_7e-88_2_650_154200_154842
land1_Camdro_NW_011591915.1_6e-93_2_650_167380_166738
R_PVJP02910308.1_259777-260428_Hippopotamus_amphibius_isolate_BS01_ScHSfme_924783HRSCAF_982583_whole_genome_shotgun_sequence
R_PVJP02910132.1_316986-317604_Hippopotamus_amphibius_isolate_BS01_ScHSfme_924629HRSCAF_982429_whole_genome_shotgun_sequence
PVJP02001603.1_1240088-1240751_Hippopotamus_amphibius_isolate_BS01_ScHSfme_1584HRSCAF_40821_whole_genome_shotgun_sequence
PVJP02910180.1_1167493-1168134_Hippopotamus_amphibius_isolate_BS01_ScHSfme_924674HRSCAF_982474_whole_genome_shotgun_sequence
R_PVJP02001579.1_410489-411103_Hippopotamus_amphibius_isolate_BS01_ScHSfme_1561HRSCAF_40704_1_whole_genome_shotgun_sequence
land1_Saitat_PVIK010002163.1_5e-41_37_305_25143_24877
land1_Nangra_SJYD01054278.1_1e-46_1_305_672362_672058
land1_Antmar_SJYB01004891.1_6e-40_1_305_286448_286144
land1_Ourour_SJXZ012020972.1_4e-41_1_295_347_53
land1_Litwal_SJYG01000233.1_1e-46_1_305_1152177_1151873
land1_Mosber_SGQJ01001549.1_6e-75_1_649_1925183_1924532
land1_Moschr_SPDY01015134.1_2e-66_107_649_62327_61780
land1_Mosmos_PVHU010007716.1_1e-76_1_649_37155_36504
land1_Rapcam_SJYW01053844.1_1e-41_1_305_642753_642449
land1_Neomos_SJYA01002910.1_9e-78_1_649_1060230_1059582
land1_Neopyg_SJYV01010468.1_6e-45_1_305_263976_263671
land1_Cephar_SJYT01021088.1_1e-51_1_305_351873_351569
land1_Sylgri_SJYH01005793.1_6e-50_1_305_221971_221667
0.02

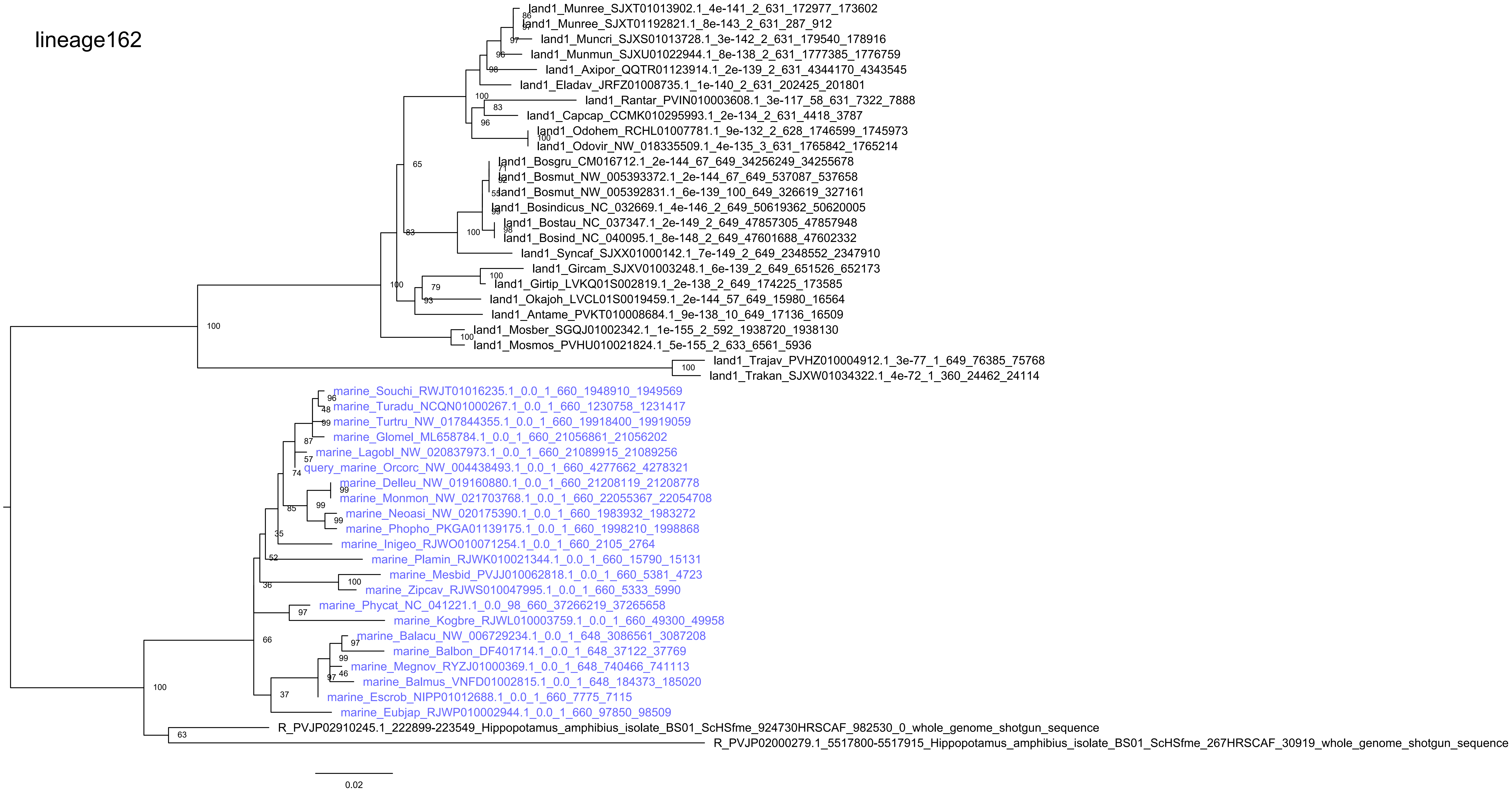
lineage162
land1_Munree_SJXT01013902.1_4e-141_2_631_172977_173602
land1_Munree_SJXT01192821.1_8e-143_2_631_287_912
land1_Muncri_SJXS01013728.1_3e-142_2_631_179540_178916
land1_Munmun_SJXU01022944.1_8e-138_2_631_1777385_1776759
land1_Axipor_QQTR01123914.1_2e-139_2_631_4344170_4343545
land1_Eladav_JRFZ01008735.1_1e-140_2_631_202425_201801
land1_Rantar_PVIN010003608.1_3e-117_58_631_7322_7888
land1_Capcap_CCMK010295993.1_2e-134_2_631_4418_3787
land1_Odohem_RCHL01007781.1_9e-132_2_628_1746599_1745973
land1_Odovir_NW_018335509.1_4e-135_3_631_1765842_1765214
land1_Bosgru_CM016712.1_2e-144_67_649_34256249_34255678
land1_Bosmut_NW_005393372.1_2e-144_67_649_537087_537658
land1_Bosmut_NW_005392831.1_6e-139_100_649_326619_327161
land1_Bosindicus_NC_032669.1_4e-146_2_649_50619362_50620005
land1_Bostau_NC_037347.1_2e-149_2_649_47857305_47857948
land1_Bosind_NC_040095.1_8e-148_2_649_47601688_47602332
land1_Syncaf_SJXX01000142.1_7e-149_2_649_2348552_2347910
land1_Gircam_SJXV01003248.1_6e-139_2_649_651526_652173
land1_Girtip_LVKQ01S002819.1_2e-138_2_649_174225_173585
land1_Okajoh_LVCL01S0019459.1_2e-144_57_649_15980_16564
land1_Antame_PVKT010008684.1_9e-138_10_649_17136_16509
land1_Mosber_SGQJ01002342.1_1e-155_2_592_1938720_1938130
land1_Mosmos_PVHU010021824.1_5e-155_2_633_6561_5936
land1_Trajav_PVHZ010004912.1_3e-77_1_649_76385_75768
land1_Trakan_SJXW01034322.1_4e-72_1_360_24462_24114
marine_Souchi_RWJT01016235.1_0.0_1_660_1948910_1949569
marine_Turadu_NCQN01000267.1_0.0_1_660_1230758_1231417
marine_Turtru_NW_017844355.1_0.0_1_660_19918400_19919059
marine_Glomel_ML658784.1_0.0_1_660_21056861_21056202
marine_Lagobl_NW_020837973.1_0.0_1_660_21089915_21089256
query_marine_Orcorc_NW_004438493.1_0.0_1_660_4277662_4278321
marine_Delleu_NW_019160880.1_0.0_1_660_21208119_21208778
marine_Monmon_NW_021703768.1_0.0_1_660_22055367_22054708
marine_Neoasi_NW_020175390.1_0.0_1_660_1983932_1983272
marine_Phopho_PKGA01139175.1_0.0_1_660_1998210_1998868
marine_Inigeo_RJWO010071254.1_0.0_1_660_2105_2764
marine_Plamin_RJWK010021344.1_0.0_1_660_15790_15131
marine_Mesbid_PVJJ010062818.1_0.0_1_660_5381_4723
marine_Zipcav_RJWS010047995.1_0.0_1_660_5333_5990
marine_Phycat_NC_041221.1_0.0_98_660_37266219_37265658
marine_Kogbre_RJWL010003759.1_0.0_1_660_49300_49958
marine_Balacu_NW_006729234.1_0.0_1_648_3086561_3087208
marine_Balbon_DF401714.1_0.0_1_648_37122_37769
marine_Megnov_RYZJ01000369.1_0.0_1_648_740466_741113
marine_Balmus_VNFD01002815.1_0.0_1_648_184373_185020
marine_Escrob_NIPP01012688.1_0.0_1_660_7775_7115
marine_Eubjap_RJWP010002944.1_0.0_1_660_97850_98509
R_PVJP02910245.1_222899-223549_Hippopotamus_amphibius_isolate_BS01_ScHSfme_924730HRSCAF_982530_0_whole_genome_shotgun_sequence
R_PVJP02000279.1_5517800-5517915_Hippopotamus_amphibius_isolate_BS01_ScHSfme_267HRSCAF_30919_whole_genome_shotgun_sequence
0.02

lineage163

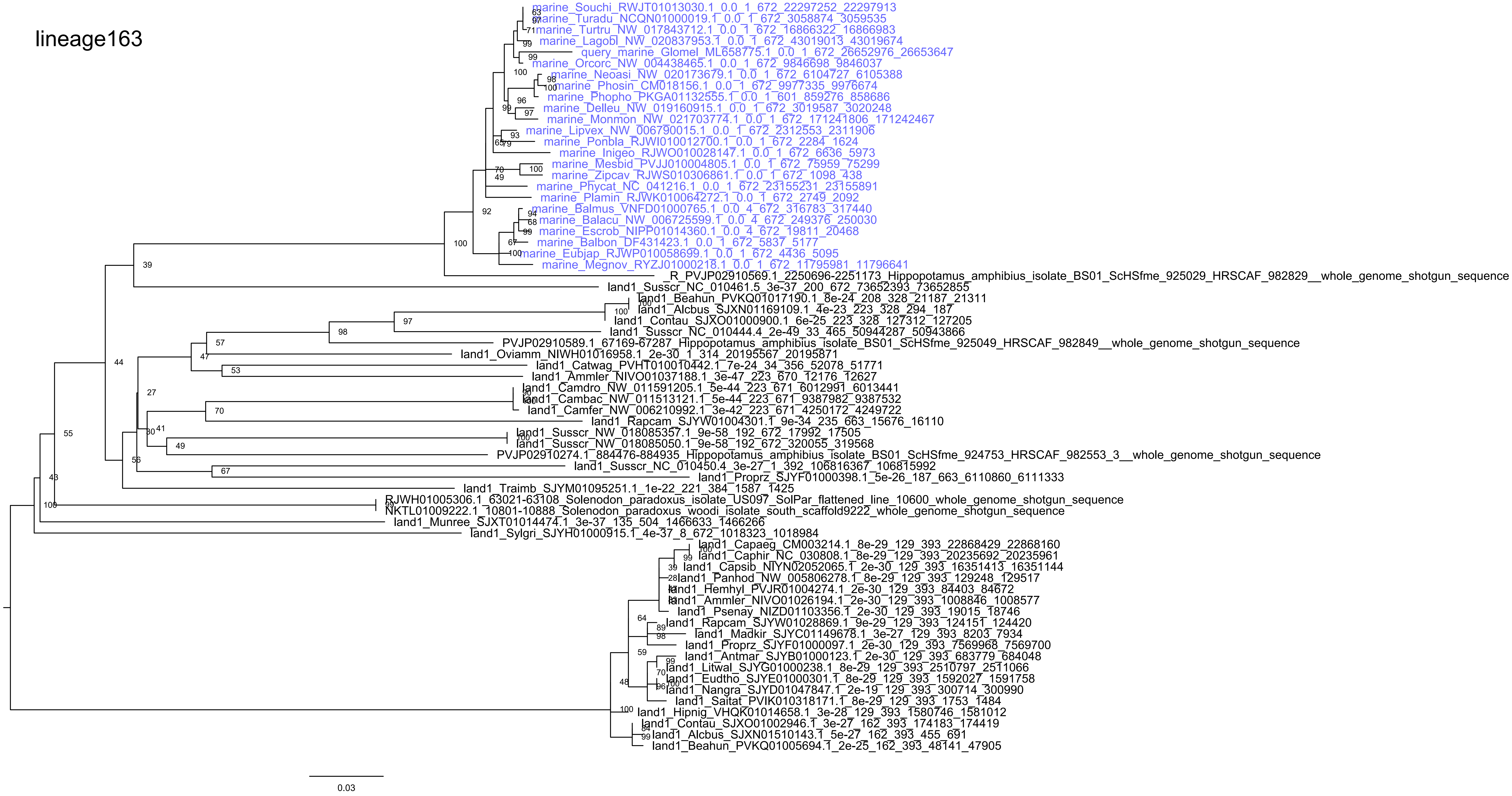
marine_Souchi_RWJT01013030.1_0.0_1_672_22297252_22297913
marine_Turadu_NCQN01000019.1_0.0_1_672_3058874_3059535
marine_Turtru_NW_017843712.1_0.0_1_672_16866322_16866983
marine_Lagobl_NW_020837953.1_0.0_1_672_43019013_43019674
query_marine_Glomel_ML658775.1_0.0_1_672_26652976_26653647
marine_Orcorc_NW_004438465.1_0.0_1_672_9846698_9846037
marine_Neoasi_NW_020173679.1_0.0_1_672_6104727_6105388
marine_Phosin_CM018156.1_0.0_1_672_9977335_9976674
marine_Phopho_PKGA01132555.1_0.0_1_601_859276_858686
marine_Delleu_NW_019160915.1_0.0_1_672_3019587_3020248
marine_Monmon_NW_021703774.1_0.0_1_672_171241806_171242467
marine_Lipvex_NW_006790015.1_0.0_1_672_2312553_2311906
marine_Ponbla_RJWI010012700.1_0.0_1_672_2284_1624
marine_Inigeo_RJWO010028147.1_0.0_1_672_6636_5973
marine_Mesbid_PVJJ010004805.1_0.0_1_672_75959_75299
marine_Zipcav_RJWS010306861.1_0.0_1_672_1098_438
marine_Phycat_NC_041216.1_0.0_1_672_23155231_23155891
marine_Plamin_RJWK010064272.1_0.0_1_672_2749_2092
marine_Balmus_VNFD01000765.1_0.0_4_672_316783_317440
marine_Balacu_NW_006725599.1_0.0_4_672_249376_250030
marine_Escrob_NIPP01014360.1_0.0_4_672_19811_20468
marine_Balbon_DF431423.1_0.0_1_672_5837_5177
marine_Eubjap_RJWP010058699.1_0.0_1_672_4436_5095
marine_Megnov_RYZJ01000218.1_0.0_1_672_11795981_11796641
_R_PVJP02910569.1_2250696-2251173_Hippopotamus_amphibius_isolate_BS01_ScHSfme_925029_HRSCAF_982829__whole_genome_shotgun_sequence
land1_Susscr_NC_010461.5_3e-37_200_672_73652393_73652855
land1_Beahun_PVKQ01017190.1_8e-24_208_328_21187_21311
land1_Alcbus_SJXN01169109.1_4e-23_223_328_294_187
land1_Contau_SJXO01000900.1_6e-25_223_328_127312_127205
land1_Susscr_NC_010444.4_2e-49_33_465_50944287_50943866
PVJP02910589.1_67169-67287_Hippopotamus_amphibius_isolate_BS01_ScHSfme_925049_HRSCAF_982849__whole_genome_shotgun_sequence
land1_Oviamm_NIWH01016958.1_2e-30_1_314_20195567_20195871
land1_Catwag_PVHT010010442.1_7e-24_34_356_52078_51771
land1_Ammler_NIVO01037188.1_3e-47_223_670_12176_12627
land1_Camdro_NW_011591205.1_5e-44_223_671_6012991_6013441
land1_Cambac_NW_011513121.1_5e-44_223_671_9387982_9387532
land1_Camfer_NW_006210992.1_3e-42_223_671_4250172_4249722
land1_Rapcam_SJYW01004301.1_9e-34_235_663_15676_16110
land1_Susscr_NW_018085357.1_9e-58_192_672_17992_17505
land1_Susscr_NW_018085050.1_9e-58_192_672_320055_319568
PVJP02910274.1_884476-884935_Hippopotamus_amphibius_isolate_BS01_ScHSfme_924753_HRSCAF_982553_3__whole_genome_shotgun_sequence
land1_Susscr_NC_010450.4_3e-27_1_392_106816367_106815992
land1_Proprz_SJYF01000398.1_5e-26_187_663_6110860_6111333
land1_Traimb_SJYM01095251.1_1e-22_221_384_1587_1425
RJWH01005306.1_63021-63108_Solenodon_paradoxus_isolate_US097_SolPar_flattened_line_10600_whole_genome_shotgun_sequence
NKTL01009222.1_10801-10888_Solenodon_paradoxus_woodi_isolate_south_scaffold9222_whole_genome_shotgun_sequence
land1_Munree_SJXT01014474.1_3e-37_135_504_1466633_1466266
land1_Sylgri_SJYH01000915.1_4e-37_8_672_1018323_1018984
land1_Capaeg_CM003214.1_8e-29_129_393_22868429_22868160
land1_Caphir_NC_030808.1_8e-29_129_393_20235692_20235961
land1_Capsib_NIYN02052065.1_2e-30_129_393_16351413_16351144
land1_Panhod_NW_005806278.1_8e-29_129_393_129248_129517
land1_Hemhyl_PVJR01004274.1_2e-30_129_393_84403_84672
land1_Ammler_NIVO01026194.1_2e-30_129_393_1008846_1008577
land1_Psenay_NIZD01103356.1_2e-30_129_393_19015_18746
land1_Rapcam_SJYW01028869.1_9e-29_129_393_124151_124420
land1_Madkir_SJYC01149678.1_3e-27_129_393_8203_7934
land1_Proprz_SJYF01000097.1_2e-30_129_393_7569968_7569700
land1_Antmar_SJYB01000123.1_2e-30_129_393_683779_684048
land1_Litwal_SJYG01000238.1_8e-29_129_393_2510797_2511066
land1_Eudtho_SJYE01000301.1_8e-29_129_393_1592027_1591758
land1_Nangra_SJYD01047847.1_2e-19_129_393_300714_300990
land1_Saitat_PVIK010318171.1_8e-29_129_393_1753_1484
land1_Hipnig_VHQK01014658.1_3e-28_129_393_1580746_1581012
land1_Contau_SJXO01002946.1_3e-27_162_393_174183_174419
land1_Alcbus_SJXN01510143.1_5e-27_162_393_455_691
land1_Beahun_PVKQ01005694.1_2e-25_162_393_48141_47905
0.03

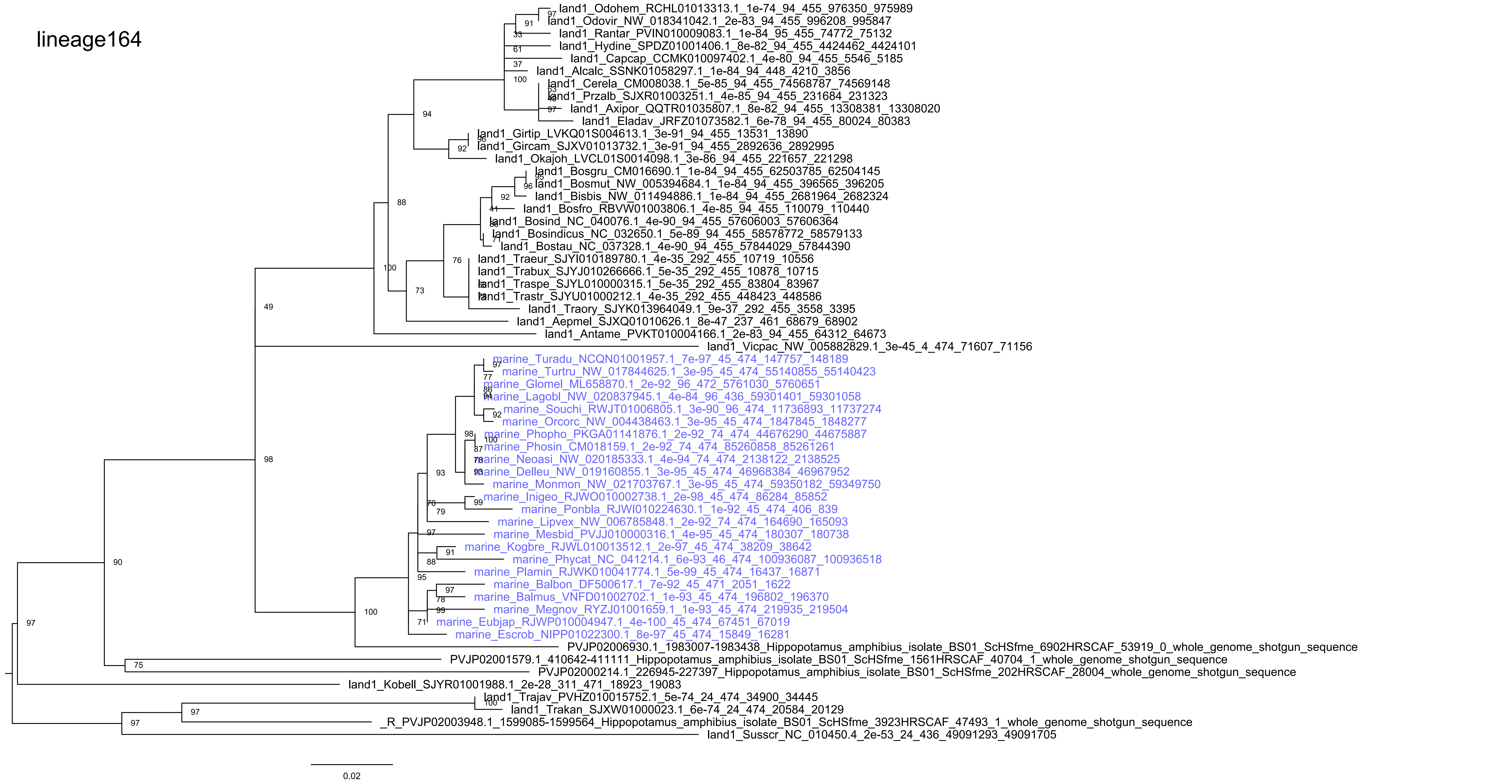
lineage164
land1_Odohem_RCHL01013313.1_1e-74_94_455_976350_975989
land1_Odovir_NW_018341042.1_2e-83_94_455_996208_995847
land1_Rantar_PVIN010009083.1_1e-84_95_455_74772_75132
land1_Hydine_SPDZ01001406.1_8e-82_94_455_4424462_4424101
land1_Capcap_CCMK010097402.1_4e-80_94_455_5546_5185
land1_Alcalc_SSNK01058297.1_1e-84_94_448_4210_3856
land1_Cerela_CM008038.1_5e-85_94_455_74568787_74569148
land1_Przalb_SJXR01003251.1_4e-85_94_455_231684_231323
land1_Axipor_QQTR01035807.1_8e-82_94_455_13308381_13308020
land1_Eladav_JRFZ01073582.1_6e-78_94_455_80024_80383
land1_Girtip_LVKQ01S004613.1_3e-91_94_455_13531_13890
land1_Gircam_SJXV01013732.1_3e-91_94_455_2892636_2892995
land1_Okajoh_LVCL01S0014098.1_3e-86_94_455_221657_221298
land1_Bosgru_CM016690.1_1e-84_94_455_62503785_62504145
land1_Bosmut_NW_005394684.1_1e-84_94_455_396565_396205
land1_Bisbis_NW_011494886.1_1e-84_94_455_2681964_2682324
land1_Bosfro_RBVW01003806.1_4e-85_94_455_110079_110440
land1_Bosind_NC_040076.1_4e-90_94_455_57606003_57606364
land1_Bosindicus_NC_032650.1_5e-89_94_455_58578772_58579133
land1_Bostau_NC_037328.1_4e-90_94_455_57844029_57844390
land1_Traeur_SJYI010189780.1_4e-35_292_455_10719_10556
land1_Trabux_SJYJ010266666.1_5e-35_292_455_10878_10715
land1_Traspe_SJYL010000315.1_5e-35_292_455_83804_83967
land1_Trastr_SJYU01000212.1_4e-35_292_455_448423_448586
land1_Traory_SJYK013964049.1_9e-37_292_455_3558_3395
land1_Aepmel_SJXQ01010626.1_8e-47_237_461_68679_68902
land1_Antame_PVKT010004166.1_2e-83_94_455_64312_64673
land1_Vicpac_NW_005882829.1_3e-45_4_474_71607_71156
marine_Turadu_NCQN01001957.1_7e-97_45_474_147757_148189
marine_Turtru_NW_017844625.1_3e-95_45_474_55140855_55140423
marine_Glomel_ML658870.1_2e-92_96_472_5761030_5760651
marine_Lagobl_NW_020837945.1_4e-84_96_436_59301401_59301058
marine_Souchi_RWJT01006805.1_3e-90_96_474_11736893_11737274
marine_Orcorc_NW_004438463.1_3e-95_45_474_1847845_1848277
marine_Phopho_PKGA01141876.1_2e-92_74_474_44676290_44675887
marine_Phosin_CM018159.1_2e-92_74_474_85260858_85261261
marine_Neoasi_NW_020185333.1_4e-94_74_474_2138122_2138525
marine_Delleu_NW_019160855.1_3e-95_45_474_46968384_46967952
marine_Monmon_NW_021703767.1_3e-95_45_474_59350182_59349750
marine_Inigeo_RJWO010002738.1_2e-98_45_474_86284_85852
marine_Ponbla_RJWI010224630.1_1e-92_45_474_406_839
marine_Lipvex_NW_006785848.1_2e-92_74_474_164690_165093
marine_Mesbid_PVJJ010000316.1_4e-95_45_474_180307_180738
marine_Kogbre_RJWL010013512.1_2e-97_45_474_38209_38642
marine_Phycat_NC_041214.1_6e-93_46_474_100936087_100936518
marine_Plamin_RJWK010041774.1_5e-99_45_474_16437_16871
marine_Balbon_DF500617.1_7e-92_45_471_2051_1622
marine_Balmus_VNFD01002702.1_1e-93_45_474_196802_196370
marine_Megnov_RYZJ01001659.1_1e-93_45_474_219935_219504
marine_Eubjap_RJWP010004947.1_4e-100_45_474_67451_67019
marine_Escrob_NIPP01022300.1_8e-97_45_474_15849_16281
PVJP02006930.1_1983007-1983438_Hippopotamus_amphibius_isolate_BS01_ScHSfme_6902HRSCAF_53919_0_whole_genome_shotgun_sequence
PVJP02001579.1_410642-411111_Hippopotamus_amphibius_isolate_BS01_ScHSfme_1561HRSCAF_40704_1_whole_genome_shotgun_sequence
PVJP02000214.1_226945-227397_Hippopotamus_amphibius_isolate_BS01_ScHSfme_202HRSCAF_28004_whole_genome_shotgun_sequence
land1_Kobell_SJYR01001988.1_2e-28_311_471_18923_19083
land1_Trajav_PVHZ010015752.1_5e-74_24_474_34900_34445
land1_Trakan_SJXW01000023.1_6e-74_24_474_20584_20129
_R_PVJP02003948.1_1599085-1599564_Hippopotamus_amphibius_isolate_BS01_ScHSfme_3923HRSCAF_47493_1_whole_genome_shotgun_sequence
land1_Susscr_NC_010450.4_2e-53_24_436_49091293_49091705
0.02

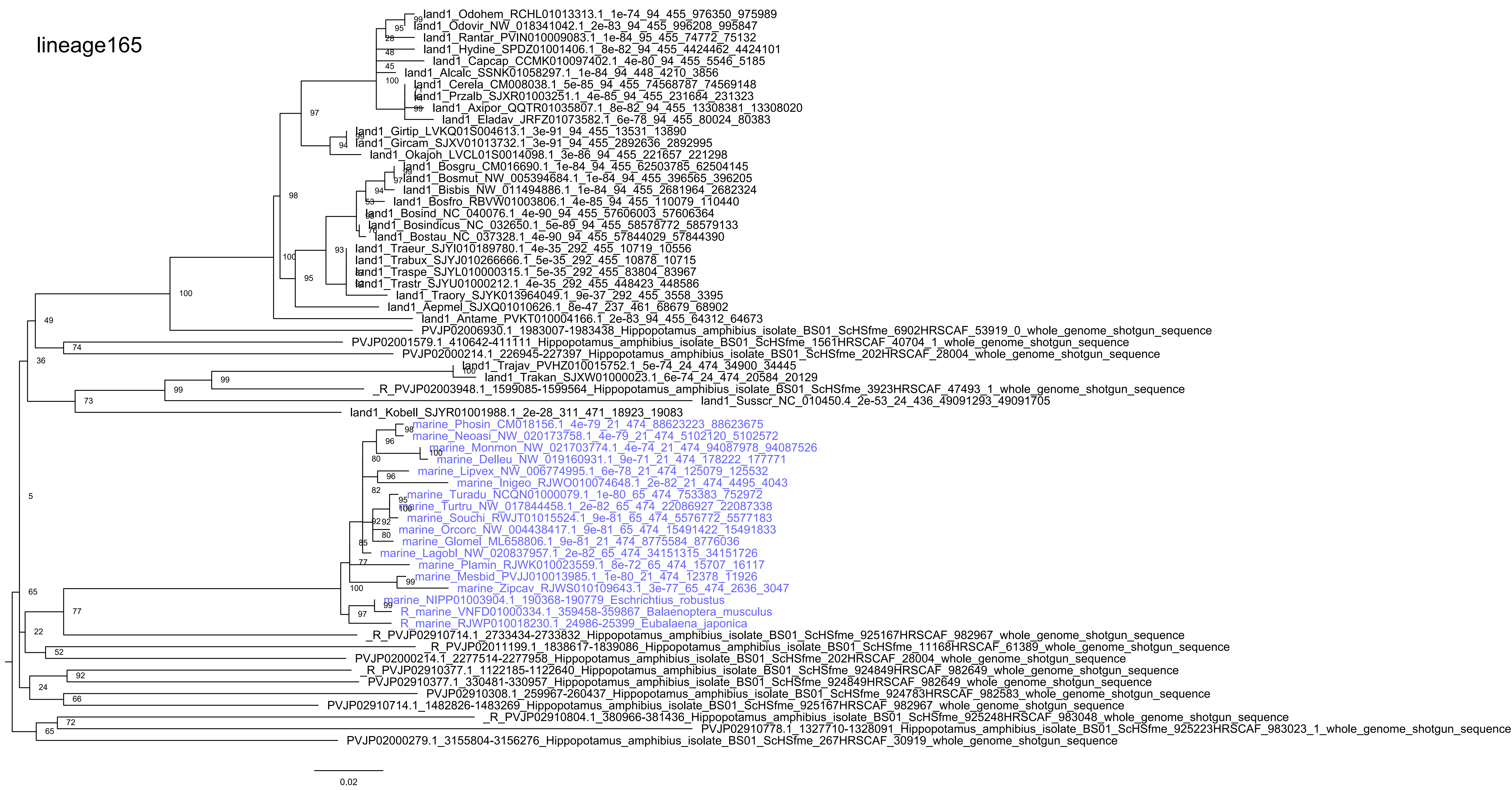

lineage165
land1_Odohem_RCHL01013313.1_1e-74_94_455_976350_975989
land1_Odovir_NW_018341042.1_2e-83_94_455_996208_995847
land1_Rantar_PVIN010009083.1_1e-84_95_455_74772_75132
land1_Hydine_SPDZ01001406.1_8e-82_94_455_4424462_4424101
land1_Capcap_CCMK010097402.1_4e-80_94_455_5546_5185
land1_Alcalc_SSNK01058297.1_1e-84_94_448_4210_3856
land1_Cerela_CM008038.1_5e-85_94_455_74568787_74569148
land1_Przalb_SJXR01003251.1_4e-85_94_455_231684_231323
land1_Axipor_QQTR01035807.1_8e-82_94_455_13308381_13308020
land1_Eladav_JRFZ01073582.1_6e-78_94_455_80024_80383
land1_Girtip_LVKQ01S004613.1_3e-91_94_455_13531_13890
land1_Gircam_SJXV01013732.1_3e-91_94_455_2892636_2892995
land1_Okajoh_LVCL01S0014098.1_3e-86_94_455_221657_221298
land1_Bosgru_CM016690.1_1e-84_94_455_62503785_62504145
land1_Bosmut_NW_005394684.1_1e-84_94_455_396565_396205
land1_Bisbis_NW_011494886.1_1e-84_94_455_2681964_2682324
land1_Bosfro_RBVW01003806.1_4e-85_94_455_110079_110440
land1_Bosind_NC_040076.1_4e-90_94_455_57606003_57606364
land1_Bosindicus_NC_032650.1_5e-89_94_455_58578772_58579133
land1_Bostau_NC_037328.1_4e-90_94_455_57844029_57844390
land1_Traeur_SJYI010189780.1_4e-35_292_455_10719_10556
land1_Trabux_SJYJ010266666.1_5e-35_292_455_10878_10715
land1_Traspe_SJYL010000315.1_5e-35_292_455_83804_83967
land1_Trastr_SJYU01000212.1_4e-35_292_455_448423_448586
land1_Traory_SJYK013964049.1_9e-37_292_455_3558_3395
land1_Aepmel_SJXQ01010626.1_8e-47_237_461_68679_68902
land1_Antame_PVKT010004166.1_2e-83_94_455_64312_64673
PVJP02006930.1_1983007-1983438_Hippopotamus_amphibius_isolate_BS01_ScHSfme_6902HRSCAF_53919_0_whole_genome_shotgun_sequence
PVJP02001579.1_410642-411111_Hippopotamus_amphibius_isolate_BS01_ScHSfme_1561HRSCAF_40704_1_whole_genome_shotgun_sequence
PVJP02000214.1_226945-227397_Hippopotamus_amphibius_isolate_BS01_ScHSfme_202HRSCAF_28004_whole_genome_shotgun_sequence
land1_Trajav_PVHZ010015752.1_5e-74_24_474_34900_34445
land1_Trakan_SJXW01000023.1_6e-74_24_474_20584_20129
_R_PVJP02003948.1_1599085-1599564_Hippopotamus_amphibius_isolate_BS01_ScHSfme_3923HRSCAF_47493_1_whole_genome_shotgun_sequence
land1_Susscr_NC_010450.4_2e-53_24_436_49091293_49091705
land1_Kobell_SJYR01001988.1_2e-28_311_471_18923_19083
marine_Phosin_CM018156.1_4e-79_21_474_88623223_88623675
marine_Neoasi_NW_020173758.1_4e-79_21_474_5102120_5102572
marine_Monmon_NW_021703774.1_4e-74_21_474_94087978_94087526
marine_Delleu_NW_019160931.1_9e-71_21_474_178222_177771
marine_Lipvex_NW_006774995.1_6e-78_21_474_125079_125532
marine_Inigeo_RJWO010074648.1_2e-82_21_474_4495_4043
marine_Turadu_NCQN01000079.1_1e-80_65_474_753383_752972
marine_Turtru_NW_017844458.1_2e-82_65_474_22086927_22087338
marine_Souchi_RWJT01015524.1_9e-81_65_474_5576772_5577183
marine_Orcorc_NW_004438417.1_9e-81_65_474_15491422_15491833
marine_Glomel_ML658806.1_9e-81_21_474_8775584_8776036
marine_Lagobl_NW_020837957.1_2e-82_65_474_34151315_34151726
marine_Plamin_RJWK010023559.1_8e-72_65_474_15707_16117
marine_Mesbid_PVJJ010013985.1_1e-80_21_474_12378_11926
marine_Zipcav_RJWS010109643.1_3e-77_65_474_2636_3047
marine_NIPP01003904.1_190368-190779_Eschrichtius_robustus
R_marine_VNFD01000334.1_359458-359867_Balaenoptera_musculus
R_marine_RJWP010018230.1_24986-25399_Eubalaena_japonica
_R_PVJP02910714.1_2733434-2733832_Hippopotamus_amphibius_isolate_BS01_ScHSfme_925167HRSCAF_982967_whole_genome_shotgun_sequence
_R_PVJP02011199.1_1838617-1839086_Hippopotamus_amphibius_isolate_BS01_ScHSfme_11168HRSCAF_61389_whole_genome_shotgun_sequence
PVJP02000214.1_2277514-2277958_Hippopotamus_amphibius_isolate_BS01_ScHSfme_202HRSCAF_28004_whole_genome_shotgun_sequence
_R_PVJP02910377.1_1122185-1122640_Hippopotamus_amphibius_isolate_BS01_ScHSfme_924849HRSCAF_982649_whole_genome_shotgun_sequence
PVJP02910377.1_330481-330957_Hippopotamus_amphibius_isolate_BS01_ScHSfme_924849HRSCAF_982649_whole_genome_shotgun_sequence
PVJP02910308.1_259967-260437_Hippopotamus_amphibius_isolate_BS01_ScHSfme_924783HRSCAF_982583_whole_genome_shotgun_sequence
PVJP02910714.1_1482826-1483269_Hippopotamus_amphibius_isolate_BS01_ScHSfme_925167HRSCAF_982967_whole_genome_shotgun_sequence
_R_PVJP02910804.1_380966-381436_Hippopotamus_amphibius_isolate_BS01_ScHSfme_925248HRSCAF_983048_whole_genome_shotgun_sequence
PVJP02910778.1_1327710-1328091_Hippopotamus_amphibius_isolate_BS01_ScHSfme_925223HRSCAF_983023_1_whole_genome_shotgun_sequence
PVJP02000279.1_3155804-3156276_Hippopotamus_amphibius_isolate_BS01_ScHSfme_267HRSCAF_30919_whole_genome_shotgun_sequence
0.02

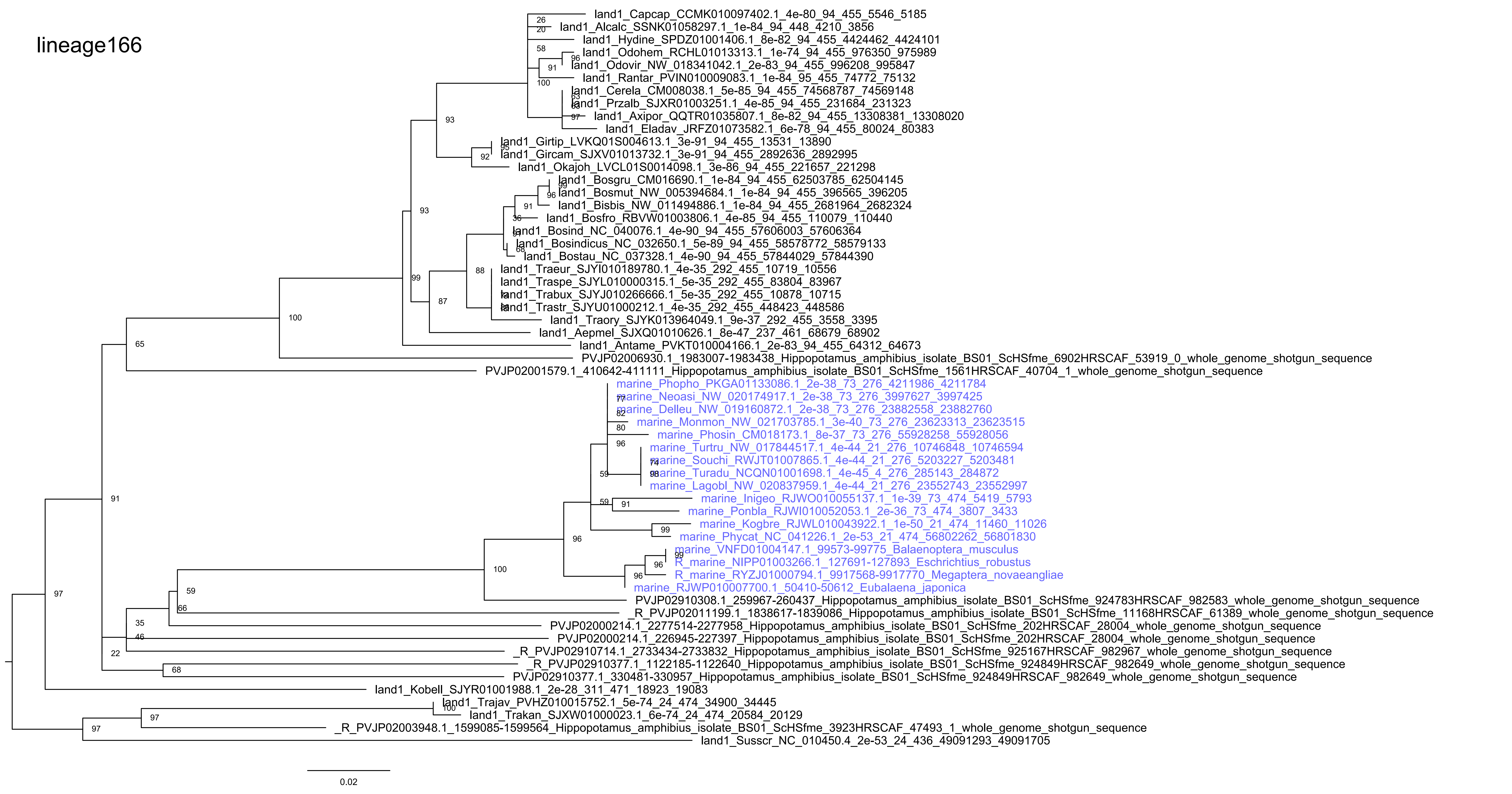
lineage166
land1_Capcap_CCMK010097402.1_4e-80_94_455_5546_5185
land1_Alcalc_SSNK01058297.1_1e-84_94_448_4210_3856
land1_Hydine_SPDZ01001406.1_8e-82_94_455_4424462_4424101
land1_Odohem_RCHL01013313.1_1e-74_94_455_976350_975989
land1_Odovir_NW_018341042.1_2e-83_94_455_996208_995847
land1_Rantar_PVIN010009083.1_1e-84_95_455_74772_75132
land1_Cerela_CM008038.1_5e-85_94_455_74568787_74569148
land1_Przalb_SJXR01003251.1_4e-85_94_455_231684_231323
land1_Axipor_QQTR01035807.1_8e-82_94_455_13308381_13308020
land1_Eladav_JRFZ01073582.1_6e-78_94_455_80024_80383
land1_Girtip_LVKQ01S004613.1_3e-91_94_455_13531_13890
land1_Gircam_SJXV01013732.1_3e-91_94_455_2892636_2892995
land1_Okajoh_LVCL01S0014098.1_3e-86_94_455_221657_221298
land1_Bosgru_CM016690.1_1e-84_94_455_62503785_62504145
land1_Bosmut_NW_005394684.1_1e-84_94_455_396565_396205
land1_Bisbis_NW_011494886.1_1e-84_94_455_2681964_2682324
land1_Bosfro_RBVW01003806.1_4e-85_94_455_110079_110440
land1_Bosind_NC_040076.1_4e-90_94_455_57606003_57606364
land1_Bosindicus_NC_032650.1_5e-89_94_455_58578772_58579133
land1_Bostau_NC_037328.1_4e-90_94_455_57844029_57844390
land1_Traeur_SJYI010189780.1_4e-35_292_455_10719_10556
land1_Traspe_SJYL010000315.1_5e-35_292_455_83804_83967
land1_Trabux_SJYJ010266666.1_5e-35_292_455_10878_10715
land1_Trastr_SJYU01000212.1_4e-35_292_455_448423_448586
land1_Traory_SJYK013964049.1_9e-37_292_455_3558_3395
land1_Aepmel_SJXQ01010626.1_8e-47_237_461_68679_68902
land1_Antame_PVKT010004166.1_2e-83_94_455_64312_64673
PVJP02006930.1_1983007-1983438_Hippopotamus_amphibius_isolate_BS01_ScHSfme_6902HRSCAF_53919_0_whole_genome_shotgun_sequence
PVJP02001579.1_410642-411111_Hippopotamus_amphibius_isolate_BS01_ScHSfme_1561HRSCAF_40704_1_whole_genome_shotgun_sequence
marine_Phopho_PKGA01133086.1_2e-38_73_276_4211986_4211784
marine_Neoasi_NW_020174917.1_2e-38_73_276_3997627_3997425
marine_Delleu_NW_019160872.1_2e-38_73_276_23882558_23882760
marine_Monmon_NW_021703785.1_3e-40_73_276_23623313_23623515
marine_Phosin_CM018173.1_8e-37_73_276_55928258_55928056
marine_Turtru_NW_017844517.1_4e-44_21_276_10746848_10746594
marine_Souchi_RWJT01007865.1_4e-44_21_276_5203227_5203481
marine_Turadu_NCQN01001698.1_4e-45_4_276_285143_284872
marine_Lagobl_NW_020837959.1_4e-44_21_276_23552743_23552997
marine_Inigeo_RJWO010055137.1_1e-39_73_474_5419_5793
marine_Ponbla_RJWI010052053.1_2e-36_73_474_3807_3433
marine_Kogbre_RJWL010043922.1_1e-50_21_474_11460_11026
marine_Phycat_NC_041226.1_2e-53_21_474_56802262_56801830
marine_VNFD01004147.1_99573-99775_Balaenoptera_musculus
R_marine_NIPP01003266.1_127691-127893_Eschrichtius_robustus
R_marine_RYZJ01000794.1_9917568-9917770_Megaptera_novaeangliae
marine_RJWP010007700.1_50410-50612_Eubalaena_japonica
PVJP02910308.1_259967-260437_Hippopotamus_amphibius_isolate_BS01_ScHSfme_924783HRSCAF_982583_whole_genome_shotgun_sequence
_R_PVJP02011199.1_1838617-1839086_Hippopotamus_amphibius_isolate_BS01_ScHSfme_11168HRSCAF_61389_whole_genome_shotgun_sequence
PVJP02000214.1_2277514-2277958_Hippopotamus_amphibius_isolate_BS01_ScHSfme_202HRSCAF_28004_whole_genome_shotgun_sequence
PVJP02000214.1_226945-227397_Hippopotamus_amphibius_isolate_BS01_ScHSfme_202HRSCAF_28004_whole_genome_shotgun_sequence
_R_PVJP02910714.1_2733434-2733832_Hippopotamus_amphibius_isolate_BS01_ScHSfme_925167HRSCAF_982967_whole_genome_shotgun_sequence
_R_PVJP02910377.1_1122185-1122640_Hippopotamus_amphibius_isolate_BS01_ScHSfme_924849HRSCAF_982649_whole_genome_shotgun_sequence
PVJP02910377.1_330481-330957_Hippopotamus_amphibius_isolate_BS01_ScHSfme_924849HRSCAF_982649_whole_genome_shotgun_sequence
land1_Kobell_SJYR01001988.1_2e-28_311_471_18923_19083
land1_Trajav_PVHZ010015752.1_5e-74_24_474_34900_34445
land1_Trakan_SJXW01000023.1_6e-74_24_474_20584_20129
_R_PVJP02003948.1_1599085-1599564_Hippopotamus_amphibius_isolate_BS01_ScHSfme_3923HRSCAF_47493_1_whole_genome_shotgun_sequence
land1_Susscr_NC_010450.4_2e-53_24_436_49091293_49091705
0.02

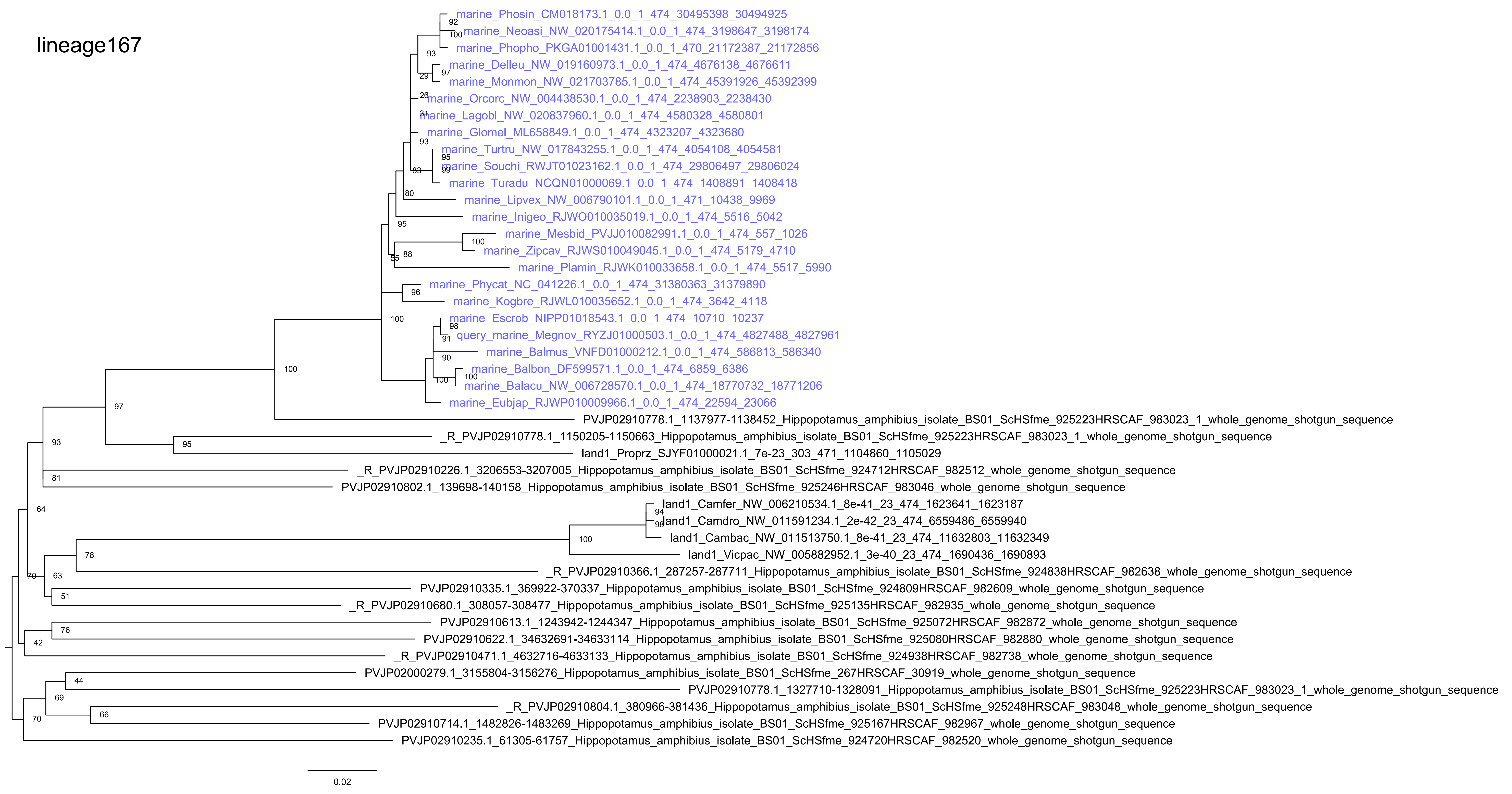
lineage167
marine_Phosin_CM018173.1_0.0_1_474_30495398_30494925
marine_Neoasi_NW_020175414.1_0.0_1_474_3198647_3198174
marine_Phopho_PKGA01001431.1_0.0_1_470_21172387_21172856
marine_Delleu_NW_019160973.1_0.0_1_474_4676138_4676611
marine_Monmon_NW_021703785.1_0.0_1_474_45391926_45392399
marine_Orcorc_NW_004438530.1_0.0_1_474_2238903_2238430
marine_Lagobl_NW_020837960.1_0.0_1_474_4580328_4580801
marine_Glomel_ML658849.1_0.0_1_474_4323207_4323680
marine_Turtru_NW_017843255.1_0.0_1_474_4054108_4054581
marine_Souchi_RWJT01023162.1_0.0_1_474_29806497_29806024
marine_Turadu_NCQN01000069.1_0.0_1_474_1408891_1408418
marine_Lipvex_NW_006790101.1_0.0_1_471_10438_9969
marine_Inigeo_RJWO010035019.1_0.0_1_474_5516_5042
marine_Mesbid_PVJJ010082991.1_0.0_1_474_557_1026
marine_Zipcav_RJWS010049045.1_0.0_1_474_5179_4710
marine_Plamin_RJWK010033658.1_0.0_1_474_5517_5990
marine_Phycat_NC_041226.1_0.0_1_474_31380363_31379890
marine_Kogbre_RJWL010035652.1_0.0_1_474_3642_4118
marine_Escrob_NIPP01018543.1_0.0_1_474_10710_10237
query_marine_Megnov_RYZJ01000503.1_0.0_1_474_4827488_4827961
marine_Balmus_VNFD01000212.1_0.0_1_474_586813_586340
marine_Balbon_DF599571.1_0.0_1_474_6859_6386
marine_Balacu_NW_006728570.1_0.0_1_474_18770732_18771206
marine_Eubjap_RJWP010009966.1_0.0_1_474_22594_23066
PVJP02910778.1_1137977-1138452_Hippopotamus_amphibius_isolate_BS01_ScHSfme_925223HRSCAF_983023_1_whole_genome_shotgun_sequence
_R_PVJP02910778.1_1150205-1150663_Hippopotamus_amphibius_isolate_BS01_ScHSfme_925223HRSCAF_983023_1_whole_genome_shotgun_sequence
land1_Proprz_SJYF01000021.1_7e-23_303_471_1104860_1105029
_R_PVJP02910226.1_3206553-3207005_Hippopotamus_amphibius_isolate_BS01_ScHSfme_924712HRSCAF_982512_whole_genome_shotgun_sequence
PVJP02910802.1_139698-140158_Hippopotamus_amphibius_isolate_BS01_ScHSfme_925246HRSCAF_983046_whole_genome_shotgun_sequence
land1_Camfer_NW_006210534.1_8e-41_23_474_1623641_1623187
land1_Camdro_NW_011591234.1_2e-42_23_474_6559486_6559940
land1_Cambac_NW_011513750.1_8e-41_23_474_11632803_11632349
land1_Vicpac_NW_005882952.1_3e-40_23_474_1690436_1690893
_R_PVJP02910366.1_287257-287711_Hippopotamus_amphibius_isolate_BS01_ScHSfme_924838HRSCAF_982638_whole_genome_shotgun_sequence
PVJP02910335.1_369922-370337_Hippopotamus_amphibius_isolate_BS01_ScHSfme_924809HRSCAF_982609_whole_genome_shotgun_sequence
_R_PVJP02910680.1_308057-308477_Hippopotamus_amphibius_isolate_BS01_ScHSfme_925135HRSCAF_982935_whole_genome_shotgun_sequence
PVJP02910613.1_1243942-1244347_Hippopotamus_amphibius_isolate_BS01_ScHSfme_925072HRSCAF_982872_whole_genome_shotgun_sequence
PVJP02910622.1_34632691-34633114_Hippopotamus_amphibius_isolate_BS01_ScHSfme_925080HRSCAF_982880_whole_genome_shotgun_sequence
_R_PVJP02910471.1_4632716-4633133_Hippopotamus_amphibius_isolate_BS01_ScHSfme_924938HRSCAF_982738_whole_genome_shotgun_sequence
PVJP02000279.1_3155804-3156276_Hippopotamus_amphibius_isolate_BS01_ScHSfme_267HRSCAF_30919_whole_genome_shotgun_sequence
PVJP02910778.1_1327710-1328091_Hippopotamus_amphibius_isolate_BS01_ScHSfme_925223HRSCAF_983023_1_whole_genome_shotgun_sequence
_R_PVJP02910804.1_380966-381436_Hippopotamus_amphibius_isolate_BS01_ScHSfme_925248HRSCAF_983048_whole_genome_shotgun_sequence
PVJP02910714.1_1482826-1483269_Hippopotamus_amphibius_isolate_BS01_ScHSfme_925167HRSCAF_982967_whole_genome_shotgun_sequence
PVJP02910235.1_61305-61757_Hippopotamus_amphibius_isolate_BS01_ScHSfme_924720HRSCAF_982520_whole_genome_shotgun_sequence
0.02

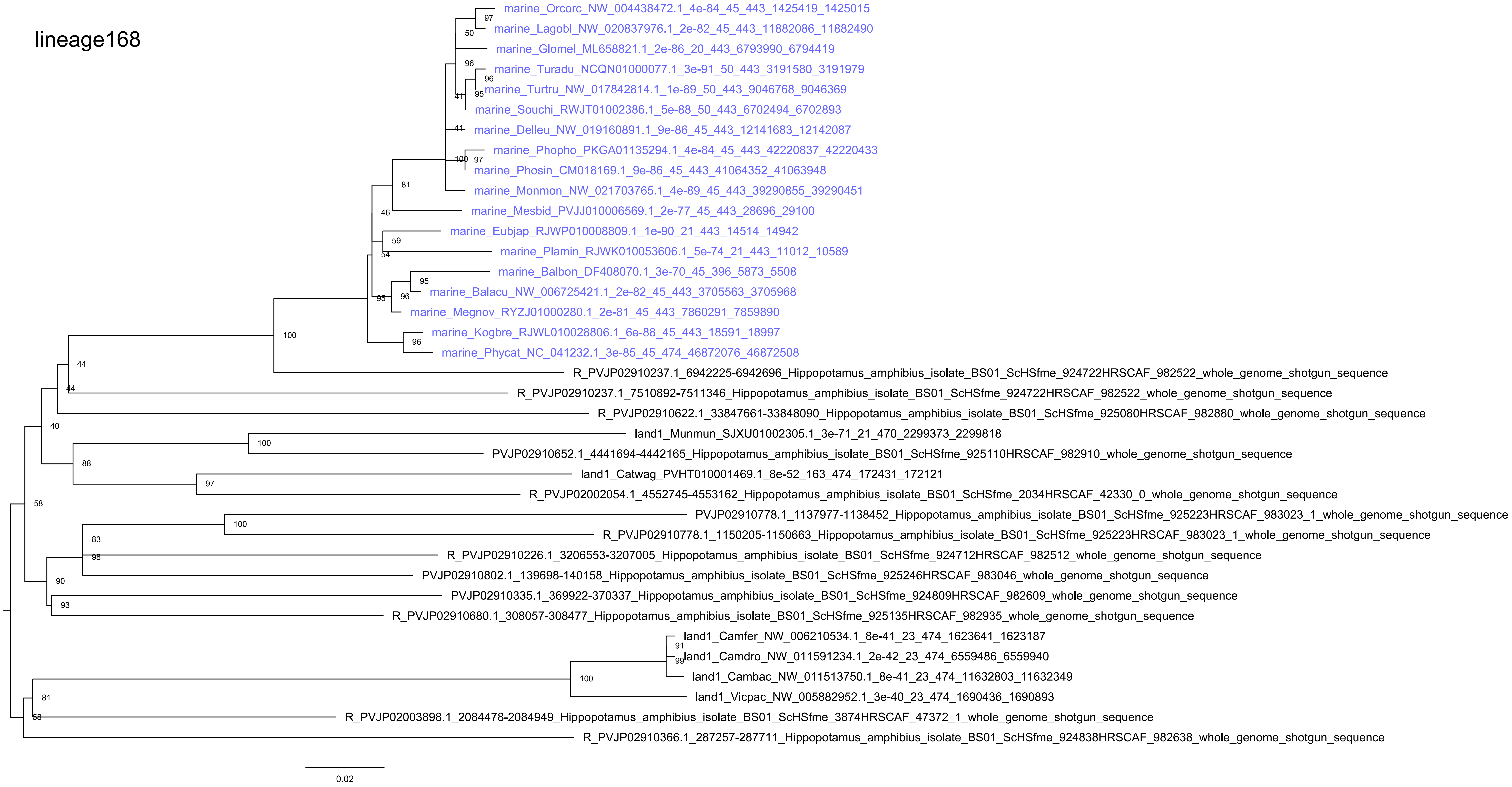

lineage168
marine_Orcorc_NW_004438472.1_4e-84_45_443_1425419_1425015
marine_Lagobl_NW_020837976.1_2e-82_45_443_11882086_11882490
marine_Glomel_ML658821.1_2e-86_20_443_6793990_6794419
marine_Turadu_NCQN01000077.1_3e-91_50_443_3191580_3191979
marine_Turtru_NW_017842814.1_1e-89_50_443_9046768_9046369
marine_Souchi_RWJT01002386.1_5e-88_50_443_6702494_6702893
marine_Delleu_NW_019160891.1_9e-86_45_443_12141683_12142087
marine_Phopho_PKGA01135294.1_4e-84_45_443_42220837_42220433
marine_Phosin_CM018169.1_9e-86_45_443_41064352_41063948
marine_Monmon_NW_021703765.1_4e-89_45_443_39290855_39290451
marine_Mesbid_PVJJ010006569.1_2e-77_45_443_28696_29100
marine_Eubjap_RJWP010008809.1_1e-90_21_443_14514_14942
marine_Plamin_RJWK010053606.1_5e-74_21_443_11012_10589
marine_Balbon_DF408070.1_3e-70_45_396_5873_5508
marine_Balacu_NW_006725421.1_2e-82_45_443_3705563_3705968
marine_Megnov_RYZJ01000280.1_2e-81_45_443_7860291_7859890
marine_Kogbre_RJWL010028806.1_6e-88_45_443_18591_18997
marine_Phycat_NC_041232.1_3e-85_45_474_46872076_46872508
R_PVJP02910237.1_6942225-6942696_Hippopotamus_amphibius_isolate_BS01_ScHSfme_924722HRSCAF_982522_whole_genome_shotgun_sequence
R_PVJP02910237.1_7510892-7511346_Hippopotamus_amphibius_isolate_BS01_ScHSfme_924722HRSCAF_982522_whole_genome_shotgun_sequence
R_PVJP02910622.1_33847661-33848090_Hippopotamus_amphibius_isolate_BS01_ScHSfme_925080HRSCAF_982880_whole_genome_shotgun_sequence
land1_Munmun_SJXU01002305.1_3e-71_21_470_2299373_2299818
PVJP02910652.1_4441694-4442165_Hippopotamus_amphibius_isolate_BS01_ScHSfme_925110HRSCAF_982910_whole_genome_shotgun_sequence
land1_Catwag_PVHT010001469.1_8e-52_163_474_172431_172121
R_PVJP02002054.1_4552745-4553162_Hippopotamus_amphibius_isolate_BS01_ScHSfme_2034HRSCAF_42330_0_whole_genome_shotgun_sequence
PVJP02910778.1_1137977-1138452_Hippopotamus_amphibius_isolate_BS01_ScHSfme_925223HRSCAF_983023_1_whole_genome_shotgun_sequence
R_PVJP02910778.1_1150205-1150663_Hippopotamus_amphibius_isolate_BS01_ScHSfme_925223HRSCAF_983023_1_whole_genome_shotgun_sequence
R_PVJP02910226.1_3206553-3207005_Hippopotamus_amphibius_isolate_BS01_ScHSfme_924712HRSCAF_982512_whole_genome_shotgun_sequence
PVJP02910802.1_139698-140158_Hippopotamus_amphibius_isolate_BS01_ScHSfme_925246HRSCAF_983046_whole_genome_shotgun_sequence
PVJP02910335.1_369922-370337_Hippopotamus_amphibius_isolate_BS01_ScHSfme_924809HRSCAF_982609_whole_genome_shotgun_sequence
R_PVJP02910680.1_308057-308477_Hippopotamus_amphibius_isolate_BS01_ScHSfme_925135HRSCAF_982935_whole_genome_shotgun_sequence
land1_Camfer_NW_006210534.1_8e-41_23_474_1623641_1623187
land1_Camdro_NW_011591234.1_2e-42_23_474_6559486_6559940
land1_Cambac_NW_011513750.1_8e-41_23_474_11632803_11632349
land1_Vicpac_NW_005882952.1_3e-40_23_474_1690436_1690893
R_PVJP02003898.1_2084478-2084949_Hippopotamus_amphibius_isolate_BS01_ScHSfme_3874HRSCAF_47372_1_whole_genome_shotgun_sequence
R_PVJP02910366.1_287257-287711_Hippopotamus_amphibius_isolate_BS01_ScHSfme_924838HRSCAF_982638_whole_genome_shotgun_sequence
0.02

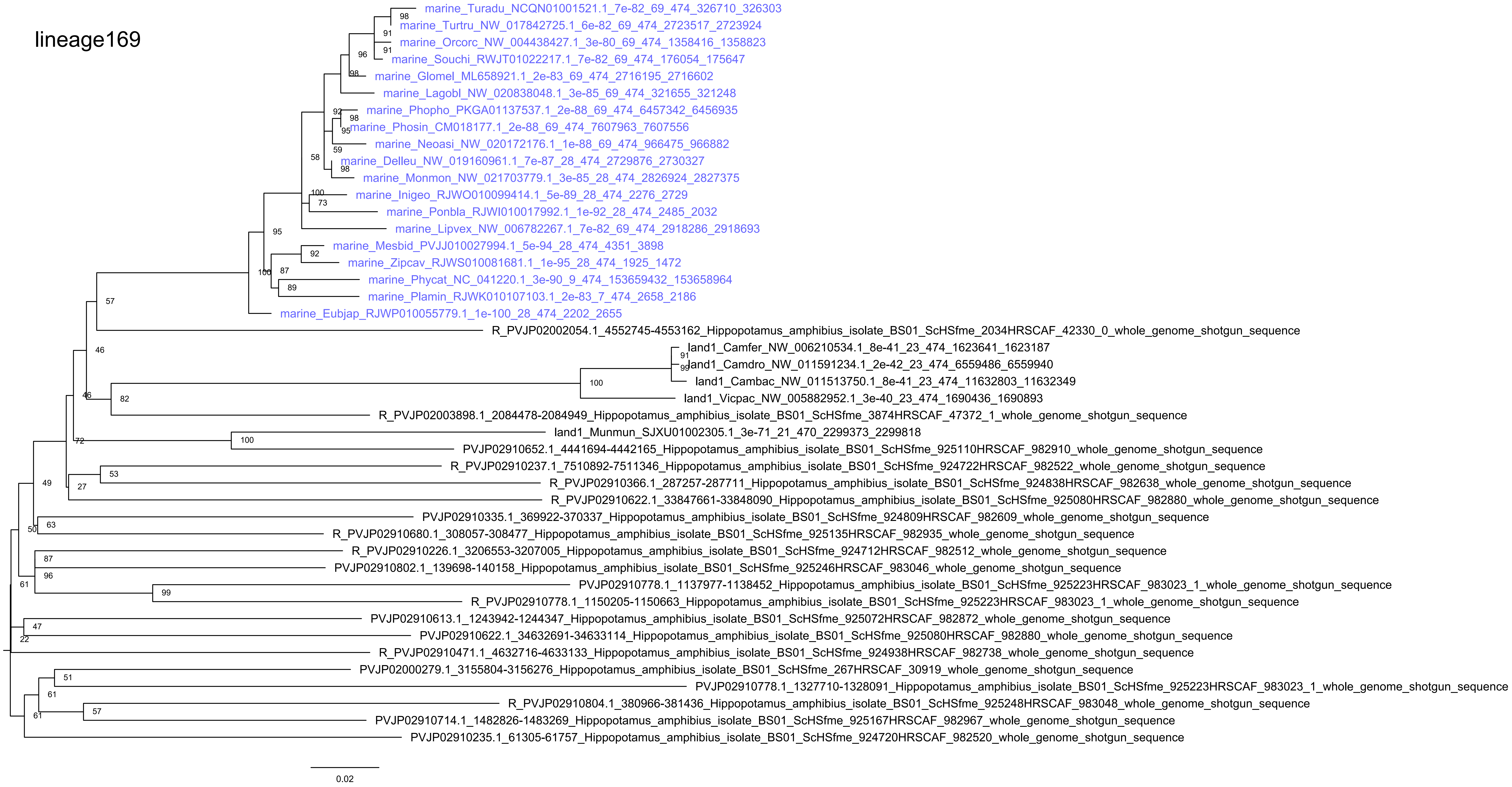

lineage169
marine_Turadu_NCQN01001521.1_7e-82_69_474_326710_326303
marine_Turtru_NW_017842725.1_6e-82_69_474_2723517_2723924
marine_Orcorc_NW_004438427.1_3e-80_69_474_1358416_1358823
marine_Souchi_RWJT01022217.1_7e-82_69_474_176054_175647
marine_Glomel_ML658921.1_2e-83_69_474_2716195_2716602
marine_Lagobl_NW_020838048.1_3e-85_69_474_321655_321248
marine_Phopho_PKGA01137537.1_2e-88_69_474_6457342_6456935
marine_Phosin_CM018177.1_2e-88_69_474_7607963_7607556
marine_Neoasi_NW_020172176.1_1e-88_69_474_966475_966882
marine_Delleu_NW_019160961.1_7e-87_28_474_2729876_2730327
marine_Monmon_NW_021703779.1_3e-85_28_474_2826924_2827375
marine_Inigeo_RJWO010099414.1_5e-89_28_474_2276_2729
marine_Ponbla_RJWI010017992.1_1e-92_28_474_2485_2032
marine_Lipvex_NW_006782267.1_7e-82_69_474_2918286_2918693
marine_Mesbid_PVJJ010027994.1_5e-94_28_474_4351_3898
marine_Zipcav_RJWS010081681.1_1e-95_28_474_1925_1472
marine_Phycat_NC_041220.1_3e-90_9_474_153659432_153658964
marine_Plamin_RJWK010107103.1_2e-83_7_474_2658_2186
marine_Eubjap_RJWP010055779.1_1e-100_28_474_2202_2655
R_PVJP02002054.1_4552745-4553162_Hippopotamus_amphibius_isolate_BS01_ScHSfme_2034HRSCAF_42330_0_whole_genome_shotgun_sequence
land1_Camfer_NW_006210534.1_8e-41_23_474_1623641_1623187
land1_Camdro_NW_011591234.1_2e-42_23_474_6559486_6559940
land1_Cambac_NW_011513750.1_8e-41_23_474_11632803_11632349
land1_Vicpac_NW_005882952.1_3e-40_23_474_1690436_1690893
R_PVJP02003898.1_2084478-2084949_Hippopotamus_amphibius_isolate_BS01_ScHSfme_3874HRSCAF_47372_1_whole_genome_shotgun_sequence
land1_Munmun_SJXU01002305.1_3e-71_21_470_2299373_2299818
PVJP02910652.1_4441694-4442165_Hippopotamus_amphibius_isolate_BS01_ScHSfme_925110HRSCAF_982910_whole_genome_shotgun_sequence
R_PVJP02910237.1_7510892-7511346_Hippopotamus_amphibius_isolate_BS01_ScHSfme_924722HRSCAF_982522_whole_genome_shotgun_sequence
R_PVJP02910366.1_287257-287711_Hippopotamus_amphibius_isolate_BS01_ScHSfme_924838HRSCAF_982638_whole_genome_shotgun_sequence
R_PVJP02910622.1_33847661-33848090_Hippopotamus_amphibius_isolate_BS01_ScHSfme_925080HRSCAF_982880_whole_genome_shotgun_sequence
PVJP02910335.1_369922-370337_Hippopotamus_amphibius_isolate_BS01_ScHSfme_924809HRSCAF_982609_whole_genome_shotgun_sequence
R_PVJP02910680.1_308057-308477_Hippopotamus_amphibius_isolate_BS01_ScHSfme_925135HRSCAF_982935_whole_genome_shotgun_sequence
R_PVJP02910226.1_3206553-3207005_Hippopotamus_amphibius_isolate_BS01_ScHSfme_924712HRSCAF_982512_whole_genome_shotgun_sequence
PVJP02910802.1_139698-140158_Hippopotamus_amphibius_isolate_BS01_ScHSfme_925246HRSCAF_983046_whole_genome_shotgun_sequence
PVJP02910778.1_1137977-1138452_Hippopotamus_amphibius_isolate_BS01_ScHSfme_925223HRSCAF_983023_1_whole_genome_shotgun_sequence
R_PVJP02910778.1_1150205-1150663_Hippopotamus_amphibius_isolate_BS01_ScHSfme_925223HRSCAF_983023_1_whole_genome_shotgun_sequence
PVJP02910613.1_1243942-1244347_Hippopotamus_amphibius_isolate_BS01_ScHSfme_925072HRSCAF_982872_whole_genome_shotgun_sequence
PVJP02910622.1_34632691-34633114_Hippopotamus_amphibius_isolate_BS01_ScHSfme_925080HRSCAF_982880_whole_genome_shotgun_sequence
R_PVJP02910471.1_4632716-4633133_Hippopotamus_amphibius_isolate_BS01_ScHSfme_924938HRSCAF_982738_whole_genome_shotgun_sequence
PVJP02000279.1_3155804-3156276_Hippopotamus_amphibius_isolate_BS01_ScHSfme_267HRSCAF_30919_whole_genome_shotgun_sequence
PVJP02910778.1_1327710-1328091_Hippopotamus_amphibius_isolate_BS01_ScHSfme_925223HRSCAF_983023_1_whole_genome_shotgun_sequence
R_PVJP02910804.1_380966-381436_Hippopotamus_amphibius_isolate_BS01_ScHSfme_925248HRSCAF_983048_whole_genome_shotgun_sequence
PVJP02910714.1_1482826-1483269_Hippopotamus_amphibius_isolate_BS01_ScHSfme_925167HRSCAF_982967_whole_genome_shotgun_sequence
PVJP02910235.1_61305-61757_Hippopotamus_amphibius_isolate_BS01_ScHSfme_924720HRSCAF_982520_whole_genome_shotgun_sequence
0.02

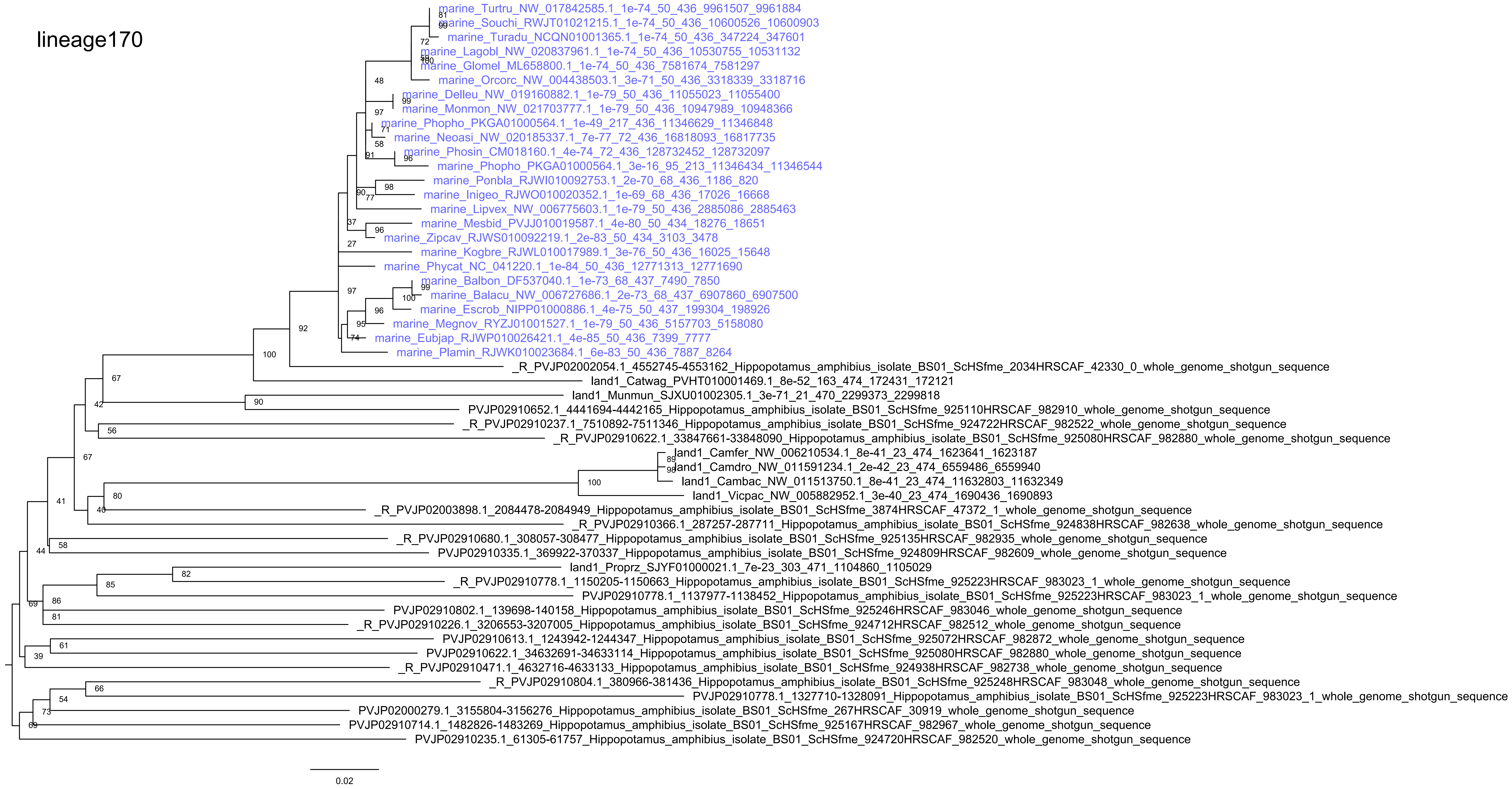
lineage170
marine_Turtru_NW_017842585.1_1e-74_50_436_9961507_9961884
marine_Souchi_RWJT01021215.1_1e-74_50_436_10600526_10600903
marine_Turadu_NCQN01001365.1_1e-74_50_436_347224_347601
marine_Lagobl_NW_020837961.1_1e-74_50_436_10530755_10531132
marine_Glomel_ML658800.1_1e-74_50_436_7581674_7581297
marine_Orcorc_NW_004438503.1_3e-71_50_436_3318339_3318716
marine_Delleu_NW_019160882.1_1e-79_50_436_11055023_11055400
marine_Monmon_NW_021703777.1_1e-79_50_436_10947989_10948366
marine_Phopho_PKGA01000564.1_1e-49_217_436_11346629_11346848
marine_Neoasi_NW_020185337.1_7e-77_72_436_16818093_16817735
marine_Phosin_CM018160.1_4e-74_72_436_128732452_128732097
marine_Phopho_PKGA01000564.1_3e-16_95_213_11346434_11346544
marine_Ponbla_RJWI010092753.1_2e-70_68_436_1186_820
marine_Inigeo_RJWO010020352.1_1e-69_68_436_17026_16668
marine_Lipvex_NW_006775603.1_1e-79_50_436_2885086_2885463
marine_Mesbid_PVJJ010019587.1_4e-80_50_434_18276_18651
marine_Zipcav_RJWS010092219.1_2e-83_50_434_3103_3478
marine_Kogbre_RJWL010017989.1_3e-76_50_436_16025_15648
marine_Phycat_NC_041220.1_1e-84_50_436_12771313_12771690
marine_Balbon_DF537040.1_1e-73_68_437_7490_7850
marine_Balacu_NW_006727686.1_2e-73_68_437_6907860_6907500
marine_Escrob_NIPP01000886.1_4e-75_50_437_199304_198926
marine_Megnov_RYZJ01001527.1_1e-79_50_436_5157703_5158080
marine_Eubjap_RJWP010026421.1_4e-85_50_436_7399_7777
marine_Plamin_RJWK010023684.1_6e-83_50_436_7887_8264
_R_PVJP02002054.1_4552745-4553162_Hippopotamus_amphibius_isolate_BS01_ScHSfme_2034HRSCAF_42330_0_whole_genome_shotgun_sequence
land1_Catwag_PVHT010001469.1_8e-52_163_474_172431_172121
land1_Munmun_SJXU01002305.1_3e-71_21_470_2299373_2299818
PVJP02910652.1_4441694-4442165_Hippopotamus_amphibius_isolate_BS01_ScHSfme_925110HRSCAF_982910_whole_genome_shotgun_sequence
_R_PVJP02910237.1_7510892-7511346_Hippopotamus_amphibius_isolate_BS01_ScHSfme_924722HRSCAF_982522_whole_genome_shotgun_sequence
_R_PVJP02910622.1_33847661-33848090_Hippopotamus_amphibius_isolate_BS01_ScHSfme_925080HRSCAF_982880_whole_genome_shotgun_sequence
land1_Camfer_NW_006210534.1_8e-41_23_474_1623641_1623187
land1_Camdro_NW_011591234.1_2e-42_23_474_6559486_6559940
land1_Cambac_NW_011513750.1_8e-41_23_474_11632803_11632349
land1_Vicpac_NW_005882952.1_3e-40_23_474_1690436_1690893
_R_PVJP02003898.1_2084478-2084949_Hippopotamus_amphibius_isolate_BS01_ScHSfme_3874HRSCAF_47372_1_whole_genome_shotgun_sequence
_R_PVJP02910366.1_287257-287711_Hippopotamus_amphibius_isolate_BS01_ScHSfme_924838HRSCAF_982638_whole_genome_shotgun_sequence
_R_PVJP02910680.1_308057-308477_Hippopotamus_amphibius_isolate_BS01_ScHSfme_925135HRSCAF_982935_whole_genome_shotgun_sequence
PVJP02910335.1_369922-370337_Hippopotamus_amphibius_isolate_BS01_ScHSfme_924809HRSCAF_982609_whole_genome_shotgun_sequence
land1_Proprz_SJYF01000021.1_7e-23_303_471_1104860_1105029
_R_PVJP02910778.1_1150205-1150663_Hippopotamus_amphibius_isolate_BS01_ScHSfme_925223HRSCAF_983023_1_whole_genome_shotgun_sequence
PVJP02910778.1_1137977-1138452_Hippopotamus_amphibius_isolate_BS01_ScHSfme_925223HRSCAF_983023_1_whole_genome_shotgun_sequence
PVJP02910802.1_139698-140158_Hippopotamus_amphibius_isolate_BS01_ScHSfme_925246HRSCAF_983046_whole_genome_shotgun_sequence
_R_PVJP02910226.1_3206553-3207005_Hippopotamus_amphibius_isolate_BS01_ScHSfme_924712HRSCAF_982512_whole_genome_shotgun_sequence
PVJP02910613.1_1243942-1244347_Hippopotamus_amphibius_isolate_BS01_ScHSfme_925072HRSCAF_982872_whole_genome_shotgun_sequence
PVJP02910622.1_34632691-34633114_Hippopotamus_amphibius_isolate_BS01_ScHSfme_925080HRSCAF_982880_whole_genome_shotgun_sequence
_R_PVJP02910471.1_4632716-4633133_Hippopotamus_amphibius_isolate_BS01_ScHSfme_924938HRSCAF_982738_whole_genome_shotgun_sequence
_R_PVJP02910804.1_380966-381436_Hippopotamus_amphibius_isolate_BS01_ScHSfme_925248HRSCAF_983048_whole_genome_shotgun_sequence
PVJP02910778.1_1327710-1328091_Hippopotamus_amphibius_isolate_BS01_ScHSfme_925223HRSCAF_983023_1_whole_genome_shotgun_sequence
PVJP02000279.1_3155804-3156276_Hippopotamus_amphibius_isolate_BS01_ScHSfme_267HRSCAF_30919_whole_genome_shotgun_sequence
PVJP02910714.1_1482826-1483269_Hippopotamus_amphibius_isolate_BS01_ScHSfme_925167HRSCAF_982967_whole_genome_shotgun_sequence
PVJP02910235.1_61305-61757_Hippopotamus_amphibius_isolate_BS01_ScHSfme_924720HRSCAF_982520_whole_genome_shotgun_sequence
0.02

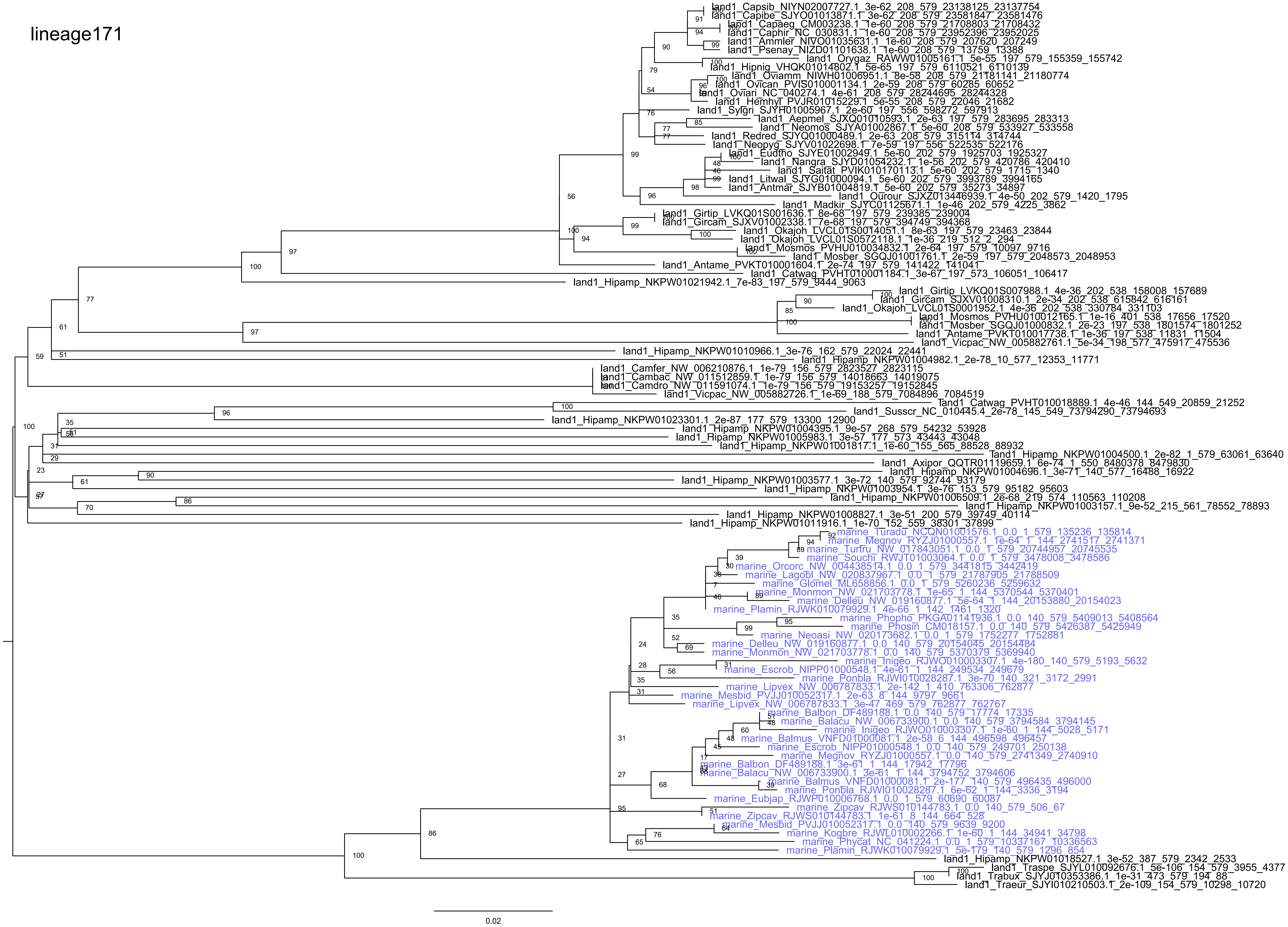
lineage171
land1_Capsib_NIYN02007727.1_3e-62_208_579_23138125_23137754
land1_Capibe_SJYO01013871.1_3e-62_208_579_23581847_23581476
land1_Capaeg_CM003238.1_1e-60_208_579_21708803_21708432
land1_Caphir_NC_030831.1_1e-60_208_579_23952396_23952025
land1_Ammler_NIVO01035631.1_1e-60_208_579_207620_207249
land1_Psenay_NIZD01101638.1_1e-60_208_579_13759_13388
land1_Orygaz_RAWW01005161.1_5e-55_197_579_155359_155742
land1_Hipnig_VHQK01014802.1_5e-65_197_579_6110521_6110139
land1_Oviamm_NIWH01006951.1_8e-58_208_579_21181141_21180774
land1_Ovican_PVIS010001134.1_2e-59_208_579_60285_60652
land1_Oviari_NC_040274.1_4e-61_208_579_28244695_28244328
land1_Hemhyl_PVJR01015229.1_5e-55_208_579_22046_21682
land1_Sylgri_SJYH01005967.1_2e-60_197_556_598272_597913
land1_Aepmel_SJXQ01010593.1_2e-63_197_579_283695_283313
land1_Neomos_SJYA01002867.1_5e-60_208_579_533927_533558
land1_Redred_SJYQ01000489.1_2e-63_208_579_315114_314744
land1_Neopyg_SJYV01022698.1_7e-59_197_556_522535_522176
land1_Eudtho_SJYE01002949.1_5e-60_202_579_1925703_1925327
land1_Nangra_SJYD01054232.1_1e-56_202_579_420786_420410
land1_Saitat_PVIK010170113.1_5e-60_202_579_1715_1340
land1_Litwal_SJYG01000094.1_5e-60_202_579_3993789_3994165
land1_Antmar_SJYB01004819.1_5e-60_202_579_35273_34897
land1_Ourour_SJXZ013446939.1_4e-50_202_579_1420_1795
land1_Madkir_SJYC01125671.1_1e-46_202_579_4225_3862
land1_Girtip_LVKQ01S001636.1_8e-68_197_579_239385_239004
land1_Gircam_SJXV01002338.1_7e-68_197_579_394749_394368
land1_Okajoh_LVCL01S0014051.1_8e-63_197_579_23463_23844
land1_Okajoh_LVCL01S0572118.1_1e-36_219_512_2_294
land1_Mosmos_PVHU010034832.1_2e-64_197_579_10097_9716
land1_Mosber_SGQJ01001761.1_2e-59_197_579_2048573_2048953
land1_Antame_PVKT010001604.1_2e-74_197_579_141422_141041
land1_Catwag_PVHT010001184.1_3e-67_197_573_106051_106417
land1_Hipamp_NKPW01021942.1_7e-83_197_579_9444_9063
land1_Girtip_LVKQ01S007988.1_4e-36_202_538_158008_157689
land1_Gircam_SJXV01008310.1_2e-34_202_538_615842_616161
land1_Okajoh_LVCL01S0001952.1_4e-36_202_538_330784_331103
land1_Mosmos_PVHU010012165.1_1e-16_401_538_17656_17520
land1_Mosber_SGQJ01000832.1_2e-23_197_538_1801574_1801252
land1_Antame_PVKT010017738.1_1e-36_197_538_11831_11504
land1_Vicpac_NW_005882761.1_5e-34_198_577_475917_475536
land1_Hipamp_NKPW01010966.1_3e-76_162_579_22024_22441
land1_Hipamp_NKPW01004982.1_2e-78_10_577_12353_11771
land1_Camfer_NW_006210876.1_1e-79_156_579_2823527_2823115
land1_Cambac_NW_011512859.1_1e-79_156_579_14018663_14019075
land1_Camdro_NW_011591074.1_1e-79_156_579_19153257_19152845
land1_Vicpac_NW_005882726.1_1e-69_188_579_7084896_7084519
land1_Catwag_PVHT010018889.1_4e-46_144_549_20859_21252
land1_Susscr_NC_010445.4_2e-78_145_549_73794290_73794693
land1_Hipamp_NKPW01023301.1_2e-87_177_579_13300_12900
land1_Hipamp_NKPW01004395.1_9e-57_268_579_54232_53928
land1_Hipamp_NKPW01005983.1_3e-57_177_573_43443_43048
land1_Hipamp_NKPW01001817.1_1e-60_155_565_88528_88932
land1_Hipamp_NKPW01004500.1_2e-82_1_579_63061_63640
land1_Axipor_QQTR01119659.1_6e-74_1_550_8480378_8479830
land1_Hipamp_NKPW01004696.1_3e-71_140_577_16488_16922
land1_Hipamp_NKPW01003577.1_3e-72_140_579_92744_93179
land1_Hipamp_NKPW01003954.1_3e-76_153_579_95182_95603
land1_Hipamp_NKPW01006509.1_2e-68_219_574_110563_110208
land1_Hipamp_NKPW01003157.1_9e-52_215_561_78552_78893
land1_Hipamp_NKPW01008827.1_3e-51_200_579_39749_40114
land1_Hipamp_NKPW01011916.1_1e-70_152_559_38301_37899
marine_Turadu_NCQN01001576.1_0.0_1_579_135236_135814
marine_Megnov_RYZJ01000557.1_1e-64_1_144_2741517_2741371
marine_Turtru_NW_017843051.1_0.0_1_579_20744957_20745535
marine_Souchi_RWJT01003064.1_0.0_1_579_3478008_3478586
marine_Orcorc_NW_004438514.1_0.0_1_579_3441815_3442419
marine_Lagobl_NW_020837967.1_0.0_1_579_21787905_21788509
marine_Glomel_ML658856.1_0.0_1_579_5260236_5259632
marine_Monmon_NW_021703778.1_1e-65_1_144_5370544_5370401
marine_Delleu_NW_019160877.1_5e-64_1_144_20153880_20154023
marine_Plamin_RJWK010079929.1_4e-66_1_142_1461_1320
marine_Phopho_PKGA01141936.1_0.0_140_579_5409013_5408564
marine_Phosin_CM018157.1_0.0_140_579_5426387_5425949
marine_Neoasi_NW_020173682.1_0.0_1_579_1752277_1752881
marine_Delleu_NW_019160877.1_0.0_140_579_20154045_20154484
marine_Monmon_NW_021703778.1_0.0_140_579_5370379_5369940
marine_Inigeo_RJWO010003307.1_4e-180_140_579_5193_5632
marine_Escrob_NIPP01000548.1_4e-61_1_144_249534_249679
marine_Ponbla_RJWI010028287.1_3e-70_140_321_3172_2991
marine_Lipvex_NW_006787833.1_2e-142_1_410_763306_762877
marine_Mesbid_PVJJ010052317.1_2e-63_8_144_9797_9661
marine_Lipvex_NW_006787833.1_3e-47_469_579_762877_762767
marine_Balbon_DF489188.1_0.0_140_579_17774_17335
marine_Balacu_NW_006733900.1_0.0_140_579_3794584_3794145
marine_Inigeo_RJWO010003307.1_1e-60_1_144_5028_5171
marine_Balmus_VNFD01000081.1_2e-58_6_144_496598_496457
marine_Escrob_NIPP01000548.1_0.0_140_579_249701_250138
marine_Megnov_RYZJ01000557.1_0.0_140_579_2741349_2740910
marine_Balbon_DF489188.1_3e-61_1_144_17942_17796
marine_Balacu_NW_006733900.1_3e-61_1_144_3794752_3794606
marine_Balmus_VNFD01000081.1_2e-177_140_579_496435_496000
marine_Ponbla_RJWI010028287.1_6e-62_1_144_3336_3194
marine_Eubjap_RJWP010006768.1_0.0_1_579_60690_60087
marine_Zipcav_RJWS010144783.1_0.0_140_579_506_67
marine_Zipcav_RJWS010144783.1_1e-61_8_144_664_528
marine_Mesbid_PVJJ010052317.1_0.0_140_579_9639_9200
marine_Kogbre_RJWL010002266.1_1e-60_1_144_34941_34798
marine_Phycat_NC_041224.1_0.0_1_579_10337167_10336563
marine_Plamin_RJWK010079929.1_5e-179_140_579_1296_854
land1_Hipamp_NKPW01018527.1_3e-52_387_579_2342_2533
land1_Traspe_SJYL010092676.1_5e-106_154_579_3955_4377
land1_Trabux_SJYJ010353386.1_1e-31_473_579_194_88
land1_Traeur_SJYI010210503.1_2e-109_154_579_10298_10720
0.02

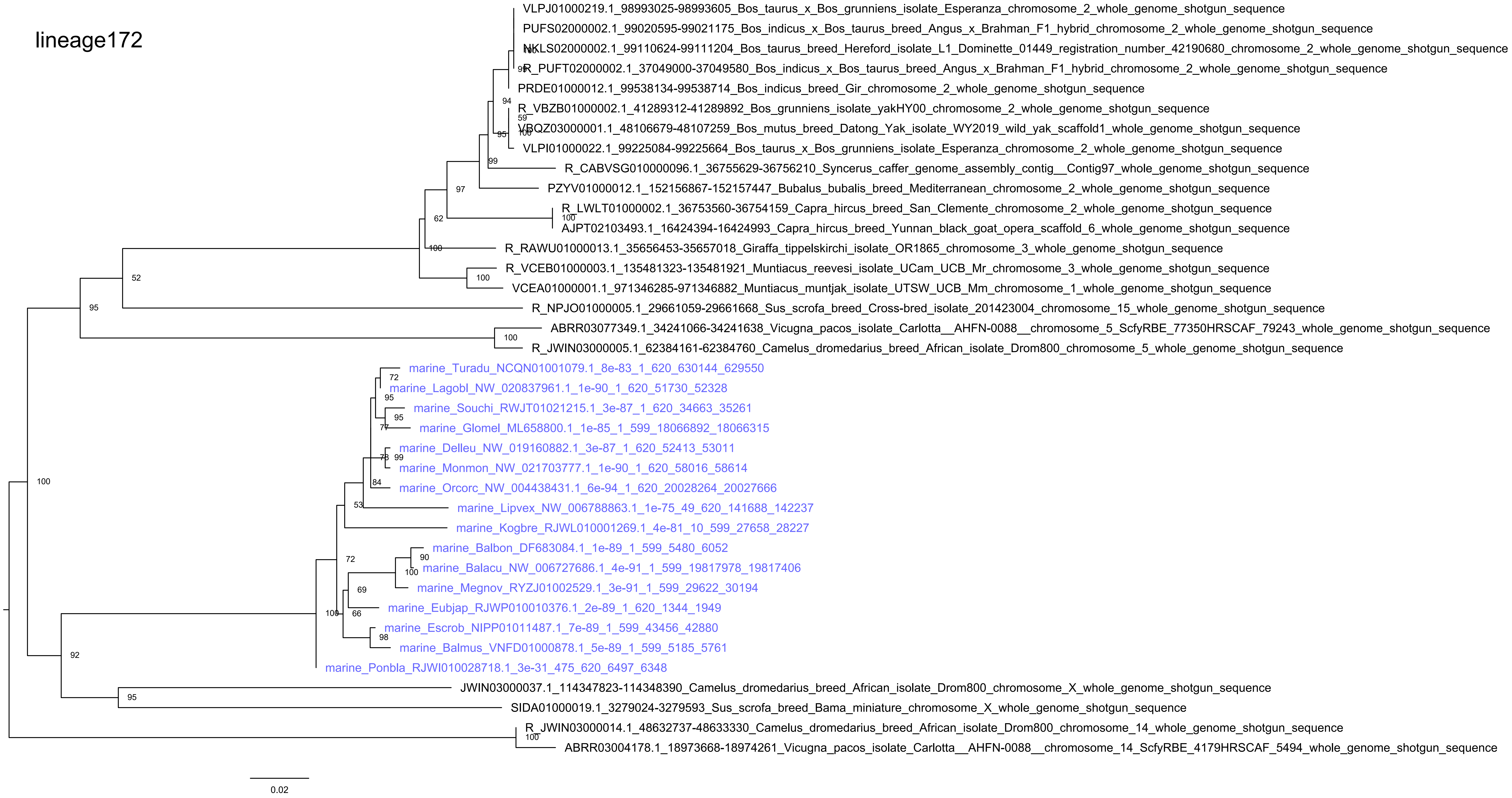
lineage172
VLPJ01000219.1_98993025-98993605_Bos_taurus_x_Bos_grunniens_isolate_Esperanza_chromosome_2_whole_genome_shotgun_sequence
PUFS02000002.1_99020595-99021175_Bos_indicus_x_Bos_taurus_breed_Angus_x_Brahman_F1_hybrid_chromosome_2_whole_genome_shotgun_sequence
NKLS02000002.1_99110624-99111204_Bos_taurus_breed_Hereford_isolate_L1_Dominette_01449_registration_number_42190680_chromosome_2_whole_genome_shotgun_sequence
R_PUFT02000002.1_37049000-37049580_Bos_indicus_x_Bos_taurus_breed_Angus_x_Brahman_F1_hybrid_chromosome_2_whole_genome_shotgun_sequence
PRDE01000012.1_99538134-99538714_Bos_indicus_breed_Gir_chromosome_2_whole_genome_shotgun_sequence
R_VBZB01000002.1_41289312-41289892_Bos_grunniens_isolate_yakHY00_chromosome_2_whole_genome_shotgun_sequence
VBQZ03000001.1_48106679-48107259_Bos_mutus_breed_Datong_Yak_isolate_WY2019_wild_yak_scaffold1_whole_genome_shotgun_sequence
VLPI01000022.1_99225084-99225664_Bos_taurus_x_Bos_grunniens_isolate_Esperanza_chromosome_2_whole_genome_shotgun_sequence
R_CABVSG010000096.1_36755629-36756210_Syncerus_caffer_genome_assembly_contig__Contig97_whole_genome_shotgun_sequence
PZYV01000012.1_152156867-152157447_Bubalus_bubalis_breed_Mediterranean_chromosome_2_whole_genome_shotgun_sequence
R_LWLT01000002.1_36753560-36754159_Capra_hircus_breed_San_Clemente_chromosome_2_whole_genome_shotgun_sequence
AJPT02103493.1_16424394-16424993_Capra_hircus_breed_Yunnan_black_goat_opera_scaffold_6_whole_genome_shotgun_sequence
R_RAWU01000013.1_35656453-35657018_Giraffa_tippelskirchi_isolate_OR1865_chromosome_3_whole_genome_shotgun_sequence
R_VCEB01000003.1_135481323-135481921_Muntiacus_reevesi_isolate_UCam_UCB_Mr_chromosome_3_whole_genome_shotgun_sequence
VCEA01000001.1_971346285-971346882_Muntiacus_muntjak_isolate_UTSW_UCB_Mm_chromosome_1_whole_genome_shotgun_sequence
R_NPJO01000005.1_29661059-29661668_Sus_scrofa_breed_Cross-bred_isolate_201423004_chromosome_15_whole_genome_shotgun_sequence
ABRR03077349.1_34241066-34241638_Vicugna_pacos_isolate_Carlotta__AHFN-0088__chromosome_5_ScfyRBE_77350HRSCAF_79243_whole_genome_shotgun_sequence
R_JWIN03000005.1_62384161-62384760_Camelus_dromedarius_breed_African_isolate_Drom800_chromosome_5_whole_genome_shotgun_sequence
marine_Turadu_NCQN01001079.1_8e-83_1_620_630144_629550
marine_Lagobl_NW_020837961.1_1e-90_1_620_51730_52328
marine_Souchi_RWJT01021215.1_3e-87_1_620_34663_35261
marine_Glomel_ML658800.1_1e-85_1_599_18066892_18066315
marine_Delleu_NW_019160882.1_3e-87_1_620_52413_53011
marine_Monmon_NW_021703777.1_1e-90_1_620_58016_58614
marine_Orcorc_NW_004438431.1_6e-94_1_620_20028264_20027666
marine_Lipvex_NW_006788863.1_1e-75_49_620_141688_142237
marine_Kogbre_RJWL010001269.1_4e-81_10_599_27658_28227
marine_Balbon_DF683084.1_1e-89_1_599_5480_6052
marine_Balacu_NW_006727686.1_4e-91_1_599_19817978_19817406
marine_Megnov_RYZJ01002529.1_3e-91_1_599_29622_30194
marine_Eubjap_RJWP010010376.1_2e-89_1_620_1344_1949
marine_Escrob_NIPP01011487.1_7e-89_1_599_43456_42880
marine_Balmus_VNFD01000878.1_5e-89_1_599_5185_5761
marine_Ponbla_RJWI010028718.1_3e-31_475_620_6497_6348
JWIN03000037.1_114347823-114348390_Camelus_dromedarius_breed_African_isolate_Drom800_chromosome_X_whole_genome_shotgun_sequence
SIDA01000019.1_3279024-3279593_Sus_scrofa_breed_Bama_miniature_chromosome_X_whole_genome_shotgun_sequence
R_JWIN03000014.1_48632737-48633330_Camelus_dromedarius_breed_African_isolate_Drom800_chromosome_14_whole_genome_shotgun_sequence
ABRR03004178.1_18973668-18974261_Vicugna_pacos_isolate_Carlotta__AHFN-0088__chromosome_14_ScfyRBE_4179HRSCAF_5494_whole_genome_shotgun_sequence
0.02

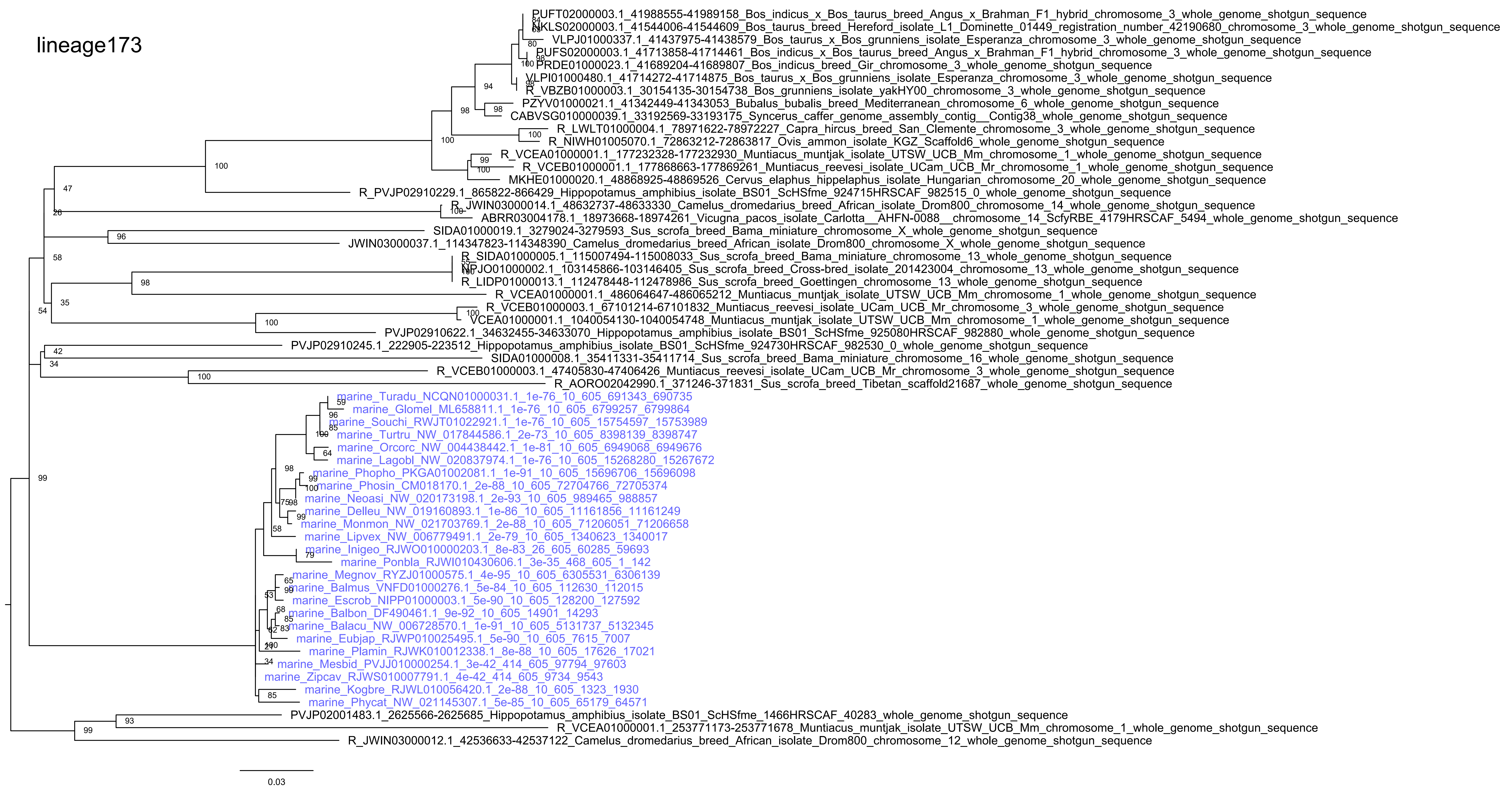

lineage173
PUFT02000003.1_41988555-41989158_Bos_indicus_x_Bos_taurus_breed_Angus_x_Brahman_F1_hybrid_chromosome_3_whole_genome_shotgun_sequence
NKLS02000003.1_41544006-41544609_Bos_taurus_breed_Hereford_isolate_L1_Dominette_01449_registration_number_42190680_chromosome_3_whole_genome_shotgun_sequence
VLPJ01000337.1_41437975-41438579_Bos_taurus_x_Bos_grunniens_isolate_Esperanza_chromosome_3_whole_genome_shotgun_sequence
PUFS02000003.1_41713858-41714461_Bos_indicus_x_Bos_taurus_breed_Angus_x_Brahman_F1_hybrid_chromosome_3_whole_genome_shotgun_sequence
PRDE01000023.1_41689204-41689807_Bos_indicus_breed_Gir_chromosome_3_whole_genome_shotgun_sequence
VLPI01000480.1_41714272-41714875_Bos_taurus_x_Bos_grunniens_isolate_Esperanza_chromosome_3_whole_genome_shotgun_sequence
R_VBZB01000003.1_30154135-30154738_Bos_grunniens_isolate_yakHY00_chromosome_3_whole_genome_shotgun_sequence
PZYV01000021.1_41342449-41343053_Bubalus_bubalis_breed_Mediterranean_chromosome_6_whole_genome_shotgun_sequence
CABVSG010000039.1_33192569-33193175_Syncerus_caffer_genome_assembly_contig__Contig38_whole_genome_shotgun_sequence
R_LWLT01000004.1_78971622-78972227_Capra_hircus_breed_San_Clemente_chromosome_3_whole_genome_shotgun_sequence
R_NIWH01005070.1_72863212-72863817_Ovis_ammon_isolate_KGZ_Scaffold6_whole_genome_shotgun_sequence
R_VCEA01000001.1_177232328-177232930_Muntiacus_muntjak_isolate_UTSW_UCB_Mm_chromosome_1_whole_genome_shotgun_sequence
R_VCEB01000001.1_177868663-177869261_Muntiacus_reevesi_isolate_UCam_UCB_Mr_chromosome_1_whole_genome_shotgun_sequence
MKHE01000020.1_48868925-48869526_Cervus_elaphus_hippelaphus_isolate_Hungarian_chromosome_20_whole_genome_shotgun_sequence
R_PVJP02910229.1_865822-866429_Hippopotamus_amphibius_isolate_BS01_ScHSfme_924715HRSCAF_982515_0_whole_genome_shotgun_sequence
R_JWIN03000014.1_48632737-48633330_Camelus_dromedarius_breed_African_isolate_Drom800_chromosome_14_whole_genome_shotgun_sequence
ABRR03004178.1_18973668-18974261_Vicugna_pacos_isolate_Carlotta__AHFN-0088__chromosome_14_ScfyRBE_4179HRSCAF_5494_whole_genome_shotgun_sequence
SIDA01000019.1_3279024-3279593_Sus_scrofa_breed_Bama_miniature_chromosome_X_whole_genome_shotgun_sequence
JWIN03000037.1_114347823-114348390_Camelus_dromedarius_breed_African_isolate_Drom800_chromosome_X_whole_genome_shotgun_sequence
R_SIDA01000005.1_115007494-115008033_Sus_scrofa_breed_Bama_miniature_chromosome_13_whole_genome_shotgun_sequence
NPJO01000002.1_103145866-103146405_Sus_scrofa_breed_Cross-bred_isolate_201423004_chromosome_13_whole_genome_shotgun_sequence
R_LIDP01000013.1_112478448-112478986_Sus_scrofa_breed_Goettingen_chromosome_13_whole_genome_shotgun_sequence
R_VCEA01000001.1_486064647-486065212_Muntiacus_muntjak_isolate_UTSW_UCB_Mm_chromosome_1_whole_genome_shotgun_sequence
R_VCEB01000003.1_67101214-67101832_Muntiacus_reevesi_isolate_UCam_UCB_Mr_chromosome_3_whole_genome_shotgun_sequence
VCEA01000001.1_1040054130-1040054748_Muntiacus_muntjak_isolate_UTSW_UCB_Mm_chromosome_1_whole_genome_shotgun_sequence
PVJP02910622.1_34632455-34633070_Hippopotamus_amphibius_isolate_BS01_ScHSfme_925080HRSCAF_982880_whole_genome_shotgun_sequence
PVJP02910245.1_222905-223512_Hippopotamus_amphibius_isolate_BS01_ScHSfme_924730HRSCAF_982530_0_whole_genome_shotgun_sequence
SIDA01000008.1_35411331-35411714_Sus_scrofa_breed_Bama_miniature_chromosome_16_whole_genome_shotgun_sequence
R_VCEB01000003.1_47405830-47406426_Muntiacus_reevesi_isolate_UCam_UCB_Mr_chromosome_3_whole_genome_shotgun_sequence
R_AORO02042990.1_371246-371831_Sus_scrofa_breed_Tibetan_scaffold21687_whole_genome_shotgun_sequence
marine_Turadu_NCQN01000031.1_1e-76_10_605_691343_690735
marine_Glomel_ML658811.1_1e-76_10_605_6799257_6799864
marine_Souchi_RWJT01022921.1_1e-76_10_605_15754597_15753989
marine_Turtru_NW_017844586.1_2e-73_10_605_8398139_8398747
marine_Orcorc_NW_004438442.1_1e-81_10_605_6949068_6949676
marine_Lagobl_NW_020837974.1_1e-76_10_605_15268280_15267672
marine_Phopho_PKGA01002081.1_1e-91_10_605_15696706_15696098
marine_Phosin_CM018170.1_2e-88_10_605_72704766_72705374
marine_Neoasi_NW_020173198.1_2e-93_10_605_989465_988857
marine_Delleu_NW_019160893.1_1e-86_10_605_11161856_11161249
marine_Monmon_NW_021703769.1_2e-88_10_605_71206051_71206658
marine_Lipvex_NW_006779491.1_2e-79_10_605_1340623_1340017
marine_Inigeo_RJWO010000203.1_8e-83_26_605_60285_59693
marine_Ponbla_RJWI010430606.1_3e-35_468_605_1_142
marine_Megnov_RYZJ01000575.1_4e-95_10_605_6305531_6306139
marine_Balmus_VNFD01000276.1_5e-84_10_605_112630_112015
marine_Escrob_NIPP01000003.1_5e-90_10_605_128200_127592
marine_Balbon_DF490461.1_9e-92_10_605_14901_14293
marine_Balacu_NW_006728570.1_1e-91_10_605_5131737_5132345
marine_Eubjap_RJWP010025495.1_5e-90_10_605_7615_7007
marine_Plamin_RJWK010012338.1_8e-88_10_605_17626_17021
marine_Mesbid_PVJJ010000254.1_3e-42_414_605_97794_97603
marine_Zipcav_RJWS010007791.1_4e-42_414_605_9734_9543
marine_Kogbre_RJWL010056420.1_2e-88_10_605_1323_1930
marine_Phycat_NW_021145307.1_5e-85_10_605_65179_64571
PVJP02001483.1_2625566-2625685_Hippopotamus_amphibius_isolate_BS01_ScHSfme_1466HRSCAF_40283_whole_genome_shotgun_sequence
R_VCEA01000001.1_253771173-253771678_Muntiacus_muntjak_isolate_UTSW_UCB_Mm_chromosome_1_whole_genome_shotgun_sequence
R_JWIN03000012.1_42536633-42537122_Camelus_dromedarius_breed_African_isolate_Drom800_chromosome_12_whole_genome_shotgun_sequence
0.03

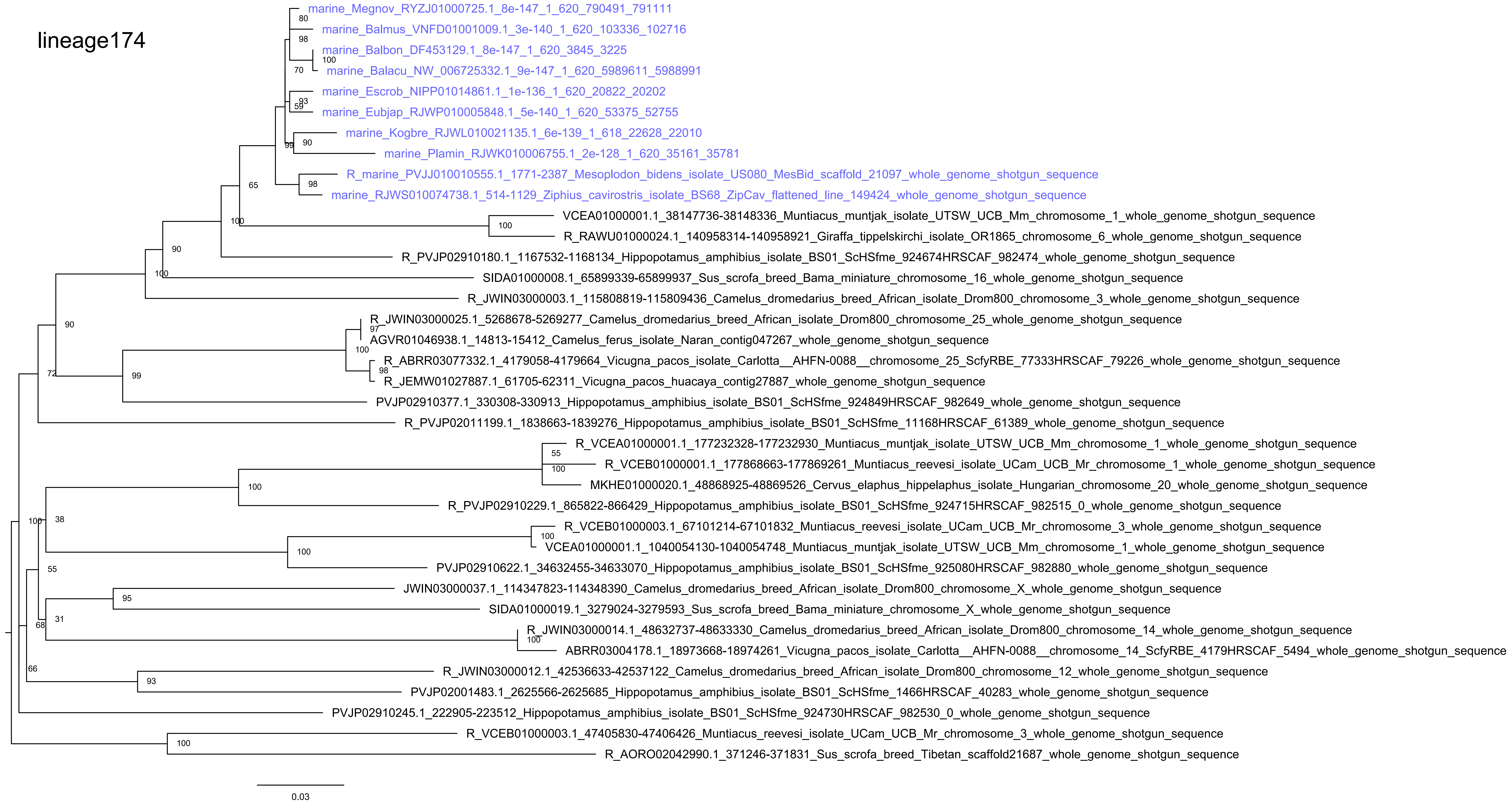
lineage174
marine_Megnov_RYZJ01000725.1_8e-147_1_620_790491_791111
marine_Balmus_VNFD01001009.1_3e-140_1_620_103336_102716
marine_Balbon_DF453129.1_8e-147_1_620_3845_3225
marine_Balacu_NW_006725332.1_9e-147_1_620_5989611_5988991
marine_Escrob_NIPP01014861.1_1e-136_1_620_20822_20202
marine_Eubjap_RJWP010005848.1_5e-140_1_620_53375_52755
marine_Kogbre_RJWL010021135.1_6e-139_1_618_22628_22010
marine_Plamin_RJWK010006755.1_2e-128_1_620_35161_35781
R_marine_PVJJ010010555.1_1771-2387_Mesoplodon_bidens_isolate_US080_MesBid_scaffold_21097_whole_genome_shotgun_sequence
marine_RJWS010074738.1_514-1129_Ziphius_cavirostris_isolate_BS68_ZipCav_flattened_line_149424_whole_genome_shotgun_sequence
VCEA01000001.1_38147736-38148336_Muntiacus_muntjak_isolate_UTSW_UCB_Mm_chromosome_1_whole_genome_shotgun_sequence
R_RAWU01000024.1_140958314-140958921_Giraffa_tippelskirchi_isolate_OR1865_chromosome_6_whole_genome_shotgun_sequence
R_PVJP02910180.1_1167532-1168134_Hippopotamus_amphibius_isolate_BS01_ScHSfme_924674HRSCAF_982474_whole_genome_shotgun_sequence
SIDA01000008.1_65899339-65899937_Sus_scrofa_breed_Bama_miniature_chromosome_16_whole_genome_shotgun_sequence
R_JWIN03000003.1_115808819-115809436_Camelus_dromedarius_breed_African_isolate_Drom800_chromosome_3_whole_genome_shotgun_sequence
R_JWIN03000025.1_5268678-5269277_Camelus_dromedarius_breed_African_isolate_Drom800_chromosome_25_whole_genome_shotgun_sequence
AGVR01046938.1_14813-15412_Camelus_ferus_isolate_Naran_contig047267_whole_genome_shotgun_sequence
R_ABRR03077332.1_4179058-4179664_Vicugna_pacos_isolate_Carlotta__AHFN-0088__chromosome_25_ScfyRBE_77333HRSCAF_79226_whole_genome_shotgun_sequence
R_JEMW01027887.1_61705-62311_Vicugna_pacos_huacaya_contig27887_whole_genome_shotgun_sequence
PVJP02910377.1_330308-330913_Hippopotamus_amphibius_isolate_BS01_ScHSfme_924849HRSCAF_982649_whole_genome_shotgun_sequence
R_PVJP02011199.1_1838663-1839276_Hippopotamus_amphibius_isolate_BS01_ScHSfme_11168HRSCAF_61389_whole_genome_shotgun_sequence
R_VCEA01000001.1_177232328-177232930_Muntiacus_muntjak_isolate_UTSW_UCB_Mm_chromosome_1_whole_genome_shotgun_sequence
R_VCEB01000001.1_177868663-177869261_Muntiacus_reevesi_isolate_UCam_UCB_Mr_chromosome_1_whole_genome_shotgun_sequence
MKHE01000020.1_48868925-48869526_Cervus_elaphus_hippelaphus_isolate_Hungarian_chromosome_20_whole_genome_shotgun_sequence
R_PVJP02910229.1_865822-866429_Hippopotamus_amphibius_isolate_BS01_ScHSfme_924715HRSCAF_982515_0_whole_genome_shotgun_sequence
R_VCEB01000003.1_67101214-67101832_Muntiacus_reevesi_isolate_UCam_UCB_Mr_chromosome_3_whole_genome_shotgun_sequence
VCEA01000001.1_1040054130-1040054748_Muntiacus_muntjak_isolate_UTSW_UCB_Mm_chromosome_1_whole_genome_shotgun_sequence
PVJP02910622.1_34632455-34633070_Hippopotamus_amphibius_isolate_BS01_ScHSfme_925080HRSCAF_982880_whole_genome_shotgun_sequence
JWIN03000037.1_114347823-114348390_Camelus_dromedarius_breed_African_isolate_Drom800_chromosome_X_whole_genome_shotgun_sequence
SIDA01000019.1_3279024-3279593_Sus_scrofa_breed_Bama_miniature_chromosome_X_whole_genome_shotgun_sequence
R_JWIN03000014.1_48632737-48633330_Camelus_dromedarius_breed_African_isolate_Drom800_chromosome_14_whole_genome_shotgun_sequence
ABRR03004178.1_18973668-18974261_Vicugna_pacos_isolate_Carlotta__AHFN-0088__chromosome_14_ScfyRBE_4179HRSCAF_5494_whole_genome_shotgun_sequence
R_JWIN03000012.1_42536633-42537122_Camelus_dromedarius_breed_African_isolate_Drom800_chromosome_12_whole_genome_shotgun_sequence
PVJP02001483.1_2625566-2625685_Hippopotamus_amphibius_isolate_BS01_ScHSfme_1466HRSCAF_40283_whole_genome_shotgun_sequence
PVJP02910245.1_222905-223512_Hippopotamus_amphibius_isolate_BS01_ScHSfme_924730HRSCAF_982530_0_whole_genome_shotgun_sequence
R_VCEB01000003.1_47405830-47406426_Muntiacus_reevesi_isolate_UCam_UCB_Mr_chromosome_3_whole_genome_shotgun_sequence
R_AORO02042990.1_371246-371831_Sus_scrofa_breed_Tibetan_scaffold21687_whole_genome_shotgun_sequence
0.03

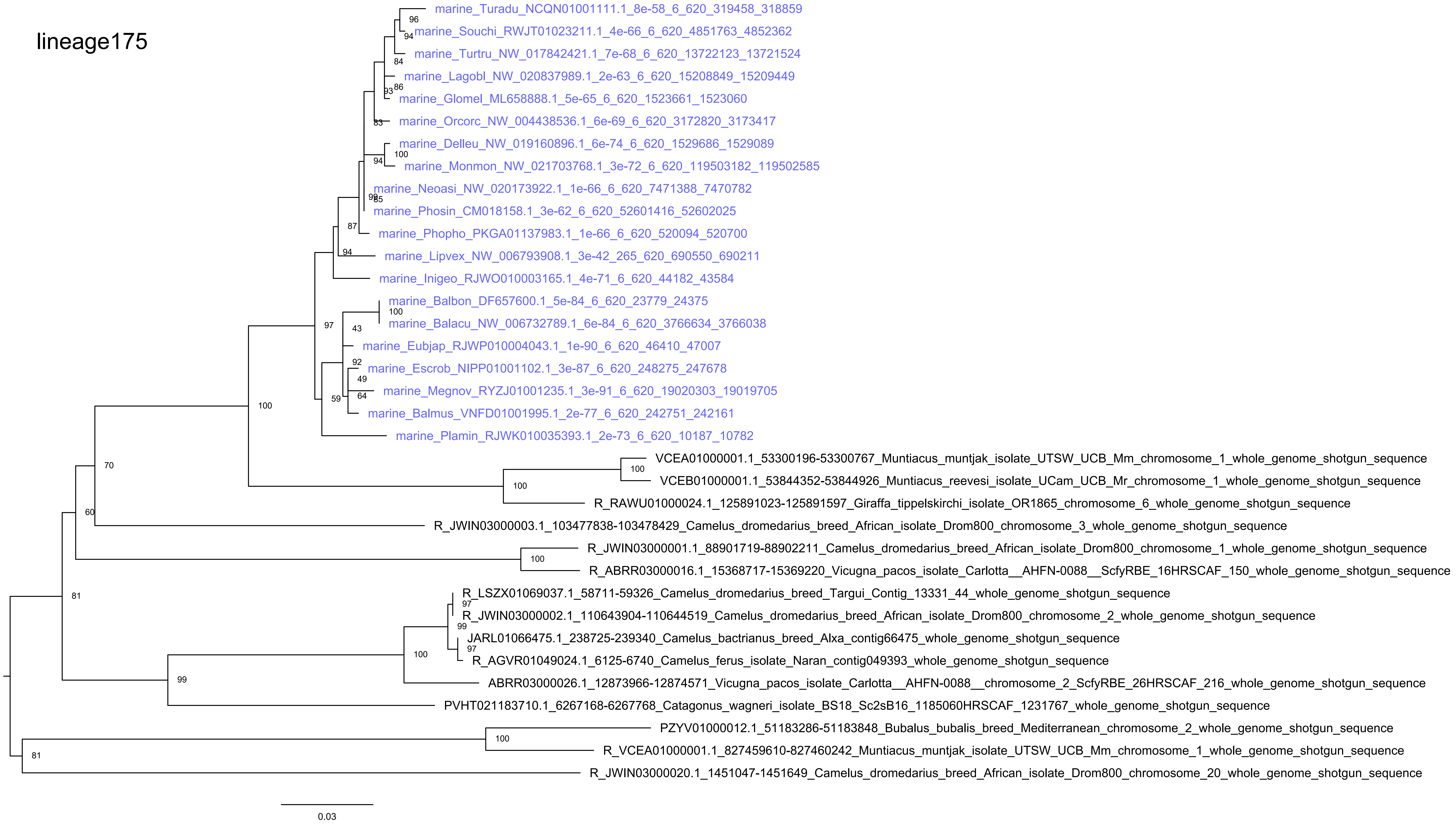
lineage175
marine_Turadu_NCQN01001111.1_8e-58_6_620_319458_318859
marine_Souchi_RWJT01023211.1_4e-66_6_620_4851763_4852362
marine_Turtru_NW_017842421.1_7e-68_6_620_13722123_13721524
marine_Lagobl_NW_020837989.1_2e-63_6_620_15208849_15209449
marine_Glomel_ML658888.1_5e-65_6_620_1523661_1523060
marine_Orcorc_NW_004438536.1_6e-69_6_620_3172820_3173417
marine_Delleu_NW_019160896.1_6e-74_6_620_1529686_1529089
marine_Monmon_NW_021703768.1_3e-72_6_620_119503182_119502585
marine_Neoasi_NW_020173922.1_1e-66_6_620_7471388_7470782
marine_Phosin_CM018158.1_3e-62_6_620_52601416_52602025
marine_Phopho_PKGA01137983.1_1e-66_6_620_520094_520700
marine_Lipvex_NW_006793908.1_3e-42_265_620_690550_690211
marine_Inigeo_RJWO010003165.1_4e-71_6_620_44182_43584
marine_Balbon_DF657600.1_5e-84_6_620_23779_24375
marine_Balacu_NW_006732789.1_6e-84_6_620_3766634_3766038
marine_Eubjap_RJWP010004043.1_1e-90_6_620_46410_47007
marine_Escrob_NIPP01001102.1_3e-87_6_620_248275_247678
marine_Megnov_RYZJ01001235.1_3e-91_6_620_19020303_19019705
marine_Balmus_VNFD01001995.1_2e-77_6_620_242751_242161
marine_Plamin_RJWK010035393.1_2e-73_6_620_10187_10782
VCEA01000001.1_53300196-53300767_Muntiacus_muntjak_isolate_UTSW_UCB_Mm_chromosome_1_whole_genome_shotgun_sequence
VCEB01000001.1_53844352-53844926_Muntiacus_reevesi_isolate_UCam_UCB_Mr_chromosome_1_whole_genome_shotgun_sequence
R_RAWU01000024.1_125891023-125891597_Giraffa_tippelskirchi_isolate_OR1865_chromosome_6_whole_genome_shotgun_sequence
R_JWIN03000003.1_103477838-103478429_Camelus_dromedarius_breed_African_isolate_Drom800_chromosome_3_whole_genome_shotgun_sequence
R_JWIN03000001.1_88901719-88902211_Camelus_dromedarius_breed_African_isolate_Drom800_chromosome_1_whole_genome_shotgun_sequence
R_ABRR03000016.1_15368717-15369220_Vicugna_pacos_isolate_Carlotta__AHFN-0088__ScfyRBE_16HRSCAF_150_whole_genome_shotgun_sequence
R_LSZX01069037.1_58711-59326_Camelus_dromedarius_breed_Targui_Contig_13331_44_whole_genome_shotgun_sequence
R_JWIN03000002.1_110643904-110644519_Camelus_dromedarius_breed_African_isolate_Drom800_chromosome_2_whole_genome_shotgun_sequence
JARL01066475.1_238725-239340_Camelus_bactrianus_breed_Alxa_contig66475_whole_genome_shotgun_sequence
R_AGVR01049024.1_6125-6740_Camelus_ferus_isolate_Naran_contig049393_whole_genome_shotgun_sequence
ABRR03000026.1_12873966-12874571_Vicugna_pacos_isolate_Carlotta__AHFN-0088__chromosome_2_ScfyRBE_26HRSCAF_216_whole_genome_shotgun_sequence
PVHT021183710.1_6267168-6267768_Catagonus_wagneri_isolate_BS18_Sc2sB16_1185060HRSCAF_1231767_whole_genome_shotgun_sequence
PZYV01000012.1_51183286-51183848_Bubalus_bubalis_breed_Mediterranean_chromosome_2_whole_genome_shotgun_sequence
R_VCEA01000001.1_827459610-827460242_Muntiacus_muntjak_isolate_UTSW_UCB_Mm_chromosome_1_whole_genome_shotgun_sequence
R_JWIN03000020.1_1451047-1451649_Camelus_dromedarius_breed_African_isolate_Drom800_chromosome_20_whole_genome_shotgun_sequence
0.03

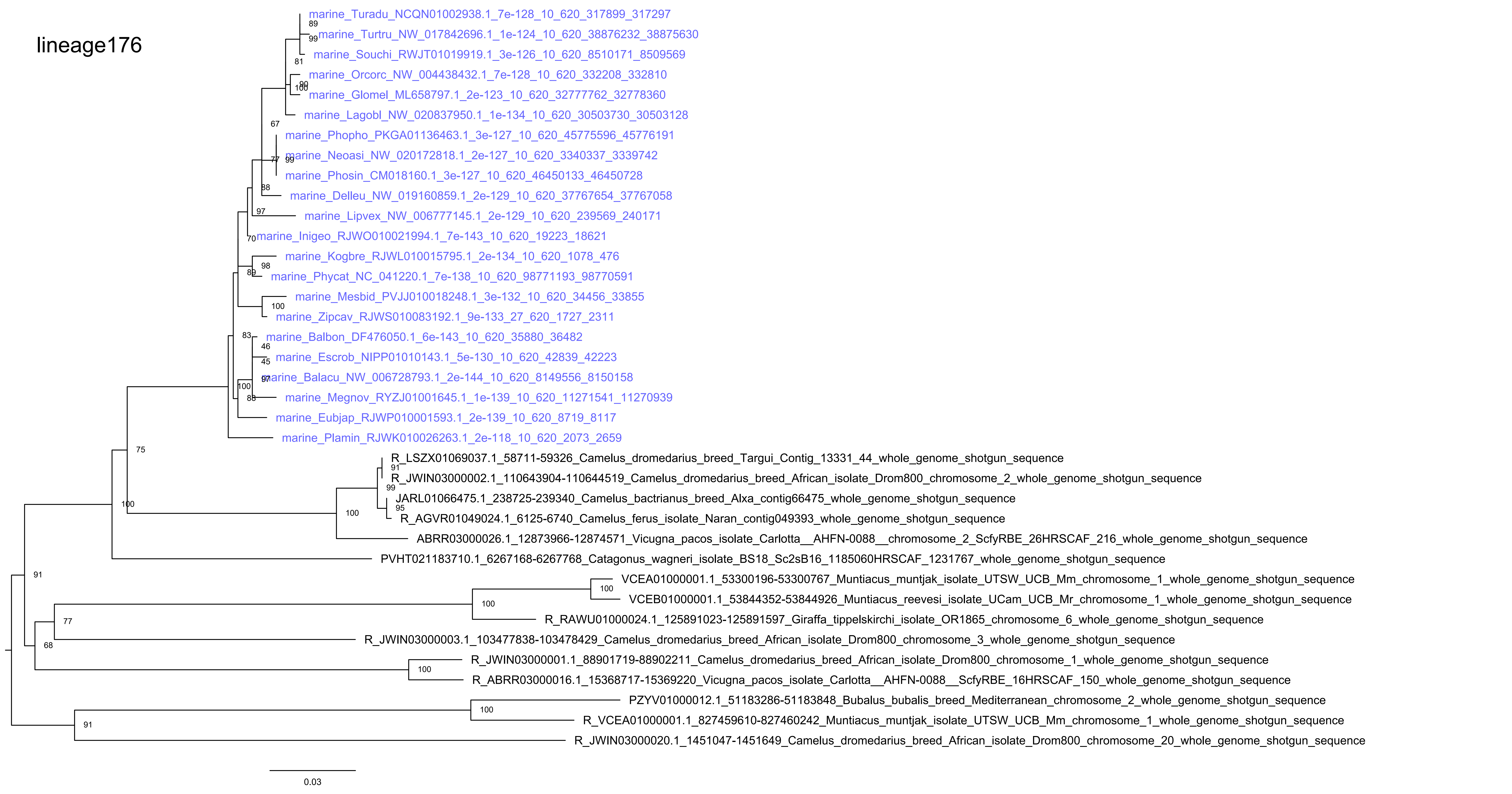

lineage176
marine_Turadu_NCQN01002938.1_7e-128_10_620_317899_317297
marine_Turtru_NW_017842696.1_1e-124_10_620_38876232_38875630
marine_Souchi_RWJT01019919.1_3e-126_10_620_8510171_8509569
marine_Orcorc_NW_004438432.1_7e-128_10_620_332208_332810
marine_Glomel_ML658797.1_2e-123_10_620_32777762_32778360
marine_Lagobl_NW_020837950.1_1e-134_10_620_30503730_30503128
marine_Phopho_PKGA01136463.1_3e-127_10_620_45775596_45776191
marine_Neoasi_NW_020172818.1_2e-127_10_620_3340337_3339742
marine_Phosin_CM018160.1_3e-127_10_620_46450133_46450728
marine_Delleu_NW_019160859.1_2e-129_10_620_37767654_37767058
marine_Lipvex_NW_006777145.1_2e-129_10_620_239569_240171
marine_Inigeo_RJWO010021994.1_7e-143_10_620_19223_18621
marine_Kogbre_RJWL010015795.1_2e-134_10_620_1078_476
marine_Phycat_NC_041220.1_7e-138_10_620_98771193_98770591
marine_Mesbid_PVJJ010018248.1_3e-132_10_620_34456_33855
marine_Zipcav_RJWS010083192.1_9e-133_27_620_1727_2311
marine_Balbon_DF476050.1_6e-143_10_620_35880_36482
marine_Escrob_NIPP01010143.1_5e-130_10_620_42839_42223
marine_Balacu_NW_006728793.1_2e-144_10_620_8149556_8150158
marine_Megnov_RYZJ01001645.1_1e-139_10_620_11271541_11270939
marine_Eubjap_RJWP010001593.1_2e-139_10_620_8719_8117
marine_Plamin_RJWK010026263.1_2e-118_10_620_2073_2659
R_LSZX01069037.1_58711-59326_Camelus_dromedarius_breed_Targui_Contig_13331_44_whole_genome_shotgun_sequence
R_JWIN03000002.1_110643904-110644519_Camelus_dromedarius_breed_African_isolate_Drom800_chromosome_2_whole_genome_shotgun_sequence
JARL01066475.1_238725-239340_Camelus_bactrianus_breed_Alxa_contig66475_whole_genome_shotgun_sequence
R_AGVR01049024.1_6125-6740_Camelus_ferus_isolate_Naran_contig049393_whole_genome_shotgun_sequence
ABRR03000026.1_12873966-12874571_Vicugna_pacos_isolate_Carlotta__AHFN-0088__chromosome_2_ScfyRBE_26HRSCAF_216_whole_genome_shotgun_sequence
PVHT021183710.1_6267168-6267768_Catagonus_wagneri_isolate_BS18_Sc2sB16_1185060HRSCAF_1231767_whole_genome_shotgun_sequence
VCEA01000001.1_53300196-53300767_Muntiacus_muntjak_isolate_UTSW_UCB_Mm_chromosome_1_whole_genome_shotgun_sequence
VCEB01000001.1_53844352-53844926_Muntiacus_reevesi_isolate_UCam_UCB_Mr_chromosome_1_whole_genome_shotgun_sequence
R_RAWU01000024.1_125891023-125891597_Giraffa_tippelskirchi_isolate_OR1865_chromosome_6_whole_genome_shotgun_sequence
R_JWIN03000003.1_103477838-103478429_Camelus_dromedarius_breed_African_isolate_Drom800_chromosome_3_whole_genome_shotgun_sequence
R_JWIN03000001.1_88901719-88902211_Camelus_dromedarius_breed_African_isolate_Drom800_chromosome_1_whole_genome_shotgun_sequence
R_ABRR03000016.1_15368717-15369220_Vicugna_pacos_isolate_Carlotta__AHFN-0088__ScfyRBE_16HRSCAF_150_whole_genome_shotgun_sequence
PZYV01000012.1_51183286-51183848_Bubalus_bubalis_breed_Mediterranean_chromosome_2_whole_genome_shotgun_sequence
R_VCEA01000001.1_827459610-827460242_Muntiacus_muntjak_isolate_UTSW_UCB_Mm_chromosome_1_whole_genome_shotgun_sequence
R_JWIN03000020.1_1451047-1451649_Camelus_dromedarius_breed_African_isolate_Drom800_chromosome_20_whole_genome_shotgun_sequence
0.03

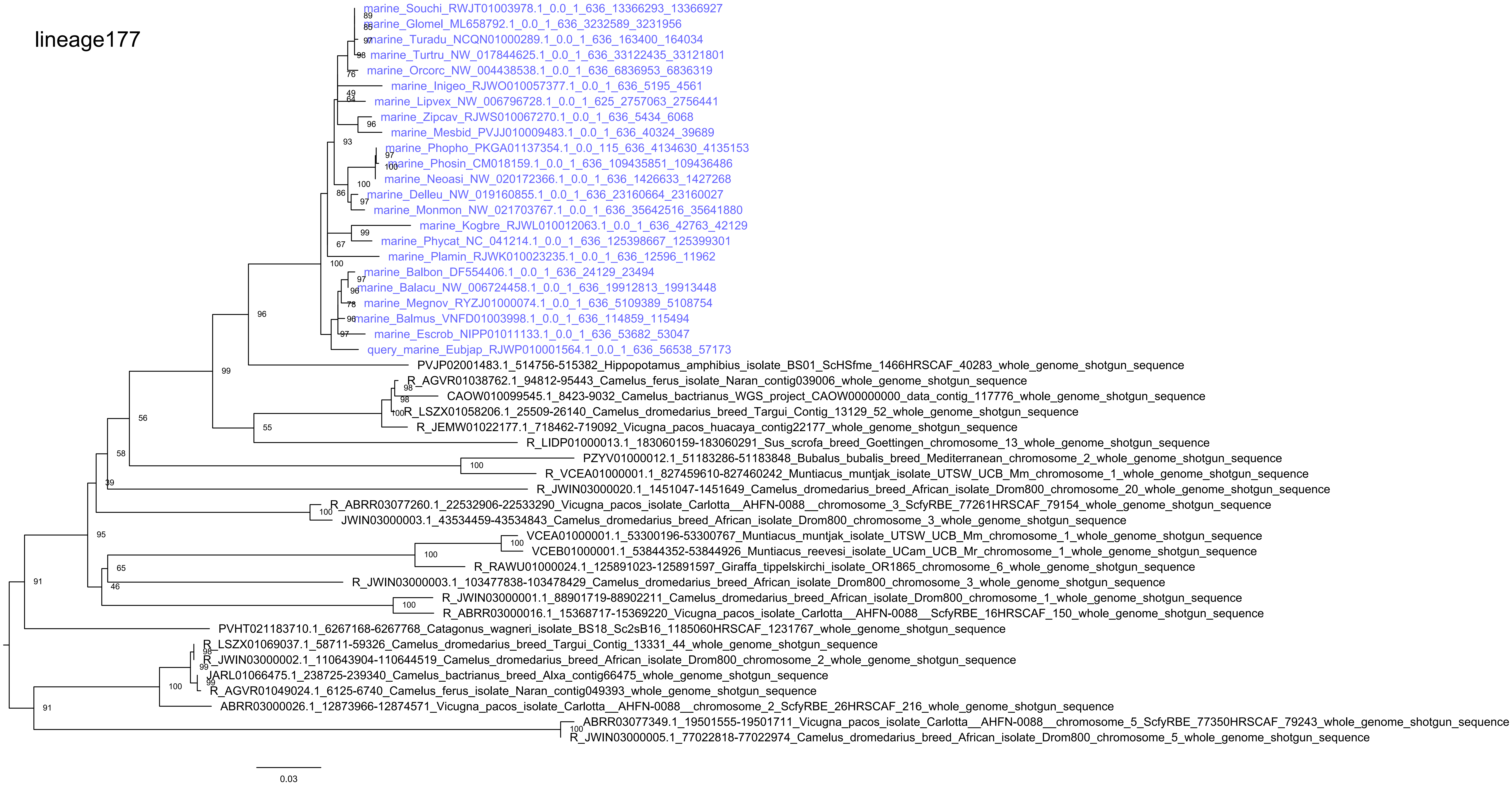

lineage177
marine_Souchi_RWJT01003978.1_0.0_1_636_13366293_13366927
marine_Glomel_ML658792.1_0.0_1_636_3232589_3231956
marine_Turadu_NCQN01000289.1_0.0_1_636_163400_164034
marine_Turtru_NW_017844625.1_0.0_1_636_33122435_33121801
marine_Orcorc_NW_004438538.1_0.0_1_636_6836953_6836319
marine_Inigeo_RJWO010057377.1_0.0_1_636_5195_4561
marine_Lipvex_NW_006796728.1_0.0_1_625_2757063_2756441
marine_Zipcav_RJWS010067270.1_0.0_1_636_5434_6068
marine_Mesbid_PVJJ010009483.1_0.0_1_636_40324_39689
marine_Phopho_PKGA01137354.1_0.0_115_636_4134630_4135153
marine_Phosin_CM018159.1_0.0_1_636_109435851_109436486
marine_Neoasi_NW_020172366.1_0.0_1_636_1426633_1427268
marine_Delleu_NW_019160855.1_0.0_1_636_23160664_23160027
marine_Monmon_NW_021703767.1_0.0_1_636_35642516_35641880
marine_Kogbre_RJWL010012063.1_0.0_1_636_42763_42129
marine_Phycat_NC_041214.1_0.0_1_636_125398667_125399301
marine_Plamin_RJWK010023235.1_0.0_1_636_12596_11962
marine_Balbon_DF554406.1_0.0_1_636_24129_23494
marine_Balacu_NW_006724458.1_0.0_1_636_19912813_19913448
marine_Megnov_RYZJ01000074.1_0.0_1_636_5109389_5108754
marine_Balmus_VNFD01003998.1_0.0_1_636_114859_115494
marine_Escrob_NIPP01011133.1_0.0_1_636_53682_53047
query_marine_Eubjap_RJWP010001564.1_0.0_1_636_56538_57173
PVJP02001483.1_514756-515382_Hippopotamus_amphibius_isolate_BS01_ScHSfme_1466HRSCAF_40283_whole_genome_shotgun_sequence
R_AGVR01038762.1_94812-95443_Camelus_ferus_isolate_Naran_contig039006_whole_genome_shotgun_sequence
CAOW010099545.1_8423-9032_Camelus_bactrianus_WGS_project_CAOW00000000_data_contig_117776_whole_genome_shotgun_sequence
R_LSZX01058206.1_25509-26140_Camelus_dromedarius_breed_Targui_Contig_13129_52_whole_genome_shotgun_sequence
R_JEMW01022177.1_718462-719092_Vicugna_pacos_huacaya_contig22177_whole_genome_shotgun_sequence
R_LIDP01000013.1_183060159-183060291_Sus_scrofa_breed_Goettingen_chromosome_13_whole_genome_shotgun_sequence
PZYV01000012.1_51183286-51183848_Bubalus_bubalis_breed_Mediterranean_chromosome_2_whole_genome_shotgun_sequence
R_VCEA01000001.1_827459610-827460242_Muntiacus_muntjak_isolate_UTSW_UCB_Mm_chromosome_1_whole_genome_shotgun_sequence
R_JWIN03000020.1_1451047-1451649_Camelus_dromedarius_breed_African_isolate_Drom800_chromosome_20_whole_genome_shotgun_sequence
R_ABRR03077260.1_22532906-22533290_Vicugna_pacos_isolate_Carlotta__AHFN-0088__chromosome_3_ScfyRBE_77261HRSCAF_79154_whole_genome_shotgun_sequence
JWIN03000003.1_43534459-43534843_Camelus_dromedarius_breed_African_isolate_Drom800_chromosome_3_whole_genome_shotgun_sequence
VCEA01000001.1_53300196-53300767_Muntiacus_muntjak_isolate_UTSW_UCB_Mm_chromosome_1_whole_genome_shotgun_sequence
VCEB01000001.1_53844352-53844926_Muntiacus_reevesi_isolate_UCam_UCB_Mr_chromosome_1_whole_genome_shotgun_sequence
R_RAWU01000024.1_125891023-125891597_Giraffa_tippelskirchi_isolate_OR1865_chromosome_6_whole_genome_shotgun_sequence
R_JWIN03000003.1_103477838-103478429_Camelus_dromedarius_breed_African_isolate_Drom800_chromosome_3_whole_genome_shotgun_sequence
R_JWIN03000001.1_88901719-88902211_Camelus_dromedarius_breed_African_isolate_Drom800_chromosome_1_whole_genome_shotgun_sequence
R_ABRR03000016.1_15368717-15369220_Vicugna_pacos_isolate_Carlotta__AHFN-0088__ScfyRBE_16HRSCAF_150_whole_genome_shotgun_sequence
PVHT021183710.1_6267168-6267768_Catagonus_wagneri_isolate_BS18_Sc2sB16_1185060HRSCAF_1231767_whole_genome_shotgun_sequence
R_LSZX01069037.1_58711-59326_Camelus_dromedarius_breed_Targui_Contig_13331_44_whole_genome_shotgun_sequence
R_JWIN03000002.1_110643904-110644519_Camelus_dromedarius_breed_African_isolate_Drom800_chromosome_2_whole_genome_shotgun_sequence
JARL01066475.1_238725-239340_Camelus_bactrianus_breed_Alxa_contig66475_whole_genome_shotgun_sequence
R_AGVR01049024.1_6125-6740_Camelus_ferus_isolate_Naran_contig049393_whole_genome_shotgun_sequence
ABRR03000026.1_12873966-12874571_Vicugna_pacos_isolate_Carlotta__AHFN-0088__chromosome_2_ScfyRBE_26HRSCAF_216_whole_genome_shotgun_sequence
ABRR03077349.1_19501555-19501711_Vicugna_pacos_isolate_Carlotta__AHFN-0088__chromosome_5_ScfyRBE_77350HRSCAF_79243_whole_genome_shotgun_sequence
R_JWIN03000005.1_77022818-77022974_Camelus_dromedarius_breed_African_isolate_Drom800_chromosome_5_whole_genome_shotgun_sequence
0.03

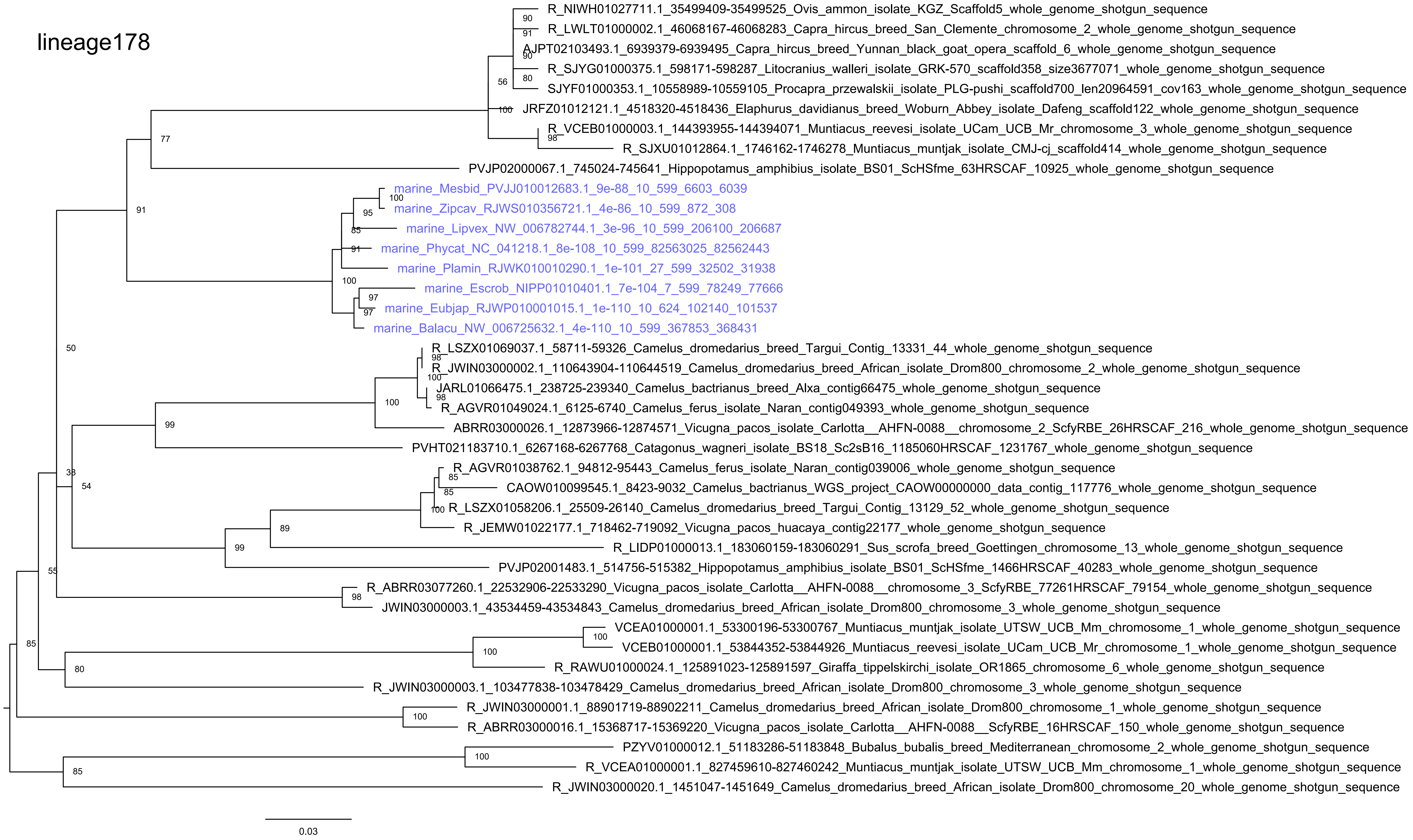
lineage178
R_NIWH01027711.1_35499409-35499525_Ovis_ammon_isolate_KGZ_Scaffold5_whole_genome_shotgun_sequence
R_LWLT01000002.1_46068167-46068283_Capra_hircus_breed_San_Clemente_chromosome_2_whole_genome_shotgun_sequence
AJPT02103493.1_6939379-6939495_Capra_hircus_breed_Yunnan_black_goat_opera_scaffold_6_whole_genome_shotgun_sequence
R_SJYG01000375.1_598171-598287_Litocranius_walleri_isolate_GRK-570_scaffold358_size3677071_whole_genome_shotgun_sequence
SJYF01000353.1_10558989-10559105_Procapra_przewalskii_isolate_PLG-pushi_scaffold700_len20964591_cov163_whole_genome_shotgun_sequence
JRFZ01012121.1_4518320-4518436_Elaphurus_davidianus_breed_Woburn_Abbey_isolate_Dafeng_scaffold122_whole_genome_shotgun_sequence
R_VCEB01000003.1_144393955-144394071_Muntiacus_reevesi_isolate_UCam_UCB_Mr_chromosome_3_whole_genome_shotgun_sequence
R_SJXU01012864.1_1746162-1746278_Muntiacus_muntjak_isolate_CMJ-cj_scaffold414_whole_genome_shotgun_sequence
PVJP02000067.1_745024-745641_Hippopotamus_amphibius_isolate_BS01_ScHSfme_63HRSCAF_10925_whole_genome_shotgun_sequence
marine_Mesbid_PVJJ010012683.1_9e-88_10_599_6603_6039
marine_Zipcav_RJWS010356721.1_4e-86_10_599_872_308
marine_Lipvex_NW_006782744.1_3e-96_10_599_206100_206687
marine_Phycat_NC_041218.1_8e-108_10_599_82563025_82562443
marine_Plamin_RJWK010010290.1_1e-101_27_599_32502_31938
marine_Escrob_NIPP01010401.1_7e-104_7_599_78249_77666
marine_Eubjap_RJWP010001015.1_1e-110_10_624_102140_101537
marine_Balacu_NW_006725632.1_4e-110_10_599_367853_368431
R_LSZX01069037.1_58711-59326_Camelus_dromedarius_breed_Targui_Contig_13331_44_whole_genome_shotgun_sequence
R_JWIN03000002.1_110643904-110644519_Camelus_dromedarius_breed_African_isolate_Drom800_chromosome_2_whole_genome_shotgun_sequence
JARL01066475.1_238725-239340_Camelus_bactrianus_breed_Alxa_contig66475_whole_genome_shotgun_sequence
R_AGVR01049024.1_6125-6740_Camelus_ferus_isolate_Naran_contig049393_whole_genome_shotgun_sequence
ABRR03000026.1_12873966-12874571_Vicugna_pacos_isolate_Carlotta__AHFN-0088__chromosome_2_ScfyRBE_26HRSCAF_216_whole_genome_shotgun_sequence
PVHT021183710.1_6267168-6267768_Catagonus_wagneri_isolate_BS18_Sc2sB16_1185060HRSCAF_1231767_whole_genome_shotgun_sequence
R_AGVR01038762.1_94812-95443_Camelus_ferus_isolate_Naran_contig039006_whole_genome_shotgun_sequence
CAOW010099545.1_8423-9032_Camelus_bactrianus_WGS_project_CAOW00000000_data_contig_117776_whole_genome_shotgun_sequence
R_LSZX01058206.1_25509-26140_Camelus_dromedarius_breed_Targui_Contig_13129_52_whole_genome_shotgun_sequence
R_JEMW01022177.1_718462-719092_Vicugna_pacos_huacaya_contig22177_whole_genome_shotgun_sequence
R_LIDP01000013.1_183060159-183060291_Sus_scrofa_breed_Goettingen_chromosome_13_whole_genome_shotgun_sequence
PVJP02001483.1_514756-515382_Hippopotamus_amphibius_isolate_BS01_ScHSfme_1466HRSCAF_40283_whole_genome_shotgun_sequence
R_ABRR03077260.1_22532906-22533290_Vicugna_pacos_isolate_Carlotta__AHFN-0088__chromosome_3_ScfyRBE_77261HRSCAF_79154_whole_genome_shotgun_sequence
JWIN03000003.1_43534459-43534843_Camelus_dromedarius_breed_African_isolate_Drom800_chromosome_3_whole_genome_shotgun_sequence
VCEA01000001.1_53300196-53300767_Muntiacus_muntjak_isolate_UTSW_UCB_Mm_chromosome_1_whole_genome_shotgun_sequence
VCEB01000001.1_53844352-53844926_Muntiacus_reevesi_isolate_UCam_UCB_Mr_chromosome_1_whole_genome_shotgun_sequence
R_RAWU01000024.1_125891023-125891597_Giraffa_tippelskirchi_isolate_OR1865_chromosome_6_whole_genome_shotgun_sequence
R_JWIN03000003.1_103477838-103478429_Camelus_dromedarius_breed_African_isolate_Drom800_chromosome_3_whole_genome_shotgun_sequence
R_JWIN03000001.1_88901719-88902211_Camelus_dromedarius_breed_African_isolate_Drom800_chromosome_1_whole_genome_shotgun_sequence
R_ABRR03000016.1_15368717-15369220_Vicugna_pacos_isolate_Carlotta__AHFN-0088__ScfyRBE_16HRSCAF_150_whole_genome_shotgun_sequence
PZYV01000012.1_51183286-51183848_Bubalus_bubalis_breed_Mediterranean_chromosome_2_whole_genome_shotgun_sequence
R_VCEA01000001.1_827459610-827460242_Muntiacus_muntjak_isolate_UTSW_UCB_Mm_chromosome_1_whole_genome_shotgun_sequence
R_JWIN03000020.1_1451047-1451649_Camelus_dromedarius_breed_African_isolate_Drom800_chromosome_20_whole_genome_shotgun_sequence
0.03

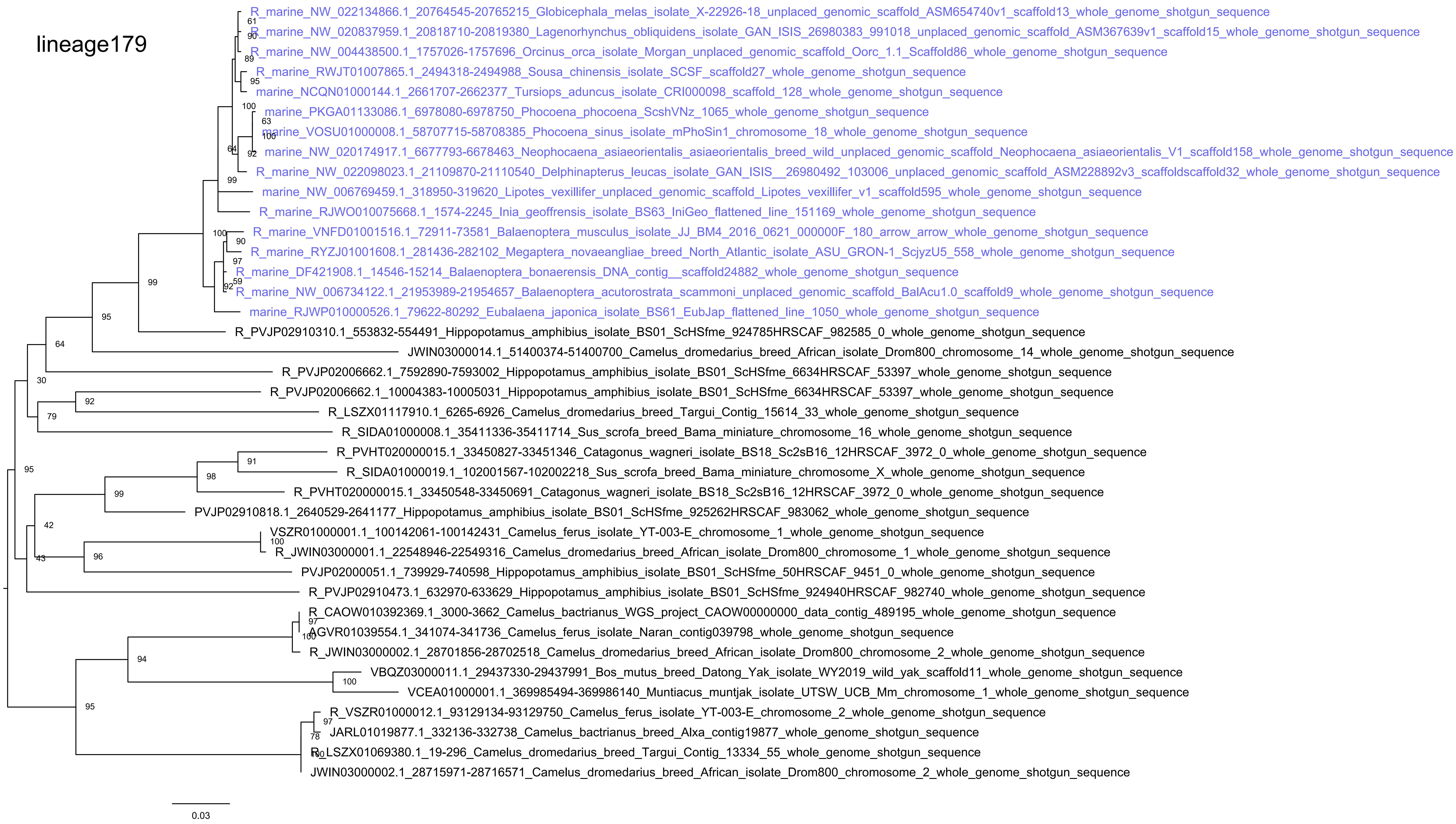
lineage179
R_marine_NW_022134866.1_20764545-20765215_Globicephala_melas_isolate_X-22926-18_unplaced_genomic_scaffold_ASM654740v1_scaffold13_whole_genome_shotgun_sequence
R_marine_NW_020837959.1_20818710-20819380_Lagenorhynchus_obliquidens_isolate_GAN_ISIS_26980383_991018_unplaced_genomic_scaffold_ASM367639v1_scaffold15_whole_genome_shotgun_sequence
R_marine_NW_004438500.1_1757026-1757696_Orcinus_orca_isolate_Morgan_unplaced_genomic_scaffold_Oorc_1.1_Scaffold86_whole_genome_shotgun_sequence
R_marine_RWJT01007865.1_2494318-2494988_Sousa_chinensis_isolate_SCSF_scaffold27_whole_genome_shotgun_sequence
marine_NCQN01000144.1_2661707-2662377_Tursiops_aduncus_isolate_CRI000098_scaffold_128_whole_genome_shotgun_sequence
marine_PKGA01133086.1_6978080-6978750_Phocoena_phocoena_ScshVNz_1065_whole_genome_shotgun_sequence
marine_VOSU01000008.1_58707715-58708385_Phocoena_sinus_isolate_mPhoSin1_chromosome_18_whole_genome_shotgun_sequence
marine_NW_020174917.1_6677793-6678463_Neophocaena_asiaeorientalis_asiaeorientalis_breed_wild_unplaced_genomic_scaffold_Neophocaena_asiaeorientalis_V1_scaffold158_whole_genome_shotgun_sequence
R_marine_NW_022098023.1_21109870-21110540_Delphinapterus_leucas_isolate_GAN_ISIS__26980492_103006_unplaced_genomic_scaffold_ASM228892v3_scaffoldscaffold32_whole_genome_shotgun_sequence
marine_NW_006769459.1_318950-319620_Lipotes_vexillifer_unplaced_genomic_scaffold_Lipotes_vexillifer_v1_scaffold595_whole_genome_shotgun_sequence
R_marine_RJWO010075668.1_1574-2245_Inia_geoffrensis_isolate_BS63_IniGeo_flattened_line_151169_whole_genome_shotgun_sequence
R_marine_VNFD01001516.1_72911-73581_Balaenoptera_musculus_isolate_JJ_BM4_2016_0621_000000F_180_arrow_arrow_whole_genome_shotgun_sequence
R_marine_RYZJ01001608.1_281436-282102_Megaptera_novaeangliae_breed_North_Atlantic_isolate_ASU_GRON-1_ScjyzU5_558_whole_genome_shotgun_sequence
R_marine_DF421908.1_14546-15214_Balaenoptera_bonaerensis_DNA_contig__scaffold24882_whole_genome_shotgun_sequence
R_marine_NW_006734122.1_21953989-21954657_Balaenoptera_acutorostrata_scammoni_unplaced_genomic_scaffold_BalAcu1.0_scaffold9_whole_genome_shotgun_sequence
marine_RJWP010000526.1_79622-80292_Eubalaena_japonica_isolate_BS61_EubJap_flattened_line_1050_whole_genome_shotgun_sequence
R_PVJP02910310.1_553832-554491_Hippopotamus_amphibius_isolate_BS01_ScHSfme_924785HRSCAF_982585_0_whole_genome_shotgun_sequence
JWIN03000014.1_51400374-51400700_Camelus_dromedarius_breed_African_isolate_Drom800_chromosome_14_whole_genome_shotgun_sequence
R_PVJP02006662.1_7592890-7593002_Hippopotamus_amphibius_isolate_BS01_ScHSfme_6634HRSCAF_53397_whole_genome_shotgun_sequence
R_PVJP02006662.1_10004383-10005031_Hippopotamus_amphibius_isolate_BS01_ScHSfme_6634HRSCAF_53397_whole_genome_shotgun_sequence
R_LSZX01117910.1_6265-6926_Camelus_dromedarius_breed_Targui_Contig_15614_33_whole_genome_shotgun_sequence
R_SIDA01000008.1_35411336-35411714_Sus_scrofa_breed_Bama_miniature_chromosome_16_whole_genome_shotgun_sequence
R_PVHT020000015.1_33450827-33451346_Catagonus_wagneri_isolate_BS18_Sc2sB16_12HRSCAF_3972_0_whole_genome_shotgun_sequence
R_SIDA01000019.1_102001567-102002218_Sus_scrofa_breed_Bama_miniature_chromosome_X_whole_genome_shotgun_sequence
R_PVHT020000015.1_33450548-33450691_Catagonus_wagneri_isolate_BS18_Sc2sB16_12HRSCAF_3972_0_whole_genome_shotgun_sequence
PVJP02910818.1_2640529-2641177_Hippopotamus_amphibius_isolate_BS01_ScHSfme_925262HRSCAF_983062_whole_genome_shotgun_sequence
VSZR01000001.1_100142061-100142431_Camelus_ferus_isolate_YT-003-E_chromosome_1_whole_genome_shotgun_sequence
R_JWIN03000001.1_22548946-22549316_Camelus_dromedarius_breed_African_isolate_Drom800_chromosome_1_whole_genome_shotgun_sequence
PVJP02000051.1_739929-740598_Hippopotamus_amphibius_isolate_BS01_ScHSfme_50HRSCAF_9451_0_whole_genome_shotgun_sequence
R_PVJP02910473.1_632970-633629_Hippopotamus_amphibius_isolate_BS01_ScHSfme_924940HRSCAF_982740_whole_genome_shotgun_sequence
R_CAOW010392369.1_3000-3662_Camelus_bactrianus_WGS_project_CAOW00000000_data_contig_489195_whole_genome_shotgun_sequence
AGVR01039554.1_341074-341736_Camelus_ferus_isolate_Naran_contig039798_whole_genome_shotgun_sequence
R_JWIN03000002.1_28701856-28702518_Camelus_dromedarius_breed_African_isolate_Drom800_chromosome_2_whole_genome_shotgun_sequence
VBQZ03000011.1_29437330-29437991_Bos_mutus_breed_Datong_Yak_isolate_WY2019_wild_yak_scaffold11_whole_genome_shotgun_sequence
VCEA01000001.1_369985494-369986140_Muntiacus_muntjak_isolate_UTSW_UCB_Mm_chromosome_1_whole_genome_shotgun_sequence
R_VSZR01000012.1_93129134-93129750_Camelus_ferus_isolate_YT-003-E_chromosome_2_whole_genome_shotgun_sequence
JARL01019877.1_332136-332738_Camelus_bactrianus_breed_Alxa_contig19877_whole_genome_shotgun_sequence
R_LSZX01069380.1_19-296_Camelus_dromedarius_breed_Targui_Contig_13334_55_whole_genome_shotgun_sequence
JWIN03000002.1_28715971-28716571_Camelus_dromedarius_breed_African_isolate_Drom800_chromosome_2_whole_genome_shotgun_sequence
0.03

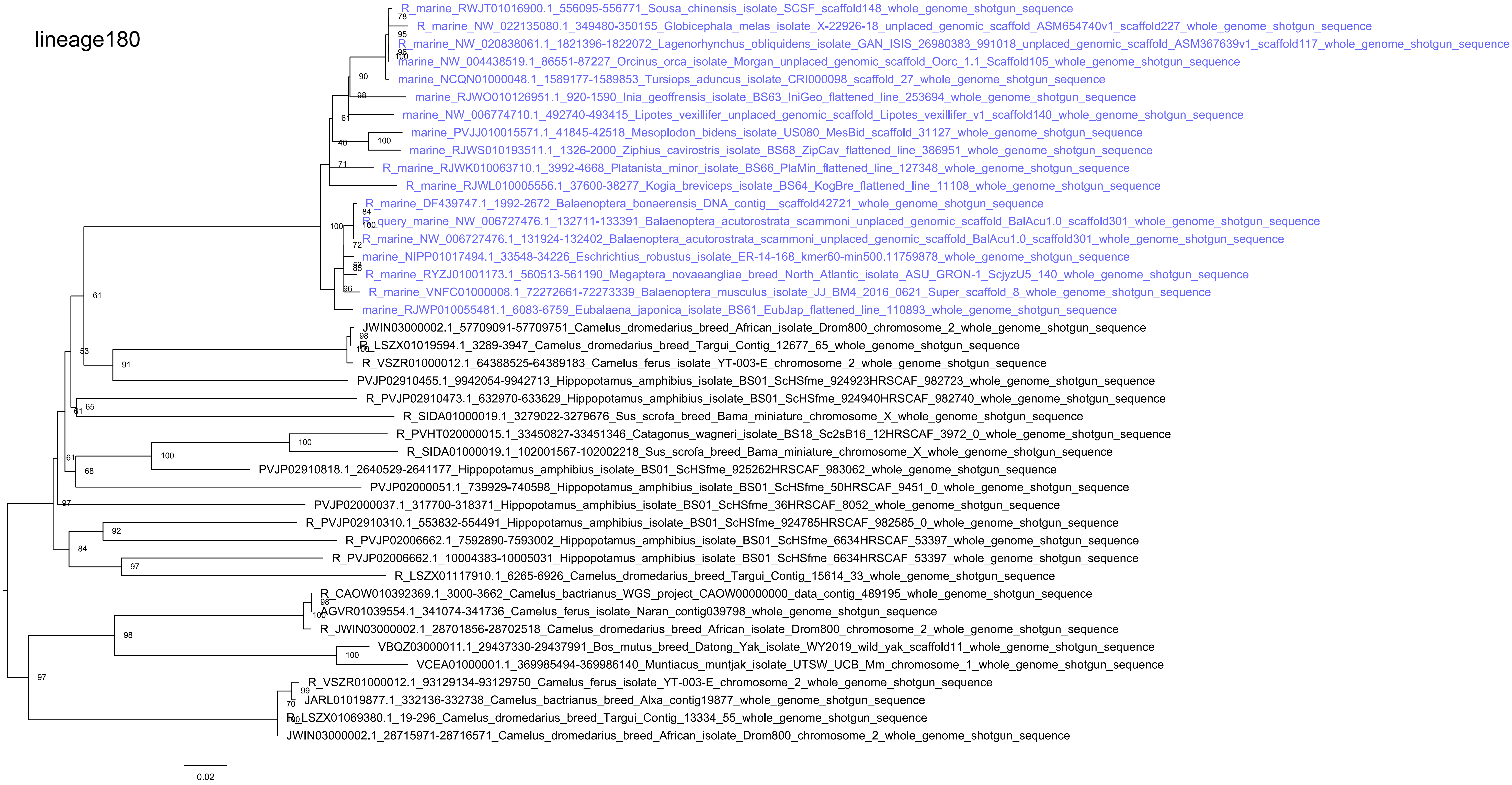

lineage180
R_marine_RWJT01016900.1_556095-556771_Sousa_chinensis_isolate_SCSF_scaffold148_whole_genome_shotgun_sequence
R_marine_NW_022135080.1_349480-350155_Globicephala_melas_isolate_X-22926-18_unplaced_genomic_scaffold_ASM654740v1_scaffold227_whole_genome_shotgun_sequence
R_marine_NW_020838061.1_1821396-1822072_Lagenorhynchus_obliquidens_isolate_GAN_ISIS_26980383_991018_unplaced_genomic_scaffold_ASM367639v1_scaffold117_whole_genome_shotgun_sequence
marine_NW_004438519.1_86551-87227_Orcinus_orca_isolate_Morgan_unplaced_genomic_scaffold_Oorc_1.1_Scaffold105_whole_genome_shotgun_sequence
marine_NCQN01000048.1_1589177-1589853_Tursiops_aduncus_isolate_CRI000098_scaffold_27_whole_genome_shotgun_sequence
marine_RJWO010126951.1_920-1590_Inia_geoffrensis_isolate_BS63_IniGeo_flattened_line_253694_whole_genome_shotgun_sequence
marine_NW_006774710.1_492740-493415_Lipotes_vexillifer_unplaced_genomic_scaffold_Lipotes_vexillifer_v1_scaffold140_whole_genome_shotgun_sequence
marine_PVJJ010015571.1_41845-42518_Mesoplodon_bidens_isolate_US080_MesBid_scaffold_31127_whole_genome_shotgun_sequence
marine_RJWS010193511.1_1326-2000_Ziphius_cavirostris_isolate_BS68_ZipCav_flattened_line_386951_whole_genome_shotgun_sequence
R_marine_RJWK010063710.1_3992-4668_Platanista_minor_isolate_BS66_PlaMin_flattened_line_127348_whole_genome_shotgun_sequence
R_marine_RJWL010005556.1_37600-38277_Kogia_breviceps_isolate_BS64_KogBre_flattened_line_11108_whole_genome_shotgun_sequence
R_marine_DF439747.1_1992-2672_Balaenoptera_bonaerensis_DNA_contig__scaffold42721_whole_genome_shotgun_sequence
R_query_marine_NW_006727476.1_132711-133391_Balaenoptera_acutorostrata_scammoni_unplaced_genomic_scaffold_BalAcu1.0_scaffold301_whole_genome_shotgun_sequence
R_marine_NW_006727476.1_131924-132402_Balaenoptera_acutorostrata_scammoni_unplaced_genomic_scaffold_BalAcu1.0_scaffold301_whole_genome_shotgun_sequence
marine_NIPP01017494.1_33548-34226_Eschrichtius_robustus_isolate_ER-14-168_kmer60-min500.11759878_whole_genome_shotgun_sequence
R_marine_RYZJ01001173.1_560513-561190_Megaptera_novaeangliae_breed_North_Atlantic_isolate_ASU_GRON-1_ScjyzU5_140_whole_genome_shotgun_sequence
R_marine_VNFC01000008.1_72272661-72273339_Balaenoptera_musculus_isolate_JJ_BM4_2016_0621_Super_scaffold_8_whole_genome_shotgun_sequence
marine_RJWP010055481.1_6083-6759_Eubalaena_japonica_isolate_BS61_EubJap_flattened_line_110893_whole_genome_shotgun_sequence
JWIN03000002.1_57709091-57709751_Camelus_dromedarius_breed_African_isolate_Drom800_chromosome_2_whole_genome_shotgun_sequence
R_LSZX01019594.1_3289-3947_Camelus_dromedarius_breed_Targui_Contig_12677_65_whole_genome_shotgun_sequence
R_VSZR01000012.1_64388525-64389183_Camelus_ferus_isolate_YT-003-E_chromosome_2_whole_genome_shotgun_sequence
PVJP02910455.1_9942054-9942713_Hippopotamus_amphibius_isolate_BS01_ScHSfme_924923HRSCAF_982723_whole_genome_shotgun_sequence
R_PVJP02910473.1_632970-633629_Hippopotamus_amphibius_isolate_BS01_ScHSfme_924940HRSCAF_982740_whole_genome_shotgun_sequence
R_SIDA01000019.1_3279022-3279676_Sus_scrofa_breed_Bama_miniature_chromosome_X_whole_genome_shotgun_sequence
R_PVHT020000015.1_33450827-33451346_Catagonus_wagneri_isolate_BS18_Sc2sB16_12HRSCAF_3972_0_whole_genome_shotgun_sequence
R_SIDA01000019.1_102001567-102002218_Sus_scrofa_breed_Bama_miniature_chromosome_X_whole_genome_shotgun_sequence
PVJP02910818.1_2640529-2641177_Hippopotamus_amphibius_isolate_BS01_ScHSfme_925262HRSCAF_983062_whole_genome_shotgun_sequence
PVJP02000051.1_739929-740598_Hippopotamus_amphibius_isolate_BS01_ScHSfme_50HRSCAF_9451_0_whole_genome_shotgun_sequence
PVJP02000037.1_317700-318371_Hippopotamus_amphibius_isolate_BS01_ScHSfme_36HRSCAF_8052_whole_genome_shotgun_sequence
R_PVJP02910310.1_553832-554491_Hippopotamus_amphibius_isolate_BS01_ScHSfme_924785HRSCAF_982585_0_whole_genome_shotgun_sequence
R_PVJP02006662.1_7592890-7593002_Hippopotamus_amphibius_isolate_BS01_ScHSfme_6634HRSCAF_53397_whole_genome_shotgun_sequence
R_PVJP02006662.1_10004383-10005031_Hippopotamus_amphibius_isolate_BS01_ScHSfme_6634HRSCAF_53397_whole_genome_shotgun_sequence
R_LSZX01117910.1_6265-6926_Camelus_dromedarius_breed_Targui_Contig_15614_33_whole_genome_shotgun_sequence
R_CAOW010392369.1_3000-3662_Camelus_bactrianus_WGS_project_CAOW00000000_data_contig_489195_whole_genome_shotgun_sequence
AGVR01039554.1_341074-341736_Camelus_ferus_isolate_Naran_contig039798_whole_genome_shotgun_sequence
R_JWIN03000002.1_28701856-28702518_Camelus_dromedarius_breed_African_isolate_Drom800_chromosome_2_whole_genome_shotgun_sequence
VBQZ03000011.1_29437330-29437991_Bos_mutus_breed_Datong_Yak_isolate_WY2019_wild_yak_scaffold11_whole_genome_shotgun_sequence
VCEA01000001.1_369985494-369986140_Muntiacus_muntjak_isolate_UTSW_UCB_Mm_chromosome_1_whole_genome_shotgun_sequence
R_VSZR01000012.1_93129134-93129750_Camelus_ferus_isolate_YT-003-E_chromosome_2_whole_genome_shotgun_sequence
JARL01019877.1_332136-332738_Camelus_bactrianus_breed_Alxa_contig19877_whole_genome_shotgun_sequence
R_LSZX01069380.1_19-296_Camelus_dromedarius_breed_Targui_Contig_13334_55_whole_genome_shotgun_sequence
JWIN03000002.1_28715971-28716571_Camelus_dromedarius_breed_African_isolate_Drom800_chromosome_2_whole_genome_shotgun_sequence
0.02

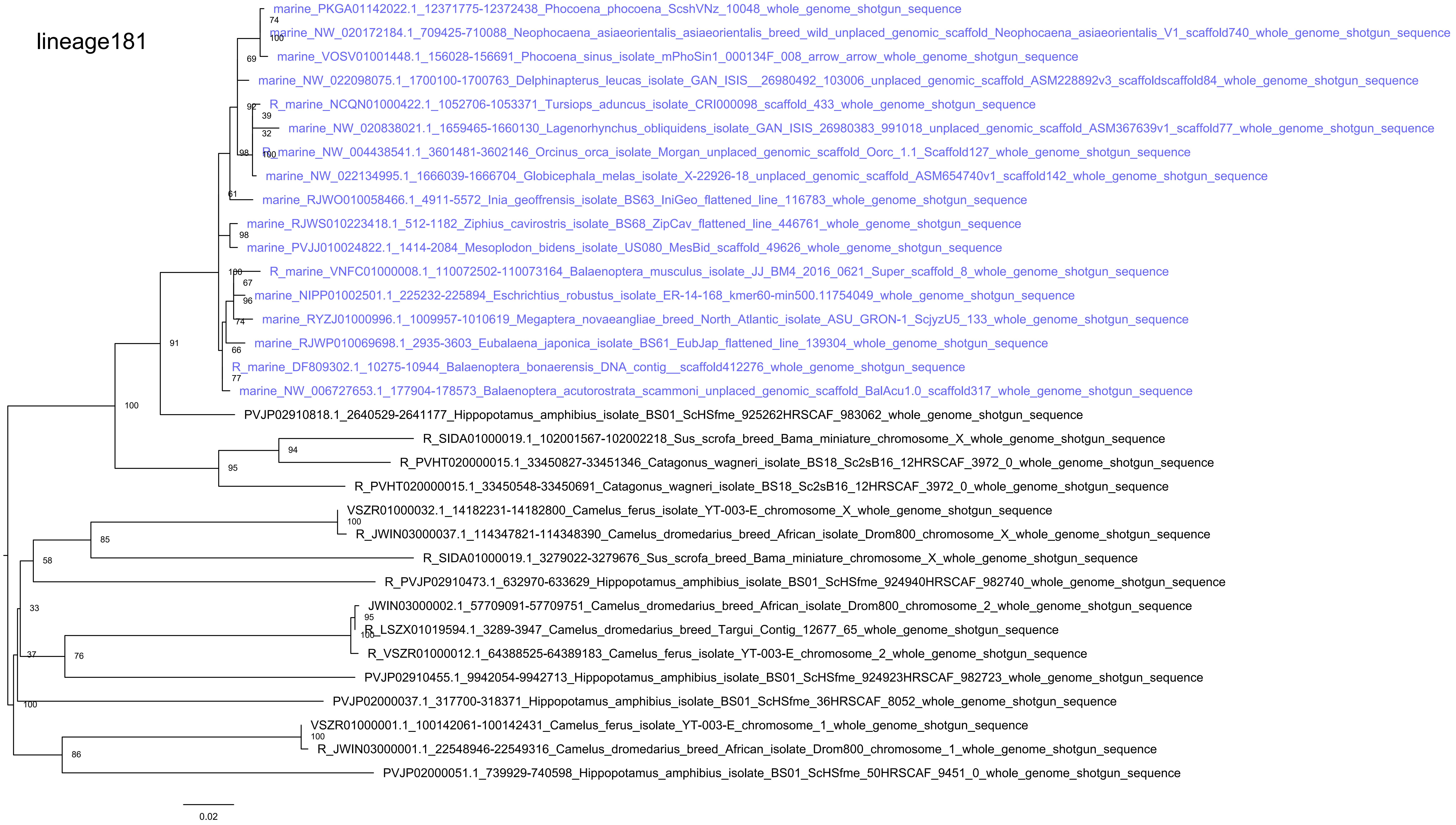
lineage181
marine_PKGA01142022.1_12371775-12372438_Phocoena_phocoena_ScshVNz_10048_whole_genome_shotgun_sequence
marine_NW_020172184.1_709425-710088_Neophocaena_asiaeorientalis_asiaeorientalis_breed_wild_unplaced_genomic_scaffold_Neophocaena_asiaeorientalis_V1_scaffold740_whole_genome_shotgun_sequence
marine_VOSV01001448.1_156028-156691_Phocoena_sinus_isolate_mPhoSin1_000134F_008_arrow_arrow_whole_genome_shotgun_sequence
marine_NW_022098075.1_1700100-1700763_Delphinapterus_leucas_isolate_GAN_ISIS__26980492_103006_unplaced_genomic_scaffold_ASM228892v3_scaffoldscaffold84_whole_genome_shotgun_sequence
R_marine_NCQN01000422.1_1052706-1053371_Tursiops_aduncus_isolate_CRI000098_scaffold_433_whole_genome_shotgun_sequence
marine_NW_020838021.1_1659465-1660130_Lagenorhynchus_obliquidens_isolate_GAN_ISIS_26980383_991018_unplaced_genomic_scaffold_ASM367639v1_scaffold77_whole_genome_shotgun_sequence
R_marine_NW_004438541.1_3601481-3602146_Orcinus_orca_isolate_Morgan_unplaced_genomic_scaffold_Oorc_1.1_Scaffold127_whole_genome_shotgun_sequence
marine_NW_022134995.1_1666039-1666704_Globicephala_melas_isolate_X-22926-18_unplaced_genomic_scaffold_ASM654740v1_scaffold142_whole_genome_shotgun_sequence
marine_RJWO010058466.1_4911-5572_Inia_geoffrensis_isolate_BS63_IniGeo_flattened_line_116783_whole_genome_shotgun_sequence
marine_RJWS010223418.1_512-1182_Ziphius_cavirostris_isolate_BS68_ZipCav_flattened_line_446761_whole_genome_shotgun_sequence
marine_PVJJ010024822.1_1414-2084_Mesoplodon_bidens_isolate_US080_MesBid_scaffold_49626_whole_genome_shotgun_sequence
R_marine_VNFC01000008.1_110072502-110073164_Balaenoptera_musculus_isolate_JJ_BM4_2016_0621_Super_scaffold_8_whole_genome_shotgun_sequence
marine_NIPP01002501.1_225232-225894_Eschrichtius_robustus_isolate_ER-14-168_kmer60-min500.11754049_whole_genome_shotgun_sequence
marine_RYZJ01000996.1_1009957-1010619_Megaptera_novaeangliae_breed_North_Atlantic_isolate_ASU_GRON-1_ScjyzU5_133_whole_genome_shotgun_sequence
marine_RJWP010069698.1_2935-3603_Eubalaena_japonica_isolate_BS61_EubJap_flattened_line_139304_whole_genome_shotgun_sequence
R_marine_DF809302.1_10275-10944_Balaenoptera_bonaerensis_DNA_contig__scaffold412276_whole_genome_shotgun_sequence
marine_NW_006727653.1_177904-178573_Balaenoptera_acutorostrata_scammoni_unplaced_genomic_scaffold_BalAcu1.0_scaffold317_whole_genome_shotgun_sequence
PVJP02910818.1_2640529-2641177_Hippopotamus_amphibius_isolate_BS01_ScHSfme_925262HRSCAF_983062_whole_genome_shotgun_sequence
R_SIDA01000019.1_102001567-102002218_Sus_scrofa_breed_Bama_miniature_chromosome_X_whole_genome_shotgun_sequence
R_PVHT020000015.1_33450827-33451346_Catagonus_wagneri_isolate_BS18_Sc2sB16_12HRSCAF_3972_0_whole_genome_shotgun_sequence
R_PVHT020000015.1_33450548-33450691_Catagonus_wagneri_isolate_BS18_Sc2sB16_12HRSCAF_3972_0_whole_genome_shotgun_sequence
VSZR01000032.1_14182231-14182800_Camelus_ferus_isolate_YT-003-E_chromosome_X_whole_genome_shotgun_sequence
R_JWIN03000037.1_114347821-114348390_Camelus_dromedarius_breed_African_isolate_Drom800_chromosome_X_whole_genome_shotgun_sequence
R_SIDA01000019.1_3279022-3279676_Sus_scrofa_breed_Bama_miniature_chromosome_X_whole_genome_shotgun_sequence
R_PVJP02910473.1_632970-633629_Hippopotamus_amphibius_isolate_BS01_ScHSfme_924940HRSCAF_982740_whole_genome_shotgun_sequence
JWIN03000002.1_57709091-57709751_Camelus_dromedarius_breed_African_isolate_Drom800_chromosome_2_whole_genome_shotgun_sequence
R_LSZX01019594.1_3289-3947_Camelus_dromedarius_breed_Targui_Contig_12677_65_whole_genome_shotgun_sequence
R_VSZR01000012.1_64388525-64389183_Camelus_ferus_isolate_YT-003-E_chromosome_2_whole_genome_shotgun_sequence
PVJP02910455.1_9942054-9942713_Hippopotamus_amphibius_isolate_BS01_ScHSfme_924923HRSCAF_982723_whole_genome_shotgun_sequence
PVJP02000037.1_317700-318371_Hippopotamus_amphibius_isolate_BS01_ScHSfme_36HRSCAF_8052_whole_genome_shotgun_sequence
VSZR01000001.1_100142061-100142431_Camelus_ferus_isolate_YT-003-E_chromosome_1_whole_genome_shotgun_sequence
R_JWIN03000001.1_22548946-22549316_Camelus_dromedarius_breed_African_isolate_Drom800_chromosome_1_whole_genome_shotgun_sequence
PVJP02000051.1_739929-740598_Hippopotamus_amphibius_isolate_BS01_ScHSfme_50HRSCAF_9451_0_whole_genome_shotgun_sequence
0.02

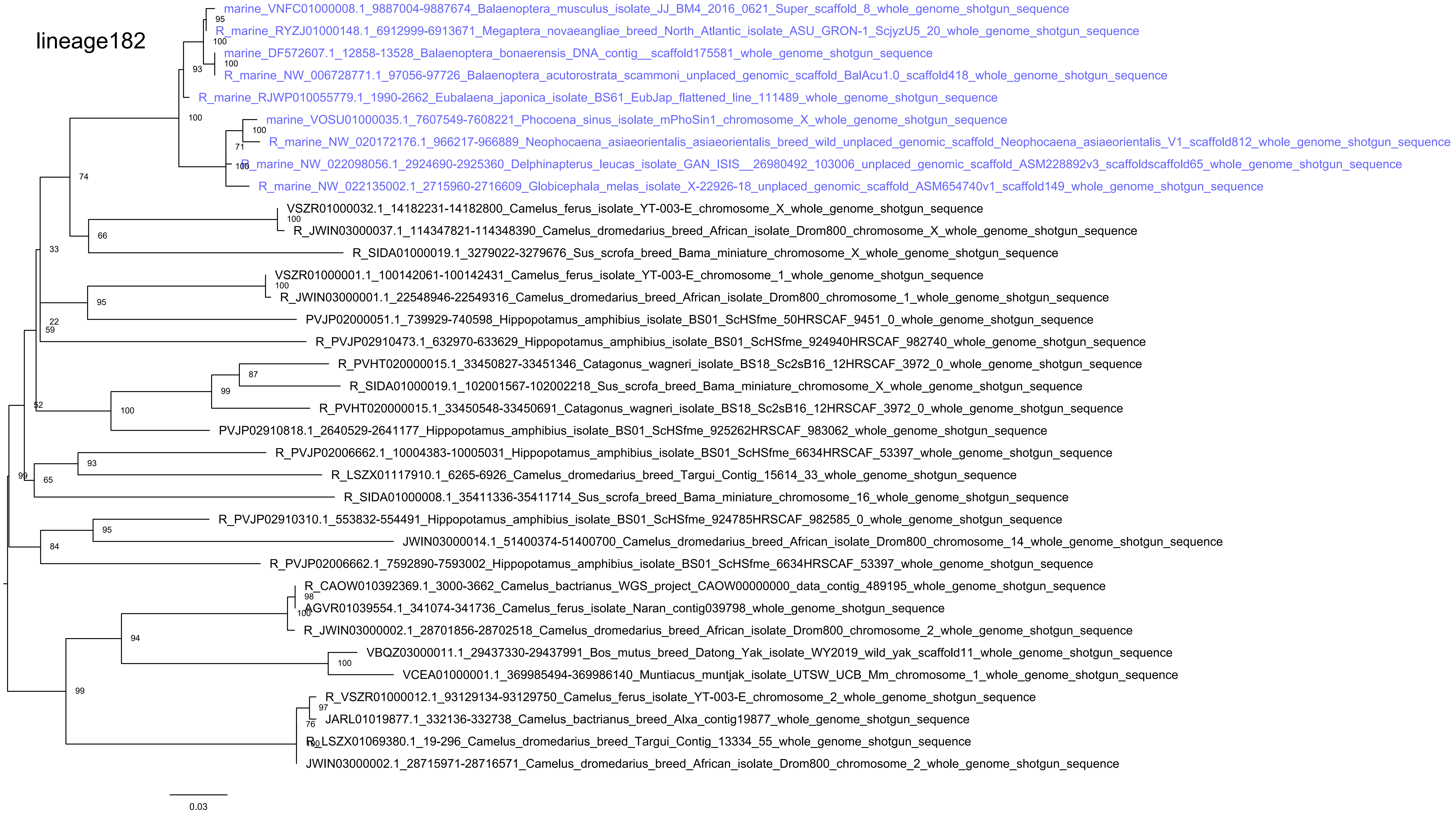
lineage182
marine_VNFC01000008.1_9887004-9887674_Balaenoptera_musculus_isolate_JJ_BM4_2016_0621_Super_scaffold_8_whole_genome_shotgun_sequence
R_marine_RYZJ01000148.1_6912999-6913671_Megaptera_novaeangliae_breed_North_Atlantic_isolate_ASU_GRON-1_ScjyzU5_20_whole_genome_shotgun_sequence
marine_DF572607.1_12858-13528_Balaenoptera_bonaerensis_DNA_contig__scaffold175581_whole_genome_shotgun_sequence
R_marine_NW_006728771.1_97056-97726_Balaenoptera_acutorostrata_scammoni_unplaced_genomic_scaffold_BalAcu1.0_scaffold418_whole_genome_shotgun_sequence
R_marine_RJWP010055779.1_1990-2662_Eubalaena_japonica_isolate_BS61_EubJap_flattened_line_111489_whole_genome_shotgun_sequence
marine_VOSU01000035.1_7607549-7608221_Phocoena_sinus_isolate_mPhoSin1_chromosome_X_whole_genome_shotgun_sequence
R_marine_NW_020172176.1_966217-966889_Neophocaena_asiaeorientalis_asiaeorientalis_breed_wild_unplaced_genomic_scaffold_Neophocaena_asiaeorientalis_V1_scaffold812_whole_genome_shotgun_sequence
R_marine_NW_022098056.1_2924690-2925360_Delphinapterus_leucas_isolate_GAN_ISIS__26980492_103006_unplaced_genomic_scaffold_ASM228892v3_scaffoldscaffold65_whole_genome_shotgun_sequence
R_marine_NW_022135002.1_2715960-2716609_Globicephala_melas_isolate_X-22926-18_unplaced_genomic_scaffold_ASM654740v1_scaffold149_whole_genome_shotgun_sequence
VSZR01000032.1_14182231-14182800_Camelus_ferus_isolate_YT-003-E_chromosome_X_whole_genome_shotgun_sequence
R_JWIN03000037.1_114347821-114348390_Camelus_dromedarius_breed_African_isolate_Drom800_chromosome_X_whole_genome_shotgun_sequence
R_SIDA01000019.1_3279022-3279676_Sus_scrofa_breed_Bama_miniature_chromosome_X_whole_genome_shotgun_sequence
VSZR01000001.1_100142061-100142431_Camelus_ferus_isolate_YT-003-E_chromosome_1_whole_genome_shotgun_sequence
R_JWIN03000001.1_22548946-22549316_Camelus_dromedarius_breed_African_isolate_Drom800_chromosome_1_whole_genome_shotgun_sequence
PVJP02000051.1_739929-740598_Hippopotamus_amphibius_isolate_BS01_ScHSfme_50HRSCAF_9451_0_whole_genome_shotgun_sequence
R_PVJP02910473.1_632970-633629_Hippopotamus_amphibius_isolate_BS01_ScHSfme_924940HRSCAF_982740_whole_genome_shotgun_sequence
R_PVHT020000015.1_33450827-33451346_Catagonus_wagneri_isolate_BS18_Sc2sB16_12HRSCAF_3972_0_whole_genome_shotgun_sequence
R_SIDA01000019.1_102001567-102002218_Sus_scrofa_breed_Bama_miniature_chromosome_X_whole_genome_shotgun_sequence
R_PVHT020000015.1_33450548-33450691_Catagonus_wagneri_isolate_BS18_Sc2sB16_12HRSCAF_3972_0_whole_genome_shotgun_sequence
PVJP02910818.1_2640529-2641177_Hippopotamus_amphibius_isolate_BS01_ScHSfme_925262HRSCAF_983062_whole_genome_shotgun_sequence
R_PVJP02006662.1_10004383-10005031_Hippopotamus_amphibius_isolate_BS01_ScHSfme_6634HRSCAF_53397_whole_genome_shotgun_sequence
R_LSZX01117910.1_6265-6926_Camelus_dromedarius_breed_Targui_Contig_15614_33_whole_genome_shotgun_sequence
R_SIDA01000008.1_35411336-35411714_Sus_scrofa_breed_Bama_miniature_chromosome_16_whole_genome_shotgun_sequence
R_PVJP02910310.1_553832-554491_Hippopotamus_amphibius_isolate_BS01_ScHSfme_924785HRSCAF_982585_0_whole_genome_shotgun_sequence
JWIN03000014.1_51400374-51400700_Camelus_dromedarius_breed_African_isolate_Drom800_chromosome_14_whole_genome_shotgun_sequence
R_PVJP02006662.1_7592890-7593002_Hippopotamus_amphibius_isolate_BS01_ScHSfme_6634HRSCAF_53397_whole_genome_shotgun_sequence
R_CAOW010392369.1_3000-3662_Camelus_bactrianus_WGS_project_CAOW00000000_data_contig_489195_whole_genome_shotgun_sequence
AGVR01039554.1_341074-341736_Camelus_ferus_isolate_Naran_contig039798_whole_genome_shotgun_sequence
R_JWIN03000002.1_28701856-28702518_Camelus_dromedarius_breed_African_isolate_Drom800_chromosome_2_whole_genome_shotgun_sequence
VBQZ03000011.1_29437330-29437991_Bos_mutus_breed_Datong_Yak_isolate_WY2019_wild_yak_scaffold11_whole_genome_shotgun_sequence
VCEA01000001.1_369985494-369986140_Muntiacus_muntjak_isolate_UTSW_UCB_Mm_chromosome_1_whole_genome_shotgun_sequence
R_VSZR01000012.1_93129134-93129750_Camelus_ferus_isolate_YT-003-E_chromosome_2_whole_genome_shotgun_sequence
JARL01019877.1_332136-332738_Camelus_bactrianus_breed_Alxa_contig19877_whole_genome_shotgun_sequence
R_LSZX01069380.1_19-296_Camelus_dromedarius_breed_Targui_Contig_13334_55_whole_genome_shotgun_sequence
JWIN03000002.1_28715971-28716571_Camelus_dromedarius_breed_African_isolate_Drom800_chromosome_2_whole_genome_shotgun_sequence
0.03

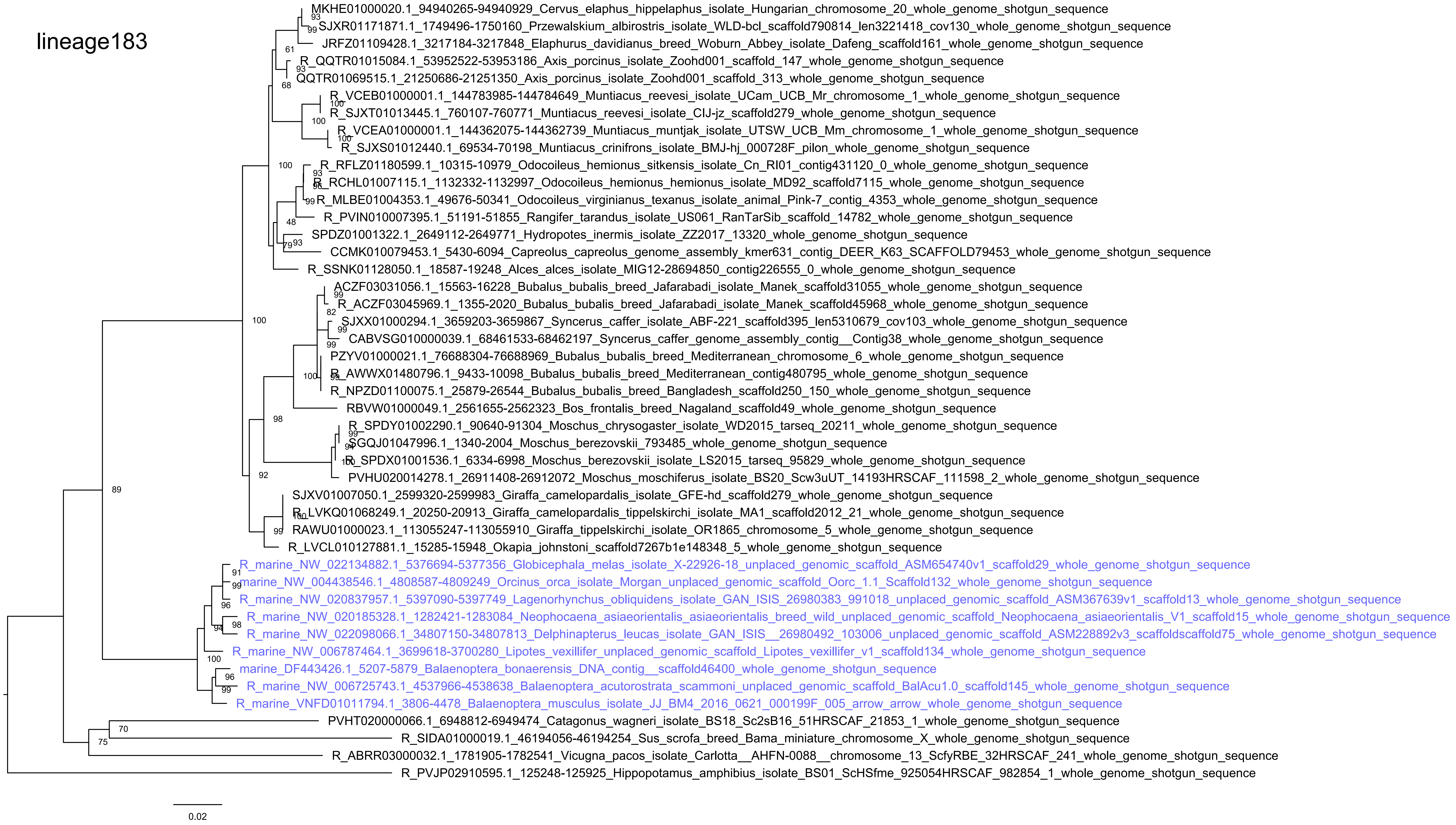

lineage183
MKHE01000020.1_94940265-94940929_Cervus_elaphus_hippelaphus_isolate_Hungarian_chromosome_20_whole_genome_shotgun_sequence
SJXR01171871.1_1749496-1750160_Przewalskium_albirostris_isolate_WLD-bcl_scaffold790814_len3221418_cov130_whole_genome_shotgun_sequence
JRFZ01109428.1_3217184-3217848_Elaphurus_davidianus_breed_Woburn_Abbey_isolate_Dafeng_scaffold161_whole_genome_shotgun_sequence
R_QQTR01015084.1_53952522-53953186_Axis_porcinus_isolate_Zoohd001_scaffold_147_whole_genome_shotgun_sequence
QQTR01069515.1_21250686-21251350_Axis_porcinus_isolate_Zoohd001_scaffold_313_whole_genome_shotgun_sequence
R_VCEB01000001.1_144783985-144784649_Muntiacus_reevesi_isolate_UCam_UCB_Mr_chromosome_1_whole_genome_shotgun_sequence
R_SJXT01013445.1_760107-760771_Muntiacus_reevesi_isolate_CIJ-jz_scaffold279_whole_genome_shotgun_sequence
R_VCEA01000001.1_144362075-144362739_Muntiacus_muntjak_isolate_UTSW_UCB_Mm_chromosome_1_whole_genome_shotgun_sequence
R_SJXS01012440.1_69534-70198_Muntiacus_crinifrons_isolate_BMJ-hj_000728F_pilon_whole_genome_shotgun_sequence
R_RFLZ01180599.1_10315-10979_Odocoileus_hemionus_sitkensis_isolate_Cn_RI01_contig431120_0_whole_genome_shotgun_sequence
R_RCHL01007115.1_1132332-1132997_Odocoileus_hemionus_hemionus_isolate_MD92_scaffold7115_whole_genome_shotgun_sequence
R_MLBE01004353.1_49676-50341_Odocoileus_virginianus_texanus_isolate_animal_Pink-7_contig_4353_whole_genome_shotgun_sequence
R_PVIN010007395.1_51191-51855_Rangifer_tarandus_isolate_US061_RanTarSib_scaffold_14782_whole_genome_shotgun_sequence
SPDZ01001322.1_2649112-2649771_Hydropotes_inermis_isolate_ZZ2017_13320_whole_genome_shotgun_sequence
CCMK010079453.1_5430-6094_Capreolus_capreolus_genome_assembly_kmer631_contig_DEER_K63_SCAFFOLD79453_whole_genome_shotgun_sequence
R_SSNK01128050.1_18587-19248_Alces_alces_isolate_MIG12-28694850_contig226555_0_whole_genome_shotgun_sequence
ACZF03031056.1_15563-16228_Bubalus_bubalis_breed_Jafarabadi_isolate_Manek_scaffold31055_whole_genome_shotgun_sequence
R_ACZF03045969.1_1355-2020_Bubalus_bubalis_breed_Jafarabadi_isolate_Manek_scaffold45968_whole_genome_shotgun_sequence
SJXX01000294.1_3659203-3659867_Syncerus_caffer_isolate_ABF-221_scaffold395_len5310679_cov103_whole_genome_shotgun_sequence
CABVSG010000039.1_68461533-68462197_Syncerus_caffer_genome_assembly_contig__Contig38_whole_genome_shotgun_sequence
PZYV01000021.1_76688304-76688969_Bubalus_bubalis_breed_Mediterranean_chromosome_6_whole_genome_shotgun_sequence
R_AWWX01480796.1_9433-10098_Bubalus_bubalis_breed_Mediterranean_contig480795_whole_genome_shotgun_sequence
R_NPZD01100075.1_25879-26544_Bubalus_bubalis_breed_Bangladesh_scaffold250_150_whole_genome_shotgun_sequence
RBVW01000049.1_2561655-2562323_Bos_frontalis_breed_Nagaland_scaffold49_whole_genome_shotgun_sequence
R_SPDY01002290.1_90640-91304_Moschus_chrysogaster_isolate_WD2015_tarseq_20211_whole_genome_shotgun_sequence
SGQJ01047996.1_1340-2004_Moschus_berezovskii_793485_whole_genome_shotgun_sequence
R_SPDX01001536.1_6334-6998_Moschus_berezovskii_isolate_LS2015_tarseq_95829_whole_genome_shotgun_sequence
PVHU020014278.1_26911408-26912072_Moschus_moschiferus_isolate_BS20_Scw3uUT_14193HRSCAF_111598_2_whole_genome_shotgun_sequence
SJXV01007050.1_2599320-2599983_Giraffa_camelopardalis_isolate_GFE-hd_scaffold279_whole_genome_shotgun_sequence
R_LVKQ01068249.1_20250-20913_Giraffa_camelopardalis_tippelskirchi_isolate_MA1_scaffold2012_21_whole_genome_shotgun_sequence
RAWU01000023.1_113055247-113055910_Giraffa_tippelskirchi_isolate_OR1865_chromosome_5_whole_genome_shotgun_sequence
R_LVCL010127881.1_15285-15948_Okapia_johnstoni_scaffold7267b1e148348_5_whole_genome_shotgun_sequence
R_marine_NW_022134882.1_5376694-5377356_Globicephala_melas_isolate_X-22926-18_unplaced_genomic_scaffold_ASM654740v1_scaffold29_whole_genome_shotgun_sequence
marine_NW_004438546.1_4808587-4809249_Orcinus_orca_isolate_Morgan_unplaced_genomic_scaffold_Oorc_1.1_Scaffold132_whole_genome_shotgun_sequence
R_marine_NW_020837957.1_5397090-5397749_Lagenorhynchus_obliquidens_isolate_GAN_ISIS_26980383_991018_unplaced_genomic_scaffold_ASM367639v1_scaffold13_whole_genome_shotgun_sequence
R_marine_NW_020185328.1_1282421-1283084_Neophocaena_asiaeorientalis_asiaeorientalis_breed_wild_unplaced_genomic_scaffold_Neophocaena_asiaeorientalis_V1_scaffold15_whole_genome_shotgun_sequence
R_marine_NW_022098066.1_34807150-34807813_Delphinapterus_leucas_isolate_GAN_ISIS__26980492_103006_unplaced_genomic_scaffold_ASM228892v3_scaffoldscaffold75_whole_genome_shotgun_sequence
R_marine_NW_006787464.1_3699618-3700280_Lipotes_vexillifer_unplaced_genomic_scaffold_Lipotes_vexillifer_v1_scaffold134_whole_genome_shotgun_sequence
marine_DF443426.1_5207-5879_Balaenoptera_bonaerensis_DNA_contig__scaffold46400_whole_genome_shotgun_sequence
R_marine_NW_006725743.1_4537966-4538638_Balaenoptera_acutorostrata_scammoni_unplaced_genomic_scaffold_BalAcu1.0_scaffold145_whole_genome_shotgun_sequence
R_marine_VNFD01011794.1_3806-4478_Balaenoptera_musculus_isolate_JJ_BM4_2016_0621_000199F_005_arrow_arrow_whole_genome_shotgun_sequence
PVHT020000066.1_6948812-6949474_Catagonus_wagneri_isolate_BS18_Sc2sB16_51HRSCAF_21853_1_whole_genome_shotgun_sequence
R_SIDA01000019.1_46194056-46194254_Sus_scrofa_breed_Bama_miniature_chromosome_X_whole_genome_shotgun_sequence
R_ABRR03000032.1_1781905-1782541_Vicugna_pacos_isolate_Carlotta__AHFN-0088__chromosome_13_ScfyRBE_32HRSCAF_241_whole_genome_shotgun_sequence
R_PVJP02910595.1_125248-125925_Hippopotamus_amphibius_isolate_BS01_ScHSfme_925054HRSCAF_982854_1_whole_genome_shotgun_sequence
0.02

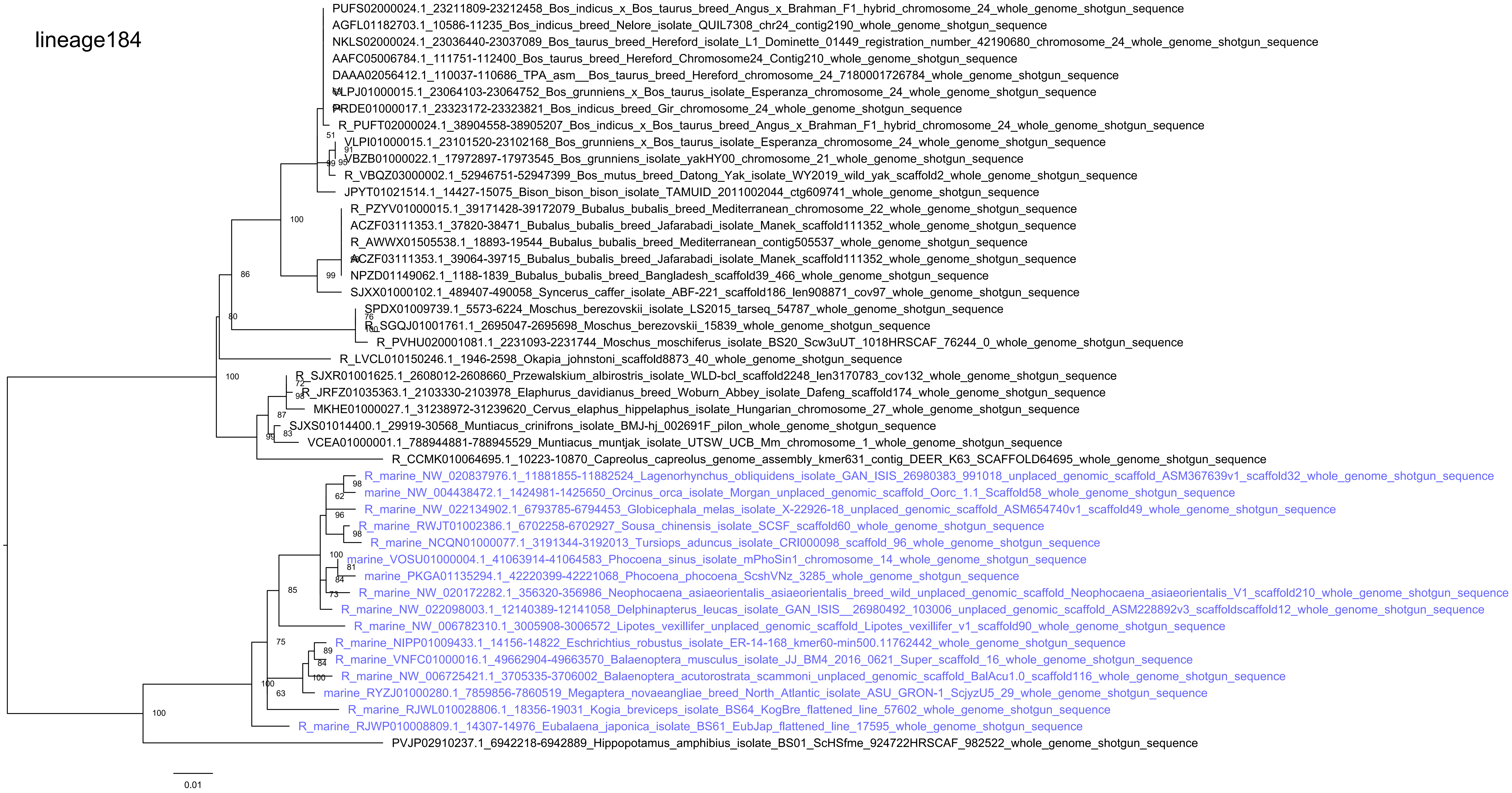

lineage184
PUFS02000024.1_23211809-23212458_Bos_indicus_x_Bos_taurus_breed_Angus_x_Brahman_F1_hybrid_chromosome_24_whole_genome_shotgun_sequence
AGFL01182703.1_10586-11235_Bos_indicus_breed_Nelore_isolate_QUIL7308_chr24_contig2190_whole_genome_shotgun_sequence
NKLS02000024.1_23036440-23037089_Bos_taurus_breed_Hereford_isolate_L1_Dominette_01449_registration_number_42190680_chromosome_24_whole_genome_shotgun_sequence
AAFC05006784.1_111751-112400_Bos_taurus_breed_Hereford_Chromosome24_Contig210_whole_genome_shotgun_sequence
DAAA02056412.1_110037-110686_TPA_asm__Bos_taurus_breed_Hereford_chromosome_24_7180001726784_whole_genome_shotgun_sequence
VLPJ01000015.1_23064103-23064752_Bos_grunniens_x_Bos_taurus_isolate_Esperanza_chromosome_24_whole_genome_shotgun_sequence
PRDE01000017.1_23323172-23323821_Bos_indicus_breed_Gir_chromosome_24_whole_genome_shotgun_sequence
R_PUFT02000024.1_38904558-38905207_Bos_indicus_x_Bos_taurus_breed_Angus_x_Brahman_F1_hybrid_chromosome_24_whole_genome_shotgun_sequence
VLPI01000015.1_23101520-23102168_Bos_grunniens_x_Bos_taurus_isolate_Esperanza_chromosome_24_whole_genome_shotgun_sequence
VBZB01000022.1_17972897-17973545_Bos_grunniens_isolate_yakHY00_chromosome_21_whole_genome_shotgun_sequence
R_VBQZ03000002.1_52946751-52947399_Bos_mutus_breed_Datong_Yak_isolate_WY2019_wild_yak_scaffold2_whole_genome_shotgun_sequence
JPYT01021514.1_14427-15075_Bison_bison_bison_isolate_TAMUID_2011002044_ctg609741_whole_genome_shotgun_sequence
R_PZYV01000015.1_39171428-39172079_Bubalus_bubalis_breed_Mediterranean_chromosome_22_whole_genome_shotgun_sequence
ACZF03111353.1_37820-38471_Bubalus_bubalis_breed_Jafarabadi_isolate_Manek_scaffold111352_whole_genome_shotgun_sequence
R_AWWX01505538.1_18893-19544_Bubalus_bubalis_breed_Mediterranean_contig505537_whole_genome_shotgun_sequence
ACZF03111353.1_39064-39715_Bubalus_bubalis_breed_Jafarabadi_isolate_Manek_scaffold111352_whole_genome_shotgun_sequence
NPZD01149062.1_1188-1839_Bubalus_bubalis_breed_Bangladesh_scaffold39_466_whole_genome_shotgun_sequence
SJXX01000102.1_489407-490058_Syncerus_caffer_isolate_ABF-221_scaffold186_len908871_cov97_whole_genome_shotgun_sequence
SPDX01009739.1_5573-6224_Moschus_berezovskii_isolate_LS2015_tarseq_54787_whole_genome_shotgun_sequence
R_SGQJ01001761.1_2695047-2695698_Moschus_berezovskii_15839_whole_genome_shotgun_sequence
R_PVHU020001081.1_2231093-2231744_Moschus_moschiferus_isolate_BS20_Scw3uUT_1018HRSCAF_76244_0_whole_genome_shotgun_sequence
R_LVCL010150246.1_1946-2598_Okapia_johnstoni_scaffold8873_40_whole_genome_shotgun_sequence
R_SJXR01001625.1_2608012-2608660_Przewalskium_albirostris_isolate_WLD-bcl_scaffold2248_len3170783_cov132_whole_genome_shotgun_sequence
R_JRFZ01035363.1_2103330-2103978_Elaphurus_davidianus_breed_Woburn_Abbey_isolate_Dafeng_scaffold174_whole_genome_shotgun_sequence
MKHE01000027.1_31238972-31239620_Cervus_elaphus_hippelaphus_isolate_Hungarian_chromosome_27_whole_genome_shotgun_sequence
SJXS01014400.1_29919-30568_Muntiacus_crinifrons_isolate_BMJ-hj_002691F_pilon_whole_genome_shotgun_sequence
VCEA01000001.1_788944881-788945529_Muntiacus_muntjak_isolate_UTSW_UCB_Mm_chromosome_1_whole_genome_shotgun_sequence
R_CCMK010064695.1_10223-10870_Capreolus_capreolus_genome_assembly_kmer631_contig_DEER_K63_SCAFFOLD64695_whole_genome_shotgun_sequence
R_marine_NW_020837976.1_11881855-11882524_Lagenorhynchus_obliquidens_isolate_GAN_ISIS_26980383_991018_unplaced_genomic_scaffold_ASM367639v1_scaffold32_whole_genome_shotgun_sequence
marine_NW_004438472.1_1424981-1425650_Orcinus_orca_isolate_Morgan_unplaced_genomic_scaffold_Oorc_1.1_Scaffold58_whole_genome_shotgun_sequence
R_marine_NW_022134902.1_6793785-6794453_Globicephala_melas_isolate_X-22926-18_unplaced_genomic_scaffold_ASM654740v1_scaffold49_whole_genome_shotgun_sequence
R_marine_RWJT01002386.1_6702258-6702927_Sousa_chinensis_isolate_SCSF_scaffold60_whole_genome_shotgun_sequence
R_marine_NCQN01000077.1_3191344-3192013_Tursiops_aduncus_isolate_CRI000098_scaffold_96_whole_genome_shotgun_sequence
marine_VOSU01000004.1_41063914-41064583_Phocoena_sinus_isolate_mPhoSin1_chromosome_14_whole_genome_shotgun_sequence
marine_PKGA01135294.1_42220399-42221068_Phocoena_phocoena_ScshVNz_3285_whole_genome_shotgun_sequence
R_marine_NW_020172282.1_356320-356986_Neophocaena_asiaeorientalis_asiaeorientalis_breed_wild_unplaced_genomic_scaffold_Neophocaena_asiaeorientalis_V1_scaffold210_whole_genome_shotgun_sequence
R_marine_NW_022098003.1_12140389-12141058_Delphinapterus_leucas_isolate_GAN_ISIS__26980492_103006_unplaced_genomic_scaffold_ASM228892v3_scaffoldscaffold12_whole_genome_shotgun_sequence
R_marine_NW_006782310.1_3005908-3006572_Lipotes_vexillifer_unplaced_genomic_scaffold_Lipotes_vexillifer_v1_scaffold90_whole_genome_shotgun_sequence
R_marine_NIPP01009433.1_14156-14822_Eschrichtius_robustus_isolate_ER-14-168_kmer60-min500.11762442_whole_genome_shotgun_sequence
R_marine_VNFC01000016.1_49662904-49663570_Balaenoptera_musculus_isolate_JJ_BM4_2016_0621_Super_scaffold_16_whole_genome_shotgun_sequence
R_marine_NW_006725421.1_3705335-3706002_Balaenoptera_acutorostrata_scammoni_unplaced_genomic_scaffold_BalAcu1.0_scaffold116_whole_genome_shotgun_sequence
marine_RYZJ01000280.1_7859856-7860519_Megaptera_novaeangliae_breed_North_Atlantic_isolate_ASU_GRON-1_ScjyzU5_29_whole_genome_shotgun_sequence
R_marine_RJWL010028806.1_18356-19031_Kogia_breviceps_isolate_BS64_KogBre_flattened_line_57602_whole_genome_shotgun_sequence
R_marine_RJWP010008809.1_14307-14976_Eubalaena_japonica_isolate_BS61_EubJap_flattened_line_17595_whole_genome_shotgun_sequence
PVJP02910237.1_6942218-6942889_Hippopotamus_amphibius_isolate_BS01_ScHSfme_924722HRSCAF_982522_whole_genome_shotgun_sequence
0.01

lineage185

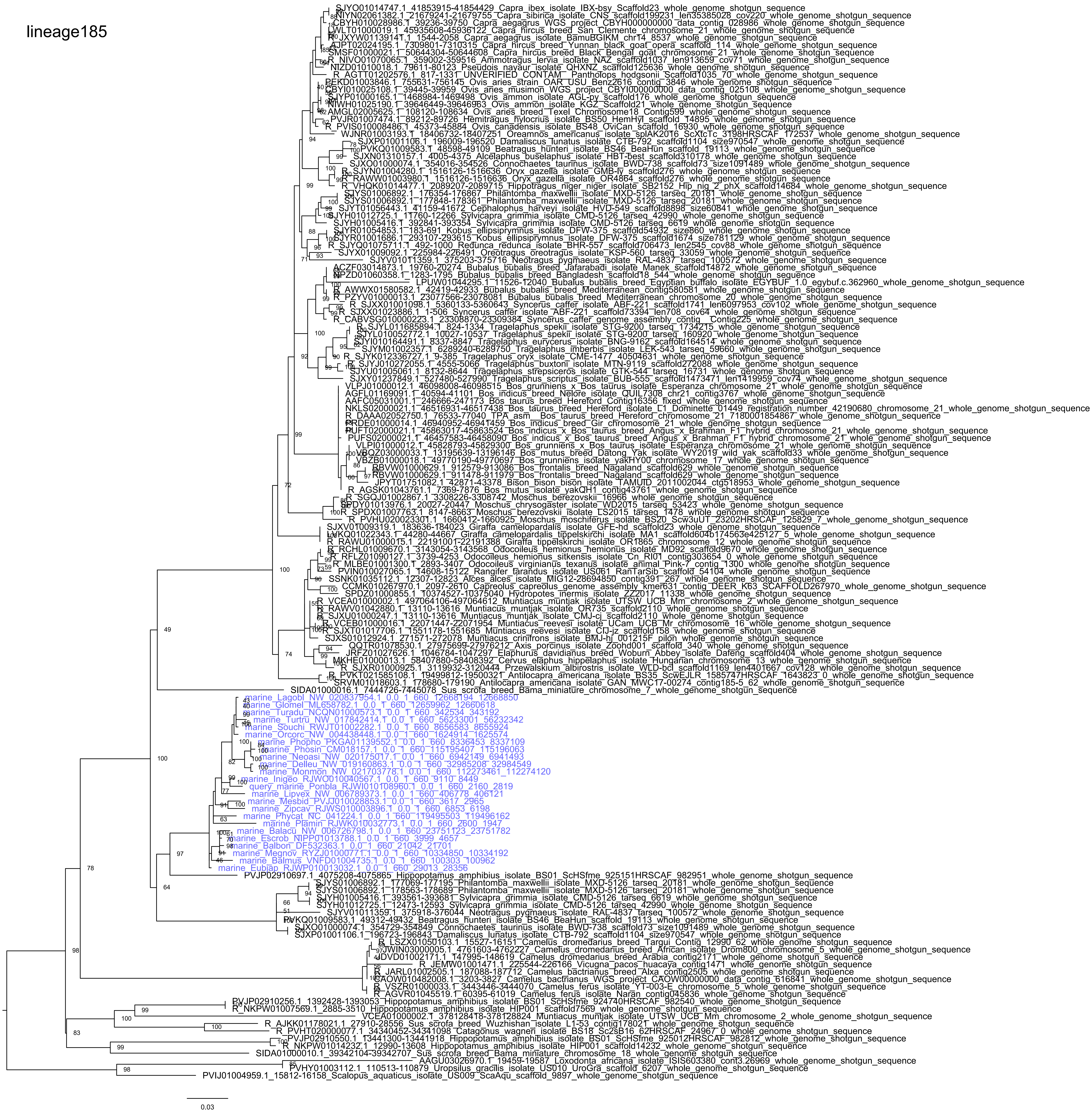

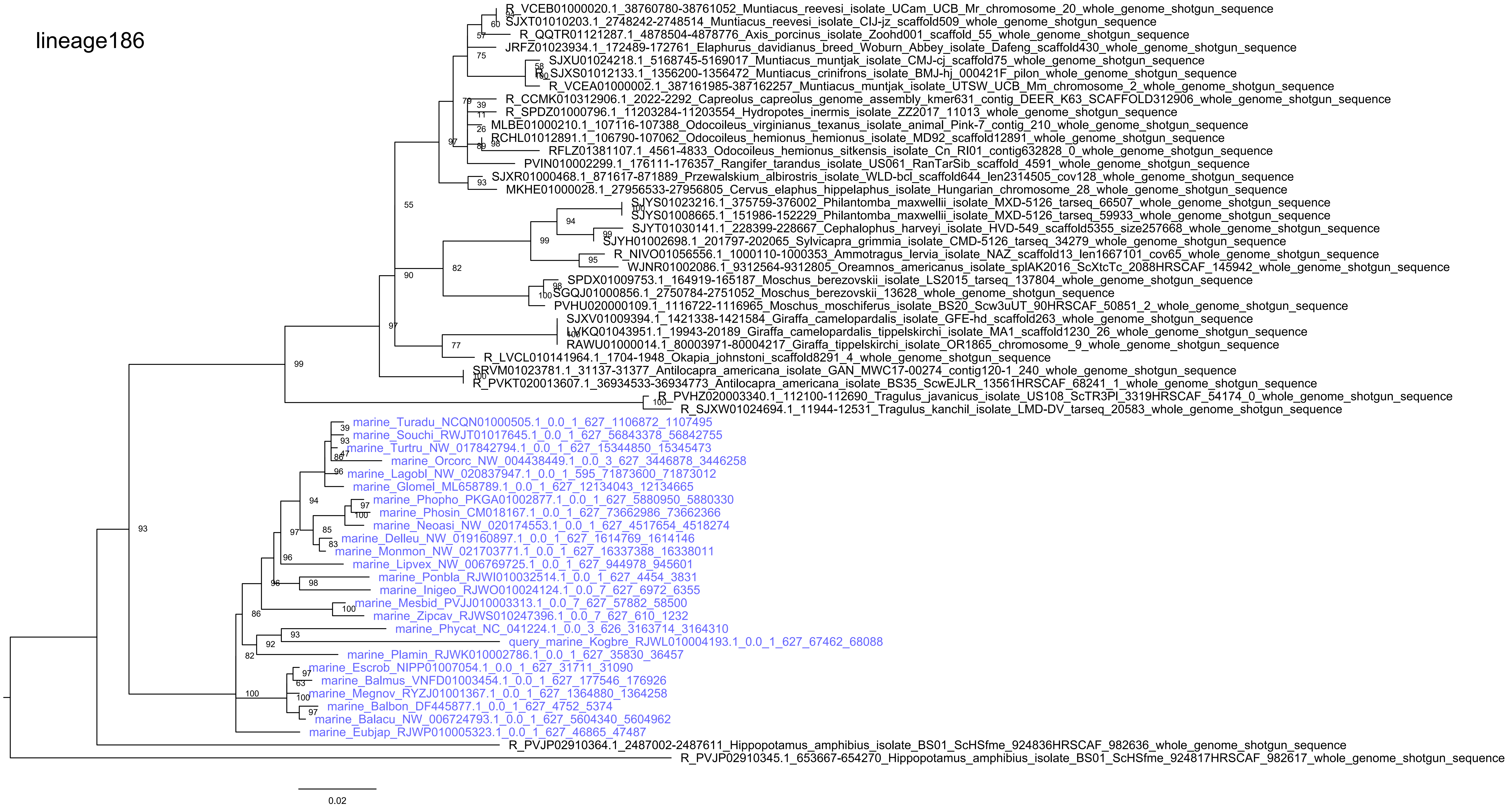
lineage186
R_VCEB01000020.1_38760780-38761052_Muntiacus_reevesi_isolate_UCam_UCB_Mr_chromosome_20_whole_genome_shotgun_sequence
SJXT01010203.1_2748242-2748514_Muntiacus_reevesi_isolate_CIJ-jz_scaffold509_whole_genome_shotgun_sequence
R_QQTR01121287.1_4878504-4878776_Axis_porcinus_isolate_Zoohd001_scaffold_55_whole_genome_shotgun_sequence
JRFZ01023934.1_172489-172761_Elaphurus_davidianus_breed_Woburn_Abbey_isolate_Dafeng_scaffold430_whole_genome_shotgun_sequence
SJXU01024218.1_5168745-5169017_Muntiacus_muntjak_isolate_CMJ-cj_scaffold75_whole_genome_shotgun_sequence
R_SJXS01012133.1_1356200-1356472_Muntiacus_crinifrons_isolate_BMJ-hj_000421F_pilon_whole_genome_shotgun_sequence
R_VCEA01000002.1_387161985-387162257_Muntiacus_muntjak_isolate_UTSW_UCB_Mm_chromosome_2_whole_genome_shotgun_sequence
R_CCMK010312906.1_2022-2292_Capreolus_capreolus_genome_assembly_kmer631_contig_DEER_K63_SCAFFOLD312906_whole_genome_shotgun_sequence
R_SPDZ01000796.1_11203284-11203554_Hydropotes_inermis_isolate_ZZ2017_11013_whole_genome_shotgun_sequence
MLBE01000210.1_107116-107388_Odocoileus_virginianus_texanus_isolate_animal_Pink-7_contig_210_whole_genome_shotgun_sequence
RCHL01012891.1_106790-107062_Odocoileus_hemionus_hemionus_isolate_MD92_scaffold12891_whole_genome_shotgun_sequence
RFLZ01381107.1_4561-4833_Odocoileus_hemionus_sitkensis_isolate_Cn_RI01_contig632828_0_whole_genome_shotgun_sequence
PVIN010002299.1_176111-176357_Rangifer_tarandus_isolate_US061_RanTarSib_scaffold_4591_whole_genome_shotgun_sequence
SJXR01000468.1_871617-871889_Przewalskium_albirostris_isolate_WLD-bcl_scaffold644_len2314505_cov128_whole_genome_shotgun_sequence
MKHE01000028.1_27956533-27956805_Cervus_elaphus_hippelaphus_isolate_Hungarian_chromosome_28_whole_genome_shotgun_sequence
SJYS01023216.1_375759-376002_Philantomba_maxwellii_isolate_MXD-5126_tarseq_66507_whole_genome_shotgun_sequence
SJYS01008665.1_151986-152229_Philantomba_maxwellii_isolate_MXD-5126_tarseq_59933_whole_genome_shotgun_sequence
SJYT01030141.1_228399-228667_Cephalophus_harveyi_isolate_HVD-549_scaffold5355_size257668_whole_genome_shotgun_sequence
SJYH01002698.1_201797-202065_Sylvicapra_grimmia_isolate_CMD-5126_tarseq_34279_whole_genome_shotgun_sequence
R_NIVO01056556.1_1000110-1000353_Ammotragus_lervia_isolate_NAZ_scaffold13_len1667101_cov65_whole_genome_shotgun_sequence
WJNR01002086.1_9312564-9312805_Oreamnos_americanus_isolate_splAK2016_ScXtcTc_2088HRSCAF_145942_whole_genome_shotgun_sequence
SPDX01009753.1_164919-165187_Moschus_berezovskii_isolate_LS2015_tarseq_137804_whole_genome_shotgun_sequence
SGQJ01000856.1_2750784-2751052_Moschus_berezovskii_13628_whole_genome_shotgun_sequence
PVHU020000109.1_1116722-1116965_Moschus_moschiferus_isolate_BS20_Scw3uUT_90HRSCAF_50851_2_whole_genome_shotgun_sequence
SJXV01009394.1_1421338-1421584_Giraffa_camelopardalis_isolate_GFE-hd_scaffold263_whole_genome_shotgun_sequence
LVKQ01043951.1_19943-20189_Giraffa_camelopardalis_tippelskirchi_isolate_MA1_scaffold1230_26_whole_genome_shotgun_sequence
RAWU01000014.1_80003971-80004217_Giraffa_tippelskirchi_isolate_OR1865_chromosome_9_whole_genome_shotgun_sequence
R_LVCL010141964.1_1704-1948_Okapia_johnstoni_scaffold8291_4_whole_genome_shotgun_sequence
SRVM01023781.1_31137-31377_Antilocapra_americana_isolate_GAN_MWC17-00274_contig120-1_240_whole_genome_shotgun_sequence
R_PVKT020013607.1_36934533-36934773_Antilocapra_americana_isolate_BS35_ScwEJLR_13561HRSCAF_68241_1_whole_genome_shotgun_sequence
R_PVHZ020003340.1_112100-112690_Tragulus_javanicus_isolate_US108_ScTR3PI_3319HRSCAF_54174_0_whole_genome_shotgun_sequence
R_SJXW01024694.1_11944-12531_Tragulus_kanchil_isolate_LMD-DV_tarseq_20583_whole_genome_shotgun_sequence
marine_Turadu_NCQN01000505.1_0.0_1_627_1106872_1107495
marine_Souchi_RWJT01017645.1_0.0_1_627_56843378_56842755
marine_Turtru_NW_017842794.1_0.0_1_627_15344850_15345473
marine_Orcorc_NW_004438449.1_0.0_3_627_3446878_3446258
marine_Lagobl_NW_020837947.1_0.0_1_595_71873600_71873012
marine_Glomel_ML658789.1_0.0_1_627_12134043_12134665
marine_Phopho_PKGA01002877.1_0.0_1_627_5880950_5880330
marine_Phosin_CM018167.1_0.0_1_627_73662986_73662366
marine_Neoasi_NW_020174553.1_0.0_1_627_4517654_4518274
marine_Delleu_NW_019160897.1_0.0_1_627_1614769_1614146
marine_Monmon_NW_021703771.1_0.0_1_627_16337388_16338011
marine_Lipvex_NW_006769725.1_0.0_1_627_944978_945601
marine_Ponbla_RJWI010032514.1_0.0_1_627_4454_3831
marine_Inigeo_RJWO010024124.1_0.0_7_627_6972_6355
marine_Mesbid_PVJJ010003313.1_0.0_7_627_57882_58500
marine_Zipcav_RJWS010247396.1_0.0_7_627_610_1232
marine_Phycat_NC_041224.1_0.0_3_626_3163714_3164310
query_marine_Kogbre_RJWL010004193.1_0.0_1_627_67462_68088
marine_Plamin_RJWK010002786.1_0.0_1_627_35830_36457
marine_Escrob_NIPP01007054.1_0.0_1_627_31711_31090
marine_Balmus_VNFD01003454.1_0.0_1_627_177546_176926
marine_Megnov_RYZJ01001367.1_0.0_1_627_1364880_1364258
marine_Balbon_DF445877.1_0.0_1_627_4752_5374
marine_Balacu_NW_006724793.1_0.0_1_627_5604340_5604962
marine_Eubjap_RJWP010005323.1_0.0_1_627_46865_47487
R_PVJP02910364.1_2487002-2487611_Hippopotamus_amphibius_isolate_BS01_ScHSfme_924836HRSCAF_982636_whole_genome_shotgun_sequence
R_PVJP02910345.1_653667-654270_Hippopotamus_amphibius_isolate_BS01_ScHSfme_924817HRSCAF_982617_whole_genome_shotgun_sequence
0.02

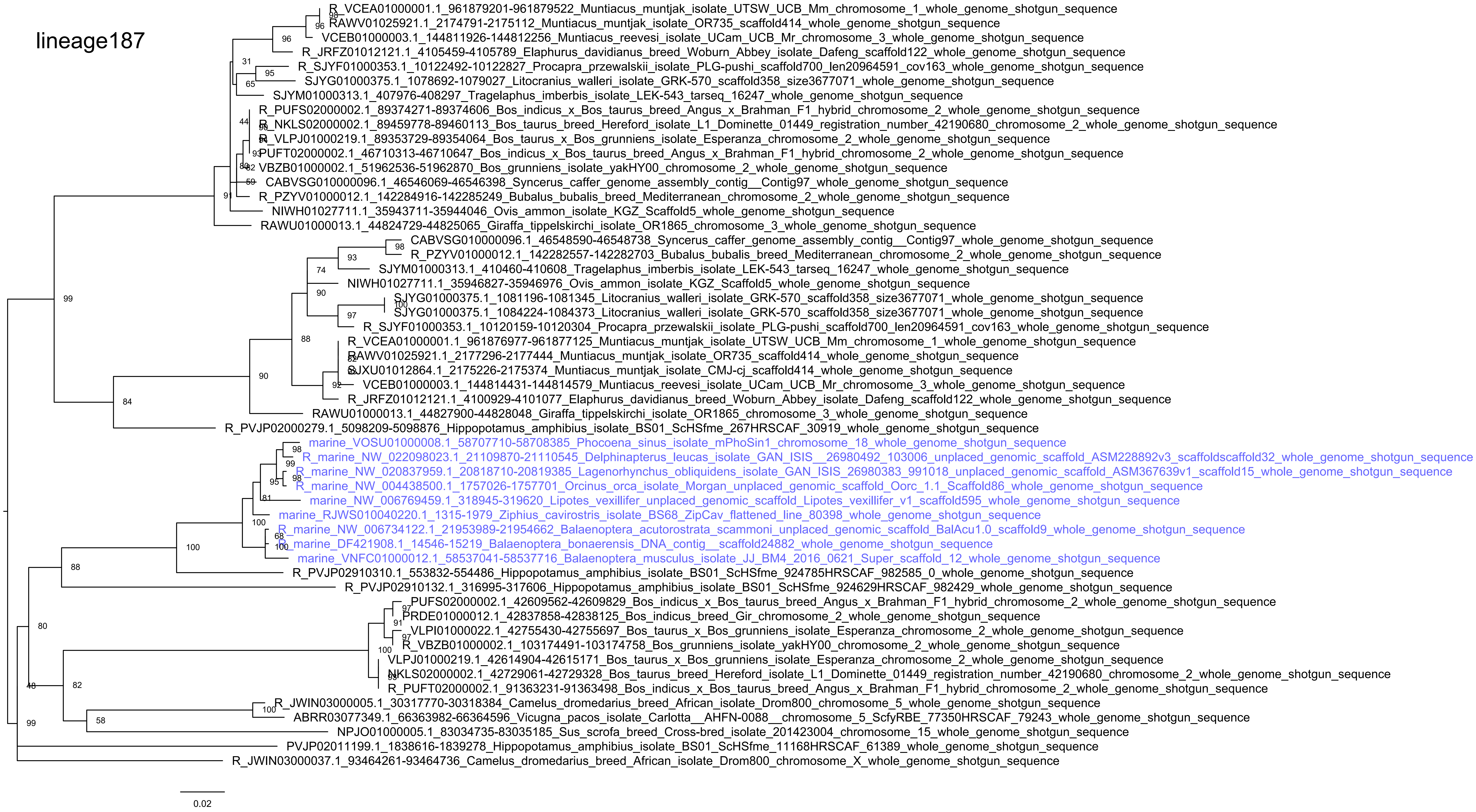
lineage187
R_VCEA01000001.1_961879201-961879522_Muntiacus_muntjak_isolate_UTSW_UCB_Mm_chromosome_1_whole_genome_shotgun_sequence
RAWV01025921.1_2174791-2175112_Muntiacus_muntjak_isolate_OR735_scaffold414_whole_genome_shotgun_sequence
VCEB01000003.1_144811926-144812256_Muntiacus_reevesi_isolate_UCam_UCB_Mr_chromosome_3_whole_genome_shotgun_sequence
R_JRFZ01012121.1_4105459-4105789_Elaphurus_davidianus_breed_Woburn_Abbey_isolate_Dafeng_scaffold122_whole_genome_shotgun_sequence
R_SJYF01000353.1_10122492-10122827_Procapra_przewalskii_isolate_PLG-pushi_scaffold700_len20964591_cov163_whole_genome_shotgun_sequence
SJYG01000375.1_1078692-1079027_Litocranius_walleri_isolate_GRK-570_scaffold358_size3677071_whole_genome_shotgun_sequence
SJYM01000313.1_407976-408297_Tragelaphus_imberbis_isolate_LEK-543_tarseq_16247_whole_genome_shotgun_sequence
R_PUFS02000002.1_89374271-89374606_Bos_indicus_x_Bos_taurus_breed_Angus_x_Brahman_F1_hybrid_chromosome_2_whole_genome_shotgun_sequence
R_NKLS02000002.1_89459778-89460113_Bos_taurus_breed_Hereford_isolate_L1_Dominette_01449_registration_number_42190680_chromosome_2_whole_genome_shotgun_sequence
R_VLPJ01000219.1_89353729-89354064_Bos_taurus_x_Bos_grunniens_isolate_Esperanza_chromosome_2_whole_genome_shotgun_sequence
PUFT02000002.1_46710313-46710647_Bos_indicus_x_Bos_taurus_breed_Angus_x_Brahman_F1_hybrid_chromosome_2_whole_genome_shotgun_sequence
VBZB01000002.1_51962536-51962870_Bos_grunniens_isolate_yakHY00_chromosome_2_whole_genome_shotgun_sequence
CABVSG010000096.1_46546069-46546398_Syncerus_caffer_genome_assembly_contig__Contig97_whole_genome_shotgun_sequence
R_PZYV01000012.1_142284916-142285249_Bubalus_bubalis_breed_Mediterranean_chromosome_2_whole_genome_shotgun_sequence
NIWH01027711.1_35943711-35944046_Ovis_ammon_isolate_KGZ_Scaffold5_whole_genome_shotgun_sequence
RAWU01000013.1_44824729-44825065_Giraffa_tippelskirchi_isolate_OR1865_chromosome_3_whole_genome_shotgun_sequence
CABVSG010000096.1_46548590-46548738_Syncerus_caffer_genome_assembly_contig__Contig97_whole_genome_shotgun_sequence
R_PZYV01000012.1_142282557-142282703_Bubalus_bubalis_breed_Mediterranean_chromosome_2_whole_genome_shotgun_sequence
SJYM01000313.1_410460-410608_Tragelaphus_imberbis_isolate_LEK-543_tarseq_16247_whole_genome_shotgun_sequence
NIWH01027711.1_35946827-35946976_Ovis_ammon_isolate_KGZ_Scaffold5_whole_genome_shotgun_sequence
SJYG01000375.1_1081196-1081345_Litocranius_walleri_isolate_GRK-570_scaffold358_size3677071_whole_genome_shotgun_sequence
SJYG01000375.1_1084224-1084373_Litocranius_walleri_isolate_GRK-570_scaffold358_size3677071_whole_genome_shotgun_sequence
R_SJYF01000353.1_10120159-10120304_Procapra_przewalskii_isolate_PLG-pushi_scaffold700_len20964591_cov163_whole_genome_shotgun_sequence
R_VCEA01000001.1_961876977-961877125_Muntiacus_muntjak_isolate_UTSW_UCB_Mm_chromosome_1_whole_genome_shotgun_sequence
RAWV01025921.1_2177296-2177444_Muntiacus_muntjak_isolate_OR735_scaffold414_whole_genome_shotgun_sequence
SJXU01012864.1_2175226-2175374_Muntiacus_muntjak_isolate_CMJ-cj_scaffold414_whole_genome_shotgun_sequence
VCEB01000003.1_144814431-144814579_Muntiacus_reevesi_isolate_UCam_UCB_Mr_chromosome_3_whole_genome_shotgun_sequence
R_JRFZ01012121.1_4100929-4101077_Elaphurus_davidianus_breed_Woburn_Abbey_isolate_Dafeng_scaffold122_whole_genome_shotgun_sequence
RAWU01000013.1_44827900-44828048_Giraffa_tippelskirchi_isolate_OR1865_chromosome_3_whole_genome_shotgun_sequence
R_PVJP02000279.1_5098209-5098876_Hippopotamus_amphibius_isolate_BS01_ScHSfme_267HRSCAF_30919_whole_genome_shotgun_sequence
marine_VOSU01000008.1_58707710-58708385_Phocoena_sinus_isolate_mPhoSin1_chromosome_18_whole_genome_shotgun_sequence
R_marine_NW_022098023.1_21109870-21110545_Delphinapterus_leucas_isolate_GAN_ISIS__26980492_103006_unplaced_genomic_scaffold_ASM228892v3_scaffoldscaffold32_whole_genome_shotgun_sequence
R_marine_NW_020837959.1_20818710-20819385_Lagenorhynchus_obliquidens_isolate_GAN_ISIS_26980383_991018_unplaced_genomic_scaffold_ASM367639v1_scaffold15_whole_genome_shotgun_sequence
R_marine_NW_004438500.1_1757026-1757701_Orcinus_orca_isolate_Morgan_unplaced_genomic_scaffold_Oorc_1.1_Scaffold86_whole_genome_shotgun_sequence
marine_NW_006769459.1_318945-319620_Lipotes_vexillifer_unplaced_genomic_scaffold_Lipotes_vexillifer_v1_scaffold595_whole_genome_shotgun_sequence
marine_RJWS010040220.1_1315-1979_Ziphius_cavirostris_isolate_BS68_ZipCav_flattened_line_80398_whole_genome_shotgun_sequence
R_marine_NW_006734122.1_21953989-21954662_Balaenoptera_acutorostrata_scammoni_unplaced_genomic_scaffold_BalAcu1.0_scaffold9_whole_genome_shotgun_sequence
R_marine_DF421908.1_14546-15219_Balaenoptera_bonaerensis_DNA_contig__scaffold24882_whole_genome_shotgun_sequence
marine_VNFC01000012.1_58537041-58537716_Balaenoptera_musculus_isolate_JJ_BM4_2016_0621_Super_scaffold_12_whole_genome_shotgun_sequence
R_PVJP02910310.1_553832-554486_Hippopotamus_amphibius_isolate_BS01_ScHSfme_924785HRSCAF_982585_0_whole_genome_shotgun_sequence
R_PVJP02910132.1_316995-317606_Hippopotamus_amphibius_isolate_BS01_ScHSfme_924629HRSCAF_982429_whole_genome_shotgun_sequence
PUFS02000002.1_42609562-42609829_Bos_indicus_x_Bos_taurus_breed_Angus_x_Brahman_F1_hybrid_chromosome_2_whole_genome_shotgun_sequence
PRDE01000012.1_42837858-42838125_Bos_indicus_breed_Gir_chromosome_2_whole_genome_shotgun_sequence
VLPI01000022.1_42755430-42755697_Bos_taurus_x_Bos_grunniens_isolate_Esperanza_chromosome_2_whole_genome_shotgun_sequence
R_VBZB01000002.1_103174491-103174758_Bos_grunniens_isolate_yakHY00_chromosome_2_whole_genome_shotgun_sequence
VLPJ01000219.1_42614904-42615171_Bos_taurus_x_Bos_grunniens_isolate_Esperanza_chromosome_2_whole_genome_shotgun_sequence
NKLS02000002.1_42729061-42729328_Bos_taurus_breed_Hereford_isolate_L1_Dominette_01449_registration_number_42190680_chromosome_2_whole_genome_shotgun_sequence
R_PUFT02000002.1_91363231-91363498_Bos_indicus_x_Bos_taurus_breed_Angus_x_Brahman_F1_hybrid_chromosome_2_whole_genome_shotgun_sequence
R_JWIN03000005.1_30317770-30318384_Camelus_dromedarius_breed_African_isolate_Drom800_chromosome_5_whole_genome_shotgun_sequence
ABRR03077349.1_66363982-66364596_Vicugna_pacos_isolate_Carlotta__AHFN-0088__chromosome_5_ScfyRBE_77350HRSCAF_79243_whole_genome_shotgun_sequence
NPJO01000005.1_83034735-83035185_Sus_scrofa_breed_Cross-bred_isolate_201423004_chromosome_15_whole_genome_shotgun_sequence
PVJP02011199.1_1838616-1839278_Hippopotamus_amphibius_isolate_BS01_ScHSfme_11168HRSCAF_61389_whole_genome_shotgun_sequence
R_JWIN03000037.1_93464261-93464736_Camelus_dromedarius_breed_African_isolate_Drom800_chromosome_X_whole_genome_shotgun_sequence
0.02

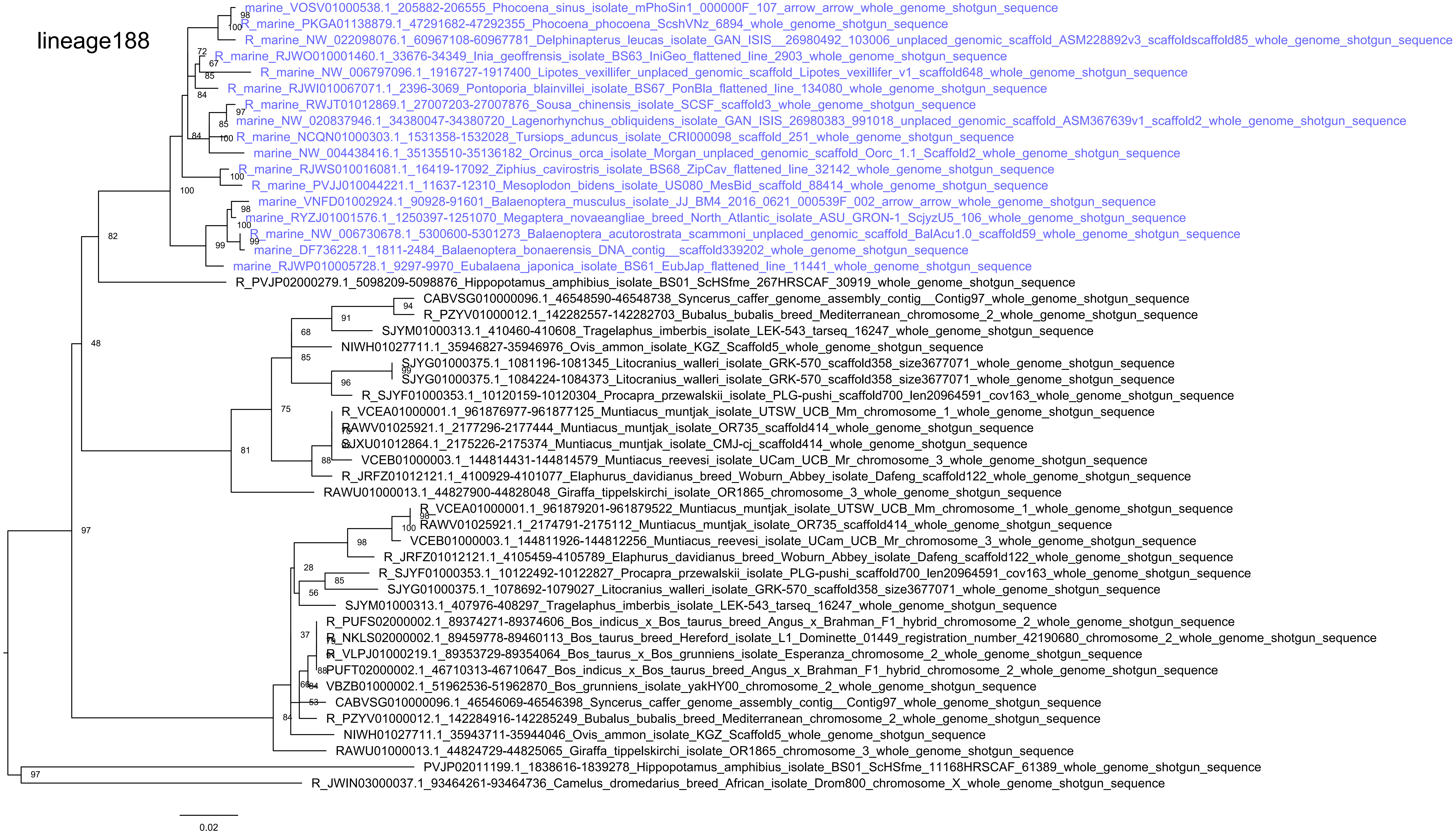
lineage188
marine_VOSV01000538.1_205882-206555_Phocoena_sinus_isolate_mPhoSin1_000000F_107_arrow_arrow_whole_genome_shotgun_sequence
R_marine_PKGA01138879.1_47291682-47292355_Phocoena_phocoena_ScshVNz_6894_whole_genome_shotgun_sequence
R_marine_NW_022098076.1_60967108-60967781_Delphinapterus_leucas_isolate_GAN_ISIS__26980492_103006_unplaced_genomic_scaffold_ASM228892v3_scaffoldscaffold85_whole_genome_shotgun_sequence
R_marine_RJWO010001460.1_33676-34349_Inia_geoffrensis_isolate_BS63_IniGeo_flattened_line_2903_whole_genome_shotgun_sequence
R_marine_NW_006797096.1_1916727-1917400_Lipotes_vexillifer_unplaced_genomic_scaffold_Lipotes_vexillifer_v1_scaffold648_whole_genome_shotgun_sequence
R_marine_RJWI010067071.1_2396-3069_Pontoporia_blainvillei_isolate_BS67_PonBla_flattened_line_134080_whole_genome_shotgun_sequence
R_marine_RWJT01012869.1_27007203-27007876_Sousa_chinensis_isolate_SCSF_scaffold3_whole_genome_shotgun_sequence
marine_NW_020837946.1_34380047-34380720_Lagenorhynchus_obliquidens_isolate_GAN_ISIS_26980383_991018_unplaced_genomic_scaffold_ASM367639v1_scaffold2_whole_genome_shotgun_sequence
R_marine_NCQN01000303.1_1531358-1532028_Tursiops_aduncus_isolate_CRI000098_scaffold_251_whole_genome_shotgun_sequence
marine_NW_004438416.1_35135510-35136182_Orcinus_orca_isolate_Morgan_unplaced_genomic_scaffold_Oorc_1.1_Scaffold2_whole_genome_shotgun_sequence
R_marine_RJWS010016081.1_16419-17092_Ziphius_cavirostris_isolate_BS68_ZipCav_flattened_line_32142_whole_genome_shotgun_sequence
R_marine_PVJJ010044221.1_11637-12310_Mesoplodon_bidens_isolate_US080_MesBid_scaffold_88414_whole_genome_shotgun_sequence
marine_VNFD01002924.1_90928-91601_Balaenoptera_musculus_isolate_JJ_BM4_2016_0621_000539F_002_arrow_arrow_whole_genome_shotgun_sequence
marine_RYZJ01001576.1_1250397-1251070_Megaptera_novaeangliae_breed_North_Atlantic_isolate_ASU_GRON-1_ScjyzU5_106_whole_genome_shotgun_sequence
R_marine_NW_006730678.1_5300600-5301273_Balaenoptera_acutorostrata_scammoni_unplaced_genomic_scaffold_BalAcu1.0_scaffold59_whole_genome_shotgun_sequence
marine_DF736228.1_1811-2484_Balaenoptera_bonaerensis_DNA_contig__scaffold339202_whole_genome_shotgun_sequence
marine_RJWP010005728.1_9297-9970_Eubalaena_japonica_isolate_BS61_EubJap_flattened_line_11441_whole_genome_shotgun_sequence
R_PVJP02000279.1_5098209-5098876_Hippopotamus_amphibius_isolate_BS01_ScHSfme_267HRSCAF_30919_whole_genome_shotgun_sequence
CABVSG010000096.1_46548590-46548738_Syncerus_caffer_genome_assembly_contig__Contig97_whole_genome_shotgun_sequence
R_PZYV01000012.1_142282557-142282703_Bubalus_bubalis_breed_Mediterranean_chromosome_2_whole_genome_shotgun_sequence
SJYM01000313.1_410460-410608_Tragelaphus_imberbis_isolate_LEK-543_tarseq_16247_whole_genome_shotgun_sequence
NIWH01027711.1_35946827-35946976_Ovis_ammon_isolate_KGZ_Scaffold5_whole_genome_shotgun_sequence
SJYG01000375.1_1081196-1081345_Litocranius_walleri_isolate_GRK-570_scaffold358_size3677071_whole_genome_shotgun_sequence
SJYG01000375.1_1084224-1084373_Litocranius_walleri_isolate_GRK-570_scaffold358_size3677071_whole_genome_shotgun_sequence
R_SJYF01000353.1_10120159-10120304_Procapra_przewalskii_isolate_PLG-pushi_scaffold700_len20964591_cov163_whole_genome_shotgun_sequence
R_VCEA01000001.1_961876977-961877125_Muntiacus_muntjak_isolate_UTSW_UCB_Mm_chromosome_1_whole_genome_shotgun_sequence
RAWV01025921.1_2177296-2177444_Muntiacus_muntjak_isolate_OR735_scaffold414_whole_genome_shotgun_sequence
SJXU01012864.1_2175226-2175374_Muntiacus_muntjak_isolate_CMJ-cj_scaffold414_whole_genome_shotgun_sequence
VCEB01000003.1_144814431-144814579_Muntiacus_reevesi_isolate_UCam_UCB_Mr_chromosome_3_whole_genome_shotgun_sequence
R_JRFZ01012121.1_4100929-4101077_Elaphurus_davidianus_breed_Woburn_Abbey_isolate_Dafeng_scaffold122_whole_genome_shotgun_sequence
RAWU01000013.1_44827900-44828048_Giraffa_tippelskirchi_isolate_OR1865_chromosome_3_whole_genome_shotgun_sequence
R_VCEA01000001.1_961879201-961879522_Muntiacus_muntjak_isolate_UTSW_UCB_Mm_chromosome_1_whole_genome_shotgun_sequence
RAWV01025921.1_2174791-2175112_Muntiacus_muntjak_isolate_OR735_scaffold414_whole_genome_shotgun_sequence
VCEB01000003.1_144811926-144812256_Muntiacus_reevesi_isolate_UCam_UCB_Mr_chromosome_3_whole_genome_shotgun_sequence
R_JRFZ01012121.1_4105459-4105789_Elaphurus_davidianus_breed_Woburn_Abbey_isolate_Dafeng_scaffold122_whole_genome_shotgun_sequence
R_SJYF01000353.1_10122492-10122827_Procapra_przewalskii_isolate_PLG-pushi_scaffold700_len20964591_cov163_whole_genome_shotgun_sequence
SJYG01000375.1_1078692-1079027_Litocranius_walleri_isolate_GRK-570_scaffold358_size3677071_whole_genome_shotgun_sequence
SJYM01000313.1_407976-408297_Tragelaphus_imberbis_isolate_LEK-543_tarseq_16247_whole_genome_shotgun_sequence
R_PUFS02000002.1_89374271-89374606_Bos_indicus_x_Bos_taurus_breed_Angus_x_Brahman_F1_hybrid_chromosome_2_whole_genome_shotgun_sequence
R_NKLS02000002.1_89459778-89460113_Bos_taurus_breed_Hereford_isolate_L1_Dominette_01449_registration_number_42190680_chromosome_2_whole_genome_shotgun_sequence
R_VLPJ01000219.1_89353729-89354064_Bos_taurus_x_Bos_grunniens_isolate_Esperanza_chromosome_2_whole_genome_shotgun_sequence
PUFT02000002.1_46710313-46710647_Bos_indicus_x_Bos_taurus_breed_Angus_x_Brahman_F1_hybrid_chromosome_2_whole_genome_shotgun_sequence
VBZB01000002.1_51962536-51962870_Bos_grunniens_isolate_yakHY00_chromosome_2_whole_genome_shotgun_sequence
CABVSG010000096.1_46546069-46546398_Syncerus_caffer_genome_assembly_contig__Contig97_whole_genome_shotgun_sequence
R_PZYV01000012.1_142284916-142285249_Bubalus_bubalis_breed_Mediterranean_chromosome_2_whole_genome_shotgun_sequence
NIWH01027711.1_35943711-35944046_Ovis_ammon_isolate_KGZ_Scaffold5_whole_genome_shotgun_sequence
RAWU01000013.1_44824729-44825065_Giraffa_tippelskirchi_isolate_OR1865_chromosome_3_whole_genome_shotgun_sequence
PVJP02011199.1_1838616-1839278_Hippopotamus_amphibius_isolate_BS01_ScHSfme_11168HRSCAF_61389_whole_genome_shotgun_sequence
R_JWIN03000037.1_93464261-93464736_Camelus_dromedarius_breed_African_isolate_Drom800_chromosome_X_whole_genome_shotgun_sequence
0.02

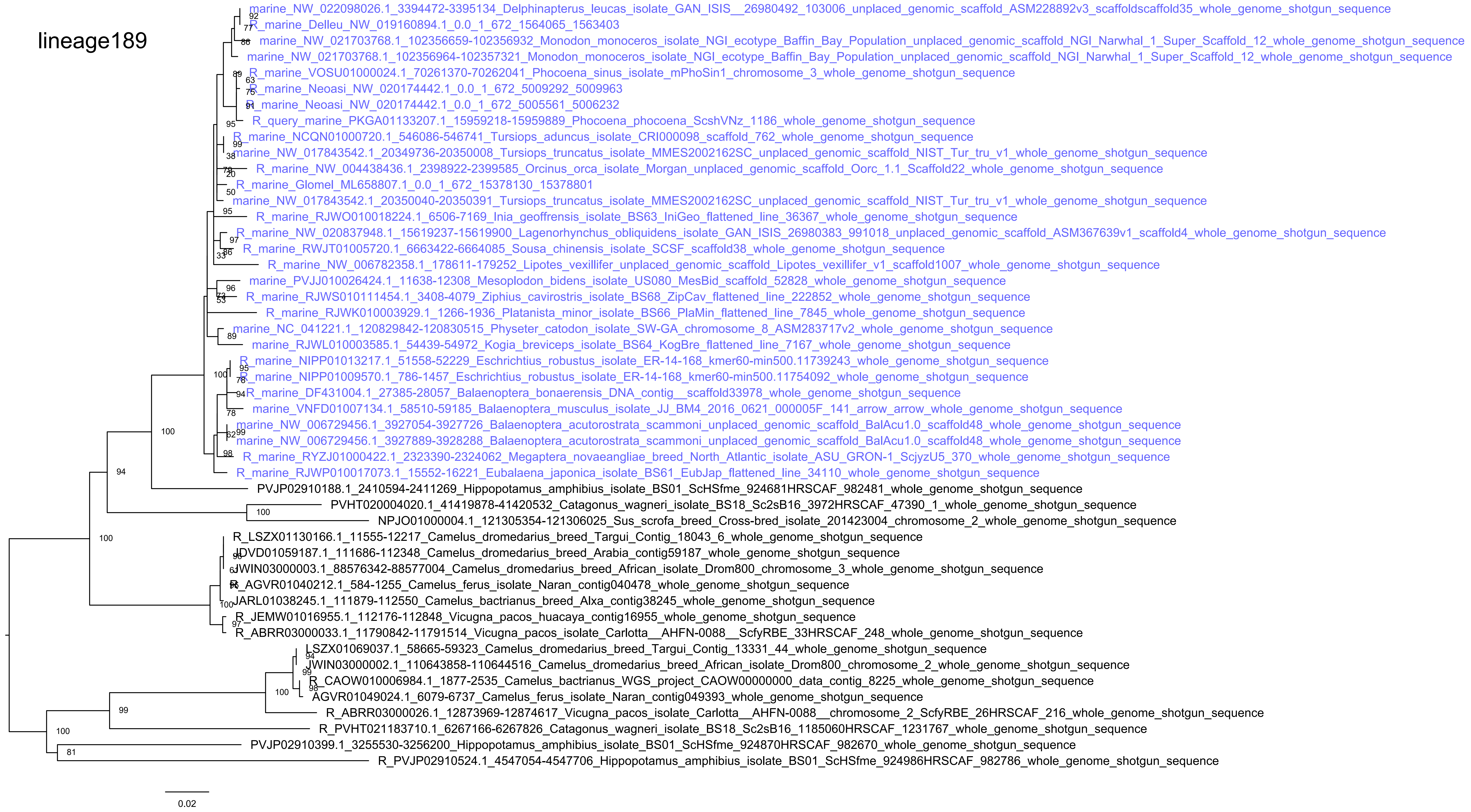
lineage189
marine_NW_022098026.1_3394472-3395134_Delphinapterus_leucas_isolate_GAN_ISIS__26980492_103006_unplaced_genomic_scaffold_ASM228892v3_scaffoldscaffold35_whole_genome_shotgun_sequence
R_marine_Delleu_NW_019160894.1_0.0_1_672_1564065_1563403
marine_NW_021703768.1_102356659-102356932_Monodon_monoceros_isolate_NGI_ecotype_Baffin_Bay_Population_unplaced_genomic_scaffold_NGI_Narwhal_1_Super_Scaffold_12_whole_genome_shotgun_sequence
marine_NW_021703768.1_102356964-102357321_Monodon_monoceros_isolate_NGI_ecotype_Baffin_Bay_Population_unplaced_genomic_scaffold_NGI_Narwhal_1_Super_Scaffold_12_whole_genome_shotgun_sequence
R_marine_VOSU01000024.1_70261370-70262041_Phocoena_sinus_isolate_mPhoSin1_chromosome_3_whole_genome_shotgun_sequence
R_marine_Neoasi_NW_020174442.1_0.0_1_672_5009292_5009963
R_marine_Neoasi_NW_020174442.1_0.0_1_672_5005561_5006232
R_query_marine_PKGA01133207.1_15959218-15959889_Phocoena_phocoena_ScshVNz_1186_whole_genome_shotgun_sequence
R_marine_NCQN01000720.1_546086-546741_Tursiops_aduncus_isolate_CRI000098_scaffold_762_whole_genome_shotgun_sequence
marine_NW_017843542.1_20349736-20350008_Tursiops_truncatus_isolate_MMES2002162SC_unplaced_genomic_scaffold_NIST_Tur_tru_v1_whole_genome_shotgun_sequence
R_marine_NW_004438436.1_2398922-2399585_Orcinus_orca_isolate_Morgan_unplaced_genomic_scaffold_Oorc_1.1_Scaffold22_whole_genome_shotgun_sequence
R_marine_Glomel_ML658807.1_0.0_1_672_15378130_15378801
marine_NW_017843542.1_20350040-20350391_Tursiops_truncatus_isolate_MMES2002162SC_unplaced_genomic_scaffold_NIST_Tur_tru_v1_whole_genome_shotgun_sequence
R_marine_RJWO010018224.1_6506-7169_Inia_geoffrensis_isolate_BS63_IniGeo_flattened_line_36367_whole_genome_shotgun_sequence
R_marine_NW_020837948.1_15619237-15619900_Lagenorhynchus_obliquidens_isolate_GAN_ISIS_26980383_991018_unplaced_genomic_scaffold_ASM367639v1_scaffold4_whole_genome_shotgun_sequence
R_marine_RWJT01005720.1_6663422-6664085_Sousa_chinensis_isolate_SCSF_scaffold38_whole_genome_shotgun_sequence
R_marine_NW_006782358.1_178611-179252_Lipotes_vexillifer_unplaced_genomic_scaffold_Lipotes_vexillifer_v1_scaffold1007_whole_genome_shotgun_sequence
marine_PVJJ010026424.1_11638-12308_Mesoplodon_bidens_isolate_US080_MesBid_scaffold_52828_whole_genome_shotgun_sequence
R_marine_RJWS010111454.1_3408-4079_Ziphius_cavirostris_isolate_BS68_ZipCav_flattened_line_222852_whole_genome_shotgun_sequence
R_marine_RJWK010003929.1_1266-1936_Platanista_minor_isolate_BS66_PlaMin_flattened_line_7845_whole_genome_shotgun_sequence
marine_NC_041221.1_120829842-120830515_Physeter_catodon_isolate_SW-GA_chromosome_8_ASM283717v2_whole_genome_shotgun_sequence
marine_RJWL010003585.1_54439-54972_Kogia_breviceps_isolate_BS64_KogBre_flattened_line_7167_whole_genome_shotgun_sequence
R_marine_NIPP01013217.1_51558-52229_Eschrichtius_robustus_isolate_ER-14-168_kmer60-min500.11739243_whole_genome_shotgun_sequence
R_marine_NIPP01009570.1_786-1457_Eschrichtius_robustus_isolate_ER-14-168_kmer60-min500.11754092_whole_genome_shotgun_sequence
R_marine_DF431004.1_27385-28057_Balaenoptera_bonaerensis_DNA_contig__scaffold33978_whole_genome_shotgun_sequence
marine_VNFD01007134.1_58510-59185_Balaenoptera_musculus_isolate_JJ_BM4_2016_0621_000005F_141_arrow_arrow_whole_genome_shotgun_sequence
marine_NW_006729456.1_3927054-3927726_Balaenoptera_acutorostrata_scammoni_unplaced_genomic_scaffold_BalAcu1.0_scaffold48_whole_genome_shotgun_sequence
marine_NW_006729456.1_3927889-3928288_Balaenoptera_acutorostrata_scammoni_unplaced_genomic_scaffold_BalAcu1.0_scaffold48_whole_genome_shotgun_sequence
R_marine_RYZJ01000422.1_2323390-2324062_Megaptera_novaeangliae_breed_North_Atlantic_isolate_ASU_GRON-1_ScjyzU5_370_whole_genome_shotgun_sequence
R_marine_RJWP010017073.1_15552-16221_Eubalaena_japonica_isolate_BS61_EubJap_flattened_line_34110_whole_genome_shotgun_sequence
PVJP02910188.1_2410594-2411269_Hippopotamus_amphibius_isolate_BS01_ScHSfme_924681HRSCAF_982481_whole_genome_shotgun_sequence
PVHT020004020.1_41419878-41420532_Catagonus_wagneri_isolate_BS18_Sc2sB16_3972HRSCAF_47390_1_whole_genome_shotgun_sequence
NPJO01000004.1_121305354-121306025_Sus_scrofa_breed_Cross-bred_isolate_201423004_chromosome_2_whole_genome_shotgun_sequence
R_LSZX01130166.1_11555-12217_Camelus_dromedarius_breed_Targui_Contig_18043_6_whole_genome_shotgun_sequence
JDVD01059187.1_111686-112348_Camelus_dromedarius_breed_Arabia_contig59187_whole_genome_shotgun_sequence
JWIN03000003.1_88576342-88577004_Camelus_dromedarius_breed_African_isolate_Drom800_chromosome_3_whole_genome_shotgun_sequence
R_AGVR01040212.1_584-1255_Camelus_ferus_isolate_Naran_contig040478_whole_genome_shotgun_sequence
JARL01038245.1_111879-112550_Camelus_bactrianus_breed_Alxa_contig38245_whole_genome_shotgun_sequence
R_JEMW01016955.1_112176-112848_Vicugna_pacos_huacaya_contig16955_whole_genome_shotgun_sequence
R_ABRR03000033.1_11790842-11791514_Vicugna_pacos_isolate_Carlotta__AHFN-0088__ScfyRBE_33HRSCAF_248_whole_genome_shotgun_sequence
LSZX01069037.1_58665-59323_Camelus_dromedarius_breed_Targui_Contig_13331_44_whole_genome_shotgun_sequence
JWIN03000002.1_110643858-110644516_Camelus_dromedarius_breed_African_isolate_Drom800_chromosome_2_whole_genome_shotgun_sequence
R_CAOW010006984.1_1877-2535_Camelus_bactrianus_WGS_project_CAOW00000000_data_contig_8225_whole_genome_shotgun_sequence
AGVR01049024.1_6079-6737_Camelus_ferus_isolate_Naran_contig049393_whole_genome_shotgun_sequence
R_ABRR03000026.1_12873969-12874617_Vicugna_pacos_isolate_Carlotta__AHFN-0088__chromosome_2_ScfyRBE_26HRSCAF_216_whole_genome_shotgun_sequence
R_PVHT021183710.1_6267166-6267826_Catagonus_wagneri_isolate_BS18_Sc2sB16_1185060HRSCAF_1231767_whole_genome_shotgun_sequence
PVJP02910399.1_3255530-3256200_Hippopotamus_amphibius_isolate_BS01_ScHSfme_924870HRSCAF_982670_whole_genome_shotgun_sequence
R_PVJP02910524.1_4547054-4547706_Hippopotamus_amphibius_isolate_BS01_ScHSfme_924986HRSCAF_982786_whole_genome_shotgun_sequence
0.02

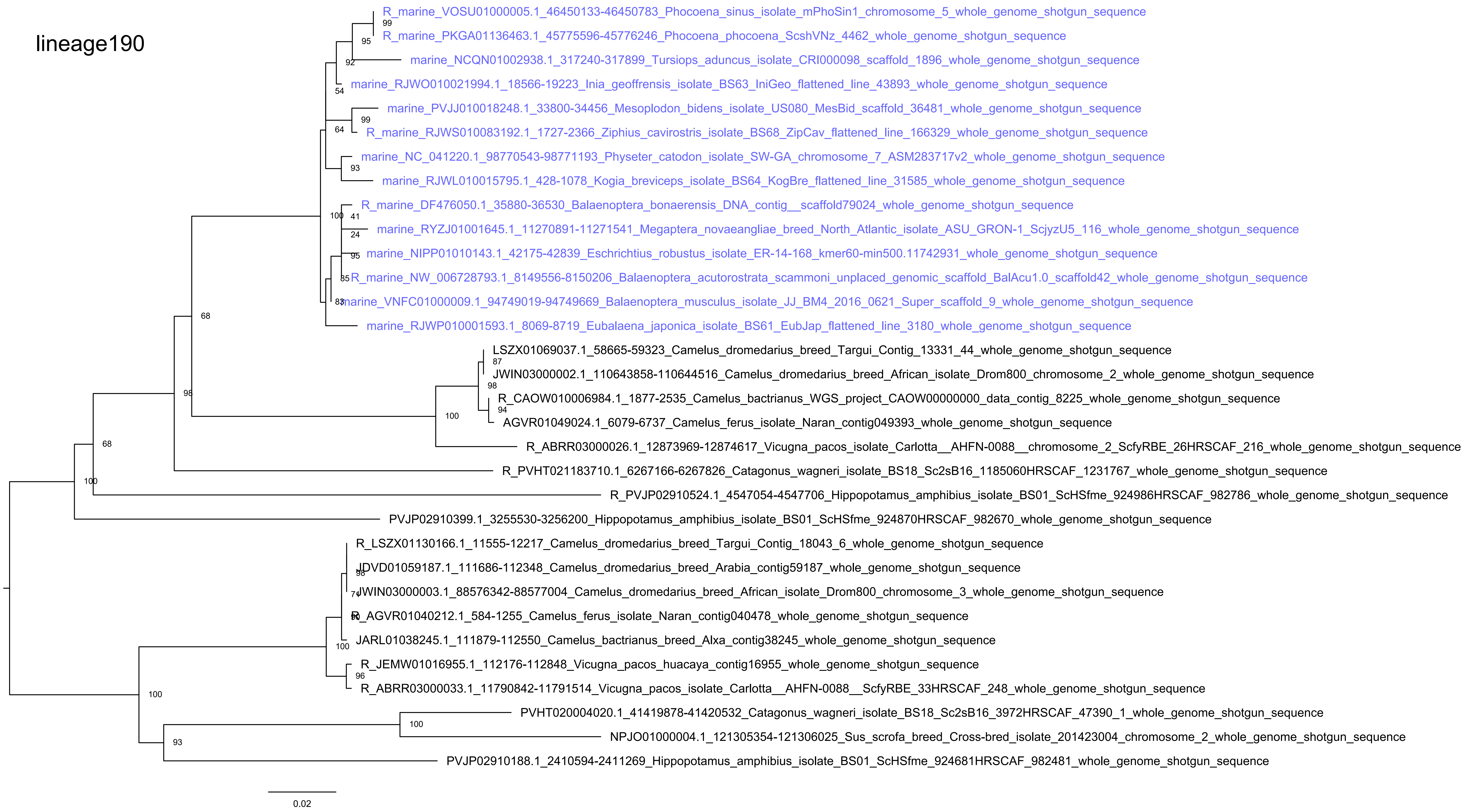
lineage190
R_marine_VOSU01000005.1_46450133-46450783_Phocoena_sinus_isolate_mPhoSin1_chromosome_5_whole_genome_shotgun_sequence
R_marine_PKGA01136463.1_45775596-45776246_Phocoena_phocoena_ScshVNz_4462_whole_genome_shotgun_sequence
marine_NCQN01002938.1_317240-317899_Tursiops_aduncus_isolate_CRI000098_scaffold_1896_whole_genome_shotgun_sequence
marine_RJWO010021994.1_18566-19223_Inia_geoffrensis_isolate_BS63_IniGeo_flattened_line_43893_whole_genome_shotgun_sequence
marine_PVJJ010018248.1_33800-34456_Mesoplodon_bidens_isolate_US080_MesBid_scaffold_36481_whole_genome_shotgun_sequence
R_marine_RJWS010083192.1_1727-2366_Ziphius_cavirostris_isolate_BS68_ZipCav_flattened_line_166329_whole_genome_shotgun_sequence
marine_NC_041220.1_98770543-98771193_Physeter_catodon_isolate_SW-GA_chromosome_7_ASM283717v2_whole_genome_shotgun_sequence
marine_RJWL010015795.1_428-1078_Kogia_breviceps_isolate_BS64_KogBre_flattened_line_31585_whole_genome_shotgun_sequence
R_marine_DF476050.1_35880-36530_Balaenoptera_bonaerensis_DNA_contig__scaffold79024_whole_genome_shotgun_sequence
marine_RYZJ01001645.1_11270891-11271541_Megaptera_novaeangliae_breed_North_Atlantic_isolate_ASU_GRON-1_ScjyzU5_116_whole_genome_shotgun_sequence
marine_NIPP01010143.1_42175-42839_Eschrichtius_robustus_isolate_ER-14-168_kmer60-min500.11742931_whole_genome_shotgun_sequence
R_marine_NW_006728793.1_8149556-8150206_Balaenoptera_acutorostrata_scammoni_unplaced_genomic_scaffold_BalAcu1.0_scaffold42_whole_genome_shotgun_sequence
marine_VNFC01000009.1_94749019-94749669_Balaenoptera_musculus_isolate_JJ_BM4_2016_0621_Super_scaffold_9_whole_genome_shotgun_sequence
marine_RJWP010001593.1_8069-8719_Eubalaena_japonica_isolate_BS61_EubJap_flattened_line_3180_whole_genome_shotgun_sequence
LSZX01069037.1_58665-59323_Camelus_dromedarius_breed_Targui_Contig_13331_44_whole_genome_shotgun_sequence
JWIN03000002.1_110643858-110644516_Camelus_dromedarius_breed_African_isolate_Drom800_chromosome_2_whole_genome_shotgun_sequence
R_CAOW010006984.1_1877-2535_Camelus_bactrianus_WGS_project_CAOW00000000_data_contig_8225_whole_genome_shotgun_sequence
AGVR01049024.1_6079-6737_Camelus_ferus_isolate_Naran_contig049393_whole_genome_shotgun_sequence
R_ABRR03000026.1_12873969-12874617_Vicugna_pacos_isolate_Carlotta__AHFN-0088__chromosome_2_ScfyRBE_26HRSCAF_216_whole_genome_shotgun_sequence
R_PVHT021183710.1_6267166-6267826_Catagonus_wagneri_isolate_BS18_Sc2sB16_1185060HRSCAF_1231767_whole_genome_shotgun_sequence
R_PVJP02910524.1_4547054-4547706_Hippopotamus_amphibius_isolate_BS01_ScHSfme_924986HRSCAF_982786_whole_genome_shotgun_sequence
PVJP02910399.1_3255530-3256200_Hippopotamus_amphibius_isolate_BS01_ScHSfme_924870HRSCAF_982670_whole_genome_shotgun_sequence
R_LSZX01130166.1_11555-12217_Camelus_dromedarius_breed_Targui_Contig_18043_6_whole_genome_shotgun_sequence
JDVD01059187.1_111686-112348_Camelus_dromedarius_breed_Arabia_contig59187_whole_genome_shotgun_sequence
JWIN03000003.1_88576342-88577004_Camelus_dromedarius_breed_African_isolate_Drom800_chromosome_3_whole_genome_shotgun_sequence
R_AGVR01040212.1_584-1255_Camelus_ferus_isolate_Naran_contig040478_whole_genome_shotgun_sequence
JARL01038245.1_111879-112550_Camelus_bactrianus_breed_Alxa_contig38245_whole_genome_shotgun_sequence
R_JEMW01016955.1_112176-112848_Vicugna_pacos_huacaya_contig16955_whole_genome_shotgun_sequence
R_ABRR03000033.1_11790842-11791514_Vicugna_pacos_isolate_Carlotta__AHFN-0088__ScfyRBE_33HRSCAF_248_whole_genome_shotgun_sequence
PVHT020004020.1_41419878-41420532_Catagonus_wagneri_isolate_BS18_Sc2sB16_3972HRSCAF_47390_1_whole_genome_shotgun_sequence
NPJO01000004.1_121305354-121306025_Sus_scrofa_breed_Cross-bred_isolate_201423004_chromosome_2_whole_genome_shotgun_sequence
PVJP02910188.1_2410594-2411269_Hippopotamus_amphibius_isolate_BS01_ScHSfme_924681HRSCAF_982481_whole_genome_shotgun_sequence
0.02

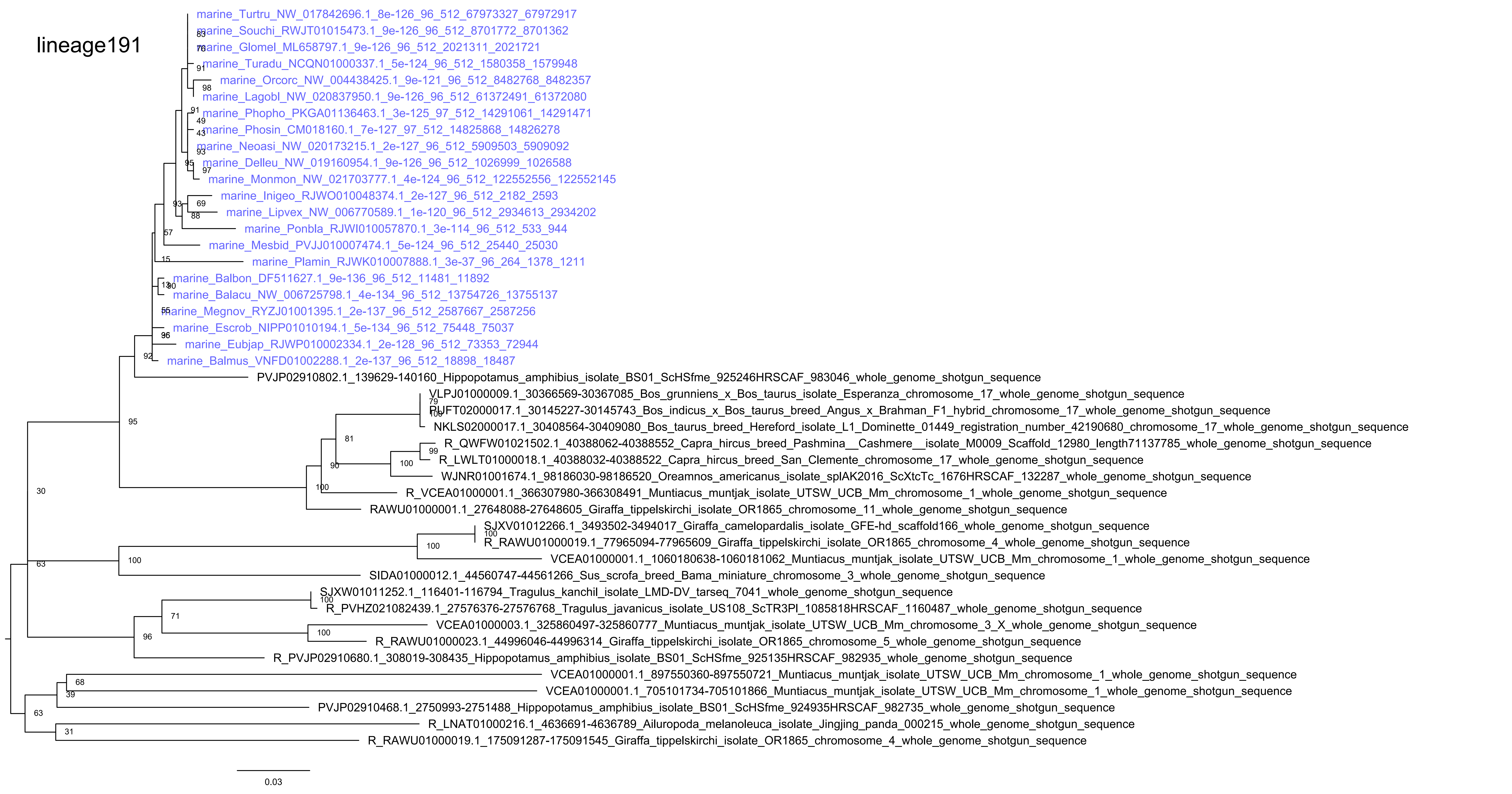
lineage191
marine_Turtru_NW_017842696.1_8e-126_96_512_67973327_67972917
marine_Souchi_RWJT01015473.1_9e-126_96_512_8701772_8701362
marine_Glomel_ML658797.1_9e-126_96_512_2021311_2021721
marine_Turadu_NCQN01000337.1_5e-124_96_512_1580358_1579948
marine_Orcorc_NW_004438425.1_9e-121_96_512_8482768_8482357
marine_Lagobl_NW_020837950.1_9e-126_96_512_61372491_61372080
marine_Phopho_PKGA01136463.1_3e-125_97_512_14291061_14291471
marine_Phosin_CM018160.1_7e-127_97_512_14825868_14826278
marine_Neoasi_NW_020173215.1_2e-127_96_512_5909503_5909092
marine_Delleu_NW_019160954.1_9e-126_96_512_1026999_1026588
marine_Monmon_NW_021703777.1_4e-124_96_512_122552556_122552145
marine_Inigeo_RJWO010048374.1_2e-127_96_512_2182_2593
marine_Lipvex_NW_006770589.1_1e-120_96_512_2934613_2934202
marine_Ponbla_RJWI010057870.1_3e-114_96_512_533_944
marine_Mesbid_PVJJ010007474.1_5e-124_96_512_25440_25030
marine_Plamin_RJWK010007888.1_3e-37_96_264_1378_1211
marine_Balbon_DF511627.1_9e-136_96_512_11481_11892
marine_Balacu_NW_006725798.1_4e-134_96_512_13754726_13755137
marine_Megnov_RYZJ01001395.1_2e-137_96_512_2587667_2587256
marine_Escrob_NIPP01010194.1_5e-134_96_512_75448_75037
marine_Eubjap_RJWP010002334.1_2e-128_96_512_73353_72944
marine_Balmus_VNFD01002288.1_2e-137_96_512_18898_18487
PVJP02910802.1_139629-140160_Hippopotamus_amphibius_isolate_BS01_ScHSfme_925246HRSCAF_983046_whole_genome_shotgun_sequence
VLPJ01000009.1_30366569-30367085_Bos_grunniens_x_Bos_taurus_isolate_Esperanza_chromosome_17_whole_genome_shotgun_sequence
PUFT02000017.1_30145227-30145743_Bos_indicus_x_Bos_taurus_breed_Angus_x_Brahman_F1_hybrid_chromosome_17_whole_genome_shotgun_sequence
NKLS02000017.1_30408564-30409080_Bos_taurus_breed_Hereford_isolate_L1_Dominette_01449_registration_number_42190680_chromosome_17_whole_genome_shotgun_sequence
R_QWFW01021502.1_40388062-40388552_Capra_hircus_breed_Pashmina__Cashmere__isolate_M0009_Scaffold_12980_length71137785_whole_genome_shotgun_sequence
R_LWLT01000018.1_40388032-40388522_Capra_hircus_breed_San_Clemente_chromosome_17_whole_genome_shotgun_sequence
WJNR01001674.1_98186030-98186520_Oreamnos_americanus_isolate_splAK2016_ScXtcTc_1676HRSCAF_132287_whole_genome_shotgun_sequence
R_VCEA01000001.1_366307980-366308491_Muntiacus_muntjak_isolate_UTSW_UCB_Mm_chromosome_1_whole_genome_shotgun_sequence
RAWU01000001.1_27648088-27648605_Giraffa_tippelskirchi_isolate_OR1865_chromosome_11_whole_genome_shotgun_sequence
SJXV01012266.1_3493502-3494017_Giraffa_camelopardalis_isolate_GFE-hd_scaffold166_whole_genome_shotgun_sequence
R_RAWU01000019.1_77965094-77965609_Giraffa_tippelskirchi_isolate_OR1865_chromosome_4_whole_genome_shotgun_sequence
VCEA01000001.1_1060180638-1060181062_Muntiacus_muntjak_isolate_UTSW_UCB_Mm_chromosome_1_whole_genome_shotgun_sequence
SIDA01000012.1_44560747-44561266_Sus_scrofa_breed_Bama_miniature_chromosome_3_whole_genome_shotgun_sequence
SJXW01011252.1_116401-116794_Tragulus_kanchil_isolate_LMD-DV_tarseq_7041_whole_genome_shotgun_sequence
R_PVHZ021082439.1_27576376-27576768_Tragulus_javanicus_isolate_US108_ScTR3PI_1085818HRSCAF_1160487_whole_genome_shotgun_sequence
VCEA01000003.1_325860497-325860777_Muntiacus_muntjak_isolate_UTSW_UCB_Mm_chromosome_3_X_whole_genome_shotgun_sequence
R_RAWU01000023.1_44996046-44996314_Giraffa_tippelskirchi_isolate_OR1865_chromosome_5_whole_genome_shotgun_sequence
R_PVJP02910680.1_308019-308435_Hippopotamus_amphibius_isolate_BS01_ScHSfme_925135HRSCAF_982935_whole_genome_shotgun_sequence
VCEA01000001.1_897550360-897550721_Muntiacus_muntjak_isolate_UTSW_UCB_Mm_chromosome_1_whole_genome_shotgun_sequence
VCEA01000001.1_705101734-705101866_Muntiacus_muntjak_isolate_UTSW_UCB_Mm_chromosome_1_whole_genome_shotgun_sequence
PVJP02910468.1_2750993-2751488_Hippopotamus_amphibius_isolate_BS01_ScHSfme_924935HRSCAF_982735_whole_genome_shotgun_sequence
R_LNAT01000216.1_4636691-4636789_Ailuropoda_melanoleuca_isolate_Jingjing_panda_000215_whole_genome_shotgun_sequence
R_RAWU01000019.1_175091287-175091545_Giraffa_tippelskirchi_isolate_OR1865_chromosome_4_whole_genome_shotgun_sequence
0.03

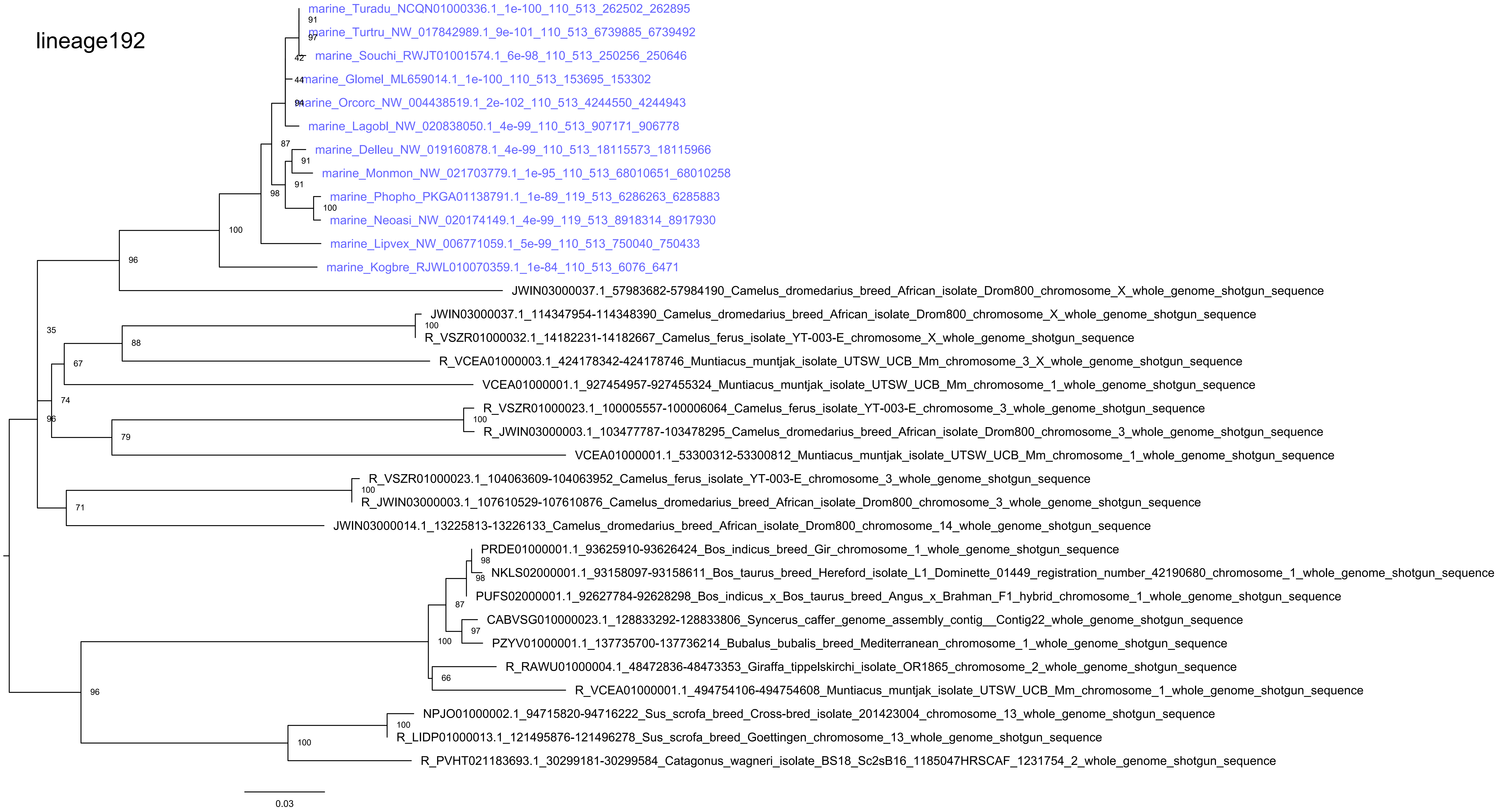
lineage192
marine_Turadu_NCQN01000336.1_1e-100_110_513_262502_262895
marine_Turtru_NW_017842989.1_9e-101_110_513_6739885_6739492
marine_Souchi_RWJT01001574.1_6e-98_110_513_250256_250646
marine_Glomel_ML659014.1_1e-100_110_513_153695_153302
marine_Orcorc_NW_004438519.1_2e-102_110_513_4244550_4244943
marine_Lagobl_NW_020838050.1_4e-99_110_513_907171_906778
marine_Delleu_NW_019160878.1_4e-99_110_513_18115573_18115966
marine_Monmon_NW_021703779.1_1e-95_110_513_68010651_68010258
marine_Phopho_PKGA01138791.1_1e-89_119_513_6286263_6285883
marine_Neoasi_NW_020174149.1_4e-99_119_513_8918314_8917930
marine_Lipvex_NW_006771059.1_5e-99_110_513_750040_750433
marine_Kogbre_RJWL010070359.1_1e-84_110_513_6076_6471
JWIN03000037.1_57983682-57984190_Camelus_dromedarius_breed_African_isolate_Drom800_chromosome_X_whole_genome_shotgun_sequence
JWIN03000037.1_114347954-114348390_Camelus_dromedarius_breed_African_isolate_Drom800_chromosome_X_whole_genome_shotgun_sequence
R_VSZR01000032.1_14182231-14182667_Camelus_ferus_isolate_YT-003-E_chromosome_X_whole_genome_shotgun_sequence
R_VCEA01000003.1_424178342-424178746_Muntiacus_muntjak_isolate_UTSW_UCB_Mm_chromosome_3_X_whole_genome_shotgun_sequence
VCEA01000001.1_927454957-927455324_Muntiacus_muntjak_isolate_UTSW_UCB_Mm_chromosome_1_whole_genome_shotgun_sequence
R_VSZR01000023.1_100005557-100006064_Camelus_ferus_isolate_YT-003-E_chromosome_3_whole_genome_shotgun_sequence
R_JWIN03000003.1_103477787-103478295_Camelus_dromedarius_breed_African_isolate_Drom800_chromosome_3_whole_genome_shotgun_sequence
VCEA01000001.1_53300312-53300812_Muntiacus_muntjak_isolate_UTSW_UCB_Mm_chromosome_1_whole_genome_shotgun_sequence
R_VSZR01000023.1_104063609-104063952_Camelus_ferus_isolate_YT-003-E_chromosome_3_whole_genome_shotgun_sequence
R_JWIN03000003.1_107610529-107610876_Camelus_dromedarius_breed_African_isolate_Drom800_chromosome_3_whole_genome_shotgun_sequence
JWIN03000014.1_13225813-13226133_Camelus_dromedarius_breed_African_isolate_Drom800_chromosome_14_whole_genome_shotgun_sequence
PRDE01000001.1_93625910-93626424_Bos_indicus_breed_Gir_chromosome_1_whole_genome_shotgun_sequence
NKLS02000001.1_93158097-93158611_Bos_taurus_breed_Hereford_isolate_L1_Dominette_01449_registration_number_42190680_chromosome_1_whole_genome_shotgun_sequence
PUFS02000001.1_92627784-92628298_Bos_indicus_x_Bos_taurus_breed_Angus_x_Brahman_F1_hybrid_chromosome_1_whole_genome_shotgun_sequence
CABVSG010000023.1_128833292-128833806_Syncerus_caffer_genome_assembly_contig__Contig22_whole_genome_shotgun_sequence
PZYV01000001.1_137735700-137736214_Bubalus_bubalis_breed_Mediterranean_chromosome_1_whole_genome_shotgun_sequence
R_RAWU01000004.1_48472836-48473353_Giraffa_tippelskirchi_isolate_OR1865_chromosome_2_whole_genome_shotgun_sequence
R_VCEA01000001.1_494754106-494754608_Muntiacus_muntjak_isolate_UTSW_UCB_Mm_chromosome_1_whole_genome_shotgun_sequence
NPJO01000002.1_94715820-94716222_Sus_scrofa_breed_Cross-bred_isolate_201423004_chromosome_13_whole_genome_shotgun_sequence
R_LIDP01000013.1_121495876-121496278_Sus_scrofa_breed_Goettingen_chromosome_13_whole_genome_shotgun_sequence
R_PVHT021183693.1_30299181-30299584_Catagonus_wagneri_isolate_BS18_Sc2sB16_1185047HRSCAF_1231754_2_whole_genome_shotgun_sequence
0.03

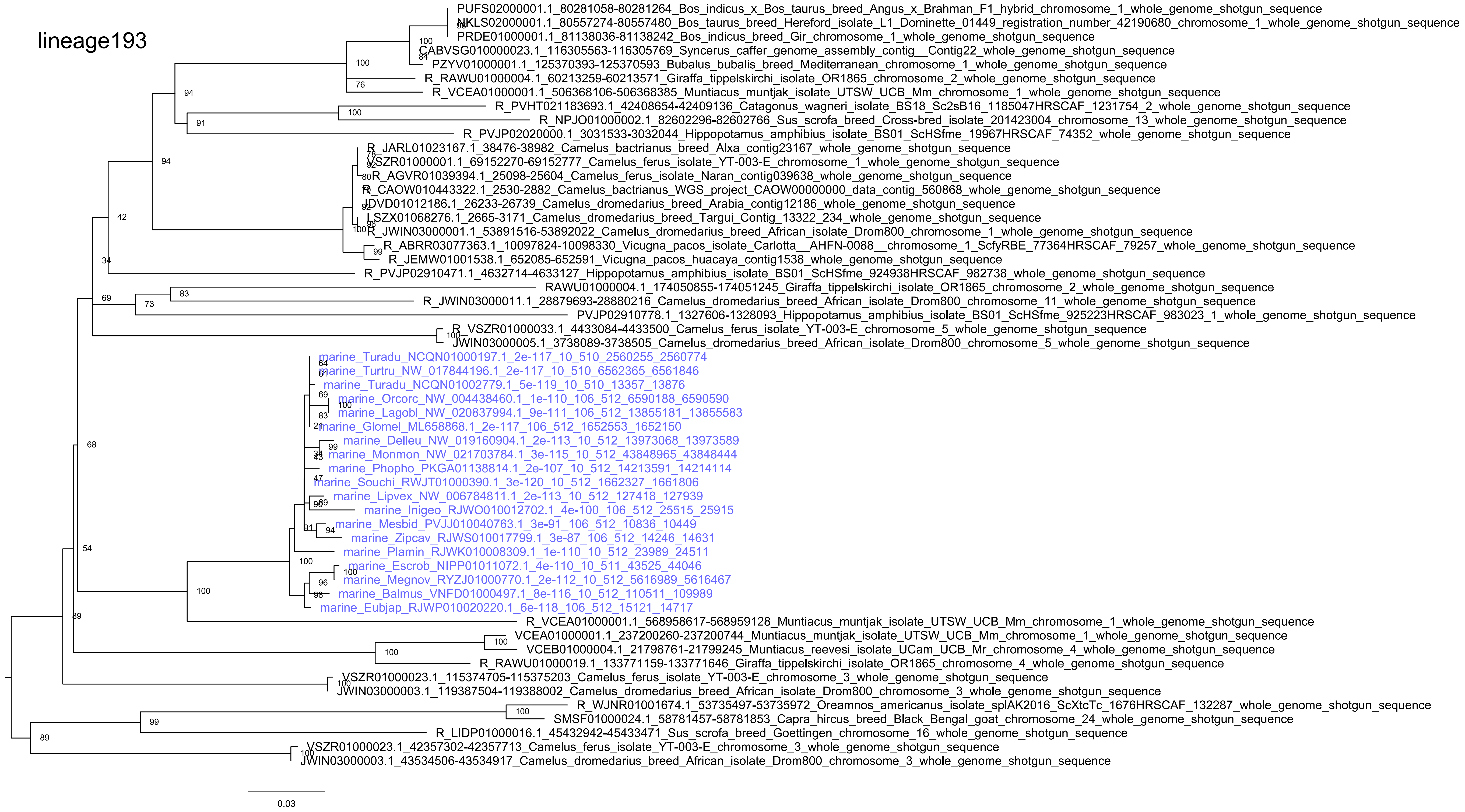
lineage193
PUFS02000001.1_80281058-80281264_Bos_indicus_x_Bos_taurus_breed_Angus_x_Brahman_F1_hybrid_chromosome_1_whole_genome_shotgun_sequence
NKLS02000001.1_80557274-80557480_Bos_taurus_breed_Hereford_isolate_L1_Dominette_01449_registration_number_42190680_chromosome_1_whole_genome_shotgun_sequence
PRDE01000001.1_81138036-81138242_Bos_indicus_breed_Gir_chromosome_1_whole_genome_shotgun_sequence
CABVSG010000023.1_116305563-116305769_Syncerus_caffer_genome_assembly_contig__Contig22_whole_genome_shotgun_sequence
PZYV01000001.1_125370393-125370593_Bubalus_bubalis_breed_Mediterranean_chromosome_1_whole_genome_shotgun_sequence
R_RAWU01000004.1_60213259-60213571_Giraffa_tippelskirchi_isolate_OR1865_chromosome_2_whole_genome_shotgun_sequence
R_VCEA01000001.1_506368106-506368385_Muntiacus_muntjak_isolate_UTSW_UCB_Mm_chromosome_1_whole_genome_shotgun_sequence
R_PVHT021183693.1_42408654-42409136_Catagonus_wagneri_isolate_BS18_Sc2sB16_1185047HRSCAF_1231754_2_whole_genome_shotgun_sequence
R_NPJO01000002.1_82602296-82602766_Sus_scrofa_breed_Cross-bred_isolate_201423004_chromosome_13_whole_genome_shotgun_sequence
R_PVJP02020000.1_3031533-3032044_Hippopotamus_amphibius_isolate_BS01_ScHSfme_19967HRSCAF_74352_whole_genome_shotgun_sequence
R_JARL01023167.1_38476-38982_Camelus_bactrianus_breed_Alxa_contig23167_whole_genome_shotgun_sequence
VSZR01000001.1_69152270-69152777_Camelus_ferus_isolate_YT-003-E_chromosome_1_whole_genome_shotgun_sequence
R_AGVR01039394.1_25098-25604_Camelus_ferus_isolate_Naran_contig039638_whole_genome_shotgun_sequence
R_CAOW010443322.1_2530-2882_Camelus_bactrianus_WGS_project_CAOW00000000_data_contig_560868_whole_genome_shotgun_sequence
JDVD01012186.1_26233-26739_Camelus_dromedarius_breed_Arabia_contig12186_whole_genome_shotgun_sequence
LSZX01068276.1_2665-3171_Camelus_dromedarius_breed_Targui_Contig_13322_234_whole_genome_shotgun_sequence
R_JWIN03000001.1_53891516-53892022_Camelus_dromedarius_breed_African_isolate_Drom800_chromosome_1_whole_genome_shotgun_sequence
R_ABRR03077363.1_10097824-10098330_Vicugna_pacos_isolate_Carlotta__AHFN-0088__chromosome_1_ScfyRBE_77364HRSCAF_79257_whole_genome_shotgun_sequence
R_JEMW01001538.1_652085-652591_Vicugna_pacos_huacaya_contig1538_whole_genome_shotgun_sequence
R_PVJP02910471.1_4632714-4633127_Hippopotamus_amphibius_isolate_BS01_ScHSfme_924938HRSCAF_982738_whole_genome_shotgun_sequence
RAWU01000004.1_174050855-174051245_Giraffa_tippelskirchi_isolate_OR1865_chromosome_2_whole_genome_shotgun_sequence
R_JWIN03000011.1_28879693-28880216_Camelus_dromedarius_breed_African_isolate_Drom800_chromosome_11_whole_genome_shotgun_sequence
PVJP02910778.1_1327606-1328093_Hippopotamus_amphibius_isolate_BS01_ScHSfme_925223HRSCAF_983023_1_whole_genome_shotgun_sequence
R_VSZR01000033.1_4433084-4433500_Camelus_ferus_isolate_YT-003-E_chromosome_5_whole_genome_shotgun_sequence
JWIN03000005.1_3738089-3738505_Camelus_dromedarius_breed_African_isolate_Drom800_chromosome_5_whole_genome_shotgun_sequence
marine_Turadu_NCQN01000197.1_2e-117_10_510_2560255_2560774
marine_Turtru_NW_017844196.1_2e-117_10_510_6562365_6561846
marine_Turadu_NCQN01002779.1_5e-119_10_510_13357_13876
marine_Orcorc_NW_004438460.1_1e-110_106_512_6590188_6590590
marine_Lagobl_NW_020837994.1_9e-111_106_512_13855181_13855583
marine_Glomel_ML658868.1_2e-117_106_512_1652553_1652150
marine_Delleu_NW_019160904.1_2e-113_10_512_13973068_13973589
marine_Monmon_NW_021703784.1_3e-115_10_512_43848965_43848444
marine_Phopho_PKGA01138814.1_2e-107_10_512_14213591_14214114
marine_Souchi_RWJT01000390.1_3e-120_10_512_1662327_1661806
marine_Lipvex_NW_006784811.1_2e-113_10_512_127418_127939
marine_Inigeo_RJWO010012702.1_4e-100_106_512_25515_25915
marine_Mesbid_PVJJ010040763.1_3e-91_106_512_10836_10449
marine_Zipcav_RJWS010017799.1_3e-87_106_512_14246_14631
marine_Plamin_RJWK010008309.1_1e-110_10_512_23989_24511
marine_Escrob_NIPP01011072.1_4e-110_10_511_43525_44046
marine_Megnov_RYZJ01000770.1_2e-112_10_512_5616989_5616467
marine_Balmus_VNFD01000497.1_8e-116_10_512_110511_109989
marine_Eubjap_RJWP010020220.1_6e-118_106_512_15121_14717
R_VCEA01000001.1_568958617-568959128_Muntiacus_muntjak_isolate_UTSW_UCB_Mm_chromosome_1_whole_genome_shotgun_sequence
VCEA01000001.1_237200260-237200744_Muntiacus_muntjak_isolate_UTSW_UCB_Mm_chromosome_1_whole_genome_shotgun_sequence
VCEB01000004.1_21798761-21799245_Muntiacus_reevesi_isolate_UCam_UCB_Mr_chromosome_4_whole_genome_shotgun_sequence
R_RAWU01000019.1_133771159-133771646_Giraffa_tippelskirchi_isolate_OR1865_chromosome_4_whole_genome_shotgun_sequence
VSZR01000023.1_115374705-115375203_Camelus_ferus_isolate_YT-003-E_chromosome_3_whole_genome_shotgun_sequence
JWIN03000003.1_119387504-119388002_Camelus_dromedarius_breed_African_isolate_Drom800_chromosome_3_whole_genome_shotgun_sequence
R_WJNR01001674.1_53735497-53735972_Oreamnos_americanus_isolate_splAK2016_ScXtcTc_1676HRSCAF_132287_whole_genome_shotgun_sequence
SMSF01000024.1_58781457-58781853_Capra_hircus_breed_Black_Bengal_goat_chromosome_24_whole_genome_shotgun_sequence
R_LIDP01000016.1_45432942-45433471_Sus_scrofa_breed_Goettingen_chromosome_16_whole_genome_shotgun_sequence
VSZR01000023.1_42357302-42357713_Camelus_ferus_isolate_YT-003-E_chromosome_3_whole_genome_shotgun_sequence
JWIN03000003.1_43534506-43534917_Camelus_dromedarius_breed_African_isolate_Drom800_chromosome_3_whole_genome_shotgun_sequence
0.03

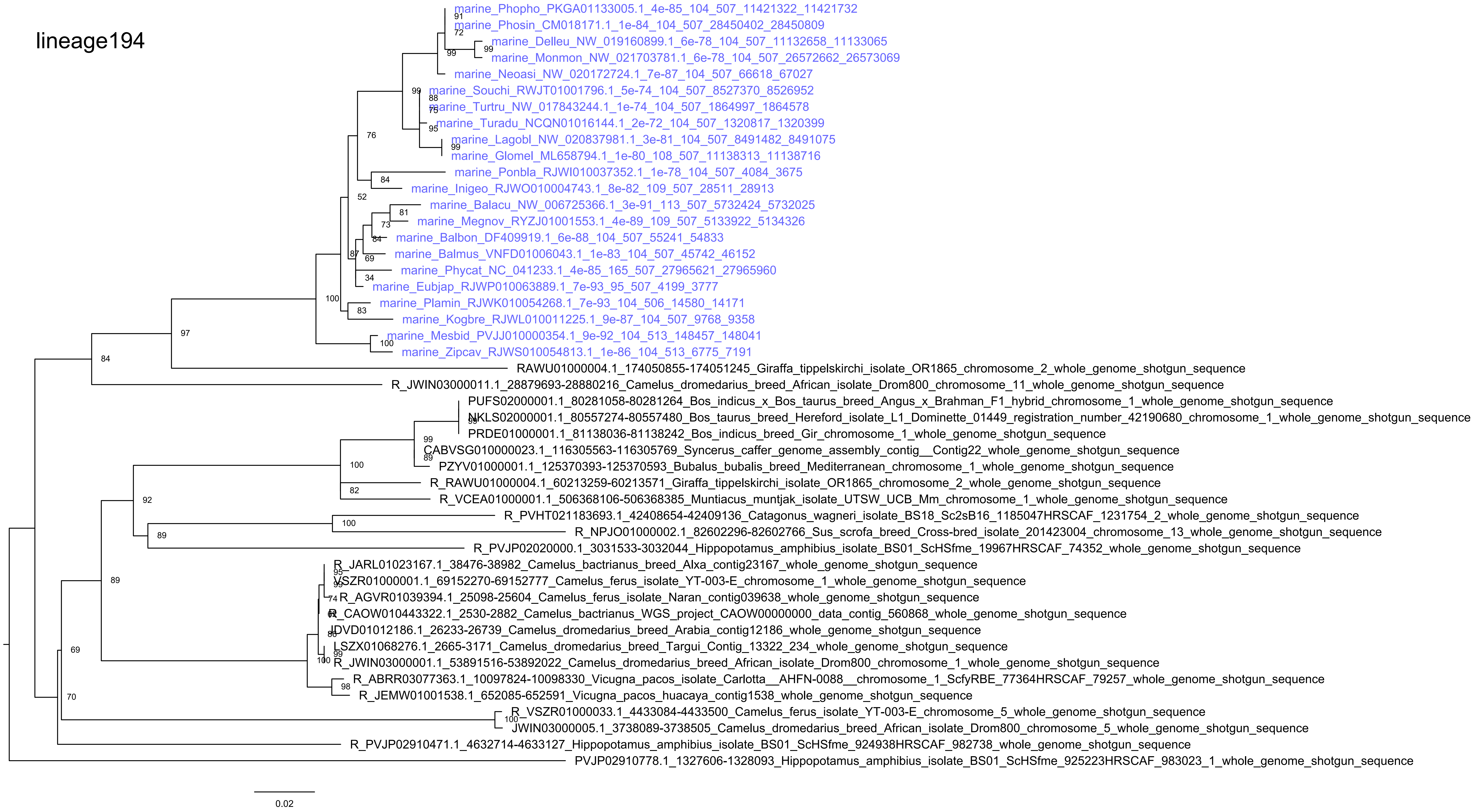
lineage194
marine_Phopho_PKGA01133005.1_4e-85_104_507_11421322_11421732
marine_Phosin_CM018171.1_1e-84_104_507_28450402_28450809
marine_Delleu_NW_019160899.1_6e-78_104_507_11132658_11133065
marine_Monmon_NW_021703781.1_6e-78_104_507_26572662_26573069
marine_Neoasi_NW_020172724.1_7e-87_104_507_66618_67027
marine_Souchi_RWJT01001796.1_5e-74_104_507_8527370_8526952
marine_Turtru_NW_017843244.1_1e-74_104_507_1864997_1864578
marine_Turadu_NCQN01016144.1_2e-72_104_507_1320817_1320399
marine_Lagobl_NW_020837981.1_3e-81_104_507_8491482_8491075
marine_Glomel_ML658794.1_1e-80_108_507_11138313_11138716
marine_Ponbla_RJWI010037352.1_1e-78_104_507_4084_3675
marine_Inigeo_RJWO010004743.1_8e-82_109_507_28511_28913
marine_Balacu_NW_006725366.1_3e-91_113_507_5732424_5732025
marine_Megnov_RYZJ01001553.1_4e-89_109_507_5133922_5134326
marine_Balbon_DF409919.1_6e-88_104_507_55241_54833
marine_Balmus_VNFD01006043.1_1e-83_104_507_45742_46152
marine_Phycat_NC_041233.1_4e-85_165_507_27965621_27965960
marine_Eubjap_RJWP010063889.1_7e-93_95_507_4199_3777
marine_Plamin_RJWK010054268.1_7e-93_104_506_14580_14171
marine_Kogbre_RJWL010011225.1_9e-87_104_507_9768_9358
marine_Mesbid_PVJJ010000354.1_9e-92_104_513_148457_148041
marine_Zipcav_RJWS010054813.1_1e-86_104_513_6775_7191
RAWU01000004.1_174050855-174051245_Giraffa_tippelskirchi_isolate_OR1865_chromosome_2_whole_genome_shotgun_sequence
R_JWIN03000011.1_28879693-28880216_Camelus_dromedarius_breed_African_isolate_Drom800_chromosome_11_whole_genome_shotgun_sequence
PUFS02000001.1_80281058-80281264_Bos_indicus_x_Bos_taurus_breed_Angus_x_Brahman_F1_hybrid_chromosome_1_whole_genome_shotgun_sequence
NKLS02000001.1_80557274-80557480_Bos_taurus_breed_Hereford_isolate_L1_Dominette_01449_registration_number_42190680_chromosome_1_whole_genome_shotgun_sequence
PRDE01000001.1_81138036-81138242_Bos_indicus_breed_Gir_chromosome_1_whole_genome_shotgun_sequence
CABVSG010000023.1_116305563-116305769_Syncerus_caffer_genome_assembly_contig__Contig22_whole_genome_shotgun_sequence
PZYV01000001.1_125370393-125370593_Bubalus_bubalis_breed_Mediterranean_chromosome_1_whole_genome_shotgun_sequence
R_RAWU01000004.1_60213259-60213571_Giraffa_tippelskirchi_isolate_OR1865_chromosome_2_whole_genome_shotgun_sequence
R_VCEA01000001.1_506368106-506368385_Muntiacus_muntjak_isolate_UTSW_UCB_Mm_chromosome_1_whole_genome_shotgun_sequence
R_PVHT021183693.1_42408654-42409136_Catagonus_wagneri_isolate_BS18_Sc2sB16_1185047HRSCAF_1231754_2_whole_genome_shotgun_sequence
R_NPJO01000002.1_82602296-82602766_Sus_scrofa_breed_Cross-bred_isolate_201423004_chromosome_13_whole_genome_shotgun_sequence
R_PVJP02020000.1_3031533-3032044_Hippopotamus_amphibius_isolate_BS01_ScHSfme_19967HRSCAF_74352_whole_genome_shotgun_sequence
R_JARL01023167.1_38476-38982_Camelus_bactrianus_breed_Alxa_contig23167_whole_genome_shotgun_sequence
VSZR01000001.1_69152270-69152777_Camelus_ferus_isolate_YT-003-E_chromosome_1_whole_genome_shotgun_sequence
R_AGVR01039394.1_25098-25604_Camelus_ferus_isolate_Naran_contig039638_whole_genome_shotgun_sequence
R_CAOW010443322.1_2530-2882_Camelus_bactrianus_WGS_project_CAOW00000000_data_contig_560868_whole_genome_shotgun_sequence
JDVD01012186.1_26233-26739_Camelus_dromedarius_breed_Arabia_contig12186_whole_genome_shotgun_sequence
LSZX01068276.1_2665-3171_Camelus_dromedarius_breed_Targui_Contig_13322_234_whole_genome_shotgun_sequence
R_JWIN03000001.1_53891516-53892022_Camelus_dromedarius_breed_African_isolate_Drom800_chromosome_1_whole_genome_shotgun_sequence
R_ABRR03077363.1_10097824-10098330_Vicugna_pacos_isolate_Carlotta__AHFN-0088__chromosome_1_ScfyRBE_77364HRSCAF_79257_whole_genome_shotgun_sequence
R_JEMW01001538.1_652085-652591_Vicugna_pacos_huacaya_contig1538_whole_genome_shotgun_sequence
R_VSZR01000033.1_4433084-4433500_Camelus_ferus_isolate_YT-003-E_chromosome_5_whole_genome_shotgun_sequence
JWIN03000005.1_3738089-3738505_Camelus_dromedarius_breed_African_isolate_Drom800_chromosome_5_whole_genome_shotgun_sequence
R_PVJP02910471.1_4632714-4633127_Hippopotamus_amphibius_isolate_BS01_ScHSfme_924938HRSCAF_982738_whole_genome_shotgun_sequence
PVJP02910778.1_1327606-1328093_Hippopotamus_amphibius_isolate_BS01_ScHSfme_925223HRSCAF_983023_1_whole_genome_shotgun_sequence
0.02

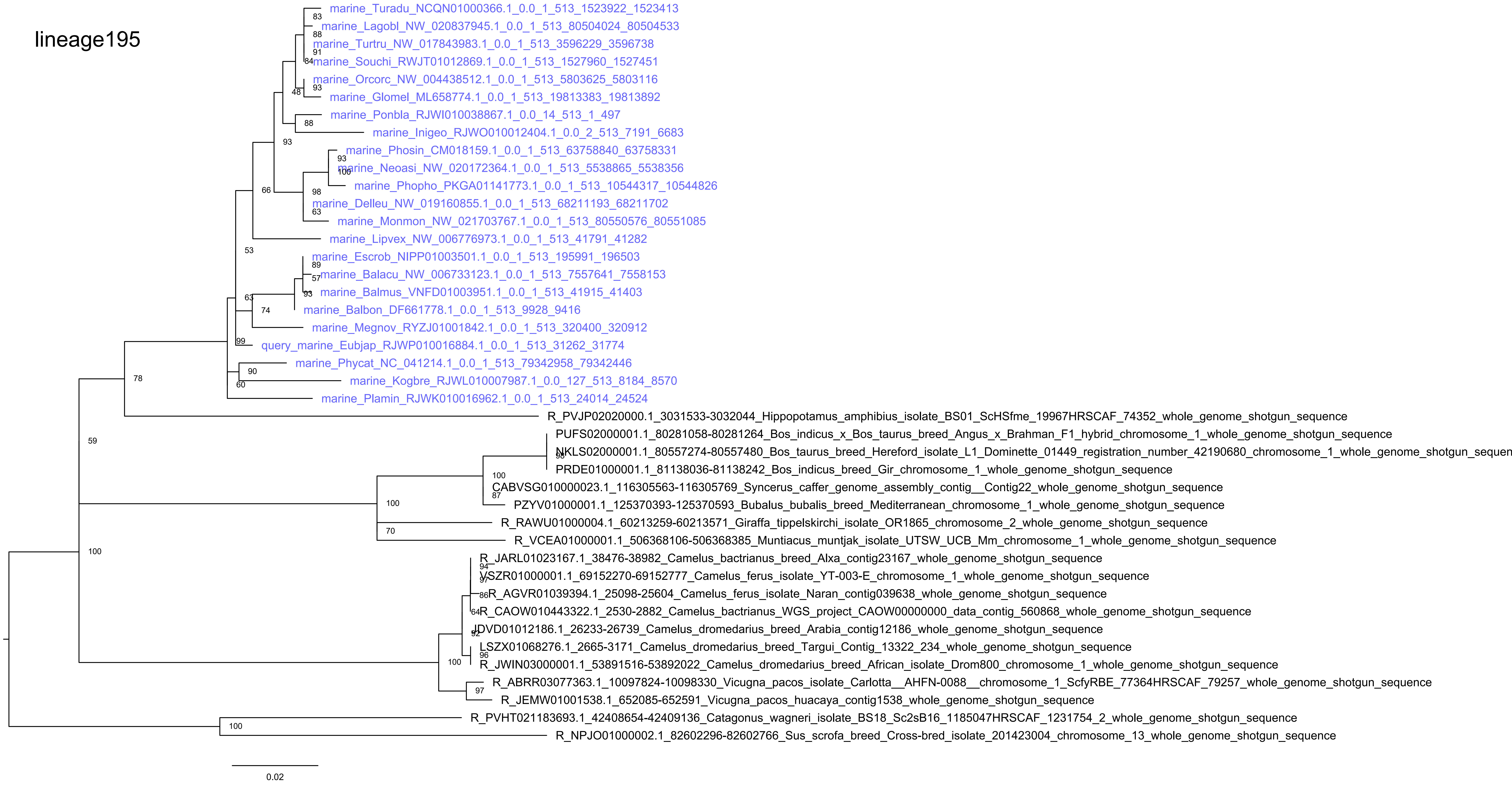

lineage195
marine_Turadu_NCQN01000366.1_0.0_1_513_1523922_1523413
marine_Lagobl_NW_020837945.1_0.0_1_513_80504024_80504533
marine_Turtru_NW_017843983.1_0.0_1_513_3596229_3596738
marine_Souchi_RWJT01012869.1_0.0_1_513_1527960_1527451
marine_Orcorc_NW_004438512.1_0.0_1_513_5803625_5803116
marine_Glomel_ML658774.1_0.0_1_513_19813383_19813892
marine_Ponbla_RJWI010038867.1_0.0_14_513_1_497
marine_Inigeo_RJWO010012404.1_0.0_2_513_7191_6683
marine_Phosin_CM018159.1_0.0_1_513_63758840_63758331
marine_Neoasi_NW_020172364.1_0.0_1_513_5538865_5538356
marine_Phopho_PKGA01141773.1_0.0_1_513_10544317_10544826
marine_Delleu_NW_019160855.1_0.0_1_513_68211193_68211702
marine_Monmon_NW_021703767.1_0.0_1_513_80550576_80551085
marine_Lipvex_NW_006776973.1_0.0_1_513_41791_41282
marine_Escrob_NIPP01003501.1_0.0_1_513_195991_196503
marine_Balacu_NW_006733123.1_0.0_1_513_7557641_7558153
marine_Balmus_VNFD01003951.1_0.0_1_513_41915_41403
marine_Balbon_DF661778.1_0.0_1_513_9928_9416
marine_Megnov_RYZJ01001842.1_0.0_1_513_320400_320912
query_marine_Eubjap_RJWP010016884.1_0.0_1_513_31262_31774
marine_Phycat_NC_041214.1_0.0_1_513_79342958_79342446
marine_Kogbre_RJWL010007987.1_0.0_127_513_8184_8570
marine_Plamin_RJWK010016962.1_0.0_1_513_24014_24524
R_PVJP02020000.1_3031533-3032044_Hippopotamus_amphibius_isolate_BS01_ScHSfme_19967HRSCAF_74352_whole_genome_shotgun_sequence
PUFS02000001.1_80281058-80281264_Bos_indicus_x_Bos_taurus_breed_Angus_x_Brahman_F1_hybrid_chromosome_1_whole_genome_shotgun_sequence
NKLS02000001.1_80557274-80557480_Bos_taurus_breed_Hereford_isolate_L1_Dominette_01449_registration_number_42190680_chromosome_1_whole_genome_shotgun_sequen
PRDE01000001.1_81138036-81138242_Bos_indicus_breed_Gir_chromosome_1_whole_genome_shotgun_sequence
CABVSG010000023.1_116305563-116305769_Syncerus_caffer_genome_assembly_contig__Contig22_whole_genome_shotgun_sequence
PZYV01000001.1_125370393-125370593_Bubalus_bubalis_breed_Mediterranean_chromosome_1_whole_genome_shotgun_sequence
R_RAWU01000004.1_60213259-60213571_Giraffa_tippelskirchi_isolate_OR1865_chromosome_2_whole_genome_shotgun_sequence
R_VCEA01000001.1_506368106-506368385_Muntiacus_muntjak_isolate_UTSW_UCB_Mm_chromosome_1_whole_genome_shotgun_sequence
R_JARL01023167.1_38476-38982_Camelus_bactrianus_breed_Alxa_contig23167_whole_genome_shotgun_sequence
VSZR01000001.1_69152270-69152777_Camelus_ferus_isolate_YT-003-E_chromosome_1_whole_genome_shotgun_sequence
R_AGVR01039394.1_25098-25604_Camelus_ferus_isolate_Naran_contig039638_whole_genome_shotgun_sequence
R_CAOW010443322.1_2530-2882_Camelus_bactrianus_WGS_project_CAOW00000000_data_contig_560868_whole_genome_shotgun_sequence
JDVD01012186.1_26233-26739_Camelus_dromedarius_breed_Arabia_contig12186_whole_genome_shotgun_sequence
LSZX01068276.1_2665-3171_Camelus_dromedarius_breed_Targui_Contig_13322_234_whole_genome_shotgun_sequence
R_JWIN03000001.1_53891516-53892022_Camelus_dromedarius_breed_African_isolate_Drom800_chromosome_1_whole_genome_shotgun_sequence
R_ABRR03077363.1_10097824-10098330_Vicugna_pacos_isolate_Carlotta__AHFN-0088__chromosome_1_ScfyRBE_77364HRSCAF_79257_whole_genome_shotgun_sequence
R_JEMW01001538.1_652085-652591_Vicugna_pacos_huacaya_contig1538_whole_genome_shotgun_sequence
R_PVHT021183693.1_42408654-42409136_Catagonus_wagneri_isolate_BS18_Sc2sB16_1185047HRSCAF_1231754_2_whole_genome_shotgun_sequence
R_NPJO01000002.1_82602296-82602766_Sus_scrofa_breed_Cross-bred_isolate_201423004_chromosome_13_whole_genome_shotgun_sequence
0.02

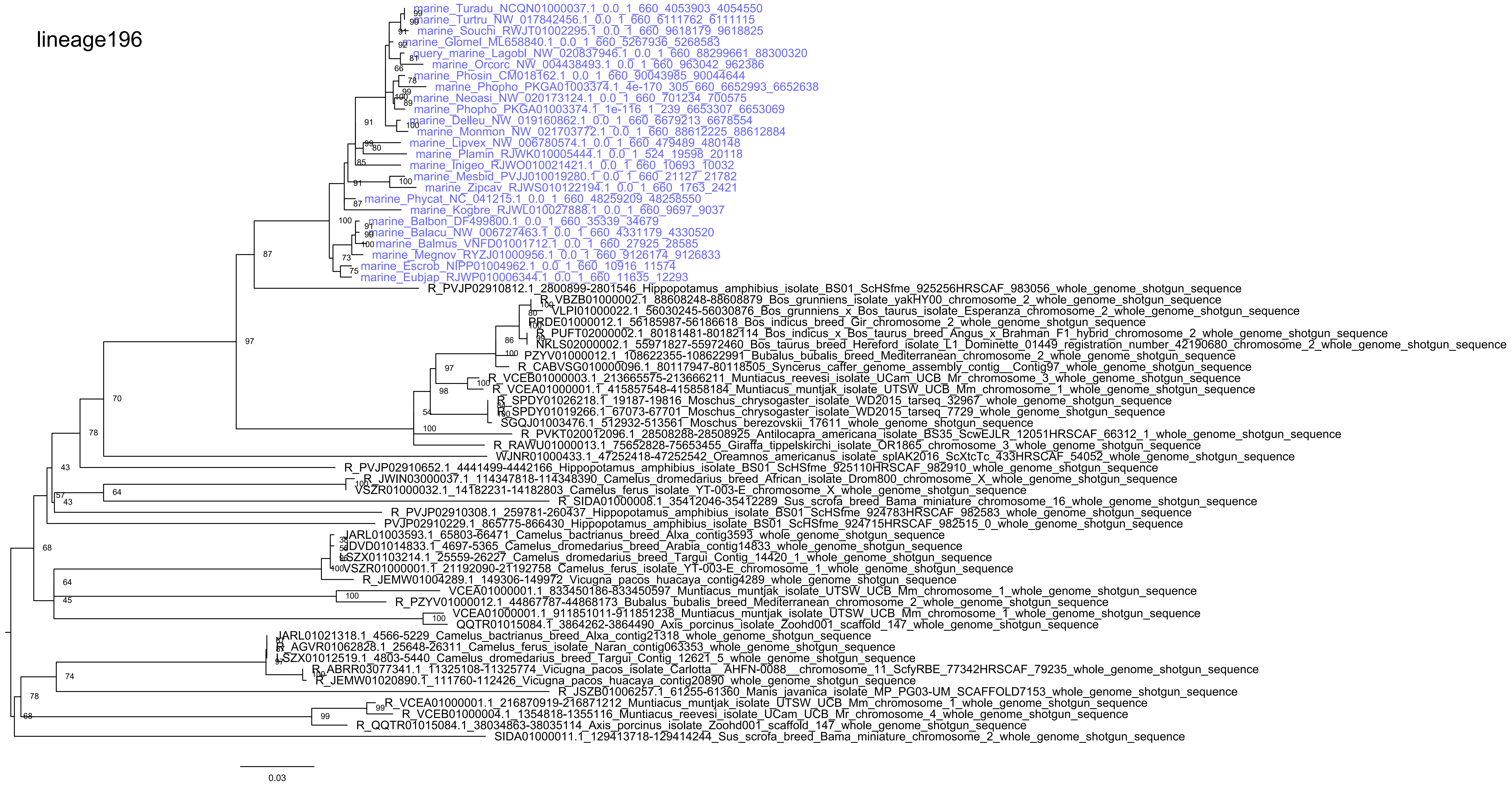
lineage196
marine_Turadu_NCQN01000037.1_0.0_1_660_4053903_4054550
marine_Turtru_NW_017842456.1_0.0_1_660_6111762_6111115
marine_Souchi_RWJT01002295.1_0.0_1_660_9618179_9618825
marine_Glomel_ML658840.1_0.0_1_660_5267936_5268583
query_marine_Lagobl_NW_020837946.1_0.0_1_660_88299661_88300320
marine_Orcorc_NW_004438493.1_0.0_1_660_963042_962386
marine_Phosin_CM018162.1_0.0_1_660_90043985_90044644
marine_Phopho_PKGA01003374.1_4e-170_305_660_6652993_6652638
marine_Neoasi_NW_020173124.1_0.0_1_660_701234_700575
marine_Phopho_PKGA01003374.1_1e-116_1_239_6653307_6653069
marine_Delleu_NW_019160862.1_0.0_1_660_6679213_6678554
marine_Monmon_NW_021703772.1_0.0_1_660_88612225_88612884
marine_Lipvex_NW_006780574.1_0.0_1_660_479489_480148
marine_Plamin_RJWK010005444.1_0.0_1_524_19598_20118
marine_Inigeo_RJWO010021421.1_0.0_1_660_10693_10032
marine_Mesbid_PVJJ010019280.1_0.0_1_660_21127_21782
marine_Zipcav_RJWS010122194.1_0.0_1_660_1763_2421
marine_Phycat_NC_041215.1_0.0_1_660_48259209_48258550
marine_Kogbre_RJWL010027888.1_0.0_1_660_9697_9037
marine_Balbon_DF499800.1_0.0_1_660_35339_34679
marine_Balacu_NW_006727463.1_0.0_1_660_4331179_4330520
marine_Balmus_VNFD01001712.1_0.0_1_660_27925_28585
marine_Megnov_RYZJ01000956.1_0.0_1_660_9126174_9126833
marine_Escrob_NIPP01004962.1_0.0_1_660_10916_11574
marine_Eubjap_RJWP010006344.1_0.0_1_660_11635_12293
R_PVJP02910812.1_2800899-2801546_Hippopotamus_amphibius_isolate_BS01_ScHSfme_925256HRSCAF_983056_whole_genome_shotgun_sequence
R_VBZB01000002.1_88608248-88608879_Bos_grunniens_isolate_yakHY00_chromosome_2_whole_genome_shotgun_sequence
VLPI01000022.1_56030245-56030876_Bos_grunniens_x_Bos_taurus_isolate_Esperanza_chromosome_2_whole_genome_shotgun_sequence
PRDE01000012.1_56185987-56186618_Bos_indicus_breed_Gir_chromosome_2_whole_genome_shotgun_sequence
R_PUFT02000002.1_80181481-80182114_Bos_indicus_x_Bos_taurus_breed_Angus_x_Brahman_F1_hybrid_chromosome_2_whole_genome_shotgun_sequence
NKLS02000002.1_55971827-55972460_Bos_taurus_breed_Hereford_isolate_L1_Dominette_01449_registration_number_42190680_chromosome_2_whole_genome_shotgun_sequence
PZYV01000012.1_108622355-108622991_Bubalus_bubalis_breed_Mediterranean_chromosome_2_whole_genome_shotgun_sequence
R_CABVSG010000096.1_80117947-80118505_Syncerus_caffer_genome_assembly_contig__Contig97_whole_genome_shotgun_sequence
R_VCEB01000003.1_213665575-213666211_Muntiacus_reevesi_isolate_UCam_UCB_Mr_chromosome_3_whole_genome_shotgun_sequence
R_VCEA01000001.1_415857548-415858184_Muntiacus_muntjak_isolate_UTSW_UCB_Mm_chromosome_1_whole_genome_shotgun_sequence
R_SPDY01026218.1_19187-19816_Moschus_chrysogaster_isolate_WD2015_tarseq_32967_whole_genome_shotgun_sequence
R_SPDY01019266.1_67073-67701_Moschus_chrysogaster_isolate_WD2015_tarseq_7729_whole_genome_shotgun_sequence
SGQJ01003476.1_512932-513561_Moschus_berezovskii_17611_whole_genome_shotgun_sequence
R_PVKT020012096.1_28508288-28508925_Antilocapra_americana_isolate_BS35_ScwEJLR_12051HRSCAF_66312_1_whole_genome_shotgun_sequence
R_RAWU01000013.1_75652828-75653455_Giraffa_tippelskirchi_isolate_OR1865_chromosome_3_whole_genome_shotgun_sequence
WJNR01000433.1_47252418-47252542_Oreamnos_americanus_isolate_splAK2016_ScXtcTc_433HRSCAF_54052_whole_genome_shotgun_sequence
R_PVJP02910652.1_4441499-4442166_Hippopotamus_amphibius_isolate_BS01_ScHSfme_925110HRSCAF_982910_whole_genome_shotgun_sequence
R_JWIN03000037.1_114347818-114348390_Camelus_dromedarius_breed_African_isolate_Drom800_chromosome_X_whole_genome_shotgun_sequence
VSZR01000032.1_14182231-14182803_Camelus_ferus_isolate_YT-003-E_chromosome_X_whole_genome_shotgun_sequence
R_SIDA01000008.1_35412046-35412289_Sus_scrofa_breed_Bama_miniature_chromosome_16_whole_genome_shotgun_sequence
R_PVJP02910308.1_259781-260437_Hippopotamus_amphibius_isolate_BS01_ScHSfme_924783HRSCAF_982583_whole_genome_shotgun_sequence
PVJP02910229.1_865775-866430_Hippopotamus_amphibius_isolate_BS01_ScHSfme_924715HRSCAF_982515_0_whole_genome_shotgun_sequence
JARL01003593.1_65803-66471_Camelus_bactrianus_breed_Alxa_contig3593_whole_genome_shotgun_sequence
JDVD01014833.1_4697-5365_Camelus_dromedarius_breed_Arabia_contig14833_whole_genome_shotgun_sequence
LSZX01103214.1_25559-26227_Camelus_dromedarius_breed_Targui_Contig_14420_1_whole_genome_shotgun_sequence
VSZR01000001.1_21192090-21192758_Camelus_ferus_isolate_YT-003-E_chromosome_1_whole_genome_shotgun_sequence
R_JEMW01004289.1_149306-149972_Vicugna_pacos_huacaya_contig4289_whole_genome_shotgun_sequence
VCEA01000001.1_833450186-833450597_Muntiacus_muntjak_isolate_UTSW_UCB_Mm_chromosome_1_whole_genome_shotgun_sequence
R_PZYV01000012.1_44867787-44868173_Bubalus_bubalis_breed_Mediterranean_chromosome_2_whole_genome_shotgun_sequence
VCEA01000001.1_911851011-911851238_Muntiacus_muntjak_isolate_UTSW_UCB_Mm_chromosome_1_whole_genome_shotgun_sequence
QQTR01015084.1_3864262-3864490_Axis_porcinus_isolate_Zoohd001_scaffold_147_whole_genome_shotgun_sequence
JARL01021318.1_4566-5229_Camelus_bactrianus_breed_Alxa_contig21318_whole_genome_shotgun_sequence
R_AGVR01062828.1_25648-26311_Camelus_ferus_isolate_Naran_contig063353_whole_genome_shotgun_sequence
LSZX01012519.1_4803-5440_Camelus_dromedarius_breed_Targui_Contig_12621_5_whole_genome_shotgun_sequence
R_ABRR03077341.1_11325108-11325774_Vicugna_pacos_isolate_Carlotta__AHFN-0088__chromosome_11_ScfyRBE_77342HRSCAF_79235_whole_genome_shotgun_sequence
R_JEMW01020890.1_111760-112426_Vicugna_pacos_huacaya_contig20890_whole_genome_shotgun_sequence
R_JSZB01006257.1_61255-61360_Manis_javanica_isolate_MP_PG03-UM_SCAFFOLD7153_whole_genome_shotgun_sequence
R_VCEA01000001.1_216870919-216871212_Muntiacus_muntjak_isolate_UTSW_UCB_Mm_chromosome_1_whole_genome_shotgun_sequence
R_VCEB01000004.1_1354818-1355116_Muntiacus_reevesi_isolate_UCam_UCB_Mr_chromosome_4_whole_genome_shotgun_sequence
R_QQTR01015084.1_38034863-38035114_Axis_porcinus_isolate_Zoohd001_scaffold_147_whole_genome_shotgun_sequence
SIDA01000011.1_129413718-129414244_Sus_scrofa_breed_Bama_miniature_chromosome_2_whole_genome_shotgun_sequence
0.03

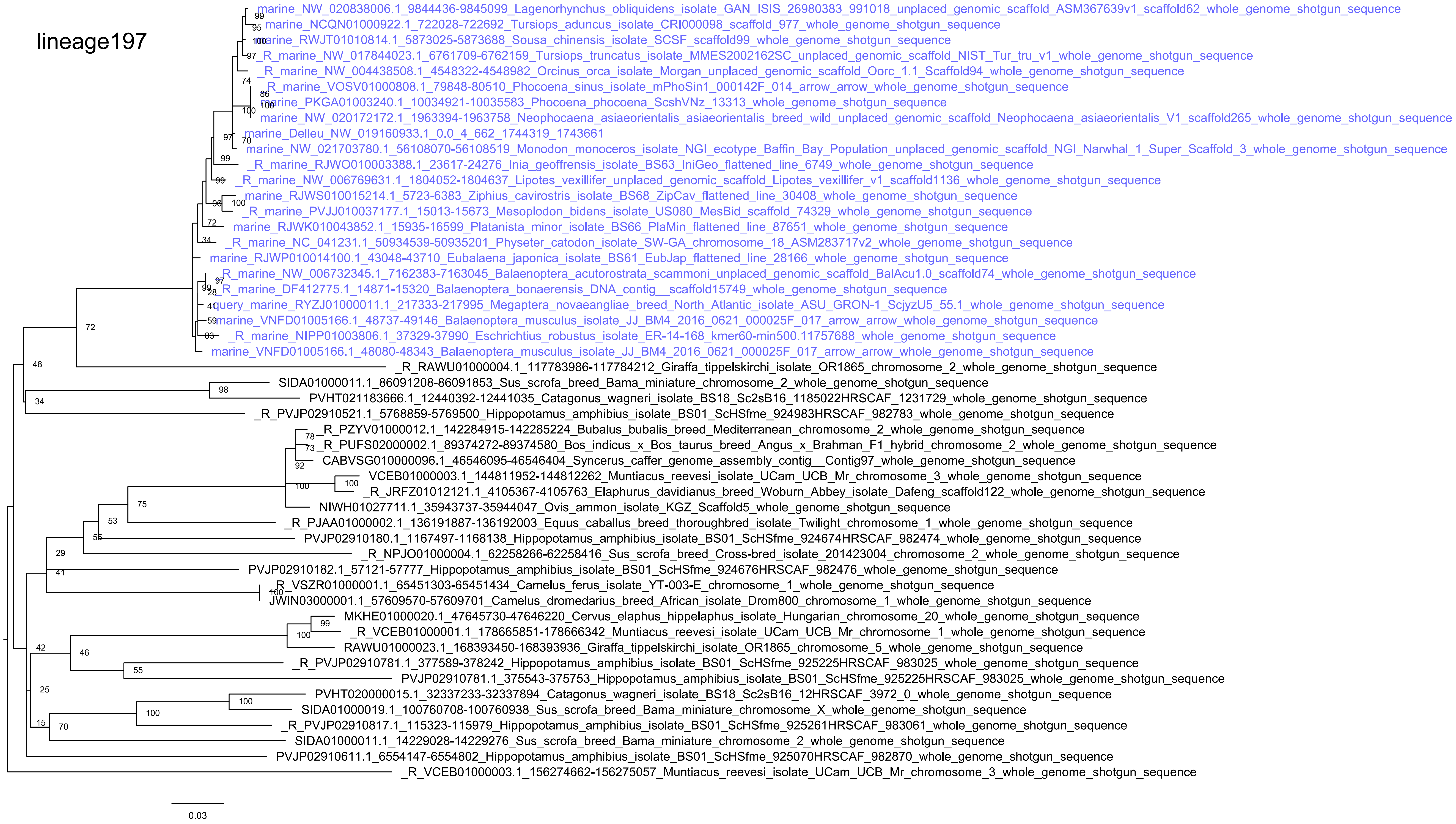
lineage197
marine_NW_020838006.1_9844436-9845099_Lagenorhynchus_obliquidens_isolate_GAN_ISIS_26980383_991018_unplaced_genomic_scaffold_ASM367639v1_scaffold62_whole_genome_shotgun_sequence
marine_NCQN01000922.1_722028-722692_Tursiops_aduncus_isolate_CRI000098_scaffold_977_whole_genome_shotgun_sequence
marine_RWJT01010814.1_5873025-5873688_Sousa_chinensis_isolate_SCSF_scaffold99_whole_genome_shotgun_sequence
_R_marine_NW_017844023.1_6761709-6762159_Tursiops_truncatus_isolate_MMES2002162SC_unplaced_genomic_scaffold_NIST_Tur_tru_v1_whole_genome_shotgun_sequence
_R_marine_NW_004438508.1_4548322-4548982_Orcinus_orca_isolate_Morgan_unplaced_genomic_scaffold_Oorc_1.1_Scaffold94_whole_genome_shotgun_sequence
_R_marine_VOSV01000808.1_79848-80510_Phocoena_sinus_isolate_mPhoSin1_000142F_014_arrow_arrow_whole_genome_shotgun_sequence
marine_PKGA01003240.1_10034921-10035583_Phocoena_phocoena_ScshVNz_13313_whole_genome_shotgun_sequence
marine_NW_020172172.1_1963394-1963758_Neophocaena_asiaeorientalis_asiaeorientalis_breed_wild_unplaced_genomic_scaffold_Neophocaena_asiaeorientalis_V1_scaffold265_whole_genome_shotgun_sequence
marine_Delleu_NW_019160933.1_0.0_4_662_1744319_1743661
marine_NW_021703780.1_56108070-56108519_Monodon_monoceros_isolate_NGI_ecotype_Baffin_Bay_Population_unplaced_genomic_scaffold_NGI_Narwhal_1_Super_Scaffold_3_whole_genome_shotgun_sequence
_R_marine_RJWO010003388.1_23617-24276_Inia_geoffrensis_isolate_BS63_IniGeo_flattened_line_6749_whole_genome_shotgun_sequence
_R_marine_NW_006769631.1_1804052-1804637_Lipotes_vexillifer_unplaced_genomic_scaffold_Lipotes_vexillifer_v1_scaffold1136_whole_genome_shotgun_sequence
marine_RJWS010015214.1_5723-6383_Ziphius_cavirostris_isolate_BS68_ZipCav_flattened_line_30408_whole_genome_shotgun_sequence
_R_marine_PVJJ010037177.1_15013-15673_Mesoplodon_bidens_isolate_US080_MesBid_scaffold_74329_whole_genome_shotgun_sequence
marine_RJWK010043852.1_15935-16599_Platanista_minor_isolate_BS66_PlaMin_flattened_line_87651_whole_genome_shotgun_sequence
_R_marine_NC_041231.1_50934539-50935201_Physeter_catodon_isolate_SW-GA_chromosome_18_ASM283717v2_whole_genome_shotgun_sequence
marine_RJWP010014100.1_43048-43710_Eubalaena_japonica_isolate_BS61_EubJap_flattened_line_28166_whole_genome_shotgun_sequence
_R_marine_NW_006732345.1_7162383-7163045_Balaenoptera_acutorostrata_scammoni_unplaced_genomic_scaffold_BalAcu1.0_scaffold74_whole_genome_shotgun_sequence
_R_marine_DF412775.1_14871-15320_Balaenoptera_bonaerensis_DNA_contig__scaffold15749_whole_genome_shotgun_sequence
query_marine_RYZJ01000011.1_217333-217995_Megaptera_novaeangliae_breed_North_Atlantic_isolate_ASU_GRON-1_ScjyzU5_55.1_whole_genome_shotgun_sequence
marine_VNFD01005166.1_48737-49146_Balaenoptera_musculus_isolate_JJ_BM4_2016_0621_000025F_017_arrow_arrow_whole_genome_shotgun_sequence
_R_marine_NIPP01003806.1_37329-37990_Eschrichtius_robustus_isolate_ER-14-168_kmer60-min500.11757688_whole_genome_shotgun_sequence
marine_VNFD01005166.1_48080-48343_Balaenoptera_musculus_isolate_JJ_BM4_2016_0621_000025F_017_arrow_arrow_whole_genome_shotgun_sequence
_R_RAWU01000004.1_117783986-117784212_Giraffa_tippelskirchi_isolate_OR1865_chromosome_2_whole_genome_shotgun_sequence
SIDA01000011.1_86091208-86091853_Sus_scrofa_breed_Bama_miniature_chromosome_2_whole_genome_shotgun_sequence
PVHT021183666.1_12440392-12441035_Catagonus_wagneri_isolate_BS18_Sc2sB16_1185022HRSCAF_1231729_whole_genome_shotgun_sequence
_R_PVJP02910521.1_5768859-5769500_Hippopotamus_amphibius_isolate_BS01_ScHSfme_924983HRSCAF_982783_whole_genome_shotgun_sequence
_R_PZYV01000012.1_142284915-142285224_Bubalus_bubalis_breed_Mediterranean_chromosome_2_whole_genome_shotgun_sequence
_R_PUFS02000002.1_89374272-89374580_Bos_indicus_x_Bos_taurus_breed_Angus_x_Brahman_F1_hybrid_chromosome_2_whole_genome_shotgun_sequence
CABVSG010000096.1_46546095-46546404_Syncerus_caffer_genome_assembly_contig__Contig97_whole_genome_shotgun_sequence
VCEB01000003.1_144811952-144812262_Muntiacus_reevesi_isolate_UCam_UCB_Mr_chromosome_3_whole_genome_shotgun_sequence
_R_JRFZ01012121.1_4105367-4105763_Elaphurus_davidianus_breed_Woburn_Abbey_isolate_Dafeng_scaffold122_whole_genome_shotgun_sequence
NIWH01027711.1_35943737-35944047_Ovis_ammon_isolate_KGZ_Scaffold5_whole_genome_shotgun_sequence
_R_PJAA01000002.1_136191887-136192003_Equus_caballus_breed_thoroughbred_isolate_Twilight_chromosome_1_whole_genome_shotgun_sequence
PVJP02910180.1_1167497-1168138_Hippopotamus_amphibius_isolate_BS01_ScHSfme_924674HRSCAF_982474_whole_genome_shotgun_sequence
_R_NPJO01000004.1_62258266-62258416_Sus_scrofa_breed_Cross-bred_isolate_201423004_chromosome_2_whole_genome_shotgun_sequence
PVJP02910182.1_57121-57777_Hippopotamus_amphibius_isolate_BS01_ScHSfme_924676HRSCAF_982476_whole_genome_shotgun_sequence
_R_VSZR01000001.1_65451303-65451434_Camelus_ferus_isolate_YT-003-E_chromosome_1_whole_genome_shotgun_sequence
JWIN03000001.1_57609570-57609701_Camelus_dromedarius_breed_African_isolate_Drom800_chromosome_1_whole_genome_shotgun_sequence
MKHE01000020.1_47645730-47646220_Cervus_elaphus_hippelaphus_isolate_Hungarian_chromosome_20_whole_genome_shotgun_sequence
_R_VCEB01000001.1_178665851-178666342_Muntiacus_reevesi_isolate_UCam_UCB_Mr_chromosome_1_whole_genome_shotgun_sequence
RAWU01000023.1_168393450-168393936_Giraffa_tippelskirchi_isolate_OR1865_chromosome_5_whole_genome_shotgun_sequence
_R_PVJP02910781.1_377589-378242_Hippopotamus_amphibius_isolate_BS01_ScHSfme_925225HRSCAF_983025_whole_genome_shotgun_sequence
PVJP02910781.1_375543-375753_Hippopotamus_amphibius_isolate_BS01_ScHSfme_925225HRSCAF_983025_whole_genome_shotgun_sequence
PVHT020000015.1_32337233-32337894_Catagonus_wagneri_isolate_BS18_Sc2sB16_12HRSCAF_3972_0_whole_genome_shotgun_sequence
SIDA01000019.1_100760708-100760938_Sus_scrofa_breed_Bama_miniature_chromosome_X_whole_genome_shotgun_sequence
_R_PVJP02910817.1_115323-115979_Hippopotamus_amphibius_isolate_BS01_ScHSfme_925261HRSCAF_983061_whole_genome_shotgun_sequence
SIDA01000011.1_14229028-14229276_Sus_scrofa_breed_Bama_miniature_chromosome_2_whole_genome_shotgun_sequence
PVJP02910611.1_6554147-6554802_Hippopotamus_amphibius_isolate_BS01_ScHSfme_925070HRSCAF_982870_whole_genome_shotgun_sequence
_R_VCEB01000003.1_156274662-156275057_Muntiacus_reevesi_isolate_UCam_UCB_Mr_chromosome_3_whole_genome_shotgun_sequence
0.03

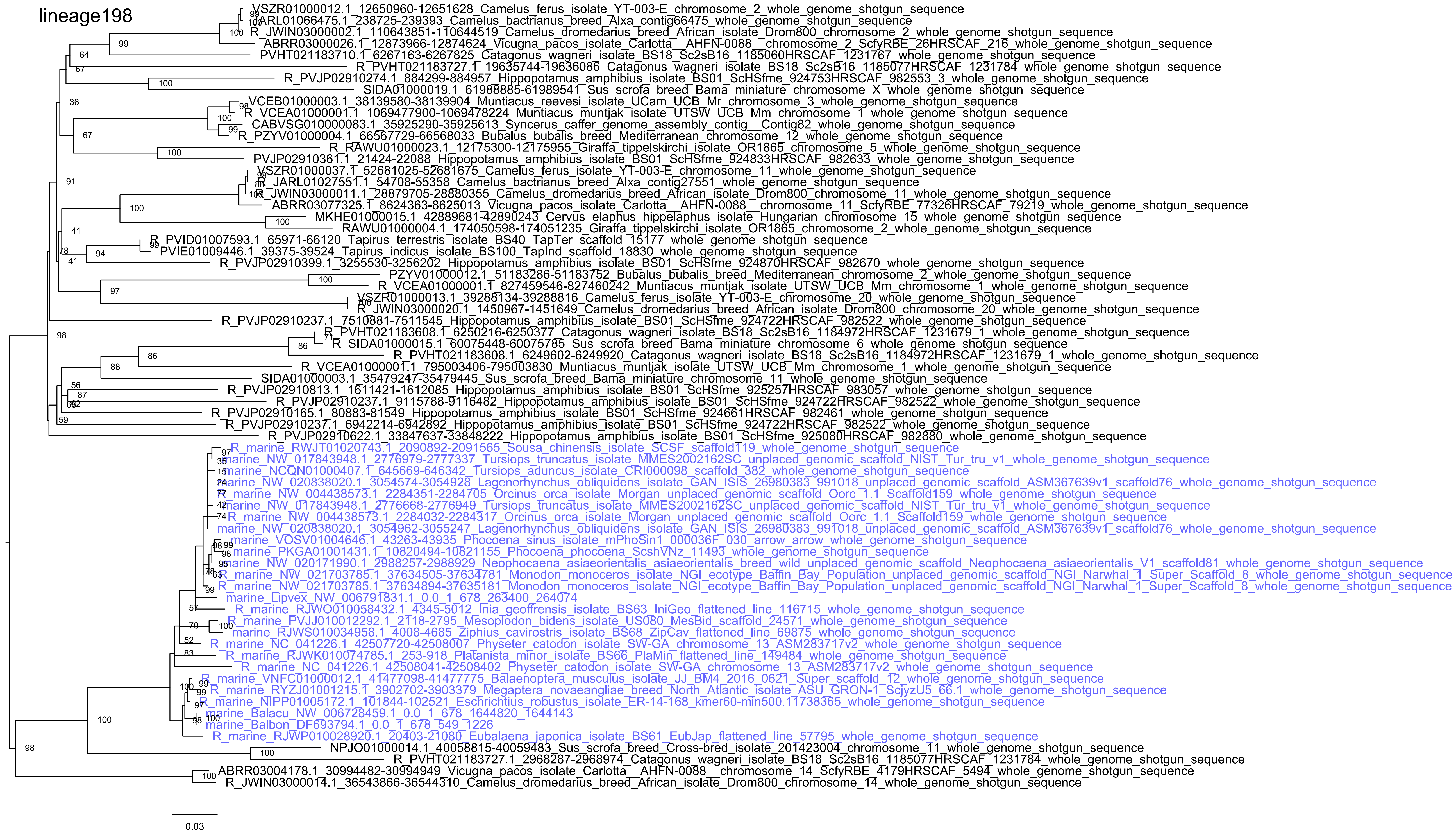
lineage198
VSZR01000012.1_12650960-12651628_Camelus_ferus_isolate_YT-003-E_chromosome_2_whole_genome_shotgun_sequence
JARL01066475.1_238725-239393_Camelus_bactrianus_breed_Alxa_contig66475_whole_genome_shotgun_sequence
R_JWIN03000002.1_110643851-110644519_Camelus_dromedarius_breed_African_isolate_Drom800_chromosome_2_whole_genome_shotgun_sequence
ABRR03000026.1_12873966-12874624_Vicugna_pacos_isolate_Carlotta__AHFN-0088__chromosome_2_ScfyRBE_26HRSCAF_216_whole_genome_shotgun_sequence
PVHT021183710.1_6267163-6267825_Catagonus_wagneri_isolate_BS18_Sc2sB16_1185060HRSCAF_1231767_whole_genome_shotgun_sequence
R_PVHT021183727.1_19635744-19636086_Catagonus_wagneri_isolate_BS18_Sc2sB16_1185077HRSCAF_1231784_whole_genome_shotgun_sequence
R_PVJP02910274.1_884299-884957_Hippopotamus_amphibius_isolate_BS01_ScHSfme_924753HRSCAF_982553_3_whole_genome_shotgun_sequence
SIDA01000019.1_61988885-61989541_Sus_scrofa_breed_Bama_miniature_chromosome_X_whole_genome_shotgun_sequence
VCEB01000003.1_38139580-38139904_Muntiacus_reevesi_isolate_UCam_UCB_Mr_chromosome_3_whole_genome_shotgun_sequence
R_VCEA01000001.1_1069477900-1069478224_Muntiacus_muntjak_isolate_UTSW_UCB_Mm_chromosome_1_whole_genome_shotgun_sequence
CABVSG010000083.1_35925290-35925613_Syncerus_caffer_genome_assembly_contig__Contig82_whole_genome_shotgun_sequence
R_PZYV01000004.1_66567729-66568033_Bubalus_bubalis_breed_Mediterranean_chromosome_12_whole_genome_shotgun_sequence
R_RAWU01000023.1_12175300-12175955_Giraffa_tippelskirchi_isolate_OR1865_chromosome_5_whole_genome_shotgun_sequence
PVJP02910361.1_21424-22088_Hippopotamus_amphibius_isolate_BS01_ScHSfme_924833HRSCAF_982633_whole_genome_shotgun_sequence
VSZR01000037.1_52681025-52681675_Camelus_ferus_isolate_YT-003-E_chromosome_11_whole_genome_shotgun_sequence
R_JARL01027551.1_54708-55358_Camelus_bactrianus_breed_Alxa_contig27551_whole_genome_shotgun_sequence
R_JWIN03000011.1_28879705-28880355_Camelus_dromedarius_breed_African_isolate_Drom800_chromosome_11_whole_genome_shotgun_sequence
ABRR03077325.1_8624363-8625013_Vicugna_pacos_isolate_Carlotta__AHFN-0088__chromosome_11_ScfyRBE_77326HRSCAF_79219_whole_genome_shotgun_sequence
MKHE01000015.1_42889681-42890243_Cervus_elaphus_hippelaphus_isolate_Hungarian_chromosome_15_whole_genome_shotgun_sequence
RAWU01000004.1_174050598-174051235_Giraffa_tippelskirchi_isolate_OR1865_chromosome_2_whole_genome_shotgun_sequence
R_PVID01007593.1_65971-66120_Tapirus_terrestris_isolate_BS40_TapTer_scaffold_15177_whole_genome_shotgun_sequence
PVIE01009446.1_39375-39524_Tapirus_indicus_isolate_BS100_TapInd_scaffold_18830_whole_genome_shotgun_sequence
R_PVJP02910399.1_3255530-3256202_Hippopotamus_amphibius_isolate_BS01_ScHSfme_924870HRSCAF_982670_whole_genome_shotgun_sequence
PZYV01000012.1_51183286-51183752_Bubalus_bubalis_breed_Mediterranean_chromosome_2_whole_genome_shotgun_sequence
R_VCEA01000001.1_827459546-827460242_Muntiacus_muntjak_isolate_UTSW_UCB_Mm_chromosome_1_whole_genome_shotgun_sequence
VSZR01000013.1_39288134-39288816_Camelus_ferus_isolate_YT-003-E_chromosome_20_whole_genome_shotgun_sequence
R_JWIN03000020.1_1450967-1451649_Camelus_dromedarius_breed_African_isolate_Drom800_chromosome_20_whole_genome_shotgun_sequence
R_PVJP02910237.1_7510881-7511545_Hippopotamus_amphibius_isolate_BS01_ScHSfme_924722HRSCAF_982522_whole_genome_shotgun_sequence
R_PVHT021183608.1_6250216-6250377_Catagonus_wagneri_isolate_BS18_Sc2sB16_1184972HRSCAF_1231679_1_whole_genome_shotgun_sequence
R_SIDA01000015.1_60075448-60075785_Sus_scrofa_breed_Bama_miniature_chromosome_6_whole_genome_shotgun_sequence
R_PVHT021183608.1_6249602-6249920_Catagonus_wagneri_isolate_BS18_Sc2sB16_1184972HRSCAF_1231679_1_whole_genome_shotgun_sequence
R_VCEA01000001.1_795003406-795003830_Muntiacus_muntjak_isolate_UTSW_UCB_Mm_chromosome_1_whole_genome_shotgun_sequence
SIDA01000003.1_35479247-35479445_Sus_scrofa_breed_Bama_miniature_chromosome_11_whole_genome_shotgun_sequence
R_PVJP02910813.1_1611421-1612085_Hippopotamus_amphibius_isolate_BS01_ScHSfme_925257HRSCAF_983057_whole_genome_shotgun_sequence
R_PVJP02910237.1_9115788-9116482_Hippopotamus_amphibius_isolate_BS01_ScHSfme_924722HRSCAF_982522_whole_genome_shotgun_sequence
R_PVJP02910165.1_80883-81549_Hippopotamus_amphibius_isolate_BS01_ScHSfme_924661HRSCAF_982461_whole_genome_shotgun_sequence
R_PVJP02910237.1_6942214-6942892_Hippopotamus_amphibius_isolate_BS01_ScHSfme_924722HRSCAF_982522_whole_genome_shotgun_sequence
R_PVJP02910622.1_33847637-33848222_Hippopotamus_amphibius_isolate_BS01_ScHSfme_925080HRSCAF_982880_whole_genome_shotgun_sequence
R_marine_RWJT01020743.1_2090892-2091565_Sousa_chinensis_isolate_SCSF_scaffold119_whole_genome_shotgun_sequence
marine_NW_017843948.1_2776979-2777337_Tursiops_truncatus_isolate_MMES2002162SC_unplaced_genomic_scaffold_NIST_Tur_tru_v1_whole_genome_shotgun_sequence
marine_NCQN01000407.1_645669-646342_Tursiops_aduncus_isolate_CRI000098_scaffold_382_whole_genome_shotgun_sequence
marine_NW_020838020.1_3054574-3054928_Lagenorhynchus_obliquidens_isolate_GAN_ISIS_26980383_991018_unplaced_genomic_scaffold_ASM367639v1_scaffold76_whole_genome_shotgun_sequence
R_marine_NW_004438573.1_2284351-2284705_Orcinus_orca_isolate_Morgan_unplaced_genomic_scaffold_Oorc_1.1_Scaffold159_whole_genome_shotgun_sequence
marine_NW_017843948.1_2776668-2776949_Tursiops_truncatus_isolate_MMES2002162SC_unplaced_genomic_scaffold_NIST_Tur_tru_v1_whole_genome_shotgun_sequence
R_marine_NW_004438573.1_2284032-2284317_Orcinus_orca_isolate_Morgan_unplaced_genomic_scaffold_Oorc_1.1_Scaffold159_whole_genome_shotgun_sequence
marine_NW_020838020.1_3054962-3055247_Lagenorhynchus_obliquidens_isolate_GAN_ISIS_26980383_991018_unplaced_genomic_scaffold_ASM367639v1_scaffold76_whole_genome_shotgun_sequence
marine_VOSV01004646.1_43263-43935_Phocoena_sinus_isolate_mPhoSin1_000036F_030_arrow_arrow_whole_genome_shotgun_sequence
marine_PKGA01001431.1_10820494-10821155_Phocoena_phocoena_ScshVNz_11493_whole_genome_shotgun_sequence
marine_NW_020171990.1_2988257-2988929_Neophocaena_asiaeorientalis_asiaeorientalis_breed_wild_unplaced_genomic_scaffold_Neophocaena_asiaeorientalis_V1_scaffold81_whole_genome_shotgun_sequence
R_marine_NW_021703785.1_37634505-37634781_Monodon_monoceros_isolate_NGI_ecotype_Baffin_Bay_Population_unplaced_genomic_scaffold_NGI_Narwhal_1_Super_Scaffold_8_whole_genome_shotgun_sequence
R_marine_NW_021703785.1_37634894-37635181_Monodon_monoceros_isolate_NGI_ecotype_Baffin_Bay_Population_unplaced_genomic_scaffold_NGI_Narwhal_1_Super_Scaffold_8_whole_genome_shotgun_sequence
marine_Lipvex_NW_006791831.1_0.0_1_678_263400_264074
R_marine_RJWO010058432.1_4345-5012_Inia_geoffrensis_isolate_BS63_IniGeo_flattened_line_116715_whole_genome_shotgun_sequence
R_marine_PVJJ010012292.1_2118-2795_Mesoplodon_bidens_isolate_US080_MesBid_scaffold_24571_whole_genome_shotgun_sequence
marine_RJWS010034958.1_4008-4685_Ziphius_cavirostris_isolate_BS68_ZipCav_flattened_line_69875_whole_genome_shotgun_sequence
R_marine_NC_041226.1_42507720-42508007_Physeter_catodon_isolate_SW-GA_chromosome_13_ASM283717v2_whole_genome_shotgun_sequence
R_marine_RJWK010074785.1_253-918_Platanista_minor_isolate_BS66_PlaMin_flattened_line_149484_whole_genome_shotgun_sequence
R_marine_NC_041226.1_42508041-42508402_Physeter_catodon_isolate_SW-GA_chromosome_13_ASM283717v2_whole_genome_shotgun_sequence
R_marine_VNFC01000012.1_41477098-41477775_Balaenoptera_musculus_isolate_JJ_BM4_2016_0621_Super_scaffold_12_whole_genome_shotgun_sequence
R_marine_RYZJ01001215.1_3902702-3903379_Megaptera_novaeangliae_breed_North_Atlantic_isolate_ASU_GRON-1_ScjyzU5_66.1_whole_genome_shotgun_sequence
R_marine_NIPP01005172.1_101844-102521_Eschrichtius_robustus_isolate_ER-14-168_kmer60-min500.11738365_whole_genome_shotgun_sequence
marine_Balacu_NW_006728459.1_0.0_1_678_1644820_1644143
marine_Balbon_DF693794.1_0.0_1_678_549_1226
R_marine_RJWP010028920.1_20403-21080_Eubalaena_japonica_isolate_BS61_EubJap_flattened_line_57795_whole_genome_shotgun_sequence
NPJO01000014.1_40058815-40059483_Sus_scrofa_breed_Cross-bred_isolate_201423004_chromosome_11_whole_genome_shotgun_sequence
R_PVHT021183727.1_2968287-2968974_Catagonus_wagneri_isolate_BS18_Sc2sB16_1185077HRSCAF_1231784_whole_genome_shotgun_sequence
ABRR03004178.1_30994482-30994949_Vicugna_pacos_isolate_Carlotta__AHFN-0088__chromosome_14_ScfyRBE_4179HRSCAF_5494_whole_genome_shotgun_sequence
R_JWIN03000014.1_36543866-36544310_Camelus_dromedarius_breed_African_isolate_Drom800_chromosome_14_whole_genome_shotgun_sequence
0.03

lineage199

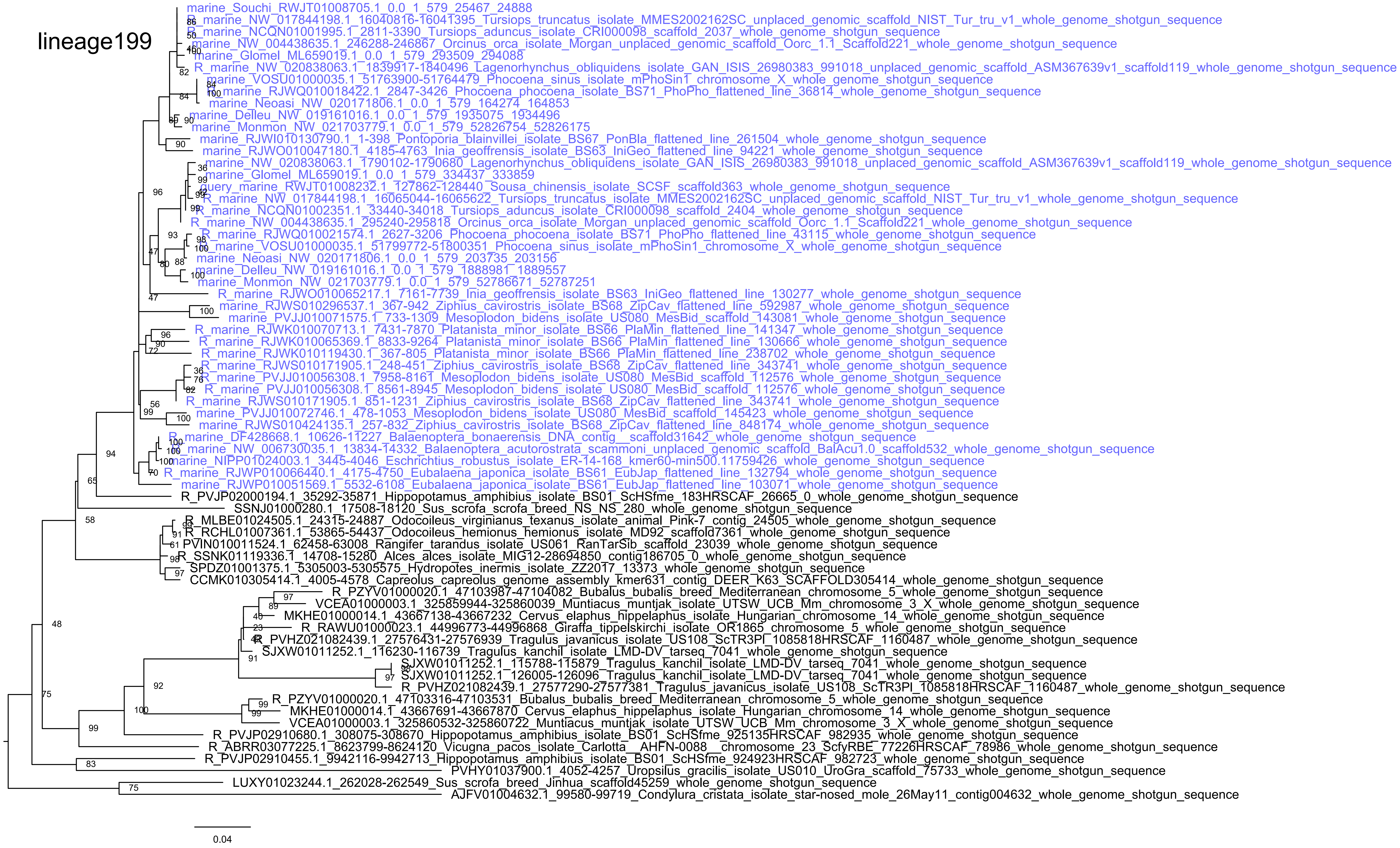

marine_Souchi_RWJT01008705.1_0.0_1_579_25467_24888
R_marine_NW_017844198.1_16040816-16041395_Tursiops_truncatus_isolate_MMES2002162SC_unplaced_genomic_scaffold_NIST_Tur_tru_v1_whole_genome_shotgun_sequence
R_marine_NCQN01001995.1_2811-3390_Tursiops_aduncus_isolate_CRI000098_scaffold_2037_whole_genome_shotgun_sequence
marine_NW_004438635.1_246288-246867_Orcinus_orca_isolate_Morgan_unplaced_genomic_scaffold_Oorc_1.1_Scaffold221_whole_genome_shotgun_sequence
marine_Glomel_ML659019.1_0.0_1_579_293509_294088
R_marine_NW_020838063.1_1839917-1840496_Lagenorhynchus_obliquidens_isolate_GAN_ISIS_26980383_991018_unplaced_genomic_scaffold_ASM367639v1_scaffold119_whole_genome_shotgun_sequence
marine_VOSU01000035.1_51763900-51764479_Phocoena_sinus_isolate_mPhoSin1_chromosome_X_whole_genome_shotgun_sequence
R_marine_RJWQ010018422.1_2847-3426_Phocoena_phocoena_isolate_BS71_PhoPho_flattened_line_36814_whole_genome_shotgun_sequence
marine_Neoasi_NW_020171806.1_0.0_1_579_164274_164853
marine_Delleu_NW_019161016.1_0.0_1_579_1935075_1934496
marine_Monmon_NW_021703779.1_0.0_1_579_52826754_52826175
marine_RJWI010130790.1_1-398_Pontoporia_blainvillei_isolate_BS67_PonBla_flattened_line_261504_whole_genome_shotgun_sequence
marine_RJWO010047180.1_4185-4763_Inia_geoffrensis_isolate_BS63_IniGeo_flattened_line_94221_whole_genome_shotgun_sequence
marine_NW_020838063.1_1790102-1790680_Lagenorhynchus_obliquidens_isolate_GAN_ISIS_26980383_991018_unplaced_genomic_scaffold_ASM367639v1_scaffold119_whole_genome_shotgun_sequence
marine_Glomel_ML659019.1_0.0_1_579_334437_333859
query_marine_RWJT01008232.1_127862-128440_Sousa_chinensis_isolate_SCSF_scaffold363_whole_genome_shotgun_sequence
R_marine_NW_017844198.1_16065044-16065622_Tursiops_truncatus_isolate_MMES2002162SC_unplaced_genomic_scaffold_NIST_Tur_tru_v1_whole_genome_shotgun_sequence
R_marine_NCQN01002351.1_33440-34018_Tursiops_aduncus_isolate_CRI000098_scaffold_2404_whole_genome_shotgun_sequence
R_marine_NW_004438635.1_295240-295818_Orcinus_orca_isolate_Morgan_unplaced_genomic_scaffold_Oorc_1.1_Scaffold221_whole_genome_shotgun_sequence
R_marine_RJWQ010021574.1_2627-3206_Phocoena_phocoena_isolate_BS71_PhoPho_flattened_line_43115_whole_genome_shotgun_sequence
marine_VOSU01000035.1_51799772-51800351_Phocoena_sinus_isolate_mPhoSin1_chromosome_X_whole_genome_shotgun_sequence
marine_Neoasi_NW_020171806.1_0.0_1_579_203735_203156
marine_Delleu_NW_019161016.1_0.0_1_579_1888981_1889557
marine_Monmon_NW_021703779.1_0.0_1_579_52786671_52787251
R_marine_RJWO010065217.1_7161-7739_Inia_geoffrensis_isolate_BS63_IniGeo_flattened_line_130277_whole_genome_shotgun_sequence
marine_RJWS010296537.1_367-942_Ziphius_cavirostris_isolate_BS68_ZipCav_flattened_line_592987_whole_genome_shotgun_sequence
marine_PVJJ010071575.1_733-1309_Mesoplodon_bidens_isolate_US080_MesBid_scaffold_143081_whole_genome_shotgun_sequence
R_marine_RJWK010070713.1_7431-7870_Platanista_minor_isolate_BS66_PlaMin_flattened_line_141347_whole_genome_shotgun_sequence
R_marine_RJWK010065369.1_8833-9264_Platanista_minor_isolate_BS66_PlaMin_flattened_line_130666_whole_genome_shotgun_sequence
R_marine_RJWK010119430.1_367-805_Platanista_minor_isolate_BS66_PlaMin_flattened_line_238702_whole_genome_shotgun_sequence
R_marine_RJWS010171905.1_248-451_Ziphius_cavirostris_isolate_BS68_ZipCav_flattened_line_343741_whole_genome_shotgun_sequence
R_marine_PVJJ010056308.1_7958-8161_Mesoplodon_bidens_isolate_US080_MesBid_scaffold_112576_whole_genome_shotgun_sequence
R_marine_PVJJ010056308.1_8561-8945_Mesoplodon_bidens_isolate_US080_MesBid_scaffold_112576_whole_genome_shotgun_sequence
R_marine_RJWS010171905.1_851-1231_Ziphius_cavirostris_isolate_BS68_ZipCav_flattened_line_343741_whole_genome_shotgun_sequence
marine_PVJJ010072746.1_478-1053_Mesoplodon_bidens_isolate_US080_MesBid_scaffold_145423_whole_genome_shotgun_sequence
marine_RJWS010424135.1_257-832_Ziphius_cavirostris_isolate_BS68_ZipCav_flattened_line_848174_whole_genome_shotgun_sequence
R_marine_DF428668.1_10626-11227_Balaenoptera_bonaerensis_DNA_contig__scaffold31642_whole_genome_shotgun_sequence
R_marine_NW_006730035.1_13834-14332_Balaenoptera_acutorostrata_scammoni_unplaced_genomic_scaffold_BalAcu1.0_scaffold532_whole_genome_shotgun_sequence
marine_NIPP01024003.1_3445-4046_Eschrichtius_robustus_isolate_ER-14-168_kmer60-min500.11759426_whole_genome_shotgun_sequence
R_marine_RJWP010066440.1_4175-4750_Eubalaena_japonica_isolate_BS61_EubJap_flattened_line_132794_whole_genome_shotgun_sequence
marine_RJWP010051569.1_5532-6108_Eubalaena_japonica_isolate_BS61_EubJap_flattened_line_103071_whole_genome_shotgun_sequence
R_PVJP02000194.1_35292-35871_Hippopotamus_amphibius_isolate_BS01_ScHSfme_183HRSCAF_26665_0_whole_genome_shotgun_sequence
SSNJ01000280.1_17508-18120_Sus_scrofa_scrofa_breed_NS_NS_280_whole_genome_shotgun_sequence
R_MLBE01024505.1_24315-24887_Odocoileus_virginianus_texanus_isolate_animal_Pink-7_contig_24505_whole_genome_shotgun_sequence
R_RCHL01007361.1_53865-54437_Odocoileus_hemionus_isolate_MD92_scaffold7361_whole_genome_shotgun_sequence
PVIN010011524.1_62458-63008_Rangifer_tarandus_isolate_US061_RanTarSib_scaffold_23039_whole_genome_shotgun_sequence
R_SSNK01119336.1_14708-15280_Alces_alces_isolate_MIG12-28694850_contig186705_0_whole_genome_shotgun_sequence
SPDZ01001375.1_5305003-5305575_Hydropotes_inermis_isolate_ZZ2017_13373_whole_genome_shotgun_sequence
CCMK010305414.1_4005-4578_Capreolus_capreolus_genome_assembly_kmer631_contig_DEER_K63_SCAFFOLD305414_whole_genome_shotgun_sequence
R_PZYV01000020.1_47103987-47104082_Bubalus_bubalis_breed_Mediterranean_chromosome_5_whole_genome_shotgun_sequence
VCEA01000003.1_325859944-325860039_Muntiacus_muntjak_isolate_UTSW_UCB_Mm_chromosome_3_X_whole_genome_shotgun_sequence
MKHE01000014.1_43667138-43667232_Cervus_elaphus_hippelaphus_isolate_Hungarian_chromosome_14_whole_genome_shotgun_sequence
R_RAWU01000023.1_44996773-44996868_Giraffa_tippelskirchi_isolate_OR1865_chromosome_5_whole_genome_shotgun_sequence
R_PVHZ021082439.1_27576431-27576939_Tragulus_javanicus_isolate_US108_ScTR3PI_1085818HRSCAF_1160487_whole_genome_shotgun_sequence
SJXW01011252.1_116230-116739_Tragulus_kanchil_isolate_LMD-DV_tarseq_7041_whole_genome_shotgun_sequence
SJXW01011252.1_115788-115879_Tragulus_kanchil_isolate_LMD-DV_tarseq_7041_whole_genome_shotgun_sequence
SJXW01011252.1_126005-126096_Tragulus_kanchil_isolate_LMD-DV_tarseq_7041_whole_genome_shotgun_sequence
R_PVHZ021082439.1_27577290-27577381_Tragulus_javanicus_isolate_US108_ScTR3PI_1085818HRSCAF_1160487_whole_genome_shotgun_sequence
R_PZYV01000020.1_47103316-47103531_Bubalus_bubalis_breed_Mediterranean_chromosome_5_whole_genome_shotgun_sequence
MKHE01000014.1_43667691-43667870_Cervus_elaphus_hippelaphus_isolate_Hungarian_chromosome_14_whole_genome_shotgun_sequence
VCEA01000003.1_325860532-325860722_Muntiacus_muntjak_isolate_UTSW_UCB_Mm_chromosome_3_X_whole_genome_shotgun_sequence
R_PVJP02910680.1_308075-308670_Hippopotamus_amphibius_isolate_BS01_ScHSfme_925135HRSCAF_982935_whole_genome_shotgun_sequence
R_ABRR03077225.1_8623799-8624120_Vicugna_pacos_isolate_Carlotta__AHFN-0088__chromosome_23_ScfyRBE_77226HRSCAF_78986_whole_genome_shotgun_sequence
R_PVJP02910455.1_9942116-9942713_Hippopotamus_amphibius_isolate_BS01_ScHSfme_924923HRSCAF_982723_whole_genome_shotgun_sequence
PVHY01037900.1_4052-4257_Uropsilus_gracilis_isolate_US010_UroGra_scaffold_75733_whole_genome_shotgun_sequence
LUXY01023244.1_262028-262549_Sus_scrofa_breed_Jinhua_scaffold45259_whole_genome_shotgun_sequence
AJFV01004632.1_99580-99719_Condylura_cristata_isolate_star-nosed_mole_26May11_contig004632_whole_genome_shotgun_sequence
0.04

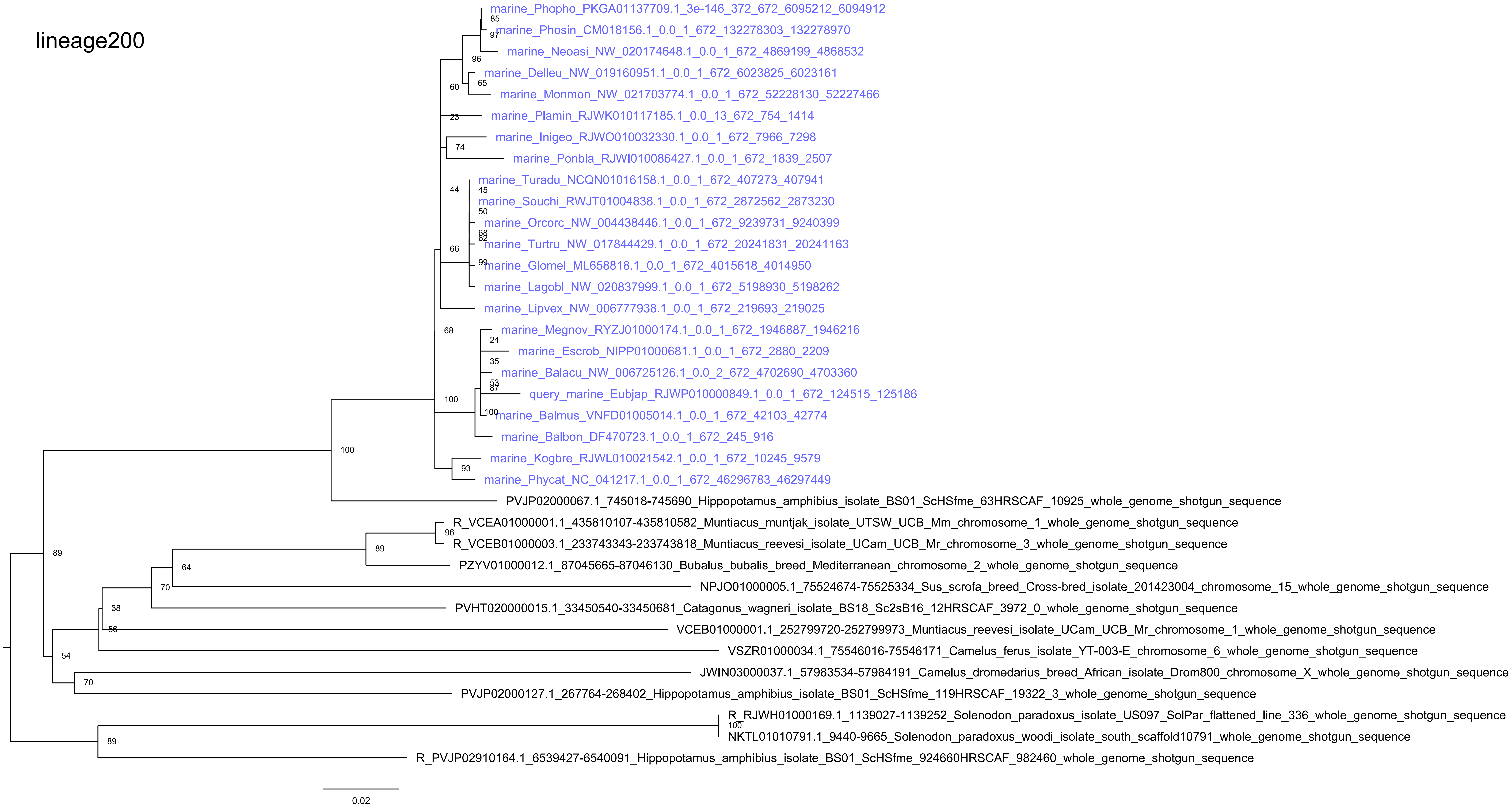
lineage200
marine_Phopho_PKGA01137709.1_3e-146_372_672_6095212_6094912
marine_Phosin_CM018156.1_0.0_1_672_132278303_132278970
marine_Neoasi_NW_020174648.1_0.0_1_672_4869199_4868532
marine_Delleu_NW_019160951.1_0.0_1_672_6023825_6023161
marine_Monmon_NW_021703774.1_0.0_1_672_52228130_52227466
marine_Plamin_RJWK010117185.1_0.0_13_672_754_1414
marine_Inigeo_RJWO010032330.1_0.0_1_672_7966_7298
marine_Ponbla_RJWI010086427.1_0.0_1_672_1839_2507
marine_Turadu_NCQN01016158.1_0.0_1_672_407273_407941
marine_Souchi_RWJT01004838.1_0.0_1_672_2872562_2873230
marine_Orcorc_NW_004438446.1_0.0_1_672_9239731_9240399
marine_Turtru_NW_017844429.1_0.0_1_672_20241831_20241163
marine_Glomel_ML658818.1_0.0_1_672_4015618_4014950
marine_Lagobl_NW_020837999.1_0.0_1_672_5198930_5198262
marine_Lipvex_NW_006777938.1_0.0_1_672_219693_219025
marine_Megnov_RYZJ01000174.1_0.0_1_672_1946887_1946216
marine_Escrob_NIPP01000681.1_0.0_1_672_2880_2209
marine_Balacu_NW_006725126.1_0.0_2_672_4702690_4703360
query_marine_Eubjap_RJWP010000849.1_0.0_1_672_124515_125186
marine_Balmus_VNFD01005014.1_0.0_1_672_42103_42774
marine_Balbon_DF470723.1_0.0_1_672_245_916
marine_Kogbre_RJWL010021542.1_0.0_1_672_10245_9579
marine_Phycat_NC_041217.1_0.0_1_672_46296783_46297449
PVJP02000067.1_745018-745690_Hippopotamus_amphibius_isolate_BS01_ScHSfme_63HRSCAF_10925_whole_genome_shotgun_sequence
R_VCEA01000001.1_435810107-435810582_Muntiacus_muntjak_isolate_UTSW_UCB_Mm_chromosome_1_whole_genome_shotgun_sequence
R_VCEB01000003.1_233743343-233743818_Muntiacus_reevesi_isolate_UCam_UCB_Mr_chromosome_3_whole_genome_shotgun_sequence
PZYV01000012.1_87045665-87046130_Bubalus_bubalis_breed_Mediterranean_chromosome_2_whole_genome_shotgun_sequence
NPJO01000005.1_75524674-75525334_Sus_scrofa_breed_Cross-bred_isolate_201423004_chromosome_15_whole_genome_shotgun_sequence
PVHT020000015.1_33450540-33450681_Catagonus_wagneri_isolate_BS18_Sc2sB16_12HRSCAF_3972_0_whole_genome_shotgun_sequence
VCEB01000001.1_252799720-252799973_Muntiacus_reevesi_isolate_UCam_UCB_Mr_chromosome_1_whole_genome_shotgun_sequence
VSZR01000034.1_75546016-75546171_Camelus_ferus_isolate_YT-003-E_chromosome_6_whole_genome_shotgun_sequence
JWIN03000037.1_57983534-57984191_Camelus_dromedarius_breed_African_isolate_Drom800_chromosome_X_whole_genome_shotgun_sequence
PVJP02000127.1_267764-268402_Hippopotamus_amphibius_isolate_BS01_ScHSfme_119HRSCAF_19322_3_whole_genome_shotgun_sequence
R_RJWH01000169.1_1139027-1139252_Solenodon_paradoxus_isolate_US097_SolPar_flattened_line_336_whole_genome_shotgun_sequence
NKTL01010791.1_9440-9665_Solenodon_paradoxus_woodi_isolate_south_scaffold10791_whole_genome_shotgun_sequence
R_PVJP02910164.1_6539427-6540091_Hippopotamus_amphibius_isolate_BS01_ScHSfme_924660HRSCAF_982460_whole_genome_shotgun_sequence
0.02

lineage201

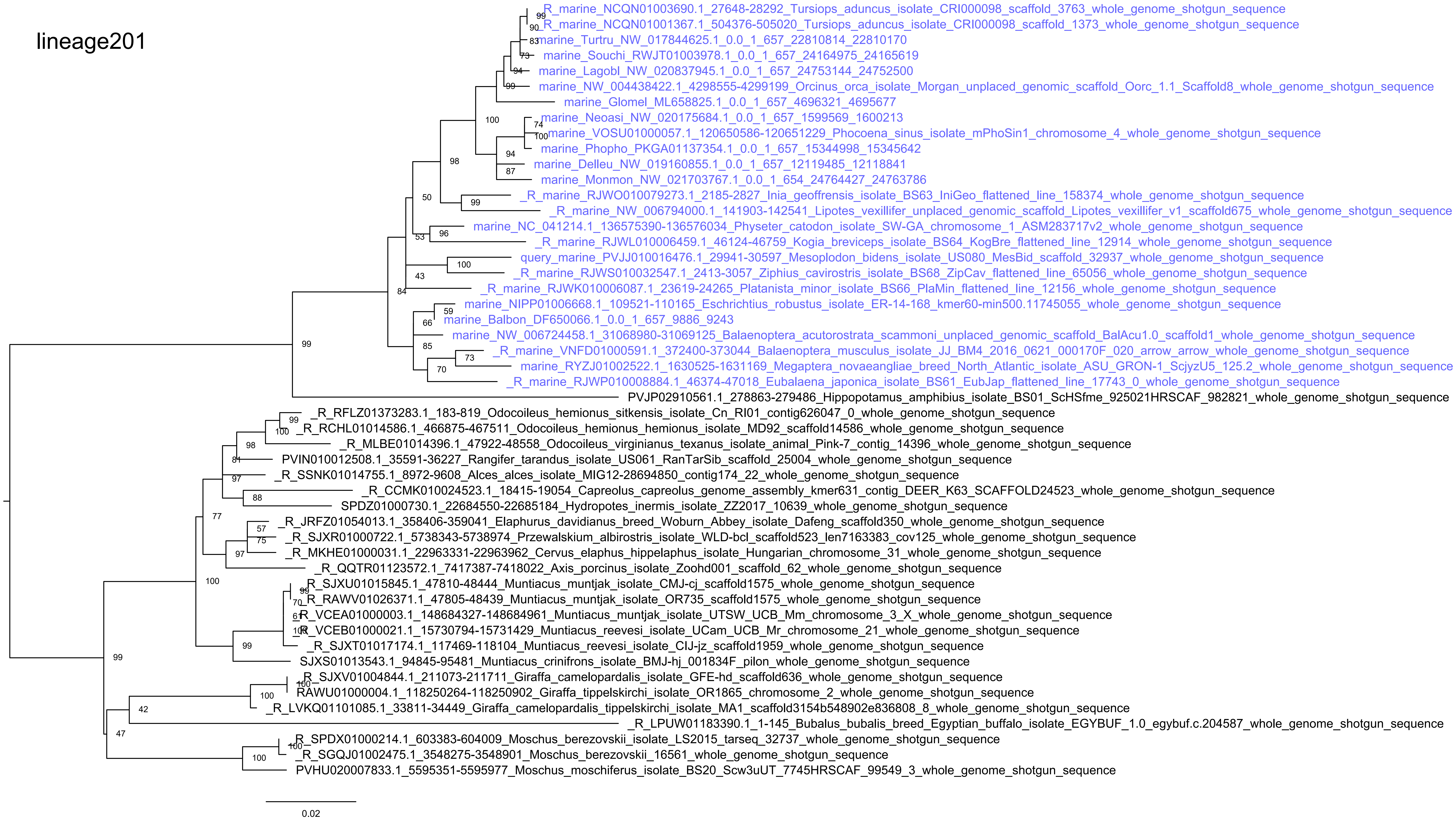
R_marine_NCQN01003690.1_27648-28292_Tursiops_aduncus_isolate_CRI000098_scaffold_3763_whole_genome_shotgun_sequence
R_marine_NCQN01001367.1_504376-505020_Tursiops_aduncus_isolate_CRI000098_scaffold_1373_whole_genome_shotgun_sequence
marine_Turtru_NW_017844625.1_0.0_1_657_22810814_22810170
marine_Souchi_RWJT01003978.1_0.0_1_657_24164975_24165619
marine_Lagobl_NW_020837945.1_0.0_1_657_24753144_24752500
marine_NW_004438422.1_4298555-4299199_Orcinus_orca_isolate_Morgan_unplaced_genomic_scaffold_Oorc_1.1_Scaffold8_whole_genome_shotgun_sequence
marine_Glomel_ML658825.1_0.0_1_657_4696321_4695677
marine_Neoasi_NW_020175684.1_0.0_1_657_1599569_1600213
marine_VOSU01000057.1_120650586-120651229_Phocoena_sinus_isolate_mPhoSin1_chromosome_4_whole_genome_shotgun_sequence
marine_Phopho_PKGA01137354.1_0.0_1_657_15344998_15345642
marine_Delleu_NW_019160855.1_0.0_1_657_12119485_12118841
marine_Monmon_NW_021703767.1_0.0_1_654_24764427_24763786
_R_marine_RJWO010079273.1_2185-2827_Inia_geoffrensis_isolate_BS63_IniGeo_flattened_line_158374_whole_genome_shotgun_sequence
_R_marine_NW_006794000.1_141903-142541_Lipotes_vexillifer_unplaced_genomic_scaffold_Lipotes_vexillifer_v1_scaffold675_whole_genome_shotgun_sequence
marine_NC_041214.1_136575390-136576034_Physeter_catodon_isolate_SW-GA_chromosome_1_ASM283717v2_whole_genome_shotgun_sequence
_R_marine_RJWL010006459.1_46124-46759_Kogia_breviceps_isolate_BS64_KogBre_flattened_line_12914_whole_genome_shotgun_sequence
query_marine_PVJJ010016476.1_29941-30597_Mesoplodon_bidens_isolate_US080_MesBid_scaffold_32937_whole_genome_shotgun_sequence
_R_marine_RJWS010032547.1_2413-3057_Ziphius_cavirostris_isolate_BS68_ZipCav_flattened_line_65056_whole_genome_shotgun_sequence
_R_marine_RJWK010006087.1_23619-24265_Platanista_minor_isolate_BS66_PlaMin_flattened_line_12156_whole_genome_shotgun_sequence
marine_NIPP01006668.1_109521-110165_Eschrichtius_robustus_isolate_ER-14-168_kmer60-min500.11745055_whole_genome_shotgun_sequence
marine_Balbon_DF650066.1_0.0_1_657_9886_9243
marine_NW_006724458.1_31068980-31069125_Balaenoptera_acutorostrata_scammoni_unplaced_genomic_scaffold_BalAcu1.0_scaffold1_whole_genome_shotgun_sequence
_R_marine_VNFD01000591.1_372400-373044_Balaenoptera_musculus_isolate_JJ_BM4_2016_0621_000170F_020_arrow_arrow_whole_genome_shotgun_sequence
marine_RYZJ01002522.1_1630525-1631169_Megaptera_novaeangliae_breed_North_Atlantic_isolate_ASU_GRON-1_ScjyzU5_125.2_whole_genome_shotgun_sequence
_R_marine_RJWP010008884.1_46374-47018_Eubalaena_japonica_isolate_BS61_EubJap_flattened_line_17743_0_whole_genome_shotgun_sequence
PVJP02910561.1_278863-279486_Hippopotamus_amphibius_isolate_BS01_ScHSfme_925021HRSCAF_982821_whole_genome_shotgun_sequence
_R_RFLZ01373283.1_183-819_Odocoileus_hemionus_sitkensis_isolate_Cn_RI01_contig626047_0_whole_genome_shotgun_sequence
_R_RCHL01014586.1_466875-467511_Odocoileus_hemionus_hemionus_isolate_MD92_scaffold14586_whole_genome_shotgun_sequence
_R_MLBE01014396.1_47922-48558_Odocoileus_virginianus_texanus_isolate_animal_Pink-7_contig_14396_whole_genome_shotgun_sequence
PVIN010012508.1_35591-36227_Rangifer_tarandus_isolate_US061_RanTarSib_scaffold_25004_whole_genome_shotgun_sequence
_R_SSNK01014755.1_8972-9608_Alces_alces_isolate_MIG12-28694850_contig174_22_whole_genome_shotgun_sequence
_R_CCMK010024523.1_18415-19054_Capreolus_capreolus_genome_assembly_kmer631_contig_DEER_K63_SCAFFOLD24523_whole_genome_shotgun_sequence
SPDZ01000730.1_22684550-22685184_Hydropotes_inermis_isolate_ZZ2017_10639_whole_genome_shotgun_sequence
_R_JRFZ01054013.1_358406-359041_Elaphurus_davidianus_breed_Woburn_Abbey_isolate_Dafeng_scaffold350_whole_genome_shotgun_sequence
_R_SJXR01000722.1_5738343-5738974_Przewalskium_albirostris_isolate_WLD-bcl_scaffold523_len7163383_cov125_whole_genome_shotgun_sequence
_R_MKHE01000031.1_22963331-22963962_Cervus_elaphus_hippelaphus_isolate_Hungarian_chromosome_31_whole_genome_shotgun_sequence
_R_QQTR01123572.1_7417387-7418022_Axis_porcinus_isolate_Zoohd001_scaffold_62_whole_genome_shotgun_sequence
R_SJXU01015845.1_47810-48444_Muntiacus_muntjak_isolate_CMJ-cj_scaffold1575_whole_genome_shotgun_sequence
R_RAWV01026371.1_47805-48439_Muntiacus_muntjak_isolate_OR735_scaffold1575_whole_genome_shotgun_sequence
R_VCEA01000003.1_148684327-148684961_Muntiacus_muntjak_isolate_UTSW_UCB_Mm_chromosome_3_X_whole_genome_shotgun_sequence
R_VCEB01000021.1_15730794-15731429_Muntiacus_reevesi_isolate_UCam_UCB_Mr_chromosome_21_whole_genome_shotgun_sequence
_R_SJXT01017174.1_117469-118104_Muntiacus_reevesi_isolate_CIJ-jz_scaffold1959_whole_genome_shotgun_sequence
SJXS01013543.1_94845-95481_Muntiacus_crinifrons_isolate_BMJ-hj_001834F_pilon_whole_genome_shotgun_sequence
R_SJXV01004844.1_211073-211711_Giraffa_camelopardalis_isolate_GFE-hd_scaffold636_whole_genome_shotgun_sequence
RAWU01000004.1_118250264-118250902_Giraffa_tippelskirchi_isolate_OR1865_chromosome_2_whole_genome_shotgun_sequence
_R_LVKQ01101085.1_33811-34449_Giraffa_camelopardalis_tippelskirchi_isolate_MA1_scaffold3154b548902e836808_8_whole_genome_shotgun_sequence
_R_LPUW01183390.1_1-145_Bubalus_bubalis_breed_Egyptian_buffalo_isolate_EGYBUF_1.0_egybuf.c.204587_whole_genome_shotgun_sequence
R_SPDX01000214.1_603383-604009_Moschus_berezovskii_isolate_LS2015_tarseq_32737_whole_genome_shotgun_sequence
_R_SGQJ01002475.1_3548275-3548901_Moschus_berezovskii_16561_whole_genome_shotgun_sequence
PVHU020007833.1_5595351-5595977_Moschus_moschiferus_isolate_BS20_Scw3uUT_7745HRSCAF_99549_3_whole_genome_shotgun_sequence
0.02

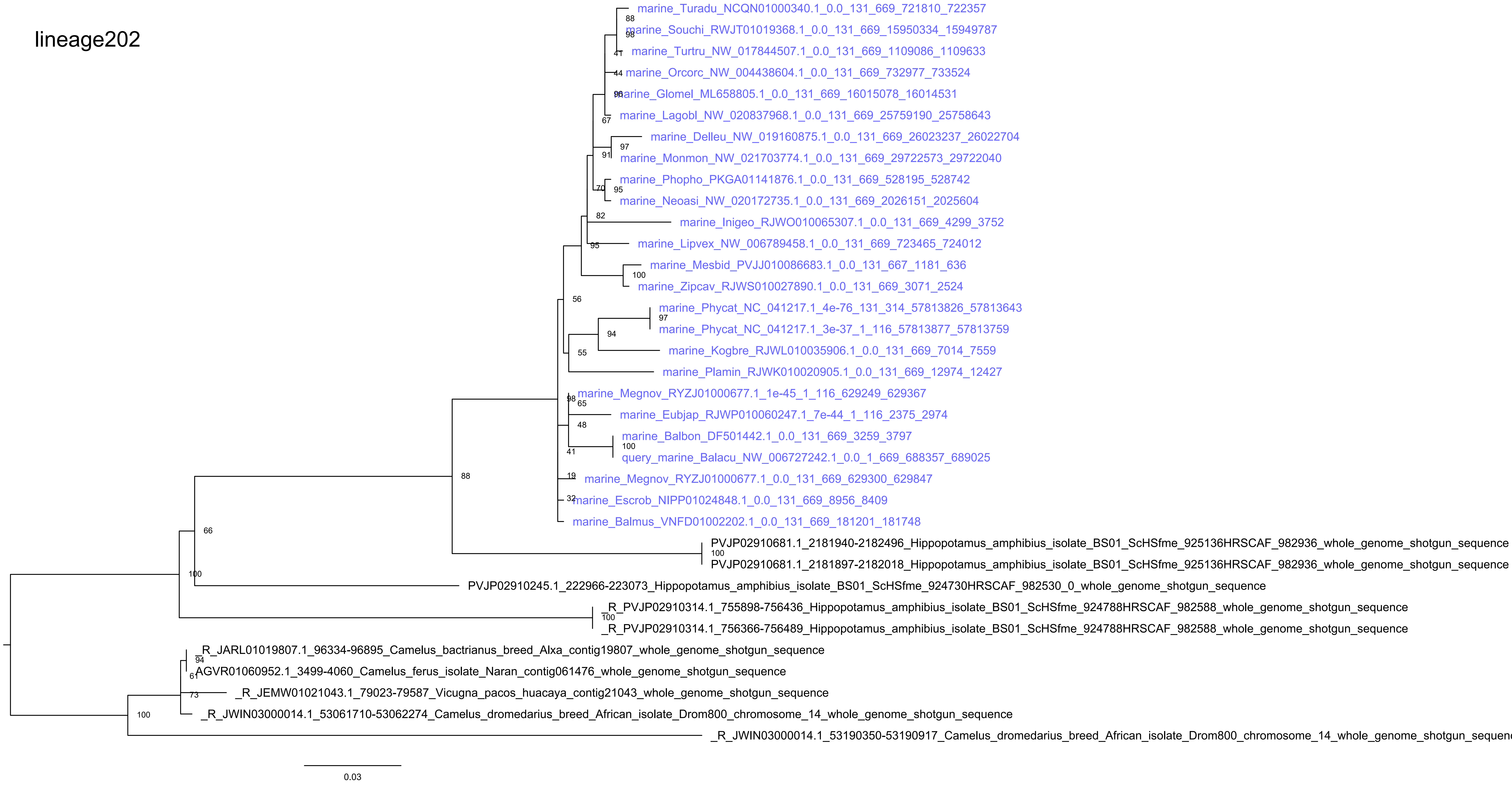
lineage202
marine_Turadu_NCQN01000340.1_0.0_131_669_721810_722357
marine_Souchi_RWJT01019368.1_0.0_131_669_15950334_15949787
marine_Turtru_NW_017844507.1_0.0_131_669_1109086_1109633
marine_Orcorc_NW_004438604.1_0.0_131_669_732977_733524
marine_Glomel_ML658805.1_0.0_131_669_16015078_16014531
marine_Lagobl_NW_020837968.1_0.0_131_669_25759190_25758643
marine_Delleu_NW_019160875.1_0.0_131_669_26023237_26022704
marine_Monmon_NW_021703774.1_0.0_131_669_29722573_29722040
marine_Phopho_PKGA01141876.1_0.0_131_669_528195_528742
marine_Neoasi_NW_020172735.1_0.0_131_669_2026151_2025604
marine_Inigeo_RJWO010065307.1_0.0_131_669_4299_3752
marine_Lipvex_NW_006789458.1_0.0_131_669_723465_724012
marine_Mesbid_PVJJ010086683.1_0.0_131_667_1181_636
marine_Zipcav_RJWS010027890.1_0.0_131_669_3071_2524
marine_Phycat_NC_041217.1_4e-76_131_314_57813826_57813643
marine_Phycat_NC_041217.1_3e-37_1_116_57813877_57813759
marine_Kogbre_RJWL010035906.1_0.0_131_669_7014_7559
marine_Plamin_RJWK010020905.1_0.0_131_669_12974_12427
marine_Megnov_RYZJ01000677.1_1e-45_1_116_629249_629367
marine_Eubjap_RJWP010060247.1_7e-44_1_116_2375_2974
marine_Balbon_DF501442.1_0.0_131_669_3259_3797
query_marine_Balacu_NW_006727242.1_0.0_1_669_688357_689025
marine_Megnov_RYZJ01000677.1_0.0_131_669_629300_629847
marine_Escrob_NIPP01024848.1_0.0_131_669_8956_8409
marine_Balmus_VNFD01002202.1_0.0_131_669_181201_181748
PVJP02910681.1_2181940-2182496_Hippopotamus_amphibius_isolate_BS01_ScHSfme_925136HRSCAF_982936_whole_genome_shotgun_sequence
PVJP02910681.1_2181897-2182018_Hippopotamus_amphibius_isolate_BS01_ScHSfme_925136HRSCAF_982936_whole_genome_shotgun_sequence
PVJP02910245.1_222966-223073_Hippopotamus_amphibius_isolate_BS01_ScHSfme_924730HRSCAF_982530_0_whole_genome_shotgun_sequence
_R_PVJP02910314.1_755898-756436_Hippopotamus_amphibius_isolate_BS01_ScHSfme_924788HRSCAF_982588_whole_genome_shotgun_sequence
_R_PVJP02910314.1_756366-756489_Hippopotamus_amphibius_isolate_BS01_ScHSfme_924788HRSCAF_982588_whole_genome_shotgun_sequence
_R_JARL01019807.1_96334-96895_Camelus_bactrianus_breed_Alxa_contig19807_whole_genome_shotgun_sequence
AGVR01060952.1_3499-4060_Camelus_ferus_isolate_Naran_contig061476_whole_genome_shotgun_sequence
_R_JEMW01021043.1_79023-79587_Vicugna_pacos_huacaya_contig21043_whole_genome_shotgun_sequence
_R_JWIN03000014.1_53061710-53062274_Camelus_dromedarius_breed_African_isolate_Drom800_chromosome_14_whole_genome_shotgun_sequence
_R_JWIN03000014.1_53190350-53190917_Camelus_dromedarius_breed_African_isolate_Drom800_chromosome_14_whole_genome_shotgun_sequen
0.03

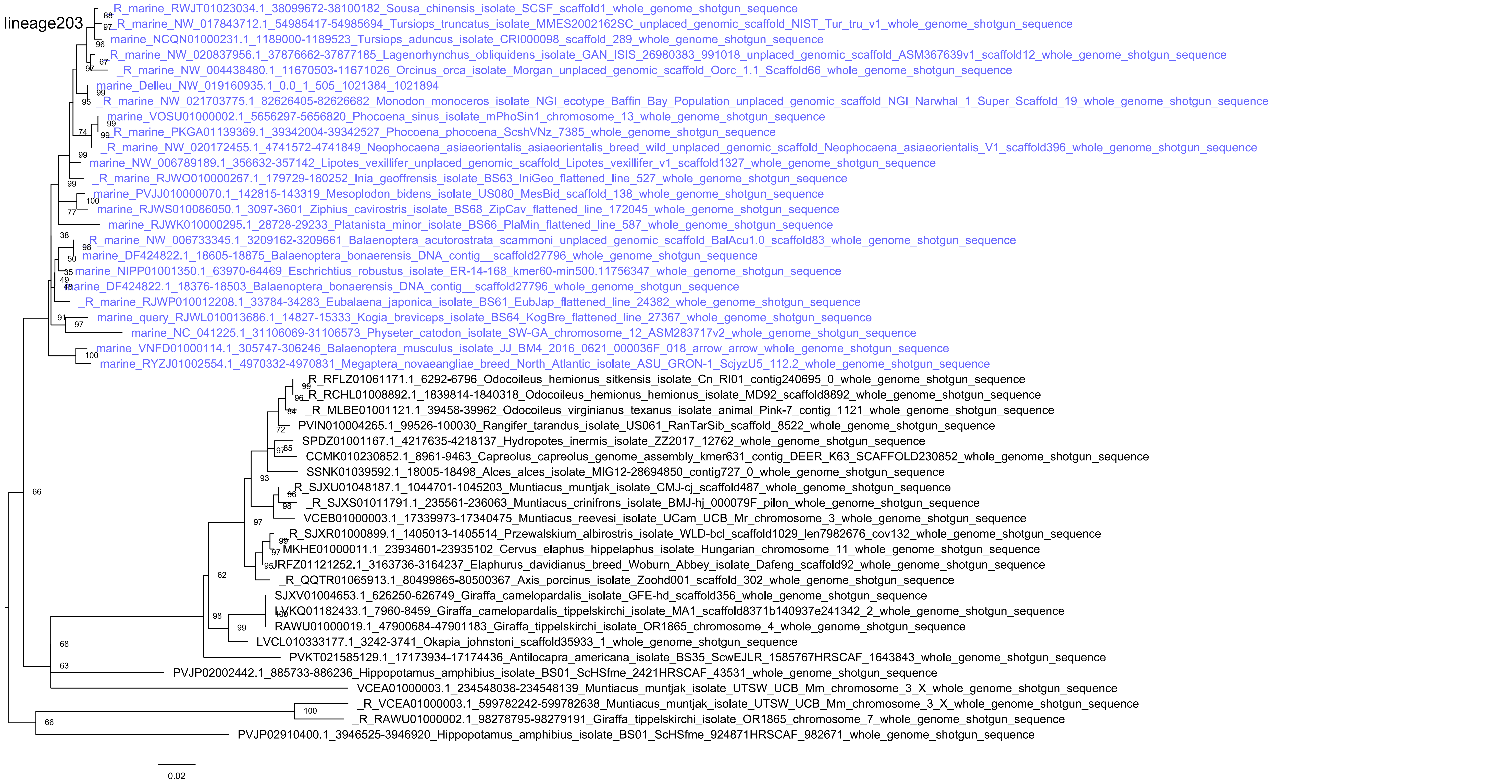
lineage203
R_marine_RWJT01023034.1_38099672-38100182_Sousa_chinensis_isolate_SCSF_scaffold1_whole_genome_shotgun_sequence
_R_marine_NW_017843712.1_54985417-54985694_Tursiops_truncatus_isolate_MMES2002162SC_unplaced_genomic_scaffold_NIST_Tur_tru_v1_whole_genome_shotgun_sequence
marine_NCQN01000231.1_1189000-1189523_Tursiops_aduncus_isolate_CRI000098_scaffold_289_whole_genome_shotgun_sequence
R_marine_NW_020837956.1_37876662-37877185_Lagenorhynchus_obliquidens_isolate_GAN_ISIS_26980383_991018_unplaced_genomic_scaffold_ASM367639v1_scaffold12_whole_genome_shotgun_sequence
_R_marine_NW_004438480.1_11670503-11671026_Orcinus_orca_isolate_Morgan_unplaced_genomic_scaffold_Oorc_1.1_Scaffold66_whole_genome_shotgun_sequence
marine_Delleu_NW_019160935.1_0.0_1_505_1021384_1021894
_R_marine_NW_021703775.1_82626405-82626682_Monodon_monoceros_isolate_NGI_ecotype_Baffin_Bay_Population_unplaced_genomic_scaffold_NGI_Narwhal_1_Super_Scaffold_19_whole_genome_shotgun_sequence
marine_VOSU01000002.1_5656297-5656820_Phocoena_sinus_isolate_mPhoSin1_chromosome_13_whole_genome_shotgun_sequence
_R_marine_PKGA01139369.1_39342004-39342527_Phocoena_phocoena_ScshVNz_7385_whole_genome_shotgun_sequence
_R_marine_NW_020172455.1_4741572-4741849_Neophocaena_asiaeorientalis_asiaeorientalis_breed_wild_unplaced_genomic_scaffold_Neophocaena_asiaeorientalis_V1_scaffold396_whole_genome_shotgun_sequence
marine_NW_006789189.1_356632-357142_Lipotes_vexillifer_unplaced_genomic_scaffold_Lipotes_vexillifer_v1_scaffold1327_whole_genome_shotgun_sequence
_R_marine_RJWO010000267.1_179729-180252_Inia_geoffrensis_isolate_BS63_IniGeo_flattened_line_527_whole_genome_shotgun_sequence
marine_PVJJ010000070.1_142815-143319_Mesoplodon_bidens_isolate_US080_MesBid_scaffold_138_whole_genome_shotgun_sequence
marine_RJWS010086050.1_3097-3601_Ziphius_cavirostris_isolate_BS68_ZipCav_flattened_line_172045_whole_genome_shotgun_sequence
marine_RJWK010000295.1_28728-29233_Platanista_minor_isolate_BS66_PlaMin_flattened_line_587_whole_genome_shotgun_sequence
R_marine_NW_006733345.1_3209162-3209661_Balaenoptera_acutorostrata_scammoni_unplaced_genomic_scaffold_BalAcu1.0_scaffold83_whole_genome_shotgun_sequence
marine_DF424822.1_18605-18875_Balaenoptera_bonaerensis_DNA_contig__scaffold27796_whole_genome_shotgun_sequence
marine_NIPP01001350.1_63970-64469_Eschrichtius_robustus_isolate_ER-14-168_kmer60-min500.11756347_whole_genome_shotgun_sequence
marine_DF424822.1_18376-18503_Balaenoptera_bonaerensis_DNA_contig__scaffold27796_whole_genome_shotgun_sequence
_R_marine_RJWP010012208.1_33784-34283_Eubalaena_japonica_isolate_BS61_EubJap_flattened_line_24382_whole_genome_shotgun_sequence
marine_query_RJWL010013686.1_14827-15333_Kogia_breviceps_isolate_BS64_KogBre_flattened_line_27367_whole_genome_shotgun_sequence
marine_NC_041225.1_31106069-31106573_Physeter_catodon_isolate_SW-GA_chromosome_12_ASM283717v2_whole_genome_shotgun_sequence
marine_VNFD01000114.1_305747-306246_Balaenoptera_musculus_isolate_JJ_BM4_2016_0621_000036F_018_arrow_arrow_whole_genome_shotgun_sequence
marine_RYZJ01002554.1_4970332-4970831_Megaptera_novaeangliae_breed_North_Atlantic_isolate_ASU_GRON-1_ScjyzU5_112.2_whole_genome_shotgun_sequence
R_RFLZ01061171.1_6292-6796_Odocoileus_hemionus_sitkensis_isolate_Cn_RI01_contig240695_0_whole_genome_shotgun_sequence
R_RCHL01008892.1_1839814-1840318_Odocoileus_hemionus_hemionus_isolate_MD92_scaffold8892_whole_genome_shotgun_sequence
_R_MLBE01001121.1_39458-39962_Odocoileus_virginianus_texanus_isolate_animal_Pink-7_contig_1121_whole_genome_shotgun_sequence
PVIN010004265.1_99526-100030_Rangifer_tarandus_isolate_US061_RanTarSib_scaffold_8522_whole_genome_shotgun_sequence
SPDZ01001167.1_4217635-4218137_Hydropotes_inermis_isolate_ZZ2017_12762_whole_genome_shotgun_sequence
CCMK010230852.1_8961-9463_Capreolus_capreolus_genome_assembly_kmer631_contig_DEER_K63_SCAFFOLD230852_whole_genome_shotgun_sequence
SSNK01039592.1_18005-18498_Alces_alces_isolate_MIG12-28694850_contig727_0_whole_genome_shotgun_sequence
R_SJXU01048187.1_1044701-1045203_Muntiacus_muntjak_isolate_CMJ-cj_scaffold487_whole_genome_shotgun_sequence
_R_SJXS01011791.1_235561-236063_Muntiacus_crinifrons_isolate_BMJ-hj_000079F_pilon_whole_genome_shotgun_sequence
VCEB01000003.1_17339973-17340475_Muntiacus_reevesi_isolate_UCam_UCB_Mr_chromosome_3_whole_genome_shotgun_sequence
R_SJXR01000899.1_1405013-1405514_Przewalskium_albirostris_isolate_WLD-bcl_scaffold1029_len7982676_cov132_whole_genome_shotgun_sequence
MKHE01000011.1_23934601-23935102_Cervus_elaphus_hippelaphus_isolate_Hungarian_chromosome_11_whole_genome_shotgun_sequence
JRFZ01121252.1_3163736-3164237_Elaphurus_davidianus_breed_Woburn_Abbey_isolate_Dafeng_scaffold92_whole_genome_shotgun_sequence
_R_QQTR01065913.1_80499865-80500367_Axis_porcinus_isolate_Zoohd001_scaffold_302_whole_genome_shotgun_sequence
SJXV01004653.1_626250-626749_Giraffa_camelopardalis_isolate_GFE-hd_scaffold356_whole_genome_shotgun_sequence
LVKQ01182433.1_7960-8459_Giraffa_camelopardalis_tippelskirchi_isolate_MA1_scaffold8371b140937e241342_2_whole_genome_shotgun_sequence
RAWU01000019.1_47900684-47901183_Giraffa_tippelskirchi_isolate_OR1865_chromosome_4_whole_genome_shotgun_sequence
LVCL010333177.1_3242-3741_Okapia_johnstoni_scaffold35933_1_whole_genome_shotgun_sequence
PVKT021585129.1_17173934-17174436_Antilocapra_americana_isolate_BS35_ScwEJLR_1585767HRSCAF_1643843_whole_genome_shotgun_sequence
PVJP02002442.1_885733-886236_Hippopotamus_amphibius_isolate_BS01_ScHSfme_2421HRSCAF_43531_whole_genome_shotgun_sequence
VCEA01000003.1_234548038-234548139_Muntiacus_muntjak_isolate_UTSW_UCB_Mm_chromosome_3_X_whole_genome_shotgun_sequence
_R_VCEA01000003.1_599782242-599782638_Muntiacus_muntjak_isolate_UTSW_UCB_Mm_chromosome_3_X_whole_genome_shotgun_sequence
_R_RAWU01000002.1_98278795-98279191_Giraffa_tippelskirchi_isolate_OR1865_chromosome_7_whole_genome_shotgun_sequence
PVJP02910400.1_3946525-3946920_Hippopotamus_amphibius_isolate_BS01_ScHSfme_924871HRSCAF_982671_whole_genome_shotgun_sequence
0.02

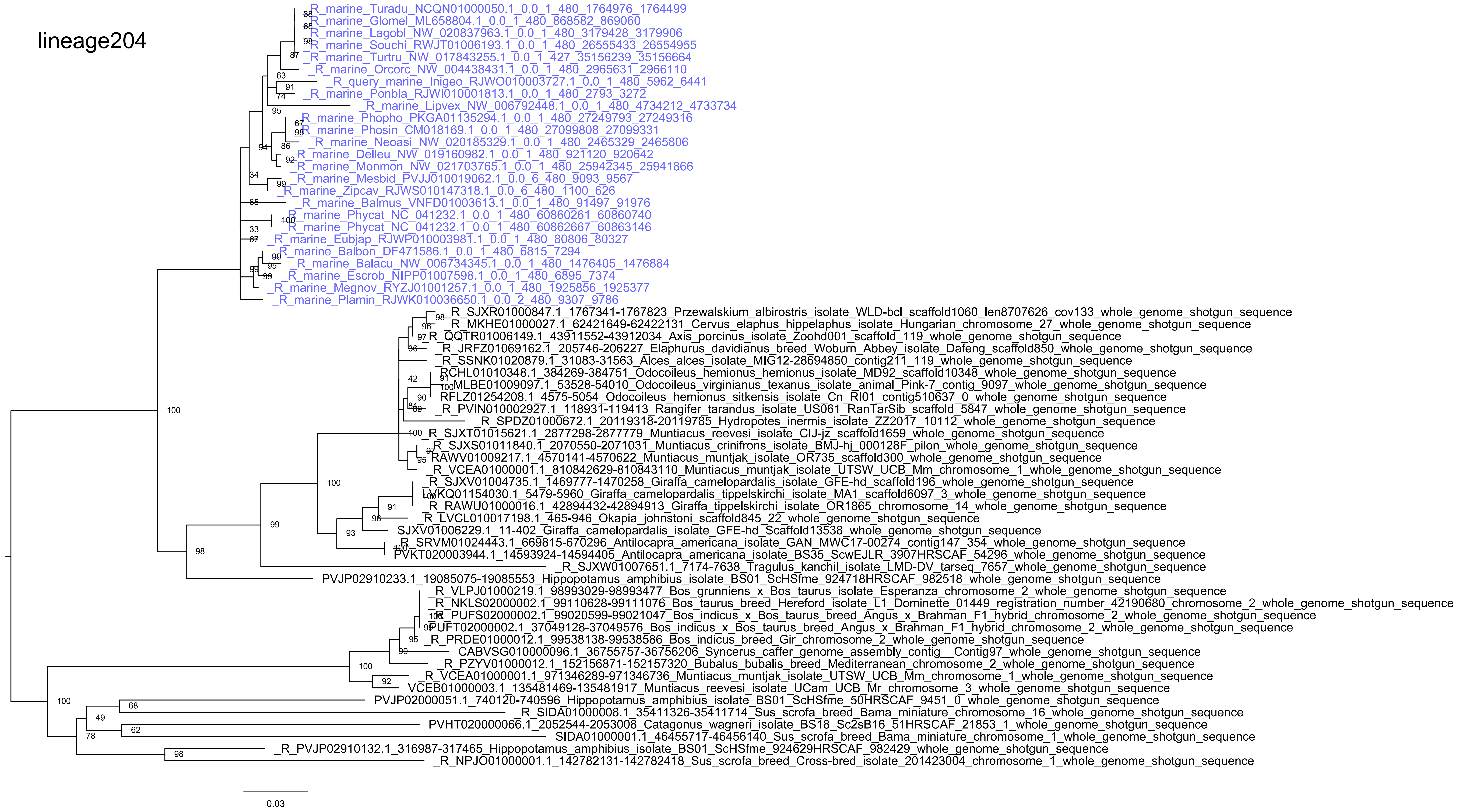

lineage204
0.03

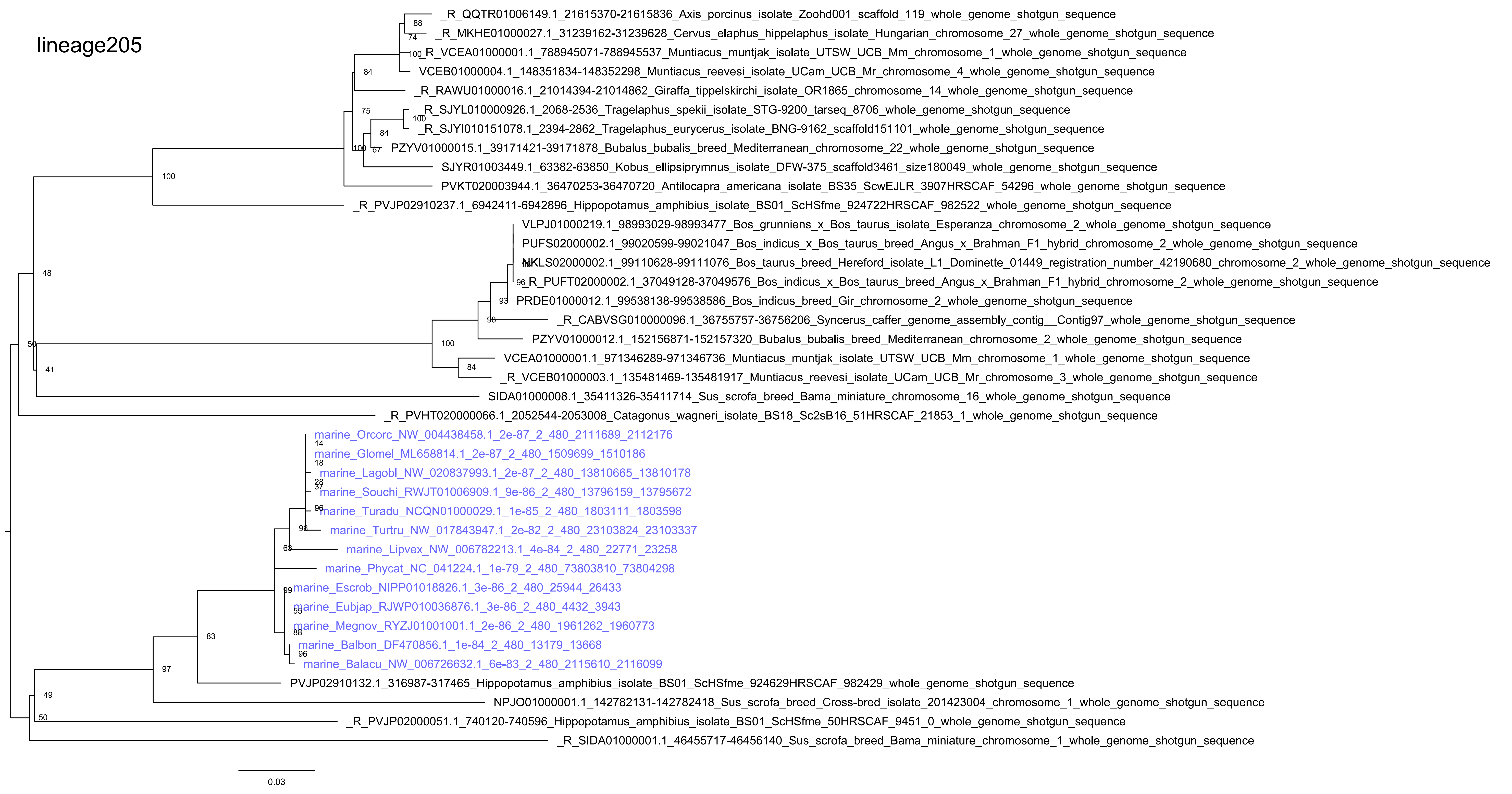
lineage205
_R_QQTR01006149.1_21615370-21615836_Axis_porcinus_isolate_Zoohd001_scaffold_119_whole_genome_shotgun_sequence
_R_MKHE01000027.1_31239162-31239628_Cervus_elaphus_hippelaphus_isolate_Hungarian_chromosome_27_whole_genome_shotgun_sequence
_R_VCEA01000001.1_788945071-788945537_Muntiacus_muntjak_isolate_UTSW_UCB_Mm_chromosome_1_whole_genome_shotgun_sequence
VCEB01000004.1_148351834-148352298_Muntiacus_reevesi_isolate_UCam_UCB_Mr_chromosome_4_whole_genome_shotgun_sequence
_R_RAWU01000016.1_21014394-21014862_Giraffa_tippelskirchi_isolate_OR1865_chromosome_14_whole_genome_shotgun_sequence
_R_SJYL010000926.1_2068-2536_Tragelaphus_spekii_isolate_STG-9200_tarseq_8706_whole_genome_shotgun_sequence
_R_SJYI010151078.1_2394-2862_Tragelaphus_eurycerus_isolate_BNG-9162_scaffold151101_whole_genome_shotgun_sequence
PZYV01000015.1_39171421-39171878_Bubalus_bubalis_breed_Mediterranean_chromosome_22_whole_genome_shotgun_sequence
SJYR01003449.1_63382-63850_Kobus_ellipsiprymnus_isolate_DFW-375_scaffold3461_size180049_whole_genome_shotgun_sequence
PVKT020003944.1_36470253-36470720_Antilocapra_americana_isolate_BS35_ScwEJLR_3907HRSCAF_54296_whole_genome_shotgun_sequence
_R_PVJP02910237.1_6942411-6942896_Hippopotamus_amphibius_isolate_BS01_ScHSfme_924722HRSCAF_982522_whole_genome_shotgun_sequence
VLPJ01000219.1_98993029-98993477_Bos_grunniens_x_Bos_taurus_isolate_Esperanza_chromosome_2_whole_genome_shotgun_sequence
PUFS02000002.1_99020599-99021047_Bos_indicus_x_Bos_taurus_breed_Angus_x_Brahman_F1_hybrid_chromosome_2_whole_genome_shotgun_sequence
NKLS02000002.1_99110628-99111076_Bos_taurus_breed_Hereford_isolate_L1_Dominette_01449_registration_number_42190680_chromosome_2_whole_genome_shotgun_sequence
_R_PUFT02000002.1_37049128-37049576_Bos_indicus_x_Bos_taurus_breed_Angus_x_Brahman_F1_hybrid_chromosome_2_whole_genome_shotgun_sequence
PRDE01000012.1_99538138-99538586_Bos_indicus_breed_Gir_chromosome_2_whole_genome_shotgun_sequence
_R_CABVSG010000096.1_36755757-36756206_Syncerus_caffer_genome_assembly_contig__Contig97_whole_genome_shotgun_sequence
PZYV01000012.1_152156871-152157320_Bubalus_bubalis_breed_Mediterranean_chromosome_2_whole_genome_shotgun_sequence
VCEA01000001.1_971346289-971346736_Muntiacus_muntjak_isolate_UTSW_UCB_Mm_chromosome_1_whole_genome_shotgun_sequence
_R_VCEB01000003.1_135481469-135481917_Muntiacus_reevesi_isolate_UCam_UCB_Mr_chromosome_3_whole_genome_shotgun_sequence
SIDA01000008.1_35411326-35411714_Sus_scrofa_breed_Bama_miniature_chromosome_16_whole_genome_shotgun_sequence
_R_PVHT020000066.1_2052544-2053008_Catagonus_wagneri_isolate_BS18_Sc2sB16_51HRSCAF_21853_1_whole_genome_shotgun_sequence
marine_Orcorc_NW_004438458.1_2e-87_2_480_2111689_2112176
marine_Glomel_ML658814.1_2e-87_2_480_1509699_1510186
marine_Lagobl_NW_020837993.1_2e-87_2_480_13810665_13810178
marine_Souchi_RWJT01006909.1_9e-86_2_480_13796159_13795672
marine_Turadu_NCQN01000029.1_1e-85_2_480_1803111_1803598
marine_Turtru_NW_017843947.1_2e-82_2_480_23103824_23103337
marine_Lipvex_NW_006782213.1_4e-84_2_480_22771_23258
marine_Phycat_NC_041224.1_1e-79_2_480_73803810_73804298
marine_Escrob_NIPP01018826.1_3e-86_2_480_25944_26433
marine_Eubjap_RJWP010036876.1_3e-86_2_480_4432_3943
marine_Megnov_RYZJ01001001.1_2e-86_2_480_1961262_1960773
marine_Balbon_DF470856.1_1e-84_2_480_13179_13668
marine_Balacu_NW_006726632.1_6e-83_2_480_2115610_2116099
PVJP02910132.1_316987-317465_Hippopotamus_amphibius_isolate_BS01_ScHSfme_924629HRSCAF_982429_whole_genome_shotgun_sequence
NPJO01000001.1_142782131-142782418_Sus_scrofa_breed_Cross-bred_isolate_201423004_chromosome_1_whole_genome_shotgun_sequence
_R_PVJP02000051.1_740120-740596_Hippopotamus_amphibius_isolate_BS01_ScHSfme_50HRSCAF_9451_0_whole_genome_shotgun_sequence
_R_SIDA01000001.1_46455717-46456140_Sus_scrofa_breed_Bama_miniature_chromosome_1_whole_genome_shotgun_sequence
0.03

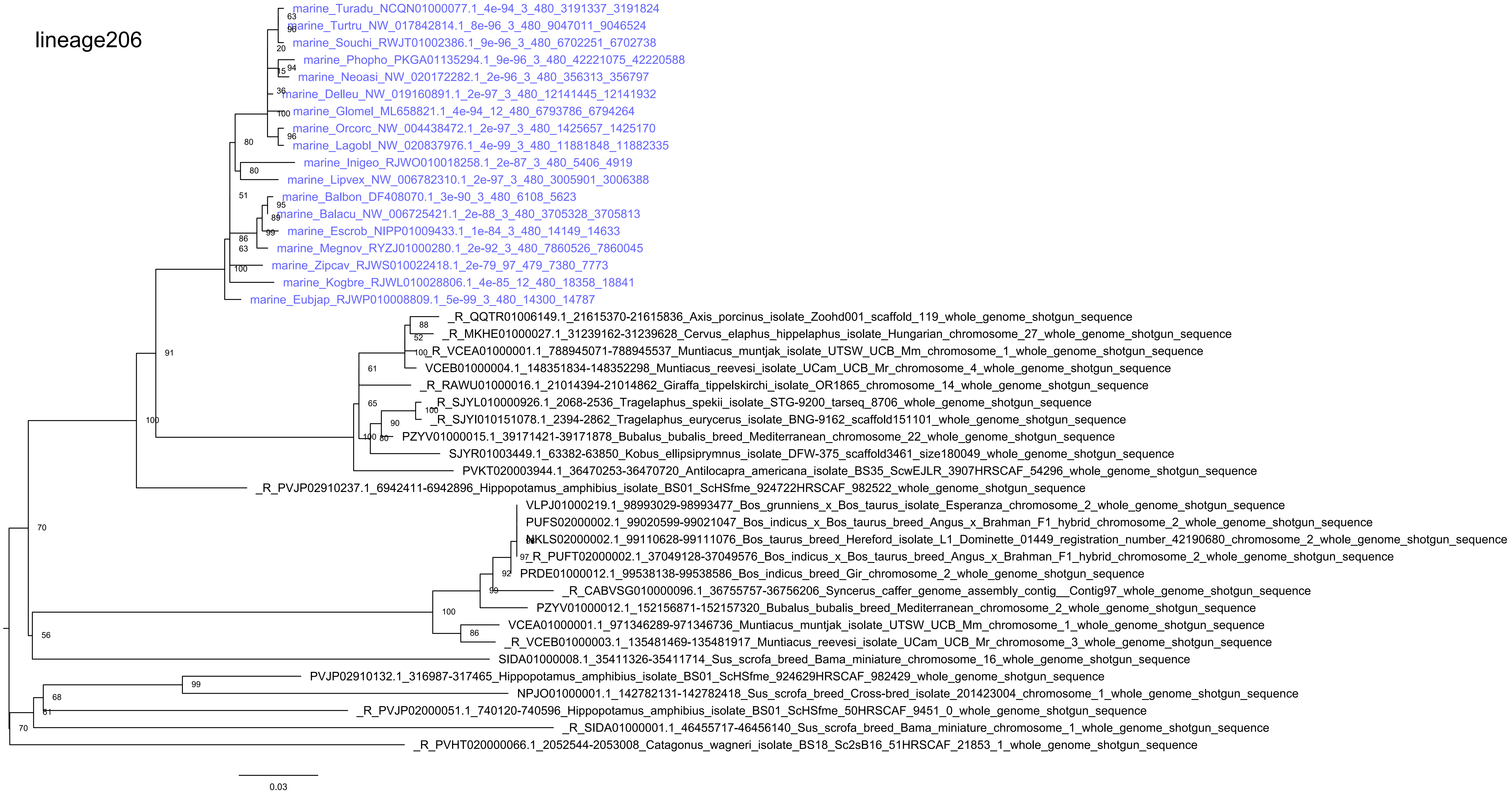

lineage206
marine_Turadu_NCQN01000077.1_4e-94_3_480_3191337_3191824
marine_Turtru_NW_017842814.1_8e-96_3_480_9047011_9046524
marine_Souchi_RWJT01002386.1_9e-96_3_480_6702251_6702738
marine_Phopho_PKGA01135294.1_9e-96_3_480_42221075_42220588
marine_Neoasi_NW_020172282.1_2e-96_3_480_356313_356797
marine_Delleu_NW_019160891.1_2e-97_3_480_12141445_12141932
marine_Glomel_ML658821.1_4e-94_12_480_6793786_6794264
marine_Orcorc_NW_004438472.1_2e-97_3_480_1425657_1425170
marine_Lagobl_NW_020837976.1_4e-99_3_480_11881848_11882335
marine_Inigeo_RJWO010018258.1_2e-87_3_480_5406_4919
marine_Lipvex_NW_006782310.1_2e-97_3_480_3005901_3006388
marine_Balbon_DF408070.1_3e-90_3_480_6108_5623
marine_Balacu_NW_006725421.1_2e-88_3_480_3705328_3705813
marine_Escrob_NIPP01009433.1_1e-84_3_480_14149_14633
marine_Megnov_RYZJ01000280.1_2e-92_3_480_7860526_7860045
marine_Zipcav_RJWS010022418.1_2e-79_97_479_7380_7773
marine_Kogbre_RJWL010028806.1_4e-85_12_480_18358_18841
marine_Eubjap_RJWP010008809.1_5e-99_3_480_14300_14787
_R_QQTR01006149.1_21615370-21615836_Axis_porcinus_isolate_Zoohd001_scaffold_119_whole_genome_shotgun_sequence
_R_MKHE01000027.1_31239162-31239628_Cervus_elaphus_hippelaphus_isolate_Hungarian_chromosome_27_whole_genome_shotgun_sequence
_R_VCEA01000001.1_788945071-788945537_Muntiacus_muntjak_isolate_UTSW_UCB_Mm_chromosome_1_whole_genome_shotgun_sequence
VCEB01000004.1_148351834-148352298_Muntiacus_reevesi_isolate_UCam_UCB_Mr_chromosome_4_whole_genome_shotgun_sequence
_R_RAWU01000016.1_21014394-21014862_Giraffa_tippelskirchi_isolate_OR1865_chromosome_14_whole_genome_shotgun_sequence
_R_SJYL010000926.1_2068-2536_Tragelaphus_spekii_isolate_STG-9200_tarseq_8706_whole_genome_shotgun_sequence
_R_SJYI010151078.1_2394-2862_Tragelaphus_eurycerus_isolate_BNG-9162_scaffold151101_whole_genome_shotgun_sequence
PZYV01000015.1_39171421-39171878_Bubalus_bubalis_breed_Mediterranean_chromosome_22_whole_genome_shotgun_sequence
SJYR01003449.1_63382-63850_Kobus_ellipsiprymnus_isolate_DFW-375_scaffold3461_size180049_whole_genome_shotgun_sequence
PVKT020003944.1_36470253-36470720_Antilocapra_americana_isolate_BS35_ScwEJLR_3907HRSCAF_54296_whole_genome_shotgun_sequence
_R_PVJP02910237.1_6942411-6942896_Hippopotamus_amphibius_isolate_BS01_ScHSfme_924722HRSCAF_982522_whole_genome_shotgun_sequence
VLPJ01000219.1_98993029-98993477_Bos_grunniens_x_Bos_taurus_isolate_Esperanza_chromosome_2_whole_genome_shotgun_sequence
PUFS02000002.1_99020599-99021047_Bos_indicus_x_Bos_taurus_breed_Angus_x_Brahman_F1_hybrid_chromosome_2_whole_genome_shotgun_sequence
NKLS02000002.1_99110628-99111076_Bos_taurus_breed_Hereford_isolate_L1_Dominette_01449_registration_number_42190680_chromosome_2_whole_genome_shotgun_sequence
_R_PUFT02000002.1_37049128-37049576_Bos_indicus_x_Bos_taurus_breed_Angus_x_Brahman_F1_hybrid_chromosome_2_whole_genome_shotgun_sequence
PRDE01000012.1_99538138-99538586_Bos_indicus_breed_Gir_chromosome_2_whole_genome_shotgun_sequence
_R_CABVSG010000096.1_36755757-36756206_Syncerus_caffer_genome_assembly_contig__Contig97_whole_genome_shotgun_sequence
PZYV01000012.1_152156871-152157320_Bubalus_bubalis_breed_Mediterranean_chromosome_2_whole_genome_shotgun_sequence
VCEA01000001.1_971346289-971346736_Muntiacus_muntjak_isolate_UTSW_UCB_Mm_chromosome_1_whole_genome_shotgun_sequence
_R_VCEB01000003.1_135481469-135481917_Muntiacus_reevesi_isolate_UCam_UCB_Mr_chromosome_3_whole_genome_shotgun_sequence
SIDA01000008.1_35411326-35411714_Sus_scrofa_breed_Bama_miniature_chromosome_16_whole_genome_shotgun_sequence
PVJP02910132.1_316987-317465_Hippopotamus_amphibius_isolate_BS01_ScHSfme_924629HRSCAF_982429_whole_genome_shotgun_sequence
NPJO01000001.1_142782131-142782418_Sus_scrofa_breed_Cross-bred_isolate_201423004_chromosome_1_whole_genome_shotgun_sequence
_R_PVJP02000051.1_740120-740596_Hippopotamus_amphibius_isolate_BS01_ScHSfme_50HRSCAF_9451_0_whole_genome_shotgun_sequence
_R_SIDA01000001.1_46455717-46456140_Sus_scrofa_breed_Bama_miniature_chromosome_1_whole_genome_shotgun_sequence
_R_PVHT020000066.1_2052544-2053008_Catagonus_wagneri_isolate_BS18_Sc2sB16_51HRSCAF_21853_1_whole_genome_shotgun_sequence
0.03

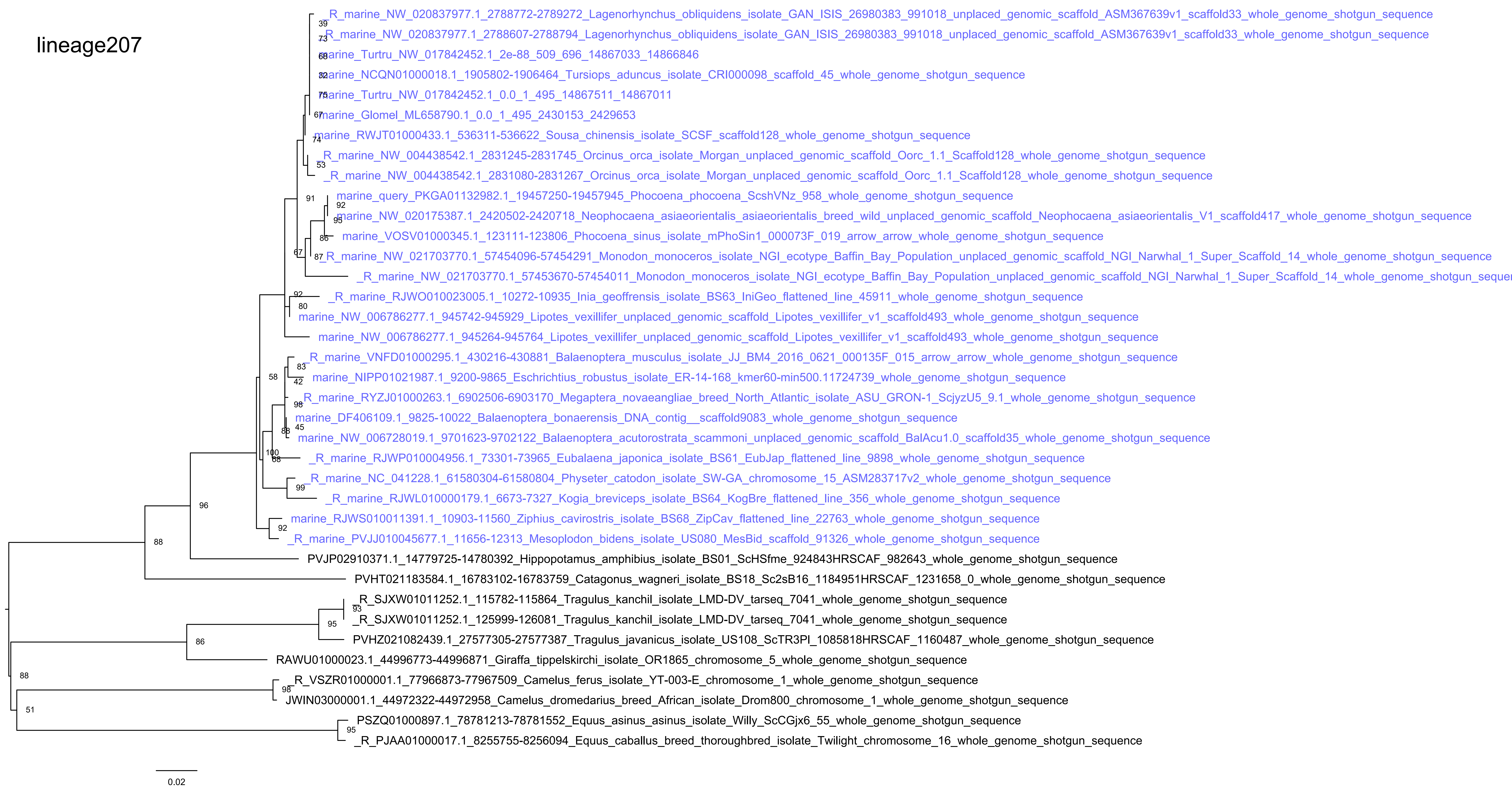
lineage207
_R_marine_NW_020837977.1_2788772-2789272_Lagenorhynchus_obliquidens_isolate_GAN_ISIS_26980383_991018_unplaced_genomic_scaffold_ASM367639v1_scaffold33_whole_genome_shotgun_sequence
_R_marine_NW_020837977.1_2788607-2788794_Lagenorhynchus_obliquidens_isolate_GAN_ISIS_26980383_991018_unplaced_genomic_scaffold_ASM367639v1_scaffold33_whole_genome_shotgun_sequence
marine_Turtru_NW_017842452.1_2e-88_509_696_14867033_14866846
marine_NCQN01000018.1_1905802-1906464_Tursiops_aduncus_isolate_CRI000098_scaffold_45_whole_genome_shotgun_sequence
marine_Turtru_NW_017842452.1_0.0_1_495_14867511_14867011
marine_Glomel_ML658790.1_0.0_1_495_2430153_2429653
marine_RWJT01000433.1_536311-536622_Sousa_chinensis_isolate_SCSF_scaffold128_whole_genome_shotgun_sequence
_R_marine_NW_004438542.1_2831245-2831745_Orcinus_orca_isolate_Morgan_unplaced_genomic_scaffold_Oorc_1.1_Scaffold128_whole_genome_shotgun_sequence
_R_marine_NW_004438542.1_2831080-2831267_Orcinus_orca_isolate_Morgan_unplaced_genomic_scaffold_Oorc_1.1_Scaffold128_whole_genome_shotgun_sequence
marine_query_PKGA01132982.1_19457250-19457945_Phocoena_phocoena_ScshVNz_958_whole_genome_shotgun_sequence
marine_NW_020175387.1_2420502-2420718_Neophocaena_asiaeorientalis_asiaeorientalis_breed_wild_unplaced_genomic_scaffold_Neophocaena_asiaeorientalis_V1_scaffold417_whole_genome_shotgun_sequence
marine_VOSV01000345.1_123111-123806_Phocoena_sinus_isolate_mPhoSin1_000073F_019_arrow_arrow_whole_genome_shotgun_sequence
_R_marine_NW_021703770.1_57454096-57454291_Monodon_monoceros_isolate_NGI_ecotype_Baffin_Bay_Population_unplaced_genomic_scaffold_NGI_Narwhal_1_Super_Scaffold_14_whole_genome_shotgun_sequence
_R_marine_NW_021703770.1_57453670-57454011_Monodon_monoceros_isolate_NGI_ecotype_Baffin_Bay_Population_unplaced_genomic_scaffold_NGI_Narwhal_1_Super_Scaffold_14_whole_genome_shotgun_sequen
_R_marine_RJWO010023005.1_10272-10935_Inia_geoffrensis_isolate_BS63_IniGeo_flattened_line_45911_whole_genome_shotgun_sequence
marine_NW_006786277.1_945742-945929_Lipotes_vexillifer_unplaced_genomic_scaffold_Lipotes_vexillifer_v1_scaffold493_whole_genome_shotgun_sequence
marine_NW_006786277.1_945264-945764_Lipotes_vexillifer_unplaced_genomic_scaffold_Lipotes_vexillifer_v1_scaffold493_whole_genome_shotgun_sequence
_R_marine_VNFD01000295.1_430216-430881_Balaenoptera_musculus_isolate_JJ_BM4_2016_0621_000135F_015_arrow_arrow_whole_genome_shotgun_sequence
marine_NIPP01021987.1_9200-9865_Eschrichtius_robustus_isolate_ER-14-168_kmer60-min500.11724739_whole_genome_shotgun_sequence
_R_marine_RYZJ01000263.1_6902506-6903170_Megaptera_novaeangliae_breed_North_Atlantic_isolate_ASU_GRON-1_ScjyzU5_9.1_whole_genome_shotgun_sequence
marine_DF406109.1_9825-10022_Balaenoptera_bonaerensis_DNA_contig__scaffold9083_whole_genome_shotgun_sequence
marine_NW_006728019.1_9701623-9702122_Balaenoptera_acutorostrata_scammoni_unplaced_genomic_scaffold_BalAcu1.0_scaffold35_whole_genome_shotgun_sequence
_R_marine_RJWP010004956.1_73301-73965_Eubalaena_japonica_isolate_BS61_EubJap_flattened_line_9898_whole_genome_shotgun_sequence
_R_marine_NC_041228.1_61580304-61580804_Physeter_catodon_isolate_SW-GA_chromosome_15_ASM283717v2_whole_genome_shotgun_sequence
_R_marine_RJWL010000179.1_6673-7327_Kogia_breviceps_isolate_BS64_KogBre_flattened_line_356_whole_genome_shotgun_sequence
marine_RJWS010011391.1_10903-11560_Ziphius_cavirostris_isolate_BS68_ZipCav_flattened_line_22763_whole_genome_shotgun_sequence
_R_marine_PVJJ010045677.1_11656-12313_Mesoplodon_bidens_isolate_US080_MesBid_scaffold_91326_whole_genome_shotgun_sequence
PVJP02910371.1_14779725-14780392_Hippopotamus_amphibius_isolate_BS01_ScHSfme_924843HRSCAF_982643_whole_genome_shotgun_sequence
PVHT021183584.1_16783102-16783759_Catagonus_wagneri_isolate_BS18_Sc2sB16_1184951HRSCAF_1231658_0_whole_genome_shotgun_sequence
_R_SJXW01011252.1_115782-115864_Tragulus_kanchil_isolate_LMD-DV_tarseq_7041_whole_genome_shotgun_sequence
_R_SJXW01011252.1_125999-126081_Tragulus_kanchil_isolate_LMD-DV_tarseq_7041_whole_genome_shotgun_sequence
PVHZ021082439.1_27577305-27577387_Tragulus_javanicus_isolate_US108_ScTR3PI_1085818HRSCAF_1160487_whole_genome_shotgun_sequence
RAWU01000023.1_44996773-44996871_Giraffa_tippelskirchi_isolate_OR1865_chromosome_5_whole_genome_shotgun_sequence
R_VSZR01000001.1_77966873-77967509_Camelus_ferus_isolate_YT-003-E_chromosome_1_whole_genome_shotgun_sequence
JWIN03000001.1_44972322-44972958_Camelus_dromedarius_breed_African_isolate_Drom800_chromosome_1_whole_genome_shotgun_sequence
PSZQ01000897.1_78781213-78781552_Equus_asinus_asinus_isolate_Willy_ScCGjx6_55_whole_genome_shotgun_sequence
_R_PJAA01000017.1_8255755-8256094_Equus_caballus_breed_thoroughbred_isolate_Twilight_chromosome_16_whole_genome_shotgun_sequence
0.02

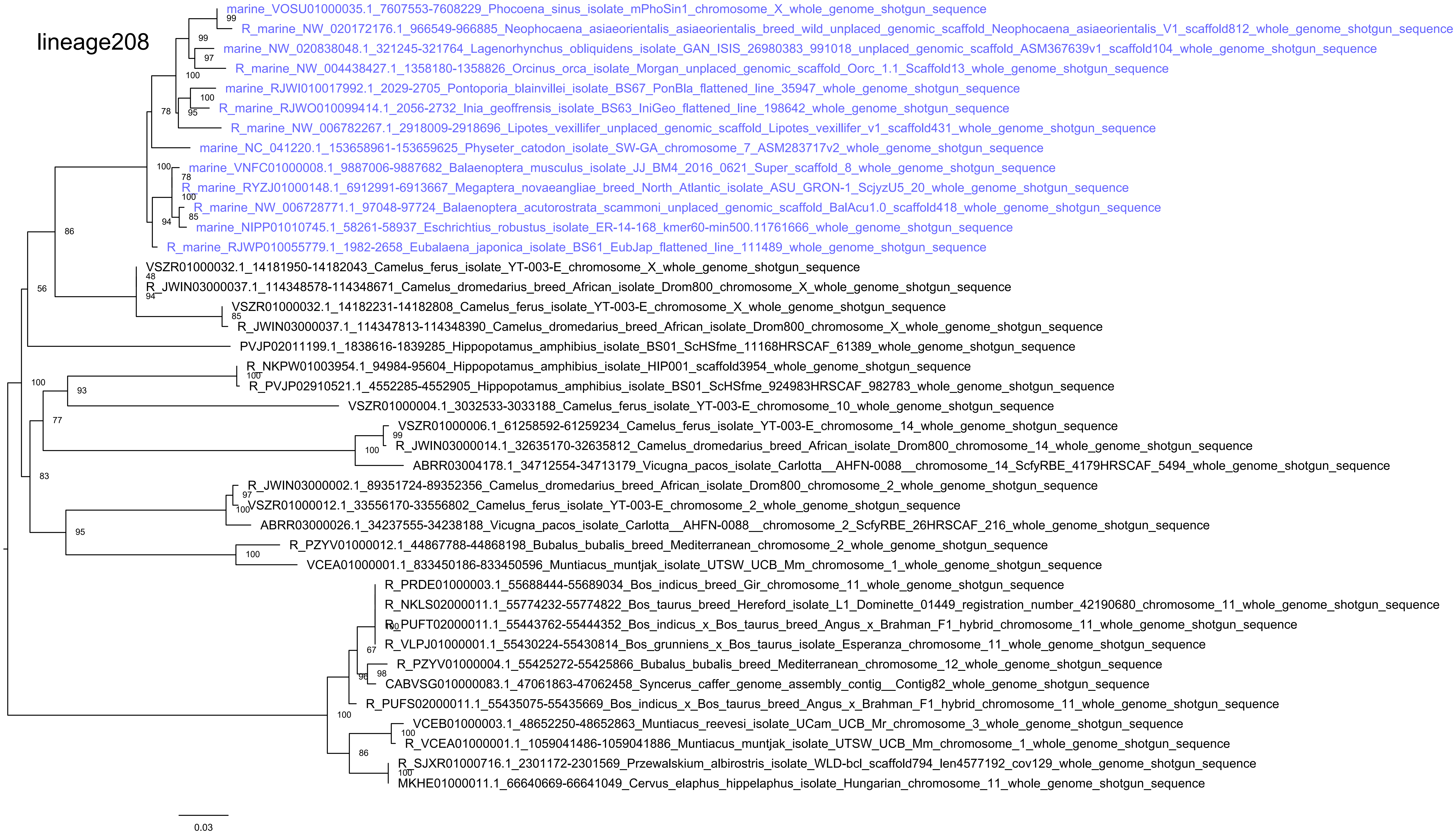
lineage208
marine_VOSU01000035.1_7607553-7608229_Phocoena_sinus_isolate_mPhoSin1_chromosome_X_whole_genome_shotgun_sequence
R_marine_NW_020172176.1_966549-966885_Neophocaena_asiaeorientalis_asiaeorientalis_breed_wild_unplaced_genomic_scaffold_Neophocaena_asiaeorientalis_V1_scaffold812_whole_genome_shotgun_sequence
marine_NW_020838048.1_321245-321764_Lagenorhynchus_obliquidens_isolate_GAN_ISIS_26980383_991018_unplaced_genomic_scaffold_ASM367639v1_scaffold104_whole_genome_shotgun_sequence
R_marine_NW_004438427.1_1358180-1358826_Orcinus_orca_isolate_Morgan_unplaced_genomic_scaffold_Oorc_1.1_Scaffold13_whole_genome_shotgun_sequence
marine_RJWI010017992.1_2029-2705_Pontoporia_blainvillei_isolate_BS67_PonBla_flattened_line_35947_whole_genome_shotgun_sequence
R_marine_RJWO010099414.1_2056-2732_Inia_geoffrensis_isolate_BS63_IniGeo_flattened_line_198642_whole_genome_shotgun_sequence
R_marine_NW_006782267.1_2918009-2918696_Lipotes_vexillifer_unplaced_genomic_scaffold_Lipotes_vexillifer_v1_scaffold431_whole_genome_shotgun_sequence
marine_NC_041220.1_153658961-153659625_Physeter_catodon_isolate_SW-GA_chromosome_7_ASM283717v2_whole_genome_shotgun_sequence
marine_VNFC01000008.1_9887006-9887682_Balaenoptera_musculus_isolate_JJ_BM4_2016_0621_Super_scaffold_8_whole_genome_shotgun_sequence
R_marine_RYZJ01000148.1_6912991-6913667_Megaptera_novaeangliae_breed_North_Atlantic_isolate_ASU_GRON-1_ScjyzU5_20_whole_genome_shotgun_sequence
R_marine_NW_006728771.1_97048-97724_Balaenoptera_acutorostrata_scammoni_unplaced_genomic_scaffold_BalAcu1.0_scaffold418_whole_genome_shotgun_sequence
marine_NIPP01010745.1_58261-58937_Eschrichtius_robustus_isolate_ER-14-168_kmer60-min500.11761666_whole_genome_shotgun_sequence
R_marine_RJWP010055779.1_1982-2658_Eubalaena_japonica_isolate_BS61_EubJap_flattened_line_111489_whole_genome_shotgun_sequence
VSZR01000032.1_14181950-14182043_Camelus_ferus_isolate_YT-003-E_chromosome_X_whole_genome_shotgun_sequence
R_JWIN03000037.1_114348578-114348671_Camelus_dromedarius_breed_African_isolate_Drom800_chromosome_X_whole_genome_shotgun_sequence
VSZR01000032.1_14182231-14182808_Camelus_ferus_isolate_YT-003-E_chromosome_X_whole_genome_shotgun_sequence
R_JWIN03000037.1_114347813-114348390_Camelus_dromedarius_breed_African_isolate_Drom800_chromosome_X_whole_genome_shotgun_sequence
PVJP02011199.1_1838616-1839285_Hippopotamus_amphibius_isolate_BS01_ScHSfme_11168HRSCAF_61389_whole_genome_shotgun_sequence
R_NKPW01003954.1_94984-95604_Hippopotamus_amphibius_isolate_HIP001_scaffold3954_whole_genome_shotgun_sequence
R_PVJP02910521.1_4552285-4552905_Hippopotamus_amphibius_isolate_BS01_ScHSfme_924983HRSCAF_982783_whole_genome_shotgun_sequence
VSZR01000004.1_3032533-3033188_Camelus_ferus_isolate_YT-003-E_chromosome_10_whole_genome_shotgun_sequence
VSZR01000006.1_61258592-61259234_Camelus_ferus_isolate_YT-003-E_chromosome_14_whole_genome_shotgun_sequence
R_JWIN03000014.1_32635170-32635812_Camelus_dromedarius_breed_African_isolate_Drom800_chromosome_14_whole_genome_shotgun_sequence
ABRR03004178.1_34712554-34713179_Vicugna_pacos_isolate_Carlotta__AHFN-0088__chromosome_14_ScfyRBE_4179HRSCAF_5494_whole_genome_shotgun_sequence
R_JWIN03000002.1_89351724-89352356_Camelus_dromedarius_breed_African_isolate_Drom800_chromosome_2_whole_genome_shotgun_sequence
VSZR01000012.1_33556170-33556802_Camelus_ferus_isolate_YT-003-E_chromosome_2_whole_genome_shotgun_sequence
ABRR03000026.1_34237555-34238188_Vicugna_pacos_isolate_Carlotta__AHFN-0088__chromosome_2_ScfyRBE_26HRSCAF_216_whole_genome_shotgun_sequence
R_PZYV01000012.1_44867788-44868198_Bubalus_bubalis_breed_Mediterranean_chromosome_2_whole_genome_shotgun_sequence
VCEA01000001.1_833450186-833450596_Muntiacus_muntjak_isolate_UTSW_UCB_Mm_chromosome_1_whole_genome_shotgun_sequence
R_PRDE01000003.1_55688444-55689034_Bos_indicus_breed_Gir_chromosome_11_whole_genome_shotgun_sequence
R_NKLS02000011.1_55774232-55774822_Bos_taurus_breed_Hereford_isolate_L1_Dominette_01449_registration_number_42190680_chromosome_11_whole_genome_shotgun_sequence
R_PUFT02000011.1_55443762-55444352_Bos_indicus_x_Bos_taurus_breed_Angus_x_Brahman_F1_hybrid_chromosome_11_whole_genome_shotgun_sequence
R_VLPJ01000001.1_55430224-55430814_Bos_grunniens_x_Bos_taurus_isolate_Esperanza_chromosome_11_whole_genome_shotgun_sequence
R_PZYV01000004.1_55425272-55425866_Bubalus_bubalis_breed_Mediterranean_chromosome_12_whole_genome_shotgun_sequence
CABVSG010000083.1_47061863-47062458_Syncerus_caffer_genome_assembly_contig__Contig82_whole_genome_shotgun_sequence
R_PUFS02000011.1_55435075-55435669_Bos_indicus_x_Bos_taurus_breed_Angus_x_Brahman_F1_hybrid_chromosome_11_whole_genome_shotgun_sequence
VCEB01000003.1_48652250-48652863_Muntiacus_reevesi_isolate_UCam_UCB_Mr_chromosome_3_whole_genome_shotgun_sequence
R_VCEA01000001.1_1059041486-1059041886_Muntiacus_muntjak_isolate_UTSW_UCB_Mm_chromosome_1_whole_genome_shotgun_sequence
R_SJXR01000716.1_2301172-2301569_Przewalskium_albirostris_isolate_WLD-bcl_scaffold794_len4577192_cov129_whole_genome_shotgun_sequence
MKHE01000011.1_66640669-66641049_Cervus_elaphus_hippelaphus_isolate_Hungarian_chromosome_11_whole_genome_shotgun_sequence
0.03

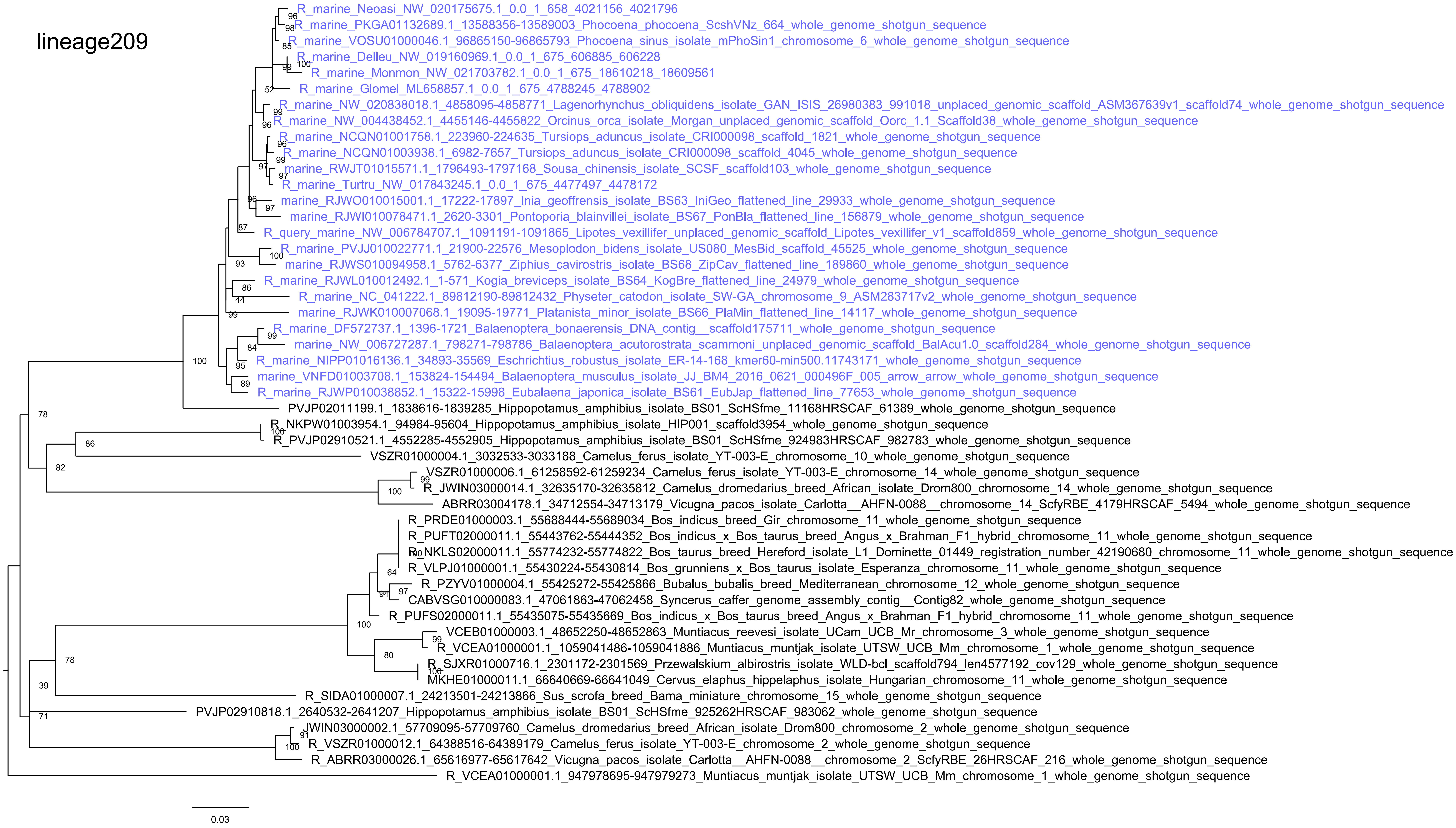
lineage209
R_marine_Neoasi_NW_020175675.1_0.0_1_658_4021156_4021796
R_marine_PKGA01132689.1_13588356-13589003_Phocoena_phocoena_ScshVNz_664_whole_genome_shotgun_sequence
R_marine_VOSU01000046.1_96865150-96865793_Phocoena_sinus_isolate_mPhoSin1_chromosome_6_whole_genome_shotgun_sequence
R_marine_Delleu_NW_019160969.1_0.0_1_675_606885_606228
R_marine_Monmon_NW_021703782.1_0.0_1_675_18610218_18609561
R_marine_Glomel_ML658857.1_0.0_1_675_4788245_4788902
R_marine_NW_020838018.1_4858095-4858771_Lagenorhynchus_obliquidens_isolate_GAN_ISIS_26980383_991018_unplaced_genomic_scaffold_ASM367639v1_scaffold74_whole_genome_shotgun_sequence
R_marine_NW_004438452.1_4455146-4455822_Orcinus_orca_isolate_Morgan_unplaced_genomic_scaffold_Oorc_1.1_Scaffold38_whole_genome_shotgun_sequence
R_marine_NCQN01001758.1_223960-224635_Tursiops_aduncus_isolate_CRI000098_scaffold_1821_whole_genome_shotgun_sequence
R_marine_NCQN01003938.1_6982-7657_Tursiops_aduncus_isolate_CRI000098_scaffold_4045_whole_genome_shotgun_sequence
marine_RWJT01015571.1_1796493-1797168_Sousa_chinensis_isolate_SCSF_scaffold103_whole_genome_shotgun_sequence
R_marine_Turtru_NW_017843245.1_0.0_1_675_4477497_4478172
marine_RJWO010015001.1_17222-17897_Inia_geoffrensis_isolate_BS63_IniGeo_flattened_line_29933_whole_genome_shotgun_sequence
marine_RJWI010078471.1_2620-3301_Pontoporia_blainvillei_isolate_BS67_PonBla_flattened_line_156879_whole_genome_shotgun_sequence
R_query_marine_NW_006784707.1_1091191-1091865_Lipotes_vexillifer_unplaced_genomic_scaffold_Lipotes_vexillifer_v1_scaffold859_whole_genome_shotgun_sequence
R_marine_PVJJ010022771.1_21900-22576_Mesoplodon_bidens_isolate_US080_MesBid_scaffold_45525_whole_genome_shotgun_sequence
marine_RJWS010094958.1_5762-6377_Ziphius_cavirostris_isolate_BS68_ZipCav_flattened_line_189860_whole_genome_shotgun_sequence
R_marine_RJWL010012492.1_1-571_Kogia_breviceps_isolate_BS64_KogBre_flattened_line_24979_whole_genome_shotgun_sequence
R_marine_NC_041222.1_89812190-89812432_Physeter_catodon_isolate_SW-GA_chromosome_9_ASM283717v2_whole_genome_shotgun_sequence
marine_RJWK010007068.1_19095-19771_Platanista_minor_isolate_BS66_PlaMin_flattened_line_14117_whole_genome_shotgun_sequence
R_marine_DF572737.1_1396-1721_Balaenoptera_bonaerensis_DNA_contig__scaffold175711_whole_genome_shotgun_sequence
marine_NW_006727287.1_798271-798786_Balaenoptera_acutorostrata_scammoni_unplaced_genomic_scaffold_BalAcu1.0_scaffold284_whole_genome_shotgun_sequence
R_marine_NIPP01016136.1_34893-35569_Eschrichtius_robustus_isolate_ER-14-168_kmer60-min500.11743171_whole_genome_shotgun_sequence
marine_VNFD01003708.1_153824-154494_Balaenoptera_musculus_isolate_JJ_BM4_2016_0621_000496F_005_arrow_arrow_whole_genome_shotgun_sequence
R_marine_RJWP010038852.1_15322-15998_Eubalaena_japonica_isolate_BS61_EubJap_flattened_line_77653_whole_genome_shotgun_sequence
PVJP02011199.1_1838616-1839285_Hippopotamus_amphibius_isolate_BS01_ScHSfme_11168HRSCAF_61389_whole_genome_shotgun_sequence
R_NKPW01003954.1_94984-95604_Hippopotamus_amphibius_isolate_HIP001_scaffold3954_whole_genome_shotgun_sequence
R_PVJP02910521.1_4552285-4552905_Hippopotamus_amphibius_isolate_BS01_ScHSfme_924983HRSCAF_982783_whole_genome_shotgun_sequence
VSZR01000004.1_3032533-3033188_Camelus_ferus_isolate_YT-003-E_chromosome_10_whole_genome_shotgun_sequence
VSZR01000006.1_61258592-61259234_Camelus_ferus_isolate_YT-003-E_chromosome_14_whole_genome_shotgun_sequence
R_JWIN03000014.1_32635170-32635812_Camelus_dromedarius_breed_African_isolate_Drom800_chromosome_14_whole_genome_shotgun_sequence
ABRR03004178.1_34712554-34713179_Vicugna_pacos_isolate_Carlotta__AHFN-0088__chromosome_14_ScfyRBE_4179HRSCAF_5494_whole_genome_shotgun_sequence
R_PRDE01000003.1_55688444-55689034_Bos_indicus_breed_Gir_chromosome_11_whole_genome_shotgun_sequence
R_PUFT02000011.1_55443762-55444352_Bos_indicus_x_Bos_taurus_breed_Angus_x_Brahman_F1_hybrid_chromosome_11_whole_genome_shotgun_sequence
R_NKLS02000011.1_55774232-55774822_Bos_taurus_breed_Hereford_isolate_L1_Dominette_01449_registration_number_42190680_chromosome_11_whole_genome_shotgun_sequence
R_VLPJ01000001.1_55430224-55430814_Bos_grunniens_x_Bos_taurus_isolate_Esperanza_chromosome_11_whole_genome_shotgun_sequence
R_PZYV01000004.1_55425272-55425866_Bubalus_bubalis_breed_Mediterranean_chromosome_12_whole_genome_shotgun_sequence
CABVSG010000083.1_47061863-47062458_Syncerus_caffer_genome_assembly_contig__Contig82_whole_genome_shotgun_sequence
R_PUFS02000011.1_55435075-55435669_Bos_indicus_x_Bos_taurus_breed_Angus_x_Brahman_F1_hybrid_chromosome_11_whole_genome_shotgun_sequence
VCEB01000003.1_48652250-48652863_Muntiacus_reevesi_isolate_UCam_UCB_Mr_chromosome_3_whole_genome_shotgun_sequence
R_VCEA01000001.1_1059041486-1059041886_Muntiacus_muntjak_isolate_UTSW_UCB_Mm_chromosome_1_whole_genome_shotgun_sequence
R_SJXR01000716.1_2301172-2301569_Przewalskium_albirostris_isolate_WLD-bcl_scaffold794_len4577192_cov129_whole_genome_shotgun_sequence
MKHE01000011.1_66640669-66641049_Cervus_elaphus_hippelaphus_isolate_Hungarian_chromosome_11_whole_genome_shotgun_sequence
R_SIDA01000007.1_24213501-24213866_Sus_scrofa_breed_Bama_miniature_chromosome_15_whole_genome_shotgun_sequence
PVJP02910818.1_2640532-2641207_Hippopotamus_amphibius_isolate_BS01_ScHSfme_925262HRSCAF_983062_whole_genome_shotgun_sequence
JWIN03000002.1_57709095-57709760_Camelus_dromedarius_breed_African_isolate_Drom800_chromosome_2_whole_genome_shotgun_sequence
R_VSZR01000012.1_64388516-64389179_Camelus_ferus_isolate_YT-003-E_chromosome_2_whole_genome_shotgun_sequence
R_ABRR03000026.1_65616977-65617642_Vicugna_pacos_isolate_Carlotta__AHFN-0088__chromosome_2_ScfyRBE_26HRSCAF_216_whole_genome_shotgun_sequence
R_VCEA01000001.1_947978695-947979273_Muntiacus_muntjak_isolate_UTSW_UCB_Mm_chromosome_1_whole_genome_shotgun_sequence
0.03

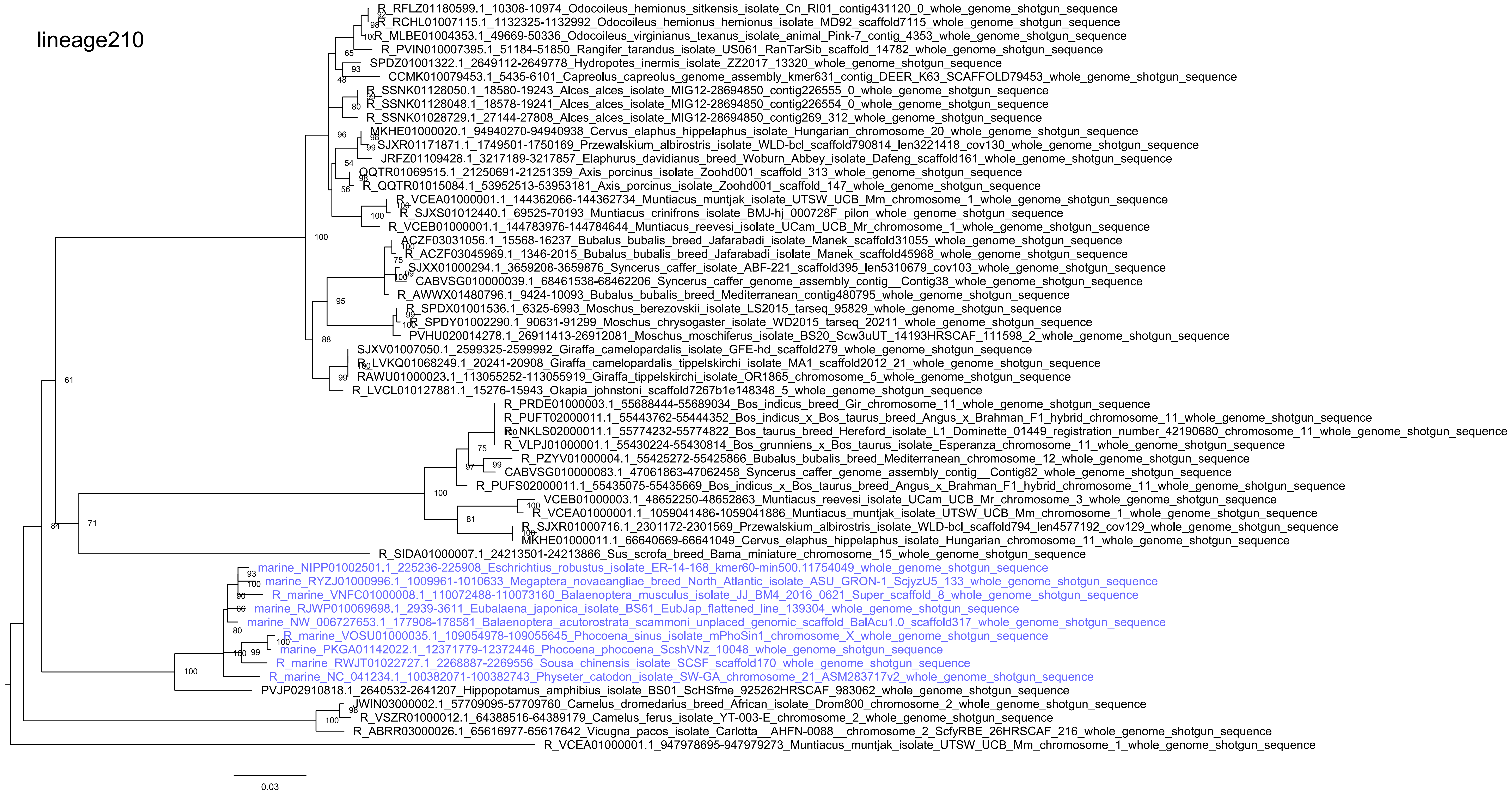
lineage210
R_RFLZ01180599.1_10308-10974_Odocoileus_hemionus_sitkensis_isolate_Cn_RI01_contig431120_0_whole_genome_shotgun_sequence
R_RCHL01007115.1_1132325-1132992_Odocoileus_hemionus_hemionus_isolate_MD92_scaffold7115_whole_genome_shotgun_sequence
R_MLBE01004353.1_49669-50336_Odocoileus_virginianus_texanus_isolate_animal_Pink-7_contig_4353_whole_genome_shotgun_sequence
R_PVIN010007395.1_51184-51850_Rangifer_tarandus_isolate_US061_RanTarSib_scaffold_14782_whole_genome_shotgun_sequence
SPDZ01001322.1_2649112-2649778_Hydropotes_inermis_isolate_ZZ2017_13320_whole_genome_shotgun_sequence
CCMK010079453.1_5435-6101_Capreolus_capreolus_genome_assembly_kmer631_contig_DEER_K63_SCAFFOLD79453_whole_genome_shotgun_sequence
R_SSNK01128050.1_18580-19243_Alces_alces_isolate_MIG12-28694850_contig226555_0_whole_genome_shotgun_sequence
R_SSNK01128048.1_18578-19241_Alces_alces_isolate_MIG12-28694850_contig226554_0_whole_genome_shotgun_sequence
R_SSNK01028729.1_27144-27808_Alces_alces_isolate_MIG12-28694850_contig269_312_whole_genome_shotgun_sequence
MKHE01000020.1_94940270-94940938_Cervus_elaphus_hippelaphus_isolate_Hungarian_chromosome_20_whole_genome_shotgun_sequence
SJXR01171871.1_1749501-1750169_Przewalskium_albirostris_isolate_WLD-bcl_scaffold790814_len3221418_cov130_whole_genome_shotgun_sequence
JRFZ01109428.1_3217189-3217857_Elaphurus_davidianus_breed_Woburn_Abbey_isolate_Dafeng_scaffold161_whole_genome_shotgun_sequence
QQTR01069515.1_21250691-21251359_Axis_porcinus_isolate_Zoohd001_scaffold_313_whole_genome_shotgun_sequence
R_QQTR01015084.1_53952513-53953181_Axis_porcinus_isolate_Zoohd001_scaffold_147_whole_genome_shotgun_sequence
R_VCEA01000001.1_144362066-144362734_Muntiacus_muntjak_isolate_UTSW_UCB_Mm_chromosome_1_whole_genome_shotgun_sequence
R_SJXS01012440.1_69525-70193_Muntiacus_crinifrons_isolate_BMJ-hj_000728F_pilon_whole_genome_shotgun_sequence
R_VCEB01000001.1_144783976-144784644_Muntiacus_reevesi_isolate_UCam_UCB_Mr_chromosome_1_whole_genome_shotgun_sequence
ACZF03031056.1_15568-16237_Bubalus_bubalis_breed_Jafarabadi_isolate_Manek_scaffold31055_whole_genome_shotgun_sequence
R_ACZF03045969.1_1346-2015_Bubalus_bubalis_breed_Jafarabadi_isolate_Manek_scaffold45968_whole_genome_shotgun_sequence
SJXX01000294.1_3659208-3659876_Syncerus_caffer_isolate_ABF-221_scaffold395_len5310679_cov103_whole_genome_shotgun_sequence
CABVSG010000039.1_68461538-68462206_Syncerus_caffer_genome_assembly_contig__Contig38_whole_genome_shotgun_sequence
R_AWWX01480796.1_9424-10093_Bubalus_bubalis_breed_Mediterranean_contig480795_whole_genome_shotgun_sequence
R_SPDX01001536.1_6325-6993_Moschus_berezovskii_isolate_LS2015_tarseq_95829_whole_genome_shotgun_sequence
R_SPDY01002290.1_90631-91299_Moschus_chrysogaster_isolate_WD2015_tarseq_20211_whole_genome_shotgun_sequence
PVHU020014278.1_26911413-26912081_Moschus_moschiferus_isolate_BS20_Scw3uUT_14193HRSCAF_111598_2_whole_genome_shotgun_sequence
SJXV01007050.1_2599325-2599992_Giraffa_camelopardalis_isolate_GFE-hd_scaffold279_whole_genome_shotgun_sequence
R_LVKQ01068249.1_20241-20908_Giraffa_camelopardalis_tippelskirchi_isolate_MA1_scaffold2012_21_whole_genome_shotgun_sequence
RAWU01000023.1_113055252-113055919_Giraffa_tippelskirchi_isolate_OR1865_chromosome_5_whole_genome_shotgun_sequence
R_LVCL010127881.1_15276-15943_Okapia_johnstoni_scaffold7267b1e148348_5_whole_genome_shotgun_sequence
R_PRDE01000003.1_55688444-55689034_Bos_indicus_breed_Gir_chromosome_11_whole_genome_shotgun_sequence
R_PUFT02000011.1_55443762-55444352_Bos_indicus_x_Bos_taurus_breed_Angus_x_Brahman_F1_hybrid_chromosome_11_whole_genome_shotgun_sequence
R_NKLS02000011.1_55774232-55774822_Bos_taurus_breed_Hereford_isolate_L1_Dominette_01449_registration_number_42190680_chromosome_11_whole_genome_shotgun_sequence
R_VLPJ01000001.1_55430224-55430814_Bos_grunniens_x_Bos_taurus_isolate_Esperanza_chromosome_11_whole_genome_shotgun_sequence
R_PZYV01000004.1_55425272-55425866_Bubalus_bubalis_breed_Mediterranean_chromosome_12_whole_genome_shotgun_sequence
CABVSG010000083.1_47061863-47062458_Syncerus_caffer_genome_assembly_contig__Contig82_whole_genome_shotgun_sequence
R_PUFS02000011.1_55435075-55435669_Bos_indicus_x_Bos_taurus_breed_Angus_x_Brahman_F1_hybrid_chromosome_11_whole_genome_shotgun_sequence
VCEB01000003.1_48652250-48652863_Muntiacus_reevesi_isolate_UCam_UCB_Mr_chromosome_3_whole_genome_shotgun_sequence
R_VCEA01000001.1_1059041486-1059041886_Muntiacus_muntjak_isolate_UTSW_UCB_Mm_chromosome_1_whole_genome_shotgun_sequence
R_SJXR01000716.1_2301172-2301569_Przewalskium_albirostris_isolate_WLD-bcl_scaffold794_len4577192_cov129_whole_genome_shotgun_sequence
MKHE01000011.1_66640669-66641049_Cervus_elaphus_hippelaphus_isolate_Hungarian_chromosome_11_whole_genome_shotgun_sequence
R_SIDA01000007.1_24213501-24213866_Sus_scrofa_breed_Bama_miniature_chromosome_15_whole_genome_shotgun_sequence
marine_NIPP01002501.1_225236-225908_Eschrichtius_robustus_isolate_ER-14-168_kmer60-min500.11754049_whole_genome_shotgun_sequence
marine_RYZJ01000996.1_1009961-1010633_Megaptera_novaeangliae_breed_North_Atlantic_isolate_ASU_GRON-1_ScjyzU5_133_whole_genome_shotgun_sequence
R_marine_VNFC01000008.1_110072488-110073160_Balaenoptera_musculus_isolate_JJ_BM4_2016_0621_Super_scaffold_8_whole_genome_shotgun_sequence
marine_RJWP010069698.1_2939-3611_Eubalaena_japonica_isolate_BS61_EubJap_flattened_line_139304_whole_genome_shotgun_sequence
marine_NW_006727653.1_177908-178581_Balaenoptera_acutorostrata_scammoni_unplaced_genomic_scaffold_BalAcu1.0_scaffold317_whole_genome_shotgun_sequence
R_marine_VOSU01000035.1_109054978-109055645_Phocoena_sinus_isolate_mPhoSin1_chromosome_X_whole_genome_shotgun_sequence
marine_PKGA01142022.1_12371779-12372446_Phocoena_phocoena_ScshVNz_10048_whole_genome_shotgun_sequence
R_marine_RWJT01022727.1_2268887-2269556_Sousa_chinensis_isolate_SCSF_scaffold170_whole_genome_shotgun_sequence
R_marine_NC_041234.1_100382071-100382743_Physeter_catodon_isolate_SW-GA_chromosome_21_ASM283717v2_whole_genome_shotgun_sequence
PVJP02910818.1_2640532-2641207_Hippopotamus_amphibius_isolate_BS01_ScHSfme_925262HRSCAF_983062_whole_genome_shotgun_sequence
JWIN03000002.1_57709095-57709760_Camelus_dromedarius_breed_African_isolate_Drom800_chromosome_2_whole_genome_shotgun_sequence
R_VSZR01000012.1_64388516-64389179_Camelus_ferus_isolate_YT-003-E_chromosome_2_whole_genome_shotgun_sequence
R_ABRR03000026.1_65616977-65617642_Vicugna_pacos_isolate_Carlotta__AHFN-0088__chromosome_2_ScfyRBE_26HRSCAF_216_whole_genome_shotgun_sequence
R_VCEA01000001.1_947978695-947979273_Muntiacus_muntjak_isolate_UTSW_UCB_Mm_chromosome_1_whole_genome_shotgun_sequence
0.03

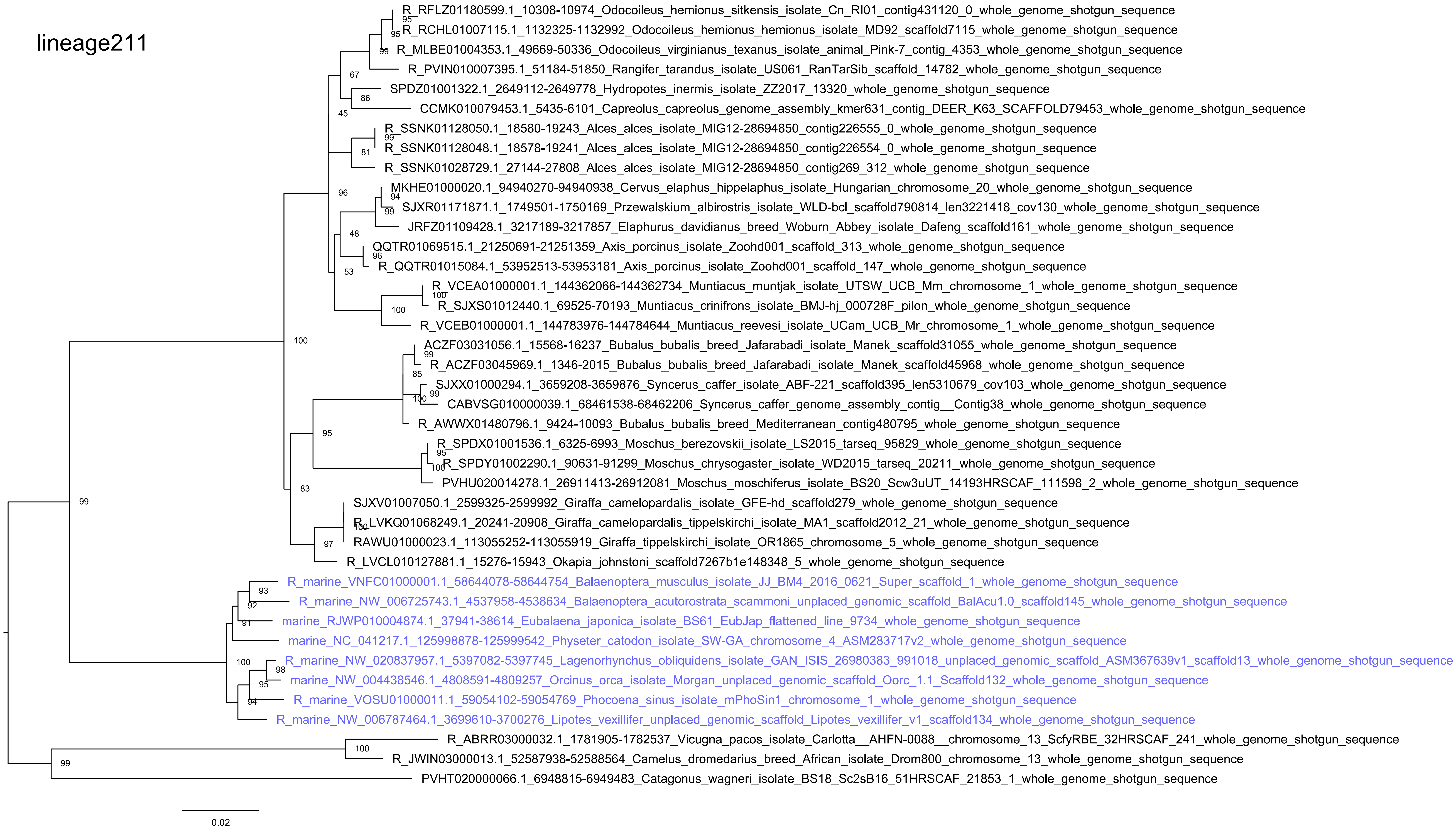
lineage211
R_RFLZ01180599.1_10308-10974_Odocoileus_hemionus_sitkensis_isolate_Cn_RI01_contig431120_0_whole_genome_shotgun_sequence
R_RCHL01007115.1_1132325-1132992_Odocoileus_hemionus_hemionus_isolate_MD92_scaffold7115_whole_genome_shotgun_sequence
R_MLBE01004353.1_49669-50336_Odocoileus_virginianus_texanus_isolate_animal_Pink-7_contig_4353_whole_genome_shotgun_sequence
R_PVIN010007395.1_51184-51850_Rangifer_tarandus_isolate_US061_RanTarSib_scaffold_14782_whole_genome_shotgun_sequence
SPDZ01001322.1_2649112-2649778_Hydropotes_inermis_isolate_ZZ2017_13320_whole_genome_shotgun_sequence
CCMK010079453.1_5435-6101_Capreolus_capreolus_genome_assembly_kmer631_contig_DEER_K63_SCAFFOLD79453_whole_genome_shotgun_sequence
R_SSNK01128050.1_18580-19243_Alces_alces_isolate_MIG12-28694850_contig226555_0_whole_genome_shotgun_sequence
R_SSNK01128048.1_18578-19241_Alces_alces_isolate_MIG12-28694850_contig226554_0_whole_genome_shotgun_sequence
R_SSNK01028729.1_27144-27808_Alces_alces_isolate_MIG12-28694850_contig269_312_whole_genome_shotgun_sequence
MKHE01000020.1_94940270-94940938_Cervus_elaphus_hippelaphus_isolate_Hungarian_chromosome_20_whole_genome_shotgun_sequence
SJXR01171871.1_1749501-1750169_Przewalskium_albirostris_isolate_WLD-bcl_scaffold790814_len3221418_cov130_whole_genome_shotgun_sequence
JRFZ01109428.1_3217189-3217857_Elaphurus_davidianus_breed_Woburn_Abbey_isolate_Dafeng_scaffold161_whole_genome_shotgun_sequence
QQTR01069515.1_21250691-21251359_Axis_porcinus_isolate_Zoohd001_scaffold_313_whole_genome_shotgun_sequence
R_QQTR01015084.1_53952513-53953181_Axis_porcinus_isolate_Zoohd001_scaffold_147_whole_genome_shotgun_sequence
R_VCEA01000001.1_144362066-144362734_Muntiacus_muntjak_isolate_UTSW_UCB_Mm_chromosome_1_whole_genome_shotgun_sequence
R_SJXS01012440.1_69525-70193_Muntiacus_crinifrons_isolate_BMJ-hj_000728F_pilon_whole_genome_shotgun_sequence
R_VCEB01000001.1_144783976-144784644_Muntiacus_reevesi_isolate_UCam_UCB_Mr_chromosome_1_whole_genome_shotgun_sequence
ACZF03031056.1_15568-16237_Bubalus_bubalis_breed_Jafarabadi_isolate_Manek_scaffold31055_whole_genome_shotgun_sequence
R_ACZF03045969.1_1346-2015_Bubalus_bubalis_breed_Jafarabadi_isolate_Manek_scaffold45968_whole_genome_shotgun_sequence
SJXX01000294.1_3659208-3659876_Syncerus_caffer_isolate_ABF-221_scaffold395_len5310679_cov103_whole_genome_shotgun_sequence
CABVSG010000039.1_68461538-68462206_Syncerus_caffer_genome_assembly_contig__Contig38_whole_genome_shotgun_sequence
R_AWWX01480796.1_9424-10093_Bubalus_bubalis_breed_Mediterranean_contig480795_whole_genome_shotgun_sequence
R_SPDX01001536.1_6325-6993_Moschus_berezovskii_isolate_LS2015_tarseq_95829_whole_genome_shotgun_sequence
R_SPDY01002290.1_90631-91299_Moschus_chrysogaster_isolate_WD2015_tarseq_20211_whole_genome_shotgun_sequence
PVHU020014278.1_26911413-26912081_Moschus_moschiferus_isolate_BS20_Scw3uUT_14193HRSCAF_111598_2_whole_genome_shotgun_sequence
SJXV01007050.1_2599325-2599992_Giraffa_camelopardalis_isolate_GFE-hd_scaffold279_whole_genome_shotgun_sequence
R_LVKQ01068249.1_20241-20908_Giraffa_camelopardalis_tippelskirchi_isolate_MA1_scaffold2012_21_whole_genome_shotgun_sequence
RAWU01000023.1_113055252-113055919_Giraffa_tippelskirchi_isolate_OR1865_chromosome_5_whole_genome_shotgun_sequence
R_LVCL010127881.1_15276-15943_Okapia_johnstoni_scaffold7267b1e148348_5_whole_genome_shotgun_sequence
R_marine_VNFC01000001.1_58644078-58644754_Balaenoptera_musculus_isolate_JJ_BM4_2016_0621_Super_scaffold_1_whole_genome_shotgun_sequence
R_marine_NW_006725743.1_4537958-4538634_Balaenoptera_acutorostrata_scammoni_unplaced_genomic_scaffold_BalAcu1.0_scaffold145_whole_genome_shotgun_sequence
marine_RJWP010004874.1_37941-38614_Eubalaena_japonica_isolate_BS61_EubJap_flattened_line_9734_whole_genome_shotgun_sequence
marine_NC_041217.1_125998878-125999542_Physeter_catodon_isolate_SW-GA_chromosome_4_ASM283717v2_whole_genome_shotgun_sequence
R_marine_NW_020837957.1_5397082-5397745_Lagenorhynchus_obliquidens_isolate_GAN_ISIS_26980383_991018_unplaced_genomic_scaffold_ASM367639v1_scaffold13_whole_genome_shotgun_sequence
marine_NW_004438546.1_4808591-4809257_Orcinus_orca_isolate_Morgan_unplaced_genomic_scaffold_Oorc_1.1_Scaffold132_whole_genome_shotgun_sequence
R_marine_VOSU01000011.1_59054102-59054769_Phocoena_sinus_isolate_mPhoSin1_chromosome_1_whole_genome_shotgun_sequence
R_marine_NW_006787464.1_3699610-3700276_Lipotes_vexillifer_unplaced_genomic_scaffold_Lipotes_vexillifer_v1_scaffold134_whole_genome_shotgun_sequence
R_ABRR03000032.1_1781905-1782537_Vicugna_pacos_isolate_Carlotta__AHFN-0088__chromosome_13_ScfyRBE_32HRSCAF_241_whole_genome_shotgun_sequence
R_JWIN03000013.1_52587938-52588564_Camelus_dromedarius_breed_African_isolate_Drom800_chromosome_13_whole_genome_shotgun_sequence
PVHT020000066.1_6948815-6949483_Catagonus_wagneri_isolate_BS18_Sc2sB16_51HRSCAF_21853_1_whole_genome_shotgun_sequence
0.02

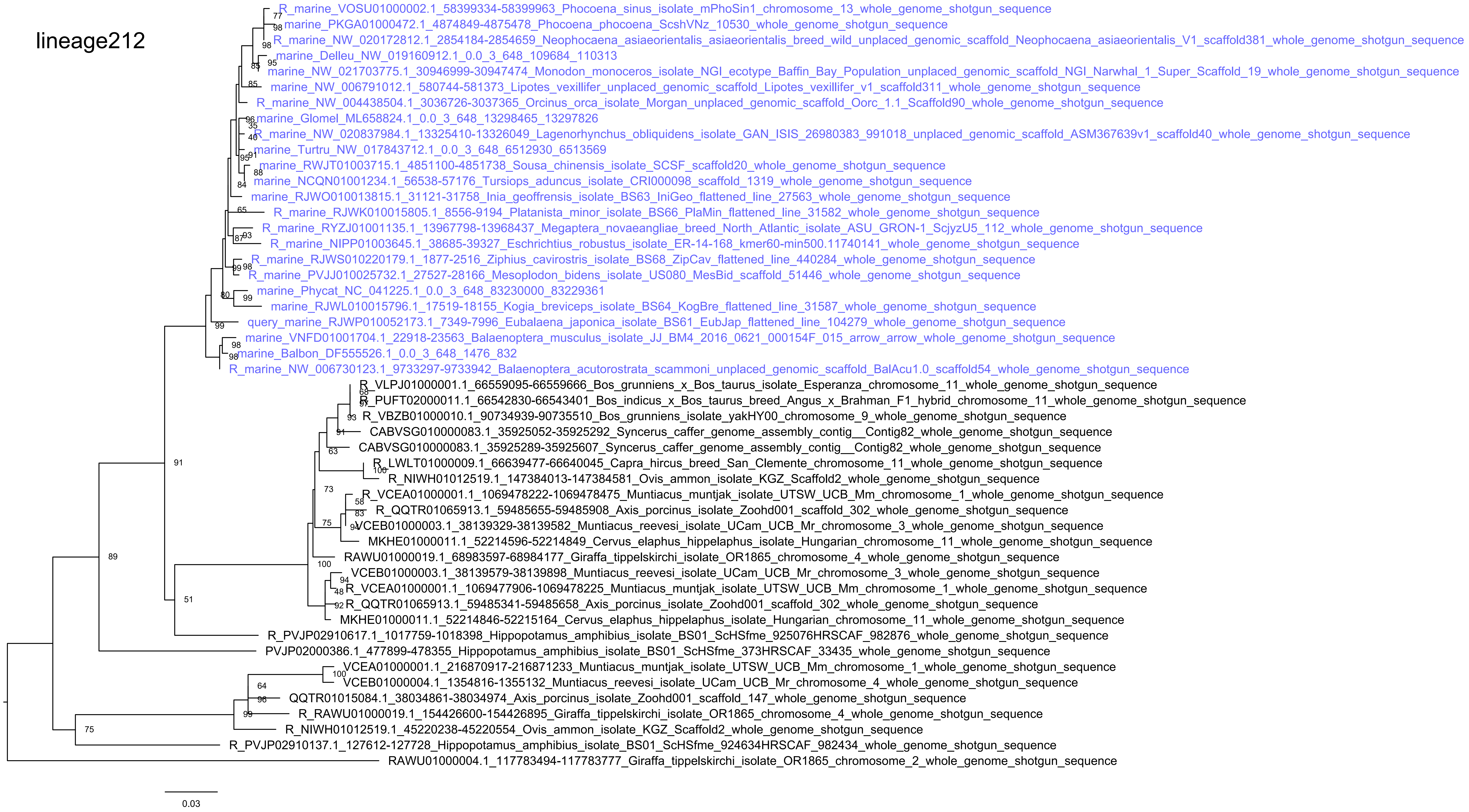
lineage212
R_marine_VOSU01000002.1_58399334-58399963_Phocoena_sinus_isolate_mPhoSin1_chromosome_13_whole_genome_shotgun_sequence
marine_PKGA01000472.1_4874849-4875478_Phocoena_phocoena_ScshVNz_10530_whole_genome_shotgun_sequence
R_marine_NW_020172812.1_2854184-2854659_Neophocaena_asiaeorientalis_asiaeorientalis_breed_wild_unplaced_genomic_scaffold_Neophocaena_asiaeorientalis_V1_scaffold381_whole_genome_shotgun_sequence
marine_Delleu_NW_019160912.1_0.0_3_648_109684_110313
marine_NW_021703775.1_30946999-30947474_Monodon_monoceros_isolate_NGI_ecotype_Baffin_Bay_Population_unplaced_genomic_scaffold_NGI_Narwhal_1_Super_Scaffold_19_whole_genome_shotgun_sequence
marine_NW_006791012.1_580744-581373_Lipotes_vexillifer_unplaced_genomic_scaffold_Lipotes_vexillifer_v1_scaffold311_whole_genome_shotgun_sequence
R_marine_NW_004438504.1_3036726-3037365_Orcinus_orca_isolate_Morgan_unplaced_genomic_scaffold_Oorc_1.1_Scaffold90_whole_genome_shotgun_sequence
marine_Glomel_ML658824.1_0.0_3_648_13298465_13297826
R_marine_NW_020837984.1_13325410-13326049_Lagenorhynchus_obliquidens_isolate_GAN_ISIS_26980383_991018_unplaced_genomic_scaffold_ASM367639v1_scaffold40_whole_genome_shotgun_sequence
marine_Turtru_NW_017843712.1_0.0_3_648_6512930_6513569
marine_RWJT01003715.1_4851100-4851738_Sousa_chinensis_isolate_SCSF_scaffold20_whole_genome_shotgun_sequence
marine_NCQN01001234.1_56538-57176_Tursiops_aduncus_isolate_CRI000098_scaffold_1319_whole_genome_shotgun_sequence
marine_RJWO010013815.1_31121-31758_Inia_geoffrensis_isolate_BS63_IniGeo_flattened_line_27563_whole_genome_shotgun_sequence
R_marine_RJWK010015805.1_8556-9194_Platanista_minor_isolate_BS66_PlaMin_flattened_line_31582_whole_genome_shotgun_sequence
R_marine_RYZJ01001135.1_13967798-13968437_Megaptera_novaeangliae_breed_North_Atlantic_isolate_ASU_GRON-1_ScjyzU5_112_whole_genome_shotgun_sequence
R_marine_NIPP01003645.1_38685-39327_Eschrichtius_robustus_isolate_ER-14-168_kmer60-min500.11740141_whole_genome_shotgun_sequence
R_marine_RJWS010220179.1_1877-2516_Ziphius_cavirostris_isolate_BS68_ZipCav_flattened_line_440284_whole_genome_shotgun_sequence
R_marine_PVJJ010025732.1_27527-28166_Mesoplodon_bidens_isolate_US080_MesBid_scaffold_51446_whole_genome_shotgun_sequence
marine_Phycat_NC_041225.1_0.0_3_648_83230000_83229361
marine_RJWL010015796.1_17519-18155_Kogia_breviceps_isolate_BS64_KogBre_flattened_line_31587_whole_genome_shotgun_sequence
query_marine_RJWP010052173.1_7349-7996_Eubalaena_japonica_isolate_BS61_EubJap_flattened_line_104279_whole_genome_shotgun_sequence
marine_VNFD01001704.1_22918-23563_Balaenoptera_musculus_isolate_JJ_BM4_2016_0621_000154F_015_arrow_arrow_whole_genome_shotgun_sequence
marine_Balbon_DF555526.1_0.0_3_648_1476_832
R_marine_NW_006730123.1_9733297-9733942_Balaenoptera_acutorostrata_scammoni_unplaced_genomic_scaffold_BalAcu1.0_scaffold54_whole_genome_shotgun_sequence
R_VLPJ01000001.1_66559095-66559666_Bos_grunniens_x_Bos_taurus_isolate_Esperanza_chromosome_11_whole_genome_shotgun_sequence
R_PUFT02000011.1_66542830-66543401_Bos_indicus_x_Bos_taurus_breed_Angus_x_Brahman_F1_hybrid_chromosome_11_whole_genome_shotgun_sequence
R_VBZB01000010.1_90734939-90735510_Bos_grunniens_isolate_yakHY00_chromosome_9_whole_genome_shotgun_sequence
CABVSG010000083.1_35925052-35925292_Syncerus_caffer_genome_assembly_contig__Contig82_whole_genome_shotgun_sequence
CABVSG010000083.1_35925289-35925607_Syncerus_caffer_genome_assembly_contig__Contig82_whole_genome_shotgun_sequence
R_LWLT01000009.1_66639477-66640045_Capra_hircus_breed_San_Clemente_chromosome_11_whole_genome_shotgun_sequence
R_NIWH01012519.1_147384013-147384581_Ovis_ammon_isolate_KGZ_Scaffold2_whole_genome_shotgun_sequence
R_VCEA01000001.1_1069478222-1069478475_Muntiacus_muntjak_isolate_UTSW_UCB_Mm_chromosome_1_whole_genome_shotgun_sequence
R_QQTR01065913.1_59485655-59485908_Axis_porcinus_isolate_Zoohd001_scaffold_302_whole_genome_shotgun_sequence
VCEB01000003.1_38139329-38139582_Muntiacus_reevesi_isolate_UCam_UCB_Mr_chromosome_3_whole_genome_shotgun_sequence
MKHE01000011.1_52214596-52214849_Cervus_elaphus_hippelaphus_isolate_Hungarian_chromosome_11_whole_genome_shotgun_sequence
RAWU01000019.1_68983597-68984177_Giraffa_tippelskirchi_isolate_OR1865_chromosome_4_whole_genome_shotgun_sequence
VCEB01000003.1_38139579-38139898_Muntiacus_reevesi_isolate_UCam_UCB_Mr_chromosome_3_whole_genome_shotgun_sequence
R_VCEA01000001.1_1069477906-1069478225_Muntiacus_muntjak_isolate_UTSW_UCB_Mm_chromosome_1_whole_genome_shotgun_sequence
R_QQTR01065913.1_59485341-59485658_Axis_porcinus_isolate_Zoohd001_scaffold_302_whole_genome_shotgun_sequence
MKHE01000011.1_52214846-52215164_Cervus_elaphus_hippelaphus_isolate_Hungarian_chromosome_11_whole_genome_shotgun_sequence
R_PVJP02910617.1_1017759-1018398_Hippopotamus_amphibius_isolate_BS01_ScHSfme_925076HRSCAF_982876_whole_genome_shotgun_sequence
PVJP02000386.1_477899-478355_Hippopotamus_amphibius_isolate_BS01_ScHSfme_373HRSCAF_33435_whole_genome_shotgun_sequence
VCEA01000001.1_216870917-216871233_Muntiacus_muntjak_isolate_UTSW_UCB_Mm_chromosome_1_whole_genome_shotgun_sequence
VCEB01000004.1_1354816-1355132_Muntiacus_reevesi_isolate_UCam_UCB_Mr_chromosome_4_whole_genome_shotgun_sequence
QQTR01015084.1_38034861-38034974_Axis_porcinus_isolate_Zoohd001_scaffold_147_whole_genome_shotgun_sequence
R_RAWU01000019.1_154426600-154426895_Giraffa_tippelskirchi_isolate_OR1865_chromosome_4_whole_genome_shotgun_sequence
R_NIWH01012519.1_45220238-45220554_Ovis_ammon_isolate_KGZ_Scaffold2_whole_genome_shotgun_sequence
R_PVJP02910137.1_127612-127728_Hippopotamus_amphibius_isolate_BS01_ScHSfme_924634HRSCAF_982434_whole_genome_shotgun_sequence
RAWU01000004.1_117783494-117783777_Giraffa_tippelskirchi_isolate_OR1865_chromosome_2_whole_genome_shotgun_sequence
0.03

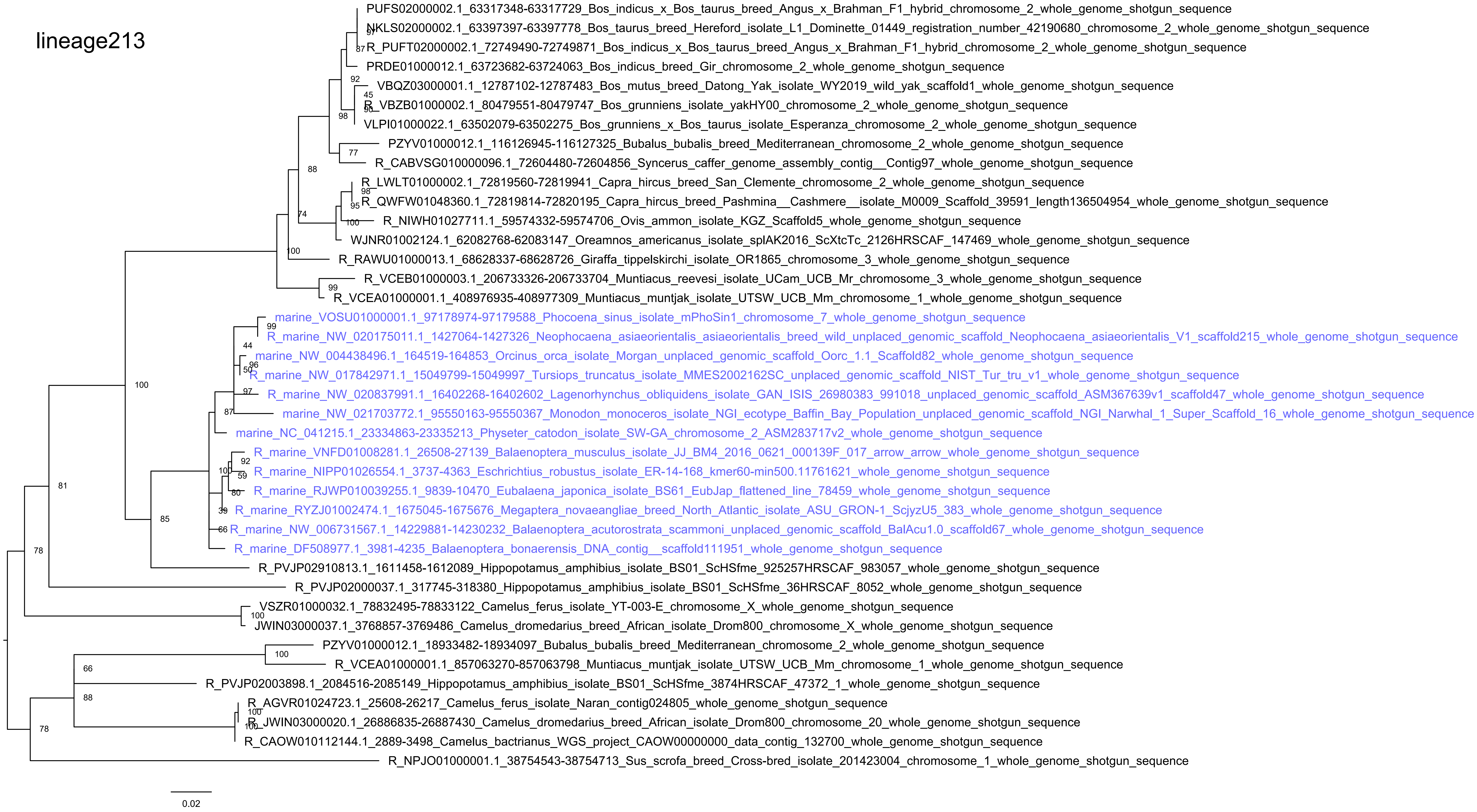
lineage213
PUFS02000002.1_63317348-63317729_Bos_indicus_x_Bos_taurus_breed_Angus_x_Brahman_F1_hybrid_chromosome_2_whole_genome_shotgun_sequence
NKLS02000002.1_63397397-63397778_Bos_taurus_breed_Hereford_isolate_L1_Dominette_01449_registration_number_42190680_chromosome_2_whole_genome_shotgun_sequence
R_PUFT02000002.1_72749490-72749871_Bos_indicus_x_Bos_taurus_breed_Angus_x_Brahman_F1_hybrid_chromosome_2_whole_genome_shotgun_sequence
PRDE01000012.1_63723682-63724063_Bos_indicus_breed_Gir_chromosome_2_whole_genome_shotgun_sequence
VBQZ03000001.1_12787102-12787483_Bos_mutus_breed_Datong_Yak_isolate_WY2019_wild_yak_scaffold1_whole_genome_shotgun_sequence
R_VBZB01000002.1_80479551-80479747_Bos_grunniens_isolate_yakHY00_chromosome_2_whole_genome_shotgun_sequence
VLPI01000022.1_63502079-63502275_Bos_grunniens_x_Bos_taurus_isolate_Esperanza_chromosome_2_whole_genome_shotgun_sequence
PZYV01000012.1_116126945-116127325_Bubalus_bubalis_breed_Mediterranean_chromosome_2_whole_genome_shotgun_sequence
R_CABVSG010000096.1_72604480-72604856_Syncerus_caffer_genome_assembly_contig__Contig97_whole_genome_shotgun_sequence
R_LWLT01000002.1_72819560-72819941_Capra_hircus_breed_San_Clemente_chromosome_2_whole_genome_shotgun_sequence
R_QWFW01048360.1_72819814-72820195_Capra_hircus_breed_Pashmina__Cashmere__isolate_M0009_Scaffold_39591_length136504954_whole_genome_shotgun_sequence
R_NIWH01027711.1_59574332-59574706_Ovis_ammon_isolate_KGZ_Scaffold5_whole_genome_shotgun_sequence
WJNR01002124.1_62082768-62083147_Oreamnos_americanus_isolate_splAK2016_ScXtcTc_2126HRSCAF_147469_whole_genome_shotgun_sequence
R_RAWU01000013.1_68628337-68628726_Giraffa_tippelskirchi_isolate_OR1865_chromosome_3_whole_genome_shotgun_sequence
R_VCEB01000003.1_206733326-206733704_Muntiacus_reevesi_isolate_UCam_UCB_Mr_chromosome_3_whole_genome_shotgun_sequence
R_VCEA01000001.1_408976935-408977309_Muntiacus_muntjak_isolate_UTSW_UCB_Mm_chromosome_1_whole_genome_shotgun_sequence
marine_VOSU01000001.1_97178974-97179588_Phocoena_sinus_isolate_mPhoSin1_chromosome_7_whole_genome_shotgun_sequence
R_marine_NW_020175011.1_1427064-1427326_Neophocaena_asiaeorientalis_asiaeorientalis_breed_wild_unplaced_genomic_scaffold_Neophocaena_asiaeorientalis_V1_scaffold215_whole_genome_shotgun_sequence
marine_NW_004438496.1_164519-164853_Orcinus_orca_isolate_Morgan_unplaced_genomic_scaffold_Oorc_1.1_Scaffold82_whole_genome_shotgun_sequence
R_marine_NW_017842971.1_15049799-15049997_Tursiops_truncatus_isolate_MMES2002162SC_unplaced_genomic_scaffold_NIST_Tur_tru_v1_whole_genome_shotgun_sequence
R_marine_NW_020837991.1_16402268-16402602_Lagenorhynchus_obliquidens_isolate_GAN_ISIS_26980383_991018_unplaced_genomic_scaffold_ASM367639v1_scaffold47_whole_genome_shotgun_sequence
marine_NW_021703772.1_95550163-95550367_Monodon_monoceros_isolate_NGI_ecotype_Baffin_Bay_Population_unplaced_genomic_scaffold_NGI_Narwhal_1_Super_Scaffold_16_whole_genome_shotgun_sequence
marine_NC_041215.1_23334863-23335213_Physeter_catodon_isolate_SW-GA_chromosome_2_ASM283717v2_whole_genome_shotgun_sequence
R_marine_VNFD01008281.1_26508-27139_Balaenoptera_musculus_isolate_JJ_BM4_2016_0621_000139F_017_arrow_arrow_whole_genome_shotgun_sequence
R_marine_NIPP01026554.1_3737-4363_Eschrichtius_robustus_isolate_ER-14-168_kmer60-min500.11761621_whole_genome_shotgun_sequence
R_marine_RJWP010039255.1_9839-10470_Eubalaena_japonica_isolate_BS61_EubJap_flattened_line_78459_whole_genome_shotgun_sequence
R_marine_RYZJ01002474.1_1675045-1675676_Megaptera_novaeangliae_breed_North_Atlantic_isolate_ASU_GRON-1_ScjyzU5_383_whole_genome_shotgun_sequence
R_marine_NW_006731567.1_14229881-14230232_Balaenoptera_acutorostrata_scammoni_unplaced_genomic_scaffold_BalAcu1.0_scaffold67_whole_genome_shotgun_sequence
R_marine_DF508977.1_3981-4235_Balaenoptera_bonaerensis_DNA_contig__scaffold111951_whole_genome_shotgun_sequence
R_PVJP02910813.1_1611458-1612089_Hippopotamus_amphibius_isolate_BS01_ScHSfme_925257HRSCAF_983057_whole_genome_shotgun_sequence
R_PVJP02000037.1_317745-318380_Hippopotamus_amphibius_isolate_BS01_ScHSfme_36HRSCAF_8052_whole_genome_shotgun_sequence
VSZR01000032.1_78832495-78833122_Camelus_ferus_isolate_YT-003-E_chromosome_X_whole_genome_shotgun_sequence
JWIN03000037.1_3768857-3769486_Camelus_dromedarius_breed_African_isolate_Drom800_chromosome_X_whole_genome_shotgun_sequence
PZYV01000012.1_18933482-18934097_Bubalus_bubalis_breed_Mediterranean_chromosome_2_whole_genome_shotgun_sequence
R_VCEA01000001.1_857063270-857063798_Muntiacus_muntjak_isolate_UTSW_UCB_Mm_chromosome_1_whole_genome_shotgun_sequence
R_PVJP02003898.1_2084516-2085149_Hippopotamus_amphibius_isolate_BS01_ScHSfme_3874HRSCAF_47372_1_whole_genome_shotgun_sequence
R_AGVR01024723.1_25608-26217_Camelus_ferus_isolate_Naran_contig024805_whole_genome_shotgun_sequence
R_JWIN03000020.1_26886835-26887430_Camelus_dromedarius_breed_African_isolate_Drom800_chromosome_20_whole_genome_shotgun_sequence
R_CAOW010112144.1_2889-3498_Camelus_bactrianus_WGS_project_CAOW00000000_data_contig_132700_whole_genome_shotgun_sequence
R_NPJO01000001.1_38754543-38754713_Sus_scrofa_breed_Cross-bred_isolate_201423004_chromosome_1_whole_genome_shotgun_sequence
0.02

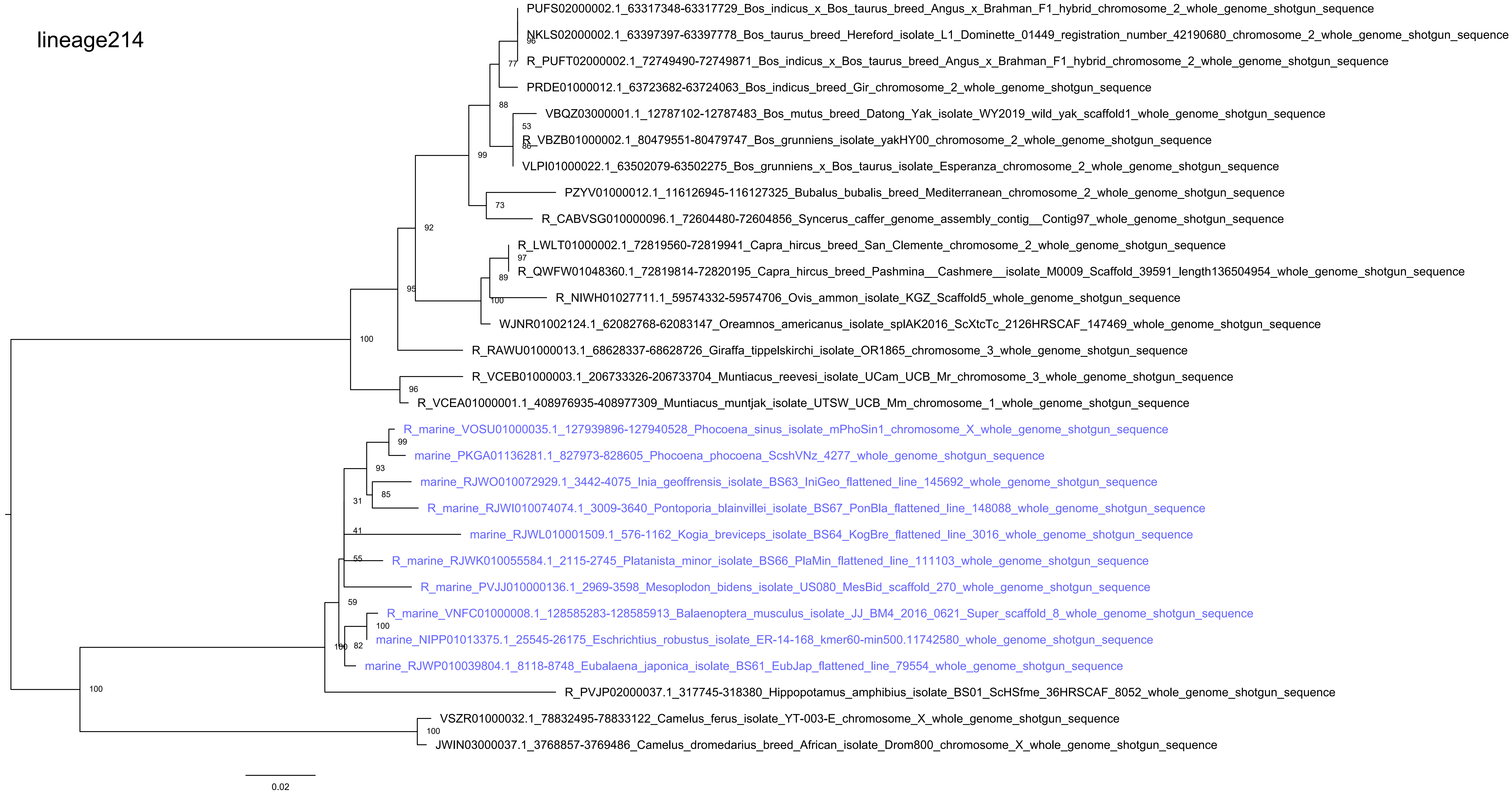
lineage214
PUFS02000002.1_63317348-63317729_Bos_indicus_x_Bos_taurus_breed_Angus_x_Brahman_F1_hybrid_chromosome_2_whole_genome_shotgun_sequence
NKLS02000002.1_63397397-63397778_Bos_taurus_breed_Hereford_isolate_L1_Dominette_01449_registration_number_42190680_chromosome_2_whole_genome_shotgun_sequence
R_PUFT02000002.1_72749490-72749871_Bos_indicus_x_Bos_taurus_breed_Angus_x_Brahman_F1_hybrid_chromosome_2_whole_genome_shotgun_sequence
PRDE01000012.1_63723682-63724063_Bos_indicus_breed_Gir_chromosome_2_whole_genome_shotgun_sequence
VBQZ03000001.1_12787102-12787483_Bos_mutus_breed_Datong_Yak_isolate_WY2019_wild_yak_scaffold1_whole_genome_shotgun_sequence
R_VBZB01000002.1_80479551-80479747_Bos_grunniens_isolate_yakHY00_chromosome_2_whole_genome_shotgun_sequence
VLPI01000022.1_63502079-63502275_Bos_grunniens_x_Bos_taurus_isolate_Esperanza_chromosome_2_whole_genome_shotgun_sequence
PZYV01000012.1_116126945-116127325_Bubalus_bubalis_breed_Mediterranean_chromosome_2_whole_genome_shotgun_sequence
R_CABVSG010000096.1_72604480-72604856_Syncerus_caffer_genome_assembly_contig__Contig97_whole_genome_shotgun_sequence
R_LWLT01000002.1_72819560-72819941_Capra_hircus_breed_San_Clemente_chromosome_2_whole_genome_shotgun_sequence
R_QWFW01048360.1_72819814-72820195_Capra_hircus_breed_Pashmina__Cashmere__isolate_M0009_Scaffold_39591_length136504954_whole_genome_shotgun_sequence
R_NIWH01027711.1_59574332-59574706_Ovis_ammon_isolate_KGZ_Scaffold5_whole_genome_shotgun_sequence
WJNR01002124.1_62082768-62083147_Oreamnos_americanus_isolate_splAK2016_ScXtcTc_2126HRSCAF_147469_whole_genome_shotgun_sequence
R_RAWU01000013.1_68628337-68628726_Giraffa_tippelskirchi_isolate_OR1865_chromosome_3_whole_genome_shotgun_sequence
R_VCEB01000003.1_206733326-206733704_Muntiacus_reevesi_isolate_UCam_UCB_Mr_chromosome_3_whole_genome_shotgun_sequence
R_VCEA01000001.1_408976935-408977309_Muntiacus_muntjak_isolate_UTSW_UCB_Mm_chromosome_1_whole_genome_shotgun_sequence
R_marine_VOSU01000035.1_127939896-127940528_Phocoena_sinus_isolate_mPhoSin1_chromosome_X_whole_genome_shotgun_sequence
marine_PKGA01136281.1_827973-828605_Phocoena_phocoena_ScshVNz_4277_whole_genome_shotgun_sequence
marine_RJWO010072929.1_3442-4075_Inia_geoffrensis_isolate_BS63_IniGeo_flattened_line_145692_whole_genome_shotgun_sequence
R_marine_RJWI010074074.1_3009-3640_Pontoporia_blainvillei_isolate_BS67_PonBla_flattened_line_148088_whole_genome_shotgun_sequence
marine_RJWL010001509.1_576-1162_Kogia_breviceps_isolate_BS64_KogBre_flattened_line_3016_whole_genome_shotgun_sequence
R_marine_RJWK010055584.1_2115-2745_Platanista_minor_isolate_BS66_PlaMin_flattened_line_111103_whole_genome_shotgun_sequence
R_marine_PVJJ010000136.1_2969-3598_Mesoplodon_bidens_isolate_US080_MesBid_scaffold_270_whole_genome_shotgun_sequence
R_marine_VNFC01000008.1_128585283-128585913_Balaenoptera_musculus_isolate_JJ_BM4_2016_0621_Super_scaffold_8_whole_genome_shotgun_sequence
marine_NIPP01013375.1_25545-26175_Eschrichtius_robustus_isolate_ER-14-168_kmer60-min500.11742580_whole_genome_shotgun_sequence
marine_RJWP010039804.1_8118-8748_Eubalaena_japonica_isolate_BS61_EubJap_flattened_line_79554_whole_genome_shotgun_sequence
R_PVJP02000037.1_317745-318380_Hippopotamus_amphibius_isolate_BS01_ScHSfme_36HRSCAF_8052_whole_genome_shotgun_sequence
VSZR01000032.1_78832495-78833122_Camelus_ferus_isolate_YT-003-E_chromosome_X_whole_genome_shotgun_sequence
JWIN03000037.1_3768857-3769486_Camelus_dromedarius_breed_African_isolate_Drom800_chromosome_X_whole_genome_shotgun_sequence
0.02

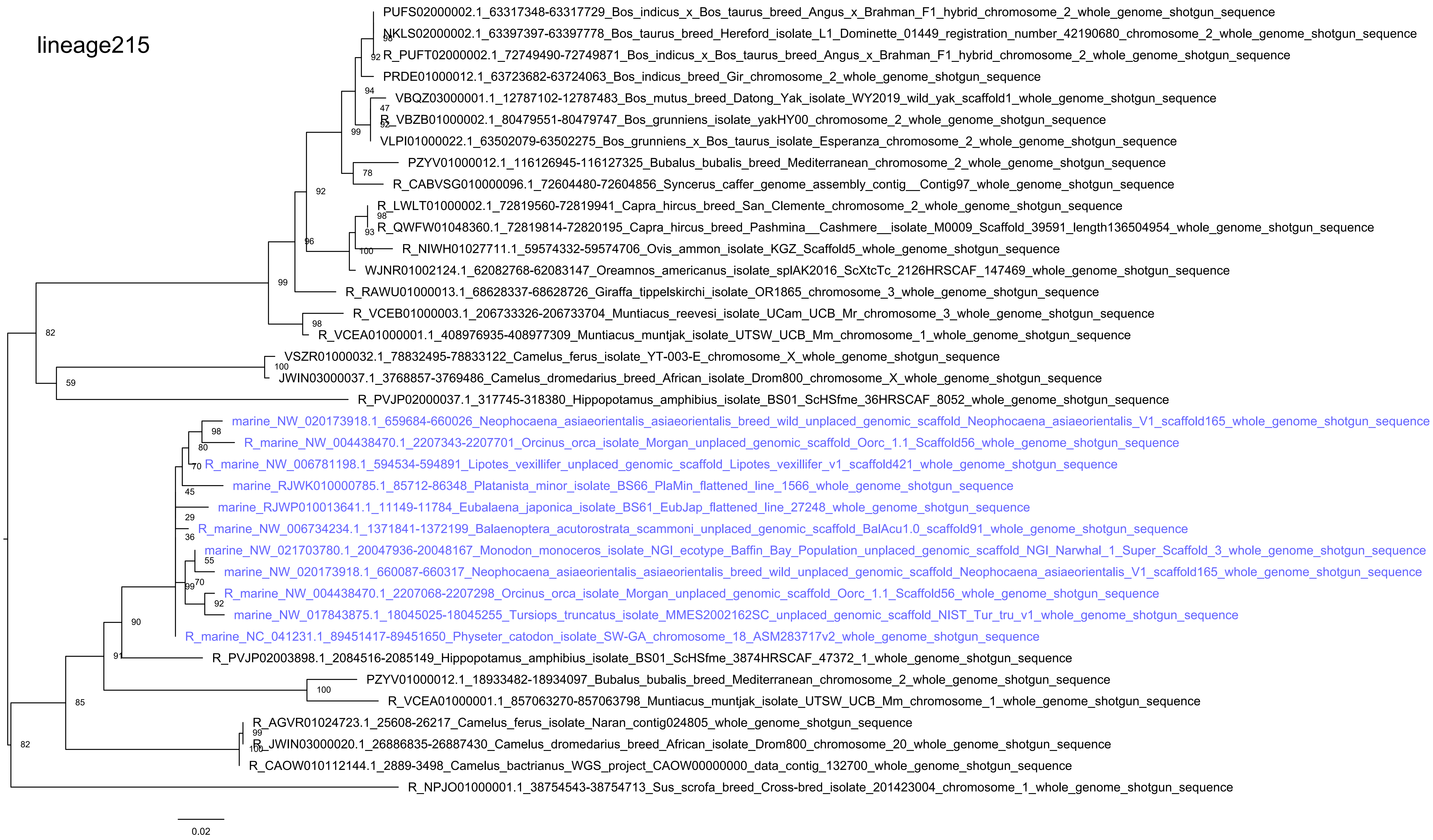
lineage215
PUFS02000002.1_63317348-63317729_Bos_indicus_x_Bos_taurus_breed_Angus_x_Brahman_F1_hybrid_chromosome_2_whole_genome_shotgun_sequence
NKLS02000002.1_63397397-63397778_Bos_taurus_breed_Hereford_isolate_L1_Dominette_01449_registration_number_42190680_chromosome_2_whole_genome_shotgun_sequence
R_PUFT02000002.1_72749490-72749871_Bos_indicus_x_Bos_taurus_breed_Angus_x_Brahman_F1_hybrid_chromosome_2_whole_genome_shotgun_sequence
PRDE01000012.1_63723682-63724063_Bos_indicus_breed_Gir_chromosome_2_whole_genome_shotgun_sequence
VBQZ03000001.1_12787102-12787483_Bos_mutus_breed_Datong_Yak_isolate_WY2019_wild_yak_scaffold1_whole_genome_shotgun_sequence
R_VBZB01000002.1_80479551-80479747_Bos_grunniens_isolate_yakHY00_chromosome_2_whole_genome_shotgun_sequence
VLPI01000022.1_63502079-63502275_Bos_grunniens_x_Bos_taurus_isolate_Esperanza_chromosome_2_whole_genome_shotgun_sequence
PZYV01000012.1_116126945-116127325_Bubalus_bubalis_breed_Mediterranean_chromosome_2_whole_genome_shotgun_sequence
R_CABVSG010000096.1_72604480-72604856_Syncerus_caffer_genome_assembly_contig__Contig97_whole_genome_shotgun_sequence
R_LWLT01000002.1_72819560-72819941_Capra_hircus_breed_San_Clemente_chromosome_2_whole_genome_shotgun_sequence
R_QWFW01048360.1_72819814-72820195_Capra_hircus_breed_Pashmina__Cashmere__isolate_M0009_Scaffold_39591_length136504954_whole_genome_shotgun_sequence
R_NIWH01027711.1_59574332-59574706_Ovis_ammon_isolate_KGZ_Scaffold5_whole_genome_shotgun_sequence
WJNR01002124.1_62082768-62083147_Oreamnos_americanus_isolate_splAK2016_ScXtcTc_2126HRSCAF_147469_whole_genome_shotgun_sequence
R_RAWU01000013.1_68628337-68628726_Giraffa_tippelskirchi_isolate_OR1865_chromosome_3_whole_genome_shotgun_sequence
R_VCEB01000003.1_206733326-206733704_Muntiacus_reevesi_isolate_UCam_UCB_Mr_chromosome_3_whole_genome_shotgun_sequence
R_VCEA01000001.1_408976935-408977309_Muntiacus_muntjak_isolate_UTSW_UCB_Mm_chromosome_1_whole_genome_shotgun_sequence
VSZR01000032.1_78832495-78833122_Camelus_ferus_isolate_YT-003-E_chromosome_X_whole_genome_shotgun_sequence
JWIN03000037.1_3768857-3769486_Camelus_dromedarius_breed_African_isolate_Drom800_chromosome_X_whole_genome_shotgun_sequence
R_PVJP02000037.1_317745-318380_Hippopotamus_amphibius_isolate_BS01_ScHSfme_36HRSCAF_8052_whole_genome_shotgun_sequence
marine_NW_020173918.1_659684-660026_Neophocaena_asiaeorientalis_asiaeorientalis_breed_wild_unplaced_genomic_scaffold_Neophocaena_asiaeorientalis_V1_scaffold165_whole_genome_shotgun_sequence
R_marine_NW_004438470.1_2207343-2207701_Orcinus_orca_isolate_Morgan_unplaced_genomic_scaffold_Oorc_1.1_Scaffold56_whole_genome_shotgun_sequence
R_marine_NW_006781198.1_594534-594891_Lipotes_vexillifer_unplaced_genomic_scaffold_Lipotes_vexillifer_v1_scaffold421_whole_genome_shotgun_sequence
marine_RJWK010000785.1_85712-86348_Platanista_minor_isolate_BS66_PlaMin_flattened_line_1566_whole_genome_shotgun_sequence
marine_RJWP010013641.1_11149-11784_Eubalaena_japonica_isolate_BS61_EubJap_flattened_line_27248_whole_genome_shotgun_sequence
R_marine_NW_006734234.1_1371841-1372199_Balaenoptera_acutorostrata_scammoni_unplaced_genomic_scaffold_BalAcu1.0_scaffold91_whole_genome_shotgun_sequence
marine_NW_021703780.1_20047936-20048167_Monodon_monoceros_isolate_NGI_ecotype_Baffin_Bay_Population_unplaced_genomic_scaffold_NGI_Narwhal_1_Super_Scaffold_3_whole_genome_shotgun_sequence
marine_NW_020173918.1_660087-660317_Neophocaena_asiaeorientalis_asiaeorientalis_breed_wild_unplaced_genomic_scaffold_Neophocaena_asiaeorientalis_V1_scaffold165_whole_genome_shotgun_sequence
R_marine_NW_004438470.1_2207068-2207298_Orcinus_orca_isolate_Morgan_unplaced_genomic_scaffold_Oorc_1.1_Scaffold56_whole_genome_shotgun_sequence
marine_NW_017843875.1_18045025-18045255_Tursiops_truncatus_isolate_MMES2002162SC_unplaced_genomic_scaffold_NIST_Tur_tru_v1_whole_genome_shotgun_sequence
R_marine_NC_041231.1_89451417-89451650_Physeter_catodon_isolate_SW-GA_chromosome_18_ASM283717v2_whole_genome_shotgun_sequence
R_PVJP02003898.1_2084516-2085149_Hippopotamus_amphibius_isolate_BS01_ScHSfme_3874HRSCAF_47372_1_whole_genome_shotgun_sequence
PZYV01000012.1_18933482-18934097_Bubalus_bubalis_breed_Mediterranean_chromosome_2_whole_genome_shotgun_sequence
R_VCEA01000001.1_857063270-857063798_Muntiacus_muntjak_isolate_UTSW_UCB_Mm_chromosome_1_whole_genome_shotgun_sequence
R_AGVR01024723.1_25608-26217_Camelus_ferus_isolate_Naran_contig024805_whole_genome_shotgun_sequence
R_JWIN03000020.1_26886835-26887430_Camelus_dromedarius_breed_African_isolate_Drom800_chromosome_20_whole_genome_shotgun_sequence
R_CAOW010112144.1_2889-3498_Camelus_bactrianus_WGS_project_CAOW00000000_data_contig_132700_whole_genome_shotgun_sequence
R_NPJO01000001.1_38754543-38754713_Sus_scrofa_breed_Cross-bred_isolate_201423004_chromosome_1_whole_genome_shotgun_sequence
0.02

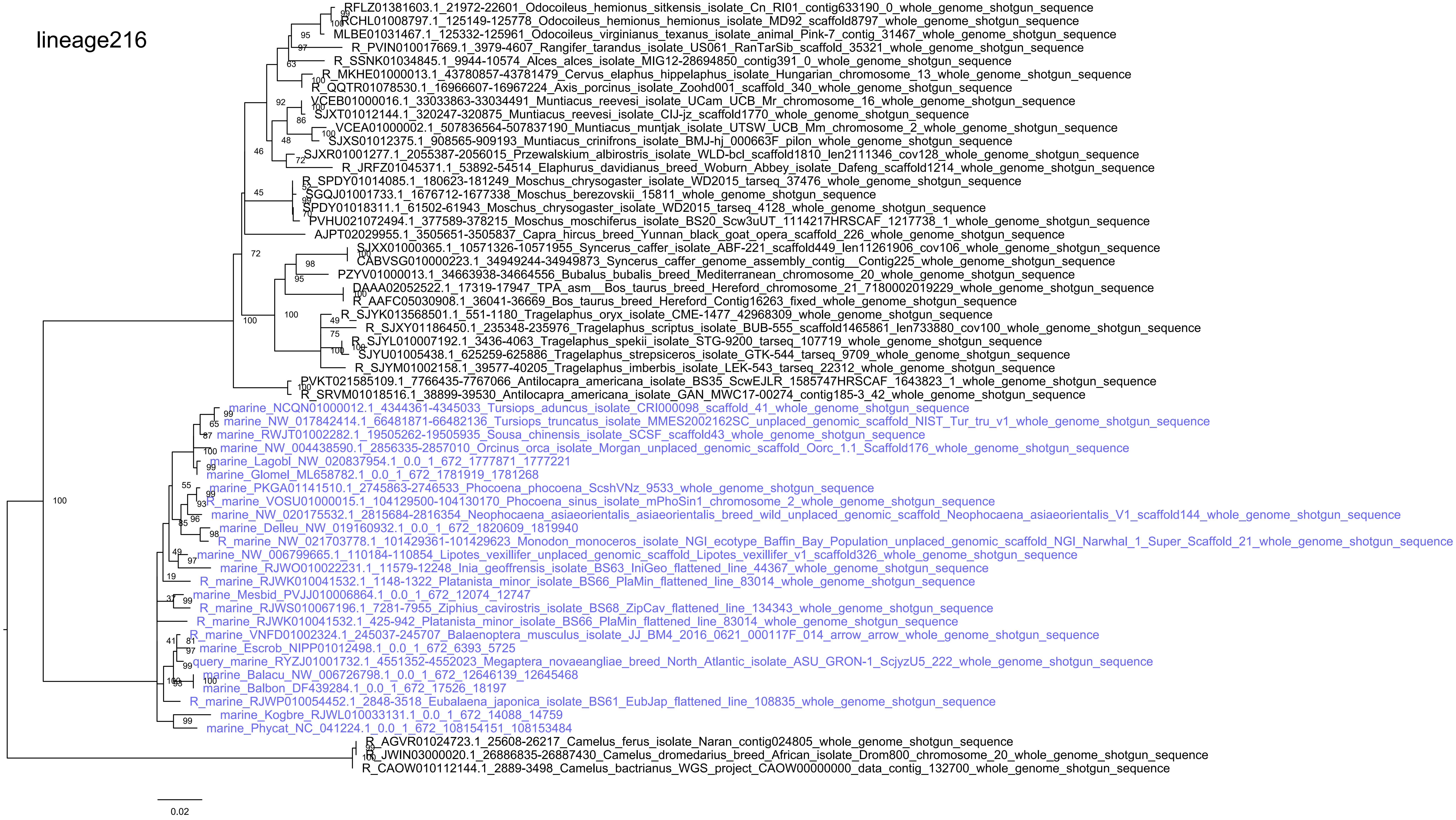
lineage216
RFLZ01381603.1_21972-22601_Odocoileus_hemionus_sitkensis_isolate_Cn_RI01_contig633190_0_whole_genome_shotgun_sequence
RCHL01008797.1_125149-125778_Odocoileus_hemionus_hemionus_isolate_MD92_scaffold8797_whole_genome_shotgun_sequence
MLBE01031467.1_125332-125961_Odocoileus_virginianus_texanus_isolate_animal_Pink-7_contig_31467_whole_genome_shotgun_sequence
R_PVIN010017669.1_3979-4607_Rangifer_tarandus_isolate_US061_RanTarSib_scaffold_35321_whole_genome_shotgun_sequence
R_SSNK01034845.1_9944-10574_Alces_alces_isolate_MIG12-28694850_contig391_0_whole_genome_shotgun_sequence
R_MKHE01000013.1_43780857-43781479_Cervus_elaphus_hippelaphus_isolate_Hungarian_chromosome_13_whole_genome_shotgun_sequence
R_QQTR01078530.1_16966607-16967224_Axis_porcinus_isolate_Zoohd001_scaffold_340_whole_genome_shotgun_sequence
VCEB01000016.1_33033863-33034491_Muntiacus_reevesi_isolate_UCam_UCB_Mr_chromosome_16_whole_genome_shotgun_sequence
SJXT01012144.1_320247-320875_Muntiacus_reevesi_isolate_CIJ-jz_scaffold1770_whole_genome_shotgun_sequence
VCEA01000002.1_507836564-507837190_Muntiacus_muntjak_isolate_UTSW_UCB_Mm_chromosome_2_whole_genome_shotgun_sequence
SJXS01012375.1_908565-909193_Muntiacus_crinifrons_isolate_BMJ-hj_000663F_pilon_whole_genome_shotgun_sequence
SJXR01001277.1_2055387-2056015_Przewalskium_albirostris_isolate_WLD-bcl_scaffold1810_len2111346_cov128_whole_genome_shotgun_sequence
R_JRFZ01045371.1_53892-54514_Elaphurus_davidianus_breed_Woburn_Abbey_isolate_Dafeng_scaffold1214_whole_genome_shotgun_sequence
R_SPDY01014085.1_180623-181249_Moschus_chrysogaster_isolate_WD2015_tarseq_37476_whole_genome_shotgun_sequence
SGQJ01001733.1_1676712-1677338_Moschus_berezovskii_15811_whole_genome_shotgun_sequence
SPDY01018311.1_61502-61943_Moschus_chrysogaster_isolate_WD2015_tarseq_4128_whole_genome_shotgun_sequence
PVHU021072494.1_377589-378215_Moschus_moschiferus_isolate_BS20_Scw3uUT_1114217HRSCAF_1217738_1_whole_genome_shotgun_sequence
AJPT02029955.1_3505651-3505837_Capra_hircus_breed_Yunnan_black_goat_opera_scaffold_226_whole_genome_shotgun_sequence
SJXX01000365.1_10571326-10571955_Syncerus_caffer_isolate_ABF-221_scaffold449_len11261906_cov106_whole_genome_shotgun_sequence
CABVSG010000223.1_34949244-34949873_Syncerus_caffer_genome_assembly_contig__Contig225_whole_genome_shotgun_sequence
PZYV01000013.1_34663938-34664556_Bubalus_bubalis_breed_Mediterranean_chromosome_20_whole_genome_shotgun_sequence
DAAA02052522.1_17319-17947_TPA_asm__Bos_taurus_breed_Hereford_chromosome_21_7180002019229_whole_genome_shotgun_sequence
R_AAFC05030908.1_36041-36669_Bos_taurus_breed_Hereford_Contig16263_fixed_whole_genome_shotgun_sequence
R_SJYK013568501.1_551-1180_Tragelaphus_oryx_isolate_CME-1477_42968309_whole_genome_shotgun_sequence
R_SJXY01186450.1_235348-235976_Tragelaphus_scriptus_isolate_BUB-555_scaffold1465861_len733880_cov100_whole_genome_shotgun_sequence
R_SJYL010007192.1_3436-4063_Tragelaphus_spekii_isolate_STG-9200_tarseq_107719_whole_genome_shotgun_sequence
SJYU01005438.1_625259-625886_Tragelaphus_strepsiceros_isolate_GTK-544_tarseq_9709_whole_genome_shotgun_sequence
R_SJYM01002158.1_39577-40205_Tragelaphus_imberbis_isolate_LEK-543_tarseq_22312_whole_genome_shotgun_sequence
PVKT021585109.1_7766435-7767066_Antilocapra_americana_isolate_BS35_ScwEJLR_1585747HRSCAF_1643823_1_whole_genome_shotgun_sequence
R_SRVM01018516.1_38899-39530_Antilocapra_americana_isolate_GAN_MWC17-00274_contig185-3_42_whole_genome_shotgun_sequence
marine_NCQN01000012.1_4344361-4345033_Tursiops_aduncus_isolate_CRI000098_scaffold_41_whole_genome_shotgun_sequence
marine_NW_017842414.1_66481871-66482136_Tursiops_truncatus_isolate_MMES2002162SC_unplaced_genomic_scaffold_NIST_Tur_tru_v1_whole_genome_shotgun_sequence
marine_RWJT01002282.1_19505262-19505935_Sousa_chinensis_isolate_SCSF_scaffold43_whole_genome_shotgun_sequence
marine_NW_004438590.1_2856335-2857010_Orcinus_orca_isolate_Morgan_unplaced_genomic_scaffold_Oorc_1.1_Scaffold176_whole_genome_shotgun_sequence
marine_Lagobl_NW_020837954.1_0.0_1_672_1777871_1777221
marine_Glomel_ML658782.1_0.0_1_672_1781919_1781268
marine_PKGA01141510.1_2745863-2746533_Phocoena_phocoena_ScshVNz_9533_whole_genome_shotgun_sequence
R_marine_VOSU01000015.1_104129500-104130170_Phocoena_sinus_isolate_mPhoSin1_chromosome_2_whole_genome_shotgun_sequence
marine_NW_020175532.1_2815684-2816354_Neophocaena_asiaeorientalis_asiaeorientalis_breed_wild_unplaced_genomic_scaffold_Neophocaena_asiaeorientalis_V1_scaffold144_whole_genome_shotgun_sequence
marine_Delleu_NW_019160932.1_0.0_1_672_1820609_1819940
R_marine_NW_021703778.1_101429361-101429623_Monodon_monoceros_isolate_NGI_ecotype_Baffin_Bay_Population_unplaced_genomic_scaffold_NGI_Narwhal_1_Super_Scaffold_21_whole_genome_shotgun_sequence
marine_NW_006799665.1_110184-110854_Lipotes_vexillifer_unplaced_genomic_scaffold_Lipotes_vexillifer_v1_scaffold326_whole_genome_shotgun_sequence
marine_RJWO010022231.1_11579-12248_Inia_geoffrensis_isolate_BS63_IniGeo_flattened_line_44367_whole_genome_shotgun_sequence
R_marine_RJWK010041532.1_1148-1322_Platanista_minor_isolate_BS66_PlaMin_flattened_line_83014_whole_genome_shotgun_sequence
marine_Mesbid_PVJJ010006864.1_0.0_1_672_12074_12747
R_marine_RJWS010067196.1_7281-7955_Ziphius_cavirostris_isolate_BS68_ZipCav_flattened_line_134343_whole_genome_shotgun_sequence
R_marine_RJWK010041532.1_425-942_Platanista_minor_isolate_BS66_PlaMin_flattened_line_83014_whole_genome_shotgun_sequence
R_marine_VNFD01002324.1_245037-245707_Balaenoptera_musculus_isolate_JJ_BM4_2016_0621_000117F_014_arrow_arrow_whole_genome_shotgun_sequence
marine_Escrob_NIPP01012498.1_0.0_1_672_6393_5725
query_marine_RYZJ01001732.1_4551352-4552023_Megaptera_novaeangliae_breed_North_Atlantic_isolate_ASU_GRON-1_ScjyzU5_222_whole_genome_shotgun_sequence
marine_Balacu_NW_006726798.1_0.0_1_672_12646139_12645468
marine_Balbon_DF439284.1_0.0_1_672_17526_18197
R_marine_RJWP010054452.1_2848-3518_Eubalaena_japonica_isolate_BS61_EubJap_flattened_line_108835_whole_genome_shotgun_sequence
marine_Kogbre_RJWL010033131.1_0.0_1_672_14088_14759
marine_Phycat_NC_041224.1_0.0_1_672_108154151_108153484
R_AGVR01024723.1_25608-26217_Camelus_ferus_isolate_Naran_contig024805_whole_genome_shotgun_sequence
R_JWIN03000020.1_26886835-26887430_Camelus_dromedarius_breed_African_isolate_Drom800_chromosome_20_whole_genome_shotgun_sequence
R_CAOW010112144.1_2889-3498_Camelus_bactrianus_WGS_project_CAOW00000000_data_contig_132700_whole_genome_shotgun_sequence
0.02

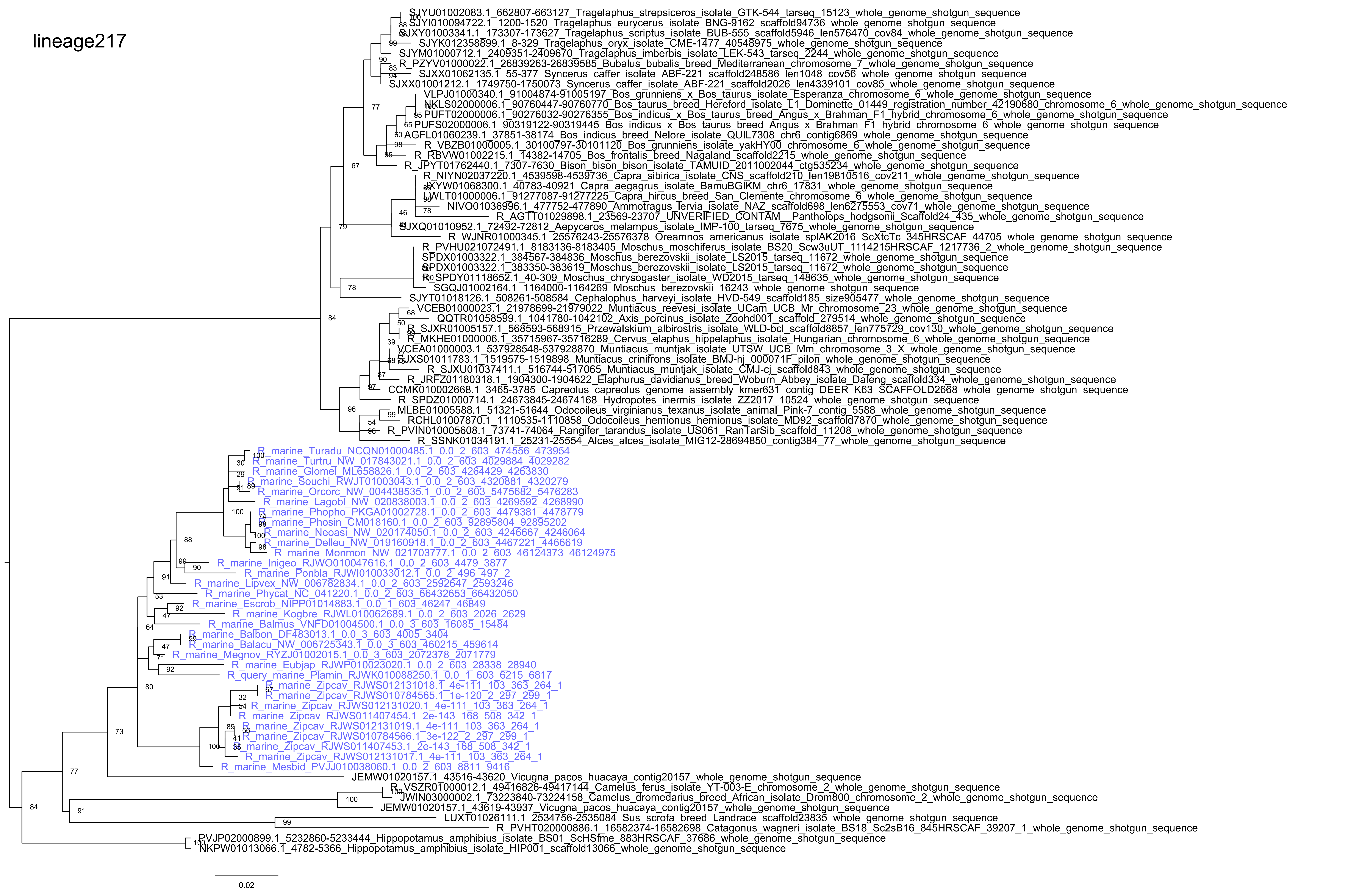
lineage217
SJYU01002083.1_662807-663127_Tragelaphus_strepsiceros_isolate_GTK-544_tarseq_15123_whole_genome_shotgun_sequence
SJYI010094722.1_1200-1520_Tragelaphus_eurycerus_isolate_BNG-9162_scaffold94736_whole_genome_shotgun_sequence
SJXY01003341.1_173307-173627_Tragelaphus_scriptus_isolate_BUB-555_scaffold5946_len576470_cov84_whole_genome_shotgun_sequence
SJYK012358899.1_8-329_Tragelaphus_oryx_isolate_CME-1477_40548975_whole_genome_shotgun_sequence
SJYM01000712.1_2409351-2409670_Tragelaphus_imberbis_isolate_LEK-543_tarseq_2244_whole_genome_shotgun_sequence
R_PZYV01000022.1_26839263-26839585_Bubalus_bubalis_breed_Mediterranean_chromosome_7_whole_genome_shotgun_sequence
SJXX01062135.1_55-377_Syncerus_caffer_isolate_ABF-221_scaffold248586_len1048_cov56_whole_genome_shotgun_sequence
SJXX01001212.1_1749750-1750073_Syncerus_caffer_isolate_ABF-221_scaffold2026_len4339101_cov85_whole_genome_shotgun_sequence
VLPJ01000340.1_91004874-91005197_Bos_grunniens_x_Bos_taurus_isolate_Esperanza_chromosome_6_whole_genome_shotgun_sequence
NKLS02000006.1_90760447-90760770_Bos_taurus_breed_Hereford_isolate_L1_Dominette_01449_registration_number_42190680_chromosome_6_whole_genome_shotgun_sequence
PUFT02000006.1_90276032-90276355_Bos_indicus_x_Bos_taurus_breed_Angus_x_Brahman_F1_hybrid_chromosome_6_whole_genome_shotgun_sequence
PUFS02000006.1_90319122-90319445_Bos_indicus_x_Bos_taurus_breed_Angus_x_Brahman_F1_hybrid_chromosome_6_whole_genome_shotgun_sequence
AGFL01060239.1_37851-38174_Bos_indicus_breed_Nelore_isolate_QUIL7308_chr6_contig6869_whole_genome_shotgun_sequence
R_VBZB01000005.1_30100797-30101120_Bos_grunniens_isolate_yakHY00_chromosome_6_whole_genome_shotgun_sequence
R_RBVW01002215.1_14382-14705_Bos_frontalis_breed_Nagaland_scaffold2215_whole_genome_shotgun_sequence
R_JPYT01762440.1_7307-7630_Bison_bison_bison_isolate_TAMUID_2011002044_ctg535234_whole_genome_shotgun_sequence
R_NIYN02037220.1_4539598-4539736_Capra_sibirica_isolate_CNS_scaffold210_len19810516_cov211_whole_genome_shotgun_sequence
JXYW01068300.1_40783-40921_Capra_aegagrus_isolate_BamuBGIKM_chr6_17831_whole_genome_shotgun_sequence
LWLT01000006.1_91277087-91277225_Capra_hircus_breed_San_Clemente_chromosome_6_whole_genome_shotgun_sequence
NIVO01036996.1_477752-477890_Ammotragus_lervia_isolate_NAZ_scaffold698_len6275553_cov71_whole_genome_shotgun_sequence
R_AGTT01029898.1_23569-23707_UNVERIFIED_CONTAM__Pantholops_hodgsonii_Scaffold24_435_whole_genome_shotgun_sequence
SJXQ01010952.1_72492-72812_Aepyceros_melampus_isolate_IMP-100_tarseq_7675_whole_genome_shotgun_sequence
R_WJNR01000345.1_25576243-25576378_Oreamnos_americanus_isolate_splAK2016_ScXtcTc_345HRSCAF_44705_whole_genome_shotgun_sequence
R_PVHU021072491.1_8183136-8183405_Moschus_moschiferus_isolate_BS20_Scw3uUT_1114215HRSCAF_1217736_2_whole_genome_shotgun_sequence
SPDX01003322.1_384567-384836_Moschus_berezovskii_isolate_LS2015_tarseq_11672_whole_genome_shotgun_sequence
SPDX01003322.1_383350-383619_Moschus_berezovskii_isolate_LS2015_tarseq_11672_whole_genome_shotgun_sequence
R_SPDY01118652.1_40-309_Moschus_chrysogaster_isolate_WD2015_tarseq_148635_whole_genome_shotgun_sequence
SGQJ01002164.1_1164000-1164269_Moschus_berezovskii_16243_whole_genome_shotgun_sequence
SJYT01018126.1_508261-508584_Cephalophus_harveyi_isolate_HVD-549_scaffold185_size905477_whole_genome_shotgun_sequence
VCEB01000023.1_21978699-21979022_Muntiacus_reevesi_isolate_UCam_UCB_Mr_chromosome_23_whole_genome_shotgun_sequence
QQTR01058599.1_1041780-1042102_Axis_porcinus_isolate_Zoohd001_scaffold_279514_whole_genome_shotgun_sequence
R_SJXR01005157.1_568593-568915_Przewalskium_albirostris_isolate_WLD-bcl_scaffold8857_len775729_cov130_whole_genome_shotgun_sequence
R_MKHE01000006.1_35715967-35716289_Cervus_elaphus_hippelaphus_isolate_Hungarian_chromosome_6_whole_genome_shotgun_sequence
VCEA01000003.1_537928548-537928870_Muntiacus_muntjak_isolate_UTSW_UCB_Mm_chromosome_3_X_whole_genome_shotgun_sequence
SJXS01011783.1_1519575-1519898_Muntiacus_crinifrons_isolate_BMJ-hj_000071F_pilon_whole_genome_shotgun_sequence
R_SJXU01037411.1_516744-517065_Muntiacus_muntjak_isolate_CMJ-cj_scaffold843_whole_genome_shotgun_sequence
R_JRFZ01180318.1_1904300-1904622_Elaphurus_davidianus_breed_Woburn_Abbey_isolate_Dafeng_scaffold334_whole_genome_shotgun_sequence
CCMK010002668.1_3465-3785_Capreolus_capreolus_genome_assembly_kmer631_contig_DEER_K63_SCAFFOLD2668_whole_genome_shotgun_sequence
R_SPDZ01000714.1_24673845-24674168_Hydropotes_inermis_isolate_ZZ2017_10524_whole_genome_shotgun_sequence
MLBE01005588.1_51321-51644_Odocoileus_virginianus_texanus_isolate_animal_Pink-7_contig_5588_whole_genome_shotgun_sequence
RCHL01007870.1_1110535-1110858_Odocoileus_hemionus_hemionus_isolate_MD92_scaffold7870_whole_genome_shotgun_sequence
R_PVIN010005608.1_73741-74064_Rangifer_tarandus_isolate_US061_RanTarSib_scaffold_11208_whole_genome_shotgun_sequence
R_SSNK01034191.1_25231-25554_Alces_alces_isolate_MIG12-28694850_contig384_77_whole_genome_shotgun_sequence
R_marine_Turadu_NCQN01000485.1_0.0_2_603_474556_473954
R_marine_Turtru_NW_017843021.1_0.0_2_603_4029884_4029282
R_marine_Glomel_ML658826.1_0.0_2_603_4264429_4263830
R_marine_Souchi_RWJT01003043.1_0.0_2_603_4320881_4320279
R_marine_Orcorc_NW_004438535.1_0.0_2_603_5475682_5476283
R_marine_Lagobl_NW_020838003.1_0.0_2_603_4269592_4268990
R_marine_Phopho_PKGA01002728.1_0.0_2_603_4479381_4478779
R_marine_Phosin_CM018160.1_0.0_2_603_92895804_92895202
R_marine_Neoasi_NW_020174050.1_0.0_2_603_4246667_4246064
R_marine_Delleu_NW_019160918.1_0.0_2_603_4467221_4466619
R_marine_Monmon_NW_021703777.1_0.0_2_603_46124373_46124975
R_marine_Inigeo_RJWO010047616.1_0.0_2_603_4479_3877
R_marine_Ponbla_RJWI010033012.1_0.0_2_496_497_2
R_marine_Lipvex_NW_006782834.1_0.0_2_603_2592647_2593246
R_marine_Phycat_NC_041220.1_0.0_2_603_66432653_66432050
R_marine_Escrob_NIPP01014883.1_0.0_1_603_46247_46849
R_marine_Kogbre_RJWL010062689.1_0.0_2_603_2026_2629
R_marine_Balmus_VNFD01004500.1_0.0_3_603_16085_15484
R_marine_Balbon_DF483013.1_0.0_3_603_4005_3404
R_marine_Balacu_NW_006725343.1_0.0_3_603_460215_459614
R_marine_Megnov_RYZJ01002015.1_0.0_3_603_2072378_2071779
R_marine_Eubjap_RJWP010023020.1_0.0_2_603_28338_28940
R_query_marine_Plamin_RJWK010088250.1_0.0_1_603_6215_6817
R_marine_Zipcav_RJWS012131018.1_4e-111_103_363_264_1
R_marine_Zipcav_RJWS010784565.1_1e-120_2_297_299_1
R_marine_Zipcav_RJWS012131020.1_4e-111_103_363_264_1
R_marine_Zipcav_RJWS011407454.1_2e-143_168_508_342_1
R_marine_Zipcav_RJWS012131019.1_4e-111_103_363_264_1
R_marine_Zipcav_RJWS010784566.1_3e-122_2_297_299_1
R_marine_Zipcav_RJWS011407453.1_2e-143_168_508_342_1
R_marine_Zipcav_RJWS012131017.1_4e-111_103_363_264_1
R_marine_Mesbid_PVJJ010038060.1_0.0_2_603_8811_9416
JEMW01020157.1_43516-43620_Vicugna_pacos_huacaya_contig20157_whole_genome_shotgun_sequence
R_VSZR01000012.1_49416826-49417144_Camelus_ferus_isolate_YT-003-E_chromosome_2_whole_genome_shotgun_sequence
JWIN03000002.1_73223840-73224158_Camelus_dromedarius_breed_African_isolate_Drom800_chromosome_2_whole_genome_shotgun_sequence
JEMW01020157.1_43619-43937_Vicugna_pacos_huacaya_contig20157_whole_genome_shotgun_sequence
LUXT01026111.1_2534756-2535084_Sus_scrofa_breed_Landrace_scaffold23835_whole_genome_shotgun_sequence
R_PVHT020000886.1_16582374-16582698_Catagonus_wagneri_isolate_BS18_Sc2sB16_845HRSCAF_39207_1_whole_genome_shotgun_sequence
PVJP02000899.1_5232860-5233444_Hippopotamus_amphibius_isolate_BS01_ScHSfme_883HRSCAF_37686_whole_genome_shotgun_sequence
NKPW01013066.1_4782-5366_Hippopotamus_amphibius_isolate_HIP001_scaffold13066_whole_genome_shotgun_sequence
0.02

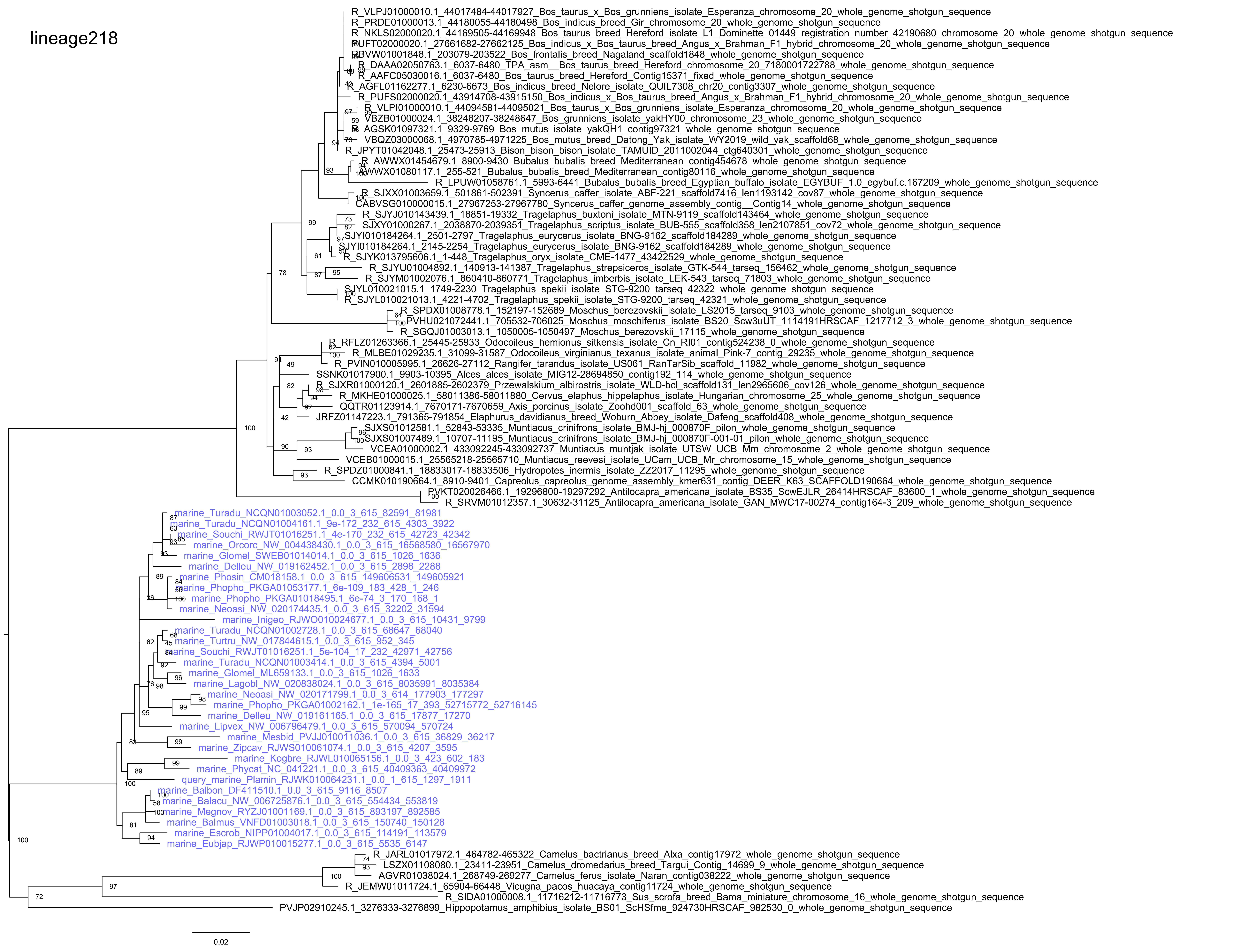

lineage218
R_VLPJ01000010.1_44017484-44017927_Bos_taurus_x_Bos_grunniens_isolate_Esperanza_chromosome_20_whole_genome_shotgun_sequence
R_PRDE01000013.1_44180055-44180498_Bos_indicus_breed_Gir_chromosome_20_whole_genome_shotgun_sequence
R_NKLS02000020.1_44169505-44169948_Bos_taurus_breed_Hereford_isolate_L1_Dominette_01449_registration_number_42190680_chromosome_20_whole_genome_shotgun_sequence
PUFT02000020.1_27661682-27662125_Bos_indicus_x_Bos_taurus_breed_Angus_x_Brahman_F1_hybrid_chromosome_20_whole_genome_shotgun_sequence
RBVW01001848.1_203079-203522_Bos_frontalis_breed_Nagaland_scaffold1848_whole_genome_shotgun_sequence
R_DAAA02050763.1_6037-6480_TPA_asm__Bos_taurus_breed_Hereford_chromosome_20_7180001722788_whole_genome_shotgun_sequence
R_AAFC05030016.1_6037-6480_Bos_taurus_breed_Hereford_Contig15371_fixed_whole_genome_shotgun_sequence
R_AGFL01162277.1_6230-6673_Bos_indicus_breed_Nelore_isolate_QUIL7308_chr20_contig3307_whole_genome_shotgun_sequence
R_PUFS02000020.1_43914708-43915150_Bos_indicus_x_Bos_taurus_breed_Angus_x_Brahman_F1_hybrid_chromosome_20_whole_genome_shotgun_sequence
R_VLPI01000010.1_44094581-44095021_Bos_taurus_x_Bos_grunniens_isolate_Esperanza_chromosome_20_whole_genome_shotgun_sequence
VBZB01000024.1_38248207-38248647_Bos_grunniens_isolate_yakHY00_chromosome_23_whole_genome_shotgun_sequence
R_AGSK01097321.1_9329-9769_Bos_mutus_isolate_yakQH1_contig97321_whole_genome_shotgun_sequence
VBQZ03000068.1_4970785-4971225_Bos_mutus_breed_Datong_Yak_isolate_WY2019_wild_yak_scaffold68_whole_genome_shotgun_sequence
R_JPYT01042048.1_25473-25913_Bison_bison_bison_isolate_TAMUID_2011002044_ctg640301_whole_genome_shotgun_sequence
R_AWWX01454679.1_8900-9430_Bubalus_bubalis_breed_Mediterranean_contig454678_whole_genome_shotgun_sequence
AWWX01080117.1_255-521_Bubalus_bubalis_breed_Mediterranean_contig80116_whole_genome_shotgun_sequence
R_LPUW01058761.1_5993-6441_Bubalus_bubalis_breed_Egyptian_buffalo_isolate_EGYBUF_1.0_egybuf.c.167209_whole_genome_shotgun_sequence
R_SJXX01003659.1_501861-502391_Syncerus_caffer_isolate_ABF-221_scaffold7416_len1193142_cov87_whole_genome_shotgun_sequence
CABVSG010000015.1_27967253-27967780_Syncerus_caffer_genome_assembly_contig__Contig14_whole_genome_shotgun_sequence
R_SJYJ010143439.1_18851-19332_Tragelaphus_buxtoni_isolate_MTN-9119_scaffold143464_whole_genome_shotgun_sequence
SJXY01000267.1_2038870-2039351_Tragelaphus_scriptus_isolate_BUB-555_scaffold358_len2107851_cov72_whole_genome_shotgun_sequence
SJYI010184264.1_2501-2797_Tragelaphus_eurycerus_isolate_BNG-9162_scaffold184289_whole_genome_shotgun_sequence
SJYI010184264.1_2145-2254_Tragelaphus_eurycerus_isolate_BNG-9162_scaffold184289_whole_genome_shotgun_sequence
R_SJYK013795606.1_1-448_Tragelaphus_oryx_isolate_CME-1477_43422529_whole_genome_shotgun_sequence
R_SJYU01004892.1_140913-141387_Tragelaphus_strepsiceros_isolate_GTK-544_tarseq_156462_whole_genome_shotgun_sequence
R_SJYM01002076.1_860410-860771_Tragelaphus_imberbis_isolate_LEK-543_tarseq_71803_whole_genome_shotgun_sequence
SJYL010021015.1_1749-2230_Tragelaphus_spekii_isolate_STG-9200_tarseq_42322_whole_genome_shotgun_sequence
R_SJYL010021013.1_4221-4702_Tragelaphus_spekii_isolate_STG-9200_tarseq_42321_whole_genome_shotgun_sequence
R_SPDX01008778.1_152197-152689_Moschus_berezovskii_isolate_LS2015_tarseq_9103_whole_genome_shotgun_sequence
PVHU021072441.1_705532-706025_Moschus_moschiferus_isolate_BS20_Scw3uUT_1114191HRSCAF_1217712_3_whole_genome_shotgun_sequence
R_SGQJ01003013.1_1050005-1050497_Moschus_berezovskii_17115_whole_genome_shotgun_sequence
R_RFLZ01263366.1_25445-25933_Odocoileus_hemionus_sitkensis_isolate_Cn_RI01_contig524238_0_whole_genome_shotgun_sequence
R_MLBE01029235.1_31099-31587_Odocoileus_virginianus_texanus_isolate_animal_Pink-7_contig_29235_whole_genome_shotgun_sequence
R_PVIN010005995.1_26626-27112_Rangifer_tarandus_isolate_US061_RanTarSib_scaffold_11982_whole_genome_shotgun_sequence
SSNK01017900.1_9903-10395_Alces_alces_isolate_MIG12-28694850_contig192_114_whole_genome_shotgun_sequence
R_SJXR01000120.1_2601885-2602379_Przewalskium_albirostris_isolate_WLD-bcl_scaffold131_len2965606_cov126_whole_genome_shotgun_sequence
R_MKHE01000025.1_58011386-58011880_Cervus_elaphus_hippelaphus_isolate_Hungarian_chromosome_25_whole_genome_shotgun_sequence
QQTR01123914.1_7670171-7670659_Axis_porcinus_isolate_Zoohd001_scaffold_63_whole_genome_shotgun_sequence
JRFZ01147223.1_791365-791854_Elaphurus_davidianus_breed_Woburn_Abbey_isolate_Dafeng_scaffold408_whole_genome_shotgun_sequence
SJXS01012581.1_52843-53335_Muntiacus_crinifrons_isolate_BMJ-hj_000870F_pilon_whole_genome_shotgun_sequence
SJXS01007489.1_10707-11195_Muntiacus_crinifrons_isolate_BMJ-hj_000870F-001-01_pilon_whole_genome_shotgun_sequence
VCEA01000002.1_433092245-433092737_Muntiacus_muntjak_isolate_UTSW_UCB_Mm_chromosome_2_whole_genome_shotgun_sequence
VCEB01000015.1_25565218-25565710_Muntiacus_reevesi_isolate_UCam_UCB_Mr_chromosome_15_whole_genome_shotgun_sequence
R_SPDZ01000841.1_18833017-18833506_Hydropotes_inermis_isolate_ZZ2017_11295_whole_genome_shotgun_sequence
CCMK010190664.1_8910-9401_Capreolus_capreolus_genome_assembly_kmer631_contig_DEER_K63_SCAFFOLD190664_whole_genome_shotgun_sequence
PVKT020026466.1_19296800-19297292_Antilocapra_americana_isolate_BS35_ScwEJLR_26414HRSCAF_83600_1_whole_genome_shotgun_sequence
R_SRVM01012357.1_30632-31125_Antilocapra_americana_isolate_GAN_MWC17-00274_contig164-3_209_whole_genome_shotgun_sequence
marine_Turadu_NCQN01003052.1_0.0_3_615_82591_81981
marine_Turadu_NCQN01004161.1_9e-172_232_615_4303_3922
marine_Souchi_RWJT01016251.1_4e-170_232_615_42723_42342
marine_Orcorc_NW_004438430.1_0.0_3_615_16568580_16567970
marine_Glomel_SWEB01014014.1_0.0_3_615_1026_1636
marine_Delleu_NW_019162452.1_0.0_3_615_2898_2288
marine_Phosin_CM018158.1_0.0_3_615_149606531_149605921
marine_Phopho_PKGA01053177.1_6e-109_183_428_1_246
marine_Phopho_PKGA01018495.1_6e-74_3_170_168_1
marine_Neoasi_NW_020174435.1_0.0_3_615_32202_31594
marine_Inigeo_RJWO010024677.1_0.0_3_615_10431_9799
marine_Turadu_NCQN01002728.1_0.0_3_615_68647_68040
marine_Turtru_NW_017844615.1_0.0_3_615_952_345
marine_Souchi_RWJT01016251.1_5e-104_17_232_42971_42756
marine_Turadu_NCQN01003414.1_0.0_3_615_4394_5001
marine_Glomel_ML659133.1_0.0_3_615_1026_1633
marine_Lagobl_NW_020838024.1_0.0_3_615_8035991_8035384
marine_Neoasi_NW_020171799.1_0.0_3_614_177903_177297
marine_Phopho_PKGA01002162.1_1e-165_17_393_52715772_52716145
marine_Delleu_NW_019161165.1_0.0_3_615_17877_17270
marine_Lipvex_NW_006796479.1_0.0_3_615_570094_570724
marine_Mesbid_PVJJ010011036.1_0.0_3_615_36829_36217
marine_Zipcav_RJWS010061074.1_0.0_3_615_4207_3595
marine_Kogbre_RJWL010065156.1_0.0_3_423_602_183
marine_Phycat_NC_041221.1_0.0_3_615_40409363_40409972
query_marine_Plamin_RJWK010064231.1_0.0_1_615_1297_1911
marine_Balbon_DF411510.1_0.0_3_615_9116_8507
marine_Balacu_NW_006725876.1_0.0_3_615_554434_553819
marine_Megnov_RYZJ01001169.1_0.0_3_615_893197_892585
marine_Balmus_VNFD01003018.1_0.0_3_615_150740_150128
marine_Escrob_NIPP01004017.1_0.0_3_615_114191_113579
marine_Eubjap_RJWP010015277.1_0.0_3_615_5535_6147
R_JARL01017972.1_464782-465322_Camelus_bactrianus_breed_Alxa_contig17972_whole_genome_shotgun_sequence
LSZX01108080.1_23411-23951_Camelus_dromedarius_breed_Targui_Contig_14699_9_whole_genome_shotgun_sequence
AGVR01038024.1_268749-269277_Camelus_ferus_isolate_Naran_contig038222_whole_genome_shotgun_sequence
R_JEMW01011724.1_65904-66448_Vicugna_pacos_huacaya_contig11724_whole_genome_shotgun_sequence
R_SIDA01000008.1_11716212-11716773_Sus_scrofa_breed_Bama_miniature_chromosome_16_whole_genome_shotgun_sequence
PVJP02910245.1_3276333-3276899_Hippopotamus_amphibius_isolate_BS01_ScHSfme_924730HRSCAF_982530_0_whole_genome_shotgun_sequence
0.02

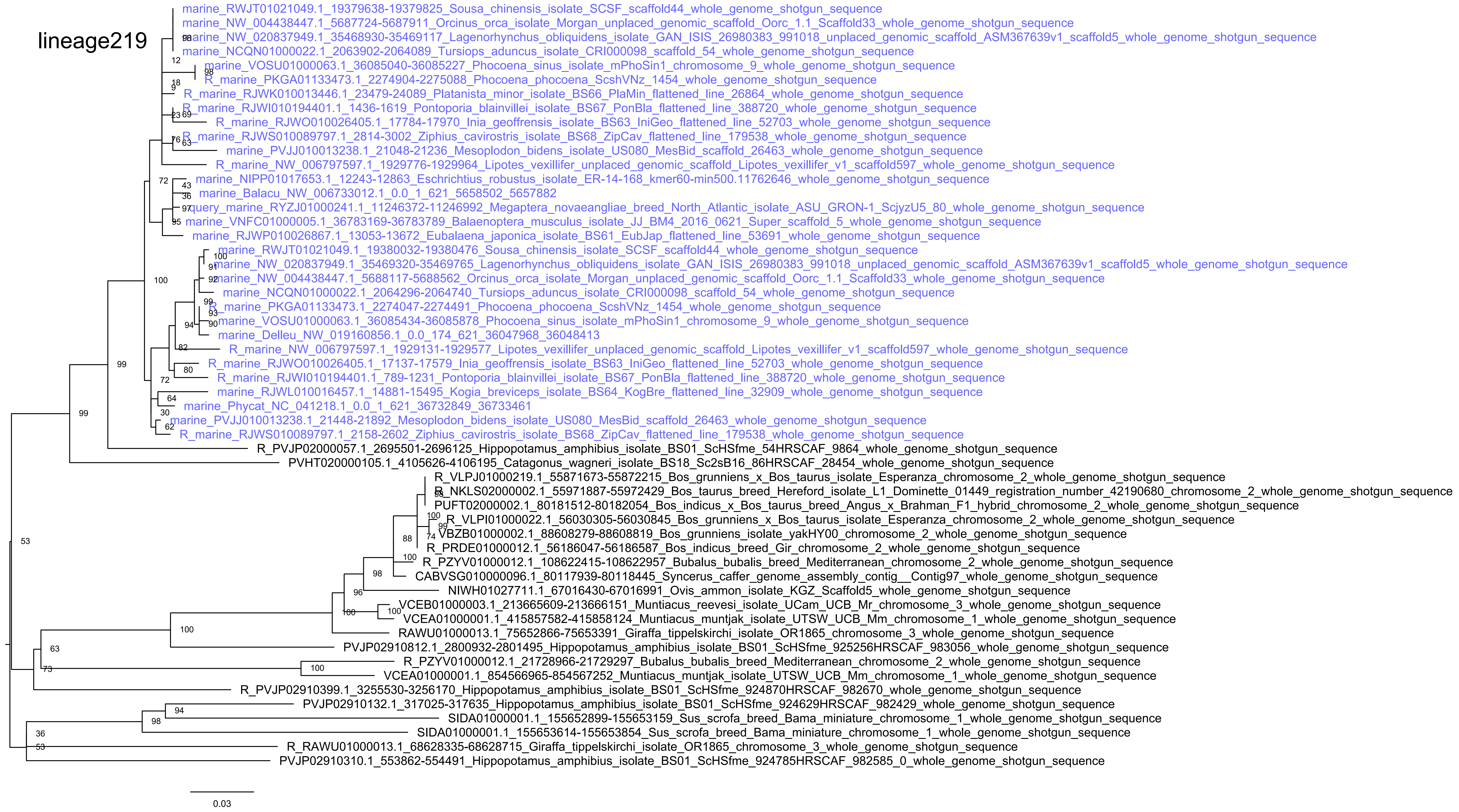
lineage219
marine_RWJT01021049.1_19379638-19379825_Sousa_chinensis_isolate_SCSF_scaffold44_whole_genome_shotgun_sequence
marine_NW_004438447.1_5687724-5687911_Orcinus_orca_isolate_Morgan_unplaced_genomic_scaffold_Oorc_1.1_Scaffold33_whole_genome_shotgun_sequence
marine_NW_020837949.1_35468930-35469117_Lagenorhynchus_obliquidens_isolate_GAN_ISIS_26980383_991018_unplaced_genomic_scaffold_ASM367639v1_scaffold5_whole_genome_shotgun_sequence
marine_NCQN01000022.1_2063902-2064089_Tursiops_aduncus_isolate_CRI000098_scaffold_54_whole_genome_shotgun_sequence
marine_VOSU01000063.1_36085040-36085227_Phocoena_sinus_isolate_mPhoSin1_chromosome_9_whole_genome_shotgun_sequence
R_marine_PKGA01133473.1_2274904-2275088_Phocoena_phocoena_ScshVNz_1454_whole_genome_shotgun_sequence
R_marine_RJWK010013446.1_23479-24089_Platanista_minor_isolate_BS66_PlaMin_flattened_line_26864_whole_genome_shotgun_sequence
R_marine_RJWI010194401.1_1436-1619_Pontoporia_blainvillei_isolate_BS67_PonBla_flattened_line_388720_whole_genome_shotgun_sequence
R_marine_RJWO010026405.1_17784-17970_Inia_geoffrensis_isolate_BS63_IniGeo_flattened_line_52703_whole_genome_shotgun_sequence
R_marine_RJWS010089797.1_2814-3002_Ziphius_cavirostris_isolate_BS68_ZipCav_flattened_line_179538_whole_genome_shotgun_sequence
marine_PVJJ010013238.1_21048-21236_Mesoplodon_bidens_isolate_US080_MesBid_scaffold_26463_whole_genome_shotgun_sequence
R_marine_NW_006797597.1_1929776-1929964_Lipotes_vexillifer_unplaced_genomic_scaffold_Lipotes_vexillifer_v1_scaffold597_whole_genome_shotgun_sequence
marine_NIPP01017653.1_12243-12863_Eschrichtius_robustus_isolate_ER-14-168_kmer60-min500.11762646_whole_genome_shotgun_sequence
marine_Balacu_NW_006733012.1_0.0_1_621_5658502_5657882
query_marine_RYZJ01000241.1_11246372-11246992_Megaptera_novaeangliae_breed_North_Atlantic_isolate_ASU_GRON-1_ScjyzU5_80_whole_genome_shotgun_sequence
marine_VNFC01000005.1_36783169-36783789_Balaenoptera_musculus_isolate_JJ_BM4_2016_0621_Super_scaffold_5_whole_genome_shotgun_sequence
marine_RJWP010026867.1_13053-13672_Eubalaena_japonica_isolate_BS61_EubJap_flattened_line_53691_whole_genome_shotgun_sequence
marine_RWJT01021049.1_19380032-19380476_Sousa_chinensis_isolate_SCSF_scaffold44_whole_genome_shotgun_sequence
marine_NW_020837949.1_35469320-35469765_Lagenorhynchus_obliquidens_isolate_GAN_ISIS_26980383_991018_unplaced_genomic_scaffold_ASM367639v1_scaffold5_whole_genome_shotgun_sequence
marine_NW_004438447.1_5688117-5688562_Orcinus_orca_isolate_Morgan_unplaced_genomic_scaffold_Oorc_1.1_Scaffold33_whole_genome_shotgun_sequence
marine_NCQN01000022.1_2064296-2064740_Tursiops_aduncus_isolate_CRI000098_scaffold_54_whole_genome_shotgun_sequence
R_marine_PKGA01133473.1_2274047-2274491_Phocoena_phocoena_ScshVNz_1454_whole_genome_shotgun_sequence
marine_VOSU01000063.1_36085434-36085878_Phocoena_sinus_isolate_mPhoSin1_chromosome_9_whole_genome_shotgun_sequence
marine_Delleu_NW_019160856.1_0.0_174_621_36047968_36048413
R_marine_NW_006797597.1_1929131-1929577_Lipotes_vexillifer_unplaced_genomic_scaffold_Lipotes_vexillifer_v1_scaffold597_whole_genome_shotgun_sequence
R_marine_RJWO010026405.1_17137-17579_Inia_geoffrensis_isolate_BS63_IniGeo_flattened_line_52703_whole_genome_shotgun_sequence
R_marine_RJWI010194401.1_789-1231_Pontoporia_blainvillei_isolate_BS67_PonBla_flattened_line_388720_whole_genome_shotgun_sequence
marine_RJWL010016457.1_14881-15495_Kogia_breviceps_isolate_BS64_KogBre_flattened_line_32909_whole_genome_shotgun_sequence
marine_Phycat_NC_041218.1_0.0_1_621_36732849_36733461
marine_PVJJ010013238.1_21448-21892_Mesoplodon_bidens_isolate_US080_MesBid_scaffold_26463_whole_genome_shotgun_sequence
R_marine_RJWS010089797.1_2158-2602_Ziphius_cavirostris_isolate_BS68_ZipCav_flattened_line_179538_whole_genome_shotgun_sequence
R_PVJP02000057.1_2695501-2696125_Hippopotamus_amphibius_isolate_BS01_ScHSfme_54HRSCAF_9864_whole_genome_shotgun_sequence
PVHT020000105.1_4105626-4106195_Catagonus_wagneri_isolate_BS18_Sc2sB16_86HRSCAF_28454_whole_genome_shotgun_sequence
R_VLPJ01000219.1_55871673-55872215_Bos_grunniens_x_Bos_taurus_isolate_Esperanza_chromosome_2_whole_genome_shotgun_sequence
R_NKLS02000002.1_55971887-55972429_Bos_taurus_breed_Hereford_isolate_L1_Dominette_01449_registration_number_42190680_chromosome_2_whole_genome_shotgun_sequence
PUFT02000002.1_80181512-80182054_Bos_indicus_x_Bos_taurus_breed_Angus_x_Brahman_F1_hybrid_chromosome_2_whole_genome_shotgun_sequence
R_VLPI01000022.1_56030305-56030845_Bos_grunniens_x_Bos_taurus_isolate_Esperanza_chromosome_2_whole_genome_shotgun_sequence
VBZB01000002.1_88608279-88608819_Bos_grunniens_isolate_yakHY00_chromosome_2_whole_genome_shotgun_sequence
R_PRDE01000012.1_56186047-56186587_Bos_indicus_breed_Gir_chromosome_2_whole_genome_shotgun_sequence
R_PZYV01000012.1_108622415-108622957_Bubalus_bubalis_breed_Mediterranean_chromosome_2_whole_genome_shotgun_sequence
CABVSG010000096.1_80117939-80118445_Syncerus_caffer_genome_assembly_contig__Contig97_whole_genome_shotgun_sequence
NIWH01027711.1_67016430-67016991_Ovis_ammon_isolate_KGZ_Scaffold5_whole_genome_shotgun_sequence
VCEB01000003.1_213665609-213666151_Muntiacus_reevesi_isolate_UCam_UCB_Mr_chromosome_3_whole_genome_shotgun_sequence
VCEA01000001.1_415857582-415858124_Muntiacus_muntjak_isolate_UTSW_UCB_Mm_chromosome_1_whole_genome_shotgun_sequence
RAWU01000013.1_75652866-75653391_Giraffa_tippelskirchi_isolate_OR1865_chromosome_3_whole_genome_shotgun_sequence
PVJP02910812.1_2800932-2801495_Hippopotamus_amphibius_isolate_BS01_ScHSfme_925256HRSCAF_983056_whole_genome_shotgun_sequence
R_PZYV01000012.1_21728966-21729297_Bubalus_bubalis_breed_Mediterranean_chromosome_2_whole_genome_shotgun_sequence
VCEA01000001.1_854566965-854567252_Muntiacus_muntjak_isolate_UTSW_UCB_Mm_chromosome_1_whole_genome_shotgun_sequence
R_PVJP02910399.1_3255530-3256170_Hippopotamus_amphibius_isolate_BS01_ScHSfme_924870HRSCAF_982670_whole_genome_shotgun_sequence
PVJP02910132.1_317025-317635_Hippopotamus_amphibius_isolate_BS01_ScHSfme_924629HRSCAF_982429_whole_genome_shotgun_sequence
SIDA01000001.1_155652899-155653159_Sus_scrofa_breed_Bama_miniature_chromosome_1_whole_genome_shotgun_sequence
SIDA01000001.1_155653614-155653854_Sus_scrofa_breed_Bama_miniature_chromosome_1_whole_genome_shotgun_sequence
R_RAWU01000013.1_68628335-68628715_Giraffa_tippelskirchi_isolate_OR1865_chromosome_3_whole_genome_shotgun_sequence
PVJP02910310.1_553862-554491_Hippopotamus_amphibius_isolate_BS01_ScHSfme_924785HRSCAF_982585_0_whole_genome_shotgun_sequence
0.03

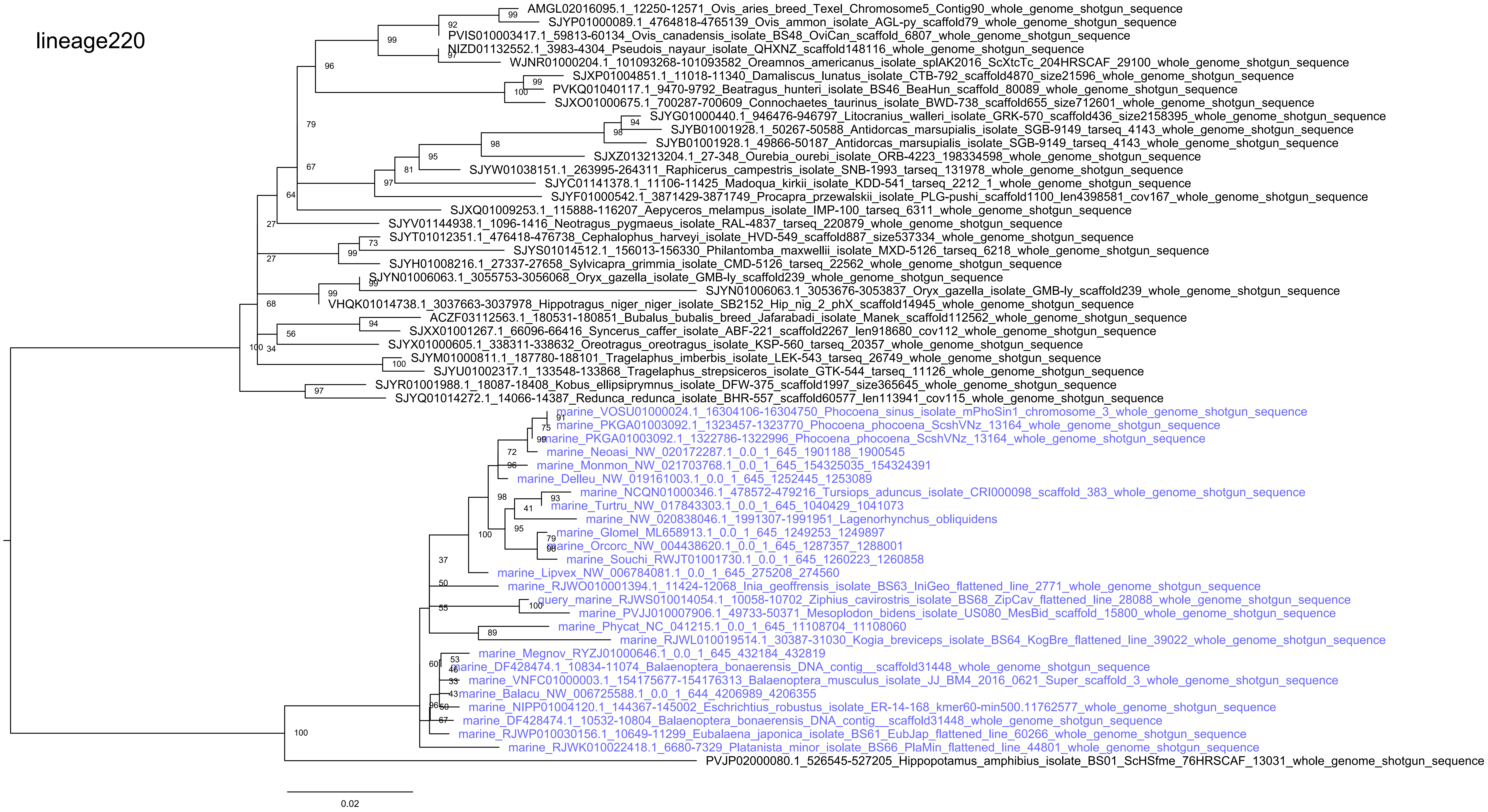

lineage220
AMGL02016095.1_12250-12571_Ovis_aries_breed_Texel_Chromosome5_Contig90_whole_genome_shotgun_sequence
SJYP01000089.1_4764818-4765139_Ovis_ammon_isolate_AGL-py_scaffold79_whole_genome_shotgun_sequence
PVIS010003417.1_59813-60134_Ovis_canadensis_isolate_BS48_OviCan_scaffold_6807_whole_genome_shotgun_sequence
NIZD01132552.1_3983-4304_Pseudois_nayaur_isolate_QHXNZ_scaffold148116_whole_genome_shotgun_sequence
WJNR01000204.1_101093268-101093582_Oreamnos_americanus_isolate_splAK2016_ScXtcTc_204HRSCAF_29100_whole_genome_shotgun_sequence
SJXP01004851.1_11018-11340_Damaliscus_lunatus_isolate_CTB-792_scaffold4870_size21596_whole_genome_shotgun_sequence
PVKQ01040117.1_9470-9792_Beatragus_hunteri_isolate_BS46_BeaHun_scaffold_80089_whole_genome_shotgun_sequence
SJXO01000675.1_700287-700609_Connochaetes_taurinus_isolate_BWD-738_scaffold655_size712601_whole_genome_shotgun_sequence
SJYG01000440.1_946476-946797_Litocranius_walleri_isolate_GRK-570_scaffold436_size2158395_whole_genome_shotgun_sequence
SJYB01001928.1_50267-50588_Antidorcas_marsupialis_isolate_SGB-9149_tarseq_4143_whole_genome_shotgun_sequence
SJYB01001928.1_49866-50187_Antidorcas_marsupialis_isolate_SGB-9149_tarseq_4143_whole_genome_shotgun_sequence
SJXZ013213204.1_27-348_Ourebia_ourebi_isolate_ORB-4223_198334598_whole_genome_shotgun_sequence
SJYW01038151.1_263995-264311_Raphicerus_campestris_isolate_SNB-1993_tarseq_131978_whole_genome_shotgun_sequence
SJYC01141378.1_11106-11425_Madoqua_kirkii_isolate_KDD-541_tarseq_2212_1_whole_genome_shotgun_sequence
SJYF01000542.1_3871429-3871749_Procapra_przewalskii_isolate_PLG-pushi_scaffold1100_len4398581_cov167_whole_genome_shotgun_sequence
SJXQ01009253.1_115888-116207_Aepyceros_melampus_isolate_IMP-100_tarseq_6311_whole_genome_shotgun_sequence
SJYV01144938.1_1096-1416_Neotragus_pygmaeus_isolate_RAL-4837_tarseq_220879_whole_genome_shotgun_sequence
SJYT01012351.1_476418-476738_Cephalophus_harveyi_isolate_HVD-549_scaffold887_size537334_whole_genome_shotgun_sequence
SJYS01014512.1_156013-156330_Philantomba_maxwellii_isolate_MXD-5126_tarseq_6218_whole_genome_shotgun_sequence
SJYH01008216.1_27337-27658_Sylvicapra_grimmia_isolate_CMD-5126_tarseq_22562_whole_genome_shotgun_sequence
SJYN01006063.1_3055753-3056068_Oryx_gazella_isolate_GMB-ly_scaffold239_whole_genome_shotgun_sequence
SJYN01006063.1_3053676-3053837_Oryx_gazella_isolate_GMB-ly_scaffold239_whole_genome_shotgun_sequence
VHQK01014738.1_3037663-3037978_Hippotragus_niger_niger_isolate_SB2152_Hip_nig_2_phX_scaffold14945_whole_genome_shotgun_sequence
ACZF03112563.1_180531-180851_Bubalus_bubalis_breed_Jafarabadi_isolate_Manek_scaffold112562_whole_genome_shotgun_sequence
SJXX01001267.1_66096-66416_Syncerus_caffer_isolate_ABF-221_scaffold2267_len918680_cov112_whole_genome_shotgun_sequence
SJYX01000605.1_338311-338632_Oreotragus_oreotragus_isolate_KSP-560_tarseq_20357_whole_genome_shotgun_sequence
SJYM01000811.1_187780-188101_Tragelaphus_imberbis_isolate_LEK-543_tarseq_26749_whole_genome_shotgun_sequence
SJYU01002317.1_133548-133868_Tragelaphus_strepsiceros_isolate_GTK-544_tarseq_11126_whole_genome_shotgun_sequence
SJYR01001988.1_18087-18408_Kobus_ellipsiprymnus_isolate_DFW-375_scaffold1997_size365645_whole_genome_shotgun_sequence
SJYQ01014272.1_14066-14387_Redunca_redunca_isolate_BHR-557_scaffold60577_len113941_cov115_whole_genome_shotgun_sequence
marine_VOSU01000024.1_16304106-16304750_Phocoena_sinus_isolate_mPhoSin1_chromosome_3_whole_genome_shotgun_sequence
marine_PKGA01003092.1_1323457-1323770_Phocoena_phocoena_ScshVNz_13164_whole_genome_shotgun_sequence
marine_PKGA01003092.1_1322786-1322996_Phocoena_phocoena_ScshVNz_13164_whole_genome_shotgun_sequence
marine_Neoasi_NW_020172287.1_0.0_1_645_1901188_1900545
marine_Monmon_NW_021703768.1_0.0_1_645_154325035_154324391
marine_Delleu_NW_019161003.1_0.0_1_645_1252445_1253089
marine_NCQN01000346.1_478572-479216_Tursiops_aduncus_isolate_CRI000098_scaffold_383_whole_genome_shotgun_sequence
marine_Turtru_NW_017843303.1_0.0_1_645_1040429_1041073
marine_NW_020838046.1_1991307-1991951_Lagenorhynchus_obliquidens
marine_Glomel_ML658913.1_0.0_1_645_1249253_1249897
marine_Orcorc_NW_004438620.1_0.0_1_645_1287357_1288001
marine_Souchi_RWJT01001730.1_0.0_1_645_1260223_1260858
marine_Lipvex_NW_006784081.1_0.0_1_645_275208_274560
marine_RJWO010001394.1_11424-12068_Inia_geoffrensis_isolate_BS63_IniGeo_flattened_line_2771_whole_genome_shotgun_sequence
query_marine_RJWS010014054.1_10058-10702_Ziphius_cavirostris_isolate_BS68_ZipCav_flattened_line_28088_whole_genome_shotgun_sequence
marine_PVJJ010007906.1_49733-50371_Mesoplodon_bidens_isolate_US080_MesBid_scaffold_15800_whole_genome_shotgun_sequence
marine_Phycat_NC_041215.1_0.0_1_645_11108704_11108060
marine_RJWL010019514.1_30387-31030_Kogia_breviceps_isolate_BS64_KogBre_flattened_line_39022_whole_genome_shotgun_sequence
marine_Megnov_RYZJ01000646.1_0.0_1_645_432184_432819
marine_DF428474.1_10834-11074_Balaenoptera_bonaerensis_DNA_contig__scaffold31448_whole_genome_shotgun_sequence
marine_VNFC01000003.1_154175677-154176313_Balaenoptera_musculus_isolate_JJ_BM4_2016_0621_Super_scaffold_3_whole_genome_shotgun_sequence
marine_Balacu_NW_006725588.1_0.0_1_644_4206989_4206355
marine_NIPP01004120.1_144367-145002_Eschrichtius_robustus_isolate_ER-14-168_kmer60-min500.11762577_whole_genome_shotgun_sequence
marine_DF428474.1_10532-10804_Balaenoptera_bonaerensis_DNA_contig__scaffold31448_whole_genome_shotgun_sequence
marine_RJWP010030156.1_10649-11299_Eubalaena_japonica_isolate_BS61_EubJap_flattened_line_60266_whole_genome_shotgun_sequence
marine_RJWK010022418.1_6680-7329_Platanista_minor_isolate_BS66_PlaMin_flattened_line_44801_whole_genome_shotgun_sequence
PVJP02000080.1_526545-527205_Hippopotamus_amphibius_isolate_BS01_ScHSfme_76HRSCAF_13031_whole_genome_shotgun_sequence
0.02

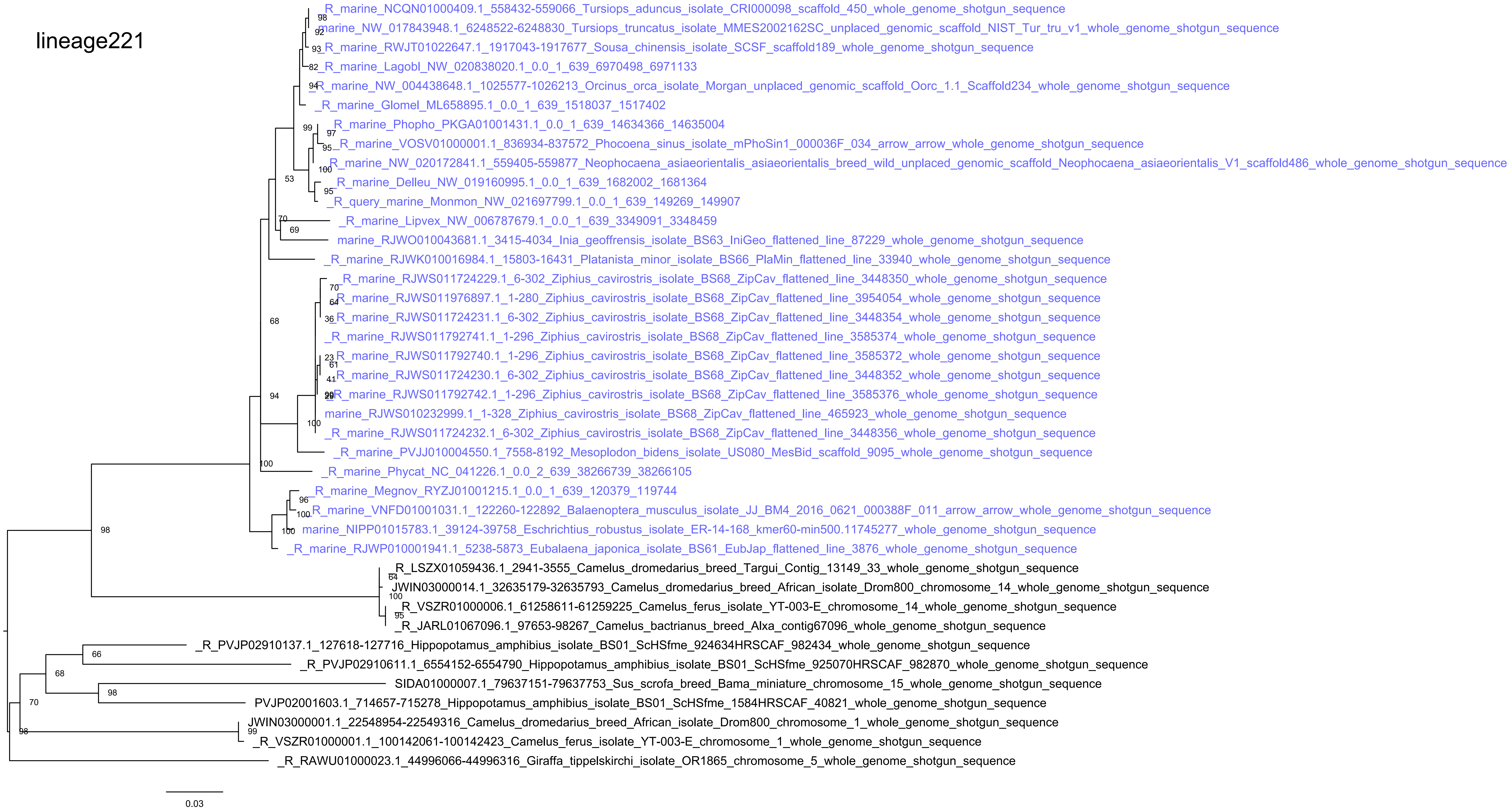

lineage221
_R_marine_NCQN01000409.1_558432-559066_Tursiops_aduncus_isolate_CRI000098_scaffold_450_whole_genome_shotgun_sequence
marine_NW_017843948.1_6248522-6248830_Tursiops_truncatus_isolate_MMES2002162SC_unplaced_genomic_scaffold_NIST_Tur_tru_v1_whole_genome_shotgun_sequence
_R_marine_RWJT01022647.1_1917043-1917677_Sousa_chinensis_isolate_SCSF_scaffold189_whole_genome_shotgun_sequence
_R_marine_Lagobl_NW_020838020.1_0.0_1_639_6970498_6971133
_R_marine_NW_004438648.1_1025577-1026213_Orcinus_orca_isolate_Morgan_unplaced_genomic_scaffold_Oorc_1.1_Scaffold234_whole_genome_shotgun_sequence
_R_marine_Glomel_ML658895.1_0.0_1_639_1518037_1517402
_R_marine_Phopho_PKGA01001431.1_0.0_1_639_14634366_14635004
_R_marine_VOSV01000001.1_836934-837572_Phocoena_sinus_isolate_mPhoSin1_000036F_034_arrow_arrow_whole_genome_shotgun_sequence
_R_marine_NW_020172841.1_559405-559877_Neophocaena_asiaeorientalis_asiaeorientalis_breed_wild_unplaced_genomic_scaffold_Neophocaena_asiaeorientalis_V1_scaffold486_whole_genome_shotgun_sequence
_R_marine_Delleu_NW_019160995.1_0.0_1_639_1682002_1681364
_R_query_marine_Monmon_NW_021697799.1_0.0_1_639_149269_149907
_R_marine_Lipvex_NW_006787679.1_0.0_1_639_3349091_3348459
marine_RJWO010043681.1_3415-4034_Inia_geoffrensis_isolate_BS63_IniGeo_flattened_line_87229_whole_genome_shotgun_sequence
_R_marine_RJWK010016984.1_15803-16431_Platanista_minor_isolate_BS66_PlaMin_flattened_line_33940_whole_genome_shotgun_sequence
_R_marine_RJWS011724229.1_6-302_Ziphius_cavirostris_isolate_BS68_ZipCav_flattened_line_3448350_whole_genome_shotgun_sequence
_R_marine_RJWS011976897.1_1-280_Ziphius_cavirostris_isolate_BS68_ZipCav_flattened_line_3954054_whole_genome_shotgun_sequence
_R_marine_RJWS011724231.1_6-302_Ziphius_cavirostris_isolate_BS68_ZipCav_flattened_line_3448354_whole_genome_shotgun_sequence
_R_marine_RJWS011792741.1_1-296_Ziphius_cavirostris_isolate_BS68_ZipCav_flattened_line_3585374_whole_genome_shotgun_sequence
_R_marine_RJWS011792740.1_1-296_Ziphius_cavirostris_isolate_BS68_ZipCav_flattened_line_3585372_whole_genome_shotgun_sequence
_R_marine_RJWS011724230.1_6-302_Ziphius_cavirostris_isolate_BS68_ZipCav_flattened_line_3448352_whole_genome_shotgun_sequence
_R_marine_RJWS011792742.1_1-296_Ziphius_cavirostris_isolate_BS68_ZipCav_flattened_line_3585376_whole_genome_shotgun_sequence
marine_RJWS010232999.1_1-328_Ziphius_cavirostris_isolate_BS68_ZipCav_flattened_line_465923_whole_genome_shotgun_sequence
_R_marine_RJWS011724232.1_6-302_Ziphius_cavirostris_isolate_BS68_ZipCav_flattened_line_3448356_whole_genome_shotgun_sequence
_R_marine_PVJJ010004550.1_7558-8192_Mesoplodon_bidens_isolate_US080_MesBid_scaffold_9095_whole_genome_shotgun_sequence
_R_marine_Phycat_NC_041226.1_0.0_2_639_38266739_38266105
_R_marine_Megnov_RYZJ01001215.1_0.0_1_639_120379_119744
_R_marine_VNFD01001031.1_122260-122892_Balaenoptera_musculus_isolate_JJ_BM4_2016_0621_000388F_011_arrow_arrow_whole_genome_shotgun_sequence
marine_NIPP01015783.1_39124-39758_Eschrichtius_robustus_isolate_ER-14-168_kmer60-min500.11745277_whole_genome_shotgun_sequence
_R_marine_RJWP010001941.1_5238-5873_Eubalaena_japonica_isolate_BS61_EubJap_flattened_line_3876_whole_genome_shotgun_sequence
_R_LSZX01059436.1_2941-3555_Camelus_dromedarius_breed_Targui_Contig_13149_33_whole_genome_shotgun_sequence
JWIN03000014.1_32635179-32635793_Camelus_dromedarius_breed_African_isolate_Drom800_chromosome_14_whole_genome_shotgun_sequence
_R_VSZR01000006.1_61258611-61259225_Camelus_ferus_isolate_YT-003-E_chromosome_14_whole_genome_shotgun_sequence
_R_JARL01067096.1_97653-98267_Camelus_bactrianus_breed_Alxa_contig67096_whole_genome_shotgun_sequence
_R_PVJP02910137.1_127618-127716_Hippopotamus_amphibius_isolate_BS01_ScHSfme_924634HRSCAF_982434_whole_genome_shotgun_sequence
_R_PVJP02910611.1_6554152-6554790_Hippopotamus_amphibius_isolate_BS01_ScHSfme_925070HRSCAF_982870_whole_genome_shotgun_sequence
SIDA01000007.1_79637151-79637753_Sus_scrofa_breed_Bama_miniature_chromosome_15_whole_genome_shotgun_sequence
PVJP02001603.1_714657-715278_Hippopotamus_amphibius_isolate_BS01_ScHSfme_1584HRSCAF_40821_whole_genome_shotgun_sequence
JWIN03000001.1_22548954-22549316_Camelus_dromedarius_breed_African_isolate_Drom800_chromosome_1_whole_genome_shotgun_sequence
_R_VSZR01000001.1_100142061-100142423_Camelus_ferus_isolate_YT-003-E_chromosome_1_whole_genome_shotgun_sequence
_R_RAWU01000023.1_44996066-44996316_Giraffa_tippelskirchi_isolate_OR1865_chromosome_5_whole_genome_shotgun_sequence
0.03

lineage222

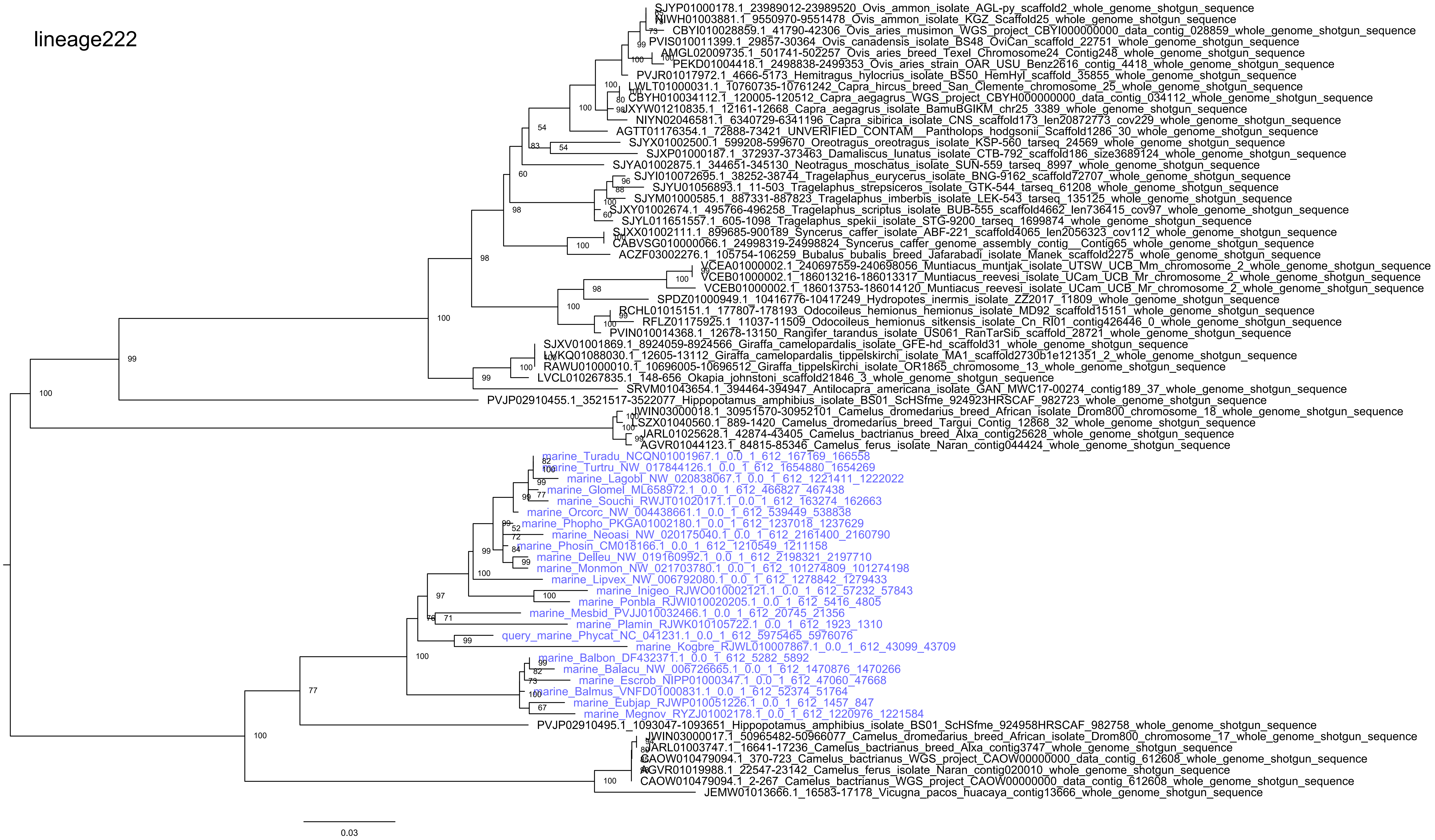
SJYP01000178.1_23989012-23989520_Ovis_ammon_isolate_AGL-py_scaffold2_whole_genome_shotgun_sequence
NIWH01003881.1_9550970-9551478_Ovis_ammon_isolate_KGZ_Scaffold25_whole_genome_shotgun_sequence
CBYI010028859.1_41790-42306_Ovis_aries_musimon_WGS_project_CBYI000000000_data_contig_028859_whole_genome_shotgun_sequence
PVIS010011399.1_29857-30364_Ovis_canadensis_isolate_BS48_OviCan_scaffold_22751_whole_genome_shotgun_sequence
AMGL02009735.1_501741-502257_Ovis_aries_breed_Texel_Chromosome24_Contig248_whole_genome_shotgun_sequence
PEKD01004418.1_2498838-2499353_Ovis_aries_strain_OAR_USU_Benz2616_contig_4418_whole_genome_shotgun_sequence
PVJR01017972.1_4666-5173_Hemitragus_hylocrius_isolate_BS50_HemHyl_scaffold_35855_whole_genome_shotgun_sequence
LWLT01000031.1_10760735-10761242_Capra_hircus_breed_San_Clemente_chromosome_25_whole_genome_shotgun_sequence
CBYH010034112.1_120005-120512_Capra_aegagrus_WGS_project_CBYH000000000_data_contig_034112_whole_genome_shotgun_sequence
JXYW01210835.1_12161-12668_Capra_aegagrus_isolate_BamuBGIKM_chr25_3389_whole_genome_shotgun_sequence
NIYN02046581.1_6340729-6341196_Capra_sibirica_isolate_CNS_scaffold173_len20872773_cov229_whole_genome_shotgun_sequence
AGTT01176354.1_72888-73421_UNVERIFIED_CONTAM__Pantholops_hodgsonii_Scaffold1286_30_whole_genome_shotgun_sequence
SJYX01002500.1_599208-599670_Oreotragus_oreotragus_isolate_KSP-560_tarseq_24569_whole_genome_shotgun_sequence
SJXP01000187.1_372937-373463_Damaliscus_lunatus_isolate_CTB-792_scaffold186_size3689124_whole_genome_shotgun_sequence
SJYA01002875.1_344651-345130_Neotragus_moschatus_isolate_SUN-559_tarseq_8997_whole_genome_shotgun_sequence
SJYI010072695.1_38252-38744_Tragelaphus_eurycerus_isolate_BNG-9162_scaffold72707_whole_genome_shotgun_sequence
SJYU01056893.1_11-503_Tragelaphus_strepsiceros_isolate_GTK-544_tarseq_61208_whole_genome_shotgun_sequence
SJYM01000585.1_887331-887823_Tragelaphus_imberbis_isolate_LEK-543_tarseq_135125_whole_genome_shotgun_sequence
SJXY01002674.1_495766-496258_Tragelaphus_scriptus_isolate_BUB-555_scaffold4662_len736415_cov97_whole_genome_shotgun_sequence
SJYL011651557.1_605-1098_Tragelaphus_spekii_isolate_STG-9200_tarseq_1699874_whole_genome_shotgun_sequence
SJXX01002111.1_899685-900189_Syncerus_caffer_isolate_ABF-221_scaffold4065_len2056323_cov112_whole_genome_shotgun_sequence
CABVSG010000066.1_24998319-24998824_Syncerus_caffer_genome_assembly_contig__Contig65_whole_genome_shotgun_sequence
ACZF03002276.1_105754-106259_Bubalus_bubalis_breed_Jafarabadi_isolate_Manek_scaffold2275_whole_genome_shotgun_sequence
VCEA01000002.1_240697559-240698056_Muntiacus_muntjak_isolate_UTSW_UCB_Mm_chromosome_2_whole_genome_shotgun_sequence
VCEB01000002.1_186013216-186013317_Muntiacus_reevesi_isolate_UCam_UCB_Mr_chromosome_2_whole_genome_shotgun_sequence
VCEB01000002.1_186013753-186014120_Muntiacus_reevesi_isolate_UCam_UCB_Mr_chromosome_2_whole_genome_shotgun_sequence
SPDZ01000949.1_10416776-10417249_Hydropotes_inermis_isolate_ZZ2017_11809_whole_genome_shotgun_sequence
RCHL01015151.1_177807-178193_Odocoileus_hemionus_hemionus_isolate_MD92_scaffold15151_whole_genome_shotgun_sequence
RFLZ01175925.1_11037-11509_Odocoileus_hemionus_sitkensis_isolate_Cn_RI01_contig426446_0_whole_genome_shotgun_sequence
PVIN010014368.1_12678-13150_Rangifer_tarandus_isolate_US061_RanTarSib_scaffold_28721_whole_genome_shotgun_sequence
SJXV01001869.1_8924059-8924566_Giraffa_camelopardalis_isolate_GFE-hd_scaffold31_whole_genome_shotgun_sequence
LVKQ01088030.1_12605-13112_Giraffa_camelopardalis_tippelskirchi_isolate_MA1_scaffold2730b1e121351_2_whole_genome_shotgun_sequence
RAWU01000010.1_10696005-10696512_Giraffa_tippelskirchi_isolate_OR1865_chromosome_13_whole_genome_shotgun_sequence
LVCL010267835.1_148-656_Okapia_johnstoni_scaffold21846_3_whole_genome_shotgun_sequence
SRVM01043654.1_394464-394947_Antilocapra_americana_isolate_GAN_MWC17-00274_contig189_37_whole_genome_shotgun_sequence
PVJP02910455.1_3521517-3522077_Hippopotamus_amphibius_isolate_BS01_ScHSfme_924923HRSCAF_982723_whole_genome_shotgun_sequence
JWIN03000018.1_30951570-30952101_Camelus_dromedarius_breed_African_isolate_Drom800_chromosome_18_whole_genome_shotgun_sequence
LSZX01040560.1_889-1420_Camelus_dromedarius_breed_Targui_Contig_12868_32_whole_genome_shotgun_sequence
JARL01025628.1_42874-43405_Camelus_bactrianus_breed_Alxa_contig25628_whole_genome_shotgun_sequence
AGVR01044123.1_84815-85346_Camelus_ferus_isolate_Naran_contig044424_whole_genome_shotgun_sequence
marine_Turadu_NCQN01001967.1_0.0_1_612_167169_166558
marine_Turtru_NW_017844126.1_0.0_1_612_1654880_1654269
marine_Lagobl_NW_020838067.1_0.0_1_612_1221411_1222022
marine_Glomel_ML658972.1_0.0_1_612_466827_467438
marine_Souchi_RWJT01020171.1_0.0_1_612_163274_162663
marine_Orcorc_NW_004438661.1_0.0_1_612_539449_538838
marine_Phopho_PKGA01002180.1_0.0_1_612_1237018_1237629
marine_Neoasi_NW_020175040.1_0.0_1_612_2161400_2160790
marine_Phosin_CM018166.1_0.0_1_612_1210549_1211158
marine_Delleu_NW_019160992.1_0.0_1_612_2198321_2197710
marine_Monmon_NW_021703780.1_0.0_1_612_101274809_101274198
marine_Lipvex_NW_006792080.1_0.0_1_612_1278842_1279433
marine_Inigeo_RJWO010002121.1_0.0_1_612_57232_57843
marine_Ponbla_RJWI010020205.1_0.0_1_612_5416_4805
marine_Mesbid_PVJJ010032466.1_0.0_1_612_20745_21356
marine_Plamin_RJWK010105722.1_0.0_1_612_1923_1310
query_marine_Phycat_NC_041231.1_0.0_1_612_5975465_5976076
marine_Kogbre_RJWL010007867.1_0.0_1_612_43099_43709
marine_Balbon_DF432371.1_0.0_1_612_5282_5892
marine_Balacu_NW_006726665.1_0.0_1_612_1470876_1470266
marine_Escrob_NIPP01000347.1_0.0_1_612_47060_47668
marine_Balmus_VNFD01000831.1_0.0_1_612_52374_51764
marine_Eubjap_RJWP010051226.1_0.0_1_612_1457_847
marine_Megnov_RYZJ01002178.1_0.0_1_612_1220976_1221584
PVJP02910495.1_1093047-1093651_Hippopotamus_amphibius_isolate_BS01_ScHSfme_924958HRSCAF_982758_whole_genome_shotgun_sequence
JWIN03000017.1_50965482-50966077_Camelus_dromedarius_breed_African_isolate_Drom800_chromosome_17_whole_genome_shotgun_sequence
JARL01003747.1_16641-17236_Camelus_bactrianus_breed_Alxa_contig3747_whole_genome_shotgun_sequence
CAOW010479094.1_370-723_Camelus_bactrianus_WGS_project_CAOW00000000_data_contig_612608_whole_genome_shotgun_sequence
AGVR01019988.1_22547-23142_Camelus_ferus_isolate_Naran_contig020010_whole_genome_shotgun_sequence
CAOW010479094.1_2-267_Camelus_bactrianus_WGS_project_CAOW00000000_data_contig_612608_whole_genome_shotgun_sequence
JEMW01013666.1_16583-17178_Vicugna_pacos_huacaya_contig13666_whole_genome_shotgun_sequence
0.03

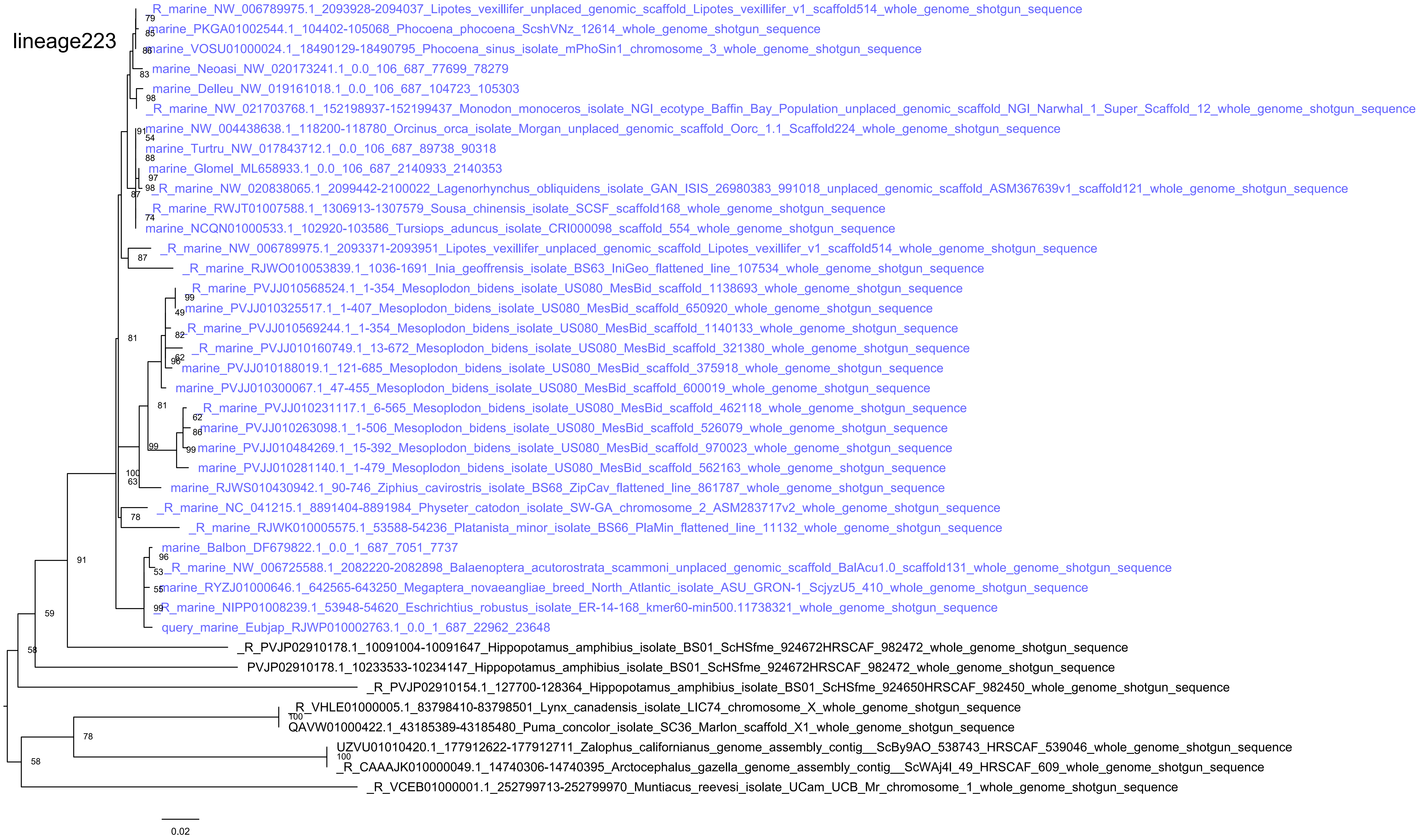

lineage223
_R_marine_NW_006789975.1_2093928-2094037_Lipotes_vexillifer_unplaced_genomic_scaffold_Lipotes_vexillifer_v1_scaffold514_whole_genome_shotgun_sequence
marine_PKGA01002544.1_104402-105068_Phocoena_phocoena_ScshVNz_12614_whole_genome_shotgun_sequence
marine_VOSU01000024.1_18490129-18490795_Phocoena_sinus_isolate_mPhoSin1_chromosome_3_whole_genome_shotgun_sequence
marine_Neoasi_NW_020173241.1_0.0_106_687_77699_78279
marine_Delleu_NW_019161018.1_0.0_106_687_104723_105303
_R_marine_NW_021703768.1_152198937-152199437_Monodon_monoceros_isolate_NGI_ecotype_Baffin_Bay_Population_unplaced_genomic_scaffold_NGI_Narwhal_1_Super_Scaffold_12_whole_genome_shotgun_sequence
marine_NW_004438638.1_118200-118780_Orcinus_orca_isolate_Morgan_unplaced_genomic_scaffold_Oorc_1.1_Scaffold224_whole_genome_shotgun_sequence
marine_Turtru_NW_017843712.1_0.0_106_687_89738_90318
marine_Glomel_ML658933.1_0.0_106_687_2140933_2140353
_R_marine_NW_020838065.1_2099442-2100022_Lagenorhynchus_obliquidens_isolate_GAN_ISIS_26980383_991018_unplaced_genomic_scaffold_ASM367639v1_scaffold121_whole_genome_shotgun_sequence
_R_marine_RWJT01007588.1_1306913-1307579_Sousa_chinensis_isolate_SCSF_scaffold168_whole_genome_shotgun_sequence
marine_NCQN01000533.1_102920-103586_Tursiops_aduncus_isolate_CRI000098_scaffold_554_whole_genome_shotgun_sequence
_R_marine_NW_006789975.1_2093371-2093951_Lipotes_vexillifer_unplaced_genomic_scaffold_Lipotes_vexillifer_v1_scaffold514_whole_genome_shotgun_sequence
_R_marine_RJWO010053839.1_1036-1691_Inia_geoffrensis_isolate_BS63_IniGeo_flattened_line_107534_whole_genome_shotgun_sequence
_R_marine_PVJJ010568524.1_1-354_Mesoplodon_bidens_isolate_US080_MesBid_scaffold_1138693_whole_genome_shotgun_sequence
marine_PVJJ010325517.1_1-407_Mesoplodon_bidens_isolate_US080_MesBid_scaffold_650920_whole_genome_shotgun_sequence
_R_marine_PVJJ010569244.1_1-354_Mesoplodon_bidens_isolate_US080_MesBid_scaffold_1140133_whole_genome_shotgun_sequence
_R_marine_PVJJ010160749.1_13-672_Mesoplodon_bidens_isolate_US080_MesBid_scaffold_321380_whole_genome_shotgun_sequence
marine_PVJJ010188019.1_121-685_Mesoplodon_bidens_isolate_US080_MesBid_scaffold_375918_whole_genome_shotgun_sequence
marine_PVJJ010300067.1_47-455_Mesoplodon_bidens_isolate_US080_MesBid_scaffold_600019_whole_genome_shotgun_sequence
_R_marine_PVJJ010231117.1_6-565_Mesoplodon_bidens_isolate_US080_MesBid_scaffold_462118_whole_genome_shotgun_sequence
marine_PVJJ010263098.1_1-506_Mesoplodon_bidens_isolate_US080_MesBid_scaffold_526079_whole_genome_shotgun_sequence
marine_PVJJ010484269.1_15-392_Mesoplodon_bidens_isolate_US080_MesBid_scaffold_970023_whole_genome_shotgun_sequence
marine_PVJJ010281140.1_1-479_Mesoplodon_bidens_isolate_US080_MesBid_scaffold_562163_whole_genome_shotgun_sequence
marine_RJWS010430942.1_90-746_Ziphius_cavirostris_isolate_BS68_ZipCav_flattened_line_861787_whole_genome_shotgun_sequence
_R_marine_NC_041215.1_8891404-8891984_Physeter_catodon_isolate_SW-GA_chromosome_2_ASM283717v2_whole_genome_shotgun_sequence
_R_marine_RJWK010005575.1_53588-54236_Platanista_minor_isolate_BS66_PlaMin_flattened_line_11132_whole_genome_shotgun_sequence
marine_Balbon_DF679822.1_0.0_1_687_7051_7737
_R_marine_NW_006725588.1_2082220-2082898_Balaenoptera_acutorostrata_scammoni_unplaced_genomic_scaffold_BalAcu1.0_scaffold131_whole_genome_shotgun_sequence
marine_RYZJ01000646.1_642565-643250_Megaptera_novaeangliae_breed_North_Atlantic_isolate_ASU_GRON-1_ScjyzU5_410_whole_genome_shotgun_sequence
_R_marine_NIPP01008239.1_53948-54620_Eschrichtius_robustus_isolate_ER-14-168_kmer60-min500.11738321_whole_genome_shotgun_sequence
query_marine_Eubjap_RJWP010002763.1_0.0_1_687_22962_23648
_R_PVJP02910178.1_10091004-10091647_Hippopotamus_amphibius_isolate_BS01_ScHSfme_924672HRSCAF_982472_whole_genome_shotgun_sequence
PVJP02910178.1_10233533-10234147_Hippopotamus_amphibius_isolate_BS01_ScHSfme_924672HRSCAF_982472_whole_genome_shotgun_sequence
_R_PVJP02910154.1_127700-128364_Hippopotamus_amphibius_isolate_BS01_ScHSfme_924650HRSCAF_982450_whole_genome_shotgun_sequence
_R_VHLE01000005.1_83798410-83798501_Lynx_canadensis_isolate_LIC74_chromosome_X_whole_genome_shotgun_sequence
QAVW01000422.1_43185389-43185480_Puma_concolor_isolate_SC36_Marlon_scaffold_X1_whole_genome_shotgun_sequence
UZVU01010420.1_177912622-177912711_Zalophus_californianus_genome_assembly_contig__ScBy9AO_538743_HRSCAF_539046_whole_genome_shotgun_sequence
_R_CAAAJK010000049.1_14740306-14740395_Arctocephalus_gazella_genome_assembly_contig__ScWAj4I_49_HRSCAF_609_whole_genome_shotgun_sequence
_R_VCEB01000001.1_252799713-252799970_Muntiacus_reevesi_isolate_UCam_UCB_Mr_chromosome_1_whole_genome_shotgun_sequence
0.02

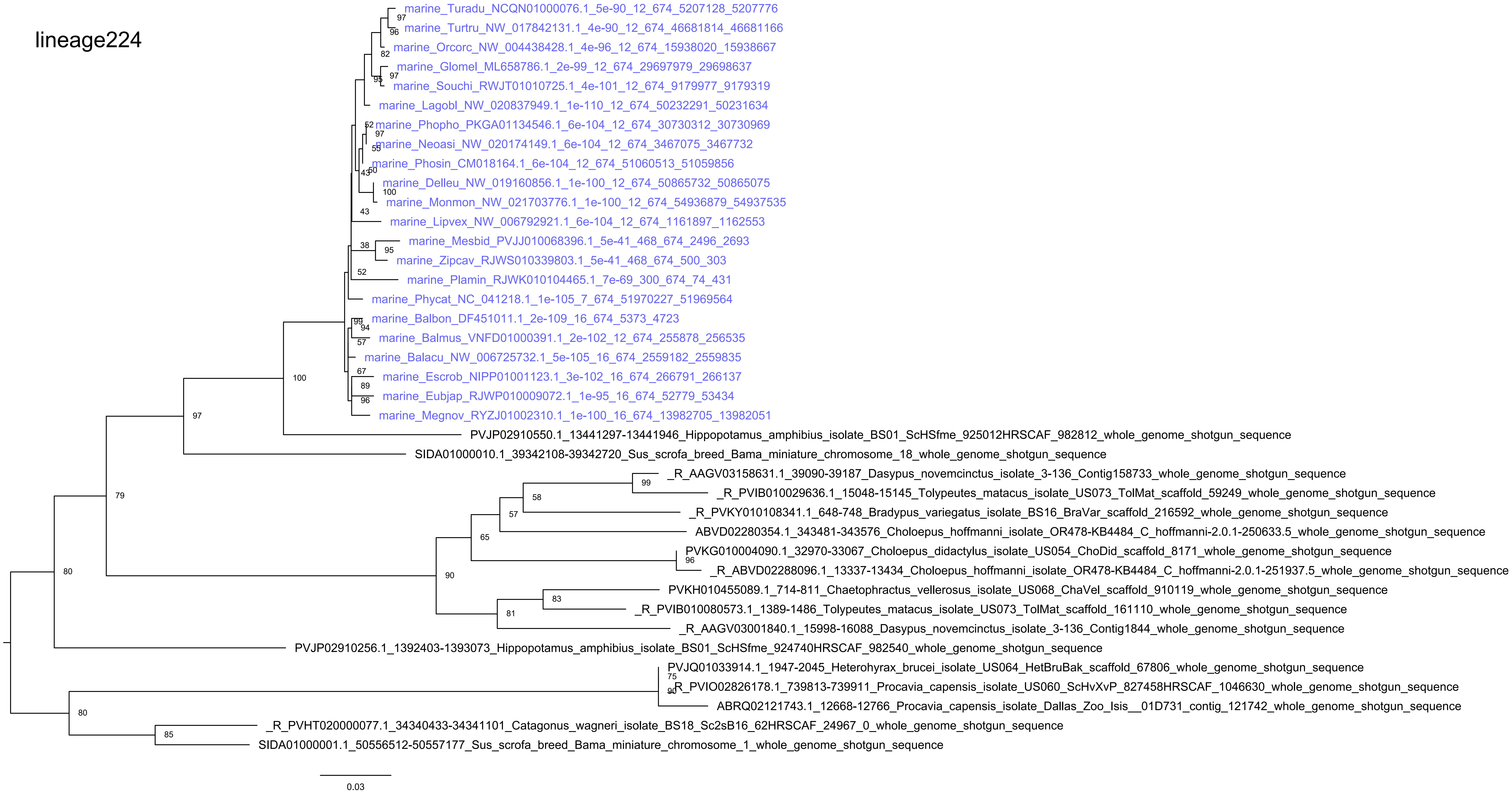

lineage224
marine_Turadu_NCQN01000076.1_5e-90_12_674_5207128_5207776
marine_Turtru_NW_017842131.1_4e-90_12_674_46681814_46681166
marine_Orcorc_NW_004438428.1_4e-96_12_674_15938020_15938667
marine_Glomel_ML658786.1_2e-99_12_674_29697979_29698637
marine_Souchi_RWJT01010725.1_4e-101_12_674_9179977_9179319
marine_Lagobl_NW_020837949.1_1e-110_12_674_50232291_50231634
marine_Phopho_PKGA01134546.1_6e-104_12_674_30730312_30730969
marine_Neoasi_NW_020174149.1_6e-104_12_674_3467075_3467732
marine_Phosin_CM018164.1_6e-104_12_674_51060513_51059856
marine_Delleu_NW_019160856.1_1e-100_12_674_50865732_50865075
marine_Monmon_NW_021703776.1_1e-100_12_674_54936879_54937535
marine_Lipvex_NW_006792921.1_6e-104_12_674_1161897_1162553
marine_Mesbid_PVJJ010068396.1_5e-41_468_674_2496_2693
marine_Zipcav_RJWS010339803.1_5e-41_468_674_500_303
marine_Plamin_RJWK010104465.1_7e-69_300_674_74_431
marine_Phycat_NC_041218.1_1e-105_7_674_51970227_51969564
marine_Balbon_DF451011.1_2e-109_16_674_5373_4723
marine_Balmus_VNFD01000391.1_2e-102_12_674_255878_256535
marine_Balacu_NW_006725732.1_5e-105_16_674_2559182_2559835
marine_Escrob_NIPP01001123.1_3e-102_16_674_266791_266137
marine_Eubjap_RJWP010009072.1_1e-95_16_674_52779_53434
marine_Megnov_RYZJ01002310.1_1e-100_16_674_13982705_13982051
PVJP02910550.1_13441297-13441946_Hippopotamus_amphibius_isolate_BS01_ScHSfme_925012HRSCAF_982812_whole_genome_shotgun_sequence
SIDA01000010.1_39342108-39342720_Sus_scrofa_breed_Bama_miniature_chromosome_18_whole_genome_shotgun_sequence
_R_AAGV03158631.1_39090-39187_Dasypus_novemcinctus_isolate_3-136_Contig158733_whole_genome_shotgun_sequence
_R_PVIB010029636.1_15048-15145_Tolypeutes_matacus_isolate_US073_TolMat_scaffold_59249_whole_genome_shotgun_sequence
_R_PVKY010108341.1_648-748_Bradypus_variegatus_isolate_BS16_BraVar_scaffold_216592_whole_genome_shotgun_sequence
ABVD02280354.1_343481-343576_Choloepus_hoffmanni_isolate_OR478-KB4484_C_hoffmanni-2.0.1-250633.5_whole_genome_shotgun_sequence
PVKG010004090.1_32970-33067_Choloepus_didactylus_isolate_US054_ChoDid_scaffold_8171_whole_genome_shotgun_sequence
_R_ABVD02288096.1_13337-13434_Choloepus_hoffmanni_isolate_OR478-KB4484_C_hoffmanni-2.0.1-251937.5_whole_genome_shotgun_sequence
PVKH010455089.1_714-811_Chaetophractus_vellerosus_isolate_US068_ChaVel_scaffold_910119_whole_genome_shotgun_sequence
_R_PVIB010080573.1_1389-1486_Tolypeutes_matacus_isolate_US073_TolMat_scaffold_161110_whole_genome_shotgun_sequence
_R_AAGV03001840.1_15998-16088_Dasypus_novemcinctus_isolate_3-136_Contig1844_whole_genome_shotgun_sequence
PVJP02910256.1_1392403-1393073_Hippopotamus_amphibius_isolate_BS01_ScHSfme_924740HRSCAF_982540_whole_genome_shotgun_sequence
PVJQ01033914.1_1947-2045_Heterohyrax_brucei_isolate_US064_HetBruBak_scaffold_67806_whole_genome_shotgun_sequence
_R_PVIO02826178.1_739813-739911_Procavia_capensis_isolate_US060_ScHvXvP_827458HRSCAF_1046630_whole_genome_shotgun_sequence
ABRQ02121743.1_12668-12766_Procavia_capensis_isolate_Dallas_Zoo_Isis__01D731_contig_121742_whole_genome_shotgun_sequence
_R_PVHT020000077.1_34340433-34341101_Catagonus_wagneri_isolate_BS18_Sc2sB16_62HRSCAF_24967_0_whole_genome_shotgun_sequence
SIDA01000001.1_50556512-50557177_Sus_scrofa_breed_Bama_miniature_chromosome_1_whole_genome_shotgun_sequence
0.03

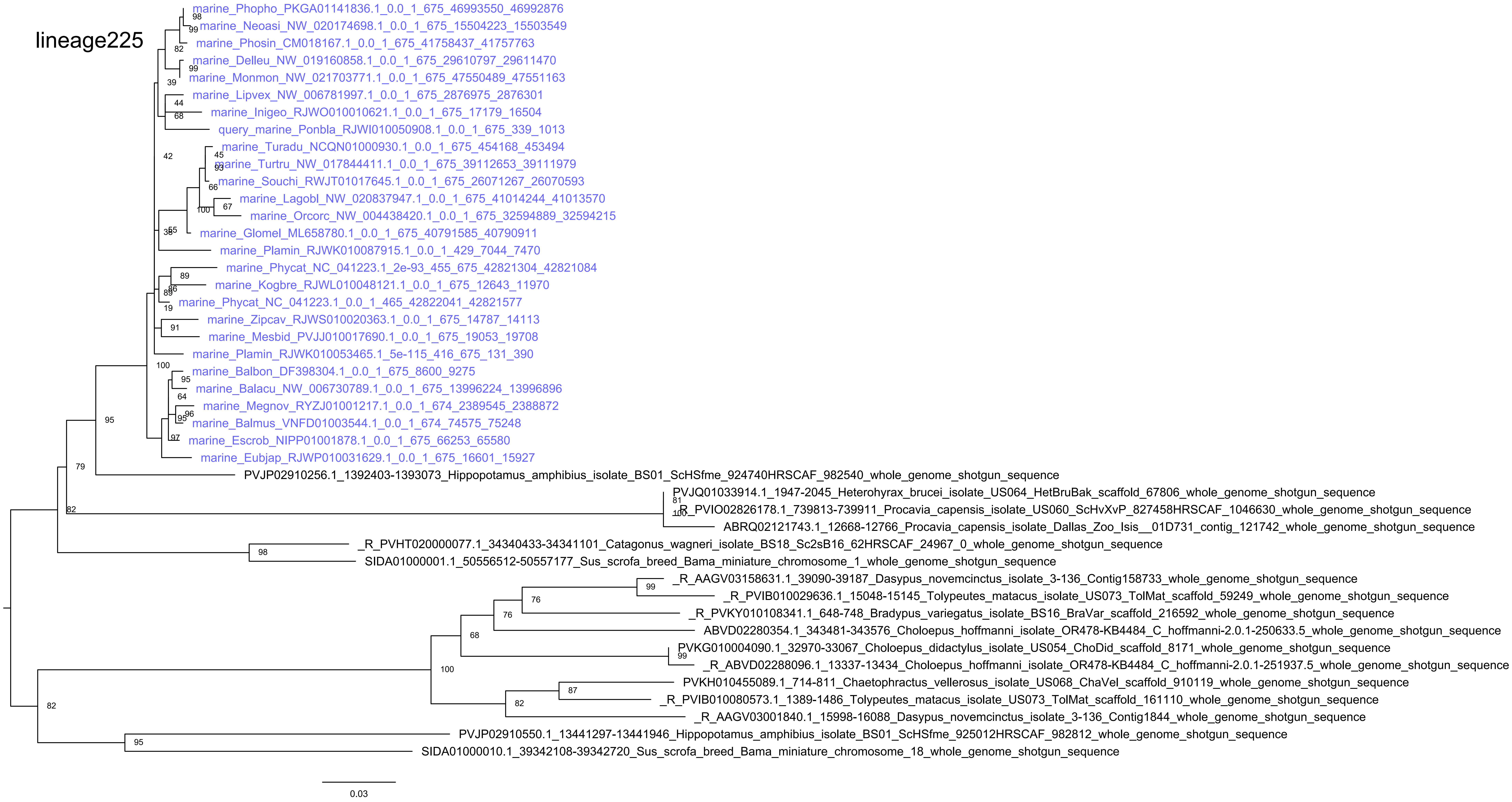
lineage225
marine_Phopho_PKGA01141836.1_0.0_1_675_46993550_46992876
marine_Neoasi_NW_020174698.1_0.0_1_675_15504223_15503549
marine_Phosin_CM018167.1_0.0_1_675_41758437_41757763
marine_Delleu_NW_019160858.1_0.0_1_675_29610797_29611470
marine_Monmon_NW_021703771.1_0.0_1_675_47550489_47551163
marine_Lipvex_NW_006781997.1_0.0_1_675_2876975_2876301
marine_Inigeo_RJWO010010621.1_0.0_1_675_17179_16504
query_marine_Ponbla_RJWI010050908.1_0.0_1_675_339_1013
marine_Turadu_NCQN01000930.1_0.0_1_675_454168_453494
marine_Turtru_NW_017844411.1_0.0_1_675_39112653_39111979
marine_Souchi_RWJT01017645.1_0.0_1_675_26071267_26070593
marine_Lagobl_NW_020837947.1_0.0_1_675_41014244_41013570
marine_Orcorc_NW_004438420.1_0.0_1_675_32594889_32594215
marine_Glomel_ML658780.1_0.0_1_675_40791585_40790911
marine_Plamin_RJWK010087915.1_0.0_1_429_7044_7470
marine_Phycat_NC_041223.1_2e-93_455_675_42821304_42821084
marine_Kogbre_RJWL010048121.1_0.0_1_675_12643_11970
marine_Phycat_NC_041223.1_0.0_1_465_42822041_42821577
marine_Zipcav_RJWS010020363.1_0.0_1_675_14787_14113
marine_Mesbid_PVJJ010017690.1_0.0_1_675_19053_19708
marine_Plamin_RJWK010053465.1_5e-115_416_675_131_390
marine_Balbon_DF398304.1_0.0_1_675_8600_9275
marine_Balacu_NW_006730789.1_0.0_1_675_13996224_13996896
marine_Megnov_RYZJ01001217.1_0.0_1_674_2389545_2388872
marine_Balmus_VNFD01003544.1_0.0_1_674_74575_75248
marine_Escrob_NIPP01001878.1_0.0_1_675_66253_65580
marine_Eubjap_RJWP010031629.1_0.0_1_675_16601_15927
PVJP02910256.1_1392403-1393073_Hippopotamus_amphibius_isolate_BS01_ScHSfme_924740HRSCAF_982540_whole_genome_shotgun_sequence
PVJQ01033914.1_1947-2045_Heterohyrax_brucei_isolate_US064_HetBruBak_scaffold_67806_whole_genome_shotgun_sequence
_R_PVIO02826178.1_739813-739911_Procavia_capensis_isolate_US060_ScHvXvP_827458HRSCAF_1046630_whole_genome_shotgun_sequence
ABRQ02121743.1_12668-12766_Procavia_capensis_isolate_Dallas_Zoo_Isis__01D731_contig_121742_whole_genome_shotgun_sequence
_R_PVHT020000077.1_34340433-34341101_Catagonus_wagneri_isolate_BS18_Sc2sB16_62HRSCAF_24967_0_whole_genome_shotgun_sequence
SIDA01000001.1_50556512-50557177_Sus_scrofa_breed_Bama_miniature_chromosome_1_whole_genome_shotgun_sequence
_R_AAGV03158631.1_39090-39187_Dasypus_novemcinctus_isolate_3-136_Contig158733_whole_genome_shotgun_sequence
_R_PVIB010029636.1_15048-15145_Tolypeutes_matacus_isolate_US073_TolMat_scaffold_59249_whole_genome_shotgun_sequence
_R_PVKY010108341.1_648-748_Bradypus_variegatus_isolate_BS16_BraVar_scaffold_216592_whole_genome_shotgun_sequence
ABVD02280354.1_343481-343576_Choloepus_hoffmanni_isolate_OR478-KB4484_C_hoffmanni-2.0.1-250633.5_whole_genome_shotgun_sequence
PVKG010004090.1_32970-33067_Choloepus_didactylus_isolate_US054_ChoDid_scaffold_8171_whole_genome_shotgun_sequence
_R_ABVD02288096.1_13337-13434_Choloepus_hoffmanni_isolate_OR478-KB4484_C_hoffmanni-2.0.1-251937.5_whole_genome_shotgun_sequence
PVKH010455089.1_714-811_Chaetophractus_vellerosus_isolate_US068_ChaVel_scaffold_910119_whole_genome_shotgun_sequence
_R_PVIB010080573.1_1389-1486_Tolypeutes_matacus_isolate_US073_TolMat_scaffold_161110_whole_genome_shotgun_sequence
_R_AAGV03001840.1_15998-16088_Dasypus_novemcinctus_isolate_3-136_Contig1844_whole_genome_shotgun_sequence
PVJP02910550.1_13441297-13441946_Hippopotamus_amphibius_isolate_BS01_ScHSfme_925012HRSCAF_982812_whole_genome_shotgun_sequence
SIDA01000010.1_39342108-39342720_Sus_scrofa_breed_Bama_miniature_chromosome_18_whole_genome_shotgun_sequence
0.03

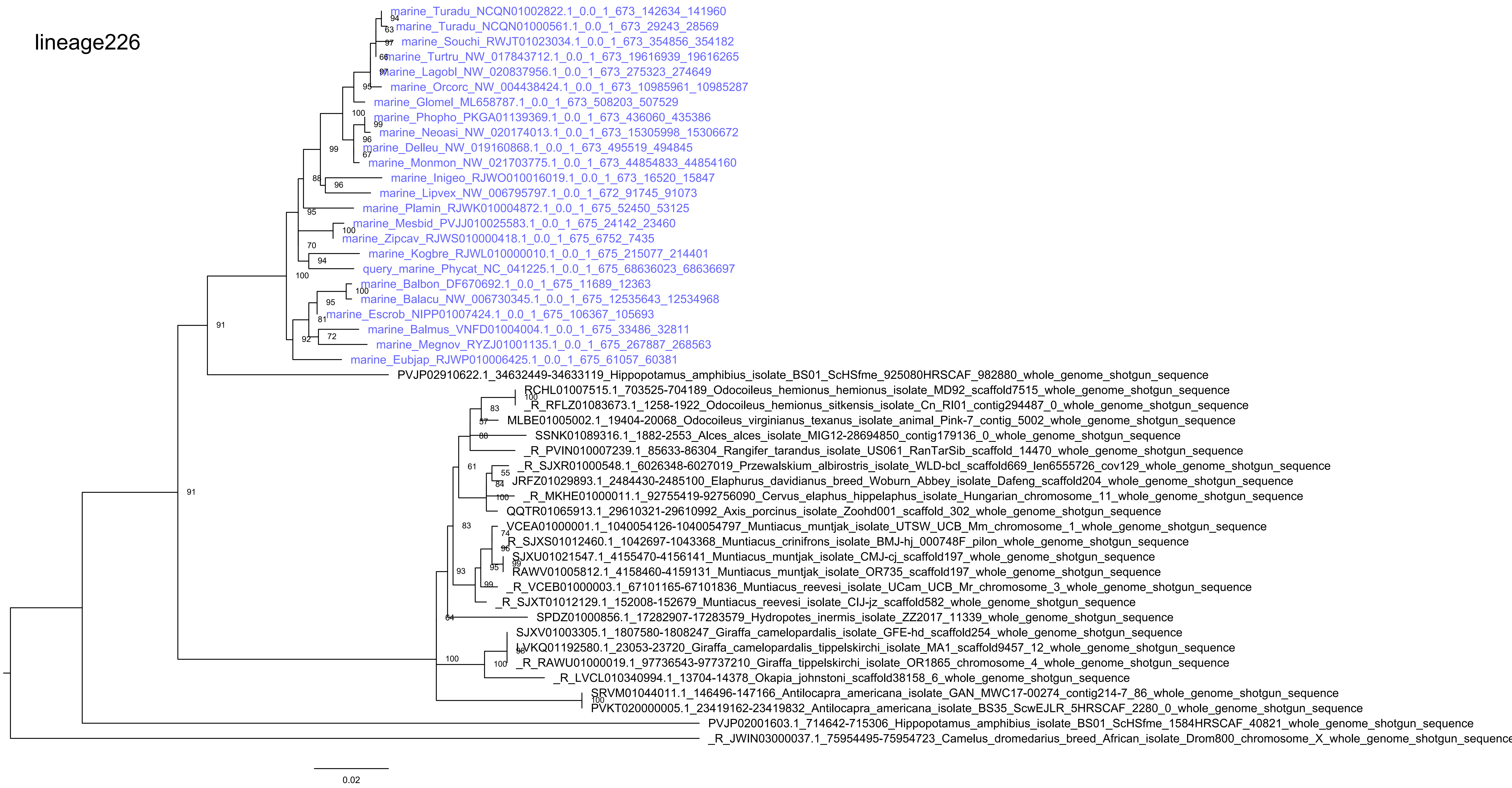
lineage226
marine_Turadu_NCQN01002822.1_0.0_1_673_142634_141960
marine_Turadu_NCQN01000561.1_0.0_1_673_29243_28569
marine_Souchi_RWJT01023034.1_0.0_1_673_354856_354182
marine_Turtru_NW_017843712.1_0.0_1_673_19616939_19616265
marine_Lagobl_NW_020837956.1_0.0_1_673_275323_274649
marine_Orcorc_NW_004438424.1_0.0_1_673_10985961_10985287
marine_Glomel_ML658787.1_0.0_1_673_508203_507529
marine_Phopho_PKGA01139369.1_0.0_1_673_436060_435386
marine_Neoasi_NW_020174013.1_0.0_1_673_15305998_15306672
marine_Delleu_NW_019160868.1_0.0_1_673_495519_494845
marine_Monmon_NW_021703775.1_0.0_1_673_44854833_44854160
marine_Inigeo_RJWO010016019.1_0.0_1_673_16520_15847
marine_Lipvex_NW_006795797.1_0.0_1_672_91745_91073
marine_Plamin_RJWK010004872.1_0.0_1_675_52450_53125
marine_Mesbid_PVJJ010025583.1_0.0_1_675_24142_23460
marine_Zipcav_RJWS010000418.1_0.0_1_675_6752_7435
marine_Kogbre_RJWL010000010.1_0.0_1_675_215077_214401
query_marine_Phycat_NC_041225.1_0.0_1_675_68636023_68636697
marine_Balbon_DF670692.1_0.0_1_675_11689_12363
marine_Balacu_NW_006730345.1_0.0_1_675_12535643_12534968
marine_Escrob_NIPP01007424.1_0.0_1_675_106367_105693
marine_Balmus_VNFD01004004.1_0.0_1_675_33486_32811
marine_Megnov_RYZJ01001135.1_0.0_1_675_267887_268563
marine_Eubjap_RJWP010006425.1_0.0_1_675_61057_60381
PVJP02910622.1_34632449-34633119_Hippopotamus_amphibius_isolate_BS01_ScHSfme_925080HRSCAF_982880_whole_genome_shotgun_sequence
RCHL01007515.1_703525-704189_Odocoileus_hemionus_hemionus_isolate_MD92_scaffold7515_whole_genome_shotgun_sequence
_R_RFLZ01083673.1_1258-1922_Odocoileus_hemionus_sitkensis_isolate_Cn_RI01_contig294487_0_whole_genome_shotgun_sequence
MLBE01005002.1_19404-20068_Odocoileus_virginianus_texanus_isolate_animal_Pink-7_contig_5002_whole_genome_shotgun_sequence
SSNK01089316.1_1882-2553_Alces_alces_isolate_MIG12-28694850_contig179136_0_whole_genome_shotgun_sequence
_R_PVIN010007239.1_85633-86304_Rangifer_tarandus_isolate_US061_RanTarSib_scaffold_14470_whole_genome_shotgun_sequence
_R_SJXR01000548.1_6026348-6027019_Przewalskium_albirostris_isolate_WLD-bcl_scaffold669_len6555726_cov129_whole_genome_shotgun_sequence
JRFZ01029893.1_2484430-2485100_Elaphurus_davidianus_breed_Woburn_Abbey_isolate_Dafeng_scaffold204_whole_genome_shotgun_sequence
_R_MKHE01000011.1_92755419-92756090_Cervus_elaphus_hippelaphus_isolate_Hungarian_chromosome_11_whole_genome_shotgun_sequence
QQTR01065913.1_29610321-29610992_Axis_porcinus_isolate_Zoohd001_scaffold_302_whole_genome_shotgun_sequence
VCEA01000001.1_1040054126-1040054797_Muntiacus_muntjak_isolate_UTSW_UCB_Mm_chromosome_1_whole_genome_shotgun_sequence
_R_SJXS01012460.1_1042697-1043368_Muntiacus_crinifrons_isolate_BMJ-hj_000748F_pilon_whole_genome_shotgun_sequence
SJXU01021547.1_4155470-4156141_Muntiacus_muntjak_isolate_CMJ-cj_scaffold197_whole_genome_shotgun_sequence
RAWV01005812.1_4158460-4159131_Muntiacus_muntjak_isolate_OR735_scaffold197_whole_genome_shotgun_sequence
_R_VCEB01000003.1_67101165-67101836_Muntiacus_reevesi_isolate_UCam_UCB_Mr_chromosome_3_whole_genome_shotgun_sequence
_R_SJXT01012129.1_152008-152679_Muntiacus_reevesi_isolate_CIJ-jz_scaffold582_whole_genome_shotgun_sequence
SPDZ01000856.1_17282907-17283579_Hydropotes_inermis_isolate_ZZ2017_11339_whole_genome_shotgun_sequence
SJXV01003305.1_1807580-1808247_Giraffa_camelopardalis_isolate_GFE-hd_scaffold254_whole_genome_shotgun_sequence
LVKQ01192580.1_23053-23720_Giraffa_camelopardalis_tippelskirchi_isolate_MA1_scaffold9457_12_whole_genome_shotgun_sequence
_R_RAWU01000019.1_97736543-97737210_Giraffa_tippelskirchi_isolate_OR1865_chromosome_4_whole_genome_shotgun_sequence
_R_LVCL010340994.1_13704-14378_Okapia_johnstoni_scaffold38158_6_whole_genome_shotgun_sequence
SRVM01044011.1_146496-147166_Antilocapra_americana_isolate_GAN_MWC17-00274_contig214-7_86_whole_genome_shotgun_sequence
PVKT020000005.1_23419162-23419832_Antilocapra_americana_isolate_BS35_ScwEJLR_5HRSCAF_2280_0_whole_genome_shotgun_sequence
PVJP02001603.1_714642-715306_Hippopotamus_amphibius_isolate_BS01_ScHSfme_1584HRSCAF_40821_whole_genome_shotgun_sequence
_R_JWIN03000037.1_75954495-75954723_Camelus_dromedarius_breed_African_isolate_Drom800_chromosome_X_whole_genome_shotgun_sequence
0.02

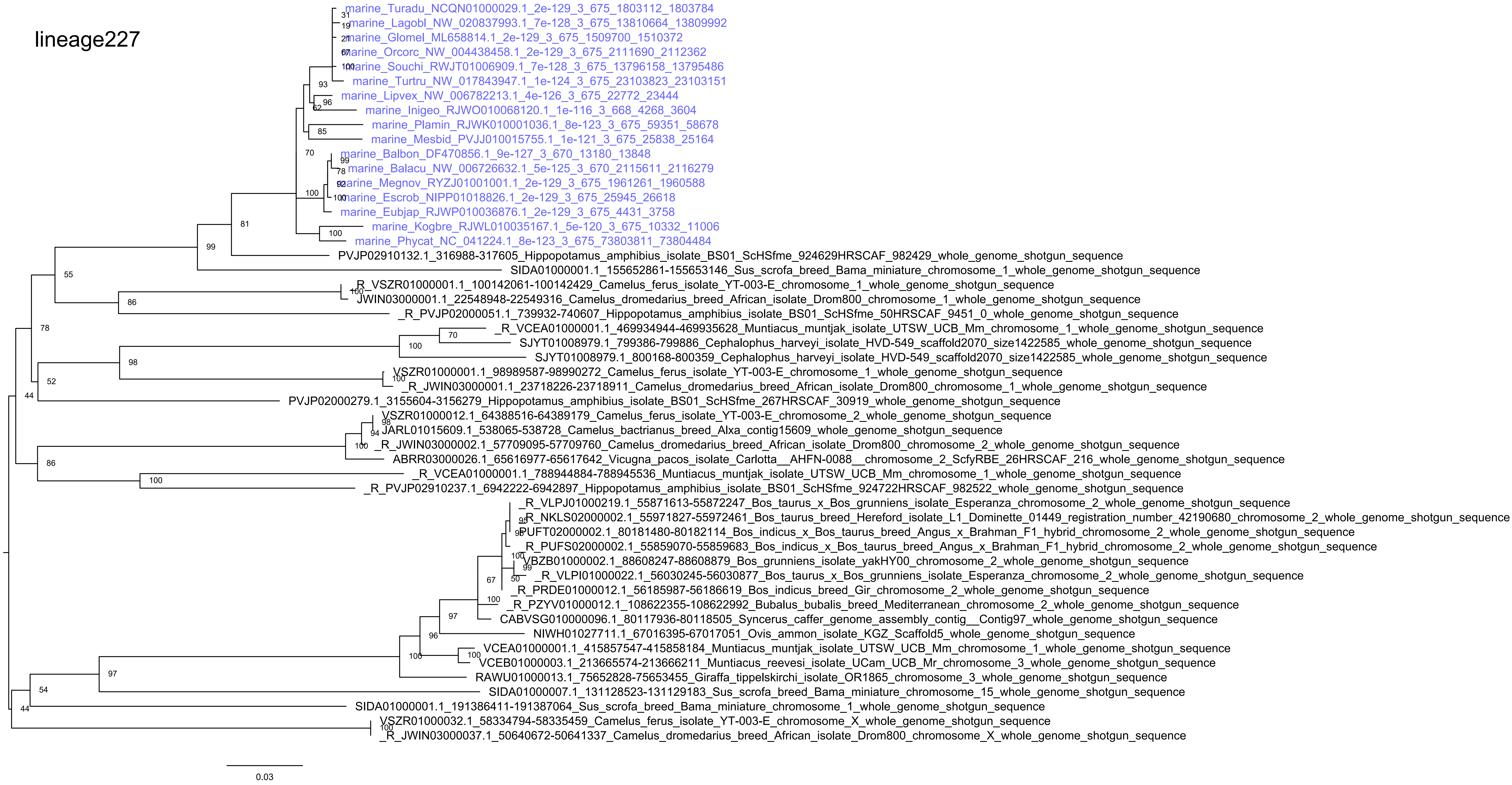
lineage227
marine_Turadu_NCQN01000029.1_2e-129_3_675_1803112_1803784
marine_Lagobl_NW_020837993.1_7e-128_3_675_13810664_13809992
marine_Glomel_ML658814.1_2e-129_3_675_1509700_1510372
marine_Orcorc_NW_004438458.1_2e-129_3_675_2111690_2112362
marine_Souchi_RWJT01006909.1_7e-128_3_675_13796158_13795486
marine_Turtru_NW_017843947.1_1e-124_3_675_23103823_23103151
marine_Lipvex_NW_006782213.1_4e-126_3_675_22772_23444
marine_Inigeo_RJWO010068120.1_1e-116_3_668_4268_3604
marine_Plamin_RJWK010001036.1_8e-123_3_675_59351_58678
marine_Mesbid_PVJJ010015755.1_1e-121_3_675_25838_25164
marine_Balbon_DF470856.1_9e-127_3_670_13180_13848
marine_Balacu_NW_006726632.1_5e-125_3_670_2115611_2116279
marine_Megnov_RYZJ01001001.1_2e-129_3_675_1961261_1960588
marine_Escrob_NIPP01018826.1_2e-129_3_675_25945_26618
marine_Eubjap_RJWP010036876.1_2e-129_3_675_4431_3758
marine_Kogbre_RJWL010035167.1_5e-120_3_675_10332_11006
marine_Phycat_NC_041224.1_8e-123_3_675_73803811_73804484
PVJP02910132.1_316988-317605_Hippopotamus_amphibius_isolate_BS01_ScHSfme_924629HRSCAF_982429_whole_genome_shotgun_sequence
SIDA01000001.1_155652861-155653146_Sus_scrofa_breed_Bama_miniature_chromosome_1_whole_genome_shotgun_sequence
_R_VSZR01000001.1_100142061-100142429_Camelus_ferus_isolate_YT-003-E_chromosome_1_whole_genome_shotgun_sequence
JWIN03000001.1_22548948-22549316_Camelus_dromedarius_breed_African_isolate_Drom800_chromosome_1_whole_genome_shotgun_sequence
_R_PVJP02000051.1_739932-740607_Hippopotamus_amphibius_isolate_BS01_ScHSfme_50HRSCAF_9451_0_whole_genome_shotgun_sequence
_R_VCEA01000001.1_469934944-469935628_Muntiacus_muntjak_isolate_UTSW_UCB_Mm_chromosome_1_whole_genome_shotgun_sequence
SJYT01008979.1_799386-799886_Cephalophus_harveyi_isolate_HVD-549_scaffold2070_size1422585_whole_genome_shotgun_sequence
SJYT01008979.1_800168-800359_Cephalophus_harveyi_isolate_HVD-549_scaffold2070_size1422585_whole_genome_shotgun_sequence
VSZR01000001.1_98989587-98990272_Camelus_ferus_isolate_YT-003-E_chromosome_1_whole_genome_shotgun_sequence
_R_JWIN03000001.1_23718226-23718911_Camelus_dromedarius_breed_African_isolate_Drom800_chromosome_1_whole_genome_shotgun_sequence
PVJP02000279.1_3155604-3156279_Hippopotamus_amphibius_isolate_BS01_ScHSfme_267HRSCAF_30919_whole_genome_shotgun_sequence
VSZR01000012.1_64388516-64389179_Camelus_ferus_isolate_YT-003-E_chromosome_2_whole_genome_shotgun_sequence
JARL01015609.1_538065-538728_Camelus_bactrianus_breed_Alxa_contig15609_whole_genome_shotgun_sequence
_R_JWIN03000002.1_57709095-57709760_Camelus_dromedarius_breed_African_isolate_Drom800_chromosome_2_whole_genome_shotgun_sequence
ABRR03000026.1_65616977-65617642_Vicugna_pacos_isolate_Carlotta__AHFN-0088__chromosome_2_ScfyRBE_26HRSCAF_216_whole_genome_shotgun_sequence
_R_VCEA01000001.1_788944884-788945536_Muntiacus_muntjak_isolate_UTSW_UCB_Mm_chromosome_1_whole_genome_shotgun_sequence
_R_PVJP02910237.1_6942222-6942897_Hippopotamus_amphibius_isolate_BS01_ScHSfme_924722HRSCAF_982522_whole_genome_shotgun_sequence
_R_VLPJ01000219.1_55871613-55872247_Bos_taurus_x_Bos_grunniens_isolate_Esperanza_chromosome_2_whole_genome_shotgun_sequence
_R_NKLS02000002.1_55971827-55972461_Bos_taurus_breed_Hereford_isolate_L1_Dominette_01449_registration_number_42190680_chromosome_2_whole_genome_shotgun_sequence
PUFT02000002.1_80181480-80182114_Bos_indicus_x_Bos_taurus_breed_Angus_x_Brahman_F1_hybrid_chromosome_2_whole_genome_shotgun_sequence
_R_PUFS02000002.1_55859070-55859683_Bos_indicus_x_Bos_taurus_breed_Angus_x_Brahman_F1_hybrid_chromosome_2_whole_genome_shotgun_sequence
VBZB01000002.1_88608247-88608879_Bos_grunniens_isolate_yakHY00_chromosome_2_whole_genome_shotgun_sequence
_R_VLPI01000022.1_56030245-56030877_Bos_taurus_x_Bos_grunniens_isolate_Esperanza_chromosome_2_whole_genome_shotgun_sequence
_R_PRDE01000012.1_56185987-56186619_Bos_indicus_breed_Gir_chromosome_2_whole_genome_shotgun_sequence
_R_PZYV01000012.1_108622355-108622992_Bubalus_bubalis_breed_Mediterranean_chromosome_2_whole_genome_shotgun_sequence
CABVSG010000096.1_80117936-80118505_Syncerus_caffer_genome_assembly_contig__Contig97_whole_genome_shotgun_sequence
NIWH01027711.1_67016395-67017051_Ovis_ammon_isolate_KGZ_Scaffold5_whole_genome_shotgun_sequence
VCEA01000001.1_415857547-415858184_Muntiacus_muntjak_isolate_UTSW_UCB_Mm_chromosome_1_whole_genome_shotgun_sequence
VCEB01000003.1_213665574-213666211_Muntiacus_reevesi_isolate_UCam_UCB_Mr_chromosome_3_whole_genome_shotgun_sequence
RAWU01000013.1_75652828-75653455_Giraffa_tippelskirchi_isolate_OR1865_chromosome_3_whole_genome_shotgun_sequence
SIDA01000007.1_131128523-131129183_Sus_scrofa_breed_Bama_miniature_chromosome_15_whole_genome_shotgun_sequence
SIDA01000001.1_191386411-191387064_Sus_scrofa_breed_Bama_miniature_chromosome_1_whole_genome_shotgun_sequence
VSZR01000032.1_58334794-58335459_Camelus_ferus_isolate_YT-003-E_chromosome_X_whole_genome_shotgun_sequence
_R_JWIN03000037.1_50640672-50641337_Camelus_dromedarius_breed_African_isolate_Drom800_chromosome_X_whole_genome_shotgun_sequence
0.03

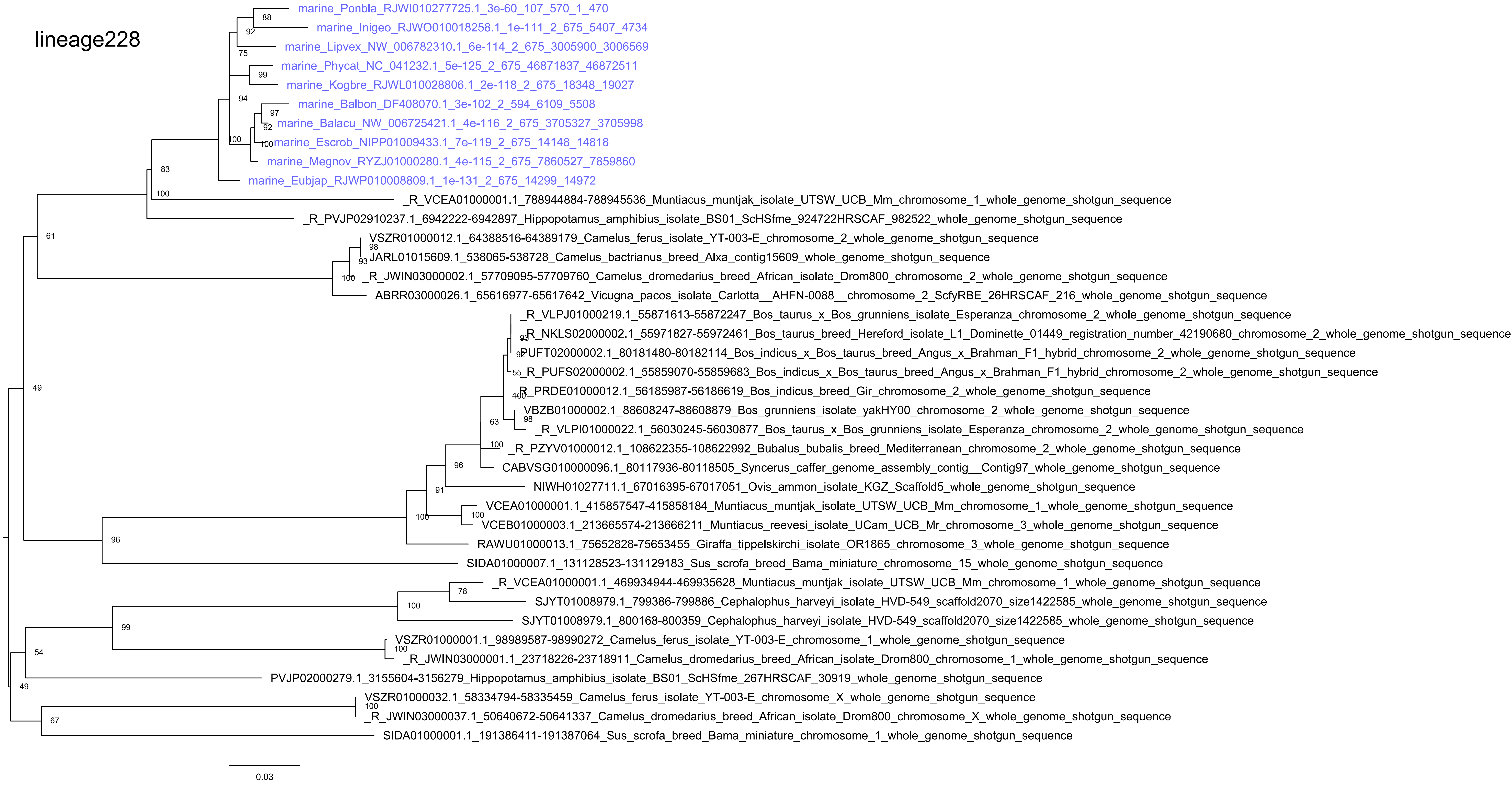
lineage228
marine_Ponbla_RJWI010277725.1_3e-60_107_570_1_470
marine_Inigeo_RJWO010018258.1_1e-111_2_675_5407_4734
marine_Lipvex_NW_006782310.1_6e-114_2_675_3005900_3006569
marine_Phycat_NC_041232.1_5e-125_2_675_46871837_46872511
marine_Kogbre_RJWL010028806.1_2e-118_2_675_18348_19027
marine_Balbon_DF408070.1_3e-102_2_594_6109_5508
marine_Balacu_NW_006725421.1_4e-116_2_675_3705327_3705998
marine_Escrob_NIPP01009433.1_7e-119_2_675_14148_14818
marine_Megnov_RYZJ01000280.1_4e-115_2_675_7860527_7859860
marine_Eubjap_RJWP010008809.1_1e-131_2_675_14299_14972
_R_VCEA01000001.1_788944884-788945536_Muntiacus_muntjak_isolate_UTSW_UCB_Mm_chromosome_1_whole_genome_shotgun_sequence
_R_PVJP02910237.1_6942222-6942897_Hippopotamus_amphibius_isolate_BS01_ScHSfme_924722HRSCAF_982522_whole_genome_shotgun_sequence
VSZR01000012.1_64388516-64389179_Camelus_ferus_isolate_YT-003-E_chromosome_2_whole_genome_shotgun_sequence
JARL01015609.1_538065-538728_Camelus_bactrianus_breed_Alxa_contig15609_whole_genome_shotgun_sequence
_R_JWIN03000002.1_57709095-57709760_Camelus_dromedarius_breed_African_isolate_Drom800_chromosome_2_whole_genome_shotgun_sequence
ABRR03000026.1_65616977-65617642_Vicugna_pacos_isolate_Carlotta__AHFN-0088__chromosome_2_ScfyRBE_26HRSCAF_216_whole_genome_shotgun_sequence
_R_VLPJ01000219.1_55871613-55872247_Bos_taurus_x_Bos_grunniens_isolate_Esperanza_chromosome_2_whole_genome_shotgun_sequence
_R_NKLS02000002.1_55971827-55972461_Bos_taurus_breed_Hereford_isolate_L1_Dominette_01449_registration_number_42190680_chromosome_2_whole_genome_shotgun_sequence
PUFT02000002.1_80181480-80182114_Bos_indicus_x_Bos_taurus_breed_Angus_x_Brahman_F1_hybrid_chromosome_2_whole_genome_shotgun_sequence
_R_PUFS02000002.1_55859070-55859683_Bos_indicus_x_Bos_taurus_breed_Angus_x_Brahman_F1_hybrid_chromosome_2_whole_genome_shotgun_sequence
_R_PRDE01000012.1_56185987-56186619_Bos_indicus_breed_Gir_chromosome_2_whole_genome_shotgun_sequence
VBZB01000002.1_88608247-88608879_Bos_grunniens_isolate_yakHY00_chromosome_2_whole_genome_shotgun_sequence
_R_VLPI01000022.1_56030245-56030877_Bos_taurus_x_Bos_grunniens_isolate_Esperanza_chromosome_2_whole_genome_shotgun_sequence
_R_PZYV01000012.1_108622355-108622992_Bubalus_bubalis_breed_Mediterranean_chromosome_2_whole_genome_shotgun_sequence
CABVSG010000096.1_80117936-80118505_Syncerus_caffer_genome_assembly_contig__Contig97_whole_genome_shotgun_sequence
NIWH01027711.1_67016395-67017051_Ovis_ammon_isolate_KGZ_Scaffold5_whole_genome_shotgun_sequence
VCEA01000001.1_415857547-415858184_Muntiacus_muntjak_isolate_UTSW_UCB_Mm_chromosome_1_whole_genome_shotgun_sequence
VCEB01000003.1_213665574-213666211_Muntiacus_reevesi_isolate_UCam_UCB_Mr_chromosome_3_whole_genome_shotgun_sequence
RAWU01000013.1_75652828-75653455_Giraffa_tippelskirchi_isolate_OR1865_chromosome_3_whole_genome_shotgun_sequence
SIDA01000007.1_131128523-131129183_Sus_scrofa_breed_Bama_miniature_chromosome_15_whole_genome_shotgun_sequence
_R_VCEA01000001.1_469934944-469935628_Muntiacus_muntjak_isolate_UTSW_UCB_Mm_chromosome_1_whole_genome_shotgun_sequence
SJYT01008979.1_799386-799886_Cephalophus_harveyi_isolate_HVD-549_scaffold2070_size1422585_whole_genome_shotgun_sequence
SJYT01008979.1_800168-800359_Cephalophus_harveyi_isolate_HVD-549_scaffold2070_size1422585_whole_genome_shotgun_sequence
VSZR01000001.1_98989587-98990272_Camelus_ferus_isolate_YT-003-E_chromosome_1_whole_genome_shotgun_sequence
_R_JWIN03000001.1_23718226-23718911_Camelus_dromedarius_breed_African_isolate_Drom800_chromosome_1_whole_genome_shotgun_sequence
PVJP02000279.1_3155604-3156279_Hippopotamus_amphibius_isolate_BS01_ScHSfme_267HRSCAF_30919_whole_genome_shotgun_sequence
VSZR01000032.1_58334794-58335459_Camelus_ferus_isolate_YT-003-E_chromosome_X_whole_genome_shotgun_sequence
_R_JWIN03000037.1_50640672-50641337_Camelus_dromedarius_breed_African_isolate_Drom800_chromosome_X_whole_genome_shotgun_sequence
SIDA01000001.1_191386411-191387064_Sus_scrofa_breed_Bama_miniature_chromosome_1_whole_genome_shotgun_sequence
0.03

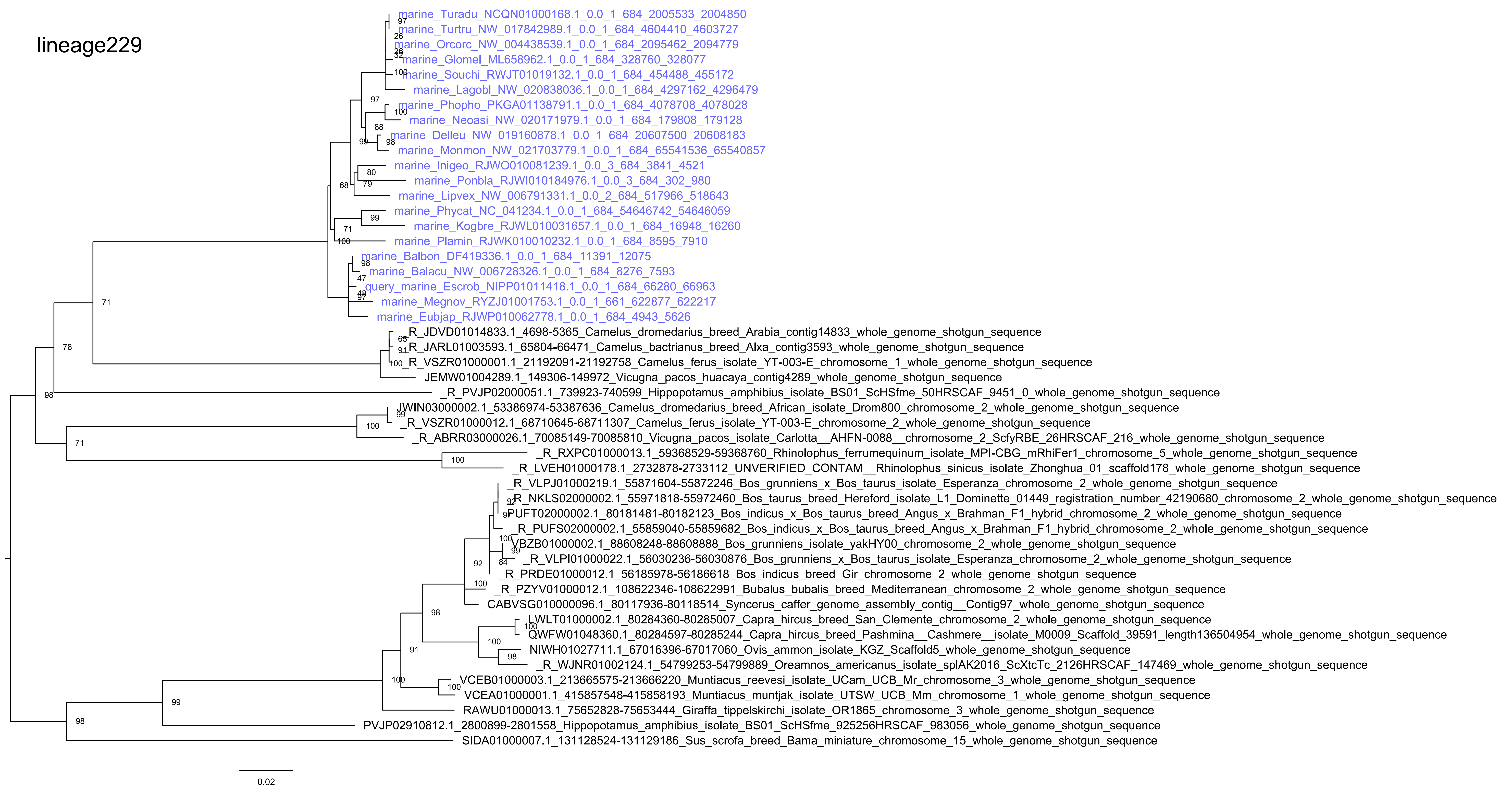
lineage229
marine_Turadu_NCQN01000168.1_0.0_1_684_2005533_2004850
marine_Turtru_NW_017842989.1_0.0_1_684_4604410_4603727
marine_Orcorc_NW_004438539.1_0.0_1_684_2095462_2094779
marine_Glomel_ML658962.1_0.0_1_684_328760_328077
marine_Souchi_RWJT01019132.1_0.0_1_684_454488_455172
marine_Lagobl_NW_020838036.1_0.0_1_684_4297162_4296479
marine_Phopho_PKGA01138791.1_0.0_1_684_4078708_4078028
marine_Neoasi_NW_020171979.1_0.0_1_684_179808_179128
marine_Delleu_NW_019160878.1_0.0_1_684_20607500_20608183
marine_Monmon_NW_021703779.1_0.0_1_684_65541536_65540857
marine_Inigeo_RJWO010081239.1_0.0_3_684_3841_4521
marine_Ponbla_RJWI010184976.1_0.0_3_684_302_980
marine_Lipvex_NW_006791331.1_0.0_2_684_517966_518643
marine_Phycat_NC_041234.1_0.0_1_684_54646742_54646059
marine_Kogbre_RJWL010031657.1_0.0_1_684_16948_16260
marine_Plamin_RJWK010010232.1_0.0_1_684_8595_7910
marine_Balbon_DF419336.1_0.0_1_684_11391_12075
marine_Balacu_NW_006728326.1_0.0_1_684_8276_7593
query_marine_Escrob_NIPP01011418.1_0.0_1_684_66280_66963
marine_Megnov_RYZJ01001753.1_0.0_1_661_622877_622217
marine_Eubjap_RJWP010062778.1_0.0_1_684_4943_5626
_R_JDVD01014833.1_4698-5365_Camelus_dromedarius_breed_Arabia_contig14833_whole_genome_shotgun_sequence
_R_JARL01003593.1_65804-66471_Camelus_bactrianus_breed_Alxa_contig3593_whole_genome_shotgun_sequence
_R_VSZR01000001.1_21192091-21192758_Camelus_ferus_isolate_YT-003-E_chromosome_1_whole_genome_shotgun_sequence
JEMW01004289.1_149306-149972_Vicugna_pacos_huacaya_contig4289_whole_genome_shotgun_sequence
_R_PVJP02000051.1_739923-740599_Hippopotamus_amphibius_isolate_BS01_ScHSfme_50HRSCAF_9451_0_whole_genome_shotgun_sequence
JWIN03000002.1_53386974-53387636_Camelus_dromedarius_breed_African_isolate_Drom800_chromosome_2_whole_genome_shotgun_sequence
_R_VSZR01000012.1_68710645-68711307_Camelus_ferus_isolate_YT-003-E_chromosome_2_whole_genome_shotgun_sequence
_R_ABRR03000026.1_70085149-70085810_Vicugna_pacos_isolate_Carlotta__AHFN-0088__chromosome_2_ScfyRBE_26HRSCAF_216_whole_genome_shotgun_sequence
_R_RXPC01000013.1_59368529-59368760_Rhinolophus_ferrumequinum_isolate_MPI-CBG_mRhiFer1_chromosome_5_whole_genome_shotgun_sequence
_R_LVEH01000178.1_2732878-2733112_UNVERIFIED_CONTAM__Rhinolophus_sinicus_isolate_Zhonghua_01_scaffold178_whole_genome_shotgun_sequence
_R_VLPJ01000219.1_55871604-55872246_Bos_grunniens_x_Bos_taurus_isolate_Esperanza_chromosome_2_whole_genome_shotgun_sequence
_R_NKLS02000002.1_55971818-55972460_Bos_taurus_breed_Hereford_isolate_L1_Dominette_01449_registration_number_42190680_chromosome_2_whole_genome_shotgun_sequence
PUFT02000002.1_80181481-80182123_Bos_indicus_x_Bos_taurus_breed_Angus_x_Brahman_F1_hybrid_chromosome_2_whole_genome_shotgun_sequence
_R_PUFS02000002.1_55859040-55859682_Bos_indicus_x_Bos_taurus_breed_Angus_x_Brahman_F1_hybrid_chromosome_2_whole_genome_shotgun_sequence
VBZB01000002.1_88608248-88608888_Bos_grunniens_isolate_yakHY00_chromosome_2_whole_genome_shotgun_sequence
_R_VLPI01000022.1_56030236-56030876_Bos_grunniens_x_Bos_taurus_isolate_Esperanza_chromosome_2_whole_genome_shotgun_sequence
_R_PRDE01000012.1_56185978-56186618_Bos_indicus_breed_Gir_chromosome_2_whole_genome_shotgun_sequence
_R_PZYV01000012.1_108622346-108622991_Bubalus_bubalis_breed_Mediterranean_chromosome_2_whole_genome_shotgun_sequence
CABVSG010000096.1_80117936-80118514_Syncerus_caffer_genome_assembly_contig__Contig97_whole_genome_shotgun_sequence
LWLT01000002.1_80284360-80285007_Capra_hircus_breed_San_Clemente_chromosome_2_whole_genome_shotgun_sequence
QWFW01048360.1_80284597-80285244_Capra_hircus_breed_Pashmina__Cashmere__isolate_M0009_Scaffold_39591_length136504954_whole_genome_shotgun_sequence
NIWH01027711.1_67016396-67017060_Ovis_ammon_isolate_KGZ_Scaffold5_whole_genome_shotgun_sequence
_R_WJNR01002124.1_54799253-54799889_Oreamnos_americanus_isolate_splAK2016_ScXtcTc_2126HRSCAF_147469_whole_genome_shotgun_sequence
VCEB01000003.1_213665575-213666220_Muntiacus_reevesi_isolate_UCam_UCB_Mr_chromosome_3_whole_genome_shotgun_sequence
VCEA01000001.1_415857548-415858193_Muntiacus_muntjak_isolate_UTSW_UCB_Mm_chromosome_1_whole_genome_shotgun_sequence
RAWU01000013.1_75652828-75653444_Giraffa_tippelskirchi_isolate_OR1865_chromosome_3_whole_genome_shotgun_sequence
PVJP02910812.1_2800899-2801558_Hippopotamus_amphibius_isolate_BS01_ScHSfme_925256HRSCAF_983056_whole_genome_shotgun_sequence
SIDA01000007.1_131128524-131129186_Sus_scrofa_breed_Bama_miniature_chromosome_15_whole_genome_shotgun_sequence
0.02

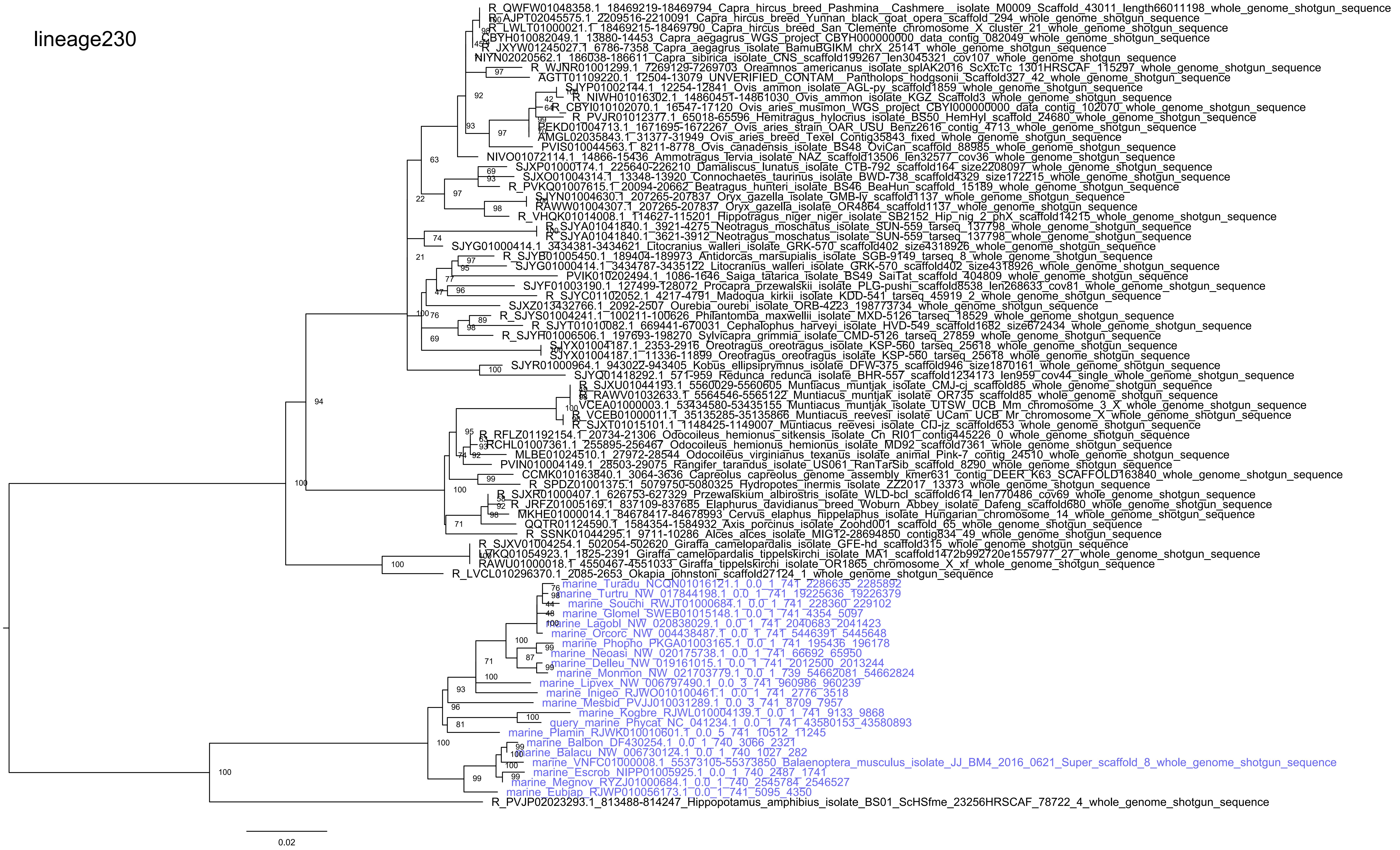
lineage230
R_QWFW01048358.1_18469219-18469794_Capra_hircus_breed_Pashmina__Cashmere__isolate_M0009_Scaffold_43011_length66011198_whole_genome_shotgun_sequence
R_AJPT02045575.1_2209516-2210091_Capra_hircus_breed_Yunnan_black_goat_opera_scaffold_294_whole_genome_shotgun_sequence
R_LWLT01000021.1_18469215-18469790_Capra_hircus_breed_San_Clemente_chromosome_X_cluster_21_whole_genome_shotgun_sequence
CBYH010082049.1_13880-14453_Capra_aegagrus_WGS_project_CBYH000000000_data_contig_082049_whole_genome_shotgun_sequence
R_JXYW01245027.1_6786-7358_Capra_aegagrus_isolate_BamuBGIKM_chrX_25141_whole_genome_shotgun_sequence
NIYN02020562.1_186038-186611_Capra_sibirica_isolate_CNS_scaffold199267_len3045321_cov107_whole_genome_shotgun_sequence
R_WJNR01001299.1_7269129-7269703_Oreamnos_americanus_isolate_splAK2016_ScXtcTc_1301HRSCAF_115297_whole_genome_shotgun_sequence
AGTT01109220.1_12504-13079_UNVERIFIED_CONTAM__Pantholops_hodgsonii_Scaffold327_42_whole_genome_shotgun_sequence
SJYP01002144.1_12254-12841_Ovis_ammon_isolate_AGL-py_scaffold1859_whole_genome_shotgun_sequence
R_NIWH01016302.1_14860451-14861030_Ovis_ammon_isolate_KGZ_Scaffold3_whole_genome_shotgun_sequence
R_CBYI010102070.1_16547-17120_Ovis_aries_musimon_WGS_project_CBYI000000000_data_contig_102070_whole_genome_shotgun_sequence
R_PVJR01012377.1_65018-65596_Hemitragus_hylocrius_isolate_BS50_HemHyl_scaffold_24680_whole_genome_shotgun_sequence
PEKD01004713.1_1671695-1672267_Ovis_aries_strain_OAR_USU_Benz2616_contig_4713_whole_genome_shotgun_sequence
AMGL02035843.1_31377-31949_Ovis_aries_breed_Texel_Contig35843_fixed_whole_genome_shotgun_sequence
PVIS010044563.1_8211-8778_Ovis_canadensis_isolate_BS48_OviCan_scaffold_88985_whole_genome_shotgun_sequence
NIVO01072114.1_14866-15436_Ammotragus_lervia_isolate_NAZ_scaffold13506_len32577_cov36_whole_genome_shotgun_sequence
SJXP01000174.1_225640-226210_Damaliscus_lunatus_isolate_CTB-792_scaffold164_size2208097_whole_genome_shotgun_sequence
SJXO01004314.1_13348-13920_Connochaetes_taurinus_isolate_BWD-738_scaffold4329_size172215_whole_genome_shotgun_sequence
R_PVKQ01007615.1_20094-20662_Beatragus_hunteri_isolate_BS46_BeaHun_scaffold_15189_whole_genome_shotgun_sequence
SJYN01004630.1_207265-207837_Oryx_gazella_isolate_GMB-ly_scaffold1137_whole_genome_shotgun_sequence
RAWW01004307.1_207265-207837_Oryx_gazella_isolate_OR4864_scaffold1137_whole_genome_shotgun_sequence
R_VHQK01014008.1_114627-115201_Hippotragus_niger_niger_isolate_SB2152_Hip_nig_2_phX_scaffold14215_whole_genome_shotgun_sequence
R_SJYA01041840.1_3921-4275_Neotragus_moschatus_isolate_SUN-559_tarseq_137798_whole_genome_shotgun_sequence
R_SJYA01041840.1_3621-3912_Neotragus_moschatus_isolate_SUN-559_tarseq_137798_whole_genome_shotgun_sequence
SJYG01000414.1_3434381-3434621_Litocranius_walleri_isolate_GRK-570_scaffold402_size4318926_whole_genome_shotgun_sequence
R_SJYB01005450.1_189404-189973_Antidorcas_marsupialis_isolate_SGB-9149_tarseq_8_whole_genome_shotgun_sequence
SJYG01000414.1_3434787-3435122_Litocranius_walleri_isolate_GRK-570_scaffold402_size4318926_whole_genome_shotgun_sequence
PVIK010202494.1_1086-1646_Saiga_tatarica_isolate_BS49_SaiTat_scaffold_404809_whole_genome_shotgun_sequence
SJYF01003190.1_127499-128072_Procapra_przewalskii_isolate_PLG-pushi_scaffold8538_len268633_cov81_whole_genome_shotgun_sequence
R_SJYC01102052.1_4217-4791_Madoqua_kirkii_isolate_KDD-541_tarseq_45919_2_whole_genome_shotgun_sequence
SJXZ013432766.1_2092-2507_Ourebia_ourebi_isolate_ORB-4223_198773734_whole_genome_shotgun_sequence
R_SJYS01004241.1_100211-100626_Philantomba_maxwellii_isolate_MXD-5126_tarseq_18529_whole_genome_shotgun_sequence
R_SJYT01010082.1_669441-670031_Cephalophus_harveyi_isolate_HVD-549_scaffold1682_size672434_whole_genome_shotgun_sequence
R_SJYH01006506.1_197693-198270_Sylvicapra_grimmia_isolate_CMD-5126_tarseq_27859_whole_genome_shotgun_sequence
SJYX01004187.1_2353-2916_Oreotragus_oreotragus_isolate_KSP-560_tarseq_25618_whole_genome_shotgun_sequence
SJYX01004187.1_11336-11899_Oreotragus_oreotragus_isolate_KSP-560_tarseq_25618_whole_genome_shotgun_sequence
SJYR01000964.1_943022-943405_Kobus_ellipsiprymnus_isolate_DFW-375_scaffold946_size1870161_whole_genome_shotgun_sequence
SJYQ01418292.1_571-959_Redunca_redunca_isolate_BHR-557_scaffold1234173_len959_cov44_single_whole_genome_shotgun_sequence
R_SJXU01044193.1_5560029-5560605_Muntiacus_muntjak_isolate_CMJ-cj_scaffold85_whole_genome_shotgun_sequence
R_RAWV01032633.1_5564546-5565122_Muntiacus_muntjak_isolate_OR735_scaffold85_whole_genome_shotgun_sequence
VCEA01000003.1_53434580-53435155_Muntiacus_muntjak_isolate_UTSW_UCB_Mm_chromosome_3_X_whole_genome_shotgun_sequence
R_VCEB01000011.1_35135285-35135866_Muntiacus_reevesi_isolate_UCam_UCB_Mr_chromosome_X_whole_genome_shotgun_sequence
R_SJXT01015101.1_1148425-1149007_Muntiacus_reevesi_isolate_CIJ-jz_scaffold653_whole_genome_shotgun_sequence
R_RFLZ01192154.1_20734-21306_Odocoileus_hemionus_sitkensis_isolate_Cn_RI01_contig445226_0_whole_genome_shotgun_sequence
RCHL01007361.1_255895-256467_Odocoileus_hemionus_hemionus_isolate_MD92_scaffold7361_whole_genome_shotgun_sequence
MLBE01024510.1_27972-28544_Odocoileus_virginianus_texanus_isolate_animal_Pink-7_contig_24510_whole_genome_shotgun_sequence
PVIN010004149.1_28503-29075_Rangifer_tarandus_isolate_US061_RanTarSib_scaffold_8290_whole_genome_shotgun_sequence
CCMK010163840.1_3064-3636_Capreolus_capreolus_genome_assembly_kmer631_contig_DEER_K63_SCAFFOLD163840_whole_genome_shotgun_sequence
R_SPDZ01001375.1_5079750-5080325_Hydropotes_inermis_isolate_ZZ2017_13373_whole_genome_shotgun_sequence
R_SJXR01000407.1_626753-627329_Przewalskium_albirostris_isolate_WLD-bcl_scaffold614_len770486_cov69_whole_genome_shotgun_sequence
R_JRFZ01005169.1_837109-837685_Elaphurus_davidianus_breed_Woburn_Abbey_isolate_Dafeng_scaffold680_whole_genome_shotgun_sequence
MKHE01000014.1_84678417-84678993_Cervus_elaphus_hippelaphus_isolate_Hungarian_chromosome_14_whole_genome_shotgun_sequence
QQTR01124590.1_1584354-1584932_Axis_porcinus_isolate_Zoohd001_scaffold_65_whole_genome_shotgun_sequence
R_SSNK01044295.1_9711-10286_Alces_alces_isolate_MIG12-28694850_contig834_49_whole_genome_shotgun_sequence
R_SJXV01004254.1_502054-502620_Giraffa_camelopardalis_isolate_GFE-hd_scaffold315_whole_genome_shotgun_sequence
LVKQ01054923.1_1825-2391_Giraffa_camelopardalis_tippelskirchi_isolate_MA1_scaffold1472b992720e1557977_27_whole_genome_shotgun_sequence
RAWU01000018.1_4550467-4551033_Giraffa_tippelskirchi_isolate_OR1865_chromosome_X_xf_whole_genome_shotgun_sequence
R_LVCL010296370.1_2085-2653_Okapia_johnstoni_scaffold27124_1_whole_genome_shotgun_sequence
marine_Turadu_NCQN01016121.1_0.0_1_741_2286635_2285892
marine_Turtru_NW_017844198.1_0.0_1_741_19225636_19226379
marine_Souchi_RWJT01000684.1_0.0_1_741_228360_229102
marine_Glomel_SWEB01015148.1_0.0_1_741_4354_5097
marine_Lagobl_NW_020838029.1_0.0_1_741_2040683_2041423
marine_Orcorc_NW_004438487.1_0.0_1_741_5446391_5445648
marine_Phopho_PKGA01003165.1_0.0_1_741_195436_196178
marine_Neoasi_NW_020175738.1_0.0_1_741_66692_65950
marine_Delleu_NW_019161015.1_0.0_1_741_2012500_2013244
marine_Monmon_NW_021703779.1_0.0_1_739_54662081_54662824
marine_Lipvex_NW_006797490.1_0.0_3_741_960986_960239
marine_Inigeo_RJWO010100461.1_0.0_1_741_2776_3518
marine_Mesbid_PVJJ010031289.1_0.0_3_741_8709_7957
marine_Kogbre_RJWL010004139.1_0.0_1_741_9133_9868
query_marine_Phycat_NC_041234.1_0.0_1_741_43580153_43580893
marine_Plamin_RJWK010010601.1_0.0_5_741_10512_11245
marine_Balbon_DF430254.1_0.0_1_740_3066_2321
marine_Balacu_NW_006730124.1_0.0_1_740_1027_282
marine_VNFC01000008.1_55373105-55373850_Balaenoptera_musculus_isolate_JJ_BM4_2016_0621_Super_scaffold_8_whole_genome_shotgun_sequence
marine_Escrob_NIPP01005925.1_0.0_1_740_2487_1741
marine_Megnov_RYZJ01000684.1_0.0_1_740_2545784_2546527
marine_Eubjap_RJWP010056173.1_0.0_1_741_5095_4350
R_PVJP02023293.1_813488-814247_Hippopotamus_amphibius_isolate_BS01_ScHSfme_23256HRSCAF_78722_4_whole_genome_shotgun_sequence
0.02

lineage231

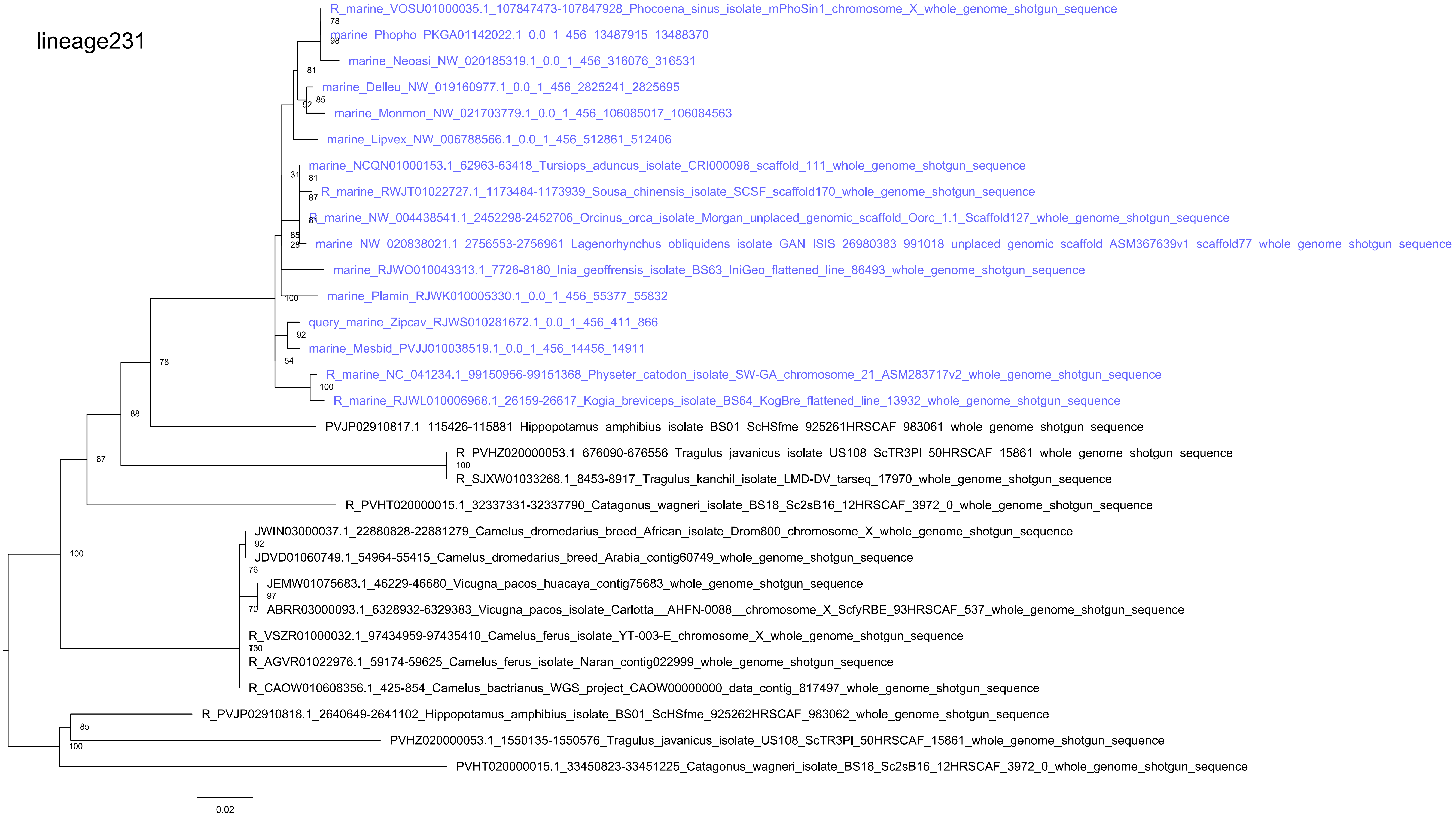
R_marine_VOSU01000035.1_107847473-107847928_Phocoena_sinus_isolate_mPhoSin1_chromosome_X_whole_genome_shotgun_sequence
marine_Phopho_PKGA01142022.1_0.0_1_456_13487915_13488370
marine_Neoasi_NW_020185319.1_0.0_1_456_316076_316531
marine_Delleu_NW_019160977.1_0.0_1_456_2825241_2825695
marine_Monmon_NW_021703779.1_0.0_1_456_106085017_106084563
marine_Lipvex_NW_006788566.1_0.0_1_456_512861_512406
marine_NCQN01000153.1_62963-63418_Tursiops_aduncus_isolate_CRI000098_scaffold_111_whole_genome_shotgun_sequence
R_marine_RWJT01022727.1_1173484-1173939_Sousa_chinensis_isolate_SCSF_scaffold170_whole_genome_shotgun_sequence
R_marine_NW_004438541.1_2452298-2452706_Orcinus_orca_isolate_Morgan_unplaced_genomic_scaffold_Oorc_1.1_Scaffold127_whole_genome_shotgun_sequence
marine_NW_020838021.1_2756553-2756961_Lagenorhynchus_obliquidens_isolate_GAN_ISIS_26980383_991018_unplaced_genomic_scaffold_ASM367639v1_scaffold77_whole_genome_shotgun_sequence
marine_RJWO010043313.1_7726-8180_Inia_geoffrensis_isolate_BS63_IniGeo_flattened_line_86493_whole_genome_shotgun_sequence
marine_Plamin_RJWK010005330.1_0.0_1_456_55377_55832
query_marine_Zipcav_RJWS010281672.1_0.0_1_456_411_866
marine_Mesbid_PVJJ010038519.1_0.0_1_456_14456_14911
R_marine_NC_041234.1_99150956-99151368_Physeter_catodon_isolate_SW-GA_chromosome_21_ASM283717v2_whole_genome_shotgun_sequence
R_marine_RJWL010006968.1_26159-26617_Kogia_breviceps_isolate_BS64_KogBre_flattened_line_13932_whole_genome_shotgun_sequence
PVJP02910817.1_115426-115881_Hippopotamus_amphibius_isolate_BS01_ScHSfme_925261HRSCAF_983061_whole_genome_shotgun_sequence
R_PVHZ020000053.1_676090-676556_Tragulus_javanicus_isolate_US108_ScTR3PI_50HRSCAF_15861_whole_genome_shotgun_sequence
R_SJXW01033268.1_8453-8917_Tragulus_kanchil_isolate_LMD-DV_tarseq_17970_whole_genome_shotgun_sequence
R_PVHT020000015.1_32337331-32337790_Catagonus_wagneri_isolate_BS18_Sc2sB16_12HRSCAF_3972_0_whole_genome_shotgun_sequence
JWIN03000037.1_22880828-22881279_Camelus_dromedarius_breed_African_isolate_Drom800_chromosome_X_whole_genome_shotgun_sequence
JDVD01060749.1_54964-55415_Camelus_dromedarius_breed_Arabia_contig60749_whole_genome_shotgun_sequence
JEMW01075683.1_46229-46680_Vicugna_pacos_huacaya_contig75683_whole_genome_shotgun_sequence
ABRR03000093.1_6328932-6329383_Vicugna_pacos_isolate_Carlotta__AHFN-0088__chromosome_X_ScfyRBE_93HRSCAF_537_whole_genome_shotgun_sequence
R_VSZR01000032.1_97434959-97435410_Camelus_ferus_isolate_YT-003-E_chromosome_X_whole_genome_shotgun_sequence
R_AGVR01022976.1_59174-59625_Camelus_ferus_isolate_Naran_contig022999_whole_genome_shotgun_sequence
R_CAOW010608356.1_425-854_Camelus_bactrianus_WGS_project_CAOW00000000_data_contig_817497_whole_genome_shotgun_sequence
R_PVJP02910818.1_2640649-2641102_Hippopotamus_amphibius_isolate_BS01_ScHSfme_925262HRSCAF_983062_whole_genome_shotgun_sequence
PVHZ020000053.1_1550135-1550576_Tragulus_javanicus_isolate_US108_ScTR3PI_50HRSCAF_15861_whole_genome_shotgun_sequence
PVHT020000015.1_33450823-33451225_Catagonus_wagneri_isolate_BS18_Sc2sB16_12HRSCAF_3972_0_whole_genome_shotgun_sequence
0.02

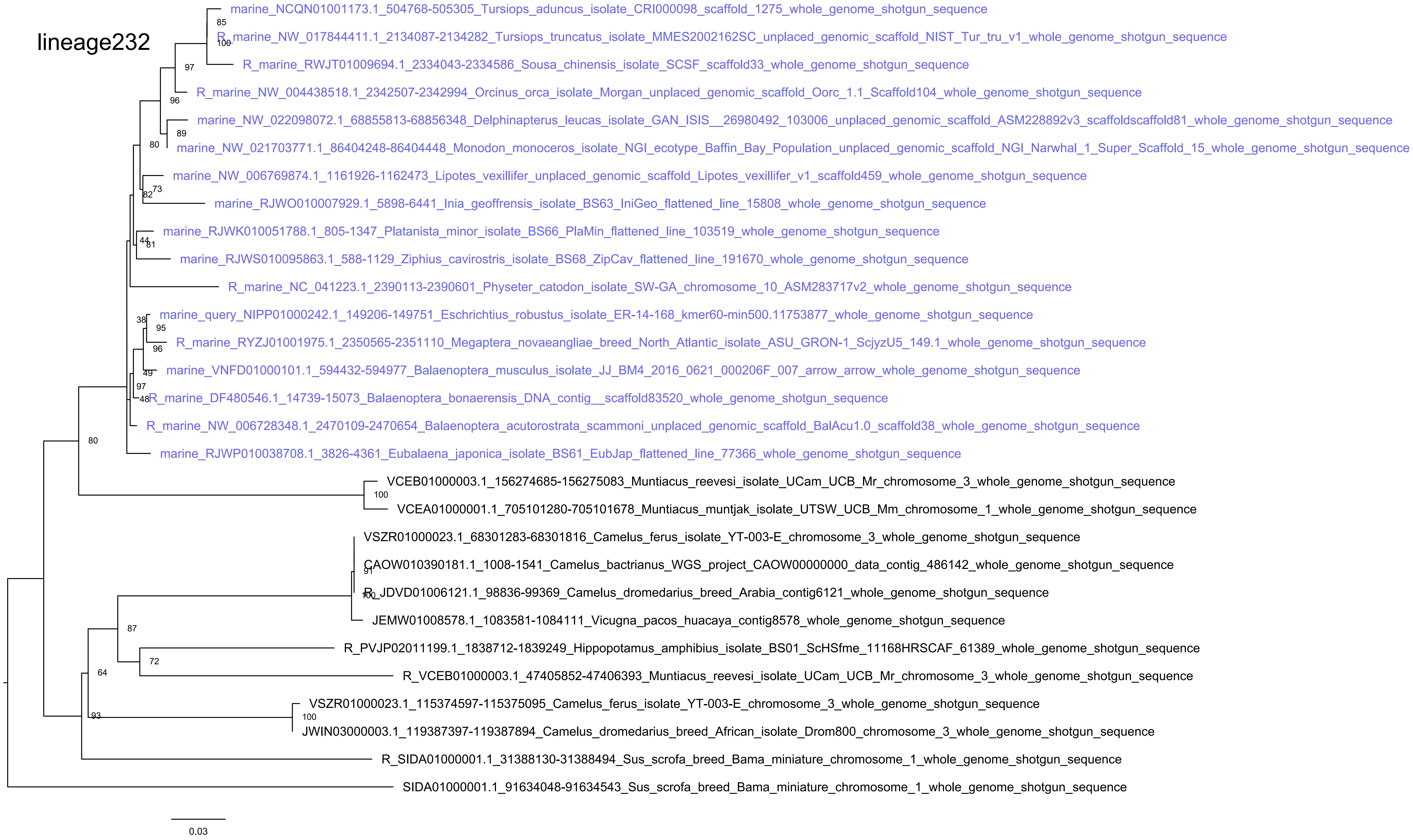
lineage232
marine_NCQN01001173.1_504768-505305_Tursiops_aduncus_isolate_CRI000098_scaffold_1275_whole_genome_shotgun_sequence
R_marine_NW_017844411.1_2134087-2134282_Tursiops_truncatus_isolate_MMES2002162SC_unplaced_genomic_scaffold_NIST_Tur_tru_v1_whole_genome_shotgun_sequence
R_marine_RWJT01009694.1_2334043-2334586_Sousa_chinensis_isolate_SCSF_scaffold33_whole_genome_shotgun_sequence
R_marine_NW_004438518.1_2342507-2342994_Orcinus_orca_isolate_Morgan_unplaced_genomic_scaffold_Oorc_1.1_Scaffold104_whole_genome_shotgun_sequence
marine_NW_022098072.1_68855813-68856348_Delphinapterus_leucas_isolate_GAN_ISIS__26980492_103006_unplaced_genomic_scaffold_ASM228892v3_scaffoldscaffold81_whole_genome_shotgun_sequence
marine_NW_021703771.1_86404248-86404448_Monodon_monoceros_isolate_NGI_ecotype_Baffin_Bay_Population_unplaced_genomic_scaffold_NGI_Narwhal_1_Super_Scaffold_15_whole_genome_shotgun_sequence
marine_NW_006769874.1_1161926-1162473_Lipotes_vexillifer_unplaced_genomic_scaffold_Lipotes_vexillifer_v1_scaffold459_whole_genome_shotgun_sequence
marine_RJWO010007929.1_5898-6441_Inia_geoffrensis_isolate_BS63_IniGeo_flattened_line_15808_whole_genome_shotgun_sequence
marine_RJWK010051788.1_805-1347_Platanista_minor_isolate_BS66_PlaMin_flattened_line_103519_whole_genome_shotgun_sequence
marine_RJWS010095863.1_588-1129_Ziphius_cavirostris_isolate_BS68_ZipCav_flattened_line_191670_whole_genome_shotgun_sequence
R_marine_NC_041223.1_2390113-2390601_Physeter_catodon_isolate_SW-GA_chromosome_10_ASM283717v2_whole_genome_shotgun_sequence
marine_query_NIPP01000242.1_149206-149751_Eschrichtius_robustus_isolate_ER-14-168_kmer60-min500.11753877_whole_genome_shotgun_sequence
R_marine_RYZJ01001975.1_2350565-2351110_Megaptera_novaeangliae_breed_North_Atlantic_isolate_ASU_GRON-1_ScjyzU5_149.1_whole_genome_shotgun_sequence
marine_VNFD01000101.1_594432-594977_Balaenoptera_musculus_isolate_JJ_BM4_2016_0621_000206F_007_arrow_arrow_whole_genome_shotgun_sequence
R_marine_DF480546.1_14739-15073_Balaenoptera_bonaerensis_DNA_contig__scaffold83520_whole_genome_shotgun_sequence
R_marine_NW_006728348.1_2470109-2470654_Balaenoptera_acutorostrata_scammoni_unplaced_genomic_scaffold_BalAcu1.0_scaffold38_whole_genome_shotgun_sequence
marine_RJWP010038708.1_3826-4361_Eubalaena_japonica_isolate_BS61_EubJap_flattened_line_77366_whole_genome_shotgun_sequence
VCEB01000003.1_156274685-156275083_Muntiacus_reevesi_isolate_UCam_UCB_Mr_chromosome_3_whole_genome_shotgun_sequence
VCEA01000001.1_705101280-705101678_Muntiacus_muntjak_isolate_UTSW_UCB_Mm_chromosome_1_whole_genome_shotgun_sequence
VSZR01000023.1_68301283-68301816_Camelus_ferus_isolate_YT-003-E_chromosome_3_whole_genome_shotgun_sequence
CAOW010390181.1_1008-1541_Camelus_bactrianus_WGS_project_CAOW00000000_data_contig_486142_whole_genome_shotgun_sequence
R_JDVD01006121.1_98836-99369_Camelus_dromedarius_breed_Arabia_contig6121_whole_genome_shotgun_sequence
JEMW01008578.1_1083581-1084111_Vicugna_pacos_huacaya_contig8578_whole_genome_shotgun_sequence
R_PVJP02011199.1_1838712-1839249_Hippopotamus_amphibius_isolate_BS01_ScHSfme_11168HRSCAF_61389_whole_genome_shotgun_sequence
R_VCEB01000003.1_47405852-47406393_Muntiacus_reevesi_isolate_UCam_UCB_Mr_chromosome_3_whole_genome_shotgun_sequence
VSZR01000023.1_115374597-115375095_Camelus_ferus_isolate_YT-003-E_chromosome_3_whole_genome_shotgun_sequence
JWIN03000003.1_119387397-119387894_Camelus_dromedarius_breed_African_isolate_Drom800_chromosome_3_whole_genome_shotgun_sequence
R_SIDA01000001.1_31388130-31388494_Sus_scrofa_breed_Bama_miniature_chromosome_1_whole_genome_shotgun_sequence
SIDA01000001.1_91634048-91634543_Sus_scrofa_breed_Bama_miniature_chromosome_1_whole_genome_shotgun_sequence
85
100
97
96
89
80
73
82
44
81
38
95
96
49
97
48
80
100
91
100
87
72
64
93
100
0.03

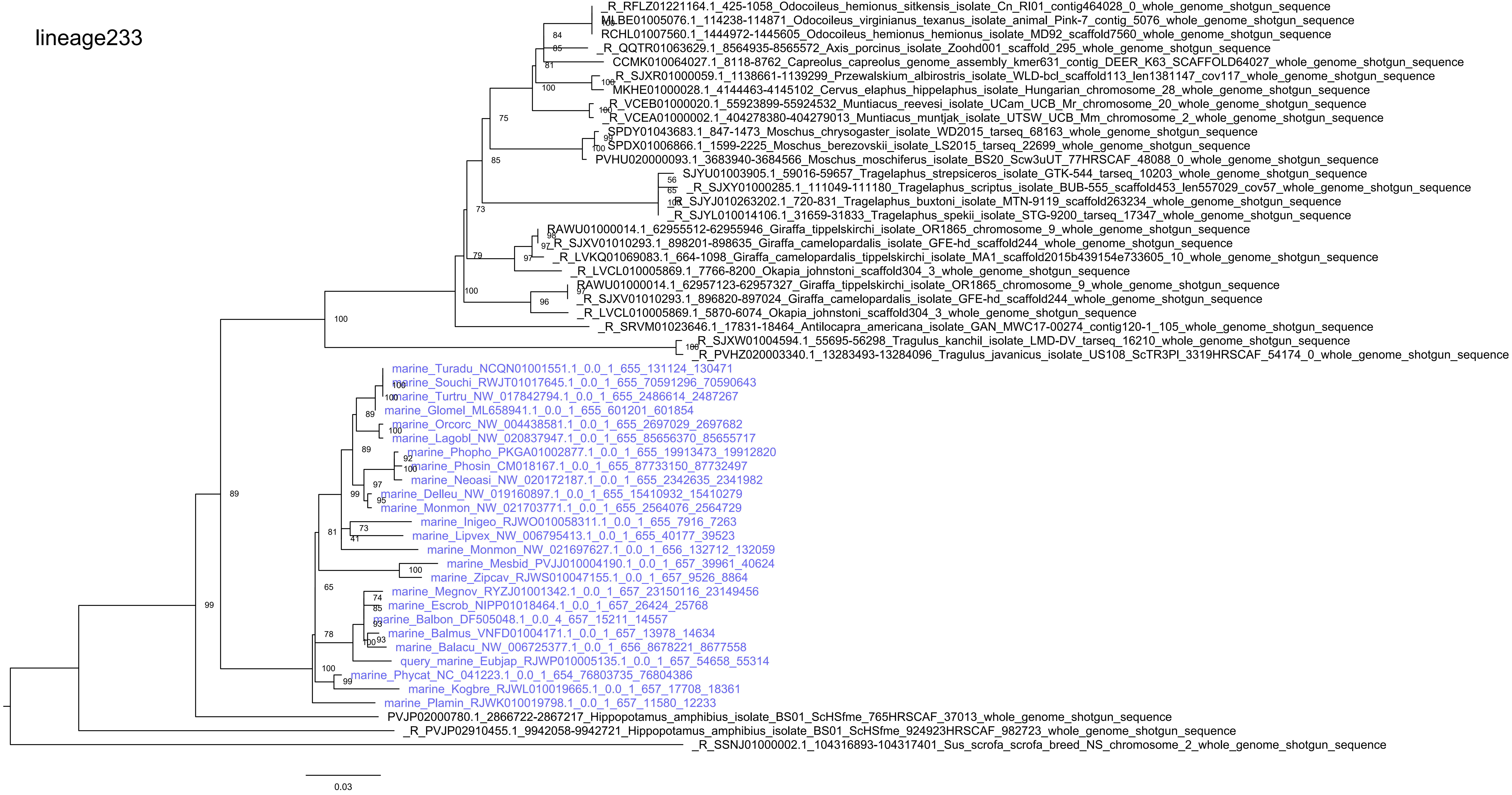
lineage233
_R_RFLZ01221164.1_425-1058_Odocoileus_hemionus_sitkensis_isolate_Cn_RI01_contig464028_0_whole_genome_shotgun_sequence
MLBE01005076.1_114238-114871_Odocoileus_virginianus_texanus_isolate_animal_Pink-7_contig_5076_whole_genome_shotgun_sequence
RCHL01007560.1_1444972-1445605_Odocoileus_hemionus_hemionus_isolate_MD92_scaffold7560_whole_genome_shotgun_sequence
_R_QQTR01063629.1_8564935-8565572_Axis_porcinus_isolate_Zoohd001_scaffold_295_whole_genome_shotgun_sequence
CCMK010064027.1_8118-8762_Capreolus_capreolus_genome_assembly_kmer631_contig_DEER_K63_SCAFFOLD64027_whole_genome_shotgun_sequence
_R_SJXR01000059.1_1138661-1139299_Przewalskium_albirostris_isolate_WLD-bcl_scaffold113_len1381147_cov117_whole_genome_shotgun_sequence
MKHE01000028.1_4144463-4145102_Cervus_elaphus_hippelaphus_isolate_Hungarian_chromosome_28_whole_genome_shotgun_sequence
_R_VCEB01000020.1_55923899-55924532_Muntiacus_reevesi_isolate_UCam_UCB_Mr_chromosome_20_whole_genome_shotgun_sequence
_R_VCEA01000002.1_404278380-404279013_Muntiacus_muntjak_isolate_UTSW_UCB_Mm_chromosome_2_whole_genome_shotgun_sequence
SPDY01043683.1_847-1473_Moschus_chrysogaster_isolate_WD2015_tarseq_68163_whole_genome_shotgun_sequence
SPDX01006866.1_1599-2225_Moschus_berezovskii_isolate_LS2015_tarseq_22699_whole_genome_shotgun_sequence
PVHU020000093.1_3683940-3684566_Moschus_moschiferus_isolate_BS20_Scw3uUT_77HRSCAF_48088_0_whole_genome_shotgun_sequence
SJYU01003905.1_59016-59657_Tragelaphus_strepsiceros_isolate_GTK-544_tarseq_10203_whole_genome_shotgun_sequence
_R_SJXY01000285.1_111049-111180_Tragelaphus_scriptus_isolate_BUB-555_scaffold453_len557029_cov57_whole_genome_shotgun_sequence
_R_SJYJ010263202.1_720-831_Tragelaphus_buxtoni_isolate_MTN-9119_scaffold263234_whole_genome_shotgun_sequence
_R_SJYL010014106.1_31659-31833_Tragelaphus_spekii_isolate_STG-9200_tarseq_17347_whole_genome_shotgun_sequence
RAWU01000014.1_62955512-62955946_Giraffa_tippelskirchi_isolate_OR1865_chromosome_9_whole_genome_shotgun_sequence
_R_SJXV01010293.1_898201-898635_Giraffa_camelopardalis_isolate_GFE-hd_scaffold244_whole_genome_shotgun_sequence
_R_LVKQ01069083.1_664-1098_Giraffa_camelopardalis_tippelskirchi_isolate_MA1_scaffold2015b439154e733605_10_whole_genome_shotgun_sequence
_R_LVCL010005869.1_7766-8200_Okapia_johnstoni_scaffold304_3_whole_genome_shotgun_sequence
RAWU01000014.1_62957123-62957327_Giraffa_tippelskirchi_isolate_OR1865_chromosome_9_whole_genome_shotgun_sequence
_R_SJXV01010293.1_896820-897024_Giraffa_camelopardalis_isolate_GFE-hd_scaffold244_whole_genome_shotgun_sequence
_R_LVCL010005869.1_5870-6074_Okapia_johnstoni_scaffold304_3_whole_genome_shotgun_sequence
_R_SRVM01023646.1_17831-18464_Antilocapra_americana_isolate_GAN_MWC17-00274_contig120-1_105_whole_genome_shotgun_sequence
_R_SJXW01004594.1_55695-56298_Tragulus_kanchil_isolate_LMD-DV_tarseq_16210_whole_genome_shotgun_sequence
_R_PVHZ020003340.1_13283493-13284096_Tragulus_javanicus_isolate_US108_ScTR3PI_3319HRSCAF_54174_0_whole_genome_shotgun_sequence
marine_Turadu_NCQN01001551.1_0.0_1_655_131124_130471
marine_Souchi_RWJT01017645.1_0.0_1_655_70591296_70590643
marine_Turtru_NW_017842794.1_0.0_1_655_2486614_2487267
marine_Glomel_ML658941.1_0.0_1_655_601201_601854
marine_Orcorc_NW_004438581.1_0.0_1_655_2697029_2697682
marine_Lagobl_NW_020837947.1_0.0_1_655_85656370_85655717
marine_Phopho_PKGA01002877.1_0.0_1_655_19913473_19912820
marine_Phosin_CM018167.1_0.0_1_655_87733150_87732497
marine_Neoasi_NW_020172187.1_0.0_1_655_2342635_2341982
marine_Delleu_NW_019160897.1_0.0_1_655_15410932_15410279
marine_Monmon_NW_021703771.1_0.0_1_655_2564076_2564729
marine_Inigeo_RJWO010058311.1_0.0_1_655_7916_7263
marine_Lipvex_NW_006795413.1_0.0_1_655_40177_39523
marine_Monmon_NW_021697627.1_0.0_1_656_132712_132059
marine_Mesbid_PVJJ010004190.1_0.0_1_657_39961_40624
marine_Zipcav_RJWS010047155.1_0.0_1_657_9526_8864
marine_Megnov_RYZJ01001342.1_0.0_1_657_23150116_23149456
marine_Escrob_NIPP01018464.1_0.0_1_657_26424_25768
marine_Balbon_DF505048.1_0.0_4_657_15211_14557
marine_Balmus_VNFD01004171.1_0.0_1_657_13978_14634
marine_Balacu_NW_006725377.1_0.0_1_656_8678221_8677558
query_marine_Eubjap_RJWP010005135.1_0.0_1_657_54658_55314
marine_Phycat_NC_041223.1_0.0_1_654_76803735_76804386
marine_Kogbre_RJWL010019665.1_0.0_1_657_17708_18361
marine_Plamin_RJWK010019798.1_0.0_1_657_11580_12233
PVJP02000780.1_2866722-2867217_Hippopotamus_amphibius_isolate_BS01_ScHSfme_765HRSCAF_37013_whole_genome_shotgun_sequence
_R_PVJP02910455.1_9942058-9942721_Hippopotamus_amphibius_isolate_BS01_ScHSfme_924923HRSCAF_982723_whole_genome_shotgun_sequence
_R_SSNJ01000002.1_104316893-104317401_Sus_scrofa_scrofa_breed_NS_chromosome_2_whole_genome_shotgun_sequence
0.03

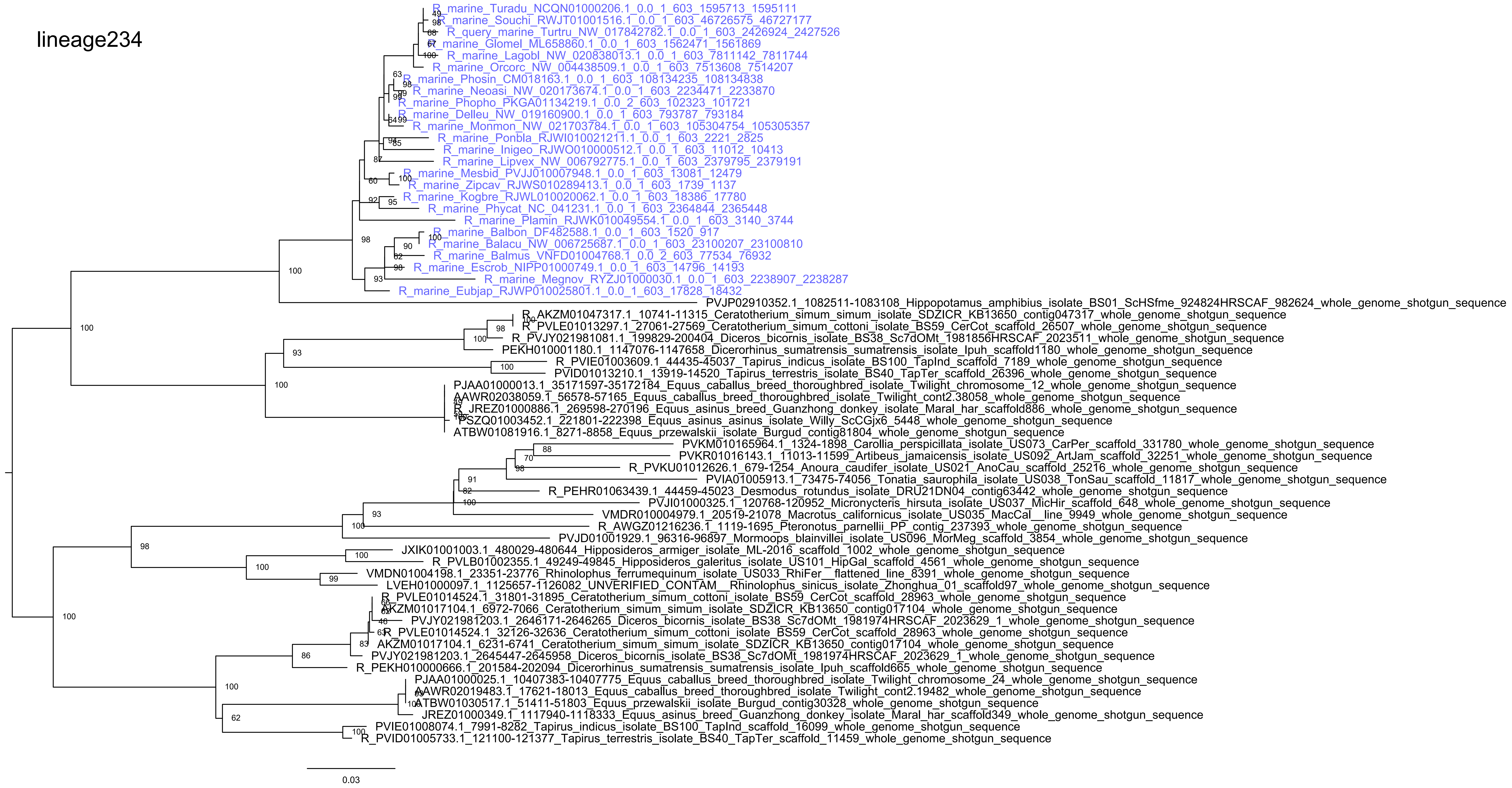
lineage234
R_marine_Turadu_NCQN01000206.1_0.0_1_603_1595713_1595111
R_marine_Souchi_RWJT01001516.1_0.0_1_603_46726575_46727177
R_query_marine_Turtru_NW_017842782.1_0.0_1_603_2426924_2427526
R_marine_Glomel_ML658860.1_0.0_1_603_1562471_1561869
R_marine_Lagobl_NW_020838013.1_0.0_1_603_7811142_7811744
R_marine_Orcorc_NW_004438509.1_0.0_1_603_7513608_7514207
R_marine_Phosin_CM018163.1_0.0_1_603_108134235_108134838
R_marine_Neoasi_NW_020173674.1_0.0_1_603_2234471_2233870
R_marine_Phopho_PKGA01134219.1_0.0_2_603_102323_101721
R_marine_Delleu_NW_019160900.1_0.0_1_603_793787_793184
R_marine_Monmon_NW_021703784.1_0.0_1_603_105304754_105305357
R_marine_Ponbla_RJWI010021211.1_0.0_1_603_2221_2825
R_marine_Inigeo_RJWO010000512.1_0.0_1_603_11012_10413
R_marine_Lipvex_NW_006792775.1_0.0_1_603_2379795_2379191
R_marine_Mesbid_PVJJ010007948.1_0.0_1_603_13081_12479
R_marine_Zipcav_RJWS010289413.1_0.0_1_603_1739_1137
R_marine_Kogbre_RJWL010020062.1_0.0_1_603_18386_17780
R_marine_Phycat_NC_041231.1_0.0_1_603_2364844_2365448
R_marine_Plamin_RJWK010049554.1_0.0_1_603_3140_3744
R_marine_Balbon_DF482588.1_0.0_1_603_1520_917
R_marine_Balacu_NW_006725687.1_0.0_1_603_23100207_23100810
R_marine_Balmus_VNFD01004768.1_0.0_2_603_77534_76932
R_marine_Escrob_NIPP01000749.1_0.0_1_603_14796_14193
R_marine_Megnov_RYZJ01000030.1_0.0_1_603_2238907_2238287
R_marine_Eubjap_RJWP010025801.1_0.0_1_603_17828_18432
PVJP02910352.1_1082511-1083108_Hippopotamus_amphibius_isolate_BS01_ScHSfme_924824HRSCAF_982624_whole_genome_shotgun_sequence
R_AKZM01047317.1_10741-11315_Ceratotherium_simum_simum_isolate_SDZICR_KB13650_contig047317_whole_genome_shotgun_sequence
R_PVLE01013297.1_27061-27569_Ceratotherium_simum_cottoni_isolate_BS59_CerCot_scaffold_26507_whole_genome_shotgun_sequence
R_PVJY021981081.1_199829-200404_Diceros_bicornis_isolate_BS38_Sc7dOMt_1981856HRSCAF_2023511_whole_genome_shotgun_sequence
PEKH010001180.1_1147076-1147658_Dicerorhinus_sumatrensis_sumatrensis_isolate_Ipuh_scaffold1180_whole_genome_shotgun_sequence
R_PVIE01003609.1_44435-45037_Tapirus_indicus_isolate_BS100_TapInd_scaffold_7189_whole_genome_shotgun_sequence
PVID01013210.1_13919-14520_Tapirus_terrestris_isolate_BS40_TapTer_scaffold_26396_whole_genome_shotgun_sequence
PJAA01000013.1_35171597-35172184_Equus_caballus_breed_thoroughbred_isolate_Twilight_chromosome_12_whole_genome_shotgun_sequence
AAWR02038059.1_56578-57165_Equus_caballus_breed_thoroughbred_isolate_Twilight_cont2.38058_whole_genome_shotgun_sequence
R_JREZ01000886.1_269598-270196_Equus_asinus_breed_Guanzhong_donkey_isolate_Maral_har_scaffold886_whole_genome_shotgun_sequence
PSZQ01003452.1_221801-222398_Equus_asinus_asinus_isolate_Willy_ScCGjx6_5448_whole_genome_shotgun_sequence
ATBW01081916.1_8271-8858_Equus_przewalskii_isolate_Burgud_contig81804_whole_genome_shotgun_sequence
PVKM010165964.1_1324-1898_Carollia_perspicillata_isolate_US073_CarPer_scaffold_331780_whole_genome_shotgun_sequence
PVKR01016143.1_11013-11599_Artibeus_jamaicensis_isolate_US092_ArtJam_scaffold_32251_whole_genome_shotgun_sequence
R_PVKU01012626.1_679-1254_Anoura_caudifer_isolate_US021_AnoCau_scaffold_25216_whole_genome_shotgun_sequence
PVIA01005913.1_73475-74056_Tonatia_saurophila_isolate_US038_TonSau_scaffold_11817_whole_genome_shotgun_sequence
R_PEHR01063439.1_44459-45023_Desmodus_rotundus_isolate_DRU21DN04_contig63442_whole_genome_shotgun_sequence
PVJI01000325.1_120768-120952_Micronycteris_hirsuta_isolate_US037_MicHir_scaffold_648_whole_genome_shotgun_sequence
VMDR010004979.1_20519-21078_Macrotus_californicus_isolate_US035_MacCal__line_9949_whole_genome_shotgun_sequence
R_AWGZ01216236.1_1119-1695_Pteronotus_parnellii_PP_contig_237393_whole_genome_shotgun_sequence
PVJD01001929.1_96316-96897_Mormoops_blainvillei_isolate_US096_MorMeg_scaffold_3854_whole_genome_shotgun_sequence
JXIK01001003.1_480029-480644_Hipposideros_armiger_isolate_ML-2016_scaffold_1002_whole_genome_shotgun_sequence
R_PVLB01002355.1_49249-49845_Hipposideros_galeritus_isolate_US101_HipGal_scaffold_4561_whole_genome_shotgun_sequence
VMDN01004198.1_23351-23776_Rhinolophus_ferrumequinum_isolate_US033_RhiFer__flattened_line_8391_whole_genome_shotgun_sequence
LVEH01000097.1_1125657-1126082_UNVERIFIED_CONTAM__Rhinolophus_sinicus_isolate_Zhonghua_01_scaffold97_whole_genome_shotgun_sequence
R_PVLE01014524.1_31801-31895_Ceratotherium_simum_cottoni_isolate_BS59_CerCot_scaffold_28963_whole_genome_shotgun_sequence
AKZM01017104.1_6972-7066_Ceratotherium_simum_simum_isolate_SDZICR_KB13650_contig017104_whole_genome_shotgun_sequence
PVJY021981203.1_2646171-2646265_Diceros_bicornis_isolate_BS38_Sc7dOMt_1981974HRSCAF_2023629_1_whole_genome_shotgun_sequence
R_PVLE01014524.1_32126-32636_Ceratotherium_simum_cottoni_isolate_BS59_CerCot_scaffold_28963_whole_genome_shotgun_sequence
AKZM01017104.1_6231-6741_Ceratotherium_simum_simum_isolate_SDZICR_KB13650_contig017104_whole_genome_shotgun_sequence
PVJY021981203.1_2645447-2645958_Diceros_bicornis_isolate_BS38_Sc7dOMt_1981974HRSCAF_2023629_1_whole_genome_shotgun_sequence
R_PEKH010000666.1_201584-202094_Dicerorhinus_sumatrensis_sumatrensis_isolate_Ipuh_scaffold665_whole_genome_shotgun_sequence
PJAA01000025.1_10407383-10407775_Equus_caballus_breed_thoroughbred_isolate_Twilight_chromosome_24_whole_genome_shotgun_sequence
AAWR02019483.1_17621-18013_Equus_caballus_breed_thoroughbred_isolate_Twilight_cont2.19482_whole_genome_shotgun_sequence
ATBW01030517.1_51411-51803_Equus_przewalskii_isolate_Burgud_contig30328_whole_genome_shotgun_sequence
JREZ01000349.1_1117940-1118333_Equus_asinus_breed_Guanzhong_donkey_isolate_Maral_har_scaffold349_whole_genome_shotgun_sequence
PVIE01008074.1_7991-8282_Tapirus_indicus_isolate_BS100_TapInd_scaffold_16099_whole_genome_shotgun_sequence
R_PVID01005733.1_121100-121377_Tapirus_terrestris_isolate_BS40_TapTer_scaffold_11459_whole_genome_shotgun_sequence
0.03

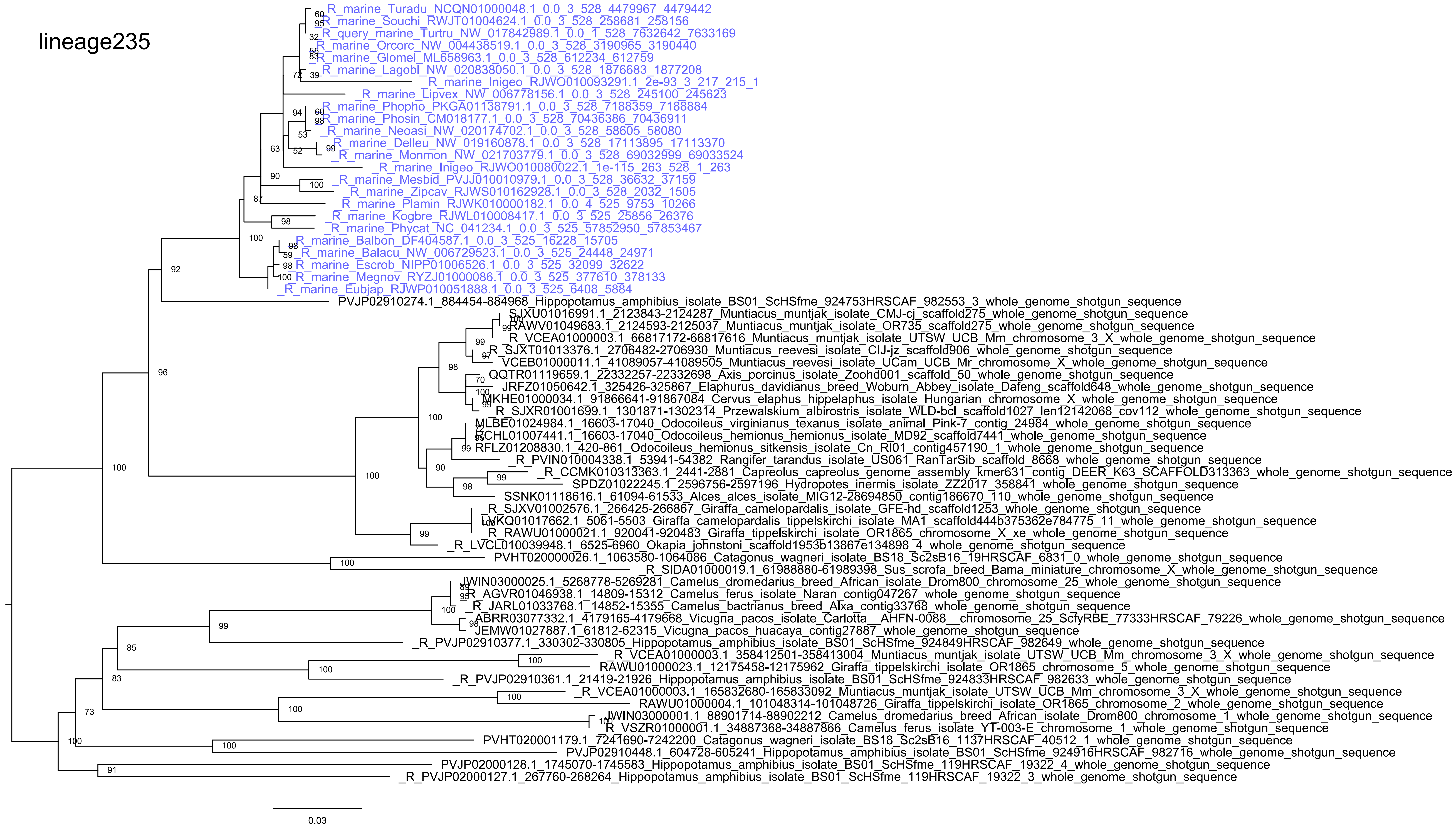
lineage235
R_marine_Turadu_NCQN01000048.1_0.0_3_528_4479967_4479442
R_marine_Souchi_RWJT01004624.1_0.0_3_528_258681_258156
R_query_marine_Turtru_NW_017842989.1_0.0_1_528_7632642_7633169
R_marine_Orcorc_NW_004438519.1_0.0_3_528_3190965_3190440
R_marine_Glomel_ML658963.1_0.0_3_528_612234_612759
R_marine_Lagobl_NW_020838050.1_0.0_3_528_1876683_1877208
_R_marine_Inigeo_RJWO010093291.1_2e-93_3_217_215_1
_R_marine_Lipvex_NW_006778156.1_0.0_3_528_245100_245623
R_marine_Phopho_PKGA01138791.1_0.0_3_528_7188359_7188884
R_marine_Phosin_CM018177.1_0.0_3_528_70436386_70436911
_R_marine_Neoasi_NW_020174702.1_0.0_3_528_58605_58080
_R_marine_Delleu_NW_019160878.1_0.0_3_528_17113895_17113370
_R_marine_Monmon_NW_021703779.1_0.0_3_528_69032999_69033524
_R_marine_Inigeo_RJWO010080022.1_1e-115_263_528_1_263
_R_marine_Mesbid_PVJJ010010979.1_0.0_3_528_36632_37159
_R_marine_Zipcav_RJWS010162928.1_0.0_3_528_2032_1505
_R_marine_Plamin_RJWK010000182.1_0.0_4_525_9753_10266
_R_marine_Kogbre_RJWL010008417.1_0.0_3_525_25856_26376
_R_marine_Phycat_NC_041234.1_0.0_3_525_57852950_57853467
R_marine_Balbon_DF404587.1_0.0_3_525_16228_15705
_R_marine_Balacu_NW_006729523.1_0.0_3_525_24448_24971
R_marine_Escrob_NIPP01006526.1_0.0_3_525_32099_32622
R_marine_Megnov_RYZJ01000086.1_0.0_3_525_377610_378133
_R_marine_Eubjap_RJWP010051888.1_0.0_3_525_6408_5884
PVJP02910274.1_884454-884968_Hippopotamus_amphibius_isolate_BS01_ScHSfme_924753HRSCAF_982553_3_whole_genome_shotgun_sequence
SJXU01016991.1_2123843-2124287_Muntiacus_muntjak_isolate_CMJ-cj_scaffold275_whole_genome_shotgun_sequence
RAWV01049683.1_2124593-2125037_Muntiacus_muntjak_isolate_OR735_scaffold275_whole_genome_shotgun_sequence
_R_VCEA01000003.1_66817172-66817616_Muntiacus_muntjak_isolate_UTSW_UCB_Mm_chromosome_3_X_whole_genome_shotgun_sequence
_R_SJXT01013376.1_2706482-2706930_Muntiacus_reevesi_isolate_CIJ-jz_scaffold906_whole_genome_shotgun_sequence
VCEB01000011.1_41089057-41089505_Muntiacus_reevesi_isolate_UCam_UCB_Mr_chromosome_X_whole_genome_shotgun_sequence
QQTR01119659.1_22332257-22332698_Axis_porcinus_isolate_Zoohd001_scaffold_50_whole_genome_shotgun_sequence
JRFZ01050642.1_325426-325867_Elaphurus_davidianus_breed_Woburn_Abbey_isolate_Dafeng_scaffold648_whole_genome_shotgun_sequence
MKHE01000034.1_91866641-91867084_Cervus_elaphus_hippelaphus_isolate_Hungarian_chromosome_X_whole_genome_shotgun_sequence
_R_SJXR01001699.1_1301871-1302314_Przewalskium_albirostris_isolate_WLD-bcl_scaffold1027_len12142068_cov112_whole_genome_shotgun_sequence
MLBE01024984.1_16603-17040_Odocoileus_virginianus_texanus_isolate_animal_Pink-7_contig_24984_whole_genome_shotgun_sequence
RCHL01007441.1_16603-17040_Odocoileus_hemionus_hemionus_isolate_MD92_scaffold7441_whole_genome_shotgun_sequence
RFLZ01208830.1_420-861_Odocoileus_hemionus_sitkensis_isolate_Cn_RI01_contig457190_1_whole_genome_shotgun_sequence
_R_PVIN010004338.1_53941-54382_Rangifer_tarandus_isolate_US061_RanTarSib_scaffold_8668_whole_genome_shotgun_sequence
_R_CCMK010313363.1_2441-2881_Capreolus_capreolus_genome_assembly_kmer631_contig_DEER_K63_SCAFFOLD313363_whole_genome_shotgun_sequence
SPDZ01022245.1_2596756-2597196_Hydropotes_inermis_isolate_ZZ2017_358841_whole_genome_shotgun_sequence
SSNK01118616.1_61094-61533_Alces_alces_isolate_MIG12-28694850_contig186670_110_whole_genome_shotgun_sequence
_R_SJXV01002576.1_266425-266867_Giraffa_camelopardalis_isolate_GFE-hd_scaffold1253_whole_genome_shotgun_sequence
LVKQ01017662.1_5061-5503_Giraffa_camelopardalis_tippelskirchi_isolate_MA1_scaffold444b375362e784775_11_whole_genome_shotgun_sequence
_R_RAWU01000021.1_920041-920483_Giraffa_tippelskirchi_isolate_OR1865_chromosome_X_xe_whole_genome_shotgun_sequence
_R_LVCL010039948.1_6525-6960_Okapia_johnstoni_scaffold1953b13867e134898_4_whole_genome_shotgun_sequence
PVHT020000026.1_1063580-1064086_Catagonus_wagneri_isolate_BS18_Sc2sB16_19HRSCAF_6831_0_whole_genome_shotgun_sequence
_R_SIDA01000019.1_61988880-61989398_Sus_scrofa_breed_Bama_miniature_chromosome_X_whole_genome_shotgun_sequence
JWIN03000025.1_5268778-5269281_Camelus_dromedarius_breed_African_isolate_Drom800_chromosome_25_whole_genome_shotgun_sequence
_R_AGVR01046938.1_14809-15312_Camelus_ferus_isolate_Naran_contig047267_whole_genome_shotgun_sequence
_R_JARL01033768.1_14852-15355_Camelus_bactrianus_breed_Alxa_contig33768_whole_genome_shotgun_sequence
ABRR03077332.1_4179165-4179668_Vicugna_pacos_isolate_Carlotta__AHFN-0088__chromosome_25_ScfyRBE_77333HRSCAF_79226_whole_genome_shotgun_sequence
JEMW01027887.1_61812-62315_Vicugna_pacos_huacaya_contig27887_whole_genome_shotgun_sequence
_R_PVJP02910377.1_330302-330805_Hippopotamus_amphibius_isolate_BS01_ScHSfme_924849HRSCAF_982649_whole_genome_shotgun_sequence
_R_VCEA01000003.1_358412501-358413004_Muntiacus_muntjak_isolate_UTSW_UCB_Mm_chromosome_3_X_whole_genome_shotgun_sequence
RAWU01000023.1_12175458-12175962_Giraffa_tippelskirchi_isolate_OR1865_chromosome_5_whole_genome_shotgun_sequence
_R_PVJP02910361.1_21419-21926_Hippopotamus_amphibius_isolate_BS01_ScHSfme_924833HRSCAF_982633_whole_genome_shotgun_sequence
_R_VCEA01000003.1_165832680-165833092_Muntiacus_muntjak_isolate_UTSW_UCB_Mm_chromosome_3_X_whole_genome_shotgun_sequence
RAWU01000004.1_101048314-101048726_Giraffa_tippelskirchi_isolate_OR1865_chromosome_2_whole_genome_shotgun_sequence
JWIN03000001.1_88901714-88902212_Camelus_dromedarius_breed_African_isolate_Drom800_chromosome_1_whole_genome_shotgun_sequence
_R_VSZR01000001.1_34887368-34887866_Camelus_ferus_isolate_YT-003-E_chromosome_1_whole_genome_shotgun_sequence
PVHT020001179.1_7241690-7242200_Catagonus_wagneri_isolate_BS18_Sc2sB16_1137HRSCAF_40512_1_whole_genome_shotgun_sequence
PVJP02910448.1_604728-605241_Hippopotamus_amphibius_isolate_BS01_ScHSfme_924916HRSCAF_982716_whole_genome_shotgun_sequence
PVJP02000128.1_1745070-1745583_Hippopotamus_amphibius_isolate_BS01_ScHSfme_119HRSCAF_19322_4_whole_genome_shotgun_sequence
_R_PVJP02000127.1_267760-268264_Hippopotamus_amphibius_isolate_BS01_ScHSfme_119HRSCAF_19322_3_whole_genome_shotgun_sequence
0.03

lineage236

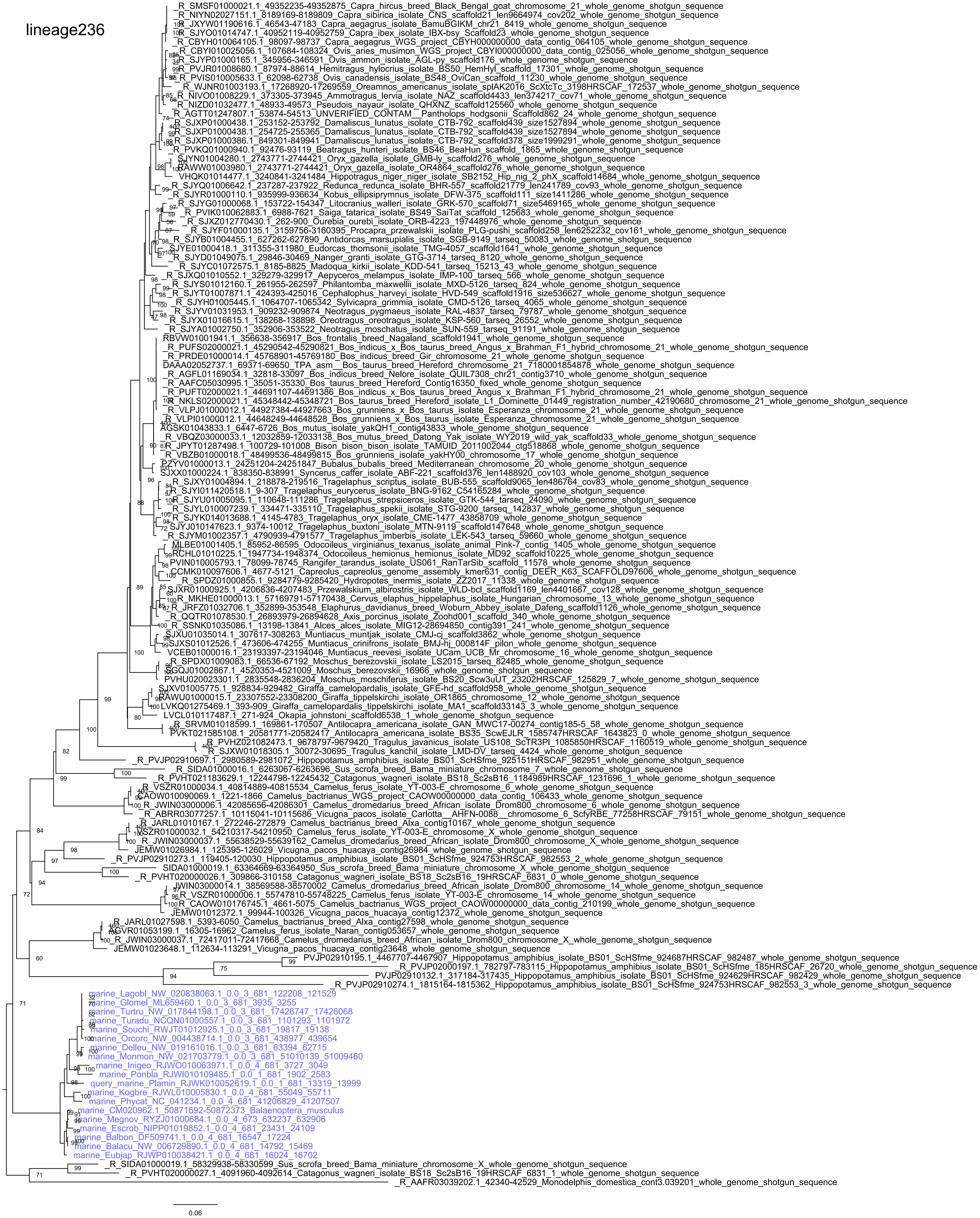
_R_SMSF01000021.1_49352235-49352875_Capra_hircus_breed_Black_Bengal_goat_chromosome_21_whole_genome_shotgun_sequence
_R_NIYN02027151.1_8189169-8189809_Capra_sibirica_isolate_CNS_scaffold21_len9664974_cov202_whole_genome_shotgun_sequence
_R_JXYW01190616.1_46543-47183_Capra_aegagrus_isolate_BamuBGIKM_chr21_8419_whole_genome_shotgun_sequence
_R_SJYO01014747.1_40952119-40952759_Capra_ibex_isolate_IBX-bsy_Scaffold23_whole_genome_shotgun_sequence
_R_CBYH010064105.1_98097-98737_Capra_aegagrus_WGS_project_CBYH000000000_data_contig_064105_whole_genome_shotgun_sequence
_R_CBYI010025056.1_107684-108324_Ovis_aries_musimon_WGS_project_CBYI000000000_data_contig_025056_whole_genome_shotgun_sequence
_R_SJYP01000165.1_345956-346591_Ovis_ammon_isolate_AGL-py_scaffold176_whole_genome_shotgun_sequence
_R_PVJR01008680.1_87974-88614_Hemitragus_hylocrius_isolate_BS50_HemHyl_scaffold_17301_whole_genome_shotgun_sequence
_R_PVIS010005633.1_62098-62738_Ovis_canadensis_isolate_BS48_OviCan_scaffold_11230_whole_genome_shotgun_sequence
_R_WJNR01003193.1_17268920-17269559_Oreamnos_americanus_isolate_splAK2016_ScXtcTc_3198HRSCAF_172537_whole_genome_shotgun_sequence
_R_NIVO01008229.1_373305-373945_Ammotragus_lervia_isolate_NAZ_scaffold4433_len374217_cov71_whole_genome_shotgun_sequence
_R_NIZD01032477.1_48933-49573_Pseudois_nayaur_isolate_QHXNZ_scaffold125560_whole_genome_shotgun_sequence
_R_AGTT01247807.1_53874-54513_UNVERIFIED_CONTAM__Pantholops_hodgsonii_Scaffold862_24_whole_genome_shotgun_sequence
_R_SJXP01000438.1_253152-253792_Damaliscus_lunatus_isolate_CTB-792_scaffold439_size1527894_whole_genome_shotgun_sequence
_R_SJXP01000438.1_254725-255365_Damaliscus_lunatus_isolate_CTB-792_scaffold439_size1527894_whole_genome_shotgun_sequence
_R_SJXP01000386.1_849301-849941_Damaliscus_lunatus_isolate_CTB-792_scaffold378_size1999291_whole_genome_shotgun_sequence
_R_PVKQ01000940.1_92476-93119_Beatragus_hunteri_isolate_BS46_BeaHun_scaffold_1865_whole_genome_shotgun_sequence
SJYN01004280.1_2743771-2744421_Oryx_gazella_isolate_GMB-ly_scaffold276_whole_genome_shotgun_sequence
RAWW01003980.1_2743771-2744421_Oryx_gazella_isolate_OR4864_scaffold276_whole_genome_shotgun_sequence
VHQK01014477.1_3240841-3241484_Hippotragus_niger_niger_isolate_SB2152_Hip_nig_2_phX_scaffold14684_whole_genome_shotgun_sequence
_R_SJYQ01006642.1_237287-237922_Redunca_redunca_isolate_BHR-557_scaffold21779_len241789_cov93_whole_genome_shotgun_sequence
_R_SJYR01000110.1_935999-936634_Kobus_ellipsiprymnus_isolate_DFW-375_scaffold111_size1411286_whole_genome_shotgun_sequence
_R_SJYG01000068.1_153722-154347_Litocranius_walleri_isolate_GRK-570_scaffold71_size5469165_whole_genome_shotgun_sequence
_R_PVIK010062883.1_6988-7621_Saiga_tatarica_isolate_BS49_SaiTat_scaffold_125683_whole_genome_shotgun_sequence
_R_SJXZ012770430.1_262-900_Ourebia_ourebi_isolate_ORB-4223_197448976_whole_genome_shotgun_sequence
_R_SJYF01000135.1_3159756-3160395_Procapra_przewalskii_isolate_PLG-pushi_scaffold258_len6252232_cov161_whole_genome_shotgun_sequence
_R_SJYB01004455.1_627262-627890_Antidorcas_marsupialis_isolate_SGB-9149_tarseq_50083_whole_genome_shotgun_sequence
SJYE01000418.1_311355-311980_Eudorcas_thomsonii_isolate_TMG-4057_scaffold1641_whole_genome_shotgun_sequence
_R_SJYD01049075.1_29846-30469_Nanger_granti_isolate_GTG-3714_tarseq_8120_whole_genome_shotgun_sequence
_R_SJYC01072575.1_8185-8825_Madoqua_kirkii_isolate_KDD-541_tarseq_15213_43_whole_genome_shotgun_sequence
_R_SJXQ01010552.1_329279-329917_Aepyceros_melampus_isolate_IMP-100_tarseq_566_whole_genome_shotgun_sequence
_R_SJYS01012160.1_261955-262597_Philantomba_maxwellii_isolate_MXD-5126_tarseq_824_whole_genome_shotgun_sequence
_R_SJYT01007871.1_424393-425016_Cephalophus_harveyi_isolate_HVD-549_scaffold1916_size536627_whole_genome_shotgun_sequence
_R_SJYH01005445.1_1064707-1065342_Sylvicapra_grimmia_isolate_CMD-5126_tarseq_4065_whole_genome_shotgun_sequence
_R_SJYV01031953.1_909232-909874_Neotragus_pygmaeus_isolate_RAL-4837_tarseq_79787_whole_genome_shotgun_sequence
_R_SJYX01016615.1_138268-138898_Oreotragus_oreotragus_isolate_KSP-560_tarseq_26552_whole_genome_shotgun_sequence
_R_SJYA01002750.1_352906-353522_Neotragus_moschatus_isolate_SUN-559_tarseq_91191_whole_genome_shotgun_sequence
RBVW01001941.1_356638-356917_Bos_frontalis_breed_Nagaland_scaffold1941_whole_genome_shotgun_sequence
_R_PUFS02000021.1_45290542-45290821_Bos_indicus_x_Bos_taurus_breed_Angus_x_Brahman_F1_hybrid_chromosome_21_whole_genome_shotgun_sequence
_R_PRDE01000014.1_45768901-45769180_Bos_indicus_breed_Gir_chromosome_21_whole_genome_shotgun_sequence
DAAA02052737.1_69371-69650_TPA_asm__Bos_taurus_breed_Hereford_chromosome_21_7180001854878_whole_genome_shotgun_sequence
_R_AGFL01169034.1_32818-33097_Bos_indicus_breed_Nelore_isolate_QUIL7308_chr21_contig3710_whole_genome_shotgun_sequence
_R_AAFC05030995.1_35051-35330_Bos_taurus_breed_Hereford_Contig16350_fixed_whole_genome_shotgun_sequence
_R_PUFT02000021.1_44691107-44691386_Bos_indicus_x_Bos_taurus_breed_Angus_x_Brahman_F1_hybrid_chromosome_21_whole_genome_shotgun_sequence
_R_NKLS02000021.1_45348442-45348721_Bos_taurus_breed_Hereford_isolate_L1_Dominette_01449_registration_number_42190680_chromosome_21_whole_genome_shotgun_sequence
_R_VLPJ01000012.1_44927384-44927663_Bos_grunniens_x_Bos_taurus_isolate_Esperanza_chromosome_21_whole_genome_shotgun_sequence
_R_VLPI01000012.1_44648249-44648528_Bos_grunniens_x_Bos_taurus_isolate_Esperanza_chromosome_21_whole_genome_shotgun_sequence
AGSK01043833.1_6447-6726_Bos_mutus_isolate_yakQH1_contig43833_whole_genome_shotgun_sequence
_R_VBQZ03000033.1_12032859-12033138_Bos_mutus_breed_Datong_Yak_isolate_WY2019_wild_yak_scaffold33_whole_genome_shotgun_sequence
_R_JPYT01287498.1_100729-101008_Bison_bison_bison_isolate_TAMUID_2011002044_ctg518868_whole_genome_shotgun_sequence
_R_VBZB01000018.1_48499536-48499815_Bos_grunniens_isolate_yakHY00_chromosome_17_whole_genome_shotgun_sequence
PZYV01000013.1_24251204-24251847_Bubalus_bubalis_breed_Mediterranean_chromosome_20_whole_genome_shotgun_sequence
SJXX01000224.1_838350-838991_Syncerus_caffer_isolate_ABF-221_scaffold376_len1488920_cov103_whole_genome_shotgun_sequence
_R_SJXY01004894.1_218878-219516_Tragelaphus_scriptus_isolate_BUB-555_scaffold9065_len486764_cov83_whole_genome_shotgun_sequence
_R_SJYI011420518.1_9-307_Tragelaphus_eurycerus_isolate_BNG-9162_C54165284_whole_genome_shotgun_sequence
_R_SJYU01005095.1_110648-111286_Tragelaphus_strepsiceros_isolate_GTK-544_tarseq_24090_whole_genome_shotgun_sequence
_R_SJYL010007239.1_334471-335110_Tragelaphus_spekii_isolate_STG-9200_tarseq_142837_whole_genome_shotgun_sequence
_R_SJYK014013688.1_4145-4783_Tragelaphus_oryx_isolate_CME-1477_43858709_whole_genome_shotgun_sequence
SJYJ010147623.1_9374-10012_Tragelaphus_buxtoni_isolate_MTN-9119_scaffold147648_whole_genome_shotgun_sequence
_R_SJYM01002357.1_4790939-4791577_Tragelaphus_imberbis_isolate_LEK-543_tarseq_59660_whole_genome_shotgun_sequence
MLBE01001405.1_85952-86595_Odocoileus_virginianus_texanus_isolate_animal_Pink-7_contig_1405_whole_genome_shotgun_sequence
RCHL01010225.1_1947734-1948374_Odocoileus_hemionus_hemionus_isolate_MD92_scaffold10225_whole_genome_shotgun_sequence
PVIN010005793.1_78099-78745_Rangifer_tarandus_isolate_US061_RanTarSib_scaffold_11578_whole_genome_shotgun_sequence
CCMK010097606.1_4677-5121_Capreolus_capreolus_genome_assembly_kmer631_contig_DEER_K63_SCAFFOLD97606_whole_genome_shotgun_sequence
_R_SPDZ01000855.1_9284779-9285420_Hydropotes_inermis_isolate_ZZ2017_11338_whole_genome_shotgun_sequence
SJXR01000925.1_4206836-4207483_Przewalskium_albirostris_isolate_WLD-bcl_scaffold1169_len4401667_cov128_whole_genome_shotgun_sequence
_R_MKHE01000013.1_57169791-57170438_Cervus_elaphus_hippelaphus_isolate_Hungarian_chromosome_13_whole_genome_shotgun_sequence
_R_JRFZ01032706.1_352899-353548_Elaphurus_davidianus_breed_Woburn_Abbey_isolate_Dafeng_scaffold1126_whole_genome_shotgun_sequence
_R_QQTR01078530.1_26893979-26894628_Axis_porcinus_isolate_Zoohd001_scaffold_340_whole_genome_shotgun_sequence
_R_SSNK01035086.1_13198-13841_Alces_alces_isolate_MIG12-28694850_contig391_241_whole_genome_shotgun_sequence
SJXU01035014.1_307617-308263_Muntiacus_muntjak_isolate_CMJ-cj_scaffold3862_whole_genome_shotgun_sequence
SJXS01012526.1_473606-474255_Muntiacus_crinifrons_isolate_BMJ-hj_000814F_pilon_whole_genome_shotgun_sequence
VCEB01000016.1_23193397-23194046_Muntiacus_reevesi_isolate_UCam_UCB_Mr_chromosome_16_whole_genome_shotgun_sequence
_R_SPDX01009083.1_66536-67192_Moschus_berezovskii_isolate_LS2015_tarseq_82485_whole_genome_shotgun_sequence
SGQJ01002867.1_4520353-4521009_Moschus_berezovskii_16966_whole_genome_shotgun_sequence
PVHU020023301.1_2835548-2836204_Moschus_moschiferus_isolate_BS20_Scw3uUT_23202HRSCAF_125829_7_whole_genome_shotgun_sequence
SJXV01005775.1_928834-929482_Giraffa_camelopardalis_isolate_GFE-hd_scaffold958_whole_genome_shotgun_sequence
RAWU01000015.1_23307552-23308200_Giraffa_tippelskirchi_isolate_OR1865_chromosome_12_whole_genome_shotgun_sequence
LVKQ01275469.1_393-909_Giraffa_camelopardalis_tippelskirchi_isolate_MA1_scaffold33143_3_whole_genome_shotgun_sequence
LVCL010117487.1_271-924_Okapia_johnstoni_scaffold6538_1_whole_genome_shotgun_sequence
_R_SRVM01018599.1_169861-170507_Antilocapra_americana_isolate_GAN_MWC17-00274_contig185-5_58_whole_genome_shotgun_sequence
PVKT021585108.1_20581771-20582417_Antilocapra_americana_isolate_BS35_ScwEJLR_1585747HRSCAF_1643823_0_whole_genome_shotgun_sequence
_R_PVHZ021082473.1_9678797-9679420_Tragulus_javanicus_isolate_US108_ScTR3PI_1085850HRSCAF_1160519_whole_genome_shotgun_sequence
_R_SJXW01018305.1_30072-30695_Tragulus_kanchil_isolate_LMD-DV_tarseq_4424_whole_genome_shotgun_sequence
_R_PVJP02910697.1_2980589-2981072_Hippopotamus_amphibius_isolate_BS01_ScHSfme_925151HRSCAF_982951_whole_genome_shotgun_sequence
_R_SIDA01000016.1_6263067-6263696_Sus_scrofa_breed_Bama_miniature_chromosome_7_whole_genome_shotgun_sequence
_R_PVHT021183629.1_12244798-12245432_Catagonus_wagneri_isolate_BS18_Sc2sB16_1184989HRSCAF_1231696_1_whole_genome_shotgun_sequence
_R_VSZR01000034.1_40814889-40815534_Camelus_ferus_isolate_YT-003-E_chromosome_6_whole_genome_shotgun_sequence
CAOW010090069.1_1221-1866_Camelus_bactrianus_WGS_project_CAOW00000000_data_contig_106433_whole_genome_shotgun_sequence
_R_JWIN03000006.1_42085656-42086301_Camelus_dromedarius_breed_African_isolate_Drom800_chromosome_6_whole_genome_shotgun_sequence
_R_ABRR03077257.1_10115041-10115686_Vicugna_pacos_isolate_Carlotta__AHFN-0088__chromosome_6_ScfyRBE_77258HRSCAF_79151_whole_genome_shotgun_sequence
_R_JARL01010167.1_272246-272879_Camelus_bactrianus_breed_Alxa_contig10167_whole_genome_shotgun_sequence
VSZR01000032.1_54210317-54210950_Camelus_ferus_isolate_YT-003-E_chromosome_X_whole_genome_shotgun_sequence
_R_JWIN03000037.1_55638529-55639162_Camelus_dromedarius_breed_African_isolate_Drom800_chromosome_X_whole_genome_shotgun_sequence
JEMW01026984.1_125395-126029_Vicugna_pacos_huacaya_contig26984_whole_genome_shotgun_sequence
_R_PVJP02910273.1_119405-120030_Hippopotamus_amphibius_isolate_BS01_ScHSfme_924753HRSCAF_982553_2_whole_genome_shotgun_sequence
SIDA01000019.1_63364669-63364950_Sus_scrofa_breed_Bama_miniature_chromosome_X_whole_genome_shotgun_sequence
_R_PVHT020000026.1_309866-310158_Catagonus_wagneri_isolate_BS18_Sc2sB16_19HRSCAF_6831_0_whole_genome_shotgun_sequence
JWIN03000014.1_38569588-38570002_Camelus_dromedarius_breed_African_isolate_Drom800_chromosome_14_whole_genome_shotgun_sequence
_R_VSZR01000006.1_55747810-55748225_Camelus_ferus_isolate_YT-003-E_chromosome_14_whole_genome_shotgun_sequence
_R_CAOW010176745.1_4661-5075_Camelus_bactrianus_WGS_project_CAOW00000000_data_contig_210199_whole_genome_shotgun_sequence
JEMW01012372.1_99944-100326_Vicugna_pacos_huacaya_contig12372_whole_genome_shotgun_sequence
_R_JARL01027598.1_5393-6050_Camelus_bactrianus_breed_Alxa_contig27598_whole_genome_shotgun_sequence
AGVR01053199.1_16305-16962_Camelus_ferus_isolate_Naran_contig053657_whole_genome_shotgun_sequence
_R_JWIN03000037.1_72417011-72417668_Camelus_dromedarius_breed_African_isolate_Drom800_chromosome_X_whole_genome_shotgun_sequence
JEMW01023648.1_112634-113291_Vicugna_pacos_huacaya_contig23648_whole_genome_shotgun_sequence
PVJP02910195.1_4467707-4467907_Hippopotamus_amphibius_isolate_BS01_ScHSfme_924687HRSCAF_982487_whole_genome_shotgun_sequence
_R_PVJP02000197.1_782797-783115_Hippopotamus_amphibius_isolate_BS01_ScHSfme_185HRSCAF_26720_whole_genome_shotgun_sequence
PVJP02910132.1_317184-317435_Hippopotamus_amphibius_isolate_BS01_ScHSfme_924629HRSCAF_982429_whole_genome_shotgun_sequence
_R_PVJP02910274.1_1815164-1815362_Hippopotamus_amphibius_isolate_BS01_ScHSfme_924753HRSCAF_982553_3_whole_genome_shotgun_sequence
marine_Lagobl_NW_020838063.1_0.0_3_681_122208_121529
marine_Glomel_ML659460.1_0.0_3_681_3935_3255
marine_Turtru_NW_017844198.1_0.0_3_681_17426747_17426068
marine_Turadu_NCQN01000557.1_0.0_3_681_1101293_1101972
marine_Souchi_RWJT01012925.1_0.0_3_681_19817_19138
marine_Orcorc_NW_004438714.1_0.0_3_681_438977_439654
marine_Delleu_NW_019161016.1_0.0_3_681_63394_62715
marine_Monmon_NW_021703779.1_0.0_3_681_51010139_51009460
marine_Inigeo_RJWO010063971.1_0.0_4_681_3727_3049
marine_Ponbla_RJWI010109485.1_0.0_1_681_1902_2583
query_marine_Plamin_RJWK010052619.1_0.0_1_681_13319_13999
marine_Kogbre_RJWL010005830.1_0.0_4_681_55049_55711
marine_Phycat_NC_041234.1_0.0_4_681_41206829_41207507
marine_CM020962.1_50871692-50872373_Balaenoptera_musculus
marine_Megnov_RYZJ01000684.1_0.0_4_673_632237_632906
marine_Escrob_NIPP01019852.1_0.0_4_681_23431_24109
marine_Balbon_DF509741.1_0.0_4_681_16547_17224
marine_Balacu_NW_006729890.1_0.0_4_681_14792_15469
marine_Eubjap_RJWP010038421.1_0.0_4_681_16024_16702
_R_SIDA01000019.1_58329938-58330599_Sus_scrofa_breed_Bama_miniature_chromosome_X_whole_genome_shotgun_sequence
_R_PVHT020000027.1_4091960-4092614_Catagonus_wagneri_isolate_BS18_Sc2sB16_19HRSCAF_6831_1_whole_genome_shotgun_sequence
_R_AAFR03039202.1_42340-42529_Monodelphis_domestica_cont3.039201_whole_genome_shotgun_sequence
0.06

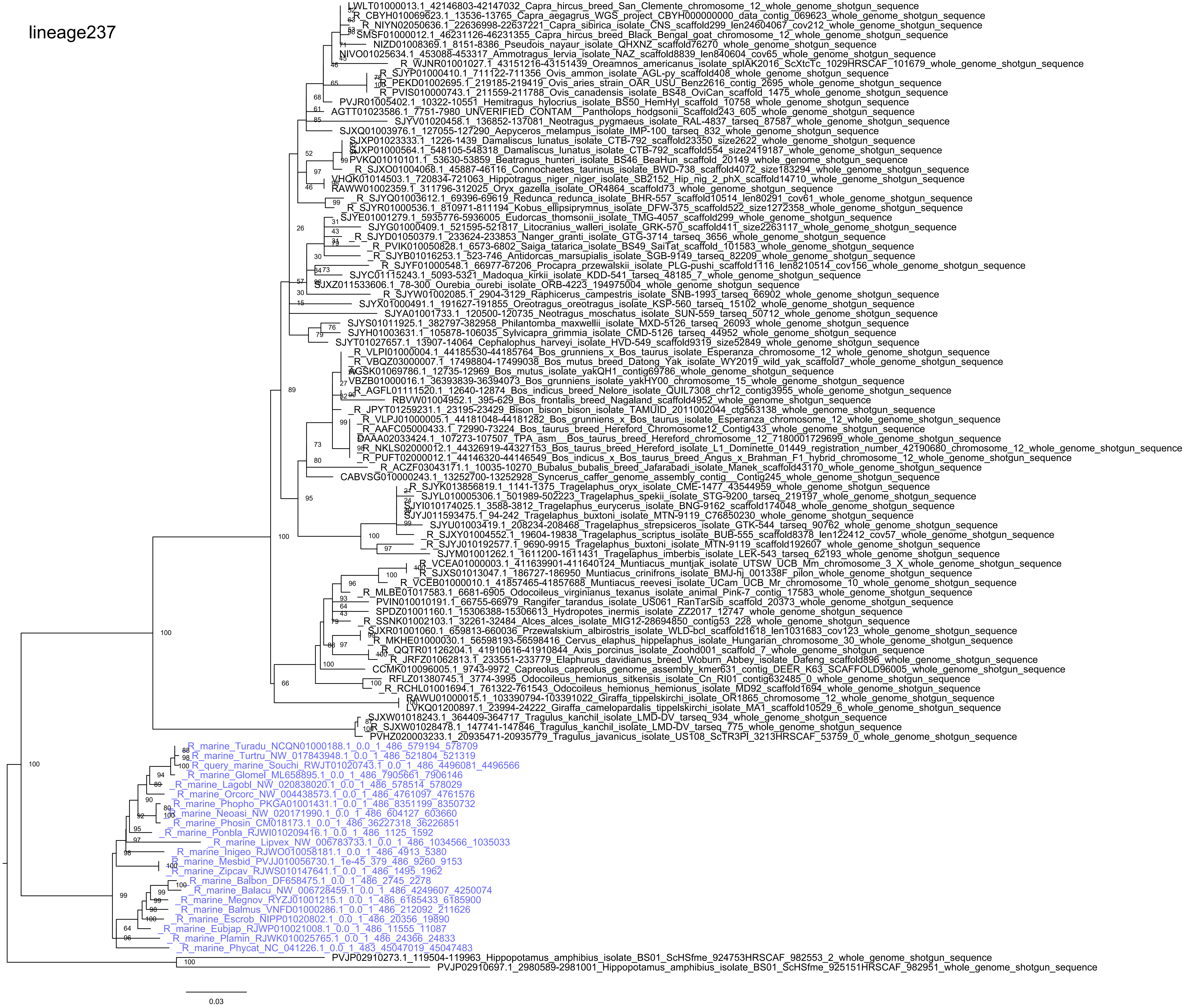

lineage237
LWLT01000013.1_42146803-42147032_Capra_hircus_breed_San_Clemente_chromosome_12_whole_genome_shotgun_sequence
_R_CBYH010069623.1_13536-13765_Capra_aegagrus_WGS_project_CBYH000000000_data_contig_069623_whole_genome_shotgun_sequence
_R_NIYN02050636.1_22636998-22637221_Capra_sibirica_isolate_CNS_scaffold299_len24604067_cov212_whole_genome_shotgun_sequence
SMSF01000012.1_46231126-46231355_Capra_hircus_breed_Black_Bengal_goat_chromosome_12_whole_genome_shotgun_sequence
NIZD01008369.1_8151-8386_Pseudois_nayaur_isolate_QHXNZ_scaffold76270_whole_genome_shotgun_sequence
NIVO01025634.1_453088-453317_Ammotragus_lervia_isolate_NAZ_scaffold8839_len840604_cov65_whole_genome_shotgun_sequence
_R_WJNR01001027.1_43151216-43151439_Oreamnos_americanus_isolate_splAK2016_ScXtcTc_1029HRSCAF_101679_whole_genome_shotgun_sequence
_R_SJYP01000410.1_711122-711356_Ovis_ammon_isolate_AGL-py_scaffold408_whole_genome_shotgun_sequence
_R_PEKD01002695.1_219185-219419_Ovis_aries_strain_OAR_USU_Benz2616_contig_2695_whole_genome_shotgun_sequence
_R_PVIS010000743.1_211559-211788_Ovis_canadensis_isolate_BS48_OviCan_scaffold_1475_whole_genome_shotgun_sequence
PVJR01005402.1_10322-10551_Hemitragus_hylocrius_isolate_BS50_HemHyl_scaffold_10758_whole_genome_shotgun_sequence
AGTT01023586.1_7751-7980_UNVERIFIED_CONTAM__Pantholops_hodgsonii_Scaffold243_605_whole_genome_shotgun_sequence
SJYV01020458.1_136852-137081_Neotragus_pygmaeus_isolate_RAL-4837_tarseq_87587_whole_genome_shotgun_sequence
SJXQ01003976.1_127055-127290_Aepyceros_melampus_isolate_IMP-100_tarseq_832_whole_genome_shotgun_sequence
SJXP01023333.1_1226-1439_Damaliscus_lunatus_isolate_CTB-792_scaffold23350_size2622_whole_genome_shotgun_sequence
SJXP01000564.1_548105-548318_Damaliscus_lunatus_isolate_CTB-792_scaffold554_size2419187_whole_genome_shotgun_sequence
PVKQ01010101.1_53630-53859_Beatragus_hunteri_isolate_BS46_BeaHun_scaffold_20149_whole_genome_shotgun_sequence
_R_SJXO01004068.1_45887-46116_Connochaetes_taurinus_isolate_BWD-738_scaffold4072_size183294_whole_genome_shotgun_sequence
VHQK01014503.1_720834-721063_Hippotragus_niger_niger_isolate_SB2152_Hip_nig_2_phX_scaffold14710_whole_genome_shotgun_sequence
RAWW01002359.1_311796-312025_Oryx_gazella_isolate_OR4864_scaffold73_whole_genome_shotgun_sequence
_R_SJYQ01003612.1_69396-69619_Redunca_redunca_isolate_BHR-557_scaffold10514_len80291_cov61_whole_genome_shotgun_sequence
_R_SJYR01000536.1_810971-811194_Kobus_ellipsiprymnus_isolate_DFW-375_scaffold522_size1272358_whole_genome_shotgun_sequence
SJYE01001279.1_5935776-5936005_Eudorcas_thomsonii_isolate_TMG-4057_scaffold299_whole_genome_shotgun_sequence
SJYG01000409.1_521595-521817_Litocranius_walleri_isolate_GRK-570_scaffold411_size2263117_whole_genome_shotgun_sequence
_R_SJYD01050379.1_233624-233853_Nanger_granti_isolate_GTG-3714_tarseq_3656_whole_genome_shotgun_sequence
_R_PVIK010050828.1_6573-6802_Saiga_tatarica_isolate_BS49_SaiTat_scaffold_101583_whole_genome_shotgun_sequence
_R_SJYB01016253.1_523-746_Antidorcas_marsupialis_isolate_SGB-9149_tarseq_82209_whole_genome_shotgun_sequence
_R_SJYF01000548.1_66977-67206_Procapra_przewalskii_isolate_PLG-pushi_scaffold1116_len8210514_cov156_whole_genome_shotgun_sequence
SJYC01115243.1_5093-5321_Madoqua_kirkii_isolate_KDD-541_tarseq_48185_7_whole_genome_shotgun_sequence
SJXZ011533606.1_78-300_Ourebia_ourebi_isolate_ORB-4223_194975004_whole_genome_shotgun_sequence
_R_SJYW01002085.1_2904-3129_Raphicerus_campestris_isolate_SNB-1993_tarseq_66902_whole_genome_shotgun_sequence
SJYX01000491.1_191627-191855_Oreotragus_oreotragus_isolate_KSP-560_tarseq_15102_whole_genome_shotgun_sequence
SJYA01001733.1_120500-120735_Neotragus_moschatus_isolate_SUN-559_tarseq_50712_whole_genome_shotgun_sequence
SJYS01011925.1_382797-382958_Philantomba_maxwellii_isolate_MXD-5126_tarseq_26093_whole_genome_shotgun_sequence
SJYH01003631.1_105878-106035_Sylvicapra_grimmia_isolate_CMD-5126_tarseq_44952_whole_genome_shotgun_sequence
SJYT01027657.1_13907-14064_Cephalophus_harveyi_isolate_HVD-549_scaffold9319_size52849_whole_genome_shotgun_sequence
_R_VLPI01000004.1_44185530-44185764_Bos_grunniens_x_Bos_taurus_isolate_Esperanza_chromosome_12_whole_genome_shotgun_sequence
_R_VBQZ03000007.1_17498804-17499038_Bos_mutus_breed_Datong_Yak_isolate_WY2019_wild_yak_scaffold7_whole_genome_shotgun_sequence
AGSK01069786.1_12735-12969_Bos_mutus_isolate_yakQH1_contig69786_whole_genome_shotgun_sequence
VBZB01000016.1_36393839-36394073_Bos_grunniens_isolate_yakHY00_chromosome_15_whole_genome_shotgun_sequence
_R_AGFL01111520.1_12640-12874_Bos_indicus_breed_Nelore_isolate_QUIL7308_chr12_contig3955_whole_genome_shotgun_sequence
RBVW01004952.1_395-629_Bos_frontalis_breed_Nagaland_scaffold4952_whole_genome_shotgun_sequence
_R_JPYT01259231.1_23195-23429_Bison_bison_bison_isolate_TAMUID_2011002044_ctg563138_whole_genome_shotgun_sequence
_R_VLPJ01000005.1_44181048-44181282_Bos_grunniens_x_Bos_taurus_isolate_Esperanza_chromosome_12_whole_genome_shotgun_sequence
_R_AAFC05000433.1_72990-73224_Bos_taurus_breed_Hereford_Chromosome12_Contig433_whole_genome_shotgun_sequence
DAAA02033424.1_107273-107507_TPA_asm__Bos_taurus_breed_Hereford_chromosome_12_7180001729699_whole_genome_shotgun_sequence
_R_NKLS02000012.1_44326919-44327153_Bos_taurus_breed_Hereford_isolate_L1_Dominette_01449_registration_number_42190680_chromosome_12_whole_genome_shotgun_sequence
_R_PUFT02000012.1_44146320-44146549_Bos_indicus_x_Bos_taurus_breed_Angus_x_Brahman_F1_hybrid_chromosome_12_whole_genome_shotgun_sequence
_R_ACZF03043171.1_10035-10270_Bubalus_bubalis_breed_Jafarabadi_isolate_Manek_scaffold43170_whole_genome_shotgun_sequence
CABVSG010000243.1_13252700-13252928_Syncerus_caffer_genome_assembly_contig__Contig245_whole_genome_shotgun_sequence
_R_SJYK013856819.1_1141-1375_Tragelaphus_oryx_isolate_CME-1477_43544959_whole_genome_shotgun_sequence
SJYL010005306.1_501989-502223_Tragelaphus_spekii_isolate_STG-9200_tarseq_219197_whole_genome_shotgun_sequence
SJYI010174025.1_3588-3812_Tragelaphus_eurycerus_isolate_BNG-9162_scaffold174048_whole_genome_shotgun_sequence
SJYJ011593475.1_94-242_Tragelaphus_buxtoni_isolate_MTN-9119_C76850230_whole_genome_shotgun_sequence
SJYU01003419.1_208234-208468_Tragelaphus_strepsiceros_isolate_GTK-544_tarseq_90762_whole_genome_shotgun_sequence
_R_SJXY01004552.1_19604-19838_Tragelaphus_scriptus_isolate_BUB-555_scaffold8378_len122412_cov57_whole_genome_shotgun_sequence
_R_SJYJ010192577.1_9690-9915_Tragelaphus_buxtoni_isolate_MTN-9119_scaffold192607_whole_genome_shotgun_sequence
SJYM01001262.1_1611200-1611431_Tragelaphus_imberbis_isolate_LEK-543_tarseq_62193_whole_genome_shotgun_sequence
_R_VCEA01000003.1_411639901-411640124_Muntiacus_muntjak_isolate_UTSW_UCB_Mm_chromosome_3_X_whole_genome_shotgun_sequence
_R_SJXS01013047.1_186727-186950_Muntiacus_crinifrons_isolate_BMJ-hj_001338F_pilon_whole_genome_shotgun_sequence
_R_VCEB01000010.1_41857465-41857688_Muntiacus_reevesi_isolate_UCam_UCB_Mr_chromosome_10_whole_genome_shotgun_sequence
_R_MLBE01017583.1_6681-6905_Odocoileus_virginianus_texanus_isolate_animal_Pink-7_contig_17583_whole_genome_shotgun_sequence
PVIN010010191.1_66755-66979_Rangifer_tarandus_isolate_US061_RanTarSib_scaffold_20373_whole_genome_shotgun_sequence
SPDZ01001160.1_15306388-15306613_Hydropotes_inermis_isolate_ZZ2017_12747_whole_genome_shotgun_sequence
_R_SSNK01002103.1_32261-32484_Alces_alces_isolate_MIG12-28694850_contig53_228_whole_genome_shotgun_sequence
SJXR01001060.1_659813-660036_Przewalskium_albirostris_isolate_WLD-bcl_scaffold1618_len1031683_cov123_whole_genome_shotgun_sequence
_R_MKHE01000030.1_56598193-56598416_Cervus_elaphus_hippelaphus_isolate_Hungarian_chromosome_30_whole_genome_shotgun_sequence
_R_QQTR01126204.1_41910616-41910844_Axis_porcinus_isolate_Zoohd001_scaffold_7_whole_genome_shotgun_sequence
_R_JRFZ01062813.1_233551-233779_Elaphurus_davidianus_breed_Woburn_Abbey_isolate_Dafeng_scaffold896_whole_genome_shotgun_sequence
CCMK010096005.1_9743-9972_Capreolus_capreolus_genome_assembly_kmer631_contig_DEER_K63_SCAFFOLD96005_whole_genome_shotgun_sequence
RFLZ01380745.1_3774-3995_Odocoileus_hemionus_sitkensis_isolate_Cn_RI01_contig632485_0_whole_genome_shotgun_sequence
_R_RCHL01001694.1_761322-761543_Odocoileus_hemionus_hemionus_isolate_MD92_scaffold1694_whole_genome_shotgun_sequence
RAWU01000015.1_103390794-103391022_Giraffa_tippelskirchi_isolate_OR1865_chromosome_12_whole_genome_shotgun_sequence
LVKQ01200897.1_23994-24222_Giraffa_camelopardalis_tippelskirchi_isolate_MA1_scaffold10529_6_whole_genome_shotgun_sequence
SJXW01018243.1_364409-364717_Tragulus_kanchil_isolate_LMD-DV_tarseq_934_whole_genome_shotgun_sequence
_R_SJXW01028478.1_147741-147846_Tragulus_kanchil_isolate_LMD-DV_tarseq_775_whole_genome_shotgun_sequence
PVHZ020003233.1_20935471-20935779_Tragulus_javanicus_isolate_US108_ScTR3PI_3213HRSCAF_53759_0_whole_genome_shotgun_sequence
_R_marine_Turadu_NCQN01000188.1_0.0_1_486_579194_578709
_R_marine_Turtru_NW_017843948.1_0.0_1_486_521804_521319
_R_query_marine_Souchi_RWJT01020743.1_0.0_1_486_4496081_4496566
_R_marine_Glomel_ML658895.1_0.0_1_486_7905661_7906146
_R_marine_Lagobl_NW_020838020.1_0.0_1_486_578514_578029
_R_marine_Orcorc_NW_004438573.1_0.0_1_486_4761097_4761576
_R_marine_Phopho_PKGA01001431.1_0.0_1_486_8351199_8350732
_R_marine_Neoasi_NW_020171990.1_0.0_1_486_604127_603660
_R_marine_Phosin_CM018173.1_0.0_1_486_36227318_36226851
_R_marine_Ponbla_RJWI010209416.1_0.0_1_486_1125_1592
_R_marine_Lipvex_NW_006783733.1_0.0_1_486_1034566_1035033
_R_marine_Inigeo_RJWO010058181.1_0.0_1_486_4913_5380
_R_marine_Mesbid_PVJJ010056730.1_1e-45_379_486_9260_9153
_R_marine_Zipcav_RJWS010147641.1_0.0_1_486_1495_1962
_R_marine_Balbon_DF658475.1_0.0_1_486_2745_2278
_R_marine_Balacu_NW_006728459.1_0.0_1_486_4249607_4250074
_R_marine_Megnov_RYZJ01001215.1_0.0_1_486_6185433_6185900
_R_marine_Balmus_VNFD01000286.1_0.0_1_486_212092_211626
_R_marine_Escrob_NIPP01020802.1_0.0_1_486_20356_19890
_R_marine_Eubjap_RJWP010021008.1_0.0_1_486_11555_11087
_R_marine_Plamin_RJWK010025765.1_0.0_1_486_24366_24833
_R_marine_Phycat_NC_041226.1_0.0_1_483_45047019_45047483
PVJP02910273.1_119504-119963_Hippopotamus_amphibius_isolate_BS01_ScHSfme_924753HRSCAF_982553_2_whole_genome_shotgun_sequence
PVJP02910697.1_2980589-2981001_Hippopotamus_amphibius_isolate_BS01_ScHSfme_925151HRSCAF_982951_whole_genome_shotgun_sequence
0.03

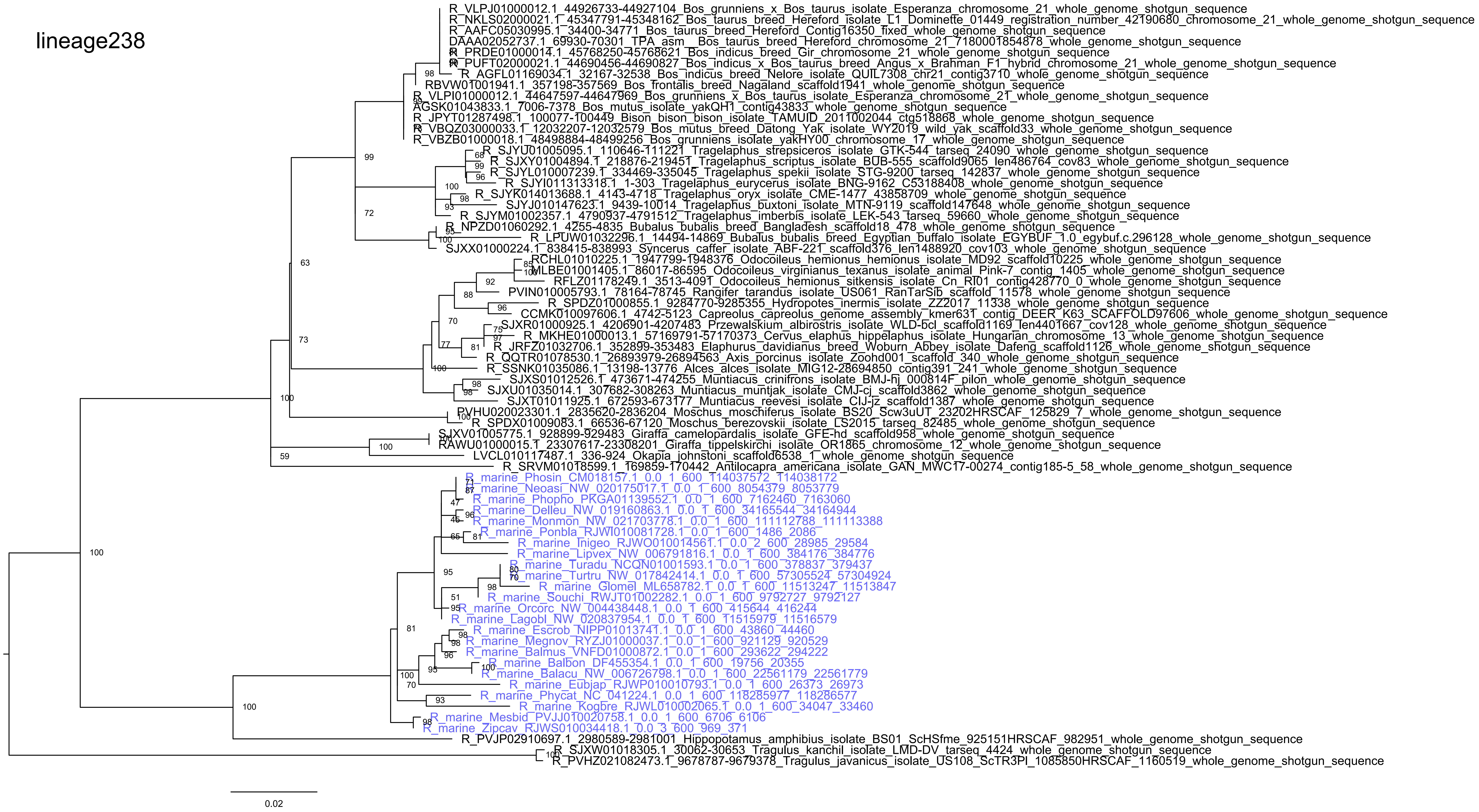
lineage238
R_VLPJ01000012.1_44926733-44927104_Bos_grunniens_x_Bos_taurus_isolate_Esperanza_chromosome_21_whole_genome_shotgun_sequence
R_NKLS02000021.1_45347791-45348162_Bos_taurus_breed_Hereford_isolate_L1_Dominette_01449_registration_number_42190680_chromosome_21_whole_genome_shotgun_sequence
R_AAFC05030995.1_34400-34771_Bos_taurus_breed_Hereford_Contig16350_fixed_whole_genome_shotgun_sequence
DAAA02052737.1_69930-70301_TPA_asm__Bos_taurus_breed_Hereford_chromosome_21_7180001854878_whole_genome_shotgun_sequence
R_PRDE01000014.1_45768250-45768621_Bos_indicus_breed_Gir_chromosome_21_whole_genome_shotgun_sequence
R_PUFT02000021.1_44690456-44690827_Bos_indicus_x_Bos_taurus_breed_Angus_x_Brahman_F1_hybrid_chromosome_21_whole_genome_shotgun_sequence
R_AGFL01169034.1_32167-32538_Bos_indicus_breed_Nelore_isolate_QUIL7308_chr21_contig3710_whole_genome_shotgun_sequence
RBVW01001941.1_357198-357569_Bos_frontalis_breed_Nagaland_scaffold1941_whole_genome_shotgun_sequence
R_VLPI01000012.1_44647597-44647969_Bos_grunniens_x_Bos_taurus_isolate_Esperanza_chromosome_21_whole_genome_shotgun_sequence
AGSK01043833.1_7006-7378_Bos_mutus_isolate_yakQH1_contig43833_whole_genome_shotgun_sequence
R_JPYT01287498.1_100077-100449_Bison_bison_bison_isolate_TAMUID_2011002044_ctg518868_whole_genome_shotgun_sequence
R_VBQZ03000033.1_12032207-12032579_Bos_mutus_breed_Datong_Yak_isolate_WY2019_wild_yak_scaffold33_whole_genome_shotgun_sequence
R_VBZB01000018.1_48498884-48499256_Bos_grunniens_isolate_yakHY00_chromosome_17_whole_genome_shotgun_sequence
R_SJYU01005095.1_110646-111221_Tragelaphus_strepsiceros_isolate_GTK-544_tarseq_24090_whole_genome_shotgun_sequence
R_SJXY01004894.1_218876-219451_Tragelaphus_scriptus_isolate_BUB-555_scaffold9065_len486764_cov83_whole_genome_shotgun_sequence
R_SJYL010007239.1_334469-335045_Tragelaphus_spekii_isolate_STG-9200_tarseq_142837_whole_genome_shotgun_sequence
R_SJYI011313318.1_1-303_Tragelaphus_eurycerus_isolate_BNG-9162_C53188408_whole_genome_shotgun_sequence
R_SJYK014013688.1_4143-4718_Tragelaphus_oryx_isolate_CME-1477_43858709_whole_genome_shotgun_sequence
SJYJ010147623.1_9439-10014_Tragelaphus_buxtoni_isolate_MTN-9119_scaffold147648_whole_genome_shotgun_sequence
R_SJYM01002357.1_4790937-4791512_Tragelaphus_imberbis_isolate_LEK-543_tarseq_59660_whole_genome_shotgun_sequence
R_NPZD01060292.1_4255-4835_Bubalus_bubalis_breed_Bangladesh_scaffold18_478_whole_genome_shotgun_sequence
R_LPUW01032296.1_14494-14869_Bubalus_bubalis_breed_Egyptian_buffalo_isolate_EGYBUF_1.0_egybuf.c.296128_whole_genome_shotgun_sequence
SJXX01000224.1_838415-838993_Syncerus_caffer_isolate_ABF-221_scaffold376_len1488920_cov103_whole_genome_shotgun_sequence
RCHL01010225.1_1947799-1948376_Odocoileus_hemionus_hemionus_isolate_MD92_scaffold10225_whole_genome_shotgun_sequence
MLBE01001405.1_86017-86595_Odocoileus_virginianus_texanus_isolate_animal_Pink-7_contig_1405_whole_genome_shotgun_sequence
RFLZ01178249.1_3513-4091_Odocoileus_hemionus_sitkensis_isolate_Cn_RI01_contig428770_0_whole_genome_shotgun_sequence
PVIN010005793.1_78164-78745_Rangifer_tarandus_isolate_US061_RanTarSib_scaffold_11578_whole_genome_shotgun_sequence
R_SPDZ01000855.1_9284770-9285355_Hydropotes_inermis_isolate_ZZ2017_11338_whole_genome_shotgun_sequence
CCMK010097606.1_4742-5123_Capreolus_capreolus_genome_assembly_kmer631_contig_DEER_K63_SCAFFOLD97606_whole_genome_shotgun_sequence
SJXR01000925.1_4206901-4207483_Przewalskium_albirostris_isolate_WLD-bcl_scaffold1169_len4401667_cov128_whole_genome_shotgun_sequence
R_MKHE01000013.1_57169791-57170373_Cervus_elaphus_hippelaphus_isolate_Hungarian_chromosome_13_whole_genome_shotgun_sequence
R_JRFZ01032706.1_352899-353483_Elaphurus_davidianus_breed_Woburn_Abbey_isolate_Dafeng_scaffold1126_whole_genome_shotgun_sequence
R_QQTR01078530.1_26893979-26894563_Axis_porcinus_isolate_Zoohd001_scaffold_340_whole_genome_shotgun_sequence
R_SSNK01035086.1_13198-13776_Alces_alces_isolate_MIG12-28694850_contig391_241_whole_genome_shotgun_sequence
SJXS01012526.1_473671-474255_Muntiacus_crinifrons_isolate_BMJ-hj_000814F_pilon_whole_genome_shotgun_sequence
SJXU01035014.1_307682-308263_Muntiacus_muntjak_isolate_CMJ-cj_scaffold3862_whole_genome_shotgun_sequence
SJXT01011925.1_672593-673177_Muntiacus_reevesi_isolate_CIJ-jz_scaffold1387_whole_genome_shotgun_sequence
PVHU020023301.1_2835620-2836204_Moschus_moschiferus_isolate_BS20_Scw3uUT_23202HRSCAF_125829_7_whole_genome_shotgun_sequence
R_SPDX01009083.1_66536-67120_Moschus_berezovskii_isolate_LS2015_tarseq_82485_whole_genome_shotgun_sequence
SJXV01005775.1_928899-929483_Giraffa_camelopardalis_isolate_GFE-hd_scaffold958_whole_genome_shotgun_sequence
RAWU01000015.1_23307617-23308201_Giraffa_tippelskirchi_isolate_OR1865_chromosome_12_whole_genome_shotgun_sequence
LVCL010117487.1_336-924_Okapia_johnstoni_scaffold6538_1_whole_genome_shotgun_sequence
R_SRVM01018599.1_169859-170442_Antilocapra_americana_isolate_GAN_MWC17-00274_contig185-5_58_whole_genome_shotgun_sequence
R_marine_Phosin_CM018157.1_0.0_1_600_114037572_114038172
R_marine_Neoasi_NW_020175017.1_0.0_1_600_8054379_8053779
R_marine_Phopho_PKGA01139552.1_0.0_1_600_7162460_7163060
R_marine_Delleu_NW_019160863.1_0.0_1_600_34165544_34164944
R_marine_Monmon_NW_021703778.1_0.0_1_600_111112788_111113388
R_marine_Ponbla_RJWI010081728.1_0.0_1_600_1486_2086
R_marine_Inigeo_RJWO010014561.1_0.0_2_600_28985_29584
R_marine_Lipvex_NW_006791816.1_0.0_1_600_384176_384776
R_marine_Turadu_NCQN01001593.1_0.0_1_600_378837_379437
R_marine_Turtru_NW_017842414.1_0.0_1_600_57305524_57304924
R_marine_Glomel_ML658782.1_0.0_1_600_11513247_11513847
R_marine_Souchi_RWJT01002282.1_0.0_1_600_9792727_9792127
R_marine_Orcorc_NW_004438448.1_0.0_1_600_415644_416244
R_marine_Lagobl_NW_020837954.1_0.0_1_600_11515979_11516579
R_marine_Escrob_NIPP01013741.1_0.0_1_600_43860_44460
R_marine_Megnov_RYZJ01000037.1_0.0_1_600_921129_920529
R_marine_Balmus_VNFD01000872.1_0.0_1_600_293622_294222
R_marine_Balbon_DF455354.1_0.0_1_600_19756_20355
R_marine_Balacu_NW_006726798.1_0.0_1_600_22561179_22561779
R_marine_Eubjap_RJWP010010793.1_0.0_1_600_26373_26973
R_marine_Phycat_NC_041224.1_0.0_1_600_118285977_118286577
R_marine_Kogbre_RJWL010002065.1_0.0_1_600_34047_33460
R_marine_Mesbid_PVJJ010020758.1_0.0_1_600_6706_6106
R_marine_Zipcav_RJWS010034418.1_0.0_3_600_969_371
R_PVJP02910697.1_2980589-2981001_Hippopotamus_amphibius_isolate_BS01_ScHSfme_925151HRSCAF_982951_whole_genome_shotgun_sequence
R_SJXW01018305.1_30062-30653_Tragulus_kanchil_isolate_LMD-DV_tarseq_4424_whole_genome_shotgun_sequence
R_PVHZ021082473.1_9678787-9679378_Tragulus_javanicus_isolate_US108_ScTR3PI_1085850HRSCAF_1160519_whole_genome_shotgun_sequence
0.02

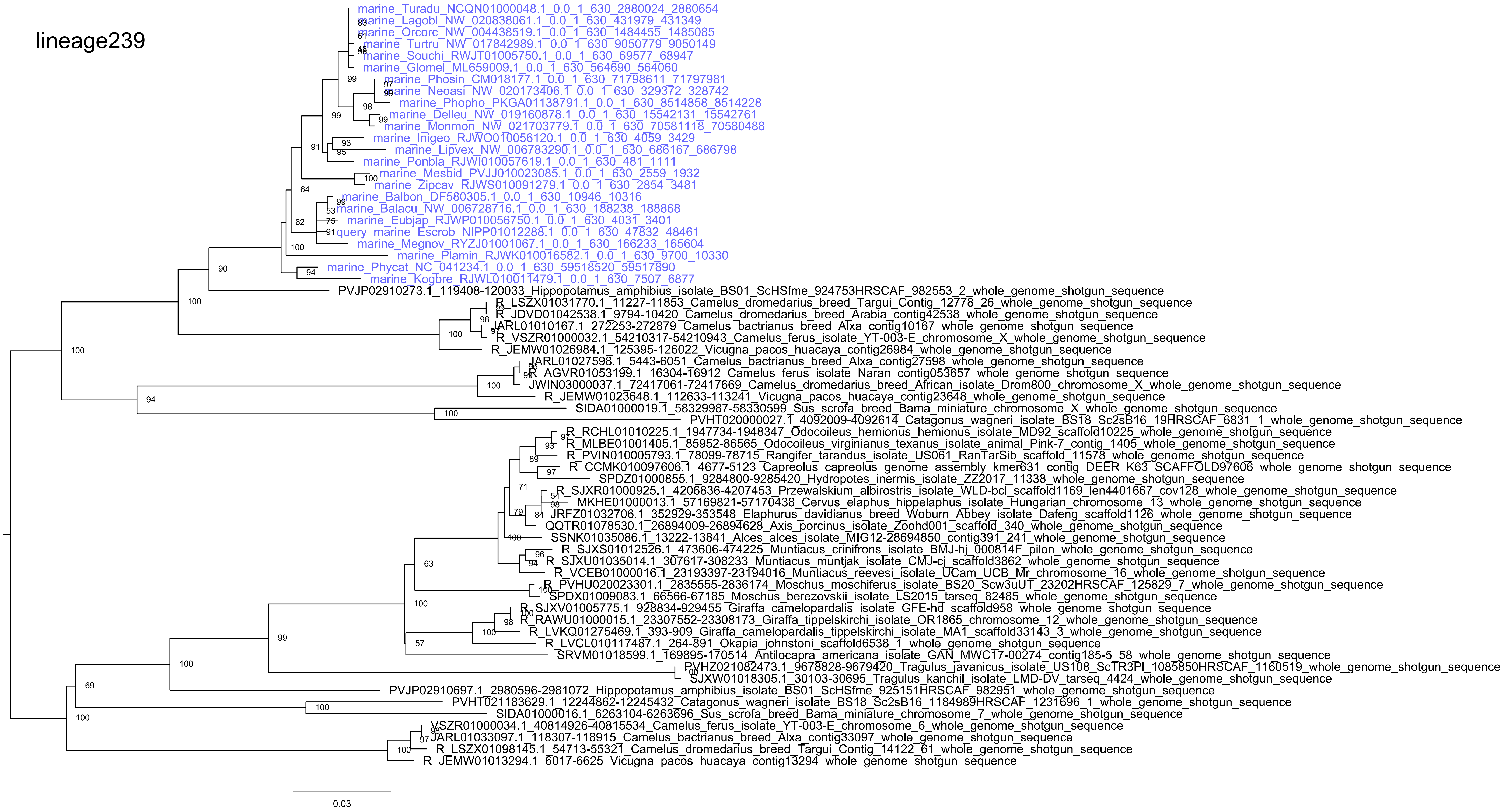

lineage239
marine_Turadu_NCQN01000048.1_0.0_1_630_2880024_2880654
marine_Lagobl_NW_020838061.1_0.0_1_630_431979_431349
marine_Orcorc_NW_004438519.1_0.0_1_630_1484455_1485085
marine_Turtru_NW_017842989.1_0.0_1_630_9050779_9050149
marine_Souchi_RWJT01005750.1_0.0_1_630_69577_68947
marine_Glomel_ML659009.1_0.0_1_630_564690_564060
marine_Phosin_CM018177.1_0.0_1_630_71798611_71797981
marine_Neoasi_NW_020173406.1_0.0_1_630_329372_328742
marine_Phopho_PKGA01138791.1_0.0_1_630_8514858_8514228
marine_Delleu_NW_019160878.1_0.0_1_630_15542131_15542761
marine_Monmon_NW_021703779.1_0.0_1_630_70581118_70580488
marine_Inigeo_RJWO010056120.1_0.0_1_630_4059_3429
marine_Lipvex_NW_006783290.1_0.0_1_630_686167_686798
marine_Ponbla_RJWI010057619.1_0.0_1_630_481_1111
marine_Mesbid_PVJJ010023085.1_0.0_1_630_2559_1932
marine_Zipcav_RJWS010091279.1_0.0_1_630_2854_3481
marine_Balbon_DF580305.1_0.0_1_630_10946_10316
marine_Balacu_NW_006728716.1_0.0_1_630_188238_188868
marine_Eubjap_RJWP010056750.1_0.0_1_630_4031_3401
query_marine_Escrob_NIPP01012288.1_0.0_1_630_47832_48461
marine_Megnov_RYZJ01001067.1_0.0_1_630_166233_165604
marine_Plamin_RJWK010016582.1_0.0_1_630_9700_10330
marine_Phycat_NC_041234.1_0.0_1_630_59518520_59517890
marine_Kogbre_RJWL010011479.1_0.0_1_630_7507_6877
PVJP02910273.1_119408-120033_Hippopotamus_amphibius_isolate_BS01_ScHSfme_924753HRSCAF_982553_2_whole_genome_shotgun_sequence
R_LSZX01031770.1_11227-11853_Camelus_dromedarius_breed_Targui_Contig_12778_26_whole_genome_shotgun_sequence
R_JDVD01042538.1_9794-10420_Camelus_dromedarius_breed_Arabia_contig42538_whole_genome_shotgun_sequence
JARL01010167.1_272253-272879_Camelus_bactrianus_breed_Alxa_contig10167_whole_genome_shotgun_sequence
R_VSZR01000032.1_54210317-54210943_Camelus_ferus_isolate_YT-003-E_chromosome_X_whole_genome_shotgun_sequence
R_JEMW01026984.1_125395-126022_Vicugna_pacos_huacaya_contig26984_whole_genome_shotgun_sequence
JARL01027598.1_5443-6051_Camelus_bactrianus_breed_Alxa_contig27598_whole_genome_shotgun_sequence
R_AGVR01053199.1_16304-16912_Camelus_ferus_breed_Naran_contig053657_whole_genome_shotgun_sequence
JWIN03000037.1_72417061-72417669_Camelus_dromedarius_breed_African_isolate_Drom800_chromosome_X_whole_genome_shotgun_sequence
R_JEMW01023648.1_112633-113241_Vicugna_pacos_huacaya_contig23648_whole_genome_shotgun_sequence
SIDA01000019.1_58329987-58330599_Sus_scrofa_breed_Bama_miniature_chromosome_X_whole_genome_shotgun_sequence
PVHT020000027.1_4092009-4092614_Catagonus_wagneri_isolate_BS18_Sc2sB16_19HRSCAF_6831_1_whole_genome_shotgun_sequence
R_RCHL01010225.1_1947734-1948347_Odocoileus_hemionus_hemionus_isolate_MD92_scaffold10225_whole_genome_shotgun_sequence
R_MLBE01001405.1_85952-86565_Odocoileus_virginianus_texanus_isolate_animal_Pink-7_contig_1405_whole_genome_shotgun_sequence
R_PVIN010005793.1_78099-78715_Rangifer_tarandus_isolate_US061_RanTarSib_scaffold_11578_whole_genome_shotgun_sequence
R_CCMK010097606.1_4677-5123_Capreolus_capreolus_genome_assembly_kmer631_contig_DEER_K63_SCAFFOLD97606_whole_genome_shotgun_sequence
SPDZ01000855.1_9284800-9285420_Hydropotes_inermis_isolate_ZZ2017_11338_whole_genome_shotgun_sequence
R_SJXR01000925.1_4206836-4207453_Przewalskium_albirostris_isolate_WLD-bcl_scaffold1169_len4401667_cov128_whole_genome_shotgun_sequence
MKHE01000013.1_57169821-57170438_Cervus_elaphus_hippelaphus_isolate_Hungarian_chromosome_13_whole_genome_shotgun_sequence
JRFZ01032706.1_352929-353548_Elaphurus_davidianus_breed_Woburn_Abbey_isolate_Dafeng_scaffold1126_whole_genome_shotgun_sequence
QQTR01078530.1_26894009-26894628_Axis_porcinus_isolate_Zoohd001_scaffold_340_whole_genome_shotgun_sequence
SSNK01035086.1_13222-13841_Alces_alces_isolate_MIG12-28694850_contig391_241_whole_genome_shotgun_sequence
R_SJXS01012526.1_473606-474225_Muntiacus_crinifrons_isolate_BMJ-hj_000814F_pilon_whole_genome_shotgun_sequence
R_SJXU01035014.1_307617-308233_Muntiacus_muntjak_isolate_CMJ-cj_scaffold3862_whole_genome_shotgun_sequence
R_VCEB01000016.1_23193397-23194016_Muntiacus_reevesi_isolate_UCam_UCB_Mr_chromosome_16_whole_genome_shotgun_sequence
R_PVHU020023301.1_2835555-2836174_Moschus_moschiferus_isolate_BS20_Scw3uUT_23202HRSCAF_125829_7_whole_genome_shotgun_sequence
SPDX01009083.1_66566-67185_Moschus_berezovskii_isolate_LS2015_tarseq_82485_whole_genome_shotgun_sequence
R_SJXV01005775.1_928834-929455_Giraffa_camelopardalis_isolate_GFE-hd_scaffold958_whole_genome_shotgun_sequence
R_RAWU01000015.1_23307552-23308173_Giraffa_tippelskirchi_isolate_OR1865_chromosome_12_whole_genome_shotgun_sequence
R_LVKQ01275469.1_393-909_Giraffa_camelopardalis_tippelskirchi_isolate_MA1_scaffold33143_3_whole_genome_shotgun_sequence
R_LVCL010117487.1_264-891_Okapia_johnstoni_scaffold6538_1_whole_genome_shotgun_sequence
SRVM01018599.1_169895-170514_Antilocapra_americana_isolate_GAN_MWC17-00274_contig185-5_58_whole_genome_shotgun_sequence
PVHZ021082473.1_9678828-9679420_Tragulus_javanicus_isolate_US108_ScTR3PI_1085850HRSCAF_1160519_whole_genome_shotgun_sequence
SJXW01018305.1_30103-30695_Tragulus_kanchil_isolate_LMD-DV_tarseq_4424_whole_genome_shotgun_sequence
PVJP02910697.1_2980596-2981072_Hippopotamus_amphibius_isolate_BS01_ScHSfme_925151HRSCAF_982951_whole_genome_shotgun_sequence
PVHT021183629.1_12244862-12245432_Catagonus_wagneri_isolate_BS18_Sc2sB16_1184989HRSCAF_1231696_1_whole_genome_shotgun_sequence
SIDA01000016.1_6263104-6263696_Sus_scrofa_breed_Bama_miniature_chromosome_7_whole_genome_shotgun_sequence
VSZR01000034.1_40814926-40815534_Camelus_ferus_isolate_YT-003-E_chromosome_6_whole_genome_shotgun_sequence
JARL01033097.1_118307-118915_Camelus_bactrianus_breed_Alxa_contig33097_whole_genome_shotgun_sequence
R_LSZX01098145.1_54713-55321_Camelus_dromedarius_breed_Targui_Contig_14122_61_whole_genome_shotgun_sequence
R_JEMW01013294.1_6017-6625_Vicugna_pacos_huacaya_contig13294_whole_genome_shotgun_sequence
0.03

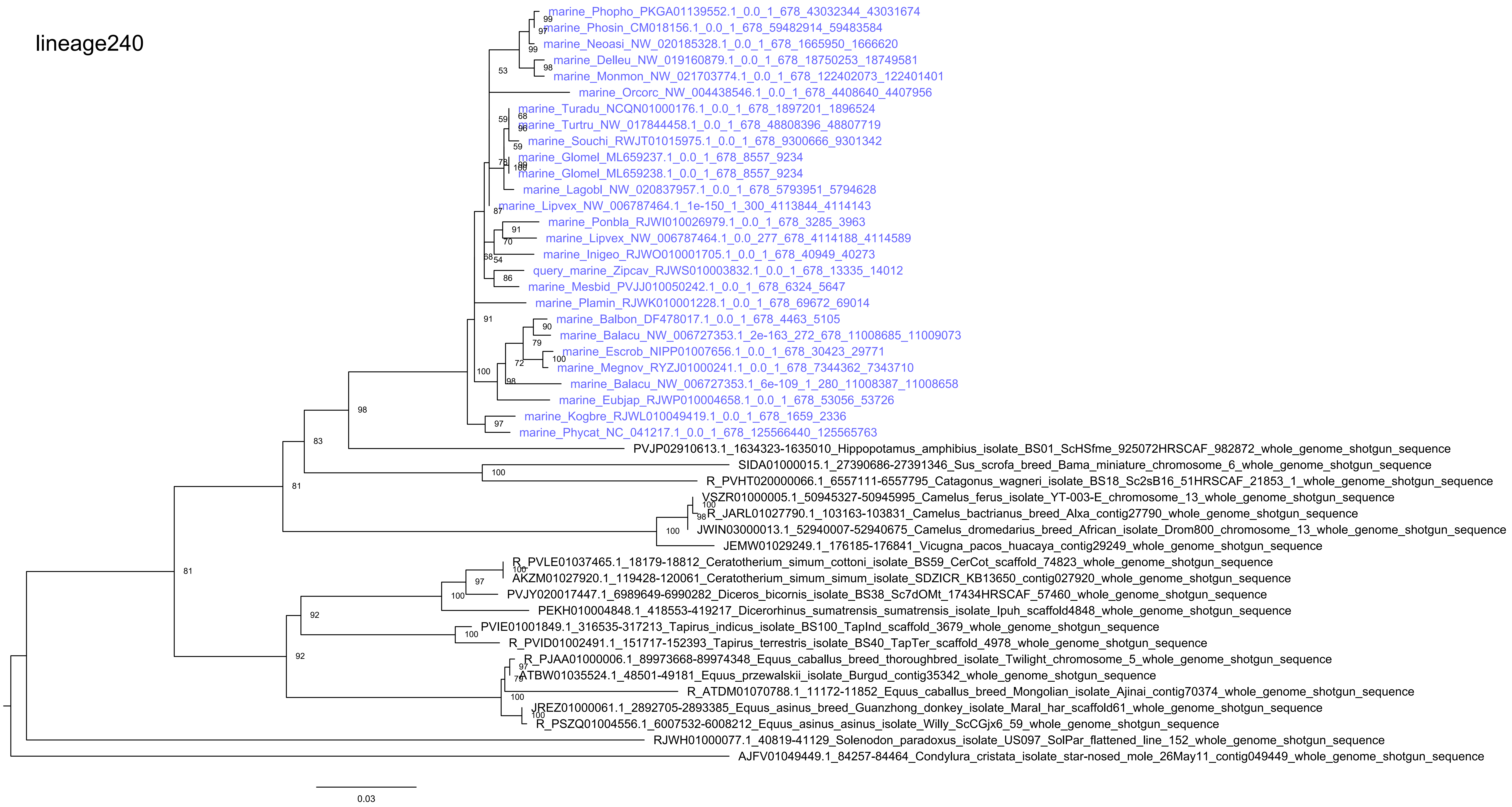

lineage240
marine_Phopho_PKGA01139552.1_0.0_1_678_43032344_43031674
marine_Phosin_CM018156.1_0.0_1_678_59482914_59483584
marine_Neoasi_NW_020185328.1_0.0_1_678_1665950_1666620
marine_Delleu_NW_019160879.1_0.0_1_678_18750253_18749581
marine_Monmon_NW_021703774.1_0.0_1_678_122402073_122401401
marine_Orcorc_NW_004438546.1_0.0_1_678_4408640_4407956
marine_Turadu_NCQN01000176.1_0.0_1_678_1897201_1896524
marine_Turtru_NW_017844458.1_0.0_1_678_48808396_48807719
marine_Souchi_RWJT01015975.1_0.0_1_678_9300666_9301342
marine_Glomel_ML659237.1_0.0_1_678_8557_9234
marine_Glomel_ML659238.1_0.0_1_678_8557_9234
marine_Lagobl_NW_020837957.1_0.0_1_678_5793951_5794628
marine_Lipvex_NW_006787464.1_1e-150_1_300_4113844_4114143
marine_Ponbla_RJWI010026979.1_0.0_1_678_3285_3963
marine_Lipvex_NW_006787464.1_0.0_277_678_4114188_4114589
marine_Inigeo_RJWO010001705.1_0.0_1_678_40949_40273
query_marine_Zipcav_RJWS010003832.1_0.0_1_678_13335_14012
marine_Mesbid_PVJJ010050242.1_0.0_1_678_6324_5647
marine_Plamin_RJWK010001228.1_0.0_1_678_69672_69014
marine_Balbon_DF478017.1_0.0_1_678_4463_5105
marine_Balacu_NW_006727353.1_2e-163_272_678_11008685_11009073
marine_Escrob_NIPP01007656.1_0.0_1_678_30423_29771
marine_Megnov_RYZJ01000241.1_0.0_1_678_7344362_7343710
marine_Balacu_NW_006727353.1_6e-109_1_280_11008387_11008658
marine_Eubjap_RJWP010004658.1_0.0_1_678_53056_53726
marine_Kogbre_RJWL010049419.1_0.0_1_678_1659_2336
marine_Phycat_NC_041217.1_0.0_1_678_125566440_125565763
PVJP02910613.1_1634323-1635010_Hippopotamus_amphibius_isolate_BS01_ScHSfme_925072HRSCAF_982872_whole_genome_shotgun_sequence
SIDA01000015.1_27390686-27391346_Sus_scrofa_breed_Bama_miniature_chromosome_6_whole_genome_shotgun_sequence
R_PVHT020000066.1_6557111-6557795_Catagonus_wagneri_isolate_BS18_Sc2sB16_51HRSCAF_21853_1_whole_genome_shotgun_sequence
VSZR01000005.1_50945327-50945995_Camelus_ferus_isolate_YT-003-E_chromosome_13_whole_genome_shotgun_sequence
R_JARL01027790.1_103163-103831_Camelus_bactrianus_breed_Alxa_contig27790_whole_genome_shotgun_sequence
JWIN03000013.1_52940007-52940675_Camelus_dromedarius_breed_African_isolate_Drom800_chromosome_13_whole_genome_shotgun_sequence
JEMW01029249.1_176185-176841_Vicugna_pacos_huacaya_contig29249_whole_genome_shotgun_sequence
R_PVLE01037465.1_18179-18812_Ceratotherium_simum_cottoni_isolate_BS59_CerCot_scaffold_74823_whole_genome_shotgun_sequence
AKZM01027920.1_119428-120061_Ceratotherium_simum_simum_isolate_SDZICR_KB13650_contig027920_whole_genome_shotgun_sequence
PVJY020017447.1_6989649-6990282_Diceros_bicornis_isolate_BS38_Sc7dOMt_17434HRSCAF_57460_whole_genome_shotgun_sequence
PEKH010004848.1_418553-419217_Dicerorhinus_sumatrensis_sumatrensis_isolate_Ipuh_scaffold4848_whole_genome_shotgun_sequence
PVIE01001849.1_316535-317213_Tapirus_indicus_isolate_BS100_TapInd_scaffold_3679_whole_genome_shotgun_sequence
R_PVID01002491.1_151717-152393_Tapirus_terrestris_isolate_BS40_TapTer_scaffold_4978_whole_genome_shotgun_sequence
R_PJAA01000006.1_89973668-89974348_Equus_caballus_breed_thoroughbred_isolate_Twilight_chromosome_5_whole_genome_shotgun_sequence
ATBW01035524.1_48501-49181_Equus_przewalskii_isolate_Burgud_contig35342_whole_genome_shotgun_sequence
R_ATDM01070788.1_11172-11852_Equus_caballus_breed_Mongolian_isolate_Ajinai_contig70374_whole_genome_shotgun_sequence
JREZ01000061.1_2892705-2893385_Equus_asinus_breed_Guanzhong_donkey_isolate_Maral_har_scaffold61_whole_genome_shotgun_sequence
R_PSZQ01004556.1_6007532-6008212_Equus_asinus_asinus_isolate_Willy_ScCGjx6_59_whole_genome_shotgun_sequence
RJWH01000077.1_40819-41129_Solenodon_paradoxus_isolate_US097_SolPar_flattened_line_152_whole_genome_shotgun_sequence
AJFV01049449.1_84257-84464_Condylura_cristata_isolate_star-nosed_mole_26May11_contig049449_whole_genome_shotgun_sequence
0.03

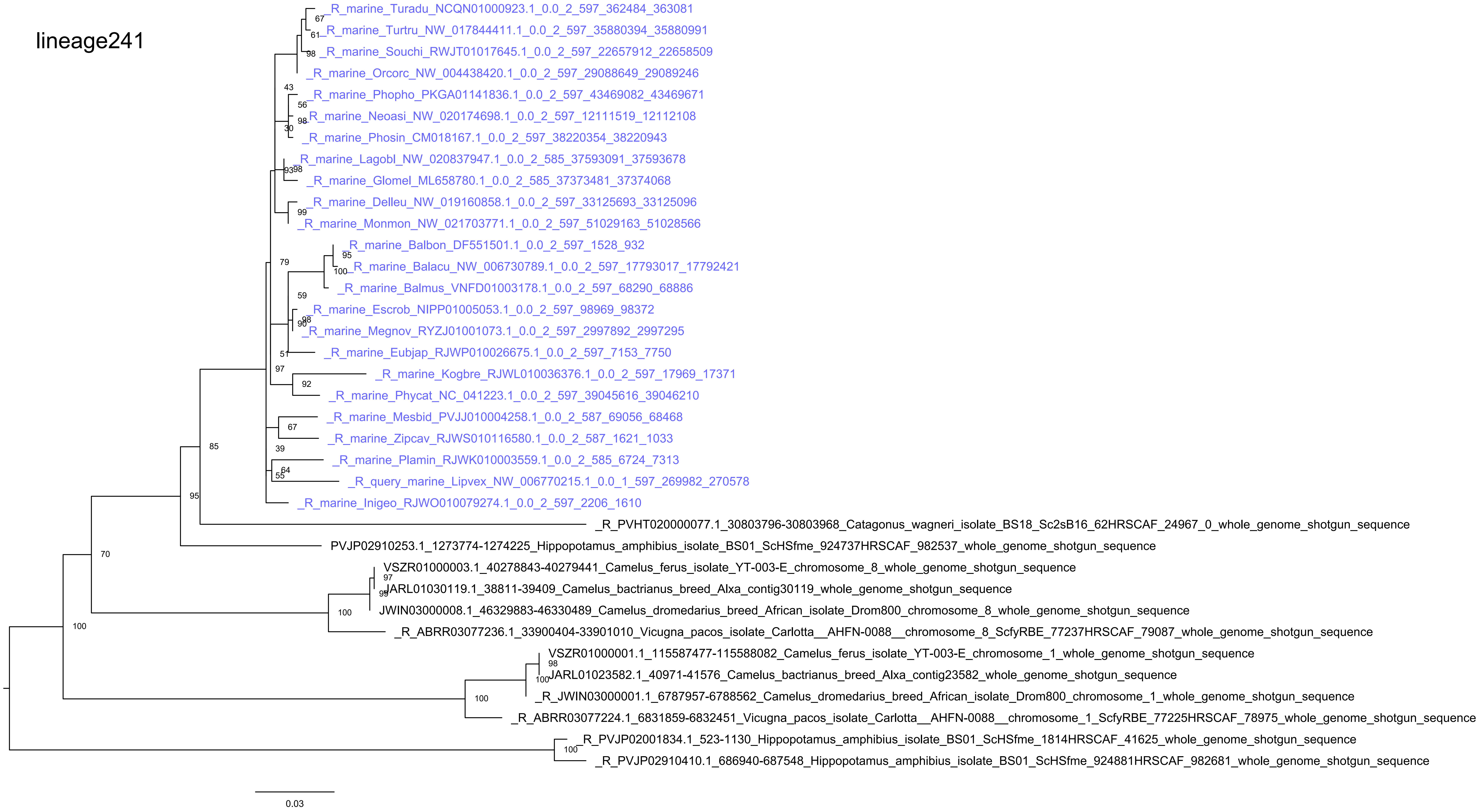

lineage241
_R_marine_Turadu_NCQN01000923.1_0.0_2_597_362484_363081
_R_marine_Turtru_NW_017844411.1_0.0_2_597_35880394_35880991
_R_marine_Souchi_RWJT01017645.1_0.0_2_597_22657912_22658509
_R_marine_Orcorc_NW_004438420.1_0.0_2_597_29088649_29089246
_R_marine_Phopho_PKGA01141836.1_0.0_2_597_43469082_43469671
_R_marine_Neoasi_NW_020174698.1_0.0_2_597_12111519_12112108
_R_marine_Phosin_CM018167.1_0.0_2_597_38220354_38220943
_R_marine_Lagobl_NW_020837947.1_0.0_2_585_37593091_37593678
_R_marine_Glomel_ML658780.1_0.0_2_585_37373481_37374068
_R_marine_Delleu_NW_019160858.1_0.0_2_597_33125693_33125096
_R_marine_Monmon_NW_021703771.1_0.0_2_597_51029163_51028566
_R_marine_Balbon_DF551501.1_0.0_2_597_1528_932
_R_marine_Balacu_NW_006730789.1_0.0_2_597_17793017_17792421
_R_marine_Balmus_VNFD01003178.1_0.0_2_597_68290_68886
_R_marine_Escrob_NIPP01005053.1_0.0_2_597_98969_98372
_R_marine_Megnov_RYZJ01001073.1_0.0_2_597_2997892_2997295
_R_marine_Eubjap_RJWP010026675.1_0.0_2_597_7153_7750
_R_marine_Kogbre_RJWL010036376.1_0.0_2_597_17969_17371
_R_marine_Phycat_NC_041223.1_0.0_2_597_39045616_39046210
_R_marine_Mesbid_PVJJ010004258.1_0.0_2_587_69056_68468
_R_marine_Zipcav_RJWS010116580.1_0.0_2_587_1621_1033
_R_marine_Plamin_RJWK010003559.1_0.0_2_585_6724_7313
_R_query_marine_Lipvex_NW_006770215.1_0.0_1_597_269982_270578
_R_marine_Inigeo_RJWO010079274.1_0.0_2_597_2206_1610
_R_PVHT020000077.1_30803796-30803968_Catagonus_wagneri_isolate_BS18_Sc2sB16_62HRSCAF_24967_0_whole_genome_shotgun_sequence
PVJP02910253.1_1273774-1274225_Hippopotamus_amphibius_isolate_BS01_ScHSfme_924737HRSCAF_982537_whole_genome_shotgun_sequence
VSZR01000003.1_40278843-40279441_Camelus_ferus_isolate_YT-003-E_chromosome_8_whole_genome_shotgun_sequence
JARL01030119.1_38811-39409_Camelus_bactrianus_breed_Alxa_contig30119_whole_genome_shotgun_sequence
JWIN03000008.1_46329883-46330489_Camelus_dromedarius_breed_African_isolate_Drom800_chromosome_8_whole_genome_shotgun_sequence
_R_ABRR03077236.1_33900404-33901010_Vicugna_pacos_isolate_Carlotta__AHFN-0088__chromosome_8_ScfyRBE_77237HRSCAF_79087_whole_genome_shotgun_sequence
VSZR01000001.1_115587477-115588082_Camelus_ferus_isolate_YT-003-E_chromosome_1_whole_genome_shotgun_sequence
JARL01023582.1_40971-41576_Camelus_bactrianus_breed_Alxa_contig23582_whole_genome_shotgun_sequence
_R_JWIN03000001.1_6787957-6788562_Camelus_dromedarius_breed_African_isolate_Drom800_chromosome_1_whole_genome_shotgun_sequence
_R_ABRR03077224.1_6831859-6832451_Vicugna_pacos_isolate_Carlotta__AHFN-0088__chromosome_1_ScfyRBE_77225HRSCAF_78975_whole_genome_shotgun_sequence
_R_PVJP02001834.1_523-1130_Hippopotamus_amphibius_isolate_BS01_ScHSfme_1814HRSCAF_41625_whole_genome_shotgun_sequence
_R_PVJP02910410.1_686940-687548_Hippopotamus_amphibius_isolate_BS01_ScHSfme_924881HRSCAF_982681_whole_genome_shotgun_sequence
0.03

lineage242

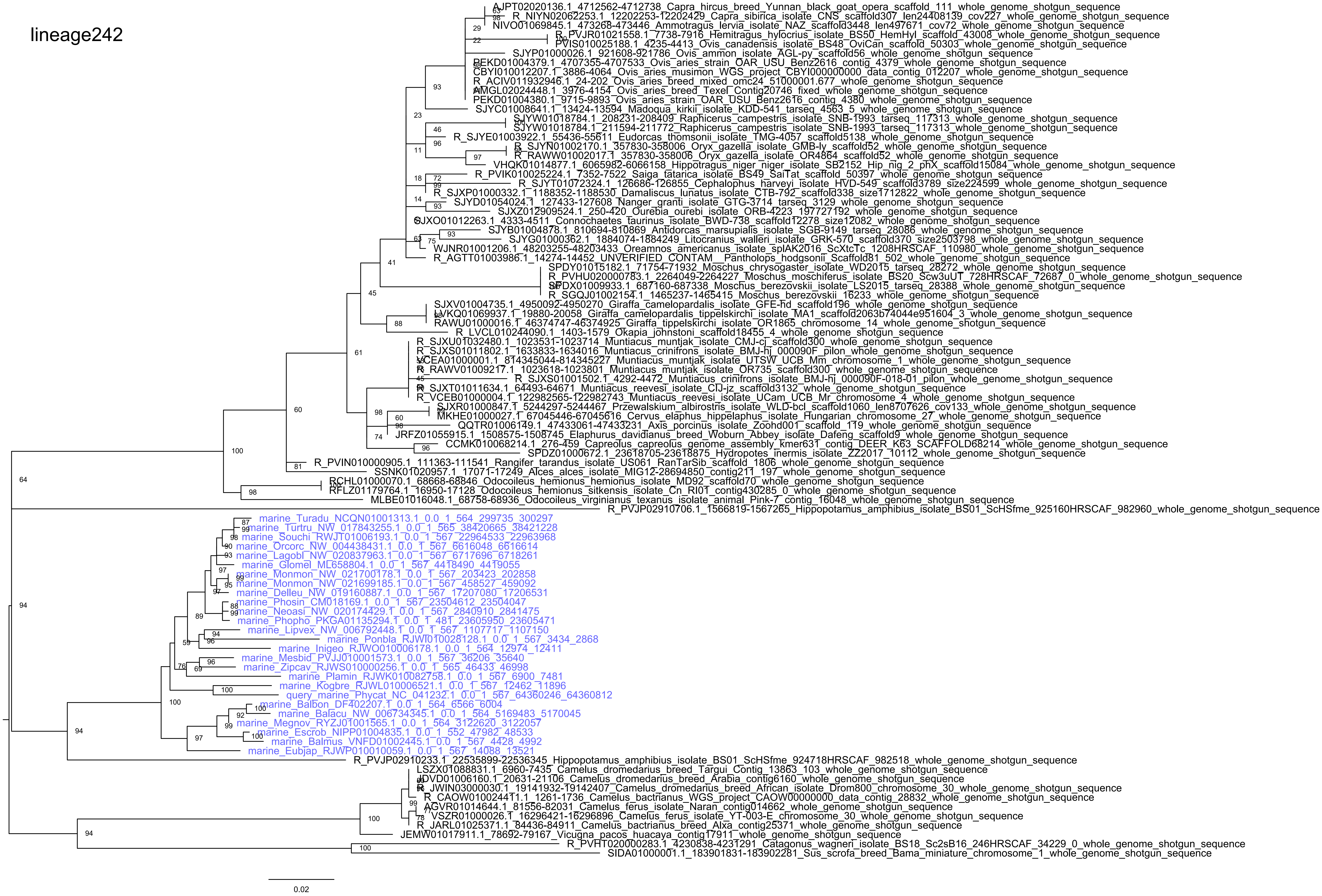

AJPT02020136.1_4712562-4712738_Capra_hircus_breed_Yunnan_black_goat_opera_scaffold_111_whole_genome_shotgun_sequence
R_NIYN02062253.1_12202253-12202429_Capra_sibirica_isolate_CNS_scaffold307_len24408139_cov227_whole_genome_shotgun_sequence
NIVO01069845.1_473268-473446_Ammotragus_lervia_isolate_NAZ_scaffold3448_len497671_cov72_whole_genome_shotgun_sequence
R_PVJR01021558.1_7738-7916_Hemitragus_hylocrius_isolate_BS50_HemHyl_scaffold_43008_whole_genome_shotgun_sequence
PVIS010025188.1_4235-4413_Ovis_canadensis_isolate_BS48_OviCan_scaffold_50303_whole_genome_shotgun_sequence
SJYP01000026.1_921608-921786_Ovis_ammon_isolate_AGL-py_scaffold56_whole_genome_shotgun_sequence
PEKD01004379.1_4707355-4707533_Ovis_aries_strain_OAR_USU_Benz2616_contig_4379_whole_genome_shotgun_sequence
CBYI010012207.1_3886-4064_Ovis_aries_musimon_WGS_project_CBYI000000000_data_contig_012207_whole_genome_shotgun_sequence
R_ACIV011932946.1_24-202_Ovis_aries_breed_mixed_omc24_51000001.677_whole_genome_shotgun_sequence
AMGL02024448.1_3976-4154_Ovis_aries_breed_Texel_Contig20746_fixed_whole_genome_shotgun_sequence
PEKD01004380.1_9715-9893_Ovis_aries_strain_OAR_USU_Benz2616_contig_4380_whole_genome_shotgun_sequence
SJYC01008641.1_13424-13594_Madoqua_kirkii_isolate_KDD-541_tarseq_4563_5_whole_genome_shotgun_sequence
SJYW01018784.1_208231-208409_Raphicerus_campestris_isolate_SNB-1993_tarseq_117313_whole_genome_shotgun_sequence
SJYW01018784.1_211594-211772_Raphicerus_campestris_isolate_SNB-1993_tarseq_117313_whole_genome_shotgun_sequence
R_SJYE01003922.1_55436-55611_Eudorcas_thomsonii_isolate_TMG-4057_scaffold5138_whole_genome_shotgun_sequence
R_SJYN01002170.1_357830-358006_Oryx_gazella_isolate_GMB-ly_scaffold52_whole_genome_shotgun_sequence
R_RAWW01002017.1_357830-358006_Oryx_gazella_isolate_OR4864_scaffold52_whole_genome_shotgun_sequence
VHQK01014877.1_6065982-6066158_Hippotragus_niger_niger_isolate_SB2152_Hip_nig_2_phX_scaffold15084_whole_genome_shotgun_sequence
R_PVIK010025224.1_7352-7522_Saiga_tatarica_isolate_BS49_SaiTat_scaffold_50397_whole_genome_shotgun_sequence
R_SJYT01072324.1_126686-126855_Cephalophus_harveyi_isolate_HVD-549_scaffold3789_size224599_whole_genome_shotgun_sequence
R_SJXP01000332.1_1188352-1188530_Damaliscus_lunatus_isolate_CTB-792_scaffold338_size1712822_whole_genome_shotgun_sequence
SJYD01054024.1_127433-127608_Nanger_granti_isolate_GTG-3714_tarseq_3129_whole_genome_shotgun_sequence
SJXZ012909524.1_250-420_Ourebia_ourebi_isolate_ORB-4223_197727192_whole_genome_shotgun_sequence
SJXO01012263.1_4333-4511_Connochaetes_taurinus_isolate_BWD-738_scaffold12278_size12082_whole_genome_shotgun_sequence
SJYB01004878.1_810694-810869_Antidorcas_marsupialis_isolate_SGB-9149_tarseq_28086_whole_genome_shotgun_sequence
SJYG01000362.1_1884074-1884249_Litocranius_walleri_isolate_GRK-570_scaffold370_size2503798_whole_genome_shotgun_sequence
WJNR01001206.1_48203255-48203433_Oreamnos_americanus_isolate_splAK2016_ScXtcTc_1208HRSCAF_110980_whole_genome_shotgun_sequence
R_AGTT01003986.1_14274-14452_UNVERIFIED_CONTAM__Pantholops_hodgsonii_Scaffold81_502_whole_genome_shotgun_sequence
SPDY01015182.1_71754-71932_Moschus_chrysogaster_isolate_WD2015_tarseq_28272_whole_genome_shotgun_sequence
R_PVHU020000783.1_2264049-2264227_Moschus_moschiferus_isolate_BS20_Scw3uUT_728HRSCAF_72687_0_whole_genome_shotgun_sequence
SPDX01009933.1_687160-687338_Moschus_berezovskii_isolate_LS2015_tarseq_28388_whole_genome_shotgun_sequence
R_SGQJ01002154.1_1465237-1465415_Moschus_berezovskii_16233_whole_genome_shotgun_sequence
SJXV01004735.1_4950092-4950270_Giraffa_camelopardalis_isolate_GFE-nd_scaffold196_whole_genome_shotgun_sequence
LVKQ01069937.1_19880-20058_Giraffa_camelopardalis_tippelskirchi_isolate_MA1_scaffold2063b74044e951604_3_whole_genome_shotgun_sequence
RAWU01000016.1_46374747-46374925_Giraffa_tippelskirchi_isolate_OR1865_chromosome_14_whole_genome_shotgun_sequence
R_LVCL010244090.1_1403-1579_Okapia_johnstoni_scaffold18455_4_whole_genome_shotgun_sequence
R_SJXU01032480.1_1023531-1023714_Muntiacus_muntjak_isolate_CMJ-cj_scaffold300_whole_genome_shotgun_sequence
R_SJXS01011802.1_1633833-1634016_Muntiacus_crinifrons_isolate_BMJ-hj_000090F_pilon_whole_genome_shotgun_sequence
VCEA01000001.1_814345044-814345227_Muntiacus_muntjak_isolate_UTSW_UCB_Mm_chromosome_1_whole_genome_shotgun_sequence
R_RAWV01009217.1_1023618-1023801_Muntiacus_muntjak_isolate_OR735_scaffold300_whole_genome_shotgun_sequence
R_SJXS01001502.1_4292-4472_Muntiacus_crinifrons_isolate_BMJ-hj_000090F-018-01_pilon_whole_genome_shotgun_sequence
R_SJXT01011634.1_64493-64671_Muntiacus_reevesi_isolate_CIJ-jz_scaffold3132_whole_genome_shotgun_sequence
R_VCEB01000004.1_122982565-122982743_Muntiacus_reevesi_isolate_UCam_UCB_Mr_chromosome_4_whole_genome_shotgun_sequence
SJXR01000847.1_5244297-5244467_Przewalskium_albirostris_isolate_WLD-bcl_scaffold1060_len8707626_cov133_whole_genome_shotgun_sequence
MKHE01000027.1_67045446-67045616_Cervus_elaphus_hippelaphus_isolate_Hungarian_chromosome_27_whole_genome_shotgun_sequence
QQTR01006149.1_47433061-47433231_Axis_porcinus_isolate_Zoohd001_scaffold_119_whole_genome_shotgun_sequence
JRFZ01055915.1_1508575-1508745_Elaphurus_davidianus_breed_Woburn_Abbey_isolate_Dafeng_scaffold9_whole_genome_shotgun_sequence
CCMK010068214.1_276-459_Capreolus_capreolus_genome_assembly_kmer631_contig_DEER_K63_SCAFFOLD68214_whole_genome_shotgun_sequence
SPDZ01000672.1_23618705-23618875_Hydropotes_inermis_isolate_ZZ2017_10112_whole_genome_shotgun_sequence
R_PVIN010000905.1_111363-111541_Rangifer_tarandus_isolate_US061_RanTarSib_scaffold_1806_whole_genome_shotgun_sequence
SSNK01020957.1_17071-17249_Alces_alces_isolate_MIG12-28694850_contig211_197_whole_genome_shotgun_sequence
RCHL01000070.1_68668-68846_Odocoileus_hemionus_hemionus_isolate_MD92_scaffold70_whole_genome_shotgun_sequence
RFLZ01179764.1_16950-17128_Odocoileus_hemionus_sitkensis_isolate_Cn_RI01_contig430285_0_whole_genome_shotgun_sequence
MLBE01016048.1_68758-68936_Odocoileus_virginianus_texanus_isolate_animal_Pink-7_contig_16048_whole_genome_shotgun_sequence
R_PVJP02910706.1_1566819-1567265_Hippopotamus_amphibius_isolate_BS01_ScHSfme_925160HRSCAF_982960_whole_genome_shotgun_sequence
marine_Turadu_NCQN01001313.1_0.0_1_564_299735_300297
marine_Turtru_NW_017843255.1_0.0_1_565_38420665_38421228
marine_Souchi_RWJT01006193.1_0.0_1_567_22964533_22963968
marine_Orcorc_NW_004438431.1_0.0_1_567_6616048_6616614
marine_Lagobl_NW_020837963.1_0.0_1_567_6717696_6718261
marine_Glomel_ML658804.1_0.0_1_567_4418490_4419055
marine_Monmon_NW_021700178.1_0.0_1_567_203423_202858
marine_Monmon_NW_021699185.1_0.0_1_567_458527_459092
marine_Delleu_NW_019160887.1_0.0_1_567_17207080_17206531
marine_Phosin_CM018169.1_0.0_1_567_23504612_23504047
marine_Neoasi_NW_020174429.1_0.0_1_567_2840910_2841475
marine_Phopho_PKGA01135294.1_0.0_1_481_23605950_23605471
marine_Lipvex_NW_006792448.1_0.0_1_567_1107717_1107150
marine_Ponbla_RJWI010028128.1_0.0_1_567_3434_2868
marine_Inigeo_RJWO010006178.1_0.0_1_564_12974_12411
marine_Mesbid_PVJJ010001573.1_0.0_1_567_36206_35640
marine_Zipcav_RJWS010000256.1_0.0_1_565_46433_46998
marine_Plamin_RJWK010082758.1_0.0_1_567_6900_7481
marine_Kogbre_RJWL010006521.1_0.0_1_567_12462_11896
query_marine_Phycat_NC_041232.1_0.0_1_567_64360246_64360812
marine_Balbon_DF402207.1_0.0_1_564_6566_6004
marine_Balacu_NW_006734345.1_0.0_1_564_5169483_5170045
marine_Megnov_RYZJ01001565.1_0.0_1_564_3122620_3122057
marine_Escrob_NIPP01004835.1_0.0_1_552_47982_48533
marine_Balmus_VNFD01002445.1_0.0_1_567_4428_4992
marine_Eubjap_RJWP010010059.1_0.0_1_567_14088_13521
R_PVJP02910233.1_22535899-22536345_Hippopotamus_amphibius_isolate_BS01_ScHSfme_924718HRSCAF_982518_whole_genome_shotgun_sequence
LSZX01088831.1_6960-7435_Camelus_dromedarius_breed_Targui_Contig_13863_103_whole_genome_shotgun_sequence
JDVD01006160.1_20631-21106_Camelus_dromedarius_breed_Arabia_contig6160_whole_genome_shotgun_sequence
R_JWIN03000030.1_19141932-19142407_Camelus_dromedarius_breed_African_isolate_Drom800_chromosome_30_whole_genome_shotgun_sequence
R_CAOW01002441.1_1261-1736_Camelus_bactrianus_WGS_project_CAOW00000000_data_contig_28832_whole_genome_shotgun_sequence
AGVR01014644.1_81556-82031_Camelus_ferus_isolate_Naran_contig014662_whole_genome_shotgun_sequence
VSZR01000026.1_16296421-16296896_Camelus_ferus_isolate_YT-003-E_chromosome_30_whole_genome_shotgun_sequence
R_JARL01025371.1_84436-84911_Camelus_bactrianus_breed_Alxa_contig25371_whole_genome_shotgun_sequence
JEMW01017911.1_78692-79167_Vicugna_pacos_huacaya_contig17911_whole_genome_shotgun_sequence
R_PVHT020000283.1_4230838-4231291_Catagonus_wagneri_isolate_BS18_Sc2sB16_246HRSCAF_34229_0_whole_genome_shotgun_sequence
SIDA01000001.1_183901831-183902281_Sus_scrofa_breed_Bama_miniature_chromosome_1_whole_genome_shotgun_sequence
0.02

lineage243

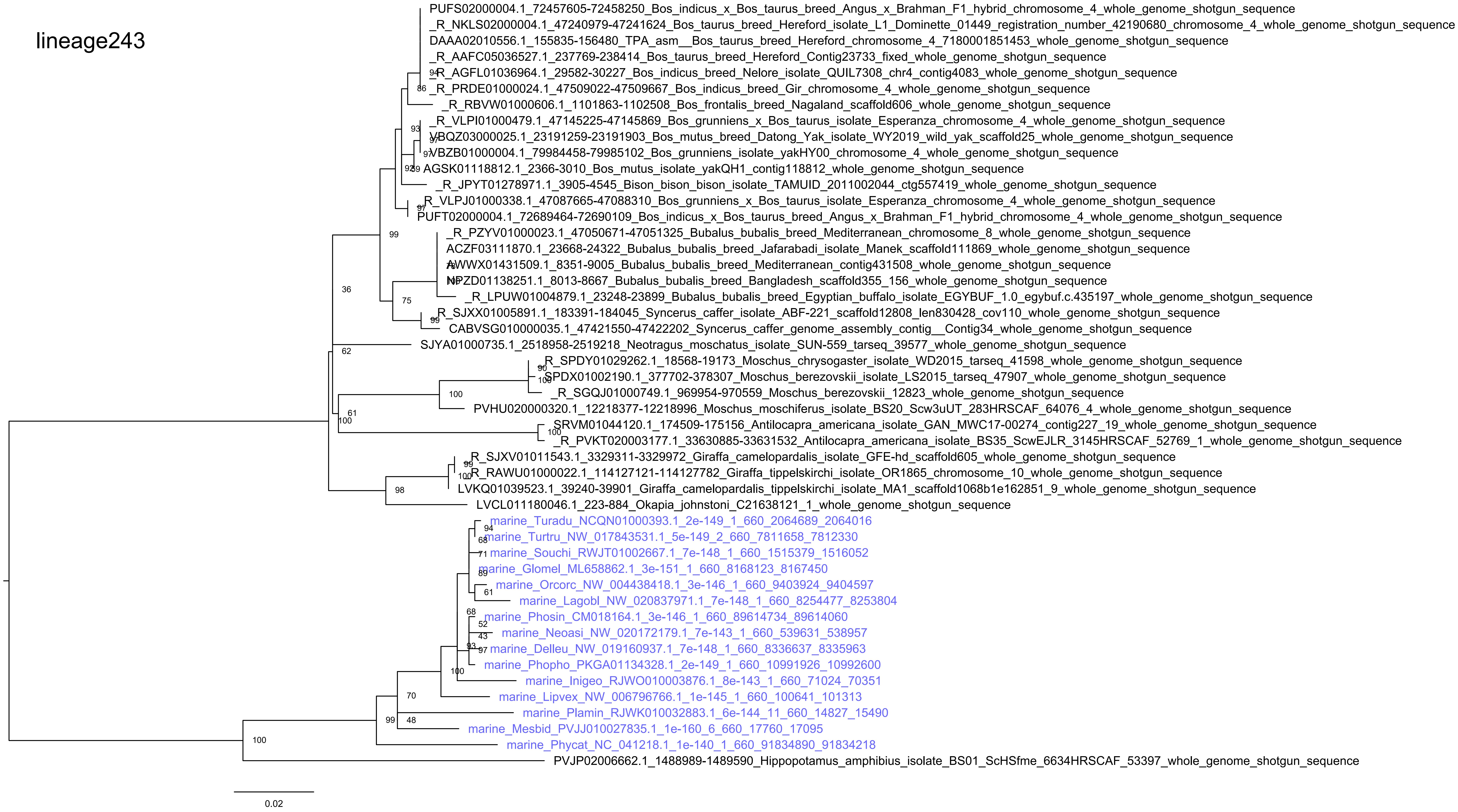
PUFS02000004.1_72457605-72458250_Bos_indicus_x_Bos_taurus_breed_Angus_x_Brahman_F1_hybrid_chromosome_4_whole_genome_shotgun_sequence
_R_NKLS02000004.1_47240979-47241624_Bos_taurus_breed_Hereford_isolate_L1_Dominette_01449_registration_number_42190680_chromosome_4_whole_genome_shotgun_sequence
DAAA02010556.1_155835-156480_TPA_asm__Bos_taurus_breed_Hereford_chromosome_4_7180001851453_whole_genome_shotgun_sequence
_R_AAFC05036527.1_237769-238414_Bos_taurus_breed_Hereford_Contig23733_fixed_whole_genome_shotgun_sequence
_R_AGFL01036964.1_29582-30227_Bos_indicus_breed_Nelore_isolate_QUIL7308_chr4_contig4083_whole_genome_shotgun_sequence
_R_PRDE01000024.1_47509022-47509667_Bos_indicus_breed_Gir_chromosome_4_whole_genome_shotgun_sequence
_R_RBVW01000606.1_1101863-1102508_Bos_frontalis_breed_Nagaland_scaffold606_whole_genome_shotgun_sequence
_R_VLPI01000479.1_47145225-47145869_Bos_grunniens_x_Bos_taurus_isolate_Esperanza_chromosome_4_whole_genome_shotgun_sequence
VBQZ03000025.1_23191259-23191903_Bos_mutus_breed_Datong_Yak_isolate_WY2019_wild_yak_scaffold25_whole_genome_shotgun_sequence
VBZB01000004.1_79984458-79985102_Bos_grunniens_isolate_yakHY00_chromosome_4_whole_genome_shotgun_sequence
AGSK01118812.1_2366-3010_Bos_mutus_isolate_yakQH1_contig118812_whole_genome_shotgun_sequence
_R_JPYT01278971.1_3905-4545_Bison_bison_bison_isolate_TAMUID_2011002044_ctg557419_whole_genome_shotgun_sequence
_R_VLPJ01000338.1_47087665-47088310_Bos_grunniens_x_Bos_taurus_isolate_Esperanza_chromosome_4_whole_genome_shotgun_sequence
PUFT02000004.1_72689464-72690109_Bos_indicus_x_Bos_taurus_breed_Angus_x_Brahman_F1_hybrid_chromosome_4_whole_genome_shotgun_sequence
_R_PZYV01000023.1_47050671-47051325_Bubalus_bubalis_breed_Mediterranean_chromosome_8_whole_genome_shotgun_sequence
ACZF03111870.1_23668-24322_Bubalus_bubalis_breed_Jafarabadi_isolate_Manek_scaffold111869_whole_genome_shotgun_sequence
AWWX01431509.1_8351-9005_Bubalus_bubalis_breed_Mediterranean_contig431508_whole_genome_shotgun_sequence
NPZD01138251.1_8013-8667_Bubalus_bubalis_breed_Bangladesh_scaffold355_156_whole_genome_shotgun_sequence
_R_LPUW01004879.1_23248-23899_Bubalus_bubalis_breed_Egyptian_buffalo_isolate_EGYBUF_1.0_egybuf.c.435197_whole_genome_shotgun_sequence
_R_SJXX01005891.1_183391-184045_Syncerus_caffer_isolate_ABF-221_scaffold12808_len830428_cov110_whole_genome_shotgun_sequence
CABVSG010000035.1_47421550-47422202_Syncerus_caffer_genome_assembly_contig__Contig34_whole_genome_shotgun_sequence
SJYA01000735.1_2518958-2519218_Neotragus_moschatus_isolate_SUN-559_tarseq_39577_whole_genome_shotgun_sequence
_R_SPDY01029262.1_18568-19173_Moschus_chrysogaster_isolate_WD2015_tarseq_41598_whole_genome_shotgun_sequence
SPDX01002190.1_377702-378307_Moschus_berezovskii_isolate_LS2015_tarseq_47907_whole_genome_shotgun_sequence
_R_SGQJ01000749.1_969954-970559_Moschus_berezovskii_12823_whole_genome_shotgun_sequence
PVHU020000320.1_12218377-12218996_Moschus_moschiferus_isolate_BS20_Scw3uUT_283HRSCAF_64076_4_whole_genome_shotgun_sequence
SRVM01044120.1_174509-175156_Antilocapra_americana_isolate_GAN_MWC17-00274_contig227_19_whole_genome_shotgun_sequence
_R_PVKT020003177.1_33630885-33631532_Antilocapra_americana_isolate_BS35_ScwEJLR_3145HRSCAF_52769_1_whole_genome_shotgun_sequence
_R_SJXV01011543.1_3329311-3329972_Giraffa_camelopardalis_isolate_GFE-hd_scaffold605_whole_genome_shotgun_sequence
_R_RAWU01000022.1_114127121-114127782_Giraffa_tippelskirchi_isolate_OR1865_chromosome_10_whole_genome_shotgun_sequence
LVKQ01039523.1_39240-39901_Giraffa_camelopardalis_tippelskirchi_isolate_MA1_scaffold1068b1e162851_9_whole_genome_shotgun_sequence
LVCL011180046.1_223-884_Okapia_johnstoni_C21638121_1_whole_genome_shotgun_sequence
marine_Turadu_NCQN01000393.1_2e-149_1_660_2064689_2064016
marine_Turtru_NW_017843531.1_5e-149_2_660_7811658_7812330
marine_Souchi_RWJT01002667.1_7e-148_1_660_1515379_1516052
marine_Glomel_ML658862.1_3e-151_1_660_8168123_8167450
marine_Orcorc_NW_004438418.1_3e-146_1_660_9403924_9404597
marine_Lagobl_NW_020837971.1_7e-148_1_660_8254477_8253804
marine_Phosin_CM018164.1_3e-146_1_660_89614734_89614060
marine_Neoasi_NW_020172179.1_7e-143_1_660_539631_538957
marine_Delleu_NW_019160937.1_7e-148_1_660_8336637_8335963
marine_Phopho_PKGA01134328.1_2e-149_1_660_10991926_10992600
marine_Inigeo_RJWO010003876.1_8e-143_1_660_71024_70351
marine_Lipvex_NW_006796766.1_1e-145_1_660_100641_101313
marine_Plamin_RJWK010032883.1_6e-144_11_660_14827_15490
marine_Mesbid_PVJJ010027835.1_1e-160_6_660_17760_17095
marine_Phycat_NC_041218.1_1e-140_1_660_91834890_91834218
PVJP02006662.1_1488989-1489590_Hippopotamus_amphibius_isolate_BS01_ScHSfme_6634HRSCAF_53397_whole_genome_shotgun_sequence
0.02

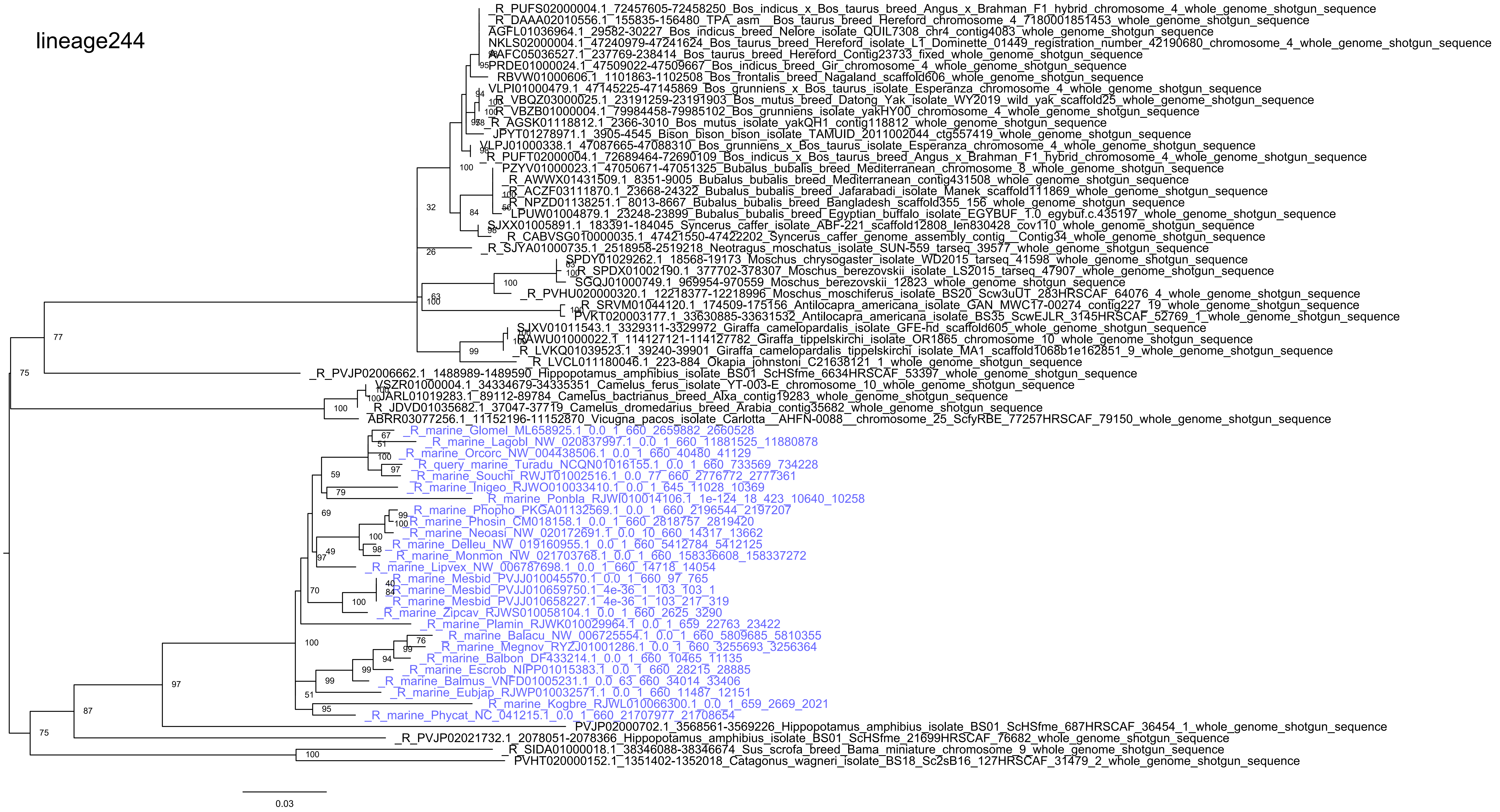
lineage244
_R_PUFS02000004.1_72457605-72458250_Bos_indicus_x_Bos_taurus_breed_Angus_x_Brahman_F1_hybrid_chromosome_4_whole_genome_shotgun_sequence
_R_DAAA02010556.1_155835-156480_TPA_asm__Bos_taurus_breed_Hereford_chromosome_4_7180001851453_whole_genome_shotgun_sequence
AGFL01036964.1_29582-30227_Bos_indicus_breed_Nelore_isolate_QUIL7308_chr4_contig4083_whole_genome_shotgun_sequence
NKLS02000004.1_47240979-47241624_Bos_taurus_breed_Hereford_isolate_L1_Dominette_01449_registration_number_42190680_chromosome_4_whole_genome_shotgun_sequence
AAFC05036527.1_237769-238414_Bos_taurus_breed_Hereford_Contig23733_fixed_whole_genome_shotgun_sequence
PRDE01000024.1_47509022-47509667_Bos_indicus_breed_Gir_chromosome_4_whole_genome_shotgun_sequence
RBVW01000606.1_1101863-1102508_Bos_frontalis_breed_Nagaland_scaffold606_whole_genome_shotgun_sequence
VLPI01000479.1_47145225-47145869_Bos_grunniens_x_Bos_taurus_isolate_Esperanza_chromosome_4_whole_genome_shotgun_sequence
_R_VBQZ03000025.1_23191259-23191903_Bos_mutus_breed_Datong_Yak_isolate_WY2019_wild_yak_scaffold25_whole_genome_shotgun_sequence
_R_VBZB01000004.1_79984458-79985102_Bos_grunniens_isolate_yakHY00_chromosome_4_whole_genome_shotgun_sequence
_R_AGSK01118812.1_2366-3010_Bos_mutus_isolate_yakQH1_contig118812_whole_genome_shotgun_sequence
JPYT01278971.1_3905-4545_Bison_bison_bison_isolate_TAMUID_2011002044_ctg557419_whole_genome_shotgun_sequence
VLPJ01000338.1_47087665-47088310_Bos_grunniens_x_Bos_taurus_isolate_Esperanza_chromosome_4_whole_genome_shotgun_sequence
_R_PUFT02000004.1_72689464-72690109_Bos_indicus_x_Bos_taurus_breed_Angus_x_Brahman_F1_hybrid_chromosome_4_whole_genome_shotgun_sequence
PZYV01000023.1_47050671-47051325_Bubalus_bubalis_breed_Mediterranean_chromosome_8_whole_genome_shotgun_sequence
_R_AWWX01431509.1_8351-9005_Bubalus_bubalis_breed_Mediterranean_contig431508_whole_genome_shotgun_sequence
_R_ACZF03111870.1_23668-24322_Bubalus_bubalis_breed_Jafarabadi_isolate_Manek_scaffold111869_whole_genome_shotgun_sequence
_R_NPZD01138251.1_8013-8667_Bubalus_bubalis_breed_Bangladesh_scaffold355_156_whole_genome_shotgun_sequence
LPUW01004879.1_23248-23899_Bubalus_bubalis_breed_Egyptian_buffalo_isolate_EGYBUF_1.0_egybuf.c.435197_whole_genome_shotgun_sequence
SJXX01005891.1_183391-184045_Syncerus_caffer_isolate_ABF-221_scaffold12808_len830428_cov110_whole_genome_shotgun_sequence
_R_CABVSG010000035.1_47421550-47422202_Syncerus_caffer_genome_assembly_contig__Contig34_whole_genome_shotgun_sequence
_R_SJYA01000735.1_2518958-2519218_Neotragus_moschatus_isolate_SUN-559_tarseq_39577_whole_genome_shotgun_sequence
SPDY01029262.1_18568-19173_Moschus_chrysogaster_isolate_WD2015_tarseq_41598_whole_genome_shotgun_sequence
_R_SPDX01002190.1_377702-378307_Moschus_berezovskii_isolate_LS2015_tarseq_47907_whole_genome_shotgun_sequence
SGQJ01000749.1_969954-970559_Moschus_berezovskii_12823_whole_genome_shotgun_sequence
_R_PVHU020000320.1_12218377-12218996_Moschus_moschiferus_isolate_BS20_Scw3uUT_283HRSCAF_64076_4_whole_genome_shotgun_sequence
_R_SRVM01044120.1_174509-175156_Antilocapra_americana_isolate_GAN_MWC17-00274_contig227_19_whole_genome_shotgun_sequence
PVKT020003177.1_33630885-33631532_Antilocapra_americana_isolate_BS35_ScwEJLR_3145HRSCAF_52769_1_whole_genome_shotgun_sequence
SJXV01011543.1_3329311-3329972_Giraffa_camelopardalis_isolate_GFE-hd_scaffold605_whole_genome_shotgun_sequence
RAWU01000022.1_114127121-114127782_Giraffa_tippelskirchi_isolate_OR1865_chromosome_10_whole_genome_shotgun_sequence
_R_LVKQ01039523.1_39240-39901_Giraffa_camelopardalis_tippelskirchi_isolate_MA1_scaffold1068b1e162851_9_whole_genome_shotgun_sequence
_R_LVCL011180046.1_223-884_Okapia_johnstoni_C21638121_1_whole_genome_shotgun_sequence
_R_PVJP02006662.1_1488989-1489590_Hippopotamus_amphibius_isolate_BS01_ScHSfme_6634HRSCAF_53397_whole_genome_shotgun_sequence
VSZR01000004.1_34334679-34335351_Camelus_ferus_isolate_YT-003-E_chromosome_10_whole_genome_shotgun_sequence
JARL01019283.1_89112-89784_Camelus_bactrianus_breed_Alxa_contig19283_whole_genome_shotgun_sequence
_R_JDVD01035682.1_37047-37719_Camelus_dromedarius_breed_Arabia_contig35682_whole_genome_shotgun_sequence
ABRR03077256.1_11152196-11152870_Vicugna_pacos_isolate_Carlotta__AHFN-0088__chromosome_25_ScfyRBE_77257HRSCAF_79150_whole_genome_shotgun_sequence
_R_marine_Glomel_ML658925.1_0.0_1_660_2659882_2660528
_R_marine_Lagobl_NW_020837997.1_0.0_1_660_11881525_11880878
_R_marine_Orcorc_NW_004438506.1_0.0_1_660_40480_41129
_R_query_marine_Turadu_NCQN01016155.1_0.0_1_660_733569_734228
_R_marine_Souchi_RWJT01002516.1_0.0_77_660_2776772_2777361
_R_marine_Inigeo_RJWO010033410.1_0.0_1_645_11028_10369
_R_marine_Ponbla_RJWI010014106.1_1e-124_18_423_10640_10258
_R_marine_Phopho_PKGA01132569.1_0.0_1_660_2196544_2197207
_R_marine_Phosin_CM018158.1_0.0_1_660_2818757_2819420
_R_marine_Neoasi_NW_020172691.1_0.0_10_660_14317_13662
_R_marine_Delleu_NW_019160955.1_0.0_1_660_5412784_5412125
_R_marine_Monmon_NW_021703768.1_0.0_1_660_158336608_158337272
_R_marine_Lipvex_NW_006787698.1_0.0_1_660_14718_14054
_R_marine_Mesbid_PVJJ010045570.1_0.0_1_660_97_765
_R_marine_Mesbid_PVJJ010659750.1_4e-36_1_103_103_1
_R_marine_Mesbid_PVJJ010658227.1_4e-36_1_103_217_319
_R_marine_Zipcav_RJWS010058104.1_0.0_1_660_2625_3290
_R_marine_Plamin_RJWK010029964.1_0.0_1_659_22763_23422
_R_marine_Balacu_NW_006725554.1_0.0_1_660_5809685_5810355
_R_marine_Megnov_RYZJ01001286.1_0.0_1_660_3255693_3256364
_R_marine_Balbon_DF433214.1_0.0_1_660_10465_11135
_R_marine_Escrob_NIPP01015383.1_0.0_1_660_28215_28885
_R_marine_Balmus_VNFD01005231.1_0.0_63_660_34014_33406
_R_marine_Eubjap_RJWP010032571.1_0.0_1_660_11487_12151
_R_marine_Kogbre_RJWL010066300.1_0.0_1_659_2669_2021
_R_marine_Phycat_NC_041215.1_0.0_1_660_21707977_21708654
PVJP02000702.1_3568561-3569226_Hippopotamus_amphibius_isolate_BS01_ScHSfme_687HRSCAF_36454_1_whole_genome_shotgun_sequence
_R_PVJP02021732.1_2078051-2078366_Hippopotamus_amphibius_isolate_BS01_ScHSfme_21699HRSCAF_76682_whole_genome_shotgun_sequence
_R_SIDA01000018.1_38346088-38346674_Sus_scrofa_breed_Bama_miniature_chromosome_9_whole_genome_shotgun_sequence
PVHT020000152.1_1351402-1352018_Catagonus_wagneri_isolate_BS18_Sc2sB16_127HRSCAF_31479_2_whole_genome_shotgun_sequence
0.03

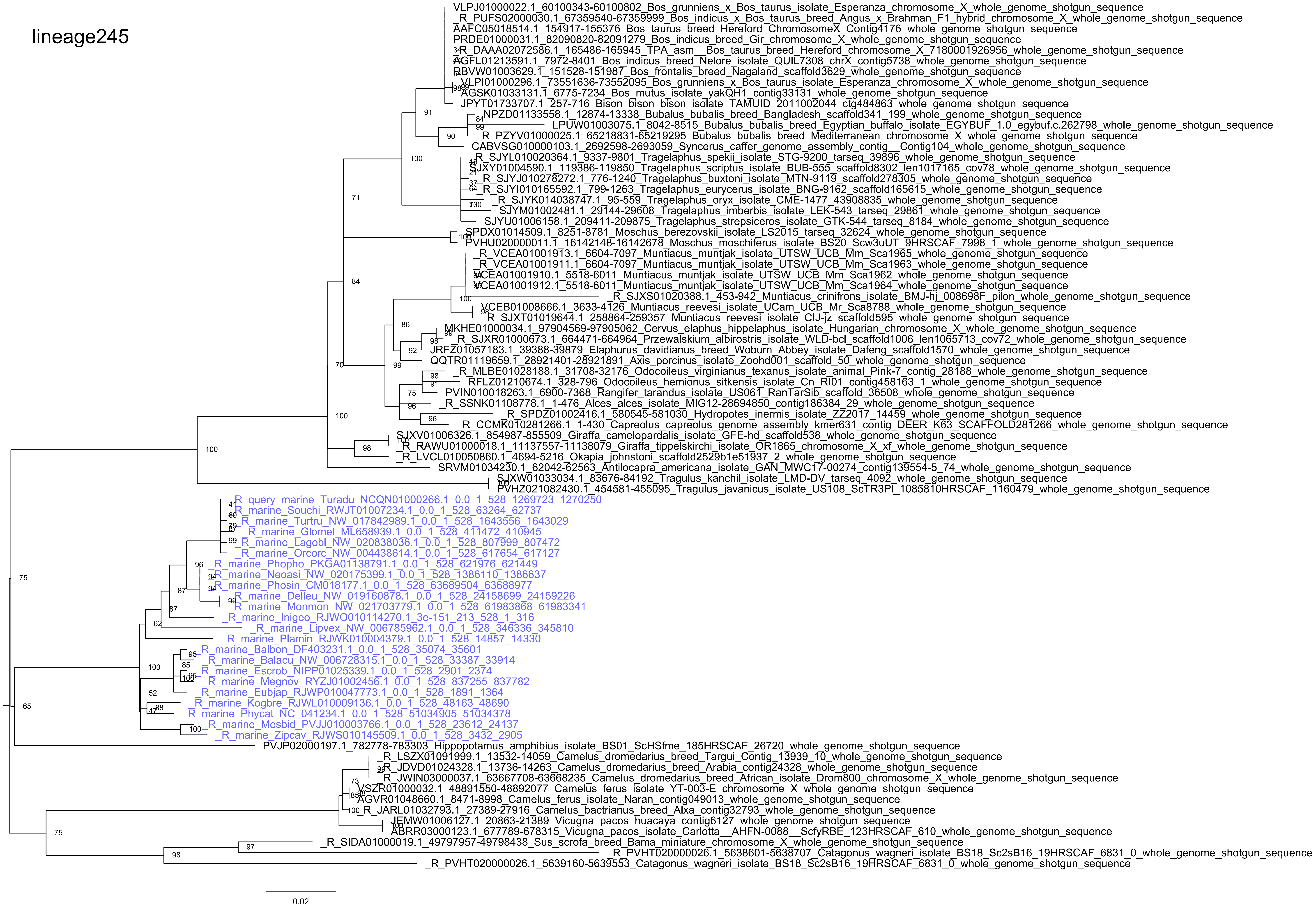
lineage245
VLPJ01000022.1_60100343-60100802_Bos_grunniens_x_Bos_taurus_isolate_Esperanza_chromosome_X_whole_genome_shotgun_sequence
_R_PUFS02000030.1_67359540-67359999_Bos_indicus_x_Bos_taurus_breed_Angus_x_Brahman_F1_hybrid_chromosome_X_whole_genome_shotgun_sequence
AAFC05018514.1_154917-155376_Bos_taurus_breed_Hereford_ChromosomeX_Contig4176_whole_genome_shotgun_sequence
PRDE01000031.1_82090820-82091279_Bos_indicus_breed_Gir_chromosome_X_whole_genome_shotgun_sequence
_R_DAAA02072586.1_165486-165945_TPA_asm__Bos_taurus_breed_Hereford_chromosome_X_7180001926956_whole_genome_shotgun_sequence
AGFL01213591.1_7972-8401_Bos_indicus_breed_Nelore_isolate_QUIL7308_chrX_contig5738_whole_genome_shotgun_sequence
RBVW01003629.1_151528-151987_Bos_frontalis_breed_Nagaland_scaffold3629_whole_genome_shotgun_sequence
VLPI01000296.1_73551636-73552095_Bos_grunniens_x_Bos_taurus_isolate_Esperanza_chromosome_X_whole_genome_shotgun_sequence
AGSK01033131.1_6775-7234_Bos_mutus_isolate_yakQH1_contig33131_whole_genome_shotgun_sequence
JPYT01733707.1_257-716_Bison_bison_bison_isolate_TAMUID_2011002044_ctg484863_whole_genome_shotgun_sequence
NPZD01133558.1_12874-13338_Bubalus_bubalis_breed_Bangladesh_scaffold341_199_whole_genome_shotgun_sequence
LPUW01003075.1_8042-8515_Bubalus_bubalis_breed_Egyptian_buffalo_isolate_EGYBUF_1.0_egybuf.c.262798_whole_genome_shotgun_sequence
_R_PZYV01000025.1_65218831-65219295_Bubalus_bubalis_breed_Mediterranean_chromosome_X_whole_genome_shotgun_sequence
CABVSG010000103.1_2692598-2693059_Syncerus_caffer_genome_assembly_contig__Contig104_whole_genome_shotgun_sequence
_R_SJYL010020364.1_9337-9801_Tragelaphus_spekii_isolate_STG-9200_tarseq_39896_whole_genome_shotgun_sequence
SJXY01004590.1_119386-119850_Tragelaphus_scriptus_isolate_BUB-555_scaffold8302_len1017165_cov78_whole_genome_shotgun_sequence
_R_SJYJ010278272.1_776-1240_Tragelaphus_buxtoni_isolate_MTN-9119_scaffold278305_whole_genome_shotgun_sequence
_R_SJYI010165592.1_799-1263_Tragelaphus_eurycerus_isolate_BNG-9162_scaffold165615_whole_genome_shotgun_sequence
_R_SJYK014038747.1_95-559_Tragelaphus_oryx_isolate_CME-1477_43908835_whole_genome_shotgun_sequence
SJYM01002481.1_29144-29608_Tragelaphus_imberbis_isolate_LEK-543_tarseq_29861_whole_genome_shotgun_sequence
SJYU01006158.1_209411-209875_Tragelaphus_strepsiceros_isolate_GTK-544_tarseq_8184_whole_genome_shotgun_sequence
SPDX01014509.1_8251-8781_Moschus_berezovskii_isolate_LS2015_tarseq_32624_whole_genome_shotgun_sequence
PVHU020000011.1_16142148-16142678_Moschus_moschiferus_isolate_BS20_Scw3uUT_9HRSCAF_7998_1_whole_genome_shotgun_sequence
_R_VCEA01001913.1_6604-7097_Muntiacus_muntjak_isolate_UTSW_UCB_Mm_Sca1965_whole_genome_shotgun_sequence
_R_VCEA01001911.1_6604-7097_Muntiacus_muntjak_isolate_UTSW_UCB_Mm_Sca1963_whole_genome_shotgun_sequence
VCEA01001910.1_5518-6011_Muntiacus_muntjak_isolate_UTSW_UCB_Mm_Sca1962_whole_genome_shotgun_sequence
VCEA01001912.1_5518-6011_Muntiacus_muntjak_isolate_UTSW_UCB_Mm_Sca1964_whole_genome_shotgun_sequence
_R_SJXS01020388.1_453-942_Muntiacus_crinifrons_isolate_BMJ-hj_008698F_pilon_whole_genome_shotgun_sequence
VCEB01008666.1_3633-4126_Muntiacus_reevesi_isolate_UCam_UCB_Mr_Sca8788_whole_genome_shotgun_sequence
_R_SJXT01019644.1_258864-259357_Muntiacus_reevesi_isolate_CIJ-jz_scaffold595_whole_genome_shotgun_sequence
MKHE01000034.1_97904569-97905062_Cervus_elaphus_hippelaphus_isolate_Hungarian_chromosome_X_whole_genome_shotgun_sequence
_R_SJXR01000673.1_664471-664964_Przewalskium_albirostris_isolate_WLD-bcl_scaffold1006_len1065713_cov72_whole_genome_shotgun_sequence
JRFZ01057183.1_39388-39879_Elaphurus_davidianus_breed_Woburn_Abbey_isolate_Dafeng_scaffold1570_whole_genome_shotgun_sequence
QQTR01119659.1_28921401-28921891_Axis_porcinus_isolate_Zoohd001_scaffold_50_whole_genome_shotgun_sequence
_R_MLBE01028188.1_31708-32176_Odocoileus_virginianus_texanus_isolate_animal_Pink-7_contig_28188_whole_genome_shotgun_sequence
RFLZ01210674.1_328-796_Odocoileus_hemionus_sitkensis_isolate_Cn_RI01_contig458163_1_whole_genome_shotgun_sequence
PVIN010018263.1_6900-7368_Rangifer_tarandus_isolate_US061_RanTarSib_scaffold_36508_whole_genome_shotgun_sequence
_R_SSNK01108778.1_1-476_Alces_alces_isolate_MIG12-28694850_contig186384_29_whole_genome_shotgun_sequence
_R_SPDZ01002416.1_580545-581030_Hydropotes_inermis_isolate_ZZ2017_14459_whole_genome_shotgun_sequence
_R_CCMK010281266.1_1-430_Capreolus_capreolus_genome_assembly_kmer631_contig_DEER_K63_SCAFFOLD281266_whole_genome_shotgun_sequence
SJXV01006326.1_854987-855509_Giraffa_camelopardalis_isolate_GFE-hd_scaffold538_whole_genome_shotgun_sequence
_R_RAWU01000018.1_11137557-11138079_Giraffa_tippelskirchi_isolate_OR1865_chromosome_X_xf_whole_genome_shotgun_sequence
_R_LVCL010050860.1_4694-5216_Okapia_johnstoni_scaffold2529b1e51937_2_whole_genome_shotgun_sequence
SRVM01034230.1_62042-62563_Antilocapra_americana_isolate_GAN_MWC17-00274_contig139554-5_74_whole_genome_shotgun_sequence
SJXW01033034.1_83676-84192_Tragulus_kanchil_isolate_LMD-DV_tarseq_4092_whole_genome_shotgun_sequence
PVHZ021082430.1_454581-455095_Tragulus_javanicus_isolate_US108_ScTR3PI_1085810HRSCAF_1160479_whole_genome_shotgun_sequence
_R_query_marine_Turadu_NCQN01000266.1_0.0_1_528_1269723_1270250
_R_marine_Souchi_RWJT01007234.1_0.0_1_528_63264_62737
_R_marine_Turtru_NW_017842989.1_0.0_1_528_1643556_1643029
_R_marine_Glomel_ML658939.1_0.0_1_528_411472_410945
_R_marine_Lagobl_NW_020838036.1_0.0_1_528_807999_807472
_R_marine_Orcorc_NW_004438614.1_0.0_1_528_617654_617127
_R_marine_Phopho_PKGA01138791.1_0.0_1_528_621976_621449
_R_marine_Neoasi_NW_020175399.1_0.0_1_528_1386110_1386637
_R_marine_Phosin_CM018177.1_0.0_1_528_63689504_63688977
_R_marine_Delleu_NW_019160878.1_0.0_1_528_24158699_24159226
_R_marine_Monmon_NW_021703779.1_0.0_1_528_61983868_61983341
_R_marine_Inigeo_RJWO010114270.1_3e-151_213_528_1_316
_R_marine_Lipvex_NW_006785962.1_0.0_1_528_346336_345810
_R_marine_Plamin_RJWK010004379.1_0.0_1_528_14857_14330
_R_marine_Balbon_DF403231.1_0.0_1_528_35074_35601
_R_marine_Balacu_NW_006728315.1_0.0_1_528_33387_33914
_R_marine_Escrob_NIPP01025339.1_0.0_1_528_2901_2374
_R_marine_Megnov_RYZJ01002456.1_0.0_1_528_837255_837782
_R_marine_Eubjap_RJWP010047773.1_0.0_1_528_1891_1364
_R_marine_Kogbre_RJWL010009136.1_0.0_1_528_48163_48690
_R_marine_Phycat_NC_041234.1_0.0_1_528_51034905_51034378
_R_marine_Mesbid_PVJJ010003766.1_0.0_1_528_23612_24137
_R_marine_Zipcav_RJWS010145509.1_0.0_1_528_3432_2905
PVJP02000197.1_782778-783303_Hippopotamus_amphibius_isolate_BS01_ScHSfme_185HRSCAF_26720_whole_genome_shotgun_sequence
_R_LSZX01091999.1_13532-14059_Camelus_dromedarius_breed_Targui_Contig_13939_10_whole_genome_shotgun_sequence
_R_JDVD01024328.1_13736-14263_Camelus_dromedarius_breed_Arabia_contig24328_whole_genome_shotgun_sequence
_R_JWIN03000037.1_63667708-63668235_Camelus_dromedarius_breed_African_isolate_Drom800_chromosome_X_whole_genome_shotgun_sequence
VSZR01000032.1_48891550-48892077_Camelus_ferus_isolate_YT-003-E_chromosome_X_whole_genome_shotgun_sequence
AGVR01048660.1_8471-8998_Camelus_ferus_isolate_Naran_contig049013_whole_genome_shotgun_sequence
_R_JARL01032793.1_27389-27916_Camelus_bactrianus_breed_Alxa_contig32793_whole_genome_shotgun_sequence
JEMW01006127.1_20863-21389_Vicugna_pacos_huacaya_contig6127_whole_genome_shotgun_sequence
ABRR03000123.1_677789-678315_Vicugna_pacos_isolate_Carlotta__AHFN-0088__ScfyRBE_123HRSCAF_610_whole_genome_shotgun_sequence
_R_SIDA01000019.1_49797957-49798438_Sus_scrofa_breed_Bama_miniature_chromosome_X_whole_genome_shotgun_sequence
_R_PVHT020000026.1_5638601-5638707_Catagonus_wagneri_isolate_BS18_Sc2sB16_19HRSCAF_6831_0_whole_genome_shotgun_sequence
_R_PVHT020000026.1_5639160-5639553_Catagonus_wagneri_isolate_BS18_Sc2sB16_19HRSCAF_6831_0_whole_genome_shotgun_sequence
0.02

lineage246

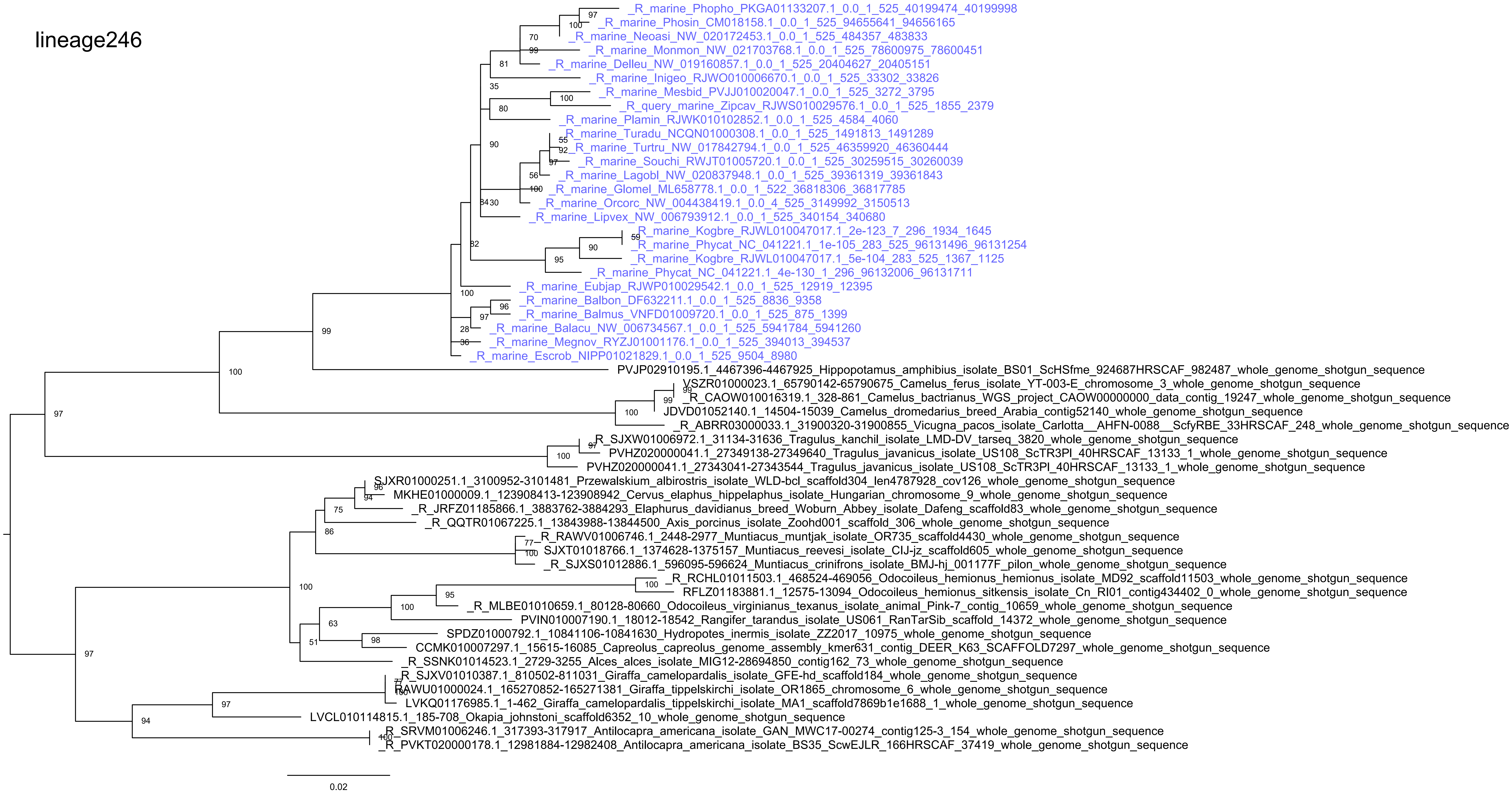

_R_marine_Phopho_PKGA01133207.1_0.0_1_525_40199474_40199998
_R_marine_Phosin_CM018158.1_0.0_1_525_94655641_94656165
_R_marine_Neoasi_NW_020172453.1_0.0_1_525_484357_483833
_R_marine_Monmon_NW_021703768.1_0.0_1_525_78600975_78600451
_R_marine_Delleu_NW_019160857.1_0.0_1_525_20404627_20405151
_R_marine_Inigeo_RJWO010006670.1_0.0_1_525_33302_33826
_R_marine_Mesbid_PVJJ010020047.1_0.0_1_525_3272_3795
_R_query_marine_Zipcav_RJWS010029576.1_0.0_1_525_1855_2379
_R_marine_Plamin_RJWK010102852.1_0.0_1_525_4584_4060
_R_marine_Turadu_NCQN01000308.1_0.0_1_525_1491813_1491289
_R_marine_Turtru_NW_017842794.1_0.0_1_525_46359920_46360444
_R_marine_Souchi_RWJT01005720.1_0.0_1_525_30259515_30260039
_R_marine_Lagobl_NW_020837948.1_0.0_1_525_39361319_39361843
_R_marine_Glomel_ML658778.1_0.0_1_522_36818306_36817785
_R_marine_Orcorc_NW_004438419.1_0.0_4_525_3149992_3150513
_R_marine_Lipvex_NW_006793912.1_0.0_1_525_340154_340680
_R_marine_Kogbre_RJWL010047017.1_2e-123_7_296_1934_1645
_R_marine_Phycat_NC_041221.1_1e-105_283_525_96131496_96131254
_R_marine_Kogbre_RJWL010047017.1_5e-104_283_525_1367_1125
_R_marine_Phycat_NC_041221.1_4e-130_1_296_96132006_96131711
_R_marine_Eubjap_RJWP010029542.1_0.0_1_525_12919_12395
_R_marine_Balbon_DF632211.1_0.0_1_525_8836_9358
_R_marine_Balmus_VNFD01009720.1_0.0_1_525_875_1399
_R_marine_Balacu_NW_006734567.1_0.0_1_525_5941784_5941260
_R_marine_Megnov_RYZJ01001176.1_0.0_1_525_394013_394537
_R_marine_Escrob_NIPP01021829.1_0.0_1_525_9504_8980
PVJP02910195.1_4467396-4467925_Hippopotamus_amphibius_isolate_BS01_ScHSfme_924687HRSCAF_982487_whole_genome_shotgun_sequence
VSZR01000023.1_65790142-65790675_Camelus_ferus_isolate_YT-003-E_chromosome_3_whole_genome_shotgun_sequence
_R_CAOW010016319.1_328-861_Camelus_bactrianus_WGS_project_CAOW00000000_data_contig_19247_whole_genome_shotgun_sequence
JDVD01052140.1_14504-15039_Camelus_dromedarius_breed_Arabia_contig52140_whole_genome_shotgun_sequence
_R_ABRR03000033.1_31900320-31900855_Vicugna_pacos_isolate_Carlotta__AHFN-0088__ScfyRBE_33HRSCAF_248_whole_genome_shotgun_sequence
_R_SJXW01006972.1_31134-31636_Tragulus_kanchil_isolate_LMD-DV_tarseq_3820_whole_genome_shotgun_sequence
PVHZ020000041.1_27349138-27349640_Tragulus_javanicus_isolate_US108_ScTR3PI_40HRSCAF_13133_1_whole_genome_shotgun_sequence
PVHZ020000041.1_27343041-27343544_Tragulus_javanicus_isolate_US108_ScTR3PI_40HRSCAF_13133_1_whole_genome_shotgun_sequence
SJXR01000251.1_3100952-3101481_Przewalskium_albirostris_isolate_WLD-bcl_scaffold304_len4787928_cov126_whole_genome_shotgun_sequence
MKHE01000009.1_123908413-123908942_Cervus_elaphus_hippelaphus_isolate_Hungarian_chromosome_9_whole_genome_shotgun_sequence
_R_JRFZ01185866.1_3883762-3884293_Elaphurus_davidianus_breed_Woburn_Abbey_isolate_Dafeng_scaffold83_whole_genome_shotgun_sequence
_R_QQTR01067225.1_13843988-13844500_Axis_porcinus_isolate_Zoohd001_scaffold_306_whole_genome_shotgun_sequence
_R_RAWV01006746.1_2448-2977_Muntiacus_muntjak_isolate_OR735_scaffold4430_whole_genome_shotgun_sequence
SJXT01018766.1_1374628-1375157_Muntiacus_reevesi_isolate_CIJ-jz_scaffold605_whole_genome_shotgun_sequence
_R_SJXS01012886.1_596095-596624_Muntiacus_crinifrons_isolate_BMJ-hj_001177F_pilon_whole_genome_shotgun_sequence
_R_RCHL01011503.1_468524-469056_Odocoileus_hemionus_hemionus_isolate_MD92_scaffold11503_whole_genome_shotgun_sequence
RFLZ01183881.1_12575-13094_Odocoileus_hemionus_sitkensis_isolate_Cn_RI01_contig434402_0_whole_genome_shotgun_sequence
_R_MLBE01010659.1_80128-80660_Odocoileus_virginianus_texanus_isolate_animal_Pink-7_contig_10659_whole_genome_shotgun_sequence
PVIN010007190.1_18012-18542_Rangifer_tarandus_isolate_US061_RanTarSib_scaffold_14372_whole_genome_shotgun_sequence
SPDZ01000792.1_10841106-10841630_Hydropotes_inermis_isolate_ZZ2017_10975_whole_genome_shotgun_sequence
CCMK010007297.1_15615-16085_Capreolus_capreolus_genome_assembly_kmer631_contig_DEER_K63_SCAFFOLD7297_whole_genome_shotgun_sequence
_R_SSNK01014523.1_2729-3255_Alces_alces_isolate_MIG12-28694850_contig162_73_whole_genome_shotgun_sequence
_R_SJXV01010387.1_810502-811031_Giraffa_camelopardalis_isolate_GFE-hd_scaffold184_whole_genome_shotgun_sequence
RAWU01000024.1_165270852-165271381_Giraffa_tippelskirchi_isolate_OR1865_chromosome_6_whole_genome_shotgun_sequence
LVKQ01176985.1_1-462_Giraffa_camelopardalis_tippelskirchi_isolate_MA1_scaffold7869b1e1688_1_whole_genome_shotgun_sequence
LVCL010114815.1_185-708_Okapia_johnstoni_scaffold6352_10_whole_genome_shotgun_sequence
_R_SRVM01006246.1_317393-317917_Antilocapra_americana_isolate_GAN_MWC17-00274_contig125-3_154_whole_genome_shotgun_sequence
_R_PVKT020000178.1_12981884-12982408_Antilocapra_americana_isolate_BS35_ScwEJLR_166HRSCAF_37419_whole_genome_shotgun_sequence
0.02

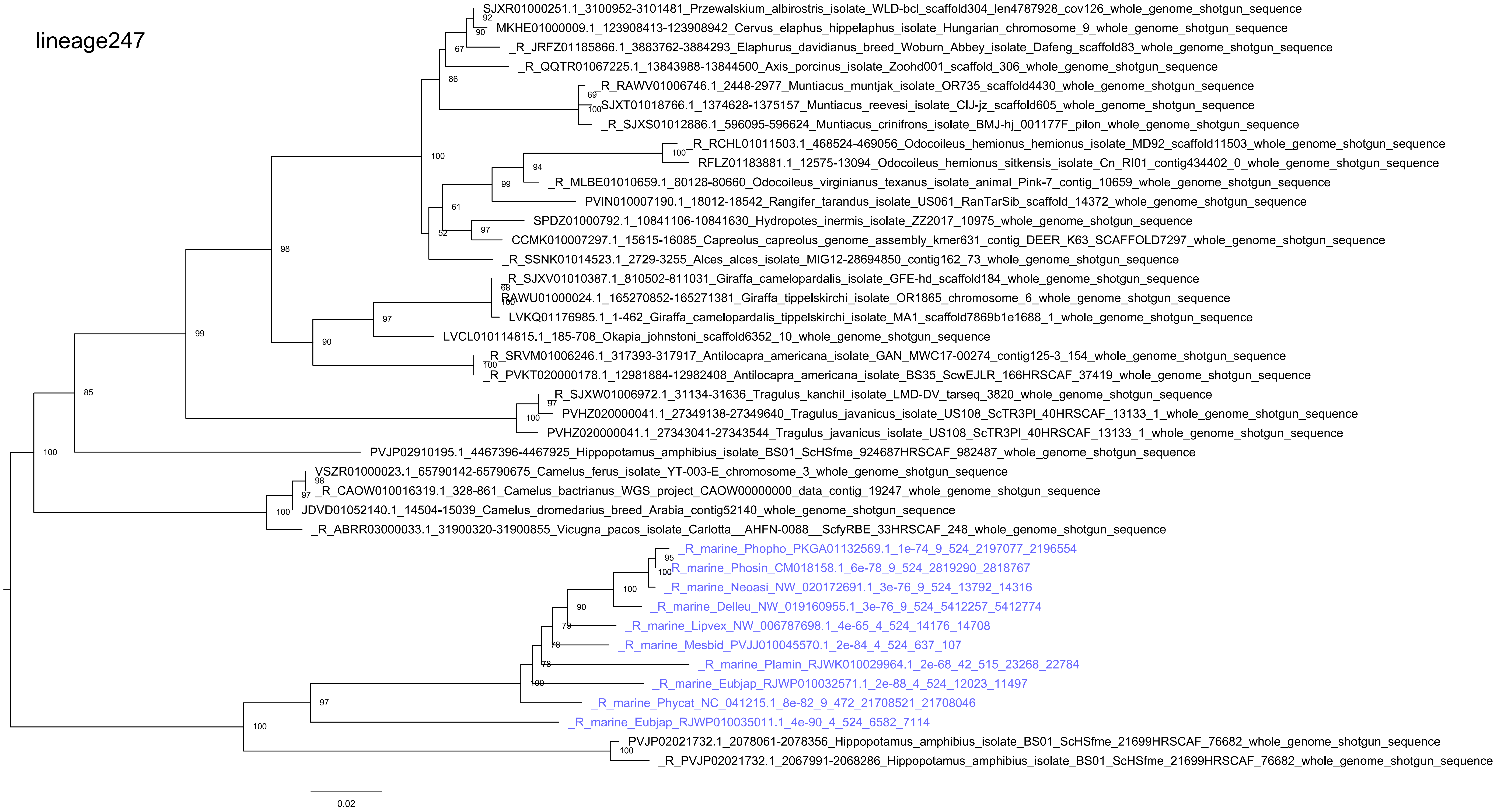
lineage247
SJXR01000251.1_3100952-3101481_Przewalskium_albirostris_isolate_WLD-bcl_scaffold304_len4787928_cov126_whole_genome_shotgun_sequence
MKHE01000009.1_123908413-123908942_Cervus_elaphus_hippelaphus_isolate_Hungarian_chromosome_9_whole_genome_shotgun_sequence
_R_JRFZ01185866.1_3883762-3884293_Elaphurus_davidianus_breed_Woburn_Abbey_isolate_Dafeng_scaffold83_whole_genome_shotgun_sequence
_R_QQTR01067225.1_13843988-13844500_Axis_porcinus_isolate_Zoohd001_scaffold_306_whole_genome_shotgun_sequence
R_RAWV01006746.1_2448-2977_Muntiacus_muntjak_isolate_OR735_scaffold4430_whole_genome_shotgun_sequence
SJXT01018766.1_1374628-1375157_Muntiacus_reevesi_isolate_CIJ-jz_scaffold605_whole_genome_shotgun_sequence
_R_SJXS01012886.1_596095-596624_Muntiacus_crinifrons_isolate_BMJ-hj_001177F_pilon_whole_genome_shotgun_sequence
R_RCHL01011503.1_468524-469056_Odocoileus_hemionus_hemionus_isolate_MD92_scaffold11503_whole_genome_shotgun_sequence
RFLZ01183881.1_12575-13094_Odocoileus_hemionus_sitkensis_isolate_Cn_RI01_contig434402_0_whole_genome_shotgun_sequence
_R_MLBE01010659.1_80128-80660_Odocoileus_virginianus_texanus_isolate_animal_Pink-7_contig_10659_whole_genome_shotgun_sequence
PVIN010007190.1_18012-18542_Rangifer_tarandus_isolate_US061_RanTarSib_scaffold_14372_whole_genome_shotgun_sequence
SPDZ01000792.1_10841106-10841630_Hydropotes_inermis_isolate_ZZ2017_10975_whole_genome_shotgun_sequence
CCMK010007297.1_15615-16085_Capreolus_capreolus_genome_assembly_kmer631_contig_DEER_K63_SCAFFOLD7297_whole_genome_shotgun_sequence
_R_SSNK01014523.1_2729-3255_Alces_alces_isolate_MIG12-28694850_contig162_73_whole_genome_shotgun_sequence
_R_SJXV01010387.1_810502-811031_Giraffa_camelopardalis_isolate_GFE-hd_scaffold184_whole_genome_shotgun_sequence
RAWU01000024.1_165270852-165271381_Giraffa_tippelskirchi_isolate_OR1865_chromosome_6_whole_genome_shotgun_sequence
LVKQ01176985.1_1-462_Giraffa_camelopardalis_tippelskirchi_isolate_MA1_scaffold7869b1e1688_1_whole_genome_shotgun_sequence
LVCL010114815.1_185-708_Okapia_johnstoni_scaffold6352_10_whole_genome_shotgun_sequence
R_SRVM01006246.1_317393-317917_Antilocapra_americana_isolate_GAN_MWC17-00274_contig125-3_154_whole_genome_shotgun_sequence
_R_PVKT020000178.1_12981884-12982408_Antilocapra_americana_isolate_BS35_ScwEJLR_166HRSCAF_37419_whole_genome_shotgun_sequence
R_SJXW01006972.1_31134-31636_Tragulus_kanchil_isolate_LMD-DV_tarseq_3820_whole_genome_shotgun_sequence
PVHZ020000041.1_27349138-27349640_Tragulus_javanicus_isolate_US108_ScTR3PI_40HRSCAF_13133_1_whole_genome_shotgun_sequence
PVHZ020000041.1_27343041-27343544_Tragulus_javanicus_isolate_US108_ScTR3PI_40HRSCAF_13133_1_whole_genome_shotgun_sequence
PVJP02910195.1_4467396-4467925_Hippopotamus_amphibius_isolate_BS01_ScHSfme_924687HRSCAF_982487_whole_genome_shotgun_sequence
VSZR01000023.1_65790142-65790675_Camelus_ferus_isolate_YT-003-E_chromosome_3_whole_genome_shotgun_sequence
_R_CAOW010016319.1_328-861_Camelus_bactrianus_WGS_project_CAOW00000000_data_contig_19247_whole_genome_shotgun_sequence
JDVD01052140.1_14504-15039_Camelus_dromedarius_breed_Arabia_contig52140_whole_genome_shotgun_sequence
_R_ABRR03000033.1_31900320-31900855_Vicugna_pacos_isolate_Carlotta__AHFN-0088__ScfyRBE_33HRSCAF_248_whole_genome_shotgun_sequence
_R_marine_Phopho_PKGA01132569.1_1e-74_9_524_2197077_2196554
_R_marine_Phosin_CM018158.1_6e-78_9_524_2819290_2818767
_R_marine_Neoasi_NW_020172691.1_3e-76_9_524_13792_14316
_R_marine_Delleu_NW_019160955.1_3e-76_9_524_5412257_5412774
_R_marine_Lipvex_NW_006787698.1_4e-65_4_524_14176_14708
_R_marine_Mesbid_PVJJ010045570.1_2e-84_4_524_637_107
_R_marine_Plamin_RJWK010029964.1_2e-68_42_515_23268_22784
_R_marine_Eubjap_RJWP010032571.1_2e-88_4_524_12023_11497
_R_marine_Phycat_NC_041215.1_8e-82_9_472_21708521_21708046
_R_marine_Eubjap_RJWP010035011.1_4e-90_4_524_6582_7114
PVJP02021732.1_2078061-2078356_Hippopotamus_amphibius_isolate_BS01_ScHSfme_21699HRSCAF_76682_whole_genome_shotgun_sequence
_R_PVJP02021732.1_2067991-2068286_Hippopotamus_amphibius_isolate_BS01_ScHSfme_21699HRSCAF_76682_whole_genome_shotgun_sequence
0.02

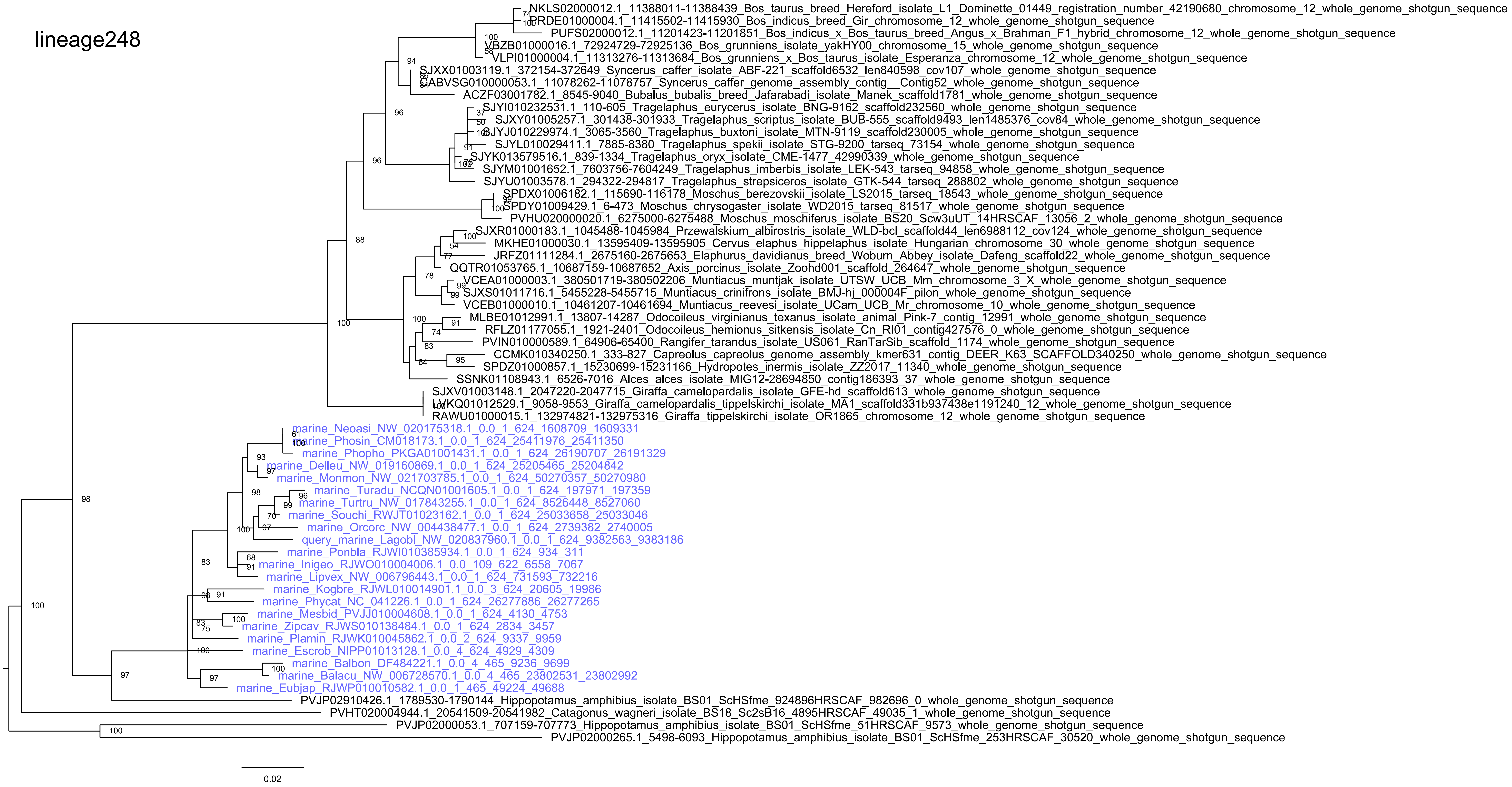

lineage248
NKLS02000012.1_11388011-11388439_Bos_taurus_breed_Hereford_isolate_L1_Dominette_01449_registration_number_42190680_chromosome_12_whole_genome_shotgun_sequence
PRDE01000004.1_11415502-11415930_Bos_indicus_breed_Gir_chromosome_12_whole_genome_shotgun_sequence
PUFS02000012.1_11201423-11201851_Bos_indicus_x_Bos_taurus_breed_Angus_x_Brahman_F1_hybrid_chromosome_12_whole_genome_shotgun_sequence
VBZB01000016.1_72924729-72925136_Bos_grunniens_isolate_yakHY00_chromosome_15_whole_genome_shotgun_sequence
VLPI01000004.1_11313276-11313684_Bos_grunniens_x_Bos_taurus_isolate_Esperanza_chromosome_12_whole_genome_shotgun_sequence
SJXX01003119.1_372154-372649_Syncerus_caffer_isolate_ABF-221_scaffold6532_len840598_cov107_whole_genome_shotgun_sequence
CABVSG010000053.1_11078262-11078757_Syncerus_caffer_genome_assembly_contig__Contig52_whole_genome_shotgun_sequence
ACZF03001782.1_8545-9040_Bubalus_bubalis_breed_Jafarabadi_isolate_Manek_scaffold1781_whole_genome_shotgun_sequence
SJYI010232531.1_110-605_Tragelaphus_eurycerus_isolate_BNG-9162_scaffold232560_whole_genome_shotgun_sequence
SJXY01005257.1_301438-301933_Tragelaphus_scriptus_isolate_BUB-555_scaffold9493_len1485376_cov84_whole_genome_shotgun_sequence
SJYJ010229974.1_3065-3560_Tragelaphus_buxtoni_isolate_MTN-9119_scaffold230005_whole_genome_shotgun_sequence
SJYL010029411.1_7885-8380_Tragelaphus_spekii_isolate_STG-9200_tarseq_73154_whole_genome_shotgun_sequence
SJYK013579516.1_839-1334_Tragelaphus_oryx_isolate_CME-1477_42990339_whole_genome_shotgun_sequence
SJYM01001652.1_7603756-7604249_Tragelaphus_imberbis_isolate_LEK-543_tarseq_94858_whole_genome_shotgun_sequence
SJYU01003578.1_294322-294817_Tragelaphus_strepsiceros_isolate_GTK-544_tarseq_288802_whole_genome_shotgun_sequence
SPDX01006182.1_115690-116178_Moschus_berezovskii_isolate_LS2015_tarseq_18543_whole_genome_shotgun_sequence
SPDY01009429.1_6-473_Moschus_chrysogaster_isolate_WD2015_tarseq_81517_whole_genome_shotgun_sequence
PVHU020000020.1_6275000-6275488_Moschus_moschiferus_isolate_BS20_Scw3uUT_14HRSCAF_13056_2_whole_genome_shotgun_sequence
SJXR01000183.1_1045488-1045984_Przewalskium_albirostris_isolate_WLD-bcl_scaffold44_len6988112_cov124_whole_genome_shotgun_sequence
MKHE01000030.1_13595409-13595905_Cervus_elaphus_hippelaphus_isolate_Hungarian_chromosome_30_whole_genome_shotgun_sequence
JRFZ01111284.1_2675160-2675653_Elaphurus_davidianus_breed_Woburn_Abbey_isolate_Dafeng_scaffold22_whole_genome_shotgun_sequence
QQTR01053765.1_10687159-10687652_Axis_porcinus_isolate_Zoohd001_scaffold_264647_whole_genome_shotgun_sequence
VCEA01000003.1_380501719-380502206_Muntiacus_muntjak_isolate_UTSW_UCB_Mm_chromosome_3_X_whole_genome_shotgun_sequence
SJXS01011716.1_5455228-5455715_Muntiacus_crinifrons_isolate_BMJ-hj_000004F_pilon_whole_genome_shotgun_sequence
VCEB01000010.1_10461207-10461694_Muntiacus_reevesi_isolate_UCam_UCB_Mr_chromosome_10_whole_genome_shotgun_sequence
MLBE01012991.1_13807-14287_Odocoileus_virginianus_texanus_isolate_animal_Pink-7_contig_12991_whole_genome_shotgun_sequence
RFLZ01177055.1_1921-2401_Odocoileus_hemionus_sitkensis_isolate_Cn_RI01_contig427576_0_whole_genome_shotgun_sequence
PVIN010000589.1_64906-65400_Rangifer_tarandus_isolate_US061_RanTarSib_scaffold_1174_whole_genome_shotgun_sequence
CCMK010340250.1_333-827_Capreolus_capreolus_genome_assembly_kmer631_contig_DEER_K63_SCAFFOLD340250_whole_genome_shotgun_sequence
SPDZ01000857.1_15230699-15231166_Hydropotes_inermis_isolate_ZZ2017_11340_whole_genome_shotgun_sequence
SSNK01108943.1_6526-7016_Alces_alces_isolate_MIG12-28694850_contig186393_37_whole_genome_shotgun_sequence
SJXV01003148.1_2047220-2047715_Giraffa_camelopardalis_isolate_GFE-hd_scaffold613_whole_genome_shotgun_sequence
LVKQ01012529.1_9058-9553_Giraffa_camelopardalis_tippelskirchi_isolate_MA1_scaffold331b937438e1191240_12_whole_genome_shotgun_sequence
RAWU01000015.1_132974821-132975316_Giraffa_tippelskirchi_isolate_OR1865_chromosome_12_whole_genome_shotgun_sequence
marine_Neoasi_NW_020175318.1_0.0_1_624_1608709_1609331
marine_Phosin_CM018173.1_0.0_1_624_25411976_25411350
marine_Phopho_PKGA01001431.1_0.0_1_624_26190707_26191329
marine_Delleu_NW_019160869.1_0.0_1_624_25205465_25204842
marine_Monmon_NW_021703785.1_0.0_1_624_50270357_50270980
marine_Turadu_NCQN01001605.1_0.0_1_624_197971_197359
marine_Turtru_NW_017843255.1_0.0_1_624_8526448_8527060
marine_Souchi_RWJT01023162.1_0.0_1_624_25033658_25033046
marine_Orcorc_NW_004438477.1_0.0_1_624_2739382_2740005
query_marine_Lagobl_NW_020837960.1_0.0_1_624_9382563_9383186
marine_Ponbla_RJWI010385934.1_0.0_1_624_934_311
marine_Inigeo_RJWO010004006.1_0.0_109_622_6558_7067
marine_Lipvex_NW_006796443.1_0.0_1_624_731593_732216
marine_Kogbre_RJWL010014901.1_0.0_3_624_20605_19986
marine_Phycat_NC_041226.1_0.0_1_624_26277886_26277265
marine_Mesbid_PVJJ010004608.1_0.0_1_624_4130_4753
marine_Zipcav_RJWS010138484.1_0.0_1_624_2834_3457
marine_Plamin_RJWK010045862.1_0.0_2_624_9337_9959
marine_Escrob_NIPP01013128.1_0.0_4_624_4929_4309
marine_Balbon_DF484221.1_0.0_4_465_9236_9699
marine_Balacu_NW_006728570.1_0.0_4_465_23802531_23802992
marine_Eubjap_RJWP010010582.1_0.0_1_465_49224_49688
PVJP02910426.1_1789530-1790144_Hippopotamus_amphibius_isolate_BS01_ScHSfme_924896HRSCAF_982696_0_whole_genome_shotgun_sequence
PVHT020004944.1_20541509-20541982_Catagonus_wagneri_isolate_BS18_Sc2sB16_4895HRSCAF_49035_1_whole_genome_shotgun_sequence
PVJP02000053.1_707159-707773_Hippopotamus_amphibius_isolate_BS01_ScHSfme_51HRSCAF_9573_whole_genome_shotgun_sequence
PVJP02000265.1_5498-6093_Hippopotamus_amphibius_isolate_BS01_ScHSfme_253HRSCAF_30520_whole_genome_shotgun_sequence
0.02

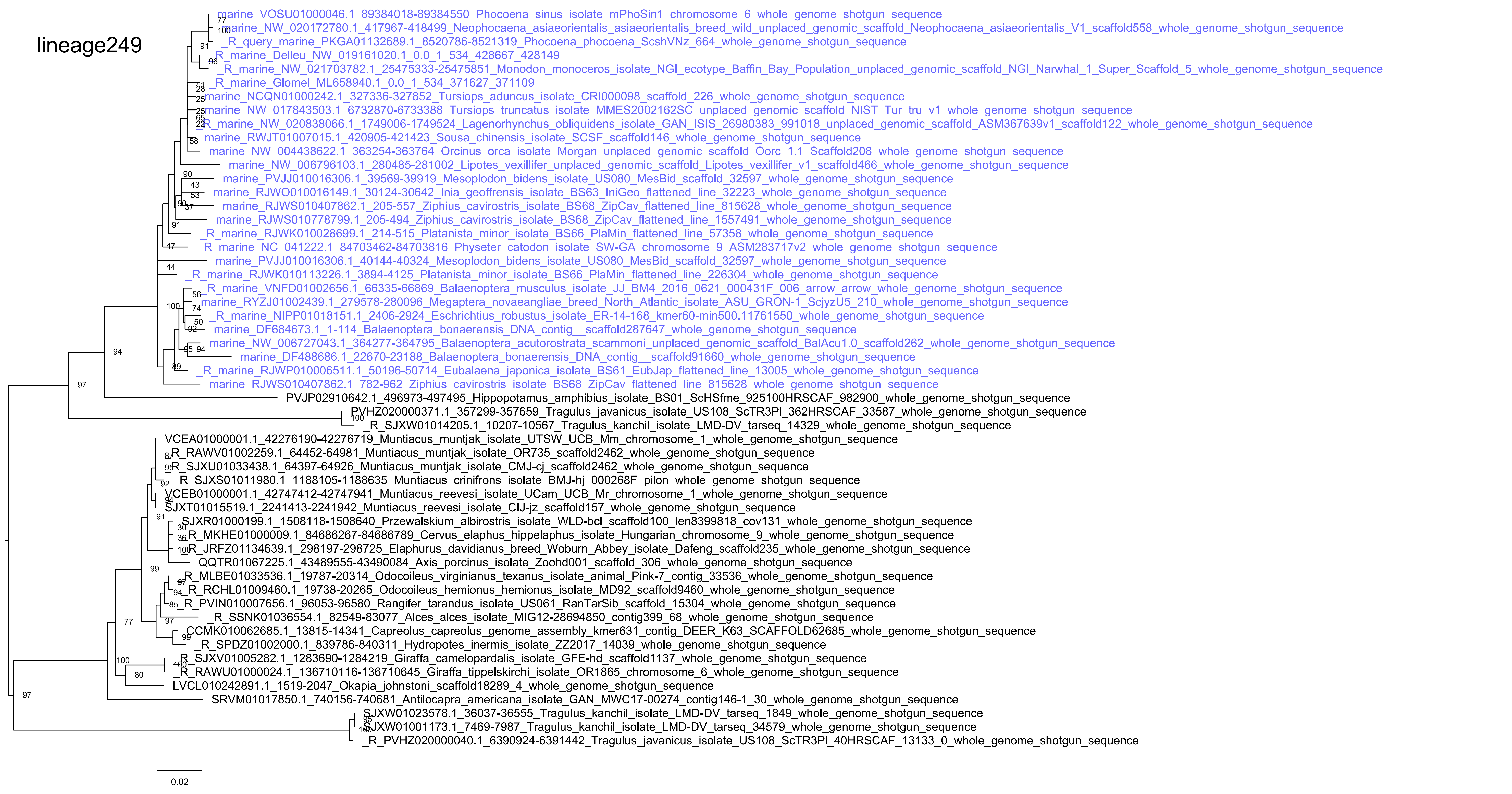
lineage249
marine_VOSU01000046.1_89384018-89384550_Phocoena_sinus_isolate_mPhoSin1_chromosome_6_whole_genome_shotgun_sequence
marine_NW_020172780.1_417967-418499_Neophocaena_asiaeorientalis_asiaeorientalis_breed_wild_unplaced_genomic_scaffold_Neophocaena_asiaeorientalis_V1_scaffold558_whole_genome_shotgun_sequence
_R_query_marine_PKGA01132689.1_8520786-8521319_Phocoena_phocoena_ScshVNz_664_whole_genome_shotgun_sequence
_R_marine_Delleu_NW_019161020.1_0.0_1_534_428667_428149
_R_marine_NW_021703782.1_25475333-25475851_Monodon_monoceros_isolate_NGI_ecotype_Baffin_Bay_Population_unplaced_genomic_scaffold_NGI_Narwhal_1_Super_Scaffold_5_whole_genome_shotgun_sequence
_R_marine_Glomel_ML658940.1_0.0_1_534_371627_371109
marine_NCQN01000242.1_327336-327852_Tursiops_aduncus_isolate_CRI000098_scaffold_226_whole_genome_shotgun_sequence
marine_NW_017843503.1_6732870-6733388_Tursiops_truncatus_isolate_MMES2002162SC_unplaced_genomic_scaffold_NIST_Tur_tru_v1_whole_genome_shotgun_sequence
_R_marine_NW_020838066.1_1749006-1749524_Lagenorhynchus_obliquidens_isolate_GAN_ISIS_26980383_991018_unplaced_genomic_scaffold_ASM367639v1_scaffold122_whole_genome_shotgun_sequence
marine_RWJT01007015.1_420905-421423_Sousa_chinensis_isolate_SCSF_scaffold146_whole_genome_shotgun_sequence
marine_NW_004438622.1_363254-363764_Orcinus_orca_isolate_Morgan_unplaced_genomic_scaffold_Oorc_1.1_Scaffold208_whole_genome_shotgun_sequence
marine_NW_006796103.1_280485-281002_Lipotes_vexillifer_unplaced_genomic_scaffold_Lipotes_vexillifer_v1_scaffold466_whole_genome_shotgun_sequence
marine_PVJJ010016306.1_39569-39919_Mesoplodon_bidens_isolate_US080_MesBid_scaffold_32597_whole_genome_shotgun_sequence
marine_RJWO010016149.1_30124-30642_Inia_geoffrensis_isolate_BS63_IniGeo_flattened_line_32223_whole_genome_shotgun_sequence
marine_RJWS010407862.1_205-557_Ziphius_cavirostris_isolate_BS68_ZipCav_flattened_line_815628_whole_genome_shotgun_sequence
marine_RJWS010778799.1_205-494_Ziphius_cavirostris_isolate_BS68_ZipCav_flattened_line_1557491_whole_genome_shotgun_sequence
_R_marine_RJWK010028699.1_214-515_Platanista_minor_isolate_BS66_PlaMin_flattened_line_57358_whole_genome_shotgun_sequence
_R_marine_NC_041222.1_84703462-84703816_Physeter_catodon_isolate_SW-GA_chromosome_9_ASM283717v2_whole_genome_shotgun_sequence
marine_PVJJ010016306.1_40144-40324_Mesoplodon_bidens_isolate_US080_MesBid_scaffold_32597_whole_genome_shotgun_sequence
_R_marine_RJWK010113226.1_3894-4125_Platanista_minor_isolate_BS66_PlaMin_flattened_line_226304_whole_genome_shotgun_sequence
_R_marine_VNFD01002656.1_66335-66869_Balaenoptera_musculus_isolate_JJ_BM4_2016_0621_000431F_006_arrow_arrow_whole_genome_shotgun_sequence
marine_RYZJ01002439.1_279578-280096_Megaptera_novaeangliae_breed_North_Atlantic_isolate_ASU_GRON-1_ScjyzU5_210_whole_genome_shotgun_sequence
_R_marine_NIPP01018151.1_2406-2924_Eschrichtius_robustus_isolate_ER-14-168_kmer60-min500.11761550_whole_genome_shotgun_sequence
marine_DF684673.1_1-114_Balaenoptera_bonaerensis_DNA_contig__scaffold287647_whole_genome_shotgun_sequence
marine_NW_006727043.1_364277-364795_Balaenoptera_acutorostrata_scammoni_unplaced_genomic_scaffold_BalAcu1.0_scaffold262_whole_genome_shotgun_sequence
marine_DF488686.1_22670-23188_Balaenoptera_bonaerensis_DNA_contig__scaffold91660_whole_genome_shotgun_sequence
_R_marine_RJWP010006511.1_50196-50714_Eubalaena_japonica_isolate_BS61_EubJap_flattened_line_13005_whole_genome_shotgun_sequence
marine_RJWS010407862.1_782-962_Ziphius_cavirostris_isolate_BS68_ZipCav_flattened_line_815628_whole_genome_shotgun_sequence
PVJP02910642.1_496973-497495_Hippopotamus_amphibius_isolate_BS01_ScHSfme_925100HRSCAF_982900_whole_genome_shotgun_sequence
PVHZ020000371.1_357299-357659_Tragulus_javanicus_isolate_US108_ScTR3PI_362HRSCAF_33587_whole_genome_shotgun_sequence
_R_SJXW01014205.1_10207-10567_Tragulus_kanchil_isolate_LMD-DV_tarseq_14329_whole_genome_shotgun_sequence
VCEA01000001.1_42276190-42276719_Muntiacus_muntjak_isolate_UTSW_UCB_Mm_chromosome_1_whole_genome_shotgun_sequence
_R_RAWV01002259.1_64452-64981_Muntiacus_muntjak_isolate_OR735_scaffold2462_whole_genome_shotgun_sequence
_R_SJXU01033438.1_64397-64926_Muntiacus_muntjak_isolate_CMJ-cj_scaffold2462_whole_genome_shotgun_sequence
_R_SJXS01011980.1_1188105-1188635_Muntiacus_crinifrons_isolate_BMJ-hj_000268F_pilon_whole_genome_shotgun_sequence
VCEB01000001.1_42747412-42747941_Muntiacus_reevesi_isolate_UCam_UCB_Mr_chromosome_1_whole_genome_shotgun_sequence
SJXT01015519.1_2241413-2241942_Muntiacus_reevesi_isolate_CIJ-jz_scaffold157_whole_genome_shotgun_sequence
SJXR01000199.1_1508118-1508640_Przewalskium_albirostris_isolate_WLD-bcl_scaffold100_len8399818_cov131_whole_genome_shotgun_sequence
_R_MKHE01000009.1_84686267-84686789_Cervus_elaphus_hippelaphus_isolate_Hungarian_chromosome_9_whole_genome_shotgun_sequence
_R_JRFZ01134639.1_298197-298725_Elaphurus_davidianus_breed_Woburn_Abbey_isolate_Dafeng_scaffold235_whole_genome_shotgun_sequence
QQTR01067225.1_43489555-43490084_Axis_porcinus_isolate_Zoohd001_scaffold_306_whole_genome_shotgun_sequence
_R_MLBE01033536.1_19787-20314_Odocoileus_virginianus_texanus_isolate_animal_Pink-7_contig_33536_whole_genome_shotgun_sequence
_R_RCHL01009460.1_19738-20265_Odocoileus_hemionus_hemionus_isolate_MD92_scaffold9460_whole_genome_shotgun_sequence
_R_PVIN010007656.1_96053-96580_Rangifer_tarandus_isolate_US061_RanTarSib_scaffold_15304_whole_genome_shotgun_sequence
_R_SSNK01036554.1_82549-83077_Alces_alces_isolate_MIG12-28694850_contig399_68_whole_genome_shotgun_sequence
CCMK010062685.1_13815-14341_Capreolus_capreolus_genome_assembly_kmer631_contig_DEER_K63_SCAFFOLD62685_whole_genome_shotgun_sequence
_R_SPDZ01002000.1_839786-840311_Hydropotes_inermis_isolate_ZZ2017_14039_whole_genome_shotgun_sequence
_R_SJXV01005282.1_1283690-1284219_Giraffa_camelopardalis_isolate_GFE-hd_scaffold1137_whole_genome_shotgun_sequence
_R_RAWU01000024.1_136710116-136710645_Giraffa_tippelskirchi_isolate_OR1865_chromosome_6_whole_genome_shotgun_sequence
LVCL010242891.1_1519-2047_Okapia_johnstoni_scaffold18289_4_whole_genome_shotgun_sequence
SRVM01017850.1_740156-740681_Antilocapra_americana_isolate_GAN_MWC17-00274_contig146-1_30_whole_genome_shotgun_sequence
SJXW01023578.1_36037-36555_Tragulus_kanchil_isolate_LMD-DV_tarseq_1849_whole_genome_shotgun_sequence
SJXW01001173.1_7469-7987_Tragulus_kanchil_isolate_LMD-DV_tarseq_34579_whole_genome_shotgun_sequence
_R_PVHZ020000040.1_6390924-6391442_Tragulus_javanicus_isolate_US108_ScTR3PI_40HRSCAF_13133_0_whole_genome_shotgun_sequence
0.02

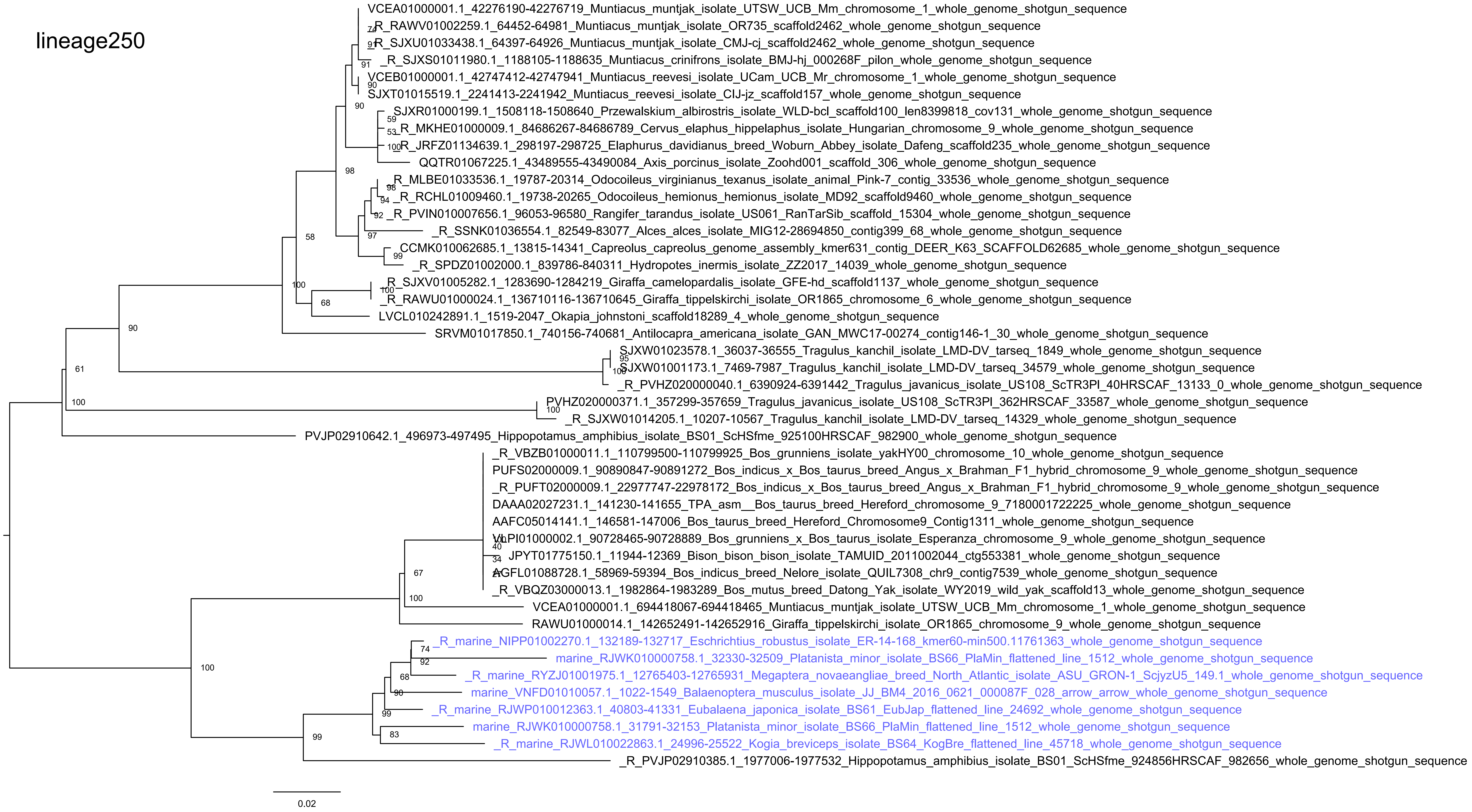

lineage250
VCEA01000001.1_42276190-42276719_Muntiacus_muntjak_isolate_UTSW_UCB_Mm_chromosome_1_whole_genome_shotgun_sequence
_R_RAWV01002259.1_64452-64981_Muntiacus_muntjak_isolate_OR735_scaffold2462_whole_genome_shotgun_sequence
_R_SJXU01033438.1_64397-64926_Muntiacus_muntjak_isolate_CMJ-cj_scaffold2462_whole_genome_shotgun_sequence
_R_SJXS01011980.1_1188105-1188635_Muntiacus_crinifrons_isolate_BMJ-hj_000268F_pilon_whole_genome_shotgun_sequence
VCEB01000001.1_42747412-42747941_Muntiacus_reevesi_isolate_UCam_UCB_Mr_chromosome_1_whole_genome_shotgun_sequence
SJXT01015519.1_2241413-2241942_Muntiacus_reevesi_isolate_CIJ-jz_scaffold157_whole_genome_shotgun_sequence
SJXR01000199.1_1508118-1508640_Przewalskium_albirostris_isolate_WLD-bcl_scaffold100_len8399818_cov131_whole_genome_shotgun_sequence
_R_MKHE01000009.1_84686267-84686789_Cervus_elaphus_hippelaphus_isolate_Hungarian_chromosome_9_whole_genome_shotgun_sequence
_R_JRFZ01134639.1_298197-298725_Elaphurus_davidianus_breed_Woburn_Abbey_isolate_Dafeng_scaffold235_whole_genome_shotgun_sequence
QQTR01067225.1_43489555-43490084_Axis_porcinus_isolate_Zoohd001_scaffold_306_whole_genome_shotgun_sequence
_R_MLBE01033536.1_19787-20314_Odocoileus_virginianus_texanus_isolate_animal_Pink-7_contig_33536_whole_genome_shotgun_sequence
_R_RCHL01009460.1_19738-20265_Odocoileus_hemionus_hemionus_isolate_MD92_scaffold9460_whole_genome_shotgun_sequence
_R_PVIN010007656.1_96053-96580_Rangifer_tarandus_isolate_US061_RanTarSib_scaffold_15304_whole_genome_shotgun_sequence
_R_SSNK01036554.1_82549-83077_Alces_alces_isolate_MIG12-28694850_contig399_68_whole_genome_shotgun_sequence
CCMK010062685.1_13815-14341_Capreolus_capreolus_genome_assembly_kmer631_contig_DEER_K63_SCAFFOLD62685_whole_genome_shotgun_sequence
_R_SPDZ01002000.1_839786-840311_Hydropotes_inermis_isolate_ZZ2017_14039_whole_genome_shotgun_sequence
_R_SJXV01005282.1_1283690-1284219_Giraffa_camelopardalis_isolate_GFE-hd_scaffold1137_whole_genome_shotgun_sequence
_R_RAWU01000024.1_136710116-136710645_Giraffa_tippelskirchi_isolate_OR1865_chromosome_6_whole_genome_shotgun_sequence
LVCL010242891.1_1519-2047_Okapia_johnstoni_scaffold18289_4_whole_genome_shotgun_sequence
SRVM01017850.1_740156-740681_Antilocapra_americana_isolate_GAN_MWC17-00274_contig146-1_30_whole_genome_shotgun_sequence
SJXW01023578.1_36037-36555_Tragulus_kanchil_isolate_LMD-DV_tarseq_1849_whole_genome_shotgun_sequence
SJXW01001173.1_7469-7987_Tragulus_kanchil_isolate_LMD-DV_tarseq_34579_whole_genome_shotgun_sequence
_R_PVHZ020000040.1_6390924-6391442_Tragulus_javanicus_isolate_US108_ScTR3PI_40HRSCAF_13133_0_whole_genome_shotgun_sequence
PVHZ020000371.1_357299-357659_Tragulus_javanicus_isolate_US108_ScTR3PI_362HRSCAF_33587_whole_genome_shotgun_sequence
_R_SJXW01014205.1_10207-10567_Tragulus_kanchil_isolate_LMD-DV_tarseq_14329_whole_genome_shotgun_sequence
PVJP02910642.1_496973-497495_Hippopotamus_amphibius_isolate_BS01_ScHSfme_925100HRSCAF_982900_whole_genome_shotgun_sequence
_R_VBZB01000011.1_110799500-110799925_Bos_grunniens_isolate_yakHY00_chromosome_10_whole_genome_shotgun_sequence
PUFS02000009.1_90890847-90891272_Bos_indicus_x_Bos_taurus_breed_Angus_x_Brahman_F1_hybrid_chromosome_9_whole_genome_shotgun_sequence
_R_PUFT02000009.1_22977747-22978172_Bos_indicus_x_Bos_taurus_breed_Angus_x_Brahman_F1_hybrid_chromosome_9_whole_genome_shotgun_sequence
DAAA02027231.1_141230-141655_TPA_asm__Bos_taurus_breed_Hereford_chromosome_9_7180001722225_whole_genome_shotgun_sequence
AAFC05014141.1_146581-147006_Bos_taurus_breed_Hereford_Chromosome9_Contig1311_whole_genome_shotgun_sequence
VLPI01000002.1_90728465-90728889_Bos_grunniens_x_Bos_taurus_isolate_Esperanza_chromosome_9_whole_genome_shotgun_sequence
JPYT01775150.1_11944-12369_Bison_bison_bison_isolate_TAMUID_2011002044_ctg553381_whole_genome_shotgun_sequence
AGFL01088728.1_58969-59394_Bos_indicus_breed_Nelore_isolate_QUIL7308_chr9_contig7539_whole_genome_shotgun_sequence
_R_VBQZ03000013.1_1982864-1983289_Bos_mutus_breed_Datong_Yak_isolate_WY2019_wild_yak_scaffold13_whole_genome_shotgun_sequence
VCEA01000001.1_694418067-694418465_Muntiacus_muntjak_isolate_UTSW_UCB_Mm_chromosome_1_whole_genome_shotgun_sequence
RAWU01000014.1_142652491-142652916_Giraffa_tippelskirchi_isolate_OR1865_chromosome_9_whole_genome_shotgun_sequence
_R_marine_NIPP01002270.1_132189-132717_Eschrichtius_robustus_isolate_ER-14-168_kmer60-min500.11761363_whole_genome_shotgun_sequence
marine_RJWK010000758.1_32330-32509_Platanista_minor_isolate_BS66_PlaMin_flattened_line_1512_whole_genome_shotgun_sequence
_R_marine_RYZJ01001975.1_12765403-12765931_Megaptera_novaeangliae_breed_North_Atlantic_isolate_ASU_GRON-1_ScjyzU5_149.1_whole_genome_shotgun_sequence
marine_VNFD01010057.1_1022-1549_Balaenoptera_musculus_isolate_JJ_BM4_2016_0621_000087F_028_arrow_arrow_whole_genome_shotgun_sequence
_R_marine_RJWP010012363.1_40803-41331_Eubalaena_japonica_isolate_BS61_EubJap_flattened_line_24692_whole_genome_shotgun_sequence
marine_RJWK010000758.1_31791-32153_Platanista_minor_isolate_BS66_PlaMin_flattened_line_1512_whole_genome_shotgun_sequence
_R_marine_RJWL010022863.1_24996-25522_Kogia_breviceps_isolate_BS64_KogBre_flattened_line_45718_whole_genome_shotgun_sequence
_R_PVJP02910385.1_1977006-1977532_Hippopotamus_amphibius_isolate_BS01_ScHSfme_924856HRSCAF_982656_whole_genome_shotgun_sequence
0.02

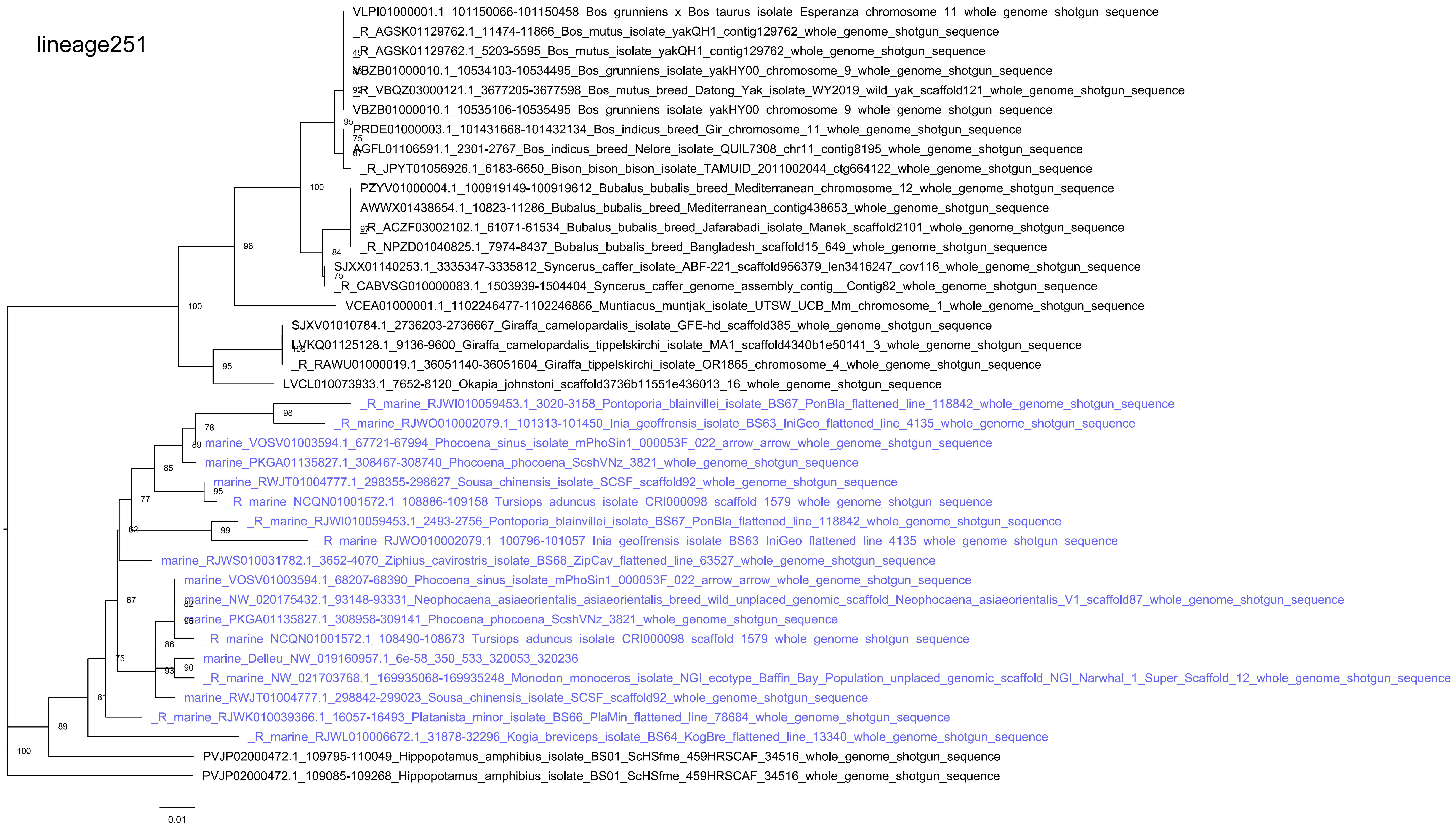
lineage251
VLPI01000001.1_101150066-101150458_Bos_grunniens_x_Bos_taurus_isolate_Esperanza_chromosome_11_whole_genome_shotgun_sequence
_R_AGSK01129762.1_11474-11866_Bos_mutus_isolate_yakQH1_contig129762_whole_genome_shotgun_sequence
_R_AGSK01129762.1_5203-5595_Bos_mutus_isolate_yakQH1_contig129762_whole_genome_shotgun_sequence
VBZB01000010.1_10534103-10534495_Bos_grunniens_isolate_yakHY00_chromosome_9_whole_genome_shotgun_sequence
_R_VBQZ03000121.1_3677205-3677598_Bos_mutus_breed_Datong_Yak_isolate_WY2019_wild_yak_scaffold121_whole_genome_shotgun_sequence
VBZB01000010.1_10535106-10535495_Bos_grunniens_isolate_yakHY00_chromosome_9_whole_genome_shotgun_sequence
PRDE01000003.1_101431668-101432134_Bos_indicus_breed_Gir_chromosome_11_whole_genome_shotgun_sequence
AGFL01106591.1_2301-2767_Bos_indicus_breed_Nelore_isolate_QUIL7308_chr11_contig8195_whole_genome_shotgun_sequence
_R_JPYT01056926.1_6183-6650_Bison_bison_bison_isolate_TAMUID_2011002044_ctg664122_whole_genome_shotgun_sequence
PZYV01000004.1_100919149-100919612_Bubalus_bubalis_breed_Mediterranean_chromosome_12_whole_genome_shotgun_sequence
AWWX01438654.1_10823-11286_Bubalus_bubalis_breed_Mediterranean_contig438653_whole_genome_shotgun_sequence
_R_ACZF03002102.1_61071-61534_Bubalus_bubalis_breed_Jafarabadi_isolate_Manek_scaffold2101_whole_genome_shotgun_sequence
_R_NPZD01040825.1_7974-8437_Bubalus_bubalis_breed_Bangladesh_scaffold15_649_whole_genome_shotgun_sequence
SJXX01140253.1_3335347-3335812_Syncerus_caffer_isolate_ABF-221_scaffold956379_len3416247_cov116_whole_genome_shotgun_sequence
_R_CABVSG010000083.1_1503939-1504404_Syncerus_caffer_genome_assembly_contig__Contig82_whole_genome_shotgun_sequence
VCEA01000001.1_1102246477-1102246866_Muntiacus_muntjak_isolate_UTSW_UCB_Mm_chromosome_1_whole_genome_shotgun_sequence
SJXV01010784.1_2736203-2736667_Giraffa_camelopardalis_isolate_GFE-hd_scaffold385_whole_genome_shotgun_sequence
LVKQ01125128.1_9136-9600_Giraffa_camelopardalis_tippelskirchi_isolate_MA1_scaffold4340b1e50141_3_whole_genome_shotgun_sequence
_R_RAWU01000019.1_36051140-36051604_Giraffa_tippelskirchi_isolate_OR1865_chromosome_4_whole_genome_shotgun_sequence
LVCL010073933.1_7652-8120_Okapia_johnstoni_scaffold3736b11551e436013_16_whole_genome_shotgun_sequence
_R_marine_RJWI010059453.1_3020-3158_Pontoporia_blainvillei_isolate_BS67_PonBla_flattened_line_118842_whole_genome_shotgun_sequence
_R_marine_RJWO010002079.1_101313-101450_Inia_geoffrensis_isolate_BS63_IniGeo_flattened_line_4135_whole_genome_shotgun_sequence
marine_VOSV01003594.1_67721-67994_Phocoena_sinus_isolate_mPhoSin1_000053F_022_arrow_arrow_whole_genome_shotgun_sequence
marine_PKGA01135827.1_308467-308740_Phocoena_phocoena_ScshVNz_3821_whole_genome_shotgun_sequence
marine_RWJT01004777.1_298355-298627_Sousa_chinensis_isolate_SCSF_scaffold92_whole_genome_shotgun_sequence
_R_marine_NCQN01001572.1_108886-109158_Tursiops_aduncus_isolate_CRI000098_scaffold_1579_whole_genome_shotgun_sequence
_R_marine_RJWI010059453.1_2493-2756_Pontoporia_blainvillei_isolate_BS67_PonBla_flattened_line_118842_whole_genome_shotgun_sequence
_R_marine_RJWO010002079.1_100796-101057_Inia_geoffrensis_isolate_BS63_IniGeo_flattened_line_4135_whole_genome_shotgun_sequence
marine_RJWS010031782.1_3652-4070_Ziphius_cavirostris_isolate_BS68_ZipCav_flattened_line_63527_whole_genome_shotgun_sequence
marine_VOSV01003594.1_68207-68390_Phocoena_sinus_isolate_mPhoSin1_000053F_022_arrow_arrow_whole_genome_shotgun_sequence
marine_NW_020175432.1_93148-93331_Neophocaena_asiaeorientalis_asiaeorientalis_breed_wild_unplaced_genomic_scaffold_Neophocaena_asiaeorientalis_V1_scaffold87_whole_genome_shotgun_sequence
marine_PKGA01135827.1_308958-309141_Phocoena_phocoena_ScshVNz_3821_whole_genome_shotgun_sequence
_R_marine_NCQN01001572.1_108490-108673_Tursiops_aduncus_isolate_CRI000098_scaffold_1579_whole_genome_shotgun_sequence
marine_Delleu_NW_019160957.1_6e-58_350_533_320053_320236
_R_marine_NW_021703768.1_169935068-169935248_Monodon_monoceros_isolate_NGI_ecotype_Baffin_Bay_Population_unplaced_genomic_scaffold_NGI_Narwhal_1_Super_Scaffold_12_whole_genome_shotgun_sequence
marine_RWJT01004777.1_298842-299023_Sousa_chinensis_isolate_SCSF_scaffold92_whole_genome_shotgun_sequence
_R_marine_RJWK010039366.1_16057-16493_Platanista_minor_isolate_BS66_PlaMin_flattened_line_78684_whole_genome_shotgun_sequence
_R_marine_RJWL010006672.1_31878-32296_Kogia_breviceps_isolate_BS64_KogBre_flattened_line_13340_whole_genome_shotgun_sequence
PVJP02000472.1_109795-110049_Hippopotamus_amphibius_isolate_BS01_ScHSfme_459HRSCAF_34516_whole_genome_shotgun_sequence
PVJP02000472.1_109085-109268_Hippopotamus_amphibius_isolate_BS01_ScHSfme_459HRSCAF_34516_whole_genome_shotgun_sequence
0.01

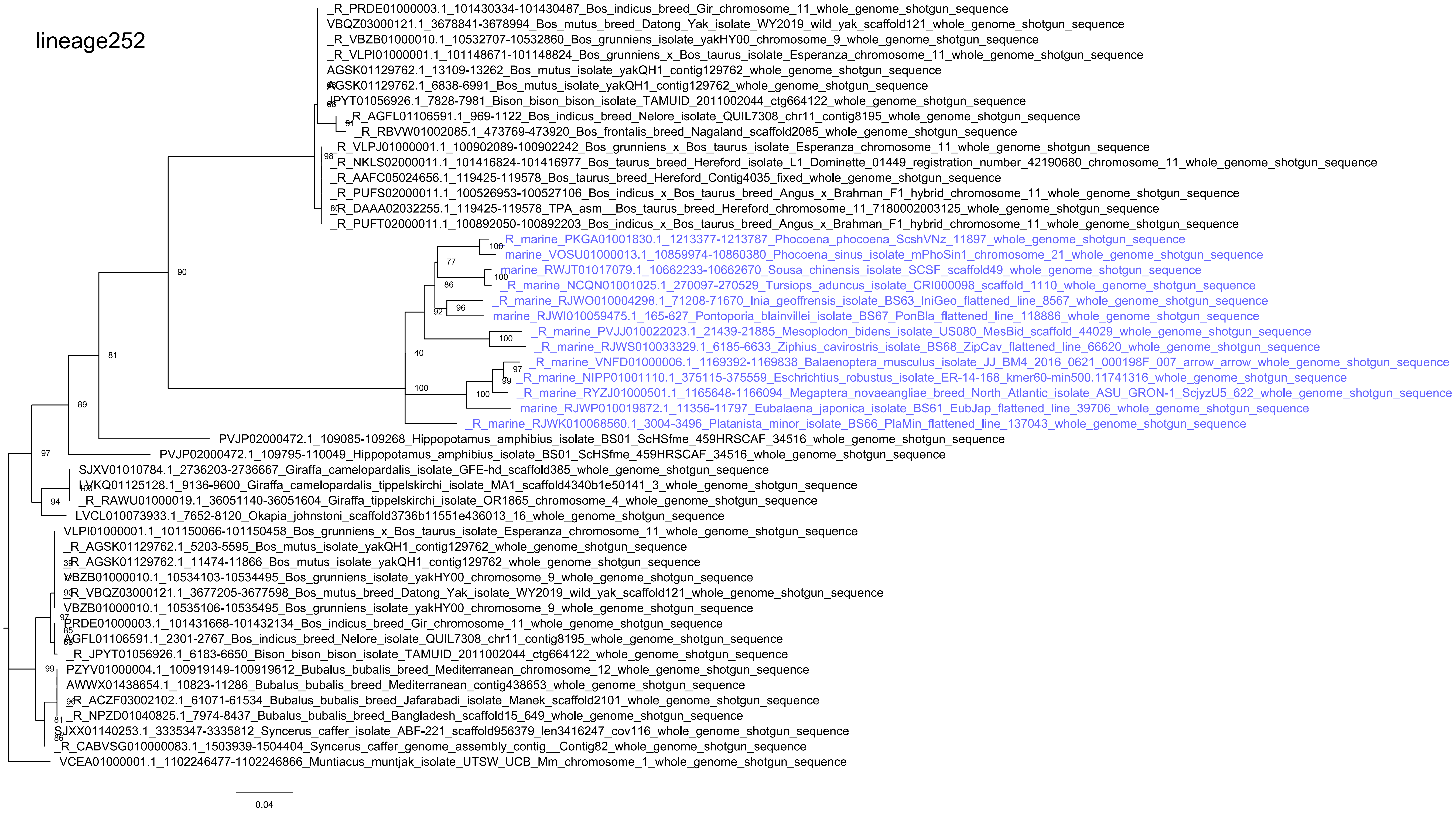
lineage252
_R_PRDE01000003.1_101430334-101430487_Bos_indicus_breed_Gir_chromosome_11_whole_genome_shotgun_sequence
VBQZ03000121.1_3678841-3678994_Bos_mutus_breed_Datong_Yak_isolate_WY2019_wild_yak_scaffold121_whole_genome_shotgun_sequence
_R_VBZB01000010.1_10532707-10532860_Bos_grunniens_isolate_yakHY00_chromosome_9_whole_genome_shotgun_sequence
_R_VLPI01000001.1_101148671-101148824_Bos_grunniens_x_Bos_taurus_isolate_Esperanza_chromosome_11_whole_genome_shotgun_sequence
AGSK01129762.1_13109-13262_Bos_mutus_isolate_yakQH1_contig129762_whole_genome_shotgun_sequence
AGSK01129762.1_6838-6991_Bos_mutus_isolate_yakQH1_contig129762_whole_genome_shotgun_sequence
JPYT01056926.1_7828-7981_Bison_bison_bison_isolate_TAMUID_2011002044_ctg664122_whole_genome_shotgun_sequence
_R_AGFL01106591.1_969-1122_Bos_indicus_breed_Nelore_isolate_QUIL7308_chr11_contig8195_whole_genome_shotgun_sequence
_R_RBVW01002085.1_473769-473920_Bos_frontalis_breed_Nagaland_scaffold2085_whole_genome_shotgun_sequence
_R_VLPJ01000001.1_100902089-100902242_Bos_grunniens_x_Bos_taurus_isolate_Esperanza_chromosome_11_whole_genome_shotgun_sequence
_R_NKLS02000011.1_101416824-101416977_Bos_taurus_breed_Hereford_isolate_L1_Dominette_01449_registration_number_42190680_chromosome_11_whole_genome_shotgun_sequence
_R_AAFC05024656.1_119425-119578_Bos_taurus_breed_Hereford_Contig4035_fixed_whole_genome_shotgun_sequence
_R_PUFS02000011.1_100526953-100527106_Bos_indicus_x_Bos_taurus_breed_Angus_x_Brahman_F1_hybrid_chromosome_11_whole_genome_shotgun_sequence
_R_DAAA02032255.1_119425-119578_TPA_asm__Bos_taurus_breed_Hereford_chromosome_11_7180002003125_whole_genome_shotgun_sequence
_R_PUFT02000011.1_100892050-100892203_Bos_indicus_x_Bos_taurus_breed_Angus_x_Brahman_F1_hybrid_chromosome_11_whole_genome_shotgun_sequence
_R_marine_PKGA01001830.1_1213377-1213787_Phocoena_phocoena_ScshVNz_11897_whole_genome_shotgun_sequence
marine_VOSU01000013.1_10859974-10860380_Phocoena_sinus_isolate_mPhoSin1_chromosome_21_whole_genome_shotgun_sequence
marine_RWJT01017079.1_10662233-10662670_Sousa_chinensis_isolate_SCSF_scaffold49_whole_genome_shotgun_sequence
_R_marine_NCQN01001025.1_270097-270529_Tursiops_aduncus_isolate_CRI000098_scaffold_1110_whole_genome_shotgun_sequence
_R_marine_RJWO010004298.1_71208-71670_Inia_geoffrensis_isolate_BS63_IniGeo_flattened_line_8567_whole_genome_shotgun_sequence
marine_RJWI010059475.1_165-627_Pontoporia_blainvillei_isolate_BS67_PonBla_flattened_line_118886_whole_genome_shotgun_sequence
_R_marine_PVJJ010022023.1_21439-21885_Mesoplodon_bidens_isolate_US080_MesBid_scaffold_44029_whole_genome_shotgun_sequence
_R_marine_RJWS010033329.1_6185-6633_Ziphius_cavirostris_isolate_BS68_ZipCav_flattened_line_66620_whole_genome_shotgun_sequence
_R_marine_VNFD01000006.1_1169392-1169838_Balaenoptera_musculus_isolate_JJ_BM4_2016_0621_000198F_007_arrow_arrow_whole_genome_shotgun_sequence
_R_marine_NIPP01001110.1_375115-375559_Eschrichtius_robustus_isolate_ER-14-168_kmer60-min500.11741316_whole_genome_shotgun_sequence
_R_marine_RYZJ01000501.1_1165648-1166094_Megaptera_novaeangliae_breed_North_Atlantic_isolate_ASU_GRON-1_ScjyzU5_622_whole_genome_shotgun_sequence
marine_RJWP010019872.1_11356-11797_Eubalaena_japonica_isolate_BS61_EubJap_flattened_line_39706_whole_genome_shotgun_sequence
_R_marine_RJWK010068560.1_3004-3496_Platanista_minor_isolate_BS66_PlaMin_flattened_line_137043_whole_genome_shotgun_sequence
PVJP02000472.1_109085-109268_Hippopotamus_amphibius_isolate_BS01_ScHSfme_459HRSCAF_34516_whole_genome_shotgun_sequence
PVJP02000472.1_109795-110049_Hippopotamus_amphibius_isolate_BS01_ScHSfme_459HRSCAF_34516_whole_genome_shotgun_sequence
SJXV01010784.1_2736203-2736667_Giraffa_camelopardalis_isolate_GFE-hd_scaffold385_whole_genome_shotgun_sequence
LVKQ01125128.1_9136-9600_Giraffa_camelopardalis_tippelskirchi_isolate_MA1_scaffold4340b1e50141_3_whole_genome_shotgun_sequence
_R_RAWU01000019.1_36051140-36051604_Giraffa_tippelskirchi_isolate_OR1865_chromosome_4_whole_genome_shotgun_sequence
LVCL010073933.1_7652-8120_Okapia_johnstoni_scaffold3736b11551e436013_16_whole_genome_shotgun_sequence
VLPI01000001.1_101150066-101150458_Bos_grunniens_x_Bos_taurus_isolate_Esperanza_chromosome_11_whole_genome_shotgun_sequence
_R_AGSK01129762.1_5203-5595_Bos_mutus_isolate_yakQH1_contig129762_whole_genome_shotgun_sequence
_R_AGSK01129762.1_11474-11866_Bos_mutus_isolate_yakQH1_contig129762_whole_genome_shotgun_sequence
VBZB01000010.1_10534103-10534495_Bos_grunniens_isolate_yakHY00_chromosome_9_whole_genome_shotgun_sequence
_R_VBQZ03000121.1_3677205-3677598_Bos_mutus_breed_Datong_Yak_isolate_WY2019_wild_yak_scaffold121_whole_genome_shotgun_sequence
VBZB01000010.1_10535106-10535495_Bos_grunniens_isolate_yakHY00_chromosome_9_whole_genome_shotgun_sequence
PRDE01000003.1_101431668-101432134_Bos_indicus_breed_Gir_chromosome_11_whole_genome_shotgun_sequence
AGFL01106591.1_2301-2767_Bos_indicus_breed_Nelore_isolate_QUIL7308_chr11_contig8195_whole_genome_shotgun_sequence
_R_JPYT01056926.1_6183-6650_Bison_bison_bison_isolate_TAMUID_2011002044_ctg664122_whole_genome_shotgun_sequence
PZYV01000004.1_100919149-100919612_Bubalus_bubalis_breed_Mediterranean_chromosome_12_whole_genome_shotgun_sequence
AWWX01438654.1_10823-11286_Bubalus_bubalis_breed_Mediterranean_contig438653_whole_genome_shotgun_sequence
_R_ACZF03002102.1_61071-61534_Bubalus_bubalis_breed_Jafarabadi_isolate_Manek_scaffold2101_whole_genome_shotgun_sequence
_R_NPZD01040825.1_7974-8437_Bubalus_bubalis_breed_Bangladesh_scaffold15_649_whole_genome_shotgun_sequence
SJXX01140253.1_3335347-3335812_Syncerus_caffer_isolate_ABF-221_scaffold956379_len3416247_cov116_whole_genome_shotgun_sequence
_R_CABVSG010000083.1_1503939-1504404_Syncerus_caffer_genome_assembly_contig__Contig82_whole_genome_shotgun_sequence
VCEA01000001.1_1102246477-1102246866_Muntiacus_muntjak_isolate_UTSW_UCB_Mm_chromosome_1_whole_genome_shotgun_sequence
0.04

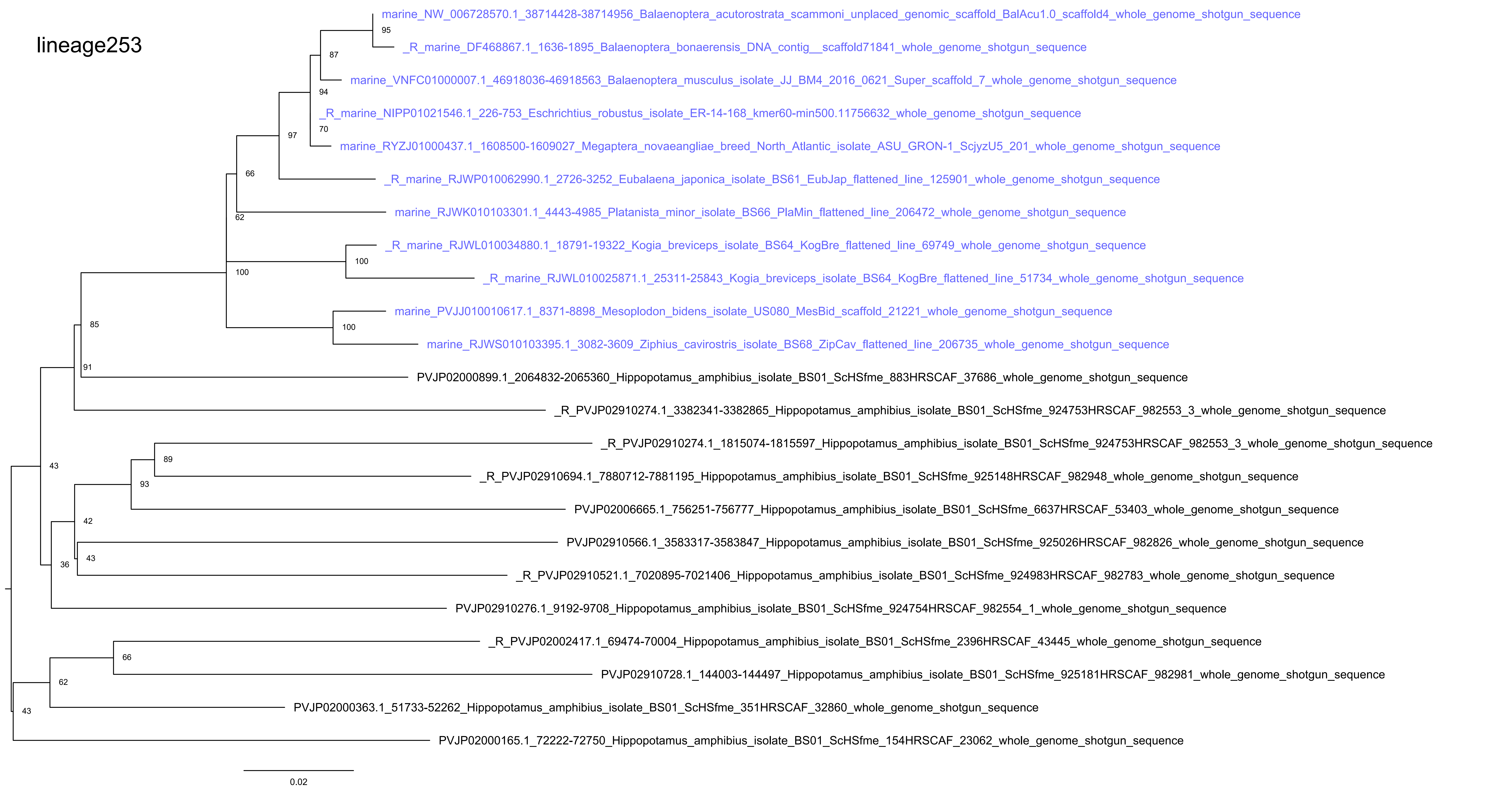
lineage253
marine_NW_006728570.1_38714428-38714956_Balaenoptera_acutorostrata_scammoni_unplaced_genomic_scaffold_BalAcu1.0_scaffold4_whole_genome_shotgun_sequence
_R_marine_DF468867.1_1636-1895_Balaenoptera_bonaerensis_DNA_contig__scaffold71841_whole_genome_shotgun_sequence
marine_VNFC01000007.1_46918036-46918563_Balaenoptera_musculus_isolate_JJ_BM4_2016_0621_Super_scaffold_7_whole_genome_shotgun_sequence
_R_marine_NIPP01021546.1_226-753_Eschrichtius_robustus_isolate_ER-14-168_kmer60-min500.11756632_whole_genome_shotgun_sequence
marine_RYZJ01000437.1_1608500-1609027_Megaptera_novaeangliae_breed_North_Atlantic_isolate_ASU_GRON-1_ScjyzU5_201_whole_genome_shotgun_sequence
_R_marine_RJWP010062990.1_2726-3252_Eubalaena_japonica_isolate_BS61_EubJap_flattened_line_125901_whole_genome_shotgun_sequence
marine_RJWK010103301.1_4443-4985_Platanista_minor_isolate_BS66_PlaMin_flattened_line_206472_whole_genome_shotgun_sequence
_R_marine_RJWL010034880.1_18791-19322_Kogia_breviceps_isolate_BS64_KogBre_flattened_line_69749_whole_genome_shotgun_sequence
_R_marine_RJWL010025871.1_25311-25843_Kogia_breviceps_isolate_BS64_KogBre_flattened_line_51734_whole_genome_shotgun_sequence
marine_PVJJ010010617.1_8371-8898_Mesoplodon_bidens_isolate_US080_MesBid_scaffold_21221_whole_genome_shotgun_sequence
marine_RJWS010103395.1_3082-3609_Ziphius_cavirostris_isolate_BS68_ZipCav_flattened_line_206735_whole_genome_shotgun_sequence
PVJP02000899.1_2064832-2065360_Hippopotamus_amphibius_isolate_BS01_ScHSfme_883HRSCAF_37686_whole_genome_shotgun_sequence
_R_PVJP02910274.1_3382341-3382865_Hippopotamus_amphibius_isolate_BS01_ScHSfme_924753HRSCAF_982553_3_whole_genome_shotgun_sequence
_R_PVJP02910274.1_1815074-1815597_Hippopotamus_amphibius_isolate_BS01_ScHSfme_924753HRSCAF_982553_3_whole_genome_shotgun_sequence
_R_PVJP02910694.1_7880712-7881195_Hippopotamus_amphibius_isolate_BS01_ScHSfme_925148HRSCAF_982948_whole_genome_shotgun_sequence
PVJP02006665.1_756251-756777_Hippopotamus_amphibius_isolate_BS01_ScHSfme_6637HRSCAF_53403_whole_genome_shotgun_sequence
PVJP02910566.1_3583317-3583847_Hippopotamus_amphibius_isolate_BS01_ScHSfme_925026HRSCAF_982826_whole_genome_shotgun_sequence
_R_PVJP02910521.1_7020895-7021406_Hippopotamus_amphibius_isolate_BS01_ScHSfme_924983HRSCAF_982783_whole_genome_shotgun_sequence
PVJP02910276.1_9192-9708_Hippopotamus_amphibius_isolate_BS01_ScHSfme_924754HRSCAF_982554_1_whole_genome_shotgun_sequence
_R_PVJP02002417.1_69474-70004_Hippopotamus_amphibius_isolate_BS01_ScHSfme_2396HRSCAF_43445_whole_genome_shotgun_sequence
PVJP02910728.1_144003-144497_Hippopotamus_amphibius_isolate_BS01_ScHSfme_925181HRSCAF_982981_whole_genome_shotgun_sequence
PVJP02000363.1_51733-52262_Hippopotamus_amphibius_isolate_BS01_ScHSfme_351HRSCAF_32860_whole_genome_shotgun_sequence
PVJP02000165.1_72222-72750_Hippopotamus_amphibius_isolate_BS01_ScHSfme_154HRSCAF_23062_whole_genome_shotgun_sequence
95
87
94
70
97
66
62
100
100
100
85
91
43
89
93
42
43
36
66
62
43
0.02

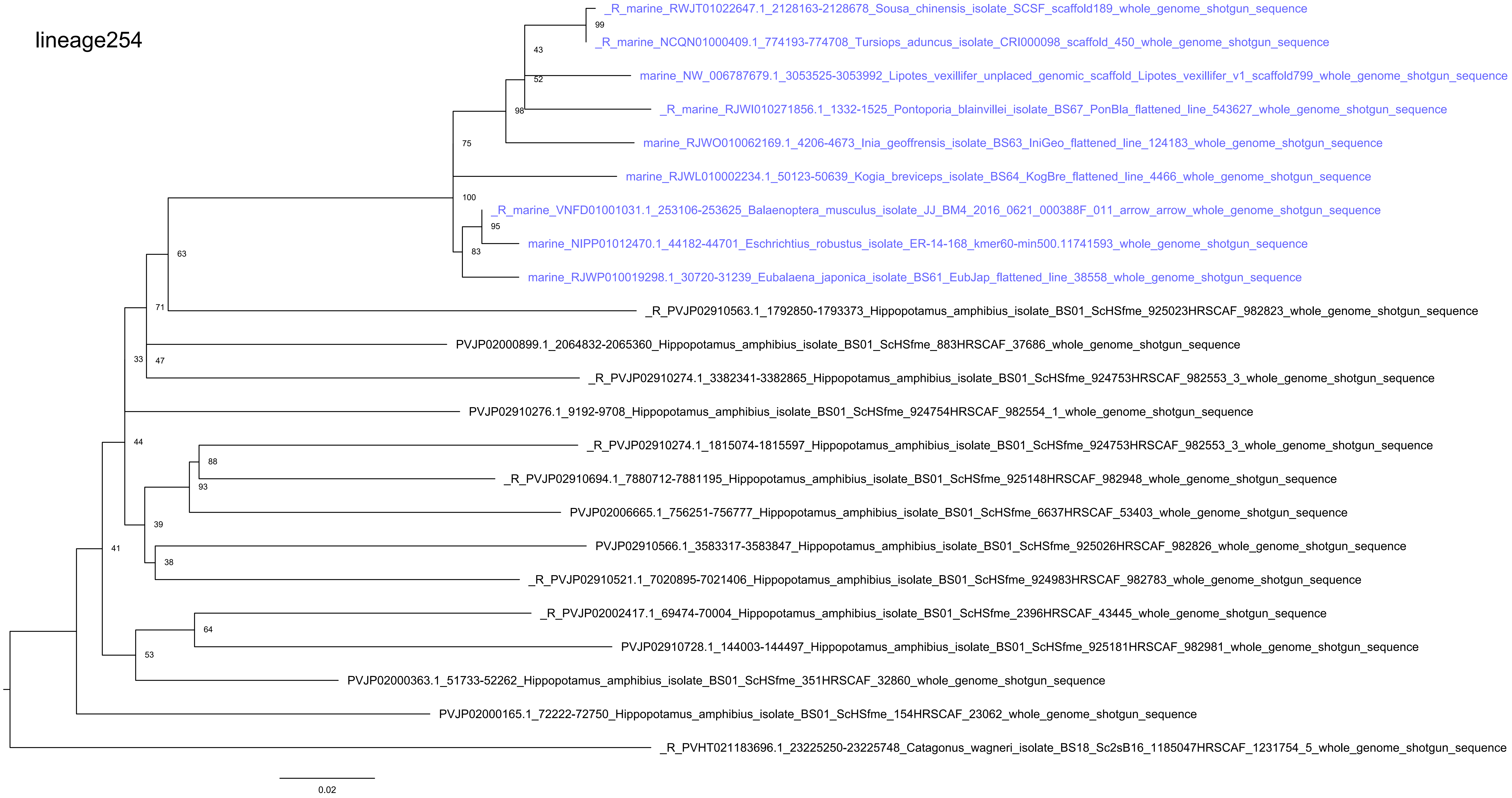
lineage254
_R_marine_RWJT01022647.1_2128163-2128678_Sousa_chinensis_isolate_SCSF_scaffold189_whole_genome_shotgun_sequence
99
_R_marine_NCQN01000409.1_774193-774708_Tursiops_aduncus_isolate_CRI000098_scaffold_450_whole_genome_shotgun_sequence
43
marine_NW_006787679.1_3053525-3053992_Lipotes_vexillifer_unplaced_genomic_scaffold_Lipotes_vexillifer_v1_scaffold799_whole_genome_shotgun_sequence
52
_R_marine_RJWI010271856.1_1332-1525_Pontoporia_blainvillei_isolate_BS67_PonBla_flattened_line_543627_whole_genome_shotgun_sequence
98
marine_RJWO010062169.1_4206-4673_Inia_geoffrensis_isolate_BS63_IniGeo_flattened_line_124183_whole_genome_shotgun_sequence
75
marine_RJWL010002234.1_50123-50639_Kogia_breviceps_isolate_BS64_KogBre_flattened_line_4466_whole_genome_shotgun_sequence
100
_R_marine_VNFD01001031.1_253106-253625_Balaenoptera_musculus_isolate_JJ_BM4_2016_0621_000388F_011_arrow_arrow_whole_genome_shotgun_sequence
95
marine_NIPP01012470.1_44182-44701_Eschrichtius_robustus_isolate_ER-14-168_kmer60-min500.11741593_whole_genome_shotgun_sequence
83
marine_RJWP010019298.1_30720-31239_Eubalaena_japonica_isolate_BS61_EubJap_flattened_line_38558_whole_genome_shotgun_sequence
63
_R_PVJP02910563.1_1792850-1793373_Hippopotamus_amphibius_isolate_BS01_ScHSfme_925023HRSCAF_982823_whole_genome_shotgun_sequence
71
PVJP02000899.1_2064832-2065360_Hippopotamus_amphibius_isolate_BS01_ScHSfme_883HRSCAF_37686_whole_genome_shotgun_sequence
33
47
_R_PVJP02910274.1_3382341-3382865_Hippopotamus_amphibius_isolate_BS01_ScHSfme_924753HRSCAF_982553_3_whole_genome_shotgun_sequence
PVJP02910276.1_9192-9708_Hippopotamus_amphibius_isolate_BS01_ScHSfme_924754HRSCAF_982554_1_whole_genome_shotgun_sequence
44
_R_PVJP02910274.1_1815074-1815597_Hippopotamus_amphibius_isolate_BS01_ScHSfme_924753HRSCAF_982553_3_whole_genome_shotgun_sequence
88
_R_PVJP02910694.1_7880712-7881195_Hippopotamus_amphibius_isolate_BS01_ScHSfme_925148HRSCAF_982948_whole_genome_shotgun_sequence
93
PVJP02006665.1_756251-756777_Hippopotamus_amphibius_isolate_BS01_ScHSfme_6637HRSCAF_53403_whole_genome_shotgun_sequence
39
PVJP02910566.1_3583317-3583847_Hippopotamus_amphibius_isolate_BS01_ScHSfme_925026HRSCAF_982826_whole_genome_shotgun_sequence
41
38
_R_PVJP02910521.1_7020895-7021406_Hippopotamus_amphibius_isolate_BS01_ScHSfme_924983HRSCAF_982783_whole_genome_shotgun_sequence
_R_PVJP02002417.1_69474-70004_Hippopotamus_amphibius_isolate_BS01_ScHSfme_2396HRSCAF_43445_whole_genome_shotgun_sequence
64
PVJP02910728.1_144003-144497_Hippopotamus_amphibius_isolate_BS01_ScHSfme_925181HRSCAF_982981_whole_genome_shotgun_sequence
53
PVJP02000363.1_51733-52262_Hippopotamus_amphibius_isolate_BS01_ScHSfme_351HRSCAF_32860_whole_genome_shotgun_sequence
PVJP02000165.1_72222-72750_Hippopotamus_amphibius_isolate_BS01_ScHSfme_154HRSCAF_23062_whole_genome_shotgun_sequence
_R_PVHT021183696.1_23225250-23225748_Catagonus_wagneri_isolate_BS18_Sc2sB16_1185047HRSCAF_1231754_5_whole_genome_shotgun_sequence
0.02

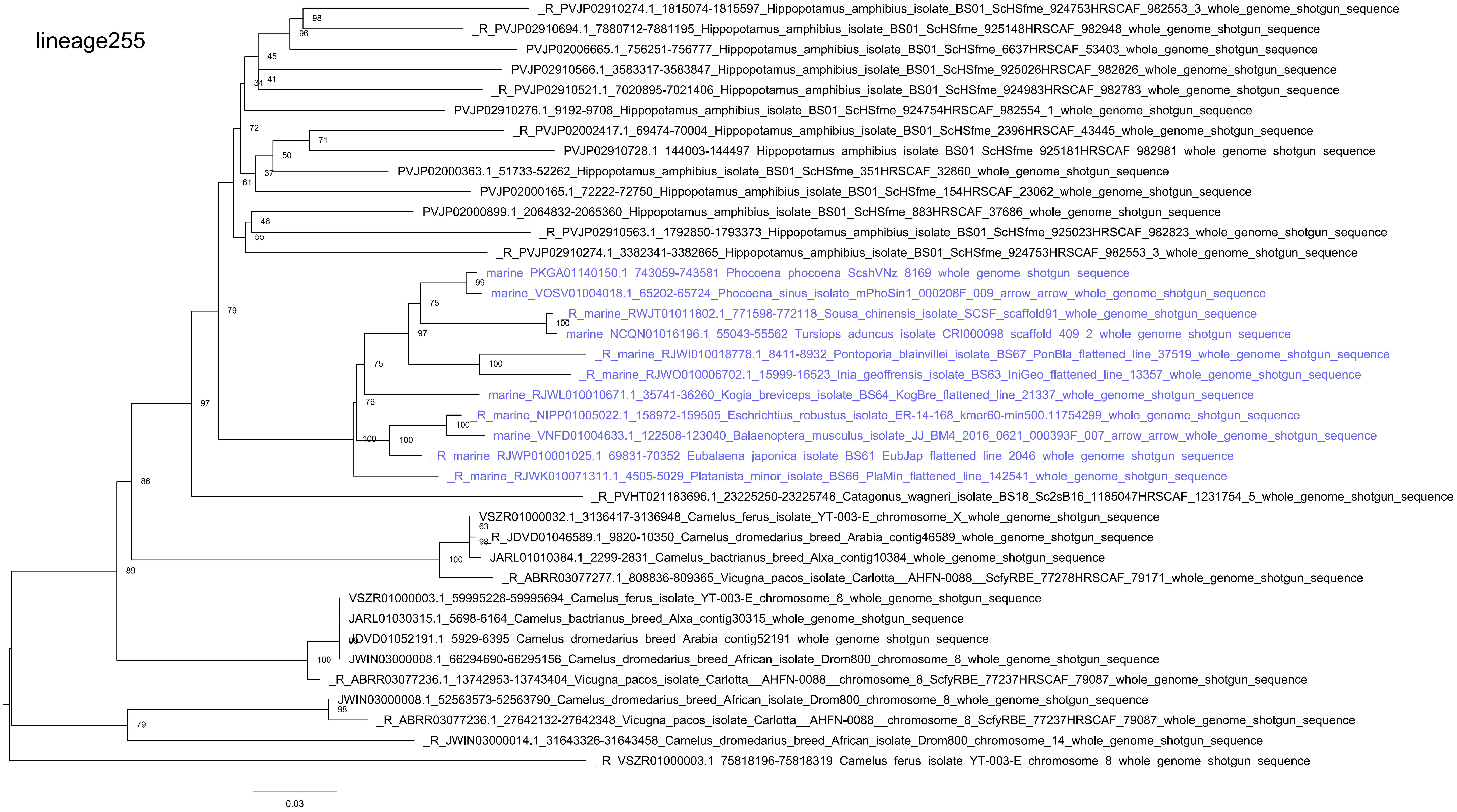
lineage255
_R_PVJP02910274.1_1815074-1815597_Hippopotamus_amphibius_isolate_BS01_ScHSfme_924753HRSCAF_982553_3_whole_genome_shotgun_sequence
_R_PVJP02910694.1_7880712-7881195_Hippopotamus_amphibius_isolate_BS01_ScHSfme_925148HRSCAF_982948_whole_genome_shotgun_sequence
PVJP02006665.1_756251-756777_Hippopotamus_amphibius_isolate_BS01_ScHSfme_6637HRSCAF_53403_whole_genome_shotgun_sequence
PVJP02910566.1_3583317-3583847_Hippopotamus_amphibius_isolate_BS01_ScHSfme_925026HRSCAF_982826_whole_genome_shotgun_sequence
_R_PVJP02910521.1_7020895-7021406_Hippopotamus_amphibius_isolate_BS01_ScHSfme_924983HRSCAF_982783_whole_genome_shotgun_sequence
PVJP02910276.1_9192-9708_Hippopotamus_amphibius_isolate_BS01_ScHSfme_924754HRSCAF_982554_1_whole_genome_shotgun_sequence
_R_PVJP02002417.1_69474-70004_Hippopotamus_amphibius_isolate_BS01_ScHSfme_2396HRSCAF_43445_whole_genome_shotgun_sequence
PVJP02910728.1_144003-144497_Hippopotamus_amphibius_isolate_BS01_ScHSfme_925181HRSCAF_982981_whole_genome_shotgun_sequence
PVJP02000363.1_51733-52262_Hippopotamus_amphibius_isolate_BS01_ScHSfme_351HRSCAF_32860_whole_genome_shotgun_sequence
PVJP02000165.1_72222-72750_Hippopotamus_amphibius_isolate_BS01_ScHSfme_154HRSCAF_23062_whole_genome_shotgun_sequence
PVJP02000899.1_2064832-2065360_Hippopotamus_amphibius_isolate_BS01_ScHSfme_883HRSCAF_37686_whole_genome_shotgun_sequence
_R_PVJP02910563.1_1792850-1793373_Hippopotamus_amphibius_isolate_BS01_ScHSfme_925023HRSCAF_982823_whole_genome_shotgun_sequence
_R_PVJP02910274.1_3382341-3382865_Hippopotamus_amphibius_isolate_BS01_ScHSfme_924753HRSCAF_982553_3_whole_genome_shotgun_sequence
marine_PKGA01140150.1_743059-743581_Phocoena_phocoena_ScshVNz_8169_whole_genome_shotgun_sequence
marine_VOSV01004018.1_65202-65724_Phocoena_sinus_isolate_mPhoSin1_000208F_009_arrow_arrow_whole_genome_shotgun_sequence
_R_marine_RWJT01011802.1_771598-772118_Sousa_chinensis_isolate_SCSF_scaffold91_whole_genome_shotgun_sequence
marine_NCQN01016196.1_55043-55562_Tursiops_aduncus_isolate_CRI000098_scaffold_409_2_whole_genome_shotgun_sequence
_R_marine_RJWI010018778.1_8411-8932_Pontoporia_blainvillei_isolate_BS67_PonBla_flattened_line_37519_whole_genome_shotgun_sequence
_R_marine_RJWO010006702.1_15999-16523_Inia_geoffrensis_isolate_BS63_IniGeo_flattened_line_13357_whole_genome_shotgun_sequence
marine_RJWL010010671.1_35741-36260_Kogia_breviceps_isolate_BS64_KogBre_flattened_line_21337_whole_genome_shotgun_sequence
_R_marine_NIPP01005022.1_158972-159505_Eschrichtius_robustus_isolate_ER-14-168_kmer60-min500.11754299_whole_genome_shotgun_sequence
marine_VNFD01004633.1_122508-123040_Balaenoptera_musculus_isolate_JJ_BM4_2016_0621_000393F_007_arrow_arrow_whole_genome_shotgun_sequence
_R_marine_RJWP010001025.1_69831-70352_Eubalaena_japonica_isolate_BS61_EubJap_flattened_line_2046_whole_genome_shotgun_sequence
_R_marine_RJWK010071311.1_4505-5029_Platanista_minor_isolate_BS66_PlaMin_flattened_line_142541_whole_genome_shotgun_sequence
_R_PVHT021183696.1_23225250-23225748_Catagonus_wagneri_isolate_BS18_Sc2sB16_1185047HRSCAF_1231754_5_whole_genome_shotgun_sequence
VSZR01000032.1_3136417-3136948_Camelus_ferus_isolate_YT-003-E_chromosome_X_whole_genome_shotgun_sequence
_R_JDVD01046589.1_9820-10350_Camelus_dromedarius_breed_Arabia_contig46589_whole_genome_shotgun_sequence
JARL01010384.1_2299-2831_Camelus_bactrianus_breed_Alxa_contig10384_whole_genome_shotgun_sequence
_R_ABRR03077277.1_808836-809365_Vicugna_pacos_isolate_Carlotta__AHFN-0088__ScfyRBE_77278HRSCAF_79171_whole_genome_shotgun_sequence
VSZR01000003.1_59995228-59995694_Camelus_ferus_isolate_YT-003-E_chromosome_8_whole_genome_shotgun_sequence
JARL01030315.1_5698-6164_Camelus_bactrianus_breed_Alxa_contig30315_whole_genome_shotgun_sequence
JDVD01052191.1_5929-6395_Camelus_dromedarius_breed_Arabia_contig52191_whole_genome_shotgun_sequence
JWIN03000008.1_66294690-66295156_Camelus_dromedarius_breed_African_isolate_Drom800_chromosome_8_whole_genome_shotgun_sequence
_R_ABRR03077236.1_13742953-13743404_Vicugna_pacos_isolate_Carlotta__AHFN-0088__chromosome_8_ScfyRBE_77237HRSCAF_79087_whole_genome_shotgun_sequence
JWIN03000008.1_52563573-52563790_Camelus_dromedarius_breed_African_isolate_Drom800_chromosome_8_whole_genome_shotgun_sequence
_R_ABRR03077236.1_27642132-27642348_Vicugna_pacos_isolate_Carlotta__AHFN-0088__chromosome_8_ScfyRBE_77237HRSCAF_79087_whole_genome_shotgun_sequence
_R_JWIN03000014.1_31643326-31643458_Camelus_dromedarius_breed_African_isolate_Drom800_chromosome_14_whole_genome_shotgun_sequence
_R_VSZR01000003.1_75818196-75818319_Camelus_ferus_isolate_YT-003-E_chromosome_8_whole_genome_shotgun_sequence
0.03

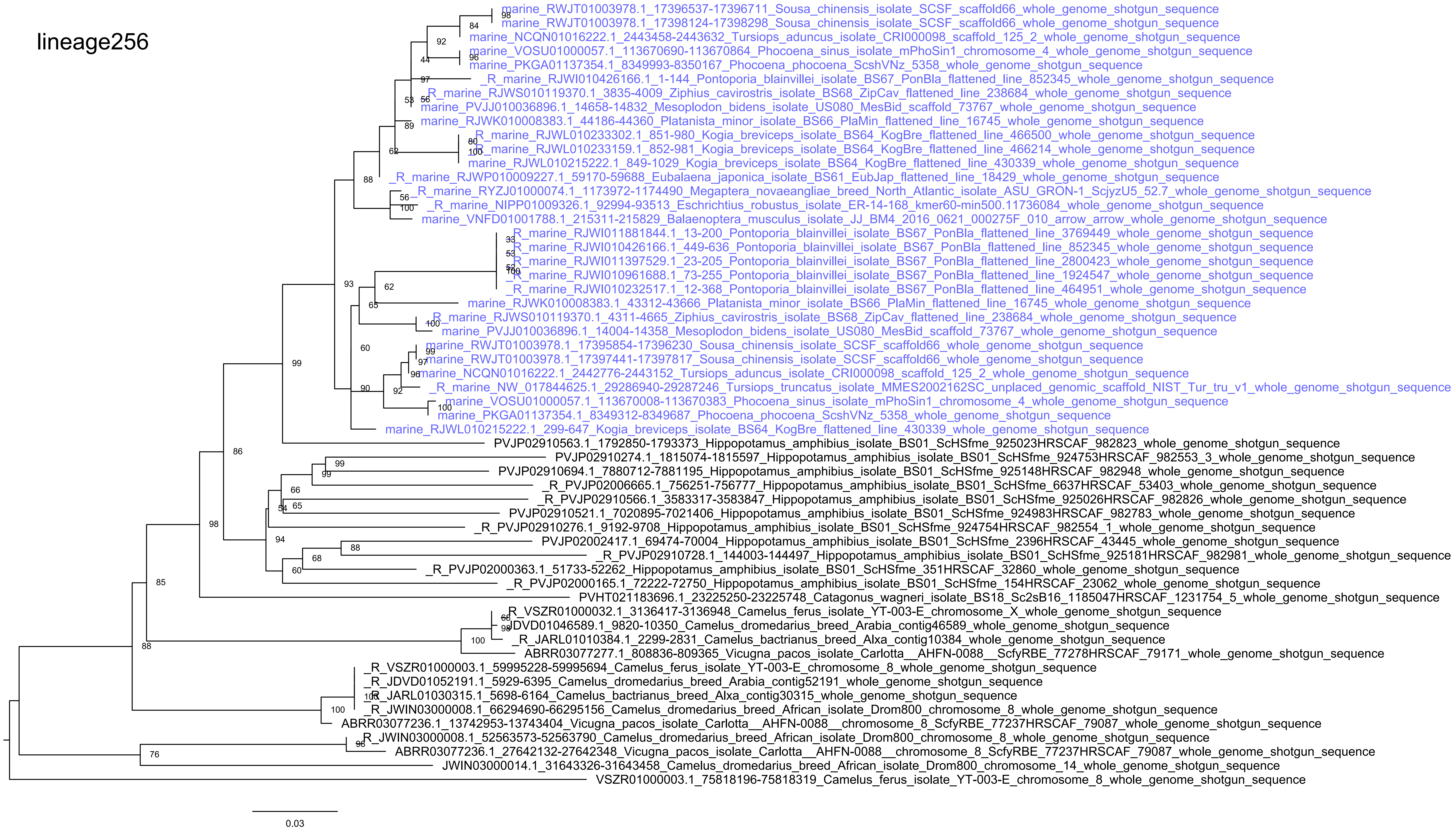
lineage256
marine_RWJT01003978.1_17396537-17396711_Sousa_chinensis_isolate_SCSF_scaffold66_whole_genome_shotgun_sequence
marine_RWJT01003978.1_17398124-17398298_Sousa_chinensis_isolate_SCSF_scaffold66_whole_genome_shotgun_sequence
marine_NCQN01016222.1_2443458-2443632_Tursiops_aduncus_isolate_CRI000098_scaffold_125_2_whole_genome_shotgun_sequence
marine_VOSU01000057.1_113670690-113670864_Phocoena_sinus_isolate_mPhoSin1_chromosome_4_whole_genome_shotgun_sequence
marine_PKGA01137354.1_8349993-8350167_Phocoena_phocoena_ScshVNz_5358_whole_genome_shotgun_sequence
_R_marine_RJWI010426166.1_1-144_Pontoporia_blainvillei_isolate_BS67_PonBla_flattened_line_852345_whole_genome_shotgun_sequence
_R_marine_RJWS010119370.1_3835-4009_Ziphius_cavirostris_isolate_BS68_ZipCav_flattened_line_238684_whole_genome_shotgun_sequence
marine_PVJJ010036896.1_14658-14832_Mesoplodon_bidens_isolate_US080_MesBid_scaffold_73767_whole_genome_shotgun_sequence
marine_RJWK010008383.1_44186-44360_Platanista_minor_isolate_BS66_PlaMin_flattened_line_16745_whole_genome_shotgun_sequence
_R_marine_RJWL010233302.1_851-980_Kogia_breviceps_isolate_BS64_KogBre_flattened_line_466500_whole_genome_shotgun_sequence
_R_marine_RJWL010233159.1_852-981_Kogia_breviceps_isolate_BS64_KogBre_flattened_line_466214_whole_genome_shotgun_sequence
marine_RJWL010215222.1_849-1029_Kogia_breviceps_isolate_BS64_KogBre_flattened_line_430339_whole_genome_shotgun_sequence
_R_marine_RJWP010009227.1_59170-59688_Eubalaena_japonica_isolate_BS61_EubJap_flattened_line_18429_whole_genome_shotgun_sequence
_R_marine_RYZJ01000074.1_1173972-1174490_Megaptera_novaeangliae_breed_North_Atlantic_isolate_ASU_GRON-1_ScjyzU5_52.7_whole_genome_shotgun_sequence
_R_marine_NIPP01009326.1_92994-93513_Eschrichtius_robustus_isolate_ER-14-168_kmer60-min500.11736084_whole_genome_shotgun_sequence
marine_VNFD01001788.1_215311-215829_Balaenoptera_musculus_isolate_JJ_BM4_2016_0621_000275F_010_arrow_arrow_whole_genome_shotgun_sequence
_R_marine_RJWI011881844.1_13-200_Pontoporia_blainvillei_isolate_BS67_PonBla_flattened_line_3769449_whole_genome_shotgun_sequence
_R_marine_RJWI010426166.1_449-636_Pontoporia_blainvillei_isolate_BS67_PonBla_flattened_line_852345_whole_genome_shotgun_sequence
_R_marine_RJWI011397529.1_23-205_Pontoporia_blainvillei_isolate_BS67_PonBla_flattened_line_2800423_whole_genome_shotgun_sequence
_R_marine_RJWI010961688.1_73-255_Pontoporia_blainvillei_isolate_BS67_PonBla_flattened_line_1924547_whole_genome_shotgun_sequence
_R_marine_RJWI010232517.1_12-368_Pontoporia_blainvillei_isolate_BS67_PonBla_flattened_line_464951_whole_genome_shotgun_sequence
marine_RJWK010008383.1_43312-43666_Platanista_minor_isolate_BS66_PlaMin_flattened_line_16745_whole_genome_shotgun_sequence
_R_marine_RJWS010119370.1_4311-4665_Ziphius_cavirostris_isolate_BS68_ZipCav_flattened_line_238684_whole_genome_shotgun_sequence
marine_PVJJ010036896.1_14004-14358_Mesoplodon_bidens_isolate_US080_MesBid_scaffold_73767_whole_genome_shotgun_sequence
marine_RWJT01003978.1_17395854-17396230_Sousa_chinensis_isolate_SCSF_scaffold66_whole_genome_shotgun_sequence
marine_RWJT01003978.1_17397441-17397817_Sousa_chinensis_isolate_SCSF_scaffold66_whole_genome_shotgun_sequence
marine_NCQN01016222.1_2442776-2443152_Tursiops_aduncus_isolate_CRI000098_scaffold_125_2_whole_genome_shotgun_sequence
_R_marine_NW_017844625.1_29286940-29287246_Tursiops_truncatus_isolate_MMES2002162SC_unplaced_genomic_scaffold_NIST_Tur_tru_v1_whole_genome_shotgun_sequence
marine_VOSU01000057.1_113670008-113670383_Phocoena_sinus_isolate_mPhoSin1_chromosome_4_whole_genome_shotgun_sequence
marine_PKGA01137354.1_8349312-8349687_Phocoena_phocoena_ScshVNz_5358_whole_genome_shotgun_sequence
marine_RJWL010215222.1_299-647_Kogia_breviceps_isolate_BS64_KogBre_flattened_line_430339_whole_genome_shotgun_sequence
PVJP02910563.1_1792850-1793373_Hippopotamus_amphibius_isolate_BS01_ScHSfme_925023HRSCAF_982823_whole_genome_shotgun_sequence
PVJP02910274.1_1815074-1815597_Hippopotamus_amphibius_isolate_BS01_ScHSfme_924753HRSCAF_982553_3_whole_genome_shotgun_sequence
PVJP02910694.1_7880712-7881195_Hippopotamus_amphibius_isolate_BS01_ScHSfme_925148HRSCAF_982948_whole_genome_shotgun_sequence
_R_PVJP02006665.1_756251-756777_Hippopotamus_amphibius_isolate_BS01_ScHSfme_6637HRSCAF_53403_whole_genome_shotgun_sequence
_R_PVJP02910566.1_3583317-3583847_Hippopotamus_amphibius_isolate_BS01_ScHSfme_925026HRSCAF_982826_whole_genome_shotgun_sequence
PVJP02910521.1_7020895-7021406_Hippopotamus_amphibius_isolate_BS01_ScHSfme_924983HRSCAF_982783_whole_genome_shotgun_sequence
_R_PVJP02910276.1_9192-9708_Hippopotamus_amphibius_isolate_BS01_ScHSfme_924754HRSCAF_982554_1_whole_genome_shotgun_sequence
PVJP02002417.1_69474-70004_Hippopotamus_amphibius_isolate_BS01_ScHSfme_2396HRSCAF_43445_whole_genome_shotgun_sequence
_R_PVJP02910728.1_144003-144497_Hippopotamus_amphibius_isolate_BS01_ScHSfme_925181HRSCAF_982981_whole_genome_shotgun_sequence
_R_PVJP02000363.1_51733-52262_Hippopotamus_amphibius_isolate_BS01_ScHSfme_351HRSCAF_32860_whole_genome_shotgun_sequence
_R_PVJP02000165.1_72222-72750_Hippopotamus_amphibius_isolate_BS01_ScHSfme_154HRSCAF_23062_whole_genome_shotgun_sequence
PVHT021183696.1_23225250-23225748_Catagonus_wagneri_isolate_BS18_Sc2sB16_1185047HRSCAF_1231754_5_whole_genome_shotgun_sequence
_R_VSZR01000032.1_3136417-3136948_Camelus_ferus_isolate_YT-003-E_chromosome_X_whole_genome_shotgun_sequence
JDVD01046589.1_9820-10350_Camelus_dromedarius_breed_Arabia_contig46589_whole_genome_shotgun_sequence
_R_JARL01010384.1_2299-2831_Camelus_bactrianus_breed_Alxa_contig10384_whole_genome_shotgun_sequence
ABRR03077277.1_808836-809365_Vicugna_pacos_isolate_Carlotta__AHFN-0088__ScfyRBE_77278HRSCAF_79171_whole_genome_shotgun_sequence
_R_VSZR01000003.1_59995228-59995694_Camelus_ferus_isolate_YT-003-E_chromosome_8_whole_genome_shotgun_sequence
_R_JDVD01052191.1_5929-6395_Camelus_dromedarius_breed_Arabia_contig52191_whole_genome_shotgun_sequence
_R_JARL01030315.1_5698-6164_Camelus_bactrianus_breed_Alxa_contig30315_whole_genome_shotgun_sequence
_R_JWIN03000008.1_66294690-66295156_Camelus_dromedarius_breed_African_isolate_Drom800_chromosome_8_whole_genome_shotgun_sequence
ABRR03077236.1_13742953-13743404_Vicugna_pacos_isolate_Carlotta__AHFN-0088__chromosome_8_ScfyRBE_77237HRSCAF_79087_whole_genome_shotgun_sequence
_R_JWIN03000008.1_52563573-52563790_Camelus_dromedarius_breed_African_isolate_Drom800_chromosome_8_whole_genome_shotgun_sequence
ABRR03077236.1_27642132-27642348_Vicugna_pacos_isolate_Carlotta__AHFN-0088__chromosome_8_ScfyRBE_77237HRSCAF_79087_whole_genome_shotgun_sequence
JWIN03000014.1_31643326-31643458_Camelus_dromedarius_breed_African_isolate_Drom800_chromosome_14_whole_genome_shotgun_sequence
VSZR01000003.1_75818196-75818319_Camelus_ferus_isolate_YT-003-E_chromosome_8_whole_genome_shotgun_sequence
0.03

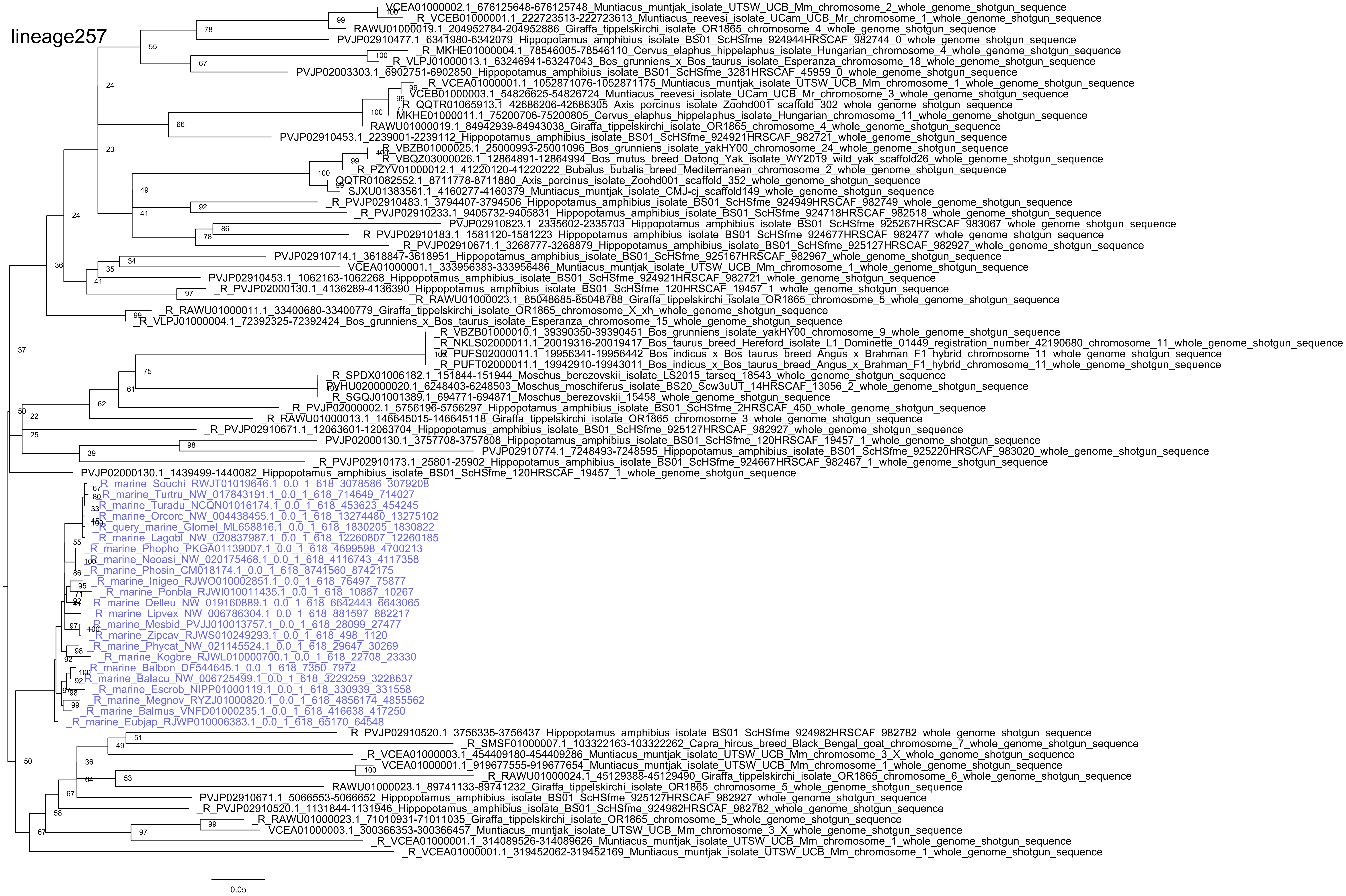

lineage257
VCEA01000002.1_676125648-676125748_Muntiacus_muntjak_isolate_UTSW_UCB_Mm_chromosome_2_whole_genome_shotgun_sequence
_R_VCEB01000001.1_222723513-222723613_Muntiacus_reevesi_isolate_UCam_UCB_Mr_chromosome_1_whole_genome_shotgun_sequence
RAWU01000019.1_204952784-204952886_Giraffa_tippelskirchi_isolate_OR1865_chromosome_4_whole_genome_shotgun_sequence
PVJP02910477.1_6341980-6342079_Hippopotamus_amphibius_isolate_BS01_ScHSfme_924944HRSCAF_982744_0_whole_genome_shotgun_sequence
_R_MKHE01000004.1_78546005-78546110_Cervus_elaphus_hippelaphus_isolate_Hungarian_chromosome_4_whole_genome_shotgun_sequence
_R_VLPJ01000013.1_63246941-63247043_Bos_grunniens_x_Bos_taurus_isolate_Esperanza_chromosome_18_whole_genome_shotgun_sequence
PVJP02003303.1_6902751-6902850_Hippopotamus_amphibius_isolate_BS01_ScHSfme_3281HRSCAF_45959_0_whole_genome_shotgun_sequence
_R_VCEA01000001.1_1052871076-1052871175_Muntiacus_muntjak_isolate_UTSW_UCB_Mm_chromosome_1_whole_genome_shotgun_sequence
VCEB01000003.1_54826625-54826724_Muntiacus_reevesi_isolate_UCam_UCB_Mr_chromosome_3_whole_genome_shotgun_sequence
_R_QQTR01065913.1_42686206-42686305_Axis_porcinus_isolate_Zoohd001_scaffold_302_whole_genome_shotgun_sequence
MKHE01000011.1_75200706-75200805_Cervus_elaphus_hippelaphus_isolate_Hungarian_chromosome_11_whole_genome_shotgun_sequence
RAWU01000019.1_84942939-84943038_Giraffa_tippelskirchi_isolate_OR1865_chromosome_4_whole_genome_shotgun_sequence
PVJP02910453.1_2239001-2239112_Hippopotamus_amphibius_isolate_BS01_ScHSfme_924921HRSCAF_982721_whole_genome_shotgun_sequence
_R_VBZB01000025.1_25000993-25001096_Bos_grunniens_isolate_yakHY00_chromosome_24_whole_genome_shotgun_sequence
_R_VBQZ03000026.1_12864891-12864994_Bos_mutus_breed_Datong_Yak_isolate_WY2019_wild_yak_scaffold26_whole_genome_shotgun_sequence
_R_PZYV01000012.1_41220120-41220222_Bubalus_bubalis_breed_Mediterranean_chromosome_2_whole_genome_shotgun_sequence
QQTR01082552.1_8711778-8711880_Axis_porcinus_isolate_Zoohd001_scaffold_352_whole_genome_shotgun_sequence
SJXU01383561.1_4160277-4160379_Muntiacus_muntjak_isolate_CMJ-cj_scaffold149_whole_genome_shotgun_sequence
_R_PVJP02910483.1_3794407-3794506_Hippopotamus_amphibius_isolate_BS01_ScHSfme_924949HRSCAF_982749_whole_genome_shotgun_sequence
_R_PVJP02910233.1_9405732-9405831_Hippopotamus_amphibius_isolate_BS01_ScHSfme_924718HRSCAF_982518_whole_genome_shotgun_sequence
PVJP02910823.1_2335602-2335703_Hippopotamus_amphibius_isolate_BS01_ScHSfme_925267HRSCAF_983067_whole_genome_shotgun_sequence
_R_PVJP02910183.1_1581120-1581223_Hippopotamus_amphibius_isolate_BS01_ScHSfme_924677HRSCAF_982477_whole_genome_shotgun_sequence
_R_PVJP02910671.1_3268777-3268879_Hippopotamus_amphibius_isolate_BS01_ScHSfme_925127HRSCAF_982927_whole_genome_shotgun_sequence
PVJP02910714.1_3618847-3618951_Hippopotamus_amphibius_isolate_BS01_ScHSfme_925167HRSCAF_982967_whole_genome_shotgun_sequence
VCEA01000001.1_333956383-333956486_Muntiacus_muntjak_isolate_UTSW_UCB_Mm_chromosome_1_whole_genome_shotgun_sequence
PVJP02910453.1_1062163-1062268_Hippopotamus_amphibius_isolate_BS01_ScHSfme_924921HRSCAF_982721_whole_genome_shotgun_sequence
_R_PVJP02000130.1_4136289-4136390_Hippopotamus_amphibius_isolate_BS01_ScHSfme_120HRSCAF_19457_1_whole_genome_shotgun_sequence
_R_RAWU01000023.1_85048685-85048788_Giraffa_tippelskirchi_isolate_OR1865_chromosome_5_whole_genome_shotgun_sequence
_R_RAWU01000011.1_33400680-33400779_Giraffa_tippelskirchi_isolate_OR1865_chromosome_X_xh_whole_genome_shotgun_sequence
_R_VLPJ01000004.1_72392325-72392424_Bos_grunniens_x_Bos_taurus_isolate_Esperanza_chromosome_15_whole_genome_shotgun_sequence
_R_VBZB01000010.1_39390350-39390451_Bos_grunniens_isolate_yakHY00_chromosome_9_whole_genome_shotgun_sequence
_R_NKLS02000011.1_20019316-20019417_Bos_taurus_breed_Hereford_isolate_L1_Dominette_01449_registration_number_42190680_chromosome_11_whole_genome_shotgun_sequence
_R_PUFS02000011.1_19956341-19956442_Bos_indicus_x_Bos_taurus_breed_Angus_x_Brahman_F1_hybrid_chromosome_11_whole_genome_shotgun_sequence
_R_PUFT02000011.1_19942910-19943011_Bos_indicus_x_Bos_taurus_breed_Angus_x_Brahman_F1_hybrid_chromosome_11_whole_genome_shotgun_sequence
_R_SPDX01006182.1_151844-151944_Moschus_berezovskii_isolate_LS2015_tarseq_18543_whole_genome_shotgun_sequence
PVHU020000020.1_6248403-6248503_Moschus_moschiferus_isolate_BS20_Scw3uUT_14HRSCAF_13056_2_whole_genome_shotgun_sequence
_R_SGQJ01001389.1_694771-694871_Moschus_berezovskii_15458_whole_genome_shotgun_sequence
_R_PVJP02000002.1_5756196-5756297_Hippopotamus_amphibius_isolate_BS01_ScHSfme_2HRSCAF_450_whole_genome_shotgun_sequence
_R_RAWU01000013.1_146645015-146645118_Giraffa_tippelskirchi_isolate_OR1865_chromosome_3_whole_genome_shotgun_sequence
_R_PVJP02910671.1_12063601-12063704_Hippopotamus_amphibius_isolate_BS01_ScHSfme_925127HRSCAF_982927_whole_genome_shotgun_sequence
PVJP02000130.1_3757708-3757808_Hippopotamus_amphibius_isolate_BS01_ScHSfme_120HRSCAF_19457_1_whole_genome_shotgun_sequence
PVJP02910774.1_7248493-7248595_Hippopotamus_amphibius_isolate_BS01_ScHSfme_925220HRSCAF_983020_whole_genome_shotgun_sequence
_R_PVJP02910173.1_25801-25902_Hippopotamus_amphibius_isolate_BS01_ScHSfme_924667HRSCAF_982467_1_whole_genome_shotgun_sequence
PVJP02000130.1_1439499-1440082_Hippopotamus_amphibius_isolate_BS01_ScHSfme_120HRSCAF_19457_1_whole_genome_shotgun_sequence
_R_marine_Souchi_RWJT01019646.1_0.0_1_618_3078586_3079208
_R_marine_Turtru_NW_017843191.1_0.0_1_618_714649_714027
_R_marine_Turadu_NCQN01016174.1_0.0_1_618_453623_454245
_R_marine_Orcorc_NW_004438455.1_0.0_1_618_13274480_13275102
_R_query_marine_Glomel_ML658816.1_0.0_1_618_1830205_1830822
_R_marine_Lagobl_NW_020837987.1_0.0_1_618_12260807_12260185
_R_marine_Phopho_PKGA01139007.1_0.0_1_618_4699598_4700213
_R_marine_Neoasi_NW_020175468.1_0.0_1_618_4116743_4117358
_R_marine_Phosin_CM018174.1_0.0_1_618_8741560_8742175
_R_marine_Inigeo_RJWO010002851.1_0.0_1_618_76497_75877
_R_marine_Ponbla_RJWI010011435.1_0.0_1_618_10887_10267
_R_marine_Delleu_NW_019160889.1_0.0_1_618_6642443_6643065
_R_marine_Lipvex_NW_006786304.1_0.0_1_618_881597_882217
_R_marine_Mesbid_PVJJ010013757.1_0.0_1_618_28099_27477
_R_marine_Zipcav_RJWS010249293.1_0.0_1_618_498_1120
_R_marine_Phycat_NW_021145524.1_0.0_1_618_29647_30269
_R_marine_Kogbre_RJWL010000700.1_0.0_1_618_22708_23330
_R_marine_Balbon_DF544645.1_0.0_1_618_7350_7972
_R_marine_Balacu_NW_006725499.1_0.0_1_618_3229259_3228637
_R_marine_Escrob_NIPP01000119.1_0.0_1_618_330939_331558
_R_marine_Megnov_RYZJ01000820.1_0.0_1_618_4856174_4855562
_R_marine_Balmus_VNFD01000235.1_0.0_1_618_416638_417250
_R_marine_Eubjap_RJWP010006383.1_0.0_1_618_65170_64548
_R_PVJP02910520.1_3756335-3756437_Hippopotamus_amphibius_isolate_BS01_ScHSfme_924982HRSCAF_982782_whole_genome_shotgun_sequence
_R_SMSF01000007.1_103322163-103322262_Capra_hircus_breed_Black_Bengal_goat_chromosome_7_whole_genome_shotgun_sequence
_R_VCEA01000003.1_454409180-454409286_Muntiacus_muntjak_isolate_UTSW_UCB_Mm_chromosome_3_X_whole_genome_shotgun_sequence
VCEA01000001.1_919677555-919677654_Muntiacus_muntjak_isolate_UTSW_UCB_Mm_chromosome_1_whole_genome_shotgun_sequence
_R_RAWU01000024.1_45129388-45129490_Giraffa_tippelskirchi_isolate_OR1865_chromosome_6_whole_genome_shotgun_sequence
RAWU01000023.1_89741133-89741232_Giraffa_tippelskirchi_isolate_OR1865_chromosome_5_whole_genome_shotgun_sequence
PVJP02910671.1_5066553-5066652_Hippopotamus_amphibius_isolate_BS01_ScHSfme_925127HRSCAF_982927_whole_genome_shotgun_sequence
_R_PVJP02910520.1_1131844-1131946_Hippopotamus_amphibius_isolate_BS01_ScHSfme_924982HRSCAF_982782_whole_genome_shotgun_sequence
_R_RAWU01000023.1_71010931-71011035_Giraffa_tippelskirchi_isolate_OR1865_chromosome_5_whole_genome_shotgun_sequence
VCEA01000003.1_300366353-300366457_Muntiacus_muntjak_isolate_UTSW_UCB_Mm_chromosome_3_X_whole_genome_shotgun_sequence
_R_VCEA01000001.1_314089526-314089626_Muntiacus_muntjak_isolate_UTSW_UCB_Mm_chromosome_1_whole_genome_shotgun_sequence
_R_VCEA01000001.1_319452062-319452169_Muntiacus_muntjak_isolate_UTSW_UCB_Mm_chromosome_1_whole_genome_shotgun_sequence
0.05

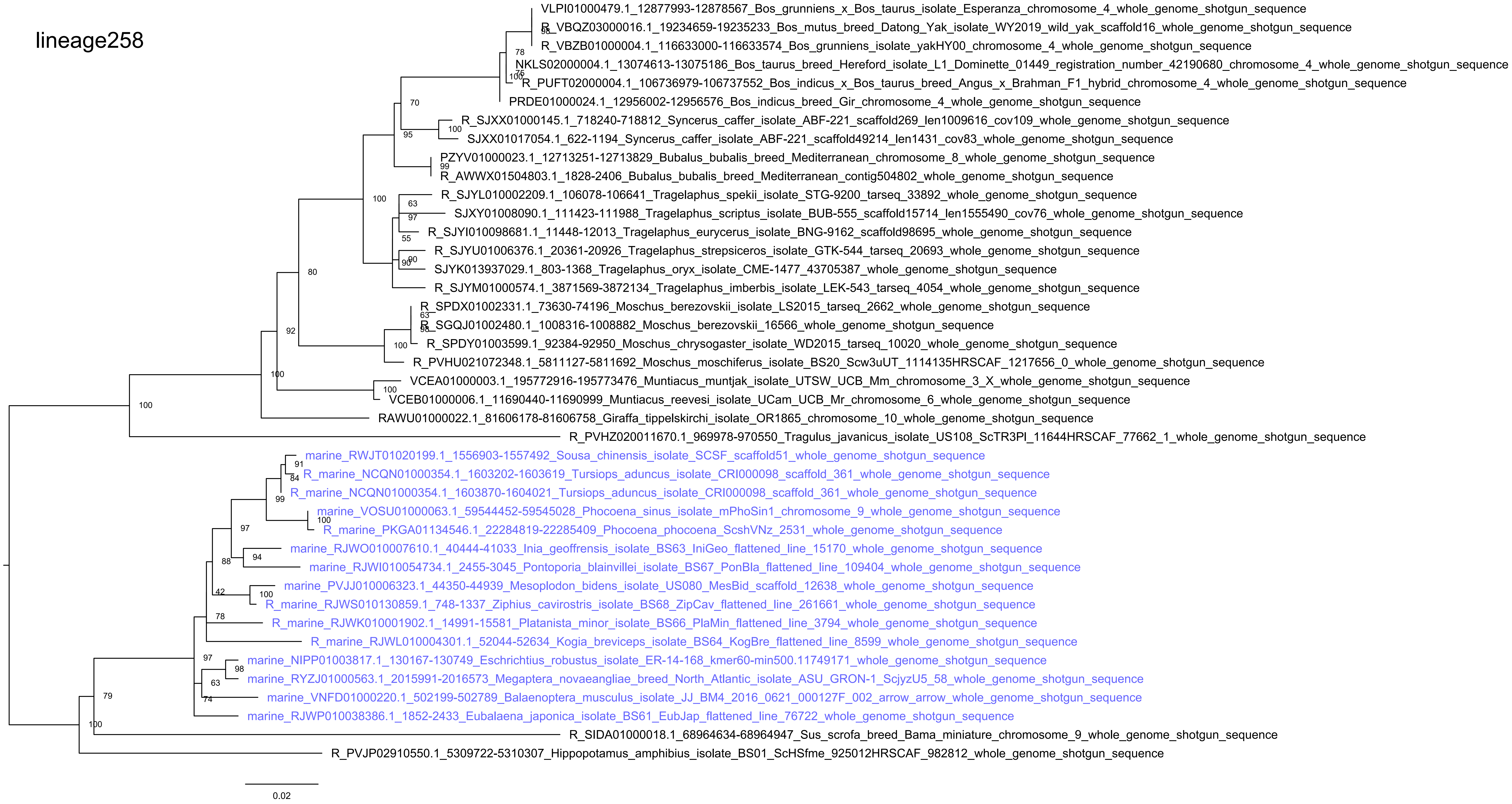
lineage258
VLPI01000479.1_12877993-12878567_Bos_grunniens_x_Bos_taurus_isolate_Esperanza_chromosome_4_whole_genome_shotgun_sequence
R_VBQZ03000016.1_19234659-19235233_Bos_mutus_breed_Datong_Yak_isolate_WY2019_wild_yak_scaffold16_whole_genome_shotgun_sequence
R_VBZB01000004.1_116633000-116633574_Bos_grunniens_isolate_yakHY00_chromosome_4_whole_genome_shotgun_sequence
NKLS02000004.1_13074613-13075186_Bos_taurus_breed_Hereford_isolate_L1_Dominette_01449_registration_number_42190680_chromosome_4_whole_genome_shotgun_sequence
R_PUFT02000004.1_106736979-106737552_Bos_indicus_x_Bos_taurus_breed_Angus_x_Brahman_F1_hybrid_chromosome_4_whole_genome_shotgun_sequence
PRDE01000024.1_12956002-12956576_Bos_indicus_breed_Gir_chromosome_4_whole_genome_shotgun_sequence
R_SJXX01000145.1_718240-718812_Syncerus_caffer_isolate_ABF-221_scaffold269_len1009616_cov109_whole_genome_shotgun_sequence
SJXX01017054.1_622-1194_Syncerus_caffer_isolate_ABF-221_scaffold49214_len1431_cov83_whole_genome_shotgun_sequence
PZYV01000023.1_12713251-12713829_Bubalus_bubalis_breed_Mediterranean_chromosome_8_whole_genome_shotgun_sequence
R_AWWX01504803.1_1828-2406_Bubalus_bubalis_breed_Mediterranean_contig504802_whole_genome_shotgun_sequence
R_SJYL010002209.1_106078-106641_Tragelaphus_spekii_isolate_STG-9200_tarseq_33892_whole_genome_shotgun_sequence
SJXY01008090.1_111423-111988_Tragelaphus_scriptus_isolate_BUB-555_scaffold15714_len1555490_cov76_whole_genome_shotgun_sequence
R_SJYI010098681.1_11448-12013_Tragelaphus_eurycerus_isolate_BNG-9162_scaffold98695_whole_genome_shotgun_sequence
R_SJYU01006376.1_20361-20926_Tragelaphus_strepsiceros_isolate_GTK-544_tarseq_20693_whole_genome_shotgun_sequence
SJYK013937029.1_803-1368_Tragelaphus_oryx_isolate_CME-1477_43705387_whole_genome_shotgun_sequence
R_SJYM01000574.1_3871569-3872134_Tragelaphus_imberbis_isolate_LEK-543_tarseq_4054_whole_genome_shotgun_sequence
R_SPDX01002331.1_73630-74196_Moschus_berezovskii_isolate_LS2015_tarseq_2662_whole_genome_shotgun_sequence
R_SGQJ01002480.1_1008316-1008882_Moschus_berezovskii_16566_whole_genome_shotgun_sequence
R_SPDY01003599.1_92384-92950_Moschus_chrysogaster_isolate_WD2015_tarseq_10020_whole_genome_shotgun_sequence
R_PVHU021072348.1_5811127-5811692_Moschus_moschiferus_isolate_BS20_Scw3uUT_1114135HRSCAF_1217656_0_whole_genome_shotgun_sequence
VCEA01000003.1_195772916-195773476_Muntiacus_muntjak_isolate_UTSW_UCB_Mm_chromosome_3_X_whole_genome_shotgun_sequence
VCEB01000006.1_11690440-11690999_Muntiacus_reevesi_isolate_UCam_UCB_Mr_chromosome_6_whole_genome_shotgun_sequence
RAWU01000022.1_81606178-81606758_Giraffa_tippelskirchi_isolate_OR1865_chromosome_10_whole_genome_shotgun_sequence
R_PVHZ020011670.1_969978-970550_Tragulus_javanicus_isolate_US108_ScTR3PI_11644HRSCAF_77662_1_whole_genome_shotgun_sequence
marine_RWJT01020199.1_1556903-1557492_Sousa_chinensis_isolate_SCSF_scaffold51_whole_genome_shotgun_sequence
R_marine_NCQN01000354.1_1603202-1603619_Tursiops_aduncus_isolate_CRI000098_scaffold_361_whole_genome_shotgun_sequence
R_marine_NCQN01000354.1_1603870-1604021_Tursiops_aduncus_isolate_CRI000098_scaffold_361_whole_genome_shotgun_sequence
marine_VOSU01000063.1_59544452-59545028_Phocoena_sinus_isolate_mPhoSin1_chromosome_9_whole_genome_shotgun_sequence
R_marine_PKGA01134546.1_22284819-22285409_Phocoena_phocoena_ScshVNz_2531_whole_genome_shotgun_sequence
marine_RJWO010007610.1_40444-41033_Inia_geoffrensis_isolate_BS63_IniGeo_flattened_line_15170_whole_genome_shotgun_sequence
marine_RJWI010054734.1_2455-3045_Pontoporia_blainvillei_isolate_BS67_PonBla_flattened_line_109404_whole_genome_shotgun_sequence
marine_PVJJ010006323.1_44350-44939_Mesoplodon_bidens_isolate_US080_MesBid_scaffold_12638_whole_genome_shotgun_sequence
R_marine_RJWS010130859.1_748-1337_Ziphius_cavirostris_isolate_BS68_ZipCav_flattened_line_261661_whole_genome_shotgun_sequence
R_marine_RJWK010001902.1_14991-15581_Platanista_minor_isolate_BS66_PlaMin_flattened_line_3794_whole_genome_shotgun_sequence
R_marine_RJWL010004301.1_52044-52634_Kogia_breviceps_isolate_BS64_KogBre_flattened_line_8599_whole_genome_shotgun_sequence
marine_NIPP01003817.1_130167-130749_Eschrichtius_robustus_isolate_ER-14-168_kmer60-min500.11749171_whole_genome_shotgun_sequence
marine_RYZJ01000563.1_2015991-2016573_Megaptera_novaeangliae_breed_North_Atlantic_isolate_ASU_GRON-1_ScjyzU5_58_whole_genome_shotgun_sequence
marine_VNFD01000220.1_502199-502789_Balaenoptera_musculus_isolate_JJ_BM4_2016_0621_000127F_002_arrow_arrow_whole_genome_shotgun_sequence
marine_RJWP010038386.1_1852-2433_Eubalaena_japonica_isolate_BS61_EubJap_flattened_line_76722_whole_genome_shotgun_sequence
R_SIDA01000018.1_68964634-68964947_Sus_scrofa_breed_Bama_miniature_chromosome_9_whole_genome_shotgun_sequence
R_PVJP02910550.1_5309722-5310307_Hippopotamus_amphibius_isolate_BS01_ScHSfme_925012HRSCAF_982812_whole_genome_shotgun_sequence
0.02

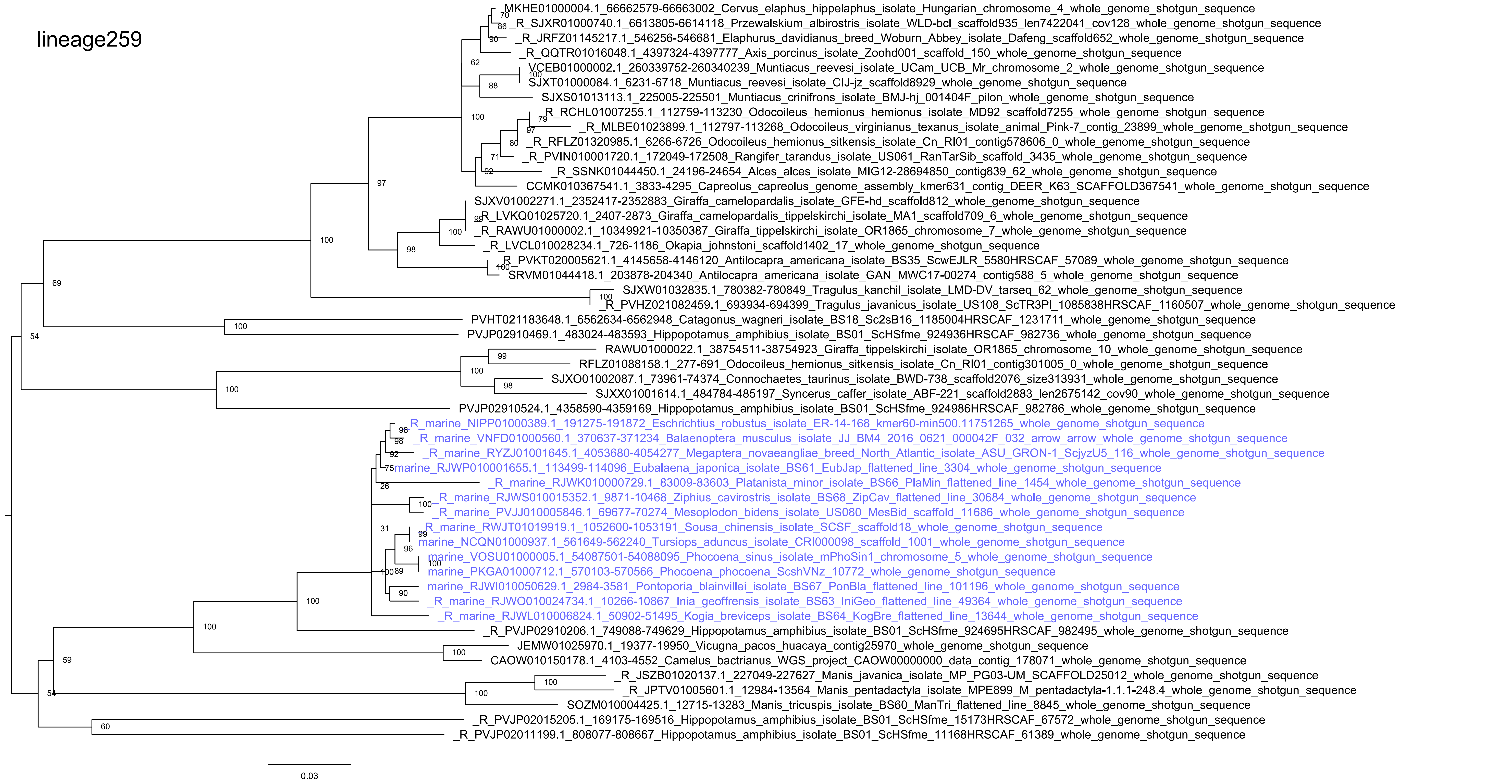
lineage259
MKHE01000004.1_66662579-66663002_Cervus_elaphus_hippelaphus_isolate_Hungarian_chromosome_4_whole_genome_shotgun_sequence
_R_SJXR01000740.1_6613805-6614118_Przewalskium_albirostris_isolate_WLD-bcl_scaffold935_len7422041_cov128_whole_genome_shotgun_sequence
_R_JRFZ01145217.1_546256-546681_Elaphurus_davidianus_breed_Woburn_Abbey_isolate_Dafeng_scaffold652_whole_genome_shotgun_sequence
_R_QQTR01016048.1_4397324-4397777_Axis_porcinus_isolate_Zoohd001_scaffold_150_whole_genome_shotgun_sequence
VCEB01000002.1_260339752-260340239_Muntiacus_reevesi_isolate_UCam_UCB_Mr_chromosome_2_whole_genome_shotgun_sequence
SJXT01000084.1_6231-6718_Muntiacus_reevesi_isolate_CIJ-jz_scaffold8929_whole_genome_shotgun_sequence
SJXS01013113.1_225005-225501_Muntiacus_crinifrons_isolate_BMJ-hj_001404F_pilon_whole_genome_shotgun_sequence
_R_RCHL01007255.1_112759-113230_Odocoileus_hemionus_hemionus_isolate_MD92_scaffold7255_whole_genome_shotgun_sequence
_R_MLBE01023899.1_112797-113268_Odocoileus_virginianus_texanus_isolate_animal_Pink-7_contig_23899_whole_genome_shotgun_sequence
_R_RFLZ01320985.1_6266-6726_Odocoileus_hemionus_sitkensis_isolate_Cn_RI01_contig578606_0_whole_genome_shotgun_sequence
_R_PVIN010001720.1_172049-172508_Rangifer_tarandus_isolate_US061_RanTarSib_scaffold_3435_whole_genome_shotgun_sequence
_R_SSNK01044450.1_24196-24654_Alces_alces_isolate_MIG12-28694850_contig839_62_whole_genome_shotgun_sequence
CCMK010367541.1_3833-4295_Capreolus_capreolus_genome_assembly_kmer631_contig_DEER_K63_SCAFFOLD367541_whole_genome_shotgun_sequence
SJXV01002271.1_2352417-2352883_Giraffa_camelopardalis_isolate_GFE-hd_scaffold812_whole_genome_shotgun_sequence
_R_LVKQ01025720.1_2407-2873_Giraffa_camelopardalis_tippelskirchi_isolate_MA1_scaffold709_6_whole_genome_shotgun_sequence
_R_RAWU01000002.1_10349921-10350387_Giraffa_tippelskirchi_isolate_OR1865_chromosome_7_whole_genome_shotgun_sequence
_R_LVCL010028234.1_726-1186_Okapia_johnstoni_scaffold1402_17_whole_genome_shotgun_sequence
_R_PVKT020005621.1_4145658-4146120_Antilocapra_americana_isolate_BS35_ScwEJLR_5580HRSCAF_57089_whole_genome_shotgun_sequence
SRVM01044418.1_203878-204340_Antilocapra_americana_isolate_GAN_MWC17-00274_contig588_5_whole_genome_shotgun_sequence
SJXW01032835.1_780382-780849_Tragulus_kanchil_isolate_LMD-DV_tarseq_62_whole_genome_shotgun_sequence
_R_PVHZ021082459.1_693934-694399_Tragulus_javanicus_isolate_US108_ScTR3PI_1085838HRSCAF_1160507_whole_genome_shotgun_sequence
PVHT021183648.1_6562634-6562948_Catagonus_wagneri_isolate_BS18_Sc2sB16_1185004HRSCAF_1231711_whole_genome_shotgun_sequence
PVJP02910469.1_483024-483593_Hippopotamus_amphibius_isolate_BS01_ScHSfme_924936HRSCAF_982736_whole_genome_shotgun_sequence
RAWU01000022.1_38754511-38754923_Giraffa_tippelskirchi_isolate_OR1865_chromosome_10_whole_genome_shotgun_sequence
RFLZ01088158.1_277-691_Odocoileus_hemionus_sitkensis_isolate_Cn_RI01_contig301005_0_whole_genome_shotgun_sequence
SJXO01002087.1_73961-74374_Connochaetes_taurinus_isolate_BWD-738_scaffold2076_size313931_whole_genome_shotgun_sequence
SJXX01001614.1_484784-485197_Syncerus_caffer_isolate_ABF-221_scaffold2883_len2675142_cov90_whole_genome_shotgun_sequence
PVJP02910524.1_4358590-4359169_Hippopotamus_amphibius_isolate_BS01_ScHSfme_924986HRSCAF_982786_whole_genome_shotgun_sequence
R_marine_NIPP01000389.1_191275-191872_Eschrichtius_robustus_isolate_ER-14-168_kmer60-min500.11751265_whole_genome_shotgun_sequence
_R_marine_VNFD01000560.1_370637-371234_Balaenoptera_musculus_isolate_JJ_BM4_2016_0621_000042F_032_arrow_arrow_whole_genome_shotgun_sequence
_R_marine_RYZJ01001645.1_4053680-4054277_Megaptera_novaeangliae_breed_North_Atlantic_isolate_ASU_GRON-1_ScjyzU5_116_whole_genome_shotgun_sequence
marine_RJWP010001655.1_113499-114096_Eubalaena_japonica_isolate_BS61_EubJap_flattened_line_3304_whole_genome_shotgun_sequence
_R_marine_RJWK010000729.1_83009-83603_Platanista_minor_isolate_BS66_PlaMin_flattened_line_1454_whole_genome_shotgun_sequence
_R_marine_RJWS010015352.1_9871-10468_Ziphius_cavirostris_isolate_BS68_ZipCav_flattened_line_30684_whole_genome_shotgun_sequence
_R_marine_PVJJ010005846.1_69677-70274_Mesoplodon_bidens_isolate_US080_MesBid_scaffold_11686_whole_genome_shotgun_sequence
R_marine_RWJT01019919.1_1052600-1053191_Sousa_chinensis_isolate_SCSF_scaffold18_whole_genome_shotgun_sequence
marine_NCQN01000937.1_561649-562240_Tursiops_aduncus_isolate_CRI000098_scaffold_1001_whole_genome_shotgun_sequence
marine_VOSU01000005.1_54087501-54088095_Phocoena_sinus_isolate_mPhoSin1_chromosome_5_whole_genome_shotgun_sequence
marine_PKGA01000712.1_570103-570566_Phocoena_phocoena_ScshVNz_10772_whole_genome_shotgun_sequence
marine_RJWI010050629.1_2984-3581_Pontoporia_blainvillei_isolate_BS67_PonBla_flattened_line_101196_whole_genome_shotgun_sequence
_R_marine_RJWO010024734.1_10266-10867_Inia_geoffrensis_isolate_BS63_IniGeo_flattened_line_49364_whole_genome_shotgun_sequence
_R_marine_RJWL010006824.1_50902-51495_Kogia_breviceps_isolate_BS64_KogBre_flattened_line_13644_whole_genome_shotgun_sequence
_R_PVJP02910206.1_749088-749629_Hippopotamus_amphibius_isolate_BS01_ScHSfme_924695HRSCAF_982495_whole_genome_shotgun_sequence
JEMW01025970.1_19377-19950_Vicugna_pacos_huacaya_contig25970_whole_genome_shotgun_sequence
CAOW010150178.1_4103-4552_Camelus_bactrianus_WGS_project_CAOW00000000_data_contig_178071_whole_genome_shotgun_sequence
_R_JSZB01020137.1_227049-227627_Manis_javanica_isolate_MP_PG03-UM_SCAFFOLD25012_whole_genome_shotgun_sequence
_R_JPTV01005601.1_12984-13564_Manis_pentadactyla_isolate_MPE899_M_pentadactyla-1.1.1-248.4_whole_genome_shotgun_sequence
SOZM010004425.1_12715-13283_Manis_tricuspis_isolate_BS60_ManTri_flattened_line_8845_whole_genome_shotgun_sequence
_R_PVJP02015205.1_169175-169516_Hippopotamus_amphibius_isolate_BS01_ScHSfme_15173HRSCAF_67572_whole_genome_shotgun_sequence
_R_PVJP02011199.1_808077-808667_Hippopotamus_amphibius_isolate_BS01_ScHSfme_11168HRSCAF_61389_whole_genome_shotgun_sequence
0.03

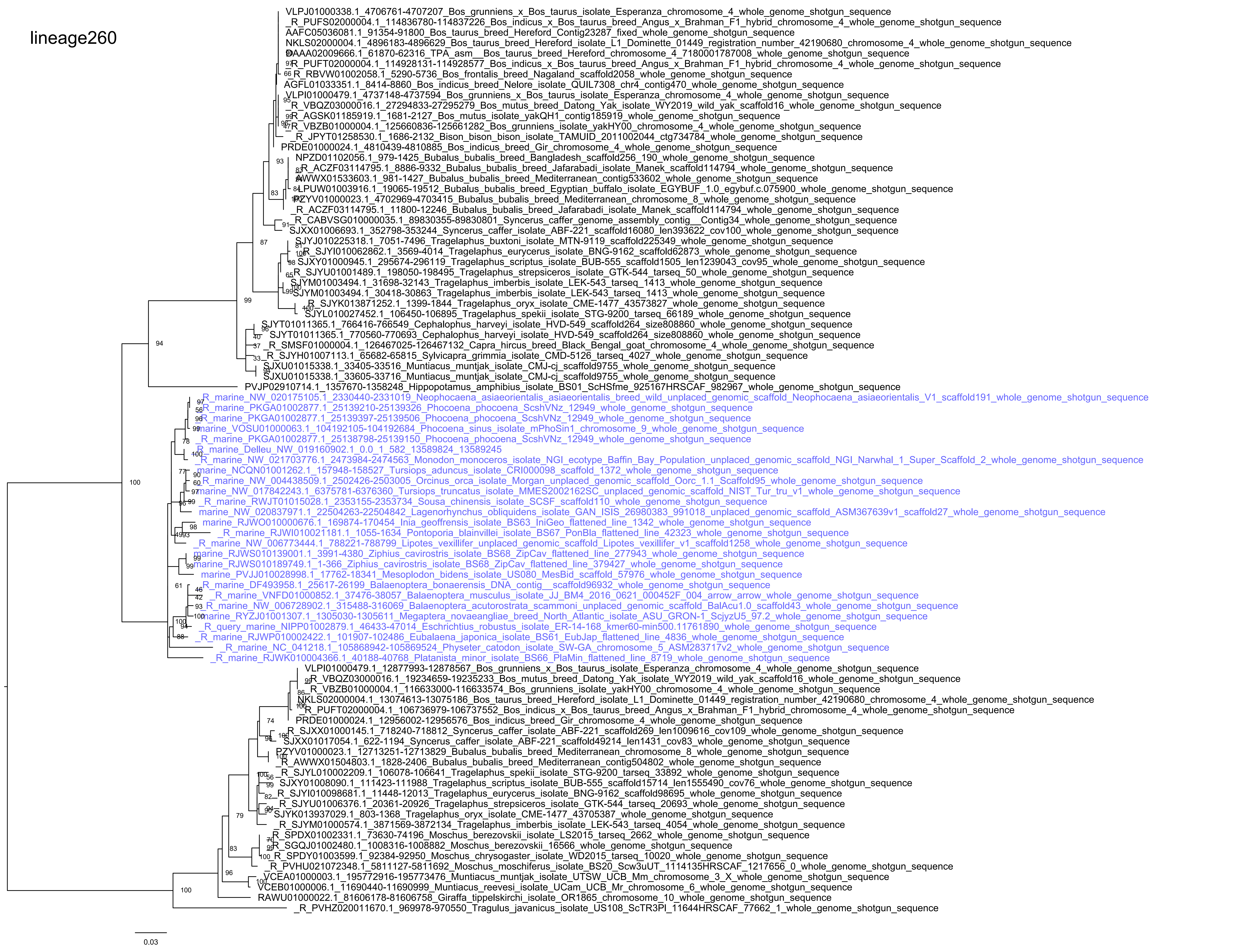

lineage260
VLPJ01000338.1_4706761-4707207_Bos_grunniens_x_Bos_taurus_isolate_Esperanza_chromosome_4_whole_genome_shotgun_sequence
_R_PUFS02000004.1_114836780-114837226_Bos_indicus_x_Bos_taurus_breed_Angus_x_Brahman_F1_hybrid_chromosome_4_whole_genome_shotgun_sequence
AAFC05036081.1_91354-91800_Bos_taurus_breed_Hereford_Contig23287_fixed_whole_genome_shotgun_sequence
NKLS02000004.1_4896183-4896629_Bos_taurus_breed_Hereford_isolate_L1_Dominette_01449_registration_number_42190680_chromosome_4_whole_genome_shotgun_sequence
DAAA02009666.1_61870-62316_TPA_asm__Bos_taurus_breed_Hereford_chromosome_4_7180001787008_whole_genome_shotgun_sequence
_R_PUFT02000004.1_114928131-114928577_Bos_indicus_x_Bos_taurus_breed_Angus_x_Brahman_F1_hybrid_chromosome_4_whole_genome_shotgun_sequence
_R_RBVW01002058.1_5290-5736_Bos_frontalis_breed_Nagaland_scaffold2058_whole_genome_shotgun_sequence
AGFL01033351.1_8414-8860_Bos_indicus_breed_Nelore_isolate_QUIL7308_chr4_contig470_whole_genome_shotgun_sequence
VLPI01000479.1_4737148-4737594_Bos_grunniens_x_Bos_taurus_isolate_Esperanza_chromosome_4_whole_genome_shotgun_sequence
_R_VBQZ03000016.1_27294833-27295279_Bos_mutus_breed_Datong_Yak_isolate_WY2019_wild_yak_scaffold16_whole_genome_shotgun_sequence
_R_AGSK01185919.1_1681-2127_Bos_mutus_isolate_yakQH1_contig185919_whole_genome_shotgun_sequence
_R_VBZB01000004.1_125660836-125661282_Bos_grunniens_isolate_yakHY00_chromosome_4_whole_genome_shotgun_sequence
_R_JPYT01258530.1_1686-2132_Bison_bison_bison_isolate_TAMUID_2011002044_ctg734784_whole_genome_shotgun_sequence
PRDE01000024.1_4810439-4810885_Bos_indicus_breed_Gir_chromosome_4_whole_genome_shotgun_sequence
NPZD01102056.1_979-1425_Bubalus_bubalis_breed_Bangladesh_scaffold256_190_whole_genome_shotgun_sequence
_R_ACZF03114795.1_8886-9332_Bubalus_bubalis_breed_Jafarabadi_isolate_Manek_scaffold114794_whole_genome_shotgun_sequence
AWWX01533603.1_981-1427_Bubalus_bubalis_breed_Mediterranean_contig533602_whole_genome_shotgun_sequence
LPUW01003916.1_19065-19512_Bubalus_bubalis_breed_Egyptian_buffalo_isolate_EGYBUF_1.0_egybuf.c.075900_whole_genome_shotgun_sequence
PZYV01000023.1_4702969-4703415_Bubalus_bubalis_breed_Mediterranean_chromosome_8_whole_genome_shotgun_sequence
_R_ACZF03114795.1_11800-12246_Bubalus_bubalis_breed_Jafarabadi_isolate_Manek_scaffold114794_whole_genome_shotgun_sequence
_R_CABVSG010000035.1_89830355-89830801_Syncerus_caffer_genome_assembly_contig__Contig34_whole_genome_shotgun_sequence
SJXX01006693.1_352798-353244_Syncerus_caffer_isolate_ABF-221_scaffold16080_len393622_cov100_whole_genome_shotgun_sequence
SJYJ010225318.1_7051-7496_Tragelaphus_buxtoni_isolate_MTN-9119_scaffold225349_whole_genome_shotgun_sequence
_R_SJYI010062862.1_3569-4014_Tragelaphus_eurycerus_isolate_BNG-9162_scaffold62873_whole_genome_shotgun_sequence
SJXY01000945.1_295674-296119_Tragelaphus_scriptus_isolate_BUB-555_scaffold1505_len1239043_cov95_whole_genome_shotgun_sequence
_R_SJYU01001489.1_198050-198495_Tragelaphus_strepsiceros_isolate_GTK-544_tarseq_50_whole_genome_shotgun_sequence
SJYM01003494.1_31698-32143_Tragelaphus_imberbis_isolate_LEK-543_tarseq_1413_whole_genome_shotgun_sequence
SJYM01003494.1_30418-30863_Tragelaphus_imberbis_isolate_LEK-543_tarseq_1413_whole_genome_shotgun_sequence
_R_SJYK013871252.1_1399-1844_Tragelaphus_oryx_isolate_CME-1477_43573827_whole_genome_shotgun_sequence
SJYL010027452.1_106450-106895_Tragelaphus_spekii_isolate_STG-9200_tarseq_66189_whole_genome_shotgun_sequence
SJYT01011365.1_766416-766549_Cephalophus_harveyi_isolate_HVD-549_scaffold264_size808860_whole_genome_shotgun_sequence
SJYT01011365.1_770560-770693_Cephalophus_harveyi_isolate_HVD-549_scaffold264_size808860_whole_genome_shotgun_sequence
_R_SMSF01000004.1_126467025-126467132_Capra_hircus_breed_Black_Bengal_goat_chromosome_4_whole_genome_shotgun_sequence
_R_SJYH01007113.1_65682-65815_Sylvicapra_grimmia_isolate_CMD-5126_tarseq_4027_whole_genome_shotgun_sequence
SJXU01015338.1_33405-33516_Muntiacus_muntjak_isolate_CMJ-cj_scaffold9755_whole_genome_shotgun_sequence
SJXU01015338.1_33605-33716_Muntiacus_muntjak_isolate_CMJ-cj_scaffold9755_whole_genome_shotgun_sequence
PVJP02910714.1_1357670-1358248_Hippopotamus_amphibius_isolate_BS01_ScHSfme_925167HRSCAF_982967_whole_genome_shotgun_sequence
_R_marine_NW_020175105.1_2330440-2331019_Neophocaena_asiaeorientalis_asiaeorientalis_breed_wild_unplaced_genomic_scaffold_Neophocaena_asiaeorientalis_V1_scaffold191_whole_genome_shotgun_sequence
_R_marine_PKGA01002877.1_25139210-25139326_Phocoena_phocoena_ScshVNz_12949_whole_genome_shotgun_sequence
_R_marine_PKGA01002877.1_25139397-25139506_Phocoena_phocoena_ScshVNz_12949_whole_genome_shotgun_sequence
marine_VOSU01000063.1_104192105-104192684_Phocoena_sinus_isolate_mPhoSin1_chromosome_9_whole_genome_shotgun_sequence
_R_marine_PKGA01002877.1_25138798-25139150_Phocoena_phocoena_ScshVNz_12949_whole_genome_shotgun_sequence
_R_marine_Delleu_NW_019160902.1_0.0_1_582_13589824_13589245
_R_marine_NW_021703776.1_2473984-2474563_Monodon_monoceros_isolate_NGI_ecotype_Baffin_Bay_Population_unplaced_genomic_scaffold_NGI_Narwhal_1_Super_Scaffold_2_whole_genome_shotgun_sequence
marine_NCQN01001262.1_157948-158527_Tursiops_aduncus_isolate_CRI000098_scaffold_1372_whole_genome_shotgun_sequence
_R_marine_NW_004438509.1_2502426-2503005_Orcinus_orca_isolate_Morgan_unplaced_genomic_scaffold_Oorc_1.1_Scaffold95_whole_genome_shotgun_sequence
marine_NW_017842243.1_6375781-6376360_Tursiops_truncatus_isolate_MMES2002162SC_unplaced_genomic_scaffold_NIST_Tur_tru_v1_whole_genome_shotgun_sequence
_R_marine_RWJT01015028.1_2353155-2353734_Sousa_chinensis_isolate_SCSF_scaffold110_whole_genome_shotgun_sequence
marine_NW_020837971.1_22504263-22504842_Lagenorhynchus_obliquidens_isolate_GAN_ISIS_26980383_991018_unplaced_genomic_scaffold_ASM367639v1_scaffold27_whole_genome_shotgun_sequence
marine_RJWO010000676.1_169874-170454_Inia_geoffrensis_isolate_BS63_IniGeo_flattened_line_1342_whole_genome_shotgun_sequence
_R_marine_RJWI010021181.1_1055-1634_Pontoporia_blainvillei_isolate_BS67_PonBla_flattened_line_42323_whole_genome_shotgun_sequence
_R_marine_NW_006773444.1_788221-788799_Lipotes_vexillifer_unplaced_genomic_scaffold_Lipotes_vexillifer_v1_scaffold1258_whole_genome_shotgun_sequence
marine_RJWS010139001.1_3991-4380_Ziphius_cavirostris_isolate_BS68_ZipCav_flattened_line_277943_whole_genome_shotgun_sequence
marine_RJWS010189749.1_1-366_Ziphius_cavirostris_isolate_BS68_ZipCav_flattened_line_379427_whole_genome_shotgun_sequence
marine_PVJJ010028998.1_17762-18341_Mesoplodon_bidens_isolate_US080_MesBid_scaffold_57976_whole_genome_shotgun_sequence
_R_marine_DF493958.1_25617-26199_Balaenoptera_bonaerensis_DNA_contig__scaffold96932_whole_genome_shotgun_sequence
_R_marine_VNFD01000852.1_37476-38057_Balaenoptera_musculus_isolate_JJ_BM4_2016_0621_000452F_004_arrow_arrow_whole_genome_shotgun_sequence
_R_marine_NW_006728902.1_315488-316069_Balaenoptera_acutorostrata_scammoni_unplaced_genomic_scaffold_BalAcu1.0_scaffold43_whole_genome_shotgun_sequence
marine_RYZJ01001307.1_1305030-1305611_Megaptera_novaeangliae_breed_North_Atlantic_isolate_ASU_GRON-1_ScjyzU5_97.2_whole_genome_shotgun_sequence
_R_query_marine_NIPP01002879.1_46433-47014_Eschrichtius_robustus_isolate_ER-14-168_kmer60-min500.11761890_whole_genome_shotgun_sequence
_R_marine_RJWP010002422.1_101907-102486_Eubalaena_japonica_isolate_BS61_EubJap_flattened_line_4836_whole_genome_shotgun_sequence
_R_marine_NC_041218.1_105868942-105869524_Physeter_catodon_isolate_SW-GA_chromosome_5_ASM283717v2_whole_genome_shotgun_sequence
_R_marine_RJWK010004366.1_40188-40768_Platanista_minor_isolate_BS66_PlaMin_flattened_line_8719_whole_genome_shotgun_sequence
VLPI01000479.1_12877993-12878567_Bos_grunniens_x_Bos_taurus_isolate_Esperanza_chromosome_4_whole_genome_shotgun_sequence
_R_VBQZ03000016.1_19234659-19235233_Bos_mutus_breed_Datong_Yak_isolate_WY2019_wild_yak_scaffold16_whole_genome_shotgun_sequence
_R_VBZB01000004.1_116633000-116633574_Bos_grunniens_isolate_yakHY00_chromosome_4_whole_genome_shotgun_sequence
NKLS02000004.1_13074613-13075186_Bos_taurus_breed_Hereford_isolate_L1_Dominette_01449_registration_number_42190680_chromosome_4_whole_genome_shotgun_sequence
_R_PUFT02000004.1_106736979-106737552_Bos_indicus_x_Bos_taurus_breed_Angus_x_Brahman_F1_hybrid_chromosome_4_whole_genome_shotgun_sequence
PRDE01000024.1_12956002-12956576_Bos_indicus_breed_Gir_chromosome_4_whole_genome_shotgun_sequence
_R_SJXX01000145.1_718240-718812_Syncerus_caffer_isolate_ABF-221_scaffold269_len1009616_cov109_whole_genome_shotgun_sequence
SJXX01017054.1_622-1194_Syncerus_caffer_isolate_ABF-221_scaffold49214_len1431_cov83_whole_genome_shotgun_sequence
PZYV01000023.1_12713251-12713829_Bubalus_bubalis_breed_Mediterranean_chromosome_8_whole_genome_shotgun_sequence
_R_AWWX01504803.1_1828-2406_Bubalus_bubalis_breed_Mediterranean_contig504802_whole_genome_shotgun_sequence
_R_SJYL010002209.1_106078-106641_Tragelaphus_spekii_isolate_STG-9200_tarseq_33892_whole_genome_shotgun_sequence
SJXY01008090.1_111423-111988_Tragelaphus_scriptus_isolate_BUB-555_scaffold15714_len1555490_cov76_whole_genome_shotgun_sequence
_R_SJYI010098681.1_11448-12013_Tragelaphus_eurycerus_isolate_BNG-9162_scaffold98695_whole_genome_shotgun_sequence
_R_SJYU01006376.1_20361-20926_Tragelaphus_strepsiceros_isolate_GTK-544_tarseq_20693_whole_genome_shotgun_sequence
SJYK013937029.1_803-1368_Tragelaphus_oryx_isolate_CME-1477_43705387_whole_genome_shotgun_sequence
_R_SJYM01000574.1_3871569-3872134_Tragelaphus_imberbis_isolate_LEK-543_tarseq_4054_whole_genome_shotgun_sequence
_R_SPDX01002331.1_73630-74196_Moschus_berezovskii_isolate_LS2015_tarseq_2662_whole_genome_shotgun_sequence
_R_SGQJ01002480.1_1008316-1008882_Moschus_berezovskii_16566_whole_genome_shotgun_sequence
_R_SPDY01003599.1_92384-92950_Moschus_chrysogaster_isolate_WD2015_tarseq_10020_whole_genome_shotgun_sequence
_R_PVHU021072348.1_5811127-5811692_Moschus_moschiferus_isolate_BS20_Scw3uUT_1114135HRSCAF_1217656_0_whole_genome_shotgun_sequence
VCEA01000003.1_195772916-195773476_Muntiacus_muntjak_isolate_UTSW_UCB_Mm_chromosome_3_X_whole_genome_shotgun_sequence
VCEB01000006.1_11690440-11690999_Muntiacus_reevesi_isolate_UCam_UCB_Mr_chromosome_6_whole_genome_shotgun_sequence
RAWU01000022.1_81606178-81606758_Giraffa_tippelskirchi_isolate_OR1865_chromosome_10_whole_genome_shotgun_sequence
_R_PVHZ020011670.1_969978-970550_Tragulus_javanicus_isolate_US108_ScTR3PI_11644HRSCAF_77662_1_whole_genome_shotgun_sequence
0.03

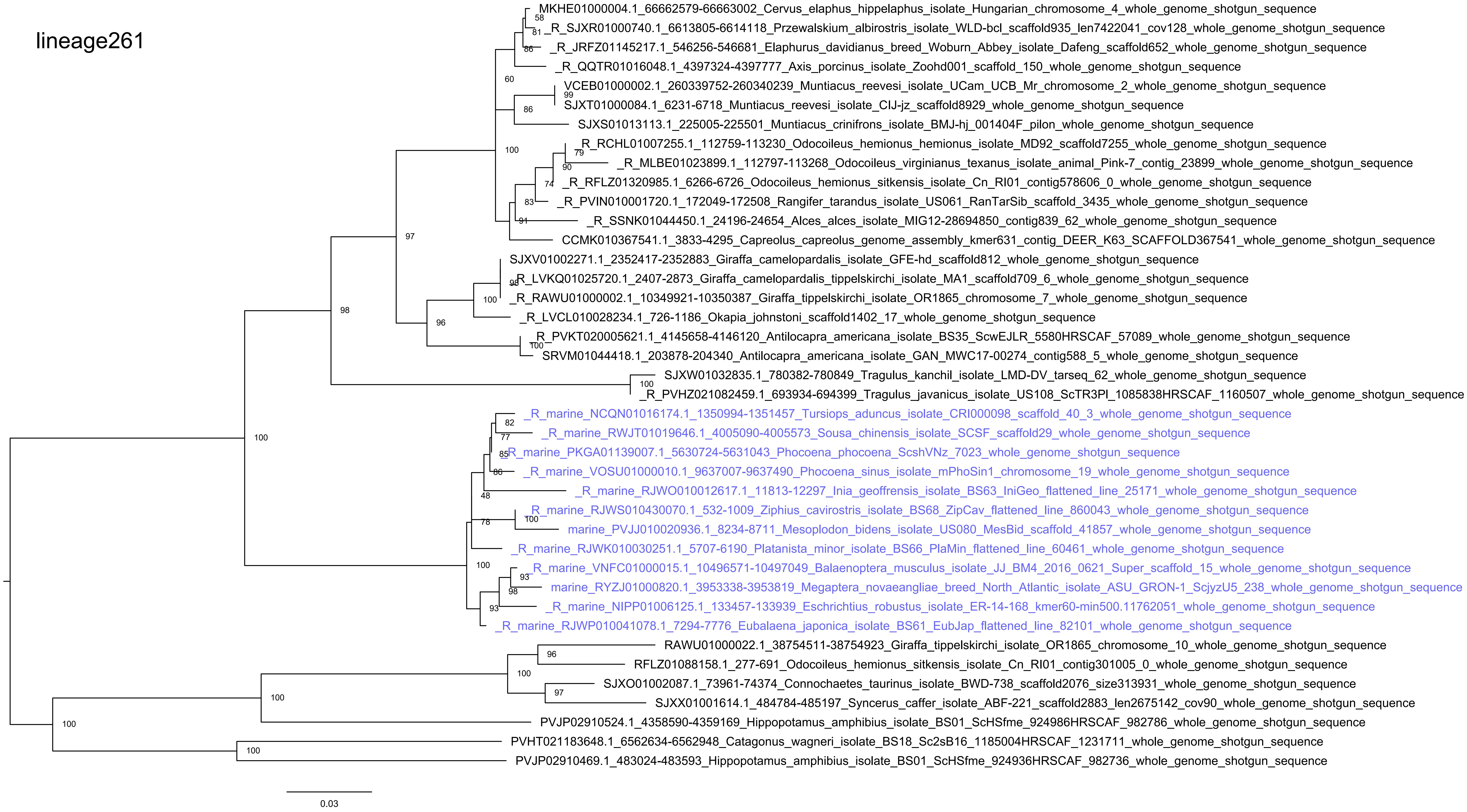
lineage261
MKHE01000004.1_66662579-66663002_Cervus_elaphus_hippelaphus_isolate_Hungarian_chromosome_4_whole_genome_shotgun_sequence
_R_SJXR01000740.1_6613805-6614118_Przewalskium_albirostris_isolate_WLD-bcl_scaffold935_len7422041_cov128_whole_genome_shotgun_sequence
_R_JRFZ01145217.1_546256-546681_Elaphurus_davidianus_breed_Woburn_Abbey_isolate_Dafeng_scaffold652_whole_genome_shotgun_sequence
_R_QQTR01016048.1_4397324-4397777_Axis_porcinus_isolate_Zoohd001_scaffold_150_whole_genome_shotgun_sequence
VCEB01000002.1_260339752-260340239_Muntiacus_reevesi_isolate_UCam_UCB_Mr_chromosome_2_whole_genome_shotgun_sequence
SJXT01000084.1_6231-6718_Muntiacus_reevesi_isolate_CIJ-jz_scaffold8929_whole_genome_shotgun_sequence
SJXS01013113.1_225005-225501_Muntiacus_crinifrons_isolate_BMJ-hj_001404F_pilon_whole_genome_shotgun_sequence
_R_RCHL01007255.1_112759-113230_Odocoileus_hemionus_hemionus_isolate_MD92_scaffold7255_whole_genome_shotgun_sequence
_R_MLBE01023899.1_112797-113268_Odocoileus_virginianus_texanus_isolate_animal_Pink-7_contig_23899_whole_genome_shotgun_sequence
_R_RFLZ01320985.1_6266-6726_Odocoileus_hemionus_sitkensis_isolate_Cn_RI01_contig578606_0_whole_genome_shotgun_sequence
_R_PVIN010001720.1_172049-172508_Rangifer_tarandus_isolate_US061_RanTarSib_scaffold_3435_whole_genome_shotgun_sequence
_R_SSNK01044450.1_24196-24654_Alces_alces_isolate_MIG12-28694850_contig839_62_whole_genome_shotgun_sequence
CCMK010367541.1_3833-4295_Capreolus_capreolus_genome_assembly_kmer631_contig_DEER_K63_SCAFFOLD367541_whole_genome_shotgun_sequence
SJXV01002271.1_2352417-2352883_Giraffa_camelopardalis_isolate_GFE-hd_scaffold812_whole_genome_shotgun_sequence
_R_LVKQ01025720.1_2407-2873_Giraffa_camelopardalis_tippelskirchi_isolate_MA1_scaffold709_6_whole_genome_shotgun_sequence
_R_RAWU01000002.1_10349921-10350387_Giraffa_tippelskirchi_isolate_OR1865_chromosome_7_whole_genome_shotgun_sequence
_R_LVCL010028234.1_726-1186_Okapia_johnstoni_scaffold1402_17_whole_genome_shotgun_sequence
_R_PVKT020005621.1_4145658-4146120_Antilocapra_americana_isolate_BS35_ScwEJLR_5580HRSCAF_57089_whole_genome_shotgun_sequence
SRVM01044418.1_203878-204340_Antilocapra_americana_isolate_GAN_MWC17-00274_contig588_5_whole_genome_shotgun_sequence
SJXW01032835.1_780382-780849_Tragulus_kanchil_isolate_LMD-DV_tarseq_62_whole_genome_shotgun_sequence
_R_PVHZ021082459.1_693934-694399_Tragulus_javanicus_isolate_US108_ScTR3PI_1085838HRSCAF_1160507_whole_genome_shotgun_sequence
_R_marine_NCQN01016174.1_1350994-1351457_Tursiops_aduncus_isolate_CRI000098_scaffold_40_3_whole_genome_shotgun_sequence
_R_marine_RWJT01019646.1_4005090-4005573_Sousa_chinensis_isolate_SCSF_scaffold29_whole_genome_shotgun_sequence
_R_marine_PKGA01139007.1_5630724-5631043_Phocoena_phocoena_ScshVNz_7023_whole_genome_shotgun_sequence
_R_marine_VOSU01000010.1_9637007-9637490_Phocoena_sinus_isolate_mPhoSin1_chromosome_19_whole_genome_shotgun_sequence
_R_marine_RJWO010012617.1_11813-12297_Inia_geoffrensis_isolate_BS63_IniGeo_flattened_line_25171_whole_genome_shotgun_sequence
_R_marine_RJWS010430070.1_532-1009_Ziphius_cavirostris_isolate_BS68_ZipCav_flattened_line_860043_whole_genome_shotgun_sequence
marine_PVJJ010020936.1_8234-8711_Mesoplodon_bidens_isolate_US080_MesBid_scaffold_41857_whole_genome_shotgun_sequence
_R_marine_RJWK010030251.1_5707-6190_Platanista_minor_isolate_BS66_PlaMin_flattened_line_60461_whole_genome_shotgun_sequence
_R_marine_VNFC01000015.1_10496571-10497049_Balaenoptera_musculus_isolate_JJ_BM4_2016_0621_Super_scaffold_15_whole_genome_shotgun_sequence
marine_RYZJ01000820.1_3953338-3953819_Megaptera_novaeangliae_breed_North_Atlantic_isolate_ASU_GRON-1_ScjyzU5_238_whole_genome_shotgun_sequence
_R_marine_NIPP01006125.1_133457-133939_Eschrichtius_robustus_isolate_ER-14-168_kmer60-min500.11762051_whole_genome_shotgun_sequence
_R_marine_RJWP010041078.1_7294-7776_Eubalaena_japonica_isolate_BS61_EubJap_flattened_line_82101_whole_genome_shotgun_sequence
RAWU01000022.1_38754511-38754923_Giraffa_tippelskirchi_isolate_OR1865_chromosome_10_whole_genome_shotgun_sequence
RFLZ01088158.1_277-691_Odocoileus_hemionus_sitkensis_isolate_Cn_RI01_contig301005_0_whole_genome_shotgun_sequence
SJXO01002087.1_73961-74374_Connochaetes_taurinus_isolate_BWD-738_scaffold2076_size313931_whole_genome_shotgun_sequence
SJXX01001614.1_484784-485197_Syncerus_caffer_isolate_ABF-221_scaffold2883_len2675142_cov90_whole_genome_shotgun_sequence
PVJP02910524.1_4358590-4359169_Hippopotamus_amphibius_isolate_BS01_ScHSfme_924986HRSCAF_982786_whole_genome_shotgun_sequence
PVHT021183648.1_6562634-6562948_Catagonus_wagneri_isolate_BS18_Sc2sB16_1185004HRSCAF_1231711_whole_genome_shotgun_sequence
PVJP02910469.1_483024-483593_Hippopotamus_amphibius_isolate_BS01_ScHSfme_924936HRSCAF_982736_whole_genome_shotgun_sequence
0.03

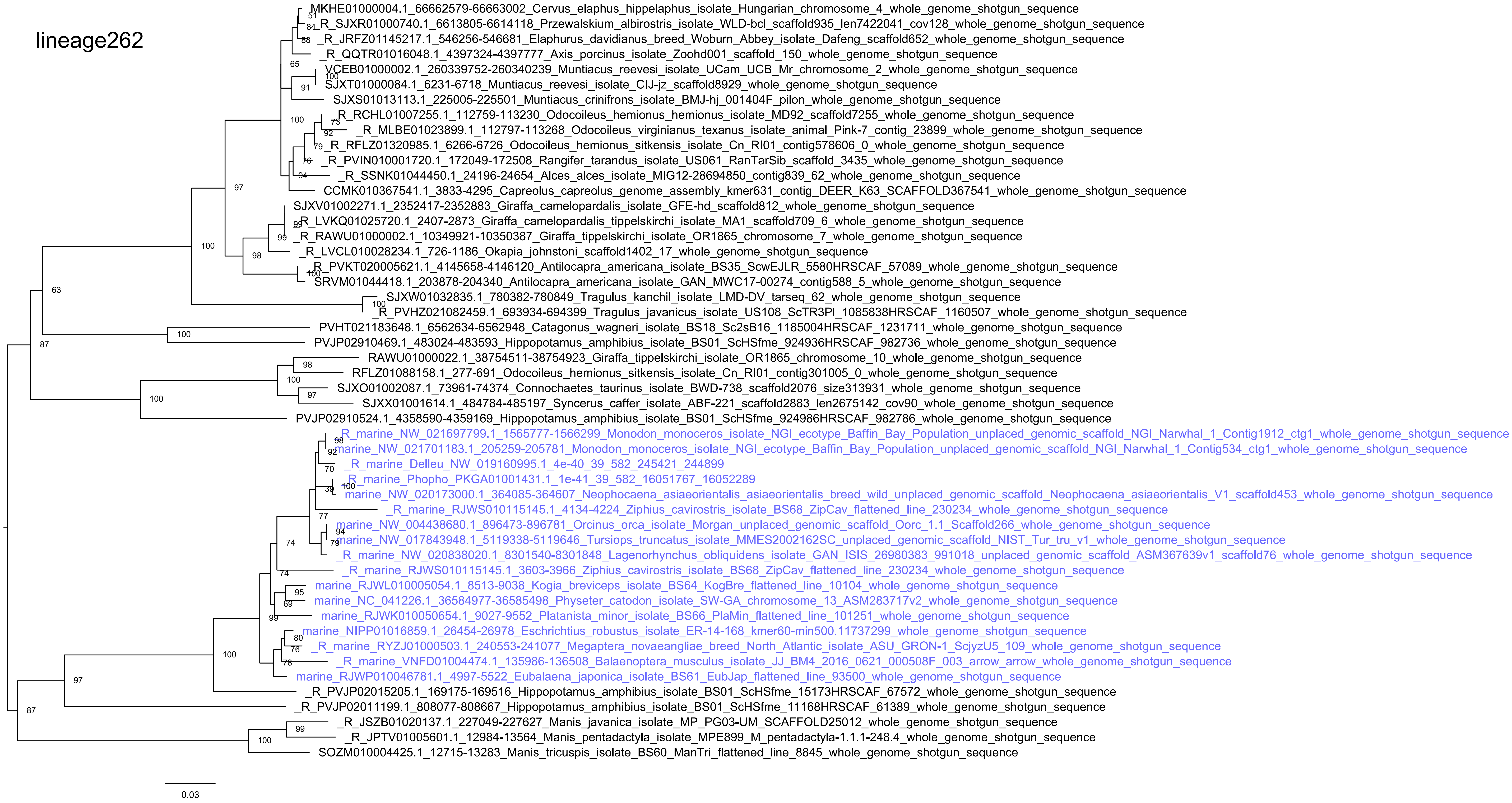
lineage262
MKHE01000004.1_66662579-66663002_Cervus_elaphus_hippelaphus_isolate_Hungarian_chromosome_4_whole_genome_shotgun_sequence
_R_SJXR01000740.1_6613805-6614118_Przewalskium_albirostris_isolate_WLD-bcl_scaffold935_len7422041_cov128_whole_genome_shotgun_sequence
_R_JRFZ01145217.1_546256-546681_Elaphurus_davidianus_breed_Woburn_Abbey_isolate_Dafeng_scaffold652_whole_genome_shotgun_sequence
_R_QQTR01016048.1_4397324-4397777_Axis_porcinus_isolate_Zoohd001_scaffold_150_whole_genome_shotgun_sequence
VCEB01000002.1_260339752-260340239_Muntiacus_reevesi_isolate_UCam_UCB_Mr_chromosome_2_whole_genome_shotgun_sequence
SJXT01000084.1_6231-6718_Muntiacus_reevesi_isolate_CIJ-jz_scaffold8929_whole_genome_shotgun_sequence
SJXS01013113.1_225005-225501_Muntiacus_crinifrons_isolate_BMJ-hj_001404F_pilon_whole_genome_shotgun_sequence
_R_RCHL01007255.1_112759-113230_Odocoileus_hemionus_hemionus_isolate_MD92_scaffold7255_whole_genome_shotgun_sequence
_R_MLBE01023899.1_112797-113268_Odocoileus_virginianus_texanus_isolate_animal_Pink-7_contig_23899_whole_genome_shotgun_sequence
_R_RFLZ01320985.1_6266-6726_Odocoileus_hemionus_sitkensis_isolate_Cn_RI01_contig578606_0_whole_genome_shotgun_sequence
_R_PVIN010001720.1_172049-172508_Rangifer_tarandus_isolate_US061_RanTarSib_scaffold_3435_whole_genome_shotgun_sequence
_R_SSNK01044450.1_24196-24654_Alces_alces_isolate_MIG12-28694850_contig839_62_whole_genome_shotgun_sequence
CCMK010367541.1_3833-4295_Capreolus_capreolus_genome_assembly_kmer631_contig_DEER_K63_SCAFFOLD367541_whole_genome_shotgun_sequence
SJXV01002271.1_2352417-2352883_Giraffa_camelopardalis_isolate_GFE-hd_scaffold812_whole_genome_shotgun_sequence
_R_LVKQ01025720.1_2407-2873_Giraffa_camelopardalis_tippelskirchi_isolate_MA1_scaffold709_6_whole_genome_shotgun_sequence
_R_RAWU01000002.1_10349921-10350387_Giraffa_tippelskirchi_isolate_OR1865_chromosome_7_whole_genome_shotgun_sequence
_R_LVCL010028234.1_726-1186_Okapia_johnstoni_scaffold1402_17_whole_genome_shotgun_sequence
_R_PVKT020005621.1_4145658-4146120_Antilocapra_americana_isolate_BS35_ScwEJLR_5580HRSCAF_57089_whole_genome_shotgun_sequence
SRVM01044418.1_203878-204340_Antilocapra_americana_isolate_GAN_MWC17-00274_contig588_5_whole_genome_shotgun_sequence
SJXW01032835.1_780382-780849_Tragulus_kanchil_isolate_LMD-DV_tarseq_62_whole_genome_shotgun_sequence
_R_PVHZ021082459.1_693934-694399_Tragulus_javanicus_isolate_US108_ScTR3PI_1085838HRSCAF_1160507_whole_genome_shotgun_sequence
PVHT021183648.1_6562634-6562948_Catagonus_wagneri_isolate_BS18_Sc2sB16_1185004HRSCAF_1231711_whole_genome_shotgun_sequence
PVJP02910469.1_483024-483593_Hippopotamus_amphibius_isolate_BS01_ScHSfme_924936HRSCAF_982736_whole_genome_shotgun_sequence
RAWU01000022.1_38754511-38754923_Giraffa_tippelskirchi_isolate_OR1865_chromosome_10_whole_genome_shotgun_sequence
RFLZ01088158.1_277-691_Odocoileus_hemionus_sitkensis_isolate_Cn_RI01_contig301005_0_whole_genome_shotgun_sequence
SJXO01002087.1_73961-74374_Connochaetes_taurinus_isolate_BWD-738_scaffold2076_size313931_whole_genome_shotgun_sequence
SJXX01001614.1_484784-485197_Syncerus_caffer_isolate_ABF-221_scaffold2883_len2675142_cov90_whole_genome_shotgun_sequence
PVJP02910524.1_4358590-4359169_Hippopotamus_amphibius_isolate_BS01_ScHSfme_924986HRSCAF_982786_whole_genome_shotgun_sequence
_R_marine_NW_021697799.1_1565777-1566299_Monodon_monoceros_isolate_NGI_ecotype_Baffin_Bay_Population_unplaced_genomic_scaffold_NGI_Narwhal_1_Contig1912_ctg1_whole_genome_shotgun_sequence
marine_NW_021701183.1_205259-205781_Monodon_monoceros_isolate_NGI_ecotype_Baffin_Bay_Population_unplaced_genomic_scaffold_NGI_Narwhal_1_Contig534_ctg1_whole_genome_shotgun_sequence
_R_marine_Delleu_NW_019160995.1_4e-40_39_582_245421_244899
_R_marine_Phopho_PKGA01001431.1_1e-41_39_582_16051767_16052289
marine_NW_020173000.1_364085-364607_Neophocaena_asiaeorientalis_asiaeorientalis_breed_wild_unplaced_genomic_scaffold_Neophocaena_asiaeorientalis_V1_scaffold453_whole_genome_shotgun_sequence
_R_marine_RJWS010115145.1_4134-4224_Ziphius_cavirostris_isolate_BS68_ZipCav_flattened_line_230234_whole_genome_shotgun_sequence
marine_NW_004438680.1_896473-896781_Orcinus_orca_isolate_Morgan_unplaced_genomic_scaffold_Oorc_1.1_Scaffold266_whole_genome_shotgun_sequence
marine_NW_017843948.1_5119338-5119646_Tursiops_truncatus_isolate_MMES2002162SC_unplaced_genomic_scaffold_NIST_Tur_tru_v1_whole_genome_shotgun_sequence
_R_marine_NW_020838020.1_8301540-8301848_Lagenorhynchus_obliquidens_isolate_GAN_ISIS_26980383_991018_unplaced_genomic_scaffold_ASM367639v1_scaffold76_whole_genome_shotgun_sequence
_R_marine_RJWS010115145.1_3603-3966_Ziphius_cavirostris_isolate_BS68_ZipCav_flattened_line_230234_whole_genome_shotgun_sequence
marine_RJWL010005054.1_8513-9038_Kogia_breviceps_isolate_BS64_KogBre_flattened_line_10104_whole_genome_shotgun_sequence
marine_NC_041226.1_36584977-36585498_Physeter_catodon_isolate_SW-GA_chromosome_13_ASM283717v2_whole_genome_shotgun_sequence
marine_RJWK010050654.1_9027-9552_Platanista_minor_isolate_BS66_PlaMin_flattened_line_101251_whole_genome_shotgun_sequence
marine_NIPP01016859.1_26454-26978_Eschrichtius_robustus_isolate_ER-14-168_kmer60-min500.11737299_whole_genome_shotgun_sequence
_R_marine_RYZJ01000503.1_240553-241077_Megaptera_novaeangliae_breed_North_Atlantic_isolate_ASU_GRON-1_ScjyzU5_109_whole_genome_shotgun_sequence
_R_marine_VNFD01004474.1_135986-136508_Balaenoptera_musculus_isolate_JJ_BM4_2016_0621_000508F_003_arrow_arrow_whole_genome_shotgun_sequence
marine_RJWP010046781.1_4997-5522_Eubalaena_japonica_isolate_BS61_EubJap_flattened_line_93500_whole_genome_shotgun_sequence
_R_PVJP02015205.1_169175-169516_Hippopotamus_amphibius_isolate_BS01_ScHSfme_15173HRSCAF_67572_whole_genome_shotgun_sequence
_R_PVJP02011199.1_808077-808667_Hippopotamus_amphibius_isolate_BS01_ScHSfme_11168HRSCAF_61389_whole_genome_shotgun_sequence
_R_JSZB01020137.1_227049-227627_Manis_javanica_isolate_MP_PG03-UM_SCAFFOLD25012_whole_genome_shotgun_sequence
_R_JPTV01005601.1_12984-13564_Manis_pentadactyla_isolate_MPE899_M_pentadactyla-1.1.1-248.4_whole_genome_shotgun_sequence
SOZM010004425.1_12715-13283_Manis_tricuspis_isolate_BS60_ManTri_flattened_line_8845_whole_genome_shotgun_sequence
0.03

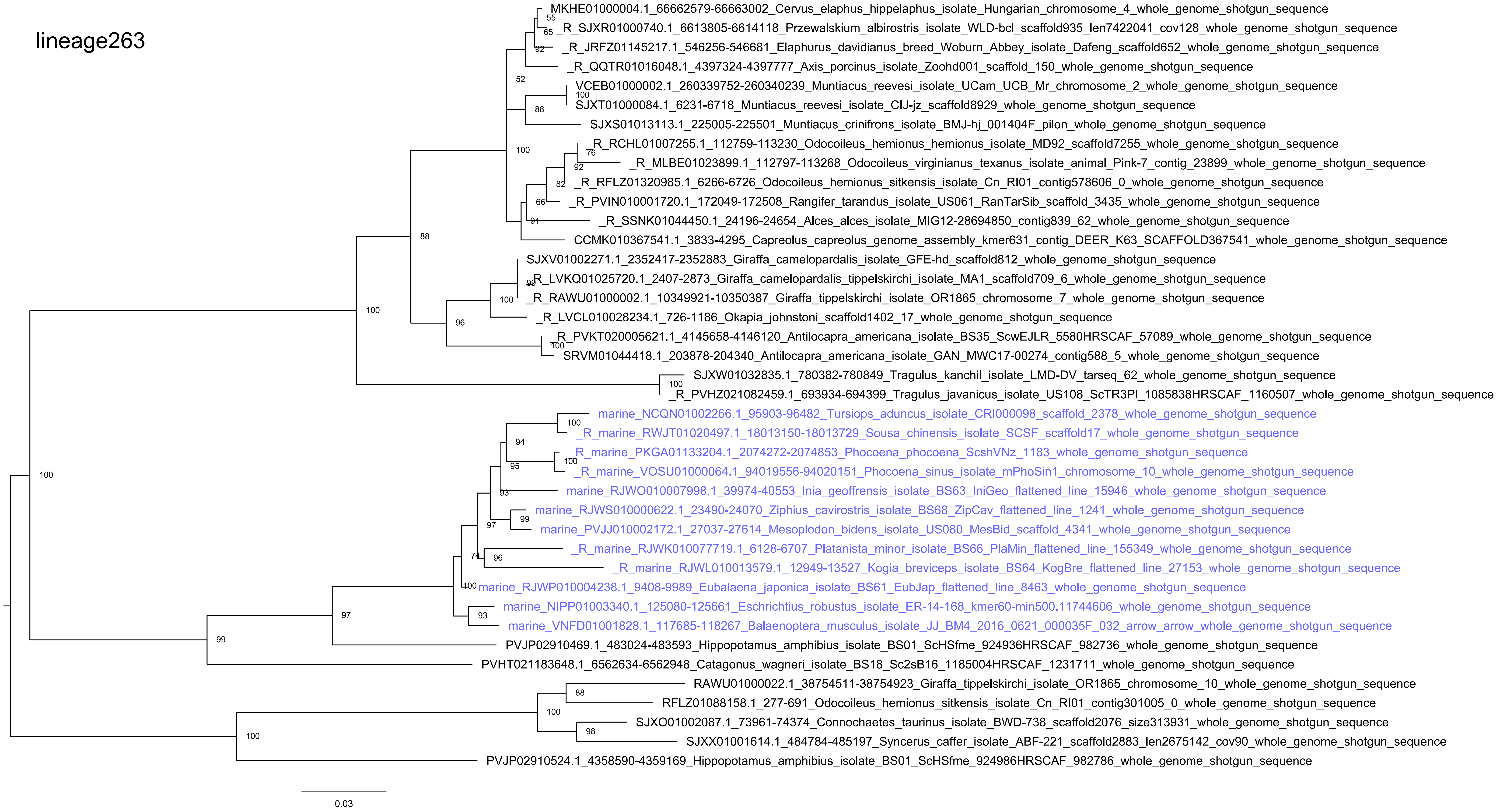
lineage263
MKHE01000004.1_66662579-66663002_Cervus_elaphus_hippelaphus_isolate_Hungarian_chromosome_4_whole_genome_shotgun_sequence
_R_SJXR01000740.1_6613805-6614118_Przewalskium_albirostris_isolate_WLD-bcl_scaffold935_len7422041_cov128_whole_genome_shotgun_sequence
_R_JRFZ01145217.1_546256-546681_Elaphurus_davidianus_breed_Woburn_Abbey_isolate_Dafeng_scaffold652_whole_genome_shotgun_sequence
_R_QQTR01016048.1_4397324-4397777_Axis_porcinus_isolate_Zoohd001_scaffold_150_whole_genome_shotgun_sequence
VCEB01000002.1_260339752-260340239_Muntiacus_reevesi_isolate_UCam_UCB_Mr_chromosome_2_whole_genome_shotgun_sequence
SJXT01000084.1_6231-6718_Muntiacus_reevesi_isolate_CIJ-jz_scaffold8929_whole_genome_shotgun_sequence
SJXS01013113.1_225005-225501_Muntiacus_crinifrons_isolate_BMJ-hj_001404F_pilon_whole_genome_shotgun_sequence
_R_RCHL01007255.1_112759-113230_Odocoileus_hemionus_hemionus_isolate_MD92_scaffold7255_whole_genome_shotgun_sequence
_R_MLBE01023899.1_112797-113268_Odocoileus_virginianus_texanus_isolate_animal_Pink-7_contig_23899_whole_genome_shotgun_sequence
_R_RFLZ01320985.1_6266-6726_Odocoileus_hemionus_sitkensis_isolate_Cn_RI01_contig578606_0_whole_genome_shotgun_sequence
_R_PVIN010001720.1_172049-172508_Rangifer_tarandus_isolate_US061_RanTarSib_scaffold_3435_whole_genome_shotgun_sequence
_R_SSNK01044450.1_24196-24654_Alces_alces_isolate_MIG12-28694850_contig839_62_whole_genome_shotgun_sequence
CCMK010367541.1_3833-4295_Capreolus_capreolus_genome_assembly_kmer631_contig_DEER_K63_SCAFFOLD367541_whole_genome_shotgun_sequence
SJXV01002271.1_2352417-2352883_Giraffa_camelopardalis_isolate_GFE-hd_scaffold812_whole_genome_shotgun_sequence
_R_LVKQ01025720.1_2407-2873_Giraffa_camelopardalis_tippelskirchi_isolate_MA1_scaffold709_6_whole_genome_shotgun_sequence
_R_RAWU01000002.1_10349921-10350387_Giraffa_tippelskirchi_isolate_OR1865_chromosome_7_whole_genome_shotgun_sequence
_R_LVCL010028234.1_726-1186_Okapia_johnstoni_scaffold1402_17_whole_genome_shotgun_sequence
_R_PVKT020005621.1_4145658-4146120_Antilocapra_americana_isolate_BS35_ScwEJLR_5580HRSCAF_57089_whole_genome_shotgun_sequence
SRVM01044418.1_203878-204340_Antilocapra_americana_isolate_GAN_MWC17-00274_contig588_5_whole_genome_shotgun_sequence
SJXW01032835.1_780382-780849_Tragulus_kanchil_isolate_LMD-DV_tarseq_62_whole_genome_shotgun_sequence
_R_PVHZ021082459.1_693934-694399_Tragulus_javanicus_isolate_US108_ScTR3PI_1085838HRSCAF_1160507_whole_genome_shotgun_sequence
marine_NCQN01002266.1_95903-96482_Tursiops_aduncus_isolate_CRI000098_scaffold_2378_whole_genome_shotgun_sequence
_R_marine_RWJT01020497.1_18013150-18013729_Sousa_chinensis_isolate_SCSF_scaffold17_whole_genome_shotgun_sequence
_R_marine_PKGA01133204.1_2074272-2074853_Phocoena_phocoena_ScshVNz_1183_whole_genome_shotgun_sequence
_R_marine_VOSU01000064.1_94019556-94020151_Phocoena_sinus_isolate_mPhoSin1_chromosome_10_whole_genome_shotgun_sequence
marine_RJWO010007998.1_39974-40553_Inia_geoffrensis_isolate_BS63_IniGeo_flattened_line_15946_whole_genome_shotgun_sequence
marine_RJWS010000622.1_23490-24070_Ziphius_cavirostris_isolate_BS68_ZipCav_flattened_line_1241_whole_genome_shotgun_sequence
marine_PVJJ010002172.1_27037-27614_Mesoplodon_bidens_isolate_US080_MesBid_scaffold_4341_whole_genome_shotgun_sequence
_R_marine_RJWK010077719.1_6128-6707_Platanista_minor_isolate_BS66_PlaMin_flattened_line_155349_whole_genome_shotgun_sequence
_R_marine_RJWL010013579.1_12949-13527_Kogia_breviceps_isolate_BS64_KogBre_flattened_line_27153_whole_genome_shotgun_sequence
marine_RJWP010004238.1_9408-9989_Eubalaena_japonica_isolate_BS61_EubJap_flattened_line_8463_whole_genome_shotgun_sequence
marine_NIPP01003340.1_125080-125661_Eschrichtius_robustus_isolate_ER-14-168_kmer60-min500.11744606_whole_genome_shotgun_sequence
marine_VNFD01001828.1_117685-118267_Balaenoptera_musculus_isolate_JJ_BM4_2016_0621_000035F_032_arrow_arrow_whole_genome_shotgun_sequence
PVJP02910469.1_483024-483593_Hippopotamus_amphibius_isolate_BS01_ScHSfme_924936HRSCAF_982736_whole_genome_shotgun_sequence
PVHT021183648.1_6562634-6562948_Catagonus_wagneri_isolate_BS18_Sc2sB16_1185004HRSCAF_1231711_whole_genome_shotgun_sequence
RAWU01000022.1_38754511-38754923_Giraffa_tippelskirchi_isolate_OR1865_chromosome_10_whole_genome_shotgun_sequence
RFLZ01088158.1_277-691_Odocoileus_hemionus_sitkensis_isolate_Cn_RI01_contig301005_0_whole_genome_shotgun_sequence
SJXO01002087.1_73961-74374_Connochaetes_taurinus_isolate_BWD-738_scaffold2076_size313931_whole_genome_shotgun_sequence
SJXX01001614.1_484784-485197_Syncerus_caffer_isolate_ABF-221_scaffold2883_len2675142_cov90_whole_genome_shotgun_sequence
PVJP02910524.1_4358590-4359169_Hippopotamus_amphibius_isolate_BS01_ScHSfme_924986HRSCAF_982786_whole_genome_shotgun_sequence
0.03

lineage264

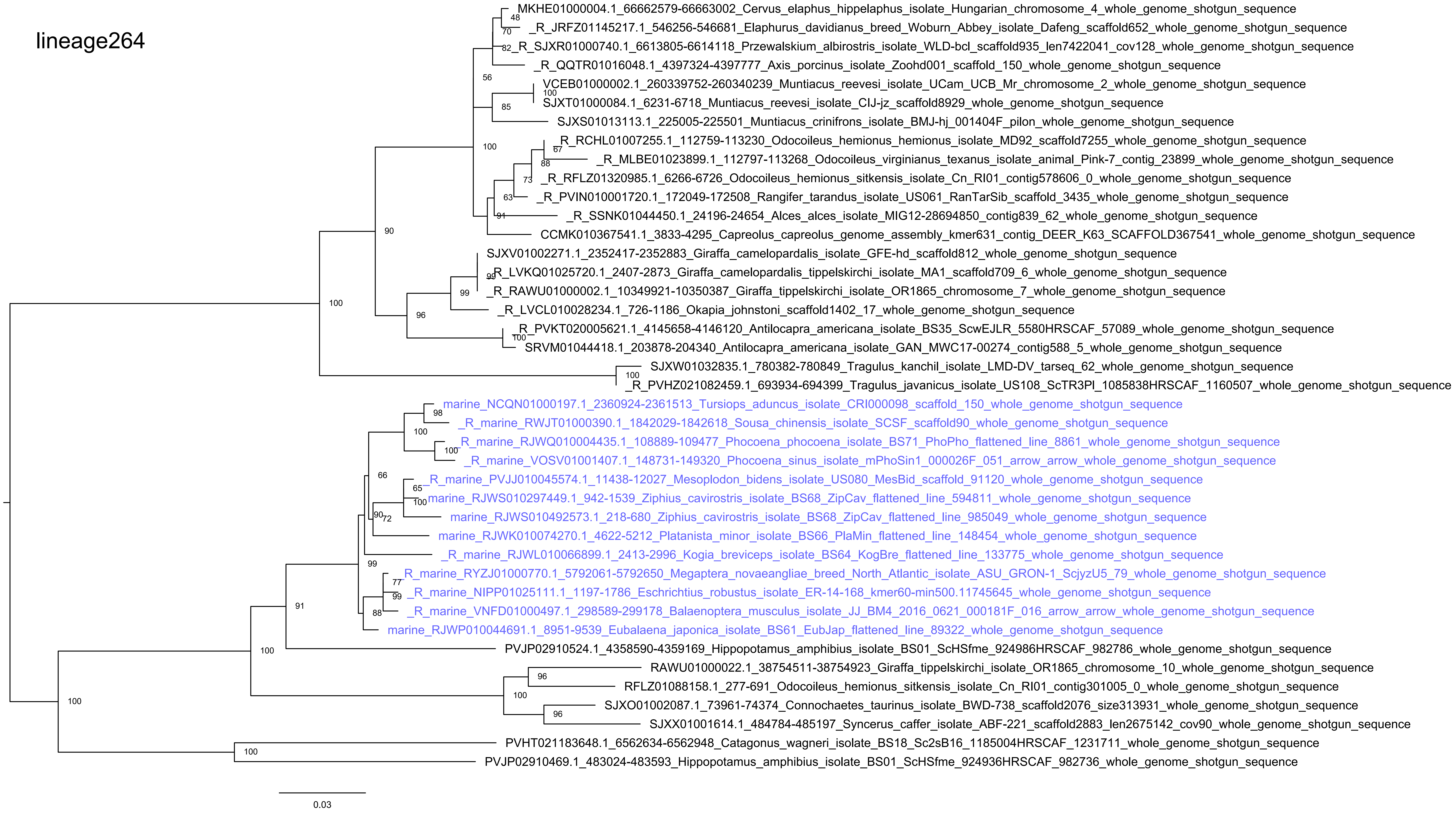
MKHE01000004.1_66662579-66663002_Cervus_elaphus_hippelaphus_isolate_Hungarian_chromosome_4_whole_genome_shotgun_sequence
_R_JRFZ01145217.1_546256-546681_Elaphurus_davidianus_breed_Woburn_Abbey_isolate_Dafeng_scaffold652_whole_genome_shotgun_sequence
_R_SJXR01000740.1_6613805-6614118_Przewalskium_albirostris_isolate_WLD-bcl_scaffold935_len7422041_cov128_whole_genome_shotgun_sequence
_R_QQTR01016048.1_4397324-4397777_Axis_porcinus_isolate_Zoohd001_scaffold_150_whole_genome_shotgun_sequence
VCEB01000002.1_260339752-260340239_Muntiacus_reevesi_isolate_UCam_UCB_Mr_chromosome_2_whole_genome_shotgun_sequence
SJXT01000084.1_6231-6718_Muntiacus_reevesi_isolate_CIJ-jz_scaffold8929_whole_genome_shotgun_sequence
SJXS01013113.1_225005-225501_Muntiacus_crinifrons_isolate_BMJ-hj_001404F_pilon_whole_genome_shotgun_sequence
_R_RCHL01007255.1_112759-113230_Odocoileus_hemionus_hemionus_isolate_MD92_scaffold7255_whole_genome_shotgun_sequence
_R_MLBE01023899.1_112797-113268_Odocoileus_virginianus_texanus_isolate_animal_Pink-7_contig_23899_whole_genome_shotgun_sequence
_R_RFLZ01320985.1_6266-6726_Odocoileus_hemionus_sitkensis_isolate_Cn_RI01_contig578606_0_whole_genome_shotgun_sequence
_R_PVIN010001720.1_172049-172508_Rangifer_tarandus_isolate_US061_RanTarSib_scaffold_3435_whole_genome_shotgun_sequence
_R_SSNK01044450.1_24196-24654_Alces_alces_isolate_MIG12-28694850_contig839_62_whole_genome_shotgun_sequence
CCMK010367541.1_3833-4295_Capreolus_capreolus_genome_assembly_kmer631_contig_DEER_K63_SCAFFOLD367541_whole_genome_shotgun_sequence
SJXV01002271.1_2352417-2352883_Giraffa_camelopardalis_isolate_GFE-hd_scaffold812_whole_genome_shotgun_sequence
_R_LVKQ01025720.1_2407-2873_Giraffa_camelopardalis_tippelskirchi_isolate_MA1_scaffold709_6_whole_genome_shotgun_sequence
_R_RAWU01000002.1_10349921-10350387_Giraffa_tippelskirchi_isolate_OR1865_chromosome_7_whole_genome_shotgun_sequence
_R_LVCL010028234.1_726-1186_Okapia_johnstoni_scaffold1402_17_whole_genome_shotgun_sequence
_R_PVKT020005621.1_4145658-4146120_Antilocapra_americana_isolate_BS35_ScwEJLR_5580HRSCAF_57089_whole_genome_shotgun_sequence
SRVM01044418.1_203878-204340_Antilocapra_americana_isolate_GAN_MWC17-00274_contig588_5_whole_genome_shotgun_sequence
SJXW01032835.1_780382-780849_Tragulus_kanchil_isolate_LMD-DV_tarseq_62_whole_genome_shotgun_sequence
_R_PVHZ021082459.1_693934-694399_Tragulus_javanicus_isolate_US108_ScTR3PI_1085838HRSCAF_1160507_whole_genome_shotgun_sequence
marine_NCQN01000197.1_2360924-2361513_Tursiops_aduncus_isolate_CRI000098_scaffold_150_whole_genome_shotgun_sequence
_R_marine_RWJT01000390.1_1842029-1842618_Sousa_chinensis_isolate_SCSF_scaffold90_whole_genome_shotgun_sequence
_R_marine_RJWQ010004435.1_108889-109477_Phocoena_phocoena_isolate_BS71_PhoPho_flattened_line_8861_whole_genome_shotgun_sequence
_R_marine_VOSV01001407.1_148731-149320_Phocoena_sinus_isolate_mPhoSin1_000026F_051_arrow_arrow_whole_genome_shotgun_sequence
_R_marine_PVJJ010045574.1_11438-12027_Mesoplodon_bidens_isolate_US080_MesBid_scaffold_91120_whole_genome_shotgun_sequence
marine_RJWS010297449.1_942-1539_Ziphius_cavirostris_isolate_BS68_ZipCav_flattened_line_594811_whole_genome_shotgun_sequence
marine_RJWS010492573.1_218-680_Ziphius_cavirostris_isolate_BS68_ZipCav_flattened_line_985049_whole_genome_shotgun_sequence
marine_RJWK010074270.1_4622-5212_Platanista_minor_isolate_BS66_PlaMin_flattened_line_148454_whole_genome_shotgun_sequence
_R_marine_RJWL010066899.1_2413-2996_Kogia_breviceps_isolate_BS64_KogBre_flattened_line_133775_whole_genome_shotgun_sequence
_R_marine_RYZJ01000770.1_5792061-5792650_Megaptera_novaeangliae_breed_North_Atlantic_isolate_ASU_GRON-1_ScjyzU5_79_whole_genome_shotgun_sequence
_R_marine_NIPP01025111.1_1197-1786_Eschrichtius_robustus_isolate_ER-14-168_kmer60-min500.11745645_whole_genome_shotgun_sequence
_R_marine_VNFD01000497.1_298589-299178_Balaenoptera_musculus_isolate_JJ_BM4_2016_0621_000181F_016_arrow_arrow_whole_genome_shotgun_sequence
marine_RJWP010044691.1_8951-9539_Eubalaena_japonica_isolate_BS61_EubJap_flattened_line_89322_whole_genome_shotgun_sequence
PVJP02910524.1_4358590-4359169_Hippopotamus_amphibius_isolate_BS01_ScHSfme_924986HRSCAF_982786_whole_genome_shotgun_sequence
RAWU01000022.1_38754511-38754923_Giraffa_tippelskirchi_isolate_OR1865_chromosome_10_whole_genome_shotgun_sequence
RFLZ01088158.1_277-691_Odocoileus_hemionus_sitkensis_isolate_Cn_RI01_contig301005_0_whole_genome_shotgun_sequence
SJXO01002087.1_73961-74374_Connochaetes_taurinus_isolate_BWD-738_scaffold2076_size313931_whole_genome_shotgun_sequence
SJXX01001614.1_484784-485197_Syncerus_caffer_isolate_ABF-221_scaffold2883_len2675142_cov90_whole_genome_shotgun_sequence
PVHT021183648.1_6562634-6562948_Catagonus_wagneri_isolate_BS18_Sc2sB16_1185004HRSCAF_1231711_whole_genome_shotgun_sequence
PVJP02910469.1_483024-483593_Hippopotamus_amphibius_isolate_BS01_ScHSfme_924936HRSCAF_982736_whole_genome_shotgun_sequence
0.03

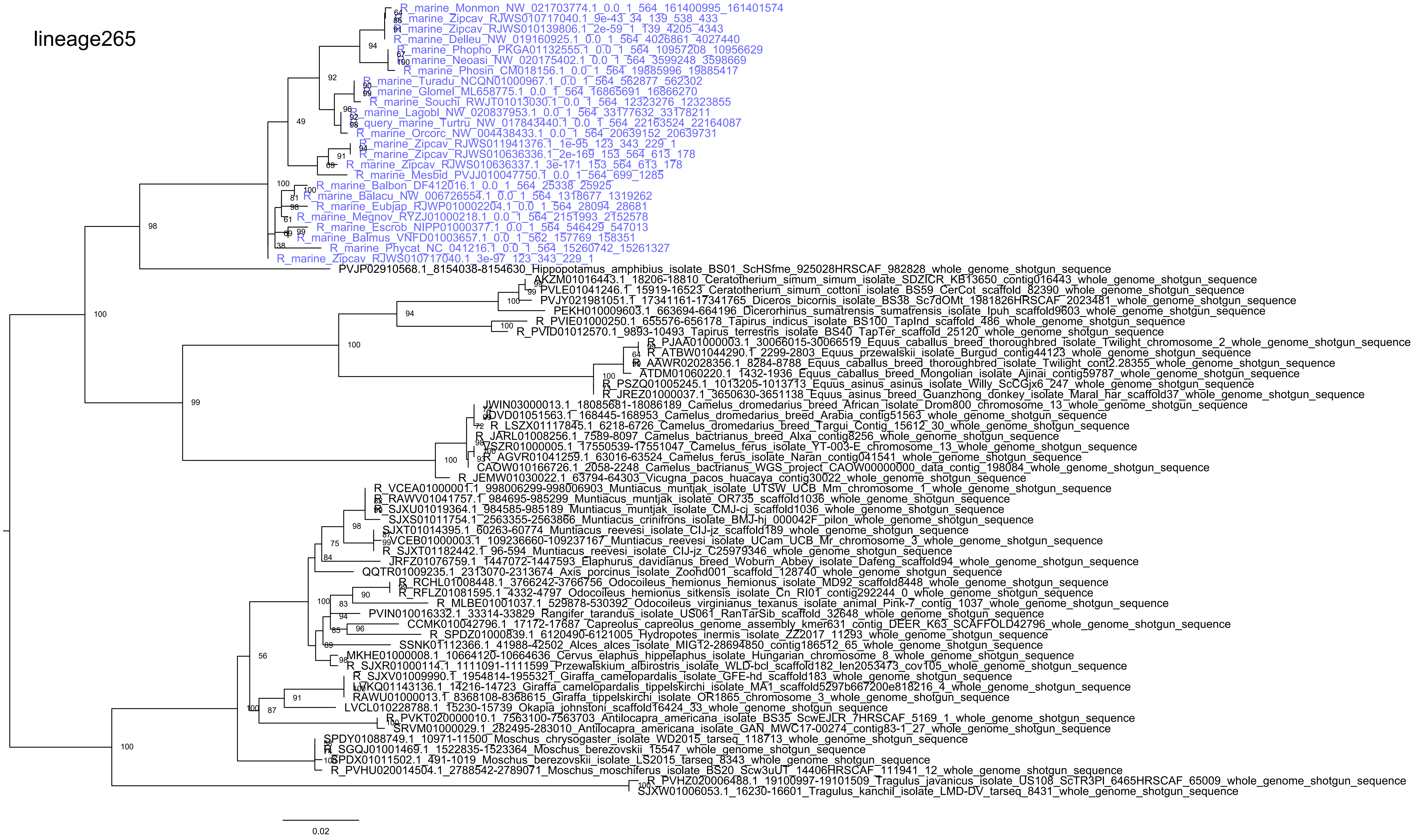
lineage265
R_marine_Monmon_NW_021703774.1_0.0_1_564_161400995_161401574
R_marine_Zipcav_RJWS010717040.1_9e-43_34_139_538_433
R_marine_Zipcav_RJWS010139806.1_2e-59_1_139_4205_4343
R_marine_Delleu_NW_019160925.1_0.0_1_564_4026861_4027440
R_marine_Phopho_PKGA01132555.1_0.0_1_564_10957208_10956629
R_marine_Neoasi_NW_020175402.1_0.0_1_564_3599248_3598669
R_marine_Phosin_CM018156.1_0.0_1_564_19885996_19885417
R_marine_Turadu_NCQN01000967.1_0.0_1_564_562877_562302
R_marine_Glomel_ML658775.1_0.0_1_564_16865691_16866270
R_marine_Souchi_RWJT01013030.1_0.0_1_564_12323276_12323855
R_marine_Lagobl_NW_020837953.1_0.0_1_564_33177632_33178211
R_query_marine_Turtru_NW_017843440.1_0.0_1_564_22163524_22164087
R_marine_Orcorc_NW_004438433.1_0.0_1_564_20639152_20639731
R_marine_Zipcav_RJWS011941376.1_1e-95_123_343_229_1
R_marine_Zipcav_RJWS010636336.1_2e-169_153_564_613_178
R_marine_Zipcav_RJWS010636337.1_3e-171_153_564_613_178
R_marine_Mesbid_PVJJ010047750.1_0.0_1_564_699_1285
R_marine_Balbon_DF412016.1_0.0_1_564_25338_25925
R_marine_Balacu_NW_006726554.1_0.0_1_564_1318677_1319262
R_marine_Eubjap_RJWP010002204.1_0.0_1_564_28094_28681
R_marine_Megnov_RYZJ01000218.1_0.0_1_564_2151993_2152578
R_marine_Escrob_NIPP01000377.1_0.0_1_564_546429_547013
R_marine_Balmus_VNFD01003657.1_0.0_1_562_157769_158351
R_marine_Phycat_NC_041216.1_0.0_1_564_15260742_15261327
R_marine_Zipcav_RJWS010717040.1_3e-97_123_343_229_1
PVJP02910568.1_8154038-8154630_Hippopotamus_amphibius_isolate_BS01_ScHSfme_925028HRSCAF_982828_whole_genome_shotgun_sequence
AKZM01016443.1_18206-18810_Ceratotherium_simum_simum_isolate_SDZICR_KB13650_contig016443_whole_genome_shotgun_sequence
PVLE01041246.1_15919-16523_Ceratotherium_simum_cottoni_isolate_BS59_CerCot_scaffold_82390_whole_genome_shotgun_sequence
PVJY021981051.1_17341161-17341765_Diceros_bicornis_isolate_BS38_Sc7dOMt_1981826HRSCAF_2023481_whole_genome_shotgun_sequence
PEKH010009603.1_663694-664196_Dicerorhinus_sumatrensis_sumatrensis_isolate_Ipuh_scaffold9603_whole_genome_shotgun_sequence
R_PVIE01000250.1_655576-656178_Tapirus_indicus_isolate_BS100_TapInd_scaffold_486_whole_genome_shotgun_sequence
R_PVID01012570.1_9893-10493_Tapirus_terrestris_isolate_BS40_TapTer_scaffold_25120_whole_genome_shotgun_sequence
R_PJAA01000003.1_30066015-30066519_Equus_caballus_breed_thoroughbred_isolate_Twilight_chromosome_2_whole_genome_shotgun_sequence
R_ATBW01044290.1_2299-2803_Equus_przewalskii_isolate_Burgud_contig44123_whole_genome_shotgun_sequence
R_AAWR02028356.1_8284-8788_Equus_caballus_breed_thoroughbred_isolate_Twilight_cont2.28355_whole_genome_shotgun_sequence
ATDM01060220.1_1432-1936_Equus_caballus_breed_Mongolian_isolate_Ajinai_contig59787_whole_genome_shotgun_sequence
R_PSZQ01005245.1_1013205-1013713_Equus_asinus_asinus_isolate_Willy_ScCGjx6_247_whole_genome_shotgun_sequence
R_JREZ01000037.1_3650630-3651138_Equus_asinus_breed_Guanzhong_donkey_isolate_Maral_har_scaffold37_whole_genome_shotgun_sequence
JWIN03000013.1_18085681-18086189_Camelus_dromedarius_breed_African_isolate_Drom800_chromosome_13_whole_genome_shotgun_sequence
JDVD01051563.1_168445-168953_Camelus_dromedarius_breed_Arabia_contig51563_whole_genome_shotgun_sequence
R_LSZX01117845.1_6218-6726_Camelus_dromedarius_breed_Targui_Contig_15612_30_whole_genome_shotgun_sequence
R_JARL01008256.1_7589-8097_Camelus_bactrianus_breed_Alxa_contig8256_whole_genome_shotgun_sequence
VSZR01000005.1_17550539-17551047_Camelus_ferus_isolate_YT-003-E_chromosome_13_whole_genome_shotgun_sequence
R_AGVR01041259.1_63016-63524_Camelus_ferus_isolate_Naran_contig041541_whole_genome_shotgun_sequence
CAOW010166726.1_2058-2248_Camelus_bactrianus_WGS_project_CAOW00000000_data_contig_198084_whole_genome_shotgun_sequence
R_JEMW01030022.1_63794-64303_Vicugna_pacos_huacaya_contig30022_whole_genome_shotgun_sequence
R_VCEA01000001.1_998006299-998006903_Muntiacus_muntjak_isolate_UTSW_UCB_Mm_chromosome_1_whole_genome_shotgun_sequence
R_RAWV01041757.1_984695-985299_Muntiacus_muntjak_isolate_OR735_scaffold1036_whole_genome_shotgun_sequence
R_SJXU01019364.1_984585-985189_Muntiacus_muntjak_isolate_CMJ-cj_scaffold1036_whole_genome_shotgun_sequence
SJXS01011754.1_2563355-2563866_Muntiacus_crinifrons_isolate_BMJ-hj_000042F_pilon_whole_genome_shotgun_sequence
SJXT01014395.1_60263-60774_Muntiacus_reevesi_isolate_CIJ-jz_scaffold189_whole_genome_shotgun_sequence
VCEB01000003.1_109236660-109237167_Muntiacus_reevesi_isolate_UCam_UCB_Mr_chromosome_3_whole_genome_shotgun_sequence
R_SJXT01182442.1_96-594_Muntiacus_reevesi_isolate_CIJ-jz_C25979346_whole_genome_shotgun_sequence
JRFZ01076759.1_1447072-1447593_Elaphurus_davidianus_breed_Woburn_Abbey_isolate_Dafeng_scaffold94_whole_genome_shotgun_sequence
QQTR01009235.1_2313070-2313674_Axis_porcinus_isolate_Zoohd001_scaffold_128740_whole_genome_shotgun_sequence
R_RCHL01008448.1_3766242-3766756_Odocoileus_hemionus_hemionus_isolate_MD92_scaffold8448_whole_genome_shotgun_sequence
R_RFLZ01081595.1_4332-4797_Odocoileus_hemionus_sitkensis_isolate_Cn_RI01_contig292244_0_whole_genome_shotgun_sequence
R_MLBE01001037.1_529878-530392_Odocoileus_virginianus_texanus_isolate_animal_Pink-7_contig_1037_whole_genome_shotgun_sequence
PVIN010016332.1_33314-33829_Rangifer_tarandus_isolate_US061_RanTarSib_scaffold_32648_whole_genome_shotgun_sequence
CCMK010042796.1_17172-17687_Capreolus_capreolus_genome_assembly_kmer631_contig_DEER_K63_SCAFFOLD42796_whole_genome_shotgun_sequence
R_SPDZ01000839.1_6120490-6121005_Hydropotes_inermis_isolate_ZZ2017_11293_whole_genome_shotgun_sequence
SSNK01112366.1_41988-42502_Alces_alces_isolate_MIG12-28694850_contig186512_65_whole_genome_shotgun_sequence
MKHE01000008.1_10664120-10664636_Cervus_elaphus_hippelaphus_isolate_Hungarian_chromosome_8_whole_genome_shotgun_sequence
R_SJXR01000114.1_1111091-1111599_Przewalskium_albirostris_isolate_WLD-bcl_scaffold182_len2053473_cov105_whole_genome_shotgun_sequence
R_SJXV01009990.1_1954814-1955321_Giraffa_camelopardalis_isolate_GFE-hd_scaffold183_whole_genome_shotgun_sequence
LVKQ01143136.1_14216-14723_Giraffa_camelopardalis_tippelskirchi_isolate_MA1_scaffold5297b667200e818216_4_whole_genome_shotgun_sequence
RAWU01000013.1_8368108-8368615_Giraffa_tippelskirchi_isolate_OR1865_chromosome_3_whole_genome_shotgun_sequence
LVCL010228788.1_15230-15739_Okapia_johnstoni_scaffold16424_33_whole_genome_shotgun_sequence
R_PVKT020000010.1_7563100-7563703_Antilocapra_americana_isolate_BS35_ScwEJLR_7HRSCAF_5169_1_whole_genome_shotgun_sequence
SRVM01000029.1_282495-283010_Antilocapra_americana_isolate_GAN_MWC17-00274_contig83-1_27_whole_genome_shotgun_sequence
SPDY01088749.1_10971-11500_Moschus_chrysogaster_isolate_WD2015_tarseq_118713_whole_genome_shotgun_sequence
R_SGQJ01001469.1_1522835-1523364_Moschus_berezovskii_15547_whole_genome_shotgun_sequence
SPDX01011502.1_491-1019_Moschus_berezovskii_isolate_LS2015_tarseq_8343_whole_genome_shotgun_sequence
R_PVHU020014504.1_2788542-2789071_Moschus_moschiferus_isolate_BS20_Scw3uUT_14406HRSCAF_111941_12_whole_genome_shotgun_sequence
R_PVHZ020006488.1_19100997-19101509_Tragulus_javanicus_isolate_US108_ScTR3PI_6465HRSCAF_65009_whole_genome_shotgun_sequence
SJXW01006053.1_16230-16601_Tragulus_kanchil_isolate_LMD-DV_tarseq_8431_whole_genome_shotgun_sequence
0.02

lineage266

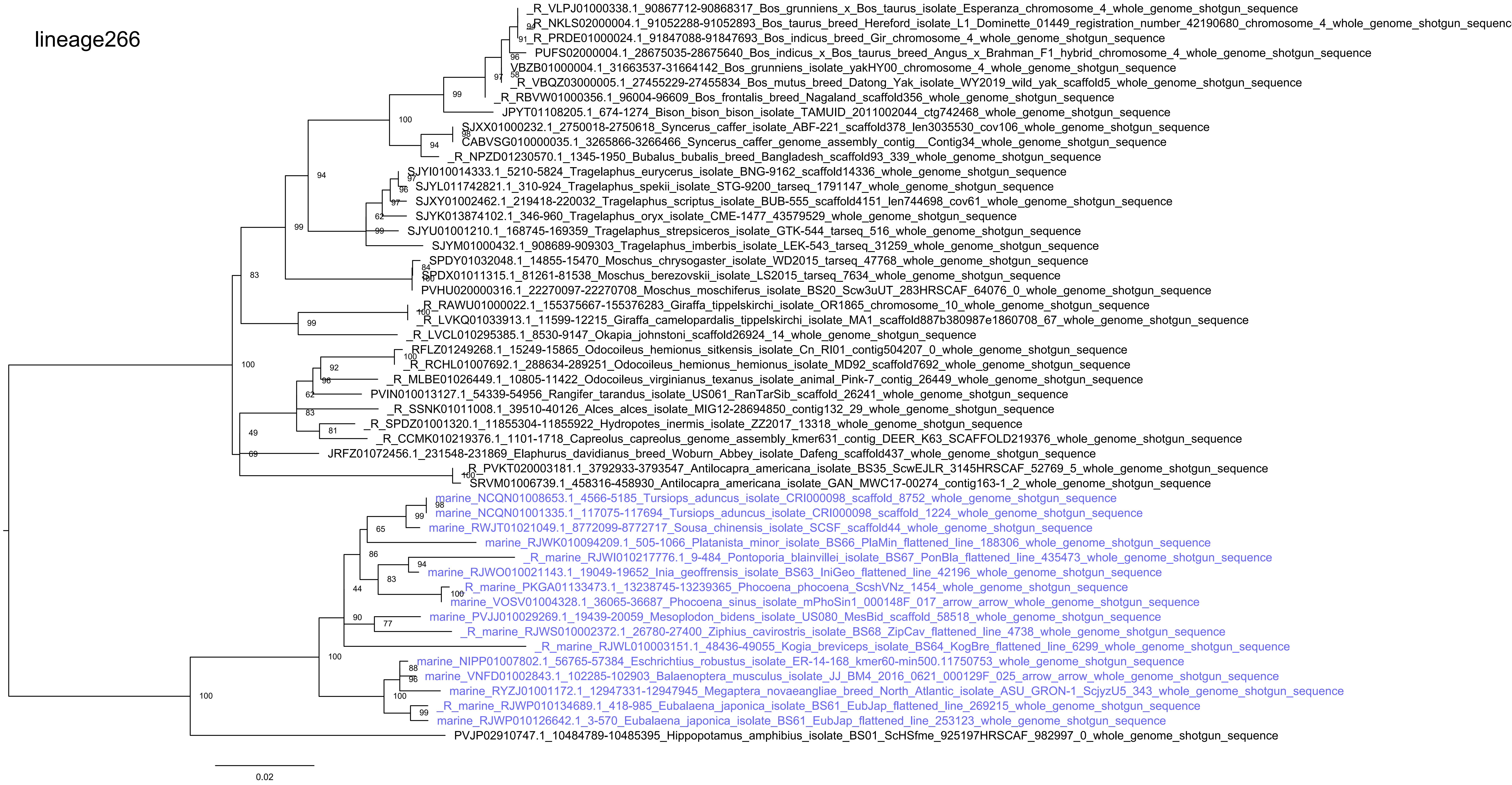
_R_VLPJ01000338.1_90867712-90868317_Bos_grunniens_x_Bos_taurus_isolate_Esperanza_chromosome_4_whole_genome_shotgun_sequence
_R_NKLS02000004.1_91052288-91052893_Bos_taurus_breed_Hereford_isolate_L1_Dominette_01449_registration_number_42190680_chromosome_4_whole_genome_shotgun_sequenc
_R_PRDE01000024.1_91847088-91847693_Bos_indicus_breed_Gir_chromosome_4_whole_genome_shotgun_sequence
PUFS02000004.1_28675035-28675640_Bos_indicus_x_Bos_taurus_breed_Angus_x_Brahman_F1_hybrid_chromosome_4_whole_genome_shotgun_sequence
VBZB01000004.1_31663537-31664142_Bos_grunniens_isolate_yakHY00_chromosome_4_whole_genome_shotgun_sequence
_R_VBQZ03000005.1_27455229-27455834_Bos_mutus_breed_Datong_Yak_isolate_WY2019_wild_yak_scaffold5_whole_genome_shotgun_sequence
_R_RBVW01000356.1_96004-96609_Bos_frontalis_breed_Nagaland_scaffold356_whole_genome_shotgun_sequence
JPYT01108205.1_674-1274_Bison_bison_bison_isolate_TAMUID_2011002044_ctg742468_whole_genome_shotgun_sequence
SJXX01000232.1_2750018-2750618_Syncerus_caffer_isolate_ABF-221_scaffold378_len3035530_cov106_whole_genome_shotgun_sequence
CABVSG010000035.1_3265866-3266466_Syncerus_caffer_genome_assembly_contig__Contig34_whole_genome_shotgun_sequence
_R_NPZD01230570.1_1345-1950_Bubalus_bubalis_breed_Bangladesh_scaffold93_339_whole_genome_shotgun_sequence
SJYI010014333.1_5210-5824_Tragelaphus_eurycerus_isolate_BNG-9162_scaffold14336_whole_genome_shotgun_sequence
SJYL011742821.1_310-924_Tragelaphus_spekii_isolate_STG-9200_tarseq_1791147_whole_genome_shotgun_sequence
SJXY01002462.1_219418-220032_Tragelaphus_scriptus_isolate_BUB-555_scaffold4151_len744698_cov61_whole_genome_shotgun_sequence
SJYK013874102.1_346-960_Tragelaphus_oryx_isolate_CME-1477_43579529_whole_genome_shotgun_sequence
SJYU01001210.1_168745-169359_Tragelaphus_strepsiceros_isolate_GTK-544_tarseq_516_whole_genome_shotgun_sequence
SJYM01000432.1_908689-909303_Tragelaphus_imberbis_isolate_LEK-543_tarseq_31259_whole_genome_shotgun_sequence
SPDY01032048.1_14855-15470_Moschus_chrysogaster_isolate_WD2015_tarseq_47768_whole_genome_shotgun_sequence
SPDX01011315.1_81261-81538_Moschus_berezovskii_isolate_LS2015_tarseq_7634_whole_genome_shotgun_sequence
PVHU020000316.1_22270097-22270708_Moschus_moschiferus_isolate_BS20_Scw3uUT_283HRSCAF_64076_0_whole_genome_shotgun_sequence
_R_RAWU01000022.1_155375667-155376283_Giraffa_tippelskirchi_isolate_OR1865_chromosome_10_whole_genome_shotgun_sequence
_R_LVKQ01033913.1_11599-12215_Giraffa_camelopardalis_tippelskirchi_isolate_MA1_scaffold887b380987e1860708_67_whole_genome_shotgun_sequence
_R_LVCL010295385.1_8530-9147_Okapia_johnstoni_scaffold26924_14_whole_genome_shotgun_sequence
RFLZ01249268.1_15249-15865_Odocoileus_hemionus_sitkensis_isolate_Cn_RI01_contig504207_0_whole_genome_shotgun_sequence
_R_RCHL01007692.1_288634-289251_Odocoileus_hemionus_hemionus_isolate_MD92_scaffold7692_whole_genome_shotgun_sequence
_R_MLBE01026449.1_10805-11422_Odocoileus_virginianus_texanus_isolate_animal_Pink-7_contig_26449_whole_genome_shotgun_sequence
PVIN010013127.1_54339-54956_Rangifer_tarandus_isolate_US061_RanTarSib_scaffold_26241_whole_genome_shotgun_sequence
_R_SSNK01011008.1_39510-40126_Alces_alces_isolate_MIG12-28694850_contig132_29_whole_genome_shotgun_sequence
_R_SPDZ01001320.1_11855304-11855922_Hydropotes_inermis_isolate_ZZ2017_13318_whole_genome_shotgun_sequence
_R_CCMK010219376.1_1101-1718_Capreolus_capreolus_genome_assembly_kmer631_contig_DEER_K63_SCAFFOLD219376_whole_genome_shotgun_sequence
JRFZ01072456.1_231548-231869_Elaphurus_davidianus_breed_Woburn_Abbey_isolate_Dafeng_scaffold437_whole_genome_shotgun_sequence
_R_PVKT020003181.1_3792933-3793547_Antilocapra_americana_isolate_BS35_ScwEJLR_3145HRSCAF_52769_5_whole_genome_shotgun_sequence
SRVM01006739.1_458316-458930_Antilocapra_americana_isolate_GAN_MWC17-00274_contig163-1_2_whole_genome_shotgun_sequence
marine_NCQN01008653.1_4566-5185_Tursiops_aduncus_isolate_CRI000098_scaffold_8752_whole_genome_shotgun_sequence
marine_NCQN01001335.1_117075-117694_Tursiops_aduncus_isolate_CRI000098_scaffold_1224_whole_genome_shotgun_sequence
marine_RWJT01021049.1_8772099-8772717_Sousa_chinensis_isolate_SCSF_scaffold44_whole_genome_shotgun_sequence
marine_RJWK010094209.1_505-1066_Platanista_minor_isolate_BS66_PlaMin_flattened_line_188306_whole_genome_shotgun_sequence
_R_marine_RJWI010217776.1_9-484_Pontoporia_blainvillei_isolate_BS67_PonBla_flattened_line_435473_whole_genome_shotgun_sequence
marine_RJWO010021143.1_19049-19652_Inia_geoffrensis_isolate_BS63_IniGeo_flattened_line_42196_whole_genome_shotgun_sequence
_R_marine_PKGA01133473.1_13238745-13239365_Phocoena_phocoena_ScshVNz_1454_whole_genome_shotgun_sequence
marine_VOSV01004328.1_36065-36687_Phocoena_sinus_isolate_mPhoSin1_000148F_017_arrow_arrow_whole_genome_shotgun_sequence
marine_PVJJ010029269.1_19439-20059_Mesoplodon_bidens_isolate_US080_MesBid_scaffold_58518_whole_genome_shotgun_sequence
_R_marine_RJWS010002372.1_26780-27400_Ziphius_cavirostris_isolate_BS68_ZipCav_flattened_line_4738_whole_genome_shotgun_sequence
_R_marine_RJWL010003151.1_48436-49055_Kogia_breviceps_isolate_BS64_KogBre_flattened_line_6299_whole_genome_shotgun_sequence
marine_NIPP01007802.1_56765-57384_Eschrichtius_robustus_isolate_ER-14-168_kmer60-min500.11750753_whole_genome_shotgun_sequence
marine_VNFD01002843.1_102285-102903_Balaenoptera_musculus_isolate_JJ_BM4_2016_0621_000129F_025_arrow_arrow_whole_genome_shotgun_sequence
marine_RYZJ01001172.1_12947331-12947945_Megaptera_novaeangliae_breed_North_Atlantic_isolate_ASU_GRON-1_ScjyzU5_343_whole_genome_shotgun_sequence
_R_marine_RJWP010134689.1_418-985_Eubalaena_japonica_isolate_BS61_EubJap_flattened_line_269215_whole_genome_shotgun_sequence
marine_RJWP010126642.1_3-570_Eubalaena_japonica_isolate_BS61_EubJap_flattened_line_253123_whole_genome_shotgun_sequence
PVJP02910747.1_10484789-10485395_Hippopotamus_amphibius_isolate_BS01_ScHSfme_925197HRSCAF_982997_0_whole_genome_shotgun_sequence
0.02

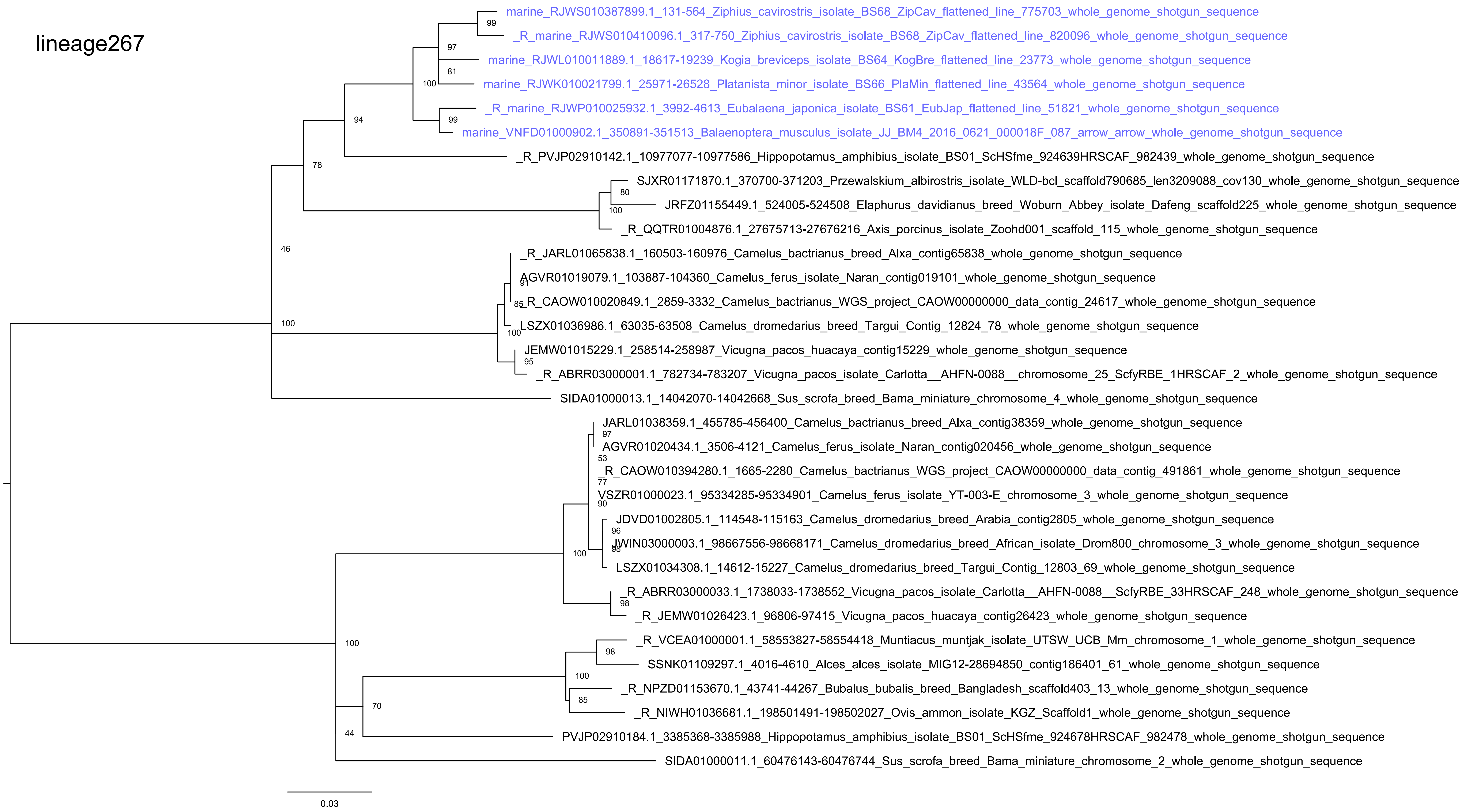
lineage267
marine_RJWS010387899.1_131-564_Ziphius_cavirostris_isolate_BS68_ZipCav_flattened_line_775703_whole_genome_shotgun_sequence
_R_marine_RJWS010410096.1_317-750_Ziphius_cavirostris_isolate_BS68_ZipCav_flattened_line_820096_whole_genome_shotgun_sequence
marine_RJWL010011889.1_18617-19239_Kogia_breviceps_isolate_BS64_KogBre_flattened_line_23773_whole_genome_shotgun_sequence
marine_RJWK010021799.1_25971-26528_Platanista_minor_isolate_BS66_PlaMin_flattened_line_43564_whole_genome_shotgun_sequence
_R_marine_RJWP010025932.1_3992-4613_Eubalaena_japonica_isolate_BS61_EubJap_flattened_line_51821_whole_genome_shotgun_sequence
marine_VNFD01000902.1_350891-351513_Balaenoptera_musculus_isolate_JJ_BM4_2016_0621_000018F_087_arrow_arrow_whole_genome_shotgun_sequence
_R_PVJP02910142.1_10977077-10977586_Hippopotamus_amphibius_isolate_BS01_ScHSfme_924639HRSCAF_982439_whole_genome_shotgun_sequence
SJXR01171870.1_370700-371203_Przewalskium_albirostris_isolate_WLD-bcl_scaffold790685_len3209088_cov130_whole_genome_shotgun_sequence
JRFZ01155449.1_524005-524508_Elaphurus_davidianus_breed_Woburn_Abbey_isolate_Dafeng_scaffold225_whole_genome_shotgun_sequence
_R_QQTR01004876.1_27675713-27676216_Axis_porcinus_isolate_Zoohd001_scaffold_115_whole_genome_shotgun_sequence
_R_JARL01065838.1_160503-160976_Camelus_bactrianus_breed_Alxa_contig65838_whole_genome_shotgun_sequence
AGVR01019079.1_103887-104360_Camelus_ferus_isolate_Naran_contig019101_whole_genome_shotgun_sequence
_R_CAOW010020849.1_2859-3332_Camelus_bactrianus_WGS_project_CAOW00000000_data_contig_24617_whole_genome_shotgun_sequence
LSZX01036986.1_63035-63508_Camelus_dromedarius_breed_Targui_Contig_12824_78_whole_genome_shotgun_sequence
JEMW01015229.1_258514-258987_Vicugna_pacos_huacaya_contig15229_whole_genome_shotgun_sequence
_R_ABRR03000001.1_782734-783207_Vicugna_pacos_isolate_Carlotta__AHFN-0088__chromosome_25_ScfyRBE_1HRSCAF_2_whole_genome_shotgun_sequence
SIDA01000013.1_14042070-14042668_Sus_scrofa_breed_Bama_miniature_chromosome_4_whole_genome_shotgun_sequence
JARL01038359.1_455785-456400_Camelus_bactrianus_breed_Alxa_contig38359_whole_genome_shotgun_sequence
AGVR01020434.1_3506-4121_Camelus_ferus_isolate_Naran_contig020456_whole_genome_shotgun_sequence
_R_CAOW010394280.1_1665-2280_Camelus_bactrianus_WGS_project_CAOW00000000_data_contig_491861_whole_genome_shotgun_sequence
VSZR01000023.1_95334285-95334901_Camelus_ferus_isolate_YT-003-E_chromosome_3_whole_genome_shotgun_sequence
JDVD01002805.1_114548-115163_Camelus_dromedarius_breed_Arabia_contig2805_whole_genome_shotgun_sequence
JWIN03000003.1_98667556-98668171_Camelus_dromedarius_breed_African_isolate_Drom800_chromosome_3_whole_genome_shotgun_sequence
LSZX01034308.1_14612-15227_Camelus_dromedarius_breed_Targui_Contig_12803_69_whole_genome_shotgun_sequence
_R_ABRR03000033.1_1738033-1738552_Vicugna_pacos_isolate_Carlotta__AHFN-0088__ScfyRBE_33HRSCAF_248_whole_genome_shotgun_sequence
_R_JEMW01026423.1_96806-97415_Vicugna_pacos_huacaya_contig26423_whole_genome_shotgun_sequence
_R_VCEA01000001.1_58553827-58554418_Muntiacus_muntjak_isolate_UTSW_UCB_Mm_chromosome_1_whole_genome_shotgun_sequence
SSNK01109297.1_4016-4610_Alces_alces_isolate_MIG12-28694850_contig186401_61_whole_genome_shotgun_sequence
_R_NPZD01153670.1_43741-44267_Bubalus_bubalis_breed_Bangladesh_scaffold403_13_whole_genome_shotgun_sequence
_R_NIWH01036681.1_198501491-198502027_Ovis_ammon_isolate_KGZ_Scaffold1_whole_genome_shotgun_sequence
PVJP02910184.1_3385368-3385988_Hippopotamus_amphibius_isolate_BS01_ScHSfme_924678HRSCAF_982478_whole_genome_shotgun_sequence
SIDA01000011.1_60476143-60476744_Sus_scrofa_breed_Bama_miniature_chromosome_2_whole_genome_shotgun_sequence
0.03

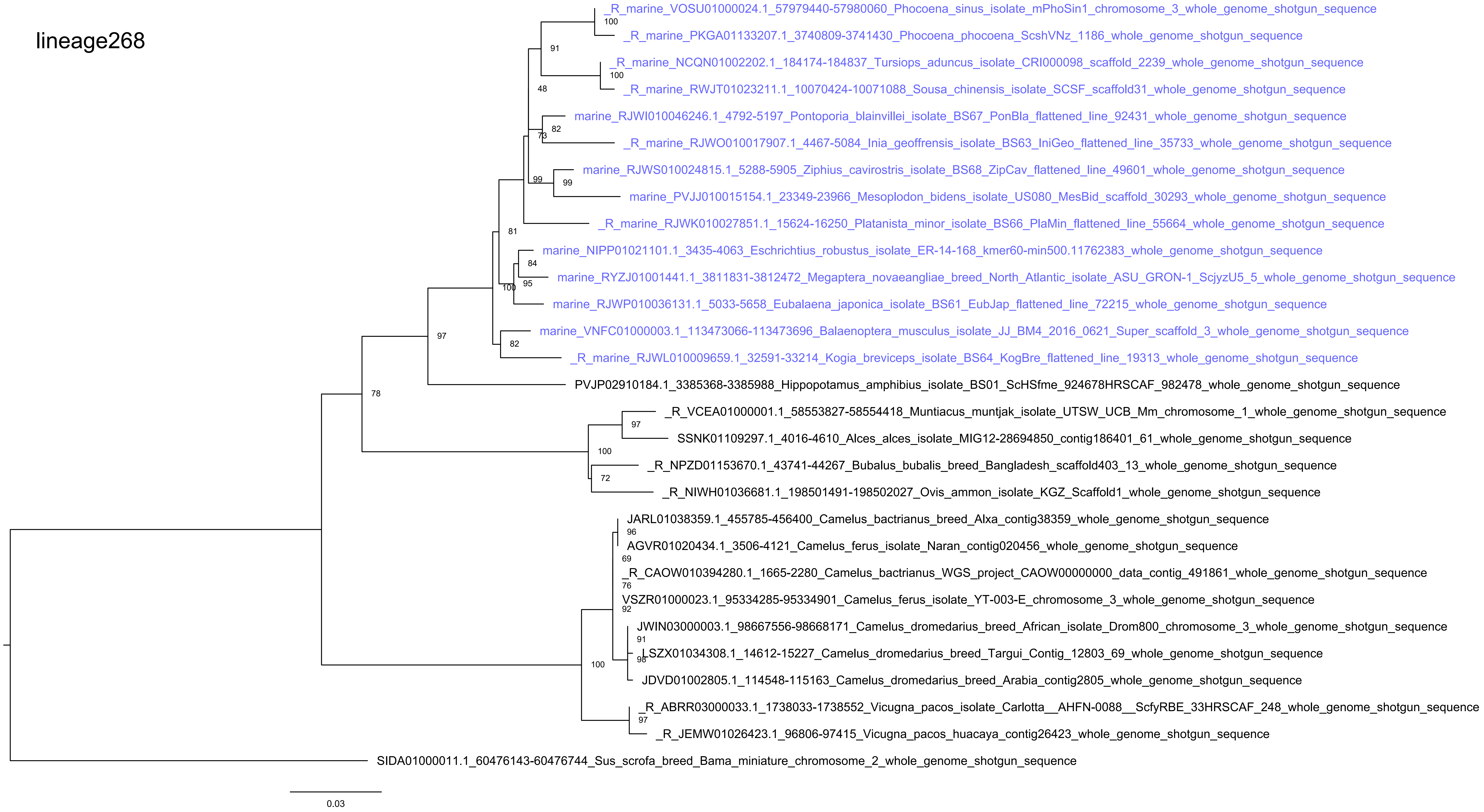

lineage268
_R_marine_VOSU01000024.1_57979440-57980060_Phocoena_sinus_isolate_mPhoSin1_chromosome_3_whole_genome_shotgun_sequence
_R_marine_PKGA01133207.1_3740809-3741430_Phocoena_phocoena_ScshVNz_1186_whole_genome_shotgun_sequence
_R_marine_NCQN01002202.1_184174-184837_Tursiops_aduncus_isolate_CRI000098_scaffold_2239_whole_genome_shotgun_sequence
_R_marine_RWJT01023211.1_10070424-10071088_Sousa_chinensis_isolate_SCSF_scaffold31_whole_genome_shotgun_sequence
marine_RJWI010046246.1_4792-5197_Pontoporia_blainvillei_isolate_BS67_PonBla_flattened_line_92431_whole_genome_shotgun_sequence
_R_marine_RJWO010017907.1_4467-5084_Inia_geoffrensis_isolate_BS63_IniGeo_flattened_line_35733_whole_genome_shotgun_sequence
marine_RJWS010024815.1_5288-5905_Ziphius_cavirostris_isolate_BS68_ZipCav_flattened_line_49601_whole_genome_shotgun_sequence
marine_PVJJ010015154.1_23349-23966_Mesoplodon_bidens_isolate_US080_MesBid_scaffold_30293_whole_genome_shotgun_sequence
_R_marine_RJWK010027851.1_15624-16250_Platanista_minor_isolate_BS66_PlaMin_flattened_line_55664_whole_genome_shotgun_sequence
marine_NIPP01021101.1_3435-4063_Eschrichtius_robustus_isolate_ER-14-168_kmer60-min500.11762383_whole_genome_shotgun_sequence
marine_RYZJ01001441.1_3811831-3812472_Megaptera_novaeangliae_breed_North_Atlantic_isolate_ASU_GRON-1_ScjyzU5_5_whole_genome_shotgun_sequence
marine_RJWP010036131.1_5033-5658_Eubalaena_japonica_isolate_BS61_EubJap_flattened_line_72215_whole_genome_shotgun_sequence
marine_VNFC01000003.1_113473066-113473696_Balaenoptera_musculus_isolate_JJ_BM4_2016_0621_Super_scaffold_3_whole_genome_shotgun_sequence
_R_marine_RJWL010009659.1_32591-33214_Kogia_breviceps_isolate_BS64_KogBre_flattened_line_19313_whole_genome_shotgun_sequence
PVJP02910184.1_3385368-3385988_Hippopotamus_amphibius_isolate_BS01_ScHSfme_924678HRSCAF_982478_whole_genome_shotgun_sequence
_R_VCEA01000001.1_58553827-58554418_Muntiacus_muntjak_isolate_UTSW_UCB_Mm_chromosome_1_whole_genome_shotgun_sequence
SSNK01109297.1_4016-4610_Alces_alces_isolate_MIG12-28694850_contig186401_61_whole_genome_shotgun_sequence
_R_NPZD01153670.1_43741-44267_Bubalus_bubalis_breed_Bangladesh_scaffold403_13_whole_genome_shotgun_sequence
_R_NIWH01036681.1_198501491-198502027_Ovis_ammon_isolate_KGZ_Scaffold1_whole_genome_shotgun_sequence
JARL01038359.1_455785-456400_Camelus_bactrianus_breed_Alxa_contig38359_whole_genome_shotgun_sequence
AGVR01020434.1_3506-4121_Camelus_ferus_isolate_Naran_contig020456_whole_genome_shotgun_sequence
_R_CAOW010394280.1_1665-2280_Camelus_bactrianus_WGS_project_CAOW00000000_data_contig_491861_whole_genome_shotgun_sequence
VSZR01000023.1_95334285-95334901_Camelus_ferus_isolate_YT-003-E_chromosome_3_whole_genome_shotgun_sequence
JWIN03000003.1_98667556-98668171_Camelus_dromedarius_breed_African_isolate_Drom800_chromosome_3_whole_genome_shotgun_sequence
LSZX01034308.1_14612-15227_Camelus_dromedarius_breed_Targui_Contig_12803_69_whole_genome_shotgun_sequence
JDVD01002805.1_114548-115163_Camelus_dromedarius_breed_Arabia_contig2805_whole_genome_shotgun_sequence
_R_ABRR03000033.1_1738033-1738552_Vicugna_pacos_isolate_Carlotta__AHFN-0088__ScfyRBE_33HRSCAF_248_whole_genome_shotgun_sequence
_R_JEMW01026423.1_96806-97415_Vicugna_pacos_huacaya_contig26423_whole_genome_shotgun_sequence
SIDA01000011.1_60476143-60476744_Sus_scrofa_breed_Bama_miniature_chromosome_2_whole_genome_shotgun_sequence
0.03

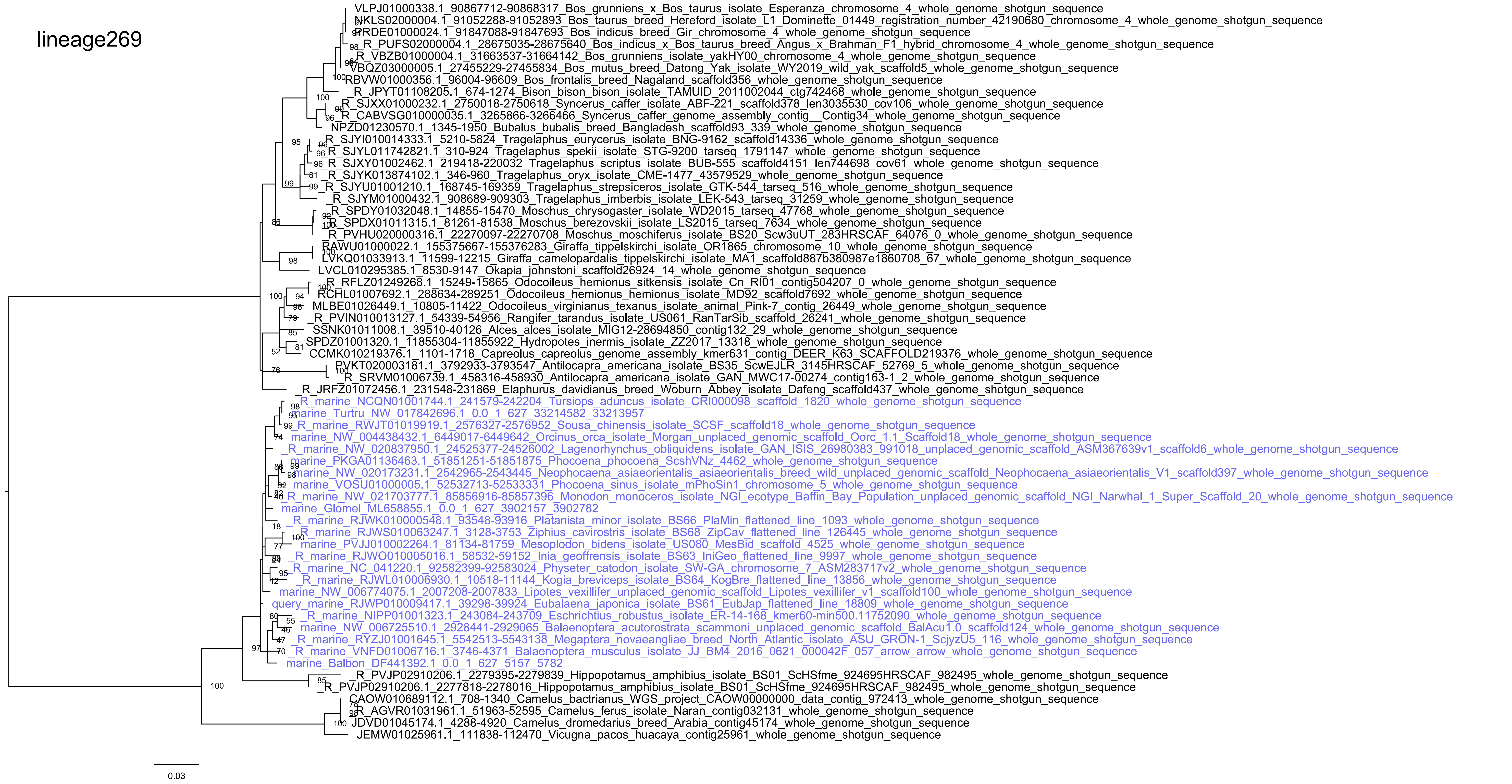

lineage269
VLPJ01000338.1_90867712-90868317_Bos_grunniens_x_Bos_taurus_isolate_Esperanza_chromosome_4_whole_genome_shotgun_sequence
NKLS02000004.1_91052288-91052893_Bos_taurus_breed_Hereford_isolate_L1_Dominette_01449_registration_number_42190680_chromosome_4_whole_genome_shotgun_sequence
PRDE01000024.1_91847088-91847693_Bos_indicus_breed_Gir_chromosome_4_whole_genome_shotgun_sequence
_R_PUFS02000004.1_28675035-28675640_Bos_indicus_x_Bos_taurus_breed_Angus_x_Brahman_F1_hybrid_chromosome_4_whole_genome_shotgun_sequence
_R_VBZB01000004.1_31663537-31664142_Bos_grunniens_isolate_yakHY00_chromosome_4_whole_genome_shotgun_sequence
VBQZ03000005.1_27455229-27455834_Bos_mutus_breed_Datong_Yak_isolate_WY2019_wild_yak_scaffold5_whole_genome_shotgun_sequence
RBVW01000356.1_96004-96609_Bos_frontalis_breed_Nagaland_scaffold356_whole_genome_shotgun_sequence
_R_JPYT01108205.1_674-1274_Bison_bison_bison_isolate_TAMUID_2011002044_ctg742468_whole_genome_shotgun_sequence
_R_SJXX01000232.1_2750018-2750618_Syncerus_caffer_isolate_ABF-221_scaffold378_len3035530_cov106_whole_genome_shotgun_sequence
_R_CABVSG010000035.1_3265866-3266466_Syncerus_caffer_genome_assembly_contig__Contig34_whole_genome_shotgun_sequence
NPZD01230570.1_1345-1950_Bubalus_bubalis_breed_Bangladesh_scaffold93_339_whole_genome_shotgun_sequence
_R_SJYI010014333.1_5210-5824_Tragelaphus_eurycerus_isolate_BNG-9162_scaffold14336_whole_genome_shotgun_sequence
_R_SJYL011742821.1_310-924_Tragelaphus_spekii_isolate_STG-9200_tarseq_1791147_whole_genome_shotgun_sequence
_R_SJXY01002462.1_219418-220032_Tragelaphus_scriptus_isolate_BUB-555_scaffold4151_len744698_cov61_whole_genome_shotgun_sequence
_R_SJYK013874102.1_346-960_Tragelaphus_oryx_isolate_CME-1477_43579529_whole_genome_shotgun_sequence
_R_SJYU01001210.1_168745-169359_Tragelaphus_strepsiceros_isolate_GTK-544_tarseq_516_whole_genome_shotgun_sequence
_R_SJYM01000432.1_908689-909303_Tragelaphus_imberbis_isolate_LEK-543_tarseq_31259_whole_genome_shotgun_sequence
_R_SPDY01032048.1_14855-15470_Moschus_chrysogaster_isolate_WD2015_tarseq_47768_whole_genome_shotgun_sequence
_R_SPDX01011315.1_81261-81538_Moschus_berezovskii_isolate_LS2015_tarseq_7634_whole_genome_shotgun_sequence
_R_PVHU020000316.1_22270097-22270708_Moschus_moschiferus_isolate_BS20_Scw3uUT_283HRSCAF_64076_0_whole_genome_shotgun_sequence
RAWU01000022.1_155375667-155376283_Giraffa_tippelskirchi_isolate_OR1865_chromosome_10_whole_genome_shotgun_sequence
LVKQ01033913.1_11599-12215_Giraffa_camelopardalis_tippelskirchi_isolate_MA1_scaffold887b380987e1860708_67_whole_genome_shotgun_sequence
LVCL010295385.1_8530-9147_Okapia_johnstoni_scaffold26924_14_whole_genome_shotgun_sequence
_R_RFLZ01249268.1_15249-15865_Odocoileus_hemionus_sitkensis_isolate_Cn_RI01_contig504207_0_whole_genome_shotgun_sequence
RCHL01007692.1_288634-289251_Odocoileus_hemionus_hemionus_isolate_MD92_scaffold7692_whole_genome_shotgun_sequence
MLBE01026449.1_10805-11422_Odocoileus_virginianus_texanus_isolate_animal_Pink-7_contig_26449_whole_genome_shotgun_sequence
_R_PVIN010013127.1_54339-54956_Rangifer_tarandus_isolate_US061_RanTarSib_scaffold_26241_whole_genome_shotgun_sequence
SSNK01011008.1_39510-40126_Alces_alces_isolate_MIG12-28694850_contig132_29_whole_genome_shotgun_sequence
SPDZ01001320.1_11855304-11855922_Hydropotes_inermis_isolate_ZZ2017_13318_whole_genome_shotgun_sequence
CCMK010219376.1_1101-1718_Capreolus_capreolus_genome_assembly_kmer631_contig_DEER_K63_SCAFFOLD219376_whole_genome_shotgun_sequence
PVKT020003181.1_3792933-3793547_Antilocapra_americana_isolate_BS35_ScwEJLR_3145HRSCAF_52769_5_whole_genome_shotgun_sequence
_R_SRVM01006739.1_458316-458930_Antilocapra_americana_isolate_GAN_MWC17-00274_contig163-1_2_whole_genome_shotgun_sequence
_R_JRFZ01072456.1_231548-231869_Elaphurus_davidianus_breed_Woburn_Abbey_isolate_Dafeng_scaffold437_whole_genome_shotgun_sequence
_R_marine_NCQN01001744.1_241579-242204_Tursiops_aduncus_isolate_CRI000098_scaffold_1820_whole_genome_shotgun_sequence
marine_Turtru_NW_017842696.1_0.0_1_627_33214582_33213957
_R_marine_RWJT01019919.1_2576327-2576952_Sousa_chinensis_isolate_SCSF_scaffold18_whole_genome_shotgun_sequence
marine_NW_004438432.1_6449017-6449642_Orcinus_orca_isolate_Morgan_unplaced_genomic_scaffold_Oorc_1.1_Scaffold18_whole_genome_shotgun_sequence
_R_marine_NW_020837950.1_24525377-24526002_Lagenorhynchus_obliquidens_isolate_GAN_ISIS_26980383_991018_unplaced_genomic_scaffold_ASM367639v1_scaffold6_whole_genome_shotgun_sequence
marine_PKGA01136463.1_51851251-51851875_Phocoena_phocoena_ScshVNz_4462_whole_genome_shotgun_sequence
marine_NW_020173231.1_2542965-2543445_Neophocaena_asiaeorientalis_asiaeorientalis_breed_wild_unplaced_genomic_scaffold_Neophocaena_asiaeorientalis_V1_scaffold397_whole_genome_shotgun_sequence
marine_VOSU01000005.1_52532713-52533331_Phocoena_sinus_isolate_mPhoSin1_chromosome_5_whole_genome_shotgun_sequence
_R_marine_NW_021703777.1_85856916-85857396_Monodon_monoceros_isolate_NGI_ecotype_Baffin_Bay_Population_unplaced_genomic_scaffold_NGI_Narwhal_1_Super_Scaffold_20_whole_genome_shotgun_sequence
marine_Glomel_ML658855.1_0.0_1_627_3902157_3902782
_R_marine_RJWK010000548.1_93548-93916_Platanista_minor_isolate_BS66_PlaMin_flattened_line_1093_whole_genome_shotgun_sequence
_R_marine_RJWS010063247.1_3128-3753_Ziphius_cavirostris_isolate_BS68_ZipCav_flattened_line_126445_whole_genome_shotgun_sequence
marine_PVJJ010002264.1_81134-81759_Mesoplodon_bidens_isolate_US080_MesBid_scaffold_4525_whole_genome_shotgun_sequence
_R_marine_RJWO010005016.1_58532-59152_Inia_geoffrensis_isolate_BS63_IniGeo_flattened_line_9997_whole_genome_shotgun_sequence
_R_marine_NC_041220.1_92582399-92583024_Physeter_catodon_isolate_SW-GA_chromosome_7_ASM283717v2_whole_genome_shotgun_sequence
_R_marine_RJWL010006930.1_10518-11144_Kogia_breviceps_isolate_BS64_KogBre_flattened_line_13856_whole_genome_shotgun_sequence
marine_NW_006774075.1_2007208-2007833_Lipotes_vexillifer_unplaced_genomic_scaffold_Lipotes_vexillifer_v1_scaffold100_whole_genome_shotgun_sequence
query_marine_RJWP010009417.1_39298-39924_Eubalaena_japonica_isolate_BS61_EubJap_flattened_line_18809_whole_genome_shotgun_sequence
_R_marine_NIPP01001323.1_243084-243709_Eschrichtius_robustus_isolate_ER-14-168_kmer60-min500.11752090_whole_genome_shotgun_sequence
marine_NW_006725510.1_2928441-2929065_Balaenoptera_acutorostrata_scammoni_unplaced_genomic_scaffold_BalAcu1.0_scaffold124_whole_genome_shotgun_sequence
_R_marine_RYZJ01001645.1_5542513-5543138_Megaptera_novaeangliae_breed_North_Atlantic_isolate_ASU_GRON-1_ScjyzU5_116_whole_genome_shotgun_sequence
_R_marine_VNFD01006716.1_3746-4371_Balaenoptera_musculus_isolate_JJ_BM4_2016_0621_000042F_057_arrow_arrow_whole_genome_shotgun_sequence
marine_Balbon_DF441392.1_0.0_1_627_5157_5782
_R_PVJP02910206.1_2279395-2279839_Hippopotamus_amphibius_isolate_BS01_ScHSfme_924695HRSCAF_982495_whole_genome_shotgun_sequence
_R_PVJP02910206.1_2277818-2278016_Hippopotamus_amphibius_isolate_BS01_ScHSfme_924695HRSCAF_982495_whole_genome_shotgun_sequence
CAOW010689112.1_708-1340_Camelus_bactrianus_WGS_project_CAOW00000000_data_contig_972413_whole_genome_shotgun_sequence
_R_AGVR01031961.1_51963-52595_Camelus_ferus_isolate_Naran_contig032131_whole_genome_shotgun_sequence
JDVD01045174.1_4288-4920_Camelus_dromedarius_breed_Arabia_contig45174_whole_genome_shotgun_sequence
JEMW01025961.1_111838-112470_Vicugna_pacos_huacaya_contig25961_whole_genome_shotgun_sequence
0.03

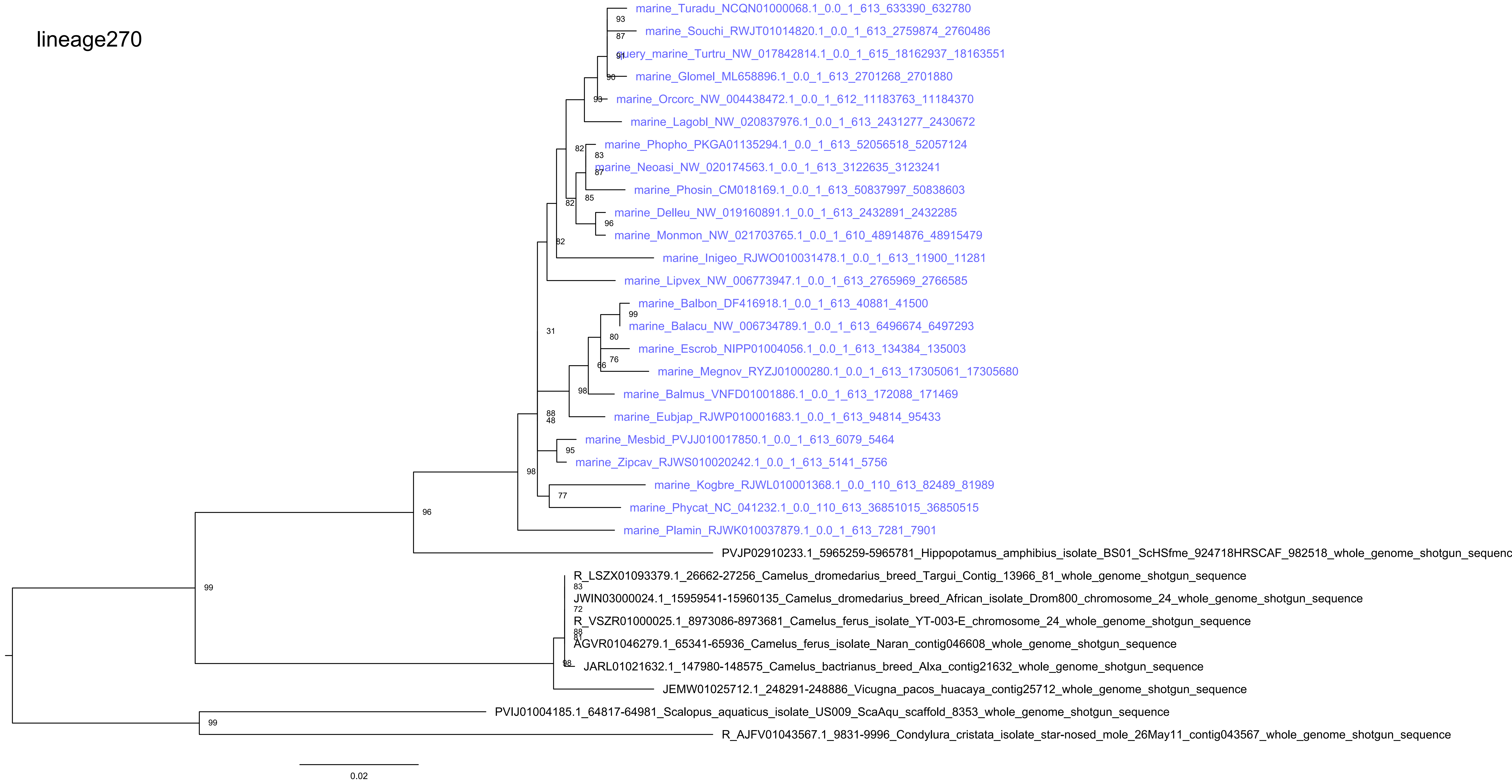
lineage270
marine_Turadu_NCQN01000068.1_0.0_1_613_633390_632780
marine_Souchi_RWJT01014820.1_0.0_1_613_2759874_2760486
query_marine_Turtru_NW_017842814.1_0.0_1_615_18162937_18163551
marine_Glomel_ML658896.1_0.0_1_613_2701268_2701880
marine_Orcorc_NW_004438472.1_0.0_1_612_11183763_11184370
marine_Lagobl_NW_020837976.1_0.0_1_613_2431277_2430672
marine_Phopho_PKGA01135294.1_0.0_1_613_52056518_52057124
marine_Neoasi_NW_020174563.1_0.0_1_613_3122635_3123241
marine_Phosin_CM018169.1_0.0_1_613_50837997_50838603
marine_Delleu_NW_019160891.1_0.0_1_613_2432891_2432285
marine_Monmon_NW_021703765.1_0.0_1_610_48914876_48915479
marine_Inigeo_RJWO010031478.1_0.0_1_613_11900_11281
marine_Lipvex_NW_006773947.1_0.0_1_613_2765969_2766585
marine_Balbon_DF416918.1_0.0_1_613_40881_41500
marine_Balacu_NW_006734789.1_0.0_1_613_6496674_6497293
marine_Escrob_NIPP01004056.1_0.0_1_613_134384_135003
marine_Megnov_RYZJ01000280.1_0.0_1_613_17305061_17305680
marine_Balmus_VNFD01001886.1_0.0_1_613_172088_171469
marine_Eubjap_RJWP010001683.1_0.0_1_613_94814_95433
marine_Mesbid_PVJJ010017850.1_0.0_1_613_6079_5464
marine_Zipcav_RJWS010020242.1_0.0_1_613_5141_5756
marine_Kogbre_RJWL010001368.1_0.0_110_613_82489_81989
marine_Phycat_NC_041232.1_0.0_110_613_36851015_36850515
marine_Plamin_RJWK010037879.1_0.0_1_613_7281_7901
PVJP02910233.1_5965259-5965781_Hippopotamus_amphibius_isolate_BS01_ScHSfme_924718HRSCAF_982518_whole_genome_shotgun_sequenc
R_LSZX01093379.1_26662-27256_Camelus_dromedarius_breed_Targui_Contig_13966_81_whole_genome_shotgun_sequence
JWIN03000024.1_15959541-15960135_Camelus_dromedarius_breed_African_isolate_Drom800_chromosome_24_whole_genome_shotgun_sequence
R_VSZR01000025.1_8973086-8973681_Camelus_ferus_isolate_YT-003-E_chromosome_24_whole_genome_shotgun_sequence
AGVR01046279.1_65341-65936_Camelus_ferus_isolate_Naran_contig046608_whole_genome_shotgun_sequence
JARL01021632.1_147980-148575_Camelus_bactrianus_breed_Alxa_contig21632_whole_genome_shotgun_sequence
JEMW01025712.1_248291-248886_Vicugna_pacos_huacaya_contig25712_whole_genome_shotgun_sequence
PVIJ01004185.1_64817-64981_Scalopus_aquaticus_isolate_US009_ScaAqu_scaffold_8353_whole_genome_shotgun_sequence
R_AJFV01043567.1_9831-9996_Condylura_cristata_isolate_star-nosed_mole_26May11_contig043567_whole_genome_shotgun_sequence
0.02

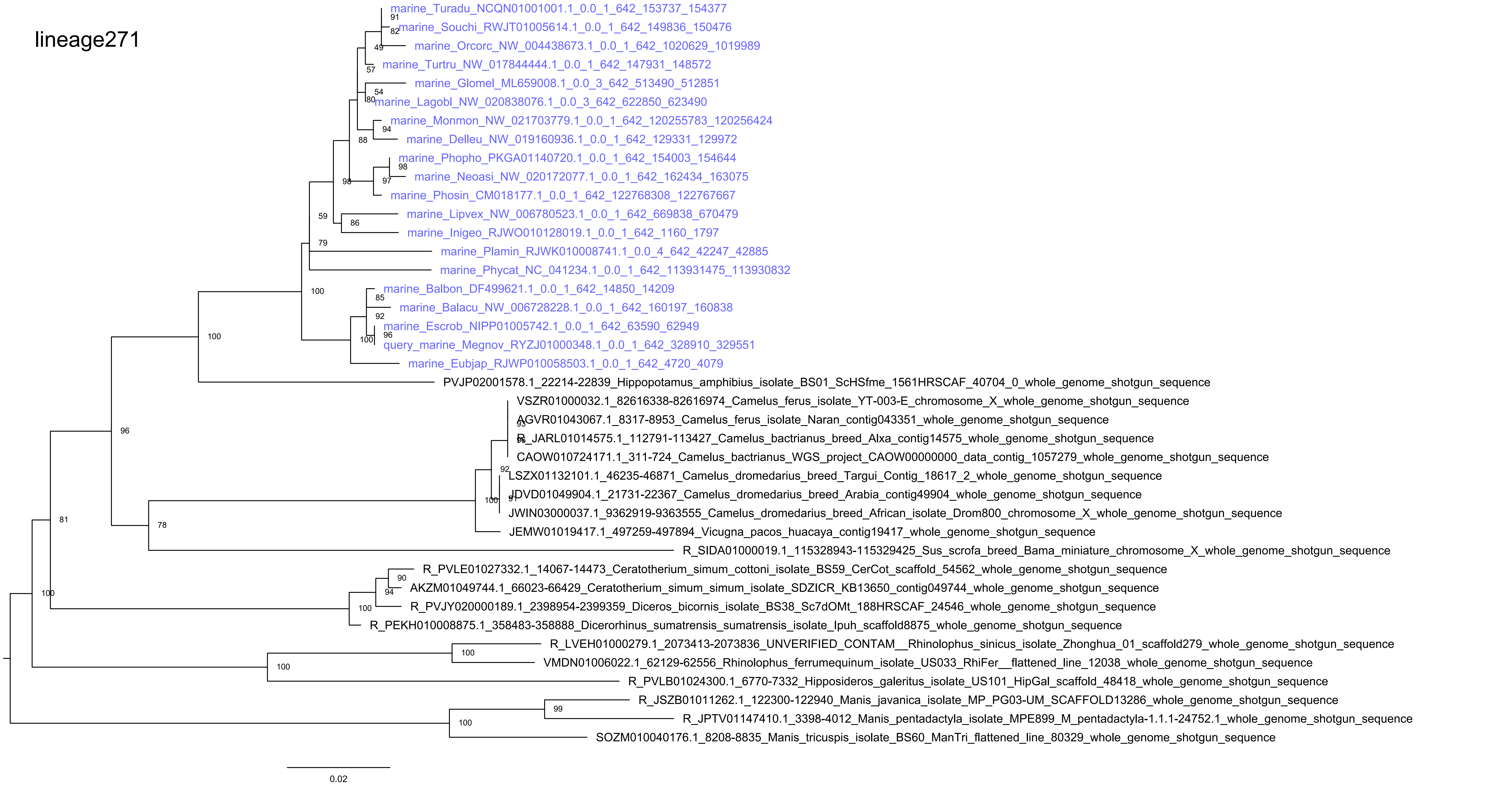
lineage271
marine_Turadu_NCQN01001001.1_0.0_1_642_153737_154377
marine_Souchi_RWJT01005614.1_0.0_1_642_149836_150476
marine_Orcorc_NW_004438673.1_0.0_1_642_1020629_1019989
marine_Turtru_NW_017844444.1_0.0_1_642_147931_148572
marine_Glomel_ML659008.1_0.0_3_642_513490_512851
marine_Lagobl_NW_020838076.1_0.0_3_642_622850_623490
marine_Monmon_NW_021703779.1_0.0_1_642_120255783_120256424
marine_Delleu_NW_019160936.1_0.0_1_642_129331_129972
marine_Phopho_PKGA01140720.1_0.0_1_642_154003_154644
marine_Neoasi_NW_020172077.1_0.0_1_642_162434_163075
marine_Phosin_CM018177.1_0.0_1_642_122768308_122767667
marine_Lipvex_NW_006780523.1_0.0_1_642_669838_670479
marine_Inigeo_RJWO010128019.1_0.0_1_642_1160_1797
marine_Plamin_RJWK010008741.1_0.0_4_642_42247_42885
marine_Phycat_NC_041234.1_0.0_1_642_113931475_113930832
marine_Balbon_DF499621.1_0.0_1_642_14850_14209
marine_Balacu_NW_006728228.1_0.0_1_642_160197_160838
marine_Escrob_NIPP01005742.1_0.0_1_642_63590_62949
query_marine_Megnov_RYZJ01000348.1_0.0_1_642_328910_329551
marine_Eubjap_RJWP010058503.1_0.0_1_642_4720_4079
PVJP02001578.1_22214-22839_Hippopotamus_amphibius_isolate_BS01_ScHSfme_1561HRSCAF_40704_0_whole_genome_shotgun_sequence
VSZR01000032.1_82616338-82616974_Camelus_ferus_isolate_YT-003-E_chromosome_X_whole_genome_shotgun_sequence
AGVR01043067.1_8317-8953_Camelus_ferus_isolate_Naran_contig043351_whole_genome_shotgun_sequence
R_JARL01014575.1_112791-113427_Camelus_bactrianus_breed_Alxa_contig14575_whole_genome_shotgun_sequence
CAOW010724171.1_311-724_Camelus_bactrianus_WGS_project_CAOW00000000_data_contig_1057279_whole_genome_shotgun_sequence
LSZX01132101.1_46235-46871_Camelus_dromedarius_breed_Targui_Contig_18617_2_whole_genome_shotgun_sequence
JDVD01049904.1_21731-22367_Camelus_dromedarius_breed_Arabia_contig49904_whole_genome_shotgun_sequence
JWIN03000037.1_9362919-9363555_Camelus_dromedarius_breed_African_isolate_Drom800_chromosome_X_whole_genome_shotgun_sequence
JEMW01019417.1_497259-497894_Vicugna_pacos_huacaya_contig19417_whole_genome_shotgun_sequence
R_SIDA01000019.1_115328943-115329425_Sus_scrofa_breed_Bama_miniature_chromosome_X_whole_genome_shotgun_sequence
R_PVLE01027332.1_14067-14473_Ceratotherium_simum_cottoni_isolate_BS59_CerCot_scaffold_54562_whole_genome_shotgun_sequence
AKZM01049744.1_66023-66429_Ceratotherium_simum_simum_isolate_SDZICR_KB13650_contig049744_whole_genome_shotgun_sequence
R_PVJY020000189.1_2398954-2399359_Diceros_bicornis_isolate_BS38_Sc7dOMt_188HRSCAF_24546_whole_genome_shotgun_sequence
R_PEKH010008875.1_358483-358888_Dicerorhinus_sumatrensis_sumatrensis_isolate_Ipuh_scaffold8875_whole_genome_shotgun_sequence
R_LVEH01000279.1_2073413-2073836_UNVERIFIED_CONTAM__Rhinolophus_sinicus_isolate_Zhonghua_01_scaffold279_whole_genome_shotgun_sequence
VMDN01006022.1_62129-62556_Rhinolophus_ferrumequinum_isolate_US033_RhiFer__flattened_line_12038_whole_genome_shotgun_sequence
R_PVLB01024300.1_6770-7332_Hipposideros_galeritus_isolate_US101_HipGal_scaffold_48418_whole_genome_shotgun_sequence
R_JSZB01011262.1_122300-122940_Manis_javanica_isolate_MP_PG03-UM_SCAFFOLD13286_whole_genome_shotgun_sequence
R_JPTV01147410.1_3398-4012_Manis_pentadactyla_isolate_MPE899_M_pentadactyla-1.1.1-24752.1_whole_genome_shotgun_sequence
SOZM010040176.1_8208-8835_Manis_tricuspis_isolate_BS60_ManTri_flattened_line_80329_whole_genome_shotgun_sequence
0.02

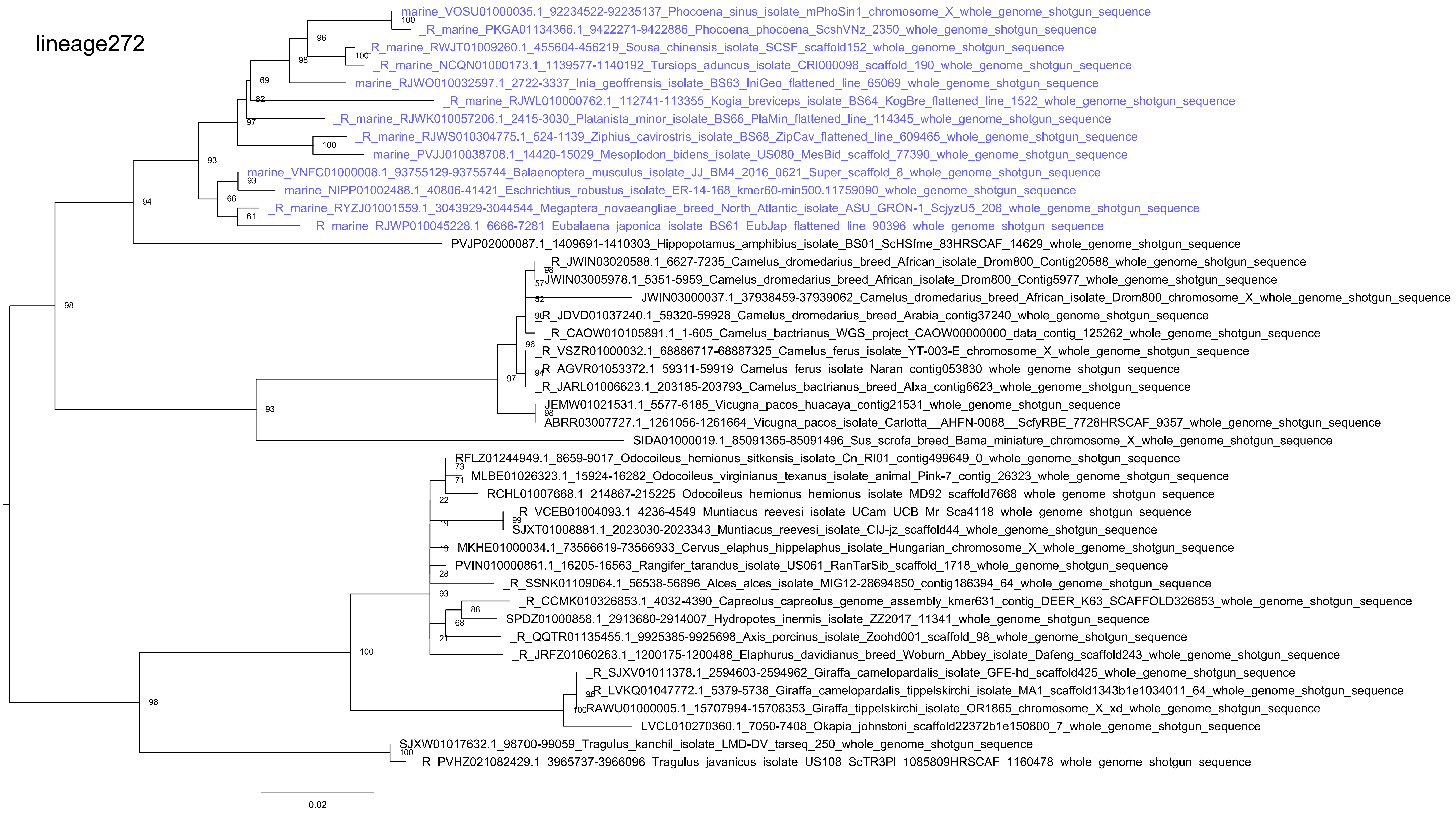
lineage272
marine_VOSU01000035.1_92234522-92235137_Phocoena_sinus_isolate_mPhoSin1_chromosome_X_whole_genome_shotgun_sequence
_R_marine_PKGA01134366.1_9422271-9422886_Phocoena_phocoena_ScshVNz_2350_whole_genome_shotgun_sequence
_R_marine_RWJT01009260.1_455604-456219_Sousa_chinensis_isolate_SCSF_scaffold152_whole_genome_shotgun_sequence
_R_marine_NCQN01000173.1_1139577-1140192_Tursiops_aduncus_isolate_CRI000098_scaffold_190_whole_genome_shotgun_sequence
marine_RJWO010032597.1_2722-3337_Inia_geoffrensis_isolate_BS63_IniGeo_flattened_line_65069_whole_genome_shotgun_sequence
_R_marine_RJWL010000762.1_112741-113355_Kogia_breviceps_isolate_BS64_KogBre_flattened_line_1522_whole_genome_shotgun_sequence
_R_marine_RJWK010057206.1_2415-3030_Platanista_minor_isolate_BS66_PlaMin_flattened_line_114345_whole_genome_shotgun_sequence
_R_marine_RJWS010304775.1_524-1139_Ziphius_cavirostris_isolate_BS68_ZipCav_flattened_line_609465_whole_genome_shotgun_sequence
marine_PVJJ010038708.1_14420-15029_Mesoplodon_bidens_isolate_US080_MesBid_scaffold_77390_whole_genome_shotgun_sequence
marine_VNFC01000008.1_93755129-93755744_Balaenoptera_musculus_isolate_JJ_BM4_2016_0621_Super_scaffold_8_whole_genome_shotgun_sequence
marine_NIPP01002488.1_40806-41421_Eschrichtius_robustus_isolate_ER-14-168_kmer60-min500.11759090_whole_genome_shotgun_sequence
_R_marine_RYZJ01001559.1_3043929-3044544_Megaptera_novaeangliae_breed_North_Atlantic_isolate_ASU_GRON-1_ScjyzU5_208_whole_genome_shotgun_sequence
_R_marine_RJWP010045228.1_6666-7281_Eubalaena_japonica_isolate_BS61_EubJap_flattened_line_90396_whole_genome_shotgun_sequence
PVJP02000087.1_1409691-1410303_Hippopotamus_amphibius_isolate_BS01_ScHSfme_83HRSCAF_14629_whole_genome_shotgun_sequence
_R_JWIN03020588.1_6627-7235_Camelus_dromedarius_breed_African_isolate_Drom800_Contig20588_whole_genome_shotgun_sequence
JWIN03005978.1_5351-5959_Camelus_dromedarius_breed_African_isolate_Drom800_Contig5977_whole_genome_shotgun_sequence
JWIN03000037.1_37938459-37939062_Camelus_dromedarius_breed_African_isolate_Drom800_chromosome_X_whole_genome_shotgun_sequence
_R_JDVD01037240.1_59320-59928_Camelus_dromedarius_breed_Arabia_contig37240_whole_genome_shotgun_sequence
_R_CAOW010105891.1_1-605_Camelus_bactrianus_WGS_project_CAOW00000000_data_contig_125262_whole_genome_shotgun_sequence
_R_VSZR01000032.1_68886717-68887325_Camelus_ferus_isolate_YT-003-E_chromosome_X_whole_genome_shotgun_sequence
_R_AGVR01053372.1_59311-59919_Camelus_ferus_isolate_Naran_contig053830_whole_genome_shotgun_sequence
_R_JARL01006623.1_203185-203793_Camelus_bactrianus_breed_Alxa_contig6623_whole_genome_shotgun_sequence
JEMW01021531.1_5577-6185_Vicugna_pacos_huacaya_contig21531_whole_genome_shotgun_sequence
ABRR03007727.1_1261056-1261664_Vicugna_pacos_isolate_Carlotta__AHFN-0088__ScfyRBE_7728HRSCAF_9357_whole_genome_shotgun_sequence
SIDA01000019.1_85091365-85091496_Sus_scrofa_breed_Bama_miniature_chromosome_X_whole_genome_shotgun_sequence
RFLZ01244949.1_8659-9017_Odocoileus_hemionus_sitkensis_isolate_Cn_RI01_contig499649_0_whole_genome_shotgun_sequence
MLBE01026323.1_15924-16282_Odocoileus_virginianus_texanus_isolate_animal_Pink-7_contig_26323_whole_genome_shotgun_sequence
RCHL01007668.1_214867-215225_Odocoileus_hemionus_hemionus_isolate_MD92_scaffold7668_whole_genome_shotgun_sequence
_R_VCEB01004093.1_4236-4549_Muntiacus_reevesi_isolate_UCam_UCB_Mr_Sca4118_whole_genome_shotgun_sequence
SJXT01008881.1_2023030-2023343_Muntiacus_reevesi_isolate_CIJ-jz_scaffold44_whole_genome_shotgun_sequence
MKHE01000034.1_73566619-73566933_Cervus_elaphus_hippelaphus_isolate_Hungarian_chromosome_X_whole_genome_shotgun_sequence
PVIN010000861.1_16205-16563_Rangifer_tarandus_isolate_US061_RanTarSib_scaffold_1718_whole_genome_shotgun_sequence
_R_SSNK01109064.1_56538-56896_Alces_alces_isolate_MIG12-28694850_contig186394_64_whole_genome_shotgun_sequence
_R_CCMK010326853.1_4032-4390_Capreolus_capreolus_genome_assembly_kmer631_contig_DEER_K63_SCAFFOLD326853_whole_genome_shotgun_sequence
SPDZ01000858.1_2913680-2914007_Hydropotes_inermis_isolate_ZZ2017_11341_whole_genome_shotgun_sequence
_R_QQTR01135455.1_9925385-9925698_Axis_porcinus_isolate_Zoohd001_scaffold_98_whole_genome_shotgun_sequence
_R_JRFZ01060263.1_1200175-1200488_Elaphurus_davidianus_breed_Woburn_Abbey_isolate_Dafeng_scaffold243_whole_genome_shotgun_sequence
_R_SJXV01011378.1_2594603-2594962_Giraffa_camelopardalis_isolate_GFE-hd_scaffold425_whole_genome_shotgun_sequence
_R_LVKQ01047772.1_5379-5738_Giraffa_camelopardalis_tippelskirchi_isolate_MA1_scaffold1343b1e1034011_64_whole_genome_shotgun_sequence
RAWU01000005.1_15707994-15708353_Giraffa_tippelskirchi_isolate_OR1865_chromosome_X_xd_whole_genome_shotgun_sequence
LVCL010270360.1_7050-7408_Okapia_johnstoni_scaffold22372b1e150800_7_whole_genome_shotgun_sequence
SJXW01017632.1_98700-99059_Tragulus_kanchil_isolate_LMD-DV_tarseq_250_whole_genome_shotgun_sequence
_R_PVHZ021082429.1_3965737-3966096_Tragulus_javanicus_isolate_US108_ScTR3Pl_1085809HRSCAF_1160478_whole_genome_shotgun_sequence
0.02

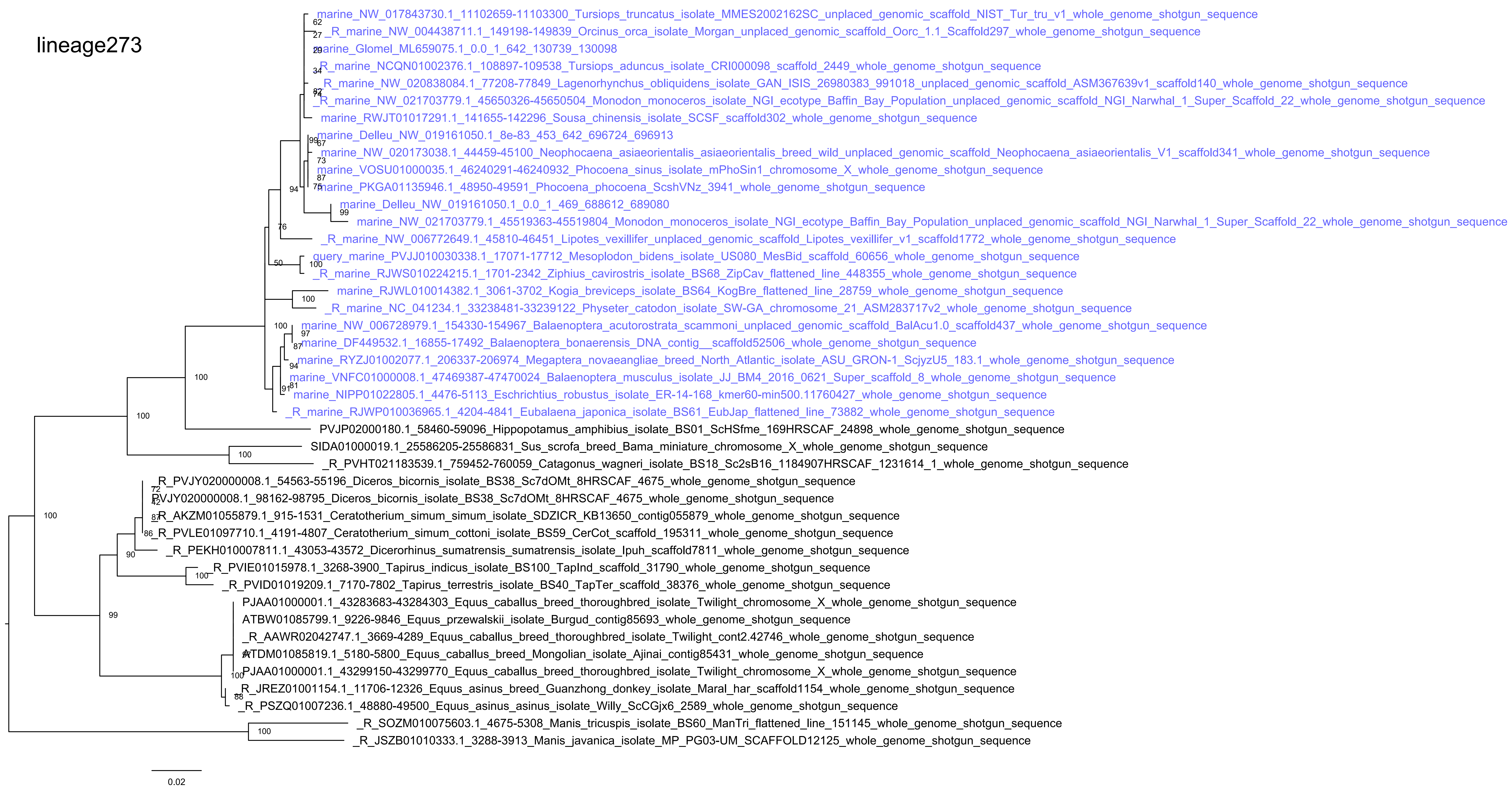

lineage273
marine_NW_017843730.1_11102659-11103300_Tursiops_truncatus_isolate_MMES2002162SC_unplaced_genomic_scaffold_NIST_Tur_tru_v1_whole_genome_shotgun_sequence
_R_marine_NW_004438711.1_149198-149839_Orcinus_orca_isolate_Morgan_unplaced_genomic_scaffold_Oorc_1.1_Scaffold297_whole_genome_shotgun_sequence
marine_Glomel_ML659075.1_0.0_1_642_130739_130098
_R_marine_NCQN01002376.1_108897-109538_Tursiops_aduncus_isolate_CRI000098_scaffold_2449_whole_genome_shotgun_sequence
_R_marine_NW_020838084.1_77208-77849_Lagenorhynchus_obliquidens_isolate_GAN_ISIS_26980383_991018_unplaced_genomic_scaffold_ASM367639v1_scaffold140_whole_genome_shotgun_sequence
_R_marine_NW_021703779.1_45650326-45650504_Monodon_monoceros_isolate_NGI_ecotype_Baffin_Bay_Population_unplaced_genomic_scaffold_NGI_Narwhal_1_Super_Scaffold_22_whole_genome_shotgun_sequence
marine_RWJT01017291.1_141655-142296_Sousa_chinensis_isolate_SCSF_scaffold302_whole_genome_shotgun_sequence
marine_Delleu_NW_019161050.1_8e-83_453_642_696724_696913
marine_NW_020173038.1_44459-45100_Neophocaena_asiaeorientalis_asiaeorientalis_breed_wild_unplaced_genomic_scaffold_Neophocaena_asiaeorientalis_V1_scaffold341_whole_genome_shotgun_sequence
marine_VOSU01000035.1_46240291-46240932_Phocoena_sinus_isolate_mPhoSin1_chromosome_X_whole_genome_shotgun_sequence
marine_PKGA01135946.1_48950-49591_Phocoena_phocoena_ScshVNz_3941_whole_genome_shotgun_sequence
marine_Delleu_NW_019161050.1_0.0_1_469_688612_689080
marine_NW_021703779.1_45519363-45519804_Monodon_monoceros_isolate_NGI_ecotype_Baffin_Bay_Population_unplaced_genomic_scaffold_NGI_Narwhal_1_Super_Scaffold_22_whole_genome_shotgun_sequence
_R_marine_NW_006772649.1_45810-46451_Lipotes_vexillifer_unplaced_genomic_scaffold_Lipotes_vexillifer_v1_scaffold1772_whole_genome_shotgun_sequence
query_marine_PVJJ010030338.1_17071-17712_Mesoplodon_bidens_isolate_US080_MesBid_scaffold_60656_whole_genome_shotgun_sequence
_R_marine_RJWS010224215.1_1701-2342_Ziphius_cavirostris_isolate_BS68_ZipCav_flattened_line_448355_whole_genome_shotgun_sequence
marine_RJWL010014382.1_3061-3702_Kogia_breviceps_isolate_BS64_KogBre_flattened_line_28759_whole_genome_shotgun_sequence
_R_marine_NC_041234.1_33238481-33239122_Physeter_catodon_isolate_SW-GA_chromosome_21_ASM283717v2_whole_genome_shotgun_sequence
marine_NW_006728979.1_154330-154967_Balaenoptera_acutorostrata_scammoni_unplaced_genomic_scaffold_BalAcu1.0_scaffold437_whole_genome_shotgun_sequence
marine_DF449532.1_16855-17492_Balaenoptera_bonaerensis_DNA_contig__scaffold52506_whole_genome_shotgun_sequence
marine_RYZJ01002077.1_206337-206974_Megaptera_novaeangliae_breed_North_Atlantic_isolate_ASU_GRON-1_ScjyzU5_183.1_whole_genome_shotgun_sequence
marine_VNFC01000008.1_47469387-47470024_Balaenoptera_musculus_isolate_JJ_BM4_2016_0621_Super_scaffold_8_whole_genome_shotgun_sequence
marine_NIPP01022805.1_4476-5113_Eschrichtius_robustus_isolate_ER-14-168_kmer60-min500.11760427_whole_genome_shotgun_sequence
_R_marine_RJWP010036965.1_4204-4841_Eubalaena_japonica_isolate_BS61_EubJap_flattened_line_73882_whole_genome_shotgun_sequence
PVJP02000180.1_58460-59096_Hippopotamus_amphibius_isolate_BS01_ScHSfme_169HRSCAF_24898_whole_genome_shotgun_sequence
SIDA01000019.1_25586205-25586831_Sus_scrofa_breed_Bama_miniature_chromosome_X_whole_genome_shotgun_sequence
_R_PVHT021183539.1_759452-760059_Catagonus_wagneri_isolate_BS18_Sc2sB16_1184907HRSCAF_1231614_1_whole_genome_shotgun_sequence
_R_PVJY020000008.1_54563-55196_Diceros_bicornis_isolate_BS38_Sc7dOMt_8HRSCAF_4675_whole_genome_shotgun_sequence
PVJY020000008.1_98162-98795_Diceros_bicornis_isolate_BS38_Sc7dOMt_8HRSCAF_4675_whole_genome_shotgun_sequence
_R_AKZM01055879.1_915-1531_Ceratotherium_simum_simum_isolate_SDZICR_KB13650_contig055879_whole_genome_shotgun_sequence
_R_PVLE01097710.1_4191-4807_Ceratotherium_simum_cottoni_isolate_BS59_CerCot_scaffold_195311_whole_genome_shotgun_sequence
_R_PEKH010007811.1_43053-43572_Dicerorhinus_sumatrensis_sumatrensis_isolate_Ipuh_scaffold7811_whole_genome_shotgun_sequence
_R_PVIE01015978.1_3268-3900_Tapirus_indicus_isolate_BS100_TapInd_scaffold_31790_whole_genome_shotgun_sequence
_R_PVID01019209.1_7170-7802_Tapirus_terrestris_isolate_BS40_TapTer_scaffold_38376_whole_genome_shotgun_sequence
PJAA01000001.1_43283683-43284303_Equus_caballus_breed_thoroughbred_isolate_Twilight_chromosome_X_whole_genome_shotgun_sequence
ATBW01085799.1_9226-9846_Equus_przewalskii_isolate_Burgud_contig85693_whole_genome_shotgun_sequence
_R_AAWR02042747.1_3669-4289_Equus_caballus_breed_thoroughbred_isolate_Twilight_cont2.42746_whole_genome_shotgun_sequence
ATDM01085819.1_5180-5800_Equus_caballus_breed_Mongolian_isolate_Ajinai_contig85431_whole_genome_shotgun_sequence
PJAA01000001.1_43299150-43299770_Equus_caballus_breed_thoroughbred_isolate_Twilight_chromosome_X_whole_genome_shotgun_sequence
_R_JREZ01001154.1_11706-12326_Equus_asinus_breed_Guanzhong_donkey_isolate_Maral_har_scaffold1154_whole_genome_shotgun_sequence
_R_PSZQ01007236.1_48880-49500_Equus_asinus_asinus_isolate_Willy_ScCGjx6_2589_whole_genome_shotgun_sequence
_R_SOZM010075603.1_4675-5308_Manis_tricuspis_isolate_BS60_ManTri_flattened_line_151145_whole_genome_shotgun_sequence
_R_JSZB01010333.1_3288-3913_Manis_javanica_isolate_MP_PG03-UM_SCAFFOLD12125_whole_genome_shotgun_sequence
0.02

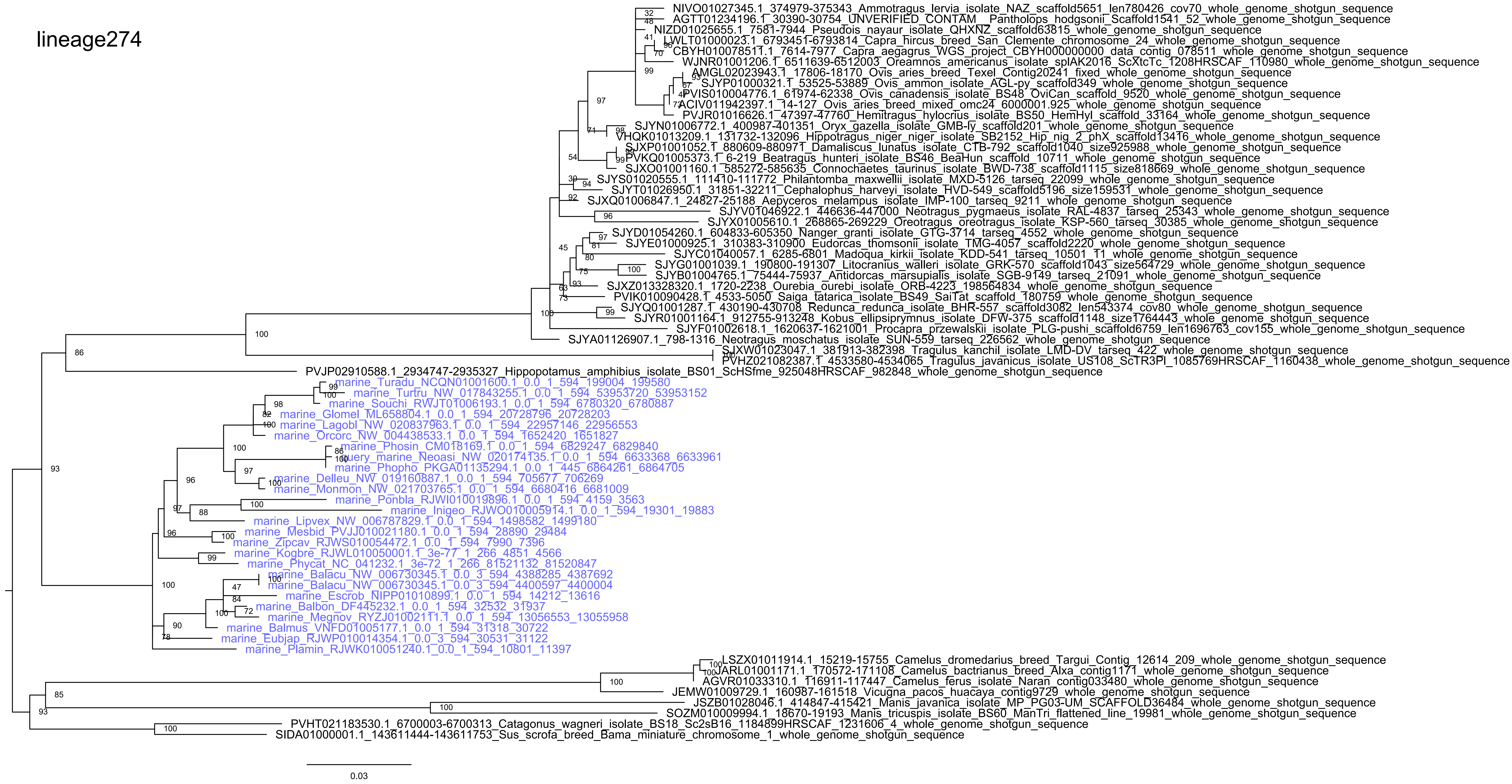
lineage274
NIVO01027345.1_374979-375343_Ammotragus_lervia_isolate_NAZ_scaffold5651_len780426_cov70_whole_genome_shotgun_sequence
AGTT01234196.1_30390-30754_UNVERIFIED_CONTAM__Pantholops_hodgsonii_Scaffold1541_52_whole_genome_shotgun_sequence
NIZD01025655.1_7581-7944_Pseudois_nayaur_isolate_QHXNZ_scaffold63815_whole_genome_shotgun_sequence
LWLT01000023.1_6793451-6793814_Capra_hircus_breed_San_Clemente_chromosome_24_whole_genome_shotgun_sequence
CBYH010078511.1_7614-7977_Capra_aegagrus_WGS_project_CBYH000000000_data_contig_078511_whole_genome_shotgun_sequence
WJNR01001206.1_6511639-6512003_Oreamnos_americanus_isolate_splAK2016_ScXtcTc_1208HRSCAF_110980_whole_genome_shotgun_sequence
AMGL02023943.1_17806-18170_Ovis_aries_breed_Texel_Contig20241_fixed_whole_genome_shotgun_sequence
SJYP01000321.1_53525-53889_Ovis_ammon_isolate_AGL-py_scaffold349_whole_genome_shotgun_sequence
PVIS010004776.1_61974-62338_Ovis_canadensis_isolate_BS48_OviCan_scaffold_9520_whole_genome_shotgun_sequence
ACIV011942397.1_14-127_Ovis_aries_breed_mixed_omc24_6000001.925_whole_genome_shotgun_sequence
PVJR01016626.1_47397-47760_Hemitragus_hylocrius_isolate_BS50_HemHyl_scaffold_33164_whole_genome_shotgun_sequence
SJYN01006772.1_400987-401351_Oryx_gazella_isolate_GMB-ly_scaffold201_whole_genome_shotgun_sequence
VHQK01013209.1_131732-132096_Hippotragus_niger_niger_isolate_SB2152_Hip_nig_2_phX_scaffold13416_whole_genome_shotgun_sequence
SJXP01001052.1_880609-880971_Damaliscus_lunatus_isolate_CTB-792_scaffold1040_size925988_whole_genome_shotgun_sequence
PVKQ01005373.1_6-219_Beatragus_hunteri_isolate_BS46_BeaHun_scaffold_10711_whole_genome_shotgun_sequence
SJXO01001160.1_585272-585635_Connochaetes_taurinus_isolate_BWD-738_scaffold1115_size818669_whole_genome_shotgun_sequence
SJYS01020555.1_111410-111772_Philantomba_maxwellii_isolate_MXD-5126_tarseq_22099_whole_genome_shotgun_sequence
SJYT01026950.1_31851-32211_Cephalophus_harveyi_isolate_HVD-549_scaffold5196_size159531_whole_genome_shotgun_sequence
SJXQ01006847.1_24827-25188_Aepyceros_melampus_isolate_IMP-100_tarseq_9211_whole_genome_shotgun_sequence
SJYV01046922.1_446636-447000_Neotragus_pygmaeus_isolate_RAL-4837_tarseq_25343_whole_genome_shotgun_sequence
SJYX01005610.1_268865-269229_Oreotragus_oreotragus_isolate_KSP-560_tarseq_30385_whole_genome_shotgun_sequence
SJYD01054260.1_604833-605350_Nanger_granti_isolate_GTG-3714_tarseq_4552_whole_genome_shotgun_sequence
SJYE01000925.1_310383-310900_Eudorcas_thomsonii_isolate_TMG-4057_scaffold2220_whole_genome_shotgun_sequence
SJYC01040057.1_6285-6801_Madoqua_kirkii_isolate_KDD-541_tarseq_10501_11_whole_genome_shotgun_sequence
SJYG01001039.1_190800-191307_Litocranius_walleri_isolate_GRK-570_scaffold1043_size564729_whole_genome_shotgun_sequence
SJYB01004765.1_75444-75937_Antidorcas_marsupialis_isolate_SGB-9149_tarseq_21091_whole_genome_shotgun_sequence
SJXZ013328320.1_1720-2238_Ourebia_ourebi_isolate_ORB-4223_198564834_whole_genome_shotgun_sequence
PVIK010090428.1_4533-5050_Saiga_tatarica_isolate_BS49_SaiTat_scaffold_180759_whole_genome_shotgun_sequence
SJYQ01001287.1_430190-430708_Redunca_redunca_isolate_BHR-557_scaffold3082_len543374_cov80_whole_genome_shotgun_sequence
SJYR01001164.1_912755-913248_Kobus_ellipsiprymnus_isolate_DFW-375_scaffold1148_size1764443_whole_genome_shotgun_sequence
SJYF01002618.1_1620637-1621001_Procapra_przewalskii_isolate_PLG-pushi_scaffold6759_len1696763_cov155_whole_genome_shotgun_sequence
SJYA01126907.1_798-1316_Neotragus_moschatus_isolate_SUN-559_tarseq_226562_whole_genome_shotgun_sequence
SJXW01023047.1_381913-382398_Tragulus_kanchil_isolate_LMD-DV_tarseq_422_whole_genome_shotgun_sequence
PVHZ021082387.1_4533580-4534065_Tragulus_javanicus_isolate_US108_ScTR3PI_1085769HRSCAF_1160438_whole_genome_shotgun_sequence
PVJP02910588.1_2934747-2935327_Hippopotamus_amphibius_isolate_BS01_ScHSfme_925048HRSCAF_982848_whole_genome_shotgun_sequence
marine_Turadu_NCQN01001600.1_0.0_1_594_199004_199580
marine_Turtru_NW_017843255.1_0.0_1_594_53953720_53953152
marine_Souchi_RWJT01006193.1_0.0_1_594_6780320_6780887
marine_Glomel_ML658804.1_0.0_1_594_20728796_20728203
marine_Lagobl_NW_020837963.1_0.0_1_594_22957146_22956553
marine_Orcorc_NW_004438533.1_0.0_1_594_1652420_1651827
marine_Phosin_CM018169.1_0.0_1_594_6829247_6829840
query_marine_Neoasi_NW_020174135.1_0.0_1_594_6633368_6633961
marine_Phopho_PKGA01135294.1_0.0_1_445_6864261_6864705
marine_Delleu_NW_019160887.1_0.0_1_594_705677_706269
marine_Monmon_NW_021703765.1_0.0_1_594_6680416_6681009
marine_Ponbla_RJWI010019896.1_0.0_1_594_4159_3563
marine_Inigeo_RJWO010005914.1_0.0_1_594_19301_19883
marine_Lipvex_NW_006787829.1_0.0_1_594_1498582_1499180
marine_Mesbid_PVJJ010021180.1_0.0_1_594_28890_29484
marine_Zipcav_RJWS010054472.1_0.0_1_594_7990_7396
marine_Kogbre_RJWL010050001.1_3e-77_1_266_4851_4566
marine_Phycat_NC_041232.1_3e-72_1_266_81521132_81520847
marine_Balacu_NW_006730345.1_0.0_3_594_4388285_4387692
marine_Balacu_NW_006730345.1_0.0_3_594_4400597_4400004
marine_Escrob_NIPP01010899.1_0.0_1_594_14212_13616
marine_Balbon_DF445232.1_0.0_1_594_32532_31937
marine_Megnov_RYZJ01002111.1_0.0_1_594_13056553_13055958
marine_Balmus_VNFD01005177.1_0.0_1_594_31318_30722
marine_Eubjap_RJWP010014354.1_0.0_3_594_30531_31122
marine_Plamin_RJWK010051240.1_0.0_1_594_10801_11397
LSZX01011914.1_15219-15755_Camelus_dromedarius_breed_Targui_Contig_12614_209_whole_genome_shotgun_sequence
JARL01001171.1_170572-171108_Camelus_bactrianus_breed_Alxa_contig1171_whole_genome_shotgun_sequence
AGVR01033310.1_116911-117447_Camelus_ferus_isolate_Naran_contig033480_whole_genome_shotgun_sequence
JEMW01009729.1_160987-161518_Vicugna_pacos_huacaya_contig9729_whole_genome_shotgun_sequence
JSZB01028046.1_414847-415421_Manis_javanica_isolate_MP_PG03-UM_SCAFFOLD36484_whole_genome_shotgun_sequence
SOZM010009994.1_18670-19193_Manis_tricuspis_isolate_BS60_ManTri_flattened_line_19981_whole_genome_shotgun_sequence
PVHT021183530.1_6700003-6700313_Catagonus_wagneri_isolate_BS18_Sc2sB16_1184899HRSCAF_1231606_4_whole_genome_shotgun_sequence
SIDA01000001.1_143611444-143611753_Sus_scrofa_breed_Bama_miniature_chromosome_1_whole_genome_shotgun_sequence
0.03

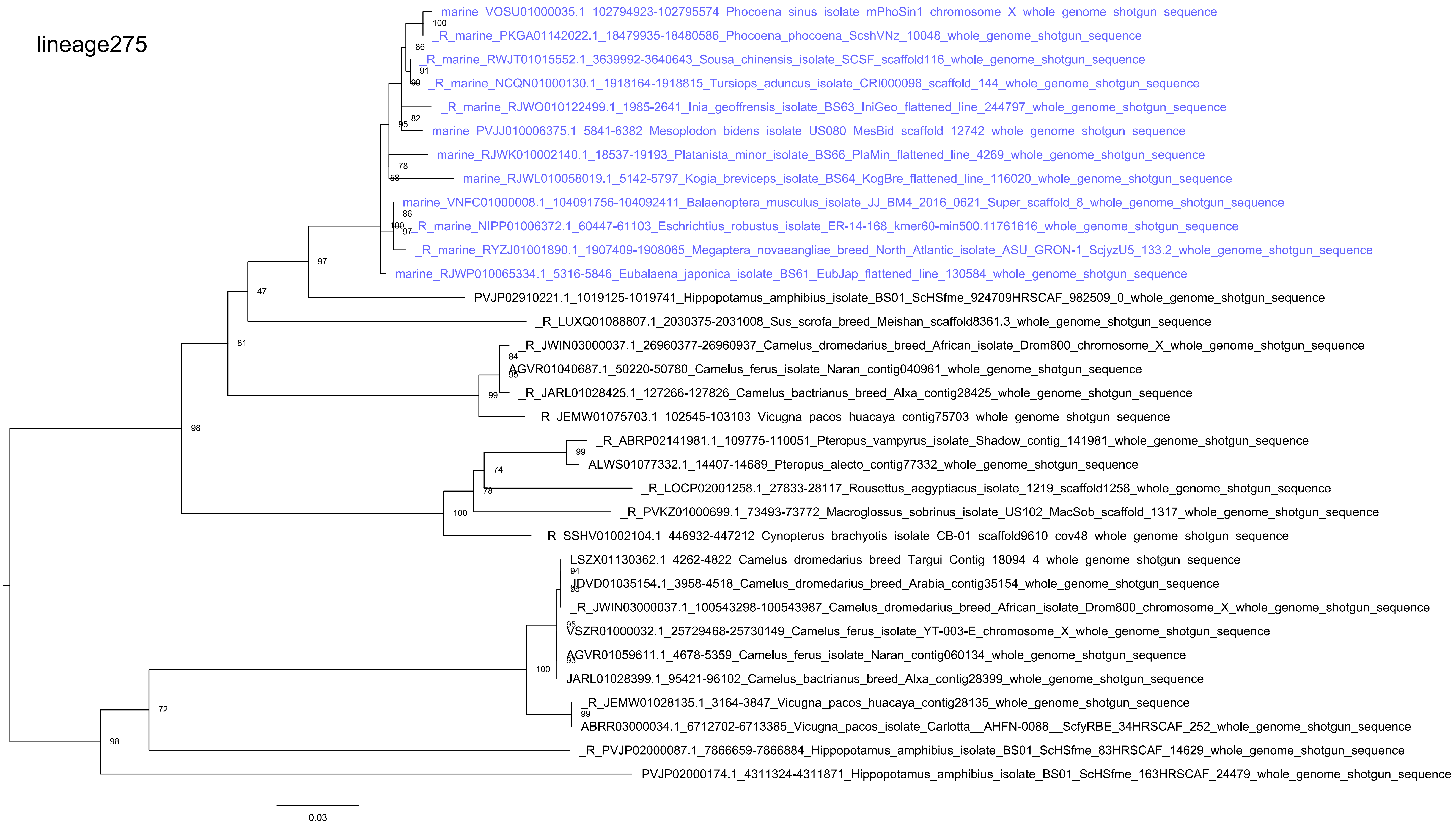
lineage275
marine_VOSU01000035.1_102794923-102795574_Phocoena_sinus_isolate_mPhoSin1_chromosome_X_whole_genome_shotgun_sequence
_R_marine_PKGA01142022.1_18479935-18480586_Phocoena_phocoena_ScshVNz_10048_whole_genome_shotgun_sequence
_R_marine_RWJT01015552.1_3639992-3640643_Sousa_chinensis_isolate_SCSF_scaffold116_whole_genome_shotgun_sequence
_R_marine_NCQN01000130.1_1918164-1918815_Tursiops_aduncus_isolate_CRI000098_scaffold_144_whole_genome_shotgun_sequence
_R_marine_RJWO010122499.1_1985-2641_Inia_geoffrensis_isolate_BS63_IniGeo_flattened_line_244797_whole_genome_shotgun_sequence
marine_PVJJ010006375.1_5841-6382_Mesoplodon_bidens_isolate_US080_MesBid_scaffold_12742_whole_genome_shotgun_sequence
marine_RJWK010002140.1_18537-19193_Platanista_minor_isolate_BS66_PlaMin_flattened_line_4269_whole_genome_shotgun_sequence
marine_RJWL010058019.1_5142-5797_Kogia_breviceps_isolate_BS64_KogBre_flattened_line_116020_whole_genome_shotgun_sequence
marine_VNFC01000008.1_104091756-104092411_Balaenoptera_musculus_isolate_JJ_BM4_2016_0621_Super_scaffold_8_whole_genome_shotgun_sequence
_R_marine_NIPP01006372.1_60447-61103_Eschrichtius_robustus_isolate_ER-14-168_kmer60-min500.11761616_whole_genome_shotgun_sequence
_R_marine_RYZJ01001890.1_1907409-1908065_Megaptera_novaeangliae_breed_North_Atlantic_isolate_ASU_GRON-1_ScjyzU5_133.2_whole_genome_shotgun_sequence
marine_RJWP010065334.1_5316-5846_Eubalaena_japonica_isolate_BS61_EubJap_flattened_line_130584_whole_genome_shotgun_sequence
PVJP02910221.1_1019125-1019741_Hippopotamus_amphibius_isolate_BS01_ScHSfme_924709HRSCAF_982509_0_whole_genome_shotgun_sequence
_R_LUXQ01088807.1_2030375-2031008_Sus_scrofa_breed_Meishan_scaffold8361.3_whole_genome_shotgun_sequence
_R_JWIN03000037.1_26960377-26960937_Camelus_dromedarius_breed_African_isolate_Drom800_chromosome_X_whole_genome_shotgun_sequence
AGVR01040687.1_50220-50780_Camelus_ferus_isolate_Naran_contig040961_whole_genome_shotgun_sequence
_R_JARL01028425.1_127266-127826_Camelus_bactrianus_breed_Alxa_contig28425_whole_genome_shotgun_sequence
_R_JEMW01075703.1_102545-103103_Vicugna_pacos_huacaya_contig75703_whole_genome_shotgun_sequence
_R_ABRP02141981.1_109775-110051_Pteropus_vampyrus_isolate_Shadow_contig_141981_whole_genome_shotgun_sequence
ALWS01077332.1_14407-14689_Pteropus_alecto_contig77332_whole_genome_shotgun_sequence
_R_LOCP02001258.1_27833-28117_Rousettus_aegyptiacus_isolate_1219_scaffold1258_whole_genome_shotgun_sequence
_R_PVKZ01000699.1_73493-73772_Macroglossus_sobrinus_isolate_US102_MacSob_scaffold_1317_whole_genome_shotgun_sequence
_R_SSHV01002104.1_446932-447212_Cynopterus_brachyotis_isolate_CB-01_scaffold9610_cov48_whole_genome_shotgun_sequence
LSZX01130362.1_4262-4822_Camelus_dromedarius_breed_Targui_Contig_18094_4_whole_genome_shotgun_sequence
JDVD01035154.1_3958-4518_Camelus_dromedarius_breed_Arabia_contig35154_whole_genome_shotgun_sequence
_R_JWIN03000037.1_100543298-100543987_Camelus_dromedarius_breed_African_isolate_Drom800_chromosome_X_whole_genome_shotgun_sequence
VSZR01000032.1_25729468-25730149_Camelus_ferus_isolate_YT-003-E_chromosome_X_whole_genome_shotgun_sequence
AGVR01059611.1_4678-5359_Camelus_ferus_isolate_Naran_contig060134_whole_genome_shotgun_sequence
JARL01028399.1_95421-96102_Camelus_bactrianus_breed_Alxa_contig28399_whole_genome_shotgun_sequence
_R_JEMW01028135.1_3164-3847_Vicugna_pacos_huacaya_contig28135_whole_genome_shotgun_sequence
ABRR03000034.1_6712702-6713385_Vicugna_pacos_isolate_Carlotta__AHFN-0088__ScfyRBE_34HRSCAF_252_whole_genome_shotgun_sequence
_R_PVJP02000087.1_7866659-7866884_Hippopotamus_amphibius_isolate_BS01_ScHSfme_83HRSCAF_14629_whole_genome_shotgun_sequence
PVJP02000174.1_4311324-4311871_Hippopotamus_amphibius_isolate_BS01_ScHSfme_163HRSCAF_24479_whole_genome_shotgun_sequence
0.03

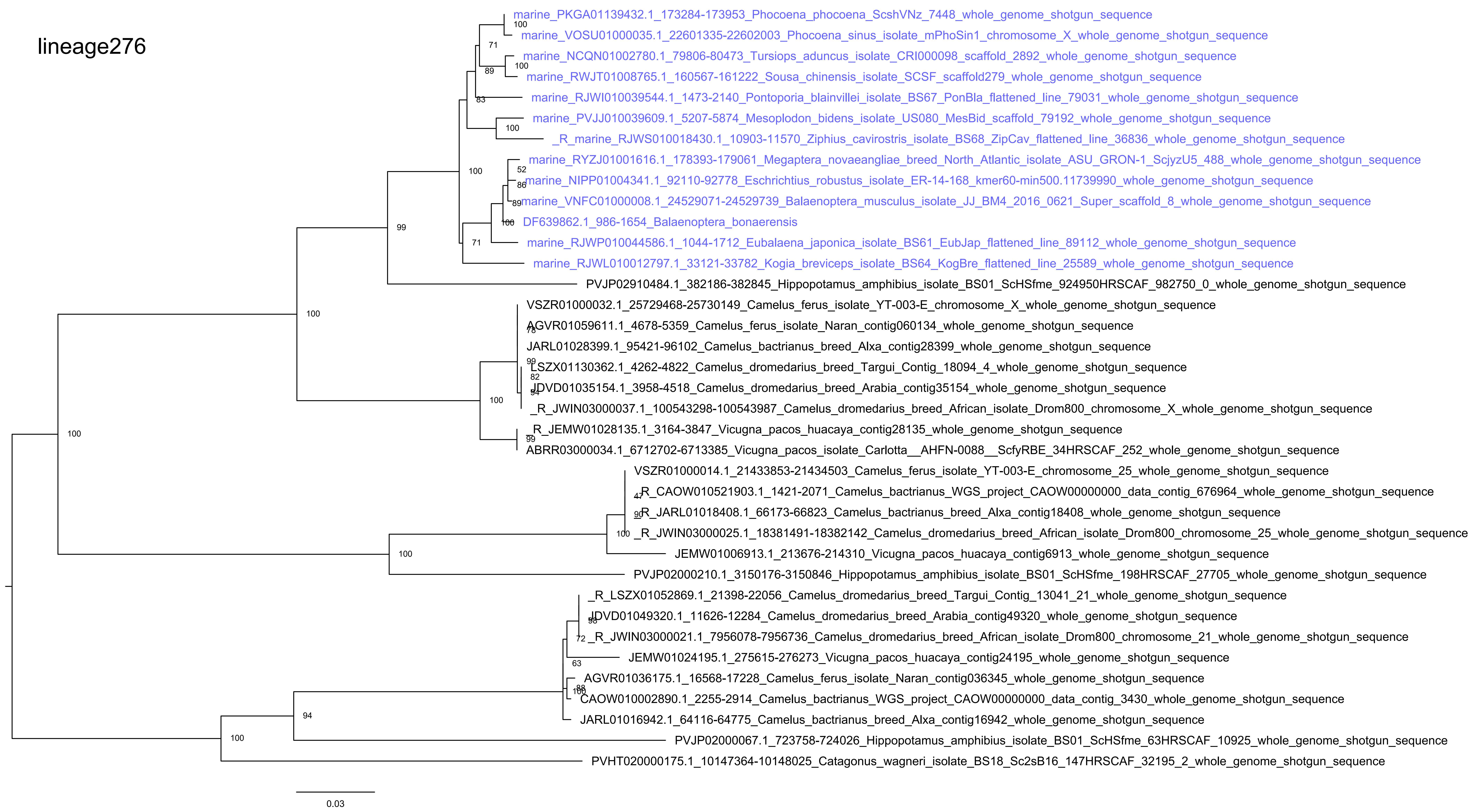
lineage276
marine_PKGA01139432.1_173284-173953_Phocoena_phocoena_ScshVNz_7448_whole_genome_shotgun_sequence
marine_VOSU01000035.1_22601335-22602003_Phocoena_sinus_isolate_mPhoSin1_chromosome_X_whole_genome_shotgun_sequence
marine_NCQN01002780.1_79806-80473_Tursiops_aduncus_isolate_CRI000098_scaffold_2892_whole_genome_shotgun_sequence
marine_RWJT01008765.1_160567-161222_Sousa_chinensis_isolate_SCSF_scaffold279_whole_genome_shotgun_sequence
marine_RJWI010039544.1_1473-2140_Pontoporia_blainvillei_isolate_BS67_PonBla_flattened_line_79031_whole_genome_shotgun_sequence
marine_PVJJ010039609.1_5207-5874_Mesoplodon_bidens_isolate_US080_MesBid_scaffold_79192_whole_genome_shotgun_sequence
_R_marine_RJWS010018430.1_10903-11570_Ziphius_cavirostris_isolate_BS68_ZipCav_flattened_line_36836_whole_genome_shotgun_sequence
marine_RYZJ01001616.1_178393-179061_Megaptera_novaeangliae_breed_North_Atlantic_isolate_ASU_GRON-1_ScjyzU5_488_whole_genome_shotgun_sequence
marine_NIPP01004341.1_92110-92778_Eschrichtius_robustus_isolate_ER-14-168_kmer60-min500.11739990_whole_genome_shotgun_sequence
marine_VNFC01000008.1_24529071-24529739_Balaenoptera_musculus_isolate_JJ_BM4_2016_0621_Super_scaffold_8_whole_genome_shotgun_sequence
DF639862.1_986-1654_Balaenoptera_bonaerensis
marine_RJWP010044586.1_1044-1712_Eubalaena_japonica_isolate_BS61_EubJap_flattened_line_89112_whole_genome_shotgun_sequence
marine_RJWL010012797.1_33121-33782_Kogia_breviceps_isolate_BS64_KogBre_flattened_line_25589_whole_genome_shotgun_sequence
PVJP02910484.1_382186-382845_Hippopotamus_amphibius_isolate_BS01_ScHSfme_924950HRSCAF_982750_0_whole_genome_shotgun_sequence
VSZR01000032.1_25729468-25730149_Camelus_ferus_isolate_YT-003-E_chromosome_X_whole_genome_shotgun_sequence
AGVR01059611.1_4678-5359_Camelus_ferus_isolate_Naran_contig060134_whole_genome_shotgun_sequence
JARL01028399.1_95421-96102_Camelus_bactrianus_breed_Alxa_contig28399_whole_genome_shotgun_sequence
LSZX01130362.1_4262-4822_Camelus_dromedarius_breed_Targui_Contig_18094_4_whole_genome_shotgun_sequence
JDVD01035154.1_3958-4518_Camelus_dromedarius_breed_Arabia_contig35154_whole_genome_shotgun_sequence
_R_JWIN03000037.1_100543298-100543987_Camelus_dromedarius_breed_African_isolate_Drom800_chromosome_X_whole_genome_shotgun_sequence
_R_JEMW01028135.1_3164-3847_Vicugna_pacos_huacaya_contig28135_whole_genome_shotgun_sequence
ABRR03000034.1_6712702-6713385_Vicugna_pacos_isolate_Carlotta__AHFN-0088__ScfyRBE_34HRSCAF_252_whole_genome_shotgun_sequence
VSZR01000014.1_21433853-21434503_Camelus_ferus_isolate_YT-003-E_chromosome_25_whole_genome_shotgun_sequence
_R_CAOW010521903.1_1421-2071_Camelus_bactrianus_WGS_project_CAOW00000000_data_contig_676964_whole_genome_shotgun_sequence
_R_JARL01018408.1_66173-66823_Camelus_bactrianus_breed_Alxa_contig18408_whole_genome_shotgun_sequence
_R_JWIN03000025.1_18381491-18382142_Camelus_dromedarius_breed_African_isolate_Drom800_chromosome_25_whole_genome_shotgun_sequence
JEMW01006913.1_213676-214310_Vicugna_pacos_huacaya_contig6913_whole_genome_shotgun_sequence
PVJP02000210.1_3150176-3150846_Hippopotamus_amphibius_isolate_BS01_ScHSfme_198HRSCAF_27705_whole_genome_shotgun_sequence
_R_LSZX01052869.1_21398-22056_Camelus_dromedarius_breed_Targui_Contig_13041_21_whole_genome_shotgun_sequence
JDVD01049320.1_11626-12284_Camelus_dromedarius_breed_Arabia_contig49320_whole_genome_shotgun_sequence
_R_JWIN03000021.1_7956078-7956736_Camelus_dromedarius_breed_African_isolate_Drom800_chromosome_21_whole_genome_shotgun_sequence
JEMW01024195.1_275615-276273_Vicugna_pacos_huacaya_contig24195_whole_genome_shotgun_sequence
AGVR01036175.1_16568-17228_Camelus_ferus_isolate_Naran_contig036345_whole_genome_shotgun_sequence
CAOW010002890.1_2255-2914_Camelus_bactrianus_WGS_project_CAOW00000000_data_contig_3430_whole_genome_shotgun_sequence
JARL01016942.1_64116-64775_Camelus_bactrianus_breed_Alxa_contig16942_whole_genome_shotgun_sequence
PVJP02000067.1_723758-724026_Hippopotamus_amphibius_isolate_BS01_ScHSfme_63HRSCAF_10925_whole_genome_shotgun_sequence
PVHT020000175.1_10147364-10148025_Catagonus_wagneri_isolate_BS18_Sc2sB16_147HRSCAF_32195_2_whole_genome_shotgun_sequence
0.03

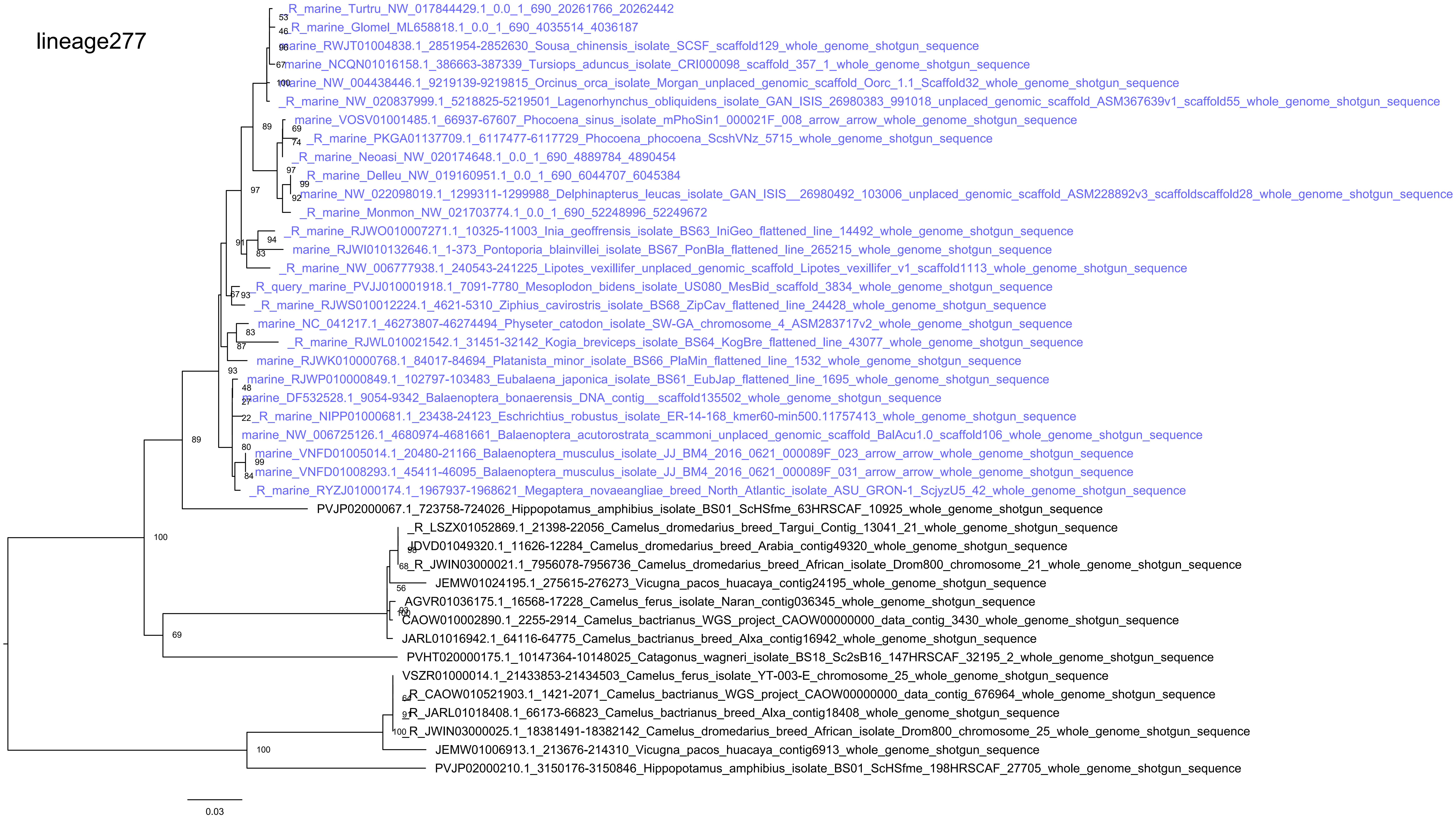
lineage277
_R_marine_Turtru_NW_017844429.1_0.0_1_690_20261766_20262442
_R_marine_Glomel_ML658818.1_0.0_1_690_4035514_4036187
marine_RWJT01004838.1_2851954-2852630_Sousa_chinensis_isolate_SCSF_scaffold129_whole_genome_shotgun_sequence
marine_NCQN01016158.1_386663-387339_Tursiops_aduncus_isolate_CRI000098_scaffold_357_1_whole_genome_shotgun_sequence
marine_NW_004438446.1_9219139-9219815_Orcinus_orca_isolate_Morgan_unplaced_genomic_scaffold_Oorc_1.1_Scaffold32_whole_genome_shotgun_sequence
_R_marine_NW_020837999.1_5218825-5219501_Lagenorhynchus_obliquidens_isolate_GAN_ISIS_26980383_991018_unplaced_genomic_scaffold_ASM367639v1_scaffold55_whole_genome_shotgun_sequence
marine_VOSV01001485.1_66937-67607_Phocoena_sinus_isolate_mPhoSin1_000021F_008_arrow_arrow_whole_genome_shotgun_sequence
_R_marine_PKGA01137709.1_6117477-6117729_Phocoena_phocoena_ScshVNz_5715_whole_genome_shotgun_sequence
_R_marine_Neoasi_NW_020174648.1_0.0_1_690_4889784_4890454
_R_marine_Delleu_NW_019160951.1_0.0_1_690_6044707_6045384
marine_NW_022098019.1_1299311-1299988_Delphinapterus_leucas_isolate_GAN_ISIS__26980492_103006_unplaced_genomic_scaffold_ASM228892v3_scaffoldscaffold28_whole_genome_shotgun_sequence
_R_marine_Monmon_NW_021703774.1_0.0_1_690_52248996_52249672
_R_marine_RJWO010007271.1_10325-11003_Inia_geoffrensis_isolate_BS63_IniGeo_flattened_line_14492_whole_genome_shotgun_sequence
marine_RJWI010132646.1_1-373_Pontoporia_blainvillei_isolate_BS67_PonBla_flattened_line_265215_whole_genome_shotgun_sequence
_R_marine_NW_006777938.1_240543-241225_Lipotes_vexillifer_unplaced_genomic_scaffold_Lipotes_vexillifer_v1_scaffold1113_whole_genome_shotgun_sequence
_R_query_marine_PVJJ010001918.1_7091-7780_Mesoplodon_bidens_isolate_US080_MesBid_scaffold_3834_whole_genome_shotgun_sequence
_R_marine_RJWS010012224.1_4621-5310_Ziphius_cavirostris_isolate_BS68_ZipCav_flattened_line_24428_whole_genome_shotgun_sequence
marine_NC_041217.1_46273807-46274494_Physeter_catodon_isolate_SW-GA_chromosome_4_ASM283717v2_whole_genome_shotgun_sequence
_R_marine_RJWL010021542.1_31451-32142_Kogia_breviceps_isolate_BS64_KogBre_flattened_line_43077_whole_genome_shotgun_sequence
marine_RJWK010000768.1_84017-84694_Platanista_minor_isolate_BS66_PlaMin_flattened_line_1532_whole_genome_shotgun_sequence
marine_RJWP010000849.1_102797-103483_Eubalaena_japonica_isolate_BS61_EubJap_flattened_line_1695_whole_genome_shotgun_sequence
marine_DF532528.1_9054-9342_Balaenoptera_bonaerensis_DNA_contig__scaffold135502_whole_genome_shotgun_sequence
_R_marine_NIPP01000681.1_23438-24123_Eschrichtius_robustus_isolate_ER-14-168_kmer60-min500.11757413_whole_genome_shotgun_sequence
marine_NW_006725126.1_4680974-4681661_Balaenoptera_acutorostrata_scammoni_unplaced_genomic_scaffold_BalAcu1.0_scaffold106_whole_genome_shotgun_sequence
marine_VNFD01005014.1_20480-21166_Balaenoptera_musculus_isolate_JJ_BM4_2016_0621_000089F_023_arrow_arrow_whole_genome_shotgun_sequence
marine_VNFD01008293.1_45411-46095_Balaenoptera_musculus_isolate_JJ_BM4_2016_0621_000089F_031_arrow_arrow_whole_genome_shotgun_sequence
_R_marine_RYZJ01000174.1_1967937-1968621_Megaptera_novaeangliae_breed_North_Atlantic_isolate_ASU_GRON-1_ScjyzU5_42_whole_genome_shotgun_sequence
PVJP02000067.1_723758-724026_Hippopotamus_amphibius_isolate_BS01_ScHSfme_63HRSCAF_10925_whole_genome_shotgun_sequence
_R_LSZX01052869.1_21398-22056_Camelus_dromedarius_breed_Targui_Contig_13041_21_whole_genome_shotgun_sequence
JDVD01049320.1_11626-12284_Camelus_dromedarius_breed_Arabia_contig49320_whole_genome_shotgun_sequence
_R_JWIN03000021.1_7956078-7956736_Camelus_dromedarius_breed_African_isolate_Drom800_chromosome_21_whole_genome_shotgun_sequence
JEMW01024195.1_275615-276273_Vicugna_pacos_huacaya_contig24195_whole_genome_shotgun_sequence
AGVR01036175.1_16568-17228_Camelus_ferus_isolate_Naran_contig036345_whole_genome_shotgun_sequence
CAOW010002890.1_2255-2914_Camelus_bactrianus_WGS_project_CAOW00000000_data_contig_3430_whole_genome_shotgun_sequence
JARL01016942.1_64116-64775_Camelus_bactrianus_breed_Alxa_contig16942_whole_genome_shotgun_sequence
PVHT020000175.1_10147364-10148025_Catagonus_wagneri_isolate_BS18_Sc2sB16_147HRSCAF_32195_2_whole_genome_shotgun_sequence
VSZR01000014.1_21433853-21434503_Camelus_ferus_isolate_YT-003-E_chromosome_25_whole_genome_shotgun_sequence
_R_CAOW010521903.1_1421-2071_Camelus_bactrianus_WGS_project_CAOW00000000_data_contig_676964_whole_genome_shotgun_sequence
_R_JARL01018408.1_66173-66823_Camelus_bactrianus_breed_Alxa_contig18408_whole_genome_shotgun_sequence
_R_JWIN03000025.1_18381491-18382142_Camelus_dromedarius_breed_African_isolate_Drom800_chromosome_25_whole_genome_shotgun_sequence
JEMW01006913.1_213676-214310_Vicugna_pacos_huacaya_contig6913_whole_genome_shotgun_sequence
PVJP02000210.1_3150176-3150846_Hippopotamus_amphibius_isolate_BS01_ScHSfme_198HRSCAF_27705_whole_genome_shotgun_sequence
0.03

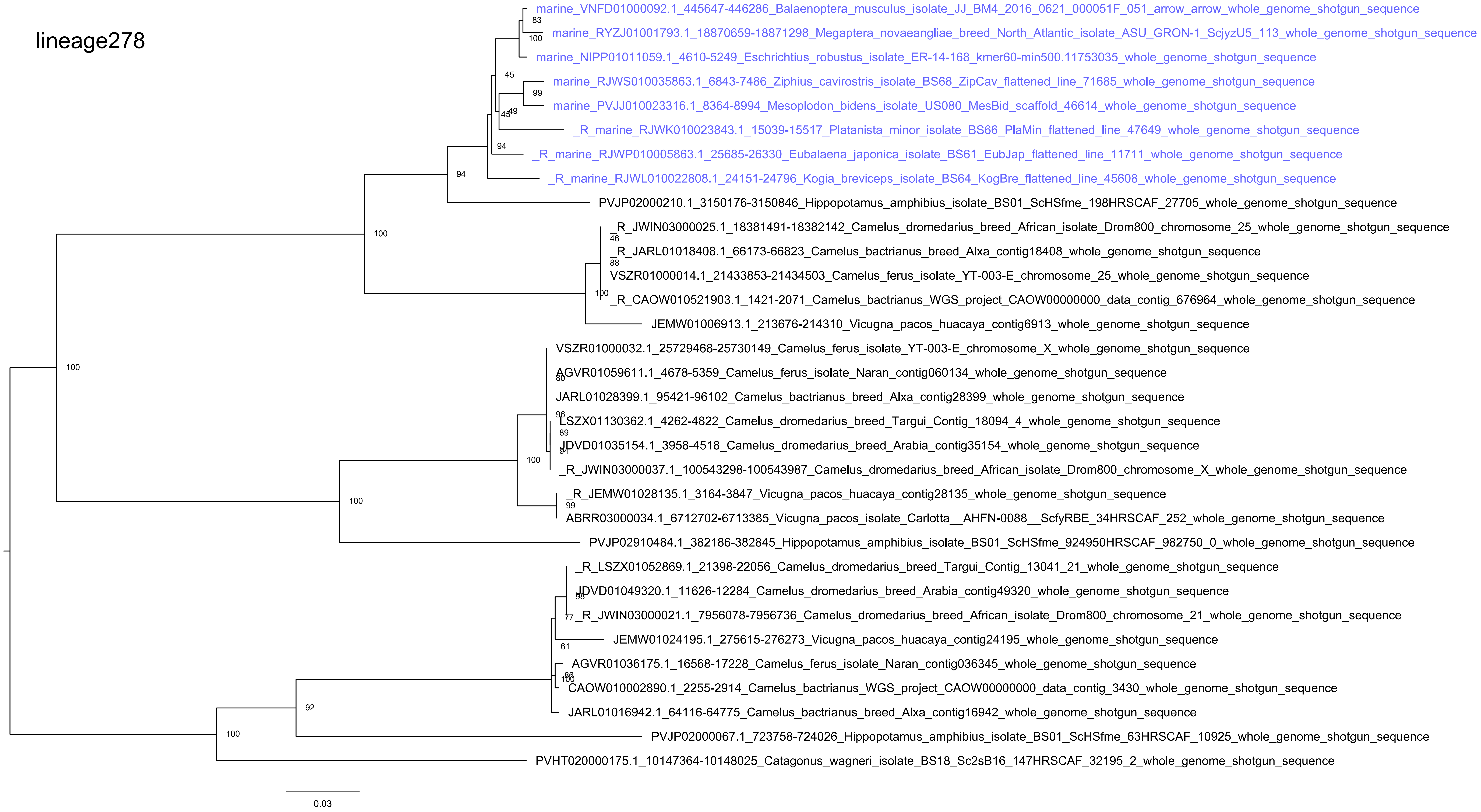

lineage278
marine_VNFD01000092.1_445647-446286_Balaenoptera_musculus_isolate_JJ_BM4_2016_0621_000051F_051_arrow_arrow_whole_genome_shotgun_sequence
marine_RYZJ01001793.1_18870659-18871298_Megaptera_novaeangliae_breed_North_Atlantic_isolate_ASU_GRON-1_ScjyzU5_113_whole_genome_shotgun_sequence
marine_NIPP01011059.1_4610-5249_Eschrichtius_robustus_isolate_ER-14-168_kmer60-min500.11753035_whole_genome_shotgun_sequence
marine_RJWS010035863.1_6843-7486_Ziphius_cavirostris_isolate_BS68_ZipCav_flattened_line_71685_whole_genome_shotgun_sequence
marine_PVJJ010023316.1_8364-8994_Mesoplodon_bidens_isolate_US080_MesBid_scaffold_46614_whole_genome_shotgun_sequence
_R_marine_RJWK010023843.1_15039-15517_Platanista_minor_isolate_BS66_PlaMin_flattened_line_47649_whole_genome_shotgun_sequence
_R_marine_RJWP010005863.1_25685-26330_Eubalaena_japonica_isolate_BS61_EubJap_flattened_line_11711_whole_genome_shotgun_sequence
_R_marine_RJWL010022808.1_24151-24796_Kogia_breviceps_isolate_BS64_KogBre_flattened_line_45608_whole_genome_shotgun_sequence
PVJP02000210.1_3150176-3150846_Hippopotamus_amphibius_isolate_BS01_ScHSfme_198HRSCAF_27705_whole_genome_shotgun_sequence
_R_JWIN03000025.1_18381491-18382142_Camelus_dromedarius_breed_African_isolate_Drom800_chromosome_25_whole_genome_shotgun_sequence
_R_JARL01018408.1_66173-66823_Camelus_bactrianus_breed_Alxa_contig18408_whole_genome_shotgun_sequence
VSZR01000014.1_21433853-21434503_Camelus_ferus_isolate_YT-003-E_chromosome_25_whole_genome_shotgun_sequence
_R_CAOW010521903.1_1421-2071_Camelus_bactrianus_WGS_project_CAOW00000000_data_contig_676964_whole_genome_shotgun_sequence
JEMW01006913.1_213676-214310_Vicugna_pacos_huacaya_contig6913_whole_genome_shotgun_sequence
VSZR01000032.1_25729468-25730149_Camelus_ferus_isolate_YT-003-E_chromosome_X_whole_genome_shotgun_sequence
AGVR01059611.1_4678-5359_Camelus_ferus_isolate_Naran_contig060134_whole_genome_shotgun_sequence
JARL01028399.1_95421-96102_Camelus_bactrianus_breed_Alxa_contig28399_whole_genome_shotgun_sequence
LSZX01130362.1_4262-4822_Camelus_dromedarius_breed_Targui_Contig_18094_4_whole_genome_shotgun_sequence
JDVD01035154.1_3958-4518_Camelus_dromedarius_breed_Arabia_contig35154_whole_genome_shotgun_sequence
_R_JWIN03000037.1_100543298-100543987_Camelus_dromedarius_breed_African_isolate_Drom800_chromosome_X_whole_genome_shotgun_sequence
_R_JEMW01028135.1_3164-3847_Vicugna_pacos_huacaya_contig28135_whole_genome_shotgun_sequence
ABRR03000034.1_6712702-6713385_Vicugna_pacos_isolate_Carlotta__AHFN-0088__ScfyRBE_34HRSCAF_252_whole_genome_shotgun_sequence
PVJP02910484.1_382186-382845_Hippopotamus_amphibius_isolate_BS01_ScHSfme_924950HRSCAF_982750_0_whole_genome_shotgun_sequence
_R_LSZX01052869.1_21398-22056_Camelus_dromedarius_breed_Targui_Contig_13041_21_whole_genome_shotgun_sequence
JDVD01049320.1_11626-12284_Camelus_dromedarius_breed_Arabia_contig49320_whole_genome_shotgun_sequence
_R_JWIN03000021.1_7956078-7956736_Camelus_dromedarius_breed_African_isolate_Drom800_chromosome_21_whole_genome_shotgun_sequence
JEMW01024195.1_275615-276273_Vicugna_pacos_huacaya_contig24195_whole_genome_shotgun_sequence
AGVR01036175.1_16568-17228_Camelus_ferus_isolate_Naran_contig036345_whole_genome_shotgun_sequence
CAOW010002890.1_2255-2914_Camelus_bactrianus_WGS_project_CAOW00000000_data_contig_3430_whole_genome_shotgun_sequence
JARL01016942.1_64116-64775_Camelus_bactrianus_breed_Alxa_contig16942_whole_genome_shotgun_sequence
PVJP02000067.1_723758-724026_Hippopotamus_amphibius_isolate_BS01_ScHSfme_63HRSCAF_10925_whole_genome_shotgun_sequence
PVHT020000175.1_10147364-10148025_Catagonus_wagneri_isolate_BS18_Sc2sB16_147HRSCAF_32195_2_whole_genome_shotgun_sequence
0.03

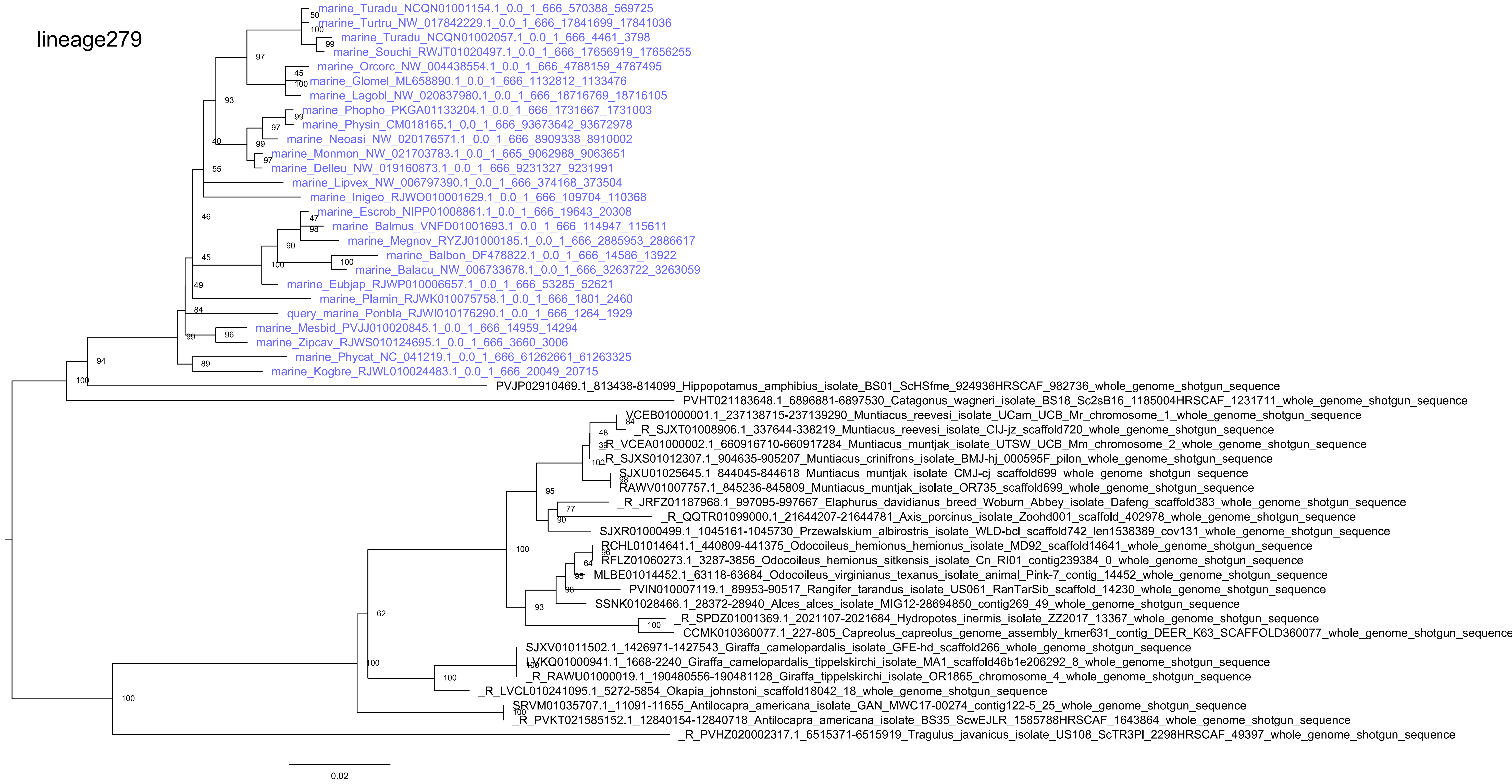
lineage279
marine_Turadu_NCQN01001154.1_0.0_1_666_570388_569725
marine_Turtru_NW_017842229.1_0.0_1_666_17841699_17841036
marine_Turadu_NCQN01002057.1_0.0_1_666_4461_3798
marine_Souchi_RWJT01020497.1_0.0_1_666_17656919_17656255
marine_Orcorc_NW_004438554.1_0.0_1_666_4788159_4787495
marine_Glomel_ML658890.1_0.0_1_666_1132812_1133476
marine_Lagobl_NW_020837980.1_0.0_1_666_18716769_18716105
marine_Phopho_PKGA01133204.1_0.0_1_666_1731667_1731003
marine_Physin_CM018165.1_0.0_1_666_93673642_93672978
marine_Neoasi_NW_020176571.1_0.0_1_666_8909338_8910002
marine_Monmon_NW_021703783.1_0.0_1_665_9062988_9063651
marine_Delleu_NW_019160873.1_0.0_1_666_9231327_9231991
marine_Lipvex_NW_006797390.1_0.0_1_666_374168_373504
marine_Inigeo_RJWO010001629.1_0.0_1_666_109704_110368
marine_Escrob_NIPP01008861.1_0.0_1_666_19643_20308
marine_Balmus_VNFD01001693.1_0.0_1_666_114947_115611
marine_Megnov_RYZJ01000185.1_0.0_1_666_2885953_2886617
marine_Balbon_DF478822.1_0.0_1_666_14586_13922
marine_Balacu_NW_006733678.1_0.0_1_666_3263722_3263059
marine_Eubjap_RJWP010006657.1_0.0_1_666_53285_52621
marine_Plamin_RJWK010075758.1_0.0_1_666_1801_2460
query_marine_Ponbla_RJWI010176290.1_0.0_1_666_1264_1929
marine_Mesbid_PVJJ010020845.1_0.0_1_666_14959_14294
marine_Zipcav_RJWS010124695.1_0.0_1_666_3660_3006
marine_Phycat_NC_041219.1_0.0_1_666_61262661_61263325
marine_Kogbre_RJWL010024483.1_0.0_1_666_20049_20715
PVJP02910469.1_813438-814099_Hippopotamus_amphibius_isolate_BS01_ScHSfme_924936HRSCAF_982736_whole_genome_shotgun_sequence
PVHT021183648.1_6896881-6897530_Catagonus_wagneri_isolate_BS18_Sc2sB16_1185004HRSCAF_1231711_whole_genome_shotgun_sequence
VCEB01000001.1_237138715-237139290_Muntiacus_reevesi_isolate_UCam_UCB_Mr_chromosome_1_whole_genome_shotgun_sequence
_R_SJXT01008906.1_337644-338219_Muntiacus_reevesi_isolate_CIJ-jz_scaffold720_whole_genome_shotgun_sequence
_R_VCEA01000002.1_660916710-660917284_Muntiacus_muntjak_isolate_UTSW_UCB_Mm_chromosome_2_whole_genome_shotgun_sequence
_R_SJXS01012307.1_904635-905207_Muntiacus_crinifrons_isolate_BMJ-hj_000595F_pilon_whole_genome_shotgun_sequence
SJXU01025645.1_844045-844618_Muntiacus_muntjak_isolate_CMJ-cj_scaffold699_whole_genome_shotgun_sequence
RAWV01007757.1_845236-845809_Muntiacus_muntjak_isolate_OR735_scaffold699_whole_genome_shotgun_sequence
_R_JRFZ01187968.1_997095-997667_Elaphurus_davidianus_breed_Woburn_Abbey_isolate_Dafeng_scaffold383_whole_genome_shotgun_sequence
_R_QQTR01099000.1_21644207-21644781_Axis_porcinus_isolate_Zoohd001_scaffold_402978_whole_genome_shotgun_sequence
SJXR01000499.1_1045161-1045730_Przewalskium_albirostris_isolate_WLD-bcl_scaffold742_len1538389_cov131_whole_genome_shotgun_sequence
RCHL01014641.1_440809-441375_Odocoileus_hemionus_hemionus_isolate_MD92_scaffold14641_whole_genome_shotgun_sequence
RFLZ01060273.1_3287-3856_Odocoileus_hemionus_sitkensis_isolate_Cn_RI01_contig239384_0_whole_genome_shotgun_sequence
MLBE01014452.1_63118-63684_Odocoileus_virginianus_texanus_isolate_animal_Pink-7_contig_14452_whole_genome_shotgun_sequence
PVIN010007119.1_89953-90517_Rangifer_tarandus_isolate_US061_RanTarSib_scaffold_14230_whole_genome_shotgun_sequence
SSNK01028466.1_28372-28940_Alces_alces_isolate_MIG12-28694850_contig269_49_whole_genome_shotgun_sequence
_R_SPDZ01001369.1_2021107-2021684_Hydropotes_inermis_isolate_ZZ2017_13367_whole_genome_shotgun_sequence
CCMK010360077.1_227-805_Capreolus_capreolus_genome_assembly_kmer631_contig_DEER_K63_SCAFFOLD360077_whole_genome_shotgun_sequence
SJXV01011502.1_1426971-1427543_Giraffa_camelopardalis_isolate_GFE-hd_scaffold266_whole_genome_shotgun_sequence
LVKQ01000941.1_1668-2240_Giraffa_camelopardalis_tippelskirchi_isolate_MA1_scaffold46b1e206292_8_whole_genome_shotgun_sequence
_R_RAWU01000019.1_190480556-190481128_Giraffa_tippelskirchi_isolate_OR1865_chromosome_4_whole_genome_shotgun_sequence
_R_LVCL010241095.1_5272-5854_Okapia_johnstoni_scaffold18042_18_whole_genome_shotgun_sequence
SRVM01035707.1_11091-11655_Antilocapra_americana_isolate_GAN_MWC17-00274_contig122-5_25_whole_genome_shotgun_sequence
_R_PVKT021585152.1_12840154-12840718_Antilocapra_americana_isolate_BS35_ScwEJLR_1585788HRSCAF_1643864_whole_genome_shotgun_sequence
_R_PVHZ020002317.1_6515371-6515919_Tragulus_javanicus_isolate_US108_ScTR3PI_2298HRSCAF_49397_whole_genome_shotgun_sequence
0.02

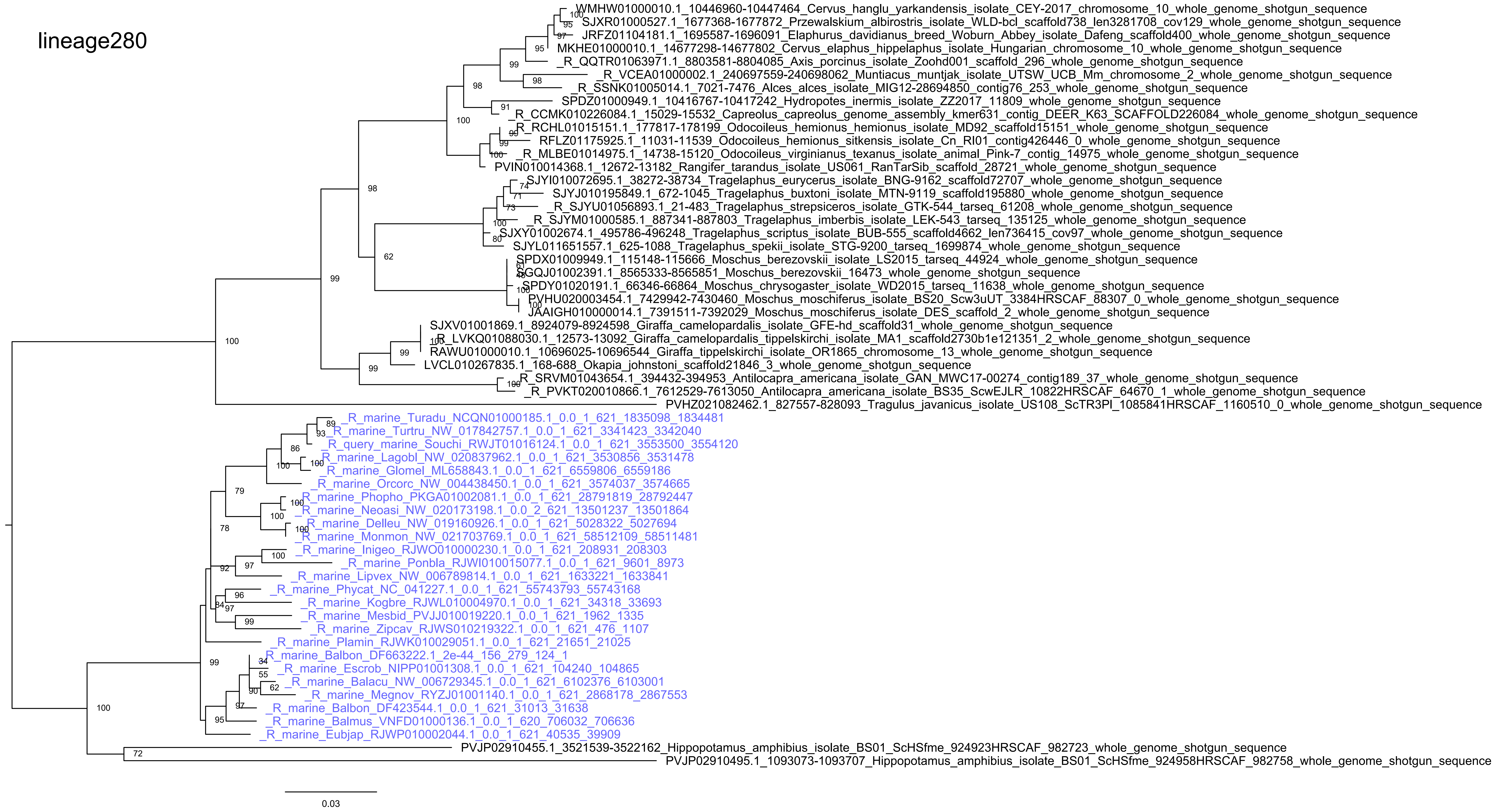
lineage280
WMHW01000010.1_10446960-10447464_Cervus_hanglu_yarkandensis_isolate_CEY-2017_chromosome_10_whole_genome_shotgun_sequence
SJXR01000527.1_1677368-1677872_Przewalskium_albirostris_isolate_WLD-bcl_scaffold738_len3281708_cov129_whole_genome_shotgun_sequence
JRFZ01104181.1_1695587-1696091_Elaphurus_davidianus_breed_Woburn_Abbey_isolate_Dafeng_scaffold400_whole_genome_shotgun_sequence
MKHE01000010.1_14677298-14677802_Cervus_elaphus_hippelaphus_isolate_Hungarian_chromosome_10_whole_genome_shotgun_sequence
_R_QQTR01063971.1_8803581-8804085_Axis_porcinus_isolate_Zoohd001_scaffold_296_whole_genome_shotgun_sequence
_R_VCEA01000002.1_240697559-240698062_Muntiacus_muntjak_isolate_UTSW_UCB_Mm_chromosome_2_whole_genome_shotgun_sequence
_R_SSNK01005014.1_7021-7476_Alces_alces_isolate_MIG12-28694850_contig76_253_whole_genome_shotgun_sequence
SPDZ01000949.1_10416767-10417242_Hydropotes_inermis_isolate_ZZ2017_11809_whole_genome_shotgun_sequence
_R_CCMK010226084.1_15029-15532_Capreolus_capreolus_genome_assembly_kmer631_contig_DEER_K63_SCAFFOLD226084_whole_genome_shotgun_sequence
_R_RCHL01015151.1_177817-178199_Odocoileus_hemionus_hemionus_isolate_MD92_scaffold15151_whole_genome_shotgun_sequence
RFLZ01175925.1_11031-11539_Odocoileus_hemionus_sitkensis_isolate_Cn_RI01_contig426446_0_whole_genome_shotgun_sequence
_R_MLBE01014975.1_14738-15120_Odocoileus_virginianus_texanus_isolate_animal_Pink-7_contig_14975_whole_genome_shotgun_sequence
PVIN010014368.1_12672-13182_Rangifer_tarandus_isolate_US061_RanTarSib_scaffold_28721_whole_genome_shotgun_sequence
SJYI010072695.1_38272-38734_Tragelaphus_eurycerus_isolate_BNG-9162_scaffold72707_whole_genome_shotgun_sequence
SJYJ010195849.1_672-1045_Tragelaphus_buxtoni_isolate_MTN-9119_scaffold195880_whole_genome_shotgun_sequence
_R_SJYU01056893.1_21-483_Tragelaphus_strepsiceros_isolate_GTK-544_tarseq_61208_whole_genome_shotgun_sequence
_R_SJYM01000585.1_887341-887803_Tragelaphus_imberbis_isolate_LEK-543_tarseq_135125_whole_genome_shotgun_sequence
SJXY01002674.1_495786-496248_Tragelaphus_scriptus_isolate_BUB-555_scaffold4662_len736415_cov97_whole_genome_shotgun_sequence
SJYL011651557.1_625-1088_Tragelaphus_spekii_isolate_STG-9200_tarseq_1699874_whole_genome_shotgun_sequence
SPDX01009949.1_115148-115666_Moschus_berezovskii_isolate_LS2015_tarseq_44924_whole_genome_shotgun_sequence
SGQJ01002391.1_8565333-8565851_Moschus_berezovskii_16473_whole_genome_shotgun_sequence
SPDY01020191.1_66346-66864_Moschus_chrysogaster_isolate_WD2015_tarseq_11638_whole_genome_shotgun_sequence
PVHU020003454.1_7429942-7430460_Moschus_moschiferus_isolate_BS20_Scw3uUT_3384HRSCAF_88307_0_whole_genome_shotgun_sequence
JAAIGH010000014.1_7391511-7392029_Moschus_moschiferus_isolate_DES_scaffold_2_whole_genome_shotgun_sequence
SJXV01001869.1_8924079-8924598_Giraffa_camelopardalis_isolate_GFE-hd_scaffold31_whole_genome_shotgun_sequence
_R_LVKQ01088030.1_12573-13092_Giraffa_camelopardalis_tippelskirchi_isolate_MA1_scaffold2730b1e121351_2_whole_genome_shotgun_sequence
RAWU01000010.1_10696025-10696544_Giraffa_tippelskirchi_isolate_OR1865_chromosome_13_whole_genome_shotgun_sequence
LVCL010267835.1_168-688_Okapia_johnstoni_scaffold21846_3_whole_genome_shotgun_sequence
_R_SRVM01043654.1_394432-394953_Antilocapra_americana_isolate_GAN_MWC17-00274_contig189_37_whole_genome_shotgun_sequence
_R_PVKT020010866.1_7612529-7613050_Antilocapra_americana_isolate_BS35_ScwEJLR_10822HRSCAF_64670_1_whole_genome_shotgun_sequence
PVHZ021082462.1_827557-828093_Tragulus_javanicus_isolate_US108_ScTR3PI_1085841HRSCAF_1160510_0_whole_genome_shotgun_sequence
_R_marine_Turadu_NCQN01000185.1_0.0_1_621_1835098_1834481
_R_marine_Turtru_NW_017842757.1_0.0_1_621_3341423_3342040
_R_query_marine_Souchi_RWJT01016124.1_0.0_1_621_3553500_3554120
_R_marine_Lagobl_NW_020837962.1_0.0_1_621_3530856_3531478
_R_marine_Glomel_ML658843.1_0.0_1_621_6559806_6559186
_R_marine_Orcorc_NW_004438450.1_0.0_1_621_3574037_3574665
_R_marine_Phopho_PKGA01002081.1_0.0_1_621_28791819_28792447
_R_marine_Neoasi_NW_020173198.1_0.0_2_621_13501237_13501864
_R_marine_Delleu_NW_019160926.1_0.0_1_621_5028322_5027694
_R_marine_Monmon_NW_021703769.1_0.0_1_621_58512109_58511481
_R_marine_Inigeo_RJWO010000230.1_0.0_1_621_208931_208303
_R_marine_Ponbla_RJWI010015077.1_0.0_1_621_9601_8973
_R_marine_Lipvex_NW_006789814.1_0.0_1_621_1633221_1633841
_R_marine_Phycat_NC_041227.1_0.0_1_621_55743793_55743168
_R_marine_Kogbre_RJWL010004970.1_0.0_1_621_34318_33693
_R_marine_Mesbid_PVJJ010019220.1_0.0_1_621_1962_1335
_R_marine_Zipcav_RJWS010219322.1_0.0_1_621_476_1107
_R_marine_Plamin_RJWK010029051.1_0.0_1_621_21651_21025
_R_marine_Balbon_DF663222.1_2e-44_156_279_124_1
_R_marine_Escrob_NIPP01001308.1_0.0_1_621_104240_104865
_R_marine_Balacu_NW_006729345.1_0.0_1_621_6102376_6103001
_R_marine_Megnov_RYZJ01001140.1_0.0_1_621_2868178_2867553
_R_marine_Balbon_DF423544.1_0.0_1_621_31013_31638
_R_marine_Balmus_VNFD01000136.1_0.0_1_620_706032_706636
_R_marine_Eubjap_RJWP010002044.1_0.0_1_621_40535_39909
PVJP02910455.1_3521539-3522162_Hippopotamus_amphibius_isolate_BS01_ScHSfme_924923HRSCAF_982723_whole_genome_shotgun_sequence
PVJP02910495.1_1093073-1093707_Hippopotamus_amphibius_isolate_BS01_ScHSfme_924958HRSCAF_982758_whole_genome_shotgun_sequence
0.03

lineage281

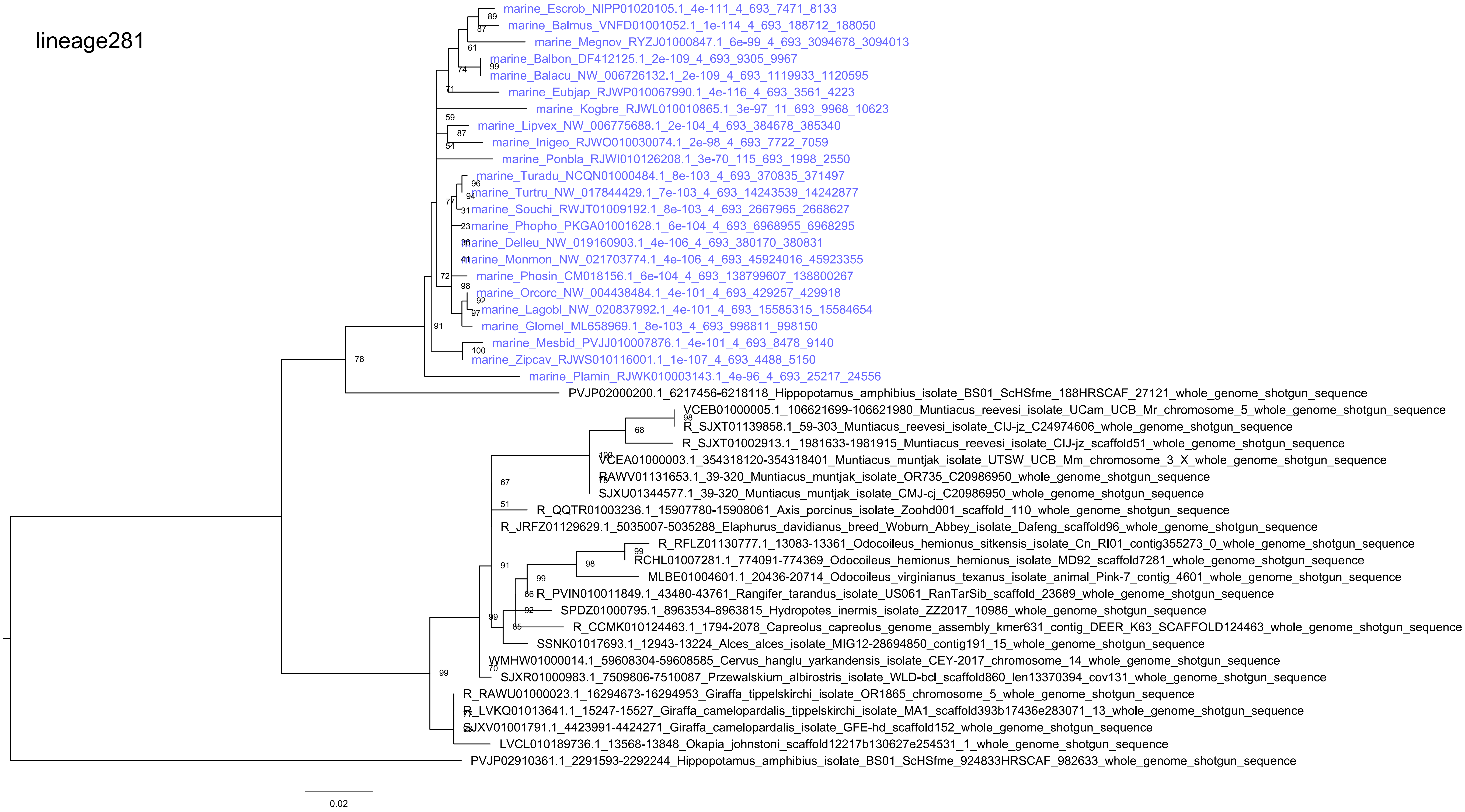

marine_Escrob_NIPP01020105.1_4e-111_4_693_7471_8133
marine_Balmus_VNFD01001052.1_1e-114_4_693_188712_188050
marine_Megnov_RYZJ01000847.1_6e-99_4_693_3094678_3094013
marine_Balbon_DF412125.1_2e-109_4_693_9305_9967
marine_Balacu_NW_006726132.1_2e-109_4_693_1119933_1120595
marine_Eubjap_RJWP010067990.1_4e-116_4_693_3561_4223
marine_Kogbre_RJWL010010865.1_3e-97_11_693_9968_10623
marine_Lipvex_NW_006775688.1_2e-104_4_693_384678_385340
marine_Inigeo_RJWO010030074.1_2e-98_4_693_7722_7059
marine_Ponbla_RJWI010126208.1_3e-70_115_693_1998_2550
marine_Turadu_NCQN01000484.1_8e-103_4_693_370835_371497
marine_Turtru_NW_017844429.1_7e-103_4_693_14243539_14242877
marine_Souchi_RWJT01009192.1_8e-103_4_693_2667965_2668627
marine_Phopho_PKGA01001628.1_6e-104_4_693_6968955_6968295
marine_Delleu_NW_019160903.1_4e-106_4_693_380170_380831
marine_Monmon_NW_021703774.1_4e-106_4_693_45924016_45923355
marine_Phosin_CM018156.1_6e-104_4_693_138799607_138800267
marine_Orcorc_NW_004438484.1_4e-101_4_693_429257_429918
marine_Lagobl_NW_020837992.1_4e-101_4_693_15585315_15584654
marine_Glomel_ML658969.1_8e-103_4_693_998811_998150
marine_Mesbid_PVJJ010007876.1_4e-101_4_693_8478_9140
marine_Zipcav_RJWS010116001.1_1e-107_4_693_4488_5150
marine_Plamin_RJWK010003143.1_4e-96_4_693_25217_24556
PVJP02000200.1_6217456-6218118_Hippopotamus_amphibius_isolate_BS01_ScHSfme_188HRSCAF_27121_whole_genome_shotgun_sequence
VCEB01000005.1_106621699-106621980_Muntiacus_reevesi_isolate_UCam_UCB_Mr_chromosome_5_whole_genome_shotgun_sequence
R_SJXT01139858.1_59-303_Muntiacus_reevesi_isolate_CIJ-jz_C24974606_whole_genome_shotgun_sequence
R_SJXT01002913.1_1981633-1981915_Muntiacus_reevesi_isolate_CIJ-jz_scaffold51_whole_genome_shotgun_sequence
VCEA01000003.1_354318120-354318401_Muntiacus_muntjak_isolate_UTSW_UCB_Mm_chromosome_3_X_whole_genome_shotgun_sequence
RAWV01131653.1_39-320_Muntiacus_muntjak_isolate_OR735_C20986950_whole_genome_shotgun_sequence
SJXU01344577.1_39-320_Muntiacus_muntjak_isolate_CMJ-cj_C20986950_whole_genome_shotgun_sequence
R_QQTR01003236.1_15907780-15908061_Axis_porcinus_isolate_Zoohd001_scaffold_110_whole_genome_shotgun_sequence
R_JRFZ01129629.1_5035007-5035288_Elaphurus_davidianus_breed_Woburn_Abbey_isolate_Dafeng_scaffold96_whole_genome_shotgun_sequence
R_RFLZ01130777.1_13083-13361_Odocoileus_hemionus_sitkensis_isolate_Cn_RI01_contig355273_0_whole_genome_shotgun_sequence
RCHL01007281.1_774091-774369_Odocoileus_hemionus_hemionus_isolate_MD92_scaffold7281_whole_genome_shotgun_sequence
MLBE01004601.1_20436-20714_Odocoileus_virginianus_texanus_isolate_animal_Pink-7_contig_4601_whole_genome_shotgun_sequence
R_PVIN010011849.1_43480-43761_Rangifer_tarandus_isolate_US061_RanTarSib_scaffold_23689_whole_genome_shotgun_sequence
SPDZ01000795.1_8963534-8963815_Hydropotes_inermis_isolate_ZZ2017_10986_whole_genome_shotgun_sequence
R_CCMK010124463.1_1794-2078_Capreolus_capreolus_genome_assembly_kmer631_contig_DEER_K63_SCAFFOLD124463_whole_genome_shotgun_sequence
SSNK01017693.1_12943-13224_Alces_alces_isolate_MIG12-28694850_contig191_15_whole_genome_shotgun_sequence
WMHW01000014.1_59608304-59608585_Cervus_hanglu_yarkandensis_isolate_CEY-2017_chromosome_14_whole_genome_shotgun_sequence
SJXR01000983.1_7509806-7510087_Przewalskium_albirostris_isolate_WLD-bcl_scaffold860_len13370394_cov131_whole_genome_shotgun_sequence
R_RAWU01000023.1_16294673-16294953_Giraffa_tippelskirchi_isolate_OR1865_chromosome_5_whole_genome_shotgun_sequence
R_LVKQ01013641.1_15247-15527_Giraffa_camelopardalis_tippelskirchi_isolate_MA1_scaffold393b17436e283071_13_whole_genome_shotgun_sequence
SJXV01001791.1_4423991-4424271_Giraffa_camelopardalis_isolate_GFE-hd_scaffold152_whole_genome_shotgun_sequence
LVCL010189736.1_13568-13848_Okapia_johnstoni_scaffold12217b130627e254531_1_whole_genome_shotgun_sequence
PVJP02910361.1_2291593-2292244_Hippopotamus_amphibius_isolate_BS01_ScHSfme_924833HRSCAF_982633_whole_genome_shotgun_sequence
0.02

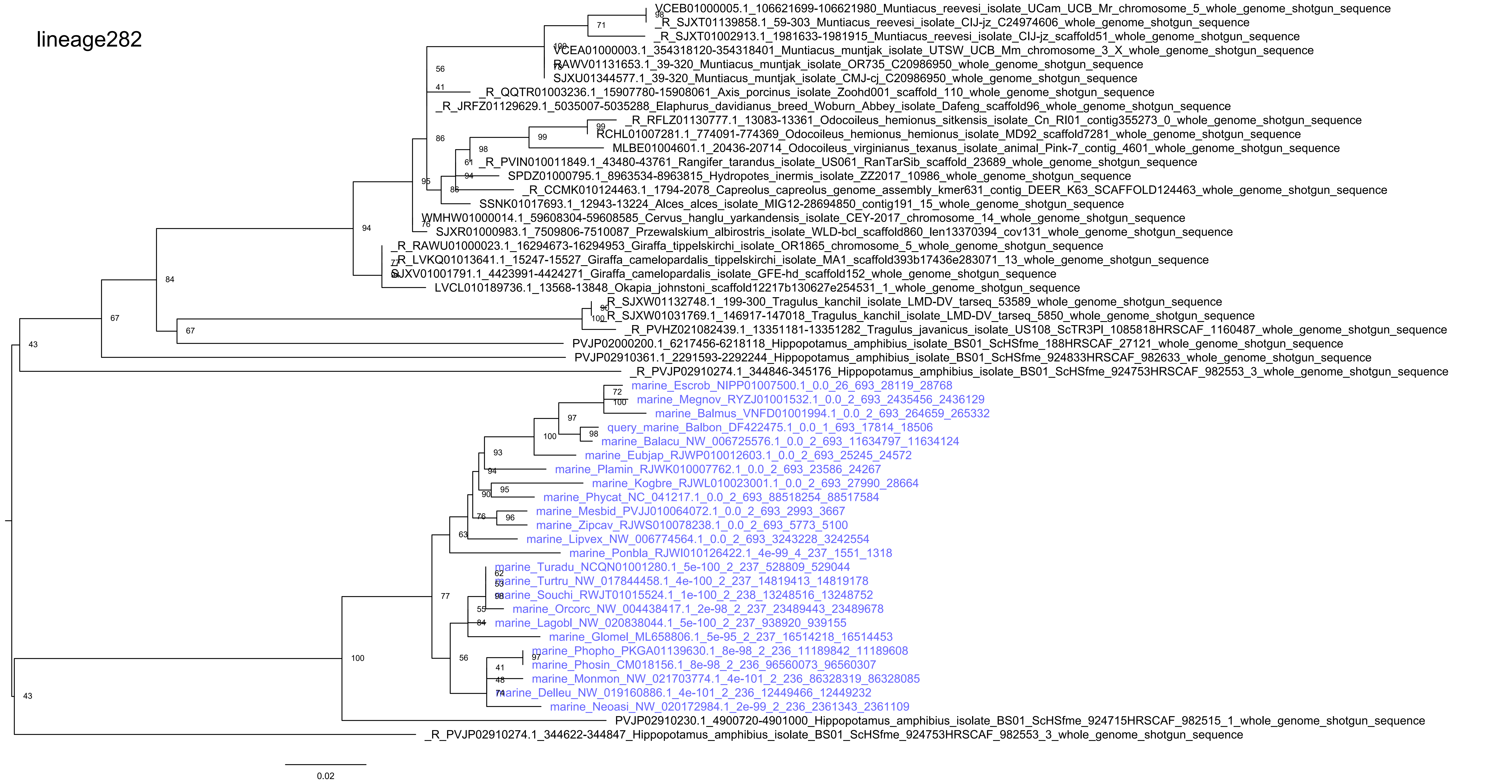
lineage282
VCEB01000005.1_106621699-106621980_Muntiacus_reevesi_isolate_UCam_UCB_Mr_chromosome_5_whole_genome_shotgun_sequence
_R_SJXT01139858.1_59-303_Muntiacus_reevesi_isolate_CIJ-jz_C24974606_whole_genome_shotgun_sequence
_R_SJXT01002913.1_1981633-1981915_Muntiacus_reevesi_isolate_CIJ-jz_scaffold51_whole_genome_shotgun_sequence
VCEA01000003.1_354318120-354318401_Muntiacus_muntjak_isolate_UTSW_UCB_Mm_chromosome_3_X_whole_genome_shotgun_sequence
RAWV01131653.1_39-320_Muntiacus_muntjak_isolate_OR735_C20986950_whole_genome_shotgun_sequence
SJXU01344577.1_39-320_Muntiacus_muntjak_isolate_CMJ-cj_C20986950_whole_genome_shotgun_sequence
_R_QQTR01003236.1_15907780-15908061_Axis_porcinus_isolate_Zoohd001_scaffold_110_whole_genome_shotgun_sequence
_R_JRFZ01129629.1_5035007-5035288_Elaphurus_davidianus_breed_Woburn_Abbey_isolate_Dafeng_scaffold96_whole_genome_shotgun_sequence
_R_RFLZ01130777.1_13083-13361_Odocoileus_hemionus_sitkensis_isolate_Cn_RI01_contig355273_0_whole_genome_shotgun_sequence
RCHL01007281.1_774091-774369_Odocoileus_hemionus_hemionus_isolate_MD92_scaffold7281_whole_genome_shotgun_sequence
MLBE01004601.1_20436-20714_Odocoileus_virginianus_texanus_isolate_animal_Pink-7_contig_4601_whole_genome_shotgun_sequence
_R_PVIN010011849.1_43480-43761_Rangifer_tarandus_isolate_US061_RanTarSib_scaffold_23689_whole_genome_shotgun_sequence
SPDZ01000795.1_8963534-8963815_Hydropotes_inermis_isolate_ZZ2017_10986_whole_genome_shotgun_sequence
_R_CCMK010124463.1_1794-2078_Capreolus_capreolus_genome_assembly_kmer631_contig_DEER_K63_SCAFFOLD124463_whole_genome_shotgun_sequence
SSNK01017693.1_12943-13224_Alces_alces_isolate_MIG12-28694850_contig191_15_whole_genome_shotgun_sequence
WMHW01000014.1_59608304-59608585_Cervus_hanglu_yarkandensis_isolate_CEY-2017_chromosome_14_whole_genome_shotgun_sequence
SJXR01000983.1_7509806-7510087_Przewalskium_albirostris_isolate_WLD-bcl_scaffold860_len13370394_cov131_whole_genome_shotgun_sequence
_R_RAWU01000023.1_16294673-16294953_Giraffa_tippelskirchi_isolate_OR1865_chromosome_5_whole_genome_shotgun_sequence
_R_LVKQ01013641.1_15247-15527_Giraffa_camelopardalis_tippelskirchi_isolate_MA1_scaffold393b17436e283071_13_whole_genome_shotgun_sequence
SJXV01001791.1_4423991-4424271_Giraffa_camelopardalis_isolate_GFE-hd_scaffold152_whole_genome_shotgun_sequence
LVCL010189736.1_13568-13848_Okapia_johnstoni_scaffold12217b130627e254531_1_whole_genome_shotgun_sequence
_R_SJXW01132748.1_199-300_Tragulus_kanchil_isolate_LMD-DV_tarseq_53589_whole_genome_shotgun_sequence
_R_SJXW01031769.1_146917-147018_Tragulus_kanchil_isolate_LMD-DV_tarseq_5850_whole_genome_shotgun_sequence
_R_PVHZ021082439.1_13351181-13351282_Tragulus_javanicus_isolate_US108_ScTR3PI_1085818HRSCAF_1160487_whole_genome_shotgun_sequence
PVJP02000200.1_6217456-6218118_Hippopotamus_amphibius_isolate_BS01_ScHSfme_188HRSCAF_27121_whole_genome_shotgun_sequence
PVJP02910361.1_2291593-2292244_Hippopotamus_amphibius_isolate_BS01_ScHSfme_924833HRSCAF_982633_whole_genome_shotgun_sequence
_R_PVJP02910274.1_344846-345176_Hippopotamus_amphibius_isolate_BS01_ScHSfme_924753HRSCAF_982553_3_whole_genome_shotgun_sequence
marine_Escrob_NIPP01007500.1_0.0_26_693_28119_28768
marine_Megnov_RYZJ01001532.1_0.0_2_693_2435456_2436129
marine_Balmus_VNFD01001994.1_0.0_2_693_264659_265332
query_marine_Balbon_DF422475.1_0.0_1_693_17814_18506
marine_Balacu_NW_006725576.1_0.0_2_693_11634797_11634124
marine_Eubjap_RJWP010012603.1_0.0_2_693_25245_24572
marine_Plamin_RJWK010007762.1_0.0_2_693_23586_24267
marine_Kogbre_RJWL010023001.1_0.0_2_693_27990_28664
marine_Phycat_NC_041217.1_0.0_2_693_88518254_88517584
marine_Mesbid_PVJJ010064072.1_0.0_2_693_2993_3667
marine_Zipcav_RJWS010078238.1_0.0_2_693_5773_5100
marine_Lipvex_NW_006774564.1_0.0_2_693_3243228_3242554
marine_Ponbla_RJWI010126422.1_4e-99_4_237_1551_1318
marine_Turadu_NCQN01001280.1_5e-100_2_237_528809_529044
marine_Turtru_NW_017844458.1_4e-100_2_237_14819413_14819178
marine_Souchi_RWJT01015524.1_1e-100_2_238_13248516_13248752
marine_Orcorc_NW_004438417.1_2e-98_2_237_23489443_23489678
marine_Lagobl_NW_020838044.1_5e-100_2_237_938920_939155
marine_Glomel_ML658806.1_5e-95_2_237_16514218_16514453
marine_Phopho_PKGA01139630.1_8e-98_2_236_11189842_11189608
marine_Phosin_CM018156.1_8e-98_2_236_96560073_96560307
marine_Monmon_NW_021703774.1_4e-101_2_236_86328319_86328085
marine_Delleu_NW_019160886.1_4e-101_2_236_12449466_12449232
marine_Neoasi_NW_020172984.1_2e-99_2_236_2361343_2361109
PVJP02910230.1_4900720-4901000_Hippopotamus_amphibius_isolate_BS01_ScHSfme_924715HRSCAF_982515_1_whole_genome_shotgun_sequence
_R_PVJP02910274.1_344622-344847_Hippopotamus_amphibius_isolate_BS01_ScHSfme_924753HRSCAF_982553_3_whole_genome_shotgun_sequence
98
71
100
56
41
99
86
99
98
61
94
95
86
76
94
77
84
67
90
100
67
43
72
100
97
98
100
93
94
95
90
76
96
63
62
53
98
77
55
84
100
56
97
41
48
74
43
0.02

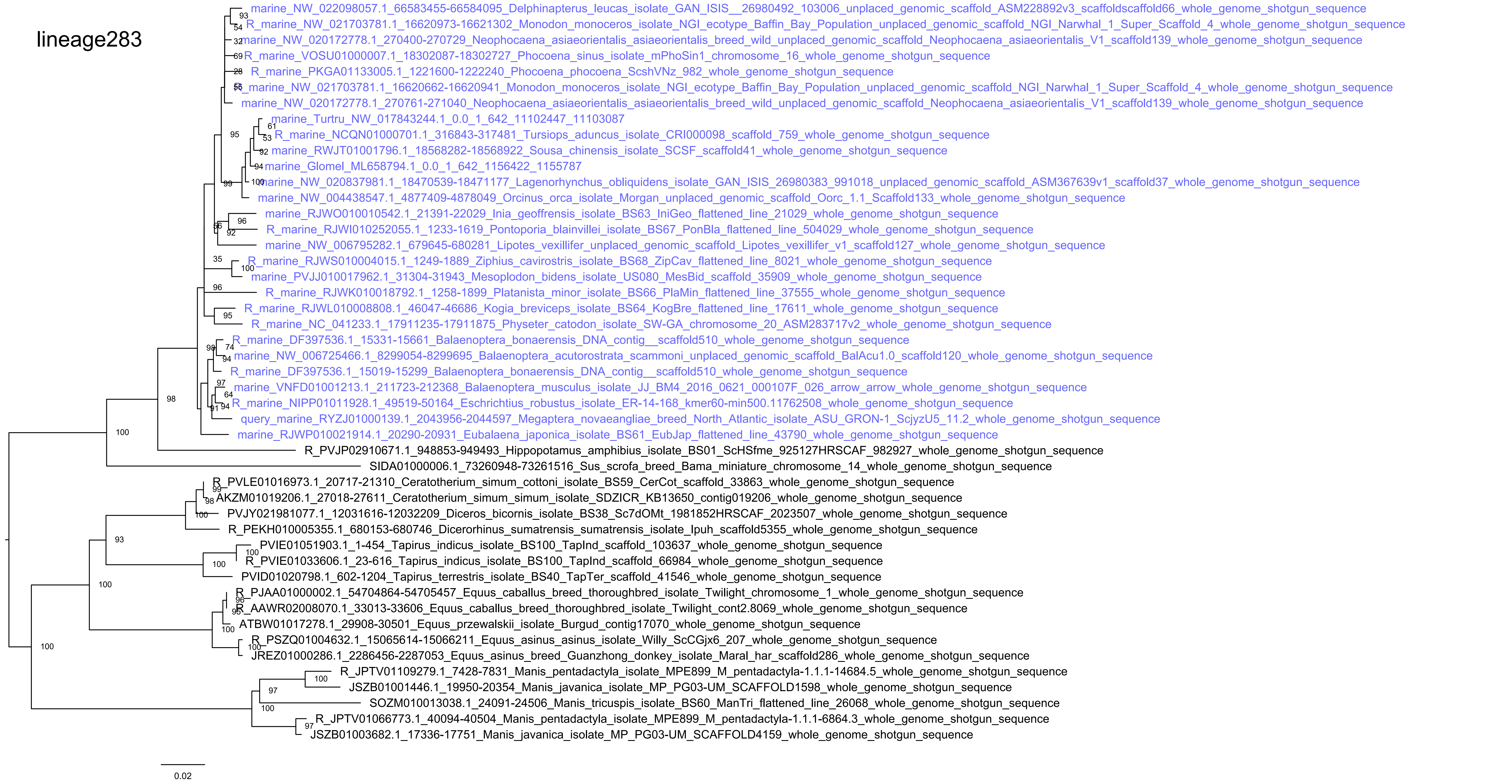

lineage283
marine_NW_022098057.1_66583455-66584095_Delphinapterus_leucas_isolate_GAN_ISIS__26980492_103006_unplaced_genomic_scaffold_ASM228892v3_scaffoldscaffold66_whole_genome_shotgun_sequence
R_marine_NW_021703781.1_16620973-16621302_Monodon_monoceros_isolate_NGI_ecotype_Baffin_Bay_Population_unplaced_genomic_scaffold_NGI_Narwhal_1_Super_Scaffold_4_whole_genome_shotgun_sequence
marine_NW_020172778.1_270400-270729_Neophocaena_asiaeorientalis_asiaeorientalis_breed_wild_unplaced_genomic_scaffold_Neophocaena_asiaeorientalis_V1_scaffold139_whole_genome_shotgun_sequence
R_marine_VOSU01000007.1_18302087-18302727_Phocoena_sinus_isolate_mPhoSin1_chromosome_16_whole_genome_shotgun_sequence
R_marine_PKGA01133005.1_1221600-1222240_Phocoena_phocoena_ScshVNz_982_whole_genome_shotgun_sequence
R_marine_NW_021703781.1_16620662-16620941_Monodon_monoceros_isolate_NGI_ecotype_Baffin_Bay_Population_unplaced_genomic_scaffold_NGI_Narwhal_1_Super_Scaffold_4_whole_genome_shotgun_sequence
marine_NW_020172778.1_270761-271040_Neophocaena_asiaeorientalis_asiaeorientalis_breed_wild_unplaced_genomic_scaffold_Neophocaena_asiaeorientalis_V1_scaffold139_whole_genome_shotgun_sequence
marine_Turtru_NW_017843244.1_0.0_1_642_11102447_11103087
R_marine_NCQN01000701.1_316843-317481_Tursiops_aduncus_isolate_CRI000098_scaffold_759_whole_genome_shotgun_sequence
marine_RWJT01001796.1_18568282-18568922_Sousa_chinensis_isolate_SCSF_scaffold41_whole_genome_shotgun_sequence
marine_Glomel_ML658794.1_0.0_1_642_1156422_1155787
marine_NW_020837981.1_18470539-18471177_Lagenorhynchus_obliquidens_isolate_GAN_ISIS_26980383_991018_unplaced_genomic_scaffold_ASM367639v1_scaffold37_whole_genome_shotgun_sequence
marine_NW_004438547.1_4877409-4878049_Orcinus_orca_isolate_Morgan_unplaced_genomic_scaffold_Oorc_1.1_Scaffold133_whole_genome_shotgun_sequence
marine_RJWO010010542.1_21391-22029_Inia_geoffrensis_isolate_BS63_IniGeo_flattened_line_21029_whole_genome_shotgun_sequence
R_marine_RJWI010252055.1_1233-1619_Pontoporia_blainvillei_isolate_BS67_PonBla_flattened_line_504029_whole_genome_shotgun_sequence
marine_NW_006795282.1_679645-680281_Lipotes_vexillifer_unplaced_genomic_scaffold_Lipotes_vexillifer_v1_scaffold127_whole_genome_shotgun_sequence
R_marine_RJWS010004015.1_1249-1889_Ziphius_cavirostris_isolate_BS68_ZipCav_flattened_line_8021_whole_genome_shotgun_sequence
marine_PVJJ010017962.1_31304-31943_Mesoplodon_bidens_isolate_US080_MesBid_scaffold_35909_whole_genome_shotgun_sequence
R_marine_RJWK010018792.1_1258-1899_Platanista_minor_isolate_BS66_PlaMin_flattened_line_37555_whole_genome_shotgun_sequence
R_marine_RJWL010008808.1_46047-46686_Kogia_breviceps_isolate_BS64_KogBre_flattened_line_17611_whole_genome_shotgun_sequence
R_marine_NC_041233.1_17911235-17911875_Physeter_catodon_isolate_SW-GA_chromosome_20_ASM283717v2_whole_genome_shotgun_sequence
R_marine_DF397536.1_15331-15661_Balaenoptera_bonaerensis_DNA_contig__scaffold510_whole_genome_shotgun_sequence
marine_NW_006725466.1_8299054-8299695_Balaenoptera_acutorostrata_scammoni_unplaced_genomic_scaffold_BalAcu1.0_scaffold120_whole_genome_shotgun_sequence
R_marine_DF397536.1_15019-15299_Balaenoptera_bonaerensis_DNA_contig__scaffold510_whole_genome_shotgun_sequence
marine_VNFD01001213.1_211723-212368_Balaenoptera_musculus_isolate_JJ_BM4_2016_0621_000107F_026_arrow_arrow_whole_genome_shotgun_sequence
R_marine_NIPP01011928.1_49519-50164_Eschrichtius_robustus_isolate_ER-14-168_kmer60-min500.11762508_whole_genome_shotgun_sequence
query_marine_RYZJ01000139.1_2043956-2044597_Megaptera_novaeangliae_breed_North_Atlantic_isolate_ASU_GRON-1_ScjyzU5_11.2_whole_genome_shotgun_sequence
marine_RJWP010021914.1_20290-20931_Eubalaena_japonica_isolate_BS61_EubJap_flattened_line_43790_whole_genome_shotgun_sequence
R_PVJP02910671.1_948853-949493_Hippopotamus_amphibius_isolate_BS01_ScHSfme_925127HRSCAF_982927_whole_genome_shotgun_sequence
SIDA01000006.1_73260948-73261516_Sus_scrofa_breed_Bama_miniature_chromosome_14_whole_genome_shotgun_sequence
R_PVLE01016973.1_20717-21310_Ceratotherium_simum_cottoni_isolate_BS59_CerCot_scaffold_33863_whole_genome_shotgun_sequence
AKZM01019206.1_27018-27611_Ceratotherium_simum_simum_isolate_SDZICR_KB13650_contig019206_whole_genome_shotgun_sequence
PVJY021981077.1_12031616-12032209_Diceros_bicornis_isolate_BS38_Sc7dOMt_1981852HRSCAF_2023507_whole_genome_shotgun_sequence
R_PEKH010005355.1_680153-680746_Dicerorhinus_sumatrensis_sumatrensis_isolate_Ipuh_scaffold5355_whole_genome_shotgun_sequence
PVIE01051903.1_1-454_Tapirus_indicus_isolate_BS100_TapInd_scaffold_103637_whole_genome_shotgun_sequence
R_PVIE01033606.1_23-616_Tapirus_indicus_isolate_BS100_TapInd_scaffold_66984_whole_genome_shotgun_sequence
PVID01020798.1_602-1204_Tapirus_terrestris_isolate_BS40_TapTer_scaffold_41546_whole_genome_shotgun_sequence
R_PJAA01000002.1_54704864-54705457_Equus_caballus_breed_thoroughbred_isolate_Twilight_chromosome_1_whole_genome_shotgun_sequence
R_AAWR02008070.1_33013-33606_Equus_caballus_breed_thoroughbred_isolate_Twilight_cont2.8069_whole_genome_shotgun_sequence
ATBW01017278.1_29908-30501_Equus_przewalskii_isolate_Burgud_contig17070_whole_genome_shotgun_sequence
R_PSZQ01004632.1_15065614-15066211_Equus_asinus_asinus_isolate_Willy_ScCGjx6_207_whole_genome_shotgun_sequence
JREZ01000286.1_2286456-2287053_Equus_asinus_breed_Guanzhong_donkey_isolate_Maral_har_scaffold286_whole_genome_shotgun_sequence
R_JPTV01109279.1_7428-7831_Manis_pentadactyla_isolate_MPE899_M_pentadactyla-1.1.1-14684.5_whole_genome_shotgun_sequence
JSZB01001446.1_19950-20354_Manis_javanica_isolate_MP_PG03-UM_SCAFFOLD1598_whole_genome_shotgun_sequence
SOZM010013038.1_24091-24506_Manis_tricuspis_isolate_BS60_ManTri_flattened_line_26068_whole_genome_shotgun_sequence
R_JPTV01066773.1_40094-40504_Manis_pentadactyla_isolate_MPE899_M_pentadactyla-1.1.1-6864.3_whole_genome_shotgun_sequence
JSZB01003682.1_17336-17751_Manis_javanica_isolate_MP_PG03-UM_SCAFFOLD4159_whole_genome_shotgun_sequence
0.02

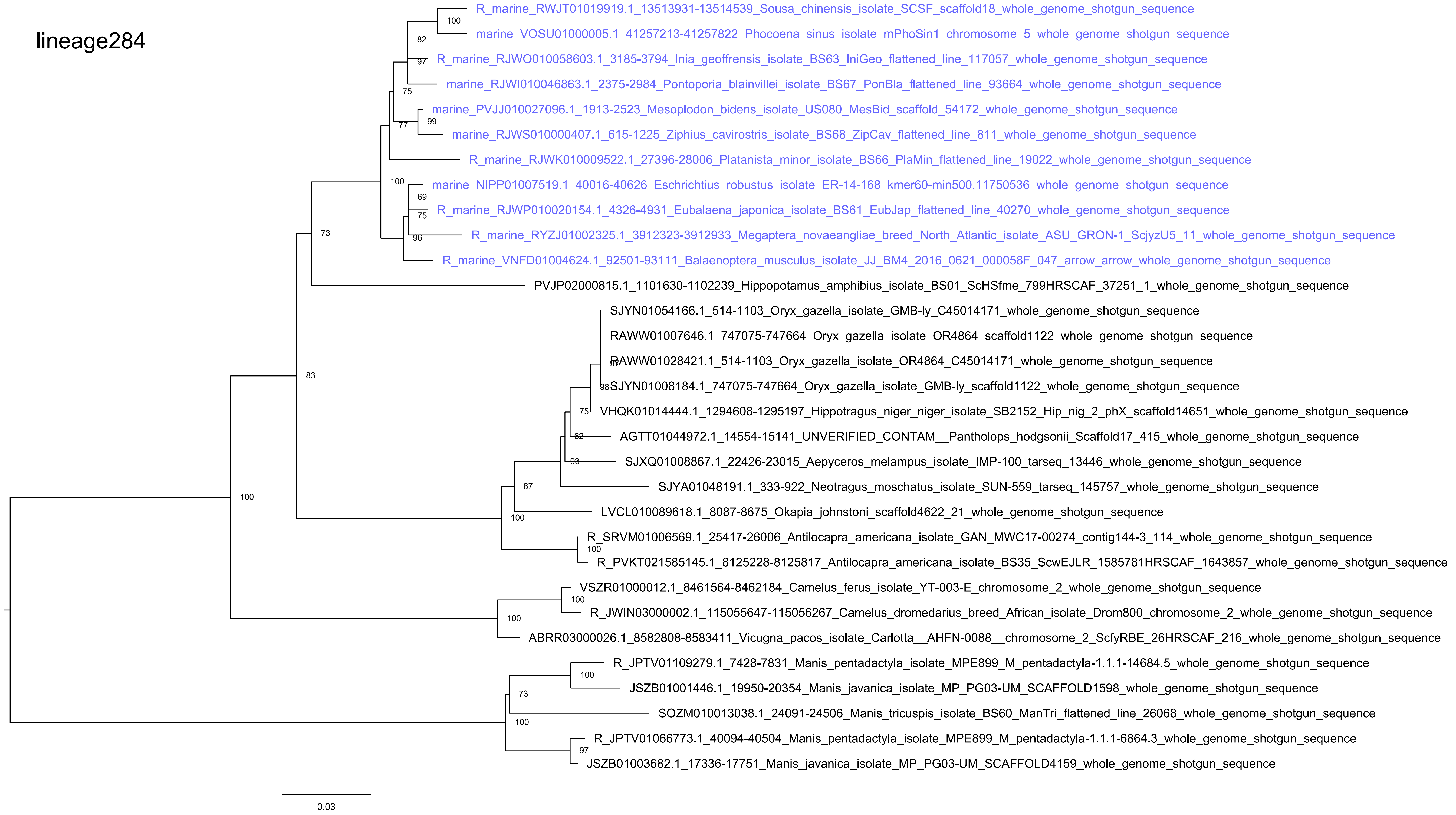
lineage284
R_marine_RWJT01019919.1_13513931-13514539_Sousa_chinensis_isolate_SCSF_scaffold18_whole_genome_shotgun_sequence
marine_VOSU01000005.1_41257213-41257822_Phocoena_sinus_isolate_mPhoSin1_chromosome_5_whole_genome_shotgun_sequence
R_marine_RJWO010058603.1_3185-3794_Inia_geoffrensis_isolate_BS63_IniGeo_flattened_line_117057_whole_genome_shotgun_sequence
marine_RJWI010046863.1_2375-2984_Pontoporia_blainvillei_isolate_BS67_PonBla_flattened_line_93664_whole_genome_shotgun_sequence
marine_PVJJ010027096.1_1913-2523_Mesoplodon_bidens_isolate_US080_MesBid_scaffold_54172_whole_genome_shotgun_sequence
marine_RJWS010000407.1_615-1225_Ziphius_cavirostris_isolate_BS68_ZipCav_flattened_line_811_whole_genome_shotgun_sequence
R_marine_RJWK010009522.1_27396-28006_Platanista_minor_isolate_BS66_PlaMin_flattened_line_19022_whole_genome_shotgun_sequence
marine_NIPP01007519.1_40016-40626_Eschrichtius_robustus_isolate_ER-14-168_kmer60-min500.11750536_whole_genome_shotgun_sequence
R_marine_RJWP010020154.1_4326-4931_Eubalaena_japonica_isolate_BS61_EubJap_flattened_line_40270_whole_genome_shotgun_sequence
R_marine_RYZJ01002325.1_3912323-3912933_Megaptera_novaeangliae_breed_North_Atlantic_isolate_ASU_GRON-1_ScjyzU5_11_whole_genome_shotgun_sequence
R_marine_VNFD01004624.1_92501-93111_Balaenoptera_musculus_isolate_JJ_BM4_2016_0621_000058F_047_arrow_arrow_whole_genome_shotgun_sequence
PVJP02000815.1_1101630-1102239_Hippopotamus_amphibius_isolate_BS01_ScHSfme_799HRSCAF_37251_1_whole_genome_shotgun_sequence
SJYN01054166.1_514-1103_Oryx_gazella_isolate_GMB-ly_C45014171_whole_genome_shotgun_sequence
RAWW01007646.1_747075-747664_Oryx_gazella_isolate_OR4864_scaffold1122_whole_genome_shotgun_sequence
RAWW01028421.1_514-1103_Oryx_gazella_isolate_OR4864_C45014171_whole_genome_shotgun_sequence
SJYN01008184.1_747075-747664_Oryx_gazella_isolate_GMB-ly_scaffold1122_whole_genome_shotgun_sequence
VHQK01014444.1_1294608-1295197_Hippotragus_niger_niger_isolate_SB2152_Hip_nig_2_phX_scaffold14651_whole_genome_shotgun_sequence
AGTT01044972.1_14554-15141_UNVERIFIED_CONTAM__Pantholops_hodgsonii_Scaffold17_415_whole_genome_shotgun_sequence
SJXQ01008867.1_22426-23015_Aepyceros_melampus_isolate_IMP-100_tarseq_13446_whole_genome_shotgun_sequence
SJYA01048191.1_333-922_Neotragus_moschatus_isolate_SUN-559_tarseq_145757_whole_genome_shotgun_sequence
LVCL010089618.1_8087-8675_Okapia_johnstoni_scaffold4622_21_whole_genome_shotgun_sequence
R_SRVM01006569.1_25417-26006_Antilocapra_americana_isolate_GAN_MWC17-00274_contig144-3_114_whole_genome_shotgun_sequence
R_PVKT021585145.1_8125228-8125817_Antilocapra_americana_isolate_BS35_ScwEJLR_1585781HRSCAF_1643857_whole_genome_shotgun_sequence
VSZR01000012.1_8461564-8462184_Camelus_ferus_isolate_YT-003-E_chromosome_2_whole_genome_shotgun_sequence
R_JWIN03000002.1_115055647-115056267_Camelus_dromedarius_breed_African_isolate_Drom800_chromosome_2_whole_genome_shotgun_sequence
ABRR03000026.1_8582808-8583411_Vicugna_pacos_isolate_Carlotta__AHFN-0088__chromosome_2_ScfyRBE_26HRSCAF_216_whole_genome_shotgun_sequence
R_JPTV01109279.1_7428-7831_Manis_pentadactyla_isolate_MPE899_M_pentadactyla-1.1.1-14684.5_whole_genome_shotgun_sequence
JSZB01001446.1_19950-20354_Manis_javanica_isolate_MP_PG03-UM_SCAFFOLD1598_whole_genome_shotgun_sequence
SOZM010013038.1_24091-24506_Manis_tricuspis_isolate_BS60_ManTri_flattened_line_26068_whole_genome_shotgun_sequence
R_JPTV01066773.1_40094-40504_Manis_pentadactyla_isolate_MPE899_M_pentadactyla-1.1.1-6864.3_whole_genome_shotgun_sequence
JSZB01003682.1_17336-17751_Manis_javanica_isolate_MP_PG03-UM_SCAFFOLD4159_whole_genome_shotgun_sequence
0.03

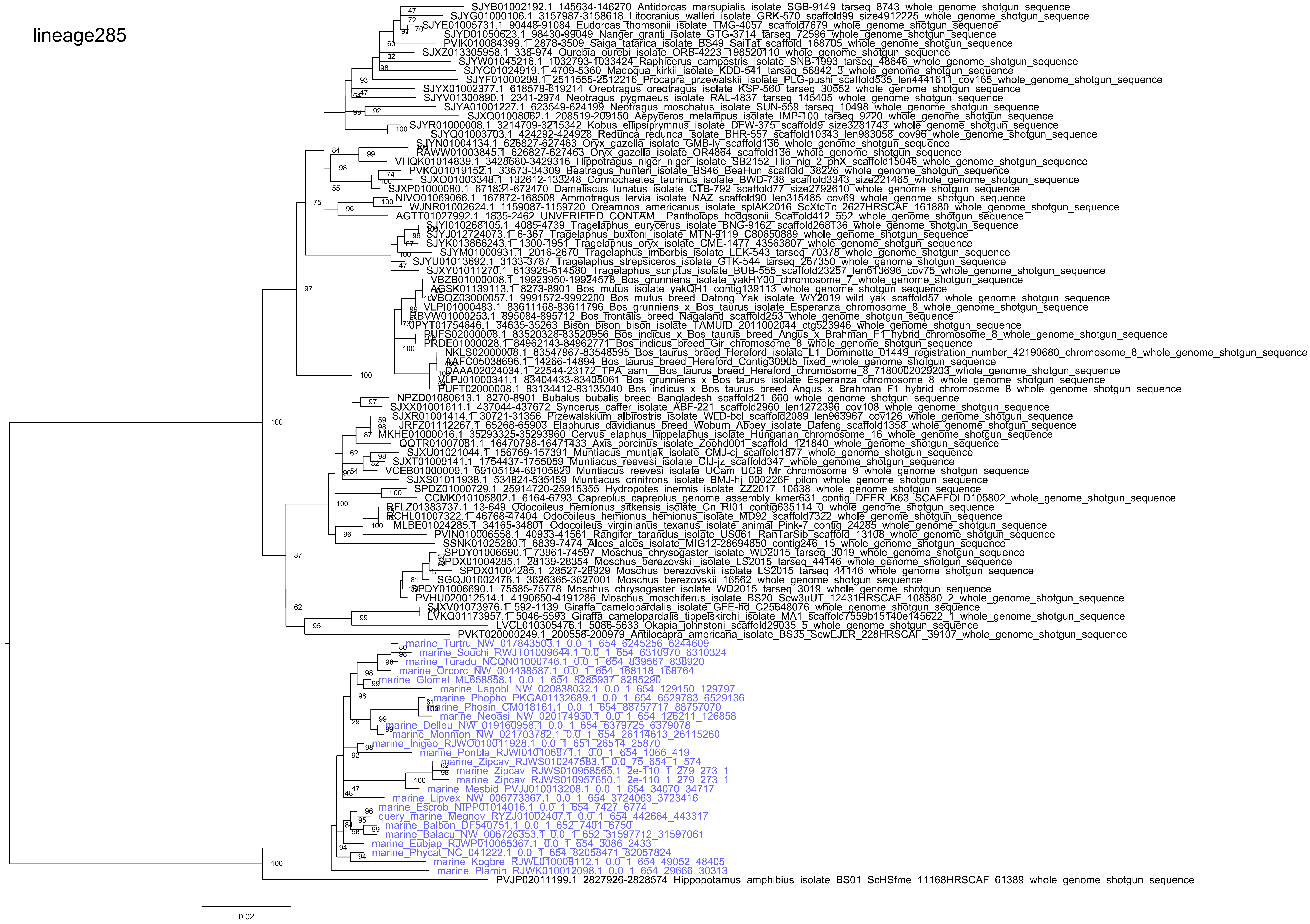
lineage285
SJYB01002192.1_145634-146270_Antidorcas_marsupialis_isolate_SGB-9149_tarseq_8743_whole_genome_shotgun_sequence
SJYG01000106.1_3157987-3158618_Litocranius_walleri_isolate_GRK-570_scaffold99_size4912225_whole_genome_shotgun_sequence
SJYE01005731.1_90448-91084_Eudorcas_thomsonii_isolate_TMG-4057_scaffold7679_whole_genome_shotgun_sequence
SJYD01050623.1_98430-99049_Nanger_granti_isolate_GTG-3714_tarseq_72596_whole_genome_shotgun_sequence
PVIK010084399.1_2878-3509_Saiga_tatarica_isolate_BS49_SaiTat_scaffold_168705_whole_genome_shotgun_sequence
SJXZ013305958.1_338-974_Ourebia_ourebi_isolate_ORB-4223_198520110_whole_genome_shotgun_sequence
SJYW01045216.1_1032793-1033424_Raphicerus_campestris_isolate_SNB-1993_tarseq_48646_whole_genome_shotgun_sequence
SJYC01024919.1_4709-5360_Madoqua_kirkii_isolate_KDD-541_tarseq_56842_3_whole_genome_shotgun_sequence
SJYF01000298.1_2511555-2512216_Procapra_przewalskii_isolate_PLG-pushi_scaffold535_len4441611_cov165_whole_genome_shotgun_sequence
SJYX01002377.1_618578-619214_Oreotragus_oreotragus_isolate_KSP-560_tarseq_30552_whole_genome_shotgun_sequence
SJYV01300890.1_2341-2974_Neotragus_pygmaeus_isolate_RAL-4837_tarseq_145405_whole_genome_shotgun_sequence
SJYA01001227.1_623549-624199_Neotragus_moschatus_isolate_SUN-559_tarseq_10498_whole_genome_shotgun_sequence
SJXQ01008062.1_208519-209150_Aepyceros_melampus_isolate_IMP-100_tarseq_9220_whole_genome_shotgun_sequence
SJYR01000008.1_3214709-3215342_Kobus_ellipsiprymnus_isolate_DFW-375_scaffold9_size3281743_whole_genome_shotgun_sequence
SJYQ01003703.1_424292-424928_Redunca_redunca_isolate_BHR-557_scaffold10343_len983058_cov96_whole_genome_shotgun_sequence
SJYN01004134.1_626827-627463_Oryx_gazella_isolate_GMB-ly_scaffold136_whole_genome_shotgun_sequence
RAWW01003845.1_626827-627463_Oryx_gazella_isolate_OR4864_scaffold136_whole_genome_shotgun_sequence
VHQK01014839.1_3428680-3429316_Hippotragus_niger_niger_isolate_SB2152_Hip_nig_2_phX_scaffold15046_whole_genome_shotgun_sequence
PVKQ01019152.1_33673-34309_Beatragus_hunteri_isolate_BS46_BeaHun_scaffold_38226_whole_genome_shotgun_sequence
SJXO01003348.1_132612-133248_Connochaetes_taurinus_isolate_BWD-738_scaffold3343_size221465_whole_genome_shotgun_sequence
SJXP01000080.1_671834-672470_Damaliscus_lunatus_isolate_CTB-792_scaffold77_size2792610_whole_genome_shotgun_sequence
NIVO01069066.1_167872-168508_Ammotragus_lervia_isolate_NAZ_scaffold90_len315485_cov69_whole_genome_shotgun_sequence
WJNR01002624.1_1159087-1159720_Oreamnos_americanus_isolate_splAK2016_ScXtcTc_2627HRSCAF_161880_whole_genome_shotgun_sequence
AGTT01027992.1_1835-2462_UNVERIFIED_CONTAM__Pantholops_hodgsonii_Scaffold412_552_whole_genome_shotgun_sequence
SJYI010268105.1_4085-4739_Tragelaphus_eurycerus_isolate_BNG-9162_scaffold268136_whole_genome_shotgun_sequence
SJYJ012724073.1_6-367_Tragelaphus_buxtoni_isolate_MTN-9119_C80650889_whole_genome_shotgun_sequence
SJYK013866243.1_1300-1951_Tragelaphus_oryx_isolate_CME-1477_43563807_whole_genome_shotgun_sequence
SJYM01000931.1_2016-2670_Tragelaphus_imberbis_isolate_LEK-543_tarseq_70378_whole_genome_shotgun_sequence
SJYU01013692.1_3133-3787_Tragelaphus_strepsiceros_isolate_GTK-544_tarseq_267350_whole_genome_shotgun_sequence
SJXY01011270.1_613926-614580_Tragelaphus_scriptus_isolate_BUB-555_scaffold23257_len613696_cov75_whole_genome_shotgun_sequence
VBZB01000008.1_19923950-19924578_Bos_grunniens_isolate_yakHY00_chromosome_7_whole_genome_shotgun_sequence
AGSK01139113.1_8273-8901_Bos_mutus_isolate_yakQH1_contig139113_whole_genome_shotgun_sequence
VBQZ03000057.1_9991572-9992200_Bos_mutus_breed_Datong_Yak_isolate_WY2019_wild_yak_scaffold57_whole_genome_shotgun_sequence
VLPI01000483.1_83611168-83611796_Bos_grunniens_x_Bos_taurus_isolate_Esperanza_chromosome_8_whole_genome_shotgun_sequence
RBVW01000253.1_895084-895712_Bos_frontalis_breed_Nagaland_scaffold253_whole_genome_shotgun_sequence
JPYT01754646.1_34635-35263_Bison_bison_bison_isolate_TAMUID_2011002044_ctg523946_whole_genome_shotgun_sequence
PUFS02000008.1_83520328-83520956_Bos_indicus_x_Bos_taurus_breed_Angus_x_Brahman_F1_hybrid_chromosome_8_whole_genome_shotgun_sequence
PRDE01000028.1_84962143-84962771_Bos_indicus_breed_Gir_chromosome_8_whole_genome_shotgun_sequence
NKLS02000008.1_83547967-83548595_Bos_taurus_breed_Hereford_isolate_L1_Dominette_01449_registration_number_42190680_chromosome_8_whole_genome_shotgun_sequence
AAFC05038696.1_14266-14894_Bos_taurus_breed_Hereford_Contig30905_fixed_whole_genome_shotgun_sequence
DAAA02024034.1_22544-23172_TPA_asm__Bos_taurus_breed_Hereford_chromosome_8_7180002029203_whole_genome_shotgun_sequence
VLPJ01000341.1_83404433-83405061_Bos_grunniens_x_Bos_taurus_isolate_Esperanza_chromosome_8_whole_genome_shotgun_sequence
PUFT02000008.1_83134412-83135040_Bos_indicus_x_Bos_taurus_breed_Angus_x_Brahman_F1_hybrid_chromosome_8_whole_genome_shotgun_sequence
NPZD01080613.1_8270-8901_Bubalus_bubalis_breed_Bangladesh_scaffold21_660_whole_genome_shotgun_sequence
SJXX01001611.1_437044-437672_Syncerus_caffer_isolate_ABF-221_scaffold2960_len1272396_cov108_whole_genome_shotgun_sequence
SJXR01001414.1_30721-31356_Przewalskium_albirostris_isolate_WLD-bcl_scaffold2089_len963967_cov126_whole_genome_shotgun_sequence
JRFZ01112267.1_65268-65903_Elaphurus_davidianus_breed_Woburn_Abbey_isolate_Dafeng_scaffold1358_whole_genome_shotgun_sequence
MKHE01000016.1_35293325-35293960_Cervus_elaphus_hippelaphus_isolate_Hungarian_chromosome_16_whole_genome_shotgun_sequence
QQTR01007081.1_16470798-16471433_Axis_porcinus_isolate_Zoohd001_scaffold_121840_whole_genome_shotgun_sequence
SJXU01021044.1_156769-157391_Muntiacus_muntjak_isolate_CMJ-cj_scaffold1877_whole_genome_shotgun_sequence
SJXT01009141.1_1754437-1755059_Muntiacus_reevesi_isolate_CIJ-jz_scaffold347_whole_genome_shotgun_sequence
VCEB01000009.1_69105194-69105829_Muntiacus_reevesi_isolate_UCam_UCB_Mr_chromosome_9_whole_genome_shotgun_sequence
SJXS01011938.1_534824-535459_Muntiacus_crinifrons_isolate_BMJ-hj_000226F_pilon_whole_genome_shotgun_sequence
SPDZ01000729.1_25914720-25915355_Hydropotes_inermis_isolate_ZZ2017_10638_whole_genome_shotgun_sequence
CCMK010105802.1_6164-6793_Capreolus_capreolus_genome_assembly_kmer631_contig_DEER_K63_SCAFFOLD105802_whole_genome_shotgun_sequence
RFLZ01383737.1_13-649_Odocoileus_hemionus_sitkensis_isolate_Cn_RI01_contig635114_0_whole_genome_shotgun_sequence
RCHL01007322.1_46768-47404_Odocoileus_hemionus_hemionus_isolate_MD92_scaffold7322_whole_genome_shotgun_sequence
MLBE01024285.1_34165-34801_Odocoileus_virginianus_texanus_isolate_animal_Pink-7_contig_24285_whole_genome_shotgun_sequence
PVIN010006558.1_40933-41561_Rangifer_tarandus_isolate_US061_RanTarSib_scaffold_13108_whole_genome_shotgun_sequence
SSNK01025280.1_6839-7474_Alces_alces_isolate_MIG12-28694850_contig246_15_whole_genome_shotgun_sequence
SPDY01006690.1_73961-74597_Moschus_chrysogaster_isolate_WD2015_tarseq_3019_whole_genome_shotgun_sequence
SPDX01004285.1_28139-28354_Moschus_berezovskii_isolate_LS2015_tarseq_44146_whole_genome_shotgun_sequence
SPDX01004285.1_28527-28929_Moschus_berezovskii_isolate_LS2015_tarseq_44146_whole_genome_shotgun_sequence
SGQJ01002476.1_3626365-3627001_Moschus_berezovskii_16562_whole_genome_shotgun_sequence
SPDY01006690.1_75585-75778_Moschus_chrysogaster_isolate_WD2015_tarseq_3019_whole_genome_shotgun_sequence
PVHU020012514.1_4190650-4191286_Moschus_moschiferus_isolate_BS20_Scw3uUT_12431HRSCAF_108580_2_whole_genome_shotgun_sequence
SJXV01073976.1_592-1139_Giraffa_camelopardalis_isolate_GFE-hd_C25648076_whole_genome_shotgun_sequence
LVKQ01173957.1_5046-5593_Giraffa_camelopardalis_tippelskirchi_isolate_MA1_scaffold7559b15140e145622_1_whole_genome_shotgun_sequence
LVCL010305476.1_5086-5633_Okapia_johnstoni_scaffold29035_5_whole_genome_shotgun_sequence
PVKT020000249.1_200558-200979_Antilocapra_americana_isolate_BS35_ScwEJLR_228HRSCAF_39107_whole_genome_shotgun_sequence
marine_Turtru_NW_017843503.1_0.0_1_654_6245256_6244609
marine_Souchi_RWJT01009644.1_0.0_1_654_6310970_6310324
marine_Turadu_NCQN01000746.1_0.0_1_654_839567_838920
marine_Orcorc_NW_004438587.1_0.0_1_654_168118_168764
marine_Glomel_ML658858.1_0.0_1_654_8285937_8285290
marine_Lagobl_NW_020838032.1_0.0_1_654_129150_129797
marine_Phopho_PKGA01132689.1_0.0_1_654_6529783_6529136
marine_Phosin_CM018161.1_0.0_1_654_88757717_88757070
marine_Neoasi_NW_020174930.1_0.0_1_654_126211_126858
marine_Delleu_NW_019160958.1_0.0_1_654_6379725_6379078
marine_Monmon_NW_021703782.1_0.0_1_654_26114613_26115260
marine_Inigeo_RJWO010011928.1_0.0_1_651_26514_25870
marine_Ponbla_RJWI010106971.1_0.0_1_654_1066_419
marine_Zipcav_RJWS010247583.1_0.0_75_654_1_574
marine_Zipcav_RJWS010958565.1_2e-110_1_279_273_1
marine_Zipcav_RJWS010957650.1_2e-110_1_279_273_1
marine_Mesbid_PVJJ010013208.1_0.0_1_654_34070_34717
marine_Lipvex_NW_006773367.1_0.0_1_654_3724063_3723416
marine_Escrob_NIPP01014016.1_0.0_1_654_7427_6774
query_marine_Megnov_RYZJ01002407.1_0.0_1_654_442664_443317
marine_Balbon_DF540751.1_0.0_1_652_7401_6750
marine_Balacu_NW_006726353.1_0.0_1_652_31597712_31597061
marine_Eubjap_RJWP010065367.1_0.0_1_654_3086_2433
marine_Phycat_NC_041222.1_0.0_1_654_82058471_82057824
marine_Kogbre_RJWL010008112.1_0.0_1_654_49052_48405
marine_Plamin_RJWK010012098.1_0.0_1_654_29666_30313
PVJP02011199.1_2827926-2828574_Hippopotamus_amphibius_isolate_BS01_ScHSfme_11168HRSCAF_61389_whole_genome_shotgun_sequence
0.02

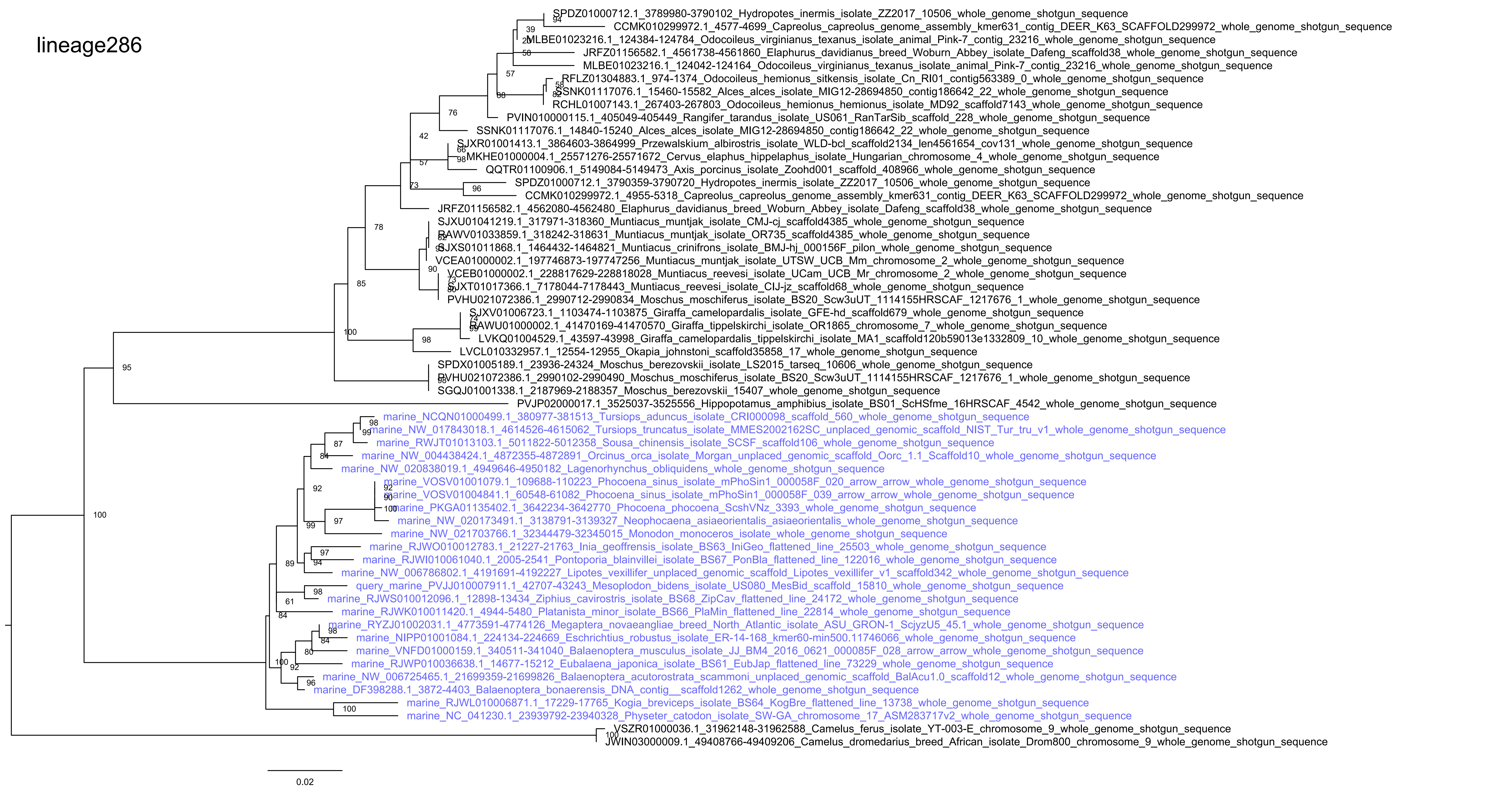
lineage286
SPDZ01000712.1_3789980-3790102_Hydropotes_inermis_isolate_ZZ2017_10506_whole_genome_shotgun_sequence
CCMK010299972.1_4577-4699_Capreolus_capreolus_genome_assembly_kmer631_contig_DEER_K63_SCAFFOLD299972_whole_genome_shotgun_sequence
MLBE01023216.1_124384-124784_Odocoileus_virginianus_texanus_isolate_animal_Pink-7_contig_23216_whole_genome_shotgun_sequence
JRFZ01156582.1_4561738-4561860_Elaphurus_davidianus_breed_Woburn_Abbey_isolate_Dafeng_scaffold38_whole_genome_shotgun_sequence
MLBE01023216.1_124042-124164_Odocoileus_virginianus_texanus_isolate_animal_Pink-7_contig_23216_whole_genome_shotgun_sequence
RFLZ01304883.1_974-1374_Odocoileus_hemionus_sitkensis_isolate_Cn_RI01_contig563389_0_whole_genome_shotgun_sequence
SSNK01117076.1_15460-15582_Alces_alces_isolate_MIG12-28694850_contig186642_22_whole_genome_shotgun_sequence
RCHL01007143.1_267403-267803_Odocoileus_hemionus_hemionus_isolate_MD92_scaffold7143_whole_genome_shotgun_sequence
PVIN010000115.1_405049-405449_Rangifer_tarandus_isolate_US061_RanTarSib_scaffold_228_whole_genome_shotgun_sequence
SSNK01117076.1_14840-15240_Alces_alces_isolate_MIG12-28694850_contig186642_22_whole_genome_shotgun_sequence
SJXR01001413.1_3864603-3864999_Przewalskium_albirostris_isolate_WLD-bcl_scaffold2134_len4561654_cov131_whole_genome_shotgun_sequence
MKHE01000004.1_25571276-25571672_Cervus_elaphus_hippelaphus_isolate_Hungarian_chromosome_4_whole_genome_shotgun_sequence
QQTR01100906.1_5149084-5149473_Axis_porcinus_isolate_Zoohd001_scaffold_408966_whole_genome_shotgun_sequence
SPDZ01000712.1_3790359-3790720_Hydropotes_inermis_isolate_ZZ2017_10506_whole_genome_shotgun_sequence
CCMK010299972.1_4955-5318_Capreolus_capreolus_genome_assembly_kmer631_contig_DEER_K63_SCAFFOLD299972_whole_genome_shotgun_sequence
JRFZ01156582.1_4562080-4562480_Elaphurus_davidianus_breed_Woburn_Abbey_isolate_Dafeng_scaffold38_whole_genome_shotgun_sequence
SJXU01041219.1_317971-318360_Muntiacus_muntjak_isolate_CMJ-cj_scaffold4385_whole_genome_shotgun_sequence
RAWV01033859.1_318242-318631_Muntiacus_muntjak_isolate_OR735_scaffold4385_whole_genome_shotgun_sequence
SJXS01011868.1_1464432-1464821_Muntiacus_crinifrons_isolate_BMJ-hj_000156F_pilon_whole_genome_shotgun_sequence
VCEA01000002.1_197746873-197747256_Muntiacus_muntjak_isolate_UTSW_UCB_Mm_chromosome_2_whole_genome_shotgun_sequence
VCEB01000002.1_228817629-228818028_Muntiacus_reevesi_isolate_UCam_UCB_Mr_chromosome_2_whole_genome_shotgun_sequence
SJXT01017366.1_7178044-7178443_Muntiacus_reevesi_isolate_CIJ-jz_scaffold68_whole_genome_shotgun_sequence
PVHU021072386.1_2990712-2990834_Moschus_moschiferus_isolate_BS20_Scw3uUT_1114155HRSCAF_1217676_1_whole_genome_shotgun_sequence
SJXV01006723.1_1103474-1103875_Giraffa_camelopardalis_isolate_GFE-hd_scaffold679_whole_genome_shotgun_sequence
RAWU01000002.1_41470169-41470570_Giraffa_tippelskirchi_isolate_OR1865_chromosome_7_whole_genome_shotgun_sequence
LVKQ01004529.1_43597-43998_Giraffa_camelopardalis_tippelskirchi_isolate_MA1_scaffold120b59013e1332809_10_whole_genome_shotgun_sequence
LVCL010332957.1_12554-12955_Okapia_johnstoni_scaffold35858_17_whole_genome_shotgun_sequence
SPDX01005189.1_23936-24324_Moschus_berezovskii_isolate_LS2015_tarseq_10606_whole_genome_shotgun_sequence
PVHU021072386.1_2990102-2990490_Moschus_moschiferus_isolate_BS20_Scw3uUT_1114155HRSCAF_1217676_1_whole_genome_shotgun_sequence
SGQJ01001338.1_2187969-2188357_Moschus_berezovskii_15407_whole_genome_shotgun_sequence
PVJP02000017.1_3525037-3525556_Hippopotamus_amphibius_isolate_BS01_ScHSfme_16HRSCAF_4542_whole_genome_shotgun_sequence
marine_NCQN01000499.1_380977-381513_Tursiops_aduncus_isolate_CRI000098_scaffold_560_whole_genome_shotgun_sequence
marine_NW_017843018.1_4614526-4615062_Tursiops_truncatus_isolate_MMES2002162SC_unplaced_genomic_scaffold_NIST_Tur_tru_v1_whole_genome_shotgun_sequence
marine_RWJT01013103.1_5011822-5012358_Sousa_chinensis_isolate_SCSF_scaffold106_whole_genome_shotgun_sequence
marine_NW_004438424.1_4872355-4872891_Orcinus_orca_isolate_Morgan_unplaced_genomic_scaffold_Oorc_1.1_Scaffold10_whole_genome_shotgun_sequence
marine_NW_020838019.1_4949646-4950182_Lagenorhynchus_obliquidens_whole_genome_shotgun_sequence
marine_VOSV01001079.1_109688-110223_Phocoena_sinus_isolate_mPhoSin1_000058F_020_arrow_arrow_whole_genome_shotgun_sequence
marine_VOSV01004841.1_60548-61082_Phocoena_sinus_isolate_mPhoSin1_000058F_039_arrow_arrow_whole_genome_shotgun_sequence
marine_PKGA01135402.1_3642234-3642770_Phocoena_phocoena_ScshVNz_3393_whole_genome_shotgun_sequence
marine_NW_020173491.1_3138791-3139327_Neophocaena_asiaeorientalis_asiaeorientalis_whole_genome_shotgun_sequence
marine_NW_021703766.1_32344479-32345015_Monodon_monoceros_isolate_whole_genome_shotgun_sequence
marine_RJWO010012783.1_21227-21763_Inia_geoffrensis_isolate_BS63_IniGeo_flattened_line_25503_whole_genome_shotgun_sequence
marine_RJWI010061040.1_2005-2541_Pontoporia_blainvillei_isolate_BS67_PonBla_flattened_line_122016_whole_genome_shotgun_sequence
marine_NW_006786802.1_4191691-4192227_Lipotes_vexillifer_unplaced_genomic_scaffold_Lipotes_vexillifer_v1_scaffold342_whole_genome_shotgun_sequence
query_marine_PVJJ010007911.1_42707-43243_Mesoplodon_bidens_isolate_US080_MesBid_scaffold_15810_whole_genome_shotgun_sequence
marine_RJWS010012096.1_12898-13434_Ziphius_cavirostris_isolate_BS68_ZipCav_flattened_line_24172_whole_genome_shotgun_sequence
marine_RJWK010011420.1_4944-5480_Platanista_minor_isolate_BS66_PlaMin_flattened_line_22814_whole_genome_shotgun_sequence
marine_RYZJ01002031.1_4773591-4774126_Megaptera_novaeangliae_breed_North_Atlantic_isolate_ASU_GRON-1_ScjyzU5_45.1_whole_genome_shotgun_sequence
marine_NIPP01001084.1_224134-224669_Eschrichtius_robustus_isolate_ER-14-168_kmer60-min500.11746066_whole_genome_shotgun_sequence
marine_VNFD01000159.1_340511-341040_Balaenoptera_musculus_isolate_JJ_BM4_2016_0621_000085F_028_arrow_arrow_whole_genome_shotgun_sequence
marine_RJWP010036638.1_14677-15212_Eubalaena_japonica_isolate_BS61_EubJap_flattened_line_73229_whole_genome_shotgun_sequence
marine_NW_006725465.1_21699359-21699826_Balaenoptera_acutorostrata_scammoni_unplaced_genomic_scaffold_BalAcu1.0_scaffold12_whole_genome_shotgun_sequence
marine_DF398288.1_3872-4403_Balaenoptera_bonaerensis_DNA_contig__scaffold1262_whole_genome_shotgun_sequence
marine_RJWL010006871.1_17229-17765_Kogia_breviceps_isolate_BS64_KogBre_flattened_line_13738_whole_genome_shotgun_sequence
marine_NC_041230.1_23939792-23940328_Physeter_catodon_isolate_SW-GA_chromosome_17_ASM283717v2_whole_genome_shotgun_sequence
VSZR01000036.1_31962148-31962588_Camelus_ferus_isolate_YT-003-E_chromosome_9_whole_genome_shotgun_sequence
JWIN03000009.1_49408766-49409206_Camelus_dromedarius_breed_African_isolate_Drom800_chromosome_9_whole_genome_shotgun_sequence
0.02

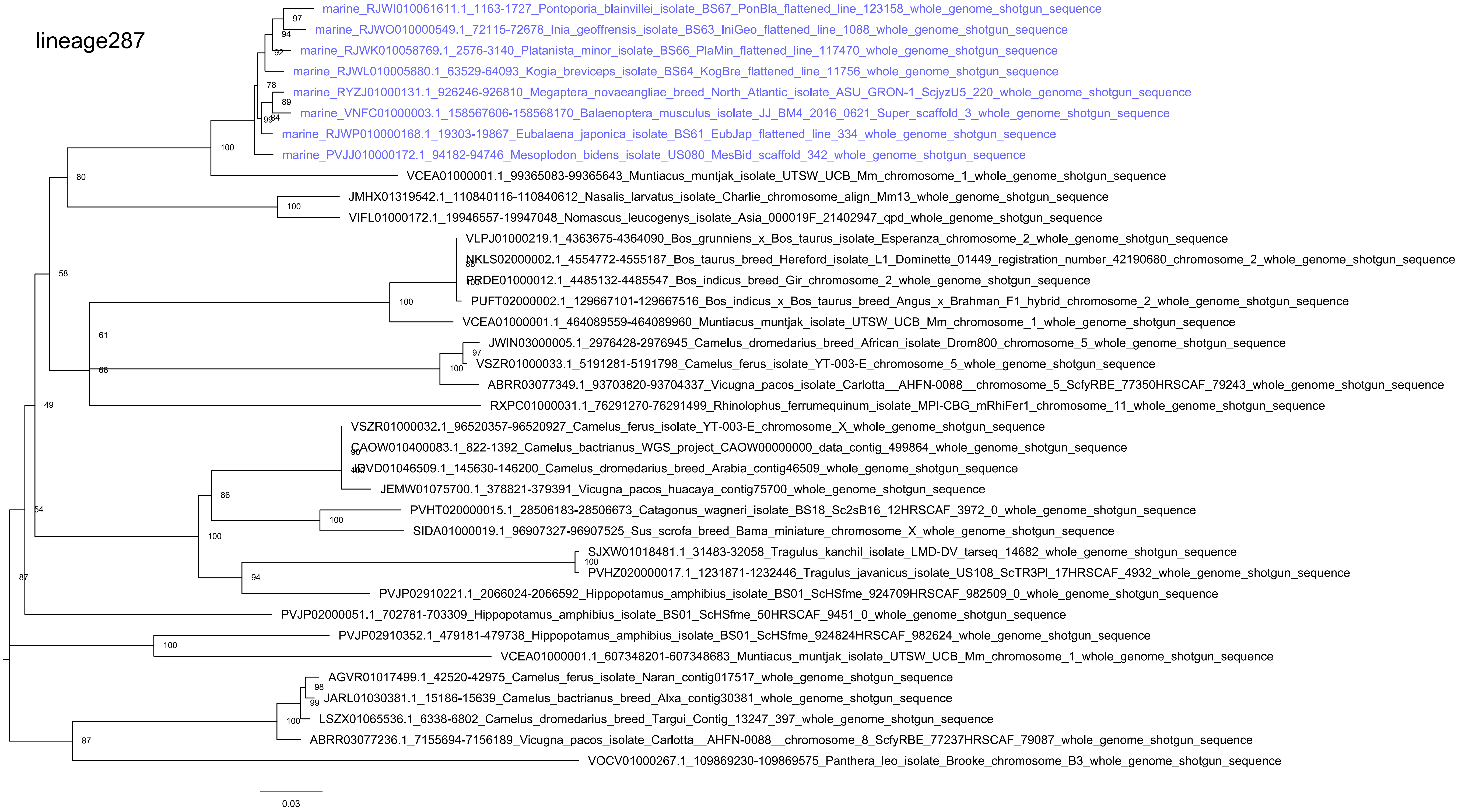
lineage287
marine_RJWI010061611.1_1163-1727_Pontoporia_blainvillei_isolate_BS67_PonBla_flattened_line_123158_whole_genome_shotgun_sequence
marine_RJWO010000549.1_72115-72678_Inia_geoffrensis_isolate_BS63_IniGeo_flattened_line_1088_whole_genome_shotgun_sequence
marine_RJWK010058769.1_2576-3140_Platanista_minor_isolate_BS66_PlaMin_flattened_line_117470_whole_genome_shotgun_sequence
marine_RJWL010005880.1_63529-64093_Kogia_breviceps_isolate_BS64_KogBre_flattened_line_11756_whole_genome_shotgun_sequence
marine_RYZJ01000131.1_926246-926810_Megaptera_novaeangliae_breed_North_Atlantic_isolate_ASU_GRON-1_ScjyzU5_220_whole_genome_shotgun_sequence
marine_VNFC01000003.1_158567606-158568170_Balaenoptera_musculus_isolate_JJ_BM4_2016_0621_Super_scaffold_3_whole_genome_shotgun_sequence
marine_RJWP010000168.1_19303-19867_Eubalaena_japonica_isolate_BS61_EubJap_flattened_line_334_whole_genome_shotgun_sequence
marine_PVJJ010000172.1_94182-94746_Mesoplodon_bidens_isolate_US080_MesBid_scaffold_342_whole_genome_shotgun_sequence
VCEA01000001.1_99365083-99365643_Muntiacus_muntjak_isolate_UTSW_UCB_Mm_chromosome_1_whole_genome_shotgun_sequence
JMHX01319542.1_110840116-110840612_Nasalis_larvatus_isolate_Charlie_chromosome_align_Mm13_whole_genome_shotgun_sequence
VIFL01000172.1_19946557-19947048_Nomascus_leucogenys_isolate_Asia_000019F_21402947_qpd_whole_genome_shotgun_sequence
VLPJ01000219.1_4363675-4364090_Bos_grunniens_x_Bos_taurus_isolate_Esperanza_chromosome_2_whole_genome_shotgun_sequence
NKLS02000002.1_4554772-4555187_Bos_taurus_breed_Hereford_isolate_L1_Dominette_01449_registration_number_42190680_chromosome_2_whole_genome_shotgun_sequence
PRDE01000012.1_4485132-4485547_Bos_indicus_breed_Gir_chromosome_2_whole_genome_shotgun_sequence
PUFT02000002.1_129667101-129667516_Bos_indicus_x_Bos_taurus_breed_Angus_x_Brahman_F1_hybrid_chromosome_2_whole_genome_shotgun_sequence
VCEA01000001.1_464089559-464089960_Muntiacus_muntjak_isolate_UTSW_UCB_Mm_chromosome_1_whole_genome_shotgun_sequence
JWIN03000005.1_2976428-2976945_Camelus_dromedarius_breed_African_isolate_Drom800_chromosome_5_whole_genome_shotgun_sequence
VSZR01000033.1_5191281-5191798_Camelus_ferus_isolate_YT-003-E_chromosome_5_whole_genome_shotgun_sequence
ABRR03077349.1_93703820-93704337_Vicugna_pacos_isolate_Carlotta__AHFN-0088__chromosome_5_ScfyRBE_77350HRSCAF_79243_whole_genome_shotgun_sequence
RXPC01000031.1_76291270-76291499_Rhinolophus_ferrumequinum_isolate_MPI-CBG_mRhiFer1_chromosome_11_whole_genome_shotgun_sequence
VSZR01000032.1_96520357-96520927_Camelus_ferus_isolate_YT-003-E_chromosome_X_whole_genome_shotgun_sequence
CAOW010400083.1_822-1392_Camelus_bactrianus_WGS_project_CAOW00000000_data_contig_499864_whole_genome_shotgun_sequence
JDVD01046509.1_145630-146200_Camelus_dromedarius_breed_Arabia_contig46509_whole_genome_shotgun_sequence
JEMW01075700.1_378821-379391_Vicugna_pacos_huacaya_contig75700_whole_genome_shotgun_sequence
PVHT020000015.1_28506183-28506673_Catagonus_wagneri_isolate_BS18_Sc2sB16_12HRSCAF_3972_0_whole_genome_shotgun_sequence
SIDA01000019.1_96907327-96907525_Sus_scrofa_breed_Bama_miniature_chromosome_X_whole_genome_shotgun_sequence
SJXW01018481.1_31483-32058_Tragulus_kanchil_isolate_LMD-DV_tarseq_14682_whole_genome_shotgun_sequence
PVHZ020000017.1_1231871-1232446_Tragulus_javanicus_isolate_US108_ScTR3PI_17HRSCAF_4932_whole_genome_shotgun_sequence
PVJP02910221.1_2066024-2066592_Hippopotamus_amphibius_isolate_BS01_ScHSfme_924709HRSCAF_982509_0_whole_genome_shotgun_sequence
PVJP02000051.1_702781-703309_Hippopotamus_amphibius_isolate_BS01_ScHSfme_50HRSCAF_9451_0_whole_genome_shotgun_sequence
PVJP02910352.1_479181-479738_Hippopotamus_amphibius_isolate_BS01_ScHSfme_924824HRSCAF_982624_whole_genome_shotgun_sequence
VCEA01000001.1_607348201-607348683_Muntiacus_muntjak_isolate_UTSW_UCB_Mm_chromosome_1_whole_genome_shotgun_sequence
AGVR01017499.1_42520-42975_Camelus_ferus_isolate_Naran_contig017517_whole_genome_shotgun_sequence
JARL01030381.1_15186-15639_Camelus_bactrianus_breed_Alxa_contig30381_whole_genome_shotgun_sequence
LSZX01065536.1_6338-6802_Camelus_dromedarius_breed_Targui_Contig_13247_397_whole_genome_shotgun_sequence
ABRR03077236.1_7155694-7156189_Vicugna_pacos_isolate_Carlotta__AHFN-0088__chromosome_8_ScfyRBE_77237HRSCAF_79087_whole_genome_shotgun_sequence
VOCV01000267.1_109869230-109869575_Panthera_leo_isolate_Brooke_chromosome_B3_whole_genome_shotgun_sequence
0.03

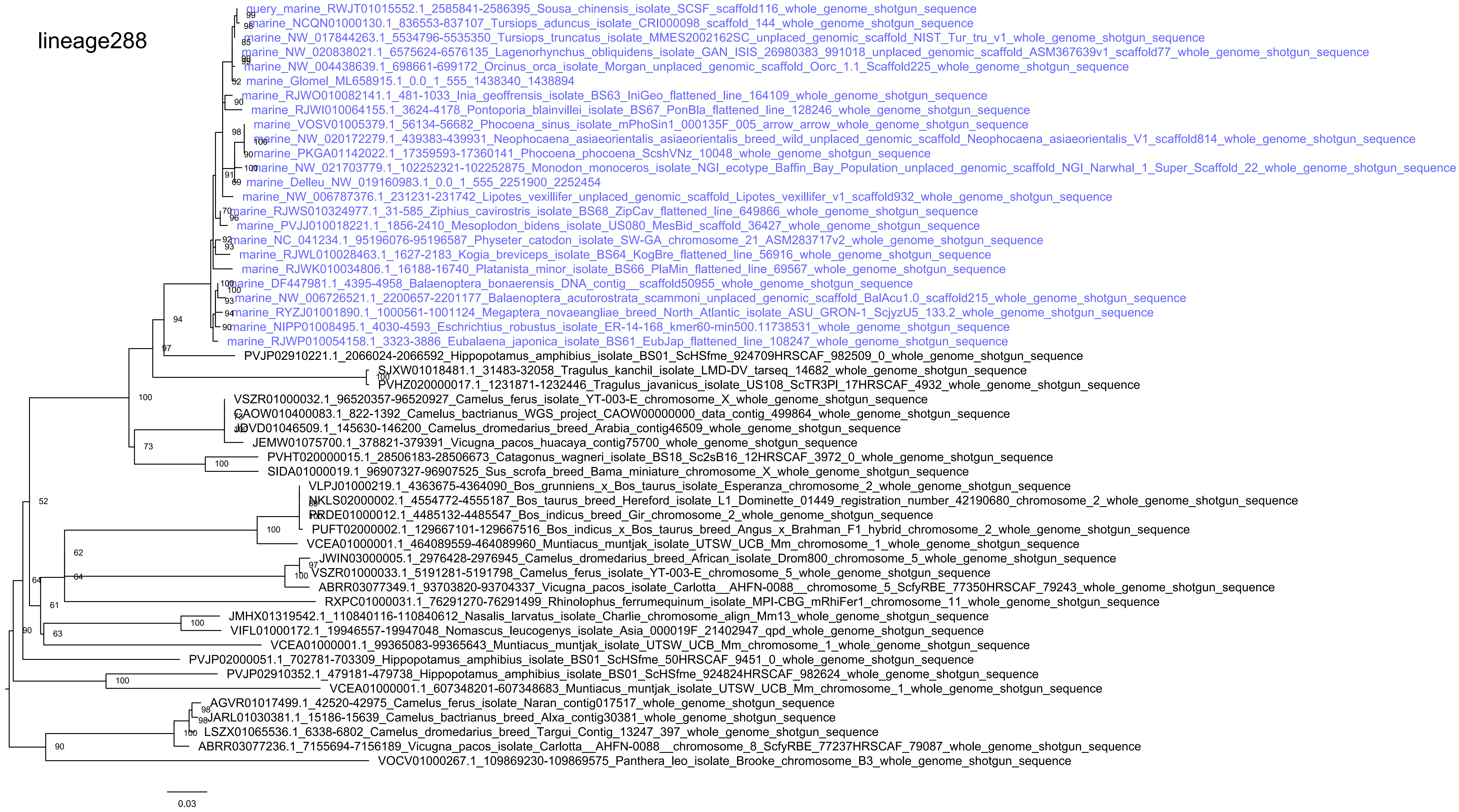
lineage288
query_marine_RWJT01015552.1_2585841-2586395_Sousa_chinensis_isolate_SCSF_scaffold116_whole_genome_shotgun_sequence
marine_NCQN01000130.1_836553-837107_Tursiops_aduncus_isolate_CRI000098_scaffold_144_whole_genome_shotgun_sequence
marine_NW_017844263.1_5534796-5535350_Tursiops_truncatus_isolate_MMES2002162SC_unplaced_genomic_scaffold_NIST_Tur_tru_v1_whole_genome_shotgun_sequence
marine_NW_020838021.1_6575624-6576135_Lagenorhynchus_obliquidens_isolate_GAN_ISIS_26980383_991018_unplaced_genomic_scaffold_ASM367639v1_scaffold77_whole_genome_shotgun_sequence
marine_NW_004438639.1_698661-699172_Orcinus_orca_isolate_Morgan_unplaced_genomic_scaffold_Oorc_1.1_Scaffold225_whole_genome_shotgun_sequence
marine_Glomel_ML658915.1_0.0_1_555_1438340_1438894
marine_RJWO010082141.1_481-1033_Inia_geoffrensis_isolate_BS63_IniGeo_flattened_line_164109_whole_genome_shotgun_sequence
marine_RJWI010064155.1_3624-4178_Pontoporia_blainvillei_isolate_BS67_PonBla_flattened_line_128246_whole_genome_shotgun_sequence
marine_VOSV01005379.1_56134-56682_Phocoena_sinus_isolate_mPhoSin1_000135F_005_arrow_arrow_whole_genome_shotgun_sequence
marine_NW_020172279.1_439383-439931_Neophocaena_asiaeorientalis_asiaeorientalis_breed_wild_unplaced_genomic_scaffold_Neophocaena_asiaeorientalis_V1_scaffold814_whole_genome_shotgun_sequence
marine_PKGA01142022.1_17359593-17360141_Phocoena_phocoena_ScshVNz_10048_whole_genome_shotgun_sequence
marine_NW_021703779.1_102252321-102252875_Monodon_monoceros_isolate_NGI_ecotype_Baffin_Bay_Population_unplaced_genomic_scaffold_NGI_Narwhal_1_Super_Scaffold_22_whole_genome_shotgun_sequence
marine_Delleu_NW_019160983.1_0.0_1_555_2251900_2252454
marine_NW_006787376.1_231231-231742_Lipotes_vexillifer_unplaced_genomic_scaffold_Lipotes_vexillifer_v1_scaffold932_whole_genome_shotgun_sequence
marine_RJWS010324977.1_31-585_Ziphius_cavirostris_isolate_BS68_ZipCav_flattened_line_649866_whole_genome_shotgun_sequence
marine_PVJJ010018221.1_1856-2410_Mesoplodon_bidens_isolate_US080_MesBid_scaffold_36427_whole_genome_shotgun_sequence
marine_NC_041234.1_95196076-95196587_Physeter_catodon_isolate_SW-GA_chromosome_21_ASM283717v2_whole_genome_shotgun_sequence
marine_RJWL010028463.1_1627-2183_Kogia_breviceps_isolate_BS64_KogBre_flattened_line_56916_whole_genome_shotgun_sequence
marine_RJWK010034806.1_16188-16740_Platanista_minor_isolate_BS66_PlaMin_flattened_line_69567_whole_genome_shotgun_sequence
marine_DF447981.1_4395-4958_Balaenoptera_bonaerensis_DNA_contig__scaffold50955_whole_genome_shotgun_sequence
marine_NW_006726521.1_2200657-2201177_Balaenoptera_acutorostrata_scammoni_unplaced_genomic_scaffold_BalAcu1.0_scaffold215_whole_genome_shotgun_sequence
marine_RYZJ01001890.1_1000561-1001124_Megaptera_novaeangliae_breed_North_Atlantic_isolate_ASU_GRON-1_ScjyzU5_133.2_whole_genome_shotgun_sequence
marine_NIPP01008495.1_4030-4593_Eschrichtius_robustus_isolate_ER-14-168_kmer60-min500.11738531_whole_genome_shotgun_sequence
marine_RJWP010054158.1_3323-3886_Eubalaena_japonica_isolate_BS61_EubJap_flattened_line_108247_whole_genome_shotgun_sequence
PVJP02910221.1_2066024-2066592_Hippopotamus_amphibius_isolate_BS01_ScHSfme_924709HRSCAF_982509_0_whole_genome_shotgun_sequence
SJXW01018481.1_31483-32058_Tragulus_kanchil_isolate_LMD-DV_tarseq_14682_whole_genome_shotgun_sequence
PVHZ020000017.1_1231871-1232446_Tragulus_javanicus_isolate_US108_ScTR3PI_17HRSCAF_4932_whole_genome_shotgun_sequence
VSZR01000032.1_96520357-96520927_Camelus_ferus_isolate_YT-003-E_chromosome_X_whole_genome_shotgun_sequence
CAOW010400083.1_822-1392_Camelus_bactrianus_WGS_project_CAOW00000000_data_contig_499864_whole_genome_shotgun_sequence
JDVD01046509.1_145630-146200_Camelus_dromedarius_breed_Arabia_contig46509_whole_genome_shotgun_sequence
JEMW01075700.1_378821-379391_Vicugna_pacos_huacaya_contig75700_whole_genome_shotgun_sequence
PVHT020000015.1_28506183-28506673_Catagonus_wagneri_isolate_BS18_Sc2sB16_12HRSCAF_3972_0_whole_genome_shotgun_sequence
SIDA01000019.1_96907327-96907525_Sus_scrofa_breed_Bama_miniature_chromosome_X_whole_genome_shotgun_sequence
VLPJ01000219.1_4363675-4364090_Bos_grunniens_x_Bos_taurus_isolate_Esperanza_chromosome_2_whole_genome_shotgun_sequence
NKLS02000002.1_4554772-4555187_Bos_taurus_breed_Hereford_isolate_L1_Dominette_01449_registration_number_42190680_chromosome_2_whole_genome_shotgun_sequence
RRDE01000012.1_4485132-4485547_Bos_indicus_breed_Gir_chromosome_2_whole_genome_shotgun_sequence
PUFT02000002.1_129667101-129667516_Bos_indicus_x_Bos_taurus_breed_Angus_x_Brahman_F1_hybrid_chromosome_2_whole_genome_shotgun_sequence
VCEA01000001.1_464089559-464089960_Muntiacus_muntjak_isolate_UTSW_UCB_Mm_chromosome_1_whole_genome_shotgun_sequence
JWIN03000005.1_2976428-2976945_Camelus_dromedarius_breed_African_isolate_Drom800_chromosome_5_whole_genome_shotgun_sequence
VSZR01000033.1_5191281-5191798_Camelus_ferus_isolate_YT-003-E_chromosome_5_whole_genome_shotgun_sequence
ABRR03077349.1_93703820-93704337_Vicugna_pacos_isolate_Carlotta__AHFN-0088__chromosome_5_ScfyRBE_77350HRSCAF_79243_whole_genome_shotgun_sequence
RXPC01000031.1_76291270-76291499_Rhinolophus_ferrumequinum_isolate_MPI-CBG_mRhiFer1_chromosome_11_whole_genome_shotgun_sequence
JMHX01319542.1_110840116-110840612_Nasalis_larvatus_isolate_Charlie_chromosome_align_Mm13_whole_genome_shotgun_sequence
VIFL01000172.1_19946557-19947048_Nomascus_leucogenys_isolate_Asia_000019F_21402947_qpd_whole_genome_shotgun_sequence
VCEA01000001.1_99365083-99365643_Muntiacus_muntjak_isolate_UTSW_UCB_Mm_chromosome_1_whole_genome_shotgun_sequence
PVJP02000051.1_702781-703309_Hippopotamus_amphibius_isolate_BS01_ScHSfme_50HRSCAF_9451_0_whole_genome_shotgun_sequence
PVJP02910352.1_479181-479738_Hippopotamus_amphibius_isolate_BS01_ScHSfme_924824HRSCAF_982624_whole_genome_shotgun_sequence
VCEA01000001.1_607348201-607348683_Muntiacus_muntjak_isolate_UTSW_UCB_Mm_chromosome_1_whole_genome_shotgun_sequence
AGVR01017499.1_42520-42975_Camelus_ferus_isolate_Naran_contig017517_whole_genome_shotgun_sequence
JARL01030381.1_15186-15639_Camelus_bactrianus_breed_Alxa_contig30381_whole_genome_shotgun_sequence
LSZX01065536.1_6338-6802_Camelus_dromedarius_breed_Targui_Contig_13247_397_whole_genome_shotgun_sequence
ABRR03077236.1_7155694-7156189_Vicugna_pacos_isolate_Carlotta__AHFN-0088__chromosome_8_ScfyRBE_77237HRSCAF_79087_whole_genome_shotgun_sequence
VOCV01000267.1_109869230-109869575_Panthera_leo_isolate_Brooke_chromosome_B3_whole_genome_shotgun_sequence
0.03

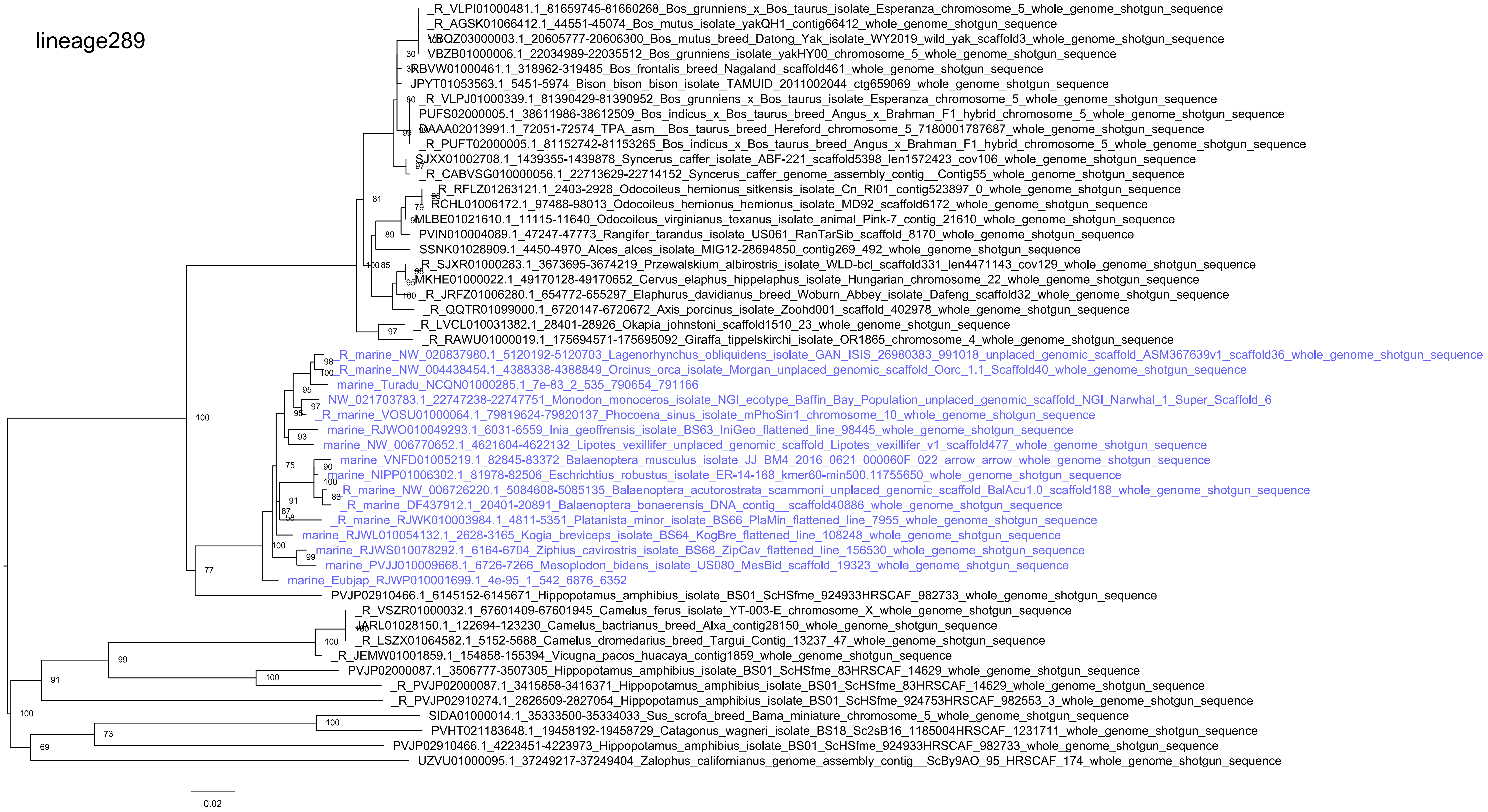
lineage289
_R_VLPI01000481.1_81659745-81660268_Bos_grunniens_x_Bos_taurus_isolate_Esperanza_chromosome_5_whole_genome_shotgun_sequence
_R_AGSK01066412.1_44551-45074_Bos_mutus_isolate_yakQH1_contig66412_whole_genome_shotgun_sequence
VBQZ03000003.1_20605777-20606300_Bos_mutus_breed_Datong_Yak_isolate_WY2019_wild_yak_scaffold3_whole_genome_shotgun_sequence
VBZB01000006.1_22034989-22035512_Bos_grunniens_isolate_yakHY00_chromosome_5_whole_genome_shotgun_sequence
RBVW01000461.1_318962-319485_Bos_frontalis_breed_Nagaland_scaffold461_whole_genome_shotgun_sequence
JPYT01053563.1_5451-5974_Bison_bison_bison_isolate_TAMUID_2011002044_ctg659069_whole_genome_shotgun_sequence
_R_VLPJ01000339.1_81390429-81390952_Bos_grunniens_x_Bos_taurus_isolate_Esperanza_chromosome_5_whole_genome_shotgun_sequence
PUFS02000005.1_38611986-38612509_Bos_indicus_x_Bos_taurus_breed_Angus_x_Brahman_F1_hybrid_chromosome_5_whole_genome_shotgun_sequence
DAAA02013991.1_72051-72574_TPA_asm__Bos_taurus_breed_Hereford_chromosome_5_7180001787687_whole_genome_shotgun_sequence
_R_PUFT02000005.1_81152742-81153265_Bos_indicus_x_Bos_taurus_breed_Angus_x_Brahman_F1_hybrid_chromosome_5_whole_genome_shotgun_sequence
SJXX01002708.1_1439355-1439878_Syncerus_caffer_isolate_ABF-221_scaffold5398_len1572423_cov106_whole_genome_shotgun_sequence
_R_CABVSG010000056.1_22713629-22714152_Syncerus_caffer_genome_assembly_contig__Contig55_whole_genome_shotgun_sequence
_R_RFLZ01263121.1_2403-2928_Odocoileus_hemionus_sitkensis_isolate_Cn_RI01_contig523897_0_whole_genome_shotgun_sequence
RCHL01006172.1_97488-98013_Odocoileus_hemionus_hemionus_isolate_MD92_scaffold6172_whole_genome_shotgun_sequence
MLBE01021610.1_11115-11640_Odocoileus_virginianus_texanus_isolate_animal_Pink-7_contig_21610_whole_genome_shotgun_sequence
PVIN010004089.1_47247-47773_Rangifer_tarandus_isolate_US061_RanTarSib_scaffold_8170_whole_genome_shotgun_sequence
SSNK01028909.1_4450-4970_Alces_alces_isolate_MIG12-28694850_contig269_492_whole_genome_shotgun_sequence
_R_SJXR01000283.1_3673695-3674219_Przewalskium_albirostris_isolate_WLD-bcl_scaffold331_len4471143_cov129_whole_genome_shotgun_sequence
MKHE01000022.1_49170128-49170652_Cervus_elaphus_hippelaphus_isolate_Hungarian_chromosome_22_whole_genome_shotgun_sequence
_R_JRFZ01006280.1_654772-655297_Elaphurus_davidianus_breed_Woburn_Abbey_isolate_Dafeng_scaffold32_whole_genome_shotgun_sequence
_R_QQTR01099000.1_6720147-6720672_Axis_porcinus_isolate_Zoohd001_scaffold_402978_whole_genome_shotgun_sequence
_R_LVCL010031382.1_28401-28926_Okapia_johnstoni_scaffold1510_23_whole_genome_shotgun_sequence
_R_RAWU01000019.1_175694571-175695092_Giraffa_tippelskirchi_isolate_OR1865_chromosome_4_whole_genome_shotgun_sequence
_R_marine_NW_020837980.1_5120192-5120703_Lagenorhynchus_obliquidens_isolate_GAN_ISIS_26980383_991018_unplaced_genomic_scaffold_ASM367639v1_scaffold36_whole_genome_shotgun_sequence
_R_marine_NW_004438454.1_4388338-4388849_Orcinus_orca_isolate_Morgan_unplaced_genomic_scaffold_Oorc_1.1_Scaffold40_whole_genome_shotgun_sequence
marine_Turadu_NCQN01000285.1_7e-83_2_535_790654_791166
NW_021703783.1_22747238-22747751_Monodon_monoceros_isolate_NGI_ecotype_Baffin_Bay_Population_unplaced_genomic_scaffold_NGI_Narwhal_1_Super_Scaffold_6
_R_marine_VOSU01000064.1_79819624-79820137_Phocoena_sinus_isolate_mPhoSin1_chromosome_10_whole_genome_shotgun_sequence
marine_RJWO010049293.1_6031-6559_Inia_geoffrensis_isolate_BS63_IniGeo_flattened_line_98445_whole_genome_shotgun_sequence
marine_NW_006770652.1_4621604-4622132_Lipotes_vexillifer_unplaced_genomic_scaffold_Lipotes_vexillifer_v1_scaffold477_whole_genome_shotgun_sequence
marine_VNFD01005219.1_82845-83372_Balaenoptera_musculus_isolate_JJ_BM4_2016_0621_000060F_022_arrow_arrow_whole_genome_shotgun_sequence
marine_NIPP01006302.1_81978-82506_Eschrichtius_robustus_isolate_ER-14-168_kmer60-min500.11755650_whole_genome_shotgun_sequence
_R_marine_NW_006726220.1_5084608-5085135_Balaenoptera_acutorostrata_scammoni_unplaced_genomic_scaffold_BalAcu1.0_scaffold188_whole_genome_shotgun_sequence
_R_marine_DF437912.1_20401-20891_Balaenoptera_bonaerensis_DNA_contig__scaffold40886_whole_genome_shotgun_sequence
_R_marine_RJWK010003984.1_4811-5351_Platanista_minor_isolate_BS66_PlaMin_flattened_line_7955_whole_genome_shotgun_sequence
marine_RJWL010054132.1_2628-3165_Kogia_breviceps_isolate_BS64_KogBre_flattened_line_108248_whole_genome_shotgun_sequence
marine_RJWS010078292.1_6164-6704_Ziphius_cavirostris_isolate_BS68_ZipCav_flattened_line_156530_whole_genome_shotgun_sequence
marine_PVJJ010009668.1_6726-7266_Mesoplodon_bidens_isolate_US080_MesBid_scaffold_19323_whole_genome_shotgun_sequence
marine_Eubjap_RJWP010001699.1_4e-95_1_542_6876_6352
PVJP02910466.1_6145152-6145671_Hippopotamus_amphibius_isolate_BS01_ScHSfme_924933HRSCAF_982733_whole_genome_shotgun_sequence
_R_VSZR01000032.1_67601409-67601945_Camelus_ferus_isolate_YT-003-E_chromosome_X_whole_genome_shotgun_sequence
JARL01028150.1_122694-123230_Camelus_bactrianus_breed_Alxa_contig28150_whole_genome_shotgun_sequence
_R_LSZX01064582.1_5152-5688_Camelus_dromedarius_breed_Targui_Contig_13237_47_whole_genome_shotgun_sequence
_R_JEMW01001859.1_154858-155394_Vicugna_pacos_huacaya_contig1859_whole_genome_shotgun_sequence
PVJP02000087.1_3506777-3507305_Hippopotamus_amphibius_isolate_BS01_ScHSfme_83HRSCAF_14629_whole_genome_shotgun_sequence
_R_PVJP02000087.1_3415858-3416371_Hippopotamus_amphibius_isolate_BS01_ScHSfme_83HRSCAF_14629_whole_genome_shotgun_sequence
_R_PVJP02910274.1_2826509-2827054_Hippopotamus_amphibius_isolate_BS01_ScHSfme_924753HRSCAF_982553_3_whole_genome_shotgun_sequence
SIDA01000014.1_35333500-35334033_Sus_scrofa_breed_Bama_miniature_chromosome_5_whole_genome_shotgun_sequence
PVHT021183648.1_19458192-19458729_Catagonus_wagneri_isolate_BS18_Sc2sB16_1185004HRSCAF_1231711_whole_genome_shotgun_sequence
PVJP02910466.1_4223451-4223973_Hippopotamus_amphibius_isolate_BS01_ScHSfme_924933HRSCAF_982733_whole_genome_shotgun_sequence
UZVU01000095.1_37249217-37249404_Zalophus_californianus_genome_assembly_contig__ScBy9AO_95_HRSCAF_174_whole_genome_shotgun_sequence
0.02

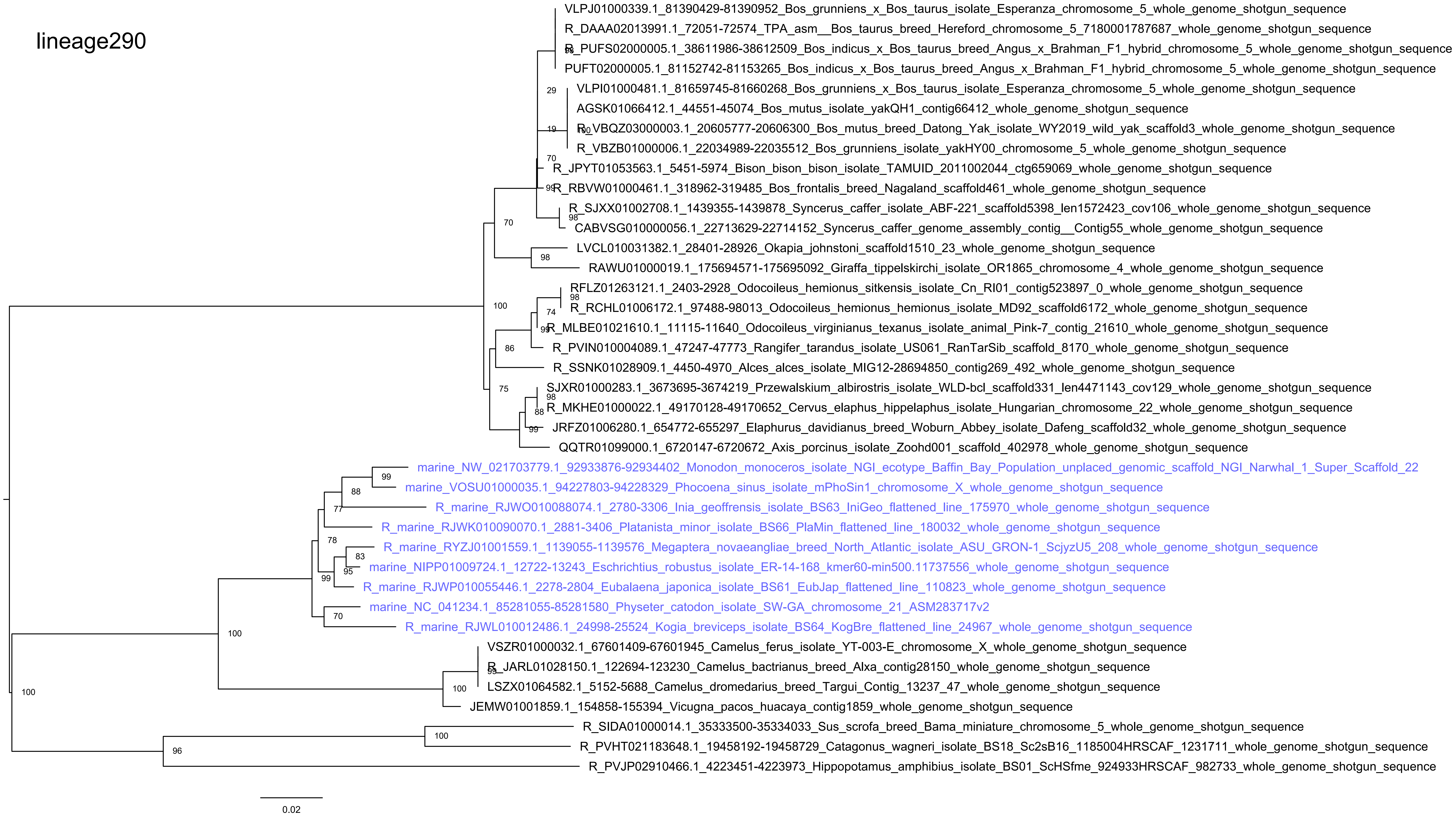
lineage290
VLPJ01000339.1_81390429-81390952_Bos_grunniens_x_Bos_taurus_isolate_Esperanza_chromosome_5_whole_genome_shotgun_sequence
R_DAAA02013991.1_72051-72574_TPA_asm__Bos_taurus_breed_Hereford_chromosome_5_7180001787687_whole_genome_shotgun_sequence
R_PUFS02000005.1_38611986-38612509_Bos_indicus_x_Bos_taurus_breed_Angus_x_Brahman_F1_hybrid_chromosome_5_whole_genome_shotgun_sequence
PUFT02000005.1_81152742-81153265_Bos_indicus_x_Bos_taurus_breed_Angus_x_Brahman_F1_hybrid_chromosome_5_whole_genome_shotgun_sequence
VLPI01000481.1_81659745-81660268_Bos_grunniens_x_Bos_taurus_isolate_Esperanza_chromosome_5_whole_genome_shotgun_sequence
AGSK01066412.1_44551-45074_Bos_mutus_isolate_yakQH1_contig66412_whole_genome_shotgun_sequence
R_VBQZ03000003.1_20605777-20606300_Bos_mutus_breed_Datong_Yak_isolate_WY2019_wild_yak_scaffold3_whole_genome_shotgun_sequence
R_VBZB01000006.1_22034989-22035512_Bos_grunniens_isolate_yakHY00_chromosome_5_whole_genome_shotgun_sequence
R_JPYT01053563.1_5451-5974_Bison_bison_bison_isolate_TAMUID_2011002044_ctg659069_whole_genome_shotgun_sequence
R_RBVW01000461.1_318962-319485_Bos_frontalis_breed_Nagaland_scaffold461_whole_genome_shotgun_sequence
R_SJXX01002708.1_1439355-1439878_Syncerus_caffer_isolate_ABF-221_scaffold5398_len1572423_cov106_whole_genome_shotgun_sequence
CABVSG010000056.1_22713629-22714152_Syncerus_caffer_genome_assembly_contig__Contig55_whole_genome_shotgun_sequence
LVCL010031382.1_28401-28926_Okapia_johnstoni_scaffold1510_23_whole_genome_shotgun_sequence
RAWU01000019.1_175694571-175695092_Giraffa_tippelskirchi_isolate_OR1865_chromosome_4_whole_genome_shotgun_sequence
RFLZ01263121.1_2403-2928_Odocoileus_hemionus_sitkensis_isolate_Cn_RI01_contig523897_0_whole_genome_shotgun_sequence
R_RCHL01006172.1_97488-98013_Odocoileus_hemionus_hemionus_isolate_MD92_scaffold6172_whole_genome_shotgun_sequence
R_MLBE01021610.1_11115-11640_Odocoileus_virginianus_texanus_isolate_animal_Pink-7_contig_21610_whole_genome_shotgun_sequence
R_PVIN010004089.1_47247-47773_Rangifer_tarandus_isolate_US061_RanTarSib_scaffold_8170_whole_genome_shotgun_sequence
R_SSNK01028909.1_4450-4970_Alces_alces_isolate_MIG12-28694850_contig269_492_whole_genome_shotgun_sequence
SJXR01000283.1_3673695-3674219_Przewalskium_albirostris_isolate_WLD-bcl_scaffold331_len4471143_cov129_whole_genome_shotgun_sequence
R_MKHE01000022.1_49170128-49170652_Cervus_elaphus_hippelaphus_isolate_Hungarian_chromosome_22_whole_genome_shotgun_sequence
JRFZ01006280.1_654772-655297_Elaphurus_davidianus_breed_Woburn_Abbey_isolate_Dafeng_scaffold32_whole_genome_shotgun_sequence
QQTR01099000.1_6720147-6720672_Axis_porcinus_isolate_Zoohd001_scaffold_402978_whole_genome_shotgun_sequence
marine_NW_021703779.1_92933876-92934402_Monodon_monoceros_isolate_NGI_ecotype_Baffin_Bay_Population_unplaced_genomic_scaffold_NGI_Narwhal_1_Super_Scaffold_22
marine_VOSU01000035.1_94227803-94228329_Phocoena_sinus_isolate_mPhoSin1_chromosome_X_whole_genome_shotgun_sequence
R_marine_RJWO010088074.1_2780-3306_Inia_geoffrensis_isolate_BS63_IniGeo_flattened_line_175970_whole_genome_shotgun_sequence
R_marine_RJWK010090070.1_2881-3406_Platanista_minor_isolate_BS66_PlaMin_flattened_line_180032_whole_genome_shotgun_sequence
R_marine_RYZJ01001559.1_1139055-1139576_Megaptera_novaeangliae_breed_North_Atlantic_isolate_ASU_GRON-1_ScjyzU5_208_whole_genome_shotgun_sequence
marine_NIPP01009724.1_12722-13243_Eschrichtius_robustus_isolate_ER-14-168_kmer60-min500.11737556_whole_genome_shotgun_sequence
R_marine_RJWP010055446.1_2278-2804_Eubalaena_japonica_isolate_BS61_EubJap_flattened_line_110823_whole_genome_shotgun_sequence
marine_NC_041234.1_85281055-85281580_Physeter_catodon_isolate_SW-GA_chromosome_21_ASM283717v2
R_marine_RJWL010012486.1_24998-25524_Kogia_breviceps_isolate_BS64_KogBre_flattened_line_24967_whole_genome_shotgun_sequence
VSZR01000032.1_67601409-67601945_Camelus_ferus_isolate_YT-003-E_chromosome_X_whole_genome_shotgun_sequence
R_JARL01028150.1_122694-123230_Camelus_bactrianus_breed_Alxa_contig28150_whole_genome_shotgun_sequence
LSZX01064582.1_5152-5688_Camelus_dromedarius_breed_Targui_Contig_13237_47_whole_genome_shotgun_sequence
JEMW01001859.1_154858-155394_Vicugna_pacos_huacaya_contig1859_whole_genome_shotgun_sequence
R_SIDA01000014.1_35333500-35334033_Sus_scrofa_breed_Bama_miniature_chromosome_5_whole_genome_shotgun_sequence
R_PVHT021183648.1_19458192-19458729_Catagonus_wagneri_isolate_BS18_Sc2sB16_1185004HRSCAF_1231711_whole_genome_shotgun_sequence
R_PVJP02910466.1_4223451-4223973_Hippopotamus_amphibius_isolate_BS01_ScHSfme_924933HRSCAF_982733_whole_genome_shotgun_sequence
0.02

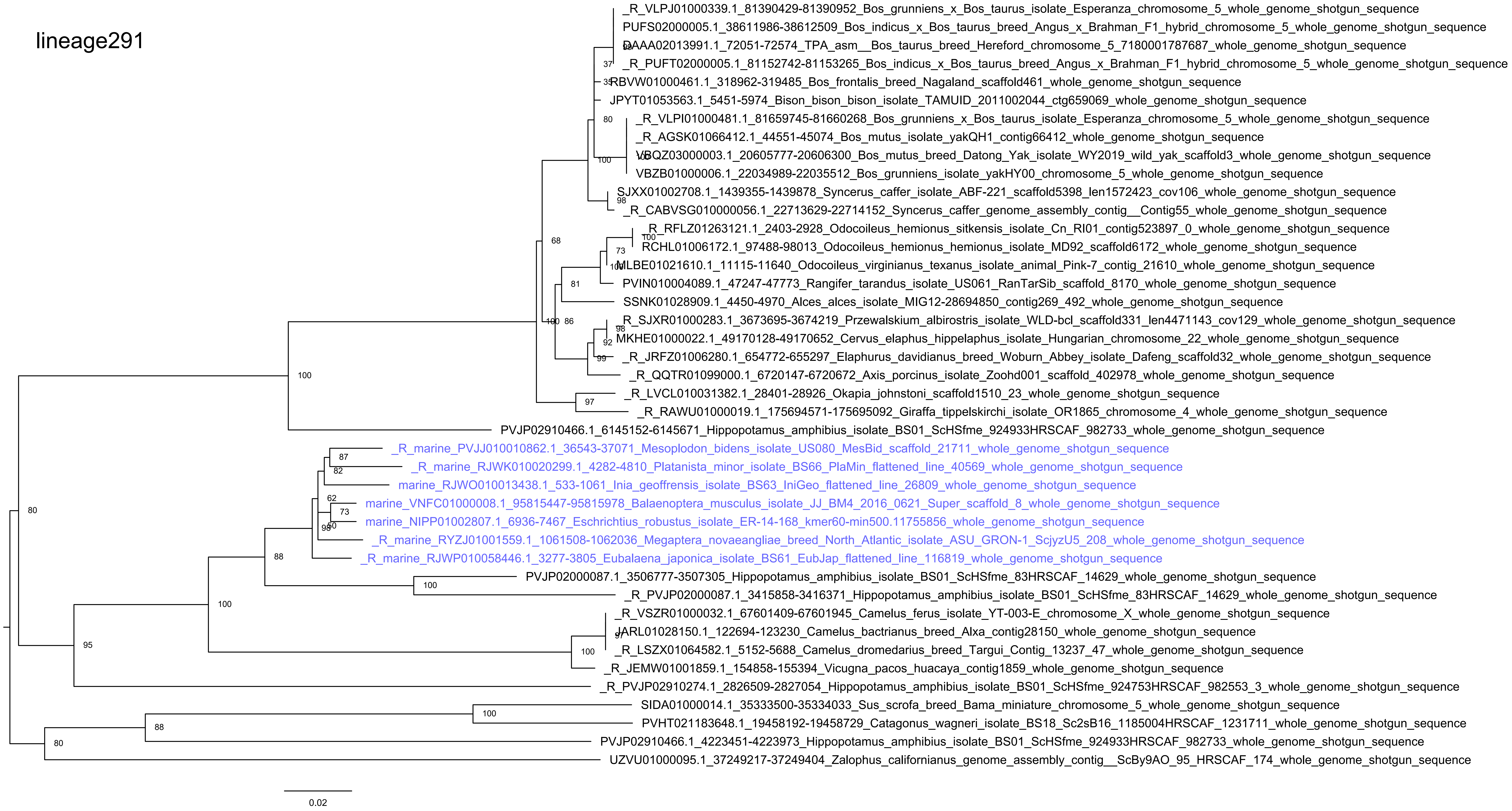
lineage291
_R_VLPJ01000339.1_81390429-81390952_Bos_grunniens_x_Bos_taurus_isolate_Esperanza_chromosome_5_whole_genome_shotgun_sequence
PUFS02000005.1_38611986-38612509_Bos_indicus_x_Bos_taurus_breed_Angus_x_Brahman_F1_hybrid_chromosome_5_whole_genome_shotgun_sequence
DAAA02013991.1_72051-72574_TPA_asm__Bos_taurus_breed_Hereford_chromosome_5_7180001787687_whole_genome_shotgun_sequence
_R_PUFT02000005.1_81152742-81153265_Bos_indicus_x_Bos_taurus_breed_Angus_x_Brahman_F1_hybrid_chromosome_5_whole_genome_shotgun_sequence
RBVW01000461.1_318962-319485_Bos_frontalis_breed_Nagaland_scaffold461_whole_genome_shotgun_sequence
JPYT01053563.1_5451-5974_Bison_bison_bison_isolate_TAMUID_2011002044_ctg659069_whole_genome_shotgun_sequence
_R_VLPI01000481.1_81659745-81660268_Bos_grunniens_x_Bos_taurus_isolate_Esperanza_chromosome_5_whole_genome_shotgun_sequence
_R_AGSK01066412.1_44551-45074_Bos_mutus_isolate_yakQH1_contig66412_whole_genome_shotgun_sequence
VBQZ03000003.1_20605777-20606300_Bos_mutus_breed_Datong_Yak_isolate_WY2019_wild_yak_scaffold3_whole_genome_shotgun_sequence
VBZB01000006.1_22034989-22035512_Bos_grunniens_isolate_yakHY00_chromosome_5_whole_genome_shotgun_sequence
SJXX01002708.1_1439355-1439878_Syncerus_caffer_isolate_ABF-221_scaffold5398_len1572423_cov106_whole_genome_shotgun_sequence
_R_CABVSG010000056.1_22713629-22714152_Syncerus_caffer_genome_assembly_contig__Contig55_whole_genome_shotgun_sequence
_R_RFLZ01263121.1_2403-2928_Odocoileus_hemionus_sitkensis_isolate_Cn_RI01_contig523897_0_whole_genome_shotgun_sequence
RCHL01006172.1_97488-98013_Odocoileus_hemionus_hemionus_isolate_MD92_scaffold6172_whole_genome_shotgun_sequence
MLBE01021610.1_11115-11640_Odocoileus_virginianus_texanus_isolate_animal_Pink-7_contig_21610_whole_genome_shotgun_sequence
PVIN010004089.1_47247-47773_Rangifer_tarandus_isolate_US061_RanTarSib_scaffold_8170_whole_genome_shotgun_sequence
SSNK01028909.1_4450-4970_Alces_alces_isolate_MIG12-28694850_contig269_492_whole_genome_shotgun_sequence
_R_SJXR01000283.1_3673695-3674219_Przewalskium_albirostris_isolate_WLD-bcl_scaffold331_len4471143_cov129_whole_genome_shotgun_sequence
MKHE01000022.1_49170128-49170652_Cervus_elaphus_hippelaphus_isolate_Hungarian_chromosome_22_whole_genome_shotgun_sequence
_R_JRFZ01006280.1_654772-655297_Elaphurus_davidianus_breed_Woburn_Abbey_isolate_Dafeng_scaffold32_whole_genome_shotgun_sequence
_R_QQTR01099000.1_6720147-6720672_Axis_porcinus_isolate_Zoohd001_scaffold_402978_whole_genome_shotgun_sequence
_R_LVCL010031382.1_28401-28926_Okapia_johnstoni_scaffold1510_23_whole_genome_shotgun_sequence
_R_RAWU01000019.1_175694571-175695092_Giraffa_tippelskirchi_isolate_OR1865_chromosome_4_whole_genome_shotgun_sequence
PVJP02910466.1_6145152-6145671_Hippopotamus_amphibius_isolate_BS01_ScHSfme_924933HRSCAF_982733_whole_genome_shotgun_sequence
_R_marine_PVJJ010010862.1_36543-37071_Mesoplodon_bidens_isolate_US080_MesBid_scaffold_21711_whole_genome_shotgun_sequence
_R_marine_RJWK010020299.1_4282-4810_Platanista_minor_isolate_BS66_PlaMin_flattened_line_40569_whole_genome_shotgun_sequence
marine_RJWO010013438.1_533-1061_Inia_geoffrensis_isolate_BS63_IniGeo_flattened_line_26809_whole_genome_shotgun_sequence
marine_VNFC01000008.1_95815447-95815978_Balaenoptera_musculus_isolate_JJ_BM4_2016_0621_Super_scaffold_8_whole_genome_shotgun_sequence
marine_NIPP01002807.1_6936-7467_Eschrichtius_robustus_isolate_ER-14-168_kmer60-min500.11755856_whole_genome_shotgun_sequence
_R_marine_RYZJ01001559.1_1061508-1062036_Megaptera_novaeangliae_breed_North_Atlantic_isolate_ASU_GRON-1_ScjyzU5_208_whole_genome_shotgun_sequence
_R_marine_RJWP010058446.1_3277-3805_Eubalaena_japonica_isolate_BS61_EubJap_flattened_line_116819_whole_genome_shotgun_sequence
PVJP02000087.1_3506777-3507305_Hippopotamus_amphibius_isolate_BS01_ScHSfme_83HRSCAF_14629_whole_genome_shotgun_sequence
_R_PVJP02000087.1_3415858-3416371_Hippopotamus_amphibius_isolate_BS01_ScHSfme_83HRSCAF_14629_whole_genome_shotgun_sequence
_R_VSZR01000032.1_67601409-67601945_Camelus_ferus_isolate_YT-003-E_chromosome_X_whole_genome_shotgun_sequence
JARL01028150.1_122694-123230_Camelus_bactrianus_breed_Alxa_contig28150_whole_genome_shotgun_sequence
_R_LSZX01064582.1_5152-5688_Camelus_dromedarius_breed_Targui_Contig_13237_47_whole_genome_shotgun_sequence
_R_JEMW01001859.1_154858-155394_Vicugna_pacos_huacaya_contig1859_whole_genome_shotgun_sequence
_R_PVJP02910274.1_2826509-2827054_Hippopotamus_amphibius_isolate_BS01_ScHSfme_924753HRSCAF_982553_3_whole_genome_shotgun_sequence
SIDA01000014.1_35333500-35334033_Sus_scrofa_breed_Bama_miniature_chromosome_5_whole_genome_shotgun_sequence
PVHT021183648.1_19458192-19458729_Catagonus_wagneri_isolate_BS18_Sc2sB16_1185004HRSCAF_1231711_whole_genome_shotgun_sequence
PVJP02910466.1_4223451-4223973_Hippopotamus_amphibius_isolate_BS01_ScHSfme_924933HRSCAF_982733_whole_genome_shotgun_sequence
UZVU01000095.1_37249217-37249404_Zalophus_californianus_genome_assembly_contig__ScBy9AO_95_HRSCAF_174_whole_genome_shotgun_sequence
0.02

lineage292

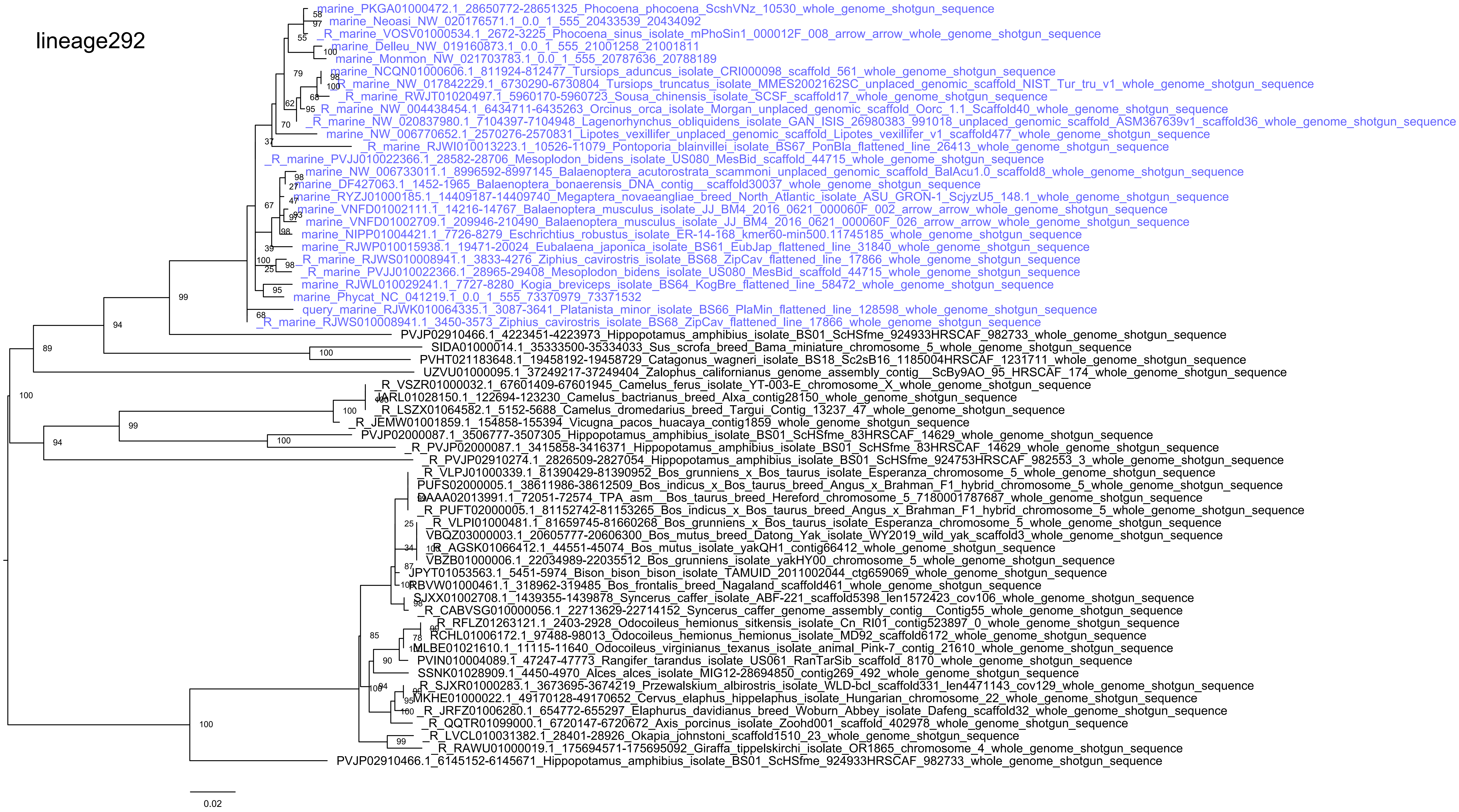
marine_PKGA01000472.1_28650772-28651325_Phocoena_phocoena_ScshVNz_10530_whole_genome_shotgun_sequence
marine_Neoasi_NW_020176571.1_0.0_1_555_20433539_20434092
_R_marine_VOSV01000534.1_2672-3225_Phocoena_sinus_isolate_mPhoSin1_000012F_008_arrow_arrow_whole_genome_shotgun_sequence
marine_Delleu_NW_019160873.1_0.0_1_555_21001258_21001811
marine_Monmon_NW_021703783.1_0.0_1_555_20787636_20788189
marine_NCQN01000606.1_811924-812477_Tursiops_aduncus_isolate_CRI000098_scaffold_561_whole_genome_shotgun_sequence
_R_marine_NW_017842229.1_6730290-6730804_Tursiops_truncatus_isolate_MMES2002162SC_unplaced_genomic_scaffold_NIST_Tur_tru_v1_whole_genome_shotgun_sequence
_R_marine_RWJT01020497.1_5960170-5960723_Sousa_chinensis_isolate_SCSF_scaffold17_whole_genome_shotgun_sequence
_R_marine_NW_004438454.1_6434711-6435263_Orcinus_orca_isolate_Morgan_unplaced_genomic_scaffold_Oorc_1.1_Scaffold40_whole_genome_shotgun_sequence
_R_marine_NW_020837980.1_7104397-7104948_Lagenorhynchus_obliquidens_isolate_GAN_ISIS_26980383_991018_unplaced_genomic_scaffold_ASM367639v1_scaffold36_whole_genome_shotgun_sequence
marine_NW_006770652.1_2570276-2570831_Lipotes_vexillifer_unplaced_genomic_scaffold_Lipotes_vexillifer_v1_scaffold477_whole_genome_shotgun_sequence
_R_marine_RJWI010013223.1_10526-11079_Pontoporia_blainvillei_isolate_BS67_PonBla_flattened_line_26413_whole_genome_shotgun_sequence
_R_marine_PVJJ010022366.1_28582-28706_Mesoplodon_bidens_isolate_US080_MesBid_scaffold_44715_whole_genome_shotgun_sequence
marine_NW_006733011.1_8996592-8997145_Balaenoptera_acutorostrata_scammoni_unplaced_genomic_scaffold_BalAcu1.0_scaffold8_whole_genome_shotgun_sequence
marine_DF427063.1_1452-1965_Balaenoptera_bonaerensis_DNA_contig__scaffold30037_whole_genome_shotgun_sequence
marine_RYZJ01000185.1_14409187-14409740_Megaptera_novaeangliae_breed_North_Atlantic_isolate_ASU_GRON-1_ScjyzU5_148.1_whole_genome_shotgun_sequence
marine_VNFD01002111.1_14216-14767_Balaenoptera_musculus_isolate_JJ_BM4_2016_0621_000060F_002_arrow_arrow_whole_genome_shotgun_sequence
marine_VNFD01002709.1_209946-210490_Balaenoptera_musculus_isolate_JJ_BM4_2016_0621_000060F_026_arrow_arrow_whole_genome_shotgun_sequence
marine_NIPP01004421.1_7726-8279_Eschrichtius_robustus_isolate_ER-14-168_kmer60-min500.11745185_whole_genome_shotgun_sequence
marine_RJWP010015938.1_19471-20024_Eubalaena_japonica_isolate_BS61_EubJap_flattened_line_31840_whole_genome_shotgun_sequence
_R_marine_RJWS010008941.1_3833-4276_Ziphius_cavirostris_isolate_BS68_ZipCav_flattened_line_17866_whole_genome_shotgun_sequence
_R_marine_PVJJ010022366.1_28965-29408_Mesoplodon_bidens_isolate_US080_MesBid_scaffold_44715_whole_genome_shotgun_sequence
marine_RJWL010029241.1_7727-8280_Kogia_breviceps_isolate_BS64_KogBre_flattened_line_58472_whole_genome_shotgun_sequence
marine_Phycat_NC_041219.1_0.0_1_555_73370979_73371532
query_marine_RJWK010064335.1_3087-3641_Platanista_minor_isolate_BS66_PlaMin_flattened_line_128598_whole_genome_shotgun_sequence
_R_marine_RJWS010008941.1_3450-3573_Ziphius_cavirostris_isolate_BS68_ZipCav_flattened_line_17866_whole_genome_shotgun_sequence
PVJP02910466.1_4223451-4223973_Hippopotamus_amphibius_isolate_BS01_ScHSfme_924933HRSCAF_982733_whole_genome_shotgun_sequence
SIDA01000014.1_35333500-35334033_Sus_scrofa_breed_Bama_miniature_chromosome_5_whole_genome_shotgun_sequence
PVHT021183648.1_19458192-19458729_Catagonus_wagneri_isolate_BS18_Sc2sB16_1185004HRSCAF_1231711_whole_genome_shotgun_sequence
UZVU01000095.1_37249217-37249404_Zalophus_californianus_genome_assembly_contig__ScBy9AO_95_HRSCAF_174_whole_genome_shotgun_sequence
_R_VSZR01000032.1_67601409-67601945_Camelus_ferus_isolate_YT-003-E_chromosome_X_whole_genome_shotgun_sequence
JARL01028150.1_122694-123230_Camelus_bactrianus_breed_Alxa_contig28150_whole_genome_shotgun_sequence
_R_LSZX01064582.1_5152-5688_Camelus_dromedarius_breed_Targui_Contig_13237_47_whole_genome_shotgun_sequence
_R_JEMW01001859.1_154858-155394_Vicugna_pacos_huacaya_contig1859_whole_genome_shotgun_sequence
PVJP02000087.1_3506777-3507305_Hippopotamus_amphibius_isolate_BS01_ScHSfme_83HRSCAF_14629_whole_genome_shotgun_sequence
_R_PVJP02000087.1_3415858-3416371_Hippopotamus_amphibius_isolate_BS01_ScHSfme_83HRSCAF_14629_whole_genome_shotgun_sequence
_R_PVJP02910274.1_2826509-2827054_Hippopotamus_amphibius_isolate_BS01_ScHSfme_924753HRSCAF_982553_3_whole_genome_shotgun_sequence
_R_VLPJ01000339.1_81390429-81390952_Bos_grunniens_x_Bos_taurus_isolate_Esperanza_chromosome_5_whole_genome_shotgun_sequence
PUFS02000005.1_38611986-38612509_Bos_indicus_x_Bos_taurus_breed_Angus_x_Brahman_F1_hybrid_chromosome_5_whole_genome_shotgun_sequence
DAAA02013991.1_72051-72574_TPA_asm__Bos_taurus_breed_Hereford_chromosome_5_7180001787687_whole_genome_shotgun_sequence
_R_PUFT02000005.1_81152742-81153265_Bos_indicus_x_Bos_taurus_breed_Angus_x_Brahman_F1_hybrid_chromosome_5_whole_genome_shotgun_sequence
_R_VLPI01000481.1_81659745-81660268_Bos_grunniens_x_Bos_taurus_isolate_Esperanza_chromosome_5_whole_genome_shotgun_sequence
VBQZ03000003.1_20605777-20606300_Bos_mutus_breed_Datong_Yak_isolate_WY2019_wild_yak_scaffold3_whole_genome_shotgun_sequence
_R_AGSK01066412.1_44551-45074_Bos_mutus_isolate_yakQH1_contig66412_whole_genome_shotgun_sequence
VBZB01000006.1_22034989-22035512_Bos_grunniens_isolate_yakHY00_chromosome_5_whole_genome_shotgun_sequence
JPYT01053563.1_5451-5974_Bison_bison_bison_isolate_TAMUID_2011002044_ctg659069_whole_genome_shotgun_sequence
RBVW01000461.1_318962-319485_Bos_frontalis_breed_Nagaland_scaffold461_whole_genome_shotgun_sequence
SJXX01002708.1_1439355-1439878_Syncerus_caffer_isolate_ABF-221_scaffold5398_len1572423_cov106_whole_genome_shotgun_sequence
_R_CABVSG010000056.1_22713629-22714152_Syncerus_caffer_genome_assembly_contig__Contig55_whole_genome_shotgun_sequence
_R_RFLZ01263121.1_2403-2928_Odocoileus_hemionus_sitkensis_isolate_Cn_RI01_contig523897_0_whole_genome_shotgun_sequence
RCHL01006172.1_97488-98013_Odocoileus_hemionus_hemionus_isolate_MD92_scaffold6172_whole_genome_shotgun_sequence
MLBE01021610.1_11115-11640_Odocoileus_virginianus_texanus_isolate_animal_Pink-7_contig_21610_whole_genome_shotgun_sequence
PVIN010004089.1_47247-47773_Rangifer_tarandus_isolate_US061_RanTarSib_scaffold_8170_whole_genome_shotgun_sequence
SSNK01028909.1_4450-4970_Alces_alces_isolate_MIG12-28694850_contig269_492_whole_genome_shotgun_sequence
_R_SJXR01000283.1_3673695-3674219_Przewalskium_albirostris_isolate_WLD-bcl_scaffold331_len4471143_cov129_whole_genome_shotgun_sequence
MKHE01000022.1_49170128-49170652_Cervus_elaphus_hippelaphus_isolate_Hungarian_chromosome_22_whole_genome_shotgun_sequence
_R_JRFZ01006280.1_654772-655297_Elaphurus_davidianus_breed_Woburn_Abbey_isolate_Dafeng_scaffold32_whole_genome_shotgun_sequence
_R_QQTR01099000.1_6720147-6720672_Axis_porcinus_isolate_Zoohd001_scaffold_402978_whole_genome_shotgun_sequence
_R_LVCL010031382.1_28401-28926_Okapia_johnstoni_scaffold1510_23_whole_genome_shotgun_sequence
_R_RAWU01000019.1_175694571-175695092_Giraffa_tippelskirchi_isolate_OR1865_chromosome_4_whole_genome_shotgun_sequence
PVJP02910466.1_6145152-6145671_Hippopotamus_amphibius_isolate_BS01_ScHSfme_924933HRSCAF_982733_whole_genome_shotgun_sequence
0.02

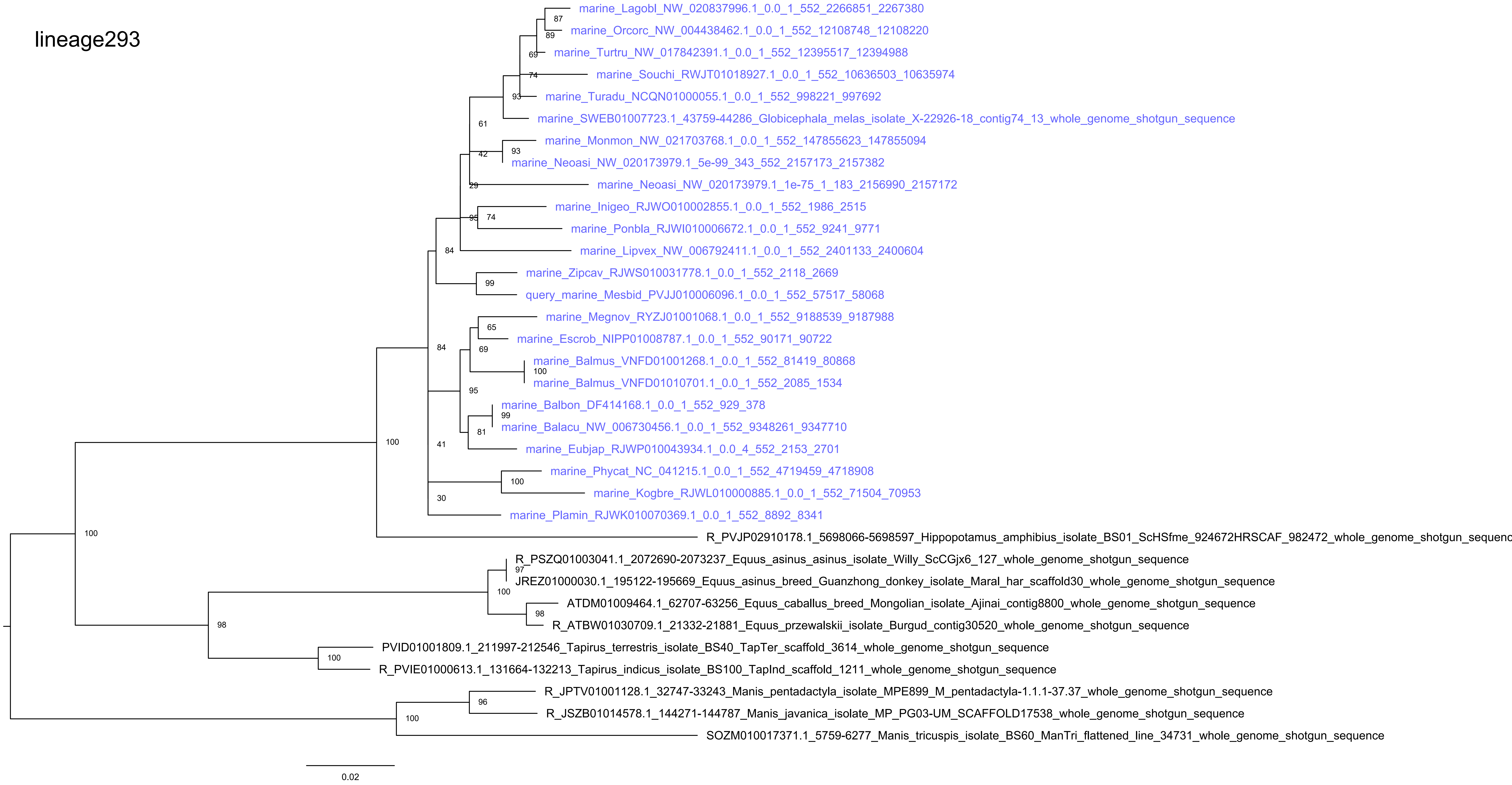
lineage293
marine_Lagobl_NW_020837996.1_0.0_1_552_2266851_2267380
marine_Orcorc_NW_004438462.1_0.0_1_552_12108748_12108220
marine_Turtru_NW_017842391.1_0.0_1_552_12395517_12394988
marine_Souchi_RWJT01018927.1_0.0_1_552_10636503_10635974
marine_Turadu_NCQN01000055.1_0.0_1_552_998221_997692
marine_SWEB01007723.1_43759-44286_Globicephala_melas_isolate_X-22926-18_contig74_13_whole_genome_shotgun_sequence
marine_Monmon_NW_021703768.1_0.0_1_552_147855623_147855094
marine_Neoasi_NW_020173979.1_5e-99_343_552_2157173_2157382
marine_Neoasi_NW_020173979.1_1e-75_1_183_2156990_2157172
marine_Inigeo_RJWO010002855.1_0.0_1_552_1986_2515
marine_Ponbla_RJWI010006672.1_0.0_1_552_9241_9771
marine_Lipvex_NW_006792411.1_0.0_1_552_2401133_2400604
marine_Zipcav_RJWS010031778.1_0.0_1_552_2118_2669
query_marine_Mesbid_PVJJ010006096.1_0.0_1_552_57517_58068
marine_Megnov_RYZJ01001068.1_0.0_1_552_9188539_9187988
marine_Escrob_NIPP01008787.1_0.0_1_552_90171_90722
marine_Balmus_VNFD01001268.1_0.0_1_552_81419_80868
marine_Balmus_VNFD01010701.1_0.0_1_552_2085_1534
marine_Balbon_DF414168.1_0.0_1_552_929_378
marine_Balacu_NW_006730456.1_0.0_1_552_9348261_9347710
marine_Eubjap_RJWP010043934.1_0.0_4_552_2153_2701
marine_Phycat_NC_041215.1_0.0_1_552_4719459_4718908
marine_Kogbre_RJWL010000885.1_0.0_1_552_71504_70953
marine_Plamin_RJWK010070369.1_0.0_1_552_8892_8341
R_PVJP02910178.1_5698066-5698597_Hippopotamus_amphibius_isolate_BS01_ScHSfme_924672HRSCAF_982472_whole_genome_shotgun_sequenc
R_PSZQ01003041.1_2072690-2073237_Equus_asinus_asinus_isolate_Willy_ScCGjx6_127_whole_genome_shotgun_sequence
JREZ01000030.1_195122-195669_Equus_asinus_breed_Guanzhong_donkey_isolate_Maral_har_scaffold30_whole_genome_shotgun_sequence
ATDM01009464.1_62707-63256_Equus_caballus_breed_Mongolian_isolate_Ajinai_contig8800_whole_genome_shotgun_sequence
R_ATBW01030709.1_21332-21881_Equus_przewalskii_isolate_Burgud_contig30520_whole_genome_shotgun_sequence
PVID01001809.1_211997-212546_Tapirus_terrestris_isolate_BS40_TapTer_scaffold_3614_whole_genome_shotgun_sequence
R_PVIE01000613.1_131664-132213_Tapirus_indicus_isolate_BS100_TapInd_scaffold_1211_whole_genome_shotgun_sequence
R_JPTV01001128.1_32747-33243_Manis_pentadactyla_isolate_MPE899_M_pentadactyla-1.1.1-37.37_whole_genome_shotgun_sequence
R_JSZB01014578.1_144271-144787_Manis_javanica_isolate_MP_PG03-UM_SCAFFOLD17538_whole_genome_shotgun_sequence
SOZM010017371.1_5759-6277_Manis_tricuspis_isolate_BS60_ManTri_flattened_line_34731_whole_genome_shotgun_sequence
0.02

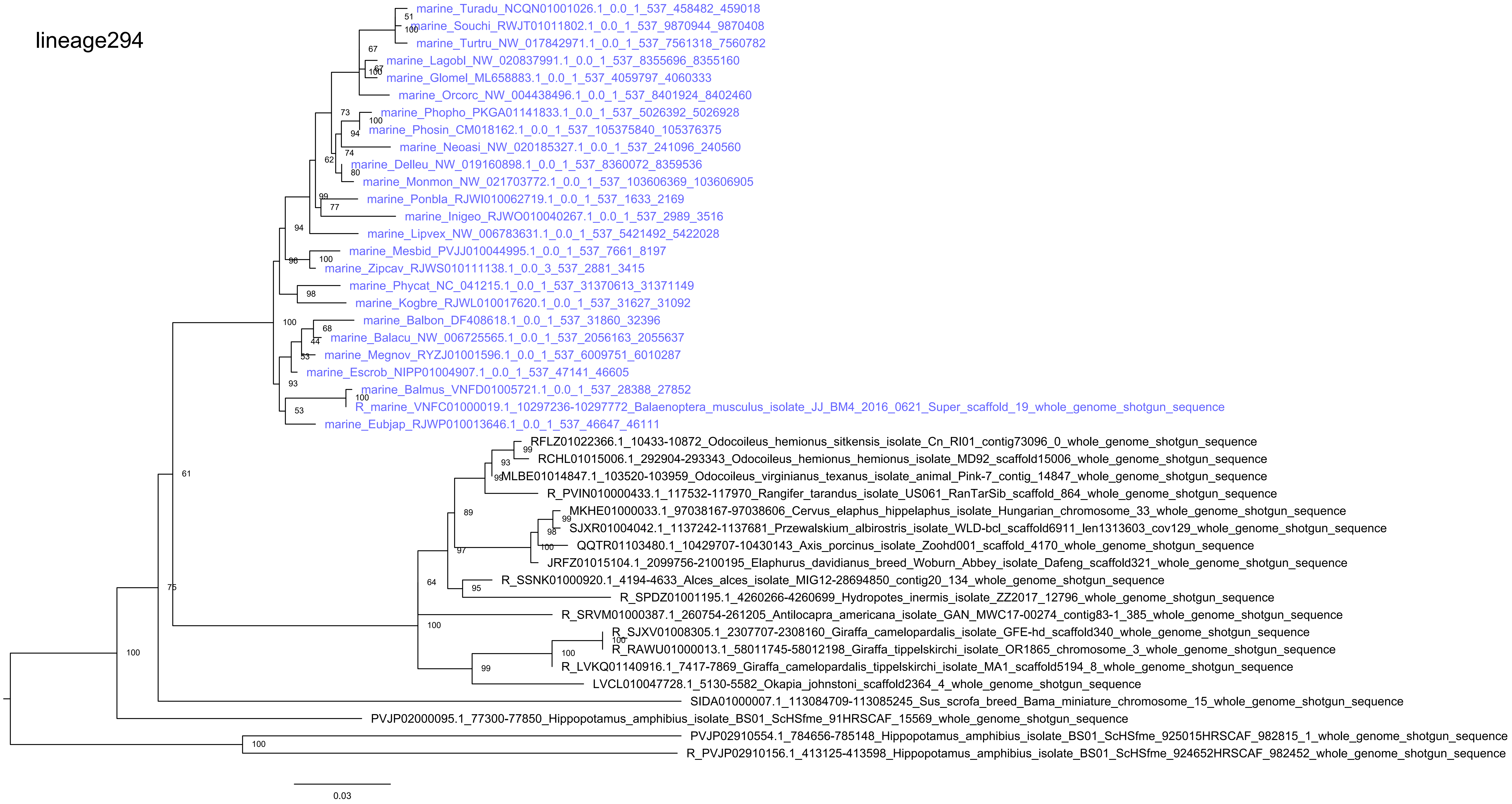
lineage294
marine_Turadu_NCQN01001026.1_0.0_1_537_458482_459018
marine_Souchi_RWJT01011802.1_0.0_1_537_9870944_9870408
marine_Turtru_NW_017842971.1_0.0_1_537_7561318_7560782
marine_Lagobl_NW_020837991.1_0.0_1_537_8355696_8355160
marine_Glomel_ML658883.1_0.0_1_537_4059797_4060333
marine_Orcorc_NW_004438496.1_0.0_1_537_8401924_8402460
marine_Phopho_PKGA01141833.1_0.0_1_537_5026392_5026928
marine_Phosin_CM018162.1_0.0_1_537_105375840_105376375
marine_Neoasi_NW_020185327.1_0.0_1_537_241096_240560
marine_Delleu_NW_019160898.1_0.0_1_537_8360072_8359536
marine_Monmon_NW_021703772.1_0.0_1_537_103606369_103606905
marine_Ponbla_RJWI010062719.1_0.0_1_537_1633_2169
marine_Inigeo_RJWO010040267.1_0.0_1_537_2989_3516
marine_Lipvex_NW_006783631.1_0.0_1_537_5421492_5422028
marine_Mesbid_PVJJ010044995.1_0.0_1_537_7661_8197
marine_Zipcav_RJWS010111138.1_0.0_3_537_2881_3415
marine_Phycat_NC_041215.1_0.0_1_537_31370613_31371149
marine_Kogbre_RJWL010017620.1_0.0_1_537_31627_31092
marine_Balbon_DF408618.1_0.0_1_537_31860_32396
marine_Balacu_NW_006725565.1_0.0_1_537_2056163_2055637
marine_Megnov_RYZJ01001596.1_0.0_1_537_6009751_6010287
marine_Escrob_NIPP01004907.1_0.0_1_537_47141_46605
marine_Balmus_VNFD01005721.1_0.0_1_537_28388_27852
R_marine_VNFC01000019.1_10297236-10297772_Balaenoptera_musculus_isolate_JJ_BM4_2016_0621_Super_scaffold_19_whole_genome_shotgun_sequence
marine_Eubjap_RJWP010013646.1_0.0_1_537_46647_46111
RFLZ01022366.1_10433-10872_Odocoileus_hemionus_sitkensis_isolate_Cn_RI01_contig73096_0_whole_genome_shotgun_sequence
RCHL01015006.1_292904-293343_Odocoileus_hemionus_hemionus_isolate_MD92_scaffold15006_whole_genome_shotgun_sequence
MLBE01014847.1_103520-103959_Odocoileus_virginianus_texanus_isolate_animal_Pink-7_contig_14847_whole_genome_shotgun_sequence
R_PVIN010000433.1_117532-117970_Rangifer_tarandus_isolate_US061_RanTarSib_scaffold_864_whole_genome_shotgun_sequence
MKHE01000033.1_97038167-97038606_Cervus_elaphus_hippelaphus_isolate_Hungarian_chromosome_33_whole_genome_shotgun_sequence
SJXR01004042.1_1137242-1137681_Przewalskium_albirostris_isolate_WLD-bcl_scaffold6911_len1313603_cov129_whole_genome_shotgun_sequence
QQTR01103480.1_10429707-10430143_Axis_porcinus_isolate_Zoohd001_scaffold_4170_whole_genome_shotgun_sequence
JRFZ01015104.1_2099756-2100195_Elaphurus_davidianus_breed_Woburn_Abbey_isolate_Dafeng_scaffold321_whole_genome_shotgun_sequence
R_SSNK01000920.1_4194-4633_Alces_alces_isolate_MIG12-28694850_contig20_134_whole_genome_shotgun_sequence
R_SPDZ01001195.1_4260266-4260699_Hydropotes_inermis_isolate_ZZ2017_12796_whole_genome_shotgun_sequence
R_SRVM01000387.1_260754-261205_Antilocapra_americana_isolate_GAN_MWC17-00274_contig83-1_385_whole_genome_shotgun_sequence
R_SJXV01008305.1_2307707-2308160_Giraffa_camelopardalis_isolate_GFE-hd_scaffold340_whole_genome_shotgun_sequence
R_RAWU01000013.1_58011745-58012198_Giraffa_tippelskirchi_isolate_OR1865_chromosome_3_whole_genome_shotgun_sequence
R_LVKQ01140916.1_7417-7869_Giraffa_camelopardalis_tippelskirchi_isolate_MA1_scaffold5194_8_whole_genome_shotgun_sequence
LVCL010047728.1_5130-5582_Okapia_johnstoni_scaffold2364_4_whole_genome_shotgun_sequence
SIDA01000007.1_113084709-113085245_Sus_scrofa_breed_Bama_miniature_chromosome_15_whole_genome_shotgun_sequence
PVJP02000095.1_77300-77850_Hippopotamus_amphibius_isolate_BS01_ScHSfme_91HRSCAF_15569_whole_genome_shotgun_sequence
PVJP02910554.1_784656-785148_Hippopotamus_amphibius_isolate_BS01_ScHSfme_925015HRSCAF_982815_1_whole_genome_shotgun_sequence
R_PVJP02910156.1_413125-413598_Hippopotamus_amphibius_isolate_BS01_ScHSfme_924652HRSCAF_982452_whole_genome_shotgun_sequence
0.03

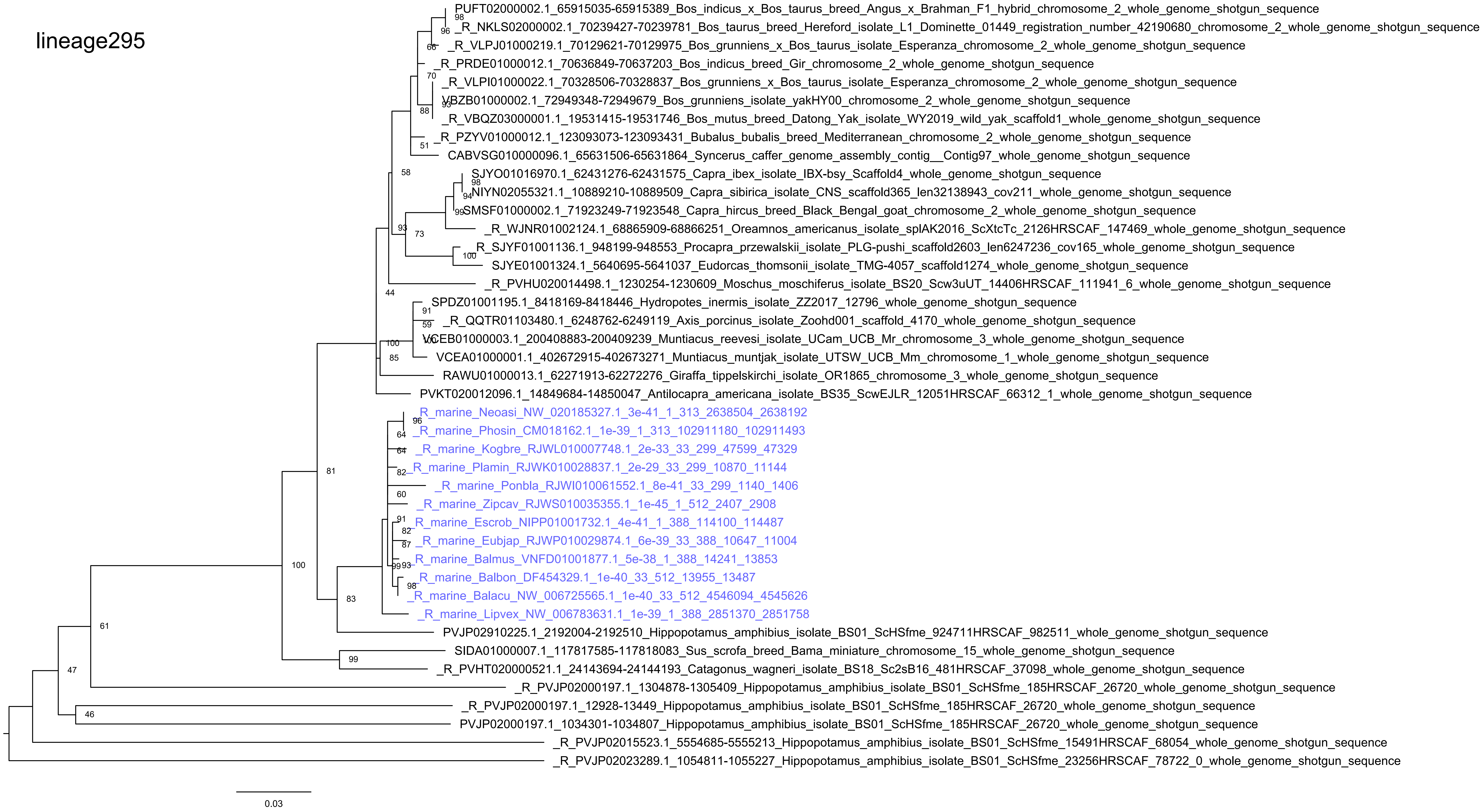
lineage295
PUFT02000002.1_65915035-65915389_Bos_indicus_x_Bos_taurus_breed_Angus_x_Brahman_F1_hybrid_chromosome_2_whole_genome_shotgun_sequence
_R_NKLS02000002.1_70239427-70239781_Bos_taurus_breed_Hereford_isolate_L1_Dominette_01449_registration_number_42190680_chromosome_2_whole_genome_shotgun_sequence
_R_VLPJ01000219.1_70129621-70129975_Bos_grunniens_x_Bos_taurus_isolate_Esperanza_chromosome_2_whole_genome_shotgun_sequence
_R_PRDE01000012.1_70636849-70637203_Bos_indicus_breed_Gir_chromosome_2_whole_genome_shotgun_sequence
_R_VLPI01000022.1_70328506-70328837_Bos_grunniens_x_Bos_taurus_isolate_Esperanza_chromosome_2_whole_genome_shotgun_sequence
VBZB01000002.1_72949348-72949679_Bos_grunniens_isolate_yakHY00_chromosome_2_whole_genome_shotgun_sequence
_R_VBQZ03000001.1_19531415-19531746_Bos_mutus_breed_Datong_Yak_isolate_WY2019_wild_yak_scaffold1_whole_genome_shotgun_sequence
_R_PZYV01000012.1_123093073-123093431_Bubalus_bubalis_breed_Mediterranean_chromosome_2_whole_genome_shotgun_sequence
CABVSG010000096.1_65631506-65631864_Syncerus_caffer_genome_assembly_contig__Contig97_whole_genome_shotgun_sequence
SJYO01016970.1_62431276-62431575_Capra_ibex_isolate_IBX-bsy_Scaffold4_whole_genome_shotgun_sequence
NIYN02055321.1_10889210-10889509_Capra_sibirica_isolate_CNS_scaffold365_len32138943_cov211_whole_genome_shotgun_sequence
SMSF01000002.1_71923249-71923548_Capra_hircus_breed_Black_Bengal_goat_chromosome_2_whole_genome_shotgun_sequence
_R_WJNR01002124.1_68865909-68866251_Oreamnos_americanus_isolate_splAK2016_ScXtcTc_2126HRSCAF_147469_whole_genome_shotgun_sequence
_R_SJYF01001136.1_948199-948553_Procapra_przewalskii_isolate_PLG-pushi_scaffold2603_len6247236_cov165_whole_genome_shotgun_sequence
SJYE01001324.1_5640695-5641037_Eudorcas_thomsonii_isolate_TMG-4057_scaffold1274_whole_genome_shotgun_sequence
_R_PVHU020014498.1_1230254-1230609_Moschus_moschiferus_isolate_BS20_Scw3uUT_14406HRSCAF_111941_6_whole_genome_shotgun_sequence
SPDZ01001195.1_8418169-8418446_Hydropotes_inermis_isolate_ZZ2017_12796_whole_genome_shotgun_sequence
_R_QQTR01103480.1_6248762-6249119_Axis_porcinus_isolate_Zoohd001_scaffold_4170_whole_genome_shotgun_sequence
VCEB01000003.1_200408883-200409239_Muntiacus_reevesi_isolate_UCam_UCB_Mr_chromosome_3_whole_genome_shotgun_sequence
VCEA01000001.1_402672915-402673271_Muntiacus_muntjak_isolate_UTSW_UCB_Mm_chromosome_1_whole_genome_shotgun_sequence
RAWU01000013.1_62271913-62272276_Giraffa_tippelskirchi_isolate_OR1865_chromosome_3_whole_genome_shotgun_sequence
PVKT020012096.1_14849684-14850047_Antilocapra_americana_isolate_BS35_ScwEJLR_12051HRSCAF_66312_1_whole_genome_shotgun_sequence
_R_marine_Neoasi_NW_020185327.1_3e-41_1_313_2638504_2638192
_R_marine_Phosin_CM018162.1_1e-39_1_313_102911180_102911493
_R_marine_Kogbre_RJWL010007748.1_2e-33_33_299_47599_47329
_R_marine_Plamin_RJWK010028837.1_2e-29_33_299_10870_11144
_R_marine_Ponbla_RJWI010061552.1_8e-41_33_299_1140_1406
_R_marine_Zipcav_RJWS010035355.1_1e-45_1_512_2407_2908
_R_marine_Escrob_NIPP01001732.1_4e-41_1_388_114100_114487
_R_marine_Eubjap_RJWP010029874.1_6e-39_33_388_10647_11004
_R_marine_Balmus_VNFD01001877.1_5e-38_1_388_14241_13853
_R_marine_Balbon_DF454329.1_1e-40_33_512_13955_13487
_R_marine_Balacu_NW_006725565.1_1e-40_33_512_4546094_4545626
_R_marine_Lipvex_NW_006783631.1_1e-39_1_388_2851370_2851758
PVJP02910225.1_2192004-2192510_Hippopotamus_amphibius_isolate_BS01_ScHSfme_924711HRSCAF_982511_whole_genome_shotgun_sequence
SIDA01000007.1_117817585-117818083_Sus_scrofa_breed_Bama_miniature_chromosome_15_whole_genome_shotgun_sequence
_R_PVHT020000521.1_24143694-24144193_Catagonus_wagneri_isolate_BS18_Sc2sB16_481HRSCAF_37098_whole_genome_shotgun_sequence
_R_PVJP02000197.1_1304878-1305409_Hippopotamus_amphibius_isolate_BS01_ScHSfme_185HRSCAF_26720_whole_genome_shotgun_sequence
_R_PVJP02000197.1_12928-13449_Hippopotamus_amphibius_isolate_BS01_ScHSfme_185HRSCAF_26720_whole_genome_shotgun_sequence
PVJP02000197.1_1034301-1034807_Hippopotamus_amphibius_isolate_BS01_ScHSfme_185HRSCAF_26720_whole_genome_shotgun_sequence
_R_PVJP02015523.1_5554685-5555213_Hippopotamus_amphibius_isolate_BS01_ScHSfme_15491HRSCAF_68054_whole_genome_shotgun_sequence
_R_PVJP02023289.1_1054811-1055227_Hippopotamus_amphibius_isolate_BS01_ScHSfme_23256HRSCAF_78722_0_whole_genome_shotgun_sequence
0.03

lineage296

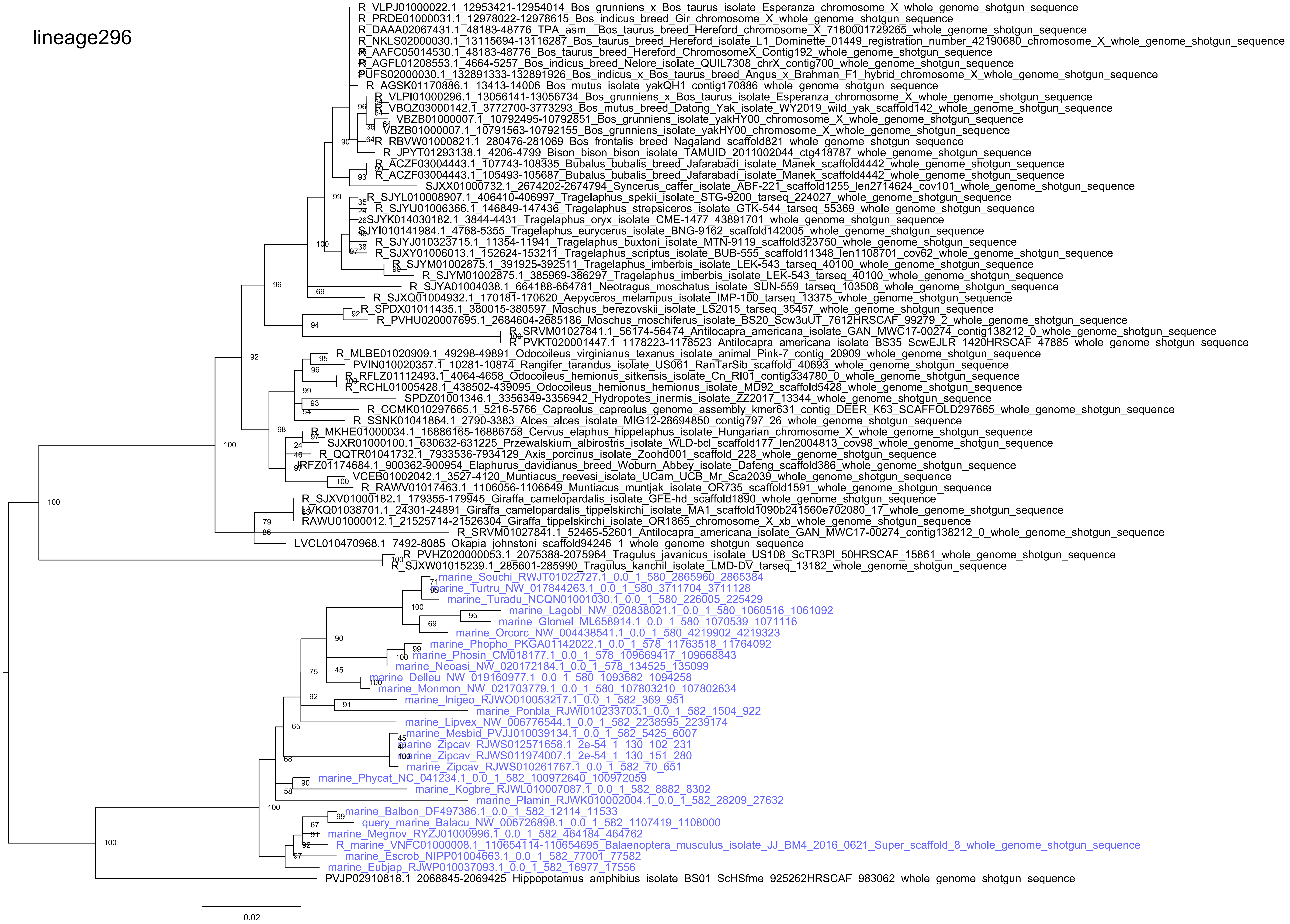
R_VLPJ01000022.1_12953421-12954014_Bos_grunniens_x_Bos_taurus_isolate_Esperanza_chromosome_X_whole_genome_shotgun_sequence
R_PRDE01000031.1_12978022-12978615_Bos_indicus_breed_Gir_chromosome_X_whole_genome_shotgun_sequence
R_DAAA02067431.1_48183-48776_TPA_asm__Bos_taurus_breed_Hereford_chromosome_X_7180001729265_whole_genome_shotgun_sequence
R_NKLS02000030.1_13115694-13116287_Bos_taurus_breed_Hereford_isolate_L1_Dominette_01449_registration_number_42190680_chromosome_X_whole_genome_shotgun_sequence
R_AAFC05014530.1_48183-48776_Bos_taurus_breed_Hereford_ChromosomeX_Contig192_whole_genome_shotgun_sequence
R_AGFL01208553.1_4664-5257_Bos_indicus_breed_Nelore_isolate_QUIL7308_chrX_contig700_whole_genome_shotgun_sequence
PUFS02000030.1_132891333-132891926_Bos_indicus_x_Bos_taurus_breed_Angus_x_Brahman_F1_hybrid_chromosome_X_whole_genome_shotgun_sequence
R_AGSK01170886.1_13413-14006_Bos_mutus_isolate_yakQH1_contig170886_whole_genome_shotgun_sequence
R_VLPI01000296.1_13056141-13056734_Bos_grunniens_x_Bos_taurus_isolate_Esperanza_chromosome_X_whole_genome_shotgun_sequence
R_VBQZ03000142.1_3772700-3773293_Bos_mutus_breed_Datong_Yak_isolate_WY2019_wild_yak_scaffold142_whole_genome_shotgun_sequence
VBZB01000007.1_10792495-10792851_Bos_grunniens_isolate_yakHY00_chromosome_X_whole_genome_shotgun_sequence
VBZB01000007.1_10791563-10792155_Bos_grunniens_isolate_yakHY00_chromosome_X_whole_genome_shotgun_sequence
R_RBVW01000821.1_280476-281069_Bos_frontalis_breed_Nagaland_scaffold821_whole_genome_shotgun_sequence
R_JPYT01293138.1_4206-4799_Bison_bison_bison_isolate_TAMUID_2011002044_ctg418787_whole_genome_shotgun_sequence
R_ACZF03004443.1_107743-108335_Bubalus_bubalis_breed_Jafarabadi_isolate_Manek_scaffold4442_whole_genome_shotgun_sequence
R_ACZF03004443.1_105493-105687_Bubalus_bubalis_breed_Jafarabadi_isolate_Manek_scaffold4442_whole_genome_shotgun_sequence
SJXX01000732.1_2674202-2674794_Syncerus_caffer_isolate_ABF-221_scaffold1255_len2714624_cov101_whole_genome_shotgun_sequence
R_SJYL010008907.1_406410-406997_Tragelaphus_spekii_isolate_STG-9200_tarseq_224027_whole_genome_shotgun_sequence
R_SJYU01006366.1_146849-147436_Tragelaphus_strepsiceros_isolate_GTK-544_tarseq_55369_whole_genome_shotgun_sequence
SJYK014030182.1_3844-4431_Tragelaphus_oryx_isolate_CME-1477_43891701_whole_genome_shotgun_sequence
SJYI010141984.1_4768-5355_Tragelaphus_eurycerus_isolate_BNG-9162_scaffold142005_whole_genome_shotgun_sequence
R_SJYJ010323715.1_11354-11941_Tragelaphus_buxtoni_isolate_MTN-9119_scaffold323750_whole_genome_shotgun_sequence
R_SJXY01006013.1_152624-153211_Tragelaphus_scriptus_isolate_BUB-555_scaffold11348_len1108701_cov62_whole_genome_shotgun_sequence
R_SJYM01002875.1_391925-392511_Tragelaphus_imberbis_isolate_LEK-543_tarseq_40100_whole_genome_shotgun_sequence
R_SJYM01002875.1_385969-386297_Tragelaphus_imberbis_isolate_LEK-543_tarseq_40100_whole_genome_shotgun_sequence
R_SJYA01004038.1_664188-664781_Neotragus_moschatus_isolate_SUN-559_tarseq_103508_whole_genome_shotgun_sequence
R_SJXQ01004932.1_170181-170620_Aepyceros_melampus_isolate_IMP-100_tarseq_13375_whole_genome_shotgun_sequence
R_SPDX01011435.1_380015-380597_Moschus_berezovskii_isolate_LS2015_tarseq_35457_whole_genome_shotgun_sequence
R_PVHU020007695.1_2684604-2685186_Moschus_moschiferus_isolate_BS20_Scw3uUT_7612HRSCAF_99279_2_whole_genome_shotgun_sequence
R_SRVM01027841.1_56174-56474_Antilocapra_americana_isolate_GAN_MWC17-00274_contig138212_0_whole_genome_shotgun_sequence
R_PVKT020001447.1_1178223-1178523_Antilocapra_americana_isolate_BS35_ScwEJLR_1420HRSCAF_47885_whole_genome_shotgun_sequence
R_MLBE01020909.1_49298-49891_Odocoileus_virginianus_texanus_isolate_animal_Pink-7_contig_20909_whole_genome_shotgun_sequence
PVIN010020357.1_10281-10874_Rangifer_tarandus_isolate_US061_RanTarSib_scaffold_40693_whole_genome_shotgun_sequence
R_RFLZ01112493.1_4064-4658_Odocoileus_hemionus_sitkensis_isolate_Cn_RI01_contig334780_0_whole_genome_shotgun_sequence
R_RCHL01005428.1_438502-439095_Odocoileus_hemionus_hemionus_isolate_MD92_scaffold5428_whole_genome_shotgun_sequence
SPDZ01001346.1_3356349-3356942_Hydropotes_inermis_isolate_ZZ2017_13344_whole_genome_shotgun_sequence
R_CCMK010297665.1_5216-5766_Capreolus_capreolus_genome_assembly_kmer631_contig_DEER_K63_SCAFFOLD297665_whole_genome_shotgun_sequence
R_SSNK01041864.1_2790-3383_Alces_alces_isolate_MIG12-28694850_contig797_26_whole_genome_shotgun_sequence
R_MKHE01000034.1_16886165-16886758_Cervus_elaphus_hippelaphus_isolate_Hungarian_chromosome_X_whole_genome_shotgun_sequence
SJXR01000100.1_630632-631225_Przewalskium_albirostris_isolate_WLD-bcl_scaffold177_len2004813_cov98_whole_genome_shotgun_sequence
R_QQTR01041732.1_7933536-7934129_Axis_porcinus_isolate_Zoohd001_scaffold_228_whole_genome_shotgun_sequence
JRFZ01174684.1_900362-900954_Elaphurus_davidianus_breed_Woburn_Abbey_isolate_Dafeng_scaffold386_whole_genome_shotgun_sequence
VCEB01002042.1_3527-4120_Muntiacus_reevesi_isolate_UCam_UCB_Mr_Sca2039_whole_genome_shotgun_sequence
R_RAWV01017463.1_1106056-1106649_Muntiacus_muntjak_isolate_OR735_scaffold1591_whole_genome_shotgun_sequence
R_SJXV01000182.1_179355-179945_Giraffa_camelopardalis_isolate_GFE-hd_scaffold1890_whole_genome_shotgun_sequence
LVKQ01038701.1_24301-24891_Giraffa_camelopardalis_tippelskirchi_isolate_MA1_scaffold1090b241560e702080_17_whole_genome_shotgun_sequence
RAWU01000012.1_21525714-21526304_Giraffa_tippelskirchi_isolate_OR1865_chromosome_X_xb_whole_genome_shotgun_sequence
R_SRVM01027841.1_52465-52601_Antilocapra_americana_isolate_GAN_MWC17-00274_contig138212_0_whole_genome_shotgun_sequence
LVCL010470968.1_7492-8085_Okapia_johnstoni_scaffold94246_1_whole_genome_shotgun_sequence
R_PVHZ020000053.1_2075388-2075964_Tragulus_javanicus_isolate_US108_ScTR3PI_50HRSCAF_15861_whole_genome_shotgun_sequence
R_SJXW01015239.1_285601-285990_Tragulus_kanchil_isolate_LMD-DV_tarseq_13182_whole_genome_shotgun_sequence
marine_Souchi_RWJT01022727.1_0.0_1_580_2865960_2865384
marine_Turtru_NW_017844263.1_0.0_1_580_3711704_3711128
marine_Turadu_NCQN01001030.1_0.0_1_580_226005_225429
marine_Lagobl_NW_020838021.1_0.0_1_580_1060516_1061092
marine_Glomel_ML658914.1_0.0_1_580_1070539_1071116
marine_Orcorc_NW_004438541.1_0.0_1_580_4219902_4219323
marine_Phopho_PKGA01142022.1_0.0_1_578_11763518_11764092
marine_Phosin_CM018177.1_0.0_1_578_109669417_109668843
marine_Neoasi_NW_020172184.1_0.0_1_578_134525_135099
marine_Delleu_NW_019160977.1_0.0_1_580_1093682_1094258
marine_Monmon_NW_021703779.1_0.0_1_580_107803210_107802634
marine_Inigeo_RJWO010053217.1_0.0_1_582_369_951
marine_Ponbla_RJWI010233703.1_0.0_1_582_1504_922
marine_Lipvex_NW_006776544.1_0.0_1_582_2238595_2239174
marine_Mesbid_PVJJ010039134.1_0.0_1_582_5425_6007
marine_Zipcav_RJWS012571658.1_2e-54_1_130_102_231
marine_Zipcav_RJWS011974007.1_2e-54_1_130_151_280
marine_Zipcav_RJWS010261767.1_0.0_1_582_70_651
marine_Phycat_NC_041234.1_0.0_1_582_100972640_100972059
marine_Kogbre_RJWL010007087.1_0.0_1_582_8882_8302
marine_Plamin_RJWK010002004.1_0.0_1_582_28209_27632
marine_Balbon_DF497386.1_0.0_1_582_12114_11533
query_marine_Balacu_NW_006726898.1_0.0_1_582_1107419_1108000
marine_Megnov_RYZJ01000996.1_0.0_1_582_464184_464762
R_marine_VNFC01000008.1_110654114-110654695_Balaenoptera_musculus_isolate_JJ_BM4_2016_0621_Super_scaffold_8_whole_genome_shotgun_sequence
marine_Escrob_NIPP01004663.1_0.0_1_582_77001_77582
marine_Eubjap_RJWP010037093.1_0.0_1_582_16977_17556
PVJP02910818.1_2068845-2069425_Hippopotamus_amphibius_isolate_BS01_ScHSfme_925262HRSCAF_983062_whole_genome_shotgun_sequence
0.02

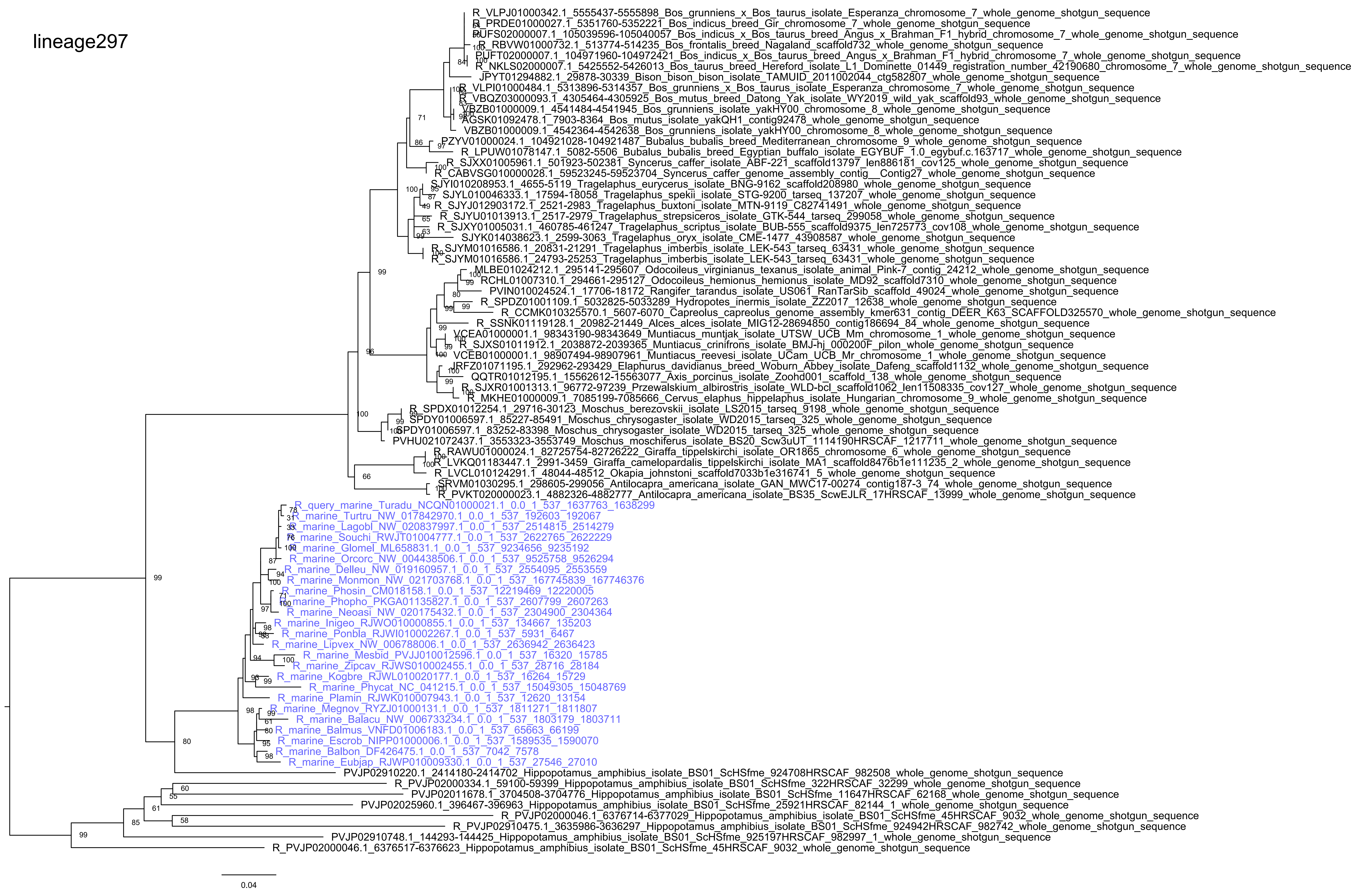
lineage297
R_VLPJ01000342.1_5555437-5555898_Bos_grunniens_x_Bos_taurus_isolate_Esperanza_chromosome_7_whole_genome_shotgun_sequence
R_PRDE01000027.1_5351760-5352221_Bos_indicus_breed_Gir_chromosome_7_whole_genome_shotgun_sequence
RUFS02000007.1_105039596-105040057_Bos_indicus_x_Bos_taurus_breed_Angus_x_Brahman_F1_hybrid_chromosome_7_whole_genome_shotgun_sequence
R_RBVW01000732.1_513774-514235_Bos_frontalis_breed_Nagaland_scaffold732_whole_genome_shotgun_sequence
PUFT02000007.1_104971960-104972421_Bos_indicus_x_Bos_taurus_breed_Angus_x_Brahman_F1_hybrid_chromosome_7_whole_genome_shotgun_sequence
R_NKLS02000007.1_5425552-5426013_Bos_taurus_breed_Hereford_isolate_L1_Dominette_01449_registration_number_42190680_chromosome_7_whole_genome_shotgun_sequence
JPYT01294882.1_29878-30339_Bison_bison_bison_isolate_TAMUID_2011002044_ctg582807_whole_genome_shotgun_sequence
R_VLPI01000484.1_5313896-5314357_Bos_grunniens_x_Bos_taurus_isolate_Esperanza_chromosome_7_whole_genome_shotgun_sequence
R_VBQZ03000093.1_4305464-4305925_Bos_mutus_breed_Datong_Yak_isolate_WY2019_wild_yak_scaffold93_whole_genome_shotgun_sequence
VBZB01000009.1_4541484-4541945_Bos_grunniens_isolate_yakHY00_chromosome_8_whole_genome_shotgun_sequence
AGSK01092478.1_7903-8364_Bos_mutus_isolate_yakQH1_contig92478_whole_genome_shotgun_sequence
VBZB01000009.1_4542364-4542638_Bos_grunniens_isolate_yakHY00_chromosome_8_whole_genome_shotgun_sequence
PZYV01000024.1_104921028-104921487_Bubalus_bubalis_breed_Mediterranean_chromosome_9_whole_genome_shotgun_sequence
R_LPUW01078147.1_5082-5506_Bubalus_bubalis_breed_Egyptian_buffalo_isolate_EGYBUF_1.0_egybuf.c.163717_whole_genome_shotgun_sequence
R_SJXX01005961.1_501923-502381_Syncerus_caffer_isolate_ABF-221_scaffold13797_len886181_cov125_whole_genome_shotgun_sequence
R_CABVSG010000028.1_59523245-59523704_Syncerus_caffer_genome_assembly_contig__Contig27_whole_genome_shotgun_sequence
SJYI010208953.1_4655-5119_Tragelaphus_eurycerus_isolate_BNG-9162_scaffold208980_whole_genome_shotgun_sequence
SJYL010046333.1_17594-18058_Tragelaphus_spekii_isolate_STG-9200_tarseq_137207_whole_genome_shotgun_sequence
R_SJYJ012903172.1_2521-2983_Tragelaphus_buxtoni_isolate_MTN-9119_C82741491_whole_genome_shotgun_sequence
R_SJYU01013913.1_2517-2979_Tragelaphus_strepsiceros_isolate_GTK-544_tarseq_299058_whole_genome_shotgun_sequence
R_SJXY01005031.1_460785-461247_Tragelaphus_scriptus_isolate_BUB-555_scaffold9375_len725773_cov108_whole_genome_shotgun_sequence
SJYK014038623.1_2599-3063_Tragelaphus_oryx_isolate_CME-1477_43908587_whole_genome_shotgun_sequence
R_SJYM01016586.1_20831-21291_Tragelaphus_imberbis_isolate_LEK-543_tarseq_63431_whole_genome_shotgun_sequence
R_SJYM01016586.1_24793-25253_Tragelaphus_imberbis_isolate_LEK-543_tarseq_63431_whole_genome_shotgun_sequence
MLBE01024212.1_295141-295607_Odocoileus_virginianus_texanus_isolate_animal_Pink-7_contig_24212_whole_genome_shotgun_sequence
RCHL01007310.1_294661-295127_Odocoileus_hemionus_hemionus_isolate_MD92_scaffold7310_whole_genome_shotgun_sequence
PVIN010024524.1_17706-18172_Rangifer_tarandus_isolate_US061_RanTarSib_scaffold_49024_whole_genome_shotgun_sequence
R_SPDZ01001109.1_5032825-5033289_Hydropotes_inermis_isolate_ZZ2017_12638_whole_genome_shotgun_sequence
R_CCMK010325570.1_5607-6070_Capreolus_capreolus_genome_assembly_kmer631_contig_DEER_K63_SCAFFOLD325570_whole_genome_shotgun_sequence
R_SSNK01119128.1_20982-21449_Alces_alces_isolate_MIG12-28694850_contig186694_84_whole_genome_shotgun_sequence
VCEA01000001.1_98343190-98343649_Muntiacus_muntjak_isolate_UTSW_UCB_Mm_chromosome_1_whole_genome_shotgun_sequence
R_SJXS01011912.1_2038872-2039365_Muntiacus_crinifrons_isolate_BMJ-hj_000200F_pilon_whole_genome_shotgun_sequence
VCEB01000001.1_98907494-98907961_Muntiacus_reevesi_isolate_UCam_UCB_Mr_chromosome_1_whole_genome_shotgun_sequence
JRFZ01071195.1_292962-293429_Elaphurus_davidianus_breed_Woburn_Abbey_isolate_Dafeng_scaffold1132_whole_genome_shotgun_sequence
QQTR01012195.1_15562612-15563077_Axis_porcinus_isolate_Zoohd001_scaffold_138_whole_genome_shotgun_sequence
R_SJXR01001313.1_96772-97239_Przewalskium_albirostris_isolate_WLD-bcl_scaffold1062_len11508335_cov127_whole_genome_shotgun_sequence
R_MKHE01000009.1_7085199-7085666_Cervus_elaphus_hippelaphus_isolate_Hungarian_chromosome_9_whole_genome_shotgun_sequence
R_SPDX01012254.1_29716-30123_Moschus_berezovskii_isolate_LS2015_tarseq_9198_whole_genome_shotgun_sequence
SPDY01006597.1_85227-85491_Moschus_chrysogaster_isolate_WD2015_tarseq_325_whole_genome_shotgun_sequence
SPDY01006597.1_83252-83398_Moschus_chrysogaster_isolate_WD2015_tarseq_325_whole_genome_shotgun_sequence
PVHU021072437.1_3553323-3553749_Moschus_moschiferus_isolate_BS20_Scw3uUT_1114190HRSCAF_1217711_whole_genome_shotgun_sequence
R_RAWU01000024.1_82725754-82726222_Giraffa_tippelskirchi_isolate_OR1865_chromosome_6_whole_genome_shotgun_sequence
R_LVKQ01183447.1_2991-3459_Giraffa_camelopardalis_tippelskirchi_isolate_MA1_scaffold8476b1e111235_2_whole_genome_shotgun_sequence
R_LVCL010124291.1_48044-48512_Okapia_johnstoni_scaffold7033b1e316741_5_whole_genome_shotgun_sequence
SRVM01030295.1_298605-299056_Antilocapra_americana_isolate_GAN_MWC17-00274_contig187-3_74_whole_genome_shotgun_sequence
R_PVKT020000023.1_4882326-4882777_Antilocapra_americana_isolate_BS35_ScwEJLR_17HRSCAF_13999_whole_genome_shotgun_sequence
R_query_marine_Turadu_NCQN01000021.1_0.0_1_537_1637763_1638299
R_marine_Turtru_NW_017842970.1_0.0_1_537_192603_192067
R_marine_Lagobl_NW_020837997.1_0.0_1_537_2514815_2514279
R_marine_Souchi_RWJT01004777.1_0.0_1_537_2622765_2622229
R_marine_Glomel_ML658831.1_0.0_1_537_9234656_9235192
R_marine_Orcorc_NW_004438506.1_0.0_1_537_9525758_9526294
R_marine_Delleu_NW_019160957.1_0.0_1_537_2554095_2553559
R_marine_Monmon_NW_021703768.1_0.0_1_537_167745839_167746376
R_marine_Phosin_CM018158.1_0.0_1_537_12219469_12220005
R_marine_Phopho_PKGA01135827.1_0.0_1_537_2607799_2607263
R_marine_Neoasi_NW_020175432.1_0.0_1_537_2304900_2304364
R_marine_Inigeo_RJWO010000855.1_0.0_1_537_134667_135203
R_marine_Ponbla_RJWI010002267.1_0.0_1_537_5931_6467
R_marine_Lipvex_NW_006788006.1_0.0_1_537_2636942_2636423
R_marine_Mesbid_PVJJ010012596.1_0.0_1_537_16320_15785
R_marine_Zipcav_RJWS010002455.1_0.0_1_537_28716_28184
R_marine_Kogbre_RJWL010020177.1_0.0_1_537_16264_15729
R_marine_Phycat_NC_041215.1_0.0_1_537_15049305_15048769
R_marine_Plamin_RJWK010007943.1_0.0_1_537_12620_13154
R_marine_Megnov_RYZJ01000131.1_0.0_1_537_1811271_1811807
R_marine_Balacu_NW_006733234.1_0.0_1_537_1803179_1803711
R_marine_Balmus_VNFD01006183.1_0.0_1_537_65663_66199
R_marine_Escrob_NIPP01000006.1_0.0_1_537_1589535_1590070
R_marine_Balbon_DF426475.1_0.0_1_537_7042_7578
R_marine_Eubjap_RJWP010009330.1_0.0_1_537_27546_27010
PVJP02910220.1_2414180-2414702_Hippopotamus_amphibius_isolate_BS01_ScHSfme_924708HRSCAF_982508_whole_genome_shotgun_sequence
R_PVJP02000334.1_59100-59399_Hippopotamus_amphibius_isolate_BS01_ScHSfme_322HRSCAF_32299_whole_genome_shotgun_sequence
PVJP02011678.1_3704508-3704776_Hippopotamus_amphibius_isolate_BS01_ScHSfme_11647HRSCAF_62168_whole_genome_shotgun_sequence
PVJP02025960.1_396467-396963_Hippopotamus_amphibius_isolate_BS01_ScHSfme_25921HRSCAF_82144_1_whole_genome_shotgun_sequence
R_PVJP02000046.1_6376714-6377029_Hippopotamus_amphibius_isolate_BS01_ScHSfme_45HRSCAF_9032_whole_genome_shotgun_sequence
R_PVJP02910475.1_3635986-3636297_Hippopotamus_amphibius_isolate_BS01_ScHSfme_924942HRSCAF_982742_whole_genome_shotgun_sequence
PVJP02910748.1_144293-144425_Hippopotamus_amphibius_isolate_BS01_ScHSfme_925197HRSCAF_982997_1_whole_genome_shotgun_sequence
R_PVJP02000046.1_6376517-6376623_Hippopotamus_amphibius_isolate_BS01_ScHSfme_45HRSCAF_9032_whole_genome_shotgun_sequence
0.04

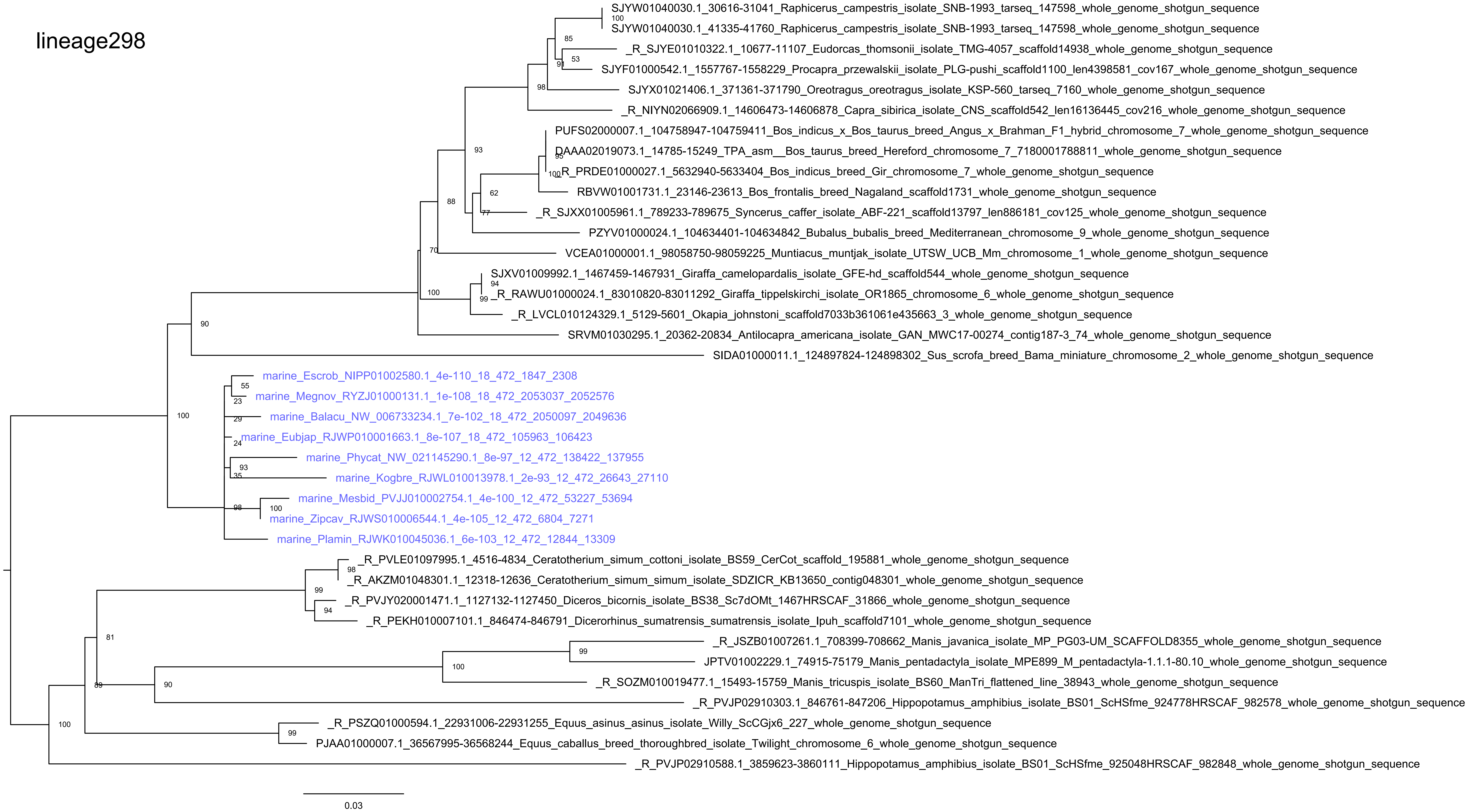

lineage298
SJYW01040030.1_30616-31041_Raphicerus_campestris_isolate_SNB-1993_tarseq_147598_whole_genome_shotgun_sequence
SJYW01040030.1_41335-41760_Raphicerus_campestris_isolate_SNB-1993_tarseq_147598_whole_genome_shotgun_sequence
_R_SJYE01010322.1_10677-11107_Eudorcas_thomsonii_isolate_TMG-4057_scaffold14938_whole_genome_shotgun_sequence
SJYF01000542.1_1557767-1558229_Procapra_przewalskii_isolate_PLG-pushi_scaffold1100_len4398581_cov167_whole_genome_shotgun_sequence
SJYX01021406.1_371361-371790_Oreotragus_oreotragus_isolate_KSP-560_tarseq_7160_whole_genome_shotgun_sequence
_R_NIYN02066909.1_14606473-14606878_Capra_sibirica_isolate_CNS_scaffold542_len16136445_cov216_whole_genome_shotgun_sequence
PUFS02000007.1_104758947-104759411_Bos_indicus_x_Bos_taurus_breed_Angus_x_Brahman_F1_hybrid_chromosome_7_whole_genome_shotgun_sequence
DAAA02019073.1_14785-15249_TPA_asm__Bos_taurus_breed_Hereford_chromosome_7_7180001788811_whole_genome_shotgun_sequence
_R_PRDE01000027.1_5632940-5633404_Bos_indicus_breed_Gir_chromosome_7_whole_genome_shotgun_sequence
RBVW01001731.1_23146-23613_Bos_frontalis_breed_Nagaland_scaffold1731_whole_genome_shotgun_sequence
_R_SJXX01005961.1_789233-789675_Syncerus_caffer_isolate_ABF-221_scaffold13797_len886181_cov125_whole_genome_shotgun_sequence
PZYV01000024.1_104634401-104634842_Bubalus_bubalis_breed_Mediterranean_chromosome_9_whole_genome_shotgun_sequence
VCEA01000001.1_98058750-98059225_Muntiacus_muntjak_isolate_UTSW_UCB_Mm_chromosome_1_whole_genome_shotgun_sequence
SJXV01009992.1_1467459-1467931_Giraffa_camelopardalis_isolate_GFE-hd_scaffold544_whole_genome_shotgun_sequence
_R_RAWU01000024.1_83010820-83011292_Giraffa_tippelskirchi_isolate_OR1865_chromosome_6_whole_genome_shotgun_sequence
_R_LVCL010124329.1_5129-5601_Okapia_johnstoni_scaffold7033b361061e435663_3_whole_genome_shotgun_sequence
SRVM01030295.1_20362-20834_Antilocapra_americana_isolate_GAN_MWC17-00274_contig187-3_74_whole_genome_shotgun_sequence
SIDA01000011.1_124897824-124898302_Sus_scrofa_breed_Bama_miniature_chromosome_2_whole_genome_shotgun_sequence
marine_Escrob_NIPP01002580.1_4e-110_18_472_1847_2308
marine_Megnov_RYZJ01000131.1_1e-108_18_472_2053037_2052576
marine_Balacu_NW_006733234.1_7e-102_18_472_2050097_2049636
marine_Eubjap_RJWP010001663.1_8e-107_18_472_105963_106423
marine_Phycat_NW_021145290.1_8e-97_12_472_138422_137955
marine_Kogbre_RJWL010013978.1_2e-93_12_472_26643_27110
marine_Mesbid_PVJJ010002754.1_4e-100_12_472_53227_53694
marine_Zipcav_RJWS010006544.1_4e-105_12_472_6804_7271
marine_Plamin_RJWK010045036.1_6e-103_12_472_12844_13309
_R_PVLE01097995.1_4516-4834_Ceratotherium_simum_cottoni_isolate_BS59_CerCot_scaffold_195881_whole_genome_shotgun_sequence
_R_AKZM01048301.1_12318-12636_Ceratotherium_simum_simum_isolate_SDZICR_KB13650_contig048301_whole_genome_shotgun_sequence
_R_PVJY020001471.1_1127132-1127450_Diceros_bicornis_isolate_BS38_Sc7dOMt_1467HRSCAF_31866_whole_genome_shotgun_sequence
_R_PEKH010007101.1_846474-846791_Dicerorhinus_sumatrensis_sumatrensis_isolate_Ipuh_scaffold7101_whole_genome_shotgun_sequence
_R_JSZB01007261.1_708399-708662_Manis_javanica_isolate_MP_PG03-UM_SCAFFOLD8355_whole_genome_shotgun_sequence
JPTV01002229.1_74915-75179_Manis_pentadactyla_isolate_MPE899_M_pentadactyla-1.1.1-80.10_whole_genome_shotgun_sequence
_R_SOZM010019477.1_15493-15759_Manis_tricuspis_isolate_BS60_ManTri_flattened_line_38943_whole_genome_shotgun_sequence
_R_PVJP02910303.1_846761-847206_Hippopotamus_amphibius_isolate_BS01_ScHSfme_924778HRSCAF_982578_whole_genome_shotgun_sequence
_R_PSZQ01000594.1_22931006-22931255_Equus_asinus_asinus_isolate_Willy_ScCGjx6_227_whole_genome_shotgun_sequence
PJAA01000007.1_36567995-36568244_Equus_caballus_breed_thoroughbred_isolate_Twilight_chromosome_6_whole_genome_shotgun_sequence
_R_PVJP02910588.1_3859623-3860111_Hippopotamus_amphibius_isolate_BS01_ScHSfme_925048HRSCAF_982848_whole_genome_shotgun_sequence
0.03

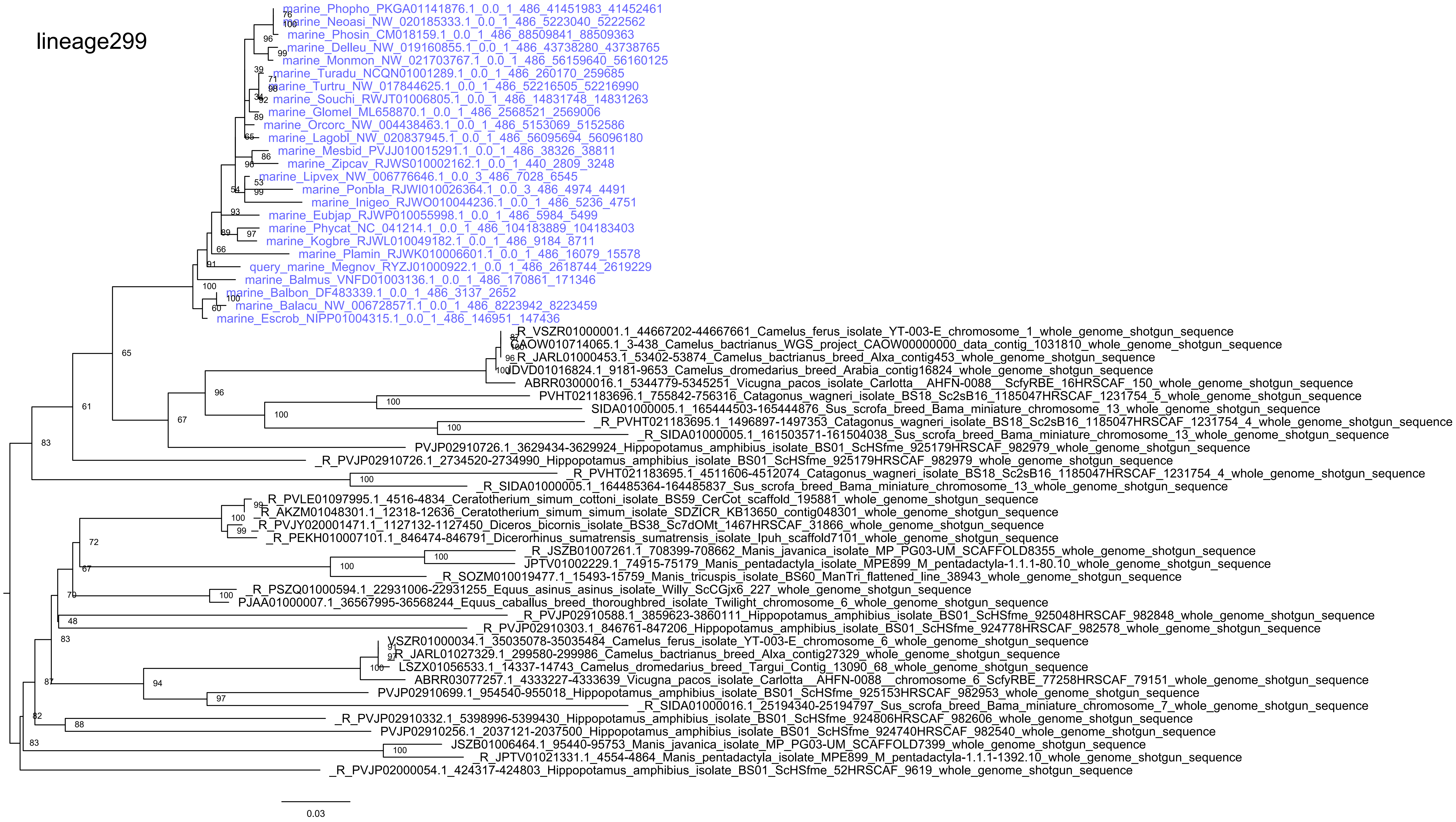
lineage299
marine_Phopho_PKGA01141876.1_0.0_1_486_41451983_41452461
marine_Neoasi_NW_020185333.1_0.0_1_486_5223040_5222562
marine_Phosin_CM018159.1_0.0_1_486_88509841_88509363
marine_Delleu_NW_019160855.1_0.0_1_486_43738280_43738765
marine_Monmon_NW_021703767.1_0.0_1_486_56159640_56160125
marine_Turadu_NCQN01001289.1_0.0_1_486_260170_259685
marine_Turtru_NW_017844625.1_0.0_1_486_52216505_52216990
marine_Souchi_RWJT01006805.1_0.0_1_486_14831748_14831263
marine_Glomel_ML658870.1_0.0_1_486_2568521_2569006
marine_Orcorc_NW_004438463.1_0.0_1_486_5153069_5152586
marine_Lagobl_NW_020837945.1_0.0_1_486_56095694_56096180
marine_Mesbid_PVJJ010015291.1_0.0_1_486_38326_38811
marine_Zipcav_RJWS010002162.1_0.0_1_440_2809_3248
marine_Lipvex_NW_006776646.1_0.0_3_486_7028_6545
marine_Ponbla_RJWI010026364.1_0.0_3_486_4974_4491
marine_Inigeo_RJWO010044236.1_0.0_1_486_5236_4751
marine_Eubjap_RJWP010055998.1_0.0_1_486_5984_5499
marine_Phycat_NC_041214.1_0.0_1_486_104183889_104183403
marine_Kogbre_RJWL010049182.1_0.0_1_486_9184_8711
marine_Plamin_RJWK010006601.1_0.0_1_486_16079_15578
query_marine_Megnov_RYZJ01000922.1_0.0_1_486_2618744_2619229
marine_Balmus_VNFD01003136.1_0.0_1_486_170861_171346
marine_Balbon_DF483339.1_0.0_1_486_3137_2652
marine_Balacu_NW_006728571.1_0.0_1_486_8223942_8223459
marine_Escrob_NIPP01004315.1_0.0_1_486_146951_147436
_R_VSZR01000001.1_44667202-44667661_Camelus_ferus_isolate_YT-003-E_chromosome_1_whole_genome_shotgun_sequence
CAOW010714065.1_3-438_Camelus_bactrianus_WGS_project_CAOW00000000_data_contig_1031810_whole_genome_shotgun_sequence
_R_JARL01000453.1_53402-53874_Camelus_bactrianus_breed_Alxa_contig453_whole_genome_shotgun_sequence
JDVD01016824.1_9181-9653_Camelus_dromedarius_breed_Arabia_contig16824_whole_genome_shotgun_sequence
ABRR03000016.1_5344779-5345251_Vicugna_pacos_isolate_Carlotta__AHFN-0088__ScfyRBE_16HRSCAF_150_whole_genome_shotgun_sequence
PVHT021183696.1_755842-756316_Catagonus_wagneri_isolate_BS18_Sc2sB16_1185047HRSCAF_1231754_5_whole_genome_shotgun_sequence
SIDA01000005.1_165444503-165444876_Sus_scrofa_breed_Bama_miniature_chromosome_13_whole_genome_shotgun_sequence
_R_PVHT021183695.1_1496897-1497353_Catagonus_wagneri_isolate_BS18_Sc2sB16_1185047HRSCAF_1231754_4_whole_genome_shotgun_sequence
_R_SIDA01000005.1_161503571-161504038_Sus_scrofa_breed_Bama_miniature_chromosome_13_whole_genome_shotgun_sequence
PVJP02910726.1_3629434-3629924_Hippopotamus_amphibius_isolate_BS01_ScHSfme_925179HRSCAF_982979_whole_genome_shotgun_sequence
_R_PVJP02910726.1_2734520-2734990_Hippopotamus_amphibius_isolate_BS01_ScHSfme_925179HRSCAF_982979_whole_genome_shotgun_sequence
_R_PVHT021183695.1_4511606-4512074_Catagonus_wagneri_isolate_BS18_Sc2sB16_1185047HRSCAF_1231754_4_whole_genome_shotgun_sequence
_R_SIDA01000005.1_164485364-164485837_Sus_scrofa_breed_Bama_miniature_chromosome_13_whole_genome_shotgun_sequence
_R_PVLE01097995.1_4516-4834_Ceratotherium_simum_cottoni_isolate_BS59_CerCot_scaffold_195881_whole_genome_shotgun_sequence
_R_AKZM01048301.1_12318-12636_Ceratotherium_simum_simum_isolate_SDZICR_KB13650_contig048301_whole_genome_shotgun_sequence
_R_PVJY020001471.1_1127132-1127450_Diceros_bicornis_isolate_BS38_Sc7dOMt_1467HRSCAF_31866_whole_genome_shotgun_sequence
_R_PEKH010007101.1_846474-846791_Dicerorhinus_sumatrensis_sumatrensis_isolate_Ipuh_scaffold7101_whole_genome_shotgun_sequence
_R_JSZB01007261.1_708399-708662_Manis_javanica_isolate_MP_PG03-UM_SCAFFOLD8355_whole_genome_shotgun_sequence
JPTV01002229.1_74915-75179_Manis_pentadactyla_isolate_MPE899_M_pentadactyla-1.1.1-80.10_whole_genome_shotgun_sequence
_R_SOZM010019477.1_15493-15759_Manis_tricuspis_isolate_BS60_ManTri_flattened_line_38943_whole_genome_shotgun_sequence
_R_PSZQ01000594.1_22931006-22931255_Equus_asinus_asinus_isolate_Willy_ScCGjx6_227_whole_genome_shotgun_sequence
PJAA01000007.1_36567995-36568244_Equus_caballus_breed_thoroughbred_isolate_Twilight_chromosome_6_whole_genome_shotgun_sequence
_R_PVJP02910588.1_3859623-3860111_Hippopotamus_amphibius_isolate_BS01_ScHSfme_925048HRSCAF_982848_whole_genome_shotgun_sequence
_R_PVJP02910303.1_846761-847206_Hippopotamus_amphibius_isolate_BS01_ScHSfme_924778HRSCAF_982578_whole_genome_shotgun_sequence
VSZR01000034.1_35035078-35035484_Camelus_ferus_isolate_YT-003-E_chromosome_6_whole_genome_shotgun_sequence
_R_JARL01027329.1_299580-299986_Camelus_bactrianus_breed_Alxa_contig27329_whole_genome_shotgun_sequence
LSZX01056533.1_14337-14743_Camelus_dromedarius_breed_Targui_Contig_13090_68_whole_genome_shotgun_sequence
ABRR03077257.1_4333227-4333639_Vicugna_pacos_isolate_Carlotta__AHFN-0088__chromosome_6_ScfyRBE_77258HRSCAF_79151_whole_genome_shotgun_sequence
PVJP02910699.1_954540-955018_Hippopotamus_amphibius_isolate_BS01_ScHSfme_925153HRSCAF_982953_whole_genome_shotgun_sequence
_R_SIDA01000016.1_25194340-25194797_Sus_scrofa_breed_Bama_miniature_chromosome_7_whole_genome_shotgun_sequence
_R_PVJP02910332.1_5398996-5399430_Hippopotamus_amphibius_isolate_BS01_ScHSfme_924806HRSCAF_982606_whole_genome_shotgun_sequence
PVJP02910256.1_2037121-2037500_Hippopotamus_amphibius_isolate_BS01_ScHSfme_924740HRSCAF_982540_whole_genome_shotgun_sequence
JSZB01006464.1_95440-95753_Manis_javanica_isolate_MP_PG03-UM_SCAFFOLD7399_whole_genome_shotgun_sequence
_R_JPTV01021331.1_4554-4864_Manis_pentadactyla_isolate_MPE899_M_pentadactyla-1.1.1-1392.10_whole_genome_shotgun_sequence
_R_PVJP02000054.1_424317-424803_Hippopotamus_amphibius_isolate_BS01_ScHSfme_52HRSCAF_9619_whole_genome_shotgun_sequence
0.03

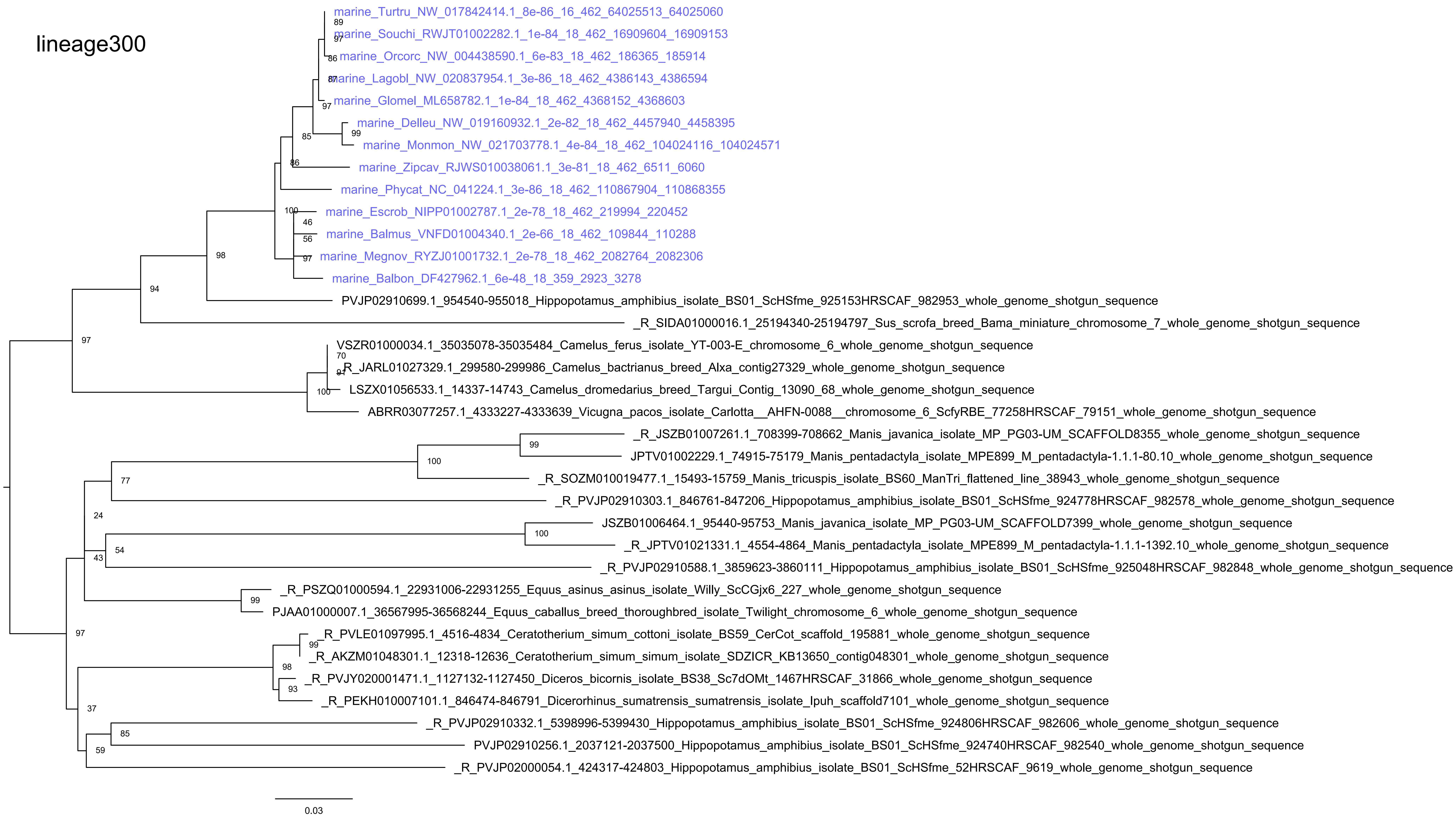
lineage300
marine_Turtru_NW_017842414.1_8e-86_16_462_64025513_64025060
marine_Souchi_RWJT01002282.1_1e-84_18_462_16909604_16909153
marine_Orcorc_NW_004438590.1_6e-83_18_462_186365_185914
marine_Lagobl_NW_020837954.1_3e-86_18_462_4386143_4386594
marine_Glomel_ML658782.1_1e-84_18_462_4368152_4368603
marine_Delleu_NW_019160932.1_2e-82_18_462_4457940_4458395
marine_Monmon_NW_021703778.1_4e-84_18_462_104024116_104024571
marine_Zipcav_RJWS010038061.1_3e-81_18_462_6511_6060
marine_Phycat_NC_041224.1_3e-86_18_462_110867904_110868355
marine_Escrob_NIPP01002787.1_2e-78_18_462_219994_220452
marine_Balmus_VNFD01004340.1_2e-66_18_462_109844_110288
marine_Megnov_RYZJ01001732.1_2e-78_18_462_2082764_2082306
marine_Balbon_DF427962.1_6e-48_18_359_2923_3278
PVJP02910699.1_954540-955018_Hippopotamus_amphibius_isolate_BS01_ScHSfme_925153HRSCAF_982953_whole_genome_shotgun_sequence
_R_SIDA01000016.1_25194340-25194797_Sus_scrofa_breed_Bama_miniature_chromosome_7_whole_genome_shotgun_sequence
VSZR01000034.1_35035078-35035484_Camelus_ferus_isolate_YT-003-E_chromosome_6_whole_genome_shotgun_sequence
_R_JARL01027329.1_299580-299986_Camelus_bactrianus_breed_Alxa_contig27329_whole_genome_shotgun_sequence
LSZX01056533.1_14337-14743_Camelus_dromedarius_breed_Targui_Contig_13090_68_whole_genome_shotgun_sequence
ABRR03077257.1_4333227-4333639_Vicugna_pacos_isolate_Carlotta__AHFN-0088__chromosome_6_ScfyRBE_77258HRSCAF_79151_whole_genome_shotgun_sequence
_R_JSZB01007261.1_708399-708662_Manis_javanica_isolate_MP_PG03-UM_SCAFFOLD8355_whole_genome_shotgun_sequence
JPTV01002229.1_74915-75179_Manis_pentadactyla_isolate_MPE899_M_pentadactyla-1.1.1-80.10_whole_genome_shotgun_sequence
_R_SOZM010019477.1_15493-15759_Manis_tricuspis_isolate_BS60_ManTri_flattened_line_38943_whole_genome_shotgun_sequence
_R_PVJP02910303.1_846761-847206_Hippopotamus_amphibius_isolate_BS01_ScHSfme_924778HRSCAF_982578_whole_genome_shotgun_sequence
JSZB01006464.1_95440-95753_Manis_javanica_isolate_MP_PG03-UM_SCAFFOLD7399_whole_genome_shotgun_sequence
_R_JPTV01021331.1_4554-4864_Manis_pentadactyla_isolate_MPE899_M_pentadactyla-1.1.1-1392.10_whole_genome_shotgun_sequence
_R_PVJP02910588.1_3859623-3860111_Hippopotamus_amphibius_isolate_BS01_ScHSfme_925048HRSCAF_982848_whole_genome_shotgun_sequence
_R_PSZQ01000594.1_22931006-22931255_Equus_asinus_asinus_isolate_Willy_ScCGjx6_227_whole_genome_shotgun_sequence
PJAA01000007.1_36567995-36568244_Equus_caballus_breed_thoroughbred_isolate_Twilight_chromosome_6_whole_genome_shotgun_sequence
_R_PVLE01097995.1_4516-4834_Ceratotherium_simum_cottoni_isolate_BS59_CerCot_scaffold_195881_whole_genome_shotgun_sequence
_R_AKZM01048301.1_12318-12636_Ceratotherium_simum_simum_isolate_SDZICR_KB13650_contig048301_whole_genome_shotgun_sequence
_R_PVJY020001471.1_1127132-1127450_Diceros_bicornis_isolate_BS38_Sc7dOMt_1467HRSCAF_31866_whole_genome_shotgun_sequence
_R_PEKH010007101.1_846474-846791_Dicerorhinus_sumatrensis_sumatrensis_isolate_Ipuh_scaffold7101_whole_genome_shotgun_sequence
_R_PVJP02910332.1_5398996-5399430_Hippopotamus_amphibius_isolate_BS01_ScHSfme_924806HRSCAF_982606_whole_genome_shotgun_sequence
PVJP02910256.1_2037121-2037500_Hippopotamus_amphibius_isolate_BS01_ScHSfme_924740HRSCAF_982540_whole_genome_shotgun_sequence
_R_PVJP02000054.1_424317-424803_Hippopotamus_amphibius_isolate_BS01_ScHSfme_52HRSCAF_9619_whole_genome_shotgun_sequence
0.03

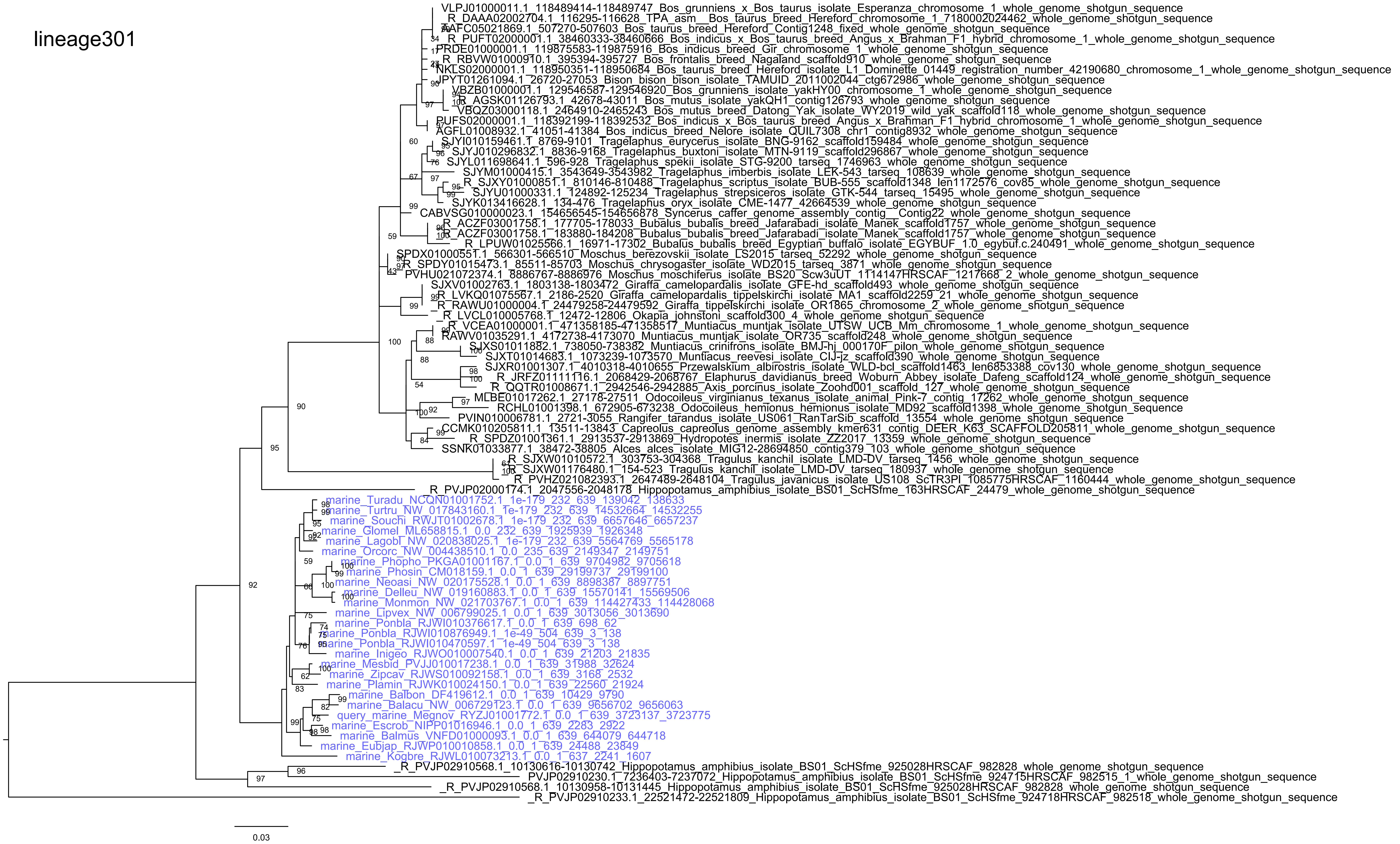

lineage301
VLPJ01000011.1_118489414-118489747_Bos_grunniens_x_Bos_taurus_isolate_Esperanza_chromosome_1_whole_genome_shotgun_sequence
_R_DAAA02002704.1_116295-116628_TPA_asm__Bos_taurus_breed_Hereford_chromosome_1_7180002024462_whole_genome_shotgun_sequence
AAFC05021869.1_507270-507603_Bos_taurus_breed_Hereford_Contig1248_fixed_whole_genome_shotgun_sequence
_R_PUFT02000001.1_38460333-38460666_Bos_indicus_x_Bos_taurus_breed_Angus_x_Brahman_F1_hybrid_chromosome_1_whole_genome_shotgun_sequence
PRDE01000001.1_119875583-119875916_Bos_indicus_breed_Gir_chromosome_1_whole_genome_shotgun_sequence
_R_RBVW01000910.1_395394-395727_Bos_frontalis_breed_Nagaland_scaffold910_whole_genome_shotgun_sequence
NKLS02000001.1_118950351-118950684_Bos_taurus_breed_Hereford_isolate_L1_Dominette_01449_registration_number_42190680_chromosome_1_whole_genome_shotgun_sequence
JPYT01261094.1_26720-27053_Bison_bison_bison_isolate_TAMUID_2011002044_ctg672986_whole_genome_shotgun_sequence
VBZB01000001.1_129546587-129546920_Bos_grunniens_isolate_yakHY00_chromosome_1_whole_genome_shotgun_sequence
_R_AGSK01126793.1_42678-43011_Bos_mutus_isolate_yakQH1_contig126793_whole_genome_shotgun_sequence
VBQZ03000118.1_2464910-2465243_Bos_mutus_breed_Datong_Yak_isolate_WY2019_wild_yak_scaffold118_whole_genome_shotgun_sequence
PUFS02000001.1_118392199-118392532_Bos_indicus_x_Bos_taurus_breed_Angus_x_Brahman_F1_hybrid_chromosome_1_whole_genome_shotgun_sequence
AGFL01008932.1_41051-41384_Bos_indicus_breed_Nelore_isolate_QUIL7308_chr1_contig8932_whole_genome_shotgun_sequence
SJYI010159461.1_8769-9101_Tragelaphus_eurycerus_isolate_BNG-9162_scaffold159484_whole_genome_shotgun_sequence
SJYJ010296832.1_8836-9168_Tragelaphus_buxtoni_isolate_MTN-9119_scaffold296867_whole_genome_shotgun_sequence
SJYL011698641.1_596-928_Tragelaphus_spekii_isolate_STG-9200_tarseq_1746963_whole_genome_shotgun_sequence
SJYM01000415.1_3543649-3543982_Tragelaphus_imberbis_isolate_LEK-543_tarseq_108639_whole_genome_shotgun_sequence
_R_SJXY01000851.1_810146-810488_Tragelaphus_scriptus_isolate_BUB-555_scaffold1348_len1172576_cov85_whole_genome_shotgun_sequence
SJYU01000331.1_124892-125234_Tragelaphus_strepsiceros_isolate_GTK-544_tarseq_15495_whole_genome_shotgun_sequence
SJYK013416628.1_134-476_Tragelaphus_oryx_isolate_CME-1477_42664539_whole_genome_shotgun_sequence
CABVSG010000023.1_154656545-154656878_Syncerus_caffer_genome_assembly_contig__Contig22_whole_genome_shotgun_sequence
_R_ACZF03001758.1_177705-178033_Bubalus_bubalis_breed_Jafarabadi_isolate_Manek_scaffold1757_whole_genome_shotgun_sequence
_R_ACZF03001758.1_183880-184208_Bubalus_bubalis_breed_Jafarabadi_isolate_Manek_scaffold1757_whole_genome_shotgun_sequence
_R_LPUW01025566.1_16971-17302_Bubalus_bubalis_breed_Egyptian_buffalo_isolate_EGYBUF_1.0_egybuf.c.240491_whole_genome_shotgun_sequence
SPDX01000551.1_566301-566510_Moschus_berezovskii_isolate_LS2015_tarseq_52292_whole_genome_shotgun_sequence
_R_SPDY01015473.1_85511-85703_Moschus_chrysogaster_isolate_WD2015_tarseq_3871_whole_genome_shotgun_sequence
PVHU021072374.1_8886767-8886976_Moschus_moschiferus_isolate_BS20_Scw3uUT_1114147HRSCAF_1217668_2_whole_genome_shotgun_sequence
SJXV01002763.1_1803138-1803472_Giraffa_camelopardalis_isolate_GFE-hd_scaffold493_whole_genome_shotgun_sequence
_R_LVKQ01075567.1_2186-2520_Giraffa_camelopardalis_tippelskirchi_isolate_MA1_scaffold2259_21_whole_genome_shotgun_sequence
_R_RAWU01000004.1_24479258-24479592_Giraffa_tippelskirchi_isolate_OR1865_chromosome_2_whole_genome_shotgun_sequence
_R_LVCL010005768.1_12472-12806_Okapia_johnstoni_scaffold300_4_whole_genome_shotgun_sequence
_R_VCEA01000001.1_471358185-471358517_Muntiacus_muntjak_isolate_UTSW_UCB_Mm_chromosome_1_whole_genome_shotgun_sequence
RAWV01035291.1_4172738-4173070_Muntiacus_muntjak_isolate_OR735_scaffold248_whole_genome_shotgun_sequence
SJXS01011882.1_738050-738382_Muntiacus_crinifrons_isolate_BMJ-hj_000170F_pilon_whole_genome_shotgun_sequence
SJXT01014683.1_1073239-1073570_Muntiacus_reevesi_isolate_CIJ-jz_scaffold390_whole_genome_shotgun_sequence
SJXR01001307.1_4010318-4010655_Przewalskium_albirostris_isolate_WLD-bcl_scaffold1463_len6853388_cov130_whole_genome_shotgun_sequence
_R_JRFZ01111116.1_2068429-2068767_Elaphurus_davidianus_breed_Woburn_Abbey_isolate_Dafeng_scaffold124_whole_genome_shotgun_sequence
_R_QQTR01008671.1_2942546-2942885_Axis_porcinus_isolate_Zoohd001_scaffold_127_whole_genome_shotgun_sequence
MLBE01017262.1_27178-27511_Odocoileus_virginianus_texanus_isolate_animal_Pink-7_contig_17262_whole_genome_shotgun_sequence
RCHL01001398.1_672905-673238_Odocoileus_hemionus_hemionus_isolate_MD92_scaffold1398_whole_genome_shotgun_sequence
PVIN010006781.1_2721-3055_Rangifer_tarandus_isolate_US061_RanTarSib_scaffold_13554_whole_genome_shotgun_sequence
CCMK010205811.1_13511-13843_Capreolus_capreolus_genome_assembly_kmer631_contig_DEER_K63_SCAFFOLD205811_whole_genome_shotgun_sequence
_R_SPDZ01001361.1_2913537-2913869_Hydropotes_inermis_isolate_ZZ2017_13359_whole_genome_shotgun_sequence
SSNK01033877.1_38472-38805_Alces_alces_isolate_MIG12-28694850_contig379_103_whole_genome_shotgun_sequence
_R_SJXW01010572.1_303753-304368_Tragulus_kanchil_isolate_LMD-DV_tarseq_1456_whole_genome_shotgun_sequence
_R_SJXW01176480.1_154-523_Tragulus_kanchil_isolate_LMD-DV_tarseq_180937_whole_genome_shotgun_sequence
_R_PVHZ021082393.1_2647489-2648104_Tragulus_javanicus_isolate_US108_ScTR3PI_1085775HRSCAF_1160444_whole_genome_shotgun_sequence
_R_PVJP02000174.1_2047556-2048178_Hippopotamus_amphibius_isolate_BS01_ScHSfme_163HRSCAF_24479_whole_genome_shotgun_sequence
marine_Turadu_NCQN01001752.1_1e-179_232_639_139042_138633
marine_Turtru_NW_017843160.1_1e-179_232_639_14532664_14532255
marine_Souchi_RWJT01002678.1_1e-179_232_639_6657646_6657237
marine_Glomel_ML658815.1_0.0_232_639_1925939_1926348
marine_Lagobl_NW_020838025.1_1e-179_232_639_5564769_5565178
marine_Orcorc_NW_004438510.1_0.0_235_639_2149347_2149751
marine_Phopho_PKGA01001167.1_0.0_1_639_9704982_9705618
marine_Phosin_CM018159.1_0.0_1_639_29199737_29199100
marine_Neoasi_NW_020175528.1_0.0_1_639_8898387_8897751
marine_Delleu_NW_019160883.1_0.0_1_639_15570141_15569506
marine_Monmon_NW_021703767.1_0.0_1_639_114427433_114428068
marine_Lipvex_NW_006799025.1_0.0_1_639_3013056_3013690
marine_Ponbla_RJWI010376617.1_0.0_1_639_698_62
marine_Ponbla_RJWI010876949.1_1e-49_504_639_3_138
marine_Ponbla_RJWI010470597.1_1e-49_504_639_3_138
marine_Inigeo_RJWO010007540.1_0.0_1_639_21203_21835
marine_Mesbid_PVJJ010017238.1_0.0_1_639_31988_32624
marine_Zipcav_RJWS010092158.1_0.0_1_639_3168_2532
marine_Plamin_RJWK010024150.1_0.0_1_639_22560_21924
marine_Balbon_DF419612.1_0.0_1_639_10429_9790
marine_Balacu_NW_006729123.1_0.0_1_639_9656702_9656063
query_marine_Megnov_RYZJ01001772.1_0.0_1_639_3723137_3723775
marine_Escrob_NIPP01016946.1_0.0_1_639_2283_2922
marine_Balmus_VNFD01000093.1_0.0_1_639_644079_644718
marine_Eubjap_RJWP010010858.1_0.0_1_639_24488_23849
marine_Kogbre_RJWL010073213.1_0.0_1_637_2241_1607
_R_PVJP02910568.1_10130616-10130742_Hippopotamus_amphibius_isolate_BS01_ScHSfme_925028HRSCAF_982828_whole_genome_shotgun_sequence
PVJP02910230.1_7236403-7237072_Hippopotamus_amphibius_isolate_BS01_ScHSfme_924715HRSCAF_982515_1_whole_genome_shotgun_sequence
_R_PVJP02910568.1_10130958-10131445_Hippopotamus_amphibius_isolate_BS01_ScHSfme_925028HRSCAF_982828_whole_genome_shotgun_sequence
_R_PVJP02910233.1_22521472-22521809_Hippopotamus_amphibius_isolate_BS01_ScHSfme_924718HRSCAF_982518_whole_genome_shotgun_sequence
0.03

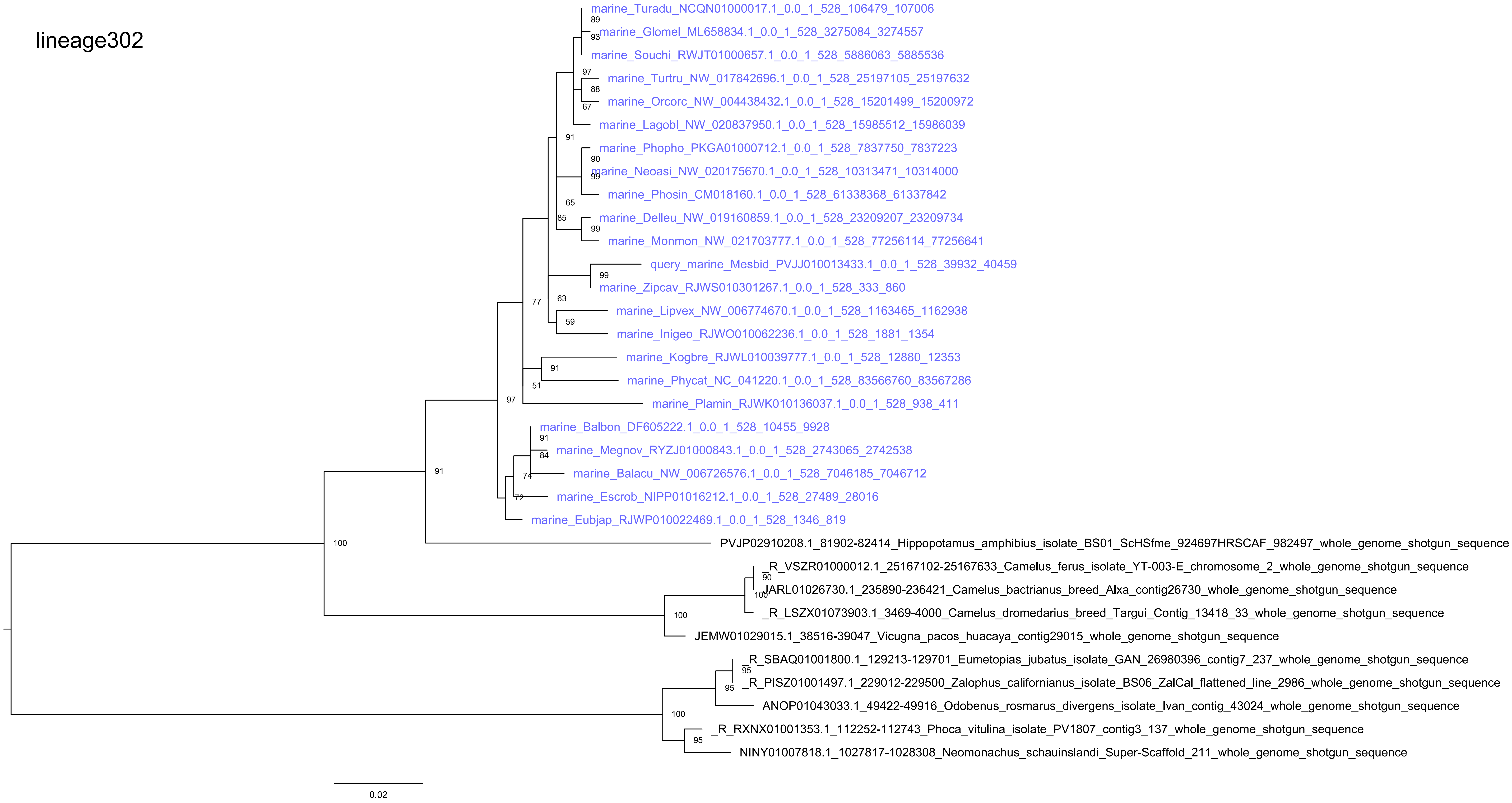
lineage302
marine_Turadu_NCQN01000017.1_0.0_1_528_106479_107006
marine_Glomel_ML658834.1_0.0_1_528_3275084_3274557
marine_Souchi_RWJT01000657.1_0.0_1_528_5886063_5885536
marine_Turtru_NW_017842696.1_0.0_1_528_25197105_25197632
marine_Orcorc_NW_004438432.1_0.0_1_528_15201499_15200972
marine_Lagobl_NW_020837950.1_0.0_1_528_15985512_15986039
marine_Phopho_PKGA01000712.1_0.0_1_528_7837750_7837223
marine_Neoasi_NW_020175670.1_0.0_1_528_10313471_10314000
marine_Phosin_CM018160.1_0.0_1_528_61338368_61337842
marine_Delleu_NW_019160859.1_0.0_1_528_23209207_23209734
marine_Monmon_NW_021703777.1_0.0_1_528_77256114_77256641
query_marine_Mesbid_PVJJ010013433.1_0.0_1_528_39932_40459
marine_Zipcav_RJWS010301267.1_0.0_1_528_333_860
marine_Lipvex_NW_006774670.1_0.0_1_528_1163465_1162938
marine_Inigeo_RJWO010062236.1_0.0_1_528_1881_1354
marine_Kogbre_RJWL010039777.1_0.0_1_528_12880_12353
marine_Phycat_NC_041220.1_0.0_1_528_83566760_83567286
marine_Plamin_RJWK010136037.1_0.0_1_528_938_411
marine_Balbon_DF605222.1_0.0_1_528_10455_9928
marine_Megnov_RYZJ01000843.1_0.0_1_528_2743065_2742538
marine_Balacu_NW_006726576.1_0.0_1_528_7046185_7046712
marine_Escrob_NIPP01016212.1_0.0_1_528_27489_28016
marine_Eubjap_RJWP010022469.1_0.0_1_528_1346_819
PVJP02910208.1_81902-82414_Hippopotamus_amphibius_isolate_BS01_ScHSfme_924697HRSCAF_982497_whole_genome_shotgun_sequence
_R_VSZR01000012.1_25167102-25167633_Camelus_ferus_isolate_YT-003-E_chromosome_2_whole_genome_shotgun_sequence
JARL01026730.1_235890-236421_Camelus_bactrianus_breed_Alxa_contig26730_whole_genome_shotgun_sequence
_R_LSZX01073903.1_3469-4000_Camelus_dromedarius_breed_Targui_Contig_13418_33_whole_genome_shotgun_sequence
JEMW01029015.1_38516-39047_Vicugna_pacos_huacaya_contig29015_whole_genome_shotgun_sequence
_R_SBAQ01001800.1_129213-129701_Eumetopias_jubatus_isolate_GAN_26980396_contig7_237_whole_genome_shotgun_sequence
_R_PISZ01001497.1_229012-229500_Zalophus_californianus_isolate_BS06_ZalCal_flattened_line_2986_whole_genome_shotgun_sequence
ANOP01043033.1_49422-49916_Odobenus_rosmarus_divergens_isolate_Ivan_contig_43024_whole_genome_shotgun_sequence
_R_RXNX01001353.1_112252-112743_Phoca_vitulina_isolate_PV1807_contig3_137_whole_genome_shotgun_sequence
NINY01007818.1_1027817-1028308_Neomonachus_schauinslandi_Super-Scaffold_211_whole_genome_shotgun_sequence
0.02

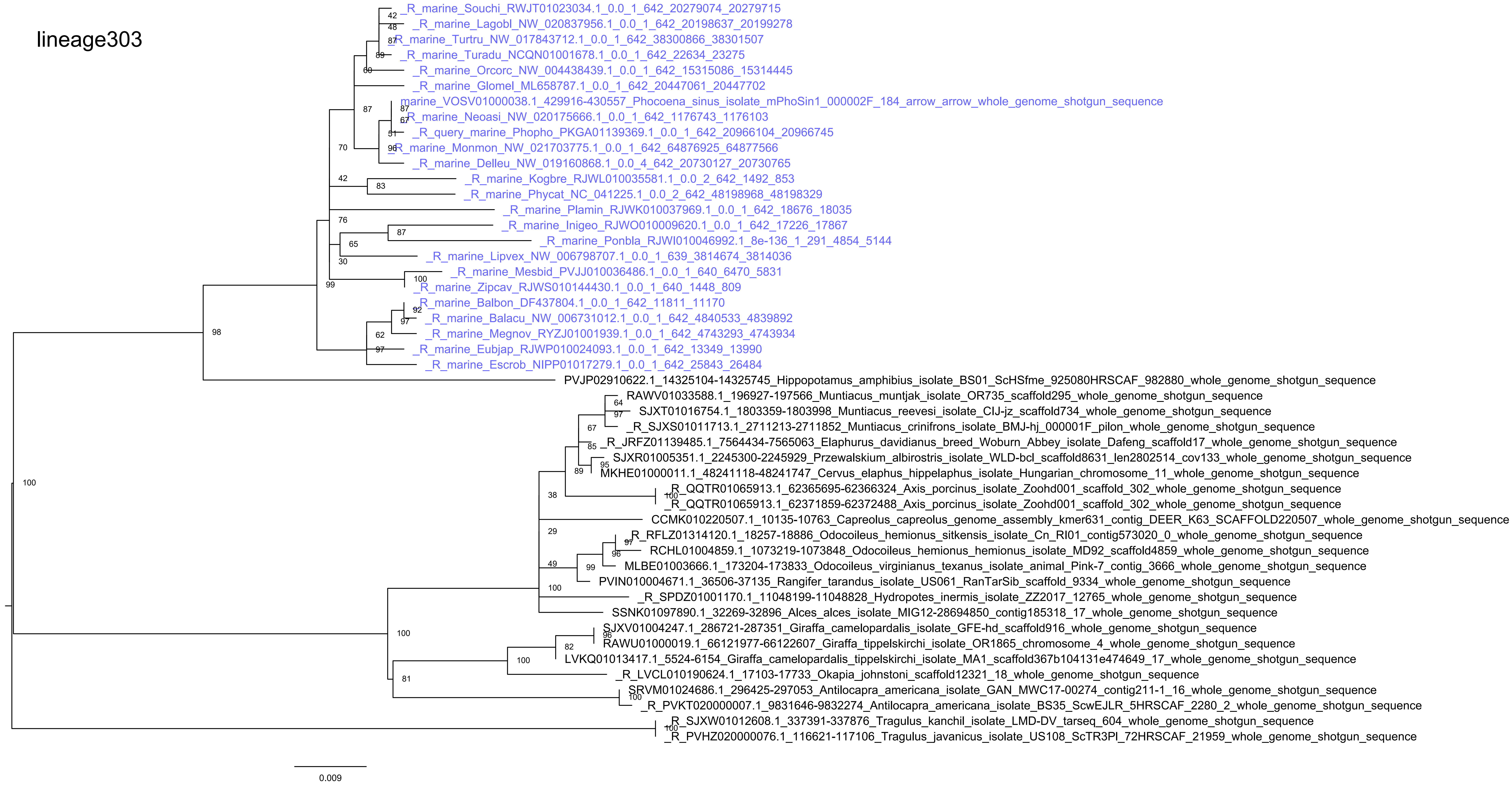
lineage303
_R_marine_Souchi_RWJT01023034.1_0.0_1_642_20279074_20279715
_R_marine_Lagobl_NW_020837956.1_0.0_1_642_20198637_20199278
_R_marine_Turtru_NW_017843712.1_0.0_1_642_38300866_38301507
_R_marine_Turadu_NCQN01001678.1_0.0_1_642_22634_23275
_R_marine_Orcorc_NW_004438439.1_0.0_1_642_15315086_15314445
_R_marine_Glomel_ML658787.1_0.0_1_642_20447061_20447702
marine_VOSV01000038.1_429916-430557_Phocoena_sinus_isolate_mPhoSin1_000002F_184_arrow_arrow_whole_genome_shotgun_sequence
_R_marine_Neoasi_NW_020175666.1_0.0_1_642_1176743_1176103
_R_query_marine_Phopho_PKGA01139369.1_0.0_1_642_20966104_20966745
_R_marine_Monmon_NW_021703775.1_0.0_1_642_64876925_64877566
_R_marine_Delleu_NW_019160868.1_0.0_4_642_20730127_20730765
_R_marine_Kogbre_RJWL010035581.1_0.0_2_642_1492_853
_R_marine_Phycat_NC_041225.1_0.0_2_642_48198968_48198329
_R_marine_Plamin_RJWK010037969.1_0.0_1_642_18676_18035
_R_marine_Inigeo_RJWO010009620.1_0.0_1_642_17226_17867
_R_marine_Ponbla_RJWI010046992.1_8e-136_1_291_4854_5144
_R_marine_Lipvex_NW_006798707.1_0.0_1_639_3814674_3814036
_R_marine_Mesbid_PVJJ010036486.1_0.0_1_640_6470_5831
_R_marine_Zipcav_RJWS010144430.1_0.0_1_640_1448_809
_R_marine_Balbon_DF437804.1_0.0_1_642_11811_11170
_R_marine_Balacu_NW_006731012.1_0.0_1_642_4840533_4839892
_R_marine_Megnov_RYZJ01001939.1_0.0_1_642_4743293_4743934
_R_marine_Eubjap_RJWP010024093.1_0.0_1_642_13349_13990
_R_marine_Escrob_NIPP01017279.1_0.0_1_642_25843_26484
PVJP02910622.1_14325104-14325745_Hippopotamus_amphibius_isolate_BS01_ScHSfme_925080HRSCAF_982880_whole_genome_shotgun_sequence
RAWV01033588.1_196927-197566_Muntiacus_muntjak_isolate_OR735_scaffold295_whole_genome_shotgun_sequence
SJXT01016754.1_1803359-1803998_Muntiacus_reevesi_isolate_CIJ-jz_scaffold734_whole_genome_shotgun_sequence
_R_SJXS01011713.1_2711213-2711852_Muntiacus_crinifrons_isolate_BMJ-hj_000001F_pilon_whole_genome_shotgun_sequence
_R_JRFZ01139485.1_7564434-7565063_Elaphurus_davidianus_breed_Woburn_Abbey_isolate_Dafeng_scaffold17_whole_genome_shotgun_sequence
SJXR01005351.1_2245300-2245929_Przewalskium_albirostris_isolate_WLD-bcl_scaffold8631_len2802514_cov133_whole_genome_shotgun_sequence
MKHE01000011.1_48241118-48241747_Cervus_elaphus_hippelaphus_isolate_Hungarian_chromosome_11_whole_genome_shotgun_sequence
_R_QQTR01065913.1_62365695-62366324_Axis_porcinus_isolate_Zoohd001_scaffold_302_whole_genome_shotgun_sequence
_R_QQTR01065913.1_62371859-62372488_Axis_porcinus_isolate_Zoohd001_scaffold_302_whole_genome_shotgun_sequence
CCMK010220507.1_10135-10763_Capreolus_capreolus_genome_assembly_kmer631_contig_DEER_K63_SCAFFOLD220507_whole_genome_shotgun_sequence
_R_RFLZ01314120.1_18257-18886_Odocoileus_hemionus_sitkensis_isolate_Cn_RI01_contig573020_0_whole_genome_shotgun_sequence
RCHL01004859.1_1073219-1073848_Odocoileus_hemionus_hemionus_isolate_MD92_scaffold4859_whole_genome_shotgun_sequence
MLBE01003666.1_173204-173833_Odocoileus_virginianus_texanus_isolate_animal_Pink-7_contig_3666_whole_genome_shotgun_sequence
PVIN010004671.1_36506-37135_Rangifer_tarandus_isolate_US061_RanTarSib_scaffold_9334_whole_genome_shotgun_sequence
_R_SPDZ01001170.1_11048199-11048828_Hydropotes_inermis_isolate_ZZ2017_12765_whole_genome_shotgun_sequence
SSNK01097890.1_32269-32896_Alces_alces_isolate_MIG12-28694850_contig185318_17_whole_genome_shotgun_sequence
SJXV01004247.1_286721-287351_Giraffa_camelopardalis_isolate_GFE-hd_scaffold916_whole_genome_shotgun_sequence
RAWU01000019.1_66121977-66122607_Giraffa_tippelskirchi_isolate_OR1865_chromosome_4_whole_genome_shotgun_sequence
LVKQ01013417.1_5524-6154_Giraffa_camelopardalis_tippelskirchi_isolate_MA1_scaffold367b104131e474649_17_whole_genome_shotgun_sequence
_R_LVCL010190624.1_17103-17733_Okapia_johnstoni_scaffold12321_18_whole_genome_shotgun_sequence
SRVM01024686.1_296425-297053_Antilocapra_americana_isolate_GAN_MWC17-00274_contig211-1_16_whole_genome_shotgun_sequence
_R_PVKT020000007.1_9831646-9832274_Antilocapra_americana_isolate_BS35_ScwEJLR_5HRSCAF_2280_2_whole_genome_shotgun_sequence
_R_SJXW01012608.1_337391-337876_Tragulus_kanchil_isolate_LMD-DV_tarseq_604_whole_genome_shotgun_sequence
_R_PVHZ020000076.1_116621-117106_Tragulus_javanicus_isolate_US108_ScTR3Pl_72HRSCAF_21959_whole_genome_shotgun_sequence
0.009

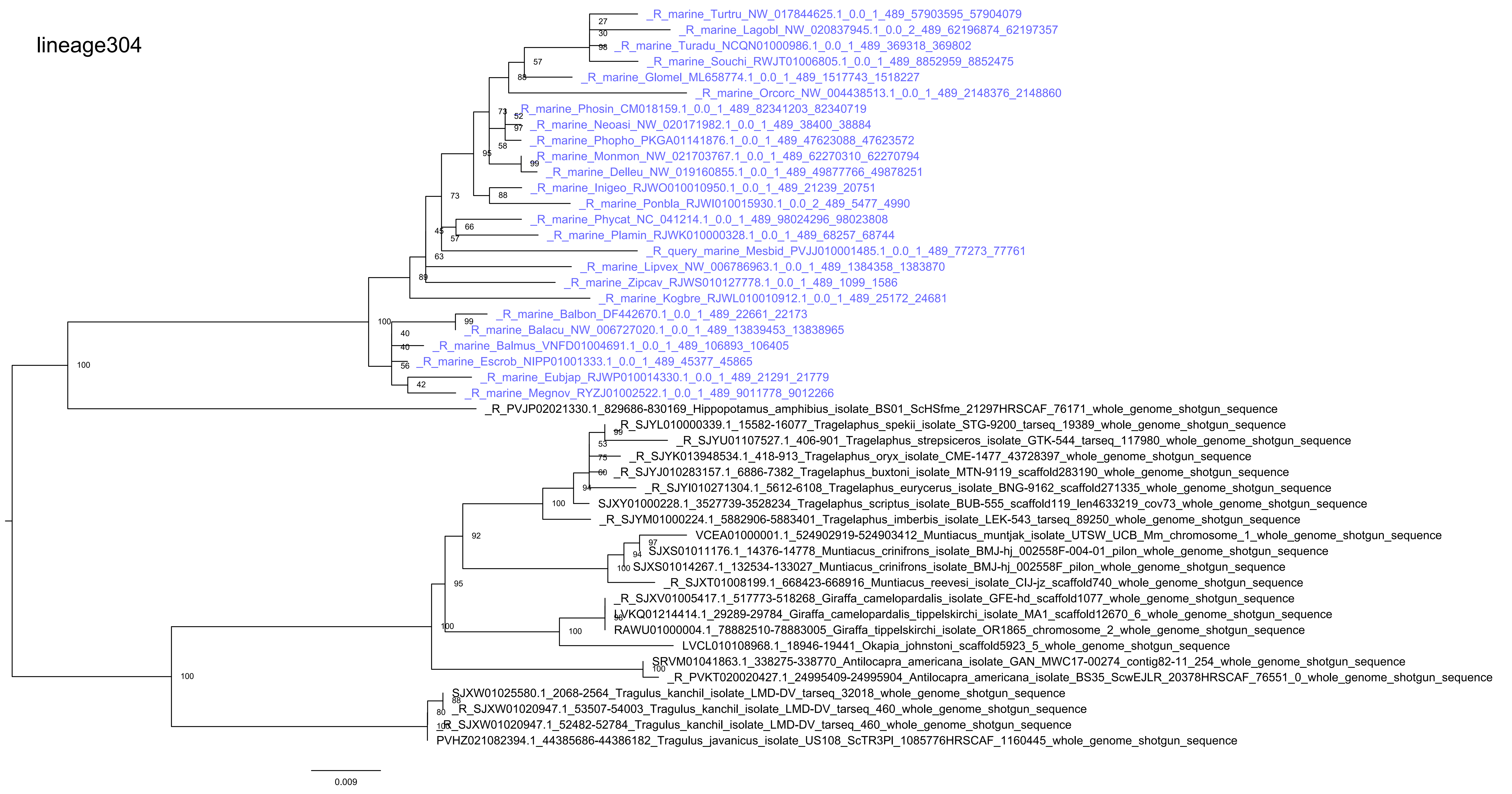
lineage304
_R_marine_Turtru_NW_017844625.1_0.0_1_489_57903595_57904079
_R_marine_Lagobl_NW_020837945.1_0.0_2_489_62196874_62197357
_R_marine_Turadu_NCQN01000986.1_0.0_1_489_369318_369802
_R_marine_Souchi_RWJT01006805.1_0.0_1_489_8852959_8852475
_R_marine_Glomel_ML658774.1_0.0_1_489_1517743_1518227
_R_marine_Orcorc_NW_004438513.1_0.0_1_489_2148376_2148860
_R_marine_Phosin_CM018159.1_0.0_1_489_82341203_82340719
_R_marine_Neoasi_NW_020171982.1_0.0_1_489_38400_38884
_R_marine_Phopho_PKGA01141876.1_0.0_1_489_47623088_47623572
_R_marine_Monmon_NW_021703767.1_0.0_1_489_62270310_62270794
_R_marine_Delleu_NW_019160855.1_0.0_1_489_49877766_49878251
_R_marine_Inigeo_RJWO010010950.1_0.0_1_489_21239_20751
_R_marine_Ponbla_RJWI010015930.1_0.0_2_489_5477_4990
_R_marine_Phycat_NC_041214.1_0.0_1_489_98024296_98023808
_R_marine_Plamin_RJWK010000328.1_0.0_1_489_68257_68744
_R_query_marine_Mesbid_PVJJ010001485.1_0.0_1_489_77273_77761
_R_marine_Lipvex_NW_006786963.1_0.0_1_489_1384358_1383870
_R_marine_Zipcav_RJWS010127778.1_0.0_1_489_1099_1586
_R_marine_Kogbre_RJWL010010912.1_0.0_1_489_25172_24681
_R_marine_Balbon_DF442670.1_0.0_1_489_22661_22173
_R_marine_Balacu_NW_006727020.1_0.0_1_489_13839453_13838965
_R_marine_Balmus_VNFD01004691.1_0.0_1_489_106893_106405
_R_marine_Escrob_NIPP01001333.1_0.0_1_489_45377_45865
_R_marine_Eubjap_RJWP010014330.1_0.0_1_489_21291_21779
_R_marine_Megnov_RYZJ01002522.1_0.0_1_489_9011778_9012266
_R_PVJP02021330.1_829686-830169_Hippopotamus_amphibius_isolate_BS01_ScHSfme_21297HRSCAF_76171_whole_genome_shotgun_sequence
_R_SJYL010000339.1_15582-16077_Tragelaphus_spekii_isolate_STG-9200_tarseq_19389_whole_genome_shotgun_sequence
_R_SJYU01107527.1_406-901_Tragelaphus_strepsiceros_isolate_GTK-544_tarseq_117980_whole_genome_shotgun_sequence
_R_SJYK013948534.1_418-913_Tragelaphus_oryx_isolate_CME-1477_43728397_whole_genome_shotgun_sequence
_R_SJYJ010283157.1_6886-7382_Tragelaphus_buxtoni_isolate_MTN-9119_scaffold283190_whole_genome_shotgun_sequence
_R_SJYI010271304.1_5612-6108_Tragelaphus_eurycerus_isolate_BNG-9162_scaffold271335_whole_genome_shotgun_sequence
SJXY01000228.1_3527739-3528234_Tragelaphus_scriptus_isolate_BUB-555_scaffold119_len4633219_cov73_whole_genome_shotgun_sequence
_R_SJYM01000224.1_5882906-5883401_Tragelaphus_imberbis_isolate_LEK-543_tarseq_89250_whole_genome_shotgun_sequence
VCEA01000001.1_524902919-524903412_Muntiacus_muntjak_isolate_UTSW_UCB_Mm_chromosome_1_whole_genome_shotgun_sequence
SJXS01011176.1_14376-14778_Muntiacus_crinifrons_isolate_BMJ-hj_002558F-004-01_pilon_whole_genome_shotgun_sequence
SJXS01014267.1_132534-133027_Muntiacus_crinifrons_isolate_BMJ-hj_002558F_pilon_whole_genome_shotgun_sequence
_R_SJXT01008199.1_668423-668916_Muntiacus_reevesi_isolate_CIJ-jz_scaffold740_whole_genome_shotgun_sequence
_R_SJXV01005417.1_517773-518268_Giraffa_camelopardalis_isolate_GFE-hd_scaffold1077_whole_genome_shotgun_sequence
LVKQ01214414.1_29289-29784_Giraffa_camelopardalis_tippelskirchi_isolate_MA1_scaffold12670_6_whole_genome_shotgun_sequence
RAWU01000004.1_78882510-78883005_Giraffa_tippelskirchi_isolate_OR1865_chromosome_2_whole_genome_shotgun_sequence
LVCL010108968.1_18946-19441_Okapia_johnstoni_scaffold5923_5_whole_genome_shotgun_sequence
SRVM01041863.1_338275-338770_Antilocapra_americana_isolate_GAN_MWC17-00274_contig82-11_254_whole_genome_shotgun_sequence
_R_PVKT020020427.1_24995409-24995904_Antilocapra_americana_isolate_BS35_ScwEJLR_20378HRSCAF_76551_0_whole_genome_shotgun_sequence
SJXW01025580.1_2068-2564_Tragulus_kanchil_isolate_LMD-DV_tarseq_32018_whole_genome_shotgun_sequence
_R_SJXW01020947.1_53507-54003_Tragulus_kanchil_isolate_LMD-DV_tarseq_460_whole_genome_shotgun_sequence
_R_SJXW01020947.1_52482-52784_Tragulus_kanchil_isolate_LMD-DV_tarseq_460_whole_genome_shotgun_sequence
PVHZ021082394.1_44385686-44386182_Tragulus_javanicus_isolate_US108_ScTR3PI_1085776HRSCAF_1160445_whole_genome_shotgun_sequence
0.009

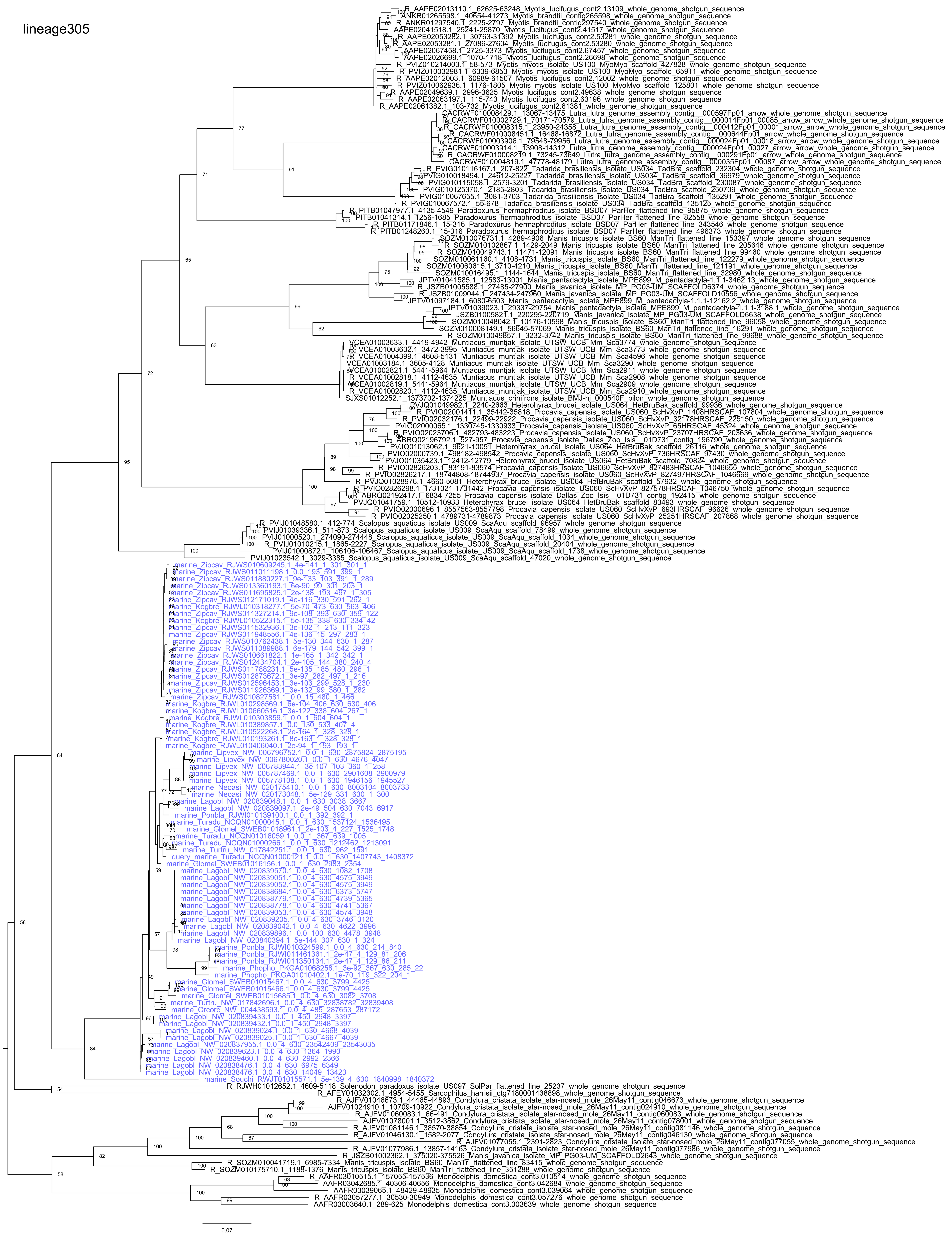
lineage305
R_AAPE02013110.1_62625-63248_Myotis_lucifugus_cont2.13109_whole_genome_shotgun_sequence
ANKR01265598.1_40654-41273_Myotis_brandtii_contig265598_whole_genome_shotgun_sequence
R_ANKR01297540.1_2225-2797_Myotis_brandtii_contig297540_whole_genome_shotgun_sequence
AAPE02041518.1_25241-25870_Myotis_lucifugus_cont2.41517_whole_genome_shotgun_sequence
R_AAPE02053282.1_30763-31392_Myotis_lucifugus_cont2.53281_whole_genome_shotgun_sequence
R_AAPE02053281.1_27086-27604_Myotis_lucifugus_cont2.53280_whole_genome_shotgun_sequence
AAPE02067458.1_2725-3373_Myotis_lucifugus_cont2.67457_whole_genome_shotgun_sequence
AAPE02026699.1_1070-1718_Myotis_lucifugus_cont2.26698_whole_genome_shotgun_sequence
R_PVIZ010214003.1_58-573_Myotis_myotis_isolate_US100_MyoMyo_scaffold_427828_whole_genome_shotgun_sequence
R_PVIZ010032981.1_6339-6853_Myotis_myotis_isolate_US100_MyoMyo_scaffold_65911_whole_genome_shotgun_sequence
R_AAPE02012003.1_60989-61507_Myotis_lucifugus_cont2.12002_whole_genome_shotgun_sequence
R_PVIZ010062936.1_1176-1805_Myotis_myotis_isolate_US100_MyoMyo_scaffold_125801_whole_genome_shotgun_sequence
R_AAPE02049639.1_2996-3625_Myotis_lucifugus_cont2.49638_whole_genome_shotgun_sequence
R_AAPE02063197.1_115-743_Myotis_lucifugus_cont2.63196_whole_genome_shotgun_sequence
R_AAPE02061382.1_103-732_Myotis_lucifugus_cont2.61381_whole_genome_shotgun_sequence
CACRWF010008429.1_13067-13475_Lutra_lutra_genome_assembly_contig__000597Fp01_arrow_whole_genome_shotgun_sequence
R_CACRWF010002729.1_70171-70579_Lutra_lutra_genome_assembly_contig__000014Fp01_00085_arrow_arrow_whole_genome_shotgun_sequence
R_CACRWF010008315.1_23950-24358_Lutra_lutra_genome_assembly_contig__000412Fp01_00001_arrow_arrow_whole_genome_shotgun_sequence
R_CACRWF010008451.1_16468-16872_Lutra_lutra_genome_assembly_contig__000644Fp01_arrow_whole_genome_shotgun_sequence
CACRWF010003906.1_79548-79956_Lutra_lutra_genome_assembly_contig__000024Fp01_00018_arrow_arrow_whole_genome_shotgun_sequence
CACRWF010003914.1_13908-14312_Lutra_lutra_genome_assembly_contig__000024Fp01_00027_arrow_arrow_whole_genome_shotgun_sequence
R_CACRWF010008219.1_73245-73649_Lutra_lutra_genome_assembly_contig__000291Fp01_arrow_whole_genome_shotgun_sequence
CACRWF010004819.1_47778-48179_Lutra_lutra_genome_assembly_contig__000035Fp01_00087_arrow_arrow_whole_genome_shotgun_sequence
R_PVIG010116167.1_207-822_Tadarida_brasiliensis_isolate_US034_TadBra_scaffold_232304_whole_genome_shotgun_sequence
PVIG010018494.1_24612-25227_Tadarida_brasiliensis_isolate_US034_TadBra_scaffold_36979_whole_genome_shotgun_sequence
PVIG010115058.1_2579-3201_Tadarida_brasiliensis_isolate_US034_TadBra_scaffold_230087_whole_genome_shotgun_sequence
PVIG010125370.1_2185-2803_Tadarida_brasiliensis_isolate_US034_TadBra_scaffold_250709_whole_genome_shotgun_sequence
PVIG010067655.1_3081-3703_Tadarida_brasiliensis_isolate_US034_TadBra_scaffold_135291_whole_genome_shotgun_sequence
R_PVIG010067572.1_55-678_Tadarida_brasiliensis_isolate_US034_TadBra_scaffold_135125_whole_genome_shotgun_sequence
R_PITB01047977.1_4135-4549_Paradoxurus_hermaphroditus_isolate_BSD07_ParHer_flattened_line_95875_whole_genome_shotgun_sequence
PITB01041314.1_1256-1685_Paradoxurus_hermaphroditus_isolate_BSD07_ParHer_flattened_line_82558_whole_genome_shotgun_sequence
R_PITB01171846.1_15-316_Paradoxurus_hermaphroditus_isolate_BSD07_ParHer_flattened_line_343546_whole_genome_shotgun_sequence
R_PITB01248260.1_15-316_Paradoxurus_hermaphroditus_isolate_BSD07_ParHer_flattened_line_496373_whole_genome_shotgun_sequence
SOZM010076731.1_4289-4906_Manis_tricuspis_isolate_BS60_ManTri_flattened_line_153397_whole_genome_shotgun_sequence
R_SOZM010102867.1_1429-2049_Manis_tricuspis_isolate_BS60_ManTri_flattened_line_205646_whole_genome_shotgun_sequence
R_SOZM010049743.1_11471-12091_Manis_tricuspis_isolate_BS60_ManTri_flattened_line_99460_whole_genome_shotgun_sequence
SOZM010061160.1_4108-4731_Manis_tricuspis_isolate_BS60_ManTri_flattened_line_122279_whole_genome_shotgun_sequence
SOZM010060615.1_3710-4210_Manis_tricuspis_isolate_BS60_ManTri_flattened_line_121191_whole_genome_shotgun_sequence
SOZM010016495.1_1144-1644_Manis_tricuspis_isolate_BS60_ManTri_flattened_line_32980_whole_genome_shotgun_sequence
JPTV01041585.1_12583-13001_Manis_pentadactyla_isolate_MPE899_M_pentadactyla-1.1.1-3462.13_whole_genome_shotgun_sequence
R_JSZB01005588.1_27485-27900_Manis_javanica_isolate_MP_PG03-UM_SCAFFOLD6374_whole_genome_shotgun_sequence
R_JSZB01009044.1_247434-247960_Manis_javanica_isolate_MP_PG03-UM_SCAFFOLD10556_whole_genome_shotgun_sequence
JPTV01097184.1_6080-6503_Manis_pentadactyla_isolate_MPE899_M_pentadactyla-1.1.1-12162.2_whole_genome_shotgun_sequence
JPTV01039023.1_29337-29754_Manis_pentadactyla_isolate_MPE899_M_pentadactyla-1.1.1-3188.1_whole_genome_shotgun_sequence
JSZB01005821.1_220295-220719_Manis_javanica_isolate_MP_PG03-UM_SCAFFOLD6638_whole_genome_shotgun_sequence
SOZM010048042.1_10176-10598_Manis_tricuspis_isolate_BS60_ManTri_flattened_line_96058_whole_genome_shotgun_sequence
SOZM010008149.1_56645-57069_Manis_tricuspis_isolate_BS60_ManTri_flattened_line_16291_whole_genome_shotgun_sequence
R_SOZM010049857.1_3232-3742_Manis_tricuspis_isolate_BS60_ManTri_flattened_line_99688_whole_genome_shotgun_sequence
VCEA01003633.1_4419-4942_Muntiacus_muntjak_isolate_UTSW_UCB_Mm_Sca3774_whole_genome_shotgun_sequence
R_VCEA01003632.1_3472-3995_Muntiacus_muntjak_isolate_UTSW_UCB_Mm_Sca3773_whole_genome_shotgun_sequence
R_VCEA01004399.1_4608-5131_Muntiacus_muntjak_isolate_UTSW_UCB_Mm_Sca4596_whole_genome_shotgun_sequence
VCEA01003184.1_3605-4128_Muntiacus_muntjak_isolate_UTSW_UCB_Mm_Sca3290_whole_genome_shotgun_sequence
VCEA01002821.1_5441-5964_Muntiacus_muntjak_isolate_UTSW_UCB_Mm_Sca2911_whole_genome_shotgun_sequence
R_VCEA01002818.1_4112-4635_Muntiacus_muntjak_isolate_UTSW_UCB_Mm_Sca2908_whole_genome_shotgun_sequence
VCEA01002819.1_5441-5964_Muntiacus_muntjak_isolate_UTSW_UCB_Mm_Sca2909_whole_genome_shotgun_sequence
R_VCEA01002820.1_4112-4635_Muntiacus_muntjak_isolate_UTSW_UCB_Mm_Sca2910_whole_genome_shotgun_sequence
SJXS01012252.1_1373702-1374225_Muntiacus_crinifrons_isolate_BMJ-hj_000540F_pilon_whole_genome_shotgun_sequence
PVJQ01049982.1_2240-2663_Heterohyrax_brucei_isolate_US064_HetBruBak_scaffold_99936_whole_genome_shotgun_sequence
R_PVIO02001411.1_35442-35818_Procavia_capensis_isolate_US060_ScHvXvP_1408HRSCAF_107804_whole_genome_shotgun_sequence
R_PVIO02032176.1_22499-22922_Procavia_capensis_isolate_US060_ScHvXvP_32178HRSCAF_225150_whole_genome_shotgun_sequence
PVIO02000065.1_1330745-1330933_Procavia_capensis_isolate_US060_ScHvXvP_65HRSCAF_45324_whole_genome_shotgun_sequence
R_PVIO02023706.1_482793-483223_Procavia_capensis_isolate_US060_ScHvXvP_23707HRSCAF_203636_whole_genome_shotgun_sequence
ABRQ02196792.1_527-957_Procavia_capensis_isolate_Dallas_Zoo_Isis__01D731_contig_196790_whole_genome_shotgun_sequence
PVJQ01013062.1_9621-10051_Heterohyrax_brucei_isolate_US064_HetBruBak_scaffold_26116_whole_genome_shotgun_sequence
PVIO02000739.1_498182-498542_Procavia_capensis_isolate_US060_ScHvXvP_736HRSCAF_97430_whole_genome_shotgun_sequence
PVJQ01035423.1_12412-12779_Heterohyrax_brucei_isolate_US064_HetBruBak_scaffold_70824_whole_genome_shotgun_sequence
R_PVIO02826203.1_83191-83574_Procavia_capensis_isolate_US060_ScHvXvP_827483HRSCAF_1046655_whole_genome_shotgun_sequence
R_PVIO02826217.1_18744808-18744937_Procavia_capensis_isolate_US060_ScHvXvP_827497HRSCAF_1046669_whole_genome_shotgun_sequence
R_PVJQ01028976.1_4660-5081_Heterohyrax_brucei_isolate_US064_HetBruBak_scaffold_57932_whole_genome_shotgun_sequence
R_PVIO02826298.1_1731021-1731442_Procavia_capensis_isolate_US060_ScHvXvP_827578HRSCAF_1046750_whole_genome_shotgun_sequence
R_ABRQ02192417.1_6834-7255_Procavia_capensis_isolate_Dallas_Zoo_Isis__01D731_contig_192415_whole_genome_shotgun_sequence
PVJQ01041759.1_10512-10933_Heterohyrax_brucei_isolate_US064_HetBruBak_scaffold_83493_whole_genome_shotgun_sequence
R_PVIO02000696.1_8557563-8557798_Procavia_capensis_isolate_US060_ScHvXvP_693HRSCAF_96626_whole_genome_shotgun_sequence
R_PVIO02025250.1_4789731-4789873_Procavia_capensis_isolate_US060_ScHvXvP_25251HRSCAF_207868_whole_genome_shotgun_sequence
R_PVIJ01048580.1_412-774_Scalopus_aquaticus_isolate_US009_ScaAqu_scaffold_96957_whole_genome_shotgun_sequence
PVIJ01039336.1_511-873_Scalopus_aquaticus_isolate_US009_ScaAqu_scaffold_78499_whole_genome_shotgun_sequence
PVIJ01000520.1_274090-274448_Scalopus_aquaticus_isolate_US009_ScaAqu_scaffold_1034_whole_genome_shotgun_sequence
R_PVIJ01010215.1_1865-2227_Scalopus_aquaticus_isolate_US009_ScaAqu_scaffold_20404_whole_genome_shotgun_sequence
PVIJ01000872.1_106106-106467_Scalopus_aquaticus_isolate_US009_ScaAqu_scaffold_1738_whole_genome_shotgun_sequence
PVIJ01023542.1_3029-3385_Scalopus_aquaticus_isolate_US009_ScaAqu_scaffold_47020_whole_genome_shotgun_sequence
marine_Zipcav_RJWS010609245.1_4e-141_1_301_301_1
marine_Zipcav_RJWS011011198.1_0.0_193_591_399_1
marine_Zipcav_RJWS011880227.1_9e-133_103_391_1_289
marine_Zipcav_RJWS013360193.1_6e-90_99_301_203_1
marine_Zipcav_RJWS011695825.1_2e-138_193_497_1_305
marine_Zipcav_RJWS012171019.1_4e-116_330_591_262_1
marine_Kogbre_RJWL010318277.1_5e-70_473_630_563_406
marine_Zipcav_RJWS011327214.1_9e-108_393_630_359_122
marine_Kogbre_RJWL010522315.1_5e-135_338_630_334_42
marine_Zipcav_RJWS011532936.1_3e-102_1_213_111_323
marine_Zipcav_RJWS011948556.1_4e-136_15_297_283_1
marine_Zipcav_RJWS010762438.1_5e-130_344_630_1_287
marine_Zipcav_RJWS011089988.1_6e-179_144_542_399_1
marine_Zipcav_RJWS010661822.1_1e-165_1_342_342_1
marine_Zipcav_RJWS012434704.1_2e-105_144_380_240_4
marine_Zipcav_RJWS011788231.1_5e-135_185_480_296_1
marine_Zipcav_RJWS012873672.1_3e-97_282_497_1_216
marine_Zipcav_RJWS012596453.1_3e-103_299_528_1_230
marine_Zipcav_RJWS011926369.1_3e-132_99_380_1_282
marine_Zipcav_RJWS010827581.1_0.0_15_480_1_466
marine_Kogbre_RJWL010298569.1_6e-104_406_630_630_406
marine_Kogbre_RJWL010660516.1_3e-122_338_604_267_1
marine_Kogbre_RJWL010303859.1_0.0_1_604_604_1
marine_Kogbre_RJWL010389857.1_0.0_130_533_407_4
marine_Kogbre_RJWL010522268.1_2e-164_1_328_328_1
marine_Kogbre_RJWL010193261.1_8e-163_1_328_328_1
marine_Kogbre_RJWL010406040.1_2e-94_1_193_193_1
marine_Lipvex_NW_006796752.1_0.0_1_630_2875824_2875195
marine_Lipvex_NW_006780020.1_0.0_1_630_4676_4047
marine_Lipvex_NW_006783944.1_3e-107_103_360_1_258
marine_Lipvex_NW_006787469.1_0.0_1_630_2901608_2900979
marine_Lipvex_NW_006778108.1_0.0_1_630_1946156_1945527
marine_Neoasi_NW_020175410.1_0.0_1_630_8003104_8003733
marine_Neoasi_NW_020173048.1_5e-129_331_630_1_300
marine_Lagobl_NW_020839048.1_0.0_1_630_3038_3667
marine_Lagobl_NW_020839097.1_2e-49_504_630_7043_6917
marine_Ponbla_RJWI010139100.1_0.0_1_392_392_1
marine_Turadu_NCQN01000045.1_0.0_1_630_1537124_1536495
marine_Glomel_SWEB01018961.1_2e-103_4_227_1525_1748
marine_Turadu_NCQN01016059.1_0.0_1_367_639_1005
marine_Turadu_NCQN01000266.1_0.0_1_630_1212462_1213091
marine_Turtru_NW_017842251.1_0.0_1_630_962_1591
query_marine_Turadu_NCQN01000121.1_0.0_1_630_1407743_1408372
marine_Glomel_SWEB01016156.1_0.0_1_630_2983_2354
marine_Lagobl_NW_020839570.1_0.0_4_630_1082_1708
marine_Lagobl_NW_020839051.1_0.0_4_630_4575_3949
marine_Lagobl_NW_020839052.1_0.0_4_630_4575_3949
marine_Lagobl_NW_020838684.1_0.0_4_630_6373_5747
marine_Lagobl_NW_020838779.1_0.0_4_630_4739_5365
marine_Lagobl_NW_020838778.1_0.0_4_630_4741_5367
marine_Lagobl_NW_020839053.1_0.0_4_630_4574_3948
marine_Lagobl_NW_020839205.1_0.0_4_630_3746_3120
marine_Lagobl_NW_020839042.1_0.0_4_630_4622_3996
marine_Lagobl_NW_020839896.1_0.0_100_630_4478_3948
marine_Lagobl_NW_020840394.1_5e-144_307_630_1_324
marine_Ponbla_RJWI010324599.1_0.0_4_630_214_840
marine_Ponbla_RJWI011461361.1_2e-47_4_129_81_206
marine_Ponbla_RJWI011350134.1_2e-47_4_129_86_211
marine_Phopho_PKGA01068258.1_3e-92_367_630_285_22
marine_Phopho_PKGA01010402.1_1e-70_119_322_204_1
marine_Glomel_SWEB01015467.1_0.0_4_630_3799_4425
marine_Glomel_SWEB01015466.1_0.0_4_630_3799_4425
marine_Glomel_SWEB01015685.1_0.0_4_630_3082_3708
marine_Turtru_NW_017842696.1_0.0_4_630_32838782_32839408
marine_Orcorc_NW_004438593.1_0.0_4_485_287653_287172
marine_Lagobl_NW_020839433.1_0.0_1_450_2948_3397
marine_Lagobl_NW_020839432.1_0.0_1_450_2948_3397
marine_Lagobl_NW_020839024.1_0.0_1_630_4668_4039
marine_Lagobl_NW_020839025.1_0.0_1_630_4667_4039
marine_Lagobl_NW_020837955.1_0.0_4_630_23542409_23543035
marine_Lagobl_NW_020839623.1_0.0_4_630_1364_1990
marine_Lagobl_NW_020839460.1_0.0_4_630_2992_2366
marine_Lagobl_NW_020838476.1_0.0_4_630_6975_6349
marine_Lagobl_NW_020838476.1_0.0_4_630_14049_13423
marine_Souchi_RWJT01015571.1_5e-139_4_630_1840998_1840372
R_RJWH01012652.1_4609-5118_Solenodon_paradoxus_isolate_US097_SolPar_flattened_line_25237_whole_genome_shotgun_sequence
R_AFEY01032302.1_4954-5455_Sarcophilus_harrisii_ctg7180001438898_whole_genome_shotgun_sequence
R_AJFV01046673.1_44465-44893_Condylura_cristata_isolate_star-nosed_mole_26May11_contig046673_whole_genome_shotgun_sequence
AJFV01024910.1_10709-10922_Condylura_cristata_isolate_star-nosed_mole_26May11_contig024910_whole_genome_shotgun_sequence
R_AJFV01060083.1_66-491_Condylura_cristata_isolate_star-nosed_mole_26May11_contig060083_whole_genome_shotgun_sequence
AJFV01078001.1_3512-3862_Condylura_cristata_isolate_star-nosed_mole_26May11_contig078001_whole_genome_shotgun_sequence
R_AJFV01081146.1_38570-38854_Condylura_cristata_isolate_star-nosed_mole_26May11_contig081146_whole_genome_shotgun_sequence
R_AJFV01046130.1_1582-2077_Condylura_cristata_isolate_star-nosed_mole_26May11_contig046130_whole_genome_shotgun_sequence
R_AJFV01077055.1_2391-2823_Condylura_cristata_isolate_star-nosed_mole_26May11_contig077055_whole_genome_shotgun_sequence
R_AJFV01077986.1_13857-14163_Condylura_cristata_isolate_star-nosed_mole_26May11_contig077986_whole_genome_shotgun_sequence
R_JSZB01002362.1_375020-375526_Manis_javanica_isolate_MP_PG03-UM_SCAFFOLD2643_whole_genome_shotgun_sequence
R_SOZM010041719.1_6985-7334_Manis_tricuspis_isolate_BS60_ManTri_flattened_line_83415_whole_genome_shotgun_sequence
R_SOZM010175710.1_1188-1376_Manis_tricuspis_isolate_BS60_ManTri_flattened_line_351288_whole_genome_shotgun_sequence
R_AAFR03010515.1_157055-157536_Monodelphis_domestica_cont3.010514_whole_genome_shotgun_sequence
AAFR03042685.1_40306-40656_Monodelphis_domestica_cont3.042684_whole_genome_shotgun_sequence
AAFR03039065.1_48429-48935_Monodelphis_domestica_cont3.039064_whole_genome_shotgun_sequence
R_AAFR03057277.1_30530-30949_Monodelphis_domestica_cont3.057276_whole_genome_shotgun_sequence
AAFR03003640.1_289-625_Monodelphis_domestica_cont3.003639_whole_genome_shotgun_sequence
0.07

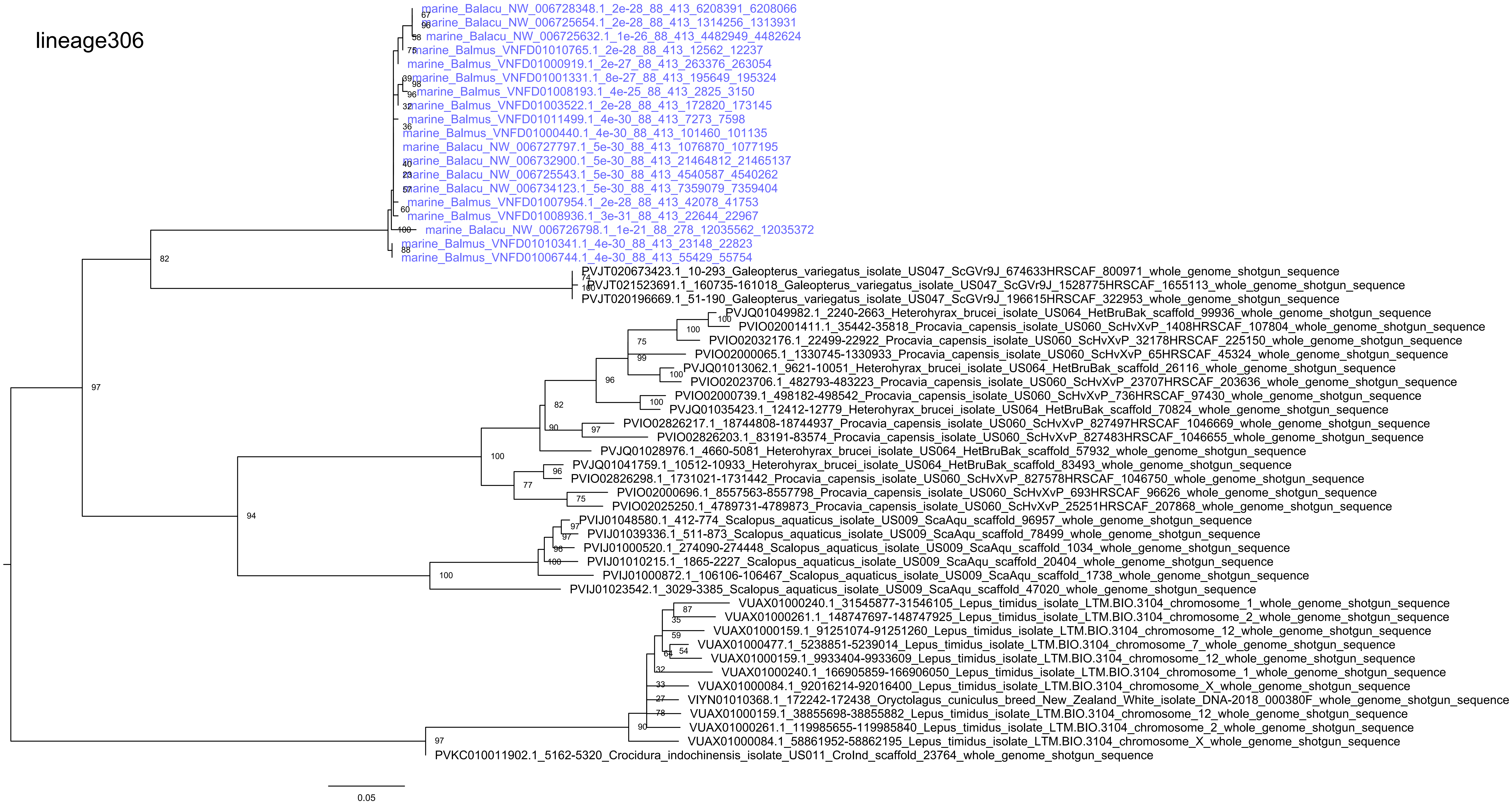

lineage306
marine_Balacu_NW_006728348.1_2e-28_88_413_6208391_6208066
marine_Balacu_NW_006725654.1_2e-28_88_413_1314256_1313931
marine_Balacu_NW_006725632.1_1e-26_88_413_4482949_4482624
marine_Balmus_VNFD01010765.1_2e-28_88_413_12562_12237
marine_Balmus_VNFD01000919.1_2e-27_88_413_263376_263054
marine_Balmus_VNFD01001331.1_8e-27_88_413_195649_195324
marine_Balmus_VNFD01008193.1_4e-25_88_413_2825_3150
marine_Balmus_VNFD01003522.1_2e-28_88_413_172820_173145
marine_Balmus_VNFD01011499.1_4e-30_88_413_7273_7598
marine_Balmus_VNFD01000440.1_4e-30_88_413_101460_101135
marine_Balacu_NW_006727797.1_5e-30_88_413_1076870_1077195
marine_Balacu_NW_006732900.1_5e-30_88_413_21464812_21465137
marine_Balacu_NW_006725543.1_5e-30_88_413_4540587_4540262
marine_Balacu_NW_006734123.1_5e-30_88_413_7359079_7359404
marine_Balmus_VNFD01007954.1_2e-28_88_413_42078_41753
marine_Balmus_VNFD01008936.1_3e-31_88_413_22644_22967
marine_Balacu_NW_006726798.1_1e-21_88_278_12035562_12035372
marine_Balmus_VNFD01010341.1_4e-30_88_413_23148_22823
marine_Balmus_VNFD01006744.1_4e-30_88_413_55429_55754
PVJT020673423.1_10-293_Galeopterus_variegatus_isolate_US047_ScGVr9J_674633HRSCAF_800971_whole_genome_shotgun_sequence
PVJT021523691.1_160735-161018_Galeopterus_variegatus_isolate_US047_ScGVr9J_1528775HRSCAF_1655113_whole_genome_shotgun_sequence
PVJT020196669.1_51-190_Galeopterus_variegatus_isolate_US047_ScGVr9J_196615HRSCAF_322953_whole_genome_shotgun_sequence
PVJQ01049982.1_2240-2663_Heterohyrax_brucei_isolate_US064_HetBruBak_scaffold_99936_whole_genome_shotgun_sequence
PVIO02001411.1_35442-35818_Procavia_capensis_isolate_US060_ScHvXvP_1408HRSCAF_107804_whole_genome_shotgun_sequence
PVIO02032176.1_22499-22922_Procavia_capensis_isolate_US060_ScHvXvP_32178HRSCAF_225150_whole_genome_shotgun_sequence
PVIO02000065.1_1330745-1330933_Procavia_capensis_isolate_US060_ScHvXvP_65HRSCAF_45324_whole_genome_shotgun_sequence
PVJQ01013062.1_9621-10051_Heterohyrax_brucei_isolate_US064_HetBruBak_scaffold_26116_whole_genome_shotgun_sequence
PVIO02023706.1_482793-483223_Procavia_capensis_isolate_US060_ScHvXvP_23707HRSCAF_203636_whole_genome_shotgun_sequence
PVIO02000739.1_498182-498542_Procavia_capensis_isolate_US060_ScHvXvP_736HRSCAF_97430_whole_genome_shotgun_sequence
PVJQ01035423.1_12412-12779_Heterohyrax_brucei_isolate_US064_HetBruBak_scaffold_70824_whole_genome_shotgun_sequence
PVIO02826217.1_18744808-18744937_Procavia_capensis_isolate_US060_ScHvXvP_827497HRSCAF_1046669_whole_genome_shotgun_sequence
PVIO02826203.1_83191-83574_Procavia_capensis_isolate_US060_ScHvXvP_827483HRSCAF_1046655_whole_genome_shotgun_sequence
PVJQ01028976.1_4660-5081_Heterohyrax_brucei_isolate_US064_HetBruBak_scaffold_57932_whole_genome_shotgun_sequence
PVJQ01041759.1_10512-10933_Heterohyrax_brucei_isolate_US064_HetBruBak_scaffold_83493_whole_genome_shotgun_sequence
PVIO02826298.1_1731021-1731442_Procavia_capensis_isolate_US060_ScHvXvP_827578HRSCAF_1046750_whole_genome_shotgun_sequence
PVIO02000696.1_8557563-8557798_Procavia_capensis_isolate_US060_ScHvXvP_693HRSCAF_96626_whole_genome_shotgun_sequence
PVIO02025250.1_4789731-4789873_Procavia_capensis_isolate_US060_ScHvXvP_25251HRSCAF_207868_whole_genome_shotgun_sequence
PVIJ01048580.1_412-774_Scalopus_aquaticus_isolate_US009_ScaAqu_scaffold_96957_whole_genome_shotgun_sequence
PVIJ01039336.1_511-873_Scalopus_aquaticus_isolate_US009_ScaAqu_scaffold_78499_whole_genome_shotgun_sequence
PVIJ01000520.1_274090-274448_Scalopus_aquaticus_isolate_US009_ScaAqu_scaffold_1034_whole_genome_shotgun_sequence
PVIJ01010215.1_1865-2227_Scalopus_aquaticus_isolate_US009_ScaAqu_scaffold_20404_whole_genome_shotgun_sequence
PVIJ01000872.1_106106-106467_Scalopus_aquaticus_isolate_US009_ScaAqu_scaffold_1738_whole_genome_shotgun_sequence
PVIJ01023542.1_3029-3385_Scalopus_aquaticus_isolate_US009_ScaAqu_scaffold_47020_whole_genome_shotgun_sequence
VUAX01000240.1_31545877-31546105_Lepus_timidus_isolate_LTM.BIO.3104_chromosome_1_whole_genome_shotgun_sequence
VUAX01000261.1_148747697-148747925_Lepus_timidus_isolate_LTM.BIO.3104_chromosome_2_whole_genome_shotgun_sequence
VUAX01000159.1_91251074-91251260_Lepus_timidus_isolate_LTM.BIO.3104_chromosome_12_whole_genome_shotgun_sequence
VUAX01000477.1_5238851-5239014_Lepus_timidus_isolate_LTM.BIO.3104_chromosome_7_whole_genome_shotgun_sequence
VUAX01000159.1_9933404-9933609_Lepus_timidus_isolate_LTM.BIO.3104_chromosome_12_whole_genome_shotgun_sequence
VUAX01000240.1_166905859-166906050_Lepus_timidus_isolate_LTM.BIO.3104_chromosome_1_whole_genome_shotgun_sequence
VUAX01000084.1_92016214-92016400_Lepus_timidus_isolate_LTM.BIO.3104_chromosome_X_whole_genome_shotgun_sequence
VIYN01010368.1_172242-172438_Oryctolagus_cuniculus_breed_New_Zealand_White_isolate_DNA-2018_000380F_whole_genome_shotgun_sequence
VUAX01000159.1_38855698-38855882_Lepus_timidus_isolate_LTM.BIO.3104_chromosome_12_whole_genome_shotgun_sequence
VUAX01000261.1_119985655-119985840_Lepus_timidus_isolate_LTM.BIO.3104_chromosome_2_whole_genome_shotgun_sequence
VUAX01000084.1_58861952-58862195_Lepus_timidus_isolate_LTM.BIO.3104_chromosome_X_whole_genome_shotgun_sequence
PVKC010011902.1_5162-5320_Crocidura_indochinensis_isolate_US011_CroInd_scaffold_23764_whole_genome_shotgun_sequence
0.05

lineage307

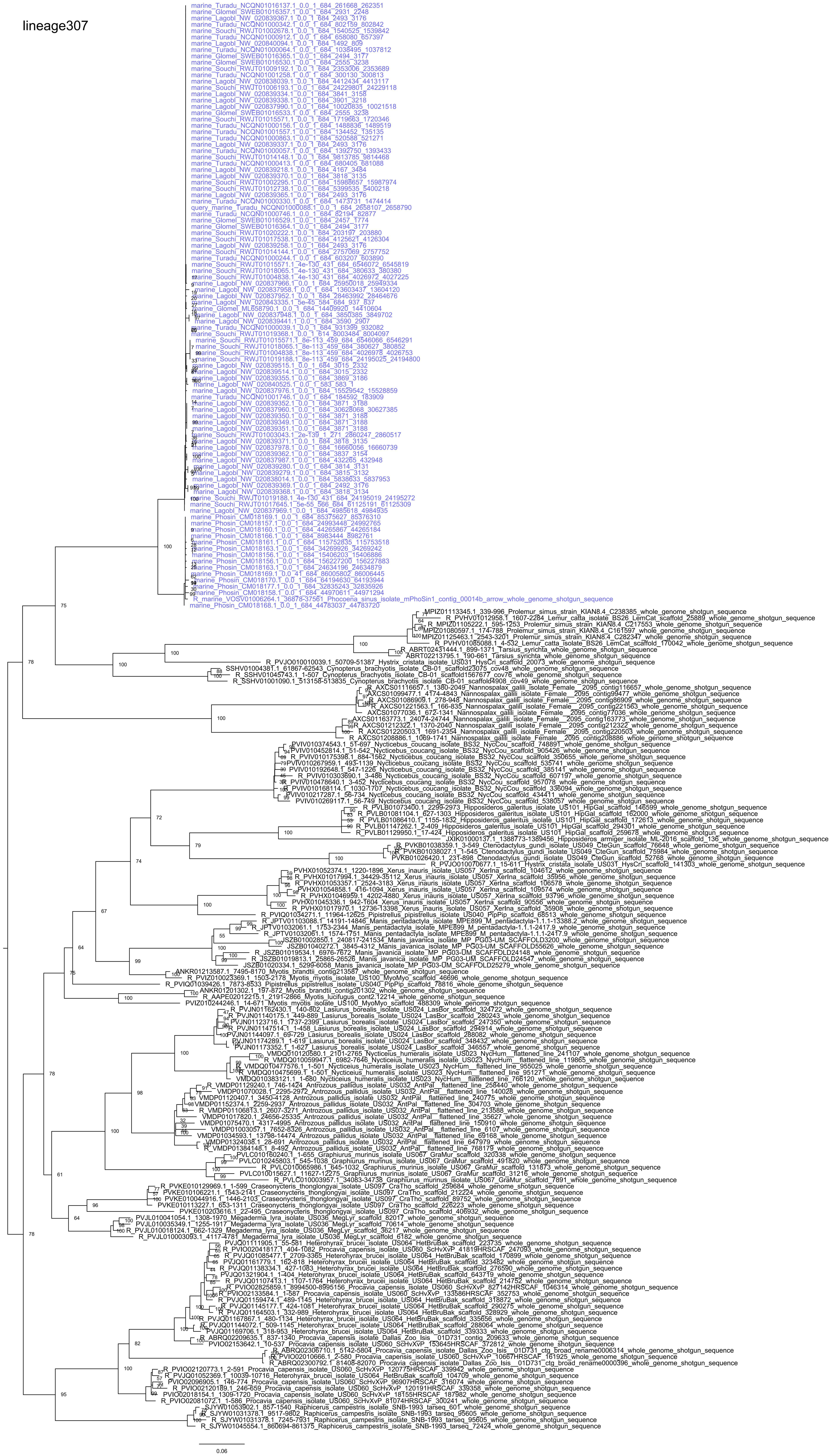

marine_Turadu_NCQN01016137.1_0.0_1_684_261668_262351
marine_Glomel_SWEB01016357.1_0.0_1_684_2931_2248
marine_Lagobl_NW_020839367.1_0.0_1_684_2493_3176
marine_Turadu_NCQN01000342.1_0.0_1_684_802159_802842
marine_Souchi_RWJT01002678.1_0.0_1_684_1540525_1539842
marine_Turadu_NCQN01000912.1_0.0_1_684_658080_657397
marine_Lagobl_NW_020840094.1_0.0_1_684_1492_809
marine_Turadu_NCQN01000064.1_0.0_1_684_1036495_1037812
marine_Glomel_SWEB01016365.1_0.0_1_684_2494_3177
marine_Glomel_SWEB01016530.1_0.0_1_684_2555_3238
marine_Souchi_RWJT01009192.1_0.0_1_684_2353006_2353689
marine_Turadu_NCQN01001258.1_0.0_1_684_300130_300813
marine_Lagobl_NW_020838039.1_0.0_1_684_4412434_4413117
marine_Souchi_RWJT01006193.1_0.0_1_684_24229801_24229118
marine_Lagobl_NW_020839334.1_0.0_1_684_3841_3158
marine_Lagobl_NW_020839338.1_0.0_1_684_3901_3218
marine_Lagobl_NW_020837990.1_0.0_1_684_10020835_10021518
marine_Glomel_SWEB01016533.1_0.0_1_684_2555_3238
marine_Souchi_RWJT01015571.1_0.0_1_684_1719663_1720346
marine_Turadu_NCQN01000156.1_0.0_1_684_1488836_1489519
marine_Turadu_NCQN01001557.1_0.0_1_684_134452_135135
marine_Turadu_NCQN01000863.1_0.0_1_684_520588_521271
marine_Lagobl_NW_020839337.1_0.0_1_684_2493_3176
marine_Turadu_NCQN01000057.1_0.0_1_684_1392750_1393433
marine_Souchi_RWJT01014148.1_0.0_1_684_9813785_9814468
marine_Turadu_NCQN01000413.1_0.0_1_684_680405_681088
marine_Lagobl_NW_020839218.1_0.0_1_684_4167_3484
marine_Lagobl_NW_020839370.1_0.0_1_684_3818_3135
marine_Souchi_RWJT01002295.1_0.0_1_684_15988657_15987974
marine_Souchi_RWJT01012738.1_0.0_1_684_5399535_5400218
marine_Lagobl_NW_020839365.1_0.0_1_684_2493_3176
marine_Turadu_NCQN01000330.1_0.0_1_684_1473731_1474414
query_marine_Turadu_NCQN01000088.1_0.0_1_684_2658107_2658790
marine_Turadu_NCQN01000746.1_0.0_1_684_82194_82877
marine_Glomel_SWEB01016529.1_0.0_1_684_2457_1774
marine_Glomel_SWEB01016364.1_0.0_1_684_2494_3177
marine_Souchi_RWJT01020222.1_0.0_1_684_203197_203880
marine_Souchi_RWJT01017538.1_0.0_1_684_4125621_4126304
marine_Lagobl_NW_020839258.1_0.0_1_684_2493_3176
marine_Souchi_RWJT01014144.1_0.0_1_684_2757069_2757752
marine_Turadu_NCQN01000244.1_0.0_1_684_603207_603890
marine_Souchi_RWJT01015571.1_4e-130_431_684_6546072_6545819
marine_Souchi_RWJT01018065.1_4e-130_431_684_380633_380380
marine_Souchi_RWJT01004838.1_4e-130_431_684_4026972_4027225
marine_Lagobl_NW_020837966.1_0.0_1_684_25950018_25949334
marine_Lagobl_NW_020837958.1_0.0_1_684_13603437_13604120
marine_Lagobl_NW_020837952.1_0.0_1_684_28463992_28464676
marine_Lagobl_NW_020843335.1_5e-45_584_684_937_837
marine_Glomel_ML658790.1_0.0_1_684_14409920_14410604
marine_Lagobl_NW_020837948.1_0.0_1_684_3850385_3849702
marine_Lagobl_NW_020839441.1_0.0_1_684_3590_2907
marine_Turadu_NCQN01000039.1_0.0_1_684_931399_932082
marine_Souchi_RWJT01019368.1_0.0_1_614_8003484_8004097
marine_Souchi_RWJT01015571.1_8e-113_459_684_6546066_6546291
marine_Souchi_RWJT01018065.1_8e-113_459_684_380627_380852
marine_Souchi_RWJT01004838.1_8e-113_459_684_4026978_4026753
marine_Souchi_RWJT01019188.1_8e-113_459_684_24195025_24194800
marine_Lagobl_NW_020839515.1_0.0_1_684_3015_2332
marine_Lagobl_NW_020839514.1_0.0_1_684_3015_2332
marine_Lagobl_NW_020839355.1_0.0_1_684_3869_3186
marine_Lagobl_NW_020840525.1_0.0_1_583_583_1
marine_Lagobl_NW_020837976.1_0.0_1_684_15529542_15528859
marine_Turadu_NCQN01001746.1_0.0_1_684_184592_183909
marine_Lagobl_NW_020839352.1_0.0_1_684_3871_3188
marine_Lagobl_NW_020837960.1_0.0_1_684_30628068_30627385
marine_Lagobl_NW_020839350.1_0.0_1_684_3871_3188
marine_Lagobl_NW_020839349.1_0.0_1_684_3871_3188
marine_Lagobl_NW_020839351.1_0.0_1_684_3871_3188
marine_Souchi_RWJT01003043.1_2e-139_1_271_2860247_2860517
marine_Lagobl_NW_020839371.1_0.0_1_684_3818_3135
marine_Lagobl_NW_020837978.1_0.0_1_684_16660056_16660739
marine_Lagobl_NW_020839362.1_0.0_1_684_3837_3154
marine_Lagobl_NW_020837987.1_0.0_1_684_432265_432948
marine_Lagobl_NW_020839280.1_0.0_1_684_3814_3131
marine_Lagobl_NW_020839279.1_0.0_1_684_3815_3132
marine_Lagobl_NW_020838014.1_0.0_1_684_5838633_5837953
marine_Lagobl_NW_020839369.1_0.0_1_684_2492_3176
marine_Lagobl_NW_020839368.1_0.0_1_684_3818_3134
marine_Souchi_RWJT01019188.1_4e-130_431_684_24195019_24195272
marine_Souchi_RWJT01017645.1_5e-55_566_684_61125191_61125309
marine_Lagobl_NW_020837969.1_0.0_1_684_4985618_4984935
marine_Phosin_CM018169.1_0.0_1_684_85375627_85376310
marine_Phosin_CM018157.1_0.0_1_684_24993448_24992765
marine_Phosin_CM018160.1_0.0_1_684_44265867_44265184
marine_Phosin_CM018166.1_0.0_1_684_8983444_8982761
marine_Phosin_CM018161.1_0.0_1_684_115752835_115753518
marine_Phosin_CM018163.1_0.0_1_684_34269926_34269242
marine_Phosin_CM018156.1_0.0_1_684_15406203_15406886
marine_Phosin_CM018156.1_0.0_1_684_156227200_156227883
marine_Phosin_CM018163.1_0.0_1_684_24634196_24634879
marine_Phosin_CM018169.1_0.0_41_684_86005802_86006445
marine_Phosin_CM018170.1_0.0_1_684_64194630_64193944
marine_Phosin_CM018177.1_0.0_1_684_32835243_32835926
marine_Phosin_CM018158.1_0.0_1_684_44970611_44971294
R_marine_VOSV01006264.1_36878-37561_Phocoena_sinus_isolate_mPhoSin1_contig_00014b_arrow_whole_genome_shotgun_sequence
marine_Phosin_CM018168.1_0.0_1_684_44783037_44783720
MPIZ01113345.1_339-996_Prolemur_simus_strain_KIAN8.4_C238385_whole_genome_shotgun_sequence
R_PVHV01012958.1_1607-2284_Lemur_catta_isolate_BS26_LemCat_scaffold_25889_whole_genome_shotgun_sequence
R_MPIZ01105222.1_595-1253_Prolemur_simus_strain_KIAN8.4_C217553_whole_genome_shotgun_sequence
MPIZ01080597.1_174-788_Prolemur_simus_strain_KIAN8.4_C161597_whole_genome_shotgun_sequence
MPIZ01125463.1_2543-3201_Prolemur_simus_strain_KIAN8.4_C282347_whole_genome_shotgun_sequence
R_PVHV01085088.1_4-532_Lemur_catta_isolate_BS26_LemCat_scaffold_170042_whole_genome_shotgun_sequence
R_ABRT02431444.1_899-1371_Tarsius_syrichta_whole_genome_shotgun_sequence
ABRT02213795.1_190-661_Tarsius_syrichta_whole_genome_shotgun_sequence
R_PVJO010010039.1_50709-51387_Hystrix_cristata_isolate_US031_HysCri_scaffold_20073_whole_genome_shotgun_sequence
SSHV01004381.1_61867-62543_Cynopterus_brachyotis_isolate_CB-01_scaffold23075_cov48_whole_genome_shotgun_sequence
R_SSHV01045743.1_1-507_Cynopterus_brachyotis_isolate_CB-01_scaffold1567677_cov76_whole_genome_shotgun_sequence
R_SSHV01001090.1_513158-513835_Cynopterus_brachyotis_isolate_CB-01_scaffold4908_cov49_whole_genome_shotgun_sequence
R_AXCS01116657.1_1380-2049_Nannospalax_galili_isolate_Female__2095_contig116657_whole_genome_shotgun_sequence
AXCS01099477.1_4174-4843_Nannospalax_galili_isolate_Female__2095_contig99477_whole_genome_shotgun_sequence
R_AXCS01086909.1_278-948_Nannospalax_galili_isolate_Female__2095_contig86909_whole_genome_shotgun_sequence
R_AXCS01221563.1_166-835_Nannospalax_galili_isolate_Female__2095_contig221563_whole_genome_shotgun_sequence
AXCS01077036.1_672-1341_Nannospalax_galili_isolate_Female__2095_contig77036_whole_genome_shotgun_sequence
AXCS01163773.1_24074-24744_Nannospalax_galili_isolate_Female__2095_contig163773_whole_genome_shotgun_sequence
R_AXCS01212322.1_1370-2040_Nannospalax_galili_isolate_Female__2095_contig212322_whole_genome_shotgun_sequence
R_AXCS01220503.1_1691-2354_Nannospalax_galili_isolate_Female__2095_contig220503_whole_genome_shotgun_sequence
R_AXCS01208886.1_1069-1741_Nannospalax_galili_isolate_Female__2095_contig208886_whole_genome_shotgun_sequence
PVIV010374543.1_51-697_Nycticebus_coucang_isolate_BS32_NycCou_scaffold_748891_whole_genome_shotgun_sequence
PVIV010452814.1_51-542_Nycticebus_coucang_isolate_BS32_NycCou_scaffold_905426_whole_genome_shotgun_sequence
R_PVIV010175398.1_884-1562_Nycticebus_coucang_isolate_BS32_NycCou_scaffold_350655_whole_genome_shotgun_sequence
PVIV010267959.1_493-1139_Nycticebus_coucang_isolate_BS32_NycCou_scaffold_535741_whole_genome_shotgun_sequence
PVIV010192648.1_547-1226_Nycticebus_coucang_isolate_BS32_NycCou_scaffold_385141_whole_genome_shotgun_sequence
R_PVIV010303690.1_3-486_Nycticebus_coucang_isolate_BS32_NycCou_scaffold_607197_whole_genome_shotgun_sequence
R_PVIV010478640.1_3-452_Nycticebus_coucang_isolate_BS32_NycCou_scaffold_957078_whole_genome_shotgun_sequence
R_PVIV010168114.1_1030-1707_Nycticebus_coucang_isolate_BS32_NycCou_scaffold_336094_whole_genome_shotgun_sequence
PVIV010217287.1_56-734_Nycticebus_coucang_isolate_BS32_NycCou_scaffold_434411_whole_genome_shotgun_sequence
PVIV010269117.1_56-749_Nycticebus_coucang_isolate_BS32_NycCou_scaffold_538057_whole_genome_shotgun_sequence
R_PVLB01073400.1_2299-2973_Hipposideros_galeritus_isolate_US101_HipGal_scaffold_146599_whole_genome_shotgun_sequence
R_PVLB01081104.1_627-1303_Hipposideros_galeritus_isolate_US101_HipGal_scaffold_162000_whole_genome_shotgun_sequence
R_PVLB01086410.1_1155-1832_Hipposideros_galeritus_isolate_US101_HipGal_scaffold_172613_whole_genome_shotgun_sequence
R_PVLB01147262.1_2-409_Hipposideros_galeritus_isolate_US101_HipGal_scaffold_294301_whole_genome_shotgun_sequence
R_PVLB01129950.1_17-424_Hipposideros_galeritus_isolate_US101_HipGal_scaffold_259678_whole_genome_shotgun_sequence
JXIK01000137.1_1388773-1389456_Hipposideros_armiger_isolate_ML-2016_scaffold_136_whole_genome_shotgun_sequence
R_PVKB01038359.1_3-549_Ctenodactylus_gundi_isolate_US049_CteGun_scaffold_76648_whole_genome_shotgun_sequence
R_PVKB01038027.1_1-545_Ctenodactylus_gundi_isolate_US049_CteGun_scaffold_75984_whole_genome_shotgun_sequence
PVKB01026420.1_231-898_Ctenodactylus_gundi_isolate_US049_CteGun_scaffold_52768_whole_genome_shotgun_sequence
R_PVJO01070677.1_15-611_Hystrix_cristata_isolate_US031_HysCri_scaffold_141303_whole_genome_shotgun_sequence
PVHX01052374.1_1220-1896_Xerus_inauris_isolate_US057_XerIna_scaffold_104612_whole_genome_shotgun_sequence
R_PVHX01017994.1_34429-35112_Xerus_inauris_isolate_US057_XerIna_scaffold_35956_whole_genome_shotgun_sequence
R_PVHX01053357.1_2524-3183_Xerus_inauris_isolate_US057_XerIna_scaffold_106578_whole_genome_shotgun_sequence
PVHX01054858.1_416-1094_Xerus_inauris_isolate_US057_XerIna_scaffold_109574_whole_genome_shotgun_sequence
R_PVHX01046959.1_4202-4880_Xerus_inauris_isolate_US057_XerIna_scaffold_93796_whole_genome_shotgun_sequence
PVHX01045336.1_942-1604_Xerus_inauris_isolate_US057_XerIna_scaffold_90556_whole_genome_shotgun_sequence
R_PVHX01017970.1_12736-13398_Xerus_inauris_isolate_US057_XerIna_scaffold_35908_whole_genome_shotgun_sequence
R_PVIQ01034271.1_11964-12625_Pipistrellus_pipistrellus_isolate_US040_PipPip_scaffold_68513_whole_genome_shotgun_sequence
R_JPTV01103088.1_14191-14846_Manis_pentadactyla_isolate_MPE899_M_pentadactyla-1.1-1-13388.2_whole_genome_shotgun_sequence
R_JPTV01032061.1_1753-2344_Manis_pentadactyla_isolate_MPE899_M_pentadactyla-1.1-1-2417.9_whole_genome_shotgun_sequence
R_JPTV01032061.1_1574-1751_Manis_pentadactyla_isolate_MPE899_M_pentadactyla-1.1-1-2417.9_whole_genome_shotgun_sequence
JSZB01002850.1_240817-241534_Manis_javanica_isolate_MP_PG03-UM_SCAFFOLD3200_whole_genome_shotgun_sequence
JSZB01004272.1_3845-4312_Manis_javanica_isolate_MP_PG03-UM_SCAFFOLD55626_whole_genome_shotgun_sequence
R_JSZB01019534.1_6976-7672_Manis_javanica_isolate_MP_PG03-UM_SCAFFOLD24148_whole_genome_shotgun_sequence
R_JSZB01019813.1_25865-26526_Manis_javanica_isolate_MP_PG03-UM_SCAFFOLD24547_whole_genome_shotgun_sequence
JSZB01020334.1_5299-6058_Manis_javanica_isolate_MP_PG03-UM_SCAFFOLD25279_whole_genome_shotgun_sequence
ANKR01213587.1_7495-8170_Myotis_brandtii_contig213587_whole_genome_shotgun_sequence
R_PVIZ01002369.1_1503-2178_Myotis_myotis_isolate_US100_MyoMyo_scaffold_46696_whole_genome_shotgun_sequence
R_PVIQ01039426.1_7873-8533_Pipistrellus_pipistrellus_isolate_US040_PipPip_scaffold_78816_whole_genome_shotgun_sequence
ANKR01201302.1_197-872_Myotis_brandtii_contig201302_whole_genome_shotgun_sequence
R_AAPE02012215.1_2191-2866_Myotis_lucifugus_cont2.12214_whole_genome_shotgun_sequence
PVIZ01024424.1_14-671_Myotis_myotis_isolate_US100_MyoMyo_scaffold_488309_whole_genome_shotgun_sequence
R_PVJN01162430.1_140-802_Lasiurus_borealis_isolate_US024_LasBor_scaffold_324722_whole_genome_shotgun_sequence
R_PVJN01140175.1_449-889_Lasiurus_borealis_isolate_US024_LasBor_scaffold_280243_whole_genome_shotgun_sequence
PVJN01123716.1_1737-2399_Lasiurus_borealis_isolate_US024_LasBor_scaffold_247350_whole_genome_shotgun_sequence
R_PVJN01147514.1_1-458_Lasiurus_borealis_isolate_US024_LasBor_scaffold_294914_whole_genome_shotgun_sequence
PVJN01144097.1_69-729_Lasiurus_borealis_isolate_US024_LasBor_scaffold_288082_whole_genome_shotgun_sequence
PVJN01174289.1_1-619_Lasiurus_borealis_isolate_US024_LasBor_scaffold_348432_whole_genome_shotgun_sequence
PVJN01173352.1_1-627_Lasiurus_borealis_isolate_US024_LasBor_scaffold_346557_whole_genome_shotgun_sequence
VMDQ010120580.1_2101-2765_Nycticeius_humeralis_isolate_US023_NycHum__flattened_line_241107_whole_genome_shotgun_sequence
R_VMDQ010059947.1_6982-7646_Nycticeius_humeralis_isolate_US023_NycHum__flattened_line_119865_whole_genome_shotgun_sequence
R_VMDQ010477576.1_1-501_Nycticeius_humeralis_isolate_US023_NycHum__flattened_line_955025_whole_genome_shotgun_sequence
R_VMDQ010475699.1_1-501_Nycticeius_humeralis_isolate_US023_NycHum__flattened_line_951271_whole_genome_shotgun_sequence
VMDQ010383121.1_1-680_Nycticeius_humeralis_isolate_US023_NycHum__flattened_line_766120_whole_genome_shotgun_sequence
R_VMDP01129240.1_746-1424_Antrozous_pallidus_isolate_US032_AntPal__flattened_line_258440_whole_genome_shotgun_sequence
VMDP01070028.1_2295-2972_Antrozous_pallidus_isolate_US032_AntPal__flattened_line_140028_whole_genome_shotgun_sequence
VMDP01120407.1_3450-4128_Antrozous_pallidus_isolate_US032_AntPal__flattened_line_240775_whole_genome_shotgun_sequence
VMDP01152374.1_2259-2937_Antrozous_pallidus_isolate_US032_AntPal__flattened_line_304703_whole_genome_shotgun_sequence
R_VMDP01106813.1_2607-3271_Antrozous_pallidus_isolate_US032_AntPal__flattened_line_213588_whole_genome_shotgun_sequence
VMDP01017820.1_24656-25335_Antrozous_pallidus_isolate_US032_AntPal__flattened_line_35627_whole_genome_shotgun_sequence
VMDP01075470.1_4317-4995_Antrozous_pallidus_isolate_US032_AntPal__flattened_line_150910_whole_genome_shotgun_sequence
VMDP01003057.1_7652-8326_Antrozous_pallidus_isolate_US032_AntPal__flattened_line_6107_whole_genome_shotgun_sequence
VMDP01034593.1_13798-14474_Antrozous_pallidus_isolate_US032_AntPal__flattened_line_69168_whole_genome_shotgun_sequence
VMDP01324038.1_28-691_Antrozous_pallidus_isolate_US032_AntPal__flattened_line_647979_whole_genome_shotgun_sequence
R_VMDP01384148.1_8-492_Antrozous_pallidus_isolate_US032_AntPal__flattened_line_768179_whole_genome_shotgun_sequence
PVLC010160240.1_1-655_Graphiurus_murinus_isolate_US067_GraMur_scaffold_320338_whole_genome_shotgun_sequence
PVLC010245803.1_545-1038_Graphiurus_murinus_isolate_US067_GraMur_scaffold_491820_whole_genome_shotgun_sequence
R_PVLC010065986.1_645-1032_Graphiurus_murinus_isolate_US067_GraMur_scaffold_131873_whole_genome_shotgun_sequence
PVLC010015627.1_11627-12275_Graphiurus_murinus_isolate_US067_GraMur_scaffold_31216_whole_genome_shotgun_sequence
R_PVLC010003957.1_34083-34738_Graphiurus_murinus_isolate_US067_GraMur_scaffold_7891_whole_genome_shotgun_sequence
R_PVKE010129969.1_1-599_Craseonycteris_thonglongyai_isolate_US097_CraTho_scaffold_259684_whole_genome_shotgun_sequence
PVKE010106221.1_1543-2141_Craseonycteris_thonglongyai_isolate_US097_CraTho_scaffold_212224_whole_genome_shotgun_sequence
PVKE010044916.1_1446-2103_Craseonycteris_thonglongyai_isolate_US097_CraTho_scaffold_89752_whole_genome_shotgun_sequence
PVKE010113227.1_653-1311_Craseonycteris_thonglongyai_isolate_US097_CraTho_scaffold_226223_whole_genome_shotgun_sequence
PVKE010203616.1_22-495_Craseonycteris_thonglongyai_isolate_US097_CraTho_scaffold_406932_whole_genome_shotgun_sequence
PVJL010041054.1_1308-1970_Megaderma_lyra_isolate_US036_MegLyr_scaffold_82017_whole_genome_shotgun_sequence
PVJL010035349.1_1255-1917_Megaderma_lyra_isolate_US036_MegLyr_scaffold_70614_whole_genome_shotgun_sequence
PVJL010018124.1_662-1329_Megaderma_lyra_isolate_US036_MegLyr_scaffold_36217_whole_genome_shotgun_sequence
R_PVJL010003093.1_4117-4781_Megaderma_lyra_isolate_US036_MegLyr_scaffold_6182_whole_genome_shotgun_sequence
PVJQ01111905.1_55-581_Heterohyrax_brucei_isolate_US064_HetBruBak_scaffold_223735_whole_genome_shotgun_sequence
R_PVIO02041817.1_404-1082_Procavia_capensis_isolate_US060_ScHvXvP_41819HRSCAF_247093_whole_genome_shotgun_sequence
R_PVJQ01085477.1_2709-3365_Heterohyrax_brucei_isolate_US064_HetBruBak_scaffold_170899_whole_genome_shotgun_sequence
PVJQ01161779.1_162-818_Heterohyrax_brucei_isolate_US064_HetBruBak_scaffold_323482_whole_genome_shotgun_sequence
R_PVJQ01138334.1_427-1083_Heterohyrax_brucei_isolate_US064_HetBruBak_scaffold_276590_whole_genome_shotgun_sequence
PVJQ01321904.1_1-404_Heterohyrax_brucei_isolate_US064_HetBruBak_scaffold_643719_whole_genome_shotgun_sequence
R_PVJQ01107413.1_1107-1764_Heterohyrax_brucei_isolate_US064_HetBruBak_scaffold_214752_whole_genome_shotgun_sequence
R_PVIO02825859.1_8994500-8995156_Procavia_capensis_isolate_US060_ScHvXvP_827142HRSCAF_1046314_whole_genome_shotgun_sequence
R_PVIO02133584.1_1-587_Procavia_capensis_isolate_US060_ScHvXvP_133586HRSCAF_352753_whole_genome_shotgun_sequence
R_PVJQ01159474.1_489-1145_Heterohyrax_brucei_isolate_US064_HetBruBak_scaffold_318872_whole_genome_shotgun_sequence
R_PVJQ01145177.1_424-1081_Heterohyrax_brucei_isolate_US064_HetBruBak_scaffold_290275_whole_genome_shotgun_sequence
PVJQ01164503.1_332-989_Heterohyrax_brucei_isolate_US064_HetBruBak_scaffold_328929_whole_genome_shotgun_sequence
R_PVJQ01167867.1_480-1134_Heterohyrax_brucei_isolate_US064_HetBruBak_scaffold_335656_whole_genome_shotgun_sequence
R_PVJQ01144072.1_509-1145_Heterohyrax_brucei_isolate_US064_HetBruBak_scaffold_288064_whole_genome_shotgun_sequence
PVJQ01169706.1_318-953_Heterohyrax_brucei_isolate_US064_HetBruBak_scaffold_339333_whole_genome_shotgun_sequence
R_ABRQ02209635.1_837-1340_Procavia_capensis_isolate_Dallas_Zoo_Isis__01D731_contig_209633_whole_genome_shotgun_sequence
PVIO02153642.1_10-537_Procavia_capensis_isolate_US060_ScHvXvP_153645HRSCAF_372812_whole_genome_shotgun_sequence
R_ABRQ02306710.1_5142-5804_Procavia_capensis_isolate_Dallas_Zoo_Isis__01D731_ctg_broad_rename0006314_whole_genome_shotgun_sequence
PVIO02010666.1_2-580_Procavia_capensis_isolate_US060_ScHvXvP_10667HRSCAF_161925_whole_genome_shotgun_sequence
R_ABRQ02300792.1_81408-82070_Procavia_capensis_isolate_Dallas_Zoo_Isis__01D731_ctg_broad_rename0000396_whole_genome_shotgun_sequence
R_PVIO02120773.1_2-591_Procavia_capensis_isolate_US060_ScHvXvP_120775HRSCAF_339942_whole_genome_shotgun_sequence
R_PVJQ01052369.1_10039-10716_Heterohyrax_brucei_isolate_US064_HetBruBak_scaffold_104709_whole_genome_shotgun_sequence
PVIO02096905.1_146-774_Procavia_capensis_isolate_US060_ScHvXvP_96907HRSCAF_316074_whole_genome_shotgun_sequence
R_PVIO02120189.1_246-859_Procavia_capensis_isolate_US060_ScHvXvP_120191HRSCAF_339358_whole_genome_shotgun_sequence
PVIO02018154.1_1309-1720_Procavia_capensis_isolate_US060_ScHvXvP_18155HRSCAF_187982_whole_genome_shotgun_sequence
R_PVIO02081072.1_1-586_Procavia_capensis_isolate_US060_ScHvXvP_81074HRSCAF_300241_whole_genome_shotgun_sequence
SJYW01053902.1_857-1540_Raphicerus_campestris_isolate_SNB-1993_tarseq_601_whole_genome_shotgun_sequence
R_SJYW01031378.1_9517-9802_Raphicerus_campestris_isolate_SNB-1993_tarseq_95605_whole_genome_shotgun_sequence
R_SJYW01031378.1_7245-7931_Raphicerus_campestris_isolate_SNB-1993_tarseq_95605_whole_genome_shotgun_sequence
R_SJYW01045554.1_860694-861375_Raphicerus_campestris_isolate_SNB-1993_tarseq_72424_whole_genome_shotgun_sequence
0.06

lineage308

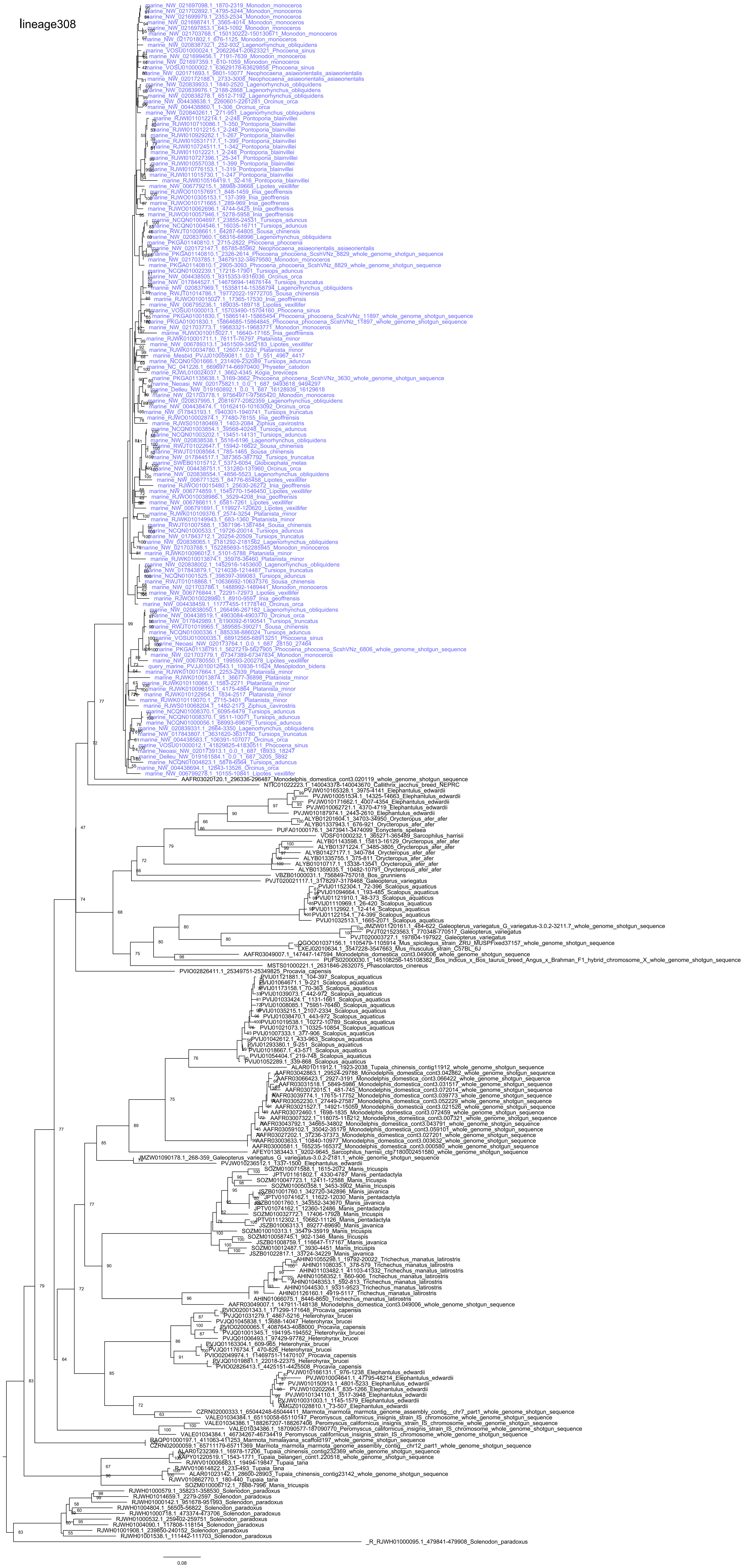

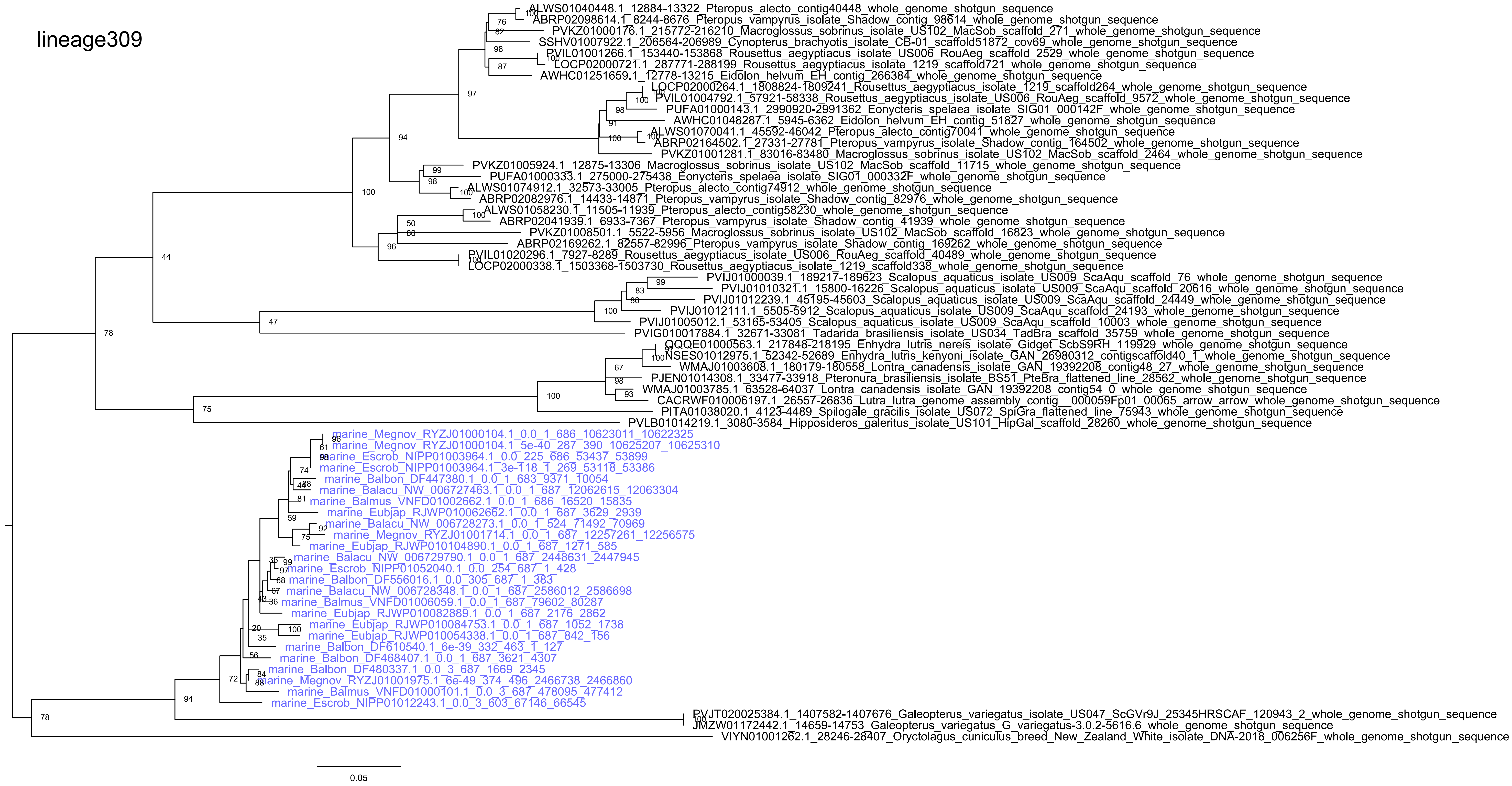

lineage309
ALWS01040448.1_12884-13322_Pteropus_alecto_contig40448_whole_genome_shotgun_sequence
ABRP02098614.1_8244-8676_Pteropus_vampyrus_isolate_Shadow_contig_98614_whole_genome_shotgun_sequence
PVKZ01000176.1_215772-216210_Macroglossus_sobrinus_isolate_US102_MacSob_scaffold_271_whole_genome_shotgun_sequence
SSHV01007922.1_206564-206989_Cynopterus_brachyotis_isolate_CB-01_scaffold51872_cov69_whole_genome_shotgun_sequence
PVIL01001266.1_153440-153868_Rousettus_aegyptiacus_isolate_US006_RouAeg_scaffold_2529_whole_genome_shotgun_sequence
LOCP02000721.1_287771-288199_Rousettus_aegyptiacus_isolate_1219_scaffold721_whole_genome_shotgun_sequence
AWHC01251659.1_12778-13215_Eidolon_helvum_EH_contig_266384_whole_genome_shotgun_sequence
LOCP02000264.1_1808824-1809241_Rousettus_aegyptiacus_isolate_1219_scaffold264_whole_genome_shotgun_sequence
PVIL01004792.1_57921-58338_Rousettus_aegyptiacus_isolate_US006_RouAeg_scaffold_9572_whole_genome_shotgun_sequence
PUFA01000143.1_2990920-2991362_Eonycteris_spelaea_isolate_SIG01_000142F_whole_genome_shotgun_sequence
AWHC01048287.1_5945-6362_Eidolon_helvum_EH_contig_51827_whole_genome_shotgun_sequence
ALWS01070041.1_45592-46042_Pteropus_alecto_contig70041_whole_genome_shotgun_sequence
ABRP02164502.1_27331-27781_Pteropus_vampyrus_isolate_Shadow_contig_164502_whole_genome_shotgun_sequence
PVKZ01001281.1_83016-83480_Macroglossus_sobrinus_isolate_US102_MacSob_scaffold_2464_whole_genome_shotgun_sequence
PVKZ01005924.1_12875-13306_Macroglossus_sobrinus_isolate_US102_MacSob_scaffold_11715_whole_genome_shotgun_sequence
PUFA01000333.1_275000-275438_Eonycteris_spelaea_isolate_SIG01_000332F_whole_genome_shotgun_sequence
ALWS01074912.1_32573-33005_Pteropus_alecto_contig74912_whole_genome_shotgun_sequence
ABRP02082976.1_14433-14871_Pteropus_vampyrus_isolate_Shadow_contig_82976_whole_genome_shotgun_sequence
ALWS01058230.1_11505-11939_Pteropus_alecto_contig58230_whole_genome_shotgun_sequence
ABRP02041939.1_6933-7367_Pteropus_vampyrus_isolate_Shadow_contig_41939_whole_genome_shotgun_sequence
PVKZ01008501.1_5522-5956_Macroglossus_sobrinus_isolate_US102_MacSob_scaffold_16823_whole_genome_shotgun_sequence
ABRP02169262.1_82557-82996_Pteropus_vampyrus_isolate_Shadow_contig_169262_whole_genome_shotgun_sequence
PVIL01020296.1_7927-8289_Rousettus_aegyptiacus_isolate_US006_RouAeg_scaffold_40489_whole_genome_shotgun_sequence
LOCP02000338.1_1503368-1503730_Rousettus_aegyptiacus_isolate_1219_scaffold338_whole_genome_shotgun_sequence
PVIJ01000039.1_189217-189623_Scalopus_aquaticus_isolate_US009_ScaAqu_scaffold_76_whole_genome_shotgun_sequence
PVIJ01010321.1_15800-16226_Scalopus_aquaticus_isolate_US009_ScaAqu_scaffold_20616_whole_genome_shotgun_sequence
PVIJ01012239.1_45195-45603_Scalopus_aquaticus_isolate_US009_ScaAqu_scaffold_24449_whole_genome_shotgun_sequence
PVIJ01012111.1_5505-5912_Scalopus_aquaticus_isolate_US009_ScaAqu_scaffold_24193_whole_genome_shotgun_sequence
PVIJ01005012.1_53165-53405_Scalopus_aquaticus_isolate_US009_ScaAqu_scaffold_10003_whole_genome_shotgun_sequence
PVIG010017884.1_32671-33081_Tadarida_brasiliensis_isolate_US034_TadBra_scaffold_35759_whole_genome_shotgun_sequence
QQQE01000563.1_217848-218195_Enhydra_lutris_nereis_isolate_Gidget_ScbS9RH_119929_whole_genome_shotgun_sequence
NSES01012975.1_52342-52689_Enhydra_lutris_kenyoni_isolate_GAN_26980312_contigscaffold40_1_whole_genome_shotgun_sequence
WMAJ01003608.1_180179-180558_Lontra_canadensis_isolate_GAN_19392208_contig48_27_whole_genome_shotgun_sequence
PJEN01014308.1_33477-33918_Pteronura_brasiliensis_isolate_BS51_PteBra_flattened_line_28562_whole_genome_shotgun_sequence
WMAJ01003785.1_63528-64037_Lontra_canadensis_isolate_GAN_19392208_contig54_0_whole_genome_shotgun_sequence
CACRWF010006197.1_26557-26836_Lutra_lutra_genome_assembly_contig__000059Fp01_00065_arrow_arrow_whole_genome_shotgun_sequence
PITA01038020.1_4123-4489_Spilogale_gracilis_isolate_US072_SpiGra_flattened_line_75943_whole_genome_shotgun_sequence
PVLB01014219.1_3080-3584_Hipposideros_galeritus_isolate_US101_HipGal_scaffold_28260_whole_genome_shotgun_sequence
marine_Megnov_RYZJ01000104.1_0.0_1_686_10623011_10622325
marine_Megnov_RYZJ01000104.1_5e-40_287_390_10625207_10625310
marine_Escrob_NIPP01003964.1_0.0_225_686_53437_53899
marine_Escrob_NIPP01003964.1_3e-118_1_269_53118_53386
marine_Balbon_DF447380.1_0.0_1_683_9371_10054
marine_Balacu_NW_006727463.1_0.0_1_687_12062615_12063304
marine_Balmus_VNFD01002662.1_0.0_1_686_16520_15835
marine_Eubjap_RJWP010062662.1_0.0_1_687_3629_2939
marine_Balacu_NW_006728273.1_0.0_1_524_71492_70969
marine_Megnov_RYZJ01001714.1_0.0_1_687_12257261_12256575
marine_Eubjap_RJWP010104890.1_0.0_1_687_1271_585
marine_Balacu_NW_006729790.1_0.0_1_687_2448631_2447945
marine_Escrob_NIPP01052040.1_0.0_254_687_1_428
marine_Balbon_DF556016.1_0.0_305_687_1_383
marine_Balacu_NW_006728348.1_0.0_1_687_2586012_2586698
marine_Balmus_VNFD01006059.1_0.0_1_687_79602_80287
marine_Eubjap_RJWP010082889.1_0.0_1_687_2176_2862
marine_Eubjap_RJWP010084753.1_0.0_1_687_1052_1738
marine_Eubjap_RJWP010054338.1_0.0_1_687_842_156
marine_Balbon_DF610540.1_6e-39_332_463_1_127
marine_Balbon_DF468407.1_0.0_1_687_3621_4307
marine_Balbon_DF480337.1_0.0_3_687_1669_2345
marine_Megnov_RYZJ01001975.1_6e-49_374_496_2466738_2466860
marine_Balmus_VNFD01000101.1_0.0_3_687_478095_477412
marine_Escrob_NIPP01012243.1_0.0_3_603_67146_66545
PVJT020025384.1_1407582-1407676_Galeopterus_variegatus_isolate_US047_ScGVr9J_25345HRSCAF_120943_2_whole_genome_shotgun_sequence
JMZW01172442.1_14659-14753_Galeopterus_variegatus_G_variegatus-3.0.2-5616.6_whole_genome_shotgun_sequence
VIYN01001262.1_28246-28407_Oryctolagus_cuniculus_breed_New_Zealand_White_isolate_DNA-2018_006256F_whole_genome_shotgun_sequence
0.05

lineage310

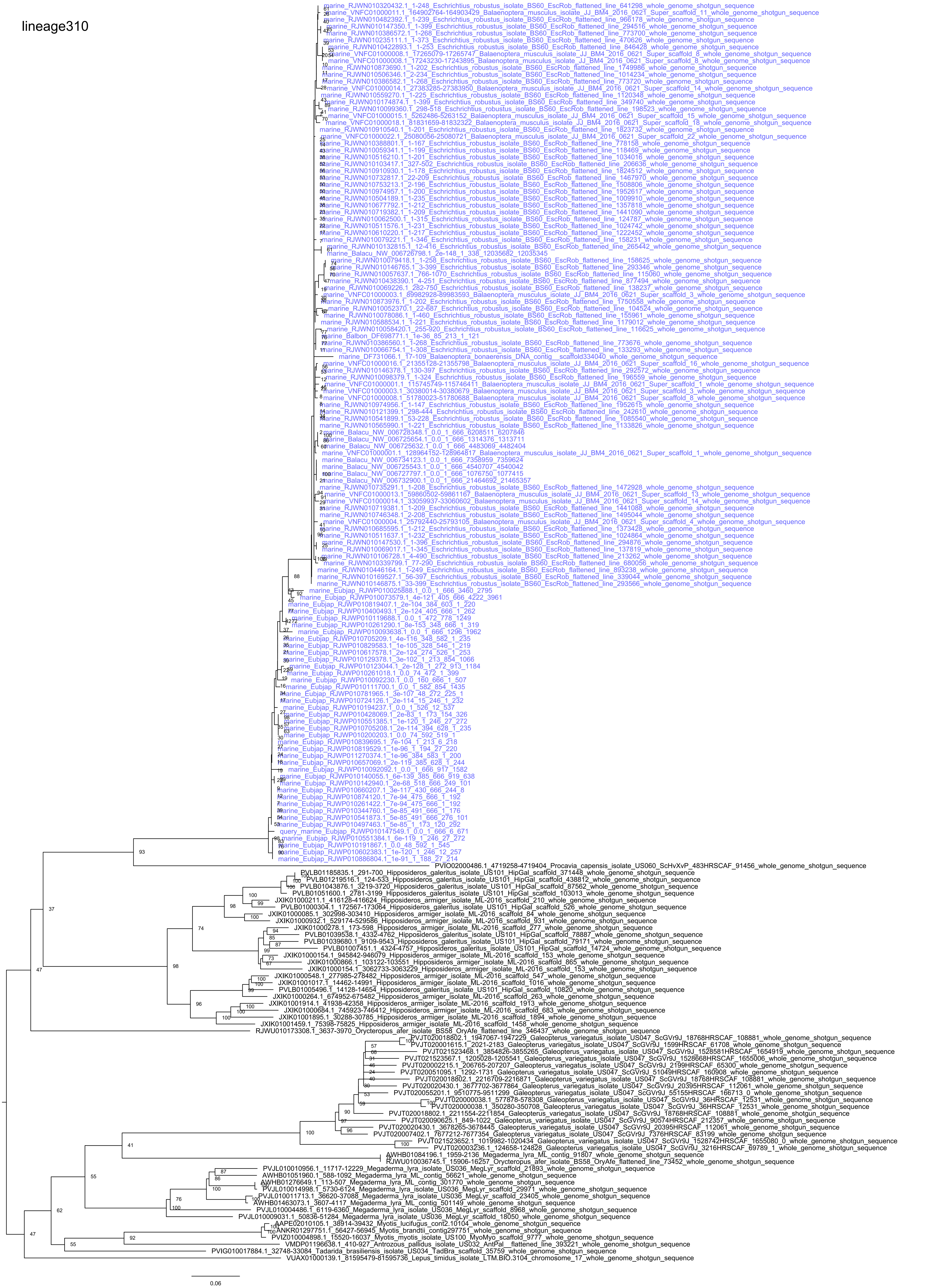

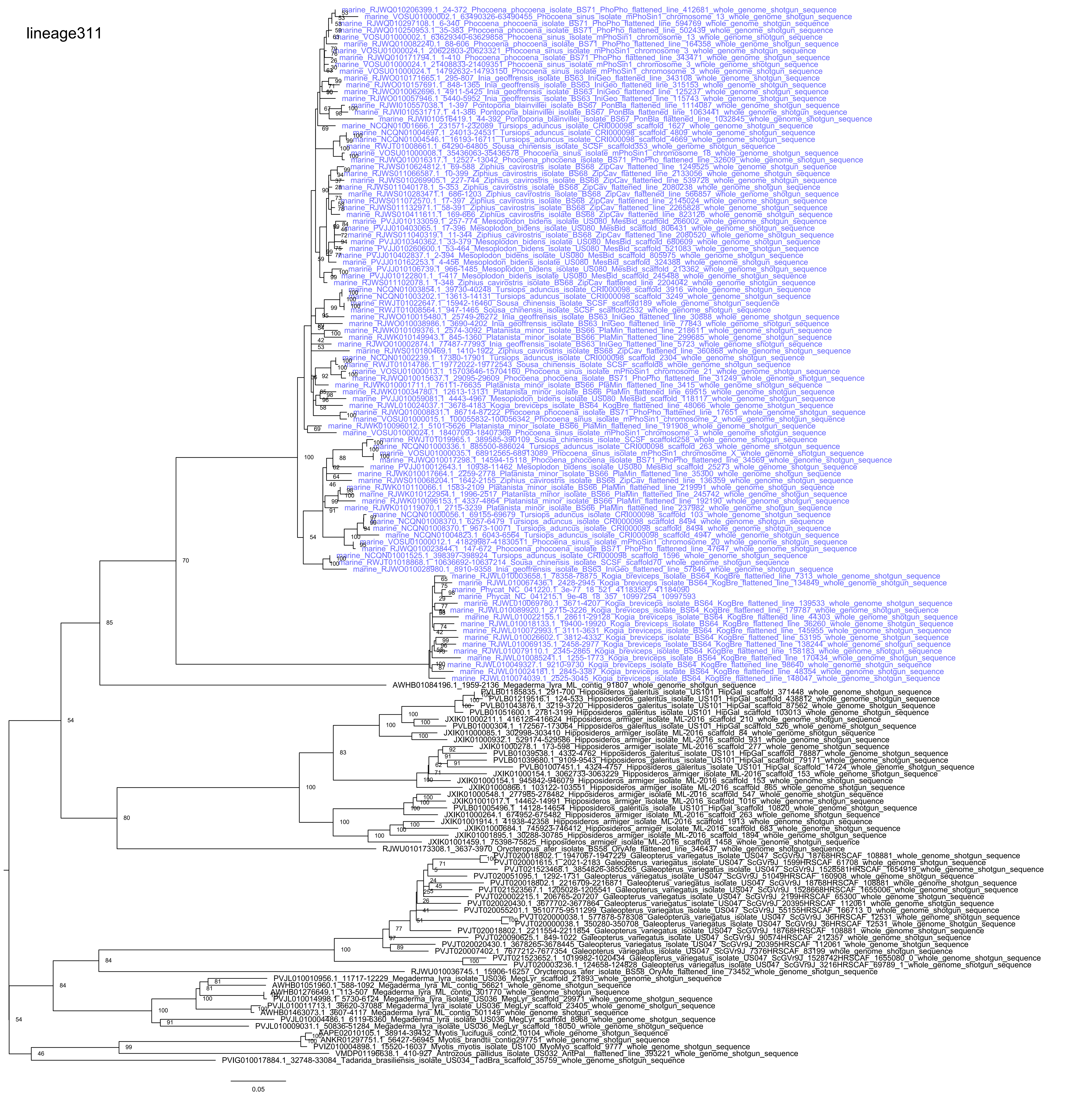
lineage311
marine_RJWQ010206399.1_24-372_Phocoena_phocoena_isolate_BS71_PhoPho_flattened_line_412681_whole_genome_shotgun_sequence
marine_VOSU01000002.1_63490326-63490455_Phocoena_sinus_isolate_mPhoSin1_chromosome_13_whole_genome_shotgun_sequence
marine_RJWQ010297108.1_6-340_Phocoena_phocoena_isolate_BS71_PhoPho_flattened_line_594769_whole_genome_shotgun_sequence
marine_RJWQ010250953.1_35-383_Phocoena_phocoena_isolate_BS71_PhoPho_flattened_line_502439_whole_genome_shotgun_sequence
marine_VOSU01000002.1_63629340-63629858_Phocoena_sinus_isolate_mPhoSin1_chromosome_13_whole_genome_shotgun_sequence
marine_RJWQ010082240.1_88-606_Phocoena_phocoena_isolate_BS71_PhoPho_flattened_line_164358_whole_genome_shotgun_sequence
marine_VOSU01000024.1_20622803-20623321_Phocoena_sinus_isolate_mPhoSin1_chromosome_3_whole_genome_shotgun_sequence
marine_RJWQ010171794.1_1-410_Phocoena_phocoena_isolate_BS71_PhoPho_flattened_line_343471_whole_genome_shotgun_sequence
marine_VOSU01000024.1_21408833-21409351_Phocoena_sinus_isolate_mPhoSin1_chromosome_3_whole_genome_shotgun_sequence
marine_VOSU01000024.1_14792632-14793150_Phocoena_sinus_isolate_mPhoSin1_chromosome_3_whole_genome_shotgun_sequence
marine_RJWO010171665.1_295-807_Inia_geoffrensis_isolate_BS63_IniGeo_flattened_line_343108_whole_genome_shotgun_sequence
marine_RJWO010157691.1_848-1365_Inia_geoffrensis_isolate_BS63_IniGeo_flattened_line_315153_whole_genome_shotgun_sequence
marine_RJWO010062696.1_4911-5425_Inia_geoffrensis_isolate_BS63_IniGeo_flattened_line_125237_whole_genome_shotgun_sequence
marine_RJWO010057946.1_5440-5952_Inia_geoffrensis_isolate_BS63_IniGeo_flattened_line_115743_whole_genome_shotgun_sequence
marine_RJWI010557038.1_1-397_Pontoporia_blainvillei_isolate_BS67_PonBla_flattened_line_1114087_whole_genome_shotgun_sequence
marine_RJWI010531717.1_41-386_Pontoporia_blainvillei_isolate_BS67_PonBla_flattened_line_1063441_whole_genome_shotgun_sequence
marine_RJWI010516419.1_44-392_Pontoporia_blainvillei_isolate_BS67_PonBla_flattened_line_1032845_whole_genome_shotgun_sequence
marine_NCQN01001666.1_231571-232089_Tursiops_aduncus_isolate_CRI000098_scaffold_1627_whole_genome_shotgun_sequence
marine_NCQN01004697.1_24013-24531_Tursiops_aduncus_isolate_CRI000098_scaffold_4809_whole_genome_shotgun_sequence
marine_NCQN01004546.1_16193-16711_Tursiops_aduncus_isolate_CRI000098_scaffold_4669_whole_genome_shotgun_sequence
marine_RWJT01008661.1_64290-64805_Sousa_chinensis_isolate_SCSF_scaffold353_whole_genome_shotgun_sequence
marine_VOSU01000008.1_35436063-35436578_Phocoena_sinus_isolate_mPhoSin1_chromosome_18_whole_genome_shotgun_sequence
marine_RJWQ010016317.1_12527-13042_Phocoena_phocoena_isolate_BS71_PhoPho_flattened_line_32609_whole_genome_shotgun_sequence
marine_RJWS010624812.1_69-588_Ziphius_cavirostris_isolate_BS68_ZipCav_flattened_line_1249525_whole_genome_shotgun_sequence
marine_RJWS011066587.1_10-399_Ziphius_cavirostris_isolate_BS68_ZipCav_flattened_line_2133056_whole_genome_shotgun_sequence
marine_RJWS010269905.1_227-744_Ziphius_cavirostris_isolate_BS68_ZipCav_flattened_line_539728_whole_genome_shotgun_sequence
marine_RJWS011040178.1_5-353_Ziphius_cavirostris_isolate_BS68_ZipCav_flattened_line_2080238_whole_genome_shotgun_sequence
marine_RJWS010283471.1_686-1203_Ziphius_cavirostris_isolate_BS68_ZipCav_flattened_line_566857_whole_genome_shotgun_sequence
marine_RJWS011072570.1_17-397_Ziphius_cavirostris_isolate_BS68_ZipCav_flattened_line_2145024_whole_genome_shotgun_sequence
marine_RJWS011132971.1_58-391_Ziphius_cavirostris_isolate_BS68_ZipCav_flattened_line_2265828_whole_genome_shotgun_sequence
marine_RJWS010411611.1_169-666_Ziphius_cavirostris_isolate_BS68_ZipCav_flattened_line_823126_whole_genome_shotgun_sequence
marine_PVJJ010133059.1_257-774_Mesoplodon_bidens_isolate_US080_MesBid_scaffold_266002_whole_genome_shotgun_sequence
marine_PVJJ010403065.1_17-396_Mesoplodon_bidens_isolate_US080_MesBid_scaffold_806431_whole_genome_shotgun_sequence
marine_RJWS011040319.1_11-344_Ziphius_cavirostris_isolate_BS68_ZipCav_flattened_line_2080520_whole_genome_shotgun_sequence
marine_PVJJ010340362.1_33-379_Mesoplodon_bidens_isolate_US080_MesBid_scaffold_680609_whole_genome_shotgun_sequence
marine_PVJJ010260600.1_53-464_Mesoplodon_bidens_isolate_US080_MesBid_scaffold_521083_whole_genome_shotgun_sequence
marine_PVJJ010402837.1_2-394_Mesoplodon_bidens_isolate_US080_MesBid_scaffold_805975_whole_genome_shotgun_sequence
marine_PVJJ010162253.1_4-456_Mesoplodon_bidens_isolate_US080_MesBid_scaffold_324388_whole_genome_shotgun_sequence
marine_PVJJ010106739.1_966-1485_Mesoplodon_bidens_isolate_US080_MesBid_scaffold_213362_whole_genome_shotgun_sequence
marine_PVJJ010122801.1_1-417_Mesoplodon_bidens_isolate_US080_MesBid_scaffold_245488_whole_genome_shotgun_sequence
marine_RJWS011102078.1_1-348_Ziphius_cavirostris_isolate_BS68_ZipCav_flattened_line_2204042_whole_genome_shotgun_sequence
marine_NCQN01003854.1_39730-40248_Tursiops_aduncus_isolate_CRI000098_scaffold_3916_whole_genome_shotgun_sequence
marine_NCQN01003202.1_13613-14131_Tursiops_aduncus_isolate_CRI000098_scaffold_3249_whole_genome_shotgun_sequence
marine_RWJT01022647.1_15942-16460_Sousa_chinensis_isolate_SCSF_scaffold189_whole_genome_shotgun_sequence
marine_RWJT01008564.1_947-1465_Sousa_chinensis_isolate_SCSF_scaffold2532_whole_genome_shotgun_sequence
marine_RJWO010015480.1_25749-26272_Inia_geoffrensis_isolate_BS63_IniGeo_flattened_line_30888_whole_genome_shotgun_sequence
marine_RJWO010038986.1_3690-4202_Inia_geoffrensis_isolate_BS63_IniGeo_flattened_line_77843_whole_genome_shotgun_sequence
marine_RJWK010109376.1_2574-3092_Platanista_minor_isolate_BS66_PlaMin_flattened_line_218611_whole_genome_shotgun_sequence
marine_RJWK010149943.1_845-1360_Platanista_minor_isolate_BS66_PlaMin_flattened_line_299685_whole_genome_shotgun_sequence
marine_RJWO010002874.1_77487-77993_Inia_geoffrensis_isolate_BS63_IniGeo_flattened_line_5723_whole_genome_shotgun_sequence
marine_RJWS010180469.1_1410-1922_Ziphius_cavirostris_isolate_BS68_ZipCav_flattened_line_360868_whole_genome_shotgun_sequence
marine_NCQN01002239.1_17380-17901_Tursiops_aduncus_isolate_CRI000098_scaffold_2304_whole_genome_shotgun_sequence
marine_RWJT01014786.1_19772022-19772543_Sousa_chinensis_isolate_SCSF_scaffold8_whole_genome_shotgun_sequence
marine_VOSU01000013.1_15703646-15704160_Phocoena_sinus_isolate_mPhoSin1_chromosome_21_whole_genome_shotgun_sequence
marine_RJWQ010015637.1_29095-29609_Phocoena_phocoena_isolate_BS71_PhoPho_flattened_line_31249_whole_genome_shotgun_sequence
marine_RJWK010001711.1_76111-76635_Platanista_minor_isolate_BS66_PlaMin_flattened_line_3415_whole_genome_shotgun_sequence
marine_RJWK010034780.1_12613-13131_Platanista_minor_isolate_BS66_PlaMin_flattened_line_69515_whole_genome_shotgun_sequence
marine_PVJJ010059081.1_4443-4967_Mesoplodon_bidens_isolate_US080_MesBid_scaffold_118117_whole_genome_shotgun_sequence
marine_RJWL010024037.1_3678-4183_Kogia_breviceps_isolate_BS64_KogBre_flattened_line_48066_whole_genome_shotgun_sequence
marine_RJWQ010008831.1_86714-87222_Phocoena_phocoena_isolate_BS71_PhoPho_flattened_line_17651_whole_genome_shotgun_sequence
marine_VOSU01000015.1_100055832-100056342_Phocoena_sinus_isolate_mPhoSin1_chromosome_2_whole_genome_shotgun_sequence
marine_RJWK010096012.1_5101-5626_Platanista_minor_isolate_BS66_PlaMin_flattened_line_191908_whole_genome_shotgun_sequence
marine_VOSU01000024.1_18407093-18407369_Phocoena_sinus_isolate_mPhoSin1_chromosome_3_whole_genome_shotgun_sequence
marine_RWJT01019965.1_389585-390109_Sousa_chinensis_isolate_SCSF_scaffold258_whole_genome_shotgun_sequence
marine_NCQN01000336.1_885500-886024_Tursiops_aduncus_isolate_CRI000098_scaffold_263_whole_genome_shotgun_sequence
marine_VOSU01000035.1_68912565-68913089_Phocoena_sinus_isolate_mPhoSin1_chromosome_X_whole_genome_shotgun_sequence
marine_RJWQ010017298.1_14594-15118_Phocoena_phocoena_isolate_BS71_PhoPho_flattened_line_34569_whole_genome_shotgun_sequence
marine_PVJJ010012643.1_10938-11462_Mesoplodon_bidens_isolate_US080_MesBid_scaffold_25273_whole_genome_shotgun_sequence
marine_RJWK010017664.1_2259-2778_Platanista_minor_isolate_BS66_PlaMin_flattened_line_35300_whole_genome_shotgun_sequence
marine_RJWS010068204.1_1642-2155_Ziphius_cavirostris_isolate_BS68_ZipCav_flattened_line_136359_whole_genome_shotgun_sequence
marine_RJWK010110066.1_1583-2109_Platanista_minor_isolate_BS66_PlaMin_flattened_line_219991_whole_genome_shotgun_sequence
marine_RJWK010122954.1_1996-2517_Platanista_minor_isolate_BS66_PlaMin_flattened_line_245742_whole_genome_shotgun_sequence
marine_RJWK010096153.1_4337-4864_Platanista_minor_isolate_BS66_PlaMin_flattened_line_192190_whole_genome_shotgun_sequence
marine_RJWK010119070.1_2715-3239_Platanista_minor_isolate_BS66_PlaMin_flattened_line_237982_whole_genome_shotgun_sequence
marine_NCQN01000056.1_69155-69679_Tursiops_aduncus_isolate_CRI000098_scaffold_103_whole_genome_shotgun_sequence
marine_NCQN01008370.1_6257-6479_Tursiops_aduncus_isolate_CRI000098_scaffold_8494_whole_genome_shotgun_sequence
marine_NCQN01008370.1_9673-10071_Tursiops_aduncus_isolate_CRI000098_scaffold_8494_whole_genome_shotgun_sequence
marine_NCQN01004823.1_6043-6564_Tursiops_aduncus_isolate_CRI000098_scaffold_4947_whole_genome_shotgun_sequence
marine_VOSU01000012.1_41829987-41830511_Phocoena_sinus_isolate_mPhoSin1_chromosome_20_whole_genome_shotgun_sequence
marine_RJWQ010023844.1_147-672_Phocoena_phocoena_isolate_BS71_PhoPho_flattened_line_47647_whole_genome_shotgun_sequence
marine_NCQN01001525.1_398397-398924_Tursiops_aduncus_isolate_CRI000098_scaffold_1596_whole_genome_shotgun_sequence
marine_RWJT01018868.1_10636692-10637214_Sousa_chinensis_isolate_SCSF_scaffold70_whole_genome_shotgun_sequence
marine_RJWO010028980.1_8910-9358_Inia_geoffrensis_isolate_BS63_IniGeo_flattened_line_57846_whole_genome_shotgun_sequence
marine_RJWL010003658.1_78358-78875_Kogia_breviceps_isolate_BS64_KogBre_flattened_line_7313_whole_genome_shotgun_sequence
marine_RJWL010067436.1_2428-2945_Kogia_breviceps_isolate_BS64_KogBre_flattened_line_134849_whole_genome_shotgun_sequence
marine_Phycat_NC_041220.1_3e-77_18_521_41183587_41184090
marine_Phycat_NC_041215.1_9e-48_18_357_10997254_10997593
marine_RJWL010069780.1_3671-4207_Kogia_breviceps_isolate_BS64_KogBre_flattened_line_139533_whole_genome_shotgun_sequence
marine_RJWL010089920.1_2715-3226_Kogia_breviceps_isolate_BS64_KogBre_flattened_line_179787_whole_genome_shotgun_sequence
marine_RJWL010022155.1_28611-29128_Kogia_breviceps_isolate_BS64_KogBre_flattened_line_44303_whole_genome_shotgun_sequence
marine_RJWL010018133.1_19400-19920_Kogia_breviceps_isolate_BS64_KogBre_flattened_line_36260_whole_genome_shotgun_sequence
marine_RJWL010072993.1_3111-3631_Kogia_breviceps_isolate_BS64_KogBre_flattened_line_145955_whole_genome_shotgun_sequence
marine_RJWL010026602.1_3812-4332_Kogia_breviceps_isolate_BS64_KogBre_flattened_line_53195_whole_genome_shotgun_sequence
marine_RJWL010069135.1_2458-2977_Kogia_breviceps_isolate_BS64_KogBre_flattened_line_138244_whole_genome_shotgun_sequence
marine_RJWL010079110.1_2345-2865_Kogia_breviceps_isolate_BS64_KogBre_flattened_line_158183_whole_genome_shotgun_sequence
marine_RJWL010085241.1_1255-1773_Kogia_breviceps_isolate_BS64_KogBre_flattened_line_170434_whole_genome_shotgun_sequence
marine_RJWL010049327.1_9210-9730_Kogia_breviceps_isolate_BS64_KogBre_flattened_line_98640_whole_genome_shotgun_sequence
marine_RJWL010024181.1_2845-3387_Kogia_breviceps_isolate_BS64_KogBre_flattened_line_48354_whole_genome_shotgun_sequence
marine_RJWL010074039.1_2525-3045_Kogia_breviceps_isolate_BS64_KogBre_flattened_line_148047_whole_genome_shotgun_sequence
AWHB01084196.1_1959-2136_Megaderma_lyra_ML_contig_91807_whole_genome_shotgun_sequence
PVLB01185835.1_291-700_Hipposideros_galeritus_isolate_US101_HipGal_scaffold_371448_whole_genome_shotgun_sequence
PVLB01219516.1_124-533_Hipposideros_galeritus_isolate_US101_HipGal_scaffold_438812_whole_genome_shotgun_sequence
PVLB01043876.1_3219-3720_Hipposideros_galeritus_isolate_US101_HipGal_scaffold_87562_whole_genome_shotgun_sequence
PVLB01051600.1_2781-3199_Hipposideros_galeritus_isolate_US101_HipGal_scaffold_103013_whole_genome_shotgun_sequence
JXIK01000211.1_416128-416624_Hipposideros_armiger_isolate_ML-2016_scaffold_210_whole_genome_shotgun_sequence
PVLB01000304.1_172567-173064_Hipposideros_galeritus_isolate_US101_HipGal_scaffold_526_whole_genome_shotgun_sequence
JXIK01000085.1_302998-303410_Hipposideros_armiger_isolate_ML-2016_scaffold_84_whole_genome_shotgun_sequence
JXIK01000932.1_529174-529586_Hipposideros_armiger_isolate_ML-2016_scaffold_931_whole_genome_shotgun_sequence
JXIK01000278.1_173-598_Hipposideros_armiger_isolate_ML-2016_scaffold_277_whole_genome_shotgun_sequence
PVLB01039538.1_4332-4762_Hipposideros_galeritus_isolate_US101_HipGal_scaffold_78887_whole_genome_shotgun_sequence
PVLB01039680.1_9109-9543_Hipposideros_galeritus_isolate_US101_HipGal_scaffold_79171_whole_genome_shotgun_sequence
PVLB01007451.1_4324-4757_Hipposideros_galeritus_isolate_US101_HipGal_scaffold_14724_whole_genome_shotgun_sequence
JXIK01000154.1_3062733-3063229_Hipposideros_armiger_isolate_ML-2016_scaffold_153_whole_genome_shotgun_sequence
JXIK01000154.1_945842-946079_Hipposideros_armiger_isolate_ML-2016_scaffold_153_whole_genome_shotgun_sequence
JXIK01000866.1_103122-103551_Hipposideros_armiger_isolate_ML-2016_scaffold_865_whole_genome_shotgun_sequence
JXIK01000548.1_277985-278482_Hipposideros_armiger_isolate_ML-2016_scaffold_547_whole_genome_shotgun_sequence
JXIK01001017.1_14462-14991_Hipposideros_armiger_isolate_ML-2016_scaffold_1016_whole_genome_shotgun_sequence
PVLB01005496.1_14128-14654_Hipposideros_galeritus_isolate_US101_HipGal_scaffold_10820_whole_genome_shotgun_sequence
JXIK01000264.1_674952-675482_Hipposideros_armiger_isolate_ML-2016_scaffold_263_whole_genome_shotgun_sequence
JXIK01001914.1_41938-42358_Hipposideros_armiger_isolate_ML-2016_scaffold_1913_whole_genome_shotgun_sequence
JXIK01000684.1_745923-746412_Hipposideros_armiger_isolate_ML-2016_scaffold_683_whole_genome_shotgun_sequence
JXIK01001895.1_30288-30785_Hipposideros_armiger_isolate_ML-2016_scaffold_1894_whole_genome_shotgun_sequence
JXIK01001459.1_75398-75825_Hipposideros_armiger_isolate_ML-2016_scaffold_1458_whole_genome_shotgun_sequence
RJWU010173308.1_3637-3970_Orycteropus_afer_isolate_BS58_OryAfe_flattened_line_346437_whole_genome_shotgun_sequence
PVJT020018802.1_1947067-1947229_Galeopterus_variegatus_isolate_US047_ScGVr9J_18768HRSCAF_108881_whole_genome_shotgun_sequence
PVJT020001615.1_2021-2183_Galeopterus_variegatus_isolate_US047_ScGVr9J_1599HRSCAF_61708_whole_genome_shotgun_sequence
PVJT021523468.1_3854826-3855265_Galeopterus_variegatus_isolate_US047_ScGVr9J_1528581HRSCAF_1654919_whole_genome_shotgun_sequence
PVJT020051095.1_1292-1731_Galeopterus_variegatus_isolate_US047_ScGVr9J_51049HRSCAF_160908_whole_genome_shotgun_sequence
PVJT020018802.1_2216709-2216871_Galeopterus_variegatus_isolate_US047_ScGVr9J_18768HRSCAF_108881_whole_genome_shotgun_sequence
PVJT021523567.1_1205028-1205541_Galeopterus_variegatus_isolate_US047_ScGVr9J_1528668HRSCAF_1655006_whole_genome_shotgun_sequence
PVJT020002215.1_206765-207207_Galeopterus_variegatus_isolate_US047_ScGVr9J_2199HRSCAF_65300_whole_genome_shotgun_sequence
PVJT020020430.1_3677702-3677864_Galeopterus_variegatus_isolate_US047_ScGVr9J_20395HRSCAF_112061_whole_genome_shotgun_sequence
PVJT020055201.1_9510775-9511299_Galeopterus_variegatus_isolate_US047_ScGVr9J_55155HRSCAF_166713_0_whole_genome_shotgun_sequence
PVJT020000038.1_577878-578308_Galeopterus_variegatus_isolate_US047_ScGVr9J_36HRSCAF_12531_whole_genome_shotgun_sequence
PVJT020000038.1_350280-350708_Galeopterus_variegatus_isolate_US047_ScGVr9J_36HRSCAF_12531_whole_genome_shotgun_sequence
PVJT020018802.1_2211554-2211854_Galeopterus_variegatus_isolate_US047_ScGVr9J_18768HRSCAF_108881_whole_genome_shotgun_sequence
PVJT020090625.1_849-1022_Galeopterus_variegatus_isolate_US047_ScGVr9J_90574HRSCAF_212357_whole_genome_shotgun_sequence
PVJT020020430.1_3678265-3678445_Galeopterus_variegatus_isolate_US047_ScGVr9J_20395HRSCAF_112061_whole_genome_shotgun_sequence
PVJT020007402.1_7677212-7677354_Galeopterus_variegatus_isolate_US047_ScGVr9J_7376HRSCAF_83199_whole_genome_shotgun_sequence
PVJT021523652.1_1019982-1020434_Galeopterus_variegatus_isolate_US047_ScGVr9J_1528742HRSCAF_1655080_0_whole_genome_shotgun_sequence
PVJT020003236.1_124658-124828_Galeopterus_variegatus_isolate_US047_ScGVr9J_3216HRSCAF_69789_1_whole_genome_shotgun_sequence
RJWU010036745.1_15906-16227_Orycteropus_afer_isolate_BS58_OryAfe_flattened_line_73452_whole_genome_shotgun_sequence
PVJL010010956.1_11717-12229_Megaderma_lyra_isolate_US036_MegLyr_scaffold_21893_whole_genome_shotgun_sequence
AWHB01051960.1_588-1092_Megaderma_lyra_ML_contig_56621_whole_genome_shotgun_sequence
AWHB01276649.1_113-507_Megaderma_lyra_ML_contig_301770_whole_genome_shotgun_sequence
PVJL010014998.1_5730-6124_Megaderma_lyra_isolate_US036_MegLyr_scaffold_29971_whole_genome_shotgun_sequence
PVJL010011713.1_36620-37088_Megaderma_lyra_isolate_US036_MegLyr_scaffold_23405_whole_genome_shotgun_sequence
AWHB01463073.1_3607-4117_Megaderma_lyra_ML_contig_501149_whole_genome_shotgun_sequence
PVJL010004486.1_6119-6360_Megaderma_lyra_isolate_US036_MegLyr_scaffold_8968_whole_genome_shotgun_sequence
PVJL010009031.1_50836-51284_Megaderma_lyra_isolate_US036_MegLyr_scaffold_18050_whole_genome_shotgun_sequence
AAPE02010105.1_38914-39432_Myotis_lucifugus_contig_10104_whole_genome_shotgun_sequence
ANKR01297751.1_56427-56945_Myotis_brandtii_contig297751_whole_genome_shotgun_sequence
PVIZ010004898.1_15520-16037_Myotis_myotis_isolate_US100_MyoMyo_scaffold_9777_whole_genome_shotgun_sequence
VMDP01196638.1_410-927_Antrozous_pallidus_isolate_US032_AntPal_flattened_line_393221_whole_genome_shotgun_sequence
PVIG010017884.1_32748-33084_Tadarida_brasiliensis_isolate_US034_TadBra_scaffold_35759_whole_genome_shotgun_sequence
0.05

lineage312

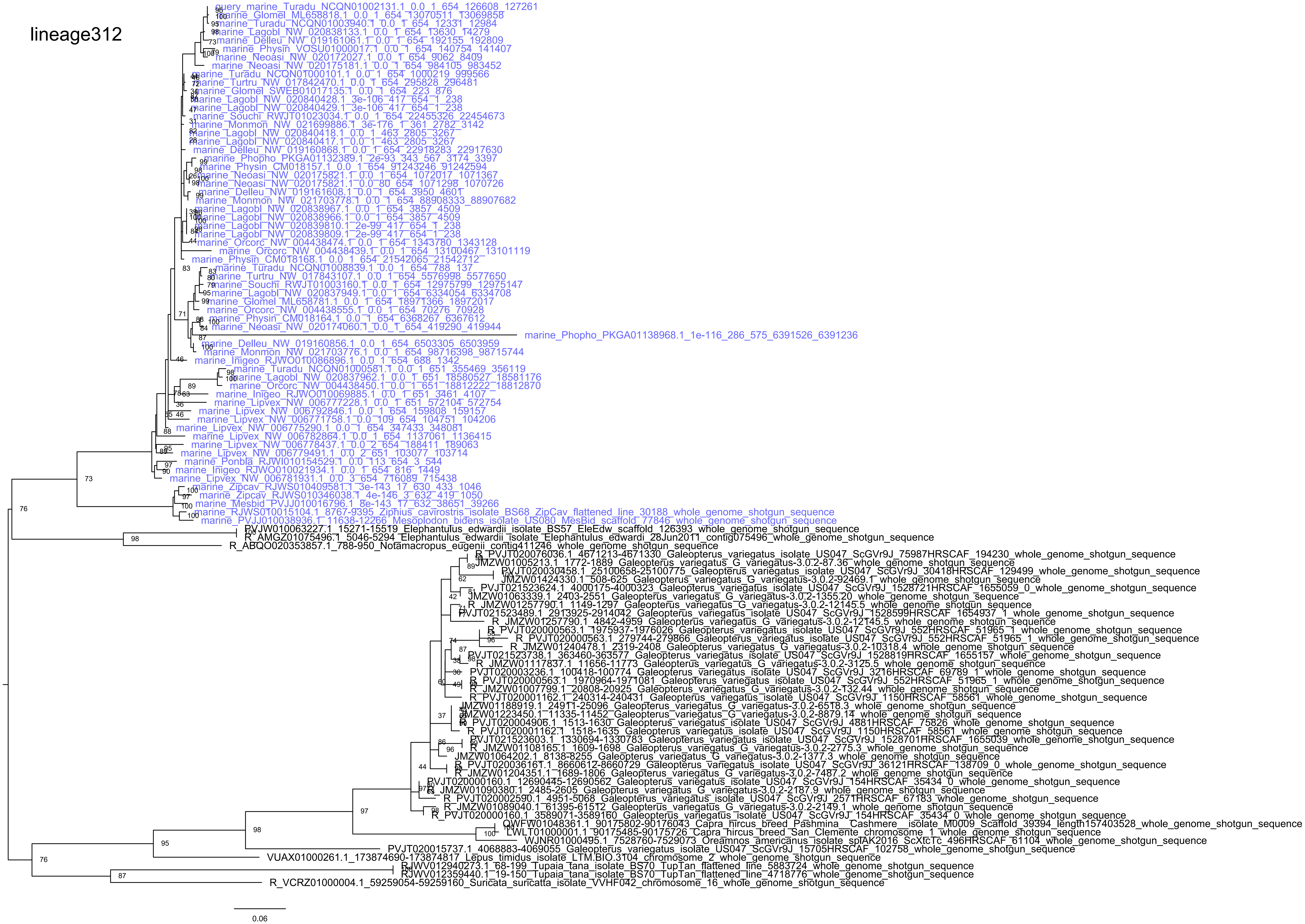

query_marine_Turadu_NCQN01002131.1_0.0_1_654_126608_127261
marine_Glomel_ML658818.1_0.0_1_654_13070511_13069858
marine_Turadu_NCQN01003940.1_0.0_1_654_12331_12984
marine_Lagobl_NW_020838133.1_0.0_1_654_13630_14279
marine_Delleu_NW_019161061.1_0.0_1_654_192155_192809
marine_Physin_VOSU01000017.1_0.0_1_654_140754_141407
marine_Neoasi_NW_020172027.1_0.0_1_654_9062_8409
marine_Neoasi_NW_020175181.1_0.0_1_654_984105_983452
marine_Turadu_NCQN01000101.1_0.0_1_654_1000219_999566
marine_Turtru_NW_017842470.1_0.0_1_654_295828_296481
marine_Glomel_SWEB01017135.1_0.0_1_654_223_876
marine_Lagobl_NW_020840428.1_3e-106_417_654_1_238
marine_Lagobl_NW_020840429.1_3e-106_417_654_1_238
marine_Souchi_RWJT01023034.1_0.0_1_654_22455326_22454673
marine_Monmon_NW_021699886.1_3e-176_1_361_2782_3142
marine_Lagobl_NW_020840418.1_0.0_1_463_2805_3267
marine_Lagobl_NW_020840417.1_0.0_1_463_2805_3267
marine_Delleu_NW_019160868.1_0.0_1_654_22918283_22917630
marine_Phopho_PKGA01132389.1_2e-93_343_567_3174_3397
marine_Physin_CM018157.1_0.0_1_654_91243246_91242594
marine_Neoasi_NW_020175821.1_0.0_1_654_1072017_1071367
marine_Neoasi_NW_020175821.1_0.0_80_654_1071298_1070726
marine_Delleu_NW_019161608.1_0.0_1_654_3950_4601
marine_Monmon_NW_021703778.1_0.0_1_654_88908333_88907682
marine_Lagobl_NW_020838967.1_0.0_1_654_3857_4509
marine_Lagobl_NW_020838966.1_0.0_1_654_3857_4509
marine_Lagobl_NW_020839810.1_2e-99_417_654_1_238
marine_Lagobl_NW_020839809.1_2e-99_417_654_1_238
marine_Orcorc_NW_004438474.1_0.0_1_654_1343780_1343128
marine_Orcorc_NW_004438439.1_0.0_1_654_13100467_13101119
marine_Physin_CM018168.1_0.0_1_654_21542065_21542712
marine_Turadu_NCQN01008839.1_0.0_1_654_788_137
marine_Turtru_NW_017843107.1_0.0_1_654_5576998_5577650
marine_Souchi_RWJT01003160.1_0.0_1_654_12975799_12975147
marine_Lagobl_NW_020837949.1_0.0_1_654_6334054_6334708
marine_Glomel_ML658781.1_0.0_1_654_18971366_18972017
marine_Orcorc_NW_004438555.1_0.0_1_654_70276_70928
marine_Physin_CM018164.1_0.0_1_654_6368267_6367612
marine_Neoasi_NW_020174060.1_0.0_1_654_419290_419944
marine_Phopho_PKGA01138968.1_1e-116_286_575_6391526_6391236
marine_Delleu_NW_019160856.1_0.0_1_654_6503305_6503959
marine_Monmon_NW_021703776.1_0.0_1_654_98716398_98715744
marine_Inigeo_RJWO010086896.1_0.0_1_654_688_1342
marine_Turadu_NCQN01000581.1_0.0_1_651_355469_356119
marine_Lagobl_NW_020837962.1_0.0_1_651_18580527_18581176
marine_Orcorc_NW_004438450.1_0.0_1_651_18812222_18812870
marine_Inigeo_RJWO010069885.1_0.0_1_651_3461_4107
marine_Lipvex_NW_006777228.1_0.0_1_651_572104_572754
marine_Lipvex_NW_006792846.1_0.0_1_654_159808_159157
marine_Lipvex_NW_006771758.1_0.0_109_654_104751_104206
marine_Lipvex_NW_006775290.1_0.0_1_654_347433_348081
marine_Lipvex_NW_006782864.1_0.0_1_654_1137061_1136415
marine_Lipvex_NW_006778437.1_0.0_2_654_188411_189063
marine_Lipvex_NW_006779491.1_0.0_2_651_103077_103714
marine_Ponbla_RJWI010154529.1_0.0_113_654_3_544
marine_Inigeo_RJWO010021934.1_0.0_1_654_816_1449
marine_Lipvex_NW_006781931.1_0.0_3_654_716089_715438
marine_Zipcav_RJWS010409581.1_3e-143_17_630_433_1046
marine_Zipcav_RJWS010346038.1_4e-146_3_632_419_1050
marine_Mesbid_PVJJ010016796.1_8e-143_17_632_38651_39266
marine_RJWS010015104.1_8767-9395_Ziphius_cavirostris_isolate_BS68_ZipCav_flattened_line_30188_whole_genome_shotgun_sequence
marine_PVJJ010038936.1_11638-12266_Mesoplodon_bidens_isolate_US080_MesBid_scaffold_77846_whole_genome_shotgun_sequence
PVJW010063227.1_15271-15519_Elephantulus_edwardii_isolate_BS57_EleEdw_scaffold_126393_whole_genome_shotgun_sequence
R_AMGZ01075496.1_5046-5294_Elephantulus_edwardii_isolate_Elephantulus_edwardi_28Jun2011_contig075496_whole_genome_shotgun_sequence
R_ABQO020353857.1_788-950_Notamacropus_eugenii_contig411246_whole_genome_shotgun_sequence
R_PVJT020076036.1_4671213-4671330_Galeopterus_variegatus_isolate_US047_ScGVr9J_75987HRSCAF_194230_whole_genome_shotgun_sequence
JMZW01005213.1_1772-1889_Galeopterus_variegatus_G_variegatus-3.0.2-87.36_whole_genome_shotgun_sequence
PVJT020030458.1_25100658-25100775_Galeopterus_variegatus_isolate_US047_ScGVr9J_30418HRSCAF_129499_whole_genome_shotgun_sequence
JMZW01424330.1_508-625_Galeopterus_variegatus_G_variegatus-3.0.2-92469.1_whole_genome_shotgun_sequence
PVJT021523624.1_4000175-4000323_Galeopterus_variegatus_isolate_US047_ScGVr9J_1528721HRSCAF_1655059_0_whole_genome_shotgun_sequence
JMZW01063339.1_2403-2551_Galeopterus_variegatus_G_variegatus-3.0.2-1355.20_whole_genome_shotgun_sequence
R_JMZW01257790.1_1149-1297_Galeopterus_variegatus_G_variegatus-3.0.2-12145.5_whole_genome_shotgun_sequence
PVJT021523489.1_2913925-2914042_Galeopterus_variegatus_isolate_US047_ScGVr9J_1528599HRSCAF_1654937_1_whole_genome_shotgun_sequence
R_JMZW01257790.1_4842-4959_Galeopterus_variegatus_G_variegatus-3.0.2-12145.5_whole_genome_shotgun_sequence
PVJT020000563.1_1975937-1976026_Galeopterus_variegatus_isolate_US047_ScGVr9J_552HRSCAF_51965_1_whole_genome_shotgun_sequence
R_PVJT020000563.1_279744-279866_Galeopterus_variegatus_isolate_US047_ScGVr9J_552HRSCAF_51965_1_whole_genome_shotgun_sequence
R_JMZW01240478.1_2319-2408_Galeopterus_variegatus_G_variegatus-3.0.2-10318.4_whole_genome_shotgun_sequence
PVJT021523738.1_363460-363577_Galeopterus_variegatus_isolate_US047_ScGVr9J_1528819HRSCAF_1655157_whole_genome_shotgun_sequence
R_JMZW01117837.1_11656-11773_Galeopterus_variegatus_G_variegatus-3.0.2-3125.5_whole_genome_shotgun_sequence
PVJT020003236.1_100418-100774_Galeopterus_variegatus_isolate_US047_ScGVr9J_3216HRSCAF_69789_1_whole_genome_shotgun_sequence
R_PVJT020000563.1_1970964-1971081_Galeopterus_variegatus_isolate_US047_ScGVr9J_552HRSCAF_51965_1_whole_genome_shotgun_sequence
R_JMZW01007799.1_20808-20925_Galeopterus_variegatus_G_variegatus-3.0.2-132.44_whole_genome_shotgun_sequence
R_PVJT020001162.1_240314-240431_Galeopterus_variegatus_isolate_US047_ScGVr9J_1150HRSCAF_58561_whole_genome_shotgun_sequence
JMZW01188919.1_24911-25096_Galeopterus_variegatus_G_variegatus-3.0.2-6518.3_whole_genome_shotgun_sequence
JMZW01223450.1_11335-11452_Galeopterus_variegatus_G_variegatus-3.0.2-8879.14_whole_genome_shotgun_sequence
R_PVJT020004906.1_1513-1630_Galeopterus_variegatus_isolate_US047_ScGVr9J_4881HRSCAF_75826_whole_genome_shotgun_sequence
R_PVJT020001162.1_1518-1635_Galeopterus_variegatus_isolate_US047_ScGVr9J_1150HRSCAF_58561_whole_genome_shotgun_sequence
PVJT021523603.1_1330694-1330783_Galeopterus_variegatus_isolate_US047_ScGVr9J_1528701HRSCAF_1655039_whole_genome_shotgun_sequence
R_JMZW01108165.1_1609-1698_Galeopterus_variegatus_G_variegatus-3.0.2-2775.3_whole_genome_shotgun_sequence
JMZW01064202.1_8138-8255_Galeopterus_variegatus_G_variegatus-3.0.2-1377.3_whole_genome_shotgun_sequence
R_PVJT020036161.1_8660612-8660729_Galeopterus_variegatus_isolate_US047_ScGVr9J_36121HRSCAF_138709_0_whole_genome_shotgun_sequence
R_JMZW01204351.1_1689-1806_Galeopterus_variegatus_G_variegatus-3.0.2-7487.2_whole_genome_shotgun_sequence
PVJT020000160.1_12690445-12690562_Galeopterus_variegatus_isolate_US047_ScGVr9J_154HRSCAF_35434_0_whole_genome_shotgun_sequence
R_JMZW01090380.1_2485-2605_Galeopterus_variegatus_G_variegatus-3.0.2-2187.9_whole_genome_shotgun_sequence
R_PVJT020002590.1_4951-5068_Galeopterus_variegatus_isolate_US047_ScGVr9J_2571HRSCAF_67183_whole_genome_shotgun_sequence
R_JMZW01089040.1_61395-61512_Galeopterus_variegatus_G_variegatus-3.0.2-2149.1_whole_genome_shotgun_sequence
R_PVJT020000160.1_3589071-3589160_Galeopterus_variegatus_isolate_US047_ScGVr9J_154HRSCAF_35434_0_whole_genome_shotgun_sequence
QWFW01048361.1_90175802-90176043_Capra_hircus_breed_Pashmina__Cashmere__isolate_M0009_Scaffold_39394_length157403528_whole_genome_shotgun_sequence
LWLT01000001.1_90175485-90175726_Capra_hircus_breed_San_Clemente_chromosome_1_whole_genome_shotgun_sequence
WJNR01000495.1_7528760-7529073_Oreamnos_americanus_isolate_spIAK2016_ScXfcTc_496HRSCAF_61104_whole_genome_shotgun_sequence
PVJT020015737.1_4068883-4069055_Galeopterus_variegatus_isolate_US047_ScGVr9J_15705HRSCAF_102758_whole_genome_shotgun_sequence
VUAX01000261.1_173874690-173874817_Lepus_timidus_isolate_LTM.BIO.3104_chromosome_2_whole_genome_shotgun_sequence
R_RJWV012940273.1_68-199_Tupaia_tana_isolate_BS70_TupTan_flattened_line_5883724_whole_genome_shotgun_sequence
RJWV012359440.1_19-150_Tupaia_tana_isolate_BS70_TupTan_flattened_line_4718776_whole_genome_shotgun_sequence
R_VCRZ01000004.1_59259054-59259160_Suricata_suricatta_isolate_VVHF042_chromosome_16_whole_genome_shotgun_sequence
0.06

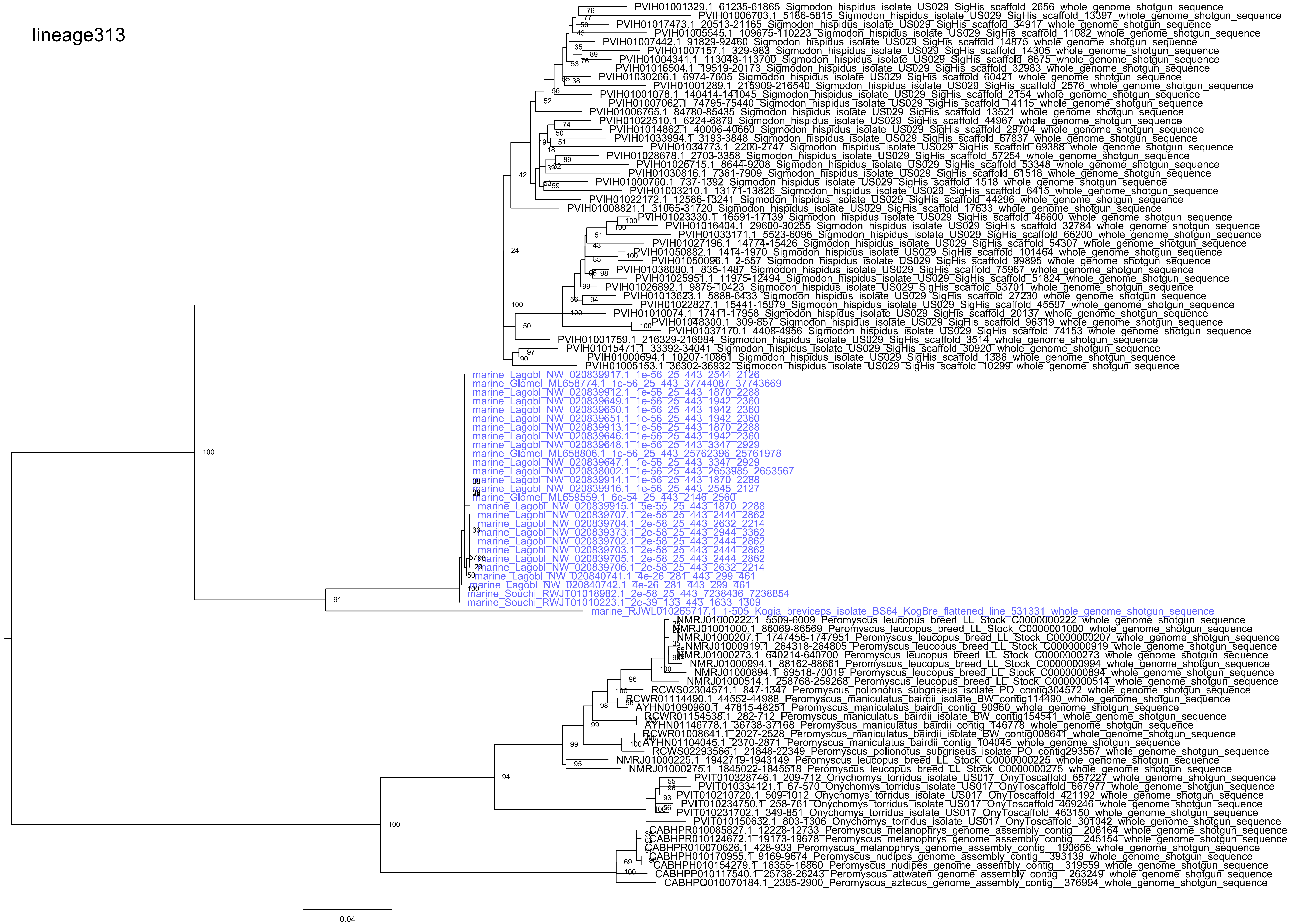
lineage313
PVIH01001329.1_61235-61865_Sigmodon_hispidus_isolate_US029_SigHis_scaffold_2656_whole_genome_shotgun_sequence
PVIH01006703.1_5186-5815_Sigmodon_hispidus_isolate_US029_SigHis_scaffold_13397_whole_genome_shotgun_sequence
PVIH01017473.1_20513-21165_Sigmodon_hispidus_isolate_US029_SigHis_scaffold_34917_whole_genome_shotgun_sequence
PVIH01005545.1_109675-110223_Sigmodon_hispidus_isolate_US029_SigHis_scaffold_11082_whole_genome_shotgun_sequence
PVIH01007442.1_91829-92460_Sigmodon_hispidus_isolate_US029_SigHis_scaffold_14875_whole_genome_shotgun_sequence
PVIH01007157.1_329-983_Sigmodon_hispidus_isolate_US029_SigHis_scaffold_14305_whole_genome_shotgun_sequence
PVIH01004341.1_113048-113700_Sigmodon_hispidus_isolate_US029_SigHis_scaffold_8675_whole_genome_shotgun_sequence
PVIH01016504.1_19519-20173_Sigmodon_hispidus_isolate_US029_SigHis_scaffold_32983_whole_genome_shotgun_sequence
PVIH01030266.1_6974-7605_Sigmodon_hispidus_isolate_US029_SigHis_scaffold_60421_whole_genome_shotgun_sequence
PVIH01001289.1_215909-216540_Sigmodon_hispidus_isolate_US029_SigHis_scaffold_2576_whole_genome_shotgun_sequence
PVIH01001078.1_140414-141045_Sigmodon_hispidus_isolate_US029_SigHis_scaffold_2154_whole_genome_shotgun_sequence
PVIH01007062.1_74795-75440_Sigmodon_hispidus_isolate_US029_SigHis_scaffold_14115_whole_genome_shotgun_sequence
PVIH01006765.1_84780-85435_Sigmodon_hispidus_isolate_US029_SigHis_scaffold_13521_whole_genome_shotgun_sequence
PVIH01022510.1_6224-6879_Sigmodon_hispidus_isolate_US029_SigHis_scaffold_44967_whole_genome_shotgun_sequence
PVIH01014862.1_40006-40660_Sigmodon_hispidus_isolate_US029_SigHis_scaffold_29704_whole_genome_shotgun_sequence
PVIH01033994.1_3193-3848_Sigmodon_hispidus_isolate_US029_SigHis_scaffold_67837_whole_genome_shotgun_sequence
PVIH01034773.1_2200-2747_Sigmodon_hispidus_isolate_US029_SigHis_scaffold_69388_whole_genome_shotgun_sequence
PVIH01028678.1_2703-3358_Sigmodon_hispidus_isolate_US029_SigHis_scaffold_57254_whole_genome_shotgun_sequence
PVIH01026715.1_8644-9208_Sigmodon_hispidus_isolate_US029_SigHis_scaffold_53348_whole_genome_shotgun_sequence
PVIH01030816.1_7361-7909_Sigmodon_hispidus_isolate_US029_SigHis_scaffold_61518_whole_genome_shotgun_sequence
PVIH01000760.1_737-1392_Sigmodon_hispidus_isolate_US029_SigHis_scaffold_1518_whole_genome_shotgun_sequence
PVIH01003210.1_13171-13826_Sigmodon_hispidus_isolate_US029_SigHis_scaffold_6415_whole_genome_shotgun_sequence
PVIH01022172.1_12586-13241_Sigmodon_hispidus_isolate_US029_SigHis_scaffold_44296_whole_genome_shotgun_sequence
PVIH01008821.1_31065-31720_Sigmodon_hispidus_isolate_US029_SigHis_scaffold_17633_whole_genome_shotgun_sequence
PVIH01023330.1_16591-17139_Sigmodon_hispidus_isolate_US029_SigHis_scaffold_46600_whole_genome_shotgun_sequence
PVIH01016404.1_29600-30255_Sigmodon_hispidus_isolate_US029_SigHis_scaffold_32784_whole_genome_shotgun_sequence
PVIH01033171.1_5523-6096_Sigmodon_hispidus_isolate_US029_SigHis_scaffold_66200_whole_genome_shotgun_sequence
PVIH01027196.1_14774-15426_Sigmodon_hispidus_isolate_US029_SigHis_scaffold_54307_whole_genome_shotgun_sequence
PVIH01050882.1_1414-1970_Sigmodon_hispidus_isolate_US029_SigHis_scaffold_101464_whole_genome_shotgun_sequence
PVIH01050096.1_2-557_Sigmodon_hispidus_isolate_US029_SigHis_scaffold_99895_whole_genome_shotgun_sequence
PVIH01038080.1_835-1487_Sigmodon_hispidus_isolate_US029_SigHis_scaffold_75967_whole_genome_shotgun_sequence
PVIH01025951.1_11975-12494_Sigmodon_hispidus_isolate_US029_SigHis_scaffold_51824_whole_genome_shotgun_sequence
PVIH01026892.1_9875-10423_Sigmodon_hispidus_isolate_US029_SigHis_scaffold_53701_whole_genome_shotgun_sequence
PVIH01013623.1_5888-6433_Sigmodon_hispidus_isolate_US029_SigHis_scaffold_27230_whole_genome_shotgun_sequence
PVIH01022827.1_15441-15979_Sigmodon_hispidus_isolate_US029_SigHis_scaffold_45597_whole_genome_shotgun_sequence
PVIH01010074.1_17411-17958_Sigmodon_hispidus_isolate_US029_SigHis_scaffold_20137_whole_genome_shotgun_sequence
PVIH01048300.1_309-857_Sigmodon_hispidus_isolate_US029_SigHis_scaffold_96319_whole_genome_shotgun_sequence
PVIH01037170.1_4408-4956_Sigmodon_hispidus_isolate_US029_SigHis_scaffold_74153_whole_genome_shotgun_sequence
PVIH01001759.1_216329-216984_Sigmodon_hispidus_isolate_US029_SigHis_scaffold_3514_whole_genome_shotgun_sequence
PVIH01015471.1_33392-34041_Sigmodon_hispidus_isolate_US029_SigHis_scaffold_30920_whole_genome_shotgun_sequence
PVIH01000694.1_10207-10861_Sigmodon_hispidus_isolate_US029_SigHis_scaffold_1386_whole_genome_shotgun_sequence
PVIH01005153.1_36302-36932_Sigmodon_hispidus_isolate_US029_SigHis_scaffold_10299_whole_genome_shotgun_sequence
marine_Lagobl_NW_020839917.1_1e-56_25_443_2544_2126
marine_Glomel_ML658774.1_1e-56_25_443_37744087_37743669
marine_Lagobl_NW_020839912.1_1e-56_25_443_1870_2288
marine_Lagobl_NW_020839649.1_1e-56_25_443_1942_2360
marine_Lagobl_NW_020839650.1_1e-56_25_443_1942_2360
marine_Lagobl_NW_020839651.1_1e-56_25_443_1942_2360
marine_Lagobl_NW_020839913.1_1e-56_25_443_1870_2288
marine_Lagobl_NW_020839646.1_1e-56_25_443_1942_2360
marine_Lagobl_NW_020839648.1_1e-56_25_443_3347_2929
marine_Glomel_ML658806.1_1e-56_25_443_25762396_25761978
marine_Lagobl_NW_020839647.1_1e-56_25_443_3347_2929
marine_Lagobl_NW_020838002.1_1e-56_25_443_2653985_2653567
marine_Lagobl_NW_020839914.1_1e-56_25_443_1870_2288
marine_Lagobl_NW_020839916.1_1e-56_25_443_2545_2127
marine_Glomel_ML659559.1_6e-54_25_443_2146_2560
marine_Lagobl_NW_020839915.1_5e-55_25_443_1870_2288
marine_Lagobl_NW_020839707.1_2e-58_25_443_2444_2862
marine_Lagobl_NW_020839704.1_2e-58_25_443_2632_2214
marine_Lagobl_NW_020839373.1_2e-58_25_443_2944_3362
marine_Lagobl_NW_020839702.1_2e-58_25_443_2444_2862
marine_Lagobl_NW_020839703.1_2e-58_25_443_2444_2862
marine_Lagobl_NW_020839705.1_2e-58_25_443_2444_2862
marine_Lagobl_NW_020839706.1_2e-58_25_443_2632_2214
marine_Lagobl_NW_020840741.1_4e-26_281_443_299_461
marine_Lagobl_NW_020840742.1_4e-26_281_443_299_461
marine_Souchi_RWJT01018982.1_2e-58_25_443_7238436_7238854
marine_Souchi_RWJT01010223.1_2e-39_133_443_1633_1309
marine_RJWL010265717.1_1-505_Kogia_breviceps_isolate_BS64_KogBre_flattened_line_531331_whole_genome_shotgun_sequence
NMRJ01000222.1_5509-6009_Peromyscus_leucopus_breed_LL_Stock_C0000000222_whole_genome_shotgun_sequence
NMRJ01001000.1_86069-86569_Peromyscus_leucopus_breed_LL_Stock_C0000001000_whole_genome_shotgun_sequence
NMRJ01000207.1_1747456-1747951_Peromyscus_leucopus_breed_LL_Stock_C0000000207_whole_genome_shotgun_sequence
NMRJ01000919.1_264318-264805_Peromyscus_leucopus_breed_LL_Stock_C0000000919_whole_genome_shotgun_sequence
NMRJ01000273.1_640214-640700_Peromyscus_leucopus_breed_LL_Stock_C0000000273_whole_genome_shotgun_sequence
NMRJ01000994.1_88162-88661_Peromyscus_leucopus_breed_LL_Stock_C0000000994_whole_genome_shotgun_sequence
NMRJ01000894.1_69518-70019_Peromyscus_leucopus_breed_LL_Stock_C0000000894_whole_genome_shotgun_sequence
NMRJ01000514.1_258768-259268_Peromyscus_leucopus_breed_LL_Stock_C0000000514_whole_genome_shotgun_sequence
RCWS02304571.1_847-1347_Peromyscus_polionotus_subgriseus_isolate_PO_contig304572_whole_genome_shotgun_sequence
RCWR01114490.1_44552-44988_Peromyscus_maniculatus_bairdii_isolate_BW_contig114490_whole_genome_shotgun_sequence
AYHN01090960.1_47815-48251_Peromyscus_maniculatus_bairdii_contig_90960_whole_genome_shotgun_sequence
RCWR01154538.1_282-712_Peromyscus_maniculatus_bairdii_isolate_BW_contig154541_whole_genome_shotgun_sequence
AYHN01146778.1_36738-37168_Peromyscus_maniculatus_bairdii_contig_146778_whole_genome_shotgun_sequence
RCWR01008641.1_2027-2528_Peromyscus_maniculatus_bairdii_isolate_BW_contig008641_whole_genome_shotgun_sequence
AYHN01104045.1_2370-2871_Peromyscus_maniculatus_bairdii_contig_104045_whole_genome_shotgun_sequence
RCWS02293566.1_21848-22349_Peromyscus_polionotus_subgriseus_isolate_PO_contig293567_whole_genome_shotgun_sequence
NMRJ01000225.1_1942719-1943149_Peromyscus_leucopus_breed_LL_Stock_C0000000225_whole_genome_shotgun_sequence
NMRJ01000275.1_1845022-1845518_Peromyscus_leucopus_breed_LL_Stock_C0000000275_whole_genome_shotgun_sequence
PVIT010328746.1_209-712_Onychomys_torridus_isolate_US017_OnyToscaffold_657227_whole_genome_shotgun_sequence
PVIT010334121.1_67-570_Onychomys_torridus_isolate_US017_OnyToscaffold_667977_whole_genome_shotgun_sequence
PVIT010210720.1_509-1012_Onychomys_torridus_isolate_US017_OnyToscaffold_421192_whole_genome_shotgun_sequence
PVIT010234750.1_258-761_Onychomys_torridus_isolate_US017_OnyToscaffold_469246_whole_genome_shotgun_sequence
PVIT010231702.1_349-851_Onychomys_torridus_isolate_US017_OnyToscaffold_463150_whole_genome_shotgun_sequence
PVIT010150632.1_803-1306_Onychomys_torridus_isolate_US017_OnyToscaffold_301042_whole_genome_shotgun_sequence
CABHPR010085827.1_12228-12733_Peromyscus_melanophrys_genome_assembly_contig__206164_whole_genome_shotgun_sequence
CABHPR010124672.1_19173-19678_Peromyscus_melanophrys_genome_assembly_contig__245154_whole_genome_shotgun_sequence
CABHPR010070626.1_428-933_Peromyscus_melanophrys_genome_assembly_contig__190656_whole_genome_shotgun_sequence
CABHPH010170955.1_9169-9674_Peromyscus_nudipes_genome_assembly_contig__393139_whole_genome_shotgun_sequence
CABHPH010154279.1_16355-16860_Peromyscus_nudipes_genome_assembly_contig__319559_whole_genome_shotgun_sequence
CABHPP010117540.1_25738-26243_Peromyscus_attwateri_genome_assembly_contig__263249_whole_genome_shotgun_sequence
CABHPQ010070184.1_2395-2900_Peromyscus_aztecus_genome_assembly_contig__376994_whole_genome_shotgun_sequence

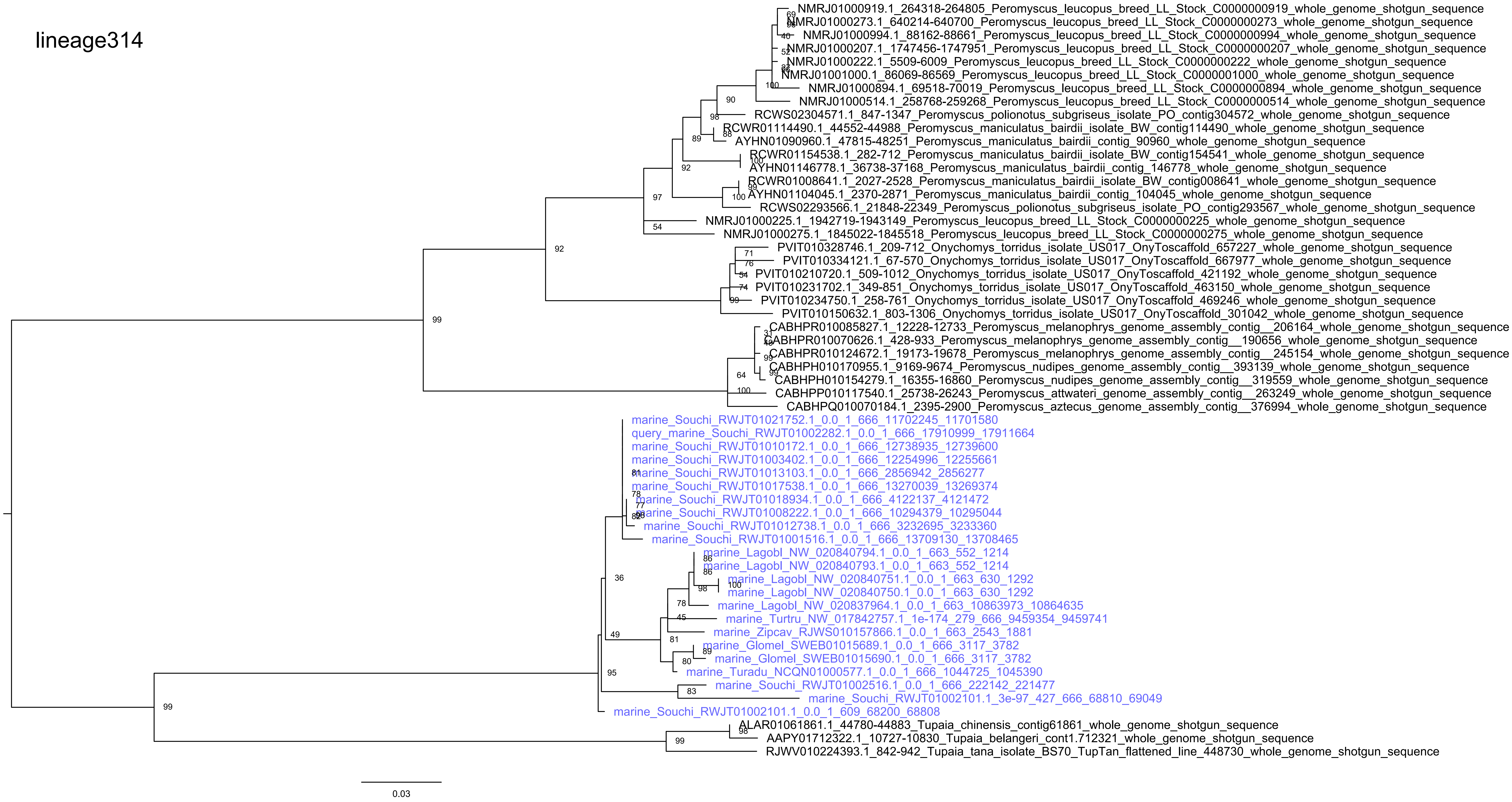
lineage314
NMRJ01000919.1_264318-264805_Peromyscus_leucopus_breed_LL_Stock_C0000000919_whole_genome_shotgun_sequence
NMRJ01000273.1_640214-640700_Peromyscus_leucopus_breed_LL_Stock_C0000000273_whole_genome_shotgun_sequence
NMRJ01000994.1_88162-88661_Peromyscus_leucopus_breed_LL_Stock_C0000000994_whole_genome_shotgun_sequence
NMRJ01000207.1_1747456-1747951_Peromyscus_leucopus_breed_LL_Stock_C0000000207_whole_genome_shotgun_sequence
NMRJ01000222.1_5509-6009_Peromyscus_leucopus_breed_LL_Stock_C0000000222_whole_genome_shotgun_sequence
NMRJ01001000.1_86069-86569_Peromyscus_leucopus_breed_LL_Stock_C0000001000_whole_genome_shotgun_sequence
NMRJ01000894.1_69518-70019_Peromyscus_leucopus_breed_LL_Stock_C0000000894_whole_genome_shotgun_sequence
NMRJ01000514.1_258768-259268_Peromyscus_leucopus_breed_LL_Stock_C0000000514_whole_genome_shotgun_sequence
RCWS02304571.1_847-1347_Peromyscus_polionotus_subgriseus_isolate_PO_contig304572_whole_genome_shotgun_sequence
RCWR01114490.1_44552-44988_Peromyscus_maniculatus_bairdii_isolate_BW_contig114490_whole_genome_shotgun_sequence
AYHN01090960.1_47815-48251_Peromyscus_maniculatus_bairdii_contig_90960_whole_genome_shotgun_sequence
RCWR01154538.1_282-712_Peromyscus_maniculatus_bairdii_isolate_BW_contig154541_whole_genome_shotgun_sequence
AYHN01146778.1_36738-37168_Peromyscus_maniculatus_bairdii_contig_146778_whole_genome_shotgun_sequence
RCWR01008641.1_2027-2528_Peromyscus_maniculatus_bairdii_isolate_BW_contig008641_whole_genome_shotgun_sequence
AYHN01104045.1_2370-2871_Peromyscus_maniculatus_bairdii_contig_104045_whole_genome_shotgun_sequence
RCWS02293566.1_21848-22349_Peromyscus_polionotus_subgriseus_isolate_PO_contig293567_whole_genome_shotgun_sequence
NMRJ01000225.1_1942719-1943149_Peromyscus_leucopus_breed_LL_Stock_C0000000225_whole_genome_shotgun_sequence
NMRJ01000275.1_1845022-1845518_Peromyscus_leucopus_breed_LL_Stock_C0000000275_whole_genome_shotgun_sequence
PVIT010328746.1_209-712_Onychomys_torridus_isolate_US017_OnyToscaffold_657227_whole_genome_shotgun_sequence
PVIT010334121.1_67-570_Onychomys_torridus_isolate_US017_OnyToscaffold_667977_whole_genome_shotgun_sequence
PVIT010210720.1_509-1012_Onychomys_torridus_isolate_US017_OnyToscaffold_421192_whole_genome_shotgun_sequence
PVIT010231702.1_349-851_Onychomys_torridus_isolate_US017_OnyToscaffold_463150_whole_genome_shotgun_sequence
PVIT010234750.1_258-761_Onychomys_torridus_isolate_US017_OnyToscaffold_469246_whole_genome_shotgun_sequence
PVIT010150632.1_803-1306_Onychomys_torridus_isolate_US017_OnyToscaffold_301042_whole_genome_shotgun_sequence
CABHPR010085827.1_12228-12733_Peromyscus_melanophrys_genome_assembly_contig__206164_whole_genome_shotgun_sequence
CABHPR010070626.1_428-933_Peromyscus_melanophrys_genome_assembly_contig__190656_whole_genome_shotgun_sequence
CABHPR010124672.1_19173-19678_Peromyscus_melanophrys_genome_assembly_contig__245154_whole_genome_shotgun_sequence
CABHPH010170955.1_9169-9674_Peromyscus_nudipes_genome_assembly_contig__393139_whole_genome_shotgun_sequence
CABHPH010154279.1_16355-16860_Peromyscus_nudipes_genome_assembly_contig__319559_whole_genome_shotgun_sequence
CABHPP010117540.1_25738-26243_Peromyscus_attwateri_genome_assembly_contig__263249_whole_genome_shotgun_sequence
CABHPQ010070184.1_2395-2900_Peromyscus_aztecus_genome_assembly_contig__376994_whole_genome_shotgun_sequence
marine_Souchi_RWJT01021752.1_0.0_1_666_11702245_11701580
query_marine_Souchi_RWJT01002282.1_0.0_1_666_17910999_17911664
marine_Souchi_RWJT01010172.1_0.0_1_666_12738935_12739600
marine_Souchi_RWJT01003402.1_0.0_1_666_12254996_12255661
marine_Souchi_RWJT01013103.1_0.0_1_666_2856942_2856277
marine_Souchi_RWJT01017538.1_0.0_1_666_13270039_13269374
marine_Souchi_RWJT01018934.1_0.0_1_666_4122137_4121472
marine_Souchi_RWJT01008222.1_0.0_1_666_10294379_10295044
marine_Souchi_RWJT01012738.1_0.0_1_666_3232695_3233360
marine_Souchi_RWJT01001516.1_0.0_1_666_13709130_13708465
marine_Lagobl_NW_020840794.1_0.0_1_663_552_1214
marine_Lagobl_NW_020840793.1_0.0_1_663_552_1214
marine_Lagobl_NW_020840751.1_0.0_1_663_630_1292
marine_Lagobl_NW_020840750.1_0.0_1_663_630_1292
marine_Lagobl_NW_020837964.1_0.0_1_663_10863973_10864635
marine_Turtru_NW_017842757.1_1e-174_279_666_9459354_9459741
marine_Zipcav_RJWS010157866.1_0.0_1_663_2543_1881
marine_Glomel_SWEB01015689.1_0.0_1_666_3117_3782
marine_Glomel_SWEB01015690.1_0.0_1_666_3117_3782
marine_Turadu_NCQN01000577.1_0.0_1_666_1044725_1045390
marine_Souchi_RWJT01002516.1_0.0_1_666_222142_221477
marine_Souchi_RWJT01002101.1_3e-97_427_666_68810_69049
marine_Souchi_RWJT01002101.1_0.0_1_609_68200_68808
ALAR01061861.1_44780-44883_Tupaia_chinensis_contig61861_whole_genome_shotgun_sequence
AAPY01712322.1_10727-10830_Tupaia_belangeri_cont1.712321_whole_genome_shotgun_sequence
RJWV010224393.1_842-942_Tupaia_tana_isolate_BS70_TupTan_flattened_line_448730_whole_genome_shotgun_sequence
0.03
